# Prediction of Multi-Type Membrane Proteins in Human by an Integrated Approach

**DOI:** 10.1371/journal.pone.0093553

**Published:** 2014-03-27

**Authors:** Guohua Huang, Yuchao Zhang, Lei Chen, Ning Zhang, Tao Huang, Yu-Dong Cai

**Affiliations:** 1 Institute of Systems Biology, Shanghai University, Shanghai, China; 2 Department of Mathematics, Shaoyang University, Shaoyang, Hunan, China; 3 Graduate School of the Chinese Academy of Sciences, Beijing, China; 4 State Key Laboratory of Medical Genomics, Institute of Health Sciences, Shanghai Institutes for Biological Sciences, Chinese Academy of Sciences, Shanghai Jiao Tong University School of Medicine, Shanghai, China; 5 College of Information Engineering, Shanghai Maritime University, Shanghai, China; 6 Department of Biomedical Engineering, Tianjin University, Tianjin Key Lab of BME Measurement, Tianjin, China; 7 Department of Genetics and Genomic Sciences, Icahn School of Medicine at Mount Sinai, New York City, New York, United States of America; University of Michigan, United States of America

## Abstract

Membrane proteins were found to be involved in various cellular processes performing various important functions, which are mainly associated to their types. However, it is very time-consuming and expensive for traditional biophysical methods to identify membrane protein types. Although some computational tools predicting membrane protein types have been developed, most of them can only recognize one kind of type. Therefore, they are not as effective as one membrane protein can have several types at the same time. To our knowledge, few methods handling multiple types of membrane proteins were reported. In this study, we proposed an integrated approach to predict multiple types of membrane proteins by employing sequence homology and protein-protein interaction network. As a result, the prediction accuracies reached 87.65%, 81.39% and 70.79%, respectively, by the leave-one-out test on three datasets. It outperformed the nearest neighbor algorithm adopting pseudo amino acid composition. The method is anticipated to be an alternative tool for identifying membrane protein types. New metrics for evaluating performances of methods dealing with multi-label problems were also presented. The program of the method is available upon request.

## Introduction

Membrane proteins are one of the three main protein classes. It is approximately estimated that 20-30% of all genes in most genomes encode membrane proteins [1]. Gao *et al*. [2] estimated the number of membrane proteins was about 8000 in human. Membrane proteins also play wide varieties of important roles invloved cellular processes [3], in the immune response, serve as enzymes, *etc*. In addition, membrane proteins constitute 60% of drug targets [4], which were crucial to new drug discovery as well as to understand the mechanism of the cellular activities [4], [5], [6]. It is reported that functions of a membrane protein are closely associated with its type [7]. However, it is time-consuming and costly to determine types of uncharacterized membrane proteins by using traditional biophysical methods [8]. Thus, there is a growing need for effective computational methods to predict the membrane protein types.

In the past decades, it was considered the importance of the predictions. Many tools were developed based on machine learning techniques, in which both features extracted from samples and the learning algorithms were equally affecting. Features used for the predictions were mainly including: amino acid composition (AAC), sequence homology information such as position-specific scoring matrices (PSSMs), pseudo amino acid composition (PseAAC), physicochemical properties of amino acids and functional domains. AAC was the simplest but often the most efficient to represent protein sequence. Cai *et al*. [9] used only AAC feature by using support vector machine (SVM) to predict membrane protein types. Reflecting the order of amino acid in the sequence and complementing AAC, PseAAC of protein sequence is considered to be the extension of it. Those features were widely applied to recognize types of uncharacterized membrane proteins [7], [10], [11], [12], [13]. Wang *et al*. [14], however, pointed out that the feature space of PseAAC was redundant, and they utilized a Supervised Locally Linear Embedding (SLLE) algorithm [15] for nonlinear dimensionality reduction. Pu *et al*. [16] proposed IAMPC, in which they extracted features from both PSSMs and protein primary sequences by using a so-called SVM fusion to identify membrane proteins types. Hayat *et al*. [17] used both the split amino acid composition (SAAC) and seven different physicochemical properties of proteins to represent protein sequences. They also employed SVM as the classifier. The method has been implemented in a web servicer called Mem-PHybrid (http://111.68.99.218/Mem-PHybrid). The hybrid features of PSSM and the SAAC of protein sequences were also used by Hayat *et al*. [18] to construct a web-based predictor. Ding *et al*. [19] used tripeptide compositions to recognize mycobacterial membrane protein types. Jia *et al*. [8] constructed a classifier based on hybrid feature space of domain profiles and physiochemical properties of membrane proteins. Hayat *et al*. [20] used features of split amino acid as well as the ensemble classifier. Chen *et al*. [21] suggested novel multiple-source features such as transmembrane segments, lipid-binding domains, signal peptides, signal anchors, GPI-anchoring signals, surface amino acids, cationic patches, and so on. On the other hand, some machine learning algorithms have been applied for the prediction of membrane protein types. For example, Fourier spectrum [22], [23], wavelet analysis and cascaded neural network [24], wavelet transform (DWT) combined with SVM [25], neighborhood preserving embedding algorithm and K-nearest neighbor algorithm [26]. However, most of these methods did not consider the fact that one membrane protein possesses multiple types, which is common for membrane proteins.

Sequence homology reflects evolutionary relationships, which in turn helps infer structures and functions of proteins. Large bodies of protein-protein interactions available provide global perspective on relationship between different proteins at system-level. It is reported that proteins share identical properties and functions with interactive proteins more probably than with none-ones. Therefore, it is possible to identify membrane protein types by means of these. In this study, we proposed an integrated method by using both the homologies between protein sequences and the similar properties between interactive proteins to predict multi-types of membrane proteins in human. To widely examine the method, three datasets were constructed from UniProt database [27]. It was suggested from the performance of the method that it could be quite effective to identify membrane protein types.

## Materials and Methods

### Datasets

3,789 sequences of experimentally verified membrane proteins of human were downloaded from the UniProt database (release 2012_09 - Oct 3, 2012) [27]. According to their intramolecular arrangements and positions in a cell, membrane proteins are generally classified into the following six types: (1) GPI (Glycosylphosphatidylinisotol)-anchor; (2) lipid-anchor; (3) multi-pass; (4) peripheral; (5) single-pass type I; (6) single-pass type II membrane proteins [28]. Correct identification of the types of membrane proteins can help distinguish proteins between different functions.

**Figure 1:**
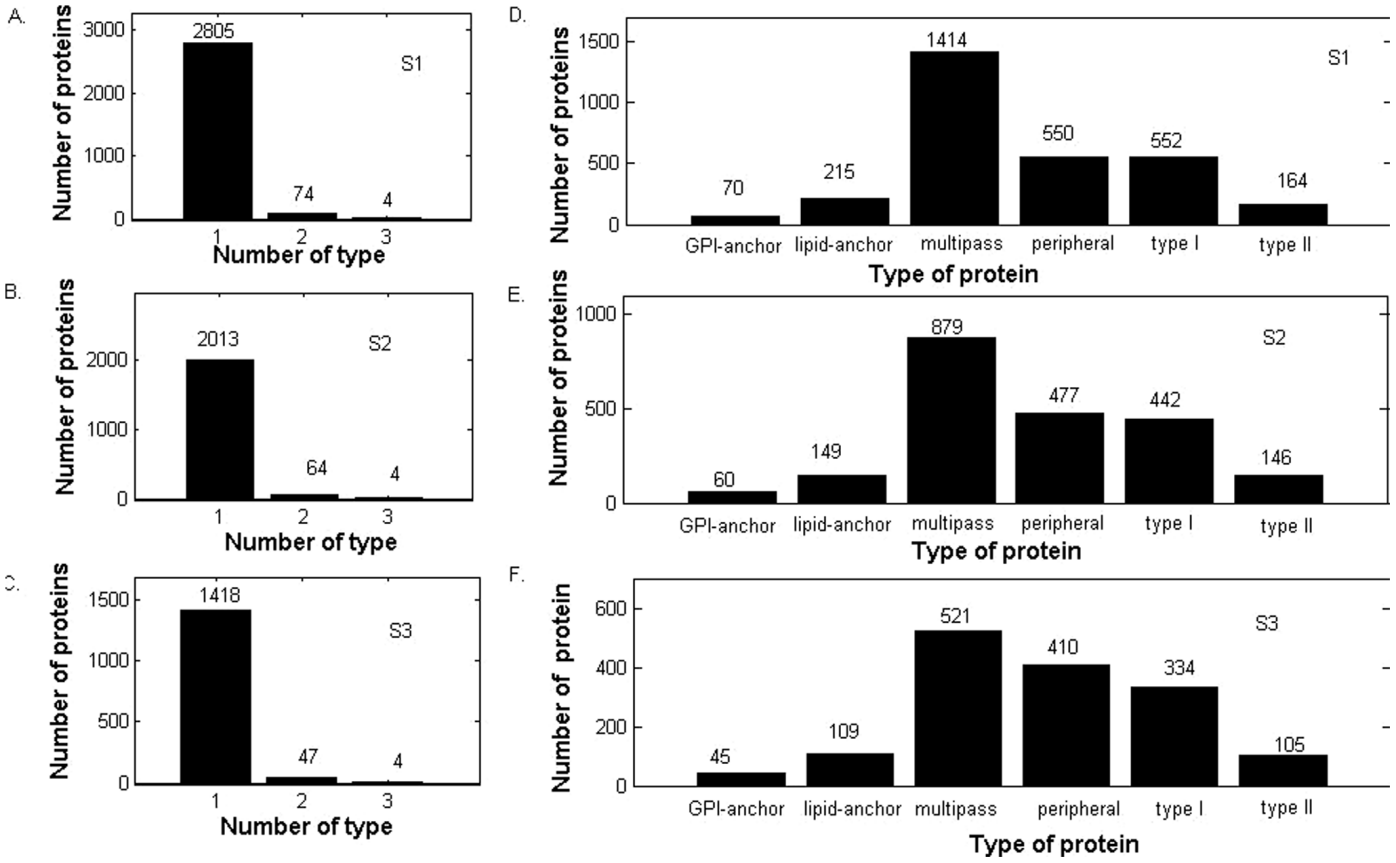
The distributions of membrane proteins in different types and in different multi-types. A, B and C depict the distribution of the type number on three datasets, D, E and F show the distribution of types of membrane proteins on three datasets, respectively.

To evaluate the perfomance of the prediction method, we employed the sequence clustering program CD-HIT (Cluster Database at High Identity with Tolerance) [29] to construct three datasets: S1, S2, and S3 from 3,789 proteins. S1 contained 2,883 protein sequences in which proteins had less than 70% sequence identity. S2 consisted of 2,081 protein sequences with sequence identity lower than 40%. S3 had 1,469 protein sequences with sequence identity less than 25%. As one membrane protein can have one or more types, **Figure 1(A, B, C)** shows the number of proteins having 1-6 types. It can be seen that no proteins have four or more types. The average numbers of types of proteins in datasets S1, S2 and S3 were 1.028, 1.035, 1.037, respectively. We also represented the distribution of membrane proteins to their types in the three datasets in **Figure 1(D, E, F)**. Detail information containing protein IDs, sequences and types were available in Table S1.

### Prediction method

In this section, the detailed procedure of the integrated prediction method was described. The integrated method included three methods: BLAST/PSI-BLAST method, network-based method and shortest-distance method.

Suppose there are *n* proteins, say 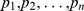, with known types in the training set *S*. For one protein 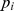 in *S*, its types were encoded into a vector
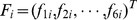
(1)where 
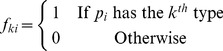
(2)


#### BLAST/PSI-BLAST method

The basic local alignment search tool (BLAST), initiated by Altschul *et al*. [30], can be used to search for local similarity between two sequences. The idea underlying BLAST is to employ similarity measure optimized by background probabilities to directly approximate sequence alignments based on dynamic programming algorithm. This method can detect weak but more biologically significant sequence similarities. Gapped BLAST and PSI-BLAST (Positional Specific Iterated BLAST) [31] are the improved versions, improving on three major aspects: consuming-time of the extension, gapped alignment, and iterative search. Here, the BLAST/PSI-BLAST program (ftp://ftp.ncbi.nlm.nih.gov/blast/executables/release/2.2.26/) was adopted to calculate the alignment score, denoted by 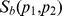, of proteins *p*_1_ and *p*_2_. For an uncharacterized membrane protein 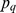, we searched a protein 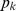 in *S* whose 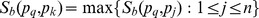. Then the types of 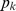 were assigned to 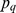 as the predicted results. However, if the protein 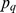 has no homologous proteins in *S*, this method cannot provide meaningful predicted results. In this case, the following two prediction methods make further predictions.

#### Network-based method

This method utilized the information of protein interactions in human retrieved from STRING [32] (http://string.embl.de/), which was a well-known database covering 5,214,234 proteins with their interactions in 1,133 organisms. These interactions consist of known and predicted protein interactions including direct (physical) and indirect (functional) associations derived from the following four sources: (1) Genomic context; (2) High-throughput experiments; (3) (Conserved) coexpression; (4) Previous knowledge. There was a confidence score for every pairwise interaction quantifying the likelihood of the interaction occurrence. Let us denote the confidence score of a pairwise interaction between two proteins *p*_1_ and *p*_2_ as 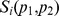. Two proteins were regarded interactive in this study if the confidence score of the interaction between them was greater than zero. For an uncharacterized protein 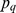, the confidence scores of interactions between 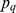 and proteins in *S* were represented by
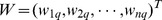
(3)where *w_jq_* denoted the confidence score of the interaction between 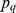 and 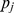 in *S*, *i.e*. 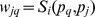. The probability of assigning the protein 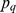 the 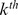 type was calculated by 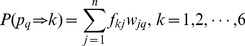
(4)The greater the probability 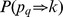 was, the more probably the query protein belonged to the 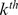
type. To handling multiple types, we took the top *t* highest probability types as the predicted results, where *t* was regarded as the smallest integer larger than or equal to the average number of types in the dataset. That is, if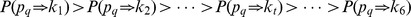, the protein would be predicted to have 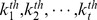 type. This method was very similar to that in our previous work [33], which was used to predict protein functions. However, this method was not always effective. If there were no interactive proteins with 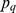 in *S*, the outputs of equation (4) were all zeros. In this case, the last method described below would make the final prediction.

#### Shortest-distance method

**Figure 2:**
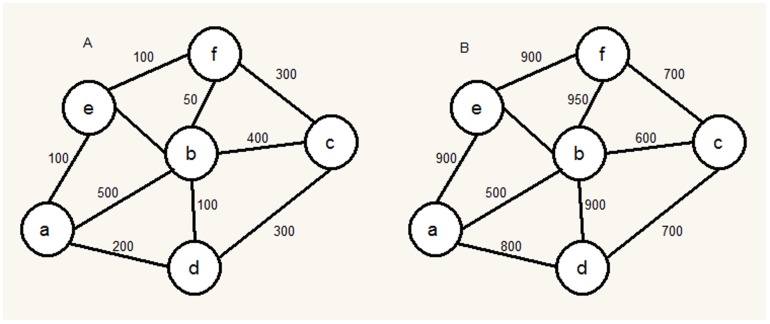
A sample of PPI interaction network and the weighted graph conversion. A represents a sample of the interaction network with edges representing confidence scores and B is the weighted graph derived from A.

As mentioned above, the higher the confidence score between two proteins, the stronger the interaction, and the more the two proteins shared identical or similar types. As shown in **Figure 2(A)**, proteins **a** and **b** were assumed to have stronger interaction with each other than any other pairs, followed by proteins **b** and **c**. In this case, protein **a** would more likely have the same types as protein **b**, and protein **b** would more likely have the same types as protein **c**. Consequently, it could be inferred that proteins **a** and **c** would more probably share the same types, however they were not interacted directly with each other. In view of this, we constructed a weighted graph *G* in which nodes represent proteins and an edge between two nodes existed if and only if the confidence score of the interaction between them was greater than zero. In addition, the weight of the edge between nodes *n*_1_ and *n*_2_ was defined as 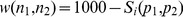
(5)where 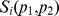 was the confidence score of the interaction between proteins *p*_1_ and *p*_2_ which are corresponding proteins of nodes *n*_1_ and *n*_2_. As shown in **Figure 2(B)**, proteins **a** and **c** were each other the shortest-distance neighbors except their direct neighbors. Therefore, the shortest-distances between proteins in *G* hold the clue to function relationship between them. This kind of weighted graph had been used in our previous work [34]. Therefore, even if two proteins had no direct interaction, it was possible to utilize the shortest distance in *G* to infer its types. By applying Dijkstra's algorithm which is one of the most well-known shortest-distance algorithms, the shortest distance between any two proteins *p*_1_ and *p*_2_ was calculated, denoted by 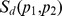
and termed as shortest-distance score. For an uncharacterized membrane protein 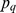, its types were predicted as the same as those of protein 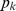 in the training set *S* whose 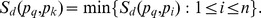


#### Integrated method for predicting membrane protein multiple types

**Figure 3:**
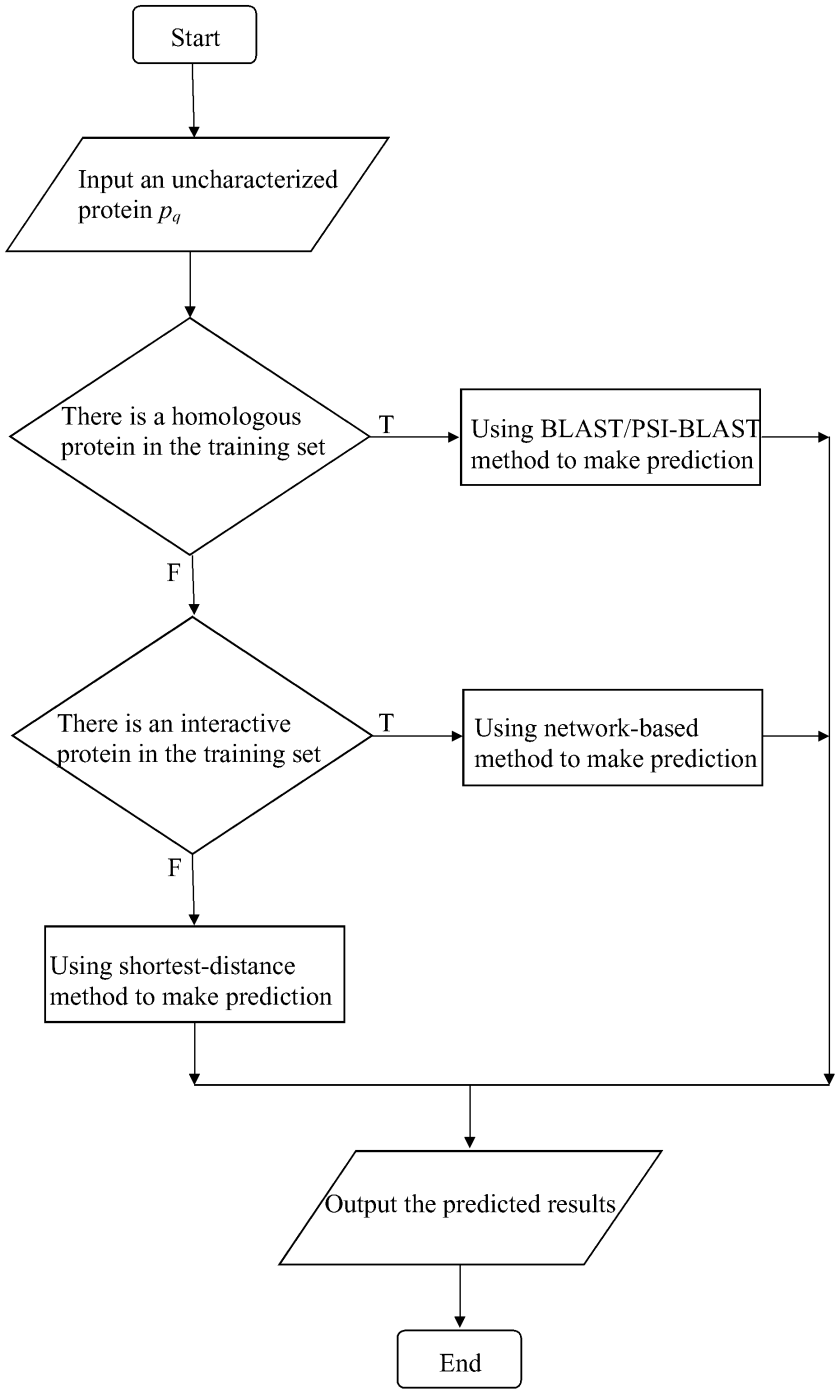
The program flow diagram of the integrated method.

The integrated method combined the BLAST/PSI-BLAST method, the network-based method and the shortest-distance method, as shown in **Figure 3**. For an uncharacterized protein 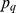, the prediction procedure by using the integrated method was as follows:

1. If there was a homologous protein with
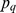
in the training set *S*, *i.e*. 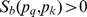 for some *k*, only the BLAST/PSI-BLAST method was applied.
2. If no homologous proteins but there was an interactive protein with 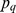 in the training set *S*, *i.e*. 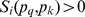 for some *k*, only the network-based method was applied.
3. If it was not the two cases above, the shortest-distance method was finally used.

### Metrics

Among the three ways examining performances of classification algorithms: cross-validation test, leave-one-out test and independent test, the leave-one-out test is considered as the best [35], [36], because the independent test requires additional datasets and the cross-validation test generates unstable results, while the leave-one-out test always provides the unique results for a given a dataset. Here, the leave-one-out test was adopted.

The commonly used mesurements *sensitivity*, *specificity*, *accuracy* and *matthew's correlation coefficient* in binary classification problems were not applicable to this study problem as one protein may simultaneously have more than one types. Deng *et al*. [37] used both concepts of *Precision* and *Recall* to measure performances of methods for multi-label classification problems. Assume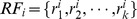 as a label set of actual types for the *i*-th protein in the dataset, and 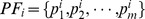 as its predicted label set of types, the *Precision* and *Recall* were defined as:
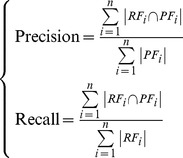
(6)where |A| denoted the number of elements in set A, and *n* refered to the total number of predicted proteins. However, neither *Precision* nor *Recall* can be used alone for the evaluation. If predicted types 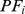 contained all the elements in actual types 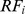, *Recall* would be equal to 1. This is of no mean, because any one can tell all possible types of a membrane protein. On the other hand, if the predicted types 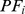 was a non-empty subset of the actual types 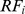, *i.e*. 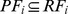, *Precision* would be equal to 1. To overcome these weaknesses, we presented a new evaluation indicator called *Accuracy* (*Acc*), which was defined as:
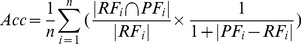
(7)The first part 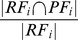 on the right of the equation was approximately equivalent to *Recall*, while 
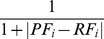
 was a penalty for 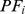 due to incorrect predictions. When 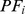 was identical to 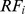, the penalty was of no role. On the contrary, the more incorrect type the 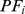 included, the lower the *Acc* was. Therefore, *Acc* reflected both *Precision* and *Recall*.

## Results and Discussion

### Choice of E-value in the BLAST/PSI-BLAST method

**Figure 4:**
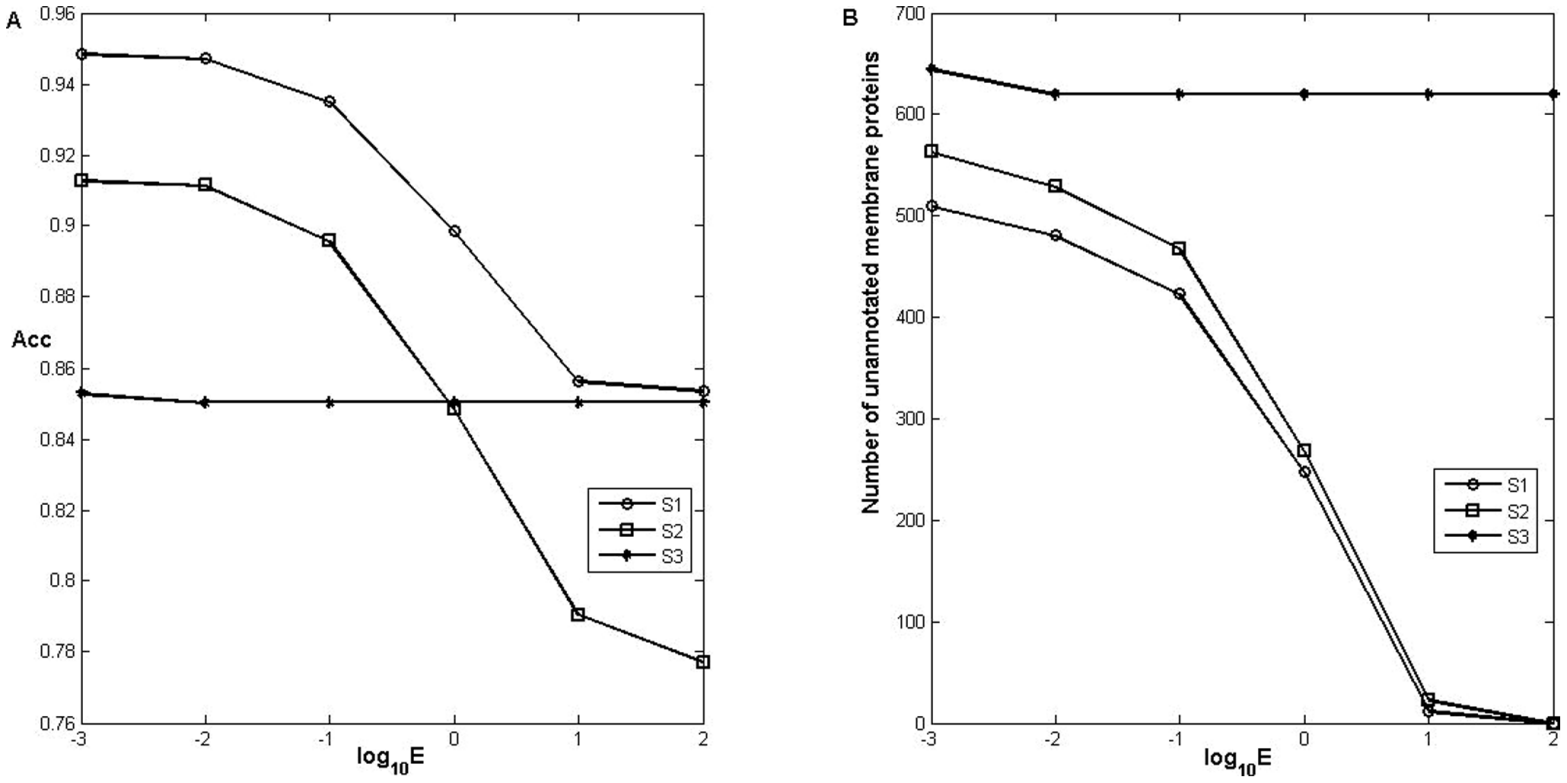
The performance of the BLAST/PSI-BLAST method under various E-values.

It was ploted in **Figure 4** the performances of the BLAST/PSI-BLAST method with different E-values. It was obviously observed from **Figure 4(A)** that the lower the threshold of E-value chosed in the BLAST program, the higher the *Acc* was, however the more the number of annotated proteins remained from **Figure 4(B)**. The commonly expected goal of prediction is of course to reach high *Acc* and simultaneously reduce the number of unannotated membrane proteins. However, this ideal objective is impossible to obtain because we are caught in the dilemma of choosing the cutoff of E-value. While lower E-values increased the statistical significance of alignment scores, it would be too strict and would accordingly lessen the number of proteins considered homologies. On the contrary, higher E-values may result in wrong homology, and thus reduce *Acc*. To balance *Acc* and the number of unannotated proteins, we set E-value 0.01 in this study.

### Performances of the individual methods and the integrated method

Three methods and the integrated method were all conducted on the three datasets S1, S2 and S3, respectively, evaluated by the leave-one-out test. Results were shown in **Tables 1** **-** **3**. It can be observed from **Table 1** that the BLAST/PSI-BLAST method achieved the best performance with the highest *Acc* 94.71%, 91.15% and 85.02% on datasets S1, S2 and S3, respectively. However, 481, 529 and 620 proteins cannot be annotated because there were no homologous proteins in the corresponding datasets. The network-based method achieved the second highest *Acc*, *i.e*. 66.68%, 62.46%, 58.75% on the three datasets, respectively. Since no interactive proteins can be found in the corresponding datasets, there were 86, 38, 41 proteins unannotated. Although *Acc* was lower, the number of unannotated proteins was much less than those in the BLAST/PSI-BLAST method. In the shortest-distance method, the lowest *Acc* achieved (54.97%, 48.75%, 44.99% on the three datasets, respectively). However, all proteins can be annotated. The shortest distance method was capable of annotating all proteins, although it was least effective.

**Table 1:** Performances of the three single methods tested on the three datasets.

	BLAST/PSI-BLASTmethod		Network-based method		Shortest-distance method	
Dataset	Acc	NU*	Acc	NU	Acc	NU
S1	94.71%	481	66.68%	86	54.97%	0
S2	91.15%	529	62.46%	38	48.75%	0
S3	85.02%	620	58.75%	41	44.99%	0

*NU represents the number of unannotated membrane proteins.

**Table 2:** Contributions of the three methods to the integrated method.

	BLAST/PSI-BLAST method		Network-based method		Shortest-distance method	
Dataset	Acc	NA*	Acc	NA	Acc	NA
S1	94.71%	2,402	53.10%	467	28.57%	14
S2	91.15%	1,552	53.51%	513	28.13%	16
S3	85.02%	849	52.60%	596	18.75%	24

*NA represents the number of annotated membrane proteins.

**Table 3:** Comparison of the integrated method with NNA based on PseACC and RWC on the three datasets.

Dataset	The integrated method	NNA based on PseACC	RWC
S1	87.65%	70.41%	81.34%
S2	81.39%	61.70%	71.40%
S3	70.79%	56.33%	56.82%

The integrated method combined the three methods above. The detail contributions of the three single methods were shown in **Table 2**. It was obvious that the BLAST/PSI-BLAST method contributed the most, annotating 2,402, 1,552 and 849 proteins and achieved *Acc* of 94.71%, 91.15% and 85.02% on datasets S1, S2 and S3, respectively. The network-based method annotated 467 of the remaining 481 unannotated proteins in S1, 513 of the remaining 529 in S2 and 596 of the remaining 620 in S3, respectively, and obtained *Acc* of 53.10%, 53.51%,52.60% on the three datasets. The rest proteins in the three datasets were predicted by the shortest-distance method with *Acc* of 28.57%, 28.13%, 18.75% on the three datasets. By combining the above predicted results for each dataset, the integrated method reached *Acc* of 87.65%, 81.39% and 70.79% on S1, S2, S3, respetively, which was listed in column 2 of **Table 3**. It was suggested that the integrated method could balance the prediction accuracy and the ratio of annotated proteins, benefiting from the high accuracy of BLAST/PSI-BLAST method and the wide range of the network-based method and shortest-distance method.

**Figure 5:**
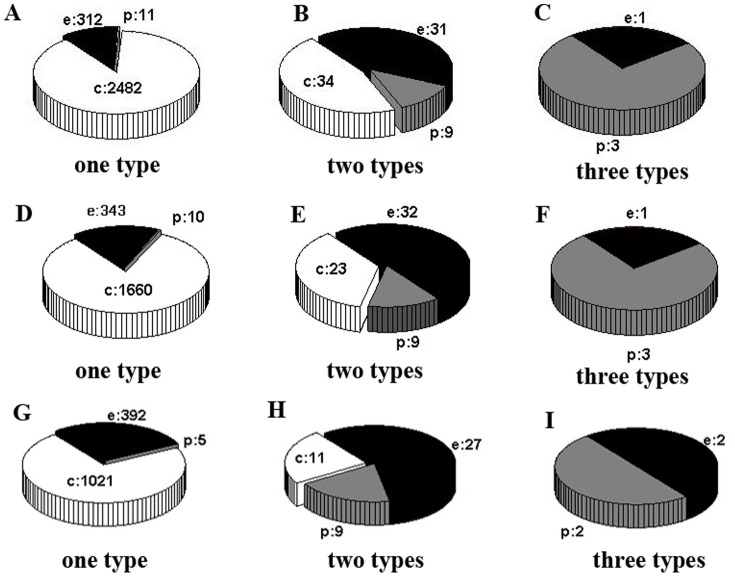
Distributions of correct and incorrect predictions for different multi-type proteins. The subfigures A, B and C were the predicted results on the dataset S1, while D, E and F were for those on the dataset S2, and G, H and I for S3. c denotes the number of completely correct predictions of membrane proteins, e is the number of incorrect predicted membrane proteins, and p represents the number of partly correct type predictions.

Membrane proteins may have multiple types. Statistical analysis were performed on the proteins having 1, 2, 3 types in each of the three datasets, respectively, with results shown in **Figure 5**. It can be seen that the prediction of 1 type proteins was the best, the 2 type ones was the second, and the 3 type ones was the least. For the 2 type proteins, nearly 50% could be correctly predicted or partly correctly predicted. It was indicated that the presented method could handle the multi-type membrane protein predictions.

### Comparison with other methods

We compared our method to two other methods: the nearest neighbor algorithm (NNA) based on pseudo amino acid composition (PseAAC) and the real weighted combination (RWC). The procedure of these two methods was described in the following two paragraphs and their performances on datasets S1, S2 and S3 were in the fourth paragraph of this section.

#### NNA based on PseAAC

The concept of PseAAC was originally initiated by Chou to predict protein subcellular localization and membrane protein types [38]. The main idea of this method was to encode each protein sequence into a numeric vector containing information of sequence order effects. Here, the brief description of this method was as follows. Detail description about this method can be found in Chou's work [38]. Without loss of generality, let 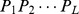 be a protein sequence with *L* amino acid residues. Accordingly, a set of discrete correlation factors can be computed by
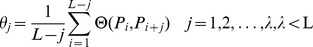
(8)where 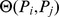 is defined by
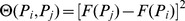
(9)
*F*(*P_i_*) is a feature value of the amino acid *P_i_*. In fact, it is converted from the 20 original feature values of 20 amino acids by 
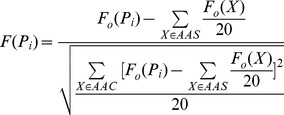
(10)where AAS is a set consisting of 20 amino acids. If the original feature values were known, 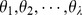 can be computed by equations (8), (9) and (10). **Table 4** listed the original feature values of 20 amino acids for some physicochemical and biochemical properties of amino acids. Then, the PseAAC of a protein sequence can be encoded into a numeric vector:
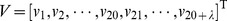
(11)where 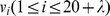 can be computed by
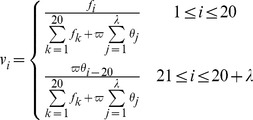
(12)where 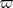 is the weight for the sequence order effects, 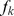
(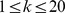) the occurrence frequency of the 20 amino acids in the protein sequence. In this study, we set 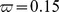, 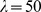 and considered the following five physicochemical and biochemical properties of amino acids: (1) Codon diversity; (2) Electrostatic charge; (3) Molecular volume; (4) Polarity; (5) Secondary structure. Their original feature values of each amino acid were listed in **Table 4**, which were retrieved from 39,40,41]. Each of the five properties contributed 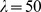 components to contain the sequence order effects. Therefore, there are totally 20+50×5 = 270 components which can comprise a 270-D vector to represent a protein sequence. The same PseAAC scheme to encode protein sequences including same parameters had been applied in Wang *et al*.'s study [42]. Given an uncharacterized protein 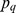 and the training set 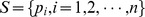, where 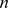 is the size of the training set, the PseAAC of these proteins can be easily obtained. The NNA based on PseAAC computed the distance between 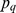 and 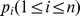 by the following equation:
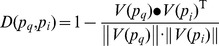
(13)where 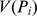 is the PseAAC of protein *P_i_*. The query protein 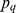 was then assigned the types of protein *p_k_* in the training set whose 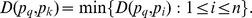


**Table 4:** Original feature values of physicochemical and biochemical properties of the 20 amino acids.

Amino acid	Polarity	Second structure	Molecular volume	Codon diversity	Electrostatic charge
A	−0.591	−1.302	−0.733	1.57	−0.146
C	−1.343	0.465	−0.862	−1.02	−0.255
D	1.05	0.302	−3.656	−0.259	−3.242
E	1.357	−1.453	1.477	0.113	−0.837
F	−1.006	−0.59	1.891	−0.397	0.412
G	−0.384	1.652	1.33	1.045	2.064
H	0.336	−0.417	−1.673	−1.474	−0.078
I	−1.239	−0.547	2.131	0.393	0.816
K	1.831	−0.561	0.533	−0.277	1.648
L	−1.019	−0.987	−1.505	1.266	−0.912
M	−0.663	−1.524	2.219	−1.005	1.212
N	0.945	0.828	1.299	−0.169	0.933
P	0.189	2.081	−1.628	0.421	−1.392
Q	0.931	−0.179	−3.005	−0.503	−1.853
R	1.538	−0.055	1.502	0.44	2.897
S	−0.228	1.399	−4.76	0.67	−2.647
T	−0.032	0.326	2.213	0.908	1.313
V	−1.337	−0.279	−0.544	1.242	−1.262
W	−0.595	0.009	0.672	−2.128	−0.184
Y	0.26	0.83	3.097	−0.838	1.512

#### RWC

RWC utilized another integrated way combining the information of BLAST alignment score, protein interaction confidence score and shortest-distance score. For two proteins *p*_1_ and *p*_2_, the score measuring their relationship was calculated by 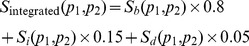
(14)Given an uncharacterized membrane protein 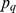, its types were predicted to be the types of protein *p_k_* in the training set whose 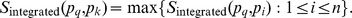


#### Performance of NNA based on PseAAC and RWC

The NNA based on PseAAC and RWC were also conducted on the three datasets S1, S2 and S3, respectively, evaluated by leave-one-out test, with results listed in **Table 3**. The *Acc* obtained by NNA based on PseAAC on S1, S2 and S3 was 70.41%, 61.70% and 56.33%, respectively, while it was 81.34%, 71.40% and 56.82% obtained by RWC. It can be seen that our method performed significantly better than the two methods. *Acc* obtained by our method was on average 17% higher than that by NNA based on PseAAC and 10% higher than that by RWC. These suggested the efficiency of the proposed integrated method in predicting membrane protein multi-types.

### Discussions

Membrane proteins are embedded in or temporarily attached to the phospholipid bi-layer of the membrane. They interact with membranes via specific structures or comformations. Therefore, the protein structures were regarded as the determinate factors in the *in vivo* types. Based on the principle of Lock and Key Theory, the affinity and specificity of molecules is strictly determined by the local binding site shape and binding pocket size [43], [44]. To date, many efforts have been made to computationally predict the protein structures [45], [46], [47], [48], [49], [50] and to identify the membrane protein types based on structures, although some of them are devised for water-soluble proteins with large molecular volumes [51], [52], [53], [54].

**Figure 6:**
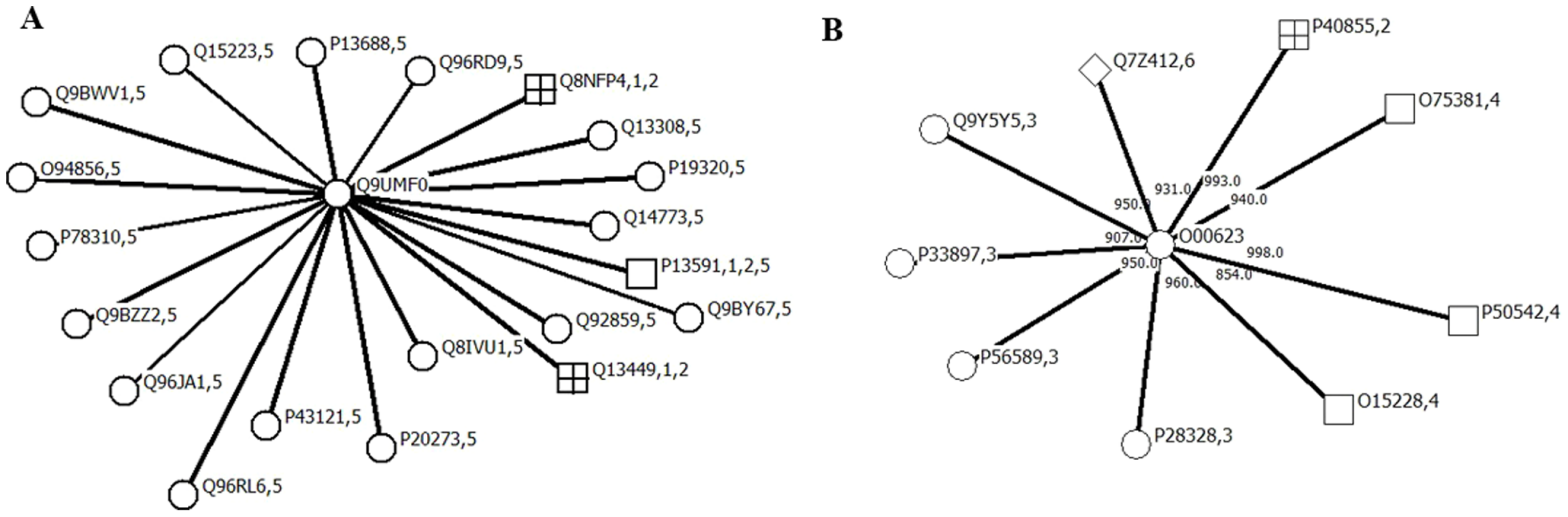
The homologous proteins of the membrane protein Q9UMF0 (A) and the interactive proteins of the membrane protein O00623 (B) in the dataset S3. The numbers represent the membrane protein types: 1 GPI-anchor, 2 lipid-anchor, 3 multi-pass, 4 peripheral, 5 single-pass type I and 6 single-pass type II.

In this paper, we employed the sequence alignment rather than protein structures for the general concept that similar amino acid sequences were prone to have similar structures. Indeed, homology modeling predictions derived from sequence alignment methods were regarded as precise and ubiquitous for analyzing structures and types [55]. Therefore, the BLAST/PS-BLAST was efficient to acquire the optimal prediction. Take the membrane protein ICAM5_HUMAN (uniprot: Q9UMF0) as an example. This protein belongs to single-pass type I. In the dataset S3, it was correctly predicted by the integrated method (via BLAST/PSI-BLAST method indeed) but wrongly by NNA based on PseAAC and RWC. The homologous proteins with Q9UMF0 in S3 and their types were shown in **Figure 6(A)**. It was obvious that the protein shared the same or similar types with most of its homologous proteins (number 5 in the **Figure 6(A)**).

Interactive proteins could share the same or similar types since they have close distance when they perform biological functions in vivo. In **Figure 6(B)**, shown were all the interactive proteins of another example membrane protein PEX12_HUMAN (uniprot: O00623) in the dataset S3, with their types. By our integrated method (via network-based method indeed), it was correctly predicted to be type of multi-pass, but wrongly to be type of peripheral by NNA based on PseAAC and to be type of single-pass single I by RWC. In the integrated method, the BLAST/PSI-BLAST can utilize the entire sequence order, and STRING can directly use the comprehensive functional-links. However, pseudo amino acid composition only can represent local information of sequence order. In RWC, the score calculated by equation (14) between membrane proteins was the weighted combination of alignment score, confidence score and shortest-distance score. The three types of scores were obtained in different ways; simple combination of them cannot characterize the complex in type relationships between membrane proteins. Experimental results also suggested that the combination of the three scores was less efficient than those by using separate ones.

However, the integrated method had some drawbacks because the BLAST/PSI-BLAST method was not primarily designed for specific proteins. Applying the “low-complexity filter” option in BLAST/ PSI-BLAST was not recommended for membrane proteins consisting of hydrophobic regions, because these regions could be the main factor determining the conformation and this would probably causes omission of the hydrophobic regions [56].

There was another problem that BLAST method would fail when membrane proteins have no homologies with known membrane proteins. However, it was still possible to discover the types according to the similarity of motifs or certain patterns with known proteins. Considering the variability of membrane proteins and the limited number of experimental high-resolution membrane protein structures, we did not further employ the similarity of motifs. This would probably reduce the prediction performances. Instead, the network-based method was implemented. Interaction of proteins might help and thus prediction would be more accurate. Although our prediction accuracy (*Acc* = 87.65%, 81.39%, 70.79%) *prima facie* suggested our integrated method performed better than individual predictions (except the BLAST method which was the most reliable one), this method come with a caveat. As the BLAST/PSI-BLAST method was suitable to annotate 2,402 of 2,883, 1,552 of 2,081, and 849 of 1,469 proteins and obtained *Acc* of 94.71%, 91.15% and 85.02%, respectively, it was quite possible that higher accuracy of the combined method was ascribed to the constitution of our dataset where most proteins have homologies and were annotated by the BLAST/PSI-BLAST method. However, in most cases, we were confronted with a dataset with few proteins having homologies with known proteins. Despite the most reliable predictions by BLAST, the network-based method was still required to annotate the remaining proteins. Here, we combined these different approaches to complement each other. Our integrated method benefited from the BLAST method and employed network-based method utilizing protein-protein interactions as complement, achieving nice performance.

## Supporting Information

Table S1 **The protein IDs, sequences and their types in three datasets.**
(PDF)
