# Supplementary material for: Prediction of Multi-Type Membrane Proteins in Human by an Integrated Approach
Source: PLoS One. 2014 Mar 27;9(3):e93553. doi: 10.1371/journal.pone.0093553 (PMC3968155; doi:10.1371/journal.pone.0093553)
Supplement: Table S1 — The protein IDs, sequences and their types in three datasets. (PDF) [file pone.0093553.s001.pdf]

Table S1: The detailed information of proteins in datasets S1, S2 and S3.

(1) Protein IDs, sequences and types in dataset S1

>P20645 single-pass type I membrane protein
MFPFYSCWRTGLLLLLLAVAVRESWQTEEKTCDLVGEKGKESEKELALVKRLKPLFNKSF
ESTVGQGSDTYIYIFRVCREAGNHTSGAGLVQINKSNGKETVVGRLNETHIFNGSNWIMLI
YKGGDEYDNHCGKEQRRAVVMISCNRHTLADNFNPVSEERGKVQDCFYLFEMDSSLACS
PEISHLSVGSILLVTFASLVAVYVVGGFLYQRLVVGAKGMEQFPHLAFWQDLGNLVADGC
DFVCRSKPRNVPAAYRGVGDDQLGEESEERDDHLLPM

>P13569 multipass membrane protein
MQRSPLEKASVVSKLFFSWTRPILRKGYRQRLELSDIYQIPSVDSADNLSEKLEREWDREL
ASKKNPKLINALRRCFFWRFMFYGIFLYLGEVTKAVQPLLLGRIIASYDPDNKEERSIAIYL
GIGLCLLFIVRTLLLHPAIFGLHHIGMQMRIAMFSLIYKKTLKLSSRVLDKISIGQLVSLLSN
NLNKFDEGLALAHFVWIAPLQVALLMGLIWELLQASAFCGLGFLIVLALFQAGLGRMMM
KYRDQRAGKISERLVITSEMIENIQSVKAYCWEEAMEKMIENLRQTELKLTRKAAYVRYF
NSSAFFFSGFFVVFLSVLPYALIKGIILRKIFTTISFCIVLRMAVTRQFPWAVQTWYDSLGAIN
KIQDFLQKQEYKTLEYNLTTTEVVMENVTAFWEEGFGELFEKAKQNNNNRKTSNGDDSL
FFSNFSLLGTPVLKDINFKIERGQLLAVAGSTGAGKTSLLMVIMGELEPSEGKIKHSGRISFC
SQFSWIMPGTIKENIIFGVSYDEYRYRSVIKACQLEEDISKFAEKDNIVLGEGGITLSGGQRA
RISLARAVYKDADLYLLDSPFGYLDVLTEKEIFESCVCKLMANKTRILVTSKMEHLKKAD
KILILHEGSSYFYGTFSELQNLQPDFSSKLMGCDSFDQFSAERRNSILTETLHRFSLEGDAP
VSWTETKKQSFKQTGEFGEKRKNSILNPINSIRKFSIVQKTPLQMNGIEEDSDEPLERRLSL
VPDSEQGEAILPRISVISTGPTLQARRRQSVLNLMTHSVNQGQNIHRKTTASTRKVSLAPQ
ANLTELDIYSRRLSQETGLEISEEINEEDLKECFFDDMESIPAVTTWNTYLRYITVHKSLIFV
LIWCLVIFLAEVAASLVVLWLLGNTPLQDKGNSTHSRNNSYAVIITSTSSYYVFYIYVGVAD
TLLAMGFFRGLPLVHTLITVSKILHHKMLHSVLQAPMSTLNTLKAGGILNRFSKDIAILDD
LLPLTIFDFIQLLLIVIGAIAVVAVLQPYIFVATVPVIVAFIMLRAYFLQTSQQLKQLESEGRSPI
FTHLVTSLKGLWTLRAFGRQPYFETLFHKALNLHTANWFLYLSTLRWFQMRIEMIFVIFFIA
VTFISILTTGEGEGRVGIILTLAMNIMSTLQWAVNSSIDVDSLMRSVSRVFKFIDMPTEGKPT
KSTKPYKNGQLSKVMIIENSHVKKDDIWPSGGQMTVKDLTAKYTEGGNAILENISFSISPG
QRVGLLGRTGSGKSTLLSAFLRLLNTEGEIQIDGVSWDSITLQQWRKAFGVIPQKVFIFSGT
FRKNLDPYEQWSDQEIWKVADEVGLRSVIEQFPGKLDFVLVDGGCVLSHGHKQLMCLAR
SVLSKAKILLLDEPSAHLDPVTYQIIRRTLKQAFADCTVILCEHRIEAMLECQQFLVIEENK
VRQYDSIQKLLNERSLFRQAISPSDRVKLFPHRNSSKCKSKPQIAALKEETEEEVQDTRL

>P11233 lipid-anchor
MAANKPKGQNSLALHKVIMVGSGGVGKSALTLQFMYDEFVEDYEPTKADSYRKKVVLD
GEEVQIDILDTAGQEDYAAIRDNYFRSGEGFLCVFSITEMESFAATADFREQILRVKEDENV
PFLLVGNKSDLEDKRQVSVEEAKNRAEQWNVNYVETSAKTRANVDKVFFDLMREIRAR
KMEDSKEKNGKKKRKSLAKRIRERCCIL

>O14641 peripheral membrane protein
MAGSSTGGGGVGETKVIYHLDEEETPYLVKIPVPAERITLGDFKSVLQRPAGAKYFFKSM

DQDFGVVKEEISDDNARLPCFNGRVVSWLVSSDNPQPEMAPPVHEPRAELAPPAPPLPPLP
PERTSGIGDSRPPSFHPNVSSSHENLEPETETESVVSLRRERPRRRDSSEHGAGGHRTGGPS
RLERHLAGYESSSTLMTSELESTSLGDSDEEDTMSRFSSSTEQSSASRLLKRHRRRRKQRP
PRLERTSSFSSVTDSTMSLNIITVTLNMEKYNFLGISIVGQSNERGDGGIYIGSIMKGGAVAA
DGRIEPGDMLLQVNDMNFENMSNDDAVRVLRDIVHKPGPIVLTVAKCWDPSPQAYFTLPR
NEPIQPIDPAAWVSHSAALTGTFPAYPGSSSMSTITSGSSLPDGCEGRGLSVHTDMASVTKA
MAAPESGLEVRDRMWLKITIPNAFLGSDVVDWLYHHVEGFPERREARKYASGLLKAGLI
RHTVNKITFSEQCYYVFGDLSGGCESYLVNLSLNDNDGSSGASDQDTLAPLPGATPWPLL
PTFSYQYPAPHPYSPQPPPYHELSSYTYGGGSASSQHSEGSRSSGSTRSDGGAGRTGRPEER
APESKSGSGSESEPSSRGGSLRRGGEASGTSDGGPPPSRGSTGGAPNLRAHPGLHPYGPPP
GMALPYNPMMVVMMPPPPPPVPPAVQPPGAPPVRDLGSVPPELTASRQSFHMAMGNPSEF
FVDVM

>P78423 single-pass type I membrane protein
MAPISLSWLLRLATFCHLTVLLAGQHHGVTKCNITCSKMTSKIPVALLIHYQQNQASCGKR
AIILETRQHRLFCADPKEQWVKDAMQHLDRQAAALTRNGGTFEKQIGEVKPRTTPAAGG
MDESVVLEPEATGESSSLEPTPSSQEAQRALGTSPELPTGVTGSSGTRLPPTPKAQDGGPV
GTELFRVPPVSTAATWQSSAPHQPGPSLWAEAKTSEAPSTQDPSTQASTASSPAPEENAPSE
GQRVWGQGQSPRPENSLEREEMGPVPAHTDAFQDWGPGSMAHVSVVPVSSEGTPSREPV
ASGSWTPKAEEPIHATMDPQRLGVLITPVPDAQAATRRQAVGLLAFLGLLFCLGVAMFTY
QSLQGCPRKMAGEMAEGLRYIPRSCGSNSYVLVPV

>Q9P299 peripheral membrane protein
MQRPEAWPRPHPGEGAAAAQAGGPAPPARAGEPSGLRLQEPSLYTIKAVFILDNDGRRLL
AKYYDDTFPSMKEQMVFEKNVFNKTSRTESEIAFFGGMTIVYKNSIDLFLYVVGSSYENE
LMLMSVLTCLFESLNHMLRKNVEKRWLLENMDGAFLVLDEIVDGGVILESDPQQVIQKV
NFRADDGGLTEQSVAQVLQSAKEQIKWSLLK

>P26006 single-pass type I membrane protein
MGPGPSRAPRAPRLMLCALALMVAAGGCVVSAFNLDTRFLVVKEAGNPGSLFGYSVALH
RQTERQQRYLLLAGAPRELAVPDGYTNRTGAVYLCPLTAHKDDCERMNITVKNDPGHHII
EDMWLGVTVASQGPAGRVLVCAHRYTQVLWSGSEDQRRMVGKCYVRGNDLELDSSDD
WQTYHNEMCNSNTDYLETGMCQLGTSGGFTQNTVYFGAPGAYNWKGNSYMIQRKEWD
LSEYSYKDPEDQGNLYIGYTMQVGSFILHPKNITIVTGAPRHRHMGAVFLLSQEAGGDLRR
RQVLEGSQVGAYFGSAIALADLNNDGWQDLLVGAPYYFERKEEVGGAIYVFMNQAGTSF
PAHPSLLLHGPSGSAFGLSVASIGDINQDGFQDIAVGAPFEGLGKVYIYHSSSKGLLRQPQQ
VIHGEKLGLPGLATFGYSLSGQMDVDENFYPDLLVGSLSDHIVLLRARPVINIVHKTLVPRP
AVLDPALCTATSCVQVELCFAYNQSAGNPNYRRNITLAYTLEADRDRRPPRLRFAGSESAV
FHGFFSMPEMRCQKLELLLMDNLRDKLRPIIISMNYSLPLRMPDRPRLGLRSLDAYPILNQ
AQALENHTEVQFQKECGPDNKCESNLQMRAAFVSEQQQKLSRLQYSRDVRKLLLSINVT
NTRTSERSGEDAHEALLTLVVPPALLLSSVRPPGACQANETIFCELGNPFKRNQRMELLIAF
EVIGVTLHTRDLQVQLQLSTSSHQDNLWPMILTLLVDYTLQTSLSMVNHRLQSFFGGTVM
GESGMKTVEDVGSPLKYEFQVGPMGEGLVGLGTLVLGLEWPYEVSNGKWLLYPTEITVH
GNGSWPCRPPGDLINPLNLTLSDPGDRPSSPQRRRRQLDPGGGQGPPPVTLAAAKKAKSE

TVLTCATGRAHCVWLECPIPDAPVVTNVTVKARVWNSTFIEDYRDFDRVRVNGWATLFLR
TSIPTINMENKTTWFSVDIDSELVEELPAEIELWLVLVAVGAGLLLLGLIILLLWKCGFFKRA
RTRALYEAKRQKAEMKSQPSETERLTDDY

>O95772 multipass membrane protein
MNHLPEDMENALTGSQSSHASLRNIHSINPTQLMARIESYEGREKKGISDVRRTFCLFVTF
DLLFVTLLWIIELNVNGGIENTLEKEVMQYDYYSSYFDIFLLAVFRFKVLILAYAVCRLRH
WWAIALTTAVTSAFLLAKVILSKLFSQGAFGYVLPIISFILAWIETWFLDFKVLPQEAEEENR
LLIVQDASERAALIPGGLSDGQFYSPPESEAGSEEAEEKQDSEKPLLEL

>P21926 multipass membrane protein
MPVKGGTKCIKYLLFGFNFIFWLAGIAVLAIGLWLRFDSQTKSIFEQETNNNNNSSFYTGVYI
LIGAGALMMLVGFLGCCGAVQESQCMLGLFFGFLLVIFAIEIAAAIWGYSHKDEVIKEVQE
FYKDTYNKLKTKDEPQRETLKAIHYALNCCGLAGGVEQFISDICPKKDVLETFTVKSCPD
AIKEVFDNKFHIIGAVGIGIAVVMIFGMIFSMILCCAIRRNREMV

>P01730 single-pass type I membrane protein
MNRGVPFRHLLLVLQLALLPAATQGKKVVLGKKGDTVELTCTASQKKSIQFHWKNSNQI
KILGNQGSFLTKGPSKLNDRADSRRSLWDQGNFPLIIKNLKIEDSDTYICEVEDQKEEVQLL
VFGLTANSDTHLLQGQSLTLTLESPPGSSPSVQCRSPRGKNIQGGKTLSVSQLELQDSGTW
TCTVLQNQKKVEFKIDIVVLAFQKASSIVYKKEGEQVEFSFPLAFTVEKLTGSGELWWQA
ERASSSKSWITFDLKNKEVSVKRVTQDPKLQMGKKLPLHLTLPQALPQYAGSGNLTLALE
AKTGKLHQEVNLVVMRATQLQKNLTCEVWGPTSPKLMLSLKLENKEAKVSKREKAVWV
LNPEAGMWQCLLSDSGQVLLESNIKVLPTWSTPVQPMALIVLGGVAGLLLFIGLGIFFCVR
CRHRRRQAERMSQIKRLLSEKKTCQCPHRFQKTCSPI

>Q9NXS2 single-pass type I membrane protein
MRSGGRGRPRLRLGERGLMEPLLPPKRRLLPRVRLLPLLLALAVGSAFYTIWSGWHRRTE
ELPLGRELRVPLIGSLPEARLRRVVGQLDPQRLWSTYLRPLLVVRTPGSPGNLQVRKFLEA
TLRSLTAGWHVELDPFTASTPLGPVDFGNVVATLDPRAARHLTLACHYDSKLFPPGSTPFV
GATDSAVPCALLLELAQALDLELSRAKKQAAPVTLQLLFLDGEEALKEWGPKDSLYGSRH
LAQLMESIPHSPGPTRIQAIELFMLLDLLGAPNPTFYSHFPRTVRWFHRLRSIEKRLHRLNL
LQSHPQEVMYFQPGEPFGSVEDDHIPFLRRGVPVLHLISTPFPAVWHTPADTEVNLHPPTV
HNLCRILAVFLAEYLGL

>Q8NFJ5 multipass membrane protein
MATTVPDGCRNGLKSKYYRLCDKAEAWGIVLETVATAGVVTSVAFMLTLPILVCKVQDSN
RRKMLPTQFLFLLGVLGIFGLTFAFIIGLDGSTGPTRFFLFGILFSICFSCLLAHAVSLTKLVR
GRKPLSLLVILGLAVGFSLVQDVIAIEYIVLTMNRTNVNVFSELSAPRRNEDFVLLLTYVLF
LMALTFLMSSFTFCGSFTGWKRHGAHIYLTMLLSIAIWVAWITLLMLPDFDRRWDDTILSS
ALAANGWVFLLAYVSPEFWLLTKQRNPMDYPVEDAFCKPQLVKKSYGVENRAYSQEEIT
QGFEETGDTLYAPYSTHFQLQNQPPQKEFSIPRAHAWPSPYKDYEVKKEGS

>Q7KZN9 multipass membrane protein

MQRLLFPPLRALKGRQYLPLLAPRAAPRAQCDCIRRPLRPGQYSTISEVALQSGRGTVSLP
SKAAERVVGRWLLVCSGTVAGAVILGGVTRLTESGLSMVDWHLIKEMKPPTSQEEWEAEF
QRYQQFPEFKILNHDMTLTEFKFIWYMEYSHRMWGRLVGLVYILPAAYFWRKGWLSRGM
KGRVLALCGLVCFQGLLGWYMVKSGLEEKSDSHDIPRVSQYRLAAHLGSALVLYCASLW
TSLSLLLPPHKLPETHQLLQLRRFAHGTAGLVFLTALSGAFVAGLDAGLVYNSFPKMGESW
IPEDLFTFSPILRNVFENPTMVQFDHRILGITSVTAITVLYFLSRRIPLPRRTKMAAVTLLALA
YTQVGLGISTLLMYVPTPLAATHQSGSLALLTGALWLMNELRRVPK

>Q9NXJ0 multipass membrane protein
MMSSKPTSHAEVNETIPNPYPPSSFMAPGFQQPLGSINLENQAQGAQRAQPYGITSPGIFAS
SQPGQGNIQMINPSVGTAVMNFKEEAKALGVIQIMVGLMHIGFGIVLCLISFSFREVLGFAS
TAVIGGYPFWGGLSFIISGSLSVSASKELSRCLVKGSLGMNIVSSILAFIGVILLLVDMCINGV
AGQDYWAVLSGKGISATLMIFSLLEFFVACATAHFANQANTTTNMSVLVIPNMYESNPVTP
ASSSAPPRCNNYSANAPK

>P47872 multipass membrane protein
MRPHLSPPLQQLLLPVLLACAAHSTGALPRLCDVLQVLWEEQDQCLQELSREQTGDLGTE
QPVPGCEGMWDNISCWPSSVPGRMVEVECPRFLRMLTSRNGSLFRNCTQDGWSETFPRP
NLACGVNVNDSSNEKRHSYLLKLKVMYTVGYSSSLVMLLVALGILCAFRRLHCTRNYIH
MHLFVSFILRALSNFIKDAVLFSSDDVTYCDAHRAGCKLVMVLFQYCIMANYSWLLVEGL
YLHTLLAISFFSERKYLQGFVAFGWGSPAIFVALWAIARHFLEDVGCWDINANASIWWIIR
GPVILSILINFILFINILRILMRKLRTQETRGNEVSHYKRLARSTLLLIPLFGIHYIVFAFSPED
AMEIQLFFELALGSFQGLVVAVLYCFLNGEVQLEVQKKWQQWHLREFPLHPVASFSNSTK
ASHLEQSQGTCRTSII

>Q15311 peripheral membrane protein
MTECFLPPTSSPSEHRRVEHGSGLTRTPSSEEISPTKFPGLYRTGEPSPPHDILHEPPDVVSDD
EKDHGKKKGKFKKKEKRTEGYAAFQEDSSGDEAESPSKMKRSKGIHVFKKPSFSKKKEK
DFKIKEKPKEEKHKEEKHKEEKHKEKKSKDLTAADVVKQWKEKKKKKKPIQEPEVPQID
VPNLKPIFGIPLADAVERTMMYDGIRLPAVFRECIDYVEKYGMKCEGIYRVSGIKSKVDEL
KAAYDREESTNLEDYEPNTVASLLKQYLRDLPENLLTKELMPRFEEACGRTTETEKVQEF
QRLLKELPECNYLLISWLIVHMDHVIAKELETKMNIQNISIVLSPTVQISNRVLYVFFTHVQ
ELFGNVVLKQVMKPLRWSNMATMPTLPETQAGIKEEIRRQEFLLNCLHRDLQGGIKDLSK
EERLWEVQRILTALKRKLREAKRQECETKIAQEIASLSKEDVSKEEMNENEEVINILLAQE
NEILTEQEELLAMEQFLRRQIASEKEEIERLRAEIAEIQSRQQHGRSETEEYSSESESESEDEE
ELQIILEDLQRQNEELEIKNNHLNQAIHEEREAIIELRVQLRLLQMQRAKAEQQAQEDEEPE
WRGGAVQPPRDGVLEPKAAKEQPKAGKEPAKPSPSRDRKETSI

>Q9UBV7 single-pass type II membrane protein
MFPSRRKAAQLPWEDGRSGLLSGGLPRKCSVFHLFVACLSLGFFSLLWLQLSCSGDVARA
VRGQGQETSGPPRACPPEPPPEHWEEDASWGPHRLAVLVPFRERFEELLVFVPHMRRFLSR
KKIRHHIYVLNQVDHFRFNRAALINVGFLESSNSTDYIAMHDVDLLPLNEELDYGFPEAGP
FHVASPELHPLYHYKTYVGGILLLSKQHYRLCNGMSNRFWGWGREDDEFYRRIKGAGLQ
LFRPSGITTGYKTFRHLHDPAWRKRDQKRIAAQKQEQFKVDREGGLNTVKYHVASRTALS

VGGAPCTVLNIMLDCDKTATPWCTFS

>Q8NFU1 multipass membrane protein
MTVTYTARVANARFGGFSQLLLLWRGSIYKLLWRELLCFLGFYMALSAAYRFVLTEGQKR
YFEKLVIYCDQYASLIPVSFVLGFYVTLVVNRWWSQYLCMPLPDALMCVVAGTVHGRDD
RGRLYRRTLMRYAGLSAVLILRSVSTAVFKRFPTIDHVVEAGFMTREERKKFENLNSSYNK
YWVPCVWFSNLAAQARREGRIRDNSALKLLLEELNVFRGKCGMLFHYDWISVPLVYTQV
VTIALYSYFLACLIGRQFLDPAQGYKDHDLDLCVPIFTLLQFFFYAGWLKVAEQLINPFGED
DDDFETNFLIDRNFQVSMLAVDEMYDDLAVLEKDLYWDAAEARAPYTAATVFQLRQPSF
QGSTFDITLAKEDMQFQRLDGLDGPMGEAPGDFLQRLLPAGAGMVAGGPLGRRLSFLLR
KNSCVSEASTGASCSCAVVPEGAAPECSCGDPLLDPGLPEPEAPPPAGPEPLTLIPGPVEPFS
IVTMPGPRGPAPPWLPSPIGEEEENLA

>Q9BZR6 GPI-anchor, lipid-anchor
MKRASAGGSRLLAWVLWLQAWQVAAPCPGACVCYNEPKVTTSCPQQGLQAVPVGIPAA
SQRIFLHGNRISHVPAASFRACRNLTILWLHSNVLARIDAAAFTGLALLEQLDLSDNAQLR
SVDPATFHGLGRLHTLHLDRCGLQELGPGLFRGLAALQYLYLQDNALQALPDDTFRDLG
NLTHLFLHGNRISSVPERAFRGLHSLDRLLLHQNRVAHVHPHAFRDLGRLMTLYLFANNLS
ALPTEALAPLRALQYLRLNDNPWVCDCRARPLWAWLQKFRGSSSEVPCSLPQRLAGRDL
KRLAANDLQGCAVATGPYHPIWTGRATDEEPLGLPKCCQPDAADKASVLEPGRPASAGNA
LKGRVPPGDSPPGNGSGPRHINDSPFGTLPGSAEPPLTAVRPEGSEPPGFPTSGPRRRPGCSR
KNRTRSHCRLGQAGSGGGGTGDSEGSGALPSLTCSLTPLGLALVLWTVLGPC

>P43080 lipid-anchor
MGNVMEGKSVEELSSTECHQWYKKFMTECPSGQLTLYEFRQFFGLKNLSPSASQYVEQM
FETFDFNKDGYIDFMEYVAALSLVLKGKVEQKLRWYFKLYDVDGNGCIDRDELLTIIQAIR
AINPCSDTTMTAEEFTDTVFSKIDVNGDGELSLEEFIEGVQKDQMLLDTLTRSLDLTRIVRR
LQNGEQDEEGADEAAEAAG

>O75508 multipass membrane protein
MVATCLQVVGFVTSFVGWIGVIVTTSTNDWVVTCGYTIPTCRKLDELGSKGLWADCVMA
TGLYHCKPLVDILILPGYVQACRALMIAASVLGLPAILLLLTVLPCIRMGQEPGVAKYRRA
QLAGVLLILLALCALVATIWFPVCAHRETTIVSFGYSLYAGWIGAVLCLVGGCVILCCAGD
AQAFGENRFYYTAGSSSPTHAKSAHV

>Q969Z4 single-pass type I membrane protein
MKPSLLCRPLSCFLMLLPWPLATLTSTTLWQCPPGEEPDLDPGQGTLCRPCPPGTFSAAWG
SSPCQPHARCSLWRRLEAQVGMATRDTLCGDCWPGWFGPWGVPRVPCQPCSWAPLGTH
GCDEWGRRARRGVEVAAGASSGGETRQPGNGTRAGGPEETAAQYAVIAIVPVFCLMGLL
GILVCNLLKRKGYHCTAHKEVGPGPGGGGSGINPAYRTEDANEDTIGVLVRLITEKKENAA
ALEELLKEYHSKQLVQTSHRPVSKLPPAPPNVPHICPHRHHLHTVQGLASLSGPCCSRCSQ
KKWPEVLLSPEAVAATTPVPSLLPNPTRVPKAGAKAGRQGEITILSVGRFRVARIPEQRTSS
MVSEVKTITEAGPSWGDLPDSPQPGLPPEQQALLGSGGSRTKWLKPPAENKAEENRYVVR
LSESNLVI

>Q9NPH3 single-pass type I membrane protein
MTLLWCVVSLYFYGILQSDASERCDDWGLDTMRQIQVFEDEPARIKCPLFEHFLKFNYST
AHSAGLTLIWYWTRQDRDLEEPINFRLPENRISKEKDVLWFRPTLLNDTGNYTCMLRNTT
YCSKVAFPLEVVQKDSCFNSPMKLPVHKLYIEYGIQRITCPNVDGYFPSSVKPTITWYMGC
YKIQNFNNVIPEGMNLSFLIALISNNGNYTCVVTYPENGRTFHLTRTLTVKVVGSPKNAVPP
VIHSPNDHVVYEKEPGEELLIPCTVYFSFLMDSRNEVWWTIDGKKPDDITIDVTINESISHS
RTEDETRTQILSIKKVTSEDLKRSYVCHARSAKGEVAKAAKVKQKVPAPRYTVELACGFG
ATVLLVVILIVVYHVYWLEMVLFYRAHFGTDETILDGKEYDIYVSYARNAEEEEFVLLTLR
GVLENEFGYKLCIFDRDSLPGGIVTDETLSFIQKSRRLLVVLSPNYVLQGTQALLELKAGL
ENMASRGNINVILVQYKAVKETKVKELKRAKTVLTVIKWKGEKSKYPQGRFWKQLQVA
MPVKKSPRRSSSDEQGLSYSSLKNV

>Q68CJ9 single-pass type II membrane protein
MNTDLAAGKMASAACSMDPIDSFELLDLLFDRQDGILRHVELGEGWGHVKDQQVLPNP
DSDDFLSSILGSGDSLPSSPLWSPEGSDSGISEDLPSDPQDTPPRSGPATSPAGCHPAQPGKG
PCLSYHPGNSCSTTTPGPVIQVPEASVTIDLEMWSPGGRICAEKPADPVDLSPRCNLTVKDL
LLSGSSGDLQQHHLGASYLLRPGAGHCQELVLTEDEKKLLAKEGITLPTQLPLTKYEERVL
KKIRRKIRNKQSAQESRKKKKEYIDGLETRMSACTAQNQELQRKVLHLEKQNLSLLEQLK
KLQAIVVQSTSKSAQTGTCVAVLLLSFALIILPSISPFGPNKTESPGDFAPVRVFSRTLHNDA
ASRVAADAVPGSEAPGPRPEADTTREESPGSPGADWGFQDTANLTNSTEELDNATLVLRNA
TEGLGQVALLDWVAPGPSTGSGRAGLEAAGDEL

>Q9NUD9 multipass membrane protein
MWPQDPSRKEVLRFAVSCRILTLMLQALFNAIIPDHHAEAFSPPRLAPSGFVDQLVEGLLG
GLSHWDAEHFLFIAEHGYLYEHNFAFFPGFPLALLVGTELLRPLRGLLSLRSCLLISVASLN
FLFFMLAAVALHDLGCLVLHCPHQSFYAALLFCLSPANVFLAAGYSEALFALLTFSAMGQL
ERGRVWTSVLLFAFATGVRSNGLVSVGFLMHSQCQGFFSSLTMLNPLRQLFKLMASLFLS
VFTLGLPFALFQYYAYTQFCLPGSARPIPEPLVQLAVDKGYRIAEGNEPPWCFWDVPLIYSY
IQDVYWNVGFLKYYELKQVPNFLLAAPVAILVAWATWTYVTTHPWLCLTLGLQRSKNNK
TLEKPDLGFLSPQVFVYVVHAAVLLLFGGLCMHVQVLTRFLGSSTPIMYWFPAHLLQDQE
PLLRSLKTVPWKPLAEDSPPGQKVPRNPIMGLLYHWKTCSPVTRYILGYFLTYWLLGLLL
HCNFLPWT

>P20273 single-pass type I membrane protein
MHLLGPWLLLLLVLEYLAFSDSSKWVFEHPETLYAWEGACVWIPCTYRALDGDLESFILFH
NPEYNKNTSKFDGTRLYESTKDGKVPSEQKRVQFLGDKNKNCTLSIHPVHLNDSGQLGLR
MESKTEKWMERIHLNVSERPFPPHIQLPPEIQESQEVTLTCLLNFSCYGYPIQLQWLLEGVP
MRQAAVTSTSLTIKSVFTRSELKFSPQWSHHGKIVTCQLQDADGKFLSNDTVQLNVKHTP
KLEIKVTPSDAIVREGDSVTMTCEVSSSNPEYTTVSWLKDGTSLKKQNTFTLNLREVTKD
QSGKYCCQVSNDVGPGRSEEVFLQVQYAPEPSTVQILHSPAVEGSQVEFLCMSLANPLPTN
YTWYHNGKEMQGRTEEKVHIPKILPWHAGTYSCVAENILGTGQRGPGAELDVQYPPKKV
TTVIQNPMPIREGDTVTLSCNYNSSNPSVTRYEWKPHGAWEEPSLGVLKIQNVGWDNTTI
ACAACNSWCSWASPVALNVQYAPRDVRVRKIKPLSEIHSGNSVSLQCDFSSSHPKEVQFF

WEKNGRLLGKESQLNFDSISPEDAGSYSCWVNNSIGQTASKAWTLEVLYAPRRLRVSMSP
GDQVMEGKSATLTCESDANPPVSHYTWFDWNNQSLPYHSQKLRLEPVKVQHSGAYWCQ
GTNSVGKGRSPLSTLTVYYSPETIGRRVAVGLGSCLAILILAICGLKLQRRWKRTQSQQGLQ
ENSSGQSFFVRNKKVRRAPLSEGPHSLGCYNPMMEDGISYTTLRFPEMNIPRTGDAESSE
MQRPPPDCDDTVTYSALHKRQVGDYENVIPDFPEDEGIHYSELIQFGVGERPQAQENVDY
VILKH

>P51787 multipass membrane protein
MAAASSPPRAERKRWGWGRLPGARRGSAGLAKKCPFSLELAEGGPAGGALYAPIAPGAP
GPAPPASPAAPAAPPVASDLGPRPPVSLDPRVSIYSTRRPVLARTHVQGRVYNFLERPTGWK
CFVYHFAVFLIVLVCLIFSVLSTIEQYAALATGTLFWMEIVLVVFFGTEYVVRLWSAGCRSK
YVGLWGRLRFARKPISIIDLIVVVASMVVLCVGSKGQVFATSAIRGIRFLQILRMLHVDRQG
GTWRLLGSVVFIHRQELITTLYIGFLGLIFSSYFVYLAEKDAVNESGRVEFGSYADALWWG
VVTVTTIGYGDKVPQTWVGKTIASCFSVFAISFFALPAGILGSGFALKVQQKQRQKHFNRQ
IPAAASLIQTAWRCYAAENPDSSTWKIYIRKAPRSHTLLSPSPKPKKSVVVKKKKFKLDKD
NGVTPGEKMLTVPHITCDPPEERRLDHFSVDGYDSSVRKSPTLLEVSMPHFMRTNSFAEDL
DLEGETLLTPITHISQLREHHRATIKVIRRMQYFVAKKKFQQARKPYDVRDVIEQYSQGHL
NLMVRIKELQRRLDQSIGKPSLFISVSEKSKDRGSNTIGARLNRVEDKVTQLDQRLALITD
MLHQLLSLHGGSTPGSGGPPREGGAHITQPCGSGGSVDPELFLPSNTLPTYEQLTVPRRGP
DEGS

>Q9GZP9 multipass membrane protein
MAYQSLRLEYLQIPPVSRAYTTACVLTTAAVQLELITPFQLYFNPELIFKHFQIWRLITNFLFF
GPVGFNFLFNMIFLYRYCRMLEEGSFRGRTADFVFMFLFGGFLMTLFGLFVSLVFLGQAFT
IMLVYVWSRRNPYVRMNFFGLLNFQAPFLPWVLMGFSLLLGNSIIVDLLGIAVGHIYFFLE
DVFPNQPGGIRILKTPSILKAIFDTPDEDPNYNPLPEERPGGFAWGEGQRLGG

>P32942 single-pass type I membrane protein
MATMVPSVLWPRACWTLLVCCLLTPGVQGQEFLLRVEPQNPVLSAGGSLFVNCSTDCPSS
EKIALETSLSKELVASGMGWAAFNLSNVTGNSRILCSVYCNGSQITGSSNITVYRLPERVEL
APLPPWQPVGQNFTLRCQVEDGSPRTSLTVVLLRWEEELSRQPAVEEPAEVTATVLASRDD
HGAPFSCRTELDMQPQGLGLFVNTSAPRQLRTFVLPVTPPRLVAPRFLEVETSWPVDCTLD
GLFPASEAQVYLALGDQMLNATVMNHGDTLTATATATARADQEGAREIVCNVTLGGERRE
ARENLTVFSFLGPIVNLSEPTAHEGSTVTVSCMAGARVQVTLDGVPAAAPGQPAQLQLNA
TESDDGRSFFCSATLEVDGEFLHRNSSVQLRVLYGPKIDRATCPQHLKWKDKTRHVLQCQ
ARGNPYPELRCLKEGSSREVPVGIPFFVNVTHNGTYQCQASSSRGKYTLVVVMDIEAGSS
HFVPVFVAVLLTLGVVTIVLALMYVFREHQRSGSYHVREESTYLPLTSMQPTEAMGEEPSR
AE

>P13688 single-pass type I membrane protein
MGHLSAPLHRVRVPWQGLLLTASLLTFWNPPTTAQLTTESMPFNVAEGKEVLLLVHNLPQ
QLFGYSWYKGERVDGNRQIVGYAIGTQQATPGPANSGRETIYPNASLLIQNVTQNDTGFY
TLQVIKSDLVNEEATGQFHVYPELPKPSISSNNSNPVEDKDAVAFTCEPETQDTTYLWWIN
NQSLPVSPRLQLSNGNRTLTLLSVTRNDTGPYECEIQNPVSANRSDPVTLNVTYGPDTPTIS

PSDTYYRPGANLSLSCYAASNPPAQYSWLINGTFQQSTQELFIPNITVNNSGSYTCHANNS
VTGCNRTTVKTIIVTELSPVVAKPQIKASKTTVTGDKDSVNLTCSTNDTGISIRWFFKNQSL
PSSERMKLSQGNTTLSINPVKREDAGTYWCEVFNPISKNQSDPIMLNVNYNALPQENGLS
PGAIAGIVIGVVALVALIAVALACFLHFGKTGRASDQRDLTEHKPSVSNHTQDHSNDPPNK
MNEVTYSTLNFEAQQPTQPTSASPSLTATEIIYSEVKKQ

>P19438 single-pass type I membrane protein
MGLSTVPDLLLPLVLLELLVGIYPSGVIGLVPHLGDREKRDSVCPQGKYIHPQNNSICCTKC
HKGTYLYNDCPGPGQDTDCRECESGSFTASENHLRHCLSCSKCRKEMGQVEISSCTVDRD
TVCGCRKNQYRHYWSENLFQCFNCSLCLNGTVHLSCQEKQNTVCTCHAGFFLRENECVS
CSNCKKSLECTKLCLPQIENVKGTEDSGTTVLLPLVIFFGLCLLSLLFIGLMYRYQRWKSKL
YSIVCGKSTPEKEGELEGTTTKPLAPNPSFSPTPGFTPTLGFSPVPSSTFTSSSTYTPGDCPNF
AAPRREVAPPYQGADPILATALASDPIPNPLQKWEDSAHKPQSLDTDDPATLYAVVENVPP
LRWKEFVRRLGLSDHEIDRLELQNGRCLREAQYSMLATWRRRTPRREATLELLGRVLRD
MDLLGCLEDIEEALCGPAALPPAPSLLR

>Q9NYQ7 multipass membrane protein
MMARRPPWRGLGGRSTPILLLLLLSLFPLSQEELGGGGHQGWDPGLAATTGPRAHIGGGA
LALCPESSGVREDGGPGLGVREPIFVGLRGRRQSARNSRGPPEQPNEELGIEHGVQPLGSR
ERETGQGPGSVLYWRPEVSSCGRTGPLQRGSLSPGALSSGVPGSGNSSPLPSDFLIRHHGP
KPVSSQRNAGTGSRKRVGTARCCGELWATGSKGQGERATTSGAERTAPRRNCLPGASGSG
PELDSAPRTARTAPASGSAPRESRTAPEPAPKRMRSRGLFRCRFLPQRPGPRPPGLPARPEAR
KVTSANRARFRRAANRHPQFPQYNYQTLVPENEAAGTAVLRVVAQDPDAGEAGRLVYSL
AALMNSRSLELFSIDPQSGLIRTAAALDRESMERHYLRVTAQDHGSPRLSATTMVAVTVAD
RNDHSPVFEQAQYRETLRENVEEGYPILQLRATDGDAPPNANLRYRFVGPPAARAAAAA
AFEIDPRSGLISTSGRVDREHMESYELVVEASDQGQEPGPRSATVRVHITVLDENDNAPQFS
EKRYVAQVREDVRPHTVVLRVTATDRDKDANGLVHYNIISGNSRGHFAIDSLTGEIQVVAP
LDFEAEREYALRIRAQDAGRPPLSNNTGLASIQVVDINDHIPIFVSTPFQVSVLENAPLGHS
VIHIQAVDADHGENARLEYSLTGVAPDTPFVINSATGWVSVSGPLDRESVEHYFFGVEARD
HGSPPLSASASVTVTVLDVNDNRPEFTMKEYHLRLNEDAAVGTSVVSVTAVDRDANSAIS
YQITGGNTRNRFAISTQGGVGLVTLALPLDYKQERYFKLVLTASDRALHDHCYVHINITDA
NTHRPVFQSAHYSVSVNEDRPMGSTIVVISASDDDVGENARITYLLEDNLPQFRIDADSGA
ITLQAPLDYEDQVTYTLAITARDNGIPQKADTTYVEVMVNDVNDNAPQFVASHYTGLVSE
DAPPFTSVLQISATDRDAHANGRVQYTFQNGEDGDGDFTIEPTSGIVRTVRRLDREAVSVY
ELTAYAVDRGVPPLRTPVSIQVMVQDVNDNAPVFPAEEFEVRVKENSIVGSVVAQITAVDP
DEGPNAHIMYQIVEGNIPELFQMDIFSGELTALIDLDYEARQEYVIVVQATSAPLVSRATVH
VRLVDQNDNSPVLNNFQILFNNYVSNRSDTFPSGIIGRIPAYDPDVSDHLFYSFERGNELQL
LVVNQTSGELRLSRKLDNNRPLVASMLVTVTDGLHSVTAQCVLRVVIITEELLANSLTVRL
ENMWQERFLSPLLGRFLEGVAAVLATPAEDVFIFNIQNDTDVGGTVLNVSFSALAPRGAG
AGAAGPWFSSEELQEQLYVRRAALAARSLLDVLPFDDNVCLREPCENYMKCVSVLRFDS
SAPFLASASTLFRPIQPIAGLRCRCPPGFTGDFCETELDLCYSNPCRNGGACARREGGYTC
VCRPRFTGEDCELDTEAGRCVPGVCRNGGTCTDAPNGGFRCQCPAGGAFEGPRCEVAAR
SFPPSSFVMFRGLRQRFHLTLSLSFATVQQSGLLFYNGRLNEKHDFLALELVAGQVRLTYST
GESNTVVSPTVPGGLSDGQWHTVHLRYYNKPRTDALGGAQGPSKDKVAVLSVDDCDVAV

ALQFGAEIGNYSCAAAGVQTSSKKSLDLTGPLLLGGVPNLPENFPVSHKDFIGCMRDLHID
GRRVDMAAFVANNGTMAGCQAKLHFCDSGPCKNSGFCSERWGSFSCDCPVGFGGKDCQ
LTMAHPHHFRGNGTLSWNFGSDMAVSVPWYLGLAFRTRATQGVLMQVQAGPHSTLLCQ
LDRGLLSVTVTRGSGRASHLLLDQVTVSDGRWHDLRLELQEEPGGRRGHHVLMVSLDFS
LFQDTMAVGSELQGLKVKQLHVGGLPPGSAEEAPQGLVGCIQGVWLGSTPSGSPALLPPS
HRVNAEPGCVVTNACASGPCPPHADCRDLWQTFSCTCQPGYYGPGCVDACLLNPCQNQ
GSCRHLPGAPHGYTCDCVGGYFGHHCEHRMDQQCPRGWWGSPTCGPCNCDVHKGFDP
NCNKTNGQCHCKEFHYRPRGSDSCLPCDCYPVGSTSRSCAPHSGQCPCRPGALGRQCNS
CDSPFAEVTASGCRVLYDACPKSLRSGVWWPQTKFGVLATVPCPRGALGAAVRLCDEAQ
GWLEPDLFNCTSPAFRELSLLLDGLELNKTALDTMEAKKLAQRLREVTGHTDHYFSQDVR
VTARLLAHLLAFESHQQGFGLTATQDAHFNENLLWAGSALLAPETGDLWAALGQRAPGG
SPGSAGLVRHLEEYAATLARNMELTYLNPMGLVTPNIMLSIDRMEHPSSPRGARRYPRYHS
NLFRGQDAWDPHTHVLLPSQSPRPSPSEVLPTSSSIENSTTSSVVPPPAPPEPEPGISIIILLVY
RTLGGLLPAQFQAERRGARLPQNPVMNSPVVSVAVFHGRNFLRGILESPISLEFRLLQTANR
SKAICVQWDPPGLAEQHGVWTARDCELVHRNGSHARCRCSRTGTFGVLMDASPRERLEG
DLELLAVFTHVVVAVSVAALVLTAAILLSLRSLKSNVRGIHANVAAALGVAELLFLLGIHRT
HNQLVCTAVAILLHYFFLSTFAWLFVQGLHLYRMQVEPRNVDRGAMRFYHALGWGVPAV
LLGLAVGLDPEGYGNPDFCWISVHEPLIWSFAGPVVLVIVMNGTMFLLAARTSCSTGQRE
AKKTSALTLRSSFLLLLLVSASWLFGLLAVNHSILAFHYLHAGLCGLQGLAVLLLFCVLNA
DARAAWMPACLGRKAAPEEARPAPGLGPGAYNNTALFEESGLIRITLGASTVSSVSSARSG
RTQDQDSQRGRSYLRDNVLVRHGSAADHTDHSLQAHAGPTDLDVAMFHRDAGADSDSD
SDLSLEEERSLSIPSSESEDNGRTRGRFQRPLCRAAQSERLLTHPKDVDGNDLLSYWPALG
ECEAAPCALQTWGSERRLGLDTSKDAANNNQPDPALTSGDETSLGRAQRQRKGILKNRL
QYPLVPQTRGAPELSWCRAATLGHRAVPAASYGRIYAGGGTGSLSQPASRYSSREQLDLLL
RRQLSRERLEEAPAPVLRPLSRPGSQECMDAAPGRLEPKDRGSTLPRRQPPRDYPGAMAG
RFGSRDALDLGAPREWLSTLPPPRRTRDLDPQPPPLPLSPQRQLSRDPLLPSRPLDSLSRSS
NSREQLDQVPSRHPSREALGPLPQLLRAREDSVSGPSHGPSTEQLDILSSILASFNSSALSSV
QSSSTPLGPHTTATPSATASVLGPSTPRSATSHSISELSPDSEVPRSEGHS

>Q92521 multipass membrane protein
MRRPLSKCGMEPGGGDASLTLHGLQNRSHGKIKLRKRKSTLYFNTQEKSARRRGDLLGE
NIYLLLFTIALRILNCFLVQTSFVPDEYWQSLEVSHHMVFNYGYLTWEWTERLRSYTYPLI
FASIYKILHLLGKDSVQLLIWIPRLAQALLSAVADVRLYSLMKQLENQEVARWVFFCQLCS
WFTWYCCTRTLTNTMETVLTIIALFYYPLEGSKSMNSVKYSSLVALAFIIRPTAVILWTPLLF
RHFCQEPRKLDLILHHFLPVGFVTLSLSLMIDRIFFGQWTLVQFNFLKFNVLQNWGTFYGS
HPWHWYFSQGFPVILGTHLPFFIHGCYLAPKRYRILLVTVLWTLLVYSMLSHKEFRFIYPV
LPFCMVFCGYSLTHLKTWKKPALSFLFLSNLFLALYTGLVHQRGTLDVMSHIQKVCYNNP
NKSSASIFIMMPCHSTPYYSHVHCPLPMRFLQCPPDLTGKSHYLDEADVFYLNPLNWLHR
EFHDDASLPTHLITFSILEEEISAFLISSNYKRTAVFFHTHLPEGRIGSHIYVVYERKLKGKFNM
KMKF

>Q9H902 multipass membrane protein
MVSWIISRLVVLIFGTLYPAYYSYKAVKSKDIKEYVKWMMYWIIFALFTTAETFTDIFLCWF
PFYYELKIAFVAWLLSPYTKGSSLLYRKFVHPTLSSKEKEIDDCLVQAKDRSYDALVHFGK

RGLNVAATAAVMAASKGQGALSERLRSFSMQDLTTIRGDGAPAPSGPPPPGSGRASGKHG
QPKMSRSASESASSSGTA

>P15907 single-pass type II membrane protein
MIHTNLKKKFSCCVLVFLLFAVICVWKEKKKGSYYDSFKLQTKEFQVLKSLGKLAMGSDS
QSVSSSSTQDPHRGRQTLGSLRGLAKAKPEASFQVWNKDSSSKNLIPRLQKIWKNYLSMN
KYKVSYKGPGPGIKFSAEALRCHLRDHVNVSMVEVTDFPFNTSEWEGYLPKESIRTKAGP
WGRCAVVSSAGSLKSSQLGREIDDHDAVLRFNGAPTANFQQDVGTKTTIRLMNSQLVTTE
KRFLKDSLYNEGILIVWDPSVYHSDIPKWYQNPDYNFFNNYKTYRKLHPNQPFYILKPQM
PWELWDILQEISPEEIQPNPPSSGMLGIIIMMTLCDQVDIYEFLPSKRKTDVCYYYQKFFDS
ACTMGAYHPLLYEKNLVKHLNQGTDEDIYLLGKATLPGFRTIHC

>P24394 single-pass type I membrane protein
MGWLCSGLLFPVSCLVLLQVASSGNMKVLQEPTCVSDYMSISTCEWKMNGPTNCSTELR
LLYQLVFLLSEAHTCIPENNGGAGCVCHLLMDDVVSADNYTLDLWAGQQLLWKGSFKPS
EHVKPRAPGNLTVHTNVSDTLLLTWSNPYPPDNYLYNHLTYAVNIWSENDPADFRIYNVT
YLEPSLRIAASTLKSGISYRARVRAWAQCYNTTWSEWSPSTKWHNSYREPFEQHLLLGVS
VSCIVILAVCLLCYVSITKIKKEWWDQIPNPARSRLVAIIIQDAQGSQWEKRSRGQEPAKCP
HWKNCLTKLLPCFLEHNMKRDEDPHKAAKEMPFQGSGKSAWCPVEISKTVLWPESISVV
RCVELFEAPVECEEEEEVEEEKGSFCASPESSRDDFQEGREGIVARLTESLFLDLLGEENGG
FCQQDMGESCLLPPSGSTSAHMPWDEFPSAGPKEAPPWGKEQPLHLEPSPPASPTQSPDNL
TCTETPLVIAGNPAYRSFSNSLSQSPCPRELGPDPLLARHLEEVEPEMPCVPQLSEPTTVPQP
EPETWEQILRRNVLQHGAAAAPVSAPTSGYQEFVHAVEQGGTQASAVVGLGPPGEAGYK
AFSSLLASSAVSPEKCGFGASSGEEGYKPFQDLIPGCPGDPAPVPVPLFTFGLDREPPRSPQS
SHLPSSSPEHLGLEPGEKVEDMPKPPLPQEQATDPLVDSLGSGIVYSALTCHLCGHLKQCH
GQEDGGQTPVMASPCCGCCCGDRSSPPTTPLRAPDPSPGGVPLEASLCPASLAPSGISEKS
KSSSSFHPAPGNAQSSSQTPKIVNFVSVGPTYMRVS

>O00398 multipass membrane protein
MANLDKYTETFKMGSNSTSTAEIYCNVTNVKFQYSLYATTYILIFIPGLLANSAALWVLCR
FISKKNKAIIFMINLSVADLAHVLSLPLRIYYYISHHWPFQRALCLLCFYLKYLNMYASICFL
TCISLQRCFFLLKPFRARDWKRRYDVGISAAIWIVVGTACLPFPILRSTDLNNNKSCFADLG
YKQMNAVALVGMITVAELAGFVIPVIIIAWCTWKTTISLRQPPMAFQGISERQKALRMVFM
CAAVFFICFTPYHINFIFYTMVKETIISSCPVVRIALYFHPFCLCLASLCCLLDPILYYFMASE
FRDQLSRHGSSVTRSRLMSKESGSSMIG

>P08138 single-pass type I membrane protein
MGAGATGRAMDGPRLLLLLLLGVSLGGAKEACPTGLYTHSGECCKACNLGEGVAQPCG
ANQTVCEPCLDSVTFSDVVSATEPCKPCTECVGLQSMSAPCVEADDAVCRCAYGYYQDE
TTGRCEACRVCEAGSGLVFSCQDKQNTVCEECPDGTYSDEANHVDPCLPCTVCEDTERQ
LRECTRWADAECEEIPGRWITRSTPPEGSDSTAPSTQEPEAPPEQDLIASTVAGVVTTVMGS
SQPVVTRGTTDNLIPVYCSILAAVVVGLVAYIAFKRWNSCKQNKQGANSRPVNQTPPPEGE
KLHSDSGISVDSQSLHDQQPHTQTASGQALKGDGGLYSSLPPAKREEVEKLLNGSAGDTW
RHLAGELGYQPEHIDSFTHEACPVRALLASWATQDSATLDALLAALRRIQRADLVESLCSE

STATSPV

>Q9H2U9 single-pass type I membrane protein
MLPGCIFLMILLIPQVKEKFILGVEGQQLVRPKKLPLIQKRDTGHTHDDDILKTYEEELLYEI
KLNRKTLVLHLLRSREFLGSNYSETFYSMKGEAFTRHPQIMDHCFYQGSIVHEYDSAASIS
TCNGLRGFFRINDQRYLIEPVKYSDEGEHLVFKYNLRVPYGANYSCTELNFTRKTVPGDN
ESEEDSKIKGIHDEKYVELFIVADDTVYRRNGHPHNKLRNRIWGMVNFVNMIYKTLNIHV
TLVGIEIWTHEDKIELYSNIETTLLRFSFWQEKILKTRKDFDHVVLLSGKWLYSHVQGISYP
GGMCLPYYSTSIIKDLLPDTNIIANRMAHQLGHNLGMQHDEFPCTCPSGKCVMDSDGSIP
ALKFSKCSQNQYHQYLKDYKPTCMLNIPFPYNFHDFQFCGNKKLDEGEECDCGPAQECT
NPCCDAHTCVLKPGFTCAEGECCESCQIKKAGSICRPAKDECDFPEMCTGHSPACPKDQFR
VNGFPCKNSEGYCFMGKCPTREDQCSELFDDEAIESHDICYKMNTKGNKFGYCKNKENR
FLPCEEKDVRCGKIYCTGGELSSLLGEDKTYHLKDPQKNATVKCKTIFLYHDSTDIGLVAS
GTKCGEGMVCNNGECLNMEKVYISTNCPSQCNENPVDGHGLQCHCEEGQAPVACEETL
HVTNITILVVVLVLVIVGIGVLILLVRYRKCIKLKQVQSPPTETLGVENKGYFGDEQQIRTEP
ILPEIHFLNKPASKDSRGIADPNQSAK

>P21917 multipass membrane protein
MGNRSTADADGLLAGRGPAAGASAGASAGLAGQGAAALVGGVLLIGAVLAGNSLVCVS
VATERALQTPTNSFIVSLAAADLLLALLVLPLFVYSEVQGGAWLLSPRLCDALMAMDVML
CTASIFNLCAISVDRFVAVAVPLRYNRQGGSRRQLLLIGATWLLSAAVAAPVLCGLNDVRG
RDPAVCRLEDRDYVVYSSVCSFFLPCPLMLLLYWATFRGLQRWEVARRAKLHGRAPRRPS
GPGPPSPTPPAPRLPQDPCGPDCAPPAPGLPRGPCGPDCAPAAPGLPPDPCGPDCAPPAPGL
PQDPCGPDCAPPAPGLPRGPCGPDCAPPAPGLPQDPCGPDCAPPAPGLPPDPCGSNCAPPD
AVRAAALPPQTPPQTRRRRRAKITGRERKAMRVLPVVVGAFLLCWTPFFVVHITQALCPA
CSVPPRLVSAVTWLGYVNSALNPVIYTVFNAEFRNVFRKALRACC

>O94804 peripheral membrane protein
MAFANFRRILRLSTFEKRKSREYEHVRRDLDPNEVWEIVGELGDGAFGKVYKAKNKETG
ALAAAKVIETKSEEELEDYIVEIEILATCDHPYIVKLLGAYYHDGKLWIMIEFCPGGAVDAI
MLELDRGLTEPQIQVVCRQMLEALNFLHSKRIIHRDLKAGNVLMTLEGDIRLADFGVSAK
NLKTLQKRDSFIGTPYWMAPEVVMCETMKDTPYDYKADIWSLGITLIEMAQIEPPHHELN
PMRVLLKIAKSDPPTLLTPSKWSVEFRDFLKIALDKNPETRPSAAQLLEHPFVSSITSNKAL
RELVAEAKAEVMEEIEDGRDEGEEEDAVDAASTLENHTQNSSEVSPPSLNADKPLEESPST
PLAPSQSQDSVNEPCSQPSGDRSLQTTSPPVVAPGNENGLAVPVPLRKSRPVSMDARIQVA
QEKQVAEQGGDLSPAANRSQKASQSRPNSSALETLGGEKLANGSLEPPAQAAPGPSKRDS
DCSSLCTSESMDYGTNLSTDLSLNKEMGSLSIKDPKLYKKTLKRTRKFVVDGVEVSITTSK
IISEDEKKDEEMRFLRRQELRELRLLQKEEHRNQTQLSNKHELQLEQMHKRFEQEINAKK
KFFDTELENLERQQKQQVEKMEQDHAVRRREEARRIRLEQDRDYTRFQEQLKLMKKEVK
NEVEKLPRQQRKESMKQKMEEHTQKKQLLDRDFVAKQKEDLELAMKRLTTDNRREICD
KERECLMKKQELLRDREAALWEMEEHQLQERHQLVKQQLKDQYFLQRHELLRKHEKER
EQMQRYNQRMIEQLKVRQQQEKARLPKIQRSEGKTRMAMYKKSLHINGGGSAAEQREKI
KQFSQQEEKRQKSERLQQQQKHENQMRDMLAQCESNMSELQQLQNEKCHLLVEHETQK
LKALDESHNQNLKEWRDKLRPRKKALEEDLNQKKREQEMFFKLSEEAECPNPSTPSKAA

KFFPYSSADAS

>O43570 single-pass type I membrane protein
MPRRSLHAAAVLLLVILKEQPSSPAPVNGSKWTYFGPDGENSWSKKYPSCGGLLQSPIDLH
SDILQYDASLTPLEFQGYNLSANKQFLLTNNGHSVKLNLPSDMHIQGLQSRYSATQLHLH
WGNPNDPHGSEHTVSGQHFAAELHIVHYNSDLYPDASTASNKSEGLAVLAVLIEMGSFNP
SYDKIFSHLQHVKYKGQEAFVPGFNIEELLPERTAEYYRYRGSLTTPPCNPTVLWTVFRNP
VQISQEQLLALETALYCTHMDDPSPREMINNFRQVQKFDERLVYTSFSQVQVCTAAGLSL
GIILSLALAGILGICIVVVVSIWLFRRKSIKKGDNKGVIYKPATKMETEAHA

>P56856 multipass membrane protein
MSTTTCQVVAFLLSILGLAGCIAATGMDMWSTQDLYDNPVTSVFQYEGLWRSCVRQSSGF
TECRPYFTILGLPAMLQAVRALMIVGIVLGAIGLLVSIFALKCIRIGSMEDSAKANMTLTSGI
MFIVSGLCAIAGVSVFANMLVTNFWMSTANMYTGMGGMVQTVQTRYTFGAALFVGWV
AGGLTLIGGVMMCIACRGLAPEETNYKAVSYHASGHSVAYKPGGFKASTGFGSNTKNKKI
YDGGARTEDEVQSYPSKHDYV

>Q9H6F2 multipass membrane protein
MELLSALSLGELALSFSRVPLFPVFDLSYFIVSILYLKYEPGAVELSRRHPIASWLCAMLHC
FGSYILADLLLGEPLIDYFSNNSSILLASAVWYLIFFCPLDLFYKCVCFLPVKLIFVAMKEVV
RVRKIAVGIHHAHHHYHHGWFVMIATGWVKGSGVALMSNFEQLLRGVWKPETNEILHM
SFPTKASLYGAILFTLQQTRWLPVSKASLIFIFTLFMVSCKVFLTATHSHSSPFDALEGYICP
VLFGSACGGDHHHDNHGGSHSGGGPGAQHSAMPAKSKEELSEGSRKKKAKKAD

>Q12884 single-pass type II membrane protein
MKTWVKIVFGVATSAVLALLVMCIVLRPSRVHNSEENTMRALTLKDILNGTFSYKTFFPN
WISGQEYLHQSADNNIVLYNIETGQSYTILSNRTMKSVNASNYGLSPDRQFVYLESDYSKL
WRYSYTATYYIYDLSNGEFVRGNELPRPIQYLCWSPVGSKLAYVYQNNIYLKQRPGDPPF
QITFNGRENKIFNGIPDWVYEEEMLATKYALWWSPNGKFLAYAEFNDTDIPVIAYSYYGDE
QYPRTINIPYPKAGAKNPVVRIFIIDTTYPAYVGPQEVPVPAMIASSDYYFSWLTWVTDERV
CLQWLKRVQNVSVLSICDFREDWQTWDCPKTQEHIEESRTGWAGGFFVSTPVFSYDAISY
YKIFSDKDGYKHIHYIKDTVENAIQITSGKWEAINIFRVTQDSLFYSSNEFEEYPGRRNIYRI
SIGSYPPSKKCVTCHLRKERCQYYTASFSDYAKYYALVCYGPGIPISTLHDGRTDQEIKILEE
NKELENALKNIQLPKEEIKKLEVDEITLWYKMILPPQFDRSKKYPLLIQVYGGPCSQSVRS
VFAVNWISYLASKEGMVIALVDGRGTAFQGDKLLYAVYRKLGVYEVEDQITAVRKFIEMG
FIDEKRIAIWGWSYGGYVSSLALASGTGLFKCGIAVAPVSSWEYYASVYTERFMGLPTKD
DNLEHYKNSTVMARAEYFRNVDYLLIHGTADDNVHFQNSAQIAKALVNAQVDFQAMW
YSDQNHGLSGLSTNHLYTHMTHFLKQCFSLSD

>Q86WC4 single-pass type I membrane protein
MEPGPTAAQRRCSLPPWLPLGLLLWSGLALGALPFGSSPHRVFHDLLSEQQLLEVEDLSLS
LLQGGGLGPLSLPPDLPDLDPECRELLLDFANSSAELTGCLVRSARPVRLCQTCYPLFQQV
VSKMDNISRAAGNTSESQSCARSLLMADRMQIVVILSEFFNTTWQEANCANCLTNNSEEL
SNSTVYFLNLFNHTLTCFEHNLQGNAHSLLQTKNYSEVCKNCREAYKTLSSLYSEMQKM

NELENKAEPGTHLCIDVEDAMNITRKLWSRTFNCSVPCSDTVPVIAVSVFILFLPVVFYLSS
FLHSEQKKRKLILPKRLKSSTSFANIQENSN

>Q9BZW2 multipass membrane protein
MKFFSYILVYRRFLFVVFTVLVLLPLPIVLHTKEAECAYTLFVVATFWLTEALPLSVTALLPS
LMLPMFGIMPSKKVASAYFKDFHLLLIGVICLATSIEKWNLHKRIALKMVMMVGVNPAWL
TLGFMSSTAFLSMWLSNTSTAAMVMPIAEAVVQQIINAEAEVEATQMTYFNGSTNHGLEI
DESVNGHEINERKEKTKPVPGYNNDTGKISSKVELEKNSGMRTKYRTKKGHVTRKLTCLC
IAYSSTIGGLTTITGTSTNLIFAEYFNTRYPDCRCLNFGSWFTFSFPAALIILLLSWIWLQWLF
LGFNFKEMFKCGKTKTVQQKACAEVIKQEYQKLGPIRYQEIVTLVLFIIMALLWFSRDPGF
VPGWSALFSEYPGFATDSTVALLIGLLFFLIPAKTLTKTTPTGEIVAFDYSPLITWKEFQSFMP
WDIAILVGGGFALADGCEESGLSKWIGNKLSPLGSLPAWLIILISSLMVTSLTEVASNPATITL
FLPILSPLAEAIHVNPLYILIPSTLCTSFAFLLPVANPPNAIVFSYGHLKVIDMVKAGLGVNIV
GVAVVMLGICTWIVPMFDLYTYPSWAPAMSNETMP

>O60641 peripheral membrane protein
MSGQTLTDRIAAAQYSVTGSAVARAVCKATTHEVMGPKKKHLDYLIQATNETNVNIPQM
ADTLFERATNSSWVVVFKALVTTHHLMVHGNERFIQYLASRNTLFNLSNFLDKSGSHGY
DMSTFIRRYSRYLNEKAFSYRQMAFDFARVKKGADGVMRTMAPEKLLKSMPILQGQIDA
LLEFDVHPNELTNGVINAAFMLLFKDLIKLFACYNDGVINLLEKFFEMKKGQCKDALEIY
KRFLTRMTRVSEFLKVAEQVGIDKGDIPDLTQAPSSLMETLEQHLNTLEGKKPGNNEGSGA
PSPLSKSSPATTVTSPNSTPAKTIDTSPPVDLFATASAAVPVSTSKPSSDLLDLQPDFSSGGAA
AAAAPAPPPPAGGATAWGDLLGEDSLAALSSVPSEAQISDPFAPEPTPPTTTAEIATASASAS
TTTTVTAVTAEVDLFGDAFAASPGEAPAASEGAAAPATPTPVAAALDACSGNDPFAPSEGS
AEAAPELDLFAMKPPETSVPVVTPTASTAPPVPATAPSPAPAVAAAAAATTAATAAATTTTT
TSAATATTAPPALDIFGDLFESTPEVAAAPKPDAAPSIDLFSTDAFSSPPQGASPVPESSLTAD
LLSVDAFAAPSPATTASPAKVDSSGVIDLFGDAFGSSASEPQPASQAASSSSASADLLAGFG
GSFMAPSPSPVTPAQNNLLQPNFEAAFGTTPSTSSSSSFDPSVFDGLGDLLMPTMAPAGQP
APVSMVPPSPAMAASKALGSDLDSSLASLVGNLGISGTTTKKGDLQWNAGEKKLTGGAN
WQPKVAPATWSAGVPPSAPLQGAVPPTSSVPPVAGAPSVGQPGAGFGMPPAGTGMPMMP
QQPVMFAQPMMRPPFGAAAVPGTQLSPSPTPASQSPKKPPAKDPLADLNIKDFL

>Q02809 peripheral membrane protein
MRPLLLLALLGWLLLAEAKGDAKPEDNLLVLTVATKETEGFRRFKRSAQFFNYKIQALGL
GEDWNVEKGTSAGGGQKVRLLKKALEKHADKEDLVILFADSYDVLFASGPRELLKKFRQ
ARSQVVFSAEELIYPDRRLETKYPVVSDGKRFLGSGGFIGYAPNLSKLVAEWEGQDSDSD
QLFYTKIFLDPEKREQINITLDHRCRIFQNLDGALDEVVLKFEMGHVRARNLAYDTLPVLI
HGNGPTKLQLNYLGNYIPRFWTFETGCTVCDEGLRSLKGIGDEALPTVLVGVFIEQPTPFV
SLFFQRLLRLHYPQKHMRLFIHNHEQHHKAQVEEFLAQHGSEYQSVKLVGPEVRMANAD
ARNMGADLCRQDRSCTYYFSVDADVALTEPNSLRLLIQQNKNVIAPLMTRHGRLWSNFW
GALSADGYYARSEDYVDIVQGRRVGVWNVPYISNIYLIKGSALRGELQSSDLFHHSKLDP
DMAFCANIRQQDVFMFLTNRHTLGHLLSLDSYRTTHLHNDLWEVFSNPEDWKEKYIHQN
YTKALAGKLVETPCPDVYWFPIFTEVACDELVEEMEHFGQWSLGNNKDNRIQGGYENVP
TIDIHMNQIGFEREWHKFLLEYIAPMTEKLYPGYYTRAQFDLAFVVRYKPDEQPSLMPHH

DASTFTINIALNRVGVDYEGGGCRFLRYNCSIRAPRKGWTLMHPGRLTHYHEGLPTTRGT
RYIAVSFVDP

>Q3SYC2 multipass membrane protein
MVEFAPLFMPWERRLQTLAVLQFVFSFLALAEICTVGFIALLFTRFWLLTVLYAAWWYLD
RDKPRQGGRHIQAIRCWTIWKYMKDYFPISLVKTAELDPSRNYIAGFHPHGVLAVGAFAN
LCTESTGFSSIFPGIRPHLMMLTLWFRAPFFRDYIMSAGLVTSEKESAAHILNRKGGGNLLG
IIVGGAQEALDARPGSFTLLLRNRKGFVRLALTHGAPLVPIFSFGENDLFDQIPNSSGSWLR
YIQNRLQKIMGISLPLFHGRGVFQYSFGLIPYRRPITTVVGKPIEVQKTLHPSEEEVNQLHQ
RYIKELCNLFEAHKLKFNIPADQHLEFC

>P41181 multipass membrane protein
MWELRSIAFSRAVFAEFLATLLFVFFGLGSALNWPQALPSVLQIAMAFGLGIGTLVQALGH
ISGAHINPAVTVACLVGCHVSVLRAAFYVAAQLLGAVAGAALLHEITPADIRGDLAVNALS
NSTTAGQAVTVELFLTLQLVLCIFASTDERRGENPGTPALSIGFSVALGHLLGIHYTGCSMN
PARSLAPAVVTGKFDDHWVFWIGPLVGAILGSLLYNYVLFPPAKSLSERLAVLKGLEPDTD
WEEREVRRRQSVELHSPQSLPRGTKA

>O43264 peripheral membrane protein
MASFVTEVLAHSGRLEKEDLGTRISRLTRRVEEIKGEVCNMISKKYSEFLPSMQSAQGLIT
QVDKLSEDIDLLKSRIESEVRRDLHVSTGEFTDLKQQLERDSVVLSLLKQLQEFSTAIEEYN
CALTEKKYVTGAQRLEEAQKCLKLLKSRKCFDLKILKSLSMELTIQKQNILYHLGEEWQK
LIVWKFPPSKDTSSLESYLQTELHLYTEQSHKEEKTPMPPISSVLLAFSVLGELHSKLKSFG
QMLLKYILRPLASCPSLHAVIESQPNIVIIRFESIMTNLEYPSPSEVFTKIRLVLEVLQKQLLD
LPLDTDLENEKTSTVPLAEMLGDMIWEDLSECLIKNCLVYSIPTNSSKLQQYEEIIQSTEEF
ENALKEMRFLKGDTTDLLKYARNINSHFANKKCQDVIVAARNLMTSEIHNTVKIIPDSKIN
VPELPTPDEDNKLEVQKVSNTQYHEVMNLEPENTLDQHSFSLPTCRISESVKKLMELAYQ
TLLEATTSSDQCAVQLFYSVRNIFHLFHDVVPTYHKENLQKLPQLAAIHHNNCMYIAHHL
LTLGHQFRLRLAPILCDGTATFVDLVPGFRRLGTECFLAQMRAQKGELLERLSSARNFSNM
DDEENYSAASKAVRQVLHQLKRLGIVWQDVLPVNIYCKAMGTLLNTAISEVIGKITALEDI
STEDGDRLYSLCKTVMDEGPQVFAPLSEESKNKKYQEEVPVYVPKWMPFKELMMMLQA
SLQEIGDRWADGKGPLAAAFSSSEVKALIRALFQNTERRAAALAKIK

>P16144 single-pass type I membrane protein
MAGPRPSPWARLLLAALISVSLSGTLANRCKKAPVKSCTECVRVDKDCAYCTDEMFRDR
RCNTQAELLAAGCQRESIVVMESSFQITEETQIDTTLRRSQMSPQGLRVRLRPGEERHFEL
EVFEPLESPVDLYILMDFSNSMSDDLDNLKKMGQNLARVLSQLTSDYTIGFGKFVDKVSV
PQTDMRPEKLKEPWPNSDPPFSFKNVISLTEDVDEFRNKLQGERISGNLDAPEGGFDAILQ
TAVCTRDIGWRPDSTHLLVFSTESAFHYEADGANVLAGIMSRNDERCHLDTTGTYTQYRT
QDYPSVPTLVRLLAKHNIIPIFAVTNYSYSYYEKLHTYFPVSSLGVLQEDSSNIVELLEEAFN
RIRSNLDIRALDSPRGLRTEVTSKMFQKTRTGSFHIRRGEVGIYQVQLRALEHVDGTHVCQ
LPEDQKGNIHLKPSFSDGLKMDAGIICDVCTCELQKEVRSARCSFNGDFVCGQCVCSEGW
SGQTCNCSTGSLSDIQPCLREGEDKPCSGRGECQCGHCVCYGEGRYEGQFCEYDNFQCPR
TSGFLCNDRGRCSMGQCVCEPGWTGPSCDCPLSNATCIDSNGGICNGRGHCECGRCHCH

QQSLYTDTICEINYSAIHPGLCEDLRSCVQCQAWGTGEKKGRTCEECNFKVKMVDELKRA
EEVVVRCSFRDEDDDCTYSYTMEGDGAPGPNSTVLVHKKKDCPPGSFWWLIPLLLLLLPL
LALLLLLCWKYCACCKACLALLPCCNRGHMVGFKEDHYMLRENLMASDHLDTPMLRS
GNLKGRDVVRWKVTNNMQRPGFATHAASINPTELVPYGLSLRLARLCTENLLKPDTREC
AQLRQEVEENLNEVYRQISGVHKLQQTKFRQQPNAGKKQDHTIVDTVLMAPRSAKPALL
KLTEKQVEQRAFHDLKVAPGYYTLTADQDARGMVEFQEGVELVDVRVPLFIRPEDDDEK
QLLVEAIDVPAGTATLGRRLVNITIIKEQARDVVSFEQPEFSVSRGDQVARIPVIRRVLDGGK
SQVSYRTQDGTAQGNRDYIPVEGELLFQPGEAWKELQVKLLELQEVDSLLRGRQVRRFH
VQLSNPKFGAHLGQPHSTTIIIRDPDELDRSFTSQMLSSQPPPHGDLGAPQNPNAKAAGSR
KIHFNWLPPSGKPMGYRVKYWIQGDSESEAHLLDSKVPSVELTNLYPYCDYEMKVCAYG
AQGEGPYSSLVSCRTHQEVPSEPGRLAFNVVSSTVTQLSWAEPAETNGEITAYEVCYGLVN
DDNRPIGPMKKVLVDNPKNRMLLIENLRESQPYRYTVKARNGAGWGPEREAIINLATQPK
RPMSIPIIPDIPIVDAQSGEDYDSFLMYSDDVLRSPSGSQRPSVSDDTGCGWKFEPLLGEEL
DLRRVTWRLPPELIPRLSASSGRSSDAEAPHGPPDDGGAGGKGGSLPRSATPGPPGEHLVN
GRMDFAFPGSTNSLHRMTTTSAAAYGTHLSPHVPHRVLSTSSTLTRDYNSLTRSEHSHSTT
LPRDYSTLTSVSSHDSRLTAGVPDTPTRLVFSALGPTSLRVSWQEPRCERPLQGYSVEYQLL
NGGELHRLNIPNPAQTSVVVEDLLPNHSYVFRVRAQSQEGWGREREGVITIESQVHPQSPL
CPLPGSAFTLSTPSAPGPLVFTALSPDSLQLSWERPRRPNGDIVGYLVTCEMAQGGGPATAF
RVDGDSPESRLTVPGLSENVPYKFKVQARTTEGFGPEREGIITIESQDGGPFPQLGSRAGLF
QHPLQSEYSSITTTHTSATEPFLVDGLTLGAQHLEAGGSLTRHVTQEFVSRTLTTSGTLSTH
MDQQFFQT

>O75078 single-pass type I membrane protein
MRLLRRWAFAALLLSLLPTPGLGTQGPAGALRWGGLPQLGGPGAPEVTEPSRLVRESSGG
EVRKQQLDTRVRQEPPGGPPVHLAQVSFVIPAFNSNFTLDLELNHHLLSSQYVERHFSREG
TTQHSTGAGDHCYYQGKLRGNPHSFAALSTCQGLHGVFSDGNLTYIVEPQEVAGPWGAP
QGPLPHLIYRTPLLPDPLGCREPGCLFAVPAQSAPPNRPRLRRKRQVRRGHPTVHSETKYV
ELIVINDHQLFEQMRQSVVLTSNFAKSVVNLADVIYKEQLNTRIVLVAMETWADGDKIQV
QDDLLETLARLMVYRREGLPEPSDATHLFSGRTFQSTSSGAAYVGGICSLSHGGGVNEYG
NMGAMAVTLAQTLGQNLGMMWNKHRSSAGDCKCPDIWLGCIMEDTGFYLPRKFSRCSI
DEYNQFLQEGGGSCLFNKPLKLLDPPECGNGFVEAGEECDCGSVQECSRAGGNCCKKCT
LTHDAMCSDGLCCRRCKYEPRGVSCREAVNECDIAETCTGDSSQCPPNLHKLDGYYCDH
EQGRCYGGRCKTRDRQCQVLWGHAAADRFCYEKLNVEGTERGSCGRKGSGWVQCSKQ
DVLCGFLLCVNISGAPRLGDLVGDISSVTFYHQGKELDCRGGHVQLADGSDLSYVEDGTA
CGPNMLCLDHRCLPASAFNFSTCPGSGERRICSHHGVCSNEGKCICQPDWTGKDCSIHNPL
PTSPPTGETERYKGPSGTNIIIGSIAGAVLVAAIVLGGTGWGFKNIRRGRSGGA

>P18627 single-pass type I membrane protein
MWEAQFLGLLFLQPLWVAPVKPLQPGAEVPVVWAQEGAPAQLPCSPTIPLQDLSLLRRAG
VTWQHQPDSGPPAAAPGHPLAPGPHPAAPSSWGPRPRRYTVLSVGPGGLRSGRLPLQPRV
QLDERGRQRGDFSLWLRPARRADAGEYRAAVHLRDRALSCRLRLRLGQASMTASPPGSL
RASDWVILNCSFSRPDRPASVHWFRNRGQGRVPVRESPHHHLAESFLFLPQVSPMDSGPW
GCILTYRDGFNVSIMYNLTVLGLEPPTPLTVYAGAGSRVGLPCRLPAGVGTRSFLTAKWTPP
GGGPDLLVTGDNGDFTLRLEDVSQAQAGTYTCHIHLQEQQLNATVTLAIITVTPKSFGSPG

SLGKLLCEVTPVSGQERFVWSSLDTPSQRSFSGPWLEAQEAQLLSQPWQCQLYQGERLLG
AAVYFTELSSPGAQRSGRAPGALPAGHLLLFLILGVLSLLLLVTGAFGFHLWRRQWRPRRF
SALEQGIHPPQAQSKIEELEQEPEPEPEPEPEPEPEPEPEQL

>Q9NZD8 peripheral membrane protein
MGEIKVSPDYNWFRGTVPLKKIIVDDDDSKIWSLYDAGPRSIRCPLIFLPPVSGTADVFFRQ
ILALTGWGYRVIALQYPVYWDHLEFCDGFRKLLDHLQLDKVHLFGASLGGFLAQKFAEY
THKSPRVHSLILCNSFSDTSIFNQTWTANSFWLMPAFMLKKIVLGNFSSGPVDPMMADAID
FMVDRLESLGQSELASRLTLNCQNSYVEPHKIRDIPVTIMDVFDQSALSTEAKEEMYKLYP
NARRAHLKTGGNFPYLCRSAEVNLYVQIHLLQFHGTKYAAIDPSMVSAEELEVQKGSLGI
SQEEQ

>P49771 single-pass type I membrane protein
MTVLAPAWSPTTYLLLLLLLSSGLSGTQDCSFQHSPISSDFAVKIRELSDYLLQDYPVTVAS
NLQDEELCGGLWRLVLAQRWMERLKTVAGSKMQGLLERVNTEIHFVTKCAFQPPPSCLR
FVQTNISRLLQETSEQLVALKPWITRQNFSRCLELQCQPDSSTLPPPWSPRPLEATAPTAPQP
PLLLLLLLPVGLLLLAAAWCLHWQRTRRRTPRPGEQVPPVPSPQDLLLVEH

>Q08378 peripheral membrane protein
MDGASAEQDGLQEDRSHSGPSSLPEAPLKPPGPLVPPDQQDKVQCAEVNRASTEGESPDG
PGQGGLCQNGPTPPFPDPPSSLDPTTSPVGPDASPGVAGFHDNLRKSQGTSAEGSVRKEAL
QSLRLSLPMQETQLCSTDSPLPLEKEEQVRLQARKWLEEQLKQYRVKRQQERSSQPATKT
RLFSTLDPELMLNPENLPRASTLAMTKEYSFLRTSVPRGPKVGSLGLPAHPREKKTSKSSKI
RSLADYRTEDSNAGNSGGNVPAPDSTKGSLKQNRSSAASVVSEISLSPDTDDRLENTSLAG
DSVSEVDGNDSDSSSYSSASTRGTYGILSKTVGTQDTPYMVNGQEIPADTLGQFPSIKDVL
QAAAAEHQDQGQEVNGEVRSRRDSICSSVSLESSAAETQEEMLQVLKEKMRLEGQLEAL
SLEASQALKEKAELQAQLAALSTKLQAQVECSHSSQQRQDSLSSEVDTLKQSCWDLERA
MTDLQNMLEAKNASLASSNNDLQVAEEQYQRLMAKVEDMQRSMLSKDNTVHDLRQQ
MTALQSQLQQVQLERTTLTSKLKASQAEISSLQSVRQWYQQQLALAQEARVRLQGEMAH
IQVGQMTQAGLLEHLKLENVSLSQQLTETQHRSMKEKGRIAAQLQGIEADMLDQEAAFM
QIQEAKTMVEEDLQRRLEEFEGERERLQRMADSAASLEQQLEQVKLTLLQRDQQLEALQ
QEHLDLMKQLTLTQEALQSREQSLDALQTHYDELQARLGELQGEAASREDTICLLQNEKII
LEAALQAAKSGKEELDRGARRLEEGTEETSETLEKLREELAIKSGQVEHLQQETAALKKQ
MQKIKEQFLQQKVMVEAYRRDATSKDQLISELKATRKRLDSELKELRQELMQVHGEKRT
AEAELSRLHREVAQVRQHMADLEGHLQSAQKERDEMETHLQSLQFDKEQMVAVTEANE
ALKKQIEELQQEARKAITEQKQKMRRLGSDLTSAQKEMKTKHKAYENAVGILSRRLQEAL
AAKEAADAELGQLRAQGGSSDSSLALHERIQALEAELQAVSHSKTLLEKELQEVIALTSQE
LEESREKVLELEDELQESRGFRKKIKRLEESNKKLALELEHEKGKLTGLGQSNAALREHNS
ILETALAKREADLVQLNLQVQAVLQRKEEEDRQMKHLVQALQASLEKEKEKVNSLKEQV
AAAKVEAGHNRRHFKAASLELSEVKKELQAKEHLVQKLQAEADDLQIREGKHSQEIAQF
QAELAEARAQLQLLQKQLDEQLSKQPVGNQEMENLKWEVDQKEREIQSLKQQLDLTEQ
QGRKELEGLQQLLQNVKSELEMAQEDLSMTQKDKFMLQAKVSELKNNMKTLLQQNQQ
LKLDLRRGAAKTRKEPKGEASSSNPATPIKIPDCPVPASLLEELLRPPPAVSKEPLKNLNSCL
QQLKQEMDSLQRQMEEHALTVHESLSSWTPLEPATASPVPPGGHAGPRGDPQRHSQSRAS

KEGPGE

>P98172 single-pass type I membrane protein
MARPGQRWLGKWLVAMVVWALCRLATPLAKNLEPVSWSSLNPKFLSGKGLVIYPKIGDK
LDIICPRAEAGRPYEYYKLYLVRPEQAAACSTVLDPNVLVTCNRPEQEIRFTIKFQEFSPNY
MGLEFKKHHDYYITSTSNGSLEGLENREGGVCRTRTMKIIMKVGQDPNAVTPEQLTTSRPS
KEADNTVKMATQAPGSRGSLGDSDGKHETVNQEEKSGPGASGGSSGDPDGFFNSKVALF
AAVGAGCVIFLLIIIFLTVLLLKLRKRHRKHTQQRAAALSLSTLASPKGGSGTAGTEPSDIIIP
LRTTENNYCPHYEKVSGDYGHPVYIVQEMPPQSPANIYYKV

>Q92896 single-pass type I membrane protein
MAACGRVRRMFRLSAALHLLLLFAAGAEKLPGQGVHSQGQGPGANFVSFVGQAGGGGP
AGQQLPQLPQSSQLQQQQQQQQQQQPQPPQPPFPAGGPPARRGGAGAGGGWKLAEEES
CREDVTRVCPKHTWSNNLAVLECLQDVREPENEISSDCNHLLWNYKLNLTTDPKFESVAR
EVCKSTITEIKECADEPVGKGYMVSCLVDHRGNITEYQCHQYITKMTAIIFSDYRLICGFM
DDCKNDINILKCGSIRLGEKDAHSQGEVVSCLEKGLVKEAEEREPKIQVSELCKKAILRVA
ELSSDDFHLDRHLYFACRDDRERFCENTQAGEGRVYKCLFNHKFEESMSEKCREALTTRQ
KLIAQDYKVSYSLAKSCKSDLKKYRCNVENLPRSREARLSYLLMCLESAVHRGRQVSSEC
QGEMLDYRRMLMEDFSLSPEIILSCRGEIEHHCSGLHRKGRTLHCLMKVVRGEKGNLGM
NCQQALQTLIQETDPGADYRIDRALNEACESVIQTACKHIRSGDPMILSCLMEHLYTEKMV
EDCEHRLLELQYFISRDWKLDPVLYRKCQGDASRLCHTHGWNETSEFMPQGAVFSCLYR
HAYRTEEQGRRLSRECRAEVQRILHQRAMDVKLDPALQDKCLIDLGKWCSEKTETGQEL
ECLQDHLDDLVVECRDIVGNLTELESEDIQIEALLMRACEPIIQNFCHDVADNQIDSGDLM
ECLIQNKHQKDMNEKCAIGVTHFQLVQMKDFRFSYKFKMACKEDVLKLCPNIKKKVDV
VICLSTTVRNDTLQEAKEHRVSLKCRRQLRVEELEMTEDIRLEPDLYEACKSDIKNFCSAV
QYGNAQIIECLKENKKQLSTRCHQKVFKLQETEMMDPELDYTLMRVCKQMIKRFCPEAD
SKTMLQCLKQNKNSELMDPKCKQMITKRQITQNTDYRLNPMLRKACKADIPKFCHGILT
KAKDDSELEGQVISCLKLRYADQRLSSDCEDQIRIIIQESALDYRLDPQLQLHCSDEISSLCA
EEAAAQEQTGQVEECLKVNLLKIKTELCKKEVLNMLKESKADIFVDPVLHTACALDIKHH
CAAITPGRGRQMSCLMEALEDKRVRLQPECKKRLNDRIEMWSYAAKVAPADGFSDLAMQ
VMTSPSKNYILSVISGSICILFLIGLMCGRITKRVTRELKDR

>Q9NX76 multipass membrane protein
MENGAVYSPTTEEDPGPARGPRSGLAAYFFMGRLPLLRRVLKGLQLLLSLLAFICEEVVSQ
CTLCGGLYFFEFVSCSAFLLSLLILIVYCTPFYERVDTTKVKSSDFYITLGTGCVFLLASIIFV
STHDRTSAEIAAIVFGFIASFMFLLDFITMLYEKRQESQLRKPENTTRAEALTEPLNA

>P03372 peripheral membrane protein, single-pass type I membrane protein
MTMTLHTKASGMALLHQIQGNELEPLNRPQLKIPLERPLGEVYLDSSKPAVYNYPEGAAY
EFNAAAAANAQVYGQTGLPYGPGSEAAAFGSNGLGGFPPLNSVSPSPLMLLHPPPQLSPF
LQPHGQQVPYYLENEPSGYTVREAGPPAFYRPNSDNRRQGGRERLASTNDKGSMAMESA
KETRYCAVCNDYASGYHYGVWSCEGCKAFFKRSIQGHNDYMCPATNQCTIDKNRRKSCQ
ACRLRKCYEVGMMKGGIRKDRRGGRMLKHKRQRDDGEGRGEVGSAGDMRAANLWPS
PLMIKRSKKNSLALSLTADQMVSALLDAEPPILYSEYDPTRPFSEASMMGLLTNLADRELV

HMINWAKRVPGFVDLTLHDQVHLLECAWLEILMIGLVWRSMEHPGKLLFAPNLLLDRNQ
GKCVEGMVEIFDMLLATSSRFRMMNLQGEEFVCLKSIILLNSGVYTFLSSTLKSLEEKDHI
HRVLDKITDTLIHLMAKAGLTLQQQHQRLAQLLLILSHIRHMSNKGMEHLYSMKCKNVV
PLYDLLLEMLDAHRLHAPTSRGGASVEETDQSHLATAGSTSSHSLQKYYITGEAEGFPATV

>Q9UGC6 lipid-anchor
MRKRQQSQNEGTPAVSQAPGNQRPNNTCCFCWCCCCSCSCLTVRNEERGENAGRPTHTT
KMESIQVLEECQNPTAEEVLSWSQNFDKMMKAPAGRNLFREFLRTEYSEENLLFWLACE
DLKKEQNKKVIEEKARMIYEDYISILSPKEVSLDSRVREVINRNLLDPNPHMYEDAQLQIY
TLMHRDSFPRFLNSQIYKSFVESTAGSSSES

>Q8WUG5 multipass membrane protein
MASDPIFTLAPPLHCHYGAFPPNASGWEQPPNASGVSVASAALAASAASRVATSTDPSCSG
FAPPDFNHCLKDWDYNGLPVLTTNAIGQWDLVCDLGWQVILEQILFILGFASGYLFLGYPA
DRFGRRGIVLLTLGLVGPCGVGGAAAGSSTGVMALRFLLGFLLAGVDLGVYLMRLELCD
PTQRLRVALAGELVGVGGHFLFLGLALVSKDWRFLQRMITAPCILFLFYGWPGLFLESAR
WLIVKRQIEEAQSVLRILAERNRPHGQMLGEEAQEALQDLENTCPLPATSSFSFASLLNYR
NIWKNLLILGFTNFIAHAIRHCYQPVGGGGSPSDFYLCSLLASGTAALACVFLGVTVDRFG
RRGILLLSMTLTGIASLVLLGLWDCEHPIFPTVWAQQGNPNRDLNEAAITTFSVLGLFSSQA
AAILSTLLAAEVIPTTVRGRGLGLIMALGALGGLSGPAQRLHMGHGAFLQHVVLAACALL
CILSIMLLPETKRKLLPEVLRDGELCRRPSLLRQPPPTRCDHVPLLATPNPAL

>P22735 lipid-anchor
MMDGPRSDVGRWGGNPLQPPTTPSPEPEPEPDGRSRRGGGRSFWARCCGCCSCRNAADD
DWGPEPSDSRGRGSSSGTRRPGSRGSDSRRPVSRGSGVNAAGDGTIREGMLVVNGVDLLS
SRSDQNRREHHTDEYEYDELIVRRGQPFHMLLLLSRTYESSDRITLELLIGNNPEVGKGTH
VIIPVGKGGSGGWKAQVVKASGQNLNLRVHTSPNAIIGKFQFTVRTQSDAGEFQLPFDPR
NEIYILFNPWCPEDIVYVDHEDWRQEYVLNESGRIYYGTEAQIGERTWNYGQFDHGVLD
ACLYILDRRGMPYGGRGDPVNVSRVISAMVNSLDDNGVLIGNWSGDYSRGTNPSAWVGS
VEILLSYLRTGYSVPYGQCWVFAGVTTTVLRCLGLATRTVTNFNSAHDTDTSLTMDIYFDE
NMKPLEHLNHDSVWNFHVWNDCWMKRPDLPSGFDGWQVVDATPQETSSGIFCCGPCSV
ESIKNGLVYMKYDTPFIFAEVNSDKVYWQRQDDGSFKIVYVEEKAIGTLIVTKAISSNMRE
DITYLYKHPEGSDAERKAVETAAAHGSKPNVYANRGSAEDVAMQVEAQDAVMGQDLMV
SVMLINHSSSRRTVKLHLYLSVTFYTGVSGTIFKETKKEVELAPGASDRVTMPVAYKEYRP
HLVDQGAMLLNVSGHVKESGQVLAKQHTFRLRTPDLSLTLLGAAVVGQECEVQIVFKNP
LPVTLTNVVFRLEGSGLQRPKILNVGDIGGNETVTLRQSFVPVRPGPRQLIASLDSPQLSQV
HGVIQVDVAPAPGDGGFFSDAGGDSHLGETIPMASRGGA

>Q70J99 peripheral membrane protein
MATLLSHPQQRPPFLRQAIKIRRRRVRDLQDPPPQMAPEIQPPSHHFSPEQRALLYEDALYT
VLHRLGHPEPNHVTEASELLRYLQEAFHVEPEEHQQTLQRVRELEKPIFCLKATVKQAKGI
LGKDVSGFSDPYCLLGIEQGVGVPGGSPGSRHRQKAVVRHTIPEEETHRTQVITQTLNPVW
DETFILEFEDITNASFHLDMWDLDTVESVRQKLGELTDLHGLRRIFKEARKDKGQDDFLG
NVVLRLQDLRCREDQWYPLEPRTETYPDRGQCHLQFQLIHKRRATSASRSQPSYTVHLHL

LQQLVSHEVTQHEAGSTSWDGSLSPQAATVLFLHATQKDLSDFHQSMAQWLAYSRLYQS
LEFPSSCLLHPITSIEYQWIQGRLKAEQQEELAASFSSLLTYGLSLIRRFRSVFPLSVSDSPAR
LQSLLRVLVQMCKMKAFGELCPNTAPLPQLVTEALQTGTTEWFHLKQQHHQPMVQGIPE
AGKALLGLVQDVIGDLHQCQRTWDKIFHNTLKIHLFSMAFRELQWLVAKRVQDHTTVVG
DVVSPEMGESLFQLYISLKELCQLRMSSSERDGVLALDNFHRWFQPAIPSWLQKTYNEAL
ARVQRAVQMDELVPLGELTKHSTSAVDLSTCFAQISHTARQLDWPDPEEAFMITVKFVEDT
CRLALVYCSLIKARARELSSGQKDQGQAANMLCVVVNDMEQLRLVIGKLPAQLAWEALE
QRVGAVLEQGQLQNTLHAQLQSALAGLGHEIRTGVRTLAEQLEVGIAKHIQKLVGVRESV
LPEDAILPLMKFLEVELCYMNTNLVQENFSSLLTLLWTHTLTVLVEAAASQRSSSLASNRL
KIALQNLEICFHAEGCGLPPKALHTATFQALQRDLELQAASSRELIRKYFCSRIQQQAETTS
EELGAVTVKASYRASEQKLRVELLSASSLLPLDSNGSSDPFVQLTLEPRHEFPELAARETQ
KHKKDLHPLFDETFEFLVPAEPCRKAGACLLLTVLDYDTLGADDLEGEAFLPLREVPGLS
GSEEPGEVPQTRLPLTYPAPNGDPILQLLEGRKGDREAQVFVRLRRHRAKQASQHALRPA
P

>O60431 multipass membrane protein
MEPEKQTEISEFFLQGLSEKPEHQTLLFTMFLSTYLVTIIGNALIILAIITDSHLHTPMYFFLF
NLSLVDTLLSSTTVPKMLANIQAQSRAIPFVGCLTQMYAFHLFGTMDSFLLAVMAIDRFVA
IVHPQRYLVLMCSPVCGLLLGASWMITNLQSLIHTCLMAQLTFCAGSEISHFFCDLMPLLK
LSGSDTHTNELVIFAFGIVVGTSPFSCILLSYIRIFWTVFKIPSTRGKWKAFSTCGLHLTVVSL
SYGTIFAVYLQPTSPSSSQKDKAAALMCGVFIPMLNPFIYSIRNKDMKAALGKLIGKVAVP
CPRPEQLLDVYHVPGSLLAARDTEMHPIPYPGGVQSLAGNRDME

>Q9ULW5 lipid-anchor
MSRKKTPKSKGASTPAASTLPTANGARPARSGTALSGPDAPPNGPLQPGRPSLGGGVDFY
DVAFKVMLVGDSGVGKTCLLVRFKDGAFLAGTFISTVGIDFRNKVLDVDGVKVKLQMW
DTAGQERFRSVTHAYYRDAHALLLLYDVTNKASFDNIQAWLTEIHEYAQHDVALMLLGN
KVDSAHERVVKREDGEKLAKEYGLPFMETSAKTGLNVDLAFTAIAKELKQRSMKAPSEP
RFRLHDYVKREGRGASCCRP

>Q9NTN9 single-pass type I membrane protein
MWGRLWPLLLSILTATAVPGPSLRRPSRELDATPRMTIPYEELSGTRHFKGQAQNYSTLLLE
EASARLLVGARGALFSLSANDIGDGAHKEIHWEASPEMQSKCHQKGKNNQTECFNHVRF
LQRLNSTHLYACGTHAFQPLCAAIDAEAFTLPTSFEEGKEKCPYDPARGFTGLIIDGGLYTA
TRYEFRSIPDIRRSRHPHSLRTEETPMHWLNDAEFVFSVLVRESKASAVGDDDKVYYFFTE
RATEEGSGSFTQSRSSHRVARVARVCKGDLGGKKILQKKWTSFLKARLICHIPLYETLRGV
CSLDAETSSRTHFYAAFTLSTQWKTLEASAICRYDLAEIQAVFAGPYMEYQDGSRRWGRY
EGGVPEPRPGSCITDSLRSQGYNSSQDLPSLVLDFVKLHPLMARPVVPTRGRPLLLKRNIR
YTHLTGTPVTTPAGPTYDLLFLGTADGWIHKAVVLGSGMHIIEETQVFRESQSVENLVISLL
QHSLYVGAPSGVIQLPLSSCSRYRSCYDCILARDPYCGWDPGTHACAAATTIANRTALIQDI
ERGNRGCESSRDTGPPPPLKTRSVLRGDDVLLPCDQPSNLARALWLLNGSMGLSDGQGG
YRVGVDGLLVTDAQPEHSGNYGCYAEENGLRTLLASYSLTVRPATPAPAPKAPATPGAQLA
PDVRLLYVLAIAALGGLCLILASSLLYVACLREGRRGRRRKYSLGRASRAGGSAVQLQTVS
GQCPGEEDEGDDEGAGGLEGSCLQIIPGEGAPAPPPPPPPPPPAELTNGLVALPSRLRRMNG

NSYVLLRQSNNGVPAGPCSFAEELSRILEKRKHTQLVEQLDESSV

>P18206 peripheral membrane protein
MPVFHTRTIESILEPVAQQISHLVIMHEEGEVDGKAIPDLTAPVAAVQAAVSNLVRVGKETV
QTTEDQILKRDMPPAFIKVENACTKLVQAAQMLQSDPYSVPARDYLIDGSRGILSGTSDLL
LTFDEAEVRKIIRVCKGILEYLTVAEVVETMEDLVTYTKNLGPGMTKMAKMIDERQQELT
HQEHRVMLVNSMNTVKELLPVLISAMKIFVTTKNSKNQGIEEALKNRNFTVEKMSAEINEI
IRVLQLTSWDEDAWASKDTEAMKRALASIDSKLNQAKGWLRDPSASPGDAGEQAIRQILD
EAGKVGELCAGKERREILGTCKMLGQMTDQVADLRARGQGSSPVAMQKAQQVSQGLDV
LTAKVENAARKLEAMTNSKQSIAKKIDAAQNWLADPNGGPEGEEQIRGALAEARKIAELC
DDPKERDDILRSLGEISALTSKLADLRRQGKGDSPEARALAKQVATALQNLQTKTNRAVA
NSRPAKAAVHLEGKIEQAQRWIDNPTVDDRGVGQAAIRGLVAEGHRLANVMMGPYRQD
LLAKCDRVDQLTAQLADLAARGEGESPQARALASQLQDSLKDLKARMQEAMTQEVSDV
FSDTTTPIKLLAVAATAPPDAPNREEVFDERAANFENHSGKLGATAEKAAAVGTANKSTVE
GIQASVKTARELTPQVVSAARILLRNPGNQAAYEHFETMKNQWIDNVEKMTGLVDEAIDT
KSLLDASEEAIKKDLDKCKVAMANIQPQMLVAGATSIARRANRILLVAKREVENSEDPKFR
EAVKAASDELSKTISPMVMDAKAVAGNISDPGLQKSFLDSGYRILGAVAKVREAFQPQEPD
FPPPPPDLEQLRLTDELAPPKPPLPEGEVPPPRPPPPEEKDEEFPEQKAGEVINQPMMMAAR
QLHDEARKWSSKPGIPAAEVGIGVVAEADAADAAGFPVPPDMEDDYEPELLLMPSNQPV
NQPILAAAQSLHREATKWSSKGNDIIAAAKRMALLMAEMSRLVRGGSGTKRALIQCAKDI
AKASDEVTRLAKEVAKQCTDKRIRTNLLQVCERIPTISTQLKILSTVKATMLGRTNISDEES
EQATEMLVHNAQNLMQSVKETVREAEAASIKIRTDAGFTLRWVRKTPWYQ

>Q9Y371 peripheral membrane protein
MNIMDFNVKKLAADAGTFLSRAVQFTEEKLGQAEKTELDAHLENLLSKAECTKIWTEKI
MKQTEVLLQPNPNARIEEFVYEKLDRKAPSRINNPELLGQYMIDAGTEFGPGTAYGNALIK
CGETQKRIGTADRELIQTSALNFLTPLRNFIEGDYKTIAKERKLLQNKRLDLDAAKTRLKK
AKAAETRNSSEQELRITQSEFDRQAEITRLLLEGISSTHAHHLRCLNDFVEAQMTYYAQCY
QYMLDLQKQLGSFPSNYLSNNNQTSVTPVPSVLPNAIGSSAMASTSGLVITSPSNLSDLKE
CSGSRKARVLYDYDAANSTELSLLADEVITVFSVVGMDSDWLMGERGNQKGKVPITYLE
LLN

>Q13445 single-pass type I membrane protein
MMAAGAALALALWLLMPPVEVGGAGPPPIQDGEFTFLLPAGRKQCFYQSAPANASLETE
YQVIGGAGLDVDFTLESPQGVLLVSESRKADGVHTVEPTEAGDYKLCFDNSFSTISEKLVF
FELIFDSLQDDEEVEGWAEAVEPEEMLDVKMEDIKESIETMRTRLERSIQMLTLLRAFEAR
DRNLQEGNLERVNFWSAVNVAVLLLVAVLQVCTLKRFFQDKRPVPT

>Q9P0N8 multipass membrane protein
MTTGDCCHLPGSLCDCSGSPAFSKVVEATGLGPPQYVAQVTSRDGRLLSTVIRALDTPSDG
PFCRICHEGANGECLLSPCGCTGTLGAVHKSCLEKWLSSSNTSYCELCHTEFAVEKRPRPL
TEWLKDPGPRTEKRTLCCDMVCFLFITPLAAISGWLCLRGAQDHLRLHSQLEAVGLIALTI
ALFTIYVLWTLVSFRYHCQLYSEWRKTNQKVRLKIREADSPEGPQHSPLAAGLLKKVAEE
TPV

>Q9NZ01 multipass membrane protein
MKHYEVEILDAKTREKLCFLDKVEPHATIAEIKNLFTKTHPQWYPARQSLRLDPKGKSLK
DEDVLQKLPVGTTATLYFRDLGAQISWVTVFLTEYAGPLFIYLLFYFRVPFIYGHKYDFTSS
RHTVVHLACICHSFHYIKRLLETLFVHRFSHGTMPLRNIFKNCTYYWGFAAWMAYYINHP
LYTPPTYGAQQVKLALAIFVICQLGNFSIHMALRDLRPAGSKTRKIPYPTKNPFTWLFLLVS
CPNYTYEVGSWIGFAIMTQCLPVALFSLVGFTQMTIWAKGKHRSYLKEFRDYPPLRMPIIPF
LL

>Q9Y5L4 peripheral membrane protein
MEGGFGSDFGGSGSGKLDPGLIMEQVKVQIAVANAQELLQRMTDKCFRKCIGKPGGSLD
NSEQKCIAMCMDRYMDAWNTVSRAYNSRLQRERANM

>Q13477 single-pass type I membrane protein
MDFGLALLLAGLLGLLLGQSLQVKPLQVEPPEPVVAVALGASRQLTCRLACADRGASVQ
WRGLDTSLGAVQSDTGRSVLTVRNASLSAAGTRVCVGSCGGRTFQHTVQLLVYAFPDQLT
VSPAALVPGDPEVACTAHKVTPVDPNALSFSLLVGGQELEGAQALGPEVQEEEEEPQGDE
DVLFRVTERWRLPPLGTPVPPALYCQATMRLPGLELSHRQAIPVLHSPTSPEPPDTTSPESP
DTTSPESPDTTSQEPPDTTSPEPPDKTSPEPAPQQGSTHTPRSPGSTRTRRPEISQAGPTQGE
VIPTGSSKPAGDQLPAALWTSSAVLGLLLLALPTYHLWKRCRHLAEDDTHPPASLRLLPQV
SAWAGLRGTGQVGISPS

>O95721 peripheral membrane protein
MSAYPKSYNPFDDDGEDEGARPAPWRDARDLPDGPDAPADRQQYLRQEVLRRAEATAAS
TSRSLALMYESEKVGVASSEELARQRGVLERTEKMVDKMDQDLKISQKHINSIKSVFGGL
VNYFKSKPVETPPEQNGTLTSQPNNRLKEAISTSKEQEAKYQASHPNLRKLDDTDPVPRG
AGSAMSTDAYPKNPHLRAYHQKIDSNLDELSMGLGRLKDIALGMQTEIEEQDDILDRLTT
KVDKLDVNIKSTERKVRQL

>P53007 multipass membrane protein
MPAPRAPRALAAAAPASGKAKLTHPGKAILAGGLAGGIEICITFPTEYVKTQLQLDERSHP
PRYRGIGDCVRQTVRSHGVLGLYRGLSSLLYGSIPKAAVRFGMFEFLSNHMRDAQGRLDS
TRGLLCGLGAGVAEAVVVVCPMETIKVKFIHDQTSPNPKYRGFFHGVREIVREQGLKGTY
QGLTATVLKQGSNQAIRFFVMTSLRNWYRGDNPNKPMNPLITGVFGAIAGAASVFGNTPL
DVIKTRMQGLEAHKYRNTWDCGLQILKKEGLKAFYKGTVPRLGRVCLDVAIVFVIYDEV
VKLLNKVWKTD

>Q96GD0 peripheral membrane protein
MARCERLRGAALRDVLGRAQGVLFDCDGVLWNGERAVPGAPELLERLARAGKAALFVS
NNSRRARPELALRFARLGFGGLRAEQLFSSALCAARLLRQRLPGPPDAPGAVFVLGGEGL
RAELRAAGLRLAGDPSAGDGAAPRVRAVLVGYDEHFSFAKLREACAHLRDPECLLVATDR
DPWHPLSDGSRTPGTGSLAAAVETASGRQALVVGKPSPYMFECITENFSIDPARTLMVGDR
LETDILFGHRCGMTTVLTLTGVSRLEEAQAYLAAGQHDLVPHYYVESIADLTEGLED

>Q9UH99 single-pass type II membrane protein
MSRRSQRLTRYSQGDDDGSSSSGGSSVAGSQSTLFKDSPLRTLKRKSSNMKRLSPAPQLGP
SSDAHTSYYSESLVHESWFPPRSSLEELHGDANWGEDLRVRRRRGTGGSESSRASGLVGR
KATEDFLGSSSGYSSEDDYVGYSDVDQQSSSSRLRSAVSRAGSLLWMVATSPGRLFRLLY
WWAGTTWYRLTTAASLLDVFVLTRRFSSLKTFLWFLLPLLLLTCLTYGAWYFYPYGLQTF
HPALVSWWAAKDSRRPDEGWEARDSSPHFQAEQRVMSRVHSLERRLEALAAEFSSNWQK
EAMRLERLELRQGAPGQGGGGGLSHEDTLALLEGLVSRREAALKEDFRRETAARIQEELS
ALRAEHQQDSEDLFKKIVRASQESEARIQQLKSEWQSMTQESFQESSVKELRRLEDQLAG
LQQELAALALKQSSVAEEVGLLPQQIQAVRDDVESQFPAWISQFLARGGGGRVGLLQREE
MQAQLRELESKILTHVAEMQGKSAREAAASLSLTLQKEGVIGVTEEQVHHIVKQALQRYS
EDRIGLADYALESGGASVISTRCSETYETKTALLSLFGIPLWYHSQSPRVILQPDVHPGNCW
AFQGPQGFAVVRLSARIRPTAVTLEHVPKALSPNSTISSAPKDFAIFGFDEDLQQEGTLLGK
FTYDQDGEPIQTFHFQAPTMATYQVVELRILTNWGHPEYTCIYRFRVHGEPAH

>Q92737 lipid-anchor
MGGSLRVAVLGAPGVGKTAIIRQFLFGDYPERHRPTDGPRLYRPAVLLDGAVYDLSIRDGD
VAGPGSSPGGPEEWPDAKDWSLQDTDAFVLVYDICSPDSFDYVKALRQRIAETRPAGAPE
APILVVGNKRDRQRLRFGPRRALAALVRRGWRCGYLECSAKYNWHVLRLFRELLRCALV
RARPAHPALRLQGALHPARCSLM

>Q9NST1 single-pass type II membrane protein
MYDAERGWSLSFAGCGFLGFYHVGATRCLSEHAPHLLRDARMLFGASAGALHCVGVLS
GIPLEQTLQVLSDLVRKARSRNIGIFHPSFNLSKFLRQGLCKCLPANVHQLISGKIGISLTRVS
DGENVLVSDFRSKDEVVDALVCSCFIPFYSGLIPPSFRGVRYVDGGVSDNVPFIDAKTTITV
SPFYGEYDICPKVKSTNFLHVDITKLSLRLCTGNLYLLSRAFVPPDLKVLGEICLRGYLDAF
RFLEEKGICNRPQPGLKSSSEGMDPEVAMPSWANMSLDSSPESAALAVRLEGDELLDHLR
LSILPWDESILDTLSPRLATALSEEMKDKGGYMSKICNLLPIRIMSYVMLPCTLPVESAIAIV
QRLVTWLPDMPDDVLWLQWVTSQVFTRVLMCLLPASRSQMPVSSQQASPCTPEQDWPC
WTPCSPKGCPAETKAEATPRSILRSSLNFFLGNKVPAGAEGLSTFPSFSLEKSL

>P14784 single-pass type I membrane protein
MAAPALSWRLPLLILLLPLATSWASAAVNGTSQFTCFYNSRANISCVWSQDGALQDTSCQ
VHAWPDRRRWNQTCELLPVSQASWACNLILGAPDSQKLTTVDIVTLRVLCREGVRWRVM
AIQDFKPFENLRLMAPISLQVVHVETHRCNISWEISQASHYFERHLEFEARTLSPGHTWEE
APLLTLKQKQEWICLETLTPDTQYEFQVRVKPLQGEFTTWSPWSQPLAFRTKPAALGKDTI
PWLGHLLVGLSGAFGFIILVYLLINCRNTGPWLKKVLKCNTPDPSKFFSQLSSEHGGDVQK
WLSSPFPSSSFSPGGLAPEISPLEVLERDKVTQLLLQQDKVPEPASLSSNHSLTSCFTNQGYF
FFHLPDALEIEACQVYFTYDPYSEEDPDEGVAGAPTGSSPQPLQPLSGEDDAYCTFPSRDD
LLLFSPSLLGGPSPPSTAPGGSGAGEERMPPSLQERVPRDWDPQPLGPPTPGVPDLVDFQPP
PELVLREAGEEVPDAGPREGVSFPWSRPPGQGEFRALNARLPLNTDAYLSLQELQGQDPT
HLV

>Q8TCT0 peripheral membrane protein
MGATGAAEPLQSVLWVKQQRCAVSLEPARALLRWWRSPGPGAGAPGADACSVPVSEIIA

VEETDVHGKHQGSGKWQKMEKPYAFTVHCVKRARRHRWKWAQVTFWCPEEQLCHLW
LQTLREMLEKLTSRPKHLLVFINPFGGKGQGKRIYERKVAPLFTLASITTDIIVTEHANQAK
ETLYEINIDKYDGIVCVGGDGMFSEVLHGLIGRTQRSAGVDQNHPRAVLVPSSLRIGIIPAG
STDCVCYSTVGTSDAETSALHIVVGDSLAMDVSSVHHNSTLLRYSVSLLGYGFYGDIIKDS
EKKRWLGLARYDFSGLKTFLSHHCYEGTVSFLPAQHTVGSPRDRKPCRAGCFVCRQSKQ
QLEEEQKKALYGLEAAEDVEEWQVVCGKFLAINATNMSCACRRSPRGLSPAAHLGDGSS
DLILIRKCSRFNFLRFLIRHTNQQDQFDFTFVEVYRVKKFQFTSKHMEDEDSDLKEGGKKR
FGHICSSHPSCCCTVSNSSWNCDGEVLHSPAIEVRVHCQLVRLFARGIEENPKPDSHS

>Q96EK6 peripheral membrane protein
MKPDETPMFDPSLLKEVDWSQNTATFSPAISPTHPGEGLVLRPLCTADLNRGFFKVLGQLT
ETGVVSPEQFMKSFEHMKKSGDYYVTVVEDVTLGQIVATATLIIEHKFIHSCAKRGRVEDV
VVSDECRGKQLGKLLLSTLTLLSKKLNCYKITLECLPQNVGFYKKFGYTVSEENYMCRRF
LK

>O95406 multipass membrane protein
MAFTFAAFCYMLALLLTAALIFFAIWHIIAFDELKTDYKNPIDQCNTLNPLVLPEYLIHAFFC
VMFLCAAEWLTLGLNMPLLAYHIWRYMSRPVMSGPGLYDPTTIMNADILAYCQKEGWC
KLAFYLLAFFYYLYGMIYVLVSS

>Q9NYT0 peripheral membrane protein
MEDGVLKEGFLVKRGHIVHNWKARWFILRQNTLVYYKLEGGRRVTPPKGRILLDGCTITC
PCLEYENRPLLIKLKTQTSTEYFLEACSREERDAWAFEITGAIHAGQPGKVQQLHSLRNSF
KLPPHISLHRIVDKMHDSNTGIRSSPNMEQGSTYKKTFLGSSLVDWLISNSFTASRLEAVTL
ASMLMEENFLRPVGVRSMGAIRSGDLAEQFLDDSTALYTFAESYKKKISPKEEISLSTVELS
GTVVKQGYLAKQGHKRKNWKVRRFVLRKDPAFLHYYDPSKEENRPVGGFSLRGSLVSA
LEDNGVPTGVKGNVQGNLFKVITKDDTHYYIQASSKAERAEWIEAIKKLT

>Q14973 multipass membrane protein
MEAHNASAPFNFTLPPNFGKRPTDLALSVILVFMLFFIMLSLGCTMEFSKIKAHLWKPKGL
AIALVAQYGIMPLTAFVLGKVFRLKNIEALAILVCGCSPGGNLSNVFSLAMKGDMNLSIVM
TTCSTFCALGMMPLLLYIYSRGIYDGDLKDKVPYKGIVISLVLVLIPCTIGIVLKSKRPQYM
RYVIKGGMIIILLCSVAVTVLSAINVGKSIMFAMTPLLIATSSLMPFIGFLLGYVLSALFCLN
GRCRRTVSMETGCQNVQLCSTILNVAFPPEVIGPLFFFPLLYMIFQLGEGLLLIAIFWCYEKF
KTPKDKTKMIYTAATTEETIPGALGNGTYKGEDCSPCTA

>P46663 multipass membrane protein
MASSWPPLELQSSNQSQLFPQNATACDNAPEAWDLLHRVLPTFIISICFFGLLGNLFVLLVF
LLPRRQLNVAEIYLANLAASDLVFVLGLPFWAENIWNQFNWPFGALLCRVINGVIKANLFI
SIFLVVAISQDRYRVLVHPMASRRQRRRQARVTCVLIWVVGGLLSIPTFLLRSIQAVPDLN
ITACILLLPHEAWHFARIVELNILGFLLPLAAIVFFNYHILASLRTREEVSRTRCGGRKDSKT
TALILTLVVAFLVCWAPYHFFAFLEFLFQVQAVRGCFWEDFIDLGLQLANFFAFTNSSLNPV
IYVFVGRLFRTKVWELYKQCTPKSLAPISSSHRKEIFQLFWRN

>Q9UNN8 single-pass type I membrane protein
MLTTLLPILLLSGWAFCSQDASDGLQRLHMLQISYFRDPYHVWYQGNASLGGHLTHVLE
GPDTNTTIIQLQPLQEPESWARTQSGLQSYLLQFHGLVRLVHQERTLAFPLTIRCFLGCELPP
EGSRAHVFFEVAVNGSSFVSFRPERALWQADTQVTSGVVTFTLQQLNAYNRTRYELREFL
EDTCVQYVQKHISAENTKGSQTSRSYTSLVLGVLVGSFIIAGVAVGIFLCTGGRRC

>Q96BD0 multipass membrane protein
MPLHQLGDKPLTFPSPNSAMENGLDHTPPSRRASPGTPLSPGSLRSAAHSPLDTSKQPLCQ
LWAEKHGARGTHEVRYVSAGQSVACGWWAFAPPCLQVLNTPKGILFFLCAAAFLQGMTV
NGFINTVITSLERRYDLHSYQSGLIASSYDIAACLCLTFVSYFGGSGHKPRWLGWGVLLMG
TGSLVFALPHFTAGRYEVELDAGVRTCPANPGAVCADSTSGLSRYQLVFMLGQFLHGVGA
TPLYTLGVTYLDENVKSSCSPVYIAIFYTAAILGPAAGYLIGGALLNIYTEMGRRTELTTESP
LWVGAWWVGFLGSGAAAFFTAVPILGYPRQLPGSQRYAVMRAAEMHQLKDSSRGEASNP
DFGKTIRDLPLSIWLLLKNPTFILLCLAGATEATLITGMSTFSPKFLESQFSLSASEAATLFGY
LVVPAGGGGTFLGGFFVNKLRLRGSAVIKFCLFCTVVSLLGILVFSLHCPSVPMAGVTASY
GGSLLPEGHLNLTAPCNAACSCQPEHYSPVCGSDGLMYFSLCHAGCPAATETNVDGQKV
YRDCSCIPQNLSSGFGHATAGKCTSTCQRKPLLLVFIFVVIFFTFLSSIPALTATLRCVRDPQR
SFALGIQWIVVRILGGIPGPIAFGWVIDKACLLWQDQCGQQGSCLVYQNSAMSRYILIMGL
LYKVLGVLFFAIACFLYKPLSESSDGLETCLPSQSSAPDSATDSQLQSSV

>Q9NQ40 multipass membrane protein
MAFLMHLLVCVFGMGSWVTINGLWVELPLLVMELPEGWYLPSYLTVVIQLANIGPLLVTL
LHHFRPSCLSEVPIIFTLLGVGTVTCIIFAFLWNMTSWVLDGHHSIAFLVLTFFLALVDCTSS
VTFLPFMSRLPTYYLTTFFVGEGLSGLLPALVALAQGSGLTTCVNVTEISDSVPSPVPTRET
DIAQGVPRALVSALPGMEAPLSHLESRYLPAHFSPLVFFLLLSIMMACCLVAFFVLQRQPRC
WEASVEDLLNDQVTLHSIRPREENDLGPAGTVDSSQGQGYLEEKAAPCCPAHLAFIYTLV
AFVNALTNGMLPSVQTYSCLSYGPVAYHLAATLSIVANPLASLVSMFLPNRSLLFLGVLSV
LGTCFGGYNMAMAVMSPCPLLQGHWGGEVLIVASWVLFSGCLSYVKVMLGVVLRDLSR
SALLWCGAAVQLGSLLGALLMFPLVNVLRLFSSADFCNLHCPA

>Q9BQL6 peripheral membrane protein
MLSSTDFTFASWELVVRVDHPNEEQQKDVTLRVSGDLHVGGVMLKLVEQINISQDWSDF
ALWWEQKHCWLLKTHWTLDKYGVQADAKLLFTPQHKMLRLRLPNLKMVRLRVSFSAV
VFKAVSDICKILNIRRSEELSLLKPSGDYFKKKKKKDKNNKEPIIEDILNLESSPTASGSSVSP
GLYSKTMTPIYDPINGTPASSTMTWFSDSPLTEQNCSILAFSQPPQSPEALADMYQPRSLVD
KAKLNAGWLDSSRSLMEQGIQEDEQLLLRFKYYSFFDLNPKYDAVRINQLYEQARWAILL
EEIDCTEEEMLIFAALQYHISKLSLSAETQDFAGESEVDEIEAALSNLEVTLEGGKADSLLE
DITDIPKLADNLKLFRPKKLLPKAFKQYWFIFKDTSIAYFKNKELEQGEPLEKLNLRGCEV
VPDVNVAGRKFGIKLLIPVADGMNEMYLRCDHENQYAQWMAACMLASKGKTMADSSY
QPEVLNILSFLRMKNRNSASQVASSLENMDMNPECFVSPRCAKRHKSKQLAARILEAHQN
VAQMPLVEAKLRFIQAWQSLPEFGLTYYLVRFKGSKKDDILGVSYNRLIKIDAATGIPVTT
WRFTNIKQWNVNWETRQVVIEFDQNVFTAFTCLSADCKIVHEYIGGYIFLSTRSKDQNET
LDEDLFHKLTGGQD

>Q13424 peripheral membrane protein
MASGRRAPRTGLLELRAGAGSGAGGERWQRVLLSLAEDVLTVSPADGDPGPEPGAPREQ
EPAQLNGAAEPGAGPPQLPEALLLQRRRVTVRKADAGGLGISIKGGRENKMPILISKIFKG
LAADQTEALFVGDAILSVNGEDLSSATHDEAVQVLKKTGKEVVLEVKYMKDVSPYFKNS
TGGTSVGWDSPPASPLQRQPSSPGPTPRNFSEAKHMSLKMAYVSKRCTPNDPEPRYLEICS
ADGQDTLFLRAKDEASARSWATAIQAQVNTLTPRVKDELQALLAATSTAGSQDIKQIGWL
TEQLPSGGTAPTLALLTEKELLLYLSLPETREALSRPARTAPLIATRLVHSGPSKGSVPYDAE
LSFALRTGTRHGVDTHLFSVESPQELAAWTRQLVDGCHRAAEGVQEVSTACTWNGRPCS
LSVHIDKGFTLWAAEPGAARAVLLRQPFEKLQMSSDDGASLLFLDFGGAEGEIQLDLHSCP
KTIVFIIHSFLSAKVTRLGLLA

>Q9HDC9 single-pass type II membrane protein
MSEADGLRQRRPLRPQVVTDDDGQAPEAKDGSSFSGRVFRVTFLMLAVSLTVPLLGAMM
LLESPIDPQPLSFKEPPLLLGVLHPNTKLRQAERLFENQLVGPESIAHIGDVMFTGTADGRV
VKLENGEIETIARFGSGPCKTRDDEPVCGRPLGIRAGPNGTLFVADAYKGLFEVNPWKRE
VKLLLSSETPIEGKNMSFVNDLTVTQDGRKIYFTDSSSKWQRRDYLLLVMEGTDDGRLLE
YDTVTREVKVLLDQLRFPNGVQLSPAEDFVLVAETTMARIRRVYVSGLMKGGADLFVEN
MPGFPDNIRPSSSGGYWVGMSTIRPNPGFSMLDFLSERPWIKRMIFKLFSQETVMKFVPRY
SLVLELSDSGAFRRSLHDPDGLVATYISEVHEHDGHLYLGSFRSPFLCRLSLQAV

>P08842 multipass membrane protein
MPLRKMKIPFLLLFFLWEAESHAASRPNIILVMADDLGIGDPGCYGNKTIRTPNIDRLASGG
VKLTQHLAASPLCTPSRAAFMTGRYPVRSGMASWSRTGVFLFTASSGGLPTDEITFAKLLK
DQGYSTALIGKWHLGMSCHSKTDFCHHPLHHGFNYFYGISLTNLRDCKPGEGSVFTTGFK
RLVFLPLQIVGVTLLTLAALNCLGLLHVPLGVFFSLLFLAALILTLFLGFLHYFRPLNCFMM
RNYEIIQQPMSYDNLTQRLTVEAAQFIQRNTETPFLLVLSYLHVHTALFSSKDFAGKSQHG
VYGDAVEEMDWSVGQILNLLDELRLANDTLIYFTSDQGAHVEEVSSKGEIHGGSNGIYKG
GKANNWEGGIRVPGILRWPRVIQAGQKIDEPTSNMDIFPTVAKLAGAPLPEDRIIDGRDLM
PLLEGKSQRSDHEFLFHYCNAYLNAVRWHPQNSTSIWKAFFFTPNFNPVGSNGCFATHVCF
CFGSYVTHHDPPLLFDISKDPRERNPLTPASEPRFYEILKVMQEAADRHTQTLPEVPDQFS
WNNFLWKPWLQLCCPSTGLSCQCDREKQDKRLSR

>P51841 single-pass type I membrane protein
MFLGLGRFSRLVLWFAAFRKLLGHHGLASAKFLWCLCLLSVMSLPQQVWTLPYKIGVVG
PWACDSLFSKALPEVAARLAIERINRDPSFDLSYSFEYVILNEDCQTSRALSSFISHHQMAS
GFIGPTNPGYCEAASLLGNSWDKGIFSWACVNYELDNKISYPTFSRTLPSPIRVLVTVMKYF
QWAHAGVISSDEDIWVHTANRVASALRSHGLPVGVVLTTGQDSQSMRKALQRIHQADRIR
IIIMCMHSALIGGETQMHLLECAHDLKMTDGTYVFVPYDALLYSLPYKHTPYRVLRNNPK
LREAYDAVLTITVESQEKTFYQAFTEAAARGEIPEKLEFDQVSPLFGTIYNSIYFIAQAMNN
AMKENGQAGAASLVQHSRNMQFHGFNQLMRTDSNGNGISEYVILDTNLKEWELHSTYT
VDMEMELLRFGGTPIHFPGGRPPRADAKCWFAEGKICHGGIDPAFAMMVCLTLLIALLSIN
GFAYFIRRRINKIQLIKGPNRILLTLEDVTFINPHFGSKRGSRASVSFQITSEVQSGRSPRLSFS
SGSLTPATYENSNIAIYEGDWVWLKKFSLGDFGDLKSIKSRASDVFEMMKDLRHENINPLL
GFFYDSGMFAIVTEFCSRGSLEDILTNQDVKLDWMFKSSLLLDLIKGMKYLHHREFVHGR

LKSRNCVVDGRFVLKVTDYGFNDILEMLRLSEEESSMEELLWTAPELLRAPRGSRLGSFA
GDVYSFAIIMQEVMVRGTPFCMMDLPAQEIINRLKKPPPVYRPVVPPEHAPPECLQLMKQ
CWAEAAEQRPTFDEIFNQFKTFNKGKKTNIIDSMLRMLEQYSSNLEDLIRERTEELEIEKQK
TEKLLTQMLPPSVAESLKKGCTVEPEGFDLVTLYFSDIVGFTTISAMSEPIEVVDLLNDLYTL
FDAIIGSHDVYKVETIGDAYMVASGLPKRNGSRHAAEIANMSLDILSSVGTFKMRHMPEV
PVRIRIGLHSGPVVAGVVGLTMPRYCLFGDTVNTASRMESTGLPYRIHVSLSTVTILQNLSE
GYEVELRGRTELKGKGTEETFWLIGKKGFMKPLPVPPPVDKDGQVGHGLQPVEIAAFQR
RKAERQLVRNKP

>P33897 multipass membrane protein
MPVLSRPRPWRGNTLKRTAVLLALAAYGAHKVYPLVRQCLAPARGLQAPAGEPTQEASG
VAAAKAGMNRVFLQRLLWLLRLLFPRVLCRETGLLALHSAALVSRTFLSVYVARLDGRLA
RCIVRKDPRAFGWQLLQWLLIALPATFVNSAIRYLEGQLALSFRSRLVAHAYRLYFSQQTY
YRVSNMDGRLRNPDQSLTEDVVAFAASVAHLYSNLTKPLLDVAVTSYTLLRAARSRGAGT
AWPSAIAGLVVFLTANVLRAFSPKFGELVAEEARRKGELRYMHSRVVANSEEIAFYGGHEV
ELALLQRSYQDLASQINLILLERLWYVMLEQFLMKYVWSASGLLMVAVPIITATGYSESDA
EAVKKAALEKKEEELVSERTEAFTIARNLLTAAADAIERIMSSYKEVTELAGYTARVHEMF
QVFEDVQRCHFKRPRELEDAQAGSGTIGRSGVRVEGPLKIRGQVVDVEQGIICENIPIVTPS
GEVVVASLNIRVEEGMHLLITGPNGCGKSSLFRILGGLWPTYGGVLYKPPPQRMFYIPQRP
YMSVGSLRDQVIYPDSVEDMQRKGYSEQDLEAILDVVHLHHILQREGGWEAMCDWKDV
LSGGEKQRIGMARMFYHRPKYALLDECTSAVSIDVEGKIFQAAKDAGIALLSITHRPSLWK
YHTHLLQFDGEGGWKFEKLDSAARLSLTEEKQRLEQQLAGIPKMQRRLQELCQILGEAVA
PAHVPAPSPQGPGGLQGAST

>Q9NSA2 multipass membrane protein
MAAGLATWLPFARAAAVGWLPLAQQPLPPAPGVKASRGDEVLVVNVSGRRFETWKNTL
DRYPDTLLGSSEKEFFYDADSGEYFFDRDPDMFRHVLNFYRTGRLHCPRQECIQAFDEEL
AFYGLVPELVGDCCLEEYRDRKKENAERLAEDEEAEQAGDGPALPAGSSLRQRLWRAFE
NPHTSTAALVFYYVTGFFIAVSVIANVVETIPCRGSARRSSREQPCGERFPQAFFCMDTACV
LIFTGEYLLRLFAAPSRCRFLRSVMSLIDVVAILPYYIGLLVPKNDDVSGAFVTLRVFRVFRI
FKFSRHSQGLRILGYTLKSCASELGFLLFSLTMAIIIFATVMFYAEKGTNKTNFTSIPAAFWY
TIVTMTTLGYGDMVPSTIAGKIFGSICSLSGVLVIALPVPVIVSNFSRIYHQNQRADKRRAQ
QKVRLARIRLAKSGTTNAFLQYKQNGGLEDSGSGEEQALCVRNRSAFEQQHHHLLHCLE
KTTCHEFTDELTFSEALGAVSPGGRTSRSTSVSSQPVGPGSLLSSCCPRRAKRRAIRLANST
ASVSRGSMQELDMLAGLRRSHAPQSRSSLNAKPHDSLDLNCDSRDFVAAIISIPTPPANTP
DESQPSSPGGGGRAGSTLRNSSLGTPCLFPETVKISSL

>Q13585 multipass membrane protein
MGPTLAVPTPYGCIGCKLPQPEYPPALIIFMFCAMVITIVVDLIGNSMVILAVTKNKKLRNS
GNIFVVSLSVADMLVAIYPYPLMLHAMSIGGWDLSQLQCQMVGFITGLSVVGSIFNIVAIAI
NRYCYICHSLQYERIFSVRNTCIYLVITWIMTVLAVLPNMYIGTIEYDPRTYTCIFNYLNNPV
FTVTIVCIHFVLPLLIVGFCYVRIWTKVLAARDPAGQNPDNQLAEVRNFLTMFVIFLLFAVC
WCPINVLTVLVAVSPKEMAGKIPNWLYLAAYFIAYFNSCLNAVIYGLLNENFRREYWTIFH
AMRHPIIFFSGLISDIREMQEARTLARARAHARDQAREQDRAHACPAVEETPMNVRNVPL

PGDAAAGHPDRASGHPKPHSRSSSAYRKSASTHHKSVFSHSKAASGHLKPVSGHSKPASG
HPKSATVYPKPASVHFKADSVHFKGDSVHFKPDSVHFKPASSNPKPITGHHVSAGSHSKSA
FSAATSHPKPTTGHIKPATSHAEPTTADYPKPATTSHPKPTAADNPELSASHCPEIPAIAHPVS
DDSDLPESASSPAAGPTKPAASQLESDTIADLPDPTVVTTSTNDYHDVVVIDVEDDPDEMA
V

>O75695 lipid-anchor
MGCFFSKRRKADKESRPENEEERPKQYSWDQREKVDPKDYMFSGLKDETVGRLPGTVA
GQQFLIQDCENCNIYIFDHSATVTIDDCTNCIIFLGPVKGSVFFRNCRDCKCTLACQQFRVR
DCRKLEVFLCCATQPIIESSSNIKFGCFQWYYPELAFQFKDAGLSIFNNTWSNIHDFTPVSG
ELNWSLLPEDAVVQDYVPIPTTEELKAVRVSTEANRSIVPISRGQRQKSSDESCLVVLFAGD
YTIANARKLIDEMVGKGFFLVQTKEVSMKAEDAQRVFREKAPDFLPLLNKGPVIALEFNG
DGAVEVCQLIVNEIFNGTKMFVSESKETASGDVDSFYNFADIQMGI

>Q9NV92 multipass membrane protein
MARRRSQRVCASGPSMLNSARGAPELLRGTATNAEVSAAAAGATGSEELPPGDRGCRNG
GGRGPAATTSSTGVAVGAEHGEDSLSRKPDPEPGRMDHHQPGTGRYQVLLNEEDNSESSA
IEQPPTSNPAPQIVQAASSAPALETDSSPPPYSSITVEVPTTSDTEVYGEFYPVPPPYSVATSL
PTYDEAEKAKAAAMAAAAAETSQRIQEEECPPRDDFSDADQLRVGNDGIFMLAFFMAFIF
NWLGFCLSFCITNTIAGRYGAICGFGLSLIKWILIVRFSDYFTGYFNGQYWLWWIFLVLGL
LLFFRGFVNYLKVRNMSESMAAAHRTRYFFLL

>O43193 multipass membrane protein
MGSPWNGSDGPEGAREPPWPALPPCDERRCSPFPLGALVPVTAVCLCLFVVGVSGNVVTV
MLIGRYRDMRTTTNLYLGSMAVSDLLILLGLPFDLYRLWRSRPWVFGPLLCRLSLYVGEG
CTYATLLHMTALSVERYLAICRPLRARVLVTRRRVRALIAVLWAVALLSAGPFLFLVGVEQ
DPGISVVPGLNGTARIASSPLASSPPLWLSRAPPPSPPSGPETAEAAALFSRECRPSPAQLGA
LRVMLWVTTAYFFLPFLCLSILYGLIGRELWSSRRPLRGPAASGRERGHRQTVRVLLVVVL
AFIICWLPFHVGRIIYINTEDSRMMYFSQYFNIVALQLFYLSASINPILYNLISKKYRAAAFK
LLLARKSRPRGFHRSRDTAGEVAGDTGGDTVGYTETSANVKTMG

>Q9Y342 multipass membrane protein
MAEFPSKVSTRTSSPAQGAEASVSALRPDLGFVRSRLGALMLLQLVLGLLVWALIADTPY
HLYPAYGWVMFVAVFLWLVTIVLFNLYLFQLHMKLYMVPWPLVLMIFNISATVLYITAFIA
CSAAVDLTSLRGTRPYNQRAAASFFACLVMIAYGVSAFFSYQAWRGVGSNAATSQMAGG
YA

>P51511 single-pass type I membrane protein
MGSDPSAPGRPGWTGSLLGDREEAARPRLLPLLLVLLGCLGLGVAAEDAEVHAENWLRL
YGYLPQPSRHMSTMRSAQILASALAEMQRFYGIPVTGVLDEETKEWMKRPRCGVPDQFG
VRVKANLRRRRKRYALTGRKWNNHHLTFSIQNYTEKLGWYHSMEAVRRAFRVWEQATPL
VFQEVPYEDIRLRRQKEADIMVLFASGFHGDSSPFDGTGGFLAHAYFPGPGLGGDTHFDA
DEPWTFSSTDLHGNNLFLVAVHELGHALGLEHSSNPNAIMAPFYQWKDVDNFKLPEDDL
RGIQQLYGTPDGQPQPTQPLPTVTPRRPGRPDHRPPRPPQPPPPGGKPERPPKPGPPVQPRA

TERPDQYGPNICDGDFDTVAMLRGEMFVFKGRWFWRVRHNRVLDNYPMPIGHFWRGLP
GDISAAYERQDGRFVFFKGDRYWLFREANLEPGYPQPLTSYGLGIPYDRIDTAIWWEPTGH
TFFFQEDRYWRFNEETQRGDPGYPKPISVWQGIPASPKGAFLSNDAAYTYFYKGTKYWKF
DNERLRMEPGYPKSILRDFMGCQEHVEPGPRWPDVARPPFNPHGGAEPGADSAEGDVGD
GDGDFGAGVNKDGGSRVVVQMEEVARTVNVVMVLVPLLLLLCVLGLTYALVQMQRKG
APRVLLYCKRSLQEWV

>Q9NY59 lipid-anchor
MVLYTTPFPNSCLSALHCVSWALIFPCYWLVDRLAASFIPTTYEKRQRADDPCCLQLLCTA
LFTPIYLALLVASLPFAFLGFLFWSPLQSARRPYIYSRLEDKGLAGGAALLSEWKGTGPGKS
FCFATANVCLLPDSLARVNNLFNTQARAKEIGQRIRNGAARPQIKIYIDSPTNTSISAASFSS
LVSPQGGDGVARAVPGSIKRTASVEYKGDGGRHPGDEAANGPASGDPVDSSSPEDACIVRI
GGEEGGRPPEADDPVPGGQARNGAGGGPRGQTPNHNQQDGDSGSLGSPSASRESLVKGR
AGPDTSASGEPGANSKLLYKASVVKKAAARRRRHPDEAFDHEVSAFFPANLDFLCLQEVF
DKRAATKLKEQLHGYFEYILYDVGVYGCQGCCSFKCLNSGLLFASRYPIMDVAYHCYPNK
CNDDALASKGALFLKVQVGSTPQDQRIVGYIACTHLHAPQEDSAIRCGQLDLLQDWLAD
FRKSTSSSSAANPEELVAFDVVCGDFNFDNCSSDDKLEQQHSLFTHYRDPCRLGPGEEKP
WAIGTLLDTNGLYDEDVCTPDNLQKVLESEEGRREYLAFPTSKSSGQKGRKELLKGNGRR
IDYMLHAEEGLCPDWKAEVEEFSFITQLSGLTDHLPVAMRLMVSSGEEEA

>Q92536 multipass membrane protein
MEAREPGRPTPTYHLVPNTSQSQVEEDVSSPPQRSSETMQLKKEISLLNGVSLVVGNMIGS
GIFVSPKGVLVHTASYGMSLIVWAIGGLFSVVGALCYAELGTTITKSGASYAYILEAFGGFI
AFIRLWVSLLVVEPTGQAIIAITFANYIIQPSFPSCDPPYLACRLLAAACICLLTFVNCAYVK
WGTRVQDTFTYAKVVALIAIIVMGLVKLCQGHSEHFQDAFEGSSWDMGNLSLALYSALFS
YSGWDTLNFVTEEIKNPERNLPLAIGISMPIVTLIYILTNVAYYTVLNISDVLSSDAVAVTFA
DQTFGMFSWTIPIAVALSCFGGLNASIFASSRLFFVGSREGHLPDLLSMIHIERFTPIPALLFN
CTMALIYLIVEDVFQLINYFSFSYWFFVGLSVVGQLYLRWKEPKRPRPLKLSVFFPIVFCIC
SVFLVIVPLFTDTINSLIGIGIALSGVPFYFMGVYLPESRRPLFIRNVLAAITRGTQQLCFCVL
TELDVAEEKKDERKTD

>Q7L5A8 multipass membrane protein
MAPAPPPAASFSPSEVQRRLAAGACWVRRGARLYDLSSFVRHHPGGEQLLRARAGQDISA
DLDGPPHRHSANARRWLEQYYVGELRGEQQGSMENEPVALEETQKTDPAMEPRFKVVD
WDKDLVDWRKPLLWQVGHLGEKYDEWVHQPVTRPIRLFHSDLIEGLSKTVWYSVPIIWV
PLVLYLSWSYYRTFAQGNVRLFTSFTTEYTVAVPKSMFPGLFMLGTFLWSLIEYLIHRFLFH
MKPPSDSYYLIMLHFVMHGQHHKAPFDGSRLVFPPVPASLVIGVFYLCMQLILPEAVGGTV
FAGGLLGYVLYDMTHYYLHFGSPHKGSYLYSLKAHHVKHHFAHQKSGFGISTKLWDYCF
HTLTPEKPHLKTQ

>P49815 peripheral membrane protein
MAKPTSKDSGLKEKFKILLGLGTPRPNPRSAEGKQTEFIITAEILRELSMECGLNNRIRMIG
QICEVAKTKKFEEHAVEALWKAVADLLQPERPLEARHAVLALLKAIVQGQGERLGVLRAL
FFKVIKDYPSNEDLHERLEVFKALTDNGRHITYLEEELADFVLQWMDVGLSSEFLLVLVNL

VKFNSCYLDEYIARMVQMICLLCVRTASSVDIEVSLQVLDAVVCYNCLPAESLPLFIVTLC
RTINVKELCEPCWKLMRNLLGTHLGHSAIYNMCHLMEDRAYMEDAPLLRGAVFFVGMA
LWGAHRLYSLRNSPTSVLPSFYQAMACPNEVVSYEIVLSITRLIKKYRKELQVVAWDILLN
IIERLLQQLQTLDSPELRTIVHDLLTTVEELCDQNEFHGSQERYFELVERCADQRPESSLLNL
ISYRAQSIHPAKDGWIQNLQALMERFFRSESRGAVRIKVLDVLSFVLLINRQFYEEELINSV
VISQLSHIPEDKDHQVRKLATQLLVDLAEGCHTHHFNSLLDIIEKVMARSLSPPPELEERDV
AAYSASLEDVKTAVLGLLVILQTKLYTLPASHATRVYEMLVSHIQLHYKHSYTLPIASSIRL
QAFDFLLLLRADSLHRLGLPNKDGVVRFSPYCVCDYMEPERGSEKKTSGPLSPPTGPPGPA
PAGPAVRLGSVPYSLLFRVLLQCLKQESDWKVLKLVLGRLPESLRYKVLIFTSPCSVDQLC
SALCSMLSGPKTLERLRGAPEGFSRTDLHLAVVPVLTALISYHNYLDKTKQREMVYCLEQ
GLIHRCASQCVVALSICSVEMPDIIIKALPVLVVKLTHISATASMAVPLLEFLSTLARLPHLYR
NFAAEQYASVFAISLPYTNPSKFNQYIVCLAHHVIAMWFIRCRLPFRKDFVPFITKGLRSNV
LLSFDDTPEKDSFRARSTSLNERPKSLRIARPPKQGLNNSPPVKEFKESSAAEAFRCRSISVS
EHVVRSRIQTSLTSASLGSADENSVAQADDSLKNLHLELTETCLDMMARYVFSNFTAVPKR
SPVGEFLLAGGRTKTWLVGNKLVTVTTSVGTGTRSLLGLDSGELQSGPESSSSPGVHVRQ
TKEAPAKLESQAGQQVSRGARDRVRSMSGGHGLRVGALDVPASQFLGSATSPGPRTAPAA
KPEKASAGTRVPVQEKTNLAAYVPLLTQGWAEILVRRPTGNTSWLMSLENPLSPFSSDINN
MPLQELSNALMAAERFKEHRDTALYKSLSVPAASTAKPPPLPRSNTVASFSSLYQSSCQGQ
LHRSVSWADSAVVMEEGSPGEVPVLVEPPGLEDVEAALGMDRRTDAYSRSSSVSSQEEKS
LHAEELVGRGIPIERVVSSEGGRPSVDLSFQPSQPLSKSSSSPELQTLQDILGDPGDKADVG
RLSPEVKARSQSGTLDGESAAWSASGEDSRGQPEGPLPSSSPRSPSGLRPRGYTISDSAPSR
RGKRVERDALKSRATASNAEKVPGINPSFVFLQLYHSPFFGDESNKPILLPNESQSFERSVQ
LLDQIPSYDTHKIAVLYVGEGQSNSELAILSNEHGSYRYTEFLTGLGRLIELKDCQPDKVYL
GGLDVCGEDGQFTYCWHDDIMQAVFHIATLMPTKDVDKHRCDKKRHLGNDFVSIVYND
SGEDFKLGTIKGQFNFVHVIVTPLDYECNLVSLQCRKDMEGLVDTSVAKIVSDRNLPFVAR
QMALHANMASQVHHSRSNPTDIYPSKWIARLRHIKRLRQRICEEAAYSNPSLPLVHPPSHS
KAPAQTPAEPTPGYEVGQRKRLISSVEDFTEFV

>O75783 multipass membrane protein
MGRVEDGGTTEELEDWDPGTSALPAPGIKQGPREQTGTGPLSQKCWEPEPDAPSQPGPAL
WSRGRARTQALAGGSSLQQLDPENTGFIGADTFTGLVHSHELPLDPAKLDMLVALAQSNE
QGQVCYQELVDLISSKRSSSFKRAIANGQRALPRDGPLDEPGLGVYKRFVRYVAYEILPCE
VDRRWYFYRHRSCPPPVFMASVTLAQIIVFLCYGARLNKWVLQTYHPEYMKSPLVYHPG
HRARAWRFLTYMFMHVGLEQLGFNALLQLMIGVPLEMVHGLLRISLLYLAGVLAGSLTV
SITDMRAPVVGGSGGVYALCSAHLANVVMNWAGMRCPYKLLRMVLALVCMSSEVGRA
VWLRFSPPLPASGPQPSFMAHLAGAVVGVSMGLTILRSYEERLRDQCGWWVVLLAYGTF
LLFAVFWNVFAYDLLGAHIPPPP

>Q05996 single-pass type I membrane protein
MACRQRGGSWSPSGWFNAGWSTYRSISLFFALVTSGNSIDVSQLVNPAFPGTVTCDEREIT
VEFPSSPGTKKWHASVVDPLGLDMPNCTYILDPEKLTLRATYDNCTRRVHGGHQMTIRV
MNNSAALRHGAVMYQFFCPAMQVEETQGLSASTICQKDFMSFSLPRVFSGLADDSKGTK
VQMGWSIEVGDGARAKTLTLPEAMKEGFSLLIDNHRMTFHVPFNATGVTHYVQGNSHLY
MVSLKLTFISPGQKVIFSSQAICAPDPVTCNATHMTLTIPEFPGKLKSVSFENQNIDVSQLHD

NGIDLEATNGMKLHFSKTLLKTKLSEKCLLHQFYLASLKLTFLLRPETVSMVIYPECLCESP
VSIVTGELCTQDGFMDVEVYSYQTQPALDLGTLRVGNSSCQPVFEAQSQGLVRFHIPLNG
CGTRYKFEDDKVVYENEIHALWTDFPPSKISRDSEFRMTVKCSYSRNDMLLNINVESLTPP
VASVKLGPFTLILQSYPDNSYQQPYGENEYPLVRFLRQPIYMEVRVLNRDDPNIKLVLDDC
WATSTMDPDSFPQWNVVVDGCAYDLDNYQTTFHPVGSSVTHPDHYQRFDMKAFAFVSE
AHVLSSLVYFHCSALICNRLSPDSPLCSVTCPVSSRHRRATGATEAEKMTVSLPGPILLLSD
DSSFRGVGSSDLKASGSSGEKSRSETGEEVGSRGAMDTKGHKTAGDVGSKAVAAVAAFA
GVVATLGFIYYLYEKRTVSNH

>O94778 multipass membrane protein
MSGEIAMCEPEFGNDKAREPSVGGRWRVSWYERFVQPCLVELLGSALFIFIGCLSVIENGT
DTGLLQPALAHGLALGLVIATLGNISGGHFNPAVSLAAMLIGGLNLVMLLPYWVSQLLGG
MLGAALAKAVSPEERFWNASGAAFVTVQEQGQVAGALVAEIILTTLLALAVCMGAINEKT
KGPLAPFSIGFAVTVDILAGGPVSGGCMNPARAFGPAVVANHWNFHWIYWLGPLLAGLLV
GLLIRCFIGDGKTRLILKAR

>O43315 multipass membrane protein
MQPEGAEKGKSFKQRLVLKSSLAKETLSEFLGTFILIVLGCGCVAQAILSRGRFGGVITINV
GFSMAVAMAIYVAGGVSGGHINPAVSLAMCLFGRMKWFKLPFYVGAQFLGAFVGAATVF
GIYYDGLMSFAGGKLLIVGENATAHIFATYPAPYLSLANAFADQVVATMILLIIVFAIFDSRN
LGAPRGLEPIAIGLLIIVIASSLGLNSGCAMNPARDLSPRLFTALAGWGFEVFRAGNNFWWI
PVVGPLVGAVIGGLIYVLVIEIHHPEPDSVFKTEQSEDKPEKYELSVIM

>Q9H223 peripheral membrane protein
MFSWMGRQAGGRERAGGADAVQTVTGGLRSLYLRKVLPLEEAYRFHEFHSPALEDADFE
NKPMILLVGQYSTGKTTFIRYLLEQDFPGMRIGPEPTTDSFIAVMYGETEGSTPGNALVVDP
KKPFRKLSRFGNAFLNRFMCSQLPNQVLKSISVIDSPGILSGEKQRISRGYDFCQVLQWFA
ERVDRIILLFDAHKLDISDEFSEAIKAFRGQDDKIRVVLNKADQVDTQQLMRVYGALMWS
LGKVINTPEVLRVYIGSFWAQPLQNTDNRRLFEAEAQDLFRDIQSLPQKAAVRKLNDLIKR
ARLAKVHAYIISYLKKEMPSVFGKENKKRELISRLPEIYIQLQREYQISAGDFPEVKAMQE
QLENYDFTKFHSLKPKLIEAVDNMLSNKISPLMNLISQEETSTPTQLVQGGAFDGTTEGPF
NQGYGEGAKEGADEEEWVVAKDKPVYDELFYTLSPINGKISGVNAKKEMVTSKLPNSVL
GKIWKLADCDCDGMLDEEEFALAKHLIKIKLDGYELPSSLPPHLVPPSHRKSLPKAD

>Q96L33 lipid-anchor
MPPRELSEAEPPPLRAPTPPPRRRSAPPELGIKCVLVGDGAVGKSSLIVSYTCNGYPARYRP
TALDTFSVQVLVDGAPVRIELWDTAGQEDFDRLRSLCYPDTDVFLACFSVVQPSSFQNITE
KWLPEIRTHNPQAPVLLVGTQADLRDDVNVLIQLDQGGREGPVPQPQAQGLAEKIRACC
YLECSALTQKNLKEVFDSAILSAIEHKARLEKKLNAKGVRTLSRCRWKKFFCFV

>Q9P253 peripheral membrane protein
MASILDEYENSLSRSAVLQPGCPSVGIPHSGYVNAQLEKEVPIFTKQRIDFTPSERITSLVVS
SNQLCMSLGKDTLLRIDLGKANEPNHVELGRKDDAKVHKMFLDHTGSHLLIALSSTEVL
YVNRNGQKVRPLARWKGQLVESVGWNKALGTESSTGPILVGTAQGHIFEAELSASEGGLF

GPAPDLYFRPLYVLNEEGGPAPVCSLEAERGPDGRSFVIATTRQRLFQFIGRAAEGAEAQGF
SGLFAAYTDHPPPFREFPSNLGYSELAFYTPKLRSAPRAFAWMMGDGVLYGALDCGRPDS
LLSEERVWEYPEGVGPGASPPLAIVLTQFHFLLLLADRVEAVCTLTGQVVLRDHFLEKFGP
LKHMVKDSSTGQLWAYTERAVFRYHVQREARDVWRTYLDMNRFDLAKEYCRERPDCLD
TVLAREADFCFRQRRYLESARCYALTQSYFEEIALKFLEARQEEALAEFLQRKLASLKPAE
RTQATLLTTWLTELYLSRLGALQGDPEALTLYRETKECFRTFLSSPRHKEWLFASRASIHEL
LASHGDTEHMVYFAVIMQDYERVVAYHCQHEAYEEALAVLARHRDPQLFYKFSPILIRHIP
RQLVDAWIEMGSRLDARQLIPALVNYSQGGEVQQVSQAIRYMEFCVNVLGETEQAIHNYL
LSLYARGRPDSLLAYLEQAGASPHRVHYDLKYALRLCAEHGHHRACVHVYKVLELYEEA
VDLALQVDVDLAKQCADLPEEDEELRKKLWLKIARHVVQEEEDVQTAMACLASCPLLKI
EDVLPFFPDFVTIDHFKEAICSSLKAYNHHIQELQREMEEATASAQRIRRDLQELRGRYGTV
EPQDKCATCDFPLLNRPFYLFLCGHMFHADCLLQAVRPGLPAYKQARLEELQRKLGAAPP
PAKGSARAKEAEGGAATAGPSREQLKADLDELVAAECVYCGELMIRSIDRPFIDPQRYEEE
QLSWL

>Q9UL45 peripheral membrane protein
MSVPGPSSPDGALTRPPYCLEAGEPTPGLSDTSPDEGLIEDLTIEDKAVEQLAEGLLSHYLP
DLQRSKQALQELTQNQVVLLDTLEQEISKFKECHSMLDINALFAEAKHYHAKLVNIRKEM
LMLHEKTSKLKKRALKLQQKRQKEELEREQQREKEFEREKQLTARPAKRM

>O43909 single-pass type II membrane protein
MTGYTMLRNGGAGNGGQTCMLRWSNRIRLTWLSFTLFVILVFFPLIAHYYLTTLDEADEA
GKRIFGPRVGNELCEVKHVLDLCRIRESVSEELLQLEAKRQELNSEIAKLNLKIEACKKSIE
NAKQDLLQLKNVISQTEHSYKELMAQNQPKLSLPIRLLPEKDDAGLPPPKATRGCRLHNC
FDYSRCPLTSGFPVYVYDSDQFVFGSYLDPLVKQAFQATARANVYVTENADIACLYVILVG
EMQEPVVLRPAELEKQLYSLPHWRTDGHNHVIINLSRKSDTQNLLYNVSTGRAMVAQSTF
YTVQYRPGFDLVVSPLVHAMSEPNFMEIPPQVPVKRKYLFTFQGEKIESLRSSLQEARSFEE
EMEGDPPADYDDRIIATLKAVQDSKLDQVLVEFTCKNQPKPSLPTEWALCGEREDRLELLK
LSTFALIITPGDPRLVISSGCATRLFEALEVGAVPVVLGEQVQLPYQDMLQWNEAALVVPK
PRVTEVHFLLRSLSDSDLLAMRRQGRFLWETYFSTADSIFNTVLAMIRTRIQIPAAPIREEAA
AEIPHRSGKAAGTDPNMADNGDLDLGPVETEPPYASPRYLRNFTLTVTDFYRSWNCAPGP
FHLFPHTPFDPVLPSEAKFLGSGTGFRPIGGGAGGSGKEFQAALGGNVPREQFTVVMLTYE
REEVLMNSLERLNGLPYLNKVVVVWNSPKLPSEDLLWPDIGVPIMVVRTEKNSLNNRFLP
WNEIETEAILSIDDDAHLRHDEIMFGFRVWREARDRIVGFPGRYHAWDIPHQSWLYNSNY
SCELSMVLTGAAFFHKYYAYLYSYVMPQAIRDMVDEYINCEDIAMNFLVSHITRKPPIKVT
SRWTFRCPGCPQALSHDDSHFHERHKCINFFVKVYGYMPLLYTQFRVDSVLFKTRLPHDK
TKCFKFI

>P37268 multipass membrane protein
MEFVKCLGHPEEFYNLVRFRIGGKRKVMPKMDQDSLSSSLKTCYKYLNQTSRSFAAVIQA
LDGEMRNAVCIFYLVLRALDTLEDDMTISVEKKVPLLHNFHSFLYQPDWRFMESKEKDRQ
VLEDFPTISLEFRNLAEKYQTVIADICRRMGIGMAEFLDKHVTSEQEWDKYCHYVAGLVG
IGLSRLFSASEFEDPLVGEDTERANSMGLFLQKTNIIRDYLEDQQGGREFWPQEVWSRYV
KKLGDFAKPENIDLAVQCLNELITNALHHIPDVITYLSRLRNQSVFNFCAIPQVMAIATLAA

CYNNQQVFKGAVKIRKGQAVTLMMDATNMPAVKAIIYQYMEEIYHRIPDSDPSSSKTRQII
STIRTQNLPNCQLISRSHYSPIYLSFVMLLAALSWQYLTTLSQVTEDYVQTGEH

>Q8TB36 multipass membrane protein
MAERQEEQRGSPPLRAEGKADAEVKLILYHWTHSFSSQKVRLVIAEKALKCEEHDVSLPL
SEHNEPWFMRLNSTGEVPVLIHGENIICEATQIIDYLEQTFLDERTPRLMPDKESMYYPRVQ
HYRELLDSLPMDAYTHGCILHPELTVDSMIPAYATTRIRSQIGNTESELKKLAEENPDLQEA
YIAKQKRLKSKLLDHDNVKYLKKILDELEKVLDQVETELQRRNEETPEEGQQPWLCGESF
TLADVSLAVTLHRLKFLGFARRNWGNGKRPNLETYYERVLKRKTFNKVLGHVNNILISAV
LPTAFRVAKKRAPKVLGTTLVVGLLAGVGYFAFMLFRKRLGSMILAFRPRPNYF

>Q93045 peripheral membrane protein
MAKTAMAYKEKMKELSMLSLICSCFYPEPRNINIYTYDDMEVKQINKRASGQAFELILKP
PSPISEAPRTLASPKKKDLSLEEIQKKLEAAEERRKSQEAQVLKQLAEKREHEREVLQKAL
EENNNFSKMAEEKLILKMEQIKENREANLAAIIERLQEKERHAAEVRRNKELQVELSG

>O00220 single-pass type I membrane protein
MAPPPARVHLGAFLAVTPNPGSAASGTEAAAATPSKVWGSSAGRIEPRGGGRGALPTSMG
QHGPSARARAGRAPGPRPAREASPRLRVHKTFKFVVVGVLLQVVPSSAATIKLHDQSIGT
QQWEHSPLGELCPPGSHRSEHPGACNRCTEGVGYTNASNNLFACLPCTACKSDEEERSPC
TTTRNTACQCKPGTFRNDNSAEMCRKCSRGCPRGMVKVKDCTPWSDIECVHKESGNGH
NIWVILVVTLVVPLLLVAVLIVCCCIGSGCGGDPKCMDRVCFWRLGLLRGPGAEDNAHNEI
LSNADSLSTFVSEQQMESQEPADLTGVTVQSPGEAQCLLGPAEAEGSQRRRLLVPANGAD
PTETLMLFFDKFANIVPFDSWDQLMRQLDLTKNEIDVVRAGTAGPGDALYAMLMKWVN
KTGRNASIHTLLDALERMEERHAREKIQDLLVDSGKFIYLEDGTGSAVSLE

>Q8IY17 single-pass type I membrane protein
MGTSSHGLATNSSGAKVAERDGFQDVLAPGEGSAGRICGAQPVPFVPQVLGVMIGAGVA
VVVTAVLILLVVRRLRVPKTPAPDGPRYRFRKRDKVLFYGRKIMRKVSQSTSSLVDTSVSA
TSRPRMRKKLKMLNIAKKILRIQKETPTLQRKEPPPAVLEADLTEGDLANSHLPSEVLYML
KNVRVLGHFEKPLFLELCRHMVFQRLGQGDYVFRPGQPDASIYVVQDGLLELCLPGPDG
KECVVKEVVPGDSVNSLLSILDVITGHQHPQRTVSARAARDSTVLRLPVEAFSAVFTKYPE
SLVRVVQIIMVRLQRVTFLALHNYLGLTNELFSHEIQPLRLFPSPGLPTRTSPVRGSKRMVS
TSATDEPRETPGRPPDPTGAPLPGPTGDPVKPTSLETPSAPLLSRCVSMPGDISGLQGGPRS
DFDMAYERGRISVSLQEEASGGSLAAPARTPTQEPREQPAGACEYSYCEDESATGGCPFGP
YQGRQTSSIFEAAKQELAKLMRIEDPSLLNSRVLLHHAKAGTIIARQGDQDVSLHFVLWG
CLHVYQRMIDKAEDVCLFVAQPGELVGQLAVLTGEPLIFTLRAQRDCTFLRISKSDFYEIM
RAQPSVVLSAAHTVAARMSPFVRQMDFAIDWTAVEAGRALYRQGDRSDCTYIVLNGRLR
SVIQRGSGKKELVGEYGRGDLIGVVEALTRQPRATTVHAVRDTELAKLPEGTLGHIKRRYP
QVVTRLIHLLSQKILGNLQQLQGPFPAGSGLGVPPHSELTNPASNLATVAILPVCAEVPMVA
FTLELQHALQAIGPTLLLNSDIIRARLGASALDSIQEFRLSGWLAQQEDAHRIVLYQTDASL
TPWTVRCLRQADCILIVGLGDQEPTLGQLEQMLENTAVRALKQLVLLHREEGAGPTRTVE
WLNMRSWCSGHLHLRCPRRLFSRRSPAKLHELYEKVFSRRADRHSDFSRLARVLTGNTIA
LVLGGGGARGCSHIGVLKALEEAGVPVDLVGGTSIGSFIGALYAEERSASRTKQRAREWA

KSMTSVLEPVLDLTYPVTSMFTGSAFNRSIHRVFQDKQIEDLWLPYFNVTTDITASAMRVH
KDGSLWRYVRASMTLSGYLPPLCDPKDGHLLMDGGYINNLPADIARSMGAKTVIAIDVG
SQDETDLSTYGDSLSGWWLLWKRLNPWADKVKVPDMAEIQSRLAYVSCVRQLEVVKSS
SYCEYLRPPIDCFKTMDFGKFDQIYDVGYQYGKAVFGGWSRGNVIEKMLTDRRSTDLNES
RRADVLAFPSSGFTDLAEIVSRIEPPTSYVSDGCADGEESDCLTEYEEDAGPDCSRDEGGSP
EGASPSTASEMEEEKSILRQRRCLPQEPPGSATDA

>Q96HA9 multipass membrane protein
MASLSGLASALESYRGRDRLIRVLGYCCQLVGGVLVEQCPARSEVGTRLLVVSTQLSHCR
TILRLFDDLAMFVYTKQYGLGAQEEDAFVRCVSVLGNLADQLYYPCEHVAWAADARVL
HVDSSRWWTLSTTLWALSLLLGVARSLWMLLKLRQRLRSPTAPFTSPLPRGKRRAMEAQ
MQSEALSLLSNLADLANAVHWLPRGVLWAGRFPPWLVGLMGTISSILSMYQAARAGGQA
EATTP

>P78329 peripheral membrane protein
MSQLSLSWLGLWPVAASPWLLLLLVGASWLLAHVLAWTYAFYDNCRRLRCFPQPPRRN
WFWGHQGMVNPTEEGMRVLTQLVATYPQGFKVWMGPISPLLSLCHPDIIRSVINASAAIAP
KDKFFYSFLEPWLGDGLLLSAGDKWSRHRRMLTPAFHFNILKPYMKIFNESVNIMHAKW
QLLASEGSACLDMFEHISLMTLDSLQKCVFSFDSHCQEKPSEYIAAILELSALVSKRHHEIL
LHIDFLYYLTPDGQRFRRACRLVHDFTDAVIQERRRTLPSQGVDDFLQAKAKSKTLDFIDV
LLLSKDEDGKKLSDEDIRAEADTFMFEGHDTTASGLSWVLYHLAKHPEYQERCRQEVQE
LLKDREPKEIEWDDLAHLPFLTMCMKESLRLHPPVPVISRHVTQDIVLPDGRVIPKGIICLIS
VFGTHHNPAVWPDPEVYDPFRFDPENIKERSPLAFIPFSAGPRNCIGQTFAMAEMKVVLAL
TLLRFRVLPDHTEPRRKPELVLRAEGGLWLRVEPLS

>P48664 multipass membrane protein
MSSHGNSLFLRESGQRLGRVGWLQRLQESLQQRALRTRLRLQTMTLEHVLRFLRRNAFIL
LTVSAVVIGVSLAFALRPYQLTYRQIKYFSFPGELLMRMLQMLVLPLIVSSLVTGMASLDN
KATGRMGMRAAVYYMVTTIIAVFIGILMVTIIHPGKGSKEGLHREGRIETIPTADAFMDLIR
NMFPPNLVEACFKQFKTQYSTRVVTRTMVRTENGSEPGASMPPPFSVENGTSFLENVTRA
LGTLQEMLSFEETVPVPGSANGINALGLVVFSVAFGLVIGGMKHKGRVLRDFFDSLNEAIM
RLVGIIIWYAPVGILFLIAGKILEMEDMAVLGGQLGMYTLTVIVGLFLHAGIVLPLIYFLVTH
RNPFPFIGGMLQALITAMGTSSSSATLPITFRCLEEGLGVDRRITRFVLPVGATVNMDGTAL
YEALAAIFIAQVNNYELNLGQITTISITATAASVGAAGIPQAGLVTMVIVLTSVGLPTEDITLI
IAVDWFLDRLRTMTNVLGDSIGAAVIEHLSQRELELQEAELTLPSLGKPYKSLMAQEKGAS
RGRGGNESAM

>P51693 single-pass type I membrane protein
MGPASPAARGLSRRPGQPPLPLLLPLLLLLLRAQPAIGSLAGGSPGAAEAPGSAQVAGLCG
RLTLHRDLRTGRWEPDPQRSRRCLRDPQRVLEYCRQMYPELQIARVEQATQAIPMERWCG
GSRSGSCAHPHHQVVPFRCLPGEFVSEALLVPEGCRFLHQERMDQCESSTRRHQEAQEAC
SSQGLILHGSGMLLPCGSDRFRGVEYVCCPPPGTPDPSGTAVGDPSTRSWPPGSRVEGAED
EEEEESFPQPVDDYFVEPPQAEEEEETVPPPSSHTLAVVGKVTPTPRPTDGVDIYFGMPGEI
SEHEGFLRAKMDLEERRMRQINEVMREWAMADNQSKNLPKADRQALNEHFQSILQTLEE

QVSGERQRLVETHATRVIALINDQRRAALEGFLAALQADPPQAERVLLALRRYLRAEQKE
QRHTLRHYQHVAAVDPEKAQQMRFQVHTHLQVIEERVNQSLGLLDQNPHLAQELRPQIQ
ELLHSEHLGPSELEAPAPGGSSEDKGGLQPPDSKDDTPMTLPKGSTEQDAASPEKEKMNP
LEQYERKVNASVPRGFPFHSSEIQRDELAPAGTGVSREAVSGLLIMGAGGGSLIVLSMLLL
RRKKPYGAISHGVVEVDPMLTLEEQQLRELQRHGYENPTYRFLEERP

>O75871 single-pass type I membrane protein
MGPPSAAPRGGHRPWQGLLITASLLTFWHPPTTVQFTIEALPSSAAEGKDVLLLACNISETI
QAYYWHKGKTAEGSPLIAGYITDIQANIPGAAYSGRETVYPNGSLLFQNITLEDAGSYTLR
TINASYDSDQATGQLHVHQNNVPGLPVGAVAGIVTGVLVGVALVAALVCFLLLSRTGRASI
QRDLREQPPPASTPGHGPSHRSTFSAPLPSPRTATPIYEELLYSDANIYCQIDHKADVVS

>O60664 peripheral membrane protein
MSADGAEADGSTQVTVEEPVQQPSVVDRVASMPLISSTCDMVSAAYASTKESYPHIKTVC
DAAEKGVRTLTAAAVSGAQPILSKLEPQIASASEYAHRGLDKLEENLPILQQPTEKVLADT
KELVSSKVSGAQEMVSSAKDTVATQLSEAVDATRGAVQSGVDKTKSVVTGGVQSVMGSR
LGQMVLSGVDTVLGKSEEWADNHLPLTDAELARIATSLDGFDVASVQQQRQEQSYFVRL
GSLSERLRQHAYEHSLGKLRATKQRAQEALLQLSQVLSLMETVKQGVDQKLVEGQEKLH
QMWLSWNQKQLQGPEKEPPKPEQVESRALTMFRDIAQQLQATCTSLGSSIQGLPTNVKD
QVQQARRQVEDLQATFSSIHSFQDLSSSILAQSRERVASAREALDHMVEYVAQNTPVTWL
VGPFAPGITEKAPEEKK

>P11912 single-pass type I membrane protein
MPGGPGVLQALPATIFLLFLLSAVYLGPGCQALWMHKVPASLMVSLGEDAHFQCPHNSSN
NANVTWWRVLHGNYTWPPEFLGPGEDPNGTLIIQNVNKSHGGIYVCRVQEGNESYQQSC
GTYLRVRQPPPRPFLDMGEGTKNRIITAEGIILLFCAVVPGTLLLFRKRWQNEKLGLDAGD
EYEDENLYEGLNLDDCSMYEDISRGLQGTYQDVGSLNIGDVQLEKP

>P55344 multipass membrane protein
MYSFMGGGLFCAWVGTILLVVAMATDHWMQYRLSGSFAHQGLWRYCLGNKCYLQTDSI
AYWNATRAFMILSALCAISGIIMGIMAFAHQPTFSRISRPFSAGIMFFSSTLFVVLALAIYTG
VTVSFLGRRFGDWRFSWSYILGWVAVLMTFFAGIFYMCAYRVHECRRLSTPR

>Q16617 multipass membrane protein
MELCRSLALLGGSLGLMFCLIALSTDFWFEAVGPTHSAHSGLWPTGHGDIISGYIHVTQTF
SIMAVLWALVSVSFLVLSCFPSLFPPGHGPLVSTTAAFAAAISMVVAMAVYTSERWDQPPHP
QIQTFFSWSFYLGWVSAILLLCTGALSLGAHCGGPRPGYETL

>Q9UMF0 single-pass type I membrane protein
MPGPSPGLRRALLGLWAALGLGLFGLSAVSQEPFWADLQPRVAFVERGGSLWLNCSTNCP
RPERGGLETSLRRNGTQRGLRWLARQLVDIREPETQPVCFFRCARRTLQARGLIRTFQRPD
RVELMPLPPWQPVGENFTLSCRVPGAGPRASLTLTLLRGAQELIRRSFAGEPPRARGAVLTA
TVLARREDHGANFSCRAELDLRPHGLGLFENSSAPRELRTFSLSPDAPRLAAPRLLEVGSE
RPVSCTLDGLFPASEARVYLALGDQNLSPDVTLEGDAFVATATATASAEQEGARQLVCNVT

LGGENRETRENVTIYSFPAPLLTLSEPSVSEGQMVTVTCAAGAQALVTLEGVPAAVPGQPA
QLQLNATENDDRRSFFCDATLDVDGETLIKNRSAELRVLYAPRLDDSDCPRSWTWPEGPE
QTLRCEARGNPEPSVHCARSDGGAVLALGLLGPVTRALSGTYRCKAANDQGEAVKDVTL
TVEYAPALDSVGCPERITWLEGTEASLSCVAHGVPPPDVICVRSGELGAVIEGLLRVAREHA
GTYRCEATNPRGSAAKNVAVTVEYGPRFEEPSCPSNWTWVEGSGRLFSCEVDGKPQPSVK
CVGSGGATEGVLLPLAPPDPSPRAPRIPRVLAPGIYVCNATNRHGSVAKTVVVSAESPPEM
DESTCPSHQTWLEGAEASALACAARGRPSPGVRCSREGIPWPEQQRVSREDAGTYHCVAT
NAHGTDSRTVTVGVEYRPVVAELAASPPGGVRPGGNFTLTCRAEAWPPAQISWRAPPGAL
NIGLSSNNSTLSVAGAMGSHGGEYECAATNAHGRHARRITVRVAGPWLWVAVGGAAGGA
ALLAAGAGLAFYVQSTACKKGEYNVQEAESSGEAVCLNGAGGGAGGAAGAEGGPEAAG
GAAESPAEGEVFAIQLTSA

>P06731 GPI-anchor, lipid-anchor
MESPSAPPHRWCIPWQRLLLTASLLTFWNPPTTAKLTIESTPFNVAEGKEVLLLVHNLPQHL
FGYSWYKGERVDGNRQIIGYVIGTQQATPGPAYSGREIIYPNASLLIQNIIQNDTGFYTLHVI
KSDLVNEEATGQFRVYPELPKPSISSNNSKPVEDKDAVAFTCEPETQDATYLWWVNNQSLP
VSPRLQLSNGNRTLTLFNVTRNDTASYKCETQNPVSARRSDSVILNVLYGPDAPTISPLNTS
YRSGENLNLSCHAASNPPAQYSWFVNGTFQQSTQELFIPNITVNNSGSYTCQAHNSDTGL
NRTTVTTITVYAEPPKPFITSNNSNPVEDEDAVALTCEPEIQNTTYLWWVNNQSLPVSPRLQ
LSNDNRTLTLLSVTRNDVGPYECGIQNKLSVDHSDPVILNVLYGPDDPTISPSYTYYRPGV
NLSLSCHAASNPPAQYSWLIDGNIQQHTQELFISNITEKNSGLYTCQANNSASGHSRTTVK
TITVSAELPKPSISSNNSKPVEDKDAVAFTCEPEAQNTTYLWWVNGQSLPVSPRLQLSNGN
RTLTLFNVTRNDARAYVCGIQNSVSANRSDPVTLDVLYGPDTPIISPPDSSYLSGANLNLSC
HSASNPSPQYSWRINGIPQQHTQVLFIAKITPNNNGTYACFVSNLATGRNNSIVKSITVSAS
GTSPGLSAGATVGIMIGVLVGVALI

>P19235 single-pass type I membrane protein
MDHLGASLWPQVGSLCLLLAGAAWAPPPNLPDPKFESKAALLAARGPEELLCFTERLEDL
VCFWEEAASAGVGPGNYSFSYQLEDEPWKLCRLHQAPTARGAVRFWCSLPTADTSSFVPL
ELRVTAASGAPRYHRVIHINEVVLLDAPVGLVARLADESGHVVLRWLPPPETPMTSHIRYE
VDVSAGNGAGSVQRVEILEGRTECVLSNLRGRTRYTFAVRARMAEPSFGGFWSAWSEPVS
LLTPSDLDPLILTLSLILVVILVLLTVLALLSHRRALKQKIWPGIPSPESEFEGLFTTHKGNFQ
LWLYQNDGCLWWSPCTPFTEDPPASLEVLSERCWGTMQAVEPGTDDEGPLLEPVGSEHA
QDTYLVLDKWLLPRNPPSEDLPGPGGSVDIVAMDEGSEASSCSSALASKPSPEGASAASFE
YTILDPSSQLLRPWTLCPELPPTPPHLKYLYLVVSDSGISTDYSSGDSQGAQGGLSDGPYSN
PYENSLIPAAEPLPPSYVACS

>Q92911 multipass membrane protein
MEAVETGERPTFGAWDYGVFALMLLVSTGIGLWVGLARGGQRSAEDFFTGGRRLAALPV
GLSLSASFMSAVQVLGVPSEAYRYGLKFLWMCLGQLLNSVLTALLFMPVFYRLGLTSTYE
YLEMRFSRAVRLCGTLQYIVATMLYTGIVIYAPALILNQVTGLDIWASLLSTGIICTFYTAVG
GMKAVVWTDVFQVVVMLSGFWVVLARGVMLVGGPRQVLTLAQNHSRINLMDFNPDPR
SRYTFWTFVVGGTLVWLSMYGVNQAQVQRYVACRTEKQAKLALLINQVGLFLIVSSAAC
CGIVMFVFYTDCDPLLLGRISAPDQYMPLLVLDIFEDLPGVPGLFLACAYSGTLSTASTSIN

AMAAVTVEDLIKPRLRSLAPRKLVIISKGLSLIYGSACLTVAALSSLLGGGVLQGSFTVMG
VISGPLLGAFILGMFLPACNTPGVLAGLGAGLALSLWVALGATLYPPSEQTMRVLPSSAAR
CVALSVNASGLLDPALLPANDSSRAPSSGMDASRPALADSFYAISYLYYGALGTLTTVLCG
ALISCLTGPTKRSTLAPGLLWWDLARQTASVAPKEEVAILDDNLVKGPEELPTGNKKPPGF
LPTNEDRLFFLGQKELEGAGSWTPCVGHDGGRDQQETNL

>Q92952 multipass membrane protein
MNSHSYNGSVGRPLGSGPGALGRDPPDPEAGHPPQPPHSPGLQVVVAKSEPARPSPGSPR
GQPQDQDDDEDDEEDEAGRQRASGKPSNVGHRLGHRRALFEKRKRLSDYALIFGMFGIV
VMVTETELSWGVYTKESLYSFALKCLISLSTAILLGLVVLYHAREIQLFMVDNGADDWRIA
MTCERVFLISLELAVCAIHPVPGHYRFTWTARLAFTYAPSVAEADVDVLLSIPMFLRLYLLG
RVMLLHSKIFTDASSRSIGALNKITFNTRFVMKTLMTICPGTVLLVFSISSWIIAAWTVRVCE
RYHDKQEVTSNFLGAMWLISITFLSIGYGDMVPHTYCGKGVCLLTGIMGAGCTALVVAVV
ARKLELTKAEKHVHNFMMDTQLTKRVKNAAANVLRETWLIYKHTRLVKKPDQARVRKH
QRKFLQAIHQAQKLRSVKIEQGKLNDQANTLTDLAKTQTVMYDLVSELHAQHEELEARL
ATLESRLDALGASLQALPGLIAQAIRPPPPPLPPRPGPGPQDQAARSSPCRWTPVAPSDCG

>Q9NZ42 multipass membrane protein
MNLERVSNEEKLNLCRKYYLGGFAFLPFLWLVNIFWFFREAFLVPAYTEQSQIKGYVWRS
AVGFLFWVIVLTSWITIFQIYRPRWGALGDYLSFTIPLGTP

>O00322 multipass membrane protein
MASAAAAEAEKGSPVVVGLLVVGNIIILLSGLSLFAETIWVTADQYRVYPLMGVSGKDDV
FAGAWIAIFCGFSFFMVASFGVGAALCRRRSMVLTYLVLMLIVYIFECASCITSYTHRDYM
VSNPSLITKQMLTFYSADTDQGQELTRLWDRVMIEQECCGTSGPMDWVNFTSAFRAATPE
VVFPWPPLCCRRTGNFIPLNEEGCRLGHMDYLFTKGCFEHIGHAIDSYTWGISWFGFAILM
WTLPVMLIAMYFYTML

>Q9BVK8 multipass membrane protein
MTLFHFGNCFALAYFPYFITYKCSGLSEYNAFWKCVQAGVTYLFVQLCKMLFLATFFPTW
EGGIYDFIGEFMKASVDVADLIGLNLVMSRNAGKGEYKIMVAALGWATAELIMSRCIPLW
VGARGIEFDWKYIQMSIDSNISLVHYIVASAQVWMITRYDLYHTFRPAVLLLMFLSVYKAF
VMETFVHLCSLGSWAALLARAVVTGLLALSTLALYVAVVNVHS

>Q9HB55 peripheral membrane protein
MDLIPNFAMETWVLVATSLVLLYIYGTHSHKLFKKLGIPGPTPLPFLGTILFYLRGLWNFDR
ECNEKYGEMWGLYEGQQPMLVIMDPDMIKTVLVKECYSVFTNQMPLGPMGFLKSALSFA
EDEEWKRIRTLLSPAFTSVKFKEMVPIISQCGDMLVRSLRQEAENSKSINLKDFFGAYTMD
VITGTLFGVNLDSLNNPQDPFLKNMKKLLKLDFLDPFLLLISLFPFLTPVFEALNIGLFPKD
VTHFLKNSIERMKESRLKDKQKHRVDFFQQMIDSQNSKETKSHKALSDLELVAQSIIIFAA
YDTTSTTLPFIMYELATHPDVQQKLQEEIDAVLPNKAPVTYDALVQMEYLDMVVNETLRL
FPVVSRVTRVCKKDIEINGVFIPKGLAVMVPIYALHHDPKYWTEPEKFCPERFSKKNKDSID
LYRYIPFGAGPRNCIGMRFALTNIKLAVIRALQNFSFKPCKETQIPLKLDNLPILQPEKPIVLK
VHLRDGITSGP

>Q15165 peripheral membrane protein
MGRLVAVGLLGIALALLGERLLALRNRLKASREVESVDLPHCHLIKGIEAGSEDIDILPNGL
AFFSVGLKFPGLHSFAPDKPGGILMMDLKEEKPRARELRISRGFDLASFNPHGISTFIDNDD
TVYLFVVNHPEFKNTVEIFKFEEAENSLLHLKTVKHELLPSVNDITAVGPAHFYATNDHYF
SDPFLKYLETYLNLHWANVVYYSPNEVKVVAEGFDSANGINISPDDKYIYVADILAHEIHV
LEKHTNMNLTQLKVLELDTLVDNLSIDPSSGDIWVGCHPNGQKLFVYDPNNPPSSEVLRIQ
NILCEKPTVTTVYANNGSVLQGSSVASVYDGKLLIGTLYHRALYCEL

>P26012 single-pass type I membrane protein
MCGSALAFFTAAFVCLQNDRRGPASFLWAAWVFSLVLGLGQGEDNRCASSNAASCARCL
ALGPECGWCVQEDFISGGSRSERCDIVSNLISKGCSVDSIEYPSVHVIIPTENEINTQVTPGE
VSIQLRPGAEANFMLKVHPLKKYPVDLYYLVDVSASMHNNIEKLNSVGNDLSRKMAFFS
RDFRLGFGSYVDKTVSPYISIHPERIHNQCSDYNLDCMPPHGYIHVLSLTENITEFEKAVHR
QKISGNIDTPEGGFDAMLQAAVCESHIGWRKEAKRLLLVMTDQTSHLALDSKLAGIVVPN
DGNCHLKNNVYVKSTTMEHPSLGQLSEKLIDNNINVIFAVQGKQFHWYKDLLPLLPGTIA
GEIESKAANLNNLVVEAYQKLISEVKVQVENQVQGIYFNITAICPDGSRKPGMEGCRNVTS
NDEVLFNVTVTMKKCDVTGGKNYAIIKPIGFNETAKIHIHRNCSCQCEDNRGPKGKCVDE
TFLDSKCFQCDENKCHFDEDQFSSESCKSHKDQPVCSGRGVCVCGKCSCHKIKLGKVYG
KYCEKDDFSCPYHHGNLCAGHGECEAGRCQCFSGWEGDRCQCPSAAAQHCVNSKGQV
CSGRGTCVCGRCECTDPRSIGRFCEHCPTCYTACKENWNCMQCLHPHNLSQAILDQCKTS
CALMEQQHYVDQTSECFSSPSYLRIFFIIFIVTFLIGLLKVLIIRQVILQWNSNKIKSSSDYRV
SASKKDKLILQSVCTRAVTYRREKPEEIKMDISKLNAHETFRCNF

>P51636 peripheral membrane protein
MGLETEKADVQLFMDDDSYSHHSGLEYADPEKFADSDQDRDPHRLNSHLKLGFEDVIAE
PVTTHSFDKVWICSHALFEISKYVMYKFLTVFLAIPLAFIAGILFATLSCLHIWILMPFVKTC
LMVLPSVQTIWKSVTDVIIAPLCTSVGRCFSSVSLQLSQD

>Q9Y5X2 peripheral membrane protein
MTGRAMDPLPAAAVGAAAEAEADEEADPPASDLPTPQAIEPQAIVQQVPAPSRMQMPQG
NPLLLSHTLQELLARDTVQVELIPEKKGLFLKHVEYEVSSQRFKSSVYRRYNDFVVFQEM
LLHKFPYRMVPALPPKRMLGADREFIEARRRALKRFVNLVARHPLFSEDVVLKLFLSFSGS
DVQNKLKESAQCVGDEFLNCKLATRAKDFLPADIQAQFAISRELIRNIYNSFHKLRDRAER
IASRAIDNAADLLIFGKELSAIGSDTTPLPSWAALNSSTWGSLKQALKGLSVEFALLADKA
AQQGKQEENDVVEKLNLFLDLLQSYKDLCERHEKGVLHKHQRALHKYSLMKRQMMSA
TAQNREPESVEQLESRIVEQENAIQTMELRNYFSLYCLHQETQLIHVYLPLTSHILRAFVNS
QIQGHKEMSKVWNDLRPKLSCLFAGPHSTLTPPCSPPEDGLCPH

>Q9UP52 single-pass type II membrane protein
MERLWGLFQRAQQLSPRSSQTVYQRVEGPRKGHLEEEEEDGEEGAETLAHFCPMELRGP
EPLGSRPRQPNLIPWAAAGRRAAPYLVLTALLIFTGAFLLGYVAFRGSCQACGDSVLVVSE
DVNYEPDLDFHQGRLYWSDLQAMFLQFLGEGRLEDTIRQTSLRERVAGSAGMAALTQDIR
AALSRQKLDHVWTDTHYVGLQFPDPAHPNTLHWVDEAGKVGEQLPLEDPDVYCPYSAI

GNVTGELVYAHYGRPEDLQDLRARGVDPVGRLLLVRVGVISFAQKVTNAQDFGAQGVLI
YPEPADFSQDPPKPSLSSQQAVYGHVHLGTGDPYTPGFPSFNQTQFPPVASSGLPSIPAQPIS
ADIASRLLRKLKGPVAPQEWQGSLLGSPYHLGPGPRLRLVVNNHRTSTPINNIFGCIEGRSE
PDHYVVIGAQRDAWGPGAAKSAVGTAILLELVRTFSSMVSNGFRPRRSLLFISWDGGDFG
SVGSTEWLEGYLSVLHLKAVVYVSLDNAVLGDDKFHAKTSPLLTSLIESVLKQVDSPNHS
GQTLYEQVVFTNPSWDAEVIRPLPMDSSAYSFTAFVGVPAVEFSFMEDDQAYPFLHTKEDT
YENLHKVLQGRLPAVAQAVAQLAGQLLIRLSHDRLLPLDFGRYGDVVLRHIGNLNEFSGD
LKARGLTLQWVYSARGDYIRAAEKLRQEIYSSEERDERLTRMYNVRIMRVEFYFLSQYVS
PADSPFRHIFMGRGDHTLGALLDHLRLLRSNSSGTPGATSSTGFQESRFRRQLALLTWTLQ
GAANALSGDVWNIDNNF

>O60568 peripheral membrane protein
MTSSGPGPRFLLLLPLLLPPAASASDRPRGRDPVNPEKLLVITVATAETEGYLRFLRSAEFFN
YTVRTLGLGEEWRGGDVARTVGGGQKVRWLKKEMEKYADREDMIIMFVDSYDVILAGS
PTELLKKFVQSGSRLLFSAESFCWPEWGLAEQYPEVGTGKRFLNSGGFIGFATTIHQIVRQ
WKYKDDDDDQLFYTRLYLDPGLREKLSLNLDHKSRIFQNLNGALDEVVLKFDRNRVRIR
NVAYDTLPIVVHGNGPTKLQLNYLGNYVPNGWTPEGGCGFCNQDRRTLPGGQPPPRVFLA
VFVEQPTPFLPRFLQRLLLLDYPPDRVTLFLHNNEVFHEPHIADSWPQLQDHFSAVKLVGP
EEALSPGEARDMAMDLCRQDPECEFYFSLDADAVLTNLQTLRILIEENRKVIAPMLSRHG
KLWSNFWGALSPDEYYARSEDYVELVQRKRVGVWNVPYISQAYVIRGDTLRMELPQRDV
FSGSDTDPDMAFCKSFRDKGIFLHLSNQHEFGRLLATSRYDTEHLHPDLWQIFDNPVDWK
EQYIHENYSRALEGEGIVEQPCPDVYWFPLLSEQMCDELVAEMEHYGQWSGGRHEDSRL
AGGYENVPTVDIHMKQVGYEDQWLQLLRTYVGPMTESLFPGYHTKARAVMNFVVRYRP
DEQPSLRPHHDSSTFTLNVALNHKGLDYEGGGCRFLRYDCVISSPRKGWALLHPGRLTHY
HEGLPTTWGTRYIMVSFVDP

>O15498 lipid-anchor
MKLYSLSVLYKGEAKVVLLKAAYDVSSFSFFQRSSVQEFMTFTSQLIVERSSKGTRASVKE
QDYLCHVYVRNDSLAGVVIADNEYPSRVAFTLLEKVLDEFSKQVDRIDWPVGSPATIHYP
ALDGHLSRYQNPREADPMTKVQAELDETKIILHNTMESLLERGEKLDDLVSKSEVLGTQS
KAFYKTARKQNSCCAIM

>P32971 single-pass type II membrane protein
MDPGLQQALNGMAPPGDTAMHVPAGSVASHLGTTSRSYFYLTTATLALCLVFTVATIMVL
VVQRTDSIPNSPDNVPLKGGNCSEDLLCILKRAPFKKSWAYLQVAKHLNKTKLSWNKDGI
LHGVRYQDGNLVIQFPGLYFIICQLQFLVQCPNNSVDLKLELLINKHIKKQALVTVCESGM
QTKHVYQNLSQFLLDYLQVNTTISVNVDTFQYIDTSTFPLENVLSIFLYSNSD

>Q9HBL7 multipass membrane protein
MGFIFSKSMNESMKNQKEFMLMNARLQLERQLIMQSEMRERQMAMQIAWSREFLKYFG
TFFGLAAISLTAGAIKKKKPAFLVPIVPLSFILTYQYDLGYGTLLERMKGEAEDILETEKSKL
QLPRGMITFESIEKARKEQSRFFIDK

>O43174 peripheral membrane protein

MGLPALLASALCTFVLPLLLFLAAIKLWDLYCVSGRDRSCALPLPPGTMGFPFFGETLQMV
LQRRKFLQMKRRKYGFIYKTHLFGRPTVRVMGADNVRRILLGEHRLVSVHWPASVRTILG
SGCLSNLHDSSHKQRKKVIMRAFSREALECYVPVITEEVGSSLEQWLSCGERGLLVYPEV
KRLMFRIAMRILLGCEPQLAGDGDSEQQLVEAFEEMTRNLFSLPIDVPFSGLYRGMKARN
LIHARIEQNIRAKICGLRASEAGQGCKDALQLLIEHSWERGERLDMQALKQSSTELLFGGH
ETTASAATSLITYLGLYPHVLQKVREELKSKGLLCKSNQDNKLDMEILEQLKYIGCVIKET
LRLNPPVPGGFRVALKTFELNGYQIPKGWNVIYSICDTHDVAEIFTNKEEFNPDRFMLPHPE
DASRFSFIPFGGGLRSCVGKEFAKILLKIFTVELARHCDWQLLNGPPTMKTSPTVYPVDNL
PARFTHFHGEI

>P36894 single-pass type I membrane protein
MPQLYIYIRLLGAYLFIISRVQGQNLDSMLHGTGMKSDSDQKKSENGVTLAPEDTLPFLKC
YCSGHCPDDAINNTCITNGHCFAIIEEDDQGETTLASGCMKYEGSDFQCKDSPKAQLRRTI
ECCRTNLCNQYLQPTLPPVVIGPFFDGSIRWLVLLISMAVCIIAMIIFSSCFCYKHYCKSISSR
RRYNRDLEQDEAFIPVGESLKDLIDQSQSSGSGSGLPLLVQRTIAKQIQMVRQVGKGRYGE
VWMGKWRGEKVAVKVFFTTEEASWFRETEIYQTVLMRHENILGFIAADIKGTGSWTQLY
LITDYHENGSLYDFLKCATLDTRALLKLAYSAACGLCHLHTEIYGTQGKPAIAHRDLKSKN
ILIKKNGSCCIADLGLAVKFNSDTNEVDVPLNTRVGTKRYMAPEVLDESLNKNHFQPYIM
ADIYSFGLIIWEMARRCITGGIVEEYQLPYYNMVPSDPSYEDMREVVCVKRLRPIVSNRW
NSDECLRAVLKLMSECWAHNPASRLTALRIKKTLAKMVESQDVKI

>Q9BWM7 multipass membrane protein
MESKMGELPLDINIQEPRWDQSTFLGRARHFFTVTDPRNLLLSGAQLEASRNIVQNYRAG
VVTPGITEDQLWRAKYVYDSAFHPDTGEKVVLIGRMSAQVPMNMTITGCMLTFYRKTPT
VVFWQWVNQSFNAIVNYSNRSGDTPITVRQLGTAYVSATTGAVATALGLKSLTKHLPPLV
GRFVPFAAVAAANCINIPLMRQRELQVGIPVADEAGQRLGYSVTAAKQGIFQVVISRICMAI
PAMAIPPLIMDTLEKKDFLKRRPWLGAPLQVGLVGFCLVFATPLCCALFPQKSSIHISNLEP
ELRAQIHEQNPSVEVVYYNKGL

>Q93086 multipass membrane protein
MGQAGCKGLCLSLFDYKTEKYVIAKNKKVGLLYRLLQASILAYLVVWVFLIKKGYQDVD
TSLQSAVITKVKGVAFTNTSDLGQRIWDVADYVIPAQGENVFFVVTNLIVTPNQRQNVCAE
NEGIPDGACSKDSDCHAGEAVTAGNGVKTGRCLRRENLARGTCEIFAWCPLETSSRPEEPF
LKEAEDFTIFIKNHIRFPKFNFSKSNVMDVKDRSFLKSCHFGPKNHYCPIFRLGSVIRWAGS
DFQDIALEGGVIGINIEWNCDLDKAASECHPHYSFSRLDNKLSKSVSSGYNFRFARYYRDA
AGVEFRTLMKAYGIRFDVMVNGKGAFFCDLVLIYLIKKREFYRDKKYEEVRGLEDSSQEA
EDEASGLGLSEQLTSGPGLLGMPEQQELQEPPEAKRGSSSQKGNGSVCPQLLEPHRST

>O14907 peripheral membrane protein
MSYIPGQPVTAVVQRVEIHKLRQGENLILGFSIGGGIDQDPSQNPFSEDKTDKGIYVTRVSE
GGPAEIAGLQIGDKIMQVNGWDMTMVTHDQARKRLTKRSEEVVRLLVTRQSLQKAVQQS
MLS

>P51575 multipass membrane protein

MARRFQEELAAFLFEYDTPRMVLVRNKKVGVIFRLIQLVVLVYVIGWVFLYEKGYQTSSG
LISSVSVKLKGLAVTQLPGLGPQVWDVADYVFPAQGDNSFVVMTNFIVTPKQTQGYCAE
HPEGGICKEDSGCTPGKAKRKAQGIRTGKCVAFNDTVKTCEIFGWCPVEVDDDIPRPALLR
EAENFTLFIKNSISFPRFKVNRRNLVEEVNAAHMKTCLFHKTLHPLCPVFQLGYVVQESGQ
NFSTLAEKGGVVGITIDWHCDLDWHVRHCRPIYEFHGLYEEKNLSPGFNFRFARHFVENG
TNYRHLFKVFGIRFDILVDGKAGKFDIIPTMTTIGSGIGIFGVATVLCDLLLLHILPKRHYYK
QKKFKYAEDMGPGAAERDLAATSSTLGLQENMRTS

>Q02978 multipass membrane protein
MAATASAGAGGIDGKPRTSPKSVKFLFGGLAGMGATVFVQPLDLVKNRMQLSGEGAKTR
EYKTSFHALTSILKAEGLRGIYTGLSAGLLRQATYTTTRLGIYTVLFERLTGADGTPPGFLL
KAVIGMTAGATGAFVGTPAEVALIRMTADGRLPADQRRGYKNVFNALIRITREEGVLTLW
RGCIPTMARAVVVNAAQLASYSQSKQFLLDSGYFSDNILCHFCASMISGLVTTAASMPVDI
AKTRIQNMRMIDGKPEYKNGLDVLFKVVRYEGFFSLWKGFTPYYARLGPHTVLTFIFLEQ
MNKAYKRLFLSG

>Q9Y272 lipid-anchor
MKLAAMIKKMCPSDSELSIPAKNCYRMVILGSSKVGKTAIVSRFLTGRFEDAYTPTIEDFH
RKFYSIRGEVYQLDILDTSGNHPFPAMRRLSILTGDVFILVFSLDNRDSFEEVQRLRQQILDT
KSCLKNKTKENVDVPLVICGNKGDRDFYREVDQREIEQLVGDDPQRCAYFEISAKKNSSL
DQMFRALFAMAKLPSEMSPDLHRKVSVQYCDVLHKKALRNKKLLRAGSGGGGGDPGD
AFGIVAPFARRPSVHSDLMYIREKASAGSQAKDKERCVIS

>O43760 multipass membrane protein
MESGAYGAAKAGGSFDLRRFLTQPQVVARAVCLVFALIVFSCIYGEGYSNAHESKQMYCV
FNRNEDACRYGSAIGVLAFLASAFFLVVDAYFPQISNATDRKYLVIGDLLFSALWTFLWFV
GFCFLTNQWAVTNPKDVLVGADSVRAAITFSFFSIFSWGVLASLAYQRYKAGVDDFIQNY
VDPTPDPNTAYASYPGASVDNYQQPPFTQNAETTEGYQPPPVY

>O00623 multipass membrane protein
MAEHGAHFTAASVADDQPSIFEVVAQDSLMTAVRPALQHVVKVLAESNPTHYGFLWRWF
DEIFTLLDLLLQQHYLSRTSASFSENFYGLKRIVMGDTHKSQRLASAGLPKQQLWKSIMFL
VLLPYLKVKLEKLVSSLREEDEYSIHPPSSRWKRFYRAFLAAYPFVNMAWEGWFLVQQLR
YILGKAQHHSPLLRLAGVQLGRLTVQDIQALEHKPAKASMMQQPARSVSEKINSALKKAV
GGVALSLSTGLSVGVFFLQFLDWWYSSENQETIKSLTALPTPPPPVHLDYNSDSPLLPKMK
TVCPLCRKTRVNDTVLATSGYVFCYRCVFHYVRSHQACPITGYPTEVQHLIKLYSPEN

>Q06432 multipass membrane protein
MSQTKMLKVRVTLFCILAGIVLAMTAVVTDHWAVLSPHMEHHNTTCEAAHFGLWRICTK
RIPMDDSKTCGPITLPGEKNCSYFRHFNPGESSEIFEFTTQKEYSISAAAIAIFSLGFIILGSLC
VLLSLGKKRDYLLRPASMFYAFAGLCILVSVEVMRQSVKRMIDSEDTVWIEYYYSWSFAC
ACAAFILLFLGGLALLLFSLPRMPRNPWESCMDAEPEH

>Q15768 single-pass type I membrane protein

MGPPHSGPGGVRVGALLLLGVLGLVSGLSLEPVYWNSANKRFQAEGGYVLYPQIGDRLD
LLCPRARPPGPHSSPNYEFYKLYLVGGAQGRRCEAPPAPNLLLTCDRPDLDLRFTIKFQEYS
PNLWGHEFRSHHDYYIIATSDGTREGLESLQGGVCLTRGMKVLLRVGQSPRGGAVPRKPV
SEMPMERDRGAAHSLEPGKENLPGDPTSNATSRGAEGPLPPPSMPAVAGAAGGLALLLLG
VAGAGGAMCWRRRRAKPSESRHPGPGSFGRGGSLGLGGGGGMGPREAEPGELGIALRG
GGAADPPFCPHYEKVSGDYGHPVYIVQDGPPQSPPNIYYKV

>P28907 single-pass type II membrane protein
MANCEFSPVSGDKPCCRLSRRAQLCLGVSILVLILVVVLAVVVPRWRQQWSGPGTTKRFP
ETVLARCVKYTEIHPEMRHVDCQSVWDAFKGAFISKHPCNITEEDYQPLMKLGTQTVPC
NKILLWSRIKDLAHQFTQVQRDMFTLEDTLLGYLADDLTWCGEFNTSKINYQSCPDWRK
DCSNNPVSVFWKTVSRRFAEAACDVVHVMLNGSRSKIFDKNSTFGSVEVHNLQPEKVQT
LEAWVIHGGREDSRDLCQDPTIKELESIISKRNIQFSCKNIYRPDKFLQCVKNPEDSSCTSEI

>P30968 multipass membrane protein
MANSASPEQNQNHCSAINNSIPLMQGNLPTLTLSGKIRVTVTFFLFLLSATFNASFLLKLQK
WTQKKEKGKKLSRMKLLLKHLTLANLLETLIVMPLDGMWNITVQWYAGELLCKVLSYL
KLFSMYAPAFMMVVISLDRSLAITRPLALKSNSKVGQSMVGLAWILSSVFAGPQLYIFRMI
HLADSSGQTKVFSQCVTHCSFSQWWHQAFYNFFTFSCLFIIPLFIMLICNAKIIFTLTRVLHQ
DPHELQLNQSKNNIPRARLKTLKMTVAFATSFTVCWTPYYVLGIWYWFDPEMLNRLSDP
VNHFFFLFAFLNPCFDPLIYGYFSL

>Q9UHA4 peripheral membrane protein
MADDLKRFLYKKLPSVEGLHAIVVSDRDGVPVIKVANDNAPEHALRPGFLSTFALATDQG
SKLGLSKNKSIICYYNTYQVVQFNRLPLVVSFIASSSANTGLIVSLEKELAPLFEELRQVVE
VS

>O76024 multipass membrane protein
MDSNTAPLGPSCPQPPPAPQPQARSRLNATASLEQERSERPRAPGPQAGPGPGVRDAAAPA
EPQAQHTRSRERADGTGPTKGDMEIPFEEVLERAKAGDPKAQTEVGKHYLQLAGDTDEE
LNSCTAVDWLVLAAKQGRREAVKLLRRCLADRRGITSENEREVRQLSSETDLERAVRKAA
LVMYWKLNPKKKKQVAVAELLENVGQVNEHDGGAQPGPVPKSLQKQRRMLERLVSSES
KNYIALDDFVEITKKYAKGVIPSSLFLQDDEDDDELAGKSPEDLPLRLKVVKYPLHAIMEI
KEYLIDMASRAGMHWLSTIIPTHHINALIFFFIVSNLTIDFFAFFIPLVIFYLSFISMVICTLKV
FQDSKAWENFRTLTDLLLRFEPNLDVEQAEVNFGWNHLEPYAHFLLSVFFVIFSFPIASKD
CIPCSELAVITGFFTVTSYLSLSTHAEPYTRRALATEVTAGLLSLLPSMPLNWPYLKVLGQT
FITVPVGHLVVLNVSVPCLLYVYLLYLFFRMAQLRNFKGTYCYLVPYLVCFMWCELSVVI
LLESTGLGLLRASIGYFLFLFALPILVAGLALVGVLQFARWFTSLELTKIAVTVAVCSVPLLL
RWWTKASFSVVGMVKSLTRSSMVKLILVWLTAIVLFCWFYVYRSEGMKVYNSTLTWQQ
YGALCGPRAWKETNMARTQILCSHLEGHRVTWTGRFKYVRVTDIDNSAESAINMLPFFIG
DWMRCLYGEAYPACSPGNTSTAEEELCRLKLLAKHPCHIKKFDRYKFEITVGMPFSSGAD
GSRSREEDDVTKDIVLRASSEFKSVLLSLRQGSLIEFSTILEGRLGSKWPVFELKAISCLNC
MAQLSPTRRHVKIEHDWRSTVHGAVKFAFDFFFFPFLSAA

>P27701 multipass membrane protein
MGSACIKVTKYFLFLFNLIFFILGAVILGFGVWILADKSSFISVLQTSSSSLRMGAYVFIGVG
AVTMLMGFLGCIGAVNEVRCLLGLYFAFLLLILIAQVTAGALFYFNMGKLKQEMGGIVTE
LIRDYNSSREDSLQDAWDYVQAQVKCCGWVSFYNWTDNAELMNRPEVTYPCSCEVKGE
EDNSLSVRKGFCEAPGNRTQSGNHPEDWPVYQEGCMEKVQAWLQENLGIILGVGVGVAII
ELLGMVLSICLCRHVHSEDYSKVPKY

>Q11206 single-pass type II membrane protein
MVSKSRWKLLAMLALVLVVMVWYSISREDRYIELFYFPIPEKKEPCLQGEAESKASKLFG
NYSRDQPIFLRLEDYFWVKTPSAYELPYGTKGSEDLLLRVLAITSSSIPKNIQSLRCRRCVV
VGNGHRLRNSSLGDAINKYDVVIRLNNAPVAGYEGDVGSKTTMRLFYPESAHFDPKVEN
NPDTLLVLVAFKAMDFHWIETILSDKKRVRKGFWKQPPLIWDVNPKQIRILNPFFMEIAAD
KLLSLPMQQPRKIKQKPTTGLLAITLALHLCDLVHIAGFGYPDAYNKKQTIHYYEQITLKS
MAGSGHNVSQEALAIKRMLEMGAIKNLTSF

>Q13651 single-pass type I membrane protein
MLPCLVVLLAALLSLRLGSDAHGTELPSPPSVWFEAEFFHHILHWTPIPNQSESTCYEVALL
RYGIESWNSISNCSQTLSYDLTAVTLDLYHSNGYRARVRAVDGSRHSNWTVTNTRFSVDE
VTLTVGSVNLEIHNGFILGKIQLPRPKMAPANDTYESIFSHFREYEIAIRKVPGNFTFTHKKV
KHENFSLLTSGEVGEFCVQVKPSVASRSNKGMWSKEECISLTRQYFTVTNVIIFFAFVLLLS
GALAYCLALQLYVRRRKKLPSVLLFKKPSPFIFISQRPSPETQDTIHPLDEEAFLKVSPELKN
LDLHGSTDSGFGSTKPSLQTEEPQFLLPDPHPQADRTLGNREPPVLGDSCSSGSSNSTDSGI
CLQEPSLSPSTGPTWEQQVGSNSRGQDDSGIDLVQNSEGRAGDTQGGSALGHHSPPEPEV
PGEEDPAAVAFQGYLRQTRCAEEKATKTGCLEEESPLTDGLGPKFGRCLVDEAGLHPPALA
KGYLKQDPLEMTLASSGAPTGQWNQPTEEWSLLALSSCSDLGISDWSFAHDLAPLGCVA
APGGLLGSFNSDLVTLPLISSLQSSE

>Q8IY34 multipass membrane protein
MPAPRAREQPRVPGERQPLLPRGARGPRRWRRAAGAAVLLVEMLERAAFFGVTANLVLY
LNSTNFNWTGEQATRAALVFLGASYLLAPVGGWLADVYLGRYRAVALSLLLYLAASGLL
PATAFPDGRSSFCGEMPASPLGPACPSAGCPRSSPSPYCAPVLYAGLLLLGLAASSVRSNLTS
FGADQVMDLGRDATRRFFNWFYWSINLGAVLSLLVVAFIQQNISFLLGYSIPVGCVGLAFF
IFLFATPVFITKPPMGSQVSSMLKLALQNCCPQLWQRHSARDRQCARVLADERSPQPGASP
QEDIANFQVLVKILPVMVTLVPYWMVYFQMQSTYVLQGLHLHIPNIFPANPANISVALRAQ
GSSYTIPEAWLLLANVVVVLILVPLKDRLIDPLLLRCKLLPSALQKMALGMFFGFTSVIVA
GVLEMERLHYIHHNETVSQQIGEVLYNAAPLSIWWQIPQYLLIGISEIFASIPGLEFAYSEAP
RSMQGAIMGIFFCLSGVGSLLGSSLVALLSLPGGWLHCPKDFGNINNCRMDLYFFLLAGIQ
AVTALLFVWIAGRYERASQGPASHSRFSRDRG

>Q96PD7 multipass membrane protein
MKTLIAAYSGVLRGERQAEADRSQRSHGGPALSREGSGRWGTGSSILSALQDLFSVTWLN
RSKVEKQLQVISVLQWVLSFLVLGVACSAILMYIFCTDCWLIAVLYFTWLVFDWNTPKKG
GRRSQWVRNWAVWRYFRDYFPIQLVKTHNLLTTRNYIFGYHPHGIMGLGAFCNFSTEATE
VSKKFPGIRPYLATLAGNFRMPVLREYLMSGGICPVSRDTIDYLLSKNGSGNAIIIVVGGA

AESLSSMPGKNAVTLRNRKGFVKLALRHGADLVPIYSFGENEVYKQVIFEEGSWGRWVQ
KKFQKYIGFAPCIFHGRGLFSSDTWGLVPYSKPITTVVGEPITIPKLEHPTQQDIDLYHTMY
MEALVKLFDKHKTKFGLPETEVLEVN

>P21583 single-pass type I membrane protein
MKKTQTWILTCIYLQLLLFNPLVKTEGICRNRVTNNVKDVTKLVANLPKDYMITLKYVPG
MDVLPSHCWISEMVVQLSDSLTDLLDKFSNISEGLSNYSIIDKLVNIVDDLVECVKENSSK
DLKKSFKSPEPRLFTPEEFFRIFNRSIDAFKDFVVASETSDCVVSSTLSPEKDSRVSVTKPFM
LPPVAASSLRNDSSSSNRKAKNPPGDSSLHWAAMALPALFSLIIGFAFGALYWKKRQPSLT
RAVENIQINEEDNEISMLQEKEREFQEV

>Q00325 multipass membrane protein
MFSSVAHLARANPFNTPHLQLVHDGLGDLRSSSPGPTGQPRRPRNLAAAAVEEQYSCDYG
SGRFFILCGLGGIISCGTTHTALVPLDLVKCRMQVDPQKYKGIFNGFSVTLKEDGVRGLAK
GWAPTFLGYSMQGLCKFGFYEVFKVLYSNMLGEENTYLWRTSLYLAASASAEFFADIALA
PMEAAKVRIQTQPGYANTLRDAAPKMYKEEGLKAFYKGVAPLWMRQIPYTMMKFACFE
RTVEALYKFVVPKPRSECSKPEQLVVTFVAGYIAGVFCAIVSHPADSVVSVLNKEKGSSAS
LVLKRLGFKGVWKGLFARIIMIGTLTALQWFIYDSVKVYFRLPRPPPPEMPESLKKKLGLT
Q

>Q07108 single-pass type II membrane protein
MSSENCFVAENSSLHPESGQENDATSPHFSTRHEGSFQVPVLCAVMNVVFITILIIALIALSV
GQYNCPGQYTFSMPSDSHVSSCSEDWVGYQRKCYFISTVKRSWTSAQNACSEHGATLAVI
DSEKDMNFLKRYAGREEHWVGLKKEPGHPWKWSNGKEFNNWFNVTGSDKCVFLKNTE
VSSMECEKNLYWICNKPYK

>Q9NZD1 multipass membrane protein
MYKDCIESTGDYFLLCDAEGPWGIILESLAILGIVVTILLLLAFLFLMRKIQDCSQWNVLPT
QLLFLLSVLGLFGLAFAFIIELNQQTAPVRYFLFGVLFALCFSCLLAHASNLVKLVRGCVSF
SWTTILCIAIGCSLLQIIIATEYVTLIMTRGMMFVNMTPCQLNVDFVVLLVYVLFLMALTFF
VSKATFCGPCENWKQHGRLIFITVLFSIIIWVVWISMLLRGNPQFQRQPQWDDPVVCIALV
TNAWVFLLLYIVPELCILYRSCRQECPLQGNACPVTAYQHSFQVENQELSRARDSDGAEED
VALTSYGTPIQPQTVDPTQECFIPQAKLSPQQDAGGV

>P37088 multipass membrane protein
MEGNKLEEQDSSPPQSTPGLMKGNKREEQGLGPEPAAPQQPTAEEEALIEFHRSYRELFEF
FCNNTTIHGAIRLVCSQHNRMKTAFWAVLWLCTFGMMYWQFGLLFGEYFSYPVSLNINL
NSDKLVFPAVTICTLNPYRYPEIKEELEELDRITEQTLFDLYKYSSFTTLVAGSRSRRDLRGT
LPHPLQRLRVPPPPHGARRARSVASSLRDNNPQVDWKDWKIGFQLCNQNKSDCFYQTYS
SGVDAVREWYRFHYINILSRLPETLPSLEEDTLGNFIFACRFNQVSCNQANYSHFHHPMYG
NCYTFNDKNNSNLWMSSMPGINNGLSLMLRAEQNDFIPLLSTVTGARVMVHGQDEPAFM
DDGGFNLRPGVETSISMRKETLDRLGGDYGDCTKNGSDVPVENLYPSKYTQQVCIHSCFQ
ESMIKECGCAYIFYPRPQNVEYCDYRKHSSWGYCYYKLQVDFSSDHLGCFTKCRKPCSVT
SYQLSAGYSRWPSVTSQEWVFQMLSRQNNYTVNNKRNGVAKVNIFFKELNYKTNSESPS

VTMVTLLSNLGSQWSLWFGSSVLSVVEMAELVFDLLVIMFLMLLRRFRSRYWSPGRGGR
GAQEVASTLASSPPSHFCPHPMSLSLSQPGPAPSPALTAPPPAYATLGPRPSPGGSAGASSST
CPLGGP

>P36941 single-pass type I membrane protein
MLLPWATSAPGLAWGPLVLGLFGLLAASQPQAVPPYASENQTCRDQEKEYYEPQHRICCS
RCPPGTYVSAKCSRIRDTVCATCAENSYNEHWNYLTICQLCRPCDPVMGLEEIAPCTSKRK
TQCRCQPGMFCAAWALECTHCELLSDCPPGTEAELKDEVGKGNNHCVPCKAGHFQNTSS
PSARCQPHTRCENQGLVEAAPGTAQSDTTCKNPLEPLPPEMSGTMLMLAVLLPLAFFLLLA
TVFSCIWKSHPSLCRKLGSLLKRRPQGEGPNPVAGSWEPPKAHPYFPDLVQPLLPISGDVSP
VSTGLPAAPVLEAGVPQQQSPLDLTREPQLEPGEQSQVAHGTNGIHVTGGSMTITGNIYIY
NGPVLGGPPGPGDLPATPEPPYPIPEEGDPGPPGLSTPHQEDGKAWHLAETEHCGATPSNR
GPRNQFITHD

>Q93070 GPI-anchor, lipid-anchor
MGPLINRCKKILLPTTVPPATMRIWLLGGLLPFLLLLSGLQRPTEGSEVAIKIDFDFAPGSFD
DQYQGCSKQVMEKLTQGDYFTKDIEAQKNYFRMWQKAHLAWLNQGKVLPQNMTTTHA
VAILFYTLNSNVHSDFTRAMASVARTPQQYERSFHFKYLHYYLTSAIQLLRKDSIMENGTL
CYEVHYRTKDVHFNAYTGATIRFGQFLSTSLLKEEAQEFGNQTLFTIFTCLGAPVQYFSLK
KEVLIPPYELFKVINMSYHPRGNWLQLRSTGNLSTYNCQLLKASSKKCIPDPIAIASLSFLT
SVIIFSKSRV

>Q9Y2I9 peripheral membrane protein
MDVLPTGGGRPGLRTELEFRGGGGEARLESQEEETIPAAPPAPRLRGAAERPRRSRDTWD
GDEDTEPGEACGGRTSRTASLVSGLLNELYSCTEEEEAAGGGRGAEGRRRRRDSLDSSTE
ASGSDVVLGGRSGAGDSRVLQELQERPSQRHQMLYLRQKDANELKTILRELKYRIGIQSA
KLLRHLKQKDRLLHKVQRNCDIVTACLQAVSQKRRVDTKLKFTLEPSLGQNGFQQWYDA
LKAVARLSTGIPKEWRRKVWLTLADHYLHSIAIDWDKTMRFTFNERSNPDDDSMGIQIVK
DLHRTGCSSYCGQEAEQDRVVLKRVLLAYARWNKTVGYCQGFNILAALILEVMEGNEGD
ALKIMIYLIDKVLPESYFVNNLRALSVDMAVFRDLLRMKLPELSQHLDTLQRTANKESGG
GYEPPLTNVFTMQWFLTLFATCLPNQTVLKIWDSVFFEGSEIILRVSLAIWAKLGEQIECCET
ADEFYSTMGRLTQEMLENDLLQSHELMQTVYSMAPFPFPQLAELREKYTYNITPFPATVK
PTSVSGRHSKARDSDEENDPDDEDAVVNAVGCLGPFSGFLAPELQKYQKQIKEPNEEQSL
RSNNIAELSPGAINSCRSEYHAAFNSMMMERMTTDINALKRQYSRIKKKQQQVHQVYI
RADKGPVTSILPSQVNSSPVINHLLLGKKMKMTNRAAKNAVIHIPGHTGGKISPVPYEDLK
TKLNSPWRTHIRVHKKNMPRTKSHPGCGDTVGLIDEQNEASKTNGLGAAEAFPSGCTATA
GREGSSPEGSTRRTIEGQSPEPVFGDADVDVSAVQAKLGALELNQRDAAAETELRVHPPC
QRHCPEPPSAPEENKATSKAPQGSNSKTPIFSPFPSVKPLRKSATARNLGLYGPTERTPTVHF
PQMSRSFSKPGGGNSGTKKR

>Q8WUD6 multipass membrane protein
MAAGAGAGSAPRWLRALSEPLSAAQLRRLEEHRYSAAGVSLLEPPLQLYWTWLLQWIPL
WMAPNSITLLGLAVNVVTTLVLISYCPTATEEAPYWTYLLCALGLFIYQSLDAIDGKQARR
TNSCSPLGELFDHGCDSLSTVFMAVGASIAARLGTYPDWFFFCSFIGMFVFYCAHWQTYV

SGMLRFGKVDVTEIQIALVIVFVLSAFGGATMWDYTIPILEIKLKILPVLGFLGGVIFSCSNY
FHVILHGGVGKNGSTIAGTSVLSPGLHIGLIIILAIMIYKKSATDVFEKHPCLYILMFGCVFA
KVSQKLVVAHMTKSELYLQDTVFLGPGLLFLDQYFNNFIDEYVVLWMAMVISSFDMVIYF
SALCLQISRHLHLNIFKTACHQAPEQVQVLSSKSHQNNMD

>Q9Y3E0 multipass membrane protein
MISLTDTQKIGMGLTGFGVFFLFFGMILFFDKALLAIGNVLFVAGLAFVIGLERTFRFFFQK
HKMKATGFFLGGVFVVLIGWPLIGMIFEIYGFFLLFRGFFPVVVGFIRRVPVLGSLLNLPGI
RSFVDKVGESNNMV

>Q15286 lipid-anchor
MARDYDHLFKLLIIGDSGVGKSSLLLRFADNTFSGSYITTIGVDFKIRTVEINGEKVKLQIW
DTAGQERFRTITSTYYRGTHGVIVVYDVTSAESFVNVKRWLHEINQNCDDVCRILVGNKN
DDPERKVVETEDAYKFAGQMGIQLFETSAKENVNVEEMFNCITELVLRAKKDNLAKQQQ
QQQNDVVKLTKNSKRKKRCC

>Q9BZM6 GPI-anchor, lipid-anchor
MAAAASPAFLLCLPLLHLLSGWSRAGWVDTHCLCYDFIITPKSRPEPQWCEVQGLVDERP
FLHYDCVNHKAKAFASLGKKVNVTKTWEEQTETLRDVVDFLKGQLLDIQVENLIPIEPLT
LQARMSCEHEAHGHGRGSWQFLFNGQKFLLFDSNNRKWTALHPGAKKMTEKWEKNRD
VTMFFQKISLGDCKMWLEEFLMYWEQMLDPTKPPSLAPGTTQPKAMATTLSPWSLLIIFL
CFILAGR

>O00294 peripheral membrane protein
MPLRDETLREVWASDSGHEEESLSPEAPRRPKQRPAPAQRLRKKRTEAPESPCPTGSKPRK
PGAGRTGRPREEPSPDPAQARAPQTVYARFLRDPEAKKRDPRETFLVARAPDAEDEEEEEE
EDEEDEEEEAEEKKEKILLPPKKPLREKSSADLKERRAKAQGPRGDLGSPDPPPKPLRVRN
KEAPAGEGTKMRKTKKKGSGEADKDPSGSPASARKSPAAMFLVGEGSPDKKALKKKGTP
KGARKEEEEEEEAATVIKKSNQKGKAKGKGKKKAKEERAPSPPVEVDEPREFVLRPAPQG
RTVRCRLTRDKKGMDRGMYPSYFLHLDTEKKVFLLAGRKRKRSKTANYLISIDPTNLSRG
GENFIGKLRSNLLGNRFTVFDNGQNPQRGYSTNVASLRQELAAVIYETNVLGFRGPRRMT
VIIPGMSAENERVPIRPRNASDGLLVRWQNKTLESLIELHNKPPVWNDDSGSYTLNFQGRV
TQASVKNFQIVHADDPDYIVLQFGRVAEDAFTLDYRYPLCALQAFAIALSSFDGKLACE

>Q9BZJ6 multipass membrane protein
MVFSAVLTAFHTGTSNTTFVVYENTYMNITLPPPFQHPDLSPLLRYSFETMAPTGLSSLTVN
STAVPTTPAAFKSLNLPLQITLSAIMIFILFVSFLGNLVVCLMVYQKAAMRSAINILLASLAF
ADMLLAVLNMPFALVTILTTRWIFGKFFCRVSAMFFWLFVIEGVAILLIISIDRFLIIVQRQDK
LNPYRAKVLIAVSWATSFCVAFPLAVGNPDLQIPSRAPQCVFGYTTNPGYQAYVILISLISFFI
PFLVILYSFMGILNTLRHNALRIHSYPEGICLSQASKLGLMSLQRPFQMSIDMGFKTRAFTTI
LILFAVFIVCWAPFTTYSLVATFSKHFYYQHNFFEISTWLLWLCYLKSALNPLIYYWRIKKF
HDACLDMMPKSFKFLPQLPGHTKRRIRPSAVYVCGEHRTVV

>Q9NPZ5 single-pass type II membrane protein

MKSALFTRFFILLPWILIVIIMLDVDTRRPVPPLTPRPYFSPYAVGRGGARLPLRRGGPAHGT
QKRNQSRPQPQPEPQLPTIYAITPTYSRPVQKAELTRLANTFRQVAQLHWILVEDAAARSEL
VSRFLARAGLPSTHLHVPTPRRYKRPGLPRATEQRNAGLAWLRQRHQHQRAQPGVLFFA
DDDNTYSLELFQEMRTTRKVSVWPVGLVGGRRYERPLVENGKVVGWYTGWRADRPFAI
DMAGFAVSLQVILSNPKAVFKRRGSQPGMQESDFLKQITTVEELEPKANNCTKVLVWHTR
TEKVNLANEPKYHLDTVKIEV

>Q86WB7 multipass membrane protein
MDRSLRNVLVVSFGFLLLFTAYGGLQSLQSSLYSEEGLGVTALSTLYGGMLLSSMFLPPLLI
ERLGCKGTIILSMCGYVAFSVGNFFASWYTLIPTSILLGLGAAPLWSAQCTYLTITGNTHAE
KAGKRGKDMVNQYFGIFFLIFQSSGVWGNLISSLVFGQTPSQETLPEEQLTSCGASDCLMA
TTTTNSTQRPSQQLVYTLLGIYTGSGVLAVLMIAAFLQPIRDVQRESEGEKKSVPFWSTLLS
TFKLYRDKRLCLLILLPLYSGLQQGFLSSEYTRSYVTCTLGIQFVGYVMICFSATDALCSVL
YGKVSQYTGRAVLYVLGAVTHVSCMIALLLWRPRADHLAVFFVFSGLWGVADAVWQTQ
NNALYGVLFEKSKEAAFANYRLWEALGFVIAFGYSMFLCVHVKLYILLGVLSLTMVAYGL
VECVESKNPIRPHAPGQVNQAEDEEIQTKM

>P23942 multipass membrane protein
MALLKVKFDQKKRVKLAQGLWLMNWFSVLAGIIIFSLGLFLKIELRKRSDVMNNSESHFV
PNSLIGMGVLSCVFNSLAGKICYDALDPAKYARWKPWLKPYLAICVLFNIILFLVALCCFLL
RGSLENTLGQGLKNGMKYYRDTDTPGRCFMKKTIDMLQIEFKCCGNNGFRDWFEIQWIS
NRYLDFSSKEVKDRIKSNVDGRYLVDGVPFSCCNPSSPRPCIQYQITNNSAHYSYDHQTEE
LNLWVRGCRAALLSYYSSLMNSMGVVTLLIWLFEVTITIGLRYLQTSLDGVSNPEESESES
EGWLLEKSVPETWKAFLESVKKLGKGNQVEAEGAGAGQAPEAG

>Q13308 single-pass type I membrane protein
MGAARGSPARPRRLPLLSVLLLPLLGGTQTAIVFIKQPSSQDALQGRRALLRCEVEAPGPV
HVYWLLDGAPVQDTERRFAQGSSLSFAAVDRLQDSGTFQCVARDDVTGEEARSANASFNI
KWIEAGPVVLKHPASEAEIQPQTQVTLRCHIDGHPRPTYQWFRDGTPLSDGQSNHTVSSK
ERNLTLRPAGPEHSGLYSCCAHSAFGQACSSQNFTLSIADESFARVVLAPQDVVVARYEEA
MFHCQFSAQPPPSLQWLFEDETPITNRSRPPHLRRATVFANGSLLLTQVRPRNAGIYRCIGQ
GQRGPPIILEATLHLAEIEDMPLFEPRVFTAGSEERVTCLPPKGLPEPSVWWEHAGVRLPTH
GRVYQKGHELVLANIAESDAGVYTCHAANLAGQRRQDVNITVATVPSWLKKPQDSQLEE
GKPGYLDCLTQATPKPTVVWYRNQMLISEDSRFEVFKNGTLRINSVEVYDGTWYRCMSS
TPAGSIEAQARVQVLEKLKFTPPPQPQQCMEFDKEATVPCSATGREKPTIKWERADGSSLP
EWVTDNAGTLHFARVTRDDAGNYTCIASNGPQGQIRAHVQLTVAVFITFKVEPERTTVYQ
GHTALLQCEAQGDPKPLIQWKGKDRILDPTKLGPRMHIFQNGSLVIHDVAPEDSGRYTCIA
GNSCNIKHTEAPLYVVDKPVPEESEGPGSPPPYKMIQTIGLSVGAAVAYIIAVLGLMFYCKK
RCKAKRLQKQPEGEEPEMECLNGGPLQNGQPSAEIQEEVALTSLGSGPAATNKRHSTSDK
MHFPRSSLQPITTLGKSEFGEVFLAKAQGLEEGVAETLVLVKSLQSKDEQQQLDFRRELEM
FGKLNHANVVRLLGLCREAEPHYMVLEYVDLGDLKQFLRISKSKDEKLKSQPLSTKQKV
ALCTQVALGMEHLSNNRFVHKDLAARNCLVSAQRQVKVSALGLSKDVYNSEYYHFRQA
WVPLRWMSPEAILEGDFSTKSDVWAFGVLMWEVFTHGEMPHGGQADDEVLADLQAGK
ARLPQPEGCPSKLYRLMQRCWALSPKDRPSFSEIASALGDSTVDSKP

>Q16819 single-pass type I membrane protein
MAWIRSTCILFFTLLFAHIAAVPIKYLPEENVHDADFGEQKDISEINLAAGLDLFQGDILLQ
KSRNGLRDPNTRWTFPIPYILADNLGLNAKGAILYAFEMFRLKSCVDFKPYEGESSYIIFQQ
FDGCWSEVGDQHVGQNISIGQGCAYKAIIEHEILHALGFYHEQSRTDRDDYVNIWWDQIL
SGYQHNFDTYDDSLITDLNTPYDYESLMHYQPFSFNKNASVPTITAKIPEFNSIIGQRLDFS
AIDLERLNRMYNCTTTHTLLDHCTFEKANICGMIQGTRDDTDWAHQDSAQAGEVDHTLL
GQCTGAGYFMQFSTSSGSAEEAALLESRILYPKRKQQCLQFFYKMTGSPSDRLVVWVRRD
DSTGNVRKLVKVQTFQGDDDHNWKIAHVVLKEEQKFRYLFQGTKGDPQNSTGGIYLDDI
TLTETPCPTGVWTVRNFSQVLENTSKGDKLQSPRFYNSEGYGFGVTLYPNSRESSGYLRL
AFHVCSGENDAILEWPVENRQVIITILDQEPDVRNRMSSSMVFTTSKSHTSPAINDTVIWD
RPSRVGTYHTDCNCFRSIDLGWSGFISHQMLKRRSFLKNDDLIIFVDFEDITHLSQTEVPTK
GKRLSPQGLILQGQEQQVSEEGSGKAMLEEALPVSLSQGQPSRQKRSVENTGPLEDHNWP
QYFRDPCDPNPCQNDGICVNVKGMASCRCISGHAFFYTGERCQAVQVHGSVLGMVIGGT
AGVIFLTFSIIAILSQRPRK

>Q99884 multipass membrane protein
MKKLQGAHLRKPVTPDLLMTPSDQGDVDLDVDFAAHRGNWTGKLDFLLSCIGYCVGLG
NVWRFPYRAYTNGGGAFLVPYFLMLAICGIPLFFLELSLGQFSSLGPLAVWKISPLFKGAG
AAMLLIVGLVAIYYNMIIAYVLFYLFASLTSDLPWEHCGNWWNTELCLEHRVSKDGNGAL
PLNLTCTVSPSEEYWSRYVLHIQGSQGIGSPGEIRWNLCLCLLLAWVIVFLCILKGVKSSGK
VVYFTATFPYLILLMLLVRGVTLPGAWKGIQFYLTPQFHHLLSSKVWIEAALQIFYSLGVGF
GGLLTFASYNTFHQNIYRDTFIVTLGNAITSILAGFAIFSVLGYMSQELGVPVDQVAKAGPG
LAFVVYPQAMTMLPLSPFWSFLFFFMLLTLGLDSQFAFLETIVTAVTDEFPYYLRPKKAVFS
GLICVAMYLMGLILTTDGGMYWLVLLDDYSASFGLMVVVITTCLAVTRVYGIQRFCRDIH
MMLGFKPGLYFRACWLFLSPATLLALMVYSIVKYQPSEYGSYRFPPWAELLGILMGLLSC
LMIPAGMLVAVLREEGSLWERLQQASRPAMDWGPSLEENRTGMYVATLAGSQSPKPLMV
HMRKYGGITSFENTAIEVDREIAEEEESMM

>P10912 single-pass type I membrane protein
MDLWQLLLTLALAGSSDAFSGSEATAAILSRAPWSLQSVNPGLKTNSSKEPKFTKCRSPER
ETFSCHWTDEVHHGTKNLGPIQLFYTRRNTQEWTQEWKECPDYVSAGENSCYFNSSFTSI
WIPYCIKLTSNGGTVDEKCFSVDEIVQPDPPIALNWTLLNVSLTGIHADIQVRWEAPRNADI
QKGWMVLEYELQYKEVNETKWKMMDPILTTSVPVYSLKVDKEYEVRVRSKQRNSGNY
GEFSEVLYVTLPQMSQFTCEEDFYFPWLLIIIFGIFGLTVMLFVFLFSKQQRIKMLILPPVPVP
KIKGIDPDLLKEGKLEEVNTILAIHDSYKPEFHSDDSWVEFIELDIDEPDEKTEESDTDRLLS
SDHEKSHSNLGVKDGDSGRTSCCEPDILETDFNANDIHEGTSEVAQPQRLKGEADLLCLD
QKNQNNSPYHDACPATQQPSVIQAEKNKPQPLPTEGAESTHQAAHIQLSNPSSLSNIDFYA
QVSDITPAGSVVLSPGQKNKAGMSQCDMHPEMVSLCQENFLMDNAYFCEADAKKCIPVA
PHIKVESHIQPSLNQEDIYITTESLTTAAGRPGTGEHVPGSEMPVPDYTSIHIVQSPQGLILNA
TALPLPDKEFLSSCGYVSTDQLNKIMP

>Q99075 single-pass type I membrane protein
MKLLPSVVLKLFLAAVLSALVTGESLERLRRGLAAGTSNPDPPTVSTDQLLPLGGGRDRK

VRDLQEADLDLLRVTLSSKPQALATPNKEEHGKRKKKGKGLGKKRDPCLRKYKDFCIHG
ECKYVKELRAPSCICHPGYHGERCHGLSLPVENRLYTYDHTTILAVVAVVLSSVCLLVIVG
LLMFRYHRRGGYDVENEEKVKLGMTNSH

>O15303 multipass membrane protein
MARPRRAREPLLVALLPLAWLAQAGLARAAGSVRLAGGLTLGGLFPVHARGAAGRACG
QLKKEQGVHRLEAMLYALDRVNADPELLPGVRLGARLLDTCSRDTYALEQALSFVQALIR
GRGDGDEVGVRCPGGVPPLRPAPPERVVAVVGASASSVSIMVANVLRLFAIPQISYASTAPE
LSDSTRYDFFSRVVPPDSYQAQAMVDIVRALGWNYVSTLASEGNYGESGVEAFVQISREA
GGVCIAQSIKIPREPKPGEFSKVIRRLMETPNARGIIIFANEDDIRRVLEAARQANLTGHFLW
VGSDSWGAKTSPILSLEDVAVGAITILPKRASIDGFDQYFMTRSLENNRRNIWFAEFWEEN
FNCKLTSSGTQSDDSTRKCTGEERIGRDSTYEQEGKVQFVIDAVYAIAHALHSMHQALCP
GHTGLCPAMEPTDGRMLLQYIRAVRFNGSAGTPVMFNENGDAPGRYDIFQYQATNGSAS
SGGYQAVGQWAETLRLDVEALQWSGDPHEVPSSLCSLPCGPGERKKMVKGVPCCWHCE
ACDGYRFQVDEFTCEACPGDMRPTPNHTGCRPTPVVRLSWSSPWAAPPLLLAVLGIVATT
TVVATFVRYNNTPIVRASGRELSYVLLTGIFLIYAITFLMVAEPGAAVCAARRLFLGLGTTLS
YSALLTKTNRIYRIFEQGKRSVTPPPFISPTSQLVITFSLTSLQVVGMIAWLGARPPHSVIDYE
EQRTVDPEQARGVLKCDMSDLSLIGCLGYSLLLMVTCTVYAIKARGVPETFNEAKPIGFT
MYTTCIIWLAFVPIFFGTAQSAEKIYIQTTTLTVSLSLSASVSLGMLYVPKTYVILFHPEQNV
QKRKRSLKATSTVAAPPKGEDAEAHK

>Q9UIQ6 single-pass type II membrane protein
MEPFTNDRLQLPRNMIENSMFEEEPDVVDLAKEPCLHPLEPDEVEYEPRGSRLLVRGLGE
HEMEEDEEDYESSAKLLGMSFMNRSSGLRNSATGYRQSPDGACSVPSARTMVVCAFVIV
VAVSVIMVIYLLPRCTFTKEGCHKKNQSIGLIQPFATNGKLFPWAQIRLPTAVVPLRYELSLH
PNLTSMTFRGSVTISVQALQVTWNIILHSTGHNISRVTFMSAVSSQEKQAEILEYAYHGQIAI
VAPEALLAGHNYTLKIEYSANISSSYYGFYGFSYTDESNEKKYFAATQFEPLAARSAFPCF
DEPAFKATFIIKIIRDEQYTALSNMPKKSSVVLDDGLVQDEFSESVKMSTYLVAFIVGEMKN
LSQDVNGTLVSIYAVPEKIGQVHYALETTVKLLEFFQNYFEIQYPLKKLDLVAIPDFEAGAM
ENWGLLTFREETLLYDSNTSSMADRKLVTKIIAHELAHQWFGNLVTMKWWNDLWLNEGF
ATFMEYFSLEKIFKELSSYEDFLDARFKTMKKDSLNSSHPISSSVQSSEQIEEMFDSLSYFK
GSSLLLMLKTYLSEDVFQHAVVLYLHNHSYASIQSDDLWDSFNEVTNQTLDVKRMMKTW
TLQKGFPLVTVQKKGKELFIQQERFFLNMKPEIQPSDTSYLWHIPLSYVTEGRNYSKYQSV
SLLDKKSGVINLTEEVLWVKVNINMNGYYIVHYADDDWEALIHQLKINPYVLSDKDRAN
LINNIFELAGLGKVPLKRAFDLINYLGNENHTAPITEALFQTDLIYNLLEKLGYMDLASRLV
TRVFKLLQNQIQQQTWTDEGTPSMRELRSALLEFACTHNLGNCSTTAMKLFDDWMASNG
TQSLPTDVMTTVFKVGAKTDKGWSFLLGKYISIGSEAEKNKILEALASSEDVRKLYWLMK
SSLNGDNFRTQKLSFIIRTVGRHFPGHLLAWDFVKENWNKLVQKFPLGSYTIQNIVAGSTY
LFSTKTHLSEVQAFFENQSEATFRLRCVQEALEVIQLNIQWMEKNLKSLTWWL

>Q9NPG4 single-pass type I membrane protein
MMQLLQLLLGLLGPGGYLFLLGDCQEVTTLTVKYQVSEEVPSGTVIGKLSQELGREERRR
QAGAAFQVLQLPQALPIQVDSEEGLLSTGRRLDREQLCRQWDPCLVSFDVLATGDLALIH
VEIQVLDINDHQPRFPKGEQELEISESASLRTRIPLDRALDPDTGPNTLHTYTLSPSEHFALD

VIVGPDETKHAELIVVKELDREIHSFFDLVLTAYDNGNPPKSGTSLVKVNVLDSNDNSPAFA
ESSLALEIQEDAAPGTLLIKLTATDPDQGPNGEVEFFLSKHMPPEVLDTFSIDAKTGQVILR
RPLDYEKNPAYEVDVQARDLGPNPIPAHCKVLIKVLDVNDNIPSIHVTWASQPSLVSEALP
KDSFIALVMADDLDSGHNGLVHCWLSQELGHFRLKRTNGNTYMLLTNATLDREQWPKYT
LTLLAQDQGLQPLSAKKQLSIQISDINDNAPVFEKSRYEVSTRENNLPSLHLITIKAHDADL
GINGKVSYRIQDSPVAHLVAIDSNTGEVTAQRSLNYEEMAGFEFQVIAEDSGQPMLASSVS
VWVSLLDANDNAPEVVQPVLSDGKASLSVLVNASTGHLLVPIETPNGLGPAGTDTPPLAT
HSSRPFLLTTIVARDADSGANGEPLYSIRSGNEAHLFILNPHTGQLFVNVTNASSLIGSEWE
LEIVVEDQGSPPLQTRALLRVMFVTSVDHLRDSARKPGALSMSMLTVICLAVLLGIFGLIL
ALFMSICRTEKKDNRAYNCREAESTYRQQPKRPQKHIQKADIHLVPVLRGQAGEPCEVGQ
SHKDVDKEAMMEAGWDPCLQAPFHLTPTLYRTLRNQGNQGAPAESREVLQDTVNLLFN
HPRQRNASRENLNLPEPQPATGQPRSRPLKVAGSPTGRLAGDQGSEEAPQRPPASSATLRR
QRHLNGKVSPEKESGPRQILRSLVRLSVAAFAERNPVEELTVDSPPVQQISQLLSLLHQGQF
QPKPNHRGNKYLAKPGGSRSAIPDTDGPSARAGGQTDPEQEEGPLDPEEDLSVKQLLEEE
LSSLLDPSTGLALDRLSAPDPAWMARLSLPLTTNYRDNVISPDAAATEEPRTFQTFGKAEA
PELSPTGTRLASTFVSEMSSLLEMLLEQRSSMPVEAASEALRRLSVCGRTLSLDLATSAAS
GMKVQGDPGGKTGTEGKSRGSSSSSRCL

>O75694 peripheral membrane protein
MPSSLLGAAMPASTSAAALQEALENAGRLIDRQLQEDRMYPDLSELLMVSAPNNPTVSG
MSDMDYPLQGPGLLSVPNLPEISSIRRVPLPPELVEQFGHMQCNCMMGVFPPISRAWLTID
SDIFMWNYEDGGDLAYFDGLSETILAVGLVKPKAGIFQPHVRHLLVLATPVDIVILGLSYA
NLQTGSGVLNDSLSGGMQLLPDPLYSLPTDNTYLLTITSTDNGRIFLAGKDGCLYEVAYQA
EAGWFSQRCRKINHSKSSLSFLVPSLLQFTFSEDDPILQIAIDNSRNILYTRSEKGVIQVYDL
GQDGQGMSRVASVSQNAIVSAAGNIARTIDRSVFKPIVQIAVIENSESLDCQLLAVTHAGVR
LYFSTCPFRQPLARPNTLTLVHVRLPPGFSASSTVEKPSKVHRALYSKGILLMAASENEDN
DILWCVNHDTFPFQKPMMETQMTAGVDGHSWALSAIDELKVDKIITPLNKDHIPITDSPVV
VQQHMLPPKKFVLLSAQGSLMFHKLRPVDQLRHLLVSNVGGDGEEIERFFKLHQEDQAC
ATCLILACSTAACDREVSAWATRAFFRYGGEAQMRFPTTLPPPSNVGPILGSPVYSSSPVPS
GSPYPNPSFLGTPSHGIQPPAMSTPVCALGNPATQATNMSCVTGPEIVYSGKHNGICIYFSRI
MGNIWDASLVVERIFKSGNREITAIESSVPCQLLESVLQELKGLQEFLDRNSQFAGGPLGNP
NTTAKVQQRLIGFMRPENGNPQQMQQELQRKFHEAQLSEKISLQAIQQLVRKSYQALAL
WKLLCEHQFTIIVAELQKELQEQLKITTFKDLVIRDKELTGALIASLINCYIRDNAAVDGISL
HLQDICPLLYSTDDAICSKANELLQRSRQVQNKTEKERMLRESLKEYQKISNQVDLSNVC
AQYRQVRFYEGVVELSLTAAEKKDPQGLGLHFYKHGEPEEDIVGLQAFQERLNSYKCITD
TLQELVNQSKAAPQSPSVPKKPGPPVLSSDPNMLSNEEAGHHFEQMLKLSQRSKDELFSIA
LYNWLIQVDLADKLLQVASPFLEPHLVRMAKVDQNRVRYMDLLWRYYEKNRSFSNAAR
VLSRLADMHSTEISLQQRLEYIARAILSAKSSTAISSIAADGEFLHELEEKMEVARIQLQIQE
TLQRQYSHHSSVQDAVSQLDSELMDITKLYGEFADPFKLAECKLAIIHCAGYSDPILVQTL
WQDIIEKELSDSVTLSSSDRMHALSLKIVLLGKIYAGTPRFFPLDFIVQFLEQQVCTLNWDV
GFVIQTMNEIGVPLPRLLEVYDQLFKSRDPFWNRMKKPLHLLDCIHVLLIRYVENPSQVLN
CERRRFTNLCLDAVCGYLVELQSMSSSVAVQAITGNFKSLQAKLERLH

>P22760 single-pass type II membrane protein

MGRKSLYLLIVGILIAYYIYTPLPDNVEEPWRMMWINAHLKTIQNLATFVELLGLHHFMDS
FKVVGSFDEVPPTSDENVTVTETKFNNILVRVYVPKRKSEALRRGLFYIHGGGWCVGSAA
LSGYDLLSRWTADRLDAVVVSTNYRLAPKYHFPIQFEDVYNALRWFLRKKVLAKYGVNP
ERIGISGDSAGGNLAAAVTQQLLDDPDVKIKLKIQSLIYPALQPLDVDLPSYQENSNFLFLS
KSLMVRFWSEYFTTDRSLEKAMLSRQHVPVESSHLFKFVNWSSLLPERFIKGHVYNNPNY
GSSELAKKYPGFLDVRAAPLLADDNKLRGLPLTYVITCQYDLLRDDGLMYVTRLRNTGV
QVTHNHVEDGFHGAFSFLGLKISHRLINQYIEWLKENL

>P30414 peripheral membrane protein
MGAQDRPQCHFDIEINREPVGRIMFQLFSDICPKTCKNFLCLCSGEKGLGKTTGKKLCYK
GSTFHRVVKNFMIQGGDFSEGNGKGGESIYGGYFKDENFILKHDRAFLLSMANRGKHTN
GSQFFITTKPAPHLDGVHVVFGLVISGFEVIEQIENLKTDAASRPYADVRVIDCGVLATKSIK
DVFEKKRKKPTHSEGSDSSSNSSSSSESSSESELEHERSRRRKHKRRPKVKRSKKRRKEAS
SSEEPRNKHAMNPKGHSERSDTNEKRSVDSSAKREKPVVRPEEIPPVPENRFLLRRDMPV
VTAEPEPKIPDVAPIVSDQKPSVSKSGRKIKGRGTIRYHTPPRSRSCSESDDDDSSETPPHWK
EEMQRLRAYRPPSGEKWSKGDKLSDPCSSRWDERSLSQRSRSWSYNGYYSDLSTARHSG
HHKKRRKEKKVKHKKKGKKQKHCRRHKQTKKRRILIPSDIESSKSSTRRMKSSCDRERSS
RSSSLSSHHSSKRDWSKSDKDVQSSLTHSSRDSYRSKSHSQSYSRGSSRSRTASKSSSHSRS
RSKSRSSSKSGHRKRASKSPRKTASQLSENKPVKTEPLRATMAQNENVVVQPVVAENIPVI
PLSDSPPPSRWKPGQKPWKPSYERIQEMKAKTTHLLPIQSTYSLANIKETGSSSSYHKREK
NSESDQSTYSKYSDRSSESSPRSRSRSSRSRSYSRSYTRSRSLASSHSRSRSPSSRSHSRNKY
SDHSQCSRSSSYTSISSDDGRRAKRRLRSSGKKNSVSHKKHSSSSEKTLHSKYVKGRDRSS
CVRKYSESRSSLDYSSDSEQSSVQATQSAQEKEKQGQMERTHNKQEKNRGEEKSKSEREC
PHSKKRTLKENLSDHLRNGSKPKRKNYAGSKWDSESNSERDVTKNSKNDSHPSSDKEEG
EATSDSESEVSEIHIKVKPTTKSSTNTSLPDDNGAWKSSKQRTSTSDSEGSCSNSENNRGKP
QKHKHGSKENLKREHTKKVKEKLKGKKDKKHKAPKRKQAFHWQPPLEFGEEEEEEIDD
KQVTQESKEKKVSENNETIKDNILKTEKSSEEDLSGKHDTVTVSSDLDQFTKDDSKLSISPT
ALNTEENVACLQNIQHVEESVPNGVEDVLQTDDNMEICTPDRSSPAKVEETSPLGNARLD
TPDINIVLKQDMATEHPQAEVVKQESSMSESKVLGEVGKQDSSSASLASAGESTGKKEVA
EKSQINLIDKKWKPLQGVGNLAAPNAATSSAVEVKVLTTVPEMKPQGLRIEIKSKNKVRP
GSLFDEVRKTARLNRRPRNQESSSDEQTPSRDDDSQSRSPSRSRSKSETKSRHRTRSVSYSH
SRSRSRSSTSSYRSRSYSRSRSRGWYSRGRTRSRSSSYRSYKSHRTSSRSRSRSSSYDPHSRS
RSYTYDSYYSRSRSRSRSQRSDSYHRGRSYNRRSRSCRSYGSDSESDRSYSHHRSPSESSR
YS

>P39210 multipass membrane protein
MALWRAYQRALAAHPWKVQVLTAGSLMGLGDIISQQLVERRGLQEHQRGRTLTMVSLG
CGFVGPVVGGWYKVLDRFIPGTTKVDALKKMLLDQGGFAPCFLGCFLPLVGALNGLSAQ
DNWAKLQRDYPDALITNYYLWPAVQLANFYLVPLHYRLAVVQCVAVIWNSYLSWKAHRL

>Q15036 peripheral membrane protein
MHFSIPETESRSGDSGGSAYVAYNIHVNGVLHCRVRYSQLLGLHEQLRKEYGANVLPAFPP
KKLFSLTPAEVEQRREQLEKYMQAVRQDPLLGSSETFNSFLRRAQQETQQVPTEEVSLEVL
LSNGQKVLVNVLTSDQTEDVLEAVAAKLDLPDDLIGYFSLFLVREKEDGAFSFVRKLQEFE

LPYVSVTSLRSQEYKIVLRKSYWDSAYDDDVMENRVGLNLLYAQTVSDIERGWILVTKEQ
HRQLKSLQEKVSKKEFLRLAQTLRHYGYLRFDACVADFPEKDCPVVVSAGNSELSLQLRL
PGQQLREGSFRVTRMRCWRVTSSVPLPSGSTSSPGRGRGEVRLELAFEYLMSKDRLQWVT
ITSPQAIMMSICLQSMVDELMVKKSGGSIRKMLRRRVGGTLRRSDSQQAVKSPPLLESPDA
TRESMVKLSSKLSAVSLRGIGSPSTDASASDVHGNFAFEGIGDEDL

>O95996 peripheral membrane protein
MASSVAPYEQLVRQVEALKAENSHLRQELRDNSSHLSKLETETSGMKEVLKHLQGKLEQ
EARVLVSSGQTEVLEQLKALQMDITSLYNLKFQPPTLGPEPAARTPEGSPVHGSGPSKDSF
GELSRATIRLLEELDRERCFLLNEIEKEEKEKLWYYSQLQGLSKRLDELPHVETQFSMQMD
LIRQQLEFEAQHIRSLMEERFGTSDEMVQRAQIRASRLEQIDKELLEAQDRVQQTEPQALL
AVKSVPVDEDPETEVPTHPEDGTPQPGNSKVEVVFWLLSMLATRDQEDTARTLLAMSSSP
ESCVAMRRSGCLPLLLQILHGTEAAAGGRAGAPGAPGAKDARMRANAALHNIVFSQPDQ
GLARKEMRVLHVLEQIRAYCETCWDWLQARDGGPEGGGAGSAPIPIEPQICQATCAVMKL
SFDEEYRRAMNELGGLQAVAELLQVDYEMHKMTRDPLNLALRRYAGMTLTNLTFGDVA
NKATLCARRGCMEAIVAQLASDSEELHQVVSSILRNLSWRADINSKKVLREAGSVTALVQ
CVLRATKESTLKSVLSALWNLSAHSTENKAAICQVDGALGFLVSTLTYKCQSNSLAIIESG
GGILRNVSSLVATREDYRQVLRDHNCLQTLLQHLTSHSLTIVSNACGTLWNLSARSARDQE
LLWDLGAVGMLRNLVHSKHKMIAMGSAAALRNLLAHRPAKHQAAATAVSPGSCVPSLYV
RKQRALEAELDARHLAQALEHLEKQGPPAAEAATKKPLPPLRHLDGLAQDYASDSGCFD
DDDAPSSLAAAAATGEPASPAALSLFLGSPFLQGQALARTPPTRRGGKEAEKDTSGEAAVA
AKAKAKLALAVARIDQLVEDISALHTSSDDSFSLSSGDPGQEAPREGRAQSCSPCRGPEGG
RREAGSRAHPLLRLKAAHASLSNDSLNSGSASDGYCPREHMLPCPLAALASRREDPRCGQ
PRPSRLDLDLPGCQAEPPAREATSADARVRTIKLSPTYQHVPLLEGASRAGAEPLAGPGISP
GARKQAWLPADHLSKVPEKLAAAPLSVASKALQKLAAQEGPLSLSRCSSLSSLSSAGRPG
PSEGGDLDDSDSSLEGLEEAGPSEAELDSTWRAPGATSLPVAIPAPRRNRGRGLGVEDATP
SSSSENYVQETPLVLSRCSSVSSLGSFESPSIASSIPSEPCSGQGSGTISPSELPDSPGQTMPPS
RSKTPPLAPAPQGPPEATQFSLQWESYVKRFLDIADCRERCRLPSELDAGSVRFTVEKPDE
NFSCASSLSALALHEHYVQQDVELRLLPSACPERGGGAGGAGLHFAGHRRREEGPAPTGS
RPRGAADQELELLRECLGAAVPARLRKVASALVPGRRALPVPVYMLVPAPAPAQEDDSCT
DSAEGTPVNFSSAASLSDETLQGPPRDQPGGPAGRQRPTGRPTSARQAMGHRHKAGGAG
RSAEQSRGAGKNRAGLELPLGRPPSAPADKDGSKPGRTRGDGALQSLCLTTPTEEAVYCF
YGNDSDEEPPAAAPTPTHRRTSAIPRAFTRERPQGRKEAPAPSKAAPAAPPPARTQPSLIAD
ETPPCYSLSSSASSLSEPEPSEPPAVHPRGREPAVTKDPGPGGGRDSSPSPRAAEELLQRCISS
ALPRRRPPVSGLRRRKPRATRLDERPAEGSRERGEEAAGSDRASDLDSVEWRAIQEGANSI
VTWLHQAAAATREASSESDSILSFVSGLSVGSTLQPPKHRKGRQAEGEMGSARRPEKRGA
ASVKTSGSPRSPAGPEKPRGTQKTTPGVPAVLRGRTVIYVPSPAPRAQPKGTPGPRATPRKV
APPCLAQPAAPAKVPSPGQQRSRSLHRPAKTSELATLSQPPRSATPPARLAKTPSSSSSQTSP
ASQPLPRKRPPVTQAAGALPGPGASPVPKTPARTLLAKQHKTQRSPVRIPFMQRPARRGPP
PLARAVPEPGPRGRAGTEAGPGARGGRLGLVRVASALSSGSESSDRSGFRRQLTFIKESPGL
RRRRSELSSAESAASAPQGASPRRGRPALPAVFLCSSRCEELRAAPRQGPAPARQRPPAARP
SPGERPARRTTSESPSRLPVRAPAARPETVKRYASLPHISVARRPDGAVPAAPASADAARRS
SDGEPRPLPRVAAPGTTWRRIRDEDVPHILRSTLPATALPLRGSTPEDAPAGPPPRKTSDAV
VQTEEVAAPKTNSSTSPSLETREPPGAPAGGQLSLLGSDVDGPSLAKAPISAPFVHEGLGVA

VGGFPASRHGSPSRSARVPPFNYVPSPMVVAATTDSAAEKAPATASATLLE

>Q13724 single-pass type II membrane protein
MARGERRRRAVPAEGVRTAERAARGGPGRRDGRGGGPRSTAGGVALAVVVLSLALGMS
GRWVLAWYRARRAVTLHSAPPVLPADSSSPAVAPDLFWGTYRPHVYFGMKTRSPKPLLTG
LMWAQQGTTPGTPKLRHTCEQGDGVGPYGWEFHDGLSFGRQHIQDGALRLTTEFVKRPG
GQHGGDWSWRVTVEPQDSGTSALPLVSLFFYVVTDGKEVLLPEVGAKGQLKFISGHTSEL
GDFRFTLLPPTSPGDTAPKYGSYNVFWTSNPGLPLLTEMVKSRLNSWFQHRPPGAPPERYL
GLPGSLKWEDRGPSGQGQGQFLIQQVTLKIPISIEFVFESGSAQAGGNQALPRLAGSLLTQ
ALESHAEGFRERFEKTFQLKEKGLSSGEQVLGQAALSGLLGGIGYFYGQGLVLPDIGVEGS
EQKVDPALFPPVPLFTAVPSRSFFPRGFLWDEGFHQLVVQRWDPSLTREALGHWLGLLNA
DGWIGREQILGDEARARVPPEFLVQRAVHANPPTLLLPVAHMLEVGDPDDLAFLRKALPR
LHAWFSWLHQSQAGPLPLSYRWRGRDPALPTLLNPKTLPSGLDDYPRASHPSVTERHLDL
RCWVALGARVLTRLAEHLGEAEVAAELGPLAASLEAAESLDELHWAPELGVFADFGNHT
KAVQLKPRPPQGLVRVVGRPQPQLQYVDALGYVSLFPLLLRLLDPTSSRLGPLLDILADSR
HLWSPFGLRSLAASSSFYGQRNSEHDPPYWRGAVWLNVNYLALGALHHYGHLEGPHQA
RAAKLHGELRANVVGNVWRQYQATGFLWEQYSDRDGRGMGCRPFHGWTSLVLLAMAE
DY

>O43813 peripheral membrane protein
MAQRAFPNPYADYNKSLAEGYFDAAGRLTPEFSQRLTNKIRELLQQMERGLKSADPRDGT
GYTGWAGIAVLYLHLYDVFGDPAYLQLAHGYVKQSLNCLTKRSITFLCGDAGPLAVAAVL
YHKMNNEKQAEDCITRLIHLNKIDPHAPNEMLYGRIGYIYALLFVNKNFGVEKIPQSHIQQI
CETILTSGENLARKRNFTAKSPLMYEWYQEYYVGAAHGLAGIYYYLMQPSLQVSQGKLH
SLVKPSVDYVCQLKFPSGNYPPCIGDNRDLLVHWCHGAPGVIYMLIQAYKVFREEKYLCD
AYQCADVIWQYGLLKKGYGLCHGSAGNAYAFLTLYNLTQDMKYLYRACKFAEWCLEYG
EHGCRTPDTPFSLFEGMAGTIYFLADLLVPTKARFPAFEL

>O60928 multipass membrane protein
MDSSNCKVIAPLLSQRYRRMVTKDGHSTLQMDGAQRGLAYLRDAWGILMDMRWRWM
MLVFSASFVVHWLVFAVLWYVLAEMNGDLELDHDAPPENHTICVKYITSFTAAFSFSLET
QLTIGYGTMFPSGDCPSAIALLAIQMLLGLMLEAFITGAFVAKIARPKNRAFSIRFTDTAVV
AHMDGKPNLIFQVANTRPSPLTSVRVSAVLYQERENGKLYQTSVDFHLDGISSDECPFFIFP
LTYYHSITPSSPLATLLQHENPSHFELVVFLSAMQEGTGEICQRRTSYLPSEIMLHHCFASLL
TRGSKGEYQIKMENFDKTVPEFPTPLVSKSPNRTDLDIHINGQSIDNFQISETGLTE

>P38435 multipass membrane protein
MAVSAGSARTSPSSDKVQKDKAELISGPRQDSRIGKLLGFEWTDLSSWRRLVTLLNRPTDP
ASLAVFRFLFGFLMVLDIPQERGLSSLDRKYLDGLDVCRFPLLDALRPLPLDWMYLVYTI
MFLGALGMMLGLCYRISCVLFLLPYWYVFLLDKTSWNNHSYLYGLLAFQLTFMDANHY
WSVDGLLNAHRRNAHVPLWNYAVLRGQIFIVYFIAGVKKLDADWVEGYSMEYLSRHWL
FSPFKLLLSEELTSLLVVHWGGLLLDLSAGFLLFFDVSRSIGLFFVSYFHCMNSQLFSIGMF
SYVMLASSPLFCSPEWPRKLVSYCPRRLQQLLPLKAAPQPSVSCVYKRSRGKSGQKPGLR
HQLGAAFTLLYLLEQLFLPYSHFLTQGYNNWTNGLYGYSWDMMVHSRSHQHVKITYRD

GRTGELGYLNPGVFTQSRRWKDHADMLKQYATCLSRLLPKYNVTEPQIYFDIWVSINDRF
QQRIFDPRVDIVQAAWSPFQRTSWVQPLLMDLSPWRAKLQEIKSSLDNHTEVVFIADFPGL
HLENFVSEDLGNTSIQLLQGEVTVELVAEQKNQTLREGEKMQLPAGEYHKVYTTSPSPSC
YMYVYVNTTELALEQDLAYLQELKEKVENGSETGPLPPELQPLLEGEVKGGPEPTPLVQT
FLRRQQRLQEIERRRNTPFHERFFRFLLRKLYVFRRSFLMTCISLRNLILGRPSLEQLAQEVT
YANLRPFEAVGELNPSNTDSSHSNPPESNPDPVHSEF

>P14778 single-pass type I membrane protein
MKVLLRLICFIALLISSLEADKCKEREEKIILVSSANEIDVRPCPLNPNEHKGTITWYKDDSK
TPVSTEQASRIHQHKEKLWFVPAKVEDSGHYYCVVRNSSYCLRIKISAKFVENEPNLCYN
AQAIFKQKLPVAGDGGLVCPYMEFFKNENNELPKLQWYKDCKPLLLDNIHFSGVKDRLIV
MNVAEKHRGNYTCHASYTYLGKQYPITRVIEFITLEENKPTRPVIVSPANETMEVDLGSQI
QLICNVTGQLSDIAYWKWNGSVIDEDDPVLGEDYYSVENPANKRRSTLITVLNISEIESRFY
KHPFTCFAKNTHGIDAAYIQLIYPVTNFQKHMIGICVTLTVIIVCSVFIYKIFKIDIVLWYRDS
CYDFLPIKASDGKTYDAYILYPKTVGEGSTSDCDIFVFKVLPEVLEKQCGYKLFIYGRDDY
VGEDIVEVINENVKKSRRLIIILVRETSGFSWLGGSSEEQIAMYNALVQDGIKVVLLELEKI
QDYEKMPESIKFIKQKHGAIRWSGDFTQGPQSAKTRFWKNVRYHMPVQRRSPSSKHQLL
SPATKEKLQREAHVPLG

>Q01638 single-pass type I membrane protein
MGFWILAILTILMYSTAAKFSKQSWGLENEALIVRCPRQGKPSYTVDWYYSQTNKSIPTQE
RNRVFASGQLLKFLPAAVADSGIYTCIVRSPTFNRTGYANVTIYKKQSDCNVPDYLMYSTV
SGSEKNSKIYCPTIDLYNWTAPLEWFKNCQALQGSRYRAHKSFLVIDNVMTEDAGDYTCK
FIHNENGANYSVTATRSFTVKDEQGFSLFPVIGAPAQNEIKEVEIGKNANLTCSACFGKGTQ
FLAAVLWQLNGTKITDFGEPRIQQEEGQNQSFSNGLACLDMVLRIADVKEEDLLLQYDCL
ALNLHGLRRHTVRLSRKNPIDHHSIYCIIAVCSVFLMLINVLVIILKMFWIEATLLWRDIAKP
YKTRNDGKLYDAYVVYPRNYKSSTDGASRVEHFVHQILPDVLENKCGYTLCIYGRDMLP
GEDVVTAVETNIRKSRRHIFILTPQITHNKEFAYEQEVALHCALIQNDAKVILIEMEALSELD
MLQAEALQDSLQHLMKVQGTIKWREDHIANKRSLNSKFWKHVRYQMPVPSKIPRKASSL
TPLAAQKQ

>Q13478 single-pass type I membrane protein
MNCRELPLTLWVLISVSTAESCTSRPHITVVEGEPFYLKHCSCSLAHEIETTTKSWYKSSGS
QEHVELNPRSSSRIALHDCVLEFWPVELNDTGSYFFQMKNYTQKWKLNVIRRNKHSCFT
ERQVTSKIVEVKKFFQITCENSYYQTLVNSTSLYKNCKKLLLENNKNPTIKKNAEFEDQGY
YSCVHFLHHNGKLFNITKTFNITIVEDRSNIVPVLLGPKLNHVAVELGKNVRLNCSALLNE
EDVIYWMFGEENGSDPNIHEEKEMRIMTPEGKWHASKVLRIENIGESNLNVLYNCTVAST
GGTDTKSFILVRKADMADIPGHVFTRGMIIAVLILVAVVCLVTVCVIYRVDLVLFYRHLTRR
DETLTDGKTYDAFVSYLKECRPENGEEHTFAVEILPRVLEKHFGYKLCIFERDVVPGGAVV
DEIHSLIEKSRRLIIVLSKSYMSNEVRYELESGLHEALVERKIKIILIEFTPVTDFTFLPQSLKL
LKSHRVLKWKADKSLSYNSRFWKNLLYLMPAKTVKPGRDEPEVLPVLSES

>Q9UBY0 multipass membrane protein
MEPLGNWRSLRAPLPPMLLLLLLQVAGPVGALAETLLNAPRAMGTSSSPPSPASVVAPGT

TLFEESRLPVFTLDYPHVQIPFEITLWILLASLAKIGFHLYHKLPTIVPESCLLIMVGLLLGGII
FGVDEKSPPAMKTDVFFLYLLPPIVLDAGYFMPTRPFFENIGTIFWYAVVGTLWNSIGIGVS
LFGICQIEAFGLSDITLLQNLLFGSLISAVDPVAVLAVFENIHVNEQLYILVFGESLLNDAVTV
VLYNLFKSFCQMKTIETIDVFAGIANFFVVGIGGVLIGIFLGFIAAFTTRFTHNIRVIEPLFVFL
YSYLSYITAEMFHLSGIMAITACAMTMNKYVEENVSQKSYTTIKYFMKMLSSVSETLIFIF
MGVSTVGKNHEWNWAFVCFTLAFCLMWRALGVFVLTQVINRFRTIPLTFKDQFIIAYGGL
RGAICFALVFLLPAAVFPRKKLFITAAIVVIFFTVFILGITIRPLVEFLDVKRSNKKQQAVSEEI
YCRLFDHVKTGIEDVCGHWGHNFWRDKFKKFDDKYLRKLLIRENQPKSSIVSLYKKLEIK
HAIEMAETGMISTVPTFASLNDCREEKIRKVTSSETDEIRELLSRNLYQIRQRTLSYNRHSLT
ADTSERQAKEILIRRRHSLRESIRKDSSLNREHRASTSTSRYLSLPKNTKLPEKLQKRRTISI
ADGNSSDSDADAGTTVLNLQPRARRFLPEQFSKKSPQSYKMEWKNEVDVDSGRDMPSTP
PTPHSREKGTQTSGLLQQPLLSKDQSGSEREDSLTEGIPPKPPPRLVWRASEPGSRKARFGS
EKP

>Q9H8Y8 lipid-anchor, peripheral membrane protein
MGSSQSVEIPGGGTEGYHVLRVQENSPGHRAGLEPFFDFIVSINGSRLNKDNDTLKDLLKA
NVEKPVKMLIYSSKTLELRETSVTPSNLWGGQGLLGVSIRFCSFDGANENVWHVLEVESN
SPAALAGLRPHSDYIIGADTVMNESEDLFSLIETHEAKPLKLYVYNTDTDNCREVIITPNSA
WGGEGSLGCGIGYGYLHRIPTRPFEEGKKISLPGQMAGTPITPLKDGFTEVQLSSVNPPSLS
PPGTTGIEQSLTGLSISSTPPAVSSVLSTGVPTVPLLPPQVNQSLTSVPPMNPATTLPGLMPLP
AGLPNLPNLNLNLPAPHIMPGVGLPELVNPGLPPLPSMPPRNLPGIAPLPLPSEFLPSFPLVP
ESSSAASSGELLSSLPPTSNAPSDPATTTAKADAASSLTVDVTPPTAKAPTTVEDRVGDSTP
VSEKPVSAAVDANASESP

>P43007 multipass membrane protein
MEKSNETNGYLDSAQAGPAAGPGAPGTAAGRARRCAGFLRRQALVLLTVSGVLAGAGL
GAALRGLSLSRTQVTYLAFPGEMLLRMLRMIILPLVVCSLVSGAASLDASCLGRLGGIAVA
YFGLTTLSASALAVALAFIIKPGSGAQTLQSSDLGLEDSGPPPVPKETVDSFLDLARNLFPSN
LVVAAFRTYATDYKVVTQNSSSGNVTHEKIPIGTEIEGMNILGLVLFALVLGVALKKLGSEG
EDLIRFFNSLNEATMVLVSWIMWYVPVGIMFLVGSKIVEMKDIIVLVTSLGKYIFASILGHV
IHGGIVLPLIYFVFTRKNPFRFLLGLLAPFATAFATCSSSATLPSMMKCIEENNGVDKRISRFI
LPIGATVNMDGAAIFQCVAAVFIAQLNNVELNAGQIFTILVTATASSVGAAGVPAGGVLTIA
IILEAIGLPTHDLPLILAVDWIVDRTTTVVNVEGDALGAGILHHLNQKATKKGEQELAEVK
VEAIPNCKSEEETSPLVTHQNPAGPVASAPELESKESVL

>Q969F8 multipass membrane protein
MHTVATSGPNASWGAPANASGCPGCGANASDGPVPSPRAVDAWLVPLFFAALMLLGLVG
NSLVIYVICRHKPMRTVTNFYIANLAATDVTFLLCCVPFTALLYPLPGWVLGDFMCKFVN
YIQQVSVQATCATLTAMSVDRWYVTVFPLRALHRRTPRLALAVSLSIWVGSAAVSAPVLA
LHRLSPGPRAYCSEAFPSRALERAFALYNLLALYLLPLLATCACYAAMLRHLGRVAVRPAP
ADSALQGQVLAERAGAVRAKVSRLVAAVVLLFAACWGPIQLFLVLQALGPAGSWHPRSY
AAYALKTWAHCMSYSNSALNPLLYAFLGSHFRQAFRRVCPCAPRRPRRPRRPGPSDPAAP
HAELLRLGSHPAPARAQKPGSSGLAARGLCVLGEDNAPL

>P15313 peripheral membrane protein
MAMEIDSRPGGLPGSSCNLGAAREHMQAVTRNYITHPRVTYRTVCSVNGPLVVLDRVKFA
QYAEIVHFTLPDGTQRSGQVLEVAGTKAIVQVFEGTSGIDARKTTCEFTGDILRTPVSEDM
LGRVFNGSGKPIDKGPVVMAEDFLDINGQPINPHSRIYPEEMIQTGISPIDVMNSIARGQKIP
IFSAAGLPHNEIAAQICRQAGLVKKSKAVLDYHDDNFAIVFAAMGVNMETARFFKSDFEQ
NGTMGNVCLFLNLANDPTIERIITPRLALTTAEFLAYQCEKHVLVILTDMSSYAEALREVSA
AREEVPGRRGFPGYMYTDLATIYERAGRVEGRGGSITQIPILTMPNDDITHPIPDLTGFITEG
QIYVDRQLHNRQIYPPINVLPSLSRLMKSAIGEGMTRKDHGDVSNQLYACYAIGKDVQAM
KAVVGEEALTSEDLLYLEFLQKFEKNFINQGPYENRSVFESLDLGWKLLRIFPKEMLKRIP
QAVIDEFYSREGALQDLAPDTAL

>Q9HB20 peripheral membrane protein
MEGVLYKWTNYLTGWQPRWFVLDNGILSYYDSQDDVCKGSKGSIKMAVCEIKVHSADN
TRMELIIPGEQHFYMKAVNAAERQRWLVALGSSKACLTDTRTKKEKEISETSESLKTKMSE
LRLYCDLLMQQVHTIQEFVHHDENHSSPSAENMNEASSLLSATCNTFITTLEECVKIANAK
FKPEMFQLHHPDPLVSPVSPSPVQMMKRSVSHPGSCSSERSSHSIKEPVSTLHRLSQRRRRT
YSDTDSCSDIPLEDPDRPVHCSKNTLNGDLASATIPEESRLMAKKQSESEDTLPSFSS

>P51797 multipass membrane protein
MAGCRGSLCCCCRWCCCCGERETRTPEELTILGETQEEEDEILPRKDYESLDYDRCINDPY
LEVLETMDNKKGRRYEAVKWMVVFAIGVCTGLVGLFVDFFVRLFTQLKFGVVQTSVEEC
SQKGCLALSLLELLGFNLTFVFLASLLVLIEPVAAGSGIPEVKCYLNGVKVPGIVRLRTLLC
KVLGVLFSVAGGLFVEKEGPMIHSGSVVGAGLPQFQSISLRKIQFNFPYFRSDRDKRDFVS
AGAAAGVAAAFGAPIGGTLFSLEEGSSFWNQGLTWKVLFCSMSATFTLNFFRSGIQFGSW
GSFQLPGLLNFGEFKCSDSDKKCHLWTAMDLGFFVVMGVIGGLLGATFNCLNKRLAKYR
MRNVHPKPKLVRVLESLLVSLVTTVVVFVASMVLGECRQMSSSSQIGNDSFQLQVTEDVN
SSIKTFFCPNDTYNDMATLFFNPQESAILQLFHQDGTFSPVTLALFFVLYFLLACWTYGISV
PSGLFVPSLLCGAAFGRLVANVLKSYIGLGHIYSGTFALIGAAAFLGGVVRMTISLTVILIES
TNEITYGLPIMVTLMVAKWTGDFFNKGIYDIHVGLRGVPLLEWETEVEMDKLRASDIMEP
NLTYVYPHTRIQSLVSILRTTVHHAFPVVTENRGNEKEFMKGNQLISNNIKFKKSSILTRAG
EQRKRSQSMKSYPSSELRNMCDEHIASEEPAEKEDLLQQMLERRYTPYPNLYPDQSPSED
WTMEERFRPLTFHGLILRSQLVTLLVRGVCYSESQSSASQPRLSYAEMAEDYPRYPDIHDL
DLTLLNPRMIVDVTPYMNPSPFTVSPNTHVSQVFNLFRTMGLRHLPVVNAVGEIVGIITRH
NLTYEFLQARLRQHYQTI

>A8K7I4 peripheral membrane protein
MGPFKSSVFILILHLLEGALSNSLIQLNNNGYEGIVVAIDPNVPEDETLIQQIKDMVTQASLY
LLEATGKRFYFKNVAILIPETWKTKADYVRPKLETYKNADVLVAESTPPGNDEPYTEQMG
NCGEKGERIHLTPDFIAGKKLAEYGPQGRAFVHEWAHLRWGVFDEYNNDEKFYLSNGRI
QAVRCSAGITGTNVVKKCQGGSCYTKRCTFNKVTGLYEKGCEFVLQSRQTEKASIMFAQ
HVDSIVEFCTEQNHNKEAPNKQNQKCNLRSTWEVIRDSEDFKKTTPMTTQPPNPTFSLLQI
GQRIVCLVLDKSGSMATGNRLNRLNQAGQLFLLQTVELGSWVGMVTFDSAAHVQNELIQ
INSGSDRDTLAKRLPAAASGGTSICSGLRSAFTVIRKKYPTDGSEIVLLTDGEDNTISGCFNE
VKQSGAIIHTVALGPSAAQELEELSKMTGGLQTYASDQVQNNGLIDAFGALSSGNGAVSQ

RSIQLESKGLTLQNSQWMNGTVIVDSTVGKDTLFLITWTMQPPQILLWDPSGQKQGGFVV
DKNTKMAYLQIPGIAKVGTWKYSLQASSQTLTLTVTSRASNATLPPITVTSKTNKDTSKFP
SPLVVYANIRQGASPILRASVTALIESVNGKTVTLELLDNGAGADATKDDGVYSRYFTTYD
TNGRYSVKVRALGGVNAARRRVIPQQSGALYIPGWIENDEIQWNPPRPEINKDDVQHKQV
CFSRTSSGGSFVASDVPNAPIPDLFPPGQITDLKAEIHGGSLINLTWTAPGDDYDHGTAHKY
IIRISTSILDLRDKFNESLQVNTTALIPKEANSEEVFLFKPENITFENGTDLFIAIQAVDKVDL
KSEISNIARVSLFIPPQTPPETPSPDETSAPCPNIHINSTIPGIHILKIMWKWIGELQLSIA

>Q9BXS4 single-pass type I membrane protein
MAAPKGSLWVRTQLGLPPLLLLTMALAGGSGTASAEAFDSVLGDTASCHRACQLTYPLHT
YPKEEELYACQRGCRLFSICQFVDDGIDLNRTKLECESACTEAYSQSDEQYACHLGCQNQL
PFAELRQEQLMSLMPKMHLLFPLTLVRSFWSDMMDSAQSFITSSWTFYLQADDGKIVIFQS
KPEIQYAPHLEQEPTNLRESSLSKMSYLQMRNSQAHRNFLEDGESDGFLRCLSLNSGWILT
TTLVLSVMVLLWICCATVATAVEQYVPSEKLSIYGDLEFMNEQKLNRYPASSLVVVRSKTE
DHEEAGPLPTKVNLAHSEI

>P41143 multipass membrane protein
MEPAPSAGAELQPPLFANASDAYPSACPSAGANASGPPGARSASSLALAIAITALYSAVCAV
GLLGNVLVMFGIVRYTKMKTATNIYIFNLALADALATSTLPFQSAKYLMETWPFGELLCK
AVLSIDYYNMFTSIFTLTMMSVDRYIAVCHPVKALDFRTPAKAKLINICIWVLASGVGVPIM
VMAVTRPRDGAVVCMLQFPSPSWYWDTVTKICVFLFAFVVPILIITVCYGLMLLRLRSVRL
LSGSKEKDRSLRRITRMVLVVVGAFVVCWAPIHIFVIVWTLVDIDRRDPLVVAALHLCIAL
GYANSSLNPVLYAFLDENFKRCFRQLCRKPCGRPDPSSFSRAREATARERVTACTPSDGPG
GGAAA

>O95140 multipass membrane protein
MSLLFSRCNSIVTVKKNKRHMAEVNASPLKHFVTAKKKINGIFEQLGAYIQESATFLEDTY
RNAELDPVTTEEQVLDVKGYLSKVRGISEVLARRHMKVAFFGRTSNGKSTVINAMLWDK
VLPSGIGHTTNCFLRVEGTDGHEAFLLTEGSEEKRSAKTVNQLAHALHQDKQLHAGSLVS
VMWPNSKCPLLKDDLVLMDSPGIDVTTELDSWIDKFCLDADVFVLVANSESTLMQTEKH
FFHKVSERLSRPNIFILNNRWDASASEPEYMEEVRRQHMERCTSFLVDELGVVDRSQAGD
RIFFVSAKEVLNARIQKAQGMPEGGGALAEGFQVRMFEFQNFERRFEECISQSAVKTKFEQ
HTVRAKQIAEAVRLIMDSLHMAAREQQVYCEEMREERQDRLKFIDKQLELLAQDYKLRI
KQITEEVERQVSTAMAEEIRRLSVLVDDYQMDFHPSPVVLKVYKNELHRHIEEGLGRNMS
DRCSTAITNSLQTMQQDMIDGLKPLLPVSVRSQIDMLVPRQCFSLNYDLNCDKLCADFQE
DIEFHFSLGWTMLVNRFLGPKNSRRALMGYNDQVQRPIPLTPANPSMPPLPQGSLTQEEFM
VSMVTGLASLTSRTSMGILVVGGVVWKAVGWRLIALSFGLYGLLYVYERLTWTTKAKER
AFKRQFVEHASEKLQLVISYTGSNCSHQVQQELSGTFAHLCQQVDVTRENLEQEIAAMNK
KIEVLDSLQSKAKLLRNKAGWLDSELNMFTHQYLQPSR

>Q9NTN3 multipass membrane protein
MAEVHRRQHARVKGEAPAKSSTLRDEEELGMASAETLTVFLKLLAAGFYGVSSFLIVVVN
KSVLTNYRFPSSLCVGLGQMVATVAVLWVGKALRVVKFPDLDRNVPRKTFPLPLLYFGNQ
ITGLFSTKKLNLPMFTVLRRFSILFTMFAEGVLLKKTFSWGIKMTVFAMIIGAFVAASSDLA

FDLEGYAFILINDVLTAANGAYVKQKLDSKELGKYGLLYYNALFMILPTLAIAYFTGDAQK
AVEFEGWADTLFLLQFTLSCVMGFILMYATVLCTQYNSALTTTIVGCIKNILITYIGMVFGG
DYIFTWTNFIGLNISIAGSLVYSYITFTEEQLSKQSEANNKLDIKGKGAV

>O14966 lipid-anchor
MGSRDHLFKVLVVGDAAVGKTSLVQRYSQDSFSKHYKSTVGVDFALKVLQWSDYEIVRL
QLWDIAGQERFTSMTRLYYRDASACVIMFDVTNATTFSNSQRWKQDLDSKLTLPNGEPVP
CLLLANKCDLSPWAVSRDQIDRFSKENGFTGWTETSVKENKNINEAMRVLIEKMMRNSTE
DIMSLSTQGDYINLQTKSSSWSCC

>O95971 GPI-anchor, lipid-anchor
MLLEPGRGCCALAILLAIVDIQSGGCINITSSASQEGTRLNLICTVWHKKEEAEGFVVFLCK
DRSGDCSPETSLKQLRLKRDPGIDGVGEISSQLMFTISQVTPLHSGTYQCCARSQKSGIRLQ
GHFFSILFTETGNYTVTGLKQRQHLEFSHNEGTLSSGFLQEKVWVMLVTSLVALQAL

>Q99437 multipass membrane protein
MTGLALLYSGVFVAFWACALAVGVCYTIFDLGFRFDVAWFLTETSPFMWSNLGIGLAISLS
VVGAAWGIYITGSSIIGGGVKAPRIKTKNLVSIIFCEAVAIYGIIMAIVISNMAEPFSATDPKAI
GHRNYHAGYSMFGAGLTVGLSNLFCGVCVGIVGSGAALADAQNPSLFVKILIVEIFGSAIG
LFGVIVAILQTSRVKMGD

>O60779 multipass membrane protein
MDVPGPVSRRAAAAAATVLLRTARVRRECWFLPTALLCAYGFFASLRPSEPFLTPYLLGPD
KNLTEREVFNEIYPVWTYSYLVLLFPVFLATDYLRYKPVVLLQGLSLIVTWFMLLYAQGLL
AIQFLEFFYGIATATEIAYYSYIYSVVDLGMYQKVTSYCRSATLVGFTVGSVLGQILVSVAG
WSLFSLNVISLTCVSVAFAVAWFLPMPQKSLFFHHIPSTCQRVNGIKVQNGGIVTDTPASNH
LPGWEDIESKIPLNMEEPPVEEPEPKPDRLLVLKVLWNDFLMCYSSRPLLCWSVWWALST
CGYFQVVNYTQGLWEKVMPSRYAAIYNGGVEAVSTLLGAVAVFAVGYIKISWSTWGEMT
LSLFSLLIAAAVYIMDTVGNIWVCYASYVVFRIIYMLLITIATFQIAANLSMERYALVFGVN
TFIALALQTLLTLIVVDASGLGLEITTQFLIYASYFALIAVVFLASGAVSVMKKCRKLEDPQS
SSQVTTS

>P14151 single-pass type I membrane protein
MIFPWKCQSTQRDLWNIFKLWGWTMLCCDFLAHHGTDCWTYHYSEKPMNWQRARRFC
RDNYTDLVAIQNKAEIEYLEKTLPFSRSYYWIGIRKIGGIWTWVGTNKSLTEEAENWGDGE
PNNKKNKEDCVEIYIKRNKDAGKWNDDACHKLKAALCYTASCQPWSCSGHGECVEIINN
YTCNCDVGYYGPQCQFVIQCEPLEAPELGTMDCTHPLGNFSFSSQCAFSCSEGTNLTGIEE
TTCGPFGNWSSPEPTCQVIQCEPLSAPDLGIMNCSHPLASFSFTSACTFICSEGTELIGKKKT
ICESSGIWSNPSPICQKLDKSFSMIKEGDYNPLFIPVAVMVTAFSGLAFIIWLARRLKKGKKS
KRSMNDPY

>Q9UPR5 multipass membrane protein
MAPLALVGVTLLLAAPPCSGAATPTPSLPPPPANDSDTSTGGCQGSYRCQPGVLLPVWEPD
DPSLGDKAARAVVYFVAMVYMFLGVSIIADRFMAAIEVITSKEKEITITKANGETSVGTVR

IWNETVSNLTLMALGSSAPEILLSVIEVCGHNFQAGELGPGTIVGSAAFNMFVVIAVCIYVI
PAGESRKIKHLRVFFVTASWSIFAYVWLYLILAVFSPGVVQVWEALLTLVFFPVCVVFAWM
ADKRLLFYKYVYKRYRTDPRSGIIIGAEGDPPKSIELDGTFVGAEAPGELGGLGPGPAEAR
ELDASRREVIQILKDLKQKHPDKDLEQLVGIANYYALLHQQKSRAFYRIQATRLMTGAGN
VLRRHAADASRRAAPAEGAGEDEDDGASRIFFEPSLYHCLENCGSVLLSVTCQGGEGNST
FYVDYRTEDGSAKAGSDYEYSEGTLVFKPGETQKELRIGIIDDDIFEEDEHFFVRLLNLRVG
DAQGMFEPDGGGRPKGRLVAPLLATVTILDDDHAGIFSFQDRLLHVSECMGTVDVRVVRS
SGARGTVRLPYRTVDGTARGGGVHYEDACGELEFGDDETMKTLQVKIVDDEEYEKKDN
FFIELGQPQWLKRGISALLLNQGDGDRKLTAEEEEARRIAEMGKPVLGENCRLEVIIEESY
DFKNTVDKLIKKTNLALVIGTHSWREQFLEAITVSAGDEEEEEDGSREERLPSCFDYVMHF
LTVFWKVLFACVPPTEYCHGWACFGVSILVIGLLTALIGDLASHFGCTVGLKDSVNAVVFV
ALGTSIPDTFASKVAALQDQCADASIGNVTGSNAVNVFLGLGVAWSVAAVYWAVQGRPFE
VRTGTLAFSVTLFTVFAFVGIAVLLYRRRPHIGGELGGPRGPKLATTALFLGLWLLYILFASL
EAYCHIRGF

>Q9H204		peripheral membrane protein
MAAPLGGMFSGQPPGPPQAPPGLPGQASLLQAAPGAPRPSSSTLVDELESSFEACFASLVS
QDYVNGTDQEEIRTGVDQCIQKFLDIARQTECFFLQKRLQLSVQKPEQVIKEDVSELRNEL
QRKDALVQKHLTKLRHWQQVLEDINVQHKKPADIPQGSLAYLEQASANIPAPLKPT

>O00237	multipass membrane protein
MWLKLFFLLLYFLVLFVLARFFEAIVWYETGIFATQLVDPVALSFKKLKTILECRGLGYSGL
PEKKDVRELVEKSGDLMEGELYSALKEEEASESVSSTNFSGEMHFYELVEDTKDGIWLVQ
VIANDRSPLVGKIHWEKMVKKVSRFGIRTGTFNCSSDPRYCRRRGWVRSTLIMSVPQTSTS
KGKVMLKEYSGRKIEVEHIFKWITAHAASRIKTIYNAEHLKEEWNKSDQYWLKIYLFANL
DQPPAFFSALSIKFTGRVEFIFVNVENWDNKSYMTDIGIYNMPSYILRTPEGIYRYGNHTGE
FISLQAMDSFLRSLQPEVNDLFVLSLVLVNLMAWMDLFITQGATIKRFVVLISTLGTYNSLL
IISWLPVLGFLQLPYLDSFYEYSLKLLRYSNTTTLASWVRADWMFYSSHPALFLSTYLGHG
LLIDYFEKKRRRNNNNDEVNANNLEWLSSLWDWYTSYLFHPIASFQNFPVESDWDEDPD
LFLERLAFPDLWLHPLIPTDYIKNLPMWRFKCLGVQSEEEMSEGSQDTENDSESENTDTLS
SEKEVFEDKQSVLHNSPGTASHCDAEACSCANKYCQTSPCERKGRSYGSYNTNEDMEPD
WLTWPADMLHCTECVVCLENFENGCLLMGLPCGHVFHQNCIVMWLAGGRHCCPVCRW
PSYKKKQPYAQHQPLSNDVPS

>Q9UNQ0		multipass membrane protein
MSSSNVEVFIPVSQGNTNGFPATASNDLKAFTEGAVLSFHNICYRVKLKSGFLPCRKPVEK
EILSNINGIMKPGLNAILGPTGGGKSSLLDVLAARKDPSGLSGDVLINGAPRPANFKCNSG
YVVQDDVVMGTLTVRENLQFSAALRLATTMTNHEKNERINRVIQELGLDKVADSKVGTQ
FIRGVSGGERKRTSIGMELITDPSILFLDEPTTGLDSSTANAVLLLLKRMSKQGRTIIFSIHQP
RYSIFKLFDSLTLLASGRLMFHGPAQEALGYFESAGYHCEAYNNPADFFLDIINGDSTAVAL
NREEDFKATEIIEPSKQDKPLIEKLAEIYVNSSFYKETKAELHQLSGGEKKKKITVFKEISYT
TSFCHQLRWVSKRSFKNLLGNPQASIAQIIVTVVLGLVIGAIYFGLKNDSTGIQNRAGVLFF
LTTNQCFSSVSAVELFVVEKKLFIHEYISGYYRVSSYFLGKLLSDLLPMRMLPSIIFTCIVYF
MLGLKPKADAFFVMMFTLMMVAYSASSMALAIAAGQSVVSVATLLMTICFVFMMIFSGL

LVNLTTIASWLSWLQYFSIPRYGFTALQHNEFLGQNFCPGLNATGNNPCNYATCTGEEYLV
KQGIDLSPWGLWKNHVALACMIVIFLTIAYLKLLFLKKYS

>O75351 peripheral membrane protein
MSSTSPNLQKAIDLASKAAQEDKAGNYEEALQLYQHAVQYFLHVVKYEAQGDKAKQSIR
AKCTEYLDRAEKLKEYLKNKEKKAQKPVKEGQPSPADEKGNDSDGEGESDDPEKKKLQ
NQLQGAIVIERPNVKWSDVAGLEGAKEALKEAVILPIKFPHLFTGKRTPWRGILLFGPPGT
GKSYLAKAVATEANNSTFFSISSSDLVSKWLGESEKLVKNLFQLARENKPSIIFIDEIDSLCG
SRSENESEAARRIKTEFLVQMQGVGVDNDGILVLGATNIPWVLDSAIRRRFEKRIYIPLPEP
HARAAMFKLHLGTTQNSLTEADFRELGRKTDGYSGADISIIVRDALMQPVRKVQSATHFK
KVRGPSRADPNHLVDDLLTPCSPGDPGAIEMTWMDVPGDKLLEPVVSMSDMLRSLSNTK
PTVNEHDLLKLKKFTEDFGQEG

>Q9UPI3 multipass membrane protein
MVNEGPNQEESDDTPVPESALQADPSVSVHPSVSVHPSVSINPSVSVHPSSSAHPSALAQP
SGLAHPSSSGPEDLSVIKVSRRRWAVVLVFSCYSMCNSFQWIQYGSINNIFMHFYGVSAFAI
DWLSMCYMLTYIPLLLPVAWLLEKFGLRTIALTGSALNCLGAWVKLGSLKPHLFPVTVVG
QLICSVAQVFILGMPSRIASVWFGANEVSTACSVAVFGNQLGIAIGFLVPPVLVPNIEDRDEL
AYHISIMFYIIGGVATLLLILVIIVFKEKPKYPPSRAQSLSYALTSPDASYLGSIARLFKNLNFV
LLVITYGLNAGAFYALSTLLNRMVIWHYPGEEVNAGRIGLTIVIAGMLGAVISGIWLDRSK
TYKETTLVVYIMTLVGMVVYTFTLNLGHLWVVFITAGTMGFFMTGYLPLGFEFAVELTYP
ESEGISSGLLNISAQVFGIIFTISQGQIIDNYGTKPGNIFLCVFLTLGAALTAFIKADLRRQKA
NKETLENKLQEEEEESNTSKVPTAVSEDHL

>Q15743 multipass membrane protein
MGNITADNSSMSCTIDHTIHQTLAPVVYVTVLVVGFPANCLSLYFGYLQIKARNELGVYLC
NLTVADLFYICSLPFWLQYVLQHDNWSHGDLSCQVCGILLYENIYISVGFLCCISVDRYLAV
AHPFRFHQFRTLKAAVGVSVVIWAKELLTSIYFLMHEEVIEDENQHRVCFEHYPIQAWQRA
INYYRFLVGFLFPICLLLASYQGILRAVRRSHGTQKSRKDQIQRLVLSTVVIFLACFLPYHVL
LLVRSVWEASCDFAKGVFNAYHFSLLLTSFNCVADPVLYCFVSETTHRDLARLRGACLAFL
TCSRTGRAREAYPLGAPEASGKSGAQGEEPELLTKLHPAFQTPNSPGSGGFPTGRLA

>Q9BSR8 multipass membrane protein
MQPPGPPPAYAPTNGDFTFVSSADAEDLSGSIASPDVKLNLGGDFIKESTATTFLRQRGYG
WLLEVEDDDPEDNKPLLEELDIDLKDIYYKIRCVLMPMPSLGFNRQVVRDNPDFWGPLAV
VLFFSMISLYGQFRVVSWIITIWIFGSLTIFLLARVLGGEVAYGQVLGVIGYSLLPLIVIAPVL
LVVGSFEVVSTLIKLFGVFWAAYSAASLLVGEEFKTKKPLLIYPIFLLYIYFLSLYTGV

>O60242 multipass membrane protein
MKAVRNLLIYIFSTYLLVMFGFNAAQDFWCSTLVKGVIYGSYSVSEMFPKNFTNCTWTLE
NPDPTKYSIYLKFSKKDLSCSNFSLLAYQFDHFSHEKIKDLLRKNHSIMQLCNSKNAFVFL
QYDKNFIQIRRVFPTNFPGLQKKGEEDQKSFFEFLVLNKVSPSQFGCHVLCTWLESCLKSE
NGRTESCGIMYTKCTCPQHLGEWGIDDQSLILLNNVVLPLNEQTEGCLTQELQTTQVCNL
TREAKRPPKEEFGMMGDHTIKSQRPRSVHEKRVPQEQADAAKFMAQTGESGVEEWSQW

STCSVTCGQGSQVRTRTCVSPYGTHCSGPLRESRVCNNTALCPVHGVWEEWSPWSLCSFT
CGRGQRTRTRSCTPPQYGGRPCEGPETHHKPCNIALCPVDGQWQEWSSWSQCSVTCSNG
TQQRSRQCTAAAHGGSECRGPWAESRECYNPECTANGQWNQWGHWSGCSKSCDGGWE
RRIRTCQGAVITGQQCEGTGEEVRRCNEQRCPAPYEICPEDYLMSMVWKRTPAGDLAFNQ
CPLNATGTTSRRCSLSLHGVAFWEQPSFARCISNEYRHLQHSIKEHLAKGQRMLAGDGMS
QVTKTLLDLTQRKNFYAGDLLMSVEILRNVTDTFKRASYIPASDGVQNFFQIVSNLLDEEN
KEKWEDAQQIYPGSIELMQVIEDFIHIVGMGMMDFQNSYLMTGNVVASIQKLPAASVLTD
INFPMKGRKGMVDWARNSEDRVVIPKSIFTPVSSKELDESSVFVLGAVLYKNLDLILPTLR
NYTVINSKIIVVTIRPEPKTTDSFLEIELAHLANGTLNPYCVLWDDSKTNESLGTWSTQGCK
TVLTDASHTKCLCDRLSTFAILAQQPREIIMESSGTPSVTLIVGSGLSCLALITLAVVYAALW
RYIRSERSIILINFCLSIISSNILILVGQTQTHNKSICTTTTAFLHFFFLASFCWVLTEAWQSYM
AVTGKIRTRLIRKRFLCLGWGLPALVVATSVGFTRTKGYGTDHYCWLSLEGGLLYAFVGPA
AAVVLVNMVIGILVFNKLVSRDGILDKKLKHRAGQMSEPHSGLTLKCAKCGVVSTTALSA
TTASNAMASLWSSCVVLPLLALTWMSAVLAMTDKRSILFQILFAVFDSLQGFVIVMVHCIL
RREVQDAFRCRLRNCQDPINADSSSSFPNGHAQIMTDFEKDVDIACRSVLHKDIGPCRAAT
ITGTLSRISLNDDEEEKGTNPEGLSYSTLPGNVISKVIIQQPTGLHMPMSMNELSNPCLKKE
NSELRRTVYLCTDDNLRGADMDIVHPQERMMESDYIVMPRSSVNNQPSMKEESKMNIG
METLPHERLLHYKVNPEFNMNPPVMDQFNMNLEQHLAPQEHMQNLPFEPRTAVKNFMA
SELDDNAGLSRSETGSTISMSSLERRKSRYSDLDFEKVMHTRKRHMELFQELNQKFQTLD
RFRDIPNTSSMENPAPNKNPWDTFKNPSEYPHYTTINVLDTEAKDALELRPAEWEKCLNLP
LDVQEGDFQTEV

>P49683 multipass membrane protein
MASSTTRGPRVSDLFSGLPPAVTTPANQSAEASAGNGSVAGADAPAVTPFQSLQLVHQLKG
LIVLLYSVVVVVGLVGNCLLVLVIARVRRLHNVTNFLIGNLALSDVLMCTACVPLTLAYAF
EPRGWVFGGGLCHLVFFLQPVTVYVSVFTLTTIAVDRYVVLVHPLRRRISLRLSAYAVLAIW
ALSAVLALPAAVHTYHVELKPHDVRLCEEFWGSQERQRQLYAWGLLLVTYLLPLLVILLS
YVRVSVKLRNRVVPGCVTQSQADWDRARRRRTFCLLVVIVVVFAVCWLPLHVFNLLRDL
DPHAIDPYAFGLVQLLCHWLAMSSACYNPFIYAWLHDSFREELRKLLVAWPRKIAPHGQN
MTVSVVI

>Q9BXI2 multipass membrane protein
MKSGPGIQAAIDLTAGAAGGTACVLTGQPFDTIKVKMQTFPDLYKGLTDCFLKTYAQVGL
RGFYKGTGPALMAYVAENSVLFMCYGFCQQFVRKVAGMDKQAKLSDLQTAAAGSFASA
FAALALCPTELVKCRLQTMYEMEMSGKIAKSHNTIWSVVKGILKKDGPLGFYHGLSSTLL
QEVPGYFFFFGGYELSRSFFASGRSKDELGPVHLMLSGGVAGICLWLVVFPVDCIKSRIQV
LSMYGKQAGFIGTLLSVVRNEGIVALYSGLKATMIRAIPANGALFVAYEYSRKMMMKQLE
AY

>O14788 single-pass type II membrane protein
MRRASRDYTKYLRGSEEMGGGPGAPHEGPLHAPPPPAPHQPPAASRSMFVALLGLGLGQ
VVCSVALFFYFRAQMDPNRISEDGTHCIYRILRLHENADFQDTTLESQDTKLIPDSCRRIKQ
AFQGAVQKELQHIVGSQHIRAEKAMVDGSWLDLAKRSKLEAQPFAHLTINATDIPSGSHK
VSLSSWYHDRGWAKISNMTFSNGKLIVNQDGFYYLYANICFRHHETSGDLATEYLQLMV

YVTKTSIKIPSSHTLMKGGSTKYWSGNSEFHFYSINVGGFFKLRSGEEISIEVSNPSLLDPD
QDATYFGAFKVRDID

>Q9NPG1 multipass membrane protein
MAMTWIVFSLWPLTVFMGHIGGHSLFSCEPITLRMCQDLPYNTTFMPNLLNHYDQQTAAL
AMEPFHPMVNLDCSRDFRPFLCALYAPICMEYGRVTLPCRRLCQRAYSECSKLMEMFGVP
WPEDMECSRFPDCDEPYPRLVDLNLAGEPTEGAPVAVQRDYGFWCPRELKIDPDLGYSFL
HVRDCSPPCPNMYFRREELSFARYFIGLISIICLSATLFTFLTFLIDVTRFRYPERPIIFYAVCY
MMVSLIFFIGFLLEDRVACNASIPAQYKASTVTQGSHNKACTMLFMILYFFTMAGSVWWV
ILTITWFLAAVPKWGSEAIEKKALLFHASAWGIPGTLTIILLAMNKIEGDNISGVCFVGLYD
VDALRYFVLAPLCLYVVVGVSLLLAGIISLNRVRIEIPLEKENQDKLVKFMIRIGVFSILYLV
PLLVVIGCYFYEQAYRGIWETTWIQERCREYHIPCPYQVTQMSRPDLILFLMKYLMALIVG
IPSVFWVGSKKTCFEWASFFHGRRKKEIVNESRQVLQEPDFAQSLLRDPNTPIIRKSRGTST
QGTSTHASSTQLAMVDDQRSKAGSIHSKVSSYHGSLHRSRDGRYTPCSYRGMEERLPHGS
MSRLTDHSRHSSSHRLNEQSRHSSIRDLSNNPMTHITHGTSMNRVIEEDGTSA

>Q15822 multipass membrane protein
MGPSCPVFLSFTKLSLWWLLLTPAGGEEAKRPPPRAPGDPLSSPSPTALPQGGSHTETEDRL
FKHLFRGYNRWARPVPNTSDVVIVRFGLSIAQLIDVDEKNQMMTTNVWLKQEWSDYKLR
WNPTDFGNITSLRVPSEMIWIPDIVLYNNADGEFAVTHMTKAHLFSTGTVHWVPPAIYKSS
CSIDVTFFPFDQQNCKMKFGSWTYDKAKIDLEQMEQTVDLKDYWESGEWAIVNATGTY
NSKKYDCCAEIYPDVTYAFVIRRLPLFYTINLIIPCLLISCLTVLVFYLPSDCGEKITLCISVLL
SLTVFLLLITEIIPSTSLVIPLIGEYLLFTMIFVTLSIVITVFVLNVHHRSPSTHTMPHWVRGAL
LGCVPRWLLMNRPPPPVELCHPLRLKLSPSYHWLESNVDAEEREVVVEEEDRWACAGHV
APSVGTLCSHGHLHSGASGPKAEALLQEGELLLSPHMQKALEGVHYIADHLRSEDADSSV
KEDWKYVAMVIDRIFLWLFIIVCFLGTIGLFLPPFLAGMI

>Q9NYW2 multipass membrane protein
MFSPADNIFIILITGEFILGILGNGYIALVNWIDWIKKKKISTVDYILTNLVIARICLISVMVVN
GIVIVLNPDVYTKNKQQIVIFTFWTFANYLNMWITTCLNVFYFLKIASSSHPLFLWLKWKI
DMVVHWILLGCFAISLLVSLIAAIVLSCDYRFHAIAKHKRNITEMFHVSKIPYFEPLTLFNLF
AIVPFIVSLISFFLLVRSLWRHTKQIKLYATGSRDPSTEVHVRAIKTMTSFIFFFFLYYISSILM
TFSYLMTKYKLAVEFGEIAAILYPLGHSLILIVLNNKLRQTFVRMLTCRKIACMI

>P26718 single-pass type II membrane protein
MGWIRGRRSRHSWEMSEFHNYNLDLKKSDFSTRWQKQRCPVVKSKCRENASPFFFCCFI
AVAMGIRFIIMVAIWSAVFLNSLFNQEVQIPLTESYCGPCPKNWICYKNNCYQFFDESKNW
YESQASCMSQNASLLKVYSKEDQDLLKLVKSYHWMGLVHIPTNGSWQWEDGSILSPNLL
TIIEMQKGDCALYASSFKGYIENCSTPNTYICMQRTV

>Q9NYW0 multipass membrane protein
MLRVVEGIFIFVVVSESVFGVLGNGFIGLVNCIDCAKNKLSTIGFILTGLAISRIFLIWIIITDG
FIQIFSPNIYASGNLIEYISYFWVIGNQSSMWFATSLSIFYFLKIANFSNYIFLWLKSRTNMVL
PFMIVFLLISSLLNFAYIAKILNDYKTKNDTVWDLNMYKSEYFIKQILLNLGVIFFFTLSLIT

CIFLIISLWRHNRQMQSNVTGLRDSNTEAHVKAMKVLISFIILFILYFIGMAIEISCFTVRENK
LLLMFGMTTTAIYPWGHSFILILGNSKLKQASLRVLQQLKCCEKRKNLRVT

>Q15842 multipass membrane protein
MLARKSIIPEEYVLARIAAENLRKPRIRDRLPKARFIAKSGACNLAHKNIREQGRFLQDIFT
TLVDLKWRHTLVIFTMSFLCSWLLFAIMWWLVAFAHGDIYAYMEKSGMEKSGLESTVCVT
NVRSFTSAFLFSIEVQVTIGFGGRMMTEECPLAITVLILQNIVGLIINAVMLGCIFMKTAQA
HRRAETLIFSRHAVIAVRNGKLCFMFRVGDLRKSMIISASVRIQVVKKTTTPEGEVVPIHQL
DIPVDNPIESNNIFLVAPLIICHVIDKRSPLYDISATDLANQDLEVIVILEGVVETTGITTQART
SYIAEEIQWGHRFVSIVTEEEGVYSVDYSKFGNTVKVAAPRCSARELDEKPSILIQTLQKSE
LSHQNSLRKRNSMRRNNSMRRNNSIRRNNSSLMVPKVQFMTPEGNQNTSES

>Q9NYW3 multipass membrane protein
MADKVQTTLLFLAVGEFSVGILGNAFIGLVNCMDWVKKRKIASIDLILTSLAISRICLLCVIL
LDCFILVLYPDVYATGKEMRIIDFFWTLTNHLSIWFATCLSIYYFFKIGNFFHPLFLWMKWRI
DRVISWILLGCVVLSVFISLPATENLNADFRFCVKAKRKTNLTWSCRVNKTQHASTKLFLN
LATLLPFCVCLMSFFLLILSLRRHIRRMQLSATGCRDPSTEAHVRALKAVISFLLLFIAYYLS
FLIATSSYFMPETELAVIFGESIALIYPSSHSFILILGNNKLRHASLKVIWKVMSILKGRKFQQ
HKQI

>Q9NYW1 multipass membrane protein
MPSAIEAIYIILIAGELTIGIWGNGFIVLVNCIDWLKRRDISLIDIILISLAISRICLLCVISLDGF
FMLLFPGTYGNSVLVSIVNVVWTFANNSSLWFTSCLSIFYLLKIANISHPFFFWLKLKINKV
MLAILLGSFLISLIISVPKNDDMWYHLFKVSHEENITWKFKVSKIPGTFKQLTLNLGVMVP
FILCLISFFLLLFSLVRHTKQIRLHATGFRDPSTEAHMRAIKAVIIFLLLLIVYYPVFLVMTSS
ALIPQGKLVLMIGDIVTVIFPSSHSFILIMGNSKLREAFLKMLRFVKCFLRRRKPFVP

>P48165 multipass membrane protein
MGDWSFLGNILEEVNEHSTVIGRVWLTVLFIFRILILGTAAEFVWGDEQSDFVCNTQQPGC
ENVCYDEAFPISHIRLWVLQIIFVSTPSLMYVGHAVHYVRMEEKRKSREAEELGQQAGTN
GGPDQGSVKKSSGSKGTKKFRLEGTLLRTYICHIIFKTLFEVGFIVGHYFLYGFRILPLYRCS
RWPCPNVVDCFVSRPTEKTIFILFMLSVASVSLFLNVMELGHLGLKGIRSALKRPVEQPLG
EIPEKSLHSIAVSSIQKAKGYQLLEEEKIVSHYFPLTEVGMVETSPLPAKPFNQFEEKISTGPL
GDLSRGYQETLPSYAQVGAQEVEGEGPPAEEGAEPEVGEKKEEAERLTTEEQEKVAVPEG
EKVETPGVDKEGEKEEPQSEKVSKQGLPAEKTPSLCPELTTDDARPLSRLSKASSRARSDD
LTV

>Q9Y5Y5 multipass membrane protein
MEKLRLLGLRYQEYVTRHPAATAQLETAVRGFSYLLAGRFADSHELSELVYSASNLLVLLN
DGILRKELRKKLPVSLSQQKLLTWLSVLECVEVFMEMGAAKVWGEVGRWLVIALVQLA
KAVLRMLLLLWFKAGLQTSPPIVPLDRETQAQPPDGDHSPGNHEQSYVGKRSNRVVRTLQ
NTPSLHSRHWGAPQQREGRQQQHHEELSATPTPLGLQETIAEFLYIARPLLHLLSLGLWGQ
RSWKPWLLAGVVDVTSLSLLSDRKGLTRRERRELRRRTILLLYYLLRSPFYDRFSEARILFL
LQLLADHVPGVGLVTRPLMDYLPTWQKIYFYSWG

>O95452 multipass membrane protein
MDWGTLHTFIGGVNKHSTSIGKVWITVIFIFRVMILVVAAQEVWGDEQEDFVCNTLQPGC
KNVCYDHFFPVSHIRLWALQLIFVSTPALLVAMHVAYYRHETTRKFRRGEKRNDFKDIEDI
KKQKVRIEGSLWWTYTSSIFFRIIFEAAFMYVFYFLYNGYHLPWVLKCGIDPCPNLVDCFIS
RPTEKTVFTIFMISASVICMLLNVAELCYLLLKVCFRRSKRAQTQKNHPNHALKESKQNE
MNELISDSGQNAITGFPS

>Q9Y6H8 multipass membrane protein
MGDWSFLGRLLENAQEHSTVIGKVWLTVLFIFRILVLGAAAEDVWGDEQSDFTCNTQQP
GCENVCYDRAFPISHIRFWALQIIFVSTPTLIYLGHVLHIVRMEEKKKEREEEEQLKRESPSP
KEPPQDNPSSRDDRGRVRMAGALLRTYVFNIIFKTLFEVGFIAGQYFLYGFELKPLYRCDR
WPCPNTVDCFISRPTEKTIFIIFMLAVACASLLLNMLEIYHLGWKKLKQGVTSRLGPDASE
APLGTADPPPLPPSSRPPAVAIGFPPYYAHTAAPLGQARAVGYPGAPPPAADFKLLALTEAR
GKGQSAKLYNGHHHLLMTEQNWANQAAERQPPALKAYPAASTPAAPSPVGSSSPPLAHE
AEAGAAPLLLDGSGSSLEGSALAGTPEEEEQAVTTAAQMHQPPLPLGDPGRASKASRASS
GRARPEDLAI

>Q92847 multipass membrane protein
MWNATPSEEPGFNLTLADLDWDASPGNDSLGDELLQLFPAPLLAGVTATCVALFVVGIAG
NLLTMLVVSRFRELRTTTNLYLSSMAFSDLLIFLCMPLDLVRLWQYRPWNFGDLLCKLFQF
VSESCTYATVLTITALSVERYFAICFPLRAKVVVTKGRVKLVIFVIWAVAFCSAGPIFVLVGV
EHENGTDPWDTNECRPTEFAVRSGLLTVMVWVSSIFFFLPVFCLTVLYSLIGRKLWRRRRG
DAVVGASLRDQNHKQTVKMLAVVVFAFILCWLPFHVGRYLFSKSFEPGSLEIAQISQYCNL
VSFVLFYLSAAINPILYNIMSKKYRVAVFRLLGFEPFSQRKLSTLKDESSRAWTESSINT

>P27037 single-pass type I membrane protein
MGAAAKLAFAVFLISCSSGAILGRSETQECLFFNANWEKDRTNQTGVEPCYGDKDKRRHC
FATWKNISGSIEIVKQGCWLDDINCYDRTDCVEKKDSPEVYFCCCEGNMCNEKFSYFPEM
EVTQPTSNPVTPKPPYYNILLYSLVPLMLIAGIVICAFWVYRHHKMAYPPVLVPTQDPGPPP
PSPLLGLKPLQLLEVKARGRFGCVWKAQLLNEYVAVKIFPIQDKQSWQNEYEVYSLPGM
KHENILQFIGAEKRGTSVDVDLWLITAFHEKGSLSDFLKANVVSWNELCHIAETMARGLA
YLHEDIPGLKDGHKPAISHRDIKSKNVLLKNNLTACIADFGLALKFEAGKSAGDTHGQVG
TRRYMAPEVLEGAINFQRDAFLRIDMYAMGLVLWELASRCTAADGPVDEYMLPFEEEIGQ
HPSLEDMQEVVVHKKKRPVLRDYWQKHAGMAMLCETIEECWDHDAEARLSAGCVGER
ITQMQRLTNIITTEDIVTVVTMVTNVDFPPKESSL

>P36888 single-pass type I membrane protein
MPALARDGGQLPLLVVFSAMIFGTITNQDLPVIKCVLINHKNNDSSVGKSSSYPMVSESPE
DLGCALRPQSSGTVYEAAAVEVDVSASITLQVLVDAPGNISCLWVFKHSSLNCQPHFDLQ
NRGVVSMVILKMTETQAGEYLLFIQSEATNYTILFTVSIRNTLLYTLRRPYFRKMENQDAL
VCISESVPEPIVEWVLCDSQGESCKEESPAVVKKEEKVLHELFGTDIRCCARNELGRECTR
LFTIDLNQTPQTTLPQLFLKVGEPLWIRCKAVHVNHGFGLTWELENKALEEGNYFEMSTY
STNRTMIRILFAFVSSVARNDTGYYTCSSSKHPSQSALVTIVEKGFINATNSSEDYEIDQYEE

FCFSVRFKAYPQIRCTWTFSRKSFPCEQKGLDNGYSISKFCNHKHQPGEYIFHAENDDAQF
TKMFTLNIRRKPQVLAEASASQASCFSDGYPLPSWTWKKCSDKSPNCTEEITEGVWNRK
ANRKVFGQWVSSSTLNMSEAIKGFLVKCCAYNSLGTSCETILLNSPGPFPFIQDNISFYATIG
VCLLFIVVLTLLICHKYKKQFRYESQLQMVQVTGSSDNEYFYVDFREYEYDLKWEFPREN
LEFGKVLGSGAFGKVMNATAYGISKTGVSIQVAVKMLKEKADSSEREALMSELKMMTQL
GSHENIVNLLGACTLSGPIYLIFEYCCYGDLLNYLRSKREKFHRTWTEIFKEHNFSFYPTFQ
SHPNSSMPGSREVQIHPDSDQISGLHGNSFHSEDEIEYENQKRLEEEEDLNVLTFEDLLCFA
YQVAKGMEFLEFKSCVHRDLAARNVLVTHGKVVKICDFGLARDIMSDSNYVVRGNARLP
VKWMAPESLFEGIYTIKSDVWSYGILLWEIFSLGVNPYPGIPVDANFYKLIQNGFKMDQPF
YATEEIYIIMQSCWAFDSRKRPSFPNLTSFLGCQLADAEEAMYQNVDGRVSECPHTYQNRR
PFSREMDLGLLSPQAQVEDS

>O60896 single-pass type I membrane protein
METGALRRPQLLPLLLLLCGGCPRAGGCNETGMLERLPLCGKAFADMMGKVDVWKWC
NLSEFIVYYESFTNCTEMEANVVGCYWPNPLAQGFITGIHRQFFSNCTVDRVHLEDPPDEV
LIPLIVIPVVLTVAMAGLVVWRSKRTDTLL

>P09496 peripheral membrane protein
MAELDPFGAPAGAPGGPALGNGVAGAGEEDPAAAFLAQQESEIAGIENDEAFAILDGGAP
GPQPHGEPPGGPDAVDGVMNGEYYQESNGPTDSYAAISQVDRLQSEPESIRKWREEQMER
LEALDANSRKQEAEWKEKAIKELEEWYARQDEQLQKTKANNRVADEAFYKQPFADVIGY
VTNINHPCYSLEQAAEEAFVNDIDESSPGTEWERVARLCDFNPKSSKQAKDVSRMRSVLIS
LKQAPLVH

>P26992 GPI-anchor, lipid-anchor
MAAPVPWACCAVLAAAAAVVYAQRHSPQEAPHVQYERLGSDVTLPCGTANWDAAVTW
RVNGTDLAPDLLNGSQLVLHGLELGHSGLYACFHRDSWHLRHQVLLHVGLPPREPVLSCR
SNTYPKGFYCSWHLPTPTYIPNTFNVTVLHGSKIMVCEKDPALKNRCHIRYMHLFSTIKYK
VSISVSNALGHNATAITFDEFTIVKPDPPENVVARPVPSNPRRLEVTWQTPSTWPDPESFPL
KFFLRYRPLILDQWQHVELSDGTAHTITDAYAGKEYIIQVAAKDNEIGTWSDWSVAAHATP
WTEEPRHLTTEAQAAETTTSTTSSLAPPPTTKICDPGELGSGGGPSAPFLVSVPITLALAAA
AATASSLLI

>P48960 multipass membrane protein
MGGRVFLAFCVWLTLPGAETQDSRGCARWCPQNSSCVNATACRCNPGFSSFSEIITTPTET
CDDINECATPSKVSCGKFSDCWNTEGSYDCVCSPGYEPVSGAKTFKNESENTCQDVDECQ
QNPRLCKSYGTCVNTLGSYTCQCLPGFKFIPEDPKVCTDVNECTSGQNPCHSSTHCLNNV
GSYQCRCRPGWQPIPGSPNGPNNTVCEDVDECSSGQHQCDSSTVCFNTVGSYSCRCRPG
WKPRHGIPNNQKDTVCEDMTFSTWTPPPGVHSQTLSRFFDKVQDLGRDSKTSSAEVTIQN
VIKLVDELMEAPGDVEALAPPVRHLIATQLLSNLEDIMRILAKSLPKGPFTYISPSNTELTL
MIQERGDKNVTMGQSSARMKLNWAVAAGAEDPGPAVAGILSIQNMTTLLANASLNLHSK
KQAELEEIYESSIRGVQLRRLSAVNSIFLSHNNTKELNSPILFAFSHLESSDGEAGRDPPAKD
VMPGPRQELLCAFWKSDSDRGGHWATEGCQVLGSKNGSTTCQCSHLSSFAILMAHYDVE
DWKLTLITRVGLALSLFCLLLCILTFLLVRPIQGSRTTIHLHLCICLFVGSTIFLAGIENEGGQ

VGLRCRLVAGLLHYCFLAAFCWMSLEGLELYFLVVRVFQGQGLSTRWLCLIGYGVPLLIV
GVSAAIYSKGYGRPRYCWLDFEQGFLWSFLGPVTFIILCNAVIFVTTVWKLTQKFSEINPD
MKKLKKARALTITAIAQLFLLGCTWVFGLFIFDDRSLVLTYVFTILNCLQGAFLYLLHCLLN
KKVREEYRKWACLVAGGSKYSEFTSTTSGTGHNQTRALRASESGI

>Q9BY08 multipass membrane protein
MGAEWELGAEAGGSLLLCAALLAAGCALGLRLGRGQGAADRGALIWLCYDALVHFALE
GPFVYLSLVGNVANSDGLIASLWKEYGKADARWVYFDPTIVSVEILTVALDGSLALFLIYAI
VKEKYYRHFLQITLCVCELYGCWMTFLPEWLTRSPNLNTSNWLYCWLYLFFFNGVWVLIP
GLLLWQSWLELKKMHQKETSSVKKFQ

>Q53FV1 multipass membrane protein
MNVGVAHSEVNPNTRVMNSRGIWLAYIILVGLLHMVLLSIPFFSIPVVWTLTNVIHNLATY
VFLHTVKGTPFETPDQGKARLLTHWEQMDYGLQFTSSRKFLSISPIVLYLLASFYTKYDAA
HFLINTASLLSVLLPKLPQFHGVRVFGINKY

>Q07954 peripheral membrane protein, single-pass type I membrane protein
MLTPPLLLLLPLLSALVAAAIDAPKTCSPKQFACRDQITCISKGWRCDGERDCPDGSDEAPE
ICPQSKAQRCQPNEHNCLGTELCVPMSRLCNGVQDCMDGSDEGPHCRELQGNCSRLGCQ
HHCVPTLDGPTCYCNSSFQLQADGKTCKDFDECSVYGTCSQLCTNTDGSFICGCVEGYLL
QPDNRSCKAKNEPVDRPPVLLIANSQNILATYLSGAQVSTITPTSTRQTTAMDFSYANETV
CWVHVGDSAAQTQLKCARMPGLKGFVDEHTINISLSLHHVEQMAIDWLTGNFYFVDDID
DRIFVCNRNGDTCVTLLDLELYNPKGIALDPAMGKVFFTDYGQIPKVERCDMDGQNRTKL
VDSKIVFPHGITLDLVSRLVYWADAYLDYIEVVDYEGKGRQTIIQGILIEHLYGLTVFENYL
YATNSDNANAQQKTSVIRVNRFNSTEYQVVTRVDKGGALHIYHQRRQPRVRSHACENDQ
YGKPGGCSDICLLANSHKARTCRCRSGFSLGSDGKSCKKPEHELFLVYGKGRPGIIRGMD
MGAKVPDEHMIPIENLMNPRALDFHAETGFIYFADTTSYLIGRQKIDGTERETILKDGIHN
VEGVAVDWMGDNLYWTDDGPKKTISVARLEKAAQTRKTLIEGKMTHPRAIVVDPLNGW
MYWTDWEEDPKDSRRGRLERAWMDGSHRDIFVTSKTVLWPNGLSLDIPAGRLYWVDAF
YDRIETILLNGTDRKIVYEGPELNHAFGLCHHGNYLFWTEYRSGSVYRLERGVGGAPPTV
TLLRSERPPIFEIRMYDAQQQQVGTNKCRVNNGGCSSLCLATPGSRQCACAEDQVLDADG
VTCLANPSYVPPPQCQPGEFACANSRCIQERWKCDGDNDCLDNSDEAPALCHQHTCPSDR
FKCENNRCIPNRWLCDGDNDCGNSEDESNATCSARTCPPNQFSCASGRCIPISWTCDLDDD
CGDRSDESASCAYPTCFPLTQFTCNNGRCININWRCDNDNDCGDNSDEAGCSHSCSSTQF
KCNSGRCIPEHWTCDGDNDCGDYSDETHANCTNQATRPPGGCHTDEFQCRLDGLCIPLR
WRCDGDTDCMDSSDEKSCEGVTHVCDPSVKFGCKDSARCISKAWVCDGDNDCEDNSDE
ENCESLACRPPSHPCANNTSVCLPPDKLCDGNDDCGDGSDEGELCDQCSLNNGGCSHNC
SVAPGEGIVCSCPLGMELGPDNHTCQIQSYCAKHLKCSQKCDQNKFSVKCSCYEGWVLEP
DGESCRSLDPFKPFIIFSNRHEIRRIDLHKGDYSVLVPGLRNTIALDFHLSQSALYWTDVVE
DKIYRGKLLDNGALTSFEVVIQYGLATPEGLAVDWIAGNIYWVESNLDQIEVAKLDGTLRT
TLLAGDIEHPRAIALDPRDGILFWTDWDASLPRIEAASMSGAGRRTVHRETGSGGWPNGL
TVDYLEKRILWIDARSDAIYSARYDGSGHMEVLRGHEFLSHPFAVTLYGGEVYWTDWRT
NTLAKANKWTGHNVTVVQRTNTQPFDLQVYHPSRQPMAPNPCEANGGQGPCSHLCLIN
YNRTVSCACPHLMKLHKDNTTCYEFKKFLLYARQMEIRGVDLDAPYYNYIISFTVPDIDN

VTVLDYDAREQRVYWSDVRTQAIKRAFINGTGVETVVSADLPNAHGLAVDWVSRNLFW
TSYDTNKKQINVARLDGSFKNAVVQGLEQPHGLVVHPLRGKLYWTDGDNISMANMDGS
NRTLLFSGQKGPVGLAIDFPESKLYWISSGNHTINRCNLDGSGLEVIDAMRSQLGKATALAI
MGDKLWWADQVSEKMGTCSKADGSGSVVLRNSTTLVMHMKVYDESIQLDHKGTNPCS
VNNGDCSQLCLPTSETTRSCMCTAGYSLRSGQQACEGVGSFLLYSVHEGIRGIPLDPNDKS
DALVPVSGTSLAVGIDFHAENDTIYWVDMGLSTISRAKRDQTWREDVVTNGIGRVEGIAV
DWIAGNIYWTDQGFDVIEVARLNGSFRYVVISQGLDKPRAITVHPEKGYLFWTEWGQYPR
IERSRLDGTERVVLVNVSISWPNGISVDYQDGKLYWCDARTDKIERIDLETGENREVVLSS
NNMDMFSVSVFEDFIYWSDRTHANGSIKRGSKDNATDSVPLRTGIGVQLKDIKVFNRDRQ
KGTNVCAVANGGCQQLCLYRGRGQRACACAHGMLAEDGASCREYAGYLLYSERTILKSI
HLSDERNLNAPVQPFEDPEHMKNVIALAFDYRAGTSPGTPNRIFFSDIHFGNIQQINDDGS
RRITIVENVGSVEGLAYHRGWDTLYWTSYTTSTITRHTVDQTRPGAFERETVITMSGDDHP
RAFVLDECQNLMFWTNWNEQHPSIMRAALSGANVLTLIEKDIRTPNGLAIDHRAEKLYFS
DATLDKIERCEYDGSHRYVILKSEPVHPFGLAVYGEHIFWTDWVRRAVQRANKHVGSNM
KLLRVDIPQQPMGIIAVANDTNSCELSPCRINNGGCQDLCLLTHQGHVNCSCRGGRILQDD
LTCRAVNSSCRAQDEFECANGECINFSLTCDGVPHCKDKSDEKPSYCNSRRCKKTFRQCS
NGRCVSNMLWCNGADDCGDGSDEIPCNKTACGVGEFRCRDGTCIGNSSRCNQFVDCEDA
SDEMNCSATDCSSYFRLGVKGVLFQPCERTSLCYAPSWVCDGANDCGDYSDERDCPGVK
RPRCPLNYFACPSGRCIPMSWTCDKEDDCEHGEDETHCNKFCSEAQFECQNHRCISKQWL
CDGSDDCGDGSDEAAHCEGKTCGPSSFSCPGTHVCVPERWLCDGDKDCADGADESIAAG
CLYNSTCDDREFMCQNRQCIPKHFVCDHDRDCADGSDESPECEYPTCGPSEFRCANGRCL
SSRQWECDGENDCHDQSDEAPKNPHCTSQEHKCNASSQFLCSSGRCVAEALLCNGQDDC
GDSSDERGCHINECLSRKLSGCSQDCEDLKIGFKCRCRPGFRLKDDGRTCADVDECSTTFP
CSQRCINTHGSYKCLCVEGYAPRGGDPHSCKAVTDEEPFLIFANRYYLRKLNLDGSNYTLL
KQGLNNAVALDFDYREQMIYWTDVTTQGSMIRRMHLNGSNVQVLHRTGLSNPDGLAVD
WVGGNLYWCDKGRDTIEVSKLNGAYRTVLVSSGLREPRALVVDVQNGYLYWTDWGDHS
LIGRIGMDGSSRSVIVDTKITWPNGLTLDYVTERIYWADAREDYIEFASLDGSNRHVVLSQ
DIPHIFALTLFEDYVYWTDWETKSINRAHKTTGTNKTLLISTLHRPMDLHVFHALRQPDVP
NHPCKVNNGGCSNLCLLSPGGGHKCACPTNFYLGSDGRTCVSNCTASQFVCKNDKCIPF
WWKCDTEDDCGDHSDEPPDCPEFKCRPGQFQCSTGICTNPAFICDGDNDCQDNSDEANC
DIHVCLPSQFKCTNTNRCIPGIFRCNGQDNCGDGEDERDCPEVTCAPNQFQCSITKRCIPRV
WVCDRDNDCVDGSDEPANCTQMTCGVDEFRCKDSGRCIPARWKCDGEDDCGDGSDEPK
EECDERTCEPYQFRCKNNRCVPGRWQCDYDNDCGDNSDEESCTPRPCSESEFSCANGRCI
AGRWKCDGDHDCADGSDEKDCTPRCDMDQFQCKSGHCIPLRWRCDADADCMDGSDEE
ACGTGVRTCPLDEFQCNNTLCKPLAWKCDGEDDCGDNSDENPEECARFVCPPNRPFRCK
NDRVCLWIGRQCDGTDNCGDGTDEEDCEPPTAHTTHCKDKKEFLCRNQRCLSSSLRCNM
FDDCGDGSDEEDCSIDPKLTSCATNASICGDEARCVRTEKAAYCACRSGFHTVPGQPGCQ
DINECLRFGTCSQLCNNTKGGHLCSCARNFMKTHNTCKAEGSEYQVLYIADDNEIRSLFP
GHPHSAYEQAFQGDESVRIDAMDVHVKAGRVYWTNWHTGTISYRSLPPAAPPTTSNRHR
RQIDRGVTHLNISGLKMPRGIAIDWVAGNVYWTDSGRDVIEVAQMKGENRKTLISGMIDE
PHAIVVDPLRGTMYWSDWGNHPKIETAAMDGTLRETLVQDNIQWPTGLAVDYHNERLY
WADAKLSVIGSIRLNGTDPIVAADSKRGLSHPFSIDVFEDYIYGVTYINNRVFKIHKFGHSPL
VNLTGGLSHASDVVLYHQHKQPEVTNPCDRKKCEWLCLLSPSGPVCTCPNGKRLDNGTC
VPVPSPTPPPDAPRPGTCNLQCFNGGSCFLNARRQPKCRCQPRYTGDKCELDQCWEHCRN

GGTCAASPSGMPTCRCPTGFTGPKCTQQVCAGYCANNSTCTVNQGNQPQCRCLPGFLGD
RCQYRQCSGYCENFGTCQMAADGSRQCRCTAYFEGSRCEVNKCSRCLEGACVVNKQSG
DVTCNCTDGRVAPSCLTCVGHCSNGGSCTMNSKMMPECQCPPHMTGPRCEEHVFSQQQP
GHIASILIPLLLLLLLVLVAGVVFWYKRRVQGAKGFQHQRMTNGAMNVEIGNPTYKMYE
GGEPDDVGGLLDADFALDPDKPTNFTNPVYATLYMGGHGSRHSLASTDEKRELLGRGPE
DEIGDPLA

>Q14627 single-pass type I membrane protein
MAFVCLAIGCLYTFLISTTFGCTSSSDTEIKVNPPQDFEIVDPGYLGYLYLQWQPPLSLDHF
KECTVEYELKYRNIGSETWKTIITKNLHYKDGFDLNKGIEAKIHTLLPWQCTNGSEVQSS
WAETTYWISPQGIPETKVQDMDCVYYNWQYLLCSWKPGIGVLLDTNYNLFYWYEGLDH
ALQCVDYIKADGQNIGCRFPYLEASDYKDFYICVNGSSENKPIRSSYFTFQLQNIVKPLPPV
YLTFTRESSCEIKLKWSIPLGPIPARCFDYEIEIREDDTTLVTATVENETYTLKTTNETRQLCF
VVRSKVNIYCSDDGIWSEWSDKQCWEGEDLSKKTLLRFWLPFGFILILVIFVTGLLLRKPN
TYPKMIPEFFCDT

>P61619 multipass membrane protein
MAIKFLEVIKPFCVILPEIQKPERKIQFKEKVLWTAITLFIFLVCCQIPLFGIMSSDSADPFYW
MRVILASNRGTLMELGISPIVTSGLIMQLLAGAKIIEVGDTPKDRALFNGAQKLFGMIITIG
QSIVYVMTGMYGDPSEMGAGICLLITIQLFVAGLIVLLLDELLQKGYGLGSGISLFIATNICE
TIVWKAFSPTTVNTGRGMEFEGAIIALFHLLATRTDKVRALREAFYRQNLPNLMNLIATIF
VFAVVIYFQGFRVDLPIKSARYRGQYNTYPIKLFYTSNIPIILQSALVSNLYVISQMLSARFSG
NLLVSLLGTWSDTSSGGPARAYPVGGLCYYLSPPESFGSVLEDPVHAVVYIVFMLGSCAFF
SKTWIEVSGSSAKDVAKQLKEQQMVMRGHRETSMVHELNRYIPTAAAFGGLCIGALSVL
ADFLGAIGSGTGILLAVTIIYQYFEIFVKEQSEVGSMGALLF

>Q9NP90 lipid-anchor
MSGKSLLLKVILLGDGGVGKSSLMNRYVTNKFDSQAFHTIGVEFLNRDLEVDGRFVTLQI
WDTAGQERFKSLRTPFYRGADCCLLTFSVDDRQSFENLGNWQKEFIYYADVKDPEHFPFV
VLGNKVDKEDRQVTTEEAQTWCMENGDYPYLETSAKDDTNVTVAFEEAVRQVLAVEEQ
LEHCMLGHTIDLNSGSKAGSSCC

>Q8NER5 single-pass type I membrane protein
MTRALCSALRQALLLLAAAAELSPGLKCVCLLCDSSNFTCQTEGACWASVMLTNGKEQV
IKSCVSLPELNAQVFCHSSNNVTKTECCFTDFCNNITLHLPTASPNAPKLGPMELAIIITVPV
CLLSIAAMLTVWACQGRQCSYRKKKRPNVEEPLSECNLVNAGKTLKDLIYDVTASGSGSG
LPLLVQRTIARTIVLQEIVGKGRFGEVWHGRWCGEDVAVKIFSSRDERSWFREAEIYQTVM
LRHENILGFIAADNKDNGTWTQLWLVSEYHEQGSLYDYLNRNIVTVAGMIKLALSIASGL
AHLHMEIVGTQGKPAIAHRDIKSKNILVKKCETCAIADLGLAVKHDSILNTIDIPQNPKVGT
KRYMAPEMLDDTMNVNIFESFKRADIYSVGLVYWEIARRCSVGGIVEEYQLPYYDMVPS
DPSIEEMRKVVCDQKFRPSIPNQWQSCEALRVMGRIMRECWYANGAARLTALRIKKTISQ
LCVKEDCKA

>P57729 lipid-anchor

MQAPHKEHLYKLLVIGDLGVGKTSIIKRYVHQNFSSHYRATIGVDFALKVLHWDPETVVR
LQLWDIAGQERFGNMTRVYYREAMGAFIVFDVTRPATFEAVAKWKNDLDSKLSLPNGKP
VSVVLLANKCDQGKDVLMNNGLKMDQFCKEHGFVGWFETSAKENINIDEASRCLVKHIL
ANECDLMESIEPDVVKPHLTSTKVASCSGCAKS

>Q9NYM4 multipass membrane protein
MVPHLLLLCLLPLVRATEPHEGRADEQSAEAALAVPNASHFFSWNNYTFSDWQNFVGRR
RYGAESQNPTVKALLIVAYSFIIVFSLFGNVLVCHVIFKNQRMHSATSLFIVNLAVADIMITL
LNTPFTLVRFVNSTWIFGKGMCHVSRFAQYCSLHVSALTLTAIAVDRHQVIMHPLKPRISIT
KGVIYIAVIWTMATFFSLPHAICQKLFTFKYSEDIVRSLCLPDFPEPADLFWKYLDLATFILL
YILPLLIISVAYARVAKKLWLCNMIGDVTTEQYFALRRKKKKTIKMLMLVVVLFALCWFPL
NCYVLLLSSKVIRTNNALYFAFHWFAMSSTCYNPFIYCWLNENFRIELKALLSMCQRPPKP
QEDRPPSPVPSFRVAWTEKNDGQRAPLANNLLPTSQLQSGKTDLSSVEPIVTMS

>P41968 multipass membrane protein
MNASCCLPSVQPTLPNGSEHLQAPFFSNQSSSAFCEQVFIKPEVFLSLGIVSLLENILVILAV
VRNGNLHSPMYFFLCSLAVADMLVSVSNALETIMIAIVHSDYLTFEDQFIQHMDNIFDSMI
CISLVASICNLLAIAVDRYVTIFYALRYHSIMTVRKALTLIVAIWVCCGVCGVVFIVYSESKM
VIVCLITMFFAMMLLMGTLYVHMFLFARLHVKRIAALPPADGVAPQQHSCMKGAVTITILL
GVFIFCWAPFFLHLVLIITCPTNPYCICYTAHFNTYLVLIMCNSVIDPLIYAFRSLELRNTFREI
LCGCNGMNLG

>Q8N2H4 multipass membrane protein
MAGQFRSYVWDPLLILSQIVLMQTVYYGSLGLWLALVDGLVRSSPSLDQMFDAEILGFST
PPGRLSMMSFILNALTCALGLLYFIRRGKQCLDFTVTVHFFHLLGCWFYSSRFPSALTWWL
VQAVCIALMAVIGEYLCMRTELKEIPLNSAPKSNV

>Q01113 single-pass type I membrane protein
MGLGRCIWEGWTLESEALRRDMGTWLLACICICTCVCLGVSVTGEGQGPRSRTFTCLTNN
ILRIDCHWSAPELGQGSSPWLLFTSNQAPGGTHKCILRGSECTVVLPPEAVLVPSDNFTITF
HHCMSGREQVSLVDPEYLPRRHVKLDPPSDLQSNISSGHCILTWSISPALEPMTTLLSYELA
FKKQEEAWEQAQHRDHIVGVTWLILEAFELDPGFIHEARLRVQMATLEDDVVEEERYTG
QWSEWSQPVCFQAPQRQGPLIPPWGWPGNTLVAVSIFLLLTGPTYLLFKLSPRVKRIFYQN
VPSPAMFFQPLYSVHNGNFQTWMGAHGAGVLLSQDCAGTPQGALEPCVQEATALLTCGP
ARPWKSVALEEEQEGPGTRLPGNLSSEDVLPAGCTEWRVQTLAYLPQEDWAPTSLTRPAPP
DSEGSRSSSSSSSSNNNNNYCALGCYGGWHLSALPGNTQSSGPIPALACGLSCDHQGLETQ
QGVAWVLAGHCQRPGLHEDLQGMLLPSVLSKARSWTF

>O95274 GPI-anchor, lipid-anchor
MDPARKAGAQAMIWTAGWLLLLLLLRGGAQALECYSCVQKADDGCSPNKMKTVKCAPG
VDVCTEAVGAVETIHGQFSLAVRGCGSGLPGKNDRGLDLHGLLAFIQLQQCAQDRCNAK
LNLTSRALDPAGNESAYPPNGVECYSCVGLSREACQGTSPPVVSCYNASDHVYKGCFDGN
VTLTAANVTVSLPVRGCVQDEFCTRDGVTGPGFTLSGSCCQGSRCNSDLRNKTYFSPRIPP
LVRLPPPEPTTVASTTSVTTSTSAPVRPTSTTKPMPAPTSQTPRQGVEHEASRDEEPRLTGG

AAGHQDRSNSGQYPAKGGPQQPHNKGCVAPTAGLAALLLAVAAGVLL

>Q13410 single-pass type I membrane protein
MAVFPSSGLPRCLLTLILLQLPKLDSAPFDVIGPPEPILAVVGEDAELPCRLSPNASAEHLEL
RWFRKKVSPAVLVHRDGREQEAEQMPEYRGRATLVQDGIAKGRVALRIRGVRVSDDGEY
TCFFREDGSYEEALVHLKVAALGSDPHISMQVQENGEICLECTSVGWYPEPQVQWRTSKG
EKFPSTSESRNPDEEGLFTVAASVIIRDTSAKNVSCYIQNLLLGQEKKVEISIPASSLPRLTPW
IVAVAVILMVLGLLTIGSIFFTWRLYNERPRERRNEFSSKERLLEELKWKKATLHAVDVTLD
PDTAHPHLFLYEDSKSVRLEDSRQKLPEKTERFDSWPCVLGRETFTSGRHYWEVEVGDRT
DWAIGVCRENVMKKGFDPMTPENGFWAVELYGNGYWALTPLRTPLPLAGPPRRVGIFLDY
ESGDISFYNMNDGSDIYTFSNVTFSGPLRPFFCLWSSGKKPLTICPIADGPERVTVIANAQDL
SKEIPLSPMGEDSAPRDADTLHSKLIPTQPSQGAP

>Q14916 multipass membrane protein
MQMDNRLPPKKVPGFCSFRYGLSFLVHCCNVIITAQRACLNLTMVVMVNSTDPHGLPNTS
TKKLLDNIKNPMYNWSPDIQGIILSSTSYGVIIIQVPVGYFSGIYSTKKMIGFALCLSSVLSL
LIPPAAGIGVAWVVVCRAVQGAAQGIVATAQFEIYVKWAPPLERGRLTSMSTSGFLLGPFIV
LLVTGVICESLGWPMVFYIFGACGCAVCLLWFVLFYDDPKDHPCISISEKEYITSSLVQQVS
SSRQSLPIKAILKSLPVWAISTGSFTFFWSHNIMTLYTPMFINSMLHVNIKENGFLSSLPYLF
AWICGNLAGQLSDFFLTRNILSVIAVRKLFTAAGFLLPAIFGVCLPYLSSTFYSIVIFLILAGA
TGSFCLGGVFINGLDIAPRYFGFIKACSTLTGMIGGLIASTLTGLILKQDPESAWFKTFILMA
AINVTGLIFYLIVATAEIQDWAKEKQHTRL

>Q5T3U5 multipass membrane protein
MERLLAQLCGSSAAWPLPLWEGDTTGHCFTQLVLSALPHALLAVLSACYLGTPRSPDYILP
CSPGWRLRLAASFLLSVFPLLDLLPVALPPGAGPGPIGLEVLAGCVAAVAWISHSLALWVL
AHSPHGHSRGPLALALVALLPAPALVLTVLWHCQRGTLLPPLLPGPMARLCLLILQLAALL
AYALGWAAPGGPREPWAQEPLLPEDQEPEVAEDGESWLSRFSYAWLAPLLARGACGELR
QPQDICRLPHRLQPTYLARVFQAHWQEGARLWRALYGAFGRCYLALGLLKLVGTMLGFS
GPLLLSLLVGFLEEGQEPLSHGLLYALGLAGGAVLGAVLQNQYGYEVYKVTLQARGAVLN
ILYCKALQLGPSRPPTGEALNLLGTDSERLLNFAGSFHEAWGLPLQLAITLYLLYQQVGVA
FVGGLILALLLVPVNKVIATRIMASNQEMLQHKDARVKLVTELLSGIRVIKFCGWEQALGA
RVEACRARELGRLRVIKYLDAACVYLWAALPVVISIVIFITYVLMGHQLTATKVFTALALV
RMLILPLNNFPWVINGLLEAKVSLDRIQLFLDLPNHNPQAYYSPDPPAEPSTVLELHGALFS
WDPVGTSLETFISHLEVKKGMLVGIVGKVGCGKSSLLAAIAGELHRLRGHVAVRGLSKGF
GLATQEPWIQFATIRDNILFGKTFDAQLYKEVLEACALNDDLSILPAGDQTEVGEKGVTLS
GGQRARIALARAVYQEKELYLLDDPLAAVDADVANHLLHRCILGMLSYTTRLLCTHRTEY
LERADAVLLMEAGRLIRAGPPSEILPLVQAVPKAWAENGQESDSATAQSVQNPEKTKEGLE
EEQSTSGRLLQEESKKEGAVALHVYQAYWKAVGQGLALAILFSLLLMQATRNAADWWLS
HWISQLKAENSSQEAQPSTSPASMGLFSPQLLLFSPGNLYIPVFPLPKAAPNGSSDIRFYLTV
YATIAGVNSLCTLLRAVLFAAGTLQAAATLHRRLLHRVLMAPVTFFNATPTGRILNRFSSDV
ACADDSLPFILNILLANAAGLLGLLAVLGSGLPWLLLLLPPLSIMYYHVQRHYRASSRELR
RLGSLTLSPLYSHLADTLAGLSVLRATGATYRFEEENLRLLELNQRCQFATSATMQWLDIR
LQLMGAAVVSAIAGIALVQHQQGLANPGLVGLSLSYALSLTGLLSGLVSSFTQTEAMLVSV

ERLEEYTCDLPQEPQGQPLQLGTGWLTQGGVEFQDVVLAYRPGLPNALDGVTFCVQPGE
KLGIVGRTGSGKSSLLLVLFRLLEPSSGRVLLDGVDTSQLELAQLRSQLAIIPQEPFLFSGTV
RENLDPQGLHKDRALWQALKQCHLSEVITSMGGLDGELGEGGRSLSLGQRQLLCLARAL
LTDAKILCIDEATASVDQKTDQLLQQTICKRFANKTVLTIAHRLNTILNSDRVLVLQAGRVV
ELDSPATLRNQPHSLFQQLLQSSQQGVPASLGGP

>P43307 single-pass type I membrane protein
MRLLPRLLLLLLLVFPATVLFRGGPRGLLAVAQDLTEDEETVEDSIIEDEDDEAEVEEDEPT
DLVEDKEEEDVSGEPEASPSADTTILFVKGEDFPANNIVKFLVGFTNKGTEDFIVESLDASF
RYPQDYQFYIQNFTALPLNTVVPPQRQATFEYSFIPAEPMGGRPFGLVINLNYKDLNGNVF
QDAVFNQTVTVIEREDGLDGETIFMYMFLAGLGLLVIVGLHQLLESRKRKRPIQKVEMGT
SSQNDVDMSWIPQETLNQINKASPRRLPRKRAQKRSVGSDE

>O14944 single-pass type I membrane protein
MTAGRRMEMLCAGRVPALLLCLGFHLLQAVLSTTVIPSCIPGESSDNCTALVQTEDNPRVA
QVSITKCSSDMNGYCLHGQCIYLVDMSQNYCRCEVGYTGVRCEHFFLTVHQPLSKEYVAL
TVILIILFLITVVGSTYYFCRWYRNRKSKEPKKEYERVTSGDPELPQV

>Q9P0I2 multipass membrane protein
MAGPELLLDSNIRLWVVLPIVIITFFVGMIRHYVSILLQSDKKLTQEQVSDSQVLIRSRVLRE
NGKYIPKQSFLTRKYYFNNPEDGFFKKTKRKVVPPSPMTDPTMLTDMMKGNVTNVLPMI
LIGGWINMTFSGFVTTKVPFPLTLRFKPMLQQGIELLTLDASWVSSASWYFLNVFGLRSIY
SLILGQDNAADQSRMMQEQMTGAAMAMPADTNKAFKTEWEALELTDHQWALDDVEEE
LMAKDLHFEGMFKKELQTSIF

>P10114 lipid-anchor
MREYKVVVLGSGGVGKSALTVQFVTGTFIEKYDPTIEDFYRKEIEVDSSPSVLEILDTAGTE
QFASMRDLYIKNGQGFILVYSLVNQQSFQDIKPMRDQIIRVKRYEKVPVILVGNKVDLESER
EVSSSEGRALAEEWGCPFMETSAKSKTMVDELFAEIVRQMNYAAQPDKDDPCCSACNIQ

>Q12908 multipass membrane protein
MNDPNSCVDNATVCSGASCVVPESNFNNILSVVLSTVLTILLALVMFSMGCNVEIKKFLGH
IKRPWGICVGFLCQFGIMPLTGFILSVAFDILPLQAVVVLIIGCCPGGTASNILAYWVDGDM
DLSVSMTTCSTLLALGMMPLCLLIYTKMWVDSGSIVIPYDNIGTSLVSLVVPVSIGMFVNH
KWPQKAKIILKIGSIAGAILIVLIAVVGGILYQSAWIIAPKLWIIGTIFPVAGYSLGFLLARIAG
LPWYRCRTVAFETGMQNTQLCSTIVQLSFTPEELNVVFTFPLIYSIFQLAFAAIFLGFYVAYK
KCHGKNKAEIPESKENGTEPESSFYKANGGFQPDEK

>P52799 single-pass type I membrane protein
MAVRRDSVWKYCWGVLMVLCRTAISKSIVLEPIYWNSSNSKFLPGQGLVLYPQIGDKLDII
CPKVDSKTVGQYEYYKVYMVDKDQADRCTIKKENTPLLNCAKPDQDIKFTIKFQEFSPNL
WGLEFQKNKDYYIISTSNGSLEGLDNQEGGVCQTRAMKILMKVGQDASSAGSTRNKDPT
RRPELEAGTNGRSSTTSPFVKPNPGSSTDGNSAGHSGNNILGSEVALFAGIASGCIIFIVIIITL
VVLLLKYRRRHRKHSPQHTTTLSLSTLATPKRSGNNNGSEPSDIIIPLRTADSVFCPHYEKV

SGDYGHPVYIVQEMPPQSPANIYYKV

>O76082 multipass membrane protein
MRDYDEVTAFLGEWGPFQRLIFFLLSASIIPNGFTGLSSVFLIATPEHRCRVPDAANLSSAW
RNHTVPLRLRDGREVPHSCRRYRLATIANFSALGLEPGRDVDLGQLEQESCLDGWEFSQD
VYLSTIVTEWNLVCEDDWKAPLTISLFFVGVLLGSFISGQLSDRFGRKNVLFVTMGMQTG
FSFLQIFSKNFEMFVVLFVLVGMGQISNYVAAFVLGTEILGKSVRIIFSTLGVCIFYAFGYM
VLPLFAYFIRDWRMLLVALTMPGVLCVALWWFIPESPRWLISQGRFEEAEVIIRKAAKANGI
VVPSTIFDPSELQDLSSKKQQSHNILDLLRTWNIRMVTIMSIMLWMTISVGYFGLSLDTPNL
HGDIFVNCFLSAMVEVPAYVLAWLLLQYLPRRYSMATALFLGGSVLLFMQLVPPDLYYLA
TVLVMVGKFGVTAAFSMVYVYTAELYPTVVRNMGVGVSSTASRLGSILSPYFVYLGAYD
RFLPYILMGSLTILTAILTLFLPESFGTPLPDTIDQMLRVKGMKHRKTPSHTRMLKDGQERP
TILKSTAF

>P43116 multipass membrane protein
MGNASNDSQSEDCETRQWLPPGESPAISSVMFSAGVLGNLIALALLARRWRGDVGCSAG
RRSSLSLFHVLVTELVFTDLLGTCLISPVVLASYARNQTLVALAPESRACTYFAFAMTFFSL
ATMLMLFAMALERYLSIGHPYFYQRRVSRSGGLAVLPVIYAVSLLFCSLPLLDYGQYVQYC
PGTWCFIRHGRTAYLQLYATLLLLLIVSVLACNFSVILNLIRMHRRSRRSRCGPSLGSGRGG
PGARRRGERVSMAEETDHLILLAIMTITFAVCSLPFTIFAYMNETSSRKEKWDLQALRFLSI
NSIIDPWVFAILRPPVLRLMRSVLCCRISLRTQDATQTSCSTQSDASKQADL

>Q9NZ52 peripheral membrane protein
MAEAEGESLESWLNKATNPSNRQEDWEYIIGFCDQINKELEGPQIAVRLLAHKIQSPQEWE
ALQALTVLEACMKNCGRRFHNEVGKFRFLNELIKVVSPKYLGDRVSEKVKTKVIELLYSW
TMALPEEAKIKDAYHMLKRQGIVQSDPPIPVDRTLIPSPPPRPKNPVFDDEEKSKLLAKLLK
SKNPDDLQEANKLIKSMVKEDEARIQKVTKRLHTLEEVNNNVRLLSEMLLHYSQEDSSD
GDRELMKELFDQCENKRRTLFKLASETEDNDNSLGDILQASDNLSRVINSYKTIIEGQVIN
GEVATLTLPDSEGNSQCSNQGTLIDLAELDTTNSLSSVLAPAPTPPSSGIPILPPPPQASGPPR
SRSSSQAEATLGPSSTSNALSWLDEELLCLGLADPAPNVPPKESAGNSQWHLLQREQSDL
DFFSPRPGTAACGASDAPLLQPSAPSSSSSQAPLPPPFPAPVVPASVPAPSAGSSLFSTGVAPA
LAPKVEPAVPGHHGLALGNSALHHLDALDQLLEEAKVTSGLVKPTTSPLIPTTTPARPLLPF
STGPGSPLFQPLSFQSQGSPPKGPELSLASIHVPLESIKPSSALPVTAYDKNGFRILFHFAKEC
PPGRPDVLVVVVSMLNTAPLPVKSIVLQAAVPKSMKVKLQPPSGTELSPFSPIQPPAAITQV
MLLANPLKEKVRLRYKLTFALGEQLSTEVGEVDQFPPVEQWGNL

>P41273 single-pass type II membrane protein
MEYASDASLDPEAPWPPAPRARACRVLPWALVAGLLLLLLLAAACAVFLACPWAVSGARA
SPGSAASPRLREGPELSPDDPAGLLDLRQGMFAQLVAQNVLLIDGPLSWYSDPGLAGVSLT
GGLSYKEDTKELVVAKAGVYYVFFQLELRRVVAGEGSGSVSLALHLQPLRSAAGAAALA
LTVDLPPASSEARNSAFGFQGRLLHLSAGQRLGVHLHTEARARHAWQLTQGATVLGLFRV
TPEIPAGLPSPRSE

>P32970 single-pass type II membrane protein

MPEEGSGCSVRRRPYGCVLRAALVPLVAGLVICLVVCIQRFAQAQQQLPLESLGWDVAEL
QLNHTGPQQDPRLYWQGGPALGRSFLHGPELDKGQLRIHRDGIYMVHIQVTLAICSSTTAS
RHHPTTLAVGICSPASRSISLLRLSFHQGCTIASQRLTPLARGDTLCTNLTGTLLPSRNTDET
FFGVQWVRP

>O43557 single-pass type II membrane protein
MEESVVRPSVFVVDGQTDIPFTRLGRSHRRQSCSVARVGLGLLLLLMGAGLAVQGWFLL
QLHWRLGEMVTRLPDGPAGSWEQLIQERRSHEVNPAAHLTGANSSLTGSGGPLLWETQL
GLAFLRGLSYHDGALVVTKAGYYYIYSKVQLGGVGCPLGLASTITHGLYKRTPRYPEELE
LLVSQQSPCGRATSSSRVWWDSSFLGGVVHLEAGEKVVVRVLDERLVRLRDGTRSYFGAF
MV

>Q92797 peripheral membrane protein
MASGSGDSVTRRSVASQFFTQEEGPGIDGMTTSERVVDLLNQAALITNDSKITVLKQVQEL
IINKDPTLLDNFLDEIIAFQADKSIEVRKFVIGFIEEACKRDIELLLKLIANLNMLLRDENVN
VVKKAILTMTQLYKVALQWMVKSRVISELQEACWDMVSAMAGDIILLLDSDNDGIRTHAI
KFVEGLIVTLSPRMADSEIPRRQEHDISLDRIPRDHPYIQYNVLWEEGKAALEQLLKFMVH
PAISSINLTTALGSLANIARQRPMFMSEVIQAYETLHANLPPTLAKSQVSSVRKNLKLHLLS
VLKHPASLEFQAQITTLLVDLGTPQAEIARNMPSSKDTRKRPRDDSDSTLKKMKLEPNLGE
DDEDKDLEPGPSGTSKASAQISGQSDTDITAEFLQPLLTPDNVANLVLISMVYLPEAMPASF
QAIYTPVESAGTEAQIKHLARLMATQMTAAGLGPGVEQTKQCKEEPKEEKVVKTESVLIK
RRLSAQGQAISVVGSLSSMSPLEEEAPQAKRRPEPIIPVTQPRLAGAGGRKKIFRLSDVLKP
LTDAQVEAMKLGAVKRILRAEKAVACSGAAQVRIKILASLVTQFNSGLKAEVLSFILEDVR
ARLDLAFAWLYQEYNAYLAAGASGSLDKYEDCLIRLLSGLQEKPDQKDGIFTKVVLEAPL
ITESALEVVRKYCEDESRTYLGMSTLRDLIFKRPSRQFQYLHVLLDLSSHEKDKVRSQALL
FIKRMYEKEQLREYVEKFALNYLQLLVHPNPPSVLFGADKDTEVAAPWTEETVKQCLYLY
LALLPQNHKLIHELAAVYTEAIADIKRTVLRVIEQPIRGMGMNSPELLLLVENCPKGAETLV
TRCLHSLTDKVPPSPELVKRVRDLYHKRLPDVRFLIPVLNGLEKKEVIQALPKLIKLNPIVV
KEVFNRLLGTQHGEGNSALSPLNPGELLIALHNIDSVKCDMKSIIKATNLCFAERNVYTSE
VLAVVMQQLMEQSPLPMLLMRTVIQSLTMYPRLGGFVMNILSRLIMKQVWKYPKVWEG
FIKCCQRTKPQSFQVILQLPPQQLGAVFDKCPELREPLLAHVRSFTPHQQAHIPNSIMTILEA
SGKQEPEAKEAPAGPLEEDDLEPLTLAPAPAPRPPQDLIGLRLAQEKALKRQLEEEQKLKP
GGVGAPSSSSPSPSPSARPGPPPSEEAMDFREEGPECETPGIFISMDDDSGLTEAALLDSSLE
GPLPKETAAGGLTLKEERSPQTLAPVGEDAMKTPSPAAEDAREPEAKGNS

>Q9NPY3 single-pass type I membrane protein
MATSMGLLLLLLLLLTQPGAGTGADTEAVVCVGTACYTAHSGKLSAAEAQNHCNQNGG
NLATVKSKEEAQHVQRVLAQLLRREAALTARMSKFWIGLQREKGKCLDPSLPLKGFSWV
GGGEDTPYSNWHKELRNSCISKRCVSLLLDLSQPLLPSRLPKWSEGPCGSPGSPGSNIEGF
VCKFSFKGMCRPLALGGPGQVTYTTPFQTTSSSLEAVPFASAANVACGEGDKDETQSHYF
LCKEKAPDVFDWGSSGPLCVSPKYGCNFNNGGCHQDCFEGGDGSFLCGCRPGFRLLDDL
VTCASRNPCSSSPCRGGATCVLGPHGKNYTCRCPQGYQLDSSQLDCVDVDECQDSPCAQ
ECVNTPGGFRCECWVGYEPGGPGEGACQDVDECALGRSPCAQGCTNTDGSFHCSCEEGY
VLAGEDGTQCQDVDECVGPGGPLCDSLCFNTQGSFHCGCLPGWVLAPNGVSCTMGPVS

LGPPSGPPDEEDKGEKEGSTVPRAATASPTRGPEGTPKATPTTSRPSLSSDAPITSAPLKML
APSGSPGVWREPSIHHATAASGPQEPAGGDSSVATQNNDGTDGQKLLLFYILGTVVAILLL
LALALGLLVYRKRRAKREEKKEKKPQNAADSYSWVPERAESRAMENQYSPTPGTDC

>Q9NUS5 peripheral membrane protein
MVHAFLIHTLRAPNTEDTGLCRVLYSCVFGAEKSPDDPRPHGAERDRLLRKEQILAVARQ
VESMCRLQQQASGRPPMDLQPQSSDEQVPLHEAPRGAFRLAAENPFQEPRTVVWLGVLS
LGFALVLDAHENLLLAEGTLRLLTRLLLDHLRLLAPSTSLLLRADRIEGILTRFLPHGQLLFL
NDQFVQGLEKEFSAAWPR

>Q9UJQ1 single-pass type I membrane protein
MDLQGRGVPSIDRLRVLLMLFHTMAQIMAEQEVENLSGLSTNPEKDIFVVRENGTTCLM
AEFAAKFIVPYDVWASNYVDLITEQADIALTRGAEVKGRCGHSQSELQVFWVDRAYALK
MLFVKESHNMSKGPEATWRLSKVQFVYDSSEKTHFKDAVSAGKHTANSHHLSALVTPAG
KSYECQAQQTISLASSDPQKTVTMILSAVHIQPFDIISDFVFSEEHKCPVDEREQLEETLPLIL
GLILGLVIMVTLAIYHVHHKMTANQVQIPRDRSQYKHMG

>O95977 multipass membrane protein
MNATGTPVAPESCQQLAAGGHSRLIVLHYNHSGRLAGRGGPEDGGLGALRGLSVAASCL
VVLENLLVLAAITSHMRSRRWVYYCLVNITLSDLLTGAAYLANVLLSGARTFRLAPAQWF
LREGLLFTALAASTFSLLFTAGERFATMVRPVAESGATKTSRVYGFIGLCWLLAALLGMLP
LLGWNCLCAFDRCSSLLPLYSKRYILFCLVIFAGVLATIMGLYGAIFRLVQASGQKAPRPAA
RRKARRLLKTVLMILLAFLVCWGPLFGLLLADVFGSNLWAQEYLRGMDWILALAVLNSA
VNPIIYSFRSREVCRAVLSFLCCGCLRLGMRGPGDCLARAVEAHSGASTTDSSLRPRDSFR
GSRSLSFRMREPLSSISSVRSI

>Q9Y5R2 single-pass type I membrane protein
MPRSRGGRAAPGPPPPPPPPGQAPRWSRWRVPGRLLLLLLPALCCLPGAARAAAAAAGA
GNRAAVAVAVARADEAEAPFAGQNWLKSYGYLLPYDSRASALHSAKALQSAVSTMQQFY
GIPVTGVLDQTTIEWMKKPRCGVPDHPHLSRRRRNKRYALTGQKWRQKHITYSIHNYTPK
VGELDTRKAIRQAFDVWQKVTPLTFEEVPYHEIKSDRKEADIMIFFASGFHGDSSPFDGEG
GFLAHAYFPGPGIGGDTHFDSDEPWTLGNANHDGNDLFLVAVHELGHALGLEHSSDPSAI
MAPFYQYMETHNFKLPQDDLQGIQKIYGPPAEPLEPTRPLPTLPVRRIHSPSERKHERQPRP
PRPPLGDRPSTPGTKPNICDGNFNTVALFRGEMFVFKDRWFWRLRNNRVQEGYPMQIEQF
WKGLPARIDAAYERADGRFVFFKGDKYWVFKEVTVEPGYPHSLGELGSCLPREGIDTALR
WEPVGKTYFFKGERYWRYSEERRATDPGYPKPITVWKGIPQAPQGAFISKEGYYTYFYKG
RDYWKFDNQKLSVEPGYPRNILRDWMGCNQKEVERRKERRLPQDDVDIMVTINDVPGS
VNAVAVVIPCILSLCILVLVYTIFQFKNKTGPQPVTYYKRPVQEWV

>Q9H665 single-pass type I membrane protein
MGPGRCLLTALLLLALAPPPEASQYCGRLEYWNPDNKCCSSCLQRFGPPPCPDYEFRENC
GLNDHGDFVTPPFRKCSSGQCNPDGAELCSPCGGGAVTPTPAAGGGRTPWRCRERPVPAK
GHCPLTPGNPGAPSSQERSSPASSIAWRTPEPVPQQAWPNFLPLVVLVLLLTLAVIAILLFILL
WHLCWPKEKADPYPYPGLVCGVPNTHTPSSSHLSSPGALETGDTWKEASLLPLLSRELSS

LASQPLSRLLDELEVLEELIVLLDPEPGPGGGMAHGTTRHLAARYGLPAAWSTFAYSLRPS
RSPLRALIEMVVAREPSASLGQLGTHLAQLGRADALRVLSKLGSSGVCWA

>O15552 multipass membrane protein
MLPDWKSSLILMAYIIIFLTGLPANLLALRAFVGRIRQPQPAPVHILLLSLTLADLLLLLLLPF
KIIEAASNFRWYLPKVVCALTSFGFYSSIYCSTWLLAGISIERYLGVAFPVQYKLSRRPLYG
VIAALVAWVMSFGHCTIVIIVQYLNTTEQVRSGNEITCYENFTDNQLDVVLPVRLELCLVL
FFIPMAVTIFCYWRFVWIMLSQPLVGAQRRRRAVGLAVVTLLNFLVCFGPYNVSHLVGYH
QRKSPWWRSIAVVFSSLNASLDPLLFYFSSSVVRRAFGRGLQVLRNQGSSLLGRRGKDTA
EGTNEDRGVGQGEGMPSSDFTTE

>Q9UBK5 single-pass type I membrane protein
MIHLGHILFLLLLPVAAAQTTPGERSSLPAFYPGTSGSCSGCGSLSLPLLAGLVAADAVASLL
IVGAVFLCARPRRSPAQEDGKVYINMPGRG

>O14842 multipass membrane protein
MDLPPQLSFGLYVAAFALGFPLNVLAIRGATAHARLRLTPSLVYALNLGCSDLLLTVSLPLK
AVEALASGAWPLPASLCPVFAVAHFFPLYAGGGFLAALSAGRYLGAAFPLGYQAFRRPCYS
WGVCAAIWALVLCHLGLVFGLEAPGGWLDHSNTSLGINTPVNGSPVCLEAWDPASAGPA
RFSLSLLLFFLPLAITAFCYVGCLRALARSGLTHRRKLRAAWVAGGALLTLLLCVGPYNAS
NVASFLYPNLGGSWRKLGLITGAWSVVLNPLVTGYLGRGPGLKTVCAARTQGGKSQK

>P32248 multipass membrane protein
MDLGKPMKSVLVVALLVIFQVCLCQDEVTDDYIGDNTTVDYTLFESLCSKKDVRNFKAW
FLPIMYSIICFVGLLGNGLVVLTYIYFKRLKTMTDTYLLNLAVADILFLLTLPFWAYSAAKS
WVFGVHFCKLIFAIYKMSFFSGMLLLLCISIDRYVAIVQAVSAHRHRARVLLISKLSCVGIW
ILATVLSIPELLYSDLQRSSSEQAMRCSLITEHVEAFITIQVAQMVIGFLVPLLAMSFCYLVII
RTLLQARNFERNKAIKVIIAVVVVFIVFQLPYNGVVLAQTVANFNITSSTCELSKQLNIAYD
VTYSLACVRCCVNPFLYAFIGVKFRNDLFKLFKDLGCLSQEQLRQWSSCRHIRRSSMSVE
AETTTTFSP

>P10301 lipid-anchor
MSSGAASGTGRGRPRGGGPGPGDPPPSETHKLVVVGGGGVGKSALTIQFIQSYFVSDYDP
TIEDSYTKICSVDGIPARLDILDTAGQEEFGAMREQYMRAGHGFLLVFAINDRQSFNEVGK
LFTQILRVKDRDDFPVVLVGNKADLESQRQVPRSEASAFGASHHVAYFEASAKLRLNVDE
AFEQLVRAVRKYQEQELPPSPPSAPRKKGGGCPCVLL

>O14669 single-pass type I membrane protein
MRGHPSLLLLYMALTTCLDTSPSEETDQEVFLGPPEAQSFLSSHTRIPRANHWDLELLTPG
NLERECLEERCSWEEAREYFEDNTLTERFWESYIYNGKGGRGRVDVASLAVGLTGGILLIV
LAGLGAFWYLRWRQHRGQQPCPQEAGLISPLSPLNPLGPPTPLPPPPPPPPGLPTYEQALA
ASGVHDAPPPPYTSLRRPH

>P78381 multipass membrane protein

MAAVGAGGSTAAPGPGAVSAGALEPGTASAAHRRLKYISLAVLVVQNASLILSIRYARTLP
GDRFFATTAVVMAEVLKGLTCLLLLFAQKRGNVKHLVLFLHEAVLVQYVDTLKLAVPSLIY
TLQNNLQYVAISNLPAATFQVTYQLKILTTALFSVLMLNRSLSRLQWASLLLLFTGVAIVQA
QQAGGGGPRPLDQNPGAGLAAVVASCLSSGFAGVYFEKILKGSSGSVWLRNLQLGLFGTA
LGLVGLWWAEGTAVATRGFFFGYTPAVWGVVLNQAFGGLLVAVVVKYADNILKGFATSLSI
VLSTVASIRLFGFHVDPLFALGAGLVIGAVYLYSLPRGAAKAIASASASASGPCVHQQPPGQ
PPPPQLSSHRGDLITEPFLPKLLTKVKGS

>Q9BX95 multipass membrane protein
MSLRQRLAQLVGRLQDPQKVARFQRLCGVEAPPRRSADRREDEKAEAPLAGDPRLRGRQ
PGAPGGPQPPGSDRNQCPAKPDGGGAPNGVRNGLAAELGPASPRRAGALRRNSLTGEEG
QLARVSNWPLYCLFCFGTELGNELFYILFFPFWIWNLDPLVGRRLVVIWVLVMYLGQCTK
DIIRWPRPASPPVVKLEVFYNSEYSMPSTHAMSGTAIPISMVLLTYGRWQYPLIYGLILIPC
WCSLVCLSRIYMGMHSILDIIAGFLYTILILAVFYPFVDLIDNFNQTHKYAPFIIIGLHLALGIF
SFTLDTWSTSRGDTAEILGSGAGIACGSHVTYNMGLVLDPSLDTLPLAGPPITVTLFGKAIL
RILIGMVFVLIIRDVMKKITIPLACKIFNIPCDDIRKARQHMEVELPYRYITYGMVGFSITFF
VPYIFFFIGIS

>P22794 single-pass type I membrane protein
MPTDMEHTGHYLHLAFLMTTVFSLSPGTKANYTRLWANSTSSWDSVIQNKTGRNQNENI
NTNPITPEVDYKGNSTNMPETSHIVALTSKSEQELYIPSVVSNSPSTVQSIENTSKSHGEIFK
KDVCAENNNNMAMLICLIIIAVLFLICTFLFLSTVVLANKVSSLRRSKQVGKRQPRSNGDF
LASGLWPAESDTWKRTKQLTGPNLVMQSTGVLTATRERKDEEGTEKLTNKQIG

>P23515 GPI-anchor, lipid-anchor
MEYQILKMSLCLFILLFLTPGILCICPLQCICTERHRHVDCSGRNLSTLPSGLQENIIHLNLSY
NHFTDLHNQLTQYTNLRTLDISNNRLESLPAHLPRSLWNMSAANNNIKLLDKSDTAYQWN
LKYLDVSKNMLEKVVLIKNTLRSLEVLNLSSNKLWTVPTNMPSKLHIVDLSNNSLTQILPG
TLINLTNLTHLYLHNNKFTFIPDQSFDQLFQLQEITLYNNRWSCDHKQNITYLLKWMMETK
AHVIGTPCSTQISSLKEHNMYPTPSGFTSSLFTVSGMQTVDTINSLSVVTQPKVTKIPKQYR
TKETTFGATLSKDTTFTSTDKAFVPYPEDTSTETINSHEAAAATLTIHLQDGMVTNTSLTSS
TKSSPTPMTLSITSGMPNNFSEMPQQSTTLNLWREETTTNVKTPLPSVANAWKVNASFLLL
LNVVVMLAV

>Q9BQI0 peripheral membrane protein
MSGELSNRFQGGKAFGLLKARQERRLAEINREFLCDQKYSDEENLPEKLTAFKEKYMEFD
LNNEGEIDLMSLKRMMEKLGVPKTHLEMKKMISEVTGGVSDTISYRDFVNMMLGKRSA
VLKLVMMFEGKANESSPKPVGPPPERDIASLP

>P27824 single-pass type I membrane protein
MEGKWLLCMLLVLGTAIVEAHDGHDDDVIDIEDDLDDVIEEVEDSKPDTTAPPSSPKVTY
KAPVPTGEVYFADSFDRGTLSGWILSKAKKDDTDDEIAKYDGKWEVEEMKESKLPGDKG
LVLMSRAKHHAISAKLNKPFLFDTKPLIVQYEVNFQNGIECGGAYVKLLSKTPELNLDQFH
DKTPYTIMFGPDKCGEDYKLHFIFRHKNPKTGIYEEKHAKRPDADLKTYFTDKKTHLYTLI

LNPDNSFEILVDQSVVNSGNLLNDMTPPVNPSREIEDPEDRKPEDWDERPKIPDPEAVKPD
DWDEDAPAKIPDEEATKPEGWLDDEPEYVPDPDAEKPEDWDEDMDGEWEAPQIANPRCE
SAPGCGVWQRPVIDNPNYKGKWKPPMIDNPSYQGIWKPRKIPNPDFFEDLEPFRMTPFSAI
GLELWSMTSDIFFDNFIICADRRIVDDWANDGWGLKKAADGAAEPGVVGQMIEAAEERP
WLWVVYILTVALPVFLVILFCCSGKKQTSGMEYKKTDAPQPDVKEEEEEKEEEKDKGDEE
EEGEEKLEEKQKSDAEEDGGTVSQEEEDRKPKAEEDEILNRSPRNRKPRRE

>P19075 multipass membrane protein
MAGVSACIKYSMFTFNFLFWLCGILILALAIWVRVSNDSQAIFGSEDVGSSSYVAVDILIAV
GAIIMILGFLGCCGAIKESRCMLLLFFIGLLLILLLQVATGILGAVFKSKSDRIVNETLYENTK
LLSATGESEKQFQEAIIVFQEEFKCCGLVNGAADWGNNFQHYPELCACLDKQRPCQSYNG
KQVYKETCISFIKDFLAKNLIIVIGISFGLAVIEILGLVFSMVLYCQIGNK

>O95178 peripheral membrane protein
MSALTRLASFARVGGRLFRSGCARTAGDGGVRHAGGGVHIEPRYRQFPQLTRSQVFQSEF
FSGLMWFWILWRFWHDSEEVLGHFPYPDPSQWTDEELGIPPDDED

>Q9NYW6 multipass membrane protein
MMGLTEGVFLILSGTQFTLGILVNCFIELVNGSSWFKTKRMSLSDFIITTLALLRIILLCIILTD
SFLIEFSPNTHDSGIIMQIIDVSWTFTNHLSIWLATCLGVLYCLKIASFSHPTFLWLKWRVSR
VMVWMLLGALLLSCGSTASLINEFKLYSVFRGIEATRNVTEHFRKKRSEYYLIHVLGTLW
YLPPLIVSLASYSLLIFSLGRHTRQMLQNGTSSRDPTTEAHKRAIRIILSFFFLFLLYFLAFLI
ASFGNFLPKTKMAKMIGEVMTMFYPAGHSFILILGNSKLKQTFVVMLRCESGHLKPGSKG
PIFS

>Q9NYW5 multipass membrane protein
MLRLFYFSAIIASVILNFVGIIMNLFITVVNCKTWVKSHRISSSDRILFSLGITRFLMLGLFLV
NTIYFVSSNTERSVYLSAFFVLCFMFLDSSSVWFVTLLNILYCVKITNFQHSVFLLLKRNISP
KIPRLLLACVLISAFTTCLYITLSQASPFPELVTTRNNTSFNISEGILSLVVSLVLSSSLQFIINV
TSASLLIHSLRRHIQKMQKNATGFWNPQTEAHVGAMKLMVYFLILYIPYSVATLVQYLPFY
AGMDMGTKSICLIFATLYSPGHSVLIIITHPKLKTTAKKILCFKK

>Q9NYW4 multipass membrane protein
MLSAGLGLLMLVAVVEFLIGLIGNGSLVVWSFREWIRKFNWSSYNLIILGLAGCRFLLQWL
IILDLSLFPLFQSSRWLRYLSIFWVLVSQASLWFATFLSVFYCKKITTFDRPAYLWLKQRAYN
LSLWCLLGYFIINLLLTVQIGLTFYHPPQGNSSIRYPFESWQYLYAFQLNSGSYLPLVVFLVS
SGMLIVSLYTHHKKMKVHSAGRRDVRAKAHITALKSLGCFLLLHLVYIMASPFSITSKTYP
PDLTSVFIWETLMAAYPSLHSLILIMGIPRVKQTCQKILWKTVCARRCWGP

>O76099 multipass membrane protein
METGNQTHAQEFLLLGFSATSEIQFILFGLFLSMYLVTFTGNLLIILAICSDSHLHTPMYFFL
SNLSFADLCFTSTTVPKMLLNILTQNKFITYAGCLSQIFFFTSFGCLDNLLLTVMAYDRFVAV
CHPLHYTVIMNPQLCGLLVLGSWCISVMGSLLETLTVLRLSFCTEMEIPHFFCDLLEVLKL
ACSDTFINNVVIYFATGVLGVISFTGIFFSYYKIVFSILRISSAGRKHKAFSTCGSHLSVVTLF

YGTGFGVYLSSAATPSSRTSLVASVMYTMVTPMLNPFIYSLRNTDMKRALGRLLSRATFFN
GDITAGLS

>Q96RI0 multipass membrane protein
MWGRLLLWPLVLGFSLSGGTQTPSVYDESGSTGGGDDSTPSILPAPRGYPGQVCANDSDT
LELPDSSRALLLGWVPTRLVPALYGLVLVVGLPANGLALWVLATQAPRLPSTMLLMNLAA
ADLLLALALPPRIAYHLRGQRWPFGEAACRLATAALYGHMYGSVLLLAAVSLDRYLALVH
PLRARALRGRRLALGLCMAAWLMAAALALPLTLQRQTFRLARSDRVLCHDALPLDAQAS
HWQPAFTCLALLGCFLPLLAMLLCYGATLHTLAASGRRYGHALRLTAVVLASAVAFFVPS
NLLLLLHYSDPSPSAWGNLYGAYVPSLALSTLNSCVDPFIYYYVSAEFRDKVRAGLFQRSP
GDTVASKASAEGGSRGMGTHSSLLQ

>O43761 multipass membrane protein
MEGASFGAGRAGAALDPVSFARRPQTLLRVASWVFSIAVFGPIVNEGYVNTDSGPELRCV
FNGNAGACRFGVALGLGAFLACAAFLLLDVRFQQISSVRDRRRAVLLDLGFSGLWSFLWF
VGFCFLTNQWQRTAPGPATTQAGDAARAAIAFSFFSILSWVALTVKALQRFRLGTDMSLFA
TEQLSTGASQAYPGYPVGSGVEGTETYQSPPFTETLDTSPKGYQVPAY

>Q96S21 lipid-anchor
MGSQGSPVKSYDYLLKFLLVGDSDVGKGEILESLQDGAAESPYAYSNGIDYKTTTILLDGR
RVKLELWDTSGQGRFCTIFRSYSRGAQGILLVYDITNRWSFDGIDRWIKEIDEHAPGVPRIL
VGNRLHLAFKRQVPTEQARAYAEKNCMTFFEVSPLCNFNVIESFTELSRIVLMRHGMEKI
WRPNRVFSLQDLCCRAIVSCTPVHLIDKLPLPVTIKSHLKSFSMANGMNAVMMHGRSYSL
ASGAGGGGSKGNSLKRSKSIRPPQSPPQNCSRSNCKIS

>Q9P2W3 lipid-anchor
MEEWDVPQMKKEVESLKYQLAFQREMASKTIPELLKWIEDGIPKDPFLNPDLMKNNPWV
EKGKCTIL

>Q9BV81 multipass membrane protein
MAAVVAKREGPPFISEAAVRGNAAVLDYCRTSVSALSGATAGILGLTGLYGFIFYLLASVLL
SLLLILKAGRRWNKYFKSRRPLFTGGLIGGLFTYVLFWTFLYGMVHVY

>P47887 multipass membrane protein
MMGQNQTSISDFLLLGLPIQPEQQNLCYALFLAMYLTTLLGNLLIIVLIRLDSHLHTPVYLF
LSNLSFSDLCFSSVTMPKLLQNMQNQDPSIPYADCLTQMYFFLYFSDLESFLLVAMAYDRY
VAICFPMHYTAICFLLHYTAIMSPMLCLSVVALSWVLTTFHAMLHTLLMARLCFCADNVIP
HFFCDMSALLKLACSDTRVNEWVIFIMGGLILVIPFLLILGSYARIVSSILKVPSSKGICKAFS
TCGSHLSVVSLFYGTVIGLYLCPSANSSTLKDTVMAMMYTVVTPMLTPFIYSLRNRDMKG
ALERVICKRKNPFLL

>P61952 lipid-anchor
MPALHIEDLPEKEKLKMEVEQLRKEVKLQRQQVSKCSEEIKNYIEERSGEDPLVKGIPEDK
NPFKEKGSCVIS

>P63211 lipid-anchor
MPVINIEDLTEKDKLKMEVDQLKKEVTLERMLVSKCCEEVRDYVEERSGEDPLVKGIPED
KNPFKELKGGCVIS

>P19440 single-pass type II membrane protein
MKKKLVVLGLLAVVLVLVIVGLCLWLPSASKEPDNHVYTRAAVAADAKQCSKIGRDALR
DGGSAVDAAIAALLCVGLMNAHSMGIGGGLFLTIYNSTTRKAEVINAREVAPRLAFATMF
NSSEQSQKGGLSVAVPGEIRGYELAHQRHGRLPWARLFQPSIQLARQGFPVGKGLAAALE
NKRTVIEQQPVLCEVFCRDRKVLREGERLTLPQLADTYETLAIEGAQAFYNGSLTAQIVKD
IQAAGGIVTAEDLNNYRAELIEHPLNISLGDVVLYMPSAPLSGPVLALILNILKGYNFSRES
VESPEQKGLTYHRIVEAFRFAYAKRTLLGDPKFVDVTEVVRNMTSEFFAAQLRAQISDDTT
HPISYYKPEFYTPDDGGTAHLSVVAEDGSAVSATSTINLYFGSKVRSPVSGILFNNEMDDFS
SPSITNEFGVPPSPANFIQPGKQPLSSMCPTIMVGQDGQVRMVVGAAGGTQITTATALAIIY
NLWFGYDVKRAVEEPRLHNQLLPNVTTVERNIDQAVTAALETRHHHTQIASTFIAVVQAIV
RTAGGWAAASDSRKGGEPAGY

>P19086 lipid-anchor
MGCRQSSEEKEAARRSRRIDRHLRSESQRQRREIKLLLLGTSNSGKSTIVKQMKIIHSGGF
NLEACKEYKPLIIYNAIDSLTRIIRALAALRIDFHNPDRAYDAVQLFALTGPAESKGEITPELL
GVMRRLWADPGAQACFSRSSEYHLEDNAAYYLNDLERIAAADYIPTVEDILRSRDMTTGI
VENKFTFKELTFKMVDVGGQRSERKKWIHCFEGVTAIIFCVELSGYDLKLYEDNQTSRMA
ESLRLFDSICNNNWFINTSLILFLNKKDLLAEKIRRIPLTICFPEYKGQNTYEEAAVYIQRQF
EDLNRNKETKEIYSHFTCATDTSNIQFVFDAVTDVIIQNNLKYIGLC

>Q00587 peripheral membrane protein
MPGPQGGRGAATMSLGKLSPVGWVSSSQGKRRLTADMISHPLGDFRHTMHVGRGGDVF
GDTSFLSNHGGSSGSTHRSPRSFLAKKLQLVRRVGAPPRRMASPPAPSPAPPAISPIIKNAISL
PQLNQAAYDSLVVGKLSFDSSPTSSTDGHSSYGLDSGFCTISRLPRSEKPHDRDRDGSFPSE
PGLRRSDSLLSFRLDLDLGPSLLSELLGVMSLPEAPAAETPAPAANPPAPTANPTGPAANPPA
TTANPPAPAANPSAPAATPTGPAANPPAPAASSTPHGHCPNGVTAGLGPVAEVKSSPVGGG
PRGPAGPALGRHWGAGWDGGHHYPEMDARQERVEVLPQARASWESLDEEWRAPQAGS
RTPVPSTVQANTFEFADAEEDDEVKV

>Q99705 multipass membrane protein
MSVGAMKKGVGRAVGLGGGSGCQATEEDPLPNCGACAPGQGGRRWRLPQPAWVEGSSA
RLWEQATGTGWMDLEASLLPTGPNASNTSDGPDNLTSAGSPPRTGSISYINIIMPSVFGTIC
LLGIIGNSTVIFAVVKKSKLHWCNNVPDIFIINLSVVDLLFLLGMPFMIHQLMGNGVWHFG
ETMCTLITAMDANSQFTSTYILTAMAIDRYLATVHPISSTKFRKPSVATLVICLLWALSFISIT
PVWLYARLIPFPGGAVGCGIRLPNPDTDLYWFTLYQFFLAFALPFVVITAAYVRILQRMTSS
VAPASQRSIRLRTKRVTRTAIAICLVFFVCWAPYYVLQLTQLSISRPTLTFVYLYNAAISLGY
ANSCLNPFVYIVLCETFRKRLVLSVKPAAQGQLRAVSNAQTADEERTESKGT

>O60755 multipass membrane protein

MADAQNISLDSPGSVGAVAVPVVFALIFLLGTVGNGLVLAVLLQPGPSAWQEPGSTTDLFIL
NLAVADLCFILCCVPFQATIYTLDAWLFGALVCKAVHLLIYLTMYASSFTLAAVSVDRYLAV
RHPLRSRALRTPRNARAAVGLVWLLAALFSAPYLSYYGTVRYGALELCVPAWEDARRRA
LDVATFAAGYLLPVAVVSLAYGRTLRFLWAAVGPAGAAAAEARRRATGRAGRAMLAVAA
LYALCWGPHHALILCFWYGRFAFSPATYACRLASHCLAYANSCLNPLVYALASRHFRARFR
RLWPCGRRRRHRARRALRRVRPASSGPPGCPGDARPSGRLLAGGGQGPEPREGPVHGGE
AARGPE

>Q9NYV7 multipass membrane protein
MIPIQLTVFFMIIYVLESLTIIVQSSLIVAVLGREWLQVRRLMPVDMILISLGISRFCLQWASM
LNNFCSYFNLNYVLCNLTITWEFFNILTFWLNSLLTVFYCIKVSSFTHHIFLWLRWRILRLFP
WILLGSLMITCVTIIPSAIGNYIQIQLLTMEHLPRNSTVTDKLENFHQYQFQAHTVALVIPFIL
FLASTIFLMASLTKQIQHHSTGHCNPSMKARFTALRSLAVLFIVFTSYFLTILITIIGTLFDKR
CWLWVWEAFVYAFILMHSTSLMLSSPTLKRILKGKC

>Q9HBW1 single-pass type I membrane protein
MKLLWQVTVHHHTWNAILLPFVYLTAQVWILCAAIAAAASAGPQNCPSVCSCSNQFSKV
VCTRRGLSEVPQGIPSNTRYLNLMENNIQMIQADTFRHLHHLEVLQLGRNSIRQIEVGAFN
GLASLNTLELFDNWLTVIPSGAFEYLSKLRELWLRNNPIESIPSYAFNRVPSLMRLDLGELK
KLEYISEGAFEGLFNLKYLNLGMCNIKDMPNLTPLVGLEELEMSGNHFPEIRPGSFHGLSSL
KKLWVMNSQVSLIERNAFDGLASLVELNLAHNNLSSLPHDLFTPLRYLVELHLHHNPWNC
DCDILWLAWWLREYIPTNSTCCGRCHAPMHMRGRYLVEVDQASFQCSAPFIMDAPRDLN
ISEGRMAELKCRTPPMSSVKWLLPNGTVLSHASRHPRISVLNDGTLNFSHVLLSDTGVYT
CMVTNVAGNSNASAYLNVSTAELNTSNYSFFTTVTVETTEISPEDTTRKYKPVPTTSTGYQ
PAYTTSTTVLIQTTRVPKQVAVPATDTTDKMQTSLDEVMKTTKIIIGCFVAVTLLAAAMLIV
FYKLRKRHQQRSTVTAARTVEIIQVDEDIPAATSAAATAAPSGVSGEGAVVLPTIHDHINYN
TYKPAHGAHWTENSLGNSLHPTVTTISEPYIIQTHTKDKVQETQI

>Q99835 multipass membrane protein
MAAARPARGPELPLLGLLLLLLLGDPGRGAASSGNATGPGPRSAGGSARRSAAVTGPPPPL
SHCGRAAPCEPLRYNVCLGSVLPYGATSTLLAGDSDSQEEAHGKLVLWSGLRNAPRCWA
VIQPLLCAVYMPKCENDRVELPSRTLCQATRGPCAIVERERGWPDFLRCTPDRFPEGCTNE
VQNIKFNSSGQCEVPLVRTDNPKSWYEDVEGCGIQCQNPLFTEAEHQDMHSYIAAFGAVT
GLCTLFTLATFVADWRNSNRYPAVILFYVNACFFVGSIGWLAQFMDGARREIVCRADGTM
RLGEPTSNETLSCVIIFVIVYYALMAGVVWFVVLTYAWHTSFKALGTTYQPLSGKTSYFHL
LTWSLPFVLTVAILAVAQVDGDSVSGICFVGYKNYRYRAGFVLAPIGLVLIVGGYFLIRGV
MTLFSIKSNHPGLLSEKAASKINETMLRLGIFGFLAFGFVLITFSCHFYDFFNQAEWERSFR
DYVLCQANVTIGLPTKQPIPDCEIKNRPSLLVEKINLFAMFGTGIAMSTWVWTKATLLIWR
RTWCRLTGQSDDEPKRIKKSKMIAKAFSKRHELLQNPGQELSFSMHTVSHDGPVAGLAFD
LNEPSADVSSAWAQHVTKMVARRGAILPQDISVTPVATPVPPEEQANLWLVEAEISPELQK
RLGRKKKRRKRKKEVCPLAPPPELHPPAPAPSTIPRLPQLPRQKCLVAAGAWGAGDSCRQ
GAWTLVSNPFCPEPSPPQDPFLPSAPAPVAWAHGRRQGLGPIHSRTNLMDTELMDADSDF

>P03999 multipass membrane protein

MRKMSEEEFYLFKNISSVGPWDGPQYHIAPVWAFYLQAAFMGTVFLIGFPLNAMVLVATL
RYKKLRQPLNYILVNVSFGGFLLCIFSVFPVFVASCNGYFVFGRHVCALEGFLGTVAGLVT
GWSLAFLAFERYIVICKPFGNFRFSSKHALTVVLATWTIGIGVSIPPFFGWSRFIPEGLQCSC
GPDWYTVGTKYRSESYTWFLFIFCFIVPLSLICFSYTQLLRALKAVAAQQQESATTQKAER
EVSRMVVVMVGSFCVCYVPYAAFAMYMVNNRNHGLDLRLVTIPSFFSKSACIYNPIIYCF
MNKQFQACIMKMVCGKAMTDESDTCSSQKTEVSTVSSTQVGPN

>O00161 lipid-anchor, peripheral membrane protein
MDNLSSEEIQQRAHQITDESLESTRRILGLAIESQDAGIKTITMLDEQKEQLNRIEEGLDQIN
KDMRETEKTLTELNKCCGLCVCPCNRTKNFESGKAYKTTWGDGGENSPCNVVSKQPGPV
TNGQLQQPTTGAASGGYIKRITNDAREDEMEENLTQVGSILGNLKDMALNIGNEIDAQNP
QIKRITDKADTNRDRIDIANARAKKLIDS

>Q9NWW5 multipass membrane protein
MEATRRRQHLGATGGPGAQLGASFLQARHGSVSADEAARTAPFHLDLWFYFTLQNWVL
DFGRPIAMLVFPLEWFPLNKPSVGDYFHMAYNVITPFLLLKLIERSPRTLPRSITYVSIIIFIM
GASIHLVGDSVNHRLLFSGYQHHLSVRENPIIKNLKPETLIDSFELLYYYDEYLGHCMWYI
PFFLILFMYFSGCFTASKAESLIPGPALLLVAPSGLYYWYLVTEGQIFILFIFTFFAMLALVLH
QKRKRLFLDSNGLFLFSSFALTLLLVALWVAWLWNDPVLRKKYPGVIYVPEPWAFYTLHV
SSRH

>Q9NPB9 multipass membrane protein
MALEQNQSTDYYYEENEMNGTYDYSQYELICIKEDVREFAKVFLPVFLTIVFVIGLAGNS
MVVAIYAYYKKQRTKTDVYILNLAVADLLLLFTLPFWAVNAVHGWVLGKIMCKITSALYT
LNFVSGMQFLACISIDRYVAVTKVPSQSGVGKPCWIICFCVWMAAILLSIPQLVFYTVNDN
ARCIPIFPRYLGTSMKALIQMLEICIGFVVPFLIMGVCYFITARTLMKMPNIKISRPLKVLLT
VVIVFIVTQLPYNIVKFCRAIDIIYSLITSCNMSKRMDIAIQVTESIALFHSCLNPILYVFMGA
SFKNYVMKVAKKYGSWRRQRQSVEEFPFDSEGPTEPTSTFSI

>P53618 peripheral membrane protein
MTAAENVCYTLINVPMDSEPPSEISLKNDLEKGDVKSKTEALKKVIIMILNGEKLPGLLMT
IIRFVLPLQDHTIKKLLLVFWEIVPKTTPDGRLLHEMILVCDAYRKDLQHPNEFIRGSTLRFL
CKLKEAELLEPLMPAIRACLEHRHSYVRRNAVLAIYTIYRNFEHLIPDAPELIHDFLVNEKD
ASCKRNAFMMLIHADQDRALDYLSTCIDQVQTFGDILQLVIVELIYKVCHANPSERARFIR
CIYNLLQSSSPAVKYEAAGTLVTLSSAPTAIKAAAQCYIDLIIKESDNNVKLIVLDRLIELKE
HPAHERVLQDLVMDILRVLSTPDLEVRKKTLQLALDLVSSRNVEELVIVLKKEVIKTNNVS
EHEDTDKYRQLLVRTLHSCSVRFPDMAANVIPVLMEFLSDNNEAAAADVLEFVREAIQRF
DNLRMLIVEKMLEVFHAIKSVKIYRGALWILGEYCSTKEDIQSVMTEIRRSLGEIPIVESEIK
KEAGELKPEEEITVGPVQKLVTEMGTYATQSALSSSRPTKKEEDRPPLRGFLLDGDFFVAA
SLATTLTKIALRYVALVQEKKKQNSFVAEAMLLMATILHLGKSSLPKKPITDDDVDRISLCL
KVLSECSPLMNDIFNKECRQSLSHMLSAKLEEEKLSQKKESEKRNVTVQPDDPISFMQLTA
KNEMNCKEDQFQLSLLAAMGNTQRKEAADPLASKLNKVTQLTGFSDPVYAEAYVHVNQ
YDIVLDVLVVNQTSDTLQNCTLELATLGDLKLVEKPSPLTLAPHDFANIKANVKVASTENG
IIFGNIVYDVSGAASDRNCVVLSDIHIDIMDYIQPATCTDAEFRQMWAEFEWENKVTVNTN

MVDLNDYLQHILKSTNMKCLTPEKALSGYCGFMAANLYARSIFGEDALANVSIEKPIHQG
PDAAVTGHIRIRAKSQGMALSLGDKINLSQKKTSI

>P34810 single-pass type I membrane protein
MRLAVLFSGALLGLLAAQGTGNDCPHKKSATLLPSFTVTPTVTESTGTTSHRTTKSHKTTT
HRTTTTGTTSHGPTTATHNPTTTSHGNVTVHPTSNSTATSQGPSTATHSPATTSHGNATVHP
TSNSTATSPGFTSSAHPEPPPPSPSPSPTSKETIGDYTWTNGSQPCVHLQAQIQIRVMYTTQG
GGEAWGISVLNPNKTKVQGSCEGAHPHLLLSFPYGHLSFGFMQDLQQKVVYLSYMAVEY
NVSFPHAAQWTFSAQNASLRDLQAPLGQSFSCSNSSIILSPAVHLDLLSLRLQAAQLPHTG
VFGQSFSCPSDRSILLPLIIGLILLGLLALVLIAFCIIRRRPSAYQAL

>P14415 single-pass type II membrane protein
MVIQKEKKSCGQVVEEWKEFVWNPRTHQFMGRTGTSWAFILLFYLVFYGFLTAMFTLTM
WVMLQTVSDHTPKYQDRLATPGLMIRPKTENLDVIVNVSDTESWDQHVQKLNKFLEPYN
DSIQAQKNDVCRPGRYYEQPDNGVLNYPKRACQFNRTQLGNCSGIGDSTHYGYSTGQPC
VFIKMNRVINFYAGANQSMNVTCAGKRDEDAENLGNFVMFPANGNIDLMYFPYYGKKF
HVNYTQPLVAVKFLNVTPNVEVNVECRINAANIATDDERDKFAGRVAFKLRINKT

>Q9Y6Q5 peripheral membrane protein
MSASAVFILDVKGKPLISRNYKGDVAMSKIEHFMPLLVQREEEGALAPLLSHGQVHFLWIK
HSNLYLVATTSKNANASLVYSFLYKTIEVFCEYFKELEEESIRDNFVIVYELLDELMDFGFP
QTTDSKILQEYITQQSNKLETGKSRVPPTVTNAVSWRSEGIKYKKNEVFIDVIESVNLLVNA
NGSVLLSEIVGTIKLKVFLSGMPELRLGLNDRVLFELTGRSKNKSVELEDVKFHQCVRLSR
FDNDRTISFIPPDGDFELMSYRLSTQVKPLIWIESVIEKFSHSRVEIMVKAKGQFKKQSVAN
GVEISVPVPSDADSPRFKTSVGSAKYVPERNVVIWSIKSFPGGKEYLMRAHFGLPSVEKEE
VEGRPPIGVKFEIPYFTVSGIQVRYMKIIEKSGYQALPWVRYITQSGDYQLRTS

>Q92843 peripheral membrane protein
MATPASAPDTRALVADFVGYKLRQKGYVCGAGPGEGPAADPLHQAMRAAGDEFETRFRR
TFSDLAAQLHVTPGSAQQRFTQVSDELFQGGPNWGRLVAFFVFGAALCAESVNKEMEPL
VGQVQEWMVAYLETQLADWIHSSGGWAEFTALYGDGALEEARRLREGNWASVRTVLTG
AVALGALVTVGAFFASK

>P52961 GPI-anchor, lipid-anchor
MQMPAMMSLLLVSVGLMEALQAQSHPITRRDLFSQEIQLDMALASFDDQYAGCAAAMT
AALPDLNHTEFQANQVYADSWTLASSQWQERQARWPEWSLSPTRPSPPPLGFRDEHGVA
LLAYTANSPLHKEFNAAVREAGRSRAHYLHHFSFKTLHFLLTEALQLLGSGQRPPRCHQV
FRGVHGLRFRPAGPRATVRLGGFASASLKHVAAQQFGEDTFFGIWTCLGAPIKGYSFFPGE
EEVLIPPFETFQVINASRLAQGPARIYLRALGKHSTYNCEYIKDKKCKSGPCHLDNSAMGQ
SPLSAVWSLLLLLWFLVVRAFPDGPGLL

>P49257 single-pass type I membrane protein
MAGSRQRGLRARVRPLFCALLLSLGRFVRGDGVGGDPAVALPHRRFEYKYSFKGPHLVQS
DGTVPFWAHAGNAIPSSDQIRVAPSLKSQRGSVWTKTKAAFENWEVEVTFRVTGRGRIGA

DGLAIWYAENQGLEGPVFGSADLWNGVGIFFDSFDNDGKKNNPAIVIIGNNGQIHYDHQN
DGASQALASCQRDFRNKPYPVRAKITYYQNTLTVMINNGFTPDKNDYEFCAKVENMIIPA
QGHFGISAATGGLADDHDVLSFLTFQLTEPGKEPPTPDKEISEKEKEKYQEEFEHFQQELD
KKKEEFQKGHPDLQGQPAEEIFESVGDRELRQVFEGQNRIHLEIKQLNRQLDMILDEQRRY
VSSLTEEISKRGAGMPGQHGQITQQELDTVVKTQHEILRQVNEMKNSMSETVRLVSGMQ
HPGSAGGVYETTQHFIDIKEHLHIVKRDIDNLVQRNMPSNEKPKCPELPPFPSCLSTVHFIIF
VVVQTVLFIGYIMYRSQQEAAAKKFF

>Q8TDJ6 peripheral membrane protein
MHLHQVLTGAVNPGDNCYSVGSVGDVPFTAYGSGCDIVILANDFECVQIIPGAKHGNIQV
SCVECSNQQGRIAASYGNAVCIFEPLGINSHKRNCQLKCQWLKTGQFFLSSVTYNLAWDP
QDNRLLTATDSIQLWAPPGDDILEEEEEIDNTVPPVLNDWKCVWQCKTSVSVHLMEWSPD
GEYFATAGKDDCLLKVWYPMTGWKSSIIPQDHHEVKRRQSSTQFSFVYLAHPRAVTGFS
WRKTSKYMPRGSVCNVLLTSCHDGVCRLWAETLLPEDCLLGEQICETTTSSIASSLSHAGR
HKDRIQHALETIHHLKNLRKGQRRSSVLVTHAELMPDQTAMHEVQRHISHHANALCHFHI
AASINPATDIPNVLVGTAFNVDDGNGGFVVHWLNNKEFHFTSSTEVFMHQLRKLSDKQV
DHENDDADREDEEHSQEDRERGLHMKLDHDLSLDRESEAGTGSSEHEDGEREGSPRTYS
RLSVPMPLPTVLLDRKIETLLTEWNKNPDMLFTIHPVDGTFLVWHVKYLDEYNPGIFRQV
QVSFSSRIPVAFPSGDASSLSKNIMMYACINATKDSHHTLLHQEGMSVGSPHGSQPHSRSH
STHMNILAPTVMMISKHIDGSLNQWAVTFADKSAFTTVLTVSHKFRYCGHRFHLNDLACH
SVLPLLLTSSHHNALLTPELDCQWDSDNKLSRLMDPVKHIKGSSKQPLRNAATRTFHDPN
AIYSELILWRVDPIGPLSYTGGVSELARINSLHTSAFSNVAWLPTLIPSYCLGTYCNSASACF
VASDGKNLRLYQAVVDARKLLDELSDPESSKLIGEVFNIVSQQSTARPGCIIELDAITNQCG
SNTQLLHVFQEDFIIGYKPHKEDMEKKETEIFFQPSQGYRPPPFSEKFFLVVIEKDSNNNSIL
HMWHLHLKSVQACLAKASEGASSESLLSVPGQKNVDSSPETSPSVSPMPHSSSIANLQTAS
KLILSSRLVYSQPLDLPESVEVIRATPSAGHLSSSSIYPVCLAPYLVVTTCSDNKVRFWKCC
MEANPECNKSDEKEIYHWKRWPLMNDEGEDNSSTVSIVGRPVAVSCSYTGRLAVAYKQPI
HHNGFVSKEFSMHVCIFECESTGGSEWVLEQTIHLDDLVKVGSVLDSRVSVDSNLFVYSK
SDALLSKDRYLIPNIKHLVHLDWVSKEDGSHILTVGVGANIFMYGRLSGIVTEQTNSKDGV
AVITLPLGGSIKQGVKSRWVLLRSIDLVSSVDGTPSLPVSLSWVRDGILVVGMDCEMHVYA
QWKHAVKFGDTEADSSNAEEAAMQDHSTFKSNMLARKSVVEGTAISDDVFCSPTVIQDG
GLFEAAHVLSPTLPQYHPTQLLELMDLGKVRRAKAILSHLVKCIAGEVAIVRDPDAGEGT
KRHLSRTISVSGSTAKETVTVGKDGTRDYTEIDSIPPLPLYALLAADQDTSYRISEESTKIPQ
SYEDQTVSQPEDQYSELFQIQDIPTDDIDLEPEKRENKSKVINLSQYGPAYFGQEHARVLSS
HLMHSSLPGLTRLEQMFLVALADTVATTSTELDESRDKSCSGRDTLDECGLRYLLAMRLH
TCLLTSLPPLYRVQLLHQGVSTCHFAWAFHSEAEEELINMIPAIQRGDPQWSELRAMGIGW
WVRNINTLRRCIEKVAKASFQRNNDALDAALFYLSMKKKAVVWGLFRSQHDEKMTTFFS
HNFNEDRWRKAALKNAFSLLGKQRFEQSAAFFLLAGSLKDAIEVCLEKMEDIQLAMVIA
RLYESEFETSSTYISILNQKILGCQKDGSGFSCKRLHPDPFLRSLAYWVMKDYTRALDTLL
EQTPKEDDEHQVIIKSCNPVAFSFYNYLRTHPLLIRRNLASPEGTLATLGLKTEKNFVDKIN
LIERKLFFTTANAHFKVGCPVLALEVLSKIPKVTKTSALSAKKDQPDFISHRMDDVPSHSK
ALSDGNGSSGIEWSNVTSSQYDWSQPIVKVDEEPLNLDWGEDHDSALDEEEDDAVGLVM
KSTDAREKDKQSDQKASDPNMLLTPQEEDDPEGDTEVDVIAEQLKFRACLKILMTELRTL
ATGYEVDGGKLRFQLYNWLEKEIAALHEICNHESVIKEYSSKTYSKVESDLLDQEEMVDK

PDIGSYERHQIERRRLQAKREHAERRKSWLQKNQDLLRVFLSYCSLHGAQGGGLASVRM
ELKFLLQESQQETTVKQLQSPLPLPTTLPLLSASIASTKTVIANPVLYLNNHIHDILYTIVQM
KTPPHPSIEDVKVHTLHSLAASLSASIYQALCDSHSYSQTEGNQFTGMAYQGLLLSDRRRL
RTESIEEHATPNSSPAQWPGVSSLINLLSSAQDEDQPKLNILLCEAVVAVYLSLLIHALATNS
SSELFRLAAHPLNNRMWAAVFGGGVKLVVKPRRQSENISAPPVLSEDIDKHRRRFNMRML
VPGRPVKDATPPPVPAERPSYKEKFIPPELSMWDYFVAKPFLPLSDSGVIYDSDESIHSDEE
DDAFFSDTQIQEHQDPNSYSWALLHLTMVKLALHNVKNFFPIAGLEFSELPVTSPLGIAVIK
NLENWEQILQEKMDQFEGPPPNYINTYPTDLSVGAGPAILRNKAMLEPENTPFKSRDSSAF
PVKRLWHFLVKQEVLQETFIRYIFTKKRKQSEVEADLGYPGGKAKVIHKESDMIMAFSVN
KANCNEIVLASTHDVQELDVTSLLACQSYIWIGEEYDRESKSSDDVDYRGSTTTLYQPSAT
SYSASQVHPPSSLPWLGTGQTSTGASVLMKRNLHNVKRMTSHPVHQYYLTGAQDGSVR
MFEWTRPQQLVCFRQAGNARVTRLYFNSQGNKCGVADGEGFLSIWQVNQTASNPKPYMS
WQCHSKATSDFAFITSSSLVATSGHSNDNRNVCLWDTLISPGNSLIHGFTCHDHGATVLQY
APKQQLLISGGRKGHVCIFDIRQRQLIHTFQAHDSAIKALALDPYEEYFTTGSAEGNIKVW
RLTGHGLIHSFKSEHAKQSIFRNIGAGVMQIDIIQGNRLFSCGADGTLKTRVLPNAFNIPNRI
LDIL

>Q14028 multipass membrane protein
MLGWVQRVLPQPPGTPRKTKMQEEEEVEPEPEMEAEVEPEPNPEEAETESESMPPEESFK
EEEVAVADPSPQETKEAALTSTISLRAQGAEISEMNSPSRRVLTWLMKGVEKVIPQPVHSIT
EDPAQILGHGSTGDTGCTDEPNEALEAQDTRPGLRLLLWLEQNLERVLPQPPKSSEVWRD
EPAVATGAASDPAPPGRPQEMGPKLQARETPSLPTPIPLQPKEEPKEAPAPEPQPGSQAQTSS
LPPTRDPARLVAWVLHRLEMALPQPVLHGKIGEQEPDSPGICDVQTISILPGGQVEPDLVLE
EVEPPWEDAHQDVSTSPQGTEVVPAYEEENKAVEKMPRELSRIEEEKEDEEEEEEEEEEEE
EEEVTEVLLDSCVVSQVGVGQSEEDGTRPQSTSDQKLWEEVGEEAKKEAEEKAKEEAEE
VAEEEAEKEPQDWAETKEEPEAEAEAASSGVPATKQHPEVQVEDTDADSCPLMAEENPPS
TVLPPPSPAKSDTLIVPSSASGTHRKKLPSEDDEAEELKALSPAESPVVAWSDPTTPKDTDG
QDRAASTASTNSAIINDRLQELVKLFKERTEKVKEKLIDPDVTSDEESPKPSPAKKAPEPAP
DTKPAEAEPVEEEHYCDMLCCKFKHRPWKKYQFPQSIDPLTNLMYVLWLFFVVMAWNW
NCWLIPVRWAFPYQTPDNIHHWLLMDYLCDLIYFLDITVFQTRLQFVRGGDIITDKKDMR
NNYLKSRRFKMDLLSLLPLDFLYLKVGVNPLLRLPRCLKYMAFFEFNSRLESILSKAYVYR
VIRTTAYLLYSLHLNSCLYYWASAYQGLGSTHWVYDGVGNSYIRCYYFAVKTLITIGGLPD
PKTLFEIVFQLLNYFTGVFAFSVMIGQMRDVVGAATAGQTYYRSCMDSTVKYMNFYKIP
KSVQNRVKTWYEYTWHSQGMLDESELMVQLPDKMRLDLAIDVNYNIVSKVALFQGCDR
QMIFDMLKRLRSVVYLPNDYVCKKGEIGREMYIIQAGQVQVLGGPDGKSVLVTLKAGSV
FGEISLLAVGGGNRRTANVVAHGFTNLFILDKKDLNEILVHYPESQKLLRKKARRMLRSNN
KPKEEKSVLILPPRAGTPKLFNAALAMTGKMGGKGAKGGKLAHLRARLKELAALEAAA
KQQELVEQAKSSQDVKGEEGSAAPDQHTHPKEAATDPPAPRTPPEPPGSPPSSPPPASLGRP
EGEEEGPAEPEEHSVRICMSPGPEPGEQILSVKMPEEREEKAE

>Q9UL51 multipass membrane protein
MDARGGGGRPGESPGATPAPGPPPPPPPAPPQQQPPPPPPPAPPPGPGPAPPQHPPRAEALPP
EAADEGGPRGRLRSRDSSCGRPGTPGAASTAKGSPNGECGRGEPQCSPAGPEGPARGPKV
SFSCRGAASGPAPGPGPAEEAGSEEAGPAGEPRGSQASFMQRQFGALLQPGVNKFSLRMF

GSQKAVEREQERVKSAGAWIIHPYSDFRFYWDFTMLLFMVGNLIIIPVGITFFKDETTAPWI
VFNVVSDTFFLMDLVLNFRTGIVIEDNTEIILDPEKIKKKYLRTWFVVDFVSSIPVDYIFLIV
EKGIDSEVYKTARALRIVRFTKILSLLRLLRLSRLIRYIHQWEEIFHMTYDLASAVMRICNLI
SMMLLLCHWDGCLQFLVPMLQDFPRNCWVSINGMVNHSWSELYSFALFKAMSHMLCIG
YGRQAPESMTDIWLTMLSMIVGATCYAMFIGHATALIQSLDSSRRQYQEKYKQVEQYMSF
HKLPADFRQKIHDYYEHRYQGKMFDEDSILGELNGPLREEIVNFNCRKLVASMPLFANAD
PNFVTAMLTKLKFEVFQPGDYIIREGTIGKKMYFIQHGVVSVLTKGNKEMKLSDGSYFGEI
CLLTRGRRTASVRADTYCRLYSLSVDNFNEVLEEYPMMRRAFETVAIDRLDRIGKKNSILL
HKVQHDLNSGVFNNQENAIIQEIVKYDREMVQQAELGQRVGLFPPPPPPPQVTSAIATLQQ
AAAMSFCPQVARPLVGPLALGSPRLVRRPPPGPAPAAASPGPPPPASPPGAPASPRAPRTSPY
GGLPAAPLAGPALPARRLSRASRPLSASQPSLPHGAPGPAASTRPASSSTPRLGPTPAARAA
APSPDRRDSASPGAAGGLDPQDSARSRLSSNL

>Q9Y2H9 peripheral membrane protein
MSDSLWTALSNFSMPSFPGGSMFRRTKSCRTSNRKSLILTSTSPTLPRPHSPLPGHLGSSPLD
SPRNFSPNTPAHFSFASSRRADGRRWSLASLPSSGYGTNTPSSTVSSSCSSQERLHQLPYQP
TVDELHFLSKHFGSTESITDEDGGRRSPAVRPRSRSLSPGRSPSSYDNEIVMMNHVYKERFP
KATAQMEEKLRDFTRAYEPDSVLPLADGVLSFIHHQIIELARDCLTKSRDGLITTVYFYELQ
ENLEKLLQDAYERSESLEVAFVTQLVKKLLIIISRPARLLECLEFNPEEFYHLLEAAEGHAK
EGHLVKTDIPRYIIRQLGLTRDPFPDVVHLEEQDSGGSNTPEQDDLSEGRSSKAKKPPGEN
DFDTIKLISNGAYGAVYLVRHRDTRQRFAMKKINKQNLILRNQIQQAFVERDILTFAENPFV
VGMFCSFETRRHLCMVMEYVEGGDCATLLKNIGALPVEMARMYFAETVLALEYLHNYGI
VHRDLKPDNLLITSMGHIKLTDFGLSKMGLMSLTTNLYEGHIEKDAREFLDKQVCGTPEYI
APEVILRQGYGKPVDWWAMGIILYEFLVGCVPFFGDTPEELFGQVISDDILWPEGDEALPT
EAQLLISSLLQTNPLVRLGAGGAFEVKQHSFFRDLDWTGLLRQKAEFIPHLESEDDTSYFD
TRSDRYHHVNSYDEDDTTEEEPVEIRQFSSCSPRFSKVYSSMEQLSQHEPKTPVAAAGSSK
REPSTKGPEEKVAGKREGLGGLTLREKTWRGGSPEIKRFSASEASFLEGEASPPLGARRRF
SALLEPSRFSAPQEDEDEARLRRPPRPSSDPAGSLDARAPKEETQGEGTSSAGDSEATDRPR
PGDLCPPSKDGDASGPRATNDLVLRRARHQQMSGDVAVEKRPSRTGGKVIKSASATALSV
MIPAVDPHGSSPLASPMSPRSLSSNPSSRDSSPSRDYSPAVSGLRSPITIQRSGKKYGFTLRAI
RVYMGDTDVYSVHHIVWHVEEGGPAQEAGLCAGDLITHVNGEPVHGMVHPEVVELILKS
GNKVAVTTTPFENTSIRIGPARRSSYKAKMARRNKRPSAKEGQESKKRSSLFRKITKQSNL
LHTSRSLSSLNRSLSSSDSLPGSPTHGLPARSPTHSYRSTPDSAYLGASSQSSSPASSTPNSPA
SSASHHIRPSTLHGLSPKLHRQYRSARCKSAGNIPLSPLAHTPSPTQASPPPLPGHTVGSSH
TTQSFPAKLHSSPPVVRPRPKSAEPPRSPLLKRVQSAEKLGASLSADKKGALRKHSLEVGH
PDFRKDFHGELALHSLAESDGETPPVEGLGAPRQVAVRRLGRQESPLSLGADPLLPEGASR
PPVSSKEKESPGGAEACTPPRATTPGGRTLERDVGCTRHQSVQTEDGTGGMARAVAKAAL
SPVQEHETGRRSSSGEAGTPLVPIVVEPARPGAKAVVPQPLGADSKGLQEPAPLAPSVPEAP
RGRERWVLEVVEERTTLSGPRSKPASPKLSPEPQTPSLAPAKCSAPSSAVTPVPPASLLGSG
TKPQVGLTSRCPAEAVPPAGLTKKGVSSPAPPGP

>A0FGR8 multipass membrane protein
MTANRDAALSSHRHPGCAQRPRTPTFASSSQRRSAFGFDDGNFPGLGERSHAPGSRLGAR
RRAKTARGLRGHRQRGAGAGLSRPGSARAPSPPRPGGPENPGGVLSVELPGLLAQLARSF

ALLLPVYALGYLGLSFSWVLLALALLAWCRRSRGLKALRLCRALALLEDEERVVRLGVR
ACDLPAWVHFPDTERAEWLNKTVKHMWPFICQFIEKLFRETIEPAVRGANTHLSTFSFTKV
DVGQQPLRINGVKVYTENVDKRQIILDLQISFVGNCEIDLEIKRYFCRAGVKSIQIHGTMRV
ILEPLIGDMPLVGALSIFFLRKPLLEINWTGLTNLLDVPGLNGLSDTIILDIISNYLVLPNRITV
PLVSEVQIAQLRFPVPKGVLRIHFIEAQDLQGKDTYLKGLVKGKSDPYGIIRVGNQIFQSRVI
KENLSPKWNEVYEALVYEHPGQELEIELFDEDPDKDDFLGSLMIDLIEVEKERLLDEWFTL
DEVPKGKLHLRLEWLTLMPNASNLDKVLTDIKADKDQANDGLSSALLILYLDSARNLPSG
KKISSNPNPVVQMSVGHKAQESKIRYKTNEPVWEENFTFFIHNPKRQDLEVEVRDEQHQC
SLGNLKVPLSQLLTSEDMTVSQRFQLSNSGPNSTIKMKIALRVLHLEKRERPPDHQHSAQV
KRPSVSKEGRKTSIKSHMSGSPGPGGSNTAPSTPVIGGSDKPGMEEKAQPPEAGPQGLHDL
GRSSSSLLASPGHISVKEPTPSIASDISLPIATQELRQRLRQLENGTTLGQSPLGQIQLTIRHSS
QRNKLIVVVHACRNLIAFSEDGSDPYVRMYLLPDKRRSGRRKTHVSKKTLNPVFDQSFDF
SVSLPEVQRRTLDVAVKNSGGFLSKDKGLLGKVLVALASEELAKGWTQWYDLTEDGTRP
QAMT

>O95219 peripheral membrane protein
MEQAPPDPERQLQPAPLEPLGSPDAGLGAAVGKEAEGAGEESSGVDTMTHNNFWLKKIEI
SVSEAEKRTGRNAMNMQETYTAYLIETRSVEHTDGQSVLTDSLWRRYSEFELLRSYLLVY
YPHIVVPPLPEKRAEFVWHKLSADNMDPDFVERRRIGLENFLLRIASHPILCRDKIFYLFLT
QEGNWKETVNETGFQLKADSRLKALNATFRVKNPDKRFTDLKHYSDELQSVISHLLRVR
ARVADRLYGVYKVHGNYGRVFSEWSAIEKEMGDGLQSAGHHMDVYASSIDDILEDEEHY
ADQLKEYLFYAEALRAVCRKHELMQYDLEMAAQDLASKKQQCEELVTGTVRTFSLKGM
TTKLFGQETPEQREARIKVLEEQINEGEQQLKSKNLEGREFVKNAWADIERFKEQKNRDL
KEALISYAVMQISMCKKGIQVWTNAKECFSKM

>Q99816 peripheral membrane protein
MAVSESQLKKMVSKYKYRDLTVRETVNVITLYKDLKPVLDSYVFNDGSSRELMNLTGTIP
VPYRGNTYNIPICLWLLDTYPYNPPICFVKPTSSMTIKTGKHVDANGKIYLPYLHEWKHPQ
SDLLGLIQVMIVVFGDEPPVFSRPISASYPPYQATGPPNTSYMPGMPGGISPYPSGYPPNPS
GYPGCPYPPGGPYPATTSSQYPSQPPVTTVGPSRDGTISEDTIRASLISAVSDKLRWRMKEE
MDRAQAELNALKRTEEDLKKGHQKLEEMVTRLDQEVAEVDKNIELLKKKDEELSSALEK
MENQSENNDIDEVIIPTAPLYKQILNLYAEENAIEDTIFYLGEALRRGVIDLDVFLKHVRLLS
RKQFQLRALMQKARKTAGLSDLY

>O43678 peripheral membrane protein
MAAAAASRGVGAKLGLREIRIHLCQRSPGSQGVRDFIEKRYVELKKANPDLPILIRECSDV
QPKLWARYAFGQETNVPLNNFSADQVTRALENVLSGKA

>P22460 multipass membrane protein
MEIALVPLENGGAMTVRGGDEARAGCGQATGGELQCPPTAGLSDGPKEPAPKGRGAQRD
ADSGVRPLPPLPDPGVRPLPPLPEELPRPRRPPPEDEEEEGDPGLGTVEDQALGTASLHHQR
VHINISGLRFETQLGTLAQFPNTLLGDPAKRLRYFDPLRNEYFFDRNRPSFDGILYYYQSGG
RLRRPVNVSLDVFADEIRFYQLGDEAMERFREDEGFIKEEEKPLPRNEFQRQVWLIFEYPE
SSGSARAIAIVSVLVILISIITFCLETLPEFRDERELLRHPPAPHQPPAPAPGANGSGVMAPPS

GPTVAPLLPRTLADPFFIVETTCVIWFTFELLVRFFACPSKAGFSRNIMNIIDVVAIFPYFITLG
TELAEQQPGGGGGGQNGQQAMSLAILRVIRLVRVFRIFKLSRHSKGLQILGKTLQASMREL
GLLIFFLFIGVILFSSAVYFAEADNQGTHFSSIPDAFWWAVVTMTTVGYGDMRPITVGGKIV
GSLCAIAGVLTIALPVPVIVSNFNYFYHRETDHEEPAVLKEEQGTQSQGPGLDRGVQRKVS
GSRGSFCKAGGTLENADSARRGSCPLEKCNVKAKSNVDLRRSLYALCLDTSRETDL

>P01130 single-pass type I membrane protein
MGPWGWKLRWTVALLLAAAGTAVGDRCERNEFQCQDGKCISYKWVCDGSAECQDGSD
ESQETCLSVTCKSGDFSCGGRVNRCIPQFWRCDGQVDCDNGSDEQGCPPKTCSQDEFRCH
DGKCISRQFVCDSDRDCLDGSDEASCPVLTCGPASFQCNSSTCIPQLWACDNDPDCEDGSD
EWPQRCRGLYVFQGDSSPCSAFEFHCLSGECIHSSWRCDGGPDCKDKSDEENCAVATCRP
DEFQCSDGNCIHGSRQCDREYDCKDMSDEVGCVNVTLCEGPNKFKCHSGECITLDKVCN
MARDCRDWSDEPIKECGTNECLDNNGGCSHVCNDLKIGYECLCPDGFQLVAQRRCEDID
ECQDPDTCSQLCVNLEGGYKCQCEEGFQLDPHTKACKAVGSIAYLFFTNRHEVRKMTLD
RSEYTSLIPNLRNVVALDTEVASNRIYWSDLSQRMICSTQLDRAHGVSSYDTVISRDIQAPD
GLAVDWIHSNIYWTDSVLGTVSVADTKGVKRKTLFRENGSKPRAIVVDPVHGFMYWTD
WGTPAKIKKGGLNGVDIYSLVTENIQWPNGITLDLLSGRLYWVDSKLHSISSIDVNGGNRK
TILEDEKRLAHPFSLAVFEDKVFWTDIINEAIFSANRLTGSDVNLLAENLLSPEDMVLFHNL
TQPRGVNWCERTTLSNGGCQYLCLPAPQINPHSPKFTCACPDGMLLARDMRSCLTEAEAA
VATQETSTVRLKVSSTAVRTQHTTTRPVPDTSRLPGATPGLTTVEIVTMSHQALGDVAGRG
NEKKPSSVRALSIVLPIVLLVFLCLGVFLLWKNWRLKNINSINFDNPVYQKTTEDEVHICH
NQDGYSYPSRQMVSLEDDVA

>Q92692 single-pass type I membrane protein
MARAAALLPSRSPPTPLLWPLLLLLLLETGAQDVRVQVLPEVRGQLGGTVELPCHLLPPV
PGLYISLVTWQRPDAPANHQNVAAFHPKMGPSFPSPKPGSERLSFVSAKQSTGQDTEAELQ
DATLALHGLTVEDEGNYTCEFATFPKGSVRGMTWLRVIAKPKNQAEAQKVTFSQDPTTVA
LCISKEGRPPARISWLSSLDWEAKETQVSGTLAGTVTVTSRFTLVPSGRADGVTVTCKVEH
ESFEEPALIPVTLSVRYPPEVSISGYDDNWYLGRTDATLSCDVRSNPEPTGYDWSTTSGTFP
TSAVAQGSQLVIHAVDSLFNTTFVCTVTNAVGMGRAEQVIFVRETPNTAGAGATGGIIGGII
AAIIATAVAATGILICRQQRKEQTLQGAEEDEDLEGPPSYKPPTPKAKLEAQEMPSQLFTLG
ASEHSPLKTPYFDAGASCTEQEMPRYHELPTLEERSGPLHPGATSLGSPIPVPPGPPAVEDV
SLDLEDEEGEEEEEYLDKINPIYDALSYSSPSDSYQGKGFVMSRAMYV

>O96008 multipass membrane protein
MGNVLAASSPPAGPPPPPAPALVGLPPPPPSPPGFTLPPLGGSLGAGTSTSRSSERTPGAATA
SASGAAEDGACGCLPNPGTFEECHRKCKELFPIQMEGVKLTVNKGLSNHFQVNHTVALST
IGESNYHFGVTYVGTKQLSPTEAFPVLVGDMDNSGSLNAQVIHQLGPGLRSKMAIQTQQS
KFVNWQVDGEYRGSDFTAAVTLGNPDVLVGSGILVAHYLQSITPCLALGGELVYHRRPGE
EGTVMSLAGKYTLNNWLATVTLGQAGMHATYYHKASDQLQVGVEFEASTRMQDTSVSF
GYQLDLPKANLLFKGSVDSNWIVGATLEKKLPPLPLTLALGAFLNHRKNKFQCGFGLTIG

>Q9BYF1 single-pass type I membrane protein
MSSSSWLLLSLVAVTAAQSTIEEQAKTFLDKFNHEAEDLFYQSSLASWNYNTNITEENVQN

MNNAGDKWSAFLKEQSTLAQMYPLQEIQNLTVKLQLQALQQNGSSVLSEDKSKRLNTIL
NTMSTIYSTGKVCNPDNPQECLLLEPGLNEIMANSLDYNERLWAWESWRSEVGKQLRPLY
EEYVVLKNEMARANHYEDYGDYWRGDYEVNGVDGYDYSRGQLIEDVEHTFEEIKPLYE
HLHAYVRAKLMNAYPSYISPIGCLPAHLLGDMWGRFWTNLYSLTVPFGQKPNIDVTDAM
VDQAWDAQRIFKEAEKFFVSVGLPNMTQGFWENSMLTDPGNVQKAVCHPTAWDLGKGD
FRILMCTKVTMDDFLTAHHEMGHIQYDMAYAAQPFLLRNGANEGFHEAVGEIMSLSAATP
KHLKSIGLLSPDFQEDNETEINFLLKQALTIVGTLPFTYMLEKWRWMVFKGEIPKDQWMK
KWWEMKREIVGVVEPVPHDETYCDPASLFHVSNDYSFIRYYTRTLYQFQFQEALCQAAK
HEGPLHKCDISNSTEAGQKLFNMLRLGKSEPWTLALENVVGAKNMNVRPLLNYFEPLFT
WLKDQNKNSFVGWSTDWSPYADQSIKVRISLKSALGDKAYEWNDNEMYLFRSSVAYAM
RQYFLKVKNQMILFGEEDVRVANLKPRISFNFFVTAPKNVSDIIPRTEVEKAIRMSRSRIND
AFRLNDNSLEFLGIQPTLGPPNQPPVSIWLIVFGVVMGVIVVGIVILIFTGIRDRKKKNKAR
SGENPYASIDISKGENNPGFQNTDDVQTSF

>Q10589 GPI-anchor, lipid-anchor, single-pass type II membrane protein
MASTSYDYCRVPMEDGDKRCKLLLGIGILVLLIIVILGVPLIIFTIKANSEACRDGLRAVME
CRNVTHLLQQELTEAQKGFQDVEAQAATCNHTVMALMASLDAEKAQGQKKVEELEGEI
TTLNHKLQDASAEVERLRRENQVLSVRIADKKYYPSSQDSSSAAAPQLLIVLLGLSALLQ

>P04201 multipass membrane protein
MDGSNVTSFVVEEPTNISTGRNASVGNAHRQIPIVHWVIMSISPVGFVENGILLWFLCFRM
RRNPFTVYITHLSIADISLLFCIFILSIDYALDYELSSGHYYTIVTLSVTFLFGYNTGLYLLTAI
SVERCLSVLYPIWYRCHRPKYQSALVCALLWALSCLVTTMEYVMCIDREEESHSRNDCRA
VIIFIAILSFLVFTPLMLVSSTILVVKIRKNTWASHSSKLYIVIMVTIIIFLIFAMPMRLLYLLYY
EYWSTFGNLHHISLLFSTINSSANPFIYFFVGSSKKKRFKESLKVVLTRAFKDEMQPRRQK
DNCNTVTVETVV

>Q5FVE4 peripheral membrane protein
MTGTPKTQEGAKDLEVDMNKTEVTPRLWTTCRDGEVLLRLSKHGPGHETPMTIPEFFRES
VNRFGTYPALASKNGKKWEILNFNQYYEACRKAAKSLIKLGLERFHGVGILGFNSAEWFI
TAVGAILAGGLCVGIYATNSAEVCQYVITHAKVNILLVENDQQLQKILSIPQSSLEPLKAIIQ
YRLPMKKNNNLYSWDDFMELGRSIPDTQLEQVIESQKANQCAVLIYTSGTTGIPKGVMLS
HDNITWIAGAVTKDFKLTDKHETVVSYLPLSHIAAQMMDIWVPIKIGALTYFAQADALKG
TLVSTLKEVKPTVFIGVPQIWEKIHEMVKKNSAKSMGLKKKAFVWARNIGFKVNSKKML
GKYNTPVSYRMAKTLVFSKVKTSLGLDHCHSFISGTAPLNQETAEFFLSLDIPIGELYGLSE
SSGPHTISNQNNYRLLSCGKILTGCKNMLFQQNKDGIGEICLWGRHIFMGYLESETETTEAI
DDEGWLHSGDLGQLDGLGFLYVTGHIKEILITAGGENVPPIPVETLVKKKIPIISNAMLVGD
KLKFLSMLLTLKCEMNQMSGEPLDKLNFEAINFCRGLGSQASTVTEIVKQQDPLVYKAIQ
QGINAVNQEAMNNAQRIEKWVILEKDFSIYGGELGPMMKLKRHFVAQKYKKQIDHMYH

>Q11128 single-pass type II membrane protein
MDPLGPAKPQWLWRRCLAGLLFQLLVAVCFFSYLRVSRDDATGSPRPGLMAVEPVTGAPN
GSRCQDSMATPAHPTLLILLWTWPFNTPVALPRCSEMVPGAADCNITADSSVYPQADAVIV
HHWDIMYNPSANLPPPTRPQGQRWIWFSMESPSNCRHLEALDGYFNLTMSYRSDSDIFTP

YGWLEPWSGQPAHPPLNLSAKTELVAWAVSNWKPDSARVRYYQSLQAHLKVDVYGRSH
KPLPKGTMMETLSRYKFYLAFENSLHPDYITEKLWRNALEAWAVPVVLGPSRSNYERFLP
PDAFIHVDDFQSPKDLARYLQELDKDHARYLSYFRWRETLRPRSFSWALAFCKACWKLQ
QESRYQTVRSIAAWFT

>O14526 peripheral membrane protein
MSYFGEHFWGEKNHGFEVLYHSVKQGPISTKELADFIRERATIEETYSKAMAKLSKLASN
GTPMGTFAPLWEVFRVSSDKLALCHLELTRKLQDLIKDVLRYGEEQLKTHKKCKEEVVST
LDAVQVLSGVSQLLPKSRENYLNRCMDQERLRRESTSQKEMDKAETKTKKAAESLRRSV
EKYNSARADFEQKMLDSALRFQAMEETHLRHMKALLGSYAHSVEDTHVQIGQVHEEFK
QNIENVSVEMLLRKFAESKGTGREKPGPLDFEAYSAAALQEAMKRLRGAKAFRLPGLSRR
EREPEPPAAVDFLEPDSGTCPEVDEEGFTVRPDVTQNSTAEPSRFSSSDSDFDDEEPRKFYV
HIKPAPARAPACSPEAAAAQLRATAGSLILPPGPGGTMKRHSSRDAAGKPQRPRSAPRTSSC
AERLQSEEQVSKNLFGPPLESAFDHEDFTGSSSLGFTSSPSPFSSSSPENVEDSGLDSPSHAA
PGPSPDSWVPRPGTPQSPPSCRAPPPEARGIRAPPLPDSPQPLASSPGPWGLEALAGGDLMP
APADPTAREGLAAPPRRLRSRKVSCPLTRSNGDLSRSLSPSPLGSSAASTALERPSFLSQTG
HGVSRGPSPVVLGSQDALPIATAFTEYVHAYFRGHSPSCLARVTGELTMTFPAGIVRVFSGT
PPPPVLSFRLVHTTAIEHFQPNADLLFSDPSQSDPETKDFWLNMAALTEALQRQAEQNPTA
SYYNVVLLRYQFSRPGPQSVPLQLSAHWQCGATLTQVSVEYGYRPGATAVPTPLTNVQILL
PVGEPVTNVRLQPAATWNLEEKRLTWRLPDVSEAGGSGRLSASWEPLSGPSTPSPVAAQF
TSEGTTLSGVDLELVGSGYRMSLVKRRFATGMYLVSC

>Q8TD43 multipass membrane protein
MVVPEKEQSWIPKIFKKKTCTTFIVDSTDPGGTLCQCGRPRTAHPAVAMEDAFGAAVVTV
WDSDAHTTEKPTDAYGELDFTGAGRKHSNFLRLSDRTDPAAVYSLVTRTWGFRAPNLVVS
VLGGSGGPVLQTWLQDLLRRGLVRAAQSTGAWIVTGGLHTGIGRHVGVAVRDHQMAST
GGTKVVAMGVAPWGVVRNRDTLINPKGSFPARYRWRGDPEDGVQFPLDYNYSAFFLVDD
GTHGCLGGENRFRLRLESYISQQKTGVGGTGIDIPVLLLLIDGDEKMLTRIENATQAQLPCL
LVAGSGGAADCLAETLEDTLAPGSGGARQGEARDRIRRFFPKGDLEVLQAQVERIMTRKE
LLTVYSSEDGSEEFETIVLKALVKACGSSEASAYLDELRLAVAWNRVDIAQSELFRGDIQW
RSFHLEASLMDALLNDRPEFVRLLISHGLSLGHFLTPMRLAQLYSAAPSNSLIRNLLDQAS
HSAGTKAPALKGGAAELRPPDVGHVLRMLLGKMCAPRYPSGGAWDPHPGQGFGESMYL
LSDKATSPLSLDAGLGQAPWSDLLLWALLLNRAQMAMYFWEMGSNAVSSALGACLLLR
VMARLEPDAEEAARRKDLAFKFEGMGVDLFGECYRSSEVRAARLLLRRCPLWGDATCLQ
LAMQADARAFFAQDGVQSLLTQKWWGDMASTTPIWALVLAFFCPPLIYTRLITFRKSEEE
PTREELEFDMDSVINGEGPVGTADPAEKTPLGVPRQSGRPGCCGGRCGGRRCLRRWFHF
WGAPVTIFMGNVVSYLLFLLLFSRVLLVDFQPAPPGSLELLLYFWAFTLLCEELRQGLSGG
GGSLASGGPGPGHASLSQRLRLYLADSWNQCDLVALTCFLLGVGCRLTPGLYHLGRTVLCI
DFMVFTVRLLHIFTVNKQLGPKIVIVSKMMKDVFFFLFFLGVWLVAYGVATEGLLRPRDS
DFPSILRRVFYRPYLQIFGQIPQEDMDVALMEHSNCSSEPGFWAHPPGAQAGTCVSQYAN
WLVVLLLVIFLLVANILLVNLLIAMFSYTFGKVQGNSDLYWKAQRYRLIREFHSRPALAPPF
IVISHLRLLLRQLCRRPRSPQPSSPALEHFRVYLSKEAERKLLTWESVHKENFLLARARDKR
ESDSERLKRTSQKVDLALKQLGHIREYEQRLKVLEREVQQCSRVLGWVAEALSRSALLPP
GGPPPPDLPGSKD

>P05181 peripheral membrane protein
MSALGVTVALLVWAAFLLLVSMWRQVHSSWNLPPGPFPLPIIGNLFQLELKNIPKSFTRLA
QRFGPVFTLYVGSQRMVVMHGYKAVKEALLDYKDEFSGRGDLPAFHAHRDRGIIFNNGP
TWKDIRRFSLTTLRNYGMGKQGNESRIQREAHFLLEALRKTQGQPFDPTFLIGCAPCNVIA
DILFRKHFDYNDEKFLRLMYLFNENFHLLSTPWLQLYNNFPSFLHYLPGSHRKVIKNVAEV
KEYVSERVKEHHQSLDPNCPRDLTDCLLVEMEKEKHSAERLYTMDGITVTVADLFFAGTE
TTSTTLRYGLLILMKYPEIEEKLHEEIDRVIGPSRIPAIKDRQEMPYMDAVVHEIQRFITLVPS
NLPHEATRDTIFRGYLIPKGTVVVPTLDSVLYDNQEFPDPEKFKPEHFLNENGKFKYSDYF
KPFSTGKRVCAGEGLARMELFLLLCAILQHFNLKPLVDPKDIDLSPIHIGFGCIPPRYKLCVI
PRS

>P48029 multipass membrane protein
MAKKSAENGIYSVSGDEKKGPLIAPGPDGAPAKGDGPVGLGTPGGRLAVPPRETWTRQM
DFIMSCVGFAVGLGNVWRFPYLCYKNGGGVFLIPYVLIALVGGIPIFFLEISLGQFMKAGSI
NVWNICPLFKGLGYASMVIVFYCNTYYIMVLAWGFYYLVKSFTTTLPWATCGHTWNTPD
CVEIFRHEDCANASLANLTCDQLADRRSPVIEFWENKVLRLSGGLEVPGALNWEVTLCLL
ACWVLVYFCVWKGVKSTGKIVYFTATFPYVVLVVLLVRGVLLPGALDGIIYYLKPDWSKL
GSPQVWIDAGTQIFFSYAIGLGALTALGSYNRFNNNCYKDAIILALINSGTSFFAGFVVFSIL
GFMAAEQGVHISKVAESGPGLAFIAYPRAVTLMPVAPLWAALFFFMLLLLGLDSQFVGVE
GFITGLLDLLPASYYFRFQREISVALCCALCFVIDLSMVTDGGMYVFQLFDYYSASGTTLL
WQAFWECVVVAWVYGADRFMDDIACMIGYRPCPWMKWCWSFFTPLVCMGIFIFNVVY
YEPLVYNNTYVYPWWGEAMGWAFALSSMLCVPLHLLGCLLRAKGTMAERWQHLTQPI
WGLHHLEYRAQDADVRGLTTLTPVSESSKVVVVESVM

>Q76EJ3 multipass membrane protein
MTAGGQAEAEGAGGEPGAARLPSRVARLLSALFYGTCSFLIVLVNKALLTTYGFPSPIFLGI
GQMAATIMILYVSKLNKIIHFPDFDKKIPVKLFPLPLLYVGNHISGLSSTSKLSLPMFTVLRK
FTIPLTLLLETIILGKQYSLNIILSVFAIILGAFIAAGSDLAFNLEGYIFVFLNDIFTAANGVYT
KQKMDPKELGKYGVLFYNACFMIIPTLIISVSTGDLQQATEFNQWKNVVFILQFLLSCFLG
FLLMYSTVLCSYYNSALTTAVVGAIKNVSVAYIGILIGGDYIFSLLNFVGLNICMAGGLRYS
FLTLSSQLKPKPVGEENICLDLKS

>Q9BY15 multipass membrane protein
MQGPLLLPGLCFLLSLFGAVTQKTKTSCAKCPPNASCVNNTHCTCNHGYTSGSGQKLFTF
PLETCNDINECTPPYSVYCGFNAVCYNVEGSFYCQCVPGYRLHSGNEQFSNSNENTCQDT
TSSKTTEGRKELQKIVDKFESLLTNQTLWRTEGRQEISSTATTILRDVESKVLETALKDPEQ
KVLKIQNDSVAIETQAITDNCSEERKTFNLNVQMNSMDIRCSDIIQGDTQGPSAIAFISYSSL
GNIINATFFEEMDKKDQVYLNSQVVSAAIGPKRNVSLSKSVTLTFQHVKMTPSTKKVFCV
YWKSTGQGSQWSRDGCFLIHVNKSHTMCNCSHLSSFAVLMALTSQEEDPVLTVITYVGLS
VSLLCLLLAALTFLLCKAIRNTSTSLHLQLSLCLFLAHLLFLVGIDRTEPKVLCSIIAGALHY
LYLAAFTWMLLEGVHLFLTARNLTVVNYSSINRLMKWIMFPVGYGVPAVTVAISAASWPH
LYGTADRCWLHLDQGFMWSFLGPVCAIFSANLVLFILVFWILKRKLSSLNSEVSTIQNTRM
LAFKATAQLFILGCTWCLGLLQVGPAAQVMAYLFTIINSLQGFFIFLVYCLLSQQVQKQYQ

KWFREIVKSKSESETYTLSSKMGPDSKPSEGDVFPGQVKRKY

>Q9H1K0 lipid-anchor
MASLDDPGEVREGFLCPLCLKDLQSFYQLHSHYEEEHSGEDRDVKGQIKSLVQKAKKAK
DRLLKREGDDRAESGTQGYESFSYGGVDPYMWEPQELGAVRSHLSDFKKHRAARIDHYV
VEVNKLIIRLEKLTAFDRTNTESAKIRAIEKSVVPWVNDQDVPFCPDCGNKFSIRNRRHHC
RLCGSIMCKKCMELISLPLANKLTSASKESLSTHTSPSQSPNSVHGSRRGSISSMSSVSSVL
DEKDDDRIRCCTHCKDTLLKREQQIDEKEHTPDIVKLYEKLRLCMEKVDQKAPEYIRMAA
SLNAGETTYSLEHASDLRVEVQKVYELIDALSKKILTLGLNQDPPPHPSNLRLQRMIRYSAT
LFVQEKLLGLMSLPTKEQFEELKKKRKEEMERKRAVERQAALESQRRLEERQSGLASRA
ANGEVASLRRGPAPLRKAEGWLPLSGGQGQSEDSDPLLQQIHNITSFIRQAKAAGRMDEV
RTLQENLRQLQDEYDQQQTEKAIELSRRQAEEEDLQREQLQMLRERELEREREQFRVASL
HTRTRSLDFREIGPFQLEPSREPRTHLAYALDLGSSPVPSSTAPKTPSLSSTQPTRVWSGPPA
VGQERLPQSSMPQQHEGPSLNPFDEEDLSSPMEEATTGPPAAGVSLDPSARILKEYNPFEEE
DEEEEAVAGNPFIQPDSPAPNPFSEEDEHPQQRLSSPLVPGNPFEEPTCINPFEMDSDSGPEA
EEPIEEELLLQQIDNIKAYIFDAKQCGRLDEVEVLTENLRELKHTLAKQKGGTD

>O60895 single-pass type I membrane protein
MASLRVERAGGPRLPRTRVGRPAALRLLLLLGAVLNPHEALAQPLPTTGTPGSEGGTVKN
YETAVQFCWNHYKDQMDPIEKDWCDWAMISRPYSTLRDCLEHFAELFDLGFPNPLAERII
FETHQIHFANCSLVQPTFSDPPEDVLLAMIIAPICLIPFLITLVVWRSKDSEAQA

>O75106 peripheral membrane protein
MHLKIVLAFLALSLITIFALAYVLLTSPGGSSQPPHCPSVSHRAQPWPHPGQSQLFADLSRE
ELTAVMRFLTQRLGPGLVDAAQAQPSDNCIFSVELQLPPKAAALAHLDRGSPPPAREALAI
VLFGGQPQPNVSELVVGPLPHPSYMRDVTVERHGGPLPYHRRPVLRAEFTQMWRHLKEV
ELPKAPIFLSSTFNYNGSTLAAVHATPRGLRSGDRATWMALYHNISGVGLFLHPVGLELLL
DHRALDPAHWTVQQVFYLGHYYADLGQLEREFKSGRLEVVRVPLPPPNGASSLRSRNSP
GPLPPLQFSPQGSQYSVQGNLVVSSLWSFTFGHGVFSGLRIFDVRFQGERIAYEVSVQECV
SIYGADSPKTMLTRYLDSSFGLGRNSRGLVRGVDCPYQATMVDIHILVGKGAVQLLPGAV
CVFEEAQGLPLRRHHNYLQNHFYGGLASSALVVRSVSSVGNYDYIWDFVLYPNGALEGR
VHATGYINTAFLKGGEEGLLFGNRVGERVLGTVHTHAFHFKLDLDVAGLKNWVVAEDVV
FKPVAAPWNPEHWLQRPQLTRQVLGKEDLTAFSLGSPLPRYLYLASNQTNAWGHQRGYRI
QIHSPLGIHIPLESDMERALSWGRYQLVVTQRKEEESQSSSIYHQNDIWTPTVTFADFINNE
TLLGEDLVAWVTASFLHIPHAEDIPNTVTLGNRVGFLLRPYNFFDEDPSIFSPGSVYFEKGQ
DAGLCSINPVACLPDLAACVPDLPPFSYHGF

>P35575 multipass membrane protein
MEEGMNVLHDFGIQSTHYLQVNYQDSQDWFILVSVIADLRNAFYVLFPIWFHLQEAVGIK
LLWVAVIGDWLNLVFKWILFGQRPYWWVLDTDYYSNTSVPLIKQFPVTCETGPGSPSGHA
MGTAGVYYVMVTSTLSIFQGKIKPTYRFRCLNVILWLGFWAVQLNVCLSRIYLAAHFPHQ
VVAGVLSGIAVAETFSHIHSIYNASLKKYFLITFFLFSFAIGFYLLLKGLGVDLLWTLEKAQR
WCEQPEWVHIDTTPFASLLKNLGTLFGLGLALNSSMYRESCKGKLSKWLPFRLSSIVASLV
LLHVFDSLKPPSQVELVFYVLSFCKSAVVPLASVSVIPYCLAQVLGQPHKKSL

>Q9BT67 multipass membrane protein
MALALAALAAVEPACGSRYQQLQNEEESGEPEQAAGDAPPPYSSISAESAAYFDYKDESG
FPKPPSYNVATTLPSYDEAERTKAEATIPLVPGRDEDFVGRDDFDDADQLRIGNDGIFMLTF
FMAFLFNWIGFFLSFCLTTSAAGRYGAISGFGLSLIKWILIVRFSTYFPGYFDGQYWLWWV
FLVLGFLLFLRGFINYAKVRKMPETFSNLPRTRVLFIY

>Q9HBG4 multipass membrane protein
MVSVFRSEEMCLSQLFLQVEAAYCCVAELGELGLVQFKDLNMNVNSFQRKFVNEVRRCE
SLERILRFLEDEMQNEIVVQLLEKSPLTPLPREMITLETVLEKLEGELQEANQNQQALKQSF
LELTELKYLLKKTQDFFETETNLADDFFTEDTSGLLELKAVPAYMTGKLGFIAGVINRERM
ASFERLLWRICRGNVYLKFSEMDAPLEDPVTKEEIQKNIFIIFYQGEQLRQKIKKICDGFRA
TVYPCPEPAVERREMLESVNVRLEDLITVITQTESHRQRLLQEAAANWHSWLIKVQKMKA
VYHILNMCNIDVTQQCVIAEIWFPVADATRIKRALEQGMELSGSSMAPIMTTVQSKTAPPT
FNRTNKFTAGFQNIVDAYGVGSYREINPAPYTIITFPFLFAVMFGDCGHGTVMLLAALWMI
LNERRLLSQKTDNEIWNTFFHGRYLILLMGIFSIYTGLIYNDCFSKSLNIFGSSWSVQPMFR
NGTWNTHVMEESLYLQLDPAIPGVYFGNPYPFGIDPIWNLASNKLTFLNSYKMKMSVILGI
VQMVFGVILSLFNHIYFRRTLNIILQFIPEMIFILCLFGYLVFMIIFKWCCFDVHVSQHAPSILI
HFINMFLFNYSDSSNAPLYKHQQEVQSFFVVMALISVPWMLLIKPFILRASHRKSQLQASR
IQEDATENIEGDSSSPSSRSGQRTSADTHGALDDHGEEFNFGDVFVHQAIHTIEYCLGCISN
TASYLRLWALSLAHAQLSEVLWTMVMNSGLQTRGWGGIVGVFIIFAVFAVLTVAILLIMEG
LSAFLHALRLHWVEFQNKFYVGDGYKFSPFSFKHILDGTAEE

>Q9BSN7 multipass membrane protein
MTVQRLVAAAVLVALVSLILNNVAAFTSNWVCQTLEDGRRRSVGLWRSCWLVDRTRGGP
SPGARAGQVDAHDCEALGWGSEAAGFQESRGTVKLQFDMMRACNLVATAALTAGQLTF
LLGLVGLPLLSPDAPCWEEAMAAAFQLASFVLVIGLVTFYRIGPYTNLSWSCYLNIGACLL
ATLAAAMLIWNILHKREDCMAPRVIVISRSLTARFRRGLDNDYVESPC

>P17540 peripheral membrane protein
MASIFSKLLTGRNASLLFATMGTSVLTTGYLLNRQKVCAEVREQPRLFPPSADYPDLRKHN
NCMAECLTPAIYAKLRNKVTPNGYTLDQCIQTGVDNPGHPFIKTVGMVAGDEESYEVFAD
LFDPVIKLRHNGYDPRVMKHTTDLDASKITQGQFDEHYVLSSRVRTGRSIRGLSLPPACTR
AERREVENVAITALEGLKGDLAGRYYKLSEMTEQDQQRLIDDHFLFDKPVSPLLTCAGMA
RDWPDARGIWHNYDKTFLIWINEEDHTRVISMEKGGNMKRVFERFCRGLKEVERLIQER
GWEFMWNERLGYILTCPSNLGTGLRAGVHVRIPKLSKDPRFSKILENLRLQKRGTGGVDT
AAVADVYDISNIDRIGRSEVELVQIVIDGVNYLVDCEKKLERGQDIKVPPPLPQFGKK

>P51828 multipass membrane protein
MPAKGRYFLNEGEEGPDQDALYEKYQLTSQHGPLLLTLLLVAATACVALIIIAFSQGDPSRH
QAILGMAFLVLAVFAALSVLMYVECLLRRWLRALALLTWACLVALGYVLVFDAWTKAAC
AWEQVPFFLFIVFVVYTLLPFSMRGAVAVGAVSTASHLLVLGSLMGGFTTPSVRVGLQLLA
NAVIFLCGNLTGAFHKHQMQDASRDLFTYTVKCIQIRRKLRIEKRQQENLLLSVLPAHISM
GMKLAIIERLKEHGDRRCMPDNNFHSLYVKRHQNVSILYADIVGFTQLASDCSPKELVVVL

NELFGKFDQIAKANECMRIKILGDCYYCVSGLPVSLPTHARNCVKMGLDMCQAIKQVRE
ATGVDINMRVGIHSGNVLCGVIGLRKWQYDVWSHDVSLANRMEAAGVPGRVHITEATLK
HLDKAYEVEDGHGQQRDPYLKEMNIRTYLVIDPRSQQPPPPSQHLPRPKGDAALKMRASV
RMTRYLESWGAARPFAHLNHRESVSSGETHVPNGRRPKSVPQRHRRTPDRSMSPKGRSE
DDSYDDEMLSAIEGLSSTRPCCSKSDDFYTFGSIFLEKGFEREYRLAPIPRARHDFACASLIF
VCILLVHVLLMPRTAALGVSFGLVACVLGLVLGLCFATKFSRCCPARGTLCTISERVETQPL
LRLTLAVLTIGSLLTVAIINLPLMPFQVPELPVGNETGLLAASSKTRALCEPLPYYTCSCVLG
FIACSVFLRMSLEPKVVLLTVALVAYLVLFNLSPCWQWDCCGQGLGNLTKPNGTTSGTPSC
SWKDLKTMTNFYLVLFYITLLTLSRQIDYYCRLDCLWKKKFKKEHEEFETMENVNRLLLE
NVLPAHVAAHFIGDKLNEDWYHQSYDCVCVMFASVPDFKVFYTECDVNKEGLECLRLLN
EIIADFDELLLKPKFSGVEKIKTIGSTYMAAAGLSVASGHENQELERQHAHIGVMVEFSIAL
MSKLDGINRHSFNSFRLRVGINHGPVIAGVIGARKPQYDIWGNTVNVASRMESTGELGKIQ
VTEETCTILQGLGYSCECRGLINVKGKGELRTYFVCTDTAKFQGLGLN

>P18827 single-pass type I membrane protein
MRRAALWLWLCALALSLQPALPQIVATNLPPEDQDGSGDDSDNFSGSGAGALQDITLSQQ
TPSTWKDTQLLTAIPTSPEPTGLEATAASTSTLPAGEGPKEGEAVVLPEVEPGLTAREQEATP
RPRETTQLPTTHLASTTTATTAQEPATSHPHRDMQPGHHETSTPAGPSQADLHTPHTEDGG
PSATERAAEDGASSQLPAAEGSGEQDFTFETSGENTAVVAVEPDRRNQSPVDQGATGASQG
LLDRKEVLGGVIAGGLVGLIFAVCLVGFMLYRMKKKDEGSYSLEEPKQANGGAYQKPTK
QEEFYA

>P48066 multipass membrane protein
MTAEKALPLGNGKAAEEARESEAPGGGCSSGGAAPARHPRVKRDKAVHERGHWNNKVE
FVLSVAGEIIGLGNVWRFPYLCYKNGGGAFLIPYVVFFICCGIPVFFLETALGQFTSEGGITC
WRKVCPLFEGIGYATQVIEAHLNVYYIIILAWAIFYLSNCFTTELPWATCGHEWNTENCVEF
QKLNVSNYSHVSLQNATSPVMEFWEHRVLAISDGIEHIGNLRWELALCLLAAWTICYFCI
WKGTKSTGKVVYVTATFPYIMLLILLIRGVTLPGASEGIKFYLYPDLSRLSDPQVWVDAGT
QIFFSYAICLGCLTALGSYNNYNNNCYRDCIMLCCLNSGTSFVAGFAIFSVLGFMAYEQGV
PIAEVAESGPGLAFIAYPKAVTMMPLSPLWATLFFMMLIFLGLDSQFVCVESLVTAVVDMY
PKVFRRGYRRELLILALSVISYFLGLVMLTEGGMYIFQLFDSYAASGMCLLFVAIFECICIG
WVYGSNRFYDNIEDMIGYRPPSLIKWCWMIMTPGICAGIFIFFLIKYKPLKYNNIYTYPAW
GYGIGWLMALSSMLCIPLWICITVWKTEGTLPEKLQKLTTPSTDLKMRGKLGVSPRMVTV
NDCDAKLKSDGTIAAITEKETHF

>Q8TEM1 single-pass type I membrane protein
MAARGRGLLLLTLSVLLAAGPSAAAAKLNIPKVLLPFTRATRVNFTLEASEGCYRWLSTRP
EVASIEPLGLDEQQCSQKAVVQARLTQPARLTSIIFAEDITTGQVLRCDAIVDLIHDIQIVSTT
RELYLEDSPLELKIQALDSEGNTFSTLAGLVFEWTIVKDSEADRFSDSHNALRILTFLESTYI
PPSYISEMEKAAKQGDTILVSGMKTGSSKLKARIQEAVYKNVRPAEVRLLILENILLNPAYD
VYLMVGTSIHYKVQKIRQGKITELSMPSDQYELQLQNSIPGPEGDPARPVAVLAQDTSMVT
ALQLGQSSLVLGHRSIRMQGASRLPNSTIYVVEPGYLGFTVHPGDRWVLETGRLYEITIEV
FDKFSNKVYVSDNIRIETVLPAEFFEVLSSSQNGSYHRIRALKRGQTAIDAALTSVVDQDG
GVHILQVPVWNQQEVEIHIPITLYPSILTFPWQPKTGAYQYTIRAHGGSGNFSWSSSSHLVA

TVTVKGVMTTGSDIGFSVIQAHDVQNPLHFGEMKVYVIEPHSMEFAPCQVEARVGQALE
LPLRISGLMPGGASEVVTLSDCSHFDLAVEVENQGVFQPLPGRLPPGSEHCSGIRVKAEAQ
GSTTLLVSYRHGHVHLSAKITIAAYLPLKAVDPSSVALVTLGSSKEMLFEGGPRPWILEPSK
FFQNVTAEDTDSIGLALFAPHSSRNYQQHWILVTCQALGEQVIALSVGNKPSLTNPFPAVEP
AVVKFVCAPPSRLTLAPVYTSPQLDMSCPLLQQNKQVVPVSSHRNPRLDLAAYDQEGRRF
DNFSSLSIQWESTRPVLASIEPELPMQLVSQDDESGQKKLHGLQAILVHEASGTTAITATAT
GYQESHLSSARTKQPHDPLVPLSASIELILVEDVRVSPEEVTIYNHPGIQAELRIREGSGYFF
LNTSTADVVKVAYQEARGVAMVHPLLPGSSTIMIHDLCLVFPAPAKAVVYYVSDIQELYIRV
VDKVEIGKTVKAYVRVLDLHKKPFLAKYFPFMDLKLRAASPIITLVALDEALDNYTITFLIR
GVAIGQTSLTASVTNKAGQRINSAPQQIEVFPPFRLMPRKVTLLIGATMQVTSEGGPQPQSN
ILFSISNESVALVSAAGLVQGLAIGNGTVSGLVQAVDAETGKVVIISQDLVQVEVLLLRAVR
IRAPIMRMRTGTQMPIYVTGITNHQNPFSFGNAVPGLTFHWSVTKRDVLDLRGRHHEASIR
LPSQYNFAMNVLGRVKGRTGLRVVVKAVDPTSGQLYGLARELSDEIQVQVFEKLQLLNPE
IEAEQILMSPNSYIKLQTNRDGAASLSYRVLDGPEKVPVVHVDEKGFLASGSMIGTSTIEVI
AQEPFGANQTIIVAVKVSPVSYLRVSMSPVLHTQNKEALVAVPLGMTVTFTVHFHDNSGD
VFHAHSSVLNFATNRDDFVQIGKGPTNNTCVVRTVSVGLTLLRVWDAEHPGLSDFMPLPV
LQAISPELSGAMVVGDVLCLATVLTSLEGLSGTWSSSANSILHIDPKTGVAVARAVGSVTV
YYEVAGHLRTYKEVVVSVPQRIMARHLHPIQTSFQEATASKVIVAVGDRSSNLRGECTPTQ
REVIQALHPETLISCQSQFKPAVFDFPSQDVFTVEPQFDTALGQYFCSITMHRLTDKQRKHL
SMKKTALVVSASLSSSHFSTEQVGAEVPFSPGLFADQAEILLSNHYTSSEIRVFGAPEVLEN
LEVKSGSPAVLAFAKEKSFGWPSFITYTVGVLDPAAGSQGPLSTTLTFSSPVTNQAIAIPVTV
AFVVDRRGPGPYGASLFQHFLDSYQVMFFTLFALLAGTAVMIIAYHTVCTPRDLAVPAALT
PRASPGHSPHYFAASSPTSPNALPPARKASPPSGLWSPAYASH

>Q9Y5J6 peripheral membrane protein
MERQQQQQQLRNLRDFLLVYNRMTELCFQRCVPSLHHRALDAEEEACLHSCAGKLIHS
NHRLMAAYVQLMPALVQRRIADYEAASAVPGVAAEQPGVSPSGS

>O60894 single-pass type I membrane protein
MARALCRLPRRGLWLLLAHHLFMTTACQEANYGALLRELCLTQFQVDMEAVGETLWCD
WGRTIRSYRELADCTWHMAEKLGCFWPNAEVDRFFLAVHGRYFRSCPISGRAVRDPPGSI
LYPFIVVPITVTLLVTALVVWQSKRTEGIV

>P23469 single-pass type I membrane protein
MEPLCPLLLVGFSLPLARALRGNETTADSNETTTTSGPPDPGASQPLLAWLLLPLLLLLLVL
LLAAYFFRFRKQRKAVVSTSDKKMPNGILEEQEQQRVMLLSRSPSGPKKYFPIPVEHLEEEI
RIRSADDCKQFREEFNSLPSGHIQGTFELANKEENREKNRYPNILPNDHSRVILSQLDGIPCS
DYINASYIDGYKEKNKFIAAQGPKQETVNDFWRMVWEQKSATIVMLTNLKERKEEKCHQ
YWPDQGCWTYGNIRVCVEDCVVLVDYTIRKFCIQPQLPDGCKAPRLVSQLHFTSWPDFG
VPFTPIGMLKFLKKVKTLNPVHAGPIVVHCSAGVGRTGTFIVIDAMMAMMHAEQKVDVF
EFVSRIRNQRPQMVQTDMQYTFIYQALLEYYLYGDTELDVSSLEKHLQTMHGTTTHFDKI
GLEEEFRKLTNVRIMKENMRTGNLPANMKKARVIQIIPYDFNRVILSMKRGQEYTDYINAS
FIDGYRQKDYFIATQGPLAHTVEDFWRMIWEWKSHTIVMLTEVQEREQDKCYQYWPTEG
SVTHGEITIEIKNDTLSEAISIRDFLVTLNQPQARQEEQVRVVRQFHFHGWPEIGIPAEGKG

MIDLIAAVQKQQQQTGNHPITVHCSAGAGRTGTFIALSNILERVKAEGLLDVFQAVKSLRL
QRPHMVQTLEQYEFCYKVVQDFIDIFSDYANFK

>Q9NWF4 multipass membrane protein
MAAPTLGRLVLTHLLVALFGMGSWAAVNGIWVELPVVVKDLPEGWSLPSYLSVVVALGN
LGLLVVTLWRQLAPGKGEQVPIQVVQVLSVVGTALLAPLWHHVAPVAGQLHSVAFLTLAL
VLAMACCTSNVTFLPFLSHLPPPFLRSFFLGQGLSALLPCVLALVQGVGRLECPPAPTNGTS
GPPLDFPERFPASTFFWALTALLVTSAAAFRGLLLLLPSLPSVTTGGSGPELQLGSPGAEEEE
KEEEEALPLQEPPSQAAGTIPGPDPEAHQLFSAHGAFLLGLMAFTSAVTNGVLPSVQSFSC
LPYGRLAYHLAVVLGSAANPLACFLAMGVLCRSLAGLVGLSLLGMLFGAYLMALAILSPC
PPLVGTTAGVVLVVLSWVLCLCVFSYVKVAASSLLHGGGRPALLAAGVAIQVGSLLGAGA
MFPPTSIYHVFQSRKDCVDPCGP

>Q02846 single-pass type I membrane protein
MTACARRAGGLPDPGLCGPAWWAPSLPRLPRALPRLPLLLLLLLLQPPALSAVFTVGVLGP
WACDPIFSRARPDLAARLAAARLNRDPGLAGGPRFEVALLPEPCRTPGSLGAVSSALARVS
GLVGPVNPAACRPAELLAEEAGIALVPWGCPWTQAEGTTAPAVTPAADALYALLRAFGWA
RVALVTAPQDLWVEAGRSLSTALRARGLPVASVTSMEPLDLSGAREALRKVRDGPRVTAVI
MVMHSVLLGGEEQRYLLEAAEELGLTDGSLVFLPFDTIHYALSPGPEALAALANSSQLRR
AHDAVLTLTRHCPSEGSVLDSLRRAQERRELPSDLNLQQVSPLFGTIYDAVFLLARGVAEA
RAAAGGRWVSGAAVARHIRDAQVPGFCGDLGGDEEPPFVLLDTDAAGDRLFATYMLDPA
RGSFLSAGTRMHFPRGGSAPGPDPSCWFDPNNICGGGLEPGLVFLGFLLVVGMGLAGAFL
AHYVRHRLLHMQMVSGPNKIILTVDDITFLHPHGGTSRKVAQGSRSSLGARSMSDIRSGPS
QHLDSPNIGVYEGDRVWLKKFPGDQHIAIRPATKTAFSKLQELRHENVALYLGLFLARGAE
GPAALWEGNLAVVSEHCTRGSLQDLLAQREIKLDWMFKSSLLLDLIKGIRYLHHRGVAHG
RLKSRNCIVDGRFVLKITDHGHGRLLEAQKVLPEPPRAEDQLWTAPELLRDPALERRGTL
AGDVFSLAIIMQEVVCRSAPYAMLELTPEEVVQRVRSPPPLCRPLVSMDQAPVECILLMKQ
CWAEQPELRPSMDHTFDLFKNINKGRKTNIIDSMLRMLEQYSSNLEDLIRERTEELELEKQ
KTDRLLTQMLPPSVAEALKTGTPVEPEYFEQVTLYFSDIVGFTTISAMSEPIEVVDLLNDLY
TLFDAIIGSHDVYKVETIGDAYMVASGLPQRNGQRHAAEIANMSLDILSAVGTFRMRHMP
EVPVRIRIGLHSGPCVAGVVGLTMPRYCLFGDTVNTASRMESTGLPYRIHVNLSTVGILRA
LDSGYQVELRGRTELKGKGAEDTFWLVGRRGFNKPIPKPPDLQPGSSNHGISLQEIPPERR
RKLEKARPGQFS

>O75616 peripheral membrane protein
MAAPSWRGARLVQSVLRVWQVGPHVARERVIPFSSLLGFQRRCVSCVAGSAFSGPRLASA
SRSNGQGSALDHFLGFSQPDSSVTPCVPAVSMNRDEQDVLLVHHPDMPENSRVLRVVLLG
APNAGKSTLSNQLLGRKVFPVSRKVHTTRCQALGVITEKETQVILLDTPGIISPGKQKRHH
LELSLLEDPWKSMESADLVVVLVDVSDKWTRNQLSPQLLRCLTKYSQIPSVLVMNKVDC
LKQKSVLLELTAALTEGVVNGKKLKMRQAFHSHPGTHCPSPAVKDPNTQSVGNPQRIGWP
HFKEIFMLSALSQEDVKTLKQYLLTQAQPGPWEYHSAVLTSQTPEEICANIIREKLLEHLPQ
EVPYNVQQKTAVWEEGPGGELVIQQKLLVPKESYVKLLIGPKGHVISQIAQEAGHDLMDIF
LCDVDIRLSVKLLK

>P78504 single-pass type I membrane protein
MRSPRTRGRSGRPLSLLLALLCALRAKVCGASGQFELEILSMQNVNGELQNGNCCGGAR
NPGDRKCTRDECDTYFKVCLKEYQSRVTAGGPCSFGSGSTPVIGGNTFNLKASRGNDRNR
IVLPFSFAWPRSYTLLVEAWDSSNDTVQPDSIIEKASHSGMINPSRQWQTLKQNTGVAHFE
YQIRVTCDDYYYGFGCNKFCRPRDDFFGHYACDQNGNKTCMEGWMGPECNRAICRQGC
SPKHGSCKLPGDCRCQYGWQGLYCDKCIPHPGCVHGICNEPWQCLCETNWGGQLCDKD
LNYCGTHQPCLNGGTCSNTGPDKYQCSCPEGYSGPNCEIAEHACLSDPCHNRGSCKETSL
GFECECSPGWTGPTCSTNIDDCSPNNCSHGGTCQDLVNGFKCVCPPQWTGKTCQLDANE
CEAKPCVNAKSCKNLIASYYCDCLPGWMGQNCDININDCLGQCQNDASCRDLVNGYRCI
CPPGYAGDHCERDIDECASNPCLNGGHCQNEINRFQCLCPTGFSGNLCQLDIDYCEPNPCQ
NGAQCYNRASDYFCKCPEDYEGKNCSHLKDHCRTTPCEVIDSCTVAMASNDTPEGVRYIS
SNVCGPHGKCKSQSGGKFTCDCNKGFTGTYCHENINDCESNPCRNGGTCIDGVNSYKCIC
SDGWEGAYCETNINDCSQNPCHNGGTCRDLVNDFYCDCKNGWKGKTCHSRDSQCDEAT
CNNGGTCYDEGDAFKCMCPGGWEGTTCNIARNSSCLPNPCHNGGTCVVNGESFTCVCK
EGWEGPICAQNTNDCSPHPCYNSGTCVDGDNWYRCECAPGFAGPDCRININECQSSPCAF
GATCVDEINGYRCVCPPGHSGAKCQEVSGRPCITMGSVIPDGAKWDDDCNTCQCLNGRI
ACSKVWCGPRPCLLHKGHSECPSGQSCIPILDDQCFVHPCTGVGECRSSSLQPVKTKCTSD
SYYQDNCANITFTFNKEMMSPGLTTEHICSELRNLNILKNVSAEYSIYIACEPSPSANNEIHV
AISAEDIRDDGNPIKEITDKIIDLVSKRDGNSSLIAAVAEVRVQRRPLKNRTDFLVPLLSSVLT
VAWICCLVTAFYWCLRKRRKPGSHTHSASEDNTTNNVREQLNQIKNPIEKHGANTVPIKD
YENKNSKMSKIRTHNSEVEEDDMDKHQQKARFAKQPAYTLVDREEKPPNGTPTKHPNWT
NKQDNRDLESAQSLNRMEYIV

>P60880 lipid-anchor
MAEDADMRNELEEMQRRADQLADESLESTRRMLQLVEESKDAGIRTLVMLDEQGEQLE
RIEEGMDQINKDMKEAEKNLTDLGKFCGLCVCPCNKLKSSDAYKKAWGNNQDGVVASQ
PARVVDEREQMAISGGFIRRVTNDARENEMDENLEQVSGIIGNLRHMALDMGNEIDTQNR
QIDRIMEKADSNKTRIDEANQRATKMLGSG

>P31391 multipass membrane protein
MSAPSTLPPGGEEGLGTAWPSAANASSAPAEAEEAVAGPGDARAAGMVAIQCIYALVCLV
GLVGNALVIFVILRYAKMKTATNIYLLNLAVADELFMLSVPFVASSAALRHWPFGSVLCRA
VLSVDGLNMFTSVFCLTVLSVDRYVAVVHPLRAATYRRPSVAKLINLGVWLASLLVTLPIA
IFADTRPARGGQAVACNLQWPHPAWSAVFVVYTFLLGFLLPVLAIGLCYLLIVGKMRAVAL
RAGWQQRRRSEKKITRLVLMVVVVFVLCWMPFYVVQLLNLFVTSLDATVNHVSLILSYA
NSCANPILYGFLSDNFRRFFQRVLCLRCCLLEGAGGAEEEPLDYYATALKSKGGAGCMCPP
LPCQQEALQPEPGRKRIPLTRTTTF

>Q9H310 multipass membrane protein
MAGSPSRAAGRRLQLPLLCLFLQGATAVLFAVFVRYNHKTDAALWHRSNHSNADNEFYF
RYPSFQDVHAMVFVGFGFLMVFLQRYGFSSVGFTFLLAAFALQWSTLVQGFLHSFHGGHI
HVGVESMINADFCAGAVLISFGAVLGKTGPTQLLLMALLEVVLFGINEFVLLHLLGVRDA
GGSMTIHTFGAYFGLVLSRVLYRPQLEKSKHRQGSVYHSDLFAMIGTIFLWIFWPSFNAALT
ALGAGQHRTALNTYYSLAASTLGTFALSALVGEDGRLDMVHIQNAALAGGVVVGTSSEM

MLTPFGALAAGFLAGTVSTLGYKFFTPILESKFKVQDTCGVHNLHGMPGVLGALLGVLVA
GLATHEAYGDGLESVFPLIAEGQRSATSQAMHQLFGLFVTLMFASVGGGLGGLLLKLPFL
DSPPRLPALRGPSSLAGAWRA

>Q96HD9 peripheral membrane protein
MCSLPVPREPLRRVAVTGGTHGNEMSGVYLARHWLHAPAELQRASFSAVPVLANPAATSG
CRRYVDHDLNRTFTSSFLNSRPTPDDPYEVTRARELNQLLGPKASGQAFDFVLDLHNTTA
NMGTCLIAKSSHEVFAMHLCRHLQLQYPELSCQVFLYQRSGEESYNLDSVAKNGLGLELG
PQPQGVLRADIFSRMRTLVATVLDFIELFNQGTAFPAFEMEAYRPVGVVDFPRTEAGHLAG
TVHPQLQDRDFQPLQPGAPIFQMFSGEDLLYEGESTVYPVFINEAAYYEKGVAFVQTEKFT
FTVPAMPALTPAPSPAS

>O00203 peripheral membrane protein
MSSNSFPYNEQSGGGEATELGQEATSTISPSGAFGLFSSDLKKNEDLKQMLESNKDSAKLD
AMKRIVGMIAKGKNASELFPAVVKNVASKNIEIKKLVYVYLVRYAEEQQDLALLSISTFQR
ALKDPNQLIRASALRVLSSIRVPIIVPIMMLAIKEASADLSPYVRKNAAHAIQKLYSLDPEQ
KEMLIEVIEKLLKDKSTLVAGSVVMAFEEVCPDRIDLIHKNYRKLCNLLVDVEEWGQVVII
HMLTRYARTQFVSPWKEGDELEDNGKNFYESDDDQKEKTDKKKKPYTMDPDHRLLIRNT
KPLLQSRNAAVVMAVAQLYWHISPKSEAGIISKSLVRLLRSNREVQYIVLQNIATMSIQRKG
MFEPYLKSFYVRSTDPTMIKTLKLEILTNLANEANISTLLREFQTYVKSQDKQFAAATIQTI
GRCATNILEVTDTCLNGLVCLLSNRDEIVVAESVVVIKKLLQMQPAQHGEIIKHMAKLLDS
ITVPVARASILWLIGENCERVPKIAPDVLRKMAKSFTSEDDLVKLQILNLGAKLYLTNSKQT
KLLTQYILNLGKYDQNYDIRDRTRFIRQLIVPNVKSGALSKYAKKIFLAQKPAPLLESPFKD
RDHFQLGTLSHTLNIKATGYLELSNWPEVAPDPSVRNVEVIELAKEWTPAGKAKQENSAK
KFYSESEEEEDSSDSSSDSESESGSESGEQGESGEEGDSNEDSSEDSSSEQDSESGRESGLEN
KRTAKRNSKAKGKSDSEDGEKENEKSKTSDSSNDESSSIEDSSSDSESESEPESESESRRVT
KEKEKKTKQDRTPLTKDVSLLDLDDFNPVSTPVALPTPALSPSLMADLEGLHLSTSSSVISV
STPAFVPTKTHVLLHRMSGKGLAAHYFFPRQPCIFGDKMVSIQITLNNTTDRKIENIHIGEK
KLPIGMKMHVFNPIDSLEPEGSITVSMGIDFCDSTQTASFQLCTKDDCFNVNIQPPVGELLL
PVAMSEKDFKKEQGVLTGMNETSAVIIAAPQNFTPSVIFQKVVNVANVGAVPSGQDNIHRF
AAKTVHSGSLMLVTVELKEGSTAQLIINTEKTVIGSVLLRELKPVLSQG

>Q15849 multipass membrane protein
MSDPHSSPLLPEPLSSRYKLYEAEFTSPSWPSTSPDTHPALPLLEMPEEKDLRSSNEDSHIVK
IEKLNERSKRKDDGVAHRDSAGQRCICLSKAVGYLTGDMKEYRIWLKDKHLALQFIDWV
LRGTAQVMFINNPLSGLIIFIGLLIQNPWWTITGGLGTVVSTLTALALGQDRSAIASGLHGY
NGMLVGLLMAVFSEKLDYYWWLLFPVTFTAMSCPVLSSALNSIFSKWDLPVFTLPFNIAV
TLYLAATGHYNLFFPTTLVEPVSSVPNITWTEMEMPLLLQAIPVGVGQVYGCDNPWTGGV
FLVALFISSPLICLHAAIGSIVGLLAALSVATPFETIYTGLWSYNCVLSCIAIGGMFYALTWQT
HLLALICALFCAYMEAAISNIMSVVGVPPGTWAFCLATIIFLLLTTNNPAIFRLPLSKVTYPE
ANRIYYLTVKSGEEEKAPSGGGGEHPPTAGPKVEEGSEAVLSKHRSVFHIEWSSIRRRSKV
FGKGEHQERQNKDPFPYRYRKPTVELLDLDTMEESSEIKVETNISKTSWIRSSMAASGKRV
SKALSYITGEMKECGEGLKDKSPVFQFFDWVLRGTSQVMFVNNPLSGILIILGLFIQNPWW
AISGCLGTIMSTLTALILSQDKSAIAAGFHGYNGVLVGLLMAVFSDKGDYYWWLLLPVIIM

SMSCPILSSALGTIFSKWDLPVFTLPFNITVTLYLAATGHYNLFFPTTLLQPASAMPNITWSE
VQVPLLLRAIPVGIGQVYGCDNPWTGGIFLIALFISSPLICLHAAIGSTMGMLAALTIATPFD
SIYFGLCGFNSTLACIAIGGMFYVITWQTHLLAIACALFAAYLGAALANMLSVFGLPPCTW
PFCLSALTFLLLTTNNPAIYKLPLSKVTYPEANRIYYLSQERNRRASIITKYQAYDVS

>Q9GZQ4 multipass membrane protein
MSGMEKLQNASWIYQQKLEDPFQKHLNSTEEYLAFLCGPRRSHFFLPVSVVYVPIFVVGV
IGNVLVCLVILQHQAMKTPTNYYLFSLAVSDLLVLLLGMPLEVYEMWRNYPFLFGPVGCY
FKTALFETVCFASILSITTVSVERYVAILHPFRAKLQSTRRRALRILGIVWGFSVLFSLPNTSI
HGIKFHYFPNGSLVPGSATCTVIKPMWIYNFIIQVTSFLFYLLPMTVISVLYYLMALRLKKD
KSLEADEGNANIQRPCRKSVNKMLFVLVLVFAICWAPFHIDRLFFSFVEEWSESLAAVFNL
VHVVSGVFFYLSSAVNPIIYNLLSRRFQAAFQNVISSFHKQWHSQHDPQLPPAQRNIFLTEC
HFVELTEDIGPQFPCQSSMHNSHLPAALSSEQMSRTNYQSFHFNKT

>P16499 lipid-anchor
MGEVTAEEVEKFLDSNIGFAKQYYNLHYRAKLISDLLGAKEAAVDFSNYHSPSSMEESEII
FDLLRDFQENLQTEKCIFNVMKKLCFLLQADRMSLFMYRTRNGIAELATRLFNVHKDAVL
EDCLVMPDQEIVFPLDMGIVGHVAHSKKIANVPNTEEDEHFCDFVDILTEYKTKNILASPI
MNGKDVVAIIMAVNKVDGSHFTKRDEEILLKYLNFANLIMKVYHLSYLHNCETRRGQILL
WSGSKVFEELTDIERQFHKALYTVRAFLNCDRYSVGLLDMTKQKEFFDVWPVLMGEVPP
YSGPRTPDGREINFYKVIDYILHGKEDIKVIPNPPPDHWALVSGLPAYVAQNGLICNIMNAP
AEDFFAFQKEPLDESGWMIKNVLSMPIVNKKEEIVGVATFYNRKDGKPFDEMDETLMESL
TQFLGWSVLNPDTYESMNKLENRKDIFQDIVKYHVKCDNEEIQKILKTREVYGKEPWECE
EEELAEILQAELPDADKYEINKFHFSDLPLTELELVKCGIQMYYELKVVDKFHIPQEALVRF
MYSLSKGYRKITYHNWRHGFNVGQTMFSLLVTGKLKRYFTDLEALAMVTAAFCHDIDHR
GTNNLYQMKSQNPLAKLHGSSILERHHLEFGKTLLRDESLNIFQNLNRRQHEHAIHMMDI
AIIATDLALYFKKRTMFQKIVDQSKTYESEQEWTQYMMLEQTRKEIVMAMMMTACDLSA
ITKPWEVQSQVALLVAAEFWEQGDLERTVLQQNPIPMMDRNKADELPKLQVGFIDFVCTF
VYKEFSRFHEEITPMLDGITNNRKEWKALADEYDAKMKVQEEKKQKQQSAKSAAAGNQ
PGGNPSPGGATTSKSCCIQ

>P20309 multipass membrane protein
MTLHNNSTTSPLFPNISSSWIHSPSDAGLPPGTVTHFGSYNVSRAAGNFSSPDGTTDDPLG
GHTVWQVVFIAFLTGILALVTIIGNILVIVSFKVNKQLKTVNNYFLLSLACADLIIGVISMNL
FTTYIIMNRWALGNLACDLWLAIDYVASNASVMNLLVISFDRYFSITRPLTYRAKRTTKRA
GVMIGLAWVISFVLWAPAILFWQYFVGKRTVPPGECFIQFLSEPTITFGTAIAAFYMPVTIM
TILYWRIYKETEKRTKELAGLQASGTEAETENFVHPTGSSRSCSSYELQQQSMKRSNRRK
YGRCHFWFTTKSWKPSSEQMDQDHSSSDSWNNNDAAASLENSASSDEEDIGSETRAIYSI
VLKLPGHSTILNSTKLPSSDNLQVPEEELGMVDLERKADKLQAQKSVDDGGSFPKSFSKL
PIQLESAVDTAKTSDVNSSVGKSTATLPLSFKEATLAKRFALKTRSQITKRKRMSLVKEKKA
AQTLSAILLAFIITWTPYNIMVLVNTFCDSCIPKTFWNLGYWLCYINSTVNPVCYALCNKT
FRTTFKMLLLCQCDKKKRRKQQYQQRQSVIFHKRAPEQAL

>P01116 lipid-anchor

MTEYKLVVVGAGGVGKSALTIQLIQNHFVDEYDPTIEDSYRKQVVIDGETCLLDILDTAG
QEEYSAMRDQYMRTGEGFLCVFAINNTKSFEDIHHYREQIKRVKDSEDVPMVLVGNKCD
LPSRTVDTKQAQDLARSYGIPFIETSAKTRQRVEDAFYTLVREIRQYRLKKISKEEKTPGCV
KIKKCIIM

>Q9Y5Y7 single-pass type I membrane protein
MARCFSLVLLLTSIWTTRLLVQGSLRAEELSIQVSCRIMGITLVSKKANQQLNFTEAKEACR
LLGLSLAGKDQVETALKASFETCSYGWVGDGFVVISRISPNPKCGKNGVGVLIWKVPVSR
QFAAYCYNSSDTWTNSCIPEIITTKDPIFNTQTATQTTEFIVSDSTYSVASPYSTIPAPTTTPPA
PASTSIPRRKKLICVTEVFMETSTMSTETEPFVENKAAFKNEAAGFGGVPTALLVLALLFFG
AAAGLGFCYVKRYVKAFPFTNKNQQKEMIETKVVKEEKANDSNPNEESKKTDKNPEESK
SPSKTTVRCLEAEV

>Q86WG5 peripheral membrane protein
MARLADYFIVVGYDHEKPGSGEGLGKIIQRFPQKDWDDTPFPQGIELFCQPGGWQLSRER
KQPTFFVVVLTDIDSDRHYCSCLTFYEAEINLQGTKKEEIEGEAKVSGLIQPAEVFAPKSLVL
VSRLYYPEIFRACLGLIYTVYVDSLNVSLESLIANLCACLVPAAGGSQKLFSLGAGDRQLIQ
TPLHDSLPITGTSVALLFQQLGIQNVLSLFCAVLTENKVLFHSASFQRLSDACRALESLMFP
LKYSYPYIPILPAQLLEVLSSPTPFIIGVHSVFKTDVHELLDVIIADLDGGTIKIPECIHLSSLP
EPLLHQTQSALSLILHPDLEVADHAFPPPRTALSHSKMLDKEVRAVFLRLFAQLFQGYRSC
LQLIRIHAEPVIHFHKTAFLGQRGLVENDFLTKVLSGMAFAGFVSERGPPYRSCDLFDELVA
FEVERIKVEENNPVKMIKHVRELAEQLFKNENPNPHMAFQKVPRPTEGSHLRVHILPFPEI
NEARVQELIQENVAKNQNAPPATRIEKKCVVPAGPPVVSIMDKVTTVFNSAQRLEVVRNCI
SFIFENKILETEKTLPAALRALKGKAARQCLTDELGLHVQQNRAILDHQQFDYIIRMMNCT
LQDCSSLEEYNIAAALLPLTSAFYRKLAPGVSQFAYTCVQDHPIWTNQQFWETTFYNAVQ
EQVRSLYLSAKEDNHAPHLKQKDKLPDDHYQEKTAMDLAAEQLRLWPTLSKSTQQELVQ
HEESTVFSQAIHFANLMVNLLVPLDTSKNKLLRTSAPGDWESGSNSIVTNSIAGSVAESYD
TESGFEDSENTDIANSVVRFITRFIDKVCTESGVTQDHIKSLHCMIPGIVAMHIETLEAVHRE
SRRLPPIQKPKILRPALLPGEEIVCEGLRVLLDPDGREEATGGLLGGPQLLPAEGALFLTTYR
ILFRGTPHDQLVGEQTVVRSFPIASITKEKKITMQNQLQQNMQEGLQITSASFQLIKVAFDE
EVSPEVVEIFKKQLMKFRYPQSIFSTFAFAAGQTTPQIILPKQKEKNTSFRTFSKTIVKGAKR
AGKMTIGRQYLLKKKTGTIVEERVNRPGWNEDDDVSVSDESELPTSTTLKASEKSTMEQL
VEKACFRDYQRLGLGTISGSSSRSRPEYFRITASNRMYSLCRSYPGLLVVPQAVQDSSLPRV
ARCYRHNRLPVVCWKNSRSGTLLLRSGGFHGKGVVGLFKSQNSPQAAPTSSLESSSSIEQ
EKYLQALLNAVSVHQKLRGNSTLTVRPAFALSPGVWASLRSSTRLISSPTSFIDVGARLAG
KDHSASFSNSSYLQNQLLKRQAALYIFGEKSQLRNFKVEFALNCEFVPVEFHEIRQVKASF
KKLMRACIPSTIPTDSEVTFLKALGDSEWFPQLHRIMQLAVVVSEVLENGSSVLVCLEEGW
DITAQVTSLVQLLSDPFYRTLEGFQMLVEKEWLSFGHKFSQRSSLTLNCQGSGFAPVFLQF
LDCVHQVHNQYPTEFEFNLYYLKFLAFHYVSNRFKTFLLDSDYERLEHGTLFDDKGEKH
AKKGVCIWECIDRMHKRSPIFFNYLYSPLEIEALKPNVNVSSLKKWDYYIEETLSTGPSYD
WMMLTPKHFPSEDSDLAGEAGPRSQRRTVWPCYDDVSCTQPDALTSLFSEIEKLEHKLNQ
APEKWQQLWERVTVDLKEEPRTDRSQRHLSRSPGIVSTNLPSYQKRSLLHLPDSSMGEEQ
NSSISPSNGVERRAATLYSQYTSKNDENRSFEGTLYKRGALLKGWKPRWFVLDVTKHQLR
YYDSGEDTSCKGHIDLAEVEMVIPAGPSMGAPKHTSDKAFFDLKTSKRVYNFCAQDGQS

AQQWMDKIQSCISDA

>P62070 lipid-anchor
MAAAGWRDGSGQEKYRLVVVGGGGVGKSALTIQFIQSYFVTDYDPTIEDSYTKQCVIDD
RAARLDILDTAGQEEFGAMREQYMRTGEGFLLVFSVTDRGSFEEIYKFQRQILRVKDRDEF
PMILIGNKADLDHQRQVTQEEGQQLARQLKVTYMEASAKIRMNVDQAFHELVRVIRKFQ
EQECPPSPEPTRKEKDKKGCHCVIF

>Q96BY9 single-pass type I membrane protein
MAAACGPGAAGYCLLLGLHLFLLTAGPALGWNDPDRMLLRDVKALTLHYDRYTTSRRL
DPIPQLKCVGGTAGCDSYTPKVIQCQNKGWDGYDVQWECKTDLDIAYKFGKTVVSCEGY
ESSEDQYVLRGSCGLEYNLDYTELGLQKLKESGKQHGFASFSDYYYKWSSADSCNMSGL
ITIVVLLGIAFVVYKLFLSDGQYSPPPYSEYPPFSHRYQRFTNSAGPPPPGFKSEFTGPQNTG
HGATSGFGSAFTGQQGYENSGPGFWTGLGTGGILGYLFGSNRAATPFSDSWYYPSYPPSY
PGTWNRAYSPLHGGSGSYSVCSNSDTKTRTASGYGGTRRR

>O43506 single-pass type I membrane protein
MAVGEPLVHIRVTLLLLWFGMFLSISGHSQARPSQYFTSPEVVIPLKVISRGRGAKAPGWL
SYSLRFGGQRYIVHMRVNKLLFAAHLPVFTYTEQHALLQDQPFIQDDCYYHGYVEGVPES
LVALSTCSGGFLGMLQINDLVYEIKPISVSATFEHLVYKIDSDDTQFPPMRCGLTEEKIAHQ
MELQLSYNFTLKQSSFVGWWTHQRFVELVVVVDNIRYLFSQSNATTVQHEVFNVVNIVD
SFYHPLEVDVILTGIDIWTASNPLPTSGDLDNVLEDFSIWKNYNLNNRLQHDVAHLFIKDT
QGMKLGVAYVKGICQNPFNTGVDVFEDNRLVVFAITLGHELGHNLGMQHDTQWCVCEL
QWCIMHAYRKVTTKFSNCSYAQYWDSTISSGLCIQPPPYPGNIFRLKYCGNLVVEEGEECD
CGTIRQCAKDPCCLLNCTLHPGAACAFGICCKDCKFLPSGTLCRQQVGECDLPEWCNGTS
HQCPDDVYVQDGISCNVNAFCYEKTCNNHDIQCKEIFGQDARSASQSCYQEINTQGNRFG
HCGIVGTTYVKCWTPDIMCGRVQCENVGVIPNLIEHSTVQQFHLNDTTCWGTDYHLGMA
IPDIGEVKDGTVCGPEKICIRKKCASMVHLSQACQPKTCNMRGICNNKQHCHCNHEWAPP
YCKDKGYGGSADSGPPPKNNMEGLNVMGKLRYLSLLCLLPLVAFLLFCLHVLFKKRTKS
KEDEEG

>Q9Y5U9 multipass membrane protein
MAFTLYSLLQAALLCVNAIAVLHEERFLKNIGWGTDQGIGGFGEEPGIKSQLMNLIRSVRT
VMRVPLIIVNSIAIVLLLLFG

>Q99467 single-pass type I membrane protein
MAFDVSCFFWVVLFSAGCKVITSWDQMCIEKEANKTYNCENLGLSEIPDTLPNTTEFLEFS
FNFLPTIHNRTFSRLMNLTFLDLTRCQINWIHEDTFQSHHQLSTLVLTGNPLIFMAETSLNGP
KSLKHLFLIQTGISNLEFIPVHNLENLESLYLGSNHISSIKFPKDFPARNLKVLDFQNNAIHYI
SREDMRSLEQAINLSLNFNGNNVKGIELGAFDSTIFQSLNFGGTPNLSVIFNGLQNSTTQSL
WLGTFEDIDDEDISSAMLKGLCEMSVESLNLQEHRFSDISSTTFQCFTQLQELDLTATHLKG
LPSGMKGLNLLKKLVLSVNHFDQLCQISAANFPSLTHLYIRGNVKKLHLGVGCLEKLGNL
QTLDLSHNDIEASDCCSLQLKNLSHLQTLNLSHNEPLGLQSQAFKECPQLELLDLAFTRLH
INAPQSPFQNLHFLQVLNLTYCFLDTSNQHLLAGLPVLRHLNLKGNHFQDGTITKTNLLQT

VGSLEVLILSSCGLLSIDQQAFHSLGKMSHVDLSHNSLTCDSIDSLSHLKGIYLNLAANSINI
ISPRLLPILSQQSTINLSHNPLDCTCSNIHFLTWYKENLHKLEGSEETTCANPPSLRGVKLSD
VKLSCGITAIGIFFLIVFLLLLAILLFFAVKYLLRWKYQHI

>Q01344 single-pass type I membrane protein
MIIVAHVLLILLGATEILQADLLPDEKISLLPPVNFTIKVTGLAQVLLQWKPNPDQEQRNVN
LEYQVKINAPKEDDYETRITESKCVTILHKGFSASVRTILQNDHSLLASSWASAELHAPPGS
PGTSIVNLTCTTNTTEDNYSRLRSYQVSLHCTWLVGTDAPEDTQYFLYYRYGSWTEECQE
YSKDTLGRNIACWFPRTFILSKGRDWLAVLVNGSSKHSAIRPFDQLFALHAIDQINPPLNVT
AEIEGTRLSIQWEKPVSAFPIHCFDYEVKIHNTRNGYLQIEKLMTNAFISIIDDLSKYDVQV
RAAVSSMCREAGLWSEWSQPIYVGNDEHKPLREWFVIVIMATICFILLILSLICKICHLWIKL
FPPIPAPKSNIKDLFVTTNYEKAGSSETEIEVICYIEKPGVETLEDSVF

>P40337 peripheral membrane protein
MPRRAENWDEAEVGAEEAGVEEYGPEEDGGEESGAEESGPEESGPEELGAEEEMEAGRP
RPVLRSVNSREPSQVIFCNRSPRVVLPVWLNFDGEPQPYPTLPPGTGRRIHSYRGHLWLFR
DAGTHDGLLVNQTELFVPSLNVDGQPIFANITLPVYTLKERCLQVVRSLVKPENYRRLDIV
RSLYEDLEDHPNVQKDLERLTQERIAHQRMGD

>Q92611 single-pass type II membrane protein
MQWRALVLGLVLLRLGLHGVLWLVFGLGPSMGFYQRFPLSFGFQRLRSPDGPASPTSGPV
GRPGGVSGPSWLQPPGTGAAQSPRKAPRRPGPGMCGPANWGYVLGGRGRGPDEYEKRY
SGAFPPQLRAQMRDLARGMFVFGYDNYMAHAFPQDELNPIHCRGRGPDRGDPSNLNIND
VLGNYSLTLVDALDTLAIMGNSSEFQKAVKLVINTVSFDKDSTVQVFEATIRVLGSLLSAH
RIITDSKQPFGDMTIKDYDNELLYMAHDLAVRLLPAFENTKTGIPYPRVNLKTGVPPDTNN
ETCTAGAGSLLVEFGILSRLLGDSTFEWVARRAVKALWNLRSNDTGLLGNVVNIQTGHWV
GKQSGLGAGLDSFYEYLLKSYILFGEKEDLEMFNAAYQSIQNYLRRGREACNEGEGDPPL
YVNVNMFSGQLMNTWIDSLQAFFPGLQVLIGDVEDAICLHAFYYAIWKRYGALPERYNW
QLQAPDVLFYPLRPELVESTYLLYQATKNPFYLHVGMDILQSLEKYTKVKCGYATLHHVI
DKSTEDRMESFFLSETCKYLYLLFDEDNPVHKSGTRYMFTTEGHIVSVDEHLRELPWKEF
FSEEGGQDQGGKSVHRPKPHELKVINSSSNCNRVPDERRYSLPLKSIYMRQIDQMVGLI

>Q9NPA0 single-pass type I membrane protein
MAAALWGFFPVLLLLLLLSGDVQSSEVPGAAAEGSGGSGVGIGDRFKIEGRAVVPGVKPQD
WISAARVLVDGEEHVGFLKTDGSFVVHDIPSGSYVVEVVSPAYRFDPVRVDITSKGKMRA
RYVNYIKTSEVVRLPYPLQMKSSGPPSYFIKRESWGWTDFLMNPMVMMMVLPLLIFVLL
PKVVNTSDPDMRREMEQSMNMLNSNHELPDVSEFMTRLFSSKSSGKSSSGSSKTGKSGA
GKRR

>Q99523 single-pass type I membrane protein
MERPWGAADGLSRWPHGLGLLLLLQLLPPSTLSQDRLDAPPPPAAPLPRWSGPIGVSWGL
RAAAAGGAFPRGGRWRRSAPGEDEECGRVRDFVAKLANNTHQHVFDDLRGSVSLSWVG
DSTGVILVLTTFHVPLVIMTFGQSKLYRSEDYGKNFKDITDLINNTFIRTEFGMAIGPENSGK
VVLTAEVSGGSRGGRIFRSSDFAKNFVQTDLPFHPLTQMMYSPQNSDYLLALSTENGLWV

SKNFGGKWEEIHKAVCLAKWGSDNTIFFTTYANGSCKADLGALELWRTSDLGKSFKTIGV
KIYSFGLGGRFLFASVMADKDTTRRIHVSTDQGDTWSMAQLPSVGQEQFYSILAANDDM
VFMHVDEPGDTGFGTIFTSDDRGIVYSKSLDRHLYTTTGGETDFTNVTSLRGVYITSVLSE
DNSIQTMITFDQGGRWTHLRKPENSECDATAKNKNECSLHIHASYSISQKLNVPMAPLSEP
NAVGIVIAHGSVGDAISVMVPDVYISDDGGYSWTKMLEGPHYYTILDSGGIIVAIEHSSRPI
NVIKFSTDEGQCWQTYTFTRDPIYFTGLASEPGARSMNISIWGFTESFLTSQWVSYTIDFKD
ILERNCEEKDYTIWLAHSTDPEDYEDGCILGYKEQFLRLRKSSVCQNGRDYVVTKQPSICL
CSLEDFLCDFGYYRPENDSKCVEQPELKGHDLEFCLYGREEHLTTNGYRKIPGDKCQGGV
NPVREVKDLKKKCTSNFLSPEKQNSKSNSVPIILAIVGLMLVTVVAGVLIVKKYVCGGRFL
VHRYSVLQQHAEANGVDGVDALDTASHTNKSGYHDDSDEDLLE

>Q04721 single-pass type I membrane protein
MPALRPALLWALLALWLCCAAPAHALQCRDGYEPCVNEGMCVTYHNGTGYCKCPEGFL
GEYCQHRDPCEKNRCQNGGTCVAQAMLGKATCRCASGFTGEDCQYSTSHPCFVSRPCLN
GGTCHMLSRDTYECTCQVGFTGKECQWTDACLSHPCANGSTCTTVANQFSCKCLTGFTG
QKCETDVNECDIPGHCQHGGTCLNLPGSYQCQCPQGFTGQYCDSLYVPCAPSPCVNGGT
CRQTGDFTFECNCLPGFEGSTCERNIDDCPNHRCQNGGVCVDGVNTYNCRCPPQWTGQF
CTEDVDECLLQPNACQNGGTCANRNGGYGCVCVNGWSGDDCSENIDDCAFASCTPGST
CIDRVASFSCMCPEGKAGLLCHLDDACISNPCHKGALCDTNPLNGQYICTCPQGYKGADC
TEDVDECAMANSNPCEHAGKCVNTDGAFHCECLKGYAGPRCEMDINECHSDPCQNDAT
CLDKIGGFTCLCMPGFKGVHCELEINECQSNPCVNNGQCVDKVNRFQCLCPPGFTGPVCQ
IDIDDCSSTPCLNGAKCIDHPNGYECQCATGFTGVLCEENIDNCDPDPCHHGQCQDGIDSY
TCICNPGYMGAICSDQIDECYSSPCLNDGRCIDLVNGYQCNCQPGTSGVNCEINFDDCASN
PCIHGICMDGINRYSCVCSPGFTGQRCNIDIDECASNPCRKGATCINGVNGFRCICPEGPHH
PSCYSQVNECLSNPCIHGNCTGGLSGYKCLCDAGWVGINCEVDKNECLSNPCQNGGTCD
NLVNGYRCTCKKGFKGYNCQVNIDECASNPCLNQGTCFDDISGYTCHCVLPYTGKNCQT
VLAPCSPNPCENAAVCKESPNFESYTCLCAPGWQGQRCTIDIDECISKPCMNHGLCHNTQ
GSYMCECPPGFSGMDCEEDIDDCLANPCQNGGSCMDGVNTFSCLCLPGFTGDKCQTDM
NECLSEPCKNGGTCSDYVNSYTCKCQAGFDGVHCENNINECTESSCFNGGTCVDGINSFS
CLCPVGFTGSFCLHEINECSSHPCLNEGTCVDGLGTYRCSCPLGYTGKNCQTLVNLCSRSP
CKNKGTCVQKKAESQCLCPSGWAGAYCDVPNVSCDIAASRRGVLVEHLCQHSGVCINAG
NTHYCQCPLGYTGSYCEEQLDECASNPCQHGATCSDFIGGYRCECVPGYQGVNCEYEVD
ECQNQPCQNGGTCIDLVNHFKCSCPPGTRGLLCEENIDDCARGPHCLNGGQCMDRIGGYS
CRCLPGFAGERCEGDINECLSNPCSSEGSLDCIQLTNDYLCVCRSAFTGRHCETFVDVCPQ
MPCLNGGTCAVASNMPDGFICRCPPGFSGARCQSSCGQVKCRKGEQCVHTASGPRCFCPS
PRDCESGCASSPCQHGGSCHPQRQPPYYSCQCAPPFSGSRCELYTAPPSTPPATCLSQYCAD
KARDGVCDEACNSHACQWDGGDCSLTMENPWANCSSPLPCWDYINNQCDELCNTVECL
FDNFECQGNSKTCKYDKYCADHFKDNHCDQGCNSEECGWDGLDCAADQPENLAEGTLV
IVVLMPPEQLLQDARSFLRALGTLLHTNLRIKRDSQGELMVYPYYGEKSAAMKKQRMTR
RSLPGEQEQEVAGSKVFLEIDNRQCVQDSDHCFKNTDAAAALLASHAIQGTLSYPLVSVV
SESLTPERTQLLYLLAVAVVIILFIILLGVIMAKRKRKHGSLWLPEGFTLRRDASNHKRREPV
GQDAVGLKNLSVQVSEANLIGTGTSEHWVDDEGPQPKKVKAEDEALLSEEDDPIDRRPW
TQQHLEAADIRRTPSLALTPPQAEQEVDVLDVNVRGPDGCTPLMLASLRGGSSDLSDEDE
DAEDSSANIITDLVYQGASLQAQTDRTGEMALHLAARYSRADAAKRLLDAGADANAQD

NMGRCPLHAAVAADAQGVFQILIRNRVTDLDARMNDGTTPLILAARLAVEGMVAELINCQ
ADVNAVDDHGKSALHWAAAVNNVEATLLLLKNGANRDMQDNKEETPLFLAAREGSYEA
AKILLDHFANRDITDHMDRLPRDVARDRMHHDIVRLLDEYNVTPSPPGTVLTSALSPVICG
PNRSFLSLKHTPMGKKSRRPSAKSTMPTSLPNLAKEAKDAKGSRRKKSLSEKVQLSESSV
TLSPVDSLESPHTYVSDTTSSPMITSPGILQASPNPMLATAAPPAPVHAQHALSFSNLHEMQ
PLAHGASTVLPSVSQLLSHHHIVSPGSGSAGSLSRLHPVPVPADWMNRMEVNETQYNEM
FGMVLAPAEGTHPGIAPQSRPPEGKHITTPREPLPPIVTFQLIPKGSIAQPAGAPQPQSTCPPA
VAGPLPTMYQIPEMARLPSVAFPTAMMPQQDGQVAQTILPAYHPFPASVGKYPTPPSQHSY
ASSNAAERTPSHSGHLQGEHPYLTPSPESPDQWSSSSPHSASDWSDVTTSPTPGGAGGGQR
GPGTHMSEPPHNNMQVYA

>Q9BVX2 multipass membrane protein
MGSQHSAAARPSSCRRKQEDDRDGLLAEREQEEAIAQFPYVEFTGRDSITCLTCQGTGYIP
TEQVNELVALIPHSDQRLRPQRTKQYVLLSILLCLLASGLVVFFLFPHSVLVDDDGIKVVKV
TFNKQDSLVILTIMATLKIRNSNFYTVAVTSLSSQIQYMNTVVSTYVTTNVSLIPPRSEQLVN
FTGKAEMGGPFSYVYFFCTVPEILVHNIVIFMRTSVKISYIGLMTQSSLETHHYVDCGGNST
AI

>Q96QD8 multipass membrane protein
MKKAEMGRFSISPDEDSSSYSSNSDFNYSYPTKQAALKSHYADVDPENQNFLLESNLGKK
KYETEFHPGTTSFGMSVFNLSNAIVGSGILGLSYAMANTGIALFIILLTFVSIFSLYSVHLLLK
TANEGGSLLYEQLGYKAFGLVGKLAASGSITMQNIGAMSSYLFIVKYELPLVIQALTNIED
KTGLWYLNGNYLVLLVSLVVILPLSLFRNLGYLGYTSGLSLLCMVFFLIVVICKKFQVPCP
VEAALIINETINTTLTQPTALVPALSHNVTENDSCRPHYFIFNSQTVYAVPILIFSFVCHPAVLP
IYEELKDRSRRRMMNVSKISFFAMFLMYLLAALFGYLTFYEHVESELLHTYSSILGTDILLL
IVRLAVLMAVTLTVPVVIFPIRSSVTHLLCASKDFSWWRHSLITVSILAFTNLLVIFVPTIRDI
FGFIGASAASMLIFILPSAFYIKLVKKEPMKSVQKIGALFFLLSGVLVMTGSMALIVLDWV
HNAPGGGH

>Q76MJ5 single-pass type I membrane protein
MASAVRGSRPWPRLGLQLQFAALLLGTLSPQVHTLRPENLLLVSTLDGSLHALSKQTGDL
KWTLRDDPVIEGPMYVTEMAFLSDPADGSLYILGTQKQQGLMKLPFTIPELVHASPCRSSD
GVFYTGRKQDAWFVVDPESGETQMTLTTEGPSTPRLYIGRTQYTVTMHDPRAPALRWNT
TYRRYSAPPMDGSPGKYMSHLASCGMGLLLTVDPGSGTVLWTQDLGVPVMGVYTWHQ
DGLRQLPHLTLARDTLHFLALRWGHIRLPASGPRDTATLFSTLDTQLLMTLYVGKDETGFY
VSKALVHTGVALVPRGLTLAPADGPTTDEVTLQVSGEREGSPSTAVRYPSGSVALPSQWLLI
GHHELPPVLHTTMLRVHPTLGSGTAETRPPENTQAPAFFLELLSLSREKLWDSELHPEEKT
PDSYLGLGPQDLLAASLTAVLLGGWILFVMRQQQPQVVEKQQETPLAPADFAHISQDAQS
LHSGASRRSQKRLQSPSKQAQPLDDPEAEQLTVVGKISFNPKDVLGRGAGGTFVFRGQFE
GRAVAVKRLLRECFGLVRREVQLLQESDRHPNVLRYFCTERGPQFHYIALELCRASLQEYV
ENPDLDRGGLEPEVVLQQLMSGLAHLHSLHIVHRDLKPGNILITGPDSQGLGRVVLSDFGL
CKKLPAGRCSFSLHSGIPGTEGWMAPELLQLLPPDSPTSAVDIFSAGCVFYYVLSGGSHPF
GDSLYRQANILTGAPCLAHLEEEVHDKVVARDLVGAMLSPLPQPRPSAPQVLAHPFFWSR
AKQLQFFQDVSDWLEKESEQEPLVRALEAGGCAVVRDNWHEHISMPLQTDLRKFRSYKG

TSVRDLLRAVRNKKHHYRELPVEVRQALGQVPDGFVQYFTNRFPRLLLHTHRAMRSCAS
ESLFLPYYPPDSEARRPCPGATGR

>Q9H3N8 multipass membrane protein
MPDTNSTINLSLSTRVTLAFFMSLVAFAIMLGNALVILAFVVDKNLRHRSSYFFLNLAISDF
FVGVISIPLYIPHTLFEWDFGKEICVFWLTTDYLLCTASVYNIVLISYDRYLSVSNAVSYRTQ
HTGVLKIVTLMVAVWVLAFLVNGPMILVSESWKDEGSECEPGFFSEWYILAITSFLEFVIPV
ILVAYFNMNIYWSLWKRDHLSRCQSHPGLTAVSSNICGHSFRGRLSSRRSLSASTEVPASFH
SERQRRKSSLMFSSRTKMNSNTIASKMGSFSQSDSVALHQREHVELLRARRLAKSLAILLG
VFAVCWAPYSLFTIVLSFYSSATGPKSVWYRIAFWLQWFNSFVNPLLYPLCHKRFQKAFLK
IFCIKKQPLPSQHSRSVSS

>Q92608 peripheral membrane protein
MAPWRKADKERHGVAIYNFQGSGAPQLSLQIGDVVRIQETCGDWYRGYLIKHKMLQGIF
PKSFIHIKEVTVEKRRNTENIIPAEIPLAQEVTTTLWEWGSIWKQLYVASKKERFLQVQSM
MYDLMEWRSQLLSGTLPKDELKELKQKVTSKIDYGNKILELDLIVRDEDGNILDPDNTSVI
SLFHAHEEATDKITERIKEEMSKDQPDYAMYSRISSSPTHSLYVFVRNFVCRIGEDAELFMS
LYDPNKQTVISENYLVRWGSRGFPKEIEMLNNLKVVFTDLGNKDLNRDKIYLICQIVRVG
KMDLKDTGAKKCTQGLRRPFGVAVMDITDIIKGKAESDEEKQHFIPFHPVTAENDFLHSLL
GKVIASKGDSGGQGLWVTMKMLVGDIIQIRKDYPHLVDRTTVVARKLGFPEIIMPGDVRN
DIYITLLQGDFDKYNKTTQRNVEVIMCVCAEDGKTLPNAICVGAGDKPMNEYRSVVYYQ
VKQPRWMETVKVAVPIEDMQRIHLRFMFRHRSSLESKDKGEKNFAMSYVKLMKEDGTTL
HDGFHDLVVLKGDSKKMEDASAYLTLPSYRHHVENKGATLSRSSSSVGGLSVSSRDVFSIS
TLVCSTKLTQNVGLLGLLKWRMKPQLLQENLEKLKIVDGEEVVKFLQDTLDALFNIMME
HSQSDEYDILVFDALIYIIGLIADRKFQHFNTVLEAYIQQHFSATLAYKKLMTVLKTYLDTS
SRGEQCEPILRTLKALEYVFKFIVRSRTLFSQLYEGKEQMEFEESMRRLFESINNLMKSQYK
TTILLQVAALKYIPSVLHDVEMVFDAKLLSQLLYEFYTCIPPVKLQKQKVQSMNEIVQSNL
FKKQECRDILLPVITKELKELLEQKDDMQHQVLERKYCVELLNSILEVLSYQDAAFTYHHI
QEIMVQLLRTVNRTVITMGRDHILISHFVACMTAILNQMGDQHYSFYIETFQTSSELVDFL
METFIMFKDLIGKNVYPGDWMAMSMVQNRVFLRAINKFAETMNQKFLEHTNFEFQLWN
NYFHLAVAFITQDSLQLEQFSHAKYNKILNKYGDMRRLIGFSIRDMWYKLGQNKICFIPG
MVGPILEMTLIPEAELRKATIPIFFDMMLCEYQRSGDFKKFENEIILKLDHEVEGGRGDEQ
YMQLLESILMECAAEHPTIAKSVENFVNLVKGLLEKLLDYRGVMTDESKDNRMSCTVNL
LNFYKDNNREEMYIRYLYKLRDLHLDCDNYTEAAYTLLLHTWLLKWSDEQCASQVMQT
GQQHPQTHRQLKETLYETIIGYFDKGKMWEEAISLCKELAEQYEMEIFDYELLSQNLIQQA
KFYESIMKILRPKPDYFAVGYYGQGFPSFLRNKVFIYRGKEYERREDFQMQLMTQFPNAE
KMNTTSAPGDDVKNAPGQYIQCFTVQPVLDEHPRFKNKPVPDQIINFYKSNYVQRFHYSR
PVRRGTVDPENEFASMWIERTSFVTAYKLPGILRWFEVVHMSQTTISPLENAIETMSTANE
KILMMINQYQSDETLPINPLSMLLNGIVDPAVMGGFAKYEKAFFTEEYVRDHPEDQDKLT
HLKDLIAWQIPFLGAGIKIHEKRVSDNLRPFHDRMEECFKNLKMKVEKEYGVREMPDFDD
RRVGRPRSMLRSYRQMSIISLASMNSDCSTPSKPTSESFDLELASPKTPRVEQEEPISPGSTL
PEVKLRRSKKRTKRSSVVFADEKAAAESDLKRLSRKHEFMSDTNLSEHAAIPLKASVLSQ
MSFASQSMPTIPALALSVAGIPGLDEANTSPRLSQTFLQLSDGDKKTLTRKKVNQFFKTML
ASKSAEEGKQIPDSLSTDL

>P54849 multipass membrane protein
MLVLLAGIFVVHIATVIMLFVSTIANVWLVSNTVDASVGLWKNCTNISCSDSLSYASEDAL
KTVQAFMILSIIFCVIALLVFVFQLFTMEKGNRFFLSGATTLVCWLCILVGVSIYTSHYANR
DGTQYHHGYSYILGWICFCFSFIIGVLYLVLRKK

>Q04609 single-pass type II membrane protein
MWNLLHETDSAVATARRPRWLCAGALVLAGGFFLLGFLFGWFIKSSNEATNITPKHNMKA
FLDELKAENIKKFLYNFTQIPHLAGTEQNFQLAKQIQSQWKEFGLDSVELAHYDVLLSYP
NKTHPNYISIINEDGNEIFNTSLFEPPPPGYENVSDIVPPFSAFSPQGMPEGDLVYVNYARTE
DFFKLERDMKINCSGKIVIARYGKVFRGNKVKNAQLAGAKGVILYSDPADYFAPGVKSYP
DGWNLPGGGVQRGNILNLNGAGDPLTPGYPANEYAYRRGIAEAVGLPSIPVHPIGYYDAQ
KLLEKMGGSAPPDSSWRGSLKVPYNVGPGFTGNFSTQKVKMHIHSTNEVTRIYNVIGTLR
GAVEPDRYVILGGHRDSWVFGGIDPQSGAAVVHEIVRSFGTLKKEGWRPRRTILFASWDA
EEFGLLGSTEWAEENSRLLQERGVAYINADSSIEGNYTLRVDCTPLMYSLVHNLTKELKSP
DEGFEGKSLYESWTKKSPSPEFSGMPRISKLGSGNDFEVFFQRLGIASGRARYTKNWETNK
FSGYPLYHSVYETYELVEKFYDPMFKYHLTVAQVRGGMVFELANSIVLPFDCRDYAVVLR
KYADKIYSISMKHPQEMKTYSVSFDSLFSAVKNFTEIASKFSERLQDFDKSNPIVLRMMND
QLMFLERAFIDPLGLPDRPFYRHVIYAPSSHNKYAGESFPGIYDALFDIESKVDPSKAWGEV
KRQIYVAAFTVQAAAETLSEVA

>Q14088 lipid-anchor
MAQPILGHGSLQPASAAGLASLELDSSLDQYVQIRIFKIIVIGDSNVGKTCLTFRFCGGTFPD
KTEATIGVDFREKTVEIEGEKIKVQVWDTAGQERFRKSMVEHYYRNVHAVVFVYDVTKM
TSFTNLKMWIQECNGHAVPPLVPKVLVGNKCDLREQIQVPSNLALKFADAHNMLLFETSA
KDPKESQNVESIFMCLACRLKAQKSLLYRDAERQQGKVQKLEFPQEANSKTSCPC

>P49286 multipass membrane protein
MSENGSFANCCEAGGWAVRPGWSGAGSARPSRTPRPPWVAPALSAVLIVTTAVDVVGNLL
VILSVLRNRKLRNAGNLFLVSLALADLVVAFYPYPLILVAIFYDGWALGEEHCKASAFVMG
LSVIGSVFNITAIAINRYCYICHSMAYHRIYRRWHTPLHICLIWLLTVVALLPNFFVGSLEYD
PRIYSCTFIQTASTQYTAAVVVIHFLLPIAVVSFCYLRIWVLVLQARRKAKPESRLCLKPSDL
RSFLTMFVVFVIFAICWAPLNCIGLAVAINPQEMAPQIPEGLFVTSYLLAYFNSCLNAIVYGL
LNQNFRREYKRILLALWNPRHCIQDASKGSHAEGLQSPAPPIIGVQHQADAL

>Q08554 single-pass type I membrane protein
MALASAAPGSIFCKQLLFSLLVLTLLCDACQKVYLRVPSHLQAETLVGKVNLEECLKSASL
IRSSDPAFRILEDGSIYTTHDLILSSERKSFSIFLSDGQRREQQEIKVVLSARENKSPKKRHTK
DTALKRSKRRWAPIPASLMENSLGPFPQHVQQIQSDAAQNYTIFYSISGPGVDKEPFNLFYI
EKDTGDIFCTRSIDREKYEQFALYGYATTADGYAPEYPLPLIIKIEDDNDNAPYFEHRVTIFT
VPENCRSGTSVGKVTATDLDEPDTLHTRLKYKILQQIPDHPKHFSIHPDTGVITTTTPFLDR
EKCDTYQLIMEVRDMGGQPFGLFNTGTITISLEDENDNPPSFTETSYVTEVEENRIDVEILR
MKVQDQDLPNTPHSKAVYKILQGNENGNFIISTDPNTNEGVLCVVKPLNYEVNRQVILQV
GVINEAQFSKAASSQTPTMCTTTVTVKIIDSDEGPECHPPVKVIQSQDGFPAGQELLGYKA

LDPEISSGEGLRYQKLGDEDNWFEINQHTGDLRTLKVLDRESKFVKNNQYNISVVAVDAV
GRSCTGTLVVHLDDYNDHAPQIDKEVTICQNNEDFAVLKPVDPDGPENGPPFQFFLDNSAS
KNWNIEEKDGKTAILRQRQNLDYNYYSVPIQIKDRHGLVATHMLTVRVCDCSTPSECRMK
DKSTRDVRPNVILGRWAILAMVLGSVLLLCILFTCFCVTAKRTVKKCFPEDIAQQNLIVSN
TEGPGEEVTEANIRLPMQTSNICDTSMSVGTVGGQGIKTQQSFEMVKGGYTLDSNKGGG
HQTLESVKGVGQGDTGRYAYTDWQSFTQPRLGEKVYLCGQDEEHKHCEDYVCSYNYEG
KGSLAGSVGCCSDRQEEEGLEFLDHLEPKFRTLAKTCIKK

>Q9Y4D2 multipass membrane protein
MPGIVVFRRRWSVGSDDLVLPAIFLFLLHTTWFVILSVVLFGLVYNPHEACSLNLVDHGRG
YLGILLSCMIAEMAIIWLSMRGGILYTEPRDSMQYVLYVRLAILVIEFIYAIVGIVWLTQYYT
SCNDLTAKNVTLGMVVCNWVVILSVCITVLCVFDPTGRTFVKLRATKRRQRNLRTYNLRH
RLEEGQATSWSRRLKVFLCCTRTKDSQSDAYSEIAYLFAEFFRDLDIVPSDIIAGLVLLRQR
QRAKRNAVLDEANNDILAFLSGMPVTRNTKYLDLKNSQEMLRYKEVCYYMLFALAAYG
WPMYLMRKPACGLCQLARSCSCCLCPARPRFAPGVTIEEDNCCGCNAIAIRRHFLDENMT
AVDIVYTSCHDAVYETPFYVAVDHDKKKVVISIRGTLSPKDALTDLTGDAERLPVEGHHGT
WLGHKGMVLSAEYIKKKLEQEMVLSQAFGRDLGRGTKHYGLIVVGHSLGAGTAAILSFL
LRPQYPTLKCFAYSPPGGLLSEDAMEYSKEFVTAVVLGKDLVPRIGLSQLEGFRRQLLDVL
QRSTKPKWRIIVGATKCIPKSELPEEVEVTTLASTRLWTHPSDLTIALSASTPLYPPGRIIHVV
HNHPAEQCCCCEQEEPTYFAIWGDNKAFNEVIISPAMLHEHLPYVVMEGLNKVLENYNK
GKTALLSAAKVMVSPTEVDLTPELIFQQQPLPTGPPMPTGLALELPTADHRNSSVRSKSQS
EMSLEGFSEGRLLSPVVAAAARQDPVELLLLSTQERLAAELQARRAPLATMESLSDTESLY
SFDSRRSSGFRSIRGSPSLHAVLERDEGHLFYIDPAIPEENPSLSSRTELLAADSLSKHSQDT
QPLEAALGSGGVTPERPPSAAANDEEEEVGGGGGGPASRGELALHNGRLGDSPSPQVLEF
AEFIDSLFNLDSKSSSFQDLYCMVVPESPTSDYAEGPKSPSQQEILLRAQFEPNLVPKPPRLF
AGSADPSSGISLSPSFPLSSSGELMDLTPTGLSSQECLAADKIRTSTPTGHGASPAKQDELVI
SAR

>P62072 peripheral membrane protein
MDPLRAQQLAAELEVEMMADMYNRMTSACHRKCVPPHYKEAELSKGESVCLDRCVSK
YLDIHERMGKKLTELSMQDEELMKRVQQSSGPA

>P35414 multipass membrane protein
MEEGGDFDNYYGADNQSECEYTDWKSSGALIPAIYMLVFLLGTTGNGLVLWTVFRSSRE
KRRSADIFIASLAVADLTFVVTLPLWATYTYRDYDWPFGTFFCKLSSYLIFVNMYASVFCLT
GLSFDRYLAIVRPVANARLRLRVSGAVATAVLWVLAALLAMPVMVLRTTGDLENTTKVQC
YMDYSMVATVSSEWAWEVGLGVSSTTVGFVVPFTIMLTCYFFIAQTIAGHFRKERIEGLRK
RRRLLSIIVVLVVTFALCWMPYHLVKTLYMLGSLLHWPCDFDLFLMNIFPYCTCISYVNSC
LNPFLYAFFDPRFRQACTSMLCCGQSRCAGTSHSSSGEKSASYSSGHSQGPGPNMGKGGE
QMHEKSIPYSQETLVVD

>Q9P296 multipass membrane protein
MGNDSVSYEYGDYSDLSDRPVDCLDGACLAIDPLRVAPLPLYAAIFLVGVPGNAMVAWVA
GKVARRRVGATWLLHLAVADLLCCLSLPILAVPIARGGHWPYGAVGCRALPSIILLTMYAS

VLLLAALSADLCFLALGPAWWSTVQRACGVQVACGAAWTLALLLTVPSAIYRRLHQEHF
PARLQCVVDYGGSSSTENAVTAIRFLFGFLGPLVAVASCHSALLCWAARRCRPLGTAIVVGF
FVCWAPYHLLGLVLTVAAPNSALLARALRAEPLIVGLALAHSCLNPMLFLYFGRAQLRRS
LPAACHWALRESQGQDESVDSKKSTSHDLVSEMEV

>P16234 single-pass type I membrane protein
MGTSHPAFLVLGCLLTGLSLILCQLSLPSILPNENEKVVQLNSSFSLRCFGESEVSWQYPMS
EEESSDVEIRNEENNSGLFVTVLEVSSASAAHTGLYTCYYNHTQTEENELEGRHIYIYVPD
PDVAFVPLGMTDYLVIVEDDDSAIIPCRTTDPETPVTLHNSEGVVPASYDSRQGFNGTFTV
GPYICEATVKGKKFQTIPFNVYALKATSELDLEMEALKTVYKSGETIVVTCAVFNNEVVDL
QWTYPGEVKGKGITMLEEIKVPSIKLVYTLTVPEATVKDSGDYECAARQATREVKEMKKV
TISVHEKGFIEIKPTFSQLEAVNLHEVKHFVVEVRAYPPPRISWLKNNLTLIENLTEITTDVE
KIQEIRYRSKLKLIRAKEEDSGHYTIVAQNEDAVKSYTFELLTQVPSSILDLVDDHHGSTGG
QTVRCTAEGTPLPDIEWMICKDIKKCNNETSWTILANNVSNIITEIHSRDRSTVEGRVTFAK
VEETIAVRCLAKNLLGAENRELKLVAPTLRSELTVAAAVLVLLVIVIISLIVLVVIWKQKPRY
EIRWRVIESISPDGHEYIYVDPMQLPYDSRWEFPRDGLVLGRVLGSGAFGKVVEGTAYGLS
RSQPVMKVAVKMLKPTARSSEKQALMSELKIMTHLGPHLNIVNLLGACTKSGPIYIITEYC
FYGDLVNYLHKNRDSFLSHHPEKPKKELDIFGLNPADESTRSYVILSFENNGDYMDMKQA
DTTQYVPMLERKEVSKYSDIQRSLYDRPASYKKKSMLDSEVKNLLSDDNSEGLTLLDLLS
FTYQVARGMEFLASKNCVHRDLAARNVLLAQGKIVKICDFGLARDIMHDSNYVSKGSTF
LPVKWMAPESIFDNLYTTLSDVWSYGILLWEIFSLGGTPYPGMMVDSTFYNKIKSGYRMA
KPDHATSEVYEIMVKCWNSEPEKRPSFYHLSEIVENLLPGQYKKSYEKIHLDFLKSDHPAV
ARMRVDSDNAYIGVTYKNEEDKLKDWEGGLDEQRLSADSGYIIPLPDIDPVPEEEDLGKR
NRHSSQTSEESAIETGSSSSTFIKREDETIEDIDMMDDIGIDSSDLVEDSFL

>Q9H013 single-pass type I membrane protein
MPGGAGAARLCLLAFALQPLRPRAAREPGWTRGSEEGSPKLQHELIIPQWKTSESPVREK
HPLKAELRVMAEGRELILDLEKNEQLFAPSYTETHYTSSGNPQTTTRKLEDHCFYHGTVR
ETELSSVTLSTCRGIRGLITVSSNLSYVIEPLPDSKGQHLIYRSEHLKPPPGNCGFEHSKPTT
RDWALQFTQQTKKRPRRMKREDLNSMKYVELYLVADYLEFQKNRRDQDATKHKLIEIAN
YVDKFYRSLNIRIALVGLEVWTHGNMCEVSENPYSTLWSFLSWRRKLLAQKYHDNAQLI
TGMSFHGTTIGLAPLMAMCSVYQSGGVNMDHSENAIGVAATMAHEMGHNFGMTHDSA
DCCSASAADGGCIMAAATGHPFPKVFNGCNRRELDRYLQSGGGMCLSNMPDTRMLYGG
RRCGNGYLEDGEECDCGEEEECNNPCCNASNCTLRPGAECAHGSCCHQCKLLAPGTLCR
EQARQCDLPEFCTGKSPHCPTNFYQMDGTPCEGGQAYCYNGMCLTYQEQCQQLWGPGA
RPAPDLCFEKVNVAGDTFGNCGKDMNGEHRKCNMRDAKCGKIQCQSSEARPLESNAVPI
DTTIIMNGRQIQCRGTHVYRGPEEEGDMLDPGLVMTGTKCGYNHICFEGQCRNTSFFETE
GCGKKCNGHGVCNNNQNCHCLPGWAPPFCNTPGHGGSIDSGPMPPESVGPVVAGVLVAIL
VLAVLMLMYYCCRQNNKLGQLKPSALPSKLRQQFSCPFRVSQNSGTGHANPTFKLQTPQ
GKRKVINTPEILRKPSQPPPRPPPDYLRGGSPPAPLPAHLSRAARNSPGPGSQIERTESSRRPP
PSRPIPPAPNCIVSQDFSRPRPPQKALPANPVPGRRSLPRPGGASPLRPPGAGPQQSRPLAAL
APKVSPREALKVKAGTRGLQGGRCRVEKTKQFMLLVVWTELPEQKPRAKHSCFLVPA

>Q6NUQ1 peripheral membrane protein

MLPAGEIGASPAAPCCSESGDERKNLEEKSDINVTVLIGSKQVSEGTDNGDLPSYVSAFIEK
EVGNDLKSLKKLDKLIEQRTVSKMQLEEQVLTISSEIPKRIRSALKNAEESKQFLNQFLEQE
THLFSAINSHLLTAQPWMDDLGTMISQIEEIERHLAYLKWISQIEELSDNIQQYLMTNNVPE
AASTLVSMAELDIKLQESSCTHLLGFMRATVKFWHKILKDKLTSDFEEILAQLHWPFIAPP
QSQTVGLSRPASAPEIYSYLETLFCQLLKLQTSDELLTEPKQLPEKYSLPASPSVILPIQVML
TPLQKRFRYHFRGNRQTNVLSKPEWYLAQVLMWIGNHTEFLDEKIQPILDKVGSLVNARL
EFSRGLMMLVLEKLATDIPCLLYDDNLFCHLVDEVLLFERELHSVHGYPGTFASCMHILSE
ETCFQRWLTVERKFALQKMDSMLSSEAAWVSQYKDITDVDEMKVPDCAETFMTLLLVIT
DRYKNLPTASRKLQFLELQKDLVDDFRIRLTQVMKEETRASLGFRYCAILNAVNYISTVLA
DWADNVFFLQLQQAALEVFAENNTLSKLQLGQLASMESSVFDDMINLLERLKHDMLTRQ
VDHVFREVKDAAKLYKKERWLSLPSQSEQAVMSLSSSACPLLLTLRDHLLQLEQQLCFSL
FKIFWQMLVEKLDVYIYQEIILANHFNEGGAAQLQFDMTRNLFPLFSHYCKRPENYFKHIK
EACIVLNLNVGSALLLKDVLQSASGQLPATAALNEVGIYKLAQQDVEILLNLRTNWPNTG
K

>P21589 GPI-anchor, lipid-anchor
MCPRAARAPATLLLALGAVLWPAAGAWELTILHTNDVHSRLEQTSEDSSKCVNASRCMG
GVARLFTKVQQIRRAEPNVLLLDAGDQYQGTIWFTVYKGAEVAHFMNALRYDAMALGN
HEFDNGVEGLIEPLLKEAKFPILSANIKAKGPLASQISGLYLPYKVLPVGDEVVGIVGYTSK
ETPFLSNPGTNLVFEDEITALQPEVDKLKTLNVNKIIALGHSGFEMDKLIAQKVRGVDVVV
GGHSNTFLYTGNPPSKEVPAGKYPFIVTSDDGRKVPVVQAYAFGKYLGYLKIEFDERGNVI
SSHGNPILLNSSIPEDPSIKADINKWRIKLDNYSTQELGKTIVYLDGSSQSCRFRECNMGNLI
CDAMINNNLRHTDEMFWNHVSMCILNGGGIRSPIDERNNGTITWENLAAVLPFGGTFDLV
QLKGSTLKKAFEHSVHRYGQSTGEFLQVGGIHVVYDLSRKPGDRVVKLDVLCTKCRVPS
YDPLKMDEVYKVILPNFLANGGDGFQMIKDELLRHDSGDQDINVVSTYISKMKVIYPAVE
GRIKFSTGSHCHGSFSLIFLSLWAVIFVLYQ

>Q9BZD6 single-pass type I membrane protein
MFTLLVLLSQLPTVTLGFPHCARGPKASKHAGEEVFTSKEEANFFIHRRLLYNRFDLELFTP
GNLERECNEELCNYEEAREIFVDEDKTIAFWQEYSAKGPTTKSDGNREKIDVMGLLTGLI
AAGVFLVIFGLLGYYLCITKCNRLQHPCSSAVYERGRHTPSIIFRRPEEAALSPLPPSVEDAG
LPSYEQAVALTRKHSVSPPPPYPGHTKGFRVFKKSMSLPSH

>P08962 multipass membrane protein
MAVEGGMKCVKFLLYVLLLAFCACAVGLIAVGVGAQLVLSQTIIQGATPGSLLPVVIIAVG
VFLFLVAFVGCCGACKENYCLMITFAIFLSLIMLVEVAAAIAGYVFRDKVMSEFNNNFRQQ
MENYPKNNHTASILDRMQADFKCCGAANYTDWEKIPSMSKNRVPDSCCINVTVGCGINF
NEKAIHKEGCVEKIGGWLRKNVLVVAAAALGIAFVEVLGIVFACCLVKSIRSGYEVM

>Q16671 single-pass type I membrane protein
MLGSLGLWALLPTAVEAPPNRRTCVFFEAPGVRGSTKTLGELLDTGTELPRAIRCLYSRCC
FGIWNLTQDRAQVEMQGCRDSDEPGCESLHCDPSPRAHPSPGSTLFTCSCGTDFCNANYS
HLPPPGSPGTPGSQGPQAAPGESIWMALVLLGLFLLLLLLLGSIILALLQRKNYRVRGEPVP
EPRPDSGRDWSVELQELPELCFSQVIREGGHAVVWAGQLQGKLVAIKAFPPRSVAQFQAE

RALYELPGLQHDHIVRFITASRGGPGRLLSGPLLVLELHPKGSLCHYLTQYTSDWGSSLRM
ALSLAQGLAFLHEERWQNGQYKPGIAHRDLSSQNVLIREDGSCAIGDLGLALVLPGLTQPP
AWTPTQPQGPAAIMEAGTQRYMAPELLDKTLDLQDWGMALRRADIYSLALLLWEILSRC
PDLRPDSSPPPFQLAYEAELGNTPTSDELWALAVQERRRPYIPSTWRCFATDPDGLRELLED
CWDADPEARLTAECVQQRLAALAHPQESHPFPESCPRGCPPLCPEDCTSIPAPTILPCRPQR
SACHFSVQQGPCSRNPQPACTLSPV

>Q13683 single-pass type I membrane protein
MAGARSRDPWGASGICYLFGSLLVELLFSRAVAFNLDVMGALRKEGEPGSLFGFSVALHR
QLQPRPQSWLLVGAPQALALPGQQANRTGGLFACPLSLEETDCYRVDIDQGADMQKESK
ENQWLGVSVRSQGPGGKIVTCAHRYEARQRVDQILETRDMIGRCFVLSQDLAIRDELDGG
EWKFCEGRPQGHEQFGFCQQGTAAAFSPDSHYLLFGAPGTYNWKGTARVELCAQGSADL
AHLDDGPYEAGGEKEQDPRLIPVPANSYFGLLFVTNIDSSDPDQLVYKTLDPADRLPGPAG
DLALNSYLGFSIDSGKGLVRAEELSFVAGAPRANHKGAVVILRKDSASRLVPEVMLSGERL
TSGFGYSLAVADLNSDGWPDLIVGAPYFFERQEELGGAVYVYLNQGGHWAGISPLRLCGS
PDSMFGISLAVLGDLNQDGFPDIAVGAPFDGDGKVFIYHGSSLGVVAKPSQVLEGEAVGIK
SFGYSLSGSLDMDGNQYPDLLVGSLADTAVLFRARPILHVSHEVSIAPRSIDLEQPNCAGG
HSVCVDLRVCFSYIAVPSSYSPTVALDYVLDADTDRRLRGQVPRVTFLSRNLEEPKHQASG
TVWLKHQHDRVCGDAMFQLQENVKDKLRAIVVTLSYSLQTPRLRRQAPGQGLPPVAPIL
NAHQPSTQRAEIHFLKQGCGEDKICQSNLQLVRARFCTRVSDTEFQPLPMDVDGTTALFAL
SGQPVIGLELMVTNLPSDPAQPQADGDDAHEAQLLVMLPDSLHYSGVRALDPAEKPLCLS
NENASHVECELGNPMKRGAQVTFYLILSTSGISIETTELEVELLLATISEQELHPVSARARV
FIELPLSIAGMAIPQQLFFSGVVRGERAMQSERDVGSKVKYEVTVSNQGQSLRTLGSAFLN
IMWPHEIANGKWLLYPMQVELEGGQGPGQKGLCSPRPNILHLDVDSRDRRRRELEPPEQQ
EPGERQEPSMSWWPVSSAEKKKNITLDCARGTANCVVFSCPLYSFDRAAVLHVWGRLWN
STFLEEYSAVKSLEVIVRANITVKSSIKNLMLRDASTVIPVMVYLDPMAVVAEGVPWWVIL
LAVLAGLLVLALLVLLLWKMGFFKRAKHPEATVPQYHAVKIPREDRQQFKEEKTGTILRN
NWGSPRREGPDAHPILAADGHPELGPDGHPGPGTA

>Q92781 peripheral membrane protein
MWLPLLLGALLWAVLWLLRDRQSLPASNAFVFITGCDSGFGRLLALQLDQRGFRVLASCL
TPSGAEDLQRVASSRLHTTLLDITDPQSVQQAAKWVEMHVKEAGLFGLVNNAGVAGIIGP
TPWLTRDDFQRVLNVNTMGPIGVTLALLPLLQQARGRVINITSVLGRLAANGGGYCVSKF
GLEAFSDSLRRDVAHFGIRVSIVEPGFFRTPVTNLESLEKTLQACWARLPPATQAHYGGAFL
TKYLKMQQRIMNLICDPDLTKVSRCLEHALTARHPRTRYSPGWDAKLLWLPASYLPASLV
DAVLTWVLPKPAQAVY

>Q12999 multipass membrane protein
MVCGGFACSKNALCALNVVYMLVSLLLIGVAAWGKGLGLVSSIHIIGGVIAVGVFLLLIAV
AGLVGAVNHHQVLLFFYMIILGLVFIFQFVISCSCLAINRSKQTDVINASWWVMSNKTRDE
LERSFDCCGLFNLTTLYQQDYDFCTAICKSQSPTCQMCGEKFLKHSDEALKILGGVGLFFS
FTEILGVWLAMRFRNQKDPRANPSAFL

>P30301 multipass membrane protein

MWELRSASFWRAIFAEFFATLFYVFFGLGSSLRWAPGPLHVLQVAMAFGLALATLVQSVG
HISGAHVNPAVTFAFLVGSQMSLLRAFCYMAAQLLGAVAGAAVLYSVTPPAVRGNLALNT
LHPAVSVGQATTVEIFLTLQFVLCIFATYDERRNGQLGSVALAVGFSLALGHLFGMYYTGA
GMNPARSFAPAILTGNFTNHWVYWVGPIIGGGLGSLLYDFLLFPRLKSISERLSVLKGAKP
DVSNGQPEVTGEPVELNTQAL

>Q9ULD8 multipass membrane protein
MPAMRGLLAPQNTFLDTIATRFDGTHSNFVLGNAQVAGLFPVVYCSDGFCDLTGFSRAEV
MQRGCACSFLYGPDTSELVRQQIRKALDEHKEFKAELILYRKSGLPFWCLLDVIPIKNEKG
EVALFLVSHKDISETKNRGGPDRWKETGGGRRRYGRARSKGFNANRRRSRAVLYHLSGH
LQKQPKGKHKLNKGVFGEKPNLPEYKVAAIRKSPFILLHCGALRATWDGFILLATLYVAVT
VPYSVCVSTAREPSAARGPPSVCDLAVEVLFILDIVLNFRTTFVSKSGQVVFAPKSICLHYV
TTWFLLDVIAALPFDLLHAFKVNVYFGAHLLKTVRLLRLLRLLPRLDRYSQYSAVVLTLL
MAVFALLAHWVACVWFYIGQREIESSESELPEIGWLQELARRLETPYYLVGRRPAGGNSSG
QSDNCSSSSEANGTGLELLGGPSLRSAYITSLYFALSSLTSVGFGNVSANTDTEKIFSICTML
IGALMHAVVFGNVTAIIQRMYARRFLYHSRTRDLRDYIRIHRIPKPLKQRMLEYFQATWAV
NNGIDTTELLQSLPDELRADIAMHLHKEVLQLPLFEAASRGCLRALSLALRPAFCTPGEYLI
HQGDALQALYFVCSGSMEVLKGGTVLAILGKGDLIGCELPRREQVVKANADVKGLTYCV
LQCLQLAGLHDSLALYPEFAPRFSRGLRGELSYNLGAGGGSAEVDTSSLSGDNTLMSTLE
EKETDGEQGPTVSPAPADEPSSPLLSPGCTSSSSAAKLLSPRRTAPRPRLGGRGRPGRAGAL
KAEAGPSAPPRALEGLRLPPMPWNVPPDLSPRVVDGIEDGCGSDQPKFSFRVGQSGPECSS
SPSPGPESGLLTVPHGPSEARNTDTLDKLRQAVTELSEQVLQMREGLQSLRQAVQLVLAPH
REGPCPRASGEGPCPASTSGLLQPLCVDTGASSYCLQPPAGSVLSGTWPHPRPGPPPLMAP
WPWGPPASQSSPWPRATAFWTSTSDSEPPASGDLCSEPSTPASPPPSEEGARTGPAEPVSQA
EATSTGEPPPGSGGLALPWDPHSLEMVLIGCHGSGTVQWTQEEGTGV

>O14804 multipass membrane protein
MRAVFIQGAEEHPAAFCYQVNGSCPRTVHTLGIQLVIYLACAAGMLIIVLGNVFVAFAVSY
FKALHTPTNFLLLSLALADMFLGLLVLPLSTIRSVESCWFFGDFLCRLHTYLDTLFCLTSIF
HLCFISIDRHCAICDPLLYPSKFTVRVALRYILAGWGVPAAYTSLFLYTDVVETRLSQWLEE
MPCVGSCQLLLNKFWGWLNFPLFFVPCLIMISLYVKIFVVATRQAQQITTLSKSLAGAAKH
ERKAAKTLGIAVGIYLLCWLPFTIDTMVDSLLHFITPPLVFDIFIWFAYFNSACNPIIYVFSYQ
WFRKALKLTLSQKVFSPQTRTVDLYQE

>P28336 multipass membrane protein
MPSKSLSNLSVTTGANESGSVPEGWERDFLPASDGTTTELVIRCVIPSLYLLIITVGLLGNIM
LVKIFITNSAMRSVPNIFISNLAAGDLLLLLTCVPVDASRYFFDEWMFGKVGCKLIPVIQLT
SVGVSVFTLTALSADRYRAIVNPMDMQTSGALLRTCVKAMGIWVVSVLLAVPEAVFSEVA
RISSLDNSSFTACIPYPQTDELHPKIHSVLIFLVYFLIPLAIISIYYYHIAKTLIKSAHNLPGEYN
EHTKKQMETRKRLAKIVLVFVGCFIFCWFPNHILYMYRSFNYNEIDPSLGHMIVTLVARVL
SFGNSCVNPFALYLLSESFRRHFNSQLCCGRKSYQERGTSYLLSSSAVRMTSLKSNAKNM
VTNSVLLNGHSMKQEMAL

>O60906 multipass membrane protein

MKPNFSLRLRIFNLNCWGIPYLSKHRADRMRRLGDFLNQESFDLALLEEVWSEQDFQYLR
QKLSPTYPAAHHFRSGIIGSGLCVFSKHPIQELTQHIYTLNGYPYMIHHGDWFSGKAVGLL
VLHLSGMVLNAYVTHLHAEYNRQKDIYLAHRVAQAWELAQFIHHTSKKADVVLLCGDL
NMHPEDLGCCLLKEWTGLHDAYLETRDFKGSEEGNTMVPKNCYVSQQELKPFPFGVRID
YVLYKAVSGFYISCKSFETTTGFDPHRGTPLSDHEALMATLFVRHSPPQQNPSSTHGPAERS
PLMCVLKEAWTELGLGMAQARWWATFASYVIGLGLLLLALLCVLAAGGGAGEAAILLW
TPSVGLVLWAGAFYLFHVQEVNGLYRAQAELQHVLGRAREAQDLGPEPQPALLLGQQEG
DRTKEQ

>Q9BXF6 peripheral membrane protein
MALVRGAEPAAGPSRWLPTHVQVTVLRARGLRGKSSGAGSTSDAYTVIQVGREKYSTSV
VEKTHGCPEWREECSFELPPGALDGLLRAQEADAGPAPWAASSAAACELVLTTMHRSLIG
VDKFLGQATVALDEVFGAGRAQHTQWYKLHSKPGKKEKERGEIEVTIQFTRNNLSASMF
DLSMKDKPRSPFSKIRDKMKGKKKYDLESASAILPSSAIEDPDLGSLGKMGKAKGFFLRN
KLRKSSLTQSNTSLGSDSTLSSASGSLAYQGPGAELLTRSPSRSSWLSTEGGRDSAQSPKLF
THKRTYSDEANQMRVAPPRALLDLQGHLDAASRSSLCVNGSHIYNEEPQGPVRHRSSISGS
LPSSGSLQAVSSRFSEEGPRSTDDTWPRGSRSNSSSEAVLGQEELSAQAKVLAPGASHPGE
EEGARLPEGKPVQVATPIVASSEAVAEKEGARKEERKPRMGLFHHHHQGLSRSELGRRSSL
GEKGGPILGASPHHSSSGEEKAKSSWFGLREAKDPTQKPSPHPVKPLSAAPVEGSPDRKQS
RSSLSIALSSGLEKLKTVTSGSIQPVTQAPQAGQMVDTKRLKDSAVLDQSAKYYHLTHDE
LISLLLQRERELSQRDEHVQELESYIDRLLVRIMETSPTLLQIPPGPPK

>Q86W47 multipass membrane protein
MAKLRVAYEYTEAEDKSIRLGLFLIISGVVSLFIFGFCWLSPALQDLQATEANCTVLSVQQI
GEVFECTFTCGADCRGTSQYPCVQVYVNNSESNSRALLHSDEHQLLTNPKCSYIPPCKRE
NQKNLESVMNWQQYWKDEIGSQPFTCYFNQHQRPDDVLLHRTHDEIVLLHCFLWPLVTF
VVGVLIVVLTICAKSLAVKAEAMKKRKFS

>Q07001 multipass membrane protein
MEGPVLTLGLLAALAVCGSWGLNEEERLIRHLFQEKGYNKELRPVAHKEESVDVALALTL
SNLISLKEVEETLTTNVWIEHGWTDNRLKWNAEEFGNISVLRLPPDMVWLPEIVLENNND
GSFQISYSCNVLVYHYGFVYWLPPAIFRSSCPISVTYFPFDWQNCSLKFSSLKYTAKEITLSL
KQDAKENRTYPVEWIIIDPEGFTENGEWEIVHRPARVNVDPRAPLDSPSRQDITFYLIIRRKP
LFYIINILVPCVLISFMVNLVFYLPADSGEKTSVAISVLLAQSVFLLLISKRLPATSMAIPLIGK
FLLFGMVLVTMVVVICVIVLNIHFRTPSTHVLSEGVKKLFLETLPELLHMSRPAEDGPSPGA
LVRRSSSLGYISKAEEYFLLKSRSDLMFEKQSERHGLARRLTTARRPPASSEQAQQELFNEL
KPAVDGANFIVNHMRDQNNYNEEKDSWNRVARTVDRLCLFVVTPVMVVGTAWIFLQGV
YNQPPPQPFPGDPYSYNVQDKRFI

>P41595 multipass membrane protein
MALSYRVSELQSTIPEHILQSTFVHVISSNWSGLQTESIPEEMKQIVEEQGNKLHWAALLIL
MVIIPTIGGNTLVILAVSLEKKLQYATNYFLMSLAVADLLVGLFVMPIALLTIMFEAMWPLP
LVLCPAWLFLDVLFSTASIMHLCAISVDRYIAIKKPIQANQYNSRATAFIKITVVWLISIGIAIP
VPIKGIETDVDNPNNITCVLTKERFGDFMLFGSLAAFFTPLAIMIVTYFLTIHALQKKAYLV

KNKPPQRLTWLTVSTVFQRDETPCSSPEKVAMLDGSRKDKALPNSGDETLMRRTSTIGKK
SVQTISNEQRASKVLGIVFFLFLLMWCPFFITNITLVLCDSCNQTTLQMLLEIFVWIGYVSS
GVNPLVYTLFNKTFRDAFGRYITCNYRATKSVKTLRKRSSKIYFRNPMAENSKFFKKHGIR
NGINPAMYQSPMRLRSSTIQSSSIILLDTLLLTENEGDKTEEQVSYV

>O75204 multipass membrane protein
MYAPGGAGLPGGRRRRSPGGSALPKQPERSLASALPGALSITALCTALAEPAWLHIHGGTC
SRQELGVSDVLGYVHPDLLKDFCMNPQTVLLLRVIAAFCFLGILCSLSAFLLDVFGPKHPA
LKITRRYAFAHILTVLQCATVIGFSYWASELILAQQQQHKKYHGSQVYVTFAVSFYLVAGA
GGASILATAANLLRHYPTEEEEQALELLSEMEENEPYPAEYEVINQFQPPPAYTP

>Q9Y5Y3 multipass membrane protein
MACNSTSLEAYTYLLLNTSNASDSGSTQLPAPLRISLAIVMLLMTVVGFLGNTVVCIIVYQ
RPAMRSAINLLLATLAFSDIMLSLCCMPFTAVTLITVRWHFGDHFCRLSATLYWFFVLEGV
AILLIISVDRFLIIVQRQDKLNPRRAKVIIAVSWVLSFCIAGPSLTGWTLVEVPARAPQCVLG
YTELPADRAYVVTLVVAVFFAPFGVMLCAYMCILNTVRKNAVRVHNQSDSLDLRQLTRAG
LRRLQRQQQVSVDLSFKTKAFTTILILFVGFSLCWLPHSVYSLLSVFSQRFYCGSSFYATST
CVLWLSYLKSVFNPIVYCWRIKKFREACIELLPQTFQILPKVPERIRRRIQPSTVYVCNENQ
SAV

>Q8NEU8 peripheral membrane protein
MPAVDKLLLEEALQDSPQTRSLLSVFEEDAGTLTDYTNQLLQAMQRVYGAQNEMCLATQ
QLSKQLLAYEKQNFALGKGDEEVISTLHYFSKVVDELNLLHTELAKQLADTMVLPIIQFRE
KDLTEVSTLKDLFGLASNEHDLSMAKYSRLPKKKENEKVKTEVGKEVAAARRKQHLSSL
QYYCALNALQYRKQMAMMEPMIGFAHGQINFFKKGAEMFSKRMDSFLSSVADMVQSIQ
VELEAEAEKMRVSQQELLSVDESVYTPDSDVAAPQINRNLIQKAGYLNLRNKTGLVTTTW
ERLYFFTQGGNLMCQPRGAVAGGLIQDLDNCSVMAVDCEDRRYCFQITTPNGKSGIILQAE
SRKENEEWICAINNISRQIYLTDNPEAVAIKLNQTALQAVTPITSFGKKQESSCPSQNLKNSE
MENENDKIVPKATASLPEAEELIAPGTPIQFDIVLPATEFLDQNRGSRRTNPFGETEDESFPE
AEDSLLQQMFIVRFLGSMAVKTDSTTEVIYEAMRQVLAARAIHNIFRMTESHLMVTSQSL
RLIDPQTQVSRANFELTSVTQFAAHQENKRLVGFVIRVPESTGEESLSTYIFESNSEGEKICY
AINLGKEIIEVQKDPEALAQLMLSIPLTNDGKYVLLNDQPDDDDGNPNEHRGAESEA

>Q8N682 multipass membrane protein
MLCFLRGMAFVPFLLVTWSSAAFIISYVVAVLSGHVNPFLPYISDTGTTPPESGIFGFMINFS
AFLGAATMYTRYKIVQKQNQTCYFSTPVFNLVSLVLGLVGCFGMGIVANFQELAVPVVHD
GGALLAFVCGVVYTLLQSIISYKSCPQWNSLSTCHIRMVISAVSCAAVIPMIVCASLISITKL
EWNPREKDYVYHVVSAICEWTVAFGFIFYFLTFIQDFQSVTLRISTEINGDI

>Q96JW4 multipass membrane protein
MTNSKGRSITDKTSGGPSSGGGFVDWTLRLNTIQSDKFLNLLLSMVPVIYQKNQEDRHKK
ANGIWQDGLSTAVQTFSNRSEQHMEYHSFSEQSFHANNGHASSSCSQKYDDYANYNYCD
GRETSETTAMLQDEDISSDGDEDAIVEVTPKLPKESSGIMALQILVPFLLAGFGTVSAGMV
LDIVQHWEVFRKVTEVFILVPALLGLKGNLEMTLASRLSTAVNIGKMDSPIEKWNLIIGNL

ALKQVQATVVGFLAAVAAIILGWIPEGKYYLDHSILLCSSSVATAFIASLLQGIIMVGVIVGS
KKTGINPDNVATPIAASFGDLITLAILAWISQGLYSCLETYYYISPLVGVFFLALTPIWIIIAAK
HPATRTVLHSGWEPVITAMVISSIGGLILDTTVSDPNLVGIVVYTPVINGIGGNLVAIQASRIS
TYLHLHSIPGELPDEPKGCYYPFRTFFGPGVNNKSAQVLLLLVIPGHLIFLYTIHLMKSGHT
SLTIIFIVVYLFGAVLQVFTLLWIADWMVHHFWRKGKDPDSFSIPYLTALGDLLGTALLALS
FHFLWLIGDRDGDVGD

>Q96JB2 peripheral membrane protein
MAEAALLLLPEAAAERDAREKLALWDRRPDTTAPLTDRQTDSVLELKAAAENLPVPAELP
IEDLCSLTSQSLPIELTSVVPESTEDILLKGFTSLGMEEERIETAQQFFSWFAKLQTQMDQDE
GTKYRQMRDYLSGFQEQCDAILNDVNSALQHLESLQKQYLFVSNKTGTLHEACEQLLKE
QSELVDLAENIQQKLSYFNELETINTKLNSPTLSVNSDGFIPMLAKLDDCITYISSHPNFKD
YPIYLLKFKQCLSKALHLMKTYTVNTLQTLTSQLLKRDPSSVPNADNAFTLFYVKFRAAA
PKVRTLIEQIELRSEKIPEYQQLLNDIHQCYLDQRELLLGPSIACTVAELTSQNNRDHCALV
RSGCAFMVHVCQDEHQLYNEFFTKPTSKLDELLEKLCVSLYDVFRPLIIHVIHLETLSELCG
ILKNEVLEDHVQNNAEQLGAFAAGVKQMLEDVQERLVYRTHIYIQTDITGYKPAPGDLAY
PDKLVMMEQIAQSLKDEQKKVPSEASFSDVHLEEGESNSLTKSGSTESLNPRPQTTISPADL
HGMWYPTVRRTLVCLSKLYRCIDRAVFQGLSQEALSACIQSLLGASESISKNKTQIDGQLF
LIKHLLILREQIAPFHTEFTIKEISLDLKKTRDAAFKILNPMTVPRFFRLNSNNALIEFLLEGT
PEIREHYLDSKKDVDRHLKSACEQFIQQQTKLFVEQLEEFMTKVSALKTMASQGGPKYTL
SQQPWAQPAKVNDLAATAYKTIKTKLPVTLRSMSLYLSNKDTEFILFKPVRNNIQQVFQKF
HALLKEEFSPEDIQIIACPSMEQLSLLLLVSK

>Q96JA3 peripheral membrane protein
MEGVLYKWTNYLSGWQPRWFLLCGGILSYYDSPEDAWKGCKGSIQMAVCEIQVHSVDN
TRMDLIIPGEQYFYLKARSVAERQRWLVALGSAKACLTDSRTQKEKEFAENTENLKTKMS
ELRLYCDLLVQQVDKTKEVTTTGVSNSEEGIDVGTLLKSTCNTFLKTLEECMQIANAAFTS
ELLYRTPPGSPQLAMLKSSKMKHPIIPIHNSLERQMELSTCENGSLNMEINGEEEILMKNKN
SLYLKSAEIDCSISSEENTDDNITVQGEIRKEDGMENLKNHDNNLTQSGSDSSCSPECLWEE
GKEVIPTFFSTMNTSFSDIELLEDSGIPTEAFLASCYAVVPVLDKLGPTVFAPVKMDLVGNI
KKVNQKYITNKEEFTTLQKIVLHEVEADVAQVRNSATEALLWLKRGLKFLKGFLTEVKNG
EKDIQTALNNAYGKTLRQHHGWVVRGVFALALRAAPSYEDFVAALTVKEGDHQKEAFSI
GMQRDLSLYLPAMEKQLAILDTLYEVHGLESDEVV

>Q13554 peripheral membrane protein
MATTVTCTRFTDEYQLYEDIGKGAFSVVRRCVKLCTGHEYAAKIINTKKLSARDHQKLER
EARICRLLKHSNIVRLHDSISEEGFHYLVFDLVTGGELFEDIVAREYYSEADASHCIQQILEA
VLHCHQMGVVHRDLKPENLLLASKCKGAAVKLADFGLAIEVQGDQQAWFGFAGTPGYL
SPEVLRKEAYGKPVDIWACGVILYILLVGYPPFWDEDQHKLYQQIKAGAYDFPSPEWDTV
TPEAKNLINQMLTINPAKRITAHEALKHPWVCQRSTVASMMHRQETVECLKKFNARRKLK
GAILTTMLATRNFSVGRQTTAPATMSTAASGTTMGLVEQAKSLLNKKADGVKPQTNSTKN
SAAATSPKGTLPPAALEPQTTVIHNPVDGIKESSDSANTTIEDEDAKAPRVPDILSSVRRGS
GAPEAEGPLPCPSPAPFSPLPAPSPRISDILNSVRRGSGTPEAEGPLSAGPPPCLSPALLGPLSS
PSPRISDILNSVRRGSGTPEAEGPSPVGPPPCPSPTIPGPLPTPSRKQEIIKTTEQLIEAVNNGD

FEAYAKICDPGLTSFEPEALGNLVEGMDFHRFYFENLLAKNSKPIHTTILNPHVHVIGEDAA
CIAYIRLTQYIDGQGRPRTSQSEETRVWHRRDGKWQNVHFHCSGAPVAPLQ

>Q2M3G0 multipass membrane protein
MVDENDIRALNVRHYRDHIGVVSQEPVLFGTTISNNIKYGRDDVTDEEMERAAREANAY
DFIMEFPNKFNTLVGEKGAQMSGGQKQRIAIARALVRNPKILILDEATSALDSESKSAVQA
ALEKASKGRTTIVVAHRLSTIRSADLIVTLKDGMLAEKGAHAELMAKRGLYYSLVMSQDI
KKADEQMESMTYSTERKTNSLPLHSVKSIKSDFIDKAEESTQSKEISLPEVSLLKILKLNKP
EWPFVVLGTLASVLNGTVHPVFSIIFAKIITMFGNNDKTTLKHDAEIYSMIFVILGVICFVSY
FMQGLFYGRAGEILTMRLRHLAFKAMLYQDIAWFDEKENSTGGLTTILAIDIAQIQGATGS
RIGVLTQNATNMGLSVIISFIYGWEMTFLILSIAPVLAVTGMIETAAMTGFANKDKQELKH
AGKIATEALENIRTIVSLTREKAFEQMYEEMLQTQHRNTSKKAQIIGSCYAFSHAFIYFAYA
AGFRFGAYLIQAGRMTPEGMFIVFTAIAYGAMAIGETLVLAPEYSKAKSGAAHLFALLEKK
PNIDSRSQEGKKPDTCEGNLEFREVSFFYPCRPDVFILRGLSLSIERGKTVAFVGSSGCGKS
TSVQLLQRLYDPVQGQVLFDGVDAKELNVQWLRSQIAIVPQEPVLFNCSIAENIAYGDNSR
VVPLDEIKEAANAANIHSFIEGLPEKYNTQVGLKGAQLSGGQKQRLAIARALLQKPKILLL
DEATSALDNDSEKVVQHALDKARTGRTCLVVTHRLSAIQNADLIVVLHNGKIKEQGTHQE
LLRNRDIYFKLVNAQSVQ

>O15504 peripheral membrane protein
MAICQFFLQGRCRFGDRCWNEHPGARGAGGGRQQPQQQPSGNNRRGWNTTSQRYSNVI
QPSSFSKSTPWGGSRDQEKPYFSSFDSGASTNRKEGFGLSENPFASLSPDEQKDEKKLLEGI
VKDMEVWESSGQWMFSVYSPVKKKPNISGFTDISPEELRLEYHNFLTSNNLQSYLNSVQR
LINQWRNRVNELKSLNISTKVALLSDVKDGVNQAAPAFGFGSSQAATFMSPGFPVNNSSS
DNAQNFSFKTNSGFAAASSGSPAGFGSSPAFGAAASTSSGISTSAPAFGFGKPEVTSAASFS
FKSPAASSFGSPGFSGLPASLATGPVRAPVAPAFGGGSSVAGFGSPGSHSHTAFSKPSSDTFG
NSSISTSLSASSSIIATDNVLFTPRDKLTVEELEQFQSKKFTLGKIPLKPPPLELLNV

>B0I1T2 peripheral membrane protein
MEDEEGPEYGKPDFVLLDQVTMEDFMRNLQLRFEKGRIYTYIGEVLVSVNPYQELPLYGP
EAIARYQGRELYERPPHLYAVANAAYKAMKHRSRDTCIVISGESGAGKTEASKHIMQYIAA
VTNPSQRAEVERVKDVLLKSTCVLEAFGNARTNRNHNSSRFGKYMDINFDFKGDPIGGHI
HSYLLEKSRVLKQHVGERNFHAFYQLLRGSEDKQLHELHLERNPAVYNFTHQGAGLNMT
VHSALDSDEQSHQAVTEAMRVIGFSPEEVESVHRILAAILHLGNIEFVETEEGGLQKEGLAV
AEEALVDHVAELTATPRDLVLRSLLARTVASGGRELIEKGHTAAEASYARDACAKAVYQRL
FEWVVNRINSVMEPRGRDPRRDGKDTVIGVLDIYGFEVFPVNSFEQFCINYCNEKLQQLFI
QLILKQEQEEYEREGITWQSVEYFNNATIVDLVERPHRGILAVLDEACSSAGTITDRIFLQT
LDMHHRHHLHYTSRQLCPTDKTMEFGRDFRIKHYAGDVTYSVEGFIDKNRDFLFQDFKR
LLYNSTDPTLRAMWPDGQQDITEVTKRPLTAGTLFKNSMVALVENLASKEPFYVRCIKPN
EDKVAGKLDENHCRHQVAYLGLLENVRVRRAGFASRQPYSRFLLRYKMTCEYTWPNHLL
GSDKAAVSALLEQHGLQGDVAFGHSKLFIRSPRTLVTLEQSRARLIPIIVLLLQKAWRGTLA
RWRCRRLRAIYTIMRWFRRHKVRAHLAELQRRFQAARQPPLYGRDLVWPLPPAVLQPFQ
DTCHALFCRWRARQLVKNIPPSDMPQIKAKVAAMGALQGLRQDWGCRRAWARDYLSSA
TDNPTASSLFAQRLKTLQDKDGFGAVLFSSHVRKVNRFHKIRNRALLLTDQHLYKLDPDR

QYRVMRAVPLEAVTGLSVTSGGDQLVVLHARGQDDLVVCLHRSRPPLDNRVGELVGVLA
AHCQGEGRTLEVRVSDCIPLSHRGVRRLISVEPRPEQPEPDFRCARGSFTLLWPSR

>Q9C0H2 multipass membrane protein
MAGVSYAAPWWVSLLHRLPHFDLSWEATSSQFRPEDTDYQQALLLLGAAALACLALDLL
FLLFYSFWLCCRRRKSEEHLDADCCCTAWCVIIATLVCSAGIAVGFYGNGETSDGIHRATY
SLRHANRTVAGVQDRVWDTAVGLNHTAEPSLQTLERQLAGRPEPLRAVQRLQGLLETLLG
YTAAIPFWRNTAVSLEVLAEQVDLYDWYRWLGYLGLLLLDVIICLLVLVGLIRSSKGILVG
VCLLGVLALVISWGALGLELAVSVGSSDFCVDPDAYVTKMVEEYSVLSGDILQYYLACSP
RAANPFQQKLSGSHKALVEMQDVVAELLRTVPWEQPATKDPLLRVQEVLNGTEVNLQHL
TALVDCRSLHLDYVQALTGFCYDGVEGLIYLALFSFVTALMFSSIVCSVPHTWQQKRGPD
EDGEEEAAPGPRQAHDSLYRVHMPSLYSCGSSYGSETSIPAAAHTVSNAPVTEYMSQNAN
FQNPRCENTPLIGRESPPPSYTSSMRAKYLATSQPRPDSSGSH

>P0CG37 GPI-anchor, lipid-anchor
MTWRHHVRLLFTVSLALQIINLGNSYQREKHNGGREEVTKVATQKHRQSPLNWTSSHFG
EVTGSAEGWGPEEPLPYSRAFGEGASARPRCCRNGGTCVLGSFCVCPAHFTGRYCEHDQR
RSECGALEHGAWTLRACHLCRCIFGALHCLPLQTPDRCDPKDFLASHAHGPSAGGAPSLL
LLLPCALLHRLLRPDAPAHPRSLVPSVLQRERRPCGRPGLGHRL

>Q05329 lipid-anchor, peripheral membrane protein
MASPGSGFWSFGSEDGSGDSENPGTARAWCQVAQKFTGGIGNKLCALLYGDAEKPAESG
GSQPPRAAARKAACACDQKPCSCSKVDVNYAFLHATDLLPACDGERPTLAFLQDVMNIL
LQYVVKSFDRSTKVIDFHYPNELLQEYNWELADQPQNLEEILMHCQTTLKYAIKTGHPRY
FNQLSTGLDMVGLAADWLTSTANTNMFTYEIAPVFVLLEYVTLKKMREIIGWPGGSGDGI
FSPGGAISNMYAMMIARFKMFPEVKEKGMAALPRLIAFTSEHSHFSLKKGAAALGIGTDS
VILIKCDERGKMIPSDLERRILEAKQKGFVPFLVSATAGTTVYGAFDPLLAVADICKKYKIW
MHVDAAWGGGLLMSRKHKWKLSGVERANSVTWNPHKMMGVPLQCSALLVREEGLMQ
NCNQMHASYLFQQDKHYDLSYDTGDKALQCGRHVDVFKLWLMWRAKGTTGFEAHVD
KCLELAEYLYNIIKNREGYEMVFDGKPQHTNVCFWYIPPSLRTLEDNEERMSRLSKVAPVI
KARMMEYGTTMVSYQPLGDKVNFFRMVISNPAATHQDIDFLIEEIERLGQDL

>Q8NGS3 multipass membrane protein
MSPENQSSVSEFLLLGLPIRPEQQAVFFALFLGMYLTTVLGNLLIMLLIQLDSHLHTPMYFF
LSHLALTDISFSSVTVPKMLMNMQTQHLAVFYKGCISQTYFFIFFADLDSFLITSMAYDRYV
AICHPLHYATIMTQSQCVMLVAGSWVIACACALLHTLLLAQLSFCADHIIPHYFCDLGALL
KLSCSDTSLNQLAIFTAALTAIMLPFLCILVSYGHIGVTILQIPSTKGICKALSTCGSHLSVVTI
YYRTIIGLYFLPPSSNTNDKNIIASVIYTAVTPMLNPFIYSLRNKDIKGALRKLLSRSGAVAH
ACNLNTLGG

>Q8NGT0 multipass membrane protein
MEWENQTILVEFFLKGHSVHPRLELLFFVLIFIMYVVILLGNGTLILISILDPHLHTPMYFFL
GNLSFLDICYTTTSIPSTLVSFLSERKTISFSGCAVQMFLGLAMGTTECVLLGMMAFDRYVA
ICNPLRYPIIMSKNAYVPMAVGSWFAGIVNSAVQTTFVVQLPFCRKNVINHFSCEILAVMKL

ACADISGNEFLMLVATILFTLMPLLLIVISYSLIISSILKIHSSEGRSKAFSTCSAHLTVVIIFYG
TILFMYMKPKSKETLNSDDLDATDKIISMFYGVMTPMMNPLIYSLRNKDVKEAVKHLPNR
RFFSK

>O75899 multipass membrane protein
MASPRSSGQPGPPPPPPPPPARLLLLLLLPLLLPLAPGAWGWARGAPRPPPSSPPLSIMGLM
PLTKEVAKGSIGRGVLPAVELAIEQIRNESLLRPYFLDLRLYDTECDNAKGLKAFYDAIKYG
PNHLMVFGGVCPSVTSIIAESLQGWNLVQLSFAATTPVLADKKKYPYFFRTVPSDNAVNPA
ILKLLKHYQWKRVGTLTQDVQRFSEVRNDLTGVLYGEDIEISDTESFSNDPCTSVKKLKGN
DVRIILGQFDQNMAAKVFCCAYEENMYGSKYQWIIPGWYEPSWWEQVHTEANSSRCLR
KNLLAAMEGYIGVDFEPLSSKQIKTISGKTPQQYEREYNNKRSGVGPSKFHGYAYDGIWVI
AKTLQRAMETLHASSRHQRIQDFNYTDHTLGRIILNAMNETNFFGVTGQVVFRNGERMG
TIKFTQFQDSREVKVGEYNAVADTLEIINDTIRFQGSEPPKDKTIILEQLRKISLPLYSILSALT
ILGMIMASAFLFFNIKNRNQKLIKMSSPYMNNLIILGGMLSYASIFLFGLDGSFVSEKTFETL
CTVRTWILTVGYTTAFGAMFAKTWRVHAIFKNVKMKKKIIKDQKLLVIVGGMLLIDLCILI
CWQAVDPLRRTVEKYSMEPDPAGRDISIRPLLEHCENTHMTIWLGIVYAYKGLLMLFGCF
LAWETRNVSIPALNDSKYIGMSVYNVGIMCIIGAAVSFLTRDQPNVQFCIVALVIIFCSTITL
CLVFVPKLITLRTNPDAATQNRRFQFTQNQKKEDSKTSTSVTSVNQASTSRLEGLQSENHR
LRMKITELDKDLEEVTMQLQDTPEKTTYIKQNHYQELNDILNLGNFTESTDGGKAILKNH
LDQNPQLQWNTTEPSRTCKDPIEDINSPEHIQRRLSLQLPILHHAYLPSIGGVDASCVSPCVS
PTASPRHRHVPPSFRVMVSGL

>Q9BUN8 multipass membrane protein
MSDIGDWFRSIPAITRYWFAATVAVPLVGKLGLISPAYLFLWPEAFLYRFQIWRPITATFYFP
VGPGTGFLYLVNLYFLYQYSTRLETGAFDGRPADYLFMLLFNWICIVITGLAMDMQLLMIP
LIMSVLYVWAQLNRDMIVSFWFGTRFKACYLPWVILGFNYIIGGSVINELIGNLVGHLYFF
LMFRYPMDLGGRNFLSTPQFLYRWLPSRRGGVSGFGVPPASMRRAADQNGGGGRHNWG
QGFRLGDQ

>Q9Y3P8 single-pass type I membrane protein
MNQADPRLRAVCLWTLTSAAMSRGDNCTDLLALGIPSITQAWGLWVLLGAVTLLFLISLA
AHLSQWTRGRSRSHPGQGRSGESVEEVPLYGNLHYLQTGRLSQDPEPDQQDPTLGGPAR
AAEEVMCYTSLQLRPPQGRIPGPGTPVKYSEVVLDSEPKSQASGPEPELYASVCAQTRRAR
ASFPDQAYANSQPAAS

>P21854 single-pass type II membrane protein
MAEAITYADLRFVKAPLKKSISSRLGQDPGADDDGEITYENVQVPAVLGVPSSLASSVLGD
KAAVKSEQPTASWRAVTSPAVGRILPCRTTCLRYLLLGLLLTCLLLGVTAICLGVRYLQVSQ
QLQQTNRVLEVTNSSLRQQLRLKITQLGQSAEDLQGSRRELAQSQEALQVEQRAHQAAE
GQLQACQADRQKTKETLQSEEQQRRALEQKLSNMENRLKPFFTCGSADTCCPSGWIMHQ
KSCFYISLTSKNWQESQKQCETLSSKLATFSEIYPQSHSYYFLNSLLPNGGSGNSYWTGLSS
NKDWKLTDDTQRTRTYAQSSKCNKVHKTWSWWTLESESCRSSLPYICEMTAFRFPD

>Q9GZM5 multipass membrane protein

MATTAAPAGGARNGAGPEWGGFEENIQGGGSAVIDMENMDDTSGSSFEDMGELHQRLRE
EEVDADAADAAAAEEEDGEFLGMKGFKGQLSRQVADQMWQAGKRQASRAFSLYANIDI
LRPYFDVEPAQVRSRLLESMIPIKMVNFPQKIAGELYGPLMLVFTLVAILLHGMKTSDTIIRE
GTLMGTAIGTCFGYWLGVSSFIYFLAYLCNAQITMLQMLALLGYGLFGHCIVLFITYNIHL
HALFYLFWLLVGGLSTLRMVAVLVSRTVGPTQRLLLCGTLAALHMLFLLYLHFAYHKVVE
GILDTLEGPNIPPIQRVPRDIPAMLPAARLPTTVLNATAKAVAVTLQSH

>O43559 lipid-anchor
MGSCCSCLNRDSVPDNHPTKFKVTNVDDEGVELGSGVMELTQSELVLHLHRREAVRWPY
LCLRRYGYDSNLFSFESGRRCQTGQGIFAFKCSRAEEIFNLLQDLMQCNSINVMEEPVIITR
NSHPAELDLPRAPQPPNALGYTVSSFSNGCPGEGPRFSAPRRLSTSSLRHPSLGEESTHALI
APDEQSHTYVNTPASEDDHRRGRHCLQPLPEGQAPFLPQARGPDQRDPQVFLQPGQVKF
VLGPTPARRHMVKCQGLCPSLHDPPHHNNNNEAPSECPAQPKCTYENVTGGLWRGAGW
RLSPEEPGWNGLAHRRAALLHYENLPPLPPVWESQAQQLGGEAGDDGDSRDGLTPSSNG
FPDGEEDETPLQKPTSTRAAIRSHGSFPVPLTRRRGSPRVFNFDFRRPGPEPPRQLNYIQVEL
KGWGGDRPKGPQNPSSPQAPMPTTHPARSSDSYAVIDLKKTVAMSNLQRALPRDDGTAR
KTRHNSTDLPL

>Q14512 peripheral membrane protein
MKICSLTLLSFLLLAAQVLLVEGKKKVKNGLHSKVVSEQKDTLGNTQIKQKSRPGNKGKF
VTKDQANCRWAATEQEEGISLKVECTQLDHEFSCVFAGNPTSCLKLKDERVYWKQVARN
LRSQKDICRYSKTAVKTRVCRKDFPESSLKLVSSTLFGNTKPRKEKTEMSPREHIKGKETTP
SSLAVTQTMATKAPECVEDPDMANQRKTALEFCGETWSSLCTFFLSIVQDTSC

>Q15771 lipid-anchor
MSMEDYDFLFKIVLIGNAGVGKTCLVRRFTQGLFPPGQGATIGVDFMIKTVEINGEKVKL
QIWDTAGQERFRSITQSYYRSANALILTYDITCEESFRCLPEWLREIEQYASNKVITVLVGN
KIDLAERREVSQQRAEEFSEAQDMYYLETSAKESDNVEKLFLDLACRLISEARQNTLVNN
VSSPLPGEGKSISYLTCCNFN

>Q14392 single-pass type I membrane protein
MRPQILLLLALLTLGLAAQHQDKVPCKMVDKKVSCQVLGLLQVPSVLPPDTETLDLSGN
QLRSILASPLGFYTALRHLDLSTNEISFLQPGAFQALTHLEHLSLAHNRLAMATALSAGGL
GPLPRVTSLDLSGNSLYSGLLERLLGEAPSLHTLSLAENSLTRLTRHTFRDMPALEQLDLHS
NVLMDIEDGAFEGLPRLTHLNLSRNSLTCISDFSLQQLRVLDLSCNSIEAFQTASQPQAEFQ
LTWLDLRENKLLHFPDLAALPRLIYLNLSNNLIRLPTGPPQDSKGIHAPSEGWSALPLSAPS
GNASGRPLSQLLNLDLSYNEIELIPDSFLEHLTSLCFLNLSRNCLRTFEARRLGSLPCLMLL
DLSHNALETLELGARALGSLRTLLLQGNALRDLPPYTFANLASLQRLNLQGNRVSPCGGP
DEPGPSGCVAFSGITSLRSLSLVDNEIELLRAGAFLHTPLTELDLSSNPGLEVATGALGGLEA
SLEVLALQGNGLMVLQVDLPCFICLKRLNLAENRLSHLPAWTQAVSLEVLDLRNNSFSLL
PGSAMGGLETSLRRLYLQGNPLSCCGNGWLAAQLHQGRVDVDATQDLICRFSSQEEVSLS
HVRPEDCEKGGLKNINLIIILTFILVSAILLTTLAACCCVRRQKFNQQYKA

>Q9H2Y9 multipass membrane protein

MDEGTGLQPGAGEQLEAPATAEAVQERCEPETLRSKSLPVLSSASCRPSLSPTSGDANPAF
GCVDSSGHQELKQGPNPLAPSPSAPSTSAGLGDCNHRVDLSKTFSVSSALAMLQERRCLY
VVLTDSRCFLVCMCFLTFIQALMVSGYLSSVITTIERRYSLKSSESGLLVSCFDIGNLVVVVF
VSYFGGRGRRPLWLAVGGLLIAFGAALFALPHFISPPYQIQELNASAPNDGLCQGGNSTAT
LEPPACPKDSGGNNHWVYVALFICAQILIGMGSTPIYTLGPTYLDDNVKKENSSLYLAIMY
VMGALGPAVGYLLGGLLIGFYVDPRNPVHLDQNDPRFIGNWWSGFLLCAIAMFLVIFPMF
TFPKKLPPRHKKKKKKKFSVDAVSDDDVLKEKSNNSEQADKKVSSMGFGKDVRDLPRAA
VRILSNMTFLFVSLSYTAESAIVTAFITFIPKFIESQFGIPASNASIYTGVIIVPSAGVGIVLGG
YIIKKLKLGARESAKLAMICSGVSLLCFSTLFIVGCESINLGGINIPYTTGPSLTMPHRNLTG
SCNVNCGCKIHEYEPVCGSDGITYFNPCLAGCVNSGNLSTGIRNYTECTCVQSRQVITPPT
VGQRSQLRVVIVKTYLNENGYAVSGKCKRTCNTLIPFLVFLFIVTFITACAQPSAIIVTLRSV
EDEERPFALGMQFVLLRTLAYIPTPIYFGAVIDTTCMLWQQECGVQGSCWEYNVTSFRFV
YFGLAAGLKFVGFIFIFLAWYSIKYKEDGLQRRRQREFPLSTVSERVGHPDNARTRSCPAFS
TQGEFHEETGLQKGIQCAAQTYPGPFPEAISSSADPGLEESPAALEPPS

>O00560	peripheral membrane protein
MSLYPSLEDLKVDKVIQAQTAFSANPANPAILSEASAPIPHDGNLYPRLYPELSQYMGLSLN
EEEIRANVAVVSGAPLQGQLVARPSSINYMVAPVTGNDVGIRRAEIKQGIREVILCKDQDG
KIGLRLKSIDNGIFVQLVQANSPASLVGLRFGDQVLQINGENCAGWSSDKAHKVLKQAFG
EKITMTIRDRPFERTITMHKDSTGHVGFIFKNGKITSIVKDSSAARNGLLTEHNICEINGQNV
IGLKDSQIADILSTSGTVVTITIMPAFIFEHIIKRMAPSIMKSLMDHTIPEV

>O95264	multipass membrane protein
MLSSVMAPLWACILVAAGILATDTHHPQDSALYHLSKQLLQKYHKEVRPVYNWTKATTV
YLDLFVHAILDVDAENQILKTSVWYQEVWNDEFLSWNSSMFDEIREISLPLSAIWAPDIIIN
EFVDIERYPDLPYVYVNSSGTIENYKPIQVVSACSLETYAFPFDVQNCSLTFKSILHTVEDV
DLAFLRSPEDIQHDKKAFLNDSEWELLSVSSTYSILQSSAGGFAQIQFNVVMRRHPLVYVV
SLLIPSIFLMLVDLGSFYLPPNCRARIVFKTSVLVGYTVFRVNMSNQVPRSVGSTPLIGHFFT
ICMAFLVLSLAKSIVLVKFLHDEQRGGQEQPFLCLRGDTDADRPRVEPRAQRAVVTESSLY
GEHLAQPGTLKEVWSQLQSISNYLQTQDQTDQQEAEWLVLLSRFDRLLFQSYLFMLGIYT
ITLCSLWALWGGV

>O14672	single-pass type I membrane protein
MVLLRVLILLLLSWAAGMGGQYGNPLNKYIRHYEGLSYNVDSLHQKHQRAKRAVSHEDQ
FLRLDFHAHGRHFNLRMKRDTSLFSDEFKVETSNKVLDYDTSHIYTGHIYGEEGSFSHGS
VIDGRFEGFIQTRGGTFYVEPAERYIKDRTLPFHSVIYHEDDINYPHKYGPQGGCADHSVFE
RMRKYQMTGVEEVTQIPQEEHAANGPELLRKKRTTSAEKNTCQLYIQTDHLFFKYYGTR
EAVIAQISSHVKAIDTIYQTTDFSGIRNISFMVKRIRINTTADEKDPTNPFRFPNIGVEKFLEL
NSEQNHDDYCLAYVFTDRDFDDGVLGLAWVGAPSGSSGGICEKSKLYSDGKKKSLNTGII
TVQNYGSHVPPKVSHITFAHEVGHNFGSPHDSGTECTPGESKNLGQKENGNYIMYARATS
GDKLNNNKFSLCSIRNISQVLEKKRNNCFVESGQPICGNGMVEQGEECDCGYSDQCKDEC
CFDANQPEGRKCKLKPGKQCSPSQGPCCTAQCAFKSKSEKCRDDSDCAREGICNGFTALC
PASDPKPNFTDCNRHTQVCINGQCAGSICEKYGLEECTCASSDGKDDKELCHVCCMKKM
DPSTCASTGSVQWSRHFSGRTITLQPGSPCNDFRGYCDVFMRCRLVDADGPLARLKKAIF

SPELYENIAEWIVAHWWAVLLMGIALIMLMAGFIKICSVHTPSSNPKLPPPKPLPGTLKRRR
PPQPIQQPQRQRPRESYQMGHMRR

>P11511 peripheral membrane protein
MVLEMLNPIHYNITSIVPEAMPAATMPVLLLTGLFLLVWNYEGTSSIPGPGYCMGIGPLISH
GRFLWMGIGSACNYYNRVYGEFMRVWISGEETLIISKSSSMFHIMKHNHYSSRFGSKLGL
QCIGMHEKGIIFNNNPELWKTTRPFFMKALSGPGLVRMVTVCAESLKTHLDRLEEVTNES
GYVDVLTLLRRVMLDTSNTLFLRIPLDESAIVVKIQGYFDAWQALLIKPDIFFKISWLYKKY
EKSVKDLKDAIEVLIAEKRRRISTEEKLEECMDFATELILAEKRGDLTRENVNQCILEMLIA
APDTMSVSLFFMLFLIAKHPNVEEAIIKEIQTVIGERDIKIDDIQKLKVMENFIYESMRYQPV
VDLVMRKALEDDVIDGYPVKKGTNIILNIGRMHRLEFFPKPNEFTLENFAKNVPYRYFQPF
GFGPRGCAGKYIAMVMMKAILVTLLRRFHVKTLQGQCVESIQKIHDLSLHPDETKNMLE
MIFTPRNSDRCLEH

>O60266 multipass membrane protein
MPRNQGFSEPEYSAEYSAEYSVSLPSDPDRGVGRTHEISVRNSGSCLCLPRFMRLTFVPESL
ENLYQTYFKRQRHETLLVLVVFAALFDCYVVVMCAVVFSSDKLASLAVAGIGLVLDIILFV
LCKKGLLPDRVTRRVLPYVLWLLITAQIFSYLGLNFARAHAASDTVGWQVFFVFSFFITLP
LSLSPIVIISVVSCVVHTLVLGVTVAQQQQEELKGMQLLREILANVFLYLCAIAVGIMSYYM
ADRKHRKAFLEARQSLEVKMNLEEQSQQQENLMLSILPKHVADEMLKDMKKDESQKDQ
QQFNTMYMYRHENVSILFADIVGFTQLSSACSAQELVKLLNELFARFDKLAAKYHQLRIKI
LGDCYYCICGLPDYREDHAVCSILMGLAMVEAISYVREKTKTGVDMRVGVHTGTVLGGV
LGQKRWQYDVWSTDVTVANKMEAGGIPGRVHISQSTMDCLKGEFDVEPGDGGSRCDYL
EEKGIETYLIIASKPEVKKTATQNGLNGSALPNGAPASSKSSSPALIETKEPNGSAHSSGSTS
EKPEEQDAQADNPSFPNPRRRLRLQDLADRVVDASEDEHELNQLLNEALLERESAQVVK
KRNTFLLSMRFMDPEMETRYSVEKEKQSGAAFSCSCVVLLCTALVEILIDPWLMTNYVTF
MVGEILLLILTICSLAAIFPRAFPKKLVAFSTWIDRTRWARNTWAMLAIFILVMANVVDMLS
CLQYYTGPSNATAGMETEGSCLENPKYYNYVAVLSLIATIMLVQVSHMVKLTLMLLVAGA
VATINLYAWRPVFDEYDHKRFREHDLPMVALEQMQGFNPGLNGTDRLPLVPSKYSMTVM
VFLMMLSFYYFSRHVEKLARTLFLWKIEVHDQKERVYEMRRWNEALVTNMLPEHVARHF
LGSKKRDEELYSQTYDEIGVMFASLPNFADFYTEESINNGGIECLRFLNEIISDFDSLLDNPK
FRVITKIKTIGSTYMAASGVTPDVNTNGFASSNKEDKSERERWQHLADLADFALAMKDTL
TNINNQSFNNFMLRIGMNKGGVLAGVIGARKPHYDIWGNTVNVASRMESTGVMGNIQV
VEETQVILREYGFRFVRRGPIFVKGKGELLTFFLKGRDKLATFPNGPSVTLPHQVVDNS

>Q16678 peripheral membrane protein
MGTSLSPNDPWPLNPLSIQQTTLLLLLSVLATVHVGQRLLRQRRRQLRSAPPGPFAWPLIG
NAAAVGQAAHLSFARLARRYGDVFQIRLGSCPIVVLNGERAIHQALVQQGSAFADRPAFA
SFRVVSGGRSMAFGHYSEHWKVQRRAAHSMMRNFFTRQPRSRQVLEGHVLSEARELVAL
LVRGSADGAFLDPRPLTVVAVANVMSAVCFGCRYSHDDPEFRELLSHNEEFGRTVGAGSL
VDVMPWLQYFPNPVRTVFREFEQLNRNFSNFILDKFLRHCESLRPGAAPRDMMDAFILSA
EKKAAGDSHGGGARLDLENVPATITDIFGASQDTLSTALQWLLLLFTRYPDVQTRVQAEL
DQVVGRDRLPCMGDQPNLPYVLAFLYEAMRFSSFVPVTIPHATTANTSVLGYHIPKDTVV
FVNQWSVNHDPLKWPNPENFDPARFLDKDGLINKDLTSRVMIFSVGKRRCIGEELSKMQL

FLFISILAHQCDFRANPNEPAKMNFSYGLTIKPKSFKVNVTLRESMELLDSAVQNLQAKET
CQ

>P11712	peripheral membrane protein
MDSLVVLVLCLSCLLLLSLWRQSSGRGKLPPGPTPLPVIGNILQIGIKDISKSLTNLSKVYGP
VFTLYFGLKPIVVLHGYEAVKEALIDLGEEFSGRGIFPLAERANRGFGIVFSNGKKWKEIRR
FSLMTLRNFGMGKRSIEDRVQEEARCLVEELRKTKASPCDPTFILGCAPCNVICSIIFHKRF
DYKDQQFLNLMEKLNENIKILSSPWIQICNNFSPIIDYFPGTHNKLLKNVAFMKSYILEKVK
EHQESMDMNNPQDFIDCFLMKMEKEKHNQPSEFTIESLENTAVDLFGAGTETTSTTLRYA
LLLLLKHPEVTAKVQEEIERVIGRNRSPCMQDRSHMPYTDAVVHEVQRYIDLLPTSLPHAV
TCDIKFRNYLIPKGTTILISLTSVLHDNKEFPNPEMFDPHHFLDEGGNFKKSKYFMPFSAGK
RICVGEALAGMELFLFLTSILQNFNLKSLVDPKNLDTTPVVNGFASVPPFYQLCFIPV

>P36405	peripheral membrane protein
MGLLSILRKLKSAPDQEVRILLLGLDNAGKTTLLKQLASEDISHITPTQGFNIKSVQSQGFK
LNVWDIGGQRKIRPYWKNYFENTDILIYVIDSADRKRFEETGQELAELLEEEKLSCVPVLIF
ANKQDLLTAAPASEIAEGLNLHTIRDRVWQIQSCSALTGEGVQDGMNWVCKNVNAKKK

>P22607	single-pass type I membrane protein
MGAPACALALCVAVAIVAGASSESLGTEQRVVGRAAEVPGPEPGQQEQLVFGSGDAVELS
CPPPGGGPMGPTVWVKDGTGLVPSERVLVGPQRLQVLNASHEDSGAYSCRQRLTQRVLC
HFSVRVTDAPSSGDDEDGEDEAEDTGVDTGAPYWTRPERMDKKLLAVPAANTVRFRCPA
AGNPTPSISWLKNGREFRGEHRIGGIKLRHQQWSLVMESVVPSDRGNYTCVVENKFGSIR
QTYTLDVLERSPHRPILQAGLPANQTAVLGSDVEFHCKVYSDAQPHIQWLKHVEVNGSKV
GPDGTPYVTVLKTAGANTTDKELEVLSLHNVTFEDAGEYTCLAGNSIGFSHHSAWLVVLP
AEEELVEADEAGSVYAGILSYGVGFFLFILVVAAVTLCRLRSPPKKGLGSPTVHKISRFPLK
RQVSLESNASMSSNTPLVRIARLSSGEGPTLANVSELELPADPKWELSRARLTLGKPLGEG
CFGQVVMAEAIGIDKDRAAKPVTVAVKMLKDDATDKDLSDLVSEMEMMKMIGKHKNIIN
LLGACTQGGPLYVLVEYAAKGNLREFLRARRPPGLDYSFDTCKPPEEQLTFKDLVSCAYQV
ARGMEYLASQKCIHRDLAARNVLVTEDNVMKIADFGLARDVHNLDYYKKTTNGRLPVK
WMAPEALFDRVYTHQSDVWSFGVLLWEIFTLGGSPYPGIPVEELFKLLKEGHRMDKPAN
CTHDLYMIMRECWHAAPSQRPTFKQLVEDLDRVLTVTSTDEYLDLSAPFEQYSPGGQDTP
SSSSSGDDSVFAHDLLPPAPPSSGGSRT

>P02708	multipass membrane protein
MEPWPLLLLFSLCSAGLVLGSEHETRLVAKLFKDYSSVVRPVEDHRQVVEVTVGLQLIQLI
NVDEVNQIVTTNVRLKQGDMVDLPRPSCVTLGVPLFSHLQNEQWVDYNLKWNPDDYG
GVKKIHIPSEKIWRPDLVLYNNADGDFAIVKFTKVLLQYTGHITWTPPAIFKSYCEIIVTHFP
FDEQNCSMKLGTWTYDGSVVAINPESDQPDLSNFMESGEWVIKESRGWKHSVTYSCCPD
TPYLDITYHFVMQRLPLYFIVNVIIPCLLFSFLTGLVFYLPTDSGEKMTLSISVLLSLTVFLLVI
VELIPSTSSAVPLIGKYMLFTMVFVIASIIITVIVINTHHRSPSTHVMPNWVRKVFIDTIPNIM
FFSTMKRPSREKQDKKIFTEDIDISDISGKPGPPPMGFHSPLIKHPEVKSAIEGIKYIAETMKS
DQESNNAAAEWKYVAMVMDHILLGVFMLVCIIGTLAVFAGRLIELNQQG

>P06756 single-pass type I membrane protein
MAFPPRRRLRLGPRGLPLLLSGLLLPLCRAFNLDVDSPAEYSGPEGSYFGFAVDFFVPSASS
RMFLLVGAPKANTTQPGIVEGGQVLKCDWSSTRRCQPIEFDATGNRDYAKDDPLEFKSHQ
WFGASVRSKQDKILACAPLYHWRTEMKQEREPVGTCFLQDGTKTVEYAPCRSQDIDADG
QGFCQGGFSIDFTKADRVLLGGPGSFYWQGQLISDQVAEIVSKYDPNVYSIKYNNQLATRT
AQAIFDDSYLGYSVAVGDFNGDGIDDFVSGVPRAARTLGMVYIYDGKNMSSLYNFTGEQ
MAAYFGFSVAATDINGDDYADVFIGAPLFMDRGSDGKLQEVGQVSVSLQRASGDFQTTK
LNGFEVFARFGSAIAPLGDLDQDGFNDIAIAAPYGGEDKKGIVYIFNGRSTGLNAVPSQILE
GQWAARSMPPSFGYSMKGATDIDKNGYPDLIVGAFGVDRAILYRARPVITVNAGLEVYPS
ILNQDNKTCSLPGTALKVSCFNVRFCLKADGKGVLPRKLNFQVELLLDKLKQKGAIRRAL
FLYSRSPSHSKNMTISRGGLMQCEELIAYLRDESEFRDKLTPITIFMEYRLDYRTAADTTGL
QPILNQFTPANISRQAHILLDCGEDNVCKPKLEVSVDSDQKKIYIGDDNPLTLIVKAQNQGE
GAYEAELIVSIPLQADFIGVVRNNEALARLSCAFKTENQTRQVVCDLGNPMKAGTQLLAG
LRFSVHQQSEMDTSVKFDLQIQSSNLFDKVSPVVSHKVDLAVLAAVEIRGVSSPDHVFLPI
PNWEHKENPETEEDVGPVVQHIYELRNNGPSSFSKAMLHLQWPYKYNNNTLLYILHYDI
DGPMNCTSDMEINPLRIKISSLQTTEKNDTVAGQGERDHLITKRDLALSEGDIHTLGCGVA
QCLKIVCQVGRLDRGKSAILYVKSLLWTETFMNKENQNHSYSLKSSASFNVIEFPYKNLPI
EDITNSTLVTTNVTWGIQPAPMPVPVWVIILAVLAGLLLLAVLVFVMYRMGFFKRVRPPQE
EQEREQLQPHENGEGNSET

>Q9NP59 multipass membrane protein
MTRAGDHNRQRGCCGSLADYLTSAKFLLYLGHSLSTWGDRMWHFAVSVFLVELYGNSLL
LTAVYGLVVAGSVLVLGAIIGDWVDKNARLKVAQTSLVVQNVSVILCGIILMMVFLHKHE
LLTMYHGWVLTSCYILIITIANIANLASTATAITIQRDWIVVVAGEDRSKLANMNATIRRIDQ
LTNILAPMAVGQIMTFGSPVIGCGFISGWNLVSMCVEYVLLWKVYQKTPALAVKAGLKEE
ETELKQLNLHKDTEPKPLEGTHLMGVKDSNIHELEHEQEPTCASQMAEPFRTFRDGWVSY
YNQPVFLAGMGLAFLYMTVLGFDCITTGYAYTQGLSGSILSILMGASAITGIMGTVAFTWL
RRKCGLVRTGLISGLAQLSCLILCVISVFMPGSPLDLSVSPFEDIRSRFIQGESITPTKIPEITT
EIYMSNGSNSANIVPETSPESVPIISVSLLFAGVIAARIGLWSFDLTVTQLLQENVIESERGIIN
GVQNSMNYLLDLLHFIMVILAPNPEAFGLLVLISVSFVAMGHIMYFRFAQNTLGNKLFACG
PDAKEVRKENQANTSVV

>Q96SL1 multipass membrane protein
MGSRWSSEEERQPLLGPGLGPGLGASWRSREAAAAALPAAVPGPGRVYGRRWLVLLLFS
LLAFVQGLVWNTWGPIQNSARQAYGFSSWDIALLVLWGPIGFLPCFAFMWLLDKRGLRIT
VLLTSFLMVLGTGLRCIPISDLILKRRLIHGGQMLNGLAGPTVMNAAPFLSTTWFSADERA
TATAIASMLSYLGGACAFLVGPLVVPAPNGTSPLLAAESSRAHIKDRIEAVLYAEFGVVCLIF
SATLAYFPPRPPLPPSVAAASQRLSYRRSVCRLLSNFRFLMIALAYAIPLGVFAGWSGVLDLI
LTPAHVSQVDAGWIGFWSIVGGCVVGIAMARFADFIRGMLKLILLLLFSGATLSSTWFTLT
CLNSITHLPLTTVTLYASCILLGVFLNSSVPIFFELFVETVYPVPEGITCGVVTFLSNMFMGV
LLFFLTFYHTELSWFNWCLPGSCLLSLLLILCFRESYDRLYLDVVVSV

>P25092 single-pass type I membrane protein
MKTLLLDLALWSLLFQPGWLSFSSQVSQNCHNGSYEISVLMMGNSAFAEPLKNLEDAVN

EGLEIVRGRLQNAGLNVTVNATFMYSDGLIHNSGDCRSSTCEGLDLLRKISNAQRMGCVL
IGPSCTYSTFQMYLDTELSYPMISAGSFGLSCDYKETLTRLMSPARKLMYFLVNFWKTND
LPFKTYSWSTSYVYKNGTETEDCFWYLNALEASVSYFSHELGFKVVLRQDKEFQDILMD
HNRKSNVIIMCGGPEFLYKLKGDRAVAEDIVIILVDLFNDQYFEDNVTAPDYMKNVLVLTL
SPGNSLLNSSFSRNLSPTKRDFALAYLNGILLFGHMLKIFLENGENITTPKFAHAFRNLTFEG
YDGPVTLDDWGDVDSTMVLLYTSVDTKKYKVLLTYDTHVNKTYPVDMSPTFTWKNSKL
PNDITGRGPQILMIAVFTLTGAVVLLLLVALLMLRKYRKDYELRQKKWSHIPPENIFPLETN
ETNHVSLKIDDDKRRDTIQRLRQCKYDKKRVILKDLKHNDGNFTEKQKIELNKLLQIDYY
NLTKFYGTVKLDTMIFGVIEYCERGSLREVLNDTISYPDGTFMDWEFKISVLYDIAKGMSY
LHSSKTEVHGRLKSTNCVVDSRMVVKITDFGCNSILPPKKDLWTAPEHLRQANISQKGDV
YSYGIIAQEIILRKETFYTLSCRDRNEKIFRVENSNGMKPFRPDLFLETAEEKELEVYLLVKN
CWEEDPEKRPDFKKIETTLAKIFGLFHDQKNESYMDTLIRRLQLYSRNLEHLVEERTQLYK
AERDRADRLNFMLLPRLVVKSLKEKGFVEPELYEEVTIYFSDIVGFTTICKYSTPMEVVDM
LNDIYKSFDHIVDHHDVYKVETIGDAYMVASGLPKRNGNRHAIDIAKMALEILSFMGTFE
LEHLPGLPIWIRIGVHSGPCAAGVVGIKMPRYCLFGDTVNTASRMESTGLPLRIHVSGSTIA
ILKRTECQFLYEVRGETYLKGRGNETTYWLTGMKDQKFNLPTPPTVENQQRLQAEFSDMI
ANSLQKRQAAGIRSQKPRRVASYKKGTLEYLQLNTTDKESTYF

>P20020 multipass membrane protein
MGDMANNSVAYSGVKNSLKEANHDGDFGITLAELRALMELRSTDALRKIQESYGDVYGI
CTKLKTSPNEGLSGNPADLERREAVFGKNFIPPKKPKTFLQLVWEALQDVTLIILEIAAIVSL
GLSFYQPPEGDNALCGEVSVGEEEGEGETGWIEGAAILLSVVCVVLVTAFNDWSKEKQFR
GLQSRIEQEQKFTVIRGGQVIQIPVADITVGDIAQVKYGDLLPADGILIQGNDLKIDESSLTG
ESDHVKKSLDKDPLLLSGTHVMEGSGRMVVTAVGVNSQTGIIFTLLGAGGEEEEKKDEK
KKEKKNKKQDGAIENRNKAKAQDGAAMEMQPLKSEEGGDGDEKDKKKANLPKKEKSV
LQGKLTKLAVQIGKAGLLMSAITVIILVLYFVIDTFWVQKRPWLAECTPIYIQYFVKFFIIGV
TVLVVAVPEGLPLAVTISLAYSVKKMMKDNNLVRHLDACETMGNATAICSDKTGTLTMNR
MTVVQAYINEKHYKKVPEPEAIPPNILSYLVTGISVNCAYTSKILPPEKEGGLPRHVGNKTE
CALLGLLLDLKRDYQDVRNEIPEEALYKVYTFNSVRKSMSTVLKNSDGSYRIFSKGASEII
LKKCFKILSANGEAKVFRPRDRDDIVKTVIEPMASEGLRTICLAFRDFPAGEPEPEWDNEN
DIVTGLTCIAVVGIEDPVRPEVPDAIKKCQRAGITVRMVTGDNINTARAIATKCGILHPGED
FLCLEGKDFNRRIRNEKGEIEQERIDKIWPKLRVLARSSPTDKHTLVKGIIDSTVSDQRQVV
AVTGDGTNDGPALKKADVGFAMGIAGTDVAKEASDIILTDDNFTSIVKAVMWGRNVYDSI
SKFLQFQLTVNVVAVIVAFTGACITQDSPLKAVQMLWVNLIMDTLASLALATEPPTESLLLR
KPYGRNKPLISRTMMKNILGHAFYQLVVVFTLLFAGEKFFDIDSGRNAPLHAPPSEHYTIV
FNTFVLMQLFNEINARKIHGERNVFEGIFNNAIFCTIVLGTFVVQIIIVQFGGKPFSCSELSIE
QWLWSIFLGMGTLLWGQLISTIPTSRLKFLKEAGHGTQKEEIPEEELAEDVEEIDHAERELR
RGQILWFRGLNRIQTQMDVVNAFQSGSSIQGALRRQPSIASQHHDVTNISTPTHIRVVNAF
RSSLYEGLEKPESRSSIHNFMTHPEFRIEDSEPHIPLIDDTDAEDDAPTKRNSSPPPSPNKNN
NAVDSGIHLTIEMNKSATSSSPGSPLHSLETSL

>Q9UKU6 single-pass type II membrane protein
MGEDDAALRAGSRGLSDPWADSVGVRPRTTERHIAVHKRLVLAFAVSLVALLAVTMLAVL
LSLRFDECGASATPGADGGPSGFPERGGNGSLPGSARRNHHAGGDSWQPEAGGVASPGTT

SAQPPSEEEREPWEPWTQLRLSGHLKPLHYNLMLTAFMENFTFSGEVNVEIACRNATRYV
VLHASRVAVEKVQLAEDRAFGAVPVAGFFLYPQTQVLVVVLNRTLDAQRNYNLKIIYNALI
ENELLGFFRSSYVLHGERRFLGVTQFSPTHARKAFPCFDEPIYKATFKISIKHQATYLSLSN
MPVETSVFEEDGWVTDHFSQTPLMSTYYLAWAICNFTYRETTTKSGVVVRLYARPDAIRR
GSGDYALHITKRLIEFYEDYFKVPYSLPKLDLLAVPKHPYAAMENWGLSIFVEQRILLDPS
VSSISYLLDVTMVIVHEICHQWFGDLVTPVWWEDVWLKEGFAHYFEFVGTDYLYPGWN
MEKQRFLTDVLHEVMLLDGLASSHPVSQEVLQATDIDRVFDWIAYKKGAALIRMLANFM
GHSVFQRGLQDYLTIHKYGNAARNDLWNTLSEALKRNGKYVNIQEVMDQWTLQMGYP
VITILGNTTAENRIIITQQHFIYDISAKTKALKLQNNSYLWQIPLTIVVGNRSHVSSEAIIWVS
NKSEHHRITYLDKGSWLLGNINQTGYFRVNYDLRNWRLLIDQLIRNHEVLSVSNRAGLID
DAFSLARAGYLPQNIPLEIIRYLSEEKDFLPWHAASRALYPLDKLLDRMENYNIFNEYILKQ
VATTYIKLGWPKNNFNGSLVQASYQHEELRREVIMLACSFGNKHCHQQASTLISDWISSN
RNRIPLNVRDIVYCTGVSLLDEDVWEFIWMKFHSTTAVSEKKILLEALTCSDDRNLLNRLL
NLSLNSEVVLDQDAIDVIIHVARNPHGRDLAWKFFRDKWKILNTRYGEALFMNSKLISGV
TEFLNTEGELKELKNFMKNYDGVAAASFSRAVETVEANVRWKMLYQDELFQWLGKALR
H

>O60669 multipass membrane protein
MPPMPSAPPVHPPPDGGWGWIVVGAAFISIGFSYAFPKAVTVFFKEIQQIFHTTYSEIAWISS
IMLAVMYAGGPVSSVLVNKYGSRPVVIAGGLLCCLGMVLASFSSSVVQLYLTMGFITGLG
LAFNLQPALTIIGKYFYRKRPMANGLAMAGSPVFLSSLAPFNQYLFNTFGWKGSFLILGSL
LLNACVAGSLMRPLGPNQTTSKSKNKTGKTEDDSSPKKIKTKKSTWEKVNKYLDFSLFKH
RGFLIYLSGNVIMFLGFFAPIIFLAPYAKDQGIDEYSAAFLLSVMAFVDMFARPSVGLIANS
KYIRPRIQYFFSFAIMFNGVCHLLCPLAQDYTSLVLYAVFFGLGFGSVSSVLFETLMDLVGA
PRFSSAVGLVTIVECGPVLLGPPLAGKLVDLTGEYKYMYMSCGAIVVAASVWLLIGNAINY
RLLAKERKEENARQKTRESEPLSKSKHSEDVNVKVSNAQSVTSERETNI

>Q9NPD5 multipass membrane protein
MDQHQHLNKTAESASSEKKKTRRCNGFKMFLAALSFSYIAKALGGIIMKISITQIERRFDIS
SSLAGLIDGSFEIGNLLVIVFVSYFGSKLHRPKLIGIGCLLMGTGSILTSLPHFFMGYYRYSK
ETHINPSENSTSSLSTCLINQTLSFNGTSPEIVEKDCVKESGSHMWIYVFMGNMLRGIGETP
IVPLGISYIDDFAKEGHSSLYLGSLNAIGMIGPVIGFALGSLFAKMYVDIGYVDLSTIRITPK
DSRWVGAWWLGFLVSGLFSIISSIPFFFLPKNPNKPQKERKISLSLHVLKTNDDRNQTANLT
NQGKNVTKNVTGFFQSLKSILTNPLYVIFLLLTLLQVSSFIGSFTYVFKYMEQQYGQSASH
ANFLLGIITIPTVATGMFLGGFIIKKFKLSLVGIAKFSFLTSMISFLFQLLYFPLICESKSVAGLT
LTYDGNNSVASHVDVPLSYCNSECNCDESQWEPVCGNNGITYLSPCLAGCKSSSGIKKHT
VFYNCSCVEVTGLQNRNYSAHLGECPRDNTCTRKFFIYVAIQVINSLFSATGGTTFILLTVK
IVQPELKALAMGFQSMVIRTLGGILAPIYFGALIDKTCMKWSTNSCGAQGACRIYNSVFFG
RVYLGLSIALRFPALVLYIVFIFAMKKKFQGKDTKASDNERKVMDEANLEFLNNGEHFVPS
AGTDSKTCNLDMQDNAAAN

>O14910 peripheral membrane protein
MLKPSVTSAPTADMATLTVVQPLTLDRDVARAIELLEKLQESGEVPVHKLQSLKKVLQSEF
CTAIREVYQYMHETITVNGCPEFRARATAKATVAAFAASEGHSHPRVVELPKTDEGLGFN

VMGGKEQNSPIYISRIIPGGVAERHGGLKRGDQLLSVNGVSVEGEHHEKAVELLKAAKDS
VKLVVRYTPKVLEEMEARFEKLRTARRRQQQLLIQQQQQQQQQTQQNHMS

>Q9UHP7 single-pass type II membrane protein
MHDSNNVEKDITPSELPANPGCLHSKEHSIKATLIWRLFFLIMFLTIIVCGMVAALSAIRANC
HQEPSVCLQAACPESWIGFQRKCFYFSDDTKNWTSSQRFCDSQDADLAQVESFQELNFLL
RYKGPSDHWIGLSREQGQPWKWINGTEWTRQFPILGAGECAYLNDKGASSARHYTERK
WICSKSDIHV

>P20700 lipid-anchor
MATATPVPPRMGSRAGGPTTPLSPTRLSRLQEKEELRELNDRLAVYIDKVRSLETENSALQ
LQVTEREEVRGRELTGLKALYETELADARRALDDTARERAKLQIELGKCKAEHDQLLLNY
AKKESDLNGAQIKLREYEAALNSKDAALATALGDKKSLEGDLEDLKDQIAQLEASLAAA
KKQLADETLLKVDLENRCQSLTEDLEFRKSMYEEEINETRRKHETRLVEVDSGRQIEYEY
KLAQALHEMREQHDAQVRLYKEELEQTYHAKLENARLSSEMNTSTVNSAREELMESRM
RIESLSSQLSNLQKESRACLERIQELEDLLAKEKDNSRRMLTDKEREMAEIRDQMQQQLN
DYEQLLDVKLALDMEISAYRKLLEGEEERLKLSPSPSSRVTVSRASSSRSVRTTRGKRKRV
DVEESEASSSVSISHSASATGNVCIEEIDVDGKFIRLKNTSEQDQPMGGWEMIRKIGDTSVS
YKYTSRYVLKAGQTVTIWAANAGVTASPPTDLIWKNQNSWGTGEDVKVILKNSQGEEVA
QRSTVFKTTIPEEEEEEEEAAGVVVEEELFHQQGTPRASNRSCAIM

>Q9Y343 peripheral membrane protein
MEVYIPSFRYEESDLERGYTVFKIEVLMNGRKHFVEKRYSEFHALHKKLKKCIKTPEIPSK
HVRNWVPKVLEQRRQGLETYLQAVILENEELPKLFLDFLNVRHLPSLPKAESCGSFDETES
EESSKLSHQPVLLFLRDPYVLPAASDFPNVVIEGVLHGIFYPHLQPR

>Q6P1A2 multipass membrane protein
MASSAEGDEGTVVALAGVLQSGFQELSLNKLATSLGASEQALRLIISIFLGYPFALFYRHYL
FYKETYLIHLFHTFTGLSIAYFNFGNQLYHSLLCIVLQFLILRLMGRTITAVLTTFCFQMAYL
LAGYYYTATGNYDIKWTMPHCVLTLKLIGLAVDYFDGGKDQNSLSSEQQKYAIRGVPSLL
EVAGFSYFYGAFLVGPQFSMNHYMKLVQGELIDIPGKIPNSIIPALKRLSLGLFYLVGYTLL
SPHITEDYLLTEDYDNHPFWFRCMYMLIWGKFVLYKYVTCWLVTEGVCILTGLGFNGFEE
KGKAKWDACANMKVWLFETNPRFTGTIASFNINTNAWVARYIFKRLKFLGNKELSQGLS
LLFLALWHGLHSGYLVCFQMEFLIVIVERQAARLIQESPTLSKLAAITVLQPFYYLVQQTIH
WLFMGYSMTAFCLFTWDKWLKVYKSIYFLGHIFFLSLLFILPYIHKAMVPRKEKLKKME

>P28845 single-pass type II membrane protein
MAFMKKYLLPILGLFMAYYYYSANEEFRPEMLQGKKVIVTGASKGIGREMAYHLAKMG
AHVVVTARSKETLQKVVSHCLELGAASAHYIAGTMEDMTFAEQFVAQAGKLMGGLDML
ILNHITNTSLNLFHDDIHHVRKSMEVNFLSYVVLTVAALPMLKQSNGSIVVVSSLAGKVAY
PMVAAYSASKFALDGFFSSIRKEYSVSRVNVSITLCVLGLIDTETAMKAVSGIVHMQAAPK
EECALEIIKGGALRQEEVYYDSSLWTTLLIRNPCRKILEFLYSTSYNMDRFINK

>P51790 multipass membrane protein

MESEQLFHRGYYRNSYNSITSASSDEELLDGAGVIMDFQTSEDDNLLDGDTAVGTHYTMT
NGGSINSSTHLLDLLDEPIPGVGTYDDFHTIDWVREKCKDRERHRRINSKKKESAWEMTK
SLYDAWSGWLVVTLTGLASGALAGLIDIAADWMTDLKEGICLSALWYNHEQCCWGSNET
TFEERDKCPQWKTWAELIIGQAEGPGSYIMNYIMYIFWALSFAFLAVSLVKVFAPYACGSGI
PEIKTILSGFIIRGYLGKWTLMIKTITLVLAVASGLSLGKEGPLVHVACCCGNIFSYLFPKYST
NEAKKREVLSAASAAGVSVAFGAPIGGVLFSLEEVSYYFPLKTLWRSFFAALVAAFVLRSI
NPFGNSRLVLFYVEYHTPWYLFELFPFILLGVFGGLWGAFFIRANIAWCRRRKSTKFGKYP
VLEVIIVAAITAVIAFPNPYTRLNTSELIKELFTDCGPLESSSLCDYRNDMNASKIVDDIPDRP
AGIGVYSAIWQLCLALIFKIIMTVFTFGIKVPSGLFIPSMAIGAIAGRIVGIAVEQLAYYHHD
WFIFKEWCEVGADCITPGLYAMVGAAACLGGVTRMTVSLVVIVFELTGGLEYIVPLMAAV
MTSKWVGDAFGREGIYEAHIRLNGYPFLDAKEEFTHTTLAADVMRPRRNDPPLAVLTQD
NMTVDDIENMINETSYNGFPVIMSKESQRLVGFALRRDLTIAIESARKKQEGIVGSSRVCFA
QHTPSLPAESPRPLKLRSILDMSPFTVTDHTPMEIVVDIFRKLGLRQCLVTHNGRLLGIITKK
DILRHMAQTANQDPASIMFN

>Q9UKY4 multipass membrane protein
MPPATGGGLAESELRPRRGRCGPQAARAAGRDVAAEAVARSPKRPAWGSRRFEAVGWWA
LLALVTLLSFATRFHRLDEPPHICWDETHFGKMGSYYINRTFFFDVHPPLGKMLIGLAGYL
SGYDGTFLFQKPGDKYEHHSYMGMRGFCAFLGSWLVPFAYLTVLDLSKSLSAALLTAALL
TFDTGCLTLSQYILLDPILMFFIMAAMLSMVKYNSCADRPFSAPWWFWLSLTGVSLAGAL
GVKFVGLFIILQVGLNTIADLWYLFGDLSLSLVTVGKHLTARVLCLIVLPLALYTATFAVHF
MVLSKSGPGDGFFSSAFQARLSGNNLHNASIPEHLAYGSVITVKNLRMAIGYLHSHRHLYP
EGIGARQQQVTTYLHKDYNNLWIIKKHNTNSDPLDPSFPVEFVRHGDIIRLEHKETSRNLH
SHYHEAPMTRKHYQVTGYGINGTGDSNDFWRIEVVNRKFGNRIKVLRSRIRFIHLVTGCV
LGSSGKVLPKWGWEQLEVTCTPYLKETLNSIWNVEDHINPKLPNISLDVLQPSFPEILLES
HMVMIRGNSGLKPKDNEFTSKPWHWPINYQGLRFSGVNDTDFRVYLLGNPVVWWLNLL
SIALYLLSGSIIAVAMQRGARLPAEVAGLSQVLLRGGGQVLLGWTLHYFPFFLMGRVLYFH
HYFPAMLFSSMLTGILWDTLLRLCAWGLASWPLARGIHVAGILSLLLGTAYSFYLFHPLAY
GMVGPLAQDPQSPMAGLRWLDSWDF

>Q9H0R1 peripheral membrane protein
MAQRAVWLISHEPGTPLCGTVRFSRRYPTVEKRARVFNGASYVPVPEDGPFLKALLFELR
LLDDDKDFVESRDSCSRINKTSIYGLLIGGEELWPVVAFLKNDMIYACVPLVEQTLSPRPPL
ISVSGVSQGFEFLFGIQDFLYSGQKNDSELNTKLSQLPDLLLQACPFGTLLDANLQNSLDN
TNFASVTQPQKQPAWKTGTYKGKPQVSISITEKVKSMQYDKQGIADTWQVVGTVTCKCD
LEGIMPNVTISLSLPTNGSPLQDILVHPCVTSLDSAILTSSSIDAMDDSAFSGPYKFPFTPPLE
SFNLCFYTSQVPVPPILGFYQMKEEEVQLRITINLKLHESVKNNFEFCEAHIPFYNRGPITHL
EYKTSFGQLEVFREKSLLIWIIGQKFPKSMEISLSGTVTFGAKSHEKQPFDPICTGETAYLKL
HFRILDYTLTGCYADQHSVQVFASGKPKISAHRKLISSDYYIWNSKAPAPVTYGSLLL

>Q14126 single-pass type I membrane protein
MARSPGRAYALLLLLICFNVGSGLHLQVLSTRNENKLLPKHPHLVRQKRAWITAPVALRE
GEDLSKKNPIAKIHSDLAEERGLKITYKYTGKGITEPPFGIFVFNKDTGELNVTSILDREETP
FFLLTGYALDARGNNVEKPLELRIKVLDINDNEPVFTQDVFVGSVEELSAAHTLVMKINAT

DADEPNTLNSKISYRIVSLEPAYPPVFYLNKDTGEIYTTSVTLDREEHSSYTLTVEARDGNG
EVTDKPVKQAQVQIRILDVNDNIPVVENKVLEGMVEENQVNVEVTRIKVFDADEIGSDN
WLANFTFASGNEGGYFHIETDAQTNEGIVTLIKEVDYEEMKNLDFSVIVANKAAFHKSIRS
KYKPTPIPIKVKVKNVKEGIHFKSSVISIYVSESMDRSSKGQIIGNFQAFDEDTGLPAHARY
VKLEDRDNWISVDSVTSEIKLAKLPDFESRYVQNGTYTVKIVAISEDYPRKTITGTVLINVE
DINDNCPTLIEPVQTICHDAEYVNVTAEDLDGHPNSGPFSFSVIDKPPGMAEKWKIARQES
TSVLLQQSEKKLGRSEIQFLISDNQGFSCPEKQVLTLTVCECLHGSGCREAQHDSYVGLGP
AAIALMILAFLLLLLVPLLLLMCHCGKGAKGFTPIPGTIEMLHPWNNEGAPPEDKVVPSFL
PVDQGGSLVGRNGVGGMAKEATMKGSSSASIVKGQHEMSEMDGRWEEHRSLLSGRATQ
FTGATGAIMTTETTKTARATGASRDMAGAQAAAVALNEEFLRNYFTDKAASYTEEDENHT
AKDCLLVYSQEETESLNASIGCCSFIEGELDDRFLDDLGLKFKTLAEVCLGQKIDINKEIEQ
RQKPATETSMNTASHSLCEQTMVNSENTYSSGSSFPVPKSLQEANAEKVTQEIVTERSVSS
RQAQKVATPLPDPMASRNVIATETSYVTGSTMPPTTVILGPSQPQSLIVTERVYAPASTLVD
QPYANEGTVVVTERVIQPHGGGSNPLEGTQHLQDVPYVMVRERESFLAPSSGVQPTLAMP
NIAVGQNVTVTERVLAPASTLQSSYQIPTENSMTARNTTVSGAGVPGPLPDFGLEESGHSN
STITTSSTRVTKHSTVQHSYS

>P54578 peripheral membrane protein
MPLYSVTVKWGKEKFEGVELNTDEPPMVFKAQLFALTGVQPARQKVMVKGGTLKDDD
WGNIKIKNGMTLLMMGSADALPEEPSAKTVFVEDMTEEQLASAMELPCGLTNLGNTCY
MNATVQCIRSVPELKDALKRYAGALRASGEMASAQYITAALRDLFDSMDKTSSSIPPIILLQ
FLHMAFPQFAEKGEQGQYLQQDANECWIQMMRVLQQKLEAIEDDSVKETDSSSASAATP
SKKKSLIDQFFGVEFETTMKCTESEEEEVTKGKENQLQLSCFINQEVKYLFTGLKLRLQEE
ITKQSPTLQRNALYIKSSKISRLPAYLTIQMVRFFYKEKESVNAKVLKDVKFPLMLDMYEL
CTPELQEKMVSFRSKFKDLEDKKVNQQPNTSDKKSSPQKEVKYEPFSFADDIGSNNCGYY
DLQAVLTHQGRSSSSGHYVSWVKRKQDEWIKFDDDKVSIVTPEDILRLSGGGDWHIAYVL
LYGPRRVEIMEEESEQ

>P16444 GPI-anchor, lipid-anchor
MWSGWWLWPLVAVCTADFFRDEAERIMRDSPVIDGHNDLPWQLLDMFNNRLQDERANL
TTLAGTHTNIPKLRAGFVGGQFWSVYTPCDTQNKDAVRRTLEQMDVVHRMCRMYPETFL
YVTSSAGIRQAFREGKVASLIGVEGGHSIDSSLGVLRALYQLGMRYLTLTHSCNTPWADN
WLVDTGDSEPQSQGLSPFGQRVVKELNRLGVLIDLAHVSVATMKATLQLSRAPVIFSHSSA
YSVCASRRNVPDDVLRLVKQTDSLVMVNFYNNYISCTNKANLSQVADHLDHIKEVAGAR
AVGFGGDFDGVPRVPEGLEDVSKYPDLIAELLRRNWTEAEVKGALADNLLRVFEAVEQAS
NLTQAPEEEPIPLDQLGGSCRTHYGYSSGASSLHRHWGLLLASLAPLVLCLSLL

>Q01650 multipass membrane protein
MAGAGPKRRALAAPAAEEKEEAREKMLAAKSADGSAPAGEGEGVTLQRNITLLNGVAII
VGTIIGSGIFVTPTGVLKEAGSPGLALVVWAACGVFSIVGALCYAELGTTISKSGGDYAYM
LEVYGSLPAFLKLWIELLIIRPSSQYIVALVFATYLLKPLFPTCPVPEEAAKLVACLCVLLLTA
VNCYSVKAATRVQDAFAAAKLLALALIILLGFVQIGKGDVSNLDPNFSFEGTKLDVGNIVL
ALYSGLFAYGGWNYLNFVTEEMINPYRNLPLAIIISLPIVTLVYVLTNLAYFTTLSTEQMLSS
EAVAVDFGNYHLGVMSWIIPVFVGLSCFGSVNGSLFTSSRLFFVGSREGHLPSILSMIHPQL

LTPVPSLVFTCVMTLLYAFSKDIFSVINFFSFFNWLCVALAIIGMIWLRHRKPELERPIKVNL
ALPVFFILACLFLIAVSFWKTPVECGIGFTIILSGLPVYFFGVWWKNKPKWLLQGIFSTTVL
CQKLMQVVPQET

>P40616 peripheral membrane protein
MGGFFSSIFSSLFGTREMRILILGLDGAGKTTILYRLQVGEVVTTIPTIGFNVETVTYKNLKF
QVWDLGGQTSIRPYWRCYYSNTDAVIYVVDSCDRDRIGISKSELVAMLEEEELRKAILVVF
ANKQDMEQAMTSSEMANSLGLPALKDRKWQIFKTSATKGTGLDEAMEWLVETLKSRQ

>Q12887 multipass membrane protein
MAASPHTLSSRLLTGCVGGSVWYLERRTIQDSPHKFLHLLRNVNKQWITFQHFSFLKRMY
VTQLNRSHNQQVRPKPEPVASPFLEKTSSGQAKAEIYEMRPLSPPSLSLSRKPNEKELIELE
PDSVIEDSIDVGKETKEEKRWKEMKLQVYDLPGILARLSKIKLTALVVSTTAAGFALAPGP
FDWPCFLLTSVGTGLASCAANSINQFFEVPFDSNMNRTKNRPLVRGQISPLLAVSFATCCAV
PGVAILTLGVNPLTGALGLFNIFLYTCCYTPLKRISIANTWVGAVVGAIPPVMGWTAATGSL
DAGAFLLGGILYSWQFPHFNALSWGLREDYSRGGYCMMSVTHPGLCRRVALRHCLALLV
LSAAAPVLDITTWTFPIMALPINAYISYLGFRFYVDADRRSSRRLFFCSLWHLPLLLLLMLT
CKRPSGGGDAGPPPS

>Q6QNK2 multipass membrane protein
MEKLLRLCCWYSWLLLFYYNFQVRGVYSRSQDHPGFQVLASASHYWPLENVDGIHELQ
DTTGDIVEGKVNKGIYLKEEKGVTLLYYGRYNSSCISKPEQCGPEGVTFSFFWKTQGEQSR
PIPSAYGGQVISNGFKVCSSGGRGSVELYTRDNSMTWEASFSPPGPYWTHVLFTWKSKEG
LKVYVNGTLSTSDPSGKVSRDYGESNVNLVIGSEQDQAKCYENGAFDEFIIWERALTPDEI
AMYFTAAIGKHALLSSTLPSLFMTSTASPVMPTDAYHPIITNLTEERKTFQSPGVILSYLQN
VSLSLPSKSLSEQTALNLTKTFLKAVGEILLLPGWIALSEDSAVVLSLIDTIDTVMGHVSSNL
HGSTPQVTVEGSSAMAEFSVAKILPKTVNSSHYRFPAHGQSFIQIPHEAFHRHAWSTVVGL
LYHSMHYYLNNIWPAHTKIAEAMHHQDCLLFATSHLISLEVSPPPTLSQNLSGSPLITVHLK
HRLTRKQHSEATNSSNRVFVYCAFLDFSSGEGVWSNHGCALTRGNLTYSVCRCTHLTNFAI
LMQVVPLELARGHQVALSSISYVGCSLSVLCLVATLVTFAVLSSVSTIRNQRYHIHANLSFA
VLVAQVLLLISFRLEPGTTPCQVMAVLLHYFFLSAFAWMLVEGLHLYSMVIKVFGSEDSKH
RYYYGMGWGFPLLICIISLSFAMDSYGTSNNCWLSLASGAIWAFVAPALFVIVVNIGILIAV
TRVISQISADNYKIHGDPSAFKLTAKAVAVLLPILGTSWVFGVLAVNGCAVVFQYMFATLNS
LQGLFIFLFHCLLNSEVRAAFKHKTKVWSLTSSSARTSNAKPFHSDLMNGTRPGMASTKL
SPWDKSSHSAHRVDLSAV

>Q9NZS9 multipass membrane protein
MEEPQKSYVNTMDLERDEPLKSTGPQISVSEFSCHCCYDILVNPTTLNCGHSFCRHCLAL
WWASSKKTECPECREKWEGFPKVSILLRDAIEKLFPDAIRLRFEDIQQNNDIVQSLAAFQK
YGNDQIPLAPNTGRANQQMGGGFFSGVLTALTGVAVVLLVYHWSSRESEHDLLVHKAVA
KWTAEEVVLWLEQLGPWASLYRERFLSERVNGRLLLTLTEEEFSKTPYTIENSSHRRAILM
ELERVKALGVKPPQNLWEYKAVNPGRSLFLLYALKSSPRLSLLYLYLFDYTDTFLPFIHTIC
PLQEDSSGEDIVTKLLDLKEPTWKQWREFLVKYSFLPYQLIAEFAWDWLEVHYWTSRFLII
NAMLLSVLELFSFWRIWSRSELKTVPQRMWSHFWKVSTQGLFVAMFWPLIPQFVCNCLF

YWALYFNPIINIDLVVKELRRLETQVL

>Q8N3R9 peripheral membrane protein
MTTSHMNGHVTEESDSEVKNVDLASPEEHQKHREMAVDCPGDLGTRMMPIRRSAQLERI
RQQQEDMRRRREEEGKKQELDLNSSMRLKKLAQIPPKTGIDNPMFDTEEGIVLESPHYAV
KILEIEDLFSSLKHIQHTLVDSQSQEDISLLLQLVQNKDFQNAFKIHNAITVHMNKASPPFPL
ISNAQDLAQEVQTVLKPVHHKEGQELTALLNTPHIQALLLAHDKVAEQEMQLEPITDERV
YESIGQYGGETVKIVRIEKARDIPLGATVRNEMDSVIISRIVKGGAAEKSGLLHEGDEVLEI
NGIEIRGKDVNEVFDLLSDMHGTLTFVLIPSQQIKPPPAKETVIHVKAHFDYDPSDDPYVPC
RELGLSFQKGDILHVISQEDPNWWQAYREGDEDNQPLAGLVPGKSFQQQREAMKQTIEE
DKEPEKSGKLWCAKKNKKKRKKVLYNANKNDDYDNEEILTYEEMSLYHQPANRKRPIILI
GPQNCGQNELRQRLMNKEKDRFASAVPHTTRSRRDQEVAGRDYHFVSRQAFEADIAAGK
FIEHGEFEKNLYGTSIDSVRQVINSGKICLLSLRTQSLKTLRNSDLKPYIIFIAPPSQERLRAL
LAKEGKNPKPEELREIIEKTREMEQNNGHYFDTAIVNSDLDKAYQELLRLINKLDTEPQW
VPSTWLR

>Q8WTV0 multipass membrane protein
MGCSAKARWAAGALGVAGLLCAVLGAVMIVMVPSLIKQQVLKNVRIDPSSLSFNMWKEI
PIPFYLSVYFFDVMNPSEILKGEKPQVRERGPYVYREFRHKSNITFNNNDTVSFLEYRTFQF
QPSKSHGSESDYIVMPNILVLGAAVMMENKPMTLKLIMTLAFTTLGERAFMNRTVGEIM
WGYKDPLVNLINKYFPGMFPFKDKFGLFAELNNSDSGLFTVFTGVQNISRIHLVDKWNGL
SKVDFWHSDQCNMINGTSGQMWPPFMTPESSLEFYSPEACRSMKLMYKESGVFEGIPTY
RFVAPKTLFANGSIYPPNEGFCPCLESGIQNVSTCRFSAPLFLSHPHFLNADPVLAEAVTGL
HPNQEAHSLFLDIHPVTGIPMNCSVKLQLSLYMKSVAGIGQTGKIEPVVLPLLWFAESGAM
EGETLHTFYTQLVLMPKVMHYAQYVLLALGCVLLLVPVICQIRSQVGAGQRAARADSHS
LACWGKGASDRTLWPTAAWSPPPAAVLRLCRSGSGHCWGLRSTLASFACRVATTLPVLEG
LGPSLGGGTGS

>P31645 multipass membrane protein
METTPLNSQKQLSACEDGEDCQENGVLQKVVPTPGDKVESGQISNGYSAVPSPGAGDDT
RHSIPATTTTLVAELHQGERETWGKKVDFLLSVIGYAVDLGNVWRFPYICYQNGGGAFLLP
YTIMAIFGGIPLFYMELALGQYHRNGCISIWRKICPIFKGIGYAICIIAFYIASYYNTIMAWAL
YYLISSFTDQLPWTSCKNSWNTGNCTNYFSEDNITWTLHSTSPAEEFYTRHVLQIHRSKGL
QDLGGISWQLALCIMLIFTVIYFSIWKGVKTSGKVVWVTATFPYIILSVLLVRGATLPGAWR
GVLFYLKPNWQKLLETGVWIDAAAQIFFSLGPGFGVLLAFASYNKFNNNCYQDALVTSV
VNCMTSFVSGFVIFTVLGYMAEMRNEDVSEVAKDAGPSLLFITYAEAIANMPASTFFAIIFF
LMLITLGLDSTFAGLEGVITAVLDEFPHVWAKRRERFVLAVVITCFFGSLVTLTFGGAYVVK
LLEEYATGPAVLTVALIEAVAVSWFYGITQFCRDVKEMLGFSPGWFWRICWVAISPLFLLFII
CSFLMSPPQLRLFQYNYPYWSIILGYCIGTSSFICIPTYIAYRLIITPGTFKERIIKSITPETPTEI
PCGDIRLNAV

>Q13367 peripheral membrane protein
MSAAPAYSEDKGGSAGPGEPEYGHDPASGGIFSSDYKRHDDLKEMLDTNKDSLKLEAMK
RIVAMIARGKNASDLFPAVVKNVACKNIEVKKLVYVYLVRYAEEQQDLALLSISTFQRGLK

DPNQLIRASALRVLSSIRVPIIVPIMMLAIKEAASDMSPYVRKTAAHAIPKLYSLDSDQKDQ
LIEVIEKLLADKTTLVAGSVVMAFEEVCPERIDLIHKNYRKLCNLLIDVEEWGQVVIISMLT
RYARTQFLSPTQNESLLEENAEKAFYGSEEDEAKGAGSEETAAAAAPSRKPYVMDPDHRL
LLRNTKPLLQSRSAAVVMAVAQLYFHLAPKAEVGVIAKALVRLLRSHSEVQYVVLQNVAT
MSIKRRGMFEPYLKSFYIRSTDPTQIKILKLEVLTNLANETNIPTVLREFQTYIRSMDKDFV
AATIQAIGRCATNIGRVRDTCLNGLVQLLSNRDELVVAESVVVIKKLLQMQPAQHGEIIKHL
AKLTDNIQVPMARASILWLIGEYCEHVPRIAPDVLRKMAKSFTAEEDIVKLQVINLAAKLY
LTNSKQTKLLTQYVLSLAKYDQNYDIRDRARFTRQLIVPSEQGGALSRHAKKLFLAPKPAP
VLESSFKDRDHFQLGSLSHLLNAKATGYQELPDWPEEAPDPSVRNVEVPEWTKCSNREK
RKEKEKPFYSDSEGESGPTESADSDPESESESDSKSSSESGSGESSSESDNEDQDEDEEKGR
GSESEQSEEDGKRKTKKKVPERKGEASSSDEGSDSSSSSSESEMTSESEEEQLEPASWSRK
TPPSSKSAPATKEISLLDLEDFTPPSVQPVSPPAIVSTSLAADLEGLTLTDSTLVPSLLSPVSGV
GRQELLHRVAGEGLAVDYTFSRQPFSGDPHMVSVHIHFSNSSDTPIKGLHVGTPKLPAGISI
QEFPEIESLAPGESATAVMGINFCDSTQAANFQLCTQTRQFYVSIQPPVGELMAPVFMSEN
EFKKEQGKLMGMNEITEKLMLPDTCRSDHIVVQKVTATANLGRVPCGTSDEYRFAGRTLT
GGSLVLLTLDARPAGAAQLTVNSEKMVIGTMLVKDVIQALTQ

>P30926 multipass membrane protein
MRRAPSLVLFFLVALCGRGNCRVANAEEKLMDDLLNKTRYNNLIRPATSSSQLISIKLQLSL
AQLISVNEREQIMTTNVWLKQEWTDYRLTWNSSRYEGVNILRIPAKRIWLPDIVLYNNAD
GTYEVSVYTNLIVRSNGSVLWLPPAIYKSACKIEVKYFPFDQQNCTLKFRSWTYDHTEID
MVLMTPTASMDDFTPSGEWDIVALPGRRTVNPQDPSYVDVTYDFIIKRKPLFYTINLIIPCV
LTTLLAILVFYLPSDCGEKMTLCISVLLALTFFLLLISKIVPPTSLDVPLIGKYLMFTMVLVTF
SIVTSVCVLNVHHRSPSTHTMAPWVKRCFLHKLPTFLFMKRPGPDSSPARAFPPSKSCVTK
PEATATSTSPSNFYGNSMYFVNPASAASKSPAGSTPVAIPRDFWLRSSGRFRQDVQEALEG
VSFIAQHMKNDDEDQSVVEDWKYVAMVVDRLFLWVFMFVCVLGTVGLFLPPLFQTHAA
SEGPYAAQRD

>P12830 single-pass type I membrane protein
MGPWSRSLSALLLLLQVSSWLCQEPEPCHPGFDAESYTFTVPRRHLERGRVLGRVNFEDC
TGRQRTAYFSLDTRFKVGTDGVITVKRPLRFHNPQIHFLVYAWDSTYRKFSTKVTLNTVGH
HHRPPPHQASVSGIQAELLTFPNSSPGLRRQKRDWVIPPISCPENEKGPFPKNLVQIKSNKD
KEGKVFYSITGQGADTPPVGVFIIERETGWLKVTEPLDRERIATYTLFSHAVSSNGNAVEDP
MEILITVTDQNDNKPEFTQEVFKGSVMEGALPGTSVMEVTATDADDDVNTYNAAIAYTIL
SQDPELPDKNMFTINRNTGVISVVTTGLDRESFPTYTLVVQAADLQGEGLSTTATAVITVT
DTNDNPPIFNPTTYKGQVPENEANVVITTLKVTDADAPNTPAWEAVYTILNDDGGQFVVT
TNPVNNDGILKTAKGLDFEAKQQYILHVAVTNVVPFEVSLTTSTATVTVDVLDVNEAPIFV
PPEKRVEVSEDFGVGQEITSYTAQEPDTFMEQKITYRIWRDTANWLEINPDTGAISTRAEL
DREDFEHVKNSTYTALIIATDNGSPVATGTGTLLLILSDVNDNAPIPEPRTIFFCERNPKPQVI
NIIDADLPPNTSPFTAELTHGASANWTIQYNDPTQESIILKPKMALEVGDYKINLKLMDNQ
NKDQVTTLEVSVCDCEGAAGVCRKAQPVEAGLQIPAILGILGGILALLILILLLLLFLRRRA
VVKEPLLPPEDDTRDNVYYYDEEGGGEEDQDFDLSQLHRGLDARPEVTRNDVAPTLMSV
PRYLPRPANPDEIGNFIDENLKAADTDPTAPPYDSLLVFDYEGSGSEAASLSSLNSSESDKD
QDYDYLNEWGNRFKKLADMYGGGEDD

>O15321 multipass membrane protein
MTVVGNPRSWSCQWLPILILLLGTGHGPGVEGVTHYKAGDPVILYVNKVGPYHNPQETY
HYYQLPVCCPEKIRHKSLSLGEVLDGDRMAESLYEIRFRENVEKRILCHMQLSSAQVEQL
RQAIEELYYFEFVVDDLPIRGFVGYMEESGFLPHSHKIGLWTHLDFHLEFHGDRIIFANVSV
RDVKPHSLDGLRPDEFLGLTHTYSVRWSETSVERRSDRRRGDDGGFFPRTLEIHWLSIINS
MVLVFLLVGFVAVILMRVLRNDLARYNLDEETTSAGSGDDFDQGDNGWKIIHTDVFRFPP
YRGLLCAVLGVGAQFLALGTGIIVMALLGMFNVHRHGAINSAAILLYALTCCISGYVSSHF
YRQIGGERWVWNIILTTSLFSVPFFLTWSVVNSVHWANGSTQALPATTILLLLTVWLLVGFP
LTVIGGIFGKNNASPFDAPCRTKNIAREIPPQPWYKSTVIHMTVGGFLPFSAISVELYYIFAT
VWGREQYTLYGILFFVFAILLSVGACISIALTYFQLSGEDYRWWWRSVLSVGSTGLFIFLYS
VFYYARRSNMSGAVQTVEFFGYSLLTGYVFFLMLGTISFFSSLKFIRYIYVNLKMD

>P52848 single-pass type II membrane protein
MPALACLRRLCRHVSPQAVLFLLFIFCLFSVFISAYYLYGWKRGLEPSADAPEPDCGDPPPV
APSRLLPLKPVQAATPSRTDPLVLVFVESLYSQLGQEVVAILESSRFKYRTEIAPGKGDMPTL
TDKGRGRFALIIYENILKYVNLDAWNRELLDKYCVAYGVGIIGFFKANENSLLSAQLKGFP
LFLHSNLGLKDCSINPKSPLLYVTRPSEVEKGVLPGEDWTVFQSNHSTYEPVLLAKTRSSE
SIPHLGADAGLHAALHATVVQDLGLHDGIQRVLFGNNLNFWLHKLVFVDAVAFLTGKRLS
LPLDRYILVDIDDIFVGKEGTRMKVEDVKALFDTQNELRAHIPNFTFNLGYSGKFFHTGTN
AEDAGDDLLLSYVKEFWWFPHMWSHMQPHLFHNQSVLAEQMALNKKFAVEHGIPTDM
GYAVAPHHSGVYPVHVQLYEAWKQVWSIRVTSTEEYPHLKPARYRRGFIHNGIMVLPRQT
CGLFTHTIFYNEYPGGSSELDKIINGGELFLTVLLNPISIFMTHLSNYGNDRLGLYTFKHLVR
FLHSWTNLRLQTLPPVQLAQKYFQIFSEEKDPLWQDPCEDKRHKDIWSKEKTCDRFPKLLI
IGPQKTGTTALYLFLGMHPDLSSNYPSSETFEEIQFFNGHNYHKGIDWYMEFFPIPSNTTSD
FYFEKSANYFDSEVAPRRAAALLPKAKVLTILINPADRAYSWYQHQRAHDDPVALKYTFH
EVITAGSDASSKLRALQNRCLVPGWYATHIERWLSAYHANQILVLDGKLLRTEPAKVMDM
VQKFLGVTNTIDYHKTLAFDPKKGFWCQLLEGGKTKCLGKSKGRKYPEMDLDSRAFLK
DYYRDHNIELSKLLYKMGQTLPTWLREDLQNTR

>P09619 single-pass type I membrane protein
MRLPGAMPALALKGELLLLSLLLLLEPQISQGLVVTPPGPELVLNVSSTFVLTCSGSAPVV
WERMSQEPPQEMAKAQDGTFSSVLTLTNLTGLDTGEYFCTHNDSRGLETDERKRLYIFVP
DPTVGFLPNDAEELFIFLTEITEITIPCRVTDPQLVVTLHEKKGDVALPVPYDHQRGFSGIFE
DRSYICKTTIGDREVDSDAYYVYRLQVSSINVSVNAVQTVVRQGENITLMCIVIGNEVVNF
EWTYPRKESGRLVEPVTDFLLDMPYHIRSILHIPSAELEDSGTYTCNVTESVNDHQDEKAI
NITVVESGYVRLLGEVGTLQFAELHRSRTLQVVFEAYPPPTVLWFKDNRTLGDSSAGEIAL
STRNVSETRYVSELTLVRVKVAEAGHYTMRAFHEDAEVQLSFQLQINVPVRVLELSESHPD
SGEQTVRCRGRGMPQPNIIWSACRDLKRCPRELPPTLLGNSSEEESQLETNVTYWEEEQEF
EVVSTLRLQHVDRPLSVRCTLRNAVGQDTQEVIVVPHSLPFKVVVISAILALVVLTIISLIILI
MLWQKKPRYEIRWKVIESVSSDGHEYIYVDPMQLPYDSTWELPRDQLVLGRTLGSGAFG
QVVEATAHGLSHSQATMKVAVKMLKSTARSSEKQALMSELKIMSHLGPHLNVVNLLGAC
TKGGPIYIITEYCRYGDLVDYLHRNKHTFLQHHSDKRRPPSAELYSNALPVGLPLPSHVSLT
GESDGGYMDMSKDESVDYVPMLDMKGDVKYADIESSNYMAPYDNYVPSAPERTCRATL

INESPVLSYMDLVGFSYQVANGMEFLASKNCVHRDLAARNVLICEGKLVKICDFGLARDI
MRDSNYISKGSTFLPLKWMAPESIFNSLYTTLSDVWSFGILLWEIFTLGGTPYPELPMNEQF
YNAIKRGYRMAQPAHASDEIYEIMQKCWEEKFEIRPPFSQLVLLLERLLGEGYKKKYQQV
DEEFLRSDHPAILRSQARLPGFHGLRSPLDTSSVLYTAVQPNEGDNDYIIPLPDPKPEVADEG
PLEGSPSLASSTLNEVNTSSTISCDSPLEPQDEPEPEPQLELQVEPEPELEQLPDSGCPAPRAE
AEDSFL

>Q99572 multipass membrane protein
MPACCSCSDVFQYETNKVTRIQSMNYGTIKWFFHVIIFSYVCFALVSDKLYQRKEPVISSV
HTKVKGIAEVKEEIVENGVKKLVHSVFDTADYTFPLQGNSFFVMTNFLKTEGQEQRLCPE
YPTRRTLCSSDRGCKKGWMDPQSKGIQTGRCVVYEGNQKTCEVSAWCPIEAVEEAPRPAL
LNSAENFTVLIKNNIDFPGHNYTTRNILPGLNITCTFHKTQNPQCPIFRLGDIFRETGDNFSD
VAIQGGIMGIEIYWDCNLDRWFHHCRPKYSFRRLDDKTTNVSLYPGYNFRYAKYYKENN
VEKRTLIKVFGIRFDILVFGTGGKFDIIQLVVYIGSTLSYFGLAAVFIDFLIDTYSSNCCRSHI
YPWCKCCQPCVVNEYYYRKKCESIVEPKPTLKYVSFVDESHIRMVNQQLLGRSLQDVKG
QEVPRPAMDFTDLSRLPLALHDTPPIPGQPEEIQLLRKEATPRSRDSPVWCQCGSCLPSQLP
ESHRCLEELCCRKKPGACITTSELFRKLVLSRHVLQFLLLYQEPLLALDVDSTNSRLRHCA
YRCYATWRFGSQDMADFAILPSCCRWRIRKEFPKSEGQYSGFKSPY

>Q9P035 multipass membrane protein
MENQVLTPHVYWAQRHRELYLRVELSDVQNPAISITENVLHFKAQGHGAKGDNVYEFHL
EFLDLVKPEPVYKLTQRQVNITVQKKVSQWWERLTKQEKRPLFLAPDFDRWLDESDAEM
ELRAKEEERLNKLRLESEGSPETLTNLRKGYLFMYNLVQFLGFSWIFVNLTVRFCILGKESF
YDTFHTVADMMYFCQMLAVVETINAAIGVTTSPVLPSLIQLLGRNFILFIIFGTMEEMQNK
AVVFFVFYLWSAIEIFRYSFYMLTCIDMDWKVLTWLRYTLWIPLYPLGCLAEAVSVIQSIPIF
NETGRFSFTLPYPVKIKVRFSFFLQIYLIMIFLGLYINFRHLYKQRRRRYGQKKKKIH

>O60721 multipass membrane protein
MGKLIRMGPQERWLLRTKRLHWSRLLFLLGMLIIGSTYQHLRRPRGLSSLWAAVSSHQPIK
LASRDLSSEEMMMMSSSPSKPSSEMGGKMLVPQASVGSDEATLSMTVENIPSMPKRTAK
MIPTTTKNNYSPTAAGTERRKEDTPTSSRTLTYYTSTSSRQIVKKYTPTPRGEMKSYSPTQV
REKVKYTPSPRGRRVGTYVPSTFMTMETSHAITPRTTVKDSDITATYKILETNSLKRIMEET
TPTTLKGMFDSTPTFLTHEVEANVLTSPRSVMEKNNLFPPRRVESNSSAHPWGLVGKSNPK
TPQGTVLLHTPATSEGQVTISTMTGSSPAETKAFTAAWSLRNPSPRTSVSAIKTAPAIVWRL
AKKPSTAPSTSTTPTVRAKLTMQVHHCVVVKPTPAMLTTPSPSLTTALLPEELSPSPSVLPPS
LPDLHPKGEYPPDLFSVEERRQGWVVLHVFGMMYVFVALAIVCDEYFVPALGVITDKLQI
SEDVAGATFMAAGGSAPELFTSLIGVFISHSNVGIGTIVGSAVFNILFVIGTCSLFSREILNLT
WWPLFRDVSFYILDLIMLILFFLDSLIAWWESLLLLLAYAFYVFTMKWNKHIEVWVKEQL
SRRPVAKVMALEDLSKPGDGAIAVDELQDNKKLKLPSLLTRGSSSTSLHNSTIRSTIYQLM
LHSLDPLREVRLAKEKEEESLNQGARAQPQAKAESKPEEEEPAKLPAVTVTPAPVPDIKGD
QKENPGGQEDVAEAESTGEMPGEEGETAGEGETEEKSGGETQPEGEGETETQGKGEECED
ENEAEGKGDNEGEDEGEIHAEDGEMKGNEGETESQELSAENHGEAKNDEKGVEDGGGS
DGGDSEEEEEEEEEQEEEEEEEEQEEEEEEEEEEEEKGNEEPLSLDWPETRQKQAIYLFLLP
IVFPLWLTVPDVRRQESRKFFVFTFLGSIMWIAMFSYLMVWWAHQVGETIGISEEIMGLTIL

AAGTSIPDLITSVIVARKGLGDMAVSSSVGSNIFDITVGLPVPWLLFSLINGLQPVPVSSNGL
FCAIVLLFLMLLFVISSIASCKWRMNKILGFTMFLLYFVFLIISVMLEDRIISCPVSV

>Q92859 single-pass type I membrane protein
MAAERGARRLLSTPSFWLYCLLLLGRRAPGAAAARSGSAPQSPGASIRTFTPFYFLVEPVD
TLSVRGSSVILNCSAYSEPSPKIEWKKDGTFLNLVSDDRRQLLPDGSLFISNVVHSKHNKPD
EGYYQCVATVESLGTIISRTAKLIVAGLPRFTSQPEPSSVYAGNNAILNCEVNADLVPFVRW
EQNRQPLLLDDRVIKLPSGMLVISNATEGDGGLYRCVVESGGPPKYSDEVELKVLPDPEVI
SDLVFLKQPSPLVRVIGQDVVLPCVASGLPTPTIKWMKNEEALDTESSERLVLLAGGSLEIS
DVTEDDAGTYFCIADNGNETIEAQAELTVQAQPEFLKQPTNIYAHESMDIVFECEVTGKPT
PTVKWVKNGDMVIPSDYFKIVKEHNLQVLGLVKSDEGFYQCIAENDVGNAQAGAQLIILE
HAPATTGPLPSAPRDVVASLVSTRFIKLTWRTPASDPHGDNLTYSVFYTKEGIARERVENTS
HPGEMQVTIQNLMPATVYIFRVMAQNKHGSGESSAPLRVETQPEVQLPGPAPNLRAYAAS
PTSITVTWETPVSGNGEIQNYKLYYMEKGTDKEQDVDVSSHSYTINGLKKYTEYSFRVVA
YNKHGPGVSTPDVAVRTLSDVPSAAPQNLSLEVRNSKSIMIHWQPPAPATQNGQITGYKIR
YRKASRKSDVTETLVSGTQLSQLIEGLDRGTEYNFRVAALTINGTGPATDWLSAETFESDL
DETRVPEVPSSLHVRPLVTSIVVSWTPPENQNIVVRGYAIGYGIGSPHAQTIKVDYKQRYYT
IENLDPSSHYVITLKAFNNVGEGIPLYESAVTRPHTDTSEVDLFVINAPYTPVPDPTPMMPP
VGVQASILSHDTIRITWADNSLPKHQKITDSRYYTVRWKTNIPANTKYKNANATTLSYLVT
GLKPNTLYEFSVMVTKGRRSSTWSMTAHGTTFELVPTSPPKDVTVVSKEGKPKTIIVNWQ
PPSEANGKITGYIIYYSTDVNAEIHDWVIEPVVGNRLTHQIQELTLDTPYYFKIQARNSKGM
GPMSEAVQFRTPKADSSDKMPNDQASGSGGKGSRLPDLGSDYKPPMSGSNSPHGSPTSPL
DSNMLLVIIVSVGVITIVVVVIIAVFCTRRTTSHQKKKRAACKSVNGSHKYKGNSKDVKPP
DLWIHHERLELKPIDKSPDPNPIMTDTPIPRNSQDITPVDNSMDSNIHQRRNSYRGHESEDS
MSTLAGRRGMRPKMMMPFDSQPPQPVISAHPIHSLDNPHHHFHSSSLASPARSHLYHPGSP
WPIGTSMSLSDRANSTESVRNTPSTDTMPASSSQTCCTDHQDPEGATSSSYLASSQEEDSG
QSLPTAHVRPSHPLKSFAVPAIPPPGPPTYDPALPSTPLLSQQALNHHIHSVKTASIGTLGRSR
PPMPVVVPSAPEVQETTRMLEDSESSYEPDELTKEMAHLEGLMKDLNAITTA

>Q9Y3Q4 multipass membrane protein
MDKLPPSMRKRLYSLPQQVGAKAWIMDEEEDAEEEGAGGRQDPSRRSIRLRPLPSPSPSA
AAGGTESRSSALGAADSEGPARGAGKSSTNGDCRRFRGSLASLGSRGGGSGGTGSGSSHG
HLHDSAEERRLIAEGDASPGEDRTPPGLAAEPERPGASAQPAASPPPPQQPPQPASASCEQP
SVDTAIKVEGGAAAGDQILPEAEVRLGQAGFMQRQFGAMLQPGVNKFSLRMFGSQKAVE
REQERVKSAGFWIIHPYSDFRFYWDLTMLLLMVGNLIIIPVGITFFKDENTTPWIVFNVVSD
TFFLIDLVLNFRTGIVVEDNTEIILDPQRIKMKYLKSWFMVDFISSIPVDYIFLIVETRIDSEV
YKTARALRIVRFTKILSLLRLLRLSRLIRYIHQWEEIFHMTYDLASAVVRIVNLIGMMLLLC
HWDGCLQFLVPMLQDFPDDCWVSINNMVNNSWGKQYSYALFKAMSHMLCIGYGRQAP
VGMSDVWLTMLSMIVGATCYAMFIGHATALIQSLDSSRRQYQEKYKQVEQYMSFHKLPP
DTRQRIHDYYEHRYQGKMFDEESILGELSEPLREEIINFNCRKLVASMPLFANADPNFVTS
MLTKLRFEVFQPGDYIIREGTIGKKMYFIQHGVVSVLTKGNKETKLADGSYFGEICLLTRG
RRTASVRADTYCRLYSLSVDNFNEVLEEYPMMRRAFETVALDRLDRIGKKNSILLHKVQH
DLNSGVFNYQENEIIQQIVQHDREMAHCAHRVQAAASATPTPTPVIWTPLIQAPLQAAAAT
TSVAIALTHHPRLPAAIFRPPPGSGLGNLGAGQTPRHLKRLQSLIPSALGSASPASSPSQVDT

PSSSSFHIQQLAGFSAPAGLSPLLPSSSSSPPPGACGSPSAPTPSAGVAATTIAGFGHFHKALG
GSLSSSDSPLLTPLQPGARSPQAAQPSPAPPGARGGLGLPEHFLPPPPSSRSPSSSPGQLGQP
PGELSLGLATGPLSTPETPPRQPEPPSLVAGASGGASPVGFTPRGGLSPPGHSPGPPRTFPSA
PPRASGSHGSLLLPPASSPPPPQVPQRRGTPPLTPGRLTQDLKLISASQPALPQDGAQTLRRA
SPHSSGESMAAFPLFPRAGGGSGGSGSSGGLGPPGRPYGAIPGQHVTLPRKTSSGSLPPPLS
LFGARATSSGGPPLTAGPQREPGARPEPVRSKLPSNL

>O75326 GPI-anchor, lipid-anchor
MTPPPPGRAAPSAPRARVPGPPARLGLPLRLRLLLLLWAAAASAQGHLRSGPRIFAVWKG
HVGQDRVDFGQTEPHTVLFHEPGSSSVWVGGRGKVYLFDFPEGKNASVRTVNIGSTKGS
CLDKRDCENYITLLERRSEGLLACGTNARHPSCWNLVNGTVVPLGEMRGYAPFSPDENSL
VLFEGDEVYSTIRKQEYNGKIPRFRRIRGESELYTSDTVMQNPQFIKATIVHQDQAYDDKIY
YFFREDNPDKNPEAPLNVSRVAQLCRGDQGGESSLSVSKWNTFLKAMLVCSDAATNKNF
NRLQDVFLLPDPSGQWRDTRVYGVFSNPWNYSAVCVYSLGDIDKVFRTSSLKGYHSSLPN
PRPGKCLPDQQPIPTETFQVADRHPEVAQRVEPMGPLKTPLFHSKYHYQKVAVHRMQASH
GETFHVLYLTTDRGTIHKVVEPGEQEHSFAFNIMEIQPFRRAAAIQTMSLDAERRKLYVSS
QWEVSQVPLDLCEVYGGGCHGCLMSRDPYCGWDQGRCISIYSSERSVLQSINPAEPHKEC
PNPKPDKAPLQKVSLAPNSRYYLSCPMESRHATYSWRHKENVEQSCEPGHQSPNCILFIEN
LTAQQYGHYFCEAQEGSYFREAQHWQLLPEDGIMAEHLLGHACALAASLWLGVLPTLTL
GLLVH

>P35916 single-pass type I membrane protein
MQRGAALCLRLWLCLGLLDGLVSGYSMTPPTLNITEESHVIDTGDSLSISCRGQHPLEWA
WPGAQEAPATGDKDSEDTGVVRDCEGTDARPYCKVLLLHEVHANDTGSYVCYYKYIKA
RIEGTTAASSYVFVRDFEQPFINKPDTLLVNRKDAMWVPCLVSIPGLNVTLRSQSSVLWPD
GQEVVWDDRRGMLVSTPLLHDALYLQCETTWGDQDFLSNPFLVHITGNELYDIQLLPRKS
LELLVGEKLVLNCTVWAEFNSGVTFDWDYPGKQAERGKWVPERRSQQTHTELSSILTIHN
VSQHDLGSYVCKANNGIQRFRESTEVIVHENPFISVEWLKGPILEATAGDELVKLPVKLAA
YPPPEFQWYKDGKALSGRHSPHALVLKEVTEASTGTYTLALWNSAAGLRRNISLELVVNV
PPQIHEKEASSPSIYSRHSRQALTCTAYGVPLPLSIQWHWRPWTPCKMFAQRSLRRRQQQD
LMPQCRDWRAVTTQDAVNPIESLDTWTEFVEGKNKTVSKLVIQNANVSAMYKCVVSNK
VGQDERLIYFYVTTIPDGFTIESKPSEELLEGQPVLLSCQADSYKYEHLRWYRLNLSTLHD
AHGNPLLLDCKNVHLFATPLAASLEEVAPGARHATLSLSIPRVAPEHEGHYVCEVQDRRSH
DKHCHKKYLSVQALEAPRLTQNLTDLLVNVSDSLEMQCLVAGAHAPSIVWYKDERLLEE
KSGVDLADSNQKLSIQRVREEDAGRYLCSVCNAKGCVNSSASVAVEGSEDKGSMEIVILV
GTGVIAVFFWVLLLLIFCNMRRPAHADIKTGYLSIIMDPGEVPLEEQCEYLSYDASQWEFP
RERLHLGRVLGYGAFGKVVEASAFGIHKGSSCDTVAVKMLKEGATASEHRALMSELKILI
HIGNHLNVVNLLGACTKPQGPLMVIVEFCKYGNLSNFLRAKRDAFSPCAEKSPEQRGRFR
AMVELARLDRRRPGSSDRVLFARFSKTEGGARRASPDQEAEDLWLSPLTMEDLVCYSFQV
ARGMEFLASRKCIHRDLAARNILLSESDVVKICDFGLARDIYKDPDYVRKGSARLPLKWM
APESIFDKVYTTQSDVWSFGVLLWEIFSLGASPYPGVQINEEFCQRLRDGTRMRAPELATP
AIRRIMLNCWSGDPKARPAFSELVEILGDLLQGRGLQEEEEVCMAPRSSQSSEEGSFSQVS
TMALHIAQADAEDSPPSLQRHSLAARYYNWVSFPGCLARGAETRGSSRMKTFEEFPMTPT
TYKGSVDNQTDSGMVLASEEFEQIESRHRQESGFSCKGPGQNVAVTRAHPDSQGRRRRPE

RGARGGQVFYNSEYGELSEPSEEDHCSPSARVTFFTDNSY

>Q9BYE9 single-pass type I membrane protein
MAQLWLSCFLLPALVVSVAANVAPKFLANMTSVILPEDLPVGAQAFWLVAEDQDNDPLTY
GMSGPNAYFFAVTPKTGEVKLASALDYETLYTFKVTISVSDPYIQVQREMLVIVEDRNDNA
PVFQNTAFSTSINETLPVGSVVFSVLAVDKDMGSAGMVVYSIEKVIPSTGDSEHLFRILAN
GSIVLNGSLSYNNKSAFYQLELKACDLGGMYHNTFTIQCSLPVFLSISVVDQPDLDPQFVR
EFYSASVAEDAAKGTSVLTVEAVDGDKGINDPVIYSISYSTRPGWFDIGADGVIRVNGSLD
REQLLEADEEVQLQVTATETHLNIYGQEAKVSIWVTVRVMDVNDHKPEFYNCSLPACTFT
PEEAQVNFTGYVDEHASPRIPIDDLTMVVYDPDKGSNGTFLLSLGGPDAEAFSVSPERAVG
SASVQVLVRVSALVDYERQTAMAVQVVATDSVSQNFSVAMVTIHLRDINDHRPTFPQSLY
VLTVPEHSATGSVVTDSIHATDPDTGAWGQITYSLLPGNGADLFQVDPVSGTVTVRNGEL
LDRESQAVYYLTLQATDGGNLSSSTTLQIHLLDINDNAPVVSGSYNIFVQEEEGNVSVTIQ
AHDNDEPGTNNSRLLFNLLPGPYSHNFSLDPDTGLLRNLGPLDREAIDPALEGRIVLTVLVS
DCGEPVLGTKVNVTITVEDINDNLPIFNQSSYNFTVKEEDPGVLVGVVKAWDADQTEANN
RISFSLSGSGANYFMIRGLVLGAGWAEGYLRLPPDVSLDYETQPVFNLTVSAENPDPQGGE
TIVDVCVNVKDVNDNPPTLDVASLRGIRVAENGSQHGQVAVVVASDVDTSAQLEIQLVNIL
CTKAGVDVGSLCWGWFSVAANGSVYINQSKAIDYEACDLVTLVVRACDLATDPGFQAYS
NNGSLLITIEDVNDNAPYFLPENKTFVIIPELVLPNREVASVRARDDDSGNNGVILFSILRVD
FISKDGATIPFQGVFSIFTSSEADVFAGSIQPVTSLDSTLQGTYQVTVQARDRPSLGPFLEAT
TTLNLFTVDQSYRSRLQFSTPKEEVGANRQAINAALTQATRTTVYIVDIQDIDSAARARPH
SYLDAYFVFPNGSALTLDELSVMIRNDQDSLTQLLQLGLVVLGSQESQESDLSKQLISVIIG
LGVALLLVLVIMTMAFVCVRKSYNRKLQAMKAAKEARKTAAGVMPSAPAIPGTNMYNT
ERANPMLNLPNKDLGLEYLSPSNDLDSVSVNSLDDNSVDVDKNSQEIKEHRPPHTPPEPD
PEPLSVVLLGRQAGASGQLEGPSYTNAGLDTTDL

>P05106 single-pass type I membrane protein
MRARPRPRPLWATVLALGALAGVGVGGPNICTTRGVSSCQQCLAVSPMCAWCSDEALPL
GSPRCDLKENLLKDNCAPESIEFPVSEARVLEDRPLSDKGSGDSSQVTQVSPQRIALRLRPD
DSKNFSIQVRQVEDYPVDIYYLMDLSYSMKDDLWSIQNLGTKLATQMRKLTSNLRIGFGA
FVDKPVSPYMYISPPEALENPCYDMKTTCLPMFGYKHVLTLTDQVTRFNEEVKKQSVSRN
RDAPEGGFDAIMQATVCDEKIGWRNDASHLLVFTTDAKTHIALDGRLAGIVQPNDGQCH
VGSDNHYSASTTMDYPSLGLMTEKLSQKNINLIFAVTENVVNLYQNYSELIPGTTVGVLS
MDSSNVLQLIVDAYGKIRSKVELEVRDLPEELSLSFNATCLNNEVIPGLKSCMGLKIGDTV
SFSIEAKVRGCPQEKEKSFTIKPVGFKDSLIVQVTFDCDCACQAQAEPNSHRCNNGNGTFE
CGVCRCGPGWLGSQCECSEEDYRPSQQDECSPREGQPVCSQRGECLCGQCVCHSSDFGKI
TGKYCECDDFSCVRYKGEMCSGHGQCSCGDCLCDSDWTGYYCNCTTRTDTCMSSNGLL
CSGRGKCECGSCVCIQPGSYGDTCEKCPTCPDACTFKKECVECKKFDRGALHDENTCNR
YCRDEIESVKELKDTGKDAVNCTYKNEDDCVVRFQYYEDSSGKSILYVVEEPECPKGPDIL
VVLLSVMGAILLIGLAALLIWKLLITIHDRKEFAKFEEERARAKWDTANNPLYKEATSTFT
NITYRGT

>P49281 multipass membrane protein
MVLGPEQKMSDDSVSGDHGESASLGNINPAYSNPSLSQSPGDSEEYFATYFNEKISIPEEEY

SCFSFRKLWAFTGPGFLMSIAYLDPGNIESDLQSGAVAGFKLLWILLLATLVGLLLQRLAAR
LGVVTGLHLAEVCHRQYPKVPRVILWLMVELAIIGSDMQEVIGSAIAINLLSVGRIPLWGG
VLITIADTFVFLFLDKYGLRKLEAFFGFLITIMALTFGYEYVTVKPSQSQVLKGMFVPSCSG
CRTPQIEQAVGIVGAVIMPHNMYLHSALVKSRQVNRNNKQEVREANKYFFIESCIALFVSFI
INVFVVSVFAEAFFGKTNEQVVEVCTNTSSPHAGLFPKDNSTLAVDIYKGGVVLGCYFGP
AALYIWAVGILAAGQSSTMTGTYSGQFVMEGFLNLKWSRFARVVLTRSIAIIPTLLVAVFQD
VEHLTGMNDFLNVLQSLQLPFALIPILTFTSLRPVMSDFANGLGWRIAGGILVLIICSINMYF
VVVYVRDLGHVALYVVAAVVSVAYLGFVFYLGWQCLIALGMSFLDCGHTCHLGLTAQPE
LYLLNTMDADSLVSR

>P21757 single-pass type II membrane protein
MEQWDHFHNQQEDTDSCSESVKFDARSMTALLPPNPKNSPSLQEKLKSFKAALIALYLLV
FAVLIPLIGIVAAQLLKWETKNCSVSSTNANDITQSLTGKGNDSEEEMRFQEVFMEHMSNM
EKRIQHILDMEANLMDTEHFQNFSMTTDQRFNDILLQLSTLFSSVQGHGNAIDEISKSLISL
NTTLLDLQLNIENLNGKIQENTFKQQEEISKLEERVYNVSAEIMAMKEEQVHLEQEIKGEV
KVLNNITNDLRLKDWEHSQTLRNITLIQGPPGPPGEKGDRGPTGESGPRGFPGPIGPPGLK
GDRGAIGFPGSRGLPGYAGRPGNSGPKGQKGEKGSGNTLTPFTKVRLVGGSGPHEGRVEIL
HSGQWGTICDDRWEVRVGQVVCRSLGYPGVQAVHKAAHFGQGTGPIWLNEVFCFGRES
SIEECKIRQWGTRACSHSEDAGVTCTL

>Q9H159 single-pass type I membrane protein
MNCYLLLRFMLGIPLLWPCLGATENSQTKKVKQPVRSHLRVKRGWVWNQFFVPEEMNT
TSHHIGQLRSDLDNGNNSFQYKLLGAGAGSTFIIDERTGDIYAIQKLDREERSLYILRAQVI
DIATGRAVEPESEFVIKVSDINDNEPKFLDEPYEAIVPEMSPEGTLVIQVTASDADDPSSGNN
ARLLYSLLQGQPYFSVEPTTGVIRISSKMDRELQDEYWVIIQAKDMIGQPGALSGTTSVLIK
LSDVNDNKPIFKESLYRLTVSESAPTGTSIGTIMAYDNDIGENAEMDYSIEEDDSQTFDIITN
HETQEGIVILKKKVDFEHQNHYGIRAKVKNHHVPEQLMKYHTEASTTFIKIQVEDVDEPP
LFLLPYYVFEVFEETPQGSFVGVVSATDPDNRKSPIRYSITRSKVFNINDNGTITTSNSLDRE
ISAWYNLSITATEKYNIEQISSIPLYVQVLNINDHAPEFSQYYETYVCENAGSGQVIQTISAV
DRDESIEEHHFYFNLSVEDTNNSSFTIIDNQDNTAVILTNRTGFNLQEEPVFYISILIADNGIPS
LTSTNTLTIHVCDCGDSGSTQTCQYQELVLSMGFKTEVIIAILICIMIIFGFIFLTLGLKQRRK
QILFPEKSEDFRENIFQYDDEGGGEEDTEAFDIAELRSSTIMRERKTRKTTSAEIRSLYRQSL
QVGPDSAIFRKFILEKLEEANTDPCAPPFDSLQTYAFEGTGSLAGSLSSLESAVSDQDESYD
YLNELGPRFKRLACMFGSAVQSNN

>P41587 multipass membrane protein
MRTLLPPALLTCWLLAPVNSIHPECRFHLEIQEEETKCAELLRSQTEKHKACSGVWDNITC
WRPANVGETVTVPCPKVFSNFYSKAGNISKNCTSDGWSETFPDFVDACGYSDPEDESKIT
FYILVKAIYTLGYSVSLMSLATGSIILCLFRKLHCTRNYIHLNLFLSFILRAISVLVKDDVLYS
SSGTLHCPDQPSSWVGCKLSLVFLQYCIMANFFWLLVEGLYLHTLLVAMLPPRRCFLAYLL
IGWGLPTVCIGAWTAARLYLEDTGCWDTNDHSVPWWVIRIPILISIIVNFVLFISIIRILLQKL
TSPDVGGNDQSQYKRLAKSTLLLIPLFGVHYMVFAVFPISISSKYQILFELCLGSFQGLVVA
VLYCFLNSEVQCELKRKWRSRCPTPSASRDYRVCGSSFSRNGSEGALQFHRGSRAQSFLQ
TETSVI

>Q12809 multipass membrane protein
MPVRRGHVAPQNTFLDTIIRKFEGQSRKFIIANARVENCAVIYCNDGFCELCGYSRAEVMQ
RPCTCDFLHGPRTQRRAAAQIAQALLGAEERKVEIAFYRKDGSCFLCLVDVVPVKNEDGA
VIMFILNFEVVMEKDMVGSPAHDTNHRGPPTSWLAPGRAKTFRLKLPALLALTARESSVR
SGGAGGAGAPGAVVVDVDLTPAAPSSESLALDEVTAMDNHVAGLGPAEERRALVGPGSPP
RSAPGQLPSPRAHSLNPDASGSSCSLARTRSRESCASVRRASSADDIEAMRAGVLPPPPRH
ASTGAMHPLRSGLLNSTSDSDLVRYRTISKIPQITLNFVDLKGDPFLASPTSDREIIAPKIKER
THNVTEKVTQVLSLGADVLPEYKLQAPRIHRWTILHYSPFKAVWDWLILLLVIYTAVFTPY
SAAFLLKETEEGPPATECGYACQPLAVVDLIVDIMFIVDILINFRTTYVNANEEVVSHPGRIA
VHYFKGWFLIDMVAAIPFDLLIFGSGSEELIGLLKTARLLRLVRVARKLDRYSEYGAAVLFL
LMCTFALIAHWLACIWYAIGNMEQPHMDSRIGWLHNLGDQIGKPYNSSGLGGPSIKDKY
VTALYFTFSSLTSVGFGNVSPNTNSEKIFSICVMLIGSLMYASIFGNVSAIIQRLYSGTARYHT
QMLRVREFIRFHQIPNPLRQRLEEYFQHAWSYTNGIDMNAVLKGFPECLQADICLHLNRSL
LQHCKPFRGATKGCLRALAMKFKTTHAPPGDTLVHAGDLLTALYFISRGSIEILRGDVVVA
ILGKNDIFGEPLNLYARPGKSNGDVRALTYCDLHKIHRDDLLEVLDMYPEFSDHFWSSLEI
TFNLRDTNMIPGSPGSTELEGGFSRQRKRKLSFRRRTDKDTEQPGEVSALGPGRAGAGPSS
RGRPGGPWGESPSSGPSSPESSEDEGPGRSSSPLRLVPFSSPRPPGEPPGGEPLMEDCEKSSD
TCNPLSGAFSGVSNIFSFWGDSRGRQYQELPRCPAPTPSLLNIPLSSPGRRPRGDVESRLDA
LQRQLNRLETRLSADMATVLQLLQRQMTLVPPAYSAVTTPGPGPTSTSPLLPVSPLPTLTLD
SLSQVSQFMACEELPPGAPELPQEGPTRRLSLPGQLGALTSQPLHRHGSDPGS

>Q15382 lipid-anchor
MPQSKSRKIAILGYRSVGKSSLTIQFVEGQFVDSYDPTIENTFTKLITVNGQEYHLQLVDTA
GQDEYSIFPQTYSIDINGYILVYSVTSIKSFEVIKVIHGKLLDMVGKVQIPIMLVGNKKDLH
MERVISYEEGKALAESWNAAFLESSAKENQTAVDVFRRIILEAEKMDGAASQGKSSCSVM

>Q15629 multipass membrane protein
MAIRKKSTKSPPVLSHEFVLQNHADIVSCVAMVFLLGLMFEITAKASIIFVTLQYNVTLPAT
EEQATESVSLYYYGIKDLATVFFYMLVAIIIHAVIQEYMLDKINRRMHFSKTKHSKFNESGQ
LSAFYLFACVWGTFILISENYISDPTILWRAYPHNLMTFQMKFFYISQLAYWLHAFPELYFQ
KTKKEDIPRQLVYIGLYLFHIAGAYLLNLNHLGLVLLVLHYFVEFLFHISRLFYFSNEKYQK
GFSLWAVLFVLGRLLTLILSVLTVGFGLARAENQKLDFSTGNFNVLAVRIAVLASICVTQAF
MMWKFINFQLRRWREHSAFQAPAVKKKPTVTKGRSSKKGTENGVNGTLTSNVADSPRNK
KEKSS

>Q15363 single-pass type I membrane protein
MVTLAELLVLLAALLATVSGYFVSIDAHAEECFFERVTSGTKMGLIFEVAEGGFLDIDVEIT
GPDNKGIYKGDRESSGKYTFAAHMDGTYKFCFSNRMSTMTPKIVMFTIDIGEAPKGQDM
ETEAHQNKLEEMINELAVAMTAVKHEQEYMEVRERIHRAINDNTNSRVVLWSFFEALVLV
AMTLGQIYYLKRFFEVRRVV

>P20138 single-pass type I membrane protein
MPLLLLLPLLWAGALAMDPNFWLQVQESVTVQEGLCVLVPCTFFHPIPYYDKNSPVHGY

WFREGAIISRDSPVATNKLDQEVQEETQGRFRLLGDPSRNNCSLSIVDARRRDNGSYFFRM
ERGSTKYSYKSPQLSVHVTDLTHRPKILIPGTLEPGHSKNLTCSVSWACEQGTPPIFSWLSA
APTSLGPRTTHSSVLIITPRPQDHGTNLTCQVKFAGAGVTTERTIQLNVTYVPQNPTTGIFPG
DGSGKQETRAGVVHGAIGGAGVTALLALCLCLIFFIVKTHRRKAARTAVGRNDTHPTTGS
ASPKHQKKSKLHGPTETSSCSGAAPTVEMDEELHYASLNFHGMNPSKDTSTEYSEVRTQ

>Q96GC9 multipass membrane protein
MAENGKNCDQRRVAMNKEHHNGNFTDPSSVNEKKRREREERQNIVLWRQPLITLQYFSL
EILVILKEWTSKLWHRQSIVVSFLLLLAVLIATYYVEGVHQQYVQRIEKQFLLYAYWIGLGI
LSSVGLGTGLHTFLLYLGPHIASVTLAAYECNSVNFPEPPYPDQIICPDEEGTEGTISLWSIIS
KVRIEACMWGIGTAIGELPPYFMARAARLSGAEPDDEEYQEFEEMLEHAESAQDFASRAK
LAVQKLVQKVGFFGILACASIPNPLFDLAGITCGHFLVPFWTFFGATLIGKAIIKMHIQKIFVI
ITFSKHIVEQMVAFIGAVPGIGPSLQKPFQEYLEAQRQKLHHKSEMGTPQGENWLSWMFE
KLVVVMVCYFILSIINSMAQSYAKRIQQRLNSEEKTK

>Q99640 peripheral membrane protein
MLERPPALAMPMPTEGTPPPLSGTPIPVPAYFRHAEPGFSLKRPRGLSRSLPPPPAKGSIPIS
RLFPPRTPGWHQLQPRRVSFRGEASETLQSPGYDPSRPESFFQQSFQRLSRLGHGSYGEVF
KVRSKEDGRLYAVKRSMSPFRGPKDRARKLAEVGSHEKVGQHPCCVRLEQAWEEGGILY
LQTELCGPSLQQHCEAWGASLPEAQVWGYLRDTLLALAHLHSQGLVHLDVKPANIFLGP
RGRCKLGDFGLLVELGTAGAGEVQEGDPRYMAPELLQGSYGTAADVFSLGLTILEVACN
MELPHGGEGWQQLRQGYLPPEFTAGLSSELRSVLVMMLEPDPKLRATAEALLALPVLRQP
RAWGVLWCMAAEALSRGWALWQALLALLCWLWHGLAHPASWLQPLGPPATPPGSPPCS
LLLDSSLSSNWDDDSLGPSLSPEAVLARTVGSTSTPRSRCTPRDALDLSDINSEPPRGSFPSF
EPRNLLSLFEDTLDPT

>Q96S06 multipass membrane protein
MRPDSPTMAAPAESLRRRKTGYSDPEPESPPAPGRGPAGSPAHLHTGTFWLTRIVLLKALA
FVYFVAFLVAFHQNKQLIGDRGLLPCRVFLKNFQQYFQDRTSWEVFSYMPTILWLMDWSD
MNSNLDLLALLGLGISSFVLITGCANMLLMAALWGLYMSLVNVGHVWYSFGWESQLLET
GFLGIFLCPLWTLSRLPQHTPTSRIVLWGFRWLIFRIMLGAGLIKIRGDRCWRDLTCMDFH
YETQPMPNPVAYYLHHSPWWFHRFETLSNHFIELLVPFFLFLGRRACIIHGVLQILFQAVLI
VSGNLSFLNWLTMVPSLACFDDATLGFLFPSGPGSLKDRVLQMQRDIRGARPEPRFGSVV
RRAANVSLGVLLAWLSVPVVLNLLSSRQVMNTHFNSLHIVNTYGAFGSITKERAEVILQG
TASSNASAPDAMWEDYEFKCKPGDPSRRPCLISPYHYRLDWLMWFAAFQTYEHNDWIIH
LAGKLLASDAEALSLLAHNPFAGRPPPRWVRGEHYRYKFSRPGGRHAAEGKWWVRKRI
GAYFPPLSLEELRPYFRDRGWPLPGPL

>P98161 multipass membrane protein
MPPAAPARLALALGLGLWLGALAGGPGRGCGPCEPPCLCGPAPGAACRVNCSGRGLRTL
GPALRIPADATALDVSHNLLRALDVGLLANLSALAELDISNNKISTLEEGIFANLFNLSEINL
SGNPFECDCGLAWLPRWAEEQQVRVVQPEAATCAGPGSLAGQPLLGIPLLDSGCGEEYVA
CLPDNSSGTVAAVSFSAAHEGLLQPEACSAFCFSTGQGLAALSEQGWCLCGAAQPSSASF
ACLSLCSGPPPPPAPTCRGPTLLQHVFPASPGATLVGPHGPLASGQLAAFHIAAPLPVTATR

WDFGDGSAEVDAAGPAASHRYVLPGRYHVTAVLALGAGSALLGTDVQVEAAPAALELV
CPSSVQSDESLDLSIQNRGGSGLEAAYSIVALGEEPARAVHPLCPSDTEIFPGNGHCYRLVV
EKAAWLQAQEQCQAWAGAALAMVDSPAVQRFLVSRVTRSLDVWIGFSTVQGVEVGPAP
QGEAFSLESCQNWLPGEPHPATAEHCVRLGPTGWCNTDLCSAPHSYVCELQPGGPVQDA
ENLLVGAPSGDLQGPLTPLAQQDGLSAPHEPVEVMVFPGLRLSREAFLTTAEFGTQELRRP
AQLRLQVYRLLSTAGTPENGSEPESRSPDNRTQLAPACMPGGRWCPGANICLPLDASCHP
QACANGCTSGPGLPGAPYALWREFLFSVPAGPPAQYSVTLHGQDVLMLPGDLVGLQHDA
GPGALLHCSPAPGHPGPRAPYLSANASSWLPHLPAQLEGTWACPACALRLLAATEQLTVL
LGLRPNPGLRLPGRYEVRAEVGNGVSRHNLSCSFDVVSPVAGLRVIYPAPRDGRLYVPTN
GSALVLQVDSGANATATARWPGGSVSARFENVCPALVATFVPGCPWETNDTLFSVVALPW
LSEGEHVVDVVVENSASRANLSLRVTAEEPICGLRATPSPEARVLQGVLVRYSPVVEAGSD
MVFRWTINDKQSLTFQNVVFNVIYQSAAVFKLSLTASNHVSNVTVNYNVTVERMNRMQG
LQVSTVPAVLSPNATLALTAGVLVDSAVEVAFLWTFGDGEQALHQFQPPYNESFPVPDPSV
AQVLVEHNVMHTYAAPGEYLLTVLASNAFENLTQQVPVSVRASLPSVAVGVSDGVLVAG
RPVTFYPHPLPSPGGVLYTWDFGDGSPVLTQSQPAANHTYASRGTYHVRLEVNNTVSGAA
AQADVRVFEELRGLSVDMSLAVEQGAPVVVSAAVQTGDNITWTFDMGDGTVLSGPEATV
EHVYLRAQNCTVTVGAASPAGHLARSLHVLVFVLEVLRVEPAACIPTQPDARLTAYVTGN
PAHYLFDWTFGDGSSNTTVRGCPTVTHNFTRSGTFPLALVLSSRVNRAHYFTSICVEPEVG
NVTLQPERQFVQLGDEAWLVACAWPPFPYRYTWDFGTEEAAPTRARGPEVTFIYRDPGSY
LVTVTASNNISAANDSALVEVQEPVLVTSIKVNGSLGLELQQPYLFSAVGRGRPASYLWDL
GDGGWLEGPEVTHAYNSTGDFTVRVAGWNEVSRSEAWLNVTVKRRVRGLVVNASRTVV
PLNGSVSFSTSLEAGSDVRYSWVLCDRCTPIPGGPTISYTFRSVGTFNIIVTAENEVGSAQD
SIFVYVLQLIEGLQVVGGGRYFPTNHTVQLQAVVRDGTNVSYSWTAWRDRGPALAGSGK
GFSLTVLEAGTYHVQLRATNMLGSAWADCTMDFVEPVGWLMVAASPNPAAVNTSVTLSA
ELAGGSGVVYTWSLEEGLSWETSEPFTTHSFPTPGLHLVTMTAGNPLGSANATVEVDVQV
PVSGLSIRASEPGGSFVAAGSSVPFWGQLATGTNVSWCWAVPGGSSKRGPHVTMVFPDAG
TFSIRLNASNAVSWVSATYNLTAEEPIVGLVLWASSKVVAPGQLVHFQILLAAGSAVTFRLQ
VGGANPEVLPGPRFSHSFPRVGDHVVSVRGKNHVSWAQAQVRIVVLEAVSGLQVPNCCE
PGIATGTERNFTARVQRGSRVAYAWYFSLQKVQGDSLVILSGRDVTYTPVAAGLLEIQVRA
FNALGSENRTLVLEVQDAVQYVALQSGPCFTNRSAQFEAATSPSPRRVAYHWDFGDGSPG
QDTDEPRAEHSYLRPGDYRVQVNASNLVSFFVAQATVTVQVLACREPEVDVVLPLQVLM
RRSQRNYLEAHVDLRDCVTYQTEYRWEVYRTASCQRPGRPARVALPGVDVSRPRLVLPRL
ALPVGHYCFVFVVSFGDTPLTQSIQANVTVAPERLVPIIEGGSYRVWSDTRDLVLDGSESY
DPNLEDGDQTPLSFHWACVASTQREAGGCALNFGPRGSSTVTIPRERLAAGVEYTFSLTV
WKAGRKEEATNQTVLIRSGRVPIVSLECVSCKAQAVYEVSRSSYVYLEGRCLNCSSGSKR
GRWAARTFSNKTLVLDETTTSTGSAGMRLVLRRGVLRDGEGYTFTLTVLGRSGEEEGCAS
IRLSPNRPPLGGSCRLFPLGAVHALTTKVHFECTGWHDAEDAGAPLVYALLLRRCRQGHC
EEFCVYKGSLSSYGAVLPPGFRPHFEVGLAVVVQDQLGAAVVALNRSLAITLPEPNGSATG
LTVWLHGLTASVLPGLLRQADPQHVIEYSLALVTVLNEYERALDVAAEPKHERQHRAQIR
KNITETLVSLRVHTVDDIQQIAAALAQCMGPSRELVCRSCLKQTLHKLEAMMLILQAETTA
GTVTPTAIGDSILNITGDLIHLASSDVRAPQPSELGAESPSRMVASQAYNLTSALMRILMRS
RVLNEEPLTLAGEEIVAQGKRSDPRSLLCYGGAPGPGCHFSIPEAFSGALANLSDVVQLIFL
VDSNPFPFGYISNYTVSTKVASMAFQTQAGAQIPIERLASERAITVKVPNNSDWAARGHRS
SANSANSVVVQPQASVGAVVTLDSSNPAAGLHLQLNYTLLDGHYLSEEPEPYLAVYLHSE

PRPNEHNCSASRRIRPESLQGADHRPYTFFISPGSRDPAGSYHLNLSSHFRWSALQVSVGLY
TSLCQYFSEEDMVWRTEGLLPLEETSPRQAVCLTRHLTAFGASLFVPPSHVRFVFPEPTADV
NYIVMLTCAVCLVTYMVMAAILHKLDQLDASRGRAIPFCGQRGRFKYEILVKTGWGRGS
GTTAHVGIMLYGVDSRSGHRHLDGDRAFHRNSLDIFRIATPHSLGSVWKIRVWHDNKGLS
PAWFLQHVIVRDLQTARSAFFLVNDWLSVETEANGGLVEKEVLAASDAALLRFRRLLVAE
LQRGFFDKHIWLSIWDRPPRSRFTRIQRATCCVLLICLFLGANAVWYGAVGDSAYSTGHVS
RLSPLSVDTVAVGLVSSVVVYPVYLAILFLFRMSRSKVAGSPSPTPAGQQVLDIDSCLDSSV
LDSSFLTFSGLHAEQAFVGQMKSDLFLDDSKSLVCWPSGEGTLSWPDLLSDPSIVGSNLRQ
LARGQAGHGLGPEEDGFSLASPYSPAKSFSASDEDLIQQVLAEGVSSPAPTQDTHMETDLL
SSLSSTPGEKTETLALQRLGELGPPSPGLNWEQPQAARLSRTGLVEGLRKRLLPAWCASLA
HGLSLLLVAVAVAVSGWVGASFPPGVSVAWLLSSSASFLASFLGWEPLKVLLEALYFSLVAK
RLHPDEDDTLVESPAVTPVSARVPRVRPPHGFALFLAKEEARKVKRLHGMLRSLLVYMLF
LLVTLLASYGDASCHGHAYRLQSAIKQELHSRAFLAITRSEELWPWMAHVLLPYVHGNQS
SPELGPPRLRQVRLQEALYPDPPGPRVHTCSAAGGFSTSDYDVGWESPHNGSGTWAYSAP
DLLGAWSWGSCAVYDSGGYVQELGLSLEESRDRLRFLQLHNWLDNRSRAVFLELTRYSPA
VGLHAAVTLRLEFPAAGRALAALSVRPFALRRLSAGLSLPLLTSVCLLLFAVHFAVAEART
WHREGRWRVLRLGAWARWLLVALTAATALVRLAQLGAADRQWTRFVRGRPRRFTSFDQ
VAQLSSAARGLAASLLFLLLVKAAQQLRFVRQWSVFGKTLCRALPELLGVTLGLVVLGVA
YAQLAILLVSSCVDSLWSVAQALLVLCPGTGLSTLCPAESWHLSPLLCVGLWALRLWGAL
RLGAVILRWRYHALRGELYRPAWEPQDYEMVELFLRRLRLWMGLSKVKEFRHKVRFEG
MEPLPSRSSRGSKVSPDVPPPSAGSDASHPSTSSSQLDGLSVSLGRLGTRCEPEPSRLQAVF
EALLTQFDRLNQATEDVYQLEQQLHSLQGRRSSRAPAGSSRGPSPGLRPALPSRLARASRG
VDLATGPSRTPLRAKNKVHPSST

>O75154 peripheral membrane protein
MASAPPASPPGSEPPGPDPEPGGPDGPGAAQLAPGPAELRLGAPVGGPDPQSPGLDEPAPG
AAADGGARWSAGPAPGLEGGPRDPGPSAPPPRSGPRGQLASPDAPGPGPRSEAPLPELDPL
FSWTEEPEECGPASCPESAPFRLQGSSSSHRARGEVDVFSPFPAPTAGELALEQGPGSPPQP
SDLSQTHPLPSEPVGSQEDGPRLRAVFDALDGDGDGFVRIEDFIQFATVYGAEQVKDLTKY
LDPSGLGVISFEDFYQGITAIRNGDPDGQCYGGVASAQDEEPLACPDEFDDFVTYEANEVT
DSAYMGSESTYSECETFTDEDTSTLVHPELQPEGDADSAGGSAVPSECLDAMEEPDHGAL
LLLPGRPHPHGQSVITVIGGEEHFEDYGEGSEAELSPETLCNGQLGCSDPAFLTPSPTKRLSS
KKVARYLHQSGALTMEALEDPSPELMEGPEEDIADKVVFLERRVLELEKDTAATGEQHSR
LRQENLQLVHRANALEEQLKEQELRACEMVLEETRRQKELLCKMEREKSIEIENLQTRLQ
QLDEENSELRSCTPCLKANIERLEEEKQKLLDEIESLTLRLSEEQENKRRMGDRLSHERHQ
FQRDKEATQELIEDLRKQLEHLQLLKLEAEQRRGRSSSMGLQEYHSRARESELEQEVRRL
KQDNRNLKEQNEELNGQIITLSIQGAKSLFSTAFSESLAAEISSVSRDELMEAIQKQEEINFR
LQDYIDRIIVAIMETNPSILEVK

>Q96CC6 multipass membrane protein
MSEARRDSTSSLQRKKPPWLKLDIPSAVPLTAEEPSFLQPLRRQAFLRSVSMPAETAHISSP
HHELRRPVLQRQTSITQTIRRGTADWFGVSKDSDSTQKWQRKSIRHCSQRYGKLKPQVLR
ELDLPSQDNVSLTSTETPPPLYVGPCQLGMQKIIDPLARGRAFRVADDTAEGLSAPHTPVTP
GAASLCSFSSSRSGFHRLPRRRKRESVAKMSFRAAAALMKGRSVRDGTFRRAQRRSFTPA

SFLEEDTTDFPDELDTSFFAREGILHEELSTYPDEVFESPSEAALKDWEKAPEQADLTGGAL
DRSELERSHLMLPLERGWRKQKEGAAAPQPKVRLRQEVVSTAGPRRGQRIAVPVRKLFA
REKRPYGLGMVGRLTNRTYRKRIDSFVKRQIEDMDDHRPFFTYWLTFVHSLVTILAVCIYG
IAPVGFSQHETVDSVLRNRGVYENVKYVQQENFWIGPSSEALIHLGAKFSPCMRQDPQVH
SFIRSAREREKHSACCVRNDRSGCVQTSEEECSSTLAVWVKWPIHPSAPELAGHKRQFGS
VCHQDPRVCDEPSSEDPHEWPEDITKWPICTKNSAGNHTNHPHMDCVITGRPCCIGTKGR
CEITSREYCDFMRGYFHEEATLCSQVHCMDDVCGLLPFLNPEVPDQFYRLWLSLFLHAGI
LHCLVSICFQMTVLRDLEKLAGWHRIAIIYLLSGVTGNLASAIFLPYRAEVGPAGSQFGILA
CLFVELFQSWQILARPWRAFFKLLAVVLFLFTFGLLPWIDNFAHISGFISGLFLSFAFLPYISF
GKFDLYRKRCQIIIFQVVFLGLLAGLVVLFYVYPVRCEWCEFLTCIPFTDKFCEKYELDAQL
H

>Q99665 single-pass type I membrane protein
MAHTFRGCSLAFMFIITWLLIKAKIDACKRGDVTVKPSHVILLGSTVNITCSLKPRQGCFH
YSRRNKLILYKFDRRINFHHGHSLNSQVTGLPLGTTLFVCKLACINSDEIQICGAEIFVGVAP
EQPQNLSCIQKGEQGTVACTWERGRDTHLYTEYTLQLSGPKNLTWQKQCKDIYCDYLDF
GINLTPESPESNFTAKVTAVNSLGSSSSLPSTFTFLDIVRPLPPWDIRIKFQKASVSRCTLYWR
DEGLVLLNRLRYRPSNSRLWNMVNVTKAKGRHDLLDLKPFTEYEFQISSKLHLYKGSWS
DWSESLRAQTPEEEPTGMLDVWYMKRHIDYSRQQISLFWKNLSVSEARGKILHYQVTLQ
ELTGGKAMTQNITGHTSWTTVIPRTGNWAVAVSAANSKGSSLPTRINIMNLCEAGLLAPRQ
VSANSEGMDNILVTWQPPRKDPSAVQEYVVEWRELHPGGDTQVPLNWLRSRPYNVSALI
SENIKSYICYEIRVYALSGDQGGCSSILGNSKHKAPLSGPHINAITEEKGSILISWNSIPVQEQ
MGCLLHYRIYWKERDSNSQPQLCEIPYRVSQNSHPINSLQPRVTYVLWMTALTAAGESSH
GNEREFCLQGKANWMAFVAPSICIAIIMVGIFSTHYFQQKVFVLLAALRPQWCSREIPDPA
NSTCAKKYPIAEEKTQLPLDRLLIDWPTPEDPEPLVISEVLHQVTPVFRHPPCSNWPQREK
GIQGHQASEKDMMHSASSPPPPRALQAESRQLVDLYKVLESRGSDPKPENPACPWTVLPA
GDLPTHDGYLPSNIDDLPSHEAPLADSLEELEPQHISLSVFPSSSLHPLTFSCGDKLTLDQLK
MRCDSLML

>P43005 multipass membrane protein
MGKPARKGCEWKRFLKNNWVLLSTVAAVVLGITTGVLVREHSNLSTLEKFYFAFPGEILM
RMLKLIILPLIISSMITGVAALDSNVSGKIGLRAVVYYFCTTLIAVILGIVLVVSIKPGVTQKV
GEIARTGSTPEVSTVDAMLDLIRNMFPENLVQACFQQYKTKREEVKPPSDPEMNMTEESF
TAVMTTAISKNKTKEYKIVGMYSDGINVLGLIVFCLVFGLVIGKMGEKGQILVDFFNALSD
ATMKIVQIIMCYMPLGILFLIAGKIIEVEDWEIFRKLGLYMATVLTGLAIHSIVILPLIYFIVVR
KNPFRFAMGMAQALLTALMISSSSATLPVTFRCAEENNQVDKRITRFVLPVGATINMDGTA
LYEAVAAVFIAQLNDLDLGIGQIITISITATSASIGAAGVPQAGLVTMVIVLSAVGLPAEDVTL
IIAVDWLLDRFRTMVNVLGDAFGTGIVEKLSKKELEQMDVSSEVNIVNPFALESTILDNED
SDTKKSYVNGGFAVDKSDTISFTQTSQF

>Q9BUZ4 peripheral membrane protein
MPGFDYKFLEKPKRRLLCPLCGKPMREPVQVSTCGHRFCDTCLQEFLSEGVFKCPEDQLP
LDYAKIYPDPELEVQVLGLPIRCIHSEEGCRWSGPLRHLQGHLNTCSFNVIPCPNRCPMKLS
RRDLPAHLQHDCPKRRLKCEFCGCDFSGEAYESHEGMCPQESVYCENKCGARMMRRLL

AQHATSECPKRTQPCTYCTKEFVFDTIQSHQYQCPRLPVACPNQCGVGTVAREDLPGHLK
DSCNTALVLCPFKDSGCKHRCPKLAMARHVEESVKPHLAMMCALVSRQRQELQELRREL
EELSVGSDGVLIWKIGSYGRRLQEAKAKPNLECFSPAFYTHKYGYKLQVSAFLNGNGSGE
GTHLSLYIRVLPGAFDNLLEWPFARRVTFSLLDQSDPGLAKPQHVTETFHPDPNWKNFQK
PGTWRGSLDESSLGFGYPKFISHQDIRKRNYVRDDAVFIRAAVELPRKILS

>O75916 peripheral membrane protein
MTIRHQGQQYRPRMAFLQKIEALVKDMQNPETGVRMQNQRVLVTSVPHAMTGSDVLQW
IVQRLWISSLEAQNLGNFIVRYGYIYPLQDPKNLILKPDGSLYRFQTPYFWPTQQWPAEDT
DYAIYLAKRNIKKKGILEEYEKENYNFLNQKMNYKWDFVIMQAKEQYRAGKERNKADR
YALDCQEKAYWLVHRCPPGMDNVLDYGLDRVTNPNEVKVNQKQTVVAVKKEIMYYQQ
ALMRSTVKSSVSLGGIVKYSEQFSSNDAIMSGCLPSNPWITDDTQFWDLNAKLVEIPTKM
RVERWAFNFSELIRDPKGRQSFQYFLKKEFSGENLGFWEACEDLKYGDQSKVKEKAEEIY
KLFLAPGARRWINIDGKTMDITVKGLKHPHRYVLDAAQTHIYMLMKKDSYARYLKSPIY
KDMLAKAIEPQETTKKSSTLPFMRRHLRSSPSPVILRQLEEEAKAREAANTVDITQPGQHM
APSPHLTVYTGTCMPPSPSSPFSSSCRSPRKPFASPSRFIRRPSTTICPSPIRVALESSSGLEQK
GECSGSMAPRGPSVTESSEASLDTSWPRSRPRAPPKARMALSFSRFLRRGCLASPVFARLS
PKCPAVSHGRVQPLGDVGQQLPRLKSKRVANFFQIKMDVPTGSGTCLMDSEDAGTGESGD
RATEKEVICPWESL

>P08514 single-pass type I membrane protein
MARALCPLQALWLLEWVLLLLGPCAAPPAWALNLDPVQLTFYAGPNGSQFGFSLDFHKD
SHGRVAIVVGAPRTLGPSQEETGGVFLCPWRAEGGQCPSLLFDLRDETRNVGSQTLQTFK
ARQGLGASVVSWSDVIVACAPWQHWNVLEKTEEAEKTPVGSCFLAQPESGRRAEYSPCR
GNTLSRIYVENDFSWDKRYCEAGFSSVVTQAGELVLGAPGGYYFLGLLAQAPVADIFSSY
RPGILLWHVSSQSLSFDSSNPEYFDGYWGYSVAVGEFDGDLNTTEYVVGAPTWSWTLGA
VEILDSYYQRLHRLRGEQMASYFGHSVAVTDVNGDGRHDLLVGAPLYMESRADRKLAEV
GRVYLFLQPRGPHALGAPSLLLTGTQLYGRFGSAIAPLGDLDRDGYNDIAVAAPYGGPSGR
GQVLVFLGQSEGLRSRPSQVLDSPFPTGSAFGFSLRGAVDIDDNGYPDLIVGAYGANQVAV
YRAQPVVKASVQLLVQDSLNPAVKSCVLPQTKTPVSCFNIQMCVGATGHNIPQKLSLNAE
LQLDRQKPRQGRRVLLLGSQQAGTTLNLDLGGKHSPICHTTMAFLRDEADFRDKLSPIVL
SLNVSLPPTEAGMAPAVVLHGDTHVQEQTRIVLDCGEDDVCVPQLQLTASVTGSPLLVGA
DNVLELQMDAANEGEGAYEAELAVHLPQGAHYMRALSNVEGFERLICNQKKENETRVV
LCELGNPMKKNAQIGIAMLVSVGNLEEAGESVSFQLQIRSKNSQNPNSKIVLLDVPVRAE
AQVELRGNSFPASLVVAAEEGEREQNSLDSWGPKVEHTYELHNNGPGTVNGLHLSIHLPG
QSQPSDLLYILDIQPQGGLQCFPQPPVNPLKVDWGLPIPSPSPIHPAHHKRDRRQIFLPEPEQ
PSRLQDPVLVSCDSAPCTVVQCDLQEMARGQRAMVTVLAFLWLPSLYQRPLDQFVLQSH
AWFNVSSLPYAVPPLSLPRGEAQVWTQLLRALEERAIPIWWVLVGVLGGLLLLTILVLAM
WKVGFFKRNRPPLEEDDEEGE

>P02730 multipass membrane protein
MEELQDDYEDMMEENLEQEEYEDPDIPESQMEEPAAHDTEATATDYHTTSHPGTHKVYV
ELQELVMDEKNQELRWMEAARWVQLEENLGENGAWGRPHLSHLTFWSLLELRRVFTKG
TVLLDLQETSLAGVANQLLDRFIFEDQIRPQDREELLRALLLKHSHAGELEALGGVKPAVL

TRSGDPSQPLLPQHSSLETQLFCEQGDGGTEGHSPSGILEKIPPDSEATLVLVGRADFLEQP
VLGFVRLQEAAELEAVELPVPIRFLFVLLGPEAPHIDYTQLGRAAATLMSERVFRIDAYMA
QSRGELLHSLEGFLDCSLVLPPTDAPSEQALLSLVPVQRELLRRRYQSSPAKPDSSFYKGLD
LNGGPDDPLQQTGQLFGGLVRDIRRRYPYYLSDITDAFSPQVLAAVIFIYFAALSPAITFGGL
LGEKTRNQMGVSELLISTAVQGILFALLGAQPLLVVGFSGPLLVFEEAFFSFCETNGLEYIV
GRVWIGFWLILLVVLVVAFEGSFLVRFISRYTQEIFSFLISLIFIYETFSKLIKIFQDHPLQKTY
NYNVLMVPKPQGPLPNTALLSLVLMAGTFFFAMMLRKFKNSSYFPGKLRRVIGDFGVPISI
LIMVLVDFFIQDTYTQKLSVPDGFKVSNSSARGWVIHPLGLRSEFPIWMMFASALPALLVFI
LIFLESQITTLIVSKPERKMVKGSGFHLDLLLVVGMGGVAALFGMPWLSATTVRSVTHAN
ALTVMGKASTPGAAAQIQEVKEQRISGLLVAVLVGLSILMEPILSRIPLAVLFGIFLYMGVTS
LSGIQLFDRILLLFKPPKYHPDVPYVKRVKTWRMHLFTGIQIICLAVLWVVKSTPASLALPF
VLILTVPLRRVLLPLIFRNVELQCLDADDAKATFDEEEGRDEYDEVAMPV

>O95838 multipass membrane protein
MKLGSSRAGPGRGSAGLLPGVHELPMGIPAPWGTSPLSFHRKCSLWAPGRPFLTLVLLVSI
KQVTGSLLEETTRKWAQYKQACLRDLLKEPSGIFCNGTFDQYVCWPHSSPGNVSVPCPSY
LPWWSEESSGRAYRHCLAQGTWQTIENATDIWQDDSECSENHSFKQNVDRYALLSTLQL
MYTVGYSFSLISLFLALTLLLFLRKLHCTRNYIHMNLFASFILRTLAVLVKDVVFYNSYSKR
PDNENGWMSYLSEMSTSCRSVQVLLHYFVGANYLWLLVEGLYLHTLLEPTVLPERRLWP
RYLLLGWAFPVLFVVPWGFARAHLENTGCWTTNGNKKIWWIIRGPMMLCVTVNFFIFLKI
LKLLISKLKAHQMCFRDYKYRLAKSTLVLIPLLGVHEILFSFITDDQVEGFAKLIRLFIQLTL
SSFHGFLVALQYGFANGEVKAELRKYWVRFLLARHSGCRACVLGKDFRFLGKCPKKLSE
GDGAEKLRKLQPSLNSGRLLHLAMRGLGELGAQPQQDHARWPRGSSLSECSEGDVTMA
NTMEEILEESEI

>P55011 multipass membrane protein
MEPRPTAPSSGAPGLAGVGETPSAAALAAARVELPGTAVPSVPEDAAPASRDGGGVRDEG
PAAAGDGLGRPLGPTPSQSRFQVDLVSENAGRAAAAAAAAAAAAAAAAGAGAGAKQTPA
DGEASGESEPAKGSEEAKGRFRVNFVDPAASSSAEDSLSDAAGVGVDGPNVSFQNGGDT
VLSEGSSLHSGGGGGSGHHQHYYYDTHTNTYYLRTFGHNTMDAVPRIDHYRHTAAQLGE
KLLRPSLAELHDELEKEPFEDGFANGEESTPTRDAVVTYTAESKGVVKFGWIKGVLVRCM
LNIWGVMLFIRLSWIVGQAGIGLSVLVIMMATVVTTITGLSTSAIATNGFVRGGGAYYLISR
SLGPEFGGAIGLIFAFANAVAVAMYVVGFAETVVELLKEHSILMIDEINDIRIIGAITVVILLGI
SVAGMEWEAKAQIVLLVILLLAIGDFVIGTFIPLESKKPKGFFGYKSEIFNENFGPDFREEET
FFSVFAIFFPAATGILAGANISGDLADPQSAIPKGTLLAILITTLVYVGIAVSVGSCVVRDATG
NVNDTIVTELTNCTSAACKLNFDFSSCESSPCSYGLMNNFQVMSMVSGFTPLISAGIFSATL
SSALASLVSAPKIFQALCKDNIYPAFQMFAKGYGKNNEPLRGYILTFLIALGFILIAELNVIA
PIISNFFLASYALINFSVFHASLAKSPGWRPAFKYYNMWISLLGAILCCIVMFVINWWAALL
TYVIVLGLYIYVTYKKPDVNWGSSTQALTYLNALQHSIRLSGVEDHVKNFRPQCLVMTGA
PNSRPALLHLVHDFTKNVGLMICGHVHMGPRRQAMKEMSIDQAKYQRWLIKNKMKAFY
APVHADDLREGAQYLMQAAGLGRMKPNTLVLGFKKDWLQADMRDVDMYINLFHDAF
DIQYGVVVIRLKEGLDISHLQGQEELLSSQEKSPGTKDVVVSVEYSKKSDLDTSKPLSEKPI
THKVEEEDGKTATQPLLKKESKGPIVPLNVADQKLLEASTQFQKKQGKNTIDVWWLFDD
GGLTLLIPYLLTTKKKWKDCKIRVFIGGKINRIDHDRRAMATLLSKFRIDFSDIMVLGDINT

KPKKENIIAFEEIIEPYRLHEDDKEQDIADKMKEDEPWRITDNELELYKTKTYRQIRLNELL
KEHSSTANIIVMSLPVARKGAVSSALYMAWLEALSKDLPPILLVRGNHQSVLTFYS

>P55017 multipass membrane protein
MAELPTTETPGDATLCSGRFTISTLLSSDEPSPPAAYDSSHPSHLTHSSTFCMRTFGYNTIDV
VPTYEHYANSTQPGEPRKVRPTLADLHSFLKQEGRHLHALAFDSRPSHEMTDGLVEGEAG
TSSEKNPEEPVRFGWVKGVMIRCMLNIWGVILYLRLPWITAQAGIVLTWIIILLSVTVTSIT
GLSISAISTNGKVKSGGTYFLISRSLGPELGGSIGLIFAFANAVGVAMHTVGFAETVRDLLQ
EYGAPIVDPINDIRIIAVVSVTVLLAISLAGMEWESKAQVLFFLVIMVSFANYLVGTLIPPSE
DKASKGFFSYRADIFVQNLVPDWRGPDGTFFGMFSIFFPSATGILAGANISGDLKDPAIAIP
KGTLMAIFWTTISYLAISATIGSCVVRDASGVLNDTVTPGWGACEGLACSYGWNFTECTQ
QHSCHYGLINYYQTMSMVSGFAPLITAGIFGATLSSALACLVSAAKVFQCLCEDQLYPLIGF
FGKGYGKNKEPVRGYLLAYAIAVAFIIIAELNTIAPIISNFFLCSYALINFSCFHASITNSPGWR
PSFQYYNKWAALFGAIISVVIMFLLTWWAALIAIGVVLFLLLYVIYKKPEVNWGSSVQAGS
YNLALSYSVGLNEVEDHIKNYRPQCLVLTGPPNFRPALVDFVGTFTRNLSLMICGHVLIGP
HKQRMPELQLIANGHTKWLNKRKIKAFYSDVIAEDLRRGVQILMQAAGLGRMKPNILVV
GFKKNWQSAHPATVEDYIGILHDAFDFNYGVCVMRMREGLNVSKMMQAHINPVFDPAE
DGKEASARVDPKALVKEEQATTIFQSEQGKKTIDIYWLFDDGGLTLLIPYLLGRKRRWSKC
KIRVFVGGQINRMDQERKAIISLLSKFRLGFHEVHILPDINQNPRAEHTKRFEDMIAPFRLN
DGFKDEATVNEMRRDCPWKISDEEITKNRVKSLRQVRLNEIVLDYSRDAALIVITLPIGRK
GKCPSSLYMAWLETLSQDLRPPVILIRGNQENVLTFYCQ

>P26045 peripheral membrane protein
MTSRLRALGGRINNIRTSELPKEKTRSEVICSIHFLDGVVQTFKVTKQDTGQVLLDMVHN
HLGVTEKEYFGLQHDDDSVDSPRWLEASKAIRKQLKGGFPCTLHFRVRFFIPDPNTLQQE
QTRHLYFLQLKMDICEGRLTCPLNSAVVLASYAVQSHFGDYNSSIHHPGYLSDSHFIPDQN
EDFLTKVESLHEQHSGLKQSEAESCYINIARTLDFYGVELHSGRDLHNLDLMIGIASAGVA
VYRKYICTSFYPWVNILKISFKRKKFFIHQRQKQAESREHIVAFNMLNYRSCKNLWKSCVE
HHTFFQAKKLLPQEKNVLSQYWTMGSRNTKKSVNNQYCKKVIGGMVWNPAMRRSLSV
EHLETKSLPSRSPPITPNWRSPRLRHEIRKPRHSSADNLANEMTYITETEDVFYTYKGSLAP
QDSDSEVSQNRSPHQESLSENNPAQSYLTQKSSSSVSPSSNAPGSCSPDGVDQQLLDDFHR
VTKGGSTEDASQYYCDKNDNGDSYLVLIRITPDEDGKFGFNLKGGVDQKMPLVVSRINPE
SPADTCIPKLNEGDQIVLINGRDISEHTHDQVVMFIKASRESHSRELALVIRRRAVRSFADF
KSEDELNQLFPEAIFPMCPEGGDTLEGSMAQLKKGLESGTVLIQFEQLYRKKPGLAITFAK
LPQNLDKNRYKDVLPYDTTRVLLQGNEDYINASYVNMEIPAANLVNKYIATQGPLPHTCA
QFWQVVWDQKLSLIVMLTTLTERGRTKCHQYWPDPPDVMNHGGFHIQCQSEDCTIAYVS
REMLVTNTQTGEEHTVTHLQYVAWPDHGVPDDSSDFLEFVNYVRSLRVDSEPVLVHCSA
GIGRTGVLVTMETAMCLTERNLPIYPLDIVRKMRDQRAMMVQTSSQYKFVCEAILRVYEE
GLVQMLDPS

>Q9NX63 lipid-anchor
MGGTTSTRRVTFEADENENITVVKGIRLSENVIDRMKESSPSGSKSQRYSGAYGASVSDEE
LKRRVAEELALEQAKKESEDQKRLKQAKELDRERAAANEQLTRAILRERICSEEERAKAK
HLARQLEEKDRVLKKQDAFYKEQLARLEERSSEFYRVTTEQYQKAAEEVEAKFKRYESH

PVCADLQAKILQCYRENTHQTLKCSALATQYMHCVNHAKQSMLEKGG

>O14745 peripheral membrane protein
MSADAAAGAPLPRLCCLEKGPNGYGFHLHGEKGKLGQYIRLVEPGSPAEKAGLLAGDRL
VEVNGENVEKETHQQVVSRIRAALNAVRLLVVDPETDEQLQKLGVQVREELLRAQEAPG
QAEPPAAAEVQGAGNENEPREADKSHPEQRELRPRLCTMKKGPSGYGFNLHSDKSKPGQ
FIRSVDPDSPAEASGLRAQDRIVEVNGVCMEGKQHGDVVSAIRAGGDETKLLVVDRETDE
FFKKCRVIPSQEHLNGPLPVPFTNGEIQKENSREALAEAALESPRPALVRSASSDTSEELNS
QDSPPKQDSTAPSSTSSSDPILDFNISLAMAKERAHQKRSSKRAPQMDWSKKNELFSNL

>P20648 multipass membrane protein
MGKAENYELYSVELGPGPGGDMAAKMSKKKKAGGGGGKRKEKLENMKKEMEINDHQ
LSVAELEQKYQTSATKGLSASLAAELLLRDGPNALRPPRGTPEYVKFARQLAGGLQCLM
WVAAAICLIAFAIQASEGDLTTDDNLYLAIALIAVVVVTGCFGYYQEFKSTNIIASFKNLVP
QQATVIRDGDKFQINADQLVVGDLVEMKGGDRVPADIRILAAQGCKVDNSSLTGESEPQT
RSPECTHESPLETRNIAFFSTMCLEGTVQGLVVNTGDRTIIGRIASLASGVENEKTPIAIEIEH
FVDIIAGLAILFGATFFIVAMCIGYTFLRAMVFFMAIVVAYVPEGLLATVTVCLSLTAKRLAS
KNCVVKNLEAVETLGSTSVICSDKTGTLTQNRMTVSHLWFDNHIHTADTTEDQSGQTFDQ
SSETWRALCRVLTLCNRAAFKSGQDAVPVPKRIVIGDASETALLKFSELTLGNAMGYRDRF
PKVCEIPFNSTNKFQLSIHTLEDPRDPRHLLVMKGAPERVLERCSSILIKGQELPLDEQWRE
AFQTAYLSLGGLGERVLGFCQLYLNEKDYPPGYAFDVEAMNFPSSGLCFAGLVSMIDPPRA
TVPDAVLKCRTAGIRVIMVTGDHPITAKAIAASVGIISEGSETVEDIAARLRVPVDQVNRKD
ARACVINGMQLKDMDPSELVEALRTHPEMVFARTSPQQKLVIVESCQRLGAIVAVTGDGV
NDSPALKKADIGVAMGIAGSDAAKNAADMILLDDNFASIVTGVEQGRLIFDNLKKSIAYTL
TKNIPELTPYLIYITVSVPLPLGCITILFIELCTDIFPSVSLAYEKAESDIMHLRPRNPKRDRLV
NEPLAAYSYFQIGAIQSFAGFTDYFTAMAQEGWFPLLCVGLRAQWEDHHLQDLQDSYGQ
EWTFGQRLYQQYTCYTVFFISIEVCQIADVLIRKTRRLSAFQQGFFRNKILVIAIVFQVCIGC
FLCYCPGMPNIFNFMPIRFQWWLVPLPYGILIFVYDEIRKLGVRCCPGSWWDQELYY

>P05981 single-pass type II membrane protein
MAQKEGGRTVPCCSRPKVAALTAGTLLLLTAIGAASWAIVAVLLRSDQEPLYPVQVSSADA
RLMVFDKTEGTWRLLCSSRSNARVAGLSCEEMGFLRALTHSELDVRTAGANGTSGFFCVD
EGRLPHTQRLLEVISVCDCPRGRFLAAICQDCGRRKLPVDRIVGGRDTSLGRWPWQVSLR
YDGAHLCGGSLLSGDWVLTAAHCFPERNRVLSRWRVFAGAVAQASPHGLQLGVQAVVYH
GGYLPFRDPNSEENSNDIALVHLSSPLPLTEYIQPVCLPAAGQALVDGKICTVTGWGNTQY
YGQQAGVLQEARVPIISNDVCNGADFYGNQIKPKMFCAGYPEGGIDACQGDSGGPFVCE
DSISRTPRWRLCGIVSWGTGCALAQKPGVYTKVSDFREWIFQAIKTHSEASGMVTQL

>O43914 single-pass type I membrane protein
MGGLEPCSRLLLLPLLLAVSGLRPVQAQAQSDCSCSTVSPGVLAGIVMGDLVLTVLIALAV
YFLGRLVPRGRGAAEAATRKQRITETESPYQELQGQRSDVYSDLNTQRPYYK

>Q9NX62 single-pass type II membrane protein
MAPMGIRLSPLGVAVFCLLGLGVLYHLYSGFLAGRFSLFGLGGEPGGGAAGPAAAADGGT

VDLREMLAVSVLAAVRGGDEVRRVRESNVLHEKSKGKTREGAEDKMTSGDVLSNRKMF
YLLKTAFPSVQINTEEHVDAADQEVILWDHKIPEDILKEVTTPKEVPAESVTVWIDPLDAT
QEYTEDLRKYVTTMVCVAVNGKPMLGVIHKPFSEYTAWAMVDGGSNVKARSSYNEKTP
RIVVSRSHSGMVKQVALQTFGNQTTIIPAGGAGYKVLALLDVPDKSQEKADLYIHVTYIK
KWDICAGNAILKALGGHMTTLSGEEISYTGSDGIEGGLLASIRMNHQALVRKLPDLEKTG
HK

>P61019 lipid-anchor
MAYAYLFKYIIIGDTGVGKSCLLLQFTDKRFQPVHDLTIGVEFGARMITIDGKQIKLQIWDT
AGQESFRSITRSYYRGAAGALLVYDITRRDTFNHLTTWLEDARQHSNSNMVIMLIGNKSD
LESRREVKKEEGEAFAREHGLIFMETSAKTASNVEEAFINTAKEIYEKIQEGVFDINNEANG
IKIGPQHAATNATHAGNQGGQQAGGGCC

>P23352 peripheral membrane protein
MVPGVPGAVLTLCLWLAASSGCLAAGPGAAAARRLDESLSAGSVQRARCASRCLSLQITR
ISAFFQHFQNNGSLVWCQNHKQCSKCLEPCKESGDLRKHQCQSFCEPLFPKKSYECLTSCE
FLKYILLVKQGDCPAPEKASGFAAACVESCEVDNECSGVKKCCSNGCGHTCQVPKTLYKG
VPLKPRKELRFTELQSGQLEVKWSSKFNISIEPVIYVVQRRWNYGIHPSEDDATHWQTVAQ
TTDERVQLTDIRPSRWYQFRVAAVNVHGTRGFTAPSKHFRSSKDPSAPPAPANLRLANSTV
NSDGSVTVTIVWDLPEEPDIPVHHYKVFWSWMVSSKSLVPTKKKRRKTTDGFQNSVILEK
LQPDCDYVVELQAITYWGQTRLKSAKVSLHFTSTHATNNKEQLVKTRKGGIQTQLPFQRR
RPTRPLEVGAPFYQDGQLQVKVYWKKTEDPTVNRYHVRWFPEACAHNRTTGSEASSGM
THENYIILQDLSFSCKYKVTVQPIRPKSHSKAEAVFFTTPPCSALKGKSHKPVGCLGEAGH
VLSKVLAKPENLSASFIVQDVNITGHFSWKMAKANLYQPMTGFQVTWAEVTTESRQNSL
PNSIISQSQILPSDHYVLTVPNLRPSTLYRLEVQVLTPGGEGPATIKTFRTPELPPSSAHRSHL
KHRHPHHYKPSPERY

>Q9HBT6 single-pass type I membrane protein
MWTSGRMSNAKNWLGLGMSLYFWGLMDLTTTVLSDTPTPQGELEALLSDKPQSHQRTK
RSWVWNQFFVLEEYTGTDPLYVGKLHSDMDRGDGSIKYILSGEGAGIVFTIDDTTGDIHAI
QRLDREERAQYTLRAQALDRRTGRPMEPESEFIIKIQDINDNEPKFLDGPYVATVPEMSPV
GTSVIQVTATDADDPTYGNSARVVYSILQGQPYFSVDSKTGVIRTALMNMDREAKEYYEV
IIQAKDMGGQLGGLAGTTTVNITLSDVNDNPPRFPQKHYQMSVLESAPISSTVGRVFAKDL
DEGINAEMKYTIVDGDGADAFDISTDPNFQVGIITVKKPLSFESKKSYTLKVEGANPHLEM
RFLNLGPFQDTTTVHISVEDVDEPPVFEPGFYFVEVPEDVAIGTTIQIISAKDPDVTNNSIRY
SIDRSSDPGRFFYVDITTGALMTARPLDREEFSWHNITVLAMEMNNPSQVGSVPVTIKVLD
VNDNAPEFPRFYEAFVCENAKAGQLIQTVSAVDQDDPRNGQHFYYSLAPEAANNPNFTIR
DNQDNTARILTRRSGFRQQEQSVFHLPILIADSGQPVLSSTGTLTIQVCSCDDDGHVMSCSP
EAYMLPVSLSRGALIAILACIFVLLVLVLLILSMRRHRKQPYIIDDEENIHENIVRYDDEGGG
EEDTEAFDIAAMWNPREAQAGAAPKTRQDMLPEIESLSRYVPQTCAVNSTVHSYVLAKLY
EADMDLWAPPFDSLQTYMFEGDGSVAGSLSSLQSATSDSEQSFDFLTDWGPRFRKLAELY
GASEGPAPLW

>Q9NYQ6 multipass membrane protein

MAPPPPPVLPVLLLLAAAAALPAMGLRAAAWEPRVPGGTRAFALRPGCTYAVGAACTPR
APRELLDVGRDGRLAGRRRVSGAGRPLPLQVRLVARSAPTALSRRLRARTHLPGCGARAR
LCGTGARLCGALCFPVPGGCAAAQHSALAAPTTLPACRCPPRPRPRCPGRPICLPPGGSVR
LRLLCALRRAAGAVRVGLALEAATAGTPSASPSPSPPLPPNLPEARAGPARRARRGTSGRG
SLKFPMPNYQVALFENEPAGTLILQLHAHYTIEGEEERVSYYMEGLFDERSRGYFRIDSAT
GAVSTDSVLDRETKETHVLRVKAVDYSTPPRSATTYITVLVKDTNDHSPVFEQSEYRERVR
ENLEVGYEVLTIRASDRDSPINANLRYRVLGGAWDVFQLNESSGVVSTRAVLDREEAAEY
QLLVEANDQGRNPGPLSATATVYIEVEDENDNYPQFSEQNYVVQVPEDVGLNTAVLRVQA
TDRDQGQNAAIHYSILSGNVAGQFYLHSLSGILDVINPLDFEDVQKYSLSIKAQDGGRPPLI
NSSGVVSVQVLDVNDNEPIFVSSPFQATVLENVPLGYPVVHIQAVDADSGENARLHYRLV
DTASTFLGGGSAGPKNPAPTPDFPFQIHNSSGWITVCAELDREEVEHYSFGVEAVDHGSPP
MSSSTSVSITVLDVNDNDPVFTQPTYELRLNEDAAVGSSVLTLQARDRDANSVITYQLTGG
NTRNRFALSSQRGGGLITLALPLDYKQEQQYVLAVTASDGTRSHTAHVLINVTDANTHRP
VFQSSHYTVSVSEDRPVGTSIATLSANDEDTGENARITYVIQDPVPQFRIDPDSGTMYTMM
ELDYENQVAYTLTIMAQDNGIPQKSDTTTLEILILDANDNAPQFLWDFYQGSIFEDAPPSTS
ILQVSATDRDSGPNGRLLYTFQGGDDGDGDFYIEPTSGVIRTQRRLDRENVAVYNLWALAV
DRGSPTPLSASVEIQVTILDINDNAPMFEKDELELFVEENNPVGSVVAKIRANDPDEGPNA
QIMYQIVEGDMRHFFQLDLLNGDLRAMVELDFEVRREYVLVVQATSAPLVSRATVHILLV
DQNDNPPVLPDFQILFNNYVTNKSNSFPTGVIGCIPAHDPDVSDSLNYTFVQGNELRLLLL
DPATGELQLSRDLDNNRPLEALMEVSVSDGIHSVTAFCTLRVTIITDDMLTNSITVRLENMS
QEKFLSPLLALFVEGVAAVLSTTKDDVFVFNVQNDTDVSSNILNVTFSALLPGGVRGQFFP
SEDLQEQIYLNRTLLTTISTQRVLPFDDNICLREPCENYMKCVSVLRFDSSAPFLSSTTVLFR
PIHPINGLRCRCPPGFTGDYCETEIDLCYSDPCGANGRCRSREGGYTCECFEDFTGEHCEV
DARSGRCANGVCKNGGTCVNLLIGGFHCVCPPGEYERPYCEVTTRSFPPQSFVTFRGLRQ
RFHFTISLTFATQERNGLLLYNGRFNEKHDFIALEIVDEQVQLTFSAGETTTTVAPKVPSGVS
DGRWHSVQVQYYNKPNIGHLGLPHGPSGEKMAVVTVDDCDTTMAVRFGKDIGNYSCAA
QGTQTGSKKSLDLTGPLLLGGVPNLPEDFPVHNRQFVGCMRNLSVDGKNVDMAGFIANN
GTREGCAARRNFCDGRRCQNGGTCVNRWNMYLCECPLRFGGKNCEQAMPHPQLFSGES
VVSWSDLNIIISVPWYLGLMFRTRKEDSVLMEATSGGPTSFRLQILNNYLQFEVSHGPSDV
ESVMLSGLRVTDGEWHHLLIELKNVKEDSEMKHLVTMTLDYGMDQNKADIGGMLPGLT
VRSVVVGGASEDKVSVRRGFRGCMQGVRMGGTPTNVATLNMNNALKVRVKDGCDVDD
PCTSSPCPPNSRCHDAWEDYSCVCDKGYLGINCVDACHLNPCENMGACVRSPGSPQGYV
CECGPSHYGPYCENKLDLPCPRGWWGNPVCGPCHCAVSKGFDPDCNKTNGQCQCKENY
YKLLAQDTCLPCDCFPHGSHSRTCDMATGQCACKPGVIGRQCNRCDNPFAEVTTLGCEVI
YNGCPKAFEAGIWWPQTKFGQPAAVPCPKGSVGNAVRHCSGEKGWLPPELFNCTTISFVD
LRAMNEKLSRNETQVDGARALQLVRALRSATQHTGTLFGNDVRTAYQLLGHVLQHESW
QQGFDLAATQDADFHEDVIHSGSALLAPATRAAWEQIQRSEGGTAQLLRRLEGYFSNVAR
NVRRTYLRPFVIVTANMILAVDIFDKFNFTGARVPRFDTIHEEFPRELESSVSFPADFFRPPE
EKEGPLLRPAGRRTTPQTTRPGPGTEREAPISRRRRHPDDAGQFAVALVIIYRTLGQLLPERY
DPDRRSLRLPHRPIINTPMVSTLVYSEGAPLPRPLERPVLVEFALLEVEERTKPVCVFWNHS
LAVGGTGGWSARGCELLSRNRTHVACQCSHTASFAVLMDISRRENGEVLPLKIVTYAAVSL
SLAALLVAFVLLSLVRMLRSNLHSIHKHLAVALFLSQLVFVIGINQTENPFLCTVVAILLHYI
YMSTFAWTLVESLHVYRMLTEVRNIDTGPMRFYYVVGWGIPAIVTGLAVGLDPQGYGNP
DFCWLSLQDTLIWSFAGPIGAVIIINTVTSVLSAKVSCQRKHHYYGKKGIVSLLRTAFLLLL

LISATWLLGLLAVNRDALSFHYLFAIFSGLQGPFVLLFHCVLNQEVRKHLKGVLGGRKLH
LEDSATTRATLLTRSLNCNTTFGDGPDMLRTDLGESTASLDSIVRDEGIQKLGVSSGLVRGS
HGEPDASLMPRSCKDPPGHDSDSDSELSLDEQSSSYASSHSSDSEDDGVGAEEKWDPARG
AVHSTPKGDAVANHVPAGWPDQSLAESDSEDPSGKPRLKVETKVSVELHREEQGSHRGE
YPPDQESGGAARLASSQPPEQRKGILKNKVTYPPPLTLTEQTLKGRLREKLADCEQSPTSS
RTSSLGSGGPDCAITVKSPGREPGRDHLNGVAMNVRTGSAQADGSDSEKP

>O14579 peripheral membrane protein
MAPPAPGPASGGSGEVDELFDVKNAFYIGSYQQCINEAQRVKLSSPERDVERDVFLYRAYL
AQRKFGVVLDEIKPSSAPELQAVRMFADYLAHESRRDSIVAELDREMSRSVDVTNTTFLL
MAASIYLHDQNPDAALRALHQGDSLECTAMTVQILLKLDRLDLARKELKRMQDLDEDAT
LTQLATAWVSLATGGEKLQDAYYIFQEMADKCSPTLLLLNGQAACHMAQGRWEAAEGLL
QEALDKDSGYPETLVNLIVLSQHLGKPPEVTNRYLSQLKDAHRSHPFIKEYQAKENDFDRL
VLQYAPSA

>Q9UL62 multipass membrane protein
MAQLYYKKVNYSPYRDRIPLQIVRAETELSAEEKAFLNAVEKGDYATVKQALQEAEIYYN
VNINCMDPLGRSALLIAIENENLEIMELLLNHSVYVGDALLYAIRKEVVGAVELLLSYRRPS
GEKQVPTLMMDTQFSEFTPDITPIMLAAHTNNYEIIKLLVQKRVTIPRPHQIRCNCVECVSS
SEVDSLRHSRSRLNIYKALASPSLIALSSEDPILTAFRLGWELKELSKVENEFKAEYEELSQ
QCKLFAKDLLDQARSSRELEIILNHRDDHSEELDPQKYHDLAKLKVAIKYHQKEFVAQPN
CQQLLATLWYDGFPGWRRKHWVVKLLTCMTIGFLFPMLSIAYLISPRSNLGLFIKKPFIKFI
CHTASYLTFLFMLLLASQHIVRTDLHVQGPPPTVVEWMILPWVLGFIWGEIKEMWDGGFT
EYIHDWWNLMDFAMNSLYLATISLKIVAYVKYNGSRPREEWEMWHPTLIAEALFAISNILS
SLRLISLFTANSHLGPLQISLGRMLLDILKFLFIYCLVLLAFANGLNQLYFYYETRAIDEPNN
CKGIRCEKQNNAFSTLFETLQSLFWSVFGLLNLYVTNVKARHEFTEFVGATMFGTYNVISL
VVLLNMLIAMMNNSYQLIADHADIEWKFARTKLWMSYFDEGGTLPPPFNIIPSPKSFLYLG
NWFNNTFCPKRDPDGRRRRRNLRSFTERNADSLIQNQHYQEVIRNLVKRYVAAMIRNSKT
HEGLTEENFKELKQDISSFRYEVLDLLGNRKHPRSFSTSSTELSQRDDNNDGSGGARAKSK
SVSFNLGCKKKTCHGPPLIRTMPRSSGAQGKSKAESSSKRSFMGPSLKKLGLLFSKFNGH
MSEPSSEPMYTISDGIVQQHCMWQDIRYSQMEKGKAEACSQSEINLSEVELGEVQGAAQS
SECPLACSSSLHCASSICSSNSKLLDSSEDVFETWGEACDLLMHKWGDGQEEQVTTRL

>A7E2Y1 peripheral membrane protein
MMDVSELGESARYLRQGYQEMTKVHTIPWDGKKRVWVPDEQDAYVEAEVKSEATGGR
VTVETKDQKVLMVREAELQPMNPPRFDLLEDMAMMTHLNEASVLHNLRQRYARWMIY
TYSGLFCVTINPYKWLPVYTASVVAAYKGKRRSDSPPHIYAVADNAYNDMLRNRDNQSM
LITGESGAGKTVNTKRVIQYFAIVAALGDGPGKKAQFLATKTGGTLEDQIIEANPAMEAFG
NAKTLRNDNSSRFGKFIRIHFGPSGKLASADIDSYLLEKSRVIFQLPGERSYHVYYQILSGR
KPELQDMLLLSMNPYDYHFCSQGVITVDNMNDGEELIATDHAMDILGFSVDEKCACYKI
VGALLHFGNMKFKQKQREEQAEADGTESADKAAYLMGVSSGDLLKGLLHPRVRVGNEY
VTKGQSVEQVVFAVGALAKATYDRLFRWLVSRINQTLDTKLPRQFFIGVLDIAGFEIFEFNS
FEQLCINFTNEKLQQFFNQHMFVLEQEEYKREGIDWVFIDFGLDLQPCIDLIEKPLGILSILE
EECMFPKASDASFRAKLYDNHAGKSPNFQQPRPDKKRKYQAHFEVVHYAGVVPYSIVG

WLEKNKDPLNETVVPIFQKSQNRLLATLYENYAGSCSTEPPKSGVKEKRKKAASFQTVSQ
LHKENLNKLMTNLRATQPHFVRCIVPNENKTPGVMDAFLVLHQLRCNGVLEGIRICRQGF
PNRLLYTDFRQRYRILNPSAIPDDTFMDSRKATEKLLGSLDLDHTQYQFGHTKVFFKAGLL
GVLEELRDQRLAKVLTLLQARSRGRLMRLEYQRLLGGRDALFTIQWNIRAFNAVKNWSW
MKLFFKMKPLLRSAQAEEELAALRAELRGLRGALAAAEAKRQELEETHVSITQEKNDLA
LQLQAEQDNLADAEERCHLLIKSKVQLEGKVKELSERLEDEEEVNADLAARRRKLEDEC
TELKKDIDDLKLTLAKAEKEKQATENKVKNLTEEMAALDESVARLTKEKKALQEAHQQA
LGDLQAEEDRVSALTKAKLRLEQQVEDLECSLEQEKKLRMDTERAKRKLEGDLKLTQES
VADAAQDKQQLEEKLKKKDSELSQLSLRVEDEQLLGAQMQKKIKELQARAEELEEELEA
ERAARARVEKQRAEAARELEELSERLEEAGGASAGQREGCRKREAELGRLRRELEEAAL
RHEATVAALRRKQAEGAAELGEQVDSLQRVRQKLEKEKSELRMEVDDLAANVETLTRAK
ASAEKLCRTYEDQLSEAKIKVEELQRQLADASTQRGRLQTESGELSRLLEEKECLISQLSR
GKALAAQSLEELRRQLEEESKAKSALAHAVQALRHDCDLLREQHEEEAEAQAELQRLLS
KANAEVAQWRSKYEADAIQRTEELEEAKKKLALRLQEAEEGVEAANAKCSSLEKAKLRL
QTESEDVTLELERATSAAAALDKKQRHLERALEERRRQEEEMQRELEAAQRESRGLGTEL
FRLRHGHEEALEALETLKRENKNLQEEISDLTDQVSLSGKSIQELEKTKKALEGEKSEIQA
ALEEAEGALELEETKTLRIQLELSQVKAEVDRKLAEKDEECANLRRNHQRAVESLQASLD
AETRARNEALRLKKKMEGDLNDLELQLGHATRQATEAQAATRLMQAQLKEEQAGRDEE
QRLAAELHEQAQALERRASLLAAELEELRAALEQGERSRRLAEQELLEATERLNLLHSQN
TGLLNQKKKLEADLAQLSGEVEEAAQERREAEEKAKKAITDAAMMAEELKKEQDTSAH
LERMKKTLEQTVRELQARLEEAEQAALRGGKKQVQKLEAKVRELEAELDAEQKKHAEA
LKGVRKHERRVKELAYQAEEDRKNLARMQDLVDKLQSKVKSYKRQFEEAEQQANTNLA
KYRKAQHELDDAEERADMAETQANKLRARTRDALGPKHKE

>O15554 multipass membrane protein
MGGDLVLGLGALRRRKRLLEQEKSLAGWALVLAGTGIGLMVLHAEMLWFGGCSWALYL
FLVKCTISISTFLLLCLIVAFHAKEVQLFMTDNGLRDWRVALTGRQAAQIVLELVVCGLHPA
PVRGPPCVQDLGAPLTSPQPWPGFLGQGEALLSLAMLLRLYLVPRAVLLRSGVLLNASYR
SIGALNQVRFRHWFVAKLYMNTHPGRLLLGLTLGLWLTTAWVLSVAERQAVNATGHLSDT
LWLIPITFLTIGYGDVVPGTMWGKIVCLCTGVMGVCCTALLVAVVARKLEFNKAEKHVHN
FMMDIQYTKEMKESAARVLQEAWMFYKHTRRKESHAARRHQRKLLAAINAFRQVRLKH
RKLREQVNSMVDISKMHMILYDLQQNLSSSHRALEKQIDTLAGKLDALTELLSTALGPRQ
LPEPSQQSK

>Q16478 multipass membrane protein
MPAELLLLLIVAFASPSCQVLSSLRMAAILDDQTVCGRGERLALALAREQINGIIEVPAKAR
VEVDIFELQRDSQYETTDTMCQILPKGVVSVLGPSSSPASASTVSHICGEKEIPHIKVGPEET
PRLQYLRFASVSLYPSNEDVSLAVSRILKSFNYPSASLICAKAECLLRLEELVRGFLISKETL
SVRMLDDSRDPTPLLKEIRDDKVSTIIIDANASISHLILRKASELGMTSAFYKYILTTMDFPI
LHLDGIVEDSSNILGFSMFNTSHPFYPEFVRSLNMSWRENCEASTYLGPALSAALMFDAVH
VVVSAVRELNRSQEIGVKPLACTSANIWPHGTSLMNYLRMVEYDGLTGRVEFNSKGQRT
NYTLRILEKSRQGHREIGVWYSNRTLAMNATTLDINLSQTLANKTLVVTTILENPYVMRRP
NFQALSGNERFEGFCVDMLRELAELLRFRYRLRLVEDGLYGAPEPNGSWTGMVGELINR
KADLAVAAFTITAEREKVIDFSKPFMTLGISILYRVHMGRKPGYFSFLDPFSPAVWLFMLLA

YLAVSCVLFLAARLSPYEWYNPHPCLRARPHILENQYTLGNSLWFPVGGFMQQGSEIMPR
ALSTRCVSGVWWAFTLIIISSYTANLAAFLTVQRMEVPVESADDLADQTNIEYGTIHAGST
MTFFQNSRYQTYQRMWNYMQSKQPSVFVKSTEEGIARVLNSRYAFLLESTMNEYHRRLN
CNLTQIGGLLDTKGYGIGMPLGSPFRDEITLAILQLQENNRLEILKRKWWEGGRCPKEEDH
RAKGLGMENIGGIFIVLICGLIIAVFVAVMEFIWSTRRSAESEEVSVCQEMLQELRHAVSCR
KTSRSRRRRRPGGPSRALLSLRAVREMRLSNGKLYSAGAGGDAGSAHGGPQRLLDDPGPP
SGARPAAPTPCTHVRVCQECRRIQALRASGAGAPPRGLGVPAEATSPPRPRPGPAGPRELA
EHE

>Q11203 single-pass type II membrane protein
MGLLVFVRNLLLALCLFLVLGFLYYSAWKLHLLQWEEDSNSVVLSFDSAGQTLGSEYDRL
GFLLNLDSKLPAELATKYANFSEGACKPGYASALMTAIFPRFSKPAPMFLDDSFRKWARIR
EFVPPFGIKGQDNLIKAILSVTKEYRLTPALDSLRCRRCIIVGNGGVLANKSLGSRIDDYDIV
VRLNSAPVKGFEKDVGSKTTLRITYPEGAMQRPEQYERDSLFVLAGFKWQDFKWLKYIV
YKERVSASDGFWKSVATRVPKEPPEIRILNPYFIQEAAFTLIGLPFNNGLMGRGNIPTLGSVA
VTMALHGCDEVAVAGFGYDMSTPNAPLHYYETVRMAAIKESWTHNIQREKEFLRKLVKA
RVITDLSSGI

>P56696 multipass membrane protein
MAEAPPRRLGLGPPPGDAPRAELVALTAVQSEQGEAGGGGSPRRLGLLGSPLPPGAPLPGP
GSGSGSACGQRSSAAHKRYRRLQNWVYNVLERPRGWAFVYHVFIFLLVFSCLVLSVLSTI
QEHQELANECLLILEFVMIVVFGLEYIVRVWSAGCCCRYRGWQGRFRFARKPFCVIDFIVF
VASVAVIAAGTQGNIFATSALRSMRFLQILRMVRMDRRGGTWKLLGSVVYAHSKELITAW
YIGFLVLIFASFLVYLAEKDANSDFSSYADSLWWGTITLTTIGYGDKTPHTWLGRVLAAGF
ALLGISFFALPAGILGSGFALKVQEQHRQKHFEKRRMPAANLIQAAWRLYSTDMSRAYLTA
TWYYYDSILPSFRELALLFEHVQRARNGGLRPLEVRRAPVPDGAPSRYPPVATCHRPGSTS
FCPGESSRMGIKDRIRMGSSQRRTGPSKQHLAPPTMPTSPSSEQVGEATSPTKVQKSWSFN
DRTRFRASLRLKPRTSAEDAPSEEVAEEKSYQCELTVDDIMPAVKTVIRSIRILKFLVAKRKF
KETLRPYDVKDVIEQYSAGHLDMLGRIKSLQTRVDQIVGRGPGDRKAREKGDKGPSDAE
VVDEISMMGRVVKVEKQVQSIEHKLDLLLGFYSRCLRSGTSASLGAVQVPLFDPDITSDY
HSPVDHEDISVSAQTLSISRSVSTNMD

>O75882 single-pass type I membrane protein
MVAAAAATEARLRRRTAATAALAGRSGGPHWDWDVTRAGRPGLGAGLRLPRLLSPPLRP
RLLLLLLLLSPPLLLLLLPCEAEAAAAAAAVSGSAAAEAKECDRPCVNGGRCNPGTGQCV
CPAGWVGEQCQHCGGRFRLTGSSGFVTDGPGNYKYKTKCTWLIEGQPNRIMRLRFNHFA
TECSWDHLYVYDGDSIYAPLVAAFSGLIVPERDGNETVPEVVATSGYALLHFFSDAAYNLT
GFNITYSFDMCPNNCSGRGECKISNSSDTVECECSENWKGEACDIPHCTDNCGFPHRGICN
SSDVRGCSCFSDWQGPGCSVPVPANQSFWTREEYSNLKLPRASHKAVVNGNIMWVVGG
YMFNHSDYNMVLAYDLASREWLPLNRSVNNVVVRYGHSLALYKDKIYMYGGKIDSTGN
VTNELRVFHIHNESWVLLTPKAKEQYAVVGHSAHIVTLKNGRVVMLVIFGHCPLYGYISNV
QEYDLDKNTWSILHTQGALVQGGYGHSSVYDHRTRALYVHGGYKAFSANKYRLADDLY
RYDVDTQMWTILKDSRFFRYLHTAVIVSGTMLVFGGNTHNDTSMSHGAKCFSSDFMAYDI
ACDRWSVLPRPDLHHDVNRFGHSAVLHNSTMYVFGGFNSLLLSDILVFTSEQCDAHRSEA

ACLAAGPGIRCVWNTGSSQCISWALATDEQEEKLKSECFSKRTLDHDRCDQHTDCYSCTA
NTNDCHWCNDHCVPRNHSCSEGQISIFRYENCPKDNPMYYCNKKTSCRSCALDQNCQW
EPRNQECIALPENICGIGWHLVGNSCLKITTAKENYDNAKLFCRNHNALLASLTTQKKVEF
VLKQLRIMQSSQSMSKLTLTPWVGLRKINVSYWCWEDMSPFTNSLLQWMPSEPSDAGFC
GILSEPSTRGLKAATCINPLNGSVCERPANHSAKQCRTPCALRTACGDCTSGSSECMWCSN
MKQCVDSNAYVASFPFGQCMEWYTMSTCPPENCSGYCTCSHCLEQPGCGWCTDPSNTG
KGKCIEGSYKGPVKMPSQAPTGNFYPQPLLNSSMCLEDSRYNWSFIHCPACQCNGHSKCI
NQSICEKCENLTTGKHCETCISGFYGDPTNGGKCQPCKCNGHASLCNTNTGKCFCTTKGV
KGDECQLCEVENRYQGNPLRGTCYYTLLIDYQFTFSLSQEDDRYYTAINFVATPDEQNRDL
DMFINASKNFNLNITWAASFSAGTQAGEEMPVVSKTNIKEYKDSFSNEKFDFRNHPNITFF
VYVSNFTWPIKIQIAFSQHSNFMDLVQFFVTFFSCFLSLLLVAAVVWKIKQSCWASRRREQ
LLREMQQMASRPFASVNVALETDEEPPDLIGGSIKTVPKPIALEPCFGNKAAVLSVFVRLPR
GLGGIPPPGQSGLAVASALVDISQQMPIVYKEKSGAVRNRKQQPPAQPGTCI

>O43374 peripheral membrane protein
MAKRSSLYIRIVEGKNLPAKDITGSSDPYCIVKVDNEPIIRTATVWKTLCPFWGEEYQVHLP
PTFHAVAFYVMDEDALSRDDVIGKVCLTRDTIASHPKGFSGWAHLTEVDPDEEVQGEIHL
RLEVWPGARACRLRCSVLEARDLAPKDRNGTSDPFVRVRYKGRTRETSIVKKSCYPRWN
ETFEFELQEGAMEALCVEAWDWDLVSRNDFLGKVVIDVQRLRVVQQEEGWFRLQPDQS
KSRRHDEGNLGSLQLEVRLRDETVLPSSYYQPLVHLLCHEVKLGMQGPGQLIPLIEETTST
ECRQDVATNLLKLFLGQGLAKDFLDLLFQLELSRTSETNTLFRSNSLASKSMESFLKVAGM
QYLHGVLGPIINKVFEEKKYVELDPSKVEVKDVGCSGLHRPQTEAEVLEQSAQTLRAHLG
ALLSALSRSVRACPAVVRATFRQLFRRVRERFPGAQHENVPFIAVTSFLCLRFFSPAIMSPKL
FHLRERHADARTSRTLLLLAKAVQNVGNMDTPASRAKEAWMEPLQPTVRQGVAQLKDFI
TKLVDIEEKDELDLQRTLSLQAPPVKEGPLFIHRTKGKGPLMSSSFKKLYFSLTTEALSFAK
TPSSKKSALIKLANIRAAEKVEEKSFGGSHVMQVIYTDDAGRPQTAYLQCKCVNELNQWL
SALRKVSINNTGLLGSYHPGVFRGDKWSCCHQKEKTGQGCDKTRSRVTLQEWNDPLDH
DLEAQLIYRHLLGVEAMLWERHRELSGGAEAGTVPTSPGKVPEDSLARLLRVLQDLREA
HSSSPAGSPPSEPNCLLELQT

>O95049 peripheral membrane protein
MEELTIWEQHTATLSKDPRRGFGIAISGGRDRPGGSMVVSDVVPGGPAEGRLQTGDHIVM
VNGVSMENATSAFAIQILKTCTKMANITVKRPRRIHLPATKASPSSPGRQDSDEDDGPQRV
EEVDQGRGYDGDSSSGSGRSWDERSRRPRPGRRGRAGSHGRRSPGGGSEANGLALVSGF
KRLPRQDVQMKPVKSVLVKRRDSEEFGVKLGSQIFIKHITDSGLAARHRGLQEGDLILQIN
GVSSQNLSLNDTRRLIEKSEGKLSLLVLRDRGQFLVNIPPAVSDSDSSPLEEGVTMADEMSS
PPADISDLASELSQAPPSHIPPPPRHAQRSPEASQTDSPVESPRLRRESSVDSRTISEPDEQRS
ELPRESSYDIYRVPSSQSMEDRGYSPDTRVVRFLKGKSIGLRLAGGNDVGIFVSGVQAGSP
ADGQGIQEGDQILQVNDVPFQNLTREEAVQFLLGLPPGEEMELVTQRKQDIFWKMVQSRV
GDSFYIRTHFELEPSPPSGLGFTRGDVFHVLDTLHPGPGQSHARGGHWLAVRMGRDLREQ
ERGIIPNQSRAEQLASLEAAQRAVGVGPGSSAGSNARAEFWRLRGLRRGAKKTTQRSRED
LSALTRQGRYPPYERVVLREASFKRPVVILGPVADIAMQKLTAEMPDQFEIAETVSRTDSPS
KIIKLDTVRVIAEKDKHALLDVTPSAIERLNYVQYYPIVVFFIPESRPALKALRQWLAPASR
RSTRRLYAQAQKLRKHSSHLFTATIPLNGTSDTWYQELKAIIREQQTRPIWTAEDQLDGSLE

DNLDLPHHGLADSSADLSCDSRVNSDYETDGEGGAYTDGEGYTDGEGGPYTDVDDEPPA
PALARSSEPVQADESQSPRDRGRISAHQGAQVDSRHPQGQWRQDSMRTYEREALKKKFM
RVHDAESSDEDGYDWGPATDL

>P25874 multipass membrane protein
MGGLTASDVHPTLGVQLFSAGIAACLADVITFPLDTAKVRLQVQGECPTSSVIRYKGVLGT
ITAVVKTEGRMKLYSGLPAGLQRQISSASLRIGLYDTVQEFLTAGKETAPSLGSKILAGLTTG
GVAVFIGQPTEVVKVRLQAQSHLHGIKPRYTGTYNAYRIIATTEGLTGLWKGTTPNLMRSV
IINCTELVTYDLMKEAFVKNNILADDVPCHLVSALIAGFCATAMSSPVDVVKTRFINSPPGQ
YKSVPNCAMKVFTNEGPTAFFKGLVPSFLRLGSWNVIMFVCFEQLKRELSKSRQTMDCAT

>P38570 single-pass type I membrane protein
MWLFHTLLCIASLALLAAFNVDVARPWLTPKGGAPFVLSSLLHQDPSTNQTWLLVTSPRT
KRTPGPLHRCSLVQDEILCHPVEHVPIPKGRHRGVTVVRSHHGVLICIQVLVRRPHSLSSEL
TGTCSLLGPDLRPQAQANFFDLENLLDPDARVDTGDCYSNKEGGGEDDVNTARQRRALE
KEEEEDKEEEEDEEEEEAGTEIAIILDGSGSIDPPDFQRAKDFISNMMRNFYEKCFECNFAL
VQYGGVIQTEFDLRDSQDVMASLARVQNITQVGSVTKTASAMQHVLDSIFTSSHGSRRKA
SKVMVVLTDGGIFEDPLNLTTVINSPKMQGVERFAIGVGEEFKSARTARELNLIASDPDETH
AFKVTNYMALDGLLSKLRYNIISMEGTVGDALHYQLAQIGFSAQILDERQVLLGAVGAFD
WSGGALLYDTRSRRGRFLNQTAAAAADAEAAQYSYLGYAVAVLHKTCSLSYIAGAPRYK
HHGAVFELQKEGREASFLPVLEGEQMGSYFGSELCPVDIDMDGSTDFLLVAAPFYHVHGE
EGRVYVYRLSEQDGSFSLARILSGHPGFTNARFGFAMAAMGDLSQDKLTDVAIGAPLEGF
GADDGASFGSVYIYNGHWDGLSASPSQRIRASTVAPGLQYFGMSMAGGFDISGDGLADIT
VGTLGQAVVFRSRPVVRLKVSMAFTPSALPIGFNGVVNVRLCFEISSVTTASESGLREALL
NFTLDVDVGKQRRRLQCSDVRSCLGCLREWSSGSQLCEDLLLMPTEGELCEEDCFSNAS
VKVSYQLQTPEGQTDHPQPILDRYTEPFAIFQLPYEKACKNKLFCVAELQLATTVSQQELV
VGLTKELTLNINLTNSGEDSYMTSMALNYPRNLQLKRMQKPPSPNIQCDDPQPVASVLIM
NCRIGHPVLKRSSAHVSVVWQLEENAFPNRTADITVTVTNSNERRSLANETHTLQFRHGF
VAVLSKPSIMYVNTGQGLSHHKEFLFHVHGENLFGAEYQLQICVPTKLRGLQVVAVKKLT
RTQASTVCTWSQERACAYSSVQHVEEWHSVSCVIASDKENVTVAAEISWDHSEELLKDV
TELQILGEISFNKSLYEGLNAENHRTKITVVFLKDEKYHSLPIIIKGSVGGLLVLIVILVILFKC
GFFKRKYQQLNLESIRKAQLKSENLLEEEN

>Q9Y2P5 multipass membrane protein
MGVRQQLALLLLLLLLLWGLGQPVWPVAVALTLRWLLGDPTCCVLLGLAMLARPWLGP
WVPHGLSLAAAALALTLLPARLPPGLRWLPADVIFLAKILHLGLKIRGCLSRQPPDTFVDA
FERRARAQPGRALLVWTGPGAGSVTFGELDARACQAAWALKAELGDPASLCAGEPTALL
VLASQAVPALCMWLGLAKLGCPTAWINPHGRGMPLAHSVLSSGARVLVVDPDLRESLEEI
LPKLQAENIRCFYLSHTSPTPGVGALGAALDAAPSHPVPADLRAGITWRSPALFIYTSGTTG
LPKPAILTHERVLQMSKMLSLSGATADDVVYTVLPLYHVMGLVVGILGCLDLGATCVLAP
KFSTSCFWDDCRQHGVTVILYVGELLRYLCNIPQQPEDRTHTVRLAMGNGLRADVWETF
QQRFGPIRIWEVYGSTEGNMGLVNYVGRCGALGKMSCLLRMLSPFELVQFDMEAAEPVR
DNQGFCIPVGLGEPGLLLTKVVSQQPFVGYRGPRELSERKLVRNVRQSGDVYYNTGDVL
AMDREGFLYFRDRLGDTFRWKGENVSTHEVEGVLSQVDFLQQVNVYGVCVPGCEGKVG

MAAVQLAPGQTFDGEKLYQHVRAWLPAYATPHFIRIQDAMEVTSTFKLMKTRLVREGFNV
GIVVDPLFVLDNRAQSFRPLTAEMYQAVCEGTWRL

>Q8IZY2 multipass membrane protein
MAFWTQLMLLLWKNFMYRRRQPVQLLVELLWPLFLFFILVAVRHSHPPLEHHECHFPNKP
LPSAGTVPWLQGLICNVNNTCFPQLTPGEEPGRLSNFNDSLVSRLLADARTVLGGASAHRT
LAGLGKLIATLRAARSTAQPQPTKQSPLEPPMLDVAELLTSLLRTESLGLALGQAQEPLHSL
LEAAEDLAQELLALRSLVELRALLQRPRGTSGPLELLSEALCSVRGPSSTVGPSLNWYEAS
DLMELVGQEPESALPDSSLSPACSELIGALDSHPLSRLLWRRLKPLILGKLLFAPDTPFTRKL
MAQVNRTFEELTLLRDVREVWEMLGPRIFTFMNDSSNVAMLQRLLQMQDEGRRQPRPG
GRDHMEALRSFLDPGSGGYSWQDAHADVGHLVGTLGRVTECLSLDKLEAAPSEAALVSR
ALQLLAEHRFWAGVVFLGPEDSSDPTEHPTPDLGPGHVRIKIRMDIDVVTRTNKIRDRFW
DPGPAADPLTDLRYVWGGFVYLQDLVERAAVRVLSGANPRAGLYLQQMPYPCYVDDVFL
RVLSRSLPLFLTLAWIYSVTLTVKAVVREKETRLRDTMRAMGLSRAVLWLGWFLSCLGPF
LLSAALLVLVLKLGDILPYSHPGVVFLFLAAFAVATVTQSFLLSAFFSRANLAAACGGLAYF
SLYLPYVLCVAWRDRLPAGGRVAASLLSPVAFGFGCESLALLEEQGEGAQWHNVGTRPTA
DVFSLAQVSGLLLLDAALYGLATWYLEAVCPGQYGIPEPWNFPFRRSYWCGPRPPKSPAP
CPTPLDPKVLVEEAPPGLSPGVSVRSLEKRFPGSPQPALRGLSLDFYQGHITAFLGHNGAG
KTTTLSILSGLFPPSGGSAFILGHDVRSSMAAIRPHLGVCPQYNVLFDMLTVDEHVWFYGR
LKGLSAAVVGPEQDRLLQDVGLVSKQSVQTRHLSGGMQRKLSVAIAFVGGSQVVILDEPT
AGVDPASRRGIWELLLKYREGRTLILSTHHLDEAELLGDRVAVVAGGRLCCCGSPLFLRRH
LGSGYYLTLVKARLPLTTNEKADTDMEGSVDTRQEKKNGSQGSRVGTPQLLALVQHWVP
GARLVEELPHELVLVLPYTGAHDGSFATLFRELDTRLAELRLTGYGISDTSLEEIFLKVVEE
CAADTDMEDGSCGQHLCTGIAGLDVTLRLKMPPQETALENGEPAGSAPETDQGSGPDAV
GRVQGWALTRQQLQALLLKRFLLARRSRRGLFAQIVLPALFVGLALVFSLIVPPFGHYPAL
RLSPTMYGAQVSFFSEDAPGDPGRARLLEALLQEAGLEEPPVQHSSHRFSAPEVPAEVAK
VLASGNWTPESPSPACQCSRPGARRLLPDCPAAAGGPPPPQAVTGSGEVVQNLTGRNLSDF
LVKTYPRLVRQGLKTKKWVNEVRYGGFSLGGRDPGLPSGQELGRSVEELWALLSPLPGG
ALDRVLKNLTAWAHSLDAQDSLKIWFNNKGWHSMVAFVNRASNAILRAHLPPGPARHAH
SITTLNHPLNLTKEQLSEGALMASSVDVLVSICVVFAMSFVPASFTLVLIEERVTRAKHLQL
MGGLSPTLYWLGNFLWDMCNYLVPACIVVLIFLAFQQRAYVAPANLPALLLLLLLYGWSIT
PLMYPASFFFSVPSTAYVVLTCINLFIGINGSMATFVLELFSDQKLQEVSRILKQVFLIFPHFC
LGRGLIDMVRNQAMADAFERLGDRQFQSPLRWEVVGKNLLAMVIQGPLFLLFTLLLQHR
SQLLPQPRVRSLPLLGEEDEDVARERERVVQGATQGDVLVLRNLTKVYRGQRMPAVDRLC
LGIPPGECFGLLGVNGAGKTSTFRMVTGDTLASRGEAVLAGHSVAREPSAAHLSMGYCPQ
SDAIFELLTGREHLELLARLRGVPEAQVAQTAGSGLARLGLSWYADRPAGTYSGGNKRKL
ATALALVGDPAVVFLDEPTTGMDPSARRFLWNSLLAVVREGRSVMLTSHSMEECEALCSR
LAIMVNGRFRCLGSPQHLKGRFAAGHTLTLRVPAARSQPAAAFVAAEFPGAELREAHGGR
LRFQLPPGGRCALARVFGELAVHGAEHGVEDFSVSQTMLEEVFLYFSKDQGKDEDTEEQK
EAGVGVDPAPGLQHPKRVSQFLDDPSTAETVL

>O95755 lipid-anchor
MVIAGASWMLGRAAASPTQTPPTTSTIRVARRSRVALVAMVIAAAGSGGPGRAEPQLSQPS
LDCGRMRSSLTPLGPPVSRDRVIASFPKWYTPEACLQLREHFHGQVSAACQRRNTGTVGL

KLSKVVVVGDLYVGKTSLIHRFCKNVFDRDYKATIGVDFEIERFEIAGIPYSLQIWDTAGQ
EKFKCIASAYYRGAQVIITAFDLTDVQTLEHTRQWLEDALRENEAGSCFIFLVGTKKDLLS
GAACEQAEADAVHLAREMQAEYWSVSAKTGENVKAFFSRVAALAFEQSVLQDLERQSS
ARLQVGNGDLIQMEGSPPETQESKRPSSLGCC

>Q04759 peripheral membrane protein
MSPFLRIGLSNFDCGSCQSCQGEAVNPYCAVLVKEYVESENGQMYIQKKPTMYPPWDSTF
DAHINKGRVMQIIVKGKNVDLISETTVELYSLAERCRKNNGKTEIWLELKPQGRMLMNAR
YFLEMSDTKDMNEFETEGFFALHQRRGAIKQAKVHHVKCHEFTATFFPQPTFCSVCHEFV
WGLNKQGYQCRQCNAAIHKKCIDKVIAKCTGSAINSRETMFHKERFKIDMPHRFKVYNY
KSPTFCEHCGTLLWGLARQGLKCDACGMNVHHRCQTKVANLCGINQKLMAEALAMIES
TQQARCLRDTEQIFREGPVEIGLPCSIKNEARPPCLPTPGKREPQGISWESPLDEVDKMCHL
PEPELNKERPSLQIKLKIEDFILHKMLGKGSFGKVFLAEFKKTNQFFAIKALKKDVVLMDD
DVECTMVEKRVLSLAWEHPFLTHMFCTFQTKENLFFVMEYLNGGDLMYHIQSCHKFDLS
RATFYAAEIILGLQFLHSKGIVYRDLKLDNILLDKDGHIKIADFGMCKENMLGDAKTNTFC
GTPDYIAPEILLGQKYNHSVDWWSFGVLLYEMLIGQSPFHGQDEEELFHSIRMDNPFYPR
WLEKEAKDLLVKLFVREPEKRLGVRGDIRQHPLFREINWEELERKEIDPPFRPKVKSPFDC
SNFDKEFLNEKPRLSFADRALINSMDQNMFRNFSFMNPGMERLIS

>P98153 single-pass type I membrane protein
MVPKADSGAFLLLFLLVLTVTEPLRPELRCNPGQFACRSGTIQCIPLPWQCDGWATCEDES
DEANCPEVTGEVRPHHGKEAVDPRQGRARGGDPSHFHAVNVAQPVRFSSFLGKCPTGWH
HYEGTASCYRVYLSGENYWDAAQTCQRLNGSLATFSTDQELRFVLAQEWDQPERSFGW
KDQRKLWVGYQYVITGRNRSLEGRWEVAFKGSSEVFLPPDPIFASAMSENDNVFCAQLQC
FHFPTLRHHDLHSWHAESCYEKSSFLCKRSQTCVDIKDNVVDEGFYFTPKGDDPCLSCTC
HGGEPEMCVAALCERPQGCQQYRKDPKECCKFMCLDPDGNSLFDSMASGMRLVVSCISS
FLILSLLLFMVHRLRQRRRERIESLIGANLHHFNLGRRIPGFDYGPDGFGTGLTPLHLSDDG
EGGTFHFHDPPPPYTAYKYPDIGQPDDPPPPYEASIHPDSVFYDPADDDAFEPVEVSLPAPG
DGGSEGALLRRLEQPLPTAGASLADLEDSADSSSALLVPPDPAQSGSTPAAEALPGGGRHS
RSSLNTVV

>P08247 multipass membrane protein
MLLLADMDVVNQLVAGGQFRVVKEPLGFVKVLQWVFAIFAFATCGSYSGELQLSVDCAN
KTESDLSIEVEFEYPFRLHQVYFDAPTCRGGTTKVFLVGDYSSSAEFFVTVAVFAFLYSMG
ALATYIFLQNKYRENNKGPMLDFLATAVFAFMWLVSSSAWAKGLSDVKMATDPENIIKEM
PVCRQTGNTCKELRDPVTSGLNTSVVFGFLNLVLWVGNLWFVFKETGWAAPFLRAPPGA
PEKQPAPGDAYGDAGYGQGPGGYGPQDSYGPQGGYQPDYGQPAGSGGSGYGPQGDYGQ
QGYGPQGAPTSFSNQM

>Q9NP61 peripheral membrane protein
MGDPSKQDILTIFKRLRSVPTNKVCFDCGAKNPSWASITYGVFLCIDCSGSHRSLGVHLSFI
RSTELDSNWSWFQLRCMQVGGNASASSFFHQHGCSTNDTNAKYNSRAAQLYREKIKSLA
SQATRKHGTDLWLDSCVVPPLSPPPKEEDFFASHVSPEVSDTAWASAIAEPSSLTSRPVETT
LENNEGGQEQGPSVEGLNVPTKATLEVSSIIKKKPNQAKKGLGAKKGSLGAQKLANTCFN

EIEKQAQAADKMKEQEDLAKVVSKEESIVSSLRLAYKDLEIQMKKDEKMNISGKKNVDS
DRLGMGFGNCRSVISHSVTSDMQTIEQESPIMAKPRKKYNDDSDDSYFTSSSSYFDEPVEL
RSSSFSSWDDSSDSYWKKETSKDTETVLKTTGYSDRPTARRKPDYEPVENTDEAQKKFGN
VKAISSDMYFGRQSQADYETRARLERLSASSSISSADLFEEPRKQPAGNYSLSSVLPNAPD
MAQFKQGVRSVAGKLSVFANGVVTSIQDRYGS

>O15399 multipass membrane protein
MRGAGGPRGPRGPAKMLLLLALACASPFPEEAPGPGGAGGPGGGLGGARPLNVALVFSGP
AYAAEAARLGPAVAAAVRSPGLDVRPVALVLNGSDPRSLVLQLCDLLSGLRVHGVVFEDD
SRAPAVAPILDFLSAQTSLPIVAVHGGAALVLTPKEKGSTFLQLGSSTEQQLQVIFEVLEEYD
WTSFVAVTTRAPGHRAFLSYIEVLTDGSLVGWEHRGALTLDPGAGEAVLSAQLRSVSAQIR
LLFCAREEAEPVFRAAEEAGLTGSGYVWFMVGPQLAGGGGSGAPGEPPLLPGGAPLPAGL
FAVRSAGWRDDLARRVAAGVAVVARGAQALLRDYGFLPELGHDCRAQNRTHRGESLHRY
FMNITWDNRDYSFNEDGFLVNPSLVVISLTRDRTWEVVGSWEQQTLRLKYPLWSRYGRFL
QPVDDTQHLTVATLEERPFVIVEPADPISGTCIRDSVPCRSQLNRTHSPPPDAPRPEKRCCKG
FCIDILKRLAHTIGFSYDLYLVTNGKHGKKIDGVWNGMIGEVFYQRADMAIGSLTINEERS
EIVDFSVPFVETGISVMVARSNGTVSPSAFLEPYSPAVWVMMFVMCLTVVAVTVFIFEYLSP
VGYNRSLATGKRPGGSTFTIGKSIWLLWALVFNNSVPVENPRGTTSKIMVLVWAFFAVIFL
ASYTANLAAFMIQEEYVDTVSGLSDRKFQRPQEQYPPLKFGTVPNGSTEKNIRSNYPDMH
SYMVRYNQPRVEEALTQLKAGKLDAFIYDAAVLNYMARKDEGCKLVTIGSGKVFATTGY
GIALHKGSRWKRPIDLALLQFLGDDEIEMLERLWLSGICHNDKIEVMSSKLDIDNMAGVF
YMLLVAMGLSLLVFAWEHLVYWRLRHCLGPTHRMDFLLAFSRGMYSCCSAEAAPPPAKP
PPPPQPLPSPAYPAPRPAPGPAPFVPRERASVDRWRRTKGAGPPGGAGLADGFHRYYGPIEP
QGLGLGLGEARAAPRGAAGRPLSPPAAQPPQKPPPSYFAIVRDKEPAEPPAGAFPGFPSPPA
PPAAAATAVGPPLCRLAFEDESPPAPARWPRSDPESQPLLGPGAGGAGGTGGAGGGAPAAP
PPCRAAPPPCPYLDLEPSPSDSEDSESLGGASLGGLEPWWFADFPYPYAERLGPPPGRYWS
VDKLGGWRAGSWDYLPPRSGPAAWHCRHCASLELLPPPRHLSCSHDGLDGGWWAPPPPP
WAAGPLPRRRARCGCPRSHPHRPRASHRTPAAAAPHHHRHRRAAGGWDLPPPAPTSRSLE
DLSSCPRAAPARRLTGPSRHARRCPHAAHWGPPLPTASHRRHRGGDLGTRRGSAHFSSLE
SEV

>P53680 peripheral membrane protein
MIRFILIQNRAGKTRLAKWYMQFDDDEKQKLIEEVHAVVTVRDAKHTNFVEFRNFKIIYR
RYAGLYFCICVDVNDNNLAYLEAIHNFVEVLNEYFHNVCELDLVFNFYKVYTVVDEMFL
AGEIRETSQTKVLKQLLMLQSLE

>Q02818 peripheral membrane protein
MPPSGPRGTLLLLPLLLLLLLRAVLAVPLERGAPNKEETPATESPDTGLYYHRYLQEVIDVL
ETDGHFREKLQAANAEDIKSGKLSRELDFVSHHVRTKLDELKRQEVSRLRMLLKAKMDA
EQDPNVQVDHLNLLKQFEHLDPQNQHTFEARDLELLIQTATRDLAQYDAAHHEEFKRYE
MLKEHERRRYLESLGEEQRKEAERKLEEQQRRHREHPKVNVPGSQAQLKEVWEELDGL
DPNRFNPKTFFILHDINSDGVLDEQELEALFTKELEKVYDPKNEEDDMREMEEERLRMRE
HVMKNVDTNQDRLVTLEEFLASTQRKEFGDTGEGWETVEMHPAYTEEELRRFEEELAAR
EAELNAKAQRLSQETEALGRSQGRLEAQKRELQQAVLHMEQRKQQQQQQQGHKAPAAH

PEGQLKFHPDTDDVPVPAPAGDQKEVDTSEKKLLERLPEVEVPQHL

>Q9NZN4 peripheral membrane protein
MFSWLKRGGARGQQPEAIRTVTSALKELYRTKLLPLEEHYRFGAFHSPALEDADFDGKPM
VLVAGQYSTGKTSFIQYLLEQEVPGSRVGPEPTTDCFVAVMHGDTEGTVPGNALVVDPDK
PFRKLNPFGNTFLNRFMCAQLPNQVLESISIIDTPGILSGAKQRVSRGYDFPAVLRWFAERV
DLIILLFDAHKLEISDEFSEAIGALRGHEDKIRVVLNKADMVETQQLMRVYGALMWALGK
VVGTPEVLRVYIGSFWSQPLLVPDNRRLFELEEQDLFRDIQGLPRHAALRKLNDLVKRARL
VRVHAYIISYLKKEMPSVFGKENKKKQLILKLPVIFAKIQLEHHISPGDFPDCQKMQELLM
AHDFTKFHSLKPKLLEALDEMLTHDIAKLMPLLRQEELESTEVGVQGGAFEGTHMGPFVE
RGPDEAMEDGEEGSDDEAEWVVTKDKSKYDEIFYNLAPADGKLSGSKAKTWMVGTKLP
NSVLGRIWKLSDVDRDGMLDDEEFALASHLIEAKLEGHGLPANLPRRLVPPSKRRHKGSA
E

>P48546 multipass membrane protein
MTTSPILQLLLRLSLCGLLLQRAETGSKGQTAGELYQRWERYRRECQETLAAAEPPSGLAC
NGSFDMYVCWDYAAPNATARASCPWYLPWHHHVAAGFVLRQCGSDGQWGLWRDHTQ
CENPEKNEAFLDQRLILERLQVMYTVGYSLSLATLLLALLILSLFRRLHCTRNYIHINLFTS
FMLRAAAILSRDRLLPRPGPYLGDQALALWNQALAACRTAQIVTQYCVGANYTWLLVEG
VYLHSLLVLVGGSEEGHFRYYLLLGWGAPALFVIPWVIVRYLYENTQCWERNEVKAIWWI
IRTPILMTILINFLIFIRILGILLSKLRTRQMRCRDYRLRLARSTLTLVPLLGVHEVVFAPVTEE
QARGALRFAKLGFEIFLSSFQGFLVSVLYCFINKEVQSEIRRGWHHCRLRRSLGEEQRQLPE
RAFRALPSGSGPGEVPTSRGLSSGTLPGPGNEASRELESYC

>P56373 multipass membrane protein
MNCISDFFTYETTKSVVVKSWTIGIINRVVQLLIISYFVGWVFLHEKAYQVRDTAIESSVVT
KVKGSGLYANRVMDVSDYVTPPQGTSVFVIITKMIVTENQMQGFCPESEEKYRCVSDSQC
GPERLPGGGILTGRCVNYSSVLRTCEIQGWCPTEVDTVETPIMMEAENFTIFIKNSIRFPLFN
FEKGNLLPNLTARDMKTCRFHPDKDPFCPILRVGDVVKFAGQDFAKLARTGGVLGIKIGW
VCDLDKAWDQCIPKYSFTRLDSVSEKSSVSPGYNFRFAKYYKMENGSEYRTLLKAFGIRF
DVLVYGNAGKFNIIPTIISSVAAFTSVGVGTVLCDIILLNFLKGADQYKAKKFEEVNETTLK
IAALTNPVYPSDQTTAEKQSTDSGAFSIGH

>Q9NPH5 multipass membrane protein
MAVSWRSWLANEGVKHLCLFIWLSMNVLLFWKTFLLYNQGPEYHYLHQMLGLGLCLSR
ASASVLNLNCSLILLPMCRTLLAYLRGSQKVPSRRTRRLLDKSRTFHITCGVTICIFSGVHV
AAHLVNALNFSVNYSEDFVELNAARYRDEDPRKLLFTTVPGLTGVCMVVVLFLMITASTY
AIRVSNYDIFWYTHNLFFVFYMLLTLHVSGGLLKYQTNLDTHPPGCISLNRTSSQNISLPEY
FSEHFHEPFPEGFSKPAEFTQHKFVKICMEEPRFQANFPQTWLWISGPLCLYCAERLYRYIR
SNKPVTIISVMSHPSDVMEIRMVKENFKARPGQYITLHCPSVSALENHPFTLTMCPTETKA
TFGVHLKIVGDWTERFRDLLLPPSSQDSEILPFIQSRNYPKLYIDGPFGSPFEESLNYEVSLC
VAGGIGVTPFASILNTLLDDWKPYKLRRLYFIWVCRDIQSFRWFADLLCMLHNKFWQENR
PDYVNIQLYLSQTDGIQKIIGEKYHALNSRLFIGRPRWKLLFDEIAKYNRGKTVGVFCCGP
NSLSKTLHKLSNQNNSYGTRFEYNKESFS

>P14679 single-pass type I membrane protein
MLLAVLYCLLWSFQTSAGHFPRACVSSKNLMEKECCPPWSGDRSPCGQLSGRGSCQNILL
SNAPLGPQFPFTGVDDRESWPSVFYNRTCQCSGNFMGFNCGNCKFGFWGPNCTERRLLV
RRNIFDLSAPEKDKFFAYLTLAKHTISSDYVIPIGTYGQMKNGSTPMFNDINIYDLFVWMH
YYVSMDALLGGSEIWRDIDFAHEAPAFLPWHRLFLLRWEQEIQKLTGDENFTIPYWDWRD
AEKCDICTDEYMGGQHPTNPNLLSPASFFSSWQIVCSRLEEYNSHQSLCNGTPEGPLRRNP
GNHDKSRTPRLPSSADVEFCLSLTQYESGSMDKAANFSFRNTLEGFASPLTGIADASQSSM
HNALHIYMNGTMSQVQGSANDPIFLLHHAFVDSIFEQWLRRHRPLQEVYPEANAPIGHNR
ESYMVPFIPLYRNGDFFISSKDLGYDYSYLQDSDPDSFQDYIKSYLEQASRIWSWLLGAAM
VGAVLTALLAGLVSLLCRHKRKQLPEEKQPLLMEKEDYHSLYQSHL

>P54920 peripheral membrane protein
MDNSGKEAEAMALLAEAERKVKNSQSFFSGLFGGSSKIEEACEIYARAANMFKMAKNWS
AAGNAFCQAAQLHLQLQSKHDAATCFVDAGNAFKKADPQEAINCLMRAIEIYTDMGRFT
IAAKHHISIAEIYETELVDIEKAIAHYEQSADYYKGEESNSSANKCLLKVAGYAALLEQYQ
KAIDIYEQVGTNAMDSPLLKYSAKDYFFKAALCHFCIDMLNAKLAVQKYEELFPAFSDSR
ECKLMKKLLEAHEEQNVDSYTESVKEYDSISRLDQWLTTMLLRIKKTIQGDEEDLR

>Q6UWB1 single-pass type I membrane protein
MRGGRGAPFWLWPLPKLALLPLLWVLFQRTRPQGSAGPLQCYGVGPLGDLNCSWEPLG
DLGAPSELHLQSQKYRSNKTQTVAVAAGRSWVAIPREQLTMSDKLLVWGTKAGQPLWPP
VFVNLETQMKPNAPRLGPDVDFSEDDPLEATVHWAPPTWPSHKVLICQFHYRRCQEAAW
TLLEPELKTIPLTPVEIQDLELATGYKVYGRCRMEKEEDLWGEWSPILSFQTPPSAPKDVW
VSGNLCGTPGGEEPLLLWKAPGPCVQVSYKVWFWVGGRELSPEGITCCCSLIPSGAEWAR
VSAVNATSWEPLTNLSLVCLDSASAPRSVAVSSIAGSTELLVTWQPGPGEPLEHVVDWARD
GDPLEKLNWVRLPPGNLSALLPGNFTVGVPYRITVTAVSASGLASASSVWGFREELAPLV
GPTLWRLQDAPPGTPAIAWGEVPRHQLRGHLTHYTLCAQSGTSPSVCMNVSGNTQSVTLP
DLPWGPCELWVTASTIAGQGPPGPILRLHLPDNTLRWKVLPGILFLWGLFLLGCGLSLATS
GRCYHLRHKVLPRWVWEKVPDPANSSSGQPHMEQVPEAQPLGDLPILEVEEMEPPPVME
SSQPAQATAPLDSGYEKHFLPTPEELGLLGPPRPQVLA

>Q9UM47 single-pass type I membrane protein
MGPGARGRRRRRRPMSPPPPPPPVRALPLLLLLAGPGAAAPPCLDGSPCANGGRCTQLPS
REAACLCPPGWVGERCQLEDPCHSGPCAGRGVCQSSVVAGTARFSCRCPRGFRGPDCSLP
DPCLSSPCAHGARCSVGPDGRFLCSCPPGYQGRSCRSDVDECRVGEPCRHGGTCLNTPGS
FRCQCPAGYTGPLCENPAVPCAPSPCRNGGTCRQSGDLTYDCACLPGFEGQNCEVNVDDC
PGHRCLNGGTCVDGVNTYNCQCPPEWTGQFCTEDVDECQLQPNACHNGGTCFNTLGGH
SCVCVNGWTGESCSQNIDDCATAVCFHGATCHDRVASFYCACPMGKTGLLCHLDDACVS
NPCHEDAICDTNPVNGRAICTCPPGFTGGACDQDVDECSIGANPCEHLGRCVNTQGSFLC
QCGRGYTGPRCETDVNECLSGPCRNQATCLDRIGQFTCICMAGFTGTYCEVDIDECQSSPC
VNGGVCKDRVNGFSCTCPSGFSGSTCQLDVDECASTPCRNGAKCVDQPDGYECRCAEGF
EGTLCDRNVDDCSPDPCHHGRCVDGIASFSCACAPGYTGTRCESQVDECRSQPCRHGGK
CLDLVDKYLCRCPSGTTGVNCEVNIDDCASNPCTFGVCRDGINRYDCVCQPGFTGPLCNV

EINECASSPCGEGGSCVDGENGFRCLCPPGSLPPLCLPPSHPCAHEPCSHGICYDAPGGFRC
VCEPGWSGPRCSQSLARDACESQPCRAGGTCSSDGMGFHCTCPPGVQGRQCELLSPCTPN
PCEHGGRCESAPGQLPVCSCPQGWQGPRCQQDVDECAGPAPCGPHGICTNLAGSFSCTCH
GGYTGPSCDQDINDCDPNPCLNGGSCQDGVGSFSCSCLPGFAGPRCARDVDECLSNPCGP
GTCTDHVASFTCTCPPGYGGFHCEQDLPDCSPSSCFNGGTCVDGVNSFSCLCRPGYTGAH
CQHEADPCLSRPCLHGGVCSAAHPGFRCTCLESFTGPQCQTLVDWCSRQPCQNGGRCVQ
TGAYCLCPPGWSGRLCDIRSLPCREAAAQIGVRLEQLCQAGGQCVDEDSSHYCVCPEGRT
GSHCEQEVDPCLAQPCQHGGTCRGYMGGYMCECLPGYNGDNCEDDVDECASQPCQHG
GSCIDLVARYLCSCPPGTLGVLCEINEDDCGPGPPLDSGPRCLHNGTCVDLVGGFRCTCPP
GYTGLRCEADINECRSGACHAAHTRDCLQDPGGGFRCLCHAGFSGPRCQTVLSPCESQPC
QHGGQCRPSPGPGGGLTFTCHCAQPFWGPRCERVARSCRELQCPVGVPCQQTPRGPRCAC
PPGLSGPSCRSFPGSPPGASNASCAAAPCLHGGSCRPAPLAPFFRCACAQGWTGPRCEAPA
AAPEVSEEPRCPRAACQAKRGDQRCDRECNSPGCGWDGGDCSLSVGDPWRQCEALQCW
RLFNNSRCDPACSSPACLYDNFDCHAGGRERTCNPVYEKYCADHFADGRCDQGCNTEEC
GWDGLDCASEVPALLARGVLVLTVLLPPEELLRSSADFLQRLSAILRTSLRFRLDAHGQA
MVFPYHRPSPGSEPRARRELAPEVIGSVVMLEIDNRLCLQSPENDHCFPDAQSAADYLGA
LSAVERLDFPYPLRDVRGEPLEPPEPSVPLLPLLVAGAVLLLVILVLGVMVARRKREHSTLW
FPEGFSLHKDVASGHKGRREPVGQDALGMKNMAKGESLMGEVATDWMDTECPEAKRLK
VEEPGMGAEEAVDCRQWTQHHLVAADIRVAPAMALTPPQGDADADGMDVNVRGPDGFT
PLMLASFCGGALEPMPTEEDEADDTSASIISDLICQGAQLGARTDRTGETALHLAARYARA
DAAKRLLDAGADTNAQDHSGRTPLHTAVTADAQGVFQILIRNRSTDLDARMADGSTALIL
AARLAVEGMVEELIASHADVNAVDELGKSALHWAAAVNNVEATLALLKNGANKDMQDS
KEETPLFLAAREGSYEAAKLLLDHFANREITDHLDRLPRDVAQERLHQDIVRLLDQPSGPR
SPPGPHGLGPLLCPPGAFLPGLKAAQSGSKKSRRPPGKAGLGPQGPRGRGKKLTLACPGPL
ADSSVTLSPVDSLDSPRPFGGPPASPGGFPLEGPYAAATATAVSLAQLGGPGRAGLGRQPPG
GCVLSLGLLNPVAVPLDWARLPPPAPPGPSFLLPLAPGPQLLNPGTPVSPQERPPPYLAVPG
HGEEYPAAGAHSSPPKARFLRVPSEHPYLTPSPESPEHWASPSPPSLSDWSESTPSPATATGA
MATTTGALPAQPLPLSVPSSLAQAQTQLGPQPEVTPKRQVLA

>P02748 multipass membrane protein
MSACRSFAVAICILEISILTAQYTTSYDPELTESSGSASHIDCRMSPWSEWSQCDPCLRQMFR
SRSIEVFGQFNGKRCTDAVGDRRQCVPTEPCEDAEDDCGNDFQCSTGRCIKMRLRCNGD
NDCGDFSDEDDCESEPRPPCRDRVVEESELARTAGYGINILGMDPLSTPFDNEFYNGLCNR
DRDGNTLTYYRRPWNVASLIYETKGEKNFRTEHYEEQIEAFKSIIQEKTSNFNAAISLKFTP
TETNKAEQCCEETASSISLHGKGSFRFSYSKNETYQLFLSYSSKKEKMFLHVKGEIHLGRF
VMRNRDVVLTTTFVDDIKALPTTYEKGEYFAFLETYGTHYSSSGSLGGLYELIYVLDKAS
MKRKGVELKDIKRCLGYHLDVSLAFSEISVGAEFNKDDCVKRGEGRAVNITSENLIDDVV
SLIRGGTRKYAFELKEKLLRGTVIDVTDFVNWASSINDAPVLISQKLSPIYNLVPVKMKNA
HLKKQNLERAIEDYINEFSVRKCHTCQNGGTVILMDGKCLCACPFKFEGIACEISKQKISE
GLPALEFPNEK

>P42702 single-pass type I membrane protein
MMDIYVCLKRPSWMVDNKRMRTASNFQWLLSTFILLYLMNQVNSQKKGAPHDLKCVTN
NLQVWNCSWKAPSGTGRGTDYEVCIENRSRSCYQLEKTSIKIPALSHGDYEITINSLHDFG

SSTSKFTLNEQNVSLIPDTPEILNLSADFSTSTLYLKWNDRGSVFPHRSNVIWEIKVLRKES
MELVKLVTHNTTLNGKDTLHHWSWASDMPLECAIHFVEIRCYIDNLHFSGLEEWSDWSP
VKNISWIPDSQTKVFPQDKVILVGSDITFCCVSQEKVLSALIGHTNCPLIHLDGENVAIKIRN
ISVSASSGTNVVFTTEDNIFGTVIFAGYPPDTPQQLNCETHDLKEIICSWNPGRVTALVGPRA
TSYTLVESFSGKYVRLKRAEAPTNESYQLLFQMLPNQEIYNFTLNAHNPLGRSQSTILVNIT
EKVYPHTPTSFKVKDINSTAVKLSWHLPGNFAKINFLCEIEIKKSNSVQEQRNVTIKGVENS
SYLVALDKLNPYTLYTFRIRCSTETFWKWSKWSNKKQHLTTEASPSKGPDTWREWSSDG
KNLIIYWKPLPINEANGKILSYNVSCSSDEETQSLSEIPDPQHKAEIRLDKNDYIISVVAKNS
VGSSPPSKIASMEIPNDDLKIEQVVGMGKGILLTWHYDPNMTCDYVIKWCNSSRSEPCLM
DWRKVPSNSTETVIESDEFRPGIRYNFFLYGCRNQGYQLLRSMIGYIEELAPIVAPNFTVED
TSADSILVKWEDIPVEELRGFLRGYLFYFGKGERDTSKMRVLESGRSDIKVKNITDISQKTL
RIADLQGKTSYHLVLRAYTDGGVGPEKSMYVVTKENSVGLIIAILIPVAVAVIVGVVTSILC
YRKREWIKETFYPDIPNPENCKALQFQKSVCEGSSALKTLEMNPCTPNNVEVLETRSAFPK
IEDTEIISPVAERPEDRSDAEPENHVVVSYCPPIIEEEIPNPAADEAGGTAQVIYIDVQSMYQP
QAKPEEEQENDPVGGAGYKPQMHLPINSTVEDIAAEEDLDKTAGYRPQANVNTWNLVSP
DSPRSIDSNSEIVSFGSPCSINSRQFLIPPKDEDSPKSNGGGWSFTNFFQNKPND

>P24557 multipass membrane protein
MEALGFLKLEVNGPMVTVALSVALLALLKWYSTSAFSRLEKLGLRHPKPSPFIGNLTFFRQ
GFWESQMELRKLYGPLCGYYLGRRMFIVISEPDMIKQVLVENFSNFTNRMASGLEFKSVA
DSVLFLRDKRWEEVRGALMSAFSPEKLNEMVPLISQACDLLLAHLKRYAESGDAFDIQRC
YCNYTTDVVASVAFGTPVDSWQAPEDPFVKHCKRFFEFCIPRPILVLLLSFPSIMVPLARILP
NKNRDELNGFFNKLIRNVIALRDQQAAEERRRDFLQMVLDARHSASPMGVQDFDIVRDV
FSSTGCKPNPSRQHQPSPMARPLTVDEIVGQAFIFLIAGYEIITNTLSFATYLLATNPDCQEK
LLREVDVFKEKHMAPEFCSLEEGLPYLDMVIAETLRMYPPAFRFTREAAQDCEVLGQRIP
AGAVLEMAVGALHHDPEHWPSPETFNPERFTAEARQQHRPFTYLPFGAGPRSCLGVRLGL
LEVKLTLLHVLHKFRFQACPETQVPLQLESKSALGPKNGVYIKIVSR

>O75436 peripheral membrane protein
MSFLGGFFGPICEIDIVLNDGETRKMAEMKTEDGKVEKHYLFYDGESVSGKVNLAFKQP
GKRLEHQGIRIEFVGQIELFNDKSNTHEFVNLVKELALPGELTQSRSYDFEFMQVEKPYES
YIGANVRLRYFLKVTIVRRLTDLVKEYDLIVHQLATYPDVNNSIKMEVGIEDCLHIEFEYN
KSKYHLKDVIVGKIYFLLVRIKIQHMELQLIKKEITGIGPSTTTETETIAKYEIMDGAPVKGE
SIPIRLFLAGYDPTPTMRDVNKKFSVRYFLNLVLVDEEDRRYFKQQEIILWRKAPEKLRKQ
RTNFHQRFESPESQASAEQPEM

>Q04771 single-pass type I membrane protein
MVDGVMILPVLIMIALPSPSMEDEKPKVNPKLYMCVCEGLSCGNEDHCEGQQCFSSLSIN
DGFHVYQKGCFQVYEQGKMTCKTPPSPGQAVECCQGDWCNRNITAQLPTKGKSFPGTQN
FHLEVGLIILSVVFAVCLLACLLGVALRKFKRRNQERLNPRDVEYGTIEGLITTNVGDSTLA
DLLDHSCTSGSGSGLPFLVQRTVARQITLLECVGKGRYGEVWRGSWQGENVAVKIFSSRD
EKSWFRETELYNTVMLRHENILGFIASDMTSRHSSTQLWLITHYHEMGSLYDYLQLTTLDT
VSCLRIVLSIASGLAHLHIEIFGTQGKPAIAHRDLKSKNILVKKNGQCCIADLGLAVMHSQS
TNQLDVGNNPRVGTKRYMAPEVLDETIQVDCFDSYKRVDIWAFGLVLWEVARRMVSNGI

VEDYKPPFYDVVPNDPSFEDMRKVVCVDQQRPNIPNRWFSDPTLTSLAKLMKECWYQNP
SARLTALRIKKTLTKIDNSLDKLKTDC

>P60033 multipass membrane protein
MGVEGCTKCIKYLLFVFNFVFWLAGGVILGVALWLRHDPQTTNLLYLELGDKPAPNTFYV
GIYILIAVGAVMMFVGFLGCYGAIQESQCLLGTFFTCLVILFACEVAAGIWGFVNKDQIAK
DVKQFYDQALQQAVVDDDANNAKAVVKTFHETLDCCGSSTLTALTTSVLKNNLCPSGSNI
ISNLFKEDCHQKIDDLFSGKLYLIGIAAIVVAVIMIFEMILSMVLCCGIRNSSVY

>P16109 single-pass type I membrane protein
MANCQIAILYQRFQRVVFGISQLLCFSALISELTNQKEVAAWTYHYSTKAYSWNISRKYCQ
NRYTDLVAIQNKNEIDYLNKVLPYYSSYYWIGIRKNNKTWTWVGTKKALTNEAENWADN
EPNNKRNNEDCVEIYIKSPSAPGKWNDEHCLKKKHALCYTASCQDMSCSKQGECLETIGN
YTCSCYPGFYGPECEYVRECGELELPQHVLMNCSHPLGNFSFNSQCSFHCTDGYQVNGPS
KLECLASGIWTNKPPQCLAAQCPPLKIPERGNMTCLHSAKAFQHQSSCSFSCEEGFALVGP
EVVQCTASGVWTAPAPVCKAVQCQHLEAPSEGTMDCVHPLTAFAYGSSCKFECQPGYRVR
GLDMLRCIDSGHWSAPLPTCEAISCEPLESPVHGSMDCSPSLRAFQYDTNCSFRCAEGFML
RGADIVRCDNLGQWTAPAPVCQALQCQDLPVPNEARVNCSHPFGAFRYQSVCSFTCNEG
LLLVGASVLQCLATGNWNSVPPECQAIPCTPLLSPQNGTMTCVQPLGSSSYKSTCQFICDE
GYSLSGPERLDCTRSGRWTDSPPMCEAIKCPELFAPEQGSLDCSDTRGEFNVGSTCHFSCD
NGFKLEGPNNVECTTSGRWSATPPTCKGIASLPTPGLQCPALTTPGQGTMYCRHHPGTFGF
NTTCYFGCNAGFTLIGDSTLSCRPSGQWTAVTPACRAVKCSELHVNKPIAMNCSNLWGNF
SYGSICSFHCLEGQLLNGSAQTACQENGHWSTTVPTCQAGPLTIQEALTYFGGAVASTIGLI
MGGTLLALLRKRFRQKDDGKCPLNPHSHLGTYGVFTNAAFDPSP

>P29074 peripheral membrane protein
MTSRFRLPAGRTYNVRASELARDRQHTEVVCNILLLDNTVQAFKVNKHDQGQVLLDVVF
KHLDLTEQDYFGLQLADDSTDNPRWLDPNKPIRKQLKRGSPYSLNFRVKFFVSDPNKLQE
EYTRYQYFLQIKQDILTGRLPCPSNTAALLASFAVQSELGDYDQSENLSGYLSDYSFIPNQP
QDFEKEIAKLHQQHIGLSPAEAEFNYLNTARTLELYGVEFHYARDQSNNEIMIGVMSGGILI
YKNRVRMNTFPWLKIVKISFKCKQFFIQLRKELHESRETLLGFNMVNYRACKNLWKACV
EHHTFFRLDRPLPPQKNFFAHYFTLGSKFRYCGRTEVQSVQYGKEKANKDRVFARSPSKP
LARKLMDWEVVSRNSISDDRLETQSLPSRSPPGTPNHRNSTFTQEGTRLRPSSVGHLVDH
MVHTSPSEVFVNQRSPSSTQANSIVLESSPSQETPGDGKPPALPPKQSKKNSWNQIHYSHS
QQDLESHINETFDIPSSPEKPTPNGGIPHDNLVLIRMKPDENGRFGFNVKGGYDQKMPVIV
SRVAPGTPADLCVPRLNEGDQVVLINGRDIAEHTHDQVVLFIKASCERHSGELMLLVRPNA
VYDVVEEKLENEPDFQYIPEKAPLDSVHQDDHSLRESMIQLAEGLITGTVLTQFDQLYRKK
PGMTMSCAKLPQNISKNRYRDISPYDATRVILKGNEDYINANYINMEIPSSSIINQYIACQGP
LPHTCTDFWQMTWEQGSSMVVMLTTQVERGRVKCHQYWPEPTGSSSYGCYQVTCHSEE
GNTAYIFRKMTLFNQEKNESRPLTQIQYIAWPDHGVPDDSSDFLDFVCHVRNKRAGKEEP
VVVHCSAGIGRTGVLITMETAMCLIECNQPVYPLDIVRTMRDQRAMMIQTPSQYRFVCEA
ILKVYEEGFVKPLTTSTNK

>O75063 single-pass type II membrane protein

MKLKQRVVLLAILLVIFIFTKVFLIDNLDTSAANREDQRAFHRMMTGLRVELAPKLDHTL
QSPWEIAAQWVVPREVYPEETPELGAVMHAMATKKIIKADVGYKGTQLKALLILEGGQK
VVFKPKRYSRDHVVEGEPYAGYDRHNAEVAAFHLDRILGFHRAPLVVGRFVNLRTEIKPV
ATEQLLSTFLTVGNNTCFYGKCYYCRETEPACADGDIMEGSVTLWLPDVWPLQKHRHPW
GRTYREGKLARWEYDESYCDAVKKTSPYDSGPRLLDIIDTAVFDYLIGNADRHHYESFQD
DEGASMLILLDNAKSFGNPSLDERSILAPLYQCCIIRVSTWNRLNYLKNGVLKSALKSAMA
HDPISPVLSDPHLDAVDQRLLSVLATVKQCTDQFGMDTVLVEDRMPLSHL

>P16422 single-pass type I membrane protein
MAPPQVLAFGLLLAAATATFAAAQEECVCENYKLAVNCFVNNNRQCQCTSVGAQNTVIC
SKLAAKCLVMKAEMNGSKLGRRAKPEGALQNNDGLYDPDCDESGLFKAKQCNGTSMC
WCVNTAGVRRTDKDTEITCSERVRTYWIIIELKHKAREKPYDSKSLRTALQKEITTRYQLD
PKFITSILYENNVITIDLVQNSSQKTQNDVDIADVAYYFEKDVKGESLFHSKKMDLTVNGE
QLDLDPGQTLIYYVDEKAPEFSMQGLKAGVIAVIVVVVIAVVAGIVVLVISRKKRMAKYE
KAEIKEMGEMHRELNA

>Q06418 single-pass type I membrane protein
MALRRSMGRPGLPPLPLPPPPRLGLLLAALASLLLPESAAAGLKLMGAPVKLTVSQGQPV
KLNCSVEGMEEPDIQWVKDGAVVQNLDQLYIPVSEQHWIGFLSLKSVERSDAGRYWCQV
EDGGETEISQPVWLTVEGVPFFTVEPKDLAVPPNAPFQLSCEAVGPPEPVTIVWWRGTTKI
GGPAPSPSVLNVTGVTQSTMFSCEAHNLKGLASSRTATVHLQALPAAPFNITVTKLSSSNA
SVAWMPGADGRALLQSCTVQVTQAPGGWEVLAVVVPVPPFTCLLRDLVPATNYSLRVRC
ANALGPSPYADWVPFQTKGLAPASAPQNLHAIRTDSGLILEWEEVIPEAPLEGPLGPYKLS
WVQDNGTQDELTVEGTRANLTGWDPQKDLIVRVCVSNAVGCGPWSQPLVVSSHDRAGQ
QGPPHSRTSWVPVVLGVLTALVTAAALALILLRKRRKETRFGQAFDSVMARGEPAVHFRA
ARSFNRERPERIEATLDSLGISDELKEKLEDVLIPEQQFTLGRMLGKGEFGSVREAQLKQE
DGSFVKVAVKMLKADIIASSDIEEFLREAACMKEFDHPHVAKLVGVSLRSRAKGRLPIPMV
ILPFMKHGDLHAFLLASRIGENPFNLPLQTLIRFMVDIACGMEYLSSRNFIHRDLAARNCM
LAEDMTVCVADFGLSRKIYSGDYYRQGCASKLPVKWLALESLADNLYTVQSDVWAFGV
TMWEIMTRGQTPYAGIENAEIYNYLIGGNRLKQPPECMEDVYDLMYQCWSADPKQRPSF
TCLRMELENILGQLSVLSASQDPLYINIERAEEPTAGGSLELPGRDQPYSGAGDGSGMGAV
GGTPSDCRYILTPGGLAEQPGQAEHQPESPLNETQRLLLLQQGLLPHSSC

>P29376 single-pass type I membrane protein
MGCWGQLLVWFGAAGAILCSSPGSQETFLRSSPLPLASPSPRDPKVSAPPSILEPASPLNSP
GTEGSWLFSTCGASGRHGPTQTQCDGAYAGTSVVVTVGAAGQLRGVQLWRVPGPGQYLI
SAYGAAGGKGAKNHLSRAHGVFVSAIFSLGLGESLYILVGQQGEDACPGGSPESQLVCLG
ESRAVEEHAAMDGSEGVPGSRRWAGGGGGGGGATYVFRVRAGELEPLLVAAGGGGRAY
LRPRDRGRTQASPEKLENRSEAPGSGGRGGAAGGGGGWTSRAPSPQAGRSLQEGAEGGQ
GCSEAWATLGWAAAGGFGGGGGACTAGGGGGGYRGGDASETDNLWADGEDGVSFIHPS
SELFLQPLAVTENHGEVEIRRHLNCSHCPLRDCQWQAELQLAECLCPEGMELAVDNVTC
MDLHKPPGPLVLMVAVVATSTLSLLMVCGVLILVKQKKWQGLQEMRLPSPELELSKLRTS
AIRTAPNPYYCQVGLGPAQSWPLPPGVTEVSPANVTLLRALGHGAFGEVYEGLVIGLPGDS
SPLQVAIKTLPELCSPQDELDFLMEALIISKFRHQNIVRCVGLSLRATPRLILLELMSGGDM

KSFLRHSRPHLGQPSPLVMRDLLQLAQDIAQGCHYLEENHFIHRDIAARNCLLSCAGPSRV
AKIGDFGMARDIYRASYYRRGDRALLPVKWMPPEAFLEGIFTSKTDSWSFGVLLWEIFSL
GYMPYPGRTNQEVLDFVVGGGRMDPPRGCPGPVYRIMTQCWQHEPELRPSFASILERLQ
YCTQDPDVLNSLLPMELGPTPEEEGTSGLGNRSLECLRPPQPQELSPEKLKSWGGSPLGP
WLSSGLKPLKSRGLQPQNLWNPTYRS

>P98164 single-pass type I membrane protein
MDRGPAAVACTLLLALVACLAPASGQECDSAHFRCGSGHCIPADWRCDGTKDCSDDADEI
GCAVVTCQQGYFKCQSEGQCIPNSWVCDQDQDCDDGSDERQDCSQSTCSSHQITCSNGQ
CIPSEYRCDHVRDCPDGADENDCQYPTCEQLTCDNGACYNTSQKCDWKVDCRDSSDEIN
CTEICLHNEFSCGNGECIPRAYVCDHDNDCQDGSDEHACNYPTCGGYQFTCPSGRCIYQN
WVCDGEDDCKDNGDEDGCESGPHDVHKCSPREWSCPESGRCISIYKVCDGILDCPGRED
ENNTSTGKYCSMTLCSALNCQYQCHETPYGGACFCPPGYIINHNDSRTCVEFDDCQIWGI
CDQKCESRPGRHLCHCEEGYILERGQYCKANDSFGEASIIFSNGRDLLIGDIHGRSFRILVE
SQNRGVAVGVAFHYHLQRVFWTDTVQNKVFSVDINGLNIQEVLNVSVETPENLAVDWVN
NKIYLVETKVNRIDMVNLDGSYRVTLITENLGHPRGIAVDPTVGYLFFSDWESLSGEPKLE
RAFMDGSNRKDLVKTKLGWPAGVTLDMISKRVYWVDSRFDYIETVTYDGIQRKTVVHG
GSLIPHPFGVSLFEGQVFFTDWTKMAVLKANKFTETNPQVYYQASLRPYGVTVYHSLRQP
YATNPCKDNNGGCEQVCVLSHRTDNDGLGFRCKCTFGFQLDTDERHCIAVQNFLIFSSQV
AIRGIPFTLSTQEDVMVPVSGNPSFFVGIDFDAQDSTIFFSDMSKHMIFKQKIDGTGREILA
ANRVENVESLAFDWISKNLYWTDSHYKSISVMRLADKTRRTVVQYLNNPRSVVVHPFAG
YLFFTDWFRPAKIMRAWSDGSHLLPVINTTLGWPNGLAIDWAASRLYWVDAYFDKIEHST
FDGLDRRRLGHIEQMTHPFGLAIFGEHLFFTDWRLGAIIRVRKADGGEMTVIRSGIAYILHL
KSYDVNIQTGSNACNQPTHPNGDCSHFCFPVPNFQRVCGCPYGMRLASNHLTCEGDPTNE
PPTEQCGLFSFPCKNGRCVPNYYLCDGVDDCHDNSDEQLCGTLNNTCSSSAFTCGHGECI
PAHWRCDKRNDCVDGSDEHNCPTHAPASCLDTQYTCDNHQCISKNWVCDTDNDCGDGS
DEKNCNSTETCQPSQFNCPNHRCIDLSFVCDGDKDCVDGSDEVGCVLNCTASQFKCASG
DKCIGVTNRCDGVFDCSDNSDEAGCPTRPPGMCHSDEFQCQEDGICIPNFWECDGHPDCL
YGSDEHNACVPKTCPSSYFHCDNGNCIHRAWLCDRDNDCGDMSDEKDCPTQPFRCPSW
QWQCLGHNICVNLSVVCDGIFDCPNGTDESPLCNGNSCSDFNGGCTHECVQEPFGAKCL
CPLGFLLANDSKTCEDIDECDILGSCSQHCYNMRGSFRCSCDTGYMLESDGRTCKVTASE
SLLLLVASQNKIIADSVTSQVHNIYSLVENGSYIVAVDFDSISGRIFWSDATQGKTWSAFQN
GTDRRVVFDSSIILTETIAIDWVGRNLYWTDYALETIEVSKIDGSHRTVLISKNLTNPRGLAL
DPRMNEHLLFWSDWGHHPRIERASMDGSMRTVIVQDKIFWPCGLTIDYPNRLLYFMDSY
LDYMDFCDYNGHHRRQVIASDLIIRHPYALTLFEDSVYWTDRATRRVMRANKWHGGNQS
VVMYNIQWPLGIVAVHPSKQPNSVNPCAFSRCSHLCLLSSQGPHFYSCVCPSGWSLSPDLL
NCLRDDQPFLITVRQHIIFGISLNPEVKSNDAMVPIAGIQNGLDVEFDDAEQYIYWVENPG
EIHRVKTDGTNRTVFASISMVGPSMNLALDWISRNLYSTNPRTQSIEVLTLHGDIRYRKTLI
ANDGTALGVGFPIGITVDPARGKLYWSDQGTDSGVPAKIASANMDGTSVKTLFTGNLEHL
ECVTLDIEEQKLYWAVTGRGVIERGNVDGTDRMILVHQLSHPWGIAVHDSFLYYTDEQYE
VIERVDKATGANKIVLRDNVPNLRGLQVYHRRNAAESSNGCSNNMNACQQICLPVPGGL
FSCACATGFKLNPDNRSCSPYNSFIVVSMLSAIRGFSLELSDHSETMVPVAGQGRNALHVD
VDVSSGFIYWCDFSSSVASDNAIRRIKPDGSSLMNIVTHGIGENGVRGIAVDWVAGNLYFT
NAFVSETLIEVLRINTTYRRVLLKVTVDMPRHIVVDPKNRYLFWADYGQRPKIERSFLDCT

NRTVLVSEGIVTPRGLAVDRSDGYVYWVDDSLDIIARIRINGENSEVIRYGSRYPTPYGITV
FENSIIWVDRNLKKIFQASKEPENTEPPTVIRDNINWLRDVTIFDKQVQPRSPAEVNNNPCL
ENNGGCSHLCFALPGLHTPKCDCAFGTLQSDGKNCAISTENFLIFALSNSLRSLHLDPENHS
PPFQTINVERTVMSLDYDSVSDRIYFTQNLASGVGQISYATLSSGIHTPTVIASGIGTADGIA
FDWITRRIYYSDYLNQMINSMAEDGSNRTVIARVPKPRAIVLDPCQGYLYWADWDTHAKI
ERATLGGNFRVPIVNSSLVMPSGLTLDYEEDLLYWVDASLQRIERSTLTGVDREVIVNAAV
HAFGLTLYGQYIYWTDLYTQRIYRANKYDGSGQIAMTTNLLSQPRGINTVVKNQKQQCN
NPCEQFNGGCSHICAPGPNGAECQCPHEGNWYLANNRKHCIVDNGERCGASSFTCSNGR
CISEEWKCDNDNDCGDGSDEMESVCALHTCSPTAFTCANGRCVQYSYRCDYYNDCGDG
SDEAGCLFRDCNATTEFMCNNRRCIPREFICNGVDNCHDNNTSDEKNCPDRTCQSGYTKC
HNSNICIPRVYLCDGDNDCGDNSDENPTYCTTHTCSSSEFQCASGRCIPQHWYCDQETDC
FDASDEPASCGHSERTCLADEFKCDGGRCIPSEWICDGDNDCGDMSDEDKRHQCQNQNC
SDSEFLCVNDRPPDRRCIPQSWVCDGDVDCTDGYDENQNCTRRTCSENEFTCGYGLCIPK
IFRCDRHNDCGDYSDERGCLYQTCQQNQFTCQNGRCISKTFVCDEDNDCGDGSDELMHL
CHTPEPTCPPHEFKCDNGRCIEMMKLCNHLDDCLDNSDEKGCGINECHDPSISGCDHNCT
DTLTSFYCSCRPGYKLMSDKRTCVDIDECTEMPFVCSQKCENVIGSYICKCAPGYLREPDG
KTCRQNSNIEPYLIFSNRYYLRNLTIDGYFYSLILEGLDNVVALDFDRVEKRLYWIDTQRQV
IERMFLNKTNKETIINHRLPAAESLAVDWVSRKLYWLDARLDGLFVSDLNGGHRRMLAQ
HCVDANNTFCFDNPRGLALHPQYGYLYWADWGHRAYIGRVGMDGTNKSVIISTKLEWP
NGITIDYTNDLLYWADAHLGYIEYSDLEGHHRHTVYDGALPHPFAITIFEDTIYWTDWNTR
TVEKGNKYDGSNRQTLVNTTHRPFDIHVYHPYRQPIVSNPCGTNNGGCSHLCLIKPGGKG
FTCECPDDFRTLQLSGSTYCMPMCSSTQFLCANNEKCIPIWWKCDGQKDCSDGSDELALC
PQRFCRLGQFQCSDGNCTSPQTLCNAHQNCPDGSDEDRLLCENHHCDSNEWQCANKRCI
PESWQCDTFNDCEDNSDEDSSHCASRTCRPGQFRCANGRCIPQAWKCDVDNDCGDHSDE
PIEECMSSAHLCDNFTEFSCKTNYRCIPKWAVCNGVDDCRDNSDEQGCEERTCHPVGDFR
CKNHHCIPLRWQCDGQNDCGDNSDEENCAPRECTESEFRCVNQQCIPSRWICDHYNDCG
DNSDERDCEMRTCHPEYFQCTSGHCVHSELKCDGSADCLDASDEADCPTRFPDGAYCQA
TMFECKNHVCIPPYWKCDGDDDCGDGSDEELHLCLDVPCNSPNRFRCDNNRCIYSHEVC
NGVDDCGDGTDETEEHCRKPTPKPCTEYEYKCGNGHCIPHDNVCDDADDCGDWSDELG
CNKGKERTCAENICEQNCTQLNEGGFICSCTAGFETNVFDRTSCLDINECEQFGTCPQHCR
NTKGSYECVCADGFTSMSDRPGKRCAAEGSSPLLLLPDNVRIRKYNLSSERFSEYLQDEE
YIQAVDYDWDPKDIGLSVVYYTVRGEGSRFGAIKRAYIPNFESGRNNLVQEVDLKLKYV
MQPDGIAVDWVGRHIYWSDVKNKRIEVAKLDGRYRKWLISTDLDQPAAIAVNPKLGLMF
WTDWGKEPKIESAWMNGEDRNILVFEDLGWPTGLSIDYLNNDRIYWSDFKEDVIETIKYD
GTDRRVIAKEAMNPYSLDIFEDQLYWISKEKGEVWKQNKFGQGKKEKTLVVNPWLTQVR
IFHQLRYNKSVPNLCKQICSHLCLLRPGGYSCACPQGSSFIEGSTTECDAAIELPINLPPPCR
CMHGGNCYFDETDLPKCKCPSGYTGKYCEMAFSKGISPGTTAVAVLLTILLIVVIGALAIA
GFFHYRRTGSLLPALPKLPSLSSLVKPSENGNGVTFRSGADLNMDIGVSGFGPETAIDRSM
AMSEDFVMEMGKQPIIFENPMYSARDSAVKVVQPIQVTVSENVDNKNYGSPINPSEIVPET
NPTSPAADGTQVTKWNLFKRKSKQTTNFENPIYAQMENEQKESVAATPPPSPSLPAKPKPP
SRRDPTPTYSATEDTFKDTANLVKEDSEV

>O95342 multipass membrane protein
MSDSVILRSIKKFGEENDGFESDKSYNNDKKSRLQDEKKGDGVRVGFFQLFRFSSSTDIWL

MFVGSLCAFLHGIAQPGVLLIFGTMTDVFIDYDVELQELQIPGKACVNNTIVWTNSSLNQ
NMTNGTRCGLLNIESEMIKFASYYAGIAVAVLITGYIQICFWVIAAARQIQKMRKFYFRRIM
RMEIGWFDCNSVGELNTRFSDDINKINDAIADQMALFIQRMTSTICGFLLGFFRGWKLTLV
IISVSPLIGIGAATIGLSVSKFTDYELKAYAKAGVVADEVISSMRTVAAFGGEKREVERYEK
NLVFAQRWGIRKGIVMGFFTGFVWCLIFLCYALAFWYGSTLVLDEGEYTPGTLVQIFLSVI
VGALNLGNASPCLEAFATGRAAATSIFETIDRKPIIDCMSEDGYKLDRIKGEIEFHNVTFHY
PSRPEVKILNDLNMVIKPGEMTALVGPSGAGKSTALQLIQRFYDPCEGMVTVDGHDIRSLN
IQWLRDQIGIVEQEPVLFSTTIAENIRYGREDATMEDIVQAAKEANAYNFIMDLPQQFDTLV
GEGGGQMSGGQKQRVAIARALIRNPKILLLDMATSALDNESEAMVQEVLSKIQHGHTIISV
AHRLSTVRAADTIIGFEHGTAVERGTHEELLERKGVYFTLVTLQSQGNQALNEEDIKDATE
DDMLARTFSRGSYQDSLRASIRQRSKSQLSYLVHEPPLAVVDHKSTYEEDRKDKDIPVQEE
VEPAPVRRILKFSAPEWPYMLVGSVGAAVNGTVTPLYAFLFSQILGTFSIPDKEEQRSQING
VCLLFVAMGCVSLFTQFLQGYAFAKSGELLTKRLRKFGFRAMLGQDIAWFDDLRNSPGAL
TTRLATDASQVQGAAGSQIGMIVNSFTNVTVAMIIAFSFSWKLSLVILCFFPFLALSGATQT
RMLTGFASRDKQALEMVGQITNEALSNIRTVAGIGKERRFIEALETELEKPFKTAIQKANIY
GFCFAFAQCIMFIANSASYRYGGYLISNEGLHFSYVFRVISAVVLSATALGRAFSYTPSYAK
AKISAARFFQLLDRQPPISVYNTAGEKWDNFQGKIDFVDCKFTYPSRPDSQVLNGLSVSISP
GQTLAFVGSSGCGKSTSIQLLERFYDPDQGKVMIDGHDSKKVNVQFLRSNIGIVSQEPVLF
ACSIMDNIKYGDNTKEIPMERVIAAAKQAQLHDFVMSLPEKYETNVGSQGSQLSRGEKQR
IAIARAIVRDPKILLLDEATSALDTESEKTVQVALDKAREGRTCIVIAHRLSTIQNADIIAVM
AQGVVIEKGTHEELMAQKGAYYKLVTTGSPIS

>P22059 peripheral membrane protein
MAATELRGVVGPGPAAIAALGGGGAGPPVVGGGGGRGDAGPGSGAASGTVVAAAAGGP
GPGAGGVAAAGPAPAPPTGGSGGSGAGGSGSAREGWLFKWTNYIKGYQRRWFVLSNGL
LSYYRSKAEMRHTCRGTINLATANITVEDSCNFIISNGGAQTYHLKASSEVERQRWVTALE
LAKAKAVKMLAESDESGDEESVSQTDKTELQNTLRTLSSKVEDLSTCNDLIAKHGTALQR
SLSELESLKLPAESNEKIKQVNERATLFRITSNAMINACRDFLMLAQTHSKKWQKSLQYER
DQRIRLEETLEQLAKQHNHLERAFRGATVLPANTPGNVGSGKDQCCSGKGDMSDEDDEN
EFFDAPEIITMPENLGHKRTGSNISGASSDISLDEQYKHQLEETKKEKRTRIPYKPNYSLNL
WSIMKNCIGKELSKIPMPVNFNEPLSMLQRLTEDLEYHELLDRAAKCENSLEQLCYVAAF
TVSSYSTTVFRTSKPFNPLLGETFELDRLEENGYRSLCEQVSHHPPAAAHHAESKNGWTLR
QEIKITSKFRGKYLSIMPLGTIHCIFHATGHHYTWKKVTTTVHNIIVGKLWIDQSGEIDIVN
HKTGDKCNLKFVPYSYFSRDVARKVTGEVTDPSGKVHFALLGTWDEKMECFKVQPVIGE
NGGDARQRGHEAEESRVMLWKRNPLPKNAENMYYFSELALTLNAWESGTAPTDSRLRPD
QRLMENGRWDEANAEKQRLEEKQRLSRKKREAEAMKATEDGTPYDPYKALWFERKKDP
VTKELTHIYRGEYWECKEKQDWSSCPDIF

>Q9Y3E7 lipid-anchor
MGLFGKTQEKPPKELVNEWSLKIRKEMRVVDRQIRDIQREEEKVKRSVKDAAKKGQKDV
CIVLAKEMIRSRKAVSKLYASKAHMNSVLMGMKNQLAVLRVAGSLQKSTEVMKAMQSL
VKIPEIQATMRELSKEMMKAGIIEEMLEDTFESMDDQEEMEEEAEMEIDRILFEITAGALG
KAPSKVTDALPEPEPPGAMAASEDEEEEEEALEAMQSRLATLRS

>P61587	peripheral membrane protein
MKERRASQKLSSKSIMDPNQNVKCKIVVVGDSQCGKTALLHVFAKDCFPENYVPTVFEN
YTASFEIDTQRIELSLWDTSGSPYYDNVRPLSYPDSDAVLICFDISRPETLDSVLKKWKGEI
QEFCPNTKMLLVGCKSDLRTDVSTLVELSNHRQTPVSYDQGANMAKQIGAATYIECSALQ
SENSVRDIFHVATLACVNKTNKNVKRNKSQRATKRISHMPSRPELSAVATDLRKDKAKSCT
VM

>O75886	peripheral membrane protein
MPLFTANPFEQDVEKATNEYNTTEDWSLIMDICDKVGSTPNGAKDCLKAIMKRVNHKVP
HVALQALTLLGACVANCGKIFHLEVCSRDFATEVRAVIKNKAHPKVCEKLKSLMVEWSEE
FQKDPQFSLISATIKSMKEEGITFPPAGSQTVSAAAKNGTSSNKNKEDEDIAKAIELSLQEQ
KQQHTETKSLYPSSEIQLNNKVARKVRALYDFEAVEDNELTFKHGEIIIVLDDSDANWWK
GENHRGIGLFPSNFVTTNLNIETEAAAVDKLNVIDDDVEEIKKSEPEPVYIDEDKMDRALQ
VLQSIDPTDSKPDSQDLLDLEDICQQMGPMIDEKLEEIDRKHSELSELNVKVLEALELYNK
LVNEAPVYSVYSKLHPPAHYPPASSGVPMQTYPVQSHGGNYMGQSIHQVTVAQSYSLGPD
QIGPLRSLPPNVNSSVTAQPAQTSYLSTGQDTVSNPTYMNQNSNLQSATGTTAYTQQMGM
SVDMSSYQNTTSNLPQLAGFPVTVPAHPVAQQHTNYHQQPLL

>Q14449	peripheral membrane protein
MTTSLQDGQSAASRAAARDSPLAAQVCGAAQGRGDAHDLAPAPWLHARALLPLPDGTR
GCAADRRKKKDLDVPEMPSIPNPFPELCCSPFTSVLSADLFPKANSRKKQVIKVYSEDETS
RALDVPSDITARDVCQLLILKNHYIDDHSWTLFEHLPHIGVERTIEDHELVIEVLSNWGIEE
ENKLYFRKNYAKYEFFKNPMYFFPEHMVSFATETNGEISPTQILQMFLSSSTYPEIHGFLHA
KEQGKKSWKKIYFFLRRSGLYFSTKGTSKEPRHLQFFSEFGNSDIYVSLAGKKKHGAPTN
YGFCFKPNKAGGPRDLKMLCAEEEQSRTCWVTAIRLLKYGMQLYQNYMHPYQGRSGCS
SQSISPMRSISENSLVAMDFSGQKSRVIENPTEALSVAVEEGLAWRKKGCLRLGTHGSPTAS
SQSSATNMAIHRSQPWFHHKISRDEAQRLIIQQGLVDGVFLVRDSQSNPKTFVLSMSHGQK
IKHFQIIPVEDDGEMFHTLDDGHTRFTDLIQLVEFYQLNKGVLPCKLKHYCARIAL

>P35968	single-pass type I membrane protein
MQSKVLLAVALWLCVETRAASVGLPSVSLDLPRLSIQKDILTIKANTTLQITCRGQRDLDW
LWPNNQSGSEQRVEVTECSDGLFCKTLTIPKVIGNDTGAYKCFYRETDLASVIYVYVQDY
RSPFIASVSDQHGVVYITENKNKTVVIPCLGSISNLNVSLCARYPEKRFVPDGNRISWDSK
KGFTIPSYMISYAGMVFCEAKINDESYQSIMYIVVVVGYRIYDVVLSPSHGIELSVGEKLVL
NCTARTELNVGIDFNWEYPSSKHQHKKLVNRDLKTQSGSEMKKFLSTLTIDGVTRSDQGL
YTCAASSGLMTKKNSTFVRVHEKPFVAFGSGMESLVEATVGERVRIPAKYLGYPPPEIKWY
KNGIPLESNHTIKAGHVLTIMEVSERDTGNYTVILTNPISKEKQSHVVSLVVYVPPQIGEKS
LISPVDSYQYGTTQTLTCTVYAIPPPHHIHWYWQLEEECANEPSQAVSVTNPYPCEEWRSV
EDFQGGNKIEVNKNQFALIEGKNKTVSTLVIQAANVSALYKCEAVNKVGRGERVISFHVT
RGPEITLQPDMQPTEQESVSLWCTADRSTFENLTWYKLGPQPLPIHVGELPTPVCKNLDTL
WKLNATMFSNSTNDILIMELKNASLQDQGDYVCLAQDRKTKKRHCVVRQLTVLERVAPT
ITGNLENQTTSIGESIEVSCTASGNPPPQIMWFKDNETLVEDSGIVLKDGNRNLTIRRVRKE
DEGLYTCQACSVLGCAKVEAFFIIEGAQEKTNLEIIILVGTAVIAMFFWLLLVIILRTVKRAN
GGELKTGYLSIVMDPDELPLDEHCERLPYDASKWEFPRDRLKLGKPLGRGAFGQVIEADA

FGIDKTATCRTVAVKMLKEGATHSEHRALMSELKILIHIGHHLNVVNLLGACTKPGGPLMV
IVEFCKFGNLSTYLRSKRNEFVPYKTKGARFRQGKDYVGAIPVDLKRRLDSITSSQSSASS
GFVEEKSLSDVEEEEAPEDLYKDFLTLEHLICYSFQVAKGMEFLASRKCIHRDLAARNILLS
EKNVVKICDFGLARDIYKDPDYVRKGDARLPLKWMAPETIFDRVYTIQSDVWSFGVLLW
EIFSLGASPYPGVKIDEEFCRRLKEGTRMRAPDYTTPEMYQTMLDCWHGEPSQRPTFSEL
VEHLGNLLQANAQQDGKDYIVLPISETLSMEEDSGLSLPTSPVSCMEEEEVCDPKFHYDN
TAGISQYLQNSKRKSRPVSVKTFEDIPLEEPEVKVIPDDNQTDSGMVLASEELKTLEDRTK
LSPSFGGMVPSKSRESVASEGSNQTSGYQSGYHSDDTDTTVYSSEEAELLKLIEIGVQTGST
AQILQPDSGTTLSSPPV

>P28908 single-pass type I membrane protein
MRVLLAALGLLFLGALRAFPQDRPFEDTCHGNPSHYYDKAVRRCCYRCPMGLFPTQQCP
QRPTDCRKQCEPDYYLDEADRCTACVTCSRDDLVEKTPCAWNSSRVCECRPGMFCSTSAV
NSCARCFFHSVCPAGMIVKFPGTAQKNTVCEPASPGVSPACASPENCKEPSSGTIPQAKPTP
VSPATSSASTMPVRGGTRLAQEAASKLTRAPDSPSSVGRPSSDPGLSPTQPCPEGSGDCRK
QCEPDYYLDEAGRCTACVSCSRDDLVEKTPCAWNSSRTCECRPGMICATSATNSCARCVP
YPICAAETVTKPQDMAEKDTTFEAPPLGTQPDCNPTPENGEAPASTSPTQSLLVDSQASKT
LPIPTSAPVALSSTGKPVLDAGPVLFWVILVLVVVVGSSAFLLCHRRACRKRIRQKLHLCY
PVQTSQPKLELVDSRPRRSSTQLRSGASVTEPVAEERGLMSQPLMETCHSVGAAYLESLPL
QDASPAGGPSSPRDLPEPRVSTEHTNNKIEKIYIMKADTVIVGTVKAELPEGRGLAGPAEPE
LEEELEADHTPHYPEQETEPPLGSCSDVMLSVEEEGKEDPLPTAASGK

>Q9H8M1 peripheral membrane protein
MAARTGHTALRRVVSGCRPKSATAAGAQAPVRNGRYLASCGILMSRTLPLHTSILPKEICA
RTFFKITAPLINKRKEYSERRILGYSMQEMYDVVSGVEDYKHFVPWCKKSDVISKRSGYC
KTRLEIGFPPVLERYTSVVTLVKPHLVKASCTDGRLFNHLETIWRFSPGLPGYPRTCTLDFSI
SFEFRSLLHSQLATLFFDEVVKQMVAAFERRACKLYGPETNIPRELMLHEVHHT

>P19634 multipass membrane protein
MVLRSGICGLSPHRIFPSLLVVVALVGLLPVLRSHGLQLSPTASTIRSSEPPRERSIGDVTTAP
PEVTPESRPVNHSVTDHGMKPRKAFPVLGIDYTHVRTPFEISLWILLACLMKIGFHVIPTISS
IVPESCLLIVVGLLVGGLIKGVGETPPFLQSDVFFLFLLPPIILDAGYFLPLRQFTENLGTILIF
AVVGTLWNAFFLGGLMYAVCLVGGEQINNIGLLDNLLFGSIISAVDPVAVLAVFEEIHINELL
HILVFGESLLNDAVTVVLYHLFEEFANYEHVGIVDIFLGFLSFFVVALGGVLVGVVYGVIAA
FTSRFTSHIRVIEPLFVFLYSYMAYLSAELFHLSGIMALIASGVVMRPYVEANISHKSHTTIK
YFLKMWSSVSETLIFIFLGVSTVAGSHHWNWTFVISTLLFCLIARVLGVLGLTWFINKFRIV
KLTPKDQFIIAYGGLRGAIAFSLGYLLDKKHFPMCDLFLTAIITVIFFTVFVQGMTIRPLVDL
LAVKKKQETKRSINEEIHTQFLDHLLTGIEDICGHYGHHHWKDKLNRFNKKYVKKCLIAG
ERSKEPQLIAFYHKMEMKQAIELVESGGMGKIPSAVSTVSMQNIHPKSLPSERILPALSKDK
EEEIRKILRNNLQKTRQRLRSYNRHTLVADPYEEAWNQMLLRRQKARQLEQKINNYLTVP
AHKLDSPTMSRARIGSDPLAYEPKEDLPVITIDPASPQSPESVDLVNEELKGKVLGLSRDPA
KVAEEDEDDDGGIMMRSKETSSPGTDDVFTPAPSDSPSSQRIQRCLSDPGPHPEPGEGEPFF
PKGQ

>P22223 single-pass type I membrane protein
MGLPRGPLASLLLLQVCWLQCAASEPCRAVFREAEVTLEAGGAEQEPGQALGKVFMGCP
GQEPALFSTDNDDFTVRNGETVQERRSLKERNPLKIFPSKRILRRHKRDWVVAPISVPENG
KGPFPQRLNQLKSNKDRDTKIFYSITGPGADSPPEGVFAVEKETGWLLLNKPLDREEIAKY
ELFGHAVSENGASVEDPMNISIIVTDQNDHKPKFTQDTFRGSVLEGVLPGTSVMQVTATDE
DDAIYTYNGVVAYSIHSQEPKDPHDLMFTIHRSTGTISVISSGLDREKVPEYTLTIQATDMD
GDGSTTTAVAVVEILDANDNAPMFDPQKYEAHVPENAVGHEVQRLTVTDLDAPNSPAWR
ATYLIMGGDDGDHFTITTHPESNQGILTTRKGLDFEAKNQHTLYVEVTNEAPFVLKLPTST
ATIVVHVEDVNEAPVFVPPSKVVEVQEGIPTGEPVCVYTAEDPDKENQKISYRILRDPAGW
LAMDPDSGQVTAVGTLDREDEQFVRNNIYEVMVLAMDNGSPPTTGTGTLLLTLIDVNDH
GPVPEPRQITICNQSPVRQVLNITDKDLSPHTSPFQAQLTDDSDIYWTAEVNEEGDTVVLSL
KKFLKQDTYDVHLSLSDHGNKEQLTVIRATVCDCHGHVETCPGPWKGGFILPVLGAVLAL
LFLLLVLLLLVRKKRKIKEPLLLPEDDTRDNVFYYGEEGGGEEDQDYDITQLHRGLEARPE
VVLRNDVAPTIIPTPMYRPRPANPDEIGNFIIENLKAANTDPTAPPYDTLLVFDYEGSGSDA
ASLSSLTSSASDQDQDYDYLNEWGSRFKKLADMYGGGEDD

>Q15223 single-pass type I membrane protein
MARMGLAGAAGRWWGLALGLTAFFLPGVHSQVVQVNDSMYGFIGTDVVLHCSFANPLP
SVKITQVTWQKSTNGSKQNVAIYNPSMGVSVLAPYRERVEFLRPSFTDGTIRLSRLELEDE
GVYICEFATFPTGNRESQLNLTVMAKPTNWIEGTQAVLRAKKGQDDKVLVATCTSANGKP
PSVVSWETRLKGEAEYQEIRNPNGTVTVISRYRLVPSREAHQQSLACIVNYHMDRFKESLT
LNVQYEPEVTIEGFDGNWYLQRMDVKLTCKADANPPATEYHWTTLNGSLPKGVEAQNRT
LFFKGPINYSLAGTYICEATNPIGTRSGQVEVNITEFPYTPSPPEHGRRAGPVPTAIIGGVAGS
ILLVLIVVGGIVVALRRRRHTFKGDYSTKKHVYGNGYSKAGIPQHHPPMAQNLQYPDDSD
DEKKAGPLGGSSYEEEEEEEEGGGGGERKVGGPHPKYDEDAKRPYFTVDEAEARQDGY
GDRTLGYQYDPEQLDLAENMVSQNDGSFISKKEWYV

>P48444 peripheral membrane protein
MVLLAAAVCTKAGKAIVSRQFVEMTRTRIEGLLAAFPKLMNTGKQHTFVETESVRYVYQ
PMEKLYMVLITTKNSNILEDLETLRLFSRVIPEYCRALEENEISEHCFDLIFAFDEIVALGYR
ENVNLAQIRTFTEMDSHEEKVFRAVRETQEREAKAEMRRKAKELQQARRDAERQGKKAP
GFGGFGSSAVSGGSTAAMITETIIETDKPKVAPAPARPSGPSKALKLGAKGKEVDNFVDKL
KSEGETIMSSSMGKRTSEATKMHAPPINMESVHMKIEEKITLTCGRDGGLQNMELHGMIM
LRISDDKYGRIRLHVENEDKKGVQLQTHPNVDKKLFTAESLIGLKNPEKSFPVNSDVGVL
KWRLQTTEESFIPLTINCWPSESGNGCDVNIEYELQEDNLELNDVVITIPLPSGVGAPVIGEI
DGEYRHDSRRNTLEWCLPVIDAKNKSGSLEFSIAGQPNDFFPVQVSFVSKKNYCNIQVTK
VTQVDGNSPVRFSTETTFLVDKYEIL

>P43121 single-pass type I membrane protein
MGLPRLVCAFLLAACCCCPRVAGVPGEAEQPAPELVEVEVGSTALLKCGLSQSQGNLSHV
DWFSVHKEKRTLIFRVRQGQGQSEPGEYEQRLSLQDRGATLALTQVTPQDERIFLCQGKRP
RSQEYRIQLRVYKAPEEPNIQVNPLGIPVNSKEPEEVATCVGRNGYPIPQVIWYKNGRPLKE
EKNRVHIQSSQTVESSGLYTLQSILKAQLVKEDKDAQFYCELNYRLPSGNHMKESREVTV
PVFYPTEKVWLEVEPVGMLKEGDRVEIRCLADGNPPPHFSISKQNPSTREAEEETTNDNG

VLVLEPARKEHSGRYECQGLDLDTMISLLSEPQELLVNYVSDVRVSPAAPERQEGSSLTLTC
EAESSQDLEFQWLREETGQVLERGPVLQLHDLKREAGGGYRCVASVPSIPGLNRTQLVNV
AIFGPPWMAFKERKVWVKENMVLNLSCEASGHPRPTISWNVNGTASEQDQDPQRVLSTL
NVLVTPELLETGVECTASNDLGKNTSILFLELVNLTTLTPDSNTTTGLSTSTASPHTRANSTS
TERKLPEPESRGVVIVAVIVCILVLAVLGAVLYFLYKKGKLPCRRSGKQEITLPPSRKSELVV
EVKSDKLPEEMGLLQGSSGDKRAPGDQGEKYIDLRH

>O75443 GPI-anchor, lipid-anchor
MNYSSFLRIWVSFIFALVQHQAQPRELMYPFWQNDTKTPKVDDGSSSEIKLAIPVFFFGVP
YRTVYVNNNGVVSFNVLVSQFTPESFPLTDGRAFVAPFWADVHNGIRGEIYYRETMEPAIL
KRATKDIRKYFKDMATFSATWVFIVTWEEVTFYGGSSTTPVNTFQAVLVSDGSYTFTLFNY
YEINWTTGTASGGDPLTGLGGVMAQAGFNGGNLTNFFSLPGSRTPEIVNIQETTNVNVPGR
WAFKVDGKEIDPANGCTSRGQFLRRGEVFWDDLNCTVKCRCLDFNNEIYCQEASCSPYE
VCEPKGKFFYCSAVETSTCVVFGEPHYHTFDGFLFHFQGSCAYLLARQCLQTSSLPFFSVE
AKNEHRRGSAVSWVKELSVEVNGYKILIPKGSYGRVKVNDLVTSLPVTLDLGTVKIYQSG
ISTAVETDFGLLVTFDGQHYASISVPGSYINSTCGLCGNYNKNPLDDFLRPDGRPAMSVLD
LGESWRVYHADWKCDSGCVDNCTQCDAATEALYFGSDYCGFLNKTDGPLWECGTVVDP
TAFVHSCVYDLCSVRDNGTLLCQAIQAYALVCQALGIPIGDWRTQTGCVSTVQCPSFSHYS
VCTSSCPDTCSDLTASRNCATPCTEGCECNQGFVLSTSQCVPLHKCGCDFDGHYYTMGEF
FWATANCTVQCLCEEGGDVYCFNKTCGSGEVCAVEDGYQGCFPKRETVCLLSQNQVLHT
FDGASYAFPSEFSYTLLKTCPERPEYLEIDINKKKPDAGPAWLRGLRILVADQEVKIGGIGA
SEVKLNGQEVELPFFHPSGKLEIYRNKNSTTVESKGVVTVQYSDIGLLYIRLSTTYFNCTG
GLCGFYNANASDEFCLPNGKCTDNLAVFLESWTTFEEICNGECGDLLKACNNDSELLKFY
RSRSRCGIINDPSNSSFLECHGVVNVTAYYRTCLFRLCQSGGNESELCDSVARYASACKNA
DVEVGPWRTYDFCPLECPENSHFEECITCTETCETLTLGPICVDSCSEGCQCDEGYALLGSQ
CVTRSECGCNFEGHQLATNETFWVDLDCQIFCYCSGTDNRVHCETIPCKDDEYCMEEGGL
YYCQARTDASCIVSGYGHYLTFDGFPFDFQTSCPLILCTTGSRPSSDSFPKFVVTAKNEDRD
PSLALWVKQVDVTVFGYSIVIHRAYKHTVLVNSERLYLPLKLGQGKINIFSFGFHVVVETD
FGLKVVYDWKTFLSITVPRSMQNSTYGLCGRYNGNPDDDLEMPMGLLASSVNEFGQSW
VKRDTFCQVGCGDRCPSCAKVEGFSKVQQLCSLIPNQNAAFSKCHSKVNPTFFYKNCLFD
SCIDGGAVQTACSWLQNYASTCQTQGITVTGWRNYTSCTVTCPPNSHYESCVSVCQPRCA
AIRLKSDCSHYCVEGCHCDAGYVLNGKSCILPHSCGCYSDGKYYEPKQLFWNSDCTRRC
RCFRRNVIQCDPRQCKSDEECALRNGVRGCFSTKTSYCLAAGGGVFRTFDGAFLRFPANC
AFVLSTICQKLPDISFQLIINFDKWSAPNLTIISPVYFYINEEQILINDRNTVKVNGTQVNVPF
ITGLATKIYSSEGFLVIDTSPDIQIYYNGFNVIKISISERLQNKVCGLCGNFNGDLTDDYVTL
RGKPVVSSVVLAQSWKTNGMQKRPLAPSCNELQFSQYAAMCDNVHIQKMQGDGYCLKL
TDMKGFFQPCYGLLDPLPFYESCYLDGCYSHKKFQLCGSLAAYGEACRSFGILSTEWIEKE
NCSGVVEDPCVGADCPNRTCELGNGRELCGCIEPPPYGNNSHDIIDAEVTCKAAQMEVSIS
KCKLFQLGFEREGVRINDRQCTGIEGEDFISFQINNTKGNCGNIVQSNGTHIMYKNTLWIES
ANNTGNIITRDRTINVEFSCAYELDIKISLDSVVKPMLSVINLTVPTQEGSFITKMALYKNAS
YKHPYRQGEVVLTTRDVLYVGVFVVGADATHLILTLNKCYATPTRDSNDKLRYFIIEGGCQ
NLKDNTIGIEENAVSLTCRFHVTVFKFIGDYDEVHLHCAVSLCDSEKYSCKITCPHNSRIAT
DYTKEPKEQIISVGPIRRKRLDWCEDNGGCEQICTSRVDGPLCSCVTGTLQEDGKSCRASN
SSMELQVWTLLLIMIQISLWHFVYKSGTTS

>P35052 GPI-anchor, lipid-anchor
MELRARGWWLLCAAAALVACARGDPASKSRSCGEVRQIYGAKGFSLSDVPQAEISGEHL
RICPQGYTCCTSEMEENLANRSHAELETALRDSSRVLQAMLATQLRSFDDHFQHLLNDSE
RTLQATFPGAFGELYTQNARAFRDLYSELRLYYRGANLHLEETLAEFWARLLERLFKQLHP
QLLLPDDYLDCLGKQAEALRPFGEAPRELRLRATRAFVAARSFVQGLGVASDVVRKVAQ
VPLGPECSRAVMKLVYCAHCLGVPGARPCPDYCRNVLKGCLANQADLDAEWRNLLDSM
VLITDKFWGTSGVESVIGSVHTWLAEAINALQDNRDTLTAKVIQGCGNPKVNPQGPGPEE
KRRRGKLAPRERPPSGTLEKLVSEAKAQLRDVQDFWISLPGTLCSEKMALSTASDDRCWN
GMARGRYLPEVMGDGLANQINNPEVEVDITKPDMTIRQQIMQLKIMTNRLRSAYNGNDV
DFQDASDDGSGSGSGDGCLDDLCSRKVSRKSSSSRTPLTHALPGLSEQEGQKTSAASCPQP
PTFLLPLLLFLALTVARPRWR

>Q9GZV3 multipass membrane protein
MAFHVEGLIAIIVFYLLILLVGIWAAWRTKNSGSAEERSEAIIVGGRDIGLLVGGFTMTATW
VGGGYINGTAEAVYVPGYGLAWAQAPIGYSLSLILGGLFFAKPMRSKGYVTMLDPFQQIY
GKRMGGLLFIPALMGEMFWAAAIFSALGATISVIIDVDMHISVIISALIATLYTLVGGLYSVA
YTDVVQLFCIFVGLWISVPFALSHPAVADIGFTAVHAKYQKPWLGTVDSSEVYSWLDSFLL
LMLGGIPWQAYFQRVLSSSSATYAQVLSFLAAFGCLVMAIPAILIGAIGASTDWNQTAYGLP
DPKTTEEADMILPIVLQYLCPVYISFFGLGAVSAAVMSSADSSILSASSMFARNIYQLSFRQ
NASDKEIVWVMRITVFVFGASATAMALLTKTVYGLWYLSSDLVYIVIFPQLLCVLFVKGT
NTYGAVAGYVSGLFLRITGGEPYLYLQPLIFYPGYYPDDNGIYNQKFPFKTLAMVTSFLTNI
CISYLAKYLFESGTLPPKLDVFDAVVARHSEENMDKTILVKNENIKLDELALVKPRQSMTL
SSTFTNKEAFLDVDSSPEGSGTEDNLQ

>Q16760 peripheral membrane protein
MAAAAGAPPPGPPQPPPPPPPEESSDSEPEAEPGSPQKLIRKVSTSGQIRQKTIIKEGMLTKQ
NNSFQRSKRRYFKLRGRTLYYAKTAKSIIFDEVDLTDASVAESSTKNVNNSFTVITPCRKLIL
CADNRKEMEDWIAALKTVQNREHFEPTQYSMDHFSGMHNWYACSHARPTYCNVCREA
LSGVTSHGLSCEVCKFKAHKRCAVRATNNCKWTTLASIGKDIIEDADGIAMPHQWLEGNL
PVSAKCTVCDKTCGSVLRLQDWRCLWCKAMVHTSCKESLLTKCPLGLCKVSVIPPTALNS
IDSDGFWKASCPPSCTSPLLVFVNSKSGDNQGVKFLRRFKQLLNPAQVFDLMNGGPHLGL
RLFQKFDTFRILVCGGDGSVGWVLSEIDSLNLHKQCQLGVLPLGTGNDLARVLGWGSAC
DDDTQLPQILEKLERASTKMLDRWSVMAYEAKLPRQASSSTVTEDFSEDSEVQQILFYED
SVAAHLSKILTSDQHSVVISSAKVLCETVKDFVARVGKAYEKTTESSEESEVMAKKCSVLK
EKLDSLLKTLDDESQASSSLPNPPPTIAEEAEDGDGSGSICGSTGDRLVASACPARPQIFRPR
EQLMLRANSLKKAIRQIIEHTEKAVDEQNAQTQEQEGFVLGLSESEEKMDHRVCPPLSHSE
SFGVPKGRSQRKVSKSPCEKLISKGSLSLGSSASLPPQPGSRDGLPALNTKILYPNVRAGMS
GSLPGGSVISRLLINADPFNSEPETLEYYTEKCVMNNYFGIGLDAKISLDFNNKRDEHPEK
CRSRTKNMMWYGVLGTKELLHRTYKNLEQKVLLECDGRPIPLPSLQGIAVLNIPSYAGGT
NFWGGTKEDDTFAAPSFDDKILEVVAVFGSMQMAVSRVIRLQHHRIAQCRTVKISILGDEG
VPVQVDGEAWVQPPGYIRIVHKNRAQTLTRDRAFESTLKSWEDKQKCELPRPPSCSLHPE
MLSEEEATQMDQFGQAAGVLIHSIREIAQSHRDMEQELAHAVNASSKSMDRVYGKPRTTE
GLNCSFVLEMVNNFRALRSETELLLSGKMALQLDPPQKEQLGSALAEMDRQLRRLADTP

WLCQSAEPGDEESVMLDLAKRSRSGKFRLVTKFKKEKNNKNKEAHSSLGAPVHLWGTEE
VAAWLEHLSLCEYKDIFTRHDIRGSELLHLERRDLKDLGVTKVGHMKRILCGIKELSRSAP
AVEA

>P06734 single-pass type II membrane protein
MEEGQYSEIEELPRRRCCRRGTQIVLLGLVTAALWAGLLTLLLLWHWDTTQSLKQLEERA
ARNVSQVSKNLESHHGDQMAQKSQSTQISQELEELRAEQQRLKSQDLELSWNLNGLQAD
LSSFKSQELNERNEASDLLERLREEVTKLRMELQVSSGFVCNTCPEKWINFQRKCYYFGK
GTKQWVHARYACDDMEGQLVSIHSPEEQDFLTKHASHTGSWIGLRNLDLKGEFIWVDGS
HVDYSNWAPGEPTSRSQGEDCVMMRGSGRWNDAFCDRKLGAWVCDRLATCTPPASEGS
AESMGPDSRPDPDGRLPTPSAPLHS

>Q9GZU1 multipass membrane protein
MTAPAGPRGSETERLLTPNPGYGTQAGPSPAPPTPPEEEDLRRRLKYFFMSPCDKFRAKGR
KPCKLMLQVVKILVVTVQLILFGLSNQLAVTFREENTIAFRHLFLLGYSDGADDTFAAYTR
EQLYQAIFHAVDQYLALPDVSLGRYAYVRGGGDPWTNGSGLALCQRYYHRGHVDPANDT
FDIDPMVVTDCIQVDPPERPPPPPSDDLTLLESSSSYKNLTLKFHKLVNVTIHFRLKTINLQS
LINNEIPDCYTFSVLITFDNKAHSGRIPISLETQAHIQECKHPSVFQHGDNSFRLLFDVVVIL
TCSLSFLLCARSLLRGFLLQNEFVGFMWRQRGRVISLWERLEFVNGWYILLVTSDVLTISG
TIMKIGIEAKNLASYDVCSILLGTSTLLVWVGVIRYLTFFHNYNILIATLRVALPSVMRFCCC
VAVIYLGYCFCGWIVLGPYHVKFRSLSMVSECLFSLINGDDMFVTFAAMQAQQGRSSLVW
LFSQLYLYSFISLFIYMVLSLFIALITGAYDTIKHPGGAGAEESELQAYIAQCQDSPTSGKFR
RGSGSACSLLCCCGRDPSEEHSLLVN

>Q6NUK1 multipass membrane protein
MLRWLRDFVLPTAACQDAEQPTRYETLFQALDRNGDGVVDIGELQEGLRNLGIPLGQDA
EEKIFTTGDVNKDGKLDFEEFMKYLKDHEKKMKLAFKSLDKNNDGKIEASEIVQSLQTL
GLTISEQQAELILQSIDVDGTMTVDWNEWRDYFLFNPVTDIEEIIRFWKHSTGIDIGDSLTIP
DEFTEDEKKSGQWWRQLLAGGIAGAVSRTSTAPLDRLKIMMQVHGSKSDKMNIFGGFRQ
MVKEGGIRSLWRGNGTNVIKIAPETAVKFWAYEQYKKLLTEEGQKIGTFERFISGSMAGAT
AQTFIYPMEVMKTRLAVGKTGQYSGIYDCAKKILKHEGLGAFYKGYVPNLLGIIPYAGIDL
AVYELLKSYWLDNFAKDSVNPGVMVLLGCGALSSTCGQLASYPLALVRTRMQAQAMLE
GSPQLNMVGLFRRIISKEGIPGLYRGITPNFMKVLPAVGISYVVYENMKQTLGVTQK

>P09848 single-pass type I membrane protein
MELSWHVVFIALLSFSCWGSDWESDRNFISTAGPLTNDLLHNLSGLLGDQSSNFVAGDKD
MYVCHQPLPTFLPEYFSSLHASQITHYKVFLSWAQLLPAGSTQNPDEKTVQCYRRLLKAL
KTARLQPMVILHHQTLPASTLRRTEAFADLFADYATFAFHSFGDLVGIWFTFSDLEEVIKEL
PHQESRASQLQTLSDAHRKAYEIYHESYAFQGGKLSVVLRAEDIPELLLEPPISALAQDTV
DFLSLDLSYECQNEASLRQKLSKLQTIEPKVKVFIFNLKLPDCPSTMKNPASLLFSLFEAIN
KDQVLTIGFDINEFLSCSSSSKKSMSCSLTGSLALQPDQQQDHETTDSSPASAYQRIWEAFA
NQSRAERDAFLQDTFPEGFLWGASTGAFNVEGGWAEGGRGVSIWDPRRPLNTTEGQATL
EVASDSYHKVASDVALLCGLRAQVYKFSISWSRIFPMGHGSSPSLPGVAYYNKLIDRLQDA
GIEPMATLFHWDLPQALQDHGGWQNESVVDAFLDYAAFCFSTFGDRVKLWVTFHEPWV

MSYAGYGTGQHPPGISDPGVASFKVAHLVLKAHARTWHHYNSHHRPQQQGHVGIVLNSD
WAEPLSPERPEDLRASERFLHFMLGWFAHPVFVDGDYPATLRTQIQQMNRQCSHPVAQLP
EFTEAEKQLLKGSADFLGLSHYTSRLISNAPQNTCIPSYDTIGGFSQHVNHVWPQTSSSWIR
VVPWGIRRLLQFVSLEYTRGKVPIYLAGNGMPIGESENLFDDSLRVDYFNQYINEVLKAIK
EDSVDVRSYIARSLIDGFEGPSGYSQRFGLHHVNFSDSSKSRTPRKSAYFFTSIIEKNGFLTK
GAKRLLPPNTVNLPSKVRAFTFPSEVPSKAKVVWEKFSSQPKFERDLFYHGTFRDDFLWG
VSSSAYQIEGAWDADGKGPSIWDNFTHTPGSNVKDNATGDIACDSYHQLDADLNMLRAL
KVKAYRFSISWSRIFPTGRNSSINSHGVDYYNRLINGLVASNIFPMVTLFHWDLPQALQDIG
GWENPALIDLFDSYADFCFQTFGDRVKFWMTFNEPMYLAWLGYGSGEFPPGVKDPGWAP
YRIAHAVIKAHARVYHTYDEKYRQEQKGVISLSLSTHWAEPKSPGVPRDVEAADRMLQFS
LGWFAHPIFRNGDYPDTMKWKVGNRSELQHLATSRLPSFTEEEKRFIRATADVFCLNTYY
SRIVQHKTPRLNPPSYEDDQEMAEEEDPSWPSTAMNRAAPWGTRRLLNWIKEEYGDIPIYI
TENGVGLTNPNTEDTDRIFYHKTYINEALKAYRLDGIDLRGYVAWSLMDNFEWLNGYTV
KFGLYHVDFNNTNRPRTARASARYYTEVITNNGMPLAREDEFLYGRFPEGFIWSAASAAY
QIEGAWRADGKGLSIWDTFSHTPLRVENDAIGDVACDSYHKIAEDLVTLQNLGVSHYRFSI
SWSRILPDGTTRYINEAGLNYYVRLIDTLLAASIQPQVTIYHWDLPQTLQDVGGWENETIV
QRFKEYADVLFQRLGDKVKFWITLNEPFVIAYQGYGYGTAAPGVSNRPGTAPYIVGHNLI
KAHAEAWHLYNDVYRASQGGVISITISSDWAEPRDPSNQEDVEAARRYVQFMGGWFAHP
IFKNGDYNEVMKTRIRDRSLAAGLNKSRLPEFTESEKRRINGTYDFFGFNHYTTVLAYNL
NYATAISSFDADRGVASIADRSWPDSGSFWLKMTPFGFRRILNWLKEEYNDPPIYVTENGV
SQREETDLNDTARIYYLRTYINEALKAVQDKVDLRGYTVWSAMDNFEWATGFSERFGLH
FVNYSDPSLPRIPKASAKFYASVVRCNGFPDPATGPHACLHQPDAGPTISPVRQEEVQFLGL
MLGTTEAQTALYVLFSLVLLGVCGLAFLSYKYCKRSKQGKTQRSQQELSPVSSF

>Q969V5 multipass membrane protein
MESGGRPSLCQFILLGTTSVVTAALYSVYRQKARVSQELKGAKKVHLGEDLKSILSEAPG
KCVPYAVIEGAVRSVKETLNSQFVENCKGVIQRLTLQEHKMVWNRTTHLWNDCSKIIHQR
TNTVPFDLVPHEDGVDVAVRVLKPLDSVDLGLETVYEKFHPSIQSFTDVIGHYISGERPKGI
QETEEMLKVGATLTGVGELVLDNNSVRLQPPKQGMQYYLSSQDFDSLLQRQESSVRLWK
VLALVFGFATCATLFFILRKQYLQRQERLRLKQMQEEFQEHEAQLLSRAKPEDRESLKSAC
VVCLSSFKSCVFLECGHVCSCTECYRALPEPKKCPICRQAITRVIPLYNS

>Q8NC60 peripheral membrane protein
MLPARLPFRLLSLFLRGSAPTAARHGLREPLLERRCAAASSFQHSSSLGRELPYDPVDTEG
FGEGGDMQERFLFPEYILDPEPQPTREKQLQELQQQQEEEERQRQQRREERRQQNLRARS
REHPVVGHPDPALPPSGVNCSGCGAELHCQDAGVPGYLPREKFLRTAEADGGLARTVCQ
RCWLLSHHRRALRLQVSREQYLELVSAALRRPGPSLVLYMVDLLDLPDALLPDLPALVGP
KQLIVLGNKVDLLPQDAPGYRQRLRERLWEDCARAGLLLAPGHQGPQRPVKDEPQDGEN
PNPPNWSRTVVRDVRLISAKTGYGVEELISALQRSWRYRGDVYLVGATNAGKSTLFNTLL
ESDYCTAKGSEAIDRATISPWPGTTLNLLKFPICNPTPYRMFKRHQRLKKDSTQAEEDLSE
QEQNQLNVLKKHGYVVGRVGRTFLYSEEQKDNIPFEFDADSLAFDMENDPVMGTHKSTK
QVELTAQDVKDAHWFYDTPGITKENCILNLLTEKEVNIVLPTQSIVPRTFVLKPGMVLFLG
AIGRIDFLQGNQSAWFTVVASNILPVHITSLDRADALYQKHAGHTLLQIPMGGKERMAGF
PPLVAEDIMLKEGLGASEAVADIKFSSAGWVSVTPNFKDRLHLRGYTPEGTVLTVRPPLLP

YIVNIKGQRIKKSVAYKTKKPPSLMYNVRKKKGKINV

>O75841 multipass membrane protein
MAKDNSTVRCFQGLLIFGNVIIGCCGIALTAECIFFVSDQHSLYPLLEATDNDDIYGAAWIGI
FVGICLFCLSVLGIVGIMKSSRKILLAYFILMFIVYAFEVASCITAATQQDFFTPNLFLKQMLE
RYQNNSPPNNDDQWKNNGVTKTWDRLMLQDNCCGVNGPSDWQKYTSAFRTENNDAD
YPWPRQCCVMNNLKEPLNLEACKLGVPGFYHNQGCYELISGPMNRHAWGVAWFGFAIL
CWTFWVLLGTMFYWSRIEY

>P33681 single-pass type I membrane protein
MGHTRRQGTSPSKCPYLNFFQLLVLAGLSHFCSGVIHVTKEVKEVATLSCGHNVSVEELA
QTRIYWQKEKKMVLTMMSGDMNIWPEYKNRTIFDITNNLSIVILALRPSDEGTYECVVLK
YEKDAFKREHLAEVTLSVKADFPTPSISDFEIPTSNIRRIICSTSGGFPEPHLSWLENGEELN
AINTTVSQDPETELYAVSSKLDFNMTTNHSFMCLIKYGHLRVNQTFNWNTTKQEHFPDNL
LPSWAITLISVNGIFVICCLTYCFAPRCRERRRNERLRRESVRPV

>Q9HB29 single-pass type I membrane protein
MWSLLLCGLSIALPLSVTADGCKDIFMKNEILSASQPFAFNCTFPPITSGEVSVTWYKNSSK
IPVSKIIQSRIHQDETWILFLPMEWGDSGVYQCVIKGRDSCHRIHVNLTVFEKHWCDTSIG
GLPNLSDEYKQILHLGKDDSLTCHLHFPKSCVLGPIKWYKDCNEIKGERFTVLETRLLVSN
VSAEDRGNYACQAILTHSGKQYEVLNGITVSITERAGYGGSVPKIIYPKNHSIEVQLGTTLI
VDCNVTDTKDNTNLRCWRVNNTLVDDYYDESKRIREGVETHVSFREHNLYTVNITFLEV
KMEDYGLPFMCHAGVSTAYIILQLPAPDFRAYLIGGLIALVAVAVSVVYIYNIFKIDIVLWYR
SAFHSTETIVDGKLYDAYVLYPKPHKESQRHAVDALVLNILPEVLERQCGYKLFIFGRDEFP
GQAVANVIDENVKLCRRLIVIVVPESLGFGLLKNLSEEQIAVYSALIQDGMKVILIELEKIED
YTVMPESIQYIKQKHGAIRWHGDFTEQSQCMKTKFWKTVRYHMPPRRCRPFPPVQLLQH
TPCYRTAGPELGSRRKKCTLTTG

>P42681 peripheral membrane protein
MILSSYNTIQSVFCCCCCCSVQKRQMRTQISLSTDEELPEKYTQRRRPWLSQLSNKKQSNT
GRVQPSKRKPLPPLPPSEVAEEKIQVKALYDFLPREPCNLALRRAEEYLILEKYNPHWWKA
RDRLGNEGLIPSNYVTENKITNLEIYEWYHRNITRNQAEHLLRQESKEGAFIVRDSRHLGS
YTISVFMGARRSTEAAIKHYQIKKNDSGQWYVAERHAFQSIPELIWYHQHNAAGLMTRLR
YPVGLMGSCLPATAGFSYEKWEIDPSELAFIKEIGSGQFGVVHLGEWRSHIQVAIKAINEGS
MSEEDFIEEAKVMMKLSHSKLVQLYGVCIQRKPLYIVTEFMENGCLLNYLRENKGKLRKE
MLLSVCQDICEGMEYLERNGYIHRDLAARNCLVSSTCIVKISDFGMTRYVLDDEYVSSFG
AKFPIKWSPPEVFLFNKYSSKSDVWSFGVLMWEVFTEGKMPFENKSNLQVVEAISEGFRL
YRPHLAPMSIYEVMYSCWHEKPEGRPTFAELLRAVTEIAETW

>P48169 multipass membrane protein
MVSAKKVPAIALSAGVSFALLRFLCLAVCLNESPGQNQKEEKLCTENFTRILDSLLDGYDN
RLRPGFGGPVTEVKTDIYVTSFGPVSDVEMEYTMDVFFRQTWIDKRLKYDGPIEILRLNN
MMVTKVWTPDTFFRNGKKSVSHNMTAPNKLFRIMRNGTILYTMRLTISAECPMRLVDFP
MDGHACPLKFGSYAYPKSEMIYTWTKGPEKSVEVPKESSSLVQYDLIGQTVSSETIKSITGE

YIVMTVYFHLRRKMGYFMIQTYIPCIMTVILSQVSFWINKESVPARTVFGITTVLTMTTLSI
SARHSLPKVSYATAMDWFIAVCFAFVFSALIEFAAVNYFTNIQMEKAKRKTSKPPQEVPAA
PVQREKHPEAPLQNTNANLNMRKRTNALVHSESDVGNRTEVGNHSSKSSTVVQESSKGT
PRSYLASSPNPFSRANAAETISAARALPSASPTSIRTGYMPRKASVGSASTRHVFGSRLQRI
KTTVNTIGATGKLSATPPPSAPPPSGSGTSKIDKYARILFPVTFGAFNMVYWVVYLSKDTM
EKSESLM

>P14410 single-pass type II membrane protein
MARKKFSGLEISLIVLFVIVTIIAIALIVVLATKTPAVDEISDSTSTPATTRVTTNPSDSGKCPN
VLNDPVNVRINCIPEQFPTEGICAQRGCCWRPWNDSLIPWCFFVDNHGYNVQDMTTTSIG
VEAKLNRIPSPTLFGNDINSVLFTTQNQTPNRFRFKITDPNNRRYEVPHQYVKEFTGPTVSD
TLYDVKVAQNPFSIQVIRKSNGKTLFDTSIGPLVYSDQYLQISTRLPSDYIYGIGEQVHKRFR
HDLSWKTWPIFTRDQLPGDNNNNLYGHQTFFMCIEDTSGKSFGVFLMNSNAMEIFIQPTPI
VTYRVTGGILDFYILLGDTPEQVVQQYQQLVGLPAMPAYWNLGFQLSRWNYKSLDVVKE
VVRRNREAGIPFDTQVTDIDYMEDKKDFTYDQVAFNGLPQFVQDLHDHGQKYVIILDPAI
SIGRRANGTTYATYERGNTQHVWINESDGSTPIIGEVWPGLTVYPDFTNPNCIDWWANECS
IFHQEVQYDGLWIDMNEVSSFIQGSTKGCNVNKLNYPPFTPDILDKLMYSKTICMDAVQN
WGKQYDVHSLYGYSMAIATEQAVQKVFPNKRSFILTRSTFAGSGRHAAHWLGDNTASWE
QMEWSITGMLEFSLFGIPLVGADICGFVAETTEELCRRWMQLGAFYPFSRNHNSDGYEHQ
DPAFFGQNSLLVKSSRQYLTIRYTLLPFLYTLFYKAHVFGETVARPVLHEFYEDTNSWIEDT
EFLWGPALLITPVLKQGADTVSAYIPDAIWYDYESGAKRPWRKQRVDMYLPADKIGLHLR
GGYIIPIQEPDVTTTASRKNPLGLIVALGENNTAKGDFFWDDGETKDTIQNGNYILYTFSVS
NNTLDIVCTHSSYQEGTTLAFQTVKILGLTDSVTEVRVAENNQPMNAHSNFTYDASNQVL
LIADLKLNLGRNFSVQWNQIFSENERFNCYPDADLATEQKCTQRGCVWRTGSSLSKAPEC
YFPRQDNSYSVNSARYSSMGITADLQLNTANARIKLPSDPISTLRVEVKYHKNDMLQFKIY
DPQKKRYEVPVPLNIPTTPISTYEDRLYDVEIKENPFGIQIRRRSSGRVIWDSWLPGFAFNDQ
FIQISTRLPSEYIYGFGEVEHTAFKRDLNWNTWGMFTRDQPPGYKLNSYGFHPYYMALEE
EGNAHGVFLLNSNAMDVTFQPTPALTYRTVGGILDFYMFLGPTPEVATKQYHEVIGHPVM
PAYWALGFQLCRYGYANTSEVRELYDAMVAANIPYDVQYTDIDYMERQLDFTIGEAFQDL
PQFVDKIRGEGMRYIIILDPAISGNETKTYPAFERGQQNDVFVKWPNTNDICWAKVWPDLP
NITIDKTLTEDEAVNASRAHVAFPDFFRTSTAEWWAREIVDFYNEKMKFDGLWIDMNEPSS
FVNGTTTNQCRNDELNYPPYFPELTKRTDGLHFRTICMEAEQILSDGTSVLHYDVHNLYG
WSQMKPTHDALQKTTGKRGIVISRSTYPTSGRWGGHWLGDNYARWDNMDKSIIGMMEF
SLFGMSYTGADICGFFNNSEYHLCTRWMQLGAFYPYSRNHNIANTRRQDPASWNETFAE
MSRNILNIRYTLLPYFYTQMHEIHANGGTVIRPLLHEFFDEKPTWDIFKQFLWGPAFMVTP
VLEPYVQTVNAYVPNARWFDYHTGKDIGVRGQFQTFNASYDTINLHVRGGHILPCQEPA
QNTFYSRQKHMKLIVAADDNQMAQGSLFWDDGESIDTYERDLYLSVQFNLNQTTLTSTIL
KRGYINKSETRLGSLHVWGKGTTPVNAVTLTYNGNKNSLPFNEDTTNMILRIDLTTHNVT
LEEPIEINWS

>Q13237 lipid-anchor
MGNGSVKPKHSKHPDGHSGNLTTDALRNKVTELERELRRKDAEIQEREYHLKELREQLS
KQTVAIAELTEELQNKCIQLNKLQDVVHMQGGSPLQASPDKVPLEVHRKTSGLVSLHSRR
GAKAGVSAEPTTRTYDLNKPPEFSFEKARVRKDSSEKKLITDALNKNQFLKRLDPQQIKD

MVECMYGRNYQQGSYIIKQGEPGNHIFVLAEGRLEVFQGEKLLSSIPMWTTFGELAILYN
CTRTASVKAITNVKTWALDREVFQNIMRRTAQARDEQYRNFLRSVSLLKNLPEDKLTKIID
CLEVEYYDKGDYIIREGEEGSTFFILAKGKVKVTQSTEGHDQPQLIKTLQKGEYFGEKALI
SDDVRSANIIAEENDVACLVIDRETFNQTVGTFEELQKYLEGYVANLNRDDEKRHAKRSM
SNWKLSKALSLEMIQLKEKVARFSSSSPFQNLEIIATLGVGGFGRVELVKVKNENVAFAMK
CIRKKHIVDTKQQEHVYSEKRILEELCSPFIVKLYRTFKDNKYVYMLLEACLGGELWSILR
DRGSFDEPTSKFCVACVTEAFDYLHRLGIIYRDLKPENLILDAEGYLKLVDFGFAKKIGSGQ
KTWTFCGTPEYVAPEVILNKGHDFSVDFWSLGILVYELLTGNPPFSGVDQMMTYNLILKGI
EKMDFPRKITRRPEDLIRRLCRQNPTERLGNLKNGINDIKKHRWLNGFNWEGLKARSLPS
PLQRELKGPIDHSYFDKYPPEKGMPPDELSGWDKDF

>Q53EU6 multipass membrane protein
MEGAELAGKILSTWLTLVLGFILLPSVFGVSLGISEIYMKILVKTLEWATIRIEKGTPKESILK
NSASVGIIQRDESPMEKGLSGLRGRDFELSDVFYFSKKGLEAIVEDEVTQRFSSEELVSWN
LLTRTNVNFQYISLRLTMVWVLGVIVRYCVLLPLRVTLAFIGISLLVIGTTLVGQLPDSSLKN
WLSELVHLTCCRICVRALSGTIHYHNKQYRPQKGGICVANHTSPIDVLILTTDGCYAMVGQ
VHGGLMGIIQRAMVKACPHVWFERSEMKDRHLVTKRLKEHIADKKKLPILIFPEGTCINN
TSVMMFKKGSFEIGGTIHPVAIKYNPQFGDAFWNSSKYNMVSYLLRMMTSWAIVCDVWY
MPPMTREEGEDAVQFANRVKSAIAIQGGLTELPWDGGLKRAKVKDIFKEEQQKNYSKMI
VGNGSLS

>P48167 multipass membrane protein
MKFLLTTAFLILISLWVEEAYSKEKSSKKGKGKKKQYLCPSQQSAEDLARVPANSTSNILN
RLLVSYDPRIRPNFKGIPVDVVVNIFINSFGSIQETTMDYRVNIFLRQKWNDPRLKLPSDFR
GSDALTVDPTMYKCLWKPDLFFANEKSANFHDVTQENILLFIFRDGDVLVSMRLSITLSCP
LDLTLFPMDTQRCKMQLESFGYTTDDLRFIWQSGDPVQLEKIALPQFDIKKEDIEYGNCTK
YYKGTGYYTCVEVIFTLRRQVGFYMMGVYAPTLLIVVLSWLSFWINPDASAARVPLGIFS
VLSLASECTTLAAELPKVSYVKALDVWLIACLLFGFASLVEYAVVQVMLNNPKRVEAEKA
RIAKAEQADGKGGNVAKKNTVNGTGTPVHISTLQVGETRCKKVCTSKSDLRSNDFSIVGS
LPRDFELSNYDCYGKPIEVNNGLGKSQAKNNKKPPPAKPVIPTAAKRIDLYARALFPFCFLF
FNVIYWSIYL

>P35612 peripheral membrane protein
MSEETVPEAASPPPPQGQPYFDRFSEDDPEYMRLRNRAADLRQDFNLMEQKKRVTMILQS
PSFREELEGLIQEQMKKGNNSSNIWALRQIADFMASTSHAVFPTSSMNVSMMTPINDLHTA
DSLNLAKGERLMRCKISSVYRLLDLYGWAQLSDTYVTLRVSKEQDHFLISPKGVSCSEVT
ASSLIKVNILGEVVEKGSSCFPVDTTGFCLHSAIYAARPDVRCIIHLHTPATAAVSAMKWGL
LPVSHNALLVGDMAYYDFNGEMEQEADRINLQKCLGPTCKILVLRNHGVVALGDTVEEA
FYKIFHLQAACEIQVSALSSAGGVENLILLEQEKHRPHEVGSVQWAGSTFGPMQKSRLGE
HEFEALMRMLDNLGYRTGYTYRHPFVQEKTKHKSEVEIPATVTAFVFEEDGAPVPALRQH
AQKQQKEKTRWLNTPNTYLRVNVADEVQRSMGSPRPKTTWMKADEVEKSSSGMPIRIEN
PNQFVPLYTDPQEVLEMRNKIREQNRQDVKSAGPQSQLLASVIAEKSRSPSTESQLMSKG
DEDTKDDSEETVPNPFSQLTDQELEEYKKEVERKKLELDGEKETAPEEPGSPAKSAPASPV
QSPAKEAETKSPLVSPSKSLEEGTKKTETSKAATTEPETTQPEGVVVNGREEEQTAEEILSK

GLSQMTTSADTDVDTSKDKTESVTSGPMSPEGSPSKSPSKKKKKFRTPSFLKKSKKKEKVES

>Q9BRL7 multipass membrane protein
MSVIFFACVVRVRDGLPLSASTDFYHTQDFLEWRRRLKSLALRLAQYPGRGSAEGCDFSI
HFSSFGDVACMAICSCQCPAAMAFCFLETLWWEFTASYDTTCIGLASRPYAFLEFDSIIQKV
KWHFNYVSSSQMECSLEKIQEELKLQPPAVLTLEDTDVANGVMNGHTPMHLEPAPNFRM
EPVTALGILSLILNIMCAALNLIRGVHLAEHSLQVAHEEIGNILAFLVPFVACIFQCYLYLFYS
PARTMKVVLMLLFICLGNMYLHGLRNLWQILFHIGVAFLSSYQILTRQLQEKQSDCGV

>Q9H0T7 lipid-anchor
MAQAHRTPQPRAAPSQPRVFKLVLLGSGSVGKSSLALRYVKNDFKSILPTVGCAFFTKVV
DVGATSLKLEIWDTAGQEKYHSVCHLYFRGANAALLVYDITRKDSFLKAQQWLKDLEEE
LHPGEVLVMLVGNKTDLSQEREVTFQEGKEFADSQKLLFMETSAKLNHQVSEVFNTVAQ
ELLQRSDEEGQALRGDAAVALNKGPARQAKCCAH

>Q93050 multipass membrane protein
MGELFRSEEMTLAQLFLQSEAAYCCVSELGELGKVQFRDLNPDVNVFQRKFVNEVRRCE
EMDRKLRFVEKEIRKANIPIMDTGENPEVPFPRDMIDLEANFEKIENELKEINTNQEALKR
NFLELTELKFILRKTQQFFDEMADPDLLEESSSLLEPSEMGRGTPLRLGFVAGVINRERIPTF
ERMLWRVCRGNVFLRQAEIENPLEDPVTGDYVHKSVFIIFFQGDQLKNRVKKICEGFRASL
YPCPETPQERKEMASGVNTRIDDLQMVLNQTEDHRQRVLQAAAKNIRVWFIKVRKMKAI
YHTLNLCNIDVTQKCLIAEVWCPVTDLDSIQFALRRGTEHSGSTVPSILNRMQTNQTPPTY
NKTNKFTYGFQNIVDAYGIGTYREINPAPYTIITFPFLFAVMFGDFGHGILMTLFAVWMVLR
ESRILSQKNENEMFSTVFSGRYIILLMGVFSMYTGLIYNDCFSKSLNIFGSSWSVRPMFTYN
WTEETLRGNPVLQLNPALPGVFGGPYPFGIDPIWNIATNKLTFLNSFKMKMSVILGIIHMLF
GVSLSLFNHIYFKKPLNIYFGFIPEIIFMTSLFGYLVILIFYKWTAYDAHTSENAPSLLIHFIN
MFLFSYPESGYSMLYSGQKGIQCFLVVVALLCVPWMLLFKPLVLRRQYLRRKHLGTLNFG
GIRVGNGPTEEDAEIIQHDQLSTHSEDADEPSEDEVFDFGDTMVHQAIHTIEYCLGCISNTA
SYLRLWALSLAHAQLSEVLWTMVIHIGLSVKSLAGGLVLFFFFTAFATLTVAILLIMEGLSA
FLHALRLHWVEFQNKFYSGTGFKFLPFSFEHIREGKFEE

>Q9UQ05 multipass membrane protein
MPVMKGLLAPQNTFLDTIATRFDGTHSNFLLANAQGTRGFPIVYCSDGFCELTGYGRTEV
MQKTCSCRFLYGPETSEPALQRLHKALEGHQEHRAEICFYRKDGSAFWCLLDMMPIKNE
MGEVVLFLFSFKDITQSGSPGLGPQGGRGDSNHENSLGRRGATWKFRSARRRSRTVLHRL
TGHFGRRGQGGMKANNNVFEPKPSVPEYKVASVGGSRCLLLHYSVSKAIWDGLILLATFY
VAVTVPYNVCFSGDDDTPITSRHTLVSDIAVEMLFILDIILNFRTTYVSQSGQVISAPRSIGLH
YLATWFFIDLIAALPFDLLYIFNITVTSLVHLLKTVRLLRLLRLLQKLERYSQCSAVVLTLLM
SVFALLAHWMACIWYVIGRREMEANDPLLWDIGWLHELGKRLEVPYVNGSVGGPSRRSA
YIAALYFTLSSLTSVGFGNVCANTDAEKIFSICTMLIGALMHAVVFGNVTAIIQRMYSRRSL
YHSRMKDLKDFIRVHRLPRPLKQRMLEYFQTTWAVNSGIDANELLRDFPDELRADIAMHL
NREILQLPLFGAASRGCLRALSLHIKTSFCAPGEYLLRRGDALQAHYYVCSGSLEVLRDN
MVLAILGKGDLIGADIPEPGQEPGLGADPNFVLKTSADVKALTYCGLQQLSSRGLAEVLR

LYPEYGAAFRAGLPRDLTFNLRQGSDTSGLSRFSRSPRLSQPRSESLGSSSDKTLPSITEAES
GAEPGGGPRPRRPLLLPNLSPARPRGSLVSLLGEELPPFSALVSSPSLSPSLSPALAGQGHSA
SPHGPPRCSAAWKPPQLLIPPLGTFGPPDLSPRIVDGIEDSGSTAEAPSFRFSRRPELPRPRSQ
APPTGTRPSPELASEAEEVKEKVCRLNQEISRLNQEVSQLSRELRHIMGLLQARLGPPGHP
AGSAWTPDPPCPQLRPPCLSPCASRPPPSLQDTTLAEVHCPASVGTMETGTALLDLRPSILP
PYPSEPDPLGPSPVPEASPPTPSLLRHSFQSRSDTFH

>Q13423 multipass membrane protein
MANLLKTVVTGCSCPLLSNLGSCKGLRVKKDFLRTFYTHQELWCKAPVKPGIPYKQLTVG
VPKEIFQNEKRVALSPAGVQNLVKQGFNVVVESGAGEASKFSDDHYRVAGAQIQGAKEVL
ASDLVVKVRAPMVNPTLGVHEADLLKTSGTLISFIYPAQNPELLNKLSQRKTTVLAMDQV
PRVTIAQGYDALSSMANIAGYKAVVLAANHFGRFFTGQITAAGKVPPAKILIVGGGVAGLA
SAGAAKSMGAIVRGFDTRAAALEQFKSLGAEPLEVDLKESGEGQGGYAKEMSKEFIEAE
MKLFAQQCKEVDILISTALIPGKKAPVLFNKEMIESMKEGSVVVDLAAEAGGNFETTKPG
ELYIHKGITHIGYTDLPSRMATQASTLYSNNITKLLKAISPDKDNFYFDVKDDFDFGTMGH
VIRGTVVMKDGKVIFPAPTPKNIPQGAPVKQKTVAELEAEKAATITPFRKTMSTASAYTAG
LTGILGLGIAAPNLAFSQMVTTFGLAGIVGYHTVWGVTPALHSPLMSVTNAISGLTAVGGL
ALMGGHLYPSTTSQGLAALAAFISSVNIAGGFLVTQRMLDMFKRPTDPPEYNYLYLLPAG
TFVGGYLAALYSGYNIEQIMYLGSGLCCVGALAGLSTQGTARLGNALGMIGVAGGLAAT
LGVLKPGPELLAQMSGAMALGGTIGLTIAKRIQISDLPQLVAAFHSLVGLAAVLTCIAEYIIE
YPHFATDAAANLTKIVAYLGTYIGGVTFSGSLIAYGKLQGLLKSAPLLLPGRHLLNAGLLA
ASVGGIIPFMVDPSFTTGITCLGSVSALSAVMGVTLTAAIGGADMPVVITVLNSYSGWALC
AEGFLLNNNLLTIVGALIGSSGAILSYIMCVAMNRSLANVILGGYGTTSTAGGKPMEISGTH
TEINLDNAIDMIREANSIIITPGYGLCAAKAQYPIADLVKMLTEQGKKVRFGIHPVAGRMPG
QLNVLLAEAGVPYDIVLEMDEINHDFPDTDLVLVIGANDTVNSAAQEDPNSIIAGMPVLEV
WKSKQVIVMKRSLGVGYAAVDNPIFYKPNTAMLLGDAKKTCDALQAKVRESYQK

>Q9H3E2 peripheral membrane protein
MDKALKEVFDYSYRDYILSWYGNLSRDEGQLYHLLLEDFWEIARQLHHRLSHVDVVKV
VCNDVVRTLLTHFCDLKAANARHEEQPRPFVLHACLRNSDDEVRFLQTCSRVLVFCLLPS
KDVQSLSLRIMLAEILTTKVLKPVVELLSNPDYINQMLLAQLAYREQMNEHHKRAYTYAP
SYEDFIKLINSNSDVEFLKQLRYQIVVEIIQATTISSFPQLKRHKGKETAAMKADLLRARNM
KRYINQLTVAKKQCEKRIRILGGPAYDQQEDGALDEGEGPQSQKILQFEDILANTFYREHF
GMYMERMDKRALISFWESVEHLKNANKNEIPQLVGEIYQNFFVESKEISVEKSLYKEIQQC
LVGNKGIEVFYKIQEDVYETLKDRYYPSFIVSDLYEKLLIKEEEKHASQMISNKDEMGPRD
EAGEEAVDDGTNQINEQASFAVNKLRELNEKLEYKRQALNSIQNAPKPDKKIVSKLKDEII
LIEKERTDLQLHMARTDWWCENLGMWKASITSGEVTEENGEQLPCYFVMVSLQEVGGV
ETKNWTVPRRLSEFQNLHRKLSECVPSLKKVQLPSLSKLPFKSIDQKFMEKSKNQLNKFL
QNLLSDERLCQSEALYAFLSPSPDYLKVIDVQGKKNSFSLSSFLERLPRDFFSHQEEETEED
SDLSDYGDDVDGRKDALAEPCFMLIGEIFELRGMFKWVRRTLIALVQVTFGRTINKQIRDT
VSWIFSEQMLVYYINIFRDAFWPNGKLAPPTTIRSKEQSQETKQRAQQKLLENIPDMLQSL
VGQQNARHGIIKIFNALQETRANKHLLYALMELLLIELCPELRVHLDQLKAGQV

>P61026 lipid-anchor

MAKKTYDLLFKLLLIGDSGVGKTCVLFRFSDDAFNTTFISTIGIDFKIKTVELQGKKIKLQI
WDTAGQERFHTITTSYYRGAMGIMLVYDITNGKSFENISKWLRNIDEHANEDVERMLLGN
KCDMDDKRVVPKGKGEQIAREHGIRFFETSAKANINIEKAFLTLAEDILRKTPVKEPNSEN
VDISSGGGVTGWKSKCC

>Q8WWA1 multipass membrane protein
METSASSSQPQDNSQVHRETEDVDYGETDFHKQDGKAGLFSQEQYERNKSSSSSSSSSSS
SSSSSSSSSESNDEDQQPRATGKHRRSLGAGYPHGNGSPGPGHGEPDVLKDELQLYGDAP
GEVVPSGESGLRRRGSDPASGEVEASQLRRLNIKKDDEFFHFVLLCFAIGALLVCYHYYAD
WFMSLGVGLLTFASLETVGIYFGLVYRIHSVLQGFIPLFQKFRLTGFRKTD

>Q9Y5I7 multipass membrane protein
MTSRTPLLVTACLYYSYCNSRHLQQGVRKSKRPVFSHCQVPETQKTDTRHLSGARAGVCP
CCHPDGLLATMRDLLQYIACFFAFFSAGFLIVATWTDCWMVNADDSLEVSTKCRGLWWE
CVTNAFDGIRTCDEYDSILAEHPLKLVVTRALMITADILAGFGFLTLLLGLDCVKFLPDEPY
IKVRICFVAGATLLIAGTPGIIGSVWYAVDVYVERSTLVLHNIFLGIQYKFGWSCWLGMAG
SLGCFLAGAVLTCCLYLFKDVGPERNYPYSLRKAYSAAGVSMAKSYSAPRTETAKMYAVD
TRV

>Q13797 single-pass type I membrane protein
MGGPAAPRGAGRLRALLLALVVAGIPAGAYNLDPQRPVHFQGPADSFFGYAVLEHFHDNT
RWVLVGAPKADSKYSPSVKSPGAVFKCRVHTNPDRRCTELDMARGKNRGTSCGKTCRED
RDDEWMGVSLARQPKADGRVLACAHRWKNIYYEADHILPHGFCYIIPSNLQAKGRTLIPC
YEEYKKKYGEEHGSCQAGIAGFFTEELVVMGAPGSFYWAGTIKVLNLTDNTYLKLNDEVI
MNRRYTYLGYAVTAGHFSHPSTIDVVGGAPQDKGIGKVYIFRADRRSGTLIKIFQASGKK
MGSYFGSSLCAVDLNGDGLSDLLVGAPMFSEIRDEGQVTVYINRGNGALEEQLALTGDGA
YNAHFGESIASLDDLDNDGFPDVAIGAPKEDDFAGAVYIYHGDAGGIVPQYSMKLSGQKI
NPVLRMFGQSISGGIDMDGNGYPDVTVGAFMSDSVVLLRARPVITVDVSIFLPGSINITAP
QCHDGQQPVNCLNVTTCFSFHGKHVPGEIGLNYVLMADVAKKEKGQMPRVYFVLLGET
MGQVTEKLQLTYMEETCRHYVAHVKRRVQDVISPIVFEAAYSLSEHVTGEEERELPPLTPV
LRWKKGQKIAQKNQTVFERNCRSEDCAADLQLQGKLLLSSMDEKTLYLALGAVKNISLNI
SISNLGDDAYDANVSFNVSRELFFINMWQKEEMGISCELLESDFLKCSVGFPFMRSKSKYE
FSVIFDTSHLSGEEEVLSFIVTAQSGNTERSESLHDNTLVLMVPLMHEVDTSITGIMSPTSFV
YGESVDAANFIQLDDLECHFQPINITLQVYNTGPSTLPGSSVSISFPNRLSSGGAEMFHVQE
MVVGQEKGNCSFQKNPTPCIIPQEQENIFHTIFAFFTKSGRKVLDCEKPGISCLTAHCNFSA
LAKEESRTIDIYMLLNTEILKKDSSSVIQFMSRAKVKVDPALRVVEIAHGNPEEVTVVFEAL
HNLEPRGYVVGWIIAISLLVGILIFLLLAVLLWKMGFFRRRYKEIIEAEKNRKENEDSWDW
VQKNQ

>Q8N441 single-pass type I membrane protein
MTPSPLLLLLLPPLLLGAFPPAAAARGPPKMADKVVPRQVARLGRTVRLQCPVEGDPPPLT
MWTKDGRTIHSGWSRFRVLPQGLKVKQVEREDAGVYVCKATNGFGSLSVNYTLVVLDDI
SPGKESLGPDSSSGGQEDPASQQWARPRFTQPSKMRRRVIARPVGSSVRLKCVASGHPRPD
ITWMKDDQALTRPEAAEPRKKKWTLSLKNLRPEDSGKYTCRVSNRAGAINATYKVDVIQ

RTRSKPVLTGTHPVNTTVDFGGTTSFQCKVRSDVKPVIQWLKRVEYGAEGRHNSTIDVGG
QKFVVLPTGDVWSRPDGSYLNKLLITRARQDDAGMYICLGANTMGYSFRSAFLTVLPDP
KPPGPPVASSSSATSLPWPVVIGIPAGAVFILGTLLLWLCQAQKKPCTPAPAPPLPGHRPPGT
ARDRSGDKDLPSLAALSAGPGVGLCEEHGSPAAPQHLLGPGPVAGPKLYPKLYTDIHTHT
HTHSHTHSHVEGKVHQHIHYQC

>P35611 peripheral membrane protein
MNGDSRAAVVTSPPPTTAPHKERYFDRVDENNPEYLRERNMAPDLRQDFNMMEQKKRVS
MILQSPAFCEELESMIQEQFKKGKNPTGLLALQQIADFMTTNVPNVYPAAPQGGMAALN
MSLGMVTPVNDLRGSDSIAYDKGEKLLRCKLAAFYRLADLFGWSQLIYNHITTRVNSEQE
HFLIVPFGLLYSEVTASSLVKINLQGDIVDRGSTNLGVNQAGFTLHSAIYAARPDVKCVVHI
HTPAGAAVSAMKCGLLPISPEALSLGEVAYHDYHGILVDEEEKVLIQKNLGPKSKVLILRN
HGLVSVGESVEEAFYYIHNLVVACEIQVRTLASAGGPDNLVLLNPEKYKAKSRSPGSPVGE
GTGSPPKWQIGEQEFEALMRMLDNLGYRTGYPYRYPALREKSKKYSDVEVPASVTGYSF
ASDGDSGTCSPLRHSFQKQQREKTRWLNSGRGDEASEEGQNGSSPKSKTKWTKEDGHRT
STSAVPNLFVPLNTNPKEVQEMRNKIREQNLQDIKTAGPQSQVLCGVVMDRSLVQGELVT
ASKAIIEKEYQPHVIVSTTGPNPFTTLTDRELEEYRREVERKQKGSEENLDEAREQKEKSPP
DQPAVPHPPPSTPIKLEEDLVPEPTTGDDSDAATFKPTLPDLSPDEPSEALGFPMLEKEEEAH
RPPSPTEAPTEASPEPAPDPAPVAEEAAPSAVEEGAAADPGSDGSPGKSPSKKKKKFRTPSF
LKKSKKKSDS

>Q9H2S1 multipass membrane protein
MSSCRYNGGVMRPLSNLSASRRNLHEMDSEAQPLQPPASVGGGGGASSPSAAAAAAAAV
SSSAPEIVVSKPEHNNSNNLALYGTGGGGSTGGGGGGGGSGHGSSSGTKSSKKKNQNIGY
KLGHRRALFEKRKRLSDYALIFGMFGIVVMVIETELSWGAYDKASLYSLALKCLISLSTIIL
LGLIIVYHAREIQLFMVDNGADDWRIAMTYERIFFICLEILVCAIHPIPGNYTFTWTARLAFS
YAPSTTTADVDIILSIPMFLRLYLIARVMLLHSKLFTDASSRSIGALNKINFNTRFVMKTLMT
ICPGTVLLVFSISLWIIAAWTVRACERYHDQQDVTSNFLGAMWLISITFLSIGYGDMVPNTY
CGKGVCLLTGIMGAGCTALVVAVVARKLELTKAEKHVHNFMMDTQLTKRVKNAAANVL
RETWLIYKNTKLVKKIDHAKVRKHQRKFLQAIHQLRSVKMEQRKLNDQANTLVDLAKT
QNIMYDMISDLNERSEDFEKRIVTLETKLETLIGSIHALPGLISQTIRQQQRDFIEAQMESYD
KHVTYNAERSRSSSRRRRSSSTAPPTSSESS

>Q9NRM0 multipass membrane protein
MARKQNRNSKELGLVPLTDDTSHAGPPGPGRALLECDHLRSGVPGGRRRKDWSCSLLVA
SLAGAFGSSFLYGYNLSVVNAPTPYIKAFYNESWERRHGRPIDPDTLTLLWSVTVSIFAIGG
LVGTLIVKMIGKVLGRKHTLLANNGFAISAALLMACSLQAGAFEMLIVGRFIMGIDGGVA
LSVLPMYLSEISPKEIRGSLGQVTAIFICIGVFTGQLLGLPELLGKESTWPYLFGVIVVPAVV
QLLSLPFLPDSPRYLLLEKHNEARAVKAFQTFLGKADVSQEVEEVLAESRVQRSIRLVSVL
ELLRAPYVRWQVVTVIVTMACYQLCGLNAIWFYTNSIFGKAGIPPAKIPYVTLSTGGIETL
AAVFSGLVIEHLGRRPLLIGGFGLMGLFFGTLTITLTLQDHAPWVPYLSIVGILAIIASFCSGP
GGIPFILTGEFFQQSQRPAAFIIAGTVNWLSNFAVGLLFPFIQKSLDTYCFLVFATICITGAIYL
YFVLPETKNRTYAEISQAFSKRNKAYPPEEKIDSAVTDGKINGRP

>P05362 single-pass type I membrane protein
MAPSSPRPALPALLVLLGALFPGPGNAQTSVSPSKVILPRGGSVLVTCSTSCDQPKLLGIETP
LPKKELLLPGNNRKVYELSNVQEDSQPMCYSNCPDGQSTAKTFLTVYWTPERVELAPLPS
WQPVGKNLTLRCQVEGGAPRANLTVVLLRGEKELKREPAVGEPAEVTTTVLVRRDHHGA
NFSCRTELDLRPQGLELFENTSAPYQLQTFVLPATPPQLVSPRVLEVDTQGTVVCSLDGLFP
VSEAQVHLALGDQRLNPTVTYGNDSFSAKASVSVTAEDEGTQRLTCAVILGNQSQETLQT
VTIYSFPAPNVILTKPEVSEGTEVTVKCEAHPRAKVTLNGVPAQPLGPRAQLLLKATPEDN
GRSFSCSATLEVAGQLIHKNQTRELRVLYGPRLDERDCPGNWTWPENSQQTPMCQAWGN
PLPELKCLKDGTFPLPIGESVTVTRDLEGTYLCRARSTQGEVTRKVTVNVLSPRYEIVIITV
VAAAVIMGTAGLSTYLYNRQRKIKKYRLQQAQKGTPMKPNTQATPP

>Q7Z3B4 peripheral membrane protein
MAFNFGAPSGTSGTAAATAAPAGGFGGFGTTSTTAGSAFSFSAPTNTGTTGLFGGTQNKGF
GFGTGFGTTTGTSTGLGTGLGTGLGFGGFNTQQQQQTTLGGLFSQPTQAPTQSNQLINTAS
ALSAPTLLGDERDAILAKWNQLQAFWGTGKGYFNNNIPPVEFTQENPFCRFKAVGYSCM
PSNKDEDGLVVLVFNKKETEIRSQQQQLVESLHKVLGGNQTLTVNVEGTKTLPDDQTEVV
IYVVERSPNGTSRRVPATTLYAHFEQANIKTQLQQLGVTLSMTRTELSPAQIKQLLQNPPAG
VDPIIWEQAKVDNPDSEKLIPVPMVGFKELLRRLKVQDQMTKQHQTRLDIISEDISELQKN
QTTSVAKIAQYKRKLMDLSHRTLQVLIKQEIQRKSGYAIQADEEQLRVQLDTIQGELNAPT
QFKGRLNELMSQIRMQNHFGAVRSEERYYIDADLLREIKQHLKQQQEGLSHLISIIKDDLE
DIKLVEHGLNETIHIRGGVFS

>Q14108 multipass membrane protein
MGRCCFYTAGTLSLLLLVTSVTLLVARVFQKAVDQSIEKKIVLRNGTEAFDSWEKPPLPVY
TQFYFFNVTNPEEILRGETPRVEEVGPYTYRELRNKANIQFGDNGTTISAVSNKAYVFERD
QSVGDPKIDLIRTLNIPVLTVIEWSQVHFLREIIEAMLKAYQQKLFVTHTVDELLWGYKDEI
LSLIHVFRPDISPYFGLFYEKNGTNDGDYVFLTGEDSYLNFTKIVEWNGKTSLDWWITDK
CNMINGTDGDSFHPLITKDEVLYVFPSDFCRSVYITFSDYESVQGLPAFRYKVPAEILANTS
DNAGFCIPEGNCLGSGVLNVSICKNGAPIIMSFPHFYQADERFVSAIEGMHPNQEDHETFV
DINPLTGIILKAAKRFQINIYVKKLDDFVETGDIRTMVFPVMYLNESVHIDKETASRLKSMI
NTTLIITNIPYIIMALGVFFGLVFTWLACKGQGSMDEGTADERAPLIRT

>P31040 peripheral membrane protein
MSGVRGLSRLLSARRLALAKAWPTVLQTGTRGFHFTVDGNKRASAKVSDSISAQYPVVD
HEFDAVVVGAGGAGLRAAFGLSEAGFNTACVTKLFPTRSHTVAAQGGINAALGNMEEDN
WRWHFYDTVKGSDWLGDQDAIHYMTEQAPAAVVELENYGMPFSRTEDGKIYQRAFGGQ
SLKFGKGGQAHRCCCVADRTGHSLLHTLYGRSLRYDTSYFVEYFALDLLMENGECRGVIA
LCIEDGSIHRIRAKNTVVATGGYGRTYFSCTSAHTSTGDGTAMITRAGLPCQDLEFVQFHP
TGIYGAGCLITEGCRGEGGILINSQGERFMERYAPVAKDLASRDVVSRSMTLEIREGRGCG
PEKDHVYLQLHHLPPEQLATRLPGISETAMIFAGVDVTKEPIPVLPTVHYNMGGIPTNYKG
QVLRHVNGQDQIVPGLYACGEAACASVHGANRLGANSLLDLVVFGRACALSIEESCRPGD
KVPPIKPNAGEESVMNLDKLRFADGSIRTSELRLSMQKSMQNHAAVFRVGSVLQEGCGKI
SKLYGDLKHLKTFDRGMVWNTDLVETLELQNLMLCALQTIYGAEARKESRGAHAREDY
KVRIDEYDYSKPIQGQQKKPFEEHWRKHTLSYVDVGTGKVTLEYRPVIDKTLNEADCAT

VPPAIRSY

>P48764 multipass membrane protein
MWGLGARGPDRGLLLALALGGLARAGGVEVEPGGAHGESGGFQVVTFEWAHVQDPYVI
ALWILVASLAKIGFHLSHKVTSVVPESALLIVLGLVLGGIVWAADHIASFTLTPTVFFFYLLP
PIVLDAGYFMPNRLFFGNLGTILLYAVVGTVWNAATTGLSLYGVFLSGLMGDLQIGLLDFL
LFGSLMAAVDPVAVLAVFEEVHVNEVLFIIVFGESLLNDAVTVVLYNVFESFVALGGDNVT
GVDCVKGIVSFFVVSLGGTLVGVVFAFLLSLVTRFTKHVRIIEPGFVFIISYLSYLTSEMLSL
SAILAITFCGICCQKYVKANISEQSATTVRYTMKMLASSAETIIFMFLGISAVNPFIWTWNT
AFVLLTLVFISVYRAIGVVLQTWLLNRYRMVQLEPIDQVVLSYGGLRGAVAFALVVLLDG
DKVKEKNLFVSTTIIVVFFTVIFQGLTIKPLVQWLKVKRSEHREPRLNEKLHGRAFDHILSA
IEDISGQIGHNYLRDKWSHFDRKFLSRVLMRRSAQKSRDRILNVFHELNLKDAISYVAEGE
RRGSLAFIRSPSTDNVVNVDFTPRSSTVEASVSYLLRENVSAVCLDMQSLEQRRRSIRDAE
DMVTHHTLQQYLYKPRQEYKHLYSRHELTPTEDEKQDREIFHRTMRKRLESFKSTKLGLN
QNKKAAKLYKRERAQKRRNSSIPNGKLPMESPAQNFTIKEKDLELSDTEEPPNYDEEMSG
GIEFLASVTKDTASDSPAGIDNPVFSPDEALDRSLLARLPPWLSPGETVVPSQRARTQIPYSP
GTFCRLMPFRLSSKSVDSFLQADGPEERPPAALPESTHM

>Q8WTQ7 lipid-anchor
MVDMGALDNLIANTAYLQARKPSDCDSKELQRRRRSLALPGLQGCAELRQKLSLNFHSL
CEQQPIGRRLFRDFLATVPTFRKAATFLEDVQNWELAEEGPTKDSALQGLVATCASAPAPG
NPQPFLSQAVATKCQAATTEEERVAAVTLAKAEAMAFLQEQPFKDFVTSAFYDKFLQWKL
FEMQPVSDKYFTEFRVLGKGGFGEVCAVQVKNTGKMYACKKLDKKRLKKKGGEKMAL
LEKEILEKVSSPFIVSLAYAFESKTHLCLVMSLMNGGDLKFHIYNVGTRGLDMSRVIFYSA
QIACGMLHLHELGIVYRDMKPENVLLDDLGNCRLSDLGLAVEMKGGKPITQRAGTNGY
MAPEILMEKVSYSYPVDWFAMGCSIYEMVAGRTPFKDYKEKVSKEDLKQRTLQDEVKFQ
HDNFTEEAKDICRLFLAKKPEQRLGSREKSDDPRKHHFFKTINFPRLEAGLIEPPFVPDPSV
VYAKDIAEIDDFSEVRGVEFDDKDKQFFKNFATGAVPIAWQEEIIETGLFEELNDPNRPTGC
EEGNSSKSGVCLLL

>P43403 peripheral membrane protein
MPDPAAHLPFFYGSISRAEAEEHLKLAGMADGLFLLRQCLRSLGGYVLSLVHDVRFHHFPI
ERQLNGTYAIAGGKAHCGPAELCEFYSRDPDGLPCNLRKPCNRPSGLEPQPGVFDCLRDA
MVRDYVRQTWKLEGEALEQAIISQAPQVEKLIATTAHERMPWYHSSLTREEAERKLYSGA
QTDGKFLLRPRKEQGTYALSLIYGKTVYHYLISQDKAGKYCIPEGTKFDTLWQLVEYLKL
KADGLIYCLKEACPNSSASNASGAAAPTLPAHPSTLTHPQRRIDTLNSDGYTPEPARITSPD
KPRPMPMDTSVYESPYSDPEELKDKKLFLKRDNLLIADIELGCGNFGSVRQGVYRMRKK
QIDVAIKVLKQGTEKADTEEMMREAQIMHQLDNPYIVRLIGVCQAEALMLVMEMAGGGP
LHKFLVGKREEIPVSNVAELLHQVSMGMKYLEEKNFVHRDLAARNVLLVNRHYAKISDF
GLSKALGADDSYYTARSAGKWPLKWYAPECINFRKFSSRSDVWSYGVTMWEALSYGQK
PYKKMKGPEVMAFIEQGKRMECPPECPPELYALMSDCWIYKWEDRPDFLTVEQRMRACY
YSLASKVEGPPGSTQKAEAACA

>Q10588 GPI-anchor, lipid-anchor

MAAQGCAASRLLQLLLQLLLLLLLLAAGGARARWRGEGTSAHLRDIFLGRCAEYRALLS
PEQRNKNCTAIWEAFKVALDKDPCSVLPSDYDLFINLSRHSIPRDKSLFWENSHLLVNSFA
DNTRRFMPLSDVLYGRVADFLSWCRQKNDSGLDYQSCPTSEDCENNPVDSFWKRASIQY
SKDSSGVIHVMLNGSEPTGAYPIKGFFADYEIPNLQKEKITRIEIWVMHEIGGPNVESCGEG
SMKVLEKRLKDMGFQYSCINDYRPVKLLQCVDHSTHPDCALKSAAAATQRKAPSLYTEQ
RAGLIIPLFLVLASRTQL

>O43283 peripheral membrane protein
MANFQEHLSCSSSPHLPFSESKTFNGLQDELTAMGNHPSPKLLEDQQEKGMVRTELIESVH
SPVTTTVLTSVSEDSRDQFENSVLQLREHDESETAVSQGNSNTVDGESTSGTEDIKIQFSRS
GSGSGGFLEGLFGCLRPVWNIIGKAYSTDYKLQQQDTWEVPFEEISELQWLGSGAQGAVF
LGKFRAEEVAIKKVREQNETDIKHLRKLKHPNIIAFKGVCTQAPCYCIIMEYCAHGQLYEV
LRAGRKITPRLLVDWSTGIASGMNYLHLHKIIHRDLKSPNVLVTHTDAVKISDFGTSKELS
DKSTKMSFAGTVAWMAPEVIRNEPVSEKVDIWSFGVVLWELLTGEIPYKDVDSSAIIWGV
GSNSLHLPVPSTCPDGFKILMKQTWQSKPRNRPSFRQTLMHLDIASADVLATPQETYFKSQ
AEWREEVKKHFEKIKSEGTCIHRLDEELIRRRREELRHALDIREHYERKLERANNLYMELS
AIMLQLEMREKELIKREQAVEKKYPGTYKRHPVRPIIHPNAMEKLMKRKGVPHKSGMQT
KRPDLLRSEGIPTTEVAPTASPLSGSPKMSTSSSKSRYRSKPRHRRGNSRGSHSDFAAILKN
QPAQENSPHPTYLHQAQSQYPSLHHHNSLQQQYQQPPPAMSQSHHPRLNMHGQDIATCA
NNLRYFGPAAALRSPLSNHAQRQLPGSSPDLISTAMAADCWRSSEPDKGQAGPWGCCQA
DAYDPCLQCRPEQYGSLDIPSAEPVGRSPDLSKSPAHNPLLENAQSSEKTEENEFSGCRSES
SLGTSHLGTPPALPRKTRPLQKSGDDSSEEEEGEVDSEVEFPRRQRPHRCISSCQSYSTFSSE
NFSVSDGEEGNTSDHSNSPDELADKLEDRLAEKLDDLLSQTPEIPIDISSHSDGLSDKECAV
RRVKTQMSLGKLCVEERGYENPMQFEESDCDSSDGECSDATVRTNKHYSSATW

>Q9UNL2 multipass membrane protein
MAPKGSSKQQSEEDLLLQDFSRNLSAKSSALFFGNAFIVSAIPIWLYWRIWHMDLIQSAVL
YSVMTLVSTYLVAFAYKNVKFVLKHKVAQKREDAVSKEVTRKLSEADNRKMSRKEKDER
ILWKKNEVADYEATTFSIFYNNTLFLVVVIVASFFILKNFNPTVNYILSISASSGLIALLSTGS
K

>Q9H4A6 peripheral membrane protein
MTSLTQRSSGLVQRRTEASRNAADKERAAGGGAGSSEDDAQSRRDEQDDDDKGDSKETR
LTLMEEVLLLGLKDREGYTSFWNDCISSGLRGCMLIELALRGRLQLEACGMRRKSLLTRK
VICKSDAPTGDVLLDEALKHVKETQPPETVQNWIELLSGETWNPLKLHYQLRNVRERLA
KNLVEKGVLTTEKQNFLLFDMTTHPLTNNNIKQRLIKKVQEAVLDKWVNDPHRMDRRLL
ALIYLAHASDVLENAFAPLLDEQYDLATKRVRQLLDLDPEVECLKANTNEVLWAVVAAFT
K

>P55285 single-pass type I membrane protein
MRTYRYFLLLFWVGQPYPTLSTPLSKRTSGFPAKKRALELSGNSKNELNRSKRSWMWNQ
FFLLEEYTGSDYQYVGKLHSDQDRGDGSLKYILSGDGAGDLFIINENTGDIQATKRLDREE
KPVYILRAQAINRRTGRPVEPESEFIIKIHDINDNEPIFTKEVYTATVPEMSDVGTFVVQVTA
TDADDPTYGNSAKVVYSILQGQPYFSVESETGIIKTALLNMDRENREQYQVVIQAKDMGG

QMGGLSGTTTVNITLTDVNDNPPRFPQSTYQFKTPESSPPGTPIGRIKASDADVGENAEIEY
SITDGEGLDMFDVITDQETQEGIITVKKLLDFEKKKVYTLKVEASNPYVEPRFLYLGPFKD
SATVRIVVEDVDEPPVFSKLAYILQIREDAQINTTIGSVTAQDPDAARNPVKYSVDRHTDM
DRIFNIDSGNGSIFTSKLLDRETLLWHNITVIATEINNPKQSSRVPLYIKVLDVNDNAPEFAE
FYETFVCEKAKADQLIQTLHAVDKDDPYSGHQFSFSLAPEAASGSNFTIQDNKDNTAGILT
RKNGYNRHEMSTYLLPVVISDNDYPVQSSTGTVTVRVCACDHHGNMQSCHAEALIHPTG
LSTGALVAILLCIVILLVTVVLFAALRRQRKKEPLIISKEDIRDNIVSYNDEGGGEEDTQAFD
IGTLRNPEAIEDNKLRRDIVPEALFLPRRTPTARDNTDVRDFINQRLKENDTDPTAPPYDSL
ATYAYEGTGSVADSLSSLESVTTDADQDYDYLSDWGPRFKKLADMYGGVDSDKDS

>O14827 peripheral membrane protein
MQKSVRYNEGHALYLAFLARKEGTKRGFLSKKTAEASRWHEKWFALYQNVLFYFEGEQS
CRPAGMYLLEGCSCERTPAPPRAGAGQGGVRDALDKQYYFTVLFGHEGQKPLELRCEEE
QDGKEWMEAIHQASYADILIEREVLMQKYIHLVQIVETEKIAANQLRHQLEDQDTEIERLK
SEIIALNKTKERMRPYQSNQEDEDPDIKKIKKVQSFMRGWLCRRKWKTIVQDYICSPHAE
SMRKRNQIVFTMVEAESEYVHQLYILVNGFLRPLRMAASSKKPPISHDDVSSIFLNSETIMF
LHEIFHQGLKARIANWPTLILADLFDILLPMLNIYQEFVRNHQYSLQVLANCKQNRDFDK
LLKQYEANPACEGRMLETFLTYPMFQIPRYIITLHELLAHTPHEHVERKSLEFAKSKLEELS
RVMHDEVSDTENIRKNLAIERMIVEGCDILLDTSQTFIRQGSLIQVPSVERGKLSKVRLGSL
SLKKEGERQCFLFTKHFLICTRSSGGKLHLLKTGGVLSLIDCTLIEEPDASDDDSKGSGQVF
GHLDFKIVVEPPDAAAFTVVLLAPSRQEKAAWMSDISQCVDNIRCNGLMTIVFEENSKVT
VPHMIKSDARLHKDDTDICFSKTLNSCKVPQIRYASVERLLERLTDLRFLSIDFLNTFLHTY
RIFTTAAVVLGKLSDIYKRPFTSIPVRSLELFFATSQNNRGEHLVDGKSPRLCRKFSSPPPLA
VSRTSSPVRARKLSLTSPLNSKIGALDLTTSSSPTTTTQSPAASPPPHTGQIPLDLSRGLSSPE
QSPGTVEENVDNPRVDLCNKLKRSIQKAVLESAPADRAGVESSPAADTTELSPCRSPSTPR
HLRYRQPGGQTADNAHCSVSPASAFAIATAAAGHGSPPGFNNTERTCDKEFIIRRTATNRVL
NVLRHWVSKHAQDFELNNELKMNVLNLLEEVLRDPDLLPQERKAAANILRALSQDDQD
DIHLKLEDIIQMTDCMKAECFESLSAMELAEQITLLDHVIFRSIPYEEFLGQGWMKLDKNE
RTPYIMKTSQHFNDMSNLVASQIMNYADVSSRANAIEKWVAVADICRCLHNYNGVLEITS
ALNRSAIYRLKKTWAKVSKQTKALMDKLQKTVSSEGRFKNLRETLKNCNPPAVPYLGMY
LTDLAFIEEGTPNFTEEGLVNFSKMRMISHIIREIRQFQQTSYRIDHQPKVAQYLLDKDLIID
EDTLYELSLKIEPRLPA

>P43003 multipass membrane protein
MTKSNGEEPKMGGRMERFQQGVRKRTLLAKKKVQNITKEDVKSYLFRNAFVLLTVTAVI
VGTILGFTLRPYRMSYREVKYFSFPGELLMRMLQMLVLPLIISSLVTGMAALDSKASGKM
GMRAVVYYMTTTIIAVVIGIIIVIIIHPGKGTKENMHREGKIVRVTAADAFLDLIRNMFPPNL
VEACFKQFKTNYEKRSFKVPIQANETLVGAVINNVSEAMETLTRITEELVPVPGSVNGVNA
LGLVVFSMCFGFVIGNMKEQGQALREFFDSLNEAIMRLVAVIMWYAPVGILFLIAGKIVEM
EDMGVIGGQLAMYTVTVIVGLLIHAVIVLPLLYFLVTRKNPWVFIGGLLQALITALGTSSSS
ATLPITFKCLEENNGVDKRVTRFVLPVGATINMDGTALYEALAAIFIAQVNNFELNFGQIITI
SITATAASIGAAGIPQAGLVTMVIVLTSVGLPTDDITLIIAVDWFLDRLRTTTNVLGDSLGAG
IVEHLSRHELKNRDVEMGNSVIEENEMKKPYQLIAQDNETEKPIDSETKM

>Q07075 single-pass type II membrane protein
MNFAEREGSKRYCIQTKHVAILCAVVVGVGLIVGLAVGLTRSCDSSGDGGPGTAPAPSHLP
SSTASPSGPPAQDQDICPASEDESGQWKNFRLPDFVNPVHYDLHVKPLLEEDTYTGTVSISI
NLSAPTRYLWLHLRETRITRLPELKRPSGDQVQVRRCFEYKKQEYVVVEAEEELTPSSGD
GLYLLTMEFAGWLNGSLVGFYRTTYTENGQVKSIVATDHEPTDARKSFPCFDEPNKKATY
TISITHPKEYGALSNMPVAKEESVDDKWTRTTFEKSVPMSTYLVCFAVHQFDSVKRISNSG
KPLTIYVQPEQKHTAEYAANITKSVFDYFEEYFAMNYSLPKLDKIAIPDFGTGAMENWGLI
TYRETNLLYDPKESASSNQQRVATVVAHELVHQWFGNIVTMDWWEDLWLNEGFASFFEF
LGVNHAETDWQMRDQMLLEDVLPVQEDDSLMSSHPIIVTVTTPDEITSVFDGISYSKGSSI
LRMLEDWIKPENFQKGCQMYLEKYQFKNAKTSDFWAALEEASRLPVKEVMDTWTRQM
GYPVLNVNGVKNITQKRFLLDPRANPSQPPSDLGYTWNIPVKWTEDNITSSVLFNRSEKE
GITLNSSNPSGNAFLKINPDHIGFYRVNYEVATWDSIATALSLNHKTFSSADRASLIDDAFAL
ARAQLLDYKVALNLTKYLKREENFLPWQRVISAVTYIISMFEDDKELYPMIEEYFQGQVKP
IADSLGWNDAGDHVTKLLRSSVLGFACKMGDREALNNASSLFEQWLNGTVSLPVNLRLL
VYRYGMQNSGNEISWNYTLEQYQKTSLAQEKEKLLYGLASVKNVTLLSRYLDLLKDTNL
IKTQDVFTVIRYISYNSYGKNMAWNWIQLNWDYLVNRYTLNNRNLGRIVTIAEPFNTELQ
LWQMESFFAKYPQAGAGEKPREQVLETVKNNIEWLKQHRNTIREWFFNLLESG

>P01133 single-pass type I membrane protein
MLLTLIILLPVVSKFSFVSLSAPQHWSCPEGTLAGNGNSTCVGPAPFLIFSHGNSIFRIDTEG
TNYEQLVVDAGVSVIMDFHYNEKRIYWVDLERQLLQRVFLNGSRQERVCNIEKNVSGMA
INWINEEVIWSNQQEGIITVTDMKGNNSHILLSALKYPANVAVDPVERFIFWSSEVAGSLYR
ADLDGVGVKALLETSEKITAVSLDVLDKRLFWIQYNREGSNSLICSCDYDGGSVHISKHPT
QHNLFAMSLFGDRIFYSTWKMKTIWIANKHTGKDMVRINLHSSFVPLGELKVVHPLAQP
KAEDDTWEPEQKLCKLRKGNCSSTVCGQDLQSHLCMCAEGYALSRDRKYCEDVNECAF
WNHGCTLGCKNTPGSYYCTCPVGFVLLPDGKRCHQLVSCPRNVSECSHDCVLTSEGPLCF
CPEGSVLERDGKTCSGCSSPDNGGCSQLCVPLSPVSWECDCFPGYDLQLDEKSCAASGPQ
PFLLFANSQDIRHMHFDGTDYGTLLSQQMGMVYALDHDPVENKIYFAHTALKWIERANM
DGSQRERLIEEGVDVPEGLAVDWIGRRFYWTDRGKSLIGRSDLNGKRSKIITKENISQPRGI
AVHPMAKRLFWTDTGINPRIESSSLQGLGRLVIASSDLIWPSGITIDFLTDKLYWCDAKQSV
IEMANLDGSKRRRLTQNDVGHPFAVAVFEDYVWFSDWAMPSVMRVNKRTGKDRVRLQG
SMLKPSSLVVVHPLAKPGADPCLYQNGGCEHICKKRLGTAWCSCREGFMKASDGKTCLA
LDGHQLLAGGEVDLKNQVTPLDILSKTRVSEDNITESQHMLVAEIMVSDQDDCAPVGCSM
YARCISEGEDATCQCLKGFAGDGKLCSDIDECEMGVPVCPPASSKCINTEGGYVCRCSEGY
QGDGIHCLDIDECQLGEHSCGENASCTNTEGGYTCMCAGRLSEPGLICPDSTPPPHLREDD
HHYSVRNSDSECPLSHDGYCLHDGVCMYIEALDKYACNCVVGYIGERCQYRDLKWWEL
RHAGHGQQQKVIVVAVCVVVLVMLLLLSLWGAHYYRTQKLLSKNPKNPYEESSRDVRSR
RPADTEDGMSSCPQPWFVVIKEHQDLKNGGQPVAGEDGQAADGSMQPTSWRQEPQLCG
MGTEQGCWIPVSSDKGSCPQVMERSFHMPSYGTQTLEGGVEKPHSLLSANPLWQQRALD
PPHQMELTQ

>O00591 multipass membrane protein
MNYSLHLAFVCLSLFTERMCIQGSQFNVEVGRSDKLSLPGFENLTAGYNKFLRPNFGGEP
VQIALTLDIASISSISESNMDYTATIYLRQRWMDQRLVFEGNKSFTLDARLVEFLWVPDTYI

VESKKSFLHEVTVGNRLIRLFSNGTVLYALRITTTVACNMDLSKYPMDTQTCKLQLESWG
YDGNDVEFTWLRGNDSVRGLEHLRLAQYTIERYFTLVTRSQQETGNYTRLVLQFELRRNV
LYFILETYVPSTFLVVLSWVSFWISLDSVPARTCIGVTTVLSMTTLMIGSRTSLPNTNCFIKAI
DVYLGICFSFVFGALLEYAVAHYSSLQQMAAKDRGTTKEVEEVSITNIINSSISSFKRKISFA
SIEISSDNVDYSDLTMKTSDKFKFVFREKMGRIVDYFTIQNPSNVDHYSKLLFPLIFMLANV
FYWAYYMYF

>Q9NQA5 multipass membrane protein
MGGFLPKAEGPGSQLQKLLPSFLVREQDWDQHLDKLHMLQQKRILESPLLRASKENDLS
VLRQLLLDCTCDVRQRGALGETALHIAALYDNLEAALVLMEAAPELVFEPTTCEAFAGQT
ALHIAVVNQNVNLVRALLTRRASVSARATGTAFRRSPRNLIYFGEHPLSFAACVNSEEIVRL
LIEHGADIRAQDSLGNTVLHILILQPNKTFACQMYNLLLSYDGHGDHLQPLDLVPNHQGLT
PFKLAGVEGNTVMFQHLMQKRRHIQWTYGPLTSILYDLTEIDSWGEELSFLELVVSSDKRE
ARQILEQTPVKELVSFKWNKYGRPYFCILAALYLLYMICFTTCCVYRPLKFRGGNRTHSRD
ITILQQKLLQEAYETREDIIRLVGELVSIVGAVIILLLEIPDIFRVGASRYFGKTILGGPFHVIIIT
YASLVLVTMVMRLTNTNGEVVPMSFALVLGWCSVMYFTRGFQMLGPFTIMIQKMIFGDL
MRFCWLMAVVILGFASAFYIIFQTEDPTSLGQFYDYPMALFTTFELFLTVIDAPANYDVDL
PFMFSIVNFAFAIIATLLMLNLFIAMMGDTHWRVAQERDELWRAQVVATTVMLERKLPRC
LWPRSGICGCEFGLGDRWFLRVENHNDQNPLRVLRYVEVFKNSDKEDDQEHPSEKQPSG
AESGTLARASLALPTSSLSRTASQSSSHRGWEILRQNTLGHLNLGLNLSEGDGEEVYHF

>Q9NP58 multipass membrane protein
MVTVGNYCEAEGPVGPAWMQDGLSPCFFFTLVPSTRMALGTLALVLALPCRRRERPAGA
DSLSWGAGPRISPYVLQLLLATLQAALPLAGLAGRVGTARGAPLPSYLLLASVLESLAGA
CGLWLLVVERSQARQRLAMGIWIKFRHSPGLLLLWTVAFAAENLALVSWNSPQWWWAR
ADLGQQVQFSLWVLRYVVSGGLFVLGLWAPGLRPQSYTLQVHEEDQDVERSQVRSAAQ
QSTWRDFGRKLRLLSGYLWPRGSPALQLVVLICLGLMGLERALNVLVPIFYRNIVNLLTEK
APWNSLAWTVTSYVFLKFLQGGGTGSTGFVSNLRTFLWIRVQQFTSRRVELLIFSHLHELS
LRWHLGRRTGEVLRIADRGTSSVTGLLSYLVFNVIPTLADIIIGIIYFSMFFNAWFGLIVFLC
MSLYLTLTIVVTEWRTKFRRAMNTQENATRARAVDSLLNFETVKYYNAESYEVERYREAII
KYQGLEWKSSASLVLLNQTQNLVIGLGLLAGSLLCAYFVTEQKLQVGDYVLFGTYIIQLY
MPLNWFGTYYRMIQTNFIDMENMFDLLKEETEVKDLPGAGPLRFQKGRIEFENVHFSYA
DGRETLQDVSFTVMPGQTLALVGPSGAGKSTILRLLFRFYDISSGCIRIDGQDISQVTQASL
RSHIGVVPQDTVLFNDTIADNIRYGRVTAGNDEVEAAAQAAGIHDAIMAFPEGYRTQVGE
RGLKLSGGEKQRVAIARTILKAPGIILLDEATSALDTSNERAIQASLAKVCANRTTIVVAHR
LSTVVNADQILVIKDGCIVERGRHEALLSRGGVYADMWQLQQGQEETSEDTKPQTMER

>O14786 single-pass type I membrane protein
MERGLPLLCAVLALVLAPAGAFRNDKCGDTIKIESPGYLTSPGYPHSYHPSEKCEWLIQAP
DPYQRIMINFNPHFDLEDRDCKYDYVEVFDGENENGHFRGKFCGKIAPPPVVSSGPFLFIK
FVSDYETHGAGFSIRYEIFKRGPECSQNYTTPSGVIKSPGFPEKYPNSLECTYIVFVPKMSEI
ILEFESFDLEPDSNPPGGMFCRYDRLEIWDGFPDVGPHIGRYCGQKTPGRIRSSSGILSMVF
YTDSAIAKEGFSANYSVLQSSVSEDFKCMEALGMESGEIHSDQITASSQYSTNWSAERSRL
NYPENGWTPGEDSYREWIQVDLGLLRFVTAVGTQGAISKETKKKYYVKTYKIDVSSNGE

DWITIKEGNKPVLFQGNTNPTDVVVAVFPKPLITRFVRIKPATWETGISMRFEVYGCKITDY
PCSGMLGMVSGLISDSQITSSNQGDRNWMPENIRLVTSRSGWALPPAPHSYINEWLQIDLG
EEKIVRGIIIQGGKHRENKVFMRKFKIGYSNNGSDWKMIMDDSKRKAKSFEGNNNYDTPE
LRTFPALSTRFIRIYPERATHGGLGLRMELLGCEVEAPTAGPTTPNGNLVDECDDDQANCH
SGTGDDFQLTGGTTVLATEKPTVIDSTIQSEFPTYGFNCEFGWGSHKTFCHWEHDNHVQL
KWSVLTSKTGPIQDHTGDGNFIYSQADENQKGKVARLVSPVVYSQNSAHCMTFWYHMS
GSHVGTLRVKLRYQKPEEYDQLVWMAIGHQGDHWKEGRVLLHKSLKLYQVIFEGEIGKG
NLGGIAVDDISINNHISQEDCAKPADLDKKNPEIKIDETGSTPGYEGEGEGDKNISRKPGNV
LKTLDPILITIIAMSALGVLLGAVCGVVLYCACWHNGMSERNLSALENYNFELVDGVKLK
KDKLNTQSTYSEA

>O94766 single-pass type II membrane protein
MKLKLKNVFLAYFLVSIAGLLYALVQLGQPCDCLPPLRAAAEQLRQKDLRISQLQAELRRP
PPAPAQPPEPEALPTIYVVTPTYARLVQKAELVRLSQTLSLVPRLHWLLVEDAEGPTPLVSG
LLAASGLLFTHLVVLTPKAQRLREGEPGWVHPRGVEQRNKALDWLRGRGGAVGGEKDP
PPPGTQGVVYFADDDNTYSRELFEEMRWTRGVSVWPVGLVGGLRFEGPQVQDGRVVGF
HTAWEPSRPFPVDMAGFAVALPLLLDKPNAQFDSTAPRGHLESSLLSHLVDPKDLEPRAAN
CTRVLVWHTRTEKPKMKQEEQLQRQGRGSDPAIEV

>P41145 multipass membrane protein
MDSPIQIFRGEPGPTCAPSACLPPNSSAWFPGWAEPDSNGSAGSEDAQLEPAHISPAIPVIITA
VYSVVFVVGLVGNSLVMFVIIRYTKMKTATNIYIFNLALADALVTTTMPFQSTVYLMNSW
PFGDVLCKIVISIDYYNMFTSIFTLTMMSVDRYIAVCHPVKALDFRTPLKAKIINICIWLLSS
SVGISAIVLGGTKVREDVDVIECSLQFPDDDYSWWDLFMKICVFIFAFVIPVLIIIVCYTLMI
LRLKSVRLLSGSREKDRNLRRITRLVLVVVAVFVVCWTPIHIFILVEALGSTSHSTAALSSYY
FCIALGYTNSSLNPILYAFLDENFKRCFRDFCFPLKMRMERQSTSRVRNTVQDPAYLRDIDG
MNKPV

>P51788 multipass membrane protein
MAAAAAEEGMEPRALQYEQTLMYGRYTQDLGAFAKEEAARIRLGGPEPWKGPPSSRAA
PELLEYGRSRCARCRVCSVRCHKFLVSRVGEDWIFLVLLGLLMALVSWVMDYAIAACLQ
AQQWMSRGLNTSILLQYLAWVTYPVVLITFSAGFTQILAPQAVGSGIPEMKTILRGVVLKE
YLTLKTFIAKVIGLTCALGSGMPLGKEGPFVHIASMCAALLSKFLSLFGGIYENESRNTEML
AAACAVGVGCCFAAPIGGVLFSIEVTSTFFAVRNYWRGFFAATFSAFIFRVLAVWNRDEETI
TALFKTRFRLDFPFDLQELPAFAVIGIASGFGGALFVYLNRKIVQVMRKQKTINRFLMRKR
LLFPALVTLLISTLTFPPGFGQFMAGQLSQKETLVTLFDNRTWVRQGLVEELEPPSTSQAWN
PPRANVFLTLVIFILMKFWMSALATTIPVPCGAFMPVFVIGAAFGRLVGESMAAWFPDGIH
TDSSTYRIVPGGYAVVGAAALAGAVTHTVSTAVIVFELTGQIAHILPVMIAVILANAVAQSL
QPSLYDSIIRIKKLPYLPELGWGRHQQYRVRVEDIMVRDVPHVALSCTFRDLRLALHRTKG
RMLALVESPESMILLGSIERSQVVALLGAQLSPARRRQHMQERRATQTSPLSDQEGPPTPE
ASVCFQVNTEDSAFPAARGETHKPLKPALKRGPSVTRNLGESPTGSAESAGIALRSLFCGS
PPPEAASEKLESCEKRKLKRVRISLASDADLEGEMSPEEILEWEEQQLDEPVNFSDCKIDPA
PFQLVERTSLHKTHTIFSLLGVDHAYVTSIGRLIGIVTLKELRKAIEGSVTAQGVKVRPPLAS
FRDSATSSSDTETTEVHALWGPHSRHGLPREGSPSDSDDKCQ

>Q9UQV4 single-pass type I membrane protein
MPRQLSAAAALFASLAVILHDGSQMRAKAFPETRDYSQPTAAATVQDIKKPVQQPAKQAP
HQTLAARFMDGHITFQTAATVKIPTTTPATTKNTATTSPITYTLVTTQATPNNSHTAPPVTEV
TVGPSLAPYSLPPTITPPAHTTGTSSSTVSHTTGNTTQPSNQTTLPATLSIALHKSTTGQKPV
QPTHAPGTTAAAHNTTRTAAPASTVPGPTLAPQPSSVKTGIYQVLNGSRLCIKAEMGIQLIV
QDKESVFSPRRYFNIDPNATQASGNCGTRKSNLLLNFQGGFVNLTFTKDEESYYISEVGAY
LTVSDPETIYQGIKHAVVMFQTAVGHSFKCVSEQSLQLSAHLQVKTTDVQLQAFDFEDDH
FGNVDECSSDYTIVLPVIGAIVVGLCLMGMGVYKIRLRCQSSGYQRI

>P50416 multipass membrane protein
MAEAHQAVAFQFTVTPDGIDLRLSHEALRQIYLSGLHSWKKKFIRFKNGIITGVYPASPSS
WLIVVVGVMTTMYAKIDPSLGIIAKINRTLETANCMSSQTKNVVSGVLFGTGLWVALIVT
MRYSLKVLLSYHGWMFTEHGKMSRATKIWMGMVKIFSGRKPMLYSFQTSLPRLPVPAVK
DTVNRYLQSVRPLMKEEDFKRMTALAQDFAVGLGPRLQWYLKLKSWWATNYVSDWWE
EYIYLRGRGPLMVNSNYYAMDLLYILPTHIQAARAGNAIHAILLYRRKLDREEIKPIRLLGS
TIPLCSAQWERMFNTSRIPGEETDTIQHMRDSKHIVVYHRGRYFKVWLYHDGRLLKPREM
EQQMQRILDNTSEPQPGEARLAALTAGDRVPWARCRQAYFGRGKNKQSLDAVEKAAFFV
TLDETEEGYRSEDPDTSMDSYAKSLLHGRCYDRWFDKSFTFVVFKNGKMGLNAEHSWA
DAPIVAHLWEYVMSIDSLQLGYAEDGHCKGDINPNIPYPTRLQWDIPGECQEVIETSLNTA
NLLANDVDFHSFPFVAFGKGIIKKCRTSPDAFVQLALQLAHYKDMGKFCLTYEASMTRLF
REGRTETVRSCTTESCDFVRAMVDPAQTVEQRLKLFKLASEKHQHMYRLAMTGSGIDRH
LFCLYVVSKYLAVESPFLKEVLSEPWRLSTSQTPQQQVELFDLENNPEYVSSGGGFGPVAD
DGYGVSYILVGENLINFHISSKFSCPETDSHRFGRHLKEAMTDIITLFGLSSNSKK

>Q9Y3Q7 single-pass type I membrane protein
MFLLLALLTELGRLQAHEGSEGIFLHVTVPRKIKSNDSEVSERKMIYIITIDGQPYTLHLGK
QSFLPQNFLVYTYNETGSLHSVSPYFMMHCHYQGYAAEFPNSFVTLSICSGLRGFLQFENI
SYGIEPVESSARFEHIIYQMKNNDPNVSILAVNYSHIWQKDQPYKVPLNSQIKNLSKLLPQ
YLEIYIIVEKALYDYMGSEMMAVTQKIVQVIGLVNTMFTQFKLTVILSSLELWSNENQISTS
GDADDILQRFLAWKRDYLILRPHDIAYLLVYRKHPKYVGATFPGTVCNKSYDAGIAMYPD
AIGLEGFSVIIAQLLGLNVGLTYDDITQCFCLRATCIMNHEAVSASGRKIFSNCSMHDYRYF
VSKFETKCLQKLSNLQPLHQNQPVCGNGILESNEECDCGNKNECQFKKCCDYNTCKLKG
SVKCGSGPCCTSKCELSIAGTPCRKSIDPECDFTEYCNGTSSNCVPDTYALNGRLCKLGTA
YCYNGQCQTTDNQCAKIFGKGAQGAPFACFKEVNSLHERSENCGFKNSQPLPCERKDVL
CGKLACVQPHKNANKSDAQSTVYSYIQDHVCVSIATGSSMRSDGTDNAYVADGTMCGPE
MYCVNKTCRKVHLMGYNCNATTKCKGKGICNNFGNCQCFPGHRPPDCKFQFGSPGGSID
DGNFQKSGDFYTEKGYNTHWNNWFILSFCIFLPFFIVFTTVIFKRNEISKSCNRENAEYNR
NSSVVSESDDVGH

>Q99965 single-pass type I membrane protein
MWRVLFLLSGLGGLRMDSNFDSLPVQITVPEKIRSIIKEGIESQASYKIVIEGKPYTVNLMQ
KNFLPHNFRVYSYSGTGIMKPLDQDFQNFCHYQGYIEGYPKSVVMVSTCTGLRGVLQFE
NVSYGIEPLESSVGFEHVIYQVKHKKADVSLYNEKDIESRDLSFKLQSVEPQQDFAKYIEM

HVIVEKQLYNHMGSDTTVVAQKVFQLIGLTNAIFVSFNITIILSSLELWIDENKIATTGEANE
LLHTFLRWKTSYLVLRPHDVAFLLVYREKSNYVGATFQGKMCDANYAGGVVLHPRTISLE
SLAVILAQLLSLSMGITYDDINKCQCSGAVCIMNPEAIHFSGVKIFSNCSFEDFAHFISKQKS
QCLHNQPRLDPFFKQQAVCGNAKLEAGEECDCGTEQDCALIGETCCDIATCRFKAGSNCA
EGPCCENCLFMSKERMCRPSFEECDLPEYCNGSSASCPENHYVQTGHPCGLNQWICIDGV
CMSGDKQCTDTFGKEVEFGPSECYSHLNSKTDVSGNCGISDSGYTQCEADNLQCGKLICK
YVGKFLLQIPRATIIYANISGHLCIAVEFASDHADSQKMWIKDGTSCGSNKVCRNQRCVSSS
YLGYDCTTDKCNDRGVCNNKKHCHCSASYLPPDCSVQSDLWPGGSIDSGNFPPVAIPARL
PERRYIENIYHSKPMRWPFFLFIPFFIIFCVLIAIMVKVNFQRKKWRTEDYSSDEQPESESEP
KG

>P21439 multipass membrane protein
MDLEAAKNGTAWRPTSAEGDFELGISSKQKRKKTKTVKMIGVLTLFRYSDWQDKLFMSL
GTIMAIAHGSGLPLMMIVFGEMTDKFVDTAGNFSFPVNFSLSLLNPGKILEEEMTRYAYYY
SGLGAGVLVAAYIQVSFWTLAAGRQIRKIRQKFFHAILRQEIGWFDINDTTELNTRLTDDIS
KISEGIGDKVGMFFQAVATFFAGFIVGFIRGWKLTLVIMAISPILGLSAAVWAKILSAFSDKE
LAAYAKAGAVAEEALGAIRTVIAFGGQNKELERYQKHLENAKEIGIKKAISANISMGIAFLL
IYASYALAFWYGSTLVISKEYTIGNAMTVFFSILIGAFSVGQAAPCIDAFANARGAAYVIFDI
IDNNPKIDSFSERGHKPDSIKGNLEFNDVHFSYPSRANVKILKGLNLKVQSGQTVALVGSS
GCGKSTTVQLIQRLYDPDEGTINIDGQDIRNFNVNYLREIIGVVSQEPVLFSTTIAENICYGR
GNVTMDEIKKAVKEANAYEFIMKLPQKFDTLVGERGAQLSGGQKQRIAIARALVRNPKIL
LLDEATSALDTESEAEVQAALDKAREGRTTIVIAHRLSTVRNADVIAGFEDGVIVEQGSHS
ELMKKEGVYFKLVNMQTSGSQIQSEEFELNDEKAATRMAPNGWKSRLFRHSTQKNLKNS
QMCQKSLDVETDGLEANVPPVSFLKVLKLNKTEWPYFVVGTVCAIANGGLQPAFSVIFSE
IIAIFGPGDDAVKQQKCNIFSLIFLFLGIISFFTFFLQGFTFGKAGEILTRRLRSMAFKAMLRQ
DMSWFDDHKNSTGALSTRLATDAAQVQGATGTRLALIAQNIANLGTGIIISFIYGWQLTLL
LLAVVPIIAVSGIVEMKLLAGNAKRDKKELEAAGKIATEAIENIRTVVSLTQERKFESMYVE
KLYGPYRNSVQKAHIYGITFSISQAFMYFSYAGCFRFGAYLIVNGHMRFRDVILVFSAIVFG
AVALGHASSFAPDYAKAKLSAAHLFMLFERQPLIDSYSEEGLKPDKFEGNITFNEVVFNYP
TRANVPVLQGLSLEVKKGQTLALVGSSGCGKSTVVQLLERFYDPLAGTVFVDFGFQLLD
GQEAKKLNVQWLRAQLGIVSQEPILFDCSIAENIAYGDNSRVVSQDEIVSAAKAANIHPFIE
TLPHKYETRVGDKGTQLSGGQKQRIAIARALIRQPQILLLDEATSALDTESEKVVQEALDK
AREGRTCIVIAHRLSTIQNADLIVVFQNGRVKEHGTHQQLLAQKGIYFSMVSVQAGTQNL

>Q9P0K1 single-pass type I membrane protein
MQAAVAVSVPFLLLCVLGTCPPARCGQAGDASLMELEKRKENRFVERQSIVPLRLIYRSGG
EDESRHDALDTRVRGDLGGPQLTHVDQASFQVDAFGTSFILDVVLNHDLLSSEYIERHIEH
GGKTVEVKGGEHCYYQGHIRGNPDSFVALSTCHGLHGMFYDGNHTYLIEPEENDTTQED
FHFHSVYKSRLFEFSLDDLPSEFQQVNITPSKFILKPRPKRSKRQLRRYPRNVEEETKYIEL
MIVNDHLMFKKHRLSVVHTNTYAKSVVNMADLIYKDQLKTRIVLVAMETWATDNKFAIS
ENPLITLREFMKYRRDFIKEKSDAVHLFSGSQFESSRSGAAYIGGICSLLKGGGVNEFGKTD
LMAVTLAQSLAHNIGIISDKRKLASGECKCEDTWSGCIMGDTGYYLPKKFTQCNIEEYHD
FLNSGGGACLFNKPSKLLDPPECGNGFIETGEECDCGTPAECVLEGAECCKKCTLTQDSQC
SDGLCCKKCKFQPMGTVCREAVNDCDIRETCSGNSSQCAPNIHKMDGYSCDGVQGICFG

GRCKTRDRQCKYIWGQKVTASDKYCYEKLNIEGTEKGNCGKDKDTWIQCNKRDVLCGY
LLCTNIGNIPRLGELDGEITSTLVVQQGRTLNCSGGHVKLEEDVDLGYVEDGTPCGPQMM
CLEHRCLPVASFNFSTCLSSKEGTICSGNGVCSNELKCVCNRHWIGSDCNTYFPHNDDAK
TGITLSGNGVAGTNIIIGIIAGTILVLALILGITAWGYKNYREQRQLPQGDYVKKPGDGDSF
YSDIPPGVSTNSASSSKKRSNGLSHSWSERIPDTKHISDICENGRPRSNSWQGNLGGNKKKI
RGKRFRPRSNSTETLSPAKSPSSSTGSIASSRKYPYPMPPLPDEDKKVNRQSARLWETSI

>P30626 peripheral membrane protein
MAYPGHPGAGGGYYPGGYGGAPGGPAFPGQTQDPLYGYFAAVAGQDGQIDADELQRCLT
QSGIAGGYKPFNLETCRLMVSMLDRDMSGTMGFNEFKELWAVLNGWRQHFISFDTDRSG
TVDPQELQKALTTMGFRLSPQAVNSIAKRYSTNGKITFDDYIACCVKLRALTDSFRRRDTA
QQGVVNFPYDDFIQCVMSV

>O94921 peripheral membrane protein
MCDLIEPQPAEKIGKMKKLRRTLSESFSRIALKKDDTTFDEICVTKMSTRNCQGMDSVIKP
LDTIPEDKKVRVQRTQSTFDPFEKPANQVKRVHSENNACINFKTSSTGKESPKVRRHSSPSS
PTSPKFGKADSYEKLEKLGEGSYATVYKGKSKVNGKLVALKVIRLQEEEGTPFTAIREASL
LKGLKHANIVLLHDIIHTKETLTLVFEYVHTDLCQYMDKHPGGLHPDNVKLFLFQLLRGL
SYIHQRYILHRDLKPQNLLISDTGELKLADFGLARAKSVPSHTYSNEVVTLWYRPPDVLLG
STEYSTCLDMWGVGCIFVEMIQGVAAFPGMKDIQDQLERIFLVLGTPNEDTWPGVHSLPH
FKPERFTLYSSKNLRQAWNKLSYVNHAEDLASKLLQCSPKNRLSAQAALSHEYFSDLPPR
LWELTDMSSIFTVPNVRLQPEAGESMRAFGKNNSYGKSLSNSKH

>Q9UKQ2 single-pass type I membrane protein
MLQGLLPVSLLLSVAVSAIKELPGVKKYEVVYPIRLHPLHKREAKEPEQQEQFETELKYK
MTINGKIAVLYLKKNKNLLAPGYTETYYNSTGKEITTSPQIMDDCYYQGHILNEKVSDASI
STCRGLRGYFSQGDQRYFIEPLSPIHRDGQEHALFKYNPDEKNYDSTCGMDGVLWAHDL
QQNIALPATKLVKLKDRKVQEHEKYIEYYLVLDNGEFKRYNENQDEIRKRVFEMANYVN
MLYKKLNTHVALVGMEIWTDKDKIKITPNASFTLENFSKWRGSVLSRRKRHDIAQLITATE
LAGTTVGLAFMSTMCSPYSVGVVQDHSDNLLRVAGTMAHEMGHNFGMFHDDYSCKCPS
TICVMDKALSFYIPTDFSSCSRLSYDKFFEDKLSNCLFNAPLPTDIISTPICGNQLVEMGEDC
DCGTSEECTNICCDAKTCKIKATFQCALGECCEKCQFKKAGMVCRPAKDECDLPEMCNG
KSGNCPDDRFQVNGFPCHHGKGHCLMGTCPTLQEQCTELWGPGTEVADKSCYNRNEGG
SKYGYCRRVDDTLIPCKANDTMCGKLFCQGGSDNLPWKGRIVTFLTCKTFDPEDTSQEIG
MVANGTKCGDNKVCINAECVDIEKAYKSTNCSSKCKGHAVCDHELQCQCEEGWIPPDCD
DSSVVFHFSIVVGVLFPMAVIFVVVAMVIRHQSSREKQKKDQRPLSTTGTRPHKQKRKPQ
MVKAVQPQEMSQMKPHVYDLPVEGNEPPASFHKDTNALPPTVFKDNPVSTPKDSNPKA

>P16260 multipass membrane protein
MAAATAAAALAAADPPPAMPQAAGAGGPTTRRDFYWLRSFLAGGIAGCCAKTTVAPLDR
VKVLLQAHNHHYKHLGVFSALRAVPQKEGFLGLYKGNGAMMIRIFPYGAIQFMAFEHYK
TLITTKLGISGHVHRLMAGSMAGMTAVICTYPLDMVRVRLAFQVKGEHSYTGIIHAFKTIY
AKEGGFFGFYRGLMPTILGMAPYAGVSFFTFGTLKSVGLSHAPTLLGRPSSDNPNVLVLKT
HVNLLCGGVAGAIAQTISYPFDVTRRRMQLGTVLPEFEKCLTMRDTMKYVYGHHGIRKG

LYRGLSLNYIRCIPSQAVAFTTYELMKQFFHLN

>Q14534 multipass membrane protein
MWTFLGIATFTYFYKKFGDFITLANREVLLCVLVFLSLGLVLSYRCRHRNGGLLGRQQSGS
QFALFSDILSGLPFIGFFWAKSPPESENKEQLEARRRRKGTNISETSLIGTAACTSTSSQNDP
EVIIVGAGVLGSALAAVLSRDGRKVTVIERDLKEPDRIVGEFLQPGGYHVLKDLGLGDTV
EGLDAQVVNGYMIHDQESKSEVQIPYPLSENNQVQSGRAFHHGRFIMSLRKAAMAEPNA
KFIEGVVLQLLEEDDVVMGVQYKDKETGDIKELHAPLTVVADGLFSKFRKSLVSNKVSVS
SHFVGFLMKNAPQFKANHAELILANPSPVLIYQISSSETRVLVDIRGEMPRNLREYMVEKI
YPQIPDHLKEPFLEATDNSHLRSMPASFLPPSSVKKRGVLLLGDAYNMRHPLTGGGMTVAF
KDIKLWRKLLKGIPDLYDDAAIFEAKKSFYWARKTSHSFVVNILAQALYELFSATDDSLHQ
LRKACFLYFKLGGECVAGPVGLLSVLSPNPLVLIGHFFAVAIYAVYFCFKSEPWITKPRALLS
SGAVLYKACSVIFPLIYSEMKYMVH

>Q92543 peripheral membrane protein
MKTETVPPFQETPAGSSCHLNNLLSSRKLMAVGVLLGWLLVIHLLVNVWLLCLLSALLVV
LGGWLGSSLAGVASGRLHLERFIPLATCPPCPEAERQLEREINRTIQMIIRDFVLSWYRSVS
QEPAFEEEMEAAMKGLVQELRRRMSVMDSHAVAQSVLTLCGCHLQSYIQAKEATAGKNG
PVEPSHLWEAYCRATAPHPAVHSPSAEVTYTRGVVNLLLQGLVPKPHLETRTGRHVVVELI
TCNVILPLISRLSDPDWIHLVLVGIFSKARDPAPCPASAPEQPSVPTSLPLIAEVEQLPEGRAS
PVAAPVFLSYSEPEGSAGPSPEVEEGHEAVEGDLGGMCEERKVGNNSSHFLQPNVRGPLF
LCEDSELESPLSELGKETIMLMTPGSFLSDRIQDALCALESSQALEPKDGEASEGAEAEEGP
GTETETGLPVSTLNSCPEIHIDTADKEIEQGDVTASVTALLEGPEKTCPSRPSCLEKDLTND
VSSLDPTLPPVLLSSSPPGPLSSATFSFEPLSSPDGPVIIQNLRITGTITAREHSGTGFHPYTLY
TVKYETALDGENSSGLQQLAYHTVNRRYREFLNLQTRLEEKPDLRKFIKNVKGPKKLFPD
LPLGNMDSDRVEARKSLLESFLKQLCAIPEIANSEEVQEFLALNTDARIAFVKKPFMVSRID
KMVVSAIVDTLKTAFPRSEPQSPTEELSEAETESKPQTEGKKASKSRLRFSSSKISPALSVTE
AQDKILYCLQEGNVESETLSMSAMESFIEKQTKLLEMQPTKAPEKDPEQPPKGRVDSCVS
DAAVPAQDPSNSDPGTETELADTALDLLLLLLTEQWKWLCTENMQKFLRLIFGTLVQRWL
EVQVANLTSPQRWVQYLLLLQESIWPGGVLPKFPRPVRTQEQKLAAEKQALQSLMGVLP
DLVVEILGVNKCRLSWGLVLESLQQPLINRHLIYCLGDIILEFLDLSASVEESAATTSASDTP
GNSKRMGVSS

>Q9BPZ7 peripheral membrane protein
MAFLDNPTIILAHIRQSHVTSDDTGMCEMVLIDHDVDLEKIHPPSMPGDSGSEIQGSNGET
QGYVYAQSVDITSSWDFGIRRRSNTAQRLERLRKERQNQIKCKNIQWKERNSKQSAQELK
SLFEKKSLKEKPPISGKQSILSVRLEQCPLQLNNPFNEYSKFDGKGHVGTTATKKIDVYLPL
HSSQDRLLPMTVVTMASARVQDLIGLICWQYTSEGREPKLNDNVSAYCLHIAEDDGEVDT
DFPPLDSNEPIHKFGFSTLALVEKYSSPGLTSKESLFVRINAAHGFSLIQVDNTKVTMKEILL
KAVKRRKGSQKVSGPQYRLEKQSEPNVAVDLDSTLESQSAWEFCLVRENSSRADGVFEED
SQIDIATVQDMLSSHHYKSFKVSMIHRLRFTTDVQLGISGDKVEIDPVTNQKASTKFWIKQ
KPISIDSDLLCACDLAEEKSPSHAIFKLTYLSNHDYKHLYFESDAATVNEIVLKVNYILESR
ASTARADYFAQKQRKLNRRTSFSFQKEKKSGQQ

>P13866 multipass membrane protein
MDSSTWSPKTTAVTRPVETHELIRNAADISIIVIYFVVVMAVGLWAMFSTNRGTVGGFFLA
GRSMVWWPIGASLFASNIGSGHFVGLAGTGAASGIAIGGFEWNALVLVVVLGWLFVPIYI
KAGVVTMPEYLRKRFGGQRIQVYLSLLSLLLYIFTKISADIFSGAIFINLALGLNLYLAIFLL
LAITALYTITGGLAAVIYTDTLQTVIMLVGSLILTGFAFHEVGGYDAFMEKYMKAIPTIVSD
GNTTFQEKCYTPRADSFHIFRDPLTGDLPWPGFIFGMSILTLWYWCTDQVIVQRCLSAKN
MSHVKGGCILCGYLKLMPMFIMVMPGMISRILYTEKIACVVPSECEKYCGTKVGCTNIAY
PTLVVELMPNGLRGLMLSVMLASLMSSLTSIFNSASTLFTMDIYAKVRKRASEKELMIAGR
LFILVLIGISIAWVPIVQSAQSGQLFDYIQSITSYLGPPIAAVFLLAIFWKRVNEPGAFWGLIL
GLLIGISRMITEFAYGTGSCMEPSNCPTIICGVHYLYFAIILFAISFITIVVISLLTKPIPDVHLYR
LCWSLRNSKEERIDLDAEEENIQEGPKETIEIETQVPEKKKGIFRRAYDLFCGLEQHGAPK
MTEEEEKAMKMKMTDTSEKPLWRTVLNVNGIILVTVAVFCHAYFA

>Q13936 multipass membrane protein
MVNENTRMYIPEENHQGSNYGSPRPAHANMNANAAAGLAPEHIPTPGAALSWQAAIDA
ARQAKLMGSAGNATISTVSSTQRKRQQYGKPKKQGSTTATRPPRALLCLTLKNPIRRACISI
VEWKPFEIIILLTIFANCVALAIYIPFPEDDSNATNSNLERVEYLFLIIFTVEAFLKVIAYGLLF
HPNAYLRNGWNLLDFIIVVVGLFSAILEQATKADGANALGGKGAGFDVKALRAFRVLRPL
RLVSGVPSLQVVLNSIIKAMVPLLHIALLVLFVIIIYAIIGLELFMGKMHKTCYNQEGIADVP
AEDDPSPCALETGHGRQCQNGTVCKPGWDGPKHGITNFDNFAFAMLTVFQCITMEGWTD
VLYWVNDAVGRDWPWIYFVTLIIIGSFFVLNLVLGVLSGEFSKEREKAKARGDFQKLREK
QQLEEDLKGYLDWITQAEDIDPENEDEGMDEEKPRNMSMPTSETESVNTENVAGGDIEGE
NCGARLAHRISKSKFSRYWRRWNRFCRRKCRAAVKSNVFYWLVIFLVFLNTLTIASEHYN
QPNWLTEVQDTANKALLALFTAEMLLKMYSLGLQAYFVSLFNRFDCFVVCGGILETILVE
TKIMSPLGISVLRCVRLLRIFKITRYWNSLSNLVASLLNSVRSIASLLLLLFLFIIIFSLLGMQL
FGGKFNFDEMQTRRSTFDNFPQSLLTVFQILTGEDWNSVMYDGIMAYGGPSFPGMLVCIY
FIILFICGNYILLNVFLAIAVDNLADAESLTSAQKEEEEEKERKKLARTASPEKKQELVEKPA
VGESKEEKIELKSITADGESPPATKINMDDLQPNENEDKSPYPNPETTGEEDEEEPEMPVGP
RPRPLSELHLKEKAVPMPEASAFFIFSSNNRFRLQCHRIVNDTIFTNLILFFILLSSISLAAEDP
VQHTSFRNHILFYFDIVFTTIFTIEIALKILGNADYVFTSIFTLEIILKMTAYGAFLHKGSFCR
NYFNILDLLVVSVSLISFGIQSSAINVVKILRVLRVLRPLRAINRAKGLKHVVQCVFVAIRTI
GNIVIVTTLLQFMFACIGVQLFKGKLYTCSDSSKQTEAECKGNYITYKDGEVDHPIIQPRS
WENSKFDFDNVLAAMMALFTVSTFEGWPELLYRSIDSHTEDKGPIYNYRVEISIFFIIYIIIIA
FFMMNIFVGFVIVTFQEQGEQEYKNCELDKNQRQCVEYALKARPLRRYIPKNQHQYKVW
YVVNSTYFEYLMFVLILLNTICLAMQHYGQSCLFKIAMNILNMLFTGLFTVEMILKLIAFK
PKGYFSDPWNVFDFLIVIGSIIDVILSETNHYFCDAWNTFDALIVVGSIVDIAITEVNPAEHT
QCSPSMNAEENSRISITFFRLFRVMRLVKLLSRGEGIRTLLWTFIKSFQALPYVALLIVMLFFI
YAVIGMQVFGKIALNDTTEINRNNNFQTFPQAVLLLFRCATGEAWQDIMLACMPGKKCAP
ESEPSNSTEGETPCGSSFAVFYFISFYMLCAFLIINLFVAVIMDNFDYLTRDWSILGPHHLDE
FKRIWAEYDPEAKGRIKHLDVVTLLRRIQPPLGFGKLCPHRVACKRLVSMNMPLNSDGTV
MFNATLFALVRTALRIKTEGNLEQANEELRAIIKKIWKRTSMKLLDQVVPPAGDDEVTVGK
FYATFLIQEYFRKFKKRKEQGLVGKPSQRNALSLQAGLRTLHDIGPEIRRAISGDLTAEEEL
DKAMKEAVSAASEDDIFRRAGGLFGNHVSYYQSDGRSAFPQTFTTQRPLHINKAGSSQGD
TESPSHEKLVDSTFTPSSYSSTGSNANINNANNTALGRLPRPAGYPSTVSTVEGHGPPLSPAI

RVQEVAWKLSSNRERHVPMCEDLELRRDSGSAGTQAHCLLLRKANPSRCHSRESQAAMA
GQEETSQDETYEVKMNHDTEACSEPSLLSTEMLSYQDDENRQLTLPEEDKRDIRQSPKRG
FLRSASLGRRASFHLECLKRQKDRGGDISQKTVLPLHLVHHQALAVAGLSPLLQRSHSPAS
FPRPFATPPATPGSRGWPPQPVPTLRLEGVESSEKLNSSFPSIHCGSWAETTPGGGGSSAARR
VRPVSLMVPSQAGAPGRQFHGSASSLVEAVLISEGLGQFAQDPKFIEVTTQELADACDMTI
EEMESAADNILSGGAPQSPNGALLPFVNCRDAGQDRAGGEEDAGCVRARGRPSEEELQD
SRVYVSSL

>Q6L9W6 single-pass type II membrane protein
MGSPRAARPPLLLRPVKLLRRRFRLLLALAVVSVGLWTLYLELVASAQVGGNPLNRRYGS
WRELAKALASRNIPAVDPHLQFYHPQRLSLEDHDIDQGVSSNSSYLKWNKPVPWLSEFRG
RANLHVFEDWCGSSIQQLRRNLHFPLYPHIRTTLRKLAVSPKWTNYGLRIFGYLHPFTDGK
IQFAIAADDNAEFWLSLDDQVSGLQLLASVGKTGKEWTAPGEFGKFRSQISKPVSLSASHR
YYFEVLHKQNEEGTDHVEVAWRRNDPGAKFTIIDSLSLSLFTNETFLQMDEVGHIPQTAAS
HVDSSNALPRDEQPPADMLRPDPRDTLYRVPLIPKSHLRHVLPDCPYKPSYLVDGLPLQRY
QGLRFVHLSFVYPNDYTRLSHMETHNKCFYQENAYYQDRFSFQEYIKIDQPEKQGLEQPG
FEENLLEESQYGEVAEETPASNNQNARMLEGRQTPASTLEQDATDYRLRSLRKLLAQPRE
GLLAPFSKRNSTASFPGRTSHIPVQQPEKRKQKPSPEPSQDSPHSDKWPPGHPVKNLPQMR
GPRPRPAGDSPRKTQWLNQVESYIAEQRRGDRMRPQAPGRGWHGEEEVVAAAGQEGQV
EGEEEGEEEEEEEDMSEVFEYVPVFDPVVNWDQTFSARNLDFQALRTDWIDLSCNTSGNL
LLPEQEALEVTRVFLKKLNQRSRGRYQLQRIVNVEKRQDQLRGGRYLLELELLEQGQRVV
RLSEYVSARGWQGIDPAGGEEVEARNLQGLVWDPHNRRRQVLNTRAQEPKLCWPQGFS
WSHRAVVHFVVPVKNQARWVQQFIKDMENLFQVTGDPHFNIVITDYSSEDMDVEMALK
RSKLRSYQYVKLSGNFERSAGLQAGIDLVKDPHSIIFLCDLHIHFPAGVIDAIRKHCVEGKM
AFAPMVMRLHCGATPQWPEGYWEVNGFGLLGIYKSDLDRIGGMNTKEFRDRWGGEDW
ELLDRILQAGLDVERLSLRNFFHHFHSKRGMWSRRQMKTL

>O00429 peripheral membrane protein
MEALIPVINKLQDVFNTVGADIIQLPQIVVVGTQSSGKSSVLESLVGRDLLPRGTGIVTRRP
LILQLVHVSQEDKRKTTGEENGVEAEEWGKFLHTKNKLYTDFDEIRQEIENETERISGNNK
GVSPEPIHLKIFSPNVVNLTLVDLPGMTKVPVGDQPKDIELQIRELILRFISNPNSIILAVTAA
NTDMATSEALKISREVDPDGRRTLAVITKLDLMDAGTDAMDVLMGRVIPVKLGIIGVVNR
SQLDINNKKSVTDSIRDEYAFLQKKYPSLANRNGTKYLARTLNRLLMHHIRDCLPELKTRI
NVLAAQYQSLLNSYGEPVDDKSATLLQLITKFATEYCNTIEGTAKYIETSELCGGARICYIF
HETFGRTLESVDPLGGLNTIDILTAIRNATGPRPALFVPEVSFELLVKRQIKRLEEPSLRCVEL
VHEEMQRIIQHCSNYSTQELLRFPKLHDAIVEVVTCLLRKRLPVTNEMVHNLVAIELAYIN
TKHPDFADACGLMNNNIEEQRRNRLARELPSAVSRDKSSKVPSALAPASQEPSPAASAEAD
GKLIQDSRRETKNVASGGGGVGDGVQEPTTGNWRGMLKTSKAEELLAEEKSKPIPIMPAS
PQKGHAVNLLDVPVPVARKLSAREQRDCEVIERLIKSYFLIVRKNIQDSVPKAVMHFLVNH
VKDTLQSELVGQLYKSSLLDDLLTESEDMAQRRKEAADMLKALQGASQIIAEIRETHLW

>Q5BKT4 multipass membrane protein
MAQLEGYYFSAALSCTFLVSCLLFSAFSRALREPYMDEIFHLPQAQRYCEGHFSLSQWDP
MITTLPGLYLVSIGVIKPAIWIFGWSEHVVCSIGMLRFVNLLFSVGNFYLLYLLFCKVQPRN

KAASSIQRVLSTLTLAVFPTLYFFNFLYYTEAGSMFFTLFAYLMCLYGNHKTSAFLGFCGFM
FRQTNIIWAVFCAGNVIAQKLTEAWKTELQKKEDRLPPIKGPFAEFRKILQFLLAYSMSFKN
LSMLLLLTWPYILLGFLFCAFVVVNGGIVIGDRSSHEACLHFPQLFYFFSFTLFFSFPHLLSP
SKIKTFLSLVWKRRILFFVVTLVSVFLVWKFTYAHKYLLADNRHYTFYVWKRVFQRYETV
KYLLVPAYIFAGWSIADSLKSKSIFWNLMFFICLFTVIVPQKLLEFRYFILPYVIYRLNIPLPP
TSRLICELSCYAVVNFITFFIFLNKTFQWPNSQDIQRFMW

>Q9NYB5 multipass membrane protein
MDTSSKENIQLFCKTSVQPVGRPSFKTEYPSSEEKQPCCGELKVFLCALSFVYFAKALAEG
YLKSTITQIERRFDIPSSLVGVIDGSFEIGNLLVITFVSYFGAKLHRPKIIGAGCVIMGVGTLLI
AMPQFFMEQYKYERYSPSSNSTLSISPCLLESSSQLPVSVMEKSKSKISNECEVDTSSSMWI
YVFLGNLLRGIGETPIQPLGIAYLDDFASEDNAAFYIGCVQTVAIIGPIFGFLLGSLCAKLYV
DIGFVNLDHITITPKDPQWVGAWWLGYLIAGIISLLAAVPFWYLPKSLPRSQSREDSNSSSE
KSKFIIDDHTDYQTPQGENAKIMEMARDFLPSLKNLFGNPVYFLYLCTSTVQFNSLFGMV
TYKPKYIEQQYGQSSSRANFVIGLINIPAVALGIFSGGIVMKKFRISVCGAAKLYLGSSVFGY
LLFLSLFALGCENSDVAGLTVSYQGTKPVSYHERALFSDCNSRCKCSETKWEPMCGENGI
TYVSACLAGCQTSNRSGKNIIFYNCTCVGIAASKSGNSSGIVGRCQKDNGCPQMFLYFLVI
SVITSYTLSLGGIPGYILLLRCIKPQLKSFALGIYTLAIRVLAGIPAPVYFGVLIDTSCLKWGF
KRCGSRGSCRLYDSNVFRHIYLGLTVILGTVSILLSIAVLFILKKNYVSKHRSFITKRERTMV
STRFQKENYTTSDHLLQPNYWPGKETQL

>P26842 single-pass type I membrane protein
MARPHPWWLCVLGTLVGLSATPAPKSCPERHYWAQGKLCCQMCEPGTFLVKDCDQHRK
AAQCDPCIPGVSFSPDHHTRPHCESCRHCNSGLLVRNCTITANAECACRNGWQCRDKECT
ECDPLPNPSLTARSSQALSPHPQPTHLPYVSEMLEARTAGHMQTLADFRQLPARTLSTHWP
PQRSLCSSDFIRILVIFSGMFLVFTLAGALFLHQRRKYRSNKGESPVEPAEPCHYSCPREEEG
STIPIQEDYRKPEPACSP

>Q969I6 multipass membrane protein
MDPMELRNVNIEPDDESSSGESAPDSYIGIGNSEKAAMSSQFANEDTESQKFLTNGFLGKK
KLADYADEHHPGTTSFGMSSFNLSNAIMGSGILGLSYAMANTGIILFIIMLLAVAILSLYSVH
LLLKTAKEGGSLIYEKLGEKAFGWPGKIGAFVSITMQNIGAMSSYLFIIKYELPEVIRAFMG
LEENTGEWYLNGNYLIIFVSVGIILPLSLLKNLGYLGYTSGFSLTCMVFFVSVVIYKKFQIP
CPLPVLDHSVGNLSFNNTLPMHVVMLPNNSESSDVNFMMDYTHRNPAGLDENQAKGSL
HDSGVEYEAHSDDKCEPKYFVFNSRTAYAIPILVFAFVCHPEVLPIYSELKDRSRRKMQTVS
NISITGMLVMYLLAALFGYLTFYGEVEDELLHAYSKVYTLDIPLLMVRLAVLVAVTLTVPIV
LFPIRTSVITLLFPKRPFSWIRHFLIAAVLIALNNVLVILVPTIKYIFGFIGASSATMLIFILPAVF
YLKLVKKETFRSPQKVGALIFLVVGIFFMIGSMALIIIDWIYDPPNSKHH

>Q86YJ5 multipass membrane protein
MLKSRLRMFLNELKLLVLTGGGRPRAEPQPRGGRGGGCGWAPFAGCSTRDGDGDEEEYY
GSEPRARGLAGDKEPRAGPLPPPAPPLPPPGALDALSLSSSLDSGLRTPQCRICFQGPEQGE
LLSPCRCDGSVRCTHQPCLIRWISERGSWSCELCYFKYQVLAISTKNPLQWQAISLTVIEKV
QIAAIVLGSLFLVASISWLIWSSLSPSAKWQRQDLLFQICYGMYGFMDVVCIGLIIHEGSSV

YRIFKRWQAVNQQWKVLNYDKTKDIGGDAGGGTAGKSGPRNSRTGPTSGATSRPPAAQR
MRTLLPQRCGYTILHLLGQLRPPDARSSSHSGREVVMRVTTV

>O75473 multipass membrane protein
MDTSRLGVLLSLPVLLQLATGGSSPRSGVLLRGCPTHCHCEPDGRMLLRVDCSDLGLSEL
PSNLSVFTSYLDLSMNNISQLLPNPLPSLRFLEELRLAGNALTYIPKGAFTGLYSLKVLMLQ
NNQLRHVPTEALQNLRSLQSLRLDANHISYVPPSCFSGLHSLRHLWLDDNALTEIPVQAFR
SLSALQAMTLALNKIHHIPDYAFGNLSSLVVLHLHNNRIHSLGKKCFDGLHSLETLDLNYN
NLDEFPTAIRTLSNLKELGFHSNNIRSIPEKAFVGNPSLITIHFYDNPIQFVGRSAFQHLPELR
TLTLNGASQITEFPDLTGTANLESLTLTGAQISSLPQTVCNQLPNLQVLDLSYNLLEDLPSFS
VCQKLQKIDLRHNEIYEIKVDTFQQLLSLRSLNLAWNKIAIIHPNAFSTLPSLIKLDLSSNLL
SSFPITGLHGLTHLKLTGNHALQSLISSENFPELKVIEMPYAYQCCAFGVCENAYKISNQWN
KGDNSSMDDLHKKDAGMFQAQDERDLEDFLLDFEEDLKALHSVQCSPSPGPFKPCEHLL
DGWLIRIGVWTIAVLALTCNALVTSTVFRSPLYISPIKLLIGVIAAVNMLTGVSSAVLAGVD
AFTFGSFARHGAWWENGVGCHVIGFLSIFASESSVFLLTLAALERGFSVKYSAKFETKAPF
SSLKVIILLCALLALTMAAVPLLGGSKYGASPLCLPLPFGEPSTMGYMVALILLNSLCFLM
MTIAYTKLYCNLDKGDLENIWDCSMVKHIALLLFTNCILNCPVAFLSFSSLINLTFISPEVIK
FILLVVVPLPACLNPLLYILFNPHFKEDLVSLRKQTYVWTRSKHPSLMSINSDDVEKQSCDS
TQALVTFTSSSITYDLPPSSVPSPAYPVTESCHLSSVAFVPCL

>O43323 lipid-anchor
MALLTNLLPLCCLALLALPAQSCGPGRGPVGRRRYARKQLVPLLYKQFVPGVPERTLGAS
GPAEGRVARGSERFRDLVPNYNPDIIFKDEENSGADRLMTERCKERVNALAIAVMNMWPG
VRLRVTEGWDEDGHHAQDSLHYEGRALDITTSDRDRNKYGLLARLAVEAGFDWVYYES
RNHVHVSVKADNSLAVRAGGCFPGNATVRLWSGERKGLRELHRGDWVLAADASGRVVP
TPVLLFLDRDLQRRASFVAVETEWPPRKLLLTPWHLVFAARGPAPAPGDFAPVFARRLRAG
DSVLAPGGDALRPARVARVAREEAVGVFAPLTAHGTLLVNDVLASCYAVLESHQWAHRAF
APLRLLHALGALLPGGAVQPTGMHWYSRLLYRLAEELLG

>Q9NQS5 multipass membrane protein
MWNSSDANFSCYHESVLGYRYVAVSWGVVVAVTGTVGNVLTLLALAIQPKLRTRFNLLIA
NLTLADLLYCTLLQPFSVDTYLHLHWRTGATFCRVFGLLLFASNSVSILTLCLIALGRYLLIA
HPKLFPQVFSAKGIVLALVSTWVVGVASFAPLWPIYILVPVVCTCSFDRIRGRPYTTILMGI
YFVLGLSSVGIFYCLIHRQVKRAAQALDQYKLRQASIHSNHVARTDEAMPGRFQELDSRL
ASGGPSEGISSEPVSAATTQTLEGDSSEVGDQINSKRAKQMAEKSPPEASAKAQPIKGARR
APDSSSEFGKVTRMCFAVFLCFALSYIPFLLLNILDARVQAPRVVHMLAANLTWLNGCINP
VLYAAMNRQFRQAYGSILKRGPRSFHRLH

>P26010 single-pass type I membrane protein
MVALPMVLVLLLVLSRGESELDAKIPSTGDATEWRNPHLSMLGSCQPAPSCQKCILSHPSC
AWCKQLNFTASGEAEARRCARREELLARGCPLEELEEPRGQQEVLQDQPLSQGARGEGA
TQLAPQRVRVTLRPGEPQQLQVRFLRAEGYPVDLYYLMDLSYSMKDDLERVRQLGHALL
VRLQEVTHSVRIGFGSFVDKTVLPFVSTVPSKLRHPCPTRLERCQSPFSFHHVLSLTGDAQ
AFEREVGRQSVSGNLDSPEGGFDAILQAALCQEQIGWRNVSRLLVFTSDDTFHTAGDGKL

GGIFMPSDGHCHLDSNGLYSRSTEFDYPSVGQVAQALSAANIQPIFAVTSAALPVYQELSK
LIPKSAVGELSEDSSNVVQLIMDAYNSLSSTVTLEHSSLPPGVHISYESQCEGPEKREGKAE
DRGQCNHVRINQTVTFWVSLQATHCLPEPHLLRLRALGFSEELIVELHTLCDCNCSDTQP
QAPHCSDGQGHLQCGVCSCAPGRLGRLCECSVAELSSPDLESGCRAPNGTGPLCSGKGHC
QCGRCSCSGQSSGHLCECDDASCERHEGILCGGFGRCQCGVCHCHANRTGRACECSGDM
DSCISPEGGLCSGHGRCKCNRCQCLDGYYGALCDQCPGCKTPCERHRDCAECGAFRTGP
LATNCSTACAHTNVTLALAPILDDGWCKERTLDNQLFFFLVEDDARGTVVLRVRPQEKGA
DHTQAIVLGCVGGIVAVGLGLVLAYRLSVEIYDRREYSRFEKEQQQLNWKQDSNPLYKSA
ITTTINPRFQEADSPTL

>P21860 single-pass type I membrane protein
MRANDALQVLGLLFSLARGSEVGNSQAVCPGTLNGLSVTGDAENQYQTLYKLYERCEVV
MGNLEIVLTGHNADLSFLQWIREVTGYVLVAMNEFSTLPLPNLRVVRGTQVYDGKFAIFV
MLNYNTNSSHALRQLRLTQLTEILSGGVYIEKNDKLCHMDTIDWRDIVRDRDAEIVVKDN
GRSCPPCHEVCKGRCWGPGSEDCQTLTKTICAPQCNGHCFGPNPNQCCHDECAGGCSGP
QDTDCFACRHFNDSGACVPRCPQPLVYNKLTFQLEPNPHTKYQYGGVCVASCPHNFVVD
QTSCVRACPPDKMEVDKNGLKMCEPCGGLCPKACEGTGSGSRFQTVDSSNIDGFVNCTK
ILGNLDFLITGLNGDPWHKIPALDPEKLNVFRTVREITGYLNIQSWPPHMHNFSVFSNLTTI
GGRSLYNRGFSLLIMKNLNVTSLGFRSLKEISAGRIYISANRQLCYHHSLNWTKVLRGPTE
ERLDIKHNRPRRDCVAEGKVCDPLCSSGGCWGPGPGQCLSCRNYSRGGVCVTHCNFLNG
EPREFAHEAECFSCHPECQPMEGTATCNGSGSDTCAQCAHFRDGPHCVSSCPHGVLGAKG
PIYKYPDVQNECRPCHENCTQGCKGPELQDCLGQTLVLIGKTHLTMALTVIAGLVVIFMM
LGGTFLYWRGRRIQNKRAMRRYLERGESIEPLDPSEKANKVLARIFKETELRKLKVLGSG
VFGTVHKGVWIPEGESIKIPVCIKVIEDKSGRQSFQAVTDHMLAIGSLDHAHIVRLLGLCPG
SSLQLVTQYLPLGSLLDHVRQHRGALGPQLLLNWGVQIAKGMYYLEEHGMVHRNLAAR
NVLLKSPSQVQVADFGVADLLPPDDKQLLYSEAKTPIKWMALESIHFGKYTHQSDVWSY
GVTVWELMTFGAEPYAGLRLAEVPDLLEKGERLAQPQICTIDVYMVMVKCWMIDENIRP
TFKELANEFTRMARDPPRYLVIKRESGPGIAPGPEPHGLTNKKLEEVELEPELDLDLDLEAE
EDNLATTTLGSALSLPVGTLNRPRGSQSLLSPSSGYMPMNQGNLGESCQESAVSGSSERCP
RPVSLHPMPRGCLASESSEGHVTGSEAELQEKVSMCRSRSRSRSPRPRGDSAYHSQRHSLL
TPVTPLSPPGLEEEDVNGYVMPDTHLKGTPSSREGTLSSVGLSSVLGTEEEDEDEEYEYM
NRRRRHSPPHPPRPSSLEELGYEYMDVGSDLSASLGSTQSCPLHPVPIMPTAGTTPDEDYE
YMNRQRDGGGPGGDYAAMGACPASEQGYEEMRAFQGPGHQAPHVHYARLKTLRSLEAT
DSAFDNPDYWHSRLFPKANAQRT

>Q6UX01 multipass membrane protein
MEAPDYEVLSVREQLFHERIRECIISTLLFATLYILCHIFLTRFKKPAEFTTVDDEDATVNKIA
LELCTFTLAIALGAVLLLPFSIISNEVLLSLPRNYYIQWLNGSLIHGLWNLVFLFSNLSLIFLM
PFAYFFTESEGFAGSRKGVLGRVYETVVMLMLLTLLVLGMVWVASAIVDKNKANRESLY
DFWEYYLPYLYSCISFLGVLLLLVCTPLGLARMFSVTGKLLVKPRLLEDLEEQLYCSAFEE
AALTRRICNPTSCWLPLDMELLHRQVLALQTQRVLLEKRRKASAWQRNLGYPLAMLCLL
VLTGLSVLIVAIHILELLIDEAAMPRGMQGTSLGQVSFSKLGSFGAVIQVVLIFYLMVSSVV
GFYSSPLFRSLRPRWHDTAMTQIIGNCVCLLVLSSALPVFSRTLGLTRFDLLGDFGRFNWL
GNFYIVFLYNAAFAGLTTLCLVKTFTAAVRAELIRAFGLDRLPLPVSGFPQASRKTQHQ

>Q9BSJ8 multipass membrane protein
MERSPGEGPSPSPMDQPSAPSDPTDQPPAAHAKPDPGSGGQPAGPGAAGEALAVLTSFGRR
LLVLIPVYLAGAVGLSVGFVLFGLALYLGWRRVRDEKERSLRAARQLLDDEEQLTAKTLY
MSHRELPAWVSFPDVEKAEWLNKIVAQVWPFLGQYMEKLLAETVAPAVRGSNPHLQTFT
FTRVELGEKPLRIIGVKVHPGQRKEQILLDLNISYVGDVQIDVEVKKYFCKAGVKGMQLH
GVLRVILEPLIGDLPFVGAVSMFFIRRPTLDINWTGMTNLLDIPGLSSLSDTMIMDSIAAFLV
LPNRLLVPLVPDLQDVAQLRSPLPRGIIRIHLLAARGLSSKDKYVKGLIEGKSDPYALVRLG
TQTFCSRVIDEELNPQWGETYEVMVHEVPGQEIEVEVFDKDPDKDDFLGRMKLDVGKVL
QASVLDDWFPLQGGQGQVHLRLEWLSLLSDAEKLEQVLQWNWGVSSRPDPPSAAILVV
YLDRAQDLPLKKGNKEPNPMVQLSIQDVTQESKAVYSTNCPVWEEAFRFFLQDPQSQEL
DVQVKDDSRALTLGALTLPLARLLTAPELILDQWFQLSSSGPNSRLYMKLVMRILYLDSSEI
CFPTVPGCPGAWDVDSENPQRGSSVDAPPRPCHTTPDSQFGTEHVLRIHVLEAQDLIAKD
RFLGGLVKGKSDPYVKLKLAGRSFRSHVVREDLNPRWNEVFEVIVTSVPGQELEVEVFDK
DLDKDDFLGRCKVRLTTVLNSGFLDEWLTLEDVPSGRLHLRLERLTPRPTAAELEEVLQV
NSLIQTQKSAELAAALLSIYMERAEDLPLRKGTKHLSPYATLTVGDSSHKTKTISQTSAPV
WDESASFLIRKPHTESLELQVRGEGTGVLGSLSLPLSELLVADQLCLDRWFTLSSGQGQVL
LRAQLGILVSQHSGVEAHSHSYSHSSSSLSEEPELSGGPPHITSSAPELRQRLTHVDSPLEAP
AGPLGQVKLTLWYYSEERKLVSIVHGCRSLRQNGRDPPDPYVSLLLLPDKNRGTKRRTSQ
KKRTLSPEFNERFEWELPLDEAQRRKLDVSVKSNSSFMSRERELLGKVQLDLAETDLSQG
VARWYDLMDNKDKGSS

>Q96AX1 peripheral membrane protein
MAAHLSYGRVNLNVLREAVRRELREFLDKCAGSKAIVWDEYLTGPFGLIAQYSLLKEHEV
EKMFTLKGNRLPAADVKNIIFFVRPRLELMDIIAENVLSEDRRGPTRDFHILFVPRRSLLCE
QRLKDLGVLGSFIHREEYSLDLIPFDGDLLSMESEGAFKECYLEGDQTSLYHAAKGLMTL
QALYGTIPQIFGKGECARQVANMMIRMKREFTGSQNSIFPVFDNLLLLDRNVDLLTPLATQ
LTYEGLIDEIYGIQNSYVKLPPEKFAPKKQGDGGKDLPTEAKKLQLNSAEELYAEIRDKNF
NAVGSVLSKKAKIISAAFEERHNAKTVGEIKQFVSQLPHMQAARGSLANHTSIAELIKDVT
TSEDFFDKLTVEQEFMSGIDTDKVNNYIEDCIAQKHSLIKVLRLVCLQSVCNSGLKQKVLD
YYKREILQTYGYEHILTLHNLEKAGLLKPQTGGRNNYPTIRKTLRLWMDDVNEQNPTDIS
YVYSGYAPLSVRLAQLLSRPGWRSIEEVLRILPGPHFEERQPLPTGLQKKRQPGENRVTLIF
FLGGVTFAEIAALRFLSQLEDGGTEYVIATTKLMNGTSWIEALMEKPF

>Q9H9H4 peripheral membrane protein
MAGAGSEARFAGLSLVQLNELLEDEGQLTEMVQKMEETQNVQLNKEMTLASNRSLAEG
NLLYQPQLDTLKARLTQKYQELQVLFEAYQIKKTKLDRQSSSASLETLLALLQAEGAKIEE
DTENMAEKFLDGELPLDSFIDVYQSKRKLAHMRRVKIEKLQEMVLKGQRLPQALAPLPPR
LPELAPTAPLPYPAPEASGPPAVAPRRIPPPPPPVPAGRLATPFTAAMSSGQAVPYPGLQCPPL
PPRVGLPTQQGFSSQFVSPYPPPLPQRPPPRLPPHQPGFILQ

>Q9HBH0 lipid-anchor
MDAPGALAQTAAPGPGRKELKIVIVGDGGCGKTSLLMVYSQGSFPEHYAPSVFEKYTASV
TVGSKEVTLNLYDTAGQEDYDRLRPLSYQNTHLVLICYDVMNPTSYDNVLIKWFPEVTHF

CRGIPMVLIGCKTDLRKDKEQLRKLRAAQLEPITYMQGLSACEQIRAALYLECSAKFREN
VEDVFREAAKVALSALKKAQRQKKRRLCLLL

>P30872 multipass membrane protein
MFPNGTASSPSSSPSPSPGSCGEGGGSRGPGAGAADGMEEPGRNASQNGTLSEGQGSAILI
SFIYSVVCLVGLCGNSMVIYVILRYAKMKTATNIYILNLAIADELLMLSVPFLVTSTLLRHW
PFGALLCRLVLSVDAVNMFTSIYCLTVLSVDRYVAVVHPIKAARYRRPTVAKVVNLGVWV
LSLLVILPIVVFSRTAANSDGTVACNMLMPEPAQRWLVGFVLYTFLMGFLLPVGAICLCYV
LIIAKMRMVALKAGWQQRKRSERKITLMVMMVVMVFVICWMPFYVVQLVNVFAEQDD
ATVSQLSVILGYANSCANPILYGFLSDNFKRSFQRILCLSWMDNAAEEPVDYYATALKSRA
YSVEDFQPENLESGGVFRNGTCTSRITTL

>A5D6W6 multipass membrane protein
MERGPVVGAGLGAGARIQALLGCLLKVLLWVASALLYFGSEQAARLLGSPCLRRLYHAW
LAAVVIFGPLLQFHVNPRTIFASHGNFFNIKFVNSAWGWTCTFLGGFVLLVVFLATRRVAVT
ARHLSRLVVGAAVWRGAGRAFLLIEDLTGSCFEPLPQGLLLHELPDRRSCLAAGHQWRG
YTVSSHTFLLTFCCLLMAEEAAVFAKYLAHGLPAGAPLRLVFLLNVLLLGLWNFLLLCTVI
YFHQYTHKVVGAAVGTFAWYLTYGSWYHQPWSPGSPGHGLFPRPHSSRKHN

>Q16799 multipass membrane protein
MAAPGDPQDELLPLAGPGSQWLRHRGEGENEAVTPKGATPAPQAGEPSPGLGARAREAA
SREAGSGPARQSPVAMETASTGVAGVSSAMDHTFSTTSKDGEGSCYTSLISDICYPPQEDST
YFTGILQKENGHVTISESPEELGTPGPSLPDVPGIESRGLFSSDSGIEMTPAESTEVNKILADP
LDQMKAEAYKYIDITRPEEVKHQEQHHPELEDKDLDFKNKDTDISIKPEGVREPDKPAPVE
GKIIKDHLLEESTFAPYIDDLSEEQRRAPQITTPVKITLTEIEPSVETTTQEKTPEKQDICLKPS
PDTVPTVTVSEPEDDSPGSITPPSSGTEPSAAESQGKGSISEDELITAIKEAKGLSYETAENPR
PVGQLADRPEVKARSGPPTIPSPLDHEASSAESGDSEIELVSEDPMAAEDALPSGYVSFGH
VGGPPPSPASPSIQYSILREEREAELDSELIIESCDASSASEESPKREQDSPPMKPSALDAIRE
ETGVRAEERAPSRRGLAEPGSFLDYPSTEPQPGPELPPGDGALEPETPMLPRKPEEDSSSNQ
SPAATKGPGPLGPGAPPPLLFLNKQKAIDLLYWRDIKQTGIVFGSFLLLLFSLTQFSVVSVVA
YLALAALSATISFRIYKSVLQAVQKTDEGHPFKAYLELEITLSQEQIQKYTDCLQFYVNSTL
KELRRLFLVQDLVDSLKFAVLMWLLTYVGALFNGLTLLLMAVVSMFTLPVVYVKHQAQI
DQYLGLVRTHINAVVAKIQAKIPGAKRHAE

>Q9UKJ8 single-pass type I membrane protein
MAVDGTLVYIRVTLLLLWLGVFLSISGYCQAGPSQHFTSPEVVIPLKVISRGRSAKAPGWL
SYSLRFGGQKHVVHMRVKKLLVSRHLPVFTYTDDRALLEDQLFIPDDCYYHGYVEAAPE
SLVVFSACFGGFRGVLKISGLTYEIEPIRHSATFEHLVYKINSNETQFPAMRCGLTEKEVARQ
QLEFEEAENSALEPKSAGDWWTHAWFLELVVVVNHDFFIYSQSNISKVQEDVFLVVNIVD
SMYKQLGTYIILIGIEIWNQGNVFPMTSIEQVLNDFSQWKQISLSQLQHDAAHMFIKNSLIS
ILGLAYVAGICRPPIDCGVDNFQGDTWSLFANTVAHELGHTLGMQHDEEFCFCGERGCIM
NTFRVPAEKFTNCSYADFMKTTLNQGSCLHNPPRLGEIFMLKRCGNGVVEREEQCDCGSV
QQCEQDACCLLNCTLRPGAACAFGLCCKDCKFMPSGELCRQEVNECDLPEWCNGTSHQ
CPEDRYVQDGIPCSDSAYCYQKRCNNHDQHCREIFGKDAKSASQNCYKEINSQGNRFGH

CGINGTTYLKCHISDVFCGRVQCENVRDIPLLQDHFTLQHTHINGVTCWGIDYHLRMNISD
IGEVKDGTVCGPGKICIHKKCVSLSVLSHVCLPETCNMKGICNNKHHCHCGYGWSPPYC
QHRGYGGSIDSGPASAKRGVFLPLIVIPSLSVLTFLFTVGLLMYLRQCSGPKETKAHSSG

>P59190	lipid-anchor
MAKQYDVLFRLLLIGDSGVGKTCLLCRFTDNEFHSSHISTIGVDFKMKTIEVDGIKVRIQI
WDTAGQERYQTITKQYYRRAQGIFLVYDISSERSYQHIMKWVSDVDEYAPEGVQKILIGN
KADEEQKRQVGREQGQQLAKEYGMDFYETSACTNLNIKESFTRLTELVLQAHRKELEGL
RMRASNELALAELEEEEGKPEGPANSSKTCWC

>Q15643	peripheral membrane protein
MSSWLGGLGSGLGQSLGQVGGSLASLTGQISNFTKDMLMEGTEEVEAELPDSRTKEIEAI
HAILRSENERLKKLCTDLEEKHEASEIQIKQQSTSYRNQLQQKEVEISHLKARQIALQDQL
LKLQSAAQSVPSGAGVPATTASSSFAYGISHHPSAFHDDDMDFGDIISSQQEINRLSNEVSR
LESEVGHWRHIAQTSKAQGTDNSDQSEICKLQNIIKELKQNRSQEIDDHQHEMSVLQNAH
QQKLTEISRRHREELSDYEERIEELENLLQQGGSGVIETDLSKIYEMQKTIQVLQIEKVEST
KKMEQLEDKIKDINKKLSSAENDRDILRREQEQLNVEKRQIMEECENLKLECSKLQPSAV
KQSDTMTEKERILAQSASVEEVFRLQQALSDAENEIMRLSSLNQDNSLAEDNLKLKMRIE
VLEKEKSLLSQEKEELQMSLLKLNNEYEVIKSTATRDISLDSELHDLRLNLEAKEQELNQSI
SEKETLIAEIEELDRQNQEATKHMILIKDQLSKQQNEGDSIISKLKQDLNDEKKRVHQLED
DKMDITKELDVQKEKLIQSEVALNDLHLTKQKLEDKVENLVDQLNKSQESNVSIQKENLE
LKEHIRQNEEELSRIRNELMQSLNQDSNSNFKDTLLKEREAEVRNLKQNLSELEQLNENL
KKVAFDVKMENEKLVLACEDVRHQLEECLAGNNQLSLEKNTIVETLKMEKGEIEAELCW
AKKRLLEEANKYEKTIEELSNARNLNTSALQLEHEHLIKLNQKKDMEIAELKKNIEQMDT
DHKETKDVLSSSLEEQKQLTQLINKKEIFIEKLKERSSKLQEELDKYSQALRKNEILRQTIE
EKDRSLGSMKEENNHLQEELERLREEQSRTAPVADPKTLDSVTELASEVSQLNTIKEHLEE
EIKHHQKIIEDQNQSKMQLLQSLQEQKKEMDEFRYQHEQMNATHTQLFLEKDEEIKSLQK
TIEQIKTQLHEERQDIQTDNSDIFQETKVQSLNIENGSEKHDLSKAETERLVKGIKERELEIK
LLNEKNISLTKQIDQLSKDEVGKLTQIIQQKDLEIQALHARISSTSHTQDVVYLQQQLQAYA
MEREKVFAVLNEKTRENSHLKTEYHKMMDIVAAKEAALIKLQDENKKLSTRFESSGQDM
FRETIQNLSRIIREKDIEIDALSQKCQTLLAVLQTSSTGNEAGGVNSNQFEELLQERDKLKQ
QVKKMEEWKQQVMTTVQNMQHESAQLQEELHQLQAQVLVDSDNNSKLQVDYTGLIQS
YEQNETKLKNFGQELAQVQHSIGQLCNTKDLLLGKLDIISPQLSSASLLTPQSAECLRASKS
EVLSESSELLQQELEELRKSLQEKDATIRTLQENNHRLSDSIAATSELERKEHEQTDSEIKQL
KEKQDVLQKLLKEKDLLIKAKSDQLLSSNENFTNKVNENELLRQAVTNLKERILILEMDIG
KLKGENEKIVETYRGKETEYQALQETNMKFSMMLREKEFECHSMKEKALAFEQLLKEKE
QGKTGELNQLLNAVKSMQEKTVVFQQERDQVMLALKQKQMENTALQNEVQRLRDKEF
RSNQELERLRNHLLESEDSYTREALAAEDREAKLRKKVTVLEEKLVSSSNAMENASHQAS
VQVESLQEQLNVVSKQRDETALQLSVSQEQVKQYALSLANLQMVLEHFQQEEKAMYSA
ELEKQKQLIAEWKKNAENLEGKVISLQECLDEANAALDSASRLTEQLDVKEEQIEELKRQ
NELRQEMLDDVQKKLMSLANSSEGKVDKVLMRNLFIGHFHTPKNQRHEVLRLMGSILG
VRREEMEQLFHDDQGGVTRWMTGWLGGGSKSVPNTPLRPNQQSVVNSSFSELFVKFLET
ESHPSIPPPKLSVHDMKPLDSPGRRKRDTNAPESFKDTAESRSGRRTDVNPFLAPRSAAVPL
INPAGLGPGGPGHLLLKPISDVLPTFTPLPALPDNSAGVVLKDLLKQ

>A8MQT2 peripheral membrane protein
MAEETGQSKLAAAKKKFKEYWQRNRPGVPAAAKRNTKANGSSPETAASGGCHSSEASSS
ASSSLHARQSPCQEQAAVLNSRSIKISRLNDTIKSLKQQKKQVEHQLEEEKKANNEKQKA
ERELEGQIQRLNTEKKKLNTDLYHMKHSLRYFEEESKDLAGRLQRSSQRIGELEWSLCAV
AATQKKKPDGFSSRSKALLKRQLEQSIREQILLKGHVTQLKESLKEVQLERDQYAEQIKGE
RAQWQQRMRKMSQEVCTLKEEKKHDTHRVEELERSLSRLKNQMAEPLPPDAPAVSSEVE
LQDLRKELERVAGELQAQVENNQCISLLNRGQKERLREQEERLQEQQERLREREKRLQQL
AEPQSDLEELKHENKSALQLEQQVKELQEKLGQVMETLTSAEKEPEAAVPASGTGGESSG
LMDLLEEKADLREHVEKLELGFIQYRRERCHQKVHRLLTEPGDSAKDASPGGGHHQAGP
GQGGEEGEAAGAAGDGVAACGSYSEGHGKFLAAARNPAAEPSPGAPAPQELGAADKHG
DLCEASLTNSVEPAQGEAREGSSQDNPTAQPVLQLLGEMQDHQEHPGLGSNCCVPCFCWA
WLPRRRR

>Q5J8M3 multipass membrane protein
MTAQGGLVANRGRRFKWAIELSGPGGGSRGRSDRGSGQGDSLYPVGYLDKQVPDTSVQE
TDRILVEKRCWDIALGPLKQIPMNLFIMYMAGNTISIFPTMMVCMMAWRPIQALMAISATF
KMLESSSQKFLQGLVYLIGNLMGLALAVYKCQSMGLLPTHASDWLAFIEPPERMEFSGGG
LLL

>Q00796 peripheral membrane protein
MAAAAKPNNLSLVVHGPGDLRLENYPIPEPGPNEVLLRMHSVGICGSDVHYWEYGRIGN
FIVKKPMVLGHEASGTVEKVGSSVKHLKPGDRVAIEPGAPRENDEFCKMGRYNLSPSIFFC
ATPPDDGNLCRFYKHNAAFCYKLPDNVTFEEGALIEPLSVGIHACRRGGVTLGHKVLVCG
AGPIGMVTLLVAKAMGAAQVVVTDLSATRLSKAKEIGADLVLQISKESPQEIARKVEGQL
GCKPEVTIECTGAEASIQAGIYATRSGGNLVLVGLGSEMTTVPLLHAAIREVDIKGVFRYCN
TWPVAISMLASKSVNVKPLVTHRFPLEKALEAFETFKKGLGLKIMLKCDPSDQNP

>O14975 multipass membrane protein
MLSAIYTVLAGLLFLPLLVNLCCPYFFQDIGYFLKVAAVGRRVRSYGKRRPARTILRAFLEK
ARQTPHKPFLLFRDETLTYAQVDRRSNQVARALHDHLGLRQGDCVALLMGNEPAYVWL
WLGLVKLGCAMACLNYNIRAKSLLHCFQCCGAKVLLVSPELQAAVEEILPSLKKDDVSIY
YVSRTSNTDGIDSFLDKVDEVSTEPIPESWRSEVTFSTPALYIYTSGTTGLPKAAMITHQRI
WYGTGLTFVSGLKADDVIYITLPFYHSAALLIGIHGCIVAGATLALRTKFSASQFWDDCRK
YNVTVIQYIGELLRYLCNSPQKPNDRDHKVRLALGNGLRGDVWRQFVKRFGDICIYEFYA
ATEGNIGFMNYARKVGAVGRVNYLQKKIITYDLIKYDVEKDEPVRDENGYCVRVPKGEV
GLLVCKITQLTPFNGYAGAKAQTEKKKLRDVFKKGDLYFNSGDLLMVDHENFIYFHDRV
GDTFRWKGENVATTEVADTVGLVDFVQEVNVYGVHVPDHEGRIGMASIKMKENHEFDG
KKLFQHIADYLPSYARPRFLRIQDTIEITGTFKHRKMTLVEEGFNPAVIKDALYFLDDTAKM
YVPMTEDIYNAISAKTLKL

>P08069 single-pass type I membrane protein
MKSGSGGGSPTSLWGLLFLSAALSLWPTSGEICGPGIDIRNDYQQLKRLENCTVIEGYLHIL
LISKAEDYRSYRFPKLTVITEYLLLFRVAGLESLGDLFPNLTVIRGWKLFYNYALVIFEMTN

LKDIGLYNLRNITRGAIRIEKNADLCYLSTVDWSLILDAVSNNYIVGNKPPKECGDLCPGT
MEEKPMCEKTTINNEYNYRCWTTNRCQKMCPSTCGKRACTENNECCHPECLGSCSAPDN
DTACVACRHYYYAGVCVPACPPNTYRFEGWRCVDRDFCANILSAESSDSEGFVIHDGECM
QECPSGFIRNGSQSMYCIPCEGPCPKVCEEEKKTKTIDSVTSAQMLQGCTIFKGNLLINIRR
GNNIASELENFMGLIEVVTGYVKIRHSHALVSLSFLKNLRLILGEEQLEGNYSFYVLDNQN
LQQLWDWDHRNLTIKAGKMYFAFNPKLCVSEIYRMEEVTGTKGRQSKGDINTRNNGERA
SCESDVLHFTSTTTSKNRIIITWHRYRPPDYRDLISFTVYYKEAPFKNVTEYDGQDACGSN
SWNMVDVDLPPNKDVEPGILLHGLKPWTQYAVYVKAVTLTMVENDHIRGAKSEILYIRTN
ASVPSIPLDVLSASNSSSQLIVKWNPPSLPNGNLSYYIVRWQRQPQDGYLYRHNYCSKDKI
PIRKYADGTIDIEEVTENPKTEVCGGEKGPCCACPKTEAEKQAEKEEAEYRKVFENFLHNS
IFVPRPERKRRDVMQVANTTMSSRSRNTTAADTYNITDPEELETEYPFFESRVDNKERTVIS
NLRPFTLYRIDIHSCNHEAEKLGCSASNFVFARTMPAEGADDIPGPVTWEPRPENSIFLKWP
EPENPNGLILMYEIKYGSQVEDQRECVSRQEYRKYGGAKLNRLNPGNYTARIQATSLSGN
GSWTDPVFFYVQAKTGYENFIHLIIALPVAVLLIVGGLVIMLYVFHRKRNNSRLGNGVLYA
SVNPEYFSAADVYVPDEWEVAREKITMSRELGQGSFGMVYEGVAKGVVKDEPETRVAIK
TVNEAASMRERIEFLNEASVMKEFNCHHVVRLLGVVSQGQPTLVIMELMTRGDLKSYLR
SLRPEMENNPVLAPPSLSKMIQMAGEIADGMAYLNANKFVHRDLAARNCMVAEDFTVKI
GDFGMTRDIYETDYYRKGGKGLLPVRWMSPESLKDGVFTTYSDVWSFGVVLWEIATLAE
QPYQGLSNEQVLRFVMEGGLLDKPDNCPDMLFELMRMCWQYNPKMRPSFLEIISSIKEE
MEPGFREVSFYYSEENKLPEPEELDLEPENMESVPLDPSASSSSLPLPDRHSGHKAENGPG
PGVLVLRASFDERQPYAHMNGGRKNERALPLPQSSTC

>P29590	peripheral membrane protein
MEPAPARSPRPQQDPARPQEPTMPPPETPSEGRQPSPSPSPTERAPASEEEFQFLRCQQCQA
EAKCPKLLPCLHTLCSGCLEASGMQCPICQAPWPLGADTPALDNVFFESLQRRLSVYRQIV
DAQAVCTRCKESADFWCFECEQLLCAKCFEAHQWFLKHEARPLAELRNQSVREFLDGTR
KTNNIFCSNPNHRTPTLTSIYCRGCSKPLCCSCALLDSSHSELKCDISAEIQQRQEELDAMT
QALQEQDSAFGAVHAQMHAAVGQLGRARAETEELIRERVRQVVAHVRAQERELLEAVDA
RYQRDYEEMASRLGRLDAVLQRIRTGSALVQRMKCYASDQEVLDMHGFLRQALCRLRQE
EPQSLQAAVRTDGFDEFKVRLQDLSSCITQGKDAAVSKKASPEAASTPRDPIDVDLPEEAE
RVKAQVQALGLAEAQPMAVVQSVPGAHPVPVYAFSIKGPSYGEDVSNTTTAQKRKCSQT
QCPRKVIKMESEEGKEARLARSSPEQPRPSTSKAVSPPHLDGPPSPRSPVIGSEVFLPNSNHV
ASGAGEAEERVVVISSSEDSDAENSSSRELDDSSSESSDLQLEGPSTLRVLDENLADPQAED
RPLVFFDLKIDNETQKISQLAAVNRESKFRVVIQPEAFFSIYSKAVSLEVGLQHFLSFLSSMR
RPILACYKLWGPGLPNFFRALEDINRLWEFQEAISGFLAALPLIRERVPGASSFKLKNLAQT
YLARNMSERSAMAAVLAMRDLCRLLEVSPGPQLAQHVYPFSSLQCFASLQPLVQAAVLPR
AEARLLALHNVSFMELLSAHRRDRQGGLKKYSRYLSLQTTTLPPAQPAFNLQALGTYFEG
LLEGPALARAEGVSTPLAGRGLAERASQQS

>O15127	multipass membrane protein
MSAFDTNPFADPVDVNPFQDPSVTQLTNAPQGGLAEFNPFSETNAATTVPVTQLPGSSQPA
VLQPSVEPTQPTPQAVVSAAQAGLLRQQEELDRKAAELERKERELQNTVANLHVRQNNW
PPLPSWCPVKPCFYQDFSTEIPADYQRICKMLYYLWMLHSVTLFLNLLACLAWFSGNSSK
GVDFGLSILWFLIFTPCAFLCWYRPIYKAFRSDNSFSFFVFFFVFFCQIGIYIIQLVGIPGLGD

SGWIAALSTLDNHSLAISVIMMVVAGFFTLCAVLSVFLLQRVHSLYRRTGASFQQAQEEFS
QGIFSSRTFHRAASSAAQGAFQGN

>Q9UBD6 multipass membrane protein
MAWNTNLRWRLPLTCLLLQVIMVILFGVFVRYDFEADAHWWSERTHKNLSDMENEFYY
RYPSFQDVHVMVFVGFGFLMTFLQRYGFSAVGFNFLLAAFGIQWALLMQGWFHFLQDRY
IVVGVENLINADFCVASVCVAFGAVLGKVSPIQLLIMTFFQVTLFAVNEFILLNLLKVKDAG
GSMTIHTFGAYFGLTVTRILYRRNLEQSKERQNSVYQSDLFAMIGTLFLWMYWPSFNSAIS
YHGDSQHRAAINTYCSLAACVLTSVAISSALHKKGKLDMVHIQNATLAGGVAVGTAAEM
MLMPYGALIIGFVCGIISTLGFVYLTPFLESRLHIQDTCGINNLHGIPGIIGGIVGAVTAASAS
LEVYGKEGLVHSFDFQGFNGDWTARTQGKFQIYGLLVTLAMALMGGIIVGLILRLPFWGQ
PSDENCFEDAVYWEMPEGNSTVYIPEDPTFKPSGPSVPSVPMVSPLPMASSVPLVP

>Q08431 peripheral membrane protein
MPRPRLLAALCGALLCAPSLLVALDICSKNPCHNGGLCEEISQEVRGDVFPSYTCTCLKGY
AGNHCETKCVEPLGMENGNIANSQIAASSVRVTFLGLQHWVPELARLNRAGMVNAWTPS
SNDDNPWIQVNLLRRMWVTGVVTQGASRLASHEYLKAFKVAYSLNGHEFDFIHDVNKK
HKEFVGNWNKNAVHVNLFETPVEAQYVRLYPTSCHTACTLRFELLGCELNGCANPLGLK
NNSIPDKQITASSSYKTWGLHLFSWNPSYARLDKQGNFNAWVAGSYGNDQWLQVDLGSS
KEVTGIITQGARNFGSVQFVASYKVAYSNDSANWTEYQDPRTGSSKIFPGNWDNHSHKKN
LFETPILARYVRILPVAWHNRIALRLELLGC

>P09958 single-pass type I membrane protein
MELRPWLLWVVAATGTLVLLAADAQGQKVFTNTWAVRIPGGPAVANSVARKHGFLNLGQ
IFGDYYHFWHRGVTKRSLSPHRPRHSRLQREPQVQWLEQQVAKRRTKRDVYQEPTDPKF
PQQWYLSGVTQRDLNVKAAWAQGYTGHGIVVSILDDGIEKNHPDLAGNYDPGASFDVN
DQDPDPQPRYTQMNDNRHGTRCAGEVAAVANNGVCGVGVAYNARIGGVRMLDGEVTDA
VEARSLGLNPNHIHIYSASWGPEDDGKTVDGPARLAEEAFFRGVSQGRGGLGSIFVWASG
NGGREHDSCNCDGYTNSIYTLSISSATQFGNVPWYSEACSSTLATTYSSGNQNEKQIVTTD
LRQKCTESHTGTSASAPLAAGIIALTLEANKNLTWRDMQHLVVQTSKPAHLNANDWATN
GVGRKVSHSYGYGLLDAGAMVALAQNWTTVAPQRKCIIDILTEPKDIGKRLEVRKTVTAC
LGEPNHITRLEHAQARLTLSYNRRGDLAIHLVSPMGTRSTLLAARPHDYSADGFNDWAFM
TTHSWDEDPSGEWVLEIENTSEANNYGTLTKFTLVLYGTAPEGLPVPPESSGCKTLTSSQA
CVVCEEGFSLHQKSCVQHCPPGFAPQVLDTHYSTENDVETIRASVCAPCHASCATCQGPA
LTDCLSCPSHASLDPVEQTCSRQSQSSRESPPQQQPPRLPPEVEAGQRLRAGLLPSHLPEVV
AGLSCAFIVLVFVTVFLVLQLRSGFSFRGVKVYTMDRGLISYKGLPPEAWQEECPSDSEED
EGRGERTAFIKDQSAL

>P20702 single-pass type I membrane protein
MTRTRAALLLFTALATSLGFNLDTEELTAFRVDSAGFGDSVVQYANSWVVVGAPQKITAA
NQTGGLYQCGYSTGACEPIGLQVPPEAVNMSLGLSLASTTSPSQLLACGPTVHHECGRNM
YLTGLCFLLGPTQLTQRLPVSRQECPRQEQDIVFLIDGSGSISSRNFATMMNFVRAVISQFQ
RPSTQFSLMQFSNKFQTHFTFEEFRRSSNPLSLLASVHQLQGFTYTATAIQNVVHRLFHASY
GARRDAAKILIVITDGKKEGDSLDYKDVIPMADAAGIIRYAIGVGLAFQNRNSWKELNDIA

SKPSQEHIFKVEDFDALKDIQNQLKEKIFAIEGTETTSSSSFELEMAQEGFSAVFTPDGPVLG
AVGSFTWSGGAFLYPPNMSPTFINMSQENVDMRDSYLGYSTELALWKGVQSLVLGAPRY
QHTGKAVIFTQVSRQWRMKAEVTGTQIGSYFGASLCSVDVDSDGSTDLVLIGAPHYYEQT
RGGQVSVCPLPRGWRRWWCDAVLYGEQGHPWGRFGAALTVLGDVNGDKLTDVVIGAP
GEEENRGAVYLFHGVLGPSISPSHSQRIAGSQLSSRLQYFGQALSGGQDLTQDGLVDLAVG
ARGQVLLLRTRPVLWVGVSMQFIPAEIPRSAFECREQVVSEQTLVQSNICLYIDKRSKNLLG
SRDLQSSVTLDLALDPGRLSPRATFQETKNRSLSRVRVLGLKAHCENFNLLLPSCVEDSVT
PITLRLNFTLVGKPLLAFRNLRPMLAADAQRYFTASLPFEKNCGADHICQDNLGISFSFPGL
KSLLVGSNLELNAEVMVWNDGEDSYGTTITFSHPAGLSYRYVAEGQKQGQLRSLHLTCDS
APVGSQGTWSTSCRINHLIFRGGAQITFLATFDVSPKAVLGDRLLLTANVSSENNTPRTSKT
TFQLELPVKYAVYTVVSSHEQFTKYLNFSESEEKESHVAMHRYQVNNLGQRDLPVSINFW
VPVELNQEAVWMDVEVSHPQNPSLRCSSEKIAPPASDFLAHIQKNPVLDCSIAGCLRFRCD
VPSFSVQEELDFTLKGNLSFGWVRQILQKKVSVVSVAEITFDTSVYSQLPGQEAFMRAQTT
TVLEKYKVHNPTPLIVGSSIGGLLLLALITAVLYKVGFFKRQYKEMMEEANGQIAPENGTQ
TPSPPSEK

>P22695 peripheral membrane protein
MKLLTRAGSFSRFYSLKVAPKVKATAAPAGAPPQPQDLEFTKLPNGLVIASLENYSPVSRIG
LFIKAGSRYEDFSNLGTTHLLRLTSSLTTKGASSFKITRGIEAVGGKLSVTATRENMAYTVE
CLRGDVDILMEFLLNVTTAPEFRRWEVADLQPQLKIDKAVAFQNPQTHVIENLHAAAYRN
ALANPLYCPDYRIGKVTSEELHYFVQNHFTSARMALIGLGVSHPVLKQVAEQFLNMRGGL
GLSGAKANYRGGEIREQNGDSLVHAAFVAESAVAGSAEANAFSVLQHVLGAGPHVKRGS
NTTSHLHQAVAKATQQPFDVSAFNASYSDSGLFGIYTISQATAAGDVIKAAYNQVKTIAQG
NLSNTDVQAAKNKLKAGYLMSVESSECFLEEVGSQALVAGSYMPPSTVLQQIDSVANADI
INAAKKFVSGQKSMAASGNLGHTPFVDEL

>Q8TAZ6 multipass membrane protein
MAPKAAKGAKPEPAPAPPPPGAKPEEDKKDGKEPSDKPQKAVQDHKEPSDKPQKAVQPK
HEVGTRRGCRRYRWELKDSNKEFWLLGHAEIKIRSLGCLIAAMILLSSLTVHPILRLIITMEI
SFFSFFILLYSFAIHRYIPFILWPISDLFNDLIACAFLVGAVVFAVRSRRSMNLHYLLAVILIGA
AGVFAFIDVCLQRNHFRGKKAKKHMLVPPPGKEKGPQQGKGPEPAKPPEPGKPPGPAKGK
K

>P55287 single-pass type I membrane protein
MKENYCLQAALVCLGMLCHSHAFAPERRGHLRPSFHGHHEKGKEGQVLQRSKRGWVW
NQFFVIEEYTGPDPVLVGRLHSDIDSGDGNIKYILSGEGAGTIFVIDDKSGNIHATKTLDREE
RAQYTLMAQAVDRDTNRPLEPPSEFIVKVQDINDNPPEFLHETYHANVPERSNVGTSVIQV
TASDADDPTYGNSAKLVYSILEGQPYFSVEAQTGIIRTALPNMDREAKEEYHVVIQAKDM
GGHMGGLSGTTKVTITLTDVNDNPPKFPQSVYQMSVSEAAVPGEEVGRVKAKDPDIGEN
GLVTYNIVDGDGMESFEITTDYETQEGVIKLKKPVDFETKRAYSLKVEAANVHIDPKFISN
GPFKDTVTVKISVEDADEPPMFLAPSYIHEVQENAAAGTVVGRVHAKDPDAANSPIRYSID
RHTDLDRFFTINPEDGFIKTTKPLDREETAWLNITVFAAEIHNRHQEAKVPVAIRVLDVNDN
APKFAAPYEGFICESDQTKPLSNQPIVTISADDKDDTANGPRFIFSLPPEIIHNPNFTVRDNR
DNTAGVYARRGGFSRQKQDLYLLPIVISDGGIPPMSSTNTLTIKVCGCDVNGALLSCNAEA

YILNAGLSTGALIAILACIVILLVIVVLFVTLRRQKKEPLIVFEEEDVRENIITYDDEGGGEED
TEAFDIATLQNPDGINGFIPRKDIKPEYQYMPRPGLRPAPNSVDVDDFINTRIQEADNDPTA
PPYDSIQIYGYEGRGSVAGSLSSLESATTDSDLDYDYLQNWGPRFKKLADLYGSKDTFDD
DS

>Q9GZQ8 lipid-anchor
MPSEKTFKQRRTFEQRVEDVRLIREQHPTKIPVIIERYKGEKQLPVLDKTKFLVPDHVNMS
ELIKIIRRRLQLNANQAFFLLVNGHSMVSVSTPISEVYESEKDEDGFLYMVYASQETFGMK
LSV

>P55290 GPI-anchor, lipid-anchor
MQPRTPLVLCVLLSQVLLLTSAEDLDCTPGFQQKVFHINQPAEFIEDQSILNLTFSDCKGND
KLRYEVSSPYFKVNSDGGLVALRNITAVGKTLFVHARTPHAEDMAELVIVGGKDIQGSLQ
DIFKFARTSPVPRQKRSIVVSPILIPENQRQPFPRDVGKVVDSDRPERSKFRLTGKGVDQEP
KGIFRINENTGSVSVTRTLDREVIAVYQLFVETTDVNGKTLEGPVPLEVIVIDQNDNRPIFR
EGPYIGHVMEGSPTGTTVMRMTAFDADDPATDNALLRYNIRQQTPDKPSPNMFYIDPEKG
DIVTVVSPALLDRETLENPKYELIIEAQDMAGLDVGLTGTATATIMIDDKNDHSPKFTKKEF
QATVEEGAVGVIVNLTVEDKDDPTTGAWRAAYTIINGNPGQSFEIHTNPQTNEGMLSVVK
PLDYEISAFHTLLIKVENEDPLVPDVSYGPSSTATVHITVLDVNEGPVFYPDPMMVTRQED
LSVGSVLLTVNATDPDSLQHQTIRYSVYKDPAGWLNINPINGTVDTTAVLDRESPFVDNSV
YTALFLAIDSGNPPATGTGTLLITLEDVNDNAPFIYPTVAEVCDDAKNLSVVILGASDKDLH
PNTDPFKFEIHKQAVPDKVWKISKINNTHALVSLLQNLNKANYNLPIMVTDSGKPPMTNIT
DLRVQVCSCRNSKVDCNAAGALRFSLPSVLLLSLFSLACL

>O96000 peripheral membrane protein
MPDSWDKDVYPEPPRRTPVQPNPIVYMMKAFDLIVDRPVTLVREFIERQHAKNRYYYYH
RQYRRVPDITECKEEDIMCMYEAEMQWKRDYKVDQEIINIMQDRLKACQQREGQNYQQ
NCIKEVEQFTQVAKAYQDRYQDLGAYSSARKCLAKQRQRMLQERKAAKEAAAATS

>Q9UQ90 multipass membrane protein
MAVLLLLLRALRRGPGPGPRPLWGPGPAWSPGFPARPGRGRPYMASRPPGDLAEAGGRAL
QSLQLRLLTPTFEGINGLLLKQHLVQNPVRLWQLLGGTFYFNTSRLKQKNKEKDKSKGKA
PEEDEEERRRRERDDQMYRERLRTLLVIAVVMSLLNALSTSGGSISWNDFVHEMLAKGEV
QRVQVVPESDVVEVYLHPGAVVFGRPRLALMYRMQVANIDKFEEKLRAAEDELNIEAKD
RIPVSYKRTGFFGNALYSVGMTAVGLAILWYVFRLAGMTGREGGFSAFNQLKMARFTIVD
GKMGKGVSFKDVAGMHEAKLEVREFVDYLKSPERFLQLGAKVPKGALLLGPPGCGKTL
LAKAVATEAQVPFLAMAGPEFVEVIGGLGAARVRSLFKEARARAPCIVYIDEIDAVGKKRS
TTMSGFSNTEEEQTLNQLLVEMDGMGTTDHVIVLASTNRADILDGALMRPGRLDRHVFID
LPTLQERREIFEQHLKSLKLTQSSTFYSQRLAELTPGFSGADIANICNEAALHAAREGHTSV
HTLNFEYAVERVLAGTAKKSKILSKEEQKVVAFHESGHALVGWMLEHTEAVMKVSITPRT
NAALGFAQMLPRDQHLFTKEQLFERMCMALGGRASEALSFNEVTSGAQDDLRKVTRIAY
SMVKQFGMAPGIGPISFPEAQEGLMGIGRRPFSQGLQQMMDHEARLLVAKAYRHTEKVL
QDNLDKLQALANALLEKEVINYEDIEALIGPPPHGPKKMIAPQRWIDAQREKQDLGEEET
EETQQPPLGGEEPTWPK

>Q96S79 lipid-anchor
MVSTYRVAVLGARGVGKSAIVRQFLYNEFSEVCVPTTARRLYLPAVVMNGHVHDLQILDF
PPISAFPVNTLQEWADTCCRGLRSVHAYILVYDICCFDSFEYVKTIRQQILETRVIGTSETPIII
VGNKRDLQRGRVIPRWNVSHLVRKTWKCGYVECSAKYNWHILLLFSELLKSVGCARCK
HVHAALRFQGALRRNRCAIM

>Q9BUM1 multipass membrane protein
MESTLGAGIVIAEALQNQLAWLENVWLWITFLGDPKILFLFYFPAAYYASRRVGIAVLWISL
ITEWLNLIFKWFLFGDRPFWWVHESGYYSQAPAQVHQFPSSCETGPGSPSGHCMITGAAL
WPIMTALSSQVATRARSRWVRVMPSLAYCTFLLAVGLSRIFILAHFPHQVLAGLITGAVLG
WLMTPRVPMERELSFYGLTALALMLGTSLIYWTLFTLGLDLSWSISLAFKWCERPEWIHV
DSRPFASLSRDSGAALGLGIALHSPCYAQVRRAQLGNGQKIACLVLAMGLLGPLDWLGHP
PQISLFYIFNFLKYTLWPCLVLALVPWAVHMFSAQEAPPIHSS

>Q00610 peripheral membrane protein
MAQILPIRFQEHLQLQNLGINPANIGFSTLTMESDKFICIREKVGEQAQVVIIDMNDPSNPIR
RPISADSAIMNPASKVIALKAGKTLQIFNIEMKSKMKAHTMTDDVTFWKWISLNTVALVT
DNAVYHWSMEGESQPVKMFDRHSSLAGCQIINYRTDAKQKWLLLTGISAQQNRVVGAM
QLYSVDRKVSQPIEGHAASFAQFKMEGNAEESTLFCFAVRGQAGGKLHIIEVGTPPTGNQP
FPKKAVDVFFPPEAQNDFPVAMQISEKHDVVFLITKYGYIHLYDLETGTCIYMNRISGETIF
VTAPHEATAGIIGVNRKGQVLSVCVEEENIIPYITNVLQNPDLALRMAVRNNLAGAEELFA
RKFNALFAQGNYSEAAKVAANAPKGILRTPDTIRRFQSVPAQPGQTSPLLQYFGILLDQGQ
LNKYESLELCRPVLQQGRKQLLEKWLKEDKLECSEELGDLVKSVDPTLALSVYLRANVP
NKVIQCFAETGQVQKIVLYAKKVGYTPDWIFLLRNVMRISPDQGQQFAQMLVQDEEPLAD
ITQIVDVFMEYNLIQQCTAFLLDALKNNRPSEGPLQTRLLEMNLMHAPQVADAILGNQMF
THYDRAHIAQLCEKAGLLQRALEHFTDLYDIKRAVVHTHLLNPEWLVNYFGSLSVEDSLE
CLRAMLSANIRQNLQICVQVASKYHEQLSTQSLIELFESFKSFEGLFYFLGSIVNFSQDPDV
HFKYIQAACKTGQIKEVERICRESNCYDPERVKNFLKEAKLTDQLPLIIVCDRFDFVHDLVL
YLYRNNLQKYIEIYVQKVNPSRLPVVIGGLLDVDCSEDVIKNLILVVRGQFSTDELVAEVE
KRNRLKLLLPWLEARIHEGCEEPATHNALAKIYIDSNNNPERFLRENPYYDSRVVGKYCEK
RDPHLACVAYERGQCDLELINVCNENSLFKSLSRYLVRRKDPELWGSVLLESNPYRRPLID
QVVQTALSETQDPEEVSVTVKAFMTADLPNELIELLEKIVLDNSVFSEHRNLQNLLILTAIK
ADRTRVMEYINRLDNYDAPDIANIAISNELFEEAFAIFRKFDVNTSAVQVLIEHIGNLDRAY
EFAERCNEPAVWSQLAKAQLQKGMVKEAIDSYIKADDPSSYMEVVQAANTSGNWEELV
KYLQMARKKARESYVETELIFALAKTNRLAELEEFINGPNNAHIQQVGDRCYDEKMYDA
AKLLYNNVSNFGRLASTLVHLGEYQAAVDGARKANSTRTWKEVCFACVDGKEFRLAQM
CGLHIVVHADELEELINYYQDRGYFEELITMLEAALGLERAHMGMFTELAILYSKFKPQK
MREHLELFWSRVNIPKVLRAAEQAHLWAELVFLYDKYEEYDNAIITMMNHPTDAWKEGQ
FKDIITKVANVELYYRAIQFYLEFKPLLLNDLLMVLSPRLDHTRAVNYFSKVKQLPLVKPY
LRSVQNHNNKSVNESLNNLFITEEDYQALRTSIDAYDNFDNISLAQRLEKHELIEFRRIAAY
LFKGNNRWKQSVELCKKDSLYKDAMQYASESKDTELAEELLQWFLQEEKRECFGACLFT
CYDLLRPDVVLETAWRHNIMDFAMPYFIQVMKEYLTKVDKLDASESLRKEEEQATETQPI
VYGQPQLMLTAGPSVAVPPQAPFGYGYTAPPYGQPQPGFGYSM

>P19022 single-pass type I membrane protein
MCRIAGALRTLLPLLAALLQASVEASGEIALCKTGFPEDVYSAVLSKDVHEGQPLLNVKFS
NCNGKRKVQYESSEPADFKVDEDGMVYAVRSFPLSSEHAKFLIYAQDKETQEKWQVAVK
LSLKPTLTEESVKESAEVEEIVFPRQFSKHSGHLQRQKRDWVIPPINLPENSRGPFPQELVRI
RSDRDKNLSLRYSVTGPGADQPPTGIFIINPISGQLSVTKPLDREQIARFHLRAHAVDINGN
QVENPIDIVINVIDMNDNRPEFLHQVWNGTVPEGSKPGTYVMTVTAIDADDPNALNGML
RYRIVSQAPSTPSPNMFTINNETGDIITVAAGLDREKVQQYTLIIQATDMEGNPTYGLSNTA
TAVITVTDVNDNPPEFTAMTFYGEVPENRVDIIVANLTVTDKDQPHTPAWNAVYRISGGDP
TGRFAIQTDPNSNDGLVTVVKPIDFETNRMFVLTVAAENQVPLAKGIQHPPQSTATVSVTVI
DVNENPYFAPNPKIIRQEEGLHAGTMLTTFTAQDPDRYMQQNIRYTKLSDPANWLKIDPV
NGQITTIAVLDRESPNVKNNIYNATFLASDNGIPPMSGTGTLQIYLLDINDNAPQVLPQEAE
TCETPDPNSINITALDYDIDPNAGPFAFDLPLSPVTIKRNWTITRLNGDFAQLNLKIKFLEAG
IYEVPIIITDSGNPPKSNISILRVKVCQCDSNGDCTDVDRIVGAGLGTGAIIAILLCIIILLILVL
MFVVWMKRRDKERQAKQLLIDPEDDVRDNILKYDEEGGGEEDQDYDLSQLQQPDTVEP
DAIKPVGIRRMDERPIHAEPQYPVRSAAPHPGDIGDFINEGLKAADNDPTAPPYDSLLVFD
YEGSGSTAGSLSSLNSSSSGGEQDYDYLNDWGPRFKKLADMYGGGDD

>Q9Y4W6 multipass membrane protein
MAHRCLRLWGRGGCWPRGLQQLLVPGGVGPGEQPCLRTLYRFVTTQARASRNSLLTDIIA
AYQRFCSRPPKGFEKYFPNGKNGKKASEPKEVMGEKKESKPAATTRSSGGGGGGGGKRG
GKKDDSHWWSRFQKGDIPWDDKDFRMFFLWTALFWGGVMFYLLLKRSGREITWKDFV
NNYLSKGVVDRLEVVNKRFVRVTFTPGKTPVDGQYVWFNIGSVDTFERNLETLQQELGIE
GENRVPVVYIAESDGSFLLSMLPTVLIIAFLLYTIRRGPAGIGRTGRGMGGLFSVGETTAKV
LKDEIDVKFKDVAGCEEAKLEIMEFVNFLKNPKQYQDLGAKIPKGAILTGPPGTGKTLLA
KATAGEANVPFITVSGSEFLEMFVGVGPARVRDLFALARKNAPCILFIDEIDAVGRKRGRG
NFGGQSEQENTLNQLLVEMDGFNTTTNVVILAGTNRPDILDPALLRPGRFDRQIFIGPPDIK
GRASIFKVHLRPLKLDSTLEKDKLARKLASLTPGFSGADVANVCNEAALIAARHLSDSINQ
KHFEQAIERVIGGLEKKTQVLQPEEKKTVAYHEAGHAVAGWYLEHADPLLKVSIIPRGKGL
GYAQYLPKEQYLYTKEQLLDRMCMTLGGRVSEEIFFGRITTGAQDDLRKVTQSAYAQIVQ
FGMNEKVGQISFDLPRQGDMVLEKPYSEATARLIDDEVRILINDAYKRTVALLTEKKADVE
KVALLLLEKEVLDKNDMVELLGPRPFAEKSTYEEFVEGTGSLDEDTSLPEGLKDWNKERE
KEKEEPPGEKVAN

>Q13433 multipass membrane protein
MARKLSVILILTFALSVTNPLHELKAAAFPQTTEKISPNWESGINVDLAISTRQYHLQQLFY
RYGENNSLSVEGFRKLLQNIGIDKIKRIHIHHDHDHHSDHEHHSDHERHSDHEHHSEHEH
HSDHDHHSHHNHAASGKNKRKALCPDHDSDSSGKDPRNSQGKGAHRPEHASGRRNVKD
SVSASEVTSTVYNTVSEGTHFLETIETPRPGKLFPKDVSSSTPPSVTSKSRVSRLAGRKTNE
SVSEPRKGFMYSRNTNENPQECFNASKLLTSHGMGIQVPLNATEFNYLCPAIINQIDARSCL
IHTSEKKAEIPPKTYSLQIAWVGGFIAISIISFLSLLGVILVPLMNRVFFKFLLSFLVALAVGTL
SGDAFLHLLPHSHASHHHSHSHEEPAMEMKRGPLFSHLSSQNIEESAYFDSTWKGLTALG
GLYFMFLVEHVLTLIKQFKDKKKKNQKKPENDDDVEIKKQLSKYESQLSTNEEKVDTDDR
TEGYLRADSQEPSHFDSQQPAVLEEEEVMIAHAHPQEVYNEYVPRGCKNKCHSHFHDTL

GQSDDLIHHHHDYHHILHHHHHQNHHPHSHSQRYSREELKDAGVATLAWMVIMGDGLH
NFSDGLAIGAAFTEGLSSGLSTSVAVFCHELPHELGDFAVLLKAGMTVKQAVLYNALSAM
LAYLGMATGIFIGHYAENVSMWIFALTAGLFMYVALVDMVPEMLHNDASDHGCSRWGYF
FLQNAGMLLGFGIMLLISIFEHKIVFRINF

>Q10472 single-pass type II membrane protein
MRKFAYCKVVLATSLIWVLLDMFLLLYFSECNKCDEKKERGLPAGDVLEPVQKPHEGPGE
MGKPVVIPKEDQEKMKEMFKINQFNLMASEMIALNRSLPDVRLEGCKTKVYPDNLPTTS
VVIVFHNEAWSTLLRTVHSVINRSPRHMIEEIVLVDDASERDFLKRPLESYVKKLKVPVHV
IRMEQRSGLIRARLKGAAVSKGQVITFLDAHCECTVGWLEPLLARIKHDRRTVVCPIIDVIS
DDTFEYMAGSDMTYGGFNWKLNFRWYPVPQREMDRRKGDRTLPVRTPTMAGGLFSIDR
DYFQEIGTYDAGMDIWGGENLEISFRIWQCGGTLEIVTCSHVGHVFRKATPYTFPGGTGQI
INKNNRRLAEVWMDEFKNFFYIISPGVTKVDYGDISSRVGLRHKLQCKPFSWYLENIYPDS
QIPRHYFSLGEIRNVETNQCLDNMARKENEKVGIFNCHGMGGNQVFSYTANKEIRTDDLC
LDVSKLNGPVTMLKCHHLKGNQLWEYDPVKLTLQHVNSNQCLDKATEEDSQVPSIRDCN
GSRSQQWLLRNVTLPEIF

>Q16820 single-pass type I membrane protein
MDLWNLSWFLFLDALLVISGLATPENFDVDGGMDQDIFDINEGLGLDLFEGDIRLDRAQIR
NSIIGEKYRWPHTIPYVLEDSLEMNAKGVILNAFERYRLKTCIDFKPWAGETNYISVFKGS
GCWSSVGNRRVGKQELSIGANCDRIATVQHEFLHALGFWHEQSRSDRDDYVRIMWDRIL
SGREHNFNTYSDDISDSLNVPYDYTSVMHYSKTAFQNGTEPTIVTRISDFEDVIGQRMDFS
DSDLLKLNQLYNCSSSLSFMDSCSFELENVCGMIQSSGDNADWQRVSQVPRGPESDHSNM
GQCQGSGFFMHFDSSSVNVGATAVLESRTLYPKRGFQCLQFYLYNSGSESDQLNIYIREYS
ADNVDGNLTLVEEIKEIPTGSWQLYHVTLKVTKKFRVVFEGRKGSGASLGGLSIDDINLSE
TRCPHHIWHIRNFTQFIGSPNGTLYSPPFYSSKGYAFQIYLNLAHVTNAGIYFHLISGANDD
QLQWPCPWQQATMTLLDQNPDIRQRMSNQRSITTDPFMTTDNGNYFWDRPSKVGTVALF
SNGTQFRRGGGYGTSAFITHERLKSRDFIKGDDVYILLTVEDISHLNSTQIQLTPAPSVQDL
CSKTTCKNDGVCTVRDGKAECRCQSGEDWWYMGERCEKRGSTRDTIVIAVSSTVAVFAL
MLIITLVSVYCTRKKYRERMSSNRPNLTPQNQHAF

>O15118 multipass membrane protein
MTARGLALGLLLLLLCPAQVFSQSCVWYGECGIAYGDKRYNCEYSGPPKPLPKDGYDLV
QELCPGFFFGNVSLCCDVRQLQTLKDNLQLPLQFLSRCPSCFYNLLNLFCELTCSPRQSQF
LNVTATEDYVDPVTNQTKTNVKELQYYVGQSFANAMYNACRDVEAPSSNDKALGLLCG
KDADACNATNWIEYMFNKDNGQAPFTITPVFSDFPVHGMEPMNNATKGCDESVDEVTAP
CSCQDCSIVCGPKPQPPPPPAPWTILGLDAMYVIMWITYMAFLLVFFGAFFAVWCYRKRYF
VSEYTPIDSNIAFSVNASDKGEASCCDPVSAAFEGCLRRLFTRWGSFCVRNPGCVIFFSLVF
ITACSSGLVFVRVTTNPVDLWSAPSSQARLEKEYFDQHFGPFFRTEQLIIRAPLTDKHIYQPY
PSGADVPFGPPLDIQILHQVLDLQIAIENITASYDNETVTLQDICLAPLSPYNTNCTILSVLN
YFQNSHSVLDHKKGDDFFVYADYHTHFLYCVRAPASLNDTSLLHDPCLGTFGGPVFPWL
VLGGYDDQNYNNATALVITFPVNNYYNDTEKLQRAQAWEKEFINFVKNYKNPNLTISFTA
ERSIEDELNRESDSDVFTVVISYAIMFLYISLALGHMKSCRRLLVDSKVSLGIAGILIVLSSV
ACSLGVFSYIGLPLTLIVIEVIPFLVLAVGVDNIFILVQAYQRDERLQGETLDQQLGRVLGEV

APSMFLSSFSETVAFFLGALSVMPAVHTFSLFAGLAVFIDFLLQITCFVSLLGLDIKRQEKNR
LDIFCCVRGAEDGTSVQASESCLFRFFKNSYSPLLLKDWMRPIVIAIFVGVLSFSIAVLNKV
DIGLDQSLSMPDDSYMVDYFKSISQYLHAGPPVYFVLEEGHDYTSSKGQNMVCGGMGC
NNDSLVQQIFNAAQLDNYTRIGFAPSSWIDDYFDWVKPQSSCCRVDNITDQFCNASVVDP
ACVRCRPLTPEGKQRPQGGDFMRFLPMFLSDNPNPKCGKGGHAAYSSAVNILLGHGTRV
GATYFMTYHTVLQTSADFIDALKKARLIASNVTETMGINGSAYRVFPYSVFYVFYEQYLTI
IDDTIFNLGVSLGAIFLVTMVLLGCELWSAVIMCATIAMVLVNMFGVMWLWGISLNAVSL
VNLVMSCGISVEFCSHITRAFTVSMKGSRVERAEEALAHMGSSVFSGITLTKFGGIVVLAF
AKSQIFQIFYFRMYLAMVLLGATHGLIFLPVLLSYIGPSVNKAKSCATEERYKGTERERLL
NF

>P07306 single-pass type II membrane protein
MTKEYQDLQHLDNEESDHHQLRKGPPPPQPLLQRLCSGPRLLLLSLGLSLLLLVVVCVIGS
QNSQLQEELRGLRETFSNFTASTEAQVKGLSTQGGNVGRKMKSLESQLEKQQKDLSEDH
SSLLLHVKQFVSDLRSLSCQMAALQGNGSERTCCPVNWVEHERSCYWFSRSGKAWADA
DNYCRLEDAHLVVVTSWEEQKFVQHHIGPVNTWMGLHDQNGPWKWVDGTDYETGFK
NWRPEQPDDWYGHGLGGGEDCAHFTDDGRWNDDVCQRPYRWVCETELDKASQEPPLL

>P04626 single-pass type I membrane protein
MELAALCRWGLLLALLPPGAASTQVCTGTDMKLRLPASPETHLDMLRHLYQGCQVVQG
NLELTYLPTNASLSFLQDIQEVQGYVLIAHNQVRQVPLQRLRIVRGTQLFEDNYALAVLDN
GDPLNNTTPVTGASPGGLRELQLRSLTEILKGGVLIQRNPQLCYQDTILWKDIFHKNNQLA
LTLIDTNRSRACHPCSPMCKGSRCWGESSEDCQSLTRTVCAGGCARCKGPLPTDCCHEQC
AAGCTGPKHSDCLACLHFNHSGICELHCPALVTYNTDTFESMPNPEGRYTFGASCVTACP
YNYLSTDVGSCTLVCPLHNQEVTAEDGTQRCEKCSKPCARVCYGLGMEHLREVRAVTSA
NIQEFAGCKKIFGSLAFLPESFDGDPASNTAPLQPEQLQVFETLEEITGYLYISAWPDSLPDL
SVFQNLQVIRGRILHNGAYSLTLQGLGISWLGLRSLRELGSGLALIHHNTHLCFVHTVPWD
QLFRNPHQALLHTANRPEDECVGEGLACHQLCARGHCWGPGPTQCVNCSQFLRGQECVE
ECRVLQGLPREYVNARHCLPCHPECQPQNGSVTCFGPEADQCVACAHYKDPPFCVARCPS
GVKPDLSYMPIWKFPDEEGACQPCPINCTHSCVDLDDKGCPAEQRASPLTSIISAVVGILLV
VVLGVVFGILIKRRQQKIRKYTMRRLLQETELVEPLTPSGAMPNQAQMRILKETELRKVK
VLGSGAFGTVYKGIWIPDGENVKIPVAIKVLRENTSPKANKEILDEAYVMAGVGSPYVSR
LLGICLTSTVQLVTQLMPYGCLLDHVRENRGRLGSQDLLNWCMQIAKGMSYLEDVRLVH
RDLAARNVLVKSPNHVKITDFGLARLLDIDETEYHADGGKVPIKWMALESILRRRFTHQS
DVWSYGVTVWELMTFGAKPYDGIPAREIPDLLEKGERLPQPPICTIDVYMIMVKCWMIDS
ECRPRFRELVSEFSRMARDPQRFVVIQNEDLGPASPLDSTFYRSLLEDDDMGDLVDAEEYL
VPQQGFFCPDPAPGAGGMVHHRHRSSSTRSGGGDLTLGLEPSEEEAPRSPLAPSEGAGSDV
FDGDLGMGAAKGLQSLPTHDPSPLQRYSEDPTVPLPSETDGYVAPLTCSPQPEYVNQPDV
RPQPPSPREGPLPAARPAGATLERPKTLSPGKNGVVKDVFAFGGAVENPEYLTPQGGAAPQ
PHPPPAFSPAFDNLYYWDQDPPERGAPPSTFKGTPTAENPEYLGLDVPV

>P17181 single-pass type I membrane protein
MMVVLLGATTLVLVAVAPWVLSAAAGGKNLKSPQKVEVDIIDDNFILRWNRSDESVGNV
TFSFDYQKTGMDNWIKLSGCQNITSTKCNFSSLKLNVYEEIKLRIRAEKENTSSWYEVDSF

TPFRKAQIGPPEVHLEAEDKAIVIHISPGTKDSVMWALDGLSFTYSLVIWKNSSGVEERIEN
IYSRHKIYKLSPETTYCLKVKAALLTSWKIGVYSPVHCIKTTVENELPPPENIEVSVQNQNY
VLKWDYTYANMTFQVQWLHAFLKRNPGNHLYKWKQIPDCENVKTTQCVFPQNVFQKGI
YLLRVQASDGNNTSFWSEEIKFDTEIQAFLLPPVFNIRSLSDSFHIYIGAPKQSGNTPVIQDY
PLIYEIIFWENTSNAERKIIEKKTDVTVPNLKPLTVYCVKARAHTMDEKLNKSSVFSDAVC
EKTKPGNTSKIWLIVGICIALFALPFVIYAAKVFLRCINYVFFPSLKPSSSIDEYFSEQPLKNL
LLSTSEEQIEKCFIIENISTIATVEETNQTDEDHKKYSSQTSQDSGNYSNEDESESKTSEELQ
QDFV

>P54852 multipass membrane protein
MSLLLLVVSALHILILILLFVATLDKSWWTLPGKESLNLWYDCTWNNDTKTWACSNVSEN
GWLKAVQVLMVLSLILCCLSFILFMFQLYTMRRGGLFYATGLCQLCTSVAVFTGALIYAIH
AEEILEKHPRGGSFGYCFALAWVAFPLALVSGIIYIHLRKRE

>P50895 single-pass type I membrane protein
MEPPDAPAQARGAPRLLLLAVLLAAHPDAQAEVRLSVPPLVEVMRGKSVILDCTPTGTHD
HYMLEWFLTDRSGARPRLASAEMQGSELQVTMHDTRGRSPPYQLDSQGRLVLAEAQVG
DERDYVCVVRAGAAGTAEATARLNVFAKPEATEVSPNKGTLSVMEDSAQEIATCNSRNG
NPAPKITWYRNGQRLEVPVEMNPEGYMTSRTVREASGLLSLTSTLYLRLRKDDRDASFHC
AAHYSLPEGRHGRLDSPTFHLTLHYPTEHVQFWVGSPSTPAGWVREGDTVQLLCRGDGS
PSPEYTLFRLQDEQEEVLNVNLEGNLTLEGVTRGQSGTYGCRVEDYDAADDVQLSKTLEL
RVAYLDPLELSEGKVLSLPLNSSAVVNCSVHGLPTPALRWTKDSTPLGDGPMLSLSSITFDS
NGTYVCEASLPTVPVLSRTQNFTLLVQGSPELKTAEIEPKADGSWREGDEVTLICSARGHP
DPKLSWSQLGGSPAEPIPGRQGWVSSSLTLKVTSALSRDGISCEASNPHGNKRHVFHFGTV
SPQTSQAGVAVMAVAVSVGLLLLVVAVFYCVRRKGGPCCRQRREKGAPPPGEPGLSHSGS
EQPEQTGLLMGGASGGARGGSGGFGDEC

>Q01959 multipass membrane protein
MSKSKCSVGLMSSVVAPAKEPNAVGPKEVELILVKEQNGVQLTSSTLTNPRQSPVEAQDRE
TWGKKIDFLLSVIGFAVDLANVWRFPYLCYKNGGGAFLVPYLLFMVIAGMPLFYMELAL
GQFNREGAAGVWKICPILKGVGFTVILISLYVGFFYNVIIAWALHYLFSSFTTELPWIHCNN
SWNSPNCSDAHPGDSSGDSSGLNDTFGTTPAAEYFERGVLHLHQSHGIDDLGPPRWQLTA
CLVLVIVLLYFSLWKGVKTSGKVVWITATMPYVVLTALLLRGVTLPGAIDGIRAYLSVDFY
RLCEASVWIDAATQVCFSLGVGFGVLIAFSSYNKFTNNCYRDAIVTTSINSLTSFSSGFVVF
SFLGYMAQKHSVPIGDVAKDGPGLIFIIYPEAIATLPLSSAWAVVFFIMLLTLGIDSAMGGM
ESVITGLIDEFQLLHRHRELFTLFIVLATFLLSLFCVTNGGIYVFTLLDHFAAGTSILFGVLIE
AIGVAWFYGVGQFSDDIQQMTGQRPSLYWRLCWKLVSPCFLLFVVVVSIVTFRPPHYGAY
IFPDWANALGWVIATSSMAMVPIYAAYKFCSLPGSFREKLAYAIAPEKDRELVDRGEVRQF
TLRHWLKV

>Q8NHJ6 single-pass type I membrane protein
MIPTFTALLCLGLSLGPRTHMQAGPLPKPTLWAEPGSVISWGNSVTIWCQGTLEAREYRLD
KEESPAPWDRQNPLEPKNKARFSIPSMTEDYAGRYRCYYRSPVGWSQPSDPLELVMTGAY
SKPTLSALPSPLVTSGKSVTLLCQSRSPMDTFLLIKERAAHPLLHLRSEHGAQQHQAEFPM

SPVTSVHGGTYRCFSSHGFSHYLLSHPSDPLELIVSGSLEDPRPSPTRSVSTAAGPEDQPLM
PTGSVPHSGLRRHWEVLIGVLVVSILLLSLLLFLLLQHWRQGKHRTLAQRQADFQRPPGA
AEPEPKDGGLQRRSSPAADVQGENFCAAVKNTQPEDGVEMDTRQSPHDEDPQAVTYAKV
KHSRPRREMASPPSPLSGEFLDTKDRQAEEDRQMDTEAAASEAPQDVTYAQLHSFTLRQK
ATEPPPSQEGASPAEPSVYATLAIH

>P27815 peripheral membrane protein
MEPPTVPSERSLSLSLPGPREGQATLKPPPQHLWRQPRTPIRIQQRGYSDSAERAERERQPH
RPIERADAMDTSDRPGLRTTRMSWPSSFHGTGTGSGGAGGGSSRRFEAENGPTPSPGRSPL
DSQASPGLVLHAGAATSQRRESFLYRSDSDYDMSPKTMSRNSSVTSEAHAEDLIVTPFAQV
LASLRSVRSNFSLLTNVPVPSNKRSPLGGPTPVCKATLSEETCQQLARETLEELDWCLEQL
ETMQTYRSVSEMASHKFKRMLNRELTHLSEMSRSGNQVSEYISTTFLDKQNEVEIPSPTM
KEREKQQAPRPRPSQPPPPPVPHLQPMSQITGLKKLMHSNSLNNSNIPRFGVKTDQEELLA
QELENLNKWGLNIFCVSDYAGGRSLTCIMYMIFQERDLLKKFRIPVDTMVTYMLTLEDHY
HADVAYHNSLHAADVLQSTHVLLATPALDAVFTDLEILAALFAAAIHDVDHPGVSNQFLIN
TNSELALMYNDESVLENHHLAVGFKLLQEDNCDIFQNLSKRQRQSLRKMVIDMVLATDM
SKHMTLLADLKTMVETKKVTSSGVLLLDNYSDRIQVLRNMVHCADLSNPTKPLELYRQ
WTDRIMAEFFQQGDRERERGMEISPMCDKHTASVEKSQVGFIDYIVHPLWETWADLVHP
DAQEILDTLEDNRDWYYSAIRQSPSPPPEEESRGPGHPPLPDKFQFELTLEEEEEEEISMAQI
PCTAQEALTAQGLSGVEEALDATIAWEASPAQESLEVMAQEASLEAELEAVYLTQQAQST
GSAPVAPDEFSSREEFVVAVSHSSPSALALQSPLLPAWRTLSVSEHAPGLPGLPSTAAEVEA
QREHQAAKRACSACAGTFGEDTSALPAPGGGGSGGDPT

>O14894 multipass membrane protein
MCTGKCARCVGLSLITLCLVCIVANALLLVPNGETSWTNTNHLSLQVWLMGGFIGGGLM
VLCPGIAAVRAGGKGCCGAGCCGNRCRMLRSVFSSAFGVLGAIYCLSVSGAGLRNGPRC
LMNGEWGYHFEDTAGAYLLNRTLWDRCEAPPRVVPWNVTLFSLLVAASCLEIVLCGIQLV
NATIGVFCGDCRKKQDTPH

>Q96FL8 multipass membrane protein
MEAPEEPAPVRGGPEATLEVRGSRCLRLSAFREELRALLVLAGPAFLVQLMVFLISFISSVF
CGHLGKLELDAVTLAIAVINVTGVSVGFGLSSACDTLISQTYGSQNLKHVGVILQRSALVL
LLCCFPCWALFLNTQHILLLFRQDPDVSRLTQTYVTIFIPALPATFLYMLQVKYLLNQGIVLP
QIVTGVAANLVNALANYLFLHQLHLGVIGSALANLISQYTLALLLFLYILGKKLHQATWG
GWSLECLQDWASFLRLAIPSMLMLCMEWWAYEVGSFLSGILGMVELGAQSIVYELAIIVY
MVPAGFSVAASVRVGNALGAGDMEQARKSSTVSLLITVLFAVAFSVLLLSCKDHVGYIFTT
DRDIINLVAQVVPIYAVSHLFEALACTSGGVLRGSGNQKVGAIVNTIGYYVVGLPIGIALMF
ATTLGVMGLWSGIIICTVFQAVCFLGFIIQLNWKKACQQAQVHANLKVNNVPRSGNSALP
QDPLHPGCPENLEGILTNDVGKTGEPQSDQQMRQEEPLPEHPQDGAKLSRKQLVLRRGLL
LLGVFLILLVGILVRFYVRIQ

>O75388 multipass membrane protein
MNGVSEGTRGCSDRQPGVLTRDRSCSRKMNSSGCLSEEVGSLRPLTVVILSASIVVGVLG
NGLVLWMTVFRMARTVSTVCFFHLALADFMLSLSLPIAMYYIVSRQWLLGEWACKLYITF

VFLSYFASNCLLVFISVDRCISVLYPVWALNHRTVQRASWLAFGVWLLAAALCSAHLKFR
TTRKWNGCTHCYLAFNSDNETAQIWIEGVVEGHIIGTIGHFLLGFLGPLAIIGTCAHLIRAK
LLREGWVHANRPKRLLLVLVSAFFIFWSPFNVVLLVHLWRRVMLKEIYHPRMLLILQASF
ALGCVNSSLNPFLYVFVGRDFQEKFFQSLTSALARAFGEEEFLSSCPRGNAPRE

>Q8N6P7 single-pass type I membrane protein
MRTLLTILTVGSLAAHAPEDPSDLLQHVKFQSSNFENILTWDSGPEGTPDTVYSIEYKTYGE
RDWVAKKGCQRITRKSCNLTVETGNLTELYYARVTAVSAGGRSATKMTDRFSSLQHTTLK
PPDVTCISKVRSIQMIVHPTPTPIRAGDGHRLTLEDIFHDLFYHLELQVNRTYQMHLGGKQ
REYEFFGLTPDTEFLGTIMICVPTWAKESAPYMCRVKTLPDRTWTYSFSGAFLFSMGFLVA
VLCYLSYRYVTKPPAPPNSLNVQRVLTFQPLRFIQEHVLIPVFDLSGPSSLAQPVQYSQIRVS
GPREPAGAPQRHSLSEITYLGQPDISILQPSNVPPPQILSPLSYAPNAAPEVGPPSYAPQVTPE
AQFPFYAPQAISKVQPSSYAPQATPDSWPPSYGVCMEGSGKDSPTGTLSSPKHLRPKGQLQ
KEPPAGSCMLGGLSLQEVTSLAMEESQEAKSLHQPLGICTDRTSDPNVLHSGEEGTPQYL
KGQLPLLSSVQIEGHPMSLPLQPPSRPCSPSDQGPSPWGLLESLVCPKDEAKSPAPETSDLE
QPTELDSLFRGLALTVQWES

>Q5T0N5 peripheral membrane protein
MSWGTELWDQFDSLDKHTQWGIDFLERYAKFVKERIEIEQNYAKQLRNLVKKYCPKRSS
KDEEPRFTSCVAFFNILNELNDYAGQREVVAEEMAHRVYGELMRYAHDLKTERKMHLQE
GRKAQQYLDMCWKQMDNSKKKFERECREAEKAQQSYERLDNDTNATKADVEKAKQQ
LNLRTHMADENKNEYAAQLQNFNGEQHKHFYVVIPQIYKQLQEMDERRTIKLSECYRGF
ADSERKVIPIISKCLEGMILAAKSVDERRDSQMVVDSFKSGFEPPGDFPFEDYSQHIYRTIS
DGTISASKQESGKMDAKTTVGKAKGKLWLFGKKPKPQSPPLTPTSLFTSSTPNGSQFLTFSI
EPVHYCMNEIKTGKPRIPSFRSLKRGVSLIMGPALEDFSHLPPEQRRKKLQQRIDELNRELQ
KESDQKDALNKMKDVYEKNPQMGDPGSLQPKLAETMNNIDRLRMEIHKNEAWLSEVEG
KTGGRGDRRHSSDINHLVTQGRESPEGSYTDDANQEVRGPPQQHGHHNEFDDEFEDDDP
LPAIGHCKAIYPFDGHNEGTLAMKEGEVLYIIEEDKGDGWTRARRQNGEEGYVPTSYIDV
TLEKNSKGS

>P19397 multipass membrane protein
MGMSSLKLLKYVLFFFNLLFWICGCCILGFGIYLLIHNNFGVLFHNLPSLTLGNVFVIVGSII
MVVAFLGCMGSIKENKCLLMSFFILLLIILLAEVTLAILLFVYEQKLNEYVAKGLTDSIHRY
HSDNSTKAAWDSIQSFLQCCGINGTSDWTSGPPASCPSDRKVEGCYAKARLWFHSNFLYIG
IITICVCVIEVLGMSFALTLNCQIDKTSQTIGL

>Q9HCU4 multipass membrane protein
MRSPATGVPLPTPPPPLLLLLLLLLPPPLLGDQVGPCRSLGSRGRGSSGACAPMGWLCPSS
ASNLWLYTSRCRDAGTELTGHLVPHHDGLRVWCPESEAHIPLPPAPEGCPWSCRLLGIGGH
LSPQGKLTLPEEHPCLKAPRLRCQSCKLAQAPGLRAGERSPEESLGGRRKRNVNTAPQFQP
PSYQATVPENQPAGTPVASLRAIDPDEGEAGRLEYTMDALFDSRSNQFFSLDPVTGAVTTA
EELDRETKSTHVFRVTAQDHGMPRRSALATLTILVTDTNDHDPVFEQQEYKESLRENLEV
GYEVLTVRATDGDAPPNANILYRLLEGSGGSPSEVFEIDPRSGVIRTRGPVDREEVESYQLT
VEASDQGRDPGPRSTTAAVFLSVEDDNDNAPQFSEKRYVVQVREDVTPGAPVLRVTASDR

DKGSNAVVHYSIMSGNARGQFYLDAQTGALDVVSPLDYETTKEYTLRVRAQDGGRPPLS
NVSGLVTVQVLDINDNAPIFVSTPFQATVLESVPLGYLVLHVQAIDADAGDNARLEYRLA
GVGHDFPFTINNGTGWISVAAELDREEVDFYSFGVEARDHGTPALTASASVSVTVLDVND
NNPTFTQPEYTVRLNEDAAVGTSVVTVSAVDRDAHSVITYQITSGNTRNRFSITSQSGGGL
VSLALPLDYKLERQYVLAVTASDGTRQDTAQIVVNVTDANTHRPVFQSSHYTVNVNEDR
PAGTTVVLISATDEDTGENARITYFMEDSIPQFRIDADTGAVTTQAELDYEDQVSYTLAITA
RDNGIPQKSDTTYLEILVNDVNDNAPQFLRDSYQGSVYEDVPPFTSVLQISATDRDSGLNG
RVFYTFQGGDDGDGDFIVESTSGIVRTLRRLDRENVAQYVLRAYAVDKGMPPARTPMEVT
VTVLDVNDNPPVFEQDEFDVFVEENSPIGLAVARVTATDPDEGTNAQIMYQIVEGNIPEVF
QLDIFSGELTALVDLDYEDRPEYVLVIQATSAPLVSRATVHVRLLDRNDNPPVLGNFEILFN
NYVTNRSSSFPGGAIGRVPAHDPDISDSLTYSFERGNELSLVLLNASTGELKLSRALDNNRP
LEAIMSVLVSDGVHSVTAQCALRVTIITDEMLTHSITLRLEDMSPERFLSPLLGLFIQAVAAT
LATPPDHVVVFNVQRDTDAPGGHILNVSLSVGQPPGPGGGPPFLPSEDLQERLYLNRSLLT
AISAQRVLPFDDNICLREPCENYMRCVSVLRFDSSAPFIASSSVLFRPIHPVGGLRCRCPPGF
TGDYCETEVDLCYSRPCGPHGRCRSREGGYTCLCRDGYTGEHCEVSARSGRCTPGVCKN
GGTCVNLLVGGFKCDCPSGDFEKPYCQVTTRSFPAHSFITFRGLRQRFHFTLALSFATKER
DGLLLYNGRFNEKHDFVALEVIQEQVQLTFSAGESTTTVSPFVPGGVSDGQWHTVQLKYY
NKPLLGQTGLPQGPSEQKVAVVTVDGCDTGVALRFGSVLGNYSCAAQGTQGGSKKSLDL
TGPLLLGGVPDLPESFPVRMRQFVGCMRNLQVDSRHIDMADFIANNGTVPGCPAKKNVC
DSNTCHNGGTCVNQWDAFSCECPLGFGGKSCAQEMANPQHFLGSSLVAWHGLSLPISQP
WYLSLMFRTRQADGVLLQAITRGRSTITLQLREGHVMLSVEGTGLQASSLRLEPGRANDG
DWHHAQLALGASGGPGHAILSFDYGQQRAEGNLGPRLHGLHLSNITVGGIPGPAGGVAR
GFRGCLQGVRVSDTPEGVNSLDPSHGESINVEQGCSLPDPCDSNPCPANSYCSNDWDSYS
CSCDPGYYGDNCTNVCDLNPCEHQSVCTRKPSAPHGYTCECPPNYLGPYCETRIDQPCPR
GWWGHPTCGPCNCDVSKGFDPDCNKTSGECHCKENHYRPPGSPTCLLCDCYPTGSLSRV
CDPEDGQCPCKPGVIGRQCDRCDNPFAEVTTNGCEVNYDSCPRAIEAGIWWPRTRFGLPA
AAPCPKGSFGTAVRHCDEHRGWLPPNLFNCTSITFSELKGFAERLQRNESGLDSGRSQQLA
LLLRNATQHTAGYFGSDVKVAYQLATRLLAHESTQRGFGLSATQDVHFTENLLRVGSALL
DTANKRHWELIQQTEGGTAWLLQHYEAYASALAQNMRHTYLSPFTIVTPNIVISVVRLDK
GNFAGAKLPRYEALRGEQPPDLETTVILPESVFRETPPVVRPAGPGEAQEPEELARRQRRH
PELSQGEAVASVIIYRTLAGLLPHNYDPDKRSLRVPKRPIINTPVVSISVHDDEELLPRALDK
PVTVQFRLLETEERTKPICVFWNHSILVSGTGGWSARGCEVVFRNESHVSCQCNHMTSFA
VLMDVSRRENGEILPLKTLTYVALGVTLAALLLTFFFLTLLRILRSNQHGIRRNLTAALGLA
QLVFLLGINQADLPFACTVIAILLHFLYLCTFSWALLEALHLYRALTEVRDVNTGPMRFYY
MLGWGVPAFITGLAVGLDPEGYGNPDFCWLSIYDTLIWSFAGPVAFAVSMSVFLYILAARA
SCAAQRQGFEKKGPVSGLQPSFAVLLLLSATWLLALLSVNSDTLLFHYLFATCNCIQGPFIF
LSYVVLSKEVRKALKLACSRKPSPDPALTTKSTLTSSYNCPSPYADGRLYQPYGDSAGSLH
STSRSGKSQPSYIPFLLREESALNPGQGPPGLGDPGSLFLEGQDQQHDPDTDSDSDLSLED
DQSGSYASTHSSDSEEEEEEEEEEAAFPGEQGWDSLLGPGAERLPLHSTPKDGGPGPGKAP
WPGDFGTTAKESSGNGAPEERLRENGDALSREGSLGPLPGSSAQPHKGILKKKCLPTISEK
SSLLRLPLEQCTGSSRGSSASEGSRGGPPPRPPPRQSLQEQLNGVMPIAMSIKAGTVDEDSS
GSEFLFFNFLH

>P36382 multipass membrane protein

MGDWSFLGNFLEEVHKHSTVVGKVWLTVLFIFRMLVLGTAAESSWGDEQADFRCDTIQP
GCQNVCYDQAFPISHIRYWVLQIIFVSTPSLVYMGHAMHTVRMQEKRKLREAERAKEVR
GSGSYEYPVAEKAELSCWEEGNGRIALQGTLLNTYVCSILIRTTMEVGFIVGQYFIYGIFLT
TLHVCRRSPCPHPVNCYVSRPTEKNVFIVFMLAVAALSLLLSLAELYHLGWKKIRQRFVKP
RQHMAKCQLSGPSVGIVQSCTPPPDFNQCLENGPGGKFFNPFSNNMASQQNTDNLVTEQ
VRGQEQTPGEGFIQVRYGQKPEVPNGVSPGHRLPHGYHSDKRRLSKASSKARSDDLSV

>Q8N6U8 multipass membrane protein
MSLNSSLSCRKELSNLTEEEGGEGGVIITQFIAIIVITIFVCLGNLVIVVTLYKKSYLLTLSNK
FVFSLTLSNFLLSVLVLPFVVTSSIRREWIFGVVWCNFSALLYLLISSASMLTLGVIAIDRYY
AVLYPMVYPMKITGNRAVMALVYIWLHSLIGCLPPLFGWSSVEFDEFKWMCVAAWHREP
GYTAFWQIWCALFPFLVMLVCYGFIFRVARVKARKVHCGTVVIVEEDAQRTGRKNSSTST
SSSGSRRNAFQGVVYSANQCKALITILVVLGAFMVTWGPYMVVIASEALWGKSSVSPSLE
TWATWLSFASAVCHPLIYGLWNKTVRKELLGMCFGDRYYREPFVQRQRTSRLFSISNRITD
LGLSPHLTALMAGGQPLGHSSSTGDTGFSCSQDSGTDMMLLEDYTSDDNPPSHCTCPPKR
RSSVTFEDEVEQIKEAAKNSILHVKAEVHKSLDSYAASLAKAIEAEAKINLFGEEALPGVL
VTARTVPGGGFGGRRGSRTLVSQRLQLQSIEEGDVLAAEQR

>O95563 multipass membrane protein
MSAAGARGLRATYHRLLDKVELMLPEKLRPLYNHPAGPRTVFFWAPIMKWGLVCAGLAD
MARPAEKLSTAQSAVLMATGFIWSRYSLVIIPKNWSLFAVNFFVGAAGASQLFRIWRYNQE
LKAKAHK

>P12318 single-pass type I membrane protein
MTMETQMSQNVCPRNLWLLQPLTVLLLLASADSQAAAPPKAVLKLEPPWINVLQEDSVT
LTCQGARSPESDSIQWFHNGNLIPTHTQPSYRFKANNNDSGEYTCQTGQTSLSDPVHLTVL
SEWLVLQTPHLEFQEGETIMLRCHSWKDKPLVKVTFFQNGKSQKFSHLDPTFSIPQANHSH
SGDYHCTGNIGYTLFSSKPVTITVQVPSMGSSSPMGIIVAVVIATAVAAIVAAVVALIYCRKK
RISANSTDPVKAAQFEPPGRQMIAIRKRQLEETNNDYETADGGYMTLNPRAPTDDDKNIY
LTLPPNDHVNSNN

>Q96PJ5 single-pass type I membrane protein
MLLWASLLAFAPVCGQSAAAHKPVISVHPPWTTFFKGERVTLTCNGFQFYATEKTTWYHR
HYWGEKLTLTPGNTLEVRESGLYRCQARGSPRSNPVRLLFSSDSLILQAPYSVFEGDTLVL
RCHRRRKEKLTAVKYTWNGNILSISNKSWDLLIPQASSNNNGNYRCIGYGDENDVFRSNF
KIIKIQELFPHPELKATDSQPTEGNSVNLSCETQLPPERSDTPLHFNFFRDGEVILSDWSTYP
ELQLPTVWRENSGSYWCGAETVRGNIHKHSPSLQIHVQRIPVSGVLLETQPSGGQAVEGE
MLVLVCSVAEGTGDTTFSWHREDMQESLGRKTQRSLRAELELPAIRQSHAGGYYCTADNS
YGPVQSMVLNVTVRETPGNRDGLVAAGATGGLLSALLLAVALLFHCWRRRKSGVGFLGD
ETRLPPAPGPGESSHSICPAQVELQSLYVDVHPKKGDLVYSEIQTTQLGEEEEANTSRTLLE
DKDVSVVYSEVKTQHPDNSAGKISSKDEES

>Q9UBF8 peripheral membrane protein
MGDTVVEPAPLKPTSEPTSGPPGNNGGSLLSVITEGVGELSVIDPEVAQKACQEVLEKVKL

LHGGVAVSSRGTPLELVNGDGVDSEIRCLDDPPAQIREEEDEMGAAVASGTAKGARRRRQ
NNSAKQSWLLRLFESKLFDISMAISYLYNSKEPGVQAYIGNRLFCFRNEDVDFYLPQLLNM
YIHMDEDVGDAIKPYIVHRCRQSINFSLQCALLLGAYSSDMHISTQRHSRGTKLRKLILSD
ELKPAHRKRELPSLSPAPDTGLSPSKRTHQRSKSDATASISLSSNLKRTASNPKVENEDEELS
SSTESIDNSFSSPVRLAPEREFIKSLMAIGKRLATLPTKEQKTQRLISELSLLNHKLPARVWL
PTAGFDHHVVRVPHTQAVVLNSKDKAPYLIYVEVLECENFDTTSVPARIPENRIRSTRSVE
NLPECGITHEQRAGSFSTVPNYDNDDEAWSVDDIGELQVELPEVHTNSCDNISQFSVDSIT
SQESKEPVFIAAGDIRRRLSEQLAHTPTAFKRDPEDPSAVALKEPWQEKVRRIREGSPYGHL
PNWRLLSVIVKCGDDLRQELLAFQVLKQLQSIWEQERVPLWIKPYKILVISADSGMIEPVV
NAVSIHQVKKQSQLSLLDYFLQEHGSYTTEAFLSAQRNFVQSCAGYCLVCYLLQVKDRH
NGNILLDAEGHIIHIDFGFILSSSPRNLGFETSAFKLTTEFVDVMGGLDGDMFNYYKMLML
QGLIAARKHMDKVVQIVEIMQQGSQLPCFHGSSTIRNLKERFHMSMTEEQLQLLVEQMV
DGSMRSITTKLYDGFQYLTNGIM

>Q9H4A5 peripheral membrane protein
MTTLTHRARRTEISKNSEKKMESEEDSNWEKSPDNEDSGDSKDIRLTLMEEVLLLGLKDK
EGYTSFWNDCISSGLRGGILIELAMRGRIYLEPPTMRKKRLLDRKVLLKSDSPTGDVLLDE
TLKHIKATEPTETVQTWIELLTGETWNPFKLQYQLRNVRERIAKNLVEKGILTTEKQNFLLF
DMTTHPVTNTTEKQRLVKKLQDSVLERWVNDPQRMDKRTLALLVLAHSSDVLENVFSSL
TDDKYDVAMNRAKDLVELDPEVEGTKPSATEMIWAVLAAFNKS

>O95259 multipass membrane protein
MTMAGGRRGLVAPQNTFLENIVRRSNDTNFVLGNAQIVDWPIVYSNDGFCKLSGYHRAE
VMQKSSTCSFMYGELTDKDTIEKVRQTFENYEMNSFEILMYKKNRTPVWFFVKIAPIRNE
QDKVVLFLCTFSDITAFKQPIEDDSCKGWGKFARLTRALTSSRGVLQQLAPSVQKGENVH
KHSRLAEVLQLGSDILPQYKQEAPKTPPHIILHYCVFKTTWDWIILILTFYTAILVPYNVSFK
TRQNNVAWLVVDSIVDVIFLVDIVLNFHTTFVGPAGEVISDPKLIRMNYLKTWFVIDLLSCL
PYDVINAFENVDEVSAFMGDPGKIGFADQIPPPLEGRESQGISSLFSSLKVVRLLRLGRVAR
KLDHYIEYGAAVLVLLVCVFGLAAHWMACIWYSIGDYEIFDEDTKTIRNNSWLYQLAMDI
GTPYQFNGSGSGKWEGGPSKNSVYISSLYFTMTSLTSVGFGNIAPSTDIEKIFAVAIMMIGSL
LYATIFGNVTTIFQQMYANTNRYHEMLNSVRDFLKLYQVPKGLSERVMDYIVSTWSMSRG
IDTEKVLQICPKDMRADICVHLNRKVFKEHPAFRLASDGCLRALAMEFQTVHCAPGDLIY
HAGESVDSLCFVVSGSLEVIQDDEVVAILGKGDVFGDVFWKEATLAQSCANVRALTYCD
LHVIKRDALQKVLEFYTAFSHSFSRNLILTYNLRKRIVFRKISDVKREEEERMKRKNEAPLI
LPPDHPVRRLFQRFRQQKEARLAAERGGRDLDDLDVEKGNVLTEHASANHSLVKASVVT
VRESPATPVSFQAASTSGVPDHAKLQAPGSECLGPKGGGGDCAKRKSWARFKDACGKSE
DWNKVSKAESMETLPERTKASGEATLKKTDSCDSGITKSDLRLDNVGEARSPQDRSPILA
EVKHSFYPIPEQTLQATVLEVRHELKEDIKALNAKMTNIEKQLSEILRILTSRRSSQSPQELF
EISRPQSPESERDIFGAS

>Q8TEY5 single-pass type II membrane protein
MDLGIPDLLDAWLEPPEDIFSTGSVLELGLHCPPPEVPVTRLQEQGLQGWKSGGDRGCGL
QESEPEDFLKLFIDPNEVYCSEASPGSDSGISEDPCHPDSPPAPRATSSPMLYEVVYEAGALE
RMQGETGPNVGLISIQLDQWSPAFMVPDSCMVSELPFDAHAHILPRAGTVAPVPCTTLLPC

QTLFLTDEEKRLLGQEGVSLPSHLPLTKAEERVLKKVRRKIRNKQSAQDSRRRKKEYIDGL
ESRVAACSAQNQELQKKVQELERHNISLVAQLRQLQTLIAQTSNKAAQTSTCVLILLFSLA
LIILPSFSPFQSRPEAGSEDYQPHGVTSRNILTHKDVTENLETQVVESRLREPPGAKDANGS
TRTLLEKMGGKPRPSGRIRSVLHADEM

>Q9UGI6 multipass membrane protein
MDTSGHFHDSGVGDLDEDPKCPCPSSGDEQQQQQQQQQQQQPPPPAPPAAPQQPLGPSLQ
PQPPQLQQQQQQQQQQQQQQQQQQPPHPLSQLAQLQSQPVHPGLLHSSPTAFRAPPSSN
STAILHPSSRQGSQLNLNDHLLGHSPSSTATSGPGGGSRHRQASPLVHRRDSNPFTEIAMSS
CKYSGGVMKPLSRLSASRRNLIEAETEGQPLQLFSPSNPPEIVISSREDNHAHQTLLHHPNA
THNHQHAGTTASSTTFPKANKRKNQNIGYKLGHRRALFEKRKRLSDYALIFGMFGIVVM
VIETELSWGLYSKDSMFSLALKCLISLSTIILLGLIIAYHTREVQLFVIDNGADDWRIAMTYE
RILYISLEMLVCAIHPIPGEYKFFWTARLAFSYTPSRAEADVDIILSIPMFLRLYLIARVMLLH
SKLFTDASSRSIGALNKINFNTRFVMKTLMTICPGTVLLVFSISLWIIAAWTVRVCERYHDQ
QDVTSNFLGAMWLISITFLSIGYGDMVPHTYCGKGVCLLTGIMGAGCTALVVAVVARKLE
LTKAEKHVHNFMMDTQLTKRIKNAAANVLRETWLIYKHTKLLKKIDHAKVRKHQRKFL
QAIHQLRSVKMEQRKLSDQANTLVDLSKMQNVMYDLITELNDRSEDLEKQIGSLESKLEH
LTASFNSLPLLIADTLRQQQQQLLSAIIEARGVSVAVGTTHTPISDSPIGVSSTSFPTPYTSSSS
C

>Q14739 multipass membrane protein
MPSRKFADGEVVRGRWPGSSLYYEVEILSHDSTSQLYTVKYKDGTELELKENDIKPLTSFR
QRKGGSTSSSPSRRRGSRSRSRSRSPGRPPKSARRSASASHQADIKEARREVEVKLTPLILK
PFGNSISRYNGEPEHIERNDAPHKNTQEKFSLSQESSYIATQYSLRPRREEVKLKEIDSKEEK
YVAKELAVRTFEVTPIRAKDLEFGGVPGVFLIMFGLPVFLFLLLLMCKQKDPSLLNFPPPLP
ALYELWETRVFGVYLLWFLIQVLFYLLPIGKVVEGTPLIDGRRLKYRLNGFYAFILTSAVIG
TSLFQGVEFHYVYSHFLQFALAATVFCVVLSVYLYMRSLKAPRNDLSPASSGNAVYDFFIG
RELNPRIGTFDLKYFCELRPGLIGWVVINLVMLLAEMKIQDRAVPSLAMILVNSFQLLYVV
DALWNEEALLTTMDIIHDGFGFMLAFGDLVWVPFIYSFQAFYLVSHPNEVSWPMASLIIVL
KLCGYVIFRGANSQKNAFRKNPSDPKLAHLKTIHTSTGKNLLVSGWWGFVRHPNYLGDLI
MALAWSLPCGFNHILPYFYIIYFTMLLVHREARDEYHCKKKYGVAWEKYCQRVPYRIFPYI
Y

>P07099 single-pass type II membrane protein
MWLEILLTSVLGFAIYWFISRDKEETLPLEDGWWGPGTRSAAREDDSIRPFKVETSDEEIH
DLHQRIDKFRFTPPLEDSCFHYGFNSNYLKKVISYWRNEFDWKKQVEILNRYPHFKTKIEG
LDIHFIHVKPPQLPAGHTPKPLLMVHGWPGSFYEFYKIIPLLTDPKNHGLSDEHVFEVICPSI
PGYGFSEASSKKGFNSVATARIFYKLMLRLGFQEFYIQGGDWGSLICTNMAQLVPSHVKG
LHLNMALVLSNFSTLTLLLGQRFGRFLGLTERDVELLYPVKEKVFYSLMRESGYMHIQCT
KPDTVGSALNDSPVGLAAYILEKFSTWTNTEFRYLEDGGLERKFSLDDLLTNVMLYWTTG
TIISSQRFYKENLGQGWMTQKHERMKVYVPTGFSAFPFELLHTPEKWVRFKYPKLISYSY
MVRGGHFAAFEEPELLAQDIRKFLSVLERQ

>P62745 lipid-anchor

MAAIRKKLVVVGDGACGKTCLLIVFSKDEFPEVYVPTVFENYVADIEVDGKQVELALWD
TAGQEDYDRLRPLSYPDTDVILMCFSVDSPDSLENIPEKWVPEVKHFCPNVPIILVANKKD
LRSDEHVRTELARMKQEPVRTDDGRAMAVRIQAYDYLECSAKTKEGVREVFETATRAAL
QKRYGSQNGCINCCKVL

>Q8WUM9 multipass membrane protein
MATLITSTTAATAASGPLVDYLWMLILGFIIAFVLAFSVGANDVANSFGTAVGSGVVTLKQ
ACILASIFETVGSVLLGAKVSETIRKGLIDVEMYNSTQGLLMAGSVSAMFGSAVWQLVASF
LKLPISGTHCIVGATIGFSLVAKGQEGVKWSELIKIVMSWFVSPLLSGIMSGILFFLVRAFIL
HKADPVPNGLRALPVFYACTVGINLFSIMYTGAPLLGFDKLPLWGTILISVGCAVFCALIV
WFFVCPRMKRKIEREIKCSPSESPLMEKKNSLKEDHEETKLSVGDIENKHPVSEVGPATVP
LQAVVEERTVSFKLGDLEEAPERERLPSVDLKEETSIDSTVNGAVQLPNGNLVQFSQAVSN
QINSSGHYQYHTVHKDSGLYKELLHKLHLAKVGDCMGDSGDKPLRRNNSYTSYTMAIC
GMPLDSFRAKEGEQKGEEMEKLTWPNADSKKRIRMDSYTSYCNAVSDLHSASEIDMSVK
AEMGLGDRKGSNGSLEEWYDQDKPEVSLLFQFLQILTACFGSFAHGGNDVSNAIGPLVAL
YLVYDTGDVSSKVATPIWLLLYGGVGICVGLWVWGRRVIQTMGKDLTPITPSSGFSIELAS
ALTVVIASNIGLPISTTHCKVGSVVSVGWLRSKKAVDWRLFRNIFMAWFVTVPISGVISAAI
MAIFRYVILRM

>Q16281 multipass membrane protein
MAKINTQYSHPSRTHLKVKTSDRDLNRAENGLSRAHSSSEETSSVLQPGIAMETRGLADS
GQGSFTGQGIARLSRLIFLLRRWAARHVHHQDQGPDSFPDRFRGAELKEVSSQESNAQAN
VGSQEPADRGRSAWPLAKCNTNTSNNTEEEKKTKKKDAIVVDPSSNLYYRWLTAIALPVF
YNWYLLICRACFDELQSEYLMLWLVLDYSADVLYVLDVLVRARTGFLEQGLMVSDTNRL
WQHYKTTTQFKLDVLSLVPTDLAYLKVGTNYPEVRFNRLLKFSRLFEFFDRTETRTNYPN
MFRIGNLVLYILIIIHWNACIYFAISKFIGFGTDSWVYPNISIPEHGRLSRKYIYSLYWSTLTLT
TIGETPPPVKDEEYLFVVVDFLVGVLIFATIVGNVGSMISNMNASRAEFQAKIDSIKQYMQF
RKVTKDLETRVIRWFDYLWANKKTVDEKEVLKSLPDKLKAEIAINVHLDTLKKVRIFQDC
EAGLLVELVLKLRPTVFSPGDYICKKGDIGKEMYIINEGKLAVVADDGVTQFVVLSDGSYF
GEISILNIKGSKSGNRRTANIRSIGYSDLFCLSKDDLMEALTEYPEAKKALEEKGRQILMKD
NLIDEELARAGADPKDLEEKVEQLGSSLDTLQTRFARLLAEYNATQMKMKQRLSQLESQ
VKGGGDKPLADGEVPGDATKTEDKQQ

>Q92575 peripheral membrane protein
MLWFQGAIPAAIATAKRSGAVFVVFVAGDDEQSTQMAASWEDDKVTEASSNSFVAIKIDT
KSEACLQFSQIYPVVCVPSSFFIGDSGIPLEVIAGSVSADELVTRIHKVRQMHLLKSETSVA
NGSQSESSVSTPSASFEPNNTCENSQSRNAELCEIPPTSDTKSDTATGGESAGHATSSQEPSG
CSDQRPAEDLNIRVERLTKKLEERREEKRKEEEQREIKKEIERRKTGKEMLDYKRKQEEEL
TKRMLEERNREKAEDRAARERIKQQIALDRAERAARFAKTKEEVEAAKAAALLAKQAE
MEVKRESYARERSTVARIQFRLPDGSSFTNQFPSDAPLEEARQFAAQTVGNTYGNFSLATM
FPRREFTKEDYKKKLLDLELAPSASVVLLPAGRPTASIVHSSSGDIWTLLGTVLYPFLAIWR
LISNFLFSNPPPTQTSVRVTSSEPPNPASSSKSEKREPVRKRVLEKRGDDFKKEGKIYRLRTQ
DDGEDENNTWNGNSTQQM

>Q13304 multipass membrane protein
MSKRSWWAGSRKPPREMLKLSGSDSSQSMNGLEVAPPGLITNFSLATAEQCGQETPLENM
LFASFYLLDFILALVGNTLALWLFIRDHKSGTPANVFLMHLAVADLSCVLVLPTRLVYHFS
GNHWPFGEIACRLTGFLFYLNMYASIYFLTCISADRFLAIVHPVKSLKLRRPLYAHLACAFL
WVVVAVAMAPLLVSPQTVQTNHTVVCLQLYREKASHHALVSLAVAFTFPFITTVTCYLLII
RSLRQGLRVEKRLKTKAVRMIAIVLAIFLVCFVPYHVNRSVYVLHYRSHGASCATQRILAL
ANRITSCLTSLNGALDPIMYFFVAEKFRHALCNLLCGKRLKGPPPSFEGKTNESSLSAKSEL

>P49190 multipass membrane protein
MAGLGASLHVWGWLMLGSCLLARAQLDSDGTITIEEQIVLVLKAKVQCELNITAQLQEG
EGNCFPEWDGLICWPRGTVGKISAVPCPPYIYDFNHKGVAFRHCNPNGTWDFMHSLNKT
WANYSDCLRFLQPDISIGKQEFFERLYVMYTVGYSISFGSLAVAILIIGYFRRLHCTRNYIHM
HLFVSFMLRATSIFVKDRVVHAHIGVKELESLIMQDDPQNSIEATSVDKSQYIGCKIAVVMF
IYFLATNYYWILVEGLYLHNLIFVAFFSDTKYLWGFILIGWGFPAAFVAAWAVARATLADAR
CWELSAGDIKWIYQAPILAAIGLNFILFLNTVRVLATKIWETNAVGHDTRKQYRKLAKSTL
VLVLVFGVHYIVFVCLPHSFTGLGWEIRMHCELFFNSFQGFFVSIIYCYCNGEVQAEVKKM
WSRWNLSVDWKRTPPCGSRRCGSVLTTVTHSTSSQSQVAASTRMVLISGKAAKIASRQPD
SHITLPGYVWSNSEQDCLPHSFHEETKEDSGRQGDDILMEKPSRPMESNPDTEGCQGETE
DVL

>P25106 multipass membrane protein
MDLHLFDYSEPGNFSDISWPCNSSDCIVVDTVMCPNMPNKSVLLYTLSFIYIFIFVIGMIAN
SVVVWVNIQAKTTGYDTHCYILNLAIADLWVVLTIPVWVVSLVQHNQWPMGELTCKVTH
LIFSINLFGSIFFLTCMSVDRYLSITYFTNTPSSRKKMVRRVVCILVWLLAFCVSLPDTYYLK
TVTSASNNETYCRSFYPEHSIKEWLIGMELVSVVLGFAVPFSIIAVFYFLLARAISASSDQEK
HSSRKIIFSYVVVFLVCWLPYHVAVLLDIFSILHYIPFTCRLEHALFTALHVTQCLSLVHCCV
NPVLYSFINRNYRYELMKAFIFKYSAKTGLTKLIDASRVSETEYSALEQSTK

>P48751 multipass membrane protein
MANGVIPPPGGASPLPQVRVPLEEPPLSPDVEEEDDDLGKTLAVSRFGDLISKPPAWDPEKP
SRSYSERDFEFHRHTSHHTHHPLSARLPPPHKLRRLPPTSARHTRRKRKKEKTSAPPSEGTP
PIQEEGGAGVDEEEEEEEEEEGESEAEPVEPPHSGTPQKAKFSIGSDEDDSPGLPGRAAVTK
PLPSVGPHTDKSPQHSSSSPSPRARASRLAGEKSRPWSPSASYDLRERLCPGSALGNPGGP
EQQVPTDEAEAQMLGSADLDDMKSHRLEDNPGVRRHLVKKPSRTQGGRGSPSGLAPILR
RKKKKKKLDRRPHEVFVELNELMLDRSQEPHWRETARWIKFEEDVEEETERWGKPHVAS
LSFRSLLELRRTIAHGAALLDLEQTTLPGIAHLVVETMIVSDQIRPEDRASVLRTLLLKHSH
PNDDKDSGFFPRNPSSSSMNSVLGNHHPTPSHGPDGAVPTMADDLGEPAPLWPHDPDAKE
KPLHMPGGDGHRGKSLKLLEKIPEDAEATVVLVGCVPFLEQPAAAFVRLNEAVLLESVLE
VPVPVRFLFVMLGPSHTSTDYHELGRSIATLMSDKLFHEAAYQADDRQDLLSAISEFLDGS
IVIPPSEVEGRDLLRSVAAFQRELLRKRREREQTKVEMTTRGGYTAPGKELSLELGGSEAT
PEDDPLLRTGSVFGGLVRDVRRRYPHYPSDLRDALHSQCVAAVLFIYFAALSPAITFGGLLG
EKTEGLMGVSELIVSTAVLGVLFSLLGAQPLLVVGFSGPLLVFEEAFFKFCRAQDLEYLTGR
VWVGLWLVVFVLALVAAEGSFLVRYISPFTQEIFAFLISLIFIYETFYKLYKVFTEHPLLPFYP
PEGALEGSLDAGLEPNGSALPPTEGPPSPRNQPNTALLSLILMLGTFFIAFFLRKFRNSRFLG

GKARRIIGDFGIPISILVMVLVDYSITDTYTQKLTVPTGLSVTSPDKRSWFIPPLGSARPFPP
WMMVAAAVPALLVLILIFMETQITALIVSQKARRLLKGSGFHLDLLLIGSLGGLCGLFGLP
WLTAATVRSVTHVNALTVMRTAIAPGDKPQIQEVREQRVTGVLIASLVGLSIVMGAVLRRI
PLAVLFGIFLYMGVTSLSGIQLSQRLLLILMPAKHHPEQPYVTKVKTWRMHLFTCIQLGCI
ALLWVVKSTAASLAFPFLLLLTVPLRHCLLPRLFQDRELQALDSEDAEPNFDEDGQDEYN
ELHMPV

>Q9P2E8 multipass membrane protein
MLMPLCGLLWWWWCCCSGWYCYGLCAPAPQMLRHQGLLKCRCRMLFNDLKVFLLRR
PPQAPLPMHGDPQPPGLAANNTLPALGAGGWAGWRGPREVVGREPPPVPPPPPLPPSSVE
DDWGGPATEPPASLLSSASSDDFCKEKTEDRYSLGSSLDSGMRTPLCRICFQGPEQGELLSP
CRCDGSVKCTHQPCLIKWISERGCWSCELCYYKYHVIAISTKNPLQWQAISLTVIEKVQVA
AAILGSLFLIASISWLIWSTFSPSARWQRQDLLFQICYGMYGFMDVVCIGLIIHEGPSVYRIF
KRWQAVNQQWKVLNYDKTKDLEDQKAGGRTNPRTSSSTQANIPSSEEETAGTPAPEQGPA
QAAGHPSGPLSHHHCAYTILHILSHLRPHEQRSPPGSSRELVMRVTTV

>Q8N490 peripheral membrane protein
MAAVVAATALKGRGARNARVLRGILAGATANKASHNRTRALQSHSSPEGKEEPEPLSPEL
EYIPRKRGKNPMKAVGLAWYSLYTRTWLGYLFYRQQLRRARNRYPKGHSKTQPRLFNGV
KVLPIPVLSDNYSYLIIDTQAQLAVAVDPSDPRAVQASIEKEGVTLVAILCTHKHWDHSGGN
RDLSRRHRDCRVYGSPQDGIPYLTHPLCHQDVVSVGRLQIRALATPGHTQGHLVYLLDGE
PYKGPSCLFSGDLLFLSGCGRTFEGNAETMLSSLDTVLGLGDDTLLWPGHEYAEENLGFA
GVVEPENLARERKMQWVQRQRLERKGTCPSTLGEERSYNPFLRTHCLALQEALGPGPGP
TGDDDYSRAQLLEELRRLKDMHKSK

>O00590 multipass membrane protein
MAATASPQPLATEDADSENSSFYYYDYLDEVAFMLCRKDAVVSFGKVFLPVFYSLIFVLGL
SGNLLLLMVLLRYVPRRRMVEIYLLNLAISNLLFLVTLPFWGISVAWHWVFGSFLCKMVS
TLYTINFYSGIFFISCMSLDKYLEIVHAQPYHRLRTRAKSLLLATIVWAVSLAVSIPDMVFVQ
THENPKGVWNCHADFGGHGTIWKLFLRFQQNLLGFLLPLLAMIFFYSRIGCVLVRLRPAG
QGRALKIAAALVVAFFVLWFPYNLTLFLHTLLDLQVFGNCEVSQHLDYALQVTESIAFLHC
CFSPILYAFSSHRFRQYLKAFLAAVLGWHLAPGTAQASLSSCSESSILTAQEEMTGMNDLG
ERQSENYPNKEDVGNKSA

>Q96JA1 single-pass type I membrane protein
MARPVRGGLGAPRRSPCLLLLWLLLLRLEPVTAAAGPRAPCAAACTCAGDSLDCGGRGL
AALPGDLPSWTRSLNLSYNKLSEIDPAGFEDLPNLQEVYLNNNELTAVPSLGAASSHVVSL
FLQHNKIRSVEGSQLKAYLSLEVLDLSLNNITEVRNTCFPHGPPIKELNLAGNRIGTLELGA
FDGLSRSLLTLRLSKNRITQLPVRAFKLPRLTQLDLNRNRIRLIEGLTFQGLNSLEVLKLQR
NNISKLTDGAFWGLSKMHVLHLEYNSLVEVNSGSLYGLTALHQLHLSNNSIARIHRKGWS
FCQKLHELVLSFNNLTRLDEESLAELSSLSVLRLSHNSISHIAEGAFKGLRSLRVLDLDHNEI
SGTIEDTSGAFSGLDSLSKLTLFGNKIKSVAKRAFSGLEGLEHLNLGGNAIRSVQFDAFVK
MKNLKELHISSDSFLCDCQLKWLPPWLIGRMLQAFVTATCAHPESLKGQSIFSVPPESFVC
DDFLKPQIITQPETTMAMVGKDIRFTCSAASSSSSPMTFAWKKDNEVLTNADMENFVHVH

AQDGEVMEYTTILHLRQVTFGHEGRYQCVITNHFGSTYSHKARLTVNVLPSFTKTPHDITI
RTTTMARLECAATGHPNPQIAWQKDGGTDFPAARERRMHVMPDDDVFFITDVKIDDAGV
YSCTAQNSAGSISANATLTVLETPSLVVPLEDRVVSVGETVALQCKATGNPPPRITWFKGD
RPLSLTERHHLTPDNQLLVVQNVVAEDAGRYTCEMSNTLGTERAHSQLSVLPAAGCRKDG
TTVGIFTIAVVSSIVLTSLVWVCIIYQTRKKSEEYSVTNTDETVVPPDVPSYLSSQGTLSDRQ
ETVVRTEGGPQANGHIESNGVCPRDASHFPEPDTHSVACRQPKLCAGSAYHKEPWKAME
KAEGTPGPHKMEHGGRVVCSDCNTEVDCYSRGQAFHPQPVSRDSAQPSAPNGPEPGGSD
QEHSPHHQCSRTAAGSCPECQGSLYPSNHDRMLTAVKKKPMASLDGKGDSSWTLARLYH
PDSTELQPASSLTSGSPERAEAQYLLVSNGHLPKACDASPESTPLTGQLPGKQRVPLLLAPK
S

>Q96K78 multipass membrane protein
MASCRAWNLRVLVAVVCGLLTGIILGLGIWRIVIRIQRGKSTSSSSTPTEFCRNGGTWENGR
CICTEEWKGLRCTIANFCENSTYMGFTFARIPVGRYGPSLQTCGKDTPNAGNPMAVRLCSL
SLYGEIELQKVTIGNCNENLETLEKQVKDVTAPLNNISSEVQILTSDANKLTAENITSATRVV
GQIFNTSRNASPEAKKVAIVTVSQLLDASEDAFQRVAATANDDALTTLIEQMETYSLSLGN
QSVVEPNIAIQSANFSSENAVGPSNVRFSVQKGASSSLVSSSTFIHTNVDGLNPDAQTELQV
LLNMTKNYTKTCGFVVVYQNDKLFQSKTFTAKSDFSQKIISSKTDENEQDQSASVDMVFSP
KYNQKEFQLYSYACVYWNLSAKDWDTYGCQKDKGTDGFLRCRCNHTTNFAVLMTFKK
DYQYPKSLDILSNVGCALSVTGLALTVIFQIVTRKVRKTSVTWVLVNLCISMLIFNLLFVFG
IENSNKNLQTSDGDINNIDFDNNDIPRTDTINIPNPMCTAIAALLHYFLLVTFTWNALSAAQ
LYYLLIRTMKPLPRHFILFISLIGWGVPAIVVAITVGVIYSQNGNNPQWELDYRQEKICWLA
IPEPNGVIKSPLLWSFIVPVTIILISNVVMFITISIKVLWKNNQNLTSTKKVSSMKKIVSTLSVA
VVFGITWILAYLMLVNDDSIRIVFSYIFCLFNTTQGLQIFILYTVRTKVFQSEASKVLMLLSSI
GRRKSLPSVTRPRLRVKMYNFLRSLPTLHERFRLLETSPSTEEITLSESDNAKESI

>P30556 multipass membrane protein
MILNSSTEDGIKRIQDDCPKAGRHNYIFVMIPTLYSIIFVVGIFGNSLVVIVIYFYMKLKTVA
SVFLLNLALADLCFLLTLPLWAVYTAMEYRWPFGNYLCKIASASVSFNLYASVFLLTCLSID
RYLAIVHPMKSRLRRTMLVAKVTCIIIWLLAGLASLPAIIHRNVFFIENTNITVCAFHYESQN
STLPIGLGLTKNILGFLFPFLIILTSYTLIWKALKKAYEIQKNKPRNDDIFKIIMAIVLFFFFSW
IPHQIFTFLDVLIQLGIIRDCRIADIVDTAMPITICIAYFNNCLNPLFYGFLGKKFKRYFLQLL
KYIPPKAKSHSNLSTKMSTLSYRPSDNVSSSTKKPAPCFEVE

>Q86SU0 single-pass type I membrane protein
MAWPKLPAPWLLLCTWLPAGCLSLLVTVQHTERYVTLFASIILKCDYTTSAQLQDVVVTW
RFKSFCKDPIFDYYSASYQAALSLGQDPSNDCNDNQREVRIVAQRRGQNEPVLGVDYRQR
KITIQNRADLVINEVMWWDHGVYYCTIEAPGDTSGDPDKEVKLIVLHWLTVIFIILGALLL
LLLIGVCWCQCCPQYCCCYIRCPCCPAHCCCPEEALARHRYMKQAQALGPQMMGKPLY
WGADRSSQVSSYPMHPLLQRDLSLPSSLPQMPMTQTTNQPPIANGVLEYLEKELRNLNLA
QPLPPDLKGRFGHPCSMLSSLGSEVVERRIIHLPPLIRDLSSSRRTSDSLHQQWLTPIPSRPW
DLREGRSHHHYPDFHQELQDRGPKSWALERRELDPSWSGRHRSSRLNGSPIHWSDRDSLS
DVPSSSEARWRPSHPPFRSRCQERPRRPSPRESTQRHGRRRRHRSYSPPLPSGLSSWSSEED
KERQPQSWRAHRRGSHSPHWPEEKPPSYRSLDITPGKNSRKKGSVERRSEKDSSHSGRSV

VI

>Q9Y5Q5 single-pass type II membrane protein
MKQSPALAPEERCRRAGSPKPVLRADDNNMGNGCSQKLATANLLRFLLLVLIPCICALVLL
LVILLSYVGTLQKVYFKSNGSEPLVTDGEIQGSDVILTNTIYNQSTVVSTAHPDQHVPAWTT
DASLPGDQSHRNTSACMNITHSQCQMLPYHATLTPLLSVVRNMEMEKFLKFFTYLHRLSC
YQHIMLFGCTLAFPECIIDGDDSHGLLPCRSFCEAAKEGCESVLGMVNYSWPDFLRCSQF
RNQTESSNVSRICFSPQQENGKQLLCGRGENFLCASGICIPGKLQCNGYNDCDDWSDEAH
CNCSENLFHCHTGKCLNYSLVCDGYDDCGDLSDEQNCDCNPTTEHRCGDGRCIAMEWV
CDGDHDCVDKSDEVNCSCHSQGLVECRNGQCIPSTFQCDGDEDCKDGSDEENCSVIQTSC
QEGDQRCLYNPCLDSCGGSSLCDPNNSLNNCSQCEPITLELCMNLPYNSTSYPNYFGHRT
QKEASISWESSLFPALVQTNCYKYLMFFSCTILVPKCDVNTGEHIPPCRALCEHSKERCESV
LGIVGLQWPEDTDCSQFPEENSDNQTCLMPDEYVEECSPSHFKCRSGQCVLASRRCDGQA
DCDDDSDEENCGCKERDLWECPSNKQCLKHTVICDGFPDCPDYMDEKNCSFCQDDELEC
ANHACVSRDLWCDGEADCSDSSDEWDCVTLSINVNSSSFLMVHRAATEHHVCADGWQE
ILSQLACKQMGLGEPSVTKLIQEQEKEPRWLTLHSNWESLNGTTLHELLVNGQSCESRSKI
SLLCTKQDCGRRPAARMNKRILGGRTSRPGRWPWQCSLQSEPSGHICGCVLIAKKWVLTV
AHCFEGRENAAVWKVVLGINNLDHPSVFMQTRFVKTIILHPRYSRAVVDYDISIVELSEDIS
ETGYVRPVCLPNPEQWLEPDTYCYITGWGHMGNKMPFKLQEGEVRIISLEHCQSYFDMK
TITTRMICAGYESGTVDSCMGDSGGPLVCEKPGGRWTLFGLTSWGSVCFSKVLGPGVYSN
VSYFVEWIKRQIYIQTFLLN

>Q9P241 multipass membrane protein
MTEALQWARYHWRRLIRGATRDDDSGPYNYSSLLACGRKSSQTPKLSGRHRIVVPHIQPF
KDEYEKFSGAYVNNRIRTTKYTLLNFVPRNLFEQFHRAANLYFLFLVVLNWVPLVEAFQK
EITMLPLVVVLTIIAIKDGLEDYRKYKIDKQINNLITKVYSRKEKKYIDRCWKDVTVGDFIR
LSCNEVIPADMVLLFSTDPDGICHIETSGLDGESNLKQRQVVRGYAEQDSEVDPEKFSSRIE
CESPNNDLSRFRGFLEHSNKERVGLSKENLLLRGCTIRNTEAVVGIVVYAGHETKAMLNN
SGPRYKRSKLERRANTDVLWCVMLLVIMCLTGAVGHGIWLSRYEKMHFFNVPEPDGHIIS
PLLAGFYMFWTMIILLQVLIPISLYVSIEIVKLGQIYFIQSDVDFYNEKMDSIVQCRALNIAE
DLGQIQYLFSDKTGTLTENKMVFRRCSVAGFDYCHEENARRLESYQEAVSEDEDFIDTVS
GSLSNMAKPRAPSCRTVHNGPLGNKPSNHLAGSSFTLGSGEGASEVPHSRQAAFSSPIETD
VVPDTRLLDKFSQITPRLFMPLDETIQNPPMETLYIIDFFIALAICNTVVVSAPNQPRQKIRH
PSLGGLPIKSLEEIKSLFQRWSVRRSSSPSLNSGKEPSSGVPNAFVSRLPLFSRMKPASPVEE
EVSQVCESPQCSSSSACCTETEKQHGDAGLLNGKAESLPGQPLACNLCYEAESPDEAALV
YAARAYQCTLRSRTPEQVMVDFAALGPLTFQLLHILPFDSVRKRMSVVVRHPLSNQVVVY
TKGADSVIMELLSVASPDGASLEKQQMIVREKTQKHLDDYAKQGLRTLCIAKKVMSDTEY
AEWLRNHFLAETSIDNREELLLESAMRLENKLTLLGATGIEDRLQEGVPESIEALHKAGIKI
WMLTGDKQETAVNIAYACKLLEPDDKLFILNTQSKDACGMLMSTILKELQKKTQALPEQV
SLSEDLLQPPVPRDSGLRAGLIITGKTLEFALQESLQKQFLELTSWCQAVVCCRATPLQKSE
VVKLVRSHLQVMTLAIGDGANDVSMIQVADIGIGVSGQEGMQAVMASDFAVSQFKHLSK
LLLVHGHWCYTRLSNMILYFFYKNVAYVNLLFWYQFFCGFSGTSMTDYWVLIFFNLLFTS
APPVIYGVLEKDVSAETLMQLPELYRSGQKSEAYLPHTFWITLLDAFYQSLVCFFVPYFTY
QGSDTDIFAFGNPLNTAALFIVLLHLVIESKSLTWIHLLVIIGSILSYFLFAIVFGAMCVTCNP

PSNPYWIMQEHMLDPVFYLVCILTTSIALLPRFVYRVLQGSLFPSPILRAKHFDRLTPEERTK
ALKKWRGAGKMNQVTSKYANQSAGKSGRRPMPGPSAVFAMKSASSCAIEQGNLSLCETA
LDQGYSETKAFEMAGPSKGKES

>O75311 multipass membrane protein
MAHVRHFRTLVSGFYFWEAALLLSLVATKETDSARSRSAPMSPSDFLDKLMGRTSGYDAR
IRPNFKGPPVNVTCNIFINSFGSIAETTMDYRVNIFLRQKWNDPRLAYSEYPDDSLDLDPSM
LDSIWKPDLFFANEKGANFHEVTTDNKLLRIFKNGNVLYSIRLTLTLSCPMDLKNFPMDVQ
TCIMQLESFGYTMNDLIFEWQDEAPVQVAEGLTLPQFLLKEEKDLRYCTKHYNTGKFTCI
EVRFHLERQMGYYLIQMYIPSLLIVILSWVSFWINMDAAPARVALGITTVLTMTTQSSGSR
ASLPKVSYVKAIDIWMAVCLLFVFSALLEYAAVNFVSRQHKELLRFRRKRKNKTEAFALE
KFYRFSDMDDEVRESRFSFTAYGMGPCLQAKDGMTPKGPNHPVQVMPKSPDEMRKVFID
RAKKIDTISRACFPLAFLIFNIFYWVIYKILRHEDIHQQQD

>O75380 peripheral membrane protein
MAAAMTFCRLLNRCGEAARSLPLGARCFGVRVSPTGEKVTHTGQVYDDKDYRRIRFVGR
QKEVNENFAIDLIAEQPVSEVETRVIACDGGGGALGHPKVYINLDKETKTGTCGYCGLQF
RQHHH

>O60337 multipass membrane protein
MDTAEEDICRVCRSEGTPEKPLYHPCVCTGSIKFIHQECLVQWLKHSRKEYCELCKHRFAF
TPIYSPDMPSRLPIQDIFAGLVTSIGTAIRYWFHYTLVAFAWLGVVPLTACRIYKCLFTGSVSS
LLTLPLDMLSTENLLADCLQGCFVVTCTLCAFISLVWLREQIVHGGAPIWLEHAAPPFNAA
GHHQNEAPAGGNGAENVAADQPANPPAENAVVGENPDAQDDQAEEEEEDNEEEDDAGV
EDAADANNGAQDDMNWNALEWDRAAEELTWERMLGLDGSLVFLEHVFWVVSLNTLFI
LVFAFCPYHIGHFSLVGLGFEEHVQASHFEGLITTIVGYILLAITLIICHGLATLVKFHRSRRL
LGVCYIVVKVSLLVVVEIGVFPLICGWWLDICSLEMFDATLKDRELSFQSAPGTTMFLHWL
VGMVYVFYFASFILLLREVLRPGVLWFLRNLNDPDFNPVQEMIHLPIYRHLRRFILSVIVFG
SIVLLMLWLPIRIIKSVLPNFLPYNVMLYSDAPVSELSLELLLLQVVLPALLEQGHTRQWLK
GLVRAWTVTAGYLLDLHSYLLGDQEENENSANQQVNNNQHARNNNAIPVVGEGLHAAH
QAILQQGGPVGFQPYRRPLNFPLRIFLLIVFMCITLLIASLICLTLPVFAGRWLMSFWTGTAK
IHELYTAACGLYVCWLTIRAVTVMVAWMPQGRRVIFQKVKEWSLMIMKTLIVAVLLAGVV
PLLLGLLFELVIVAPLRVPLDQTPLFYPWQDWALGVLHAKIIAAITLMGPQWWLKTVIEQV
YANGIRNIDLHYIVRKLAAPVISVLLLSLCVPYVIASGVVPLLGVTAEMQNLVHRRIYPFLL
MVVVLMAILSFQVRQFKRLYEHIKNDKYLVGQRLVNYERKSGKQGSSPPPPQSSQE

>Q13634 single-pass type I membrane protein
MKITSTSCICPVLVCLCFVQRCYGTAHHSSIKVMRNQTKHIEGETEVHHRPKRGWVWNQF
FVLEEHMGPDPQYVGKLHSNSDKGDGSVKYILTGEGAGTIFIIDDTTGDIHSTKSLDREQK
THYVLHAQAIDRRTNKPLEPESEFIIKVQDINDNAPKFTDGPYIVTVPEMSDMGTSVLQVT
ATDADDPTYGNSARVVYSILQGQPYFSVDPKTGVIRTALHNMDREAREHYSVVIQAKDM
AGQVGGLSGSTTVNITLTDVNDNPPRFPQKHYQLYVPESAQVGSAVGKIKANDADTGSNA
DMTYSIINGDGMGIFSISTDKETREGILSLKKPLNYEKKKSYTLNIEGANTHLDFRFSHLGP
FKDATMLKIIVGDVDEPPLFSMPSYLMEVYENAKIGTVVGTVLAQDPDSTNSLVRYFINYN

VEDDRFFNIDANTGTIRTTKVLDREETPWYNITVTASEIDNPDLLSHVTVGIRVLDVNDNPP
ELAREYDIIVCENSKPGQVIHTISATDKDDFANGPRFNFFLDERLPVNPNFTLKDNEDNTAS
ILTRRRRFSRTVQDVYYLPIMISDGGIPSLSSSSTLTIRVCACERDGRVRTCHAEAFLSSAGLS
TGALIAILLCVLILLAIVVLFITLRRSKKEPLIISEEDVRENVVTYDDEGGGEEDTEAFDITAL
RNPSAAEELKYRRDIRPEVKLTPRHQTSSTLESIDVQEFIKQRLAEADLDPSVPPYDSLQTY
AYEGQRSEAGSISSLDSATTQSDQDYHYLGDWGPEFKKLAELYGEIESERTT

>P18405 multipass membrane protein
MATATGVAEERLLAALAYLQCAVGCAVFARNRQTNSVYGRHALPSHRLRVPARAAWVVQ
ELPSLALPLYQYASESAPRLRSAPNCILLAMFLVHYGHRCLIYPFLMRGGKPMPLLACTMA
IMFCTCNGYLQSRYLSHCAVYADDWVTDPRFLIGFGLWLTGMLINIHSDHILRNLRKPGDT
GYKIPRGGLFEYVTAANYFGEIMEWCGYALASWSVQGAAFAFFTFCFLSGRAKEHHEWY
LRKFEEYPKFRKIIIPFLF

>Q96KG7 single-pass type I membrane protein
MVISLNSCLSFICLLLCHWIGTASPLNLEDPNVCSHWESYSVTVQESYPHPFDQIYYTSCTD
ILNWFKCTRHRVSYRTAYRHGEKTMYRRKSQCCPGFYESGEMCVPHCADKCVHGRCIAP
NTCQCEPGWGGTNCSSACDGDHWGPHCTSRCQCKNGALCNPITGACHCAAGFRGWRCE
DRCEQGTYGNDCHQRCQCQNGATCDHVTGECRCPPGYTGAFCEDLCPPGKHGPQCEQR
CPCQNGGVCHHVTGECSCPSGWMGTVCGQPCPEGRFGKNCSQECQCHNGGTCDAATGQ
CHCSPGYTGERCQDECPVGTYGVLCAETCQCVNGGKCYHVSGACLCEAGFAGERCEAR
LCPEGLYGIKCDKRCPCHLENTHSCHPMSGECACKPGWSGLYCNETCSPGFYGEACQQIC
SCQNGADCDSVTGKCTCAPGFKGIDCSTPCPLGTYGINCSSRCGCKNDAVCSPVDGSCTC
KAGWHGVDCSIRCPSGTWGFGCNLTCQCLNGGACNTLDGTCTCAPGWRGEKCELPCQD
GTYGLNCAERCDCSHADGCHPTTGHCRCLPGWSGVHCDSVCAEGRWGPNCSLPCYCKN
GASCSPDDGICECAPGFRGTTCQRICSPGFYGHRCSQTCPQCVHSSGPCHHITGLCDCLPG
FTGALCNEVCPSGRFGKNCAGICTCTNNGTCNPIDRSCQCYPGWIGSDCSQPCPPAHWGP
NCIHTCNCHNGAFCSAYDGECKCTPGWTGLYCTQRCPLGFYGKDCALICQCQNGADCDH
ISGQCTCRTGFMGRHCEQKCPSGTYGYGCRQICDCLNNSTCDHITGTCYCSPGWKGARC
DQAGVIIVGNLNSLSRTSTALPADSYQIGAIAGIIILVLVVLFLLALFIIYRHKQKGKESSMPA
VTYTPAMRVVNADYTISGTLPHSNGGNANSHYFTNPSYHTLTQCATSPHVNNRDRMTVT
KSKNNQLFVNLKNVNPGKRGPVGDCTGTLPADWKHGGYLNELGAFGLDRSYMGKSLK
DLGKNSEYNSSNCSLSSSENPYATIKDPPVLIPKSSECGYVEMKSPARRDSPYAEINNSTSA
NRNVYEVEPTVSVVQGVFSNNGRLSQDPYDLPKNSHIPCHYDLLPVRDSSSSPKQEDSGG
SSSNSSSSSE

>Q969M3 multipass membrane protein
MSGFENLNTDFYQTSYSIDDQSQQSYDYGGSGGPYSKQYAGYDYSQQGRFVPPDMMQP
QQPYTGQIYQPTQAYTPASPQPFYGNNFEDEPPLLEELGINFDHIWQKTLTVLHPLKVADG
SIMNETDLAGPMVFCLAFGATLLLAGKIQFGYVYGISAIGCLGMFCLLNLMSMTGVSFGC
VASVLGYCLLPMILLSSFAVIFSLQGMVGIILTAGIIGWCSFSASKIFISALAMEGQQLLVAYP
CALLYGVFALISVF

>Q16445 multipass membrane protein

MASSLPWLCIILWLENALGKLEVEGNFYSENVSRILDNLLEGYDNRLRPGFGGAVTEVKT
DIYVTSFGPVSDVEMEYTMDVFFRQTWTDERLKFGGPTEILSLNNLMVSKIWTPDTFFRN
GKKSIAHNMTTPNKLFRIMQNGTILYTMRLTINADCPMRLVNFPMDGHACPLKFGSYAYP
KSEIIYTWKKGPLYSVEVPEESSSLLQYDLIGQTVSSETIKSNTGEYVIMTVYFHLQRKMG
YFMIQIYTPCIMTVILSQVSFWINKESVPARTVFGITTVLTMTTLSISARHSLPKVSYATAMD
WFIAVCFAFVFSALIEFAAVNYFTNLQTQKAKRKAQFAAPPTVTISKATEPLEAEIVLHPDS
KYHLKKRITSLSLPIVSSSEANKVLTRAPILQSTPVTPPPLSPAFGGTSKIDQYSRILFPVAFA
GFNLVYWVVYLSKDTMEVSSSVE

>P47870 multipass membrane protein
MWRVRKRGYFGIWSFPLIIAAVCAQSVNDPSNMSLVKETVDRLLKGYDIRLRPDFGGPPV
AVGMNIDIASIDMVSEVNMDYTLTMYFQQAWRDKRLSYNVIPLNLTLDNRVADQLWVPD
TYFLNDKKSFVHGVTVKNRMIRLHPDGTVLYGLRITTTAACMMDLRRYPLDEQNCTLEIE
SYGYTTDDIEFYWRGDDNAVTGVTKIELPQFSIVDYKLITKKVVFSTGSYPRLSLSFKLKR
NIGYFILQTYMPSILITILSWVSFWINYDASAARVALGITTVLTMTTINTHLRETLPKIPYVK
AIDMYLMGCFVFVFMALLEYALVNYIFFGRGPQRQKKAAEKAASANNEKMRLDVNKIFY
KDIKQNGTQYRSLWDPTGNLSPTRRTTNYDFSLYTMDPHENILLSTLEIKNEMATSEAVMG
LGDPRSTMLAYDASSIQYRKAGLPRHSFGRNALERHVAQKKSRLRRRASQLKITIPDLTDV
NAIDRWSRIFFPVVFSFFNIVYWLYYVN

>O15049 peripheral membrane protein
MATAPGPAGIAMGSVGSLLERQDFSPEELRAALAGSRGSRQPDGLLRKGLGQREFLSYLH
LPKKDSKSTKNTKRAPRNEPADYATLYYREHSRAGDFSKTSLPERGRFDKCRIRPSVFKPT
AGNGKGFLSMQSLASHKGQKLWRSNGSLHTLACHPPLSPGPRASQARAQLLHALSLDEG
GPEPEPSLSDSSSGGSFGRSPGTGPSPFSSSLGHLNHLGGSLDRASQGPKEAGPPAVLSCLPE
PPPPYEFSCSSAEEMGAVLPETCEELKRGLGDEDGSNPFTQVLEERQRLWLAELKRLYVER
LHEVTQKAERSERNLQLQLFMAQQEQRRLRKELRAQQGLAPEPRAPGTLPEADPSARPEE
EARWEVCQKTAEISLLKQQLREAQAELAQKLAEIFSLKTQLRGSRAQAQAQDAELVRLRE
AVRSLQEQAPREEAPGSCETDDCKSRGLLGEAGGSEARDSAEQLRAELLQERLRGQEQAL
RFEQERRTWQEEKERVLRYQREIQGGYMDMYRRNQALEQELRALREPPTPWSPRLESSKI

>Q16558 multipass membrane protein
MVKKLVMAQKRGETRALCLGVTMVVCAVITYYILVTTVLPLYQKSVWTQESKCHLIETNI
RDQEELKGKKVPQYPCLWVNVSAAGRWAVLYHTEDTRDQNQQCSYIPGSVDNYQTARA
DVEKVRAKFQEQQVFYCFSAPRGNETSVLFQRLYGPQALLFSLFWPTFLLTGGLLIIAMVK
SNQYLSILAAQK

>O43300 single-pass type I membrane protein
MGLHFKWPLGAPMLAAIYAMSMVLKMLPALGMACPPKCRCEKLLFYCDSQGFHSVPNA
TDKGSLGLSLRHNHITELERDQFASFSQLTWLHLDHNQISTVKEDAFQGLYKLKELILSSN
KIFYLPNTTFTQLINLQNLDLSFNQLSSLHPELFYGLRKLQTLHLRSNSLRTIPVRLFWDCR
SLEFLDLSTNRLRSLARNGFAGLIKLRELHLEHNQLTKINFAHFLRLSSLHTLFLQWNKISN
LTCGMEWTWGTLEKLDLTGNEIKAIDLTVFETMPNLKILLMDNNKLNSLDSKILNSLRSLT
TVGLSGNLWECSARICALASWLGSFQGRWEHSILCHSPDHTQGEDILDAVHGFQLCWNLS

TTVTVMATTYRDPTTEYTKRISSSSYHVGDKEIPTTAGIAVTTEEHFPEPDNAIFTQRVITGT
MALLFSFFFIIFIVFISRKCCPPTLRRIRQCSMVQNHRQLRSQTRLHMSNMSDQGPYNEYEP
THEGPFIIINGYGQCKCQQLPYKECEV

>O60609 GPI-anchor, lipid-anchor
MVRPLNPRPLPPVVLMLLLLLPPSPLPLAAGDPLPTESRLMNSCLQARRKCQADPTCSAA
YHHLDSCTSSISTPLPSEEPSVPADCLEAAQQLRNSSLIGCMCHRRMKNQVACLDIYWTVH
RARSLGNYELDVSPYEDTVTSKPWKMNLSKLNMLKPDSDLCLKFAMLCTLNDKCDRLR
KAYGEACSGPHCQRHVCLRQLLTFFEKAAEPHAQGLLLCPCAPNDRGCGERRRNTIAPNC
ALPPVAPNCLELRRLCFSDPLCRSRLVDFQTHCHPMDILGTCATEQSRCLRAYLGLIGTAM
TPNFVSNVNTSVALSCTCRGSGNLQEECEMLEGFFSHNPCLTEAIAAKMRFHSQLFSQDW
PHPTFAVMAHQNENPAVRPQPWVPSLFSCTLPLILLLSLW

>Q6IWH7 multipass membrane protein
MRMAATAWAGLQGPPLPTLCPAVRTGLYCRDQAHAERWAMTSETSSGSHCARSRMLRRR
AQEEDSTVLIDVSPPEAEKRGSYGSTAHASEPGGQQAAACRAGSPAKPRIADFVLVWEED
LKLDRQQDSAARDRTDMHRTWRETFLDNLRAAGLCVDQQDVQDGNTTVHYALLSASWA
VLCYYAEDLRLKLPLQELPNQASNWSAGLLAWLGIPNVLLEVVPDVPPEYYSCRFRVNKL
PRFLGSDNQDTFFTSTKRHQILFEILAKTPYGHEKKNLLGIHQLLAEGVLSAAFPLHDGPF
KTPPEGPQAPRLNQRQVLFQHWARWGKWNKYQPLDHVRRYFGEKVALYFAWLGFYTG
WLLPAAVVGTLVFLVGCFLVFSDIPTQELCGSKDSFEMCPLCLDCPFWLLSSACALAQAGR
LFDHGGTVFFSLFMALWAVLLLEYWKRKSATLAYRWDCSDYEDTEERPRPQFAASAPMT
APNPITGEDEPYFPERSRARRMLAGSVVIVVMVAVVVMCLVSIILYRAIMAIVVSRSGNTLL
AAWASRIASLTGSVVNLVFILILSKIYVSLAHVLTRWEMHRTQTKFEDAFTLKVFIFQFVNF
YSSPVYIAFFKGRFVGYPGNYHTLFGVRNEECAAGGCLIELAQELLVIMVGKQVINNMQE
VLIPKLKGWWQKFRLRSKKRKAGASAGASQGPWEDDYELVPCEGLFDEYLEMVLQFGF
VTIFVAACPLAPLFALLNNWVEIRLDARKFVCEYRRPVAERAQDIGIWFHILAGLTHLAVIS
NAFLLAFSSDFLPRAYYRWTRAHDLRGFLNFTLARAPSSFAAAHNRTCRYRAFRDDDGHY
SQTYWNLLAIRLAFVIVFEHVVFSVGRLLDLLVPDIPESVEIKVKREYYLAKQALAENEVL
FGTNGTKDEQPEGSELSSHWTPFTVPKASQLQQ

>Q9NYL5 peripheral membrane protein
MELISPTVIIILGCLALFLLLQRKNLRRPPCIKGWIPWIGVGFEFGKAPLEFIEKARIKYGPIF
TVFAMGNRMTFVTEEEGINVFLKSKKVDFELAVQNIVYRTASIPKNVFLALHEKLYIMLKG
KMGTVNLHQFTGQLTEELHEQLENLGTHGTMDLNNLVRHLLYPVTVNMLFNKSLFSTNK
KKIKEFHQYFQVYDEDFEYGSQLPECLLRNWSKSKKWFLELFEKNIPDIKACKSAKDNSM
TLLQATLDIVETETSKENSPNYGLLLLWASLSNAVPVAFWTLAYVLSHPDIHKAIMEGISSV
FGKAGKDKIKVSEDDLENLLLIKWCVLETIRLKAPGVITRKVVKPVEILNYIIPSGDLLMLS
PFWLHRNPKYFPEPELFKPERWKKANLEKHSFLDCFMAFGSGKFQCPARWFALLEVQMCI
ILILYKYDCSLLDPLPKQSYLHLVGVPQPEGQCRIEYKQRI

>Q96RI8 multipass membrane protein
MSSNSSLLVAVQLCYANVNGSCVKIPFSPGSRVILYIVFGFGAVLAVFGNLLVMISILHFKQL
HSPTNFLVASLACADFLVGVTVMPFSMVRTVESCWYFGRSFCTFHTCCDVAFCYSSLFHL

CFISIDRYIAVTDPLVYPTKFTVSVSGICISVSWILPLMYSGAVFYTGVYDDGLEELSDALNC
IGGCQTVVNQNWVLTDFLSFFIPTFIMIILYGNIFLVARRQAKKIENTGSKTESSSESYKARV
ARRERKAAKTLGVTVVAFMISWLPYSIDSLIDAFMGFITPACIYEICCWCAYYNSAMNPLIY
ALFYPWFRKAIKVIVTGQVLKNSSATMNLFSEHI

>Q96RJ0 multipass membrane protein
MMPFCHNIINISCVKNNWSNDVRASLYSLMVLIILTTLVGNLIVIVSISHFKQLHTPTNWLIH
SMATVDFLLGCLVMPYSMVRSAEHCWYFGEVFCKIHTSTDIMLSSASIFHLSFISIDRYYAV
CDPLRYKAKMNILVICVMIFISWSVPAVFAFGMIFLELNFKGAEEIYYKHVHCRGGCSVFFS
KISGVLTFMTSFYIPGSIMLCVYYRIYLIAKEQARLISDANQKLQIGLEMKNGISQSKERKA
VKTLGIVMGVFLICWCPFFICTVMDPFLHYIIPPTLNDVLIWFGYLNSTFNPMVYAFFYPW
FRKALKMMLFGKIFQKDSSRCKLFLELSS

>O75751 multipass membrane protein
MPSFDEALQRVGEFGRFQRRVFLLLCLTGVTFAFLFVGVVFLGTQPDHYWCRGPSAAALA
ERCGWSPEEEWNRTAPASRGPEPPERRGRCQRYLLEAANDSASATSALSCADPLAAFPNR
SAPLVPCRGGWRYAQAHSTIVSEFDLVCVNAWMLDLTQAILNLGFLTGAFTLGYAADRYG
RIVIYLLSCLGVGVTGVVVAFAPNFPVFVIFRFLQGVFGKGTWMTCYVIVTEIVGSKQRRI
VGIVIQMFFTLGIIILPGIAYFIPNWQGIQLAITLPSFLFLLYYWVVPESPRWLITRKKGDKAL
QILRRIAKCNGKYLSSNYSEITVTDEEVSNPSFLDLVRTPQMRKCTLILMFAWFTSAVVYQ
GLVMRLGIIGGNLYIDFFISGVVELPGALLILLTIERLGRRLPFAASNIVAGVACLVTAFLPEGI
AWLRTTVATLGRLGITMAFEIVYLVNSELYPTTLRNFGVSLCSGLCDFGGIIAPFLLFRLAAV
WLELPLIIFGILASICGGLVMLLPETKGIALPETVDDVEKLGSPHSCKCGRNKKTPVSRSHL

>Q03113 lipid-anchor
MSGVVRTLSRCLLPAEAGGARERRAGSGARDAEREARRRSRDIDALLARERRAVRRLVKI
LLLGAGESGKSTFLKQMRIIHGREFDQKALLEFRDTIFDNILKGSRVLVDARDKLGIPWQY
SENEKHGMFLMAFENKAGLPVEPATFQLYVPALSALWRDSGIREAFSRRSEFQLGESVKYF
LDNLDRIGQLNYFPSKQDILLARKATKGIVEHDFVIKKIPFKMVDVGGQRSQRQKWFQCF
DGITSILFMVSSSEYDQVLMEDRRTNRLVESMNIFETIVNNKLFFNVSIILFLNKMDLLVEK
VKTVSIKKHFPDFRGDPHRLEDVQRYLVQCFDRKRRNRSKPLFHHFTTAIDTENVRFVFHA
VKDTILQENLKDIMLQ

>P00533 single-pass type I membrane protein
MRPSGTAGAALLALLAALCPASRALEEKKVCQGTSNKLTQLGTFEDHFLSLQRMFNNCE
VVLGNLEITYVQRNYDLSFLKTIQEVAGYVLIALNTVERIPLENLQIIRGNMYYENSYALAV
LSNYDANKTGLKELPMRNLQEILHGAVRFSNNPALCNVESIQWRDIVSSDFLSNMSMDFQ
NHLGSCQKCDPSCPNGSCWGAGEENCQKLTKIICAQQCSGRCRGKSPSDCCHNQCAAGC
TGPRESDCLVCRKFRDEATCKDTCPPLMLYNPTTYQMDVNPEGKYSFGATCVKKCPRNY
VVTDHGSCVRACGADSYEMEEDGVRKCKKCEGPCRKVCNGIGIGEFKDSLSINATNIKHF
KNCTSISGDLHILPVAFRGDSFTHTPPLDPQELDILKTVKEITGFLLIQAWPENRTDLHAFEN
LEIIRGRTKQHGQFSLAVVSLNITSLGLRSLKEISDGDVIISGNKNLCYANTINWKKLFGTSG
QKTKIISNRGENSCKATGQVCHALCSPEGCWGPEPRDCVSCRNVSRGRECVDKCNLLEGE
PREFVENSECIQCHPECLPQAMNITCTGRGPDNCIQCAHYIDGPHCVKTCPAGVMGENNTL

VWKYADAGHVCHLCHPNCTYGCTGPGLEGCPTNGPKIPSIATGMVGALLLLLVVALGIGL
FMRRRHIVRKRTLRRLLQERELVEPLTPSGEAPNQALLRILKETEFKKIKVLGSGAFGTVY
KGLWIPEGEKVKIPVAIKELREATSPKANKEILDEAYVMASVDNPHVCRLLGICLTSTVQLI
TQLMPFGCLLDYVREHKDNIGSQYLLNWCVQIAKGMNYLEDRRLVHRDLAARNVLVKT
PQHVKITDFGLAKLLGAEEKEYHAEGGKVPIKWMALESILHRIYTHQSDVWSYGVTVWE
LMTFGSKPYDGIPASEISSILEKGERLPQPPICTIDVYMIMVKCWMIDADSRPKFRELIIEFSK
MARDPQRYLVIQGDERMHLPSPTDSNFYRALMDEEDMDDVVDADEYLIPQQGFFSSPSTS
RTPLLSSLSATSNNSTVACIDRNGLQSCPIKEDSFLQRYSSDPTGALTEDSIDDTFLPVPEYIN
QSVPKRPAGSVQNPVYHNQPLNPAPSRDPHYQDPHSTAVGNPEYLNTVQPTCVNSTFDSP
AHWAQKGSHQISLDNPDYQQDFFPKEAKPNGIFKGSTAENAEYLRVAPQSSEFIGA

>Q9BXP2 multipass membrane protein
MASESSPLLAYRLLGEEGVALPANGAGGPGGASARKLSTFLGVVVPTVLSMFSIVVFLRIG
FVVGHAGLLQALAMLLVAYFILALTVLSVCAIATNGAVQGGGAYFMISRTLGPEVGGSIGL
MFYLANVCGCAVSLLGLVESVLDVFGADATGPSGLRVLPQGYGWNLLYGSLLLGLVGGV
CTLGAGLYARASFLTFLLVSGSLASVLISFVAVGPRDIRLTPRPGPNGSSLPPRFGHFTGFNSS
TLKDNLGAGYAEDYTTGAVMNFASVFAVLFNGCTGIMAGANMSGELKDPSRAIPLGTIVA
VAYTFFVYVLLFFLSSFTCDRTLLQEDYGFFRAISLWPPLVLIGIYATALSASMSSLIGASRIL
HALARDDLFGVILAPAKVVSRGGNPWAAVLYSWGLVQLVLLAGKLNTLAAVVTVFYLVA
YAAVDLSCLSLEWASAPNFRPTFSLFSWHTCLLGVASCLLMMFLISPGAAGGSLLLMGLLA
ALLTARGGPSSWGYVSQALLFHQVRKYLLRLDVRKDHVKFWRPQLLLLVGNPRGALPLL
RLANQLKKGGLYVLGHVTLGDLDSLPSDPVQPQYGAWLSLVDRAQVKAFVDLTLSPSVR
QGAQHLLRISGLGGMKPNTLVLGFYDDAPPQDHFLTDPAFSEPADSTREGSSPALSTLFPPP
RAPGSPRALNPQDYVATVADALKMNKNVVLARASGALPPERLSRGSGGTSQLHHVDVWP
LNLLRPRGGPGYVDVCGLFLLQMATILGMVPAWHSARLRIFLCLGPREAPGAAEGRLRAL
LSQLRIRAEVQEVVWGEGAGAGEPEAEEEGDFVNSGRGDAEAEALARSANALVRAQQG
RGTGGGPGGPEGGDAEGPITALTFLYLPRPPADPARYPRYLALLETLTRDLGPTLLVHGVTP
VTCTDL

>P21709 single-pass type I membrane protein
MERRWPLGLGLVLLLCAPLPPGARAKEVTLMDTSKAQGELGWLLDPPKDGWSEQQQILN
GTPLYMYQDCPMQGRRDTDHWLRSNWIYRGEEASRVHVELQFTVRDCKSFPGGAGPLG
CKETFNLLYMESDQDVGIQLRRPLFQKVTTVAADQSFTIRDLVSGSVKLNVERCSLGRLTR
RGLYLAFHNPGACVALVSVRVFYQRCPETLNGLAQFPDTLPGPAGLVEVAGTCLPHARASP
RPSGAPRMHCSPDGEWLVPVGRCHCEPGYEEGGSGEACVACPSGSYRMDMDTPHCLTCP
QQSTAESEGATICTCESGHYRAPGEGPQVACTGPPSAPRNLSFSASGTQLSLRWEPPADTG
GRQDVRYSVRCSQCQGTAQDGGPCQPCGVGVHFSPGARGLTTPAVHVNGLEPYANYTFN
VEAQNGVSGLGSSGHASTSVSISMGHAESLSGLSLRLVKKEPRQLELTWAGSRPRSPGANL
TYELHVLNQDEERYQMVLEPRVLLTELQPDTTYIVRVRMLTPLGPGPFSPDHEFRTSPPVSR
GLTGGEIVAVIFGLLLGAALLLGILVFRSRRAQRQRQQRQRDRATDVDREDKLWLKPYVD
LQAYEDPAQGALDFTRELDPAWLMVDTVIGEGEFGEVYRGTLRLPSQDCKTVAIKTLKDT
SPGGQWWNFLREATIMGQFSHPHILHLEGVVTKRKPIMIITEFMENGALDAFLREREDQLV
PGQLVAMLQGIASGMNYLSNHNYVHRDLAARNILVNQNLCCKVSDFGLTRLLDDFDGTY
ETQGGKIPIRWTAPEAIAHRIFTTASDVWSFGIVMWEVLSFGDKPYGEMSNQEVMKSIEDG

YRLPPPVDCPAPLYELMKNCWAYDRARRPHFQKLQAHLEQLLANPHSLRTIANFDPRMTL
RLPSLSGSDGIPYRTVSEWLESIRMKRYILHFHSAGLDTMECVLELTAEDLTQMGITLPGHQ
KRILCSIQGFKD

>Q8N0W4 single-pass type I membrane protein
MSRPQGLLWLPLLFTPVCVMLNSNVLLWLTALAIKFTLIDSQAQYPVVNTNYGKIRGLRTP
LPNEILGPVEQYLGVPYASPPTGERRFQPPEPPSSWTGIRNTTQFAAVCPQHLDERSLLHDM
LPIWFTANLDTLMTYVQDQNEDCLYLNIYVPTEDDIHDQNSKKPVMVYIHGGSYMEGTG
NMIDGSILASYGNVIVITINYRLGILGFLSTGDQAAKGNYGLLDQIQALRWIEENVGAFGG
DPKRVTIFGSGAGASCVSLLTLSHYSEGLFQKAIIQSGTALSSWAVNYQPAKYTRILADKVG
CNMLDTTDMVECLRNKNYKELIQQTITPATYHIAFGPVIDGDVIPDDPQILMEQGEFLNYD
IMLGVNQGEGLKFVDGIVDNEDGVTPNDFDFSVSNFVDNLYGYPEGKDTLRETIKFMYTD
WADKENPETRRKTLVALFTDHQWVAPAVATADLHAQYGSPTYFYAFYHHCQSEMKPSWA
DSAHGDEVPYVFGIPMIGPTELFSCNFSKNDVMLSAVVMTYWTNFAKTGDPNQPVPQDT
KFIHTKPNRFEEVAWSKYNPKDQLYLHIGLKPRVRDHYRATKVAFWLELVPHLHNLNEIFQ
YVSTTTKVPPPDMTSFPYGTRRSPAKIWPTTKRPAITPANNPKHSKDPHKTGPEDTTVLIET
KRDYSTELSVTIAVGASLLFLNILAFAALYYKKDKRRHETHRRPSPQRNTTNDIAHIQNEEI
MSLQMKQLEHDHECESLQAHDTLRLTCPPDYTLTLRRSPDDIPLMTPNTITMIPNTLTGMQ
PLHTFNTFSGGQNSTNLPHGHSTTRV

>A4D1S5 lipid-anchor
MHFSSSARAADENFDYLFKIILIGDSNVGKTCVVQHFKSGVYTETQQNTIGVDFTVRSLDI
DGKKVKMQVWDTAGQERFRTITQSYYRSAHAAIIAYDLTRRSTFESIPHWIHEIEKYGAAN
VVIMLIGNKCDLWEKRHVLFEDACTLAEKYGLLAVLETSAKESKNIEEVFVLMAKELIAR
NSLHLYGESALNGLPLDSSPVLMAQGPSEKTHCTC

>Q9BXC1 multipass membrane protein
MPANYTCTRPDGDNTDFRYFIYAVTYTVILVPGLIGNILALWVFYGYMKETKRAVIFMINL
AIADLLQVLSLPLRIFYYLNHDWPFGPGLCMFCFYLKYVNMYASIYFLVCISVRRFWFLM
YPFRFHDCKQKYDLYISIAGWLIICLACVLFPLLRTSDDTSGNRTKCFVDLPTRNVNLAQS
VVMMTIGELIGFVTPLLIVLYCTWKTVLSLQDKYPMAQDLGEKQKALKMILTCAGVFLIC
FAPYHFSFPLDFLVKSNEIKSCLARRVILIFHSVALCLASLNSCLDPVIYYFSTNEFRRRLSRQ
DLHDSIQLHAKSFVSNHTASTMTPELC

>P28335 multipass membrane protein
MVNLRNAVHSFLVHLIGLLVWQCDISVSPVAAIVTDIFNTSDGGRFKFPDGVQNWPALSIVI
IIIMTIGGNILVIMAVSMEKKLHNATNYFLMSLAIADMLVGLLVMPLSLLAILYDYVWPLPR
YLCPVWISLDVLFSTASIMHLCAISLDRYVAIRNPIEHSRFNSRTKAIMKIAIVWAISIGVSVP
IPVIGLRDEEKVFVNNTTCVLNDPNFVLIGSFVAFFIPLTIMVITYCLTIYVLRRQALMLLHG
HTEEPPGLSLDFLKCCKRNTAEEENSANPNQDQNARRRKKKERRPRGTMQAINNERKAS
KVLGIVFFVFLIMWCPFFITNILSVLCEKSCNQKLMEKLLNVFVWIGYVCSGINPLVYTLFN
KIYRRAFSNYLRCNYKVEKKPPVRQIPRVAATALSGRELNVNIYRHTNEPVIEKASDNEPGI
EMQVENLELPVNPSSVVSERISSV

>Q8TDV5 multipass membrane protein
MESSFSFGVILAVLASLIIATNTLVAVAVLLLIHKNDGVSLCFTLNLAVADTLIGVAISGLLTD
QLSSPSRPTQKTLCSLRMAFVTSSAAASVLTVMLITFDRYLAIKQPFRYLKIMSGFVAGACI
AGLWLVSYLIGFLPLGIPMFQQTAYKGQCSFFAVFHPHFVLTLSCVGFFPAMLLFVFFYCD
MLKIASMHSQQIRKMEHAGAMAGGYRSPRTPSDFKALRTVSVLIGSFALSWTPFLITGIVQ
VACQECHLYLVLERYLWLLGVGNSLLNPLIYAYWQKEVRLQLYHMALGVKKVLTSFLLFL
SARNCGPERPRESSCHIVTISSSEFDG

>P54219 multipass membrane protein
MLRTILDAPQRLLKEGRASRQLVLVVVFVALLLDNMLFTVVVPIVPTFLYDMEFKEVNSSL
HLGHAGSSPHALASPAFSTIFSFFNNNTVAVEESVPSGIAWMNDTASTIPPPATEAISAHKNN
CLQGTGFLEEEITRVGVLFASKAVMQLLVNPFVGPLTNRIGYHIPMFAGFVIMFLSTVMFAF
SGTYTLLFVARTLQGIGSSFSSVAGLGMLASVYTDDHERGRAMGTALGGLALGLLVGAPF
GSVMYEFVGKSAPFLILAFLALLDGALQLCILQPSKVSPESAKGTPLFMLLKDPYILVAAGS
ICFANMGVAILEPTLPIWMMQTMCSPKWQLGLAFLPASVSYLIGTNLFGVLANKMGRWL
CSLIGMLVVGTSLLCVPLAHNIFGLIGPNAGLGLAIGMVDSSMMPIMGHLVDLRHTSVYG
SVYAIADVAFCMGFAIGPSTGGAIVKAIGFPWLMVITGVINIVYAPLCYYLRSPPAKEEKLAI
LSQDCPMETRMYATQKPTKEFPLGEDSDEEPDHEE

>Q15825 multipass membrane protein
MLTSKGQGFLHGGLCLWLCVFTPFFKGCVGCATEERLFHKLFSHYNQFIRPVENVSDPVT
VHFEVAITQLANVDEVNQIMETNLWLRHIWNDYKLRWDPMEYDGIETLRVPADKIWKPDI
VLYNNAVGDFQVEGKTKALLKYNGMITWTPPAIFKSSCPMDITFFPFDHQNCSLKFGSWT
YDKAEIDLLIIGSKVDMNDFWENSEWEIIDASGYKHDIKYNCCEEIYTDITYSFYIRRLPMF
YTINLIIPCLFISFLTVLVFYLPSDCGEKVTLCISVLLSLTVFLLVITETIPSTSLVVPLVGEYLL
FTMIFVTLSIVVTVFVLNIHYRTPTTHTMPRWVKTVFLKLLPQVLLMRWPLDKTRGTGSD
AVPRGLARRPAKGKLASHGEPRHLKECFHCHKSNELATSKRRLSHQPLQWVVENSEHSPE
VEDVINSVQFIAENMKSHNETKEVEDDWKYVAMVVDRVFLWVFIIVCVFGTAGLFLQPLL
GNTGKS

>O14763 single-pass type I membrane protein
MEQRGQNAPAASGARKRHGPGPREARGARPGPRVPKTLVLVVAAVLLLVSAESALITQQD
LAPQQRAAPQQKRSSPSEGLCPPGHHISEDGRDCISCKYGQDYSTHWNDLLFCLRCTRCD
SGEVELSPCTTTRNTVCQCEEGTFREEDSPEMCRKCRTGCPRGMVKVGDCTPWSDIECVH
KESGTKHSGEVPAVEETVTSSPGTPASPCSLSGIIIGVTVAAVVLIVAVFVCKSLLWKKVLPY
LKGICSGGGGDPERVDRSSQRPGAEDNVLNEIVSILQPTQVPEQEMEVQEPAEPTGVNMLS
PGESEHLLEPAEAERSQRRRLLVPANEGDPTETLRQCFDDFADLVPFDSWEPLMRKLGLM
DNEIKVAKAEAAGHRDTLYTMLIKWVNKTGRDASVHTLLDALETLGERLAKQKIEDHLL
SSGKFMYLEGNADSAMS

>Q7Z5G4 lipid-anchor
MRPQQAPVSGKVFIQRDYSSGTRCQFQTKFPAELENRIDRQQFEETVRTLNNLYAEAEKLG
GQSYLEGCLACLTAYTIFLCMETHYEKVLKKVSKYIQEQNEKIYAPQGLLLTDPIERGLRVI
EITIYEDRGMSSGR

>Q9UF47 lipid-anchor
MACNIPNQRQRTLSTTGEALYEILGLHKGASNEEIKKTYRKLALKHHPDKNPDDPAATEKF
KEINNAHAILTDISKRSIYDKYGSLGLYVAEQFGDENVNTYFMLSSWWAKALFVIVGLLTG
CYFCCCLCCCCNCCCGHCRPESSVPEEDFYVSPEDLEEQIKSDMEKDVDFPVFLQPTNAN
EKTQLIKEGSRSYCTDS

>Q9Y561 single-pass type I membrane protein
MACRWSTKESPRWRSALLLLFLAGVYGNGALAEHSENVHISGVSTACGETPEQIRAPSGII
TSPGWPSEYPAKINCSWFIRANPGEIITISFQDFDIQGSRRCNLDWLTIETYKNIESYRACGS
TIPPPYISSQDHIWIRFHSDDNISRKGFRLAYFSGKSEEPNCACDQFRCGNGKCIPEAWKCN
NMDECGDSSDEEICAKEANPPTAAAFQPCAYNQFQCLSRFTKVYTCLPESLKCDGNIDCL
DLGDEIDCDVPTCGQWLKYFYGTFNSPNYPDFYPPGSNCTWLIDTGDHRKVILRFTDFKL
DGTGYGDYVKIYDGLEENPHKLLRVLTAFDSHAPLTVVSSSGQIRVHFCADKVNAARGFN
ATYQVDGFCLPWEIPCGGNWGCYTEQQRCDGYWHCPNGRDETNCTMCQKEEFPCSRNG
VCYPRSDRCNYQNHCPNGSDEKNCFFCQPGNFHCKNNRCVFESWVCDSQDDCGDGSDE
ENCPVIVPTRVITAAVIGSLICGLLLVIALGCTCKLYSLRMFERRSFETQLSRVEAELLRREAP
PSYGQLIAQGLIPPVEDFPVCSPNQASVLENLRLAVRSQLGFTSVRLPMAGRSSNIWNRIFN
FARSRHSGSLALVSADGDEVVPSQSTSREPERNHTHRSLFSVESDDTDTENERRDMAGAS
GGVAAPLPQKVPPTTAVEATVGACASSSTQSTRGGHADNGRDVTSVEPPSVSPARHQLTSA
LSRMTQGLRWVRFTLGRSSSLSQNQSPLRQLDNGVSGREDDDDVEMLIPISDGSSDFDVN
DCSRPLLDLASDQGQGLRQPYNATNPGVRPSNRDGPCERCGIVHTAQIPDTCLEVTLKNET
SDDEALLLC

>Q9Y6M9 peripheral membrane protein
MAFLASGPYLTHQQKVLRLYKRALRHLESWCVQRDKYRYFACLMRARFEEHKNEKDMA
KATQLLKEAEEEFWYRQHPQPYIFPDSPGGTSYERYDCYKVPEWCLDDWHPSEKAMYPD
YFAKREQWKKLRRESWEREVKQLQEETPPGGPLTEALPPARKEGDLPPLWWYIVTRPRER
PM

>Q99541 peripheral membrane protein
MASVAVDPQPSVVTRVVNLPLVSSTYDLMSSAYLSTKDQYPYLKSVCEMAENGVKTITSV
AMTSALPIIQKLEPQIAVANTYACKGLDRIEERLPILNQPSTQIVANAKGAVTGAKDAVTTT
VTGAKDSVASTITGVMDKTKGAVTGSVEKTKSVVSGSINTVLGSRMMQLVSSGVENALT
KSELLVEQYLPLTEEELEKEAKKVEGFDLVQKPSYYVRLGSLSTKLHSRAYQQALSRVKE
AKQKSQQTISQLHSTVHLIEFARKNVYSANQKIQDAQDKLYLSWVEWKRSIGYDDTDESH
CAEHIESRTLAIARNLTQQLQTTCHTLLSNIQGVPQNIQDQAKHMGVMAGDIYSVFRNAA
SFKEVSDSLLTSSKGQLQKMKESLDDVMDYLVNNTPLNWLVGPFYPQLTESQNAQDQGA
EMDKSSQETQRSEHKTH

>Q16620 single-pass type I membrane protein
MSSWIRWHGPAMARLWGFCWLVVGFWRAAFACPTSCKCSASRIWCSDPSPGIVAFPRLEP
NSVDPENITEIFIANQKRLEIINEDDVEAYVGLRNLTIVDSGLKFVAHKAFLKNSNLQHINFT
RNKLTSLSRKHFRHLDLSELILVGNPFTCSCDIMWIKTLQEAKSSPDTQDLYCLNESSKNIP

LANLQIPNCGLPSANLAAPNLTVEEGKSITLSCSVAGDPVPNMYWDVGNLVSKHMNETSH
TQGSLRITNISSDDSGKQISCVAENLVGEDQDSVNLTVHFAPTITFLESPTSDHHWCIPFTVK
GNPKPALQWFYNGAILNESKYICTKIHVTNHTEYHGCLQLDNPTHMNNGDYTLIAKNEY
GKDEKQISAHFMGWPGIDDGANPNYPDVIYEDYGTAANDIGDTTNRSNEIPSTDVTDKTG
REHLSVYAVVVIASVVGFCLLVMLFLLKLARHSKFGMKGPASVISNDDDSASPLHHISNGS
NTPSSSEGGPDAVIIGMTKIPVIENPQYFGITNSQLKPDTFVQHIKRHNIVLKRELGEGAFGK
VFLAECYNLCPEQDKILVAVKTLKDASDNARKDFHREAELLTNLQHEHIVKFYGVCVEGD
PLIMVFEYMKHGDLNKFLRAHGPDAVLMAEGNPPTELTQSQMLHIAQQIAAGMVYLASQ
HFVHRDLATRNCLVGENLLVKIGDFGMSRDVYSTDYYRVGGHTMLPIRWMPPESIMYRK
FTTESDVWSLGVVLWEIFTYGKQPWYQLSNNEVIECITQGRVLQRPRTCPQEVYELMLGC
WQREPHMRKNIKGIHTLLQNLAKASPVYLDILG

>Q9NZB2 peripheral membrane protein
MGVQGFQDYIEKHCPSAVVPVELQKLARGSLVGGGRQRPPQTPLRLLVDADNCLHRLYG
GFYTDWVSGGQWNHMLGYLAALAKACFGGNIELFVFFNGALEKARLHEWVKRQGNER
QTAQQIVSHVQNKGTPPPKVWFLPPVCMAHCIRLALIRFHVKVAQSIEDHHQEVIGFCREN
GFHGLVAYDSDYALCNIPYYFSAHALKLSRNGKSLTTSQYLMHEVAKQLDLNPNRFPIFAA
LLGNHILPDEDLASFHWSLLGPEHPLASLKVRAHQLVLPPCDVVIKAVADYVRNIQDTSDL
DAIAKDVFQHSQSRTDDKVIRFKRAIGYYSATSKPMSFHPPHYLAARPGPFGMPGMVPPH
VPPQMLNIPQTSLQAKPVAPQVPSPGGAPGQGPYPYSLSEPAPLTLDTSGKNLTEQNSYSNI
PHEGKHTPLYERSSPINPAQSGSPNHVDSAYFPGSSTSSSSDNDEGSGGATNHISGNKIGWE
KTGSHSEPQARGDPGDQTKAEGSSTASSGSQLAEGKGSQMGTVQPIPCLLSMPTRNHMDI
TTPPLPPVAPEVLRVAEHRHKKGLMYPYIFHVLTKGEIKIAVSIEDEANKDLPPAALLYRPV
RQYVYGVLFSLAESRKKTERLAFRKNRLPPEFSPVIIKEWAAYKGKSPQTPELVEALAFRE
WTCPNLKRLWLGKAVEDKNRRMRAFLACMRSDTPAMLNPANVPTHLMVLCCVLRYMV
QWPGARILRRQELDAFLAQALSPKLYEPDQLQELKIENLDPRGIQLSALFMSGVDMALFA
NDACGQPIPWEHCCPWMYFDGKLFQSKLLKASREKTPLIDLCDGQADQAAKVEKMRQS
VLEGLSFSRQSHTLPFPPPPALPFYPASAYPRHFGPVPPSQGRGRGFAGVCGFGGPYGETVA
TGPYRAFRVAAASGHCGAFSGSDSSRTSKSQGGVQPIPSQGGKLEIAGTVVGHWAGSRRG
RGGRGPFPLQVVSVGGPARGRPRGVISTPVIRTFGRGGRYYGRGYKNQAAIQGRPPYAAS
AEEVAKELKSKSGESKSSAMSSDGSLAENGVMAEEKPAPQMNGSTGDARAPSHSESALN
NDSKTCNTNPHLNALSTDSACRREAALEAAVLNKEE

>Q8NGR3 multipass membrane protein
MEAANESSEGISFVLLGLTTSPGQQRPLFVLFLLLYVASLLGNGLIVAAIQASPALHAPMYF
LLAHLSFADLCFASVTVPKMLANLLAHDHSISLAGCLTQMYFFFALGVTDSCLLAAMAYD
CYVAIRHPLPYATRMSRAMCAALVGMAWLVSHVHSLLYILLMARLSFCASHQVPHFFCDH
QPLLRLSCSDTHHIQLLIFTEGAAVVVTPFLLILASYGAIAAAVLQLPSASGRLRAVSTCGSH
LAVVSLFYGTVIAVYFQATSRREAEWGRVATVMYTVVTPMLNPIIYSLWNRDVQGALRAL
LIGRRISASDS

>Q9GZQ6 multipass membrane protein
MEGEPSQPPNSSWPLSQNGTNTEATPATNLTFSSYYQHTSPVAAMFIVAYALIFLLCMVGNT
LVCFIVLKNRHMHTVTNMFILNLAVSDLLVGIFCMPTTLVDNLITGWPFDNATCKMSGLV

QGMSVSASVFTLVAIAVERFRCIVHPFREKLTLRKALVTIAVIWALALLIMCPSAVTLTVTRE
EHHFMVDARNRSYPLYSCWEAWPEKGMRRVYTTVLFSHIYLAPLALIVVMYARIARKLC
QAPGPAPGGEEAADPRASRRRARVVHMLVMVALFFTLSWLPLWALLLLIDYGQLSAPQLH
LVTVYAFPFAHWLAFFNSSANPIIYGYFNENFRRGFQAAFRARLCPRPSGSHKEAYSERPG
GLLHRRVFVVVRPSDSGLPSESGPSSGAPRPGRLPLRNGRVAHHGLPREGPGCSHLPLTIPA
WDI

>Q9HCJ2 single-pass type I membrane protein
MLNKMTLHPQQIMIGPRFNRALFDPLLVVLLALQLLVVAGLVRAQTCPSVCSCSNQFSKVI
CVRKNLREVPDGISTNTRLLNLHENQIQIIKVNSFKHLRHLEILQLSRNHIRTIEIGAFNGLA
NLNTLELFDNRLTTIPNGAFVYLSKLKELWLRNNPIESIPSYAFNRIPSLRRLDLGELKRLSY
ISEGAFEGLSNLRYLNLAMCNLREIPNLTPLIKLDELDLSGNHLSAIRPGSFQGLMHLQKL
WMIQSQIQVIERNAFDNLQSLVEINLAHNNLTLLPHDLFTPLHHLERIHLHHNPWNCNCDI
LWLSWWIKDMAPSNTACCARCNTPPNLKGRYIGELDQNYFTCYAPVIVEPPADLNVTEG
MAAELKCRASTSLTSVSWITPNGTVMTHGAYKVRIAVLSDGTLNFTNVTVQDTGMYTCM
VSNSVGNTTASATLNVTAATTTPFSYFSTVTVETMEPSQDEARTTDNNVGPTPVVDWETT
NVTTSLTPQSTRSTEKTFTIPVTDINSGIPGIDEVMKTTKIIIGCFVAITLMAAVMLVIFYKMR
KQHHRQNHHAPTRTVEIINVDDEITGDTPMESHLPMPAIEHEHLNHYNSYKSPFNHTTTVN
TINSIHSSVHEPLLIRMNSKDNVQETQI

>Q53GQ0 multipass membrane protein
MESALPAAGFLYWVGAGTVAYLALRISYSLFTALRVWGVGNEAGVGPGLGEWAVVTGST
DGIGKSYAEELAKHGMKVVLISRSKDKLDQVSSEIKEKFKVETRTIAVDFASEDIYDKIKTG
LAGLEIGILVNNVGMSYEYPEYFLDVPDLDNVIKKMININILSVCKMTQLVLPGMVERSKG
AILNISSGSGMLPVPLLTIYSATKTFVDFFSQCLHEEYRSKGVFVQSVLPYFVATKLAKIRKP
TLDKPSPETFVKSAIKTVGLQSRTNGYLIHALMGSIISNLPSWIYLKIVMNMNKSTRAHYL
KKTKKN

>P43004 multipass membrane protein
MASTEGANNMPKQVEVRMHDSHLGSEEPKHRHLGLRLCDKLGKNLLLTLTVFGVILGAV
CGGLLRLASPIHPDVVMLIAFPGDILMRMLKMLILPLIISSLITGLSGLDAKASGRLGTRAM
VYYMSTTIIAAVLGVILVLAIHPGNPKLKKQLGPGKKNDEVSSLDAFLDLIRNLFPENLVQ
ACFQQIQTVTKKVLVAPPPDEEANATSAVVSLLNETVTEVPEETKMVIKKGLEFKDGMNV
LGLIGFFIAFGIAMGKMGDQAKLMVDFFNILNEIVMKLVIMIMWYSPLGIACLICGKIIAIK
DLEVVARQLGMYMVTVIIGLIIHGGIFLPLIYFVVTRKNPFSFFAGIFQAWITALGTASSAGT
LPVTFRCLEENLGIDKRVTRFVLPVGATINMDGTALYEAVAAIFIAQMNGVVLDGGQIVTV
SLTATLASVGAASIPSAGLVTMLLILTAVGLPTEDISLLVAVDWLLDRMRTSVNVVGDSFGA
GIVYHLSKSELDTIDSQHRVHEDIEMTKTQSIYDDMKNHRESNSNQCVYAAHNSVIVDEC
KVTLAANGKSADCSVEEEPWKREK

>P16070 single-pass type I membrane protein
MDKFWWHAAWGLCLVPLSLAQIDLNITCRFAGVFHVEKNGRYSISRTEAADLCKAFNSTL
PTMAQMEKALSIGFETCRYGFIEGHVVIPRIHPNSICAANNTGVYILTSNTSQYDTYCFNAS
APPEEDCTSVTDLPNAFDGPITITIVNRDGTRYVQKGEYRTNPEDIYPSNPTDDDVSSGSSS

ERSSTSGGYIFYTFSTVHPIPDEDSPWITDSTDRIPATTLMSTSATATETATKRQETWDWFSW
LFLPSESKNHLHTTTQMAGTSSNTISAGWEPNEENEDERDRHLSFSGSGIDDDEDFISSTIS
TTPRAFDHTKQNQDWTQWNPSHSNPEVLLQTTTRMTDVDRNGTTAYEGNWNPEAHPPLI
HHEHHEEEETPHSTSTIQATPSSTTEETATQKEQWFGNRWHEGYRQTPKEDSHSTTGTAAA
SAHTSHPMQGRTTPSPEDSSWTDFFNPISHPMGRGHQAGRRMDMDSSHSITLQPTANPNT
GLVEDLDRTGPLSMTTQQSNSQSFSTSHEGLEEDKDHPTTSTLTSSNRNDVTGGRRDPNHS
EGSTTLLEGYTSHYPHTKESRTFIPVTSAKTGSFGVTAVTVGDSNSNVNRSLSGDQDTFHP
SGGSHTTHGSESDGHSHGSQEGGANTTSGPIRTPQIPEWLIILASLLALALILAVCIAVNSRR
RCGQKKKLVINSGNGAVEDRKPSGLNGEASKSQEMVHLVNKESSETPDQFMTADETRNL
QNVDMKIGV

>O95221 multipass membrane protein
MTRKNYTSLTEFVLLGLADTLELQIILFLFFLVIYTLTVLGNLGMILLIRIDSQLHTPMYFFL
ANLSFVDVCNSTTITPKMLADLLSEKKTISFAGCFLQMYFFISLATTECILFGLMAYDRYAA
ICRPLLYSLIMSRTVYLKMAAGAFAAGLLNFMVNTSHVSSLSFCDSNVIHHFFCDSPPLFKL
SCSDTILKESISSILAGVNIVGTLLVILSSYSYVLFSIFSMHSGEGRHRAFSTCASHLTAIILFY
ATCIYTYLRPSSSYSLNQDKVASVFYTVVIPMLNPLIYSLRSKEVKKALANVISRKRTSSFL

>Q9NUN7 multipass membrane protein
MAPAADREGYWGPTTSTLDWCEENYSVTWYIAEFWNTVSNLIMIIPPMFGAVQSVRDGL
EKRYIASYLALTVVGMGSWCFHMTLKYEMQLLDELPMIYSCCIFVYCMFECFKIKNSVNY
HLLFTLVLFSLIVTTVYLKVKEPIFHQVMYGMLVFTLVLRSIYIVTWVYPWLRGLGYTSLG
IFLLGFLFWNIDNIFCESLRNFRKKVPPIIGITTQFHAWWHILTGLGSYLHILFSLYTRTLYLR
YRPKVKFLFGIWPVILFEPLRKH

>Q6IAA8 lipid-anchor
MGCCYSSENEDSDQDREERKLLLDPSSPPTKALNGAEPNYHSLPSARTDEQALLSSILAKT
ASNIIDVSAADSQGMEQHEYMDRARQYSTRLAVLSSSLTHWKKLPPLPSLTSQPHQVLAS
EPIPFSDLQQVSRIAAYAYSALSQIRVDAKEELVVQFGIP

>Q03395 multipass membrane protein
MAPVLPLVLPLQPRIRLAQGLWLLSWLLALAGGVILLCSGHLLVQLRHLGTFLAPSCQFPV
LPQAALAAGAVALGTGLVGVGASRASLNAALYPPWRGVLGPLLVAGTAGGGGLLVVGLG
LALALPGSLDEALEEGLVTALAHYKDTEVPGHCQAKRLVDELQLRYHCCGRHGYKDWF
GVQWVSSRYLDPGDRDVADRIQSNVEGLYLTDGVPFSCCNPHSPRPCLQNRLSDSYAHPL
FDPRQPNQNLWAQGCHEVLLEHLQDLAGTLGSMLAVTFLLQALVLLGLRYLQTALEGLG
GVIDAGGETQGYLFPSGLKDMLKTAWLQGGVACRPAPEEAPPGEAPPKEDLSEA

>P60852 single-pass type I membrane protein
MAGGSATTWGYPVALLLLVATLGLGRWLQPDPGLPGLRHSYDCGIKGMQLLVFPRPGQTL
RFKVVDEFGNRFDVNNCSICYHWVTSRPQEPAVFSADYRGCHVLEKDGRFHLRVFMEAV
LPNGRVDVAQDATLICPKPDPSRTLDSQLAPPAMFSVSTPQTLSFLPTSGHTSQGSGHAFPS
PLDPGHSSVHPTPALPSPGPGPTLATLAQPHWGTLEHWDVNKRDYIGTHLSQEQCQVASG
HLPCIVRRTSKEACQQAGCCYDNTREVPCYYGNTATVQCFRDGYFVLVVSQEMALTHRIT

LANIHLAYAPTSCSPTQHTEAFVVFYFPLTHCGTTMQVAGDQLIYENWLVSGIHIQKGPQG
SITRDSTFQLHVRCVFNASDFLPIQASIFPPPSPAPMTQPGPLRLELRIAKDETFSSYYGEDD
YPIVRLLREPVHVEVRLLQRTDPNLVLLLHQCWGAPSANPFQQPQWPILSDGCPFKGDSY
RTQMVALDGATPFQSHYQRFTVATFALLDSGSQRALRGLVYLFCSTSACHTSGLETCSTAC
STGTTRQRRSSGHRNDTARPQDIVSSPGPVGFEDSYGQEPTLGPTDSNGNSSLRPLLWAVL
LLPAVALVLGFGVFVGLSQTWAQKLWESNRQ

>Q01362 multipass membrane protein
MDTESNRRANLALPQEPSSVPAFEVLEISPQEVSSGRLLKSASSPPLHTWLTVLKKEQEFLG
VTQILTAMICLCFGTVVCSVLDISHIEGDIFSSFKAGYPFWGAIFFSISGMLSIISERRNATYL
VRGSLGANTASSIAGGTGITILIINLKKSLAYIHIHSCQKFFETKCFMASFSTEIVVMMLFLTI
LGLGSAVSLTICGAGEELKGNKVPEDRVYEELNIYSATYSELEDPGEMSPPIDL

>O14681 multipass membrane protein
MADSVKTFLQDLARGIKDSIWGICTISKLDARIQQKREEQRRRRASSVLAQRRAQSIERKQ
ESEPRIVSRIFQCCAWNGGVFWFSLLLFYRVFIPVLQSVTARIIGDPSLHGDVWSWLEFFLT
SIFSALWVLPLFVLSKVVNAIWFQDIADLAFEVSGRKPHPFPSVSKIIADMLFNLLLQALFLI
QGMFVSLFPIHLVGQLVSLLHMSLLYSLYCFEYRWFNKGIEMHQRLSNIERNWPYYFGFG
LPLAFLTAMQSSYIISGCLFSILFPLFIISANEAKTPGKAYLFQLRLFSLVVFLSNRLFHKTVY
LQSALSSSTSAEKFPSPHPSPAKLKATAGH

>O60939 single-pass type I membrane protein
MHRDAWLPRPAFSLTGLSLFFSLVPPGRSMEVTVPATLNVLNGSDARLPCTFNSCYTVNHK
QFSLNWTYQECNNCSEEMFLQFRMKIINLKLERFQDRVEFSGNPSKYDVSVMLRNVQPED
EGIYNCYIMNPPDRHRGHGKIHLQVLMEEPPERDSTVAVIVGASVGGFLAVVILVLMVVK
CVRRKKEQKLSTDDLKTEEEGKTDGEGNPDDGAK

>Q8WWT9 multipass membrane protein
MAALAAAAKKVWSARRLLVLLFTPLALLPVVFALPPKEGRCLFVILLMAVYWCTEALPLS
VTALLPIVLFPFMGILPSNKVCPQYFLDTNFLFLSGLIMASAIEEWNLHRRIALKILMLVGV
QPARLILGMMVTTSFLSMWLSNTASTAMMLPIANAILKSLFGQKEVRKDPSQESEENTAA
VRRNGLHTVPTEMQFLASTEAKDHPGETEVPLDLPADSRKEDEYRRNIWKGFLISIPYSAS
IGGTATLTGTAPNLILLGQLKSFFPQCDVVNFGSWFIFAFPLMLLFLLAGWLWISFLYGGLS
FRGWRKNKSEIRTNAEDRARAVIREEYQNLGPIKFAEQAVFILFCMFAILLFTRDPKFIPGW
ASLFNPGFLSDAVTGVAIVTILFFFPSQRPSLKWWFDFKAPNTETEPLLTWKKAQETVPWN
IILLLGGGFAMAKGCEESGLSVWIGGQLHPLENVPPALAVLLITVVIAFFTEFASNTATIIIFL
PVLAELAIRLRVHPLYLMIPGTVGCSFAFMLPVSTPPNSIAFASGHLLVKDMVRTGLLMNL
MGVLLLSLAMNTWAQTIFQLGTFPDWADMYSVNVTALPPTLANDTFRTL

>Q969N2 single-pass type I membrane protein
MAAAMPLALLVLLLLGPGGWCLAEPPRDSLREELVITPLPSGDVAATFQFRTRWDSELQRE
GVSHYRLFPKALGQLISKYSLRELHLSFTQGFWRTRYWGPPFLQAPSGAELWVWFQDTVT
DVDKSWKELSNVLSGIFCASLNFIDSTNTVTPTASFKPLGLANDTDHYFLRYAVLPREVVC
TENLTPWKKLLPCSSKAGLSVLLKADRLFHTSYHSQAVHIRPVCRNARCTSISWELRQTLS

VVFDAFITGQGKKDWSLFRMFSRTLTEPCPLASESRVYVDITTYNQDNETLEVHPPPTTTY
QDVILGTRKTYAIYDLLDTAMINNSRNLNIQLKWKRPPENEAPPVPFLHAQRYVSGYGLQ
KGELSTLLYNTHPYRAFPVLLLDTVPWYLRLYVHTLTITSKGKENKPSYIHYQPAQDRLQP
HLLEMLIQLPANSVTKVSIQFERALLKWTEYTPDPNHGFYVSPSVLSALVPSMVAAKPVD
WEESPLFNSLFPVSDGSNYFVRLYTEPLLVNLPTPDFSMPYNVICLTCTVVAVCYGSFYNLL
TRTFHIEEPRTGGLAKRLANLIRRARGVPPL

>Q01970 peripheral membrane protein
MAGAQPGVHALQLEPPTVVETLRRGSKFIKWDEETSSRNLVTLRVDPNGFFLYWTGPNM
EVDTLDISSIRDTRTGRYARLPKDPKIREVLGFGGPDARLEEKLMTVVSGPDPVNTVFLNF
MAVQDDTAKVWSEELFKLAMNILAQNASRNTFLRKAYTKLKLQVNQDGRIPVKNILKMF
SADKKRVETALESCGLKFNRSESIRPDEFSLEIFERFLNKLCLRPDIDKILLEIGAKGKPYLTL
EQLMDFINQKQRDPRLNEVLYPPLRPSQARLLIEKYEPNQQFLERDQMSMEGFSRYLGGE
ENGILPLEALDLSTDMTQPLSAYFINSSHNTYLTAGQLAGTSSVEMYRQALLWGCRCVEL
DVWKGRPPEEEPFITHGFTMTTEVPLRDVLEAIAETAFKTSPYPVILSFENHVDSAKQQAK
MAEYCRSIFGDALLIEPLDKYPLAPGVPLPSPQDLMGRILVKNKKRHRPSAGGPDSAGRK
RPLEQSNSALSESSAATEPSSPQLGSPSSDSCPGLSNGEEVGLEKPSLEPQKSLGDEGLNRG
PYVLGPADREDEEEDEEEEEQTDPKKPTTDEGTASSEVNATEEMSTLVNYIEPVKFKSFEA
ARKRNKCFEMSSFVETKAMEQLTKSPMEFVEYNKQQLSRIYPKGTRVDSSNYMPQLFWN
VGCQLVALNFQTLDVAMQLNAGVFEYNGRSGYLLKPEFMRRPDKSFDPFTEVIVDGIVAN
ALRVKVISGQFLSDRKVGIYVEVDMFGLPVDTRRKYRTRTSQGNSFNPVWDEEPFDFPKV
VLPTLASLRIAAFEEGGKFVGHRILPVSAIRSGYHYVCLRNEANQPLCLPALLIYTEASDYI
PDDHQDYAEALINPIKHVSLMDQRARQLAALIGESEAQAGQETCQDTQSQQLGSQPSSNP
TPSPLDASPRRPPGPTTSPASTSLSSPGQRDDLIASILSEVAPTPLDELRGHKALVKLRSRQE
RDLRELRKKHQRKAVTLTRRLLDGLAQAQAEGRCRLRPGALGGAADVEDTKEGEDEAK
RYQEFQNRQVQSLLELREAQVDAEAQRRLEHLRQALQRLREVVLDANTTQFKRLKEMN
EREKKELQKILDRKRHNSISEAKMRDKHKKEAELTEINRRHITESVNSIRRLEEAQKQRHD
RLVAGQQQVLQQLAEEEPKLLAQLAQECQEQRARLPQEIRRSLLGEMPEGLGDGPLVACA
SNGHAPGSSGHLSGADSESQEENTQL

>O14613 peripheral membrane protein
MSTKVPIYLKRGSRKGKKEKLRDLLSSDMISPPLGDFRHTIHIGSGGGSDMFGDISFLQGK
FHLLPGTMVEGPEEDGTFDLPFQFTRTATVCGRELPDGPSPLLKNAISLPVIGGPQALTLPT
AQAPPKPPRLHLETPQPSPQEGGSVDIWRIPETGSPNSGLTPESGAEEPFLSNASSLLSLHVD
LGPSILDDVLQIMDQDLDSMQIPT

>O76062 multipass membrane protein
MAPTQGPRAPLEFGGPLGAAALLLLLPATMFHLLLAARSGPARLLGPPASLPGLEVLWSPR
ALLLWLAWLGLQAALYLLPARKVAEGQELKDKSRLRYPINGFQALVLTALLVGLGMSAGL
PLGALPEMLLPLAFVATLTAFIFSLFLYMKAQVAPVSALAPGGNSGNPIYDFFLGRELNPRIC
FFDFKYFCELRPGLIGWVLINLALLMKEAELRGSPSLAMWLVNGFQLLYVGDALWHEEA
VLTTMDITHDGFGFMLAFGDMAWVPFTYSLQAQFLLHHPQPLGLPMASVICLINATGYYI
FRGANSQKNTFRKNPSDPRVAGLETISTATGRKLLVSGWWGMVRHPNYLGDLIMALAWS
LPCGVSHLLPYFYLLYFTALLVHREARDERQCLQKYGLAWQEYCRRVPYRIMPYIY

>Q13224 multipass membrane protein
MKPRAECCSPKFWLVLAVLAVSGSRARSQKSPPSIGIAVILVGTSDEVAIKDAHEKDDFHHL
SVVPRVELVAMNETDPKSIITRICDLMSDRKIQGVVFADDTDQEAIAQILDFISAQTLTPILGI
HGGSSMIMADKDESSMFFQFGPSIEQQASVMLNIMEEYDWYIFSIVTTYFPGYQDFVNKIR
STIENSFVGWELEEVLLLDMSLDDGDSKIQNQLKKLQSPIILLYCTKEEATYIFEVANSVGL
TGYGYTWIVPSLVAGDTDTVPAEFPTGLISVSYDEWDYGLPARVRDGIAIITTAASDMLSEH
SFIPEPKSSCYNTHEKRIYQSNMLNRYLINVTFEGRNLSFSEDGYQMHPKLVIILLNKERKW
ERVGKWKDKSLQMKYYVWPRMCPETEEQEDDHLSIVTLEEAPFVIVESVDPLSGTCMRN
TVPCQKRIVTENKTDEEPGYIKKCCKGFCIDILKKISKSVKFTYDLYLVTNGKHGKKINGT
WNGMIGEVVMKRAYMAVGSLTINEERSEVVDFSVPFIETGISVMVSRSNGTVSPSAFLEPF
SADVWVMMFVMLLIVSAVAVFVFEYFSPVGYNRCLADGREPGGPSFTIGKAIWLLWGLVF
NNSVPVQNPKGTTSKIMVSVWAFFAVIFLASYTANLAAFMIQEEYVDQVSGLSDKKFQRP
NDFSPPFRFGTVPNGSTERNIRNNYAEMHAYMGKFNQRGVDDALLSLKTGKLDAFIYDAA
VLNYMAGRDEGCKLVTIGSGKVFASTGYGIAIQKDSGWKRQVDLAILQLFGDGEMEELE
ALWLTGICHNEKNEVMSSQLDIDNMAGVFYMLGAAMALSLITFICEHLFYWQFRHCFMG
VCSGKPGMVFSISRGIYSCIHGVAIEERQSVMNSPTATMNNTHSNILRLLRTAKNMANLSG
VNGSPQSALDFIRRESSVYDISEHRRSFTHSDCKSYNNPPCEENLFSDYISEVERTFGNLQL
KDSNVYQDHYHHHHRPHSIGSASSIDGLYDCDNPPFTTQSRSISKKPLDIGLPSSKHSQLSD
LYGKFSFKSDRYSGHDDLIRSDVSDISTHTVTYGNIEGNAAKRRKQQYKDSLKKRPASAK
SRREFDEIELAYRRRPPRSPDHKRYFRDKEGLRDFYLDQFRTKENSPHWEHVDLTDIYKER
SDDFKRDSVSGGGPCTNRSHIKHGTGDKHGVVSGVPAPWEKNLTNVEWEDRSGGNFCRS
CPSKLHNYSTTVTGQNSGRQACIRCEACKKAGNLYDISEDNSLQELDQPAAPVAVTSNAST
TKYPQSPTNSKAQKKNRNKLRRQHSYDTFVDLQKEEAALAPRSVSLKDKGRFMDGSPYA
HMFEMSAGESTFANNKSSVPTAGHHHHNNPGGGYMLSKSLYPDRVTQNPFIPTFGDDQCL
LHGSKSYFFRQPTVAGASKARPDFRALVTNKPVVSALHGAVPARFQKDICIGNQSNPCVPN
NKNPRAFNGSSNGHVYEKLSSIESDV

>Q8NGG5 multipass membrane protein
MNHVVKHNHTAVTKVTEFILMGITDNPGLQAPLFGLFLIIYLVTVIGNLGMVILTYLDSKL
HTPMYFFLRHLSITDLGYSTVIAPKMLVNFIVHKNTISYNWYATQLAFFEIFIISELFILSAMA
YDRYVAICKPLLYVIIMAEKVLWVLVIVPYLYSTFVSLFLTIKLFKLSFCGSNIISYFYCDCIP
LMSILCSDTNELELIILIFSGCNLLFSLSIVLISYMFILVAILRMNSRKGRYKAFSTCSSHLTVV
IMFYGTLLFIYLQPKSSHTLAIDKMASVFYTLLIPMLNPLIYSLRNKEVKDALKRTLTNRFK
IPI

>Q8NGP3 multipass membrane protein
MPNFTDVTEFTLLGLTCRQELQVLFFVVFLAVYMITLLGNIGMIILISISPQLQSPMYFFLSH
LSFADVCFSSNVTPKMLENLLSETKTISYVGCLVQCYFFIAVVHVEVYILAVMAFDRYMAG
CNPLLYGSKMSRTVCVRLISVPYVYGFSVSLICTLWTYGLYFCGNFEINHFYCADPPLIQIA
CGRVHIKEITMIVIAGINFTYSLSVVLISYTLIVVAVLRMRSADGRRKAFSTCGSHLTAVSMF
YGTPIFMYLRRPTEESVEQGKMVAVFYTTVIPMLNPMIYSLRNKDVKEAVNKAITKTYVR
Q

>P08913 multipass membrane protein
MGSLQPDAGNASWNGTEAPGGGARATPYSLQVTLTLVCLAGLLMLLTVFGNVLVIIAVFT
SRALKAPQNLFLVSLASADILVATLVIPFSLANEVMGYWYFGKAWCEIYLALDVLFCTSSIV
HLCAISLDRYWSITQAIEYNLKRTPRRIKAIIITVWVISAVISFPPLISIEKKGGGGGPQPAEPR
CEINDQKWYVISSCIGSFFAPCLIMILVYVRIYQIAKRRTRVPPSRRGPDAVAAPPGGTERRP
NGLGPERSAGPGGAEAEPLPTQLNGAPGEPAPAGPRDTDALDLEESSSSDHAERPPGPRRP
ERGPRGKGKARASQVKPGDSLPRRGPGATGIGTPAAGPGEERVGAAKASRWRGRQNREK
RFTFVLAVVIGVFVVCWFPFFFTYTLTAVGCSVPRTLFKFFFWFGYCNSSLNPVIYTIFNHD
FRRAFKKILCRGDRKRIV

>Q5SZK8 single-pass type I membrane protein
MHSAGTPGLSSRRTGNSTSFQPGPPPPPRLLLLLLLLLSLVSRVPAQPAAFGRALLSPGLAG
AAGVPAEEAIVLANRGLRVPFGREVWLDPLHDLVLQVQPGDRCAVSVLDNDALAQRPGR
LSPKRFPCDFGPGEVRYSHLGARSPSRDRVRLQLRYDAPGGAVVLPLVLEVEVVFTQLEV
VTRNLPLVVEELLGTSNALDARSLEFAFQPETEECRVGILSGLGALPRYGELLHYPQVPGG
AREGGAPETLLMDCKAFQELGVRYRHTAASRSPNRDWIPMVVELRSRGAPVGSPALKRE
HFQVLVRIRGGAENTAPKPSFVAMMMMEVDQFVLTALTPDMLAAEDAESPSDLLIFNLTSP
FQPGQGYLVSTDDRSLPLSSFTQRDLRLLKIAYQPPSEDSDQERLFELELEVVDLEGAASDP
FAFMVVVKPMNTMAPVVTRNTGLILYEGQSRPLTGPAGSGPQNLVISDEDDLEAVRLEVV
AGLRHGHLVILGASSGSSAPKSFTVAELAAGQVVYQHDDRDGSLSDNLVLRMVDGGGRH
QVQFLFPITLVPVDDQPPVLNANTGLTLAEGETVPILPLSLSATDMDSDDSLLLFVLESPFLT
TGHLLLRQTHPPHEKQELLRGLWRKEGAFYERTVTEWQQQDITEGRLFYRHSGPHSPGPV
TDQFTFRVQDNHDPPNQSGLQRFVIRIHPVDRLPPELGSGCPLRMVVQESQLTPLRKKWLR
YTDLDTDDRELRYTVTQSPTDTDENHLPAPLGTLVLTDNPSVVVTHFTQAQINHHKIAYRP
PGQELGVATRVAQFQFQVEDRAGNVAPGTFTLYLHPVDNQPPEILNTGFTIQEKGHHILSET
ELHVNDVDTDVAHISFTLTQAPKHGHMRVSGQILHVGGLFHLEDIKQGRVSYAHNGDKSL
TDSCSLEVSDRHHVVPITLRVNVRPVDDEVPILSHPTGTLESYLDVLENGATEITANVIKGT
NEETDDLMLTFLLEDPPLYGEILVNGIPAEQFTQRDILEGSVVYTHTSGEIGLLPKADSFNLS
LSDMSQEWRIGGNTIQGVTIWVTILPVDSQAPEIFVGEQLIVMEGDKSVITSVHISAEDVDS
LNDDILCTIVIQPTSGYVENISPAPGSEKSRAGIAISAFNLKDLRQGHINYVQSVHKGVEPV
EDRFVFRCSDGINFSERQFFPIVIIPTNDEQPEMFMREFMVMEGMSLVIDTPILNAADADVP
LDDLTFTITQFPTHGHIMNQLINGTVLVESFTLDQIIESSSIIYEHDDSETQEDSFVIKLTDGK
HSVEKTVLIIVIPVDDETPRMTINNGLEIEIGDTKIINNKILMATDLDSEDKSLVYIIRYGPGH
GLLQRRKPTGAFENITLGMNFTQDEVDRNLIQYVHLGQEGIRDLIKFDVTDGINPLIDRYF
YVSIGSIDIVFPDVISKGVSLKEGGKVTLTTDLLSTSDLNSPDENLVFTITRAPMRGHLECTD
QPGVSITSFTQLQLAGNKIYYIHTADDEVKMDSFEFQVTDGRNPVFRTFRISISDVDNKKPV
VTIHKLVVSESENKLITPFELTVEDRDTPDKLLKFTITQVPIHGHLLFNNTRPVMVFTKQDL
NENLISYKHDGTESSEDSFSFTVTDGTHTDFYVFPDTVFETRRPQVMKIQVLAVDNSVPQI
AVNKGASTLRTLATGHLGFMITSKILKVEDRDSLHISLRFIVTEAPQHGYLLNLDKGNHSIT
QFTQADIDDMKICYVLREGANATSDMFYFAVEDGGGNKLTYQNFRLNWAWISFEKEYYL
VNEDSKFLDVVLKRRGYLGETSFISIGTRDRTAEKDKDFKGKAQKQVQFNPGQTRATWR
VRILSDGEHEQSETFQVVLSEPVLAALEFPTVATVEIVDPGDEPTVFIPQSKYSVEEDVGEL
FIPIRRSGDVSQELMVVCYTQQGTATGTVPTSVLSYSDYISRPEDHTSVVRFDKDEREKLC
RIVIIDDSLYEEEETFHVLLSMPMGGRIGSEFPGAQVTIVPDKDDEPIFYFGDVEYSVDESA

GYVEVQVWRTGTDLSKSSSVTVRSRKTDPPSADAGTDYVGISRNLDFAPGVNMQPVRVV
ILDDLGQPALEGIEKFELVLRMPMNAALGEPSKATVSINDSVSDLPKMQFKERIYTGSESD
GQIVTMIHRTGDVQYRSSVRCYTRQGSAQVMMDFEERPNTDTSIITFLPGETEKPCILELM
DDVLYEEVEELRLVLGTPQSNSPFGAAVGEQNETLIRIRDDADKTVIKFGETKFSVTEPKEP
GESVVIRIPVIRQGDTSKVSIVRVHTKDGSATSGEDYHPVSEEIEFKEGETQHVVEIEVTFD
GVREMREAFTVHLKPDENMIAEMQLTKAIVYIEEMSSMADVTFPSVPQIVSLLMYDDTSK
AKESAEPMSGYPVICITACNPKYSDYDKTGSICASENINDTLTRYRWLISAPAGPDGVTSPM
REVDFDTFFTSSKMVTLDSIYFQPGSRVQCAARAVNTNGDEGLELMSPIVTISREEGLCQPR
VPGVVGAEPFSAKLRYTGPEDADYTNLIKLTVTMPHIDGMLPVISTRELSNFELTLSPDGTR
VGNHKCSNLLDYTEVKTHYGFLTDATKNPEIIGETYPYQYSLSIRGSTTLRFYRNLNLEAC
LWEFVSYYDMSELLADCGGTIGTDGQVLNLVQSYVTLRVPLYVSYVFHSPVGVGGWQHF
DLKSELRLTFVYDTAILWNDGIGSPPEAELQGSLYPTSMRIGDEGRLAVHFKTEAQFHGLF
VLSHPASFTSSVIMSADHPGLTFSLRLIRSEPTYNQPVQQWSFVSDFAVRDYSGTYTVKLVP
CTAPSHQEYRLPVTCNPREPVTFDLDIRFQQVSDPVAAEFSLNTQMYLLSKKSLWLSDGS
MGFGQESDVAFAEGDIIYGRVMVDPVQNLGDSFYCSIEKVFLCTGADGYVPKYSPMNAE
YGCLADSPSLLYRFKIVDKAQPETQATSFGNVLFNAKLAVDDPEAILLVNQPGSDGFKVDS
TPLFQVALGREWYIHTIYTVRSKDNANRGIGKRSVEYHSLVSQGKPQSTTKSRKKREIRST
PSLAWEIGAENSRGTNIQHIALDRTKRQIPHGRAPPDGILPWELNSPSSAVSLVTVVGGTTV
GLLTICLTVIAVLMCRGKESFRGKDAPKGSSSSEPMVPPQSHHNDSSEV

>Q9NZV1 single-pass type I membrane protein
MYLVAGDRGLAGCGHLLVSLLGLLLLLARSGTRALVCLPCDESKCEEPRNCPGSIVQGVC
GCCYTCASQRNESCGGTFGIYGTCDRGLRCVIRPPLNGDSLTEYEAGVCEDENWTDDQLL
GFKPCNENLIAGCNIINGKCECNTIRTCSNPFEFPSQDMCLSALKRIEEEKPDCSKARCEVQ
FSPRCPEDSVLIEGYAPPGECCPLPSRCVCNPAGCLRKVCQPGNLNILVSKASGKPGECCDL
YECKPVFGVDCRTVECPPVQQTACPPDSYETQVRLTADGCCTLPTRCECLSGLCGFPVCEV
GSTPRIVSRGDGTPGKCCDVFECVNDTKPACVFNNVEYYDGDMFRMDNCRFCRCQGGV
AICFTAQCGEINCERYYVPEGECCPVCEDPVYPFNNPAGCYANGLILAHGDRWREDDCTF
CQCVNGERHCVATVCGQTCTNPVKVPGECCPVCEEPTIITVDPPACGELSNCTLTGKDCIN
GFKRDHNGCRTCQCINTEELCSERKQGCTLNCPFGFLTDAQNCEICECRPRPKKCRPIICDK
YCPLGLLKNKHGCDICRCKKCPELSCSKICPLGFQQDSHGCLICKCREASASAGPPILSGTC
LTVDGHHHKNEESWHDGCRECYCLNGREMCALITCPVPACGNPTIHPGQCCPSCADDFV
VQKPELSTPSICHAPGGEYFVEGETWNIDSCTQCTCHSGRVLCETEVCPPLLCQNPSRTQD
SCCPQCTDQPFRPSLSRNNSVPNYCKNDEGDIFLAAESWKPDVCTSCICIDSVISCFSESCPS
VSCERPVLRKGQCCPYCIEDTIPKKVVCHFSGKAYADEERWDLDSCTHCYCLQGQTLCST
VSCPPLPCVEPINVEGSCCPMCPEMYVPEPTNIPIEKTNHRGEVDLEVPLWPTPSENDIVHL
PRDMGHLQVDYRDNRLHPSEDSSLDSIASVVVPIIICLSIIIAFLFINQKKQWIPLLCWYRTP
TKPSSLNNQLVSVDCKKGTRVQVDSSQRMLRIAEPDARFSGFYSMQKQNHLQADNFYQT
V

>Q9NP78 multipass membrane protein
MRLWKAVVVTLAFMSVDICVTTAIYVFSHLDRSLLEDIRHFNIFDSVLDLWAACLYRSCLL
LGATIGVAKNSALGPRRLRASWLVITLVCLFVGIYAMVKLLLFSEVRRPIRDPWFWALFVW
TYISLGASFLLWWLLSTVRPGTQALEPGAATEAEGFPGSGRPPPEQASGATLQKLLSYTKP

DVAFLVAASFFLIVAALGETFLPYYTGRAIDGIVIQKSMDQFSTAVVIVCLLAIGSSFAAGIR
GGIFTLIFARLNIRLRNCLFRSLVSQETSFFDENRTGDLISRLTSDTTMVSDLVSQNINVFLRN
TVKVTGVVVFMFSLSWQLSLVTFMGFPIIMMVSNIYGKYYKRLSKEVQNALARASNTAE
ETISAMKTVRSFANEEEEAEVYLRKLQQVYKLNRKEAAAYMYYVWGSGLTLLVVQVSIL
YYGGHLVISGQMTSGNLIAFIIYEFVLGDCMESVGSVYSGLMQGVGAAEKVFEFIDRQPT
MVHDGSLAPDHLEGRVDFENVTFTYRTRPHTQVLQNVSFSLSPGKVTALVGPSGSGKSSC
VNILENFYPLEGGRVLLDGKPISAYDHKYLHRVISLVSQEPVLFARSITDNISYGLPTVPFEM
VVEAAQKANAHGFIMELQDGYSTETGEKGAQLSGGQKQRVAMARALVRNPPVLILDEAT
SALDAESEYLIQQAIHGNLQKHTVLIIAHRLSTVEHAHLIVVLDKGRVVQQGTHQQLLAQ
GGLYAKLVQRQMLGLQPAADFTAGHNEPVANGSHKA

>Q9UPY5 multipass membrane protein
MVRKPVVSTISKGGYLQGNVNGRLPSLGNKEPPGQEKVQLKRKVTLLRGVSIIIGTIIGAGI
FISPKGVLQNTGSVGMSLTIWTVCGVLSLFGALSYAELGTTIKKSGGHYTYILEVFGPLPAF
VRVWVELLIIRPAATAVISLAFGRYILEPFFIQCEIPELAIKLITAVGITVVMVLNSMSVSWSA
RIQIFLTFCKLTAILIIIVPGVMQLIKGQTQNFKDAFSGRDSSITRLPLAFYYGMYAYAGWFY
LNFVTEEVENPEKTIPLAICISMAIVTIGYVLTNVAYFTTINAEELLLSNAVAVTFSERLLGNF
SLAVPIFVALSCFGSMNGGVFAVSRLFYVASREGHLPEILSMIHVRKHTPLPAVIVLHPLTMI
MLFSGDLDSLLNFLSFARWLFIGLAVAGLIYLRYKCPDMHRPFKVPLFIPALFSFTCLFMVA
LSLYSDPFSTGIGFVITLTGVPAYYLFIIWDKKPRWFRIMSEKITRTLQIILEVVPEEDKL

>P17658 multipass membrane protein
MRSEKSLTLAAPGEVRGPEGEQQDAGDFPEAGGGGGCCSSERLVINISGLRFETQLRTLSL
FPDTLLGDPGRRVRFFDPLRNEYFFDRNRPSFDAILYYYQSGGRLRRPVNVPLDIFLEEIRF
YQLGDEALAAFREDEGCLPEGGEDEKPLPSQPFQRQVWLLFEYPESSGPARGIAIVSVLVIL
ISIVIFCLETLPQFRVDGRGGNNGGVSRVSPVSRGSQEEEEDEDDSYTFHHGITPGEMGTGG
SSSLSTLGGSFFTDPFFLVETLCIVWFTFELLVRFSACPSKPAFFRNIMNIIDLVAIFPYFITLG
TELVQQQEQQPASGGGGQNGQQAMSLAILRVIRLVRVFRIFKLSRHSKGLQILGKTLQASM
RELGLLIFFLFIGVILFSSAVYFAEADDDDSLFPSIPDAFWWAVVTMTTVGYGDMYPMTVG
GKIVGSLCAIAGVLTIALPVPVIVSNFNYFYHRETEQEEQGQYTHVTCGQPAPDLRATDNG
LGKPDFPEANRERRPSYLPTPHRAYAEKRMLTEV

>Q96QE2 multipass membrane protein
MSRKASENVEYTLRSLSSLMGERRRKQPEPDAASAAGECSLLAAAESSTSLQSAGAGGG
GVGDLERAARRQFQQDETPAFVYVVAVFSALGGFLFGYDTGVVSGAMLLLKRQLSLDAL
WQELLVSSTVGAAAVSALAGGALNGVFGRRAAILLASALFTAGSAVLAAANNKETLLAG
RLVVGLGIGIASMTVPVYIAEVSPPNLRGRLVTINTLFITGGQFFASVVDGAFSYLQKDGW
RYMLGLAAVPAVIQFFGFLFLPESPRWLIQKGQTQKARRILSQMRGNQTIDEEYDSIKNNIE
EEEKEVGSAGPVICRMLSYPPTRRALIVGCGLQMFQQLSGINTIMYYSATILQMSGVEDDR
LAIWLASVTAFTNFIFTLVGVWLVEKVGRRKLTFGSLAGTTVALIILALGFVLSAQVSPRITF
KPIAPSGQNATCTRYSYCNECMLDPDCGFCYKMNKSTVIDSSCVPVNKASTNEAAWGRC
ENETKFKTEDIFWAYNFCPTPYSWTALLGLILYLVFFAPGMGPMPWTVNSEIYPLWARSTG
NACSSGINWIFNVLVSLTFLHTAEYLTYYGAFFLYAGFAAVGLLFIYGCLPETKGKKLEEIES
LFDNRLCTCGTSDSDEGRYIEYIRVKGSNYHLSDNDASDVE

>Q02487 single-pass type I membrane protein
MEAARPSGSWNGALCRLLLLTLAILIFASDACKNVTLHVPSKLDAEKLVGRVNLKECFTA
ANLIHSSDPDFQILEDGSVYTTNTILLSSEKRSFTILLSNTENQEKKKIFVFLEHQTKVLKKR
HTKEKVLRRAKRRWAPIPCSMLENSLGPFPLFLQQVQSDTAQNYTIYYSIRGPGVDQEPRN
LFYVERDTGNLYCTRPVDREQYESFEIIAFATTPDGYTPELPLPLIIKIEDENDNYPIFTEETY
TFTIFENCRVGTTVGQVCATDKDEPDTMHTRLKYSIIGQVPPSPTLFSMHPTTGVITTTSSQ
LDRELIDKYQLKIKVQDMDGQYFGLQTTSTCIINIDDVNDHLPTFTRTSYVTSVEENTVDV
EILRVTVEDKDLVNTANWRANYTILKGNENGNFKIVTDAKTNEGVLCVVKPLNYEEKQQ
MILQIGVVNEAPFSREASPRSAMSTATVTVNVEDQDEGPECNPPIQTVRMKENAEVGTTS
NGYKAYDPETRSSSGIRYKKLTDPTGWVTIDENTGSIKVFRSLDREAETIKNGIYNITVLAS
DQGGRTCTGTLGIILQDVNDNSPFIPKKTVIICKPTMSSAEIVAVDPDEPIHGPPFDFSLESST
SEVQRMWRLKAINDTAARLSYQNDPPFGSYVVPITVRDRLGMSSVTSLDVTLCDCITEND
CTHRVDPRIGGGGVQLGKWAILAILLGIALLFCILFTLVCGASGTSKQPKVIPDDLAQQNLI
VSNTEAPGDDKVYSANGFTTQTVGASAQGVCGTVGSGIKNGGQETIEMVKGGHQTSESC
RGAGHHHTLDSCRGGHTEVDNCRYTYSEWHSFTQPRLGEKVYLCNQDENHKHAQDYVL
TYNYEGRGSVAGSVGCCSERQEEDGLEFLDNLEPKFRTLAEACMKR

>Q96B42 multipass membrane protein
MPSAFSVSSFPVSIPAVLTQTDWTEPWLMGLATFHALCVLLTCLSSRSYRLQIGHFLCLVIL
VYCAEYINEAAAMNWRLFSKYQYFDSRGMFISIVFSAPLLVNAMIIVVMWVWKTLNVMT
DLKNAQERRKEKKRRRKED

>P16591 peripheral membrane protein
MGFGSDLKNSHEAVLKLQDWELRLLETVKKFMALRIKSDKEYASTLQNLCNQVDKESTV
QMNYVSNVSKSWLLMIQQTEQLSRIMKTHAEDLNSGPLHRLTMMIKDKQQVKKSYIGVH
QQIEAEMIKVTKTELEKLKCSYRQLIKEMNSAKEKYKEALAKGKETEKAKERYDKATMK
LHMLHNQYVLALKGAQLHQNQYYDITLPLLLDSLQKMQEEMIKALKGIFDEYSQITSLVT
EEIVNVHKEIQMSVEQIDPSTEYNNFIDVHRTTAAKEQEIEFDTSLLEENENLQANEIMWN
NLTAESLQVMLKTLAEELMQTQQMLLNKEEAVLELEKRIEESSETCEKKSDIVLLLSQKQA
LEELKQSVQQLRCTEAKFSAQKELLEQKVQENDGKEPPPVVNYEEDARSVTSMERKERL
SKFESIRHSIAGIIRSPKSALGSSALSDMISISEKPLAEQDWYHGAIPRIEAQELLKKQGDFLV
RESHGKPGEYVLSVYSDGQRRHFIIQYVDNMYRFEGTGFSNIPQLIDHHYTTKQVITKKSG
VVLLNPIPKDKKWILSHEDVILGELLGKGNFGEVYKGTLKDKTSVAVKTCKEDLPQELKIK
FLQEAKILKQYDHPNIVKLIGVCTQRQPVYIIMELVSGGDFLTFLRRKKDELKLKQLVKFSL
DAAAGMLYLESKNCIHRDLAARNCLVGENNVLKISDFGMSRQEDGGVYSSSGLKQIPIKW
TAPEALNYGRYSSESDVWSFGILLWETFSLGVCPYPGMTNQQAREQVERGYRMSAPQHC
PEDISKIMMKCWDYKPENRPKFSELQKELTIIKRKLT

>Q9H0C2 multipass membrane protein
MHREPAKKKAEKRLFDASSFGKDLLAGGVAAAVSKTAVAPIERVKLLLQVQASSKQISPEA
RYKGMVDCLVRIPREQGFFSFWRGNLANVIRYFPTQALNFAFKDKYKQLFMSGVNKEKQ
FWRWFLANLASGGAAGATSLCVVYPLDFARTRLGVDIGKGPEERQFKGLGDCIMKIAKSD
GIAGLYQGFGVSVQGIIVYRASYFGAYDTVKGLLPKPKKTPFLVSFFIAQVVTTCSGILSYP

FDTVRRRMMMQSGEAKRQYKGTLDCFVKIYQHEGISSFFRGAFSNVLRGTGGALVLVLY
DKIKEFFHIDIGGR

>Q16827 single-pass type I membrane protein
MGHLPTGIHGARRLLPLLWLFVLFKNATAFHVTVQDDNNIVVSLEASDVISPASVYVVKIT
GESKNYFFEFEEFNSTLPPPVIFKASYHGLYYIITLVVVNGNVVTKPSRSITVLTKPLPVTSV
SIYDYKPSPETGVLFEIHYPEKYNVFTRVNISYWEGKDFRTMLYKDFFKGKTVFNHWLPG
MCYSNITFQLVSEATFNKSTLVEYSGVSHEPKQHRTAPYPPQNISVRIVNLNKNNWEEQSG
NFPEESFMRSQDTIGKEKLFHFTEETPEIPSGNISSGWPDFNSSDYETTSQPYWWDSASAAP
ESEDEFVSVLPMEYENNSTLSETEKSTSGSFSFFPVQMILTWLPPKPPTAFDGFHIHIEREEN
FTEYLMVDEEAHEFVAELKEPGKYKLSVTTFSSSGSCETRKSQSAKSLSFYISPSGEWIEEL
TEKPQHVSVHVLSSTTALMSWTSSQENYNSTIVSVVSLTCQKQKESQRLEKQYCTQVNSS
KPIIENLVPGAQYQVVIYLRKGPLIGPPSDPVTFAIVPTGIKDLMLYPLGPTAVVLSWTRPYL
GVFRKYVVEMFYFNPATMTSEWTTYYEIAATVSLTASVRIANLLPAWYYNFRVTMVTWG
DPELSCCDSSTISFITAPVAPEITSVEYFNSLLYISWTYGDDTTDLSHSRMLHWMVVAEGKK
KIKKSVTRNVMTAILSLPPGDIYNLSVTACTERGSNTSMLRLVKLEPAPPKSLFAVNKTQTS
VTLLWVEEGVADFFEVFCQQVGSSQKTKLQEPVAVSSHVVTISSLLPATAYNCSVTSFSHDS
PSVPTFIAVSTMVTEMNPNVVVISVLAILSTLLIGLLLVTLIILRKKHLQMARECGAGTFVNF
ASLERDGKLPYNWRRSIFAFLTLLPSCLWTDYLLAFYINPWSKNGLKKRKLTNPVQLDDF
DAYIKDMAKDSDYKFSLQFEELKLIGLDIPHFAADLPLNRCKNRYTNILPYDFSRVRLVSM
NEEEGADYINANYIPGYNSPQEYIATQGPLPETRNDFWKMVLQQKSQIIVMLTQCNEKRR
VKCDHYWPFTEEPIAYGDITVEMISEEEQDDWACRHFRINYADEMQDVMHFNYTAWPDH
GVPTANAAESILQFVHMVRQQATKSKGPMIIHCSAGVGRTGTFIALDRLLQHIRDHEFVDI
LGLVSEMRSYRMSMVQTEEQYIFIHQCVQLMWMKKKQQFCISDVIYENVSKS

>Q4G0F5 peripheral membrane protein
MSFFGFGQSVEVEILLNDAESRKRAEHKTEDGKKEKYFLFYDGETVSGKVSLALKNPNK
RLEHQGIKIEFIGQIELYYDRGNHHEFVSLVKDLARPGEITQSQAFDFEFTHVEKPYESYTG
QNVKLRYFLRATISRRLNDVVKEMDIVVHTLSTYPELNSSIKMEVGIEDCLHIEFEYNKSK
YHLKDVIVGKIYFLLVRIKIKHMEIDIIKRETTGTGPNVYHENDTIAKYEIMDGAPVRGESIP
IRLFLAGYELTPTMRDINKKFSVRYYLNLVLIDEEERRYFKQQEVVLWRKGDIVRKSMSH
QAAIASQRFEGTTSLGEVRTPSQLSDNNCRQ

>O43150 peripheral membrane protein
MPDQISVSEFVAETHEDYKAPTASSFTTRTAQCRNTVAAIEEALDVDRMVLYKMKKSVKA
INSSGLAHVENEEQYTQALEKFGGNCVCRDDPDLGSAFLKFSVFTKELTALFKNLIQNMN
NIISFPLDSLLKGDLKGVKGDLKKPFDKAWKDYETKITKIEKEKKEHAKLHGMIRTEISGA
EIAEEMEKERRFFQLQMCEYLLKVNEIKIKKGVDLLQNLIKYFHAQCNFFQDGLKAVESL
KPSIETLSTDLHTIKQAQDEERRQLIQLRDILKSALQVEQKEDSQIRQSTAYSLHQPQGNKE
HGTERNGSLYKKSDGIRKVWQKRKCSVKNGFLTISHGTANRPPAKLNLLTCQVKTNPEEK
KCFDLISHDRTYHFQAEDEQECQIWMSVLQNSKEEALNNAFKGDDNTGENNIVQELTKEII
SEVQRMTGNDVCCDCGAPDPTWLSTNLGILTCIECSGIHRELGVHYSRMQSLTLDVLGTS
ELLLAKNIGNAGFNEIMECCLPAEDSVKPNPGSDMNARKDYITAKYIERRYARKKHADNA
AKLHSLCEAVKTRDIFGLLQAYADGVDLTEKIPLANGHEPDETALHLAVRSVDRTSLHIVD

FLVQNSGNLDKQTGKGSTALHYCCLTDNAECLKLLLRGKASIEIANESGETPLDIAKRLKH
EHCEELLTQALSGRFNSHVHVEYEWRLLHEDLDESDDDMDEKLQPSPNRREDRPISFYQL
GSNQLQSNAVSLARDAANLAKEKQRAFMPSILQNETYGALLSGSPPPAQPAAPSTTSAPPL
PPRNVGKVQTASSANTLWKTNSVSVDGGSRQRSSSDPPAVHPPLPPLRVTSTNPLTPTPPPP
VAKTPSVMEALSQPSKPAPPGISQIRPPPLPPQPPSRLPQKKPAPGADKSTPLTNKGQPRGPV
DLSATEALGPLSNAMVLQPPAPMPRKSQATKLKPKRVKALYNCVADNPDELTFSEGDVIIV
DGEEDQEWWIGHIDGDPGRKGAFPVSFVHFIAD

>Q07157 peripheral membrane protein
MSARAAAAKSTAMEETAIWEQHTVTLHRAPGFGFGIAISGGRDNPHFQSGETSIVISDVLK
GGPAEGQLQENDRVAMVNGVSMDNVEHAFAVQQLRKSGKNAKITIRRKKKVQIPVSRPD
PEPVSDNEEDSYDEEIHDPRSGRSGVVNRRSEKIWPRDRSASRERSLSPRSDRRSVASSQPA
KPTKVTLVKSRKNEEYGLRLASHIFVKEISQDSLAARDGNIQEGDVVLKINGTVTENMSLT
DAKTLIERSKGKLKMVVQRDERATLLNVPDLSDSIHSANASERDDISEIQSLASDHSGRSH
DRPPRRSRSRSPDQRSEPSDHSRHSPQQPSNGSLRSRDEERISKPGAVSTPVKHADDHTPKT
VEEVTVERNEKQTPSLPEPKPVYAQVGQPDVDLPVSPSDGVLPNSTHEDGILRPSMKLVKF
RKGDSVGLRLAGGNDVGIFVAGVLEDSPAAKEGLEEGDQILRVNNVDFTNIIREEAVLFLL
DLPKGEEVTILAQKKKDVYRRIVESDVGDSFYIRTHFEYEKESPYGLSFNKGEVFRVVDTL
YNGKLGSWLAIRIGKNHKEVERGIIPNKNRAEQLASVQYTLPKTAGGDRADFWRFRGLRS
SKRNLRKSREDLSAQPVQTKFPAYERVVLREAGFLRPVTIFGPIADVAREKLAREEPDIYQI
AKSEPRDAGTDQRSSGIIRLHTIKQIIDQDKHALLDVTPNAVDRLNYAQWYPIVVFLNPDS
KQGVKTMRMRLCPESRKSARKLYERSHKLRKNNHHLFTTTINLNSMNDGWYGALKEAI
QQQQNQLVWVSEGKADGATSDDLDLHDDRLSYLSAPGSEYSMYSTDSRHTSDYEDTDTE
GGAYTDQELDETLNDEVGTPPESAITRSSEPVREDSSGMHHENQTYPPYSPQAQPQPIHRI
DSPGFKPASQQKAEASSPVPYLSPETNPASSTSAVNHNVNLTNVRLEEPTPAPSTSYSPQAD
SLRTPSTEAAHIMLRDQEPSLSSHVDPTKVYRKDPYPEEMMRQNHVLKQPAVSHPGHRPD
KEPNLTYEPQLPYVEKQASRDLEQPTYRYESSSYTDQFSRNYEHRLRYEDRVPMYEEQWS
YYDDKQPYPSRPPFDNQHSQDLDSRQHPEESSERGYFPRFEEPAPLSYDSRPRYEQAPRAS
ALRHEEQPAPGYDTHGRLRPEAQPHPSAGPKPAESKQYFEQYSRSYEQVPPQGFTSRAGH
FEPLHGAAAVPPLIPSSQHKPEALPSNTKPLPPPPTQTEEEEDPAMKPQSVLTRVKMFENKR
SASLETKKDVNDTGSFKPPEVASKPSGAPIIGPKPTSQNQFSEHDKTLYRIPEPQKPQLKPPE
DIVRSNHYDPEEDEEYYRKQLSYFDRRSFENKPPAHIAASHLSEPAKPAHSQNQSNFSSYSS
KGKPPEADGVDRSFGEKRYEPIQATPPPPPLPSQYAQPSQPVTSASLHIHSKGAHGEGNSVS
LDFQNSLVSKPDPPPSQNKPATFRPPNREDTAQAAFYPQKSFPDKAPVNGTEQTQKTVTPA
YNRFTPKPYTSSARPFERKFESPKFNHNLLPSETAHKPDLSSKTPTSPKTLVKSHSLAQPPEF
DSGVETFSIHAEKPKYQINNISTVPKAIPVSPSAVEEDEDEDGHTVVATARGIFNSNGGVLSS
IETGVSIIIPQGAIPEGVEQEIYFKVCRDNSILPPLDKEKGETLLSPLVMCGPHGLKFLKPVEL
RLPHCDPKTWQNKCLPGDPNYLVGANCVSVLIDHF

>Q8N442 peripheral membrane protein
MWTLVGRGWGCARALAPRATGAALLVAPGPRSAPTLGAAPESWATDRLYSSAEFKEKLD
MSRFPVENIRNFSIVAHVDHGKSTLADRLLELTGTIDKTKNNKQVLDKLQVERERGITVKA
QTASLFYNCEGKQYLLNLIDTPGHVDFSYEVSRSLSACQGVLLVVDANEGIQAQTVANFF
LAFEAQLSVIPVINKIDLKNADPERVENQIEKVFDIPSDECIKISAKLGTNVESVLQAIIERIPP

PKVHRKNPLRALVFDSTFDQYRGVIANVALFDGVVSKGDKIVSAHTQKTYEVNEVGVLN
PNEQPTHKLYAGQVGYLIAGMKDVTEAQIGDTLCLHKQPVEPLPGFKSAKPMVFAGMYP
LDQSEYNNLKSAIEKLTLNDSSVTVHRDSSLALGAGWRLGFLGLLHMEVFNQRLEQEYN
ASVILTTPTVPYKAVLSSSKLIKEHREKEITIINPAQFPDKSKVTEYLEPVVLGTIITPDEYTG
KIMMLCEARRAVQKNMIFIDQNRVMLKYLFPLNEIVVDFYDSLKSLSSGYASFDYEDAGY
QTAELVKMDILLNGNTVEELVTVVHKDKAHSIGKAICERLKDSLPRQLFEIAIQAAIGSKII
ARETVKAYRKNVLAKCYGGDITRKMKLLKRQAEGKKKLRKIGNVEVPKDAFIKVLKTQS
SK

>Q969V1 multipass membrane protein
MNPFHASCWNTSAELLNKSWNKEFAYQTASVVDTVILPSMIGIICSTGLVGNILIVFTIIRSR
KKTVPDIYICNLAVADLVHIVGMPFLIHQWARGGEWVFGGPLCTIITSLDTCNQFACSAIMT
VMSVDRYFALVQPFRLTRWRTRYKTIRINLGLWAASFILALPVWVYSKVIKFKDGVESCAF
DLTSPDDVLWYTLYLTITTFFFPLPLILVCYILILCYTWEMYQQNKDARCCNPSVPKQRVM
KLTKMVLVLVVVFILSAAPYHVIQLVNLQMEQPTLAFYVGYYLSICLSYASSSINPFLYILLS
GNFQKRLPQIQRRATEKEINNMGNTLKSHF

>Q8WWG9 single-pass type I membrane protein
MLKMEPLNSTHPGTAASSSPLESRAAGGGSGNGNEYFYILVVMSFYGIFLIGIMLGYMKS
KRREKKSSLLLLYKDEERLWGEAMKPLPVVSGLRSVQVPLMLNMLQESVAPALSCTLCS
MEGDSVSSESSSPDVHLTIQEEGADDELEETSETPLNESSEGSSENIHQNS

>P23510 single-pass type II membrane protein
MERVQPLEENVGNAARPRFERNKLLLVASVIQGLGLLLCFTYICLHFSALQVSHRYPRIQSI
KVQFTEYKKEKGFILTSQKEDEIMKVQNNSVIINCDGFYLISLKGYFSQEVNISLHYQKDEE
PLFQLKKVRSVNSLMVASLTYKDKVYLNVTTDNTSLDDFHVNGGELILIHQNPGEFCVL

>Q09328 single-pass type II membrane protein
MALFTPWKLSSQKLGFFLVTFGFIWGMMLLHFTIQQRTQPESSSMLREQILDLSKRYIKAL
AEENRNVVDGPYAGVMTAYDLKKTLAVLLDNILQRIGKLESKVDNLVVNGTGTNSTNSTT
AVPSLVALEKINVADIINGAQEKCVLPPMDGYPHCEGKIKWMKDMWRSDPCYADYGVDG
STCSFFIYLSEVENWCPHLPWRAKNPYEEADHNSLAEIRTDFNILYSMMKKHEEFRWMRL
RIRRMADAWIQAIKSLAEKQNLEKRKRKKVLVHLGLLTKESGFKIAETAFSGGPLGELVQ
WSDLITSLYLLGHDIRISASLAELKEIMKKVVGNRSGCPTVGDRIVELIYIDIVGLAQFKKT
LGPSWVHYQCMLRVLDSFGTEPEFNHANYAQSKGHKTPWGKWNLNPQQFYTMFPHTPD
NSFLGFVVEQHLNSSDIHHINEIKRQNQSLVYGKVDSFWKNKKIYLDIIHTYMEVHATVYG
SSTKNIPSYVKNHGILSGRDLQFLLRETKLFVGLGFPYEGPAPLEAIANGCAFLNPKFNPPK
SSKNTDFFIGKPTLRELTSQHPYAEVFIGRPHVWTVDLNNQEEVEDAVKAILNQKIEPYMP
YEFTCEGMLQRINAFIEKQDFCHGQVMWPPLSALQVKLAEPGQSCKQVCQESQLICEPSF
FQHLNKDKDMLKYKVTCQSSELAKDILVPSFDPKNKHCVFQGDLLLFSCAGAHPRHQRV
CPCRDFIKGQVALCKDCL

>Q9NS75 multipass membrane protein
MERKFMSLQPSISVSEMEPNGTFSNNNSRNCTIENFKREFFPIVYLIIFFWGVLGNGLSIYVF

LQPYKKSTSVNVFMLNLAISDLLFISTLPFRADYYLRGSNWIFGDLACRIMSYSLYVNMYS
SIYFLTVLSVVRFLAMVHPFRLLHVTSIRSAWILCGIIWILIMASSIMLLDSGSEQNGSVTSC
LELNLYKIAKLQTMNYIALVVGCLLPFFTLSICYLLIIRVLLKVEVPESGLRVSHRKALTTIII
TLIIFFLCFLPYHTLRTVHLTTWKVGLCKDRLHKALVITLALAAANACFNPLLYYFAGENF
KDRLKSALRKGHPQKAKTKCVFPVSVWLRKETRV

>P25705 peripheral membrane protein
MLSVRVAAAVVRALPRRAGLVSRNALGSSFIAARNFHASNTHLQKTGTAEMSSILEERILG
ADTSVDLEETGRVLSIGDGIARVHGLRNVQAEEMVEFSSGLKGMSLNLEPDNVGVVVFG
NDKLIKEGDIVKRTGAIVDVPVGEELLGRVVDALGNAIDGKGPIGSKTRRRVGLKAPGIIP
RISVREPMQTGIKAVDSLVPIGRGQRELIIGDRQTGKTSIAIDTIINQKRFNDGSDEKKKLYCI
YVAIGQKRSTVAQLVKRLTDADAMKYTIVVSATASDAAPLQYLAPYSGCSMGEYFRDNG
KHALIIYDDLSKQAVAYRQMSLLLRRPPGREAYPGDVFYLHSRLLERAAKMNDAFGGGSL
TALPVIETQAGDVSAYIPTNVISITDGQIFLETELFYKGIRPAINVGLSVSRVGSAAQTRAMK
QVAGTMKLELAQYREVAAFAQFGSDLDAATQQLLSRGVRLTELLKQGQYSPMAIEEQVAV
IYAGVRGYLDKLEPSKITKFENAFLSHVVSQHQALLGTIRADGKISEQSDAKLKEIVTNFL
AGFEA

>P17948 single-pass type I membrane protein
MVSYWDTGVLLCALLSCLLLTGSSSGSKLKDPELSLKGTQHIMQAGQTLHLQCRGEAAH
KWSLPEMVSKESERLSITKSACGRNGKQFCSTLTLNTAQANHTGFYSCKYLAVPTSKKKE
TESAIYIFISDTGRPFVEMYSEIPEIIHMTEGRELVIPCRVTSPNITVTLKKFPLDTLIPDGKRII
WDSRKGFIISNATYKEIGLLTCEATVNGHLYKTNYLTHRQTNTIIDVQISTPRPVKLLRGHTL
VLNCTATTPLNTRVQMTWSYPDEKNKRASVRRRIDQSNSHANIFYSVLTIDKMQNKDKGL
YTCRVRSGPSFKSVNTSVHIYDKAFITVKHRKQQVLETVAGKRSYRLSMKVKAFPSPEVV
WLKDGLPATEKSARYLTRGYSLIIKDVTEEDAGNYTILLSIKQSNVFKNLTATLIVNVKPQI
YEKAVSSFPDPALYPLGSRQILTCTAYGIPQPTIKWFWHPCNHNHSEARCDFCSNNEESFIL
DADSNMGNRIESITQRMAIIEGKNKMASTLVVADSRISGIYICIASNKVGTVGRNISFYITDV
PNGFHVNLEKMPTEGEDLKLSCTVNKFLYRDVTWILLRTVNNRTMHYSISKQKMAITKEH
SITLNLTIMNVSLQDSGTYACRARNVYTGEEILQKKEITIRDQEAPYLLRNLSDHTVAISSST
TLDCHANGVPEPQITWFKNNHKIQQEPGIILGPGSSTLFIERVTEEDEGVYHCKATNQKGS
VESSAYLTVQGTSDKSNLELITLTCTCVAATLFWLLLTLFIRKMKRSSSEIKTDYLSIIMDPD
EVPLDEQCERLPYDASKWEFARERLKLGKSLGRGAFGKVVQASAFGIKKSPTCRTVAVK
MLKEGATASEYKALMTELKILTHIGHHLNVVNLLGACTKQGGPLMVIVEYCKYGNLSNY
LKSKRDLFFLNKDAALHMEPKKEKMEPGLEQGKKPRLDSVTSSESFASSGFQEDKSLSDV
EEEEDSDGFYKEPITMEDLISYSFQVARGMEFLSSRKCIHRDLAARNILLSENNVVKICDFG
LARDIYKNPDYVRKGDTRLPLKWMAPESIFDKIYSTKSDVWSYGVLLWEIFSLGGSPYPG
VQMDEDFCSRLREGMRMRAPEYSTPEIYQIMLDCWHRDPKERPRFAELVEKLGDLLQAN
VQQDGKDYIPINAILTGNSGFTYSTPAFSEDFFKESISAPKFNSGSSDDVRYVNAFKFMSLE
RIKTFEELLPNATSMFDDYQGDSSTLLASPMLKRFTWTDSKPKASLKIDLRVTSKSKESGL
SDVSRPSFCHSSCGHVSEGKRRFTYDHAELERKIACCSPPPDYNSVVLYSTPPI

>P48058 multipass membrane protein
MRIISRQIVLLFSGFWGLAMGAFPSSVQIGGLFIRNTDQEYTAFRLAIFLHNTSPNASEAPFN

LVPHVDNIETANSFAVTNAFCSQYSRGVFAIFGLYDKRSVHTLTSFCSALHISLITPSFPTEGE
SQFVLQLRPSLRGALLSLLDHYEWNCFVFLYDTDRGYSILQAIMEKAGQNGWHVSAICVE
NFNDVSYRQLLEELDRRQEKKFVIDCEIERLQNILEQIVSVGKHVKGYHYIIANLGFKDISL
ERFIHGGANVTGFQLVDFNTPMVIKLMDRWKKLDQREYPGSETPPKYTSALTYDGVLVM
AETFRSLRRQKIDISRRGNAGDCLANPAAPWGQGIDMERTLKQVRIQGLTGNVQFDHYGR
RVNYTMDVFELKSTGPRKVGYWNDMDKLVLIQDVPTLGNDTAAIENRTVVVTTIMESPY
VMYKKNHEMFEGNDKYEGYCVDLASEIAKHIGIKYKIAIVPDGKYGARDADTKIWNGM
VGELVYGKAEIAIAPLTITLVREEVIDFSKPFMSLGISIMIKKPQKSKPGVFSFLDPLAYEIW
MCIVFAYIGVSVVLFLVSRFSPYEWHTEEPEDGKEGPSDQPPNEFGIFNSLWFSLGAFMQQ
GCDISPRSLSGRIVGGVWWFFTLIIISSYTANLAAFLTVERMVSPIESAEDLAKQTEIAYGTL
DSGSTKEFFRRSKIAVYEKMWTYMRSAEPSVFTRTTAEGVARVRKSKGKFAFLLESTMNE
YIEQRKPCDTMKVGGNLDSKGYGVATPKGSSLRTPVNLAVLKLSEAGVLDKLKNKWWY
DKGECGPKDSGSKDKTSALSLSNVAGVFYILVGGLGLAMLVALIEFCYKSRAEAKRMKLT
FSEAIRNKARLSITGSVGENGRVLTPDCPKAVHTGTAIRQSSGLAVIASDLP

>P17302 multipass membrane protein
MGDWSALGKLLDKVQAYSTAGGKVWLSVLFIFRILLLGTAVESAWGDEQSAFRCNTQQP
GCENVCYDKSFPISHVRFWVLQIIFVSVPTLLYLAHVFYVMRKEEKLNKKEEELKVAQTD
GVNVDMHLKQIEIKKFKYGIEEHGKVKMRGGLLRTYIISILFKSIFEVAFLLIQWYIYGFSLS
AVYTCKRDPCPHQVDCFLSRPTEKTIFIIFMLVVSLVSLALNIIELFYVFFKGVKDRVKGKS
DPYHATSGALSPAKDCGSQKYAYFNGCSSPTAPLSPMSPPGYKLVTGDRNNSSCRNYNKQ
ASEQNWANYSAEQNRMGQAGSTISNSHAQPFDFPDDNQNSKKLAAGHELQPLAIVDQRP
SSRASSRASSRPRPDDLEI

>Q6NXT4 multipass membrane protein
MGTIHLFRKPQRSFFGKLLREFRLVAADRRSWKILLFGVINLICTGFLLMWCSSTNSIALTA
YTYLTIFDLFSLMTCLISYWVTLRKPSPVYSFGFERLEVLAVFASTVLAQLGALFILKESAE
RFLEQPEIHTGRLLVGTFVALCFNLFTMLSIRNKPFAYVSEAASTSWLQEHVADLSRSLCGII
PGLSSIFLPRMNPFVLIDLAGAFALCITYMLIEINNYFAVDTASAIAIALMTFGTMYPMSVYS
GKVLLQTTPPHVIGQLDKLIREVSTLDGVLEVRNEHFWTLGFGSLAGSVHVRIRRDANEQ
MVLAHVTNRLYTLVSTLTVQIFKDDWIRPALLSGPVAANVLNFSDHHVIPMPLLKGTDDL
NPVTSTPAKPSSPPPEFSFNTPGKNVNPVILLNTQTRPYGFGLNHGHTPYSSMLNQGLGVP
GIGATQGLRTGFTNIPSRYGTNNRIGQPRP

>P56199 single-pass type I membrane protein
MAPRPRARPGVAVACCWLLTVVLRCCVSFNVDVKNSMTFSGPVEDMFGYTVQQYENEE
GKWVLIGSPLVGQPKNRTGDVYKCPVGRGESLPCVKLDLPVNTSIPNVTEVKENMTFGST
LVTNPNGGFLACGPLYAYRCGHLHYTTGICSDVSPTFQVVNSIAPVQECSTQLDIVIVLDGS
NSIYPWDSVTAFLNDLLERMDIGPKQTQVGIVQYGENVTHEFNLNKYSSTEEVLVAAKKI
VQRGGRQTMTALGIDTARKEAFTEARGARRGVKKVMVIVTDGESHDNHRLKKVIQDCE
DENIQRFSIAILGSYNRGNLSTEKFVEEIKSIASEPTEKHFFNVSDELALVTIVKTLGERIFAL
EATADQSAASFEMEMSQTGFSAHYSQDWVMLGAVGAYDWNGTVVMQKASQIIIPRNTTF
NVESTKKNEPLASYLGYTVNSATASSGDVLYIAGQPRYNHTGQVIIYRMEDGNIKILQTLS
GEQIGSYFGSILTTTDIDKDSNTDILLVGAPMYMGTEKEEQGKVYVYALNQTRFEYQMSL

EPIKQTCCSSRQHNSCTTENKNEPCGARFGTAIAAVKDLNLDGFNDIVIGAPLEDDHGGAV
YIYHGSGKTIRKEYAQRIPSGGDGKTLKFFGQSIHGEMDLNGDGLTDVTIGGLGGAALFW
SRDVAVVKVTMNFEPNKVNIQKKNCHMEGKETVCINATVCFDVKLKSKEDTIYEADLQY
RVTLDSLRQISRSFFSGTQERKVQRNITVRKSECTKHSFYMLDKHDFQDSVRITLDFNLTD
PENGPVLDDSLPNSVHEYIPFAKDCGNKEKCISDLSLHVATTEKDLLIVRSQNDKFNVSLTV
KNTKDSAYNTRTIVHYSPNLVFSGIEAIQKDSCESNHNITCKVGYPFLRRGEMVTFKILFQF
NTSYLMENVTIYLSATSDSEEPPETLSDNVVNISIPVKYEVGLQFYSSASEYHISIAANETVP
EVINSTEDIGNEINIFYLIRKSGSFPMPELKLSISFPNMTSNGYPVLYPTGLSSSENANCRPHI
FEDPFSINSGKKMTTSTDHLKRGTILDCNTCKFATITCNLTSSDISQVNVSLILWKPTFIKSYF
SSLNLTIRGELRSENASLVLSSSNQKRELAIQISKDGLPGRVPLWVILLSAFAGLLLLMLLIL
ALWKIGFFKRPLKKKMEK

>Q86XQ3 multipass membrane protein
MSQHRHQRHSRVISSSPVDTTSVGFCPTFKKFKRNDDECRAFVKRVIMSRFFKIIMISTVTS
NAFFMALWTSYDIRYRLFRLLEFSEIFFVSICTSELSMKVYVDPINYWKNGYNLLDVIIIIV
MFLPYALRQLMGKQFTYLYIADGMQSLRILKLIGYSQGIRTLITAVGQTVYTVASVLLLLFL
LMYIFAILGFCLFGSPDNGDHDNWGNLAAAFFTLFSLATVDGWTDLQKQLDNREFALSRA
FTIIFILLASFIFLNMFVGVMIMHTEDSIRKFERELMLEQQEMLMGEKQVILQRQQEEISRL
MHIQKNADCTSFSELVENFKKTLSHTDPMVLDDFGTSLPFIDIYFSTLDYQDTTVHKLQEL
YYEIVHVLSLMLEDLPQEKPQSLEKVDEK

>Q13255 multipass membrane protein
MVGLLLFFFPAIFLEVSLLPRSPGRKVLLAGASSQRSVARMDGDVIIGALFSVHHQPPAEKV
PERKCGEIREQYGIQRVEAMFHTLDKINADPVLLPNITLGSEIRDSCWHSSVALEQSIEFIRD
SLISIRDEKDGINRCLPDGQSLPPGRTKKPIAGVIGPGSSSVAIQVQNLLQLFDIPQIAYSATSI
DLSDKTLYKYFLRVVPSDTLQARAMLDIVKRYNWTYVSAVHTEGNYGESGMDAFKELA
AQEGLCIAHSDKIYSNAGEKSFDRLLRKLRERLPKARVVVCFCEGMTVRGLLSAMRRLG
VVGEFSLIGSDGWADRDEVIEGYEVEANGGITIKLQSPEVRSFDDYFLKLRLDTNTRNPWF
PEFWQHRFQCRLPGHLLENPNFKRICTGNESLEENYVQDSKMGFVINAIYAMAHGLQNM
HHALCPGHVGLCDAMKPIDGSKLLDFLIKSSFIGVSGEEVWFDEKGDAPGRYDIMNLQYT
EANRYDYVHVGTWHEGVLNIDDYKIQMNKSGVVRSVCSEPCLKGQIKVIRKGEVSCCWI
CTACKENEYVQDEFTCKACDLGWWPNADLTGCEPIPVRYLEWSNIESIIAIAFSCLGILVTL
FVTLIFVLYRDTPVVKSSSRELCYIILAGIFLGYVCPFTLIAKPTTTSCYLQRLLVGLSSAMC
YSALVTKTNRIARILAGSKKKICTRKPRFMSAWAQVIIASILISVQLTLVVTLIIMEPPMPILS
YPSIKEVYLICNTSNLGVVAPLGYNGLLIMSCTYYAFKTRNVPANFNEAKYIAFTMYTTCII
WLAFVPIYFGSNYKIITTCFAVSLSVTVALGCMFTPKMYIIIAKPERNVRSAFTTSDVVRMH
VGDGKLPCRSNTFLNIFRRKKAGAGNANSNGKSVSWSEPGGGQVPKGQHMWHRLSVHV
KTNETACNQTAVIKPLTKSYQGSGKSLTFSDTSTKTLYNVEEEEDAQPIRFSPPGSPSMVVH
RRVPSAATTPPLPSHLTAEETPLFLAEPALPKGLPPPLQQQQQPPPQQKSLMDQLQGVVSNF
STAIPDFHAVLAGPGGPGNGLRSLYPPPPPPQHLQMLPLQLSTFGEELVSPPADDDDDSERF
KLLQEYVYEHEREGNTEEDELEEEEEDLQAASKLTPDDSPALTPPSPFRDSVASGSSVPSSP
VSESVLCTPPNVSYASVILRDYKQSSSTL

>Q96E17 lipid-anchor

MRHEAPMQMASAQDARYGQKDSSDQNFDYMFKLLIIGNSSVGKTSFLFRYADDSFTSAF
VSTVGIDFKVKTVFKNEKRIKLQIWDTAGQERYRTITTAYYRGAMGFILMYDITNEESFNA
VQDWSTQIKTYSWDNAQVILVGNKCDMEDERVISTERGQHLGEQLGFEFFETSAKDNINV
KQTFERLVDIICDKMSESLETDPAITAAKQNTRLKETPPPPQPNCAC

>O00469 peripheral membrane protein
MGGCTVKPQLLLLALVLHPWNPCLGADSEKPSSIPTDKLLVITVATKESDGFHRFMQSAK
YFNYTVKVLGQGEEWRGGDGINSIGGGQKVRLMKEVMEHYADQDDLVVMFTECFDVIF
AGGPEEVLKKFQKANHKVVFAADGILWPDKRLADKYPVVHIGKRYLNSGGFIGYAPYVN
RIVQQWNLQDNDDDQLFYTKVYIDPLKREAINITLDHKCKIFQTLNGAVDEVVLKFENGK
ARAKNTFYETLPVAINGNGPTKILLNYFGNYVPNSWTQDNGCTLCEFDTVDLSAVDVHPN
VSIGVFIEQPTPFLPRFLDILLTLDYPKEALKLFIHNKEVYHEKDIKVFFDKAKHEIKTIKIVG
PEENLSQAEARNMGMDFCRQDEKCDYYFSVDADVVLTNPRTLKILIEQNRKIIAPLVTRH
GKLWSNFWGALSPDGYYARSEDYVDIVQGNRVGVWNVPYMANVYLIKGKTLRSEMNE
RNYFVRDKLDPDMALCRNAREMGVFMYISNRHEFGRLLSTANYNTSHYNNDLWQIFENP
VDWKEKYINRDYSKIFTENIVEQPCPDVFWFPIFSEKACDELVEEMEHYGKWSGGKHHDS
RISGGYENVPTDDIHMKQVDLENVWLHFIREFIAPVTLKVFAGYYTKGFALLNFVVKYSP
ERQRSLRPHHDASTFTINIALNNVGEDFQGGGCKFLRYNCSIESPRKGWSFMHPGRLTHLH
EGLPVKNGTRYIAVSFIDP

>Q8NGZ2 multipass membrane protein
MTNQTQMMEFLLVRFTENWVLLRLHALLFSLIYLTAVLMNLVIILLMILDHRLHMAMYFF
LRHLSFLDLCLISATVPKSILNSVASTDSISFLGCVLQLFLVVLLAGSEIGILTAMSYDRYAAI
CCPLHCEAVMSRGLCVQLMALSWLNRGALGLLYTAGTFSLNFYGSDELHQFFCDVPALL
KLTCSKEHAIISVSVAIGVCYAFSCLVCIVVSYVYIFSAVLRISQRQRQSKAFSNCVPHLIVV
TVFLVTGAVAYLKPGSDAPSILDLLVSVFYSVAPPTLNPVIYCLKNKDIKSALSKVLWNVRS
SGVMKR

>Q15256 single-pass type I membrane protein
MRRAVCFPALCLLLNLHAAGCFSGNNDHFLAINQKKSGKPVFIYKHSQDIEKSLDIAPQKI
YRHSYHSSSEAQVSKRHQIVNSAFPRPAYDPSLNLLAMDGQDLEVENLPIPAANVIVVTLQ
MDVNKLNITLLRIFRQGVAAALGLLPQQVHINRLIGKKNSIELFVSPINRKTGISDALPSEEV
LRSLNINVLHQSLSQFGITEVSPEKNVLQGQHEADKIWSKEGFYAVVIFLSIFVIIVTCLMIL
YRLKERFQLSLRQDKEKNQEIHLSPITLQPALSEAKTVHSMVQPEQAPKVLNVVVDPQGR
GAPEIKATTATSVCPSPFKMKPIGLQERRGSNVSLTLDMSSLGNIEPFVSIPTPREKVAMEYL
QSASRILTRSQLRDVVASSHLLQSEFMEIPMNFVDPKEIDIPRHGTKNRYKTILPNPLSRVCL
RPKNVTDSLSTYINANYIRGYSGKEKAFIATQGPMINTVDDFWQMVWQEDSPVIVMITKL
KEKNEKCVLYWPEKRGIYGKVEVLVISVNECDNYTIRNLVLKQGSHTQHVKHYWYTSWP
DHKTPDSAQPLLQLMLDVEEDRLASQGRGPVVVHCSAGIGRTGCFIATSIGCQQLKEEGV
VDALSIVCQLRMDRGGMVQTSEQYEFVHHALCLYESRLSAETVQ

>P18564 single-pass type I membrane protein
MGIELLCLFFLFLGRNDHVQGGCALGGAETCEDCLLIGPQCAWCAQENFTHPSGVGERCD
TPANLLAKGCQLNFIENPVSQVEILKNKPLSVGRQKNSSDIVQIAPQSLILKLRPGGAQTLQ

VHVRQTEDYPVDLYYLMDLSASMDDDLNTIKELGSRLSKEMSKLTSNFRLGFGSFVEKPV
SPFVKTTPEEIANPCSSIPYFCLPTFGFKHILPLTNDAERFNEIVKNQKISANIDTPEGGFDAI
MQAAVCKEKIGWRNDSLHLLVFVSDADSHFGMDSKLAGIVIPNDGLCHLDSKNEYSMST
VLEYPTIGQLIDKLVQNNVLLIFAVTQEQVHLYENYAKLIPGATVGLLQKDSGNILQLIISAY
EELRSEVELEVLGDTEGLNLSFTAICNNGTLFQHQKKCSHMKVGDTASFSVTVNIPHCERR
SRHIIIKPVGLGDALELLVSPECNCDCQKEVEVNSSKCHHGNGSFQCGVCACHPGHMGPR
CECGEDMLSTDSCKEAPDHPSCSGRGDCYCGQCICHLSPYGNIYGPYCQCDNFSCVRHKG
LLCGGNGDCDCGECVCRSGWTGEYCNCTTSTDSCVSEDGVLCSGRGDCVCGKCVCTNP
GASGPTCERCPTCGDPCNSKRSCIECHLSAAGQAREECVDKCKLAGATISEEEDFSKDGSV
SCSLQGENECLITFLITTDNEGKTIIHSINEKDCPKPPNIPMIMLGVSLAILLIGVVLLCIWKL
LVSFHDRKEVAKFEAERSKAKWQTGTNPLYRGSTSTFKNVTYKHREKQKVDLSTDC

>P40200 single-pass type I membrane protein
MEKKWKYCAVYYIIQIHFVKGVWEKTVNTEENVYATLGSDVNLTCQTQTVGFFVQMQW
SKVTNKIDLIAVYHPQYGFYCAYGRPCESLVTFTETPENGSKWTLHLRNMSCSVSGRYEC
MLVLYPEGIQTKIYNLLIQTHVTADEWNSNHTIEIEINQTLEIPCFQNSSSKISSEFTYAWSVE
NSSTDSWVLLSKGIKEDNGTQETLISQNHLISNSTLLKDRVKLGTDYRLHLSPVQIFDDGR
KFSCHIRVGPNKILRSSTTVKVFAKPEIPVIVENNSTDVLVERRFTCLLKNVFPKANITWFID
GSFLHDEKEGIYITNEERKGKDGFLELKSVLTRVHSNKPAQSDNLTIWCMALSPVPGNKV
WNISSEKITFLLGSEISSTDPPLSVTESTLDTQPSPASSVSPARYPATSSVTLVDVSALRPNTTP
QPSNSSMTTRGFNYPWTSSGTDTKKSVSRIPSETYSSSPSGAGSTLHDNVFTSTARAFSEVP
TTANGSTKTNHVHITGIVVNKPKDGMSWPVIVAALLFCCMILFGLGVRKWCQYQKEIME
RPPPFKPPPPPIKYTCIQEPNESDLPYHEMETL

>Q8IZF3 multipass membrane protein
MKMKSQATMICCLVFFLSTECSHYRSKIHLKAGDKLQSPEGKPKTGRIQEKCEGPCISSSN
CSQPCAKDFHGEIGFTCNQKKWQKSAETCTSLSVEKLFKDSTGASRLSVAAPSIPLHILDFR
APETIESVAQGIRKNCPFDYACITDMVKSSETTSGNIAFIVELLKNISTDLSDNVTREKMKS
YSEVANHILDTAAISNWAFIPNKNASSDLLQSVNLFARQLHIHNNSENIVNELFIQTKGFHIN
HNTSEKSLNFSMSMNNTTEDILGMVQIPRQELRKLWPNASQAISIAFPTLGAILREAHLQN
VSLPRQVNGLVLSVVLPERLQEIILTFEKINKTRNARAQCVGWHSKKRRWDEKACQMML
DIRNEVKCRCNYTSVVMSFSILMSSKSMTDKVLDYITCIGLSVSILSLVLCLIIEATVWSRV
VVTEISYMRHVCIVNIAVSLLTANVWFIIGSHFNIKAQDYNMCVAVTFFSHFFYLSLFFWML
FKALLIIYGILVIFRRMMKSRMMVIGFAIGYGCPLIIAVTTVAITEPEKGYMRPEACWLNWD
NTKALLAFAIPAFVIVAVNLIVVLVVAVNTQRPSIGSSKSQDVVIIMRISKNVAILTPLLGLTW
GFGIATLIEGTSLTFHIIFALLNAFQGFFILLFGTIMDHKIRDALRMRMSSLKGKSRAAENAS
LGPTNGSKLMNRQG

>P98196 multipass membrane protein
MDCSLVRTLVHRYCAGEENWVDSRTIYVGHREPPPGAEAYIPQRYPDNRIVSSKYTFWNFI
PKNLFEQFRRVANFYFLIIFLVQLIIDTPTSPVTSGLPLFFVITVTAIKQGYEDWLRHKADNA
MNQCPVHFIQHGKLVRKQSRKLRVGDIVMVKEDETFPCDLIFLSSNRGDGTCHVTTASLD
GESSHKTHYAVQDTKGFHTEEDIGGLHATIECEQPQPDLYKFVGRINVYSDLNDPVVRPLG
SENLLLRGATLKNTEKIFGVAIYTGMETKMALNYQSKSQKRSAVEKSMNAFLIVYLCILIS

KALINTVLKYMWQSEPFRDEPWYNQKTESERQRNLFLKAFTDFLAFMVLFNYIIPVSMYV
TVEMQKFLGSYFITWDEDMFDEETGEGPLVNTSDLNEELGQVEYIFTDKTGTLTENNMEF
KECCIEGHVYVPHVICNGQVLPESSGIDMIDSSPSVNGREREELFFRALCLCHTVQVKDDD
SVDGPRKSPDGGKSCVYISSSPDEVALVEGVQRLGFTYLRLKDNYMEILNRENHIERFELL
EILSFDSVRRRMSVIVKSATGEIYLFCKGADSSIFPRVIEGKVDQIRARVERNAVEGLRTLCV
AYKRLIQEEYEGICKLLQAAKVALQDREKKLAEAYEQIEKDLTLLGATAVEDRLQEKAAD
TIEALQKAGIKVWVLTGDKMETAAATCYACKLFRRNTQLLELTTKRIEEQSLHDVLFELSK
TVLRHSGSLTRDNLSGLSADMQDYGLIIDGAALSLIMKPREDGSSGNYRELFLEICRSCSA
VLCCRMAPLQKAQIVKLIKFSKEHPITLAIGDGANDVSMILEAHVGIGVIGKEGRQAARNS
DYAIPKFKHLKKMLLVHGHFYYIRISELVQYFFYKNVCFIFPQFLYQFFCGFSQQTLYDTAY
LTLYNISFTSLPILLYSLMEQHVGIDVLKRDPTLYRDVAKNALLRWRVFIYWTLLGLFDALV
FFFGAYFVFENTTVTSNGQIFGNWTFGTLVFTVMVFTVTLKLALDTHYWTWINHFVIWGS
LLFYVVFSLLWGGVIWPFLNYQRMYYVFIQMLSSGPAWLAIVLLVTISLLPDVLKKVLCR
QLWPTATERVQTKSQCLSVEQSTIFMLSQTSSSLSF

>P01732	single-pass type I membrane protein
MALPVTALLLPLALLLHAARPSQFRVSPLDRTWNLGETVELKCQVLLSNPTSGCSWLFQP
RGAAASPTFLLYLSQNKPKAAEGLDTQRFSGKRLGDTFVLTLSDFRRENEGYYFCSALSNS
IMYFSHFVPVFLPAKPTTTPAPRPPTPAPTIASQPLSLRPEACRPAAGGAVHTRGLDFACDIYI
WAPLAGTCGVLLLSLVITLYCNHRNRRRVCKCPRPVVKSGDKPSLSARYV

>O43520	multipass membrane protein
MSTERDSETTFDEDSQPNDEVVPYSDDETEDELDDQGSAVEPEQNRVNREAEENREPFRK
ECTWQVKANDRKYHEQPHFMNTKFLCIKESKYANNAIKTYKYNAFTFIPMNLFEQFKRA
ANLYFLALLILQAVPQISTLAWYTTLVPLLVVLGVTAIKDLVDDVARHKMDKEINNRTCEVI
KDGRFKVAKWKEIQVGDVIRLKKNDFVPADILLLSSSEPNSLCYVETAELDGETNLKFKM
SLEITDQYLQREDTLATFDGFIECEEPNNRLDKFTGTLFWRNTSFPLDADKILLRGCVIRNT
DFCHGLVIFAGADTKIMKNSGKTRFKRTKIDYLMNYMVYTIFVVLILLSAGLAIGHAYWE
AQVGNSSWYLYDGEDDTPSYRGFLIFWGYIIVLNTMVPISLYVSVEVIRLGQSHFINWDLQ
MYYAEKDTPAKARTTTLNEQLGQIHYIFSDKTGTLTQNIMTFKKCCINGQIYGDHRDASQ
HNHNKIEQVDFSWNTYADGKLAFYDHYLIEQIQSGKEPEVRQFFFLLAVCHTVMVDRTDG
QLNYQAASPDEGALVNAARNFGFAFLARTQNTITISELGTERTYNVLAILDFNSDRKRMSII
VRTPEGNIKLYCKGADTVIYERLHRMNPTKQETQDALDIFANETLRTLCLCYKEIEEKEFT
EWNKKFMAASVASTNRDEALDKVYEEIEKDLILLGATAIEDKLQDGVPETISKLAKADIKI
WVLTGDKKETAENIGFACELLTEDTTICYGEDINSLLHARMENQRNRGGVYAKFAPPVQE
SFFPPGGNRALIITGSWLNEILLEKKTKRNKILKLKFPRTEEERRMRTQSKRRLEAKKEQRQ
KNFVDLACECSAVICCRVTPKQKAMVVDLVKRYKKAITLAIGDGANDVNMIKTAHIGVGI
SGQEGMQAVMSSDYSFAQFRYLQRLLLVHGRWSYIRMCKFLRYFFYKNFAFTLVHFWYSF
FNGYSAQTAYEDWFITLYNVLYTSLPVLLMGLLDQDVSDKLSLRFPGLYIVGQRDLLFNY
KRFFVSLLHGVLTSMILFFIPLGAYLQTVGQDGEAPSDYQSFAVTIASALVITVNFQIGLDTS
YWTFVNAFSIFGSIALYFGIMFDFHSAGIHVLFPSAFQFTGTASNALRQPYIWLTIILAVAVC
LLPVVAIRFLSMTIWPSESDKIQKHRKRLKAEEQWQRRQQVFRRGVSTRRSAYAFSHQRG
YADLISSGRSIRKKRSPLDAIVADGTAEYRRTGDS

>O60235 single-pass type II membrane protein
MYRPARVTSTSRFLNPYVVCFIVVAGVVILAVTIALLVYFLAFDQKSYFYRSSFQLLNVEY
NSQLNSPATQEYRTLSGRIESLITKTFKESNLRNQFIRAHVAKLRQDGSGVRADVVMKFQF
TRNNNGASMKSRIESVLRQMLNNSGNLEINPSTEITSLTDQAAANWLINECGAGPDLITLS
EQRILGGTEAEEGSWPWQVSLRLNNAHHCGGSLINNMWILTAAHCFRSNSNPRDWIATSG
ISTTFPKLRMRVRNILIHNNYKSATHENDIALVRLENSVTFTKDIHSVCLPAATQNIPPGSTA
YVTGWGAQEYAGHTVPELRQGQVRIISNDVCNAPHSYNGAILSGMLCAGVPQGGVDAC
QGDSGGPLVQEDSRRLWFIVGIVSWGDQCGLPDKPGVYTRVTAYLDWIRQQTGI

>Q9NPI9 multipass membrane protein
MSYYGSSYHIINADAKYPGYPPEHIIAEKRRARRRLLHKDGSCNVYFKHIFGEWGSYVVD
IFTTLVDTKWRHMFVIFSLSYILSWLIFGSVFWLIAFHHGDLLNDPDITPCVDNVHSFTGAF
LFSLETQTTIGYGYRCVTEECSVAVLMVILQSILSCIINTFIIGAALAKMATARKRAQTIRFSY
FALIGMRDGKLCLMWRIGDFRPNHVVEGTVRAQLLRYTEDSEGRMTMAFKDLKLVNDQI
ILVTPVTIVHEIDHESPLYALDRKAVAKDNFEILVTFIYTGDSTGTSHQSRSSYVPREILWGH
RFNDVLEVKRKYYKVNCLQFEGSVEVYAPFCSAKQLDWKDQQLHIEKAPPVRESCTSDT
KARRRSFSAVAIVSSCENPEETTTSATHEYRETPYQKALLTLNRISVESQM

>Q8NGG7 multipass membrane protein
MGFLSPMHPCRPPTQRRMAAGNHSTVTEFILKGLTKRADLQLPLFLLFLGIYLVTIVGNLG
MITLICLNSQLHTPMYYFLSNLSLMDLCYSSVITPKMLVNFVSEKNIISYAGCMSQLYFFLV
FVIAECYMLTVMAYDRYVAICHPLLYNIIMSHHTCLLLVAVVYAIGLIGSTIETGLMLKLPY
CEHLISHYFCDILPLMKLSCSSTYDVEMTVFFSAGFNIIVTSLTVLVSYTFILSSILGISTTEGR
SKAFSTCSSHLAAVGMFYGSTAFMYLKPSTISSLTQENVASVFYTTVIPMLNPLIYSLRNKE
VKAAVQKTLRGKLF

>P49685 multipass membrane protein
MDPEETSVYLDYYYATSPNSDIRETHSHVPYTSVFLPVFYTAVFLTGVLGNLVLMGALHFK
PGSRRLIDIFIINLAASDFIFLVTLPLWVDKEASLGLWRTGSFLCKGSSYMISVNMHCSVLLL
TCMSVDRYLAIVWPVVSRKFRRTDCAYVVCASIWFISCLLGLPTLLSRELTLIDDKPYCAE
KKATPIKLIWSLVALIFTFFVPLLSIVTCYCCIARKLCAHYQQSGKHNKKLKKSIKIIFIVVAA
FLVSWLPFNTFKFLAIVSGLRQEHYLPSAILQLGMEVSGPLAFANSCVNPFIYYIFDSYIRRA
IVHCLCPCLKNYDFGSSTETSDSHLTKALSTFIHAEDFARRRKRSVSL

>P17252 peripheral membrane protein
MADVFPGNDSTASQDVANRFARKGALRQKNVHEVKDHKFIARFFKQPTFCSHCTDFIWG
FGKQGFQCQVCCFVVHKRCHEFVTFSCPGADKGPDTDDPRSKHKFKIHTYGSPTFCDHC
GSLLYGLIHQGMKCDTCDMNVHKQCVINVPSLCGMDHTEKRGRIYLKAEVADEKLHVTV
RDAKNLIPMDPNGLSDPYVKLKLIPDPKNESKQKTKTIRSTLNPQWNESFTFKLKPSDKDR
RLSVEIWDWDRTTRNDFMGSLSFGVSELMKMPASGWYKLLNQEEGEYYNVPIPEGDEEG
NMELRQKFEKAKLGPAGNKVISPSEDRKQPSNNLDRVKLTDFNFLMVLGKGSFGKVMLA
DRKGTEELYAIKILKKDVVIQDDDVECTMVEKRVLALLDKPPFLTQLHSCFQTVDRLYFV
MEYVNGGDLMYHIQQVGKFKEPQAVFYAAEISIGLFFLHKRGIIYRDLKLDNVMLDSEGH
IKIADFGMCKEHMMDGVTTRTFCGTPDYIAPEIIAYQPYGKSVDWWAYGVLLYEMLAGQ

PPFDGEDEDELFQSIMEHNVSYPKSLSKEAVSVCKGLMTKHPAKRLGCGPEGERDVREHA
FFRRIDWEKLENREIQPPFKPKVCGKGAENFDKFFTRGQPVLTPPDQLVIANIDQSDFEGFS
YVNPQFVHPILQSAV

>P09936 lipid-anchor
MQLKPMEINPEMLNKVLSRLGVAGQWRFVDVLGLEEESLGSVPAPACALLLLFPLTAQHE
NFRKKQIEELKGQEVSPKVYFMKQTIGNSCGTIGLIHAVANNQDKLGFEDGSVLKQFLSET
EKMSPEDRAKCFEKNEAIQAAHDAVAQEGQCRVDDKVNFHFILFNNVDGHLYELDGRMP
FPVNHGASSEDTLLKDAAKVCREFTEREQGEVRFSAVALCKAA

>Q8NDV2 multipass membrane protein
MNSWDAGLAGLLVGTMGVSLLSNALVLLCLLHSADIRRQAPALFTLNLTCGNLLCTVVN
MPLTLAGVVAQRQPAGDRLCRLAAFLDTFLAANSMLSMAALSIDRWVAVVFPLSYRAKM
RLRDAALMVAYTWLHALTFPAAALALSWLGFHQLYASCTLCSRRPDERLRFAVFTGAFHA
LSFLLSFVVLCCTYLKVLKVARFHCKRIDVITMQTLVLLVDLHPSVRERCLEEQKRRRQRA
TKKISTFIGTFLVCFAPYVITRLVELFSTVPIGSHWGVLSKCLAYSKAASDPFVYSLLRHQY
RKSCKEILNRLLHRRSIHSSGLTGDSHSQNILPVSE

>P48201 multipass membrane protein
MFACAKLACTPSLIRAGSRVAYRPISASVLSRPEASRTGEGSTVFNGAQNGVSQLIQREFQT
SAISRDIDTAAKFIGAGAATVGVAGSGAGIGTVFGSLIIGYARNPSLKQQLFSYAILGFALSE
AMGLFCLMVAFLILFAM

>P78310 single-pass type I membrane protein
MALLLCFVLLCGVVDFARSLSITTPEEMIEKAKGETAYLPCKFTLSPEDQGPLDIEWLISPA
DNQKVDQVIILYSGDKIYDDYYPDLKGRVHFTSNDLKSGDASINVTNLQLSDIGTYQCKV
KKAPGVANKKIHLVVLVKPSGARCYVDGSEEIGSDFKIKCEPKEGSLPLQYEWQKLSDSQ
KMPTSWLAEMTSSVISVKNASSEYSGTYSCTVRNRVGSDQCLLRLNVVPPSNKAGLIAGA
IIGTLLALALIGLIIFCCRKKRREEKYEKEVHHDIREDVPPPKSRTSTARSYIGSNHSSLGSMS
PSNMEGYSKTQYNQVPSEDFERTPQSPTLPPAKVAAPNLSRMGAIPVMIPAQSKDGSIV

>Q5TEA6 single-pass type I membrane protein
MKPLSLLIEILIILGVTIKTIKAEEHNKRQKERNVTTQVSVNEIKQYLSHILEQRTSSNVINK
RENLLEKKKNQRKIRIKGIQNKDILKRNKNHLQKQAEKNFTDEGDQLFKMGIKVLQQSKS
QKQKEEAYLLFAKAADMGNLKAMEKMADALLFGNFGVQNITAAIQLYESLAKEGSCKA
QNALGFLSSYGIGMEYDQAKALIYYTFGSAGGNMMSQMILGYRYLSGINVLQNCEVALS
YYKKVADYIADTFEKSEGVPVEKVRLTERPENLSSNSEILDWDIYQYYKFLAERGDVQIQ
VSLGQLHLIGRKGLDQDYYKALHYFLKAAKAGSANAMAFIGKMYLEGNAAVPQNNATA
FKYFSMAASKGNAIGLHGLGLLYFHGKGVPLNYAEALKYFQKAAEKGWPDAQFQLGFM
YYSGSGIWKDYKLAFKYFYLASQSGQPLAIYYLAKMYATGTGVVRSCRTAVELYKGVCE
LGHWAEKFLTAYFAYKDGDIDSSLVQYALLAEMGYEVAQSNSAFILESKKANILEKEKMYP
MALLLWNRAAIQGNAFARVKIGDYHYYGYGTKKDYQTAATHYSIAANKYHNAQAMFNL
AYMYEHGLGITKDIHLARRLYDMAAQTSPDAHIPVLFAVMKLETTHLLRDILFFNFTTRW
NWLKLDNTIGPHWDLFVIGLIVPGLILLLRNHHG

>P05067 single-pass type I membrane protein
MLPGLALLLLAAWTARALEVPTDGNAGLLAEPQIAMFCGRLNMHMNVQNGKWDSDPS
GTKTCIDTKEGILQYCQEVYPELQITNVVEANQPVTIQNWCKRGRKQCKTHPHFVIPYRCL
VGEFVSDALLVPDKCKFLHQERMDVCETHLHWHTVAKETCSEKSTNLHDYGMLLPCGID
KFRGVEFVCCPLAEESDNVDSADAEEDDSDVWWGGADTDYADGSEDKVVEVAEEEEVA
EVEEEEADDDEDDEDGDEVEEEAEEPYEEATERTTSIATTTTTTTESVEEVVREVCSEQAE
TGPCRAMISRWYFDVTEGKCAPFFYGGCGGNRNNFDTEEYCMAVCGSAMSQSLLKTTQE
PLARDPVKLPTTAASTPDAVDKYLETPGDENEHAHFQKAKERLEAKHRERMSQVMREW
EEAERQAKNLPKADKKAVIQHFQEKVESLEQEAANERQQLVETHMARVEAMLNDRRRL
ALENYITALQAVPPRPRHVFNMLKKYVRAEQKDRQHTLKHFEHVRMVDPKKAAQIRSQV
MTHLRVIYERMNQSLSLLYNVPAVAEEIQDEVDELLQKEQNYSDDVLANMISEPRISYGND
ALMPSLTETKTTVELLPVNGEFSLDDLQPWHSFGADSVPANTENEVEPVDARPAADRGLT
TRPGSGLTNIKTEEISEVKMDAEFRHDSGYEVHHQKLVFFAEDVGSNKGAIIGLMVGGVVI
ATVIVITLVMLKKKQYTSIHHGVVEVDAAVTPEERHLSKMQQNGYENPTYKFFEQMQN

>Q9NRW1 lipid-anchor
MSAGGDFGNPLRKFKLVFLGEQSVGKTSLITRFMYDSFDNTYQATIGIDFLSKTMYLEDRT
VRLQLWDTAGQERFRSLIPSYIRDSTVAVVVYDITNLNSFQQTSKWIDDVRTERGSDVIIML
VGNKTDLADKRQITIEEGEQRAKELSVMFIETSAKTGYNVKQLFRRVASALPGMENVQEK
SKEGMIDIKLDKPQEPPASEGGCSC

>O15438 multipass membrane protein
MDALCGSGELGSKFWDSNLSVHTENPDLTPCFQNSLLAWVPCIYLWVALPCYLLYLRHHC
RGYIILSHLSKLKMVLGVLLWCVSWADLFYSFHGLVHGRAPAPVFFVTPLVVGVTMLLAT
LLIQYERLQGVQSSGVLIIFWFLCVVCAIVPFRSKILLAKAEGEISDPFRFTTFYIHFALVLSA
LILACFREKPPFFSAKNVDPNPYPETSAGFLSRLFFWWFTKMAIYGYRHPLEEKDLWSLKE
EDRSQMVVQQLLEAWRKQEKQTARHKASAAPGKNASGEDEVLLGARPRPRKPSFLKAL
LATFGSSFLISACFKLIQDLLSFINPQLLSILIRFISNPMAPSWWGFLVAGLMFLCSMMQSLIL
QHYYHYIFVTGVKFRTGIMGVIYRKALVITNSVKRASTVGEIVNLMSVDAQRFMDLAPFL
NLLWSAPLQIILAIYFLWQNLGPSVLAGVAFMVLLIPLNGAVAVKMRAFQVKQMKLKDSR
IKLMSEILNGIKVLKLYAWEPSFLKQVEGIRQGELQLLRTAAYLHTTTTFTWMCSPFLVTLI
TLWVYVYVDPNNVLDAEKAFVSVSLFNILRLPLNMLPQLISNLTQASVSLKRIQQFLSQEE
LDPQSVERKTISPGYAITIHSGTFTWAQDLPPTLHSLDIQVPKGALVAVVGPVGCGKSSLVS
ALLGEMEKLEGKVHMKGSVAYVPQQAWIQNCTLQENVLFGKALNPKRYQQTLEACALL
ADLEMLPGGDQTEIGEKGINLSGGQRQRVSLARAVYSDADIFLLDDPLSAVDSHVAKHIFD
HVIGPEGVLAGKTRVLVTHGISFLPQTDFIIVLADGQVSEMGPYPALLQRNGSFANFLCNY
APDEDQGHLEDSWTALEGAEDKEALLIEDTLSNHTDLTDNDPVTYVVQKQFMRQLSALS
SDGEGQGRPVPRRHLGPSEKVQVTEAKADGALTQEEKAAIGTVELSVFWDYAKAVGLCT
TLAICLLYVGQSAAAIGANVWLSAWTNDAMADSRQNNTSLRLGVYAALGILQGFLVMLA
AMAMAAGGIQAARVLHQALLHNKIRSPQSFFDTTPSGRILNCFSKDIYVVDEVLAPVILML
LNSFFNAISTLVVIMASTPLFTVVILPLAVLYTLVQRFYAATSRQLKRLESVSRSPIYSHFSET
VTGASVIRAYNRSRDFEIISDTKVDANQRSCYPYIISNRWLSIGVEFVGNCVVLFAALFAVI
GRSSLNPGLVGLSVSYSLQVTFALNWMIRMMSDLESNIVAVERVKEYSKTETEAPWVVEG

SRPPEGWPPRGEVEFRNYSVRYRPGLDLVLRDLSLHVHGGEKVGIVGRTGAGKSSMTLCL
FRILEAAKGEIRIDGLNVADIGLHDLRSQLTIIPQDPILFSGTLRMNLDPFGSYSEEDIWWAL
ELSHLHTFVSSQPAGLDFQCSEGGENLSVGQRQLVCLARALLRKSRILVLDEATAAIDLET
DNLIQATIRTQFDTCTVLTIAHRLNTIMDYTRVLVLDKGVVAEFDSPANLIAARGIFYGMAR
DAGLA

>Q96AW1 single-pass type I membrane protein
MRRQPAKVAALLLGLLLECTEAKKHCWYFEGLYPTYYICRSYEDCCGSRCCVRALSIQRL
WYFWFLLMMGVLFCCGAGFFIRRRMYPPPLIEEPAFNVSYTRQPPNPGPGAQQPGPPYYT
DPGGPGMNPVGNSMAMAFQVPPNSPQGSVACPPPPAYCNTPPPPYEQVVKAK

>Q8N4L2 multipass membrane protein
MAADGVDERSPLLSASHSGNVTPTAPPYLQESSPRAELPPPYTAIASPDASGIPVINCRVCQ
SLINLDGKLHQHVVKCTVCNEATPIKNPPTGKKYVRCPCNCLLICKDTSRRIGCPRPNCRRI
INLGPVMLISEEQPAQPALPIQPEGTRVVCGHCGNTFLWMELRFNTLAKCPHCKKISSVGS
ALPRRRCCAYITIGMICIFIGVGLTVGTPDFARRFRATYVSWAIAYLLGLICLIRACYWGAIR
VSYPEHSFA

>Q8NGD4 multipass membrane protein
MAHTNESMVSEFVLLGLSNSWGLQLFFFAIFSIVYVTSVLGNVLIIVIISFDSHLNSPMYFLL
SNLSFIDICQSNFATPKMLVDFFIERKTISFEGCMAQIFVLHSFVGSEMMLLVAMAYDRFIAI
CKPLHYSTIMNRRLCVIFVSISWAVGVLHSVSHLAFTVDLPFCGPNEVDSFFCDLPLVIELA
CMDTYEMEIMTLTNSGLISLSCFLALIISYTIILIGVRCRSSSGSSKALSTLTAHITVVILFFGP
CIYFYIWPFSRLPVDKFLSVFYTVCTPLLNPIIYSLRNEDVKAAMWKLRNRHVNSWKN

>Q15070 multipass membrane protein
MAMGLMCGRRELLRLLQSGRRVHSVAGPSQWLGKPLTTRLLFPVAPCCCRPHYLFLAAS
GPRSLSTSAISFAEVQVQAPPVVAATPSPTAVPEVASGETADVVQTAAEQSFAELGLGSYTP
VGLIQNLLEFMHVDLGLPWWGAIAACTVFARCLIFPLIVTGQREAARIHNHLPEIQKFSSRI
REAKLAGDHIEYYKASSEMALYQKKHGIKLYKPLILPVTQAPIFISFFIALREMANLPVPSL
QTGGLWWFQDLTVSDPIYILPLAVTATMWAVLELGAETGVQSSDLQWMRNVIRMMPLITL
PITMHFPTAVFMYWLSSNLFSLVQVSCLRIPAVRTVLKIPQRVVHDLDKLPPREGFLESFKK
GWKNAEMTRQLREREQRMRNQLELAARGPLRQTFTHNPLLQPGKDNPPNIPSSSSKPKSK
YPWHDTLG

>P42261 multipass membrane protein
MQHIFAFFCTGFLGAVVGANFPNNIQIGGLFPNQQSQEHAAFRFALSQLTEPPKLLPQIDIV
NISDSFEMTYRFCSQFSKGVYAIFGFYERRTVNMLTSFCGALHVCFITPSFPVDTSNQFVLQ
LRPELQDALISIIDHYKWQKFVYIYDADRGLSVLQKVLDTAAEKNWQVTAVNILTTTEEG
YRMLFQDLEKKKERLVVVDCESERLNAILGQIIKLEKNGIGYHYILANLGFMDIDLNKFKE
SGANVTGFQLVNYTDTIPAKIMQQWKNSDARDHTRVDWKRPKYTSALTYDGVKVMAEA
FQSLRRQRIDISRRGNAGDCLANPAVPWGQGIDIQRALQQVRFEGLTGNVQFNEKGRRTN
YTLHVIEMKHDGIRKIGYWNEDDKFVPAATDAQAGGDNSSVQNRTYIVTTILEDPYVMLK
KNANQFEGNDRYEGYCVELAAEIAKHVGYSYRLEIVSDGKYGARDPDTKAWNGMVGEL

VYGRADVAVAPLTITLVREEVIDFSKPFMSLGISIMIKKPQKSKPGVFSFLDPLAYEIWMCIV
FAYIGVSVVLFLVSRFSPYEWHSEEFEEGRDQTTSDQSNEFGIFNSLWFSLGAFMQQGCDIS
PRSLSGRIVGGVWWFFTLIIISSYTANLAAFLTVERMVSPIESAEDLAKQTEIAYGTLEAGST
KEFFRRSKIAVFEKMWTYMKSAEPSVFVRTTEEGMIRVRKSKGKYAYLLESTMNEYIEQR
KPCDTMKVGGNLDSKGYGIATPKGSALRNPVNLAVLKLNEQGLLDKLKNKWWYDKGEC
GSGGGDSKDKTSALSLSNVAGVFYILIGGLGLAMLVALIEFCYKSRSESKRMKGFCLIPQQ
SINEAIRTSTLPRNSGAGASSGGSGENGRVVSHDFPKSMQSIPCMSHSSGMPLGATGL

>P50443 multipass membrane protein
MSSESKEQHNVSPRDSAEGNDSYPSGIHLELQRESSTDFKQFETNDQCRPYHRILIERQEKS
DTNFKEFVIKKLQKNCQCSPAKAKNMILGFLPVLQWLPKYDLKKNILGDVMSGLIVGILL
VPQSIAYSLLAGQEPVYGLYTSFFASIIYFLLGTSRHISVGIFGVLCLMIGETVDRELQKAGY
DNAHSAPSLGMVSNGSTLLNHTSDRICDKSCYAIMVGSTVTFIAGVYQVAMGFFQVGFVS
VYLSDALLSGFVTGASFTILTSQAKYLLGLNLPRTNGVGSLITTWIHVFRNIHKTNLCDLIT
SLLCLLVLLPTKELNEHFKSKLKAPIPIELVVVVAATLASHFGKLHENYNSSIAGHIPTGFMP
PKVPEWNLIPSVAVDAIAISIIGFAITVSLSEMFAKKHGYTVKANQEMYAIGFCNIIPSFFHCF
TTSAALAKTLVKESTGCHTQLSGVVTALVLLLVLLVIAPLFYSLQKSVLGVITIVNLRGALR
KFRDLPKMWSISRMDTVIWFVTMLSSALLSTEIGLLVGVCFSIFCVILRTQKPKSSLLGLVE
ESEVFESVSAYKNLQIKPGIKIFRFVAPLYYINKECFKSALYKQTVNPILIKVAWKKAAKRKI
KEKVVTLGGIQDEMSVQLSHDPLELHTIVIDCSAIQFLDTAGIHTLKEVRRDYEAIGIQVLL
AQCNPTVRDSLTNGEYCKKEEENLLFYSVYEAMAFAEVSKNQKGVCVPNGLSLSSD

>P07333 single-pass type I membrane protein
MGPGVLLLLLVATAWHGQGIPVIEPSVPELVVKPGATVTLRCVGNGSVEWDGPPSPHWTL
YSDGSSSILSTNNATFQNTGTYRCTEPGDPLGGSAAIHLYVKDPARPWNVLAQEVVVFED
QDALLPCLLTDPVLEAGVSLVRVRGRPLMRHTNYSFSPWHGFTIHRAKFIQSQDYQCSAL
MGGRKVMSISIRLKVQKVIPGPPALTLVPAELVRIRGEAAQIVCSASSVDVNFDVFLQHNN
TKLAIPQQSDFHNNRYQKVLTLNLDQVDFQHAGNYSCVASNVQGKHSTSMFFRVVESAY
LNLSSEQNLIQEVTVGEGLNLKVMVEAYPGLQGFNWTYLGPFSDHQPEPKLANATTKDT
YRHTFTLSLPRLKPSEAGRYSFLARNPGGWRALTFELTLRYPPEVSVIWTFINGSGTLLCAA
SGYPQPNVTWLQCSGHTDRCDEAQVLQVWDDPYPEVLSQEPFHKVTVQSLLTVETLEHN
QTYECRAHNSVGSGSWAFIPISAGAHTHPPDEFLFTPVVVACMSIMALLLLLLLLLLYKYK
QKPKYQVRWKIIESYEGNSYTFIDPTQLPYNEKWEFPRNNLQFGKTLGAGAFGKVVEATA
FGLGKEDAVLKVAVKMLKSTAHADEKEALMSELKIMSHLGQHENIVNLLGACTHGGPVL
VITEYCCYGDLLNFLRRKAEAMLGPSLSPGQDPEGGVDYKNIHLEKKYVRRDSGFSSQGV
DTYVEMRPVSTSSNDSFSEQDLDKEDGRPLELRDLLHFSSQVAQGMAFLASKNCIHRDVA
ARNVLLTNGHVAKIGDFGLARDIMNDSNYIVKGNARLPVKWMAPESIFDCVYTVQSDVW
SYGILLWEIFSLGLNPYPGILVNSKFYKLVKDGYQMAQPAFAPKNIYSIMQACWALEPTHR
PTFQQICSFLQEQAQEDRRERDYTNLPSSSRSGGSGSSSSELEEESSSEHLTCCEQGDIAQPL
LQPNNYQFC

>Q9NYJ8 peripheral membrane protein
MAQGSHQIDFQVLHDLRQKFPEVPEVVVSRCMLQNNNNLDACCAVLSQESTRYLYGEGD
LNFSDDSGISGLRNHMTSLNLDLQSQNIYHHGREGSRMNGSRTLTHSISDGQLQGGQSNSE

LFQQEPQTAPAQVPQGFNVFGMSSSSGASNSAPHLGFHLGSKGTSSLSQQTPRFNPIMVTL
APNIQTGRNTPTSLHIHGVPPPVLNSPQGNSIYIRPYITTPGGTTRQTQQHSGWVSQFNPMN
PQQVYQPSQPGPWTTCPASNPLSHTSSQQPNQQGHQTSHVYMPISSPTTSQPPTIHSSGSSQ
SSAHSQYNIQNISTGPRKNQIEIKLEPPQRNNSSKLRSSGPRTSSTSSSVNSQTLNRNQPTVY
IAASPPNTDELMSRSQPKVYISANAATGDEQVMRNQPTLFISTNSGASAASRNMSGQVSM
GPAFIHHHPPKSRAIGNNSATSPRVVVTQPNTKYTFKITVSPNKPPAVSPGVVSPTFELTNLL
NHPDHYVETENIQHLTDPTLAHVDRISETRKLSMGSDDAAYTQALLVHQKARMERLQRE
LEIQKKKLDKLKSEVNEMENNLTRRRLKRSNSISQIPSLEEMQQLRSCNRQLQIDIDCLTKE
IDLFQARGPHFNPSAIHNFYDNIGFVGPVPPKPKDQRSIIKTPKTQDTEDDEGAQWNCTAC
TFLNHPALIRCEQCEMPRHF

>P40145 multipass membrane protein
MELSDVRCLTGSEELYTIHPTPPAGDGRSASRPQRLLWQTAVRHITEQRFIHGHRGGSGSGS
GGSGKASDPAGGGPNHHAPQLSGDSALPLYSLGPGERAHSTCGTKVFPERSGSGSASGSG
GGGDLGFLHLDCAPSNSDFFLNGGYSYRGVIFPTLRNSFKSRDLERLYQRYFLGQRRKSEV
VMNVLDVLTKLTLLVLHLSLASAPMDPLKGILLGFFTGIEVVICALVVVRKDTTSHTYLQY
SGVVTWVAMTTQILAAGLGYGLLGDGIGYVLFTLFATYSMLPLPLTWAILAGLGTSLLQVI
LQVVIPRLAVISINQVVAQAVLFMCMNTAGIFISYLSDRAQRQAFLETRRCVEARLRLETEN
QRQERLVLSVLPRFVVLEMINDMTNVEDEHLQHQFHRIYIHRYENVSILFADVKGFTNLST
TLSAQELVRMLNELFARFDRLAHEHHCLRIKILGDCYYCVSGLPEPRQDHAHCCVEMGLS
MIKTIRYVRSRTKHDVDMRIGIHSGSVLCGVLGLRKWQFDVWSWDVDIANKLESGGIPGR
IHISKATLDCLNGDYNVEEGHGKERNEFLRKHNIETYLIKQPEDSLLSLPEDIVKESVSSSD
RRNSGATFTEGSWSPELPFDNIVGKQNTLAALTRNSINLLPNHLAQALHVQSGPEEINKRIE
HTIDLRSGDKLRREHIKPFSLMFKDSSLEHKYSQMRDEVFKSNLVCAFIVLLFITAIQSLLPS
SRVMPMTIQFSILIMLHSALVLITTAEDYKCLPLILRKTCCWINETYLARNVIIFASILINFLG
AILNILWCDFDKSIPLKNLTFNSSAVFTDICSYPEYFVFTGVLAMVTCAVFLRLNSVLKLAV
LLIMIAIYALLTETVYAGLFLRYDNLNHSGEDFLGTKEVSLLLMAMFLLAVFYHGQQLEYT
ARLDFLWRVQAKEEINEMKELREHNENMLRNILPSHVARHFLEKDRDNEELYSQSYDAVG
VMFASIPGFADFYSQTEMNNQGVECLRLLNEIIADFDELLGEDRFQDIEKIKTIGSTYMAVS
GLSPEKQQCEDKWGHLCALADFSLALTESIQEINKHSFNNFELRIGISHGSVVAGVIGAKKP
QYDIWGKTVNLASRMDSTGVSGRIQVPEETYLILKDQGFAFDYRGEIYVKGISEQEGKIKT
YFLLGRVQPNPFILPPRRLPGQYSLAAVVLGLVQSLNRQRQKQLLNENNNTGIIKGHYNRR
TLLSPSGTEPGAQAEGTDKSDLP

>P54709 single-pass type II membrane protein
MTKNEKKSLNQSLAEWKLFIYNPTTGEFLGRTAKSWGLILLFYLVFYGFLAALFSFTMWV
MLQTLNDEVPKYRDQIPSPGLMVFPKPVTALEYTFSRSDPTSYAGYIEDLKKFLKPYTLEE
QKNLTVCPDGALFEQKGPVYVACQFPISLLQACSGMNDPDFGYSQGNPCILVKMNRIIGLK
PEGVPRIDCVSKNEDIPNVAVYPHNGMIDLKYFPYYGKKLHVGYLQPLVAVQVSFAPNNT
GKEVTVECKIDGSANLKSQDDRDKFLGRVMFKITARA

>P51512 single-pass type I membrane protein
MILLTFSTGRRLDFVHHSGVFFLQTLLWILCATVCGTEQYFNVEVWLQKYGYLPPTDPRM
SVLRSAETMQSALAAMQQFYGINMTGKVDRNTIDWMKKPRCGVPDQTRGSSKFHIRRK

RYALTGQKWQHKHITYSIKNVTPKVGDPETRKAIRRAFDVWQNVTPLTFEEVPYSELENG
KRDVDITIIFASGFHGDSSPFDGEGGFLAHAYFPGPGIGGDTHFDSDEPWTLGNPNHDGND
LFLVAVHELGHALGLEHSNDPTAIMAPFYQYMETDNFKLPNDDLQGIQKIYGPPDKIPPPT
RPLPTVPPHRSIPPADPRKNDRPKPPRPPTGRPSYPGAKPNICDGNFNTLAILRREMFVFKD
QWFWRVRNNRVMDGYPMQITYFWRGLPPSIDAVYENSDGNFVFFKGNKYWVFKDTTLQ
PGYPHDLITLGSGIPPHGIDSAIWWEDVGKTYFFKGDRYWRYSEEMKTMDPGYPKPITVW
KGIPESPQGAFVHKENGFTYFYKGKEYWKFNNQILKVEPGYPRSILKDFMGCDGPTDRVK
EGHSPPDDVDIVIKLDNTASTVKAIAIVIPCILALCLLVLVYTVFQFKRKGTPRHILYCKRSM
QEWV

>Q12791 multipass membrane protein
MANGGGGGGGSSGGGGGGGGSSLRMSSNIHANHLSLDASSSSSSSSSSSSSSSSSSSSSSVH
EPKMDALIIPVTMEVPCDSRGQRMWWAFLASSMVTFFGGLFIILLWRTLKYLWTVCCHCG
GKTKEAQKINNGSSQADGTLKPVDEKEEAVAAEVGWMTSVKDWAGVMISAQTLTGRVL
VVLVFALSIGALVIYFIDSSNPIESCQNFYKDFTLQIDMAFNVFFLLYFGLRFIAANDKLWF
WLEVNSVVDFFTVPPVFVSVYLNRSWLGLRFLRALRLIQFSEILQFLNILKTSNSIKLVNLL
SIFISTWLTAAGFIHLVENSGDPWENFQNNQALTYWECVYLLMVTMSTVGYGDVYAKTTL
GRLFMVFFILGGLAMFASYVPEIIELIGNRKKYGGSYSAVSGRKHIVVCGHITLESVSNFLK
DFLHKDRDDVNVEIVFLHNISPNLELEALFKRHFTQVEFYQGSVLNPHDLARVKIESADAC
LILANKYCADPDAEDASNIMRVISIKNYHPKIRIITQMLQYHNKAHLLNIPSWNWKEGDD
AICLAELKLGFIAQSCLAQGLSTMLANLFSMRSFIKIEEDTWQKYYLEGVSNEMYTEYLSS
AFVGLSFPTVCELCFVKLKLLMIAIEYKSANRESRILINPGNHLKIQEGTLGFFIASDAKEV
KRAFFYCKACHDDITDPKRIKKCGCKRPKMSIYKRMRRACCFDCGRSERDCSCMSGRVR
GNVDTLERAFPLSSVSVNDCSTSFRAFEDEQPSTLSPKKKQRNGGMRNSPNTSPKLMRHD
PLLIPGNDQIDNMDSNVKKYDSTGMFHWCAPKEIEKVILTRSEAAMTVLSGHVVVCIFGD
VSSALIGLRNLVMPLRASNFHYHELKHIVFVGSIEYLKREWETLHNFPKVSILPGTPLSRAD
LRAVNINLCDMCVILSANQNNIDDTSLQDKECILASLNIKSMQFDDSIGVLQANSQGFTPP
GMDRSSPDNSPVHGMLRQPSITTGVNIPIITELVNDTNVQFLDQDDDDDPDTELYLTQPFA
CGTAFAVSVLDSLMSATYFNDNILTLIRTLVTGGATPELEALIAEENALRGGYSTPQTLANR
DRCRVAQLALLDGPFADLGDGGCYGDLFCKALKTYNMLCFGIYRLRDAHLSTPSQCTKR
YVITNPPYEFELVPTDLIFCLMQFDHNAGQSRASLSHSSHSSQSSSKKSSSVHSIPSTANRQN
RPKSRESRDKQKYVQEERL

>Q6UX65 multipass membrane protein
MWWFQQGLSFLPSALVIWTSAAFIFSYITAVTLHHIDPALPYISDTGTVAPEKCLFGAMLNI
AAVLCIATIYVRYKQVHALSPEENVIIKLNKAGLVLGILSCLGLSIVANFQKTTLFAAHVSG
AVLTFGMGSLYMFVQTILSYQMQPKIHGKQVFWIRLLLVIWCGVSALSMLTCSSVLHSGN
FGTDLEQKLHWNPEDKGYVLHMITTAAEWSMSFSFFGFFLTYIRDFQKISLRVEANLHGLT
LYDTAPCPINNERTRLLSRDI

>P27105 lipid-anchor, peripheral membrane protein
MAEKRHTRDSEAQRLPDSFKDSPSKGLGPCGWILVAFSFLFTVITFPISIWMCIKIIKEYERA
IIFRLGRILQGGAKGPGLFFILPCTDSFIKVDMRTISFDIPPQEILTKDSVTISVDGVVYYRVQ
NATLAVANITNADSATRLLAQTTLRNVLGTKNLSQILSDREEIAHNMQSTLDDATDAWGIK

VERVEIKDVKLPVQLQRAMAAEAEASREARAKVIAAEGEMNASRALKEASMVITESPAA
LQLRYLQTLTTIAAEKNSTIVFPLPIDMLQGIIGAKHSHLG

>O00337 multipass membrane protein
MENDPSRRRESISLTPVAKGLENMGADFLESLEEGQLPRSDLSPAEIRSSWSEAAPKPFSRW
RNLQPALRARSFCREHMQLFRWIGTGLLCTGLSAFLLVACLLDFQRALALFVLTCVVLTFL
GHRLLKRLLGPKLRRFLKPQGHPRLLLWFKRGLALAAFLGLVLWLSLDTSQRPEQLVSFA
GICVFVALLFACSKHHCAVSWRAVSWGLGLQFVLGLLVIRTEPGFIAFEWLGEQIRIFLSYT
KAGSSFVFGEALVKDVFAFQVLPIIVFFSCVISVLYHVGLMQWVILKIAWLMQVTMGTTAT
ETLSVAGNIFVSQTEAPLLIRPYLADMTLSEVHVVMTGGYATIAGSLLGAYISFGIDATSLIA
ASVMAAPCALALSKLVYPEVEESKFRREEGVKLTYGDAQNLIEAASTGAAISVKVVANIA
ANLIAFLAVLDFINAALSWLGDMVDIQGLSFQLICSYILRPVAFLMGVAWEDCPVVAELLGI
KLFLNEFVAYQDLSKYKQRRLAGAEEWVGDRKQWISVRAEVLTTFALCGFANFSSIGIML
GGLTSMVPQRKSDFSQIVLRALFTGACVSLVNACMAGILYMPRGAEVDCMSLLNTTLSSS
SFEIYQCCREAFQSVNPEFSPEALDNCCRFYNHTICAQ

>P56750 multipass membrane protein
MAFYPLQIAGLVLGFLGMVGTLATTLLPQWRVSAFVGSNIIVFERLWEGLWMNCIRQARV
RLQCKFYSSLLALPPALETARALMCVAVALSLIALLIGICGMKQVQCTGSNERAKAYLLGT
SGVLFILTGIFVLIPVSWTANIIIRDFYNPAIHIGQKRELGAALFLGWASAAVLFIGGGLLCGF
CCCNRKKQGYRYPVPGYRVPHTDKRRNTTMLSKTSTSYV

>P56748 multipass membrane protein
MATHALEIAGLFLGGVGMVGTVAVTVMPQWRVSAFIENNIVVFENFWEGLWMNCVRQA
NIRMQCKIYDSLLALSPDLQAARGLMCAASVMSFLAFMMAILGMKCTRCTGDNEKVKA
HILLTAGIIFIITGMVVLIPVSWVANAIIRDFYNSIVNVAQKRELGEALYLGWTTALVLIVGG
ALFCCVFCCNEKSSSYRYSIPSHRTTQKSYHTGKKSPSVYSRSQYV

>Q6YHK3 GPI-anchor, lipid-anchor
MQGPPLLTAAHLLCVCTAALAVAPGPRFLVTAPGIIRPGGNVTIGVELLEHCPSQVTVKAEL
LKTASNLTVSVLEAEGVFEKGSFKTLTLPSLPLNSADEIYELRVTGRTQDEILFSNSTRLSFE
TKRISVFIQTDKALYKPKQEVKFRIVTLFSDFKPYKTSLNILIKDPKSNLIQQWLSQQSDLG
VISKTFQLSSHPILGDWSIQVQVNDQTYYQSFQVSEYVLPKFEVTLQTPLYCSMNSKHLNG
TITAKYTYGKPVKGDVTLTFLPLSFWGKKKNITKTFKINGSANFSFNDEEMKNVMDSSNG
LSEYLDLSSPGPVEILTTVTESVTGISRNVSTNVFFKQHDYIIEFFDYTTVLKPSLNFTATVK
VTRADGNQLTLEERRNNVVITVTQRNYTEYWSGSNSGNQKMEAVQKINYTVPQSGTFKI
EFPILEDSSELQLKAYFLGSKSSMAVHSLFKSPSKTYIQLKTRDENIKVGSPFELVVSGNKR
LKELSYMVVSRGQLVAVGKQNSTMFSLTPENSWTPKACVIVYYIEDDGEIISDVLKIPVQL
VFKNKIKLYWSKVKAEPSEKVSLRISVTQPDSIVGIVAVDKSVNLMNASNDITMENVVHEL
ELYNTGYYLGMFMNSFAVFQECGLWVLTDANLTKDYIDGVYDNAEYAERFMEENEGHIV
DIHDFSLGSSPHVRKHFPETWIWLDTNMGYRIYQEFEVTVPDSITSWVATGFVISEDLGLGL
TTTPVELQAFQPFFIFLNLPYSVIRGEEFALEITIFNYLKDATEVKVIIEKSDKFDILMTSNEIN
ATGHQQTLLVPSEDGATVLFPIRPTHLGEIPITVTALSPTASDAVTQMILVKAEGIEKSYSQSI
LLDLTDNRLQSTLKTLSFSFPPNTVTGSERVQITAIGDVLGPSINGLASLIRMPYGCGEQNMI

NFAPNIYILDYLTKKKQLTDNLKEKALSFMRQGYQRELLYQREDGSFSAFGNYDPSGSTW
LSAFVLRCFLEADPYIDIDQNVLHRTYTWLKGHQKSNGEFWDPGRVIHSELQGGNKSPVT
LTAYIVTSLLGYRKYQPNIDVQESIHFLESEFSRGISDNYTLALITYALSSVGSPKAKEALNM
LTWRAEQEGGMQFWVSSESKLSDSWQPRSLDIEVAAYALLSHFLQFQTSEGIPIMRWLSRQ
RNSLGGFASTQDTTVALKALSEFAALMNTERTNIQVTVTGPSSPSPVKFLIDTHNRLLLQTA
ELAVVQPTAVNISANGFGFAICQLNVVYNVKASGSSRRRRSIQNQEAFDLDVAVKENKDD
LNHVDLNVCTSFSGPGRSGMALMEVNLLSGFMVPSEAISLSETVKKVEYDHGKLNLYLD
SVNETQFCVNIPAVRNFKVSNTQDASVSIVDYYEPRRQAVRSYNSEVKLSSCDLCSDVQGC
RPCEDGASGSHHHSSVIFIFCFKLLYFMELWL

>P11215 single-pass type I membrane protein
MALRVLLLTALTLCHGFNLDTENAMTFQENARGFGQSVVQLQGSRVVVGAPQEIVAANQ
RGSLYQCDYSTGSCEPIRLQVPVEAVNMSLGLSLAATTSPPQLLACGPTVHQTCSENTYVK
GLCFLFGSNLRQQPQKFPEALRGCPQEDSDIAFLIDGSGSIIPHDFRRMKEFVSTVMEQLKK
SKTLFSLMQYSEEFRIHFTFKEFQNNPNPRSLVKPITQLLGRTHTATGIRKVVRELFNITNGA
RKNAFKILVVITDGEKFGDPLGYEDVIPEADREGVIRYVIGVGDAFRSEKSRQELNTIASKP
PRDHVFQVNNFEALKTIQNQLREKIFAIEGTQTGSSSSFEHEMSQEGFSAAITSNGPLLSTV
GSYDWAGGVFLYTSKEKSTFINMTRVDSDMNDAYLGYAAAIILRNRVQSLVLGAPRYQHI
GLVAMFRQNTGMWESNANVKGTQIGAYFGASLCSVDVDSNGSTDLVLIGAPHYYEQTRG
GQVSVCPLPRGRARWQCDAVLYGEQGQPWGRFGAALTVLGDVNGDKLTDVAIGAPGEE
DNRGAVYLFHGTSGSGISPSHSQRIAGSKLSPRLQYFGQSLSGGQDLTMDGLVDLTVGAQ
GHVLLLRSQPVLRVKAIMEFNPREVARNVFECNDQVVKGKEAGEVRVCLHVQKSTRDRL
REGQIQSVVTYDLALDSGRPHSRAVFNETKNSTRRQTQVLGLTQTCETLKLQLPNCIEDPV
SPIVLRLNFSLVGTPLSAFGNLRPVLAEDAQRLFTALFPFEKNCGNDNICQDDLSITFSFMS
LDCLVVGGPREFNVTVTVRNDGEDSYRTQVTFFFPLDLSYRKVSTLQNQRSQRSWRLACE
SASSTEVSGALKSTSCSINHPIFPENSEVTFNITFDVDSKASLGNKLLLKANVTSENNMPRT
NKTEFQLELPVKYAVYMVVTSHGVSTKYLNFTASENTSRVMQHQYQVSNLGQRSLPISLV
FLVPVRLNQTVIWDRPQVTFSENLSSTCHTKERLPSHSDFLAELRKAPVVNCSIAVCQRIQC
DIPFFGIQEEFNATLKGNLSFDWYIKTSHNHLLIVSTAEILFNDSVFTLLPGQGAFVRSQTET
KVEPFEVPNPLPLIVGSSVGGLLLLALITAALYKLGFFKRQYKDMMSEGGPPGAEPQ

>P30531 multipass membrane protein
MATNGSKVADGQISTEVSEAPVANDKPKTLVVKVQKKAADLPDRDTWKGRFDFLMSCV
GYAIGLGNVWRFPYLCGKNGGGAFLIPYFLTLIFAGVPLFLLECSLGQYTSIGGLGVWKLA
PMFKGVGLAAAVLSFWLNIYYIVIISWAIYYLYNSFTTTLPWKQCDNPWNTDRCFSNYSM
VNTTNMTSAVVEFWERNMHQMTDGLDKPGQIRWPLAITLAIAWILVYFCIWKGVGWTG
KVVYFSATYPYIMLIILFFRGVTLPGAKEGILFYITPNFRKLSDSEVWLDAATQIFFSYGLGL
GSLIALGSYNSFHNNVYRDSIIVCCINSCTSMFAGFVIFSIVGFMAHVTKRSIADVAASGPG
LAFLAYPEAVTQLPISPLWAILFFSMLLMLGIDSQFCTVEGFITALVDEYPRLLRNRRELFIA
AVCIISYLIGLSNITQGGIYVFKLFDYYSASGMSLLFLVFFECVSISWFYGVNRFYDNIQEM
VGSRPCIWWKLCWSFFTPIIVAGVFIFSAVQMTPLTMGNYVFPKWGQGVGWLMALSSMV
LIPGYMAYMFLTLKGSLKQRIQVMVQPSEDIVRPENGPEQPQAGSSTSKEAYI

>P47898 multipass membrane protein

MDLPVNLTSFSLSTPSPLETNHSLGKDDLRPSSPLLSVFGVLILTLLGFLVAATFAWNLLVLA
TILRVRTFHRVPHNLVASMAVSDVLVAALVMPLSLVHELSGRRWQLGRRLCQLWIACDVL
CCTASIWNVTAIALDRYWSITRHMEYTLRTRKCVSNVMIALTWALSAVISLAPLLFGWGET
YSEGSEECQVSREPSYAVFSTVGAFYLPLCVVLFVYWKIYKAAKFRVGSRKTNSVSPISEA
VEVKDSAKQPQMVFTVRHATVTFQPEGDTWREQKEQRAALMVGILIGVFVLCWIPFFLTE
LISPLCSCDIPAIWKSIFLWLGYSNSFFNPLIYTAFNKNYNSAFKNFFSRQH

>Q8NFT2 multipass membrane protein
MESISMMGSPKSLSETFLPNGINGIKDARKVTVGVIGSGDFAKSLTIRLIRCGYHVVIGSRN
PKFASEFFPHVVDVTHHEDALTKTNIIFVAIHREHYTSLWDLRHLLVGKILIDVSNNMRINQ
YPESNAEYLASLFPDSLIVKGFNVVSAWALQLGPKDASRQVYICSNNIQARQQVIELARQL
NFIPIDLGSLSSAREIENLPLRLFTLWRGPVVVAISLATFFFLYSFVRDVIHPYARNQQSDFYK
IPIEIVNKTLPIVAITLLSLVYLAGLLAAAYQLYYGTKYRRFPPWLETWLQCRKQLGLLSFFF
AMVHVAYSLCLPMRRSERYLFLNMAYQQVHANIENSWNEEEVWRIEMYISFGIMSLGLLS
LLAVTSIPSVSNALNWREFSFIQSTLGYVALLISTFHVLIYGWKRAFEEEYYRFYTPPNFVL
ALVLPSIVILGKIILFLPCISRKLKRIKKGWEKSQFLEEGMGGTIPHVSPERVTVM

>P56749 multipass membrane protein
MGCRDVHAATVLSFLCGIASVAGLFAGTLLPNWRKLRLITFNRNEKNLTVYTGLWVKCAR
YDGSSDCLMYDTTWYSSVDQLDLRVLQFALPLSMLIAMGALLLCLIGMCNTAFRSSVPNI
KLAKCLVNSAGCHLVAGLLFFLAGTVSLSPSIWVIFYNIHLNKKFEPVFSFDYAVYVTIASA
GGLFMTSLILFIWYCTCKSLPSPFWQPLYSHPPSMHTYSQPYSARSRLSAIEIDIPVVSHTT

>Q9UP38 multipass membrane protein
MAEEEAPKKSRAAGGGASWELCAGALSARLAEEGSGDAGGRRRPPVDPRRLARQLLLLL
WLLEAPLLLGVRAQAAGQGPGQGPGPGQQPPPPPQQQQSGQQYNGERGISVPDHGYCQP
ISIPLCTDIAYNQTIMPNLLGHTNQEDAGLEVHQFYPLVKVQCSAELKFFLCSMYAPVCTV
LEQALPPCRSLCERARQGCEALMNKFGFQWPDTLKCEKFPVHGAGELCVGQNTSDKGTP
TPSLLPEFWTSNPQHGGGGHRGGFPGGAGASERGKFSCPRALKVPSYLNYHFLGEKDCG
APCEPTKVYGLMYFGPEELRFSRTWIGIWSVLCCASTLFTVLTYLVDMRRFSYPERPIIFLS
GCYTAVAVAYIAGFLLEDRVVCNDKFAEDGARTVAQGTKKEGCTILFMMLYFFSMASSIW
WVILSLTWFLAAGMKWGHEAIEANSQYFHLAAWAVPAIKTITILALGQVDGDVLSGVCFV
GLNNVDALRGFVLAPLFVYLFIGTSFLLAGFVSLFRIRTIMKHDGTKTEKLEKLMVRIGVF
SVLYTVPATIVIACYFYEQAFRDQWERSWVAQSCKSYAIPCPHLQAGGGAPPHPPMSPDFT
VFMIKYLMTLIVGITSGFWIWSGKTLNSWRKFYTRLTNSKQGETTV

>P04035 multipass membrane protein
MLSRLFRMHGLFVASHPWEVIVGTVTLTICMMSMNMFTGNNKICGWNYECPKFEEDVLS
SDIIILTITRCIAILYIYFQFQNLRQLGSKYILGIAGLFTIFSSFVFSTVVIHFLDKELTGLNEAL
PFFLLLIDLSRASTLAKFALSSNSQDEVRENIARGMAILGPTFTLDALVECLVIGVGTMSGV
RQLEIMCCFGCMSVLANYFVFMTFFPACVSLVLELSRESREGRPIWQLSHFARVLEEEENK
PNPVTQRVKMIMSLGLVLVHAHSRWIADPSPQNSTADTSKVSLGLDENVSKRIEPSVSLWQ
FYLSKMISMDIEQVITLSLALLLAVKYIFFEQTETESTLSLKNPITSPVVTQKKVPDNCCRRE
PMLVRNNQKCDSVEEETGINRERKVEVIKPLVAETDTPNRATFVVGNSSLLDTSSVLVTQE

PEIELPREPRPNEECLQILGNAEKGAKFLSDAEIIQLVNAKHIPAYKLETLMETHERGVSIRR
QLLSKKLSEPSSLQYLPYRDYNYSLVMGACCENVIGYMPIPVGVAGPLCLDEKEFQVPMA
TTEGCLVASTNRGCRAIGLGGGASSRVLADGMTRGPVVRLPRACDSAEVKAWLETSEGFA
VIKEAFDSTSRFARLQKLHTSIAGRNLYIRFQSRSGDAMGMNMISKGTEKALSKLHEYFPE
MQILAVSGNYCTDKKPAAINWIEGRGKSVVCEAVIPAKVVREVLKTTTEAMIEVNINKNLV
GSAMAGSIGGYNAHAANIVTAIYIACGQDAAQNVGSSNCITLMEASGPTNEDLYISCTMPS
IEIGTVGGGTNLLPQQACLQMLGVQGACKDNPGENARQLARIVCGTVMAGELSLMAAL
AAGHLVKSHMIHNRSKINLQDLQGACTKKTA

>Q9UMR2 peripheral membrane protein
MATDSWALAVDEQEAAAESLSNLHLKEEKIKPDTNGAVVKTNANAEKTDEEEKEDRAAQ
SLLNKLIRSNLVDNTNQVEVLQRDPNSPLYSVKSFEELRLKPQLLQGVYAMGFNRPSKIQE
NALPLMLAEPPQNLIAQSQSGTGKTAAFVLAMLSQVEPANKYPQCLCLSPTYELALQTGK
VIEQMGKFYPELKLAYAVRGNKLERGQKISEQIVIGTPGTVLDWCSKLKFIDPKKIKVFVL
DEADVMIATQGHQDQSIRIQRMLPRNCQMLLFSATFEDSVWKFAQKVVPDPNVIKLKREE
ETLDTIKQYYVLCSSRDEKFQALCNLYGAITIAQAMIFCHTRKTASWLAAELSKEGHQVAL
LSGEMMVEQRAAVIERFREGKEKVLVTTNVCARGIDVEQVSVVINFDLPVDKDGNPDNE
TYLHRIGRTGRFGKRGLAVNMVDSKHSMNILNRIQEHFNKKIERLDTDDLDEIEKIAN

>P10721 single-pass type I membrane protein
MRGARGAWDFLCVLLLLLRVQTGSSQPSVSPGEPSPPSIHPGKSDLIVRVGDEIRLLCTDPG
FVKWTFEILDETNENKQNEWITEKAEATNTGKYTCTNKHGLSNSIYVFVRDPAKLFLVDR
SLYGKEDNDTLVRCPLTDPEVTNYSLKGCQGKPLPKDLRFIPDPKAGIMIKSVKRAYHRLC
LHCSVDQEGKSVLSEKFILKVRPAFKAVPVVSVSKASYLLREGEEFTVTCTIKDVSSSVYST
WKRENSQTKLQEKYNSWHHGDFNYERQATLTISSARVNDSGVFMCYANNTFGSANVTTT
LEVVDKGFINIFPMINTTVFVNDGENVDLIVEYEAFPKPEHQQWIYMNRTFTDKWEDYPK
SENESNIRYVSELHLTRLKGTEGGTYTFLVSNSDVNAAIAFNVYVNTKPEILTYDRLVNGM
LQCVAAGFPEPTIDWYFCPGTEQRCSASVLPVDVQTLNSSGPPFGKLVVQSSIDSSAFKHN
GTVECKAYNDVGKTSAYFNFAFKGNNKEQIHPHTLFTPLLIGFVIVAGMMCIIVMILTYKY
LQKPMYEVQWKVVEEINGNNYVYIDPTQLPYDHKWEFPRNRLSFGKTLGAGAFGKVVE
ATAYGLIKSDAAMTVAVKMLKPSAHLTEREALMSELKVLSYLGNHMNIVNLLGACTIGGP
TLVITEYCCYGDLLNFLRRKRDSFICSKQEDHAEAALYKNLLHSKESSCSDSTNEYMDMK
PGVSYVVPTKADKRRSVRIGSYIERDVTPAIMEDDELALDLEDLLSFSYQVAKGMAFLAS
KNCIHRDLAARNILLTHGRITKICDFGLARDIKNDSNYVVKGNARLPVKWMAPESIFNCV
YTFESDVWSYGIFLWELFSLGSSPYPGMPVDSKFYKMIKEGFRMLSPEHAPAEMYDIMKT
CWDADPLKRPTFKQIVQLIEKQISESTNHIYSNLANCSPNRQKPVVDHSVRINSVGSTASSS
QPLLVHDDV

>Q01668 multipass membrane protein
MMMMMMMKKMQHQRQQQADHANEANYARGTRLPLSGEGPTSQPNSSKQTVLSWQAA
IDAARQAKAAQTMSTSAPPPVGSLSQRKRQQYAKSKKQGNSSNSRPARALFCLSLNNPIR
RACISIVEWKPFDIFILLAIFANCVALAIYIPFPEDDSNSTNHNLEKVEYAFLIIFTVETFLKIIA
YGLLLHPNAYVRNGWNLLDFVIVIVGLFSVILEQLTKETEGGNHSSGKSGGFDVKALRAF
RVLRPLRLVSGVPSLQVVLNSIIKAMVPLLHIALLVLFVIIIYAIIGLELFIGKMHKTCFFADS

DIVAEEDPAPCAFSGNGRQCTANGTECRSGWVGPNGGITNFDNFAFAMLTVFQCITMEGW
TDVLYWMNDAMGFELPWVYFVSLVIFGSFFVLNLVLGVLSGEFSKEREKAKARGDFQKL
REKQQLEEDLKGYLDWITQAEDIDPENEEEGGEEGKRNTSMPTSETESVNTENVSGEGEN
RGCCGSLCQAISKSKLSRRWRRWNRFNRRRCRAAVKSVTFYWLVIVLVFLNTLTISSEHYN
QPDWLTQIQDIANKVLLALFTCEMLVKMYSLGLQAYFVSLFNRFDCFVVCGGITETILVEL
EIMSPLGISVFRCVRLLRIFKVTRHWTSLSNLVASLLNSMKSIASLLLLLFLFIIIFSLLGMQL
FGGKFNFDETQTKRSTFDNFPQALLTVFQILTGEDWNAVMYDGIMAYGGPSSSGMIVCIYF
IILFICGNYILLNVFLAIAVDNLADAESLNTAQKEEAEEKERKKIARKESLENKKNNKPEVN
QIANSDNKVTIDDYREEDEDKDPYPPCDVPVGEEEEEEEEEDEPEVPAGPRPRRISELNMKE
KIAPIPEGSAFFILSKTNPIRVGCHKLINHHIFTNLILVFIMLSSAALAAEDPIRSHSFRNTILG
YFDYAFTAIFTVEILLKMTTFGAFLHKGAFCRNYFNLLDMLVVGVSLVSFGIQSSAISVVKI
LRVLRVLRPLRAINRAKGLKHVVQCVFVAIRTIGNIMIVTTLLQFMFACIGVQLFKGKFYR
CTDEAKSNPEECRGLFILYKDGDVDSPVVRERIWQNSDFNFDNVLSAMMALFTVSTFEG
WPALLYKAIDSNGENIGPIYNHRVEISIFFIIYIIIVAFFMMNIFVGFVIVTFQEQGEKEYKNCE
LDKNQRQCVEYALKARPLRRYIPKNPYQYKFWYVVNSSPFEYMMFVLIMLNTLCLAMQ
HYEQSKMFNDAMDILNMVFTGVFTVEMVLKVIAFKPKGYFSDAWNTFDSLIVIGSIIDVA
LSEADPTESENVPVPTATPGNSEESNRISITFFRLFRVMRLVKLLSRGEGIRTLLWTFIKSFQA
LPYVALLIAMLFFIYAVIGMQMFGKVAMRDNNQINRNNNFQTFPQAVLLLFRCATGEAWQ
EIMLACLPGKLCDPESDYNPGEEYTCGSNFAIVYFISFYMLCAFLIINLFVAVIMDNFDYLTR
DWSILGPHHLDEFKRIWSEYDPEAKGRIKHLDVVTLLRRIQPPLGFGKLCPHRVACKRLVA
MNMPLNSDGTVMFNATLFALVRTALKIKTEGNLEQANEELRAVIKKIWKKTSMKLLDQV
VPPAGDDEVTVGKFYATFLIQDYFRKFKKRKEQGLVGKYPAKNTTIALQAGLRTLHDIGPE
IRRAISCDLQDDEPEETKREEEDDVFKRNGALLGNHVNHVNSDRRDSLQQTNTTHRPLHV
QRPSIPPASDTEKPLFPPAGNSVCHNHHNHNSIGKQVPTSTNANLNNANMSKAAHGKRPSI
GNLEHVSENGHHSSHKHDREPQRRSSVKRTRYYETYIRSDSGDEQLPTICREDPEIHGYFR
DPHCLGEQEYFSSEECYEDDSSPTWSRQNYGYYSRYPGRNIDSERPRGYHHPQGFLEDDD
SPVCYDSRRSPRRRLLPPTPASHRRSSFNFECLRRQSSQEEVPSSPIFPHRTALPLHLMQQQI
MAVAGLDSSKAQKYSPSHSTRSWATPPATPPYRDWTPCYTPLIQVEQSEALDQVNGSLPSL
HRSSWYTDEPDISYRTFTPASLTVPSSFRNKNSDKQRSADSLVEAVLISEGLGRYARDPKFV
SATKHEIADACDLTIDEMESAASTLLNGNVRPRANGDVGPLSHRQDYELQDFGPGYSDEE
PDPGRDEEDLADEMICITTL

>Q9NRM6 single-pass type I membrane protein
MSLVLLSLAALCRSAVPREPTVQCGSETGPSPEWMLQHDLIPGDLRDLRVEPVTTSVATGD
YSILMNVSWVLRADASIRLLKATKICVTGKSNFQSYSCVRCNYTEAFQTQTRPSGGKWTF
SYIGFPVELNTVYFIGAHNIPNANMNEDGPSMSVNFTSPGCLDHIMKYKKKCVKAGSLWD
PNITACKKNEETVEVNFTTTPLGNRYMALIQHSTIIGFSQVFEPHQKKQTRASVVIPVTGDS
EGATVQLTPYFPTCGSDCIRHKGTVVLCPQTGVPFPLDNNKSKPGGWLPLLLLSLLVATWV
LVAGIYLMWRHERIKKTSFSTTTLLPPIKVLVVYPSEICFHHTICYFTEFLQNHCRSEVILEK
WQKKKIAEMGPVQWLATQKKAADKVVFLLSNDVNSVCDGTCGKSEGSPSENSQDLFPL
AFNLFCSDLRSQIHLHKYVVVYFREIDTKDDYNALSVCPKYHLMKDATAFCAELLHVKQ
QVSAGKRSQACHDGCCSL

>Q9UKG1 peripheral membrane protein

MPGIDKLPIEETLEDSPQTRSLLGVFEEDATAISNYMNQLYQAMHRIYDAQNELSAATHLT
SKLLKEYEKQRFPLGGDDEVMSSTLQQFSKVIDELSSCHAVLSTQLADAMMFPITQFKER
DLKEILTLKEVFQIASNDHDAAINRYSRLSKKRENDKVKYEVTEDVYTSRKKQHQTMMH
YFCALNTLQYKKKIALLEPLLGYMQAQISFFKMGSENLNEQLEEFLANIGTSVQNVRREM
DSDIETMQQTIEDLEVASDPLYVPDPDPTKFPVNRNLTRKAGYLNARNKTGLVSSTWDRQ
FYFTQGGNLMSQARGDVAGGLAMDIDNCSVMAVDCEDRRYCFQITSFDGKKSSILQAES
KKDHEEWICTINNISKQIYLSENPEETAARVNQSALEAVTPSPSFQQRHESLRPAAGQSRPPT
ARTSSSGSLGSESTNLAALSLDSLVAPDTPIQFDIISPVCEDQPGQAKAFGQGGRRTNPFGES
GGSTKSETEDSILHQLFIVRFLGSMEVKSDDHPDVVYETMRQILAARAIHNIFRMTESHLL
VTCDCLKLIDPQTQVTRLTFPLPCVVLYATHQENKRLFGFVLRTSSGRSESNLSSVCYIFES
NNEGEKICDSVGLAKQIALHAELDRRASEKQKEIERVKEKQQKELNKQKQIEKDLEEQSR
LIAASSRPNQASSEGQFVVLSSSQSEESDLGEGGKKRESEA

>Q96BA8 single-pass type II membrane protein
MDAVLEPFPADRLFPGSSFLDLGDLNESDFLNNAHFPEHLDHFTENMEDFSNDLFSSFFDD
PVLDEKSPLLDMELDSPTPGIQAEHSYSLSGDSAPQSPLVPIKMEDTTQDAEHGAWALGH
KLCSIMVKQEQSPELPVDPLAAPSAMAAAAAMATTPLLGLSPLSRLPIPHQAPGEMTQLP
VIKAEPLEVNQFLKVTPEDLVQMPPTPPSSHGSDSDGSQSPRSLPPSSPVRPMARSSTAISTS
PLLTAPHKLQGTSGPLLLTEEEKRTLIAEGYPIPTKLPLTKAEEKALKRVRRKIKNKISAQES
RRKKKEYVECLEKKVETFTSENNELWKKVETLENANRTLLQQLQKLQTLVTNKISRPYK
MAATQTGTCLMVAALCFVLVLGSLVPCLPEFSSGSQTVKEDPLAADGVYTASQMPSRSLL
FYDDGAGLWEDGRSTLLPMEPPDGWEINPGGPAEQRPRDHLQHDHLDSTHETTKYLSEA
WPKDGGNGTSPDFSHSKEWFHDRDLGPNTTIKLS

>Q495T6 single-pass type II membrane protein
MGKSEGPVGMVESAGRAGQKRPGFLEGGLLLLLLLVTAALVALGVLYADRRGKQLPRLA
SRLCFLQEERTFVKRKPRGIPEAQEVSEVCTTPGCVIAAARILQNMDPTTEPCDDFYQFAC
GGWLRRHVIPETNSRYSIFDVLRDELEVILKAVLENSTAKDRPAVEKARTLYRSCMNQSVIE
KRGSQPLLDILEVVGGWPVAMDRWNETVGLEWELERQLALMNSQFNRRVLIDLFIWNDD
QNSSRHIIYIDQPTLGMPSREYYFNGGSNRKVREAYLQFMVSVATLLREDANLPRDSCLVQ
EDMVQVLELETQLAKATVPQEERHDVIALYHRMGLEELQSQFGLKGFNWTLFIQTVLSSV
KIKLLPDEEVVVYGIPYLQNLENIIDTYSARTIQNYLVWRLVLDRIGSLSQRFKDTRVNYRK
ALFGTMVEEVRWRECVGYVNSNMENAVGSLYVREAFPGDSKSMVRELIDKVRTVFVETL
DELGWMDEESKKKAQEKAMSIREQIGHPDYILEEMNRRLDEEYSNLNFSEDLYFENSLQN
LKVGAQRSLRKLREKVDPNLWIIGAAVVNAFYSPNRNQIVFPAGILQPPFFSKEQPQALNF
GGIGMVIGHEITHGFDDNGRNFDKNGNMMDWWSNFSTQHFREQSECMIYQYGNYSWDL
ADEQNVNGFNTLGENIADNGGVRQAYKAYLKWMAEGGKDQQLPGLDLTHEQLFFINYA
QVWCGSYRPEFAIQSIKTDVHSPLKYRVLGSLQNLAAFADTFHCARGTPMHPKERCRVW

>Q9Y4P8 peripheral membrane protein
MNLASQSGEAGAGQLLFANFNQDNTEVKGASRAAGLGRRAVVWSLAVGSKSGYKFFSLS
SVDKLEQIYECTDTEDVCIVERLFSSSLVAIVSLKAPRKLKVCHFKKGTEICNYSYSNTILAV
KLNRQRLIVCLEESLYIHNIRDMKVLHTIRETPPNPAGLCALSINNDNCYLAYPGSATIGEV
QVFDTINLRAANMIPAHDSPLAALAFDASGTKLATASEKGTVIRVFSIPEGQKLFEFRRGVK

RCVSICSLAFSMDGMFLSASSNTETVHIFKLETVKEKPPEEPTTWTGYFGKVLMASTSYLP
SQVTEMFNQGRAFATVRLPFCGHKNICSLATIQKIPRLLVGAADGYLYMYNLDPQEGGEC
ALMKQHRLDGSLETTNEILDSASHDCPLVTQTYGAAAGKGTYVPSSPTRLAYTDDLGAVG
GACLEDEASALRLDEDSEHPPMILRTD

>O14807 lipid-anchor
MATSAVPSDNLPTYKLVVVGDGGVGKSALTIQFFQKIFVPDYDPTIEDSYLKHTEIDNQWA
ILDVLDTAGQEEFSAMREQYMRTGDGFLIVYSVTDKASFEHVDRFHQLILRVKDRESFPMI
LVANKVDLMHLRKITREQGKEMATKHNIPYIETSAKDPPLNVDKAFHDLVRVIRQQIPEKS
QKKKKKTKWRGDRATGTHKLQCVIL

>Q9NX52 multipass membrane protein
MAAVHDLEMESMNLNMGREMKEELEEEEKMREDGGGKDRAKSKKVHRIVSKWMLPEK
SRGTYLERANCFPPPVFIISISLAELAVFIYYAVWKPQKQWITLDTGILESPFIYSPEKREEAW
RFISYMLVHAGVQHILGNLCMQLVLGIPLEMVHKGLRVGLVYLAGVIAGSLASSIFDPLRY
LVGASGGVYALMGGYFMNVLVNFQEMIPAFGIFRLLIIILIIVLDMGFALYRRFFVPEDGSP
VSFAAHIAGGFAGMSIGYTVFSCFDKALLKDPRFWIAIAAYLACVLFAVFFNIFLSPAN

>Q8WV92 peripheral membrane protein
MAKSGLRQDPQSTAAATVLKRAVELDSESRYPQALVCYQEGIDLLLQVLKGTKDNTKRC
NLREKISKYMDRAENIKKYLDQEKEDGKYHKQIKIEENATGFSYESLFREYLNETVTEVWI
EDPYIRHTHQLYNFLRFCEMLIKRPCKVKTIHLLTSLDEGIEQVQQSRGLQEIEESLRSHGV
LLEVQYSSSIHDREIRFNNGWMIKIGRGLDYFKKPQSRFSLGYCDFDLRPCHETTVDIFHK
KHTKNI

>P06126 single-pass type I membrane protein
MLFLLLPLLAVLPGDGNADGLKEPLSFHVTWIASFYNHSWKQNLVSGWLSDLQTHTWDS
NSSTIVFLCPWSRGNFSNEEWKELETLFRIRTIRSFEGIRRYAHELQFEYPFEIQVTGGCELH
SGKVSGSFLQLAYQGSDFVSFQNNSWLPYPVAGNMAKHFCKVLNQNQHENDITHNLLSD
TCPRFILGLLDAGKAHLQRQVKPEAWLSHGPSPGPGHLQLVCHVSGFYPKPVWVMWMR
GEQEQQGTQRGDILPSADGTWYLRATLEVAAGEAADLSCRVKHSSLEGQDIVLYWEHHSS
VGFIILAVIVPLLLLIGLALWFRKRCFC

>Q8NGX5 multipass membrane protein
MEQVNKTVVREFVVLGFSSLARLQQLLFVIFLLLYLFTLGTNAIIISTIVLDRALHTPMYFF
LAILSCSEICYTFVIVPKMLVDLLSQKKTISFLGCAIQMFSFLFFGSSHSFLLAAMGYDRYM
AICNPLRYSVLMGHGVCMGLMAAACACGFTVSLVTTSLVFHLPFHSSNQLHHFFCDISPV
LKLASQHSGFSQLVIFMLGVFALVIPLLLILVSYIRIISAILKIPSSVGRYKTFSTCASHLIVVT
VHYSCASFIYLRPKTNYTSSQDTLISVSYTILTPLFNPMIYSLRNKEFKSALRRTIGQTFYPLS

>Q9UHC9 multipass membrane protein
MAEAGLRGWLLWALLLRLAQSEPYTTIHQPGYCAFYDECGKNPELSGSLMTLSNVSCLS
NTPARKITGDHLILLQKICPRLYTGPNTQACCSAKQLVSLEASLSITKALLTRCPACSDNFV
NLHCHNTCSPNQSLFINVTRVAQLGAGQLPAVVAYEAFYQHSFAEQSYDSCSRVRVPAAAT

LAVGTMCGVYGSALCNAQRWLNFQGDTGNGLAPLDITFHLLEPGQAVGSGIQPLNEGVA
RCNESQGDDVATCSCQDCAASCPAIARPQALDSTFYLGQMPGSLVLIIILCSVFAVVTILLVG
FRVAPARDKSKMVDPKKGTSLSDKLSFSTHTLLGQFFQGWGTWVASWPLTILVLSVIPVVA
LAAGLVFTELTTDPVELWSAPNSQARSEKAFHDQHFGPFFRTNQVILTAPNRSSYRYDSLL
LGPKNFSGILDLDLLLELLELQERLRHLQVWSPEAQRNISLQDICYAPLNPDNTSLYDCCIN
SLLQYFQNNRTLLLLTANQTLMGQTSQVDWKDHFLYCANAPLTFKDGTALALSCMADYG
APVFPFLAIGGYKGKDYSEAEALIMTFSLNNYPAGDPRLAQAKLWEEAFLEEMRAFQRR
MAGMFQVTFMAERSLEDEINRTTAEDLPIFATSYIVIFLYISLALGSYSSWSRVMVDSKATL
GLGGVAVVLGAVMAAMGFFSYLGIRSSLVILQVVPFLVLSVGADNIFIFVLEYQRLPRRPGE
PREVHIGRALGRVAPSMLLCSLSEAICFFLGALTPMPAVRTFALTSGLAVILDFLLQMSAFVA
LLSLDSKRQEASRLDVCCCVKPQELPPPGQGEGLLLGFFQKAYAPFLLHWITRGVVLLLFL
ALFGVSLYSMCHISVGLDQELALPKDSYLLDYFLFLNRYFEVGAPVYFVTTLGYNFSSEA
GMNAICSSAGCNNFSFTQKIQYATEFPEQSYLAIPASSWVDDFIDWLTPSSCCRLYISGPNK
DKFCPSTVNSLNCLKNCMSITMGSVRPSVEQFHKYLPWFLNDRPNIKCPKGGLAAYSTSV
NLTSDGQVLDTVAILSPRLEYSGTISAHCNLYLLDSASRFMAYHKPLKNSQDYTEALRAAR
ELAANITADLRKVPGTDPAFEVFPYTITNVFYEQYLTILPEGLFMLSLCLVPTFAVSCLLLGL
DLRSGLLNLLSIVMILVDTVGFMALWGISYNAVSLINLVSAVGMSVEFVSHITRSFAISTKPT
WLERAKEATISMGSAVFAGVAMTNLPGILVLGLAKAQLIQIFFFRLNLLITLLGLLHGLVFL
PVILSYVGPDVNPALALEQKRAEEAVAAVMVASCPNHPSRVSTADNIYVNHSFEGSIKGAG
AISNFLPNNGRQF

>O94956 multipass membrane protein
MGPRIGPAGEVPQVPDKETKATMGTENTPGGKASPDPQDVRPSVFHNIKLFVLCHSLLQL
AQLMISGYLKSSISTVEKRFGLSSQTSGLLASFNEVGNTALIVFVSYFGSRVHRPRMIGYGA
ILVALAGLLMTLPHFISEPYRYDNTSPEDMPQDFKASLCLPTTSAPASAPSNGNCSSYTETQ
HLSVVGIMFVAQTLLGVGGVPIQPFGISYIDDFAHNSNSPLYLGILFAVTMMGPGLAFGLGS
LMLRLYVDINQMPEGGISLTIKDPRWVGAWWLGFLIAAGAVALAAIPYFFFPKEMPKEKRE
LQFRRKVLAVTDSPARKGKDSPSKQSPGESTKKQDGLVQIAPNLTVIQFIKVFPRVLLQTLR
HPIFLLVVLSQVCLSSMAAGMAIFLPKFLERQFSITASYANLLIGCLSFPSVIVGIVVGGVLV
KRLHLGPVGCGALCLLGMLLCLFFSLPLFFIGCSSHQIAGITHQTSAHPGLELSPSCMEACS
CPLDGFNPVCDPSTRVEYITPCHAGCSSWVVQDALDNSQVFYTNCSCVVEGNPVLAGSC
DSTCSHLVVPFLLLVSLGSALACLTHTPSFMLILRGVKKEDKTLAVGIQFMFLRILAWMPSP
VIHGSAIDTTCVHWALSCGRRAVCRYYNNDLLRNRFIGLQFFFKTGSVICFALVLAVLRQQ
DKEARTKESRSSPAVEQQLLVSGPGKKPEDSRV

>Q9P0V8 single-pass type I membrane protein
MVMRPLWSLLLWEALLPITVTGAQVLSKVGGSVLLVAARPPGFQVREAIWRSLWPSEELL
ATFFRGSLETLYHSRFLGRAQLHSNLSLELGPLESGDSGNFSVLMVDTRGQPWTQTLQLK
VYDAVPRPVVQVFIAVERDAQPSKTCQVFLSCWAPNISEITYSWRRETTMDFGMEPHSLFT
DGQVLSISLGPGDRDVAYSCIVSNPVSWDLATVTPWDSCHHEAAPGKASYKDVLLVVVPV
SLLLMLVTLFSAWHWCPCSGKKKKDVHADRVGPETENPLVQDLP

>P55291 single-pass type I membrane protein
MDAAFLLVLGLLAQSLCLSLGVPGWRRPTTLYPWRRAPALSRVRRAWVIPPISVSENHKR

LPYPLVQIKSDKQQLGSVIYSIQGPGVDEEPRGVFSIDKFTGKVFLNAMLDREKTDRFRLR
AFALDLGGSTLEDPTDLEIVVVDQNDNRPAFLQEAFTGRVLEGAVPGTYVTRAEATDADD
PETDNAALRFSILQQGSPELFSIDELTGEIRTVQVGLDREVVAVYNLTLQVADMSGDGLTAT
ASAIITLDDINDNAPEFTRDEFFMEAIEAVSGVDVGRLEVEDRDLPGSPNWVARFTILEGDP
DGQFTIRTDPKTNEGVLSIVKALDYESCEHYELKVSVQNEAPLQAAALRAERGQAKVRV
HVQDTNEPPVFQENPLRTSLAEGAPPGTLVATFSARDPDTEQLQRLSYSKDYDPEDWLQV
DAATGRIQTQHVLSPASPFLKGGWYRAIVLAQDDASQPRTATGTLSIEILEVNDHAPVLAPP
PPGSLCSEPHQGPGLLLGATDEDLPPHGAPFHFQLSPRLPELGRNWSLSQVNVSHARLRPR
HQVPEGLHRLSLLLRDSGQPPQQREQPLNVTVCRCGKDGVCLPGAAALLAGGTGLSLGA
LVIVLASALLLLVLVLLVALRARFWKQSRGKGLLHGPQDDLRDNVLNYDEQGGGEEDQD
AYDISQLRHPTALSLPLGPPPLRRDAPQGRLHPQPPRVLPTSPLDIADFINDGLEAADSDPSV
PPYDTALIYDYEGDGSVAGTLSSILSSQGDEDQDYDYLRDWGPRFARLADMYGHPCGLEY
GARWDHQAREGLSPGALLPRHRGRTA

>P50406 multipass membrane protein
MVPEPGPTANSTPAWGAGPPSAPGGSGWVAAALCVVIALTAAANSLLIALICTQPALRNTS
NFFLVSLFTSDLMVGLVVMPPAMLNALYGRWVLARGLCLLWTAFDVMCCSASILNLCLIS
LDRYLLILSPLRYKLRMTPLRALALVLGAWSLAALASFLPLLLGWHELGHARPPVPGQCR
LLASLPFVLVASGLTFFLPSGAICFTYCRILLAARKQAVQVASLTTGMASQASETLQVPRTP
RPGVESADSRRLATKHSRKALKASLTLGILLGMFFVTWLPFFVANIVQAVCDCISPGLFDVL
TWLGYCNSTMNPIIYPLFMRDFKRALGRFLPCPRCPRERQASLASPSLRTSHSGPRPGLSL
QQVLPLPLPPDSDSDSDAGSGGSSGLRLTAQLLLPGEATQDPPLPTRAAAAVNFFNIDPAEP
ELRPHPLGIPTN

>Q9Y624 single-pass type I membrane protein
MGTKAQVERKLLCLFILAILLCSLALGSVTVHSSEPEVRIPENNPVKLSCAYSGFSSPRVEW
KFDQGDTTRLVCYNNKITASYEDRVTFLPTGITFKSVTREDTGTYTCMVSEEGGNSYGEV
KVKLIVLVPPSKPTVNIPSSATIGNRAVLTCSEQDGSPPSEYTWFKDGIVMPTNPKSTRAFSN
SSYVLNPTTGELVFDPLSASDTGEYSCEARNGYGTPMTSNAVRMEAVERNVGVIVAAVLV
TLILLGILVFGIWFAYSRGHFDRTKKGTSSKKVIYSQPSARSEGEFKQTSSFLV

>P30273 single-pass type I membrane protein
MIPAVVLLLLLLLVEQAAALGEPQLCYILDAILFLYGIVLTLLYCRLKIQVRKAAITSYEKSDG
VYTGLSTRNQETYETLKHEKPPQ

>Q05901 multipass membrane protein
MLPDFMLVLIVLGIPSSATTGFNSIAENEDALLRHLFQGYQKWVRPVLHSNDTIKVYFGLK
ISQLVDVDEKNQLMTTNVWLKQEWTDHKLRWNPDDYGGIHSIKVPSESLWLPDIVLFEN
ADGRFEGSLMTKVIVKSNGTVVWTPPASYKSSCTMDVTFFPFDRQNCSMKFGSWTYDGT
MVDLILINENVDRKDFFDNGEWEILNAKGMKGNRRDGVYSYPFITYSFVLRRLPLFYTLF
LIIPCLGLSFLTVLVFYLPSDEGEKLSLSTSVLVSLTVFLLVIEEIIPSSSKVIPLIGEYLLFIMIF
VTLSIIVTVFVINVHHRSSSTYHPMAPWVKRLFLQKLPKLLCMKDHVDRYSSPEKEESQPV
VKGKVLEKKKQKQLSDGEKVLVAFLEKAADSIRYISRHVKKEHFISQVVQDWKFVAQVL
DRIFLWLFLIVSVTGSVLIFTPALKMWLHSYH

>Q68EM7 peripheral membrane protein
MKKQFNRMKQLANQTVGRAEKTEVLSEDLLQIERRLDTVRSICHHSHKRLVACFQGQHG
TDAERRHKKLPLTALAQNMQEASTQLEDSLLGKMLETCGDAENQLALELSQHEVFVEKEI
VDPLYGIAEVEIPNIQKQRKQLARLVLDWDSVRARWNQAHKSSGTNFQGLPSKIDTLKEE
MDEAGNKVEQCKDQLAADMYNFMAKEGEYGKFFVTLLEAQADYHRKALAVLEKTLPE
MRAHQDKWAEKPAFGTPLEEHLKRSGREIALPIEACVMLLLETGMKEEGLFRIGAGASKL
KKLKAALDCSTSHLDEFYSDPHAVAGALKSYLRELPEPLMTFNLYEEWTQVASVQDQDK
KLQDLWRTCQKLPPQNFVNFRYLIKFLAKLAQTSDVNKMTPSNIAIVLGPNLLWARNEGT
LAEMAAATSVHVVAVIEPIIQHADWFFPEEVEFNVSEAFVPLTTPSSNHSFHTGNDSDSGTL
ERKRPASMAVMEGDLVKKESFGVKLMDFQAHRRGGTLNRKHISPAFQPPLPPTDGSTVVP
AGPEPPPQSSRAESSSGGGTVPSSAGILEQGPSPGDGSPPKPKDPVSAAVPAPGRNNSQIAS
GQNQPQAAAGSHQLSMGQPHNAAGPSPHTLRRAVKKPAPAPPKPGNPPPGHPGGQSSSGT
SQHPPSLSPKPPTRSPSPPTQHTGQPPGQPSAPSQLSAPRRYSSSLSPIQAPNHPPPQPPTQAT
PLMHTKPNSQGPPNPMALPSEHGLEQPSHTPPQTPTPPSTPPLGKQNPSLPAPQTLAGGNPE
TAQPHAGTLPRPRPVPKPRNRPSVPPPPQPPGVHSAGDSSLTNTAPTASKIVTDSNSRVSEP
HRSIFPEMHSDSASKDVPGRILLDIDNDTESTAL

>Q08334 single-pass type I membrane protein
MAWSLGSWLGGCLLVSALGMVPPPENVRMNSVNFKNILQWESPAFAKGNLTFTAQYLSY
RIFQDKCMNTTLTECDFSSLSKYGDHTLRVRAEFADEHSDWVNITFCPVDDTIIGPPGMQV
EVLADSLHMRFLAPKIENEYETWTMKNVYNSWTYNVQYWKNGTDEKFQITPQYDFEVL
RNLEPWTTYCVQVRGFLPDRNKAGEWSEPVCEQTTHDETVPSWMVAVILMASVFMVCL
ALLGCFALLWCVYKKTKYAFSPRNSLPQHLKEFLGHPHHNTLLFFSFPLSDENDVFDKLSV
IAEDSESGKQNPGDSCSLGTPPGQGPQS

>P38484 single-pass type I membrane protein
MRPTLLWSLLLLLGVFAAAAAAPPDPLSQLPAPQHPKIRLYNAEQVLSWEPVALSNSTRPV
VYQVQFKYTDSKWFTADIMSIGVNCTQITATECDFTAASPSAGFPMDFNVTLRLRAELGA
LHSAWVTMPWFQHYRNVTVGPPENIEVTPGEGSLIIRFSSPFDIADTSTAFFCYYVHYWEK
GGIQQVKGPFRSNSISLDNLKPSRVYCLQVQAQLLWNKSNIFRVGHLSNISCYETMADAST
ELQQVILISVGTFSLLSVLAGACFFLVLKYRGLIKYWFHTPPSIPLQIEEYLKDPTQPILEAL
DKDSSPKDDVWDSVSIISFPEKEQEDVLQTL

>Q9Y6J6 single-pass type I membrane protein
MSTLSNFTQTLEDVFRRIFITYMDNWRQNTTAEQEALQAKVDAENFYYVILYLMVMIGM
FSFIIVAILVSTVKSKRREHSNDPYHQYIVEDWQEKYKSQILNLEESKATIHENIGAAGFKM
SP

>Q9UKL4 multipass membrane protein
MGEWTILERLLEAAVQQHSTMIGRILLTVVVIFRILIVAIVGETVYDDEQTMFVCNTLQPGC
NQACYDRAFPISHIRYWVFQIIMVCTPSLCFITYSVHQSAKQRERRYSTVFLALDRDPPESI
GGPGGTGGGGSGGGKREDKKLQNAIVNGVLQNTENTSKETEPDCLEVKELTPHPSGLRTA
SKSKLRRQEGISRFYIIQVVFRNALEIGFLVGQYFLYGFSVPGLYECNRYPCIKEVECYVSRP

TEKTVFLVFMFAVSGICVVLNLAELNHLGWRKIKLAVRGAQAKRKSIYEIRNKDLPRVSVP
NFGRTQSSDSAYV

>Q86XP0 peripheral membrane protein
MESLSPGGPPGHPYQGEASTCWQLTVRVLEARNLRWADLLSEADPYVILQLSTAPGMKFK
TKTLTDTSHPVWNEAFRFLIQSQVKNVLELSIYDEDSVTEDDICFKVLYDISEVLPGKLLRK
TFSQSPQGEEELDVEFLMEETSDRPENLITNKVIVARELSCLDVHLDSTGSTAVVADQDKLE
LELVLKGSYEDTQTSFLGTASAFRFHYMAALETELSGRLRSSRSNGWNGDNSAGYLTVPL
RPLTIGKEVTMDVPAPNAPGVRLQLKAEGCPEELAVHLGFNLCAEEQAFLSRRKQVVAKA
LKQALQLDRDLQEDEVPVVGIMATGGGARAMTSLYGHLLALQKLGLLDCVTYFSGISGS
TWTMAHLYGDPEWSQRDLEGPIRYAREHLAKSKLEVFSPERLASYRRELELRAEQGHPTT
FVDLWALVLESMLHGQVMDQKLSGQRAALERGQNPLPLYLSLNVKENNLETLDFKEWV
EFSPYEVGFLKYGAFVPPELFGSEFFMGRLMRRIPEPRICFLEAIWSNIFSLNLLDAWYDLT
SSGESWKQHIKDKTRSLEKEPLTTSGTSSRLEASWLQPGTALAQAFKGFLTGRPLHQRSPN
FLQGLQLHQDYCSHKDFSTWADYQLDSMPSQLTPKEPRLCLVDAAYFINTSSPSMFRPGR
RLDLILSFDYSLSAPFEALQQTELYCRARGLPFPRVEPSPQDQHQPRECHLFSDPACPEAPIL
LHFPLVNASFKDHSAPGVQRSPAELQGGQVDLTGATCPYTLSNMTYKEEDFERLLRLSDY
NVQTSQGAILQALRTALKHRTLEARPPRAQT

>Q9UKV5 multipass membrane protein
MPLLFLERFPWPSLRTYTGLSGLALLGTIISAYRALSQPEAGPGEPDQLTASLQPEPPAPARP
SAGGPRARDVAQYLLSDSLFVWVLVNTACCVLMLVAKLIQCIVFGPLRVSERQHLKDKFW
NFIFYKFIFIFGVLNVQTVEEVVMWCLWFAGLVFLHLMVQLCKDRFEYLSFSPTTPMSSHG
RVLSLLVAMLLSCCGLAAVCSITGYTHGMHTLAFMAAESLLVTVRTAHVILRYVIHLWDL
NHEGTWEGKGTYVYYTDFVMELTLLSLDLMHHIHMLLFGNIWLSMASLVIFMQLRYLFH
EVQRRIRRHKNYLRVVGNMEARFAVATPEELAVNNDDCAICWDSMQAARKLPCGHLFHN
SCLRSWLEQDTSCPTCRMSLNIADNNRVREEHQGENLDENLVPVAAAEGRPRLNQHNHFF
HFDGSRIASWLPSFSVEVMHTTNILGITQASNSQLNAMAHQIQEMFPQVPYHLVLQDLQLT
RSVEITTDNILEGRIQVPFPTQRSDSIRPALNSPVERPSSDQEEGETSAQTERVPLDLSPRLEE
TLDFGEVEVEPSEVEDFEARGSRFSKSADERQRMLVQRKDELLQQARKRFLNKSSEDDAA
SESFLPSEGASSDPVTLRRRMLAAAAERRLQKQQTS

>P12821 single-pass type I membrane protein
MGAASGRRGPGLLLPLPLLLLLPPQPALALDPGLQPGNFSADEAGAQLFAQSYNSSAEQV
LFQSVAASWAHDTNITAENARRQEEAALLSQEFAEAWGQKAKELYEPIWQNFTDPQLRRII
GAVRTLGSANLPLAKRQQYNALLSNMSRIYSTAKVCLPNKTATCWSLDPDLTNILASSRSY
AMLLFAWEGWHNAAGIPLKPLYEDFTALSNEAYKQDGFTDTGAYWRSWYNSPTFEDDLE
HLYQQLEPLYLNLHAFVRRALHRRYGDRYINLRGPIPAHLLGDMWAQSWENIYDMVVPFP
DKPNLDVTSTMLQQGWNATHMFRVAEEFFTSLELSPMPPEFWEGSMLEKPADGREVVCH
ASAWDFYNRKDFRIKQCTRVTMDQLSTVHHEMGHIQYYLQYKDLPVSLRRGANPGFHE
AIGDVLALSVSTPEHLHKIGLLDRVTNDTESDINYLLKMALEKIAFLPFGYLVDQWRWGV
FSGRTPPSRYNFDWWYLRTKYQGICPPVTRNETHFDAGAKFHVPNVTPYIRYFVSFVLQF
QFHEALCKEAGYEGPLHQCDIYRSTKAGAKLRKVLQAGSSRPWQEVLKDMVGLDALDA
QPLLKYFQPVTQWLQEQNQQNGEVLGWPEYQWHPPLPDNYPEGIDLVTDEAEASKFVEE

YDRTSQVVWNEYAEANWNYNTNITTETSKILLQKNMQIANHTLKYGTQARKFDVNQLQ
NTTIKRIIKKVQDLERAALPAQELEEYNKILLDMETTYSVATVCHPNGSCLQLEPDLTNVM
ATSRKYEDLLWAWEGWRDKAGRAILQFYPKYVELINQAARLNGYVDAGDSWRSMYETP
SLEQDLERLFQELQPLYLNLHAYVRRALHRHYGAQHINLEGPIPAHLLGNMWAQTWSNIY
DLVVPFPSAPSMDTTEAMLKQGWTPRRMFKEADDFFTSLGLLPVPPEFWNKSMLEKPTD
GREVVCHASAWDFYNGKDFRIKQCTTVNLEDLVVAHHEMGHIQYFMQYKDLPVALREG
ANPGFHEAIGDVLALSVSTPKHLHSLNLLSSEGGSDEHDINFLMKMALDKIAFIPFSYLVD
QWRWRVFDGSITKENYNQEWWSLRLKYQGLCPPVPRTQGDFDPGAKFHIPSSVPYIRYFV
SFIIQFQFHEALCQAAGHTGPLHKCDIYQSKEAGQRLATAMKLGFSRPWPEAMQLITGQP
NMSASAMLSYFKPLLDWLRTENELHGEKLGWPQYNWTPNSARSEGPLPDSGRVSFLGLD
LDAQQARVGQWLLLFLGIALLVATLGLSQRLFSIRHRSLHRHSHGPQFGSEVELRHS

>P11801 lipid-anchor
MGCGTSKVLPEPPKDVQLDLVKKVEPFSGTKSDVYKHFITEVDSVGPVKAGFPAASQYAH
PCPGPPTAGHTEPPSEPPRRARVAKYRAKFDPRVTAKYDIKALIGRGSFSRVVRVEHRATRQ
PYAIKMIETKYREGREVCESELRVLRRVRHANIIQLVEVFETQERVYMVMELATGGELFDR
IIAKGSFTERDATRVLQMVLDGVRYLHALGITHRDLKPENLLYYHPGTDSKIIITDFGLASA
RKKGDDCLMKTTCGTPEYIAPEVLVRKPYTNSVDMWALGVIAYILLSGTMPFEDDNRTRL
YRQILRGKYSYSGEPWPSVSNLAKDFIDRLLTVDPGARMTALQALRHPWVVSMAASSSM
KNLHRSISQNLLKRASSRCQSTKSAQSTRSSRSTRSNKSRRVRERELRELNLRYQQQYNG

>P47888 multipass membrane protein
MSLQKLMEPEAGTNRTAVAEFILLGLVQTEEMQPVVFVLLLFAYLVTTGGNLSILAAVLVE
PKLHAPMYFFLGNLSVLDVGCITVTVPAMLGRLLSHKSTISYDACLSQLFFFHLLAGMDC
FLLTAMAYDRLLAICQPLTYSTRMSQTVQRMLVAASWACAFTNALTHTVAMSTLNFCGPN
EVNHFYCDLPQLFQLSCSSTQLNELLLFVAAAFMAVAPLVFISVSYAHVVAAVLQIRSAEGR
KKAFSTCGSHLTVVGIFYGTGVFSYMRLGSVESSDKDKGVGVFMTVINPMLNPLIYSLRN
TDVQGALCQLLVGKRSLT

>P43119 multipass membrane protein
MADSCRNLTYVRGSVGPATSTLMFVAGVVGNGLALGILSARRPARPSAFAVLVTGLAATD
LLGTSFLSPAVFVAYARNSSLLGLARGGPALCDAFAFAMTFFGLASMLILFAMAVERCLALS
HPYLYAQLDGPRCARLALPAIYAFCVLFCALPLLGLGQHQQYCPGSWCFLRMRWAQPGG
AAFSLAYAGLVALLVAAIFLCNGSVTLSLCRMYRQQKRHQGSLGPRPRTGEDEVDHLILLA
LMTVVMAVCSLPLTIRCFTQAVAPDSSSEMGDLLAFRFYAFNPILDPWVFILFRKAVFQRLK
LWVCCLCLGPAHGDSQTPLSQLASGRRDPRAPSAPVGKEGSCVPLSAWGEGQVEPLPPTQ
QSSGSAVGTSSKAEASVACSLC

>Q8IZJ3 peripheral membrane protein
MSGALLWPLLPLLLLLLSARDGVRAAQPQAPGYLIAAPSVFRAGVEEVISVTIFNSPREVT
VQAQLVAQGEPVVQSQGAILDKGTIKLKVPTGLRGQALLKVWGRGWQAEEGPLFHNQTS
VTVDGRGASVFIQTDKPVYRPQHRVLISIFTVSPNLRPVNEKLEAYILDPRGSRMIEWRHL
KPFCCGITNMSFPLSDQPVLGEWFIFVEMQGHAYNKSFEVQKYVLPKFELLIDPPRYIQDL
DACETGTVRARYTFGKPVAGALMINMTVNGVGYYSHEVGRPVLRTTKILGSRDFDICVR

DMIPADVPEHFRGRVSIWAMVTSVDGSQQVAFDDSTPVQRQLVDIRYSKDTRKQFKPGLA
YVGKVELSYPDGSPAEGVTVQIKAELTPKDNIYTSEVVSQRGLVGFEIPSIPTSAQHVWLET
KVMALNGKPVGAQYLPSYLSLGSWYSPSQCYLQLQPPSHPLQVGEEAYFSVKSTCPCNFT
LYYEVAARGNIVLSGQQPAHTTQQRSKRAAPALEKPIRLTHLSETEPPPAPEAEVDVCVTSL
HLAVTPSMVPLGRLLVFYVRENGEGVADSLQFAVETFFENQVSVTYSANETQPGEVVDLRI
RAARGSCVCVAAVDKSVYLLRSGFRLTPAQVFQELEDYDVSDSFGVSREDGPFWWAGLT
AQRRRRSSVFPWPWGITKDSGFAFTETGLVVMTDRVSLNHRQDGGLYTDEAVPAFQPHTG
SLVAVAPSRHPPRTEKRKRTFFPETWIWHCLNISDPSGEGTLSVKVPDSITSWVGEAVALSTS
QGLGIAEPSLLKTFKPFFVDFMLPALIIRGEQVKIPLSVYNYMGTCAEVYMKLSVPKGIQF
VGHPGKRHVTKKMCVAPGEAEPIWVVLSFSDLGLNNITAKALAYGDTNCCRDGRSSKHP
EENHADRRVPIGVDHVRRSVMVEAEGVPRAYTYSAFFCPSERVHISTPNKYEFQYVQRPL
RLTRFDVAVRAHNDARVALSSGPQDTAGMIEIVLGGHQNTRSWISTSKMGEPVASAHTAKI
LSWDEFRTFWISWRGGLIQVGHGPEPSNESVIVAWTLPRPPEVQFIGFSTGWGSMGEFRIW
RKMEVDESYSEAFTLGVPHGAIPGSERATASIIGDVMGPTLNHLNNLLRLPFGCGEQNMIH
FAPNVFVLKYLQKTQQLSPEVERETTDYLVQGYQRQLTYKRQDGSYSAFGERDASGSMW
LTAFVLKSFAQARSFIFVDPRELAAAKSWIIQQQQADGSFLAVGRVLNKDIQGGIHGTVPLT
AYVVVALLETGTASEEERGSTDKARHFLESAAPLAMDPYSCALTTYALTLLRSPAAPEALR
KLRSLAIMRDGVTHWSLSNSWDVDKGTFLSFSDRVSQSVVSAEVEMTAYALLTYTLLGDV
AAALPVVKWLSQQRNALGGFSSTQDTCVALQALAEYAILSYAGGINLTVSLASTNLDYQE
TFELHRTNQKVLQTAAIPSLPTGLFVSAKGDGCCLMQIDVTYNVPDPVAKPAFQLLVSLQE
PEAQGRPPPMPASAAEGSRGDWPPADDDDPAADQHHQEYKVMLEVCTRWLHAGSSNMA
VLEVPLLSGFRADIESLEQLLLDKHMGMKRYEVAGRRVLFYFDEIPSRCLTCVRFRALREC
VVGRTSALPVSVYDYYEPAFEATRFYNVSTHSPLARELCAGPACNEVERAPARGPGWFPG
ESGPAVAPEEGAAIARCGCDHDCGAQGNPVCGSDGVVYASACRLREAACRQAAPLEPAPP
SCCALEQRLPASSSSTYGDDLASVAPGPLQQDVKLNGAGLEVEDSDPEPEGEAEDRVTAG
PRPPVSSGNLESSTQSASPFHRWGQTPAPQRHSGRVVGAHRPGLLSPVFVYSPAFQSGGEE
GLWMSNTCTLR

>P57727 single-pass type II membrane protein
MGENDPPAVEAPFSFRSLFGLDDLKISPVAPDADAVAAQILSLLPLKFFPIIVIGIIALILALAI
GLGIHFDCSGKYRCRSSFKCIELIARCDGVSDCKDGEDEYRCVRVGGQNAVLQVFTAASW
KTMCSDDWKGHYANVACAQLGFPSYVSSDNLRVSSLEGQFREEFVSIDHLLPDDKVTALH
HSVYVREGCASGHVVTLQCTACGHRRGYSSRIVGGNMSLLSQWPWQASLQFQGYHLCG
GSVITPLWIITAAHCVYDLYLPKSWTIQVGLVSLLDNPAPSHLVEKIVYHSKYKPKRLGNDI
ALMKLAGPLTFNEMIQPVCLPNSEENFPDGKVCWTSGWGATEDGAGDASPVLNHAAVPL
ISNKICNHRDVYGGIISPSMLCAGYLTGGVDSCQGDSGGPLVCQERRLWKLVGATSFGIGC
AEVNKPGVYTRVTSFLDWIHEQMERDLKT

>Q9NRZ7 multipass membrane protein
MGLLAFLKTQFVLHLLVGFVFVVSGLVINFVQLCTLALWPVSKQLYRRLNCRLAYSLWSQ
LVMLLEWWSCTECTLFTDQATVERFGKEHAVIILNHNFEIDFLCGWTMCERFGVLGSSKV
LAKKELLYVPLIGWTWYFLEIVFCKRKWEEDRDTVVEGLRRLSDYPEYMWFLLYCEGTR
FTETKHRVSMEVAAAKGLPVLKYHLLPRTKGFTTAVKCLRGTVAAVYDVTLNFRGNKNPS
LLGILYGKKYEADMCVRRFPLEDIPLDEKEAAQWLHKLYQEKDALQEIYNQKGMFPGEQ

FKPARRPWTLLNFLSWATILLSPLFSFVLGVFASGSPLLILTFLGFVGAASFGVRRLIGVTEIE
KGSSYGNQEFKKKE

>P59901 single-pass type I membrane protein
MTLILTSLLFFGLSLGPRTRVQAENLPKPILWAEPGPVITWHNPVTIWCQGTLEAQGYRLD
KEGNSMSRHILKTLESENKVKLSIPSMMWEHAGRYHCYYQSPAGWSEPSDPLELVVTAYS
RPTLSALPSPVVTSGVNVTLRCASRLGLGRFTLIEEGDHRLSWTLNSHQHNHGKFQALFP
MGPLTFSNRGTFRCYGYENNTPYVWSEPSDPLQLLVSGVSRKPSLLTLQGPVVTPGENLTL
QCGSDVGYIRYTLYKEGADGLPQRPGRQPQAGLSQANFTLSPVSRSYGGQYRCYGAHNV
SSEWSAPSDPLDILIAGQISDRPSLSVQPGPTVTSGEKVTLLCQSWDPMFTFLLTKEGAAHP
PLRLRSMYGAHKYQAEFPMSPVTSAHAGTYRCYGSRSSNPYLLSHPSEPLELVVSGATET
LNPAQKKSDSKTAPHLQDYTVENLIRMGVAGLVLLFLGILLFEAQHSQRSPPRCSQEANSR
KDNAPFRVVEPWEQI

>P09693 single-pass type I membrane protein
MEQGKGLAVLILAIILLQGTLAQSIKGNHLVKVYDYQEDGSVLLTCDAEAKNITWFKDGK
MIGFLTEDKKKWNLGSNAKDPRGMYQCKGSQNKSKPLQVYYRMCQNCIELNAATISGFL
FAEIVSIFVLAVGVYFIAGQDGVRQSRASDKQTLLPNDQLYQPLKDREDDQYSHLQGNQL
RRN

>P32302 multipass membrane protein
MNYPLTLEMDLENLEDLFWELDRLDNYNDTSLVENHLCPATEGPLMASFKAVFVPVAYSL
IFLLGVIGNVLVLVILERHRQTRSSTETFLFHLAVADLLLVFILPFAVAEGSVGWVLGTFLCK
TVIALHKVNFYCSSLLLACIAVDRYLAIVHAVHAYRHRRLLSIHITCGTIWLVGFLLALPEIL
FAKVSQGHHNNSLPRCTFSQENQAETHAWFTSRFLYHVAGFLLPMLVMGWCYVGVVHR
LRQAQRRPQRQKAVRVAILVTSIFFLCWSPYHIVIFLDTLARLKAVDNTCKLNGSLPVAITM
CEFLGLAHCCLNPMLYTFAGVKFRSDLSRLLTKLGCTGPASLCQLFPSWRRSSLSESENATS
LTTF

>P41597 multipass membrane protein
MLSTSRSRFIRNTNESGEEVTTFFDYDYGAPCHKFDVKQIGAQLLPPLYSLVFIFGFVGNML
VVLILINCKKLKCLTDIYLLNLAISDLLFLITLPLWAHSAANEWVFGNAMCKLFTGLYHIGY
FGGIFFIILLTIDRYLAIVHAVFALKARTVTFGVVTSVITWLVAVFASVPGIIFTKCQKEDSVY
VCGPYFPRGWNNFHTIMRNILGLVLPLLIMVICYSGILKTLLRCRNEKKRHRAVRVIFTIMI
VYFLFWTPYNIVILLNTFQEFFGLSNCESTSQLDQATQVTETLGMTHCCINPIIYAFVGEKF
RSLFHIALGCRIAPLQKPVCGGPGVRPGKNVKVTTQGLLDGRGKGKSIGRAPEASLQDKE
GA

>P51681 multipass membrane protein
MDYQVSSPIYDINYYTSEPCQKINVKQIAARLLPPLYSLVFIFGFVGNMLVILILINCKRLKS
MTDIYLLNLAISDLFFLLTVPFWAHYAAAQWDFGNTMCQLLTGLYFIGFFSGIFFIILLTIDR
YLAVVHAVFALKARTVTFGVVTSVITWVVAVFASLPGIIFTRSQKEGLHYTCSSHFPYSQY
QFWKNFQTLKIVILGLVLPLLVMVICYSGILKTLLRCRNEKKRHRAVRLIFTIMIVYFLFWA
PYNIVLLLNTFQEFFGLNNCSSSNRLDQAMQVTETLGMTHCCINPIIYAFVGEKFRNYLLV

FFQKHIAKRFCKCCSIFQQEAPERASSVYTRSTGEQEISVGL

>Q8N158 GPI-anchor, lipid-anchor
MSALRPLLLLLLPLCPGPGPGPGSEAKVTRSCAETRQVLGARGYSLNLIPPALISGEHLRVC
PQEYTCCSSETEQRLIRETEATFRGLVEDSGSFLVHTLAARHRKFDEFFLEMLSVAQHSLTQ
LFSHSYGRLYAQHALIFNGLFSRLRDFYGESGEGLDDTLADFWAQLLERVFPLLHPQYSFP
PDYLLCLSRLASSTDGSLQPFGDSPRRLRLQITRTLVAARAFVQGLETGRNVVSEALKVPV
SEGCSQALMRLIGCPLCRGVPSLMPCQGFCLNVVRGCLSSRGLEPDWGNYLDGLLILADK
LQGPFSFELTAESIGVKISEGLMYLQENSAKVSAQVFQECGPPDPVPARNRRAPPPREEAG
RLWSMVTEEERPTTAAGTNLHRLVWELRERLARMRGFWARLSLTVCGDSRMAADASLE
AAPCWTGAGRGRYLPPVVGGSPAEQVNNPELKVDASGPDVPTRRRRLQLRAATARMKTA
ALGHDLDGQDADEDASGSGGGQQYADDWMAGAVAPPARPPRPPYPPRRDGSGGKGGGG
SARYNQGRSRSGGASIGFHTQTILILSLSALALLGPR

>P22455 single-pass type I membrane protein
MRLLLALLGVLLSVPGPPVLSLEASEEVELEPCLAPSLEQQEQELTVALGQPVRLCCGRAE
RGGHWYKEGSRLAPAGRVRGWRGRLEIASFLPEDAGRYLCLARGSMIVLQNLTLITGDSL
TSSNDDEDPKSHRDPSNRHSYPQQAPYWTHPQRMEKKLHAVPAGNTVKFRCPAAGNPTP
TIRWLKDGQAFHGENRIGGIRLRHQHWSLVMESVVPSDRGTYTCLVENAVGSIRYNYLLD
VLERSPHRPILQAGLPANTTAVVGSDVELLCKVYSDAQPHIQWLKHIVINGSSFGADGFPY
VQVLKTADINSSEVEVLYLRNVSAEDAGEYTCLAGNSIGLSYQSAWLTVLPEEDPTWTAA
APEARYTDIILYASGSLALAVLLLLAGLYRGQALHGRHPRPPATVQKLSRFPLARQFSLESG
SSGKSSSSLVRGVRLSSSGPALLAGLVSLDLPLDPLWEFPRDRLVLGKPLGEGCFGQVVRA
EAFGMDPARPDQASTVAVKMLKDNASDKDLADLVSEMEVMKLIGRHKNIINLLGVCTQE
GPLYVIVECAAKGNLREFLRARRPPGPDLSPDGPRSSEGPLSFPVLVSCAYQVARGMQYLE
SRKCIHRDLAARNVLVTEDNVMKIADFGLARGVHHIDYYKKTSNGRLPVKWMAPEALF
DRVYTHQSDVWSFGILLWEIFTLGGSPYPGIPVEELFSLLREGHRMDRPPHCPPELYGLMR
ECWHAAPSQRPTFKQLVEALDKVLLAVSEEYLDLRLTFGPYSPSGGDASSTCSSSDSVFSH
DPLPLGSSSFPFGSGVQT

>P34995 multipass membrane protein
MSPCGPLNLSLAGEATTCAAPWVPNTSAVPPSGASPALPIFSMTLGAVSNLLALALLAQAA
GRLRRRRSAATFLLFVASLLATDLAGHVIPGALVLRLYTAGRAPAGGACHFLGGCMVFFG
LCPLLLGCGMAVERCVGVTRPLLHAARVSVARARLALAAVAAVALAVALLPLARVGRYEL
QYPGTWCFIGLGPPGGWRQALLAGLFASLGLVALLAALVCNTLSGLALLRARWRRRSRRP
PPASGPDSRRRWGAHGPRSASASSASSIASASTFFGGSRSSGSARRARAHDVEMVGQLVGI
MVVSCICWSPMLVLVALAVGGWSSTSLQRPLFLAVRLASWNQILDPWVYILLRQAVLRQL
LRLLPPRAGAKGGPAGLGLTPSAWEASSLRSSRHSGLSHF

>Q16873 multipass membrane protein
MKDEVALLAAVTLLGVLLQAYFSLQVISARRAFRVSPPLTTGPPEFERVYRAQVNCSEYFP
LFLATLWVAGIFFHEGAAALCGLVYLFARLRYFQGYARSAQLRLAPLYASARALWLLVALA
ALGLLAHFLPAALRAALLGRLRTLLPWA

>Q96CW1 peripheral membrane protein
MIGGLFIYNHKGEVLISRVYRDDIGRNAVDAFRVNVIHARQQVRSPVTNIARTSFFHVKRS
NIWLAAVTKQNVNAAMVFEFLYKMCDVMAAYFGKISEENIKNNFVLIYELLDEILDFGYP
QNSETGALKTFITQQGIKSQHQTKEEQSQITSQVTGQIGWRREGIKYRRNELFLDVLESVN
LLMSPQGQVLSAHVSGRVVMKSYLSGMPECKFGMNDKIVIEKQGKGTADETSKSGKQSI
AIDDCTFHQCVRLSKFDSERSISFIPPDGEFELMRYRTTKDIILPFRVIPLVREVGRTKLEVKV
VIKSNFKPSLLAQKIEVRIPTPLNTSGVQVICMKGKAKYKASENAIVWKIKRMAGMKESQI
SAEIELLPTNDKKKWARPPISMNFEVPFAPSGLKVRYLKVFEPKLNYSDHDVIKWVRYIGR
SGIYETRC

>Q14957 multipass membrane protein
MGGALGPALLLTSLFGAWAGLGPGQGEQGMTVAVVFSSSGPPQAQFRARLTPQSFLDLPL
EIQPLTVGVNTTNPSSLLTQICGLLGAAHVHGIVFEDNVDTEAVAQILDFISSQTHVPILSISG
GSAVVLTPKEPGSAFLQLGVSLEQQLQVLFKVLEEYDWSAFAVITSLHPGHALFLEGVRAV
ADASHVSWRLLDVVTLELGPGGPRARTQRLLRQLDAPVFVAYCSREEAEVLFAEAAQAG
LVGPGHVWLVPNLALGSTDAPPATFPVGLISVVTESWRLSLRQKVRDGVAILALGAHSYW
RQHGTLPAPAGDCRVHPGPVSPAREAFYRHLLNVTWEGRDFSFSPGGYLVQPTMVVIALN
RHRLWEMVGRWEHGVLYMKYPVWPRYSASLQPVVDSRHLTVATLEERPFVIVESPDPGT
GGCVPNTVPCRRQSNHTFSSGDVAPYTKLCCKGFCIDILKKLARVVKFSYDLYLVTNGKH
GKRVRGVWNGMIGEVYYKRADMAIGSLTINEERSEIVDFSVPFVETGISVMVARSNGTVS
PSAFLEPYSPAVWVMMFVMCLTVVAITVFMFEYFSPVSYNQNLTRGKKSGGPAFTIGKSV
WLLWALVFNNSVPIENPRGTTSKIMVLVWAFFAVIFLASYTANLAAFMIQEQYIDTVSGLS
DKKFQRPQDQYPPFRFGTVPNGSTERNIRSNYRDMHTHMVKFNQRSVEDALTSLKMGKL
DAFIYDAAVLNYMAGKDEGCKLVTIGSGKVFATTGYGIAMQKDSHWKRAIDLALLQFLG
DGETQKLETVWLSGICQNEKNEVMSSKLDIDNMAGVFYMLLVAMGLALLVFAWEHLVY
WKLRHSVPNSSQLDFLLAFSRGIYSCFSGVQSLASPPRQASPDLTASSAQASVLKMLQAAR
DMVTTAGVSSSLDRATRTIENWGGGRRAPPPSPCPTPRSGPSPCLPTPDPPPEPSPTGWGPP
DGGRAALVRRAPQPPGRPPTPGPPLSDVSRVSRRPAWEARWPVRTGHCGRHLSASERPLSP
ARCHYSSFPRADRSGRPFLPLFPELEDLPLLGPEQLARREALLHAAWARGSRPRHASLPSS
VAEAFARPSSLPAGCTGPACARPDGHSACRRLAQAQSMCLPIYREACQEGEQAGAPAWQH
RQHVCLHAHAHLPFCWGAVCPHLPPCASHGSWLSGAWGPLGHRGRTLGLGTGYRDSGG
LDEISRVARGTQGFPGPCTWRRISSLESEV

>P08648 single-pass type I membrane protein
MGSRTPESPLHAVQLRWGPRRRPPLLPLLLLLLPPPPRVGGFNLDAEAPAVLSGPPGSFFGF
SVEFYRPGTDGVSVLVGAPKANTSQPGVLQGGAVYLCPWGASPTQCTPIEFDSKGSRLLE
SSLSSSEGEEPVEYKSLQWFGATVRAHGSSILACAPLYSWRTEKEPLSDPVGTCYLSTDNF
TRILEYAPCRSDFSWAAGQGYCQGGFSAEFTKTGRVVLGGPGSYFWQGQILSATQEQIAES
YYPEYLINLVQGQLQTRQASSIYDDSYLGYSVAVGEFSGDDTEDFVAGVPKGNLTYGYVTI
LNGSDIRSLYNFSGEQMASYFGYAVAATDVNGDGLDDLLVGAPLLMDRTPDGRPQEVGRV
YVYLQHPAGIEPTPTLTLTGHDEFGRFGSSLTPLGDLDQDGYNDVAIGAPFGGETQQGVVF
VFPGGPGGLGSKPSQVLQPLWAASHTPDFFGSALRGGRDLDGNGYPDLIVGSFGVDKAVV
YRGRPIVSASASLTIFPAMFNPEERSCSLEGNPVACINLSFCLNASGKHVADSIGFTVELQLD
WQKQKGGVRRALFLASRQATLTQTLLIQNGAREDCREMKIYLRNESEFRDKLSPIHIALNF

SLDPQAPVDSHGLRPALHYQSKSRIEDKAQILLDCGEDNICVPDLQLEVFGEQNHVYLGD
KNALNLTFHAQNVGEGGAYEAELRVTAPPEAEYSGLVRHPGNFSSLSCDYFAVNQSRLLV
CDLGNPMKAGASLWGGLRFTVPHLRDTKKTIQFDFQILSKNLNNSQSDVVSFRLSVEAQA
QVTLNGVSKPEAVLFPVSDWHPRDQPQKEEDLGPAVHHVYELINQGPSSISQGVLELSCPQ
ALEGQQLLYVTRVTGLNCTTNHPINPKGLELDPEGSLHHQQKREAPSRSSASSGPQILKCP
EAECFRLRCELGPLHQQESQSLQLHFRVWAKTFLQREHQPFSLQCEAVYKALKMPYRILP
RQLPQKERQVATAVQWTKAEGSYGVPLWIIILAILFGLLLLGLLIYILYKLGFFKRSLPYGTA
MEKAQLKPPATSDA

>P55064 multipass membrane protein
MKKEVCSVAFLKAVFAEFLATLIFVFFGLGSALKWPSALPTILQIALAFGLAIGTLAQALGP
VSGGHINPAITLALLVGNQISLLRAFFYVAAQLVGAIAGAGILYGVAPLNARGNLAVNALN
NNTTQGQAMVVELILTFQLALCIFASTDSRRTSPVGSPALSIGLSVTLGHLVGIYFTGCSMN
PARSFGPAVVMNRFSPAHWVFWVGPIVGAVLAAILYFYLLFPNSLSLSERVAIIKGTYEPDE
DWEEQREERKKTMELTTR

>Q7Z6J2 peripheral membrane protein
MTLRRLRKLQQKEEAAATPDPAARTPDSEVAPAAPVPTPGPPAAAATPGPPADELYAALED
YHPAELYRALAVSGGTLPRRKGSGFRWKNLSQSPEQQRKVLTLEKEDNQTFGFEIQTYGL
HHREEQRVEMVTFVCRVHESSPAQLAGLTPGDTIASVNGLNVEGIRHREIVDIIKASGNVL
RLETLYGTSIRKAELEARLQYLKQTLYEKWGEYRSLMVQEQRLVHGLVVKDPSIYDTLES
VRSCLYGAGLLPGSLPFGPLLAVPGRPRGGARRARGDADDAVYHTCFFGDSEPPALPPPPP
PARAFGPGPAETPAVGPGPGPRAALSRSASVRCAGPGGGGGGGAPGALWTEAREQALCGP
GLRKTKYRSFRRRLLKFIPGLNRSLEEEESQL

>Q99941 single-pass type II membrane protein
MAELMLLSEIADPTRFFTDNLLSPEDWGLQNSTLYSGLDEVAEEQTQLFRCPEQDVPFDGS
SLDVGMDVSPSEPPWELLPIFPDLQVKSEPSSPCSSSSLSSESSRLSTEPSSEALGVGEVLHV
KTESLAPPLCLLGDDPTSSFETVQINVIPTSDDSSDVQTKIEPVSPCSSVNSEASLLSADSSS
QAFIGEEVLEVKTESLSPSGCLLWDVPAPSLGAVQISMGPSLDGSSGKALPTRKPPLQPKPV
VLTTVPMPSRAVPPSTTVLLQSLVQPPPVSPVVLIQGAIRVQPEGPAPSLPRPERKSIVPAPM
PGNSCPPEVDAKLLKRQQRMIKNRESACQSRRKKKEYLQGLEARLQAVLADNQQLRREN
AALRRRLEALLAENSELKLGSGNRKVVCIMVFLLFIAFNFGPVSISEPPSAPISPRMNKGEP
QPRRHLLGFSEQEPVQGVEPLQGSSQGPKEPQPSPTDQPSFSNLTAFPGGAKELLLRDLDQ
LFLSSDCRHFNRTESLRLADELSGWVQRHQRGRRKIPQRAQERQKSQPRKKSPPVKAVPIQ
PPGPPERDSVGQLQLYRHPDRSQPAFLDAIDRREDTFYVVSFRRDHLLLPAISHNKTSRPK
MSLVMPAMAPNETLSGRGAPGDYEEMMQIECEVMDTRVIHIKTSTVPPSLRKQPSPTPGN
ATGGPLPVSAASQAHQASHQPLYLNHP

>Q8NC56 multipass membrane protein
MAGLSDLELRRELQALGFQPGPITDTTRDVYRNKLRRLRGEARLRDEERLREEARPRGEE
RLREEARLREDAPLRARPAAASPRAEPWLSQPASGSAYATPGAYGDIRPSAASWVGSRGLA
YPARPAQLRRRASVRGSSEEDEDARTPDRATQGPGLAARRWWAASPAPARLPSSLLGPDP
RPGLRATRAGPAGAARARPEVGRRLERWLSRLLLWASLGLLLVFLGILWVKMGKPSAPQE

AEDNMKLLPVDCERKTDEFCQAKQKAALLELLHELYNFLAIQAGNFECGNPENLKSKCIP
VMEAQEYIANVTSSSSAKFEAALTWILSSNKDVGIWLKGEDQSELVTTVDKVVCLESAHP
RMGVGCRLSRALLTAVTNVLIFFWCLAFLWGLLILLKYRWRKLEEEEQAMYEMVKKIIDV
VQDHYVDWEQDMERYPYVGILHVRDSLIPPQSRRRMKRVWDRAVEFLASNESRIQTESH
RVAGEDMLVWRWTKPSSFSDSER

>Q04844 multipass membrane protein
MARAPLGVLLLLGLLGRGVGKNEELRLYHHLFNNYDPGSRPVREPEDTVTISLKVTLTNLI
SLNEKEETLTTSVWIGIDWQDYRLNYSKDDFGGIETLRVPSELVWLPEIVLENNIDGQFGV
AYDANVLVYEGGSVTWLPPAIYRSVCAVEVTYFPFDWQNCSLIFRSQTYNAEEVEFTFAV
DNDGKTINKIDIDTEAYTENGEWAIDFCPGVIRRHHGGATDGPGETDVIYSLIIRRKPLFYVI
NIIVPCVLISGLVLLAYFLPAQAGGQKCTVSINVLLAQTVFLFLIAQKIPETSLSVPLLGRFLI
FVMVVATLIVMNCVIVLNVSQRTPTTHAMSPRLRHVLLELLPRLLGSPPPPEAPRAASPPR
RASSVGLLLRAEELILKKPRSELVFEGQRHRQGTWTAAFCQSLGAAAPEVRCCVDAVNFV
AESTRDQEATGEEVSDWVRMGNALDNICFWAALVLFSVGSSLIFLGAYFNRVPDLPYAPCI
QP

>Q86YT5 multipass membrane protein
MASALSYVSKFKSFVILFVTPLLLLPLVILMPAKFVRCAYVIILMAIYWCTEVIPLAVTSLMP
VLLFPLFQILDSRQVCVQYMKDTNMLFLGGLIVAVAVERWNLHKRIALRTLLWVGAKPAR
LMLGFMGVTALLSMWISNTATTAMMVPIVEAILQQMEATSAATEAGLELVDKGKAKELP
GSQVIFEGPTLGQQEDQERKRLCKAMTLCICYAASIGGTATLTGTGPNVVLLGQMNELFPD
SKDLVNFASWFAFAFPNMLVMLLFAWLWLQFVYMRFNFKKSWGCGLESKKNEKAALKV
LQEEYRKLGPLSFAEINVLICFFLLVILWFSRDPGFMPGWLTVAWVEGETKYVSDATVAIFV
ATLLFIVPSQKPKFNFRSQTEEERKTPFYPPPLLDWKVTQEKVPWGIVLLLGGGFALAKGS
EASGLSVWMGKQMEPLHAVPPAAITLILSLLVAVFTECTSNVATTTLFLPIFASMSRSIGLNP
LYIMLPCTLSASFAFMLPVATPPNAIVFTYGHLKVADMVKTGVIMNIIGVFCVFLAVNTWG
RAIFDLDHFPDWANVTHIET

>P78352 peripheral membrane protein
MDCLCIVTTKKYRYQDEDTPPLEHSPAHLPNQANSPPVIVNTDTLEAPGYELQVNGTEGE
MEYEEITLERGNSGLGFSIAGGTDNPHIGDDPSIFITKIIPGGAAAQDGRLRVNDSILFVNEV
DVREVTHSAAVEALKEAGSIVRLYVMRRKPPAEKVMEIKLIKGPKGLGFSIAGGVGNQHIP
GDNSIYVTKIIEGGAAHKDGRLQIGDKILAVNSVGLEDVMHEDAVAALKNTYDVVYLKVA
KPSNAYLSDSYAPPDITTSYSQHLDNEISHSSYLGTDYPTAMTPTSPRRYSPVAKDLLGEEDI
PREPRRIVIHRGSTGLGFNIVGGEDGEGIFISFILAGGPADLSGELRKGDQILSVNGVDLRNA
SHEQAAIALKNAGQTVTIIAQYKPEEYSRFEAKIHDLREQLMNSSLGSGTASLRSNPKRGF
YIRALFDYDKTKDCGFLSQALSFRFGDVLHVIDASDEEWWQARRVHSDSETDDIGFIPSKR
RVERREWSRLKAKDWGSSSGSQGREDSVLSYETVTQMEVHYARPIIILGPTKDRANDDLL
SEFPDKFGSCVPHTTRPKREYEIDGRDYHFVSSREKMEKDIQAHKFIEAGQYNSHLYGTSV
QSVREVAEQGKHCILDVSANAVRRLQAAHLHPIAIFIRPRSLENVLEINKRITEEQARKAFD
RATKLEQEFTECFSAIVEGDSFEEIYHKVKRVIEDLSGPYIWVPARERL

>P35346 multipass membrane protein

MEPLFPASTPSWNASSPGAASGGGDNRTLVGPAPSAGARAVLVPVLYLLVCAAGLGGNTL
VIYVVLRFAKMKTVTNIYILNLAVADVLYMLGLPFLATQNAASFWPFGPVLCRLVMTLDG
VNQFTSVFCLTVMSVDRYLAVVHPLSSARWRRPRVAKLASAAAWVLSLCMSLPLLVFADV
QEGGTCNASWPEPVGLWGAVFIIYTAVLGFFAPLLVICLCYLLIVVKVRAAGVRVGCVRRR
SERKVTRMVLVVVLVFAGCWLPFFTVNIVNLAVALPQEPASAGLYFFVVILSYANSCANPV
LYGFLSDNFRQSFQKVLCLRKGSGAKDADATEPRPDRIRQQQEATPPAHRAAANGLMQTS
KL

>O60503 multipass membrane protein
MASPPHQQLLHHHSTEVSCDSSGDSNSVRVKINPKQLSSNSHPKHCKYSISSSCSSSGDSG
GVPRRVGGGGRLRRQKKLPQLFERASSRWWDPKFDSVNLEEACLERCFPQTQRRFRYALF
YIGFACLLWSIYFAVHMRSRLIVMVAPALCFLLVCVGFFLFTFTKLYARHYAWTSLALTLLV
FALTLAAQFQVLTPVSGRGDSSNLTATARPTDTCLSQVGSFSMCIEVLFLLYTVMHLPLYLS
LCLGVAYSVLFETFGYHFRDEACFPSPGAGALHWELLSRGLLHGCIHAIGVHLFVMSQVR
SRSTFLKVGQSIMHGKDLEVEKALKERMIHSVMPRIIADDLMKQGDEESENSVKRHATSS
PKNRKKKSSIQKAPIAFRPFKMQQIEEVSILFADIVGFTKMSANKSAHALVGLLNDLFGRF
DRLCEETKCEKISTLGDCYYCVAGCPEPRADHAYCCIEMGLGMIKAIEQFCQEKKEMVN
MRVGVHTGTVLCGILGMRRFKFDVWSNDVNLANLMEQLGVAGKVHISEATAKYLDDRY
EMEDGKVIERLGQSVVADQLKGLKTYLISGQRAKESRCSCAEALLSGFEVIDGSQVSSGPR
GQGTASSGNVSDLAQTVKTFDNLKTCPSCGITFAPKSEAGAEGGAPQNGCQDEHKNSTK
ASGGPNPKTQNGLLSPPQEEKLTNSQTSLCEILQEKGRWAGVSLDQSALLPLRFKNIREKT
DAHFVDVIKEDSLMKDYFFKPPINQFSLNFLDQELERSYRTSYQEEVIKNSPVKTFASPTFS
SLLDVFLSTTVFLTLSTTCFLKYEAATVPPPPAALAVFSAALLLEVLSLAVSIRMVFFLEDV
MACTKRLLEWIAGWLPRHCIGAILVSLPALAVYSHVTSEYETNIHFPVFTGSAALIAVVHY
CNFCQLSSWMRSSLATVVGAGPLLLLYVSLCPDSSVLTSPLDAVQNFSSERNPCNSSVPRD
LRRPASLIGQEVVLVFFLLLLLVWFLNREFEVSYRLHYHGDVEADLHRTKIQSMRDQADW
LLRNIIPYHVAEQLKVSQTYSKNHDSGGVIFASIVNFSEFYEENYEGGKECYRVLNELIGDF
DELLSKPDYSSIEKIKTIGATYMAASGLNTAQAQDGSHPQEHLQILFEFAKEMMRVVDDFN
NNMLWFNFKLRVGFNHGPLTAGVIGTTKLLYDIWGDTVNIASRMDTTGVECRIQVSEESY
RVLSKMGYDFDYRGTVNVKGKGQMKTYLYPKCTDHRVIPQHQLSISPDIRVQVDGSIGRS
PTDEIANLVPSVQYVDKTSLGSDSSTQAKDAHLSPKRPWKEPVKAEERGRFGKAIEKDDC
DETGIEEANELTKLNVSKSV

>Q9UQ49 peripheral membrane protein
MEEVTTCSFNSPLFRQEDDRGITYRIPALLYIPPTHTFLAFAEKRSTRRDEDALHLVLRRGL
RIGQLVQWGPLKPLMEATLPGHRTMNPCPVWEQKSGCVFLFFICVRGHVTERQQIVSGRN
AARLCFIYSQDAGCSWSEVRDLTEEVIGSELKHWATFAVGPGHGIQLQSGRLVIPAYTYYIP
SWFFCFQLPCKTRPHSLMIYSDDLGVTWHHGRLIRPMVTVECEVAEVTGRAGHPVLYCSA
RTPNRCRAEALSTDHGEGFQRLALSRQLCEPPHGCQGSVVSFRPLEIPHRCQDSSSKDAPTI
QQSSPGSSLRLEEEAGTPSESWLLYSHPTSRKQRVDLGIYLNQTPLEAACWSRPWILHCGP
CGYSDLAALEEEGLFGCLFECGTKQECEQIAFRLFTHREILSHLQGDCTSPGRNPSQFKSN

>Q8NBI2 multipass membrane protein
MVSGRFYLSCLLLGSLGSMCILFTIYWMQYWRGGFAWNGSIYMFNWHPVLMVAGMVVF

YGGASLVYRLPQSWVGPKLPWKLLHAALHLMAFVLTVVGLVAVFTFHNHGRTANLYSLH
SWLGITTVFLFACQWFLGFAVFLLPWASMWLRSLLKPIHVFFGAAILSLSIASVISGINEKLF
FSLKNTTRPYHSLPSEAVFANSTGMLVVAFGLLVLYILLASSWKRPEPGILTDRQPLLHDGE

>P63215 lipid-anchor
MKGETPVNSTMSIGQARKMVEQLKIEASLCRIKVSKAAADLMTYCDAHACEDPLITPVPT
SENPFREKKFFCALL

>Q9NPB3 lipid-anchor
MGNCAKRPWRRGPKDPLQWLGSPPRGSCPSPSSSPKEQGDPAPGVQGYSVLNSLVGPACI
FLRPSIAATQLDRELRPEEIEELQVAFQEFDRDRDGYIGCRELGACMRTLGYMPTEMELIEI
SQQISGGKVDFEDFVELMGPKLLAETADMIGVRELRDAFREFDTNGDGRISVGELRAALK
ALLGERLSQREVDEILQDVDLNGDGLVDFEEFVRMMSR

>O75197 single-pass type I membrane protein
MEAAPPGPPWPLLLLLLLLLALCGCPAPAAASPLLLFANRRDVRLVDAGGVKLESTIVVSG
LEDAAAVDFQFSKGAVYWTDVSEEAIKQTYLNQTGAAVQNVVISGLVSPDGLACDWVGK
KLYWTDSETNRIEVANLNGTSRKVLFWQDLDQPRAIALDPAHGYMYWTDWGETPRIERA
GMDGSTRKIIVDSDIYWPNGLTIDLEEQKLYWADAKLSFIHRANLDGSFRQKVVEGSLTHP
FALTLSGDTLYWTDWQTRSIHACNKRTGGKRKEILSALYSPMDIQVLSQERQPFFHTRCEE
DNGGCSHLCLLSPSEPFYTCACPTGVQLQDNGRTCKAGAEEVLLLARRTDLRRISLDTPDF
TDIVLQVDDIRHAIAIDYDPLEGYVYWTDDEVRAIRRAYLDGSGAQTLVNTEINDPDGIAV
DWVARNLYWTDTGTDRIEVTRLNGTSRKILVSEDLDEPRAIALHPVMGLMYWTDWGENP
KIECANLDGQERRVLVNASLGWPNGLALDLQEGKLYWGDAKTDKIEVINVDGTKRRTLL
EDKLPHIFGFTLLGDFIYWTDWQRRSIERVHKVKASRDVIIDQLPDLMGLKAVNVAKVVG
TNPCADRNGGCSHLCFFTPHATRCGCPIGLELLSDMKTCIVPEAFLVFTSRAAIHRISLETN
NNDVAIPLTGVKEASALDFDVSNNHIYWTDVSLKTISRAFMNGSSVEHVVEFGLDYPEGM
AVDWMGKNLYWADTGTNRIEVARLDGQFRQVLVWRDLDNPRSLALDPTKGYIYWTEWG
GKPRIVRAFMDGTNCMTLVDKVGRANDLTIDYADQRLYWTDLDTNMIESSNMLGQERVV
IADDLPHPFGLTQYSDYIYWTDWNLHSIERADKTSGRNRTLIQGHLDFVMDILVFHSSRQD
GLNDCMHNNGQCGQLCLAIPGGHRCGCASHYTLDPSSRNCSPPTTFLLFSQKSAISRMIPD
DQHSPDLILPLHGLRNVKAIDYDPLDKFIYWVDGRQNIKRAKDDGTQPFVLTSLSQGQNP
DRQPHDLSIDIYSRTLFWTCEATNTINVHRLSGEAMGVVLRGDRDKPRAIVVNAERGYLY
FTNMQDRAAKIERAALDGTEREVLFTTGLIRPVALVVDNTLGKLFWVDADLKRIESCDLS
GANRLTLEDANIVQPLGLTILGKHLYWIDRQQQMIERVEKTTGDKRTRIQGRVAHLTGIHA
VEEVSLEEFSAHPCARDNGGCSHICIAKGDGTPRCSCPVHLVLLQNLLTCGEPPTCSPDQFA
CATGEIDCIPGAWRCDGFPECDDQSDEEGCPVCSAAQFPCARGQCVDLRLRCDGEADCQ
DRSDEADCDAICLPNQFRCASGQCVLIKQQCDSFPDCIDGSDELMCEITKPPSDDSPAHSSA
IGPVIGIILSLFVMGGVYFVCQRVVCQRYAGANGPFPHEYVSGTPHVPLNFIAPGGSQHGPF
TGIACGKSMMSSVSLMGGRGGVPLYDRNHVTGASSSSSSSTKATLYPPILNPPPSPATDPSL
YNMDMFYSSNIPATARPYRPYIIRGMAPPTTPCSTDVCDSDYSASRWKASKYYLDLNSDS
DPYPPPPTPHSQYLSAEDSCPPSPATERSYFHLFPPPPSPCTDSS

>P18577 multipass membrane protein

MSSKYPRSVRRCLPLWALTLEAALILLFYFFTHYDASLEDQKGLVASYQVGQDLTVMAAL
GLGFLTSNFRRHSWSSVAFNLFMLALGVQWAILLDGFLSQFPPGKVVITLFSIRLATMSAM
SVLISAGAVLGKVNLAQLVVMVLVEVTALGTLRMVISNIFNTDYHMNLRHFYVFAAYFGL
TVAWCLPKPLPKGTEDNDQRATIPSLSAMLGALFLWMFWPSVNSPLLRSPIQRKNAMFNT
YYALAVSVVTAISGSSLAHPQRKISMTYVHSAVLAGGVAVGTSCHLIPSPWLAMVLGLVAG
LISIGGAKCLPVCCNRVLGIHHISVMHSIFSLLGLLGEITYIVLLVLHTVWNGNGMIGFQVL
LSIGELSLAIVIALTSGLLTGLLLNLKIWKAPHVAKYFDDQVFWKFPHLAVGF

>Q13571 multipass membrane protein
MDPRLSTVRQTCCCFNVRIATTALAIYHVIMSVLLFIEHSVEVAHGKASCKLSQMGYLRIA
DLISSFLLITMLFIISLSLLIGVVKNREKYLLPFLSLQIMDYLLCLLTLLGSYIELPAYLKLASR
SRASSSKFPLMTLQLLDFCLSILTLCSSYMEVPTYLNFKSMNHMNYLPSQEDMPHNQFIK
MMIIFSIAFITVLIFKVYMFKCVWRCYRLIKCMNSVEEKRNSKMLQKVVLPSYEEALSLPS
KTPEGGPAPPPYSEV

>P19320 single-pass type I membrane protein
MPGKMVVILGASNILWIMFAASQAFKIETTPESRYLAQIGDSVSLTCSTTGCESPFFSWRTQ
IDSPLNGKVTNEGTTSTLTMNPVSFGNEHSYLCTATCESRKLEKGIQVEIYSFPKDPEIHLSG
PLEAGKPITVKCSVADVYPFDRLEIDLLKGDHLMKSQEFLEDADRKSLETKSLEVTFTPVIE
DIGKVLVCRAKLHIDEMDSVPTVRQAVKELQVYISPKNTVISVNPSTKLQEGGSVTMTCSS
EGLPAPEIFWSKKLDNGNLQHLSGNATLTLIAMRMEDSGIYVCEGVNLIGKNRKEVELIVQ
EKPFTVEISPGPRIAAQIGDSVMLTCSVMGCESPSFSWRTQIDSPLSGKVRSEGTNSTLTLSP
VSFENEHSYLCTVTCGHKKLEKGIQVELYSFPRDPEIEMSGGLVNGSSVTVSCKVPSVYPL
DRLEIELLKGETILENIEFLEDTDMKSLENKSLEMTFIPTIEDTGKALVCQAKLHIDDMEFEP
KQRQSTQTLYVNVAPRDTTVLVSPSSILEEGSSVNMTCLSQGFPAPKILWSRQLPNGELQPL
SENATLTLISTKMEDSGVYLCEGINQAGRSRKEVELIIQVTPKDIKLTAFPSESVKEGDTVIIS
CTCGNVPETWIILKKKAETGDTVLKSIDGAYTIRKAQLKDAGVYECESKNKVGSQLRSLT
LDVQGRENNKDYFSPELLVLYFASSLIIPAIGMIIYFARKANMKGSYSLVEAQKSKV

>O75015 GPI-anchor, lipid-anchor
MWQLLLPTALLLLVSAGMRTEDLPKAVVFLEPQWYSVLEKDSVTLKCQGAYSPEDNSTQ
WFHNESLISSQASSYFIDAATVNDSGEYRCQTNLSTLSDPVQLEVHIGWLLLQAPRWVFKE
EDPIHLRCHSWKNTALHKVTYLQNGKDRKYFHHNSDFHIPKATLKDSGSYFCRGLVGSK
NVSSETVNITITQGLAVSTISSFSPPGYQVSFCLVMVLLFAVDTGLYFSVKTNI

>P22888 multipass membrane protein
MKQRFSALQLLKLLLLLQPPLPRALREALCPEPCNCVPDGALRCPGPTAGLTRLSLAYLPV
KVIPSQAFRGLNEVIKIEISQIDSLERIEANAFDNLLNLSEILIQNTKNLRYIEPGAFINLPRLK
YLSICNTGIRKFPDVTKVFSSESNFILEICDNLHITTIPGNAFQGMNNESVTLKLYGNGFEEV
QSHAFNGTTLTSLELKENVHLEKMHNGAFRGATGPKTLDISSTKLQALPSYGLESIQRLIAT
SSYSLKKLPSRETFVNLLEATLTYPSHCCAFRNLPTKEQNFSHSISENFSKQCESTVRKVNN
KTLYSSMLAESELSGWDYEYGFCLPKTPRCAPEPDAFNPCEDIMGYDFLRVLIWLINILAI
MGNMTVLFVLLTSRYKLTVPRFLMCNLSFADFCMGLYLLLIASVDSQTKGQYYNHAIDW
QTGSGCSTAGFFTVFASELSVYTLTVITLERWHTITYAIHLDQKLRLRHAILIMLGGWLFSS

LIAMLPLVGVSNYMKVSICFPMDVETTLSQVYILTILILNVVAFFIICACYIKIYFAVRNPELM
ATNKDTKIAKKMAILIFTDFTCMAPISFFAISAAFKVPLITVTNSKVLLVLFYPINSCANPFLY
AIFTKTFQRDFFLLLSKFGCCKRRAELYRRKDFSAYTSNCKNGFTGSNKPSQSTLKLSTLH
CQGTALLDKTRYTEC

>Q9H3M0 multipass membrane protein
MDGSGERSLPEPGSQSSAASDDIEIVVNVGGVRQVLYGDLLSQYPETRLAELINCLAGGY
DTIFSLCDDYDPGKREFYFDRDPDAFKCVIEVYYFGEVHMKKGICPICFKNEMDFWKVDL
KFLDDCCKSHLSEKREELEEIARRVQLILDDLGVDAAEGRWRRCQKCVWKFLEKPESSCP
ARVVAVLSFLLILVSSVVMCMGTIPELQVLDAEGNRVEHPTLENVETACIGWFTLEYLLRL
FSSPNKLHFALSFMNIVDVLAILPFYVSLTLTHLGARMMELTNVQQAVQALRIMRIARIFKL
ARHSSGLQTLTYALKRSFKELGLLLMYLAVGIFVFSALGYTMEQSHPETLFKSIPQSFWWA
IITMTTVGYGDIYPKTTLGKLNAAISFLCGVIAIALPIHPIINNFVRYYNKQRVLETAAKHEL
ELMELNSSSGGEGKTGGSRSDLDNLPPEPAGKEAPSCSSRLKLSHSDTFIPLLTEEKHHRTR
LQSCK

>Q53QZ3 peripheral membrane protein
MQKSTNSDTSVETLNSTRQGTGAVQMRIKNANSHHDRLSQSKSMILTDVGKVTEPISRHR
RNHSQHILKDVIPPLEQLMVEKEGYLQKAKIADGGKKLRKNWSTSWIVLSSRRIEFYKES
KQQALSNMKTGHKPESVDLCGAHIEWAKEKSSRKNVFQITTVSGNEFLLQSDIDFIILDWF
HAIKNAIDRLPKDSSCPSRNLELFKIQRSSSTELLSHYDSDIKEQKPEHRKSLMFRLHHSAS
DTSDKNRVKSRLKKFITRRPSLKTLQEKGLIKDQIFGSHLHKVCERENSTVPWFVKQCIEA
VEKRGLDVDGIYRVSGNLATIQKLRFIVNQEEKLNLDDSQWEDIHVVTGALKMFFRELPE
PLFPYSFFEQFVEAIKKQDNNTRIEAVKSLVQKLPPPNRDTMKVLFGHLTKIVAKASKNLM
STQSLGIVFGPTLLRAENETGNMAIHMVYQNQIAELMLSEYSKIFGSEED

>P48549 multipass membrane protein
MSALRRKFGDDYQVVTTSSSGSGLQPQGPGQDPQQQLVPKKKRQRFVDKNGRCNVQHG
NLGSETSRYLSDLFTTLVDLKWRWNLFIFILTYTVAWLFMASMWWVIAYTRGDLNKAHV
GNYTPCVANVYNFPSAFLFFIETEATIGYGYRYITDKCPEGIILFLFQSILGSIVDAFLIGCMFI
KMSQPKKRAETLMFSEHAVISMRDGKLTLMFRVGNLRNSHMVSAQIRCKLLKSRQTPEG
EFLPLDQLELDVGFSTGADQLFLVSPLTICHVIDAKSPFYDLSQRSMQTEQFEIVVILEGIVE
TTGMTCQARTSYTEDEVLWGHRFFPVISLEEGFFKVDYSQFHATFEVPTPPYSVKEQEEML
LMSSPLIAPAITNSKERHNSVECLDGLDDITTKLPSKLQKITGREDFPKKLLRMSSTTSEKA
YSLGDLPMKLQRISSVPGNSEEKLVSKTTKMLSDPMSQSVADLPPKLQKMAGGAARMEG
NLPAKLRKMNSDRFT

>Q8NFH5 peripheral membrane protein
MAAFAVEPQGPALGSEPMMLGSPTSPKPGVNAQFLPGFLMGDLPAPVTPQPRSISGPSVGV
MEMRSPLLAGGSPPQPVVPAHKDKSGAPPVRSIYDDISSPGLGSTPLTSRRQPNISVMQSPL
VGVTSTPGTGQSMFSPASIGQPRKTTLSPAQLDPFYTQGDSLTSEDHLDDSWVTVFGFPQA
SASYILLQFAQYGNILKHVMSNTGNWMHIRYQSKLQARKALSKDGRIFGESIMIGVKPCID
KSVMESSDRCALSSPSLAFTPPIKTLGTPTQPGSTPRISTMRPLATAYKASTSDYQVISDRQT
PKKDESLVSKAMEYMFGW

>Q9UKI2 peripheral membrane protein
MPAKTPIYLKAANNKKGKKFKLRDILSPDMISPPLGDFRHTIHIGKEGQHDVFGDISFLQG
NYELLPGNQEKAHLGQFPGHNEFFRANSTSDSVFTETPSPVLKNAISLPTIGGSQALMLPL
LSPVTFNSKQESFGPAKLPRLSCEPVMEEKAQEKSSLLENGTVHQGDTSWGSSGSASQSSQ
GRDSHSSSLSEQYPDWPAEDMFDHPTPCELIKGKTKSEESLSDLTGSLLSLQLDLGPSLLDE
VLNVMDKNK

>P01135 single-pass type I membrane protein
MVPSAGQLALFALGIVLAACQALENSTSPLSADPPVAAAVVSHFNDCPDSHTQFCFHGTC
RFLVQEDKPACVCHSGYVGARCEHADLLAVVAASQKKQAITALVVVSIVALAVLIITCVLI
HCCQVRKHCEWCRALICRHEKPSALLKGRTACCHSETVV

>Q8N1C3 multipass membrane protein
MGPLKAFLFSPFLLRSQSRGVRLVFLLLTLHLGNCVDKADDEDDEDLTVNKTWVLAPKIH
EGDITQILNSLLQGYDNKLRPDIGVRPTVIETDVYVNSIGPVDPINMEYTIDIIFAQTWFDSR
LKFNSTMKVLMLNSNMVGKIWIPDTFFRNSRKSDAHWITTPNRLLRIWNDGRVLYTLRLT
INAECYLQLHNFPMDEHSCPLEFSSYGYPKNEIEYKWKKPSVEVADPKYWRLYQFAFVGL
RNSTEITHTISGDYVIMTIFFDLSRRMGYFTIQTYIPCILTVVLSWVSFWINKDAVPARTSLGI
TTVLTMTTLSTIARKSLPKVSYVTAMDLFVSVCFIFVFAALMEYGTLHYFTSNQKGKTATK
DRKLKNKASMTPGLHPGSTLIPMNNISVPQEDDYGYQCLEGKDCASFFCCFEDCRTGSWR
EGRIHIRIAKIDSYSRIFFPTAFALFNLVYWVGYLYL

>P18505 multipass membrane protein
MWTVQNRESLGLLSFPVMITMVCCAHSTNEPSNMSYVKETVDRLLKGYDIRLRPDFGGP
PVDVGMRIDVASIDMVSEVNMDYTLTMYFQQSWKDKRLSYSGIPLNLTLDNRVADQLWV
PDTYFLNDKKSFVHGVTVKNRMIRLHPDGTVLYGLRITTTAACMMDLRRYPLDEQNCTL
EIESYGYTTDDIEFYWNGGEGAVTGVNKIELPQFSIVDYKMVSKKVEFTTGAYPRLSLSFR
LKRNIGYFILQTYMPSTLITILSWVSFWINYDASAARVALGITTVLTMTTISTHLRETLPKIP
YVKAIDIYLMGCFVFVFLALLEYAFVNYIFFGKGPQKKGASKQDQSANEKNKLEMNKVQ
VDAHGNILLSTLEIRNETSGSEVLTSVSDPKATMYSYDSASIQYRKPLSSREAYGRALDRH
GVPSKGRIRRRASQLKVKIPDLTDVNSIDKWSRMFFPITFSLFNVVYWLYYVH

>Q7Z3F1 multipass membrane protein
MNSNLPAENLTIAVNMTKTLPTAVTHGFNSTNDPPSMSITRLFPALLECFGIVLCGYIAGRA
NVITSTQAKGLGNFVSRFALPALLFKNMVVLNFSNVDWSFLYSILIAKASVFFIVCVLTLLV
ASPDSRFSKAGLFPIFATQSNDFALGYPIVEALYQTTYPEYLQYIYLVAPISLMMLNPIGFIFC
EIQKWKDTQNASQNKIKIVGLGLLRVLQNPIVFMVFIGIAFNFILDRKVPVYVENFLDGLG
NSFSGSALFYLGLTMVGKIKRLKKSAFVVLILLITAKLLVLPLLCREMVELLDKGDSVVNH
TSLSNYAFLYGVFPVAPGVAIFATQFNMEVEIITSGMVISTFVSAPIMYVSAWLLTFPTMDPK
PLAYAIQNVSFDISIVSLISLIWSLAILLLSKKYKQLPHMLTTNLLIAQSIVCAGMMIWNFVK
EKNFVGQILVFVLLYSSLYSTYLWTGLLAISLFLLKKRERVQIPVGIIIISGWGIPALLVGVLLI
TGKHNGDSIDSAFFYGKEQMITTAVTLFCSILIAGISLMCMNQTAQAGSYEGFDQSQSHKV
VEPGNTAFEESPAPVNEPELFTSSIPETSCCSCSMGNGELHCPSIEPIANTSTSEPVIPSFEKN

NHCVSRCNSQSCILAQEEEQYLQSGDQQLTRHVLLCLLLIIGLFANLSSCLWWLFNQEPGR
LYVELQFFCAVFNFGQGFISFGIFGLDKHLIILPFKRRLEFLWNNKDTAENRDSPVSEEIKMT
CQQFIHYHRDLCIRNIVKERRCGAKTSAGTFCGCDLVSWLIEVGLASDRGEAVIYGDRLVQ
GGVIQHITNEYEFRDEYLFYRFLQKSPEQSPPAINANTLQQERYKEIEHSSPPSHSPKT

>P32238 multipass membrane protein
MDVVDSLLVNGSNITPPCELGLENETLFCLDQPRPSKEWQPAVQILLYSLIFLLSVLGNTLVI
TVLIRNKRMRTVTNIFLLSLAVSDLMLCLFCMPFNLIPNLLKDFIFGSAVCKTTTYFMGTSV
SVSTFNLVAISLERYGAICKPLQSRVWQTKSHALKVIAATWCLSFTIMTPYPIYSNLVPFTK
NNNQTANMCRFLLPNDVMQQSWHTFLLLILFLIPGIVMMVAYGLISLELYQGIKFEASQKK
SAKERKPSTTSSGKYEDSDGCYLQKTRPPRKLELRQLSTGSSSRANRIRSNSSAANLMAK
KRVIRMLIVIVVLFFLCWMPIFSANAWRAYDTASAERRLSGTPISFILLLSYTSSCVNPIIYCF
MNKRFRLGFMATFPCCPNPGPPGARGEVGEEEEGGTTGASLSRFSYSHMSASVPPQ

>P43308 single-pass type I membrane protein
MRLLSFVVLALFAVTQAEEGARLLASKSLLNRYAVEGRDLTLQYNIYNVGSSAALDVELS
DDSFPPEDFGIVSGMLNVKWDRIAPASNVSHTVVLRPLKAGYFNFTSATITYLAQEDGPVV
IGSTSAPGQGGILAQREFDRRFSPHFLDWAAFGVMTLPSIGIPLLLWYSSKRKYDTPKTKK
N

>Q16849 single-pass type I membrane protein
MRRPRRPGGLGGSGGLRLLLCLLLLSSRPGGCSAVSAHGCLFDRRLCSHLEVCIQDGLFG
QCQVGVGQARPLLQVTSPVLQRLQGVLRQLMSQGLSWHDDLTQYVISQEMERIPRLRPP
EPRPRDRSGLAPKRPGPAGELLLQDIPTGSAPAAQHRLPQPPVGKGGAGASSSLSPLQAEL
LPPLLEHLLLPPQPPHPSLSYEPALLQPYLFHQFGSRDGSRVSEGSPGMVSVGPLPKAEAPA
LFSRTASKGIFGDHPGHSYGDLPGPSPAQLFQDSGLLYLAQELPAPSRARVPRLPEQGSSSR
AEDSPEGYEKEGLGDRGEKPASPAVQPDAALQRLAAVLAGYGVELRQLTPEQLSTLLTLL
QLLPKGAGRNPGGVVNVGADIKKTMEGPVEGRDTAELPARTSPMPGHPTASPTSSEVQQV
PSPVSSEPPKAARPPVTPVLLEKKSPLGQSQPTVAGQPSARPAAEEYGYIVTDQKPLSLAA
GVKLLEILAEHVHMSSGSFINISVVGPALTFRIRHNEQNLSLADVTQQAGLVKSELEAQTG
LQILQTGVGQREEAAAVLPQTAHSTSPMRSVLLTLVALAGVAGLLVALAVALCVRQHARQ
QDKERLAALGPEGAHGDTTFEYQDLCRQHMATKSLFNRAEGPPEPSRVSSVSSQFSDAAQ
ASPSSHSSTPSWCEEPAQANMDISTGHMILAYMEDHLRNRDRLAKEWQALCAYQAEPNT
CATAQGEGNIKKNRHPDFLPYDHARIKLKVESSPSRSDYINASPIIEHDPRMPAYIATQGPLS
HTIADFWQMVWESGCTVIVMLTPLVEDGVKQCDRYWPDEGASLYHVYEVNLVSEHIWC
EDFLVRSFYLKNVQTQETRTLTQFHFLSWPAEGTPASTRPLLDFRRKVNKCYRGRSCPIIVH
CSDGAGRTGTYILIDMVLNRMAKGVKEIDIAATLEHVRDQRPGLVRSKDQFEFALTAVAEE
VNAILKALPQ

>Q14623 lipid-anchor
MSPARLRPRLHFCLVLLLLLVVPAAWGCGPGRVVGSRRRPPRKLVPLAYKQFSPNVPEKTL
GASGRYEGKIARSSERFKELTPNYNPDIIFKDEENTGADRLMTQRCKDRLNSLAISVMNQ
WPGVKLRVTEGWDEDGHHSEESLHYEGRAVDITTSDRDRNKYGLLARLAVEAGFDWVY
YESKAHVHCSVKSEHSAAAKTGGCFPAGAQVRLESGARVALSAVRPGDRVLAMGEDGSP

TFSDVLIFLDREPHRLRAFQVIETQDPPRRLALTPAHLLFTADNHTEPAARFRATFASHVQP
GQYVLVAGVPGLQPARVAAVSTHVALGAYAPLTKHGTLVVEDVVASCFAAVADHHLAQLA
FWPLRLFHSLAWGSWTPGEGVHWYPQLLYRLGRLLLEEGSFHPLGMSGAGS

>Q9Y6M7 multipass membrane protein
MERFRLEKKLPGPDEEAVVDLGKTSSTVNTKFEKEELESHRAVYIGVHVPFSKESRRRHR
HRGHKHHHRRRKDKESDKEDGRESPSYDTPSQRVQFILGTEDDDEEHIPHDLFTEMDELC
YRDGEEYEWKETARWLKFEEDVEDGGDRWSKPYVATLSLHSLFELRSCILNGTVMLDMR
ASTLDEIADMVLDNMIASGQLDESIRENVREALLKRHHHQNEKRFTSRIPLVRSFADIGKK
HSDPHLLERNGEGLSASRHSLRTGLSASNLSLRGESPLSLLLGHLLPSSRAGTPAGSRCTTP
VPTPQNSPPSSPSISRLTSRSSQESQRQAPELLVSPASDDIPTVVIHPPEEDLEAALKGEEQKN
EENVDLTPGILASPQSAPGNLDNSKSGEIKGNGSGGSRENSTVDFSKVDMNFMRKIPTGAE
ASNVLVGEVDFLERPIIAFVRLAPAVLLTGLTEVPVPTRFLFLLLGPAGKAPQYHEIGRSIAT
LMTDEIFHDVAYKAKDRNDLLSGIDEFLDQVTVLPPGEWDPSIRIEPPKSVPSQEKRKIPVF
HNGSTPTLGETPKEAAHHAGPELQRTGRLFGGLILDIKRKAPFFLSDFKDALSLQCLASILF
LYCACMSPVITFGGLLGEATEGRISAIESLFGASLTGIAYSLFAGQPLTILGSTGPVLVFEKIL
YKFCRDYQLSYLSLRTSIGLWTSFLCIVLVATDASSLVCYITRFTEEAFAALICIIFIYEALEKL
FDLGETYAFNMHNNLDKLTSYSCVCTEPPNPSNETLAQWKKDNITAHNISWRNLTVSECK
KLRGVFLGSACGHHGPYIPDVLFWCVILFFTTFFLSSFLKQFKTKRYFPTKVRSTISDFAVFL
TIVIMVTIDYLVGVPSPKLHVPEKFEPTHPERGWIISPLGDNPWWTLLIAAIPALLCTILIFM
DQQITAVIINRKEHKLKKGAGYHLDLLMVGVMLGVCSVMGLPWFVAATVLSISHVNSLK
VESECSAPGEQPKFLGIREQRVTGLMIFILMGLSVFMTSVLKFIPMPVLYGVFLYMGVSSL
KGIQLFDRIKLFGMPAKHQPDLIYLRYVPLWKVHIFTVIQLTCLVLLWVIKVSAAAVVFPM
MVLALVFVRKLMDLCFTKRELSWLDDLMPESKKKKEDDKKKKEKEEAERMLQDDDDT
VHLPFEGGSLLQIPVKALKYSPDKPVSVKISFEDEPRKKYVDAETSL

>Q8TCJ2 multipass membrane protein
MAEPSAPESKHKSSLNSSPWSGLMALGNSRHGHHGPGAQCAHKAAGGAAPPKPAPAGLS
GGLSQPAGWQSLLSFTILFLAWLAGFSSRLFAVIRFESIIHEFDPWFNYRSTHHLASHGFYEF
LNWFDERAWYPLGRIVGGTVYPGLMITAGLIHWILNTLNITVHIRDVCVFLAPTFSGLTSIS
TFLLTRELWNQGAGLLAACFIAIVPGYISRSVAGSFDNEGIAIFALQFTYYLWVKSVKTGSV
FWTMCCCLSYFYMVSAWGGYVFIINLIPLHVFVLLLMQRYSKRVYIAYSTFYIVGLILSMQ
IPFVGFQPIRTSEHMAAAGVFALLQAYAFLQYLRDRLTKQEFQTLFFLGVSLAAGAVFLSVI
YLTYTGYIAPWSGRFYSLWDTGYAKIHIPIIASVSEHQPTTWVSFFFDLHILVCTFPAGLWFC
IKNINDERVFVALYAISAVYFAGVMVRLMLTLTPVVCMLSAIAFSNVFEHYLGDDMKRENP
PVEDSSDEDDKRNQGNLYDKAGKVRKHATEQEKTEEGLGPNIKSIVTMLMLMLLMMFAV
HCTWVTSNAYSSPSVVLASYNHDGTRNILDDFREAYFWLRQNTDEHARVMSWWDYGYQ
IAGMANRTTLVDNNTWNNSHIALVGKAMSSNETAAYKIMRTLDVDYVLVIFGGVIGYSGD
DINKFLWMVRIAEGEHPKDIRESDYFTPQGEFRVDKAGSPTLLNCLMYKMSYYRFGEMQ
LDFRTPPGFDRTRNAEIGNKDIKFKHLEEAFTSEHWLVRIYKVKAPDNRETLDHKPRVTNI
FPKQKYLSKKTTKRKRGYIKNKLVFKKGKKISKKTV

>Q8IZQ1 peripheral membrane protein
MNMVKRIMGRPRQEECSPQDNALGLMHLRRLFTELCHPPRHMTQKEQEEKLYMMLPVF

NRVFGNAPPNTMTEKFSDLLQFTTQVSRLMVTEIRRRASNKSTEAASRAIVQFLEINQSEE
ASRGWMLLTTINLLASSGQKTVDCMTTMSVPSTLVKCLYLFFDLPHVPEAVGGAQNELPL
AERRGLLQKVFVQILVKLCSFVSPAEELAQKDDLQLLFSAITSWCPPYNLPWRKSAGEVL
MTISRHGLSVNVVKYIHEKECLSTCVQNMQQSDDLSPLEIVEMFAGLSCFLKDSSDVSQTL
LDDFRIWQGYNFLCDLLLRLEQAKEAESKDALKDLVNLITSLTTYGVSELKPAGITTGAPF
LLPGFAVPQPAGKGHSVRNVQAFAVLQNAFLKAKTSFLAQIILDAITNIYMADNANYFILES
QHTLSQFAEKISKLPEVQNKYFEMLEFVVFSLNYIPCKELISVSILLKSSSSYHCSIIAMKTL
LKFTRHDYIFKDVFREVGLLEVMVNLLHKYAALLKDPTQALNEQGDSRNNSSVEDQKHL
ALLVMETLTVLLQGSNTNAGIFREFGGARCAHNIVKYPQCRQHALMTIQQLVLSPNGDDD
MGTLLGLMHSAPPTELQLKTDILRALLSVLRESHRSRTVFRKVGGFVYITSLLVAMERSLS
CPPKNGWEKVNQNQVFELLHTVFCTLTAAMRYEPANSHFFKTEIQYEKLADAVRFLGCFS
DLRKISAMNVFPSNTQPFQRLLEEDVISIESVSPTLRHCSKLFIYLYKVATDSFDSRAEQIPPC
LTSESSLPSPWGTPALSRKRHAYHSVSTPPVYPPKNVADLKLHVTTSSLQSSDAVIIHPGAM
LAMLDLLASVGSVTQPEHALDLQLAVANILQSLVHTERNQQVMCEAGLHARLLQRCSAA
LADEDHSLHPPLQRMFERLASQALEPMVLREFLRLASPLNCGAWDKKLLKQYRVHKPSS
LSYEPEMRSSMITSLEGLGTDNVFSLHEDNHYRISKSLVKSAEGSTVPLTRVKCLVSMTTP
HDIRLHGSSVTPAFVEFDTSLEGFGCLFLPSLAPHNAPTNNTVTTGLIDGAVVSGIGSGERF
FPPPSGLSYSSWFCIEHFSSPPNNHPVRLLTVVRRANSSEQHYVCLAIVLSAKDRSLIVSTK
EELLQNYVDDFSEESSFYEILPCCARFRCGELIIEGQWHHLVLVMSKGMLKNSTAALYIDG
QLVNTVKLHYVHSTPGGSGSANPPVVSTVYAYIGTPPAQRQIASLVWRLGPTHFLEEVLPS
SNVTTIYELGPNYVGSFQAVCMPCKDAKSEGVVPSPVSLVPEEKVSFGLYALSVSSLTVARI
RKVYNKLDSKAIAKQLGISSHENATPVKLIHNSAGHLNGSARTIGAALIGYLGVRTFVPKP
VATTLQYVGGAAAILGLVAMASDVEGLYAAVKALVCVVKSNPLASKEMERIKGYQLLAM
LLKKKRSLLNSHILHLTFSLVGTVDSGHETSIIPNSTAFQDLLCDFEVWLHAPYELHLSLFE
HFIELLTESSEASKNAKLMREFQLIPKLLLTLRDMSLSQPTIAAISNVLSFLLQGFPSSNDLL
RFGQFISSTLPTFAVCEKFVVMEINNEEKLDTGTEEEFGGLVSANLILLRNRLLDILLKLIYT
SKEKTSINLQACEELVKTLGFDWIMMFMEEHLHSTTVTAAMRILVVLLSNQSILIKFKEGL
SGGGWLEQTDSVLTNKIGTVLGFNVGRSAGGRSTVREINRDACHFPGFPVLQSFLPKHTN
VPALYFLLMALFLQQPVSELPENLQVSVPVISCRSKQGCQFDLDSIWTFIFGVPASSGTVVS
SIHNVCTEAVFLLLGMLRSMLTSPWQSEEEGSWLREYPVTLMQFFRYLYHNVPDLASMW
MSPDFLCALAATVFPFNIRPYSEMVTDLDDEVGSPAEEFKAFAADTGMNRSQSEYCNVGT
KTYLTNHPAKKFVFDFMRVLIIDNLCLTPASKQTPLIDLLLEASPERSTRTQQKEFQTYILDS
VMDHLLAADVLLGEDASLPITSGGSYQVLVNNVFYFTQRVVDKLWQGMFNKESKLLIDFI
IQLIAQSKRRSQGLSLDAVYHCLNRTILYQFSRAHKTVPQQVALLDSLRVLTVNRNLILGPG
NHDQEFISCLAHCLINLHVGSNVDGFGLEAEARMTTWHIMIPSDIEPDGSYSQDISEGRQL
LIKAVNRVWTELIHSKKQVLEELFKVTLPVNERGHVDIATARPLIEEAALKCWQNHLAHE
KKCISRGEALAPTTQSKLSRVSSGFGLSKLTGSRRNRKESGLNKHSLSTQEISQWMFTHIAV
VRDLVDTQYKEYQERQQNALKYVTEEWCQIECELLRERGLWGPPIGSHLDKWMLEMTE
GPCRMRKKMVRNDMFYNHYPYVPETEQETNVASEIPSKQPETPDDIPQKKPARYRRAVSY
DSKEYYMRLASGNPAIVQDAIVESSEGEAAQQEPEHGEDTIAKVKGLVKPPLKRSRSAPD
GGDEENQEQLQDQIAEGSSIEEEEKTDNATLLRLLEEGEKIQHMYRCARVQGLDTSEGLLL
FGKEHFYVIDGFTMTATREIRDIETLPPNMHEPIIPRGARQGPSQLKRTCSIFAYEDIKEVHK
RRYLLQPIAVEVFSGDGRNYLLAFQKGIRNKVYQRFLAVVPSLTDSSESVSGQRPNTSVEQ
GSGLLSTLVGEKSVTQRWERGEISNFQYLMHLNTLAGRSYNDLMQYPVFPWILADYDSE

EVDLTNPKTFRNLAKPMGAQTDERLAQYKKRYKDWEDPNGETPAYHYGTHYSSAMIVAS
YLVRMEPFTQIFLRLQGGHFDLADRMFHSVREAWYSASKHNMADVKELIPEFFYLPEFLF
NSNNFDLGCKQNGTKLGDVILPPWAKGDPREFIRVHREALECDYVSAHLHEWIDLIFGYK
QQGPAAVEAVNVFHHLFYEGQVDIYNINDPLKETATIGFINNFGQIPKQLFKKPHPPKRVRS
RLNGDNAGISVLPGSTSDKIFFHHLDNLRPSLTPVKELKEPVGQIVCTDKGILAVEQNKVLI
PPTWNKTFAWGYADLSCRLGTYESDKAMTVYECLSEWGQILCAICPNPKLVITGGTSTVV
CVWEMGTSKEKAKTVTLKQALLGHTDTVTCATASLAYHIIVSGSRDRTCIIWDLNKLSFLT
QLRGHRAPVSALCINELTGDIVSCAGTYIHVWSINGNPIVSVNTFTGRSQQIICCCMSEMNE
WDTQNVIVTGHSDGVVRFWRMEFLQVPETPAPEPAEVLEMQEDCPEAQIGQEAQDEDSS
DSEADEQSISQDPKDTPSQPSSTSHRPRAASCRATAAWCTDSGSDDSRRWSDQLSLDEKD
GFIFVNYSEGQTRAHLQGPLSHPHPNPIEVRNYSRLKPGYRWERQLVFRSKLTMHTAFDR
KDNAHPAEVTALGISKDHSRILVGDSRGRVFSWSVSDQPGRSAADHWVKDEGGDSCSGC
SVRFSLTERRHHCRNCGQLFCQKCSRFQSEIKRLKISSPVRVCQNCYYNLQHERGSEDGPR
NC

>Q8N8Q8 multipass membrane protein
MLCRLGGRWLRPLPALQLWARDLPLAPVPTSGAKRPTLPVWAVAPVSAVHANGWYEALA
ASSPVRVAEEVLLGVHAATGLPWWGSILLSTVALRGAVTLPLAAYQHYILAKVENLQPEIK
TIARHLNQEVAVRANQLGWSKRDARLTYLKNMRRLISELYVRDNCHPFKATVLVWIQLP
MWIFMSFALRNLSTGAAHSEGFSVQEQLATGGILWFPDLTAPDSTWILPISVGVINLLIVEIC
ALQKIGMSRFQTYITYFVRAMSVLMIPIAATVPSSIVLYWLCSSFVGLSQNLLLRSPGFRQL
CRIPSTKSDSETPYKDIFAAFNTKFISRK

>Q8NAC3 single-pass type I membrane protein
MPVPWFLLSLALGRSPVVLSLERLVGPQDATHCSPVSLEPWGDEERLRVQFLAQQSLSLA
PVTAATARTALSGLSGADGRREERGRGKSWVCLSLGGSGNTEPQKKGLSCRLWDSDILCL
PGDIVPAPGPVLAPTHLQTELVLRCQKETDCDLCLRVAVHLAVHGHWEEPEDEEKFGGAA
DSGVEEPRNASLQAQVVLSFQAYPTARCVLLEVQVPAALVQFGQSVGSVVYDCFEAALGS
EVRIWSYTQPRYEKELNHTQQLPDCRGLEVWNSIPSCWALPWLNVSADGDNVHLVLNVS
EEQHFGLSLYWNQVQGPPKPRWHKNLTGPQIITLNHTDLVPCLCIQVWPLEPDSVRTNICP
FREDPRAHQNLWQAARLQLLTLQSWLLDAPCSLPAEAALCWRAPGGDPCQPLVPPLSWE
NVTVDKVLEFPLLKGHPNLCVQVNSSEKLQLQECLWADSLGPLKDDVLLLETRGPQDNR
SLCALEPSGCTSLPSKASTRAARLGEYLLQDLQSGQCLQLWDDDLGALWACPMDKYIHK
RWALVWLACLLFAAALSLILLLKKDHAKGWLRLLKQDVRSGAAARGRAALLLYSADDS
GFERLVGALASALCQLPLRVAVDLWSRRELSAQGPVAWFHAQRRQTLQEGGVVVLLFSPG
AVALCSEWLQDGVSGPGAHGPHDAFRASLSCVLPDFLQGRAPGSYVGACFDRLLHPDAVP
ALFRTVPVFTLPSQLPDFLGALQQPRAPRSGRLQERAEQVSRALQPALDSYFHPPGTPAPG
RGVGPGAGPGAGDGT

>P32246 multipass membrane protein
METPNTTEDYDTTTEFDYGDATPCQKVNERAFGAQLLPPLYSLVFVIGLVGNILVVLVLVQ
YKRLKNMTSIYLLNLAISDLLFLFTLPFWIDYKLKDDWVFGDAMCKILSGFYYTGLYSEIF
FIILLTIDRYLAIVHAVFALRARTVTFGVITSIIIWALAILASMPGLYFSKTQWEFTHHTCSLH
FPHESLREWKLFQALKLNLFGLVLPLLVMIICYTGIIKILLRRPNEKKSKAVRLIFVIMIIFFLF

WTPYNLTILISVFQDFLFTHECEQSRHLDLAVQVTEVIAYTHCCVNPVIYAFVGERFRKYLR
QLFHRRVAVHLVKWLPFLSVDRLERVSSTSPSTGEHELSAGF

>P13385 GPI-anchor, lipid-anchor
MDCRKMARFSYSVIWIMAISKVFELGLVAGLGHQEFARPSRGYLAFRDDSIWPQEEPAIRP
RSSQRVPPMGIQHSKELNRTCCLNGGTCMLGSFCACPPSFYGRNCEHDVRKENCGSVPHD
TWLPKKCSLCKCWHGQLRCFPQAFLPGCDGLVMDEHLVASRTPELPPSARTTTFMLVGIC
LSIQSYY

>P41180 multipass membrane protein
MAFYSCCWVLLALTWHTSAYGPDQRAQKKGDIILGGLFPIHFGVAAKDQDLKSRPESVEC
IRYNFRGFRWLQAMIFAIEEINSSPALLPNLTLGYRIFDTCNTVSKALEATLSFVAQNKIDSL
NLDEFCNCSEHIPSTIAVVGATGSGVSTAVANLLGLFYIPQVSYASSSRLLSNKNQFKSFLRT
IPNDEHQATAMADIIEYFRWNWVGTIAADDDYGRPGIEKFREEAEERDICIDFSELISQYSD
EEEIQHVVEVIQNSTAKVIVVFSSGPDLEPLIKEIVRRNITGKIWLASEAWASSSLIAMPQYF
HVVGGTIGFALKAGQIPGFREFLKKVHPRKSVHNGFAKEFWEETFNCHLQEGAKGPLPVD
TFLRGHEESGDRFSNSSTAFRPLCTGDENISSVETPYIDYTHLRISYNVYLAVYSIAHALQDI
YTCLPGRGLFTNGSCADIKKVEAWQVLKHLRHLNFTNNMGEQVTFDECGDLVGNYSIIN
WHLSPEDGSIVFKEVGYYNVYAKKGERLFINEEKILWSGFSREVPFSNCSRDCLAGTRKGII
EGEPTCCFECVECPDGEYSDETDASACNKCPDDFWSNENHTSCIAKEIEFLSWTEPFGIALT
LFAVLGIFLTAFVLGVFIKFRNTPIVKATNRELSYLLLFSLLCCFSSSLFFIGEPQDWTCRLRQ
PAFGISFVLCISCILVKTNRVLLVFEAKIPTSFHRKWWGLNLQFLLVFLCTFMQIVICVIWLY
TAPPSSYRNQELEDEIIFITCHEGSLMALGFLIGYTCLLAAICFFFAFKSRKLPENFNEAKFIT
FSMLIFFIVWISFIPAYASTYGKFVSAVEVIAILAASFGLLACIFFNKIYIILFKPSRNTIEEVRC
STAAHAFKVAARATLRRSNVSRKRSSSLGGSTGSTPSSSISSKSNSEDPFPQPERQKQQQPL
ALTQQEQQQQPLTLPQQQRSQQQPRCKQKVIFGSGTVTFSLSFDEPQKNAMAHRNSTHQN
SLEAQKSSDTLTRHQPLLPLQCGETDLDLTVQETGLQGPVGGDQRPEVEDPEELSPALVVS
SSQSFVISGGGSTVTENVVNS

>P18084 single-pass type I membrane protein
MPRAPAPLYACLLGLCALLPRLAGLNICTSGSATSCEECLLIHPKCAWCSKEDFGSPRSITSR
CDLRANLVKNGCGGEIESPASSFHVLRSLPLSSKGSGSAGWDVIQMTPQEIAVNLRPGDKT
TFQLQVRQVEDYPVDLYYLMDLSLSMKDDLDNIRSLGTKLAEEMRKLTSNFRLGFGSFV
DKDISPFSYTAPRYQTNPCIGYKLFPNCVPSFGFRHLLPLTDRVDSFNEEVRKQRVSRNRDA
PEGGFDAVLQAAVCKEKIGWRKDALHLLVFTTDDVPHIALDGKLGGLVQPHDGQCHLNE
ANEYTASNQMDYPSLALLGEKLAENNINLIFAVTKNHYMLYKNFTALIPGTTVEILDGDSK
NIIQLIINAYNSIRSKVELSVWDQPEDLNLFFTATCQDGVSYPGQRKCEGLKIGDTASFEVSL
EARSCPSRHTEHVFALRPVGFRDSLEVGVTYNCTCGCSVGLEPNSARCNGSGTYVCGLCE
CSPGYLGTRCECQDGENQSVYQNLCREAEGKPLCSGRGDCSCNQCSCFESEFGKIYGPFC
ECDNFSCARNKGVLCSGHGECHCGECKCHAGYIGDNCNCSTDISTCRGRDGQICSERGHC
LCGQCQCTEPGAFGEMCEKCPTCPDACSTKRDCVECLLLHSGKPDNQTCHSLCRDEVIT
WVDTIVKDDQEAVLCFYKTAKDCVMMFTYVELPSGKSNLTVLREPECGNTPNAMTILLA
VVGSILLVGLALLAIWKLLVTIHDRREFAKFQSERSRARYEMASNPLYRKPISTHTVDFTFN
KFNKSYNGTVD

>Q8WWY8 peripheral membrane protein
MLRFYLFISLLCLSRSDAEETCPSFTRLSFHSAVVGTGLNVRLMLYTRKNLTCAQTINSSAF
GNLNVTKKTTFIVHGFRPTGSPPVWMDDLVKGLLSVEDMNVVVVDWNRGATTLIYTHAS
SKTRKVAMVLKEFIDQMLAEGASLDDIYMIGVSLGAHISGFVGEMYDGWLGRITGLDPA
GPLFNGKPHQDRLDPSDAQFVDVIHSDTDALGYKEPLGNIDFYPNGGLDQPGCPKTILGG
FQYFKCDHQRSVYLYLSSLRESCTITAYPCDSYQDYRNGKCVSCGTSQKESCPLLGYYAD
NWKDHLRGKDPPMTKAFFDTAEESPFCMYHYFVDIITWNKNVRRGDITIKLRDKAGNTT
ESKINHEPTTFQKYHQVSLLARFNQDLDKVAAISLMFSTGSLIGPRYKLRILRMKLRSLAHP
ERPQLCRYDLVLMENVETVFQPILCPELQL

>P04843 single-pass type I membrane protein
MEAPAAGLFLLLLLGTWAPAPGSASSEAPPLINEDVKRTVDLSSHLAKVTAEVVLAHLGG
GSTSRATSFLLALEPELEARLAHLGVQVKGEDEEENNLEVRETKIKGKSGRFFTVKLPVAL
DPGAKISVIVETVYTHVLHPYPTQITQSEKQFVVFEGNHYFYSPYPTKTQTMRVKLASRNV
ESYTKLGNPTRSEDLLDYGPFRDVPAYSQDTFKVHYENNSPFLTITSMTRVIEVSHWGNIA
VEENVDLKHTGAVLKGPFSRYDYQRQPDSGISSIRSFKTILPAAAQDVYYRDEIGNVSTSH
LLILDDSVEMEIRPRFPLFGGWKTHYIVGYNLPSYEYLYNLGDQYALKMRFVDHVFDEQV
IDSLTVKIILPEGAKNIEIDSPYEISRAPDELHYTYLDTFGRPVIVAYKKNLVEQHIQDIVVHY
TFNKVLMLQEPLLVVAAFYILFFTVIIYVRLDFSITKDPAAEARMKVACITEQVLTLVNKRIG
LYRHFDETVNRYKQSRDISTLNSGKKSLETEHKALTSEIALLQSRLKTEGSDLCDRVSEMQ
KLDAQVKELVLKSAVEAERLVAGKLKKDTYIENEKLISGKRQELVTKIDHILDAL

>Q9HC62 peripheral membrane protein
MYRWLVRILGTIFRFCDRSVPPARALLKRRRSDSTLFSTVDTDEIPAKRPRLDCFIHQVKNS
LYNAASLFGFPFQLTTKPMVTSACNGTRNVAPSGEVFSNSSSCELTGSGSWNNMLKLGNK
SPNGISDYPKIRVTVTRDQPRRVLPSFGFTLNSEGCNRRPGGRRHSKGNPESSLMWKPQEQ
AVTEMISEESGKGLRRPHCTVEEGVQKEEREKYRKLLERLKESGHGNSVCPVTSNYHSSQ
RSQMDTLKTKGWGEEQNHGVKTTQFVPKQYRLVETRGPLCSLRSEKRCSKGKITDTETM
VGIRFENESRRGYQLEPDLSEEVSARLRLGSGSNGLLRRKVSIIETKEKNCSGKERDRRTD
DLLELTEDMEKEISNALGHGPQDEILSSAFKLRITRGDIQTLKNYHWLNDEVINFYMNLLV
ERNKKQGYPALHVFSTFFYPKLKSGGYQAVKRWTKGVNLFEQEIILVPIHRKVHWSLVVID
LRKKCLKYLDSMGQKGHRICEILLQYLQDESKTKRNSDLNLLEWTHHSMKPHEIPQQLN
GSDCGMFTCKYADYISRDKPITFTQHQMPLFRKKMVWEILHQQLL

>P08100 multipass membrane protein
MNGTEGPNFYVPFSNATGVVRSPFEYPQYYLAEPWQFSMLAAYMFLLIVLGFPINFLTLYV
TVQHKKLRTPLNYILLNLAVADLFMVLGGFTSTLYTSLHGYFVFGPTGCNLEGFFATLGGE
IALWSLVVLAIERYVVVCKPMSNFRFGENHAIMGVAFTWVMALACAAPPLAGWSRYIPE
GLQCSCGIDYYTLKPEVNNESFVIYMFVVHFTIPMIIIFFCYGQLVFTVKEAAAQQQESATT
QKAEKEVTRMVIIMVIAFLICWVPYASVAFYIFTHQGSNFGPIFMTIPAFFAKSAAIYNPVIYI
MMNKQFRNCMLTTICCGKNPLGDDEASATVSKTETSQVAPA

>Q8NFM7 single-pass type I membrane protein

MAPWLQLCSVFFTVNACLNGSQLAVAAGGSGRARGADTCGWRGVGPASRNSGLYNITFK
YDNCTTYLNPVGKHVIADAQNITISQYACHDQVAVTILWSPGALGIEFLKGFRVILEELKSE
GRQCQQLILKDPKQLNSSFKRTGMESQPFLNMKFETDYFVKVVPFPSIKNESNYHPFFFRT
RACDLLLQPDNLACKPFWKPRNLNISQHGSDMQVSFDHAPHNFGFRFFYLHYKLKHEGP
FKRKTCKQEQTTETTSCLLQNVSPGDYIIELVDDTNTTRKVMHYALKPVHSPWAGPIRAVA
ITVPLVVISAFATLFTVMCRKKQQENIYSHLDEESSESSTYTAALPRERLRPRPKVFLCYSSK
DGQNHMNVVQCFAYFLQDFCGCEVALDLWEDFSLCREGQREWVIQKIHESQFIIVVCSKG
MKYFVDKKNYKHKGGGRGSGKGELFLVAVSAIAEKLRQAKQSSSAALSKFIAVYFDYSCE
GDVPGILDLSTKYRLMDNLPQLCSHLHSRDHGLQEPGQHTRQGSRRNYFRSKSGRSLYVA
ICNMHQFIDEEPDWFEKQFVPFHPPPLRYREPVLEKFDSGLVLNDVMCKPGPESDFCLKVE
AAVLGATGPADSQHESQHGGLDQDGEARPALDGSAALQPLLHTVKAGSPSDMPRDSGIY
DSSVPSSELSLPLMEGLSTDQTETSSLTESVSSSSGLGEEEPPALPSKLLSSGSCKADLGCRS
YTDELHAVAPL

>Q86UW1 multipass membrane protein
MEPGRTQIKLDPRYTADLLEVLKTNYGIPSACFSQPPTAAQLLRALGPVELALTSILTLLAL
GSIAIFLEDAVYLYKNTLCPIKRRTLLWKSSAPTVVSVLCCFGLWIPRSLVLVEMTITSFYAV
CFYLLMLVMVEGFGGKEAVLRTLRDTPMMVHTGPCCCCCPCCPRLLLTRKKLQLLMLGP
FQYAFLKITLTLVGLFLVPDGIYDPADISEGSTALWINTFLGVSTLLALWTLGIISRQARLHL
GEQNMGAKFALFQVLLILTALQPSIFSVLANGGQIACSPPYSSKTRSQVMNCHLLILETFLM
TVLTRMYYRRKDHKVGYETFSSPDLDLNLKA

>P08582 GPI-anchor, lipid-anchor
MRGPSGALWLLLALRTVLGGMEVRWCATSDPEQHKCGNMSEAFREAGIQPSLLCVRGTS
ADHCVQLIAAQEADAITLDGGAIYEAGKEHGLKPVVGEVYDQEVGTSYYAVAVVRRSSH
VTIDTLKGVKSCHTGINRTVGWNVPVGYLVESGRLSVMGCDVLKAVSDYFGGSCVPGAG
ETSYSESLCRLCRGDSSGEGVCDKSPLERYYDYSGAFRCLAEGAGDVAFVKHSTVLENTD
GKTLPSWGQALLSQDFELLCRDGSRADVTEWRQCHLARVPAHAVVVRADTDGGLIFRLL
NEGQRLFSHEGSSFQMFSSEAYGQKDLLFKDSTSELVPIATQTYEAWLGHEYLHAMKGLL
CDPNRLPPYLRWCVLSTPEIQKCGDMAVAFRRQRLKPEIQCVSAKSPQHCMERIQAEQVD
AVTLSGEDIYTAGKTYGLVPAAGEHYAPEDSSNSYYVVAVVRRDSSHAFTLDELRGKRSC
HAGFGSPAGWDVPVGALIQRGFIRPKDCDVLTAVSEFFNASCVPVNNPKNYPSSLCALCV
GDEQGRNKCVGNSQERYYGYRGAFRCLVENAGDVAFVRHTTVFDNTNGHNSEPWAAEL
RSEDYELLCPNGARAEVSQFAACNLAQIPPHAVMVRPDTNIFTVYGLLDKAQDLFGDDHN
KNGFKMFDSSNYHGQDLLFKDATVRAVPVGEKTTYRGWLGLDYVAALEGMSSQQCSGA
AAPAPGAPLLPLLLPALAARLLPPAL

>Q7RTM1 multipass membrane protein
MLEGLGSPASPRAAASASVAGSSGPAACSPPSSSAPRSPESPAPRRGGVRASVPQKLAEML
SSQYGLIVFVAGLLLLLAWAVHAAGVSKSDLLCFLTALMLLQLLWMLWYVGRSSAHRRL
FRLKDTHAGAGWLRGSITLFAVITVILGCLKIGYFIGFSECLSATEGVFPVTHSVHTLLQVY
FLWGHAKDIIQSFKTLERFGVIHSVFTNLLLWANGVLNESKHQLNEHKERLITLGFGNITT
VLDDHTPQCNCTPPTLCTAISHGIYYLYPFNIEYQILASTMLYVLWKNIGRKVDSHQHQKM
QFKSDGVMVGAVLGLTVLAATIAVVVVYLIHIGRSKTKSESALIMFYLYAITLLMLMGAAG

LAGIRIYRIDEKSLDESKNPARKLDSDLLVGTASGSWLISWGSILAILCAEGHPRYTWYNLP
YSILAIVEKYIQNLFIFESIHREPEKLSEDIQTLRVVTVCNGNTMPLASSCPKSGGVARDVAP
QGKDMPPAANGNVCMRESHDKEEEKQEESSWGGSPSPVRLPRFLQGNAKRKVLRNIAAF
LFLCNISLWIPPAFGCRPEYDNGLEEIVFGFEPWIIVVNLAMPFSIFYRMHAAASLFEVYCKI

>Q8N6F1 multipass membrane protein
MANSGLQLLGYFLALGGWVGIIASTALPQWKQSSYAGDAIITAVGLYEGLWMSCASQSTG
QVQCKLYDSLLALDGHIQSARALMVVAVLLGFVAMVLSVVGMKCTRVGDSNPIAKGRVA
IAGGALFILAGLCTLTAVSWYATLVTQEFFNPSTPVNARYEFGPALFVGWASAGLAVLGGSF
LCCTCPEPERPNSSPQPYRPGPSAAAREPVVKLPASAKGPLGV

>Q9UN19 peripheral membrane protein
MGRAELLEGKMSTQDPSDLWSRSDGEAELLQDLGWYHGNLTRHAAEALLLSNGCDGSY
LLRDSNETTGLYSLSVRAKDSVKHFHVEYTGYSFKFGFNEFSSLKDFVKHFANQPLIGSET
GTLMVLKHPYPRKVEEPSIYESVRVHTAMQTGRTEDDLVPTAPSLGTKEGYLTKQGGLVK
TWKTRWFTLHRNELKYFKDQMSPEPIRILDLTECSAVQFDYSQERVNCFCLVFPFRTFYLC
AKTGVEADEWIKILRWKLSQIRKQLNQGEGTIRSRSFIFK

>Q9ULC0 single-pass type I membrane protein
MELLQVTILFLLPSICSSNSTGVLEAANNSLVVTTTKPSITTPNTESLQKNVVTPTTGTTPKG
TITNELLKMSLMSTATFLTSKDEGLKATTTDVRKNDSIISNVTVTSVTLPNAVSTLQSSKPK
TETQSSIKTTEIPGSVLQPDASPSKTGTLTSIPVTIPENTSQSQVIGTEGGKNASTSATSRSYS
SIILPVVIALIVITLSVFVLVGLYRMCWKADPGTPENGNDQPQSDKESVKLLTVKTISHESG
EHSAQGKTKN

>O43157 single-pass type I membrane protein
MPALGPALLQALWAGWVLTLQPLPPTAFTPNGTYLQHLARDPTSGTLYLGATNFLFQLSPG
LQLEATVSTGPVLDSRDCLPPVMPDECPQAQPTNNPNQLLLVSPGALVVCGSVHQGVCEQ
RRLGQLEQLLLRPERPGDTQYVAANDPAVSTVGLVAQGLAGEPLLFVGRGYTSRGVGGGI
PPITTRALWPPDPQAAFSYEETAKLAVGRLSEYSHHFVSAFARGASAYFLFLRRDLQAQSR
AFRAYVSRVCLRDQHYYSYVELPLACEGGRYGLIQAAAVATSREVAHGEVLFAAFSSAAPP
TVGRPPSAAAGASGASALCAFPLDEVDRLANRTRDACYTREGRAEDGTEVAYIEYDVNS
DCAQLPVDTLDAYPCGSDHTPSPMASRVPLEATPILEWPGIQLTAVAVTMEDGHTIAFLGDS
QGQLHRVYLGPGSDGHPYSTQSIQQGSAVSRDLTFDGTFEHLYVMTQSTLLKVPVASCAQ
HLDCASCLAHRDPYCGWCVLLGRCSRRSECSRGQGPEQWLWSFQPELGCLQVAAMSPAN
ISREETREVFLSVPDLPPLWPGESYSCHFGEHQSPALLTGSGVMCPSPDPSEAPVLPRGADY
VSVSVELRFGAVVIAKTSLSFYDCVAVTELRPSAQCQACVSSRWGCNWCVWQHLCTHKA
SCDAGPMVASHQSPLVSPDPPARGGPSPSPPTAPKALATPAPDTLPVEPGAPSTATASDISPG
ASPSLLSPWGPWAGSGSISSPGSTGSPLHEEPSPPSPQNGPGTAVPAPTDFRPSATPEDLLASP
LSPSEVAAVPPADPGPEALHPTVPLDLPPATVPATTFPGAMGSVKPALDWLTREGGELPEAD
EWTGGDAPAFSTSTLLSGDGDSAELEGPPAPLILPSSLDYQYDTPGLWELEEATLGASSCPC
VESVQGSTLMPVHVEREIRLLGRNLHLFQDGPGDNECVMELEGLEVVVEARVECEPPPDT
QCHVTCQQHQLSYEALQPELRVGLFLRRAGRLRVDSAEGLHVVLYDCSVGHGDCSRCQT
AMPQYGCVWCEGERPRCVTREACGEAEAVATQCPAPLIHSVEPLTGPVDGGTRVTIRGSN

LGQHVQDVLGMVTVAGVPCAVDAQEYEVSSSLVCITGASGEEVAGATAVEVPGRGRGVS
EHDFAYQDPKVHSIFPARGPRAGGTRLTLNGSKLLTGRLEDIRVVVGDQPCHLLPEQQSEQ
LRCETSPRPTPATLPVAVWFGATERRLQRGQFKYTLDPNITSAGPTKSFLSGGREICVRGQN
LDVVQTPRIRVTVVSRMLQPSQGLGRRRRVVPETACSLGPSCSSQQFEEPCHVNSSQLITCR
TPALPGLPEDPWVRVEFILDNLVFDFATLNPTPFSYEADPTLQPLNPEDPTMPFRHKPGSVF
SVEGENLDLAMSKEEVVAMIGDGPCVVKTLTRHHLYCEPPVEQPLPRHHALREAPDSLPE
FTVQMGNLRFSLGHVQYDGESPGAFPVAAQVGLGVGTSLLALGVIIIVLMYRRKSKQALR
DYKKVQIQLENLESSVRDRCKKEFTDLMTEMTDLTSDLLGSGIPFLDYKVYAERIFFPGHR
ESPLHRDLGVPESRRPTVEQGLGQLSNLLNSKLFLTKFIHTLESQRTFSARDRAYVASLLTV
ALHGKLEYFTDILRTLLSDLVAQYVAKNPKLMLRRTETVVEKLLTNWMSICLYTFVRDSV
GEPLYMLFRGIKHQVDKGPVDSVTGKAKYTLNDNRLLREDVEYRPLTLNALLAVGPGAG
EAQGVPVKVLDCDTISQAKEKMLDQLYKGVPLTQRPDPRTLDVEWRSGVAGHLILSDED
VTSEVQGLWRRLNTLQHYKVPDGATVALVPCLTKHVLRENQDYVPGERTPMLEDVDEGG
IRPWHLVKPSDEPEPPRPRRGSLRGGERERAKAIPEIYLTRLLSMKGTLQKFVDDLFQVILS
TSRPVPLAVKYFFDLLDEQAQQHGISDQDTIHIWKTNSLPLRFWINIIKNPQFVFDVQTSDN
MDAVLLVIAQTFMDACTLADHKLGRDSPINKLLYARDIPRYKRMVERYYADIRQTVPASD
QEMNSVLAELSWNYSGDLGARVALHELYKYINKYYDQIITALEEDGTAQKMQLGYRLQQ
IAAAVENKVTDL

>Q8N114 single-pass type I membrane protein
MTAPVPAPRILLPLLLLLLLLTPPPGARGEVCMASRGLSLFPESCPDFCCGTCDDQYCCSDVL
KKFVWSEERCAVPEASVPASVEPVEQLGSALRFRPGYNDPMSGFGATLAVGLTIFVLSVVT
IIICFTCSCCCLYKTCRRPRPVVTTTTSTTVVHAPYPQPPSVPPSYPGPSYQGYHTMPPQPG
MPAAPYPMQYPPPYPAQPMGPPAYHETLAGGAAAPYPASQPPYNPAYMDAPKAAL

>Q8NHS3 multipass membrane protein
MAGLRNESEQEPLLGDTPGSREWDILETEEHYKSRWRSIRILYLTMFLSSVGFSVVMMSIW
PYLQKIDPTADTSFLGWVIASYSLGQMVASPIFGLWSNYRPRKEPLIVSILISVAANCLYAYL
HIPASHNKYYMLVARGLLGIGAGNVAVVRSYTAGATSLQERTSSMANISMCQALGFILGPV
FQTCFTFLGEKGVTWDVIKLQINMYTTPVLLSAFLGILNIILILAILREHRVDDSGRQCKSIN
FEEASTDEAQVPQGNIDQVAVVAINVLFFVTLFIFALFETIITPLTMDMYAWTQEQAVLYNGI
ILAALGVEAVVIFLGVKLLSKKIGERAILLGGLIVVWVGFFILLPWGNQFPKIQWEDLHNN
SIPNTTFGEIIIGLWKSPMEDDNERPTGCSIEQAWCLYTPVIHLAQFLTSAVLIGLGYPVCNL
MSYTLYSKILGPKPQGVYMGWLTASGSGARILGPMFISQVYAHWGPRWAFSLVCGIIVLTI
TLLGVVYKRLIALSVRYGRIQE

>Q04912 single-pass type I membrane protein
MELLPPLPQSFLLLLLLPAKPAAGEDWQCPRTPYAASRDFDVKYVVPSFSAGGLVQAMVT
YEGDRNESAVFVAIRNRLHVLGPDLKSVQSLATGPAGDPGCQTCAACGPGPHGPPGDTDT
KVLVLDPALPALVSCGSSLQGRCFLHDLEPQGTAVHLAAPACLFSAHHNRPDDCPDCVASP
LGTRVTVVEQGQASYFYVASSLDAAVAASFSPRSVSIRRLKADASGFAPGFVALSVLPKHL
VSYSIEYVHSFHTGAFVYFLTVQPASVTDDPSALHTRLARLSATEPELGDYRELVLDCRFAP
KRRRRGAPEGGQPYPVLRVAHSAPVGAQLATELSIAEGQEVLFGVFVTGKDGGPGVGPNS
VVCAFPIDLLDTLIDEGVERCCESPVHPGLRRGLDFFQSPSFCPNPPGLEALSPNTSCRHFPL

LVSSSFSRVDLFNGLLGPVQVTALYVTRLDNVTVAHMGTMDGRILQVELVRSLNYLLYVS
NFSLGDSGQPVQRDVSRLGDHLLFASGDQVFQVPIQGPGCRHFLTCGRCLRAWHFMGCG
WCGNMCGQQKECPGSWQQDHCPPKLTEFHPHSGPLRGSTRLTLCGSNFYLHPSGLVPEGT
HQVTVGQSPCRPLPKDSSKLRPVPRKDFVEEFECELEPLGTQAVGPTNVSLTVTNMPPGK
HFRVDGTSVLRGFSFMEPVLIAVQPLFGPRAGGTCLTLEGQSLSVGTSRAVLVNGTECLLA
RVSEGQLLCATPPGATVASVPLSLQVGGAQVPGSWTFQYREDPVVLSISPNCGYINSHITIC
GQHLTSAWHLVLSFHDGLRAVESRCERQLPEQQLCRLPEYVVRDPQGWVAGNLSARGDG
AAGFTLPGFRFLPPPHPPSANLVPLKPEEHAIKFEYIGLGAVADCVGINVTVGGESCQHEFR
GDMVVCPLPPSLQLGQDGAPLQVCVDGECHILGRVVRPGPDGVPQSTLLGILLPLLLLVAA
LATALVFSYWWRRKQLVLPPNLNDLASLDQTAGATPLPILYSGSDYRSGLALPAIDGLDST
TCVHGASFSDSEDESCVPLLRKESIQLRDLDSALLAEVKDVLIPHERVVTHSDRVIGKGHF
GVVYHGEYIDQAQNRIQCAIKSLSRITEMQQVEAFLREGLLMRGLNHPNVLALIGIMLPPE
GLPHVLLPYMCHGDLLQFIRSPQRNPTVKDLISFGLQVARSMEYLAEQKFVHRDLAARNC
MLDESFTVKVADFGLARDILDREYYSVQQHRHARLPVKWMALESLQTYRFTTKSDVWSF
GVLLWELLTRGAPPYRHIDPFDLTHFLAQGRRLPQPEYCPDSLYQVMQQCWEADPAVRPT
FRVLVGEVEQIVSALLGDHYVQLPATYMNLGPSTSHEMNVRPEQPQFSPMPGNVRRPRPL
SEPPRPT

>P17301 single-pass type I membrane protein
MGPERTGAAPLPLLLVLALSQGILNCCLAYNVGLPEAKIFSGPSSEQFGYAVQQFINPKGN
WLLVGSPWSGFPENRMGDVYKCPVDLSTATCEKLNLQTSTSIPNVTEMKTNMSLGLILTR
NMGTGGFLTCGPLWAQQCGNQYYTTGVCSDISPDFQLSASFSPATQPCPSLIDVVVVCDES
NSIYPWDAVKNFLEKFVQGLDIGPTKTQVGLIQYANNPRVVFNLNTYKTKEEMIVATSQTS
QYGGDLTNTFGAIQYARKYAYSAASGGRRSATKVMVVVTDGESHDGSMLKAVIDQCNHD
NILRFGIAVLGYLNRNALDTKNLIKEIKAIASIPTERYFFNVSDEAALLEKAGTLGEQIFSIEG
TVQGGDNFQMEMSQVGFSADYSSQNDILMLGAVGAFGWSGTIVQKTSHGHLIFPKQAFD
QILQDRNHSSYLGYSVAAISTGESTHFVAGAPRANYTGQIVLYSVNENGNITVIQAHRGDQ
IGSYFGSVLCSVDVDKDTITDVLLVGAPMYMSDLKKEEGRVYLFTIKKGILGQHQFLEGPE
GIENTRFGSAIAALSDINMDGFNDVIVGSPLENQNSGAVYIYNGHQGTIRTKYSQKILGSDG
AFRSHLQYFGRSLDGYGDLNGDSITDVSIGAFGQVVQLWSQSIADVAIEASFTPEKITLVNK
NAQIILKLCFSAKFRPTKQNNQVAIVYNITLDADGFSSRVTSRGLFKENNERCLQKNMVVN
QAQSCPEHIIYIQEPSDVVNSLDLRVDISLENPGTSPALEAYSETAKVFSIPFHKDCGEDGLCI
SDLVLDVRQIPAAQEQPFIVSNQNKRLTFSVTLKNKRESAYNTGIVVDFSENLFFASFSLPV
DGTEVTCQVAASQKSVACDVGYPALKREQQVTFTINFDFNLQNLQNQASLSFQALSESQE
ENKADNLVNLKIPLLYDAEIHLTRSTNINFYEISSDGNVPSIVHSFEDVGPKFIFSLKVTTGS
VPVSMATVIIHIPQYTKEKNPLMYLTGVQTDKAGDISCNADINPLKIGQTSSSVSFKSENFR
HTKELNCRTASCSNVTCWLKDVHMKGEYFVNVTTRIWNGTFASSTFQTVQLTAAAEINT
YNPEIYVIEDNTVTIPLMIMKPDEKAEVPTGVIIGSIIAGILLLLALVAILWKLGFFKRKYEK
MTKNPDEIDETTELSS

>Q9UMX9 multipass membrane protein
MGSNSGQAGRHIYKSLADDGPFDSVEPPKRPTSRLIMHSMAMFGREFCYAVEAAYVTPVL
LSVGLPSSLYSIVWFLSPILGFLLQPVVGSASDHCRSRWGRRRPYILTLGVMMLVGMALYL
NGATVVAALIANPRRKLVWAISVTMIGVVLFDFAADFIDGPIKAYLFDVCSHQDKEKGLHY

HALFTGFGGALGYLLGAIDWAHLELGRLLGTEFQVMFFFSALVLTLCFTVHLCSISEAPLT
EVAKGIPPQQTPQDPPLSSDGMYEYGSIEKVKNGYVNPELAMQGAKNKNHAEQTRRAMT
LKSLLRALVNMPPHYRYLCISHLIGWTAFLSNMLFFTDFMGQIVYRGDPYSAHNSTEFLIY
ERGVEVGCWGLCINSVFSSLYSYFQKVLVSYIGLKGLYFTGYLLFGLGTGFIGLFPNVYSTL
VLCSLFGVMSSTLYTVPFNLITEYHREEEKERQQAPGGDPDNSVRGKGMDCATLTCMVQL
AQILVGGGLGFLVNTAGTVVVVVITASAVALIGCCFVALFVRYVD

>O00254 multipass membrane protein
MKALIFAAAGLLLLLPTFCQSGMENDTNNLAKPTLPIKTFRGAPPNSFEEFPFSALEGWTG
ATITVKIKCPEESASHLHVKNATMGYLTSSLSTKLIPAIYLLVFVVGVPANAVTLWMLFFRT
RSICTTVFYTNLAIADFLFCVTLPFKIAYHLNGNNWVFGEVLCRATTVIFYGNMYCSILLLA
CISINRYLAIVHPFTYRGLPKHTYALVTCGLVWATVFLYMLPFFILKQEYYLVQPDITTCHD
VHNTCESSSPFQLYYFISLAFFGFLIPFVLIIYCYAAIIRTLNAYDHRWLWYVKASLLILVIFTI
CFAPSNIILIIHHANYYYNNTDGLYFIYLIALCLGSLNSCLDPFLYFLMSKTRNHSTAYLTK

>P55085 multipass membrane protein
MRSPSAAWLLGAAILLAASLSCSGTIQGTNRSSKGRSLIGKVDGTSHVTGKGVTVETVFSV
DEFSASVLTGKLTTVFLPIVYTIVFVVGLPSNGMALWVFLFRTKKKHPAVIYMANLALADL
LSVIWFPLKIAYHIHGNNWIYGEALCNVLIGFFYGNMYCSILFMTCLSVQRYWVIVNPMG
HSRKKANIAIGISLAIWLLILLVTIPLYVVKQTIFIPALNITTCHDVLPEQLLVGDMFNYFLSL
AIGVFLFPAFLTASAYVLMIRMLRSSAMDENSEKKRKRAIKLIVTVLAMYLICFTPSNLLLV
VHYFLIKSQGQSHVYALYIVALCLSTLNSCIDPFVYYFVSHDFRDHAKNALLCRSVRTVKQ
MQVSLTSKKHSRKSSSYSSSSTTVKTSY

>O43181 peripheral membrane protein
MAAVSMSVVLRQTLWRRRAVAVAALSVSRVPTRSLRTSTWRLAQDQTQDTQLITVDEKL
DITTLTGVPEEHIKTRKVRIFVPARNNMQSGVNNTKKWKMEFDTRERWENPLMGWASTA
DPLSNMVLTFSTKEDAVSFAEKNGWSYDIEERKVPKPKSKSYGANFSWNKRTRVSTK

>O15455 single-pass type I membrane protein
MRQTLPCIYFWGGLLPFGMLCASSTTKCTVSHEVADCSHLKLTQVPDDLPTNITVLNLTH
NQLRRLPAANFTRYSQLTSLDVGFNTISKLEPELCQKLPMLKVLNLQHNELSQLSDKTFAF
CTNLTELHLMSNSIQKIKNNPFVKQKNLITLDLSHNGLSSTKLGTQVQLENLQELLLSNNK
IQALKSEELDIFANSSLKKLELSSNQIKEFSPGCFHAIGRLFGLFLNNVQLGPSLTEKLCLEL
ANTSIRNLSLSNSQLSTTSNTTFLGLKWTNLTMLDLSYNNLNVVGNDSFAWLPQLEYFFLE
YNNIQHLFSHSLHGLFNVRYLNLKRSFTKQSISLASLPKIDDFSFQWLKCLEHLNMEDNDI
PGIKSNMFTGLINLKYLSLSNSFTSLRTLTNETFVSLAHSPLHILNLTKNKISKIESDAFSWL
GHLEVLDLGLNEIGQELTGQEWRGLENIFEIYLSYNKYLQLTRNSFALVPSLQRLMLRRVA
LKNVDSSPSPFQPLRNLTILDLSNNNIANINDDMLEGLEKLEILDLQHNNLARLWKHANPG
GPIYFLKGLSHLHILNLESNGFDEIPVEVFKDLFELKIIDLGLNNLNTLPASVFNNQVSLKSL
NLQKNLITSVEKKVFGPAFRNLTELDMRFNPFDCTCESIAWFVNWINETHTNIPELSSHYLC
NTPPHYHGFPVRLFDTSSCKDSAPFELFFMINTSILLIFIFIVLLIHFEGWRISFYWNVSVHRV
LGFKEIDRQTEQFEYAAYIIHAYKDKDWVWEHFSSMEKEDQSLKFCLEERDFEAGVFELE
AIVNSIKRSRKIIFVITHHLLKDPLCKRFKVHHAVQQAIEQNLDSIILVFLEEIPDYKLNHAL

CLRRGMFKSHCILNWPVQKERIGAFRHKLQVALGSKNSVH

>Q8NCG7 multipass membrane protein
MPGMVLFGRRWAIASDDLVFPGFFELVVRVLWWIGILTLYLMHRGKLDCAGGALLSSYLI
VLMILLAVVICTVSAIMCVSMRGTICNPGPRKSMSKLLYIRLALFFPEMVWASLGAAWVA
DGVQCDRTVVNGIIATVVVSWIIIAATVVSIIIVFDPLGGKMAPYSSAGPSHLDSHDSSQLL
NGLKTAATSVWETRIKLLCCCIGKDDHTRVAFSSTAELFSTYFSDTDLVPSDIAAGLALLHQ
QQDNIRNNQEPAQVVCHAPGSSQEADLDAELENCHHYMQFAAAAYGWPLYIYRNPLTGL
CRIGGDCCRSRTTDYDLVGGDQLNCHFGSILHTTGLQYRDFIHVSFHDKVYELPFLVALDH
RKESVVVAVRGTMSLQDVLTDLSAESEVLDVECEVQDRLAHKGISQAARYVYQRLINDGI
LSQAFSIAPEYRLVIVGHSLGGGAAALLATMLRAAYPQVRCYAFSPPRGLWSKALQEYSQS
FIVSLVLGKDVIPRLSVTNLEDLKRRILRVVAHCNKPKYKILLHGLWYELFGGNPNNLPTE
LDGGDQEVLTQPLLGEQSLLTRWSPAYSFSSDSPLDSSPKYPPLYPPGRIIHLQEEGASGRFG
CCSAAHYSAKWSHEAEFSKILIGPKMLTDHMPDILMRALDSVVSDRAACVSCPAQGVSSV
DVA

>Q9UP83 peripheral membrane protein
MGWVGGRRRDSASPPGRSRSAADDINPAPANMEGGGGSVAVAGLGARGSGAAAATVREL
LQDGCYSDFLNEDFDVKTYTSQSIHQAVIAEQLAKLAQGISQLDRELHLQVVARHEDLLA
QATGIESLEGVLQMMQTRIGALQGAVDRIKAKIVEPYNKIVARTAQLARLQVACDLLRRIIR
ILNLSKRLQGQLQGGSREITKAAQSLNELDYLSQGIDLSGIEVIENDLLFIARARLEVENQA
KRLLEQGLETQNPTQVGTALQVFYNLGTLKDTITSVVDGYCATLEENINSALDIKVLTQPS
QSAVRGGPGRSTMPTPGNTAALRASFWTNMEKLMDHIYAVCGQVQHLQKVLAKKRDPV
SHICFIEEIVKDGQPEIFYTFWNSVTQALSSQFHMATNSSMFLKQAFEGEYPKLLRLYNDL
WKRLQQYSQHIQGNFNASGTTDLYVDLQHMEDDAQDIFIPKKPDYDPEKALKDSLQPYE
AAYLSKSLSRLFDPINLVFPPGGRNPPSSDELDGIIKTIASELNVAAVDTNLTLAVSKNVAKTI
QLYSVKSEQLLSTQGDASQVIGPLTEGQRRNVAVVNSLYKLHQSVTKAIHALMENAVQPL
LTSVGDAIEAIIITMHQEDFSGSLSSSGKPDVPCSLYMKELQGFIARVMSDYFKHFECLDFV
FDNTEAIAQRAVELFIRHASLIRPLGEGGKMRLAADFAQMELAVGPFCRRVSDLGKSYRM
LRSFRPLLFQASEHVASSPALGDVIPFSIIIQFLFTRAPAELKSPFQRAEWSHTRFSQWLDDH
PSEKDRLLLIRGALEAYVQSVRSREGKEFAPVYPIMVQLLQKAMSALQ

>Q8NFP4 GPI-anchor, lipid-anchor
MEVTCLLLLALIPFHCRGQGVYAPAQAQIVHAGQACVVKEDNISERVYTIREGDTLMLQC
LVTGHPRPQVRWTKTAGSASDKFQETSVFNETLRIERIARTQGGRYYCKAENGVGVPAIKS
IRVDVQYLDEPMLTVHQTVSDVRGNFYQEKTVFLRCTVNSNPPARFIWKRGSDTLSHSQD
NGVDIYEPLYTQGETKVLKLKNLRPQDYASYTCQVSVRNVCGIPDKAITFRLTNTTAPPAL
KLSVNETLVVNPGENVTVQCLLTGGDPLPQLQWSHGPGPLPLGALAQGGTLSIPSVQARD
SGYYNCTATNNVGNPAKKTVNLLVRSMKNATFQITPDVIKESENIQLGQDLKLSCHVDAV
PQEKVTYQWFKNGKPARMSKRLLVTRNDPELPAVTSSLELIDLHFSDYGTYLCMASFPGA
PVPDLSVEVNISSETVPPTISVPKGRAVVTVREGSPAELQCEVRGKPRPPVLWSRVDKEAA
LLPSGLPLEETPDGKLRLERVSRDMSGTYRCQTARYNGFNVRPREAQVQLNVQFPPEVEP
SSQDVRQALGRPVLLRCSLLRGSPQRIASAVWRFKGQLLPPPPVVPAAAEAPDHAELRLDA
VTRDSSGSYECSVSNDVGSAACLFQVSAKAYSPEFYFDTPNPTRSHKLSKNYSYVLQWTQ

REPDAVDPVLNYRLSIRQLNQHNAVVKAIPVRRVEKGQLLEYILTDLRVPHSYEVRLTPYT
TFGAGDMASRIIHYTEPINSPNLSDNTCHFEDEKICGYTQDLTDNFDWTRQNALTQNPKRS
PNTGPPTDISGTPEGYYMFIETSRPRELGDRARLVSPLYNASAKFYCVSFFYHMYGKHIGS
LNLLVRSRNKGALDTHAWSLSGNKGNVWQQAHVPISPSGPFQIIFEGVRGPGYLGDIAIDD
VTLKKGECPRKQTDPNKVVVMPGSGAPCQSSPQLWGPMAIFLLALQR

>P49069 multipass membrane protein
MESMAVATDGGERPGVPAGSGLSASQRRAELRRRKLLMNSEQRINRIMGFHRPGSGAEEE
SQTKSKQQDSDKLNSLSVPSVSKRVVLGDSVSTGTTDQQGGVAEVKGTQLGDKLDSFIKP
PECSSDVNLELRQRNRGDLTADSVQRGSRHGLEQYLSRFEEAMKLRKQLISEKPSQEDGN
TTEEFDSFRIFRLVGCALLALGVRAFVCKYLSIFAPFLTLQLAYMGLYKYFPKSEKKIKTTV
LTAALLLSGIPAEVINRSMDTYSKMGEVFTDLCVYFFTFIFCHELLDYWGSEVP

>Q9UKG4 multipass membrane protein
MGLLQGLLRVRKLLLVVCVPLLLLPLPVLHPSSEASCAYVLIVTAVYWVSEAVPLGAAALV
PAFLYPFFGVLRSNEVAAEYFKNTTLLLVGVICVAAAVEKWNLHKRIALRMVLMAGAKPG
MLLLCFMCCTTLLSMWLSNTSTTAMVMPIVEAVLQELVSAEDEQLVAGNSNTEEAEPISL
DVKNSQPSLELIFVNEESNADLTTLMHNENLNGVPSITNPIKTANQHQGKKQHPSQEKPQV
LTPSPRKQKLNRKYRSHHDQMICKCLSLSISYSATIGGLTTIIGTSTSLIFLEHFNNQYPAAE
VVNFGTWFLFSFPISLIMLVVSWFWMHWLFLGCNFKETCSLSKKKKTKREQLSEKRIQEE
YEKLGDISYPEMVTGFFFILMTVLWFTREPGFVPGWDSFFEKKGYRTDATVSVFLGFLLFL
IPAKKPCFGKKNDGENQEHSLGTEPIITWKDFQKTMPWEIVILVGGGYALASGSKSSGLST
WIGNQMLSLSSLPPWAVTLLACILVSIVTEFVSNPATITIFLPILCSLSETLHINPLYTLIPVTM
CISFAVMLPVGNPPNAIVFSYGHCQIKDMVKAGLGVNVIGLVIVMVAINTWGVSLFHLDT
YPAWARVSNITDQA

>O76081 lipid-anchor
MPQLSQDNQECLQKHFSRPSIWTQFLPLFRAQRYNTDIHQITENEGDLRAVPDIKSFPPAQL
PDSPAAPKLFGLLSSPLSSLARFFSHLLRRPPPEAPRRRLDFSPLLPALPAARLSRGHEELPG
RLSLLLGAALALPGRPSGGRPLRPPHPVAKPREEDATAGQSSPMPQMGSERMEMRKRQMP
AAQDTPGAAPGQPGAGSRGSNACCFCWCCCCSCSCLTVRNQEDQRPTIASHELRADLPT
WEESPAPTLEEVNAWAQSFDKLMVTPAGRNAFREFLRTEFSEENMLFWMACEELKKEAN
KNIIEEKARIIYEDYISILSPKEVSLDSRVREVINRNMVEPSQHIFDDAQLQIYTLMHRDSYP
RFMNSAVYKDLLQSLSEKSIEA

>Q08828 multipass membrane protein
MAGAPRGGGGGGGGAGEPGGAERAAGTSRRRGLRACDEEFACPELEALFRGYTLRLEQ
AATLKALAVLSLLAGALALAELLGAPGPAPGLAKGSHPVHCVLFLALLVVTNVRSLQVPQ
LQQVGQLALLFSLTFALLCCPFALGGPARGSAGAAGGPATAEQGVWQLLLVTFVSYALLP
VRSLLAIGFGLVVAASHLLVTATLVPAKRPRLWRTLGANALLFVGVNMYGVFVRILTERSQ
RKAFLQARSCIEDRLRLEDENEKQERLLMSLLPRNVAMEMKEDFLKPPERIFHKIYIQRHD
NVSILFADIVGFTGLASQCTAQELVKLLNELFGKFDELATENHCRRIKILGDCYYCVSGLTQ
PKTDHAHCCVEMGLDMIDTITSVAEATEVDLNMRVGLHTGRVLCGVLGLRKWQYDVWS
NDVTLANVMEAAGLPGKVHITKTTLACLNGDYEVEPGYGHERNSFLKTHNIETFFIVPSH

RRKIFPGLILSDIKPAKRMKFKTVCYLLVQLMHCRKMFKAEIPFSNVMTCEDDDKRRALR
TASEKLRNRSSFSTNVVYTTPGTRVNRYISRLLEARQTELEMADLNFFTLKYKHVEREQK
YHQLQDEYFTSAVVLTLILAALFGLVYLLIFPQSVVVLLLLVFCICFLVACVLYLHITRVQCF
PGCLTIQIRTVLCIFIVVLIYSVAQGCVVGCLPWAWSSKPNSSLVVLSSGGQRTALPTLPCES
THHALLCCLVGTLPLAIFFRVSSLPKMILLSGLTTSYILVLELSGYTRTGGGAVSGRSYEPIV
AILLFSCALALHARQVDIRLRLDYLWAAQAEEEREDMEKVKLDNRRILFNLLPAHVAQHF
LMSNPRNMDLYYQSYSQVGVMFASIPNFNDFYIELDGNNMGVECLRLLNEIIADFDELME
KDFYKDIEKIKTIGSTYMAAVGLAPTSGTKAKKSISSHLSTLADFAIEMFDVLDEINYQSYN
DFVLRVGINVGPVVAGVIGARRPQYDIWGNTVNVASRMDSTGVQGRIQVTEEVHRLLRR
CPYHFVCRGKVSVKGKGEMLTYFLEGRTDGNGSQIRSLGLDRKMCPFGRAGLQGRRPPV
CPMPGVSVRAGLPPHSPGQYLPSAAAGKEA

>Q6PIU1 multipass membrane protein
MPSSGRALLDSPLDSGSLTSLDSSVFCSEGEGEPLALGDCFTVNVGGSRFVLSQQALSCFP
HTRLGKLAVVVASYRRPGALAAVPSPLELCDDANPVDNEYFFDRSSQAFRYVLHYYRTGR
LHVMEQLCALSFLQEIQYWGIDELSIDSCCRDRYFRRKELSETLDFKKDTEDQESQHESEQ
DFSQGPCPTVRQKLWNILEKPGSSTAARIFGVISIIFVVVSIINMALMSAELSWLDLQLLEIL
EYVCISWFTGEFVLRFLCVRDRCRFLRKVPNIIDLLAILPFYITLLVESLSGSQTTQELENVG
RIVQVLRLLRALRMLKLGRHSTGLRSLGMTITQCYEEVGLLLLFLSVGISIFSTVEYFAEQS
IPDTTFTSVPCAWWWATTSMTTVGYGDIRPDTTTGKIVAFMCILSGILVLALPIAIINDRFSA
CYFTLKLKEAAVRQREALKKLTKNIATDSYISVNLRDVYARSIMEMLRLKGRERASTRSSG
GDDFWF

>Q96NL1 multipass membrane protein
MELHYLAKKSNQADLCDARDWSSRGLPGDQADTAATRAALCCQKQCASTPRATEMEGS
KLSSSPASPSSSLQNSTLQPDAFPPGLLHSGNNQITAERKVCNCCSQELETSFTYVDKNINL
EQRNRSSPSAKGHNHPGELGWENPNEWSQEAAISLISEEEDDTSSEATSSGKSIDYGFISAI
LFLVTGILLVIISYIVPREVTVDPNTVAAREMERLEKESARLGAHLDRCVIAGLCLLTLGGVI
LSCLLMMSMWKGELYRRNRFASSKESAKLYGSFNFRMKTSTNENTLELSLVEEDALAVQS

>Q96CH1 multipass membrane protein
MWSCSWFNGTGLVEELPACQDLQLGLSLLSLLGLVVGVPVGLCYNALLVLANLHSKASM
TMPDVYFVNMAVAGLVLSALAPVHLLGPPSSRWALWSVGGEVHVALQIPFNVSSLVAMYS
TALLSLDHYIERALPRTYMASVYNTRHVCGFVWGGALLTSFSSLLFYICSHVSTRALECAK
MQNAEAADATLVFIGYVVPALATLYALVLLSRVRREDTPLDRDTGRLEPSAHRLLVATVCT
QFGLWTPHYLILLGHTVIISRGKPVDAHYLGLLHFVKDFSKLLAFSSSFVTPLLYRYMNQS
FPSKLQRLMKKLPCGDRHCSPDHMGVQQVLA

>Q99527 multipass membrane protein
MDVTSQARGVGLEMYPGTAQPAAPNTTSPELNLSHPLLGTALANGTGELSEHQQYVIGLF
LSCLYTIFLFPIGFVGNILILVVNISFREKMTIPDLYFINLAVADLILVADSLIEVFNLHERYYDI
AVLCTFMSLFLQVNMYSSVFFLTWMSFDRYIALARAMRCSLFRTKHHARLSCGLIWMAS
VSATLVPFTAVHLQHTDEACFCFADVREVQWLEVTLGFIVPFAIIGLCYSLIVRVLVRAHRH
RGLRPRRQKALRMILAVVLVFFVCWLPENVFISVHLLQRTQPGAAPCKQSFRHAHPLTGHI

VNLAAFSNSCLNPLIYSFLGETFRDKLRLYIEQKTNLPALNRFCHAALKAVIPDSTEQSDVR
FSSAV

>Q00535 peripheral membrane protein
MQKYEKLEKIGEGTYGTVFKAKNRETHEIVALKRVRLDDDDEGVPSSALREICLLKELKH
KNIVRLHDVLHSDKKLTLVFEFCDQDLKKYFDSCNGDLDPEIVKSFLFQLLKGLGFCHSRN
VLHRDLKPQNLLINRNGELKLADFGLARAFGIPVRCYSAEVVTLWYRPPDVLFGAKLYST
SIDMWSAGCIFAELANAGRPLFPGNDVDDQLKRIFRLLGTPTEEQWPSMTKLPDYKPYPM
YPATTSLVNVVPKLNATGRDLLQNLLKCNPVQRISAEEALQHPYFSDFCPP

>Q9H2D1 multipass membrane protein
MTGQGQSASGSSAWSTVFRHVRYENLIAGVSGGVLSNLALHPLDLVKIRFAVSDGLELRP
KYNGILHCLTTIWKLDGLRGLYQGVTPNIWGAGLSWGLYFFFYNAIKSYKTEGRAERLEA
TEYLVSAAEAGAMTLCITNPLWVTKTRLMLQYDAVVNSPHRQYKGMFDTLVKIYKYEGV
RGLYKGFVPGLFGTSHGALQFMAYELLKLKYNQHINRLPEAQLSTVEYISVAALSKIFAVA
ATYPYQVVRARLQDQHMFYSGVIDVITKTWRKEGVGGFYKGIAPNLIRVTPACCITFVVY
ENVSHFLLDLREKRK

>Q9H295 multipass membrane protein
MGIWTSGTDIFLSLWEIYVSPRSPGWMDFIQHLGVCCLVALISVGLLSVAACWFLPSIIAAA
ASWIITCVLLCCSKHARCFILLVFLSCGLREGRNALIAAGTGIVILGHVENIFHNFKGLLDG
MTCNLRAKSFSIHFPLLKKYIEAIQWIYGLATPLSVFDDLVSWNQTLAVSLFSPSHVLEAQL
NDSKGEVLSVLYQMATTTEVLSSLGQKLLAFAGLSLVLLGTGLFMKRFLGPCGWKYENIY
ITRQFVQFDERERHQQRPCVLPLNKEERRKYVIIPTFWPTPKERKNLGLFFLPILIHLCIWVL
FAAVDYLLYRLIFSVSKQFQSLPGFEVHLKLHGEKQGTQDIIHDSSFNISVFEPNCIPKPKFL
LSETWVPLSVILLILVMLGLLSSILMQLKILVSASFYPSVERKRIQYLHAKLLKKRSKQPLG
EVKRRLSLYLTKIHFWLPVLKMIRKKQMDMASADKS

>P55040 peripheral membrane protein
MTLNNVTMRQGTVGMQPQQQRWSIPADGRHLMVQKEPHQYSHRNRHSATPEDHCRRS
WSSDSTDSVISSESGNTYYRVVLIGEQGVGKSTLANIFAGVHDSMDSDCEVLGEDTYERTL
MVDGESATIILLDMWENKGENEWLHDHCMQVGDAYLIVYSITDRASFEKASELRIQLRRA
RQTEDIPIILVGNKSDLVRCREVSVSEGRACAVVFDCKFIETSAAVQHNVKELFEGIVRQVR
LRRDSKEKNERRLAYQKRKESMPRKARRFWGKIVAKNNKNMAFKLKSKSCHDLSVL

>Q9H2F3 multipass membrane protein
MADSAQAQKLVYLVTGGCGFLGEHVVRMLLQREPRLGELRVFDQHLGPWLEELKTGPV
RVTAIQGDVTQAHEVAAAVAGAHVVIHTAGLVDVFGRASPKTIHEVNVQGTRNVIEACVQ
TGTRFLVYTSSMEVVGPNTKGHPFYRGNEDTPYEAVHRHPYPCSKALAEWLVLEANGRK
VRGGLPLVTCALRPTGIYGEGHQIMRDFYRQGLRLGGWLFRAIPASVEHGRVYVGNVAW
MHVLAARELEQRATLMGGQVYFCYDGSPYRSYEDFNMEFLGPCGLRLVGARPLLPYWLL
VFLAALNALLQWLLRPLVLYAPLLNPYTLAVANTTFTVSTDKAQRHFGYEPLFSWEDSRTR
TILWVQAATGSAQ

>Q15612 multipass membrane protein
MDNSNWTSVSHFVLLGISTHPEEQIPLFLVFSLMYAINISGNLAIITLILSAPRLHIPMYIFLS
NLALTDICFTSTTVPKMLQIIFSPTKVISYTGCLAQTYFFICFAVMENFILAVMAYDRYIAICH
PFHYTMILTRMLCVKMVVMCHALSHLHAMLHTFLIGQLIFCADNRIPHFFCDLYALMKIS
CTSTYLNTLMIHTEGAVVISGALAFITASYACIILVVLRIPSAKGRWKTFSTCGSHLTVVAIF
YGTLSWVYFRPLSSYSVTKGRIITVVYTVVTPMLNPFIYSLRNGDVKGGFMKWMSRMQT
FFFR

>O14520 multipass membrane protein
MVQASGHRRSTRGSKMVSWSVIAKIQEILQRKMVREFLAEFMSTYVMMVFGLGSVAHM
VLNKKYGSYLGVNLGFGFGVTMGVHVAGRISGAHMNAAVTFANCALGRVPWRKFPVYV
LGQFLGSFLAAATIYSLFYTAILHFSGGQLMVTGPVATAGIFATYLPDHMTLWRGFLNEAW
LTGMLQLCLFAITDQENNPALPGTEALVIGILVVIIGVSLGMNTGYAINPSRDLPPRIFTFIAG
WGKQVFSNGENWWWVPVVAPLLGAYLGGIIYLVFIGSTIPREPLKLEDSVAYEDHGITVLP
KMGSHEPTISPLTPVSVSPANRSSVHPAPPLHESMALEHF

>Q92482 multipass membrane protein
MGRQKELVSRCGEMLHIRYRLLRQALAECLGTLILVMFGCGSVAQVVLSRGTHGGFLTIN
LAFGFAVTLGILIAGQVSGAHLNPAVTFAMCFLAREPWIKLPIYTLAQTLGAFLGAGIVFGL
YYDAIWHFADNQLFVSGPNGTAGIFATYPSGHLDMINGFFDQFIGTASLIVCVLAIVDPYN
NPVPRGLEAFTVGLVVLVIGTSMGFNSGYAVNPARDFGPRLFTALAGWGSAVFTTGQHW
WWVPIVSPLLGSIAGVFVYQLMIGCHLEQPPPSNEEENVKLAHVKHKEQI

>Q14680 peripheral membrane protein
MKDYDELLKYYELHETIGTGGFAKVKLACHILTGEMVAIKIMDKNTLGSDLPRIKTEIEAL
KNLRHQHICQLYHVLETANKIFMVLEYCPGGELFDYIISQDRLSEEETRVVFRQIVSAVAYV
HSQGYAHRDLKPENLLFDEYHKLKLIDFGLCAKPKGNKDYHLQTCCGSLAYAAPELIQGK
SYLGSEADVWSMGILLYVLMCGFLPFDDDNVMALYKKIMRGKYDVPKWLSPSSILLLQQ
MLQVDPKKRISMKNLLNHPWIMQDYNYPVEWQSKNPFIHLDDDCVTELSVHHRNNRQT
MEDLISLWQYDHLTATYLLLLAKKARGKPVRLRLSSFSCGQASATPFTDIKSNNWSLEDVT
ASDKNYVAGLIDYDWCEDDLSTGAATPRTSQFTKYWTESNGVESKSLTPALCRTPANKLK
NKENVYTPKSAVKNEEYFMFPEPKTPVNKNQHKREILTTPNRYTTPSKARNQCLKETPIKI
PVNSTGTDKLMTGVISPERRCRSVELDLNQAHMEETPKRKGAKVFGSLERGLDKVITVLT
RSKRKGSARDGPRRLKLHYNVTTTRLVNPDQLLNEIMSILPKKHVDFVQKGYTLKCQTQS
DFGKVTMQFELEVCQLQKPDVVGIRRQRLKGDAWVYKRLVEDILSSCKV

>Q8WY07 multipass membrane protein
MPWQAFRRFGQKLVRRRTLESGMAETRLARCLSTLDLVALGVGSTLGAGVYVLAGEVAK
DKAGPSIVICFLVAALSSVLAGLCYAEFGARVPRSGSAYLYSYVTVGELWAFTTGWNLILS
YVIGTASVARAWSSAFDNLIGNHISKTLQGSIALHVPHVLAEYPDFFALGLVLLLTGLLALG
ASESALVTKVFTGVNLLVLGFVMISGFVKGDVHNWKLTEEDYELAMAELNDTYSLGPLG
SGGFVPFGFEGILRGAATCFYAFVGFDCIATTGEEAQNPQRSIPMGIVISLSVCFLAYFAVSS
ALTLMMPYYQLQPESPLPEAFLYIGWAPARYVVAVGSLCALSTSLLGSMFPMPRVIYAMAE
DGLLFRVLARIHTGTRTPIIATVVSGIIAAFMAFLFKLTDLVDLMSIGTLLAYSLVSICVLILR

YQPDQETKTGEEVELQEEAITTESEKLTLWGLFFPLNSIPTPLSGQIVYVCSSLLAVLLTALC
LVLAQWSVPLLSGDLLWTAVVVLLLLLIIGIIVVIWRQPQSSTPLHFKVPALPLLPLMSIFVN
IYLMMQMTAGTWARFGVWMLIGFAIYFGYGIQHSLEEIKSNQPSRKSRAKTVDLDPGTLY
VHSV

>Q96P66 multipass membrane protein
MTSTCTNSTRESNSSHTCMPLSKMPISLAHGIIRSTVLVIFLAASFVGNIVLALVLQRKPQLL
QVTNRFIFNLLVTDLLQISLVAPWVVATSVPLFWPLNSHFCTALVSLTHLFAFASVNTIVVVS
VDRYLSIIHPLSYPSKMTQRRGYLLLYGTWIVAILQSTPPLYGWGQAAFDERNALCSMIWG
ASPSYTILSVVSFIVIPLIVMIACYSVVFCAARRQHALLYNVKRHSLEVRVKDCVENEDEEG
AEKKEEFQDESEFRRQHEGEVKAKEGRMEAKDGSLKAKEGSTGTSESSVEARGSEEVRE
SSTVASDGSMEGKEGSTKVEENSMKADKGRTEVNQCSIDLGEDDMEFGEDDINFSEDDVE
AVNIPESLPPSRRNSNSNPPLPRCYQCKAAKVIFIIIFSYVLSLGPYCFLAVLAVWVDVETQV
PQWVITIIIWLFFLQCCIHPYVYGYMHKTIKKEIQDMLKKFFCKEKPPKEDSHPDLPGTEG
GTEGKIVPSYDSATFP

>P16473 multipass membrane protein
MRPADLLQLVLLLDLPRDLGGMGCSSPPCECHQEEDFRVTCKDIQRIPSLPPSTQTLKLIET
HLRTIPSHAFSNLPNISRIYVSIDVTLQQLESHSFYNLSKVTHIEIRNTRNLTYIDPDALKELP
LLKFLGIFNTGLKMFPDLTKVYSTDIFFILEITDNPYMTSIPVNAFQGLCNETLTLKLYNNGF
TSVQGYAFNGTKLDAVYLNKNKYLTVIDKDAFGGVYSGPSLLDVSQTSVTALPSKGLEHL
KELIARNTWTLKKLPLSLSFLHLTRADLSYPSHCCAFKNQKKIRGILESLMCNESSMQSLR
QRKSVNALNSPLHQEYEENLGDSIVGYKEKSKFQDTHNNAHYYVFFEEQEDEIIGFGQEL
KNPQEETLQAFDSHYDYTICGDSEDMVCTPKSDEFNPCEDIMGYKFLRIVVWFVSLLALL
GNVFVLLILLTSHYKLNVPRFLMCNLAFADFCMGMYLLLIASVDLYTHSEYYNHAIDWQT
GPGCNTAGFFTVFASELSVYTLTVITLERWYAITFAMRLDRKIRLRHACAIMVGGWVCCFL
LALLPLVGISSYAKVSICLPMDTETPLALAYIVFVLTLNIVAFVIVCCCYVKIYITVRNPQYN
PGDKDTKIAKRMAVLIFTDFICMAPISFYALSAILNKPLITVSNSKILLVLFYPLNSCANPFLY
AIFTKAFQRDVFILLSKFGICKRQAQAYRGQRVPPKNSTDIQVQKVTHDMRQGLHNMEDV
YELIENSHLTPKKQGQISEEYMQTVL

>O15357 peripheral membrane protein
MASACGAPGPGGALGSQAPSWYHRDLSRAAAEELLARAGRDGSFLVRDSESVAGAFALC
VLYQKHVHTYRILPDGEDFLAVQTSQGVPVRRFQTLGELIGLYAQPNQGLVCALLLPVEGE
REPDPPDDRDASDGEDEKPPLPPRSGSTSISAPTGPSSPLPAPETPTAPAAESAPNGLSTVSH
DYLKGSYGLDLEAVRGGASHLPHLTRTLATSCRRLHSEVDKVLSGLEILSKVFDQQSSPM
VTRLLQQQNLPQTGEQELESLVLKLSVLKDFLSGIQKKALKALQDMSSTAPPAPQPSTRKA
KTIPVQAFEVKLDVTLGDLTKIGKSQKFTLSVDVEGGRLVLLRRQRDSQEDWTTFTHDRIR
QLIKSQRVQNKLGVVFEKEKDRTQRKDFIFVSARKREAFCQLLQLMKNKHSKQDEPDMIS
VFIGTWNMGSVPPPKNVTSWFTSKGLGKTLDEVTVTIPHDIYVFGTQENSVGDREWLDLL
RGGLKELTDLDYRPIAMQSLWNIKVAVLVKPEHENRISHVSTSSVKTGIANTLGNKGAVGV
SFMFNGTSFGFVNCHLTSGNEKTARRNQNYLDILRLLSLGDRQLNAFDISLRFTHLFWFGD
LNYRLDMDIQEILNYISRKEFEPLLRVDQLNLEREKHKVFLRFSEEEISFPPTYRYERGSRD
TYAWHKQKPTGVRTNVPSWCDRILWKSYPETHIICNSYGCTDDIVTSDHSPVFGTFEVGVT

SQFISKKGLSKTSDQAYIEFESIEAIVKTASRTKFFIEFYSTCLEEYKKSFENDAQSSDNINFL
KVQWSSRQLPTLKPILADIEYLQDQHLLLTVKSMDGYESYGECVVALKSMIGSTAQQFLTF
LSHRGEETGNIRGSMKVRVPTERLGTRERLYEWISIDKDEAGAKSKAPSVSRGSQEPRSGS
RKPAFTEASCPLSRLFEEPEKPPPTGRPPAPPRAAPREEPLTPRLKPEGAPEPEGVAAPPPKN
SFNNPAYYVLEGVPHQLLPPEPPSPARAPVPSATKNKVAITVPAPQLGHHRHPRVGEGSSSD
EESGGTLPPPDFPPPPLPDSAIFLPPSLDPLPGPVVRGRGGAEARGPPPPKAHPRPPLPPGPSP
ASTFLGEVASGDDRSCSVLQMAKTLSEVDYAPAGPARSALLPGPLELQPPRGLPSDYGRPL
SFPPPRIRESIQEDLAEEAPCLQGGRASGLGEAGMSAWLRAIGLERYEEGLVHNGWDDLEF
LSDITEEDLEEAGVQDPAHKRLLLDTLQLSK

>Q14CZ8 single-pass type I membrane protein
MKRERGALSRASRALRLAPFVYLLLIQTDPLEGVNITSPVRLIHGTVGKSALLSVQYSSTSS
DRPVVKWQLKRDKPVTVVQSIGTEVIGTLRPDYRDRIRLFENGSLLLSDLQLADEGTYEV
EISITDDTFTGEKTINLTVDVPISRPQVLVASTTVLELSEAFTLNCSHENGTKPSYTWLKDG
KPLLNDSRMLLSPDQKVLTITRVLMEDDDLYSCMVENPISQGRSLPVKITVYRRSSLYIILS
TGGIFLLVTLVTVCACWKPSKRKQKKLEKQNSLEYMDQNDDRLKPEADTLPRSGEQERK
NPMALYILKDKDSPETEENPAPEPRSATEPGPPGYSVSPAVPGRSPGLPIRSARRYPRSPARSP
ATGRTHSSPPRAPSSPGRSRSASRTLRTAGVHIIREQDEAGPVEISA

>P62330 lipid-anchor
MGKVLSKIFGNKEMRILMLGLDAAGKTTILYKLKLGQSVTTIPTVGFNVETVTYKNVKFN
VWDVGGQDKIRPLWRHYYTGTQGLIFVVDCADRDRIDEARQELHRIINDREMRDAIILIFA
NKQDLPDAMKPHEIQEKLGLTRIRDRNWYVQPSCATSGDGLYEGLTWLTSNYKS

>Q8WXD0 multipass membrane protein
MIVFLVFKHLFSLRLITMFFLLHFIVLINVKDFALTQGSMITPSCQKGYFPCGNLTKCLPRAF
HCDGKDDCGNGADEENCGDTSGWATIFGTVHGNANSVALTQECFLKQYPQCCDCKETEL
ECVNGDLKSVPMISNNVTLLSLKKNKIHSLPDKVFIKYTKLKKIFLQHNCIRHISRKAFFGL
CNLQILYLNHNCITTLRPGIFKDLHQLTWLILDDNPITRISQRLFTGLNSLFFLSMVNNYLEA
LPKQMCAQMPQLNWVDLEGNRIKYLTNSTFLSCDSLTVLFLPRNQIGFVPEKTFSSLKNLG
ELDLSSNTITELSPHLFKDLKLLQKLNLSSNPLMYLHKNQFESLKQLQSLDLERIEIPNINTR
MFQPMKNLSHIYFKNFRYCSYAPHVRICMPLTDGISSFEDLLANNILRIFVWVIAFITCFGN
LFVIGMRSFIKAENTTHAMSIKILCCADCLMGVYLFFVGIFDIKYRGQYQKYALLWMESV
QCRLMGFLAMLSTEVSVLLLTYLTLEKFLVIVFPFSNIRPGKRQTSVILICIWMAGFLIAVIPF
WNKDYFGNFYGKNGVCFPLYYDQTEDIGSKGYSLGIFLGVNLLAFLIIVFSYITMFCSIQKT
ALQTTEVRNCFGREVAVANRFFFIVFSDAICWIPVFVVKILSLFRVEIPDTMTSWIVIFFLPV
NSALNPILYTLTTNFFKDKLKQLLHKHQRKSIFKIKKKSLSTSIVWIEDSSSLKLGVLNKITL
GDSIMKPVS

>Q96P68 multipass membrane protein
MNEPLDYLANASDFPDYAAAFGNCTDENIPLKMHYLPVIYGIIFLVGFPGNAVVISTYIFK
MRPWKSSTIIMLNLACTDLLYLTSLPFLIHYYASGENWIFGDFMCKFIRFSFHFNLYSSILFL
TCFSIFRYCVIIHPMSCFSIHKTRCAVVACAVVWIISLVAVIPMTFLITSTNRTNRSACLDLTSS
DELNTIKWYNLILTATTFCLPLVIVTLCYTTIIHTLTHGLQTDSCLKQKARRLTILLLLAFYV

CFLPFHILRVIRIESRLLSISCSIENQIHEAYIVSRPLAALNTFGNLLLYVVVSDNFQQAVCST
VRCKVSGNLEQAKKISYSNNP

>Q05940 multipass membrane protein
MALSELALVRWLQESRRSRKLILFIVFLALLLDNMLLTVVVPIIPSYLYSIKHEKNATEIQTA
RPVHTASISDSFQSIFSYYDNSTMVTGNATRDLTLHQTATQHMVTNASAVPSDCPSEDKDL
LNENVQVGLLFASKATVQLITNPFIGLLTNRIGYPIPIFAGFCIMFVSTIMFAFSSSYAFLLIAR
SLQGIGSSCSSVAGMGMLASVYTDDEERGNVMGIALGGLAMGVLVGPPFGSVLYEFVGK
TAPFLVLAALVLLDGAIQLFVLQPSRVQPESQKGTPLTTLLKDPYILIAAGSICFANMGIAML
EPALPIWMMETMCSRKWQLGVAFLPASISYLIGTNIFGILAHKMGRWLCALLGMIIVGVSI
LCIPFAKNIYGLIAPNFGVGFAIGMVDSSMMPIMGYLVDLRHVSVYGSVYAIADVAFCMGY
AIGPSAGGAIAKAIGFPWLMTIIGIIDILFAPLCFFLRSPPAKEEKMAILMDHNCPIKTKMYT
QNNIQSYPIGEDEESESD

>Q92574 peripheral membrane protein
MAQQANVGELLAMLDSPMLGVRDDVTAVFKENLNSDRGPMLVNTLVDYYLETSSQPAL
HILTTLQEPHDKHLLDRINEYVGKAATRLSILSLLGHVIRLQPSWKHKLSQAPLLPSLLKCL
KMDTDVVVLTTGVLVLITMLPMIPQSGKQHLLDFFDIFGRLSSWCLKKPGHVAEVYLVHL
HASVYALFHRLYGMYPCNFVSFLRSHYSMKENLETFEEVVKPMMEHVRIHPELVTGSKD
HELDPRRWKRLETHDVVIECAKISLDPTEASYEDGYSVSHQISARFPHRSADVTTSPYADT
QNSYGCATSTPYSTSRLMLLNMPGQLPQTLSSPSTRLITEPPQATLWSPSMVCGMTTPPTSP
GNVPPDLSHPYSKVFGTTAGGKGTPLGTPATSPPPAPLCHSDDYVHISLPQATVTPPRKEER
MDSARPCLHRQHHLLNDRGSEEPPGSKGSVTLSDLPGFLGDLASEEDSIEKDKEEAAISRE
LSEITTAEAEPVVPRGGFDSPFYRDSLPGSQRKTHSAASSSQGASVNPEPLHSSLDKLGPDT
PKQAFTPIDLPCGSADESPAGDRECQTSLETSIFTPSPCKIPPPTRVGFGSGQPPPYDHLFEVA
LPKTAHHFVIRKTEELLKKAKGNTEEDGVPSTSPMEVLDRLIQQGADAHSKELNKLPLPS
KSVDWTHFGGSPPSDEIRTLRDQLLLLHNQLLYERFKRQQHALRNRRLLRKVIKAAALEE
HNAAMKDQLKLQEKDIQMWKVSLQKEQARYNQLQEQRDTMVTKLHSQIRQLQHDREE
FYNQSQELQTKLEDCRNMIAELRIELKKANNKVCHTELLLSQVSQKLSNSESVQQQMEFL
NRQLLVLGEVNELYLEQLQNKHSDTTKEVEMMKAAYRKELEKNRSHVLQQTQRLDTSQ
KRILELESHLAKKDHLLLEQKKYLEDVKLQARGQLQAAESRYEAQKRITQVFELEILDLY
GRLEKDGLLKKLEEEKAEAAEAAEERLDCCNDGCSDSMVGHNEEASGHNGETKTPRPSS
ARGSSGSRGGGGSSSSSSELSTPEKPPHQRAGPFSSRWETTMGEASASIPTTVGSLPSSKSF
LGMKARELFRNKSESQCDEDGMTSSLSESLKTELGKDLGVEAKIPLNLDGPHPSPPTPDSV
GQLHIMDYNETHHEHS

>Q96E35 peripheral membrane protein
MTDFKLGIVRLGRVAGKTKYTLIDEQDIPLVESYSFEARMEVDADGNGAKIFAYAFDKNR
GRGSGRLLHELLWERHRGGVAPGFQVVHLNAVTVDNRLDNLQLVPWGWRPKAEETSSK
QREQSLYWLAIQQLPTDPIEEQFPVLNVTRYYNANGDVVEEEENSCTYYECHYPPCTVIEK
QLREFNICGRCQVARYCGSQCQQKDWPAHKKHCRERKRPFQHELEPER

>Q8NGD2 multipass membrane protein
MDVGNKSTMSEFVLLGLSNSWELQMFFFMVFSLLYVATMVGNSLIVITVIVDPHLHSPMY

FLLTNLSIIDMSLASFATPKMITDYLTGHKTISFDGCLTQIFFLHLFTGTEIILLMAMSFDRYI
AICKPLHYASVISPQVCVALVVASWIMGVMHSMSQVIFALTLPFCGPYEVDSFFCDLPVVF
QLACVDTYVLGLFMISTSGIIALSCFIVLFNSYVIVLVTVKHHSSRGSSKALSTCTAHFIVVF
LFFGPCIFIYMWPLSSFLTDKILSVFYTIFTPTLNPIIYTLRNQEVKIAMRKLKNRFLNFNKA
MPS

>Q9NP94 multipass membrane protein
MEQLLGIKLGCLFALLALTLGCGLTPICFKWFQIDAARGHHRLVLRLLGCISAGVFLGAGF
MHMTAEALEEIESQIQKFMVQNRSASERNSSGDADSAHMEYPYGELIISLGFFFVFFLESL
ALQCCPGAAGGSTVQDEEWGGAHIFELHSHGHLPSPSKGPLRALVLLLSLSFHSVFEGLAV
GLQPTVAATVQLCLAVLAHKGLVVFGVGMRLVHLGTSSRWAVFSILLLALMSPLGLAVGL
AVTGGDSEGGRGLAQAVLEGVAAGTFLYVTFLEILPRELASPEAPLAKWSCVAAGFAFMA
FIALWA

>Q9UKS6 peripheral membrane protein
MAPEEDAGGEALGGSFWEAGNYRRTVQRVEDGHRLCGDLVSCFQERARIEKAYAQQLA
DWARKWRGTVEKGPQYGTLEKAWHAFFTAAERLSALHLEVREKLQGQDSERVRAWQR
GAFHRPVLGGFRESRAAEDGFRKAQKPWLKRLKEVEASKKSYHAARKDEKTAQTRESH
AKADSAVSQEQLRKLQERVERCAKEAEKTKAQYEQTLAELHRYTPRYMEDMEQAFETC
QAAERQRLLFFKDMLLTLHQHLDLSSSEKFHELHRDLHQGIEAASDEEDLRWWRSTHGP
GMAMNWPQFEEWSLDTQRTISRKEKGGRSPDEVTLTSIVPTRDGTAPPPQSPGSPGTGQDE
EWSDEESPRKAATGVRVRALYDYAGQEADELSFRAGEELLKMSEEDEQGWCQGQLQSGR
IGLYPANYVECVGA

>Q13702 peripheral membrane protein
MGQDQTKQQIEKGLQLYQSNQTEKALQVWTKVLEKSSDLMGRFRVLGCLVTAHSEMGR
YKEMLKFAVVQIDTARELEDADFLLESYLNLARSNEKLCEFHKTISYCKTCLGLPGTRAGA
QLGGQVSLSMGNAFLGLSVFQKALESFEKALRYAHNNDDAMLECRVCCSLGSFYAQVKD
YEKALFFPCKAAELVNNYGKGWSLKYRAMSQYHMAVAYRLLGRLGSAMECCEESMKIA
LQHGDRPLQALCLLCFADIHRSRGDLETAFPRYDSAMSIMTEIGNRLGQVQALLGVAKCW
VARKALDKALDAIERAQDLAEEVGNKLSQLKLHCLSESIYRSKGLQRELRAHVVRFHECV
EETELYCGLCGESIGEKNSRLQALPCSHIFHLRCLQNNGTRSCPNCRRSSMKPGFV

>Q8NFF2 multipass membrane protein
MALRGTLRPLKVRRRREMLPQQVGFVCAVLALVCCASGLFGSLGHKTASASKRVLPDTW
RNRKLMAPVNGTQTAKNCTDPAIHEFPTDLFSNKERQHGAVLLHILGALYMFYALAIVCD
DFFVPSLEKICERLHLSEDVAGATFMAAGSSTPELFASVIGVFITHGDVGVGTIVGSAVFNIL
CIIGVCGLFAGQVVRLTWWAVCRDSVYYTISVIVLIVFIYDEQIVWWEGLVLIILYVFYILIM
KYNVKMQAFFTVKQKSIANGNPVNSELEAGNDFYDGSYDDPSVPLLGQVKEKPQYGKN
PVVMVDEIMSSSPPKFTFPEAGLRIMITNKFGPRTRLRMASRIIINERQRLINSANGVSSKPL
QNGRHENIENGNVPVENPEDPQQNQEQQPPPQPPPPEPEPVEADFLSPFSVPEARGDKVK
WVFTWPLIFLLCVTIPNCSKPRWEKFFMVTFITATLWIAVFSYIMVWLVTIIGYTLGIPDVIM
GITFLAAGTSVPDCMASLIVARQGLGDMAVSNTIGSNVFDILVGLGVPWGLQTMVVNYGS
TVKINSRGLVYSVVLLLGSVALTVLGIHLNKWRLDRKLGVYVLVLYAIFLCFSIMIEFNVFT

FVNLPMCREDD

>Q5S007 peripheral membrane protein
MASGSCQGCEEDEETLKKLIVRLNNVQEGKQIETLVQILEDLLVFTYSERASKLFQGKNIH
VPLLIVLDSYMRVASVQQVGWSLLCKLIEVCPGTMQSLMGPQDVGNDWEVLGVHQLILK
MLTVHNASVNLSVIGLKTLDLLLTSGKITLLILDEESDIFMLIFDAMHSFPANDEVQKLGCK
ALHVLFERVSEEQLTEFVENKDYMILLSALTNFKDEEEIVLHVLHCLHSLAIPCNNVEVLM
SGNVRCYNIVVEAMKAFPMSERIQEVSCCLLHRLTLGNFFNILVLNEVHEFVVKAVQQYP
ENAALQISALSCLALLTETIFLNQDLEEKNENQENDDEGEEDKLFWLEACYKALTWHRKN
KHVQEAACWALNNLLMYQNSLHEKIGDEDGHFPAHREVMLSMLMHSSSKEVFQASANA
LSTLLEQNVNFRKILLSKGIHLNVLELMQKHIHSPEVAESGCKMLNHLFEGSNTSLDIMAA
VVPKILTVMKRHETSLPVQLEALRAILHFIVPGMPEESREDTEFHHKLNMVKKQCFKNDIH
KLVLAALNRFIGNPGIQKCGLKVISSIVHFPDALEMLSLEGAMDSVLHTLQMYPDDQEIQC
LGLSLIGYLITKKNVFIGTGHLLAKILVSSLYRFKDVAEIQTKGFQTILAILKLSASFSKLLVH
HSFDLVIFHQMSSNIMEQKDQQFLNLCCKCFAKVAMDDYLKNVMLERACDQNNSIMVEC
LLLLGADANQAKEGSSLICQVCEKESSPKLVELLLNSGSREQDVRKALTISIGKGDSQIISLL
LRRLALDVANNSICLGGFCIGKVEPSWLGPLFPDKTSNLRKQTNIASTLARMVIRYQMKSA
VEEGTASGSDGNFSEDVLSKFDEWTFIPDSSMDSVFAQSDDLDSEGSEGSFLVKKKSNSIS
VGEFYRDAVLQRCSPNLQRHSNSLGPIFDHEDLLKRKRKILSSDDSLRSSKLQSHMRHSDS
ISSLASEREYITSLDLSANELRDIDALSQKCCISVHLEHLEKLELHQNALTSFPQQLCETLKS
LTHLDLHSNKFTSFPSYLLKMSCIANLDVSRNDIGPSVVLDPTVKCPTLKQFNLSYNQLSF
VPENLTDVVEKLEQLILEGNKISGICSPLRLKELKILNLSKNHISSLSENFLEACPKVESFSA
RMNFLAAMPFLPPSMTILKLSQNKFSCIPEAILNLPHLRSLDMSSNDIQYLPGPAHWKSLN
LRELLFSHNQISILDLSEKAYLWSRVEKLHLSHNKLKEIPPEIGCLENLTSLDVSYNLELRSF
PNEMGKLSKIWDLPLDELHLNFDFKHIGCKAKDIIRFLQQRLKKAVPYNRMKLMIVGNTG
SGKTTLLQQLMKTKKSDLGMQSATVGIDVKDWPIQIRDKRKRDLVLNVWDFAGREEFYS
THPHFMTQRALYLAVYDLSKGQAEVDAMKPWLFNIKARASSSPVILVGTHLDVSDEKQR
KACMSKITKELLNKRGFPAIRDYHFVNATEESDALAKLRKTIINESLNFKIRDQLVVGQLIP
DCYVELEKIILSERKNVPIEFPVIDRKRLLQLVRENQLQLDENELPHAVHFLNESGVLLHFQ
DPALQLSDLYFVEPKWLCKIMAQILTVKVEGCPKHPKGIISRRDVEKFLSKKRKFPKNYMS
QYFKLLEKFQIALPIGEEYLLVPSSLSDHRPVIELPHCENSEIIIRLYEMPYFPMGFWSRLINR
LLEISPYMLSGRERALRPNRMYWRQGIYLNWSPEAYCLVGSEVLDNHPESFLKITVPSCRK
GCILLGQVVDHIDSLMEEWFPGLLEIDICGEGETLLKKWALYSFNDGEEHQKILLDDLMK
KAEEGDLLVNPDQPRLTIPISQIAPDLILADLPRNIMLNNDELEFEQAPEFLLGDGSFGSVYR
AAYEGEEVAVKIFNKHTSLRLLRQELVVLCHLHHPSLISLLAAGIRPRMLVMELASKGSLD
RLLQQDKASLTRTLQHRIALHVADGLRYLHSAMIIYRDLKPHNVLLFTLYPNAAIIAKIADY
GIAQYCCRMGIKTSEGTPGFRAPEVARGNVIYNQQADVYSFGLLLYDILTTGGRIVEGLKF
PNEFDELEIQGKLPDPVKEYGCAPWPMVEKLIKQCLKENPQERPTSAQVFDILNSAELVCL
TRRILLPKNVIVECMVATHHNSRNASIWLGCGHTDRGQLSFLDLNTEGYTSEEVADSRILC
LALVHLPVEKESWIVSGTQSGTLLVINTEDGKKRHTLEKMTDSVTCLYCNSFSKQSKQKN
FLLVGTADGKLAIFEDKTVKLKGAAPLKILNIGNVSTPLMCLSESTNSTERNVMWGGCGT
KIFSFSNDFTIQKLIETRTSQLFSYAAFSDSNIITVVVDTALYIAKQNSPVVEVWDKKTEKLC
GLIDCVHFLREVMVKENKESKHKMSYSGRVKTLCLQKNTALWIGTGGGHILLLDLSTRRL
IRVIYNFCNSVRVMMTAQLGSLKNVMLVLGYNRKNTEGTQKQKEIQSCLTVWDINLPHEV

QNLEKHIEVRKELAEKMRRTSVE

>Q9Y345 multipass membrane protein
MDCSAPKEMNKLPANSPEAAAAQGHPDGPCAPRTSPEQELPAAAAPPPPRVPRSASTGAQ
TFQSADARACEAERPGVGSCKLSSPRAQAASAALRDLREAQGAQASPPPGSSGPGNALHC
KIPFLRGPEGDANVSVGKGTLERNNTPVVGWVNMSQSTVVLATDGITSVLPGSVATVATQ
EDEQGDENKARGNWSSKLDFILSMVGYAVGLGNVWRFPYLAFQNGGGAFLIPYLMMLA
LAGLPIFFLEVSLGQFASQGPVSVWKAIPALQGCGIAMLIISVLIAIYYNVIICYTLFYLFASF
VSVLPWGSCNNPWNTPECKDKTKLLLDSCVISDHPKIQIKNSTFCMTAYPNVTMVNFTSQ
ANKTFVSGSEEYFKYFVLKISAGIEYPGEIRWPLALCLFLAWVIVYASLAKGIKTSGKVVY
FTATFPYVVLVILLIRGVTLPGAGAGIWYFITPKWEKLTDATVWKDAATQIFFSLSAAWGG
LITLSSYNKFHNNCYRDTLIVTCTNSATSIFAGFVIFSVIGFMANERKVNIENVADQGPGIAF
VVYPEALTRLPLSPFWAIIFFLMLLTLGLDTMFATIETIVTSISDEFPKYLRTHKPVFTLGCCI
CFFIMGFPMITQGGIYMFQLVDTYAASYALVIIAIFELVGISYVYGLQRFCEDIEMMIGFQPN
IFWKVCWAFVTPTILTFILCFSFYQWEPMTYGSYRYPNWSMVLGWLMLACSVIWIPIMFVI
KMHLAPGRFIERLKLVCSPQPDWGPFLAQHRGERYKNMIDPLGTSSLGLKLPVKDLELGT
QC

>Q7Z699 peripheral membrane protein
MSEETATSDNDNSYARVRAVVMTRDDSSGGWLPLGGSGLSSVTVFKVPHQEENGCADFFI
RGERLRDKMVVLECMLKKDLIYNKVTPTFHHWKIDDKKFGLTFQSPADARAFDRGIRRAI
EDISQGCPESKNEAEGADDLQANEEDSSSSLVKDHLFQQETVVTSEPYRSSNIRPSPFEDLN
ARRVYMQSQANQITFGQPGLDIQSRSMEYVQRQISKECGSLKSQNRVPLKSIRHVSFQDED
EIVRINPRDILIRRYADYRHPDMWKNDLERDDADSSIQFSKPDSKKSDYLYSCGDETKLSSP
KDSVVFKTQPSSLKIKKSKRRKEDGERSRCVYCQERFNHEENVRGKCQDAPDPIKRCIYQ
VSCMLCAESMLYHCMSDSEGDFSDPCSCDTSDDKFCLRWLALVALSFIVPCMCCYVPLR
MCHRCGEACGCCGGKHKAAG

>Q14439 multipass membrane protein
MGHNGSWISPNASEPHNASGAEAAGVNRSALGEFGEAQLYRQFTTTVQVVIFIGSLLGNF
MVLWSTCRTTVFKSVTNRFIKNLACSGICASLVCVPFDIILSTSPHCCWWIYTMLFCKVVK
FLHKVFCSVTILSFPAIALDRYYSVLYPLERKISDAKSRELVMYIWAHAVVASVPVFAVTNV
ADIYATSTCTEVWSNSLGHLVYVLVYNITTVIVPVVVVFLFLILIRRALSASQKKKVIIAALR
TPQNTISIPYASQREAELHATLLSMVMVFILCSVPYATLVVYQTVLNVPDTSVFLLLTAVWL
PKVSLLANPVLFLTVNKSVRKCLIGTLVQLHHRYSRRNVVSTGSGMAEASLEPSIRSGSQL
LEMFHIGQQQIFKPTEDEEESEAKYIGSADFQAKEIFSTCLEGEQGPQFAPSAPPLSTVDSVS
QVAPAAPVEPETFPDKYSLQFGFGPFELPPQWLSETRNSKKRLLPPLGNTPEELIQTKVPKV
GRVERKMSRNNKVSIFPKVDS

>Q96QK1 peripheral membrane protein
MPTTQQSPQDEQEKLLDEAIQAVKVQSFQMKRCLDKNKLMDALKHASNMLGELRTSML
SPKSYYELYMAISDELHYLEVYLTDEFAKGRKVADLYELVQYAGNIIPRLYLLITVGVVYV
KSFPQSRKDILKDLVEMCRGVQHPLRGLFLRNYLLQCTRNILPDEGEPTDEETTGDISDSM
DFVLLNFAEMNKLWVRMQHQGHSRDREKRERERQELRILVGTNLVRLSQLEGVNVERYK

QIVLTGILEQVVNCRDALAQEYLMECIIQVFPDEFHLQTLNPFLRACAELHQNVNVKNIIIA
LIDRLALFAHREDGPGIPADIKLFDIFSQQVATVIQSRQDMPSEDVVSLQVSLINLAMKCYP
DRVDYVDKVLETTVEIFNKLNLEHIATSSAVSKELTRLLKIPVDTYNNILTVLKLKHFHPLF
EYFDYESRKSMSCYVLSNVLDYNTEIVSQDQVDSIMNLVSTLIQDQPDQPVEDPDPEDFA
DEQSLVGRFIHLLRSEDPDQQYLILNTARKHFGAGGNQRIRFTLPPLVFAAYQLAFRYKENS
KVDDKWEKKCQKIFSFAHQTISALIKAELAELPLRLFLQGALAAGEIGFENHETVAYEFMS
QAFSLYEDEISDSKAQLAAITLIIGTFERMKCFSEENHEPLRTQCALAASKLLKKPDQGRAV
STCAHLFWSGRNTDKNGEELHGGKRVMECLKKALKIANQCMDPSLQVQLFIEILNRYIYF
YEKENDAVTIQVLNQLIQKIREDLPNLESSEETEQINKHFHNTLEHLRLRRESPESEGPIYEG
LIL

>P37288 multipass membrane protein
MRLSAGPDAGPSGNSSPWWPLATGAGNTSREAEALGEGNGPPRDVRNEELAKLEIAVLA
VTFAVAVLGNSSVLLALHRTPRKTSRMHLFIRHLSLADLAVAFFQVLPQMCWDITYRFRGP
DWLCRVVKHLQVFGMFASAYMLVVMTADRYIAVCHPLKTLQQPARRSRLMIAAAWVLSF
VLSTPQYFVFSMIEVNNVTKARDCWATFIQPWGSRAYVTWMTGGIFVAPVVILGTCYGFI
CYNIWCNVRGKTASRQSKGAEQAGVAFQKGFLLAPCVSSVKSISRAKIRTVKMTFVIVTA
YIVCWAPFFIIQMWSVWDPMSVWTESENPTITITALLGSLNSCCNPWIYMFFSGHLLQDCV
QSFPCCQNMKEKFNKEDTDSMSRRQTFYSNNRSPTNSTGMWKDSPKSSKSIKFIPVST

>Q3T906 single-pass type I membrane protein, single-pass type II membrane protein
MLFKLLQRQTYTCLSHRYGLYVCFLGVVVTIVSAFQFGEVVLEWSRDQYHVLFDSYRDNI
AGKSFQNRLCLPMPIDVVYTWVNGTDLELLKELQQVREQMEEEQKAMREILGKNTTEPT
KKSEKQLECLLTHCIKVPMLVLDPALPANITLKDLPSLYPSFHSASDIFNVAKPKNPSTNVS
VVVFDSTKDVEDAHSGLLKGNSRQTVWRGYLTTDKEVPGLVLMQDLAFLSGFPPTFKET
NQLKTKLPENLSSKVKLLQLYSEASVALLKLNNPKDFQELNKQTKKNMTIDGKELTISPAY
LLWDLSAISQSKQDEDISASRFEDNEELRYSLRSIERHAPWVRNIFIVTNGQIPSWLNLDNP
RVTIVTHQDVFRNLSHLPTFSSPAIESHIHRIEGLSQKFIYLNDDVMFGKDVWPDDFYSHSK
GQKVYLTWPVPNCAEGCPGSWIKDGYCDKACNNSACDWDGGDCSGNSGGSRYIAGGG
GTGSIGVGQPWQFGGGINSVSYCNQGCANSWLADKFCDQACNVLSCGFDAGDCGQDHF
HELYKVILLPNQTHYIIPKGECLPYFSFAEVAKRGVEGAYSDNPIIRHASIANKWKTIHLIMH
SGMNATTIHFNLTFQNTNDEEFKMQITVEVDTREGPKLNSTAQKGYENLVSPITLLPEAEIL
FEDIPKEKRFPKFKRHDVNSTRRAQEEVKIPLVNISLLPKDAQLSLNTLDLQLEHGDITLKG
YNLSKSALLRSFLMNSQHAKIKNQAIITDETNDSLVAPQEKQVHKSILPNSLGVSERLQRLT
FPAVSVKVNGHDQGQNPPLDLETTARFRVETHTQKTIGGNVTKEKPPSLIVPLESQMTKEK
KITGKEKENSRMEENAENHIGVTEVLLGRKLQHYTDSYLGFLPWEKKKYFQDLLDEEESL
KTQLAYFTDSKNTGRQLKDTFADSLRYVNKILNSKFGFTSRKVPAHMPHMIDRIVMQELQ
DMFPEEFDKTSFHKVRHSEDMQFAFSYFYYLMSAVQPLNISQVFDEVDTDQSGVLSDREI
RTLATRIHELPLSLQDLTGLEHMLINCSKMLPADITQLNNIPPTQESYYDPNLPPVTKSLVTN
CKPVTDKIHKAYKDKNKYRFEIMGEEEIAFKMIRTNVSHVVGQLDDIRKNPRKFVCLNDN
IDHNHKDAQTVKAVLRDFYESMFPIPSQFELPREYRNRFLHMHELQEWRAYRDKLKFWT
HCVLATLIMFTIFSFFAEQLIALKRKIFPRRRIHKEASPNRIRV

>Q9NY72 single-pass type I membrane protein

MPAFNRLFPLASLVLIYWVSVCFPVCVEVPSETEAVQGNPMKLRCISCMKREEVEATTVVE
WFYRPEGGKDFLIYEYRNGHQEVESPFQGRLQWNGSKDLQDVSITVLNVTLNDSGLYTC
NVSREFEFEAHRPFVKTTRLIPLRVTEEAGEDFTSVVSEIMMYILLVFLTLWLLIEMIYCYR
KVSKAEEAAQENASDYLAIPSENKENSAVPVEE

>P78369 multipass membrane protein
MASTASEIIAFMVSISGWVLVSSTLPTDYWKVSTIDGTVITTATYWANLWKACVTDSTGVS
NCKDFPSMLALDGYIQACRGLMIAAVSLGFFGSIFALFGMKCTKVGGSDKAKAKIACLAG
IVFILSGLCSMTGCSLYANKITTEFFDPLFVEQKYELGAALFIGWAGASLCIIGGVIFCFSISD
NNKTPRYTYNGATSVMSSRTKYHGGEDFKTTNPSKQFDKNAYV

>P55286 single-pass type I membrane protein
MPERLAEMLLDLWTPLIILWITLPPCIYMAPMNQSQVLMSGSPLELNSLGEEQRILNRSKR
GWVWNQMFVLEEFSGPEPILVGRLHTDLDPGSKKIKYILSGDGAGTIFQINDVTGDIHAIK
RLDREEKAEYTLTAQAVDWETSKPLEPPSEFIIKVQDINDNAPEFLNGPYHATVPEMSILGT
SVTNVTATDADDPVYGNSAKLVYSILEGQPYFSIEPETAIIKTALPNMDREAKEEYLVVIQA
KDMGGHSGGLSGTTTLTVTLTDVNDNPPKFAQSLYHFSVPEDVVLGTAIGRVKANDQDIG
ENAQSSYDIIDGDGTALFEITSDAQAQDGIIRLRKPLDFETKKSYTLKVEAANVHIDPRFSG
RGPFKDTATVKIVVEDADEPPVFSSPTYLLEVHENAALNSVIGQVTARDPDITSSPIRFSIDR
HTDLERQFNINADDGKITLATPLDRELSVWHNITIIATEIRNHSQISRVPVAIKVLDVNDNAP
EFASEYEAFLCENGKPGQVIQTVSAMDKDDPKNGHYFLYSLLPEMVNNPNFTIKKNEDNS
LSILAKHNGFNRQKQEVYLLPIIISDSGNPPLSSTSTLTIRVCGCSNDGVVQSCNVEAYVLPI
GLSMGALIAILACIILLLVIVVLFVTLRRHKNEPLIIKDDEDVRENIIRYDDEGGGEEDTEAF
DIATLQNPDGINGFLPRKDIKPDLQFMPRQGLAPVPNGVDVDEFINVRLHEADNDPTAPPY
DSIQIYGYEGRGSVAGSLSSLESTTSDSDQNFDYLSDWGPRFKRLGELYSVGESDKET

>Q13418 peripheral membrane protein
MDDIFTQCREGNAVAVRLWLDNTENDLNQGDDHGFSPLHWACREGRSAVVEMLIMRGA
RINVMNRGDDTPLHLAASHGHRDIVQKLLQYKADINAVNEHGNVPLHYACFWGQDQVA
EDLVANGALVSICNKYGEMPVDKAKAPLRELLRERAEKMGQNLNRIPYKDTFWKGTTRT
RPRNGTLNKHSGIDFKQLNFLTKLNENHSGELWKGRWQGNDIVVKVLKVRDWSTRKSR
DFNEECPRLRIFSHPNVLPVLGACQSPPAPHPTLITHWMPYGSLYNVLHEGTNFVVDQSQA
VKFALDMARGMAFLHTLEPLIPRHALNSRSVMIDEDMTARISMADVKFSFQCPGRMYAPA
WVAPEALQKKPEDTNRRSADMWSFAVLLWELVTREVPFADLSNMEIGMKVALEGLRPTIP
PGISPHVCKLMKICMNEDPAKRPKFDMIVPILEKMQDK

>Q86YR6 peripheral membrane protein
MVAEVCSMPTASTVKKPFDLRSKMGKWCHHRFPCCRGSGKSNMGTSGDHDDSFMKML
RSKMGKCCRHCFPCCRGSGTSNVGTSGDHENSFMKMLRSKMGKWCCHCFPCCRGSGKS
NVGAWGDYDHSAFMEPRYHIRREDLDKLHRAAWWGKVPRKDLIVMLRDTDMNKRDKE
KRTALHLASANGNSEVVQLLLDRRCQLNVLDNKKRTALIKAIQCQEDECVLMLLEHGAD
RNIPDEYGNTALHYAIYNEDKLMAKALLLYGADIESKNKCGLTPLLLGVHEQKQQVVKFL
IKKKANLNVLDRYGRTALILAVCCGSASIVNLLLEQNVDVSSQDLSGQTAREYAVSSHHHV
ICELLSDYKEKQMLKISSENSNPEQDLKLTSEEESQRLKVSENSQPEKMSQEPEINKDCDRE

VEEEIKKHGSNPVGLPENLTNGASAGNGDDGLIPQRRSRKPENQQFPDTENEEYHSDEQN
DTRKQLSEEQNTGISQDEILTNKQKQIEVAEQKMNSELSLSHKKEEDLLRENSVLQEEIAM
LRLELDETKHQNQLRENKILEEIESVKEKTDKLLRAMQLNEEALTKTNI

>Q9H207 multipass membrane protein
MAIGNWTEISEFILMSFSSLPTEIQSLLFLTFLTIYLVTLKGNSLIILVTLADPMLHSPMYFFL
RNLSFLEIGFNLVIVPKMLGTLLAQDTTISFLGCATQMYFFFFFGVAECFLLATMAYDRYVA
ICSPLHYPVIMNQRTRAKLAAASWFPGFPVATVQTTWLFSFPFCGTNKVNHFFCDSPPVLK
LVCADTALFEIYAIVGTILVVMIPCLLILCSYTRIAAAILKIPSAKGKHKAFSTCSSHLLVVSL
FYISSSLTYFWPKSNNSPESKKLLSLSYTVVTPMLNPIIYSLRNSEVKNALSRTFHKVLALR
NCIP

>Q9H210 multipass membrane protein
MRQINQTQVTEFLLLGLSDGPHTEQLLFIVLLGVYLVTVLGNLLLISLVHVDSQLHTPMYF
FLCNLSLADLCFSTNIVPQALVHLLSRKKVIAFTLCAARLLFFLIFGCTQCALLAVMSYDRY
VAICNPLRYPNIMTWKVCVQLATGSWTSGILVSVVDTTFILRLPYRGSNSIAHFFCEAPALLI
LASTDTHASEMAIFLMGVVILLIPVFLILVSYGRIIVTVVKMKSTVGSLKAFSTCGSHLMVV
ILFYGSAIITYMTPKSSKQQEKSVSVFYAIVTPMLNPLIYSLRNKDVKAALRKVATRNFP

>P30532 multipass membrane protein
MAARGSGPRALRLLLLVQLVAGRCGLAGAAGGAQRGLSEPSSIAKHEDSLLKDLFQDYER
WVRPVEHLNDKIKIKFGLAISQLVDVDEKNQLMTTNVWLKQEWIDVKLRWNPDDYGGI
KVIRVPSDSVWTPDIVLFDNADGRFEGTSTKTVIRYNGTVTWTPPANYKSSCTIDVTFFPFD
LQNCSMKFGSWTYDGSQVDIILEDQDVDKRDFFDNGEWEIVSATGSKGNRTDSCCWYPY
VTYSFVIKRLPLFYTLFLIIPCIGLSFLTVLVFYLPSNEGEKICLCTSVLVSLTVFLLVIEEIIPSS
SKVIPLIGEYLVFTMIFVTLSIMVTVFAINIHHRSSSTHNAMAPLVRKIFLHTLPKLLCMRSH
VDRYFTQKEETESGSGPKSSRNTLEAALDSIRYITRHIMKENDVREVVEDWKFIAQVLDR
MFLWTFLFVSIVGSLGLFVPVIYKWANILIPVHIGNANK

>Q9Y3Q3 single-pass type I membrane protein
MGSTVPRSASVLLLLLLLRRAEQPCGAELTFELPDNAKQCFHEEVEQGVKFSLDYQVITG
GHYDVDCYVEDPQGNTIYRETKKQYDSFTYRAEVKGVYQFCFSNEFSTFSHKTVYFDFQ
VGDEPPILPDMGNRVTALTQMESACVTIHEALKTVIDSQTHYRLREAQDRARAEDLNSRV
SYWSVGETIALFVVSFSQVLLLKSFFTEKRPISRAVHS

>Q7Z7G2 lipid-anchor
MAFLMKSMISNQVKNLGFGGGSEENKEEGGASDPAAAQGMTREEYEEYQKQMIEEKME
RDAAFTQKKAERACLRVHLREKYRLPKSEMDENQIQMAGDDVDLPEDLRKMVDEDQEE
EEDKDSILGQIQNLQNMDLDTIKEKAQATFTEIKQTAEQKCSVM

>P47211 multipass membrane protein
MELAVGNLSEGNASWPEPPAPEPGPLFGIGVENFVTLVVFGLIFALGVLGNSLVITVLARSK
PGKPRSTTNLFILNLSIADLAYLLFCIPFQATVYALPTWVLGAFICKFIHYFFTVSMLVSIFTL
AAMSVDRYVAIVHSRRSSSLRVSRNALLGVGCIWALSIAMASPVAYHQGLFHPRASNQTF

CWEQWPDPRHKKAYVVCTFVFGYLLPLLLICFCYAKVLNHLHKKLKNMSKKSEASKKKT
AQTVLVVVVVFGISWLPHHIIHLWAEFGVFPLTPASFLFRITAHCLAYSNSSVNPIIYAFLSEN
FRKAYKQVFKCHIRKDSHLSDTKESKSRIDTPPSTNCTHV

>P32245 multipass membrane protein
MVNSTHRGMHTSLHLWNRSSYRLHSNASESLGKGYSDGGCYEQLFVSPEVFVTLGVISLL
ENILVIVAIAKNKNLHSPMYFFICSLAVADMLVSVSNGSETIVITLLNSTDTDAQSFTVNIDN
VIDSVICSSLLASICSLLSIAVDRYFTIFYALQYHNIMTVKRVGIIISCIWAACTVSGILFIIYSD
SSAVIICLITMFFTMLALMASLYVHMFLMARLHIKRIAVLPGTGAIRQGANMKGAITLTILI
GVFVVCWAPFFLHLIFYISCPQNPYCVCFMSHFNLYLILIMCNSIIDPLIYALRSQELRKTFK
EIICCYPLGGLCDLSSRY

>Q14940 multipass membrane protein
MLRAALSLLALPLAGAAEEPTQKPESPGEPPPGLELFRWQWHEVEAPYLVALWILVASLA
KIVFHLSRKVTSLVPESCLLILLGLVLGGIVLAVAKKAEYQLEPGTFFLFLLPPIVLDSGYFM
PSRLFFDNLGAILTYAVVGTLWNAFTTGAALWGLQQAGLVAPRVQAGLLDFLLFGSLISAV
DPVAVLAVFEEVHVNETLFIIVFGESLLNDAVTVVLYKVCNSFVEMGSANVQATDYLKGV
ASLFVVSLGGAAVGLVFAFLLALTTRFTKRVRIIEPLLVFLLAYAAYLTAEMASLSAILAVTM
CGLGCKKYVEANISHKSRTTVKYTMKTLASCAETVIFMLLGISAVDSSKWAWDSGLVLGT
LIFILFFRALGVVLQTWVLNQFRLVPLDKIDQVVMSYGGLRGAVAFALVILLDRTKVPAKD
YFVATTIVVVFFTVIVQGLTIKPLVKWLKVKRSEHHKPTLNQELHEHTFDHILAAVEDVVG
HHGYHYWRDRWEQFDKKYLSQLLMRRSAYRIRDQIWDVYYRLNIRDAISFVDQGGHVL
SSTGLTLPSMPSRNSVAETSVTNLLRESGSGACLDLQVIDTVRSGRDREDAVMHHLLCGGL
YKPRRRYKASCSRHFISEDAQERQDKEVFQQNMKRRLESFKSTKHNICFTKSKPRPRKTG
RRKKDGVANAEATNGKHRGLGFQDTAAVILTVESEEEEEESDSSETEKEDDEGIIFVARATS
EVLQEGKVSGSLEVCPSPRIIPPSPTCAEKELPWKSGQGDLAVYVSSETTKIVPVDMQTGW
NQSISSLESLASPPCNQAPILTCLPPHPRGTEEPQVPLHLPSDPRSSFAFPPSLAKAGRSRSES
SADLPQQQELQPLMGHKDHTHLSPGTATSHWCIQFNRGSRL

>Q8WTW3 peripheral membrane protein
MATAATSPALKRLDLRDPAALFETHGAEEIRGLERQVRAEIEHKKEELRQMVGERYRDLIE
AADTIGQMRRCAVGLVDAVKATDQYCARLRQAGSAAPRPPRAQQPQQPSQEKFYSMAA
QIKLLLEIPEKIWSSMEASQCLHATQLYLLCCHLHSLLQLDSSSSRYSPVLSRFPILIRQVAA
ASHFRSTILHESKMLLKCQGVSDQAVAEALCSIMLLEESSPRQALTDFLLARKATIQKLLNQ
PHHGAGIKAQICSLVELLATTLKQAHALFYTLPEGLLPDPALPCGLLFSTLETITGQHPAGK
GTGVLQEEMKLCSWFKHLPASIVEFQPTLRTLAHPISQEYLKDTLQKWIHMCNEDIKNGIT
NLLMYVKSMKGLAGIRDAMWELLTNESTNHSWDVLCRRLLEKPLLFWEDMMQQLFLD
RLQTLTKEGFDSISSSSKELLVSALQELESSTSNSPSNKHIHFEYNMSLFLWSESPNDLPSDA
AWVSVANRGQFASSGLSMKAQAISPCVQNFCSALDSKLKVKLDDLLAYLPSDDSSLPKDV
SPTQAKSSAFDRYADAGTVQEMLRTQSVACIKHIVDCIRAELQSIEEGVQGQQDALNSAKL
HSVLFMARLCQSLGELCPHLKQCILGKSESSEKPAREFRALRKQGKVKTQEIIPTQAKWQE
VKEVLLQQSVMGYQVWSSAVVKVLIHGFTQSLLLDDAGSVLATATSWDELEIQEEAESGS
SVTSKIRLPAQPSWYVQSFLFSLCQEINRVGGHALPKVTLQEMLKSCMVQVVAAYEKLSE
EKQIKKEGAFPVTQNRALQLLYDLRYLNIVLTAKGDEVKSGRSKPDSRIEKVTDHLEALID

PFDLDVFTPHLNSNLHRLVQRTSVLFGLVTGTENQLAPRSSTFNSQEPHNILPLASSQIRFGL
LPLSMTSTRKAKSTRNIETKAQVVPPARSTAGDPTVPGSLFRQLVSEEDNTSAPSLFKLGW
LSSMTK

>O75192 multipass membrane protein
MDAFTRFTNQTQGRDRLFRATQYTCMLLRYLLEPKAGKEKVVMKLKKLESSVSTGRKW
FRLGNVVHAIQATEQSIHATDLVPRLCLTLANLNRVIYFICDTILWVRSVGLTSGINKEKWR
TRAAHHYYYSLLLSLVRDLYEISLQMKRVTCDRAKKEKSASQDPLWFSVAEEETEWLQSF
LLLLFRSLKQHPPLLLDTVKNLCDILNPLDQLGIYKSNPGIIGLGGLVSSIAGMITVAYPQM
KLKTR

>P15144 single-pass type II membrane protein
MAKGFYISKSLGILGILLGVAAVCTIIALSVVYSQEKNKNANSSPVASTTPSASATTNPASAT
TLDQSKAWNRYRLPNTLKPDSYRVTLRPYLTPNDRGLYVFKGSSTVRFTCKEATDVIIIHSK
KLNYTLSQGHRVVLRGVGGSQPPDIDKTELVEPTEYLVVHLKGSLVKDSQYEMDSEFEGE
LADDLAGFYRSEYMEGNVRKVVATTQMQAADARKSFPCFDEPAMKAEFNITLIHPKDLTA
LSNMLPKGPSTPLPEDPNWNVTEFHTTPKMSTYLLAFIVSEFDYVEKQASNGVLIRIWARP
SAIAAGHGDYALNVTGPILNFFAGHYDTPYPLPKSDQIGLPDFNAGAMENWGLVTYRENS
LLFDPLSSSSSNKERVVTVIAHELAHQWFGNLVTIEWWNDLWLNEGFASYVEYLGADYAE
PTWNLKDLMVLNDVYRVMAVDALASSHPLSTPASEINTPAQISELFDAISYSKGASVLRML
SSFLSEDVFKQGLASYLHTFAYQNTIYLNLWDHLQEAVNNRSIQLPTTVRDIMNRWTLQM
GFPVITVDTSTGTLSQEHFLLDPDSNVTRPSEFNYVWIVPITSIRDGRQQQDYWLIDVRAQ
NDLFSTSGNEWVLLNLNVTGYYRVNYDEENWRKIQTQLQRDHSAIPVINRAQIINDAFNL
ASAHKVPVTLALNNTLFLIEERQYMPWEAALSSLSYFKLMFDRSEVYGPMKNYLKKQVT
PLFIHFRNNTNNWREIPENLMDQYSEVNAISTACSNGVPECEEMVSGLFKQWMENPNNNP
IHPNLRSTVYCNAIAQGGEEEWDFAWEQFRNATLVNEADKLRAALACSKELWILNRYLSY
TLNPDLIRKQDATSTIISITNNVIGQGLVWDFVQSNWKKLFNDYGGGSFSFSNLIQAVTRRF
STEYELQQLEQFKKDNEETGFGSGTRALEQALEKTKANIKWVKENKEVVLQWFTENSK

>P51170 multipass membrane protein
MAPGEKIKAKIKKNLPVTGPQAPTIKELMRWYCLNTNTHGCRRIVVSRGRLRRLLWIGFT
LTAVALILWQCALLVFSFYTVSVSIKVHFRKLDFPAVTICNINPYKYSTVRHLLADLEQETRE
ALKSLYGFPESRKRREAESWNSVSEGKQPRFSHRIPLLIFDQDEKGKARDFFTGRKRKVGG
SIIHKASNVMHIESKQVVGFQLCSNDTSDCATYTFSSGINAIQEWYKLHYMNIMAQVPLE
KKINMSYSAEELLVTCFFDGVSCDARNFTLFHHPMHGNCYTFNNRENETILSTSMGGSEY
GLQVILYINEEEYNPFLVSSTGAKVIIHRQDEYPFVEDVGTEIETAMVTSIGMHLTESFKLSE
PYSQCTEDGSDVPIRNIYNAAYSLQICLHSCFQTKMVEKCGCAQYSQPLPPAANYCNYQQ
HPNWMYCYYQLHRAFVQEELGCQSVCKEACSFKEWTLTTSLAQWPSVVSEKWLLPVLT
WDQGRQVNKKLNKTDLAKLLIFYKDLNQRSIMESPANSIEMLLSNFGGQLGLWMSCSVV
CVIEIIEVFFIDFFSIIARRQWQKAKEWWAWKQAPPCPEAPRSPQGQDNPALDIDDDLPTFN
SALHLPPALGTQVPGTPPPKYNTLRLERAFSNQLTDTQMLDEL

>O15218 multipass membrane protein
MSVKPSWGPGPSEGVTAVPTSDLGEIHNWTELLDLFNHTLSECHVELSQSTKRVVLFALYL

AMFVVGLVENLLVICVNWRGSGRAGLMNLYILNMAIADLGIVLSLPVWMLEVTLDYTWL
WGSFSCRFTHYFYFVNMYSSIFFLVCLSVDRYVTLTSASPSWQRYQHRVRRAMCAGIWVL
SAIIPLPEVVHIQLVEGPEPMCLFMAPFETYSTWALAVALSTTILGFLLPFPLITVFNVLTACR
LRQPGQPKSRRHCLLLCAYVAVFVMCWLPYHVTLLLLTLHGTHISLHCHLVHLLYFFYDVI
DCFSMLHCVINPILYNFLSPHFRGRLLNAVVHYLPKDQTKAGTCASSSSCSTQHSIIITKGD
SQPAAAAPHPEPSLSFQAHHLLPNTSPISPTQPLTPS

>Q9Y698 multipass membrane protein
MGLFDRGVQMLLTTVGAFAAFSLMTIAVGTDYWLYSRGVCKTKSVSENETSKKNEEVMT
HSGLWRTCCLEGNFKGLCKQIDHFPEDADYEADTAEYFLRAVRASSIFPILSVILLFMGGLC
IAASEFYKTRHNIILSAGIFFVSAGLSNIIGIIVYISANAGDPSKSDSKKNSYSYGWSFYFGAL
SFIIAEMVGVLAVHMFIDRHKQLRATARATDYLQASAITRIPSYRYRYQRRSRSSSRSTEPS
HSRDASPVGIKGFNTLPSTEISMYTLSRDPLKAATTPTATYNSDRDNSFLQVHNCIQKENK
DSLHSNTANRRTTPV

>Q8NGJ1 multipass membrane protein
MDQINHTNVKEFFFLELTRSRELEFFLFVVFFAVYVATVLGNALIVVTITCESRLHTPMYFL
LRNKSVLDIVFSSITVPKFLVDLLSDRKTISYNDCMAQIFFFHFAGGADIFFLSVMAYDRYL
AIAKPLHYVTMMRKEVWVALVVASWVSGGLHSIIQVILMLPFPFCGPNTLDAFYCYVLQV
VKLACTDTFALELFMISNNGLVTLLWFLLLLGSYTVILVMLRSHSGEGRNKALSTCTSHML
VVTLHFVPCVYIYCRPFMTLPMDTTISINNTVITPMLNPIIYSLRNQEMKSAMQRLQRRLG
PSESRKWG

>Q96DS6 multipass membrane protein
MTSQPISNETIIMLPSNVINFSQAEKPEPTNQGQDSLKKRLQAKVKVIGVHSSLAGSILSAL
SALVGFILLSVNPAALNPASLQCKLDEKDIPTRLLLSYDYHSPYTMDCHRAKASLAGTLSL
MLVSTVLEFCLAVLTAVLQWKQTV

>Q9GZW8 multipass membrane protein
MLLQSQTMGVSHSFTPKGITIPQREKPGHMYQNEDYLQNGLPTETTVLGTVQILCCLLISS
LGAILVFAPYPSHFNPAISTTLMSGYPFLGALCFGITGSLSIISGKQSTKPFDLSSLTSNAVSSV
TAGAGLFLLADSMVALRTASQHCGSEMDYLSSLPYSEYYYPIYEIKDCLLTSVSLTGVLVV
MLIFTVLELLLAAYSSVFWWKQLYSNNPGSSFSSTQSQDHIQQVKKSSSRSWI

>Q9H3V2 multipass membrane protein
MDSSTAHSPVFLVFPPEITASEYESTELSATTFSTQSPLQKLFARKMKILGTIQILFGIMTFSF
GVIFLFTLLKPYPRFPFIFLSGYPFWGSVLFINSGAFLIAVKRKTTETLIILSRIMNFLSALGAI
AGIILLTFGFILDQNYICGYSHQNSQCKAVTVLFLGILITLMTFSIIELFISLPFSILGCHSEDC
DCEQCC

>P16452 lipid-anchor
MGQALGIKSCDFQAARNNEEHHTKALSSRRLFVRRGQPFTIILYFRAPVRAFLPALKKVAL
TAQTGEQPSKINRTQATFPISSLGDRKWWSAVVEERDAQSWTISVTTPADAVIGHYSLLLQ
VSGRKQLLLGQFTLLFNPWNREDAVFLKNEAQRMEYLLNQNGLIYLGTADCIQAESWDF

GQFEGDVIDLSLRLLSKDKQVEKWSQPVHVARVLGALLHFLKEQRVLPTPQTQATQEGAL
LNKRRGSVPILRQWLTGRGRPVYDGQAWVLAAVACTVLRCLGIPARVVTTFASAQGTGGR
LLIDEYYNEEGLQNGEGQRGRIWIFQTSTECWMTRPALPQGYDGWQILHPSAPNGGGVL
GSCDLVPVRAVKEGTLGLTPAVSDLFAAINASCVVWKCCEDGTLELTDSNTKYVGNNISTK
GVGSDRCEDITQNYKYPEGSLQEKEVLERVEKEKMEREKDNGIRPPSLETASPLYLLLKAP
SSLPLRGDAQISVTLVNHSEQEKAVQLAIGVQAVHYNGVLAAKLWRKKLHLTLSANLEKII
TIGLFFSNFERNPPENTFLRLTAMATHSESNLSCFAQEDIAICRPHLAIKMPEKAEQYQPLTA
SVSLQNSLDAPMEDCVISILGRGLIHRERSYRFRSVWPENTMCAKFQFTPTHVGLQRLTVE
VDCNMFQNLTNYKSVTVVAPELSA

>Q9BY19 multipass membrane protein
MNSMTSAVPVANSVLVVAPHNGYPVTPGIMSHVPLYPNSQPQVHLVPGNPPSLVSNVNGQ
PVQKALKEGKTLGAIQIIIGLAHIGLGSIMATVLVGEYLSISFYGGFPFWGGLWFIISGSLSV
AAENQPYSYCLLSGSLGLNIVSAICSAVGVILFITDLSIPHPYAYPDYYPYAWGVNPGMAIS
GVLLVFCLLEFGIACASSHFGCQLVCCQSSNVSVIYPNIYAANPVITPEPVTSPPSYSSEIQA
NK

>Q9H8T0 peripheral membrane protein
MNPFWSMSTSSVRKRSEGEEKTLTGDVKTSPPRTAPKKQLPSIPKNALPITKPTSPAPAAQS
TNGTHASYGPFYLEYSLLAEFTLVVKQKLPGVYVQPSYRSALMWFGVIFIRHGLYQDGVF
KFTVYIPDNYPDGDCPRLVFDIPVFHPLVDPTSGELDVKRAFAKWRRNHNHIWQVLMYAR
RVFYKIDTASPLNPEAAVLYEKDIQLFKSKVVDSVKVCTARLFDQPKIEDPYAISFSPWNPS
VHDEAREKMLTQKKPEEQHNKSVHVAGLSWVKPGSVQPFSKEEKTVAT

>Q15011 multipass membrane protein
MESETEPEPVTLLVKSPNQRHRDLELSGDRGWSVGHLKAHLSRVYPERPRPEDQRLIYSG
KLLLDHQCLRDLLPKQEKRHVLHLVCNVKSPSKMPEINAKVAESTEEPAGSNRGQYPEDS
SSDGLRQREVLRNLSSPGWENISRPEAAQQAFQGLGPGFSGYTPYGWLQLSWFQQIYARQ
YYMQYLAATAASGAFVPPPSAQEIPVVSAPAPAPIHNQFPAENQPANQNAAPQVVVNPGA
NQNLRMNAQGGPIVEEDDEINRDWLDWTYSAATFSVFLSILYFYSSLSRFLMVMGATVV
MYLHHVGWFPFRPRPVQNFPNDGPPPDVVNQDPNNNLQEGTDPETEDPNHLPPDRDVLD
GEQTSPSFMSTAWLVFKTFFASLLPEGPPAIAN

>O14610 lipid-anchor
MAQDLSEKDLLKMEVEQLKKEVKNTRIPISKAGKEIKEYVEAQAGNDPFLKGIPEDKNPF
KEKGGCLIS

>Q9Y3A0 peripheral membrane protein
MATLLRPVLRRLCGLPGLQRPAAEMPLRARSDGAGPLYSHHLPTSPLQKGLLAAGSAAM
ALYNPYRHDMVAVLGETTGHRTLKVLRDQMRRDPEGAQILQERPRISTSTLDLGKLQSLP
EGSLGREYLRFLDVNRVSPDTRAPTRFVDDEELAYVIQRYREVHDMLHTLLGMPTNILGEI
VVKWFEAVQTGLPMCILGAFFGPIRLGAQSLQVLVSELIPWAVQNGRRAPCVLNLYYERR
WEQSLRALREELGITAPPMHVQGLA

>O94759 multipass membrane protein
MEPSALRKAGSEQEEGFEGLPRRVTDLGMVSNLRRSNSSLFKSWRLQCPFGNNDKQESLS
SWIPENIKKKECVYFVESSKLSDAGKVVCQCGYTHEQHLEEATKPHTFQGTQWDPKKHV
QEMPTDAFGDIVFTGLSQKVKKYVRVSQDTPSSVIYHLMTQHWGLDVPNLLISVTGGAK
NFNMKPRLKSIFRRGLVKVAQTTGAWIITGGSHTGVMKQVGEAVRDFSLSSSYKEGELITI
GVATWGTVHRREGLIHPTGSFPAEYILDEDGQGNLTCLDSNHSHFILVDDGTHGQYGVEIP
LRTRLEKFISEQTKERGGVAIKIPIVCVVLEGGPGTLHTIDNATTNGTPCVVVEGSGRVADVI
AQVANLPVSDITISLIQQKLSVFFQEMFETFTESRIVEWTKKIQDIVRRRQLLTVFREGKDG
QQDVDVAILQALLKASRSQDHFGHENWDHQLKLAVAWNRVDIARSEIFMDEWQWKPSD
LHPTMTAALISNKPEFVKLFLENGVQLKEFVTWDTLLYLYENLDPSCLFHSKLQKVLVEDP
ERPACAPAAPRLQMHHVAQVLRELLGDFTQPLYPRPRHNDRLRLLLPVPHVKLNVQGVSL
RSLYKRSSGHVTFTMDPIRDLLIWAIVQNRRELAGIIWAQSQDCIAAALACSKILKELSKEE
EDTDSSEEMLALAEEYEHRAIGVFTECYRKDEERAQKLLTRVSEAWGKTTCLQLALEAKD
MKFVSHGGIQAFLTKVWWGQLSVDNGLWRVTLCMLAFPLLLTGLISFREKRLQDVGTPA
ARARAFFTAPVVVFHLNILSYFAFLCLFAYVLMVDFQPVPSWCECAIYLWLFSLVCEEMRQ
LFYDPDECGLMKKAALYFSDFWNKLDVGAILLFVAGLTCRLIPATLYPGRVILSLDFILFCL
RLMHIFTISKTLGPKIIIVKRMMKDVFFFLFLLAVWVVSFGVAKQAILIHNERRVDWLFRGA
VYHSYLTIFGQIPGYIDGVNFNPEHCSPNGTDPYKPKCPESDATQQRPAFPEWLTVLLLCLY
LLFTNILLLNLLIAMFNYTFQQVQEHTDQIWKFQRHDLIEEYHGRPAAPPPFILLSHLQLFIK
RVVLKTPAKRHKQLKNKLEKNEEAALLSWEIYLKENYLQNRQFQQKQRPEQKIEDISNKV
DAMVDLLDLDPLKRSGSMEQRLASLEEQVAQTAQALHWIVRTLRASGFSSEADVPTLAS
QKAAEEPDAEPGGRKKTEEPGDSYHVNARHLLYPNCPVTRFPVPNEKVPWETEFLIYDPP
FYTAERKDAAAMDPMGDTLEPLSTIQYNVVDGLRDRRSFHGPYTVQAGLPLNPMGRTGL
RGRGSLSCFGPNHTLYPMVTRWRRNEDGAICRKSIKKMLEVLVVKLPLSEHWALPGGSRE
PGEMLPRKLKRILRQEHWPSFENLLKCGMEVYKGYMDDPRNTDNAWIETVAVSVHFQDQ
NDVELNRLNSNLHACDSGASIRWQVVDRRIPLYANHKTLLQKAAAEFGAHY

>P12110 peripheral membrane protein
MLQGTCSVLLLWGILGAIQAQQQEVISPDTTERNNNCPEKTDCPIHVYFVLDTSESVTMQS
PTDILLFHMKQFVPQFISQLQNEFYLDQVALSWRYGGLHFSDQVEVFSPPGSDRASFIKNL
QGISSFRRGTFTDCALANMTEQIRQDRSKGTVHFAVVITDGHVTGSPCGGIKLQAERAREE
GIRLFAVAPNQNLKEQGLRDIASTPHELYRNDYATMLPDSTEIDQDTINRIIKVMKHEAYGE
CYKVSCLEIPGPSGPKGYRGQKGAKGNMGEPGEPGQKGRQGDPGIEGPIGFPGPKGVPGF
KGEKGEFGADGRKGAPGLAGKNGTDGQKGKLGRIGPPGCKGDPGNRGPDGYPGEAGSP
GERGDQGGKGDPGRPGRRGPPGEIGAKGSKGYQGNSGAPGSPGVKGAKGGPGPRGPKG
EPGRRGDPGTKGSPGSDGPKGEKGDPGPEGPRGLAGEVGNKGAKGDRGLPGPRGPQGAL
GEPGKQGSRGDPGDAGPRGDSGQPGPKGDPGRPGFSYPGPRGAPGEKGEPGPRGPEGGR
GDFGLKGEPGRKGEKGEPADPGPPGEPGPRGPRGVPGPEGEPGPPGDPGLTECDVMTYVR
ETCGCCDCEKRCGALDVVFVIDSSESIGYTNFTLEKNFVINVVNRLGAIAKDPKSETGTRV
GVVQYSHEGTFEAIQLDDERIDSLSSFKEAVKNLEWIAGGTWTPSALKFAYDRLIKESRRQ
KTRVFAVVITDGRHDPRDDDLNLRALCDRDVTVTAIGIGDMFHEKHESENLYSIACDKPQ
QVRNMTLFSDLVAEKFIDDMEDVLCPDPQIVCPDLPCQTELSVAQCTQRPVDIVFLLDGSE
RLGEQNFHKARRFVEQVARRLTLARRDDDPLNARVALLQFGGPGEQQVAFPLSHNLTAIH
EALETTQYLNSFSHVGAGVVHAINAIVRSPRGGARRHAELSFVFLTDGVTGNDSLHESAH

SMRKQNVVPTVLALGSDVDMDVLTTLSLGDRAAVFHEKDYDSLAQPGFFDRFIRWIC

>Q9NZH0 multipass membrane protein
MFVASERKMRAHQVLTFLLLFVITSVASENASTSRGCGLDLLPQYVSLCDLDAIWGIVVEA
VAGAGALITLLLMLILLVRLPFIKEKEKKSPVGLHFLFLLGTLGLFGLTFAFIIQEDETICSVR
RFLWGVLFALCFSCLLSQAWRVRRLVRHGTGPAGWQLVGLALCLMLVQVIIAVEWLVLTV
LRDTRPACAYEPMDFVMALIYDMVLLVVTLGLALFTLCGKFKRWKLNGAFLLITAFLSVL
IWVAWMTMYLFGNVKLQQGDAWNDPTLAITLAASGWVFVIFHAIPEIHCTLLPALQENTP
NYFDTSQPRMRETAFEEDVQLPRAYMENKAFSMDEHNAALRTAGFPNGSLGKRPSGSLG
KRPSAPFRSNVYQPTEMAVVLNGGTIPTAPPSHTGRHLW

>P04234 single-pass type I membrane protein
MEHSTFLSGLVLATLLSQVSPFKIPIEELEDRVFVNCNTSITWVEGTVGTLLSDITRLDLGKR
ILDPRGIYRCNGTDIYKDKESTVQVHYRMCQSCVELDPATVAGIIVTDVIATLLLALGVFCF
AGHETGRLSGAADTQALLRNDQVYQPLRDRDDAQYSHLGGNWARNK

>Q13586 single-pass type I membrane protein
MDVCVRLALWLLWGLLLHQGQSLSHSHSEKATGTSSGANSEESTAAEFCRIDKPLCHSED
EKLSFEAVRNIHKLMDDDANGDVDVEESDEFLREDLNYHDPTVKHSTFHGEDKLISVEDL
WKAWKSSEVYNWTVDEVVQWLITYVELPQYEETFRKLQLSGHAMPRLAVTNTTMTGTV
LKMTDRSHRQKLQLKALDTVLFGPPLLTRHNHLKDFMLVVSIVIGVGGCWFAYIQNRYSK
EHMKKMMKDLEGLHRAEQSLHDLQERLHKAQEEHRTVEVEKVHLEKKLRDEINLAKQE
AQRLKELREGTENERSRQKYAEEELEQVREALRKAEKELESHSSWYAPEALQKWLQLTH
EVEVQYYNIKKQNAEKQLLVAKEGAEKIKKKRNTLFGTFHVAHSSSLDDVDHKILTAKQA
LSEVTAALRERLHRWQQIEILCGFQIVNNPGIHSLVAALNIDPSWMGSTRPNPAHFIMTDDV
DDMDEEIVSPLSMQSPSLQSSVRQRLTEPQHGLGSQRDLTHSDSESSLHMSDRQRVAPKPP
QMSRAADEALNAMTSNGSHRLIEGVHPGSLVEKLPDSPALAKKALLALNHGLDKAHSLM
ELSPSAPPGGSPHLDSSRSHSPSSPDPDTPSPVGDSRALQASRNTRIPHLAGKKAVAEEDNG
SIGEETDSSPGRKKFPLKIFKKPLKK

>Q9H339 multipass membrane protein
MSSSGSSHPFLLTGFPGLEEAHHWISVFFLFMYISILFGNGTLLLLIKEDHNLHEPMYFFLA
MLAATDLGLALTTMPTVLGVLWLDHREIGSAACFSQAYFIHSLSFLESGILLAMAYDRFIAI
CNPLRYTSVLTNTRVVKIGLGVLMRGFVSVVPPIRPLYFFLYCHSHVLSHAFCLHQDVIKL
ACADTTFNRLYPAVLVVFIFVLDYLIIFISYVLILKTVLSIASREERAKALITCVSHICCVLVF
YVTVIGLSLIHRFGKQVPHIVHLIMSYAYFLFPPLMNPITYSVKTKQIQNAILHLFTTHRIGT

>Q8NH59 multipass membrane protein
MSQVTNTTQEGIYFILTDIPGFEASHIWISIPVCCLYTISIMGNTTILTVIRTEPSVHQRMYLFL
SMLALTDLGLTLTTLPTVMQLLWFNVRRISSEACFAQFFFLHGFSFMESSVLLAMSVDCYV
AICCPLHYASILTNEVIGRTGLAIICCCVLAVLPSLFLLKRLPFCHSHLLSRSYCLHQDMIRLV
CADIRLNSWYGFALALLIIIVDPLLIVISYTLILKNILGTATWAERLRALNNCLSHILAVLVLY
IPMVGVSMTHRFAKHASPLVHVIMANIYLLAPPVMNPIIYSVKNKQIQWGMLNFLSLKNM
HSR

>Q9UK08 lipid-anchor
MSNNMAKIAEARKTVEQLKLEVNIDRMKVSQAAAELLAFCETHAKDDPLVTPVPAAENP
FRDKRLFCVLL

>P22748 GPI-anchor, lipid-anchor
MRMLLALLALSAARPSASAESHWCYEVQAESSNYPCLVPVKWGGNCQKDRQSPINIVTT
KAKVDKKLGRFFFSGYDKKQTWTVQNNGHSVMMLLENKASISGGGLPAPYQAKQLHLH
WSDLPYKGSEHSLDGEHFAMEMHIVHEKEKGTSRNVKEAQDPEDEIAVLAFLVEAGTQV
NEGFQPLVEALSNIPKPEMSTTMAESSLLDLLPKEEKLRHYFRYLGSLTTPTCDEKVVWTV
FREPIQLHREQILAFSQKLYYDKEQTVSMKDNVRPLQQLGQRTVIKSGAPGRPLPWALPAL
LGPMLACLLAGFLR

>P61006 lipid-anchor
MAKTYDYLFKLLLIGDSGVGKTCVLFRFSEDAFNSTFISTIGIDFKIRTIELDGKRIKLQIWD
TAGQERFRTITTAYYRGAMGIMLVYDITNEKSFDNIRNWIRNIEEHASADVEKMILGNKCD
VNDKRQVSKERGEKLALDYGIKFMETSAKANINVENAFFTLARDIKAKMDKKLEGNSPQ
GSNQGVKITPDQQKRSSFFRCVLL

>Q92508 multipass membrane protein
MEPHVLGAVLYWLLLPCALLAACLLRFSGLSLVYLLFLLLLPWFPGPTRCGLQGHTGRLL
RALLGLSLLFLVAHLALQICLHIVPRLDQLLGPSCSRWETLSRHIGVTRLDLKDIPNAIRLVA
PDLGILVVSSVCLGICGRLARNTRQSPHPRELDDDERDVDASPTAGLQEAATLAPTRRSRL
AARFRVTAHWLLVAAGRVLAVTLLALAGIAHPSALSSVYLLLFLALCTWWACHFPISTRGF
SRLCVAVGCFGAGHLICLYCYQMPLAQALLPPAGIWARVLGLKDFVGPTNCSSPHALVLNT
GLDWPVYASPGVLLLLCYATASLRKLRAYRPSGQRKEAAKGYEARELELAELDQWPQER
ESDQHVVPTAPDTEADNCIVHELTGQSSVLRRPVRPKRAEPREASPLHSLGHLIMDQSYVC
ALIAMMVWSITYHSWLTFVLLLWACLIWTVRSRHQLAMLCSPCILLYGMTLCCLRYVWA
MDLRPELPTTLGPVSLRQLGLEHTRYPCLDLGAMLLYTLTFWLLLRQFVKEKLLKWAESP
AALTEVTVADTEPTRTQTLLQSLGELVKGVYAKYWIYVCAGMFIVVSFAGRLVVYKIVYM
FLFLLCLTLFQVYYSLWRKLLKAFWWLVVAYTMLVLIAVYTFQFQDFPAYWRNLTGFTDE
QLGDLGLEQFSVSELFSSILVPGFFLLACILQLHYFHRPFMQLTDMEHVSLPGTRLPRWAHR
QDAVSGTPLLREEQQEHQQQQQEEEEEEEDSRDEGLGVATPHQATQVPEGAAKWGLVAE
RLLELAAGFSDVLSRVQVFLRRLLELHVFKLVALYTVWVALKEVSVMNLLLVVLWAFALP
YPRFRPMASCLSTVWTCVIIVCKMLYQLKVVNPQEYSSNCTEPFPNSTNLLPTEISQSLLYR
GPVDPANWFGVRKGFPNLGYIQNHLQVLLLLVFEAIVYRRQEHYRRQHQLAPLPAQAVFA
SGTRQQLDQDLLGCLKYFINFFFYKFGLEICFLMAVNVIGQRMNFLVTLHGCWLVAILTRR
HRQAIARLWPNYCLFLALFLLYQYLLCLGMPPALCIDYPWRWSRAVPMNSALIKWLYLPD
FFRAPNSTNLISDFLLLLCASQQWQVFSAERTEEWQRMAGVNTDRLEPLRGEPNPVPNFIH
CRSYLDMLKVAVFRYLFWLVLVVVFVTGATRISIFGLGYLLACFYLLLFGTALLQRDTRAR
LVLWDCLILYNVTVIISKNMLSLLACVFVEQMQTGFCWVIQLFSLVCTVKGYYDPKEMM
DRDQDCLLPVEEAGIIWDSVCFFFLLLQRRVFLSHYYLHVRADLQATALLASRGFALYNA
ANLKSIDFHRRIEEKSLAQLKRQMERIRAKQEKHRQGRVDRSRPQDTLGPKDPGLEPGPD
SPGGSSPPRRQWWRPWLDHATVIHSGDYFLFESDSEEEEEAVPEDPRPSAQSAFQLAYQA

WVTNAQAVLRRRQQEQEQARQEQAGQLPTGGGPSQEVEPAEGPEEAAAGRSHVVQRVL
STAQFLWMLGQALVDELTRWLQEFTRHHGTMSDVLRAERYLLTQELLQGGEVHRGVLD
QLYTSQAEATLPGPTEAPNAPSTVSSGLGAEEPLSSMTDDMGSPLSTGYHTRSGSEEAVTD
PGEREAGASLYQGLMRTASELLLDRRLRIPELEEAELFAEGQGRALRLLRAVYQCVAAHSE
LLCYFIIILNHMVTASAGSLVLPVLVFLWAMLSIPRPSKRFWMTAIVFTEIAVVVKYLFQFGF
FPWNSHVVLRRYENKPYFPPRILGLEKTDGYIKYDLVQLMALFFHRSQLLCYGLWDHEED
SPSKEHDKSGEEEQGAEEGPGVPAATTEDHIQVEARVGPTDGTPEPQVELRPRDTRRISLRF
RRRKKEGPARKGAAAIEAEDREEEEGEEEKEAPTGREKRPSRSGGRVRAAGRRLQGFCLS
LAQGTYRPLRRFFHDILHTKYRAATDVYALMFLADVVDFIIIIFGFWAFGKHSAATDITSSL
SDDQVPEAFLVMLLIQFSTMVVDRALYLRKTVLGKLAFQVALVLAIHLWMFFILPAVTER
MFNQNVVAQLWYFVKCIYFALSAYQIRCGYPTRILGNFLTKKYNHLNLFLFQGFRLVPFLV
ELRAVMDWVWTDTTLSLSSWMCVEDIYANIFIIKCSRETEKKYPQPKGQKKKKIVKYGM
GGLIILFLIAIIWFPLLFMSLVRSVVGVVNQPIDVTVTLKLGGYEPLFTMSAQQPSIIPFTAQA
YEELSRQFDPQPLAMQFISQYSPEDIVTAQIEGSSGALWRISPPSRAQMKRELYNGTADITL
RFTWNFQRDLAKGGTVEYANEKHMLALAPNSTARRQLASLLEGTSDQSVVIPNLFPKYIR
APNGPEANPVKQLQPNEEADYLGVRIQLRREQGAGATGFLEWWVIELQECRTDCNLLPM
VIFSDKVSPPSLGFLAGYGIMGLYVSIVLVIGKFVRGFFSEISHSIMFEELPCVDRILKLCQDI
FLVRETRELELEEELYAKLIFLYRSPETMIKWTREKE

>Q8NBR0 single-pass type I membrane protein
MAPPPPSPQLLLLAALARLLGPSEVMAGPAEEAGAHCPESLWPLPPQVSPRVTYTRVSPGQ
AEDVTFLYHPCAHPWLKLQLALLAYACMANPSLTPDFSLTQDRPLVLTAWGLALEMAWV
EPAWAAHWLMRRRRRKQRKKKAWIYCESLSGPAPSEPTPGRGRLCRRGCVQALALAFAL
RSWRPPGTEVTSQGPRQPSSSGAKRRRLRAALGPQPTRSALRFPSASPGSLKAKQSMAGIP
GRESNAPSVPTVSLLPGAPGGNASSRTEAQVPNGQGSPGGCVCSSQASPAPRAAAPPRAA
RGPTPRTEEAAWAAMALTFLLVLLTLATLCTRLHRNFRRGESIYWGPTADSQDTVAAVLKR
RLLQPSRRVKRSRRRPLLPPTPDSGPEGESSE

>Q8TAI7 lipid-anchor
MPLVRYRKVVILGYRCVGKTSLAHQFVEGEFSEGYDPTVENTYSKIVTLGKDEFHLHLVD
TAGQDEYSILPYSFIIGVHGYVLVYSVTSLHSFQVIESLYQKLHEGHGKTRVPVVLVGNKA
DLSPEREVQAVEGKKLAESWGATFMESSARENQLTQGIFTKVIQEIARVENSYGQERRCHL
M

>P11509 peripheral membrane protein
MLASGMLLVALLVCLTVMVLMSVWQQRKSKGKLPPGPTPLPFIGNYLQLNTEQMYNSLM
KISERYGPVFTIHLGPRRVVVLCGHDAVREALVDQAEEFSGRGEQATFDWVFKGYGVVFS
NGERAKQLRRFSIATLRDFGVGKRGIEERIQEEAGFLIDALRGTGGANIDPTFFLSRTVSNVI
SSIVFGDRFDYKDKEFLSLLRMMLGIFQFTSTSTGQLYEMFSSVMKHLPGPQQQAFQLLQ
GLEDFIAKKVEHNQRTLDPNSPRDFIDSFLIRMQEEEKNPNTEFYLKNLVMTTLNLFIGGTE
TVSTTLRYGFLLLMKHPEVEAKVHEEIDRVIGKNRQPKFEDRAKMPYMEAVIHEIQRFGD
VIPMSLARRVKKDTKFRDFFLPKGTEVFPMLGSVLRDPSFFSNPQDFNPQHFLNEKGQFKK
SDAFVPFSIGKRNCFGEGLARMELFLFFTTVMQNFRLKSSQPKDIDVSPKHVGFATIPRNY
TMSFLPR

>P30530 single-pass type I membrane protein
MAWRCPRMGRVPLAWCLALCGWACMAPRGTQAEESPFVGNPGNITGARGLTGTLRCQL
QVQGEPPEVHWLRDGQILELADSTQTQVPLGEDEQDDWIVVSQLRITSLQLSDTGQYQCL
VFLGHQTFVSQPGYVGLEGLPYFLEEPEDRTVAANTPFNLSCQAQGPPEPVDLLWLQDAV
PLATAPGHGPQRSLHVPGLNKTSSFSCEAHNAKGVTTSRTATITVLPQQPRNLHLVSRQPTE
LEVAWTPGLSGIYPLTHCTLQAVLSNDGMGIQAGEPDPPEEPLTSQASVPPHQLRLGSLHPH
TPYHIRVACTSSQGPSSWTHWLPVETPEGVPLGPPENISATRNGSQAFVHWQEPRAPLQGT
LLGYRLAYQGQDTPEVLMDIGLRQEVTLELQGDGSVSNLTVCVAAYTAAGDGPWSLPVP
LEAWRPGQAQPVHQLVKEPSTPAFSWPWWYVLLGAVVAAACVLILALFLVHRRKKETRY
GEVFEPTVERGELVVRYRVRKSYSRRTTEATLNSLGISEELKEKLRDVMVDRHKVALGKT
LGEGEFGAVMEGQLNQDDSILKVAVKTMKIAICTRSELEDFLSEAVCMKEFDHPNVMRLI
GVCFQGSERESFPAPVVILPFMKHGDLHSFLLYSRLGDQPVYLPTQMLVKFMADIASGME
YLSTKRFIHRDLAARNCMLNENMSVCVADFGLSKKIYNGDYYRQGRIAKMPVKWIAIESL
ADRVYTSKSDVWSFGVTMWEIATRGQTPYPGVENSEIYDYLRQGNRLKQPADCLDGLYA
LMSRCWELNPQDRPSFTELREDLENTLKALPPAQEPDEILYVNMDEGGGYPEPPGAAGGA
DPPTQPDPKDSCSCLTAAEVHPAGRYVLCPSTTPSPAQPADRGSPAAPGQEDGA

>A6NI73 single-pass type I membrane protein
MAPWSHPSAQLQPVGGDAVSPALMVLLCLGLSLGPRTHVQAGNLSKATLWAEPGSVISRG
NSVTIRCQGTLEAQEYRLVKEGSPEPWDTQNPLEPKNKARFSIPSMTEHHAGRYRCYYYS
PAGWSEPSDPLELVVTGFYNKPTLSALPSPVVTSGENVTLQCGSRLRFDRFILTEEGDHKLS
WTLDSQLTPSGQFQALFPVGPVTPSHRWMLRCYGSRRHILQVWSEPSDLLEIPVSGAADN
LSPSQNKSDSGTASHLQDYAVENLIRMGMAGLILVVLGILIFQDWHSQRSPQAAAGR

>O43653 GPI-anchor, lipid-anchor
MKAVLLALLMAGLALQPGTALLCYSCKAQVSNEDCLQVENCTQLGEQCWTARIRAVGLL
TVISKGCSLNCVDDSQDYYVGKKNITCCDTDLCNASGAHALQPAAAILALLPALGLLLWG
PGQL

>Q14210 GPI-anchor, lipid-anchor
MRTALLLLAALAVATGPALTLRCHVCTSSSNCKHSVVCPASSRFCKTTNTVEPLRGNLVKK
DCAESCTPSYTLQGQVSSGTSSTQCCQEDLCNEKLHNAAPTRTALAHSALSLGLALSLLAV
ILAPSL

>Q96BF3 single-pass type I membrane protein
MGSPGMVLGLLVQIWALQEASSLSVQQGPNLLQVRQGSQATLVCQVDQATAWERLRVK
WTKDGAILCQPYITNGSLSLGVCGPQGRLSWQAPSHLTLQLDPVSLNHSGAYVCWAAVEI
PELEEAEGNITRLFVDPDDPTQNRNRIASFPGFLFVLLGVGSMGVAAIVWGAWFWGRRSC
QQRDSGNSPGNAFYSNVLYRPRGAPKKSEDCSGEGKDQRGQSIYSTSFPQPAPRQPHLASR
PCPSPRPCPSPRPGHPVSMVRVSPRPSPTQQPRPKGFPKVGEE

>Q9H3T3 single-pass type I membrane protein
MQTPRASPPRPALLLLLLLLGGAHGLFPEEPPPLSVAPRDYLNHYPVFVGSGPGRLTPAEG

ADDLNIQRVLRVNRTLFIGDRDNLYRVELEPPTSTELRYQRKLTWRSNPSDINVCRMKGKQ
EGECRNFVKVLLLRDESTLFVCGSNAFNPVCANYSIDTLQPVGDNISGMARCPYDPKHAN
VALFSDGMLFTATVTDFLAIDAVIYRSLGDRPTLRTVKHDSKWFKEPYFVHAVEWGSHVY
FFFREIAMEFNYLEKVVVSRVARVCKNDVGGSPRVLEKQWTSFLKARLNCSVPGDSHFYF
NVLQAVTGVVSLGGRPVVLAVFSTPSNSIPGSAVCAFDLTQVAAVFEGRFREQKSPESIWTP
VPEDQVPRPRPGCCAAPGMQYNASSALPDDILNFVKTHPLMDEAVPSLGHAPWILRTLMR
HQLTRVAVDVGAGPWGNQTVVFLGSEAGTVLKFLVRPNASTSGTSGLSVFLEEFETYRPD
RCGRPGGGETGQRLLSLELDAASGGLLAAFPRCVVRVPVARCQQYSGCMKNCIGSQDPY
CGWAPDGSCIFLSPGTRAAFEQDVSGASTSGLGDCTGLLRASLSEDRAGLVSVNLLVTSSV
AAFVVGAVVSGFSVGWFVGLRERRELARRKDKEAILAHGAGEAVLSVSRLGERRAQGPG
GRGGGGGGGAGVPPEALLAPLMQNGWAKATLLQGGPHDLDSGLLPTPEQTPLPQKRLPT
PHPHPHALGPRAWDHGHPLLPASASSSLLLLAPARAPEQPPAPGEPTPDGRLYAARPGRAS
HGDFPLTPHASPDRRRVVSAPTGPLDPASAADGLPRPWSPPPTGSLRRPLGPHAPPAATLRR
THTFNSGEARPGDRHRGCHARPGTDLAHLLPYGGADRTAPPVP

>Q6P5W5 multipass membrane protein
MASLVSLELGLLLAVLVVTATASPPAGLLSLLTSGQGALDQEALGGLLNTLADRVHCANGP
CGKCLSVEDALGLGEPEGSGLPPGPVLEARYVARLSAAAVLYLSNPEGTCEDARAGLWAS
HADHLLALLESPKALTPGLSWLLQRMQARAAGQTPKTACVDIPQLLEEAVGAGAPGSAG
GVLAALLDHVRSGSCFHALPSPQYFVDFVFQQHSSEVPMTLAELSALMQRLGVGREAHS
DHSHRHRGASSRDPVPLISSSNSSSVWDTVCLSARDVMAAYGLSEQAGVTPEAWAQLSPA
LLQQQLSGACTSQSRPPVQDQLSQSERYLYGSLATLLICLCAVFGLLLLTCTGCRGVTHYIL
QTFLSLAVGALTGDAVLHLTPKVLGLHTHSEEGLSPQPTWRLLAMLAGLYAFFLFENLFNL
LLPRDPEDLEDGPCGHSSHSHGGHSHGVSLQLAPSELRQPKPPHEGSRADLVAEESPELLN
PEPRRLSPELRLLPYMITLGDAVHNFADGLAVGAAFASSWKTGLATSLAVFCHELPHELGD
FAALLHAGLSVRQALLLNLASALTAFAGLYVALAVGVSEESEAWILAVATGLFLYVALCDM
LPAMLKVRDPRPWLLFLLHNVGLLGGWTVLLLLSLYEDDITF

>Q8NET8 multipass membrane protein
MKAHPKEMVPLMGKRVAAPSGNPAILPEKRPAEITPTKKSAHFFLEIEGFEPNPTVAKTSPP
VFSKPMDSNIRQCISGNCDDMDSPQSPQDDVTETPSNPNSPSAQLAKEEQRRKKRRLKKRI
FAAVSEGCVEELVELLVELQELCRRRHDEDVPDFLMHKLTASDTGKTCLMKALLNINPNT
KEIVRILLAFAEENDILGRFINAEYTEEAYEGQTALNIAIERRQGDIAALLIAAGADVNAHA
KGAFFNPKYQHEGFYFGETPLALAACTNQPEIVQLLMEHEQTDITSRDSRGNNILHALVTV
AEDFKTQNDFVKRMYDMILLRSGNWELETTRNNDGLTPLQLAAKMGKAEILKYILSREIK
EKRLRSLSRKFTDWAYGPVSSSLYDLTNVDTTTDNSVLEITVYNTNIDNRHEMLTLEPLHT
LLHMKWKKFAKHMFFLSFCFYFFYNITLTLVSYYRPREEEAIPHPLALTHKMGWLQLLGR
MFVLIWAMCISVKEGIAIFLLRPSDLQSILSDAWFHFVFFIQAVLVILSVFLYLFAYKEYLAC
LVLAMALGWANMLYYTRGFQSMGMYSVMIQKVILHDVLKFLFVYIVFLLGFGVALASLI
EKCPKDNKDCSSYGSFSDAVLELFKLTIGLGDLNIQQNSKYPILFLFLLITYVILTFVLLLNM
LIALMGETVENVSKESERIWRLQRARTILEFEKMLPEWLRSRFRMGELCKVAEDDFRLCL
RINEVKWTEWKTHVSFLNEDPGPVRRTDFNKIQDSSRNNSKTTLNAFEEVEEFPETSV

>Q9BV35 multipass membrane protein

MRGSPGDAERRQRWGRLFEELDSNKDGRVDVHELRQGLARLGGGNPDPGAQQGISSEG
DADPDGGLDLEEFSRYLQEREQRLLLMFHSLDRNQDGHIDVSEIQQSFRALGISISLEQAE
KILHSMDRDGTMTIDWQEWRDHFLLHSLENVEDVLYFWKHSTVLDIGECLTVPDEFSKQ
EKLTGMWWKQLVAGAVAGAVSRTGTAPLDRLKVFMQVHASKTNRLNILGGLRSMVLEG
GIRSLWRGNGINVLKIAPESAIKFMAYEQIKRAILGQQETLHVQERFVAGSLAGATAQTIIY
PMEVLKTRLTLRRTGQYKGLLDCARRILEREGPRAFYRGYLPNVLGIIPYAGIDLAVYETL
KNWWLQQYSHDSADPGILVLLACGTISSTCGQIASYPLALVRTRMQAQASIEGGPQLSML
GLLRHILSQEGMRGLYRGIAPNFMKVIPAVSISYVVYENMKQALGVTSR

>O95182 peripheral membrane protein
MASATRLIQRLRNWASGHDLQGKLQLRYQEISKRTQPPPKLPVGPSHKLSNNYYCTRDGR
RESVPPSIIMSSQKALVSGKPAESSAVAATEKKAVTPAPPIKRWELSSDQPYL

>O75908 multipass membrane protein
MEPGGARLRLQRTEGLGGERERQPCGDGNTETHRAPDLVQWTRHMEAVKAQLLEQAQG
QLRELLDRAMREAIQSYPSQDKPLPPPPPGSLSRTQEPSLGKQKVFIIRKSLLDELMEVQHF
RTIYHMFIAGLCVFIISTLAIDFIDEGRLLLEFDLLIFSFGQLPLALVTWVPMFLSTLLAPYQA
LRLWARGTWTQATGLGCALLAAHAVVLCALPVHVAVEHQLPPASRCVLVFEQVRFLMKS
YSFLREAVPGTLRARRGEGIQAPSFSSYLYFLFCPTLIYRETYPRTPYVRWNYVAKNFAQAL
GCVLYACFILGRLCVPVFANMSREPFSTRALVLSILHATLPGIFMLLLIFFAFLHCWLNAFAE
MLRFGDRMFYRDWWNSTSFSNYYRTWNVVVHDWLYSYVYQDGLRLLGARARGVAML
GVFLVSAVAHEYIFCFVLGFFYPVMLILFLVIGGMLNFMMHDQRTGPAWNVLMWTMLFL
GQGIQVSLYCQEWYARRHCPLPQATFWGLVTPRSWSCHT

>Q13606 multipass membrane protein
MEFTDRNYTLVTEFILLGFPTRPELQIVLFLMFLTLYAIILIGNIGLMLLIRIDPHLQTPMYFFL
SNLSFVDLCYFSDIVPKMLVNFLSENKSISYYGCALQFYFFCTFADTESFILAAMAYDRYVA
ICNPLLYTVVMSRGICMRLIVLSYLGGNMSSLVHTSFAFILKYCDKNVINHFFCDLPPLLKL
SCTDTTINEWLLSTYGSSVEIICFIIIIISYFFILLSVLKIRSFSGRKKTFSTCASHLTSVTIYQGT
LLFIYSRPSYLYSPNTDKIISVFYTIFIPVLNPLIYSLRNKDVKDAAEKVLRSKVDSS

>P22680 peripheral membrane protein
MMTTSLIWGIAIAACCCLWLILGIRRRQTGEPPLENGLIPYLGCALQFGANPLEFLRANQR
KHGHVFTCKLMGKYVHFITNPLSYHKVLCHGKYFDWKKFHFATSAKAFGHRSIDPMDGN
TTENINDTFIKTLQGHALNSLTESMMENLQRIMRPPVSSNSKTAAWVTEGMYSFCYRVMF
EAGYLTIFGRDLTRRDTQKAHILNNLDNFKQFDKVFPALVAGLPIHMFRTAHNAREKLAES
LRHENLQKRESISELISLRMFLNDTLSTFDDLEKAKTHLVVLWASQANTIPATFWSLFQMIR
NPEAMKAATEEVKRTLENAGQKVSLEGNPICLSQAELNDLPVLDSIIKESLRLSSASLNIRT
AKEDFTLHLEDGSYNIRKDDIIALYPQLMHLDPEIYPDPLTFKYDRYLDENGKTKTTFYCN
GLKLKYYYMPFGSGATICPGRLFAIHEIKQFLILMLSYFELELIEGQAKCPPLDQSRAGLGIL
PPLNDIEFKYKFKHL

>Q9NSA0 multipass membrane protein
MAFSKLLEQAGGVGLFQTLQVLTFILPCLMIPSQMLLENFSAAIPGHRCWTHMLDNGSAV

STNMTPKALLTISIPPGPNQGPHQCRRFRQPQWQLLDPNATATSWSEADTEPCVDGWVYD
RSVFTSTIVAKWDLVCSSQGLKPLSQSIFMSGILVGSFIWGLLSYRFGRKPMLSWCCLQLAV
AGTSTIFAPTFVIYCGLRFVAAFGMAGIFLSSLTLMVEWTTTSRRAVTMTVVGCAFSAGQA
ALGGLAFALRDWRTLQLAASVPFFAISLISWWLPESARWLIIKGKPDQALQELRKVARING
HKEAKNLTIEVLMSSVKEEVASAKEPRSVLDLFCVPVLRWRSCAMLVVNFSLLISYYGLVF
DLQSLGRDIFLLQALFGAVDFLGRATTALLLSFLGRRTIQAGSQAMAGLAILANMLVPQDL
QTLRVVFAVLGKGCFGISLTCLTIYKAELFPTPVRMTADGILHTVGRLGAMMGPLILMSRQ
ALPLLPPLLYGVISIASSLVVLFFLPETQGLPLPDTIQDLESQKSTAAQGNRQEAVTVESTSL

>Q6AZY7 single-pass type II membrane protein
MKVRSAGGDGDALCVTEEDLAGDDEDMPTFPCTQKGRPGPRCSRCQKNLSLHTSVRILY
LFLALLLVAVAVLASLVFRKVDSLSEDISLTQSIYDKKLVLMQKNLQGLDPKALNNCSFCH
EAGQLGPEIRKLQEELEGIQKLLLAQEVQLDQTLQAQEVLSTTSRQISQEMGSCSFSIHQV
NQSLGLFLAQVRGWQATTAGLDLSLKDLTQECYDVKAAVHQINFTVGQTSEWIHGIQRKT
DEETLTLQKIVTDWQNYTRLFSGLRTTSTKTGEAVKNIQATLGASSQRISQNSESMHDLVL
QVMGLQLQLDNISSFLDDHEENMHDLQYHTHYAQNRTVERFESLEGRMASHEIEIGTIFT
NINATDNHVHSMLKYLDDVRLSCTLGFHTHAEELYYLNKSVSIMLGTTDLLRERFSLLSA
RLDLNVRNLSMIVEEMKAVDTQHGEILRNVTILRGAPGPPGPRGFKGDMGVKGPVGGRG
PKGDPGSLGPLGPQGPQGQPGEAGPVGERGPVGPRGFPGLKGSKGSFGTGGPRGQPGPKG
DIGPPGPEGPPGSPGPSGPQGKPGIAGKTGSPGQRGAMGPKGEPGIQGPPGLPGPPGPPGSQ
SFY

>O00155 multipass membrane protein
MAPTEPWSPSPGSAPWDYSGLDGLEELELCPAGDLPYGYVYIPALYLAAFAVGLLGNAFV
VWLLAGRRGPRRLVDTFVLHLAAADLGFVLTLPLWAAAAALGGRWPFGDGLCKLSSFAL
AGTRCAGALLLAGMSVDRYLAVVKLLEARPLRTPRCALASCCGVWAVALLAGLPSLVYR
GLQPLPGGQDSQCGEEPSHAFQGLSLLLLLLTFVLPLVVTLFCYCRISRRLRRPPHVGRARR
NSLRIIFAIESTFVGSWLPFSALRAVFHLARLGALPLPCPLLLALRWGLTIATCLAFVNSCAN
PLIYLLLDRSFRARALDGACGRTGRLARRISSASSLSRDDSSVFRCRAQAANTASASW

>Q9H6X2 single-pass type I membrane protein
MATAERRALGIGFQWLSLATLVLICAGQGGRREDGGPACYGGFDLYFILDKSGSVLHHWN
EIYYFVEQLAHKFISPQLRMSFIVFSTRGTTLMKLTEDREQIRQGLEELQKVLPGGDTYMH
EGFERASEQIYYENRQGYRTASVIIALTDGELHEDLFFYSEREANRSRDLGAIVYCVGVKD
FNETQLARIADSKDHVFPVNDGFQALQGIIHSILKKSCIEILAAEPSTICAGESFQVVVRGN
GFRHARNVDRVLCSFKINDSVTLNEKPFSVEDTYLLCPAPILKEVGMKAALQVSMNDGLS
FISSSVIITTTHCSDGSILAIALLILFLLLALALLWWFWPLCCTVIIKEVPPPPAEESEEEDDDG
LPKKKWPTVDASYYGGRGVGGIKRMEVRWGEKGSTEEGAKLEKAKNARVKMPEQEYE
FPEPRNLNNNMRRPSSPRKWYSPIKGKLDALWVLLRKGYDRVSVMRPQPGDTGRCINFTR
VKNNQPAKYPLNNAYHTSSPPPAPIYTPPPPAPHCPPPPPSAPTPPIPSPPSTLPPPPQAPPPNR
APPPSRPPPRPSV

>P25105 multipass membrane protein
MEPHDSSHMDSEFRYTLFPIVYSIIFVLGVIANGYVLWVFARLYPCKKFNEIKIFMVNLTMA

DMLFLITLPLWIVYYQNQGNWILPKFLCNVAGCLFFINTYCSVAFLGVITYNRFQAVTRPIK
TAQANTRKRGISLSLVIWVAIVGAASYFLILDSTNTVPDSAGSGNVTRCFEHYEKGSVPVLI
IHIFIVFSFFLVFLIILFCNLVIIRTLLMQPVQQQRNAEVKRRALWMVCTVLAVFIICFVPHHV
VQLPWTLAELGFQDSKFHQAINDAHQVTLCLLSTNCVLDPVIYCFLTKKFRKHLTEKFYS
MRSSRKCSRATTDTVTEVVVPFNQIPGNSLKN

>Q8NGC0 multipass membrane protein
MTEFHLQSQMPSIRLIFRRLSLGRIKPSQSPRCSTSFMVVPSFSIAEHWRRMKGANLSQGM
EFELLGLTTDPQLQRLLFVVFLGMYTATLLGNLVMFLLIHVSATLHTPMYSLLKSLSFLDFC
YSSTVVPQTLVNFLAKRKVISYFGCMTQMFFYAGFATSECYLIAAMAYDRYAAICNPLLYS
TIMSPEVCASLIVGSYSAGFLNSLIHTGCIFSLKFCGAHVVTHFFCDGPPILSLSCVDTSLCEI
LLFIFAGFNLLSCTLTILISYFLILNTILKMSSAQGRFKAFSTCASHLTAICLFFGTTLFMYLRP
RSSYSLTQDRTVAVIYTVVIPVLNPLMYSLRNKDVKKALIKVWGRKTME

>Q16581 multipass membrane protein
MASFSAETNSTDLLSQPWNEPPVILSMVILSLTFLLGLPGNGLVLWVAGLKMQRTVNTIWF
LHLTLADLLCCLSLPFSLAHLALQGQWPYGRFLCKLIPSIIVLNMFASVFLLTAISLDRCLVV
FKPIWCQNHRNVGMACSICGCIWVVAFVMCIPVFVYREIFTTDNHNRCGYKFGLSSSLDY
PDFYGDPLENRSLENIVQPPGEMNDRLDPSSFQTNDHPWTVPTVFQPQTFQRPSADSLPRG
SARLTSQNLYSNVFKPADVVSPKIPSGFPIEDHETSPLDNSDAFLSTHLKLFPSASSNSFYES
ELPQGFQDYYNLGQFTDDDQVPTPLVAITITRLVVGFLLPSVIMIACYSFIVFRMQRGRFAK
SQSKTFRVAVVVVAVFLVCWTPYHIFGVLSLLTDPETPLGKTLMSWDHVCIALASANSCFN
PFLYALLGKDFRKKARQSIQGILEAAFSEELTRSTHCPSNNVISERNSTTV

>O15258 multipass membrane protein
MSEGDSVGESVHGKPSVVYRFFTRLGQIYQSWLDKSTPYTAVRWVVTLGLSFVYMIRVYL
LQGWYIVTYALGIYHLNLFIAFLSPKVDPSLMEDSDDGPSLPTKQNEEFRPFIRRLPEFKFW
HAATKGILVAMVCTFFDAFNVPVFWPILVMYFIMLFCITMKRQIKHMIKYRYIPFTHGKRR
YRGKEDAGKAFAS

>Q8NGI7 multipass membrane protein
MEGINKTAKMQFFFRPFSPDPEVQMLIFVVFLMMYLTSLGGNATIAVIVQINHSLHTPMYF
FLANLAVLEIFYTSSITPLALANLLSMGKTPVSITGCGTQMFFFVFLGGADCVLLVVMAYD
QFIAICHPLRYRLIMSWSLCVELLVGSLVLGFLLSLPLTILIFHLPFCHNDEIYHFYCDMPAV
MRLACADTRVHKTALYIISFIVLSIPLSLISISYVFIVVAILRIRSAEGRQQAYSTCSSHILVVLL
QYGCTSFIYLSPSSSYSPEMGRVVSVAYTFITPILNPLIYSLRNKELKDALRKALRKF

>P40818 peripheral membrane protein
MPAVASVPKELYLSSSLKDLNKKTEVKPEKISTKSYVHSALKIFKTAEECRLDRDEERAYVL
YMKYVTVYNLIKKRPDFKQQQDYFHSILGPGNIKKAVEEAERLSESLKLRYEEAEVRKKL
EEKDRQEEAQRLQQKRQETGREDGGTLAKGSLENVLDSKDKTQKSNGEKNEKCETKEK
GAITAKELYTMMTDKNISLIIMDARRMQDYQDSCILHSLSVPEEAISPGVTASWIEAHLPD
DSKDTWKKRGNVEYVVLLDWFSSAKDLQIGTTLRSLKDALFKWESKTVLRNEPLVLEGG
YENWLLCYPQYTTNAKVTPPPRRQNEEVSISLDFTYPSLEESIPSKPAAQTPPASIEVDENIE

LISGQNERMGPLNISTPVEPVAASKSDVSPIIQPVPSIKNVPQIDRTKKPAVKLPEEHRIKSES
TNHEQQSPQSGKVIPDRSTKPVVFSPTLMLTDEEKARIHAETALLMEKNKQEKELRERQQ
EEQKEKLRKEEQEQKAKKKQEAEENEITEKQQKAKEEMEKKESEQAKKEDKETSAKRG
KEITGVKRQSKSEHETSDAKKSVEDRGKRCPTPEIQKKSTGDVPHTSVTGDSGSGKPFKIK
GQPESGILRTGTFREDTDDTERNKAQREPLTRARSEEMGRIVPGLPSGWAKFLDPITGTFRY
YHSPTNTVHMYPPEMAPSSAPPSTPPTHKAKPQIPAERDREPSKLKRSYSSPDITQAIQEEE
KRKPTVTPTVNRENKPTCYPKAEISRLSASQIRNLNPVFGGSGPALTGLRNLGNTCYMNSI
LQCLCNAPHLADYFNRNCYQDDINRSNLLGHKGEVAEEFGIIMKALWTGQYRYISPKDFK
ITIGKINDQFAGYSQQDSQELLLFLMDGLHEDLNKADNRKRYKEENNDHLDDFKAAEHA
WQKHKQLNESIIVALFQGQFKSTVQCLTCHKKSRTFEAFMYLSLPLASTSKCTLQDCLRLF
SKEEKLTDNNRFYCSHCRARRDSLKKIEIWKLPPVLLVHLKRFSYDGRWKQKLQTSVDFP
LENLDLSQYVIGPKNNLKKYNLFSVSNHYGGLDGGHYTAYCKNAARQRWFKFDDHEVS
DISVSSVKSSAAYILFYTSLGPRVTDVAT

>Q8NGK4 multipass membrane protein
MLPSNITSTHPAVFLLVGIPGLEHLHAWISIPFCFAYTLALLGNCTLLFIIQADAALHEPMYL
FLAMLATIDLVLSSTTLPKMLAIFWFRDQEINFFACLVQMFFLHSFSIMESAVLLAMAFDRY
VAICKPLHYTTVLTGSLITKIGMAAVARAVTLMTPLPFLLRRFHYCRGPVIAHCYCEHMAV
VRLACGDTSFNNIYGIAVAMFIVVLDLLFVILSYVFILQAVLQLASQEARYKAFGTCVSHIG
AILSTYTPVVISSVMHRVARHAAPRVHILLAIFYLLFPPMVNPIIYGVKTKQIREYVLSLFQR
KNM

>Q8NGC4 multipass membrane protein
MERINSTLLTAFILTGIPYPLRLRTLFFVFFFLIYILTQLGNLLILITVWADPRLHARPMYIFLG
VLSVIDMSISSIIVPRLMMNFTLGVKPIPFGGCVAQLYFYHFLGSTQCFLYTLMAYDRYLAI
CQPLRYPVLMTAKLSALLVAGAWMAGSIHGALQAILTFRLPYCGPNQVDYFFCDIPAVLRL
ACADTTVNELVTFVDIGVVVASCFSLILLSYIQIIQAILRIHTADGRRRAFSTCGAHVTVVTV
YYVPCAFIYLRPETNSPLDGAAALVPTAITPFLNPLIYTLRNQEVKLALKRMLRSPRTPSEV

>P79483 single-pass type I membrane protein
MVCLKLPGGSSLAALTVTLMVLSSRLAFAGDTRPRFLELRKSECHFFNGTERVRYLDRYF
HNQEEFLRFDSDVGEYRAVTELGRPVAESWNSQKDLLEQKRGRVDNYCRHNYGVGESFT
VQRRVHPQVTVYPAKTQPLQHHNLLVCSVSGFYPGSIEVRWFRNGQEEKAGVVSTGLIQN
GDWTFQTLVMLETVPRSGEVYTCQVEHPSVTSALTVEWRARSESAQSKMLSGVGGFVLG
LLFLGAGLFIYFRNQKGHSGLQPTGFLS

>Q9BXN2 single-pass type II membrane protein
MEYHPDLENLDEDGYTQLHFDSQSNTRIAVVSEKGSCAASPPWRLIAVILGILCLVILVIAV
VLGTMAIWRSNSGSNTLENGYFLSRNKENHSQPTQSSLEDSVTPTKAVKTTGVLSSPCPPN
WIIYEKSCYLFSMSLNSWDGSKRQCWQLGSNLLKIDSSNELGFIVKQVSSQPDNSFWIGLS
RPQTEVPWLWEDGSTFSSNLFQIRTTATQENPSPNCVWIHVSVIYDQLCSVPSYSICEKKFS
M

>Q8NGQ6 multipass membrane protein

MAKNNLTRVTEFILMGFMDHPKLEIPLFLVFLSFYLVTLLGNVGMIMLIQVDVKLYTPMYF
FLSHLSLLDACYTSVITPQILATLATGKTVISYGHCAAQFFLFTICAGTECFLLAVMAYDRY
AAIRNPLLYTVAMNPRLCWSLVVGAYVCGVSGAILRTTCTFTLSFCKDNQINFFFCDLPPLL
KLACSDTANIEIVIIFFGNFVILANASVILISYLLIIKTILKVKSSGGRAKTFSTCASHITAVALF
FGALIFMYLQSGSGKSLEEDKVVSVFYTVVIPMLNPLIYSLRNKDVKDAFRKVARRLQVS
LSM

>Q8NGP9 multipass membrane protein
MDKENSSMVTEFIFMGITQDPQMEIIFFVVFLIVYLVNVVGNIGMIILITTDTQLHTPMYFFL
CNLSFVDLGYSSAIAPRMLADFLTNHKVISFSSCATQFAFFVGFVDAECYVLAAMAYGRFV
AICRPLHYSTFMSKQVCLALMLGSYLAGLVSLVAHTTLTFSLSYCGSNIINHFFCEIPPLLAL
SCSDTYISEILLFSLCGFIEFSTILIIFISYTFILVAIIRMRSAEGRLKAFSTCGSHLTGITLFYGT
VMFMYLRPTSSYSLDQDKWASVFYTVIIPMLNPLIYSLRNKDVKAAFKKLIGKKSQ

>Q99680 multipass membrane protein
MCFSPILEINMQSESNITVRDDIDDINTNMYQPLSYPLSFQVSLTGFLMLEIVLGLGSNLTVL
VLYCMKSNLINSVSNIITMNLHVLDVIICVGCIPLTIVILLLSLESNTALICCFHEACVSFASV
STAINVFAITLDRYDISVKPANRILTMGRAVMLMISIWIFSFFSFLIPFIEVNFFSLQSGNTWE
NKTLLCVSTNEYYTELGMYYHLLVQIPIFFFTVVVMLITYTKILQALNIRIGTRFSTGQKKK
ARKKKTISLTTQHEATDMSQSSGGRNVVFGVRTSVSVIIALRRAVKRHRERRERQKRVFR
MSLLIISTFLLCWTPISVLNTTILCLGPSDLLVKLRLCFLVMAYGTTIFHPLLYAFTRQKFQK
VLKSKMKKRVVSIVEADPLPNNAVIHNSWIDPKRNKKITFEDSEIREKCLVPQVVTD

>P21462 multipass membrane protein
METNSSLPTNISGGTPAVSAGYLFLDIITYLVFAVTFVLGVLGNGLVIWVAGFRMTHTVTTI
SYLNLAVADFCFTSTLPFFMVRKAMGGHWPFGWFLCKFVFTIVDINLFGSVFLIALIALDR
CVCVLHPVWTQNHRTVSLAKKVIIGPWVMALLLTLPVIIRVTTVPGKTGTVACTFNFSPWT
NDPKERINVAVAMLTVRGIIRFIIGFSAPMSIVAVSYGLIATKIHKQGLIKSSRPLRVLSFVAA
AFFLCWSPYQVVALIATVRIRELLQGMYKEIGIAVDVTSALAFFNSCLNPMLYVFMGQDFR
ERLIHALPASLERALTEDSTQTSDTATNSTLPSAEVELQAK

>P48039 multipass membrane protein
MQGNGSALPNASQPVLRGDGARPSWLASALACVLIFTIVVDILGNLLVILSVYRNKKLRN
AGNIFVVSLAVADLVVAIYPYPLVLMSIFNNGWNLGYLHCQVSGFLMGLSVIGSIFNITGIAI
NRYCYICHSLKYDKLYSSKNSLCYVLLIWLLTLAAVLPNLRAGTLQYDPRIYSCTFAQSVS
SAYTIAVVVFHFLVPMIIVIFCYLRIWILVLQVRQRVKPDRKPKLKPQDFRNFVTMFVVFVL
FAICWAPLNFIGLAVASDPASMVPRIPEWLFVASYYMAYFNSCLNAIIYGLLNQNFRKEYRR
IIVSLCTARVFFVDSSNDVADRVKWKPSPLMTNNNVVKVDSV

>P35408 multipass membrane protein
MSTPGVNSSASLSPDRLNSPVTIPAVMFIFGVVGNLVAIVVLCKSRKEQKETTFYTLVCGLA
VTDLLGTLLVSPVTIATYMKGQWPGGQPLCEYSTFILLFFSLSGLSIICAMSVERYLAINHA
YFYSHYVDKRLAGLTLFAVYASNVLFCALPNMGLGSSRLQYPDTWCFIDWTTNVTAHAA
YSYMYAGFSSFLILATVLCNVLVCGALLRMHRQFMRRTSLGTEQHHAAAAASVASRGHP

AASPALPRLSDFRRRRSFRRIAGAEIQMVILLIATSLVVLICSIPLVVRVFVNQLYQPSLEREV
SKNPDLQAIRIASVNPILDPWIYILLRKTVLSKAIEKIKCLFCRIGGSRRERSGQHCSDSQRT
SSAMSGHSRSFISRELKEISSTSQTLLPDLSLPDLSENGLGGRNLLPGVPGMGLAQEDTTSL
RTLRISETSDSSQGQDSESVLLVDEAGGSGRAGPAPKGSSLQVTFPSETLNLSEKCI

>Q9UHI7 multipass membrane protein
MRAQEDLEGRTQHETTRDPSTPLPTEPKFDMLYKIEDVPPWYLCILLGFQHYLTCFSGTIA
VPFLLAEALCVGHDQHMVSQLIGTIFTCVGITTLIQTTVGIRLPLFQASAFAFLVPAKAILAL
ERWKCPPEEEIYGNWSLPLNTSHIWHPRIREVQGAIMVSSVVEVVIGLLGLPGALLNYIGP
LTVTPTVSLIGLSVFQAAGDRAGSHWGISACSILLIILFSQYLRNLTFLLPVYRWGKGLTLL
RIQIFKMFPIMLAIMTVWLLCYVLTLTDVLPTDPKAYGFQARTDARGDIMAIAPWIRIPYPC
QWGLPTVTAAAVLGMFSATLAGIIESIGDYYACARLAGAPPPPVHAINRGIFTEGICCIIAGL
LGTGNGSTSSSPNIGVLGITKVGSRRVVQYGAAIMLVLGTIGKFTALFSSLPDPILGGMFCT
LFGMITAVGLSNLQFVDMNSSRNLFVLGFSMFFGLTLPNYLESNPGAINTGILEVDQILIVL
LTTEMFVGGCLAFILDNTVPGSPEERGLIQWKAGAHANSDMSSSLKSYDFPIGMGIVKRIT
FLKYIPICPVFKGFSSSSKDQIAIPEDTPENTETASVCTKV

>Q8NG95 multipass membrane protein
MKAGNFSDTPEFFLLGLSGDPELQPILFMLFLSMYLATMLGNLLIILAVNSDSHLHTPMYF
LLSILSLVDICFTSTTMPKMLVNIQAQAQSINYTGCLTQICFVLVFVGLENGILVMMAYDRF
VAICHPLRYNVIMNPKLCGLLLLLSFIVSVLDALLHTLMVLQLTFCIDLEIPHFFCELAHILK
LACSDVLINNILVYLVTSLLGVVPLSGIIFSYTRIVSSVMKIPSAGGKYKAFSICGSHLIVVSL
FYGTGFGVYLSSGATHSSRKGAIASVMYTVVTPMLNPLIYSLRNKDMLKALRKLISRIPSF
H

>O43610 peripheral membrane protein
MDAAVTDDFQQILPIEQLRSTHASNDYVERPPAPCKQALSSPSLIVQTHKSDWSLATMPTS
LPRSLSQCHQLQPLPQHLSQSSIASSMSHSTTASDQRLLASITPSPSGQSIIRTQPGAGVHPK
ADGALKGEAEQSAGHPSEHLFICEECGRCKCVPCTAARPLPSCWLCNQRCLCSAESLLDY
GTCLCCVKGLFYHCSTDDEDNCADEPCSCGPSSCFVRWAAMSLISLFLPCLCCYLPTRGCL
HLCQQGYDSLRRPGCRCKRHTNTVCRKISSGSAPFPKAQEKSV

>Q8NGJ0 multipass membrane protein
MSITKAWNSSSVTMFILLGFTDHPELQALLFVTFLGIYLTTLAWNLALIFLIRGDTHLHTPM
YFFLSNLSFIDICYSSAVAPNMLTDFFWEQKTISFVGCAAQFFFFVGMGLSECLLLTAMAYD
RYAAISSPLLYPTIMTQGLCTRMVVGAYVGGFLSSLIQASSIFRLHFCGPNIINHFFCDLPPV
LALSCSDTFLSQVVNFLVVVTVGGTSFLQLLISYGYIVSAVLKIPSAEGRWKACNTCASHL
MVVTLLFGTALFVYLRPSSSYLLGRDKVVSVFYSLVIPMLNPLIYSLRNKEIKDALWKVLE
RKKVFS

>Q8NGF4 multipass membrane protein
MRLMKEVRGRNQTEVTEFLLLGLSDNPDLQGVLFALFLLIYMANMVGNLGMIVLIKIDLC
LHTPMYFFLSSLSFVDASYSSSVTPKMLVNLMAENKAISFHGCAAQFYFFGSFLGTECFLL
AMMAYDRYAAIWNPLLYPVLVSGRICFLLIATSFLAGCGNAAIHTGMTFRLSFCGSNRINH

FYCDTPPLLKLSCSDTHFNGIVIMAFSSFIVISCVMIVLISYLCIFIAVLKMPSLEGRHKAFST
CASYLMAVTIFFGTILFMYLRPTSSYSMEQDKVVSVFYTVIIPVLNPLIYSLKNKDVKKAL
KKILWKHIL

>P49755 single-pass type I membrane protein
MSGLSGPPARRGPFPLALLLLFLLGPRLVLAISFHLPINSRKCLREEIHKDLLVTGAYEISDQ
SGGAGGLRSHLKITDSAGHILYSKEDATKGKFAFTTEDYDMFEVCFESKGTGRIPDQLVIL
DMKHGVEAKNYEEIAKVEKLKPLEVELRRLEDLSESIVNDFAYMKKREEEMRDTNESTN
TRVLYFSIFSMFCLIGLATWQVFYLRRFFKAKKLIE

>Q8NGR6 multipass membrane protein
MMSFAPNASHSPVFLLLGFSRANISYTLLFFLFLAIYLTTILGNVTLVLLISWDSRLHSPMY
YLLRGLSVIDMGLSTVTLPQLLAHLVSHYPTIPAARCLAQFFFFYAFGVTDTLVIAVMALDR
YVAICDPLHYALVMNHQRCACLLALSWVVSILHTMLRVGLVLPLCWTGDAGGNVNLPHF
FCDHRPLLRASCSDIHSNELAIFFEGGFLMLGPCALIVLSYVRIGAAILRLPSAAGRRRAVST
CGSHLTMVGFLYGTIICVYFQPPFQNSQYQDMVASVMYTAITPLANPFVYSLHNKDVKGA
LCRLLEWVKVDP

>P22303 GPI-anchor, lipid-anchor
MRPPQCLLHTPSLASPLLLLLLWLLGGGVGAEGREDAELLVTVRGGRLRGIRLKTPGGPV
SAFLGIPFAEPPMGPRRFLPPEPKQPWSGVVDATTFQSVCYQYVDTLYPGFEGTEMWNPN
RELSEDCLYLNVWTPYPRPTSPTPVLVWIYGGGFYSGASSLDVYDGRFLVQAERTVLVSM
NYRVGAFGFLALPGSREAPGNVGLLDQRLALQWVQENVAAFGGDPTSVTLFGESAGAAS
VGMHLLSPPSRGLFHRAVLQSGAPNGPWATVGMGEARRRATQLAHLVGCPPGGTGGNDT
ELVACLRTRPAQVLVNHEWHVLPQESVFRFSFVPVVDGDFLSDTPEALINAGDFHGLQVL
VGVVKDEGSYFLVYGAPGFSKDNESLISRAEFLAGVRVGVPQVSDLAAEAVVLHYTDWL
HPEDPARLREALSDVVGDHNVVCPVAQLAGRLAAQGARVYAYVFEHRASTLSWPLWMG
VPHGYEIEFIFGIPLDPSRNYTAEEKIFAQRLMRYWANFARTGDPNEPRDPKAPQWPPYTA
GAQQYVSLDLRPLEVRRGLRAQACAFWNRFLPKLLSATDTLDEAERQWKAEFHRWSSY
MVHWKNQFDHYSKQDRCSDL

>P05107 single-pass type I membrane protein
MLGLRPPLLALVGLLSLGCVLSQECTKFKVSSCRECIESGPGCTWCQKLNFTGPGDPDSIR
CDTRPQLLMRGCAADDIMDPTSLAETQEDHNGGQKQLSPQKVTLYLRPGQAAAFNVTFR
RAKGYPIDLYYLMDLSYSMLDDLRNVKKLGGDLLRALNEITESGRIGFGSFVDKTVLPFV
NTHPDKLRNPCPNKEKECQPPFAFRHVLKLTNNSNQFQTEVGKQLISGNLDAPEGGLDAM
MQVAACPEEIGWRNVTRLLVFATDDGFHFAGDGKLGAILTPNDGRCHLEDNLYKRSNEFD
YPSVGQLAHKLAENNIQPIFAVTSRMVKTYEKLTEIIPKSAVGELSEDSSNVVQLIKNAYNK
LSSRVFLDHNALPDTLKVTYDSFCSNGVTHRNQPRGDCDGVQINVPITFQVKVTATECIQE
QSFVIRALGFTDIVTVQVLPQCECRCRDQSRDRSLCHGKGFLECGICRCDTGYIGKNCECQ
TQGRSSQELEGSCRKDNNSIICSGLGDCVCGQCLCHTSDVPGKLIYGQYCECDTINCERYN
GQVCGGPGRGLCFCGKCRCHPGFEGSACQCERTTEGCLNPRRVECSGRGRCRCNVCECH
SGYQLPLCQECPGCPSPCGKYISCAECLKFEKGPFGKNCSAACPGLQLSNNPVKGRTCKE
RDSEGCWVAYTLEQQDGMDRYLIYVDESRECVAGPNIAAIVGGTVAGIVLIGILLLVIWKA

LIHLSDLREYRRFEKEKLKSQWNNDNPLFKSATTTVMNPKFAES

>Q9HBX9 multipass membrane protein
MTSGSVFFYILIFGKYFSHGGGQDVKCSLGYFPCGNITKCLPQLLHCNGVDDCGNQADED
NCGDNNGWSLQFDKYFASYYKMTSQYPFEAETPECLVGSVPVQCLCQGLELDCDETNLR
AVPSVSSNVTAMSLQWNLIRKLPPDCFKNYHDLQKLYLQNNKITSISIYAFRGLNSLTKLYL
SHNRITFLKPGVFEDLHRLEWLIIEDNHLSRISPPTFYGLNSLILLVLMNNVLTRLPDKPLCQ
HMPRLHWLDLEGNHIHNLRNLTFISCSNLTVLVMRKNKINHLNENTFAPLQKLDELDLGS
NKIENLPPLIFKDLKELSQLNLSYNPIQKIQANQFDYLVKLKSLSLEGIEISNIQQRMFRPLM
NLSHIYFKKFQYCGYAPHVRSCKPNTDGISSLENLLASIIQRVFVWVVSAVTCFGNIFVICM
RPYIRSENKLYAMSIISLCCADCLMGIYLFVIGGFDLKFRGEYNKHAQLWMESTHCQLVGS
LAILSTEVSVLLLTFLTLEKYICIVYPFRCVRPGKCRTITVLILIWITGFIVAFIPLSNKEFFKN
YYGTNGVCFPLHSEDTESIGAQIYSVAIFLGINLAAFIIIVFSYGSMFYSVHQSAITATEIRNQ
VKKEMILAKRFFFIVFTDALCWIPIFVVKFLSLLQVEIPGTITSWVVIFILPINSALNPILYTLT
TRPFKEMIHRFWYNYRQRKSMDSKGQKTYAPSFIWVEMWPLQEMPPELMKPDLFTYPCE
MSLISQSTRLNSYS

>P29371 multipass membrane protein
MATLPAAETWIDGGGGVGADAVNLTASLAAGAATGAVETGWLQLLDQAGNLSSSPSALG
LPVASPAPSQPWANLTNQFVQPSWRIALWSLAYGVVVAVAVLGNLIVIWIILAHKRMRTVT
NYFLVNLAFSDASMAAFNTLVNFIYALHSEWYFGANYCRFQNFFPITAVFASIYSMTAIAVD
RYMAIIDPLKPRLSATATKIVIGSIWILAFLLAFPQCLYSKTKVMPGRTLCFVQWPEGPKQH
FTYHIIVIILVYCFPLLIMGITYTIVGITLWGGEIPGDTCDKYHEQLKAKRKVVKMMIIVVM
TFAICWLPYHIYFILTAIYQQLNRWKYIQQVYLASFWLAMSSTMYNPIIYCCLNKRFRAGF
KRAFRWCPFIKVSSYDELELKTTRFHPNRQSSMYTVTRMESMTVVFDPNDADTTRSSRKK
RATPRDPSFNGCSRRNSKSASATSSFISSPYTSVDEYS

>Q12907 single-pass type I membrane protein
MAAEGWIWRWGWGRRCLGRPGLLGPGPGPTTPLFLLLLLGSVTADITDGNSEHLKREHS
LIKPYQQGVGSSSMPLWDFQGSTMLTSQYVRLTPDERSKEGSIWNHQPCFLKDWEMHVHF
KVHGTGKKNLHGDGIALWYTRDRLVPGPVFGSKDNFHGLAIFLDTYPNDETTERVFPYIS
VMVNNGSLSYDHSKDGRWTELAGCTADFRNRDHDTFLAVRYSRGRLTVMTDLEDKNEW
KNCIDITGVRLPTGYYFGASAGTGDLSDNHDIISMKLFQLMVEHTPDEESIDWTKIEPSVN
FLKSPKDNVDDPTGNFRSGPLTGWRVFLLLLCALLGIVVCAVVGAVVFQKRQERNKRFY

>Q9H2C5 multipass membrane protein
MPTFNGSVFMPSAFILIGIPGLESVQCWIGIPFSAMYLIGVIGNSLILVIIKYENSLHIPMYIFL
AMLAATDIALNTCILPKMLGIFWFHLPEISFDACLFQMWLIHSFQAIESGILLAMALDRYVA
ICIPLRHATIFSQQFLTHIGLGVTLRAAILIIPSLGLIKCCLKHYRTTVISHSYCEHMAIVKLAT
EDIRVNKIYGLFVAFAILGFDIIFITLSYVQIFITVFQLPQKEARFKAFNTCIAHICVFLQFYLL
AFFSFFTHRFGSHIPPYIHILLSNLYLLVPPFLNPIVYGVKTKQIRDHIVKVFFFKKVT

>Q9Y4G6 peripheral membrane protein
MVALSLKICVRHCNVVKTMQFEPSTAVYDACRVIRERVPEAQTGQASDYGLFLSDEDPRK

GIWLEAGRTLDYYMLRNGDILEYKKKQRPQKIRMLDGSVKTVMVDDSKTVGELLVTICS
RIGITNYEEYSLIQETIEEKKEEGTGTLKKDRTLLRDERKMEKLKAKLHTDDDLNWLDHS
RTFREQGVDENETLLLRRKFFYSDQNVDSRDPVQLNLLYVQARDDILNGSHPVSFEKACE
FGGFQAQIQFGPHVEHKHKPGFLDLKEFLPKEYIKQRGAEKRIFQEHKNCGEMSEIEAKV
KYVKLARSLRTYGVSFFLVKEKMKGKNKLVPRLLGITKDSVMRVDEKTKEVLQEWPLTT
VKRWAASPKSFTLDFGEYQESYYSVQTTEGEQISQLIAGYIDIILKKKQSKDRFGLEGDEES
TMLEESVSPKKSTILQQQFNRTGKAEHGSVALPAVMRSGSSGPETFNVGSMPSPQQQVMV
GQMHRGHMPPLTSAQQALMGTINTSMHAVQQAQDDLSELDSLPPLGQDMASRVWVQNK
VDESKHEIHSQVDAITAGTASVVNLTAGDPADTDYTAVGCAITTISSNLTEMSKGVKLLAA
LMDDEVGSGEDLLRAARTLAGAVSDLLKAVQPTSGEPRQTVLTAAGSIGQASGDLLRQIG
ENETDERFQDVLMSLAKAVANAAAMLVLKAKNVAQVAEDTVLQNRVIAAATQCALSTSQ
LVACAKVVSPTISSPVCQEQLIEAGKLVDRSVENCVRACQAATTDSELLKQVSAAASVVSQ
ALHDLLQHVRQFASRGEPIGRYDQATDTIMCVTESIFSSMGDAGEMVRQARVLAQATSDL
VNAMRSDAEAEIDMENSKKLLAAAKLLADSTARMVEAAKGAAANPENEDQQQRLREA
AEGLRVATNAAAQNAIKKKIVNRLEVAAKQAAAAATQTIAASQNAAVSNKNPAAQQQLV
QSCKAVADHIPQLVQGVRGSQAQAEDLSAQLALIISSQNFLQPGSKMVSSAKAAVPTVSDQ
AAAMQLSQCAKNLATSLAELRTASQKAHEACGPMEIDSALNTVQTLKNELQDAKMAAV
ESQLKPLPGETLEKCAQDLGSTSKAVGSSMAQLLTCAAQGNEHYTGVAARETAQALKTL
AQAARGVAASTTDPAAAHAMLDSARDVMEGSAMLIQEAKQALIAPGDAERQQRLAQVA
KAVSHSLNNCVNCLPGQKDVDVALKSIGESSKKLLVDSLPPSTKPFQEAQSELNQAAADL
NQSAGEVVHATRGQSGELAAASGKFSDDFDEFLDAGIEMAGQAQTKEDQIQVIGNLKNIS
MASSKLLLAAKSLSVDPGAPNAKNLLAAAARAVTESINQLITLCTQQAPGQKECDNALRE
LETVKGMLDNPNEPVSDLSYFDCIESVMENSKVLGESMAGISQNAKTGDLPAFGECVGIA
SKALCGLTEAAAQAAYLVGISDPNSQAGHQGLVDPIQFARANQAIQMACQNLVDPGSSPS
QVLSAATIVAKHTSALCNACRIASSKTANPVAKRHFVQSAKEVANSTANLVKTIKALDGDF
SEDNRNKCRIATAPLIEAVENLTAFASNPEFVSIPAQISSEGSQAQEPILVSAKTMLESSSYLIR
TARSLAINPKDPPTWSVLAGHSHTVSDSIKSLITSIRDKAPGQRECDYSIDGINRCIRDIEQA
SLAAVSQSLATRDDISVEALQEQLTSVVQEIGHLIDPIATAARGEAAQLGHKVTQLASYFEP
LILAAVGVASKILDHQQQMTVLDQTKTLAESALQMLYAAKEGGGNPKAQHTHDAITEAA
QLMKEAVDDIMVTLNEAASEVGLVGGMVDAIAEAMSKLDEGTPPEPKGTFVDYQTTVV
KYSKAIAVTAQEMMTKSVTNPEELGGLASQMTSDYGHLAFQGQMAAATAEPEEIGFQIRT
RVQDLGHGCIFLVQKAGALQVCPTDSYTKRELIECARAVTEKVSLVLSALQAGNKGTQAC
ITAATAVSGIIADLDTTIMFATAGTLNAENSETFADHRENILKTAKALVEDTKLLVSGAASTP
DKLAQAAQSSAATITQLAEVVKLGAASLGSDDPETQVVLINAIKDVAKALSDLISATKGAA
SKPVDDPSMYQLKGAAKVMVTNVTSLLKTVKAVEDEATRGTRALEATIECIKQELTVFQS
KDVPEKTSSPEESIRMTKGITMATAKAVAAGNSCRQEDVIATANLSRKAVSDMLTACKQAS
FHPDVSDEVRTRALRFGTECTLGYLDLLEHVLVILQKPTPEFKQQLAAFSKRVAGAVTELI
QAAEAMKGTEWVDPEDPTVIAETELLGAAASIEAAAKKLEQLKPRAKPKQADETLDFEE
QILEAAKSIAAATSALVKSASAAQRELVAQGKVGSIPANAADDGQWSQGLISAARMVAAA
TSSLCEAANASVQGHASEEKLISSAKQVAASTAQLLVACKVKADQDSEAMRRLQAAGNA
VKRASDNLVRAAQKAAFGKADDDDVVVKTKFVGGIAQIIAAQEEMLKKERELEEARKK
LAQIRQQQYKFLPTELREDEG

>P25103 multipass membrane protein

MDNVLPVDSDLSPNISTNTSEPNQFVQPAWQIVLWAAAYTVIVVTSVVGNVVVMWIILAH
KRMRTVTNYFLVNLAFAEASMAAFNTVVNFTYAVHNEWYYGLFYCKFHNFFPIAAVFASI
YSMTAVAFDRYMAIIHPLQPRLSATATKVVICVIWVLALLLAFPQGYYSTTETMPSRVVCM
IEWPEHPNKIYEKVYHICVTVLIYFLPLLVIGYAYTVVGITLWASEIPGDSSDRYHEQVSAK
RKVVKMMIVVVCTFAICWLPFHIFFLLPYINPDLYLKKFIQQVYLAIMWLAMSSTMYNPII
YCCLNDRFRLGFKHAFRCCPFISAGDYEGLEMKSTRYLQTQGSVYKVSRLETTISTVVGA
HEEEPEDGPKATPSSLDLTSNCSSRSDSKTMTESFSFSSNVLS

>Q96P67 multipass membrane protein
MNNNTTCIQPSMISSMALPIIYILLCIVGVFGNTLSQWIFLTKIGKKTSTHIYLSHLVTANLLV
CSAMPFMSIYFLKGFQWEYQSAQCRVVNFLGTLSMHASMFVSLLILSWIAISRYATLMQK
DSSQETTSCYEKIFYGHLLKKFRQPNFARKLCIYIWGVVLGIIIPVTVYYSVIEATEGEESLC
YNRQMELGAMISQIAGLIGTTFIGFSFLVVLTSYYSFVSHLRKIRTCTSIMEKDLTYSSVKRH
LLVIQILLIVCFLPYSIFKPIFYVLHQRDNCQQLNYLIETKNILTCLASARSSTDPIIFLLLDKT
FKKTLYNLFTKSNSAHMQSYG

>O95665 multipass membrane protein
METSSPRPPRPSSNPGLSLDARLGVDTRLWAKVLFTALYALIWALGAAGNALSAHVVLKA
RAGRAGRLRHHVLSLALAGLLLLLVGVPVELYSFVWFHYPWVFGDLGCRGYYFVHELCA
YATVLSVAGLSAERCLAVCQPLRARSLLTPRRTRWLVALSWAASLGLALPMAVIMGQKHE
LETADGEPEPASRVCTVLVSRTALQVFIQVNVLVSFVLPLALTAFLNGVTVSHLLALCSQVP
STSTPGSSTPSRLELLSEEGLLSFIVWKKTFIQGGQVSLVRHKDVRRIRSLQRSVQVLRAIV
VMYVICWLPYHARRLMYCYVPDDAWTDPLYNFYHYFYMVTNTLFYVSSAVTPLLYNAV
SSSFRKLFLEAVSSLCGEHHPMKRLPPKPQSPTLMDTASGFGDPPETRT

>P36544 multipass membrane protein
MRCSPGGVWLALAASLLHVSLQGEFQRKLYKELVKNYNPLERPVANDSQPLTVYFSLSLL
QIMDVDEKNQVLTTNIWLQMSWTDHYLQWNVSEYPGVKTVRFPDGQIWKPDILLYNSA
DERFDATFHTNVLVNSSGHCQYLPPGIFKSSCYIDVRWFPFDVQHCKLKFGSWSYGGWSL
DLQMQEADISGYIPNGEWDLVGIPGKRSERFYECCKEPYPDVTFTVTMRRRTLYYGLNLLI
PCVLISALALLVFLLPADSGEKISLGITVLLSLTVFMLLVAEIMPATSDSVPLIAQYFASTMIIV
GLSVVVTVIVLQYHHHDPDGGKMPKWTRVILLNWCAWFLRMKRPGEDKVRPACQHKQ
RRCSLASVEMSAVAPPPASNGNLLYIGFRGLDGVHCVPTPDSGVVCGRMACSPTHDEHLL
HGGQPPEGDPDLAKILEEVRYIANRFRCQDESEAVCSEWKFAACVVDRLCLMAFSVFTIIC
TIGILMSAPNFVEAVSKDFA

>Q8TCW9 multipass membrane protein
METTMGFMDDNATNTSTSFLSVLNPHGAHATSFPFNFSYSDYDMPLDEDEDVTNSRTFFA
AKIVIGMALVGIMLVCGIGNFIFIAALVRYKKLRNLTNLLIANLAISDFLVAIVCCPFEMDYY
VVRQLSWEHGHVLCTSVNYLRTVSLYVSTNALLAIAIDRYLAIVHPLRPRMKCQTATGLIA
LVWTVSILIAIPSAYFTTETVLVIVKSQEKIFCGQIWPVDQQLYYKSYFLFIFGIEFVGPVVT
MTLCYARISRELWFKAVPGFQTEQIRKRLRCRRKTVLVLMCILTAYVLCWAPFYGFTIVRD
FFPTVFVKEKHYLTAFYIVECIAMSNSMINTLCFVTVKNDTVKYFKKIMLLHWKASYNGG
KSSADLDLKTIGMPATEEVDCIRLK

>Q8NG99 multipass membrane protein
MEARNQTAISKFLLLGLIEDPELQPVLFSLFLSMYLVTILGNLLILLAVISDSHLHTPMYFFL
SNLSFLDICLSTTTIPKMLVNIQAQNRSITYSGCLTQICFVLFFAGLENCLLAAMAYDRYVAI
CHPLRYTVIMNPRLCGLLILLSLLTSVVNALLLSLMVLRLSFCTDLEIPLFFCELAQVIQLTC
SDTLINNILIYFAACIFGGVPLSGIILSYTQITSCVLRMPSASGKHKAVSTCGSHLSIVLLFYG
AGLGVYISSVVTDSPRKTAVASVMYSVFPQMVNPFIYSLRNKDMKGTLRKFIGRIPSLLWC
AICFGFRFLE

>P06213 single-pass type I membrane protein
MATGGRRGAAAAPLLVAVAALLLGAAGHLYPGEVCPGMDIRNNLTRLHELENCSVIEGHL
QILLMFKTRPEDFRDLSFPKLIMITDYLLLFRVYGLESLKDLFPNLTVIRGSRLFFNYALVIFE
MVHLKELGLYNLMNITRGSVRIEKNNELCYLATIDWSRILDSVEDNYIVLNKDDNEECGDI
CPGTAKGKTNCPATVINGQFVERCWTHSHCQKVCPTICKSHGCTAEGLCCHSECLGNCSQ
PDDPTKCVACRNFYLDGRCVETCPPPYYHFQDWRCVNFSFCQDLHHKCKNSRRQGCHQ
YVIHNNKCIPECPSGYTMNSSNLLCTPCLGPCPKVCHLLEGEKTIDSVTSAQELRGCTVIN
GSLIINIRGGNNLAAELEANLGLIEEISGYLKIRRSYALVSLSFFRKLRLIRGETLEIGNYSFY
ALDNQNLRQLWDWSKHNLTITQGKLFFHYNPKLCLSEIHKMEEVSGTKGRQERNDIALK
TNGDQASCENELLKFSYIRTSFDKILLRWEPYWPPDFRDLLGFMLFYKEAPYQNVTEFDG
QDACGSNSWTVVDIDPPLRSNDPKSQNHPGWLMRGLKPWTQYAIFVKTLVTFSDERRTY
GAKSDIIYVQTDATNPSVPLDPISVSNSSSQIILKWKPPSDPNGNITHYLVFWERQAEDSELF
ELDYCLKGLKLPSRTWSPPFESEDSQKHNQSEYEDSAGECCSCPKTDSQILKELEESSFRK
TFEDYLHNVVFVPRKTSSGTGAEDPRPSRKRRSLGDVGNVTVAVPTVAAFPNTSSTSVPTS
PEEHRPFEKVVNKESLVISGLRHFTGYRIELQACNQDTPEERCSVAAYVSARTMPEAKADD
IVGPVTHEIFENNVVHLMWQEPKEPNGLIVLYEVSYRRYGDEELHLCVSRKHFALERGCR
LRGLSPGNYSVRIRATSLAGNGSWTEPTYFYVTDYLDVPSNIAKIIIGPLIFVFLFSVVIGSIY
LFLRKRQPDGPLGPLYASSNPEYLSASDVFPCSVYVPDEWEVSREKITLLRELGQGSFGMV
YEGNARDIIKGEAETRVAVKTVNESASLRERIEFLNEASVMKGFTCHHVVRLLGVVSKGQ
PTLVVMELMAHGDLKSYLRSLRPEAENNPGRPPPTLQEMIQMAAEIADGMAYLNAKKFV
HRDLAARNCMVAHDFTVKIGDFGMTRDIYETDYYRKGGKGLLPVRWMAPESLKDGVFT
TSSDMWSFGVVLWEITSLAEQPYQGLSNEQVLKFVMDGGYLDQPDNCPERVTDLMRMC
WQFNPKMRPTFLEIVNLLKDDLHPSFPEVSFFHSEENKAPESEELEMEFEDMENVPLDRSS
HCQREEAGGRDGGSSLGFKRSYEEHIPYTHMNGGKKNGRILTLPRSNPS

>Q8NGI9 multipass membrane protein
MAVGRNNTIVTKFILLGLSDHPQMKIFLFMLFLGLYLLTLAWNLSLIALIKMDSHLHMPMY
FFLSNLSFLDICYVSSTAPKMLSDIITEQKTISFVGCATQYFVFCGMGLTECFLLAAMAYDR
YAAICNPLLYTVLISHTLCLKMVVGAYVGGFLSSFIETYSVYQHDFCGPYMINHFFCDLPP
VLALSCSDTFTSEVVTFIVSVVVGIVSVLVVLISYGYIVAAVVKISSATGRTKAFSTCASHLT
AVTLFYGSGFFMYMRPSSSYSLNRDKVVSIFYALVIPVVNPIIYSFRNKEIKNAMRKAMER
DPGISHGGPFIFMTLG

>Q8N0Y5 multipass membrane protein
MAGNNFTEVTVFILSGFANHPELQVSLFLMFLFIYLFTVLGNLGLITLIRMDSQLHTPMYFF

LSNLAFIDIFYSSTVTPKALVNFQSNRRSISFVGCFVQMYFFVGLVCCECFLLGSMAYNRYI
AICNPLLYSVVMSQKVSNWLGVMPYVIGFTSSLISVWVISSLAFCDSSINHFFCDTTALLAL
SCVDTFGTEMVSFVLAGFTLLSSLLIITVTYIIIISAILRIQSAAGRQKAFSTCASHLMAVTIF
YGSLIFTYLQPDNTSSLTQAQVASVFYTIVIPMLNPLIYSLRNKDVKNALLRVIHRKLFP

>P16410 single-pass type I membrane protein
MACLGFQRHKAQLNLATRTWPCTLLFFLLFIPVFCKAMHVAQPAVVLASSRGIASFVCEYA
SPGKATEVRVTVLRQADSQVTEVCAATYMMGNELTFLDDSICTGTSSGNQVNLTIQGLRA
MDTGLYICKVELMYPPPYYLGIGNGTQIYVIDPEPCPDSDFLLWILAAVSSGLFFYSFLLTAV
SLSKMLKKRSPLTTGVYVKMPPTEPECEKQFQPYFIPIN

>P14770 single-pass type I membrane protein
MPAWGALFLLWATAEATKDCPSPCTCRALETMGLWVDCRGHGLTALPALPARTRHLLLAN
NSLQSVPPGAFDHLPQLQTLDVTQNPWHCDCSLTYLRLWLEDRTPEALLQVRCASPSLAA
HGPLGRLTGYQLGSCGWQLQASWVRPGVLWDVALVAVAALGLALLAGLLCATTEALD

>Q9BTV4 multipass membrane protein
MAANYSSTSTRREHVKVKTSSQPGFLERLSETSGGMFVGLMAFLLSFYLIFTNEGRALKTA
TSLAEGLSLVVSPDSIHSVAPENEGRLVHIIGALRTSKLLSDPNYGVHLPAVKLRRHVEMYQ
WVETEESREYTEDGQVKKETRYSYNTEWRSEIINSKNFDREIGHKNPSAMAVESFMATAP
FVQIGRFFLSSGLIDKVDNFKSLSLSKLEDPHVDIIRRGDFFYHSENPKYPEVGDLRVSFSYA
GLSGDDPDLGPAHVVTVIARQRGDQLVPFSTKSGDTLLLLHHGDFSAEEVFHRELRSNSM
KTWGLRAAGWMAMFMGLNLMTRILYTLVDWFPVFRDLVNIGLKAFAFCVATSLTLLTVA
AGWLFYRPLWALLIAGLALVPILVARTRVPAKKLE

>Q8WU17 multipass membrane protein
MAAVGPPQQQVRMAHQQVWAALEVALRVPCLYIIDAIFNSYPDSSQSRFCIVLQIFLRLFG
VFASSIVLILSQRSLFKFYTYSSAFLLAATSVLVNYYASLHIDFYGAYNTSAFGIELLPRKGP
SLWMALIVLQLTFGIGYVTLLQIHSIYSQLIILDLLVPVIGLITELPLHIRETLLFTSSLILTLNT
VFVLAVKLKWFYYSTRYVYLLVRHMYRIYGLQLLMEDTWKRIRFPDILRVFWLTRVTAQA
TVLMYILRMANETDSFFISWDDFWDLICNLIISGCDSTLTVLGMSAVISSVAHYLGLGILAFI
GSTEEDDRRLGFVAPVLFFILALQTGLSGLRPEERLIRLSRNMCLLLTAVLHFIHGMTDPVL
MSLSASHVSSFRRHFPVLFVSACLFILPVLLSYVLWHHYALNTWLFAVTAFCVELCLKVIV
SLTVYTLFMIDGYYNVLWEKLDDYVYYVRSTGSIIEFIFGVVMFGNGAYTMMFESGSKIR
AFMMCLHAYFNIYLQAKNGWKTFMNRRTAVKKINSLPEIKGSRLQEINDVCAICYHEFTT
SARITPCNHYFHALCLRKWLYIQDTCPMCHQKVYIEDDIKDNSNVSNNNGFIPPNETPEEA
VREAAAESDRELNEDDSTDCDDDVQRERNGVIQHTGAAAEEFNDDTD

>Q8NGP2 multipass membrane protein
MAPENFTRVTEFILTGVSSCPELQIPLFLVFLVLYGLTMAGNLGIITLTSVDSRLQTPMYFFL
QHLALINLGNSTVIAPKMLINFLVKKKTTSFYECATQLGGFLFFIVSEVIMLALMAYDRYV
AICNPLLYMVVVSRRLCLLLVSLTYLYGFSTAIVVSSYVFSVSYCSSNIINHFYCDNVPLLAL
SCSDTYLPETVVFISAATNVVGSLIIVLVSYFNIVLSILKICSSEGRKKAFSTCASHMMAVTIF
YGTLLFMYVQPRSNHSLDTDDKMASVFYTLVIPMLNPLIYSLRNKDVKTALQRFMTNLC

YSFKTM

>Q8NH41 multipass membrane protein
MLTSLTDLCFSPIQVAEIKSLPKSMNETNHSRVTEFVLLGLSSSRELQPFLFLTFSLLYLAILL
GNFLIILTVTSDSRLHTPMYFLLANLSFIDVCVASFATPKMIADFLVERKTISFDACLAQIFFV
HLFTGSEMVLLVSMAYDRYVAICKPLHYMTVMSRRVCVVLVLISWFVGFIHTTSQLAFTV
NLPFCGPNKVDSFFCDLPLVTKLACIDTYVVSLLIVADSGFLSLSSFLLLVVSYTVILVTVRN
RSSASMAKARSTLTAHITVVTLFFGPCIFIYVWPFSSYSVDKVLAVFYTIFTLILNPVIYTLR
NKEVKAAMSKLKSRYLKPSQVSVVIRNVLFLETK

>Q8TAE7 multipass membrane protein
MTFGRSGAASVVLNVGGARYSLSRELLKDFPLRRVSRLHGCRSERDVLEVCDDYDRERN
EYFFDRHSEAFGFILLYVRGHGKLRFAPRMCELSFYNEMIYWGLEGAHLEYCCQRRLDDR
MSDTYTFYSADEPGVLGRDEARPGGAEAAPSRRWLERMRRTFEEPTSSLAAQILASVSVV
FVIVSMVVLCASTLPDWRNAAADNRSLDDRSRYSAGPGREPSGIIEAICIGWFTAECIVRFI
VSKNKCEFVKRPLNIIDLLAITPYYISVLMTVFTGENSQLQRAGVTLRVLRMMRIFWVIKL
ARHFIGLQTLGLTLKRCYREMVMLLVFICVAMAIFSALSQLLEHGLDLETSNKDFTSIPAAC
WWVIISMTTVGYGDMYPITVPGRILGGVCVVSGIVLLALPITFIYHSFVQCYHELKFRSAR
YSRSLSTEFLN

>Q8NH10 multipass membrane protein
MAHINCTQATEFILVGLTDHQELKMPLFVLFLSIYLFTVVGNLGLILLIRADTSLNTPMYFF
LSNLAFVDFCYSSVITPKMLGNFLYKQNVISFDACATQLGCFLTFMISESLLLASMAYDRYV
AICNPLLYMVVMTPGICIQLVAVPYSYSFLMALFHTILTFRLSYCHSNIVNHFYCDDMPLLR
LTCSDTRFKQLWIFACAGIMFISSLLIVFVSYMFIISAILRMHSAEGRQKAFSTCGSHMLAVT
IFYGTLIFMYLQPSSSHALDTDKMASVFYTVIIPMLNPLIYSLQNKEVKEALKKIIINKN

>P08571 GPI-anchor, lipid-anchor
MERASCLLLLLLLPLVHVSATTPEPCELDDEDFRCVCNFSEPQPDWSEAFQCVSAVEVEIHA
GGLNLEPFLKRVDADADPRQYADTVKALRVRRLTVGAAQVPAQLLVGALRVLAYSRLKE
LTLEDLKITGTMPPLPLEATGLALSSLRLRNVSWATGRSWLAELQQWLKPGLKVLSIAQA
HSPAFSCEQVRAFPALTSLDLSDNPGLGERGLMAALCPHKFPAIQNLALRNTGMETPTGVC
AALAAAGVQPHSLDLSHNSLRATVNPSAPRCMWSSALNSLNLSFAGLEQVPKGLPAKLRV
LDLSCNRLNRAPQPDELPEVDNLTLDGNPFLVPGTALPHEGSMNSGVVPACARSTLSVGV
SGTLVLLQGARGFA

>Q13319 lipid-anchor
MGTVLSLSPASSAKGRRPGGLPEEKKKAPPAGDEALGGYGAPPVGKGGKGESRLKRPSVL
ISALTWKRLVAASAKKKKGSKKVTPKPASTGPDPLVQQRNRENLLRKGRDPPDGGGTAKP
LAVPVPTVPAAAATCEPPSGGSAAAQPPGSGGGKPPPPPPPAPQVAPPVPGGSPRRVIVQAS
TGELLRCLGDFVCRRCYRLKELSPGELVGWFRGVDRSLLLQGWQDQAFITPANLVFVYLL
CRESLRGDELASAAELQAAFLTCLYLAYSYMGNEISYPLKPFLVEPDKERFWQRCLRLIQR
LSPQMLRLNADPHFFTQVFQDLKNEGEAAASGGGPPSGGAPAASSAARDSCAAGTKHWT
MNLDR

>P30408 multipass membrane protein
MCYGKCARCIGHSLVGLALLCIAANILLYFPNGETKYASENHLSRFVWFFSGIVGGGLLML
LPAFVFIGLEQDDCCGCCGHENCGKRCAMLSSVLAALIGIAGSGYCVIVAALGLAEGPLCL
DSLGQWNYTFASTEGQYLLDTSTWSECTEPKHIVEWNVSLFSILLALGGIEFILCLIQVING
VLGGICGFCCSHQQQYDC

>P11230 multipass membrane protein
MTPGALLMLLGALGAPLAPGVRGSEAEGRLREKLFSGYDSSVRPAREVGDRVRVSVGLIL
AQLISLNEKDEEMSTKVYLDLEWTDYRLSWDPAEHDGIDSLRITAESVWLPDVVLLNNND
GNFDVALDISVVVSSDGSVRWQPPGIYRSSCSIQVTYFPFDWQNCTMVFSSYSYDSSEVSL
QTGLGPDGQGHQEIHIHEGTFIENGQWEIIHKPSRLIQPPGDPRGGREGQRQEVIFYLIIRRK
PLFYLVNVIAPCILITLLAIFVFYLPPDAGEKMGLSIFALLTLTVFLLLLADKVPETSLSVPIII
KYLMFTMVLVTFSVILSVVVLNLHHRSPHTHQMPLWVRQIFIHKLPLYLRLKRPKPERDL
MPEPPHCSSPGSGWGRGTDEYFIRKPPSDFLFPKPNRFQPELSAPDLRRFIDGPNRAVALLP
ELREVVSSISYIARQLQEQEDHDALKEDWQFVAMVVDRLFLWTFIIFTSVGTLVIFLDATY
HLPPPDPFP

>O00574 multipass membrane protein
MAEHDYHEDYGFSSFNDSSQEEHQDFLQFSKVFLPCMYLVVFVCGLVGNSLVLVISIFYHK
LQSLTDVFLVNLPLADLVFVCTLPFWAYAGIHEWVFGQVMCKSLLGIYTINFYTSMLILTCI
TVDRFIVVVKATKAYNQQAKRMTWGKVTSLLIWVISLLVSLPQIIYGNVFNLDKLICGYH
DEAISTVVLATQMTLGFFLPLLTMIVCYSVIIKTLLHAGGFQKHRSLKIIFLVMAVFLLTQMP
FNLMKFIRSTHWEYYAMTSFHYTIMVTEAIAYLRACLNPVLYAFVSLKFRKNFWKLVKDI
GCLPYLGVSHQWKSSEDNSKTFSASHNVEATSMFQL

>Q9GZK3 multipass membrane protein
MNWVNKSVPQEFILLVFSDQPWLEIPPFVMFLFSYILTIFGNLTIILVSHVDFKLHTPMYFFL
SNLSLLDLCYTTSTVPQMLVNICNTRKVISYGGCVAQLFIFLALGSTECLLLAVMCFDRFVA
ICRPLHYSIIMHQRLCFQLAAASWISGFSNSVLQSTWTLKMPLCGHKEVDHFFCEVPALLK
LSCVDTTANEAELFFISVLFLLIPVTLILISYAFIVQAVLRIQSAEGQRKAFGTCGSHLIVVSL
FYGTAISMYLQPPSPSSKDRGKMVSLFCGIIAPMLNPLIYTLRNKEVKEAFKRLVAKSLLNQ
EIRNMQMISFAKDTVLTYLTNFSASCPIFVITIENYCNLPQRKFP

>Q9UNH6 peripheral membrane protein
MDMNSFSPMMPTSPLSMINQIKFEDEPDLKDLFITVDEPESHVTTIETFITYRIITKTSRGEF
DSSEFEVRRRYQDFLWLKGKLEEAHPTLIIPPLPEKFIVKGMVERFNDDFIETRRKALHKFL
NRIADHPTLTFNEDFKIFLTAQAWELSSHKKQGPGLLSRMGQTVRAVASSMRGVKNRPEEF
MEMNNFIELFSQKINLIDKISQRIYKEEREYFDEMKEYGPIHILWSASEEDLVDTLKDVASCI
DRCCKATEKRMSGLSEALLPVVHEYVLYSEMLMGVMKRRDQIQAELDSKVEVLTYKKA
DTDLLPEEIGKLEDKVECANNALKADWERWKQNMQNDIKLAFTDMAEENIHYYEQCLA
TWESFLTSQTNLHLEEASEDKP

>O43861 multipass membrane protein

MADQIPLYPVRSAAAAAANRKRAAYYSAAGPRPGADRHSRYQLEDESAHLDEMPLMMS
EEGFENEESDYHTLPRARIMQRKRGLEWFVCDGWKFLCTSCCGWLINICRRKKELKART
VWLGCPEKCEEKHPRNSIKNQKYNVFTFIPGVLYEQFKFFLNLYFLVISCSQFVPALKIGYL
YTYWAPLGFVLAVTMTREAIDEFRRFQRDKEVNSQLYSKLTVRGKVQVKSSDIQVGDLIIV
EKNQRIPSDMVFLRTSEKAGSCFIRTDQLDGETDWKLKVAVSCTQQLPALGDLFSISAYVY
AQKPQMDIHSFEGTFTREDSDPPIHESLSIENTLWASTIVASGTVIGVVIYTGKETRSVMNTS
NPKNKVGLLDLELNRLTKALFLALVALSIVMVTLQGFVGPWYRNLFRFLLLFSYIIPISLRV
NLDMGKAVYGWMMMKDENIPGTVVRTSTIPEELGRLVYLLTDKTGTLTQNEMIFKRLHL
GTVSYGADTMDEIQSHVRDSYSQMQSQAGGNNTGSTPLRKAQSSAPKVRKSVSSRIHEA
VKAIVLCHNVTPVYESRAGVTEETEFAEADQDFSDENRTYQASSPDEVALVQWTESVGLT
LVSRDLTSMQLKTPSGQVLSFCILQLFPFTSESKRMGVIVRDESTAEITFYMKGADVAMSPI
VQYNDWLEEECGNMAREGLRTLVVAKKALTEEQYQDFESRYTQAKLSMHDRSLKVAAV
VESLEREMELLCLTGVEDQLQADVRPTLEMLRNAGIKIWMLTGDKLETATCIAKSSHLVSR
TQDIHIFRQVTSRGEAHLELNAFRRKHDCALVISGDSLEVCLKYYEHEFVELACQCPAVVC
CRCSPTQKARIVTLLQQHTGRRTCAIGDGGNDVSMIQAADCGIGIEGKEGKQASLAADFSI
TQFRHIGRLLMVHGRNSYKRSAALGQFVMHRGLIISTMQAVFSSVFYFASVPLYQGFLMV
GYATIYTMFPVFSLVLDQDVKPEMAMLYPELYKDLTKGRSLSFKTFLIWVLISIYQGGILM
YGALVLFESEFVHVVAISFTALILTELLMVALTVRTWHWLMVVAEFLSLGCYVSSLAFLNE
YFGIGRVSFGAFLDVAFITTVTFLWKVSAITVVSCLPLYVLKYLRRKLSPPSYCKLAS

>P29275 multipass membrane protein
MLLETQDALYVALELVIAALSVAGNVLVCAAVGTANTLQTPTNYFLVSLAAADVAVGLFAI
PFAITISLGFCTDFYGCLFLACFVLVLTQSSIFSLLAVAVDRYLAICVPLRYKSLVTGTRARGV
IAVLWVLAFGIGLTPFLGWNSKDSATNNCTEPWDGTTNESCCLVKCLFENVVPMSYMVYF
NFFGCVLPPLLIMLVIYIKIFLVACRQLQRTELMDHSRTTLQREIHAAKSLAMIVGIFALCWL
PVHAVNCVTLFQPAQGKNKPKWAMNMAILLSHANSVVNPIVYAYRNRDFRYTFHKIISRY
LLCQADVKSGNGQAGVQPALGVGL

>Q13636 lipid-anchor
MAIRELKVCLLGDTGVGKSSIVCRFVQDHFDHNISPTIGASFMTKTVPCGNELHKFLIWDT
AGQERFHSLAPMYYRGSAAAVIVYDITKQDSFYTLKKWVKELKEHGPENIVMAIAGNKC
DLSDIREVPLKDAKEYAESIGAIVVETSAKNAINIEELFQGISRQIPPLDPHENGNNGTIKVE
KPTMQASRRCC

>Q5TCQ9 peripheral membrane protein
MSKTLKKKKHWLSKVQECAVSWAGPPGDFGAEIRGGAERGEFPYLGRLREEPGGGTCCV
VSGKAPSPGDVLLEVNGTPVSGLTNRDTLAVIRHFREPIRLKTVKPGKVINKDLRHYLSLQ
FQKGSIDHKLQQVIRDNLYLRTIPCTTRAPRDGEVPGVDYNFISVEQFKALEESGALLESGT
YDGNFYGTPKPPAEPSPFQPDPVDQVLFDNEFDAESQRKRTTSVSKMERMDSSLPEEEEDE
DKEAINGSGNAENRERHSESSDWMKTVPSYNQTNSSMDFRNYMMRDETLEPLPKNWEM
AYTDTGMIYFIDHNTKTTTWLDPRLCKKAKAPEDCEDGELPYGWEKIEDPQYGTYYVDF
TLVAQAGVQWHDLGSLQPPPPGFNHLNQKTQFENPVEEAKRKKQLGQVEIGSSKPDMEK
SHFTRDPSQLKGVLVRASLKKSTMGFGFTIIGGDRPDEFLQVKNVLKDGPAAQDGKIAPG
DVIVDINGNCVLGHTHADVVQMFQLVPVNQYVNLTLCRGYPLPDDSEDPVVDIVAATPVI

NGQSLTKGETCMNPQDFKPGAMVLEQNGKSGHTLTGDGLNGPSDASEQRVSMASSGSSQ
PELVTIPLIKGPKGFGFAIADSPTGQKVKMILDSQWCQGLQKGDIIKEIYHQNVQNLTHLQ
VVEVLKQFPVGADVPLLILRGGPPSPTKTAKMKTDKKENAGSLEAINEPIPQPMPFPPSIIRS
GSPKLDPSEVYLKSKTLYEDKPPNTKDLDVFLRKQESGFGFRVLGGDGPDQSIYIGAIIPLG
AAEKDGRLRAADELMCIDGIPVKGKSHKQVLDLMTTAARNGHVLLTVRRKIFYGEKQPE
DDSSQAFISTQNGSPRLNRAEVPARPAPQEPYDVVLQRKENEGFGFVILTSKNKPPPGVIPH
KIGRVIEGSPADRCGKLKVGDHISAVNGQSIVELSHDNIVQLIKDAGVTVTLTVIAEEEHHG
PPSGTNSARQSPALQHRPMGQSQANHIPGDRSALEGEIGKDVSTSYRHSWSDHKHLAQPD
TAVISVVGSRHNQNLGCYPVELERGPRGFGFSLRGGKEYNMGLFILRLAEDGPAIKDGRIH
VGDQIVEINGEPTQGITHTRAIELIQAGGNKVLLLLRPGTGLIPDHGDWDINNPSSSNVIYD
EQSPLPPSSHFASIFEESHVPVIEESLRVQICEKAEELKDIVPEKKSTLNENQPEIKHQSLLQK
NVSKRDPPSSHGHSNKKNLLKVENGVTRRGRSVSPKKPASQHSEEHLDKIPSPLKNNPKR
RPRDQSLSPSKGENKSCQVSTRAGSGQDQCRKSRGRSASPKKQQKIEGSKAPSNAEAKLL
EGKSRRIAGYTGSNAEQIPDGKEKSDVIRKDAKQNQLEKSRTRSPEKKIKRMVEKSLPSK
MTNKTTSKEVSENEKGKKVTTGETSSSNDKIGENVQLSEKRLKQEPEEKVVSNKTEDHK
GKELEAADKNKETGRFKPESSSPVKKTLITPGPWKVPSGNKVTGTIGMAEKRQ

>Q9H5J4 multipass membrane protein
MNMSVLTLQEYEFEKQFNENEAIQWMQENWKKSFLFSALYAAFIFGGRHLMNKRAKFEL
RKPLVLWSLTLAVFSIFGALRTGAYMVYILMTKGLKQSVCDQGFYNGPVSKFWAYAFVLS
KAPELGDTIFIILRKQKLIFLHWYHHITVLLYSWYSYKDMVAGGGWFMTMNYGVHAVMY
SYYALRAAGFRVSRKFAMFITLSQITQMLMGCVVNYLVFCWMQHDQCHSHFQNIFWSSL
MYLSYLVLFCHFFFEAYIGKMRKTTKAE

>P47900 multipass membrane protein
MTEVLWPAVPNGTDAAFLAGPGSSWGNSTVASTAAVSSSFKCALTKTGFQFYYLPAVYILV
FIIGFLGNSVAIWMFVFHMKPWSGISVYMFNLALADFLYVLTLPALIFYYFNKTDWIFGDA
MCKLQRFIFHVNLYGSILFLTCISAHRYSGVVYPLKSLGRLKKKNAICISVLVWLIVVVAISP
ILFYSGTGVRKNKTITCYDTTSDEYLRSYFIYSMCTTVAMFCVPLVLILGCYGLIVRALIYK
DLDNSPLRRKSIYLVIIVLTVFAVSYIPFHVMKTMNLRARLDFQTPAMCAFNDRVYATYQV
TRGLASLNSCVDPILYFLAGDTFRRRLSRATRKASRRSEANLQSKSEDMTLNILPEFKQNG
DTSL

>Q8NGX8 multipass membrane protein
MTTIILEVDNHTVTTRFILLGFPTRPAFQLLFFSIFLATYLLTLLENLLIILAIHSDGQLHKPM
YFFLSHLSFLEMWYVTVISPKMLVDFLSHDKSISFNGCMTQLYFFVTFVCTEYILLAIMAF
DRYVAICNPLRYPVIMTNQLCGTLAGGCWFCGLMTAMIKMVFIAQLHYCGMPQINHYFC
DISPLLNVSCEDASQAEMVDFFLALMVIAIPLCVVVASYAAILATILRIPSAQGRQKAFSTC
ASHLTVVILFYSMTLFTYARPKLMYAYNSNKVVSVLYTVIVPLLNPIIYCLRNHEVKAALR
KTIHCRGSGPQGNGAFSS

>A6NND4 multipass membrane protein
MDATACNESVDGSPVFYLLGIPSLPETFFLPVFFIFLLFYLLILMGNALILVAVVAEPSLHKP
MYFFLINLSTLDILFTTTTVPKMLSLFLLGDRFLSFSSCLLQMYLFQSFTCSEAFILVVMAY

DRYVAICHPLHYPVLMNPQTNATLAASAWLTALLLPIPAVVRTSQMAYNSIAYIYHCFCDH
LAVVQASCSDTTPQTLMGFCIAMVVSFLPLLLVLLSYVHILASVLRISSLEGRAKAFSTCSS
HLLVVGTYYSSIAIAYVAYRADLPLDFHIMGNVVYAILTPILNPLIYTLRNRDVKAAITKIMS
QDPGCDRSI

>Q8NGD5 multipass membrane protein
MDPQNYSLVSEFVLHGLCTSRHLQNFFFIFFFGVYVAIMLGNLLILVTVISDPCLHSSPMYF
LLGNLAFLDMWLASFATPKMIRDFLSDQKLISFGGCMAQIFFLHFTGGAEMVLLVSMAYD
RYVAICKPLHYMTLMSWQTCIRLVLASWVVGFVHSISQVAFTVNLPYCGPNEVDSFFCDL
PLVIKLACMDTYVLGIIMISDSGLLSLSCFLLLLISYTVILLAIRQRAAGSTSKALSTCSAHIM
VVTLFFGPCIFVYVRPFSRFSVDKLLSVFYTIFTPLLNPIIYTLRNEEMKAAMKKLQNRRVT
FQ

>Q8NGT1 multipass membrane protein
MNITLYSFLYVLRLNEGNSTGRNIDERKKMQGENFTIWSIFFLEGFSQYPGLEVVLFVFSLV
MYLTTLLGNSTLILITILDSRLKTPMYLFLGNLSFMDICYTSASVPTLLVNLLSSQKTIIFSGC
AVQMYLSLAMGSTECVLLAVMAYDRYVAICNPLRYSIIMNRCVCARMATVSWVTGCLTA
LLETSFALQIPLCGNLIDHFTCEILAVLKLACTSSLLMNTIMLVVSILLLPIPMLLVCISYIFILS
TILRITSAEGRNKAFSTCGAHLTVVILYYGAALSMYLKPSSSNAQKIDKIISLLYGVLTPML
NPIIYSLRNKEVKDAMKKLLGKITLHQTHEHL

>Q8IY95 multipass membrane protein
MAAGGRMEDGSLDITQSIEDDPLLDAQLLPHHSLQAHFRPRFHPLPTVIIVNLLWFIHLVFV
VLAFLTGVLCSYPNPNEDKCPGNYTNPLKVQTVIILGKVILWILHLLLECYIQYHHSKIRNR
GYNLIYRSTRHLKRLALMIQSSGNTVLLLILCMQHSFPEPGRLYLDLILAILALELICSLICL
LIYTVKIRRFNKAKPEPDILEEEKIYAYPSNITSETGFRTISSLEEIVEKQGDTIEYLKRHNAL
LSKRLLALTSSDLGCQPSRT

>P60201 multipass membrane protein
MGLLECCARCLVGAPFASLVATGLCFFGVALFCGCGHEALTGTEKLIETYFSKNYQDYEYL
INVIHAFQYVIYGTASFFFLYGALLLAEGFYTTGAVRQIFGDYKTTICGKGLSATVTGGQKG
RGSRGQHQAHSLERVCHCLGKWLGHPDKFVGITYALTVVWLLVFACSAVPVYIYFNTWT
TCQSIAFPSKTSASIGSLCADARMYGVLPWNAFPGKVCGSNLLSICKTAEFQMTFHLFIAA
FVGAAATLVSLLTFMIAATYNFAVLKLMGRGTKF

>Q9NZN1 single-pass type I membrane protein
MKAPIPHLILLYATFTQSLKVVTKRGSADGCTDWSIDIKKYQVLVGEPVRIKCALFYGYIRT
NYSLAQSAGLSLMWYKSSGPGDFEEPIAFDGSRMSKEEDSIWFRPTLLQDSGLYACVIRNS
TYCMKVSISLTVGENDTGLCYNSKMKYFEKAELSKSKEISCRDIEDFLLPTREPEILWYKE
CRTKTWRPSIVFKRDTLLIREVREDDIGNYTCELKYGGFVVRRTTELTVTAPLTDKPPKLLY
PMESKLTIQETQLGDSANLTCRAFFGYSGDVSPLIYWMKGEKFIEDLDENRVWESDIRILK
EHLGEQEVSISLIVDSVEEGDLGNYSCYVENGNGRRHASVLLHKRELMYTVELAGGLGAI
LLLLVCLVTIYKCYKIEIMLFYRNHFGAEELDGDNKDYDAYLSYTKVDPDQWNQETGEEE
RFALEILPDMLEKHYGYKLFIPDRDLIPTGTYIEDVARCVDQSKRLIIVMTPNYVVRRGWSI

FELETRLRNMLVTGEIKVILIECSELRGIMNYQEVEALKHTIKLLTVIKWHGPKCNKLNSKF
WKRLQYEMPFKRIEPITHEQALDVSEQGPFGELQTVSAISMAAATSTALATAHPDLRSTFH
NTYHSQMRQKHYYRSYEYDVPPTGTLPLTSIGNQHTYCNIPMTLINGQRPQTKSSREQNP
DEAHTNSAILPLLPRETSISSVIW

>Q9P1Q5 multipass membrane protein
MRENNQSSTLEFILLGVTGQQEQEDFFYILFLFIYPITLIGNLLIVLAICSDVRLHNPMYFLL
ANLSLVDIFFSSVTIPKMLANHLLGSKSISFGGCLTQMYFMIALGNTDSYILAAMAYDRAV
AISRPLHYTTIMSPRSCIWLIAGSWVIGNANALPHTLLTASLSFCGNQEVANFYCDITPLLKL
SCSDIHFHVKMMYLGVGIFSVPLLCIIVSYIRVFSTVFQVPSTKGVLKAFSTCGSHLTVVSL
YYGTVMGTYFRPLTNYSLKDAVITVMYTAVTPMLNPFIYSLRNRDMKAALRKLFNKRISS

>P07550 multipass membrane protein
MGQPGNGSAFLLAPNGSHAPDHDVTQERDEVWVVGMGIVMSLIVLAIVFGNVLVITAIAK
FERLQTVTNYFITSLACADLVMGLAVVPFGAAHILMKMWTFGNFWCEFWTSIDVLCVTA
SIETLCVIAVDRYFAITSPFKYQSLLTKNKARVIILMVWIVSGLTSFLPIQMHWYRATHQEAI
NCYANETCCDFFTNQAYAIASSIVSFYVPLVIMVFVYSRVFQEAKRQLQKIDKSEGRFHVQ
NLSQVEQDGRTGHGLRRSSKFCLKEHKALKTLGIIMGTFTLCWLPFFIVNIVHVIQDNLIRK
EVYILLNWIGYVNSGFNPLIYCRSPDFRIAFQELLCLRRSSLKAYGNGYSSNGNTGEQSGY
HVEQEKENKLLCEDLPGTEDFVGHQGTVPSDNIDSQGRNCSTNDSLL

>Q8NGG3 multipass membrane protein
MDSTFTGYNLYNLQVKTEMDKLSSGLDIYRNPLKNKTEVTMFILTGFTDDFELQVFLFLLF
FAIYLFTLIGNLGLVVLVIEDSWLHNPMYYFLSVLSFLDACYSTVVTPKMLVNFLAKNKSIS
FIGCATQMLLFVTFGTTECFLLAAMAYDHYVAIYNPLLYSVSMSPRVYVPLITASYVAGILH
ATIHIVATFSLSFCGSNEIRHVFCDMPPLLAISCSDTHTNQLLLFYFVGSIEIVTILIVLISCDFI
LLSILKMHSAKGRQKAFSTCGSHLTGVTIYHGTILVSYMRPSSSYASDHDIIVSIFYTIVIPKL
NPIIYSLRNKEVKKAVKKMLKLVYK

>P21453 multipass membrane protein
MGPTSVPLVKAHRSSVSDYVNYDIIVRHYNYTGKLNISADKENSIKLTSVVFILICCFIILENI
FVLLTIWKTKKFHRPMYYFIGNLALSDLLAGVAYTANLLLSGATTYKLTPAQWFLREGSM
FVALSASVFSLLAIAIERYITMLKMKLHNGSNNFRLFLLISACWVISLILGGLPIMGWNCISA
LSSCSTVLPLYHKHYILFCTTVFTLLLLSIVILYCRIYSLVRTRSRRLTFRKNISKASRSSEKSL
ALLKTVIIVLSVFIACWAPLFILLLLDVGCKVKTCDILFRAEYFLVLAVLNSGTNPIIYTLTN
KEMRRAFIRIMSCCKCPSGDSAGKFKRPIIAGMEFSRSKSDNSSHPQKDEGDNPETIMSSG
NVNSSS

>O43749 multipass membrane protein
MSGTNQSSVSEFLLLGLSRQPQQQHLLFVFFLSMYLATVLGNLLIILSVSIDSCLHTPMYFF
LSNLSFVDICFSFTTVPKMLANHILETQTISFCGCLTQMYFVFMFVDMDNFLLAVMAYDHF
VAVCHPLHYTAKMTHQLCALLVAGLWVVANLNVLLHTLLMAPLSFCADNAITHFFCDVT
PLLKLSCSDTHLNEVIILSEGALVMITPFLCILASYMHITCTVLKVPSTKGRWKAFSTCGSH
LAVVLLFYSTIIAVYFNPLSSHSAEKDTMATVLYTVVTPMLNPFIYSLRNRYLKGALKKVV

GRVVFSV

>P50607 peripheral membrane protein
MTSKPHSDWIPYSVLDDEGRNLRQQKLDRQRALLEQKQKKKRQEPLMVQANADGRPRS
RRARQSEEQAPLVESYLSSSGSTSYQVQEADSLASVQLGATRPTAPASAKRTKAAATAGGQ
GGAARKEKKGKHKGTSGPAALAEDKSEAQGPVQILTVGQSDHAQDAGETAAGGGERPS
GQDLRATMQRKGISSSMSFDEDEEDEEENSSSSSQLNSNTRPSSATSRKSVREAASAPSPTA
PEQPVDVEVQDLEEFALRPAPQGITIKCRITRDKKGMDRGMYPTYFLHLDREDGKKVFLL
AGRKRKKSKTSNYLISVDPTDLSRGGDSYIGKLRSNLMGTKFTVYDNGVNPQKASSSTLE
SGTLRQELAAVCYETNVLGFKGPRKMSVIVPGMNMVHERVSIRPRNEHETLLARWQNKN
TESIIELQNKTPVWNDDTQSYVLNFHGRVTQASVKNFQIIHGNDPDYIVMQFGRVAEDVFT
MDYNYPLCALQAFAIALSSFDSKLACE

>P83436 peripheral membrane protein
MDFSKFLADDFDVKEWINAAFRAGSKEAASGKADGHAATLVMKLQLFIQEVNHAVEETS
HQALQNMPKVLRDVEALKQEASFLKEQMILVKEDIKKFEQDTSQSMQVLVEIDQVKSRM
QLAAESLQEADKWSTLSADIEETFKTQDIAVISAKLTGMQNSLMMLVDTPDYSEKCVHLE
ALKNRLEALASPQIVAAFTSQAVDQSKVFVKVFTEIDRMPQLLAYYYKCHKVQLLAAWQ
ELCQSDLSLDRQLTGLYDALLGAWHTQIQWATQVFQKPHEVVMVLLIQTLGALMPSLPSC
LSNGVERAGPEQELTRLLEFYDATAHFAKGLEMALLPHLHEHNLVKVTELVDAVYDPYKP
YQLKYGDMEESNLLIQMSAVPLEHGEVIDCVQELSHSVNKLFGLASAAVDRCVRFTNGLG
TCGLLSALKSLFAKYVSDFTSTLQSIRKKCKLDHIPPNSLFQEDWTAFQNSIRIIATCGELLR
HCGDFEQQLANRILSTAGKYLSDSCSPRSLAGFQESILTDKKNSAKNPWQEYNYLQKDNP
AEYASLMEILYTLKEKGSSNHNLLAAPRAALTRLNQQAHQLAFDSVFLRIKQQLLLISKMD
SWNTAGIGETLTDELPAFSLTPLEYISNIGQYIMSLPLNLEPFVTQEDSALELALHAGKLPFP
PEQGDELPELDNMADNWLGSIARATMQTYCDAILQIPELSPHSAKQLATDIDYLINVMDA
LGLQPSRTLQHIVTLLKTRPEDYRQVSKGLPRRLATTVATMRSVNY

>Q96MW5 peripheral membrane protein
MATAATIPSVATATAAALGEVEDEGLLASLFRDRFPEAQWRERPDVGRYLRELSGSGLERL
RREPERLAEERAQLLQQTRDLAFANYKTFIRGAECTERIHRLFGDVEASLGRLLDRLPSFQ
QSCRNFVKEAEEISSNRRMNSLTLNRHTEILEILEIPQLMDTCVRNSYYEEALELAAYVRRL
ERKYSSIPVIQGIVNEVRQSMQLMLSQLIQQLRTNIQLPACLRVIGYLRRMDVFTEAELRVK
FLQARDAWLRSILTAIPNDDPYFHITKTIEASRVHLFDIITQYRAIFSDEDPLLPPAMGEHTV
NESAIFHGWVLQKVSQFLQVLETDLYRGIGGHLDSLLGQCMYFGLSFSRVGADFRGQLAP
VFQRVAISTFQKAIQETVEKFQEEMNSYMLISAPAILGTSNMPAAVPATQPGTLQPPMVLLD
FPPLACFLNNILVAFNDLRLCCPVALAQDVTGALEDALAKVTKIILAFHRAEEAAFSSGEQE
LFVQFCTVFLEDLVPYLNRCLQVLFPPAQIAQTLGIPPTQLSKYGNLGHVNIGAIQEPLAFIL
PKRETLFTLDDQALGPELTAPAPEPPAEEPRLEPAGPACPEGGRAETQAEPPSVGP

>Q8NGQ3 multipass membrane protein
MKTLCSFLQISRNMHQENQTTITEFILLGLSNQAEHQNLLFVLFLSMYVVTVVGNGLIIVAI
SLDIYLHTPMYLFLAYLSFADISSISNSVPKMLVNIQTNSQSISYESCITQMYFSIVFVVTDNL
LLGTMAFDHFVAICHPLNYTTFMRARFGTLLTVISWFLSNIIALTHTLLLIQLLFCDHNTLP

HFFCDLAPLLKLSCSDTMINELVLFIVGLSVIIFPFVLIFFSYVCIIRAVLGVSSTQGKWKAFS
TCGSHLTIALLFYGTTVGVYFFPSSTHPEDTDKIGAVLFTVVTPMMNPFIYSLRNKDMKGA
LRKLINRKISSL

>Q8NGZ6 multipass membrane protein
MDTGNKTLPQDFLLLGFPGSQTLQLSLFMLFLVMYILTVSGNVAILMLVSTSHQLHTPMYF
FLSNLSFLEIWYTTAAVPKALAILLGRSQTISFTSCLLQMYFVFSLGCTEYFLLAAMAYDRC
LAICYPLHYGAIMSSLLSAQLALGSWVCGFVAIAVPTALISGLSFCGPRAINHFFCDIAPWIA
LACTNTQAVELVAFVIAVVVILSSCLITFVSYVYIISTILRIPSASGRSKAFSTCSSHLTVVLIW
YGSTVFLHVRTSIKDALDLIKAVHVLNTVVTPVLNPFIYTLRNKEVRETLLKKWKGK

>Q9P2D0 peripheral membrane protein
MSSPMPDCTSKCRSLKHALDVLSVVTKGSENQIKAFLSSHCYNAATIKDVFGRNALHLVS
SCGKKGVLDWLIQKGVDLLVKDKESGWTALHRSIFYGHIDCVWSLLKHGVSLYIQDKEG
LSALDLVMKDRPTHVVFKNTDPTDVYTWGDNTNFTLGHGSQNSKHHPELVDLFSRSGIYI
KQVVLCKFHSVFLSQKGQVYTCGHGPGGRLGHGDEQTCLVPRLVEGLNGHNCSQVAAA
KDHTVVLTEDGCVYTFGLNIFHQLGIIPPPSSCNVPRQIQAKYLKGRTIIGVAAGRFHTVLW
TREAVYTMGLNGGQLGCLLDPNGEKCVTAPRQVSALHHKDIALSLVAASDGATVCVTTR
GDIYLLADYQCKKMASKQLNLKKVLVSGGHMEYKVDPEHLKENGGQKICILAMDGAGR
VFCWRSVNSSLKQCRWAYPRQVFISDIALNRNEILFVTQDGEGFRGRWFEEKRKSSEKKEI
LSNLHNSSSDVSYVSDINSVYERIRLEKLTFAHRAVSVSTDPSGCNFAILQSDPKTSLYEIPA
VSSSSFFEEFGKLLREADEMDSIHDVTFQVGNRLFPAHKYILAVHSDFFQKLFLSDGNTSEF
TDIYQKDEDSAGCHLFVVEKVHPDMFEYLLQFIYTDTCDFLTHGFKPRIHLNKNPEEYQG
TLNSHLNKVNFHEDDNQKSAFEVYKSNQAQTVSERQKSKPKSCKKGKNIREDDPVRMLQ
TVAKKFDFSNLSSRLDGVRFENEKINVIAKNTGNKLKLSQKKCSFLCDVTMKSVDGKEFP
CHKCVLCARLEYFHSMLSSSWIEASSCAALEMPIHSDILKVILDYLYTDEAVVIKESQNVD
FICSVLVVADQLLITRLKEICEVALTEKLTLKNAAMLLEFAAMYSAKQLKLSCLQFIGLNM
AALLEARSLDVLSDGVLKDLSEFYRKMIPAMDRRVITPYQDGPDISYLEVEDGDIFLKEEI
NMEQNHSETMFKKAKTKAKKKPRKRSDSSGGYNLSDIIQSPSSTGLLKSGKTNSVESLPE
LLTSDSEGSYAGVGSPRDLQSPDFTTGFHSDKIEAKVKPYVNGTSPVYSREDLKPWEKSPI
LKISAPQPIPSNRIDTTSSASWVAGSFSPVSPPVVDLRTIMEIEESRQKCGATPKSHLGKTVS
HGVKLSQKQRKMIALTTKENNSGMNSMETVLFTPSKAPKPVNAWASSLHSVSSKSFRDFL
LEEKKSVTSHSSGDHVKKVSFKGIENSQAPKIVRCSTHGTPGPEGNHISDLPLLDSPNPWL
SSSVTAPSMVAPVTFASIVEEELQQEAALIRSREKPLALIQIEEHAIQDLLVFYEAFGNPEEF
VIVERTPQGPLAVPMWNKHGC

>Q96LB2 multipass membrane protein
MDPTISTLDTELTPINGTEETLCYKQTLSLTVLTCIVSLVGLTGNAVVLWLLGCRMRRNAFS
IYILNLAAADFLFLSGRLIYSLLSFISIPHTISKILYPVMMFSYFAGLSFLSAVSTERCLSVLWP
IWYRCHRPTHLSAVVCVLLWALSLLRSILEWMLCGFLFSGADSAWCQTSDFITVAWLIFLC
VVLCGSSLVLLIRILCGSRKIPLTRLYVTILLTVLVFLLCGLPFGIQFFLFLWIHVDREVLFCH
VHLVSIFLSALNSSANPIIYFFVGSFRQRQNRQNLKLVLQRALQDASEVDEGGGQLPEEILE
LSGSRLEQ

>Q9BQ31 multipass membrane protein
MVFGEFFHRPGQDEELVNLNVGGFKQSVDQSTLLRFPHTRLGKLLTCHSEEAILELCDDYS
VADKEYYFDRNPSLFRYVLNFYYTGKLHVMEELCVFSFCQEIEYWGINELFIDSCCSNRYQ
ERKEENHEKDWDQKSHDVSTDSSFEESSLFEKELEKFDTLRFGQLRKKIWIRMENPAYCLS
AKLIAISSLSVVLASIVAMCVHSMSEFQNEDGEVDDPVLEGVEIACIAWFTGELAVRLAAA
PCQKKFWKNPLNIIDFVSIIPFYATLAVDTKEEESEDIENMGKVVQILRLMRIFRILKLARHS
VGLRSLGATLRHSYHEVGLLLLFLSVGISIFSVLIYSVEKDDHTSSLTSIPICWWWATISMTT
VGYGDTHPVTLAGKLIASTCIICGILVVALPITIIFNKFSKYYQKQKDIDVDQCSEDAPEKCH
ELPYFNIRDIYAQRMHTFITSLSSVGIVVSDPDSTDASSIEDNEDICNTTSLENCTAK

>P48230 multipass membrane protein
MCTGGCARCLGGTLIPLAFFGFLANILLFFPGGKVIDDNDHLSQEIWFFGGILGSGVLMIFP
ALVFLGLKNNDCCGCCGNEGCGKRFAMFTSTIFAVVGFLGAGYSFIISAISINKGPKCLMA
NSTWGYPFHDGDYLNDEALWNKCREPLNVVPWNLTLFSILLVVGGIQMVLCAIQVVNGL
LGTLCGDCQCCGCCGGDGPV

>Q9HB89 multipass membrane protein
MTPLCLNCSVLPGDLYPGGARNPMACNGSAARGHFDPEDLNLTDEALRLKYLGPQQTEL
FMPICATYLLIFVVGAVGNGLTCLVILRHKAMRTPTNYYLFSLAVSDLLVLLVGLPLELYEM
WHNYPFLLGVGGCYFRTLLFEMVCLASVLNVTALSVERYVAVVHPLQARSMVTRAHVRR
VLGAVWGLAMLCSLPNTSLHGIRQLHVPCRGPVPDSAVCMLVRPRALYNMVVQTTALLF
FCLPMAIMSVLYLLIGLRLRRERLLLMQEAKGRGSAAARSRYTCRLQQHDRGRRQVTKM
LFVLVVVFGICWAPFHADRVMWSVVSQWTDGLHLAFQHVHVISGIFFYLGSAANPVLYSL
MSSRFRETFQEALCLGACCHRLRPRHSSHSLSRMTTGSTLCDVGSLGSWVHPLAGNDGPE
AQQETDPS

>O75445 single-pass type I membrane protein
MNCPVLSLGSGFLFQVIEMLIFAYFASISLTESRGLFPRLENVGAFKKVSIVPTQAVCGLPDR
STFCHSSAAAESIQFCTQRFCIQDCPYRSSHPTYTALFSAGLSSCITPDKNDLHPNAHSNSAS
FIFGNHKSCFSSPPSPKLMASFTLAVWLKPEQQGVMCVIEKTVDGQIVFKLTISEKETMFY
YRTVNGLQPPIKVMTLGRILVKKWIHLSVQVHQTKISFFINGVEKDHTPFNARTLSGSITDF
ASGTVQIGQSLNGLEQFVGRMQDFRLYQVALTNREILEVFSGDLLRLHAQSHCRCPGSHPR
VHPLAQRYCIPNDAGDTADNRVSRLNPEAHPLSFVNDNDVGTSWVSNVFTNITQLNQGV
TISVDLENGQYQVFYIIIQFFSPQPTEIRIQRKKENSLDWEDWQYFARNCGAFGMKNNGDL
EKPDSVNCLQLSNFTPYSRGNVTFSILTPGPNYRPGYNNFYNTPSLQEFVKATQIRFHFHGQ
YYTTETAVNLRHRYYAVDEITISGRCQCHGHADNCDTTSQPYRCLCSQESFTEGLHCDRCL
PLYNDKPFRQGDQVYAFNCKPCQCNSHSKSCHYNISVDPFPFEHFRGGGGVCDDCEHNTT
GRNCELCKDYFFRQVGADPSAIDVCKPCDCDTVGTRNGSILCDQIGGQCNCKRHVSGRQ
CNQCQNGFYNLQELDPDGCSPCNCNTSGTVDGDITCHQNSGQCKCKANVIGLRCDHCNF
GFKFLRSFNDVGCEPCQCNLHGSVNKFCNPHSGQCECKKEAKGLQCDTCRENFYGLDVT
NCKACDCDTAGSLPGTVCNAKTGQCICKPNVEGRQCNKCLEGNFYLRQNNSFLCLPCNC
DKTGTINGSLLCNKSTGQCPCKLGVTGLRCNQCEPHRYNLTIDNFQHCQMCECDSLGTLP
GTICDPISGQCLCVPNRQGRRCNQCQPGFYISPGNATGCLPCSCHTTGAVNHICNSLTGQC
VCQDASIAGQRCDQCKDHYFGFDPQTGRCQPCNCHLSGALNETCHLVTGQCFCKQFVTG

SKCDACVPSASHLDVNNLLGCSKTPFQQPPPRGQVQSSSAINLSWSPPDSPNAHWLTYSLL
RDGFEIYTTEDQYPYSIQYFLDTDLLPYTKYSYYIETTNVHGSTRSVAVTYKTKPGVPEGN
LTLSYIIPIGSDSVTLTWTTLSNQSGPIEKYILSCAPLAGGQPCVSYEGHETSATIWNLVPFAK
YDFSVQACTSGGCLHSLPITVTTAQAPPQRLSPPKMQKISSTELHVEWSPPAELNGIIIRYEL
YMRRLRSTKETTSEESRVFQSSGWLSPHSFVESANENALKPPQTMTTITGLEPYTKYEFRV
LAVNMAGSVSSAWVSERTGESAPVFMIPPSVFPLSSYSLNISWEKPADNVTRGKVVGYDIN
MLSEQSPQQSIPMAFSQLLHTAKSQELSYTVEGLKPYRIYEFTITLCNSVGCVTSASGAGQ
TLAAAPAQLRPPLVKGINSTTIHLRWFPPEELNGPSPIYQLERRESSLPALMTTMMKGIRFIG
NGYCKFPSSTHPVNTDFTGIKASFRTKVPEGLIVFAASPGNQEEYFALQLKKGRLYFLFDPQ
GSPVEVTTTNDHGKQYSDGKWHEIIAIRHQAFGQITLDGIYTGSSAILNGSTVIGDNTGVF
LGGLPRSYTILRKDPEIIQKGFVGCLKDVHFMKNYNPSAIWEPLDWQSSEEQINVYNSWE
GCPASLNEGAQFLGAGFLELHPYMFHGGMNFEISFKFRTDQLNGLLLFVYNKDGPDFLA
MELKSGILTFRLNTSLAFTQVDLLLGLSYCNGKWNKVIIKKEGSFISASVNGLMKHASESG
DQPLVVNSPVYVGGIPQELLNSYQHLCLEQGFGGCMKDVKFTRGAVVNLASVSSGAVRV
NLDGCLSTDSAVNCRGNDSILVYQGKEQSVYEGGLQPFTEYLYRVIASHEGGSVYSDWSR
GRTTGAAPQSVPTPSRVRSLNGYSIEVTWDEPVVRGVIEKYILKAYSEDSTRPPRMPSASA
EFVNTSNLTGILTGLLPFKNYAVTLTACTLAGCTESSHALNISTPQEAPQEVQPPVAKSLPSS
LLLSWNPPKKANGIITQYCLYMDGRLIYSGSEENYIVTDLAVFTPHQFLLSACTHVGCTNS
SWVLLYTAQLPPEHVDSPVLTVLDSRTIHIQWKQPRKISGILERYVLYMSNHTHDFTIWSVI
YNSTELFQDHMLQYVLPGNKYLIKLGACTGGGCTVSEASEALTDEDIPEGVPAPKAHSYS
PDSFNVSWTEPEYPNGVITSYGLYLDGILIHNSSELSYRAYGFAPWSLHSFRVQACTAKGC
ALGPLVENRTLEAPPEGTVNVFVKTQGSRKAHVRWEAPFRPNGLLTHSVLFTGIFYVDPV
GNNYTLLNVTKVMYSGEETNLWVLIDGLVPFTNYTVQVNISNSQGSLITDPITIAMPPGAP
DGVLPPRLSSATPTSLQVVWSTPARNNAPGSPRYQLQMRSGDSTHGFLELFSNPSASLSYE
VSDLQPYTEYMFRLVASNGFGSAHSSWIPFMTAEDKPGPVVPPILLDVKSRMMLVTWQHP
RKSNGVITHYNIYLHGRLYLRTPGNVTNCTVMHLHPYTAYKFQVEACTSKGCSLSPESQT
VWTLPGAPEGIPSPELFSDTPTSVIISWQPPTHPNGLVENFTIERRVKGKEEVTTLVTLPRSH
SMRFIDKTSALSPWTKYEYRVLMSTLHGGTNSSAWVEVTTRPSRPAGVQPPVVTVLEPDA
VQVTWKPPLIQNGDILSYEIHMPDPHITLTNVTSAVLSQKVTHLIPFTNYSVTIVACSGGNG
YLGGCTESLPTYVTTHPTVPQNVGPLSVIPLSESYVVISWQPPSKPNGPNLRYELLRRKIQQ
PLASNPPEDLNRWHNIYSGTQWLYEDKGLSRFTTYEYMLFVHNSVGFTPSREVTVTTLAG
LPERGANLTASVLNHTAIDVRWAKPTVQDLQGEVEYYTLFWSSATSNDSLKILPDVNSHVI
GHLKPNTEYWIFISVFNGVHSINSAGLHATTCDGEPQGMLPPEVVIINSTAVRVIWTSPSNP
NGVVTEYSIYVNNKLYKTGMNVPGSFILRDLSPFTIYDIQVEVCTIYACVKSNGTQITTVED
TPSDIPTPTIRGITSRSLQIDWVSPRKPNGIILGYDLLWKTWYPCAKTQKLVQDQSDELCKA
VRCQKPESICGHICYSSEAKVCCNGVLYNPKPGHRCCEEKYIPFVLNSTGVCCGGRIQEAQ
PNHQCCSGYYARILPGEVCCPDEQHNRVSVGIGDSCCGRMPYSTSGNQICCAGRLHDGHG
QKCCGRQIVSNDLECCGGEEGVVYNRLPGMFCCGQDYVNMSDTICCSASSGESKAHIKK
NDPVPVKCCETELIPKSQKCCNGVGYNPLKYVCSDKISTGMMMKETKECRILCPASMEAT
EHCGRCDFNFTSHICTVIRGSHNSTGKASIEEMCSSAEETIHTGSVNTYSYTDVNLKPYMT
YEYRISAWNSYGRGLSKAVRARTKEDVPQGVSPPTWTKIDNLEDTIVLNWRKPIQSNGPII
YYILLRNGIERFRGTSLSFSDKEGIQPFQEYSYQLKACTVAGCATSSKVVAATTQGVPESILP
PSITALSAVALHLSWSVPEKSNGVIKEYQIRQVGKGLIHTDTTDRRQHTVTGLQPYTNYSF
TLTACTSAGCTSSEPFLGQTLQAAPEGVWVTPRHIIINSTTVELYWSLPEKPNGLVSQYQLS

RNGNLLFLGGSEEQNFTDKNLEPNSRYTYKLEVKTGGGSSASDDYIVQTPMSTPEEIYPPY
NITVIGPYSIFVAWIPPGILIPEIPVEYNVLLNDGSVTPLAFSVGHHQSTLLENLTPFTQYEIRI
QACQNGSCGVSSRMFVKTPEAAPMDLNSPVLKALGSACIEIKWMPPEKPNGIIINYFIYRR
PAGIEEESVLFVWSEGALEFMDEGDTLRPFTLYEYRVRACNSKGSVESLWSLTQTLEAPPQ
DFPAPWAQATSAHSVLLNWTKPESPNGIISHYRVVYQERPDDPTFNSPTVHAFTVKGTSHQ
AHLYGLEPFTTYRIGVVAANHAGEILSPWTLIQTLESSPSGLRNFIVEQKENGRALLLQWSE
PMRTNGVIKTYNIFSDGFLEYSGLNRQFLFRRLDPFTLYTLTLEACTRAGCAHSAPQPLWT
DEAPPDSQLAPTVHSVKSTSVELSWSEPVNPNGKIIRYEVIRRCFEGKAWGNQTIQADEKI
VFTEYNTERNTFMYNDTGLQPWTQCEYKIYTWNSAGHTCSSWNVVRTLQAPPEGLSPPV
ISYVSMNPQKLLISWIPPEQSNGIIQSYRLQRNEMLYPFSFDPVTFNYTDEELLPFSTYSYAL
QACTSGGCSTSKPTSITTLEAAPSEVSPPDLWAVSATQMNVCWSPPTVQNGKITKYLVRYD
NKESLAGQGLCLLVSHLQPYSQYNFSLVACTNGGCTASVSKSAWTMEALPENMDSPTLQ
VTGSESIEITWKPPRNPNGQIRSYELRRDGTIVYTGLETRYRDFTLTPGVEYSYTVTASNSQ
GGILSPLVKDRTSPSAPSGMEPPKLQARGPQEILVNWDPPVRTNGDIINYTLFIRELFERETK
IIHINTTHNSFGMQSYIVNQLKPFHRYEIRIQACTTLGCASSDWTFIQTPEIAPLMQPPPHLE
VQMAPGGFQPTVSLLWTGPLQPNGKVLYYELYRRQIATQPRKSNPVLIYNGSSTSFIDSEL
LPFTEYEYQVWAVNSAGKAPSSWTWCRTGPAPPEGLRAPTFHVISSTQAVVNISAPGKPNG
IVSLYRLFSSSAHGAETVLSEGMATQQTLHGLQAFTNYSIGVEACTCFNCCSKGPTAELRT
HPAPPSGLSSPQIGTLASRTASFRWSPPMFPNGVIHSYELQFHVACPPDSALPCTPSQIETKY
TGLGQKASLGGLQPYTTYKLRVVAHNEVGSTASEWISFTTQKELPQYRAPFSVDSNLSVV
CVNWSDTFLLNGQLKEYVLTDGGRRVYSGLDTTLYIPRTADKTFFFQVICTTDEGSVKTPL
IQYDTSTGLGLVLTTPGKKKGSRSKSTEFYSELWFIVLMAMLGLILLAIFLSLILQRKIHKEP
YIRERPPLVPLQKRMSPLNVYPPGENHMGLADTKIPRSGTPVSIRSNRSACVLRIPSQNQTS
LTYSQGSLHRSVSQLMDIQDKKVLMDNSLWEAIMGHNSGLYVDEEDLMNAIKDFSSVTK
ERTTFTDTHL

>Q8NGN0 multipass membrane protein
MNPANHSQVAGFVLLGLSQVWELRFVFFTVFSAVYFMTVVGNLLIVVIVTSDPHLHTTMY
FLLGNLSFLDFCYSSITAPRMLVDLLSGNPTISFGGCLTQLFFFHFIGGIKIFLLTVMAYDRYI
AISQPLHYTLIMNQTVCALLMAASWVGGFIHSIVQIALTIQLPFCGPDKLDNFYCDVPQLIK
LACTDTFVLELLMVSNNGLVTLMCFLVLLGSYTALLVMLRSHSREGRSKALSTCASHIAV
VTLIFVPCIYVYTRPFRTFPMDKAVSVLYTIVTPMLNPAIYTLRNKEVIMAMKKLWRRKKD
PIGPLEHRPLH

>P46721 multipass membrane protein
MGETEKRIETHRIRCLSKLKMFLLAITCAFVSKTLSGSYMNSMLTQIERQFNIPTSLVGFIN
GSFEIGNLLLIIFVSYFGTKLHRPIMIGIGCVVMGLGCFLKSLPHFLMNQYEYESTVSVSGN
LSSNSFLCMENGTQILRPTQDPSECTKEVKSLMWVYVLVGNIVRGMGETPILPLGISYIEDF
AKFENSPLYIGLVETGAIIGPLIGLLLASFCANVYVDTGFVNTDDLIITPTDTRWVGAWWFG
FLICAGVNVLTAIPFFFLPNTLPKEGLETNADIIKNENEDKQKEEVKKEKYGITKDFLPFMK
SLSCNPIYMLFILVSVIQFNAFVNMISFMPKYLEQQYGISSSDAIFLMGIYNLPPICIGYIIGG
LIMKKFKITVKQAAHIGCWLSLLEYLLYFLSFLMTCENSSVVGINTSYEGIPQDLYVENDIF
ADCNVDCNCPSKIWDPVCGNNGLSYLSACLAGCETSIGTGINMVFQNCSCIQTSGNSSAV
LGLCDKGPDCSLMLQYFLILSAMSSFIYSLAAIPGYMVLLRCMKSEEKSLGVGLHTFCTRV

FAGIPAPIYFGALMDSTCLHWGTLKCGESGACRIYDSTTFRYIYLGLPAALRGSSFVPALIILI
LLRKCHLPGENASSGTELIETKVKGKENECKDIYQKSTVLKDDELKTKL

>Q13740 single-pass type I membrane protein
MESKGASSCRLLFCLLISATVFRPGLGWYTVNSAYGDTIIIPCRLDVPQNLMFGKWKYEKP
DGSPVFIAFRSSTKKSVQYDDVPEYKDRLNLSENYTLSISNARISDEKRFVCMLVTEDNVF
EAPTIVKVFKQPSKPEIVSKALFLETEQLKKLGDCISEDSYPDGNITWYRNGKVLHPLEGA
VVIIFKKEMDPVTQLYTMTSTLEYKTTKADIQMPFTCSVTYYGPSGQKTIHSEQAVFDIYY
PTEQVTIQVLPPKNAIKEGDNITLKCLGNGNPPPEEFLFYLPGQPEGIRSSNTYTLTDVRRN
ATGDYKCSLIDKKSMIASTAITVHYLDLSLNPSGEVTRQIGDALPVSCTISASRNATVVVWM
KDNIRLRSSPSFSSLHYQDAGNYVCETALQEVEGLKKRESLTLIVEGKPQIKMTKKTDPSG
LSKTIICHVEGFPKPAIQWTITGSGSVINQTEESPYINGRYYSKIIISPEENVTLTCTAENQLER
TVNSLNVSAISIPEHDEADEISDENREKVNDQAKLIVGIVVGLLLAALVAGVVYWLYMKK
SKTASKHVNKDLGNMEENKKLEENNHKTEA

>P19021 single-pass type I membrane protein
MAGRVPSLLVLLVFPSSCLAFRSPLSVFKRFKETTRPFSNECLGTTRPVVPIDSSDFALDIRM
PGVTPKQSDTYFCMSMRIPVDEEAFVIDFKPRASMDTVHHMLLFGCNMPSSTGSYWFCD
EGTCTDKANILYAWARNAPPTRLPKGVGFRVGGETGSKYFVLQVHYGDISAFRDNNKDCS
GVSLHLTRLPQPLIAGMYLMMSVDTVIPAGEKVVNSDISCHYKNYPMHVFAYRVHTHHL
GKVVSGYRVRNGQWTLIGRQSPQLPQAFYPVGHPVDVSFGDLLAARCVFTGEGRTEATHI
GGTSSDEMCNLYIMYYMEAKHAVSFMTCTQNVAPDMFRTIPPEANIPIPVKSDMVMMHE
HHKETEYKDKIPLLQQPKREEEEVLDQGDFYSLLSKLLGEREDVVHVHKYNPTEKAESES
DLVAEIANVVQKKDLGRSDAREGAEHERGNAILVRDRIHKFHRLVSTLRPPESRVFSLQQP
PPGEGTWEPEHTGDFHMEEALDWPGVYLLPGQVSGVALDPKNNLVIFHRGDHVWDGNS
FDSKFVYQQIGLGPIEEDTILVIDPNNAAVLQSSGKNLFYLPHGLSIDKDGNYWVTDVALH
QVFKLDPNNKEGPVLILGRSMQPGSDQNHFCQPTDVAVDPGTGAIYVSDGYCNSRIVQFS
PSGKFITQWGEESSGSSPLPGQFTVPHSLALVPLLGQLCVADRENGRIQCFKTDTKEFVREI
KHSSFGRNVFAISYIPGLLFAVNGKPHFGDQEPVQGFVMNFSNGEIIDIFKPVRKHFDMPHD
IVASEDGTVYIGDAHTNTVWKFTLTEKLEHRSVKKAGIEVQEIKEAEAVVETKMENKPTSS
ELQKMQEKQKLIKEPGSGVPVVLITTLLVIPVVVLLAIAIFIRWKKSRAFGDSEHKLETSSG
RVLGRFRGKGSGGLNLGNFFASRKGYSRKGFDRLSTEGSDQEKEDDGSESEEEYSAPLPA
LAPSSS

>P21918 multipass membrane protein
MLPPGSNGTAYPGQFALYQQLAQGNAVGGSAGAPPLGPSQVVTACLLTLLIIWTLLGNVLV
CAAIVRSRHLRANMTNVFIVSLAVSDLFVALLVMPWKAVAEVAGYWPFGAFCDVWVAFD
IMCSTASILNLCVISVDRYWAISRPFRYKRKMTQRMALVMVGLAWTLSILISFIPVQLNWH
RDQAASWGGLDLPNNLANWTPWEEDFWEPDVNAENCDSSLNRTYAISSSLISFYIPVAIMI
VTYTRIYRIAQVQIRRISSLERAAEHAQSCRSSAACAPDTSLRASIKKETKVLKTLSVIMGV
FVCCWLPFFILNCMVPFCSGHPEGPPAGFPCVSETTFDVFVWFGWANSSLNPVIYAFNADF
QKVFAQLLGCSHFCSRTPVETVNISNELISYNQDIVFHKEIAAAYIHMMPNAVTPGNREVD
NDEEEGPFDRMFQIYQTSPDGDPVAESVWELDCEGEISLDKITPFTPNGFH

>P41594 multipass membrane protein
MVLLLILSVLLLKEDVRGSAQSSERRVVAHMPGDIIIGALFSVHHQPTVDKVHERKCGAVR
EQYGIQRVEAMLHTLERINSDPTLLPNITLGCEIRDSCWHSAVALEQSIEFIRDSLISSEEEEG
LVRCVDGSSSSFRSKKPIVGVIGPGSSSVAIQVQNLLQLFNIPQIAYSATSMDLSDKTLFKYF
MRVVPSDAQQARAMVDIVKRYNWTYVSAVHTEGNYGESGMEAFKDMSAKEGICIAHSY
KIYSNAGEQSFDKLLKKLTSHLPKARVVACFCEGMTVRGLLMAMRRLGLAGEFLLLGSD
GWADRYDVTDGYQREAVGGITIKLQSPDVKWFDDYYLKLRPETNHRNPWFQEFWQHRF
QCRLEGFPQENSKYNKTCNSSLTLKTHHVQDSKMGFVINAIYSMAYGLHNMQMSLCPGY
AGLCDAMKPIDGRKLLESLMKTNFTGVSGDTILFDENGDSPGRYEIMNFKEMGKDYFDYI
NVGSWDNGELKMDDDEVWSKKSNIIRSVCSEPCEKGQIKVIRKGEVSCCWTCTPCKENE
YVFDEYTCKACQLGSWPTDDLTGCDLIPVQYLRWGDPEPIAAVVFACLGLLATLFVTVVFI
IYRDTPVVKSSSRELCYIILAGICLGYLCTFCLIAKPKQIYCYLQRIGIGLSPAMSYSALVTKT
NRIARILAGSKKKICTKKPRFMSACAQLVIAFILICIQLGIIVALFIMEPPDIMHDYPSIREVYL
ICNTTNLGVVTPLGYNGLLILSCTFYAFKTRNVPANFNEAKYIAFTMYTTCIIWLAFVPIYF
GSNYKIITMCFSVSLSATVALGCMFVPKVYIILAKPERNVRSAFTTSTVVRMHVGDGKSSS
AASRSSSLVNLWKRRGSSGETLRYKDRRLAQHKSEIECFTPKGSMGNGGRATMSSSNGKS
VTWAQNEKSSRGQHLWQRLSIHINKKENPNQTAVIKPFPKSTESRGLGAGAGAGGSAGGV
GATGGAGCAGAGPGGPESPDAGPKALYDVAEAEEHFPAPARPRSPSPISTLSHRAGSASRT
DDDVPSLHSEPVARSSSSQGSLMEQISSVVTRFTANISELNSMMLSTAAPSPGVGAPLCSSY
LIPKEIQLPTTMTTFAEIQPLPAIEVTGGAQPAAGAQAAGDAARESPAAGPEAAAAKPDLEE
LVALTPPSPFRDSVDSGSTTPNSPVSESALCIPSSPKYDTLIIRDYTQSSSSL

>P16871 single-pass type I membrane protein
MTILGTTFGMVFSLLQVVSGESGYAQNGDLEDAELDDYSFSCYSQLEVNGSQHSLTCAFE
DPDVNTTNLEFEICGALVEVKCLNFRKLQEIYFIETKKFLLIGKSNICVKVGEKSLTCKKIDL
TTIVKPEAPFDLSVIYREGANDFVVTFNTSHLQKKYVKVLMHDVAYRQEKDENKWTHVN
LSSTKLTLLQRKLQPAAMYEIKVRSIPDHYFKGFWSEWSPSYYFRTPEINNSSGEMDPILLTI
SILSFFSVALLVILACVLWKKRIKPIVWPSLPDHKKTLEHLCKKPRKNLNVSFNPESFLDCQI
HRVDDIQARDEVEGFLQDTFPQQLEESEKQRLGGDVQSPNCPSEDVVITPESFGRDSSLTC
LAGNVSACDAPILSSSRSLDCRESGKNGPHVYQDLLLSLGTTNSTLPPPFSLQSGILTLNPV
AQGQPILTSLGSNQEEAYVTMSSFYQNQ

>P58335 single-pass type I membrane protein
MVAERSPARSPGSWLFPGLWLLVLSGPGGLLRAQEQPSCRRAFDLYFVLDKSGSVANNWI
EIYNFVQQLAERFVSPEMRLSFIVFSSQATIILPLTGDRGKISKGLEDLKRVSPVGETYIHEG
LKLANEQIQKAGGLKTSSIIIALTDGKLDGLVPSYAEKEAKISRSLGASVYCVGVLDFEQAQ
LERIADSKEQVFPVKGGFQALKGIINSILAQSCTEILELQPSSVCVGEEFQIVLSGRGFMLGS
RNGSVLCTYTVNETYTTSVKPVSVQLNSMLCPAPILNKAGETLDVSVSFNGGKSVISGSLI
VTATECSNGIAAIIVILVLLLLLGIGLMWWFWPLCCKVVIKDPPPPPAPAPKEEEEEPLPTKK
WPTVDASYYGGRGVGGIKRMEVRWGDKGSTEEGARLEKAKNAVVKIPEETEEPIRPRPPR
PKPTHQPPQTKWYTPIKGRLDALWALLRRQYDRVSLMRPQEGDEVCIWECIEKELTA

>Q13291 single-pass type I membrane protein
MDPKGLLSLTFVLFLSLAFGASYGTGGRMMNCPKILRQLGSKVLLPLTYERINKSMNKSIH

IVVTMAKSLENSVENKIVSLDPSEAGPPRYLGDRYKFYLENLTLGIRESRKEDEGWYLMTL
EKNVSVQRFCLQLRLYEQVSTPEIKVLNKTQENGTCTLILGCTVEKGDHVAYSWSEKAGT
HPLNPANSSHLLSLTLGPQHADNIYICTVSNPISNNSQTFSPWPGCRTDPSETKPWAVYAGL
LGGVIMILIMVVILQLRRRGKTNHYQTTVEKKSLTIYAQVQKPGPLQKKLDSFPAQDPCTTI
YVAATEPVPESVQETNSITVYASVTLPES

>Q8N4V1 single-pass type I membrane protein
MAPSLWKGLVGIGLFALAHAAFSAAQHRSYMRLTEKEDESLPIDIVLQTLLAFAVTCYGIV
HIAGEFKDMDATSELKNKTFDTLRNHPSFYVFNHRGRVLFRPSDTANSSNQDALSSNTSLK
LRKLESLRR

>P07911 GPI-anchor, lipid-anchor
MGQPSLTWMLMVVVASWFITTAATDTSEARWCSECHSNATCTEDEAVTTCTCQEGFTGD
GLTCVDLDECAIPGAHNCSANSSCVNTPGSFSCVCPEGFRLSPGLGCTDVDECAEPGLSHC
HALATCVNVVGSYLCVCPAGYRGDGWHCECSPGSCGPGLDCVPEGDALVCADPCQAHR
TLDEYWRSTEYGEGYACDTDLRGWYRFVGQGGARMAETCVPVLRCNTAAPMWLNGTH
PSSDEGIVSRKACAHWSGHCCLWDASVQVKACAGGYYVYNLTAPPECHLAYCTDPSSVE
GTCEECSIDEDCKSNNGRWHCQCKQDFNITDISLLEHRLECGANDMKVSLGKCQLKSLGF
DKVFMYLSDSRCSGFNDRDNRDWVSVVTPARDGPCGTVLTRNETHATYSNTLYLADEIII
RDLNIKINFACSYPLDMKVSLKTALQPMVSALNIRVGGTGMFTVRMALFQTPSYTQPYQG
SSVTLSTEAFLYVGTMLDGGDLSRFALLMTNCYATPSSNATDPLKYFIIQDRCPHTRDSTIQ
VVENGESSQGRFSVQMFRFAGNYDLVYLHCEVYLCDTMNEKCKPTCSGTRFRSGSVIDQ
SRVLNLGPITRKGVQATVSRAFSSLGLLKVWLPLLLSATLTLTFQ

>Q9BSK2 multipass membrane protein
MATGGQQKENTLLHLFAGGCGGTVGAIFTCPLEVIKTRLQSSRLALRTVYYPQVHLGTISG
AGMVRPTSVTPGLFQVLKSILEKEGPKSLFRGLGPNLVGVAPSRAVYFACYSKAKEQFNGI
FVPNSNIVHIFSAGSAAFITNSLMNPIWMVKTRMQLEQKVRGSKQMNTLQCARYVYQTE
GIRGFYRGLTASYAGISETIICFAIYESLKKYLKEAPLASSANGTEKNSTSFFGLMAAAALSK
GCASCIAYPHEVIRTRLREEGTKYKSFVQTARLVFREEGYLAFYRGLFAQLIRQIPNTAIVLS
TYELIVYLLEDRTQ

>O15354 multipass membrane protein
MRAPGALLARMSRLLLLLLLKVSASSALGVAPASRNETCLGESCAPTVIQRRGRDAWGPG
NSARDVLRARAPREEQGAAFLAGPSWDLPAAPGRDPAAGRGAEASAAGPPGPPTRPPGP
WRWKGARGQEPSETLGRGNPTALQLFLQISEEEEKGPRGAGISGRSQEQSVKTVPGASDL
FYWPRRAGKLQGSHHKPLSKTANGLAGHEGWTIALPGRALAQNGSLGEGIHEPGGPRRG
NSTNRRVRLKNPFYPLTQESYGAYAVMCLSVVIFGTGIIGNLAVMCIVCHNYYMRSISNSLL
ANLAFWDFLIIFFCLPLVIFHELTKKWLLEDFSCKIVPYIEVASLGVTTFTLCALCIDRFRAA
TNVQMYYEMIENCSSTTAKLAVIWVGALLLALPEVVLRQLSKEDLGFSGRAPAERCIIKISP
DLPDTIYVLALTYDSARLWWYFGCYFCLPTLFTITCSLVTARKIRKAEKACTRGNKRQIQL
ESQMNCTVVALTILYGFCIIPENICNIVTAYMATGVSQQTMDLLNIISQFLLFFKSCVTPVLL
FCLCKPFSRAFMECCCCCCEECIQKSSTVTSDDNDNEYTTELELSPFSTIRREMSTFASVGT
HC

>P11229 multipass membrane protein
MNTSAPPAVSPNITVLAPGKGPWQVAFIGITTGLLSLATVTGNLLVLISFKVNTELKTVNNY
FLLSLACADLIIGTFSMNLYTTYLLMGHWALGTLACDLWLALDYVASNASVMNLLLISFD
RYFSVTRPLSYRAKRTPRRAALMIGLAWLVSFVLWAPAILFWQYLVGERTVLAGQCYIQFL
SQPIITFGTAMAAFYLPVTVMCTLYWRIYRETENRARELAALQGSETPGKGGGSSSSSERS
QPGAEGSPETPPGRCCRCCRAPRLLQAYSWKEEEEEDEGSMESLTSSEGEEPGSEVVIKMP
MVDPEAQAPTKQPPRSSPNTVKRPTKKGRDRAGKGQKPRGKEQLAKRKTFSLVKEKKAA
RTLSAILLAFILTWTPYNIMVLVSTFCKDCVPETLWELGYWLCYVNSTINPMCYALCNKAF
RDTFRLLLLCRWDKRRWRKIPKRPGSVHRTPSRQC

>P48050 multipass membrane protein
MHGHSRNGQAHVPRRKRRNRFVKKNGQCNVYFANLSNKSQRYMADIFTTCVDTRWRY
MLMIFSAAFLVSWLFFGLLFWCIAFFHGDLEASPGVPAAGGPAAGGGGAAPVAPKPCIMH
VNGFLGAFLFSVETQTTIGYGFRCVTEECPLAVIAVVVQSIVGCVIDSFMIGTIMAKMARPK
KRAQTLLFSHHAVISVRDGKLCLMWRVGNLRKSHIVEAHVRAQLIKPYMTQEGEYLPLD
QRDLNVGYDIGLDRIFLVSPIIIVHEIDEDSPLYGMGKEELESEDFEIVVILEGMVEATAMTT
QARSSYLASEILWGHRFEPVVFEEKSHYKVDYSRFHKTYEVAGTPCCSARELQESKITVLP
APPPPPSAFCYENELALMSQEEEEMEEEAAAAAAVAAGLGLEAGSKEEAGIIRMLEFGSHL
DLERMQASLPLDNISYRRESAI

>Q8NGR8 multipass membrane protein
MERINHTSSVSEFILLGLSSRPEDQKTLFVLFLIVYLVTITGNLLIILAIRFNPHLQTPMYFFL
SFLSLTDICFTTSVVPKMLMNFLSEKKTISYAGCLTQMYFLYALGNSDSCLLAVMAFDRYV
AVCDPFHYVTTMSHHHCVLLVAFSCSFPHLHSLLHTLLLNRLTFCDSNVIHHFLCDLSPVL
KLSCSSIFVNEIVQMTEAPIVLVTRFLCIAFSYIRILTTVLKIPSTSGKRKAFSTCGFYLTVVT
LFYGSIFCVYLQPPSTYAVKDHVATIVYTVLSSMLNPFIYSLRNKDLKQGLRKLMSKRS

>P41586 multipass membrane protein
MAGVVHVSLAALLLLPMAPAMHSDCIFKKEQAMCLEKIQRANELMGFNDSSPGCPGMW
DNITCWKPAHVGEMVLVSCPELFRIFNPDQVWETETIGESDFGDSNSLDLSDMGVVSRNC
TEDGWSEPFPHYFDACGFDEYESETGDQDYYYLSVKALYTVGYSTSLVTLTTAMVILCRF
RKLHCTRNFIHMNLFVSFMLRAISVFIKDWILYAEQDSNHCFISTVECKAVMVFFHYCVVS
NYFWLFIEGLYLFTLLVETFFPERRYFYWYTIIGWGTPTVCVTVWATLRLYFDDTGCWDM
NDSTALWWVIKGPVVGSIMVNFVLFIGIIVILVQKLQSPDMGGNESSIYLRLARSTLLLIPLF
GIHYTVFAFSPENVSKRERLVFELGLGSFQGFVVAVLYCFLNGEVQAEIKRKWRSWKVNR
YFAVDFKHRHPSLASSGVNGGTQLSILSKSSSQIRMSGLPADNLAT

>Q9NYP7 multipass membrane protein
MEHFDASLSTYFKALLGPRDTRVKGWFLLDNYIPTFICSVIYLLIVWLGPKYMRNKQPFSC
RGILVVYNLGLTLLSLYMFCELVTGVWEGKYNFFCQGTRTAGESDMKIIRVLWWYYFSKL
IEFMDTFFFILRKNNHQITVLHVYHHASMLNIWWFVMNWVPCGHSYFGATLNSFIHVLM
YSYYGLSSVPSMRPYLWWKKYITQGQLLQFVLTIIQTSCGVIWPCTFPLGWLYFQIGYMIS
LIALFTNFYIQTYNKKGASRRKDHLKDHQNGSMAAVNGHTNSFSPLENNVKPRKLRKD

>Q6IEV9 multipass membrane protein
MQQNNSVPEFILLGLTQDPLRQKIVFVIFLIFYMGTVVGNMLIIVTIKSSRTLGSPMYFFLFY
LSFADSCFSTSTAPRLIVDALSEKKIITYNECMTQVFALHLFGCMEIFVLILMAVDRYVAICK
PLRYPTIMSQQVCIILIVLAWIGSLIHSTAQIILALRLPFCGPYLIDHYCCDLQPLLKLACMDT
YMINLLLVSNSGAICSSSFMILIISYIVILHSLRNHSAKGKKKALSACTSHIIVVILFFGPCIFIY
TRPPTTFPMDKMVAVFYTIGTPFLNPLIYTLRNAEVKNAMRKLWHGKIISENKG

>Q96R08 multipass membrane protein
MENNTEVTEFILVGLTDDPELQIPLFIVFLFIYLITLVGNLGMIELILLDSCLHTPMYFFLSNL
SLVDFGYSSAVTPKVMVGFLTGDKFILYNACATQFFFFVAFITAESFLLASMAYDRYAALCK
PLHYTTTMTTNVCACLAIGSYICGFLNASIHTGNTFRLSFCRSNVVEHFFCDAPPLLTLSCS
DNYISEMVIFFVVGFNDLFSILVILISYLFIFITIMKMRSPEGRQKAFSTCASHLTAVSIFYGTG
IFMYLRPNSSHFMGTDKMASVFYAIVIPMLNPLVYSLRNKEVKSAFKKTVGKAKASIGFIF

>P35368 multipass membrane protein
MNPDLDTGHNTSAPAHWGELKNANFTGPNQTSSNSTLPQLDITRAISVGLVLGAFILFAIV
GNILVILSVACNRHLRTPTNYFIVNLAMADLLLSFTVLPFSAALEVLGYWVLGRIFCDIWAA
VDVLCCTASILSLCAISIDRYIGVRYSLQYPTLVTRRKAILALLSVWVLSTVISIGPLLGWKE
PAPNDDKECGVTEEPFYALFSSLGSFYIPLAVILVMYCRVYIVAKRTTKNLEAGVMKEMSN
SKELTLRIHSKNFHEDTLSSTKAKGHNPRSSIAVKLFKFSREKKAAKTLGIVVGMFILCWLP
FFIALPLGSLFSTLKPPDAVFKVVFWLGYFNSCLNPIIYPCSSKEFKRAFVRILGCQCRGRGR
RRRRRRRRLGGCAYTYRPWTRGGSLERSQSRKDSLDDSGSCLSGSQRTLPSASPSPGYLG
RGAPPPVELCAFPEWKAPGALLSLPAPEPPGRRGRHDSGPLFTFKLLTEPESPGTDGGASN
GGCEAAADVANGQPGFKSNMPLAPGQF

>Q15041 multipass membrane protein
MAEGDNRSTNLLAAETASLEEQLQGWGEVMLMADKVLRWERAWFPPAIMGVVSLVFLII
YYLDPSVLSGVSCFVMFLCLADYLVPILAPRIFGSNKWTTEQQQRFHEICSNLVKTRRRAV
GWWKRLFTLKEEKPKMYFMTMIVSLAAVAWVGQQVHNLLLTYLIVTSLLLLPGLNQHGII
LKYIGMAKREINKLLKQKEKKNE

>Q9NQS1 peripheral membrane protein
MQAERGARGGRGRRPGRGRPGGDRHSERPGAAAAVARGGGGGGGGDGGGRRGRGRGR
GFRGARGGRGGGGAPRGSRREPGGWGAGASAPVEDDSDAETYGEENDEQGNYSKRKIV
SNWDRYQDIEKEVNNESGESQRGTDFSVLLSSAGDSFSQFRFAEEKEWDSEASCPKQNSA
FYVDSELLVRALQELPLCLRLNVAAELVQGTVPLEVPQVKPKRTDDGKGLGMQLKGPLG
PGGRGPIFELKSVAAGCPVLLGKDNPSPGPSRDSQKPTSPLQSAGDHLEEELDLLLNLDAPI
KEGDNILPDQTSQDLKSKEDGEVVQEEEVCAKPSVTEEKNMEPEQPSTSKNVTEEELEDW
LDSMIS

>Q15436 peripheral membrane protein
MTTYLEFIQQNEERDGVRFSWNVWPSSRLEATRMVVPVAALFTPLKERPDLPPIQYEPVLC
SRTTCRAVLNPLCQVDYRAKLWACNFCYQRNQFPPSYAGISELNQPAELLPQFSSIEYVVL

RGPQMPLIFLYVVDTCMEDEDLQALKESMQMSLSLLPPTALVGLITFGRMVQVHELGCEG
ISKSYVFRGTKDLSAKQLQEMLGLSKVPLTQATRGPQVQQPPPSNRFLQPVQKIDMNLTDL
LGELQRDPWPVPQGKRPLRSSGVALSIAVGLLECTFPNTGARIMMFIGGPATQGPGMVVG
DELKTPIRSWHDIDKDNAKYVKKGTKHFEALANRAATTGHVIDIYACALDQTGLLEMKC
CPNLTGGYMVMGDSFNTSLFKQTFQRVFTKDMHGQFKMGFGGTLEIKTSREIKISGAIGP
CVSLNSKGPCVSENEIGTGGTCQWKICGLSPTTTLAIYFEVVNQHNAPIPQGGRGAIQFVT
QYQHSSGQRRIRVTTIARNWADAQTQIQNIAASFDQEAAAILMARLAIYRAETEEGPDVLR
WLDRQLIRLCQKFGEYHKDDPSSFRFSETFSLYPQFMFHLRRSSFLQVFNNSPDESSYYRH
HFMRQDLTQSLIMIQPILYAYSFSGPPEPVLLDSSSILADRILLMDTFFQILIYHGETIAQWRK
SGYQDMPEYENFRHLLQAPVDDAQEILHSRFPMPRYIDTEHGGSQARFLLSKVNPSQTHN
NMYAWGQESGAPILTDDVSLQVFMDHLKKLAVSSAA

>Q8NGS0 multipass membrane protein
MENQSSISEFFLRGISAPPEQQQSLFGIFLCMYLVTLTGNLLIILAIGSDLHLHTPMYFFLAN
LSFVDMGLTSSTVTKMLVNIQTRHHTISYTGCLTQMYFFLMFGDLDSFFLAAMAYDRYVA
ICHPLCYSTVMRPQVCALMLALCWVLTNIVALTHTFLMARLSFCVTGEIAHFFCDITPVLK
LSCSDTHINEMMVFVLGGTVLIVPFLCIVTSYIHIVPAILRVRTRGGVGKAFSTCSSHLCVV
CVFYGTLFSAYLCPPSIASEEKDIAAAAMYTIVTPMLNPFIYSLRNKDMKGALKRLFSHRSI
VSS

>P34741 single-pass type I membrane protein
MRRAWILLTLGLVACVSAESRAELTSDKDMYLDNSSIEEASGVYPIDDDDYASASGSGADE
DVESPELTTSRPLPKILLTSAAPKVETTTLNIQNKIPAQTKSPEETDKEKVHLSDSERKMDPA
EEDTNVYTEKHSDSLFKRTEVLAAVIAGGVIGFLFAIFLILLLVYRMRKKDEGSYDLGERK
PSSAAYQKAPTKEFYA

>Q9NZJ5 single-pass type I membrane protein
MERAISPGLLVRALLLLLLLLLGLAARTVAAGRARGLPAPTAEAAFGLGAAAAPTSATRVPA
AGAVAAAEVTVEDAEALPAAAGEQEPRGPEPDDETELRPRGRSLVIISTLDGRIAALDPEN
HGKKQWDLDVGSGSLVSSSLSKPEVFGNKMIIPSLDGALFQWDQDRESMETVPFTVESLL
ESSYKFGDDVVLVGGKSLTTYGLSAYSGKVRYICSALGCRQWDSDEMEQEEDILLLQRTQ
KTVRAVGPRSGNEKWNFSVGHFELRYIPDMETRAGFIESTFKPNENTEESKIISDVEEQEAA
IMDIVIKVSVADWKVMAFSKKGGHLEWEYQFCTPIASAWLLKDGKVIPISLFDDTSYTSN
DDVLEDEEDIVEAARGATENSVYLGMYRGQLYLQSSVRISEKFPSSPKALESVTNENAIIPL
PTIKWKPLIHSPSRTPVLVGSDEFDKCLSNDKFSHEEYSNGALSILQYPYDNGYYLPYYKR
ERNKRSTQITVRFLDNPHYNKNIRKKDPVLLLHWWKEIVATILFCIIATTFIVRRLFHPHPHR
QRKESETQCQTENKYDSVSGEANDSSWNDIKNSGYISRYLTDFEPIQCLGRGGFGVVFEA
KNKVDDCNYAIKRIRLPNRELAREKVMREVKALAKLEHPGIVRYFNAWLEAPPEKWQEK
MDEIWLKDESTDWPLSSPSPMDAPSVKIRRMDPFATKEHIEIIAPSPQRSRSFSVGISCDQTS
SSESQFSPLEFSGMDHEDISESVDAAYNLQDSCLTDCDVEDGTMDGNDEGHSFELCPSEAS
PYVRSRERTSSSIVFEDSGCDNASSKEEPKTNRLHIGNHCANKLTAFKPTSSKSSSEATLSIS
PPRPTTLSLDLTKNTTEKLQPSSPKVYLYIQMQLCRKENLKDWMNGRCTIEERERSVCLHI
FLQIAEAVEFLHSKGLMHRDLKPSNIFFTMDDVVKVGDFGLVTAMDQDEEEQTVLTPMPA
YARHTGQVGTKLYMSPEQIHGNSYSHKVDIFSLGLILFELLYPFSTQMERVRTLTDVRNLK

FPPLFTQKYPCEYVMVQDMLSPSPMERPEAINIIENAVFEDLDFPGKTVLRQRSRSLSSSGT
KHSRQSNNSHSPLPSN

>Q9H244 multipass membrane protein
MQAVDNLTSAPGNTSLCTRDYKITQVLFPLLYTVLFFVGLITNGLAMRIFFQIRSKSNFIIFL
KNTVISDLLMILTFPFKILSDAKLGTGPLRTFVCQVTSVIFYFTMYISISFLGLITIDRYQKTT
RPFKTSNPKNLLGAKILSVVIWAFMFLLSLPNMILTNRQPRDKNVKKCSFLKSEFGLVWHE
IVNYICQVIFWINFLIVIVCYTLITKELYRSYVRTRGVGKVPRKKVNVKVFIIIAVFFICFVPF
HFARIPYTLSQTRDVFDCTAENTLFYVKESTLWLTSLNACLDPFIYFFLCKSFRNSLISMLK
CPNSATSLSQDNRKKEQDGGDPNEETPM

>O14828 multipass membrane protein
MAQSRDGGNPFAEPSELDNPFQDPAVIQHRPSRQYATLDVYNPFETREPPPAYEPPAPAPLP
PPSAPSLQPSRKLSPTEPKNYGSYSTQASAAAATAELLKKQEELNRKAEELDRRERELQHA
ALGGTATRQNNWPPLPSFCPVQPCFFQDISMEIPQEFQKTVSTMYYLWMCSTLALLLNFL
ACLASFCVETNNGAGFGLSILWVLLFTPCSFVCWYRPMYKAFRSDSSFNFFVFFFIFFVQD
VLFVLQAIGIPGWGFSGWISALVVPKGNTAVSVLMLLVALLFTGIAVLGIVMLKRIHSLYRR
TGASFQKAQQEFAAGVFSNPAVRTAAANAAAGAAENAFRAP

>O60741 multipass membrane protein
MEGGGKPNSSSNSRDDGNSVFPAKASATGAGPAAAEKRLGTPPGGGGAGAKEHGNSVCF
KVDGGGGGGGGGGGEEPAGGFEDAEGPRRQYGFMQRQFTSMLQPGVNKFSLRMFGSQ
KAVEKEQERVKTAGFWIIHPYSDFRFYWDLIMLIMMVGNLVIIPVGITFFTEQTTTPWIIFNV
ASDTVFLLDLIMNFRTGTVNEDSSEIILDPKVIKMNYLKSWFVVDFISSIPVDYIFLIVEKGM
DSEVYKTARALRIVRFTKILSLLRLLRLSRLIRYIHQWEEIFHMTYDLASAVVRIFNLIGMM
LLLCHWDGCLQFLVPLLQDFPPDCWVSLNEMVNDSWGKQYSYALFKAMSHMLCIGYGA
QAPVSMSDLWITMLSMIVGATCYAMFVGHATALIQSLDSSRRQYQEKYKQVEQYMSFHK
LPADMRQKIHDYYEHRYQGKIFDEENILNELNDPLREEIVNFNCRKLVATMPLFANADPNF
VTAMLSKLRFEVFQPGDYIIREGAVGKKMYFIQHGVAGVITKSSKEMKLTDGSYFGEICLL
TKGRRTASVRADTYCRLYSLSVDNFNEVLEEYPMMRRAFETVAIDRLDRIGKKNSILLQKF
QKDLNTGVFNNQENEILKQIVKHDREMVQAIAPINYPQMTTLNSTSSTTTPTSRMRTQSPP
VYTATSLSHSNLHSPSPSTQTPQPSAILSPCSYTTAVCSPPVQSPLAARTFHYASPTASQLSL
MQQQPQQQVQQSQPPQTQPQQPSPQPQTPGSSTPKNEVHKSTQALHNTNLTREVRPLSAS
QPSLPHEVSTLISRPHPTVGESLASIPQPVTAVPGTGLQAGGRSTVPQRVTLFRQMSSGAIPP
NRGVPPAPPPPAAALPRESSSVLNTDPDAEKPRFASNL

>Q10469 single-pass type II membrane protein
MRFRIYKRKVLILTLVVAACGFVLWSSNGRQRKNEALAPPLLDAEPARGAGGRGGDHPSV
AVGIRRVSNVSAASLVPAVPQPEADNLTLRYRSLVYQLNFDQTLRNVDKAGTWAPRELVL
VVQVHNRPEYLRLLLDSLRKAQGIDNVLVIFSHDFWSTEINQLIAGVNFCPVLQVFFPFSIQ
LYPNEFPGSDPRDCPRDLPKNAALKLGCINAEYPDSFGHYREAKFSQTKHHWWWKLHFV
WERVKILRDYAGLILFLEEDHYLAPDFYHVFKKMWKLKQQECPECDVLSLGTYSASRSFY
GMADKVDVKTWKSTEHNMGLALTRNAYQKLIECTDTFCTYDDYNWDWTLQYLTVSCL
PKFWKVLVPQIPRIFHAGDCGMHHKKTCRPSTQSAQIESLLNNNKQYMFPETLTISEKFTV

VAISPPRKNGGWGDIRDHELCKSYRRLQ

>Q15722 multipass membrane protein
MNTTSSAAPPSLGVEFISLLAIILLSVALAVGLPGNSFVVWSILKRMQKRSVTALMVLNLA
LADLAVLLTAPFFLHFLAQGTWSFGLAGCRLCHYVCGVSMYASVLLITAMSLDRSLAVAR
PFVSQKLRTKAMARRVLAGIWVLSFLLATPVLAYRTVVPWKTNMSLCFPRYPSEGHRAFH
LIFEAVTGFLLPFLAVVASYSDIGRRLQARRFRRSRRTGRLVVLIILTFAAFWLPYHVVNLAE
AGRALAGQAAGLGLVGKRLSLARNVLIALAFLSSSVNPVLYACAGGGLLRSAGVGFVAK
LLEGTGSEASSTRRGGSLGQTARSGPAALEPGPSESLTASSPLKLNELN

>Q9H205 multipass membrane protein
MELWNFTLGSGFILVGILNDSGSPELLCATITILYLLALISNGLLLLAITMEARLHMPMYLLL
GQLSLMDLLFTSVVTPKALADFLRRENTISFGGCALQMFLALTMGGAEDLLLAFMAYDR
YVAICHPLTYMTLMSSRACWLMVATSWILASLSALIYTVYTMHYPFCRAQEIRHLLCEIPH
LLKVACADTSRYELMVYVMGVTFLIPSLAAILASYTQILLTVLHMPSNEGRKKALVTCSSH
LTVVGMFYGAATFMYVLPSSFHSTRQDNIISVFYTIVTPALNPLIYSLRNKEVMRALRRVL
GKYMLPAHSTL

>Q9UBG0 single-pass type I membrane protein
MGPGRPAPAPWPRHLLRCVLLLGCLHLGRPGAPGDAALPEPNVFLIFSHGLQGCLEAQGG
QVRVTPACNTSLPAQRWKWVSRNRLFNLGTMQCLGTGWPGTNTTASLGMYECDREALN
LRWHCRTLGDQLSLLLGARTSNISKPGTLERGDQTRSGQWRIYGSEEDLCALPYHEVYTIQ
GNSHGKPCTIPFKYDNQWFHGCTSTGREDGHLWCATTQDYGKDERWGFCPIKSNDCETF
WDKDQLTDSCYQFNFQSTLSWREAWASCEQQGADLLSITEIHEQTYINGLLTGYSSTLWIG
LNDLDTSGGWQWSDNSPLKYLNWESDQPDNPSEENCGVIRTESSGGWQNRDCSIALPYV
CKKKPNATAEPTPPDRWANVKVECEPSWQPFQGHCYRLQAEKRSWQESKKACLRGGGD
LVSIHSMAELEFITKQIKQEVEELWIGLNDLKLQMNFEWSDGSLVSFTHWHPFEPNNFRDS
LEDCVTIWGPEGRWNDSPCNQSLPSICKKAGQLSQGAAEEDHGCRKGWTWHSPSCYWL
GEDQVTYSEARRLCTDHGSQLVTITNRFEQAFVSSLIYNWEGEYFWTALQDLNSTGSFFW
LSGDEVMYTHWNRDQPGYSRGGCVALATGSAMGLWEVKNCTSFRARYICRQSLGTPVTP
ELPGPDPTPSLTGSCPQGWASDTKLRYCYKVFSSERLQDKKSWVQAQGACQELGAQLLSL
ASYEEEHFVANMLNKIFGESEPEIHEQHWFWIGLNRRDPRGGQSWRWSDGVGFSYHNFD
RSRHDDDDIRGCAVLDLASLQWVAMQCDTQLDWICKIPRGTDVREPDDSPQGRREWLRF
QEAEYKFFEHHSTWAQAQRICTWFQAELTSVHSQAELDFLSHNLQKFSRAQEQHWWIGL
HTSESDGRFRWTDGSIINFISWAPGKPRPVGKDKKCVYMTASREDWGDQRCLTALPYICK
RSNVTKETQPPDLPTTALGGCPSDWIQFLNKCFQVQGQEPQSRVKWSEAQFSCEQQEAQL
VTITNPLEQAFITASLPNVTFDLWIGLHASQRDFQWVEQEPLMYANWAPGEPSGPSPAPSG
NKPTSCAVVLHSPSAHFTGRWDDRSCTEETHGFICQKGTDPSLSPSPAALPPAPGTELSYLN
GTFRLLQKPLRWHDALLLCESRNASLAYVPDPYTQAFLTQAARGLRTPLWIGLAGEEGSR
RYSWVSEEPLNYVGWQDGEPQQPGGCTYVDVDGAWRTTSCDTKLQGAVCGVSSGPPPP
RRISYHGSCPQGLADSAWIPFREHCYSFHMELLLGHKEARQRCQRAGGAVLSILDEMENV
FVWEHLQSYEGQSRGAWLGMNFNPKGGTLVWQDNTAVNYSNWGPPGLGPSMLSHNSC
YWIQSNSGLWRPGACTNITMGVVCKLPRAEQSSFSPSALPENPAALVVVLMAVLLLLALLT
AALILYRRRQSIERGAFEGARYSRSSSPTEATEKNILVSDMEMNEQQE

>Q8NGQ1 multipass membrane protein
MIFPSHDSQAFTSVDMEVGNCTILTEFILLGFSADSQWQPILFGVFLMLYLITLSGNMTLVIL
IRTDSHLHTPMYFFIGNLSFLDFWYTSVYTPKILASCVSEDKRISLAGCGAQLFFSCVVAYT
ECYLLAAMAYDRHAAICNPLLYSGTMSTALCTGLVAGSYIGGFLNAIAHTANTFRLHFCG
KNIIDHFFCDAPPLVKMSCTNTRVYEKVLLGVVGFTVLSSILAILISYVNILLAILRIHSASGR
HKAFSTCASHLISVMLFYGSLLFMYSRPSSTYSLERDKVAALFYTVINPLLNPLIYSLRNKD
IKEAFRKATQTIQPQT

>Q9NZG7 multipass membrane protein
MESARENIDLQPGSSDPRSQPINLNHYATKKSVAESMLDVALFMSNAMRLKAVLEQGPSS
HYYTTLVTLISLSLLLQVVIGVLLVVIARLNLNEVEKQWRLNQLNNAATILVFFTVVINVFI
TAFGAHKTGFLAARASRNPL

>Q8NGE7 multipass membrane protein
MLGSKPRVHLYILPCASQQVSTMGDRGTSNHSEMTDFILAGFRVRPELHILLFLLFLFVYA
MILLGNVGMMTIIMTDPRLNTPMYFFLGNLSFIDLFYSSVIEPKAMINFWSENKSISFAGCV
AQLFLFALLIVTEGFLLAAMAYDRFIAICNPLLYSVQMSTRLCTQLVAGSYFCGCISSVIQTS
MTFTLSFCASRAVDHFYCDSRPLQRLSCSDLFIHRMISFSLSCIIILPTIIVIIVSYMYIVSTVL
KIHSTEGHKKAFSTCSSHLGVVSVLYGAVFFMYLTPDRFPELSKVASLCYSLVTPMLNPLIY
SLRNKDVQEALKKFLEKKNIIL

>Q8WVQ1 single-pass type II membrane protein
MPVQLSEHPEWNESMHSLRISVGGLPVLASMTKAADPRFRPRWKVILTFFVGAAILWLLC
SHRPAPGRPPTHNAHNWRLGQAPANWYNDTYPLSPPQRTPAGIRYRIAVIADLDTESRAQE
ENTWFSYLKKGYLTLSDSGDKVAVEWDKDHGVLESHLAEKGRGMELSDLIVFNGKLYSV
DDRTGVVYQIEGSKAVPWVILSDGDGTVEKGFKAEWLAVKDERLYVGGLGKEWTTTTG
DVVNENPEWVKVVGYKGSVDHENWVSNYNALRAAAGIQPPGYLIHESACWSDTLQRW
FFLPRRASQERYSEKDDERKGANLLLSASPDFGDIAVSHVGAVVPTHGFSSFKFIPNTDDQI
IVALKSEEDSGRVASYIMAFTLDGRFLLPETKIGSVKYEGIEFI

>Q96KK3 multipass membrane protein
MLMLLVRGTHYENLRSKVVLPTPLGGRSTETFVSEFPGPDTGIRWRRSDEALRVNVGGVR
RQLSARALARFPGTRLGRLQAAASEEQARRLCDDYDEAAREFYFDRHPGFFLSLLHFYRT
GHLHVLDELCVFAFGQEADYWGLGENALAACCRARYLERRLTQPHAWDEDSDTPSSVDP
CPDEISDVQRELARYGAARCGRLRRRLWLTMENPGYSLPSKLFSCVSISVVLASIAAMCIH
SLPEYQAREAAAAVAAVAAGRSPEGVRDDPVLRRLEYFCIAWFSFEVSSRLLLAPSTRNFF
CHPLNLIDIVSVLPFYLTLLAGVALGDQGGKEFGHLGKVVQVFRLMRIFRVLKLARHSTGL
RSLGATLKHSYREVGILLLYLAVGVSVFSGVAYTAEKEEDVGFNTIPACWWWGTVSMTTV
GYGDVVPVTVAGKLAASGCILGGILVVALPITIIFNKFSHFYRRQKALEAAVRNSNHQEFED
LLSSIDGVSEASLETSRETSQEGQSADLESQAPSEPPHPQMY

>P30411 multipass membrane protein
MFSPWKISMFLSVREDSVPTTASFSADMLNVTLQGPTLNGTFAQSKCPQVEWLGWLNTIQ

PPFLWVLFVLATLENIFVLSVFCLHKSSCTVAEIYLGNLAAADLILACGLPFWAITISNNFD
WLFGETLCRVVNAIISMNLYSSICFLMLVSIDRYLALVKTMSMGRMRGVRWAKLYSLVIW
GCTLLLSSPMLVFRTMKEYSDEGHNVTACVISYPSLIWEVFTNMLLNVVGFLLPLSVITFC
TMQIMQVLRNNEMQKFKEIQTERRATVLVLVVLLLFIICWLPFQISTFLDTLHRLGILSSCQ
DERIIDVITQIASFMAYSNSCLNPLVYVIVGKRFRKKSWEVYQGVCQKGGCRSEPIQMENS
MGTLRTSISVERQIHKLQDWAGSRQ

>O95371 multipass membrane protein
MDGVNDSSLQGFVLMGISDHPQLEMIFFIAILFSYLLTLLGNSTIILLSRLEARLHTPMYFFL
SNLSSLDLAFATSSVPQMLINLWGPGKTISYGGCITQLYVFLWLGATECILLVVMAFDRYVA
VCRPLRYTAIMNPQLCWLLAVIACLGGLGNSVIQSTFTLQLPLCGHRRVEGFLCEVPAMIK
LACGDTSLNQAVLNGVCTFFTAVPLSIIVISYCLIAQAVLKIRSAEGRRKAFNTCLSHLLVVF
LFYGSASYGYLLPAKNSKQDQGKFISLFYSLVTPMVNPLIYTLRNMEVKGALRRLLGKGR
EVG

>Q8NGQ2 multipass membrane protein
MQPYTKNWTQVTEFVMMGFAGIHEAHLLFFILFLTMYLFTLVENLAIILVVGLDHRLRRP
MYFFLTHLSCLEIWYTSVTVPKMLAGFIGVDGGKNISYADCLSQLFIFTFLGATECFLLAA
MAYDRYVAICMPLHYGAFVSWGTCIRLAAACWLVGFLTPILPIYLLSQLTFYGPNVIDHFS
CDASPLLALSCSDVTWKETVDFLVSLAVLLASSMVIAVSYGNIVWTLLHIRSAAERWKAFS
TCAAHLTVVSLFYGTLFFMYVQTKVTSSINFNKVVSVFYSVVTPMLNPLIYSLRNKEVKG
ALGRVFSLNFWKGQ

>Q8IZV2 multipass membrane protein
MEEPQRARSHTVTTTASSFAENFSTSSSSFAYDREFLRTLPGFLIVAEIVLGLLVWTLIAGTE
YFRVPAFGWVMFVAVFYWVLTVFFLIIYITMTYTRIPQVPWTTVGLCFNGSAFVLYLSAAV
VDASSVSPERDSHNFNSWAASSFFAFLVTICYAGNTYFSFIAWRSRTIQ

>Q8NGF9 multipass membrane protein
MTEFIFLVLSPNQEVQRVCFVIFLFLYTAIVLGNFLIVLTVMTSRSLGSPMYFFLSYLSFMEI
CYSSATAPKLISDLLAERKVISWWGCMAQLFFLHFFGGTEIFLLTVMAYDHYVAICKPLSY
TTIMNWQVCTVLVGIAWVGGFMHSFAQILLIFHLLFCGPNVINHYFCDLVPLLKLACSDTF
LIGLLIVANGGTLSVISFGVLLASYMVILLHLRTWSSEGWCKALSTCGSHFAVVILFFGPCV
FNSLRPSTTLPIDKMVAVFYTVITAILNPVIYSLRNAEMRKAMKRLWIRTLRLNEK

>P28566 multipass membrane protein
MNITNCTTEASMAIRPKTITEKMLICMTLVVITTLTTLLNLAVIMAIGTTKKLHQPANYLICS
LAVTDLLVAVLVMPLSIIYIVMDRWKLGYFLCEVWLSVDMTCCTCSILHLCVIALDRYWAI
TNAIEYARKRTAKRAALMILTVWTISIFISMPPLFWRSHRRLSPPPSQCTIQHDHVIYTIYSTL
GAFYIPLTLILILYYRIYHAAKSLYQKRGSSRHLSNRSTDSQNSFASCKLTQTFCVSDFSTSD
PTTEFEKFHASIRIPPFDNDLDHPGERQQISSTRERKAARILGLILGAFILSWLPFFIKELIVGL
SIYTVSSEVADFLTWLGYVNSLINPLLYTSFNEDFKLAFKKLIRCREHT

>Q9Y5X5 multipass membrane protein

MNSFFGTPAASWCLLESDVSSAPDKEAGRERRALSVQQRGGPAWSGSLEWSRQSAGDRR
RLGLSRQTAKSSWSRSRDRTCCCRRAWWILVPAADRARRERFIMNEKWDTNSSENWHPI
WNVNDTKHHLYSDINITYVNYYLHQPQVAAIFIISYFLIFFLCMMGNTVVCFIVMRNKHM
HTVTNLFILNLAISDLLVGIFCMPITLLDNIIAGWPFGNTMCKISGLVQGISVAASVFTLVAIA
VDRFQCVVYPFKPKLTIKTAFVIIMIIWVLAITIMSPSAVMLHVQEEKYYRVRLNSQNKTSP
VYWCREDWPNQEMRKIYTTVLFANIYLAPLSLIVIMYGRIGISLFRAAVPHTGRKNQEQW
HVVSRKKQKIIKMLLIVALLFILSWLPLWTLMMLSDYADLSPNELQIINIYIYPFAHWLAFG
NSSVNPIIYGFFNENFRRGFQEAFQLQLCQKRAKPMEAYALKAKSHVLINTSNQLVQESTF
QNPHGETLLYRKSAEKPQQELVMEELKETTNSSEI

>Q8IZF5 multipass membrane protein
MVCSAAPLLLLATTLPLLGSPVAQASQPVSETGVRPREGLQRRQWGPLIGRDKAWNERID
RPFPACPIPLSSSFGRWPKGQTMWAQTSTLTLTEEELGQSQAGGESGSGQLLDQENGAGES
ALVSVYVHLDFPDKTWPPELSRTLTLPAASASSSPRPLLTGLRLTTECNVNHKGNFYCACL
SGYQWNTSICLHYPPCQSLHNHQPCGCLVFSHPEPGYCQLLPPGSPVTCLPAVPGILNLNSQ
LQMPGDTLSLTLHLSQEATNLSWFLRHPGSPSPILLQPGTQVSVTSSHGQAALSVSNMSHH
WAGEYMSCFEAQGFKWNLYEVVRVPLKATDVARLPYQLSISCATSPGFQLSCCIPSTNLAY
TAAWSPGEGSKASSFNESGSQCFVLAVQRCPMADTTYACDLQSLGLAPLRVPISITIIQDGD
ITCPEDASVLTWNVTKAGHVAQAPCPESKRGIVRRLCGADGVWGPVHSSCTDARLLALFT
RTKLLQAGQGSPAEEVPQILAQLPGQAAEASSPSDLLTLLSTMKYVAKVVAEARIQLDRRA
LKNLLIATDKVLDMDTRSLWTLAQARKPWAGSTLLLAVETLACSLCPQDHPFAFSLPNVL
LQSQLFGPTFPADYSISFPTRPPLQAQIPRHSLAPLVRNGTEISITSLVLRKLDHLLPSNYGQG
LGDSLYATPGLVLVISIMAGDRAFSQGEVIMDFGNTDGSPHCVFWDHSLFQGRGGWSKEG
CQAQVASASPTAQCLCQHLTAFSVLMSPHTVPEEPALALLTQVGLGASILALLVCLGVYWL
VWRVVVRNKISYFRHAALLNMVFCLLAADTCFLGAPFLSPGPRSPLCLAAAFLCHFLYLA
TFFWMLAQALVLAHQLLFVFHQLAKHRVLPLMVLLGYLCPLGLAGVTLGLYLPQGQYLR
EGECWLDGKGGALYTFVGPVLAIIGVNGLVLAMAMLKLLRPSLSEGPPAEKRQALLGVIK
ALLILTPIFGLTWGLGLATLLEEVSTVPHYIFTILNTLQGVFILLFGCLMDRKIQEALRKRFC
RAQAPSSTISLVSCCLQILSCASKSMSEGIPWPSSEDMGTARS

>Q9P2W7 single-pass type II membrane protein
MPKRRDILAIVLIVLPWTLLITVWHQSTLAPLLAVHKDEGSDPRRETPPGADPREYCTSDR
DIVEVVRTEYVYTRPPPWSDTLPTIHVVTPTYSRPVQKAELTRMANTLLHVPNLHWLVVE
DAPRRTPLTARLLRDTGLNYTHLHVETPRNYKLRGDARDPRIPRGTMQRNLALRWLRETF
PRNSSQPGVVYFADDDNTYSLELFEEMRSTRRVSVWPVAFVGGLRYEAPRVNGAGKVVG
WKTVFDPHRPFAIDMAGFAVNLRLILQRSQAYFKLRGVKGGYQESSLLRELVTLNDLEPK
AANCTKILVWHTRTEKPVLVNEGKKGFTDPSVEI

>Q9BRK3 single-pass type I membrane protein
MALPSRILLWKLVLLQSSAVLLHSGSSVPAAAGSSVVSESAVSWEAGARAVLRCQSPRMV
WTQDRLHDRQRVLHWDLRGPGGGPARRLLDLYSAGEQRVYEARDRGRLELSASAFDDG
NFSLLIRAVEETDAGLYTCNLHHHYCHLYESLAVRLEVTDGPPATPAYWDGEKEVLAVARG
APALLTCVNRGHVWTDRHVEEAQQVVHWDRQPPGVPHDRADRLLDLYASGERRAYGPL
FLRDRVAVGADAFERGDFSLRIEPLEVADEGTYSCHLHHHYCGLHERRVFHLTVAEPHAEP

PPRGSPGNGSSHSGAPGPDPTLARGHNVINVIVPESRAHFFQQLGYVLATLLLFILLLVTVL
LAARRRRGGYEYSDQKSGKSKGKDVNLAEFAVAAGDQMLYRSEDIQLDYKNNILKERAE
LAHSPLPAKYIDLDKGFRKENCK

>Q8IWJ2 peripheral membrane protein
MEDLVQDGVASPATPGTGKSKLETLPKEDLIKFAKKQMMLIQKAKSRCTELEKEIEELRSK
PVTEGTGDIIKALTERLDALLLEKAETEQQCLSLKKENIKMKQEVEDSVTKMGDAHKELE
QSHINYVKEIENLKNELMAVRSKYSEDKANLQKQLEEAMNTQLELSEQLKFQNNSEDNV
KKLQEEIEKIRPGFEEQILYLQKQLDATTDEKKETVTQLQNIIEANSQHYQKNINSLQEELL
QLKAIHQEEVKELMCQIEASAKEHEAEINKLNELKENLVKQCEASEKNIQKKYECELENL
RKATSNANQDNQICSILLQENTFVEQVVNEKVKHLEDTLKELESQHSILKDEVTYMNNLK
LKLEMDAQHIKDEFFHEREDLEFKINELLLAKEEQGCVIEKLKSELAGLNKQFCYTVEQH
NREVQSLKEQHQKEISELNETFLSDSEKEKLTLMFEIQGLKEQCENLQQEKQEAILNYESL
REIMEILQTELGESAGKISQEFESMKQQQASDVHELQQKLRTAFTEKDALLETVNRLQGE
NEKLLSQQELVPELENTIKNLQEKNGVYLLSLSQRDTMLKELEGKINSLTEEKDDFINKLK
NSHEEMDNFHKKCEREERLILELGKKVEQTIQYNSELEQKVNELTGGLEETLKEKDQNDQ
KLEKLMVQMKVLSEDKEVLSAEVKSLYEENNKLSSEKKQLSRDLEVFLSQKEDVILKEHI
TQLEKKLQLMVEEQDNLNKLLENEQVQKLFVKTQLYGFLKEMGSEVSEDSEEKDVVNV
LQAVGESLAKINEEKCNLAFQRDEKVLELEKEIKCLQEESVVQCEELKSLLRDYEQEKVLL
RKELEEIQSEKEALQSDLLEMKNANEKTRLENQNLLIQVEEVSQTCSKSEIHNEKEKCFIK
EHENLKPLLEQKELRDRRAELILLKDSLAKSPSVKNDPLSSVKELEEKIENLEKECKEKEE
KINKIKLVAVKAKKELDSSRKETQTVKEELESLRSEKDQLSASMRDLIQGAESYKNLLLEY
EKQSEQLDVEKERANNFEHRIEDLTRQLRNSTLQCETINSDNEDLLARIETLQSNAKLLEV
QILEVQRAKAMVDKELEAEKLQKEQKIKEHATTVNELEELQVQLQKQKKQLQKTMQELE
LVKKDAQQTTLMNMEIADYERLMKELNQKLTNKNNKIEDLEQEIKIQKQKQETLQEEITS
LQSSVQQYEEKNTKIKQLLVKTKKELADSKQAETDHLILQASLKGELEASQQQVEVYKIQ
LAEITSEKHKIHEHLKTSAEQHQRTLSAYQQRVTALQEECRAAKAEQATVTSEFESYKVRV
HNVLKQQKNKSMSQAETEGAKQEREHLEMLIDQLKIKLQDSQNNLQINVSELQTLQSEH
DTLLERHNKMLQETVSKEAELREKLCSIQSENMMMKSEHTQTVSQLTSQNEVLRNSFRD
QVRHLQEEHRKTVETLQQQLSKMEAQLFQLKNEPTTRSPVSSQQSLKNLRERRNTDLPLL
DMHTVTREEGEGMETTDTESVSSASTYTQSLEQLLNSPETKLEPPLWHAEFTKEELVQKL
SSTTKSADHLNGLLRETEATNAILMEQIKLLKSEIRRLERNQEREKSAANLEYLKNVLLQFI
FLKPGSERERLLPVINTMLQLSPEEKGKLAAVAQGEEENASRSSGWASYLHSWSGLR

>Q96SQ9 peripheral membrane protein
MEATGTWALLLLALALLLLLLTLALSGTRARGHLPPGPTPLPLLGNLLQLRPGALYSGLMRLS
KKYGPVFTIYLGPWRPVVVLVGQEAVREALGGQAEEFSGRGTVAMLEGTFDGHGVFFSN
GERWRQLRKFTMLALRDLGMGKREGEELIQAEARCLVETFQGTEGRPFDPSLLLAQATSN
VVCSLLFGLRFSYEDKEFQAVVRAAGGTLLGVSSQGGQTYEMFSWFLRPLPGPHKQLLH
HVSTLAAFTVRQVQQHQGNLDASGPARDLVDAFLLKMAQEEQNPGTEFTNKNMLMTVI
YLLFAGTMTVSTTVGYTLLLLMKYPHVQKWVREELNRELGAGQAPSLGDRTRLPYTDAV
LHEAQRLLALVPMGIPRTLMRTTRFRGYTLPQGTEVFPLLGSILHDPNIFKHPEEFNPDRFL
DADGRFRKHEAFLPFSLGKRVCLGEGLAKAELFLFFTTILQAFSLESPCPPDTLSLKPTVSG
LFNIPPAFQLQVRPTDLHSTTQTR

>Q8NGE3 multipass membrane protein
MAGENHTTLPEFLLLGFSDLKALQGPLFWVVLLVYLVTLLGNSLIILLTQVSPALHSPMYF
FLRQLSVVELFYTTDIVPRTLANLGSPHPQAISFQGCAAQMYVFIVLGISECCLLTAMAYDR
YVAICQPLRYSTLLSPRACMAMVGTSWLTGIITATTHASLIFSLPFRSHPIIPHFLCDILPVLR
LASAGKHRSEISVMTATIVFIMIPFSLIVTSYIRILGAILAMASTQSRRKVFSTCSSHLLVVSL
FFGTASITYIRPQAGSSVTTDRVLSLFYTVITPMLNPIIYTLRNKDVRRALRHLVKRQRPSP

>Q9Y251 peripheral membrane protein
MLLRSKPALPPPLMLLLLGPLGPLSPGALPRPAQAQDVVDLDFFTQEPLHLVSPSFLSVTID
ANLATDPRFLILLGSPKLRTLARGLSPAYLRFGGTKTDFLIFDPKKESTFEERSYWQSQVNQ
DICKYGSIPPDVEEKLRLEWPYQEQLLLREHYQKKFKNSTYSRSSVDVLYTFANCSGLDLI
FGLNALLRTADLQWNSSNAQLLLDYCSSKGYNISWELGNEPNSFLKKADIFINGSQLGEDF
IQLHKLLRKSTFKNAKLYGPDVGQPRRKTAKMLKSFLKAGGEVIDSVTWHHYYLNGRTA
TKEDFLNPDVLDIFISSVQKVFQVVESTRPGKKVWLGETSSAYGGGAPLLSDTFAAGFMW
LDKLGLSARMGIEVVMRQVFFGAGNYHLVDENFDPLPDYWLSLLFKKLVGTKVLMASV
QGSKRRKLRVYLHCTNTDNPRYKEGDLTLYAINLHNVTKYLRLPYPFSNKQVDKYLLRPL
GPHGLLSKSVQLNGLTLKMVDDQTLPPLMEKPLRPGSSLGLPAFSYSFFVIRNAKVAACI

>P15328 GPI-anchor, lipid-anchor
MAQRMTTQLLLLLVWVAVVGEAQTRIAWARTELLNVCMNAKHHKEKPGPEDKLHEQCR
PWRKNACCSTNTSQEAHKDVSYLYRFNWNHCGEMAPACKRHFIQDTCLYECSPNLGPWI
QQVDQSWRKERVLNVPLCKEDCEQWWEDCRTSYTCKSNWHKGWNWTSGFNKCAVGA
ACQPFHFYFPTPTVLCNEIWTHSYKVSNYSRGSGRCIQMWFDPAQGNPNEEVARFYAAA
MSGAGPWAAWPFLLSLALMLLWLLS

>P16671 multipass membrane protein
MGCDRNCGLIAGAVIGAVLAVFGGILMPVGDLLIQKTIKKQVVLEEGTIAFKNWVKTGTE
VYRQFWIFDVQNPQEVMMNSSNIQVKQRGPYTYRVRFLAKENVTQDAEDNTVSFLQPNG
AIFEPSLSVGTEADNFTVLNLAVAAASHIYQNQFVQMILNSLINKSKSSMFQVRTLRELLW
GYRDPFLSLVPYPVTTTVGLFYPYNNTADGVYKVFNGKDNISKVAIIDTYKGKRNLSYWE
SHCDMINGTDAASFPPFVEKSQVLQFFSSDICRSIYAVFESDVNLKGIPVYRFVLPSKAFASP
VENPDNYCFCTEKIISKNCTSYGVLDISKCKEGRPVYISLPHFLYASPDVSEPIDGLNPNEEE
HRTYLDIEPITGFTLQFAKRLQVNLLVKPSEKIQVLKNLKRNYIVPILWLNETGTIGDEKAN
MFRSQVTGKINLLGLIEMILLSVGVVMFVAFMISYCACRSKTIK

>Q06187 peripheral membrane protein
MAAVILESIFLKRSQQKKKTSPLNFKKRLFLLTVHKLSYYEYDFERGRRGSKKGSIDVEKIT
CVETVVPEKNPPPERQIPRRGEESSEMEQISIIERFPYPFQVVYDEGPLYVFSPTEELRKRWI
HQLKNVIRYNSDLVQKYHPCFWIDGQYLCCSQTAKNAMGCQILENRNGSLKPGSSHRKT
KKPLPPTPEEDQILKKPLPPEPAAAPVSTSELKKVVALYDYMPMNANDLQLRKGDEYFILE
ESNLPWWRARDKNGQEGYIPSNYVTEAEDSIEMYEWYSKHMTRSQAEQLLKQEGKEGG
FIVRDSSKAGKYTVSVFAKSTGDPQGVIRHYVVCSTPQSQYYLAEKHLFSTIPELINYHQH
NSAGLISRLKYPVSQQNKNAPSTAGLGYGSWEIDPKDLTFLKELGTGQFGVVKYGKWRG

QYDVAIKMIKEGSMSEDEFIEEAKVMMNLSHEKLVQLYGVCTKQRPIFIITEYMANGCLLN
YLREMRHRFQTQQLLEMCKDVCEAMEYLESKQFLHRDLAARNCLVNDQGVVKVSDFGL
SRYVLDDEYTSSVGSKFPVRWSPPEVLMYSKFSSKSDIWAFGVLMWEIYSLGKMPYERFT
NSETAEHIAQGLRLYRPHLASEKVYTIMYSCWHEKADERPTFKILLSNILDVMDEES

>Q15391 multipass membrane protein
MINSTSTQPPDESCSQNLLITQQIIPVLYCMVFIAGILLNGVSGWIFFYVPSSKSFIIYLKNIVI
ADFVMSLTFPFKILGDSGLGPWQLNVFVCRVSAVLFYVNMYVSIVFFGLISFDRYYKIVKP
LWTSFIQSVSYSKLLSVIVWMLMLLLAVPNIILTNQSVREVTQIKCIELKSELGRKWHKASN
YIFVAIFWIVFLLLIVFYTAITKKIFKSHLKSSRNSTSVKKKSSRNIFSIVFVFFVCFVPYHIAR
IPYTKSQTEAHYSCQSKEILRYMKEFTLLLSAANVCLDPIIYFFLCQPFREILCKKLHIPLKA
QNDLDISRIKRGNTTLESTDTL

>Q9Y2U8 multipass membrane protein
MAAAAASAPQQLSDEELFSQLRRYGLSPGPVTESTRPVYLKKLKKLREEEQQQHRSGGR
GNKTRNSNNNNTAAATVAAAGPAAAAAAGMGVRPVSGDLSYLRTPGGLCRISASGPESL
LGGPGGASAAPAAGSKVLLGFSSDESDVEASPRDQAGGGGRKDRASLQYRGLKAPPAPL
AASEVTNSNSAERRKPHSWWGARRPAGPELQTPPGKDGAVEDEEGEGEDGEERDPETEE
PLWASRTVNGSRLVPYSCRENYSDSEEEDDDDVASSRQVLKDDSLSRHRPRRTHSKPLPPL
TAKSAGGRLETSVQGGGGLAMNDRAAAAGSLDRSRNLEEAAAAEQGGGCDQVDSSPVP
RYRVNAKKLTPLLPPPLTDMDSTLDSSTGSLLKTNNHIGGGAFSVDSPRIYSNSLPPSAAVA
ASSSLRINHANHTGSNHTYLKNTYNKPKLSEPEEELLQQFKREEVSPTGSFSAHYLSMFLL
TAACLFFLILGLTYLGMRGTGVSEDGELSIENPFGETFGKIQESEKTLMMNTLYKLHDRLA
QLAGDHECGSSSQRTLSVQEAAAYLKDLGPEYEGIFNTSLQWILENGKDVGIRCVGFGPE
EELTNITDVQFLQSTRPLMSFWCRFRRAFVTVTHRLLLLCLGVVMVCVVLRYMKYRWTK
EEEETRQMYDMVVKIIDVLRSHNEACQENKDLQPYMPIPHVRDSLIQPHDRKKMKKVW
DRAVDFLAANESRVRTETRRIGGADFLVWRWIQPSASCDKILVIPSKVWQGQAFHLDRRN
SPPNSLTPCLKIRNMFDPVMEIGDQWHLAIQEAILEKCSDNDGIVHIAVDKNSREGCVYVK
CLSPEYAGKAFKALHGSWFDGKLVTVKYLRLDRYHHRFPQALTSNTPLKPSNKHMNSMS
HLRLRTGLTNSQGSS

>P33241 peripheral membrane protein
MAEASSDPGAEEREELLGPTAQWSVEDEEEAVHEQCQHERDRQLQAQDEEGGGHVPERP
KQEMLLSLKPSEAPELDEDEGFGDWSQRPEQRQQHEGAQGALDSGEPPQCRSPEGEQED
RPGLHAYEKEDSDEVHLEELSLSKEGPGPEDTVQDNLGAAGAEEEQEEHQKCQQPRTPSP
LVLEGTIEQSSPPLSPTTKLIDRTESLNRSIEKSNSVKKSQPDLPISKIDQWLEQYTQAIETAG
RTPKLARQASIELPSMAVASTKSRWETGEVQAQSAAKTPSCKDIVAGDMSKKSLWEQKGG
SKTSSTIKSTPSGKRYKFVATGHGKYEKVLVEGGPAP

>Q92730 lipid-anchor
MKERRAPQPVVARCKLVLVGDVQCGKTAMLQVLAKDCYPETYVPTVFENYTACLETEEQ
RVELSLWDTSGSPYYDNVRPLCYSDSDAVLLCFDISRPETVDSALKKWRTEILDYCPSTRV
LLIGCKTDLRTDLSTLMELSHQKQAPISYEQGCAIAKQLGAEIYLEGSAFTSEKSIHSIFRTA
SMLCLNKPSPLPQKSPVRSLSKRLLHLPSRSELISSTFKKEKAKSCSIM

>O14626	multipass membrane protein
MTNSSFFCPVYKDLEPFTYFFYLVFLVGIIGSCFATWAFIQKNTNHRCVSIYLINLLTADFLLT
LALPVKIVVDLGVAPWKLKIFHCQVTACLIYINMYLSIIFLAFVSIDRCLQLTHSCKIYRIQE
PGFAKMISTVVWLMVLLIMVPNMMIPIKDIKEKSNVGCMEFKKEFGRNWHLLTNFICVAIF
LNFSAIILISNCLVIRQLYRNKDNENYPNVKKALINILLVTTGYIICFVPYHIVRIPYTLSQTE
VITDCSTRISLFKAKEATLLLAVSNLCFDPILYYHLSKAFRSKVTETFASPKETKAQKEKLR
CENNA

>P30542	multipass membrane protein
MPPSISAFQAAYIGIEVLIALVSVPGNVLVIWAVKVNQALRDATFCFIVSLAVADVAVGALVI
PLAILINIGPQTYFHTCLMVACPVLILTQSSILALLAIAVDRYLRVKIPLRYKMVVTPRRAAV
AIAGCWILSFVVGLTPMFGWNNLSAVERAWAANGSMGEPVIKCEFEKVISMEYMVYFNFF
VWVLPPLLLMVLIYLEVFYLIRKQLNKKVSASSGDPQKYYGKELKIAKSLALILFLFALSW
LPLHILNCITLFCPSCHKPSILTYIAIFLTHGNSAMNPIVYAFRIQKFRVTFLKIWNDHFRCQP
APPIDEDLPEERPDD

>O00212	lipid-anchor
MTAAQAAGEEAPPGVRSVKVVLVGDGGCGKTSLLMVFADGAFPESYTPTVFERYMVNL
QVKGKPVHLHIWDTAGQDDYDRLRPLFYPDASVLLLCFDVTSPNSFDNIFNRWYPEVNHF
CKKVPIIVVGCKTDLCKDKSLVNKLRRNGLEPVTYHRGQEMARSVGAVAYLECSARLHD
NVHAVFQEAAEVALSSRGRNFWRRITQGFCVVT

>Q8NH85	multipass membrane protein
MAEVNIIYVTVFILKGITNRPELQAPCFGVFLVIYLVTVLGNLGLITLIKIDTRLHTPMYYFL
SHLAFVDLCYSSAITPKMMVNFVVERNTIPFHACATQLGCFLTFMITECFLLASMAYDCYV
AICSPLHYSTLMSRRVCIQLVAVPYIYSFLVALFHTVITFRLTYCGPNLINHFYCDDLPFLALS
CSDTHMKEILIFAFAGFDMISSSSIVLTSYIFIIAAILRIRSTQGQHKAISTCGSHMVTVTIFYG
TLIFMYLQPKSNHSLDTDKMASVFYTVVIPMLNPLIYSLRNKEVKDASKKALDKGCENLQ
ILTFLKIRKLY

>Q8NGE0	multipass membrane protein
MLRNGSIVTEFILVGFQQSSTSTRALLFALFLALYSLTMAMNGLIIFITSWTDPKLNSPMYFF
LGHLSLLDVCFITTTIPQMLIHLVVRDHIVSFVCCMTQMYFVFCVGVAECILLAFMAYDRY
VAICYPLNYVPIISQKVCVRLVGTAWFFGLINGIFLEYISFREPFRRDNHIESFFCEAPIVIGLS
CGDPQFSLWAIFADAIVVILSPMVLTVTSYVHILATILSKASSSGRGKTFSTCASHLTVVIFLY
TSAMFSYMNPHSTHGPDKDKPFSLLYTIITPMCNPIIYSFRNKEIKEAMVRALGRTRLAQPQ
SV

>Q8NGE9	multipass membrane protein
MAERNYTVVTEFFLTAFTEHLQWRVPLFLIFLSFYLATMLGNTGMILLIRGDRRLHTPMYF
FLSHLSLVDICYSSAIIPQMLAVLWEHGTTISQARCAAQFFLFTFFASIDCYLLAIMAYDRYT
AVCQPLLYVTIITEKARWGLVTGAYVAGFFSAFVRTVTAFTLSFCGNNEINFIFCDLPPLLKL
SCGDSYTQEVVIIVFALFVMPACILVILVSYLFIIVAILQIHSAGGRAKTFSTCASHLTAVALFF

GTLIFMYLRDNTGQSSEGDRVVSVLYTVVTPMLNPLIYSLRNKEVKEATRKALSKSKPAR
RP

>O60279 single-pass type I membrane protein
MTAEGPSPPARWHRRLPGLWAAALLLLGLPRLSVRADGKFFVLESQNGSQGLQLEAARLS
CKSRGAHLASADELRRVVQDCSFAVCTTGWLADGTLGTTVCSKGSGEQQIMRAVDVRIES
NPVPGGTYSALCIKDEEKPCGDPPSFPHTILQGRTGLEMGDELLYVCAPGHIMGHRETAFT
LLCNSCGEWYGLVQACGKDEAEAHIDYEDNFPDDRSVSFRELMEDSRTEADEDRGQGDS
SEEAPKQDRLVSISVGRENIARDKVFVPTTGLPGAGSSVPADSPGSRLLQKHLFWFPAEAF
HKPGLEKEVDDDTKKQFSAGDNHSGVKLVPGEPETKVIYGNTDGPSGPFVGKNDSKAGD
PVVSSSDESWLDGYPVTEGAWRKTEAEEEEDGDRGDGSVGLDENVLVTPDQPILVEVKK
PKSSTLTPSEGMTHSSVLPSQMLDVEALALRPVNASETEGIGDGDLTKYQSTLPWRFITEES
PMATLSYELTSSTLEILTVNTVKQTPNHIPSTIMATTQPPVETTVPEIQDSFPYLLSEDFFGQE
GPGPGASEELHPTLESCVGDGCPGLSRGPVIATIVTVLCLLLLLLAGVGMVWGYRKCQHKS
SVYKLNVGQRQARHYHQQIEMEKV

>Q8TDV0 multipass membrane protein
MLAAAFADSNSSSMNVSFAHLHFAGGYLPSDSQDWRTIIPALLVAVCLVGFVGNLCVIGILL
HNAWKGKPSMIHSLILNLSLADLSLLLFSAPIRATAYSKSVWDLGWFVCKSSDWFIHTCM
AAKSLTIVVVAKVCFMYASDPAKQVSIHNYTIWSVLVAIWTVASLLPLPEWFFSTIRHHEG
VEMCLVDVPAVAEEFMSMFGKLYPLLAFGLPLFFASFYFWRAYDQCKKRGTKTQNLRNQI
RSKQVTVMLLSIAIISALLWLPEWVAWLWVWHLKAAGPAPPQGFIALSQVLMFSISSANPL
IFLVMSEEFREGLKGVWKWMITKKPPTVSESQETPAGNSEGLPDKVPSPESPASIPEKEKPS
SPSSGKGKTEKAEIPILPDVEQFWHERDTVPSVQDNDPIPWEHEDQETGEGVK

>Q8NH67 multipass membrane protein
MCQQILRDCILLIHHLCINRKKVSLVMLGPAYNHTMETPASFLLVGIPGLQSSHLWLAISLS
AMYIIALLGNTIIVTAIWMDSTRHEPMYCFLCVLAAVDIVMASSVVPKMVSIFCSGDSSISF
SACFTQMFFVHLATAVETGLLLTMAFDRYVAICKPLHYKRILTPQVMLGMSMAITIRAIIAI
TPLSWMVSHLPFCGSNVVVHSYCEHIALARLACADPVPSSLYSLIGSSLMVGSDVAFIAAS
YILILKAVFGLSSKTAQLKALSTCGSHVGVMALYYLPGMASIYAAWLGQDVVPLHTQVLL
ADLYVIIPATLNPIIYGMRTKQLRERIWSYLMHVLFDHSNLGS

>P56746 multipass membrane protein
MSMAVETFGFFMATVGLLMLGVTLPNSYWRVSTVHGNVITTNTIFENLWFSCATDSLGVY
NCWEFPSMLALSGYIQACRALMITAILLGFLGLLLGIAGLRCTNIGGLELSRKAKLAATAG
ALHILAGICGMVAISWYAFNITRDFFDPLYPGTKYELGPALYLGWSASLISILGGLCLCSAC
CCGSDEDPAASARRPYQAPVSVMPVATSDQEGDSSFGKYGRNAYV

>P41440 multipass membrane protein
MVPSSPAVEKQVPVEPGPDPELRSWRHLVCYLCFYGFMAQIRPGESFITPYLLGPDKNFTR
EQVTNEITPVLSYSYLAVLVPVFLLTDYLRYTPVLLLQGLSFVSVWLLLLLGHSVAHMQL
MELFYSVTMAARIAYSSYIFSLVRPARYQRVAGYSRAAVLLGVFTSSVLGQLLVTVGRVSF
STLNYISLAFLTFSVVLALFLKRPKRSLFFNRDDRGRCETSASELERMNPGPGGKLGHALR

VACGDSVLARMLRELGDSLRRPQLRLWSLWWVFNSAGYYLVVYYVHILWNEVDPTTNS
ARVYNGAADAASTLLGAITSFAAGFVKIRWARWSKLLIAGVTATQAGLVFLLAHTRHPSSI
WLCYAAFVLFRGSYQFLVPIATFQIASSLSKELCALVFGVNTFFATIVKTIITFIVSDVRGLGL
PVRKQFQLYSVYFLILSIIYFLGAMLDGLRHCQRGHHPRQPPAQGLRSAAEEKAAQALSV
QDKGLGGLQPAQSPPLSPEDSLGAVGPASLEQRQSDPYLAQAPAPQAAEFLSPVTTPSPCTL
CSAQASGPEAADETCPQLAVHPPGVSKLGLQCLPSDGVQNVNQ

>Q8TDV2 multipass membrane protein
MGDELAPCPVGTTAWPALIQLISKTPCMPQAASNTSLGLGDLRVPSSMLYWLFLPSSLLAA
ATLAVSPLLLVTILRNQRLRQEPHYLLPANILLSDLAYILLHMLISSSSLGGWELGRMACGIL
TDAVFAACTSTILSFTAIVLHTYLAVIHPLRYLSFMSHGAAWKAVALIWLVACCFPTFLIWLS
KWQDAQLEEQGASYILPPSMGTQPGCGLLVIVTYTSILCVLFLCTALIANCFWRIYAEAKT
SGIWGQGYSRARGTLLIHSVLITLYVSTGVVFSLDMVLTRYHHIDSGTHTWLLAANSEVL
MMLPRAMLTYLYLLRYRQLLGMVRGHLPSRRHQAIFTIS

>Q9NQC7 peripheral membrane protein
MSSGLWSQEKVTSPYWEERIFYLLLQECSVTDKQTQKLLKVPKGSIGQYIQDRSVGHSRIP
SAKGKKNQIGLKILEQPHAVLFVDEKDVVEINEKFTELLLAITNCEERFSLFKNRNRLSKGL
QIDVGCPVKVQLRSGEEKFPGVVRFRGPLLAERTVSGIFFGVELLEEGRGQGFTDGVYQG
KQLFQCDEDCGVFVALDKLELIEDDDTALESDYAGPGDTMQVELPPLEINSRVSLKVGETI
ESGTVIFCDVLPGKESLGYFVGVDMDNPIGNWDGRFDGVQLCSFACVESTILLHINDIIPAL
SESVTQERRPPKLAFMSRGVGDKGSSSHNKPKATGSTSDPGNRNRSELFYTLNGSSVDSQ
PQSKSKNTWYIDEVAEDPAKSLTEISTDFDRSSPPLQPPPVNSLTTENRFHSLPFSLTKMPNT
NGSIGHSPLSLSAQSVMEELNTAPVQESPPLAMPPGNSHGLEVGSLAEVKENPPFYGVIRW
IGQPPGLNEVLAGLELEDECAGCTDGTFRGTRYFTCALKKALFVKLKSCRPDSRFASLQPV
SNQIERCNSLAFGGYLSEVVEENTPPKMEKEGLEIMIGKKKGIQGHYNSCYLDSTLFCLFA
FSSVLDTVLLRPKEKNDVEYYSETQELLRTEIVNPLRIYGYVCATKIMKLRKILEKVEAAS
GFTSEEKDPEEFLNILFHHILRVEPLLKIRSAGQKVQDCYFYQIFMEKNEKVGVPTIQQLLE
WSFINSNLKFAEAPSCLIIQMPRFGKDFKLFKKIFPSLELNITDLLEDTPRQCRICGGLAMYE
CRECYDDPDISAGKIKQFCKTCNTQVHLHPKRLNHKYNPVSLPKDLPDWDWRHGCIPCQ
NMELFAVLCIETSHYVAFVKYGKDDSAWLFFDSMADRDGGQNGFNIPQVTPCPEVGEYL
KMSLEDLHSLDSRRIQGCARRLLCDAYMCMYQSPTMSLYK

>Q01453 multipass membrane protein
MLLLLLSIIVLHVAVLVLLFVSTIVSQWIVGNGHATDLWQNCSTSSSGNVHHCFSSSPNEW
LQSVQATMILSIIFSILSLFLFFCQLFTLTKGGRFYITGIFQILAGLCVMSAAAIYTVRHPEWH
LNSDYSYGFAYILAWVAFPLALLSGVIYVILRKRE

>Q8NH87 multipass membrane protein
MQRSNHTVTEFILLGFTTDPGMQLGLFVVFLGVYSLTVVGNSTLIVLICNDSCLHTPMYFF
TGNLSFLDLWYSSVYTPKILVTCISEDKSISFAGCLCQFFFSAGLAYSECYLLAAVAYDRYV
AISKPLLYAQAMSIKLCALLVAVSYCGGFINSSIITKKTFSFNFCRENIIDDFFCDLLPLVELA
CGEKGGYKIMMYFLLASNVICPAVLILASYLFIITSVLRISSSKGYLKAFSTCSSHLTSVTLY
YGSILYIYALPRSSYSFDMDKIVSTFYTVVFPMLNLMIYSLRNKDVKEALKKLLP

>Q8NEC5 multipass membrane protein
MDQNSVPEKAQNEADTNNADRFFRSHSSPPHHRPGHSRALHHYELHHHGVPHQRGESH
HPPEFQDFHDQALSSHVHQSHHHSEARNHGRAHGPTGFGLAPSQGAVPSHRSYGEDYHD
ELQRDGRRHHDGSQYGGFHQQSDSHYHRGSHHGRPQYLGENLSHYSSGVPHHGEASHH
GGSYLPHGPNPYSESFHHSEASHLSGLQHDESQHHQVPHRGWPHHHQVHHHGRSRHHE
AHQHGKSPHHGETISPHSSVGSYQRGISDYHSEYHQGDHHPSEYHHGDHPHHTQHHYHQ
THRHRDYHQHQDHHGAYHSSYLHGDYVQSTSQLSIPHTSRSLIHDAPGPAASRTGVFPYH
VAHPRGSAHSMTRSSSTIRSRVTQMSKKVHTQDISTKHSEDWGKEEGQFQKRKTGRLQRT
RKKGHSTNLFQWLWEKLTFLIQGFREMIRNLTQSLAFETFIFFVVCLNTVMLVAQTFAEVEI
RGEWYFMALDSIFFCIYVVEALLKIIALGLSYFFDFWNNLDFFIMAMAVLDFLLMQTHSFA
IYHQSLFRILKVFKSLRALRAIRVLRRLSFLTSVQEVTGTLGQSLPSIAAILILMFTCLFLFSA
VLRALFRKSDPKRFQNIFTTIFTLFTLLTLDDWSLIYMDSRAQGAWYIIPILVIYIIIQYFIFLN
LVITVLVDSFQTALFKGLEKAKQERAARIQEKLLEDSLTELRAAEPKEVASEGTMLKRLIEK
KFGTMTEKQQELLFHYLQLVASVEQEQQKFRSQAAVIDEIVDTTFEAGEEDFRN

>O43505 single-pass type II membrane protein
MQMSYAIRCAFYQLLLAALMLVAMLQLLYLSLLSGLHGQEEQDQYFEFFPPSPRSVDQVK
AQLRTALASGGVLDASGDYRVYRGLLKTTMDPNDVILATHASVDNLLHLSGLLERWEGP
LSVSVFAATKEEAQLATVLAYALSSHCPDMRARVAMHLVCPSRYEAAVPDPREPGEFALLR
SCQEVFDKLARVAQPGINYALGTNVSYPNNLLRNLAREGANYALVIDVDMVPSEGLWRG
LREMLDQSNQWGGTALVVPAFEIRRARRMPMNKNELVQLYQVGEVRPFYYGLCTPCQAP
TNYSRWVNLPEESLLRPAYVVPWQDPWEPFYVAGGKVPTFDERFRQYGFNRISQACELHV
AGFDFEVLNEGFLVHKGFKEALKFHPQKEAENQHNKILYRQFKQELKAKYPNSPRRC

>P78380 single-pass type II membrane protein
MTFDDLKIQTVKDQPDEKSNGKKAKGLQFLYSPWWCLAAATLGVLCLGLVVTIMVLGM
QLSQVSDLLTQEQANLTHQKKKLEGQISARQQAEEASQESENELKEMIETLARKLNEKSK
EQMELHHQNLNLQETLKRVANCSAPCPQDWIWHGENCYLFSSGSFNWEKSQEKCLSLDA
KLLKINSTADLDFIQQAISYSSFPFWMGLSRRNPSYPWLWEDGSPLMPHLFRVRGAVSQTY
PSGTCAYIQRGAVYAENCILAAFSICQKKANLRAQ

>Q86UD3 multipass membrane protein
MTTSRCSHLPEVLPDCTSSAAPVVKTVEDCGSLVNGQPQYVMQVSAKDGQLLSTVVRTL
ATQSPFNDRPMCRICHEGSSQEDLLSPCECTGTLGTIHRSCLEHWLSSSNTSYCELCHFRFA
VERKPRPLVEWLRNPGPQHEKRTLFGDMVCFLFITPLATISGWLCLRGAVDHLHFSSRLEA
VGLIALTVALFTIYLFWTLVSFRYHCRLYNEWRRTNQRVILLIPKSVNVPSNQPSLLGLHSV
KRNSKETVV

>Q04900 single-pass type I membrane protein
MSRLSRSLLWAATCLGVLCVLSADKNTTQHPNVTTLAPISNVTSAPVTSLPLVTTPAPETCE
GRNSCVSCFNVSVVNTTCFWIECKDESYCSHNSTVSDCQVGNTTDFCSVSTATPVPTANST
AKPTVQPSPSTTSKTVTTSGTTNNTVTPTSQPVRKSTFDAASFIGGIVLVLGVQAVIFFLYKF
CKSKERNYHTL

>P09497 peripheral membrane protein
MADDFGFFSSSESGAPEAAEEDPAAAFLAQQESEIAGIENDEGFGAPAGSHAAPAQPGPTS
GAGSEDMGTTVNGDVFQEANGPADGYAAIAQADRLTQEPESIRKWREEQRKRLQELDAA
SKVTEQEWREKAKKDLEEWNQRQSEQVEKNKINNRIADKAFYQQPDADIIGYVASEEAF
VKESKEETPGTEWEKVAQLCDFNPKSSKQCKDVSRLRSVLMSLKQTPLSR

>Q96S52 multipass membrane protein
MAAAGAAATHLEVARGKRAALFFAAVAIVLGLPLWWKTTETYRASLPYSQISGLNALQLR
LMVPVTVVFTRESVPLDDQEKLPFTVVHEREIPLKYKMKIKCRFQKAYRRALDHEEEALS
SGSVQEAEAMLDEPQEQAEGSLTVYVISEHSSLLPQDMMSYIGPKRTAVVRGIMHREAFNI
IGRRIVQVAQAMSLTEDVLAAALADHLPEDKWSAEKRRPLKSSLGYEITFSLLNPDPKSHD
VYWDIEGAVRRYVQPFLNALGAAGNFSVDSQILYYAMLGVNPRFDSASSSYYLDMHSLP
HVINPVESRLGSSAASLYPVLNFLLYVPELAHSPLYIQDKDGAPVATNAFHSPRWGGIMVY
NVDSKTYNASVLPVRVEVDMVRVMEVFLAQLRLLFGIAQPQLPPKCLLSGPTSEGLMTW
ELDRLLWARSVENLATATTTLTSLAQLLGKISNIVIKDDVASEVYKAVAAVQKSAEELASGH
LASAFVASQEAVTSSELAFFDPSLLHLLYFPDDQKFAIYIPLFLPMAVPILLSLVKIFLETRKS
WRKPEKTD

>Q8TE54 multipass membrane protein
MTGAKRKKKSMLWSKMHTPQCEDIIQWCRRRLPILDWAPHYNLKENLLPDTVSGIMLAV
QQVTQGLAFAVLSSVHPVFGLYGSLFPAIIYAIFGMGHHVATGTFALTSLISANAVERIVPQN
MQNLTTQSNTSVLGLSDFEMQRIHVAAAVSFLGGVIQVAMFVLQLGSATFVVTEPVISAMT
TGAATHVVTSQVKYLLGMKMPYISGPLGFFYIYAYVFENIKSVRLEALLLSLLSIVVLVLV
KELNEQFKRKIKVVLPVDLVLIIAASFACYCTNMENTYGLEVVGHIPQGIPSPRAPPMNILS
AVITEAFGVALVGYVASLALAQGSAKKFKYSIDDNQEFLAHGLSNIVSSFFFCIPSAAAMGR
TAGLYSTGAKTQVACLISCIFVLIVIYAIGPLLYWLPMCVLASIIVVGLKGMLIQFRDLKKY
WNVDKIDWGIWVSTYVFTICFAANVGLLFGVVCTIAIVIGRFPRAMTVSIKNMKEMEFKV
KTEMDSETLQQVKIISINNPLVFLNAKKFYTDLMNMIQKENACNQPLDDISKCEQNTLLNS
LSNGNCNEEASQSCPNEKCYLILDCSGFTFFDYSGVSMLVEVYMDCKGRSVDVLLAHCT
ASLIKAMTYYGNLDSEKPIFFESVSAAISHIHSNKNLSKLSDHSEV

>P23528 peripheral membrane protein
MASGVAVSDGVIKVFNDMKVRKSSTPEEVKKRKKAVLFCLSEDKKNIILEEGKEILVGDV
GQTVDDPYATFVKMLPDKDCRYALYDATYETKESKKEDLVFIFWAPESAPLKSKMIYASSK
DAIKKKLTGIKHELQANCYEEVKDRCTLAEKLGGSAVISLEGKPL

>Q8NBI6 single-pass type II membrane protein
MGLLRGGLPCARAMARLGAVRSHYCALLLAAALAVCAFYYLGSGRETFSSATKRLKEAR
AGAPAAPSPPALELARGSVAPAPGAKAKSLEGGGAGPVDYHLLMMFTKAEHNAALQAKA
RVALRSLLRLAKFEAHEVLNLHFVSEEASREVAKGLLRELLPPAAGFKCKVIFHDVAVLTD
KLFPIVEAMQKHFSAGLGTYYSDSIFFLSVAMHQIMPKEILQIIQLDLDLKFKTNIRELFEEF
DSFLPGAIIGIAREMQPVYRHTFWQFRHENPQTRVGGPPPEGLPGFNSGVMLLNLEAMRQ
SPLYSRLLEPAQVQQLADKYHFRGHLGDQDFFTMIGMEHPKLFHVLDCTWNRQLCTWW

RDHGYSDVFEAYFRCEGHVKIYHGNCNTPIPED

>Q6IF63 multipass membrane protein
MAETLQLNSTFLHPNFFILTGFPGLGSAQTWLTLVFGPIYLLALLGNGALPAVVWIDSTLHQ
PMFLLLAILAATDLGLATSIAPGLLAVLWLGPRSVPYAVCLVQMFFVHALTAMESGVLLAM
ACDRAAAIGRPLHYPVLVTKACVGYAALALALKAVAIVVPFPLLVAKFEHFQAKTIGHTYC
AHMAVVELVVGNTQATNLYGLALSLAISGMDILGITGSYGLIAHAVLQLPTREAHAKAFG
TCSSHICVILAFYIPGLFSYLTHRFGHHTVPKPVHILLSNIYLLLPPALNPLIYGARTKQIRDR
LLETFTFRKSPL

>Q9NY26 multipass membrane protein
MGPWGEPELLVWRPEAVASEPPVPVGLEVKLGALVLLLVLTLLCSLVPICVLRRPGANHEG
SASRQKALSLVSCFAGGVFLATCLLDLLPDYLAAIDEALAALHVTLQFPLQEFILAMGFFL
VLVMEQITLAYKEQSGPSPLEETRALLGTVNGGPQHWHDGPGVPQASGAPATPSALRACV
LVFSLALHSVFEGLAVGLQRDRARAMELCLALLLHKGILAVSLSLRLLQSHLRAQVVAGC
GILFSCMTPLGIGLGAALAESAGPLHQLAQSVLEGMAAGTFLYITFLEILPQELASSEQRIL
KVILLLAGFALLTGLLFIQI

>Q15077 multipass membrane protein
MEWDNGTGQALGLPPTTCVYRENFKQLLLPPVYSAVLAAGLPLNICVITQICTSRRALTRT
AVYTLNLALADLLYACSLPLLIYNYAQGDHWPFGDFACRLVRFLFYANLHGSILFLTCISFQ
RYLGICHPLAPWHKRGGRRAAWLVCVAVWLAVTTQCLPTAIFAATGIQRNRTVCYDLSPP
ALATHYMPYGMALTVIGFLLPFAALLACYCLLACRLCRQDGPAEPVAQERRGKAARMAV
VVAAAFAISFLPFHITKTAYLAVRSTPGVPCTVLEAFAAAYKGTRPFASANSVLDPILFYFTQ
KKFRRRPHELLQKLTAKWQRQGR

>Q96AM1 multipass membrane protein
MAGNCSWEAHPGNRNKMCPGLSEAPELYSRGFLTIEQIAMLPPPAVMNYIFLLLCLCGLV
GNGLVLWFFGFSIKRNPFSIYFLHLASADVGYLFSKAVFSILNTGGFLGTFADYIRSVCRVL
GLCMFLTGVSLLPAVSAERCASVIFPAWYWRRRPKRLSAVVCALLWVLSLLVTCLHNYFC
VFLGRGAPGAACRHMDIFLGILLFLLCCPLMVLPCLALILHVECRARRRQRSAKLNHVILA
MVSVFLVSSIYLGIDWFLFWVFQIPAPFPEYVTDLCICINSSAKPIVYFLAGRDKSQRLWEP
LRVVFQRALRDGAELGEAGGSTPNTVTMEMQCPPGNAS

>P34981 multipass membrane protein
MENETVSELNQTQLQPRAVVALEYQVVTILLVLIICGLGIVGNIMVVLVVMRTKHMRTPTN
CYLVSLAVADLMVLVAAGLPNITDSIYGSWVYGYVGCLCITYLQYLGINASSCSITAFTIER
YIAICHPIKAQFLCTFSRAKKIIIFVWAFTSLYCMLWFFLLDLNISTYKDAIVISCGYKISRNY
YSPIYLMDFGVFYVVPMILATVLYGFIARILFLNPIPSDPKENSKTWKNDSTHQNTNLNVN
TSNRCFNSTVSSRKQVTKMLAVVVILFALLWMPYRTLVVVNSFLSSPFQENWFLLFCRICI
YLNSAINPVIYNLMSQKFRAAFRKLCNCKQKPTEKPANYSVALNYSVIKESDHFSTELDDI
TVTDTYLSATKVSFDDTCLASEVSFSQS

>Q9H2V7 multipass membrane protein

MAGSDTAPFLSQADDPDDGPVPGTPGLPGSTGNPKSEEPEVPDQEGLQRITGLSPGRSALI
VAVLCYINLLNYMDRFTVAGVLPDIEQFFNIGDSSSGLIQTVFISSYMVLAPVFGYLGDRYN
RKYLMCGGIAFWSLVTLGSSFIPGEHFWLLLLTRGLVGVGEASYSTIAPTLIADLFVADQRS
RMLSIFYFAIPVGSGLGYIAGSKVKDMAGDWHWALRVTPGLGVVAVLLLFLVVREPPRGA
VERHSDLPPLNPTSWWADLRALARNPSFVLSSLGFTAVAFVTGSLALWAPAFLLRSRVVLG
ETPPCLPGDSCSSSDSLIFGLITCLTGVLGVGLGVEISRRLRHSNPRADPLVCATGLLGSAPF
LFLSLACARGSIVATYIFIFIGETLLSMNWAIVADILLYVVIPTRRSTAEAFQIVLSHLLGDAG
SPYLIGLISDRLRRNWPPSFLSEFRALQFSLMLCAFVGALGGAAFLGTAIFIEADRRRAQLH
VQGLLHEAGSTDDRIVVPQRGRSTRVPVASVLI

>P78536 single-pass type I membrane protein
MRQSLLFLTSVVPFVLAPRPPDDPGFGPHQRLEKLDSLLSDYDILSLSNIQQHSVRKRDLQ
TSTHVETLLTFSALKRHFKLYLTSSTERFSQNFKVVVVDGKNESEYTVKWQDFFTGHVVG
EPDSRVLAHIRDDDVIIRINTDGAEYNIEPLWRFVNDTKDKRMLVYKSEDIKNVSRLQSPK
VCGYLKVDNEELLPKGLVDREPPEELVHRVKRRADPDPMKNTCKLLVVADHRFYRYMGR
GEESTTTNYLIELIDRVDDIYRNTSWDNAGFKGYGIQIEQIRILKSPQEVKPGEKHYNMAK
SYPNEEKDAWDVKMLLEQFSFDIAEEASKVCLAHLFTYQDFDMGTLGLAYVGSPRANSH
GGVCPKAYYSPVGKKNIYLNSGLTSTKNYGKTILTKEADLVTTHELGHNFGAEHDPDGLA
ECAPNEDQGGKYVMYPIAVSGDHENNKMFSNCSKQSIYKTIESKAQECFQERSNKVCGNS
RVDEGEECDPGIMYLNNDTCCNSDCTLKEGVQCSDRNSPCCKNCQFETAQKKCQEAINAT
CKGVSYCTGNSSECPPPGNAEDDTVCLDLGKCKDGKCIPFCEREQQLESCACNETDNSCK
VCCRDLSGRCVPYVDAEQKNLFLRKGKPCTVGFCDMNGKCEKRVQDVIERFWDFIDQLS
INTFGKFLADNIVGSVLVFSLIFWIPFSILVHCVDKKLDKQYESLSLFHPSNVEMLSSMDSA
SVRIIKPFPAPQTPGRLQPAPVIPSAPAAPKLDHQRMDTIQEDPSTDSHMDEDGFEKDPFPN
SSTAAKSFEDLTDHPVTRSEKAASFKLQRQNRVDSKETEC

>P28906 single-pass type I membrane protein
MLVRRGARAGPRMPRGWTALCLLSLLPSGFMSLDNNGTATPELPTQGTFSNVSTNVSYQE
TTTPSTLGSTSLHPVSQHGNEATTNITETTVKFTSTSVITSVYGNTNSSVQSQTSVISTVFTT
PANVSTPETTLKPSLSPGNVSDLSTTSTSLATSPTKPYTSSSPILSDIKAEIKCSGIREVKLTQG
ICLEQNKTSSCAEFKKDRGEGLARVLCGEEQADADAGAQVCSLLLAQSEVRPQCLLLVLA
NRTEISSKLQLMKKHQSDLKKLGILDFTEQDVASHQSYSQKTLIALVTSGALLAVLGITGYF
LMNRRSWSPTGERLGEDPYYTENGGGQGYSSGPGTSPEAQGKASVNRGAQENGTGQATS
RNGHSARQHVVADTEL

>Q9Y289 multipass membrane protein
MSVGVSTSAPLSPTSGTSVGMSTFSIMDYVVFVLLLVLSLAIGLYHACRGWGRHTVGELL
MADRKMGCLPVALSLLATFQSAVAILGVPSEIYRFGTQYWFLGCCYFLGLLIPAHIFIPVFY
RLHLTSAYEYLELRFNKTVRVCGTVTFIFQMVIYMGVVLYAPSLALNAVTGFDLWLSVLA
LGIVCTVYTALGGLKAVIWTDVFQTLVMFLGQLAVIIVGSAKVGGLGRVWAVASQHGRIS
GFELDPDPFVRHTFWTLAFGGVFMMLSLYGVNQAQVQRYLSSRTEKAAVLSCYAVFPFQQ
VSLCVGCLIGLVMFAYYQEYPMSIQQAQAAPDQFVLYFVMDLLKGLPGLPGLFIACLFSGS
LSTISSAFNSLATVTMEDLIRPWFPEFSEARAIMLSRGLAFGYGLLCLGMAYISSQMGPVLQ
AAISIFGMVGGPLLGLFCLGMFFPCANPPGAVVGLLAGLVMAFWIGIGSIVTSMGSSMPPS

PSNGSSFSLPTNLTVATVTTLMPLTTFSKPTGLQRFYSLSYLWYSAHNSTTVIVVGLIVSLLT
GRMRGRSLNPATIYPVLPKLLSLLPLSCQKRLHCRSYGQDHLDTGLFPEKPRNGVLGDSR
DKEAMALDGTAYQGSSSTCILQETSL

>Q9H0U4 lipid-anchor
MNPEYDYLFKLLLIGDSGVGKSCLLLRFADDTYTESYISTIGVDFKIRTIELDGKTIKLQIW
DTAGQERFRTITSSYYRGAHGIIVVYDVTDQESYANVKQWLQEIDRYASENVNKLLVGNK
SDLTTKKVVDNTTAKEFADSLGIPFLETSAKNATNVEQAFMTMAAEIKKRMGPGAASGGE
RPNLKIDSTPVKPAGGGCC

>O95267 peripheral membrane protein
MGTLGKAREAPRKPSHGCRAASKARLEAKPANSPFPSHPSLAHITQFRMMVSLGHLAKG
ASLDDLIDSCIQSFDADGNLCRSNQLLQVMLTMHRIVISSAELLQKVITLYKDALAKNSPG
LCLKICYFVRYWITEFWVMFKMDASLTDTMEEFQELVKAKGEELHCRLIDTTQINARDWS
RKLTQRIKSNTSKKRKVSLLFDHLEPEELSEHLTYLEFKSFRRISFSDYQNYLVNSCVKENP
TMERSIALCNGISQWVQLMVLSRPTPQLRAEVFIKFIQVAQKLHQLQNFNTLMAVIGGLCH
SSISRLKETSSHVPHEINKVLGEMTELLSSSRNYDNYRRAYGECTDFKIPILGVHLKDLISLY
EAMPDYLEDGKVNVHKLLALYNHISELVQLQEVAPPLEANKDLVHLLTLSLDLYYTEDEI
YELSYAREPRNHRAPPLTPSKPPVVVDWASGVSPKPDPKTISKHVQRMVDSVFKNYDHDQ
DGYISQEEFEKIAASFPFSFCVMDKDREGLISRDEITAYFMRASSIYSKLGLGFPHNFQETTY
LKPTFCDNCAGFLWGVIKQGYRCKDCGMNCHKQCKDLVVFECKKRAKNPVAPTENNTS
VGPVSNLCSLGAKDLLHAPEEGPFTFPNGEAVEHGEESKDRTIMLMGVSSQKISLRLKRAV
AHKATQTESQPWIGSEGPSGPFVLSSPRKTAQDTLYVLPSPTSPCPSPVLVRKRAFVKWEN
KDSLIKSKEELRHLRLPTYQELEQEINTLKADNDALKIQLKYAQKKIESLQLEKSNHVLAQ
MEQGDCS

>Q8TDT2 multipass membrane protein
MDTTMEADLGATGHRPRTELDDEDSYPQGGWDTVFLVALLLLGLPANGLMAWLAGSQA
RHGAGTRLALLLLSLALSDFLFLAAAAFQILEIRHGGHWPLGTAACRFYYFLWGVSYSSG
LFLLAALSLDRCLLALCPHWYPGHRPVRLPLWVCAGVWVLATLFSVPWLVFPEAAVWW
YDLVICLDFWDSEELSLRMLEVLGGFLPFLLLLVCHVLTQATACRTCHRQQQPAACRGFAR
VARTILSAYVVLRLPYQLAQLLYLAFLWDVYSGYLLWEALVYSDYLILLNSCLSPFLCLMA
SADLRTLLRSVLSSFAAALCEERPGSFTPTEPQTQLDSEGPTLPEPMAEAQSQMDPVAQPQ
VNPTLQPRSDPTAQPQLNPTAQPQSDPTAQPQLNLMAQPQSDSVAQPQADTNVQTPAPAA
SSVPSPCDEASPTPSSHPTPGALEDPATPPASEGESPSSTPPEAAPGAGPT

>Q9UBN6 single-pass type I membrane protein
MGLWGQSVPTASSARAGRYPGARTASGTRPWLLDPKILKFVVFIVAVLLPVRVDSATIPRQ
DEVPQQTVAPQQQRRSLKEEECPAGSHRSEYTGACNPCTEGVDYTIASNNLPSCLLCTVC
KSGQTNKSSCTTTRDTVCQCEKGSFQDKNSPEMCRTCRTGCPRGMVKVSNCTPRSDIKCK
NESAASSTGKTPAAEETVTTILGMLASPYHYLIIIVVLVIILAVVVVGFSCRKKFISYLKGICS
GGGGGPERVHRVLFRRRSCPSRVPGAEDNARNETLSNRYLQPTQVSEQEIQGQELAELTG
VTVESPEEPQRLLEQAEAEGCQRRRLLVPVNDADSADISTLLDASATLEEGHAKETIQDQL
VGSEKLFYEEDEAGSATSCL

>P41231 multipass membrane protein
MAADLGPWNDTINGTWDGDELGYRCRFNEDFKYVLLPVSYGVVCVPGLCLNAVALYIFL
CRLKTWNASTTYMFHLAVSDALYAASLPLLVYYYARGDHWPFSTVLCKLVRFLFYTNLYC
SILFLTCISVHRCLGVLRPLRSLRWGRARYARRVAGAVWVLVLACQAPVLYFVTTSARGGR
VTCHDTSAPELFSRFVAYSSVMLGLLFAVPFAVILVCYVLMARRLLKPAYGTSGGLPRAKR
KSVRTIAVVLAVFALCFLPFHVTRTLYYSFRSLDLSCHTLNAINMAYKVTRPLASANSCLDP
VLYFLAGQRLVRFARDAKPPTGPSPATPARRRLGLRRSDRTDMQRIEDVLGSSEDSRRTEST
PAGSENTKDIRL

>Q8NH73 multipass membrane protein
MEKINNVTEFIFWGLSQSPEIEKVCFVVFSFFYIIILLGNLLIMLTVCLSNLFKSPMYFFLSFL
SFVDICYSSVTAPKMIVDLLAKDKTISYVGCMLQLFGVHFFGCTEIFILTVMAYDRYVAICK
PLHYMTIMNRETCNKMLLGTWVGGFLHSIIQVALVVQLPFCGPNEIDHYFCDVHPVLKLA
CTETYIVGVVVTANSGTIALGSFVILLISYSIILVSLRKQSAEGRRKALSTCGSHIAMVVIFFG
PCTFMYMRPDTTFSEDKMVAVFYTIITPMLNPLIYTLRNAEVKNAMKKLWGRNVFLEAK
GK

>P46094 multipass membrane protein
MESSGNPESTTFFYYDLQSQPCENQAWVFATLATTVLYCLVFLLSLVGNSLVLWVLVKYES
LESLTNIFILNLCLSDLVFACLLPVWISPYHWGWVLGDFLCKLLNMIFSISLYSSIFFLTIMTI
HRYLSVVSPLSTLRVPTLRCRVLVTMAVWVASILSSILDTIFHKVLSSGCDYSELTWYLTSV
YQHNLFFLLSLGIILFCYVEILRTLFRSRSKRRHRTVKLIFAIVVAYFLSWGPYNFTLFLQTLF
RTQIIRSCEAKQQLEYALLICRNLAFSHCCFNPVLYVFVGVKFRTHLKHVLRQFWFCRLQA
PSPASIPHSPGAFAYEGASFY

>O43633 peripheral membrane protein
MDLLFGRRKTPEELLRQNQRALNRAMRELDRERQKLETQEKKIIADIKKMAKQGQMDAV
RIMAKDLVRTRRYVRKFVLMRANIQAVSLKIQTLKSNNSMAQAMKGVTKAMGTMNRQL
KLPQIQKIMMEFERQAEIMDMKEEMMNDAIDDAMGDEEDEEESDAVVSQVLDELGLSLT
DELSNLPSTGGSLSVAAGGKKAEAAASALADADADLEERLKNLRRD

>Q8NG98 multipass membrane protein
MEAENLTELSKFLLLGLSDDPELQPVLFGLFLSMYLVTVLGNLLIILAVSSDSHLHTPMYFF
LSNLSFVDICFISTTVPKMLVSIQARSKDISYMGCLTQVYFLMMFAGMDTFLLAVMAYDRF
VAICHPLHYTVIMNPCLCGLLVLASWFIIFWFSLVHILLMKRLTFSTGTEIPHFFCEPAQVLK
VACSNTLLNNIVLYVATALLGVFPVAGILFSYSQIVSSLMGMSSTKGKYKAFSTCGSHLCV
VSLFYGTGLGVYLSSAVTHSSQSSSTASVMYAMVTPMLNPFIYSLRNKDVKGALERLLSR
ADSCP

>Q6UWP7 multipass membrane protein
MHSRGREIVVLLNPWSINEAVSSYCTYFIKQDSKSFGIMVSWKGIYFILTLFWGSFFGSIFM
LSPFLPLMFVNPSWYRWINNRLVATWLTLPVALLETMFGVKVIITGDAFVPGERSVIIMNH
RTRMDWMFLWNCLMRYSYLRLEKICLKASLKGVPGFGWAMQAAAYIFIHRKWKDDKSH

FEDMIDYFCDIHEPLQLLIFPEGTDLTENSKSRSNAFAEKNGLQKYEYVLHPRTTGFTFVVD
RLREGKNLDAVHDITVAYPHNIPQSEKHLLQGDFPREIHFHVHRYPIDTLPTSKEDLQLWC
HKRWEEKEERLRSFYQGEKNFYFTGQSVIPPCKSELRVLVVKLLSILYWTLFSPAMCLLIYL
YSLVKWYFIITIVIFVLQERIFGGLEIIELACYRLLHKQPHLNSKKNE

>Q9Y6H6 single-pass type I membrane protein
METTNGTETWYESLHAVLKALNATLHSNLLCRPGPGLGPDNQTEERRASLPGRDDNSYM
YILFVMFLFAVTVGSLILGYTRSRKVDKRSDPYHVYIKNRVSMI

>Q8TDS7 multipass membrane protein
MNQTLNSSGTVESALNYSRGSTVHTAYLVLSSLAMFTCLCGMAGNSMVIWLLGFRMHRN
PFCIYILNLAAADLLFLFSMASTLSLETQPLVNTTDKVHELMKRLMYFAYTVGLSLLTAIST
QRCLSVLFPIWFKCHRPRHLSAWVCGLLWTLCLLMNGLTSSFCSKFLKFNEDRCFRVDMV
QAALIMGVLTPVMTLSSLTLFVWVRRSSQQWRRQPTRLFVVVLASVLVFLICSLPLSIYWF
VLYWLSLPPEMQVLCFSLSRLSSSVSSSANPVIYFLVGSRRSHRLPTRSLGTVLQQALREEP
ELEGGETPTVGTNEMGA

>Q8NH09 multipass membrane protein
MALGNHSTITEFLLLGLSADPNIRALLFVLFLGIYLLTIMENLMLLLMIRADSCLHKPMYFF
LSHLSFVDLCFSSVIVPKMLENLLSQRKTISVEGCLAQVFFVFVTAGTEACLLSGMAYDRH
AAICRPLLYGQIMGKQLYMHLVWGSWGLGFLDALINVLLAVNMVFCEAKIIHHYSYEMPS
LLPLSCSDISRSLIALLCSTLLHGLGNFLLVFLSYTRIISTILSISSTSGRSKAFSTCSAHLTAVT
LYYGSGLLRHLMPNSGSPIELIFSVQYTVVTPMLNSLIYSLKNKEVKGERSLRDSSHLPQL
HKGQARWKRPAFTEGRREPGHPELSIPVTPQPQGACACSALRAAPTALP

>Q9UBJ2 multipass membrane protein
MTHMLNAAADRVKWTRSSAAKRAACLVAAAYALKTLYPIIGKRLKQSGHGKKKAAAYP
AAENTEILHCTETICEKPSPGVNADFFKQLLELRKILFPKLVTTETGWLCLHSVALISRTFLSI
YVAGLDGKIVKSIVEKKPRTFIIKLIKWLMIAIPATFVNSAIRYLECKLALAFRTRLVDHAYE
TYFTNQTYYKVINMDGRLANPDQSLTEDIMMFSQSVAHLYSNLTKPILDVMLTSYTLIQTA
TSRGASPIGPTLLAGLVVYATAKVLKACSPKFGKLVAEEAHRKGYLRYVHSRIIANVEEIAF
YRGHKVEMKQLQKSYKALADQMNLILSKRLWYIMIEQFLMKYVWSSSGLIMVAIPIITAT
GFADGEDGQKQVMVSERTEAFTTARNLLASGADAIERIMSSYKEVTELAGYTARVYNMF
WVFDEVKRGIYKRTAVIQESESHSKNGAKVELPLSDTLAIKGKVIDVDHGIICENVPIITPAG
EVVASRLNFKVEEGMHLLITGPNGCGKSSLFRILSGLWPVYEGVLYKPPPQHMFYIPQRPY
MSLGSLRDQVIYPDSVDDMHDKGYTDQDLERILHNVHLYHIVQREGGWDAVMDWKDV
LSGGEKQRMGMARMFYHKPKYALLDECTSAVSIDVEGKIFQAAKGAGISLLSITHRPSLW
KYHTHLLQFDGEGGWRFEQLDTAIRLTLSEEKQKLESQLAGIPKMQQRLNELCKILGEDS
VLKTIKNEDETS

>O75881 peripheral membrane protein
MAGEVSAATGRFSLERLGLPGLALAAALLLLALCLLVRRTRRPGEPPLIKGWLPYLGVVL
NLRKDPLRFMKTLQKQHGDTFTVLLGGKYITFILDPFQYQLVIKNHKQLSFRVFSNKLLEK
AFSISQLQKNHDMNDELHLCYQFLQGKSLDILLESMMQNLKQVFEPQLLKTTSWDTAELY

PFCSSIIFEITFTTIYGKVIVCDNNKFISELRDDFLKFDDKFAYLVSNIPIELLGNVKSIREKIIK
CFSSEKLAKMQGWSEVFQSRQDVLEKYYVHEDLEIGAHHLGFLWASVANTIPTMFWAMY
YLLRHPEAMAAVRDEIDRLLQSTGQKKGSGFPIHLTREQLDSLICLESSIFEALRLSSYSTTI
RFVEEDLTLSSETGDYCVRKGDLVAIFPPVLHGDPEIFEAPEEFRYDRFIEDGKKKTTFFKR
GKKLKCYLMPFGTGTSKCPGRFFALMEIKQLLVILLTYFDLEIIDDKPIGLNYSRLLFGIQYP
DSDVLFRYKVKS

>Q14451 peripheral membrane protein
MELDLSPPHLSSSPEDLCPAPGTPPGTPRPPDTPLPEEVKRSQPLLIPTTGRKLREEERRATS
LPSIPNPFPELCSPPSQSPILGGPSSARGLLPRDASRPHVVKVYSEDGACRSVEVAAGATAR
HVCEMLVQRAHALSDETWGLVECHPHLALERGLEDHESVVEVQAAWPVGGDSRFVFRK
NFAKYELFKSSPHSLFPEKMVSSCLDAHTGISHEDLIQNFLNAGSFPEIQGFLQLRGSGRKL
WKRFFCFLRRSGLYYSTKGTSKDPRHLQYVADVNESNVYVVTQGRKLYGMPTDFGFCVK
PNKLRNGHKGLRIFCSEDEQSRTCWLAAFRLFKYGVQLYKNYQQAQSRHLHPSCLGSPPL
RSASDNTLVAMDFSGHAGRVIENPREALSVALEEAQAWRKKTNHRLSLPMPASGTSLSAAI
HRTQLWFHGRISREESQRLIGQQGLVDGLFLVRESQRNPQGFVLSLCHLQKVKHYLILPSE
EEGRLYFSMDDGQTRFTDLLQLVEFHQLNRGILPCLLRHCCTRVAL

>Q8NH18 multipass membrane protein
MADDNFTVVTEFILLGLTDHAELKAVLFVVFLVIYAITLLRNLGMILLIQITSKLHTPMYFL
LSCLSFVDACYSSAIAPKMLVNLLVVKATISFSACMVQHLCFGVFITTEGFLLSVMAYDRY
VAIVSPLLYTVAMSDRKCVELVTGSWIGGIVNTLIHTISLRRLSFCRLNAVSHFFCDIPSLLK
LSCSDTSMNELLLLTFSGVIAMATFLTVIISYIFIAFASLRIHSASGRQQAFSTCASHLTAVTIF
YGTLIFSYIQPSSQYFVEQEKVVSMFYTLGIPMLNLLIHSLRNKDVKEAVKRAIEMKHFLC

>P21145 multipass membrane protein
MAPAAATGGSTLPSGFSVFTTLPDLLFIFEFIFGGLVWILVASSLVPWPLVQGWVMFVSVFC
FVATTTLIILYIIGAHGGETSWVTLDAAYHCTAALFYLSASVLEALATITMQDGFTYRHYHE
NIAAVVFSYIATLLYVVHAVFSLIRWKSS

>Q8WXG6 multipass membrane protein
MVQKKKFCPRLLDYLVIVGARHPSSDSVAQTPELLRRYPLEDHTEFPLPPDVVFFCQPEGC
LSVRQRRMSLRDDTSFVFTLTDKDTGVTRYGICVNFYRSFQKRISKEKGEGGAGSRGKEG
THATCASEEGGTESSESGSSLQPLSADSTPDVNQSPRGKRRAKAGSRSRNSTLTSLCVLSH
YPFFSTFRECLYTLKRLVDCCSERLLGKKLGIPRGVQRDTMWRIFTGSLLVEEKSSALLHD
LREIEAWIYRLLRSPVPVSGQKRVDIEVLPQELQPALTFALPDPSRFTLVDFPLHLPLELLGV
DACLQVLTCILLEHKVVLQSRDYNALSMSVMAFVAMIYPLEYMFPVIPLLPTCMASAEQL
LLAPTPYIIGVPASFFLYKLDFKMPDDVWLVDLDSNRVIAPTNAEVLPILPEPESLELKKHL
KQALASMSLNTQPILNLEKFHEGQEIPLLLGRPSNDLQSTPSTEFNPLIYGNDVDSVDVATR
VAMVRFFNSANVLQGFQMHTRTLRLFPRPVVAFQAGSFLASRPRQTPFAEKLARTQAVEY
FGEWILNPTNYAFQRIHNNMFDPALIGDKPKWYAHQLQPIHYRVYDSNSQLAEALSVPPE
RDSDSEPTDDSGSDSMDYDDSSSSYSSLGDFVSEMMKCDINGDTPNVDPLTHAALGDASE
VEIDELQNQKEAEEPGPDSENSQENPPLRSSSSTTASSSPSTVIHGANSEPADSTEMDDKAA
VGVSKPLPSVPPSIGKSNVDRRQAEIGEGSVRRRIYDNPYFEPQYGFPPEEDEDEQGESYTP

RFSQHVSGNRAQKLLRPNSLRLASDSDAESDSRASSPNSTVSNTSTEGFGGIMSFASSLYR
NHSTSFSLSNLTLPTKGAREKATPFPSLKVFGLNTLMEIVTEAGPGSGEGNRRALVDQKSS
VIKHSPTVKREPPSPQGRSSNSSENQQFLKEVVHSVLDGQGVGWLNMKKVRRLLESEQLR
VFVLSKLNRMVQSEDDARQDIIPDVEISRKVYKGMLDLLKCTVLSLEQSYAHAGLGGMA
SIFGLLEIAQTHYYSKEPDKRKRSPTESVNTPVGKDPGLAGRGDPKAMAQLRVPQLGPRA
PSATGKGPKELDTRSLKEENFIASIELWNKHQEVKKQKALEKQRPEVIKPVFDLGETEEKK
SQISADSGVSLTSSSQRTDQDSVIGVSPAVMIRSSSQDSEVSTVVSNSSGETLGADSDLSSNA
GDGPGGEGSVHLASSRGTLSDSEIETNSATSTIFGKAHSLKPSIKEKLAGSPIRTSEDVSQRV
YLYEGLLGRDKGSMWDQLEDAAMETFSISKERSTLWDQMQFWEDAFLDAVMLEREGM
GMDQGPQEMIDRYLSLGEHDRKRLEDDEDRLLATLLHNLISYMLLMKVNKNDIRKKVRR
LMGKSHIGLVYSQQINEVLDQLANLNGRDLSIWSSGSRHMKKQTFVVHAGTDTNGDIFF
MEVCDDCVVLRSNIGTVYERWWYEKLINMTYCPKTKVLCLWRRNGSETQLNKFYTKKC
RELYYCVKDSMERAAARQQSIKPGPELGGEFPVQDLKTGEGGLLQVTLEGINLKFMHNQ
VFIELNHIKKCNTVRGVFVLEEFVPEIKEVVSHKYKTPMAHEICYSVLCLFSYVAAVHSSEE
DLRTPPRPVSS

>Q9UK23 single-pass type I membrane protein
MATSTGRWLLLRLALFGFLWEASGGLDSGASRDDDLLLPYPRARARLPRDCTRVRAGNR
EHESWPPPPATPGAGGLAVRTFVSHFRDRAVAGHLTRAVEPLRTFSVLEPGGPGGCAARRR
ATVEETARAADCRVAQNGGFFRMNSGECLGNVVSDERRVSSSGGLQNAQFGIRRDGTLVT
GYLSEEEVLDTENPFVQLLSGVVWLIRNGSIYINESQATECDETQETGSFSKFVNVISARTAI
GHDRKGQLVLFHADGQTEQRGINLWEMAEFLLKQDVVNAINLDGGGSATFVLNGTLASY
PSDHCQDNMWRCPRQVSTVVCVHEPRCQPPDCHGHGTCVDGHCQCTGHFWRGPGCDE
LDCGPSNCSQHGLCTETGCRCDAGWTGSNCSEECPLGWHGPGCQRPCKCEHHCPCDPKT
GNCSVSRVKQCLQPPEATLRAGELSFFTRTAWLALTLALAFLLLISTAANLSLLLSRAERNR
RLHGDYAYHPLQEMNGEPLAAEKEQPGGAHNPFKD

>Q8TD46 single-pass type I membrane protein
MLCPWRTANLGLLLILTIFLVAASSSLCMDEKQITQNYSKVLAEVNTSWPVKMATNAVLC
CPPIALRNLIIITWEIILRGQPSCTKAYRKETNETKETNCTDERITWVSRPDQNSDLQIRPVAI
THDGYYRCIMVTPDGNFHRGYHLQVLVTPEVTLFQNRNRTAVCKAVAGKPAAQISWIPEG
DCATKQEYWSNGTVTVKSTCHWEVHNVSTVTCHVSHLTGNKSLYIELLPVPGAKKSAKL
YIPYIILTIIILTIVGFIWLLKVNGCRKYKLNKTESTPVVEEDEMQPYASYTEKNNPLYDTTN
KVKASEALQSEVDTDLHTL

>Q8NGM8 multipass membrane protein
MGNWSTVTEITLIAFPALLEIRISLFVVLVVTYTLTATGNITIISLIWIDHRLQTPMYFFLSNLS
FLDILYTTVITPKLLACLLGEEKTISFAGCMIQTYFYFFLGTVEFILLAVMSFDRYMAICDPL
HYTVIMNSRACLLLVLGCWVGAFLSVLFPTIVVTRLPYCRKEINHFFCDIAPLLQVACINTH
LIEKINFLLSALVILSSLAFTTGSYVYIISTILRIPSTQGRQKAFSTCASHITVVSIAHGSNIFVY
VRPNQNSSLDYDKVAAVLITVVTPLLNPFIYSLRNEKVQEVLRETVNRIMTLIQRKT

>Q02928 peripheral membrane protein
MSVSVLSPSRLLGDVSGILQAASLLILLLLLIKAVQLYLHRQWLLKALQQFPCPPSHWLFG

HIQELQQDQELQRIQKWVETFPSACPHWLWGGKVRVQLYDPDYMKVILGRSDPKSHGSY
RFLAPWIGYGLLLLNGQTWFQHRRMLTPAFHYDILKPYVGLMADSVRVMLDKWEELLG
QDSPLEVFQHVSLMTLDTIMKCAFSHQGSIQVDRNSQSYIQAISDLNNLVFSRVRNAFHQN
DTIYSLTSAGRWTHRACQLAHQHTDQVIQLRKAQLQKEGELEKIKRKRHLDFLDILLLAK
MENGSILSDKDLRAEVDTFMFEGHDTTASGISWILYALATHPKHQERCREEIHSLLGDGASI
TWNHLDQMPYTTMCIKEALRLYPPVPGIGRELSTPVTFPDGRSLPKGIMVLLSIYGLHHNP
KVWPNPEVFDPFRFAPGSAQHSHAFLPFSGGSRNCIGKQFAMNELKVATALTLLRFELLPD
PTRIPIPIARLVLKSKNGIHLRLRRLPNPCEDKDQL

>P14923 peripheral membrane protein
MEVMNLMEQPIKVTEWQQTYTYDSGIHSGANTCVPSVSSKGIMEEDEACGRQYTLKKTT
TYTQGVPPSQGDLEYQMSTTARAKRVREAMCPGVSGEDSSLLLATQVEGQATNLQRLAE
PSQLLKSAIVHLINYQDDAELATRALPELTKLLNDEDPVVVTKAAMIVNQLSKKEASRRA
LMGSPQLVAAVVRTMQNTSDLDTARCTTSILHNLSHHREGLLAIFKSGGIPALVRMLSSPVE
SVLFYAITTLHNLLLYQEGAKMAVRLADGLQKMVPLLNKNNPKFLAITTDCLQLLAYGNQ
ESKLIILANGGPQALVQIMRNYSYEKLLWTTSRVLKVLSVCPSNKPAIVEAGGMQALGKH
LTSNSPRLVQNCLWTLRNLSDVATKQEGLESVLKILVNQLSVDDVNVLTCATGTLSNLTCN
NSKNKTLVTQNSGVEALIHAILRAGDKDDITEPAVCALRHLTSRHPEAEMAQNSVRLNYGI
PAIVKLLNQPNQWPLVKATIGLIRNLALCPANHAPLQEAAVIPRLVQLLVKAHQDAQRHVA
AGTQQPYTDGVRMEEIVEGCTGALHILARDPMNRMEIFRLNTIPLFVQLLYSSVENIQRVA
AGVLCELAQDKEAADAIDAEGASAPLMELLHSRNEGTATYAAAVLFRISEDKNPDYRKRV
SVELTNSLFKHDPAAWEAAQSMIPINEPYGDDMDATYRPMYSSDVPLDPLEMHMDMDGD
YPIDTYSDGLRPPYPTADHMLA

>P29972 multipass membrane protein
MASEFKKKLFWRAVVAEFLATTLFVFISIGSALGFKYPVGNNQTAVQDNVKVSLAFGLSIA
TLAQSVGHISGAHLNPAVTLGLLLSCQISIFRALMYIIAQCVGAIVATAILSGITSSLTGNSLG
RNDLADGVNSGQGLGIEIIGTLQLVLCVLATTDRRRRDLGGSAPLAIGLSVALGHLLAIDY
TGCGINPARSFGSAVITHNFSNHWIFWVGPFIGGALAVLIYDFILAPRSSDLTDRVKVWTSG
QVEEYDLDADDINSRVEMKPK

>Q53F39 multipass membrane protein
MAMIELGFGRQNFHPLKRKSSLLLKLIAVVFAVLLFCEFLIYYLAIFQCNWPEVKTTASDGE
QTTREPVLKAMFLADTHLLGEFLGHWLDKLRREWQMERAFQTALWLLQPEVVFILGDIF
DEGKWSTPEAWADDVERFQKMFRHPSHVQLKVVAGNHDIGFHYEMNTYKVERFEKVFS
SERLFSWKGINFVMVNSVALNGDGCGICSETEAELIEVSHRLNCSREARGSSRCGPGPLLP
TSAPVLLQHYPLYRRSDANCSGEDAAPAEERDIPFKENYDVLSREASQKLLWWLQPRLVL
SGHTHSACEVHHGGRVPELSVPSFSWRNRNNPSFIMGSITPTDYTLSKCYLPREDVVLIIYC
GVVGFLVVLTLTHFGLLASPFLSGLNLLGKRKTR

>Q92959 multipass membrane protein
MGLLPKLGASQGSDTSTSRAGRCARSVFGNIKVFVLCQGLLQLCQLLYSAYFKSSLTTIEK
RFGLSSSSSGLISSLNEISNAILIIFVSYFGSRVHRPRLIGIGGLFLAAGAFILTLPHFLSEPYQY
TLASTGNNSRLQAELCQKHWQDLPPSKCHSTTQNPQKETSSMWGLMVVAQLLAGIGTVP

IQPFGISYVDDFSEPSNSPLYISILFAISVFGPAFGYLLGSVMLQIFVDYGRVNTAAVNLVPGD
PRWIGAWWLGLLISSALLVLTSFPFFFFPRAMPIGAKRAPATADEARKLEEAKSRGSLVDFI
KRFPCIFLRLLMNSLFVLVVLAQCTFSSVIAGLSTFLNKFLEKQYGTSAAYANFLIGAVNLP
AAALGMLFGGILMKRFVFSLQAIPRIATTIITISMILCVPLFFMGCSTPTVAEVYPPSTSSSIH
PQSPACRRDCSCPDSIFHPVCGDNGIEYLSPCHAGCSNINMSSATSKQLIYLNCSCVTGGSA
SAKTGSCPVPCAHFLLPAIFLISFVSLIACISHNPLYMMVLRVVNQEEKSFAIGVQFLLMRLL
AWLPSPALYGLTIDHSCIRWNSLCLGRRGACAYYDNDALRDRYLGLQMGYKALGMLLLC
FISWRVKKNKEYNVQKAAGLI

>O60423 multipass membrane protein
MGTGPAQTPRSTRAGPEPSPAPPGPGDTGDSDVTQEGSGPAGIRGGETVIRAGMGDSPGR
GAPERRHKAQPGRARKYEWRPEGPTSMGSLGQREDLQDEDRNSAFTWKVQANNRAYN
GQFKEKVILCWQRKKYKTNVIRTAKYNFYSFLPLNLYEQFHRVSNLFFLIIIILQSIPDISTLP
WFSLSTPMVCLLFIRATRDLVDDMGRHKSDRAINNRPCQILMGKSFKQKKWQDLCVGDV
VCLRKDNIVPADMLLLASTEPSSLCYVETVDIDGETNLKFRQALMVTHKELATIKKMASF
QGTVTCEAPNSRMHHFVGCLEWNDKKYSLDIGNLLLRGCRIRNTDTCYGLVIYAGFDTKI
MKNCGKIHLKRTKLDLLMNKLVVVIFISVVLVCLVLAFGFGFSVKEFKDHHYYLSGVHGS
SVAAESFFVFWSFLILLSVTIPMSMFILSEFIYLGNSVFIDWDVQMYYKPQDVPAKARSTSL
NDHLGQVEYIFSDKTGTLTQNILTFNKCCISGRVYGPDSEATTRPKENPYLWNKFADGKLL
FHNAALLHLVRTNGDEAVREFWRLLAICHTVMVRESPRERPDQLLYQAASPDEGALVTAA
RNFGYVFLSRTQDTVTIMELGEERVYQVLAIMDFNSTRKRMSVLVRKPEGAICLYTKGAD
TVIFERLHRRGAMEFATEEALAAFAQETLRTLCLAYREVAEDIYEDWQQRHQEASLLLQN
RAQALQQLLGATAIEDRLQDGVPETIKCLKKSNIKIWVLTGDKQETAVNIGFACELLSENM
LILEEKEISRILETYWENSNNLLTRESLSQVKLALVINGDFLDKLLVSLRKEPRALAQNVNM
DEAWQELGQSRRDFLYARRLSLLCRRFGLPLAAPPAQDSRARRSSEVLQERAFVDLASKC
QAVICCRVTPKQKALIVALVKKYHQVVTLAIGDGANDINMIKTADVGVGLAGQEGMQAV
QNSDFVLGQFCFLQRLLLVHGRWSYVRICKFLRYFFYKSMASMMVQVWFACYNGFTGQ
PLYEGWFLALFNLLYSTLPVLYIGLFEQDVSAEQSLEKPELYVVGQKDELFNYWVFVQAIA
HGVTTSLVNFFMTLWISRDTAGPASFSDHQSFAVVVALSCLLSITMEVILIIKYWTALCVATI
LLSLGFYAIMTTTTQSFWLFRVSPTTFPFLYADLSVMSSPSILLVVLLSVSINTFPVLALRVIF
PALKELRAKEEKVEEGPSEEIFTMEPLPHVHRESRARRSSYAFSHREGYANLITQGTILRRG
PGVSSDIASESLDPSDEEAASSPKESQ

>P04920 multipass membrane protein
MSSAPRRPAKGADSFCTPEPESLGPGTPGFPEQEEDELHRTLGVERFEEILQEAGSRGGEEP
GRSYGEEDFEYHRQSSHHIHHPLSTHLPPDARRRKTPQGPGRKPRRRPGASPTGETPTIEE
GEEDEDEASEAEGARALTQPSPVSTPSSVQFFLQEDDSADRKAERTSPSSPAPLPHQEATPR
ASKGAQAGTQVEEAEAEAVAVASGTAGGDDGGASGRPLPKAQPGHRSYNLQERRRIGSM
TGAEQALLPRVPTDEIEAQTLATADLDLMKSHRFEDVPGVRRHLVRKNAKGSTQSGREGR
EPGPTPRARPRAPHKPHEVFVELNELLLDKNQEPQWRETARWIKFEEDVEEETERWGKPH
VASLSFRSLLELRRTLAHGAVLLDLDQQTLPGVAHQVVEQMVISDQIKAEDRANVLRALL
LKHSHPSDEKDFSFPRNISAGSLGSLLGHHHGQGAESDPHVTEPLMGGVPETRLEVERERE
LPPPAPPAGITRSKSKHELKLLEKIPENAEATVVLVGCVEFLSRPTMAFVRLREAVELDAVL
EVPVPVRFLFLLLGPSSANMDYHEIGRSISTLMSDKQFHEAAYLADEREDLLTAINAFLDCS

VVLPPSEVQGEELLRSVAHFQRQMLKKREEQGRLLPTGAGLEPKSAQDKALLQMVEAAG
AAEDDPLRRTGRPFGGLIRDVRRRYPHYLSDFRDALDPQCLAAVIFIYFAALSPAITFGGLL
GEKTQDLIGVSELIMSTALQGVVFCLLGAQPLLVIGFSGPLLVFEEAFFSFCSSNHLEYLVG
RVWIGFWLVFLALLMVALEGSFLVRFVSRFTQEIFAFLISLIFIYETFYKLVKIFQEHPLHGCS
ASNSSEVDGGENMTWAGARPTLGPGNRSLAGQSGQGKPRGQPNTALLSLVLMAGTFFIA
FFLRKFKNSRFFPGRIRRVIGDFGVPIAILIMVLVDYSIEDTYTQKLSVPSGFSVTAPEKRGW
VINPLGEKSPFPVWMMVASLLPAILVFILIFMETQITTLIISKKERMLQKGSGFHLDLLLIVA
MGGICALFGLPWLAAATVRSVTHANALTVMSKAVAPGDKPKIQEVKEQRVTGLLVALLVG
LSIVIGDLLRQIPLAVLFGIFLYMGVTSLNGIQFYERLHLLLMPPKHHPDVTYVKKVRTLRM
HLFTALQLLCLALLWAVMSTAASLAFPFILILTVPLRMVVLTRIFTDREMKCLDANEAEPVF
DEREGVDEYNEMPMPV

>O43306 multipass membrane protein
MSWFSGLLVPKVDERKTAWGERNGQKRSRRRGTRAGGFCTPRYMSCLRDAEPPSPTPAG
PPRCPWQDDAFIRRGGPGKGKELGLRAVALGFEDTEVTTTAGGTAEVAPDAVPRSGRSCW
RRLVQVFQSKQFRSAKLERLYQRYFFQMNQSSLTLLMAVLVLLTAVLLAFHAAPARPQPAY
VALLACAAALFVGLMVVCNRHSFRQDSMWVVSYVVLGILAAVQVGGALAADPRSPSAG
LWCPVFFVYIAYTLLPIRMRAAVLSGLGLSTLHLILAWQLNRGDAFLWKQLGANVLLFLC
TNVIGICTHYPAEVSQRQAFQETRGYIQARLHLQHENRQQERLLLSVLPQHVAMEMKEDI
NTKKEDMMFHKIYIQKHDNVSILFADIEGFTSLASQCTAQELVMTLNELFARFDKLAAEN
HCLRIKILGDCYYCVSGLPEARADHAHCCVEMGVDMIEAISLVREVTGVNVNMRVGIHSG
RVHCGVLGLRKWQFDVWSNDVTLANHMEAGGRAGRIHITRATLQYLNGDYEVEPGRGG
ERNAYLKEQHIETFLILGASQKRKEEKAMLAKLQRTRANSMEGLMPRWVPDRAFSRTKD
SKAFRQMGIDDSSKDNRGTQDALNPEDEVDEFLSRAIDARSIDQLRKDHVRRFLLTFQRE
DLEKKYSRKVDPRFGAYVACALLVFCFICFIQLLIFPHSTLMLGIYASIFLLLLITVLICAVYS
CGSLFPKALQRLSRSIVRSRAHSTAVGIFSVLLVFTSAIANMFTCNHTPIRSCAARMLNLTPA
DITACHLQQLNYSLGLDAPLCEGTMPTCSFPEYFIGNMLLSLLASSVFLHISSIGKLAMIFV
LGLIYLVLLLLGPPATIFDNYDLLLGVHGLASSNETFDGLDCPAAGRVALKYMTPVILLVFA
LALYLHAQQVESTARLDFLWKLQATGEKEEMEELQAYNRRLLHNILPKDVAAHFLARERR
NDELYYQSCECVAVMFASIANFSEFYVELEANNEGVECLRLLNEIIADFDEIISEERFRQLEK
IKTIGSTYMAASGLNASTYDQVGRSHITALADYAMRLMEQMKHINEHSFNNFQMKIGLN
MGPVVAGVIGARKPQYDIWGNTVNVSSRMDSTGVPDRIQVTTDLYQVLAAKGYQLECR
GVVKVKGKGEMTTYFLNGGPSS

>Q8NH19 multipass membrane protein
MEFVLLGFSDIPNLHWMLFSIFLLMYLMILMCNGIIILLIKIHPALQTPMYFFLSNFSLLEICY
VTIIIPRMLMDIWTQKGNISLFACATQMCFFLMLGGTECLLLTVMAYDRYVAICKPLQYPL
VMNHKVCIQLIIASWTITIPVVIGETCQIFLLPFCGTNTINHFFCDIPPILKLACGNIFVNEITV
HVVAVVFITVPFLLIVVSYGKIISNILKLSSARGKAKAFSTCSSHLIVVILFFGAGTITYLQPK
PHQFQRMGKLISLFYTILIPTLNPIIYTLRNKDIMVALRKLLAKLLT

>Q14246 multipass membrane protein
MRGFNLLLFWGCCVMHSWEGHIRPTRKPNTKGNNCRDSTLCPAYATCTNTVDSYYCACK
QGFLSSNGQNHFKDPGVRCKDIDECSQSPQPCGPNSSCKNLSGRYKCSCLDGFSSPTGND

WVPGKPGNFSCTDINECLTSSVCPEHSDCVNSMGSYSCSCQVGFISRNSTCEDVDECADPR
ACPEHATCNNTVGNYSCFCNPGFESSSGHLSFQGLKASCEDIDECTEMCPINSTCTNTPGS
YFCTCHPGFAPSNGQLNFTDQGVECRDIDECRQDPSTCGPNSICTNALGSYSCGCIAGFHP
NPEGSQKDGNFSCQRVLFKCKEDVIPDNKQIQQCQEGTAVKPAYVSFCAQINNIFSVLDKV
CENKTTVVSLKNTTESFVPVLKQISTWTKFTKEETSSLATVFLESVESMTLASFWKPSANIT
PAVRTEYLDIESKVINKECSEENVTLDLVAKGDKMKIGCSTIEESESTETTGVAFVSFVGME
SVLNERFFKDHQAPLTTSEIKLKMNSRVVGGIMTGEKKDGFSDPIIYTLENIQPKQKFERPI
CVSWSTDVKGGRWTSFGCVILEASETYTICSCNQMANLAVIMASGELTMDFSLYIISHVGII
ISLVCLVLAIATFLLCRSIRNHNTYLHLHLCVCLLLAKTLFLAGIHKTDNKMGCAIIAGFLH
YLFLACFFWMLVEAVILFLMVRNLKVVNYFSSRNIKMLHICAFGYGLPMLVVVISASVQP
QGYGMHNRCWLNTETGFIWSFLGPVCTVIVINSLLLTWTLWILRQRLSSVNAEVSTLKDT
RLLTFKAFAQLFILGCSWVLGIFQIGPVAGVMAYLFTIINSLQGAFIFLIHCLLNGQVREEYK
RWITGKTKPSSQSQTSRILLSSMPSASKTG

>O95271 peripheral membrane protein
MAASRRSQHHHHHHQQQLQPAPGASAPPPPPPPPLSPGLAPGTTPASPTASGLAPFASPRH
GLALPEGDGSRDPPDRPRSPDPVDGTSCCSTTSTICTVAAAPVVPAVSTSSAAGVAPNPAGS
GSNNSPSSSSSPTSSSSSSPSSPGSSLAESPEAAGVSSTAPLGPGAAGPGTGVPAVSGALREL
LEACRNGDVSRVKRLVDAANVNAKDMAGRKSSPLHFAAGFGRKDVVEHLLQMGANVH
ARDDGGLIPLHNACSFGHAEVVSLLLCQGADPNARDNWNYTPLHEAAIKGKIDVCIVLLQ
HGADPNIRNTDGKSALDLADPSAKAVLTGEYKKDELLEAARSGNEEKLMALLTPLNVNC
HASDGRKSTPLHLAAGYNRVRIVQLLLQHGADVHAKDKGGLVPLHNACSYGHYEVTELL
LKHGACVNAMDLWQFTPLHEAASKNRVEVCSLLLSHGADPTLVNCHGKSAVDMAPTPEL
RERLTYEFKGHSLLQAAREADLAKVKKTLALEIINFKQPQSHETALHCAVASLHPKRKQVT
ELLLRKGANVNEKNKDFMTPLHVAAERAHNDVMEVLHKHGAKMNALDTLGQTALHRA
ALAGHLQTCRLLLSYGSDPSIISLQGFTAAQMGNEAVQQILSESTPIRTSDVDYRLLEASKA
GDLETVKQLCSSQNVNCRDLEGRHSTPLHFAAGYNRVSVVEYLLHHGADVHAKDKGGL
VPLHNACSYGHYEVAELLVRHGASVNVADLWKFTPLHEAAAKGKYEICKLLLKHGADPT
KKNRDGNTPLDLVKEGDTDIQDLLRGDAALLDAAKKGCLARVQKLCTPENINCRDTQGR
NSTPLHLAAGYNNLEVAEYLLEHGADVNAQDKGGLIPLHNAASYGHVDIAALLIKYNTC
VNATDKWAFTPLHEAAQKGRTQLCALLLAHGADPTMKNQEGQTPLDLATADDIRALLID
AMPPEALPTCFKPQATVVSASLISPASTPSCLSAASSIDNLTGPLAELAVGGASNAGDGAAG
TERKEGEVAGLDMNISQFLKSLGLEHLRDIFETEQITLDVLADMGHEELKEIGINAYGHRH
KLIKGVERLLGGQQGTNPYLTFHCVNQGTILLDLAPEDKEYQSVEEEMQSTIREHRDGGN
AGGIFNRYNVIRIQKVVNKKLRERFCHRQKEVSEENHNHHNERMLFHGSPFINAIIHKGFD
ERHAYIGGMFGAGIYFAENSSKSNQYVYGIGGGTGCPTHKDRSCYICHRQMLFCRVTLGK
SFLQFSTMKMAHAPPGHHSVIGRPSVNGLAYAEYVIYRGEQAYPEYLITYQIMKPEAPSQT
ATAAEQKT

>Q8NGF8 multipass membrane protein
MASTSNVTELIFTGLFQDPAVQSVCFVVFLPVYLATVVGNGLIVLTVSISKSLDSPMYFFLS
CLSLVEISYSSTIAPKFIIDLLAKIKTISLEGCLTQIFFFHFFGVAEILLIVVMAYDCYVAICKPL
HYMNIISRQLCHLLVAGSWLGGFCHSIIQILVIIQLPFCGPNVIDHYFCDLQPLFKLACTDTF
MEGVIVLANSGLFSVFSFLILVSSYIVILVNLRNHSAEGRHKALSTCASHITVVILFFGPAIFL

YMRPSSTFTEDKLVAVFYTVITPMLNPIIYTLRNAEVKIAIRRLWSKKENPGRE

>Q99788 multipass membrane protein
MRMEDEDYNTSISYGDEYPDYLDSIVVLEDLSPLEARVTRIFLVVVYSIVCFLGILGNGLVII
IATFKMKKTVNMVWFLNLAVADFLFNVFLPIHITYAAMDYHWVFGTAMCKISNFLLIHNM
FTSVFLLTIISSDRCISVLLPVWSQNHRSVRLAYMACMVIWVLAFFLSSPSLVFRDTANLHG
KISCFNNFSLSTPGSSSWPTHSQMDPVGYSRHMVVTVTRFLCGFLVPVLIITACYLTIVCKL
QRNRLAKTKKPFKIIVTIIITFFLCWCPYHTLNLLELHHTAMPGSVFSLGLPLATALAIANSC
MNPILYVFMGQDFKKFKVALFSRLVNALSEDTGHSSYPSHRSFTKMSSMNERTSMNERET
GML

>Q86Y39 multipass membrane protein
MAPKVFRQYWDIPDGTDCHRKAYSTTSIASVAGLTAAAYRVTLNPPGTFLEGVAKVGQYT
FTAAAVGAVFGLTTCISAHVREKPDDPLNYFLGGCAGGLTLGARTHNYGIGAAACVYFGI
AASLVKMGRLEGWEVFAKPKV

>Q5T9A4 peripheral membrane protein
MSWLFGVNKGPKGEGAGPPPPLPPAQPGAEGGGDRGLGDRPAPKDKWSNFDPTGLERAA
KAARELEHSRYAKEALNLAQMQEQTLQLEQQSKLKEYEAAVEQLKSEQIRAQAEERRKT
LSEETRQHQARAQYQDKLARQRYEDQLKQQQLLNEENLRKQEESVQKQEAMRRATVER
EMELRHKNEMLRVETEARARAKAERENADIIREQIRLKASEHRQTVLESIRTAGTLFGEGF
RAFVTDRDKVTATVAGLTLLAVGVYSAKNATAVTGRFIEARLGKPSLVRETSRITVLEALR
HPIQVSRRLLSRPQDVLEGVVLSPSLEARVRDIAIATRNTKKNRGLYRHILLYGPPGTGKTL
FAKKLALHSGMDYAIMTGGDVAPMGREGVTAMHKLFDWANTSRRGLLLFMDEADAFLR
KRATEEISKDLRATLNAFLYHMGQHSNKFMLVLASNLPEQFDCAINSRIDVMVHFDLPQQ
EERERLVRLHFDNCVLKPATEGKRRLKLAQFDYGRKCSEVARLTEGMSGREIAQLAVSWQ
ATAYASKDGVLTEAMMDACVQDAVQQYRQKMRWLKAEGPGRGVEHPLSGVQGETLTS
WSLATDPSYPCLAGPCTFRICSWMGTGLCPGPLSPRMSCGGGRPFCPPGHPLL

>Q9NY99 peripheral membrane protein
MGTEGPPPPAASRGRQGCLLVPARTKTTIALLYDEESENAYDIRLKLTKEVLTIQKQDVVC
VGGSHQGRNRRTVTLRRQPVGGLGLSIKGGSEHNVPVVISKIFEDQAADQTGMLFVGDAV
LQVNGIHVENATHEEVVHLLRNAGDEVTITVEYLREAPAFLKLPLGSPGPSSDHSSGASSP
LFDSGLHLNGNSSTTAPSSPSSPIAKDPRYEKRWLDTLSVPLSMARISRYKAGTEKLRWNA
FEVLALDGVSSGILRFYTAQDGTDWLRAVSANIRELTLQNMKMANKCCSPSDQVVHMG
WVNEKLQGADSSQTFRPKFLALKGPSFYVFSTPPVSTFDWVRAERTYHLCEVLFKVHKF
WLTEDCWLQANLYLGLQDFDFEDQRPYCFSIVAGHGKSHVFNVELGSELAMWEKSFQRA
TFMEVQRTGSRTYMCSWQGEMLCFTVDFALGFTCFESKTKNVLWRFKFSQLKGSSDDGK
TRVKLLFQNLDTKQIETKELEFQDLRAVLHCIHSFIAAKVASVDPGFMDSQSLARKYMYSS

>P26572 single-pass type II membrane protein
MLKKQSAGLVLWGAILFVAWNALLLLFFWTRPAPGRPPSVSALDGDPASLTREVIRLAQD
AEVELERQRGLLQQIGDALSSQRGRVPTAAPPAQPRVPVTPAPAVIPILVIACDRSTVRRCLD
KLLHYRPSAELFPIIVSQDCGHEETAQAIASYGSAVTHIRQPDLSSIAVPPDHRKFQGYYKIA

RHYRWALGQVFRQFRFPAAVVVEDDLEVAPDFFEYFRATYPLLKADPSLWCVSAWNDNG
KEQMVDASRPELLYRTDFFPGLGWLLLAELWAELEPKWPKAFWDDWMRRPEQRQGRAC
IRPEISRTMTFGRKGVSHGQFFDQHLKFIKLNQQFVHFTQLDLSYLQREAYDRDFLARVYG
APQLQVEKVRTNDRKELGEVRVQYTGRDSFKAFAKALGVMDDLKSGVPRAGYRGIVTF
QFRGRRVHLAPPLTWEGYDPSWN

>Q9UJY4 peripheral membrane protein
MAATAVAAAVAGTESAQGPPGPAASLELWLNKATDPSMSEQDWSAIQNFCEQVNTDPNGP
THAPWLLAHKIQSPQEKEALYALTVLEMCMNHCGEKFHSEVAKFRFLNELIKVLSPKYLG
SWATGKVKGRVIEILFSWTVWFPEDIKIRDAYQMLKKQGIIKQDPKLPVDKILPPPSPWPKS
SIFDADEEKSKLLTRLLKSNHPEDLQAANRLIKNLVKEEQEKSEKVSKRVSAVEEVRSHVK
VLQEMLSMYRRPGQAPPDQEALQVVYERCEKLRPTLFRLASDTTDDDDALAEILQANDL
LTQGVLLYKQVMEGRVTFGNRVTSSLGDIPVSRVFQNPAGCMKTCPLIDLEVDNGPAQMG
TVVPSLLHQDLAALGISDAPVTGMVSGQNCCEEKRNPSSSTLPGGGVQNPSADRNLLDLL
SAQPAPCPLNYVSQKSVPKEVPPGTKSSPGWSWEAGPLAPSPSSQNTPLAQVFVPLESVKP
SSLPPLIVYDRNGFRILLHFSQTGAPGHPEVQVLLLTMMSTAPQPVWDIMFQVAVPKSMRV
KLQPASSSKLPAFSPLMPPAVISQMLLLDNPHKEPIRLRYKLTFNQGGQPFSEVGEVKDFPD
LAVLGAA

>Q8TBR7 multipass membrane protein
MLLTLAGGALFFPGLFALCTWALRRSQPGWSRTDCVMISTRLVSSVHAVLATGSGIVIIRSC
DDVITGRHWLAREYVWFLIPYMIYDSYAMYLCEWCRTRDQNRAPSLTLRNFLSRNRLMIT
HHAVILFVLVPVAQRLRGDLGDFFVGCIFTAELSTPFVSLGRVLIQLKQQHTLLYKVNGILT
LATFLSCRILLFPFMYWSYGRQQGLSLLQVPFSIPFYCNVANAFLVAPQIYWFCLLCRKAVR
LFDTPQAKKDG

>P19526 single-pass type II membrane protein
MWLRSHRQLCLAFLLVCVLSVIFFLHIHQDSFPHGLGLSILCPDRRLVTPPVAIFCLPGTAM
GPNASSSCPQHPASLSGTWTVYPNGRFGNQMGQYATLLALAQLNGRRAFILPAMHAALA
PVFRITLPVLAPEVDSRTPWRELQLHDWMSEEYADLRDPFLKLSGFPCSWTFFHHLREQIR
REFTLHDHLREEAQSVLGQLRLGRTGDRPRTFVGVHVRRGDYLQVMPQRWKGVVGDSA
YLRQAMDWFRARHEAPVFVVTSNGMEWCKENIDTSQGDVTFAGDGQEATPWKDFALLT
QCNHTIMTIGTFGFWAAYLAGGDTVYLANFTLPDSEFLKIFKPEAAFLPEWVGINADLSPL
WTLAKP

>P09564 single-pass type I membrane protein
MAGPPRLLLLPLLLALARGLPGALAAQEVQQSPHCTTVPVGASVNITCSTSGGLRGIYLR
QLGPQPQDIIYYEDGVVPTTDRRFRGRIDFSGSQDNLTITMHRLQLSDTGTYTCQAITEVN
VYGSGTLVLVTEEQSQGWHRCSDAPPRASALPAPPTGSALPDPQTASALPDPPAASALPAA
LAVISFLLGLGLGVACVLARTQIKKLCSWRDKNSAACVVYEDMSHSRCNTLSSPNQYQ

>P55851 multipass membrane protein
MVGFKATDVPPTATVKFLGAGTAACIADLITFPLDTAKVRLQIQGESQGPVRATASAQYRG
VMGTILTMVRTEGPRSLYNGLVAGLQRQMSFASVRIGLYDSVKQFYTKGSEHASIGSRLLA

GSTTGALAVAVAQPTDVVKVRFQAQARAGGGRRYQSTVNAYKTIAREEGFRGLWKGTSP
NVARNAIVNCAELVTYDLIKDALLKANLMTDDLPCHFTSAFGAGFCTTVIASPVDVVKTR
YMNSALGQYSSAGHCALTMLQKEGPRAFYKGFMPSFLRLGSWNVVMFVTYEQLKRAL
MAACTSREAPF

>P55735 peripheral membrane protein
MVSVINTVDTSHEDMIHDAQMDYYGTRLATCSSDRSVKIFDVRNGGQILIADLRGHEGPV
WQVAWAHPMYGNILASCSYDRKVIIWREENGTWEKSHEHAGHDSSVNSVCWAPHDYGL
ILACGSSDGAISLLTYTGEGQWEVKKINNAHTIGCNAVSWAPAVVPGSLIDHPSGQKPNYI
KRFASGGCDNLIKLWKEEEDGQWKEEQKLEAHSDWVRDVAWAPSIGLPTSTIASCSQDGR
VFIWTCDDASSNTWSPKLLHKFNDVVWHVSWSITANILAVSGGDNKVTLWKESVDGQW
VCISDVNKGQGSVSASVTEGQQNEQ

>Q8NFM4 multipass membrane protein
MARLFSPRPPPSEDLFYETYYSLSQQYPLLLLLLGIVLCALAALLAVAWASGRELTSDPSFL
TTVLCALGGFSLLLGLASREQRLQRWTRPLSGLVWVALLALGHAFLFTGGVVSAWDQVS
YFLFVIFTAYAMLPLGMRDAAVAGLASSLSHLLVLGLYLGPQPDSRPALLPQLAANAVLFL
CGNVAGVYHKALMERALRATFREALSSLHSRRRLDTEKKHQEHLLLSILPAYLAREMKAE
IMARLQAGQGSRPESTNNFHSLYVKRHQGVSVLYADIVGFTRLASECSPKELVLMLNELFG
KFDQIAKEHECMRIKILGDCYYCVSGLPLSLPDHAINCVRMGLDMCRAIRKLRAATGVDI
NMRVGVHSGSVLCGVIGLQKWQYDVWSHDVTLANHMEAGGVPGRVHITGATLALLAG
AYAVEDAGMEHRDPYLRELGEPTYLVIDPRAEEEDEKGTAGGLLSSLEGLKMRPSLLMTR
YLESWGAAKPFAHLSHGDSPVSTSTPLPEKTLASFSTQWSLDRSRTPRGLDDELDTGDAK
FFQVIEQLNSQKQWKQSKDFNPLTLYFREKEMEKEYRLSAIPAFKYYEACTFLVFLSNFIIQ
MLVTNRPPALAITYSITFLLFLLILFVCFSEDLMRCVLKGPKMLHWLPALSGLVATRPGLRI
ALGTATILLVFAMAITSLFFFPTSSDCPFQAPNVSSMISNLSWELPGSLPLISVPYSMHCCTL
GFLSCSLFLHMSFELKLLLLLLWLAASCSLFLHSHAWLSECLIVRLYLGPLDSRPGVLKEPK
LMGAISFFIFFFTLLVLARQNEYYCRLDFLWKKKLRQEREETETMENLTRLLLENVLPAHV
APQFIGQNRRNEDLYHQSYECVCVLFASVPDFKEFYSESNINHEGLECLRLLNEIIADFDEL
LSKPKFSGVEKIKTIGSTYMAATGLNATSGQDAQQDAERSCSHLGTMVEFAVALGSKLDVI
NKHSFNNFRLRVGLNHGPVVAGVIGAQKPQYDIWGNTVNVASRMESTGVLGKIQVTEET
AWALQSLGYTCYSRGVIKVKGKGQLCTYFLNTDLTRTGPPSATLG

>Q8TDN1 multipass membrane protein
MPMPSRDGGLHPRHHHYGSHSPWSQLLSSPMETPSIKGLYYRRVRKVGALDASPVDLKK
EILINVGGRRYLLPWSTLDRFPLSRLSKLRLCRSYEEIVQLCDDYDEDSQEFFFDRSPSAFG
VIVSFLAAGKLVLLQEMCALSFQEELAYWGIEEAHLERCCLRKLLRKLEELEELAKLHRE
DVLRQQRETRRPASHSSRWGLCMNRLREMVENPQSGLPGKVFACLSILFVATTAVSLCVST
MPDLRAEEDQGECSRKCYYIFIVETICVAWFSLEFCLRFVQAQDKCQFFQGPLNIIDILAISP
YYVSLAVSEEPPEDGERPSGSSYLEKVGLVLRVLRALRILYVMRLARHSLGLQTLGLTVRR
CTREFGLLLLFLAVAITLFSPLVYVAEKESGRVLEFTSIPASYWWAIISMTTVGYGDMVPRS
VPGQMVALSSILSGILIMAFPATSIFHTFSHSYLELKKEQEQLQARLRHLQNTGPASECELLD
PHVASEHELMNDVNDLILEGPALPIMHM

>Q92523 multipass membrane protein
MAEAHQAVAFQFTVTPDGVDFRLSREALKHVYLSGINSWKKRLIRIKNGILRGVYPGSPTS
WLVVIMATVGSSFCNVDISLGLVSCIQRCLPQGCGPYQTPQTRALLSMAIFSTGVWVTGIF
FFRQTLKLLLCYHGWMFEMHGKTSNLTRIWAMCIRLLSSRHPMLYSFQTSLPKLPVPRVSA
TIQRYLESVRPLLDDEEYYRMELLAKEFQDKTAPRLQKYLVLKSWWASNYVSDWWEEYI
YLRGRSPLMVNSNYYVMDLVLIKNTDVQAARLGNIIHAMIMYRRKLDREEIKPVMALGI
VPMCSYQMERMFNTTRIPGKDTDVLQHLSDSRHVAVYHKGRFFKLWLYEGARLLKPQDL
EMQFQRILDDPSPPQPGEEKLAALTAGGRVEWAQARQAFFSSGKNKAALEAIERAAFFVA
LDEESYSYDPEDEASLSLYGKALLHGNCYNRWFDKSFTLISFKNGQLGLNAEHAWADAPII
GHLWEFVLGTDSFHLGYTETGHCLGKPNPALAPPTRLQWDIPKQCQAVIESSYQVAKALA
DDVELYCFQFLPFGKGLIKKCRTSPDAFVQIALQLAHFRDRGKFCLTYEASMTRMFREGRT
ETVRSCTSESTAFVQAMMEGSHTKADLRDLFQKAAKKHQNMYRLAMTGAGIDRHLFCL
YLVSKYLGVSSPFLAEVLSEPWRLSTSQIPQSQIRMFDPEQHPNHLGAGGGFGPVADDGY
GVSYMIAGENTIFFHISSKFSSSETNAQRFGNHIRKALLDIADLFQVPKAYS

>Q9H3R2 single-pass type I membrane protein
MKAIIHLTLLALLSVNTATNQGNSADAVTTTETATSGPTVAAADTTETNFPETASTTANTPF
PTATSPAPPIISTHSSSTIPTPAPPIISTHSSSTIPIPTAADSESTTNVNSLATSDIITASSPNDGLIT
MVPSETQSNNEMSPTTEDNQSSGPPTGTALLETSTLNSTGPSNPCQDDPCADNSLCVKLH
NTSFCLCLEGYYYNSSTCKKGKVFPGKISVTVSETFDPEEKHSMAYQDLHSEITSLFKDVF
GTSVYGQTVILTVSTSLSPRSEMRADDKFVNVTIVTILAETTSDNEKTVTEKINKAIRSSSS
NFLNYDLTLRCDYYGCNQTADDCLNGLACDCKSDLQRPNPQSPFCVASSLKCPDACNAQ
HKQCLIKKSGGAPECACVPGYQEDANGNCQKCAFGYSGLDCKDKFQLILTIVGTIAGIVIL
SMIIALIVTARSNNKTKHIEEENLIDEDFQNLKLRSTGFTNLGAEGSVFPKVRITASRDSQM
QNPYSRHSSMPRPDY

>Q13470 peripheral membrane protein
MLPEAGSLWLLKLLRDIQLAQFYWPILEELNVTRPEHFDFVKPEDLDGIGMGRPAQRRLS
EALKRLRSGPKSKNWVYKILGGFAPEHKEPTLPSDSPRHLPEPEGGLKCLIPEGAVCRGEL
LGSGCFGVVHRGLWTLPSGKSVPVAVKSLRVGPEGPMGTELGDFLREVSVMMNLEHPHV
LRLHGLVLGQPLQMVMELAPLGSLHARLTAPAPTPPLLVALLCLFLRQLAGAMAYLGARG
LVHRDLATRNLLLASPRTIKVADFGLVRPLGGARGRYVMGGPRPIPYAWCAPESLRHGAFS
SASDVWMFGVTLWEMFSGGEEPWAGVPPYLILQRLEDRARLPRPPLCSRALYSLALRCW
APHPADRPSFSHLEGLLQEAGPSEACCVRDVTEPGALRMETGDPITVIEGSSSFHSPDSTIW
KGQNGRTFKVGSFPASAVTLADAGGLPATRPVHRGTPARGDQHPGSIDGDRKKANLWDA
PPARGQRRNMPLERMKGISRSLESVLSLGPRPTGGGSSPPEIRQARAVPQGPPGLPPRPPLS
SSSPQPSQPSRERLPWPKRKPPHNHPMGMPGARKAAALSGGLLSDPELQRKIMEVELSVH
GVTHQECQTALGATGGDVVSAIRNLKVDQLFHLSSRSRADCWRILEHYQWDLSAASRYV
LARP

>Q16853 single-pass type II membrane protein
MNQKTILVLLILAVITIFALVCVLLVGRGGDGGEPSQLPHCPSVSPSAQPWTHPGQSQLFAD
LSREELTAVMRFLTQRLGPGLVDAAQARPSDNCVFSVELQLPPKAAALAHLDRGSPPPARE
ALAIVFFGRQPQPNVSELVVGPLPHPSYMRDVTVERHGGPLPYHRRPVLFQEYLDIDQMIF

NRELPQASGLLHHCCFYKHRGRNLVTMTTAPRGLQSGDRATWFGLYYNISGAGFFLHHV
GLELLVNHKALDPARWTIQKVFYQGRYYDSLAQLEAQFEAGLVNVVLIPDNGTGGSWSL
KSPVPPGPAPPLQFYPQGPRFSVQGSRVASSLWTFSFGLGAFSGPRIFDVRFQGERLVYEISL
QEALAIYGGNSPAAMTTRYVDGGFGMGKYTTPLTRGVDCPYLATYVDWHFLLESQAPKT
IRDAFCVFEQNQGLPLRRHHSDLYSHYFGGLAETVLVVRSMSTLLNYDYVWDTVFHPSG
AIEIRFYATGYISSAFLFGATGKYGNQVSEHTLGTVHTHSAHFKVDLDVAGLENWVWAED
MVFVPMAVPWSPEHQLQRLQVTRKLLEMEEQAAFLVGSATPRYLYLASNHSNKWGHPR
GYRIQMLSFAGEPLPQNSSMARGFSWERYQLAVTQRKEEEPSSSSVFNQNDPWAPTVDFS
DFINNETIAGKDLVAWVTAGFLHIPHAEDIPNTVTVGNGVGFFLRPYNFFDEDPSFYSADSI
YFRGDQDAGACEVNPLACLPQAAACAPDLPAFSHGGFSHN

>Q8NGV0 multipass membrane protein
MGSFNTSFEDGFILVGFSDWPQLEPILFVFIFIFYSLTLFGNTIIIALSWLDLRLHTPMYFFLS
HLSLLDLCFTTSTVPQLLINLCGVDRTITRGGCVAQLFIYLALGSTECVLLVVMAFDRYAAV
CRPLHYMAIMHPHLCQTLAIASWGAGFVNSLIQTGLAMAMPLCGHRLNHFFCEMPVFLK
LACADTEGTEAKMFVARVIVVAVPAALILGSYVHIAHAVLRVKSTAGRRKAFGTCGSHLLV
VFLFYGSAIYTYLQSIHNYSEREGKFVALFYTIITPILNPLIYTLRNKDVKGALWKVLWRGR
DSG

>Q14118 single-pass type I membrane protein
MRMSVGLSLLLPLSGRTFLLLLSVVMAQSHWPSEPSEAVRDWENQLEASMHSVLSDLHE
AVPTVVGIPDGTAVVGRSFRVTIPTDLIASSGDIIKVSAAGKEALPSWLHWDSQSHTLEGLP
LDTDKGVHYISVSATRLGANGSHIPQTSSVFSIEVYPEDHSELQSVRTASPDPGEVVSSACA
ADEPVTVLTVILDADLTKMTPKQRIDLLHRMRSFSEVELHNMKLVPVVNNRLFDMSAFM
AGPGNAKKVVENGALLSWKLGCSLNQNSVPDIHGVEAPAREGAMSAQLGYPVVGWHIA
NKKPPLPKRVRRQIHATPTPVTAIGPPTTAIQEPPSRIVPTPTSPAIAPPTETMAPPVRDPVPG
KPTVTIRTRGAIIQTPTLGPIQPTRVSEAGTTVPGQIRPTMTIPGYVEPTAVATPPTTTTKKPR
VSTPKPATPSTDSTTTTTRRPTKKPRTPRPVPRVTTKVSITRLETASPPTRIRTTTSGVPRGGE
PNQRPELKNHIDRVDAWVGTYFEVKIPSDTFYDHEDTTTDKLKLTLKLREQQLVGEKSWV
QFNSNSQLMYGLPDSSHVGKHEYFMHATDKGGLSAVDAFEIHVHRRPQGDRAPARFKAK
FVGDPALVLNDIHKKIALVKKLAFAFGDRNCSTITLQNITRGSIVVEWTNNTLPLEPCPKEQ
IAGLSRRIAEDDGKPRPAFSNALEPDFKATSITVTGSGSCRHLQFIPVVPPRRVPSEAPPTEV
PDRDPEKSSEDDVYLHTVIPAVVVAAILLIAGIIAMICYRKKRKGKLTLEDQATFIKKGVPIIF
ADELDDSKPPPSSSMPLILQEEKAPLPPPEYPNQSVPETTPLNQDTMGEYTPLRDEDPNAPP
YQPPPPFTAPMEGKGSRPKNMTPYRSPPPYVPP

>O75843 peripheral membrane protein
MVVPSLKLQDLIEEIRGAKTQAQEREVIQKECAHIRASFRDGDPVHRHRQLAKLLYVHML
GYPAHFGQMECLKLIASSRFTDKRVGYLGAMLLLDERHDAHLLITNSIKNDLSQGIQPVQ
GLALCTLSTMGSAEMCRDLAPEVEKLLLQPSPYVRKKAILTAVHMIRKVPELSSVFLPPCA
QLLHERHHGILLGTITLITELCERSPAALRHFRKVVPQLVHILRTLVTMGYSTEHSISGVSDP
FLQVQILRLLRILGRNHEESSETMNDLLAQVATNTDTSRNAGNAVLFETVLTIMDIRSAAG
LRVLAVNILGRFLLNSDRNIRYVALTSLLRLVQSDHSAVQRHRPTVVECLRETDASLSRRAL
ELSLALVNSSNVRAMMQELQAFLESCPPDLRADCASGILLAAERFAPTKRWHIDTILHVLT

TAGTHVRDDAVANLTQLIGGAQELHAYSVRRLYNALAEDISQQPLVQVAAWCIGEYGDLL
LAGNCEEIEPLQVDEEEVLALLEKVLQSHMSLPATRGYALTALMKLSTRLCGDNNRIRQV
VSIYGSCLDVELQQRAVEYDTLFRKYDHMRAAILEKMPLVERDGPQADEEAKESKEAAQ
LSEAAPVPTEPQASQLLDLLDLLDGASGDVQHPPHLDPSPGGALVHLLDLPCVPPPPAPIPD
LKVFEREGVQLNLSFIRPPENPALLLITITATNFSEGDVTHFICQAAVPKSLQLQLQAPSGNT
VPARGGLPITQLFRILNPNKAPLRLKLRLTYDHFHQSVQEIFEVNNLPVESWQ

>Q8NH74 multipass membrane protein
MERQNQSCVVEFILLGFSNYPELQGQLFVAFLVIYLVTLIGNAIIIVIVSLDQSLHVPMYLFL
LNLSVVDLSFSAVIMPEMLVVLSTEKTTISFGGCFAQMYFILLFGGAECFLLGAMAYDRFA
AICHPLNYQMIMNKGVFMKLIIFSWALGFMLGTVQTSWVSSFPFCGLNEINHISCETPAVL
ELACADTFLFEIYAFTGTFLIILVPFLLILLSYIRVLFAILKMPSTTGRQKAFSTCAAHLTSVTL
FYGTASMTYLQPKSGYSPETKKVMSLSYSLLTPLLNLLIYSLRNSEMKRALMKLWRRRVV
LHTI

>Q6UVK1 single-pass type I membrane protein
MQSGPRPPLPAPGLALALTLTMLARLASAASFFGENHLEVPVATALTDIDLQLQFSTSQPEA
LLLLAAGPADHLLLQLYSGRLQVRLVLGQEELRLQTPAETLLSDSIPHTVVLTVVEGWATL
SVDGFLNASSAVPGAPLEVPYGLFVGGTGTLGLPYLRGTSRPLRGCLHAATLNGRSLLRPL
TPDVHEGCAEEFSASDDVALGFSGPHSLAAFPAWGTQDEGTLEFTLTTQSRQAPLAFQAG
GRRGDFIYVDIFEGHLRAVVEKGQGTVLLHNSVPVADGQPHEVSVHINAHRLEISVDQYP
THTSNRGVLSYLEPRGSLLLGGLDAEASRHLQEHRLGLTPEATNASLLGCMEDLSVNGQR
RGLREALLTRNMAAGCRLEEEEYEDDAYGHYEAFSTLAPEAWPAMELPEPCVPEPGLPPV
FANFTQLLTISPLVVAEGGTAWLEWRHVQPTLDLMEAELRKSQVLFSVTRGARHGELELDI
PGAQARKMFTLLDVVNRKARFIHDGSEDTSDQLVLEVSVTARVPMPSCLRRGQTYLLPIQ
VNPVNDPPHIIFPHGSLMVILEHTQKPLGPEVFQAYDPDSACEGLTFQVLGTSSGLPVERRD
QPGEPATEFSCRELEAGSLVYVHRGGPAQDLTFRVSDGLQASPPATLKVVAIRPAIQIHRSTG
LRLAQGSAMPILPANLSVETNAVGQDVSVLFRVTGALQFGELQKQGAGGVEGAEWWATQ
AFHQRDVEQGRVRYLSTDPQHHAYDTVENLALEVQVGQEILSNLSFPVTIQRATVWMLRL
EPLHTQNTQQETLTTAHLEATLEEAGPSPPTFHYEVVQAPRKGNLQLQGTRLSDGQGFTQ
DDIQAGRVTYGATARASEAVEDTFRFRVTAPPYFSPLYTFPIHIGGDPDAPVLTNVLLVVPE
GGEGVLSADHLFVKSLNSASYLYEVMERPRHGRLAWRGTQDKTTMVTSFTNEDLLRGRL
VYQHDDSETTEDDIPFVATRQGESSGDMAWEEVRGVFRVAIQPVNDHAPVQTISRIFHVAR
GGRRLLTTDDVAFSDADSGFADAQLVLTRKDLLFGSIVAVDEPTRPIYRFTQEDLRKRRVLF
VHSGADRGWIQLQVSDGQHQATALLEVQASEPYLRVANGSSLVVPQGGQGTIDTAVLHLD
TNLDIRSGDEVHYHVTAGPRWGQLVRAGQPATAFSQQDLLDGAVLYSHNGSLSPRDTMAF
SVEAGPVHTDATLQVTIALEGPLAPLKLVRHKKIYVFQGEAAEIRRDQLEAAQEAVPPADI
VFSVKSPPSAGYLVMVSRGALADEPPSLDPVQSFSQEAVDTGRVLYLHSRPEAWSDAFSLD
VASGLGAPLEGVLVELEVLPAAIPLEAQNFSVPEGGSLTLAPPLLRVSGPYFPTLLGLSLQV
LEPPQHGALQKEDGPQARTLSAFSWRMVEEQLIRYVHDGSETLTDSFVLMANASEMDRQ
SHPVAFTVTVLPVNDQPPILTTNTGLQMWEGATAPIPAEALRSTDGDSGSEDLVYTIEQPSN
GRVVLRGAPGTEVRSFTQAQLDGGLVLFSHRGTLDGGFRFRLSDGEHTSPGHFFRVTAQK
QVLLSLKGSQTLTVCPGSVQPLSSQTLRASSSAGTDPQLLLYRVVRGPQLGRLFHAQQDST
GEALVNFTQAEVYAGNILYEHEMPPEPFWEAHDTLELQLSSPPARDVAATLAVAVSFEAAC

PQRPSHLWKNKGLWVPEGQRARITVAALDASNLLASVPSPQRSEHDVLFQVTQFPSRGQL
LVSEEPLHAGQPHFLQSQLAAGQLVYAHGGGGTQQDGFHFRAHLQGPAGASVAGPQTSE
AFAITVRDVNERPPQPQASVPLRLTRGSRAPISRAQLSVVDPDSAPGEIEYEVQRAPHNGFL
SLVGGGLGPVTRFTQADVDSGRLAFVANGSSVAGIFQLSMSDGASPPLPMSLAVDILPSAIE
VQLRAPLEVPQALGRSSLSQQQLRVVSDREEPEAAYRLIQGPQYGHLLVGGRPTSAFSQFQ
IDQGEVVFAFTNFSSSHDHFRVLALARGVNASAVVNVTVRALLHVWAGGPWPQGATLRL
DPTVLDAGELANRTGSVPRFRLLEGPRHGRVVRVPRARTEPGGSQLVEQFTQQDLEDGRL
GLEVGRPEGRAPGPAGDSLTLELWAQGVPPAVASLDFATEPYNAARPYSVALLSVPEAART
EAGKPESSTPTGEPGPMASSPEPAVAKGGFLSFLEANMFSVIIPMCLVLLLLALILPLLFYLR
KRNKTGKHDVQVLTAKPRNGLAGDTETFRKVEPGQAIPLTAVPGQGPPPGGQPDPELLQF
CRTPNPALKNGQYWV

>Q6UXD5 single-pass type I membrane protein
MGTPRAQHPPPPQLLFLILLSCPWIQGLPLKEEEILPEPGSETPTVASEALAELLHGALLRRG
PEMGYLPGSDRDPTLATPPAGQTLAVPSLPRATEPGTGPLTTAVTPNGVRGAGPTAPELLTP
PPGTTAPPPPSPASPGPPLGPEGGEEETTTTIITTTTVTTTVTSPVLCNNNISEGEGYVESPDL
GSPVSRTLGLLDCTYSIHVYPGYGIEIQVQTLNLSQEEELLVLAGGGSPGLAPRLLANSSML
GEGQVLRSPTNRLLLHFQSPRVPRGGGFRIHYQAYLLSCGFPPRPAHGDVSVTDLHPGGTA
TFHCDSGYQLQGEETLICLNGTRPSWNGETPSCMASCGGTIHNATLGRIVSPEPGGAVGPN
LTCRWVIEAAEGRRLHLHFERVSLDEDNDRLMVRSGGSPLSPVIYDSDMDDVPERGLISD
AQSLYVELLSETPANPLLLSLRFEAFEEDRCFAPFLAHGNVTTTDPEYRPGALATFSCLPGY
ALEPPGPPNAIECVDPTEPHWNDTEPACKAMCGGELSEPAGVVLSPDWPQSYSPGQDCV
WGVHVQEEKRILLQVEILNVREGDMLTLFDGDGPSARVLAQLRGPQPRRRLLSSGPDLTL
QFQAPPGPPNPGLGQGFVLHFKEVPRNDTCPELPPPEWGWRTASHGDLIRGTVLTYQCEP
GYELLGSDILTCQWDLSWSAAPPACQKIMTCADPGEIANGHRTASDAGFPVGSHVQYRCL
PGYSLEGAAMLTCYSRDTGTPKWSDRVPKCALKYEPCLNPGVPENGYQTLYKHHYQAGE
SLRFFCYEGFELIGEVTITCVPGHPSQWTSQPPLCKVTQTTDPSRQLEGGNLALAILLPLGL
VIVLGSGVYIYYTKLQGKSLFGFSGSHSYSPITVESDFSNPLYEAGDTREYEVSI

>Q96MF6 peripheral membrane protein
MAWAGSRRVPAGTRAAAERCCRLSLSPGAQPAPPPGPLPPPRPMRFLTSCSLLLPRAAQIL
AAEAGLPSSRSFMGFAAPFTNKRKAYSERRIMGYSMQEMYEVVSNVQEYREFVPWCKKS
LVVSSRKGHLKAQLEVGFPPVMERYTSAVSMVKPHMVKAVCTDGKLFNHLETIWRFSPGI
PAYPRTCTVDFSISFEFRSLLHSQLATMFFDEVVKQNVAAFERRAATKFGPETAIPRELMFH
EVHQT

>Q9BUB7 multipass membrane protein
MLFLALGSPWAVELPLCGRRTALCAAAALRGPRASVSRASSSSGPSGPVAGWSTGPSGAA
RLLRRPGRAQIPVYWEGYVRFLNTPSDKSEDGRLIYTGNMARAVFGVKCFSYSTSLIGLTF
LPYIFTQNNAISESVPLPIQIIFYGIMGSFTVITPVLLHFITKGYVIRLYHEATTDTYKAITYNA
MLAETSTVFHQNDVKIPDAKHVFTTFYAKTKSLLVNPVLFPNREDYIHLMGYDKEEFILY
MEETSEEKRHKDDK

>Q8TDM5 GPI-anchor, lipid-anchor

MVLCWLLLLVMALPPGTTGVKDCVFCELTDSMQCPGTYMHCGDDEDCFTGHGVAPGTG
PVINKGCLRATSCGLEEPVSYRGVTYSLTTNCCTGRLCNRAPSSQTVGATTSLALGLGMLL
PPRLL

>Q86YS3 peripheral membrane protein
MAGGAGWSGAPAALLRSVRRLREVFEVCGRDPDGFLRVERVAALGLRFGQGEEVEKLVK
YLDPNDLGRINFKDFCRGVFAMKGCEELLKDVLSVESAGTLPCAPEIPDCVEQGSEVTGPT
FADGELIPREPGFFPEDEEEAMTLAPPEGPQELYTDSPMESTQSLEGSVGSPAEKDGGLGGL
FLPEDKSLVHTPSMTTSDLSTHSTTSLISNEEQFEDYGEGDDVDCAPSSPCPDDETRTNVYS
DLGSSVSSSAGQTPRKMRHVYNSELLDVYCSQCCKKINLLNDLEARLKNLKANSPNRKIS
STAFGRQLMHSSNFSSSNGSTEDLFRDSIDSCDNDITEKVSFLEKKVTELENDSLTNGDLKS
KLKQENTQLVHRVHELEEMVKDQETTAEQALEEEARRHREAYGKLEREKATEVELLNAR
VQQLEEENTELRTTVTRLKSQTEKLDEERQRMSDRLEDTSLRLKDEMDLYKRMMDKLRQ
NRLEFQKEREATQELIEDLRKELEHLQMYKLDCERPGRGRSASSGLGEFNARAREVELEH
EVKRLKQENYKLRDQNDDLNGQILSLSLYEAKNLFAAQTKAQSLAAEIDTASRDELMEAL
KEQEEINFRLRQYMDKIILAILDHNPSILEIKH

>O14514 multipass membrane protein
MRGQAAAPGPVWILAPLLLLLLLLGRRARAAAGADAGPGPEPCATLVQGKFFGYFSAAA
VFPANASRCSWTLRNPDPRRYTLYMKVAKAPVPCSGPGRVRTYQFDSFLESTRTYLGVESF
DEVLRLCDPSAPLAFLQASKQFLQMRRQQPPQHDGLRPRAGPPGPTDDFSVEYLVVGNR
NPSRAACQMLCRWLDACLAGSRSSHPCGIMQTPCACLGGEAGGPAAGPLAPRGDVCLRD
AVAGGPENCLTSLTQDRGGHGATGGWKLWSLWGECTRDCGGGLQTRTRTCLPAPGVEGG
GCEGVLEEGRQCNREACGPAGRTSSRSQSLRSTDARRREELGDELQQFGFPAPQTGDPAA
EEWSPWSVCSSTCGEGWQTRTRFCVSSSYSTQCSGPLREQRLCNNSAVCPVHGAWDEWS
PWSLCSSTCGRGFRDRTRTCRPPQFGGNPCEGPEKQTKFCNIALCPGRAVDGNWNEWSS
WSACSASCSQGRQQRTRECNGPSYGGAECQGHWVETRDCFLQQCPVDGKWQAWASWG
SCSVTCGAGSQRRERVCSGPFFGGAACQGPQDEYRQCGTQRCPEPHEICDEDNFGAVIWK
ETPAGEVAAVRCPRNATGLILRRCELDEEGIAYWEPPTYIRCVSIDYRNIQMMTREHLAKA
QRGLPGEGVSEVIQTLVEISQDGTSYSGDLLSTIDVLRNMTEIFRRAYYSPTPGDVQNFVQI
LSNLLAEENRDKWEEAQLAGPNAKELFRLVEDFVDVIGFRMKDLRDAYQVTDNLVLSIH
KLPASGATDISFPMKGWRATGDWAKVPEDRVTVSKSVFSTGLTEADEASVFVVGTVLYRN
LGSFLALQRNTTVLNSKVISVTVKPPPRSLRTPLEIEFAHMYNGTTNQTCILWDETDVPSSS
APPQLGPWSWRGCRTVPLDALRTRCLCDRLSTFAILAQLSADANMEKATLPSVTLIVGCG
VSSLTLLMLVIIYVSVWRYIRSERSVILINFCLSIISSNALILIGQTQTRNKVMCTLVAAFLHFF
FLSSFCWVLTEAWQSYMAVTGHLRNRLIRKRFLCLGWGLPALVVAISVGFTKAKGYSTMN
YCWLSLEGGLLYAFVGPAAAVVLVNMVIGILVFNKLVSKDGITDKKLKERAGASLWSSCV
VLPLLALTWMSAVLAVTDRRSALFQILFAVFDSLEGFVIVMVHCILRREVQDAVKCRVVDR
QEEGNGDSGGSFQNGHAQLMTDFEKDVDLACRSVLNKDIAACRTATITGTLKRPSLPEEE
KLKLAHAKGPPTNFNSLPANVSKLHLHGSPRYPGGPLPDFPNHSLTLKRDKAPKSSFVGD
GDIFKKLDSELSRAQEKALDTSYVILPTATATLRPKPKEEPKYSIHIDQMPQTRLIHLSTAPE
ASLPARSPPSRQPPSGGPPEAPPAQPPPPPPPPPPPPQQPLPPPPNLEPAPPSLGDPGEPAAHPG
PSTGPSTKNENVATLSVSSLERRKSRYAELDFEKIMHTRKRHQDMFQDLNRKLQHAAEKD
KEVLGPDSKPEKQQTPNKRPWESLRKAHGTPTWVKKELEPLQPSPLELRSVEWERSGATI

PLVGQDIIDLQTEV

>Q8NH40 multipass membrane protein
MSPDGNHSSDPTEFVLAGLPNLNSARVELFSVFLLVYLLNLTGNVLIVGVVRADTRLQTP
MYFFLGNLSCLEILLTSVIIPKMLSNFLSRQHTISFAACITQFYFYFFLGASEFLLLAVMSAD
RYLAICHPLRYPLLMSGAVCFRVALACWVGGLVPVLGPTVAVALLPFCKQGAVVQHFFCD
SGPLLRLACTNTKKLEETDFVLASLVIVSSLLITAVSYGLIVLAVLSIPSASGRQKAFSTCTS
HLIVVTLFYGSAIFLYVRPSQSGSVDTNWAVTVITTFVTPLLNPFIYALRNEQVKEALKDMF
RKVVAGVLGNLLLDKCLSEKAVK

>Q96A29 multipass membrane protein
MNRAPLKRSRILHMALTGASDPSAEAEANGEKPFLLRALQIALVVSLYWVTSISMVFLNK
YLLDSPSLRLDTPIFVTFYQCLVTTLLCKGLSALAACCPGAVDFPSLRLDLRVARSVLPLSV
VFIGMITFNNLCLKYVGVAFYNVGRSLTTVFNVLLSYLLLKQTTSFYALLTCGIIIGGFWLG
VDQEGAEGTLSWLGTVFGVLASLCVSLNAIYTTKVLPAVDGSIWRLTFYNNVNACILFLPL
LLLLGELQALRDFAQLGSAHFWGMMTLGGLFGFAIGYVTGLQIKFTSPLTHNVSGTAKAC
AQTVLAVLYYEETKSFLWWTSNMMVLGGSSAYTWVRGWEMKKTPEEPSPKDSEKSAMG
V

>Q8IUW5 single-pass type I membrane protein
MAPRALPGSAVLAAAVFVGGAVSSPLVAPDNGSSRTLHSRTETTPSPSNDTGNGHPEYIAY
ALVPVFFIMGLFGVLICHLLKKKGYRCTTEAEQDIEEEKVEKIELNDSVNENSDTVGQIVH
YIMKNEANADVLKAMVADNSLYDPESPVTPSTPGSPPVSPGPLSPGGTPGKHVCGHHLHT
VGGVVERDVCHRCRHKRWHFIKPTNKSRESRPRRQGEVTVLSVGRFRVTKVEHKSNQKE
RRSLMSVSGAETVNGEVPATPVKRERSGTE

>Q7Z403 multipass membrane protein
MAQPLAFILDVPETPGDQGQGPSPYDESEVHDSFQQLIQEQSQCTAQEGLELQQREREVT
GSSQQTLWRPEGTQSTATLRILASMPSRTIGRSRGAIISQYYNRTVQLRCRSSRPLLGNFVR
SAWPSLRLYDLELDPTALEEEEKQSLLVKELQSLAVAQRDHMLRGMPLSLAEKRSLREKSR
TPRGKWRGQPGSGGVCSCCGRLRYACVLALHSLGLALLSALQALMPWRYALKRIGGQFG
SSVLSYFLFLKTLLAFNALLLLLLVAFIMGPQVAFPPALPGPAPVCTGLELLTGAGCFTHTV
MYYGHYSNATLNQPCGSPLDGSQCTPRVGGLPYNMPLAYLSTVGVSFFITCITLVYSMAH
SFGESYRVGSTSGIHAITVFCSWDYKVTQKRASRLQQDNIRTRLKELLAEWQLRHSPRSVC
GRLRQAAVLGLVWLLCLGTALGCAVAVHVFSEFMIQSPEAAGQEAVLLVLPLVVGLLNLG
APYLCRVLAALEPHDSPVLEVYVAICRNLILKLAILGTLCYHWLGRRVGVLQGQCWEDFV
GQELYRFLVMDFVLMLLDTLFGELVWRIISEKKLKRRRKPEFDIARNVLELIYGQTLTWLG
VLFSPLLPAVQIIKLLLVFYVKKTSLLANCQAPRRPWLASHMSTVFLTLLCFPAFLGAAVFL
CYAVWQVKPSSTCGPFRTLDTMYEAGRVWVRHLEAAGPRVSWLPWVHRYLMENTFFVF
LVSALLLAVIYLNIQVVRGQRKVICLLKEQISNEGEDKIFLINKLHSIYERKEREERSRVGTT
EEAAAPPALLTDEQDA

>P15391 single-pass type I membrane protein
MPPPRLLFFLLFLTPMEVRPEEPLVVKVEEGDNAVLQCLKGTSDGPTQQLTWSRESPLKPF

LKLSLGLPGLGIHMRPLAIWLFIFNVSQQMGGFYLCQPGPPSEKAWQPGWTVNVEGSGEL
FRWNVSDLGGLGCGLKNRSSEGPSSPSGKLMSPKLYVWAKDRPEIWEGEPPCLPPRDSLN
QSLSQDLTMAPGSTLWLSCGVPPDSVSRGPLSWTHVHPKGPKSLLSLELKDDRPARDMW
VMETGLLLPRATAQDAGKYYCHRGNLTMSFHLEITARPVLWHWLLRTGGWKVSAVTLAY
LIFCLCSLVGILHLQRALVLRRKRKRMTDPTRRFFKVTPPPGSGPQNQYGNVLSLPTPTSGL
GRAQRWAAGLGGTAPSYGNPSSDVQADGALGSRSPPGVGPEEEEGEGYEEPDSEEDSEFY
ENDSNLGQDQLSQDGSGYENPEDEPLGPEDEDSFSNAESYENEDEELTQPVARTMDFLSP
HGSAWDPSREATSLGSQSYEDMRGILYAAPQLRSIRGQPGPNHEEDADSYENMDNPDGPD
PAWGGGGRMGTWSTR

>Q9GZZ7 GPI-anchor, lipid-anchor
MVRCLGPALLLLLLLGSASSVGGNRCVDAAEACTADARCQRLRSEYVAQCLGRAAQGGC
PRARCRRALRRFFARGPPALTHALLFCPCAGPACAERRRQTFVPSCAFSGPGPAPPSCLEPL
NFCERSRVCRCARAAAGPWRGWGRGLSPAHRPPAAQASPPGLSGLVHPSAQRPRRLPAGP
GRPLPARLRGPRGVPAGTAVTPNYVDNVSARVAPWCDCGASGNRREDCEAFRGLFTRNR
CLDGAIQAFASGWPPVLLDQLNPQGDPEHSLLQVSSTGRALERRSLLSILPVLALPALL

>Q93100 lipid-anchor
MAGAAGLTAEVSWKVLERRARTKRSGSVYEPLKSINLPRPDNETLWDKLDHYYRIVKSTL
LLYQSPTTGLFPTKTCGGDQKAKIQDSLYCAAGAWALALAYRRIDDDKGRTHELEHSAIK
CMRGILYCYMRQADKVQQFKQDPRPTTCLHSVFNVHTGDELLSYEEYGHLQINAVSLYLL
YLVEMISSGLQIIYNTDEVSFIQNLVFCVERVYRVPDFGVWERGSKYNNGSTELHSSSVGL
AKAALEAINGFNLFGNQGCSWSVIFVDLDAHNRNRQTLCSLLPRESRSHNTDAALLPCIS
YPAFALDDEVLFSQTLDKVVRKLKGKYGFKRFLRDGYRTSLEDPNRCYYKPAEIKLFDGIE
CEFPIFFLYMMIDGVFRGNPKQVQEYQDLLTPVLHHTTEGYPVVPKYYYVPADFVEYEKN
NPGSQKRFPSNCGRDGKLFLWGQALYIIAKLLADELISPKDIDPVQRYVPLKDQRNVSMRF
SNQGPLENDLVVHVALIAESQRLQVFLNTYGIQTQTPQQVEPIQIWPQQELVKAYLQLGIN
EKLGLSGRPDRPIGCLGTSKIYRILGKTVVCYPIIFDLSDFYMSQDVFLLIDDIKNALQFIKQ
YWKMHGRPLFLVLIREDNIRGSRFNPILDMLAALKKGIIGGVKVHVDRLQTLISGAVVEQL
DFLRISDTEELPEFKSFEELEPPKHSKVKRQSSTPSAPELGQQPDVNISEWKDKPTHEILQK
LNDCSCLASQAILLGILLKREGPNFITKEGTVSDHIERVYRRAGSQKLWLAVRYGAAFTQK
FSSSIAPHITTFLVHGKQVTLGAFGHEEEVISNPLSPRVIQNIIYYKCNTHDEREAVIQQELVI
HIGWIISNNPELFSGMLKIRIGWIIHAMEYELQIRGGDKPALDLYQLSPSEVKQLLLDILQPQ
QNGRCWLNRRQIDGSLNRTPTGFYDRVWQILERTPNGIIVAGKHLPQQPTLSDMTMYEMN
FSLLVEDTLGNIDQPQYRQIVVELLMVVSIVLERNPELEFQDKVDLDRLVKEAFNEFQKDQ
SRLKEIEKQDDMTSFYNTPPLGKRGTCSYLTKAVMNLLLEGEVKPNNDDPCLIS

>O94823 multipass membrane protein
MALSVDSSWHRWQWRVRDGFPHCPSETTPLLSPEKGRQSYNLTQQRVVFPNNSIFHQDW
EEVSRRYPGNRTCTTKYTLFTFLPRNLFEQFHRWANLYFLFLVILNWMPSMEVFHREITML
PLAIVLFVIMIKDGMEDFKRHRFDKAINCSNIRIYERKEQTYVQKCWKDVRVGDFIQMKC
NEIVPADILLLFSSDPNGICHLETASLDGETNLKQRCVVKGFSQQEVQFEPELFHNTIVCEK
PNNHLNKFKGYMEHPDQTRTGFGCESLLLRGCTIRNTEMAVGIVIYAGHETKAMLNNSGP
RYKRSKIERRMNIDIFFCIGILILMCLIGAVGHSIWNGTFEEHPPFDVPDANGSFLPSALGGF

YMFLTMIILLQVLIPISLYVSIELVKLGQVFFLSNDLDLYDEETDLSIQCRALNIAEDLGQIQY
IFSDKTGTLTENKMVFRRCTIMGSEYSHQENAKRLETPKELDSDGEEWTQYQCLSFSARW
AQDPATMRSQKGAQPLRRSQSARVPIQGHYRQRSMGHRESSQPPVAFSSSIEKDVTPDKNL
LTKVRDAALWLETLSDSRPAKASLSTTSSIADFFLALTICNSVMVSTTTEPRQRVTIKPSSKA
LGTSLEKIQQLFQKLKLLSLSQSFSSTAPSDTDLGESLGANVATTDSDERDDASVCSGGDST
DDGGYRSSMWDQGDILESGSGTSLEEALEAPATDLARPEFCYEAESPDEAALVHAAHAYS
FTLVSRTPEQVTVRLPQGTCLTFSLLCTLGFDSVRKRMSVVVRHPLTGEIVVYTKGADSVI
MDLLEDPACVPDINMEKKLRKIRARTQKHLDLYARDGLRTLCIAKKVVSEEDFRRWASFR
REAEASLDNRDELLMETAQHLENQLTLLGATGIEDRLQEGVPDTIATLREAGIQLWVLTGD
KQETAVNIAHSCRLLNQTDTVYTINTENQETCESILNCALEELKQFRELQKPDRKLFGFRLP
SKTPSITSEAVVPEAGLVIDGKTLNAIFQGKLEKKFLELTQYCRSVLCCRSTPLQKSMIVKL
VRDKLRVMTLSIGDGANDVSMIQAADIGIGISGQEGMQAVMSSDFAITRFKHLKKLLLVH
GHWCYSRLARMVVYYLYKNVCYVNLLFWYQFFCGFSSSTMIDYWQMIFFNLFFTSLPPL
VFGVLDKDISAETLLALPELYKSGQNSECYNLSTFWISMVDAFYQSLICFFIPYLAYKGSDI
DVFTFGTPINTISLTTILLHQAMEMKTWTIFHGVVLLGSFLMYFLVSLLYNATCVICNSPTN
PYWVMEGQLSNPTFYLVCFLTPVVALLPRYFFLSLQGTCGKSLISKAQKIDKLPPDKRNLEI
QSWRSRQRPAPVPEVARPTHHPVSSITGQDFSASTPKSSNPPKRKHVEESVLHEQRCGTEC
MRDDSCSGDSSAQLSSGEHLLGPNRIMAYSRGQTDMCRCSKRSSHRRSQSSLTI

>P28221 multipass membrane protein
MSPLNQSAEGLPQEASNRSLNATETSEAWDPRTLQALKISLAVVLSVITLATVLSNAFVLTT
ILLTRKLHTPANYLIGSLATTDLLVSILVMPISIAYTITHTWNFGQILCDIWLSSDITCCTASIL
HLCVIALDRYWAITDALEYSKRRTAGHAATMIAIVWAISICISIPPLFWRQAKAQEEMSDCL
VNTSQISYTIYSTCGAFYIPSVLLIILYGRIYRAARNRILNPPSLYGKRFTTAHLITGSAGSSL
CSLNSSLHEGHSHSAGSPLFFNHVKIKLADSALERKRISAARERKATKILGIILGAFIICWLP
FFVVSLVLPICRDSCWIHPALFDFFTWLGYLNSLINPIIYTVFNEEFRQAFQKIVPFRKAS

>Q8N3E9 peripheral membrane protein
MLCGRWRRCRRPPEEPPVAAQVAAQVAAPVALPSPPTPSDGGTKRPGLRALKKMGLTEDE
DVRAMLRGSRLRKIRSRTWHKERLYRLQEDGLSVWFQRRIPRAPSQHIFFVQHIEAVREGH
QSEGLRRFGGAFAPARCLTIAFKGRRKNLDLAAPTAEEAQRWVRGLTKLRARLDAMSQRE
RLDHWIHSYLHRADSNQDSKMSFKEIKSLLRMVNVDMNDMYAYLLFKECDHSNNDRLE
GAEIEEFLRRLLKRPELEEIFHQYSGEDRVLSAPELLEFLEDQGEEGATLARAQQLIQTYEL
NETAKQHELMTLDGFMMYLLSPEGAALDNTHTCVFQDMNQPLAHYFISSSHNTYLTDSQI
GGPSSTEAYVRAFAQGCRCVELDCWEGPGGEPVIYHGHTLTSKILFRDVVQAVRDHAFTL
SPYPVILSLENHCGLEQQAAMARHLCTILGDMLVTQALDSPNPEELPSPEQLKGRVLVKG
KKLPAARSEDGRALSDREEEEEDDEEEEEEVEAAAQRRLAKQISPELSALAVYCHATRLRT
LHPAPNAPQPCQVSSLSERKAKKLIREAGNSFVRHNARQLTRVYPLGLRMNSANYSPQEM
WNSGCQLVALNFQTPGYEMDLNAGRFLVNGQCGYVLKPACLRQPDSTFDPEYPGPPRTTL
SIQVLTAQQLPKLNAEKPHSIVDPLVRIEIHGVPADCARQETDYVLNNGFNPRWGQTLQFQ
LRAPELALVRFVVEDYDATSPNDFVGQFTLPLSSLKQGYRHIHLLSKDGASLSPATLFIQIRI
QRS

>Q9BQQ3 peripheral membrane protein

MGLGVSAEQPAGGAEGFHLHGVQENSPAQQAGLEPYFDFIITIGHSRLNKENDTLKALLK
ANVEKPVKLEVFNMKTMRVREVEVVPSNMWGGQGLLGASVRFCSFRRASEQVWHVLD
VEPSSPAALAGLRPYTDYVVGSDQILQESEDFFTLIESHEGKPLKLMVYNSKSDSCREVTV
TPNAAWGGEGSLGCGIGYGYLHRIPTQPPSYHKKPPGTPPPSALPLGAPPPDALPPGPTPED
SPSLETGSRQSDYMEALLQAPGSSMEDPLPGPGSPSHSAPDPDGLPHFMETPLQPPPPVQR
VMDPGFLDVSGISLLDNSNASVWPSLPSSTELTTTAVSTSGPEDICSSSSSHERGGEATWSG
SEFEVSFLDSPGAQAQADHLPQLTLPDSLTSAASPEDGLSAELLEAQAEEEPASTEGLDTGT
EAEGLDSQAQISTTE

>P15529 single-pass type I membrane protein
MEPPGRRECPFPSWRFPGLLLAAMVLLLYSFSDACEEPPTFEAMELIGKPKPYYEIGERVD
YKCKKGYFYIPPLATHTICDRNHTWLPVSDDACYRETCPYIRDPLNGQAVPANGTYEFGY
QMHFICNEGYYLIGEEILYCELKGSVAIWSGKPPICEKVLCTPPPKIKNGKHTFSEVEVFEY
LDAVTYSCDPAPGPDPFSLIGESTIYCGDNSVWSRAAPECKVVKCRFPVVENGKQISGFGK
KFYYKATVMFECDKGFYLDGSDTIVCDSNSTWDPPVPKCLKVLPPSSTKPPALSHSVSTSS
TTKSPASSASGPRPTYKPPVSNYPGYPKPEEGILDSLDVWVIAVIVIAIVVGVAVICVVPYRY
LQRRKKKGTYLTDETHREVKFTSL

>Q9UJ68 lipid-anchor
MLSATRRACQLLLLHSLFPVPRMGNSASNIVSPQEALPGRKEQTPVAAKHHVNGNRTVEP
FPEGTQMAVFGMGCFWGAERKFWVLKGVYSTQVGFAGGYTSNPTYKEVCSEKTGHAEV
VRVVYQPEHMSFEELLKVFWENHDPTQGMRQGNDHGTQYRSAIYPTSAKQMEAALSSK
ENYQKVLSEHGFGPITTDIREGQTFYYAEDYHQQYLSKNPNGYCGLGGTGVSCPVGIKK

>Q8NHA4 multipass membrane protein
MWQKNQTSLADFILEGLFDDSLTHLFLFSLTMVVFLIAVSGNTLTILLICIDPQLHTPMYFLL
SQLSLMDLMHVSTIILKMATNYLSGKKSISFVGCATQHFLYLCLGGAECFLLAVMSYDRYV
AICHPLRYAVLMNKKVGLMMAVMSWLGASVNSLIHMAILMHFPFCGPRKVYHFYCEFPA
VVKLVCGDITVYETTVYISSILLLLPIFLISTSYVFILQSVIQMRSSGSKRNAFATCGSHLTVV
SLWFGACIFSYMRPRSQCTLLQNKVGSVFYSIITPTLNSLIYTLRNKDVAKALRRVLRRDVI
TQCIQRLQLWLPRV

>Q8NBJ9 multipass membrane protein
MFALGLPFLVLLVASVESHLGVLGPKNVSQKDAEFERTYVDEVNSELVNIYTFNHTVTRNR
TEGVRVSVNVLNKQKGAPLLFVVRQKEAVVSFQVPLILRGMFQRKYLYQKVERTLCQPPT
KNESEIQFFYVDVSTLSPVNTTYQLRVSRMDDFVLRTGEQFSFNTTAAQPQYFKYEFPEGV
DSVIVKVTSNKAFPCSVISIQDVLCPVYDLDNNVAFIGMYQTMTKKAAITVQRKDFPSNSF
YVVVVVVKTEDQACGGSLPFYPFAEDEPVDQGHRQKTLSVLVSQAVTSEAYVSGMLFCLGI
FLSFYLLTVLLACWENWRQKKKTLLVAIDRACPESGHPRVLADSFPGSSPYEGYNYGSFE
NVSGSTDGLVDSAGTGDLSYGYQGRSFEPVGTRPRVDSMSSVEEDDYDTLTDIDSDKNVI
RTKQYLYVADLARKDKRVLRKKYQIYFWNIATIAVFYALPVVQLVITYQTVVNVTGNQDI
CYYNFLCAHPLGNLSAFNNILSNLGYILLGLLFLLIILQREINHNRALLRNDLCALECGIPK
HFGLFYAMGTALMMEGLLSACYHVCPNYTNFQFDTSFMYMIAGLCMLKLYQKRHPDIN
ASAYSAYACLAIVIFFSVLGVVFGKGNTAFWIVFSIIHIIATLLLSTQLYYMGRWKLDSGIFR

RILHVLYTDCIRQCSGPLYVDRMVLLVMGNVINWSLAAYGLIMRPNDFASYLLAIGICNLL
LYFAFYIIMKLRSGERIKLIPLLCIVCTSVVWGFALFFFFQGLSTWQKTPAESREHNRDCILL
DFFDDHDIWHFLSSIAMFGSFLVLLTLDDDLDTVQRDKIYVF

>Q13177 lipid-anchor
MSDNGELEDKPPAPPVRMSSTIFSTGGKDPLSANHSLKPLPSVPEEKKPRHKIISIFSGTEKG
SKKKEKERPEISPPSDFEHTIHVGFDAVTGEFTGMPEQWARLLQTSNITKLEQKKNPQAVL
DVLKFYDSNTVKQKYLSFTPPEKDGFPSGTPALNAKGTEAPAVVTEEEDDDEETAPPVIAP
RPDHTKSIYTRSVIDPVPAPVGDSHVDGAAKSLDKQKKKTKMTDEEIMEKLRTIVSIGDPK
KKYTRYEKIGQGASGTVFTATDVALGQEVAIKQINLQKQPKKELIINEILVMKELKNPNIVN
FLDSYLVGDELFVVMEYLAGGSLTDVVTETCMDEAQIAAVCRECLQALEFLHANQVIHR
DIKSDNVLLGMEGSVKLTDFGFCAQITPEQSKRSTMVGTPYWMAPEVVTRKAYGPKVDI
WSLGIMAIEMVEGEPPYLNENPLRALYLIATNGTPELQNPEKLSPIFRDFLNRCLEMDVEK
RGSAKELLQHPFLKLAKPLSSLTPLIMAAKEAMKSNR

>Q9GZN0 multipass membrane protein
MTNSSSTSTSSTTGGSLLLLCEEEESWAGRRIPVSLLYSGLAIGGTLANGMVIYLVSSFRKL
QTTSNAFIVNGCAADLSVCALWMPQEAVLGLLPTGSAEPPADWDGAGGSYRLLRGGLLG
LGLTVSLLSHCLVALNRYLLITRAPATYQALYQRRHTAGMLALSWALALGLVLLLPPWAPR
PGAAPPRVHYPALLAAAALLAQTALLLHCYLGIVRRVRVSVKRVSVLNFHLLHQLPGCAA
AAAAFPGAQHAPGPGGAAHPAQAQPLPPALHPRRAQRRLSGLSVLLLCCVFLLATQPLVW
VSLASGFSLPVPWGVQAASWLLCCALSALNPLLYTWRNEEFRRSVRSVLPGVGDAAAAA
VAATAVPAVSQAQLGTRAAGQHW

>Q8NGQ4 multipass membrane protein
MPVGKLVFNQSEPTEFVFRAFTTATEFQVLLFLLFLLLYLMILCGNTAIIWVVCTHSTLRTP
MYFFLSNLSFLELCYTTVVVPLMLSNILGAQKPISLAGCGAQMFFFVTLGSTDCFLLAIMA
YDRYVAICHPLHYTLIMTRELCTQMLGGALGLALFPSLQLTALIFTLPFCGHHQEINHFLCD
VPPVLRLACADIRVHQAVLYVVSILVLTIPFLLICVSYVFITCAILSIRSAEGRRRAFSTCSFHL
TVVLLQYGCCSLVYLRPRSSTSEDEDSQIALVYTFVTPLLNPLLYSLRNKDVKGALRSAIIR
KAASDAN

>P42701 single-pass type I membrane protein
MEPLVTWVVPLLFLFLLSRQGAACRTSECCFQDPPYPDADSGSASGPRDLRCYRISSDRYE
CSWQYEGPTAGVSHFLRCCLSSGRCCYFAAGSATRLQFSDQAGVSVLYTVTLWVESWAR
NQTEKSPEVTLQLYNSVKYEPPLGDIKVSKLAGQLRMEWETPDNQVGAEVQFRHRTPSSP
WKLGDCGPQDDDTESCLCPLEMNVAQEFQLRRRQLGSQGSSWSKWSSPVCVPPENPPQP
QVRFSVEQLGQDGRRRLTLKEQPTQLELPEGCQGLAPGTEVTYRLQLHMLSCPCKAKATR
TLHLGKMPYLSGAAYNVAVISSNQFGPGLNQTWHIPADTHTEPVALNISVGTNGTTMYWP
ARAQSMTYCIEWQPVGQDGGLATCSLTAPQDPDPAGMATYSWSRESGAMGQEKCYYITIF
ASAHPEKLTLWSTVLSTYHFGGNASAAGTPHHVSVKNHSLDSVSVDWAPSLLSTCPGVLK
EYVVRCRDEDSKQVSEHPVQPTETQVTLSGLRAGVAYTVQVRADTAWLRGVWSQPQRFS
IEVQVSDWLIFFASLGSFLSILLVGVLGYLGLNRAARHLCPPLPTPCASSAIEFPGGKETWQ
WINPVDFQEEASLQEALVVEMSWDKGERTEPLEKTELPEGAPELALDTELSLEDGDRCKA

KM

>P04062 peripheral membrane protein
MEFSSPSREECPKPLSRVSIMAGSLTGLLLLQAVSWASGARPCIPKSFGYSSVVCVCNATYC
DSFDPPTFPALGTFSRYESTRSGRRMELSMGPIQANHTGTGLLLTLQPEQKFQKVKGFGGA
MTDAAALNILALSPPAQNLLLKSYFSEEGIGYNIIRVPMASCDFSIRTYTYADTPDDFQLHN
FSLPEEDTKLKIPLIHRALQLAQRPVSLLASPWTSPTWLKTNGAVNGKGSLKGQPGDIYHQ
TWARYFVKFLDAYAEHKLQFWAVTAENEPSAGLLSGYPFQCLGFTPEHQRDFIARDLGPTL
ANSTHHNVRLLMLDDQRLLLPHWAKVVLTDPEAAKYVHGIAVHWYLDFLAPAKATLGE
THRLFPNTMLFASEACVGSKFWEQSVRLGSWDRGMQYSHSIITNLLYHVVGWTDWNLAL
NPEGGPNWVRNFVDSPIIVDITKDTFYKQPMFYHLGHFSKFIPEGSQRVGLVASQKNDLDA
VALMHPDGSAVVVVLNRSSKDVPLTIKDPAVGFLETISPGYSIHTYLWRRQ

>P11836 multipass membrane protein
MTTPRNSVNGTFPAEPMKGPIAMQSGPKPLFRRMSSLVGPTQSFFMRESKTLGAVQIMNG
LFHIALGGLLMIPAGIYAPICVTVWYPLWGGIMYIISGSLLAATEKNSRKCLVKGKMIMNSL
SLFAAISGMILSIMDILNIKISHFLKMESLNFIRAHTPYINIYNCEPANPSEKNSPSTQYCYSIQ
SLFLGILSVMLIFAFFQELVIAGIVENEWKRTCSRPKSNIVLLSAEEKKEQTIEIKEEVVGLTE
TSSQPKNEEDIEIIPIQEEEEEETETNFPEPPQDQESSPIENDSSP

>O43868 multipass membrane protein
MEKASGRQSIALSTVETGTVNPGLELMEKEVEPEGSKRTDAQGHSLGDGLGPSTYQRRSR
WPFSKARSFCKTHASLFKKILLGLLCLAYAAYLLAACILNFQRALALFVITCLVIFVLVHSF
LKKLLGKKLTRCLKPFENSRLRLWTKWVFAGVSLVGLILWLALDTAQRPEQLIPFAGICMF
ILILFACSKHHSAVSWRTVFSGLGLQFVFGILVIRTDLGYTVFQWLGEQVQIFLNYTVAGSS
FVFGDTLVKDVFAFQALPIIIFFGCVVSILYYLGLVQWVVQKVAWFLQITMGTTATETLAVA
GNIFVGMTEAPLLIRPYLGDMTLSEIHAVMTGGFATISGTVLGAFIAFGVDASSLISASVMA
APCALASSKLAYPEVEESKFKSEEGVKLPRGKERNVLEAASNGAVDAIGLATNVAANLIAF
LAVLAFINAALSWLGELVDIQGLTFQVICSYLLRPMVFMMGVEWTDCPMVAEMVGIKFFI
NEFVAYQQLSQYKNKRLSGMEEWIEGEKQWISVRAEIITTFSLCGFANLSSIGITLGGLTSIV
PHRKSDLSKVVVRALFTGACVSLISACMAGILYVPRGAEADCVSFPNTSFTNRTYETYMC
CRGLFQSTSLNGTNPPSFSGPWEDKEFSAMALTNCCGFYNNTVCA

>P25101 multipass membrane protein
METLCLRASFWLALVGCVISDNPERYSTNLSNHVDDFTTFRGTELSFLVTTHQPTNLVLPS
NGSMHNYCPQQTKITSAFKYINTVISCTIFIVGMVGNATLLRIIYQNKCMRNGPNALIASLA
LGDLIYVVIDLPINVFKLLAGRWPFDHNDFGVFLCKLFPFLQKSSVGITVLNLCALSVDRY
RAVASWSRVQGIGIPLVTAIEIVSIWILSFILAIPEAIGFVMVPFEYRGEQHKTCMLNATSKF
MEFYQDVKDWWLFGFYFCMPLVCTAIFYTLMTCEMLNRRNGSLRIALSEHLKQRREVAK
TVFCLVVIFALCWFPLHLSRILKKTVYNEMDKNRCELLSFLLLMDYIGINLATMNSCINPIA
LYFVSKKFKNCFQSCLCCCCYQSKSLMTSVPMNGTSIQWKNHDQNNHNTDRSSHKDSM
N

>P43155 peripheral membrane protein

MLAFAARTVVKPLGFLKPFSLMKASSRFKAHQDALPRLPVPPLQQSLDHYLKALQPIVSE
EEWAHTKQLVDEFQASGGVGERLQKGLERRARKTENWLSEWWLKTAYLQYRQPVVIYS
SPGVMLPKQDFVDLQGQLRFAAKLIEGVLDFKVMIDNETLPVEYLGGKPLCMNQYYQILS
SCRVPGPKQDTVSNFSKTKKPPTHITVVHNYQFFELDVYHSDGTPLTADQIFVQLEKIWNS
SLQTNKEPVGILTSNHRNSWAKAYNTLIKDKVNRDSVRSIQKSIFTVCLDATMPRVSEDVY
RSHVAGQMLHGGGSRLNSGNRWFDKTLQFIVAEDGSCGLVYEHAAAEGPPIVTLLDYVIE
YTKKPELVRSPLVPLPMPKKLRFNITPEIKSDIEKAKQNLSIMIQDLDITVMVFHHFGKDFP
KSEKLSPDAFIQMALQLAYYRIYGQACATYESASLRMFHLGRTDTIRSASMDSLTFVKAM
DDSSVTEHQKVELLRKAVQAHRGYTDRAIRGEAFDRHLLGLKLQAIEDLVSMPDIFMDTS
YAIAMHFHLSTSQVPAKTDCVMFFGPVVPDGYGVCYNPMEAHINFSLSAYNSCAETNAA
RLAHYLEKALLDMRALLQSHPRAKL

>Q8NGA8 multipass membrane protein
MVTEFIFLGLSDSQGLQTFLFMLFFVFYGGIVFGNLLIVITVVSDSHLHSPMYFLLANLSLI
DLSLSSVTAPKMITDFFSQRKVISFKGCLVQIFLLHFFGGSEMVILIAMGFDRYIAICKPLHY
TTIMCGNACVGIMAVAWGIGFLHSVSQLAFAVHLPFCGPNEVDSFYCDLPRVIKLACTDTY
RLDIMVIANSGVLTVCSFVLLIISYTIILMTIQHRPLDKSSKALSTLTAHITVVLLFFGPCVFIY
AWPFPIKSLDKFLAVFYSVITPLLNPIIYTLRNKDMKTAIRQLRKWDAHSSVKF

>P10909 peripheral membrane protein
MMKTLLLFVGLLLTWESGQVLGDQTVSDNELQEMSNQGSKYVNKEIQNAVNGVKQIKT
LIEKTNEERKTLLSNLEEAKKKKEDALNETRESETKLKELPGVCNETMMALWEECKPCLK
QTCMKFYARVCRSGSGLVGRQLEEFLNQSSPFYFWMNGDRIDSLLENDRQQTHMLDVM
QDHFSRASSIIDELFQDRFFTREPQDTYHYLPFSLPHRRPHFFFPKSRIVRSLMPFSPYEPLN
FHAMFQPFLEMIHEAQQAMDIHFHSPAFQHPPTEFIREGDDDRTVCREIRHNSTGCLRMKD
QCDKCREILSVDCSTNNPSQAKLRRELDESLQVAERLTRKYNELLKSYQWKMLNTSSLLE
QLNEQFNWVSRLANLTQGEDQYYLRVTTVASHTSDSDVPSGVTEVVVKLFDSDPITVTVP
VEVSRKNPKFMETVAEKALQEYRKKHREE

>Q8IWX5 multipass membrane protein
MAELLRSLQDSQLVARFQRRCGLFPAPDEGPRENGADPTERAARVPGVEHLPAANGKGGE
APANGLRRAAAPEAYVQKYVVKNYFYYYLFQFSAALGQEVFYITFLPFTHWNIDPYLSRR
LIIIWVLVMYIGQVAKDVLKWPRPSSPPVVKLEKRLIAEYGMPSTHAMAATAIAFTLLISTM
DRYQYPFVLGLVMAVVFSTLVCLSRLYTGMHTVLDVLGGVLITALLIVLTYPAWTFIDCLD
SASPLFPVCVIVVPFFLCYNYPVSDYYSPTRADTTTILAAGAGVTIGFWINHFFQLVSKPAE
SLPVIQNIPPLTTYMLVLGLTKFAVGIVLILLVRQLVQNLSLQVLYSWFKVVTRNKEARRRL
EIEVPYKFVTYTSVGICATTFVPMLHRFLGLP

>Q75V66 multipass membrane protein
MGDPDLLEVLAEEGEKVNKHIDYSFQMSEQSLSSRETSFLINEETMPAKRFNLFLRRRLMF
QKNQQSKDSIFFRDGIRQIDFVLSYVDDVKKDAELKAERRKEFETNLRKTGLELEIEDKRD
SEDGRTYFVKIHAPWEVLVTYAEVLGIKMPIKESDIPRPKHTPISYVLGPVRLPLSVKYPHP
EYFTAQFSRHRQELFLIEDQATFFPSSSRNRIVYYILSRCPFGIEDGKKRFGIERLLNSNTYSS
AYPLHDGQYWKPSEPPNPTNERYTLHQNWARFSYFYKEQPLDLIKNYYGEKIGIYFVFLG

FYTEMLFFAAVVGLACFIYGLLSMEHNTSSTEICDPEIGGQMIMCPLCDQVCDYWRLNST
CLASKFSHLFDNESTVFFAIFMGIWVTLFLEFWKQRQARLEYEWDLVDFEEEQQQLQLRP
EFEAMCKHRKLNAVTKEMEPYMPLYTRIPWYFLSGATVTLWMSLVVTSMVAVIVYRLSVF
ATFASFMESDASLKQVKSFLTPQITTSLTGSCLNFIVILILNFFYEKISAWITKMEIPRTYQEY
ESSLTLKMFLFQFVNFYSSCFYVAFFKGKFVGYPGKYTYLFNEWRSEECDPGGCLIELTTQ
LTIIMTGKQIFGNIKEAIYPLALNWWRRRKARTNSEKLYSRWEQDHDLESFGPLGLFYEYL
ETVTQFGFVTLFVASFPLAPLLALINNIVEIRVDAWKLTTQYRRTVASKAHSIGVWQDILYG
MAVLSVATNAFIVAFTSDIIPRLVYYYAYSTNATQPMTGYVNNSLSVFLIADFPNHTAPSEK
RDFITCRYRDYRYPPDDENKYFHNMQFWHVLAAKMTFIIVMEHVVFLVKFLLAWMIPDV
PKDVVERIKREKLMTIKILHDFELNKLKENLGINSNEFAKHVMIEENKAQLAKSTL

>P32297 multipass membrane protein
MGSGPLSLPLALSPPRLLLLLLLSLLPVARASEAEHRLFERLFEDYNEIIRPVANVSDPVIIHF
EVSMSQLVKVDEVNQIMETNLWLKQIWNDYKLKWNPSDYGGAEFMRVPAQKIWKPDIV
LYNNAVGDFQVDDKTKALLKYTGEVTWIPPAIFKSSCKIDVTYFPFDYQNCTMKFGSWSY
DKAKIDLVLIGSSMNLKDYWESGEWAIIKAPGYKHDIKYNCCEEIYPDITYSLYIRRLPLFY
TINLIIPCLLISFLTVLVFYLPSDCGEKVTLCISVLLSLTVFLLVITETIPSTSLVIPLIGEYLLFT
MIFVTLSIVITVFVLNVHYRTPTTHTMPSWVKTVFLNLLPRVMFMTRPTSNEGNAQKPRPL
YGAELSNLNCFSRAESKGCKEGYPCQDGMCGYCHHRRIKISNFSANLTRSSSSESVDAVLS
LSALSPEIKEAIQSVKYIAENMKAQNEAKEIQDDWKYVAMVIDRIFLWVFTLVCILGTAGL
FLQPLMAREDA

>P24588 lipid-anchor
METTISEIHVENKDEKRSAEGSPGAERQKEKASMLCFKRRKKAAKALKPKAGSEAADVA
RKCPQEAGASDQPEPTRGAWASLKRLVTRRKRSESSKQQKPLEGEMQPAINAEDADLSKK
KAKSRLKIPCIKFPRGPKRSNHSKIIEDSDCSIKVQEEAEILDIQTQTPLNDQATKAKSTQDL
SEGISRKDGDEVCESNVSNSTTSGEKVISVELGLDNGHSAIQTGTLILEEIETIKEKQDVQP
QQASPLETSETDHQQPVLSDVPPLPAIPDQQIVEEASNSTLESAPNGKDYESTEIVAEETKP
KDTELSQESDFKENGITEEKSKSEESKRMEPIAIIITDTEISEFDVTKSKNVPKQFLISAENEQ
VGVFANDNGFEDRTSEQYETLLIETASSLVKNAIQLSIEQLVNEMASDDNKINNLLQ

>Q9UJ96 multipass membrane protein
MEPWPCSPGGGGGTRARHVIINVGGCRVRLAWAALARCPLARLERLRACRGHDDLLRVC
DDYDVSRDEFFFDRSPCAFRAIVALLRAGKLRLLRGPCALAFRDELAYWGIDEARLERCC
LRRLRRREEEAAEARAGPTERGAQGSPARALGPRGRLQRGRRRLRDVVDNPHSGLAGKL
FACVSVSFVAVTAVGLCLSTMPDIRAEEERGECSPKCRSLFVLETVCVAWFSFEFLLRSLQA
ESKCAFLRAPLNIIDILALLPFYVSLLLGLAAGPGGTKLLERAGLVLRLLRALRVLYVMRL
ARHSLGLRSLGLTMRRCAREFGLLLLFLCVAMALFAPLVHLAERELGARRDFSSVPASYW
WAVISMTTVGYGDMVPRSLPGQVVALSSILSGILLMAFPVTSIFHTFSRSYSELKEQQQRA
ASPEPALQEDSTHSATATEDSSQGPDSAGLADDSADALWVRAGR

>Q9Y3D7 peripheral membrane protein
MAKYLAQIIVMGVQVVGRAFARALRQEFAASRAAADARGRAGHRSAAASNLSGLSLQE
AQQILNVSKLSPEEVQKNYEHLFKVNDKSVGGSFYLQSKVVRAKERLDEELKIQAQEDRE

KGQMPHT

>Q9Y3A3 peripheral membrane protein
MVMAEGTAVLRRNRPGTKAQDFYNWPDESFDEMDSTLAVQQYIQQNIRADCSNIDKILEP
PEGQDEGVWKYEHLRQFCLELNGLAVKLQSECHPDTCTQMTATEQWIFLCAAHKTPKEC
PAIDYTRHTLDGAACLLNSNKYFPSRVSIKESSVAKLGSVCRRIYRIFSHAYFHHRQIFDEY
ENETFLCHRFTKFVMKYNLMSKDNLIVPILEEEVQNSVSGESEA

>P13796 peripheral membrane protein
MARGSVSDEEMMELREAFAKVDTDGNGYISFNELNDLFKAACLPLPGYRVREITENLMA
TGDLDQDGRISFDEFIKIFHGLKSTDVAKTFRKAINKKEGICAIGGTSEQSSVGTQHSYSEE
EKYAFVNWINKALENDPDCRHVIPMNPNTNDLFNAVGDGIVLCKMINLSVPDTIDERTINK
KKLTPFTIQENLNLALNSASAIGCHVVNIGAEDLKEGKPYLVLGLLWQVIKIGLFADIELSR
NEALIALLREGESLEDLMKLSPEELLLRWANYHLENAGCNKIGNFSTDIKDSKAYYHLLEQ
VAPKGDEEGVPAVVIDMSGLREKDDIQRAECMLQQAERLGCRQFVTATDVVRGNPKLNL
AFIANLFNRYPALHKPENQDIDWGALEGETREERTFRNWMNSLGVNPRVNHLYSDLSDAL
VIFQLYEKIKVPVDWNRVNKPPYPKLGGNMKKLENCNYAVELGKNQAKFSLVGIGGQDL
NEGNRTLTLALIWQLMRRYTLNILEEIGGGQKVNDDIIVNWVNETLREAKKSSSISSFKDP
KISTSLPVLDLIDAIQPGSINYDLLKTENLNDDEKLNNAKYAISMARKIGARVYALPEDLVE
VNPKMVMTVFACLMGKGMKRV

>Q9H9E3 peripheral membrane protein
MADLDSPPKLSGVQQPSEGVGGGRCSEISAELIRSLTELQELEAVYERLCGEEKVVERELD
ALLEQQNTIESKMVTLHRMGPNLQLIEGDAKQLAGMITFTCNLAENVSSKVRQLDLAKN
RLYQAIQRADDILDLKFCMDGVQTALRSEDYEQAAAHTHRYLCLDKSVIELSRQGKEGS
MIDANLKLLQEAEQRLKAIVAEKFAIATKEGDLPQVERFFKIFPLLGLHEEGLRKFSEYLCK
QVASKAEENLLMVLGTDMSDRRAAVIFADTLTLLFEGIARIVETHQPIVETYYGPGRLYTLI
KYLQVECDRQVEKVVDKFIKQRDYHQQFRHVQNNLMRNSTTEKIEPRELDPILTEVTLM
NARSELYLRFLKKRISSDFEVGDSMASEEVKQEHQKCLDKLLNNCLLSCTMQELIGLYVT
MEEYFMRETVNKAVALDTYEKGQLTSSMVDDVFYIVKKCIGRALSSSSIDCLCAMINLATT
ELESDFRDVLCNKLRMGFPATTFQDIQRGVTSAVNIMHSSLQQGKFDTKGIESTDEAKMSF
LVTLNNVEVCSENISTLKKTLESDCTKLFSQGIGGEQAQAKFDSCLSDLAAVSNKFRDLLQ
EGLTELNSTAIKPQVQPWINSFFSVSHNIEEEEFNDYEANDPWVQQFILNLEQQMAEFKAS
LSPVIYDSLTGLMTSLVAVELEKVVLKSTFNRLGGLQFDKELRSLIAYLTTVTTWTIRDKFA
RLSQMATILNLERVTEILDYWGPNSGPLTWRLTPAEVRQVLALRIDFRSEDIKRLRL

>Q9H2W1 multipass membrane protein
MTSQPVPNETIIVLPSNVINFSQAEKPEPTNQGQDSLKKHLHAEIKVIGTIQILCGMMVLSL
GIILASASFSPNFTQVTSTLLNSAYPFIGPFFFIISGSLSIATEKRLTKLLVHSSLVGSILSALSAL
VGFIILSVKQATLNPASLQCELDKNNIPTRSYVSYFYHDSLYTTDCYTAKASLAGTLSLMLI
CTLLEFCLAVLTAVLRWKQAYSDFPGSVLFLPHSYIGNSGMSSKMTHDCGYEELLTS

>Q8NHL6 single-pass type I membrane protein
MTPILTVLICLGLSLGPRTHVQAGHLPKPTLWAEPGSVITQGSPVTLRCQGGQETQEYRLY

REKKTALWITRIPQELVKKGQFPIPSITWEHAGRYRCYYGSDTAGRSESSDPLELVVTGAYI
KPTLSAQPSPVVNSGGNVILQCDSQVAFDGFSLCKEGEDEHPQCLNSQPHARGSSRAIFSV
GPVSPSRRWWYRCYAYDSNSPYEWSLPSDLLELLVLGVSKKPSLSVQPGPIVAPEETLTLQ
CGSDAGYNRFVLYKDGERDFLQLAGAQPQAGLSQANFTLGPVSRSYGGQYRCYGAHNL
SSEWSAPSDPLDILIAGQFYDRVSLSVQPGPTVASGENVTLLCQSQGWMQTFLLTKEGAA
DDPWRLRSTYQSQKYQAEFPMGPVTSAHAGTYRCYGSQSSKPYLLTHPSDPLELVVSGPS
GGPSSPTTGPTSTSGPEDQPLTPTGSDPQSGLGRHLGVVIGILVAVILLLLLLLLLFLILRHRR
QGKHWTSTQRKADFQHPAGAVGPEPTDRGLQWRSSPAADAQEENLYAAVKHTQPEDGVE
MDTRSPHDEDPQAVTYAEVKHSRPRREMASPPSPLSGEFLDTKDRQAEEDRQMDTEAAA
SEAPQDVTYAQLHSLTLRREATEPPPSQEGPSPAVPSIYATLAIH

>Q969E2	multipass membrane protein
MSEKENNFPPLPKFIPVKPCFYQNFSDEIPVEHQVLVKRIYRLWMFYCATLGVNLIACLAW
WIGGGSGTNFGLAFVWLLLFTPCGYVCWFRPVYKAFRADSSFNFMAFFFIFGAQFVLTVI
QAIGFSGWGACGWLSAIGFFQYSPGAAVVMLLPAIMFSVSAAMMAIAIMKVHRIYRGAG
GSFQKAQTEWNTGTWRNPPSREAQYNNFSGNSLPEYPTVPSYPGSGQWP

>P52948	peripheral membrane protein
MFNKSFGTPFGGGTGGFGTTSTFGQNTGFGTTSGGAFGTSAFGSSNNTGGLFGNSQTKPG
GLFGTSSFSQPATSTSTGFGFGTSTGTANTLFGTASTGTSLFSSQNNAFAQNKPTGFGNFGT
STSSGGLFGTTNTTSNPFGSTSGSLFGPSSFTAAPTGTTIKFNPPTGTDTMVKAGVSTNISTK
HQCITAMKEYESKSLEELRLEDYQANRKGPQNQVGAGTTTGLFGSSPATSSATGLFSSSTT
NSGFAYGQNKTAFGTSTTGFGTNPGGLFGQQNQQTTSLFSKPFGQATTTQNTGFSFGNTST
IGQPSTNTMGLFGVTQASQPGGLFGTATNTSTGTAFGTGTGLFGQTNTGFGAVGSTLFGNN
KLTTFGSSTTSAPSFGTTSGGLFGNKPTLTLGTNTNTSNFGFGTNTSGNSIFGSKPAPGTLGT
GLGAGFGTALGAGQASLFGNNQPKIGGPLGTGAFGAPGFNTTTATLGFGAPQAPVALTDP
NASAAQQAVLQQHINSLTYSPFGDSPLFRNPMSDPKKKEERLKPTNPAAQKALTTPTHYKL
TPRPATRVRPKALQTTGTAKSHLFDGLDDDEPSLANGAFMPKKSIKKLVLKNLNNSNLFSP
VNRDSENLASPSEYPENGERFSFLSKPVDENHQQDGDEDSLVSHFYTNPIAKPIPQTPESAG
NKHSNSNSVDDTIVALNMRAALRNGLEGSSEETSFHDESLQDDREEIENNSYHMHPAGIIL
TKVGYYTIPSMDDLAKITNEKGECIVSDFTIGRKGYGSIYFEGDVNLTNLNLDDIVHIRRKE
VVVYLDDNQKPPVGEGLNRKAEVTLDGVWPTDKTSRCLIKSPDRLADINYEGRLEAVSR
KQGAQFKEYRPETGSWVFKVSHFSKYGLQDSDEEEEEHPSKTSTKKLKTAPLPPASQTTPL
QMALNGKPAPPPQSQSPEVEQLGRVVELDSDMVDITQEPVLDTMLEESMPEDQEPVSAST
HIASSLGINPHVLQIMKASLLTDEEDVDMALDQRFSRLPSKADTSQEICSPRLPISASHSSKT
RSLVGGLLQSKFTSGAFLSPSVSVQECRTPRAASLMNIPSTSSWSVPPPLTSVFTMPSPAPEV
PLKTVGTRRQLGLVPREKSVTYGKGKLLMDMALFMGRSFRVGWGPNWTLANSGEQLNG
SHELENHQIADSMEFGFLPNPVAVKPLTESPFKVHLEKLSLRQRKPDEDMKLYQTPLELKL
KHSTVHVDELCPLIVPNLGVAVIHDYADWVKEASGDLPEAQIVKHWSLTWTLCEALWGH
LKELDSQLNEPREYIQILERRRAFSRWLSCTATPQIEEEVSLTQKNSPVEAVFSYLTGKRISE
ACSLAQQSGDHRLALLLSQFVGSQSVRELLTMQLVDWHQLQADSFIQDERLRIFALLAGK
PVWQLSEKKQINVCSQLDWKRSLAIHLWYLLPPTASISRALSMYEEAFQNTSDSDRYACSP
LPSYLEGSGCVIAEEQNSQTPLRDVCFHLLKLYSDRHYDLNQLLEPRSITADPLDYRLSWH
LWEVLRALNYTHLSAQCEGVLQASYAGQLESEGLWEWAIFVLLHIDNSGIREKAVRELLT

RHCQLLETPESWAKETFLTQKLRVPAKWIHEAKAVRAHMESDKHLEALCLFKAEHWNRC
HKLIIRHLASDAIINENYDYLKGFLEDLAPPERSSLIQDWETSGLVYLDYIRVIEMLRHIQQV
DCSGNDLEQLHIKVTSLCSRIEQIQCYSAKDRLAQSDMAKRVANLLRVVLSLHHPPDRTSD
STPDPQRVPLRLLAPHIGRLPMPEDYAMDELRSLTQSYLRELAVGSL

>P48048 multipass membrane protein
MNASSRNVFDTLIRVLTESMFKHLRKWVVTRFFGHSRQRARLVSKDGRCNIEFGNVEAQS
RFIFFVDIWTTVLDLKWRYKMTIFITAFLGSWFFFGLLWYAVAYIHKDLPEFHPSANHTPCV
ENINGLTSAFLFSLETQVTIGYGFRCVTEQCATAIFLLIFQSILGVIINSFMCGAILAKISRPKK
RAKTITFSKNAVISKRGGKLCLLIRVANLRKSLLIGSHIYGKLLKTTVTPEGETIILDQININF
VVDAGNENLFFISPLTIYHVIDHNSPFFHMAAETLLQQDFELVVFLDGTVESTSATCQVRTS
YVPEEVLWGYRFAPIVSKTKEGKYRVDFHNFSKTVEVETPHCAMCLYNEKDVRARMKRG
YDNPNFILSEVNETDDTKM

>P08908 multipass membrane protein
MDVLSPGQGNNTTSPPAPFETGGNTTGISDVTVSYQVITSLLLGTLIFCAVLGNACVVAAIA
LERSLQNVANYLIGSLAVTDLMVSVLVLPMAALYQVLNKWTLGQVTCDLFIALDVLCCTS
SILHLCAIALDRYWAITDPIDYVNKRTPRRAAALISLTWLIGFLISIPPMLGWRTPEDRSDPD
ACTISKDHGYTIYSTFGAFYIPLLLMLVLYGRIFRAARFRIRKTVKKVEKTGADTRHGASPA
PQPKKSVNGESGSRNWRLGVESKAGGALCANGAVRQGDDGAALEVIEVHRVGNSKEHL
PLPSEAGPTPCAPASFERKNERNAEAKRKMALARERKTVKTLGIIMGTFILCWLPFFIVALV
LPFCESSCHMPTLLGAIINWLGYSNSLLNPVIYAYFNKDFQNAFKKIIKCKFCRQ

>Q8NG97 multipass membrane protein
MGDVNQSVASDFILVGLFSHSGSRQLLFSLVAVMFVIGLLGNTVLLFLIRVDSRLHTPMYFL
LSQLSLFDIGCPMVTIPKMASDFLRGEGATSYGGGAAQIFFLTLMGVAEGVLLVLMSYDRY
VAVCQPLQYPVLMRRQVCLLMMGSSWVVGVLNASIQTSITLHFPYCASRIVDHFFCEVPA
LLKLSCADTCAYEMALSTSGVLILMLPLSLIATSYGHVLQAVLSMRSEEARHKAVTTCSSH
ITVVGLFYGAAVFMYMVPCAYHSPQQDNVVSLFYSLVTPTLNPLIYSLRNPEVWMALVKV
LSRAGLRQMC

>Q86SK9 multipass membrane protein
MPGPATDAGKIPFCDAKEEIRAGLESSEGGGGPERPGARGQRQNIVWRNVVLMSLLHLGA
VYSLVLIPKAKPLTLLWAYFCFLLAALGVTAGAHRLWSHRSYRAKLPLRIFLAVANSMAFQ
NDIFEWSRDHRAHHKYSETDADPHNARRGFFFSHIGWLFVRKHRDVIEKGRKLDVTDLL
ADPVVRIQRKYYKISVVLMCFVVPTLVPWYIWGESLWNSYFLASILRYTISLNISWLVNSA
AHMYGNRPYDKHISPRQNPLVALGAIGEGFHNYHHTFPFDYSASEFGLNFNPTTWFIDFM
CWLGLATDRKRATKPMIEARKARTGDSSA

>P08174 GPI-anchor, lipid-anchor, single-pass type I membrane protein
MTVARPSVPAALPLLGELPRLLLLVLLCLPAVWGDCGLPPDVPNAQPALEGRTSFPEDTVIT
YKCEESFVKIPGEKDSVICLKGSQWSDIEEFCNRSCEVPTRLNSASLKQPYITQNYFPVGTV
VEYECRPGYRREPSLSPKLTCLQNLKWSTAVEFCKKKSCPNPGEIRNGQIDVPGGILFGATI
SFSCNTGYKLFGSTSSFCLISGSSVQWSDPLPECREIYCPAPPQIDNGIIQGERDHYGYRQSV

TYACNKGFTMIGEHSIYCTVNNDEGEWSGPPPECRGKSLTSKVPPTVQKPTTVNVPTTEVS
PTSQKTTTKTTTPNAQATRSTPVSRTTKHFHETTPNKGSGTTSGTTRLLSGHTCFTLTGLLG
TLVTMGLLT

>Q9UQB8 peripheral membrane protein
MSLSRSEEMHRLTENVYKTIMEQFNPSLRNFIAMGKNYEKALAGVTYAAKGYFDALVKM
GELASESQGSKELGDVLFQMAEVHRQIQNQLEEMLKSFHNELLTQLEQKVELDSRYLSAA
LKKYQTEQRSKGDALDKCQAELKKLRKKSQGSKNPQKYSDKELQYIDAISNKQGELENY
VSDGYKTALTEERRRFCFLVEKQCAVAKNSAAYHSKGKELLAQKLPLWQQACADPSKIPE
RAVQLMQQVASNGATLPSALSASKSNLVISDPIPGAKPLPVPPELAPFVGRMSAQESTPIMN
GVTGPDGEDYSPWADRKAAQPKSLSPPQSQSKLSDSYSNTLPVRKSVTPKNSYATTENKT
LPRSSSMAAGLERNGRMRVKAIFSHAAGDNSTLLSFKEGDLITLLVPEARDGWHYGESEK
TKMRGWFPFSYTRVLDSDGSDRLHMSLQQGKSSSTGNLLDKDDLAIPPPDYGAASRAFPA
QTASGFKQRPYSVAVPAFSQGLDDYGARSMSRNPFAHVQLKPTVTNDRCDLSAQGPEGRE
HGDGSARTLAGR

>Q8IYJ3 peripheral membrane protein
MPQRGHPSQEGLWALPSLPMAHGPKPETEGLLDLSFLTEEEQEAIAGVLQRDARLRQLEE
GRVSKLRASVADPGQLKILTGDWFQEARSQRHHNAHFGSDLVRASMRRKKSTRGDQAPG
HDREAEAAVKEKEEGPEPRLTIDEAPQERLRETEGPDFPSPSVPLKASDPEEASQAQEDPG
QGDQQVCAEEADPELEPASGGEQEPRPQQAQTKAASQILENGEEAPGPDPSLDRMLSSSSS
VSSLNSSTLSGSQMSLSGDAEAVQVRGSVHFALHYEPGAAELRVHVIQCQGLAAARRRRS
DPYVKSYLLPDKQSKRKTAVKKRNLNPVFNETLRYSVPQAELQGRVLSLSVWHRESLGR
NIFLGEVEVPLDTWDWGSEPTWLPLQPRVPPSPDDLPSRGLLALSLKYVPAGSEGAGLPPS
GELHFWVKEARDLLPLRAGSLDTYVQCFVLPDDSQASRQRTRVVRRSLSPVFNHTMVYD
GFGPADLRQACAELSLWDHGALANRQLGGTRLSLGTGSSYGLQVPWMDSTPEEKQLWQ
ALLEQPCEWVDGLLPLRTNLAPRT

>O15121 multipass membrane protein
MGSRVSREDFEWVYTDQPHADRRREILAKYPEIKSLMKPDPNLIWIIIMMVLTQLGAFYIV
KDLDWKWVIFGAYAFGSCINHSMTLAIHEIAHNAAFGNCKAMWNRWFGMFANLPIGIPY
SISFKRYHMDHHRYLGADGVDVDIPTDFEGWFFCTAFRKFIWVILQPLFYAFRPLFINPKPI
TYLEVINTVAQVTFDILIYYFLGIKSLVYMLAASLLGLGLHPISGHFIAEHYMFLKGHETYS
YYGPLNLLTFNVGYHNEHHDFPNIPGKSLPLVRKIAAEYYDNLPHYNSWIKVLYDFVMDD
TISPYSRMKRHQKGEMVLE

>Q8NGT5 multipass membrane protein
MMDNHSSATEFHLLGFPGSQGLHHILFAIFFFFYLVTLMGNTVIIVIVCVDKRLQSPMYFFL
SHLSTLEILVTTIIVPMMLWGLLFLGCRQYLSLHVSLNFSCGTMEFALLGVMAVDRYVAVC
NPLRYNIIMNSSTCIWVVIVSWVFGFLSEIWPIYATFQFTFRKSNSLDHFYCDRGQLLKLSC
DNTLLTEFILFLMAVFILIGSLIPTIVSYTYIISTILKIPSASGRRKAFSTFASHFTCVVIGYGSC
LFLYVKPKQTQGVEYNKIVSLLVSVLTPFLNPFIFTLRNDKVKEALRDGMKRCCQLLKD

>Q70E73 peripheral membrane protein

MEQLSDEEIDHGAEEDSDKEDQDLDKMFGAWLGELDKLTQSLDSDKPMEPVKRSPLRQE
TNMANFSYRFSIYNLNEALNQGETVDLDALMADLCSIEQELSSIGSGNSKRQITETKATQK
LPVSRHTLKHGTLKGLSSSSNRIAKPSHASYSLDDVTAQLEQASLSMDEAAQQSVLEDTK
PLVTNQHRRTASAGTVSDAEVHSISNSSHSSITSAASSMDSLDIDKVTRPQELDLTHQGQPI
TEEEQAAKLKAEKIRVALEKIKEAQVKKLVIRVHMSDDSSKTMMVDERQTVRQVLDNLM
DKSHCGYSLDWSLVETVSELQMERIFEDHENLVENLLNWTRDSQNKLIFMERIEKYALFK
NPQNYLLGKKETAEMADRNKEVLLEECFCGSSVTVPEIEGVLWLKDDGKKSWKKRYFLL
RASGIYYVPKGKAKVSRDLVCFLQLDHVNVYYGQDYRNKYKAPTDYCLVLKHPQIQKKS
QYIKYLCCDDVRTLHQWVNGIRIAKYGKQLYMNYQEALKRTESAYDWTSLSSSSIKSGSS
SSSIPESQSNHSNQSDSGVSDTQPAGHVRSQSIVSSVFSEAWKRGTQLEESSKARMESMNR
PYTSLVPPLSPQPKIVTPYTASQPSPPLPPPPPPPPPPPPPPPPPPPPLPSQSAPSAGSAAPMFV
KYSTITRLQNASQHSGALFKPPTPPVMQSQSVKPQILVPPNGVVPPPPPPPPPPTPGSAMAQ
LKPAPCAPSLPQFSAPPPPLKIHQVQHITQVAPPTPPPPPPIPAPLPPQAPPKPLVTIPAPTSTKT
VAPVVTQAAPPTPTPPVPPAKKQPAFPASYIPPSPPTPPVPVPPPTLPKQQSFCAKPPPSPLSP
VPSVVKQIASQFPPPPTPPAMESQPLKPVPANVAPQSPPAVKAKPKWQPSSIPVPSPDFPPPP
PESSLVFPPPPPSPVPAPPPPPPPTASPTPDKSGSPGKKTSKTSSPGGKKPPPTPQRNSSIKSSS
GAEHPEPKRPSVDSLVSKFTPPAESGSPSKETLPPPAAPPKPGKLNLSGVNLPGVLQQGCVS
AKAPVLSGRGKDSVVEFPSPPSDSDFPPPPPETELPLPPIEIPAVFSGNTSPKVAVVNPQPQQ
WSKMSVKKAPPPTRPKRNDSTRLTQAEISEQPTMATVVPQVPTSPKSSLSVQPGFLADLNR
TLQRKSITRHGSLSSRMSRAEPTATMDDMALPPPPPELLSDQQKAGYGGSHISGYATLRRG
PPPAPPKRDQNTKLSRDW

>Q9NQW8 multipass membrane protein
MFKSLTKVNKVKPIGENNENEQSSRRNEEGSHPSNQSQQTTAQEENKGEEKSLKTKSTPV
TSEEPHTNIQDKLSKKNSSGDLTTNPDPQNAAEPTGTVPEQKEMDPGKEGPNSPQNKPPA
APVINEYADAQLHNLVKRMRQRTALYKKKLVEGDLSSPEASPQTAKPTAVPPVKESDDKP
TEHYYRLLWFKVKKMPLTEYLKRIKLPNSIDSYTDRLYLLWLLLVTLAYNWNCCFIPLRLV
FPYQTADNIHYWLIADIICDIIYLYDMLFIQPRLQFVRGGDIIVDSNELRKHYRTSTKFQLDV
ASIIPFDICYLFFGFNPMFRANRMLKYTSFFEFNHHLESIMDKAYIYRVIRTTGYLLFILHINA
CVYYWASNYEGIGTTRWVYDGEGNEYLRCYYWAVRTLITIGGLPEPQTLFEIVFQLLNFFS
GVFVFSSLIGQMRDVIGAATANQNYFRACMDDTIAYMNNYSIPKLVQKRVRTWYEYTWD
SQRMLDESDLLKTLPTTVQLALAIDVNFSIISKVDLFKGCDTQMIYDMLLRLKSVLYLPGD
FVCKKGEIGKEMYIIKHGEVQVLGGPDGTKVLVTLKAGSVFGEISLLAAGGGNRRTANVV
AHGFANLLTLDKKTLQEILVHYPDSERILMKKARVLLKQKAKTAEATPPRKDLALLFPPKE
ETPKLFKTLLGGTGKASLARLLKLKREQAAQKKENSEGGEEEGKENEDKQKENEDKQKE
NEDKGKENEDKDKGREPEEKPLDRPECTASPIAVEEEPHSVRRTVLPRGTSRQSLIISMAPS
AEGGEEVLTIEVKEKAKQ

>Q9H4E5 lipid-anchor
MNCKEGTDSSCGCRGNDEKKMLKCVVVGDGAVGKTCLLMSYANDAFPEEYVPTVFDHY
AVTVTVGGKQHLLGLYDTAGQEDYNQLRPLSYPNTDVFLICFSVVNPASYHNVQEEWVP
ELKDCMPHVPYVLIGTQIDLRDDPKTLARLLYMKEKPLTYEHGVKLAKAIGAQCYLECSA
LTQKGLKAVFDEAILTIFHPKKKKKRCSEGHSCCSII

>P14222 multipass membrane protein
MAARLLLLGILLLLLPLPVPAPCHTAARSECKRSHKFVPGAWLAGEGVDVTSLRRSGSFPV
DTQRFLRPDGTCTLCENALQEGTLQRLPLALTNWRAQGSGCQRHVTRAKVSSTEAVARD
AARSIRNDWKVGLDVTPKPTSNVHVSVAGSHSQAANFAAQKTHQDQYSFSTDTVECRFY
SFHVVHTPPLHPDFKRALGDLPHHFNASTQPAYLRLISNYGTHFIRAVELGGRISALTALRT
CELALEGLTDNEVEDCLTVEAQVNIGIHGSISAEAKACEEKKKKHKMTASFHQTYRERHS
EVVGGHHTSINDLLFGIQAGPEQYSAWVNSLPGSPGLVDYTLEPLHVLLDSQDPRREALR
RALSQYLTDRARWRDCSRPCPPGRQKSPRDPCQCVCHGSAVTTQDCCPRQRGLAQLEVT
FIQAWGLWGDWFTATDAYVKLFFGGQELRTSTVWDNNNPIWSVRLDFGDVLLATGGPLR
LQVWDQDSGRDDDLLGTCDQAPKSGSHEVRCNLNHGHLKFRYHARCLPHLGGGTCLDY
VPQMLLGEPPGNRSGAVW

>Q9NR80 peripheral membrane protein
MPWEEPAGEKPSCSHSQKAFHMEPAQKPCFTTDMVTWALLCISAETVRGEAPSQPRGIPH
RSPVSVDDLWLEKTQRKKLQKQAHVERRLHIGAVHKDGVKCWRKTIITSPESLNLPRRSH
PLSQSAPTGLNHMGWPEHTPGTAMPDGALDTAVCADEVGSEEDLYDDLHSSSHHYSHPG
GGGEQLAINELISDGSVVCAEALWDHVTMDDQELGFKAGDVIEVMDATNREWWWGRVA
DGEGWFPASFVRLRVNQDEPADDDAPLAGNSGAEDGGAEAQSSKDQMRTNVINEILSTER
DYIKHLRDICEGYVRQCRKRADMFSEEQLRTIFGNIEDIYRCQKAFVKALEQRFNRERPHL
SELGACFLEHQADFQIYSEYCNNHPNACVELSRLTKLSKYVYFFEACRLLQKMIDISLDGF
LLTPVQKICKYPLQLAELLKYTHPQHRDFKDVEAALHAMKNVAQLINERKRRLENIDKIA
QWQSSIEDWEGEDLLVRSSELIYSGELTRVTQPQAKSQQRMFFLFDHQLIYCKKDLLRRD
VLYYKGRLDMDGLEVVDLEDGKDRDLHVSIKNAFRLHRGATGDSHLLCTRKPEQKQRW
LKAFAREREQVQLDQETGFSITELQRKQAMLNASKQQVTGKPKAVGRPCYLTRQKHPALP
SNRPQQQVLVLAEPRRKPSTFWHSISRLAPFRK

>Q15622 multipass membrane protein
MEPGNDTQISEFLLLGFSQEPGLQPFLFGLFLSMYLVTVLGNLLIILATISDSHLHTPMYFFL
SNLSFADICVTSTTIPKMLMNIQTQNKVITYIACLMQMYFFILFAGFENFLLSVMAYDRFVA
ICHPLHYMVIMNPHLCGLLVLASWTMSALYSLLQILMVVRLSFCTALEIPHFFCELNQVIQ
LACSDSFLNHMVIYFTVALLGGGPLTGILYSYSKIISSIHAISSAQGKYKAFSTCASHLSVVS
LFYGAILGVYLSSAATRNSHSSATASVMYTVVTPMLNPFIYSLRNKDIKRALGIHLLWGTM
KGQFFKKCP

>Q5T0T0 multipass membrane protein
MSMPLHQISAIPSQDAISARVYRSKTKEKEREEQNEKTLGHFMSHSSNISKAGSPPSASAPA
PVSSFSRTSITPSSQDICRICHCEGDDESPLITPCHCTGSLHFVHQACLQQWIKSSDTRCCEL
CKYEFIMETKLKPLRKWEKLQMTSSERRKIMCSVTFHVIAITCVVWSLYVLIDRTAEEIKQ
GQATGILEWPFWTKLVVVAIGFTGGLLFMYVQCKVYVQLWKRLKAYNRVIYVQNCPETS
KKNIFEKSPLTEPNFENKHGYGICHSDTNSSCCTEPEDTGAEIIHV

>P08581 single-pass type I membrane protein
MKAPAVLAPGILVLLFTLVQRSNGECKEALAKSEMNVNMKYQLPNFTAETPIQNVILHEH
HIFLGATNYIYVLNEEDLQKVAEYKTGPVLEHPDCFPCQDCSSKANLSGGVWKDNINMAL

VVDTYYDDQLISCGSVNRGTCQRHVFPHNHTADIQSEVHCIFSPQIEEPSQCPDCVVSALG
AKVLSSVKDRFINFFVGNTINSSYFPDHPLHSISVRRLKETKDGFMFLTDQSYIDVLPEFRD
SYPIKYVHAFESNNFIYFLTVQRETLDAQTFHTRIIRFCSINSGLHSYMEMPLECILTEKRKK
RSTKKEVFNILQAAYVSKPGAQLARQIGASLNDDILFGVFAQSKPDSAEPMDRSAMCAFPI
KYVNDFFNKIVNKNNVRCLQHFYGPNHEHCFNRTLLRNSSGCEARRDEYRTEFTTALQRV
DLFMGQFSEVLLTSISTFIKGDLTIANLGTSEGRFMQVVVSRSGPSTPHVNFLLDSHPVSPE
VIVEHTLNQNGYTLVITGKKITKIPLNGLGCRHFQSCSQCLSAPPFVQCGWCHDKCVRSEE
CLSGTWTQQICLPAIYKVFPNSAPLEGGTRLTICGWDFGFRRNNKFDLKKTRVLLGNESCT
LTLSESTMNTLKCTVGPAMNKHFNMSIIISNGHGTTQYSTFSYVDPVITSISPKYGPMAGGT
LLTLTGNYLNSGNSRHISIGGKTCTLKSVSNSILECYTPAQTISTEFAVKLKIDLANRETSIFS
YREDPIVYEIHPTKSFISGGSTITGVGKNLNSVSVPRMVINVHEAGRNFTVACQHRSNSEIIC
CTTPSLQQLNLQLPLKTKAFFMLDGILSKYFDLIYVHNPVFKPFEKPVMISMGNENVLEIK
GNDIDPEAVKGEVLKVGNKSCENIHLHSEAVLCTVPNDLLKLNSELNIEWKQAISSTVLGK
VIVQPDQNFTGLIAGVVSISTALLLLLGFFLWLKKRKQIKDLGSELVRYDARVHTPHLDRL
VSARSVSPTTEMVSNESVDYRATFPEDQFPNSSQNGSCRQVQYPLTDMSPILTSGDSDISSP
LLQNTVHIDLSALNPELVQAVQHVVIGPSSLIVHFNEVIGRGHFGCVYHGTLLDNDGKKIH
CAVKSLNRITDIGEVSQFLTEGIIMKDFSHPNVLSLLGICLRSEGSPLVVLPYMKHGDLRNFI
RNETHNPTVKDLIGFGLQVAKGMKYLASKKFVHRDLAARNCMLDEKFTVKVADFGLAR
DMYDKEYYSVHNKTGAKLPVKWMALESLQTQKFTTKSDVWSFGVLLWELMTRGAPPY
PDVNTFDITVYLLQGRRLLQPEYCPDPLYEVMLKCWHPKAEMRPSFSELVSRISAIFSTFIG
EHYVHVNATYVNVKCVAPYPSLLSSEDNADDEVDTRPASFWETS

>Q643R3 multipass membrane protein
MSQGSPGDWAPLDPTPGPPASPNPFVHELHLSRLQRVKFCLLGALLAPIRVLLAFIVLFLLW
PFAWLQVAGLSEEQLQEPITGWRKTVCHNGVLGLSRLLFFLLGFLRIRVRGQRASRLQAPV
LVAAPHSTFFDPIVLLPCDLPKVVSRAENLSVPVIGALLRFNQAILVSRHDPASRRRVVEEV
RRRATSGGKWPQVLFFPEGTCSNKKALLKFKPGAFIAGVPVQPVLIRYPNSLDTTSWAWR
GPGVLKVLWLTASQPCSIVDVEFLPVYHPSPEESRDPTLYANNVQRVMAQALGIPATECEF
VGSLPVIVVGRLKVALEPQLWELGKVLRKAGLSAGYVDAGAEPGRSRMISQEEFARQLQL
SDPQTVAGAFGYFQQDTKGLVDFRDVALALAALDGGRSLEELTRLAFELFAEEQAEGPNR
LLYKDGFSTILHLLLGSPHPAATALHAELCQAGSSQGLSLCQFQNFSLHDPLYGKLFSTYLR
PPHTSRGTSQTPNASSPGNPTALANGTVQAPKQKGD

>Q9NZU7 lipid-anchor
MGGGDGAAFKRPGDGARLQRVLGLGSRREPRSLPAGGPAPRRTAPPPPGHASAGPAAMSS
HIAKSESKTSLLKAAAAAASGGSRAPRHGPARDPGLPSRRLPGSCPATPQSSGDPSSRRPLC
RPAPREEGARGSQRVLPQAHCRPREALPAAASRPSPSSPLPPARGRDGEERGLSPALGLRGS
LRARGRGDSVPAAASEADPFLHRLRPMLSSAFGQDRSLRPEEIEELREAFREFDKDKDGYI
NCRDLGNCMRTMGYMPTEMELIELSQQINMNLGGHVDFDDFVELMGPKLLAETADMIG
VKELRDAFREFDTNGDGEISTSELREAMRKLLGHQVGHRDIEEIIRDVDLNGDGRVDFEEF
VRMMSR

>P51571 single-pass type I membrane protein
MAAMASLGALALLLLSSLSRCSAEACLEPQITPSYYTTSDAVISTETVFIVEISLTCKNRVQ

NMALYADVGGKQFPVTRGQDVGRYQVSWSLDHKSAHAGTYEVRFFDEESYSLLRKAQR
NNEDISIIPPLFTVSVDHRGTWNGPWVSTEVLAAAIGLVIYYLAFSAKSHIQA

>Q96RF0 peripheral membrane protein
MALRARALYDFRSENPGEISLREHEVLSLCSEQDIEGWLEGVNSRGDRGLFPASYVQVIRA
PEPGPAGDGGPGAPARYANVPPGGFEPLPVAPPASFKPPPDAFQALLQPQQAPPPSTFQPPG
AGFPYGGGALQPSPQQLYGGYQASQGSDDDWDDEWDDSSTVADEPGALGSGAYPDLDG
SSSAGVGAAGRYRLSTRSDLSLGSRGGSVPPQHHPSGPKSSATVSRNLNRFSTFVKSGGEA
FVLGEASGFVKDGDKLCVVLGPYGPEWQENPYPFQCTIDDPTKQTKFKGMKSYISYKLVP
THTQVPVHRRYKHFDWLYARLAEKFPVISVPHLPEKQATGRFEEDFISKRRKGLIWWMNH
MASHPVLAQCDVFQHFLTCPSSTDEKAWKQGKRKAEKDEMVGANFFLTLSTPPAAALDL
QEVESKIDGFKCFTKKMDDSALQLNHTANEFARKQVTGFKKEYQKVGQSFRGLSQAFEL
DQQAFSVGLNQAIAFTGDAYDAIGELFAEQPRQDLDPVMDLLALYQGHLANFPDIIHVQK
GKAWPLEQVIWSVLCRLKGATLTAVPLWVSESYSTGEEASRDVDAWVFSLECKLDCSTGS
FLLEYLALGNEYSFSKVQRVPLMTVLSF

>Q8NGV5 multipass membrane protein
MYRFTDFDVSNISIYLNHVLFYTTQQAGDLEHMETRNYSAMTEFFLVGLSQYPELQLFLF
LLCLIMYMIILLGNSLLIIITILDSRLHTPMYFFLGNLSFLDICYTSSSIPPMLIIFMSERKSISFI
GCALQMVVSLGLGSTECVLLAVMAYDHYVAICNPLRYSIIMNGVLYVQMAAWSWIIGCLT
SLLQTVLTMMLPFCGNNVIDHITCEILALLKLVCSDITINVLIMTVTNIVSLVILLLLIFISYVF
ILSSILRINCAEGRKKAFSTCSAHSIVVILFYGSALFMYMKPKSKNTNTSDEIIGLSYGVVSP
MLNPIIYSLRNKEVKEAVKKVLSRHLHLLKM

>Q96FZ7 lipid-anchor
MGNLFGRKKQSRVTEQDKAILQLKQQRDKLRQYQKRIAQQLERERALARQLLRDGRKER
AKLLLKKKRYQEQLLDRTENQISSLEAMVQSIEFTQIEMKVMEGLQFGNECLNKMHQVM
SIEEVERILDETQEAVEYQRQIDELLAGSFTQEDEDAILEELSAITQEQIELPEVPSEPLPEKIP
ENVPVKARPRQAELVAAS

>Q15075 peripheral membrane protein
MLRRILQRTPGRVGSQGSDLDSSATPINTVDVNNESSSEGFICPQCMKSLGSADELFKHYE
AVHDAGNDSGHGGESNLALKRDDVTLLRQEVQDLQASLKEEKWYSEELKKELEKYQGL
QQQEAKPDGLVTDSSAELQSLEQQLEEAQTENFNIKQMKDLFEQKAAQLATEIADIKSKY
DEERSLREAAEQKVTRLTEELNKEATVIQDLKTELLQRPGIEDVAVLKKELVQVQTLMDN
MTLERERESEKLKDECKKLQSQYASSEATISQLRSELAKGPQEVAVYVQELQKLKSSVNEL
TQKNQTLTENLLKKEQDYTKLEEKHNEESVSKKNIQATLHQKDLDCQQLQSRLSASETSL
HRIHVELSEKGEATQKLKEELSEVETKYQHLKAEFKQLQQQREEKEQHGLQLQSEINQLH
SKLLETERQLGEAHGRLKEQRQLSSEKLMDKEQQVADLQLKLSRLEEQLKEKVTNSTELQ
HQLDKTKQQHQEQQALQQSTTAKLREAQNDLEQVLRQIGDKDQKIQNLEALLQKSKENI
SLLEKEREDLYAKIQAGEGETAVLNQLQEKNHTLQEQVTQLTEKLKNQSESHKQAQENLH
DQVQEQKAHLRAAQDRVLSLETSVNELNSQLNESKEKVSQLDIQIKAKTELLLSAEAAKT
AQRADLQNHLDTAQNALQDKQQELNKITTQLDQVTAKLQDKQEHCSQLESHLKEYKEK
YLSLEQKTEELEGQIKKLEADSLEVKASKEQALQDLQQQRQLNTDLELRATELSKQLEME

KEIVSSTRLDLQKKSEALESIKQKLTKQEEEKKILKQDFETLSQETKIQHEELNNRIQTTVT
ELQKVKMEKEALMTELSTVKDKLSKVSDSLKNSKSEFEKENQKGKAAILDLEKTCKELK
HQLQVQMENTLKEQKELKKSLEKEKEASHQLKLELNSMQEQLIQAQNTLKQNEKEEQQL
QGNINELKQSSEQKKKQIEALQGELKIAVLQKTELENKLQQQLTQAAQELAAEKEKISVLQ
NNYEKSQETFKQLQSDFYGRESELLATRQDLKSVEEKLSLAQEDLISNRNQIGNQNKLIQE
LKTAKATLEQDSAKKEQQLQERCKALQDIQKEKSLKEKELVNEKSKLAEIEEIKCRQEKEI
TKLNEELKSHKLESIKEITNLKDAKQLLIQQKLELQGKADSLKAAVEQEKRNQQILKDQV
KKEEEELKKEFIEKEAKLHSEIKEKEVGMKKHEENEAKLTMQITALNENLGTVKKEWQSS
QRRVSELEKQTDDLRGEIAVLEATVQNNQDERRALLERCLKGEGEIEKLQTKVLELQRKL
DNTTAAVQELGRENQSLQIKHTQALNRKWAEDNEVQNCMACGKGFSVTVRRHHCRQCG
NIFCAECSAKNALTPSSKKPVRVCDACFNDLQG

>Q9NRD9 multipass membrane protein
MGFCLALAWTLLVGAWTPLGAQNPISWEVQRFDGWYNNLMEHRWGSKGSRLQRLVPAS
YADGVYQPLGEPHLPNPRDLSNTISRGPAGLASLRNRTVLGVFFGYHVLSDLVSVETPGCP
AEFLNIRIPPGDPMFDPDQRGDVVLPFQRSRWDPETGRSPSNPRDPANQVTGWLDGSAIY
GSSHSWSDALRSFSRGQLASGPDPAFPRDSQNPLLMWAAPDPATGQNGPRGLYAFGAERG
NREPFLQALGLLWFRYHNLWAQRLARQHPDWEDEELFQHARKRVIATYQNIAVYEWLPS
FLQKTLPEYTGYRPFLDPSISSEFVAASEQFLSTMVPPGVYMRNASCHFQGVINRNSSVSR
ALRVCNSYWSREHPSLQSAEDVDALLLGMASQIAEREDHVLVEDVRDFWPGPLKFSRTD
HLASCLQRGRDLGLPSYTKARAALGLSPITRWQDINPALSRSNDTVLEATAALYNQDLSW
LELLPGGLLESHRDPGPLFSTIVLEQFVRLRDGDRYWFENTRNGLFSKKEIEEIRNTTLQDV
LVAVINIDPSALQPNVFVWHKGDPCPQPRQLSTEGLPACAPSVVRDYFEGSGFGFGVTIGT
LCCFPLVSLLSAWIVARLRMRNFKRLQGQDRQSIVSEKLVGGMEALEWQGHKEPCRPVLV
YLQPGQIRVVDGRLTVLRTIQLQPPQKVNFVLSSNRGRRTLLLKIPKEYDLVLLFNLEEERQ
ALVENLRGALKESGLSIQEWELREQELMRAAVTREQRRHLLETFFRHLFSQVLDINQADA
GTLPLDSSQKVREALTCELSRAEFAESLGLKPQDMFVESMFSLADKDGNGYLSFREFLDIL
VVFMKGSPEEKSRLMFRMYDFDGNGLISKDEFIRMLRSFIEISNNCLSKAQLAEVVESMFR
ESGFQDKEELTWEDFHFMLRDHNSELRFTQLCVKGVEVPEVIKDLCRRASYISQDMICPSP
RVSARCSRSDIETELTPQRLQCPMDTDPPQEIRRRFGKKVTSFQPLLFTEAHREKFQRSCLH
QTVQQFKRFIENYRRHIGCVAVFYAIAGGLFLERAYYYAFAAHHTGITDTTRVGIILSRGTA
ASISFMFSYILLTMCRNLITFLRETFLNRYVPFDAAVDFHRLIASTAIVLTVLHSVGHVVNV
YLFSISPLSVLSCLFPGLFHDDGSELPQKYYWWFFQTVPGLTGVVLLLILAIMYVFASHHF
RRRSFRGFWLTHHLYILLYVLLIIHGSFALIQLPRFHIFFLVPAIIYGGDKLVSLSRKKVEISVV
KAELLPSGVTHLRFQRPQGFEYKSGQWVRIACLALGTTEYHPFTLTSAPHEDTLSLHIRAA
GPWTTRLREIYSAPTGDRCARYPKLYLDGPFGEGHQEWHKFEVSVLVGGGIGVTPFASILK
DLVFKSSVSCQVFCKKIYFIWVTRTQRQFEWLADIIREVEENDHQDLVSVHIYITQLAEKFD
LRTTMLYICERHFQKVLNRSLFTGLRSITHFGRPPFEPFFNSLQEVHPQVRKIGVFSCGPPG
MTKNVEKACQLINRQDRTHFSHHYENF

>P33032 multipass membrane protein
MNSSFHLHFLDLNLNATEGNLSGPNVKNKSSPCEDMGIAVEVFLTLGVISLLENILVIGAIV
KNKNLHSPMYFFVCSLAVADMLVSMSSAWETITIYLLNNKHLVIADAFVRHIDNVFDSMIC
ISVVASMCSLLAIAVDRYVTIFYALRYHHIMTARRSGAIIAGIWAFCTGCGIVFILYSESTYVI

LCLISMFFAMLFLLVSLYIHMFLLARTHVKRIAALPGASSARQRTSMQGAVTVTMLLGVFT
VCWAPFFLHLTLMLSCPQNLYCSRFMSHFNMYLILIMCNSVMDPLIYAFRSQEMRKTFKEI
ICCRGFRIACSFPRRD

>Q11130 single-pass type II membrane protein
MNNAGHGPTRRLRGLGVLAGVALLAALWLLWLLGSAPRGTPAPQPTITILVWHWPFTDQ
PPELPSDTCTRYGIARCHLSANRSLLASADAVVFHHRELQTRRSHLPLAQRPRGQPWVWA
SMESPSHTHGLSHLRGIFNWVLSYRRDSDIFVPYGRLEPHWGPSPPLPAKSRVAAWVVSNF
QERQLRARLYRQLAPHLRVDVFGRANGRPLCASCLVPTVAQYRFYLSFENSQHRDYITEK
FWRNALVAGTVPVVLGPPRATYEAFVPADAFVHVDDFGSARELAAFLTGMNESRYQRFFA
WRDRLRVRLFTDWRERFCAICDRYPHLPRSQVYEDLEGWFQA

>Q9H252 multipass membrane protein
MPVRRGHVAPQNTYLDTIIRKFEGQSRKFLIANAQMENCAIIYCNDGFCELFGYSRVEVM
QQPCTCDFLTGPNTPSSAVSRLAQALLGAEECKVDILYYRKDASSFRCLVDVVPVKNEDG
AVIMFILNFEDLAQLLAKCSSRSLSQRLLSQSFLGSEGSHGRPGGPGPGTGRGKYRTISQIP
QFTLNFVEFNLEKHRSSSTTEIEIIAPHKVVERTQNVTEKVTQVLSLGADVLPEYKLQAPRI
HRWTILHYSPFKAVWDWLILLLVIYTAVFTPYSAAFLLSDQDESRRGACSYTCSPLTVVDLI
VDIMFVVDIVINFRTTYVNTNDEVVSHPRRIAVHYFKGWFLIDMVAAIPFDLLIFRTGSDET
TTLIGLLKTARLLRLVRVARKLDRYSEYGAAVLFLLMCTFALIAHWLACIWYAIGNVERPY
LEHKIGWLDSLGVQLGKRYNGSDPASGPSVQDKYVTALYFTFSSLTSVGFGNVSPNTNSE
KVFSICVMLIGSLMYASIFGNVSAIIQRLYSGTARYHTQMLRVKEFIRFHQIPNPLRQRLEEY
FQHAWSYTNGIDMNAVLKGFPECLQADICLHLHRALLQHCPAFSGAGKGCLRALAVKFK
TTHAPPGDTLVHLGDVLSTLYFISRGSIEILRDDVVVAILGKNDIFGEPVSLHAQPGKSSAD
VRALTYCDLHKIQRADLLEVLDMYPAFAESFWSKLEVTFNLRDAAGGLHSSPRQAPGSQD
HQGFFLSDNQSGSPHELGPQFPSKGYSLLGPGSQNSMGAGPCAPGHPDAAPPLSISDASGL
WPELLQEMPPRHSPQSPQEDPDCWPLKLGSRLEQLQAQMNRLESRVSSDLSRILQLLQKP
MPQGHASYILEAPASNDLALVPIASETTSPGPRLPQGFLPPAQTPSYGDLDDCSPKHRNSSP
RMPHLAVATDKTLAPSSEQEQPEGLWPPLASPLHPLEVQGLICGPCFSSLPEHLGSVPKQLD
FQRHGSDPGFAGSWGH

>Q96PS8 multipass membrane protein
MVFTQAPAEIMGHLRIRSLLARQCLAEFLGVFVLMLLTQGAVAQAVTSGETKGNFFTMFL
AGSLAVTIAIYVGGNVSGAHLNPAFSLAMCIVGRLPWVKLPIYILVQLLSAFCASGATYVL
YHDALQNYTGGNLTVTGPKETASIFATYPAPYLSLNNGFLDQVLGTGMLIVGLLAILDRRN
KGVPAGLEPVVVGMLILALGLSMGANCGIPLNPARDLGPRLFTYVAGWGPEVFSAGNGW
WWVPVVAPLVGATVGTATYQLLVALHHPEGPEPAQDLVSAQHKASELETPASAQMLECKL

>Q11201 single-pass type II membrane protein
MVTLRKRTLKVLTFLVLFIFLTSFFLNYSHTMVATTWFPKQMVLELSENLKRLIKHRPCTCT
HCIGQRKLSAWFDERFNQTMQPLLTAQNALLEDDTYRWWLRLQREKKPNNLNDTIKELF
RVVPGNVDPMLEKRSVGCRRCAVVGNSGNLRESSYGPEIDSHDFVLRMNKAPTAGFEAD
VGTKTTHHLVYPESFRELGDNVSMILVPFKTIDLEWVVSAITTGTISHTYIPVPAKIRVKQD
KILIYHPAFIKYVFDNWLQGHGRYPSTGILSVIFSMHVCDEVDLYGFGADSKGNWHHYWE

NNPSAGAFRKTGVHDADFESNVTATLASINKIRIFKGR

>P13591 GPI-anchor, lipid-anchor, single-pass type I membrane protein
MLQTKDLIWTLFFLGTAVSLQVDIVPSQGEISVGESKFFLCQVAGDAKDKDISWFSPNGEK
LTPNQQRISVVWNDDSSSTLTIYNANIDDAGIYKCVVTGEDGSESEATVNVKIFQKLMFKN
APTPQEFREGEDAVIVCDVVSSLPPTIIWKHKGRDVILKKDVRFIVLSNNYLQIRGIKKTDE
GTYRCEGRILARGEINFKDIQVIVNVPPTIQARQNIVNATANLGQSVTLVCDAEGFPEPTMS
WTKDGEQIEQEEDDEKYIFSDDSSQLTIKKVDKNDEAEYICIAENKAGEQDATIHLKVFAK
PKITYVENQTAMELEEQVTLTCEASGDPIPSITWRTSTRNISSEEKASWTRPEKQETLDGHM
VVRSHARVSSLTLKSIQYTDAGEYICTASNTIGQDSQSMYLEVQYAPKLQGPVAVYTWEG
NQVNITCEVFAYPSATISWFRDGQLLPSSNYSNIKIYNTPSASYLEVTPDSENDFGNYNCTA
VNRIGQESLEFILVQADTPSSPSIDQVEPYSSTAQVQFDEPEATGGVPILKYKAEWRAVGEE
VWHSKWYDAKEASMEGIVTIVGLKPETTYAVRLAALNGKGLGEISAASEFKTQPVQGEPS
APKLEGQMGEDGNSIKVNLIKQDDGGSPIRHYLVRYRALSSEWKPEIRLPSGSDHVMLKS
LDWNAEYEVYVVAENQQGKSKAAHFVFRTSAQPTAIPANGSPTSGLSTGAIVGILIVIFVLL
LVVVDITCYFLNKCGLFMCIAVNLCGKAGPGAKGKDMEEGKAAFSKDESKEPIVEVRTEE
ERTPNHDGGKHTEPNETTPLTEPEKGPVEAKPECQETETKPAPAEVKTVPNDATQTKENES
KA

>Q15078 lipid-anchor
MGTVLSLSPSYRKATLFEDGAATVGHYTAVQNSKNAKDKNLKRHSIISVLPWKRIVAVSA
KKKNSKKVQPNSSYQNNITHLNNENLKKSLSCANLSTFAQPPPAQPPAPPASQLSGSQTGG
SSSVKKAPHPAVTSAGTPKRVIVQASTSELLRCLGEFLCRRCYRLKHLSPTDPVLWLRSVD
RSLLLQGWQDQGFITPANVVFLYMLCRDVISSEVGSDHELQAVLLTCLYLSYSYMGNEISY
PLKPFLVESCKEAFWDRCLSVINLMSSKMLQINADPHYFTQVFSDLKNESGQEDKKRLLL
GLDR

>Q9UP95 multipass membrane protein
MPHFTVVPVDGPRRGDYDNLEGLSWVDYGERAELDDSDGHGNHRESSPFLSPLEASRGI
DYYDRNLALFEEELDIRPKVSSLLGKLVSYTNLTQGAKEHEEAESGEGTRRRAAEAPSMG
TLMGVYLPCLQNIFGVILFLRLTWMVGTAGVLQALLIVLICCCCTLLTAISMSAIATNGVVP
AGGSYFMISRSLGPEFGGAVGLCFYLGTTFAAAMYILGAIEILLTYIAPPAAIFYPSGAHDTS
NATLNNMRVYGTIFLTFMTLVVFVGVKYVNKFASLFLACVIISILSIYAGGIKSIFDPPVFPV
CMLGNRTLSRDQFDICAKTAVVDNETVATQLWSFFCHSPNLTTDSCDPYFMLNNVTEIPGI
PGAAAGVLQENLWSAYLEKGDIVEKHGLPSADAPSLKESLPLYVVADIATSFTVLVGIFFPS
VTGIMAGSNRSGDLRDAQKSIPVGTILAIITTSLVYFSSVVLFGACIEGVVLRDKYGDGVSR
NLVVGTLAWPSPWVIVIGSFFSTCGAGLQSLTGAPRLLQAIAKDNIIPFLRVFGHGKVNGEP
TWALLLTALIAELGILIASLDMVAPILSMFFLMCYLFVNLACAVQTLLRTPNWRPRFKYYH
WALSFLGMSLCLALMFVSSWYYALVAMLIAGMIYKYIEYQGAEKEWGDGIRGLSLSAAR
YALLRLEEGPPHTKNWRPQLLVLLKLDEDLHVKYPRLLTFASQLKAGKGLTIVGSVIQGSF
LESYGEAQAAEQTIKNMMEIEKVKGFCQVVVASKVREGLAHLIQSCGLGGMRHNSVVLG
WPYGWRQSEDPRAWKTFIDTVRCTTAAHLALLVPKNIAFYPSNHERYLEGHIDVWWIVH
DGGMLMLLPFLLRQHKVWRKCRMRIFTVAQMDDNSIQMKKDLAVFLYHLRLEAEVEVV
EMHNSDISAYTYERTLMMEQRSQMLRQMRLTKTEREREAQLVKDRHSALRLESLYSDEE

DESAVGADKIQMTWTRDKYMTETWDPSHAPDNFRELVHIKPDQSNVRRMHTAVKLNEVI
VTRSHDARLVLLNMPGPPRNSEGDENYMEFLEVLTEGLERVLLVRGGGREVITIYS

>P56817 single-pass type I membrane protein
MAQALPWLLLWMGAGVLPAHGTQHGIRLPLRSGLGGAPLGLRLPRETDEEPEEPGRRGS
FVEMVDNLRGKSGQGYYVEMTVGSPPQTLNILVDTGSSNFAVGAAPHPFLHRYYQRQLSS
TYRDLRKGVYVPYTQGKWEGELGTDLVSIPHGPNVTVRANIAAITESDKFFINGSNWEGIL
GLAYAEIARPDDSLEPFFDSLVKQTHVPNLFSLQLCGAGFPLNQSEVLASVGGSMIIGGIDH
SLYTGSLWYTPIRREWYYEVIIVRVEINGQDLKMDCKEYNYDKSIVDSGTTNLRLPKKVFE
AAVKSIKAASSTEKFPDGFWLGEQLVCWQAGTTPWNIFPVISLYLMGEVTNQSFRITILPQ
QYLRPVEDVATSQDDCYKFAISQSSTGTVMGAVIMEGFYVVFDRARKRIGFAVSACHVHD
EFRTAAVEGPFVTLDMEDCGYNIPQTDESTLMTIAYVMAAICALFMLPLCLMVCQWCCLR
CLRQQHDDFADDISLLK

>Q8NEZ2 peripheral membrane protein
MSWLFPLTKSASSSAAGSPGGLTSLQQQKQRLIESLRNSHSSIAEIQKDVEYRLPFTINNLTI
NINILLPPQFPQEKPVISVYPPIRHHLMDKQGVYVTSPLVNNFTMHSDLGKIIQSLLDEFWK
NPPVLAPTSTAFPYLYSNPSGMSPYASQGFPFLPPYPPQEANRSITSLSVADTVSSSTTSHTT
AKPAAPSFGVLSNLPLPIPTVDASIPTSQNGFGYKMPDVPDAFPELSELSVSQLTDMNEQEE
VLLEQFLTLPQLKQIITDKDDLVKSIEELARKNLLLEPSLEAKRQTVLDKYELLTQMKSTFE
KKMQRQHELSESCSASALQARLKVAAHEAEEESDNIAEDFLEGKMEIDDFLSSFMEKRTIC
HCRRAKEEKLQQAIAMHSQFHAPL

>O14756 peripheral membrane protein
MWLYLAAFVGLYYLLHWYRERQVVSHLQDKYVFITGCDSGFGNLLARQLDARGLRVLA
ACLTEKGAEQLRGQTSDRLETVTLDVTKMESIAAATQWVKEHVGDRGLWGLVNNAGILT
PITLCEWLNTEDSMNMLKVNLIGVIQVTLSMLPLVRRARGRIVNVSSILGRVAFFVGGYCV
SKYGVEAFSDILRREIQHFGVKISIVEPGYFRTGMTNMTQSLERMKQSWKEAPKHIKETYG
QQYFDALYNIMKEGLLNCSTNLNLVTDCMEHALTSVHPRTRYSAGWDAKFFFIPLSYLPTS
LADYILTRSWPKPAQAV

>Q8IVI9 peripheral membrane protein
MRDPLTDCPYNKVYKNLKEFSQNGENFCKQVTSVLQQRANLEISYAKGLQKLASKLSKA
LQNTRKSCVSSAWAWASEGMKSTADLHQKLGKAIELEAIKPTYQVLNVQEKKRKSLDNE
VEKTANLVISNWNQQIKAKKKLMVSTKKHEALFQLVESSKQSMTEKEKRKLLNKLTKSTE
KLEKEDENYYQKNMAGYSTRLKWENTLENCYQSILELEKERIQLLCNNLNQYSQHISLFG
QTLTTCHTQIHCAISKIDIEKDIQAVMEETAILSTENKSEFLLTDYFEEDPNSAMDKERRKSL
LKPKLLRLQRDIEKASKDKEGLERMLKTYSSTSSFSDAKSQKDTAALMDENNLKLDLLEA
NSYKLSSMLAELEQRPQPSHPCSNSIFRWREKEHTHSYVKISRPFLMKRLENIVSKASSGG
QSNPGSSTPAPGAAQLSSRLCKALYSFQARQDDELNLEKGDIVIIHEKKEGGWWFGSLNG
KKGHFPAAYVEELPSNAGNTATKA

>Q8NGC9 multipass membrane protein
MSFFFVDLRPMNRSATHIVTEFILLGFPGCWKIQIFLFSLFLVIYVLTLLGNGAIIYAVRCNPL

LHTPMYFLLGNFAFLEIWYVSSTIPNMLVNILSKTKAISFSGCFLQFYFFFSLGTTECLFLAV
MAYDRYLAICHPLQYPAIMTVRFCGKLVSFCWLIGFLGYPIPIFYISQLPFCGPNIIDHFLCD
MDPLMALSCAPAPITECIFYTQSSLVLFFTSMYILRSYILLLTAVFQVPSAAGRRKAFSTCGS
HLVVVSLFYGTVMVMYVSPTYGIPTLLQKILTLVYSVTTPLFNPLIYTLRNKDMKLALRNV
LFGMRIRQNS

>Q13555 peripheral membrane protein
MATTATCTRFTDDYQLFEELGKGAFSVVRRCVKKTSTQEYAAKIINTKKLSARDHQKLER
EARICRLLKHPNIVRLHDSISEEGFHYLVFDLVTGGELFEDIVAREYYSEADASHCIHQILES
VNHIHQHDIVHRDLKPENLLLASKCKGAAVKLADFGLAIEVQGEQQAWFGFAGTPGYLSP
EVLRKDPYGKPVDIWACGVILYILLVGYPPFWDEDQHKLYQQIKAGAYDFPSPEWDTVTP
EAKNLINQMLTINPAKRITADQALKHPWVCQRSTVASMMHRQETVECLRKFNARRKLKG
AILTTMLVSRNFSAAKSLLNKKSDGGVKKRKSSSSVHLMPQSNNKNSLVSPAQEPAPLQTA
MEPQTTVVHNATDGIKGSTESCNTTTEDEDLKARSPEGRSSRDRTAPSAGMQPQPSLCSSA
MRKQEIIKITEQLIEAINNGDFEAYTKICDPGLTSFEPEALGNLVEGMDFHKFYFENLLSKN
SKPIHTTILNPHVHVIGEDAACIAYIRLTQYIDGQGRPRTSQSEETRVWHRRDGKWLNVHY
HCSGAPAAPLQ

>Q8NGC7 multipass membrane protein
MFFIIHSLVTSVFLTALGPQNRTMHFVTEFVLLGFHGQREMQSCFFSFILVLYLLTLLGNGAI
VCAVKLDRRLHTPMYILLGNFAFLEIWYISSTVPNMLVNILSEIKTISFSGCFLQFYFFFSLG
TTECFFLSVMAYDRYLAICRPLHYPSIMTGKFCIILVCVCWVGGFLCYPVPIVLISQLPFCGP
NIIDHLVCDPGPLFALACISAPSTELICYTFNSMIIFGPFLSILGSYTLVIRAVLCIPSGAGRTK
AFSTCGSHLMVVSLFYGTLMVMYVSPTSGNPAGMQKIITLVYTAMTPFLNPLIYSLRNKD
MKDALKRVLGLTVSQN

>Q53TN4 multipass membrane protein
MAMEGYWRFLALLGSALLVGFLSVIFALVWVLHYREGLGWDGSALEFNWHPVLMVTGF
VFIQGIAIIVYRLPWTWKCSKLLMKSIHAGLNAVAAILAIISVVAVFENHNVNNIANMYSLH
SWVGLIAVICYLLQLLSGFSVFLLPWAPLSLRAFLMPIHVYSGIVIFGTVIATALMGLTEKLI
FSLRDPAYSTFPPEGVFVNTLGLLILVFGALIFWIVTRPQWKRPKEPNSTILHPNGGTEQGA
RGSMPAYSGNNMDKSDSELNSEVAARKRNLALDEAGQRSTM

>Q9ULB5 single-pass type I membrane protein
MKLGKVEFCHFLQLIALFLCFSGMSQAELSRSRSKPYFQSGRSRTKRSWVWNQFFVLEEY
MGSDPLYVGKLHSDVDKGDGSIKYILSGEGASSIFIIDENTGDIHATKRLDREEQAYYTLRA
QALDRLTNKPVEPESEFVIKIQDINDNEPKFLDGPYTAGVPEMSPVGTSVVQVTATDADDP
TYGNSARVVYSILQGQPYFSVEPKTGVIKTALPNMDREAKDQYLLVIQAKDMVGQNGGL
SGTTSVTVTLTDVNDNPPRFPRRSYQYNVPESLPVASVVARIKAADADIGANAEMEYKIVD
GDGLGIFKISVDKETQEGIITIQKELDFEAKTSYTLRIEAANKDADPRFLSLGPFSDTTTVKII
VEDVDEPPVFSSPLYPMEVSEATQVGNIIGTVAAHDPDSSNSPVRYSIDRNTDLERYFNIDA
NSGVITTAKSLDRETNAIHNITVLAMESQNPSQVGRGYVAITILDINDNAPEFAMDYETTV
CENAQPGQVIQKISAVDKDEPSNGHQFYFSLTTDATNNNHNFSLKDNKDNTASILTRRNGFR
RQEQSVYYLPIFIVDSGSPSLSSTNTLTIRVCDCDADGVAQTCNAEAYVLPAGLSTGALIAIL

ACVLTLLVLILLIVTMRRRKKEPLIFDEERDIRENIVRYDDEGGGEEDTEAFDMAALRNLN
VIRDTKTRRDVTPEIQFLSRPAFKSIPDNVIFREFIWERLKEADVDPGAPPYDSLQTYAFEG
NGSVAESLSSLDSISSNSDQNYDYLSDWGPRFKRLADMYGTGQESLYS

>Q8NGC6 multipass membrane protein
MEAMKLLNQSQVSEFILLGLTSSQDVEFLLFALFSVIYVVTVLGNLLIIVTVFNTPNLNTPM
YFLLGNLSFVDMTLASFATPKVILNLLKKQKVISFAGCFTQIFLLHLLGGVEMVLLVSMAF
DRYVAICKPLHYMTIMNKKVCVLLVVTSWLLGLLHSGFQIPFAVNLPFCGPNVVDSIFCDL
PLVTKLACIDIYFVQVVIVANSGIISLSCFIILLISYSLILITIKNHSPTGQSKARSTLTAHITVVI
LFFGPCIFIYIWPFGNHSVDKFLAVFYTIITPILNPIIYTLRNKEMKISMKKLWRAFVNSREDT

>Q8NH43 multipass membrane protein
MDLKNGSLVTEFILLGFFGRWELQIFFFVTFSLIYGATVMGNILIMVTVTCRSTLHSPLYFLL
GNLSFLDMCLSTATTPKMIIDLLTDHKTISVWGCVTQMFFMHFFGGAEMTLLIIMAFDRYV
AICKPLHYRTIMSHKLLKGFAILSWIIGFLHSISQIVLTMNLPFCGHNVINNIFCDLPLVIKLA
CIETYTLELFVIADSGLLSFTCFILLLVSYIVILVSVPKKSSHGLSKALSTLSAHIIVVTLFFGP
CIFIYVWPFSSLASNKTLAVFYTVITPLLNPSIYTLRNKKMQEAIRKLRFQYVSSAQNF

>Q9BZJ7 multipass membrane protein
MANSTGLNASEVAGSLGLILAAVVEVGALLGNGALLVVVLRTPGLRDALYLAHLCVVDL
LAAASIMPLGLLAAPPPGLGRVRLGPAPCRAARFLSAALLPACTLGVAALGLARYRLIVHP
LRPGSRPPPVLVLTAVWAAAGLLGALSLLGTPPAPPPAPARCSVLAGGLGPFRPLWALLAFA
LPALLLLGAYGGIFVVARRAALRPPRPARGSRLHSDSLDSRLSILPPLRPRLPGGKAALAPA
LAVGQFAACWLPYGCACLAPAARAAEAEAAVTWVAYSAFAAHPFLYGLLQRPVRLALGR
LSRRALPGPVRACTPQAWHPRALLQCLQRPPEGPAVGPSEAPEQTPELAGGRSPAYQGPPE
SSLS

>P80723 lipid-anchor
MGGKLSKKKKGYNVNDEKAKEKDKKAEGAATEEEGTPKESEPQAAAEPAEAKEGKEKP
DQDAEGKAEEKEGEKDAAAAKEEAPKAEPEKTEGAAEAKAEPPKAPEQEQAAPGPAAG
GEAPKAAEAAAAPAESAAPAAGEEPSKEEGEPKKTEAPAAPAAQETKSDGAPASDSKPGS
SEAAPSSKETPAATEAPSSTPKAQGPAASAEEPKPVEAPAANSDQTVTVKE

>P40197 single-pass type I membrane protein
MLRGTLLCAVLGLLRAQPFPCPPACKCVFRDAAQCSGGDVARISALGLPTNLTHILLFGMG
RGVLQSQSFSGMTVLQRLMISDSHISAVAPGTFSDLIKLKTLRLSRNKITHLPGALLDKMV
LLEQLFLDHNALRGIDQNMFQKLVNLQELALNQNQLDFLPASLFTNLENLKLLDLSGNNL
THLPKGLLGAQAKLERLLLHSNRLVSLDSGLLNSLGALTELQFHRNHIRSIAPGAFDRLPN
LSSLTLSRNHLAFLPSALFLHSHNLTLLTLFENPLAELPGVLFGEMGGLQELWLNRTQLRTL
PAAAFRNLSRLRYLGVTLSPRLSALPQGAFQGLGELQVLALHSNGLTALPDGLLRGLGKL
RQVSLRRNRLRALPRALFRNLSSLESVQLDHNQLETLPGDVFGALPRLTEVLLGHNSWRC
DCGLGPFLGWLRQHLGLVGGEEPPRCAGPGAHAGLPLWALPGGDAECPGPRGPPPRPAA
DSSSEAPVHPALAPNSSEPWVWAQPVTTGKGQDHSPFWGFYFLLLAVQAMITVIIVFAMIK
IGQLFRKLIRERALG

>Q8NH42 multipass membrane protein
MERANHSVVSEFILLGLSKSQNLQILFFLGFSVVFVGIVLGNLLILVTVTFDSLLHTPMYFL
LSNLSCIDMILASFATPKMIVDFLRERKTISWWGCYSQMFFMHLLGGSEMMLLVAMAIDR
YVAICKPLHYMTIMSPRVLTGLLLSSYAVGFVHSSSQMAFMLTLPFCGPNVIDSFFCDLPLVI
KLACKDTYILQLLVIADSGLLSLVCFLLLLVSYGVIIFSVRYRAASRSSKAFSTLSAHITVVT
LFFAPCVFIYVWPFSRYSVDKILSVFYTIFTPLLNPIIYTLRNQEVKAAIKKRLCI

>Q9NPA1 multipass membrane protein
MDFSPSSELGFHFVAFILLTRHRTAFPASGKKRETDYSDGDPLDVHKRLPSSAGEDRAVML
GFAMMGFSVLMFFLLGTTILKPFMLSIQREESTCTAIHTDIMDDWLDCAFTCGVHCHGQG
KYPCLQVFVNLSHPGQKALLHYNEEAVQINPKCFYTPKCHQDRNDLLNSALDIKEFFDHK
NGTPFSCFYSPASQSEDVILIKKYDQMAIFHCLFWPSLTLLGGALIVGMVRLTQHLSLLCEK
YSTVVRDEVGGKVPYIEQHQFKLCIMRRSKGRAEKS

>Q8NGD3 multipass membrane protein
MDKSNSSVVSEFVLLGLCSSQKLQLFYFCFFSVLYTVIVLGNLLIILTVTSDTSLHSPMYFLL
GNLSFVDICQASFATPKMIADFLSAHETISFSGCIAQIFFIHLFTGGEMVLLVSMAYDRYVAI
CKPLYYVVIMSRRTCTVLVMISWAVSLVHTLSQLSFTVNLPFCGPNVVDSFFCDLPRVTKL
ACLDSYIIEILIVVNSGILSLSTFSLLVSSYIIILVTVWLKSSAAMAKAFSTLASHIAVVILFFG
PCIFIYVWPFTISPLDKFLAIFYTVFTPVLNPIIYTLRNRDMKAAVRKIVNHYLRPRRISEMSL
VVRTSFH

>O95047 multipass membrane protein
MGDNITSIREFLLLGFPVGPRIQMLLFGLFSLFYVFTLLGNGTILGLISLDSRLHAPMYFFLS
HLAVVDIAYACNTVPRMLVNLLHPAKPISFAGRMMQTFLFSTFAVTECLLLVVMSYDLYVA
ICHPLRYLAIMTWRVCITLAVTSWTTGVLLSLIHLVLLLPLPFCRPQKIYHFFCEILAVLKLA
CADTHINENMVLAGAISGLVGPLSTIVVSYMCILCAILQIQSREVQRKAFRTCFSHLCVIGL
VYGTAIIMYVGPRYGNPKEQKKYLLLFHSLFNPMLNPLICSLRNSEVKNTLKRVLGVERA
L

>Q9HC21 multipass membrane protein
MVGYDPKPDGRNNTKFQVAVAGSVSGLVTRALISPFDVIKIRFQLQHERLSRSDPSAKYHG
ILQASRQILQEEGPTAFWKGHVPAQILSIGYGAVQFLSFEMLTELVHRGSVYDAREFSVHFV
CGGLAACMATLTVHPVDVLRTRFAAQGEPKVYNTLRHAVGTMYRSEGPQVFYKGLAPTL
IAIFPYAGLQFSCYSSLKHLYKWAIPAEGKKNENLQNLLCGSGAGVISKTLTYPLDLFKKRL
QVGGFEHARAAFGQVRRYKGLMDCAKQVLQKEGALGFFKGLSPSLLKAALSTGFMFFS
YEFFCNVFHCMNRTASQR

>Q9UK17 multipass membrane protein
MAAGVAAWLPFARAAAIGWMPVANCPMPLAPADKNKRQDELIVLNVSGRRFQTWRTTL
ERYPDTLLGSTEKEFFFNEDTKEYFFDRDPEVFRCVLNFYRTGKLHYPRYECISAYDDELA
FYGILPEIIGDCCYEEYKDRKRENAERLMDDNDSENNQESMPSLSFRQTMWRAFENPHTS
TLALVFYYVTGFFIAVSVITNVVETVPCGTVPGSKELPCGERYSVAFFCLDTACVMIFTVEY

LLRLFAAPSRYRFIRSVMSIIDVVAIMPYYIGLVMTNNEDVSGAFVTLRVFRVFRIFKFSRHS
QGLRILGYTLKSCASELGFLLFSLTMAIIIFATVMFYAEKGSSASKFTSIPASFWYTIVTMTTL
GYGDMVPKTIAGKIFGSICSLSGVLVIALPVPVIVSNFSRIYHQNQRADKRRAQKKARLARI
RVAKTGSSNAYLHSKRNGLLNEALELTGTPEEEHMGKTTSLIESQHHHLLHCLEKTTGLSY
LVDDPLLSVRTSTIKNHEFIDEQMFEQNCMESSMQNYPSTRSPSLSSHPGLTTTCCSRRSKK
TTHLPNSNLPATRLRSMQELSTIHIQGSEQPSLTTSRSSLNLKADDGLRPNCKTSQITTAIISIP
TPPALTPEGESRPPPASPGPNTNIPSIASNVVKVSAL

>P25025 multipass membrane protein
MEDFNMESDSFEDFWKGEDLSNYSYSSTLPPFLLDAAPCEPESLEINKYFVVIIYALVFLLS
LLGNSLVMLVILYSRVGRSVTDVYLLNLALADLLFALTLPIWAASKVNGWIFGTFLCKVVS
LLKEVNFYSGILLLACISVDRYLAIVHATRTLTQKRYLVKFICLSIWGLSLLLALPVLLFRRT
VYSSNVSPACYEDMGNNTANWRMLLRILPQSFGFIVPLLIMLFCYGFTLRTLFKAHMGQK
HRAMRVIFAVVLIFLLCWLPYNLVLLADTLMRTQVIQETCERRNHIDRALDATEILGILHSC
LNPLIYAFIGQKFRHGLLKILAIHGLISKDSLPKDSRPSFVGSSSGHTSTTL

>Q8NGD0 multipass membrane protein
METANYTKVTEFVLTGLSQTREVQLVLFVIFLSFYLFILPGNILIICTIRLDPHLTSPMYFLLA
NLALLDIWYSSITAPKMLIDFFVERKIISFGGCIAQLFFLHFVGASEMFLLTVMAYDRYAAIC
RPLHYATIMNRRLCCILVALSWMGGFIHSIIQVALIVRLPFCGPNELDSYFCDITQVVRIACA
NTFPEELVMICSSGLISVVCFIALLMSYAFLLALLKKHSGSGENTNRAMSTCYSHITIVVLM
FGPSIYIYARPFDSFSLDKVVSVFHTVIFPLLNPIIYTLRNKEVKAAMRKVVTKYILCEEK

>Q1HG44 multipass membrane protein
MTLWNGVLPFYPQPRHAAGFSVPLLIVILVFLALAASFLLILPGIRGHSRWFWLVRVLLSLF
IGAEIVAVHFSAEWFVGTVNTNTSYKAFSAARVTARVRLLVGLEGINITLTGTPVHQLNETI
DYNEQFTWRLKENYAAEYANALEKGLPDPVLYLAEKFTPSSPCGLYHQYHLAGHYASATL
WVAFCFWLLSNVLLSTPAPLYGGLALLTTGAFALFGVFALASISSVPLCPLRLGSSALTTQY
GAAFWVTLATGVLCLFLGGAVVSLQYVRPSALRTLLDQSAKDCSQERGGSPLILGDPLHK
QAALPDLKCITTNL

>Q6RW13 multipass membrane protein
MELPAVNLKVILLGHWLLTTWGCIVFSGSYAWANFTILALGVWAVAQRDSIDAISMFLGGL
LATIFLDIVHISIFYPRVSLTDTGRFGVGMAILSLLLKPLSCCFVYHMYRERGGELLVHTGFL
GSSQDRSAYQTIDSAEAPADPFAVPEGRSQDARGY

>P46093 multipass membrane protein
MGNHTWEGCHVDSRVDHLFPPSLYIFVIGVGLPTNCLALWAAYRQVQQRNELGVYLMNL
SIADLLYICTLPLWVDYFLHHDNWIHGPGSCKLFGFIFYTNIYISIAFLCCISVDRYLAVAHP
LRFARLRRVKTAVAVSSVVWATELGANSAPLFHDELFRDRYNHTFCFEKFPMEGWVAWM
NLYRVFVGFLFPWALMLLSYRGILRAVRGSVSTERQEKAKIKRLALSLIAIVLVCFAPYHVL
LLSRSAIYLGRPWDCGFEERVFSAYHSSLAFTSLNCVADPILYCLVNEGARSDVAKALHNL
LRFLASDKPQEMANASLTLETPLTSKRNSTAKAMTGSWAATPPSQGDQVQLKMLPPAQ

>Q63HR2 peripheral membrane protein
MKSSGPVERLLRALGRRDSSRAASRPRKAEPHSFREKVFRKKPPVCAVCKVTIDGTGVSC
RVCKVATHRKCEAKVTSACQALPPVELRRNTAPVRRIEHLGSTKSLNHSKQRSTLPRSFSL
DPLMERRWDLDLTYVTERILAAAFPARPDEQRHRGHLRELAHVLQSKHRDKYLLFNLSE
KRHDLTRLNPKVQDFGWPELHAPPLDKLCSICKAMETWLSADPQHVVVLYCKGNKGKL
GVIVSAYMHYSKISAGADQALATLTMRKFCEDKVATELQPSQRRYISYFSGLLSGSIRMNS
SPLFLHYVLIPMLPAFEPGTGFQPFLKIYQSMQLVYTSGVYHIAGPGPQQLCISLEPALLLK
GDVMVTCYHKGGRGTDRTLVFRVQFHTCTIHGPQLTFPKDQLDEAWTDERFPFQASVEFV
FSSSPEKIKGSTPRNDPSVSVDYNTTEPAVRWDSYENFNQHHEDSVDGSLTHTRGPLDGSP
YAQVQRPPRQTPPAPSPEPPPPPMLSVSSDSGHSSTLTTEPAAESPGRPPPTAAERQELDRLL
GGCGVASGGRGAGRETAILDDEEQPTVGGGPHLGVYPGHRPGLSRHCSCRQGYREPCGV
PNGGYYRPEGTLERRRLAYGGYEGSPQGYAEASMEKRRLCRSLSEGLYPYPPEMGKPATG
DFGYRAPGYREVVILEDPGLPALYPCPACEEKLALPTAALYGLRLEREAGEGWASEAGKPL
LHPVRPGHPLPLLLPACGHHHAPMPDYSCLKPPKAGEEGHEGCSYTMCPEGRYGHPGYPA
LVTYSYGGAVPSYCPAYGRVPHSCGSPGEGRGYPSPGAHSPRAGSISPGSPPYPQSRKLSYE
IPTEEGGDRYPLPGHLASAGPLASAESLEPVSWREGPSGHSTLPRSPRDAPCSASSELSGPS
TPLHTSSPVQGKESTRRQDTRSPTSAPTQRLSPGEALPPVSQAGTGKAPELPSGSGPEPLAP
SPVSPTFPPSSPSDWPQERSPGGHSDGASPRSPVPTTLPGLRHAPWQGPRGPPDSPDGSPLT
PVPSQMPWLVASPEPPQSSPTPAFPLAASYDTNGLSQPPLPEKRHLPGPGQQPGPWGPEQA
SSPARGISHHVTFAPLLSDNVPQTPEPPTQESQSNVKFVQDTSKFWYKPHLSRDQAIALLK
DKDPGAFLIRDSHSFQGAYGLALKVATPPPSAQPWKGDPVEQLVRHFLIETGPKGVKIKGC
PSEPYFGSLSALVSQHSISPISLPCCLRIPSKDPLEETPEAPVPTNMSTAADLLRQGAACSVL
YLTSVETESLTGPQAVARASSAALSCSPRPTPAVVHFKVSAQGITLTDNQRKLFFRRHYPVN
SITFSSTDPQDRRWTNPDGTTSKIFGFVAKKPGSPWENVCHLFAELDPDQPAGAIVTFITKV
LLGQRK

>Q86YS6 lipid-anchor
MAGPGPGPGDPDEQYDFLFKLVLVGDASVGKTCVVQRFKTGAFSERQGSTIGVDFTMKT
LEIQGKRVKLQIWDTAGQERFRTITQSYYRSANGAILAYDITKRSSFLSVPHWIEDVRKYA
GSNIVQLLIGNKSDLSELREVSLAEAQSLAEHYDILCAIETSAKDSSNVEEAFLRVATELIM
RHGGPLFSEKSPDHIQLNSKDIGEGWGCGC

>Q9UHX3 multipass membrane protein
MGGRVFLVFLAFCVWLTLPGAETQDSRGCARWCPQDSSCVNATACRCNPGFSSFSEIITTP
METCDDINECATLSKVSCGKFSDCWNTEGSYDCVCSPGYEPVSGAKTFKNESENTCQDV
DECQQNPRLCKSYGTCVNTLGSYTCQCLPGFKLKPEDPKLCTDVNECTSGQNPCHSSTHC
LNNVGSYQCRCRPGWQPIPGSPNGPNNTVCEDVDECSSGQHQCDSSTVCFNTVGSYSCR
CRPGWKPRHGIPNNQKDTVCEDMTFSTWTPPPGVHSQTLSRFFDKVQDLGRDYKPGLAN
NTIQSILQALDELLEAPGDLETLPRLQQHCVASHLLDGLEDVLRGLSKNLSNGLLNFSYPA
GTELSLEVQKQVDRSVTLRQNQAVMQLDWNQAQKSGDPGPSVVGLVSIPGMGKLLAEA
PLVLEPEKQMLLHETHQGLLQDGSPILLSDVISAFLSNNDTQNLSSPVTFTFSHRSVIPRQK
VLCVFWEHGQNGCGHWATTGCSTIGTRDTSTICRCTHLSSFAVLMAHYDVQEEDPVLTVI
TYMGLSVSLLCLLLAALTFLLCKAIQNTSTSLHLQLSLCLFLAHLLFLVAIDQTGHKVLCSII
AGTLHYLYLATLTWMLLEALYLFLTARNLTVVNYSSINRFMKKLMFPVGYGVPAVTVAISA

ASRPHLYGTPSRCWLQPEKGFIWGFLGPVCAIFSVNLVLFLVTLWILKNRLSSLNSEVSTLR
NTRMLAFKATAQLFILGCTWCLGILQVGPAARVMAYLFTIINSLQGVFIFLVYCLLSQQVRE
QYGKWSKGIRKLKTESEMHTLSSSAKADTSKPSTVN

>P08172 multipass membrane protein
MNNSTNSSNNSLALTSPYKTFEVVFIVLVAGSLSLVTIIGNILVMVSIKVNRHLQTVNNYFL
FSLACADLIIGVFSMNLYTLYTVIGYWPLGPVVCDLWLALDYVVSNASVMNLLIISFDRYF
CVTKPLTYPVKRTTKMAGMMIAAAWVLSFILWAPAILFWQFIVGVRTVEDGECYIQFFSN
AAVTFGTAIAAFYLPVIIMTVLYWHISRASKSRIKKDKKEPVANQDPVSPSLVQGRIVKPNN
NNMPSSDDGLEHNKIQNGKAPRDPVTENCVQGEEKESSNDSTSVSAVASNMRDDEITQDE
NTVSTSLGHSKDENSKQTCIRIGTKTPKSDSCTPTNTTVEVVGSSGQNGDEKQNIVARKIV
KMTKQPAKKKPPPSREKKVTRTILAILLAFIITWAPYNVMVLINTFCAPCIPNTVWTIGYWL
CYINSTINPACYALCNATFKKTFKHLLMCHYKNIGATR

>O15403 multipass membrane protein
MTQNKLKLCSKANVYTEVPDGGWGWAVAVSFFFVEVFTYGIIKTFGVFFNDLMDSFNESN
SRISWIISICVFVLTFSAPLATVLSNRFGHRLVVMLGGLLVSTGMVAASFSQEVSHMYVAIGI
ISGLGYCFSFLPTVTILSQYFGKRRSIVTAVASTGECFAVFAFAPAIMALKERIGWRYSLLFV
GLLQLNIVIFGALLRPIFIRGPASPKIVIQENRKEAQYMLENEKTRTSIDSIDSGVELTTSPKN
VPTHTNLELEPKADMQQVLVKTSPRPSEKKAPLLDFSILKEKSFICYALFGLFATLGFFAPSL
YIIPLGISLGIDQDRAAFLLSTMAIAEVFGRIGAGFVLNREPIRKIYIELICVILLTVSLFAFTFA
TEFWGLMSCSIFFGFMVGTIGGTHIPLLAEDDVVGIEKMSSAAGVYIFIQSIAGLAGPPLAG
LLVDQSKIYSRAFYSCAAGMALAAVCLALVRPCKMGLCQHHHSGETKVVSHRGKTLQDI
PEDFLEMDLAKNEHRVHVQMEPV

>P17152 multipass membrane protein
MAAWGRRRLGPGSSGGSARERVSLSATDCYIVHEIYNGENAQDQFEYELEQALEAQYKYI
VIEPTRIGDETARWITVGNCLHKTAVLAGTACLFTPLALPLDYSHYISLPAGVLSLACCTLY
GISWQFDPCCKYQVEYDAYKLSRLPLHTLTSSTPVVLVRKDDLHRKRLHNTIALAALVYC
VKKIYELYAV

>Q08289 peripheral membrane protein
MVQRDMSKSPPTAAAAVAQEIQMELLENVAPAGALGAAAQSYGKGARRKNRFKGSDGS
TSSDTTSNSFVRQGSADSYTSRPSDSDVSLEEDREAVRREAERQAQAQLEKAKTKPVAFAV
RTNVSYSAAHEDDVPVPGMAISFEAKDFLHVKEKFNNDWWIGRLVKEGCEIGFIPSPVKL
ENMRLQHEQRAKQGKFYSSKSGGNSSSSLGDIVPSSRKSTPPSSAIDIDATGLDAEENDIPA
NHRSPKPSANSVTSPHSKEKRMPFFKKTEHTPPYDVVPSMRPVVLVGPSLKGYEVTDMM
QKALFDFLKHRFEGRISITRVTADISLAKRSVLNNPSKHAIIERSNTRSSLAEVQSEIERIFEL
ARTLQLVVLDADTINHPAQLSKTSLAPIIVYVKISSPKVLQRLIKSRGKSQAKHLNVQMVA
ADKLAQCPPELFDVILDENQLEDACEHLADYLEAYWKATHPPSSSLPNPLLSRTLATSSLPL
SPTLASNSQGSQGDQRTDRSAPIRSASQAEEEPSVEPVKKSQHRSSSSAPHHNHRSGTSRG
LSRQETFDSETQESRDSAYVEPKEDYSHDHVDHYASHRDHNHRDETHGSSDHRHRESRH
RSRDVDREQDHNECNKQRSRHKSKDRYCEKDGEVISKKRNEAGEWNRDVYIRQ

>P80303 peripheral membrane protein
MRWRTILLQYCFLLITCLLTALEAVPIDIDKTKVQNIHPVESAKIEPPDTGLYYDEYLKQVID
VLETDKHFREKLQKADIEEIKSGRLSKELDLVSHHVRTKLDELKRQEVGRLRMLIKAKLD
SLQDIGMDHQALLKQFDHLNHLNPDKFESTDLDMLIKAATSDLEHYDKTRHEEFKKYEM
MKEHERREYLKTLNEEKRKEEESKFEEMKKKHENHPKVNHPGSKDQLKEVWEETDGLD
PNDFDPKTFFKLHDVNSDGFLDEQELEALFTKELEKVYDPKNEEDDMVEMEEERLRMRE
HVMSEVDTNKDRLVTLEEFLKATEKKEFLEPDSWETLDQQQFFTEEELKEYENIIALQENE
LKKKADELQKQKEELQRQHDQLEAQKLEYHQVIQQMEQKKLQQGIPPSGPAGELKFEPHI

>P14317 peripheral membrane protein
MWKSVVGHDVSVSVETQGDDWDTDPDFVNDISEKEQRWGAKTIEGSGRTEHINIHQLRN
KVSEEHDVLRKKEMESGPKASHGYGGRFGVERDRMDKSAVGHEYVAEVEKHSSQTDAA
KGFGGKYGVERDRADKSAVGFDYKGEVEKHTSQKDYSRGFGGRYGVEKDKWDKAALG
YDYKGETEKHESQRDYAKGFGGQYGIQKDRVDKSAVGFNEMEAPTTAYKKTTPIEAASS
GTRGLKAKFESMAEEKRKREEEEKAQQVARRQQERKAVTKRSPEAPQPVIAMEEPAVPAP
LPKKISSEAWPPVGTPPSSESEPVRTSREHPVPLLPIRQTLPEDNEEPPALPPRTLEGLQVEEE
PVYEAEPEPEPEPEPEPENDYEDVEEMDRHEQEDEPEGDYEEVLEPEDSSFSSALAGSSGC
PAGAGAGAVALGISAVAVYDYQGEGSDELSFDPDDVITDIEMVDEGWWRGRCHGHFGLFP
ANYVKLLE

>Q02643 multipass membrane protein
MDRRMWGAHVFCVLSPLPTVLGHMHPECDFITQLREDESACLQAAEEMPNTTLGCPATW
DGLLCWPTAGSGEWVTLPCPDFFSHFSSESGAVKRDCTITGWSEPFPPYPVACPVPLELLA
EEESYFSTVKIIYTVGHSISIVALFVAITILVALRRLHCPRNYVHTQLFTTFILKAGAVFLKDA
ALFHSDDTDHCSFSTVLCKVSVAASHFATMTNFSWLLAEAVYLNCLLASTSPSSRRAFWW
LVLAGWGLPVLFTGTWVSCKLAFEDIACWDLDDTSPYWWIIKGPIVLSVGVNFGLFLNIIR
ILVRKLEPAQGSLHTQSQYWRLSKSTLFLIPLFGIHYIIFNFLPDNAGLGIRLPLELGLGSFQG
FIVAILYCFLNQEVRTEISRKWHGHDPELLPAWRTRAKWTTPSRSAAKVLTSMC

>Q8IVB4 multipass membrane protein
MERQSRVMSEKDEYQFQHQGAVELLVFNFLLILTILTIWLFKNHRFRFLHETGGAMVYGLI
MGLILRYATAPTDIESGTVYDCVKLTFSPSTLLVNITDQVYEYKYKREISQHNINPHQGNAI
LEKMTFDPEIFFNVLLPPIIFHAGYSLKKRHFFQNLGSILTYAFLGTAISCIVIGLIMYGFVKA
MIHAGQLKNGDFHFTDCLFFGSLMSATDPVTVLAIFHELHVDPDLYTLLFGESVLNDAVAI
VLTYSISIYSPKENPNAFDAAAFFQSVGNFLGIFAGSFAMGSAYAIITALLTKFTKLCEFPML
ETGLFFLLSWSAFLSAEAAGLTGIVAVLFCGVTQAHYTYNNLSSDSKIRTKQLFEFMNFLA
ENVIFCYMGLALFTFQNHIFNALFILGAFLAIFVARACNIYPLSFLLNLGRKQKIPWNFQHM
MMFSGLRGAIAFALAIRNTESQPKQMMFTTTLLLVFFTVWVFGGGTTPMLTWLQIRVGVD
LDENLKEDPSSQHQEANNLDKNMTKAESARLFRMWYSFDHKYLKPILTHSGPPLTTTLPE
WCGPISRLLTSPQAYGEQLKEDDVECIVNQDELAINYQEQASSPCSPPARLGLDQKASPQT
PGKENIYEGDLGLGGYELKLEQTLGQSQLN

>Q8NGI8 multipass membrane protein
MTGGGNITEITYFILLGFSDFPRIIKVLFTIFLVIYITSLAWNLSLIVLIRMDSHLHTPMYFFLS

NLSFIDVCYISSTVPKMLSNLLQEQQTITFVGCIIQYFIFSTMGLSESCLMTAMAYDRYAAIC
NPLLYSSIMSPTLCVWMVLGAYMTGLTASLFQIGALLQLHFCGSNVIRHFFCDMPQLLILS
CTDTFFVQVMTAILTMFFGIASALVIMISYGYIGISIMKITSAKGRSKAFNTCASHLTAVSLF
YTSGIFVYLSSSSGGSSSFDRFASVFYTVVIPMLNPLIYSLRNKEIKDALKRLQKRKCC

>Q9BPV8 multipass membrane protein
MTAAIRRQRELSILPKVTLEAMNTTVMQGFNRSERCPRDTRIVQLVFPALYTVVFLTGILLN
TLALWVFVHIPSSSTFIIYLKNTLVADLIMTLMLPFKILSDSHLAPWQLRAFVCRFSSVIFYE
TMYVGIVLLGLIAFDRFLKIIRPLRNIFLKKPVFAKTVSIFIWFFLFFISLPNTILSNKEATPSS
VKKCASLKGPLGLKWHQMVNNICQFIFWTVFILMLVFYVVIAKKVYDSYRKSKSKDRKN
NKKLEGKVFVVVAVFFVCFAPFHFARVPYTHSQTNNKTDCRLQNQLFIAKETTLFLAATNI
CMDPLIYIFLCKKFTEKLPCMQGRKTTASSQENHSSQTDNITLG

>Q9UHI5 multipass membrane protein
MEEGARHRNNTEKKHPGGGESDASPEAGSGGGGVALKKEIGLVSACGIIVGNIIGSGIFVS
PKGVLENAGSVGLALIVWIVTGFITVVGALCYAELGVTIPKSGGDYSYVKDIFGGLAGFLR
LWIAVLVIYPTNQAVIALTFSNYVLQPLFPTCFPPESGLRLLAAICLLLLTWVNCSSVRWATR
VQDIFTAGKLLALALIIIMGIVQICKGEYFWLEPKNAFENFQEPDIGLVALAFLQGSFAYGG
WNFLNYVTEELVDPYKNLPRAIFISIPLVTFVYVFANVAYVTAMSPQELLASNAVAVTFGEK
LLGVMAWIMPISVALSTFGGVNGSLFTSSRLFFAGAREGHLPSVLAMIHVKRCTPIPALLFT
CISTLLMLVTSDMYTLINYVGFINYLFYGVTVAGQIVLRWKKPDIPRPIKINLLFPIIYLLFW
AFLLVFSLWSEPVVCGIGLAIMLTGVPVYFLGVYWQHKPKCFSDFIELLTLVSQKMCVVV
YPEVERGSGTEEANEDMEEQQQPMYQPTPTKDKDVAGQPQP

>Q9ULC3 lipid-anchor
MLEEDMEVAIKMVVVGNGAVGKSSMIQRYCKGIFTKDYKKTIGVDFLERQIQVNDEDVR
LMLWDTAGQEEFDAITKAYYRGAQACVLVFSTTDRESFEAVSSWREKVVAEVGDIPTVLV
QNKIDLLDDSCIKNEEAEALAKRLKLRFYRTSVKEDLNVNEVFKYLAEKYLQKLKQQIAE
DPELTHSSSNKIGVFNTSGGSHSGQNSGTLNGGDVINLRPNKQRTKKNRNPFSSCSIP

>Q9UBT7 peripheral membrane protein
MAASPGPAGVGGAGAVYGSGSSGFALDSGLEIKTRSVEQTLLPLVSQITTLINHKDNTKKS
DKTLQAIQRVGQAVNLAVGRFVKVGEAIANENWDLKEEINIACIEAKQAGETIAALTDITN
LNHLESDGQITIFTDKTGVIKAARLLLSSVTKVLLLADRVVIKQIITSRNKVLATMERLEKV
NSFQEFVQIFSQFGNEMVEFAHLSGDRQNDLKDEKKKAKMAAARAVLEKCTMMLLTASK
TCLRHPNCESAHKNKEGVFDRMKVALDKVIEIVTDCKPNGETDISSISIFTGIKEFKMNIEA
LRENLYFQSKENLSVTLEVILERMEDFTDSAYTSHEHRERILELSTQARMELQQLISVWIQA
QSKKTKSIAEELELSILKISHSLNELKKELHSTATQLAADLLKYHADHVVLKALKLTGVEG
NLEALAEYACKLSEQKEQLVETCRLLRHISGTEPLEITCIHAEETFQVTGQQIISAAETLTLH
PSSKIAKENLDVFCEAWESQISDMSTLLREINDVFEGRRGEKYGYLSLPKPMKNNANLKS
LKPDKPDSEEQAKIAKLGLKLGLLTSDADCEIEKWEDQENEIVQYGRNMSSMAYSLYLFT
RGEGPLKTSQDLIHQLEVFAAEGLKLTSSVQAFSKQLKDDDKLMLLLEINKLIPLCHQLQT
VTKTSLQNKVFLKVDKCITKTRSMMALLVQLLSLCYKLLKKLQMENNGWVSVTNKDTM
DSKT

>Q96GF1 multipass membrane protein
MASKGPSASASPENSSAGGPSGSSNGAGESGGQDSTFECNICLDTAKDAVISLCGHLFCWP
CLHQWLETRPNRQVCPVCKAGISRDKVIPLYGRGSTGQQDPREKTPPRPQGQRPEPENRG
GFQGFGFGDGGFQMSFGIGAFPFGIFATAFNINDGRPPPAVPGTPQYVDEQFLSRLFLFVAL
VIMFWLLIA

>Q8NGH3 multipass membrane protein
MCSFFLCQTGKQAKISMGEENQTFVSKFIFLGLSQDLQTQILLFILFLIIYLLTVLGNQLIIILI
FLDSRLHTPMYFFLRNLSFADLCFSTSIVPQVLVHFLVKRKTISFYGCMTQIIVFLLVGCTEC
ALLAVMSYDRYVAVCKPLYYSTIMTQRVCLWLSFRSWASGALVSLVDTSFTFHLPYWGQN
IINHYFCEPPALLKLASIDTYSTEMAIFSMGVVILLAPVSLILGSYWNIISTVIQMQSGEGRL
KAFSTCGSHLIVVVLFYGSGIFTYMRPNSKTTKELDKMISVFYTAVTPMLNPIIYSLRNKDV
KGALRKLVGRKCFSHRQ

>Q9UIG8 multipass membrane protein
MQGKKPGGSSGGGRSGELQGDEAQRNKKKKKKVSCFSNIKIFLVSECALMLAQGTVGAY
LVSVLTTLERRFNLQSADVGVIASSFEIGNLALILFVSYFGARGHRPRLIGCGGIVMALGAL
LSALPEFLTHQYKYEAGEIRWGAEGRDVCAANGSGGDEGPDPDLICRNRTATNMMYLLLI
GAQVLLGIGATPVQPLGVSYIDDHVRRKDSSLYIGILFTMLVFGPACGFILGSFCTKIYVDA
VFIDTSNLDITPDDPRWIGAWWGGFLLCGALLFFSSLLMFGFPQSLPPHSEPAMESEQAML
SEREYERPKPSNGVLRHPLEPDSSASCFQQLRVIPKVTKHLLSNPVFTCIILAACMEIAVVA
GFAAFLGKYLEQQFNLTTSSANQLLGMTAIPCACLGIFLGGLLVKKLSLSALGAIRMAMLV
NLVSTACYVSFLFLGCDTGPVAGVTVPYGNSTAPGSALDPYSPCNNNCECQTDSFTPVCG
ADGITYLSACFAGCNSTNLTGCACLTTVPAENATVVPGKCPSPGCQEAFLTFLCVMCICSLI
GAMAQTPSVIILIRTVSPELKSYALGVLFLLLRLLGFIPPPLIFGAGIDSTCLFWSTFCGEQGA
CVLYDNVVYRYLYVSIAIALKSFAFILYTTTWQCLRKNYKRYIKNHEGGLSTSEFFASTLTL
DNLGRDPVPANQTHRTKFIYNLEDHEWCENMESVL

>P29475 peripheral membrane protein
MEDHMFGVQQIQPNVISVRLFKRKVGGLGFLVKERVSKPPVIISDLIRGGAAEQSGLIQAG
DIILAVNGRPLVDLSYDSALEVLRGIASETHVVLILRGPEGFTTHLETTFTGDGTPKTIRVTQ
PLGPPTKAVDLSHQPPAGKEQPLAVDGASGPGNGPQHAYDDGQEAGSLPHANGLAPRPPG
QDPAKKATRVSLQGRGENNELLKEIEPVLSLLTSGSRGVKGGAPAKAEMKDMGIQVDRDL
DGKSHKPLPLGVENDRVFNDLWGKGNVPVVLNNPYSEKEQPPTSGKQSPTKNGSPSKCP
RFLKVKNWETEVVLTDTLHLKSTLETGCTEYICMGSIMHPSQHARRPEDVRTKGQLFPLA
KEFIDQYYSSIKRFGSKAHMERLEEVNKEIDTTSTYQLKDTELIYGAKHAWRNASRCVGRI
QWSKLQVFDARDCTTAHGMFNYICNHVKYATNKGNLRSAITIFPQRTDGKHDFRVWNSQ
LIRYAGYKQPDGSTLGDPANVQFTEICIQQGWKPPRGRFDVLPLLLQANGNDPELFQIPPEL
VLEVPIRHPKFEWFKDLGLKWYGLPAVSNMLLEIGGLEFSACPFSGWYMGTEIGVRDYCD
NSRYNILEEVAKKMNLDMRKTSSLWKDQALVEINIAVLYSFQSDKVTIVDHHSATESFIKH
MENEYRCRGGCPADWVWIVPPMSGSITPVFHQEMLNYRLTPSFEYQPDPWNTHVWKGT
NGTPTKRRAIGFKKLAEAVKFSAKLMGQAMAKRVKATILYATETGKSQAYAKTLCEIFKH
AFDAKVMSMEEYDIVHLEHETLVLVVTSTFGNGDPPENGEKFGCALMEMRHPNSVQEER

KSYKVRFNSVSSYSDSQKSSGDGPDLRDNFESAGPLANVRFSVFGLGSRAYPHFCAFGHA
VDTLLEELGGERILKMREGDELCGQEEAFRTWAKKVFKAACDVFCVGDDVNIEKANNSL
ISNDRSWKRNKFRLTFVAEAPELTQGLSNVHKKRVSAARLLSRQNLQSPKSSRSTIFVRLH
TNGSQELQYQPGDHLGVFPGNHEDLVNALIERLEDAPPVNQMVKVELLEERNTALGVISN
WTDELRLPPCTIFQAFKYYLDITTPPTPLQLQQFASLATSEKEKQRLLVLSKGLQEYEEWK
WGKNPTIVEVLEEFPSIQMPATLLLTQLSLLQPRYYSISSSPDMYPDEVHLTVAIVSYRTRDG
EGPIHHGVCSSWLNRIQADELVPCFVRGAPSFHLPRNPQVPCILVGPGTGIAPFRSFWQQR
QFDIQHKGMNPCPMVLVFGCRQSKIDHIYREETLQAKNKGVFRELYTAYSREPDKPKKYV
QDILQEQLAESVYRALKEQGGHIYVCGDVTMAADVLKAIQRIMTQQGKLSAEDAGVFIS
RMRDDNRYHEDIFGVTLRTYEVTNRLRSESIAFIEESKKDTDEVFSS

>P14672 multipass membrane protein
MPSGFQQIGSEDGEPPQQRVTGTLVLAVFSAVLGSLQFGYNIGVINAPQKVIEQSYNETWL
GRQGPEGPSSIPPGTLTTLWALSVAIFSVGGMISSFLIGIISQWLGRKRAMLVNNVLAVLGGS
LMGLANAAASYEMLILGRFLIGAYSGLTSGLVPMYVGEIAPTHLRGALGTLNQLAIVIGILI
AQVLGLESLLGTASLWPLLLGLTVLPALLQLVLLPFCPESPRYLYIIQNLEGPARKSLKRLTG
WADVSGVLAELKDEKRKLERERPLSLLQLLGSRTHRQPLIIAVVLQLSQQLSGINAVFYYS
TSIFETAGVGQPAYATIGAGVVNTVFTLVSVLLVERAGRRTLHLLGLAGMCGCAILMTVAL
LLLERVPAMSYVSIVAIFGFVAFFEIGPGPIPWFIVAELFSQGPRPAAMAVAGFSNWTSNFIIG
MGFQYVAEAMGPYVFLLFAVLLLGFFIFTFLRVPETRGRTFDQISAAFHRTPSLLEQEVKPS
TELEYLGPDEND

>O60512 single-pass type II membrane protein
MLRRLLERPCTLALLVGSQLAVMMYLSLGGFRSLSALFGRDQGPTFDYSHPRDVYSNLSH
LPGAPGGPPAPQGLPYCPERSPLLVGPVSVSFSPVPSLAEIVERNPRVEPGGRYRPAGCEPRS
RTAIIVPHRAREHHLRLLLYHLHPFLQRQQLAYGIYVIHQAGNGTFNRAKLLNVGVREALR
DEEWDCLFLHDVDLLPENDHNLYVCDPRGPRHVAVAMNKFGYSLPYPQYFGGVSALTPD
QYLKMNGFPNEYWGWGGEDDDIATRVRLAGMKISRPPTSVGHYKMVKHRGDKGNEEN
PHRFDLLVRTQNSWTQDGMNSLTYQLLARELGPLYTNITADIGTDPRGPRAPSGPRYPPGS
SQAFRQEMLQRRPPARPGPLSTANHTALRGSH

>Q6UXG3 single-pass type I membrane protein
MRLLVLLWGCLLLPGYEALEGPEEISGFEGDTVSLQCTYREELRDHRKYWCRKGGILFSR
CSGTIYAEEEGQETMKGRVSIRDSRQELSLIVTLWNLTLQDAGEYWCGVEKRGPDESLLIS
LFVFPGPCCPPSPSPTFQPLATTRLQPKAKAQQTQPPGLTSPGLYPAATTAKQGKTGAEAPP
LPGTSQYGHERTSQYTGTSPHPATSPPAGSSRPPMQLDSTSAEDTSPALSSGSSKPRVSIPMV
RILAPVLVLLSLLSAAGLIAFCSHLLLWRKEAQQATETQRNEKFCLSRLTAEEKEAPSQAPE
GDVISMPPLHTSEEELGFSKFVSA

>Q9NYZ4 single-pass type I membrane protein
MLLLLLLLPLLWGTKGMEGDRQYGDGYLLQVQELVTVQEGLCVHVPCSFSYPQDGWTD
SDPVHGYWFRAGDRPYQDAPVATNNPDREVQAETQGRFQLLGDIWSNDCSLSIRDARKR
DKGSYFFRLERGSMKWSYKSQLNYKTKQLSVFVTALTHRPDILILGTLESGHSRNLTCSVP
WACKQGTPPMISWIGASVSSPGPTTARSSVLTLTPKPQDHGTSLTCQVTLPGTGVTTTSTVR

LDVSYPPWNLTMTVFQGDATASTALGNGSSLSVLEGQSLRLVCAVNSNPPARLSWTRGSL
TLCPSRSSNPGLLELPRVHVRDEGEFTCRAQNAQGSQHISLSLSLQNEGTGTSRPVSQVTL
AAVGGAGATALAFLSFCIIFIIVRSCRKKSARPAAGVGDTGMEDAKAIRGSASQGPLTESW
KDGNPLKKPPPAVAPSSGEEGELHYATLSFHKVKPQDPQGQEATDSEYSEIKIHKRETAETQ
ACLRNHNPSSKEVRG

>Q9Y2G3 multipass membrane protein
MWRWIRQQLGFDPPHQSDTRTIYVANRFPQNGLYTPQKFIDNRIISSKYTVWNFVPKNLFE
QFRRVANFYFLIIFLVQLMIDTPTSPVTSGLPLFFVITVTAIKQGYEDWLRHNSDNEVNGAP
VYVVRSGGLVKTRSKNIRVGDIVRIAKDEIFPADLVLLSSDRLDGSCHVTTASLDGETNLK
THVAVPETALLQTVANLDTLVAVIECQQPEADLYRFMGRMIITQQMEEIVRPLGPESLLLRG
ARLKNTKEIFGVAVYTGMETKMALNYKSKSQKRSAVEKSMNTFLIIYLVILISEAVISTILK
YTWQAEEKWDEPWYNQKTEHQRNSSKILRFISDFLAFLVLYNFIIPISLYVTVEMQKFLGSF
FIGWDLDLYHEESDQKAQVNTSDLNEELGQVEYVFTDKTGTLTENEMQFRECSINGMKY
QEINGRLVPEGPTPDSSEGNLSYLSSLSHLNNLSHLTTSSSFRTSPENETELIKEHDLFFKAVS
LCHTVQISNVQTDCTGDGPWQSNLAPSQLEYYASSPDEKALVEAAARIGIVFIGNSEETME
VKTLGKLERYKLLHILEFDSDRRRMSVIVQAPSGEKLLFAKGAESSILPKCIGGEIEKTRIH
VDEFALKGLRTLCIAYRKFTSKEYEEIDKRIFEARTALQQREEKLAAVFQFIEKDLILLGATA
VEDRLQDKVRETIEALRMAGIKVWVLTGDKHETAVSVSLSCGHFHRTMNILELINQKSDS
ECAEQLRQLARRITEDHVIQHGLVVDGTSLSLALREHEKLFMEVCRNCSAVLCCRMAPLQ
KAKVIRLIKISPEKPITLAVGDGANDVSMIQEAHVGIGIMGKEGRQAARNSDYAIARFKFLS
KLLFVHGHFYYIRIATLVQYFFYKNVCFITPQFLYQFYCLFSQQTLYDSVYLTLYNICFTSLP
ILIYSLLEQHVDPHVLQNKPTLYRDISKNRLLSIKTFLYWTILGFSHAFIFFFGSYLLIGKDTS
LLGNGQMFGNWTFGTLVFTVMVITVTVKMALETHFWTWINHLVTWGSIIFYFVFSLFYG
GILWPFLGSQNMYFVFIQLLSSGSAWFAIILMVVTCLFLDIIKKVFDRHLHPTSTEKAQLTE
TNAGIKCLDSMCCFPEGEAACASVGRMLERVIGRCSPTHISRSWSASDPFYTNDRSILTLST
MDSSTC

>Q8NH76 multipass membrane protein
MDTSTSVTYDSSLQISQFILMGLPGIHEWQHWLSLPLTLLYLLALGANLLIIITIQHETVLHE
PMYHLLGILAVVDIGLATTIMPKILAIFWFDAKAISLPMCFAQIYAIHCFFCIESGIFLCMAVD
RYIAICRPLQYPSIVTKAFVFKATGFIMLRNGLLTIPVPILAAQRHYCSRNEIEHCLCSNLGVI
SLACDDITVNKFYQLMLAWVLVGSDMALVFSSYAVILHSVLRLNSAEAMSKALSTCSSHLI
LILFHTGIIVLSVTHLAEKKIPLIPVFLNVLHNVIPPALNPLACALRMHKLRLGFQRLLGLGQ
DVSK

>Q8NGH5 multipass membrane protein
MIQPMASPSNSSTVPVSEFLLICFPNFQSWQHWLSLPLSLLFLLAMGANTTLLITIQLEASL
HQPLYYLLSLLSLLDIVLCLTVIPKVLAIFWYDLRSISFPACFLQMFIMNSFLPMESCTFMV
MAYDRYVAICHPLRYPSIITNQFVAKASVFIVVRNALLTAPIPILTSLLHYCGENVIENCICAN
LSVSRLSCDNFTLNRIYQFVAGWTLLGSDLFLIFLSYTFILRAVLRFKAEGAAVKALSTCGS
HFILILFFSTILLVVVLTNVARKKVPMDILILLNVLHHLIPPALNPIVYGVRTKEIKQGIQKLL
QRGR

>P25116 multipass membrane protein
MGPRRLLLVAACFSLCGPLLSARTRARRPESKATNATLDPRSFLLRNPNDKYEPFWEDEEK
NESGLTEYRLVSINKSSPLQKQLPAFISEDASGYLTSSWLTLFVPSVYTGVFVVSLPLNIMAI
VVFILKMKVKKPAVVYMLHLATADVLFVSVLPFKISYYFSGSDWQFGSELCRFVTAAFYC
NMYASILLMTVISIDRFLAVVYPMQSLSWRTLGRASFTCLAIWALAIAGVVPLLLKEQTIQ
VPGLNITTCHDVLNETLLEGYYAYYFSAFSAVFFFVPLIISTVCYVSIIRCLSSSAVANRSKKS
RALFLSAAVFCIFIICFGPTNVLLIAHYSFLSHTSTTEAAYFAYLLCVCVSSISCCIDPLIYYYA
SSECQRYVYSILCCKESSDPSSYNSSGQLMASKMDTCSSNLNNSIYKKLLT

>Q8NGB2 multipass membrane protein
MYVSNCNPCAIHRKINYPNTKLDFEQVNNITEFILLGLTQNAEAQKLLFAVFTLIYFLTMVD
NLIIVVTITTSPALDSPVYFFLSFFSFIDGCSSSTMAPKMIFDLLTEKKTISFSGCMTQLFVEH
FFGGVEIILLVVMAYDCYVAICKPLYYLITMNRQVCGLLVAMAWVGGFLHALIQMLLIVW
LPFCGPNVIDHFICDLFPLLKLSCTDTHVFGLFVAANSGLMCMLIFSILITSYVLILCSQRKA
LSTCAFHITVVVLFFVPCILVYLRPMITFPIDKAVSVFYTVVTPMLNPLIYTLRNTEVKNAM
KQLWSQIIWGNNLCD

>Q5VWK5 single-pass type I membrane protein
MNQVTIQWDAVIALYILFSWCHGGITNINCSGHIWVEPATIFKMGMNISIYCQAAIKNCQPR
KLHFYKNGIKERFQITRINKTTARLWYKNFLEPHASMYCTAECPKHFQETLICGKDISSGYP
PDIPDEVTCVIYEYSGNMTCTWNAGKLTYIDTKYVVHVKSLETEEEQQYLTSSYINISTDS
LQGGKKYLVWVQAANALGMEESKQLQIHLDDIVIPSAAVISRAETINATVPKTIIYWDSQT
TIEKVSCEMRYKATTNQTWNVKEFDTNFTYVQQSEFYLEPNIKYVFQVRCQETGKRYWQ
PWSSLFFHKTPETVPQVTSKAFQHDTWNSGLTVASISTGHLTSDNRGDIGLLLGMIVFAVM
LSILSLIGIFNRSFRTGIKRRILLLIPKWLYEDIPNMKNSNVVKMLQENSELMNNNSSEQVLY
VDPMITEIKEIFIPEHKPTDYKKENTGPLETRDYPQNSLFDNTTVVYIPDLNTGYKPQISNFL
PEGSHLSNNNEITSLTLKPPVDSLDSGNNPRLQKHPNFAFSVSSVNSLSNTIFLGELSLILNQ
GECSSPDIQNSVEEETTMLLENDSPSETIPEQTLLPDEFVSCLGIVNEELPSINTYFPQNILES
HFNRISLLEK

>Q8NH37 multipass membrane protein
MDIPQNITEFFMLGLSQNSEVQRVLFVVFLLIYVVTVCGNMLIVVTITSSPTLASPVYFFLA
NLSFIDTFYSSSMAPKLIADSLYEGRTISYECCMAQLFGAHFLGGVEIILLTVMAYDRYVAI
CKPLHNTTIMTRHLCAMLVGVAWLGGFLHSLVQLLLVLWLPFCGPNVINHFACDLYPLLE
VACTNTYVIGLLVVANSGLICLLNFLMLAASYIVILYSLRSHSADGRCKALSTCGAHFIVVA
LFFVPCIFTYVHPFSTLPIDKNMALFYGILTPMLNPLIYTLRNEEVKNAMRKLFTW

>Q06495 multipass membrane protein
MLSYGERLGSPAVSPLPVRGGHVMRGTAFAYVPSPQVLHRIPGTSAYAFPSLGPVALAEHT
CPCGEVLERHEPLPAKLALEEEQKPESRLVPKLRQAGAMLLKVPLMLTFLYLFVCSLDML
SSAFQLAGGKVAGDIFKDNAILSNPVAGLVVGILVTVLVQSSSTSTSIIVSMVSSGLLEVSSA
IPIIMGSNIGTSVTNTIVALMQAGDRTDFRRAFAGATVHDCFNWLSVLVLLPLEAATGYLH
HITRLVVASFNIHGGRDAPDLLKIITEPFTKLIIQLDESVITSIATGDESLRNHSLIQIWCHPDS
LQAPTSMSRAEANSSQTLGNATMEKCNHIFVDTGLPDLAVGLILLAGSLVLLCTCLILLVK

MLNSLLKGQVAKVIQKVINTDFPAPFTWVTGYFAMVVGASMTFVVQSSSVFTSAITPLIGL
GVISIERAYPLTLGSNIGTTTTAILAALASPREKLSSAFQIALCHFFFNISGILLWYPVPCTRLP
IRMAKALGKRTAKYRWFAVLYLLVCFLLLPSLVFGISMAGWQVMVGVGTPFGALLAFVV
LINVLQSRSPGHLPKWLQTWDFLPRWMHSLKPLDHLITRATLCCARPEPRSPPLPPRVFLE
ELPPATPSPRLALPAHHNATRL

>Q8NGH9 multipass membrane protein
MPSINDTHFYPPFFLLLGIPGLDTLHIWISFPFCIVYLIAIVGNMTILFVIKTEHSLHQPMFYF
LAMLSMIDLGLSTSTIPKMLGIFWFNLQEISFGGCLLQMFFIHMFTGMETVLLVVMAYDRF
VAICNPLQYTMILTNKTISILASVVVGRNLVLVTPFVFLILRLPFCGHNIVPHTYCEHRGLAG
LACAPIKINIIYGLMVISYIIVDVILIASSYVLILRAVFRLPSQDVRLKAFNTCGSHVCVMLCF
YTPAFFSFMTHRFGQNIPHYIHILLANLYVVVPPALNPVIYGVRTKQIREQIVKIFVQKE

>Q8NCM2 multipass membrane protein
MPGGKRGLVAPQNTFLENIVRRSSESSFLLGNAQIVDWPVVYSNDGFCKLSGYHRADVM
QKSSTCSFMYGELTDKKTIEKVRQTFDNYESNCFEVLLYKKNRTPVWFYMQIAPIRNEHE
KVVLFLCTFKDITLFKQPIEDDSTKGWTKFARLTRALTNSRSVLQQLTPMNKTEVVHKHSR
LAEVLQLGSDILPQYKQEAPKTPPHIILHYCAFKTTWDWVILILTFYTAIMVPYNVSFKTKQ
NNIAWLVLDSVVDVIFLVDIVLNFHTTFVGPGGEVISDPKLIRMNYLKTWFVIDLLSCLPY
DIINAFENVDEGISSLFSSLKVVRLLRLGRVARKLDHYLEYGAAVLVLLVCVFGLVAHWLA
CIWYSIGDYEVIDEVTNTIQIDSWLYQLALSIGTPYRYNTSAGIWEGGPSKDSLYVSSLYFT
MTSLTTIGFGNIAPTTDVEKMFSVAMMMVGSLLYATIFGNVTTIFQQMYANTNRYHEMLN
NVRDFLKLYQVPKGLSERVMDYIVSTWSMSKGIDTEKVLSICPKDMRADICVHLNRKVFN
EHPAFRLASDGCLRALAVEFQTIHCAPGDLIYHAGESVDALCFVVSGSLEVIQDDEVVAIL
GKGDVFGDIFWKETTLAHACANVRALTYCDLHIIKREALLKVLDFYTAFANSFSRNLTLTC
NLRKRIIFRKISDVKKEEEERLRQKNEVTLSIPVDHPVRKLFQKFKQQKELRNQGSTQGDP
ERNQLQVESRSLQNGASITGTSVVTVSQITPIQTSLAYVKTSESLKQNNRDAMELKPNGGA
DQKCLKVNSPIRMKNGNGKGWLRLKNNMGAHEEKKEDWNNVTKAESMGLLSEDPKSS
DSENSVTKNPLRKTDSCDSGITKSDLRLDKAGEARSPLEHSPIQADAKHPFYPIPEQALQTT
LQEVKHELKEDIQLLSCRMTALEKQVAEILKILSEKSVPQASSPKSQMPLQVPPQIPCQDIFS
VSRPESPESDKDEIHF

>Q8NGB4 multipass membrane protein
MGAKNNVTEFVLFGLFESREMQHTCFVVFFLFHVLTVLGNLLVIITINARKTLKSPMYFFL
SQLSFADICYPSTTIPKMIADTFVEHKIISFNGCMTQLFSAHFFGGTEIFLLTAMAYDRYVAI
CRPLHYTAIMDCRKCGLLAGASWLAGFLHSILQTLLTVQLPFCGPNEIDNFFCDVHPLLKL
ACADTYMVGLIVVANSGMISLASFFILIISYVIILLNLRSQSSEDRRKAVSTCGSHVITVLLVL
MPPMFMYIRPSTTLAADKLIILFNIVMPPLLNPLIYTLRNNDVKNAMRKLFRVKRSLGEK

>Q8NH49 multipass membrane protein
MVATNNVTEIIFVGFSQNWSEQRVISVMFLLMYTAVVLGNGLIVVTILASKVLTSPMYFFLS
YLSFVEICYCSVMAPKLIFDSFIKRKVISLKGCLTQMFSLHFFGGTEAFLLMVMAYDRYVAI
CKPLHYMAIMNQRMCGLLVRIAWGGGLLHSVGQTFLIFQLPFCGPNIMDHYFCDVHPVL
ELACADTFFISLLIITNGGSISVVSFFVLMASYLIILHFLRSHNLEGQHKALSTCASHVTVVD

LFFIPCSLVYIRPCVTLPADKIVAVFYTVVTPLLNPVIYSFRNAEVKNAMRRFIGGKVI

>Q96PD2 single-pass type I membrane protein
MASRAVVRARRCPQCPQVRAAAAAPAWAALPLSRSLPPCSNSSSFSMPLFLLLLLVLLLLL
EDAGAQQGDGCGHTVLGPESGTLTSINYPQTYPNSTVCEWEIRVKMGERVRIKFGDFDIE
DSDSCHFNYLRIYNGIGVSRTEIGKYCGLGLQMNHSIESKGNEITLLFMSGIHVSGRGFLAS
YSVIDKQDLITCLDTASNFLEPEFSKYCPAGCLLPFAEISGTIPHGYRDSSPLCMAGVHAGV
VSNTLGGQISVVISKGIPYYESSLANNVTSVVGHLSTSLFTFKTSGCYGTLGMESGVIADP
QITASSVLEWTDHTGQENSWKPKKARLKKPGPPWAAFATDEYQWLQIDLNKEKKITGIIT
TGSTMVEHNYYVSAYRILYSDDGQKWTVYREPGVEQDKIFQGNKDYHQDVRNNFLPPII
ARFIRVNPTQWQQKIAMKMELLGCQFIPKGRPPKLTQPPPPRNSNDLKNTTAPPKIAKGRA
PKFTQPLQPRSSNEFPAQTEQTTASPDIRNTTVTPNVTKDVALAAVLVPVLVMVLTTLILILV
CAWHWRNRKKKTEGTYDLPYWDRAGWWKGMKQFLPAKAVDHEETPVRYSSSEVNHLS
PREVTTVLQADSAEYAQPLVGGIVGTLHQRSTFKPEEGKEAGYADLDPYNSPGQEVYHAY
AEPLPITGPEYATPIIMDMSGHPTTSVGQPSTSTFKATGNQPPPLVGTYNTLLSRTDSCSSAQ
AQYDTPKAGKPGLPAPDELVYQVPQSTQEVSGAGRDGECDVFKEIL

>P51172 multipass membrane protein
MAEHRSMDGRMEAATRGGSHLQAAAQTPPRPGPPSAPPPPPKEGHQEGLVELPASFRELL
TFFCTNATIHGAIRLVCSRGNRLKTTSWGLLSLGALVALCWQLGLLFERHWHRPVLMAVS
VHSERKLLPLVTLCDGNPRRPSPVLRHLELLDEFARENIDSLYNVNLSKGRAALSATVPRH
EPPFHLDREIRLQRLSHSGSRVRVGFRLCNSTGGDCFYRGYTSGVAAVQDWYHFHYVDIL
ALLPAAWEDSHGSQDGHFVLSCSYDGLDCQARQFRTFHHPTYGSCYTVDGVWTAQRPGI
THGVGLVLRVEQQPHLPLLSTLAGIRVMVHGRNHTPFLGHHSFSVRPGTEATISIREDEVH
RLGSPYGHCTAGGEGVEVELLHNTSYTRQACLVSCFQQLMVETCSCGYYLHPLPAGAEY
CSSARHPAWGHCFYRLYQDLETHRLPCTSRCPRPCRESAFKLSTGTSRWPSAKSAGWTLA
TLGEQGLPHQSHRQRSSLAKINIVYQELNYRSVEEAPVYSVPQLLSAMGSLCSLWFGASV
LSLLELLELLLDASALTLVLGGRRLRRAWFSWPRASPASGASSIKPEASQMPPPAGGTSDD
PEPSGPHLPRVMLPGVLAGVSAEESWAGPQPLETLDT

>Q9H2C8 multipass membrane protein
MFLSSRMITSVSPSTSTNSSFLLTGFSGMEQQYPWLSIPFSSIYAMVLLGNCMVLHVIWTEP
SLHQPMFYFLSMLALTDLCMGLSTVYTVLGILWGIIREISLDSCIAQSYFIHGLSFMESSVLL
TMAFDRYIAICNPLRYSSILTNSRIIKIGLTIIGRSFFFITPPIICLKFFNYCHFHILSHSFCLHQD
LLRLACSDIRFNSYYALMLVICILLLDAILILFSYILILKSVLAVASQEERHKLFQTCISHICAV
LVFYIPIISLTMVHRFGKHLSPVAHVLIGNIYILFPPLMNPIIYSVKTQQIHTRMLRLFSLKRY

>O95907 multipass membrane protein
MGAGGPRRGEGPPDGGWGWVVLGACFVVTGFAYGFPKAVSVFFRALMRDFDAGYSDTA
WVSSIMLAMLYGTGPVSSILVTRFGCRPVMLAGGLLASAGMILASFATRLLELYLTAGVLT
GLGLALNFQPSLIMLGLYFERRRPLANGLAAAGSPVFLSALSPLGQQLLERFGWRGGFLLL
GGLLLHCCACGAVMRPPPGPGPRPRRDSAGDRAGDAPGEAEADGAGLQLREASPRVRPR
RRLLDLAVCTDRAFAVYAVTKFLMALGLFVPAILLVNYAKDAGVPDTDAAFLLSIVGFVDI
VARPACGALAGLARLRPHVPYLFSLALLANGLTDLSSARARSYGALVAFCVAFGLSYGMV

GALQFEVLMAAVGAPRFPSALGLVLLVEAAAVLIGPPSAGRLVDVLKNYEIIFYLAGSEVA
LAGVFMAVATNCCLRCAKAAPSGPGTEGGASDTEDAEAEGDSEPLPVVAEEPGNLEALEV
LSARGEPTEPEIEARPRLAAESV

>Q03431 multipass membrane protein
MGTARIAPGLALLLCCPVLSSAYALVDADDVMTKEEQIFLLHRAQAQCEKRLKEVLQRPA
SIMESDKGWTSASTSGKPRKDKASGKLYPESEEDKEAPTGSRYRGRPCLPEWDHILCWPL
GAPGEVVAVPCPDYIYDFNHKGHAYRRCDRNGSWELVPGHNRTWANYSECVKFLTNETR
EREVFDRLGMIYTVGYSVSLASLTVAVLILAYFRRLHCTRNYIHMHLFLSFMLRAVSIFVKD
AVLYSGATLDEAERLTEEELRAIAQAPPPPATAAAGYAGCRVAVTFFLYFLATNYYWILVEG
LYLHSLIFMAFFSEKKYLWGFTVFGWGLPAVFVAVWVSVRATLANTGCWDLSSGNKKWII
QVPILASIVLNFILFINIVRVLATKLRETNAGRCDTRQQYRKLLKSTLVLMPLFGVHYIVFM
ATPYTEVSGTLWQVQMHYEMLFNSFQGFFVAIIYCFCNGEVQAEIKKSWSRWTLALDFKR
KARSGSSSYSYGPMVSHTSVTNVGPRVGLGLPLSPRLLPTATTNGHPQLPGHAKPGTPALE
TLETTPPAMAAPKDDGFLNGSCSGLDEEASGPERPPALLQEEWETVM

>Q8NGJ4 multipass membrane protein
MFLPNDTQFHPSSFLLLGIPGLETLHIWIGFPFCAVYMIALIGNFTILLVIKTDSSLHQPMFYF
LAMLATTDVGLSTATIPKMLGIFWINLRGIIFEACLTQMFFIHNFTLMESAVLVAMAYDSYV
AICNPLQYSAILTNKVVSVIGLGVFVRALIFVIPSILLILRLPFCGNHVIPHTYCEHMGLAHL
SCASIKINIIYGLCAICNLVFDITVIALSYVHILCAVFRLPTHEARLKSLSTCGSHVCVILAFY
TPALFSFMTHRFGRNVPRYIHILLANLYVVVPPMLNPVIYGVRTKQIYKCVKKILLQEQGM
EKEEYLIHTRF

>Q8NGJ5 multipass membrane protein
MGDWNNSDAVEPIFILRGFPGLEYVHSWLSILFCLAYLVAFMGNVTILSVIWIESSLHQPM
YYFISILAVNDLGMSLSTLPTMLAVLWLDAPEIQASACYAQLFFIHTFTFLESSVLLAMAFD
RFVAICHPLHYPTILTNSVIGKIGLACLLRSLGVVLPTPLLLRHYHYCHGNALSHAFCLHQD
VLRLSCTDARTNSIYGLCVVIATLGVDSIFILLSYVLILNTVLDIASREEQLKALNTCVSHIC
VVLIFFVPVIGVSMVHRFGKHLSPIVHILMADIYLLLPPVLNPIVYSVRTKQIRLGILHKFVL
RRRF

>O94777 multipass membrane protein
MATGTDQVVGLGLVAVSLIIFTYYTAWVILLPFIDSQHVIHKYFLPRAYAVAIPLAAGLLLLL
FVGLFISYVMLKTKRVTKKAQ

>Q9BZ72 peripheral membrane protein
MIIKEYRIPLPMTVEEYRIAQLYMIQKKSRNETYGEGSGVEILENRPYTDGPGGSGQYTHK
VYHVGMHIPSWFRSILPKAALRVVEESWNAYPYTRTRFTCPFVEKFSIDIETFYKTDAGEN
PDVFNLSPVEKNQLTIDFIDIVKDPVPHNEYKTEEDPKLFQSTKTQRGPLSENWIEEYKKQ
VFPIMCAYKLCKVEFRYWGMQSKIERFIHDTGLRRVMVRAHRQAWCWQDEWYGLSME
NIRELEKEAQLMLSRKMAQFNEDGEEATELVKHEAVSDQTSGEPPEPSSSNGEPLVGRGLK
KQWSTSSKSSRSSKRGASPSRHSISEWRMQSIARDSDESSDDEFFDAHEDLSDTEEMFPKD
ITKWSSNDLMDKIESPEPEDTQDGLYRQGAPEFRVASSVEQLNIIEDEVSQPLAAPPSKIHV

LLLVLHGGTILDTGAGDPSSKKGDANTIANVFDTVMRVHYPSALGRLAIRLVPCPPVCSDA
FALVSNLSPYSHDEGCLSSSQDHIPLAALPLLATSSPQYQEAVATVIQRANLAYGDFIKSQEG
MTFNGQVCLIGDCVGGILAFDALCYSNQPVSESQSSSRRGSVVSMQDNDLLSPGILMNAA
HCCGGGGGGGGGGGSSGGGGSSGGSSLESSRHLSRSNVDIPRSNGTEDPKRQLPRKRSDS
STYELDTIQQHQAFLSSLHASVLRTEPCSRHSSSSTMLDGTGALGRFDFEITDLFLFGCPLG
LVLALRKTVIPALDVFQLRPACQQVYNLFHPADPSASRLEPLLERRFHALPPFSVPRYQRYP
LGDGCSTLLADVLQTHNAAFQEHGAPSSPGTAPASRGFRRASEISIASQVSGMAESYTASSI
AQKAPDALSHTPSVRRLSLLALPAPSPTTPGPHPPARKASPGLERAPGLPELDIGEVAAKW
WGQKRIDYALYCPDALTAFPTVALPHLFHASYWESTDVVSFLLRQVMRHDNSSILELDGK
EVSVFTPSKPREKWQRKRTHVKLRNVTANHRINDALANEDGPQVLTGRFMYGPLDMVTL
TGEKVDVHIMTQPPSGEWLYLDTLVTNNSGRVSYTIPESHRLGVGVYPIKMVVRGDHTFA
DSYITVLPKGTEFVVFSIDGSFAASVSIMGSDPKVRAGAVDVVRHWQDLGYLIIYVTGRPD
MQKQRVVAWLAQHNFPHGVVSFCDGLVHDPLRHKANFLKLLISELHLRVHAAYGSTKDV
AVYSAISLSPMQIYIVGRPTKKLQQQCQFITDGYAAHLAQLKYSHRARPARNTATRMALR
KGSFGLPGQGDFLRSRNHLLRTISAQPSGPSHRHERTQSQADGEQRGQRSMSVAAGCWGR
AMTGRLEPGAAAGPK

>O95490 multipass membrane protein
MVSSGCRMRSLWFIIVISFLPNTEGFSRAALPFGLVRRELSCEGYSIDLRCPGSDVIMIESAN
YGRTDDKICDADPFQMENTDCYLPDAFKIMTQRCNNRTQCIVVTGSDVFPDPCPGTYKYL
EVQYECVPYIFVCPGTLKAIVDSPCIYEAEQKAGAWCKDPLQAADKIYFMPWTPYRTDTL
IEYASLEDFQNSRQTTTYKLPNRVDGTGFVVYDGAVFFNKERTRNIVKFDLRTRIKSGEAII
NYANYHDTSPYRWGGKTDIDLAVDENGLWVIYATEQNNGMIVISQLNPYTLRFEATWETV
YDKRAASNAFMICGVLYVVRSVYQDNESETGKNSIDYIYNTRLNRGEYVDVPFPNQYQYI
AAVDYNPRDNQLYVWNNNFILRYSLEFGPPDPAQVPTTAVTITSSAELFKTIISTTSTTSQKG
PMSTTVAGSQEGSKGTKPPPAVSTTKIPPITNIFPLPERFCEALDSKGIKWPQTQRGMMVER
PCPKGTRGTASYLCMISTGTWNPKGPDLSNCTSHWVNQLAQKIRSGENAASLANELAKH
TKGPVFAGDVSSSVRLMEQLVDILDAQLQELKPSEKDSAGRSYNKLQKREKTCRAYLKAI
VDTVDNLLRPEALESWKHMNSSEQAHTATMLLDTLEEGAFVLADNLLEPTRVSMPTENIV
LEVAVLSTEGQIQDFKFPLGIKGAGSSIQLSANTVKQNSRNGLAKLVFIIYRSLGQFLSTENA
TIKLGADFIGRNSTIAVNSHVISVSINKESSRVYLTDPVLFTLPHIDPDNYFNANCSFWNYSE
RTMMGYWSTQGCKLVDTNKTRTTCACSHLTNFAILMAHREIAYKDGVHELLLTVITWVGI
VISLVCLAICIFTFCFFRGLQSDRNTIHKNLCINLFIAEFIFLIGIDKTKYAIACPIFAGLLHFFF
LAAFAWMCLEGVQLYLMLVEVFESEYSRKKYYYVAGYLFPATVVGVSAAIDYKSYGTEK
ACWLHVDNYFIWSFIGPVTFIILLNIIFLVITLCKMVKHSNTLKPDSSRLENIKSWVLGAFAL
LCLLGLTWSFGLLFINEETIVMAYLFTIFNAFQGVFIFIFHCALQKKVRKEYGKCFRHSYCC
GGLPTESPHSSVKASTTRTSARYSSGTQSRIRRMWNDTVRKQSESSFISGDINSTSTLNQG
MTGNYLLTNPLLRPHGTNNPYNTLLAETVVCNAPSAPVFNSPGHSLNNARDTSAMDTLPL
NGNFNNSYSLHKGDYNDSVQVVDCGLSLNDTAFEKMIISELVHNNLRGSSKTHNLELTLP
VKPVIGGSSSEDDAIVADASSLMHSDNPGLELHHKELEAPLIPQRTHSLLYQPQKKVKSEG
TDSYVSQLTAEAEDHLQSPNRDSLYTSMPNLRDSPYPESSPDMEEDLSPSRRSENEDIYYK
SMPNLGAGHQLQMCYQISRGNSDGYIIPINKEGCIPEGDVREGQMQLVTSL

>Q99807 peripheral membrane protein

MSCAGAAAAPRLWRLRPGARRSLSAYGRRTSVRFRSSGMTLDNISRAAVDRIIRVDHAGE
YGANRIYAGQMAVLGRTSVGPVIQKMWDQEKDHLKKFNELMVTFRVRPTVLMPLWNVL
GFALGAGTALLGKEGAMACTVAVEESIAHHYNNQIRTLMEEDPEKYEELLQLIKKFRDEE
LEHHDIGLDHDAELAPAYAVLKSIIQAGCRVAIYLSERL

>Q9GZP7 multipass membrane protein
MVGDTLKLLSPLMTRYFFLLFYSTDSSDLNENQHPLDFDEMAFGKVKSGISFLIQTGVGIL
GNSFLLCFYNLILFTGHKLRPTDLILSQLALANSMVLFFKGIPQTMAAFGLKYLLNDTGCK
FVFYYHRVGTRVSLSTICLLNGFQAIKLNPSICRWMEIKIRSPRFIDFCCLLCWAPHVLMNA
SVLLLVNGPLNSKNSSAKNNYGYCSYKASKRFSSLHAVLYFSPDFMSLGFMVWASGSMV
FFLYRHKQQVQHNHSNRLSCRPSQEARATHTIMVLVSSFFVFYSVHSFLTIWTTVVANPGQ
WIVTNSVLVASCFPARSPFVLIMSDTHISQFCFACRTRKTLFPNLVVMP

>Q8NGW1 multipass membrane protein
MSGENVTRVGTFILVGFPTAPGLQYLLFLLFLLTYLFVLVENLAIILTVWSSTSLHRPMYYF
LSSMSFLEIWYVSDITPKMLEGFLLQQKRISFVGCMTQLYFFSSLVCTECVLLASMAYDRY
VAICHPLRYHVLVTPGLCLQLVGFSFVSGFTISMIKVCFISSVTFCGSNVLNHFFCDISPILKL
ACTDFSTAELVDFILAFIILVFPLLATMLSYAHITLAVLRIPSATGCWRAFFTCASHLTVVTVF
YTALLFMYVRPQAIDSRSSNKLISVLYTVITPILNPLIYCLRNKEFKNALKKAFGLTSCAVEG
RLSSLLELHLQIHSQPL

>P49821 peripheral membrane protein
MLATRRLLGWSLPARVSVRFSGDTTAPKKTSFGSLKDEDRIFTNLYGRHDWRLKGSLSRG
DWYKTKEILLKGPDWILGEIKTSGLRGRGGAGFPTGLKWSFMNKPSDGRPKYLVVNADE
GEPGTCKDREILRHDPHKLLEGCLVGGRAMGARAAYIYIRGEFYNEASNLQVAIREAYEA
GLIGKNACGSGYDFDVFVVRGAGAYICGEETALIESIEGKQGKPRLKPPFPADVGVFGCPT
TVANVETVAVSPTICRRGGTWFAGFGRERNSGTKLFNISGHVNHPCTVEEEMSVPLKELIE
KHAGGVTGGWDNLLAVIPGGSSTPLIPKSVCETVLMDFDALVQAQTGLGTAAVIVMDRST
DIVKAIARLIEFYKHESCGQCTPCREGVDWMNKVMARFVRGDARPAEIDSLWEISKQIEG
HTICALGDGAAWPVQGLIRHFRPELEERMQRFAQQHQARQAAS

>Q8NGK1 multipass membrane protein
MTILLNSSLQRATFFLTGFQGLEGLHGWISIPFCFIYLTVILGNLTILHVICTDATLHGPMYYF
LGMLAVTDLGLCLSTLPTVLGIFWFDTREIGIPACFTQLFFIHTLSSMESSVLLSMSIDRYVA
VCNPLHDSTVLTPACIVKMGLSSVLRSALLILPLPFLLKRFQYCHSHVLAHAYCLHLEIMK
LACSSIIVNHIYGLFVVACTVGVDSLLIFLSYALILRTVLSIASHQERLRALNTCVSHICAVLL
FYIPMIGLSLVHRFGEHLPRVVHLFMSYVYLLVPPLMNPIIYSIKTKQIRQRIIKKFQFIKSLR
CFWKD

>Q8NGK0 multipass membrane protein
MTLGSLGNSSSSVSATFLLSGIPGLERMHIWISIPLCFMYLVSIPGNCTILFIIKTERSLHEPM
YLFLSMLALIDLGLSLCTLPTVLGIFWVGAREISHDACFAQLFFIHCFSFLESSVLLSMAFD
RFVAICHPLHYVSILTNTVIGRIGLVSLGRSVALIFPLPFMLKRFPYCGSPVLSHSYCLHQEV
MKLACADMKANSIYGMFVIVSTVGIDSLLILFSYALILRTVLSIASRAERFKALNTCVSHIC

AVLLFYTPMIGLSVIHRFGKQAPHLVQVVMGFMYLLFPPVMNPIVYSVKTKQIRDRVTHA
FCY

>Q14964 lipid-anchor
METIWIYQFRLIVIGDSTVGKSCLLHRFTQGRFPGLRSPACDPTVGVDFFSRLLEIEPGKRIK
LQLWDTAGQERFRSITRSYYRNSVGGFLVFDITNRRSFEHVKDWLEEAKMYVQPFRIVFLL
VGHKCDLASQRQVTREEAEKLSADCGMKYIETSAKDATNVEESFTILTRDIYELIKKGEICI
QDGWEGVKSGFVPNTVHSSEEAVKPRKECFC

>P57768 peripheral membrane protein
MATPYVPVPMPIGNSASSFTTNRNQRSSSFGSVSTSSNSSKGQLEDSNMGNFKQTSVPDQ
MDNTSSVCSSPLIRTKFTGTASSIEYSTRPRDTEEQNPETVNWEDRPSTPTILGYEVMEERA
KFTVYKILVKKTPEESWVVFRRYTDFSRLNDKLKEMFPGFRLALPPKRWFKDNYNADFLE
DRQLGLQAFLQNLVAHKDIANCLAVREFLCLDDPPGPFDSLEESRAFCETLEETNYRLQKE
LLEKQKEMESLKKLLSEKQLHIDTLENRIRTLSLEPEESLDVSETEGEQILKVESSALEVDQ
DVLDEESRADNKPCLSFSEPENAVSEIEVAEVAYDAEED

>Q8NH63 multipass membrane protein
MTNLNASQANHRNFILTGIPGTPDKNPWLAFPLGFLYTLTLLGNGTILAVIKVEPSLHEPTY
YFLSILALTDVSLSMSTLPSMLSIYWFNAPQIVFDACIMQMFFIHVFGIVESGVLVSMAFDR
FVAIRNPLHYVSILTHDVIRKTGIAVLTRAVCVVFPVPFLIKCLPFCHSNVLSHSYCLHQNM
MRLACASTRINSLYGLIVVIFTLGLDVLLTLLSYVLTLKTVLGIVSRGERLKTLSTCLSHMS
TVLLFYVPFMGAASMIHRFWEHLSPVVHMVMADIYLLLPPVLNPIVYSVKTKQI

>Q9NQX7 single-pass type II membrane protein
MVKISFQPAVAGIKGDKADKASASAPAPASATEILLTPAREEQPPQHRSKRGGSVGGVCYLS
MGMVVLLMGLVFASVYIYRYFFLAQLARDNFFRCGVLYEDSLSSQVRTQMELEEDVKIYL
DENYERINVPVPQFGGGDPADIIHDFQRGLTAYHDISLDKCYVIELNTTIVLPPRNFWELLM
NVKRGTYLPQTYIIQEEMVVTEHVSDKEALGSFIYHLCNGKDTYRLRRRATRRRINKRGA
KNCNAIRHFENTFVVETLICGVV

>Q9HC97 multipass membrane protein
MNGTYNTCGSSDLTWPPAIKLGFYAYLGVLLVLGLLLNSLALWVFCCRMQQWTETRIYM
TNLAVADLCLLCTLPFVLHSLRDTSDTPLCQLSQGIYLTNRYMSISLVTAIAVDRYVAVRHP
LRARGLRSPRQAAAVCAVLWVLVIGSLVARWLLGIQEGGFCFRSTRHNFNSMAFPLLGFYL
PLAVVVFCSLKVVTALAQRPPTDVGQAEATRKAARMVWANLLVFVVCFLPLHVGLTVRL
AVGWNACALLETIRRALYITSKLSDANCCLDAICYYYMAKEFQEASALAVAPSAKAHKSQ
DSLCVTLA

>Q8NGJ8 multipass membrane protein
MSTLPTQIAPNSSTSMAPTFLLVGMPGLSGAPSWWTLPLIAVYLLSALGNGTILWIIALQPA
LHRPMHFFLFLLSVSDIGLVTALMPTLLGIALAGAHTVPASACLLQMVFIHVFSVMESSVL
LAMSIDRALAICRPLHYPALLTNGVISKISLAISFRCLGLHLPLPFLLAYMPYCLPQVLTHSY
CLHPDVARLACPEAWGAAYSLFVVLSAMGLDPLLIFFSYGLIGKVLQGVESREDRWKAGQ

TCAAHLSAVLLFYIPMILLALINHPELPITQHTHTLLSYVHFLLPPLINPILYSVKMKEIRKRI
LNRLQPRKVGGAQ

>Q8NH90 multipass membrane protein
MTLGNSTEVTEFYLLGFGAQHEFWCILFIVFLLIYVTSIMGNSGIILLINTDSRFQTLTYFFL
QHLAFVDICYTSAITPKMLQSFTEEKNLMLFQGCVIQFLVYATFATSDCYLLAMMAVDPYV
AICKPLHYTVIMSRTVCIRLVAGSYIMGSINASVQTGFTCSLSFCKSNSINHFFCDVPPILALS
CSNVDINIMLLVVFVGSNLIFTGLVVIFSYIYIMATILKMSSSAGRKKSFSTCASHLTAVTIFY
GTLSYMYLQSHSNNSQENMKVAFIFYGTVIPMLNPLIYSLRNKEVKEALKVIGKKLF

>Q8NH56 multipass membrane protein
MPLFNSLCWFPTIHVTPPSFILNGIPGLERVHVWISLPLCTMYIIFLVGNLGLVYLIYYEESL
HHPMYFFFGHALSLIDLLTCTTTLPNALCIFWFSLKEINFNACLAQMFFVHGFTGVESGVL
MLMALDRYVAICYPLRYATTLTNPIIAKAELATFLRGVLLMIPFPFLVKRLPFCQSNIISHTY
CDHMSVVKLSCASIKVNVIYGLMVALLIGVFDICCISLSYTLILKAAISLSSSDARQKAFSTC
TAHISAIIITYVPAFFTFFAHRFGGHTIPPSLHIIVANLYLLLPPTLNPIVYGVKTKQIRKSVIKF
FQGDKGAG

>P30939 multipass membrane protein
MDFLNSSDQNLTSEELLNRMPSKILVSLTLSGLALMTTTINSLVIAAIIVTRKLHHPANYLIC
SLAVTDFLVAVLVMPFSIVYIVRESWIMGQVVCDIWLSVDITCCTCSILHLSAIALDRYRAIT
DAVEYARKRTPKHAGIMITIVWIISVFISMPPLFWRHQGTSRDDECIIKHDHIVSTIYSTFGA
FYIPLALILILYYKIYRAAKTLYHKRQASRIAKEEVNGQVLLESGEKSTKSVSTSYVLEKSL
SDPSTDFDKIHSTVRSLRSEFKHEKSWRRQKISGTRERKAATTLGLILGAFVICWLPFFVKE
LVVNVCDKCKISEEMSNFLAWLGYLNSLINPLIYTIFNEDFKKAFQKLVRCRC

>Q8NGI3 multipass membrane protein
MNHMSASLKISNSSKFQVSEFILLGFPGIHSWQHWLSLPLALLYLSALAANTLILIIIWQNPS
LQQPMYIFLGILCMVDMGLATTIIPKILAIFWFDAKVISLPECFAQIYAIHFFVGMESGILLC
MAFDRYVAICHPLRYPSIVTSSLILKATLFMVLRNGLFVTPVPVLAAQRDYCSKNEIEHCLC
SNLGVTSLACDDRRPNSICQLVLAWLGMGSDLSLIILSYILILYSVLRLNSAEAAAKALSTC
SSHLTLILFFYTIVVVISVTHLTEMKATLIPVLLNVLHNIIPPSLNPTVYALQTKELRAAFQKV
LFALTKEIRS

>P15309 single-pass type I membrane protein
MRAAPLLLARAASLSLGFLFLLFFWLDRSVLAKELKFVTLVFRHGDRSPIDTFPTDPIKESS
WPQGFGQLTQLGMEQHYELGEYIRKRYRKFLNESYKHEQVYIRSTDVDRTLMSAMTNLA
ALFPPEGVSIWNPILLWQPIPVHTVPLSEDQLLYLPFRNCPRFQELESETLKSEEFQKRLHPY
KDFIATLGKLSGLHGQDLFGIWSKVYDPLYCESVHNFTLPSWATEDTMTKLRELSELSLLS
LYGIHKQKEKSRLQGGVLVNEILNHMKRATQIPSYKKLIMYSAHDTTVSGLQMALDVYN
GLLPPYASCHLTELYFEKGEYFVEMYYRNETQHEPYPLMLPGCSPSCPLERFAELVGPVIPQ
DWSTECMTTNSHQGTEDSTD

>Q8NGI2 multipass membrane protein

MLTLNKTDLIPASFILNGVPGLEDTQLWISFPFCSMYVVAMVGNCGLLYLIHYEDALHKPM
YYFLAMLSFTDLVMCSSTIPKALCIFWFHLKDIGFDECLVQMFFTHTFTGMESGVLMLMA
LDRYVAICYPLRYSTILTNPVIAKVGTATFLRGVLLIIPFTFLTKLLPYCRGNILPHTYCDHMS
VAKLSCGNVKVNAIYGLMVALLIWGFDILCITNSYTMILRAVVSLSSADARQKAFNTCTAH
ICAIVFSYTPAFFSFFSHRFGEHIIPPSCHIIVANIYLLLPPTMNPIVYGVKTKQIRDCVIRILSG
SKDTKSYSM

>Q8N4S9 multipass membrane protein
MSNDGRSRNRDRRYDEVPSDLPYQDTTIRTHPTLHDSERAVSADPLPPPPLPLQPPFGPDFY
SSDTEEPAIAPDLKPVRRFVPDSWKNFFRGKKKDPEWDKPVSDIRYISDGVECSPPASPARP
NHRSPLNSCKDPYGGSEGTFSSRKEADAVFPRDPYGSLDRHTQTVRTYSEKVEEYNLRYS
YMKSWAGLLRILGVVELLLGAGVFACVTAYIHKDSEWYNLFGYSQPYGMGGVGGLGSM
YGGYYYTGPKTPFVLVVAGLAWITTIIILVLGMSMYYRTILLDSNWWPLTEFGINVALFILY
MAAAIVYVNDTNRGGLCYYPLFNTPVNAVFCRVEGGQIAAMIFLFVTMIVYLISALVCLK
LWRHEAARRHREYMEQQEINEPSLSSKRKMCEMATSGDRQRDSEVNFKELRTAKMKPEL
LSGHIPPGHIPKPIVMPDYVAKYPVIQTDDERERYKAVFQDQFSEYKELSAEVQAVLRKFD
ELDAVMSRLPHHSESRQEHERISRIHEEFKKKKNDPTFLEKKERCDYLKNKLSHIKQRIQE
YDKVMNWDVQGYS

>P30203 single-pass type I membrane protein
MWLFFGITGLLTAALSGHPSPAPPDQLNTSSAESELWEPGERLPVRLTNGSSSCSGTVEVRL
EASWEPACGALWDSRAAEAVCRALGCGGAEAASQLAPPTPELPPPPAAGNTSVAANATLA
GAPALLCSGAEWRLCEVVEHACRSDGRRARVTCAENRALRLVDGGGACAGRVEMLEHG
EWGSVCDDTWDLEDAHVVCRQLGCGWAVQALPGLHFTPGRGPIHRDQVNCSGAEAYLW
DCPGLPGQHYCGHKEDAGAVCSEHQSWRLTGGADRCEGQVEVHFRGVWNTVCDSEWY
PSEAKVLCQSLGCGTAVERPKGLPHSLSGRMYYSCNGEELTLSNCSWRFNNSNLCSQSLA
ARVLCSASRSLHNLSTPEVPASVQTVTIESSVTVKIENKESRELMLLIPSIVLGILLLGSLIFIA
FILLRIKGKYALPVMVNHQHLPTTIPAGSNSYQPVPITIPKEVFMLPIQVQAPPPEDSDSGSD
SDYEHYDFSAQPPVALTTFYNSQRHRVTDEEVQQSRFQMPPLEEGLEELHASHIPTANPGH
CITDPPSLGPQYHPRSNSESSTSSGEDYCNSPKSKLPPWNPQVFSSERSSFLEQPPNLELAGT
QPAFSAGPPADDSSSTSSGEWYQNFQPPPQPPSEEQFGCPGSPSPQPDSTDNDDYDDISAA

>Q8NGP6 multipass membrane protein
MRRNCTLVTEFILLGLTSRRELQILLFTLFLAIYMVTVAGNLGMIVLIQANAWLHMPMYFF
LSHLSFVDLCFSSNVTPKMLEIFLSEKKSISYPACLVQCYLFIALVHVEIYILAVMAFDRYM
AICNPLLYGSRMSKSVCSFLITVPYVYGALTGLMETMWTYNLAFCGPNEINHFYCADPPLI
KLACSDTYNKELSMFIVAGWNLSFSLFIICISYLYIFPAILKIRSTEGRQKAFSTCGSHLTAVTI
FYATLFFMYLRPPSKESVEQGKMVAVFYTTVIPMLNLIIYSLRNKNVKEALIKELSMKIYFS

>Q8NGE2 multipass membrane protein
MKNKTVLTEFILLGLTDVPELQVAVFTFLFLAYLLSILGNLTILILTLLDSHLQTPMYFFLRNF
SFLEISFTNIFIPRVLISITTGNKSISFAGCFTQYFFAMFLGATEFYLLAAMSYDRYVAICKPLH
YTTIMSSRICIQLIFCSWLGGLMAIIPTITLMSQQDFCASNRLNHYFCDYEPLLELSCSDTSLI
EKVVFLVASVTLVVTLVLVILSYAFIIKTILKLPSAQQRTKAFSTCSSHMIVISLSYGSCMFM

YINPSAKEGDTFNKGVALLITSVAPLLNPFIYTLRNQQVKQPFKDMVKKLLNL

>Q96L94 peripheral membrane protein
MLEVHIPSVGPEAEGPRQSPEKSHMVFRVEVLCSGRRHTVPRRYSEFHALHKRIKKLYKVP
DFPSKRLPNWRTRGLEQRRQGLEAYIQGILYLNQEVPKELLEFLRLRHFPTDPKASNWGTL
REFLPGDSSSQQHQRPVLSFHVDPYVCNPSPESLPNVVVNGVLQGLYSFSISPDKAQPKAA
CHPAPLPPMP

>O75752 single-pass type II membrane protein
MASALWTVLPSRMSLRSLKWSLLLLSLLSFFVMWYLSLPHYNVIERVNWMYFYEYEPIY
RQDFHFTLREHSNCSHQNPFLVILVTSHPSDVKARQAIRVTWGEKKSWWGYEVLTFFLLG
QEAEKEDKMLALSLEDEHLLYGDIIRQDFLDTYNNLTLKTIMAFRWVTEFCPNAKYVMKT
DTDVFINTGNLVKYLLNLNHSEKFFTGYPLIDNYSYRGFYQKTHISYQEYPFKVFPPYCSG
LGYIMSRDLVPRIYEMMGHVKPIKFEDVYVGICLNLLKVNIHIPEDTNLFFLYRIHLDVCQL
RRVIAAHGFSSKEIITFWQVMLRNTTCHY

>Q9UKP6 multipass membrane protein
MALTPESPSSFPGLAATGSSVPEPPGGPNATLNSSWASPTEPSSLEDLVATGTIGTLLSAMGV
VGVVGNAYTLVVTCRSLRAVASMYVYVVNLALADLLYLLSIPFIVATYVTKEWHFGDVGC
RVLFGLDFLTMHASIFTLTVMSSERYAAVLRPLDTVQRPKGYRKLLALGTWLLALLLTLPV
MLAMRLVRRGPKSLCLPAWGPRAHRAYLTLLFATSIAGPGLLIGLLYARLARAYRRSQRAS
FKRARRPGARALRLVLGIVLLFWACFLPFWLWQLLAQYHQAPLAPRTARIVNYLTTCLTY
GNSCANPFLYTLLTRNYRDHLRGRVRGPGSGGGRGPVPSLQPRARFQRCSGRSLSSCSPQP
TDSLVLAPAAPARPAPEGPRAPA

>Q8NH51 multipass membrane protein
MEQHNLTTVNEFILTGITDIAELQAPLFALFLMIYVISVMGNLGMIVLTKLDSRLQTPMYFF
LRHLAFMDLGYSTTVGPKMLVNFVVDKNIISYYFCATQLAFFLVFIGSELFILSAMSYDLYV
AICNPLLYTVIMSRRVCQVLVAIPYLYCTFISLLVTIKIFTLSFCGYNVISHFYCDSLPLLPLLC
SNTHEIELIILIFAAIDLISSLLIVLLSYLLILVAILRMNSAGRQKAFSTCGAHLTVVIVFYGTL
LFMYVQPKSSHSFDTDKVASIFYTLVIPMLNPLIYSLRNKDVKYALRRTWNNLCNIFV

>P11168 multipass membrane protein
MTEDKVTGTLVFTVITAVLGSFQFGYDIGVINAPQQVIISHYRHVLGVPLDDRKAINNYVIN
STDELPTISYSMNPKPTPWAEEETVAAAQLITMLWSLSVSSFAVGGMTASFFGGWLGDTLG
RIKAMLVANILSLVGALLMGFSKLGPSHILIIAGRSISGLYCGLISGLVPMYIGEIAPTALRGA
LGTFHQLAIVTGILISQIIGLEFILGNYDLWHILLGLSGVRAILQSLLLFFCPESPRYLYIKLDE
EVKAKQSLKRLRGYDDVTKDINEMRKEREEASSEQKVSIIQLFTNSSYRQPILVALMLHVA
QQFSGINGIFYYSTSIFQTAGISKPVYATIGVGAVNMVFTAVSVFLVEKAGRRSLFLIGMSG
MFVCAIFMSVGLVLLNKFSWMSYVSMIAIFLFVSFFEIGPGPIPWFMVAEFFSQGPRPAALA
IAAFSNWTCNFIVALCFQYIADFCGPYVFFLFAGVLLAFTLFTFFKVPETKGKSFEEIAAEFQ
KKSGSAHRPKAAVEMKFLGATETV

>Q8WWA0 GPI-anchor, lipid-anchor

MNQLSFLLFLIATTRGWSTDEANTYFKEWTCSSSPSLPRSCKEIKDECPSAFDGLYFLRTEN
GVIYQTFCDMTSGGGGWTLVASVHENDMRGKCTVGDRWSSQQGSKAVYPEGDGNWAN
YNTFGSAEAATSDDYKNPGYYDIQAKDLGIWHVPNKSPMQHWRNSSLLRYRTDTGFLQT
LGHNLFGIYQKYPVKYGEGKCWTDNGPVIPVVYDFGDAQKTASYYSPYGQREFTAGFVQ
FRVFNNERAANALCAGMRVTGCNTEHHCIGGGGYFPEASPQQCGDFSGFDWSGYGTHV
GYSSSREITEAAVLLFYR

>Q9NZP2 multipass membrane protein
MKNHTVIRTFILLGLTGDPHLQVLLFIFLFLTYMLSVTGNLTIITLTLVDHHLKTPMYFFLRN
FSFLEVSFTTVCIPRFLYNISMGDNTITYNACASQIFFVILFGATEFFLLAAMSYDRYVAICK
PLHYVVIMNNRVCTLLVLCCWVAGLMIIVPPLSLGLQLEFCDSNAIDHFSCDAGPLLKISCS
DTWVIEQMVILMAVFALIITLVCVILSYLYIVRTILKFPSVQQRKKAFSTCSSHMIVVSIAYG
SCIFIYIKPSAKDEVAINKGVSVLTTSVAPLLNPFIYTLRNKQVKQAFSDSIKRIAFLSKK

>Q8NGG2 multipass membrane protein
MSYSIYKSTVNIPLSHGVVHSFCHNMNCNFMHIFKFVLDFNMKNVTEVTLFVLKGFTDNL
ELQTIFFFLFLAIYLFTLMGNLGLILVVIRDSQLHKPMYYFLSMLSSVDACYSSVITPNMLV
DFTTKNKVISFLGCVAQVFLACSFGTTECFLLAAMAYDRYVAIYNPLLYSVSMSPRVYMPL
INASYVAGILHATIHTVATFSLSFCGANEIRRVFCDIPPLLAISYSDTHTNQLLLFYFVGSIELV
TILIVLISYGLILLAILKMYSAEGRRKVFSTCGAHLTGVSIYYGTILFMYVRPSSSYASDHD
MIVSIFYTIVIPLLNPVIYSLRNKDVKDSMKKMFGKNQVINKVYFHTKK

>Q96G91 multipass membrane protein
MAANVSGAKSCPANFLAAADDKLSGFQGDFLWPILVVEFLVAVASNGLALYRFSIRKQRP
WHPAVVFSVQLAVSDLLCALTLPPLAAYLYPPKHWRYGEAACRLERFLFTCNLLGSVIFIT
CISLNRYLGIVHPFFARSHLRPKHAWAVSAAGWVLAALLAMPTLSFSHLKRPQQGAGNCS
VARPEACIKCLGTADHGLAAYRAYSLVLAGLGCGLPLLLTLAAYGALGRAVLRSPGMTVA
EKLRVAALVASGVALYASSYVPYHIMRVLNVDARRRWSTRCPSFADIAQATAALELGPYVG
YQVMRGLMPLAFCVHPLLYMAAVPSLGCCCRHCPGYRDSWNPEDAKSTGQALPLNATA
APKPSEPQSRELSQ

>Q8NHG8 peripheral membrane protein
MGAKQSGPAAANGRTRAYSGSDLPSSSSGGANGTAGGGGGARAAAAGRFPAQVPSAHQP
SASGGAAAAAAAPAAPAAPRSRSLGGAVGSVASGARAAQSPFSIPNSSSGPYGSQDSVHSS
PEDGGGGRDRPVGGSPGGPRLVIGSLPAHLSPHMFGGFKCPVCSKFVSSDEMDLHLVMCL
TKPRITYNEDVLSKDAGECAICLEELQQGDTIARLPCLCIYHKGCIDEWFEVNRSCPEHPS
D

>Q8IUK5 single-pass type I membrane protein
MRGELWLLVLVLREAARALSPQPGAGHDEGPGSGWAAKGTVRGWNRRARESPGHVSEP
DRTQLSQDLGGGTLAMDTLPDNRTRVVEDNHSYYVSRLYGPSEPHSRELWVDVAEANRS
QVKIHTILSNTHRQASRVVLSFDFPFYGHPLRQITIATGGFIFMGDVIHRMLTATQYVAPLM
ANFNPGYSDNSTVVYFDNGTVFVVQWDHVYLQGWEDKGSFTFQAALHHDGRIVFAYKE
IPMSVPEISSSQHPVKTGLSDAFMILNPSPDVPESRRRSIFEYHRIELDPSKVTSMSAVEFTPL

PTCLQHRSCDACMSSDLTFNCSWCHVLQRCSSGFDRYRQEWMDYGCAQEAEGRMCEDF
QDEDHDSASPDTSFSPYDGDLTTTSSSLFIDSLTTEDDTKLNPYAGGDGLQNNLSPKTKGTP
VHLGTIVGIVLAVLLVAAIILAGIYINGHPTSNAALFFIERRPHHWPAMKFRSHPDHSTYAE
VEPSGHEKEGFMEAEQC

>Q8N146 multipass membrane protein
MMGRRNDTNVADFILTGLSDSEEVQMALFMLFLLIYLITMLGNVGMLLIIRLDLQLHTPM
YFFLTHLSFIDLSYSTVVTPKTLANLLTSNYISFTGCFAQMFCFVFLGTAECYLLSSMAYDR
YAAICSPLHYTVIMPKRLCLALITGPYVIGFMDSFVNVVSMSRLHFCDSNIIHHFFCDTSPIL
ALSCTDTDNTEMLIFIIAGSTLMVSLITISASYVSILSTILKINSTSGKQKAFSTCVSHLLGVTI
FYGTMIFTYLKPRKSYSLGRDQVAPVFYTIVIPMLNPLIYSLRNREVKNALIRVMQRRQDS
R

>Q8NH61 multipass membrane protein
MTETSLSSQCFPMSVLNNTIAEPLIFLLMGIPGLKATQYWISIPFCLLYVVAVSGNSMILFVV
LCERSLHKPMYYFLSMLSATDLSLSLCTLSTTLGVFWFEAREINLNACIAQMFFLHGFTFM
ESGVLLAMAFDRFVAICYPLRYTTILTNARIAKIGMSMLIRNVAVMLPVMLFVKRLSFCSS
MVLSHSYCYHVDLIQLSCTDNRINSILGLFALLSTTGFDCPCILLSYILIIRSVLSIASSEERRK
AFNTCTSHISAVSIFYLPLISLSLVHRYGHSAPPFVHIIMANVFLLIPPVLNPIIYSVKIKQIQK
AIIKVLIQKHSKSNHQLFLIRDKAIYE

>P48509 multipass membrane protein
MGEFNEKKTTCGTVCLKYLLFTYNCCFWLAGLAVMAVGIWTLALKSDYISLLASGTYLAT
AYILVVAGTVVMVTGVLGCCATFKERRNLLRLYFILLLIIFLLEIIAGILAYAYYQQLNTELK
ENLKDTMTKRYHQPGHEAVTSAVDQLQQEFHCCGSNNSQDWRDSEWIRSQEAGGRVVP
DSCCKTVVALCGQRDHASNIYKVEGGCITKLETFIQEHLRVIGAVGIGIACVQVFGMIFTCC
LYRSLKLEHY

>Q8N127 multipass membrane protein
MLESNYTMPTEFLFVGFTDYLPLRVTLFLVFLLVYTLTMVGNILLIILVNINSSLQIPMYYFL
SNLSFLDISCSTAITPKMLANFLASRKSISPYGCALQMFFFASFADAECLILAAMAYDRYAAI
CNPLLYTTLMSRRVCVCFIVLAYFSGSTTSLVHVCLTFRLSFCGSNIVNHFFCDIPPLLALSC
TDTQINQLLLFALCSFIQTSTFVVIFISYFCILITVLSIKSSGGRSKTFSTCASHLIAVTLFYGA
LLFMYLQPTTSYSLDTDKVVAVFYTVVFPMFNPIIYSFRNKDVKNALKKLLERIGYSNEW
YLNRLRIVNI

>Q01814 multipass membrane protein
MGDMTNSDFYSKNQRNESSHGGEFGCTMEELRSLMELRGTEAVVKIKETYGDTEAICRR
LKTSPVEGLPGTAPDLEKRKQIFGQNFIPPKKPKTFLQLVWEALQDVTLIILEIAAIISLGLSF
YHPPGEGNEGCATAQGGAEDEGEAEAGWIEGAAILLSVICVVLVTAFNDWSKEKQFRGLQ
SRIEQEQKFTVVRAGQVVQIPVAEIVVGDIAQVKYGDLLPADGLFIQGNDLKIDESSLTGES
DQVRKSVDKDPMLLSGTHVMEGSGRMLVTAVGVNSQTGIIFTLLGAGGEEEEKKDKKGV
KKGDGLQLPAADGAAASNAADSANASLVNGKMQDGNVDASQSKAKQQDGAAAMEMQ
PLKSAEGGDADDRKKASMHKKEKSVLQGKLTKLAVQIGKAGLVMSAITVIILVLYFTVDT

FVVNKKPWLPECTPVYVQYFVKFFIIGVTVLVVAVPEGLPLAVTISLAYSVKKMMKDNNL
VRHLDACETMGNATAICSDKTGTLTTNRMTVVQAYVGDVHYKEIPDPSSINTKTMELLIN
AIAINSAYTTKILPPEKEGALPRQVGNKTECGLLGFVLDLKQDYEPVRSQMPEEKLYKVYT
FNSVRKSMSTVIKLPDESFRMYSKGASEIVLKKCCKILNGAGEPRVFRPRDRDEMVKKVIE
PMACDGLRTICVAYRDFPSSPEPDWDNENDILNELTCICVVGIEDPVRPEVPEAIRKCQRAG
ITVRMVTGDNINTARAIAIKCGIIHPGEDFLCLEGKEFNRRIRNEKGEIEQERIDKIWPKLRV
LARSSPTDKHTLVKGIIDSTHTEQRQVVAVTGDGTNDGPALKKADVGFAMGIAGTDVAKE
ASDIILTDDNFSSIVKAVMWGRNVYDSISKFLQFQLTVNVVAVIVAFTGACITQDSPLKAVQ
MLWVNLIMDTFASLALATEPPTETLLLRKPYGRNKPLISRTMMKNILGHAVYQLALIFTLL
FVGEKMFQIDSGRNAPLHSPPSEHYTIIFNTFVMMQLFNEINARKIHGERNVFDGIFRNPIF
CTIVLGTFAIQIVIVQFGGKPFSCSPLQLDQWMWCIFIGLGELVWGQVIATIPTSRLKFLKEA
GRLTQKEEIPEEELNEDVEEIDHAERELRRGQILWFRGLNRIQTQIRVVKAFRSSLYEGLEK
PESRTSIHNFMAHPEFRIEDSQPHIPLIDDTDLEEDAALKQNSSPPSSLNKNNSAIDSGINLTT
DTSKSATSSSPGSPIHSLETSL

>P30559 multipass membrane protein
MEGALAANWSAEAANASAAPPGAEGNRTAGPPRRNEALARVEVAVLCLILLLALSGNAC
VLLALRTTRQKHSRLFFFMKHLSIADLVVAVFQVLPQLLWDITFRFYGPDLLCRLVKYLQV
VGMFASTYLLLLMSLDRCLAICQPLRSLRRRTDRLAVLATWLGCLVASAPQVHIFSLREVA
DGVFDCWAVFIQPWGPKAYITWITLAVYIVPVIVLAACYGLISFKIWQNLRLKTAAAAAAE
APEGAAAGDGGRVALARVSSVKLISKAKIRTVKMTFIIVLAFIVCWTPFFFVQMWSVWDA
NAPKEASAFIIVMLLASLNSCCNPWIYMLFTGHLFHELVQRFLCCSASYLKGRRLGETSAS
KKSNSSSFVLSHRSSSQRSCSQPSTA

>Q8NFX7 peripheral membrane protein
MSAKSAISKEIFAPLDERMLGAVQVKRRTKKKIPFLATGGQGEYLTYICLSVTNKKPTQASI
TKVKQFEGSTSFVRRSQWMLEQLRQVNGIDPNGDSAEFDLLFENAFDQWVASTASEKCTF
FQILHHTCQRYLTDRKPEFINCQSKIMGGNSILHSAADSVTSAVQKASQALNERGERLGRA
EEKTEDLKNSAQQFAETAHKLAMKHKC

>O14817 multipass membrane protein
MARACLQAVKYLMFAFNLLFWLGGCGVLGVGIWLAATQGSFATLSSSFPSLSAANLLIITG
AFVMAIGFVGCLGAIKENKCLLLTFFLLLLLVFLLEATIAILFFAYTDKIDRYAQQDLKKGL
HLYGTQGNVGLTNAWSIIQTDFRCCGVSNYTDWFEVYNATRVPDSCCLEFSESCGLHAPG
TWWKAPCYETVKVWLQENLLAVGIFGLCTALVQILGLTFAMTMYCQVVKADTYCA

>Q8NHC7 multipass membrane protein
MPNSTTVMEFLLMRFSDVWTLQILHSASFFMLYLVTLMGNILIVTVTTCDSSLHMPMYFF
LRNLSILDACYISVTVPTSCVNSLLDSTTISKAGCVAQVFLVVFFVYVELLFLTIMAHDRYV
AVCQPLHYPVIVNSRICIQMTLASLLSGLVYAGMHTGSTFQLPFCRSNVIHQFFCDIPSLLK
LSCSDTFSNEVMIVVSALGVGGGCFIFIIRSYIHIFSTVLGFPRGADRTKAFSTCIPHILVVSV
FLSSCSSVYLRPPAIPAATQDLILSGFYSIMPPLFNPIIYSLRNKQIKVAIKKIMKRIFYSENV

>Q8NFN8 multipass membrane protein

MEPEINCSELCDSFPGQELDRRPLHDLCKTTITSSHHSSKTISSLSPVLLGIVWTFLSCGLLLI
LFFLAFTIHCRKNRIVKMSSPNLNIVTLLGSCLTYSSAYLFGIQDVLVGSSMETLIQTRLSML
CIGTSLVFGPILGKSWRLYKVFTQRVPDKRVIIKDLQLLGLVAALLMADVILLMTWVLTDPI
QCLQILSVSMTVTGKDVSCTSTSTHFCASRYSDVWIALIWGCKGLLLLYGAYLAGLTGHV
SSPPVNQSLTIMVGVNLLVLAAGLLFVVTRYLHSWPNLVFGLTSGGIFVCTTTINCFIFIPQL
KQWKAFEEENQTIRRMAKYFSTPNKSFHTQYGEEENCHPRGEKSSMERLLTEKNAVIESL
QEQVNNAKEKIVRLMSAECTYDLPEGAAPPASSPNKDVQAVASVHTLAAAQGPSGHLSDF
QNDPGMAARDSQCTSGPSSYAQSLEGPGKDSSFSPGKEEKISDSKDFSDHLDSGCSQKPW
TEQSLGPERGDQVPMNPSQSLLPERGGSDPQRQRHLENSEEPPERRSRVSSVIREKLQEVL
QDLGLGPEASLSTAPSCHQQTWKNSAAFSPQKMPLSKELGFSPYMVRRRRAAQRARSHF
PGSAPSSVGHRANRTVPGAHSRLHVQNGDSPSLAPQTTDSRVRRPSSRKPSLPSDPQDRPG
TLEGSKQSQTEPEGARGSKAAFLRQPSGSGRAPSPAAPCLSKASPDLPEQWQLWPPVPSGC
ASLSSQHSYFDTESSSSDEFFCRCHRPYCEICFQSSSDSSDSGTSDTDPEPTGGLASWEKLW
ARSKPIVNFKDDLKPTLV

>Q8NG77 multipass membrane protein
MEMRNTTPDFILLGLFNHTRAHQVLFMMLLATVLTSLFSNALMILLIHWDHRLHRPMYFL
LSQLSLMDMMLVSTTVPKMAADYLTGNKAISRAGCGVQIFFLPTLGGGECFLLAAMAYD
RYAAVCHPLRYPTLMSWQLCLRMTMSSWLLGAADGLLQAVATLSFPYCGAHEIDHFFCE
APVLVRLACADTSVFENAMYICCVLMLLVPFSLILSSYGLILAAVLLMRSTEARKKAFATC
SSHVAVVGLFYGAGIFTYMRPKSHRSTNHDKVVSAFYTMFTPLLNPLIYSVRNSEVKEAL
KRWLGTCVNLKHQQNEAHRSR

>P20813 peripheral membrane protein
MELSVLLFLALLTGLLLLLVQRHPNTHDRLPPGPRPLPLLGNLLQMDRRGLLKSFLRFREK
YGDVFTVHLGPRPVVMLCGVEAIREALVDKAEAFSGRGKIAMVDPFFRGYGVIFANGNR
WKVLRRFSVTTMRDFGMGKRSVEERIQEEAQCLIEELRKSKGALMDPTFLFQSITANIICSI
VFGKRFHYQDQEFLKMLNLFYQTFSLISSVFGQLFELFSGFLKYFPGAHRQVYKNLQEINA
YIGHSVEKHRETLDPSAPKDLIDTYLLHMEKEKSNAHSEFSHQNLNLNTLSLFFAGTETTS
TTLRYGFLLMLKYPHVAERVYREIEQVIGPHRPPELHDRAKMPYTEAVIYEIQRFSDLLPM
GVPHIVTQHTSFRGYIIPKDTEVFLILSTALHDPHYFEKPDAFNPDHFLDANGALKKTEAFI
PFSLGKRICLGEGIARAELFLFFTTILQNFSMASPVAPEDIDLTPQECGVGKIPPTYQIRFLPR

>Q8NH72 multipass membrane protein
MENQNNVTEFILLGLTENLELWKIFSAVFLVMYVATVLENLLIVVTIITSQSLRSPMYFFLTF
LSLLDVMFSSVVAPKVIVDTLSKSTTISLKGCLTQLFVEHFFGGVGIILLTVMAYDRYVAIC
KPLHYTIIMSPRVCCLMVGGAWVGGFMHAMIQLLFMYQIPFCGPNIIDHFICDLFQLLTLA
CTDTHILGLLVTLNSGMMCVAIFLILIASYTVILCSLKSYSSKGRHKALSTCSSHLTVVVLFF
VPCIFLYMRPVVTHPIDKAMAVSDSIITPMLNPLIYTLRNAEVKSAMKKLWMKWEALAGK

>Q8NGL7 multipass membrane protein
MEKSNNSTLFILLGFSQNKNIEVLCFVLFLFCYIAIWMGNLLIMISITCTQLIHQPMYFFLNY
LSLSDLCYTSTVTPKLMVDLLAERKTISYNNCMIQLFTTHFFGGIEIFILTGMAYDRYVAIC
KPLHYTIIMSRQKCNTIIIVCCTGGFIHSASQFLLTIFVPFCGPNEIDHYFCDVYPLLKLACSN

IHMIGLLVIANSGLIALVTFVVLLLSYVFILYTIRAYSAERRSKALATCSSHVIVVVLFFAPAL
FIYIRPVTTFSEDKVFALFYTIIAPMFNPLIYTLRNTEMKNAMRKVWCCQILLKRNQLF

>Q8NFY4 single-pass type I membrane protein
MRVFLLCAYILLLMVSQLRAVSFPEDDEPLNTVDYHYSRQYPVFRGRPSGNESQHRLDFQ
LMLKIRDTLYIAGRDQVYTVNLNEMPKTEVIPNKKLTWRSRQQDRENCAMKGKHKDEC
HNFIKVFVPRNDEMVFVCGTNAFNPMCRYYRLSTLEYDGEEISGLARCPFDARQTNVALF
ADGKLYSATVADFLASDAVIYRSMGDGSALRTIKYDSKWIKEPHFLHAIEYGNYVYFFFRE
IAVEHNNLGKAVYSRVARICKNDMGGSQRVLEKHWTSFLKARLNCSVPGDSFFYFDVLQS
ITDIIQINGIPTVVGVFTTQLNSIPGSAVCAFSMDDIEKVFKGRFKEQKTPDSVWTAVPEDK
VPKPRPGCCAKHGLAEAYKTSIDFPDETLSFIKSHPLMDSAVPPIADEPWFTKTRVRYRLTA
ISVDHSAGPYQNYTVIFVGSEAGMVLKVLAKTSPFSLNDSVLLEEIEAYNHAKCSAENEED
KKVISLQLDKDHHALYVAFSSCIIRIPLSRCERYGSCKKSCIASRDPYCGWLSQGSCGRVTP
GMLAEGYEQDTEFGNTAHLGDCHEILPTSTTPDYKIFGGPTSDMEVSSSSVTTMASIPEITP
KVIDTWRPKLTSSRKFVVQDDPNTSDFTDPLSGIPKGVRWEVQSGESNQMVHMNVLITCV
FAAFVLGAFIAGVAVYCYRDMFVRKNRKIHKDAESAQSCTDSSGSFAKLNGLFDSPVKEY
QQNIDSPKLYSNLLTSRKELPPNGDTKSMVMDHRGQPPELAALPTPESTPVLHQKTLQAM
KSHSEKAHGHGASRKETPQFFPSSPPPHSPLSHGHIPSAIVLPNATHDYNTSFSNSNAHKAE
KKLQNIDHPLTKSSSKRDHRRSVDSRNTLNDLLKHLNDPNSNPKAIMGDIQMAHQNLML
DPMGSMSEVPPKVPNREASLYSPPSTLPRNSPTKRVDVPTTPGVPMTSLERQRGYHKNSSQ
RHSISAMPKNLNSPNGVLLSRQPSMNRGGYMPTPTGAKVDYIQGTPVSVHLQPSLSRQSS
YTSNGTLPRTGLKRTPSLKPDVPPKPSFVPQTPSVRPLNKYTY

>P10747 single-pass type I membrane protein
MLRLLLALNLFPSIQVTGNKILVKQSPMLVAYDNAVNLSCKYSYNLFSREFRASLHKGLDS
AVEVCVVYGNYSQQLQVYSKTGFNCDGKLGNESVTFYLQNLYVNQTDIYFCKIEVMYPP
PYLDNEKSNGTIIHVKGKHLCPSPLFPGPSKPFWVLVVVGGVLACYSLLVTVAFIIFWVRS
KRSRLLHSDYMNMTPRRPGPTRKHYQPYAPPRDFAAYRS

>Q8NGL9 multipass membrane protein
MQLNNNVTEFILLGLTQDPFWKKIVFVIFLRLYLGTLLGNLLIIISVKTSQALKNPMFFFLFY
LSLSDTCLSTSITPRMIVDALLKKTTISFSECMIQVFSSHVFGCLEIFILILTAVDRYVDICKPL
HYMTIISQWVCGVLMAVAWVGSCVHSLVQIFLALSLPFCGPNVINHCFCDLQPLLKQACS
ETYVVNLLLVSNSGAICAVSYVMLIFSYVIFLHSLRNHSAEVIKKALSTCVSHIIVVILFFGP
CIFMYTCLATVFPMDKMIAVFYTVGTSFLNPVIYTLKNTEVKSAMRKLWSKKLITDDKR

>Q8NGM1 multipass membrane protein
MQNQSFVTEFVLLGLSQNPNVQEIVFVVFLFVYIATVGGNMLIVVTILSSPALLVSPMYFFL
GFLSFLDACFSSVITPKMIVDSLYVTKTISFEGCMMQLFAEHFFAGVEVIVLTAMAYDRYVA
ICKPLHYSSIMNRRLCGILMGVAWTGGLLHSMIQILFTFQLPFCGPNVINHFMCDLYPLLEL
ACTDTHIFGLMVVINSGFICIINFSLLLVSYAVILLSLRTHSSEGRWKALSTCGSHIAVVILFF
VPCIFVYTRPPSAFSLDKMAAIFYIILNPLLNPLIYTFRNKEVKQAMRRIWNRLMVVSDEK
ENIKL

>Q9Y678 peripheral membrane protein
MLKKFDKKDEESGGGSNPFQHLEKSAVLQEARVFNETPINPRKCAHILTKILYLINQGEHL
GTTEATEAFFAMTKLFQSNDPTLRRMCYLTIKEMSCIAEDVIIVTSSLTKDMTGKEDNYRG
PAVRALCQITDSTMLQAIERYMKQAIVDKVPSVSSSALVSSLHLLKCSFDVVKRWVNEAQ
EAASSDNIMVQYHALGLLYHVRKNDRLAVNKMISKVTRHGLKSPFAYCMMIRVASKQLE
EEDGSRDSPLFDFIESCLRNKHEMVVYEAASAIVNLPGCSAKELAPAVSVLQLFCSSPKAA
LRYAAVRTLNKVAMKHPSAVTACNLDLENLVTDSNRSIATLAITTLLKTGSESSIDRLMKQI
SSFMSEISDEFKVVVVQAISALCQKYPRKHAVLMNFLFTMLREEGGFEYKRAIVDCIISIIEE
NSESKETGLSHLCEFIEDCEFTVLATRILHLLGQEGPKTTNPSKYIRFIYNRVVLEHEEVRA
GAVSALAKFGAQNEEMLPSILVLLKRCVMDDDNEVRDRATFYLNVLEQKQKALNAGYIL
NGLTVSIPGLERALQQYTLEPSEKPFDLKSVPLATAPMAEQRTESTPITAVKQPEKVAATRQ
EIFQEQLAAVPEFRGLGPLFKSSPEPVALTESETEYVIRCTKHTFTNHMVFQFDCTNTLNDQ
TLENVTVQMEPTEAYEVLCYVPARSLPYNQPGTCYTLVALPKEDPTAVACTFSCMMKFTV
KDCDPTTGETDDEGYEDEYVLEDLEVTVADHIQKVMKLNFEAAWDEVGDEFEKEETFTL
STIKTLEEAVGNIVKFLGMHPCERSDKVPDNKNTHTLLLAGVFRGGHDILVRSRLLLLDTV
TMQVTARSLEELPVDIILASVG

>Q8NGL6 multipass membrane protein
MELLTNNLKFITDPFVCRLRHLSPTPSEEHMKNKNNVTEFILLGLTQNPEGQKVLFVTFLLI
YMVTIMGNLLIIVTIMASQSLGSPMYFFLASLSFIDTVYSTAFAPKMIVDLLSEKKTISFQGC
MAQLFMDHLFAGAEVILLVVMAYDRYMAICKPLHELITMNRRVCVLMLLAAWIGGFLHS
LVQFLFIYQLPFCGPNVIDNFLCDLYPLLKLACTNTYVTGLSMIANGGAICAVTFFTILLSY
GVILHSLKTQSLEGKRKAFYTCASHVTVVILFFVPCIFLYARPNSTFPIDKSMTVVLTFITPM
LNPLIYTLKNAEMKSAMRKLWSKKVSLAGKWLYHS

>Q8NGN3 multipass membrane protein
MSNASLVTAFILTGLPHAPGLDALLFGIFLVVYVLTVLGNLLILLVIRVDSHLHTPMYYFLT
NLSFIDMWFSTVTVPKMLMTLVSPSGRAISFHSCVAQLYFFHFLGSTECFLYTVMSYDRYL
AISYPLRYTSMMSGSRCALLATGTWLSGSLHSAVQTILTFHLPYCGPNQIQHYFCDAPPILK
LACADTSANVMVIFVDIGIVASGCFVLIVLSYVSIVCSILRIRTSDGRRRAFQTCASHCIVVL
CFFVPCVVIYLRPGSMDAMDGVVAIFYTVLTPLLNPVVYTLRNKEVKKAVLKLRDKVAHP
QRK

>Q8NGZ0 multipass membrane protein
MGHQNHTFSSDFILLGLFSSSPTSVVFFLVLFVIFIMSVTENTLMILLIRSDSRLHTPMYFLLS
HLSLMDILHVSNIVPKMVTNFLSGSRTISFAGCGFQVFLSLTLLGGECLLLAAMSCDRYVAI
CHPLRYPILMKEYASALMAGGSWLIGVFNSTVHTAYALQFPFCGSRAIDHFFCEVPAMLKL
SCADTTRYERGVCVSAVIFLLIPFSLISASYGQIILTVLQMKSSEARKKSFSTCSFHMIVVTM
YYGPFIFTYMRPKSYHTPGQDKFLAIFYTILTPTLNPFIYSFRNKDVLAVMKNMLKSNFLH
KKMNRKIPECVFCLFLC

>Q8NH70 multipass membrane protein
MRPSSNVTEFVLLGLTQDPDVKKTLFVMFLLIYIVTMVGNLLIWVTTIGSPSLGSLMYFFL
AYLSLMDAIYSTAMSPKLMIDLLCDKIAISLSACMGQLFIEHLLGGAEVFLLVVMAYDRYV

AISKPLHYLNIMNRLVCILLLVVAMIGGFVHSVVQIVFLYSLPICGPNVIDHSVCDMYPLLE
LLCLDTYFIGLTVVANGGIICMVIFTFLLISCGVILNFLKTYSQEERHKALPTCISHIIVVALVF
VPCIFMYVRPVSNFPFDKLMTVFYSIITLMLNPLIYSLRQSEMKNAMKNLWCEKLSIVRKR
VSPTLNIFIPSSKATNRR

>Q8NGN1 multipass membrane protein
MNPENWTQVTSFVLLGFPSSHLIQFLVFLGLMVTYIVTATGKLLIIVLSWIDQRLHIQMYFF
LRNFSFLELLLVTVVVPKMLVVILTGDHTISFVSCIIQSYLYFFLGTTDFFLLAVMSLDRYLA
ICRPLRYETLMNGHVCSQLVLASWLAGFLWVLCPTVLMASLPFCGPNGIDHFFRDSWPLL
RLSCGDTHLLKLVAFMLSTLVLLGSLALTSVSYACILATVLRAPTAAERRKAFSTCASHLTV
VVIIYGSSIFLYIRMSEAQSKLLNKGASVLSCIITPLLNPFIFTLRNDKVQQALREALGWPRL
TAVMKLRVTSQRK

>Q9NYA4 peripheral membrane protein
MGEEGPPSLEYIQAKDLFPPKELVKEEENLQVPFTVLQGEGVEFLGRAADALIAISNYRLHI
KFKDSVINVPLRMIDSVESRDMFQLHISCKDSKVVRCHFSTFKQCQEWLSRLSRATARPAK
PEDLFAFAYHAWCLGLTEEDQHTHLCQPGEHIRCRQEAELARMGFDLQNVWRVSHINSN
YKLCPSYPQKLLVPVWITDKELENVASFRSWKRIPVVVYRHLRNGAAIARCSQPEISWWG
WRNADDEYLVTSIAKACALDPGTRATGGSLSTGNNDTSEACDADFDSSLTACSGVESTAA
PQKLLILDARSYTAAVANRAKGGGCECEEYYPNCEVVFMGMANIHAIRNSFQYLRAVCSQ
MPDPSNWLSALESTKWLQHLSVMLKAAVLVANTVDREGRPVLVHCSDGWDRTPQIVALA
KILLDPYYRTLEGFQVLVESDWLDFGHKFGDRCGHQENVEDQNEQCPVFLQWLDSVHQL
LKQFPCLFEFNEAFLVKLVQHTYSCLYGTFLANNPCEREKRNIYKRTCSVWALLRAGNKN
FHNFLYTPSSDMVLHPVCHVRALHLWTAVYLPASSPCTLGEENMDLYLSPVAQSQEFSGRS
LDRLPKTRSMDDLLSACDTSSPLTRTSSDPNLNNHCQEVRVGLEPWHSNPEGSETSFVDSG
VGGPQQTVGEVGLPPPLPSSQKDYLSNKPFKSHKSCSPSYKLLNTAVPREMKSNTSDPEIK
VLEETKGPAPDPSAQDELGRTLDGIGEPPEHCPETEAVSALSKVISNKCDGVCNFPESSQNS
PTGTPQQAQPDSMLGVPSKCVLDHSLSTVCNPPSAACQTPLDPSTDFLNQDPSGSVASISH
QEQLSSVPDLTHGEEDIGKRGNNRNGQLLENPRFGKMPLELVRKPISQSQISEFSFLGSNW
DSFQGMVTSFPSGEATPRRLLSYGCCSKRPNSKQMRATGPCFGGQWAQREGVKSPVCSSH
SNGHCTGPGGKNQMWLSSHPKQVSSTKPVPLNCPSPVPPLYLDDDGLPFPTDVIQHRLRQI
EAGYKQEVEQLRRQVRELQMRLDIRHCCAPPAEPPMDYEDDFTCLKESDGSDTEDFGSD
HSEDCLSEASWEPVDKKETEVTRWVPDHMASHCYNCDCEFWLAKRRHHCRNCGNVFC
AGCCHLKLPIPDQQLYDPVLVCNSCYEHIQVSRARELMSQQLKKPIATASS

>Q92572 peripheral membrane protein
MIKAILIFNNHGKPRLSKFYQPYSEDTQQQIIRETFHLVSKRDENVCNFLEGGLLIGGSDNK
LIYRHYATLYFVFCVDSSESELGILDLIQVFVETLDKCFENVCELDLIFHVDKVHNILAEMV
MGGMVLETNMNEIVTQIDAQNKLEKSEAGLAGAPARAVSAVKNMNLPEIPRNINIGDISIK
VPNLPSFK

>Q8NGM9 multipass membrane protein
MGVKNHSTVTEFLLSGLTEQAELQLPLFCLFLGIYTVTVVGNLSMISIIRLNRQLHTPMYY
FLSSLSFLDFCYSSVITPKMLSGFLCRDRSISYSGCMIQLFFFCVCVISECYMLAAMACDRY

VAICSPLLYRVIMSPRVCSLLVAAVFSVGFTDAVIHGGCILRLSFCGSNIIKHYFCDIVPLIKLS
CSSTYIDELLIFVIGGFNMVATSLTIIISYAFILTSILRIHSKKGRCKAFSTCSSHLTAVLMFYGS
LMSMYLKPASSSSLTQEKVSSVFYTTVILMLNPLIYSLRNNEVRNALMKLLRRKISLSPG

>Q9H300 multipass membrane protein
MAWRGWAQRGWGCGQAWGASVGGRSCEELTAVLTPPQLLGRRFNFFIQQKCGFRKAPR
KVEPRRSDPGTSGEAYKRSALIPPVEETVFYPSPYPIRSLIKPLFFTVGFTGCAFGSAAIWQY
ESLKSRVQSYFDGIKADWLDSIRPQKEGDFRKEINKWWNNLSDGQRTVTGIIAANVLVFC
LWRVPSLQRTMIRYFTSNPASKVLCSPMLLSTFSHFSLFHMAANMYVLWSFSSSIVNILGQ
EQFMAVYLSAGVISNFVSYVGKVATGRYGPSLGASGAIMTVLAAVCTKIPEGRLAIIFLPMF
TFTAGNALKAIIAMDTAGMILGWKFFDHAAHLGGALFGIWYVTYGHELIWKNREPLVKI
WHEIRTNGPKKGGGSK

>Q15046 peripheral membrane protein
MAAVQAAEVKVDGSEPKLSKNELKRRLKAEKKVAEKEAKQKELSEKQLSQATAAATNHT
TDNGVGPEEESVDPNQYYKIRSQAIHQLKVNGEDPYPHKFHVDISLTDFIQKYSHLQPGDH
LTDITLKVAGRIHAKRASGGKLIFYDLRGEGVKLQVMANSRNYKSEEEFIHINNKLRRGDII
GVQGNPGKTKKGELSIIPYEITLLSPCLHMLPHLHFGLKDKETRYRQRYLDLILNDFVRQK
FIIRSKIITYIRSFLDELGFLEIETPMMNIIPGGAVAKPFITYHNELDMNLYMRIAPELYHKML
VVGGIDRVYEIGRQFRNEGIDLTHNPEFTTCEFYMAYADYHDLMEITEKMVSGMVKHITG
SYKVTYHPDGPEGQAYDVDFTPPFRRINMVEELEKALGMKLPETNLFETEETRKILDDICV
AKAVECPPPRTTARLLDKLVGEFLEVTCINPTFICDHPQIMSPLAKWHRSKEGLTERFELFV
MKKEICNAYTELNDPMRQRQLFEEQAKAKAAGDDEAMFIDENFCTALEYGLPPTAGWG
MGIDRVAMFLTDSNNIKEVLLFPAMKPEDKKENVATTDTLESTTVGTSV

>Q8IUQ0 peripheral membrane protein
MGPVSLLPKYQKLNTWNGDLAKMTHLQAGLSPETIEKARLELNENPDVLHQDIQQVRD
MIITRPDIGFLRTDDAFILRFLRARKFHQADAFRLLAQYFQYRQLNLDMFKNFKADDPGIK
RALIDGFPGVLENRDHYGRKILLLFAANWDQSRNSFTDILRAILLSLEVLIEDPELQINGFIL
IIDWSNFSFKQASKLTPSILKLAIEGLQDSFPARFGGVHFVNQPWYIHALYTLIKPFLKDKTR
KRIFLHGNNLNSLHQLIHPEFLPSEFGGTLPPYDMGTWARTLLGPDYSDENDYTHTSYNA
MHVKHTSSNLERECSPKLMKRSQSVVEAGTLKHEEKGENENTQPLLALD

>P43630 single-pass type I membrane protein
MSLTVVSMACVGFFLLQGAWPLMGGQDKPFLSARPSTVVPRGGHVALQCHYRRGFNNF
MLYKEDRSHVPIFHGRIFQESFIMGPVTPAHAGTYRCRGSRPHSLTGWSAPSNPLVIMVTG
NHRKPSLLAHPGPLLKSGETVILQCWSDVMFEHFFLHREGISEDPSRLVGQIHDGVSKANF
SIGPLMPVLAGTYRCYGSVPHSPYQLSAPSDPLDIVITGLYEKPSLSAQPGPTVQAGENVTL
SCSSWSSYDIYHLSREGEAHERRLRAVPKVNRTFQADFPLGPATHGGTYRCFGSFRALPCV
WSNSSDPLLVSVTGNPSSSWPSPTEPSSKSGICRHLHVLIGTSVVIFLFILLLFFLLYRWCSN
KKNAAVMDQEPAGDRTVNRQDSDEQDPQEVTYAQLDHCVFIQRKISRPSQRPKTPLTDTS
VYTELPNAEPRSKVVSCPRAPQSGLEGVF

>Q8IU68 multipass membrane protein

MLLPRSVSSERAPGVPEPEELWEAEMERLRGSGTPVRGLPYAMMDKRLIWQLREPAGVQ
TLRWQRWQRRRQTVERRLREAAQRLARGLGLWEGALYEIGGLFGTGIRSYFTFLRFLLLL
NLLSLLLTASFVLLPLVWLRPPDPGPTLNLTLQCPGSRQSPPGVLRFHNQLWHVLTGRAFT
NTYLFYGAYRVGPESSSVYSIRLAYLLSPLACLLLCFCGTLRRMVKGLPQKTLLGQGYQA
PLSAKVFSSWDFCIRVQEAATIKKHEISNEFKVELEEGRRFQLMQQQTRAQTACRLLSYLR
VNVLNGLLVVGAISAIFWATKYSQDNKEESLFLLLQYLPPGVIALVNFLGPLLFTFLVQLEN
YPPNTEVNLTLIWCVVLKLASLGMFSVSLGQTILCIGRDKSSCESYGYNVCDYQCWENSV
GEELYKLSIFNFLLTVAFAFLVTLPRRLLVDRFSGRFWAWLEREEFLVPKNVLDIVAGQTVT
WMGLFYCPLLPLLNSVFLFLTFYIKKYTLLKNSRASSRPFRASSSTFFFQLVLLLGLLLAAV
PLGYVVSSIHSSWDCGLFTNYSAPWQVVPELVALGLPPIGQRALHYLGSHAFSFPLLIMLSL
VLTVCVSQTQANARAIHRLRKQLVWQVQEKWHLVEDLSRLLPEPGPSDSPGPKYPASQAS
RPQSFCPGCPCPGSPGHQAPRPGPSVVDAAGLRSPCPGQHGAPASARRFRFPSGAEL

>Q0VAF6 peripheral membrane protein
MSPLRPLLLALALASVPCAQGACPASADLKHSDGTRTCAKLYDKSDPYYENCCGGAELSL
ESGADLPYLPSNWANTASSLVVAPRCELTVWSRQGKAGKTHKFSAGTYPRLEEYRRGILG
DWSNAISALYCRCS

>Q12860 GPI-anchor, lipid-anchor
MKMWLLVSHLVIISITTCLAEFTWYRRYGHGVSEEDKGFGPIFEEQPINTIYPEESLEGKVS
LNCRARASPFPVYKWRMNNGDVDLTSDRYSMVGGNLVINNPDKQKDAGIYYCLASNNY
GMVRSTEATLSFGYLDPFPPEERPEVRVKEGKGMVLLCDPPYHFPDDLSYRWLLNEFPVFI
TMDKRRFVSQTNGNLYIANVEASDKGNYSCFVSSPSITKSVFSKFIPLIPIPERTTKPYPADI
VVQFKDVYALMGQNVTLECFALGNPVPDIRWRKVLEPMPSTAEISTSGAVLKIFNIQLEDE
GIYECEAENIRGKDKHQARIYVQAFPEWVEHINDTEVDIGSDLYWPCVATGKPIPTIRWLK
NGYAYHKGELRLYDVTFENAGMYQCIAENTYGAIYANAELKILALAPTFEMNPMKKKILA
AKGGRVIIECKPKAAPKPKFSWSKGTEWLVNSSRILIWEDGSLEINNITRNDGGIYTCFAEN
NRGKANSTGTLVITDPTRIILAPINADITVGENATMQCAASFDPALDLTFVWSFNGYVIDFN
KENIHYQRNFMLDSNGELLIRNAQLKHAGRYTCTAQTIVDNSSASADLVVRGPPGPPGGL
RIEDIRATSVALTWSRGSDNHSPISKYTIQTKTILSDDWKDAKTDPPIIEGNMEAARAVDLIP
WMEYEFRVVATNTLGRGEPSIPSNRIKTDGAAPNVAPSDVGGGGGRNRELTITWAPLSREY
HYGNNFGYIVAFKPFDGEEWKKVTVTNPDTGRYVHKDETMSPSTAFQVKVKAFNNKGD
GPYSLVAVINSAQDAPSEAPTEVGVKVLSSSEISVHWEHVLEKIVESYQIRYWAAHDKEEA
ANRVQVTSQEYSARLENLLPDTQYFIEVGACNSAGCGPPSDMIEAFTKKAPPSQPPRIISSV
RSGSRYIITWDHVVALSNESTVTGYKVLYRPDGQHDGKLYSTHKHSIEVPIPRDGEYVVEV
RAHSDGGDGVVSQVKISGAPTLSPSLLGLLLPAFGILVYLEF

>Q5JQS5 multipass membrane protein
MKSDNHSFLGDSPKAFILLGVSDRPWLELPLFVVLLLSYVLAMLGNVAIILASRVDPQLHS
PMYIFLSHLSFLDLCYTTTTVPQMLVNMGSSQKTISYGGCTVQYAVFHWLGCTECIVLAA
MALDRYVAICKPLHYAVLMHRALCQQLVALAWLSGFGNSFVQVVLTVQLPFCGRQVLNN
FFCEVPAVIKLSCADTAVNDTILAVLVAFFVLVPLALILLSYGFIARAVLRIQSSKGRHKAFG
TCSSHLMIVSLFYLPAIYMYLQPPSSYSQEQGKFISLFYSIITPTLNPFTYTLRNKDMKGALR
RLLARIWRLCG

>P11049	multipass membrane protein
MSAQESCLSLIKYFLFVFNLFFFVLGSLIFCFGIWILIDKTSFVSFVGLAFVPLQIWSKVLAIS
GIFTMGIALLGCVGALKELRCLLGLYFGMLLLLFATQITLGILISTQRAQLERSLRDVVEKTI
QKYGTNPEETAAEESWDYVQFQLRCCGWHYPQDWFQVLILRGNGSEAHRVPCSCYNLSA
TNDSTILDKVILPQLSRLGHLARSRHSADICAVPAESHIYREGCAQGLQKWLHNNLISIVGI
CLGVGLLELGFMTLSIFLCRNLDHVYNRLARYR

>O15027	peripheral membrane protein
MPGLDRPLSRQNPHDGVVTPAASPSLPQPGLQMPGQWGPVQGGPQPSGQHRSPCPEGPV
PSGVPCATSVPHFPTPSILHQGPGHEQHSPLVAPPAALPSDGRDEVSHLQSGSHLANNSDPE
STFRQNPRIVNHWASPELRQNPGVKNEHRPASALVNPLARGDSPENRTHHPLGAGAGSGC
APLEADSGASGALAMFFQGGETENEENLSSEKAGLSGQADFDDFCSSPGLGRPPAPTHVG
AGSLCQALLPGPSNEAAGDVWGDTASTGVPDASGSQYENVENLEFVQNQEVLPSEPLNL
DPSSPSDQFRYGPLPGPAVPRHGAVCHTGAPDATLHTVHPDSVSSSYSSRSHGRLSGSARP
QELVGTFIQQEVGKPEDEASGSFFKQIDSSPVGGETDETTVSQNYRGSVSQPSTPSPPKPTG
IFQTSANSSFEPVKSHLVGVKPFEADRANVVGEVRETCVRQKQCRPAAALPDASPGNLEQ
PPDNMETLCAPQVCPLPLNSTTEAVHMLPHAGAPPLDTVYPAPEKRPSARTQGPVKCESPA
TTLWAQSELPDFGGNVLLAPAAPALYVCAKPQPPVVQPPEEAMSGQQSRNPSSAAPVQSR
GGIGASENLENPPKMGEEEALQSQASSGYASLLSSPPTESLQNPPVLIAQPDHSYNLAQPIN
FSVSLSNSHEKNQSWREALVGDRPAVSSWALGGDSGENTSLSGIPTSSVLSLSLPSSVAQSN
FPQGSGASEMVSNQPANLLVQPPSQPVPENLVPESQKDRKAGSALPGFANSPAGSTSVVLV
PPAHGTLVPDGNKANHSSHQEDTYGALDFTLSRTLENPVNVYNPSHSDSLASQQSVASHP
RQSGPGAPNLDRFYQQVTKDAQGQPGLERAQQELVPPQQQASPPQLPKAMFSELSNPESL
PAQGQAQNSAQSPASLVLVDAGQQLPPRPPQSSSVSLVSSGSGQAAVPSEQPWPQPVPALA
PGPPPQDLAAYYYYRPLYDAYQPQYSLPYPPEPGAASLYYQDVYSLYEPRYRPYDGAASA
YAQNYRYPEPERPSSRASHSSERPPPRQGYPEGYYSSKSGWSSQSDYYASYYSSQYDYGD
PGHWDRYHYSARVRDPRTYDRRYWCDAEYDAYRREHSAFGDRPEKRDNNWRYDPRFTG
SFDDDPDPHRDPYGEEVDRRSVHSEHSARSLHSAHSLASRRSSLSSHSHQSQIYRSHNVAA
GSYEAPLPPGSFHGDFAYGTYRSNFSSGPGFPEYGYPADTVWPAMEQVSSRPTSPEKFSVP
HVCARFGPGGQLIKVIPNLPSEGQPALVEVHSMEALLQHTSEQEEMRAFPGPLAKDDTHK
VDVINFAQNKAMKCLQNENLIDKESASLLWNFIVLLCRQNGTVVGTDIAELLLRDHRTV
WLPGKSPNEANLIDFTNEAVEQVEEEESGEAQLSFLTGGPAAAASSLERETERFRELLLYG
RKKDALESAMKNGLWGHALLLASKMDSRTHARVMTRFANSLPINDPLQTVYQLMSGRM
PAASTCCGDEKWGDWRPHLAMVLSNLNNNMDVESRTMATMGDTLASRGLLDAAHFCY
LMAQAGFGVYTKKTTKLVLIGSNHSLPFLKFATNEAIQRTEAYEYAQSLGAETCPLPSFQV
FKFIYSCRLAEMGLATQAFHYCEAIAKSILTQPHLYSPVLISQLVQMASQLRLFDPQLKEKP
EEESLAAPTWLVHLQQVERQIKEGAGVWHQDGALPQQCPGTPSSEMEQLDRPGLSQPGA
LGIANPLLAVPAPSPEHSSPSVRLLPSAPQTLPDGPLASPARVPMFPVPLPPGPLEPGPGCVT
PGPALGFLEPSGPGLPPGVPPLQERRHLLQEARSPDPGIVPQEAPVGNSLSELSEENFDGKF
ANLTPSRTVPDSEAPPGWDRADSGPTQPPLSLSPAPETKRPGQAAKKETKEPKKGESWFFR
WLPGKKKTEAYLPDDKNKSIVWDEKKNQWVNLNEPEEEKKAPPPPPTSMPKTVQAAPPA
LPGPPGAPVNMYSRRAAGTRARYVDVLNPSGTQRSEPALAPADFVAPLAPLPIPSNLFVPT
PDAEEPQLPDGTGREGPAAARGLANPEPAPEPKVLSSAASLPGSELPSSRPEGSQGGELSRC

SSMSSLSREVSQHFNQAPGDLPAAGGPPSGAMPFYNPAQLAQACATSGSSRLGRIGQRKH
LVLN

>Q96GZ6 multipass membrane protein
MDGTETRQRRLDSCGKPGELGLPHPLSTGGLPVASEDGALRAPESQSVTPKPLETEPSRET
TWSIGLQVTVPFMFAGLGLSWAGMLLDYFQHWPVFVEVKDLLTLVPPLVGLKGNLEMTL
ASRLSTAANTGQIDDPQEQHRVISSNLALIQVQATVVGLLAAVAALLLGVVSREEVDVAKV
ELLCASSVLTAFLAAFALGVLMVCIVIGARKLGVNPDNIATPIAASLGDLITLSILALVSSFF
YRHKDSRYLTPLVCLSFAALTPVWVLIAKQSPPIVKILKFGWFPIILAMVISSFGGLILSKTVS
KQQYKGMAIFTPVICGVGGNLVAIQTSRISTYLHMWSAPGVLPLQMKKFWPNPCSTFCTS
EINSMSARVLLLLVVPGHLIFFYIIYLVEGQSVINSQTFVVLYLLAGLIQVTILLYLAEVMVR
LTWHQALDPDNHCIPYLTGLGDLLGSSSVGHTAAVPRRCTASPGWGLIQPFICTQHLIVSLL
SFYFPFCLLAKTSI

>Q8WZ73 peripheral membrane protein
MWATCCNWFCLDGQPEEVPPPQGARMQAYSNPGYSSFPSPTGLEPSCKSCGAHFANTARK
QTCLDCKKNFCMTCSSQVGNGPRLCLLCQRFRATAFQREELMKMKVKDLRDYLSLHDIS
TEMCREKEELVLLVLGQQPVISQEDRTRASTLSPDFPEQQAFLTQPHSSMVPPTSPNLPSSS
AQATSVPPAQVQENQQANGHVSQDQEEPVYLESVARVPAEDETQSIDSEDSFVPGRRASLS
DLTDLEDIEGLTVRQLKEILARNFVNYKGCCEKWELMERVTRLYKDQKGLQHLVSGAED
QNGGAVPSGLEENLCKICMDSPIDCVLLECGHMVTCTKCGKRMNECPICRQYVIRAVHVF
RS

>Q9H346 multipass membrane protein
MSDSNLSDNHLPDTFFLTGIPGLEAAHFWIAIPFCAMYLVALVGNAALILVIAMDNALHAP
MYLFLCLLSLTDLALSSTTVPKMLAILWLHAGEISFGGCLAQMFCVHSIYALESSILLAMA
FDRYVAICNPLRYTTILNHAVIGRIGFVGLFRSVAIVSPFIFLLRRLPYCGHRVMTHTYCEHM
GIARLACANITVNIVYGLTVALLAMGLDSILIAISYGFILHAVFHLPSHDAQHKALSTCGSHI
GIILVFYIPAFFSFLTHRFGHHEVPKHVHIFLANLYVLVPPVLNPILYGARTKEIRSRLLKLLH
LGKTSI

>Q8NGJ2 multipass membrane protein
MPSASAMIIFNLSSYNPGPFILVGIPGLEQFHVWIGIPFCIIYIVAVVGNCILLYLIVVEHSLHE
PMFFFLSMLAMTDLILSTAGVPKALSIFWLGAREITFPGCLTQMFFLHYNFVLDSAILMAM
AFDHYVAICSPLRYTTILTPKTIIKSAMGISFRSFCIILPDVFLLTCLPFCRTRIIPHTYCEHIGV
AQLACADISINFWYGFCVPIMTVISDVILIAVSYAHILCAVFGLPSQDACQKALGTCGSHVC
VILMFYTPAFFSILAHRFGHNVSRTFHIMFANLYIVIPPALNPMVYGVKTKQIRDKVILLFSK
GTG

>Q8NGZ4 multipass membrane protein
MGLGNESSLMDFILLGFSDHPRLEAVLFVFVLFFYLLTLVGNFTIIIISYLDPPLHTPMYFFLS
NLSLLDICFTTSLAPQTLVNLQRPKKTITYGGCVAQLYISLALGSTECILLADMALDRYIAV
CKPLHYVVIMNPRLCQQLASISWLSGLASSLIHATFTLQLPLCGNHRLDHFICEVPALLKLA
CVDTTVNELVLFVVSVLFVVIPPALISISYGFITQAVLRIKSVEARHKAFSTCSSHLTVVIIFY

GTIIYVYLQPSDSYAQDQGKFISLFYTMVTPTLNPIIYTLRNKDMKEALRKLLSGKL

>O43772 multipass membrane protein
MADQPKPISPLKNLLAGGFGGVCLVFVGHPLDTVKVRLQTQPPSLPGQPPMYSGTFDCFR
KTLFREGITGLYRGMAAPIIGVTPMFAVCFFGFGLGKKLQQKHPEDVLSYPQLFAAGMLSG
VFTTGIMTPGERIKCLLQIQASSGESKYTGTLDCAKKLYQEFGIRGIYKGTVLTLMRDVPAS
GMYFMTYEWLKNIFTPEGKRVSELSAPRILVAGGIAGIFNWAVAIPPDVLKSRFQTAPPGKY
PNGFRDVLRELIRDEGVTSLYKGFNAVMIRAFPANAACFLGFEVAMKFLNWATPNL

>Q8NGZ5 multipass membrane protein
MGMVRHTNESNLAGFILLGFSDYPQLQKVLFVLILILYLLTILGNTTIILVSRLEPKLHMPM
YFFLSHLSFLYRCFTSSVIPQLLVNLWEPMKTIAYGGCLVHLYNSHALGSTECVLPAVMSCD
RYVAVCRPLHYTVLMHIHLCMALASMAWLSGIATTLVQSTLTLQLPFCGHRQVDHFICEVP
VLIKLACVGTTFNEAELFVASILFLIVPVSFILVSSGYIAHAVLRIKSATRRQKAFGTCFSHLT
VVTIFYGTIIFMYLQPAKSRSRDQGKFVSLFYTVVTRMLNPLIYTLRIKEVKGALKKVLAK
ALGVNIL

>P49768 multipass membrane protein
MTELPAPLSYFQNAQMSEDNHLSNTVRSQNDNRERQEHNDRRSLGHPEPLSNGRPQGNS
RQVVEQDEEEDEELTLKYGAKHVIMLFVPVTLCMVVVVATIKSVSFYTRKDGQLIYTPFT
EDTETVGQRALHSILNAAIMISVIVVMTILLVVLYKYRCYKVIHAWLIISSLLLLFFFSFIYL
GEVFKTYNVAVDYITVALLIWNFGVVGMISIHWKGPLRLQQAYLIMISALMALVFIKYLPE
WTAWLILAVISVYDLVAVLCPKGPLRMLVETAQERNETLFPALIYSSTMVWLVNMAEGDP
EAQRRVSKNSKYNAESTERESQDTVAENDDGGFSEEWEAQRDSHLGPHRSTPESRAAVQE
LSSSILAGEDPEERGVKLGLGDFIFYSVLVGKASATASGDWNTTIACFVAILIGLCLTLLLLA
IFKKALPALPISITFGLVFYFATDYLVQPFMDQLAFHQFYI

>P51685 multipass membrane protein
MDYTLDLSVTTVTDYYYPDIFSSPCDAELIQTNGKLLLAVFYCLLFVFSLLGNSLVILVLVV
CKKLRSITDVYLLNLALSDLLFVFSFPFQTYYLLDQWVFGTVMCKVVSGFYYIGFYSSMF
FITLMSVDRYLAVVHAVYALKVRTIRMGTTLCLAVWLTAIMATIPLLVFYQVASEDGVLQC
YSFYNQQTLKWKIFTNFKMNILGLLIPFTIFMFCYIKILHQLKRCQNHNKTKAIRLVLIVVI
ASLLFWVPFNVVLFLTSLHSMHILDGCSISQQLTYATHVTEIISFTHCCVNPVIYAFVGEKFK
KHLSEIFQKSCSQIFNYLGRQMPRESCEKSSSCQQHSSRSSSVDYIL

>Q14626 single-pass type I membrane protein
MSSSCSGLSRVLVAVATALVSASSPCPQAWGPPGVQYGQPGRSVKLCCPGVTAGDPVSWF
RDGEPKLLQGPDSGLGHELVLAQADSTDEGTYICQTLDGALGGTVTLQLGYPPARPVVSC
QAADYENFSCTWSPSQISGLPTRYLTSYRKKTVLGADSQRRSPSTGPWPCPQDPLGAARC
VVHGAEFWSQYRINVTEVNPLGASTRLLDVSLQSILRPDPPQGLRVESVPGYPRRLRASW
TYPASWPCQPHFLLKFRLQYRPAQHPAWSTVEPAGLEEVITDAVAGLPHAVRVSARDFLDA
GTWSTWSPEAWGTPSTGTIPKEIPAWGQLHTQPEVEPQVDSPAPPRPSLQPHPRLLDHRDS
VEQVAVLASLGILSFLGLVAGALALGLWLRLRRGGKDGSPKPGFLASVIPVDRRPGAPNL

>Q96JC1 peripheral membrane protein
MHDAFEPVPILEKLPLQIDCLAAWEEWLLVGTKQGHLLLYRIRKDVVPADVASPESGSCN
RFEVTLEKSNKNFSKKIQQIHVVSQFKILVSLLENNIYVHDLLTFQQITTVSKAKGASLFTC
DLQHTETGEEVLRMCVAVKKKLQLYFWKDREFHELQGDFSVPDVPKSMAWCENSICVGF
KRDYYLIRVDGKGSIKELFPTGKQLEPLVAPLADGKVAVGQDDLTVVLNEEGICTQKCALN
WTDIPVAMEHQPPYIIAVLPRYVEIRTFEPRLLVQSIELQRPRFITSGGSNIIYVASNHFVWRL
IPVPMATQIQQLLQDKQFELALQLAEMKDDSDSEKQQQIHHIKNLYAFNLFCQKRFDESM
QVFAKLGTDPTHVMGLYPDLLPTDYRKQLQYPNPLPVLSGAELEKAHLALIDYLTQKRSQ
LVKKLNDSDHQSSTSPLMEGTPTIKSKKKLLQIIDTTLLKCYLHTNVALVAPLLRLENNHC
HIEESEHVLKKAHKYSELIILYEKKGLHEKALQVLVDQSKKANSPLKGHERTVQYLQHLG
TENLHLIFSYSVWVLRDFPEDGLKIFTEDLPEVESLPRDRVLGFLIENFKGLAIPYLEHIIHV
WEETGSRFHNCLIQLYCEKVQGLMKEYLLSFPAGKTPVPAGEEEGELGEYRQKLLMFLEIS
SYYDPGRLICDFPFDGLLEERALLLGRMGKHEQALFIYVHILKDTRMAEEYCHKHYDRN
KDGNKDVYLSLLRMYLSPPSIHCLGPIKLELLEPKANLQAALQVLELHHSKLDTTKALNL
LPANTQINDIRIFLEKVLEENAQKKRFNQVLKNLLHAEFLRVQEERILHQQVKCIITEEKVC
MVCKKKIGNSAFARYPNGVVVHYFCSKEVNPADT

>Q9H9S5 single-pass type II membrane protein
MRLTRCQAALAAAITLNLLVLFYVSWLQHQPRNSRARGPRRASAAGPRVTVLVREFEAFD
NAVPELVDSFLQQDPAQPVVVAADTLPYPPLALPRIPNVRLALLQPALDRPAAASRPETYVA
TEFVALVPDGARAEAPGLLERMVEALRAGSARLVAAPVATANPARCLALNVSLREWTARY
GAAPAAPRCDALDGDAVVLLRARDLFNLSAPLARPVGTSLFLQTALRGWAVQLLDLTFAA
ARQPPLATAHARWKAEREGRARRAALLRALGIRLVSWEGGRLEWFGCNKETTRCFGTVV
GDTPAYLYEERWTPPCCLRALRETARYVVGVLEAAGVRYWLEGGSLLGAARHGDIIPWD
YDVDLGIYLEDVGNCEQLRGAEAGSVVDERGFVWEKAVEGDFFRVQYSESNHLHVDLW
PFYPRNGVMTKDTWLDHRQDVEFPEHFLQPLVPLPFAGFVAQAPNNYRRFLELKFGPGVI
ENPQYPNPALLSLTGSG

>O14967 single-pass type I membrane protein
MHFQAFWLCLGLLFISINAEFMDDDVETEDFEENSEEIDVNESELSSEIKYKTPQPIGEVYF
AETFDSGRLAGWVLSKAKKDDMDEEISIYDGRWEIEELKENQVPGDRGLVLKSRAKHHA
ISAVLAKPFIFADKPLIVQYEVNFQDGIDCGGAYIKLLADTDDLILENFYDKTSYIIMFGPDK
CGEDYKLHFIFRHKHPKTGVFEEKHAKPPDVDLKKFFTDRKTHLYTLVMNPDDTFEVLVD
QTVVNKGSLLEDVVPPIKPPKEIEDPNDKKPEEWDERAKIPDPSAVKPEDWDESEPAQIED
SSVVKPAGWLDDEPKFIPDPNAEKPDDWNEDTDGEWEAPQILNPACRIGCGEWKPPMIDN
PKYKGVWRPPLVDNPNYQGIWSPRKIPNPDYFEDDHPFLLTSFSALGLELWSMTSDIYFDN
FIICSEKEVADHWAADGWRWKIMIANANKPGVLKQLMAAAEGHPWLWLIYLVTAGVPIA
LITSFCWPRKVKKKHKDTEYKKTDICIPQTKGVLEQEEKEEKAALEKPMDLEEEKKQNDG
EMLEKEEESEPEEKSEEEIEIIEGQEESNQSNKSGSEDEMKEADESTGSGDGPIKSVRKRRV
RKD

>Q8NGE5 multipass membrane protein
MICENHTRVTEFILLGFTNNPEMQVSLFIFFLAIYTVTLLGNFLIVTVTSVDLALQTPMYFFL
QNLSLLEVCFTLVMVPKMLVDLVSPRKIISFVGCGTQMYFFFFFGSSECFLLSMMAYDRFV

AICNPLHYSVIMNRSLCLWMAIGSWMSGVPVSMLQTAWMMALPFCGPNAVDHFFCDGPP
VLKLVTVDTTMYEMQALASTLLFIMFPFCLILVSYTRIIITILRMSSATGRQKAFSTCSSHLI
VVSLFYGTASLTYLRPKSNQSPESKKLVSLSYTVITPMLNPIIYGLRNNEVKGAVKRTITQK
VLQKLDVF

>Q9NP84 single-pass type I membrane protein
MARGSLRRLLRLLVLGLWLALLRSVAGEQAPGTAPCSRGSSWSADLDKCMDCASCRARP
HSDFCLGCAAAPPAPFRLLWPILGGALSLTFVLGLLSGFLVWRRCRRREKFTTPIEETGGEG
CPAVALIQ

>Q7Z4I7 peripheral membrane protein
MTGSNMSDALANAVCQRCQARFSPAERIVNSNGELYHEHCFVCAQCFRPFPEGLFYEFEG
RKYCEHDFQMLFAPCCGSCGEFIIGRVIKAMNNNWHPGCFRCELCDVELADLGFVKNAG
RHLCRPCHNREKAKGLGKYICQRCHLVIDEQPLMFRSDAYHPDHFNCTHCGKELTAEARE
LKGELYCLPCHDKMGVPICGACRRPIEGRVVNALGKQWHVEHFVCAKCEKPFLGHRHYE
KKGLAYCETHYNQLFGDVCYNCSHVIEGDVVSALNKAWCVSCFSCSTCNSKLTLKNKFV
EFDMKPVCKRCYEKFPLELKKRLKKLSELTSRKAQPKATDLNSA

>Q03252 lipid-anchor
MATPLPGRAGGPATPLSPTRLSRLQEKEELRELNDRLAHYIDRVRALELENDRLLLKISEKE
EVTTREVSGIKALYESELADARRVLDETARERARLQIEIGKLRAELDEVNKSAKKREGELT
VAQGRVKDLESLFHRSEVELAAALSDKRGLESDVAELRAQLAKAEDGHAVAKKQLEKET
LMRVDLENRCQSLQEELDFRKSVFEEEVRETRRRHERRLVEVDSSRQQEYDFKMAQALE
ELRSQHDEQVRLYKLELEQTYQAKLDSAKLSSDQNDKAASAAREELKEARMRLESLSYQ
LSGLQKQASAAEDRIRELEEAMAGERDKFRKMLDAKEQEMTEMRDVMQQQLAEYQEL
LDVKLALDMEINAYRKLLEGEEERLKLSPSPSSRVTVSRATSSSSGSLSATGRLGRSKRKRL
EVEEPLGSGPSVLGTGTGGSGGFHLAQQASASGSVSIEEIDLEGKFVQLKNNSDKDQSLG
NWRIKRQVLEGEEIAYKFTPKYILRAGQMVTVWAAGAGVAHSPPSTLVWKGQSSWGTGE
SFRTVLVNADGEEVAMRTVKKSSVMRENENGEEEEEEAEFGEEDLFHQQGDPRTTSRGCY
VM

>Q86UT5 peripheral membrane protein
MVTPSPPGNHSLSLEAPRLHTASDLLGNHSLGLPLITALVGSRDRRGRVFSPVPVPLPTNPT
TQHPTRQKLPSTLSGHRVCQAHGEPVLGLCPLLPLFCCPPHPPDPWSLERPRFCLLSKEEG
KSFGFHLQQELGRAGHVVCRVDPGTSAQRQGLQEGDRILAVNNDVVEHEDYAVVVRRIR
ASSPRVLLTVLARHAHDVARAQLGEDAHLCPTLGPGVRPRLCHIVKDEGGFGFSVTHGNQ
GPFWLVLSTGGAAERAGVPPGARLLEVNGVSVEKFTHNQLTRKLWQSGQQVTLLVAGPE
VEEQCRQLGLPLAAPLAEGWALPTKPRCLHLEKGPQGFGFLLREEKGLDGRPGQFLWEV
DPGLPAKKAGMQAGDRLVAVAGESVEGLGHEETVSRIQGQGSCVSLTVVDPEADRFFSMV
RLSPLLFLENTEAPASPRGSSSASLVETEDPSLEDTSVPSVPLGSRQCFLYPGPGGSYGFRLS
CVASGPRLFISQVTPGGSAARAGLQVGDVILEVNGYPVGGQNDLERLQQLPEAEPPLCLK
LAARSLRGLEAWIPPGAAEDWALASDLL

>Q14315 peripheral membrane protein

MMNNSGYSDAGLGLGDETDEMPSTEKDLAEDAPWKKIQQNTFTRWCNEHLKCVGKRLT
DLQRDLSDGLRLIALLEVLSQKRMYRKFHPRPNFRQMKLENVSVALEFLEREHIKLVSIDS
KAIVDGNLKLILGLIWTLILHYSISMPMWEDEDDEDARKQTPKQRLLGWIQNKVPQLPITN
FNRDWQDGKALGALVDNCAPGLCPDWEAWDPNQPVENAREAMQQADDWLGVPQVIAP
EEIVDPNVDEHSVMTYLSQFPKAKLKPGAPVRSKQLNPKKAIAYGPGIEPQGNTVLQPAHF
TVQTVDAGVGEVLVYIEDPEGHTEEAKVVPNNDKDRTYAVSYVPKVAGLHKVTVLFAGQ
NIERSPFEVNVGMALGDANKVSARGPGLEPVGNVANKPTYFDIYTAGAGTGDVAVVIVDP
QGRRDTVEVALEDKGDSTFRCTYRPAMEGPHTVHVAFAGAPITRSPFPVHVSEACNPNAC
RASGRGLQPKGVRVKEVADFKVFTKGAGSGELKVTVKGPKGTEEPVKVREAGDGVFECE
YYPVVPGKYVVTITWGGYAIPRSPFEVQVSPEAGVQKVRAWGPGLETGQVGKSADFVVE
AIGTEVGTLGFSIEGPSQAKIECDDKGDGSCDVRYWPTEPGEYAVHVICDDEDIRDSPFIAH
ILPAPPDCFPDKVKAFGPGLEPTGCIVDKPAEFTIDARAAGKGDLKLYAQDADGCPIDIKVI
PNGDGTFRCSYVPTKPIKHTIIISWGGVNVPKSPFRVNVGEGSHPERVKVYGPGVEKTGLK
ANEPTYFTVDCSEAGQGDVSIGIKCAPGVVGPAEADIDFDIIKNDNDTFTVKYTPPGAGRY
TIMVLFANQEIPASPFHIKVDPSHDASKVKAEGPGLNRTGVEVGKPTHFTVLTKGAGKAKL
DVQFAGTAKGEVVRDFEIIDNHDYSYTVKYTAVQQGNMAVTVTYGGDPVPKSPFVVNVA
PPLDLSKIKVQGLNSKVAVGQEQAFSVNTRGAGGQGQLDVRMTSPSRRPIPCKLEPGGGA
EAQAVRYMPPEEGPYKVDITYDGHPVPGSPFAVEGVLPPDPSKVCAYGPGLKGGLVGTPA
PFSIDTKGAGTGGLGLTVEGPCEAKIECQDNGDGSCAVSYLPTEPGEYTINILFAEAHIPGSP
FKATIRPVFDPSKVRASGPGLERGKVGEAATFTVDCSEAGEAELTIEILSDAGVKAEVLIHN
NADGTYHITYSPAFPGTYTITIKYGGHPVPKFPTRVHVQPAVDTSGVKVSGPGVEPHGVLR
EVTTEFTVDARSLTATGGNHVTARVLNPSGAKTDTYVTDNGDGTYRVQYTAYEEGVHLV
EVLYDEVAVPKSPFRVGVTEGCDPTRVRAFGPGLEGGLVNKANRFTVETRGAGTGGLGLA
IEGPSEAKMSCKDNKDGSCTVEYIPFTPGDYDVNITFGGRPIPGSPFRVPVKDVVDPGKVK
CSGPGLGAGVRARVPQTFTVDCSQAGRAPLQVAVLGPTGVAEPVEVRDNGDGTHTVHYT
PATDGPYTVAVKYADQEVPRSPFKIKVLPAHDASKVRASGPGLNASGIPASLPVEFTIDARD
AGEGLLTVQILDPEGKPKKANIRDNGDGTYTVSYLPDMSGRYTITIKYGGDEIPYSPFRIHA
LPTGDASKCLVTVSIGGHGLGACLGPRIQIGQETVITVDAKAAGEGKVTCTVSTPDGAELD
VDVVENHDGTFDIYYTAPEPGKYVITIRFGGEHIPNSPFHVLACDPLPHEEEPSEVPQLRQP
YAPPRPGARPTHWATEEPVVPVEPMESMLRPFNLVIPFAVQKGELTGEVRMPSGKTARPNI
TDNKDGTITVRYAPTEKGLHQMGIKYDGNHIPGSPLQFYVDAINSRHVSAYGPGLSHGMV
NKPATFTIVTKDAGEGGLSLAVEGPSKAEITCKDNKDGTCTVSYLPTAPGDYSIIVRFDDKH
IPGSPFTAKITGDDSMRTSQLNVGTSTDVSLKITESDLSQLTASIRAPSGNEEPCLLKRLPNR
HIGISFTPKEVGEHVVSVRKSGKHVTNSPFKILVGPSEIGDASKVRVWGKGLSEGHTFQVA
EFIVDTRNAGYGGLGLSIEGPSKVDINCEDMEDGTCKVTYCPTEPGTYIINIKFADKHVPGS
PFTVKVTGEGRMKESITRRRQAPSIATIGSTCDLNLKIPGNWFQMVSAQERLTRTFTRSSHT
YTRTERTEISKTRGGETKREVRVEESTQVGGDPFPAVFGDFLGRERLGSFGSITRQQEGEAS
SQDMTAQVTSPSGKVEAAEIVEGEDSAYSVRFVPQEMGPHTVAVKYRGQHVPGSPFQFTV
GPLGEGGAHKVRAGGTGLERGVAGVPAEFSIWTREAGAGGLSIAVEGPSKAEIAFEDRKD
GSCGVSYVVQEPGDYEVSIKFNDEHIPDSPFVVPVASLSDDARRLTVTSLQETGLKVNQPA
SFAVQLNGARGVIDARVHTPSGAVEECYVSELDSDKHTIRFIPHENGVHSIDVKFNGAHIPG
SPFKIRVGEQSQAGDPGLVSAYGPGLEGGTTGVSSEFIVNTLNAGSGALSVTIDGPSKVQL
DCRECPEGHVVTYTPMAPGNYLIAIKYGGPQHIVGSPFKAKVTGPRLSGGHSLHETSTVL
VETVTKSSSSRGSSYSSIPKFSSDASKVVTRGPGLSQAFVGQKNSFTVDCSKAGTNMMMV

GVHGPKTPCEEVYVKHMGNRVYNVTYTVKEKGDYILIVKWGDESVPGSPFKVKVP

>P32241 multipass membrane protein
MRPPSPLPARWLCVLAGALAWALGPAGGQAARLQEECDYVQMIEVQHKQCLEEAQLEN
ETIGCSKMWDNLTCWPATPRGQVVVLACPLIFKLFSSIQGRNVSRSCTDEGWTHLEPGPYP
IACGLDDKAASLDEQQTMFYGSVKTGYTIGYGLSLATLLVATAILSLFRKLHCTRNYIHMH
LFISFILRAAAVFIKDLALFDSGESDQCSEGSVGCKAAMVFFQYCVMANFFWLLVEGLYLY
TLLAVSFFSERKYFWGYILIGWGVPSTFTMVWTIARIHFEDYGCWDTINSSLWWIIKGPILT
SILVNFILFICIIRILLQKLRPPDIRKSDSSPYSRLARSTLLLIPLFGVHYIMFAFFPDNFKPEVK
MVFELVVGSFQGFVVAILYCFLNGEVQAELRRKWRRWHLQGVLGWNPKYRHPSGGSNG
ATCSTQVSMLTRVSPGARRSSSFQAEVSLV

>Q9UKX5 single-pass type I membrane protein
MDLPRGLVVAWALSLWPGFTDTFNMDTRKPRVIPGSRTAFFGYTVQQHDISGNKWLVVG
APLETNGYQKTGDVYKCPVIHGNCTKLNLGRVTLSNVSERKDNMRLGLSLATNPKDNSF
LACSPLWSHECGSSYYTTGMCSRVNSNFRFSKTVAPALQRCQTYMDIVIVLDGSNSIYPW
VEVQHFLINILKKFYIGPGQIQVGVVQYGEDVVHEFHLNDYRSVKDVVEAASHIEQRGGT
ETRTAFGIEFARSEAFQKGGRKGAKKVMIVITDGESHDSPDLEKVIQQSERDNVTRYAVAV
LGYYNRRGINPETFLNEIKYIASDPDDKHFFNVTDEAALKDIVDALGDRIFSLEGTNKNET
SFGLEMSQTGFSSHVVEDGVLLGAVGAYDWNGAVLKETSAGKVIPLRESYLKEFPEELKN
HGAYLGYTVTSVVSSRQGRVYVAGAPRFNHTGKVILFTMHNNRSLTIHQAMRGQQIGSYF
GSEITSVDIDGDGVTDVLLVGAPMYFNEGRERGKVYVYELRQNLFVYNGTLKDSHSYQN
ARFGSSIASVRDLNQDSYNDVVVGAPLEDNHAGAIYIFHGFRGSILKTPKQRITASELATGL
QYFGCSIHGQLDLNEDGLIDLAVGALGNAVILWSRPVVQINASLHFEPSKINIFHRDCKRSG
RDATCLAAFLCFTPIFLAPHFQTTTVGIRYNATMDERRYTPRAHLDEGGDRFTNRAVLLSS
GQELCERINFHVLDTADYVKPVTFSVEYSLEDPDHGPMLDDGWPTTLRVSVPFWNGCNE
DEHCVPDLVLDARSDLPTAMEYCQRVLRKPAQDCSAYTLSFDTTVFIIESTRQRVAVEATLE
NRGENAYSTVLNISQSANLQFASLIQKEDSDGSIECVNEERRLQKQVCNVSYPFFRAKAKV
AFRLDFEFSKSIFLHHLEIELAAGSDSNERDSTKEDNVAPLRFHLKYEADVLFTRSSSLSHY
EVKPNSSLERYDGIGPPFSCIFRIQNLGLFPIHGMMMKITIPIATRSGNRLLKLRDFLTDEAN
TSCNIWGNSTEYRPTPVEEDLRRAPQLNHSNSDVVSINCNIRLVPNQEINFHLLGNLWLRS
LKALKYKSMKIMVNAALQRQFHSPFIFREEDPSRQIVFEISKQEDWQVPIWIIVGSTLGGLL
LLALLVLALWKLGFFRSARRRREPGLDPTPKVLE

>Q8N6H7 peripheral membrane protein
MAAEPNKTEIQTLFKRLRAVPTNKACFDCGAKNPSWASITYGVFLCIDCSGVHRSLGVHL
SFIRSTELDSNWNWFQLRCMQVGGNANATAFFRQHGCTANDANTKYNSRAAQMYREKI
RQLGSAALARHGTDLWIDNMSSAVPNHSPEKKDSDFFTEHTQPPAWDAPATEPSGTQQPA
PSTESSGLAQPEHGPNTDLLGTSPKASLELKSSIIGKKKPAAAKKGLGAKKGLGAQKVSSQ
SFSEIERQAQVAEKLREQQAADAKKQAEESMVASMRLAYQELQIDRKKEEKKLQNLEGK
KREQAERLGMGLVSRSSVSHSVLSEMQVIEQETPVSAKSSRSQLDLFDDVGTFASGPPKY
KDNPFSLGESFGSRWDTDAAWGMDRVEEKEPEVTISSIRPISERATNRREVESRSSGLESSE
ARQKFAGAKAISSDMFFGREVDAEYEARSRLQQLSGSSAISSSDLFGDMDGAHGAGSVSL
GNVLPTADIAQFKQGVKSVAGKMAVLANGVMNSLQDRYGSY

>O43194 multipass membrane protein
MASPSLPGSDCSQIIDHSHVPEFEVATWIKITLILVYLIIFVMGLLGNSATIRVTQVLQKKGY
LQKEVTDHMVSLACSDILVFLIGMPMEFYSIIWNPLTTSSYTLSCKLHTFLFEACSYATLLH
VLTLSFERYIAICHPFRYKAVSGPCQVKLLIGFVWVTSALVALPLLFAMGTEYPLVNVPSHR
GLTCNRSSTRHHEQPETSNMSICTNLSSRWTVFQSSIFGAFVVYLVVLLSVAFMCWNMMQ
VLMKSQKGSLAGGTRPPQLRKSESEESRTARRQTIIFLRLIVVTLAVCWMPNQIRRIMAAA
KPKHDWTRSYFRAYMILLPFSETFFYLSSVINPLLYTVSSQQFRRVFVQVLCCRLSLQHAN
HEKRLRVHAHSTTDSARFVQRPLLFASRRQSSARRTEKIFLSTFQSEAEPQSKSQSLSLESL
EPNSGAKPANSAAENGFQEHEV

>Q8NGB9 multipass membrane protein
MDEANHSVVSEFVFLGLSDSRKIQLLLFLFFSVFYVSSLMGNLLIVLTVTSDPRLQSPMYFL
LANLSIINLVFCSSTAPKMIYDLFRKHKTISFGGCVVQIFFIHAVGGTEMVLLIAMAFDRYV
AICKPLHYLTIMNPQRCILFLVISWIIGIIHSVIQLAFVVDLLFCGPNELDSFFCDLPRFIKLAC
IETYTLGFMVTANSGFISLASFLILIISYIFILVTVQKKSSGGIFKAFSMLSAHVIVVVLVFGPL
IFFYIFPFPTSHLDKFLAIFDAVITPVLNPVIYTFRNKEMMVAMRRRCSQFVNYSKIF

>Q9Y5P1 multipass membrane protein
MWPNITAAPFLLTGFPGLEAAHHWISIPFFAVYVCILLGNGMLLYLIKHDHSLHEPMYYFLT
MLAGTDLMVTLTTMPTVMGILWVNHREISSVGCFLQAYFIHSLSVVESGSLLAMAYDCFI
AIRNPLRYASILTNTRVIALGVGVFLRGFVSILPVILRLFSFSYCKSHVITRAFCLHQEIMRLA
CADITFNRLYPVILISLTIFLDCLIILFSYILILNTVIGIASGEERAKALNTCISHISCVLIFYVTV
MGLTFIYRFGKNVPEVVHIIMSYIYFLFPPLMNPVIYSIKTKQIQYGIIRLLSKHRFSS

>Q6PP77 multipass membrane protein
MDRVYEIPEEPNVDPVSSLEEDVIRGANPRFTFPFSILFSTFLYCGEAASALYMVRIYRKNS
ETYWMTYTFSFFMFSSIMVQLTLIFVHRDLAKDKPLSLFMHLILLGPVIRCLEAMIKYLTL
WKKEEQEEPYVSLTRKKMLIDGEEVLIEWEVGHSIRTLAMHRNAYKRMSQIQAFLGSVPQ
LTYQLYVSLISAEVPLGRVVLMVFSLVSVTYGATLCNMLAIQIKYDDYKIRLGPLEVLCITI
WRTLEITSRLLILVLFSATLKLKAVPFLVLNFLIILFEPWIKFWRSGAQMPNNIEKNFSRVGT
LVVLISVTILYAGINFSCWSALQLRLADRDLVDKGQNWGHMGLHYSVRLVENVIMVLVFK
FFGVKVLLNYCHSLIALQLIIAYLISIGFMLLFFQYLHPLRSLFTHNVVDYLHCVCCHQHPR
TRVENSEPPFETEARQSVV

>P34982 multipass membrane protein
MDGGNQSEGSEFLLLGMSESPEQQRILFWMFLSMYLVTVVGNVLIILAISSDSRLHTPVYF
FLANLSFTDLFFVTNTIPKMLVNLQSHNKAISYAGCLTQLYFLVSLVALDNLILAVMAYDRY
VAICCPLHYTTAMSPKLCILLLSLCWVLSVLYGLIHTLLMTRVTFCGSRKIHYIFCEMYVLL
RMACSNIQINHTVLIATGCFIFLIPFGFVIISYVLIIRAILRIPSVSKKYKAFSTCASHLGAVSLF
YGTLCMVYLKPLHTYSVKDSVATVMYAVVTPMMNPFIYSLRNKDMHGALGRLLDKHFK
RLT

>Q9H267 peripheral membrane protein

MAFPHRPDAPELPDFSMLKRLARDQLIYLLEQLPGKKDLFIEADLMSPLDRIANVSILKQH
EVDKLYKVENKPALSSNEQLCFLVRPRIKNMRYIASLVNADKLAGRTRKYKVIFSPQKFYA
CEMVLEEEGIYGDVSCDEWAFSLLPLDVDLLSMELPEFFRDYFLEGDQRWINTVAQALHL
LSTLYGPFPNCYGIGRCAKMAYELWRNLEEEEDGETKGRRPEIGHIFLLDRDVDFVTALCS
QVVYEGLVDDTFRIKCGSVDFGPEVTSSDKSLKVLLNAEDKVFNEIRNEHFSNVFGFLSQK
ARNLQAQYDRRRGMDIKQMKNFVSQELKGLKQEHRLLSLHIGACESIMKKKTKQDFQEL
IKTEHALLEGFNIRESTSYIEEHIDRQVSPIESLRLMCLLSITENGLIPKDYRSLKTQYLQSYG
PEHLLTFSNLRRAGLLTEQAPGDTLTAVESKVSKLVTDKAAGKITDAFSSLAKRSNFRAISK
KLNLIPRVDGEYDLKVPRDMAYVFGGAYVPLSCRIIEQVLERRSWQGLDEVVRLLNCSDF
AFTDMTKEDKASSESLRLILVVFLGGCTFSEISALRFLGREKGYRFIFLTTAVTNSARLMEA
MSEVKA

>O00258 multipass membrane protein
MSSAAADHWAWLLVLSFVFGCNVLRILLPSFSSFMSRVLQKDAEQESQMRAEIQDMKQE
LSTVNMMDEFARYARLERKINKMTDKLKTHVKARTAQLAKIKWVISVAFYVLQAALMIS
LIWKYYSVPVAVVPSKWITPLDRLVAFPTRVAGGVGITCWILVCNKVVAIVLHPFS

>P59551 multipass membrane protein
MNGDHMVLGSSVTDKKAIILVTILLLLRLVAIAGNGFITAALGVEWVLRRMLLPCDKLLVS
LGASRFCLQSVVMGKTIYVFLHPMAFPYNPVLQFLAFQWDFLNAATLWSSTWLSVFYCV
KIATFTHPVFFWLKHKLSGWLPWMLFSSVGLSSFTTILFFIGNHRMYQNYLRNHLQPWNV
TGDSIRSYCEKFYLFPLKMITWTMPTAVFFICMILLITSLGRHRKKALLTTSGFREPSVQAHI
KALLALLSFAMLFISYFLSLVFSAAGIFPPLDFKFWVWESVIYLCAAVHPIILLFSNCRLRAV
LKSRRSSRCGTP

>Q9H1C0 multipass membrane protein
MLANSSSTNSSVLPCPDYRPTHRLHLVVYSLVLAAGLPLNALALWVFLRALRVHSVVSVY
MCNLAASDLLFTLSLPVRLSYYALHHWPFPDLLCQTTGAIFQMNMYGSCIFLMLINVDRY
AAIVHPLRLRHLRRPRVARLLCLGVWALILVFAVPAARVHRPSRCRYRDLEVRLCFESFSDE
LWKGRLLPLVLLAEALGFLLPLAAVVYSSGRVFWTLARPDATQSQRRRKTVRLLLANLVIF
LLCFVPYNSTLAVYGLLRSKLVAASVPARDRVRGVLMVMVLLAGANCVLDPLVYYFSAE
GFRNTLRGLGTPHRARTSATNGTRAALAQSERSAVTTDATRPDAASQGLLRPSDSHSLSSF
TQCPQDSAL

>P26951 single-pass type I membrane protein
MVLLWLTLLLIALPCLLQTKEDPNPPITNLRMKAKAQQLTWDLNRNVTDIECVKDADYS
MPAVNNSYCQFGAISLCEVTNYTVRVANPPFSTWILFPENSGKPWAGAENLTCWIHDVDFL
SCSWAVGPGAPADVQYDLYLNVANRRQQYECLHYKTDAQGTRIGCRFDDISRLSSGSQSS
HILVRGRSAAFGIPCTDKFVVFSQIEILTPPNMTAKCNKTHSFMHWKMRSHFNRKFRYELQ
IQKRMQPVITEQVRDRTSFQLLNPGTYTVQIRARERVYEFLSAWSTPQRFECDQEEGANTR
AWRTSLLIALGTLLALVCVFVICRRYLVMQRLFPRIPHMKDPIGDSFQNDKLVVWEAGKA
GLEECLVTEVQVVQKT

>P31639 multipass membrane protein

MEEHTEAGSAPEMGAQKALIDNPADILVIAAYFLLVIGVGLWSMCRTNRGTVGGYFLAGR
SMVWWPVGASLFASNIGSGHFVGLAGTGAASGLAVAGFEWNALFVVLLLGWLFAPVYLT
AGVITMPQYLRKRFGGRRIRLYLSVLSLFLYIFTKISVDMFSGAVFIQQALGWNIYASVIAL
LGITMIYTVTGGLAALMYTDTVQTFVILGGACILMGYAFHEVGGYSGLFDKYLGAATSLT
VSEDPAVGNISSFCYRPRPDSYHLLRHPVTGDLPWPALLLGLTIVSGWYWCSDQVIVQRCL
AGKSLTHIKAGCILCGYLKLTPMFLMVMPGMISRILYPDEVACVVPEVCRRVCGTEVGCSN
IAYPRLVVKLMPNGLRGLMLAVMLAALMSSLASIFNSSSTLFTMDIYTRLRPRAGDRELLL
VGRLWVVFIVVVSVAWLPVVQAAQGGQLFDYIQAVSSYLAPPVSAVFVLALFVPRVNEQG
AFWGLIGGLLMGLARLIPEFSFGSGSCVQPSACPAFLCGVHYLYFAIVLFFCSGLLTLTVSL
CTAPIPRKHLHRLVFSLRHSKEEREDLDADEQQGSSLPVQNGCPESAMEMNEPQAPAPSLF
RQCLLWFCGMSRGGVGSPPPLTQEEAAAAARRLEDISEDPSWARVVNLNALLMMAVAVF
LWGFYA

>P08240 peripheral membrane protein
MLDFFTIFSKGGLVLWCFQGVSDSCTGPVNALIRSVLLQERGGNNSFTHEALTLKYKLDN
QFELVFVVGFQKILTLTYVDKLIDDVHRLFRDKYRTEIQQQSALSLLNGTFDFQNDFLRLL
REAEESSKIRAPTTMKKFEDSEKAKKPVRSMIETRGEKPKEKAKNSKKKGAKKEGSDGPL
ATSKPVPAEKSGLPVGPENGVELSKEELIRRKREEFIQKHGRGMEKSNKSTKSDAPKEKGK
KAPRVWELGGCANKEVLDYSTPTTNGTPEAALSEDINLIRGTGSGGQLQDLDCSSSDDEG
AAQNSTKPSATKGTLGGMFGMLKGLVGSKSLSREDMESVLDKMRDHLIAKNVAADIAVQ
LCESVANKLEGKVMGTFSTVTSTVKQALQESLVQILQPQRRVDMLRDIMDAQRRQRPYV
VTFCGVNGVGKSTNLAKISFWLLENGFSVLIAACDTFRAGAVEQLRTHTRRLSALHPPEK
HGGRTMVQLFEKGYGKDAAGIAMEAIAFARNQGFDVVLVDTAGRMQDNAPLMTALAKL
ITVNTPDLVLFVGEALVGNEAVDQLVKFNRALADHSMAQTPRLIDGIVLTKFDTIDDKVGA
AISMTYITSKPIVFVGTGQTYCDLRSLNAKAVVAALMKA

>Q6ZPD8 multipass membrane protein
MAFFSRLNLQEGLQTFFVLQWIPVYIFLGAIPILLIPYFLLFSKFWPLAVLSLAWLTYDWNT
HSQGGRRSAWVRNWTLWKYFRNYFPVKLVKTHDLSPKHNYIIANHPHGILSFGVFINFAT
EATGIARIFPSITPFVGTLERIFWIPIVREYVMSMGVCPVSSSALKYLLTQKGSGNAVVIVVG
GAAEALLCRPGASTLFLKQRKGFVKMALQTGAYLVPSYSFGENEVFNQETFPEGTWLRLF
QKTFQDTFKKILGLNFCTFHGRGFTRGSWGFLPFNRPITTVVGEPLPIPRIKRPNQKTVDKY
HALYISALRKLFDQHKVEYGLPETQELTIT

>Q14500 multipass membrane protein
MTAASRANPYSIVSSEEDGLHLVTMSGANGFGNGKVHTRRRCRNRFVKKNGQCNIEFAN
MDEKSQRYLADMFTTCVDIRWRYMLLIFSLAFLASWLLFGIIFWVIAVAHGDLEPAEGRGR
TPCVMQVHGFMAAFLFSIETQTTIGYGLRCVTEECPVAVFMVVAQSIVGCIIDSFMIGAIMA
KMARPKKRAQTLLFSHNAVVALRDGKLCLMWRVGNLRKSHIVEAHVRAQLIKPRVTEEG
EYIPLDQIDIDVGFDKGLDRIFLVSPITILHEIDEASPLFGISRQDLETDDFEIVVILEGMVEAT
AMTTQARSSYLANEILWGHRFEPVLFEEKNQYKIDYSHFHKTYEVPSTPRCSAKDLVENK
FLLPSANSFCYENELAFLSRDEEDEADGDQDGRSRDGLSPQARHDFDRLQAGGGVLEQRP
YRRESEI

>Q9Y219 single-pass type I membrane protein
MRAQGRGRLPRRLLLLLALWVQAARPMGYFELQLSALRNVNGELLSGACCDGDGRTTR
AGGCGHDECDTYVRVCLKEYQAKVTPTGPCSYGHGATPVLGGNSFYLPPAGAAGDRAR
ARARAGGDQDPGLVVIPFQFAWPRSFTLIVEAWDWDNDTTPNEELLIERVSHAGMINPED
RWKSLHFSGHVAHLELQIRVRCDENYYSATCNKFCRPRNDFFGHYTCDQYGNKACMDG
WMGKECKEAVCKQGCNLLHGGCTVPGECRCSYGWQGRFCDECVPYPGCVHGSCVEPW
QCNCETNWGGLLCDKDLNYCGSHHPCTNGGTCINAEPDQYRCTCPDGYSGRNCEKAEH
ACTSNPCANGGSCHEVPSGFECHCPSGWSGPTCALDIDECASNPCAAGGTCVDQVDGFE
CICPEQWVGATCQLDANECEGKPCLNAFSCKNLIGGYYCDCIPGWKGINCHINVNDCRGQ
CQHGGTCKDLVNGYQCVCPRGFGGRHCELERDECASSPCHSGGLCEDLADGFHCHCPQG
FSGPLCEVDVDLCEPSPCRNGARCYNLEGDYYCACPDDFGGKNCSVPREPCPGGACRVID
GCGSDAGPGMPGTAASGVCGPHGRCVSQPGGNFSCICDSGFTGTYCHENIDDCLGQPCRN
GGTCIDEVDAFRCFCPSGWEGELCDTNPNDCLPDPCHSRGRCYDLVNDFYCACDDGWKG
KTCHSREFQCDAYTCSNGGTCYDSGDTFRCACPPGWKGSTCAVAKNSSCLPNPCVNGGT
CVGSGASFSCICRDGWEGRTCTHNTNDCNPLPCYNGGICVDGVNWFRCECAPGFAGPDC
RINIDECQSSPCAYGATCVDEINGYRCSCPPGRAGPRCQEVIGFGRSCWSRGTPFPHGSSW
VEDCNSCRCLDGRRDCSKVWCGWKPCLLAGQPEALSAQCPLGQRCLEKAPGQCLRPPCE
AWGECGAEEPPSTPCLPRSGHLDNNCARLTLHFNRDHVPQGTTVGAICSGIRSLPATRAVA
RDRLLVLLCDRASSGASAVEVAVSFSPARDLPDSSLIQGAAHAIVAAITQRGNSSLLLAVTE
VKVETVVTGGSSTGLLVPVLCGAFSVLWLACVVLCVWWTRKRRKERERSRLPREESANN
QWAPLNPIRNPIERPGGHKDVLYQCKNFTPPPRRADEALPGPAGHAAVREDEEDEDLGRG
EEDSLEAEKFLSHKFTKDPGRSPGRPAHWASGPKVDNRAVRSINEARYAGKE

>Q9UBY8 multipass membrane protein
MNPASDGGTSESIFDLDYASWGIRSTLMVAGFVFYLGVFVVCHQLSSSLNATYRSLVAREK
VFWDLAATRAVFGVQSTAAGLWALLGDPVLHADKARGQQNWCWFHITTATGFFCFENVA
VHLSNLIFRTFDLFLVIHHLFAFLGFLGCLVNLQAGHYLAMTTLLLEMSTPFTCVSWMLLK
AGWSESLFWKLNQWLMIHMFHCRMVLTYHMWWVCFWHWDGLVSSLYLPHLTLFLVGL
ALLTLIINPYWTHKKTQQLLNPVDWNFAQPEAKSRPEGNGQLLRKKRP

>Q9Y5U5 single-pass type I membrane protein
MAQHGAMGAFRALCGLALLCALSLGQRPTGGPGCGPGRLLLGTGTDARCCRVHTTRCC
RDYPGEECCSEWDCMCVQPEFHCGDPCCTTCRHHPCPPGQGVQSQGKFSFGFQCIDCAS
GTFSGGHEGHCKPWTDCTQFGFLTVFPGNKTHNAVCVPGSPPAEPLGWLTVVLLAVAACV
LLLTSAQLGLHIWQLRSQCMWPRETQLLLEVPPSTEDARSCQFPEEERGERSAEEKGRLG
DLWV

>Q96D31 multipass membrane protein
MHPEPAPPPSRSSPELPPSGGSTTSGSRRSRRRSGDGEPPGAPPPPPSAVTYPDWIGQSYSEV
MSLNEHSMQALSWRKLYLSRAKLKASSRTSALLSGFAMVAMVEVQLDADHDYPPGLLIA
FSACTTVLVAVHLFALMISTCILPNIEAVSNVHNLNSVKESPHERMHRHIELAWAFSTVIGTL
LFLAEVVLLCWVKFLPLKKQPGQPRPTSKPPASGAAANVSTSGITPGQAAAIASTTIMVPF
GLIFIVFAVHFYRSLVSHKTDRQFQELNELAEFARLQDQLDHRGDHPLTPGSHYA

>A6NM76 multipass membrane protein
MKNRTSVTDFILLGLTDNPQLQVVIFSFLFLTYVLSVTGNLTIISLTLLDSHLKTPMYFFLRN
FSLEISFTSVCNPRFLISILTGDKSISYNACAAQLFFFIFLGSTEFFLLASMSYDCYVAICKPLH
YTTIMSDRICYQLIISSWLAGFLVIFPPLAMGLQLDFCDSNVIDHFTCDSAPLLQISCTDTST
LELMSFILALFTLISTLILVILSYTYIIRTILRIPSAQQRKKAFSTCSSHVIVVSISYGSCIFMYV
KTSAKEGVALTKGVAILNTSVAPMLNPFIYTLRNQQVKQAFKDVLRKISHKKKKH

>Q9H228 multipass membrane protein
MESGLLRPAPVSEVIVLHYNYTGKLRGARYQPGAGLRADAVVCLAVCAFIVLENLAVLLV
LGRHPRFHAPMFLLLGSLTLSDLLAGAAYAANILLSGPLTLKLSPALWFAREGGVFVALTAS
VLSLLAIALERSLTMARRGPAPVSSRGRTLAMAAAAWGVSLLLGLLPALGWNCLGRLDA
CSTVLPLYAKAYVLFCVLAFVGILAAICALYARIYCQVRANARRLPARPGTAGTTSTRARR
KPRSLALLRTLSVVLLAFVACWGPLFLLLLLDVACPARTCPVLLQADPFLGLAMANSLLNP
IIYTLTNRDLRHALLRLVCCGRHSCGRDPSGSQQSASAAEASGGLRRCLPPGLDGSFSGSE
RSSPQRDGLDTSGSTGSPGAPTAARTLVSEPAAD

>Q16280 multipass membrane protein
MTEKTNGVKSSPANNHNHHAPPAIKANGKDDHRTSSRPHSAADDDTSSELQRLADVDAP
QQGRSGFRRIVRLVGIIREWANKNFREEEPRPDSFLERFRGPELQTVTTQEGDGKGDKDGE
DKGTKKKFELFVLDPAGDWYYCWLFVIAMPVLYNWCLLVARACFSDLQKGYYLVWLVL
DYVSDVVYIADLFIRLRTGFLEQGLLVKDTKKLRDNYIHTLQFKLDVASIIPTDLIYFAVDIH
SPEVRFNRLLHFARMFEFFDRTETRTNYPNIFRISNLVLYILVIIHWNACIYYAISKSIGFGVD
TWVYPNITDPEYGYLAREYIYCLYWSTLTLTTIGETPPPVKDEEYLFVIFDFLIGVLIFATIVG
NVGSMISNMNATRAEFQAKIDAVKHYMQFRKVSKGMEAKVIRWFDYLWTNKKTVDERE
ILKNLPAKLRAEIAINVHLSTLKKVRIFHDCEAGLLVELVLKLRPQVFSPGDYICRKGDIGK
EMYIIKEGKLAVVADDGVTQYALLSAGSCFGEISILNIKGSKMGNRRTANIRSLGYSDLFCL
SKDDLMEAVTEYPDAKKVLEERGREILMKEGLLDENEVATSMEVDVQEKLGQLETNMET
LYTRFGRLLAEYTGAQQKLKQRITVLETKMKQNNEDDYLSDGMNSPELAAADEP

>P22459 multipass membrane protein
MEVAMVSAESSGCNSHMPYGYAAQARARERERLAHSRAAAAAAVAAATAAVEGSGGSG
GGSHHHHQSRGACTSHDPQSSRGSRRRRRQRSEKKKAHYRQSSFPHCSDLMPSGSEEKIL
RELSEEEEDEEEEEEEEEEGRFYYSEDDHGDECSYTDLLPQDEGGGGYSSVRYSDCCERV
VINVSGLRFETQMKTLAQFPETLLGDPEKRTQYFDPLRNEYFFDRNRPSFDAILYYYQSGG
RLKRPVNVPFDIFTEEVKFYQLGEEALLKFREDEGFVREEEDRALPENEFKKQIWLLFEYP
ESSSPARGIAIVSVLVILISIVIFCLETLPEFRDDRDLVMALSAGGHGGLLNDTSAPHLENSG
HTIFNDPFFIVETVCIVWFSFEFVVRCFACPSQALFFKNIMNIIDIVSILPYFITLGTDLAQQQ
GGGNGQQQQAMSFAILRIIRLVRVFRIFKLSRHSKGLQILGHTLRASMRELGLLIFFLFIGVI
LFSSAVYFAEADEPTTHFQSIPDAFWWAVVTMTTVGYGDMKPITVGGKIVGSLCAIAGVLT
IALPVPVIVSNFNYFYHRETENEEQTQLTQNAVSCPYLPSNLLKKFRSSTSSSLGDKSEYLE
MEEGVKESLCAKEEKCQGKGDDSETDKNNCSNAKAVETDV

>P51157 lipid-anchor
MSDSEEESQDRQLKIVVLGDGASGKTSLTTCFAQETFGKQYKQTIGLDFFLRRITLPGNLN

VTLQIWDIGGQTIGGKMLDKYIYGAQGVLLVYDITNYQSFENLEDWYTVVKKVSEESET
QPLVALVGNKIDLEHMRTIKPEKHLRFCQENGFSSHFVSAKTGDSVFLCFQKVAAEILGIKL
NKAEIEQSQRVVKADIVNYNQEPMSRTVNPPRSSMCAVQ

>Q8NGE8 multipass membrane protein
MDQRNYTRVKEFTFLGITQSRELSQVLFTFLFLVYMTTLMGNFLIMVTVTCESHLHTPMY
FLLRNLSILDICFSSITAPKVLIDLLSETKTISFSGCVTQMFFFHLLGGADVFSLSVMAFDRYI
AISKPLHYMTIMSRGRCTGLIVASWVGGFVHSIAQISLLLPLPFCGPNVLDTFYCDVPQVL
KLACTDTFTLELLMISNNGLVSWFVFFFLLISYTVILMMLRSHTGEGRRKAISTCTSHITVV
TLHFVPCIYVYARPFTALPTDTAISVTFTVISPLLNPIIYTLRNQEMKLAMRKLKRRLGQSER
ILIQ

>Q9ULT6 single-pass type I membrane protein
MRPRSGGRPGATGRRRRRLRRRPRGLRCSRLPPPPPLPLLLGLLLAAAGPGAARAKETAFV
EVVLFESSPSGDYTTYTTGLTGRFSRAGATLSAEGEIVQMHPLGLCNNNDEEDLYEYGWV
GVVKLEQPELDPKPCLTVLGKAKRAVQRGATAVIFDVSENPEAIDQLNQGSEDPLKRPVV
YVKGADAIKLMNIVNKQKVARARIQHRPPRQPTEYFDMGIFLAFFVVVSLVCLILLVKIKL
KQRRSQNSMNRLAVQALEKMETRKFNSKSKGRREGSCGALDTLSSSSTSDCAICLEKYID
GEELRVIPCTHRFHRKCVDPWLLQHHTCPHCRHNIIEQKGNPSAVCVETSNLSRGRQQRVT
LPVHYPGRVHRTNAIPAYPTRTSMDSHGNPVTLLTMDRHGEQSLYSPQTPAYIRSYPPLHLD
HSLAAHRCGLEHRAYSPAHPFRRPKLSGRSFSKAACFSQYETMYQHYYFQGLSYPEQEGQ
SPPSLAPRGPARAFPPSGSGSLLFPTVVHVAPPSHLESGSTSSFSCYHGHRSVCSGYLADCP
GSDSSSSSSSGQCHCSSSDSVVDCTEVSNQGVYGSCSTFRSSLSSDYDPFIYRSRSPCRASE
AGGSGSSGRGPALCFEGSPPPEELPAVHSHGAGRGEPWPGPASPSGDQVSTCSLEMNYSSN
SSLEHRGPNSSTSEVGLEASPGAAPDLRRTWKGGHELPSCACCCEPQPSPAGPSAGAAGSS
TLFLGPHLYEGSGPAGGEPQSGSSQGLYGLHPDHLPRTDGVKYEGLPCCFYEEKQVARGG
GGGSGCYTEDYSVSVQYTLTEEPPPGCYPGARDLSQRIPIIPEDVDCDLGLPSDCQGTHSL
GSWGGTRGPDTPRPHRGLGATREEERALCCQARALLRPGCPPEEAGAVRANFPSALQDTQ
ESSTTATEAAGPRSHSADSSSPGA

>Q9NSD7 multipass membrane protein
MQMADAATIATMNKAAGGDKLAELFSLVPDLLEAANTSGNASLQLPDLWWELGLELPD
GAPPGHPPGSGGAESADTEARVRILISVVYWVVCALGLAGNLLVLYLMKSMQGWRKSSI
NLFVTNLALTDFQFVLTLPFWAVENALDFKWPFGKAMCKIVSMVTSMNMYASVFFLTAM
SVTRYHSVASALKSHRTRGHGRGDCCGRSLGDSCCFSAKALCVWIWALAALASLPSAIFS
TTVKVMGEELCLVRFPDKLLGRDRQFWLGLYHSQKVLLGFVLPLGIIILCYLLLVRFIADR
RAAGTKGGAAVAGGRPTGASARRLSKVTKSVTIVVLSFFLCWLPNQALTTWSILIKFNAVP
FSQEYFLCQVYAFPVSVCLAHSNSCLNPVLYCLVRREFRKALKSLLWRIASPSITSMRPFTA
TTKPEHEDQGLQAPAPPHAAAEPDLLYYPPGVVVYSGGRYDLLPSSSAY

>P52803 GPI-anchor, lipid-anchor
MLHVEMLTLVFLVLWMCVFSQDPGSKAVADRYAVYWNSSNPRFQRGDYHIDVCINDYLD
VFCPHYEDSVPEDKTERYVLYMVNFDGYSACDHTSKGFKRWECNRPHSPNGPLKFSEKF
QLFTPFSLGFEFRPGREYFYISSAIPDNGRRSCLKLKVFVRPTNSCMKTIGVHDRVFDVND

KVENSLEPADDTVHESAEPSRGENAAQTPRIPSRLLAILLFLLAMLLTL

>Q96L42 multipass membrane protein
MPVMKGLLAPQNTFLDTIATRFDGTHSNFILANAQVAKGFPIVYCSDGFCELAGFARTEV
MQKSCSCKFLFGVETNEQLMLQIEKSLEEKTEFKGEIMFYKKNGSPFWCLLDIVPIKNEKG
DVVLFLASFKDITDTKVKITPEDKKEDKVKGRSRAGTHFDSARRRSRAVLYHISGHLQRRE
KNKLKINNNVFVDKPAFPEYKVSDAKKSKFILLHFSTFKAGWDWLILLATFYVAVTVPYN
VCFIGNDDLSTTRSTTVSDIAVEILFIIDIILNFRTTYVSKSGQVIFEARSICIHYVTTWFIIDLI
AALPFDLLYAFNVTVVSLVHLLKTVRLLRLLRLLQKLDRYSQHSTIVLTLLMSMFALLAH
WMACIWYVIGKMEREDNSLLKWEVGWLHELGKRLESPYYGNNTLGGPSIRSAYIAALYF
TLSSLTSVGFGNVSANTDAEKIFSICTMLIGALMHALVFGNVTAIIQRMYSRWSLYHTRTK
DLKDFIRVHHLPQQLKQRMLEYFQTTWSVNNGIDSNELLKDFPDELRSDITMHLNKEILQ
LSLFECASRGCLRSLSLHIKTSFCAPGEYLLRQGDALQAIYFVCSGSMEVLKDSMVLAILG
KGDLIGANLSIKDQVIKTNADVKALTYCDLQCIILKGLFEVLDLYPEYAHKFVEDIQHDLT
YNLREGHESDVISRLSNKSMVSQSEPKGNGNINKRLPSIVEDEEEEEEGEEEEAVSLSPICT
RGSSSRNKKVGSNKAYLGLSLKQLASGTVPFHSPIRVSRSNSPKTKQEIDPPNHNKRKEKN
LKLQLSTLNNAGPPDLSPRIVDGIEDGNSSEESQTFDFGSERIRSEPRISPPLGDPEIGAAVLF
IKAEETKQQINKLNSEVTTLTQEVSQLGKDMRNVIQLLENVLSPQQPSRFCSLHSTSVCPSR
ESLQTRTSWSAHQPCLHLQTGGAAYTQAQLCSSNITSDIWSVDPSSVGSSPQRTGAHEQNP
ADSELYHSPSLDYSPSHYQVVQEGHLQFLRCISPHSDSTLTPLQSISATLSSSVCSSSETSLHL
VLPSRSEEGSFSQGTVSSFSLENLPGSWNQEGMASASTKPLENLPLEVVTSTAEVKDNKAI
NV

>Q9BVI4 multipass membrane protein
MEREPGAAGVRRALGRRLEAVLASRSEANAVFDILAVLQSEDQEEIQEAVRTCSRLFGALL
ERGELFVGQLPSEEMVMTGSQGATRKYKVWMRHRYHSCCNRLGELLGHPSFQVKELAL
SALLKFVQLEGAHPLEKSKWEGNYLFPRELFKLVVGGLLSPEEDQSLLLSQFREYLDYDD
TRYHTMQAAVDAVARVTGQHPEVPPAFWNNAFTLLSAVSLPRREPTVSSFYVKRAELWDT
WKVAHLKEHRRVFQAMWLSFLKHKLPLSLYKKVLLIVHDAILPQLAQPTLMIDFLTRACD
LGGALSLLALNGLFILIHKHNLEYPDFYRKLYGLLDPSVFHVKYRARFFHLADLFLSSSHLP
AYLVAAFAKRLARLALTAPPEALLMVLPFICNLLRRHPACRVLVHRPHGPELDADPYDPGE
EDPAQSRALESSLWELQALQRHYHPEVSKAASVINQALSMPEVSIAPLLELTAYEIFERDLK
KKGPEPVPLEFIPAQGLLGRPGELCAQHFTLS

>Q96RD3 multipass membrane protein
MPIANDTQFHTSSFLLLGIPGLEDVHIWIGFPFFSVYLIALLGNAAIFFVIQTEQSLHEPMYY
CLAMLDSIDLSLSTATIPKMLGIFWFNIKEISFGGYLSQMFFIHFFTVMESIVLVAMAFDRYI
AICKPLWYTMILTSKIISLIAGIAVLRSLYMVIPLVFLLLRLPFCGHRIIPHTYCEHMGIARLA
CASIKVNIMFGLGSISLLLLDVLLIILSHIRILYAVFCLPSWEARLKALNTCGSHIGVILAFSTP
AFFSFFTHCFGHDIPQYIHIFLANLYVVVPPTLNPVIYGVRTKHIRETVLRIFFKTDH

>Q8NH01 multipass membrane protein
MTNTSSSDFTLLGLLVNSEAAGIVFTVILAVFLGAVTANLVMIFLIQVDSRLHTPMYFLLSQ
LSIMDTLFICTTVPKLLADMVSKEKIISFVACGIQIFLYLTMIGSEFFLLGLMAYDCYVAVCN

PLRYPVLMNRKKCLLLAAGAWFGGSLDGFLLTPITMNVPYCGSRSINHFFCEIPAVLKLAC
ADTSLYETLMYICCVLMLLIPISIISTSYSLILLTIHRMPSAEGRKKAFTTCSSHLTVVSIFYG
AAFYTYVLPQSFHTPEQDKVVSAFYTIVTPMLNPLIYSLRNKDVIGAFKKVFACCSSAQKV
ATSDA

>Q14524 multipass membrane protein
MANFLLPRGTSSFRRFTRESLAAIEKRMAEKQARGSTTLQESREGLPEEEAPRPQLDLQAS
KKLPDLYGNPPQELIGEPLEDLDPFYSTQKTFIVLNKGKTIFRFSATNALYVLSPFHPIRRAA
VKILVHSLFNMLIMCTILTNCVFMAQHDPPPWTKYVEYTFTAIYTFESLVKILARGFCLHAF
TFLRDPWNWLDFSVIIMAYTTEFVDLGNVSALRTFRVLRALKTISVISGLKTIVGALIQSVK
KLADVMVLTVFCLSVFALIGLQLFMGNLRHKCVRNFTALNGTNGSVEADGLVWESLDLY
LSDPENYLLKNGTSDVLLCGNSSDAGTCPEGYRCLKAGENPDHGYTSFDSFAWAFLALFR
LMTQDCWERLYQQTLRSAGKIYMIFFMLVIFLGSFYLVNLILAVVAMAYEEQNQATIAETE
EKEKRFQEAMEMLKKEHEALTIRGVDTVSRSSLEMSPLAPVNSHERRSKRRKRMSSGTEE
CGEDRLPKSDSEDGPRAMNHLSLTRGLSRTSMKPRSSRGSIFTFRRRDLGSEADFADDENS
TAGESESHHTSLLVPWPLRRTSAQGQPSPGTSAPGHALHGKKNSTVDCNGVVSLLGAGDP
EATSPGSHLLRPVMLEHPPDTTTPSEEPGGPQMLTSQAPCVDGFEEPGARQRALSAVSVLT
SALEELEESRHKCPPCWNRLAQRYLIWECCPLWMSIKQGVKLVVMDPFTDLTITMCIVLN
TLFMALEHYNMTSEFEEMLQVGNLVFTGIFTAEMTFKIIALDPYYYFQQGWNIFDSIIVILS
LMELGLSRMSNLSVLRSFRLLRVFKLAKSWPTLNTLIKIIGNSVGALGNLTLVLAIIVFIFAV
VGMQLFGKNYSELRDSDSGLLPRWHMMDFFHAFLIIFRILCGEWIETMWDCMEVSGQSL
CLLVFLLVMVIGNLVVLNLFLALLLSSFSADNLTAPDEDREMNNLQLALARIQRGLRFVKR
TTWDFCCGLLRQRPQKPAALAAQGQLPSCIATPYSPPPPETEKVPPTRKETRFEEGEQPGQ
GTPGDPEPVCVPIAVAESDTDDQEEDEENSLGTEEESSKQQESQPVSGGPEAPPDSRTWSQ
VSATASSEAEASASQADWRQQWKAEPQAPGCGETPEDSCSEGSTADMTNTAELLEQIPDL
GQDVKDPEDCFTEGCVRRCPCCAVDTTQAPGKVWWRLRKTCYHIVEHSWFETFIIFMILL
SSGALAFEDIYLEERKTIKVLLEYADKMFTYVFVLEMLLKWVAYGFKKYFTNAWCWLDF
LIVDVSLVSLVANTLGFAEMGPIKSLRTLRALRPLRALSRFEGMRVVVNALVGAIPSIMNVL
LVCLIFWLIFSIMGVNLFAGKFGRCINQTEGDLPLNYTIVNNKSQCESLNLTGELYWTKVK
VNFDNVGAGYLALLQVATFKGWMDIMYAAVDSRGYEEQPQWEYNLYMYIYFVIFIIFGSF
FTLNLFIGVIIDNFNQQKKKLGGQDIFMTEEQKKYYNAMKKLGSKKPQKPIPRPLNKYQG
FIFDIVTKQAFDVTIMFLICLNMVTMMVETDDQSPEKINILAKINLLFVAIFTGECIVKLAAL
RHYYFTNSWNIFDFVVVILSIVGTVLSDIIQKYFFSPTLFRVIRLARIGRILRLIRGAKGIRTLL
FALMMSLPALFNIGLLLFLVMFIYSIFGMANFAYVKWEAGIDDMFNFQTFANSMLCLFQIT
TSAGWDGLLSPILNTGPPYCDPTLPNSNGSRGDCGSPAVGILFFTTYIIISFLIVVNMYIAIILE
NFSVATEESTEPLSEDDFDMFYEIWEKFDPEATQFIEYSVLSDFADALSEPLRIAKPNQISLIN
MDLPMVSGDRIHCMDILFAFTKRVLGESGEMDALKIQMEEKFMAANPSKISYEPITTTLRR
KHEEVSAMVIQRAFRRHLLQRSLKHASFLFRQQAGSGLSEEDAPEREGLIAYVMSENFSRP
LGPPSSSSISSTSFPPSYDSVTRATSDNLQVRGSDYSHSEDLADFPPSPDRDRESIV

>P30536 multipass membrane protein
MAPPWVPAMGFTLAPSLGCFVGSRFVHGEGLRWYAGLQKPSWHPPHWVLGPVWGTLYS
AMGYGSYLVWKELGGFTEKAVVPLGLYTGQLALNWAWPPIFFGARQMGWALVDLLLVS
GAAAATTVAWYQVSPLAARLLYPYLAWLAFTTTLNYCVWRDNHGWRGGRRLPE

>A6NHA9 multipass membrane protein
MENRNNMTEFVLLGLTENPKMQKIIFVVFFVIYIITVVGYVLIVVTITASPSLGSPMYLSLA
YLSFIDACYSSVNTPNLITHSLYGKKAILFNGCMTQVFGEHFFGGAEGILLTVMAYDHYVA
ICKPLHYMTIMNQCVCALLMGVVWMGGFLHATIQILFIFQLPFCGPNVIDHFMCDLNPLL
NLACTDTHMLELFIAANSGFICLLNFALLLVSYVVILCSLRTHSLEARHKALSTCVSHITVVI
LFFVPCIFVYMRPAATLPIDKAVAIFYTMITPMLNPLIYTLKNAQMKNAIRKLCSRKDISGD
K

>Q5JTC6 peripheral membrane protein
METQKDEAAQAKGAAASGSTREQTAEKGAKNKAAEATEGPTSEPSSSGPGRLKKTAMKL
FGGKKGICTLPSFFGGGRSKGSGKGSSKKGLSKSKTHDGLSEAAHGPEDVVSEGTGFSLPL
PELPCQFPSSQSAHGALETGSRCKTSVAGATEKAVAEKFPSMPKPKKGLKGFFSSIRRHRKS
KVTGAEQSEPGAKGPERVRARPHEHVSSAPQVPCFEETFQAPRKENANPQDAPGPKVSPT
PEPSPPATEKMACKDPEKPMEACASAHVQPKPAPEASSLEEPHSPETGEKVVAGEVNPPNG
PVGDPLSLLFGDVTSLKSFDSLTGCGDIIAEQDMDSMTDSMASGGQRANRDGTKRSSCLV
TYQGGGEEMALPDDDDEEEEEEEEVELEEEEEEVKEEEEDDDLEYLWETAQMYPRPNMN
LGYHPTTSPGHHGYMLLDPVRSYPGLAPGELLTPQSDQQESAPNSDEGYYDSTTPGFEDD
SGEALGLVRRDCLPRDSYSGDALYEFYEPDDSLENSPPGDDCLYDLHGRSSEMFDPFLNFE
PFLSSRPPGAMETEEERLVTIQKQLLYWELRREQLEAQEARAREAHAREAHAREAYTREA
YGREAYAREAHTWEAHGREARTREAQAREVRCRETQVRETQARQEKPVLEYQMRPLGP
SVMGLAAGVSGTSQISHRGITSAFPTTASSEPDWRDFRPLEKRYEGTCSKKDQSTCLMQLF
QSDAMFEPDMQEANFGGSPRRAYPTYSPPEDPEEEEVEKEGNATVSFSQALVEFTSNGNLF
SSMSCSSDSDSSFTQNLPELPPMVTFDIADVERDGEGKCEENPEFHNDEDLAASLEAFELG
YYHKHAFNNYHSRFYQGLPWGVSSLPRYLGLPGLHPRPPPAAMALNRRSRSLDTAETLE
MELSNSHLVQGYLESDELQAQQEDSDEEDEEEEEGEWSRDSPLSLYTEPPGAYDWPAWAP
CPLPVGPGPAWISPNQLDRPSSQSPYRQATCCIPPMTMSISLSVPESRAPGESGPQLARPSHL
HLPMGPCYNLQPQASQSMRARPRDVLLPVDEPSCSSSSGGFSPSPLPQAKPVGITHGIPQLP
RVRPEHPQPQPTHYGPSSLDLSKERAEQGASLATSYSSTAMNGNLAK

>Q70SY1 single-pass type II membrane protein
MEVLESGEQGVLQWDRKLSELSEPGDGEALMYHTHFSELLDEFSQNVLGQLLNDPFLSE
KSVSMEVEPSPTSPAPLIQAEHSYSLCEEPRAQSPFTHITTSDSFNDDEVESEKWYLSTDFPS
TSIKTEPVTDEPPPGLVPSVTLTITAISTPLEKEEPPLEMNTGVDSSCQTIIPKIKLEPHEVDQF
LNFSPKEAPVDHLHLPPTPPSSHGSDSEGSLSPNPRLHPFSLPQTHSPSRAAPRAPSALSSSP
LLTAPHKLQGSGPLVLTEEEKRTLIAEGYPIPTKLPLSKSEEKALKKIRRKIKNKISAQESRR
KKKEYMDSLEKKVESCSTENLELRKKVEVLENTNRTLLQQLQKLQTLVMGKVSRTCKLA
GTQTGTCLMVVVLCFAVAFGSFFQGYGPYPSATKMALPSQHSLQEPYTASVVRSRNLLIYE
EHSPPEESSSPGSAGELGGWDRGSSLLRVSGLESRPDVDLPHFIISNETSLEKSVLLELQQHL
VSAKLEGNETLKVVELDRRVNTTF

>O95466 lipid-anchor
MGNAAGSAEQPAGPAAPPPKQPAPPKQPMPAAGELEERFNRALNCMNLPPDKVQLLSQY
DNEKKWELICDQERFQVKNPPAAYIQKLKSYVDTGGVSRKVAADWMSNLGFKRRVQEST

QVLRELETSLRTNHIGWVQEFLNEENRGLDVLLEYLAFAQCSVTYDMESTDNGASNSEK
NKPLEQSVEDLSKGPPSSVPKSRHLTIKLTPAHSRKALRNSRIVSQKDDVHVCIMCLRAIM
NYQSGFSLVMNHPACVNEIALSLNNKNPRTKALVLELLAAVCLVRGGHDIILAAFDNFKEV
CGEQHRFEKLMEYFRNEDSNIDFMVACMQFINIVVHSVENMNFRVFLQYEFTHLGLDLYL
ERLRLTESDKLQVQIQAYLDNIFDVGALLEDTETKNAVLEHMEELQEQVALLTERLRDAE
NESMAKIAELEKQLSQARKELETLRERFSESTAMGPSRRPPEPEKAPPAAPTRPSALELKVE
ELEEKGLIRILRGPGDAVSIEILPVAVATPSGGDAPTPGVPTGSPSPDLAPAAEPAPGAAPPPP
PPLPGLPSPQEAPPSAPPQAPPLPGSPEPPPAPPLPGDLPPPPPPPPPPGTDGPVPPPPPPPPP
PGGPPDALGRRDSELGPGVKAKKPIQTKFRMPLLNWVALKPSQITGTVFTELNDEKVLQE
LDMSDFEEQFKTKSQGPSLDLSALKSKAAQKAPSKATLIEANRAKNLAITLRKGNLGAERI
CQAIEAYDLQALGLDFLELLMRFLPTEYERSLITRFEREQRPMEELSEEDRFMLCFSRIPRL
PERMTTLTFLGNFPDTAQLLMPQLNAIIAASMSIKSSDKLRQILEIVLAFGNYMNSSKRGAA
YGFRLQSLDALLEMKSTDRKQTLLHYLVKVIAEKYPQLTGFHSDLHFLDKAGSVSLDSVL
ADVRSLQRGLELTQREFVRQDDCMVLKEFLRANSPTMDKLLADSKTAQEAFESVVEYFG
ENPKTTSPGLFFSLFSRFIKAYKKAEQEVEQWKKEAAAQEAGADTPGKGEPPAPKSPPKA
RRPQMDLISELKRRQQKEPLIYESDRDGAIEDIITVIKTVPFTARTGKRTSRLLCEASLGEEM
PL

>P07359 single-pass type I membrane protein
MPLLLLLLLLPSPLHPHPICEVSKVASHLEVNCDKRNLTALPPDLPKDTTILHLSENLLYTFS
LATLMPYTRLTQLNLDRCELTKLQVDGTLPVLGTLDLSHNQLQSLPLLGQTLPALTVLDVS
FNRLTSLPLGALRGLGELQELYLKGNELKTLPPGLLTPTPKLEKLSLANNNLTELPAGLLNG
LENLDTLLLQENSLYTIPKGFFGSHLLPFAFLHGNPWLCNCEILYFRRWLQDNAENVYVW
KQGVDVKAMTSNVASVQCDNSDKFPVYKYPGKGCPTLGDEGDTDLYDYYPEEDTEGDK
VRATRTVVKFPTKAHTTPWGLFYSWSTASLDSQMPSSLHPTQESTKEQTTFPPRWTPNFTL
HMESITFSKTPKSTTEPTPSPTTSEPVPEPAPNMTTLEPTPSPTTPEPTSEPAPSPTTPEPTPIPT
IATSPTILVSATSLITPKSTFLTTTKPVSLLESTKKTIPELDQPPKLRGVLQGHLESSRNDPFLH
PDFCCLLPLGFYVLGLFWLLFASVVLILLLSWVGHVKPQALDSGQGAALTTATQTTHLEL
QRGRQVTVPRAWLLFLRGSLPTFRSSLFLWVRPNGRVGPLVAGRRPSALSQGRGQDLLST
VSIRYSGHSL

>P35606 peripheral membrane protein
MPLRLDIKRKLTARSDRVKSVDLHPTEPWMLASLYNGSVCVWNHETQTLVKTFEVCDLP
VRAAKFVARKNWVVTGADDMQIRVFNYNTLERVHMFEAHSDYIRCIAVHPTQPFILTSSD
DMLIKLWDWDKKWSCSQVFEGHTHYVMQIVINPKDNNQFASASLDRTIKVWQLGSSSPN
FTLEGHEKGVNCIDYYSGGDKPYLISGADDRLVKIWDYQNKTCVQTLEGHAQNVSCASF
HPELPIIITGSEDGTVRIWHSSTYRLESTLNYGMERVWCVASLRGSNNVALGYDEGSIIVKL
GREEPAMSMDANGKIIWAKHSEVQQANLKAMGDAEIKDGERLPLAVKDMGSCEIYPQTI
QHNPNGRFVVVCGDGEYIIYTAMALRNKSFGSAQEFAWAHDSSEYAIRESNSIVKIFKNFK
EKKSFKPDFGAESIYGGFLLGVRSVNGLAFYDWDNTELIRRIEIQPKHIFWSDSGELVCIAT
EESFFILKYLSEKVLAAQETHEGVTEDGIEDAFEVLGEIQEIVKTGLWVGDCFIYTSSVNRL
NYYVGGEIVTIAHLDRTMYLLGYIPKDNRLYLGDKELNIISYSLLVSVLEYQTAVMRRDFS
MADKVLPTIPKEQRTRVAHFLEKQGFKQQALTVSTDPEHRFELALQLGELKIAYQLAVEAE
SEQKWKQLAELAISKCQFGLAQECLHHAQDYGGLLLLATASGNANMVNKLAEGAERDG

KNNVAFMSYFLQGKVDACLELLIRTGRLPEAAFLARTYLPSQVSRVVKLWRENLSKVNQK
AAESLADPTEYENLFPGLKEAFVVEEWVKETHADLWPAKQYPLVTPNEERNVMEEGKDF
QPSRSTAQQELDGKPASPTPVIVASHTANKEEKSLLELEVDLDNLELEDIDTTDINLDEDIL
DD

>Q9BQT8 multipass membrane protein
MSAKPEVSLVREASRQIVAGGSAGLVEICLMHPLDVVKTRFQIQRCATDPNSYKSLVDSFR
MIFQMEGLFGFYKGILPPILAETPKRAVKFFTFEQYKKLLGYVSLSPALTFAIAGLGSGLTEA
IVVNPFEVVKVGLQANRNTFAEQPSTVGYARQIIKKEGWGLQGLNKGLTATLGRHGVFN
MVYFGFYYNVKNMIPVNKDPILEFWRKFGIGLLSGTIASVINIPFDVAKSRIQGPQPVPGEI
KYRTCFKTMATVYQEEGILALYKGLLPKIMRLGPGGAVMLLVYEYTYSWLQENW

>O43603 multipass membrane protein
MNVSGCPGAGNASQAGGGGGWHPEAVIVPLLFALIFLVGTVGNTLVLAVLLRGGQAVSTT
NLFILNLGVADLCFILCCVPFQATIYTLDGWVFGSLLCKAVHFLIFLTMHASSFTLAAVSLD
RYLAIRYPLHSRELRTPRNALAAIGLIWGLSLLFSGPYLSYYRQSQLANLTVCHPAWSAPR
RRAMDICTFVFSYLLPVLVLGLTYARTLRYLWRAVDPVAAGSGARRAKRKVTRMILIVAA
LFCLCWMPHHALILCVWFGQFPLTRATYALRILSHLVSYANSCVNPIVYALVSKHFRKGFR
TICAGLLGRAPGRASGRVCAAARGTHSGSVLERESSDLLHMSEAAGALRPCPGASQPCILE
PCPGPSWQGPKAGDSILTVDVA

>O43688 multipass membrane protein
MQRRWVFVLLDVLCLLVASLPFAILTLVNAPYKRGFYCGDDSIRYPYRPDTITHGLMAGVT
ITATVILVSAGEAYLVYTDRLYSRSDFNNYVAAVYKVLGTFLFGAAVSQSLTDLAKYMIGR
LRPNFLAVCDPDWSRVNCSVYVQLEKVCRGNPADVTEARLSFYSGHSSFGMYCMVFLAL
YVQARLCWKWARLLRPTVQFFLVAFALYVGYTRVSDYKHHWSDVLVGLLQGALVAALT
VCYISDFFKARPPQHCLKEEELERKPSLSLTLTLGEADHNHYGYPHSSS

>P27449 multipass membrane protein
MSESKSGPEYASFFAVMGASAAMVFSALGAAYGTAKSGTGIAAMSVMRPEQIMKSIIPVV
MAGIIAIYGLVVAVLIANSLNDDISLYKSFLQLGAGLSVGLSGLAAGFAIGIVGDAGVRGTA
QQPRLFVGMILILIFAEVLGLYGLIVALILSTK

>Q9BY67 single-pass type I membrane protein
MASVVLPSGSQCAAAAAAAAPPGLRLRLLLLLFSAAALIPTGDGQNLFTKDVTVIEGEVA
TISCQVNKSDDSVIQLLNPNRQTIYFRDFRPLKDSRFQLLNFSSSELKVSLTNVSISDEGRYF
CQLYTDPPQESYTTITVLVPPRNLMIDIQKDTAVEGEEIEVNCTAMASKPATTIRWFKGNTE
LKGKSEVEEWSDMYTVTSQLMLKVHKEDDGVPVICQVEHPAVTGNLQTQRYLEVQYKP
QVHIQMTYPLQGLTREGDALELTCEAIGKPQPVMVTWVRVDDEMPQHAVLSGPNLFINNL
NKTDNGTYRCEASNIVGKAHSDYMLYVYDPPTTIPPPTTTTTTTTTTTTILTIITDSRAGEE
GSIRAVDHAVIGGVVAVVVFAMLCLLIILGRYFARHKGTYFTHEAKGADDAADADTAIINA
EGGQNNSEEKKEYFI

>P07202 single-pass type I membrane protein

MRALAVLSVTLVMACTEAFFPFISRGKELLWGKPEESRVSSVLEESKRLVDTAMYATMQR
NLKKRGILSPAQLLSFSKLPEPTSGVIARAAEIMETSIQAMKRKVNLKTQQSQHPTDALSE
DLLSIIANMSGCLPYMLPPKCPNTCLANKYRPITGACNNRDHPRWGASNTALARWLPPVY
EDGFSQPRGWNPGFLYNGFPLPPVREVTRHVIQVSNEVVTDDDRYSDLLMAWGQYIDHDI
AFTPQSTSKAAFGGGADCQMTCENQNPCFPIQLPEEARPAAGTACLPFYRSSAACGTGDQ
GALFGNLSTANPRQQMNGLTSFLDASTVYGSSPALERQLRNWTSAEGLLRVHARLRDSGR
AYLPFVPPRAPAACAPEPGIPGETRGPCFLAGDGRASEVPSLTALHTLWLREHNRLAAALK
ALNAHWSADAVYQEARKVVGALHQIITLRDYIPRILGPEAFQQYVGPYEGYDSTANPTVS
NVFSTAAFRFGHATIHPLVRRLDASFQEHPDLPGLWLHQAFFSPWTLLRGGGLDPLIRGLL
ARPAKLQVQDQLMNEELTERLFVLSNSSTLDLASINLQRGRDHGLPGYNEWREFCGLPRL
ETPADLSTAIASRSVADKILDLYKHPDNIDVWLGGLAENFLPRARTGPLFACLIGKQMKAL
RDGDWFWWENSHVFTDAQRRELEKHSLSRVICDNTGLTRVPMDAFQVGKFPEDFESCDSI
TGMNLEAWRETFPQDDKCGFPESVENGDFVHCEESGRRVLVYSCRHGYELQGREQLTCT
QEGWDFQPPLCKDVNECADGAHPPCHASARCRNTKGGFQCLCADPYELGDDGRTCVDS
GRLPRVTWISMSLAALLIGGFAGLTSTVICRWTRTGTKSTLPISETGGGTPELRCGKHQAVG
TSPQRAAAQDSEQESAGMEGRDTHRLPRAL

>Q8IX19 single-pass type II membrane protein
MEVEEIYKHQEVKMQAPAFRDKKQGVSAKNQGAHDPDYENITLAFKNQDHAKGGHSRP
TSQVPAQCRPPSDSTQVPCWLYRAILSLYILLALAFVLCIILSAFIMVKNAEMSKELLGFKR
ELWNVSNSVQACEERQKRGWDSVQQSITMVRSKIDRLETTLAGIKNIDTKVQKILEVLQK
MPQSSPQ

>Q8IU99 multipass membrane protein
MMDKFRMIFQFLQSNQESFMNGICGIMALASAQMYSAFDFNCPCLPGYNAAYSAGILLAP
PLVLFLLGLVMNNNVSMLAEEWKRPLGRRAKDPAVLRYMFCSMAQRALIAPVVWVAVTL
LDGKCFLCAFCTAVPVSALGNGSLAPGLPAPELARLLARVPCPEIYDGDWLLAREVAVRYL
RCISQALGWSFVLLTTLLAFVVRSVRPCFTQAAFLKSKYWSHYIDIERKLFDETCTEHAKA
FAKVCIQQFFEAMNHDLELGHTHGTLATAPASAAAPTTPDGAEEEREKLRGITDQGTMNR
LLTSWHKCKPPLRLGQEEPPLMGNGWAGGGPRPPRKEVATYFSKV

>Q496F6 single-pass type I membrane protein
MWLLPALLLLCLSGCLSLKGPGSVTGTAGDSLTVWCQYESMYKGYNKYWCRGQYDTSC
ESIVETKGEEKVERNGRVSIRDHPEALAFTVTMQNLNEDDAGSYWCKIQTVWVLDSWSR
DPSDLVRVYVSPAITTPRRTTHPATPPIFLVVNPGRNLSTGEVLTQNSGFRLSSPHFLLVVLL
KLPLLLSMLGAVFWVNRPQWAPPGR

>Q8NH05 multipass membrane protein
MKKEQDSNVTEFVLLGLSSSWELQLFLFLLFLFFYIAIVLGNLLIVVTVQAHAHLLQSPMY
YFLGHLSFIDLCLSCVTVPKMLGDFLQQGKSISFSGCLAQIYFLHFLGASEMFLLTVMAYD
RYVAICNPLRYLTVMNPQLCLWLVLACWCGGFIHSIMQVILVIQLPFCGPNELDNFYCDVP
QVIKLACMDTYVVEVLVIANSGLLSLVCFLVLLFSYAIILITLRTHFCQGQNKVFSTCASHLT
VVSLIFVPCVFIYLRPFCSFSVDKIFSLFYTVITPMLNPLIYTLRNTDMKTAMKKLRIKPCGI
PLPC

>O15375	multipass membrane protein
MPQALERADGSWAWVVLLATMVTQGLTLGFPTCIGIFFTELQWEFQASNSETSWFPSILTA
VLHMAGPLCSILVGRFGCRVTVMLGGVLASLGMVASSFSHNLSQLYFTAGFITGLGMCFSF
QSSITVLGFYFVRRRVLANALASMGVSLGITLWPLLSRYLLENLGWRGTFLVFGGIFLHCCI
CGAIIRPVATSVAPETKECPPPPPETPALGCLAACGRTIQRHLAFDILRHNTGYCVYILGVM
WSVLGFPLPQVFLVPYAMWHSVDEQQAALLISIIGFSNIFLRPLAGLMAGRPAFASHRKYL
FSLALLLNGLTNLVCAASGDFWVLVGYCLAYSVSMSGIGALIFQVLMDIVPMDQFPRALG
LFTVLDGLAFLISPPLAGLLLDATNNFSYVFYMSSFFLISAALFMGGSFYALQKKEQGKQA
VAADALERDLFLEAKDGPGKQRSPEIMCQSSRQPRPAGVNKHLWGCPASSRTSHEWLLWP
KAVLQAKQTALGWNSPT

>Q7Z410	single-pass type II membrane protein
MEPTVADVHLVPRTTKEVPALDAACCRAASIGVVATSLVVLTLGVLLAFLSTQGFHVDHTA
ELRGIRWTSSLRRETSDYHRTLTPTLEALLHFLLRPLQTLSLGLEEELLQRGIRARLREHGIS
LAAYGTIVSAELTGRHKGPLAERDFKSGRCPGNSFSCGNSQCVTKVNPECDDQEDCSDGS
DEAHCECGLQPAWRMAGRIVGGMEASPGEFPWQASLRENKEHFCGAAIINARWLVSAAH
CFNEFQDPTKWVAYVGATYLSGSEASTVRAQVVQIVKHPLYNADTADFDVAVLELTSPLPF
GRHIQPVCLPAATHIFPPSKKCLISGWGYLKEDFLVKPEVLQKATVELLDQALCASLYGHSL
TDRMVCAGYLDGKVDSCQGDSGGPLVCEEPSGRFFLAGIVSWGIGCAEARRPGVYARVT
RLRDWILEATTKASMPLAPTMAPAPAAPSTAWPTSPESPVVSTPTKSMQALSTVPLDWVT
VPKLQECGARPAMEKPTRVVGGFGAASGEVPWQVSLKEGSRHFCGATVVGDRWLLSAA
HCFNHTKVEQVRAHLGTASLLGLGGSPVKIGLRRVVLHPLYNPGILDFDLAVLELASPLAF
NKYIQPVCLPLAIQKFPVGRKCMISGWGNTQEGNATKPELLQKASVGIIDQKTCSVLYNFS
LTDRMICAGFLEGKVDSCQGDSGGPLACEEAPGVFYLAGIVSWGIGCAQVKKPGVYTRIT
RLKGWILEIMSSQPLPMSPPSTTRMLATTSPRTTAGLTVPGATPSRPTPGAASRVTGQPANS
TLSAVSTTARGQTPFPDAPEATTHTQLPDCGLAPAALTRIVGGSAAGRGEWPWQVSLWLR
RREHRCGAVLVAERWLLSAAHCFDVYGDPKQWAAFLGTPFLSGAEGQLERVARIYKHPFY
NLYTLDYDVALLELAGPVRRSRLVRPICLPEPAPRPPDGTRCVITGWGSVREGGSMARQLQ
KAAVRLLSEQTCRRFYPVQISSRMLCAGFPQGGVDSCSGDAGGPLACREPSGRWVLTGVT
SWGYGCGRPHFPGVYTRVAAVRGWIGQHIQE

>Q15620	multipass membrane protein
MAAENSSFVTQFILAGLTDQPGVQIPLFFLFLGFYVVTVVGNLGLITLIRLNSHLHTPMYFF
LYNLSFIDFCYSSVITPKMLMSFVLKKNSISYAGCMTQLFFFLFFVVSESFILSAMAYDRYV
AICNPLLYMVTMSPQVCFLLLLGVYGMGFAGAMAHTACMMGVTFCANNLVNHYMCDIL
PLLECACTSTYVNELVVFVVVGIDIGVPTVTIFISYALILSSIFHIDSTEGRSKAFSTCSSHIIA
VSLFFGSGAFMYLKPFSLLAMNQGKVSSLFYTTVVPMLNPLIYSLRNKDVKVALKKILNK
NAFS

>P48145	multipass membrane protein
MDNASFSEPWPANASGPDPALSCSNASTLAPLPAPLAVAVPVVYAVICAVGLAGNSAVLYV
LLRAPRMKTVTNLFILNLAIADELFTLVLPINIADFLLRQWPFGELMCKLIVAIDQYNTFSSL
YFLTVMSADRYLVVLATAESRRVAGRTYSAARAVSLAVWGIVTLVVLPFAVFARLDDEQG

RRQCVLVFPQPEAFWWRASRLYTLVLGFAIPVSTICVLYTTLLCRLHAMRLDSHAKALER
AKKRVTFLVVAILAVCLLCWTPYHLSTVVALTTDLPQTPLVIAISYFITSLSYANSCLNPFLY
AFLDASFRRNLRQLITCRAAA

>Q96T83 multipass membrane protein
MEPGDAARPGSGRATGAPPPRLLLLPLLLGWGLRVAAAASASSSGAAAEDSSAMEELATE
KEAEESHRQDSVSLLTFILLLTLTILTIWLFKHRRVRFLHETGLAMIYGLIVGVILRYGTPATS
GRDKSLSCTQEDRAFSTLLVNVSGKFFEYTLKGEISPGKINSVEQNDMLRKVTFDPEVFFN
ILLPPIIFHAGYSLKKRHFFRNLGSILAYAFLGTAVSCFIIGNLMYGVVKLMKIMGQLSDKF
YYTDCLFFGAIISATDPVTVLAIFNELHADVDLYALLFGESVLNDAVAIVLSSSIVAYQPAGL
NTHAFDAAAFFKSVGIFLGIFSGSFTMGAVTGVNANVTKFTKLHCFPLLETALFFLMSWST
FLLAEACGFTGVVAVLFCGITQAHYTYNNLSVESRSRTKQLFEVLHFLAENFIFSYMGLAL
FTFQKHVFSPIFIIGAFVAIFLGRAAHIYPLSFFLNLGRRHKIGWNFQHMMMFSGLRGAMA
FALAIRDTASYARQMMFTTTLLIVFFTVWIIGGGTTPMLSWLNIRVGVEEPSEEDQNEHHW
QYFRVGVDPDQDPPPNNDSFQVLQGDGPDSARGNRTKQESAWIFRLWYSFDHNYLKPILT
HSGPPLTTTLPAWCGLLARCLTSPQVYDNQEPLREEDSDFILTEGDLTLTYGDSTVTANGSS
SSHTASTSLEGSRRTKSSSEEVLERDLGMGDQKVSSRGTRLVFPLEDNA

>O15393 single-pass type II membrane protein
MALNSGSPPAIGPYYENHGYQPENPYPAQPTVVPTVYEVHPAQYYPSPVPQYAPRVLTQAS
NPVVCTQPKSPSGTVCTSKTKKALCITLTLGTFLVGAALAAGLLWKFMGSKCSNSGIECDS
SGTCINPSNWCDGVSHCPGGEDENRCVRLYGPNFILQVYSSQRKSWHPVCQDDWNENYG
RAACRDMGYKNNFYSSQGIVDDSGSTSFMKLNTSAGNVDIYKKLYHSDACSSKAVVSLR
CIACGVNLNSSRQSRIVGGESALPGAWPWQVSLHVQNVHVCGGSIITPEWIVTAAHCVEK
PLNNPWHWTAFAGILRQSFMFYGAGYQVEKVISHPNYDSKTKNNDIALMKLQKPLTFND
LVKPVCLPNPGMMLQPEQLCWISGWGATEEKGKTSEVLNAAKVLLIETQRCNSRYVYDN
LITPAMICAGFLQGNVDSCQGDSGGPLVTSKNNIWWLIGDTSWGSGCAKAYRPGVYGNV
MVFTDWIYRQMRADG

>Q8NGH7 multipass membrane protein
MTLVSFFSFLSKPLIMLLSNSSWRLSQPSFLLVGIPGLEESQHWIALPLGILYLLALVGNVTI
LFIIWMDPSLHQSMYLFLSMLAAIDLVLASSTAPKALAVLLVHAHEIGYIVCLIQMFFIHAF
SSMESGVLVAMALDCYVAICHPLHHSTILHPGVIGCIGMVVLVRGLLLLIPFPILLGKLIFCQ
ATIIGHAYCEHMAVVKLACSETTVNRAYGLTMALLVIGLDVLAIGVSYAHILQAVLKVPGS
EARLKAFSTCGSHICVILVFYVPGIFSFLTHRFGHHVPHHVHVLLATWYLLMPPALNPLVY
GVKTQQIRQRVLRVFTQKD

>O95222 multipass membrane protein
MEWRNHSGRVSEFVLLGFPAPAPLQVLLFALLLLAYVLVLTENTLIIMAIRNHSTLHKPMY
FFLANMSFLEIWYVTVTIPKMLAGFVGSKQDHGQLISFEGCMTQLYFFLGLGCTECVLLA
VMAYDRYMAICYPLHYPVIVSGRLCVQMAAGSWAGGFGISMVKVFLISGLSYCGPNIINH
FFCDVSPLLNLSCTDMSTAELTDFILAIFILLGPLSVTGASYVAITGAVMHIPSAAGRYKAFS
TCASHLTVVIIFYAASIFIYARPKALSAFDTNKLVSVLYAVIVPLLNPIIYCLRNQEVKRALCC
TLHLYQHQDPDPKKASRNV

>P48357 single-pass type I membrane protein
MICQKFCVVLLHWEFIYVITAFNLSYPITPWRFKLSCMPPNSTYDYFLLPAGLSKNTSNSN
GHYETAVEPKFNSSGTHFSNLSKTTFHCCFRSEQDRNCSLCADNIEGKTFVSTVNSLVFQQI
DANWNIQCWLKGDLKLFICYVESLFKNLFRNYNYKVHLLYVLPEVLEDSPLVPQKGSFQ
MVHCNCSVHECCECLVPVPTAKLNDTLLMCLKITSGGVIFQSPLMSVQPINMVKPDPPLG
LHMEITDDGNLKISWSSPPLVPFPLQYQVKYSENSTTVIREADKIVSATSLLVDSILPGSSYE
VQVRGKRLDGPGIWSDWSTPRVFTTQDVIYFPPKILTSVGSNVSFHCIYKKENKIVPSKEIV
WWMNLAEKIPQSQYDVVSDHVSKVTFFNLNETKPRGKFTYDAVYCCNEHECHHRYAEL
YVIDVNINISCETDGYLTKMTCRWSTSTIQSLAESTLQLRYHRSSLYCSDIPSIHPISEPKDCY
LQSDGFYECIFQPIFLLSGYTMWIRINHSLGSLDSPPTCVLPDSVVKPLPPSSVKAEITINIGL
LKISWEKPVFPENNLQFQIRYGLSGKEVQWKMYEVYDAKSKSVSLPVPDLCAVYAVQVR
CKRLDGLGYWSNWSNPAYTVVMDIKVPMRGPEFWRIINGDTMKKEKNVTLLWKPLMKN
DSLCSVQRYVINHHTSCNGTWSEDVGNHTKFTFLWTEQAHTVTVLAINSIGASVANFNLT
FSWPMSKVNIVQSLSAYPLNSSCVIVSWILSPSDYKLMYFIIEWKNLNEDGEIKWLRISSSV
KKYYIHDHFIPIEKYQFSLYPIFMEGVGKPKIINSFTQDDIEKHQSDAGLYVIVPVIISSSILLL
GTLLISHQRMKKLFWEDVPNPKNCSWAQGLNFQKPETFEHLFIKHTASVTCGPLLLEPETI
SEDISVDTSWKNKDEMMPTTVVSLLSTTDLEKGSVCISDQFNSVNFSEAEGTEVTYEDES
QRQPFVKYATLISNSKPSETGEEQGLINSSVTKCFSSKNSPLKDSFSNSSWEIEAQAFFILSD
QHPNIISPHLTFSEGLDELLKLEGNFPEENNDKKSIYYLGVTSIKKRESGVLLTDKSRVSCPF
PAPCLFTDIRVLQDSCSHFVENNINLGTSSKKTFASYMPQFQTCSTQTHKIMENKMCDLTV

>Q86SM5 multipass membrane protein
MFGLFGLWRTFDSVVFYLTLIVGLGGPVGNGLVLWNLGFRIKKGPFSIYLLHLAAADFLFL
SCRVGFSVAQAALGAQDTLYFVLTFLWFAVGLWLLAAFSVERCLSDLFPACYQGCRPRHA
SAVLCALVWTPTLPAVPLPANACGLLRNSACPLVCPRYHVASVTWFLVLARVAWTAGVVL
FVWVTCCSTRPRPRLYGIVLGALLLLFFCGLPSVFYWSLQPLLNFLLPVFSPLATLLACVNS
SSKPLIYSGLGRQPGKREPLRSVLRRALGEGAELGARGQSLPMGLL

>Q86UN2 GPI-anchor, lipid-anchor
MLRKGCCVELLLLLLVAAELPLGGGCPRDCVCYPAPMTVSCQAHNFAAIPEGIPVDSERVFL
QNNRIGLLQPGHFSPAMVTLWIYSNNITYIHPSTFEGFVHLEELDLGDNRQLRTLAPETFQG
LVKLHALYLYKCGLSALPAGVFGGLHSLQYLYLQDNHIEYLQDDIFVDLVNLSHLFLHGN
KLWSLGPGTFRGLVNLDRLLLHENQLQWVHHKAFHDLRRLTTLFLFNNSLSELQGECLAP
LGALEFLRLNGNPWDCGCRARSLWEWLQRFRGSSSAVPCVSPGLRHGQDLKLLRAEDFR
NCTGPASPHQIKSHTLTTTDRAARKEHHSPHGPTRSKGHPHGPRPGHRKPGKNCTNPRNR
NQISKAGAGKQAPELPDYAPDYQHKFSFDIMPTARPKRKGKCARRTPIRAPSGVQQASSAS
SLGASLLAWTLGLAVTLR

>Q02246 GPI-anchor, lipid-anchor
MGTATRRKPHLLLVAAVALVSSSAWSSALGSQTTFGPVFEDQPLSVLFPEESTEEQVLLACR
ARASPPATYRWKMNGTEMKLEPGSRHQLVGGNLVIMNPTKAQDAGVYQCLASNPVGTV
VSREAILRFGFLQEFSKEERDPVKAHEGWGVMLPCNPPAHYPGLSYRWLLNEFPNFIPTDG
RHFVSQTTGNLYIARTNASDLGNYSCLATSHMDFSTKSVFSKFAQLNLAAEDTRLFAPSIK

ARFPAETYALVGQQVTLECFAFGNPVPRIKWRKVDGSLSPQWTTAEPTLQIPSVSFEDEGT
YECEAENSKGRDTVQGRIIVQAQPEWLKVISDTEADIGSNLRWGCAAAGKPRPTVRWLR
NGEPLASQNRVEVLAGDLRFSKLSLEDSGMYQCVAENKHGTIYASAELAVQALAPDFRLN
PVRRLIPAARGGEILIPCQPRAAPKAVVLWSKGTEILVNSSRVTVTPDGTLIIRNISRSDEGK
YTCFAENFMGKANSTGILSVRDATKITLAPSSADINLGDNLTLQCHASHDPTMDLTFTWTL
DDFPIDFDKPGGHYRRTNVKETIGDLTILNAQLRHGGKYTCMAQTVVDSASKEATVLVRG
PPGPPGGVVVRDIGDTTIQLSWSRGFDNHSPIAKYTLQARTPPAGKWKQVRTNPANIEGNA
ETAQVLGLTPWMDYEFRVIASNILGTGEPSGPSSKIRTREAAPSVAPSGLSGGGGAPGELIV
NWTPMSREYQNGDGFGYLLSFRRQGSTHWQTARVPGADAQYFVYSNESVRPYTPFEVKI
RSYNRRGDGPESLTALVYSAEEEPRVAPTKVWAKGVSSSEMNVTWEPVQQDMNGILLGY
EIRYWKAGDKEAAADRVRTAGLDTSARVSGLHPNTKYHVTVRAYNRAGTGPASPSANAT
TMKPPPRRPPGNISWTFSSSSLSIKWDPVVPFRNESAVTGYKMLYQNDLHLTPTLHLTGKN
WIEIPVPEDIGHALVQIRTTGPGGDGIPAEVHIVRNGGTSMMVENMAVRPAPHPGTVISHSV
AMLILIGSLEL

>O14512 peripheral membrane protein
MVFRNVGRPPEEEDVEAAPEPGPSELLCPRHRCALDPKALPPGLALERTWGPAAGLEAQL
AALGLGQPAGPGVKTVGGGCCPCPCPPQPPPPQPQPPAAAPQAGEDPTETSDALLVLEGLE
SEAESLETNSCSEEELSSPGRGGGGGGRLLLQPPGPELPPVPFPLQDLVPLGRLSRGEQQQQ
QQQQPPPPPPPPPGPLRPLAGPSRKGSFKIRLSRLFRTKSCNGGSGGGDGTGKRPSGELAAS
AASLTDMGGSAGRELDAGRKPKLTRTQSAFSPVSFSPLFTGETVSLVDVDISQRGLTSPHPP
TPPPPPRRSLSLLDDISGTLPTSVLVAPMGSSLQSFPLPPPPPPHAPDAFPRIAPIRAAESLHSQ
PPQHLQCPLYRPDSSSFAASLRELEKCGWYWGPMNWEDAEMKLKGKPDGSFLVRDSSDP
RYILSLSFRSQGITHHTRMEHYRGTFSLWCHPKFEDRCQSVVEFIKRAIMHSKNGKFLYFL
RSRVPGLPPTPVQLLYPVSRFSNVKSLQHLCRFRIRQLVRIDHIPDLPLPKPLISYIRKFYYY
DPQEEVYLSLKEAQLISKQKQEVEPST

>Q9UI09 peripheral membrane protein
MELVQVLKRGLQQITGHGGLRGYLRVFFRTNDAKVGTLVGEDKYGNKYYEDNKQFFGR
HRWVVYTTEMNGKNTFWDVDGSMVPPEWHRWLHSMTDDPPTTKPLTARKFIWTNHKF
NVTGTPEQYVPYSTTRKKIQEWIPPSTPYK

>Q8NGX1 multipass membrane protein
MCSGNQTSQNQTASTDFTLTGLFAESKHAALLYTVTFLLFLMALTGNALLILLIHSEPRLHT
PMYFFISQLALMDLMYLCVTVPKMLVGQVTGDDTISPSGCGIQMFFHLTLAGAEVFLLAA
MAYDRYAAVCRPLHYPLLMNQRVCQLLVSACWVLGMVDGLLLTPITMSFPFCQSRKILSF
FCETPALLKLSCSDVSLYKMLTYLCCILMLLTPIMVISSSYTLILHLIHRMNSAAGRRKALA
TCSSHMIIVLLLFGASFYTYMLRSSYHTAEQDMMVSAFYTIFTPVLNPLIYSLRNKDVTRA
LRSMMQSRMNQEK

>Q9BVK6 single-pass type I membrane protein
MAVELGVLLVRPRPGTGLGRVMRTLLLVLWLATRGSALYFHIGETEKKCFIEEIPDETMVIG
NYRTQLYDKQREEYQPATPGLGMFVEVKDPEDKVILARQYGSEGRFTFTSHTPGEHQICL
HSNSTKFSLFAGGMLRVHLDIQVGEHANDYAEIAAKDKLSELQLRVRQLVEQVEQIQKEQ

NYQRWREERFRQTSESTNQRVLWWSILQTLILVAIGVWQMRHLKSFFEAKKLV

>P27930 single-pass type I membrane protein
MLRLYVLVMGVSAFTLQPAAHTGAARSCRFRGRHYKREFRLEGEPVALRCPQVPYWLWA
SVSPRINLTWHKNDSARTVPGEEETRMWAQDGALWLLPALQEDSGTYVCTTRNASYCDK
MSIELRVFENTDAFLPFISYPQILTLSTSGVLVCPDLSEFTRDKTDVKIQWYKDSLLLDKDN
EKFLSVRGTTHLLVHDVALEDAGYYRCVLTFAHEGQQYNITRSIELRIKKKKEETIPVIISPL
KTISASLGSRLTIPCKVFLGTGTPLTTMLWWTANDTHIESAYPGGRVTEGPRQEYSENNEN
YIEVPLIFDPVTREDLHMDFKCVVHNTLSFQTLRTTVKEASSTFSWGIVLAPLSLAFLVLGG
IWMHRRCKHRTGKADGLTVLWPHHQDFQSYPK

>Q7Z412 single-pass type II membrane protein
MKSDSSTSAAPLRGLGGPLRSSEPVRAVPARAPAVDLLEEAADLLVVHLDFRAALETCERA
WQSLANHAVAEEPAGTSLEVKCSLCVVGIQALAEMDRWQEVLSWVLQYYQVPEKLPPK
VLELCILLYSKMQEPGAVLDVVGAWLQDPANQNLPEYGALAEFHVQRVLLPLGCLSEAEE
LVVGSAAFGEERRLDVLQAIHTARQQQKQEHSGSEEAQKPNLEGSVSHKFLSLPMLVRQL
WDSAVSHFFSLPFKKSLLAALILCLLVVRFDPASPSSLHFLYKLAQLFRWIRKAAFSRLYQL
RIRD

>O14964 peripheral membrane protein
MGRGSGTFERLLDKATSQLLLETDWESILQICDLIRQGDTQAKYAVNSIKKKVNDKNPHV
ALYALEVMESVVKNCGQTVHDEVANKQTMEELKDLLKRQVEVNVRNKILYLIQAWAHA
FRNEPKYKVVQDTYQIMKVEGHVFPEFKESDAMFAAERAPDWVDAEECHRCRVQFGVM
TRKHHCRACGQIFCGKCSSKYSTIPKFGIEKEVRVCEPCYEQLNRKAEGKATSTTELPPEYL
TSPLSQQSQLPPKRDETALQEEEELQLALALSQSEAEEKERLRQKSTYTSYPKAEPMPSAS
SAPPASSLYSSPVNSSAPLAEDIDPELARYLNRNYWEKKQEEARKSPTPSAPVPLTEPAAQP
GEGHAAPTNVVENPLPETDSQPIPPSGGPFSEPQFHNGESEESHEQFLKALQNAVTTFVNR
MKSNHMRGRSITNDSAVLSLFQSINGMHPQLLELLNQLDERRLYYEGLQDKLAQIRDARG
ALSALREEHREKLRRAAEEAERQRQIQLAQKLEIMRQKKQEYLEVQRQLAIQRLQEQEKE
RQMRLEQQKQTVQMRAQMPAFPLPYAQLQAMPAAGGVLYQPSGPASFPSTFSPAGSVEGS
PMHGVYMSQPAPAAGPYPSMPSTAADPSMVSAYMYPAGATGAQAAPQAQAGPTASPAYS
SYQPTPTAGYQNVASQAPQSLPAISQPPQSSTMGYMGSQSVSMGYQPYNMQNLMTTLPS
QDASLPPQQPYIAGQQPMYQQMAPSGGPPQQQPPVAQQPQAQGPPAQGSEAQLISFD

>Q96GW7 GPI-anchor, lipid-anchor
MAQLFLPLLAALVLAQAPAALADVLEGDSSEDRAFRVRIAGDAPLQGVLGGALTIPCHVH
YLRPPPSRRAVLGSPRVKWTFLSRGREAEVLVARGVRVKVNEAYRFRVALPAYPASLTDVS
LALSELRPNDSGIYRCEVQHGIDDSSDAVEVKVKGVVFLYREGSARYAFSFSGAQEACARI
GAHIATPEQLYAAYLGGYEQCDAGWLSDQTVRYPIQTPREACYGDMDGFPGVRNYGVVD
PDDLYDVYCYAEDLNGELFLGDPPEKLTLEEARAYCQERGAEIATTGQLYAAWDGGLDHC
SPGWLADGSVRYPIVTPSQRCGGGLPGVKTLFLFPNQTGFPNKHSRFNVYCFRDSAQPSAI
PEASNPASNPASDGLEAIVTVTETLEELQLPQEATESESRGAIYSIPIMEDGGGGSSTPEDPA
EAPRTLLEFETQSMVPPTGFSEEEGKALEEEEKYEDEEEKEEEEEEEEVEDEALWAWPSEL
SSPGPEASLPTEPAAQEESLSQAPARAVLQPGASPLPDGESEASRPPRVHGPPTETLPTPRER

NLASPSPSTLVEAREVGEATGGPELSGVPRGESEETGSSEGAPSLLPATRAPEGTRELEAPSE
DNSGRTAPAGTSVQAQPVLPTDSASRGGVAVVPASGDCVPSPCHNGGTCLEEEEGVRCLC
LPGYGGDLCDVGLRFCNPGWDAFQGACYKHFSTRRSWEEAETQCRMYGAHLASISTPEE
QDFINNRYREYQWIGLNDRTIEGDFLWSDGVPLLYENWNPGQPDSYFLSGENCVVMVWH
DQGQWSDVPCNYHLSYTCKMGLVSCGPPPELPLAQVFGRPRLRYEVDTVLRYRCREGLA
QRNLPLIRCQENGRWEAPQISCVPRRPARALHPEEDPEGRQGRLLGRWKALLIPPSSPMPG
P

>Q86WV6 multipass membrane protein
MPHSSLHPSIPCPRGHGAQKAALVLLSACLVTLWGLGEPPEHTLRYLVLHLASLQLGLLLN
GVCSLAEELRHIHSRYRGSYWRTVRACLGCPLRRGALLLLSIYFYYSLPNAVGPPFTWML
ALLGLSQALNILLGLKGLAPAEISAVCEKGNFNVAHGLAWSYYIGYLRLILPELQARIRTYN
QHYNNLLRGAVSQRLYILLPLDCGVPDNLSMADPNIRFLDKLPQQTGDHAGIKDRVYSNSI
YELLENGQRAGTCVLEYATPLQTLFAMSQYSQAGFSREDRLEQAKLFCRTLEDILADAPES
QNNCRLIAYQEPADDSSFSLSQEVLRHLRQEEKEEVTVGSLKTSAVPSTSTMSQEPELLISG
MEKPLPLRTDFS

>P28827 single-pass type I membrane protein
MRGLGTCLATLAGLLLTAAGETFSGGCLFDEPYSTCGYSQSEGDDFNWEQVNTLTKPTSD
PWMPSGSFMLVNASGRPEGQRAHLLLPQLKENDTHCIDFHYFVSSKSNSPPGLLNVYVKV
NNGPLGNPIWNISGDPTRTWNRAELAISTFWPNFYQVIFEVITSGHQGYLAIDEVKVLGHP
CTRTPHFLRIQNVEVNAGQFATFQCSAIGRTVAGDRLWLQGIDVRDAPLKEIKVTSSRRFIA
SFNVVNTTKRDAGKYRCMIRTEGGVGISNYAELVVKEPPVPIAPPQLASVGATYLWIQLNA
NSINGDGPIVAREVEYCTASGSWNDRQPVDSTSYKIGHLDPDTEYEISVLLTRPGEGGTGSP
GPALRTRTKCADPMRGPRKLEVVEVKSRQITIRWEPFGYNVTRCHSYNLTVHYCYQVGG
QEQVREEVSWDTENSHPQHTITNLSPYTNVSVKLILMNPEGRKESQELIVQTDEDLPGAVP
TESIQGSTFEEKIFLQWREPTQTYGVITLYEITYKAVSSFDPEIDLSNQSGRVSKLGNETHFL
FFGLYPGTTYSFTIRASTAKGFGPPATNQFTTKISAPSMPAYELETPLNQTDNTVTVMLKPA
HSRGAPVSVYQIVVEEERPRRTKKTTEILKCYPVPIHFQNASLLNSQYYFAAEFPADSLQA
AQPFTIGDNKTYNGYWNTPLLPYKSYRIYFQAASRANGETKIDCVQVATKGAATPKPVPE
PEKQTDHTVKIAGVIAGILLFVIIFLGVVLVMKKRKLAKKRKETMSSTRQEMTVMVNSMD
KSYAEQGTNCDEAFSFMDTHNLNGRSVSSPSSFTMKTNTLSTSVPNSYYPDETHTMASDT
SSLVQSHTYKKREPADVPYQTGQLHPAIRVADLLQHITQMKCAEGYGFKEEYESFFEGQSA
PWDSAKKDENRMKNRYGNIIAYDHSRVRLQTIEGDTNSDYINGNYIDGYHRPNHYIATQG
PMQETIYDFWRMVWHENTASIIMVTNLVEVGRVKCCKYWPDDTEIYKDIKVTLIETELLA
EYVIRTFAVEKRGVHEIREIRQFHFTGWPDHGVPYHATGLLGFVRQVKSKSPPSAGPLVVH
CSAGAGRTGCFIVIDIMLDMAEREGVVDIYNCVRELRSRRVNMVQTEEQYVFIHDAILEA
CLCGDTSVPASQVRSLYYDMNKLDPQTNSSQIKEEFRTLNMVTPTLRVEDCSIALLPRNHE
KNRCMDILPPDRCLPFLITIDGESSNYINAALMDSYKQPSAFIVTQHPLPNTVKDFWRLVL
DYHCTSVVMLNDVDPAQLCPQYWPENGVHRHGPIQVEFVSADLEEDIISRIFRIYNAARPQ
DGYRMVQQFQFLGWPMYRDTPVSKRSFLKLIRQVDKWQEEYNGGEGRTVVHCLNGGG
RSGTFCAISIVCEMLRHQRTVDVFHAVKTLRNNKPNMVDLLDQYKFCYEVALEYLNSG

>P07332 peripheral membrane protein

MGFSSELCSPQGHGVLQQMQEAELRLLEGMRKWMAQRVKSDREYAGLLHHMSLQDSG
GQSRAISPDSPISQSWAEITSQTEGLSRLLRQHAEDLNSGPLSKLSLLIRERQQLRKTYSEQ
WQQLQQELTKTHSQDIEKLKSQYRALARDSAQAKRKYQEASKDKDRDKAKDKYVRSL
WKLFAHHNRYVLGVRAAQLHHQHHHQLLLPGLLRSLQDLHEEMACILKEILQEYLEISSL
VQDEVVAIHREMAAAAARIQPEAEYQGFLRQYGSAPDVPPCVTFDESLLEEGEPLEPGEL
QLNELTVESVQHTLTSVTDELAVATEMVFRRQEMVTQLQQELRNEEENTHPRERVQLLGK
RQVLQEALQGLQVALCSQAKLQAQQELLQTKLEHLGPGEPPPVLLLQDDRHSTSSSEQER
EGGRTPTLEILKSHISGIFRPKFSLPPPLQLIPEVQKPLHEQLWYHGAIPRAEVAELLVHSGD
FLVRESQGKQEYVLSVLWDGLPRHFIIQSLDNLYRLEGEGFPSIPLLIDHLLSTQQPLTKKSG
VVLHRAVPKDKWVLNHEDLVLGEQIGRGNFGEVFSGRLRADNTLVAVKSCRETLPPDLKA
KFLQEARILKQYSHPNIVRLIGVCTQKQPIYIVMELVQGGDFLTFLRTEGARLRVKTLLQM
VGDAAAGMEYLESKCCIHRDLAARNCLVTEKNVLKISDFGMSREEADGVYAASGGLRQV
PVKWTAPEALNYGRYSSESDVWSFGILLWETFSLGASPYPNLSNQQTREFVEKGGRLPCP
ELCPDAVFRLMEQCWAYEPGQRPSFSTIYQELQSIRKRHR

>P47890 multipass membrane protein
MEGKNLTSISECFLLGFSEQLEEQKPLFGSFLFMYLVTVAGNLLIILVIITDTQLHTPMYFFL
ANLSLADACFVSTTVPKMLANIQIQSQAISYSGCLLQLYFFMLFVMLEAFLLAVMAYDCY
VAICHPLHYILIMSPGLCIFLVSASWIMNALHSLLHTLLMNSLSFCANHEIPHFFCDINPLLS
LSCTDPFTNELVIFITGGLTGLICVLCLIISYTNVFSTILKIPSAQGKRKAFSTCSSHLSVVSLF
FGTSFCVDFSSPSTHSAQKDTVASVMYTVVTPMLNPFIYSLRNQEIKSSLRKLIWVRKIHSP

>Q05655 peripheral membrane protein
MAPFLRIAFNSYELGSLQAEDEANQPFCAVKMKEALSTERGKTLVQKKPTMYPEWKSTF
DAHIYEGRVIQIVLMRAAEEPVSEVTVGVSVLAERCKKNNGKAEFWLDLQPQAKVLMSV
QYFLEDVDCKQSMRSEDEAKFPTMNRRGAIKQAKIHYIKNHEFIATFFGQPTFCSVCKDFV
WGLNKQGYKCRQCNAAIHKKCIDKIIGRCTGTAANSRDTIFQKERFNIDMPHRFKVHNY
MSPTFCDHCGSLLWGLVKQGLKCEDCGMNVHHKCREKVANLCGINQKLLAEALNQVTQ
RASRRSDSASSEPVGIYQGFEKKTGVAGEDMQDNSGTYGKIWEGSSKCNINNFIFHKVLG
KGSFGKVLLGELKGRGEYFAIKALKKDVVLIDDDVECTMVEKRVLTLAAENPFLTHLICTF
QTKDHLFFVMEFLNGGDLMYHIQDKGRFELYRATFYAAEIMCGLQFLHSKGIIYRDLKLD
NVLLDRDGHIKIADFGMCKENIFGESRASTFCGTPDYIAPEILQGLKYTFSVDWWSFGVLL
YEMLIGQSPFHGDDEDELFESIRVDTPHYPRWITKESKDILEKLFEREPTKRLGVTGNIKIHP
FFKTINWTLLEKRRLEPPFRPKVKSPRDYSNFDQEFLNEKARLSYSDKNLIDSMDQSAFAG
FSFVNPKFEHLLED

>Q99712 multipass membrane protein
MDAIHIGMSSTPLVKHTAGAGLKANRPRVMSKSGHSNVRIDKVDGIYLLYLQDLWTTVID
MKWRYKLTLFAATFVMTWFLFGVIYYAIAFIHGDLEPGEPISNHTPCIMKVDSLTGAFLFSL
ESQTTIGYGVRSITEECPHAIFLLVAQLVITTLIEIFITGTFLAKIARPKKRAETIKFSHCAVITK
QNGKLCLVIQVANMRKSLLIQCQLSGKLLQTHVTKEGERILLNQATVKFHVDSSSESPFLIL
PMTFYHVLDETSPLRDLTPQNLKEKEFELVVLLNATVESTSAVCQSRTSYIPEEIYWGFEFV
PVVSLSKNGKYVADFSQFEQIRKSPDCTFYCADSEKQQLEEKYRQEDQRERELRTLLLQQ
SNV

>Q5GH77 multipass membrane protein
METVFEEMDEESTGGVSSSKEEIVLGQRLHLSFPFSIIFSTVLYCGEVAFGLYMFEIYRKAN
DTFWMSFTISFIIVGAILDQIILMFFNKDLRRNKAALLFWHILLLGPIVRCLHTIRNYHKWL
KNLKQEKEETQVSITKRNTMLEREIAFSIRDNFMQQKAFKYMSVIQAFLGSVPQLILQMYI
SLTIREWPLNRALLMTFSLLSVTYGAIRCNILAIQISNDDTTIKLPPIEFFCVVMWRFLEVISR
VVTLAFFIASLKLKSLPVLLIIYFVSLLAPWLEFWKSGAHLPGNKENNSNMVGTVLMLFLI
TLLYAAINFSCWSAVKLQLSDDKIIDGRQRWGHRILHYSFQFLENVIMILVFRFFGGKTLLN
CCDSLIAVQLIISYLLATGFMLLFYQYLYPWQSGKVLPGRTENQPEAPYYYVNIEKTEKNK
NKQLRNYCHSCNRVGYFSIRKSMTCS

>Q9NS40 multipass membrane protein
MPVRRGHVAPQNTFLGTIIRKFEGQNKKFIIANARVQNCAIIYCNDGFCEMTGFSRPDVMQ
KPCTCDFLHGPETKRHDIAQIAQALLGSEERKVEVTYYHKNGSTFICNTHIIPVKNQEGVA
MMFIINFEYVTDNENAATPERVNPILPIKTVNRKFFGFKFPGLRVLTYRKQSLPQEDPDVVV
IDSSKHSDDSVAMKHFKSPTKESCSPSEADDTKALIQPSKCSPLVNISGPLDHSSPKRQWDR
LYPDMLQSSSQLSHSRSRESLCSIRRASSVHDIEGFGVHPKNIFRDRHASEDNGRNVKGPF
NHIKSSLLGSTSDSNLNKYSTINKIPQLTLNFSEVKTEKKNSSPPSSDKTIIAPKVKDRTHNV
TEKVTQVLSLGADVLPEYKLQTPRINKFTILHYSPFKAVWDWLILLLVIYTAIFTPYSAAFL
LNDREEQKRRECGYSCSPLNVVDLIVDIMFIIDILINFRTTYVNQNEEVVSDPAKIAIHYFKG
WFLIDMVAAIPFDLLIFGSGSDETTTLIGLLKTARLLRLVRVARKLDRYSEYGAAVLMLLM
CIFALIAHWLACIWYAIGNVERPYLTDKIGWLDSLGQQIGKRYNDSDSSSGPSIKDKYVTAL
YFTFSSLTSVGFGNVSPNTNSEKIFSICVMLIGSLMYASIFGNVSAIIQRLYSGTARYHMQML
RVKEFIRFHQIPNPLRQRLEEYFQHAWTYTNGIDMNMVLKGFPECLQADICLHLNQTLLQ
NCKAFRGASKGCLRALAMKFKTTHAPPGDTLVHCGDVLTALYFLSRGSIEILKDDIVVAIL
GKNDIFGEMVHLYAKPGKSNADVRALTYCDLHKIQREDLLEVLDMYPEFSDHFLTNLELT
FNLRHESAKADLLRSQSMNDSEGDNCKLRRRKLSFESEGEKENSTNDPEDSADTIRHYQS
SKRHFEEKKSRSSSFISSIDDEQKPLFSGIVDSSPGIGKASGLDFEETVPTSGRMHIDKRSHS
CKDITDMRSWERENAHPQPEDSSPSALQRAAWGISETESDLTYGEVEQRLDLLQEQLNRL
ESQMTTDIQTILQLLQKQTTVVPPAYSMVTAGSEYQRPIIQLMRTSQPEASIKTDRSFSPSSQ
CPEFLDLEKSKLKSKESLSSGVHLNTASEDNLTSLLKQDSDLSLELHLRQRKTYVHPIRHPS
LPDSSLSTVGIVGLHRHVSDPGLPGK

>P16581 single-pass type I membrane protein
MIASQFLSALTLVLLIKESGAWSYNTSTEAMTYDEASAYCQQRYTHLVAIQNKEEIEYLNSI
LSYSPSYYWIGIRKVNNVWVWVGTQKPLTEEAKNWAPGEPNNRQKDEDCVEIYIKREKD
VGMWNDERCSKKKLALCYTAACTNTSCSGHGECVETINNYTCKCDPGFSGLKCEQIVNC
TALESPEHGSLVCSHPLGNFSYNSSCSISCDRGYLPSSMETMQCMSSGEWSAPIPACNVVE
CDAVTNPANGFVECFQNPGSFPWNTTCTFDCEEGFELMGAQSLQCTSSGNWDNEKPTCK
AVTCRAVRQPQNGSVRCSHSPAGEFTFKSSCNFTCEEGFMLQGPAQVECTTQGQWTQQIP
VCEAFQCTALSNPERGYMNCLPSASGSFRYGSSCEFSCEQGFVLKGSKRLQCGPTGEWDN
EKPTCEAVRCDAVHQPPKGLVRCAHSPIGEFTYKSSCAFSCEEGFELHGSTQLECTSQGQW
TEEVPSCQVVKCSSLAVPGKINMSCSGEPVFGTVCKFACPEGWTLNGSAARTCGATGHWS
GLLPTCEAPTESNIPLVAGLSAAGLSLLTLAPFLLWLRKCLRKAKKFVPASSCQSLESDGSY

QKPSYIL

>Q8IZS7 single-pass type II membrane protein
MVSNFFHVIQVFEKSATLISKTEHIGFVIYSWRKSTTHLGSRRKFAISIYLSEVSLQKYDCPF
SGTSFVVFSLFLICAMAGDVVYADIKTVRTSPLELAFPLQRSVSFNFSTVHKSCPAKDWKV
HKGKCYWIAETKKSWNKSQNDCAINNSYLMVIQDITAMVRFNI

>Q5JRS4 multipass membrane protein
MPKLNSTFVTEFLFEGFSSFRRQHKLVFFVVFLTLYLLTLSGNVIIMTIIRLDHHLHTPMYFF
LCMLSISETCYTVAIIPHMLSGLLNPHQPIATQSCATQLFFYLTFGINNCFLLTVMGYDRYVA
ICNPLRYSVIMGKRACIQLASGSLGIGLGMAIVQVTSVFGLPFCDAFVISHFFCDVRHLLKL
ACTDTTVNEIINFVVSVCVLVLPMGLVFISYVLIISTILKIASAEGQKKAFATCASHLTVVIIH
YGCASIIYLKPKSQSSLGQDRLISVTYTHHSPTEPCCVQPEEQGGQRCSAQSRGAKNSVSL
MKRGCEGFSFAFINMY

>Q8WZ92 multipass membrane protein
MNSLKDGNHTALTGFILLGLTDDPILRVILFMIILSGNLSIIILIRISSQLHHPMYFFLSHLAFA
DMAYSSSVTPNMLVNFLVERNTVSYLGCAIQLGSAAFFATVECVLLAAMAYDRFVAICSPL
LYSTKMSTQVSVQLLLVVYIAGFLIAVSYTTSFYFLLFCGPNQVNHFFCDFAPLLELSCSDIS
VSTVVLSFSSGSIIVVTVCVIAVCYIYILITILKMRSTEGHHKAFSTCTSHLTVVTLFYGTITFI
YVMPNFSYSTDQNKVVSVLYTVVIPMLNPLIYSLRNKEIKGALKRELVRKILSHDACYFSR
TSNNDIT

>Q7RTX1 multipass membrane protein
MLLCTARLVGLQLLISCCWAFACHSTESSPDFTLPGDYLLAGLFPLHSGCLQVRHRPEVTL
CDRSCSFNEHGYHLFQAMRLGVEEINNSTALLPNITLGYQLYDVCSDSANVYATLRVLSLP
GQHHIELQGDLLHYSPTVLAVIGPDSTNRAATTAALLSPFLVPMISYAASSETLSVKRQYPS
FLRTIPNDKYQVETMVLLLQKFGWTWISLVGSSDDYGQLGVQALENQATGQGICIAFKDI
MPFSAQVGDERMQCLMRHLAQAGATVVVVFSSRQLARVFFESVVLTNLTGKVWVASEA
WALSRHITGVPGIQRIGMVLGVAIQKRAVPGLKAFEEAYARADKKAPRPCHKGSWCSSNQ
LCRECQAFMAHTMPKLKAFSMSSAYNAYRAVYAVAHGLHQLLGCASGACSRGRVYPWQ
LLEQIHKVHFLLHKDTVAFNDNRDPLSSYNIIAWDWNGPKWTFTVLGSSTWSPVQLNINE
TKIQWHGKDNQVPKSVCSSDCLEGHQRVVTGFHHCCFECVPCGAGTFLNKSDLYRCQPC
GKEEWAPEGSQTCFPRTVVFLALREHTSWVLLAANTLLLLLLLGTAGLFAWHLDTPVVRS
AGGRLCFLMLGSLAAGSGSLYGFFGEPTRPACLLRQALFALGFTIFLSCLTVRSFQLIIIFKFS
TKVPTFYHAWVQNHGAGLFVMISSAAQLLICLTWLVVWTPLPAREYQRFPHLVMLECTET
NSLGFILAFLYNGLLSISAFACSYLGKDLPENYNEAKCVTFSLLFNFVSWIAFFTTASVYDG
KYLPAANMMAGLSSLSSGFGGYFLPKCYVILCRPDLNSTEHFQASIQDYTRRCGST

>Q99928 multipass membrane protein
MAPKLLLLLCLFSGLHARSRKVEEDEYEDSSSNQKWVLAPKSQDTDVTLILNKLLREYDK
KLRPDIGIKPTVIDVDIYVNSIGPVSSINMEYQIDIFFAQTWTDSRLRFNSTMKILTLNSNMV
GLIWIPDTIFRNSKTAEAHWITTPNQLLRIWNDGKILYTLRLTINAECQLQLHNFPMDEHSC
PLIFSSYGYPKEEMIYRWRKNSVEAADQKSWRLYQFDFMGLRNTTEIVTTSAGDYVVMTI

YFELSRRMGYFTIQTYIPCILTVVLSWVSFWIKKDATPARTALGITTVLTMTTLSTIARKSLP
RVSYVTAMDLFVTVCFLFVFAALMEYATLNYYSSCRKPTTTKKTTSLLHPDSSRWIPERISL
QAPSNYSLLDMRPPPTAMITLNNSVYWQEFEDTCVYECLDGKDCQSFFCCYEECKSGSW
RKGRIHIDILELDSYSRVFFPTSFLLFNLVYWVGYLYL

>P42081 single-pass type I membrane protein
MDPQCTMGLSNILFVMAFLLSGAAPLKIQAYFNETADLPCQFANSQNQSLSELVVFWQDQ
ENLVLNEVYLGKEKFDSVHSKYMGRTSFDSDSWTLRLHNLQIKDKGLYQCIIHHKKPTGM
IRIHQMNSELSVLANFSQPEIVPISNITENVYINLTCSSIHGYPEPKKMSVLLRTKNSTIEYDG
VMQKSQDNVTELYDVSISLSVSFPDVTSNMTIFCILETDKTRLLSSPFSIELEDPQPPPDHIP
WITAVLPTVIICVMVFCLILWKWKKKKRPRNSYKCGTNTMEREESEQTKKREKIHIPERSD
EAQRVFKSSKTSSCDKSDTCF

>Q7Z614 peripheral membrane protein
MASPEHPGSPGCMGPITQCTARTQQEAPATGPDLPHPGPDGHLDTHSGLSSNSSMTTRELQ
QYWQNQKCRWKHVKLLFEIASARIEERKVSKFVVYQIIVIQTGSFDNNKAVLERRYSDFA
KLQKALLKTFREEIEDVEFPRKHLTGNFAEEMICERRRALQEYLGLLYAIRCVRRSREFLDF
LTRPELREAFGCLRAGQYPRALELLLRVLPLQEKLTAHCPAAAVPALCAVLLCHRDLDRPA
EAFAAGERALQRLQAREGHRYYAPLLDAMVRLAYALGKDFVTLQERLEESQLRRPTPRGI
TLKELTVREYLH

>Q8WZ94 multipass membrane protein
MGTGNDTTVVEFTLLGLSEDTTVCAILFLVFLGIYVVTLMGNISIIVLIRRSHHLHTPMYIFL
CHLAFVDIGYSSSVTPVMLMSFLRKETSLPVAGCVAQLCSVVTFGTAECFLLAAMAYDRY
VAICSPLLYSTCMSPGVCIILVGMSYLGGCVNAWTFIGCLLRLSFCGPNKVNHFFCDYSPLL
KLACSHDFTFEIIPAISSGSIIVATVCVIAISYIYILITILKMHSTKGRHKAFSTCTSHLTAVTLF
YGTITFIYVMPKSSYSTDQNKVVSVFYTVVIPMLNPLIYSLRNKEIKGALKRELRIKIFS

>P54753 single-pass type I membrane protein
MARARPPPPPSPPPGLLPLLPPLLLLPLLLLPAGCRALEETLMDTKWVTSELAWTSHPESG
WEEVSGYDEAMNPIRTYQVCNVRESSQNNWLRTGFIWRRDVQRVYVELKFTVRDCNSIP
NIPGSCKETFNLFYYEADSDVASASSPFWMENPYVKVDTIAPDESFSRLDAGRVNTKVRSF
GPLSKAGFYLAFQDQGACMSLISVRAFYKKCASTTAGFALFPETLTGAEPTSLVIAPGTCIP
NAVEVSVPLKLYCNGDGEWMVPVGACTCATGHEPAAKESQCRPCPPGSYKAKQGEGPCL
PCPPNSRTTSPAASICTCHNNFYRADSDSADSACTTVPSPPRGVISNVNETSLILEWSEPRDL
GGRDDLLYNVICKKCHGAGGASACSRCDDNVEFVPRQLGLTERRVHISHLLAHTRYTFEV
QAVNGVSGKSPLPPRYAAVNITTNQAAPSEVPTLRLHSSSGSSLTLSWAPPERPNGVILDYE
MKYFEKSEGIASTVTSQMNSVQLDGLRPDARYVVQVRARTVAGYGQYSRPAEFETTSER
GSGAQQLQEQLPLIVGSATAGLVFVVAVVVIAIVCLRKQRHGSDSEYTEKLQQYIAPGMK
VYIDPFTYEDPNEAVREFAKEIDVSCVKIEEVIGAGEFGEVCRGRLKQPGRREVFVAIKTLK
VGYTERQRRDFLSEASIMGQFDHPNIIRLEGVVTKSRPVMILTEFMENCALDSFLRLNDGQ
FTVIQLVGMLRGIAAGMKYLSEMNYVHRDLAARNILVNSNLVCKVSDFGLSRFLEDDPSD
PTYTSSLGGKIPIRWTAPEAIAYRKFTSASDVWSYGIVMWEVMSYGERPYWDMSNQDVIN
AVEQDYRLPPPMDCPTALHQLMLDCWVRDRNLRPKFSQIVNTLDKLIRNAASLKVIASAQ

SGMSQPLLDRTVPDYTTFTTVGDWLDAIKMGRYKESFVSAGFASFDLVAQMTAEDLLRIG
VTLAGHQKKILSSIQDMRLQMNQTLPVQV

>P22897 single-pass type I membrane protein
MRLPLLLVFASVIPGAVLLLDTRQFLIYNEDHKRCVDAVSPSAVQTAACNQDAESQKFRW
VSESQIMSVAFKLCLGVPSKTDWVAITLYACDSKSEFQKWECKNDTLLGIKGEDLFFNYG
NRQEKNIMLYKGSGLWSRWKIYGTTDNLCSRGYEAMYTLLGNANGATCAFPFKFENKW
YADCTSAGRSDGWLWCGTTTDYDTDKLFGYCPLKFEGSESLWNKDPLTSVSYQINSKSAL
TWHQARKSCQQQNAELLSITEIHEQTYLTGLTSSLTSGLWIGLNSLSFNSGWQWSDRSPFR
YLNWLPGSPSAEPGKSCVSLNPGKNAKWENLECVQKLGYICKKGNTTLNSFVIPSESDVP
THCPSQWWPYAGHCYKIHRDEKKIQRDALTTCRKEGGDLTSIHTIEELDFIISQLGYEPNDE
LWIGLNDIKIQMYFEWSDGTPVTFTKWLRGEPSHENNRQEDCVVMKGKDGYWADRGCE
WPLGYICKMKSRSQGPEIVEVEKGCRKGWKKHHFYCYMIGHTLSTFAEANQTCNNENAY
LTTIEDRYEQAFLTSFVGLRPEKYFWTGLSDIQTKGTFQWTIEEEVRFTHWNSDMPGRKPG
CVAMRTGIAGGLWDVLKCDEKAKFVCKHWAEGVTHPPKPTTTPEPKCPEDWGASSRTSL
CFKLYAKGKHEKKTWFESRDFCRALGGDLASINNKEEQQTIWRLITASGSYHKLFWLGLT
YGSPSEGFTWSDGSPVSYENWAYGEPNNYQNVEYCGELKGDPTMSWNDINCEHLNNWI
CQIQKGQTPKPEPTPAPQDNPPVTEDGWVIYKDYQYYFSKEKETMDNARAFCKRNFGDL
VSIQSESEKKFLWKYVNRNDAQSAYFIGLLISLDKKFAWMDGSKVDYVSWATGEPNFANE
DENCVTMYSNSGFWNDINCGYPNAFICQRHNSSINATTVMPTMPSVPSGCKEGWNFYSN
KCFKIFGFMEEERKNWQEARKACIGFGGNLVSIQNEKEQAFLTYHMKDSTFSAWTGLND
VNSEHTFLWTDGRGVHYTNWGKGYPGGRRSSLSYEDADCVVIIGGASNEAGKWMDDTC
DSKRGYICQTRSDPSLTNPPATIQTDGFVKYGKSSYSLMRQKFQWHEAETYCKLHNSLIAS
ILDPYSNAFAWLQMETSNERVWIALNSNLTDNQYTWTDKWRVRYTNWAADEPKLKSAC
VYLDLDGYWKTAHCNESFYFLCKRSDEIPATEPPQLPGRCPESDHTAWIPFHGHCYYIESS
YTRNWGQASLECLRMGSSLVSIESAAESSFLSYRVEPLKSKTNFWIGLFRNVEGTWLWIN
NSPVSFVNWNTGDPSGERNDCVALHASSGFWSNIHCSSYKGYICKRPKIIDAKPTHELLTT
KADTRKMDPSKPSSNVAGVVIIVILLILTGAGLAAYFFYKKRRVHLPQEGAFENTLYFNSQS
SPGTSDMKDLVGNIEQNEHSVI

>Q96R30 multipass membrane protein
METWVNQSYTDGFFLLGIFSHSTADLVLFSVVMAVFTVALCGNVLLIFLIYMDPHLHTPM
YFFLSQLSLMDLMLVCTNVPKMAANFLSGRKSISFVGCGIQIGLFVCLVGSEGLLLGLMAY
DRYVAISHPLHYPILMNQRVCLQITGSSWAFGIIDGLIQMVVVMNFPYCGLRKVNHFFCEM
LSLLKLACVDTSLFEKVIFACCVFMLLFPFSIIVASYAHILGTVLQMHSAQAWKKALATCSS
HLTAVTLFYGAAMFIYLRPRHYRAPSHDKVASIFYTVLTPMLNPLIYSLRNREVMGALRKG
LDRCRIGSQH

>Q9NPR2 single-pass type I membrane protein
MGLRSWLAAPWGALPPRPPLLLLLLLLLLLQPPPPTWALSPRISLPLGSEERPFLRFEAEHIS
NYTALLLSRDGRTLYVGAREALFALSSNLSFLPGGEYQELLWGADAEKKQQCSFKGKDPQ
RDCQNYIKILLPLSGSHLFTCGTAAFSPMCTYINMENFTLARDEKGNVLLEDGKGRCPFDP
NFKSTALVVDGELYTGTVSSFQGNDPAISRSQSLRPTKTESSLNWLQDPAFVASAYIPESLG
SLQGDDDKIYFFFSETGQEFEFFENTIVSRIARICKGDEGGERVLQQRWTSFLKAQLLCSRP

DDGFPFNVLQDVFTLSPSPQDWRDTLFYGVFTSQWHRGTTEGSAVCVFTMKDVQRVFSG
LYKEVNRETQQWYTVTHPVPTPRPGACITNSARERKINSSLQLPDRVLNFLKDHFLMDGQ
VRSRMLLLQPQARYQRVAVHRVPGLHHTYDVLFLGTGDGRLHKAVSVGPRVHIIEELQIFS
SGQPVQNLLLDTHRGLLYAASHSGVVQVPMANCSLYRSCGDCLLARDPYCAWSGSSCKH
VSLYQPQLATRPWIQDIEGASAKDLCSASSVVSPSFVPTGEKPCEQVQFQPNTVNTLACPL
LSNLATRLWLRNGAPVNASASCHVLPTGDLLLVGTQQLGEFQCWSLEEGFQQLVASYCPE
VVEDGVADQTDEGGSVPVIISTSRVSAPAGGKASWGADRSYWKEFLVMCTLFVLAVLLPV
LFLLYRHRNSMKVFLKQGECASVHPKTCPVVLPPETRPLNGLGPPSTPLDHRGYQSLSDSP
PGSRVFTESEKRPLSIQDSFVEVSPVCPRPRVRLGSEIRDSVV

>Q9Y487 multipass membrane protein
MGSLFRSETMCLAQLFLQSGTAYECLSALGEKGLVQFRDLNQNVSSFQRKFVGEVKRCEE
LERILVYLVQEINRADIPLPEGEASPPAPPLKQVLEMQEQLQKLEVELREVTKNKEKLRKN
LLELIEYTHMLRVTKTFVKRNVEFEPTYEEFPSLESDSLLDYSCMQRLGAKLGFVSGLINQ
GKVEAFEKMLWRVCKGYTIVSYAELDESLEDPETGEVIKWYVFLISFWGEQIGHKVKKIC
DCYHCHVYPYPNTAEERREIQEGLNTRIQDLYTVLHKTEDYLRQVLCKAAESVYSRVIQV
KKMKAIYHMLNMCSFDVTNKCLIAEVWCPEADLQDLRRALEEGSRESGATIPSFMNIIPT
KETPPTRIRTNKFTEGFQNIVDAYGVGSYREVNPALFTIITFPFLFAVMFGDFGHGFVMFLF
ALLLVLNENHPRLNQSQEIMRMFFNGRYILLLMGLFSVYTGLIYNDCFSKSVNLFGSGWN
VSAMYSSSHPPAEHKKMVLWNDSVVRHNSILQLDPSIPGVFRGPYPLGIDPIWNLATNRLT
FLNSFKMKMSVILGIIHMTFGVILGIFNHLHFRKKFNIYLVSIPELLFMLCIFGYLIFMIFYK
WLVFSAETSRVAPSILIEFINMFLFPASKTSGLYTGQEYVQRVLLVVTALSVPVLFLGKPLFL
LWLHNGRSCFGVNRSGYTLIRKDSEEEVSLLGSQDIEEGNHQVEDGCREMACEEFNFGEI
LMTQVIHSIEYCLGCISNTASYLRLWALSLAHAQLSDVLWAMLMRVGLRVDTTYGVLLLL
PVIALFAVLTIFILLIMEGLSAFLHAIRLHWVEFQNKFYVGAGTKFVPFSFSLLSSKFNNDDS
VA

>O43759 multipass membrane protein
MEGGAYGAGKAGGAFDPYTLVRQPHTILRVVSWLFSIVVFGSIVNEGYLNSASEGEEFCIY
NRNPNACSYGVAVGVLAFLTCLLYLALDVYFPQISSVKDRKKAVLSDIGVSAFWAFLWFV
GFCYLANQWQVSKPKDNPLNEGTDAARAAIAFSFFSIFTWAGQAVLAFQRYQIGADSALF
SQDYMDPSQDSSMPYAPYVEPTGPDPAGMGGTYQQPANTFDTEPQGYQSQGY

>Q13635 multipass membrane protein
MASAGNAAEPQDRGGGGSGCIGAPGRPAGGGRRRRTGGLRRAAAPDRDYLHRPSYCDA
AFALEQISKGKATGRKAPLWLRAKFQRLLFKLGCYIQKNCGKFLVVGLLIFGAFAVGLKA
ANLETNVEELWVEVGGRVSRELNYTRQKIGEEAMFNPQLMIQTPKEEGANVLTTEALLQ
HLDSALQASRVHVYMYNRQWKLEHLCYKSGELITETGYMDQIIEYLYPCLIITPLDCFWE
GAKLQSGTAYLLGKPPLRWTNFDPLEFLEELKKINYQVDSWEEMLNKAEVGHGYMDRPC
LNPADPDCPATAPNKNSTKPLDMALVLNGGCHGLSRKYMHWQEELIVGGTVKNSTGKLV
SAHALQTMFQLMTPKQMYEHFKGYEYVSHINWNEDKAAAILEAWQRTYVEVVHQSVAQ
NSTQKVLSFTTTTLDDILKSFSDVSVIRVASGYLLMLAYACLTMLRWDCSKSQGAVGLAG
VLLVALSVAAGLGLCSLIGISFNAATTQVLPFLALGVGVDDVFLLAHAFSETGQNKRIPFED
RTGECLKRTGASVALTSISNVTAFFMAALIPIPALRAFSLQAAVVVVFNFAMVLLIFPAILSM

DLYRREDRRLDIFCCFTSPCVSRVIQVEPQAYTDTHDNTRYSPPPPYSSHSFAHETQITMQS
TVQLRTEYDPHTHVYYTTAEPRSEISVQPVTVTQDTLSCQSPESTSSTRDLLSQFSDSSLHC
LEPPCTKWTLSSFAEKHYAPFLLKPKAKVVVIFLFLGLLGVSLYGTTRVRDGLDLTDIVPRE
TREYDFIAAQFKYFSFYNMYIVTQKADYPNIQHLLYDLHRSFSNVKYVMLEENKQLPKM
WLHYFRDWLQGLQDAFDSDWETGKIMPNNYKNGSDDGVLAYKLLVQTGSRDKPIDISQL
TKQRLVDADGIINPSAFYIYLTAWVSNDPVAYAASQANIRPHRPEWVHDKADYMPETRLRI
PAAEPIEYAQFPFYLNGLRDTSDFVEAIEKVRTICSNYTSLGLSSYPNGYPFLFWEQYIGLR
HWLLLFISVVLACTFLVCAVFLLNPWTAGIIVMVLALMTVELFGMMGLIGIKLSAVPVVILI
ASVGIGVEFTVHVALAFLTAIGDKNRRAVLALEHMFAPVLDGAVSTLLGVLMLAGSEFDFI
VRYFFAVLAILTILGVLNGLVLLPVLLSFFGPYPEVSPANGLNRLPTPSPEPPPSVVRFAMPP
GHTHSGSDSSDSEYSSQTTVSGLSEELRHYEAQQGAGGPAHQVIVEATENPVFAHSTVVH
PESRHHPPSNPRQQPHLDSGSLPPGRQGQQPRRDPPREGLWPPPYRPRRDAFEISTEGHSGP
SNRARWGPRGARSHNPRNPASTAMGSSVPGYCQPITTVTASASVTVAVHPPPVPGPGRNPR
GGLCPGYPETDHGLFEDPHVPFHVRCERRDSKVEVIELQDVECEERPRGSSSN

>Q8N1M1 multipass membrane protein
MTVTYSSKVANATFFGFHRLLLKWRGSIYKLLYREFIVFAVLYTAISLVYRLLLTGVQKRYF
EKLSIYCDRYAEQIPVTFVLGFYVTLVVNRWWNQFVNLPWPDRLMFLISSSVHGSDEHGR
LLRRTLMRYVNLTSLLIFRSVSTAVYKRFPTMDHVVEAGFMTTDERKLFNHLKSPHLKYW
VPFIWFGNLATKARNEGRIRDSVDLQSLMTEMNRYRSWCSLLFGYDWVGIPLVYTQVVT
LAVYTFFFACLIGRQFLDPTKGYAGHDLDLYIPIFTLLQFFFYAGWLKVAEQLINPFGEDDD
DFETNWCIDRNLQVSLLAVDEMHMSLPKMKKDIYWDDSAARPPYTLAAADYCIPSFLGS
TVQMGLSGSDFPDEEWLWDYEKHGHRHSMIRRVKRFLSAHEHPSSPRRRSYRRQTSDSS
MFLPRDDLSPARDLLDVPSRNPPRASPTWKKSCFPEGSPTLHFSMGELSTIRETSQTSTLQS
LTPQSSVRTSPIKMPLVPEVLITAAEAPVPTSGGYHHDSATSILSSEFTGVQPSKTEQQQGPM
GSILSPSEKETPPGGPSPQTVSASAEENIFNCEEDPGDTFLKRWSLPGFLGSSHTSLGNLSPD
PMSSQPALLIDTETSSEISGINIVAGSRVSSDMLYLMENLDTKETDIIELNKETEESPK

>Q9H342 multipass membrane protein
MKISNNSLGFLPTTFILVGIPGLESEHLWISVPFSLIYIIIFLGNGIILHVIRTDIALHQPMYLFL
AMLALAEVRVSASTLPTVLGIFLFGNTEISLEACLFPDVLHPFFIHDGASCAAGHVFGPLYS
HLQPTELHSYPDTAQGLWHRSYYRTEKHYAHGSVAHSLMASALLWPQCPLTFLLSAPQSY
LSCGNISVNNIYGIFIVTSTFGLDSLLIVISYGLILHTVLGIATGEGRKKALNTCGSHVCAVL
AYYVPMIGLSIVHRLGHRVSPLLQAMMANAYLFFPPVVNPIVYSIKTKEIHGAIVRMLLEK
RRRV

>Q8IUH8 multipass membrane protein
MACLGFLLPVGFLLLISTVAGGKYGVAHVVSENWSKDYCILFSSDYITLPRDLHHAPLLPL
YDGTKAPWCPGEDSPHQAQLRSPSQRPLRQTTAMVMRGNCSFHTKGWLAQGQGAHGLL
IVSRVSDQQCSDTTLAPQDPRQPLADLTIPVAMLHYADMLDILSHTRGEAVVRVAMYAPPE
PIIDYNMLVIFILAVGTVAAGGYWAGLTEANRLQRRRARRGGGSGGHHQLQEAAAAEGA
QKEDNEDIPVDFTPAMTGVVVTLSCSLMLLLYFFYDHFVYVTIGIFGLGAGIGLYSCLSPLV
CRLSLRQYQRPPHSLWASLPLPLLLLASLCATVIIFWVAYRNEDRWAWLLQDTLGISYCLF
VLHRVRLPTLKNCSSFLLALLAFDVFFVFVTPFFTKTGESIMAQVALGPAESSSHERLPMVL

KVPRLRVSALTLCSQPFSILGFGDIVVPGFLVAYCCRFDVQVCSRQIYFVACTVAYAVGLLV
TFMAMVLMQMGQPALLYLVSSTLLTSLAVAACRQELSLFWTGQGRAKMCGLGCAPSAGS
RQKQEGAADAHTASTLERGTSRGAGDLDSNPGEDTTEIVTISENEATNPEDRSDSSEGWSD
AHLDPNELPFIPPGASEELMPLMPMAMLIPLMPLMPPPSELGHVHAQAQAHETGLPWAGL
HKRKGLKVRKSMSTQAPL

>Q8N0Y3 multipass membrane protein
MKIANNTVVTEFILLGLTQSQDIQLLVFVLILIFYLIILPGNFLIIFTIRSDPGLTAPLYLFLGNL
AFLDASYSFIVAPRMLVDFLSEKKVISYRGCITQLFFLHFLGGGEGLLLVVMAFDRYIAICR
PLHCSTVMNPRACYAMMLALWLGGFVHSIIQVVLILRLPFCGPNQLDNFFCDVRQVIKLA
CTDMFVVELLMVFNSGLMTLLCFLGLLASYAVILCHVRRAASEGKNKAMSTCTTRVIIILL
MFGPAIFIYMCPFRALPADKMVSLFHTVIFPLMNPMIYTLRNQEVKTSMKRLLSRHVVCQ
VDFIIRN

>P46092 multipass membrane protein
MGTEATEQVSWGHYSGDEEDAYSAEPLPELCYKADVQAFSRAFQPSVSLTVAALGLAGN
GLVLATHLAARRAARSPTSAHLLQLALADLLLALTLPFAAAGALQGWSLGSATCRTISGLY
SASFHAGFLFLACISADRYVAIARALPAGPRPSTPGRAHLVSVIVWLLSLLLALPALLFSQD
GQREGQRRCRLIFPEGLTQTVKGASAVAQVALGFALPLGVMVACYALLGRTLLAARGPER
RRALRVVVALVAAFVVLQLPYSLALLLDTADLLAARERSCPASKRKDVALLVTSGLALAR
CGLNPVLYAFLGLRFRQDLRRLLRGGSCPSGPQPRRGCPRRPRLSSCSAPTETHSLSWDN

>Q12879 multipass membrane protein
MGRVGYWTLLVLPALLVWRGPAPSAAAEKGPPALNIAVMLGHSHDVTERELRTLWGPEQ
AAGLPLDVNVVALLMNRTDPKSLITHVCDLMSGARIHGLVFGDDTDQEAVAQMLDFISSH
TFVPILGIHGGASMIMADKDPTSTFFQFGASIQQQATVMLKIMQDYDWHVFSLVTTIFPGY
REFISFVKTTVDNSFVGWDMQNVITLDTSFEDAKTQVQLKKIHSSVILLYCSKDEAVLILSE
ARSLGLTGYDFFWIVPSLVSGNTELIPKEFPSGLISVSYDDWDYSLEARVRDGIGILTTAASS
MLEKFSYIPEAKASCYGQMERPEVPMHTLHPFMVNVTWDGKDLSFTEEGYQVHPRLVVI
VLNKDREWEKVGKWENHTLSLRHAVWPRYKSFSDCEPDDNHLSIVTLEEAPFVIVEDIDP
LTETCVRNTVPCRKFVKINNSTNEGMNVKKCCKGFCIDILKKLSRTVKFTYDLYLVTNGK
HGKKVNNVWNGMIGEVVYQRAVMAVGSLTINEERSEVVDFSVPFVETGISVMVSRSNGT
VSPSAFLEPFSASVWVMMFVMLLIVSAIAVFVFEYFSPVGYNRNLAKGKAPHGPSFTIGKA
IWLLWGLVFNNSVPVQNPKGTTSKIMVSVWAFFAVIFLASYTANLAAFMIQEEFVDQVTG
LSDKKFQRPHDYSPPFRFGTVPNGSTERNIRNNYPYMHQYMTKFNQKGVEDALVSLKTG
KLDAFIYDAAVLNYKAGRDEGCKLVTIGSGYIFATTGYGIALQKGSPWKRQIDLALLQFVG
DGEMEELETLWLTGICHNEKNEVMSSQLDIDNMAGVFYMLAAAMALSLITFIWEHLFYW
KLRFCFTGVCSDRPGLLFSISRGIYSCIHGVHIEEKKKSPDFNLTGSQSNMLKLLRSAKNISS
MSNMNSSRMDSPKRAADFIQRGSLIMDMVSDKGNLMYSDNRSFQGKESIFGDNMNELQ
TFVANRQKDNLNNYVFQGQHPLTLNESNPNTVEVAVSTESKANSRPRQLWKKSVDSIRQD
SLSQNPVSQRDEATAENRTHSLKSPRYLPEEMAHSDISETSNRATCHREPDNSKNHKTKDN
FKRSVASKYPKDCSEVERTYLKTKSSSPRDKIYTIDGEKEPGFHLDPPQFVENVTLPENVDF
PDPYQDPSENFRKGDSTLPMNRNPLHNEEGLSNNDQYKLYSKHFTLKDKGSPHSETSERY
RQNSTHCRSCLSNMPTYSGHFTMRSPFKCDACLRMGNLYDIDEDQMLQETGNPATGEQV

YQQDWAQNNALQLQKNKLRISRQHSYDNIVDKPRELDLSRPSRSISLKDRERLLEGNFYG
SLFSVPSSKLSGKKSSLFPQGLEDSKRSKSLLPDHTSDNPFLHSHRDDQRLVIGRCPSDPYK
HSLPSQAVNDSYLRSSLRSTASYCSRDSRGHNDVYISEHVMPYAANKNNMYSTPRVLNSC
SNRRVYKKMPSIESDV

>Q969R2 peripheral membrane protein
MGKAAAPSRGGGCGGRSRGLSSLFTVVPCLSCHTAAPGMSASTSGSGPEPKPQPQPVPEP
ERGPLSEQVSEAVSEAVPRSEPVSETTSEPEPGAGQPSELLQGSRPGSESSSGVGAGPFTKA
ASEPLSRAVGSATFLRPESGSLPALKPLPLLRPGQAKTPLGVPMSGTGTTSSAPLALLPLDS
FEGWLLKWTNYLKGYQRRWFVLGNGLLSYYRNQGEMAHTCRGTINLSTAHIDTEDSCGI
LLTSGARSYHLKASSEVDRQQWITALELAKAKAVRVMNTHSDDSGDDDEATTPADKSEL
HHTLKNLSLKLDDLSTCNDLIAKHGAALQRSLTELDGLKIPSESGEKLKVVNERATLFRITS
NAMINACRDFLELAEIHSRKWQRALQYEQEQRVHLEETIEQLAKQHNSLERAFHSAPGRP
ANPSKSFIEGSLLTPKGEDSEEDEDTEYFDAMEDSTSFITVITEAKEDSRKAEGSTGTSSVD
WSSADNVLDGASLVPKGSSKVKRRVRIPNKPNYSLNLWSIMKNCIGRELSRIPMPVNFNEP
LSMLQRLTEDLEYHHLLDKAVHCTSSVEQMCLVAAFSVSSYSTTVHRIAKPFNPMLGETFE
LDRLDDMGLRSLCEQVSHHPPSAAHYVFSKHGWSLWQEITISSKFRGKYISIMPLGAIHLE
FQASGNHYVWRKSTSTVHNIIVGKLWIDQSGDIEIVNHKTNDRCQLKFLPYSYFSKEAAR
KVTGVVSDSQGKAHYVLSGSWDEQMECSKVMHSSPSSPSSDGKQKTVYQTLSAKLLWK
KYPLPENAENMYYFSELALTLNEHEEGVAPTDSRLRPDQRLMEKGRWDEANTEKQRLEE
KQRLSRRRRLEACGPGSSCSSEEEKEADAYTPLWFEKRLDPLTGEMACVYKGGYWEAKE
KQDWHMCPNIF

>P49146 multipass membrane protein
MGPIGAEADENQTVEEMKVEQYGPQTTPRGELVPDPEPELIDSTKLIEVQVVLILAYCSIIL
LGVIGNSLVIHVVIKFKSMRTVTNFFIANLAVADLLVNTLCLPFTLTYTLMGEWKMGPVLC
HLVPYAQGLAVQVSTITLTVIALDRHRCIVYHLESKISKRISFLIIGLAWGISALLASPLAIFRE
YSLIEIIPDFEIVACTEKWPGEEKSIYGTVYSLSSLLILYVLPLGIISFSYTRIWSKLKNHVSPG
AANDHYHQRRQKTTKMLVCVVVVFAVSWLPLHAFQLAVDIDSQVLDLKEYKLIFTVFHII
AMCSTFANPLLYGWMNSNYRKAFLSAFRCEQRLDAIHSEVSVTFKAKKNLEVRKNSGPN
DSFTEATNV

>P51679 multipass membrane protein
MNPTDIADTTLDESIYSNYYLYESIPKPCTKEGIKAFGELFLPPLYSLVFVFGLLGNSVVVLV
LFKYKRLRSMTDVYLLNLAISDLLFVFSLPFWGYYAADQWVFGLGLCKMISWMYLVGFY
SGIFFVMLMSIDRYLAIVHAVFSLRARTLTYGVITSLATWSVAVFASLPGFLFSTCYTERNHT
YCKTKYSLNSTTWKVLSSLEINILGLVIPLGIMLFCYSMIIRTLQHCKNEKKNKAVKMIFAV
VVLFLGFWTPYNIVLFLETLVELEVLQDCTFERYLDYAIQATETLAFVHCCLNPIIYFFLGE
KFRKYILQLFKTCRGLFVLCQYCGLLQIYSADTPSSSYTQSTMDHDLHDAL

>Q8NB49 multipass membrane protein
MQMVPSLPPASECAGEEKRVGTRTVFVGNHPVSETEAYIAQRFCDNRIVSSKYTLWNFLP
KNLFEQFRRIANFYFLIIFLVQVTVDTPTSPVTSGLPLFFVITVTAIKQGYEDCLRHRADNEV
NKSTVYIIENAKRVRKESEKIKVGDVVEVQADETFPCDLILLSSCTTDGTCYVTTASLDGE

SNCKTHYAVRDTIALCTAESIDTLRAAIECEQPQPDLYKFVGRINIYSNSLEAVARSLGPENL
LLKGATLKNTEKIYGVAVYTGMETKMALNYQGKSQKRSAVEKSINAFLIVYLFILLTKAAV
CTTLKYVWQSTPYNDEPWYNQKTQKERETLKVLKMFTDFLSFMVLFNFIIPVSMYVTVE
MQKFLGSFFISWDKDFYDEEINEGALVNTSDLNEELGQVDYVFTDKTGTLTENSMEFIECC
IDGHKYKGVTQEVDGLSQTDGTLTYFDKVDKNREELFLRALCLCHTVEIKTNDAVDGATE
SAELTYISSSPDEIALVKGAKRYGFTFLGNRNGYMRVENQRKEIEEYELLHTLNFDAVRRR
MSVIVKTQEGDILLFCKGADSAVFPRVQNHEIELTKVHVERNAMDGYRTLCVAFKEIAPD
DYERINRQLIEAKMALQDREEKMEKVFDDIETNMNLIGATAVEDKLQDQAAETIEALHAA
GLKVWVLTGDKMETAKSTCYACRLFQTNTELLELTTKTIEESERKEDRLHELLIEYRKKLL
HEFPKSTRSFKKAWTEHQEYGLIIDGSTLSLILNSSQDSSSNNYKSIFLQICMKCTAVLCCR
MAPLQKAQIVRMVKNLKGSPITLSIGDGANDVSMILESHVGIGIKGKEGRQAARNSDYSV
PKFKHLKKLLLAHGHLYYVRIAHLVQYFFYKNLCFILPQFLYQFFCGFSQQPLYDAAYLTM
YNICFTSLPILAYSLLEQHINIDTLTSDPRLYMKISGNAMLQLGPFLYWTFLAAFEGTVFFFG
TYFLFQTASLEENGKVYGNWTFGTIVFTVLVFTVTLKLALDTRFWTWINHFVIWGSLAFY
VFFSFFWGGIIWPFLKQQRMYFVFAQMLSSVSTWLAIILLIFISLFPEILLIVLKNVRRRSAR
RNLSCRRASDSLSARPSVRPLLLRTFSDESNVL

>Q6P4F1 single-pass type II membrane protein
MVRIQRRKLLASCLCVTATVFLLVTLQVMVELGKFERKEFKSSSLQDGHTKMEEAPTHLN
SFLKKEGLTFNRKRKWELDSYPIMLWWSPLTGETGRLGQCGADACFFTINRTYLHHHMT
KAFLFYGTDFNIDSLPLPRKAHHDWAVFHEESPKNNYKLFHKPVITLFNYTATFSRHSHLPL
TTQYLESIEVLKSLRYLVPLQSKNKLRKRLAPLVYVQSDCDPPSDRDSYVRELMTYIEVDS
YGECLRNKDLPQQLKNPASMDADGFYRIIAQYKFILAFENAVCDDYITEKFWRPLKLGVV
PVYYGSPSITDWLPSNKSAILVSEFSHPRELASYIRRLDSDDRLYEAYVEWKLKGEISNQRL
LTALRERKWGVQDVNQDNYIDAFECMVCTKVWANIRLQEKGLPPKRWEAEDTHLSCPEP
TVFAFSPLRTPPLSSLREMWISSFEQSKKEAQALRWLVDRNQNFSSQEFWGLVFKD

>P51800 multipass membrane protein
MEELVGLREGFSGDPVTLQELWGPCPHIRRAIQGGLEWLKQKVFRLGEDWYFLMTLGVL
MALVSYAMNFAIGCVVRAHQWLYREIGDSHLLRYLSWTVYPVALVSFSSGFSQSITPSSGG
SGIPELKTMLAGVILEDYLDIKNFGAKVVGLSCTLATGSTLFLGKVGPFVHLSVMIAAYLG
RVRTTTIGEPENKSKQNEMLVAAAAVGVATVFAAPFSGVLFSIEVMSSHFSVRDYWRGFFA
ATCGAFIFRLLAVFNSEQETITSLYKTSFRVDVPFDLPEIFFFVALGGICGVLSCAYLFCQRTF
LSFIKTNRYSSKLLATSKPVYSALATLLLASITYPPGVGHFLASRLSMKQHLDSLFDNHSW
ALMTQNSSPPWPEELDPQHLWWEWYHPRFTIFGTLAFFLVMKFWMLILATTIPMPAGYFM
PIFILGAAIGRLLGEALAVAFPEGIVTGGVTNPIMPGGYALAGAAAFSGAVTHTISTALLAFE
LTGQIVHALPVLMAVLAANAIAQSCQPSFYDGTIIVKKLPYLPRILGRNIGSHHVRVEHFM
NHSITTLAKDTPLEEVVKVVTSTDVTEYPLVESTESQILVGIVQRAQLVQALQAEPPSRAPG
HQQCLQDILARGCPTEPVTLTLFSETTLHQAQNLFKLLNLQSLFVTSRGRAVGCVSWVEM
KKAISNLTNPPAPK

>Q8IVU1 single-pass type I membrane protein
MAVQRAASPRRPPAPLWPRLLLPLLLLLLPAPSEGLGHSAELAFAVEPSDDVAVPGQPIVLD
CRVEGTPPVRITWRKNGVELPESTHSTLLANGSLMIRHFRLEPGGSPSDEGDYECVAQNRF

GLVVSRKARIQAATMSDFHVHPQATVGEEGGVARFQCQIHGLPKPLITWEKNRVPIDTDNE
RYTLLPKGVLQITGLRAEDGGIFHCVASNIASIRISHGARLTVSGSGSGAYKEPAILVGPENL
TLTVHQTAVLECVATGNPRPIVSWSRLDGRPIGVEGIQVLGTGNLIISDVTVQHSGVYVCA
ANRPGTRVRRTAQGRLVVQAPAEFVQHPQSISRPAGTTAMFTCQAQGEPPPHVTWLKNGQ
VLGPGGHVRLKNNNSTLTISGIGPEDEAIYQCVAENSAGSSQASARLTVLWAEGLPGPPRN
VRAVSVSSTEVRVSWSEPLANTKEIIGYVLHIRKAADPPELEYQEAVSKSTFQHLVSDLEPS
TAYSFYIKAYTPRGASSASVPTLASTLGEAPAPPPLSVRVLGSSSLQLLWEPWPRLAQHEGG
FKLFYRPASKTSFTGPILLPGTVSSYNLSQLDPTAVYEVKLLAYNQHGDGNATVRFVSLRG
ASERTALSPPCDCRKEEAANQTSTTGIVIGIHIGVTCIIFCVLFLLFGQRGRVLLCKDVENQL
SPPQGPRSQRDPGILALNGARRGQRGQLGRDEKRVDMKELEQLFPPASAAGQPDPRPTQD
PAAPAPCEETQLSVLPLQGCGLMEGKTTEAKTTEATAPCAGLAAAPPPPDGGPGLLSEGQ
ASRPAAARVTQPAHSEQ

>Q14289 peripheral membrane protein
MSGVSEPLSRVKLGTLRRPEGPAEPMVVVPVDVEKEDVRILKVCFYSNSFNPGKNFKLVK
CTVQTEIREIITSILLSGRIGPNIRLAECYGLRLKHMKSDEIHWLHPQMTVGEVQDKYECLH
VEAEWRYDLQIRYLPEDFMESLKEDRTTLLYFYQQLRNDYMQRYASKVSEGMALQLGCL
ELRRFFKDMPHNALDKKSNFELLEKEVGLDLFFPKQMQENLKPKQFRKMIQQTFQQYAS
LREEECVMKFFNTLAGFANIDQETYRCELIQGWNITVDLVIGPKGIRQLTSQDAKPTCLAE
FKQIRSIRCLPLEEGQAVLQLGIEGAPQALSIKTSSLAEAENMADLIDGYCRLQGEHQGSLII
HPRKDGEKRNSLPQIPMLNLEARRSHLSESCSIESDIYAEIPDETLRRPGGPQYGIAREDVV
LNRILGEGFFGEVYEGVYTNHKGEKINVAVKTCKKDCTLDNKEKFMSEAVIMKNLDHPHI
VKLIGIIEEEPTWIIMELYPYGELGHYLERNKNSLKVLTLVLYSLQICKAMAYLESINCVHR
DIAVRNILVASPECVKLGDFGLSRYIEDEDYYKASVTRLPIKWMSPESINFRRFTTASDVW
MFAVCMWEILSFGKQPFFWLENKDVIGVLEKGDRLPKPDLCPPVLYTLMTRCWDYDPSD
RPRFTELVCSLSDVYQMEKDIAMEQERNARYRTPKILEPTAFQEPPPKPSRPKYRPPPQTNL
LAPKLQFQVPEGLCASSPTLTSPMEYPSPVNSLHTPPLHRHNVFKRHSMREEDFIQPSSREE
AQQLWEAEKVKMRQILDKQQKQMVEDYQWLRQEEKSLDPMVYMNDKSPLTPEKEVGY
LEFTGPPQKPPRLGAQSIQPTANLDRTDDLVYLNVMELVRAVLELKNELCQLPPEGYVVV
VKNVGLTLRKLIGSVDDLLPSLPSSSRTEIEGTQKLLNKDLAELINKMRLAQQNAVTSLSEE
CKRQMLTASHTLAVDAKNLLDAVDQAKVLANLAHPPAE

>Q86Y34 multipass membrane protein
MATPRGLGALLLLLLLLPTSGQEKPTEGPRNTCLGSNNMYDIFNLNDKALCFTKCRQSGSD
SCNVENLQRYWLNYEAHLMKEGLTQKVNTPFLKALVQNLSTNTAEDFYFSLEPSQVPRQ
VMKDEDKPPDRVRLPKSLFRSLPGNRSVVRLAVTILDIGPGTLFKGPRLGLGDGSGVLNNR
LVGLSVGQMHVTKLAEPLEIVFSHQRPPPNMTLTCVFWDVTKGTTGDWSSEGCSTEVRPE
GTVCCCDHLTFFALLLRPTLDQSTVHILTRISQAGCGVSMIFLAFTIILYAFLRLSRERFKSED
APKIHVALGGSLFLLNLAFLVNVGSGSKGSDAACWARGAVFHYFLLCAFTWMGLEAFHL
YLLAVRVFNTYFGHYFLKLSLVGWGLPALMVIGTGSANSYGLYTIRDRENRTSLELCWFR
EGTTMYALYITVHGYFLITFLFGMVVLALVVWKIFTLSRATAVKERGKNRKKVLTLLGLSS
LVGVTWGLAIFTPLGLSTVYIFALFNSLQGVFICCWFTILYLPSQSTTVSSSTARLDQAHSAS
QE

>P32418 multipass membrane protein
MYNMRRLSLSPTFSMGFHLLVTVSLLFSHVDHVIAETEMEGEGNETGECTGSYYCKKGVI
LPIWEPQDPSFGDKIARATVYFVAMVYMFLGVSIIADRFMSSIEVITSQEKEITIKKPNGETT
KTTVRIWNETVSNLTLMALGSSAPEILLSVIEVCGHNFTAGDLGPSTIVGSAAFNMFIIIALC
VYVVPDGETRKIKHLRVFFVTAAWSIFAYTWLYIILSVISPGVVEVWEGLLTFFFFPICVVFA
WVADRRLLFYKYVYKRYRAGKQRGMIIEHEGDRPSSKTEIEMDGKVVNSHVENFLDGAL
VLEVDERDQDDEEARREMARILKELKQKHPDKEIEQLIELANYQVLSQQQKSRAFYRIQA
TRLMTGAGNILKRHAADQARKAVSMHEVNTEVTENDPVSKIFFEQGTYQCLENCGTVAL
TIIRRGGDLTNTVFVDFRTEDGTANAGSDYEFTEGTVVFKPGDTQKEIRVGIIDDDIFEEDE
NFLVHLSNVKVSSEASEDGILEANHVSTLACLGSPSTATVTIFDDDHAGIFTFEEPVTHVSE
SIGIMEVKVLRTSGARGNVIVPYKTIEGTARGGGEDFEDTCGELEFQNDEIVKTISVKVIDD
EEYEKNKTFFLEIGEPRLVEMSEKKALLLNELGGFTITGKYLFGQPVFRKVHAREHPILSTV
ITIADEYDDKQPLTSKEEEERRIAEMGRPILGEHTKLEVIIEESYEFKSTVDKLIKKTNLALV
VGTNSWREQFIEAITVSAGEDDDDDECGEEKLPSCFDYVMHFLTVFWKVLFAFVPPTEYW
NGWACFIVSILMIGLLTAFIGDLASHFGCTIGLKDSVTAVVFVALGTSVPDTFASKVAATQD
QYADASIGNVTGSNAVNVFLGIGVAWSIAAIYHAANGEQFKVSPGTLAFSVTLFTIFAFINV
GVLLYRRRPEIGGELGGPRTAKLLTSCLFVLLWLLYIFFSSLEAYCHIKGF

>Q9Y5Z0 single-pass type I membrane protein
MGALARALLLPLLAQWLLRAAPELAPAPFTLPLRVAAATNRVVAPTPGPGTPAERHADGL
ALALEPALASPAGAANFLAMVDNLQGDSGRGYYLEMLIGTPPQKLQILVDTGSSNFAVAG
TPHSYIDTYFDTERSSTYRSKGFDVTVKYTQGSWTGFVGEDLVTIPKGFNTSFLVNIATIFE
SENFFLPGIKWNGILGLAYATLAKPSSSLETFFDSLVTQANIPNVFSMQMCGAGLPVAGSGT
NGGSLVLGGIEPSLYKGDIWYTPIKEEWYYQIEILKLEIGGQSLNLDCREYNADKAIVDSGT
TLLRLPQKVFDAVVEAVARASLIPEFSDGFWTGSQLACWTNSETPWSYFPKISIYLRDENSS
RSFRITILPQLYIQPMMGAGLNYECYRFGISPSTNALVIGATVMEGFYVIFDRAQKRVGFAA
SPCAEIAGAAVSEISGPFSTEDVASNCVPAQSLSEPILWIVSYALMSVCGAILLVLIVLLLLPF
RCQRRPRDPEVVNDESSLVRHRWK

>O60733 peripheral membrane protein
MQFFGRLVNTFSGVTNLFSNPFRVKEVAVADYTSSDRVREEGQLILFQNTPNRTWDCVLVN
PRNSQSGFRLFQLELEADALVNFHQYSSQLLPFYESSPQVLHTEVLQHLTDLIRNHPSWSV
AHLAVELGIRECFHHSRIISCANCAENEEGCTPLHLACRKGDGEILVELVQYCHTQMDVTD
YKGETVFHYAVQGDNSQVLQLLGRNAVAGLNQVNNQGLTPLHLACQLGKQEMVRVLLL
CNARCNIMGPNGYPIHSAMKFSQKGCAEMIISMDSSQIHSKDPRYGASPLHWAKNAEMA
RMLLKRGCNVNSTSSAGNTALHVAVMRNRFDCAIVLLTHGANADARGEHGNTPLHLAM
SKDNVEMIKALIVFGAEVDTPNDFGETPTFLASKIGRLVTRKAILTLLRTVGAEYCFPPIHG
VPAEQGSAAPHHPFSLERAQPPPISLNNLELQDLMHISRARKPAFILGSMRDEKRTHDHLL
CLDGGGVKGLIIIQLLIAIEKASGVATKDLFDWVAGTSTGGILALAILHSKSMAYMRGMYF
RMKDEVFRGSRPYESGPLEEFLKREFGEHTKMTDVRKPKVMLTGTLSDRQPAELHLFRNY
DAPETVREPRFNQNVNLRPPAQPSDQLVWRAARSSGAAPTYFRPNGRFLDGGLLANNPTL
DAMTEIHEYNQDLIRKGQANKVKKLSIVVSLGTGRSPQVPVTCVDVFRPSNPWELAKTVF
GAKELGKMVVDCCTDPDGRAVDRARAWCEMVGIQYFRLNPQLGTDIMLDEVSDTVLVN
ALWETEVYIYEHREEFQKLIQLLLSP

>P36383 multipass membrane protein
MSWSFLTRLLEEIHNHSTFVGKIWLTVLIVFRIVLTAVGGESIYYDEQSKFVCNTEQPGCEN
VCYDAFAPLSHVRFWVFQIILVATPSVMYLGYAIHKIAKMEHGEADKKAARSKPYAMRW
KQHRALEETEEDNEEDPMMYPEMELESDKENKEQSQPKPKHDGRRRIREDGLMKIYVLQ
LLARTVFEVGFLIGQYFLYGFQVHPFYVCSRLPCPHKIDCFISRPTEKTIFLLIMYGVTGLCL
LLNIWEMLHLGFGTIRDSLNSKRRELEDPGAYNYPFTWNTPSAPPGYNIAVKPDQIQYTEL
SNAKIAYKQNKANTAQEQQYGSHEENLPADLEALQREIRMAQERLDLAVQAYSHQNNPH
GPREKKAKVGSKAGSNKSTASSKSGDGKTSVWI

>P49795 lipid-anchor
MPTPHEAEKQITGPEEADRPPSMSSHDTASPAAPSRNPCCLCWCCCCSCSWNQERRRAWQ
ASRESKLQPLPSCEVCATPSPEEVQSWAQSFDKLMHSPAGRSVFRAFLRTEYSEENMLFWL
ACEELKAEANQHVVDEKARLIYEDYVSILSPKEVSLDSRVREGINKKMQEPSAHTFDDAQ
LQIYTLMHRDSYPRFLSSPTYRALLLQGPSQSSSEA

>Q9H341 multipass membrane protein
MLLSNITQFSPIFYLTSFPGLEGIKHWIFIPFFFMYMVAISGNCFILIIIKTNPRLHTPMYYLLS
LLALTDLGLCVSTLPTTMGIFWFNSQSIYFGACQIQMFCIHSFSFMESSVLLMMSFDRFVAI
CHPLRYSVIITGQQVVRAGLIVIFRGPVATIPIVLLLKAFPYCGSVVLSHSFCLHQEVIQLAC
TDTTFNNLYGLMVVVFTVMLDLVLIALSYGLILHTVAGLASQEEQRRAFQTCTAHLCAVL
VFFVPMMGLSLVHRFGKHAPPAIHLLMANVYLFVPPMLNPIIYSIKTKEIHRAIIKLLGLKK
ASK

>Q7Z449 multipass membrane protein
MSSPGPSQPPAEDPPWPARLLRAPLGLLRLDPSGGALLLCGLVALLGWSWLRRRRARGIPP
GPTPWPLVGNFGHVLLPPFLRRRSWLSSRTRAAGIDPSVIGPQVLLAHLARVYGSIFSFFIG
HYLVVVLSDFHSVREALVQQAEVFSDRPRVPLISIVTKEKGVVFAHYGPVWRQQRKFSHS
TLRHFGLGKLSLEPKIIEEFKYVKAEMQKHGEDPFCPFSIISNAVSNIICSLCFGQRFDYTNS
EFKKMLGFMSRGLEICLNSQVLLVNICPWLYYLPFGPFKELRQIEKDITSFLKKIIKDHQESL
DRENPQDFIDMYLLHMEEERKNNSNSSFDEEYLFYIIGDLFIAGTDTTTNSLLWCLLYMSL
NPDVQEKVHEEIERVIGANRAPSLTDKAQMPYTEATIMEVQRLTVVVPLAIPHMTSENTVL
QGYTIPKGTLILPNLWSVHRDPAIWEKPEDFYPNRFLDDQGQLIKKETFIPFGIGKRVCMGE
QLAKMELFLMFVSLMQSFAFALPEDSKKPLLTGRFGLTLAPHPFNITISRR

>Q3ZAQ7 multipass membrane protein
MERPDKAALNALQPPEFRNESSLASTLKTLLFFTALMITVPIGLYFTTKSYIFEGALGMSNR
DSYFYAAIVAVVAVHVVLALFVYVAWNEGSRQWREGKQD

>P11279 single-pass type I membrane protein
MAAPGSARRPLLLLLLLLLLGLMHCASAAMFMVKNGNGTACIMANFSAAFSVNYDTKS
GPKNMTFDLPSDATVVLNRSSCGKENTSDPSLVIAFGRGHTLTLNFTRNATRYSVQLMSFV
YNLSDTHLFPNASSKEIKTVESITDIRADIDKKYRCVSGTQVHMNNVTVTLHDATIQAYLS
NSSFSRGETRCEQDRPSPTTAPPAPPSPSPSPVPKSPSVDKYNVSGTNGTCLLASMGLQLNL

TYERKDNTTVTRLLNINPNKTSASGSCGAHLVTLELHSEGTTVLLFQFGMNASSSRFFLQG
IQLNTILPDARDPAFKAANGSLRALQATVGNSYKCNAEEHVRVTKAFSVNIFKVWVQAFK
VEGGQFGSVEECLLDENSMLIPIAVGGALAGLVLIVLIAYLVGRKRSHAGYQTI

>Q9NZV8 multipass membrane protein
MAAGVAAWLPFARAAAIGWMPVASGPMPAPPRQERKRTQDALIVLNVSGTRFQTWQDTL
ERYPDTLLGSSERDFFYHPETQQYFFDRDPDIFRHILNFYRTGKLHYPRHECISAYDEELAF
FGLIPEIIGDCCYEEYKDRRRENAERLQDDADTDTAGESALPTMTARQRVWRAFENPHTS
TMALVFYYVTGFFIAVSVIANVVETVPCGSSPGHIKELPCGERYAVAFFCLDTACVMIFTVE
YLLRLAAAPSRYRFVRSVMSIIDVVAILPYYIGLVMTDNEDVSGAFVTLRVFRVFRIFKFSR
HSQGLRILGYTLKSCASELGFLLFSLTMAIIIFATVMFYAEKGSSASKFTSIPAAFWYTIVTM
TTLGYGDMVPKTIAGKIFGSICSLSGVLVIALPVPVIVSNFSRIYHQNQRADKRRAQKKAR
LARIRAAKSGSANAYMQSKRNGLLSNQLQSSEDEQAFVSKSGSSFETQHHHLLHCLEKTT
NHEFVDEQVFEESCMEVATVNRPSSHSPSLSSQQGVTSTCCSRRHKKTFRIPNANVSGSHQ
GSIQELSTIQIRCVERTPLSNSRSSLNAKMEECVKLNCEQPYVTTAIISIPTPPVTTPEGDDRP
ESPEYSGGNIVRVSAL

>Q9HC58 multipass membrane protein
MRPSGDEDRARRRRRRRRRRRDLLLSQLCFLASVALLLWSLSSLREQKELDLMDLVGEDRK
WMMARKLMQVNDTLTSEDAGLRNSKNCTEPALHEFPNDIFTNEDRRQGAVVLHVLCAIY
MFYALAIVCDDFFVPSLEKICERLHLSEDVAGATFMAAGSSAPELFTSVIGVFITKGDVGVG
TIVGSAVFNILCIIGVCGLFAGQVVALSSWCLLRDSIYYTLSVIALIVFIYDEKVSWWESLVL
VLMYLIYIVIMKYNACIHQCFERRTKGAGNMVNGLANNAEIDDSSNCDATVVLLKKANF
HRKASVIMVDELLSAYPHQLSFSEAGLRIMITSHFPPKTRLSMASRMLINERQRLINSRAYT
NGESEVAIKIPIKHTVENGTGPSSAPDRGVNGTRRDDVVAEAGNETENENEDNENDEEEEE
DEDDDEGPYTPFDTPSGKLETVKWAFTWPLSFVLYFTVPNCNKPRWEKWFMVTFASSTL
WIAAFSYMMVWMVTIIGYTLGIPDVIMGITFLAAGTSVPDCMASLIVARQGMGDMAVSN
SIGSNVFDILIGLGLPWALQTLAVDYGSYIRLNSRGLIYSVGLLLASVFVTVFGVHLNKWQ
LDKKLGCGCLLLYGVFLCFSIMTEFNVFTFVNLPMCGDH

>P24903 peripheral membrane protein
MDSISTAILLLLLALVCLLLTLSSRDKGKLPPGPRPLSILGNLLLLCSQDMLTSLTKLSKEYG
SMYTVHLGPRRVVVLSGYQAVKEALVDQGEEFSGRGDYPAFFNFTKGNGIAFSSGDRWK
VLRQFSIQILRNFGMGKRSIEERILEEGSFLLAELRKTEGEPFDPTFVLSRSVSNIICSVLFGS
RFDYDDERLLTIIRLINDNFQIMSSPWGELYDIFPSLLDWVPGPHQRIFQNFKCLRDLIAHSV
HDHQASLDPRSPRDFIQCFLTKMAEEKEDPLSHFHMDTLLMTTHNLLFGGTKTVSTTLHH
AFLALMKYPKVQARVQEEIDLVVGRARLPALKDRAAMPYTDAVIHEVQRFADIIPMNLPH
RVTRDTAFRGFLIPKGTDVITLLNTVHYDPSQFLTPQEFNPEHFLDANQSFKKSPAFMPFSA
GRRLCLGESLARMELFLYLTAILQSFSLQPLGAPEDIDLTPLSSGLGNLPRPFQLCLRPR

>Q15907 lipid-anchor
MGTRDDEYDYLFKVVLIGDSGVGKSNLLSRFTRNEFNLESKSTIGVEFATRSIQVDGKTIK
AQIWDTAGQERYRAITSAYYRGAVGALLVYDIAKHLTYENVERWLKELRDHADSNIVIML
VGNKSDLRHLRAVPTDEARAFAEKNNLSFIETSALDSTNVEEAFKNILTEIYRIVSQKQIAD

RAAHDESPGNNVVDISVPPTTDGQKPNKLQCCQNL

>Q9BV57 peripheral membrane protein
MVQAWYMDDAPGDPRQPHRPDPGRPVGLEQLRRLGVLYWKLDADKYENDPELEKIRRE
RNYSWMDIITICKDKLPNYEEKIKMFYEEHLHLDDEIRYILDGSGYFDVRDKEDQWIRIFM
EKGDMVTLPAGIYHRFTVDEKNYTKAMRLFVGEPVWTAYNRPADHFEARGQYVKFLAQ
TA

>Q8NH79 multipass membrane protein
MRNGTVITEFILLGFPVIQGLQTPLFIAIFLTYILTLAGNGLIIATVWAEPRLQIPMYFFLCNLS
FLEIWYTTTVIPKLLGTFVVARTVICMSCCLLQAFFHFFVGTTEFLILTIMSFDRYLTICNPLH
HPTIMTSKLCLQLALSSWVVGFTIVFCQTMLLIQLPFCGNNVISHFYCDVGPSLKAACIDTS
ILELLGVIATILVIPGSLLFNMISYIYILSAILRIPSATGHQKTFSTCASHLTVVSLLYGAVLFM
YLRPTAHSSFKINKVVSVLNTILTPLLNPFIYTIRNKEVKGALRKAMTCPKTGHAK

>Q15760 multipass membrane protein
MVFAHRMDNSKPHLIIPTLLVPLQNRSCTETATPLPSQYLMELSEEHSWMSNQTDLHYVL
KPGEVATASIFFGILWLFSIFGNSLVCLVIHRSRRTQSTTNYFVVSMACADLLISVASTPFVLL
QFTTGRWTLGSATCKVVRYFQYLTPGVQIYVLLSICIDRFYTIVYPLSFKVSREKAKKMIA
ASWVFDAGFVTPVLFFYGSNWDSHCNYFLPSSWEGTAYTVIHFLVGFVIPSVLIILFYQKVI
KYIWRIGTDGRTVRRTMNIVPRTKVKTIKMFLILNLLFLLSWLPFHVAQLWHPHEQDYKK
SSLVFTAITWISFSSSASKPTLYSIYNANFRRGMKETFCMSSMKCYRSNAYTITTSSRMAKK
NYVGISEIPSMAKTITKDSIYDSFDREAKEKKLAWPINSNPPNTFV

>P35613 single-pass type I membrane protein
MAAALFVLLGFALLGTHGASGAAGFVQAPLSQQRWVGGSVELHCEAVGSPVPEIQWWFE
GQGPNDTCSQLWDGARLDRVHIHATYHQHAASTISIDTLVEEDTGTYECRASNDPDRNHL
TRAPRVKWVRAQAVVLVLEPGTVFTTVEDLGSKILLTCSLNDSATEVTGHRWLKGGVVLK
EDALPGQKTEFKVDSDDQWGEYSCVFLPEPMGTANIQLHGPPRVKAVKSSEHINEGETAM
LVCKSESVPPVTDWAWYKITDSEDKALMNGSESRFFVSSSQGRSELHIENLNMEADPGQY
RCNGTSSKGSDQAIITLRVRSHLAALWPFLGIVAEVLVLVTIIFIYEKRRKPEDVLDDDDAG
SAPLKSSGQHQNDKGKNVRQRNSS

>P34910 single-pass type I membrane protein
MDPKYFILILFCGHLNNTFFSKTETITTEKQSQPTLFTSSMSQVLANSQNTTGNPLGQPTQF
SDTFSGQSISPAKVTAGQPTPAVYTSSEKPEAHTSAGQPLAYNTKQPTPIANTSSQQAVFTS
ARQLPSARTSTTQPPKSFVYTFTQQSSSVQIPSRKQITVHNPSTQPTSTVKNSPRSTPGFILD
TTSNKQTPQKNNYNSIAAILIGVLLTSMLVAIIIIVLWKCLRKPVLNDQNWAGRSPFADGET
PDICMDNIRENEISTKRTSIISLTPWKPSKSTLLADDLEIKLFESSENIEDSNNPKTEKIKDQV
NGTSEDSADGSTVGTAVSSSDDADLPPPPPLLDLEGQESNQSDKPTMTIVSPLPNDSTSLPP
SLDCLNQDCGDHKSEIIQSFPPLDSLNLPLPPVDFMKNQEDSNLEIQCQEFSIPPNSDQDLN
ESLPPPPAELL

>Q96LB1 multipass membrane protein

MDPTTPAWGTESTTVNGNDQALLLLCGKETLIPVFLILFIALVGLVGNGFVLWLLGFRMRR
NAFSVYVLSLAGADFLFLCFQIINCLVYLSNFFCSISINFPSFFTTVMTCAYLAGLSMLSTVS
TERCLSVLWPIWYRCRRPRHLSAVVCVLLWALSLLLSILEGKFCGFLFSDGDSGWCQTFDF
ITAAWLIFLFMVLCGSSLALLVRILCGSRGLPLTRLYLTILLTVLVFLLCGLPFGIQWFLILWI
WKDSDVLFCHIHPVSVVLSSLNSSANPIIYFFVGSFRKQWRLQQPILKLALQRALQDIAEV
DHSEGCFRQGTPEMSRSSLV

>Q01718 multipass membrane protein
MKHIINSYENINNTARNNSDCPRVVLPEEIFFTISIVGVLENLIVLLAVFKNKNLQAPMYFFI
CSLAISDMLGSLYKILENILIILRNMGYLKPRGSFETTADDIIDSLFVLSLLGSIFSLSVIAADR
YITIFHALRYHSIVTMRRTVVVLTVIWTFCTGTGITMVIFSHHVPTVITFTSLFPLMLVFILCL
YVHMFLLARSHTRKISTLPRANMKGAITLTILLGVFIFCWAPFVLHVLLMTFCPSNPYCAC
YMSLFQVNGMLIMCNAVIDPFIYAFRSPELRDAFKKMIFCSRYW

>Q8IZR5 multipass membrane protein
MRSGEELDGFEGEASSTSMISGASSPYQPTTEPVSQRRGLAGLRCDPDYLRGALGRLKVA
QVILALIAFICIETIMACSPCEGLYFFEFVSCSAFVVTGVLLIMFSLNLHMRIPQINWNLTDL
VNTGLSAFLFFIASIVLAALNHRAGAEIAAVIFGFLATAAYAVNTFLAVQKWRVSVRQQSTN
DYIRARTESRDVDSRPEIQRLDTFSYSTNVTVRKKSPTNLLSLNHWQLA

>Q99828 lipid-anchor
MGGSGSRLSKELLAEYQDLTFLTKQEILLAHRRFCELLPQEQRSVESSLRAQVPFEQILSLP
ELKANPFKERICRVFSTSPAKDSLSFEDFLDLLSVFSDTATPDIKSHYAFRIFDFDDDGTLNR
EDLSRLVNCLTGEGEDTRLSASEMKQLIDNILEESDIDRDGTINLSEFQHVISRSPDFASSFKI
VL

>O15440 multipass membrane protein
MKDIDIGKEYIIPSPGYRSVRERTSTSGTHRDREDSKFRRTRPLECQDALETAARAEGLSLD
ASMHSQLRILDEEHPKGKYHHGLSALKPIRTTSKHQHPVDNAGLFSCMTFSWLSSLARVA
HKKGELSMEDVWSLSKHESSDVNCRRLERLWQEELNEVGPDAASLRRVVWIFCRTRLILS
IVCLMITQLAGFSGPAFMVKHLLEYTQATESNLQYSLLLVLGLLLTEIVRSWSLALTWALN
YRTGVRLRGAILTMAFKKILKLKNIKEKSLGELINICSNDGQRMFEAAAVGSLLAGGPVVA
ILGMIYNVIILGPTGFLGSAVFILFYPAMMFASRLTAYFRRKCVAATDERVQKMNEVLTYIK
FIKMYAWVKAFSQSVQKIREEERRILEKAGYFQSITVGVAPIVVVIASVVTFSVHMTLGFDL
TAAQAFTVVTVFNSMTFALKVTPFSVKSLSEASVAVDRFKSLFLMEEVHMIKNKPASPHIK
IEMKNATLAWDSSHSSIQNSPKLTPKMKKDKRASRGKKEKVRQLQRTEHQAVLAEQKGH
LLLDSDERPSPEEEEGKHIHLGHLRLQRTLHSIDLEIQEGKLVGICGSVGSGKTSLISAILGQ
MTLLEGSIAISGTFAYVAQQAWILNATLRDNILFGKEYDEERYNSVLNSCCLRPDLAILPSS
DLTEIGERGANLSGGQRQRISLARALYSDRSIYILDDPLSALDAHVGNHIFNSAIRKHLKSK
TVLFVTHQLQYLVDCDEVIFMKEGCITERGTHEELMNLNGDYATIFNNLLLGETPPVEINS
KKETSGSQKKSQDKGPKTGSVKKEKAVKPEEGQLVQLEEKGQGSVPWSVYGVYIQAAG
GPLAFLVIMALFMLNVGSTAFSTWWLSYWIKQGSGNTTVTRGNETSVSDSMKDNPHMQ
YYASIYALSMAVMLILKAIRGVVFVKGTLRASSRLHDELFRRILRSPMKFFDTTPTGRILNR
FSKDMDEVDVRLPFQAEMFIQNVILVFFCVGMIAGVFPWFLVAVGPLVILFSVLHIVSRVLI

RELKRLDNITQSPFLSHITSSIQGLATIHAYNKGQEFLHRYQELLDDNQAPFFLFTCAMRWL
AVRLDLISIALITTTGLMIVLMHGQIPPAYAGLAISYAVQLTGLFQFTVRLASETEARFTSVE
RINHYIKTLSLEAPARIKNKAPSPDWPQEGEVTFENAEMRYRENLPLVLKKVSFTIKPKEKI
GIVGRTGSGKSSLGMALFRLVELSGGCIKIDGVRISDIGLADLRSKLSIIPQEPVLFSGTVRS
NLDPFNQYTEDQIWDALERTHMKECIAQLPLKLESEVMENGDNFSVGERQLLCIARALLR
HCKILILDEATAAMDTETDLLIQETIREAFADCTMLTIAHRLHTVLGSDRIMVLAQGQVVE
FDTPSVLLSNDSSRFYAMFAAAENKVAVKG

>Q68DA7 peripheral membrane protein
MEGTHCTLQLHKPITELCYISFCLPKGEVRGFSYKGTVTLDRSNKGFHNCYQVREESDIIS
LSQEPDEHPGDIFFKQTPTKDILTELYKLTTERERLLTNLLSSDHILGITMGNQEGKLQELSV
SLAPEDDCFQSAGDWQGELPVGPLNKRSTHGNKKPRRSSGRRESFGALPQKRTKRKGRG
GRESAPLMGKDKICSSHSLPLSRTRPNLWVLEEKGNLLPNGALACSLQRRESCPPDIPKTP
DTDLGFGSFETAFKDTGLGREVLPPDCSSTEAGGDGIRRPPSGLEHQQTGLSESHQDPEKH
PEAEKDEMEKPAKRTCKQKPVSKVVAKVQDLSSQVQRVVKTHSKGKETIAIRPAAHAEFV
PKADLLTLPGAEAGAHGSRRQGKERQGDRSSQSPAGETASISSVSASAEGAVNKVPLKVIE
SEKLDEAPEGKRLGFPVHTSVPHTRPETRNKRRAGLPLGGHKSLFLDLPHKVGPDSSQPR
GDKKKPSPPAPAALGKVFNNSASQSSTHKQTSPVPSPLSPRLPSPQQHHRILRLPALPGERE
AALNDSPCRKSRVFSGCVSADTLEPPSSAKVTETKGASPAFLRAGQPRLVPGETLEKSLGP
GKTTAEPQHQSPPGISSEGFPWDGFNEQTPKDLPNRDGGAWVLGYRAGPACPFLLHEERE
KSNRSELYLDLHPDHSLTEQDDRTPGRLQAVWPPPKTKDTEEKVGLKYTEAEYQAAILHL
KREHKEEIENLQAQFELRAFHIRGEHAMITARLEETIENLKHELEHRWRGGCEERKDVCIS
TDDDCPPKTFRNVCVQTDRETFLKPCESESKTTRSNQLVPKKLNISSLSQLSPPNDHKDIHA
ALQPMEGMASNQQKALPPPPASIPPPPPLPSGLGSLSPAPPMPPVSAGPPLPPPPPPPPPLPPP
SSAGPPPPPPPPPPLPNSPAPPNPGGPPPAPPPPGLAPPPPPGLFFGLGSSSSQCPRKPAIEPSCP
MKPLYWTRIQISDRSQNATPTLWDSLEEPDIRDPSEFEYLFSKDTTQQKKKPLSETYEKKN
KVKKIIKLLDGKRSQTVGILISSLHLEMKDIQQAIFNVDDSVVDLETLAALYENRAQEDEL
VKIRKYYETSKEEELKLLDKPEQFLHELAQIPNFAERAQCIIFRSVFSEGITSLHRKVEIITRA
SKDLLHVKSVKDILALILAFGNYMNGGNRTRGQADGYSLEILPKLKDVKSRDNGINLVDY
VVKYYLRYYDQEAGTEKSVFPLPEPQDFFLASQVKFEDLIKDLRKLKRQLEASEKQMVV
VCKESPKEYLQPFKDKLEEFFQKAKKEHKMEESHLENAQKSFETTVRYFGMKPKSGEKEI
TPSYVFMVWYEFCSDFKTIWKRESKNISKERLKMAQESVSKLTSEKKVETKKINPTASLKE
RLRQKEASVTTN

>Q9NVD7 peripheral membrane protein
MATSPQKSPSVPKSPTPKSPPSRKKDDSFLGKLGGTLARRKKAKEVSELQEEGMNAINLPL
SPIPFELDPEDTMLEENEVRTMVDPNSRSDPKLQELMKVLIDWINDVLVGERIIVKDLAED
LYDGQVLQKLFEKLESEKLNVAEVTQSEIAQKQKLQTVLEKINETLKLPPRSIKWNVDSVH
AKSLVAILHLLVALSQYFRAPIRLPDHVSIQVVVVQKREGILQSRQIQEEITGNTEALSGRHE
RDAFDTLFDHAPDKLNVVKKTLITFVNKHLNKLNLEVTELETQFADGVYLVLLMGLLEG
YFVPLHSFFLTPDSFEQKVLNVSFAFELMQDGGLEKPKPRPEDIVNCDLKSTLRVLYNLFT
KYRNVE

>Q5UCC4 single-pass type I membrane protein

MAAASAGATRLLLLLLMAVAAPSRARGSGCRAGTGARGAGAEGREGEACGTVGLLLEHS
FEIDDSANFRKRGSLLWNQQDGTLSLSQRQLSEEERGRLRDVAALNGLYRVRIPRRPGALD
GLEAGGYVSSFVPACSLVESHLSDQLTLHVDVAGNVVGVSVVTHPGGCRGHEVEDVDLE
LFNTSVQLQPPTTAPGPETAAFIERLEMEQAQKAKNPQEQKSFFAKYWMYIIPVVLFLMM
SGAPDTGGQGGGGGGGGGGGSGR

>Q7RTR8 multipass membrane protein
MATELDKIFLILAIAEFIISMLGNVFIGLVNCSEGIKNQKVFSADFILTCLAISTIGQLLVILFD
SFLVGLASHLYTTYRLGKTVIMLWHMTNHLTTWLATCLSIFYFFKIAHFPHSLFLWLRWR
MNGMIVMLLILSLFLLIFDSLVLEIFIDISLNIIDKSNLTLYLDESKTLYDKLSILKTLLSLTSFI
PFSLFLTSLLFLFLSLVRHTRNLKLSSLGSRDSSTEAHRRAMKMVMSFLFLFIVHFFSLQVA
NGIFFMLWNNKYIKFVMLALNAFPSCHSFILILGNSKLRQTAVRLLWHLRNYTKTPNALPL

>Q8NGS7 multipass membrane protein
MERTNDSTSTEFFLVGLSAHPKLQTVFFVLILWMYLMILLGNGVLISVIIFDSHLHTPMYFF
LCNLSFLDVCYTSSSVPLILASFLAVKKKVSFSGCMVQMFISFAMGATECMILGTMALDRY
VAICYPLRYPVIMSKGAYVAMAAGSWVTGLVDSVVQTAFAMQLPFCANNVIKHFVCEILA
ILKLACADISINVISMTGSNLIVLVIPLLVISISYIFIVATILRIPSTEGKHKAFSTCSAHLTVVIIF
YGTIFFMYAKPESKASVDSGNEDIIEALISLFYGVMTPMLNPLIYSLRNKDVKAAVKNILCR
KNFSDGK

>Q8NGX3 multipass membrane protein
MRGFNKTTVVTQFILVGFSSLGELQLLLFVIFLLLYLTILVANVTIMAVIRFSWTLHTPMYGF
LFILSFSESCYTFVIIPQLLVHLLSDTKTISFMACATQLFFFLGFACTNCLLIAVMGYDRYVAI
CHPLRYTLIINKRLGLELISLSGATGFFIALVATNLICDMRFCGPNRVNHYFCDMAPVIKLAC
TDTHVKELALFSLSILVIMVPFLLILISYGFIVNTILKIPSAEGKKAFVTCASHLTVVFVHYG
CASIIYLRPKSKSASDKDQLVAVTYTVVTPLLNPLVYSLRNKEVKTALKRVLGMPVATKMS

>P13726 single-pass type I membrane protein
METPAWPRVPRPETAVARTLLLGWVFAQVAGASGTTNTVAAYNLTWKSTNFKTILEWEPK
PVNQVYTVQISTKSGDWKSKCFYTTDTECDLTDEIVKDVKQTYLARVFSYPAGNVESTGS
AGEPLYENSPEFTPYLETNLGQPTIQSFEQVGTKVNVTVEDERTLVRRNNTFLSLRDVFGK
DLIYTLYYWKSSSSGKKTAKTNTNEFLIDVDKGENYCFSVQAVIPSRTVNRKSTDSPVECM
GQEKGEFREIFYIIGAVVFVVIILVIILAISLHKCRKAGVGQSWKENSPLNVS

>Q14699 lipid-anchor
MGCGLNKLEKRDEKRPGNIYSTLKRPQVETKIDVSYEYRFLEFTTLSAAELPGSSAVRLAS
LRDLPAQLLELYQQGFSLAALHPFVQPTHEREKTPLEHIFRAILIKKTDRSQKTDLHNEGYI
LELDCCSSLDHPTDQKLIPEFIKKIQEAASQGLKFVGVIPQYHSSVNSAGSSAPVSTANSTE
DARDAKNARGDHASLENEKPGTGDVCSAPAGRNQSPEPSSGPRGEVPLAKQPSSPSGEGD
GGELSPQGVSKTLDGPESNPLEVHEEPLSGKMEIFTLFNKPKSHQKCRQYYPVTIPLHVSK
NGQTVSGLDANWLEHMSDHFRKGGMLVNAVFYLGIVNDSLHGLTDGVFIFEAVSTEDSK
TIQGYDAIVVEQWTVLEGVEVQTDYVPLLNSLAAYGWQLTCVLPTPVVKTTSEGSVSTK
QIVFLQRPCLPQKIKKKESKFQWRFSREEMHNRQMRKSKGKLSARDKQQAEENEKNLED

QSSKAGDMGNCVSGQQQEGGVSEEMKGPVQEDKGEQLSPGGLLCGVGVEGEAVQNGPA
SHSRALVGICTGHSNPGEDARDGDAEEVRELGTVEEN

>O95180 multipass membrane protein
MTEGARAADEVRVPLGAPPPGPAALVGASPESPGAPGREAERGSELGVSPSESPAAERGAE
LGADEEQRVPYPALAATVFFCLGQTTRPRSWCLRLVCNPWFEHVSMLVIMLNCVTLGMF
RPCEDVECGSERCNILEAFDAFIFAFFAVEMVIKMVALGLFGQKCYLGDTWNRLDFFIVVA
GMMEYSLDGHNVSLSAIRTVRVLRPLRAINRVPSMRILVTLLLDTLPMLGNVLLLCFFVFFI
FGIVGVQLWAGLLRNRCFLDSAFVRNNNLTFLRPYYQTEEGEENPFICSSRRDNGMQKCS
HIPGRRELRMPCTLGWEAYTQPQAEGVGAARNACINWNQYYNVCRSGDSNPHNGAINF
DNIGYAWIAIFQVITLEGWVDIMYYVMDAHSFYNFIYFILLIIVGSFFMINLCLVVIATQFSE
TKQRESQLMREQRARHLSNDSTLASFSEPGSCYEELLKYVGHIFRKVKRRSLRLYARWQS
RWRKKVDPSAVQGQGPGHRQRRAGRHTASVHHLVYHHHHHHHHHYHFSHGSPRRPGPE
PGACDTRLVRAGAPPSPPSPGRGPPDAESVHSIYHADCHIEGPQERARVAHAAATAAASLR
LATGLGTMNYPTILPSGVGSGKGSTSPGPKGKWAGGPPGTGGHGPLSLNSPDPYEKIPHV
VGEHGLGQAPGHLSGLSVPCPLPSPPAGTLTCELKSCPYCTRALEDPEGELSGSESGDSDG
RGVYEFTQDVRHGDRWDPTRPPRATDTPGPGPGSPQRRAQQRAAPGEPGWMGRLWVTF
SGKLRRIVDSKYFSRGIMMAILVNTLSMGVEYHEQPEELTNALEISNIVFTSMFALEMLLK
LLACGPLGYIRNPYNIFDGIIVVISVWEIVGQADGGLSVLRTFRLLRVLKLVRFLPALRRQL
VVLVKTMDNVATFCTLLMLFIFIFSILGMHLFGCKFSLKTDTGDTVPDRKNFDSLLWAIVT
VFQILTQEDWNVVLYNGMASTSSWAALYFVALMTFGNYVLFNLLVAILVEGFQAEGDANR
SDTDEDKTSVHFEEDFHKLRELQTTELKMCSLAVTPNGHLEGRGSLSPPLIMCTAATPMPT
PKSSPFLDAAPSLPDSRRGSSSSGDPPLGDQKPPASLRSSPCAPWGPSGAWSSRRSSWSSLG
RAPSLKRRGQCGERESLLSGEGKGSTDDEAEDGRAAPGPRATPLRRAESLDPRPLRPAALP
PTKCRDRDGQVVALPSDFFLRIDSHREDAAELDDDSEDSCCLRLHKVLEPYKPQWCRSRE
AWALYLFSPQNRFRVSCQKVITHKMFDHVVLVFIFLNCVTIALERPDIDPGSTERVFLSVSN
YIFTAIFVAEMMVKVVALGLLSGEHAYLQSSWNLLDGLLVLVSLVDIVVAMASAGGAKIL
GVLRVLRLLRTLRPLRVISRAPGLKLVVETLISSLRPIGNIVLICCAFFIIFGILGVQLFKGKFY
YCEGPDTRNISTKAQCRAAHYRWVRRKYNFDNLGQALMSLFVLSSKDGWVNIMYDGLD
AVGVDQQPVQNHNPWMLLYFISFLLIVSFFVLNMFVGVVVENFHKCRQHQEAEEARRRE
EKRLRRLERRRRSTFPSPEAQRRPYYADYSPTRRSIHSLCTSHYLDLFITFIICVNVITMSME
HYNQPKSLDEALKYCNYVFTIVFVFEAALKLVAFGFRRFFKDRWNQLDLAIVLLSLMGIT
LEEIEMSAALPINPTIIRIMRVLRIARVLKLLKMATGMRALLDTVVQALPQVGNLGLLFML
LFFIYAALGVELFGRLECSEDNPCEGLSRHATFSNFGMAFLTLFRVSTGDNWNGIMKDTLR
ECSREDKHCLSYLPALSPVYFVTFVLVAQFVLVNVVVAVLMKHLEESNKEAREDAELDAE
IELEMAQGPGSARRVDADRPPLPQESPGARDAPNLVARKVSVSRMLSLPNDSYMFRPVVP
ASAPHPRPLQEVEMETYGAGTPLGSVASVHSPPAESCASLQIPLAVSSPARSGEPLHALSPR
GTARSPSLSRLLCRQEAVHTDSLEGKIDSPRDTLDPAEPGEKTPVRPVTQGGSLQSPPRSPR
PASVRTRKHTFGQRCVSSRPAAPGGEEAEASDPADEEVSHITSSACPWQPTAEPHGPEASPV
AGGERDLRRLYSVDAQGFLDKPGRADEQWRPSAELGSGEPGEAKAWGPEAEPALGARR
KKKMSPPCISVEPPAEDEGSARPSAAEGGSTTLRRRTPSCEATPHRDSLEPTEGSGAGGDPA
AKGERWGQASCRAEHLTVPSFAFEPLDLGVPSGDPFLDGSHSVTPESRASSSGAIVPLEPPE
SEPPMPVGDPPEKRRGLYLTVPQCPLEKPGSPSATPAPGGGADDPV

>P51164 single-pass type II membrane protein
MAALQEKKTCGQRMEEFQRYCWNPDTGQMLGRTLSRWVWISLYYVAFYVVMTGLFALC
LYVLMQTVDPYTPDYQDQLRSPGVTLRPDVYGEKGLEIVYNVSDNRTWADLTQTLHAFL
AGYSPAAQEDSINCTSEQYFFQESFRAPNHTKFSCKFTADMLQNCSGLADPNFGFEEGKPC
FIIKMNRIVKFLPSNGSAPRVDCAFLDQPRELGQPLQVKYYPPNGTFSLHYFPYYGKKAQP
HYSNPLVAAKLLNIPRNAEVAIVCKVMAEHVTFNNPHDPYEGKVEFKLKIEK

>Q495M3 multipass membrane protein
MSVTKSTEGPQGAVAIKLDLMSPPESAKKLENKDSTFLDESPSESAGLKKTKGITVFQALI
HLVKGNMGTGILGLPLAVKNAGILMGPLSLLVMGFIACHCMHILVKCAQRFCKRLNKPFM
DYGDTVMHGLEANPNAWLQNHAHWGRHIVSFFLIITQLGFCCVYIVFLADNLKQVVEAV
NSTTNNCYSNETVILTPTMDSRLYMLSFLPFLVLLVLIRNLRILTIFSMLANISMLVSLVIIIQY
ITQEIPDPSRLPLVASWKTYPLFFGTAIFSFESIGVVLPLENKMKNARHFPAILSLGMSIVTSL
YIGMAALGYLRFGDDIKASISLNLPNCWLYQSVKLLYIAGILCTYALQFYVPAEIIIPFAISRV
STRWALPLDLSIRLVMVCLTCLLAILIPRLDLVISLVGSVSGTALALIIPPLLEVTTFYSEGMS
PLTIFKDALISILGFVGFVVGTYQALDELLKSEDSHPFSNSTTFVR

>Q86W10 single-pass type II membrane protein
MEPSWLQELMAHPFLLLILLCMSLLLFQVIRLYQRRRWMIRALHLFPAPPAHWFYGHKEF
YPVKEFEVYHKLMEKYPCAVPLWVGPFTMFFSVHDPDYAKILLKRQDPKSAVSHKILESW
VGRGLVTLDGSKWKKHRQIVKPGFNISILKIFITMMSESVRMMLNKWEEHIAQNSRLELF
QHVSLMTLDSIMKCAFSHQGSIQLDSTLDSYLKAVFNLSKISNQRMNNFLHHNDLVFKFSS
QGQIFSKFNQELHQFTEKVIQDRKESLKDKLKQDTTQKRRWDFLDILLSAKSENTKDFSE
ADLQAEVKTFMFAGHDTTSSAISWILYCLAKYPEHQQRCRDEIRELLGDGSSITWEHLSQ
MPYTTMCIKECLRLYAPVVNISRLLDKPITFPDGRSLPAGITVFINIWALHHNPYFWEDPQV
FNPLRFSRENSEKIHPYAFIPFSAGLRNCIGQHFAIIECKVAVALTLLRFKLAPDHSRPPQPVR
QVVLKSKNGIHVFAKKVC

>Q8IZJ1 single-pass type I membrane protein
MGARSGARGALLLALLLCWDPRLSQAGTDSGSEVLPDSFPSAPAEPLPYFLQEPQDAYIV
KNKPVELRCRAFPATQIYFKCNGEWVSQNDHVTQEGLDEATGLRVREVQIEVSRQQVEEL
FGLEDYWCQCVAWSSAGTTKSRRAYVRIAYLRKNFDQEPLGKEVPLDHEVLLQCRPPEG
VPVAEVEWLKNEDVIDPTQDTNFLLTIDHNLIIRQARLSDTANYTCVAKNIVAKRRSTTATV
IVYVNGGWSSWAEWSPCSNRCGRGWQKRTRTCTNPAPLNGGAFCEGQAFQKTACTTICP
VDGAWTEWSKWSACSTECAHWRSRECMAPPPQNGGRDCSGTLLDSKNCTDGLCMQNK
KTLSDPNSHLLEASGDAALYAGLVVAIFVVVAILMAVGVVVYRRNCRDFDTDITDSSAALT
GGFHPVNFKTARPSNPQLLHPSVPPDLTASAGIYRGPVYALQDSTDKIPMTNSPLLDPLPSL
KVKVYSSSTTGSGPGLADGADLLGVLPPGTYPSDFARDTHFLHLRSASLGSQQLLGLPRD
PGSSVSGTFGCLGGRLSIPGTGVSLLVPNGAIPQGKFYEMYLLINKAESTLPLSEGTQTVLS
PSVTCGPTGLLLCRPVILTMPHCAEVSARDWIFQLKTQAHQGHWEEVVTLDEETLNTPCY
CQLEPRACHILLDQLGTYVFTGESYSRSAVKRLQLAVFAPALCTSLEYSLRVYCLEDTPVAL
KEVLELERTLGGYLVEEPKPLMFKDSYHNLRLSLHDLPHAHWRSKLLAKYQEIPFYHIWS
GSQKALHCTFTLERHSLASTELTCKICVRQVEGEGQIFQLHTTLAETPAGSLDTLCSAPGST
VTTQLGPYAFKIPLSIRQKICNSLDAPNSRGNDWRMLAQKLSMDRYLNYFATKASPTGVIL

DLWEALQQDDGDLNSLASALEEMGKSEMLVAVATDGDC

>Q96R67 multipass membrane protein
MEKKKNVTEFILIGLTQNPIMEKVTFVVFLVLYMITLSGNLLIVVTITTSQALSSPMYFFLTH
LSLIDTVYSSSSAPKLIVDSFQEKKIISFNGCMAQAYAEHIFGATEIILLTVMACDCYVAICKP
LNYTTIMSHSLCILLVAVAWVGGFLHATIQILFTVWLPFCGPNVIGHFMCDLYPLLKLVCID
THTLGLFVAVNSGFICLLNFLILVVSYVIILRSLKNNSLEGRCKALSTCISHIIVVVLFFVPCIF
VYLRSVTTLPIDKAVAVFYTMVVPMLNPVVYTLRNAEVKSAIRKLWRKKVTSDND

>Q8NGS4 multipass membrane protein
MFPANWTSVKVFFFLGFFHYPKVQVIIFAVCLLMYLITLLGNIFLISITILDSHLHTPMYLFLS
NLSFLDIWYSSSALSPMLANFVSGRNTISFSGCATQMYLSLAMGSTECVLLPMMAYDRYV
AICNPLRYPVIMNRRTCVQIAAGSWMTGCLTAMVEMMSVLPLSLCGNSIINHFTCEILAIL
KLVCVDTSLVQLIMLVISVLLLPMPMLLICISYAFILASILRISSVEGRSKAFSTCTAHLMVV
VLFYGTALSMHLKPSAVDSQEIDKFMALVYAGQTPMLNPIIYSLRNKEVKVALKKLLIRNH
FNTAFISILK

>Q8NGL3 multipass membrane protein
MMMVLRNLSMEPTFALLGFTDYPKLQIPLFLVFLLMYVITVVGNLGMIIIIKINPKFHTPMY
FFLSHLSFVDFCYSSIVTPKLLENLVMADKSIFYFSCMMQYFLSCTAVVTESFLLAVMAYD
RFVAICNPLLYTVAMSQRLCALLVAGSYLWGMFGPLVLLCYALRLNFSGPNVINHFFCEYT
ALISVSGSDILIPHLLLFSFATFNEMCTLLIILTSYVFIFVTVLKIRSVSGRHKAFSTWASHLTS
ITIFHGTILFLYCVPNSKNSRQTVKVASVFYTVVNPMLNPLIYSLRNKDVKDAFWKLIHTQ
VPFH

>Q7Z602 multipass membrane protein
MPGHNTSRNSSCDPIVTPHLISLYFIVLIGGLVGVISILFLLVKMNTRSVTTMAVINLVVVHS
VFLLTVPFRLTYLIKKTWMFGLPFCKFVSAMLHIHMYLTFLFYVVILVTRYLIFFKCKDKVE
FYRKLHAVAASAGMWTLVIVIVVPLVVSRYGIHEEYNEEHCFKFHKELAYTYVKIINYMIV
IFVIAVAVILLVFQVFIIMLMVQKLRHSLLSHQEFWAQLKNLFFIGVILVCFLPYQFFRIYYLN
VVTHSNACNSKVAFYNEIFLSVTAISCYDLLLFVFGGSHWFKQKIIGLWNCVLCR

>Q6VVX0 peripheral membrane protein
MWKLWRAEEGAAALGGALFLLLFALGVRQLLKQRRPMGFPPGPPGLPFIGNIYSLAASSE
LPHVYMRKQSQVYGEIFSLDLGGISTVVLNGYDVVKECLVHQSEIFADRPCLPLFMKMTK
MGGLLNSRYGRGWVDHRRLAVNSFRYFGYGQKSFESKILEETKFFNDAIETYKGRPFDFK
QLITNAVSNITNLIIFGERFTYEDTDFQHMIELFSENVELAASASVFLYNAFPWIGILPFGKH
QQLFRNAAVVYDFLSRLIEKASVNRKPQLPQHFVDAYLDEMDQGKNDPSSTFSKENLIFS
VGELIIAGTETTTNVLRWAILFMALYPNIQGQVQKEIDLIMGPNGKPSWDDKCKMPYTEA
VLHEVLRFCNIVPLGIFHATSEDAVVRGYSIPKGTTVITNLYSVHFDEKYWRDPEVFHPERF
LDSSGYFAKKEALVPFSLGRRHCLGEHLARMEMFLFFTALLQRFHLHFPHELVPDLKPRLG
MTLQPQPYLICAERR

>Q7Z418 multipass membrane protein

MEVSGHPQARRCCPEALGKLFPGLCFLCFLVTYALVGAVVFSAIEDGQVLVAADDGEFEK
FLEELCRILNCSETVVEDRKQDLQGHLQKVKPQWFNRTTHWSFLSSLFFCCTVFSTVGYG
YIYPVTRLGKYLCMLYALFGIPLMFLVLTDTGDILATILSTSYNRFRKFPFFTRPLLSKWCPK
SLFKKKPDPKPADEAVPQIIISAEELPGPKLGTCPSRPSCSMELFERSHALEKQNTLQLPPQA
MERSNSCPELVLGRLSYSIISNLDEVGQQVERLDIPLPIIALIVFAYISCAAAILPFWETQLDF
ENAFYFCFVTLTTIGFGDTVLEHPNFFLFFSIYIIVGMEIVFIAFKLVQNRLIDIYKNVMLFFA
KGKFYHLVKK

>Q8NGX9 multipass membrane protein
MRNLSGGHVEEFVLVGFPTTPPLQLLLFVLFFAIYLLTLLENALIVFTIWLAPSLHRPMYFF
LGHLSFLELWYINVTIPRLLAAFLTQDGRVSYVGCMTQLYFFIALACTECVLLAVMAYDRY
LAICGPLLYPSLMPSSLATRLAAASWGSGFFSSMMKLLFISQLSYCGPNIINHFFCDISPLLN
LTCSDKEQAELVDFLLALVMILLPLLAVVSSYTAIIAAILRIPTSRGRHKAFSTCAAHLAVVV
IYYSSTLFTYARPRAMYTFNHNKIISVLYTIIVPFFNPAIYCLRNKEVKEAFRKTVMGRCHY
PRDVQD

>Q401N2 multipass membrane protein
MMALWSLLHLTFLGFSITLLLVHGQGFQGTAAIWPSLFNVNLSKKVQESIQIPNNGSAPLL
VDVRVFVSNVFNVDILRYTMSSMLLLRLSWLDTRLAWNTSAHPRHAITLPWESLWTPRLT
ILEALWVDWRDQSPQARVDQDGHVKLNLALATETNCNFELLHFPRDHSNCSLSFYALSNT
AMELEFQAHVVNEIVSVKREYVVYDLKTQVPPQQLVPCFQVTLRLKNTALKSIIALLVPAE
ALLLADVCGGLLPLRAIERIGYKVTLLLSYLVLHSSLVQALPSSSSCNPLLIYYFTILLLLLF
LSTIETVLLAGLLARGNLGAKSGPSPAPRGEQREHGNPGPHPAEEPSRGVKGSQRSWPETA
DRIFFLVYVVGVLCTQFVFAGIWMWAACKSDAAPGEAAPHGRRPRL

>Q15835 lipid-anchor
MDFGSLETVVANSAFIAARGSFDGSSSQPSRDKKYLAKLKLPPLSKCESLRDSLSLEFESVC
LEQPIGKKLFQQFLQSAEKHLPALELWKDIEDYDTADNDLQPQKAQTILAQYLDPQAKLF
CSFLDEGIVAKFKEGPVEIQDGLFQPLLQATLAHLGQAPFQEYLGSLYFLRFLQWKWLEAQ
PMGEDWFLDFRVLGKGGFGEVSACQMKATGKLYACKKLNKKRLKKRKGYQGAMVEKK
ILMKVHSRFIVSLAYAFETKADLCLVMTIMNGGDIRYHIYNVNEENPGFPEPRALFYTAQII
CGLEHLHQRRIVYRDLKPENVLLDNDGNVRISDLGLAVELLDGQSKTKGYAGTPGFMAPE
LLQGEEYDFSVDYFALGVTLYEMIAARGPFRARGEKVENKELKHRIISEPVKYPDKFSQAS
KDFCEALLEKDPEKRLGFRDETCDKLRAHPLFKDLNWRQLEAGMLMPPFIPDSKTVYAK
DIQDVGAFSTVKGVAFDKTDTEFFQEFATGNCPIPWQEEMIETGIFGELNVWRSDGQMPD
DMKGISGGSSSSSKSGMCLVS

>O00408 lipid-anchor
MGQACGHSILCRSQQYPAARPAEPRGQQVFLKPDEPPPPPQPCADSLQDALLSLGSVIDISG
LQRAVKEALSAVLPRVETVYTYLLDGESQLVCEDPPHELPQEGKVREAIISQKRLGCNGLG
FSDLPGKPLARLVAPLAPDTQVLVMPLADKEAGAVAAVILVHCGQLSDNEEWSLQAVEKH
TLVALRRVQVLQQRGPREAPRAVQNPPEGTAEDQKGGAAYTDRDRKILQLCGELYDLDAS
SLQLKVLQYLQQETRASRCCLLLVSEDNLQLSCKVIGDKVLGEEVSFPLTGCLGQVVEDK
KSIQLKDLTSEDVQQLQSMLGCELQAMLCVPVISRATDQVVALACAFNKLEGDLFTDEDE

HVIQHCFHYTSTVLTSTLAFQKEQKLKCECQALLQVAKNLFTHLDDVSVLLQEIITEARNL
SNAEICSVFLLDQNELVAKVFDGGVVDDESYEIRIPADQGIAGHVATTGQILNIPDAYAHPL
FYRGVDDSTGFRTRNILCFPIKNENQEVIGVAELVNKINGPWFSKFDEDLATAFSIYCGISIA
HSLLYKKVNEAQYRSHLANEMMMYHMKVSDDEYTKLLHDGIQPVAAIDSNFASFTYTPR
SLPEDDTSMAILSMLQDMNFINNYKIDCPTLARFCLMVKKGYRDPPYHNWMHAFSVSHF
CYLLYKNLELTNYLEDIEIFALFISCMCHDLDHRGTNNSFQVASKSVLAALYSSEGSVMER
HHFAQAIAILNTHGCNIFDHFSRKDYQRMLDLMRDIILATDLAHHLRIFKDLQKMAEVGY
DRNNKQHHRLLLCLLMTSCDLSDQTKGWKTTRKIAELIYKEFFSQGDLEKAMGNRPME
MMDREKAYIPELQISFMEHIAMPIYKLLQDLFPKAAELYERVASNREHWTKVSHKFTIRGL
PSNNSLDFLDEEYEVPDLDGTRAPINGCCSLDAE

>Q92729 single-pass type I membrane protein
MARAQALVLALTFQLCAPETETPAAGCTFEEASDPAVPCEYSQAQYDDFQWEQVRIHPGT
RAPADLPHGSYLMVNTSQHAPGQRAHVIFQSLSENDTHCVQFSYFLYSRDGHSPGTLGVY
VRVNGGPLGSAVWNMTGSHGRQWHQAELAVSTFWPNEYQVLFEALISPDRRGYMGLDD
ILLLSYPCAKAPHFSRLGDVEVNAGQNASFQCMAAGRAAEAERFLLQRQSGALVPAAGV
RHISHRRFLATFPLAAVSRAEQDLYRCVSQAPRGAGVSNFAELIVKEPPTPIAPPQLLRAGP
TYLIIQLNTNSIIGDGPIVRKEIEYRMARGPWAEVHAVSLQTYKLWHLDPDTEYEISVLLTR
PGDGGTGRPGPPLISRTKCAEPMRAPKGLAFAEIQARQLTLQWEPLGYNVTRCHTYTVSL
CYHYTLGSSHNQTIRECVKTEQGVSRYTIKNLLPYRNVHVRLVLTNPEGRKEGKEVTFQT
DEDVPSGIAAESLTFTPLEDMIFLKWEEPQEPNGLITQYEISYQSIESSDPAVNVPGPRRTISK
LRNETYHVFSNLHPGTTYLFSVRARTGKGFGQAALTEITTNISAPSFDYADMPSPLGESENT
ITVLLRPAQGRGAPISVYQVIVEEERARRLRREPGGQDCFPVPLTFEAALARGLVHYFGAE
LAASSLPEAMPFTVGDNQTYRGFWNPPLEPRKAYLIYFQAASHLKGETRLNCIRIARKAA
CKESKRPLEVSQRSEEMGLILGICAGGLAVLILLLGAIIVIIRKGRDHYAYSYYPKPVNMTK
ATVNYRQEKTHMMSAVDRSFTDQSTLQEDERLGLSFMDTHGYSTRGDQRSGGVTEASSL
LGGSPRRPCGRKGSPYHTGQLHPAVRVADLLQHINQMKTAEGYGFKQEYESFFEGWDATK
KKDKVKGSRQEPMPAYDRHRVKLHPMLGDPNADYINANYIDGYHRSNHFIATQGPKPEM
VYDFWRMVWQEHCSSIVMITKLVEVGRVKCSRYWPEDSDTYGDIKIMLVKTETLAEYVV
RTFALERRGYSARHEVRQFHFTAWPEHGVPYHATGLLAFIRRVKASTPPDAGPIVIHCSAG
TGRTGCYIVLDVMLDMAECEGVVDIYNCVKTLCSRRVNMIQTEEQYIFIHDAILEACLCG
ETTIPVSEFKATYKEMIRIDPQSNSSQLREEFQTLNSVTPPLDVEECSIALLPRNRDKNRSM
DVLPPDRCLPFLISTDGDSNNYINAALTDSYTRSAAFIVTLHPLQSTTPDFWRLVYDYGCTS
IVMLNQLNQSNSAWPCLQYWPEPGRQQYGLMEVEFMSGTADEDLVARVFRVQNISRLQE
GHLLVRHFQFLRWSAYRDTPDSKKAFLHLLAEVDKWQAESGDGRTIVHCLNGGGRSGTF
CACATVLEMIRCHNLVDVFFAAKTLRNYKPNMVETMDQYHFCYDVALEYLEGLESR

>Q8IWK6 multipass membrane protein
MEPPGRRRGRAQPPLLLPLSLLALLALLGGGGGGGAAALPAGCKHDGRPRGAGRAAGA
AEGKVVCSSLELAQVLPPDTLPNRTVTLILSNNKISELKNGSFSGLSLLERLDLRNNLISSID
PGAFWGLSSLKRLDLTNNRIGCLNADIFRGLTNLVRLNLSGNLFSSLSQGTFDYLASLRSLE
FQTEYLLCDCNILWMHRWVKEKNITVRDTRCVYPKSLQAQPVTGVKQELLTCDPPLELPS
FYMTPSHRQVVFEGDSLPFQCMASYIDQDMQVLWYQDGRIVETDESQGIFVEKNMIHNC
SLIASALTISNIQAGSTGNWGCHVQTKRGNNTRTVDIVVLESSAQYCPPERVVNNKGDFR

WPRTLAGITAYLQCTRNTHGSGIYPGNPQDERKAWRRCDRGGFWADDDYSRCQYANDVT
RVLYMFNQMPLNLTNAVATARQLLAYTVEAANFSDKMDVIFVAEMIEKFGRFTKEEKSKE
LGDVMVDIASNIMLADERVLWLAQREAKACSRIVQCLQRIATYRLAGGAHVYSTYSPNIA
LEAYVIKSTGFTGMTCTVFQKVAASDRTGLSDYGRRDPEGNLDKQLSFKCNVSNTFSSLA
LKNTIVEASIQLPPSLFSPKQKRELRPTDDSLYKLQLIAFRNGKLFPATGNSTNLADDGKRR
TVVTPVILTKIDGVNVDTHHIPVNVTLRRIAHGADAVAARWDFDLLNGQGGWKSDGCHIL
YSDENITTIQCYSLSNYAVLMDLTGSELYTQAASLLHPVVYTTAIILLLCLLAVIVSYIYHHS
LIRISLKSWHMLVNLCFHIFLTCVVFVGGITQTRNASICQAVGIILHYSTLATVLWVGVTAR
NIYKQVTKKAKRCQDPDEPPPPPRPMLRFYLIGGGIPIIVCGITAAANIKNYGSRPNAPYCW
MAWEPSLGAFYGPASFITFVNCMYFLSIFIQLKRHPERKYELKEPTEEQQRLAANENGEIN
HQDSMSLSLISTSALENEHTFHSQLLGASLTLLLYVALWMFGALAVSLYYPLDLVFSFVFGA
TSLSFSAFFVVHHCVNREDVRLAWIMTCCPGRSSYSVQVNVQPPNSNGTNGEAPKCPNSS
AESSCTNKSASSFKNSSQGCKLTNLQAAAAQCHANSLPLNSTPQLDNSLTEHSMDNDIKM
HVAPLEVQFRTNVHSSRHHKNRSKGHRASRLTVLREYAYDVPTSVEGSVQNGLPKSRLGN
NEGHSRSRRAYLAYRERQYNPPQQDSSDACSTLPKSSRNFEKPVSTTSKKDALRKPAVVEL
ENQQKSYGLNLAIQNGPIKSNGQEGPLLGTDSTGNVRTGLWKHETTV

>Q8IU80 single-pass type II membrane protein
MLLLFHSKRMPVAEAPQVAGGQGDGGDGEEAEPEGMFKACEDSKRKARGYLRLVPLFVL
LALLVLASAGVLLWYFLGYKAEVMVSQVYSGSLRVLNRHFSQDLTRRESSAFRSETAKAQ
KMLKELITSTRLGTYYNSSSVYSFGEGPLTCFFWFILQIPEHRRLMLSPEVVQALLVEELLS
TVNSSAAVPYRAEYEVDPEGLVILEASVKDIAALNSTLGCYRYSYVGQGQVLRLKGPDHL
ASSCLWHLQGPKDLMLKLRLEWTLAECRDRLAMYDVAGPLEKRLITSVYGCSRQEPVVE
VLASGAIMAVVWKKGLHSYYDPFVLSVQPVVFQACEVNLTLDNRLDSQGVLSTPYFPSY
YSPQTHCSWHLTVPSLDYGLALWFDAYALRRQKYDLPCTQGQWTIQNRRLCGLRILQPYA
ERIPVVATAGITINFTSQISLTGPGVRVHYGLYNQSDPCPGEFLCSVNGLCVPACDGVKDCP
NGLDERNCVCRATFQCKEDSTCISLPKVCDGQPDCLNGSDEEQCQEGVPCGTFTFQCEDR
SCVKKPNPQCDGRPDCRDGSDEEHCDCGLQGPSSRIVGGAVSSEGEWPWQASLQVRGRH
ICGGALIADRWVITAAHCFQEDSMASTVLWTVFLGKVWQNSRWPGEVSFKVSRLLLHPY
HEEDSHDYDVALLQLDHPVVRSAAVRPVCLPARSHFFEPGLHCWITGWGALREGGPISNA
LQKVDVQLIPQDLCSEVYRYQVTPRMLCAGYRKGKKDACQGDSGGPLVCKALSGRWFL
AGLVSWGLGCGRPNYFGVYTRITGVISWIQQVVT

>Q15116 single-pass type I membrane protein
MQIPQAPWPVVWAVLQLGWRPGWFLDSPDRPWNPPTFSPALLVVTEGDNATFTCSFSNTS
ESFVLNWYRMSPSNQTDKLAAFPEDRSQPGQDCRFRVTQLPNGRDFHMSVVRARRNDSG
TYLCGAISLAPKAQIKESLRAELRVTERRAEVPTAHPSPSPRPAGQFQTLVVGVVGGLLGSL
VLLVWVLAVICSRAARGTIGARRTGQPLKEDPSAVPVFSVDYGELDFQWREKTPEPPVPC
VPEQTEYATIVFPSGMGTSSPARRGSADGPRSAQPLRPEDGHCSWPL

>Q8ND25 peripheral membrane protein
MGGKQSTAARSRGPFPGVSTDDSAVPPPGGAPHFGHYRTGGGAMGLRSRSVSSVAGMGM
DPSTAGGVPFGLYTPASRGTGDSERAPGGGGSASDSTYAHGNGYQETGGGHHRDGMLYL
GSRASLADALPLHIAPRWFSSHSGFKCPICSKSVASDEMEMHFIMCLSKPRLSYNDDVLTK

DAGECVICLEELLQGDTIARLPCLCIYHKSCIDSWFEVNRSCPEHPAD

>Q7Z7M1 multipass membrane protein
MDAPWGAGERWLHGAAVDRSGVSLGPPPTPQVNQGTLGPQVAPVAAGEVVKTAGGVCK
FSGQRLSWWQAQESCEQQFGHLALQPPDGVLASRLRDPVWVGQREAPLRRPPQRRARTT
AVLVFDERTADRAARLRSPLPELAALTACTHVQWDCASPDPAALFSVAAPALPNALQLRAF
AEPGGVVRAALVVRGQHAPFLAAFRADGRWHHVCATWEQRGGRWALFSDGRRRAGAR
GLGAGHPVPSGGILVLGQDQDSLGGGFSVRHALSGNLTDFHLWARALSPAQLHRARACAP
PSEGLLFRWDPGALDVTPSLLPTVWVRLLCPVPSEECPTWNPGPRSEGSELCLEPQPFLCC
YRTEPYRRLQDAQSWPGQDVISRVNALANDIVLLPDPLSEVHGALSPAEASSFLGLLEHVL
AMEMAPLGPAALLAVVRFLKRVVALGAGDPELLLTGPWEQLSQGVVSVASLVLEEQVAD
TWLSLREVIGGPMALVASVQRLAPLLSTSMTSERPRMRIQHRHAGLSGVTVIHSWFTSRVF
QHTLEGPDLEPQAPASSEEANRVQRFLSTQVGSAIISSEVWDVTGEVNVAMTFHLQHRAQ
SPLFPPHPPSPYTGGAWATTGCSVAALYLDSTACFCNHSTSFAILLQIYEVQRGPEEESLLRT
LSFVGCGVSFCALTTTFLLFLVAGVPKSERTTVHKNLTFSLASAEGFLMTSEWAKANEVAC
VAVTVAMHFLFLVAFSWMLVEGLLLWRKVVAVSMHPGPGMRLYHATGWGVPVGIVAVTL
AMLPHDYVAPGHCWLNVHTNAIWAFVGPVLFVLTANTCILARVVMITVSSARRRARMLS
PQPCLQQQIWTQIWATVKPVLVLLPVLGLTWLAGILVHLSPAWAYAAVGLNSIQGLYIFLVY
AACNEEVRSALQRMAEKKVAEVLRALGVWGGAAKEHSLPFSVLPLFLPPKPSTPRHPLK
APA

>Q6ZMI3 single-pass type II membrane protein
MARGAEGGRGDAGWGLRGALAAVALLSALNAAGTVFALCQWRGLSSALRALEAQRGR
EQREDSALRSFLAELSRAPRGASAPPQDPASSARNKRSHSGEPAPHIRAESHDMLMMMTY
SMVPIRVMVDLCNSTKGICLTGPSGPPGPPGAGGLPGHNGLDGQPGPQGPKGEKGANGK
RGKMGIPGAAGNPGERGEKGDHGELGLQGNEGPPGQKGEKGDKGDVSNDVLLAGAKG
DQGPPGPPGPPGPPGPPGPPGSRRAKGPRQPSMFNGQCPGETCAIPNDDTLVGKADEKASE
HHSPQAESMITSIGNPVQVLKVTETFGTWIRESANKSDDRIWVTEHFSGIMVKEFKDQPSL
LNGSYTFIHLPYYFHGCGHVVYNNSLYYHKGGSNTLVRFEFGQETSQTLKLENALYFDRK
YLFANSKTYFNLAVDEKGLWIIYASSVDGSSILVAQLDERTFSVVQHVNTTYPKSKAGNAFI
ARGILYVTDTKDMRVTFAFDLLGGKQINANFDLRTSQSVLAMLAYNMRDQHLYSWEDGH
LMLYPVQFLSTTLNQ

>P24530 multipass membrane protein
MQPPPSLCGRALVALVLACGLSRIWGEERGFPPDRATPLLQTAEIMTPPTKTLWPKGSNAS
LARSLAPAEVPKGDRTAGSPPRTISPPPCQGPIEIKETFKYINTVVSCLVFVLGIIGNSTLLRII
YKNKCMRNGPNILIASLALGDLLHIVIDIPINVYKLLAEDWPFGAEMCKLVPFIQKASVGIT
VLSLCALSIDRYRAVASWSRIKGIGVPKWTAVEIVLIWVVSVVLAVPEAIGFDIITMDYKGS
YLRICLLHPVQKTAFMQFYKTAKDWWLFSFYFCLPLAITAFFYTLMTCEMLRKKSGMQIA
LNDHLKQRREVAKTVFCLVLVFALCWLPLHLSRILKLTLYNQNDPNRCELLSFLLVLDYIGI
NMASLNSCINPIALYLVSKRFKNCFKSCLCCWCQSFEEKQSLEEKQSCLKFKANDHGYDN
FRSSNKYSSS

>O60331 peripheral membrane protein

MELEVPDEAESAEAGAVPSEAAWAAESGAAAGLAQKKAAPTEVLSMTAQPGPGHGKKL
GHRGVDASGETTYKKTTSSTLKGAIQLGIGYTVGHLSSKPERDVLMQDFYVVESIFFPSEG
SNLTPAHHFQDFRFKTYAPVAFRYFRELFGIRPDDYLYSLCNEPLIELSNPGASGSLFYVTSD
DEFIIKTVMHKEAEFLQKLLPGYYMNLNQNPRTLLPKFYGLYCVQSGGKNIRVVVMNNIL
PRVVKMHLKFDLKGSTYKRRASKKEKEKSFPTYKDLDFMQDMPEGLLLDADTFSALVKT
LQRDCLVLESFKIMDYSLLLGVHNIDQHERERQAQGAQSTSDEKRPVGQKALYSTAMESI
QGGAARGEAIESDDTMGGIPAVNGRGERLLLHIGIIDILQSYRFIKKLEHTWKALVHDGDT
VSVHRPSFYAERFFKFMSNTVFRKNSSLKSSPSKKGRGGALLAVKPLGPTAAFSASQIPSER
EEAQYDLRGARSYPTLEDEGRPDLLPCTPPSFEEATTASIATTLSSTSLSIPERSPSETSEQPR
YRRRTQSSGQDGRPQEEPPAEEDLQQITVQVEPACSVEIVVPKEEDAGVEASPAGASAAVE
VETASQASDEEGAPASQASDEEDAPATDIYFPTDERSWVYSPLHYSAQAPPASDGESDT

>Q86UN3 GPI-anchor, lipid-anchor
MLPGLRRLLQAPASACLLLMLLALPLAAPSCPMLCTCYSSPPTVSCQANNFSSVPLSLPPS
TQRLFLQNNLIRTLRPGTFGSNLLTLWLFSNNLSTIYPGTFRHLQALEELDLGDNRHLRSLE
PDTFQGLERLQSLHLYRCQLSSLPGNIFRGLVSLQYLYLQENSLLHLQDDLFADLANLSHL
FLHGNRLRLLTEHVFRGLGSLDRLLLHGNRLQGVHRAAFRGLSRLTILYLFNNSLASLPGE
ALADLPSLEFLRLNANPWACDCRARPLWAWFQRARVSSSDVTCATPPERQGRDLRALREA
DFQACPPAAPTRPGSRARGNSSSNHLYGVAEAGAPPADPSTLYRDLPAEDSRGRQGGDAPT
EDDYWGGYGGEDQRGEQMCPGAACQAPPDSRGPALSAGLPSPLLCLLLLVPHHL

>A5X5Y0 multipass membrane protein
MEGSWFHRKRFSFYLLLGFLLQGRGVTFTINCSGFGQHGADPTALNSVFNRKPFRPVTNIS
VPTQVNISFAMSAILDVNEQLHLLSSFLWLEMVWDNPFISWNPEECEGITKMSMAAKNLW
LPDIFIIELMDVDKTPKGLTAYVSNEGRIRYKKPMKVDSICNLDIFYFPFDQQNCTLTFSSFL
YTVDSMLLDMEKEVWEITDASRNILQTHGEWELLGLSKATAKLSRGGNLYDQIVFYVAIR
RRPSLYVINLLVPSGFLVAIDALSFYLPVKSGNRVPFKITLLLGYNVFLLMMSDLLPTSGTPL
IGVYFALCLSLMVGSLLETIFITHLLHVATTQPPPLPRWLHSLLLHCNSPGRCCPTAPQKEN
KGPGLTPTHLPGVKEPEVSAGQMPGPAEAELTGGSEWTRAQREHEAQKQHSVELWLQFS
HAMDAMLFRLYLLFMASSIITVICLWNT

>Q8NGL2 multipass membrane protein
MGKENCTTVAEFILLGLSDVPELRVCLFLLFLLIYGVTLLANLGMIALIQVSSRLHTPMYFF
LSHLSSVDFCYSSIIVPKMLANIFNKDKAISFLGCMVQFYLFCTCVVTEVFLLAVMAYDRF
VAICNPLLYTVTMSWKVRVELASCCYFCGTVCSLIHLCLALRIPFYRSNVINHFFCDLPPVL
SLACSDITVNETLLFLVATLNESVTIMIILTSYLLILTTILKMGSAEGRHKAFSTCASHLTAITV
FHGTVLSIYCRPSSGNSGDADKVATVFYTVVIPMLNSVIYSLRNKDVKEALRKVMGSKIHS

>Q8NGY5 multipass membrane protein
MDTGNWSQVAEFIILGFPHLQGVQIYLFLLLLLIYLMTVLGNLLIFLVVCLDSRLHTPMYH
FVSILSFSELGYTAATIPKMLANLLSEKKTISFSGCLLQIYFFHSLGATECYLLTAMAYDRYL
AICRPLHYPTLMTPTLCAEIAIGCWLGGLAGPVVEISLISRLPFCGPNRIQHVFCDFPPVLSL
ACTDTSINVLVDFVINSCKILATFLLILCSYVQIICTVLRIPSAAGKRKAISTCASHFTVVLIF
YGSILSMYVQLKKSYSLDYDQALAVVYSVLTPFLNPFIYSLRNKEIKEAVRRQLKRIGILA

>P32239 multipass membrane protein
MELLKLNRSVQGTGPGPGASLCRPGAPLLNSSSVGNLSCEPPRIRGAGTRELELAIRITLYA
VIFLMSVGGNMLIIVVLGLSRRLRTVTNAFLLSLAVSDLLLAVACMPFTLLPNLMGTFIFGT
VICKAVSYLMGVSVSVSTLSLVAIALERYSAICRPLQARVWQTRSHAARVIVATWLLSGLL
MVPYPVYTVVQPVGPRVLQCVHRWPSARVRQTWSVLLLLLLFFIPGVVMAVAYGLISREL
YLGLRFDGDSDSDSQSRVRNQGGLPGAVHQNGRCRPETGAVGEDSDGCYVQLPRSRPAL
ELTALTAPGPGSGSRPTQAKLLAKKRVVRMLLVIVVLFFLCWLPVYSANTWRAFDGPGAH
RALSGAPISFIHLLSYASACVNPLVYCFMHRRFRQACLETCARCCPRPPRARPRALPDEDPP
TPSIASLSRLSYTTISTLGPG

>P31644 multipass membrane protein
MDNGMFSGFIMIKNLLLFCISMNLSSHFGFSQMPTSSVKDETNDNITIFTRILDGLLDGYDN
RLRPGLGERITQVRTDIYVTSFGPVSDTEMEYTIDVFFRQSWKDERLRFKGPMQRLPLNNL
LASKIWTPDTFFHNGKKSIAHNMTTPNKLLRLEDDGTLLYTMRLTISAECPMQLEDFPMD
AHACPLKFGSYAYPNSEVVYVWTNGSTKSVVVAEDGSRLNQYHLMGQTVGTENISTSTG
EYTIMTAHFHLKRKIGYFVIQTYLPCIMTVILSQVSFWLNRESVPARTVFGVTTVLTMTTLS
ISARNSLPKVAYATAMDWFIAVCYAFVFSALIEFATVNYFTKRGWAWDGKKALEAAKIKK
KREVILNKSTNAFTTGKMSHPPNIPKEQTPAGTSNTTSVSVKPSEEKTSESKKTYNSISKID
KMSRIVFPVLFGTFNLVYWATYLNREPVIKGAASPK

>Q9Y4A9 multipass membrane protein
MQRANHSTVTQFILVGFSVFPHLQLMLFLLFLLMYLFTLLGNLLIMATVWSERSLHTPMY
LFLCALSVSEILYTVAIIPRMLADLLSTQRSIAFLACASQMFFSFSFGFTHSFLLTVMGYDRY
VAICHPLRYNVLMSPRGCACLVGCSWAGGLVMGMVVTSAIFHLAFCGHKEIHHFACHVPP
LLKLACGDDVLVVAKGVGLVCITALLGCFLLILLSYAFIVAAILKIPSAEGRNKAFSTCASHL
TVVVVHYGFASVIYLKPKSPQSLEGDTLMGITYTVLTPFLSPIIFSLRNKELKVAMKKTFFS
KLYPEKNVMM

>Q96FZ5 multipass membrane protein
MSHGAGLVRTTCSSGSALGPGAGAAQPSASPLEGLLDLSYPRTHAALLKVAQMVTLLIAFI
CVRSSLWTNYSAYSYFEVVTICDLIMILAFYLVHLFRFYRVLTCISWPLSELLHYLIGTLLLL
IASIVAASKSYNQSGLVAGAIFGFMATFLCMASIWLSYKISCVTQSTDAAV

>Q96P65 multipass membrane protein
MQALNITPEQFSRLLRDHNLTREQFIALYRLRPLVYTPELPGRAKLALVLTGVLIFALALFG
NALVFYVVTRSKAMRTVTNIFICSLALSDLLITFFCIPVTMLQNISDNWLGGAFICKMVPFV
QSTAVVTEILTMTCIAVERHQGLVHPFKMKWQYTNRRAFTMLGVVWLVAVIVGSPMWHV
QQLEIKYDFLYEKEHICCLEEWTSPVHQKIYTTFILVILFLLPLMVMLILYSKIGYELWIKKR
VGDGSVLRTIHGKEMSKIARKKKRAVIMMVTVVALFAVCWAPFHVVHMMIEYSNFEKEY
DDVTIKMIFAIVQIIGFSNSICNPIVYAFMNENFKKNVLSAVCYCIVNKTFSPAQRHGNSGIT
MMRKKAKFSLRENPVEETKGEAFSDGNIEVKLCEQTEEKKKLKRHLALFRSELAENSPLD
SGH

>Q99571 multipass membrane protein
MAGCCAALAAFLFEYDTPRIVLIRSRKVGLMNRAVQLLILAYVIGWVFVWEKGYQETDS
VVSSVTTKVKGVAVTNTSKLGFRIWDVADYVIPAQEENSLFVMTNVILTMNQTQGLCPEIP
DATTVCKSDASCTAGSAGTHSNGVSTGRCVAFNGSVKTCEVAAWCPVEDDTHVPQPAFL
KAAENFTLLVKNNIWYPKFNFSKRNILPNITTTYLKSCIYDAKTDPFCPIFRLGKIVENAGH
SFQDMAVEGGIMGIQVNWDCNLDRAASLCLPRYSFRRLDTRDVEHNVSPGYNFRFAKYY
RDLAGNEQRTLIKAYGIRFDIIVFGKAGKFDIIPTMINIGSGLALLGMATVLCDIIVLYCMKK
RLYYREKKYKYVEDYEQGLASELDQ

>P29274 multipass membrane protein
MPIMGSSVYITVELAIAVLAILGNVLVCWAVWLNSNLQNVTNYFVVSLAAADIAVGVLAIP
FAITISTGFCAACHGCLFIACFVLVLTQSSIFSLLAIAIDRYIAIRIPLRYNGLVTGTRAKGIIAI
CWVLSFAIGLTPMLGWNNCGQPKEGKNHSQGCGEGQVACLFEDVVPMNYMVYFNFFAC
VLVPLLLMLGVYLRIFLAARRQLKQMESQPLPGERARSTLQKEVHAAKSLAIIVGLFALC
WLPLHIINCFTFFCPDCSHAPLWLMYLAIVLSHTNSVVNPFIYAYRIREFRQTFRKIIRSHVL
RQQEPFKAAGTSARVLAAHGSDGEQVSLRLNGHPPGVWANGSAPHPERRPNGYALGLVS
GGSAQESQGNTGLPDVELLSHELKGVCPEPPGLDDPLAQDGAGVS

>P61966 peripheral membrane protein
MMRFMLLFSRQGKLRLQKWYLATSDKERKKMVRELMQVVLARKPKMCSFLEWRDLKV
VYKRYASLYFCCAIEGQDNELITLELIHRYVELLDKYFGSVCELDIIFNFEKAYFILDEFLMG
GDVQDTSKKSVLKAIEQADLLQEEDESPRSVLEEMGLA

>P63241 peripheral membrane protein
MADDLDFETGDAGASATFPMQCSALRKNGFVVLKGRPCKIVEMSTSKTGKHGHAKVHL
VGIDIFTGKKYEDICPSTHNMDVPNIKRNDFQLIGIQDGYLSLLQDSGEVREDLRLPEGDL
GKEIEQKYDCGEEILITVLSAMTEEAAVAIKAMAK

>P41146 multipass membrane protein
MEPLFPAPFWEVIYGSHLQGNLSLLSPNHSLLPPHLLLNASHGAFLPLGLKVTIVGLYLAVC
VGGLLGNCLVMYVILRHTKMKTATNIYIFNLALADTLVLLTLPFQGTDILLGFWPFGNALC
KTVIAIDYYNMFTSTFTLTAMSVDRYVAICHPIRALDVRTSSKAQAVNVAIWALASVVGVP
VAIMGSAQVEDEEIECLVEIPTPQDYWGPVFAICIFLFSFIVPVLVISVCYSLMIRRLRGVRLL
SGSREKDRNLRRITRLVLVVVAVFVGCWTPVQVFVLAQGLGVQPSSETAVAILRFCTALGY
VNSCLNPILYAFLDENFKACFRKFCCASALRRDVQVSDRVRSIAKDVALACKTSETVPRPA

>Q70HW3 multipass membrane protein
MDRPGFVAALVAGGVAGVSVDLILFPLDTIKTRLQSPQGFNKAGGFHGIYAGVPSAAIGSF
PNAAAFFITYEYVKWFLHADSSSYLTPMKHMLAASAGEVVACLIRVPSEVVKQRAQVSAS
TRTFQIFSNILYEEGIQGLYRGYKSTVLREIPFSLVQFPLWESLKALWSWRQDHVVDSWQSA
VCGAFAGGFAAAVTTPLDVAKTRITLAKAGSSTADGNVLSVLHGVWRSQGLAGLFAGVFP
RMAAISLGGFIFLGAYDRTHSLLLEVGRKSP

>Q8N144 multipass membrane protein

MGEWAFLGSLLDAVQLQSPLVGRLWLVVMLIFRILVLATVGGAVFEDEQEEFVCNTLQPG
CRQTCYDRAFPVSHYRFWLFHILLLSAPPVLFVVYSMHRAGKEAGGAEAAAQCAPGLPE
AQCAPCALRARRARRCYLLSVALRLLAELTFLGGQALLYGFRVAPHFACAGPPCPHTVDC
FVSRPTEKTVFVLFYFAVGLLSALLSVAELGHLLWKGRPRAGERDNRCNRAHEEAQKLLP
PPPPPPPPPALPSRRPGPEPCAPPAYAHPAPASLRECGSGRGKASPATGRRDLAI

>Q9UBV2 single-pass type I membrane protein
MRVRIGLTLLLCAVLLSLASASSDEEGSQDESLDSKTTLTSDESVKDHTTAGRVVAGQIFLD
SEESELESSIQEEEDSLKSQEGESVTEDISFLESPNPENKDYEEPKKVRKPALTAIEGTAHGE
PCHFPFLFLDKEYDECTSDGREDGRLWCATTYDYKADEKWGFCETEEEAAKRRQMQEAE
MMYQTGMKILNGSNKKSQKREAYRYLQKAASMNHTKALERVSYALLFGDYLPQNIQAA
REMFEKLTEEGSPKGQTALGFLYASGLGVNSSQAKALVYYTFGALGGNLIAHMVLGYRY
WAGIGVLQSCESALTHYRLVANHVASDISLTGGSVVQRIRLPDEVENPGMNSGMLEEDLIQ
YYQFLAEKGDVQAQVGLGQLHLHGGRGVEQNHQRAFDYFNLAANAGNSHAMAFLGK
MYSEGSDIVPQSNETALHYFKKAADMGNPVGQSGLGMAYLYGRGVQVNYDLALKYFQK
AAEQGWVDGQLQLGSMYYNGIGVKRDYKQALKYFNLASQGGHILAFYNLAQMHASGT
GVMRSCHTAVELFKNVCERGRWSERLMTAYNSYKDGDYNAAVIQYLLLAEQGYEVAQS
NAAFILDQREASIVGENETYPRALLHWNRAASQGYTVARIKLGDYHFYGFGTDVDYETAF
IHYRLASEQQHSAQAMFNLGYMHEKGLGIKQDIHLAKRFYDMAAEASPDAQVPVFLALC
KLGVVYFLQYIRETNIRDMFTQLDMDQLLGPEWDLYLMTIIALLLGTVIAYRQRQHQDMP
APRPPGPRPAPPQQEGPPEQQPPQ

>Q7Z3T8 peripheral membrane protein
MDSYFKAAVSDLDKLLDDFEQNPDEQDYLQDVQNAYDSNHCSVSSELASSQRTSLLPKD
QECVNSCASSETSYGTNESSLNEKTLKGLTSIQNEKNVTGLDLLSSVDGGTSDEIQPLYMG
RCSKPICDLISDMGNLVHATNSEEDIKKLLPDDFKSNADSLIGLDLSSVSDTPCVSSTDHDS
DTVREQQNDISSELQNREIGGIKELGIKVDTTLSDSYNYSGTENLKDKKIFNQLESIVDFNM
SSALTRQSSKMFHAKDKLQHKSQPCGLLKDVGLVKEEVDVAVITAAECLKEEGKTSALTC
SLPKNEDLCLNDSNSRDENFKLPDFSFQEDKTVIKQSAQEDSKSLDLKDNDVIQDSSSALH
VSSKDVPSSLSCLPASGSMCGSLIESKARGDFLPQHEHKDNIQDAVTIHEEIQNSVVLGGEP
FKENDLLKQEKCKSILLQSLIEGMEDRKIDPDQTVIRAESLDGGDTSSTVVESQEGLSGTH
VPESSDCCEGFINTFSSNDMDGQDLDYFNIDEGAKSGPLISDAELDAFLTEQYLQTTNIKSF
EENVNDSKSQMNQIDMKGLDDGNINNIYFNAEAGAIGESHGINIICEIVDKQNTIENGLSL
GEKSTIPVQQGLPTSKSEITNQLSVSDINSQSVGGARPKQLFSLPSRTRSSKDLNKPDVPDTI
ESEPSTADTVVPITCAIDSTADPQVSFNSNYIDIESNSEGGSSFVTANEDSVPENTCKEGLVL
GQKQPTWVPDSEAPNCMNCQVKFTFTKRRHHCRACGKVFCGVCCNRKCKLQYLEKEAR
VCVVCYETISKAQAFERMMSPTGSNLKSNHSDECTTVQPPQENQTSSIPSPATLPVSALKQ
PGVEGLCSKEQKRVWFADGILPNGEVADTTKLSSGSKRCSEDFSPLSPDVPMTVNTVDHS
HSTTVEKPNNETGDITRNEIIQSPISQVPSVEKLSMNTGNEGLPTSGSFTLDDDVFAETEEPS
SPTGVLVNSNLPIASISDYRLLCDINKYVCNKISLLPNDEDSLPPLLVASGEKGSVPVVEEHP
SHEQIILLLEGESFHPVTFVLNANLLVNVKFIFYSSDKYWYFSTNGLHGLGQAEIIILLLCLP
NEDTIPKDIFRLFITIYKDALKGKYIENLDNITFTESFLSSKDHGGFLFITPTFQKLDDLSLPS
NPFLCGILIQKLEIPWAKVFPMRLMLRLGAEYKAYPAPLTSIRGRKPLFGEIGHTIMNLLVD
LRNYQYTLHNIDQLLIHMEMGKSCIKIPRKKYSDVMKVLNSSNEHVISIGASFSTEADSHL

VCIQNDGIYETQANSATGHPRKVTGASFVVFNGALKTSSGFLAKSSIVEDGLMVQITPETM
NGLRLALREQKDFKITCGKVDAVDLREYVDICWVDAEEKGNKGVISSVDGISLQGFPSEKI
KLEADFETDEKIVKCTEVFYFLKDQDLSILSTSYQFAKEIAMACSAALCPHLKTLKSNGMN
KIGLRVSIDTDMVEFQAGSEGQLLPQHYLNDLDSALIPVIHGGTSNSSLPLEIELVFFIIEHLF

>P15382 single-pass type I membrane protein
MILSNTTAVTPFLTKLWQETVQQGGNMSGLARRSPRSSDGKLEALYVLMVLGFFGFFTLGI
MLSYIRSKKLEHSNDPFNVYIESDAWQEKDKAYVQARVLESYRSCYVVENHLAIEQPNTH
LPETKPSP

>Q14849 multipass membrane protein
MSKLPRELTRDLERSLPAVASLGSSLSHSQSLSSHLLPPPEKRRAISDVRRTFCLFVTFDLLFI
SLLWIIELNTNTGIRKNLEQEIIQYNFKTSFFDIFVLAFFRFSGLLLGYAVLRLRHWWVIAVT
TLVSSAFLIVKVILSELLSKGAFGYLLPIVSFVLAWLETWFLDFKVLPQEAEEERWYLAAQ
VAVARGPLLFSGALSEGQFYSPPESFAGSDNESDEEVAGKKSFSAQEREYIRQGKEATAVVD
QILAQEENWKFEKNNEYGDTVYTIEVPFHGKTFILKTFLPCPAELVYQEVILQPERMVLWN
KTVTACQILQRVEDNTLISYDVSAGAAGGVVSPRDFVNVRRIERRRDRYLSSGIATSHSAK
PPTHKYVRGENGPGGFIVLKSASNPRVCTFVWILNTDLKGRLPRYLIHQSLAATMFEFAFH
LRQRISELGARA

>P29320 single-pass type I membrane protein
MDCQLSILLLLLSCSVLDSFGELIPQPSNEVNLLDSKTIQGELGWISYPSHGWEEISGVDEHY
TPIRTYQVCNVMDHSQNNWLRTNWVPRNSAQKIYVELKFTLRDCNSIPLVLGTCKETFNL
YYMESDDDHGVKFREHQFTKIDTIAADESFTQMDLGDRILKLNTEIREVGPVNKKGFYLA
FQDVGACVALVSVRVYFKKCPFTVKNLAMFPDTVPMDSQSLVEVRGSCVNNSKEEDPPR
MYCSTEGEWLVPIGKCSCNAGYEERGFMCQACRPGFYKALDGNMKCAKCPPHSSTQED
GSMNCRCENNYFRADKDPPSMACTRPPSSPRNVISNINETSVILDWSWPLDTGGRKDVTF
NIICKKCGWNIKQCEPCSPNVRFLPRQFGLTNTTVTVTDLLAHTNYTFEIDAVNGVSELSSP
PRQFAAVSITTNQAAPSPVLTIKKDRTSRNSISLSWQEPEHPNGIILDYEVKYYEKQEQETSY
TILRARGTNVTISSLKPDTIYVFQIRARTAAGYGTNSRKFEFETSPDSFSISGESSQVVMIAIS
AAVAIILLTVVIYVLIGRFCGYKSKHGADEKRLHFGNGHLKLPGLRTYVDPHTYEDPTQAV
HEFAKELDATNISIDKVVGAGEFGEVCSGRLKLPSKKEISVAIKTLKVGYTEKQRRDFLGE
ASIMGQFDHPNIIRLEGVVTKSKPVMIVTEYMENGSLDSFLRKHDAQFTVIQLVGMLRGIA
SGMKYLSDMGYVHRDLAARNILINSNLVCKVSDFGLSRVLEDDPEAAYTTRGGKIPIRWT
SPEAIAYRKFTSASDVWSYGIVLWEVMSYGERPYWEMSNQDVIKAVDEGYRLPPPMDCP
AALYQLMLDCWQKDRNNRPKFEQIVSILDKLIRNPGSLKIITSAAARPSNLLLDQSNVDITT
FRTTGDWLNGVWTAHCKEIFTGVEYSSCDTIAKISTDDMKKVGVTVVGPQKKIISSIKALE
TQSKNGPVPV

>Q96AD5 single-pass type II membrane protein
MFPREKTWNISFAGCGFLGVYYVGVASCLREHAPFLVANATHIYGASAGALTATALVTGV
CLGEAGAKFIEVSKEARKRFLGPLHPSFNLVKIIRSFLLKVLPADSHEHASGRLGISLTRVSD
GENVIISHFNSKDELIQANVCSGFIPVYCGLIPPSLQGVRYVDGGISDNLPLYELKNTITVSP
FSGESDICPQDSSTNIHELRVTNTSIQFNLRNLYRLSKALFPPEPLVLREMCKQGYRDGLRF

LQRNGLLNRPNPLLALPPARPHGPEDKDQAVESAQAEDYSQLPGEDHILEHLPARLNEALL
EACVEPTDLLTTLSNMLPVRLATAMMVPYTLPLESALSFTIRLLEWLPDVPEDIRWMKEQT
GSICQYLVMRAKRKLGRHLPSRLPEQVELRRVQSLPSVPLSCAAYREALPGWMRNNLSLG
DALAKWEECQRQLLLGLFCTNVAFPPEALRMRAPADPAPAPADPASPQHQLAGPAPLLSTP
APEARPVIGALGL

>Q9H0F7 peripheral membrane protein
MGLLDRLSVLLGLKKKEVHVLCLGLDNSGKTTIINKLKPSNAQSQNILPTIGFSIEKFKSSS
LSFTVFDMSGQGRYRNLWEHYYKEGQAIIFVIDSSDRLRMVVAKEELDTLLNHPDIKHRRI
PILFFANKMDLRDAVTSVKVSQLLCLENIKDKPWHICASDAIKGEGLQEGVDWLQDQIQT
VKT

>P51159 lipid-anchor
MSDGDYDYLIKFLALGDSGVGKTSVLYQYTDGKFNSKFITTVGIDFREKRVVYRASGPDG
ATGRGQRIHLQLWDTAGQERFRSLTTAFFRDAMGFLLLFDLTNEQSFLNVRNWISQLQMH
AYCENPDIVLCGNKSDLEDQRVVKEEEAIALAEKYGIPYFETSAANGTNISQAIEMLLDLI
MKRMERCVDKSWIPEGVVRSNGHASTDQLSEEKEKGACGC

>Q9NPA2 GPI-anchor, lipid-anchor
MRLRLRLLALLLLLLAPPARAPKPSAQDVSLGVDWLTRYGYLPPPHPAQAQLQSPEKLRD
AIKVMQRFAGLPETGRMDPGTVATMRKPRCSLPDVLGVAGLVRRRRRYALSGSVWKKRT
LTWRVRSFPQSSQLSQETVRVLMSYALMAWGMESGLTFHEVDSPQGQEPDILIDFARAFH
QDSYPFDGLGGTLAHAFFPGEHPISGDTHFDDEETWTFGSKDGEGTDLFAVAVHEFGHAL
GLGHSSAPNSIMRPFYQGPVGDPDKYRLSQDDRDGLQQLYGKAPQTPYDKPTRKPLAPPP
QPPASPTHSPSFPIPDRCEGNFDAIANIRGETFFFKGPWFWRLQPSGQLVSPRPARLHRFWE
GLPAQVRVVQAAYARHRDGRILLFSGPQFWVFQDRQLEGGARPLTELGLPPGEEVDAVFS
WPQNGKTYLVRGRQYWRYDEAAARPDPGYPRDLSLWEGAPPSPDDVTVSNAGDTYFFK
GAHYWRFPKNSIKTEPDAPQPMGPNWLDCPAPSSGPRAPRPPKATPVSETCDCQCELNQA
AGRWPAPIPLLLLPLLVGGVASR

>P06239 lipid-anchor
MGCGCSSHPEDDWMENIDVCENCHYPIVPLDGKGTLLIRNGSEVRDPLVTYEGSNPPASP
LQDNLVIALHSYEPSHDGDLGFEKGEQLRILEQSGEWWKAQSLTTGQEGFIPFNFVAKANS
LEPEPWFFKNLSRKDAERQLLAPGNTHGSFLIRESESTAGSFSLSVRDFDQNQGEVVKHYK
IRNLDNGGFYISPRITFPGLHELVRHYTNASDGLCTRLSRPCQTQKPQKPWWEDEWEVPR
ETLKLVERLGAGQFGEVWMGYYNGHTKVAVKSLKQGSMSPDAFLAEANLMKQLQHQRL
VRLYAVVTQEPIYIITEYMENGSLVDFLKTPSGIKLTINKLLDMAAQIAEGMAFIEERNYIHR
DLRAANILVSDTLSCKIADFGLARLIEDNEYTAREGAKFPIKWTAPEAINYGTFTIKSDVWS
FGILLTEIVTHGRIPYPGMTNPEVIQNLERGYRMVRPDNCPEELYQLMRLCWKERPEDRPT
FDYLRSVLEDFFTATEGQYQPQP

>Q9NQC3 multipass membrane protein
MEDLDQSPLVSSSDSPPRPQPAFKYQFVREPEDEEEEEEEEEDEDEDLEELEVLERKPAAG
LSAAPVPTAPAAGAPLMDFGNDFVPPAPRGPLPAAPPVAPERQPSWDPSPVSSTVPAPSPLS

AAAVSPSKLPEDDEPPARPPPPPPASVSPQAEPVWTPPAPAPAAPPSTPAAPKRRGSSGSVDE
TLFALPAASEPVIRSSAENMDLKEQPGNTISAGQEDFPSVLLETAASLPSLSPLSAASFKEHE
YLGNLSTVLPTEGTLQENVSEASKEVSEKAKTLLIDRDLTEFSELEYSEMGSSFSVSPKAES
AVIVANPREEIIVKNKDEEEKLVSNNILHNQQELPTALTKLVKEDEVVSSEKAKDSFNEKRV
AVEAPMREEYADFKPFERVWEVKDSKEDSDMLAAGGKIESNLESKVDKKCFADSLEQTN
HEKDSESSNDDTSFPSTPEGIKDRSGAYITCAPFNPAATESIATNIFPLLGDPTSENKTDEKKI
EEKKAQIVTEKNTSTKTSNPFLVAAQDSETDYVTTDNLTKVTEEVVANMPEGLTPDLVQE
ACESELNEVTGTKIAYETKMDLVQTSEVMQESLYPAAQLCPSFEESEATPSPVLPDIVMEAP
LNSAVPSAGASVIQPSSSPLEASSVNYESIKHEPENPPPYEEAMSVSLKKVSGIKEEIKEPENI
NAALQETEAPYISIACDLIKETKLSAEPAPDFSDYSEMAKVEQPVPDHSELVEDSSPDSEPV
DLFSDDSIPDVPQKQDETVMLVKESLTETSFESMIEYENKEKLSALPPEGGKPYLESFKLSL
DNTKDTLLPDEVSTLSKKEKIPLQMEELSTAVYSNDDLFISKEAQIRETETFSDSSPIEIIDEF
PTLISSKTDSFSKLAREYTDLEVSHKSEIANAPDGAGSLPCTELPHDLSLKNIQPKVEEKISF
SDDFSKNGSATSKVLLLPPDVSALATQAEIESIVKPKVLVKEAEKKLPSDTEKEDRSPSAIFS
AELSKTSVVDLLYWRDIKKTGVVFGASLFLLLSLTVFSIVSVTAYIALALLSVTISFRIYKGV
IQAIQKSDEGHPFRAYLESEVAISEELVQKYSNSALGHVNCTIKELRRLFLVDDLVDSLKFA
VLMWVFTYVGALFNGLTLLILALISLFSVPVIYERHQAQIDHYLGLANKNVKDAMAKIQA
KIPGLKRKAE

>Q5T2T1 peripheral membrane protein
MPALSTGSGSDTGLYELLAALPAQLQPHVDSQEDLTFLWDMFGEKSLHSLVKIHEKLHYY
EKQSPVPILHGAAALADDLAEELQNKPLNSEIRELLKLLSKPNVKALLSVHDTVAQKNYD
PVLPPMPEDIDDEEDSVKIIRLVKNREPLGATIKKDEQTGAIIVARIMRGGAADRSGLIHVG
DELREVNGIPVEDKRPEEIIQILAQSQGAITFKIIPGSKEETPSKEGKMFIKALFDYNPNEDK
AIPCKEAGLSFKKGDILQIMSQDDATWWQAKHEADANPRAGLIPSKHFQERRLALRRPEIL
VQPLKVSNRKSSGFRKSFRLSRKDKKTNKSMYECKKSDQYDTADVPTYEEVTPYRRQTN
EKYRLVVLVGPVGVGLNELKRKLLISDTQHYGVTVPHTTRARRSQESDGVEYIFISKHLFE
TDVQNNKFIEYGEYKNNYYGTSIDSVRSVLAKNKVCLLDVQPHTVKHLRTLEFKPYVIFI
KPPSIERLRETRKNAKIISSRDDQGAAKPFTEEDFQEMIKSAQIMESQYGHLFDKIIINDDLT
VAFNELKTTFDKLETETHWVPVSWLHS

>P34969 multipass membrane protein
MMDVNSSGRPDLYGHLRSFLLPEVGRGLPDLSPDGGADPVAGSWAPHLLSEVTASPAPTW
DAPPDNASGCGEQINYGRVEKVVIGSILTLITLLTIAGNCLVVISVCFVKKLRQPSNYLIVSL
ALADLSVAVAVMPFVSVTDLIGGKWIFGHFFCNVFIAMDVMCCTASIMTLCVISIDRYLGIT
RPLTYPVRQNGKCMAKMILSVWLLSASITLPPLFGWAQNVNDDKVCLISQDFGYTIYSTAV
AFYIPMSVMLFMYYQIYKAARKSAAKHKFPGFPRVEPDSVIALNGIVKLQKEVEECANLS
RLLKHERKNISIFKREQKAATTLGIIVGAFTVCWLPFFLLSTARPFICGTSCSCIPLWVERTFL
WLGYANSLINPFIYAFFNRDLRTTYRSLLQCQYRNINRKLSAAGMHEALKLAERPERPEFV
LRACTRRVLLRPEKRPPVSVWVLQSPDHHNWLADKMLTTVEKKVMIHD

>Q9HBE5 single-pass type I membrane protein
MPRGWAAPLLLLLLQGGWGCPDLVCYTDYLQTVICILEMWNLHPSTLTLTWQDQYEELK
DEATSCSLHRSAHNATHATYTCHMDVFHFMADDIFSVNITDQSGNYSQECGSFLLAESIKP

APPFNVTVTFSGQYNISWRSDYEDPAFYMLKGKLQYELQYRNRGDPWAVSPRRKLISVDS
RSVSLLPLEFRKDSSYELQVRAGPMPGSSYQGTWSEWSDPVIFQTQSEELKEGWNPHLLL
LLLLVIVFIPAFWSLKTHPLWRLWKKIWAVPSPERFFMPLYKGCSGDFKKWVGAPFTGSSL
ELGPWSPEVPSTLEVYSCHPPRSPAKRLQLTELQEPAELVESDGVPKPSFWPTAQNSGGSA
YSEERDRPYGLVSIDTVTVLDAEGPCTWPCSCEDDGYPALDLDAGLEPSPGLEDPLLDAG
TTVLSCGCVSAGSPGLGGPLGSLLDRLKPPLADGEDWAGGLPWGGRSPGGVSESEAGSPL
AGLDMDTFDSGFVGSDCSSPVECDFTSPGDEGPPRSYLRQWVVIPPPLSSPGPQAS

>P30518 multipass membrane protein
MLMASTTSAVPGHPSLPSLPSNSSQERPLDTRDPLLARAELALLSIVFVAVALSNGLVLAAL
ARRGRRGHWAPIHVFIGHLCLADLAVALFQVLPQLAWKATDRFRGPDALCRAVKYLQMV
GMYASSYMILAMTLDRHRAICRPMLAYRHGSGAHWNRPVLVAWAFSLLLSLPQLFIFAQR
NVEGGSGVTDCWACFAEPWGRRTYVTWIALMVFVAPTLGIAACQVLIFREIHASLVPGPSE
RPGGRRRGRRTGSPGEGAHVSAAVAKTVRMTLVIVVVYVLCWAPFFLVQLWAAWDPEAP
LEGAPFVLLMLLASLNSCTNPWIYASFSSSVSSELRSLLCCARGRTPPSLGPQDESCTTASSS
LAKDTSS

>Q13241 single-pass type II membrane protein
MAVFKTTLWRLISGTLGIICLSLMSTLGILLKNSFTKLSIEPAFTPGPNIELQKDSDCCSCQE
KWVGYRCNCYFISSEQKTWNESRHLCASQKSSLLQLQNTDELDFMSSSQQFYWIGLSYSE
EHTAWLWENGSALSQYLFPSFETFNTKNCIAYNPNGNALDESCEDKNRYICKQQLI

>Q13425 peripheral membrane protein
MRVAAATAAAGAGPAMAVWTRATKAGLVELLLRERWVRVVAELSGESLSLTGDAAAAEL
EPALGPAAAAFNGLPNGGGAGDSLPGSPSRGLGPPSPPAPPRGPAGEAGASPPVRRVRVVK
QEAGGLGISIKGGRENRMPILISKIFPGLAADQSRALRLGDAILSVNGTDLRQATHDQAVQ
ALKRAGKEVLLEVKFIREVTPYIKKPSLVSDLPWEGAAPQSPSFSGSEDSGSPKHQNSTKD
RKIIPLKMCFAARNLSMPDLENRLIELHSPDSRNTLILRCKDTATAHSWFVAIHTNIMALLP
QVLAELNAMLGATSTAGGSKEVKHIAWLAEQAKLDGGRQQWRPVLMAVTEKDLLLYDC
MPWTRDAWASPCHSYPLVATRLVHSGSGCRSPSLGSDLTFATRTGSRQGIEMHLFRVETHR
DLSSWTRILVQGCHAAAELIKEVSLGCMLNGQEVRLTIHYENGFTISRENGGSSSILYRYPF
ERLKMSADDGIRNLYLDFGGPEGELTMDLHSCPKPIVFVLHTFLSAKVTRMGLLV

>Q9H3Q1 peripheral membrane protein
MPILKQLVSSSVHSKRRSRADLTAEMISAPLGDFRHTMHVGRAGDAFGDTSFLNSKAGEP
DGESLDEQPSSSSSKRSLLSRKFRGSKRSQSVTRGEREQRDMLGSLRDSALFVKNAMSLP
QLNEKEAAEKGTSKLPKSLSSSPVKKANDGEGGDEEAGTEEAVPRRNGAAGPHSPDPLLD
EQAFGDLTDLPVVPKATYGLKHAESIMSFHIDLGPSMLGDVLSIMDKEEWDPEEGEGGYH
GDEGAAGTITQAPPYAVAAPPLARQEGKAGPDLPSLPSHALEDEGWAAAAPSPGSARSMG
SHTTRDSSSLSSCTSGILEERSPAFRGPDRARAAVSRQPDKEFSFMDEEEEDEIRV

>Q92629 single-pass type II membrane protein
MPQEQYTHHRSTMPGSVGPQVYKVGIYGWRKRCLYFFVLLLMILILVNLAMTIWILKVM
NFTIDGMGNLRITEKGLKLEGDSEFLQPLYAKEIQSRPGNALYFKSARNVTVNILNDQTKV

LTQLITGPKAVEAYGKKFEVKTVSGKLLFSADNNEVVVGAERLRVLGAEGTVFPKSIETPN
VRADPFKELRLESPTRSLVMEAPKGVEINAEAGNMEATCRTELRLESKDGEIKLDAAKIRL
PRLPHGSYTPTGTRQKVFEICVCANGRLFLSQAGAGSTCQINTSVCL

>P00387 lipid-anchor
MGAQLSTLGHMVLFPVWFLYSLLMKLFQRSTPAITLESPDIKYPLRLIDREIISHDTRRFRF
ALPSPQHILGLPVGQHIYLSARIDGNLVVRPYTPISSDDDKGFVDLVIKVYFKDTHPKFPAG
GKMSQYLESMQIGDTIEFRGPSGLLVYQGKGKFAIRPDKKSNPIIRTVKSVGMIAGGTGITP
MLQVIRAIMKDPDDHTVCHLLFANQTEKDILLRPELEELRNKHSARFKLWYTLDRAPEAW
DYGQGFVNEEMIRDHLPPPEEEPLVLMCGPPPMIQYACLPNLDHVGHPTERCFVF

>Q9GZN6 multipass membrane protein
MKTEAQPSTSLLANTSWTGTVISDSVPGSQTWEDKGSLTRSATSWTSEAQVSAARVAEAQ
ARTSQPKQISVLEALTASALNQKPTHEKVQMTEKKESEVLLARPFWSSKTEYILAQVGFS
MKPSCLWRFAYLWLNSGGCSFAAIYIFMLFLVGVPLLFLEMAAGQSMRQGGMGVWKIIA
PWIGGVGYSSFMVCFILGLYFNVVNSWIIFYMSQSFQFPVPWEKCPLTMNSSGFDPECERT
TPSIYFWYQQALKASDRIEDGGSPVYSLVLPFFLCWCLVGAFMINGLKSTGKVIYVLVLLP
CFIIVGFFIRTLLLEGAKFGLQQLVVAKISDVYNMSVWSLAGGQVLSNTGIGLGSVASLASY
MPQSNNCLSDAFLVSVINLLTLLVFTSFNFCVLGFWATVITHRCCERNAEILLKLINLGKLPP
DAKPPVNLLYNPTSIYNAWLSGLPQHIKSMVLREVTECNIETQFLKASEGPKFAFLSFVEA
MSFLPPSVFWSFIFFLMLLAMGLSSAIGIMQGIITPLQDTFSFFRKHTKLLIVGVFLLMFVCG
LFFTRPSGSYFIRLLSDYWIVFPIIVVVVFETMAVSWAYGARRFLADLTILLGHPISPIFGWL
WPHLCPVVLLIIFVTMMVHLCMKPITYMSWDSSTSKEVLRPYPPWALLLMITLFAIVILPIP
AYFVYCRIHRIPFRPKSGDGPMTASTSLPLSHQLTPSKEVQKEEILQVDETKYPSTCNVTS

>Q96JQ5 multipass membrane protein
MHQTYSRHCRPEESTFSAAMTTMQGMEQAMPGAGPGVPQLGNMAVIHSHLWKGLQEKF
LKGEPKVLGVVQILTALMSLSMGITMMCMASNTYGSNPISVYIGYTIWGSVMFIISGSLSIA
AGIRTTKGLVRGSLGMNITSSVLAASGILINTFSLAFYSFHHPYCNYYGNSNNCHGTMSIL
MGLDGMVLLLSVLEFCIAVSLSAFGCKVLCCTPGGVVLILPSHSHMAETASPTPLNEV

>Q9Y3M8 peripheral membrane protein
MFSQVPRTPASGCYYLNSMTPEGQEMYLRFDQTTRRSPYRMSRILARHQLVTKIQQEIEA
KEACDWLRAAGFPQYAQLYEDSQFPINIVAVKNDHDFLEKDLVEPLCRRLNTLNKCASMK
LDVNFQRKKGDDSDEEDLCISNKWTFQRTSRRWSRVDDLYTLLPRGDRNGSPGGTGMRN
TTSSESVLTDLSEPEVCSIHSESSGGSDSRSQPGQCCTDNPVMLDAPLVSSSLPQPPRDVLN
HPFHPKNEKPTRARAKSFLKRMETLRGKGAHGRHKGSGRTGGLVISGPMLQQEPESFKA
MQCIQIPNGDLQNSPPPACRKGLPCSGKSSGESSPSEHSSSGVSTPCLKERKCHEANKRGG
MYLEDLDVLAGTALPDAGDQSRMHEFHSQENLVVHIPKDHKPGTFPKALSIESLSPTDSSN
GVNWRTGSISLGREQVPGAREPRLMASCHRASRVSIYDNVPGSHLYASTGDLLDLEKDDL
FPHLDDILQHVNGLQEVVDDWSKDVLPELQTHDTLVGEPGLSTFPSPNQITLDFEGNSVSE
GRTTPSDVERDVTSLNESEPPGVRDRRDSGVGASLTRPNRRLRWNSFQLSHQPRPAPASPH
ISSQTASQLSLLQRFSLLRLTAIMEKHSMSNKHGWTWSVPKFMKRMKVPDYKDKAVFGV
PLIVHVQRTGQPLPQSIQQALRYLRSNCLDQVGLFRKSGVKSRIHALRQMNENFPENVNY

EDQSAYDVADMVKQFFRDLPEPLFTNKLSETFLHIYQYVSKEQRLQAVQAAILLLADENR
EVLQTLLCFLNDVVNLVEENQMTPMNLAVCLAPSLFHLNLLKKESSPRVIQKKYATGKPD
QKDLNENLAAAQGLAHMIMECDRLFEVPHELVAQSRNSYVEAEIHVPTLEELGTQLEESG
ATFHTYLNHLIQGLQKEAKEKFKGWVTCSSTDNTDLAFKKVGDGNPLKLWKASVEVEAP
PSVVLNRVLRERHLWDEDFVQWKVVETLDRQTEIYQYVLNSMAPHPSRDFVVLRTWKT
DLPKGMCTLVSLSVEHEEAQLLGGVRAVVMDSQYLIEPCGSGKSRLTHICRIDLKGHSPE
WYSKGFGHLCAAEVARIRNSFQPLIAEGPETKI

>P40189 single-pass type I membrane protein
MLTLQTWLVQALFIFLTTESTGELLDPCGYISPESPVVQLHSNFTAVCVLKEKCMDYFHVN
ANYIVWKTNHFTIPKEQYTIINRTASSVTFTDIASLNIQLTCNILTFGQLEQNVYGITIISGLPP
EKPKNLSCIVNEGKKMRCEWDGGRETHLETNFTLKSEWATHKFADCKAKRDTPTSCTVD
YSTVYFVNIEVWVEAENALGKVTSDHINFDPVYKVKPNPPHNLSVINSEELSSILKLTWTN
PSIKSVIILKYNIQYRTKDASTWSQIPPEDTASTRSSFTVQDLKPFTEYVFRIRCMKEDGKG
YWSDWSEEASGITYEDRPSKAPSFWYKIDPSHTQGYRTVQLVWKTLPPFEANGKILDYEV
TLTRWKSHLQNYTVNATKLTVNLTNDRYLATLTVRNLVGKSDAAVLTIPACDFQATHPVM
DLKAFPKDNMLWVEWTTPRESVKKYILEWCVLSDKAPCITDWQQEDGTVHRTYLRGNL
AESKCYLITVTPVYADGPGSPESIKAYLKQAPPSKGPTVRTKKVGKNEAVLEWDQLPVDV
QNGFIRNYTIFYRTIIGNETAVNVDSSHTEYTLSSLTSDTLYMVRMAAYTDEGGKDGPEFTF
TTPKFAQGEIEAIVVPVCLAFLLTTLLGVLFCFNKRDLIKKHIWPNVPDPSKSHIAQWSPHT
PPRHNFNSKDQMYSDGNFTDVSVVEIEANDKKPFPEDLKSLDLFKKEKINTEGHSSGIGGS
SCMSSSRPSISSSDENESSQNTSSTVQYSTVVHSGYRHQVPSVQVFSRSESTQPLLDSEERP
EDLQLVDHVDGGDGILPRQQYFKQNCSQHESSPDISHFERSKQVSSVNEEDFVRLKQQISD
HISQSCGSGQMKMFQEVSAADAFGPGTEGQVERFETVGMEAATDEGMPKSYLPQTVRQ
GGYMPQ

>Q7LDG7 peripheral membrane protein
MAGTLDLDKGCTVEELLRGCIEAFDDSGKVRDPQLVRMFLMMHPWYIPSSQLAAKLLHI
YQQSRKDNSNSLQVKTCHLVRYWISAFPAEFDLNPELAEQIKELKALLDQEGNRRHSSLID
IDSVPTYKWKRQVTQRNPVGQKKRKMSLLFDHLEPMELAEHLTYLEYRSFCKILFQDYHS
FVTHGCTVDNPVLERFISLFNSVSQWVQLMILSKPTAPQRALVITHFVHVAEKLLQLQNFN
TLMAVVGGLSHSSISRLKETHSHVSPETIKLWEGLTELVTATGNYGNYRRRLAACVGFRFPI
LGVHLKDLVALQLALPDWLDPARTRLNGAKMKQLFSILEELAMVTSLRPPVQANPDLLSL
LTVSLDQYQTEDELYQLSLQREPRSKSSPTSPTSCTPPPRPPVLEEWTSAAKPKLDQALVVE
HIEKMVESVFRNFDVDGDGHISQEEFQIIRGNFPYLSAFGDLDQNQDGCISREEMVSYFLR
SSSVLGGRMGFVHNFQESNSLRPVACRHCKALILGIYKQGLKCRACGVNCHKQCKDRLS
VECRRRAQSVSLEGSAPSPSPMHSHHHRAFSFSLPRPGRRGSRPPEIREEEVQTVEDGVFDI
HL

>P15311 peripheral membrane protein
MPKPINVRVTTMDAELEFAIQPNTTGKQLFDQVVKTIGLREVWYFGLHYVDNKGFPTWL
KLDKKVSAQEVRKENPLQFKFRAKFYPEDVAEELIQDITQKLFFLQVKEGILSDEIYCPPET
AVLLGSYAVQAKFGDYNKEVHKSGYLSSERLIPQRVMDQHKLTRDQWEDRIQVWHAEHR
GMLKDNAMLEYLKIAQDLEMYGINYFEIKNKKGTDLWLGVDALGLNIYEKDDKLTPKIG

FPWSEIRNISFNDKKFVIKPIDKKAPDFVFYAPRLRINKRILQLCMGNHELYMRRRKPDTIE
VQQMKAQAREEKHQKQLERQQLETEKKRRETVEREKEQMMREKEELMLRLQDYEEKT
KKAERELSEQIQRALQLEEERKRAQEEAERLEADRMAALRAKEELERQAVDQIKSQEQLA
AELAEYTAKIALLEEARRRKEDEVEEWQHRAKEAQDDLVKTKEELHLVMTAPPPPPPPVY
EPVSYHVQESLQDEGAEPTGYSAELSSEGIRDDRNEEKRITEAEKNERVQRQLLTLSSELSQ
ARDENKRTHNDIIHNENMRQGRDKYKTLRQIRQGNTKQRIDEFEAL

>P15941 single-pass type I membrane protein
MTPGTQSPFFLLLLLTVLTVVTGSGHASSTPGGEKETSATQRSSVPSSTEKNAVSMTSSVLS
SHSPGSGSSTTQGQDVTLAPATEPASGSAATWGQDVTSVPVTRPALGSTTPPAHDVTSAPD
NKPAPGSTAPPAHGVTSAPDTRPAPGSTAPPAHGVTSAPDTRPAPGSTAPPAHGVTSAPDTR
PAPGSTAPPAHGVTSAPDTRPAPGSTAPPAHGVTSAPDTRPAPGSTAPPAHGVTSAPDTRPA
PGSTAPPAHGVTSAPDTRPAPGSTAPPAHGVTSAPDTRPAPGSTAPPAHGVTSAPDTRPAPG
STAPPAHGVTSAPDTRPAPGSTAPPAHGVTSAPDTRPAPGSTAPPAHGVTSAPDTRPAPGST
APPAHGVTSAPDTRPAPGSTAPPAHGVTSAPDTRPAPGSTAPPAHGVTSAPDTRPAPGSTAP
PAHGVTSAPDTRPAPGSTAPPAHGVTSAPDTRPAPGSTAPPAHGVTSAPDTRPAPGSTAPPA
HGVTSAPDTRPAPGSTAPPAHGVTSAPDTRPAPGSTAPPAHGVTSAPDTRPAPGSTAPPAHG
VTSAPDTRPAPGSTAPPAHGVTSAPDTRPAPGSTAPPAHGVTSAPDTRPAPGSTAPPAHGVT
SAPDTRPAPGSTAPPAHGVTSAPDTRPAPGSTAPPAHGVTSAPDTRPAPGSTAPPAHGVTSA
PDTRPAPGSTAPPAHGVTSAPDTRPAPGSTAPPAHGVTSAPDTRPAPGSTAPPAHGVTSAPD
TRPAPGSTAPPAHGVTSAPDTRPAPGSTAPPAHGVTSAPDTRPAPGSTAPPAHGVTSAPDTR
PAPGSTAPPAHGVTSAPDTRPAPGSTAPPAHGVTSAPDTRPAPGSTAPPAHGVTSAPDTRPA
PGSTAPPAHGVTSAPDTRPAPGSTAPPAHGVTSAPDTRPAPGSTAPPAHGVTSAPDTRPAPG
STAPPAHGVTSAPDNRPALGSTAPPVHNVTSASGSASGSASTLVHNGTSARATTTPASKSTP
FSIPSHHSDTPTTLASHSTKTDASSTHHSSVPPLTSSNHSTSPQLSTGVSFFFLSFHISNLQFN
SSLEDPSTDYYQELQRDISEMFLQIYKQGGFLGLSNIKFRPGSVVVQLTLAFREGTINVHD
VETQFNQYKTEAASRYNLTISDVSVSDVPFPFSAQSGAGVPGWGIALLVLVCVLVALAIVY
LIALAVCQCRRKNYGQLDIFPARDTYHPMSEYPTYHTHGRYVPPSSTDRSPYEKVSAGNG
GSSLSYTNPAVAATSANL

>Q7Z444 lipid-anchor
MELPTKPGTFDLGLATWSPSFQGETHRAQARRRDVGRQLPEYKAVVVGASGVGKSALTI
QLNHQCFVEDHDPTIQDSYWKELTLDSGDCILNVLDTAGQAIHRALRDQCLAVCDGVLG
VFALDDPSSLIQLQQIWATWGPHPAQPLVLVGNKCDLVTTAGDAHAAAAALAHSWGAHF
VETSAKTRQGVEEAFSLLVHEIQRVQEAMAKEPMARSCREKTRHQKATCHCGCSVA

>P14384 GPI-anchor, lipid-anchor
MDFPCLWLGLLLPLVAALDFNYHRQEGMEAFLKTVAQNYSSVTHLHSIGKSVKGRNLWV
LVVGRFPKEHRIGIPEFKYVANMHGDETVGRELLLHLIDYLVTSDGKDPEITNLINSTRIHI
MPSMNPDGFEAVKKPDCYYSIGRENYNQYDLNRNFPDAFEYNNVSRQPETVAVMKWLK
TETFVLSANLHGGALVASYPFDNGVQATGALYSRSLTPDDDVFQYLAHTYASRNPNMKK
GDECKNKMNFPNGVTNGYSWYPLQGGMQDYNYIWAQCFEITLELSCCKYPREEKLPSF
WNNNKASLIEYIKQVHLGVKGQVFDQNGNPLPNVIVEVQDRKHICPYRTNKYGEYYLLL
LPGSYIINVTVPGHDPHITKVIIPEKSQNFSALKKDILLPFQGQLDSIPVSNPSCPMIPLYRNL

PDHSAATKPSLFLFLVSLLHIFFK

>Q03135 peripheral membrane protein
MSGGKYVDSEGHLYTVPIREQGNIYKPNNKAMADELSEKQVYDAHTKEIDLVNRDPKHL
NDDVVKIDFEDVIAEPEGTHSFDGIWKASFTTFTVTKYWFYRLLSALFGIPMALIWGIYFAI
LSFLHIWAVVPCIKSFLIEIQCISRVYSIYVHTVCDPLFEAVGKIFSNVRINLQKEI

>Q9NSD5 multipass membrane protein
MDSRVSGTTSNGETKPVYPVMEKKEEDGTLERGHWNNKMEFVLSVAGEIIGLGNVWRFP
YLCYKNGGGAFFIPYLVFLFTCGIPVFLLETALGQYTSQGGVTAWRKICPIFEGIGYASQMI
VILLNVYYIIVLAWALFYLFSSFTIDLPWGGCYHEWNTEHCMEFQKTNGSLNGTSENATSP
VIEFWERRVLKISDGIQHLGALRWELALCLLLAWVICYFCIWKGVKSTGKVVYFTATFPYL
MLVVLLIRGVTLPGAAQGIQFYLYPNLTRLWDPQVWMDAGTQIFFSFAICLGCLTALGSYN
KYHNNCYRDCIALCFLNSGTSFVAGFAIFSILGFMSQEQGVPISEVAESGPGLAFIAYPRAVV
MLPFSPLWACCFFFMVVLLGLDSQFVCVESLVTALVDMYPHVFRKKNRREVLILGVSVVS
FLVGLIMLTEGGMYVFQLFDYYAASGMCLLFVAIFESLCVAWVYGAKRFYDNIEDMIGYR
PWPLIKYCWLFLTPAVCTATFLFSLIKYTPLTYNKKYTYPWWGDALGWLLALSSMVCIPA
WSLYRLGTLKGPFRERIRQLMCPAEDLPQRNPAGPSAPATPRTSLLRLTELESHC

>O95500 multipass membrane protein
MASTAVQLLGFLLSFLGMVGTLITTILPHWRRTAHVGTNILTAVSYLKGLWMECVWHSTGI
YQCQIYRSLLALPQDLQAARALMVISCLLSGIACACAVIGMKCTRCAKGTPAKTTFAILGG
TLFILAGLLCMVAVSWTTNDVVQNFYNPLLPSGMKFEIGQALYLGFISSSLSLIGGTLLCLS
CQDEAPYRPYQAPPRATTTTANTAPAYQPPAAYKDNRAPSVTSATHSGYRLNDYV

>Q03405 GPI-anchor, lipid-anchor
MGHPPLLPLLLLLHTCVPASWGLRCMQCKTNGDCRVEECALGQDLCRTTIVRLWEEGEE
LELVEKSCTHSEKTNRTLSYRTGLKITSLTEVVCGLDLCNQGNSGRAVTYSRSRYLECISCG
SSDMSCERGRHQSLQCRSPEEQCLDVVTHWIQEGEEGRPKDDRHLRGCGYLPGCPGSNG
FHNNDTFHFLKCCNTTKCNEGPILELENLPQNGRQCYSCKGNSTHGCSSEETFLIDCRGPM
NQCLVATGTHEPKNQSYMVRGCATASMCQHAHLGDAFSMNHIDVSCCTKSGCNHPDLD
VQYRSGAAPQPGPAHLSLTITLLMTARLWGGTLLWT

>Q15761 multipass membrane protein
MDLELDEYYNKTLATENNTAATRNSDFPVWDDYKSSVDDLQYFLIGLYTFVSLLGFMGN
LLILMALMKKRNQKTTVNFLIGNLAFSDILVVLFCSPFTLTSVLLDQWMFGKVMCHIMPF
LQCVSVLVSTLILISIAIVRYHMIKHPISNNLTANHGYFLIATVWTLGFAICSPLPVFHSLVEL
QETFGSALLSSRYLCVESWPSDSYRIAFTISLLLVQYILPLVCLTVSHTSVCRSISCGLSNKE
NRLEENEMINLTLHPSKKSGPQVKLSGSHKWSYSFIKKHRRRYSKKTACVLPAPERPSQEN
HSRILPENFGSVRSQLSSSSKFIPGVPTCFEIKPEENSDVHELRVKRSVTRIKKRSRSVFYRLT
ILILVFAVSWMPLHLFHVVTDFNDNLISNRHFKLVYCICHLLGMMSCCLNPILYGFLNNGIK
ADLVSLIHCLHM

>P51684 multipass membrane protein

MSGESMNFSDVFDSSEDYFVSVNTSYYSVDSEMLLCSLQEVRQFSRLFVPIAYSLICVFGL
LGNILVVITFAFYKKARSMTDVYLLNMAIADILFVLTLPFWAVSHATGAWVFSNATCKLLK
GIYAINFNCGMLLLTCISMDRYIAIVQATKSFRLRSRTLPRSKIICLVVWGLSVIISSSTFVFN
QKYNTQGSDVCEPKYQTVSEPIRWKLLMLGLELLFGFFIPLMFMIFCYTFIVKTLVQAQNS
KRHKAIRVIIAVVLVFLACQIPHNMVLLVTAANLGKMNRSCQSEKLIGYTKTVTEVLAFLH
CCLNPVLYAFIGQKFRNYFLKILKDLWCVRRKYKSSGFSCAGRYSENISRQTSETADNDNA
SSFTM

>Q99706 single-pass type I membrane protein
MSMSPTVIILACLGFFLDQSVWAHVGGQDKPFCSAWPSAVVPQGGHVTLRCHYRRGFNIF
TLYKKDGVPVPELYNRIFWNSFLISPVTPAHAGTYRCRGFHPHSPTEWSAPSNPLVIMVTGL
YEKPSLTARPGPTVRAGENVTLSCSSQSSFDIYHLSREGEAHELRLPAVPSINGTFQADFPL
GPATHGETYRCFGSFHGSPYEWSDPSDPLPVSVTGNPSSSWPSPTEPSFKTGIARHLHAVIR
YSVAIILFTILPFFLLHRWCSKKKDAAVMNQEPAGHRTVNREDSDEQDPQEVTYAQLDHCI
FTQRKITGPSQRSKRPSTDTSVCIELPNAEPRALSPAHEHHSQALMGSSRETTALSQTQLAS
SNVPAAGI

>O00483 peripheral membrane protein
MLRQIIGQAKKHPSLIPLFVFIGTGATGATLYLLRLALFNPDVCWDRNNPEPWNKLGPNDQ
YKFYSVNVDYSKLKKERPDF

>A6ND48 multipass membrane protein
MDNLTKVTEFLLMEFSGIWELQVLHAGLFLLIYLAVLVGNLLIIAVITLDQHLHTPMYFFLK
NLSVLDLCYISVTVPKSIRNSLTRRSSISYLGCVAQVYFFSAFASAELAFLTVMSYDRYVAIC
HPLQYRAVMTSGGCYQMAVTTWLSCFSYAAVHTGNMFREHVCRSSVIHQFFRDIPHVLAL
VSCEVFFVEFLTLALSSCLVLGCFILMMISYFQIFSTVLRIPSGQSRAKAFSTCSPQLIVIMLF
LTTGLFAALGPIAKALSIQDLVIALTYTVLPPFLNPIIYSLRNKEIKTAMWRLFVKIYFLQK

>Q13557 peripheral membrane protein
MASTTTCTRFTDEYQLFEELGKGAFSVVRRCMKIPTGQEYAAKIINTKKLSARDHQKLER
EARICRLLKHPNIVRLHDSISEEGFHYLVFDLVTGGELFEDIVAREYYSEADASHCIQQILES
VNHCHLNGIVHRDLKPENLLLASKSKGAAVKLADFGLAIEVQGDQQAWFGFAGTPGYLS
PEVLRKDPYGKPVDMWACGVILYILLVGYPPFWDEDQHRLYQQIKAGAYDFPSPEWDTV
TPEAKDLINKMLTINPAKRITASEALKHPWICQRSTVASMMHRQETVDCLKKFNARRKLK
GAILTTMLATRNFSAAKSLLKKPDGVKESTESSNTTIEDEDVKARKQEIIKVTEQLIEAINN
GDFEAYTKICDPGLTAFEPEALGNLVEGMDFHRFYFENALSKSNKPIHTIILNPHVHLVGDD
AACIAYIRLTQYMDGSGMPKTMQSEETRVWHRRDGKWQNVHFHRSGSPTVPIKPPCIPNG
KENFSGGTSLWQNI

>P35523 multipass membrane protein
MEQSRSQQRGGEQSWWGSDPQYQYMPFEHCTSYGLPSENGGLQHRLRKDAGPRHNVHP
TQIYGHHKEQFSDREQDIGMPKKTGSSSTVDSKDEDHYSKCQDCIHRLGQVVRRKLGED
GIFLVLLGLLMALVSWSMDYVSAKSLQAYKWSYAQMQPSLPLQFLVWVTFPLVLILFSAL
FCHLISPQAVGSGIPEMKTILRGVVLKEYLTMKAFVAKVVALTAGLGSGIPVGKEGPFVHIA

SICAAVLSKFMSVFCGVYEQPYYYSDILTVGCAVGVGCCFGTPLGGVLFSIEVTSTYFAVR
NYWRGFFAATFSAFVFRVLAVWNKDAVTITALFRTNFRMDFPFDLKELPAFAAIGICCGLL
GAVFVYLHRQVMLGVRKHKALSQFLAKHRLLYPGIVTFVIASFTFPPGMGQFMAGELMP
REAISTLFDNNTWVKHAGDPESLGQSAVWIHPRVNVVIIIFLFFVMKFWMSIVATTMPIPCG
GFMPVFVLGAAFGRLVGEIMAMLFPDGILFDDIIYKILPGGYAVIGAAALTGAVSHTVSTAV
ICFELTGQIAHILPMMVAVILANMVAQSLQPSLYDSIIQVKKLPYLPDLGWNQLSKYTIFVE
DIMVRDVKFVSASYTYGELRTLLQTTTVKTLPLVDSKDSMILLGSVERSELQALLQRHLCP
ERRLRAAQEMARKLSELPYDGKARLAGEGLPGAPPGRPESFAFVDEDEDEDLSGKSELPP
SLALHPSTTAPLSPEEPNGPLPGHKQQPEAPEPAGQRPSIFQSLLHCLLGRARPTKKKTTQD
STDLVDNMSPEEIEAWEQEQLSQPVCFDSCCIDQSPFQLVEQTTLHKTHTLFSLLGLHLAY
VTSMGKLRGVLALEELQKAIEGHTKSGVQLRPPLASFRNTTSTRKSTGAPPSSAENWNLP
EDRPGATGTGDVIAASPETPVPSPSPEPPLSLAPGKVEGELEELELVESPGLEEELADILQGP
SLRSTDEEDEDELIL

>P48544 multipass membrane protein
MAGDSRNAMNQDMEIGVTPWDPKKIPKQARDYVPIATDRTRLLAEGKKPRQRYMEKSG
KCNVHHGNVQETYRYLSDLFTTLVDLKWRFNLLVFTMVYTVTWLFFGFIWWLIAYIRGD
LDHVGDQEWIPCVENLSGFVSAFLFSIETETTIGYGFRVITEKCPEGIILLLVQAILGSIVNAF
MVGCMFVKISQPKKRAETLMFSNNAVISMRDEKLCLMFRVGDLRNSHIVEASIRAKLIKS
RQTKEGEFIPLNQTDINVGFDTGDDRLFLVSPLIISHEINQKSPFWEMSQAQLHQEEFEVVV
ILEGMVEATGMTCQARSSYMDTEVLWGHRFTPVLTLEKGFYEVDYNTFHDTYETNTPSC
CAKELAEMKREGRLLQYLPSPPLLGGCAEAGLDAEAEQNEEDEPKGLGGSREARGSV

>Q99732 peripheral membrane protein
MSVPGPYQAATGPSSAPSAPPSYEETVAVNSYYPTPPAPMPGPTTGLVTGPDGKGMNPPSY
YTQPAPIPNNNPITVQTVYVQHPITFLDRPIQMCCPSCNKMIVSQLSYNAGALTWLSCGSL
CLLGCIAGCCFIPFCVDALQDVDHYCPNCRALLGTYKRL

>P13987 GPI-anchor, lipid-anchor
MGIQGGSVLFGLLLVLAVFCHSGHSLQCYNCPNPTADCKTAVNCSSDFDACLITKAGLQV
YNKCWKFEHCNFNDVTTRLRENELTYYCCKKDLCNFNEQLENGGTSLSEKTVLLLVTPFL
AAAWSLHP

>P80370 single-pass type I membrane protein
MTATEALLRVLLLLLAFGHSTYGAECFPACNPQNGFCEDDNVCRCQPGWQGPLCDQCVT
SPGCLHGLCGEPGQCICTDGWDGELCDRDVRACSSAPCANNRTCVSLDDGLYECSCAPG
YSGKDCQKKDGPCVINGSPCQHGGTCVDDEGRASHASCLCPPGFSGNFCEIVANSCTPNP
CENDGVCTDIGGDFRCRCPAGFIDKTCSRPVTNCASSPCQNGGTCLQHTQVSYECLCKPEF
TGLTCVKKRALSPQQVTRLPSGYGLAYRLTPGVHELPVQQPEHRILKVSMKELNKKTPLL
TEGQAICFTILGVLTSLVVLGTVGIVFLNKCETWVSNLRYNHMLRKKKNLLLQYNSGEDL
AVNIIFPEKIDMTTFSKEAGDEEI

>Q96PP8 lipid-anchor
MALEIHMSDPMCLIENFNEQLKVNQEALEILSAITQPVVVVAIVGLYRTGKSYLMNKLAG

KNKGFSVASTVQSHTKGIWIWCVPHPNWPNHTLVLLDTEGLGDVEKADNKNDIQIFALAL
LLSSTFVYNTVNKIDQGAIDLLHNVTELTDLLKARNSPDLDRVEDPADSASFFPDLVWTLR
DFCLGLEIDGQLVTPDEYLENSLRPKQGSDQRVQNFNLPRLCIQKFFPKKKCFIFDLPAHQK
KLAQLETLPDDELEPEFVQQVTEFCSYIFSHSMTKTLPGGIMVNGSRLKNLVLTYVNAISS
GDLPCIENAVLALAQRENSAAVQKAIAHYDQQMGQKVQLPMETLQELLDLHRTSEREAIE
VFMKNSFKDVDQSFQKELETLLDAKQNDICKRNLEASSDYCSALLKDIFGPLEEAVKQGI
YSKPGGHNLFIQKTEELKAKYYREPRKGIQAEEVLQKYLKSKESVSHAILQTDQALTETEK
KKKEAQVKAEAEKAEAQRLAAIQRQNEQMMQERERLHQEQVRQMEIAKQNWLAEQQK
MQEQQMQEQAAQLSTTFQAQNRSLLSELQHAQRTVNNDDPCVLL

>Q8TDB8 multipass membrane protein
MEFHNGGHVSGIGGFLVSLTSRMKPHTLAVTPALIFAITVATIGSFQFGYNTGVINAPETIIKE
FINKTLTDKANAPPSEVLLTNLWSLSVAIFSVGGMIGSFSVGLFVNRFGRRNSMLIVNLLAA
TGGCLMGLCKIAESVEMLILGRLVIGLFCGLCTGFVPMYIGEISPTALRGAFGTLNQLGIVI
GILVAQIFGLELILGSEELWPVLLGFTILPAILQSAALPCCPESPRFLLINRKKEENATRILQRL
WGTQDVSQDIQEMKDESARMSQEKQVTVLELFRVSSYRQPIIISIVLQLSQQLSGINAVFY
YSTGIFKDAGVQQPIYATISAGVVNTIFTLLSLFLVERAGRRTLHMIGLGGMAFCSTLMTVS
LLLKNHYNGMSFVCIGAILVFVACFEIGPGPIPWFIVAELFSQGPRPAAMAVAGCSNWTSNF
LVGLLFPSAAYYLGAYVFIIFTGFLITFLAFTFFKVPETRGRTFEDITRAFEGQAHGADRSGK
DGVMGMNSIEPAKETTTNV

>Q08357 multipass membrane protein
MAMDEYLWMVILGFIIAFILAFSVGANDVANSFGTAVGSGVVTLRQACILASIFETTGSVLL
GAKVGETIRKGIIDVNLYNETVETLMAGEVSAMVGSAVWQLIASFLRLPISGTHCIVGSTIG
FSLVAIGTKGVQWMELVKIVASWFISPLLSGFMSGLLFVLIRIFILKKEDPVPNGLRALPVFY
AATIAINVFSIMYTGAPVLGLVLPMWAIALISFGVALLFAFFVWLFVCPWMRRKITGKLQK
EGALSRVSDESLSKVQEAESPVFKELPGAKANDDSTIPLTGAAGETLGTSEGTSAGSHPRA
AYGRALSMTHGSVKSPISNGTFGFDGHTRSDGHVYHTVHKDSGLYKDLLHKIHIDRGPEE
KPAQESNYRLLRRNNSYTCYTAAICGLPVHATFRAADSSAPEDSEKLVGDTVSYSKKRLR
YDSYSSYCNAVAEAEIEAEEGGVEMKLASELADPDQPREDPAEEEKEEKDAPEVHLLFHF
LQVLTACFGSFAHGGNDVSNAIGPLVALWLIYKQGGVTQEAATPVWLLFYGGVGICTGLW
VWGRRVIQTMGKDLTPITPSSGFTIELASAFTVVIASNIGLPVSTTHCKVGSVVAVGWIRSR
KAVDWRLFRNIFVAWFVTVPVAGLFSAAVMALLMYGILPYV

>Q9NQ84 multipass membrane protein
MAIHKALVMCLGLPLFLFPGAWAQGHVPPGCSQGLNPLYYNLCDRSGAWGIVLEAVAGA
GIVTTFVLTIILVASLPFVQDTKKRSLLGTQVFFLLGTLGLFCLVFACVVKPDFSTCASRRFL
FGVLFAICFSCLAAHVFALNFLARKNHGPRGWVIFTVALLLTLVEVIINTEWLIITLVRGSGE
GGPQGNSSAGWAVASPCAIANMDFVMALIYVMLLLLGAFLGAWPALCGRYKRWRKHGV
FVLLTTATSVAIWVVWIVMYTYGNKQHNSPTWDDPTLAIALAANAWAFVLFYVIPEVSQV
TKSSPEQSYQGDMYPTRGVGYETILKEQKGQSMFVENKAFSMDEPVAAKRPVSPYSGYN
GQLLTSVYQPTEMALMHKVPSEGAYDIILPRATANSQVMGSANSTLRAEDMYSAQSHQA
ATPPKDGKNSQVFRNPYVWD

>O94910 multipass membrane protein
MARLAAVLWNLCVTAVLVTSATQGLSRAGLPFGLMRRELACEGYPIELRCPGSDVIMVEN
ANYGRTDDKICDADPFQMENVQCYLPDAFKIMSQRCNNRTQCVVVAGSDAFPDPCPGTY
KYLEVQYDCVPYKVEQKVFVCPGTLQKVLEPTSTHESEHQSGAWCKDPLQAGDRIYVMP
WIPYRTDTLTEYASWEDYVAARHTTTYRLPNRVDGTGFVVYDGAVFYNKERTRNIVKYD
LRTRIKSGETVINTANYHDTSPYRWGGKTDIDLAVDENGLWVIYATEGNNGRLVVSQLNP
YTLRFEGTWETGYDKRSASNAFMVCGVLYVLRSVYVDDDSEAAGNRVDYAFNTNANRE
EPVSLTFPNPYQFISSVDYNPRDNQLYVWNNYFVVRYSLEFGPPDPSAGPATSPPLSTTTTA
RPTPLTSTASPAATTPLRRAPLTTHPVGAINQLGPDLPPATAPVPSTRRPPAPNLHVSPELFCE
PREVRRVQWPATQQGMLVERPCPKGTRGIASFQCLPALGLWNPRGPDLSNCTSPWVNQVA
QKIKSGENAANIASELARHTRGSIYAGDVSSSVKLMEQLLDILDAQLQALRPIERESAGKN
YNKMHKRERTCKDYIKAVVETVDNLLRPEALESWKDMNATEQVHTATMLLDVLEEGAF
LLADNVREPARFLAAKENVVLEVTVLNTEGQVQELVFPQEEYPRKNSIQLSAKTIKQNSR
NGVVKVVFILYNNLGLFLSTENATVKLAGEAGPGGPGGASLVVNSQVIAASINKESSRVFL
MDPVIFTVAHLEDKNHFNANCSFWNYSERSMLGYWSTQGCRLVESNKTHTTCACSHLTN
FAVLMAHREIYQGRINELLLSVITWVGIVISLVCLAICISTFCFLRGLQTDRNTIHKNLCINLF
LAELLFLVGIDKTQYEIACPIFAGLLHYFFLAAFSWLCLEGVHLYLLLVEVFESEYSRTKYY
YLGGYCFPALVVGIAAAIDYRSYGTEKACWLRVDNYFIWSFIGPVSFVIVVNLVFLMVTLH
KMIRSSSVLKPDSSRLDNIKSWALGAIALLFLLGLTWAFGLLFINKESVVMAYLFTTFNAFQ
GVFIFVFHCALQKKVHKEYSKCLRHSYCCIRSPPGGTHGSLKTSAMRSNTRYYTGTQSRIR
RMWNDTVRKQTESSFMAGDINSTPTLNRGTMGNHLLTNPVLQPRGGTSPYNTLIAESVGF
NPSSPPVFNSPGSYREPKHPLGGREACGMDTLPLNGNFNNSYSLRSGDFPPGDGGPEPPRG
RNLADAAAFEKMIISELVHNNLRGSSSAAKGPPPPEPPVPPVPGGGGEEEAGGPGGADRA
EIELLYKALEEPLLLPRAQSVLYQSDLDESESCTAEDGATSRPLSSPPGRDSLYASGANLRDS
PSYPDSSPEGPSEALPPPPPAPPGPPEIYYTSRPPALVARNPLQGYYQVRRPSHEGYLAAPGL
EGPGPDGDGQMQLVTSL

>O14908 peripheral membrane protein
MPLGLGRRKKAPPLVENEEAEPGRGGLGVGEPGPLGGGGSGGPQMGLPPPPPALRPRLVF
HTQLAHGSPTGRIEGFTNVKELYGKIAEAFRLPTAEVMFCTLNTHKVDMDKLLGGQIGLE
DFIFAHVKGQRKEVEVFKSEDALGLTITDNGAGYAFIKRIKEGSVIDHIHLISVGDMIEAING
QSLLGCRHYEVARLLKELPRGRTFTLKLTEPRKAFDMISQRSAGGRPGSGPQLGTGRGTLR
LRSRGPATVEDLPSAFEEKAIEKVDDLLESYMGIRDTELAATMVELGKDKRNPDELAEAL
DERLGDFAFPDEFVFDVWGAIGDAKVGRY

>Q8NG92 multipass membrane protein
MAMDNVTAVFQFLLIGISNYPQWRDTFFTLVLIIYLSTLLGNGFMIFLIHFDPNLHTPIYFFL
SNLSFLDLCYGTASMPQALVHCFSTHPYLSYPRCLAQTSVSLALATAECLLLAAMAYDRV
VAISNPLRYSVVMNGPVCVCLVATSWGTSLVLTAMLILSLRLHFCGANVINHFACEILSLIKL
TCSDTSLNEFMILITSIFTLLLPFGFVLLSYIRIAMAIIRIRSLQGRLKAFTTCGSHLTVVTIFY
GSAISMYMKTQSKSYPDQDKFISVFYGALTPMLNPLIYSLRKKDVKRAIRKVMLKRT

>O95377 multipass membrane protein
MNWSIFEGLLSGVNKYSTAFGRIWLSLVFIFRVLVYLVTAERVWSDDHKDFDCNTRQPGC

SNVCFDEFFPVSHVRLWALQLILVTCPSLLVVMHVAYREVQEKRHREAHGENSGRLYLNP
GKKRGGLWWTYVCSLVFKASVDIAFLYVFHSFYPKYILPPVVKCHADPCPNIVDCFISKPS
EKNIFTLFMVATAAICILLNLVELIYLVSKRCHECLAARKAQAMCTGHHPHGTTSSCKQDD
LLSGDLIFLGSDSHPPLLPDRPRDHVKKTIL

>P10636 peripheral membrane protein
MAEPRQEFEVMEDHAGTYGLGDRKDQGGYTMHQDQEGDTDAGLKESPLQTPTEDGSEE
PGSETSDAKSTPTAEDVTAPLVDEGAPGKQAAAQPHTEIPEGTTAEEAGIGDTPSLEDEAA
GHVTQEPESGKVVQEGFLREPGPPGLSHQLMSGMPGAPLLPEGPREATRQPSGTGPEDTE
GGRHAPELLKHQLLGDLHQEGPPLKGAGGKERPGSKEEVDEDRDVDESSPQDSPPSKASP
AQDGRPPQTAAREATSIPGFPAEGAIPLPVDFLSKVSTEIPASEPDGPSVGRAKGQDAPLEFT
FHVEITPNVQKEQAHSEEHLGRAAFPGAPGEGPEARGPSLGEDTKEADLPEPSEKQPAAAP
RGKPVSRVPQLKARMVSKSKDGTGSDDKKAKTSTRSSAKTLKNRPCLSPKHPTPGSSDPL
IQPSSPAVCPEPPSSPKYVSSVTSRTGSSGAKEMKLKGADGKTKIATPRGAAPPGQKGQAN
ATRIPAKTPPAPKTPPSSGEPPKSGDRSGYSSPGSPGTPGSRSRTPSLPTPPTREPKKVAVVRT
PPKSPSSAKSRLQTAPVPMPDLKNVKSKIGSTENLKHQPGGGKVQIINKKLDLSNVQSKCG
SKDNIKHVPGGGSVQIVYKPVDLSKVTSKCGSLGNIHHKPGGGQVEVKSEKLDFKDRVQS
KIGSLDNITHVPGGGNKKIETHKLTFRENAKAKTDHGAEIVYKSPVVSGDTSPRHLSNVSS
TGSIDMVDSPQLATLADEVSASLAKQGL

>Q5JRA6 single-pass type I membrane protein
MAAAPGLLVWLLVLRLPWRVPGQLDPSTGRRFSEHKLCADDECSMLMYRGEALEDFTGP
DCRFVNFKKGDPVYVYYKLARGWPEVWAGSVGRTFGYFPKDLIQVVHEYTKEELQVPT
DETDFVCFDGGRDDFHNYNVEELLGFLELYNSAATDSEKAVEKTLQDMEKNPELSKEREP
EPEPVEANSEESDSVFSENTEDLQEQFTTQKHHSHANSQANHAQGEQASFESFEEMLQDK
LKVPESENNKTSNSSQVSNEQDKIDAYKLLKKEMTLDLKTKFGSTADALVSDDETTRLVT
SLEDDFDEELDTEYYAVGKEDEENQEDFDELPLLTFTDGEDMKTPAKSGVEKYPTDKEQN
SNEEDKVQLTVPPGIKNDDKNILTTWGDTIFSIVTGGEETRDTMDLESSSSEEEKEDDDDA
LVPDSKQGKPQSATDYSDPDNVDDGLFIVDIPKTNNDKEVNAEHHIKGKGRGVQESKRGL
VQDKTELEDENQEGMTVHSSVHSNNLNSMPAAEKGKDTLKSAYDDTENDLKGAAIHISK
GMLHEEKPGEQILEGGSESESAQKAAGNQMNDRKIQQESLGSAPLMGDDHPNASRDSVE
GDALVNGAKLHTLSVEHQREELKEELVLKTQNQPRFSSPDEIDLPRELEDEVPILGRNLPW
QQERDVAATASKQMSEKIRLSEGEAKEDSLDEEFFHHKAMQGTEVGQTDQTDSTGGPAFL
SKVEEDDYPSEELLEDENAINAKRSKEKNPGNQGRQFDVNLQVPDRAVLGTIHPDPEIEES
KQETSMILDSEKTSETAAKGVNTGGREPNTMVEKERPLADKKAQRPFERSDFSDSIKIQTP
ELGEVFQNKDSDYLKNDNPEEHLKTSGLAGEPEGELSKEDHENTEKYMGTESQGSAAAE
PEDDSFHWTPHTSVEPGHSDKREDLLIISSFFKEQQSLQRFQKYFNVHELEALLQEMSSKL
KSAQQESLPYNMEKVLDKVFRASESQILSIAEKMLDTRVAENRDLGMNENNIFEEAAVLD
DIQDLIYFVRYKHSTAEETATLVMAPPLEEGLGGAMEEMQPLHEDNFSREKTAELNVQVP
EEPTHLDQRVIGDTHASEVSQKPNTEKDLDPGPVTTEDTPMDAIDANKQPETAAEEPASVT
PLENAILLIYSFMFYLTKSLVATLPDDVQPGPDFYGLPWKPVFITAFLGIASFAIFLWRTVLV
VKDRVYQVTEQQISEKLKTIMKENTELVQKLSNYEQKIKESKKHVQETRKQNMILSDEAI
KYKDKIKTLEKNQEILDDTAKNLRVMLESEREQNVKNQDLISENKKSIEKLKDVISMNAS
EFSEVQIALNEAKLSEEKVKSECHRVQEENARLKKKKEQLQQEIEDWSKLHAELSEQIKSF

EKSQKDLEVALTHKDDNINALTNCITQLNLLECESESEGQNKGGNDSDELANGEVGGDRN
EKMKNQIKQMMDVSRTQTAISVVEEDLKLLQLKLRASVSTKCNLEDQVKKLEDDRNSLQ
AAKAGLEDECKTLRQKVEILNELYQQKEMALQKKLSQEEYERQEREHRLSAADEKAVSA
AEEVKTYKRRIEEMEDELQKTERSFKNQIATHEKKAHENWLKARAAERAIAEEKREAAN
LRHKLLELTQKMAMLQEEPVIVKPMPGKPNTQNPPRRGPLSQNGSFGPSPVSGGECSPPLT
VEPPVRPLSATLNRRDMPRSEFGSVDGPLPHPRWSAEASGKPSPSDPGSGTATMMNSSSRG
SSPTRVLDEGKVNMAPKGPPPFPGVPLMSTPMGGPVPPPIRYGPPPQLCGPFGPRPLPPPFG
PGMRPPLGLREFAPGVPPGRRDLPLHPRGFLPGHAPFRPLGSLGPREYFIPGTRLPPPTHGP
QEYPPPPAVRDLLPSGSRDEPPPASQSTSQDCSQALKQSP

>Q9UMD9 single-pass type II membrane protein
MDVTKKNKRDGTEVTERIVTETVTTRLTSLPPKGGTSNGYAKTASLGGGSRLEKQSLTHG
SSGYINSTGSTRGHASTSSYRRAHSPASTLPNSPGSTFERKTHVTRHAYEGSSSGNSSPEYP
RKEFASSSTRGRSQTRESEIRVRLQSASPSTRWTELDDVKRLLKGSRSASVSPTRNSSNTLPI
PKKGTVETKIVTASSQSVSGTYDATILDANLPSHVWSSTLPAGSSMGTYHNNMTTQSSSLL
NTNAYSAGSVFGVPNNMASCSPTLHPGLSTSSSVFGMQNNLAPSLTTLSHGTTTTSTAYGV
KKNMPQSPAAVNTGVSTSAACTTSVQSDDLLHKDCKFLILEKDNTPAKKEMELLIMTKDS
GKVFTASPASIAATSFSEDTLKKEKQAAYNADSGLKAEANGDLKTVSTKGKTTTADIHSY
GSSGGGGSGGGGGVGGAGGGPWGPAPAWCPCGSCCSWWKWLLGLLLTWLLLLGLLFGL
IALAEEVRKLKARVDELERIRRSILPYGDSMDRIEKDRLQGMAPAAGADLDKIGLHSDSQE
ELWMFVRKKLMMEQENGNLRGSPGPKGDMGSPGPKGDRGFPGTPGIPGPLGHPGPQGPK
GQKGSVGDPGMEGPMGQRGREGPMGPRGEAGPPGSGEKGERGAAGEPGPHGPPGVPGS
VGPKGSSGSPGPQGPPGPVGLQGLRGEVGLPGVKGDKGPMGPPGPKGDQGEKGPRGLTG
EPGMRGLPGAVGEPGAKGAMGPAGPDGHQGPRGEQGLTGMPGIRGPPGPSGDPGKPGLT
GPQGPQGLPGTPGRPGIKGEPGAPGKIVTSEGSSMLTVPGPPGPPGAMGPPGPPGAPGPAG
PAGLPGHQEVLNLQGPPGPPGPRGPPGPSIPGPPGPRGPPGEGLPGPPGPPGSFLSNSETFLS
GPPGPPGPPGPKGDQGPPGPRGHQGEQGLPGFSTSGSSSFGLNLQGPPGPPGPQGPKGDKG
DPGVPGALGIPSGPSEGGSSSTMYVSGPPGPPGPPGPPGSISSSGQEIQQYISEYMQSDSIRS
YLSGVQGPPGPPGPPGPVTTITGETFDYSELASHVVSYLRTSGYGVSLFSSSISSEDILAVLQ
RDDVRQYLRQYLMGPRGPPGPPGASGDGSLLSLDYAELSSRILSYMSSSGISIGLPGPPGPP
GLPGTSYEELLSLLRGSEFRGIVGPPGPPGPPGIPGNVWSSISVEDLSSYLHTAGLSFIPGPPG
PPGPPGPRGPPGVSGALATYAAENSDSFRSELISYLTSPDVRSFIVGPPGPPGPQGPPGDSRL
LSTDASHSRGSSSSSHSSSVRRGSSYSSSMSTGGGGAGSLGAGGAFGEAAGDRGPYGTDI
GPGGGYGAAAEGGMYAGNGGLLGADFAGDLDYNELAVRVSESMQRQGLLQGMAYTVQ
GPPGQPGPQGPPGISKVFSAYSNVTADLMDFFQTYGAIQGPPGQKGEMGTPGPKGDRGPA
GPPGHPGPPGPRGHKGEKGDKGDQVYAGRRRRRSIAVKP

>Q7RTX7 multipass membrane protein
MRDNEKAWWQQWTSHTGLEGWGGTQEDRMGFGGAVAALRGRPSPLQSTIHESYGRPEE
QVLINRQEITNKADAWDMQEFITHMYIKQLLRHPAFQLLLALLLVINAITIALRTNSYLDQ
KHYELFSTIDDIVLTILLCEVLLGWLNGFWIFWKDGWNILNFIIVFILLLRFFINEINIPSINYT
LRALRLVHVCMAVEPLARIIRVILQSVPDMANIMVLILFFMLVFSVFGVTLFGAFVPKHFQ
NIQVALYTLFICITQDGWVDIYSDFQTEKREYAMEIGGAIYFTIFITIGAFIGINLFVIVVTTNL
EQMMKAGEQGQQQRITFSETGAEEEEENDQLPLVHCVVARSEKSGLLQEPLAGGPLSNLS

ENTCDNFCLVLEAIQENLRQYKEIRDELNMIVEEVRAIRFNQEQESEVLNRRSSTSGSLETT
SSKDIRQMSQQQDLLSALVSMEKVHDSSSQILLKKHKSSH

>P20594 single-pass type I membrane protein
MALPSLLLLVAALAGGVRPPGARNLTLAVVLPEHNLSYAWAWPRVGPAVALAVEALGRAL
PVDLRFVSSELEGACSEYLAPLSAVDLKLYHDPDLLLGPGCVYPAASVARFASHWRLPLLT
AGAVASGFSAKNDHYRTLVRTGPSAPKLGEFVVTLHGHFNWTARAALLYLDARTDDRPH
YFTIEGVFEALQGSNLSVQHQVYAREPGGPEQATHFIRANGRIVYICGPLEMLHEILLQAQ
RENLTNGDYVFFYLDVFGESLRAGPTRATGRPWQDNRTREQAQALREAFQTVLVITYREP
PNPEYQEFQNRLLIRAREDFGVELGPSLMNLIAGCFYDGILLYAEVLNETIQEGGTREDGL
RIVEKMQGRRYHGVTGLVVMDKNNDRETDFVLWAMGDLDSGDFQPAAHYSGAEKQIW
WTGRPIPWVKGAPPSDNPPCAFDLDDPSCDKTPLSTLAIVALGTGITFIMFGVSSFLIFRKL
MLEKELASMLWRIRWEELQFGNSERYHKGAGSRLTLSLRGSSYGSLMTAHGKYQIFANTG
HFKGNVVAIKHVNKKRIELTRQVLFELKHMRDVQFNHLTRFIGACIDPPNICIVTEYCPRGS
LQDILENDSINLDWMFRYSLINDLVKGMAFLHNSIISSHGSLKSSNCVVDSRFVLKITDYGL
ASFRSTAEPDDSHALYAKKLWTAPELLSGNPLPTTGMQKADVYSFGIILQEIALRSGPFYLE
GLDLSPKEIVQKVRNGQRPYFRPSIDRTQLNEELVLLMERCWAQDPAERPDFGQIKGFIRR
FNKEGGTSILDNLLLRMEQYANNLEKLVEERTQAYLEEKRKAEALLYQILPHSVAEQLKRG
ETVQAEAFDSVTIYFSDIVGFTALSAESTPMQVVTLLNDLYTCFDAIIDNFDVYKVETIGDA
YMVVSGLPGRNGQRHAPEIARMALALLDAVSSFRIRHRPHDQLRLRIGVHTGPVCAGVV
GLKMPRYCLFGDTVNTASRMESNGQALKIHVSSTTKDALDELGCFQLELRGDVEMKGKG
KMRTYWLLGERKGPPGLL

>Q9BZZ2 single-pass type I membrane protein
MGFLPKLLLLASFFPAGQASWGVSSPQDVQGVKGSCLLIPCIFSFPADVEVPDGITAIWYY
DYSGQRQVVSHSADPKLVEARFRGRTEFMGNPEHRVCNLLLKDLQPEDSGSYNFRFEISE
VNRWSDVKGTLVTVTEEPRVPTIASPVELLEGTEVDFNCSTPYVCLQEQVRLQWQGQDPA
RSVTFNSQKFEPTGVGHLETLHMAMSWQDHGRILRCQLSVANHRAQSEIHLQVKYAPKG
VKILLSPSGRNILPGELVTLTCQVNSSYPAVSSIKWLKDGVRLQTKTGVLHLPQAAWSDAG
VYTCQAENGVGSLVSPPISLHIFMAEVQVSPAGPILENQTVTLVCNTPNEAPSDLRYSWYK
NHVLLEDAHSHTLRLHLATRADTGFYFCEVQNVHGSERSGPVSVVVNHPPLTPVLTAFLE
TQAGLVGILHCSVVSEPLATLVLSHGGHILASTSGDSDHSPRFSGTSGPNSLRLEIRDLEETD
SGEYKCSATNSLGNATSTLDFHANAARLLISPAAEVVEGQAVTLSCRSGLSPTPDARFSWY
LNGALLHEGPGSSLLLPAASSTDAGSYHCRARDGHSASGPSSPAVLTVLYPPRQPTFTTRLD
LDAAGAGAGRRGLLLCRVDSDPPARLQLLHKDRVVATSLPSGGGCSTCGGCSPRMKVTK
APNLLRVEIHNPLLEEEGLYLCEASNALGNASTSATFNGQATVLAIAPSHTLQEGTEANLTC
NVSREAAGSPANFSWFRNGVLWAQGPLETVTLLPVARTDAALYACRILTEAGAQLSTPVLL
SVLYPPDRPKLSALLDMGQGHMALFICTVDSRPLALLALFHGEHLLATSLGPQVPSHGRF
QAKAEANSLKLEVRELGLGDSGSYRCEATNVLGSSNTSLFFQVRGAWVQVSPSPELQEGQ
AVVLSCQVHTGVPEGTSYRWYRDGQPLQESTSATLRFAAITLTQAGAYHCQAQAPGSATT
SLAAPISLHVSYAPRHVTLTTLMDTGPGRLGLLLCRVDSDPPAQLRLLHGDRLVASTLQGV
GGPEGSSPRLHVAVAPNTLRLEIHGAMLEDEGVYICEASNTLGQASASADFDAQAVNVQV
WPGATVREGQLVNLTCLVWTTHPAQLTYTWYQDGQQRLDAHSIPLPNVTVRDATSYRCG
VGPPGRAPRLSRPITLDVLYAPRNLRLTYLLESHGGQLALVLCTVDSRPPAQLALSHAGRL

LASSTAASVPNTLRLELRGPQPRDEGFYSCSARSPLGQANTSLELRLEGVRVILAPEAAVPE
GAPITVTCADPAAHAPTLYTWYHNGRWLQEGPAASLSFLVATRAHAGAYSCQAQDAQGT
RSSRPAALQVLYAPQDAVLSSFRDSRARSMAVIQCTVDSEPPAELALSHDGKVLATSSGVH
SLASGTGHVQVARNALRLQVQDVPAGDDTYVCTAQNLLGSISTIGRLQVEGARVVAEPGL
DVPEGAALNLSCRLLGGPGPVGNSTFAWFWNDRRLHAEPVPTLAFTHVARAQAGMYHC
LAELPTGAAASAPVMLRVLYPPKTPTMMVFVEPEGGLRGILDCRVDSEPLASLTLHLGSRL
VASSQPQGAPAEPHIHVLASPNALRVDIEALRPSDQGEYICSASNVLGSASTSTYFGVRALH
RLHQFQQLLWVLGLLVGLLLLLLGLGACYTWRRRRVCKQSMGENSVEMAFQKETTQLID
PDAATCETSTCAPPLG

>Q05397 peripheral membrane protein
MAAAYLDPNLNHTPNSSTKTHLGTGMERSPGAMERVLKVFHYFESNSEPTTWASIIRHGD
ATDVRGIIQKIVDSHKVKHVACYGFRLSHLRSEEVHWLHVDMGVSSVREKYELAHPPEE
WKYELRIRYLPKGFLNQFTEDKPTLNFFYQQVKSDYMLEIADQVDQEIALKLGCLEIRRS
YWEMRGNALEKKSNYEVLEKDVGLKRFFPKSLLDSVKAKTLRKLIQQTFRQFANLNREE
SILKFFEILSPVYRFDKECFKCALGSSWIISVELAIGPEEGISYLTDKGCNPTHLADFTQVQTI
QYSNSEDKDRKGMLQLKIAGAPEPLTVTAPSLTIAENMADLIDGYCRLVNGTSQSFIIRPQK
EGERALPSIPKLANSEKQGMRTHAVSVSETDDYAEIIDEEDTYTMPSTRDYEIQRERIELGR
CIGEGQFGDVHQGIYMSPENPALAVAIKTCKNCTSDSVREKFLQEALTMRQFDHPHIVKLI
GVITENPVWIIMELCTLGELRSFLQVRKYSLDLASLILYAYQLSTALAYLESKRFVHRDIAA
RNVLVSSNDCVKLGDFGLSRYMEDSTYYKASKGKLPIKWMAPESINFRRFTSASDVWMF
GVCMWEILMHGVKPFQGVKNNDVIGRIENGERLPMPPNCPPTLYSLMTKCWAYDPSRRP
RFTELKAQLSTILEEEKAQQEERMRMESRRQATVSWDSGGSDEAPPKPSRPGYPSPRSSEG
FYPSPQHMVQTNHYQVSGYPGSHGITAMAGSIYPGQASLLDQTDSWNHRPQEIAMWQPN
VEDSTVLDLRGIGQVLPTHLMEERLIRQQQEMEEDQRWLEKEERFLKPDVRLSRGSIDRE
DGSLQGPIGNQHIYQPVGKPDPAAPPKKPPRPGAPGHLGSLASLSSPADSYNEGVKLQPQE
ISPPPTANLDRSNDKVYENVTGLVKAVIEMSSKIQPAPPEEYVPMVKEVGLALRTLLATVD
ETIPLLPASTHREIEMAQKLLNSDLGELINKMKLAQQYVMTSLQQEYKKQMLTAAHALAV
DAKNLLDVIDQARLKMLGQTRPH

>Q8NCG5 single-pass type II membrane protein
MLLPKKMKLLLFLVSQMAILALFFHMYSHNISSLSMKAQPERMHVLVLSSWRSGSSFVGQ
LFGQHPDVFYLMEPAWHVWMTFKQSTAWMLHMAVRDLIRAVFLCDMSVFDAYMEPGPR
RQSSLFQWENSRALCSAPACDIIPQDEIIPRAHCRLLCSQQPFEVVEKACRSYSHVVLKEVR
FFNLQSLYPLLKDPSLNLHIVHLVRDPRAVFRSRERTKGDLMIDSRIVMGQHEQKLKKEDQ
PYYVMQVICQSQLEIYKTIQSLPKALQERYLLVRYEDLARAPVAQTSRMYEFVGLEFLPHL
QTWVHNITRGKGMGDHAFHTNARDALNVSQAWRWSLPYEKVSRLQKACGDAMNLLGY
RHVRSEQEQRNLLLDLLSTWTVPEQIH

>Q5TZ20 multipass membrane protein
MEETNNSSEKGFLLLGFSDQPQLERFLFAIILYFYVLSLLGNTALILVCCLDSRLHTPMYFFL
SNLSCVDICFTTSVAPQLLVTMNKKDKTMSYGGCVAQLYVAMGLGSSECILLAVMAYDRY
AAVCRPLRYIAIMHPRFCASLAGGAWLSGLITSLIQCSLTVQLPLCGHRTLDHIFCEVPVLIK
LACVDTTFNEAELFVASVVFLIVPVLLILVSYGFITQAVLRIKSAAGRQKAFGTCSSHLVVVI

IFYGTIIFMYLQPANRRSKNQGKFVSLFYTIVTPLLNPIIYTLRNKDVKGALRTLILGSAAGQ
SHKD

>Q9UJY5 peripheral membrane protein
MEPAMEPETLEARINRATNPLNKELDWASINGFCEQLNEDFEGPPLATRLLAHKIQSPQEW
EAIQALTVLETCMKSCGKRFHDEVGKFRFLNELIKVVSPKYLGSRTSEKVKNKILELLYSW
TVGLPEEVKIAEAYQMLKKQGIVKSDPKLPDDTTFPLPPPRPKNVIFEDEEKSKMLARLLK
SSHPEDLRAANKLIKEMVQEDQKRMEKISKRVNAIEEVNNNVKLLTEMVMSHSQGGAAA
GSSEDLMKELYQRCERMRPTLFRLASDTEDNDEALAEILQANDNLTQVINLYKQLVRGEE
VNGDATAGSIPGSTSALLDLSGLDLPPAGTTYPAMPTRPGEQASPEQPSASVSLLDDELMS
LGLSDPTPPSGPSLDGTGWNSFQSSDATEPPAPALAQAPSMESRPPAQTSLPASSGLDDLDL
LGKTLLQQSLPPESQQVRWEKQQPTPRLTLRDLQNKSSSCSSPSSSATSLLHTVSPEPPRPP
QQPVPTELSLASITVPLESIKPSNILPVTVYDQHGFRILFHFARDPLPGRSDVLVVVVSMLST
APQPIRNIVFQSAVPKVMKVKLQPPSGTELPAFNPIVHPSAITQVLLLANPQKEKVRLRYKL
TFTMGDQTYNEMGDVDQFPPPETWGSL

>Q9NXG6 single-pass type II membrane protein
MAAAAVTGQRPETAAAEEASRPQWAPPDHCQAQAAAGLGDGEDAPVRPLCKPRGICSRA
YFLVLMVFVHLYLGNVLALLLFVHYSNGDESSDPGPQHRAQGPGPEPTLGPLTRLEGIKV
GHERKVQLVTDRDHFIRTLSLKPLLFEIPGFLTDEECRLIIHLAQMKGLQRSQILPTEEYEEA
MSTMQVSQLDLFRLLDQNRDGHLQLREVLAQTRLGNGWWMTPESIQEMYAAIKADPDG
DGVLSLQEFSNMDLRDFHKYMRSHKAESSELVRNSHHTWLYQGEGAHHIMRAIRQRVLR
LTRLSPEIVELSEPLQVVRYGEGGHYHAHVDSGPVYPETICSHTKLVANESVPFETSCRYM
TVLFYLNNVTGGGETVFPVADNRTYDEMSLIQDDVDLRDTRRHCDKGNLRVKPQQGTAV
FWYNYLPDGQGWVGDVDDYSLHGGCLVTRGTKWIANNWINVDPSRARQALFQQEMAR
LAREGGTDSQPEWALDRAYRDARVEL

>Q9UNX9 multipass membrane protein
MGLARALRRLSGALDSGDSRAGDEEEAGPGLCRNGWAPAPVQSPVGRRRGRFVKKDGH
CNVRFVNLGGQGARYLSDLFTTCVDVRWRWMCLLFSCSFLASWLLFGLAFWLIASLHGD
LAAPPPPAPCFSHVASFLAAFLFALETQTSIGYGVRSVTEECPAAVAAVVLQCIAGCVLDAF
VVGAVMAKMAKPKKRNETLVFSENAVVALRDHRLCLMWRVGNLRRSHLVEAHVRAQLL
QPRVTPEGEYIPLDHQDVDVGFDGGTDRIFLVSPITIVHEIDSASPLYELGRAELARADFELV
VILEGMVEATAMTTQCRSSYLPGELLWGHRFEPVLFQRGSQYEVDYRHFHRTYEVPGTPV
CSAKELDERAEQASHSLKSSFPGSLTAFCYENELALSCCQEEDEDDETEEGNGVETEDGA
ASPRVLTPTLALTLPP

>Q00973 single-pass type II membrane protein
MWLGRRALCALVLLLACASLGLLYASTRDAPGLRLPLAPWAPPQSPRRPELPDLAPEPRYA
HIPVRIKEQVVGLLAWNNCSCESSGGGLPLPFQKQVRAIDLTKAFDPAELRAASATREQEF
QAFLSRSQSPADQLLIAPANSPLQYPLQGVEVQPLRSILVPGLSLQAASGQEVYQVNLTASL
GTWDVAGEVTGVTLTGEGQADLTLVSPGLDQLNRQLQLVTYSSRSYQTNTADTVRFSTEG
HEAAFTIRIRHPPNPRLYPPGSLPQGAQYNISALVTIATKTFLRYDRLRALITSIRRFYPTVTV
VIADDSDKPERVSGPYVEHYLMPFGKGWFAGRNLAVSQVTTKYVLWVDDDFVFTARTRL

ERLVDVLERTPLDLVGGAVREISGFATTYRQLLSVEPGAPGLGNCLRQRRGFHHELVGFPG
CVVTDGVVNFFLARTDKVREVGFDPRLSRVAHLEFFLDGLGSLRVGSCSDVVVDHASKLK
LPWTSRDAGAETYARYRYPGSLDESQMAKHRLLFFKHRLQCMTSQ

>Q8NGF0 multipass membrane protein
MAQVRALHKIMALFSANSIGAMNNSDTRIAGCFLTGIPGLEQLHIWLSIPFCIMYITALEGN
GILICVILSQAILHEPMYIFLSMLASADVLLSTTTMPKALANLWLGYSLISFDGCLTQMFFIH
FLFIHSAVLLAMAFDRYVAICSPLRYVTILTSKVIGKIVTAALSHSFIIMFPSIFLLEHLHYCQI
NIIAHTFCEHMGIAHLSCSDISINVWYGLAAALLSTGLDIMLITVSYIHILQAVFRLLSQDAR
SKALSTCGSHICVILLFYVPALFSVFAYRFGGRSVPCYVHILLASLYVVIPPMLNPVIYGVRT
KPILEGAKQMFSNLAKGSK

>Q68D85 single-pass type I membrane protein
MTWRAAASTCAALLILLWALTTEGDLKVEMMAGGTQITPLNDNVTIFCNIFYSQPLNITS
MGITWFWKSLTFDKEVKVFEFFGDHQEAFRPGAIVSPWRLKSGDASLRLPGIQLEEAGEY
RCEVVVTPLKAQGTVQLEVVASPASRLLLDQVGMKENEDKYMCESSGFYPEAINITWEK
QTQKFPHPIEISEDVITGPTIKNMDGTFNVTSCLKLNSSQEDPGTVYQCVVRHASLHTPLRS
NFTLTAARHSLSETEKTDNFSIHWWPISFIGVGLVLLIVLIPWKKICNKSSSAYTPLKCILKH
WNSFDTQTLKKEHLIFFCTRAWPSYQLQDGEAWPPEGSVNINTIQQLDVFCRQEGKWSEV
PYVQAFFALRDNPDLCQCCRIDPALLTVTSGKSIDDNSTKSEKQTPREHSDAVPDAPILPVS
PIWEPPPATTSTTPVLSSQPPTLLLPLQ

>Q9H1R2 lipid-anchor
MTEGVLPGLYLGNFIDAKDLDQLGRNKITHIISIHESPQPLLQDITYLRIPVADTPEVPIKKH
FKECINFIHCCRLNGGNCLVHCFAGISRSTTIVTAYVMTVTGLGWRDVLEAIKATRPIANPN
PGFRQQLEEFGWASSQKGARHRTSKTSGAQCPPMTSATCLLAARVALLSAALVREATGRT
AQRCRLSPRAAAERLLGPPPHVAAGWSPDPKYQICLCFGEEDPGPTQHPKEQLIMADVQV
QLRPGSSSCTLSASTERPDGSSTPGNPDGITHLQCSCLHPKRAASSSCTR

>Q6ZNB7 multipass membrane protein
MKNPEAQQDVSVSQGFRMLFYTMKPSETSFQTLEEVPDYVKKATPFFISLMLLELVVSWI
LKGKPPGRLDDALTSISAGVLSRLPSLFFRSIELTSYIYIWENYRLFNLPWDSPWTWYSAFL
GVDFGYYWFHRMAHEVNIMWAGHQTHHSSEDYNLSTALRQSVLQIYTSWIFYSPLALFIP
PSVYAVHLQFNLLYQFWIHTEVINNLGPLELILNTPSHHRVHHGRNRYCIDKNYAGVLIIW
DKIFGTFEAENEKVVYGLTHPINTFEPIKVQFHHLFSIWTTFWATPGFFNKFSVIFKGPGWG
PGKPRLGLSEEIPEVTGKEVPFSSSSSQLLKIYTVVQFALMLAFYEETFADTAALSQVTLLL
RVCFIILTLTSIGFLLDQRPKAAIMETLRCLMFLMLYRFGHLKPLVPSLSSAFEIVFSICIAFW
GVRSMKQLTSHPWK

>Q96EV8 peripheral membrane protein
MLETLRERLLSVQQDFTSGLKTLSDKSREAKVKSKPRTVPFLPKYSAGLELLSRYEDTWA
ALHRRAKDCASAGELVDSEVVMLSAHWEKKKTSLVELQEQLQQLPALIADLESMTANLT
HLEASFEEVENNLLHLEDLCGQCELERCKHMQSQQLENYKKNKRKELETFKAELDAEHA
QKVLEMEHTQQMKLKERQKFFEEAFQQDMEQYLSTGYLQIAERREPIGSMSSMEVNVD

MLEQMDLMDISDQEALDVFLNSGGEENTVLSPALGPESSTCQNEITLQVPNPSELRAKPPS
SSSTCTDSATRDISEGGESPVVQSDEEEVQVDTALATSHTDREATPDGGEDSDS

>Q96A54 multipass membrane protein
MSSHKGSVVAQGNGAPASNREADTVELAELGPLLEEKGKRVIANPPKAEEEQTCPVPQEE
EEEVRVLTLPLQAHHAMEKMEEFVYKVWEGRWRVIPYDVLPDWLKDNDYLLHGHRPPM
PSFRACFKSIFRIHTETGNIWTHLLGFVLFLFLGILTMLRPNMYFMAPLQEKVVFGMFFLGA
VLCLSFSWLFHTVYCHSEKVSRTFSKLDYSGIALLIMGSFVPWLYYSFYCSPQPRLIYLSIV
CVLGISAIIVAQWDRFATPKHRQTRAGVFLGLGLSGVVPTMHFTIAEGFVKATTVGQMGW
FFLMAVMYITGAGLYAARIPERFFPGKFDIWFQSHQIFHVLVVAAAFVHFYGVSNLQEFRY
GLEGGCTDDTLL

>P25089 multipass membrane protein
METNFSIPLNETEEVLPEPAGHTVLWIFSLLVHGVTFVFGVLGNGLVIWVAGFRMTRTVNT
ICYLNLALADFSFSAILPFRMVSVAMREKWPFGSFLCKLVHVMIDINLFVSVYLITIIALDR
CICVLHPAWAQNHRTMSLAKRVMTGLWIFTIVLTLPNFIFWTTISTTNGDTYCIFNFAFWGD
TAVERLNVFITMAKVFLILHFIIGFSVPMSIITVCYGIIAAKIHRNHMIKSSRPLRVFAAVVAS
FFICWFPYELIGILMAVWLKEMLLNGKYKIILVLINPTSSLAFFNSCLNPILYVFMGRNFQER
LIRSLPTSLERALTEVPDSAQTSNTDTTSASPPEETELQAM

>Q14789 single-pass type I membrane protein
MLSRLSGLANVVLHELSGDDDTDQNMRAPLDPELHQESDMEFNNTTQEDVQERLAYAE
QLVVELKDIIRQKDVQLQQKDEALQEERKAADNKIKKLKLHAKAKLTSLNKYIEEMKAQ
GGTVLPTEPQSEEQLSKHDKSSTEEEMEIEKIKHKLQEKEELISTLQAQLTQAQAEQPAQSS
TEMEEFVMMKQQLQEKEEFISTLQAQLSQTQAEQAAQQVVREKDARFETQVRLHEDELL
QLVTQADVETEMQQKLRVLQRKLEEHEESLVGRAQVVDLLQQELTAAEQRNQILSQQLQ
QMEAEHNTLRNTVETEREESKILLEKMELEVAERKLSFHNLQEEMHHLLEQFEQAGQAQ
AELESRYSALEQKHKAEMEEKTSHILSLQKTGQELQSACDALKDQNSKLLQDKNEQAVQ
SAQTIQQLEDQLQQKSKEISQFLNRLPLQQHETASQTSFPDVYNEGTQAVTEENIASLQKR
VVELENEKGALLLSSIELEELKAENEKLSSQITLLEAQNRTGEADREVSEISIVDIANKRSSS
AEESGQDVLENTFSQKHKELSVLLLEMKEAQEEIAFLKLQLQGKRAEEADHEVLDQKEM
KQMEGEGIAPIKMKVFLEDTGQDFPLMPNEESSLPAVEKEQASTEHQSRTSEEISLNDAGV
ELKSTKQDGDKSLSAVPDIGQCHQDELERLKSQILELELNFHKAQEIYEKNLDEKAKEISN
LNQLIEEFKKNADNNSSAFTALSEERDQLLSQVKELSMVTELRAQVKQLEMNLAEAERQ
RRLDYESQTAHDNLLTEQIHSLSIEAKSKDVKIEVLQNELDDVQLQFSEQSTLIRSLQSQLQ
NKESEVLEGAERVRHISSKVEELSQALSQKELEITKMDQLLLEKKRDVETLQQTIEEKDQQ
VTEISFSMTEKMVQLNEEKFSLGVEIKTLKEQLNLLSRAEEAKKEQVEEDNEVSSGLKQN
YDEMSPAGQISKEELQHEFDLLKKENEQRKRKLQAALINRKELLQRVSRLEEELANLKDE
SKKEIPLSETERGEVEEDKENKEYSEKCVTSKCQEIEIYLKQTISEKEVELQHIRKDLEEKL
AAEEQFQALVKQMNQTLQDKTNQIDLLQAEISENQAIIQKLITSNTDASDGDSVALVKETV
VISPPCTGSSEHWKPELEEKILALEKEKEQLQKKLQEALTSRKAILKKAQEKERHLREELK
QQKDDYNRLQEQFDEQSKENENIGDQLRQLQIQVRESIDGKLPSTDQQESCSSTPGLEEPL
FKATEQHHTQPVLESNLCPDWPSHSEDASALQGGTSVAQIKAQLKEIEAEKVELELKVSST
TSELTKKSEEVFQLQEQINKQGLEIESLKTVSHEAEVHAESLQQKLESSQLQIAGLEHLREL

QPKLDELQKLISKKEEDVSYLSGQLSEKEAALTKIQTEIIEQEDLIKALHTQLEMQAKEHDE
RIKQLQVELCEMKQKPEEIGEESRAKQQIQRKLQAALISRKEALKENKSLQEELSLARGTI
ERLTKSLADVESQVSAQNKEKDTVLGRLALLQEERDKLITEMDRSLLENQSLSSSCESLKL
ALEGLTEDKEKLVKEIESLKSSKIAESTEWQEKHKELQKEYEILLQSYENVSNEAERIQHV
VEAVRQEKQELYGKLRSTEANKKETEKQLQEAEQEMEEMKEKMRKFAKSKQQKILELEE
ENDRLRAEVHPAGDTAKECMETLLSSNASMKEELERVKMEYETLSKKFQSLMSEKDSLSE
EVQDLKHQIEGNVSKQANLEATEKHDNQTNVTEEGTQSIPGETEEQDSLSMSTRPTCSESV
PSAKSANPAVSKDFSSHDEINNYLQQIDQLKERIAGLEEEKQKNKEFSQTLENEKNTLLSQI
STKDGELKMLQEEVTKMNLLNQQIQEELSRVTKLKETAEEEKDDLEERLMNQLAELNGSI
GNYCQDVTDAQIKNELLESEMKNLKKCVSELEEEKQQLVKEKTKVESEIRKEYLEKIQGA
QKEPGNKSHAKELQELLKEKQQEVKQLQKDCIRYQEKISALERTVKALEFVQTESQKDLE
ITKENLAQAVEHRKKAQAELASFKVLLDDTQSEAARVLADNLKLKKELQSNKESVKSQM
KQKDEDLERRLEQAEEKHLKEKKNMQEKLDALRREKVHLEETIGEIQVTLNKKDKEVQQ
LQENLDSTVTQLAAFTKSMSSLQDDRDRVIDEAKKWERKFSDAIQSKEEEIRLKEDNCSV
LKDQLRQMSIHMEELKINISRLEHDKQIWESKAQTEVQLQQKVCDTLQGENKELLSQLEE
TRHLYHSSQNELAKLESELKSLKDQLTDLSNSLEKCKEQKGNLEGIIRQQEADIQNSKFSY
EQLETDLQASRELTSRLHEEINMKEQKIISLLSGKEEAIQVAIAELRQQHDKEIKELENLLSQ
EEEENIVLEEENKKAVDKTNQLMETLKTIKKENIQQKAQLDSFVKSMSSLQNDRDRIVGD
YQQLEERHLSIILEKDQLIQEAAAENNKLKEEIRGLRSHMDDLNSENAKLDAELIQYREDL
NQVITIKDSQQKQLLEVQLQQNKELENKYAKLEEKLKESEEANEDLRRSFNALQEEKQDL
SKEIESLKVSISQLTRQVTALQEEGTLGLYHAQLKVKEEEVHRLSALFSSSQKRIAELEEEL
VCVQKEAAKKVGEIEDKLKKELKHLHHDAGIMRNETETAEERVAELARDLVEMEQKLLM
VTKENKGLTAQIQSFGRSMSSLQNSRDHANEELDELKRKYDASLKELAQLKEQGLLNRER
DALLSETAFSMNSTEENSLSHLEKLNQQLLSKDEQLLHLSSQLEDSYNQVQSFSKAMASL
QNERDHLWNELEKFRKSEEGKQRSAAQPSTSPAEVQSLKKAMSSLQNDRDRLLKELKNL
QQQYLQINQEITELHPLKAQLQEYQDKTKAFQIMQEELRQENLSWQHELHQLRMEKSSW
EIHERRMKEQYLMAISDKDQQLSHLQNLIRELRSSSSQTQPLKVQYQRQASPETSASPDGS
QNLVYETELLRTQLNDSLKEIHQKELRIQQLNSNFSQLLEEKNTLSIQLCDTSQSLRENQQH
YGDLLNHCAVLEKQVQELQAGPLNIDVAPGAPQEKNGVHRKSDPEELREPQQSFSEAQQ
QLCNTRQEVNELRKLLEEERDQRVAAENALSVAEEQIRRLEHSEWDSSRTPIIGSCGTQEQ
ALLIDLTSNSCRRTRSGVGWKRVLRSLCHSRTRVPLLAAIYFLMIHVLLILCFTGHL

>O75781 lipid-anchor
MEVLAAETTSQQERLQAIAEKRKRQAEIENKRRQLEDERRQLQHLKSKALRERWLLEGT
PSSASEGDEDLRRQMQDDEQKTRLLEDSVSRLEKEIEVLERGDSAPATAKENAAAPSPVR
APAPSPAKEERKTEVVMNSQQTPVGTPKDKRVSNTPLRTVDGSPMMKAAMYSVEITVEK
DKVTGETRVLSSTTLLPRQPLPLGIKVYEDETKVVHAVDGTAENGIHPLSSSEVDELIHKA
DEVTLSEAGSTAGAAETRGAVEGAARTTPSRREITGVQAQPGEATSGPPGIQPGQEPPVTM
IFMGYQNVEDEAETKKVLGLQDTITAELVVIEDAAEPKEPAPPNGSAAEPPTEAASREENQ
AGPEATTSDPQDLDMKKHRCKCCSIM

>Q9H344 multipass membrane protein
MGLFNVTHPAFFLLTGIPGLESSHSWLSGPLCVMYAVALGGNTVILQAVRVEPSLHEPMYY
FLSMLSFSDVAISMATLPTVLRTFCLNARNITFDACLIQMFLIHFFSMMESGILLAMSFDRY

VAICDPLRYATVLTTEVIAAMGLGAAARSFITLFPLPFLIKRLPICRSNVLSHSYCLHPDMM
RLACADISINSIYGLFVLVSTFGMDLFFIFLSYVLILRSVMATASREERLKALNTCVSHILAV
LAFYVPMIGVSTVHRFGKHVPCYIHVLMSNVYLFVPPVLNPLIYSAKTKEIRRAIFRMFHH
IKI

>P05177 peripheral membrane protein
MALSQSVPFSATELLLASAIFCLVFWVLKGLRPRVPKGLKSPPEPWGWPLLGHVLTLGKNP
HLALSRMSQRYGDVLQIRIGSTPVLVLSRLDTIRQALVRQGDDFKGRPDLYTSTLITDGQSL
TFSTDSGPVWAARRRLAQNALNTFSIASDPASSSSCYLEEHVSKEAKALISRLQELMAGPG
HFDPYNQVVVSVANVIGAMCFGQHFPESSDEMLSLVKNTHEFVETASSGNPLDFFPILRYL
PNPALQRFKAFNQRFLWFLQKTVQEHYQDFDKNSVRDITGALFKHSKKGPRASGNLIPQE
KIVNLVNDIFGAGFDTVTTAISWSLMYLVTKPEIQRKIQKELDTVIGRERRPRLSDRPQLPY
LEAFILETFRHSSFLPFTIPHSTTRDTTLNGFYIPKKCCVFVNQWQVNHDPELWEDPSEFRP
ERFLTADGTAINKPLSEKMMLFGMGKRRCIGEVLAKWEIFLFLAILLQQLEFSVPPGVKVD
LTPIYGLTMKHARCEHVQARRFSIN

>Q8NH04 multipass membrane protein
MEQSNYSVYADFILLGLFSNARFPWLLFALILLVFLTSIASNVVKIILIHIDSRLHTPMYFLLS
QLSLRDILYISTIVPKMLVDQVMSQRAISFAGCTAQHFLYLTLAGAEFFLLGLMSYDRYVAI
CNPLHYPVLMSRKICWLIVAAAWLGGSIDGFLLTPVTMQFPFCASREINHFFCEVPALLKLS
CTDTSAYETAMYVCCIMMLLIPFSVISGSYTRILITVYRMSEAEGRGKAVATCSSHMVVVS
LFYGAAMYTYVLPHSYHTPEQDKAVSAFYTILTPMLNPLIYSLRNKDVTGALQKVVGRC
VSSGKVTTF

>Q14773 single-pass type I membrane protein
MGSLFPLSLLFFLAAAYPGVGSALGRRTKRAQSPKGSPLAPSGTSVPFWVRMSPEFVAVQP
GKSVQLNCSNSCPQPQNSSLRTPLRQGKTLRGPGWVSYQLLDVRAWSSLAHCLVTCAGK
TRWATSRITAYKPPHSVILEPPVLKGRKYTLRCHVTQVFPVGYLVVTLRHGSRVIYSESLER
FTGLDLANVTLTYEFAAGPRDFWQPVICHARLNLDGLVVRNSSAPITLMLAWSPAPTALAS
GSIAALVGILLTVGAAYLCKCLAMKSQA

>O43889 single-pass type II membrane protein
MELELDAGDQDLLAFLLEESGDLGTAPDEAVRAPLDWALPLSEVPSDWEVDDLLCSLLSP
PASLNILSSSNPCLVHHDHTYSLPRETVSMDLGECEISLTGRTGFMGLAIHTFPFAESESCRK
EGTQMTPQHMEELAEQEIARLVLTDEEKSLLEKEGLILPETLPLTKTEEQILKRVRRKIRNK
RSAQESRRKKKVYVGGLESRVLKYTAQNMELQNKVQLLEEQNLSLLDQLRKLQAMVIEI
SNKTSSSSTCILVLLVSFCLLLVPAMYSSDTRGSLPAEHGVLSRQLRALPSEDPYQLELPALQ
SEVPKDSTHQWLDGSDCVLQAPGNTSCLLHYMPQAPSAEPPLEWPFPDLFSEPLCRGPILP
LQANLTRKGGWLPTGSPSVILQDRYSG

>Q9NRX6 single-pass type I membrane protein
MTNVYSLDGILVFGLLFVCTCAYFKKVPRLKTWLLSEKKGVWGVFYKAAVIGTRLHAAV
AIACVVMAFYVLFIK

>Q15303 single-pass type I membrane protein
MKPATGLWVWVSLLVAAGTVQPSDSQSVCAGTENKLSSLSDLEQQYRALRKYYENCEVV
MGNLEITSIEHNRDLSFLRSVREVTGYVLVALNQFRYLPLENLRIIRGTKLYEDRYALAIFLN
YRKDGNFGLQELGLKNLTEILNGGVYVDQNKFLCYADTIHWQDIVRNPWPSNLTLVSTNG
SSGCGRCHKSCTGRCWGPTENHCQTLTRTVCAEQCDGRCYGPYVSDCCHRECAGGCSGP
KDTDCFACMNFNDSGACVTQCPQTFVYNPTTFQLEHNFNAKYTYGAFCVKKCPHNFVV
DSSSCVRACPSSKMEVEENGIKMCKPCTDICPKACDGIGTGSLMSAQTVDSSNIDKFINCT
KINGNLIFLVTGIHGDPYNAIEAIDPEKLNVFRTVREITGFLNIQSWPPNMTDFSVFSNLVTI
GGRVLYSGLSLLILKQQGITSLQFQSLKEISAGNIYITDNSNLCYYHTINWTTLFSTINQRIVI
RDNRKAENCTAEGMVCNHLCSSDGCWGPGPDQCLSCRRFSRGRICIESCNLYDGEFREFE
NGSICVECDPQCEKMEDGLLTCHGPGPDNCTKCSHFKDGPNCVEKCPDGLQGANSFIFKY
ADPDRECHPCHPNCTQGCNGPTSHDCIYYPWTGHSTLPQHARTPLIAAGVIGGLFILVIVGL
TFAVYVRRKSIKKKRALRRFLETELVEPLTPSGTAPNQAQLRILKETELKRVKVLGSGAFGT
VYKGIWVPEGETVKIPVAIKILNETTGPKANVEFMDEALIMASMDHPHLVRLLGVCLSPTI
QLVTQLMPHGCLLEYVHEHKDNIGSQLLLNWCVQIAKGMMYLEERRLVHRDLAARNVL
VKSPNHVKITDFGLARLLEGDEKEYNADGGKMPIKWMALECIHYRKFTHQSDVWSYGV
TIWELMTFGGKPYDGIPTREIPDLLEKGERLPQPPICTIDVYMVMVKCWMIDADSRPKFKE
LAAEFSRMARDPQRYLVIQGDDRMKLPSPNDSKFFQNLLDEEDLEDMMDAEEYLVPQAF
NIPPPIYTSRARIDSNRSEIGHSPPPAYTPMSGNQFVYRDGGFAAEQGVSVPYRAPTSTIPEA
PVAQGATAEIFDDSCCNGTLRKPVAPHVQEDSSTQRYSADPTVFAPERSPRGELDEEGYMT
PMRDKPKQEYLNPVEENPFVSRRKNGDLQALDNPEYHNASNGPPKAEDEYVNEPLYLNT
FANTLGKAEYLKNNILSMPEKAKKAFDNPDYWNHSLPPRSTLQHPDYLQEYSTKYFYKQ
NGRIRPIVAENPEYLSEFSLKPGTVLPPPPYRHRNTVV

>Q9NS93 multipass membrane protein
MGFLQLLVVAVLASEHRVAGAAEVFGNSSEGLIEFSVGKFRYFELNRPFPEEAILHDISSNV
TFLIFQIHSQYQNTTVSFSPTLLSNSSETGTASGLVFILRPEQSTCTWYLGTSGIQPVQNMAI
LLSYSERDPVPGGCNLEFDLDIDPNIYLEYNFFETTIKFAPANLGYARGVDPPPCDAGTDQD
SRWRLQYDVYQYFLPENDLTEEMLLKHLQRMVSVPQVKASALKVVTLTANDKTSVSFSS
LPGQGVIYNVIVWDPFLNTSAAYIPAHTYACSFEAGEGSCASLGRVSSKVFFTLFALLGFFI
CFFGHRFWKTELFFIGFIIMGFFFYILITRLTPIKYDVNLILTAVTGSVGGMFLVAVWWRFGIL
SICMLCVGLVLGFLISSVTFFTPLGNLKIFHDDGVFWVTFSCIAILIPVVFMGCLRILNILTCG
VIGSYSVVLAIDSYWSTSLSYITLNVLKRALNKDFHRAFTNVPFQTNDFIILAVWGMLAVS
GITLQIRRERGRPFFPPHPYKLWKQERERRVTNILDPSYHIPPLRERLYGRLTQIKGLFQKEQ
PAGERTPLLL

>O14684 multipass membrane protein
MPAHSLVMSSPALPAFLLCSTLLVIKMYVVAIITGQVRLRKKAFANPEDALRHGGPQYCRS
DPDVERCLRAHRNDMETIYPFLFLGFVYSFLGPNPFVAWMHFLVFLVGRVAHTVAYLGKL
RAPIRSVTYTLAQLPCASMALQILWEAARHL

>Q8NH69 multipass membrane protein
MDWENCSSLTDFFLLGITNNPEMKVTLFAVFLAVYIINFSANLGMIVLIRMDYQLHTPMYF
FLSHLSFCDLCYSTATGPKMLVDLLAKNKSIPFYGCALQFLVFCIFADSECLLLSVMAFDRY

KAIINPLLYTVNMSSRVCYLLLTGVYLVGIADALIHMTLAFRLCFCGSNEINHFFCDIPPLLL
LSRSDTQVNELVLFTVFGFIELSTISGVFISYCYIILSVLEIHSAEGRFKALSTCTSHLSAVAIF
QGTLLFMYFRPSSSYSLDQDKMTSLFYTLVVPMLNPLIYSLRNKDVKEALKKLKNKILF

>Q9Y5N1 multipass membrane protein
MERAPPDGPLNASGALAGEAAAAGGARGFSAAWTAVLAALMALLIVATVLGNALVMLA
FVADSSLRTQNNFFLLNLAISDFLVGAFCIPLYVPYVLTGRWTFGRGLCKLWLVVDYLLCT
SSAFNIVLISYDRFLSVTRAVSYRAQQGDTRRAVRKMLLVWVLAFLLYGPAILSWEYLSGG
SSIPEGHCYAEFFYNWYFLITASTLEFFTPFLSVTFFNLSIYLNIQRRTRLRLDGAREAAGPE
PPPEAQPSPPPPPGCWGCWQKGHGEAMPLHRYGVGEAAVGAEAGEATLGGGGGGGSVA
SPTSSSGSSSRGTERPRSLKRGSKPSASSASLEKRMKMVSQSFTQRFRLSRDRKVAKSLAVI
VSIFGLCWAPYTLLMIIRAACHGHCVPDYWYETSFWLLWANSAVNPVLYPLCHHSFRRAF
TKLLCPQKLKIQPHSSLEHCWK

>Q5QJU3 multipass membrane protein
MGAPHWWDQLQAGSSEVDWCEDNYTIVPAIAEFYNTISNVLFFILPPICMCLFRQYATCFN
SGIYLIWTLLVVVGIGSVYFHATLSFLGQMLDELAVLWVLMCALAMWFPRRYLPKIFRND
RGRFKVVVSVLSAVTTCLAFVKPAINNISLMTLGVPCTALLIAELKRCDNMRVFKLGLFSG
LWWTLALFCWISDRAFCELLSSFNFPYLHCMWHILICLAAYLGCVCFAYFDAASEIPEQGP
VIKFWPNEKWAFIGVPYVSLLCANKKSSVKIT

>Q93063 single-pass type II membrane protein
MCASVKYNIRGPALIPRMKTKHRIYYITLFSIVLLGLIATGMFQFWPHSIESSNDWNVEKRS
IRDVPVVRLPADSPIPERGDLSCRMHTCFDVYRCGFNPKNKIKVYIYALKKYVDDFGVSVS
NTISREYNELLMAISDSDYYTDDINRACLFVPSIDVLNQNTLRIKETAQAMAQLSRWDRGT
NHLLFNMLPGGPPDYNTALDVPRDRALLAGGGFSTWTYRQGYDVSIPVYSPLSAEVDLPE
KGPGPRQYFLLSSQVGLHPEYREDLEALQVKHGESVLVLDKCTNLSEGVLSVRKRCHKH
QVFDYPQVLQEATFCVVLRGARLGQAVLSDVLQAGCVPVVIADSYILPFSEVLDWKRASV
VVPEEKMSDVYSILQSIPQRQIEEMQRQARWFWEAYFQSIKAIALATLQIINDRIYPYAAISY
EEWNDPPAVKWGSVSNPLFLPLIPPQSQGFTAIVLTYDRVESLFRVITEVSKVPSLSKLLVV
WNNQNKNPPEDSLWPKIRVPLKVVRTAENKLSNRFFPYDEIETEAVLAIDDDIIMLTSDELQ
FGYEVWREFPDRLVGYPGRLHLWDHEMNKWKYESEWTNEVSMVLTGAAFYHKYFNYL
YTYKMPGDIKNWVDAHMNCEDIAMNFLVANVTGKAVIKVTPRKKFKCPECTAIDGLSLD
QTHMVERSECINKFASVFGTMPLKVVEHRADPVLYKDDFPEKLKSFPNIGSL

>P06127 single-pass type I membrane protein
MPMGSLQPLATLYLLGMLVASCLGRLSWYDPDFQARLTRSNSKCQGQLEVYLKDGWHM
VCSQSWGRSSKQWEDPSQASKVCQRLNCGVPLSLGPFLVTYTPQSSIICYGQLGSFSNCSH
SRNDMCHSLGLTCLEPQKTTPPTTRPPPTTTPEPTAPPRLQLVAQSGGQHCAGVVEFYSGS
LGGTISYEAQDKTQDLENFLCNNLQCGSFLKHLPETEAGRAQDPGEPREHQPLPIQWKIQ
NSSCTSLEHCFRKIKPQKSGRVLALLCSGFQPKVQSRLVGGSSICEGTVEVRQGAQWAALC
DSSSARSSLRWEEVCREQQCGSVNSYRVLDAGDPTSRGLFCPHQKLSQCHELWERNSYC
KKVFVTCQDPNPAGLAAGTVASIILALVLLVVLLVVCGPLAYKKLVKKFRQKKQRQWIGP
TGMNQNMSFHRNHTATVRSHAENPTASHVDNEYSQPPRNSHLSAYPALEGALHRSSMQP

DNSSDSDYDLHGAQRL

>Q13393 lipid-anchor
MSLKNEPRVNTSALQKIAADMSNIIENLDTRELHFEGEEVDYDVSPSDPKIQEVYIPFSAIY
NTQGFKEPNIQTYLSGCPIKAQVLEVERFTSTTRVPSINLYTIELTHGEFKWQVKRKFKHFQ
EFHRELLKYKAFIRIPIPTRRHTFRRQNVREEPREMPSLPRSSENMIREEQFLGRRKQLEDY
LTKILKMPMYRNYHATTEFLDISQLSFIHDLGPKGIEGMIMKRSGGHRIPGLNCCGQGRAC
YRWSKRWLIVKDSFLLYMKPDSGAIAFVLLVDKEFKIKVGKKETETKYGIRIDNLSRTLIL
KCNSYRHARWWGGAIEEFIQKHGTNFLKDHRFGSYAAIQENALAKWYVNAKGYFEDVA
NAMEEANEEIFITDWWLSPEIFLKRPVVEGNRWRLDCILKRKAQQGVRIFIMLYKEVELAL
GINSEYTKRTLMRLHPNIKVMRHPDHVSSTVYLWAHHEKLVIIDQSVAFVGGIDLAYGRW
DDNEHRLTDVGSVKRVTSGPSLGSLPPAAMESMESLRLKDKNEPVQNLPIQKSIDDVDSK
LKGIGKPRKFSKFSLYKQLHRHHLHDADSISSIDSTSSYFNHYRSHHNLIHGLKPHFKLFHP
SSESEQGLTRPHADTGSIRSLQTGVGELHGETRFWHGKDYCNFVFKDWVQLDKPFADFID
RYSTPRMPWHDIASAVHGKAARDVARHFIQRWNFTKIMKSKYRSLSYPFLLPKSQTTAHE
LRYQVPGSVHANVQLLRSAADWSAGIKYHEESIHAAYVHVIENSRHYIYIENQFFISCADD
KVVFNKIGDAIAQRILKAHRENQKYRVYVVIPLLPGFEGDISTGGGNALQAIMHFNYRTM
CRGENSILGQLKAELGNQWINYISFCGLRTHAELEGNLVTELIYVHSKLLIADDNTVIIGSA
NINDRSMLGKRDSEMAVIVQDTETVPSVMDGKEYQAGRFARGLRLQCFRVVLGYLDDPS
EDIQDPVSDKFFKEVWVSTAARNATIYDKVFRCLPNDEVHNLIQLRDFINKPVLAKEDPIR
AEEELKKIRGFLVQFPFYFLSEESLLPSVGTKEAIVPMEVWT

>Q08462 multipass membrane protein
MWQEAMRRRRYLRDRSEEAAGGGDGLPRSRDWLYESYYCMSQQHPLIVFLLLIVMGSC
LALLAVFFALGLEVEDHVAFLITVPTALAIFFAIFILVCIESVFKKLLRLFSLVIWICLVAMGY
LFMCFGGTVSPWDQVSFFLFIIFVVYTMLPFNMRDAIIASVLTSSSHTIVLSVCLSATPGGK
EHLVWQILANVIIFICGNLAGAYHKHLMELALQQTYQDTCNCIKSRIKLEFEKRQQERLLL
SLLPAHIAMEMKAEIIQRLQGPKAGQMENTNNFHNLYVKRHTNVSILYADIVGFTRLASDC
SPGELVHMLNELFGKFDQIAKENECMRIKILGDCYYCVSGLPISLPNHAKNCVKMGLDMC
EAIKKVRDATGVDINMRVGVHSGNVLCGVIGLQKWQYDVWSHDVTLANHMEAGGVPG
RVHISSVTLEHLNGAYKVEEGDGDIRDPYLKQHLVKTYFVINPKGERRSPQHLFRPRHTLD
GAKMRASVRMTRYLESWGAAKPFAHLHHRDSMTTENGKISTTDVPMGQHNFQNRTLRT
KSQKKRFEEELNERMIQAIDGINAQKQWLKSEDIQRISLLFYNKVLEKEYRATALPAFKYY
VTCACLIFFCIFIVQILVLPKTSVLGISFGAAFLLLAFILFVCFAGQLLQCSKKASPLLMWLL
KSSGIIANRPWPRISLTIITTAIILMMAVFNMFFLSDSEETIPPTANTTNTSFSASNNQVAILRA
QNLFFLPYFIYSCILGLISCSVFLRVNYELKMLIMMVALVGYNTILLHTHAHVLGDYSQVLF
ERPGIWKDLKTMGSVSLSIFFITLLVLGRQNEYYCRLDFLWKNKFKKEREEIETMENLNRV
LLENVLPAHVAEHFLARSLKNEELYHQSYDCVCVMFASIPDFKEFYTESDVNKEGLECLRL
LNEIIADFDDLLSKPKFSGVEKIKTIGSTYMAATGLSAVPSQEHSQEPERQYMHIGTMVEFA
FALVGKLDAINKHSFNDFKLRVGINHGPVIAGVIGAQKPQYDIWGNTVNVASRMDSTGVL
DKIQVTEETSLVLQTLGYTCTCRGIINVKGKGDLKTYFVNTEMSRSLSQSNVAS

>Q8IZF4 multipass membrane protein
MDHCGALFLCLCLLTLQNATTETWEELLSYMENMQVSRGRSSVFSSRQLHQLEQMLLNT

SFPGYNLTLQTPTIQSLAFKLSCDFSGLSLTSATLKRVPQAGGQHARGQHAMQFPAELTRD
ACKTRPRELRLICIYFSNTHFFKDENNSSLLNNYVLGAQLSHGHVNNLRDPVNISFWHNQ
SLEGYTLTCVFWKEGARKQPWGGWSPEGCRTEQPSHSQVLCRCNHLTYFAVLMQLSPAL
VPAELLAPLTYISLVGCSISIVASLITVLLHFHFRKQSDSLTRIHMNLHASVLLLNIAFLLSPAF
AMSPVPGSACTALAAALHYALLSCLTWMAIEGFNLYLLLGRVYNIYIRRYVFKLGVLGWG
APALLVLLSLSVKSSVYGPCTIPVFDSWENGTGFQNMSICWVRSPVVHSVLVMGYGGLTS
LFNLVVLAWALWTLRRLRERADAPSVRACHDTVTVLGLTVLLGTTWALAFFSFGVFLLPQ
LFLFTILNSLYGFFLFLWFCSQRCRSEAEAKAQIEAFSSSQTTQ

>Q6Y288 single-pass type II membrane protein
MRPPACWWLLAPPALLALLTCSLAFGLASEDTKKEVKQSQDLEKSGISRKNDIDLKGIVFV
IQSQSNSFHAKRAEQLKKSILKQAADLTQELPSVLLLHQLAKQEGAWTILPLLPHFSVTYS
RNSSWIFFCEEETRIQIPKLLETLRRYDPSKEWFLGKALHDEEATIIHHYAFSENPTVFKYPD
FAAGWALSIPLVNKLTKRLKSESLKSDFTIDLKHEIALYIWDKGGGPPLTPVPEFCTNDVDF
YCATTFHSFLPLCRKPVKKKDIFVAVKTCKKFHGDRIPIVKQTWESQASLIEYYSDYTENSI
PTVDLGIPNTDRGHCGKTFAILERFLNRSQDKTAWLVIVDDDTLISISRLQHLLSCYDSGEP
VFLGERYGYGLGTGGYSYITGGGGMVFSREAVRRLLASKCRCYSNDAPDDMVLGMCFS
GLGIPVTHSPLFHQARPVDYPKDYLSHQVPISFHKHWNIDPVKVYFTWLAPSDEDKARQE
TQKGFREEL

>Q6IF00 multipass membrane protein
MGMEGLLQNSTNFVLTGLITHPAFPGLLFAIVFSIFVVAITANLVMILLIHMDSRLHTPMYFL
LSQLSIMDTIYICITVPKMLQDLLSKDKTISFLGCAVQIFLYLTLIGGEFFLLGLMAYDRYVA
VCNPLRYPLLMNRRVCLFMVVGSWVGGSLDGFMLTPVTMSFPFCRSREINHFFCEIPAVLK
LSCTDTSLYETLMYACCVLMLLIPLSVISVSYTHILLTVHRMNSAEGRRKAFATCSSHIMVV
SVFYGAAFYTNVLPHSYHTPEKDKVVSAFYTILTPMLNPLIYSLRNKDVAAALRKVLGRC
GSSQSIRVATVIRKG

>P23458 peripheral membrane protein
MQYLNIKEDCNAMAFCAKMRSSKKTEVNLEAPEPGVEVIFYLSDREPLRLGSGEYTAEEL
CIRAAQACRISPLCHNLFALYDENTKLWYAPNRTITVDDKMSLRLHYRMRFYFTNWHGTN
DNEQSVWRHSPKKQKNGYEKKKIPDATPLLDASSLEYLFAQGQYDLVKCLAPIRDPKTEQ
DGHDIENECLGMAVLAISHYAMMKKMQLPELPKDISYKRYIPETLNKSIRQRNLLTRMRIN
NVFKDFLKEFNNKTICDSSVSTHDLKVKYLATLETLTKHYGAEIFETSMLLISSENEMNWF
HSNDGGNVLYYEVMVTGNLGIQWRHKPNVVSVEKEKNKLKRKKLENKHKKDEEKNKIR
EEWNNFSYFPEITHIVIKESVVSINKQDNKKMELKLSSHEEALSFVSLVDGYFRLTADAHH
YLCTDVAPPLIVHNIQNGCHGPICTEYAINKLRQEGSEEGMYVLRWSCTDFDNILMTVTCF
EKSEQVQGAQKQFKNFQIEVQKGRYSLHGSDRSFPSLGDLMSHLKKQILRTDNISFMLKR
CCQPKPREISNLLVATKKAQEWQPVYPMSQLSFDRILKKDLVQGEHLGRGTRTHIYSGTL
MDYKDDEGTSEEKKIKVILKVLDPSHRDISLAFFEAASMMRQVSHKHIVYLYGVCVRDVE
NIMVEEFVEGGPLDLFMHRKSDVLTTPWKFKVAKQLASALSYLEDKDLVHGNVCTKNLL
LAREGIDSECGPFIKLSDPGIPITVLSRQECIERIPWIAPECVEDSKNLSVAADKWSFGTTLW
EICYNGEIPLKDKTLIEKERFYESRCRPVTPSCKELADLMTRCMNYDPNQRPFFRAIMRDI
NKLEEQNPDIVSEKKPATEVDPTHFEKRFLKRIRDLGEGHFGKVELCRYDPEGDNTGEQVA

VKSLKPESGGNHIADLKKEIEILRNLYHENIVKYKGICTEDGGNGIKLIMEFLPSGSLKEYL
PKNKNKINLKQQLKYAVQICKGMDYLGSRQYVHRDLAARNVLVESEHQVKIGDFGLTKA
IETDKEYYTVKDDRDSPVFWYAPECLMQSKFYIASDVWSFGVTLHELLTYCDSDSSPMAL
FLKMIGPTHGQMTVTRLVNTLKEGKRLPCPPNCPDEVYQLMRKCWEFQPSNRTSFQNLIE
GFEALLK

>Q9H596 peripheral membrane protein
MTASASSFSSSQGVQQPSIYSFSQITRSLFLSNGVAANDKLLLSSNRITAIVNASVEVVNVFF
EGIQYIKVPVTDARDSRLYDFFDPIADLIHTIDMRQGRTLLHCMAGVSRSASLCLAYLMKY
HSMSLLDAHTWTKSRRPIIRPNNGFWEQLINYEFKLFNNNTVRMINSPVGNIPDIYEKDLR
MMISM

>O00168 single-pass type I membrane protein
MASLGHILVFCVGLLTMAKAESPKEHDPFTYDYQSLQIGGLVIAGILFILGILIVLSRRCRCK
FNQQQRTGEPDEEEGTFRSSIRRLSTRRR

>Q16512 peripheral membrane protein
MASDAVQSEPRSWSLLEQLGLAGADLAAPGVQQQLELERERLRREIRKELKLKEGAENL
RRATTDLGRSLGPVELLLRGSSRRLDLLHQQLQELHAHVVLPDPAATHDGPQSPGAGGPT
CSATNLSRVAGLEKQLAIELKVKQGAENMIQTYSNGSTKDRKLLLTAQQMLQDSKTKIDII
RMQLRRALQAGQLENQAAPDDTQGSPDLGAVELRIEELRHHFRVEHAVAEGAKNVLRLL
SAAKAPDRKAVSEAQEKLTESNQKLGLLREALERRLGELPADHPKGRLLREELAAASSAA
FSTRLAGPFPATHYSTLCKPAPLTGTLEVRVVGCRDLPETIPWNPTPSMGGPGTPDSRPPFL
SRPARGLYSRSGSLSGRSSLKAEAENTSEVSTVLKLDNTVVGQTSWKPCGPNAWDQSFTL
ELERARELELAVFWRDQRGLCALKFLKLEDFLDNERHEVQLDMEPQGCLVAEVTFRNPVI
ERIPRLRRQKKIFSKQQGKAFQRARQMNIDVATWVRLLRRLIPNATGTGTFSPGASPGSEA
RTTGDISVEKLNLGTDSDSSPQKSSRDPPSSPSSLSSPIQESTAPELPSETQETPGPALCSPLR
KSPLTLEDFKFLAVLGRGHFGKVLLSEFRPSGELFAIKALKKGDIVARDEVESLMCEKRILA
AVTSAGHPFLVNLFGCFQTPEHVCFVMEYSAGGDLMLHIHSDVFSEPRAIFYSACVVLGL
QFLHEHKIVYRDLKLDNLLLDTEGYVKIADFGLCKEGMGYGDRTSTFCGTPEFLAPEVLT
DTSYTRAVDWWGLGVLLYEMLVGESPFPGDDEEEVFDSIVNDEVRYPRFLSAEAIGIMRR
LLRRNPERRLGSSERDAEDVKKQPFFRTLGWEALLARRLPPPFVPTLSGRTDVSNFDEEFT
GEAPTLSPPRDARPLTAAEQAAFLDFDFVAGGC

>Q9UBL9 multipass membrane protein
MAAAQPKYPAGATARRLARGCWSALWDYETPKVIVVRNRRLGVLYRAVQLLILLYFVWY
VFIVQKSYQESETGPESSIITKVKGITTSEHKVWDVEEYVKPPEGGSVFSIITRVEATHSQTQ
GTCPESIRVHNATCLSDADCVAGELDMLGNGLRTGRCVPYYQGPSKTCEVFGWCPVEDG
ASVSQFLGTMAPNFTILIKNSIHYPKFHFSKGNIADRTDGYLKRCTFHEASDLYCPIFKLGFI
VEKAGESFTELAHKGGVIGVIINWDCDLDLPASECNPKYSFRRLDPKHVPASSGYNFRFAK
YYKINGTTTRTLIKAYGIRIDVIVHGQAGKFSLIPTIINLATALTSVGVGSFLCDWILLTFMN
KNKVYSHKKFDKVCTPSHPSGSWPVTLARVLGQAPPEPGHRSEDQHPSPPSGQEGQQGA
ECGPAFPPLRPCPISAPSEQMVDTPASEPAQASTPTDPKGLAQL

>Q9P2R3 peripheral membrane protein
MAEEEVAKLEKHLMLLRQEYVKLQKKLAETEKRCALLAAQANKESSSESFISRLLAIVAD
LYEQEQYSDLKIKVGDRHISAHKFVLAARSDSWSLANLSSTKELDLSDANPEVTMTMLR
WIYTDELEFREDDVFLTELMKLANRFQLQLLRERCEKGVMSLVNVRNCIRFYQTAEELNA
STLMNYCAEIIASHWDDLRKEDFSSMSAQLLYKMIKSKTEYPLHKAIKVEREDVVFLYLIE
MDSQLPGKLNEADHNGDLALDLALSRRLESIATTLVSHKADVDMVDKSGWSLLHKGIQR
GDLFAATFLIKNGAFVNAATLGAQETPLHLVALYSSKKHSADVMSEMAQIAEALLQAGAN
PNMQDSKGRTPLHVSIMAGNEYVFSQLLQCKQLDLELKDHEGSTALWLAVQHITVSSDQS
VNPFEDVPVVNGTSFDENSFAARLIQRGSHTDAPDTATGNCLLQRAAGAGNEAAALFLAT
NGAHVNHRNKWGETPLHTACRHGLANLTAELLQQGANPNLQTEEALPLPKEAASLTSLA
DSVHLQTPLHMAIAYNHPDVVSVILEQKANALHATNNLQIIPDFSLKDSRDQTVLGLALW
TGMHTIAAQLLGSGAAINDTMSDGQTLLHMAIQRQDSKSALFLLEHQADINVRTQDGETA
LQLAIRNQLPLVVDAICTRGADMSVPDEKGNPPLWLALANNLEDIASTLVRHGCDATCWG
PGPGGCLQTLLHRAIDENNEPTACFLIRSGCDVNSPRQPGANGEGEEEARDGQTPLHLAAS
WGLEETVQCLLEFGANVNAQDAEGRTPIHVAISSQHGVIIQLLVSHPDIHLNVRDRQGLTPF
ACAMTFKNNKSAEAILKRESGAAEQVDNKGRNFLHVAVQNSDIESVLFLISVHANVNSRV
QDASKLTPLHLAVQAGSEIIVRNLLLAGAKVNELTKHRQTALHLAAQQDLPTICSVLLENG
VDFAAVDENGNNALHLAVMHGRLNNIRVLLTECTVDAEAFNLRGQSPLHILGQYGKENA
AAIFDLFLECMPGYPLDKPDADGSTVLLLAYMKGNANLCRAIVRSGARLGVNNNQGVNI
FNYQVATKQLLFRLLDMLSKEPPWCDGSYCYECTARFGVTTRKHHCRHCGRLLCHKCST
KEIPIIKFDLNKPVRVCNICFDVLTLGGVS

>Q92854 single-pass type I membrane protein
MRMCTPIRGLLMALAVMFGTAMAFAPIPRITWEHREVHLVQFHEPDIYNYSALLLSEDKD
TLYIGAREAVFAVNALNISEKQHEVYWKVSEDKKAKCAEKGKSKQTECLNYIRVLQPLSA
TSLYVCGTNAFQPACDHLNLTSFKFLGKNEDGKGRCPFDPAHSYTSVMVDGELYSGTSYN
FLGSEPIISRNSSHSPLRTEYAIPWLNEPSFVFADVIRKSPDSPDGEDDRVYFFFTEVSVEYEF
VFRVLIPRIARVCKGDQGGLRTLQKKWTSFLKARLICSRPDSGLVFNVLRDVFVLRSPGLK
VPVFYALFTPQLNNVGLSAVCAYNLSTAEEVFSHGKYMQSTTVEQSHTKWVRYNGPVPK
PRPGACIDSEARAANYTSSLNLPDKTLQFVKDHPLMDDSVTPIDNRPRLIKKDVNYTQIVV
DRTQALDGTVYDVMFVSTDRGALHKAISLEHAVHIIEETQLFQDFEPVQTLLLSSKKGNRF
VYAGSNSGVVQAPLAFCGKHGTCEDCVLARDPYCAWSPPTATCVALHQTESPSRGLIQEM
SGDASVCPDKSKGSYRQHFFKHGGTAELKCSQKSNLARVFWKFQNGVLKAESPKYGLM
GRKNLLIFNLSEGDSGVYQCLSEERVKNKTVFQVVAKHVLEVKVVPKPVVAPTLSVVQTE
GSRIATKVLVASTQGSSPPTPAVQATSSGAITLPPKPAPTGTSCEPKIVINTVPQLHSEKTMYL
KSSDNRLLMSLFLFFFVLFLCLFFYNCYKGYLPRQCLKFRSALLIGKKKPKSDFCDREQSL
KETLVEPGSFSQQNGEHPKPALDTGYETEQDTITSKVPTDREDSQRIDDLSARDKPFDVKC
ELKFADSDADGD

>Q14330 multipass membrane protein
MITLNNQDQPVPFNSSHPDEYKIAALVFYSCIFIIGLFVNITALWVFSCTTKKRTTVTIYMM
NVALVDLIFIMTLPFRMFYYAKDEWPFGEYFCQILGALTVFYPSIALWLLAFISADRYMAIV
QPKYAKELKNTCKAVLACVGVWIMTLTTTTPLLLLYKDPDKDSTPATCLKISDIIYLKAVN
VLNLTRLTFFFLIPLFIMIGCYLVIIHNLLHGRTSKLKPKVKEKSIRIIITLLVQVLVCFMPFHIC

FAFLMLGTGENSYNPWGAFTTFLMNLSTCLDVILYYIVSKQFQARVISVMLYRNYLRSMR
RKSFRSGSLRSLSNINSEML

>O60725 multipass membrane protein
MAGCAARAPPGSEARLSLATFLLGASVLALPLLTRAGLQGRTGLALYVAGLNALLLLLYR
PPRYQIAIRACFLGFVFGCGTLLSFSQSSWSHFGWYMCSLSLFHYSEYLVTAVNNPKSLSL
DSFLLNHSLEYTVAALSSWLEFTLENIFWPELKQITWLSVTGLLMVVFGECLRKAAMFTA
GSNFNHVVQNEKSDTHTLVTSGVYAWFRHPSYVGWFYWSIGTQVMLCNPICGVSYALTV
WRFFRDRTEEEEISLIHFFGEEYLEYKKRVPTGLPFIKGVKVDL

>P35212 multipass membrane protein
MGDWGFLEKLLDQVQEHSTVVGKIWLTVLFIFRILILGLAGESVWGDEQSDFECNTAQPG
CTNVCYDQAFPISHIRYWVLQFLFVSTPTLVYLGHVIYLSRREERLRQKEGELRALPAKDP
QVERALAAVERQMAKISVAEDGRLRIRGALMGTYVASVLCKSVLEAGFLYGQWRLYGWT
MEPVFVCQRAPCPYLVDCFVSRPTEKTIFIIFMLVVGLISLVLNLLELVHLLCRCLSRGMRA
RQGQDAPPTQGTSSDPYTDQVFFYLPVGQGPSSPPCPTYNGLSSSEQNWANLTTEERLASS
RPPLFLDPPPQNGQKPPSRPSSSASKKQYV

>Q9Y5X0 peripheral membrane protein
MFPEQQKEEFVSVWVRDPRIQKEDFWHSYIDYEICIHTNSMCFTMKTSCVRRRYREFVWL
RQRLQSNALLVQLPELPSKNLFFNMNNRQHVDQRRQGLEDFLRKVLQNALLLSDSSLHLF
LQSHLNSEDIEACVSGQTKYSVEEAIHKFALMNRRFPEEDEEGKKENDIDYDSESSSSGLG
HSSDDSSSHGCKVNTAPQES

>P13945 multipass membrane protein
MAPWPHENSSLAPWPDLPTLAPNTANTSGLPGVPWEAALAGALLALAVLATVGGNLLVIV
AIAWTPRLQTMTNVFVTSLAAADLVMGLLVVPPAATLALTGHWPLGATGCELWTSVDVL
CVTASIETLCALAVDRYLAVTNPLRYGALVTKRCARTAVVLVWVVSAAVSFAPIMSQWWR
VGADAEAQRCHSNPRCCAFASNMPYVLLSSSVSFYLPLLVMLFVYARVFVVATRQLRLLR
GELGRFPPEESPPAPSRSLAPAPVGTCAPPEGVPACGRRPARLLPLREHRALCTLGLIMGTF
TLCWLPFFLANVLRALGGPSLVPGPAFLALNWLGYANSAFNPLIYCRSPDFRSAFRRLLCR
CGRRLPPEPCAAARPALFPSGVPAARSSPAQPRLCQRLDGASWGVS

>O43609 peripheral membrane protein
MDPQNQHGSGSSLVVIQQPSLDSRQRLDYEREIQPTAILSLDQIKAIRGSNEYTEGPSVVKR
PAPRTAPRQEKHERTHEIIPINVNNNYEHRHTSHLGHAVLPSNARGPILSRSTSTGSAASSGS
NSSASSEQGLLGRSPPTRPVPGHRSERAIRTQPKQLIVDDLKGSLKEDLTQHKFICEQCGKC
KCGECTAPRTLPSCLACNRQCLCSAESMVEYGTCMCLVKGIFYHCSNDDEGDSYSDNPCS
CSQSHCCSRYLCMGAMSLFLPCLLCYPPAKGCLKLCRRCYDWIHRPGCRCKNSNTVYCK
LESCPSRGQGKPS

>P50336 peripheral membrane protein
MGRTVVVLGGGISGLAASYHLSRAPCPPKVVLVESSERLGGWIRSVRGPNGAIFELGPRGI
RPAGALGARTLLLVSELGLDSEVLPVRGDHPAAQNRFLYVGGALHALPTGLRGLLRPSPPF

SKPLFWAGLRELTKPRGKEPDETVHSFAQRRLGPEVASLAMDSLCRGVFAGNSRELSIRSC
FPSLFQAEQTHRSILLGLLLGAGRTPQPDSALIRQALAERWSQWSLRGGLEMLPQALETHL
TSRGVSVLRGQPVCGLSLQAEGRWKVSLRDSSLEADHVISAIPASVLSELLPAEAAPLARA
LSAITAVSVAVVNLQYQGAHLPVQGFGHLVPSSEDPGVLGIVYDSVAFPEQDGSPPGLRVT
VMLGGSWLQTLEASGCVLSQELFQQRAQEAAATQLGLKEMPSHCLVHLHKNCIPQYTLG
HWQKLESARQFLTAHRLPLTLAGASYEGVAVNDCIESGRQAAVSVLGTEPNS

>P48551 single-pass type I membrane protein
MLLSQNAFIFRSLNLVLMVYISLVFGISYDSPDYTDESCTFKISLRNFRSILSWELKNHSIVP
THYTLLYTIMSKPEDLKVVKNCANTTRSFCDLTDEWRSTHEAYVTVLEGFSGNTTLFSCS
HNFWLAIDMSFEPPEFEIVGFTNHINVMVKFPSIVEEELQFDLSLVIEEQSEGIVKKHKPEIK
GNMSGNFTYIIDKLIPNTNYCVSVYLEHSDEQAVIKSPLKCTLLPPGQESESAESAKIGGIIT
VFLIALVLTSTIVTLKWIGYICLRNSLPKVLNFHNFLAWPFPNLPPLEAMDMVEVIYINRKK
KVWDYNYDDESDSDTEAAPRTSGGGYTMHGLTVRPLGQASATSTESQLIDPESEEEPDLP
EVDVELPTMPKDSPQQLELLSGPCERRKSPLQDPFPEEDYSSTEGSGGRITFNVDLNSVFLR
VLDDDEDSDDLEAPLMLSSHLEEMVDPEDPDNVQSNHLLASGEGTQPTFPSPSSEGLWSED
APSDQSDTSESDVDLGDGYIMR

>Q9NQN1 multipass membrane protein
MEKANETSPVMGFVLLRLSAHPELEKTFFVLILLMYLVILLGNGVLILVTILDSRLHTPMYF
FLGNLSFLDICFTTSSVPLVLDSFLTPQETISFSACAVQMALSFAMAGTECLLLSMMAFDRY
VAICNPLRYSVIMSKAAYMPMAASSWAIGGAASVVHTSLAIQLPFCGDNVINHFTCEILAV
LKLACADISINVISMEVTNVIFLGVPVLFISFSYVFIITTILRIPSAEGRKKVFSTCSAHLTVVI
VFYGTLFFMYGKPKSKDSMGADKEDLSDKLIPLFYGVVTPMLNPIIYSLRNKDVKAAVRR
LLRPKGFTQ

>O14617 peripheral membrane protein
MALKMVKGSIDRMFDKNLQDLVRGIRNHKEDEAKYISQCIDEIKQELKQDNIAVKANAVC
KLTYLQMLGYDISWAAFNIIEVMSASKFTFKRIGYLAASQSFHEGTDVIMLTTNQIRKDLSS
PSQYDTGVALTGLSCFVTPDLARDLANDIMTLMSHTKPYIRKKAVLIMYKVFLKYPESLRP
AFPRLKEKLEDPDPGVQSAAVNVICELARRNPKNYLSLAPLFFKLMTSSTNNWVLIKIIKLF
GALTPLEPRLGKKLIEPLTNLIHSTSAMSLLYECVNTVIAVLISLSSGMPNHSASIQLCVQKL
RILIEDSDQNLKYLGLLAMSKILKTHPKSVQSHKDLILQCLDDKDESIRLRALDLLYGMVS
KKNLMEIVKKLMTHVDKAEGTTYRDELLTKIIDICSQSNYQYITNFEWYISILVELTRLEGT
RHGHLIAAQMLDVAIRVKAIRKFAVSQMSALLDSAHLLASSTQRNGICEVLYAAAWICGEF
SEHLQEPHHTLEAMLRPRVTTLPGHIQAVYVQNVVKLYASILQQKEQAGEAEGAQAVTQL
MVDRLPQFVQSADLEVQERASCILQLVKHIQKLQAKDVPVAEEVSALFAGELNPVAPKAQ
KKVPVPEGLDLDAWINEPLSDSESEDERPRAVFHEEEQRRPKHRPSEADEEELARRREARK
QEQANNPFYIKSSPSPQKRYQDTPGVEHIPVVQIDLSVPLKVPGLPMSDQYVKLEEERRHR
QKLEKDKRRKKRKEKEKKGKRRHSSLPTESDEDIAPAQQVDIVTEEMPENALPSDEDDKD
PNDPYRALDIDLDKPLADSEKLPIQKHRNTETSKSPEKDVPMVEKKSKKPKKKEKKHKEK
ERDKEKKKEKEKKKSPKPKKKKHRKEKEERTKGKKKSKKQPPGSEEAAGEPVQNGAPEE
EQLPPESSYSLLAENSYVKMTCDIRGSLQEDSQVTVAIVLENRSSSILKGMELSVLDSLNAR
MARPQGSSVHDGVPVPFQLPPGVSNEAQYVFTIQSIVMAQKLKGTLSFIAKNDEGATHEK

LDFRLHFSCSSYLITTPCYSDAFAKLLESGDLSMSSIKVDGIRMSFQNLLAKICFHHHFSVV
ERVDSCASMYSRSIQGHHVCLLVKKGENSVSVDGKCSDSTLLSNLLEEMKATLAKC

>Q8NGY6 multipass membrane protein
MDQYNHSSLAEFVFLGFASVGYVRGWLFVLLLLAYLFTICGNMLIFSVIRLDAALHTPMY
HFVSVLSFLELWYTATTIPKMLSNILSEKKTISFAGCLLQTYFFHSLGASECYLLTAMAYDR
YLAICRPLHYPIIMTTTLCAKMAAACWTCGFLCPISEVILASQLPFCAYNEIQHIFCDFPPLL
SLACKDTSANILVDFAINAFIILITFFFIMISYARIIGAVLKIKTASGRKKAFSTCASHLAVVLIF
FGSIIFMYVRLKKSYSLTLDRTLAIVYSVLTPMVNPIIYSLRNKEIIKAIKRTIFQKGDKASLA
HL

>O95197 multipass membrane protein
MAEPSAATQSHSISSSSFGAEPSAPGGGGSPGACPALGTKSCSSSCADSFVSSSSSQPVSLFS
TSQEGLSSLCSDEPSSEIMTSSFLSSSEIHNTGLTILHGEKSHVLGSQPILAKEGKDHLDLLD
MKKMEKPQGTSNNVSDSSVSLAAGVHCDRPSIPASFPEHPAFLSKKIGQVEEQIDKETKNP
NGVSSREAKTALDADDRFTLLTAQKPPTEYSKVEGIYTYSLSPSKVSGDDVIEKDSPESPFE
VIIDKAAFDKEFKDSYKESTDDFGSWSVHTDKESSEDISETNDKLFPLRNKEAGRYPMSAL
LSRQFSHTNAALEEVSRCVNDMHNFTNEILTWDLVPQVKQQTDKSSDCITKTTGLDMSEY
NSEIPVVNLKTSTHQKTPVCSIDGSTPITKSTGDWAEASLQQENAITGKPVPDSLNSTKEFSI
KGVQGNMQKQDDTLAELPGSPPEKCDSLGSGVATVKVVLPDDHLKDEMDWQSSALGEIT
EADSSGESDDTVIEDITADTSFENNKIQAEKPVSIPSAVVKTGEREIKEIPSCEREEKTSKNFE
ELVSDSELHQDQPDILGRSPASEAACSKVPDTNVSLEDVSEVAPEKPITTENPKLPSTVSPN
VFNETEFSLNVTTSAYLESLHGKNVKHIDDSSPEDLIAAFTETRDKGIVDSERNAFKAISEK
MTDFKTTPPVEVLHENESGGSEIKDIGSKYSEQSKETNGSEPLGVFPTQGTPVASLDLEQE
QLTIKALKELGERQVEKSTSAQRDAELPSEEVLKQTFTFAPESWPQRSYDILERNVKNGSD
LGISQKPITIRETTRVDAVSSLSKTELVKKHVLARLLTDFSVHDLIFWRDVKKTGFVFGTTLI
MLLSLAAFSVISVVSYLILALLSVTISFRIYKSVIQAVQKSEEGHPFKAYLDVDITLSSEAFH
NYMNAAMVHINRALKLIIRLFLVEDLVDSLKLAVFMWLMTYVGAVFNGITLLILAELLIFS
VPIVYEKYKTQIDHYVGIARDQTKSIVEKIQAKLPGIAKKKAE

>Q9H3T2 single-pass type I membrane protein
MPRAPHFMPLLLLLLLLSLPHTQAAFPQDPLPLLISDLQGTSPLSWFRGLEDDAVAAELGL
DFQRFLTLNRTLLVAARDHVFSFDLQAEEEGEGLVPNKYLTWRSQDVENCAVRGKLTDEC
YNYIRVLVPWDSQTLLACGTNSFSPVCRSYGITSLQQEGEELSGQARCPFDATQSNVAIFAE
GSLYSATAADFQASDAVVYRSLGPQPPLRSAKYDSKWLREPHFVQALEHGDHVYFFFREV
SVEDARLGRVQFSRVARVCKRDMGGSPRALDRHWTSFLKLRLNCSVPGDSTFYFDVLQA
LTGPVNLHGRSALFGVFTTQTNSIPGSAVCAFYLDEIERGFEGKFKEQRSLDGAWTPVSED
RVPSPRPGSCAGVGGAALFSSSRDLPDDVLTFIKAHPLLDPAVPPVTHQPLLTLTSRALLTQV
AVDGMAGPHSNITVMFLGSNDGTVLKVLTPGGRSGGPEPILLEEIDAYSPARCSGKRTAQT
ARRIIGLELDTEGHRLFVAFSGCIVYLPLSRCARHGACQRSCLASQDPYCGWHSSRGCVDI
RGSGGTDVDQAGNQESMEHGDCQDGATGSQSGPGDSAYGVRRDLPPASASRSVPIPLLLA
SVAAAFALGASVSGLLVSCACRRAHRRRGKDIETPGLPRPLSLRSLARLHGGGPEPPPPSK
DGDAVQTPQLYTTFLPPPEGVPPPELACLPTPESTPELPVKHLRAAGDPWEWNQNRNNAK
EGPGRSRGGHAAGGPAPRVLVRPPPPGCPGQAVEVTTLEELLRYLHGPQPPRKGAEPPAPL

TSRALPPEPAPALLGGPSPRPHECASPLRLDVPPEGRCASAPARPALSAPAPRLGVGGGRRL
PFSGHRAPPALLTRVPSGGPSRYSGGPGKHLLYLGRPEGYRGRALKRVDVEKPQLSLKPPL
VGPSSRQAVPNGGRFNF

>Q9BZC7 multipass membrane protein
MGFLHQLQLLLWKNVTLKRRSPWVLAFEIFIPLVLFFILLGLRQKKPTISVKEAFYTAAPLT
SAGILPVMQSLCPDGQRDEFGFLQYANSTVTQLLERLDRVVEEGNLFDPARPSLGSELEAL
RQHLEALSAGPGTSGSHLDRSTVSSFSLDSVARNPQELWRFLTQNLSLPNSTAQALLAARV
DPPEVYHLLFGPSSALDSQSGLHKGQEPWSRLGGNPLFRMEELLLAPALLEQLTCTPGSGE
LGRILTVPESQKGALQGYRDAVCSGQAAARARRFSGLSAELRNQLDVAKVSQQLGLDAP
NGSDSSPQAPPPRRLQALLGDLLDAQKVLQDVDVLSALALLLPQGACTGRTPGPPASGAG
GAANGTGAGAVMGPNATAEEGAPSAAALATPDTLQGQCSAFVQLWAGLQPILCGNNRTIE
PEALRRGNMSSLGFTSKEQRNLGLLVHLMTSNPKILYAPAGSEVDRVILKANETFAFVGNV
THYAQVWLNISAEIRSFLEQGRLQQHLRWLQQYVAELRLHPEALNLSLDELPPALRQDNF
SLPSGMALLQQLDTIDNAACGWIQFMSKVSVDIFKGFHDEESIVNYTLNQAYQDNVTVFA
SVIFQTRKDGSLPPHVHYKIRQNSSFTEKTNEIRRAYWRPGPNTGGRFYFLYGFVWIQDM
MERAIIDTFVGHDVVEPGSYVQMFPYPCYTRDDFLFVIEHMMPLCMVISWVYSVAMTIQH
IVAEKEHRLKEVMKTMGLNNAVHWVAWFITGFVQLSISVTALTAILKYGQVLMHSHVVII
WLFLAVYAVATIMFCFLVSVLYSKAKLASACGGIIYFLSYVPYMYVAIREEVAHDKITAFEK
CIASLMSTTAFGLGSKYFALYEVAGVGIQWHTFSQSPVEGDDFNLLLAVTMLMVDAVVYG
ILTWYIEAVHPGMYGLPRPWYFPLQKSYWLGSGRTEAWEWSWPWARTPRLSVMEEDQA
CAMESRRFEETRGMEEEPTHLPLVVCVDKLTKVYKDDKKLALNKLSLNLYENQVVSFLG
HNGAGKTTTMSILTGLFPPTSGSATIYGHDIRTEMDEIRKNLGMCPQHNVLFDRLTVEEHL
WFYSRLKSMAQEEIRREMDKMIEDLELSNKRHSLVQTLSGGMKRKLSVAIAFVGGSRAIIL
DEPTAGVDPYARRAIWDLILKYKPGRTILLSTHHMDEADLLGDRIAIISHGKLKCCGSPLFL
KGTYGDGYRLTLVKRPAEPGGPQEPGLASSPPGRAPLSSCSELQVSQFIRKHVASCLLVSDT
STELSYILPSEAAKKGAFERLFQHLERSLDALHLSSFGLMDTTLEEVFLKVSEEDQSLENSE
ADVKESRKDVLPGAEGPASGEGHAGNLARCSELTQSQASLQSASSVGSARGDEGAGYTD
VYGDYRPLFDNPQDPDNVSLQEVEAEALSRVGQGSRKLDGGWLKVRQFHGLLVKRFHC
ARRNSKALFSQILLPAFFVCVAMTVALSVPEIGDLPPLVLSPSQYHNYTQPRGNFIPYANEE
RREYRLRLSPDASPQQLVSTFRLPSGVGATCVLKSPANGSLGPTLNLSSGESRLLAARFFDS
MCLESFTQGLPLSNFVPPPPSPAPSDSPASPDEDLQAWNVSLPPTAGPEMWTSAPSLPRLVR
EPVRCTCSAQGTGFSCPSSVGGHPPQMRVVTGDILTDITGHNVSEYLLFTSDRFRLHRYGA
ITFGNVLKSIPASFGTRAPPMVRKIAVRRAAQVFYNNKGYHSMPTYLNSLNNAILRANLPK
SKGNPAAYGITVTNHPMNKTSASLSLDYLLQGTDVVIAIFIIVAMSFVPASFVVFLVAEKST
KAKHLQFVSGCNPIIYWLANYVWDMLNYLVPATCCVIILFVFDLPAYTSPTNFPAVLSLFLL
YGWSITPIMYPASFWFEVPSSAYVFLIVINLFIGITATVATFLLQLFEHDKDLKVVNSYLKSC
FLIFPNYNLGHGLMEMAYNEYINEYYAKIGQFDKMKSPFEWDIVTRGLVAMAVEGVVGFL
LTIMCQYNFLRRPQRMPVSTKPVEDDVDVASERQRVLRGDADNDMVKIENLTKVYKSRK
IGRILAVDRLCLGVRPGECFGLLGVNGAGKTSTFKMLTGDESTTGGEAFVNGHSVLKELL
QVQQSLGYCPQCDALFDELTAREHLQLYTRLRGISWKDEARVVKWALEKLELTKYADKP
AGTYSGGNKRKLSTAIALIGYPAFIFLDEPTTGMDPKARRFLWNLILDLIKTGRSVVLTSHS
MEECEALCTRLAIMVNGRLRCLGSIQHLKNRFGDGYMITVRTKSSQSVKDVVRFFNRNFP
EAMLKERHHTKVQYQLKSEHISLAQVFSKMEQVSGVLGIEDYSVSQTTLDNVFVNFAKK

QSDNLEQQETEPPSALQSPLGCLLSLLRPRSAPTELRALVADEPEDLDTEDEGLISFEEERA
QLSFNTDTLC

>O15530 peripheral membrane protein
MARTTSQLYDAVPIQSSVVLCSCPSPSMVRTQTESSTPPGIPGGSRQGPAMDGTAAEPRPGA
GSLQHAQPPPQPRKKRPEDFKFGKILGEGSFSTVVLARELATSREYAIKILEKRHIIKENKVP
YVTRERDVMSRLDHPFFVKLYFTFQDDEKLYFGLSYAKNGELLKYIRKIGSFDETCTRFYT
AEIVSALEYLHGKGIIHRDLKPENILLNEDMHIQITDFGTAKVLSPESKQARANSFVGTAQY
VSPELLTEKSACKSSDLWALGCIIYQLVAGLPPFRAGNEYLIFQKIIKLEYDFPEKFFPKARD
LVEKLLVLDATKRLGCEEMEGYGPLKAHPFFESVTWENLHQQTPPKLTAYLPAMSEDDED
CYGNYDNLLSQFGCMQVSSSSSSHSLSASDTGLPQRSGSNIEQYIHDLDSNSFELDLQFSE
DEKRLLLEKQAGGNPWHQFVENNLILKMGPVDKRKGLFARRRQLLLTEGPHLYYVDPVN
KVLKGEIPWSQELRPEAKNFKTFFVHTPNRTYYLMDPSGNAHKWCRKIQEVWRQRYQSH
PDAAVQ

>Q14703 single-pass type I membrane protein
MKLVNIWLLLLVVLLCGKKHLGDRLEKKSFEKAPCPGCSHLTLKVEFSSTVVEYEYIVAFN
GYFTAKARNSFISSALKSSEVDNWRIIPRNNPSSDYPSDFEVIQIKEKQKAGLLTLEDHPNIK
RVTPQRKVFRSLKYAESDPTVPCNETRWSQKWQSSRPLRRASLSLGSGFWHATGRHSSRR
LLRAIPRQVAQTLQADVLWQMGYTGANVRVAVFDTGLSEKHPHFKNVKERTNWTNERTL
DDGLGHGTFVAGVIASMRECQGFAPDAELHIFRVFTNNQVSYTSWFLDAFNYAILKKIDVL
NLSIGGPDFMDHPFVDKVWELTANNVIMVSAIGNDGPLYGTLNNPADQMDVIGVGGIDFE
DNIARFSSRGMTTWELPGGYGRMKPDIVTYGAGVRGSGVKGGCRALSGTSVASPVVAGA
VTLLVSTVQKRELVNPASMKQALIASARRLPGVNMFEQGHGKLDLLRAYQILNSYKPQAS
LSPSYIDLTECPYMWPYCSQPIYYGGMPTVVNVTILNGMGVTGRIVDKPDWQPYLPQNG
DNIEVAFSYSSVLWPWSGYLAISISVTKKAASWEGIAQGHVMITVASPAETESKNGAEQTS
TVKLPIKVKIIPTPPRSKRVLWDQYHNLRYPPGYFPRDNLRMKNDPLDWNGDHIHTNFRD
MYQHLRSMGYFVEVLGAPFTCFDASQYGTLLMVDSEEEYFPEEIAKLRRDVDNGLSLVIF
SDWYNTSVMRKVKFYDENTRQWWMPDTGGANIPALNELLSVWNMGFSDGLYEGEFTL
ANHDMYYASGCSIAKFPEDGVVITQTFKDQGLEVLKQETAVVENVPILGLYQIPAEGGGRI
VLYGDSNCLDDSHRQKDCFWLLDALLQYTSYGVTPPSLSHSGNRQRPPSGAGSVTPERM
EGNHLHRYSKVLEAHLGDPKPRPLPACPRLSWAKPQPLNETAPSNLWKHQKLLSIDLDKV
VLPNFRSNRPQVRPLSPGESGAWDIPGGIMPGRYNQEVGQTIPVFAFLGAMVVLAFFVVQI
NKAKSRPKRRKPRVKRPQLMQQVHPPKTPSV

>Q9UGH3 multipass membrane protein
MMGIGKNTTSKSMEAGSSTEGKYEDEAKHPAFFTLPVVINGGATSSGEQDNEDTELMAIY
TTENGIAEKSSLAETLDSTGSLDPQRSDMIYTIEDVPPWYLCIFLGLQHYLTCFSGTIAVPFL
LADAMCVGYDQWATSQLIGTIFFCVGITTLLQTTFGCRLPLFQASAFAFLAPARAILSLDK
WKCNTTDVSVANGTAELLHTEHIWYPRIREIQGAIIMSSLIEVVIGLLGLPGALLKYIGPLTI
TPTVALIGLSGFQAAGERAGKHWGIAMLTIFLVLLFSQYARNVKFPLPIYKSKKGWTAYKL
QLFKMFPIILAILVSWLLCFIFTVTDVFPPDSTKYGFYARTDARQGVLLVAPWFKVPYPFQW
GLPTVSAAGVIGMLSAVVASIIESIGDYYACARLSCAPPPPIHAINRGIFVEGLSCVLDGIFGT
GNGSTSSSPNIGVLGITKVGSRRVIQCGAALMLALGMIGKFSALFASLPDPVLGALFCTLFG

MITAVGLSNLQFIDLNSSRNLFVLGFSIFFGLVLPSYLRQNPLVTGITGIDQVLNVLLTTAMF
VGGCVAFILDNTIPGTPEERGIRKWKKGVGKGNKSLDGMESYNLPFGMNIIKKYRCFSYL
PISPTFVGYTWKGLRKSDNSRSSDEDSQATG

>P43657 multipass membrane protein
MVSVNSSHCFYNDSFKYTLYGCMFSMVFVLGLISNCVAIYIFICVLKVRNETTTYMINLAM
SDLLFVFTLPFRIFYFTTRNWPFGDLLCKISVMLFYTNMYGSILFLTCISVDRFLAIVYPFKS
KTLRTKRNAKIVCTGVWLTVIGGSAPAVFVQSTHSQGNNASEACFENFPEATWKTYLSRIV
IFIEIVGFFIPLILNVTCSSMVLKTLTKPVTLSRSKINKTKVLKMIFVHLIIFCFCFVPYNINLIL
YSLVRTQTFVNCSVVAAVRTMYPITLCIAVSNCCFDPIVYYFTSDTIQNSIKMKNWSVRRSD
FRFSEVHGAENFIQHNLQTLKSKIFDNESAA

>Q7RTX0 multipass membrane protein
MLGPAVLGLSLWALLHPGTGAPLCLSQQLRMKGDYVLGGLFPLGEAEEAGLRSRTRPSSP
VCTRFSSNGLLWALAMKMAVEEINNKSDLLPGLRLGYDLFDTCSEPVVAMKPSLMFLAK
AGSRDIAAYCNYTQYQPRVLAVIGPHSSELAMVTGKFFSFFLMPQVSYGASMELLSARETF
PSFFRTVPSDRVQLTAAAELLQEFGWNWVAALGSDDEYGRQGLSIFSALAAARGICIAHEG
LVPLPRADDSRLGKVQDVLHQVNQSSVQVVLLFASVHAAHALFNYSISSRLSPKVWVASE
AWLTSDLVMGLPGMAQMGTVLGFLQRGAQLHEFPQYVKTHLALATDPAFCSALGEREQ
GLEEDVVGQRCPQCDCITLQNVSAGLNHHQTFSVYAAVYSVAQALHNTLQCNASGCPAQ
DPVKPWQLLENMYNLTFHVGGLPLRFDSSGNVDMEYDLKLWVWQGSVPRLHDVGRFN
GSLRTERLKIRWHTSDNQKPVSRCSRQCQEGQVRRVKGFHSCCYDCVDCEAGSYRQNPD
DIACTFCGQDEWSPERSTRCFRRRSRFLAWGEPAVLLLLLLLSLALGLVLAALGLFVHHRD
SPLVQASGGPLACFGLVCLGLVCLSVLLFPGQPSPARCLAQQPLSHLPLTGCLSTLFLQAAE
IFVESELPLSWADRLSGCLRGPWAWLVVLLAMLVEVALCTWYLVAFPPEVVTDWHMLPTE
ALVHCRTRSWVSFGLAHATNATLAFLCFLGTFLVRSQPGCYNRARGLTFAMLAYFITWVS
FVPLLANVQVVLRPAVQMGALLLCVLGILAAFHLPRCYLLMRQPGLNTPEFFLGGGPGDA
QGQNDGNTGNQGKHE

>O75056 single-pass type I membrane protein
MKPGPPHRAGAAHGAGAGAGAAAGPGARGLLLPPLLLLLLAGRAAGAQRWRSENFERP
VDLEGSGDDDSFPDDELDDLYSGSGSGYFEQESGIETAMRFSPDVALAVSTTPAVLPTTNIQ
PVGTPFEELPSERPTLEPATSPLVVTEVPEEPSQRATTVSTTMATTAATSTGDPTVATVPATVA
TATPSTPAAPPFTATTAVIRTTGVRRLLPLPLTTVATARATTPEAPSPPTTAAVLDTEAPTPRLV
STATSRPRALPRPATTQEPDIPERSTLPLGTTAPGPTEVAQTPTPETFLTTIRDEPEVPVSGGPS
GDFELPEEETTQPDTANEVVAVGGAAAKASSPPGTLPKGARPGPGLLDNAIDSGSSAAQLP
QKSILERKEVLVAVIVGGVVGALFAAFLVTLLIYRMKKKDEGSYTLEEPKQASVTYQKPD
KQEEFYA

>Q2HXU8 single-pass type II membrane protein
MSEEVTYATLTFQDSAGARNNRDGNNLRKRGHPAPSPIWRHAALGLVTLCLMLLIGLVTL
GMMFLQISNDINSDSEKLSQLQKTIQQQQDNLSQQLGNSNNLSMEEEFLKSQISSVLKRQE
QMAIKLCQELIIHTSDHRCNPCPKMWQWYQNSCYYFTTNEEKTWANSRKDCIDKNSTLV
KIDSLEEKDFLMSQPLLMFSFFWLGLSWDSSGRSWFWEDGSVPSPSLFSTKELDQINGSKG

CAYFQKGNIYISRCSAEIFWICEKTAAPVKTEDLD

>O43427 peripheral membrane protein
MTSELDIFVGNTTLIDEDVYRLWLDGYSVTDAVALRVRSGILEQTGATAAVLQSDTMDHY
RTFHMLERLLHAPPKLLHQLIFQIPPSRQALLIERYYAFDEAFVREVLGKKLSKGTKKDLD
DISTKTGITLKSCRRQFDNFKRVFKVVEEMRGSLVDNIQQHFLLSDRLARDYAAIVFFANN
RFETGKKKLQYLSFGDFAFCAELMIQNWTLGAVGEAPTDPDSQMDDMDMDLDKEFLQD
LKELKVLVADKDLLDLHKSLVCTALRGKLGVFSEMEANFKNLSRGLVNVAAKLTHNKDV
RDLFVDLVEKFVEPCRSDHWPLSDVRFFLNQYSASVHSLDGFRHQALWDRYMGTLRGCL
LRLYHD

>P35240 peripheral membrane protein
MAGAIASRMSFSSLKRKQPKTFTVRIVTMDAEMEFNCEMKWKGKDLFDLVCRTLGLRET
WFFGLQYTIKDTVAWLKMDKKVLDHDVSKEEPVTFHFLAKFYPENAEEELVQEITQHLFF
LQVKKQILDEKIYCPPEASVLLASYAVQAKYGDYDPSVHKRGFLAQEELLPKRVINLYQM
TPEMWEERITAWYAEHRGRARDEAEMEYLKIAQDLEMYGVNYFAIRNKKGTELLLGVDA
LGLHIYDPENRLTPKISFPWNEIRNISYSDKEFTIKPLDKKIDVFKFNSSKLRVNKLILQLCIG
NHDLFMRRRKADSLEVQQMKAQAREEKARKQMERQRLAREKQMREEAERTRDELERR
LLQMKEEATMANEALMRSEETADLLAEKAQITEEEAKLLAQKAAEAEQEMQRIKATAIRT
EEEKRLMEQKVLEAEVLALKMAEESERRAKEADQLKQDLQEAREAERRAKQKLLEIATK
PTYPPMNPIPAPLPPDIPSFNLIGDSLSFDFKDTDMKRLSMEIEKEKVEYMEKSKHLQEQLN
ELKTEIEALKLKERETALDILHNENSDRGGSSKHNTIKKLTLQSAKSRVAFFEEL

>O00522 peripheral membrane protein
MGNPENIEDAYVAVIRPKNTASLNSREYRAKSYEILLHEVPIEGQKKKRKKVLLETKLQGN
SEITQGILDYVVETTKPISPANQGIRGKRVVLMKKFPLDGEKMGREASLFIVPSVVKDNTK
YTYTPGCPIFYCLQDIMRVCSESSTHFATLTARMLIALDKWLDERHAQSHFIPALFRPSPLER
IKTNVINPAYATESGQTENSLHMGYSALEIKSKMLALEKADTCIYNPLFGSDLQYTNRVDK
VVINPYFGLGAPDYSKIQIPKQEKWQRSMSSVTEDKERQWVDDFPLHRSACEGDSELLSR
LLSERFSVNQLDSDHWAPIHYACWYGKVEATRILLEKGKCNPNLLNGQLSSPLHFAAGGG
HAEIVQILLNHPETDRHITDQQGRSPLNICEENKQNNWEEAAKLLKEAINKPYEKVRIYRM
DGSYRSVELKHGNNTTVQQIMEGMRLSQETQQYFTIWICSENLSLQLKPYHKPLQHVRD
WPEILAELTNLDPQRETPQLFLRRDVRLPLEVEKQIEDPLAILILFDEARYNLLKGFYTAPD
AKLITLASLLLQIVYGNYESKKHKQGFLNEENLKSIVPVTKLKSKAPHWTNRILHEYKNLS
TSEGVSKEMHHLQRMFLQNCWEIPTYGAAFFTGQIFTKASPSNHKVIPVYVGVNIKGLHL
LNMETKALLISLKYGCFMWQLGDTDTCFQIHSMENKMSFIVHTKQAGLVVKLLMKLNG
QLMPTERNS

>O15503 multipass membrane protein
MPRLHDHFWSCSCAHSARRRGPPRASAAGLAAKVGEMINVSVSGPSLLAAHGAPDADPA
PRGRSAAMSGPEPGSPYPNTWHHRLLQRSLVLFSVGVVLALVLNLLQIQRNVTLFPEEVIA
TIFSSAWWVPPCCGTAAAVVGLLYPCIDSHLGEPHKFKREWASVMRCIAVFVGINHASAKL
DFANNVQLSLTLAALSLGLWWTFDRSRSGLGLGITIAFLATLITQFLVYNGVYQYTSPDFL
YIRSWLPCIFFSGGVTVGNIGRQLAMGVPEKPHSD

>O95630 peripheral membrane protein
MSDHGDVSLPPEDRVRALSQLGSAVEVNEDIPPRRYFRSGVEIIRMASIYSEEGNIEHAFILY
NKYITLFIEKLPKHRDYKSAVIPEKKDTVKKLKEIAFPKAEELKAELLKRYTKEYTEYNEE
KKKEAEELARNMAIQQELEKEKQRVAQQKQQQLEQEQFHAFEEMIRNQELEKERLKIVQ
EFGKVDPGLGGPLVPDLEKPSLDVFPTLTVSSIQPSDCHTTVRPAKPPVVDRSLKPGALSNS
ESIPTIDGLRHVVVPGRLCPQFLQLASANTARGVETCGILCGKLMRNEFTITHVLIPKQSAG
SDYCNTENEEELFLIQDQQGLITLGWIHTHPTQTAFLSSVDLHTHCSYQMMLPESVAIVCS
PKFQETGFFKLTDHGLEEISSCRQKGFHPHSKDPPLFCSCSHVTVVDRAVTITDLR

>Q8TEB9 multipass membrane protein
MQRRSRGINTGLILLLSQIFHVGINNIPPVTLATLALNIWFFLNPQKPLYSSCLSVEKCYQQK
DWQRLLLSPLHHADDWHLYFNMASMLWKGINLERRLGSRWFAYVITAFSVLTGVVYLLL
QFAVAEFMDEPDFKRSCAVGFSGVLFALKVLNNHYCPGGFVNILGFPVPNRFACWVELVAI
HLFSPGTSFAGHLAGILVGLMYTQGPLKKIMEACAGGFSSSVGYPGRQYYFNSSGSSGYQ
DYYPHGRPDHYEEAPRNYDTYTAGLSEEEQLERALQASLWDRGNTRNSPPPYGFHLSPEE
MRRQRLHRFDSQ

>Q9UI40 multipass membrane protein
MDLQQSTTITSLEKWCLDESLSGCRRHYSVKKKLKLIRVLGLFMGLVAISTVSFSISAFSET
DTQSTGEASVVSGPRVAQGYHQRTLLDLNDKILDYTPQPPLSKEGESENSTDHAQGDYPK
DIFSLEERRKGAIILHVIGMIYMFIALAIVCDEFFVPSLTVITEKLGISDDVAGATFMAAGGS
APELFTSLIGVFIAHSNVGIGTIVGSAVFNILFVIGMCALFSREILNLTWWPLFRDVSFYIVD
LIMLIIFFLDNVIMWWESLLLLTAYFCYVVFMKFNVQVEKWVKQMINRNKVVKVTAPEA
QAKPSAARDKDEPTLPAKPRLQRGGSSASLHNSLMRNSIFQLMIHTLDPLAEELGSYGKL
KYYDTMTEEGRFREKASILHKIAKKKCHVDENERQNGAANHVEKIELPNSTSTDVEMTPS
SDASEPVQNGNLSHNIEGAEAQTADEEEDQPLSLAWPSETRKQVTFLIVFPIVFPLWITLPD
VRKPSSRKFFPITFFGSITWIAVFSYLMVWWAHQVGETIGISEEIMGLTILAAGTSIPDLITSV
IVARKGLGDMAVSSSVGSNIFDITVGLPLPWLLYTVIHRFQPVAVSSNGLFCAIVLLFIMLLF
VILSIALCKWRMNKILGFIMFGLYFVFLVVSVLLEDRILTCPVSI

>Q9C004 peripheral membrane protein
MEPPIPQSAPLTPNSVMVQPLLDSRMSHSRLQHPLTILPIDQVKTSHVENDYIDNPSLALTT
GPKRTRGGAPELAPTPARCDQDVTHHWISFSGRPSSVSSSSSTSSDQRLLDHMAPPPVADQ
ASPRAVRIQPKVVHCQPLDLKGPAVPPELDKHFLLCEACGKCKCKECASPRTLPSCWVCN
QECLCSAQTLVNYGTCMCLVQGIFYHCTNEDDEGSCADHPCSCSRSNCCARWSFMGALS
VVLPCLLCYLPATGCVKLAQRGYDRLRRPGCRCKHTNSVICKAASGDAKTSRPDKPF

>Q9NP60 single-pass type I membrane protein
MKPPFLLALVVCSVVSTNLKMVSKRNSVDGCIDWSVDLKTYMALAGEPVRVKCALFYS
YIRTNYSTAQSTGLRLMWYKNKGDLEEPIIFSEVRMSKEEDSIWFHSAEAQDSGFYTCVLR
NSTYCMKVSMSLTVAENESGLCYNSRIRYLEKSEVTKRKEISCPDMDDFKKSDQEPDVVW
YKECKPKMWRSIIIQKGNALLIQEVQEEDGGNYTCELKYEGKLVRRTTELKVTALLTDKPP
KPLFPMENQPSVIDVQLGKPLNIPCKAFFGFSGESGPMIYWMKGEKFIEELAGHIREGEIRL

LKEHLGEKEVELALIFDSVVEADLANYTCHVENRNGRKHASVLLRKKDLIYKIELAGGLG
AIFLLLVLLVVIYKCYNIELMLFYRQHFGADETNDDNKEYDAYLSYTKVDQDTLDCDNPE
EEQFALEVLPDVLEKHYGYKLFIPERDLIPSGTYMEDLTRYVEQSRRLIIVLTPDYILRRGW
SIFELESRLHNMLVSGEIKVILIECTELKGKVNCQEVESLKRSIKLLSLIKWKGSKSSKLNSK
FWKHLVYEMPIKKKEMLPRCHVLDSAEQGLFGELQPIPSIAMTSTSATLVSSQADLPEFHP
SDSMQIRHCCRGYKHEIPATTLPVPSLGNHHTYCNLPLTLLNGQLPLNNTLKDTQEFHRNS
SLLPLSSKELSFTSDIW

>Q8NFT8 single-pass type I membrane protein
MQPRRAQAPGAQLLPALALLLLLLGAGPRGSSLANPVPAAPLSAPGPCAAQPCRNGGVCT
SRPEPDPQHPAPAGEPGYSCTCPAGISGANCQLVADPCASNPCHHGNCSSSSSSSSDGYLCI
CNEGYEGPNCEQALPSLPATGWTESMAPRQLQPVPATQEPDKILPRSQATVTLPTWQPKTG
QKVVEMKWDQVEVIPDIACGNASSNSSAGGRLVSFEVPQNTSVKIRQDATASLILLWKVT
ATGFQQCSLIDGRSVTPLQASGGLVLLEEMLALGNNHFIGFVNDSVTKSIVALRLTLVVKV
STCVPGESHANDLECSGKGKCTTKPSEATFSCTCEEQYVGTFCEEYDACQRKPCQNNASC
IDANEKQDGSNFTCVCLPGYTGELCQSKIDYCILDPCRNGATCISSLSGFTCQCPEGYFGSA
CEEKVDPCASSPCQNNGTCYVDGVHFTCNCSPGFTGPTCAQLIDFCALSPCAHGTCRSVG
TSYKCLCDPGYHGLYCEEEYNECLSAPCLNAATCRDLVNGYECVCLAEYKGTHCELYKD
PCANVSCLNGATCDSDGLNGTCICAPGFTGEECDIDINECDSNPCHHGGSCLDQPNGYNC
HCPHGWVGANCEIHLQWKSGHMAESLTNMPRHSLYIIIGALCVAFILMLIILIVGICRISRIE
YQGSSRPAYEEFYNCRSIDSEFSNAIASIRHARFGKKSRPAMYDVSPIAYEDYSPDDKPLVT
LIKTKDL

>Q96LC7 single-pass type I membrane protein
MLLPLLLSSLLGGSQAMDGRFWIRVQESVMVPEGLCISVPCSFSYPRQDWTGSTPAYGYW
FKAVTETTKGAPVATNHQSREVEMSTRGRFQLTGDPAKGNCSLVIRDAQMQDESQYFFRV
ERGSYVRYNFMNDGFFLKVTALTQKPDVYIPETLEPGQPVTVICVFNWAFEECPPPSFSWT
GAALSSQGTKPTTSHFSVLSFTPRPQDHNTDLTCHVDFSRKGVSAQRTVRLRVAYAPRDLV
ISISRDNTPALEPQPQGNVPYLEAQKGQFLRLLCAADSQPPATLSWVLQNRVLSSSHPWGP
RPLGLELPGVKAGDSGRYTCRAENRLGSQQRALDLSVQYPPENLRVMVSQANRTVLENL
GNGTSLPVLEGQSLCLVCVTHSSPPARLSWTQRGQVLSPSQPSDPGVLELPRVQVEHEGEF
TCHARHPLGSQHVSLSLSVHYSPKLLGPSCSWEAEGLHCSCSSQASPAPSLRWWLGEELL
EGNSSQDSFEVTPSSAGPWANSSLSLHGGLSSGLRLRCEAWNVHGAQSGSILQLPDKKGLI
STAFSNGAFLGIGITALLFLCLALIIMKILPKRRTQTETPRPRFSRHSTILDYINVVPTAGPLA
QKRNQKATPNSPRTPLPPGAPSPESKKNQKKQYQLPSFPEPKSSTQAPESQESQEELHYATL
NFPGVRPRPEARMPKGTQADYAEVKFQ

>Q9UIL1 peripheral membrane protein
MRRRVFSSQDWRASGWDGMGFFSRRTFCGRSGRSCRGQLVQVSRPEVSAGSLLLPAPQA
EDHSSRILYPRPKSLLPKMMNADMDAVDAENQVELEEKTRLINQVLELQHTLEDLSARVD
AVKEENLKLKSENQVLGQYIENLMSASSVFQTTDTKSKRK

>Q09327 single-pass type II membrane protein
MKMRRYKLFLMFCMAGLCLISFLHFFKTLSYVTFPRELASLSPNLVSSFFWNNAPVTPQAS

PEPGGPDLLRTPLYSHSPLLQPLPPSKAAEELHRVDLVLPEDTTEYFVRTKAGGVCFKPGT
KMLERPPPGRPEEKPEGANGSSARRPPRYLLSARERTGGRGARRKWVECVCLPGWHGPS
CGVPTVVQYSNLPTKERLVPREVPRRVINAINVNHEFDLLDVRFHELGDVVDAFVVCESN
FTAYGEPRPLKFREMLTNGTFEYIRHKVLYVFLDHFPPGGRQDGWIADDYLRTFLTQDGVS
RLRNLRPDDVFIIDDADEIPARDGVLFLKLYDGWTEPFAFHMRKSLYGFFWKQPGTLEVVS
GCTVDMLQAVYGLDGIRLRRRQYYTMPNFRQYENRTGHILVQWSLGSPLHFAGWHCSW
CFTPEGIYFKLVSAQNGDFPRWGDYEDKRDLNYIRGLIRTGGWFDGTQQEYPPADPSEHM
YAPKYLLKNYDRFHYLLDNPYQEPRSTAAGGWRHRGPEGRPPARGKLDEAEV

>Q9Y512 multipass membrane protein
MGTVHARSLEPLPSSGPDFGGLGEEAEFVEVEPEAKQEILENKDVVVQHVHFDGLGRTKD
DIIICEIGDVFKAKNLIEVMRKSHEAREKLLRLGIFRQVDVLIDTCQGDDALPNGLDVTFEV
TELRRLTGSYNTMVGNNEGSMVLGLKLPNLLGRAEKVTFQFSYGTKETSYGLSFFKPRPG
NFERNFSVNLYKVTGQFPWSSLRETDRGMSAEYSFPIWKTSHTVKWEGVWRELGCLSRT
ASFAVRKESGHSLKSSLSHAMVIDSRNSSILPRRGALLKVNQELAGYTGGDVSFIKEDFEL
QLNKQLIFDSVFSASFWGGMLVPIGDKPSSIADRFYLGGPTSIRGFSMHSIGPQSEGDYLGG
EAYWAGGLHLYTPLPFRPGQGGFGELFRTHFFLNAGNLCNLNYGEGPKAHIRKLAECIRW
SYGAGIVLRLGNIARLELNYCVPMGVQTGDRICDGVQFGAGIRFL

>O15162 single-pass type II membrane protein
MDKQNSQMNASHPETNLPVGYPPQYPPTAFQGPPGYSGYPGPQVSYPPPPAGHSGPGPAG
FPVPNQPVYNQPVYNQPVGAAGVPWMPAPQPPLNCPPGLEYLSQIDQILIHQQIELLEVLT
GFETNNKYEIKNSFGQRVYFAAEDTDCCTRNCCGPSRPFTLRIIDNMGQEVITLERPLRCSS
CCCPCCLQEIEIQAPPGVPIGYVIQTWHPCLPKFTIQNEKREDVLKISGPCVVCSCCGDVDF
EIKSLDEQCVVGKISKHWTGILREAFTDADNFGIQFPLDLDVKMKAVMIGACFLIDFMFFE
STGSQEQKSGVW

>Q9H2E6 single-pass type I membrane protein
MRSEALLLYFTLLHFAGAGFPEDSEPISISHGNYTKQYPVFVGHKPGRNTTQRHRLDIQMI
MIMNGTLYIAARDHIYTVDIDTSHTEEIYCSKKLTWKSRQADVDTCRMKGKHKDECHNFI
KVLLKKNDDALFVCGTNAFNPSCRNYKMDTLEPFGDEFSGMARCPYDAKHANVALFAD
GKLYSATVTDFLAIDAVIYRSLGESPTLRTVKHDSKWLKEPYFVQAVDYGDYIYFFFREIAV
EYNTMGKVVFPRVAQVCKNDMGGSQRVLEKQWTSFLKARLNCSVPGDSHFYFNILQAVT
DVIRINGRDVVLATFSTPYNSIPGSAVCAYDMLDIASVFTGRFKEQKSPDSTWTPVPDERVP
KPRPGCCAGSSSLERYATSNEFPDDTLNFIKTHPLMDEAVPSIFNRPWFLRTMVRYRLTKIA
VDTAAGPYQNHTVVFLGSEKGIILKFLARIGNSGFLNDSLFLEEMSVYNSEKCSYDGVED
KRIMGMQLDRASSSLYVAFSTCVIKVPLGRCERYGKCKKTCIASRDPYCGWIKEGGACSH
LSPNSRLTFEQDIERGNTDGLGDCHNSFVALNGHSSSLLPSTTTSDSTAQEGYESRGGMLD
WKHLLDSPDSTDPLGAVSSHNHQDKKGVIRESYLKGHDQLVPVTLLAIAVILAFVMGAVF
SGITVYCVCDHRRKDVAVVQRKEKELTHSRRGSMSSVTKLSGLFGDTQSKDPKPEAILTPL
MHNGKLATPGNTAKMLIKADQHHLDLTALPTPESTPTLQQKRKPSRGSREWERNQNLINA
CTKDMPPMGSPVIPTDLPLRASPSHIPSVVVLPITQQGYQHEYVDQPKMSEVAQMALEDQ
AATLEYKTIKEHLSSKSPNHGVNLVENLDSLPPKVPQREASLGPPGASLSQTGLSKRLEMH
HSSSYGVDYKRSYPTNSLTRSHQATTLKRNNTNSSNSSHLSRNQSFGRGDNPPPAPQRVDS

IQVHSSQPSGQAVTVSRQPSLNAYNSLTRSGLKRTPSLKPDVPPKPSFAPLSTSMKPNDACT

>Q8N357 multipass membrane protein
MAWTKYQLFLAGLMLVTGSINTLSAKWADNFMAEGCGGSKEHSFQHPFLQAVGMFLGE
FSCLAAFYLLRCRAAGQSDSSVDPQQPFNPLLFLPPALCDMTGTSLMYVALNMTSASSFQ
MLRGAVIIFTGLFSVAFLGRRLVLSQWLGILATIAGLVVVGLADLLSKHDSQHKLSEVITGD
LLIIMAQIIVAIQMVLEEKFVYKHNVHPLRAVGTEGLFGFVILSLLLVPMYYIPAGSFSGNPR
GTLEDALDAFCQVGQQPLIAVALLGNISSIAFFNFAGISVTKELSATTRMVLDSLRTVVIWA
LSLALGWEAFHALQILGFLILLIGTALYNGLHRPLLGRLSRGRPLAEESEQERLLGGTRTPI
NDAS

>Q9UBX3 multipass membrane protein
MAAEARVSRWYFGGLASCGAACCTHPLDLLKVHLQTQQEVKLRMTGMALRVVRTDGIL
ALYSGLSASLCRQMTYSLTRFAIYETVRDRVAKGSQGPLPFHEKVLLGSVSGLAGGFVGTP
ADLVNVRMQNDVKLPQGQRRNYAHALDGLYRVAREEGLRRLFSGATMASSRGALVTVG
QLSCYDQAKQLVLSTGYLSDNIFTHFVASFIAGGCATFLCQPLDVLKTRLMNSKGEYQGV
FHCAVETAKLGPLAFYKGLVPAGIRLIPHTVLTFVFLEQLRKNFGIKVPS

>Q8TCQ1 multipass membrane protein
MLGWCEAIARNPHRIPNNTRTPEISGDLADASQTSTLNEKSPGRSASRSSNISKASSPTTGT
APRSQSRLSVCPSTQDICRICHCEGDEESPLITPCRCTGTLRFVHQSCLHQWIKSSDTRCCE
LCKYDFIMETKLKPLRKWEKLQMTTSERRKIFCSVTFHVIAITCVVWSLYVLIDRTAEEIKQ
GNDNGVLEWPFWTKLVVVAIGFTGGLVFMYVQCKVYVQLWRRLKAYNRVIFVQNCPDT
AKKLEKNFSCNVNTDIKDAVVVPVPQTGANSLPSAEGGPPEVVSV

>P51148 lipid-anchor
MAGRGGAARPNGPAAGNKICQFKLVLLGESAVGKSSLVLRFVKGQFHEYQESTIGAAFLT
QTVCLDDTTVKFEIWDTAGQERYHSLAPMYYRGAQAAIVVYDITNTDTFARAKNWVKEL
QRQASPNIVIALAGNKADLASKRAVEFQEAQAYADDNSLLFMETSAKTAMNVNEIFMAIA
KKLPKNEPQNATGAPGRNRGVDLQENNPASRSQCCSN

>Q14654 multipass membrane protein
MLSRKGIIPEEYVLTRLAEDPAEPRYRARQRRARFVSKKGNCNVAHKNIREQGRFLQDVFT
TLVDLKWPHTLLIFTMSFLCSWLLFAMAWWLIAFAHGDLAPSEGTAEPCVTSIHSFSSAFL
FSIEVQVTIGFGGRMVTEECPLAILILIVQNIVGLMINAIMLGCIFMKTAQAHRRAETLIFSK
HAVIALRHGRLCFMLRVGDLRKSMIISATIHMQVVRKTTSPEGEVVPLHQVDIPMENGVG
GNSIFLVAPLIIYHVIDANSPLYDLAPSDLHHHQDLEIIVILEGVVETTGITTQARTSYLADEI
LWGQRFVPIVAEEDGRYSVDYSKFGNTIKVPTPLCTARQLDEDHSLLEALTLASARGPLRK
RSVPMAKAKPKFSISPDSLS

>Q04656 multipass membrane protein
MDPSMGVNSVTISVEGMTCNSCVWTIEQQIGKVNGVHHIKVSLEEKNATIIYDPKLQTPK
TLQEAIDDMGFDAVIHNPDPLPVLTDTLFLTVTASLTLPWDHIQSTLLKTKGVTDIKIYPQK
RTVAVTIIPSIVNANQIKELVPELSLDTGTLEKKSGACEDHSMAQAGEVVLKMKVEGMTC

HSCTSTIEGKIGKLQGVQRIKVSLDNQEATIVYQPHLISVEEMKKQIEAMGFPAFVKKQPK
YLKLGAIDVERLKNTPVKSSEGSQQRSPSYTNDSTATFIIDGMHCKSCVSNIESTLSALQYV
SSIVVSLENRSAIVKYNASSVTPESLRKAIEAVSPGLYRVSITSEVESTSNSPSSSSLQKIPLN
VVSQPLTQETVINIDGMTCNSCVQSIEGVISKKPGVKSIRVSLANSNGTVEYDPLLTSPETL
RGAIEDMGFDATLSDTNEPLVVIAQPSSEMPLLTSTNEFYTKGMTPVQDKEEGKNSSKCYI
QVTGMTCASCVANIERNLRREEGIYSILVALMAGKAEVRYNPAVIQPPMIAEFIRELGFGAT
VIENADEGDGVLELVVRGMTCASCVHKIESSLTKHRGILYCSVALATNKAHIKYDPEIIGPR
DIIHTIESLGFEASLVKKDRSASHLDHKREIRQWRRSFLVSLFFCIPVMGLMIYMMVMDHH
FATLHHNQNMSKEEMINLHSSMFLERQILPGLSVMNLLSFLLCVPVQFFGGWYFYIQAYK
ALKHKTANMDVLIVLATTIAFAYSLIILLVAMYERAKVNPITFFDTPPMLFVFIALGRWLEHI
AKGKTSEALAKLISLQATEATIVTLDSDNILLSEEQVDVELVQRGDIIKVVPGGKFPVDGRV
IEGHSMVDESLITGEAMPVAKKPGSTVIAGSINQNGSLLICATHVGADTTLSQIVKLVEEAQ
TSKAPIQQFADKLSGYFVPFIVFVSIATLLVWIVIGFLNFEIVETYFPGYNRSISRTETIIRFAF
QASITVLCIACPCSLGLATPTAVMVGTGVGAQNGILIKGGEPLEMAHKVKVVVFDKTGTIT
HGTPVVNQVKVLTESNRISHHKILAIVGTAESNSEHPLGTAITKYCKQELDTETLGTCIDFQ
VVPGCGISCKVTNIEGLLHKNNWNIEDNNIKNASLVQIDASNEQSSTSSSMIIDAQISNALN
AQQYKVLIGNREWMIRNGLVINNDVNDFMTEHERKGRTAVLVAVDDELCGLIAIADTVKP
EAELAIHILKSMGLEVVLMTGDNSKTARSIASQVGITKVFAEVLPSHKVAKVKQLQEEGKR
VAMVGDGINDSPALAMANVGIAIGTGTDVAIEAADVVLIRNDLLDVVASIDLSRETVKRIRI
NFVFALIYNLVGIPIAAGVFMPIGLVLQPWMGSAAMAASSVSVVLSSLFLKLYRKPTYESY
ELPARSQIGQKSPSEISVHVGIDDTSRNSPKLGLLDRIVNYSRASINSLLSDKRSLNSVVTSE
PDKHSLLVGDFREDDDTAL

>P51168 multipass membrane protein
MHVKKYLLKGLHRLQKGPGYTYKELLVWYCDNTNTHGPKRIICEGPKKKAMWFLLTLLF
AALVCWQWGIFIRTYLSWEVSVSLSVGFKTMDFPAVTICNASPFKYSKIKHLLKDLDELME
AVLERILAPELSHANATRNLNFSIWNHTPLVLIDERNPHHPMVLDLFGDNHNGLTSSSASE
KICNAHGCKMAMRLCSLNRTQCTFRNFTSATQALTEWYILQATNIFAQVPQQELVEMSYP
GEQMILACLFGAEPCNYRNFTSIFYPHYGNCYIFNWGMTEKALPSANPGTEFGLKLILDIG
QEDYVPFLASTAGVRLMLHEQRSYPFIRDEGIYAMSGTETSIGVLVDKLQRMGEPYSPCTV
NGSEVPVQNFYSDYNTTYSIQACLRSCFQDHMIRNCNCGHYLYPLPRGEKYCNNRDFPD
WAHCYSDLQMSVAQRETCIGMCKESCNDTQYKMTISMADWPSEASEDWIFHVLSQERD
QSTNITLSRKGIVKLNIYFQEFNYRTIEESAANNIVWLLSNLGGQFGFWMGGSVLCLIEFGE
IIIDFVWITIIKLVALAKSLRQRRAQASYAGPPPTVAELVEAHTNFGFQPDTAPRSPNTGPYPS
EQALPIPGTPPPNYDSLRLQPLDVIESDSEGDAI

>Q13614 peripheral membrane protein
MEKSSSCESLGSQPAAARPPSVDSLSSASTSHSENSVHTKSASVVSSDSISTSADNFSPDLR
VLRESNKLAEMEEPPLLPGENIKDMAKDVTYICPFTGAVRGTLTVTNYRLYFKSMERDPPF
VLDASLGVINRVEKIGGASSRGENSYGLETVCKDIRNLRFAHKPEGRTRRSIFENLMKYAF
PVSNNLPLFAFEYKEVFPENGWKLYDPLLEYRRQGIPNESWRITKINERYELCDTYPALLV
VPANIPDEELKRVASFRSRGRIPVLSWIHPESQATITRCSQPMVGVSGKRSKEDEKYLQAIM
DSNAQSHKIFIFDARPSVNAVANKAKGGGYESEDAYQNAELVFLDIHNIHVMRESLRKLK
EIVYPNIEETHWLSNLESTHWLEHIKLILAGALRIADKVESGKTSVVVHCSDGWDRTAQLT

SLAMLMLDGYYRTIRGFEVLVEKEWLSFGHRFQLRVGHGDKNHADADRSPVFLQFIDCV
WQMTRQFPTAFEFNEYFLITILDHLYSCLFGTFLCNSEQQRGKENLPKRTVSLWSYINSQLE
DFTNPLYGSYSNHVLYPVASMRHLELWVGYYIRWNPRMKPQEPIHNRYKELLAKRAELQ
KKVEELQREISNRSTSSSERASSPAQCVTPVQTVV

>P38567 GPI-anchor, lipid-anchor
MGVLKFKHIFFRSFVKSSGVSQIVFTFLLIPCCLTLNFRAPPVIPNVPFLWAWNAPSEFCLGK
FDEPLDMSLFSFIGSPRINATGQGVTIFYVDRLGYYPYIDSITGVTVNGGIPQKISLQDHLDK
AKKDITFYMPVDNLGMAVIDWEEWRPTWARNWKPKDVYKNRSIELVQQQNVQLSLTEA
TEKAKQEFEKAGKDFLVETIKLGKLLRPNHLWGYYLFPDCYNHHYKKPGYNGSCFNVEI
KRNDDLSWLWNESTALYPSIYLNTQQSPVAATLYVRNRVREAIRVSKIPDAKSPLPVFAYTR
IVFTDQVLKFLSQDELVYTFGETVALGASGIVIWGTLSIMRSMKSCLLLDNYMETILNPYII
NVTLAAKMCSQVLCQEQGVCIRKNWNSSDYLHLNPDNFAIQLEKGGKFTVRGKPTLEDL
EQFSEKFYCSCYSTLSCKEKADVKDTDAVDVCIADGVCIDAFLKPPMETEEPQIFYNASPS
TLSATMFIVSILFLIISSVASL

>Q9NTQ9 multipass membrane protein
MNWAFLQGLLSGVNKYSTVLSRIWLSVVFIFRVLVYVVAAEEVWDDEQKDFVCNTKQPG
CPNVCYDEFFPVSHVRLWALQLILVTCPSLLVVMHVAYREERERKHHLKHGPNAPSLYDN
LSKKRGGLWWTYLLSLIFKAAVDAGFLYIFHRLYKDYDMPRVVACSVEPCPHTVDCYISR
PTEKKVFTYFMVTTAAICILLNLSEVFYLVGKRCMEIFGPRHRRPRCRECLPDTCPPYVLSQ
GGHPEDGNSVLMKAGSAPVDAGGYP

>P40879 multipass membrane protein
MIEPFGNQYIVARPVYSTNAFEENHKKTGRHHKTFLDHLKVCCSCSPQKAKRIVLSLFPIA
SWLPAYRLKEWLLSDIVSGISTGIVAVLQGLAFALLVDIPPVYGLYASFFPAIIYLFFGTSRHIS
VGPFPILSMMVGLAVSGAVSKAVPDRNATTLGLPNNSNNSSLLDDERVRVAAAASVTVLS
GIIQLAFGILRIGFVVIYLSESLISGFTTAAAVHVLVSQLKFIFQLTVPSHTDPVSIFKVLYSVF
SQIEKTNIADLVTALIVLLVVSIVKEINQRFKDKLPVPIPIEFIMTVIAAGVSYGCDFKNRFKV
AVVGDMNPGFQPPITPDVETFQNTVGDCFGIAMVAFAVAFSVASVYSLKYDYPLDGNQELI
ALGLGNIVCGVFRGFAGSTALSRSAVQESTGGKTQIAGLIGAIIVLIVVLAIGFLLAPLQKSV
LAALALGNLKGMLMQFAEIGRLWRKDKYDCLIWIMTFIFTIVLGLGLGLAASVAFQLLTIV
FRTQFPKCSTLANIGRTNIYKNKKDYYDMYEPEGVKIFRCPSPIYFANIGFFRRKLIDAVGFS
PLRILRKRNKALRKIRKLQKQGLLQVTPKGFICTVDTIKDSDEELDNNQIEVLDQPINTTDL
PFHIDWNDDLPLNIEVPKISLHSLILDFSAVSFLDVSSVRGLKSILQEFIRIKVDVYIVGTDDD
FIEKLNRYEFFDGEVKSSIFFLTIHDAVLHILMKKDYSTSKFNPSQEKDGKIDFTINTNGGLR
NRVYEVPVETKF

>Q9UKX7 peripheral membrane protein
MAKRNAEKELTDRNWDQEDEAEEVGTFSMASEEVLKNRAIKKAKRRNVGFESDTGGAF
KGFKGLVVPSGGGRFSGFGSGAGGKPLEGLSNGNNITSAPPFASAKAAADPKVAFGSLAA
NGPTTLVDKVSNPKTNGDSQQPSSSGLASSKACVGNAYHKQLAALNCSVRDWIVKHVNT
NPLCDLTPIFKDYEKYLANIEQQHGNSGRNSESESNKVAAETQSPSLFGSTKLQQESTFLFH
GNKTEDTPDKKMEVASEKKTDPSSLGATSASFNFGKKVDSSVLGSLSSVPLTGFSFSPGNS

SLFGKDTTQSKPVSSPFPTKPLEGQAEGDSGECKGGDEEENDEPPKVVVTEVKEEDAFYS
KKCKLFYKKDNEFKEKGIGTLHLKPTANQKTQLLVRADTNLGNILLNVLIPPNMPCTRTG
KNNVLIVCVPNPPIDEKNATMPVTMLIRVKTSEDADELHKILLEKKDA

>Q8WY21 single-pass type I membrane protein
MGKVGAGGGSQARLSALLAGAGLLILCAPGVCGGGSCCPSPHPSSAPRSASTPRGFSHQG
RPGRAPATPLPLVVRPLFSVAPGDRALSLERARGTGASMAVAARSGRRRRSGADQEKAER
GEGASRSPRGVLRDGGQQEPGTRERDPDKATRFRMEELRLTSTTFALTGDSAHNQAMVH
WSGHNSSVILILTKLYDYNLGSITESSLWRSTDYGTTYEKLNDKVGLKTILSYLYVCPTNK
RKIMLLTDPEIESSLLISSDEGATYQKYRLNFYIQSLLFHPKQEDWILAYSQDQKLYSSAEFG
RRWQLIQEGVVPNRFYWSVMGSNKEPDLVHLEARTVDGHSHYLTCRMQNCTEANRNQP
FPGYIDPDSLIVQDHYVFVQLTSGGRPHYYVSYRRNAFAQMKLPKYALPKDMHVISTDEN
QVFAAVQEWNQNDTYNLYISDTRGVYFTLALENVQSSRGPEGNIMIDLYEVAGIKGMFLA
NKKIDNQVKTFITYNKGRDWRLLQAPDTDLRGDPVHCLLPYCSLHLHLKVSENPYTSGII
ASKDTAPSIIVASGNIGSELSDTDISMFVSSDAGNTWRQIFEEEHSVLYLDQGGVLVAMKHT
SLPIRHLWLSFDEGRSWSKYSFTSIPLFVDGVLGEPGEETLIMTVFGHFSHRSEWQLVKVD
YKSIFDRRCAEEDYRPWQLHSQGEACIMGAKRIYKKRKSERKCMQGKYAGAMESEPCVC
TEADFDCDYGYERHSNGQCLPAFWFNPSSLSKDCSLGQSYLNSTGYRKVVSNNCTDGVR
EQYTAKPQKCPGKAPRGLRIVTADGKLTAEQGHNVTLMVQLEEGDVQRTLIQVDFGDGIA
VSYVNLSSMEDGIKHVYQNVGIFRVTVQVDNSLGSDSAVLYLHVTCPLEHVHLSLPFVTT
KNKEVNATAVLWPSQVGTLTYVWWYGNNTEPLITLEGSISFRFTSEGMNTITVQVSAGNAI
LQDTKTIAVYEEFRSLRLSFSPNLDDYNPDIPEWRRDIGRVIKKSLVEATGVPGQHILVAVLP
GLPTTAELFVLPYQDPAGENKRSTDDLEQISELLIHTLNQNSVHFELKPGVRVLVHAAHLT
AAPLVDLTPTHSGSAMLMLLSVVFVGLAVFVIYKFKRRVALPSPPSPSTQPGDSSLRLQRA
RHATPPSTPKRGSAGAQYAI

>Q9ULH4 single-pass type I membrane protein
METLLGGLLAFGMAFAVVDACPKYCVCQNLSESLGTLCPSKGLLFVPPDIDRRTVELRLG
GNFIIHISRQDFANMTGLVDLTLSRNTISHIQPFSFLDLESLRSLHLDSNRLPSLGEDTLRGLV
NLQHLIVNNNQLGGIADEAFEDFLLTLEDLDLSYNNLHGLPWDSVRRMVNLHQLSLDHN
LLDHIAEGTFADLQKLARLDLTSNRLQKLPPDPIFARSQASALTATPFAPPLSFSFGGNPLHC
NCELLWLRRLERDDDLETCGSPGGLKGRYFWHVREEEFVCEPPLITQHTHKLLVLEGQAA
TLKCKAIGDPSPLIHWVAPDDRLVGNSSRTAVYDNGTLDIFITTSQDSGAFTCIAANAAGEA
TAMVEVSIVQLPHLSNSTSRTAPPKSRLSDITGSSKTSRGGGGSGGGEPPKSPPERAVLVSE
VTTTSALVKWSVSKSAPRVKMYQLQYNCSDDEVLIYRMIPASNKAFVVNNLVSGTGYDL
CVLAMWDDTATTLTATNIVGCAQFFTKADYPQCQSMHSQILGGTMILVIGGIIVATLLVFIVI
LMVRYKVCNHEAPSKMAAAVSNVYSQTNGAQPPPPSSAPAGAPPQGPPKVVVRNELLDF
TASLARASDSSSSSSLGSGEAAGLGRAPWRIPPSAPRPKPSLDRLMGAFASLDLKSQRKEE
LLDSRTPAGRGAGTSARGHHSDREPLLGPPAARARSLLPLPLEGKAKRSHSFDMGDFAAA
AAGGVVPGGYSPPRKVSNIWTKRSLSVNGMLLPFEESDLVGARGTFGSSEWVMESTV

>Q9H3H5 multipass membrane protein
MWAFSELPMPLLINLIVSLLGFVATVTLIPAFRGHFIAARLCGQDLNKTSRQQIPESQGVISG
AVFLIILFCFIPFPFLNCFVKEQCKAFPHHEFVALIGALLAICCMIFLGFADDVLNLRWRHKL

LLPTAASLPLLMVYFTNFGNTTIVVPKPFRPILGLHLDLGILYYVYMGLLAVFCTNAINILA
GINGLEAGQSLVISASIIVFNLVELEGDCRDDHVFSLYFMIPFFFTTLGLLYHNWYPSRVFVG
DTFCYFAGMTFAVVGILGHFSKTMLLFFMPQVFNFLYSLPQLLHIIPCPRHRIPRLNIKTGKL
EMSYSKFKTKSLSFLGTFILKVAESLQLVTVHQSETEDGEFTECNNMTLINLLLKVLGPIHE
RNLTLLLLLLQILGSAITFSIRYQLVRLFYDV

>P56181 peripheral membrane protein
MAAPCLLRQGRAGALKTMLQEAQVFRGLASTVSLSAESGKSEKGQPQNSKKQSPPKKPA
PVPAEPFDNTTYKNLQHHDYSTYTFLDLNLELSKFRMPQPSSGRESPRH

>Q9UP65 lipid-anchor
MGSSEVSIIPGLQKEEKAAVERRRLHVLKALKKLRIEADEAPVVAVLGSGGGLRAHIACLG
VLSEMKEQGLLDAVTYLAGVSGSTWAISSLYTNDGDMEALEADLKHRFTRQEWDLAKSL
QKTIQAARSENYSLTDFWAYMVISKQTRELPESHLSNMKKPVEEGTLPYPIFAAIDNDLQP
SWQEARAPETWFEFTPHHAGFSALGAFVSITHFGSKFKKGRLVRTHPERDLTFLRGLWGS
ALGNTEVIREYIFDQLRNLTLKGLWRRAVANAKSIGHLIFARLLRLQESSQGEHPPPEDEGG
EPEHTWLTEMLENWTRTSLEKQEQPHEDPERKGSLSNLMDFVKKTGICASKWEWGTTHN
FLYKHGGIRDKIMSSRKHLHLVDAGLAINTPFPLVLPPTREVHLILSFDFSAGDPFETIRATT
DYCRRHKIPFPQVEEAELDLWSKAPASCYILKGETGPVVMHFPLFNIDACGGDIEAWSDTY
DTFKLADTYTLDVVVLLLALAKKNVRENKKKILRELMNVAGLYYPKDSARSCCLA

>Q9UK22 peripheral membrane protein
MDGDGDPESVGQPEEASPEEQPEEASAEEERPEDQQEEEAAAAAAYLDELPEPLLLRVLA
ALPAAELVQACRLVCLRWKELVDGAPLWLLKCQQEGLVPEGGVEEERDHWQQFYFLSKR
RRNLLRNPCGEEDLEGWCDVEHGGDGWRVEELPGDSGVEFTHDESVKKYFASSFEWCR
KAQVIDLQAEGYWEELLDTTQPAIVVKDWYSGRSDAGCLYELTVKLLSEHENVLAEFSSG
QVAVPQDSDGGGWMEISHTFTDYGPGVRFVRFEHGGQDSVYWKGWFGARVTNSSVWV
EP

>Q6ZMB0 single-pass type II membrane protein
MAFPCRRSLTAKTLACLLVGVSFLALQQWFLQAPRSPREERSPQEETPEGPTDAPAADEPP
SELVPGPPCVANASANATADFEQLPARIQDFLRYRHCRHFPLLWDAPAKCAGGRGVFLLLA
VKSAPEHYERRELIRRTWGQERSYGGRPVRRLFLLGTPGPEDEARAERLAELVALEAREH
GDVLQWAFADTFLNLTLKHLHLLDWLAARCPHARFLLSGDDDVFVHTANVVRFLQAQPP
GRHLFSGQLMEGSVPIRDSWSKYFVPPQLFPGSAYPVYCSGGGFLLSGPTARALRAAARH
TPLFPIDDAYMGMCLERAGLAPSGHEGIRPFGVQLPGAQQSSFDPCMYRELLLVHRFAPYE
MLLMWKALHSPALSCDRGHRVS

>Q86YB8 peripheral membrane protein
MSQGVRRAGAGQGVAAAVQLLVTLSFLRSVVEAQVTGVLDDCLCDIDSIDNFNTYKIFPK
IKKLQERDYFRYYKVNLKRPCPFWAEDGHCSIKDCHVEPCPESKIPVGIKAGHSNKYLKM
ANNTKELEDCEQANKLGAINSTLSNQSKEAFIDWARYDDSRDHFCELDDERSPAAQYVDL
LLNPERYTGYKGTSAWRVWNSIYEENCFKPRSVYRPLNPLAPSRGEDDGESFYTWLEGLC
LEKRVFYKLISGLHASINLHLCANYLLEETWGKPSWGPNIKEFKHRFDPVETKGEGPRRLK

NLYFLYLIELRALSKVAPYFERSIVDLYTGNAEEDADTKTLLLNIFQDTKSFPMHFDEKSMF
AGDKKGAKSLKEEFRLHFKNISRIMDCVGCDKCRLWGKLQTQGLGTALKILFSEKEIQKL
PENSPSKGFQLTRQEIVALLNAFGRLSTSIRDLQNFKVLLQHSR

>Q04671 multipass membrane protein
MHLEGRDGRRYPGAPAVELLQTSVPSGLAELVAGKRRLPRGAGGADPSHSCPRGAAGQSS
WAPAGQEFASFLTKGRSHSSLPQMSSSRSKDSCFTENTPLLRNSLQEKGSRCIPVYHPEFIT
AEESWEDSSADWERRYLLSREVSGLSASASSEKGDLLDSPHIRLRLSKLRRCVQWLKVM
GLFAFVVLCSILFSLYPDQGKLWQLLALSPLENYSVNLSSHVDSTLLQVDLAGALVASGPS
RPGREEHIVVELTQADALGSRWRRPQQVTHNWTVYLNPRRSEHSVMSRTFEVLTRETVSI
SIRASLQQTQAVPLLMAHQYLRGSVETQVTIATAILAGVYALIIFEIVHRTLAAMLGSLAAL
AALAVIGDRPSLTHVVEWIDFETLALLFGMMILVAIFSETGFFDYCAVKAYRLSRGRVWAM
IIMLCLIAAVLSAFLDNVTTMLLFTPVTIRLCEVLNLDPRQVLIAEVIFTNIGGAATAIGDPP
NVIIVSNQELRKMGLDFAGFTAHMFIGICLVLLVCFPLLRLLYWNRKLYNKEPSEIVELKHE
IHVWRLTAQRISPASREETAVRRLLLGKVLALEHLLARRLHTFHRQISQEDKNWETNIQEL
QKKHRISDGILLAKCLTVLGFVIFMFFLNSFVPGIHLDLGWIAILGAIWLLILADIHDFEIILH
RVEWATLLFFAALFVLMEALAHLHLIEYVGEQTALLIKMVPEEQRLIAAIVLVVWVSALAS
SLIDNIPFTATMIPVLLNLSHDPEVGLPAPPLMYALAFGACLGGNGTLIGASANVVCAGIAE
QHGYGFSFMEFFRLGFPMMVVSCTVGMCYLLVAHVVVGWN

>Q9NXB9 multipass membrane protein
MEHLKAFDDEINAFLDNMFGPRDSRVRGWFMLDSYLPTFFLTVMYLLSIWLGNKYMKN
RPALSLRGILTLYNLGITLLSAYMLAELILSTWEGGYNLQCQDLTSAGEADIRVAKVLWWY
YFSKSVEFLDTIFFVLRKKTSQITFLHVYHHASMFNIWWCVLNWIPCGQSFFGPTLNSFIHI
LMYSYYGLSVFPSMHKYLWWKKYLTQAQLVQFVLTITHTMSAVVKPCGFPFGCLIFQSSY
MLTLVILFLNFYVQTYRKKPMKKDMQEPPAGKEVKNGFSKAYFTAANGVMNKKAQ

>Q5T9L3 multipass membrane protein
MAGAIIENMSTKKLCIVGGILLVFQIIAFLVGGLIAPGPTTAVSYMSVKCVDARKNHHKTK
WFVPWGPNHCDKIRDIEEAIPREIEANDIVFSVHIPLPHMEMSPWFQFMLFILQLDIAFKLN
NQIRENAEVSMDVSLAYRDDAFAEWTEMAHERVPRKLKCTFTSPKTPEHEGRYYECDVL
PFMEIGSVAHKFYLLNIRLPVNEKKKINVGIGEIKDIRLVGIHQNGGFTKVWFAMKTFLTPS
IFIIMVWYWRRITMMSRPPVLLEKVIFALGISMTFINIPVEWFSIGFDWTWMLLFGDIRQGI
FYAMLLSFWIIFCGEHMMDQHERNHIAGYWKQVGPIAVGSFCLFIFDMCERGVQLTNPFY
SIWTTDIGTELAMAFIIVAGICLCLYFLFLCFMVFQVFRNISGKQSSLPAMSKVRRLHYEGLI
FRFKFLMLITLACAAMTVIFFIVSQVTEGHWKWGGVTVQVNSAFFTGIYGMWNLYVFAL
MFLYAPSHKNYGEDQSNGDLGVHSGEELQLTTTITHVDGPTEIYKLTRKEAQE

>A6NMS3 multipass membrane protein
MARENHSLAAEFILIGFTNYPELKTLLFVVFSAIYLVTMVGNLGLVALIYVERRLLTPMYIF
LGNLALMDSCCSCAVTPKMLENFFSEDRIISLYECMAQFYFLCLAETTDCFLLATMAYDRY
VAICHPLQYHTMMSKTLCIRMTTGAFKAGNLHSMIHVGLLLRLTFCRSNKIHHFFCDILPL
YRLSCTDPSINELMIYIFSIPIQIFTIATVLISYLCILLTVFKMKSKEGRGKAFSTCASHFLSVSI
FYICLLMYIGPSEEGDKDTPVAIFYAIVIPLLNPFIYSLRNKEVINVLKKIMRNYNILKQTCSI

ANLFLIY

>O95461 single-pass type II membrane protein
MLGICRGRRKFLAASLSLLCIPAITWIYLFSGSFEDGKPVSLSPLESQAHSPRYTASSQRERE
SLEVRMREVEEENRALRRQLSLAQGRAPSHRRGNHSKTYSMEEGTGDSENLRAGIVAGN
SSECGQQPVVEKCETIHVAIVCAGYNASRDVVTLVKSVLFHRRNPLHFHLIADSIAEQILAT
LFQTWMVPAVRVDFYNADELKSEVSWIPNKHYSGIYGLMKLVLTKTLPANLERVIVLDTDI
TFATDIAELWAVFHKFKGQQVLGLVENQSDWYLGNLWKNHRPWPALGRGYNTGVILLLL
DKLRKMKWEQMWRLTAERELMGMLSTSLADQDIFNAVIKQNPFLVYQLPCFWNVQLSD
HTRSEQCYRDVSDLKVIHWNSPKKLRVKNKHVEFFRNLYLTFLEYDGNLLRRELFGCPSE
ADVNSENLQKQLSELDEDDLCYEFRRERFTVHRTHLYFLHYEYEPAADSTDVTLVAQLSM
DRLQMLEAICKHWEGPISLALYLSDAEAQQFLRYAQGSEVLMSRHNVGYHIVYKEGQFY
PVNLLRNVAMKHISTPYMFLSDIDFLPMYGLYEYLRKSVIQLDLANTKKAMIVPAFETLRY
RLSFPKSKAELLSMLDMGTLFTFRYHVWTKGHAPTNFAKWRTATTPYRVEWEADFEPYV
VVRRDCPEYDRRFVGFGWNKVAHIMELDVQEYEFIVLPNAYMIHMPHAPSFDITKFRSNK
QYRICLKTLKEEFQQDMSRRYGFAALKYLTAENNS

>P07307 single-pass type II membrane protein
MAKDFQDIQQLSSEENDHPFHQGEGPGTRRLNPRRGNPFLKGPPPAQPLAQRLCSMVCFS
LLALSFNILLLVVICVTGSQSEGHGGAQLQAELRSLKEAFSNFSSSTLTEVQAISTHGGSVG
DKITSLGAKLEKQQQDLKADHDALLFHLKHFPVDLRFVACQMELLHSNGSQRTCCPVNW
VEHQGSCYWFSHSGKAWAEAEKYCQLENAHLVVINSWEEQKFIVQHTNPFNTWIGLTDS
DGSWKWVDGTDYRHNYKNWAVTQPDNWHGHELGGSEDCVEVQPDGRWNDDFCLQVY
RWVCEKRRNATGEVA

>P49757 peripheral membrane protein
MNKLRQSFRRKKDVYVPEASRPHQWQTDEEGVRTGKCSFPVKYLGHVEVDESRGMHIC
EDAVKRLKAERKFFKGFFGKTGKKAVKAVLWVSADGLRVVDEKTKDLIVDQTIEKVSFC
APDRNFDRAFSYICRDGTTRRWICHCFMAVKDTGERLSHAVGCAFAACLERKQKREKEC
GVTATFDASRTTFTREGSFRVTTATEQAEREEIMKQMQDAKKAETDKIVVGSSVAPGNTAP
SPSSPTSPTSDATTSLEMNNPHAIPRRHAPIEQLARQGSFRGFPALSQKMSPFKRQLSLRINE
LPSTMQRKTDFPIKNAVPEVEGEAESISSLCSQITNAFSTPEDPFSSAPMTKPVTVVAPQSPT
FQANGTDSAFHVLAKPAHTALAPVAMPVRETNPWAHAPDAANKEIAATCSGTEWGQSSG
AASPGLFQAGHRRTPSEADRWLEEVSKSVRAQQPQASAAPLQPVLQPPPPTAISQPASPFQ
GNAFLTSQPVPVGVVPALQPAFVPAQSYPVANGMPYPAPNVPVVGITPSQMVANVFGTAG
HPQAAHPHQSPSLVRQQTFPHYEASSATTSPFFKPPAQHLNGSAAFNGVDDGRLASADRH
TEVPTGTCPVDPFEAQWAALENKSKQRTNPSPTNPFSSDLQKTFEIEL

>P21730 multipass membrane protein
MDSFNYTTPDYGHYDDKDTLDLNTPVDKTSNTLRVPDILALVIFAVVFLVGVLGNALVVW
VTAFEAKRTINAIWFLNLAVADFLSCLALPILFTSIVQHHHWPFGGAACSILPSLILLNMYAS
ILLLATISADRFLLVFKPIWCQNFRGAGLAWIACAVAWGLALLLTIPSFLYRVVREEYFPPKV
LCGVDYSHDKRRERAVAIVRLVLGFLWPLLTLTICYTFILLRTWSRRATRSTKTLKVVVAVV
ASFFIFWLPYQVTGIMMSFLEPSSPTFLLLKKLDSLCVSFAYINCCINPIIYVVAGQGFQGRL

RKSLPSLLRNVLTEESVVRESKSFTRSTVDTMAQKTQAV

>O43292 multipass membrane protein
MGLLSDPVRRRALARLVLRLNAPLCVLSYVAGIAWFLALVFPPLTQRTYMSENAMGSTM
VEEQFAGGDRARAFARDFAAHRKKSGALPVAWLERTMRSVGLEVYTQSFSRKLPFPDETH
ERYMVSGTNVYGILRAPRAASTESLVLTVPCGSDSTNSQAVGLLLALAAHFRGQIYWAKD
IVFLVTEHDLLGTEAWLEAYHDVNVTGMQSSPLQGRAGAIQAAVALELSSDVVTSLDVAV
EGLNGQLPNLDLLNLFQTFCQKGGLLCTLQGKLQPEDWTSLDGPLQGLQTLLLMVLRQA
SGRPHGSHGLFLRYRVEALTLRGINSFRQYKYDLVAVGKALEGMFRKLNHLLERLHQSFFL
YLLPGLSRFVSIGLYMPAVGFLLLVLGLKALELWMQLHEAGMGLEEPGGAPGPSVPLPPSQ
GVGLASLVAPLLISQAMGLALYVLPVLGQHVATQHFPVAEAEAVVLTLLAIYAAGLALPHN
THRVVSTQAPDRGWMALKLVALIYLALQLGCIALTNFSLGFLLATTMVPTAALAKPHGPR
TLYAALLVLTSPAATLLGSLFLWRELQEAPLSLAEGWQLFLAALAQGVLEHHTYGALLFPL
LSLGLYPCWLLFWNVLFWK

>O94979 peripheral membrane protein
MKLKEVDRTAMQAWSPAQNHPIYLATGTSAQQLDATFSTNASLEIFELDLSDPSLDMKSC
ATFSSSHRYHKLIWGPYKMDSKGDVSGVLIAGGENGNIILYDPSKIIAGDKEVVIAQNDKH
TGPVRALDVNIFQTNLVASGANESEIYIWDLNNFATPMTPGAKTQPPEDISCIAWNRQVQHI
LASASPSGRATVWDLRKNEPIIKVSDHSNRMHCSGLAWHPDVATQMVLASEDDRLPVIQ
MWDLRFASSPLRVLENHARGILAIAWSMADPELLLSCGKDAKILCSNPNTGEVLYELPTNT
QWCFDIQWCPRNPAVLSAASFDGRISVYSIMGGSTDGLRQKQVDKLSSSFGNLDPFGTGQ
PLPPLQIPQQTAQHSIVLPLKKPPKWIRRPVGASFSFGGKLVTFENVRMPSHQGAEQQQQQ
HHVFISQVVTEKEFLSRSDQLQQAVQSQGFINYCQKKIDASQTEFEKNVWSFLKVNFEDD
SRGKYLELLGYRKEDLGKKIALALNKVDGANVALKDSDQVAQSDGEESPAAEEQLLGEHI
KEEKEESEFLPSSGGTFNISVSGDIDGLITQALLTGNFESAVDLCLHDNRMADAIILAIAGG
QELLARTQKKYFAKSQSKITRLITAVVMKNWKEIVESCDLKNWREALAAVLTYAKPDEFS
ALCDLLGTRLENEGDSLLQTQACLCYICAGNVEKLVACWTKAQDGSHPLSLQDLIEKVVI
LRKAVQLTQAMDTSTVGVLLAAKMSQYANLLAAQGSIAAALAFLPDNTNQPNIMQLRDR
LCRAQGEPVAGHESPKIPYEKQQLPKGRPGPVAGHHQMPRVQTQQYYPHGENPPPPGFIM
HGNVNPNAAGQLPTSPGHMHTQVPPYPQPQPYQPAQPYPFGTGGSAMYRPQQPVAPPTS
NAYPNTPYISSASSYTGQSQLYAAQHQASSPTSSPATSFPPPPSSGASFQHGGPGAPPSSSAY
ALPPGTTGTLPAASELPASQRTGPQNGWNDPPALNRVPKKKKMPENFMPPVPITSPIMNPL
GDPQSQMLQQQPSAPVPLSSQSSFPQPHLPGGQPFHGVQQPLGQTGMPPSFSKPNIEGAPG
APIGNTFQHVQSLPTKKITKKPIPDEHLILKTTFEDLIQRCLSSATDPQTKRKLDDASKRLEF
LYDKLREQTLSPTITSGLHNIARSIETRNYSEGLTMHTHIVSTSNFSETSAFMPVLKVVLTQ
ANKLGV

>Q16625 multipass membrane protein
MSSRPLESPPPYRPDEFKPNHYAPSNDIYGGEMHVRPMLSQPAYSFYPEDEILHFYKWTSP
PGVIRILSMLIIVMCIAIFACVASTLAWDRGYGTSLLGGSVGYPYGGSGFGSYGSGYGYGY
GYGYGYGGYTDPRAAKGFMLAMAAFCFIAALVIFVTSVIRSEMSRTRRYYLSVIIVSAILGI
MVFIATIVYIMGVNPTAQSSGSLYGSQIYALCNQFYTPAATGLYVDQYLYHYCVVDPQEAI
AIVLGFMIIVAFALIIFFAVKTRRKMDRYDKSNILWDKEHIYDEQPPNVEEWVKNVSAGTQ

DVPSPPSDYVERVDSPMAYSSNGKVNDKRFYPESSYKSTPVPEVVQELPLTSPVDDFRQPR
YSSGGNFETPSKRAPAKGRAGRSKRTEQDHYETDYTTGGESCDELEEDWIREYPPITSDQQ
RQLYKRNFDTGLQEYKSLQSELDEINKELSRLDKELDDYREESEEYMAAADEYNRLKQV
KGSADYKSKKNHCKQLKSKLSHIKKMVGDYDRQKT

>P23276 single-pass type II membrane protein
MEGGDQSEEEPRERSQAGGMGTLWSQESTPEERLPVEGSRPWAVARRVLTAILILGLLLCF
SVLLFYNFQNCGPRPCETSVCLDLRDHYLASGNTSVAPCTDFFSFACGRAKETNNSFQELA
TKNKNRLRRILEVQNSWHPGSGEEKAFQFYNSCMDTLAIEAAGTGPLRQVIEELGGWRIS
GKWTSLNFNRTLRLLMSQYGHFPFFRAYLGPHPASPHTPVIQIDQPEFDVPLKQDQEQKIY
AQIFREYLTYLNQLGTLLGGDPSKVQEHSSLSISITSRLFQFLRPLEQRRAQGKLFQMVTID
QLKEMAPAIDWLSCLQATFTPMSLSPSQSLVVHDVEYLKNMSQLVEEMLLKQRDFLQSH
MILGLVVTLSPALDSQFQEARRKLSQKLRELTEQPPMPARPRWMKCVEETGTFFEPTLAAL
FVREAFGPSTRSAAMKLFTAIRDALITRLRNLPWMNEETQNMAQDKVAQLQVEMGASEW
ALKPELARQEYNDIQLGSSFLQSVLSCVRSLRARIVQSFLQPHPQHRWKVSPWDVNAYYS
VSDHVVVFPAGLLQPPFFHPGYPRAVNFGAAGSIMAHELLHIFYQLLLPGGCLACDNHAL
QEAHLCLKRHYAAFPLPSRTSFNDSLTFLENAADVGGLAIALQAYSKRLLRHHGETVLPSL
DLSPQQIFFRSYAQVMCRKPSPQDSHDTHSPPHLRVHGPLSSTPAFARYFRCARGALLNPSS
RCQLW

>Q8J025 single-pass type I membrane protein
MSWPRRLLLRYLFPALLLHGLGEGSALLHPDSRSHPRSLEKSAWRAFKESQCHHMLKHL
HNGARITVQMPPTIEGHWVSTGCEVRSGPEFITRSYRFYHNNTFKAYQFYYGSNRCTNPT
YTLIIRGKIRLRQASWIIRGGTEADYQLHNVQVICHTEAVAEKLGQQVNRTCPGFLADGGP
WVQDVAYDLWREENGCECTKAVNFAMHELQLIRVEKQYLHHNLDHLVEELFLGDIHTDA
TQRMFYRPSSYQPPLQNAKNHDHACIACRIIYRSDEHHPPILPPKADLTIGLHGEWVSQRC
EVRPEVLFLTRHFIFHDNNNTWEGHYYHYSDPVCKHPTFSIYARGRYSRGVLSSRVMGGT
EFVFKVNHMKVTPMDAATASLLNVFNGNECGAEGSWQVGIQQDVTHTNGCVALGIKLP
HTEYEIFKMEQDARGRYLLFNGQRPSDGSSPDRPEKRATSYQMPLVQCASSSPRAEDLAE
DSGSSLYGRAPGRHTWSLLLAALACLVPLLHWNIRR

>Q5XXA6 multipass membrane protein
MRVNEKYSTLPAEDRSVHIINICAIEDIGYLPSEGTLLNSLSVDPDAECKYGLYFRDGRRKV
DYILVYHHKRPSGNRTLVRRVQHSDTPSGARSVKQDHPLPGKGASLDAGSGEPPMDYHE
DDKRFRREEYEGNLLEAGLELERDEDTKIHGVGFVKIHAPWNVLCREAEFLKLKMPTKK
MYHINETRGLLKKINSVLQKITDPIQPKVAEHRPQTMKRLSYPFSREKQHLFDLSDKDSFF
DSKTRSTIVYEILKRTTCTKAKYSMGITSLLANGVYAAAYPLHDGDYNGENVEFNDRKLL
YEEWARYGVFYKYQPIDLVRKYFGEKIGLYFAWLGVYTQMLIPASIVGIIVFLYGCATMDE
NIPSMEMCDQRHNITMCPLCDKTCSYWKMSSACATARASHLFDNPATVFFSVFMALWAA
TFMEHWKRKQMRLNYRWDLTGFEEEEEAVKDHPRAEYEARVLEKSLKKESRNKEKRRHI
PEESTNKWKQRVKTAMAGVKLTDKVKLTWRDRFPAYLTNLVSIIFMIAVTFAIVLGVIIYRI
SMAAALAMNSSPSVRSNIRVTVTATAVIINLVVIILLDEVYGCIARWLTKIEVPKTEKSFEER
LIFKAFLLKFVNSYTPIFYVAFFKGRFVGRPGDYVYIFRSFRMEECAPGGCLMELCIQLSIIM
LGKQLIQNNLFEIGIPKMKKLIRYLKLKQQSPPDHEECVKRKQRYEVDYNLEPFAGLTPEY

MEMIIQFGFVTLFVASFPLAPLFALLNNIIEIRLDAKKFVTELRRPVAVRAKDIGIWYNILRGI
GKLAVIINAFVISFTSDFIPRLVYLYMYSKNGTMHGFVNHTLSSFNVSDFQNGTAPNDPLDL
GYEVQICRYKDYREPPWSENKYDISKDFWAVLAARLAFVIVFQNLVMFMSDFVDWVIPDI
PKDISQQIHKEKVLMVELFMREEQDKQQLLETWMEKERQKDEPPCNHHNTKACPDSLGS
PAPSHAYHGGVL

>Q9BWV1 single-pass type I membrane protein
MLRGTMTAWRGMRPEVTLACLLLATAGCFADLNEVPQVTVQPASTVQKPGGTVILGCVV
EPPRMNVTWRLNGKELNGSDDALGVLITHGTLVITALNNHTVGRYQCVARMPAGAVASV
PATVTLANLQDFKLDVQHVIEVDEGNTAVIACHLPESHPKAQVRYSVKQEWLEASRGNYL
IMPSGNLQIVNASQEDEGMYKCAAYNPVTQEVKTSGSSDRLRVRRSTAEAARIIYPPEAQT
IIVTKGQSLILECVASGIPPPRVTWAKDGSSVTGYNKTRFLLSNLLIDTTSEEDSGTYRCMA
DNGVGQPGAAVILYNVQVFEPPEVTMELSQLVIPWGQSAKLTCEVRGNPPPSVLWLRNAV
PLISSQRLRLSRRALRVLSMGPEDEGVYQCMAENEVGSAHAVVQLRTSRPSITPRLWQDA
ELATGTPPVSPSKLGNPEQMLRGQPALPRPPTSVGPASPQCPGEKGQGAPAEAPIILSSPRTS
KTDSYELVWRPRHEGSGRAPILYYVVKHRKVTNSSDDWTISGIPANQHRLTLTRLDPGSLY
EVEMAAYNCAGEGQTAMVTFRTGRRPKPEIMASKEQQIQRDDPGASPQSSSQPDHGRLSP
PEAPDRPTISTASETSVYVTWIPRGNGGFPIQSFRVEYKKLKKVGDWILATSAIPPSRLSVEI
TGLEKGTSYKFRVRALNMLGESEPSAPSRPYVVSGYSGRVYERPVAGPYITFTDAVNETTI
MLKWMYIPASNNNTPIHGFYIYYRPTDSDNDSDYKKDMVEGDKYWHSISHLQPETSYDI
KMQCFNEGGESEFSNVMICETKARKSSGQPGRLPPPTLAPPQPPLPETIERPVGTGAMVAR
SSDLPYLIVGVVLGSIVLIIVTFIPFCLWRAWSKQKHTTDLGFPRSALPPSCPYTMVPLGGLP
GHQASGQPYLSGISGRACANGIHMNRGCPSAAVGYPGMKPQQHCPGELQQQSDTSSLLR
QTHLGNGYDPQSHQITRGPKSSPDEGSFLYTLPDDSTHQLLQPHHDCCQRQEQPAAVGQS
GVRRAPDSPVLEAVWDPPFHSGPPCCLGLVPVEEVDSPDSCQVSGGDWCPQHPVGAYVG
QEPGMQLSPGPLVRVSFETPPLTI

>P24071 single-pass type I membrane protein
MDPKQTTLLCLVLCLGQRIQAQEGDFPMPFISAKSSPVIPLDGSVKIQCQAIREAYLTQLMII
KNSTYREIGRRLKFWNETDPEFVIDHMDANKAGRYQCQYRIGHYRFRYSDTLELVVTGLY
GKPFLSADRGLVLMPGENISLTCSSAHIPFDRFSLAKEGELSLPQHQSGEHPANFSLGPVDL
NVSGIYRCYGWYNRSPYLWSFPSNALELVVTDSIHQDYTTQNLIRMAVAGLVLVALLAILV
ENWHSHTALNKEASADVAEPSWSQQMCQPGLTFARTPSVCK

>Q9UBM7 multipass membrane protein
MAAKSQPNIPKAKSLDGVTNDRTASQGQWGRAWEVDWFSLASVIFLLLFAPFIVYYFIMA
CDQYSCALTGPVVDIVTGHARLSDIWAKTPPITRKAAQLYTLWVTFQVLLYTSLPDFCHKF
LPGYVGGIQEGAVTPAGVVNKYQINGLQAWLLTHLLWFANAHLLSWFSPTIIFDNWIPLLW
CANILGYAVSTFAMVKGYFFPTSARDCKFTGNFFYNYMMGIEFNPRIGKWFDFKLFFNGR
PGIVAWTLINLSFAAKQRELHSHVTNAMVLVNVLQAIYVIDFFWNETWYLKTIDICHDHF
GWYLGWGDCVWLPYLYTLQGLYLVYHPVQLSTPHAVGVLLLGLVGYYIFRVANHQKDLF
RRTDGRCLIWGRKPKVIECSYTSADGQRHHSKLLVSGFWGVARHFNYVGDLMGSLAYCL
ACGGGHLLPYFYIIYMAILLTHRCLRDEHRCASKYGRDWERYTAAVPYRLLPGIF

>Q86W33 multipass membrane protein
MDTLEEVTWANGSTALPPPLAPNISVPHRCLLLLYEDIGTSRVRYWDLLLLIPNVLFLIFLL
WKLPSARAKIRITSSPIFITFYILVFVVALVGIARAVVSMTVSTSNAATVADKILWEITRFFLL
AIELSVIILGLAFGHLESKSSIKRVLAITTVLSLAYSVTQGTLEILYPDAHLSAEDFNIYGHGG
RQFWLVSSCFFFLVYSLVVILPKTPLKERISLPSRRSFYVYAGILALLNLLQGLGSVLLCFDII
EGLCCVDATTFLYFSFFAPLIYVAFLRGFFGSEPKILFSYKCQVDETEEPDVHLPQPYAVARR
EGLEAAGAAGASAASYSSTQFDSAGGVAYLDDIASMPCHTGSINSTDSERWKAINA

>Q96RN1 multipass membrane protein
MAQLERSAISGFSSKSRRNSFAYDVKREVYNEETFQQEHKRKASSSGNMNINITTFRHHV
QCRCSWHRFLRCVLTIFPFLEWMCMYRLKDWLLGDLLAGISVGLVQVPQGLTLSLLARQ
LIPPLNIAYAAFCSSVIYVIFGSCHQMSIGSFFLVSALLINVLKVSPFNNGQLVMGSFVKNEF
SAPSYLMGYNKSLSVVATTTFLTGIIQLIMGVLGLGFIATYLPESAMSAYLAAVALHIMLSQ
LTFIFGIMISFHAGPISFFYDIINYCVALPKANSTSILVFLTVVVALRINKCIRISFNQYPIEFPM
ELFLIIGFTVIANKISMATETSQTLIDMIPYSFLLPVTPDFSLLPKIILQAFSLSLVSSFLLIFLG
KKIASLHNYSVNSNQDLIAIGLCNVVSSFFRSCVFTGAIARTIIQDKSGGRQQFASLVGAGV
MLLLMVKMGHFFYTLPNAVLAGIILSNVIPYLETISNLPSLWRQDQYDCALWMMTFSSSIF
LGLDIGLIISVVSAFFITTVRSHRAKILLLGQIPNTNIYRSINDYREIITIPGVKIFQCCSSITFV
NVYYLKHKLLKEVDMVKVPLKEEEIFSLFNSSDTNLQGGKICRCFCNCDDLEPLPRILYTE
RFENKLDPEASSINLIHCSHFESMNTSQTASEDQVPYTVSSVSQKNQGQQYEEVEEVWLP
NNSSRNSSPGLPDVAESQGRRSLIPYSDASLLPSVHTIILDFSMVHYVDSRGLVVLRQICNA
FQNANILILIAGCHSSIVRAFERNDFFDAGITKTQLFLSVHDAVLFALSRKVIGSSELSIDESE
TVIRETYSETDKNDNSRYKMSSSFLGSQKNVSPGFIKIQQPVEEESELDLELESEQEAGLGL
DLDLDRELEPEMEPKAETETKTQTEMEPQPETEPEMEPNPKSRPRAHTFPQQRYWPMYHP
SMASTQSQTQTRTWSVERRRHPMDSYSPEGNSNEDV

>Q7L804 peripheral membrane protein
MMLSEQAQKWFPTHVQVTVLQAKDLKPKGKSGTNDTYTIIQLGKEKYSTSVAEKTLEPV
WKEEASFELPGLLIQGSPEKYILFLIVMHRSLVGLDKFLGQVAINLNDIFEDKQRRKTEWF
RLESKQGKRIKNRGEIKVNIQFMRNNMTASMFDLSMKDKTRSPFAKLKDKMKGRKNDG
TFSDTSSAIIPSTHMPDANSEFSSGEIQMKSKPKKPFLLGPQRLSSAHSMSDLSGSHMSSEK
LKAGTIGQTHLLGHQLDSFGTVPESGSLKSPHRRTLSFDTSKMNQPDSIVDEGELCFGRQN
DPFTNVTASLPQKFATLPRKKNPFEESSETWDSSMNLFSKPIEIRKENKREKREKVSLFERV
TGKKDSRRSDKLNNGGSDSPCDLKSPNAFSENRQDYFDYESTNPFTAKFRASNIMPSSSFH
MSPTSNEDLRKIPDSNPFDATAGYRSLTYEEVLQELVKHKELLRRKDTHIRELEDYIDNLLV
RVMEETPSILRVPYEPSRKAGKFSNS

>Q99536 peripheral membrane protein
MSDEREVAEAATGEDASSPPPKTEAASDPQHPAASEGAAAAAASPPLLRCLVLTGFGGYD
KVKLQSRPAAPPAPGPGQLTLRLRACGLNFADLMARQGLYDRLPPLPVTPGMEGAGVVIA
VGEGVSDRKAGDRVMVLNRSGMWQEEVTVPSVQTFLIPEAMTFEEAAALLVNYITAYMV
LFDFGNLQPGHSVLVHMAAGGVGMAAVQLCRTVENVTVFGTASASKHEALKENGVTHPI
DYHTTDYVDEIKKISPKGVDIVMDPLGGSDTAKGYNLLKPMGKVVTYGMANLLTGPKRN
LMALARTWWNQFSVTALQLLQANRAVCGFHLGYLDGEVELVSGVVARLLALYNQGHIKP

HIDSVWPFEKVADAMKQMQEKKNVGKVLLVPGPEKEN

>P07949 single-pass type I membrane protein
MAKATSGAAGLRLLLLLLLPLLGKVALGLYFSRDAYWEKLYVDQAAGTPLLYVHALRDA
PEEVPSFRLGQHLYGTYRTRLHENNWICIQEDTGLLYLNRSLDHSSWEKLSVRNRGFPLLT
VYLKVFLSPTSLREGECQWPGCARVYFSFFNTSFPACSSLKPRELCFPETRPSFRIRENRPPG
TFHQFRLLPVQFLCPNISVAYRLLEGEGLPFRCAPDSLEVSTRWALDREQREKYELVAVCTV
HAGAREEVVMVPFPVTVYDEDDSAPTFPAGVDTASAVVEFKRKEDTVVATLRVFDADVV
PASGELVRRYTSTLLPGDTWAQQTFRVEHWPNETSVQANGSFVRATVHDYRLVLNRNLSI
SENRTMQLAVLVNDSDFQGPGAGVLLLHFNVSVLPVSLHLPSTYSLSVSRRARRFAQIGKV
CVENCQAFSGINVQYKLHSSGANCSTLGVVTSAEDTSGILFVNDTKALRRPKCAELHYM
VVATDQQTSRQAQAQLLVTVEGSYVAEEAGCPLSCAVSKRRLECEECGGLGSPTGRCEWR
QGDGKGITRNFSTCSPSTKTCPDGHCDVVETQDINICPQDCLRGSIVGGHEPGEPRGIKAG
YGTCNCFPEEEKCFCEPEDIQDPLCDELCRTVIAAAVLFSFIVSVLLSAFCIHCYHKFAHKPP
ISSAEMTFRRPAQAFPVSYSSSGARRPSLDSMENQVSVDAFKILEDPKWEFPRKNLVLGKT
LGEGEFGKVVKATAFHLKGRAGYTTVAVKMLKENASPSELRDLLSEFNVLKQVNHPHVIK
LYGACSQDGPLLLIVEYAKYGSLRGFLRESRKVGPGYLGSGGSRNSSSLDHPDERALTMG
DLISFAWQISQGMQYLAEMKLVHRDLAARNILVAEGRKMKISDFGLSRDVYEEDSYVKRS
QGRIPVKWMAIESLFDHIYTTQSDVWSFGVLLWEIVTLGGNPYPGIPPERLFNLLKTGHRM
ERPDNCSEEMYRLMLQCWKQEPDKRPVFADISKDLEKMMVKRRDYLDLAASTPSDSLIY
DDGLSEEETPLVDCNNAPLPRALPSTWIENKLYGMSDPNWPGESPVPLTRADGTNTGFPR
YPNDSVYANWMLSPSAAKLMDTFDS

>P25445 single-pass type I membrane protein
MLGIWTLLPLVLTSVARLSSKSVNAQVTDINSKGLELRKTVTTVETQNLEGLHHDGQFCH
KPCPPGERKARDCTVNGDEPDCVPCQEGKEYTDKAHFSSKCRRCRLCDEGHGLEVEINC
TRTQNTKCRCKPNFFCNSTVCEHCDPCTKCEHGIIKECTLTSNTKCKEEGSRSNLGWLCLL
LLPIPLIVWVKRKEVQKTCRKHRKENQGSHESPTLNPETVAINLSDVDLSKYITTIAGVMT
LSQVKGFVRKNGVNEAKIDEIKNDNVQDTAEQKVQLLRNWHQLHGKKEAYDTLIKDLK
KANLCTLAEKIQTIILKDITSDSENSNFRNEIQSLV

>Q16718 peripheral membrane protein
MAGVLKKTTGLVGLAVCNTPHERLRILYTKILDVLEEIPKNAAYRKYTEQITNEKLAMVK
AEPDVKKLEDQLQGGQLEEVILQAEHELNLARKMREWKLWEPLVEEPPADQWKWPI

>Q9NRA2 multipass membrane protein
MRSPVRDLARNDGEESTDRTPLLPGAPRAEAAPVCCSARYNLAILAFFGFFIVYALRVNLS
VALVDMVDSNTTLEDNRTSKACPEHSAPIKVHHNQTGKKYQWDAETQGWILGSFFYGYII
TQIPGGYVASKIGGKMLLGFGILGTAVLTLFTPIAADLGVGPLIVLRALEGLGEGVTFPAMH
AMWSSWAPPLERSKLLSISYAGAQLGTVISLPLSGIICYYMNWTYVFYFFGTIGIFWFLLWI
WLVSDTPQKHKRISHYEKEYILSSLRNQLSSQKSVPWVPILKSLPLWAIVVAHFSYNWTFY
TLLTLLPTYMKEILRFNVQENGFLSSLPYLGSWLCMILSGQAADNLRAKWNFSTLCVRRIF
SLIGMIGPAVFLVAAGFIGCDYSLAVAFLTISTTLGGFCSSGFSINHLDIAPSYAGILLGITNTF
ATIPGMVGPVIAKSLTPDNTVGEWQTVFYIAAAINVFGAIFFTLFAKGEVQNWALNDHHG

HRH

>Q8NGX0 multipass membrane protein
MEPQNTSTVTNFQLLGFQNLLEWQALLFVIFLLIYCLTIIGNVVIITVVSQGLRLHSPMYMF
LQHLSFLEVWYTSTTVPLLLANLLSWGQAISFSACMAQLYFFVFLGATECFLLAFMAYDR
YLAICSPLRYPFLMHRGLCARLVVVSWCTGVSTGFLPSLMISRLDFCGRNQINHFFCDLPP
LMQLSCSRVYITEVTIFILSIAVLCICFFLTLGPYVFIVSSILRIPSTSGRRKTFSTCGSHLAVVT
LYYGTMISMYVCPSPHLLPEINKIISVFYTVVTPLLNPVIYSLRNKDFKEAVRKVMRRKCGI
LWSTSKRKFLY

>Q13508 GPI-anchor, lipid-anchor
MKTGHFEIVTMLLATMILVDIFQVKAEVLDMADNAFDDEYLKCTDRMEIKYVPQLLKEE
KASHQQLDTVWENAKAKWAARKTQIFLPMNFKDNHGIALMAYISEAQEQTPFYHLFSEA
VKMAGQSREDYIYGFQFKAFHFYLTRALQLLRKPCEASSKTVVYRTSQGTSFTFGGLNQA
RFGHFTLAYSAKPQAANDQLTVLSIYTCLGVDIENFLDKESERITLIPLNEVFQVSQEGAGN
NLILQSINKTCSHYECAFLGGLKTENCIENLEYFQPIYVYNPGEKNQKLEDHSEKNWKLED
HGEKNQKLEDHGVKILEPTQIPGMKIPEPFPLPEDKSQGNINNPTPGPVPVPGPKSHPSASS
GKLLLPQFGMVIILISVSAINLFVAL

>P36956 multipass membrane protein
MDEPPFSEAALEQALGEPCDLDAALLTDIEDMLQLINNQDSDFPGLFDPPYAGSGAGGTDP
ASPDTSSPGSLSPPPATLSSSLEAFLSGPQAAPSPLSPPQPAPTPLKMYPSMPAFSPGPGIKEE
SVPLSILQTPTPQPLPGALLPQSFPAPAPPQFSSTPVLGYPSPPGGFSTGSPPGNTQQPLPGLP
LASPPGVPPVSLHTQVQSVVPQQLLTVTAAPTAAPVTTTVTSQIQQVPVLLQPHFIKADSLL
LTAMKTDGATVKAAGLSPLVSGTTVQTGPLPTLVSGGTILATVPLVVDAEKLPINRLAAGS
KAPASAQSRGEKRTAHNAIEKRYRSSINDKIIELKDLVVGTEAKLNKSAVLRKAIDYIRFLQ
HSNQKLKQENLSLRTAVHKSKSLKDLVSACGSGGNTDVLMEGVKTEVEDTLTPPPSDAGS
PFQSSPLSLGSRGSGSGGSGSDSEPDSPVFEDSKAKPEQRPSLHSRGMLDRSRLALCTLVFL
CLSCNPLASLLGARGLPSPSDTTSVYHSPGRNVLGTESRDGPGWAQWLLPPVVWLLNGLL
VLVSLVLLFVYGEPVTRPHSGPAVYFWRHRKQADLDLARGDFAQAAQQLWLALRALGRP
LPTSHLDLACSLLWNLIRHLLQRLWVGRWLAGRAGGLQQDCALRVDASASARDAALVY
HKLHQLHTMGKHTGGHLTATNLALSALNLAECAGDAVSVATLAEIYVAAALRVKTSLPRA
LHFLTRFFLSSARQACLAQSGSVPPAMQWLCHPVGHRFFVDGDWSVLSTPWESLYSLAG
NPVDPLAQVTQLFREHLLERALNCVTQPNPSPGSADGDKEFSDALGYLQLLNSCSDAAG
APAYSFSISSSMATTTGVDPVAKWWASLTAVVIHWLRRDEEAAERLCPLVEHLPRVLQESE
RPLPRAALHSFKAARALLGCAKAESGPASLTICEKASGYLQDSLATTPASSSIDKAVQLFLC
DLLLVVRTSLWRQQQPPAPAPAAQGTSSRPQASALELRGFQRDLSSLRRLAQSFRPAMRRV
FLHEATARLMAGASPTRTHQLLDRSLRRRAGPGGKGGAVAELEPRPTRREHAEALLLASC
YLPPGFLSAPGQRVGMLAEAARTLEKLGDRRLLHDCQQMLMRLGGGTTVTSS

>Q6UXN8 single-pass type II membrane protein
MHEEEIYTSLQWDSPAPDTYQKCLSSNKCSGACCLVMVISCVFCMGLLTASIFLGVKLLQV
STIAMQQQEKLIQQERALLNFTEWKRSCALQMKYCQAFMQNSLSSAHNSSPCPNNWIQN
RESCYYVSEIWSIWHTSQENCLKEGSTLLQIESKEEMDFITGSLRKIKGSYDYWVGLSQDG

HSGRWLWQDGSSPSPGLLPAERSQSANQVCGYVKSNSLLSSNCSTWKYFICEKYALRSSV

>O95425 peripheral membrane protein
MKRKERIARRLEGIENDTQPILLQSCTGLVTHRLLEEDTPRYMRASDPASPHIGRSNEEEET
SDSSLEKQTRSKYCTETSGVHGDSPYGSGTMDTHSLESKAERIARYKAERRRQLAEKYGL
TLDPEADSEYLSRYTKSRKEPDAVEKRGGKSDKQEESSRDASSLYPGTETMGLRTCAGES
KDYALHVGDGSSDPEVLLNIENQRRGQELSATRQAHDLSPAAESSSTFSFSGRDSSFTEVPR
SPKHAHSSSLQQAASRSPSFGDPQLSPEARPSTGKPKHEWFLQKDSEGDTPSLINWPSRVK
VREKLVKEESARNSPELASESVTQRRHQPAPVHYVSFQSEHSAFDRVPSKAAGSTRQPIRG
YVQPADTGHTAKLVTPETPENASECSWVASATQNVPKPPSLTVLEGDGRDSPVLHVCESK
AEEEEGEGEGEEKEEDVCFTEALEQSKKTLLALEGDGLVRSPEDPSRNEDFGKPAVSTVTL
EHQKELENVAQPPQAPHQPTERTGRSEMVLYIQSEPVSQDAKPTGHNREASKKRKVRTRS
LSDFTGPPQLQALKYKDPASRRELELPSSKTEGPYGEISMLDTKVSVAQLRSAFLASANAC
RRPELKSRVERSAEGPGLPTGVERERGSRKPRRYFSPGESRKTSERFRTQPITSAERKESDR
CTSHSETPTVDDEEKVDERAKLSVAAKRLLFREMEKSFDEQNVPKRRSRNTAVEQRLRRL
QDRSLTQPITTEEVVIAATEPIPASCSGGTHPVMARLPSPTVARSAVQPARLQASAHQKALA
KDQTNEGKELAEQGEPDSSTLSLAEKLALFNKLSQPVSKAISTRNRIDTRQRRMNARYQT
QPVTLGEVEQVQSGKLIPFSPAVNTSVSTVASTVAPMYAGDLRTKPPLDHNASATDYKFSS
SIENSDSPVRSILKSQAWQPLVEGSENKGMLREYGETESKRALTGRDSGMEKYGSFEEAE
ASYPILNRAREGDSHKESKYAVPRRGSLERANPPITHLGDEPKEFSMAKMNAQGNLDLRD
RLPFEEKVEVENVMKRKFSLRAAEFGEPTSEQTGTAAGKTIAQTTAPVSWKPQDSSEQPQ
EKLCKNPCAMFAAGEIKTPTGEGLLDSPSKTMSIKERLALLKKSGEEDWRNRLSRRQEGG
KAPASSLHTQEAGRSLIKKRVTESRESQMTIEERKQLITVREEAWKTRGRGAANDSTQFTV
AGRMVKKGLASPTAITPVASPICGKTRGTTPVSKPLEDIEARPDMQLESDLKLDRLETFLR
RLNNKVGGMHETVLTVTGKSVKEVMKPDDDETFAKFYRSVDYNMPRSPVEMDEDFDVI
FDPYAPKLTSSVAEHKRAVRPKRRVQASKNPLKMLAAREDLLQEYTEQRLNVAFMESKR
MKVEKMSSNSNFSEVTLAGLASKENFSNVSLRSVNLTEQNSNNSAVPYKRLMLLQIKGRR
HVQTRLVEPRASALNSGDCFLLLSPHCCFLWVGEFANVIEKAKASELATLIQTKRELGCRA
TYIQTIEEGINTHTHAAKDFWKLLGGQTSYQSAGDPKEDELYEAAIIETNCIYRLMDDKLV
PDDDYWGKIPKCSLLQPKEVLVFDFGSEVYVWHGKEVTLAQRKIAFQLAKHLWNGTFDY
ENCDINPLDPGECNPLIPRKGQGRPDWAIFGRLTEHNETILFKEKFLDWTELKRSNEKNPG
ELAQHKEDPRTDVKAYDVTRMVSMPQTTAGTILDGVNVGRGYGLVEGHDRRQFEITSVS
VDVWHILEFDYSRLPKQSIGQFHEGDAYVVKWKFMVSTAVGSRQKGEHSVRAAGKEKC
VYFFWQGRHSTVSEKGTSALMTVELDEERGAQVQVLQGKEPPCFLQCFQGGMVVHSGR
REEEEENVQSEWRLYCVRGEVPVEGNLLEVACHCSSLRSRTSMVVLNVNKALIYLWHGC
KAQAHTKEVGRTAANKIKEQCPLEAGLHSSSKVTIHECDEGSEPLGFWDALGRRDRKAY
DCMLQDPGSFNFAPRLFILSSSSGDFAATEFVYPARAPSVVSSMPFLQEDLYSAPQPALFLV
DNHHEVYLWQGWWPIENKITGSARIRWASDRKSAMETVLQYCKGKNLKKPAPKSYLIHA
GLEPLTFTNMFPSWEHREDIAEITEMDTEVSNQITLVEDVLAKLCKTIYPLADLLARPLPEG
VDPLKLEIYLTDEDFEFALDMTRDEYNALPAWKQVNLKKAKGLF

>Q9UJ42 multipass membrane protein
MTALSSENCSFQYQLRQTNQPLDVNYLLFLIILGKILLNILTLGMRRKNTCQNFMEYFCISL
AFVDLLLLVNISIILYFRDFVLLSIRFTKYHICLFTQIISFTYGFLHYPVFLTACIDYCLNFSKT

TKLSFKCQKLFYFFTVILIWISVLAYVLGDPAIYQSLKAQNAYSRHCPFYVSIQSYWLSFFM
VMILFVAFITCWEEVTTLVQAIRITSYMNETILYFPFSSHSSYTVRSKKIFLSKLIVCFLSTWL
PFVLLQVIIVLLKVQIPAYIEMNIPWLYFVNSFLIATVYWFNCHKLNLKDIGLPLDPFVNWK
CCFIPLTIPNLEQIEKPISIMIC

>P02686 peripheral membrane protein
MGNHAGKRELNAEKASTNSETNRGESEKKRNLGELSRTTSEDNEVFGEADANQNNGTSS
QDTAVTDSKRTADPKNAWQDAHPADPGSRPHLIRLFSRDAPGREDNTFKDRPSESDELQTI
QEDSAATSESLDVMASQKRPSQRHGSKYLATASTMDHARHGFLPRHRDTGILDSIGRFFG
GDRGAPKRGSGKDSHHPARTAHYGSLPQKSHGRTQDENPVVHFFKNIVTPRTPPPSQGKG
RGLSLSRFSWGAEGQRPGFGYGGRASDYKSAHKGFKGVDAQGTLSKIFKLGGRDSRSGS
PMARR

>P78324 single-pass type I membrane protein
MEPAGPAPGRLGPLLCLLLAASCAWSGVAGEEELQVIQPDKSVLVAAGETATLRCTATSLIP
VGPIQWFRGAGPGRELIYNQKEGHFPRVTTVSDLTKRNNMDFSIRIGNITPADAGTYYCVK
FRKGSPDDVEFKSGAGTELSVRAKPSAPVVSGPAARATPQHTVSFTCESHGFSPRDITLKW
FKNGNELSDFQTNVDPVGESVSYSIHSTAKVVLTREDVHSQVICEVAHVTLQGDPLRGTA
NLSETIRVPPTLEVTQQPVRAENQVNVTCQVRKFYPQRLQLTWLENGNVSRTETASTVTE
NKDGTYNWMSWLLVNVSAHRDDVKLTCQVEHDGQPAVSKSHDLKVSAHPKEQGSNTA
AENTGSNERNIYIVVGVVCTLLVALLMAALYLVRIRQKKAQGSTSSTRLHEPEKNAREITQ
DTNDITYADLNLPKGKKPAPQAAEPNNHTEYASIQTSPQPASEDTLTYADLDMVHLNRTPK
QPAPKPEPSFSEYASVQVPRK

>O75900 single-pass type II membrane protein
MGRGARVPSEAPGAGVERRWLGAALVALCLLPALVLLARLGAPAVPAWSAAQGDVAALG
LSAVPPTRVPGPLAPRRRRYTLTPARLRWDHFNLTYRILSFPRNLLSPRETRRALAAAFRM
WSDVSPFSFREVAPEQPSDLRIGFYPINHTDCLVSALHHCFDGPTGELAHAFFPPHGGIHFD
DSEYWVLGPTRYSWKKGVWLTDLVHVAAHEIGHALGLMHSQHGRALMHLNATLRGWK
ALSQDELWGLHRLYGCLDRLFVCASWARRGFCDARRRLMKRLCPSSCDFCYEFPFPTVAT
TPPPPRTKTRLVPEGRNVTFRCGQKILHKKGKVYWYKDQEPLEFSYPGYLALGEAHLSIIA
NAVNEGTYTCVVRRQQRVLTTYSWRVRVRG

>Q9UEY8 peripheral membrane protein
MSSDASQGVITTPPPPSMPHKERYFDRINENDPEYIRERNMSPDLRQDFNMMEQRKRVTQI
LQSPAFREDLECLIQEQMKKGHNPTGLLALQQIADYIMANSFSGFSSPPLSLGMVTPINDLP
GADTSSYVKGEKLTRCKLASLYRLVDLFGWAHLANTYISVRISKEQDHIIIIPRGLSFSEATA
SNLVKVNIIGEVVDQGSTNLKIDHTGFSPHAAIYSTRPDVKCVIHIHTLATAAVSSMKCGIL
PISQESLLLGDVAYYDYQGSLEEQEERIQLQKVLGPSCKVLVLRNHGVVALGETLEEAFHY
IFNVQLACEIQVQALAGAGGVDNLHVLDFQKYKAFTYTVAASGGGGVNMGSHQKWKV
GEIEFEGLMRTLDNLGYRTGYAYRHPLIREKPRHKSDVEIPATVTAFSFEDDTVPLSPLKYM
AQRQQREKTRWLNSPNTYMKVNVPEESRNGETSPRTKITWMKAEDSSKVSGGTPIKIEDP
NQFVPLNTNPNEVLEKRNKIREQNRYDLKTAGPQSQLLAGIVVDKPPSTMQFEDDDHGPP
APPNPFSHLTEGELEEYKRTIERKQQGLEDAEQELLSDDASSVSQIQSQTQSPQNVPEKLEE

NHELFSKSFISMEVPVMVVNGKDDMHDVEDELAKRVSRLSTSTTIENIEITIKSPEKIEEVL
SPEGSPSKSPSKKKKKFRTPSFLKKNKKKEKVEA

>Q96RC9 multipass membrane protein
MTLRNSSSVTEFILVGLSEQPELQLPLFLLFLGIYVFTVVGNLGLITLIGINPSLHTPMYFFLF
NLSFIDLCYSCVFTPKMLNDFVSESIISYVGCMTQLFFFCFFVNSECYVLVSMAYDRYVAIC
NPLLYMVTMSPRVCFLLMFGSYVVGFAGAMAHTGSMLRLTFCDSNVIDHYLCDVLPLLQ
LSCTSTHVSELVFFIVVGVITMLSSISIVISYALILSNILCIPSAEGRSKAFSTWGSHIIAVALFF
GSGTFTYLTTSFPGSMNHGRFASVFYTNVVPMLNPSIYSLRNKDDKLALGKTLKRVLF

>P63000 lipid-anchor
MQAIKCVVVGDGAVGKTCLLISYTTNAFPGEYIPTVFDNYSANVMVDGKPVNLGLWDTA
GQEDYDRLRPLSYPQTDVFLICFSLVSPASFENVRAKWYPEVRHHCPNTPIILVGTKLDLR
DDKDTIEKLKEKKLTPITYPQGLAMAKEIGAVKYLECSALTQRGLKTVFDEAIRAVLCPPP
VKKRKRKCLLL

>Q7Z7A4 peripheral membrane protein
MAFMEKPPAGKVLLDDTVPLTAAIEASQSLQSHTEYIIRVQRGISVENSWQIVRRYSDFDLL
NNSLQIAGLSLPLPPKKLIGNMDREFIAERQKGLQNYLNVITTNHILSNCELVKKFLDPNN
YSANYTEIALQQVSMFFRSEPKWEVVEPLKDIGWRIRKKYFLMKIKNQPKERLVLSWADL
GPDKYLSDKDFQCLIKLLPSCLHPYIYRVTFATANESSALLIRMFNEKGTLKDLIYKAKPKD
PFLKKYCNPKKIQGLELQQIKTYGRQILEVLKFLHDKGFPYGHLHASNVMLDGDTCRLLD
LENSLLGLPSFYRSYFSQFRKINTLESVDVHCFGHLLYEMTYGRPPDSVPVDSFPPAPSMAV
VAVLESTLSCEACKNGMPTISRLLQMPLFSDVLLTTSEKPQFKIPTKLKEALRIAKECIEKRL
IEEQKQIHQHRRLTRAQSHHGSEEERKKRKILARKKSKRSALENSEEHSAKYSNSNNSAGS
GASSPLTSPSSPTPPSTSGISALPPPPPPPPPPAAPLPPASTEAPAQLSSQAVNGMSRGALLSSI
QNFQKGTLRKAKTCDHSAPKIG

>Q96NE9 peripheral membrane protein
MNKLNFHNNRVMQDRRSVCIFLPNDESLNIIINVKILCHQLLVQVCDLLRLKDCHLFGLSV
IQNNEHVYMELSQKLYKYCPKEWKKEASKVRQYEVTWGIDQFGPPMIIHFRVQYYVENG
RLISDRAARYYYYWHLRKQVLHSQCVLREEAYFLLAAFALQADLGNFKRNKHYGKYFEP
EAYFPSWVVSKRGKDYILKHIPNMHKDQFALTASEAHLKYIKEAVRLDDVAVHYYRLYKD
KREIEASLTLGLTMRGIQIFQNLDEEKQLLYDFPWTNVGKLVFVGKKFEILPDGLPSARKLI
YYTGCPMRSRHLLQLLSNSHRLYMNLQPVLRHIRKLEENEEKKQYRESYISDNLDLDMD
QLEKRSRASGSSAGSMKHKRLSRHSTASHSSSHTSGIEADTKPRDTGPEDSYSSSAIHRKL
KTCSSMTSHGSSHTSGVESGGKDRLEEDLQDDEIEMLVDDPRDLEQMNEESLEVSPDMCI
YITEDMLMSRKLNGHSGLIVKEIGSSTSSSSETVVKLRGQSTDSLPQTICRKPKTSTDRHSL
SLDDIRLYQKDFLRIAGLCQDTAQSYTFGCGHELDEEGLYCNSCLAQQCINIQDAFPVKRT
SKYFSLDLTHDEVPEFVV

>P46060 peripheral membrane protein
MASEDIAKLAETLAKTQVAGGQLSFKGKSLKLNTAEDAKDVIKEIEDFDSLEALRLEGNT
VGVEAARVIAKALEKKSELKRCHWSDMFTGRLRTEIPPALISLGEGLITAGAQLVELDLSD

NAFGPDGVQGFEALLKSSACFTLQELKLNNCGMGIGGGKILAAALTECHRKSSAQGKPLA
LKVFVAGRNRLENDGATALAEAFRVIGTLEEVHMPQNGINHPGITALAQAFAVNPLLRVIN
LNDNTFTEKGAVAMAETLKTLRQVEVINFGDCLVRSKGAVAIADAIRGGLPKLKELNLSFC
EIKRDAALAVAEAMADKAELEKLDLNGNTLGEEGCEQLQEVLEGFNMAKVLASLSDDED
EEEEEEGEEEEEEAEEEEEEDEEEEEEEEEEEEEEPQQRGQGEKSATPSRKILDPNTGEPAPV
LSSPPPADVSTFLAFPSPEKLLRLGPKSSVLIAQQTDTSDPEKVVSAFLKVSSVFKDEATVR
MAVQDAVDALMQKAFNSSSFNSNTFLTRLLVHMGLLKSEDKVKAIANLYGPLMALNHM
VQQDYFPKALAPLLLAFVTKPNSALESCSFARHSLLQTLYKV

>Q5T4F4 multipass membrane protein
MQTSEREGSGPELSPSVMPEAPLESPPFPTKSPAFDLFNLVLSYKRLEIYLEPLKDAGDGVR
YLLRWQMPLCSLLTCLGLNVLFLTLNEGAWYSVGALMISVPALLGYLQEVCRARLPDSEL
MRRKYHSVRQEDLQRGRLSRPEAVAEVKSFLIQLEAFLSRLCCTCEAAYRVLHWENPVVS
SQFYGALLGTVCMLYLLPLCWVLTLLNSTLFLGNVEFFRVVSEYRASLQQRMNPKQEEH
AFESPPPPDVGGKDGLMDSTPALTPTEDLTPGSVEEAEEAEPDEEFKDAIEETHLVVLEDDE
GAPCPAEDELALQDNGFLSKNEVLRSKVSRLTERLRKRYPTNNFGNCTGCSATFSVLKKR
RSCSNCGNSFCSRCCSFKVPKSSMGATAPEAQRETVFVCASCNQTLSK

>P49418 peripheral membrane protein
MADIKTGIFAKNVQKRLNRAQEKVLQKLGKADETKDEQFEEYVQNFKRQEAEGTRLQRE
LRGYLAAIKGMQEASMKLTESLHEVYEPDWYGREDVKMVGEKCDVLWEDFHQKLVDG
SLLTLDTYLGQFPDIKNRIAKRSRKLVDYDSARHHLEALQSSKRKDESRISKAEEEFQKAQ
KVFEEFNVDLQEELPSLWSRRVGFYVNTFKNVSSLEAKFHKEIAVLCHKLYEVMTKLGDQ
HADKAFTIQGAPSDSGPLRIAKTPSPPEEPSPLPSPTASPNHTLAPASPAPARPRSPSQTRKGP
PVPPLPKVTPTKELQQENIISFFEDNFVPEISVTTPSQNEVPEVKKEETLLDLDFDPFKPEVT
PAGSAGVTHSPMSQTLPWDLWTTSTDLVQPASGGSFNGFTQPQDTSLFTMQTDQSMICNL
AESEQAPPTEPKAEEPLAAVTPAVGLDLGMDTRAEEPVEEAVIIPGADADAAVGTLVSAAE
GAPGEEAEAEKATVPAGEGVSLEEAKIGTETTEGAESAQPEAEELEATVPQEKVIPSVVIEP
ASNHEEEGENEITIGAEPKETTEDAAPPGPTSETPELATEQKPIQDPQPTPSAPAMGAADQL
ASAREASQELPPGFLYKVETLHDFEAANSDELTLQRGDVVLVVPSDSEADQDAGWLVGV
KESDWLQYRDLATYKGLFPENFTRRLD

>Q6ZVD8 peripheral membrane protein
MKRNGSRNCLNRRSRFGSRERDWLREDVKRGCVYLYGADTTTATTTTTTSSSSSSSSSSD
LHLVLCTVETPASEICAGEGRESLYLQLHGDLVRRLEPTERPLQIVYDYLSRLGFDDPVRIQ
EEATNPDLGCMIRFYGEKPCHMDRLDRILLSGIYNVRKGKTQLHKWAERLVVLCGTCLIV
SSVKDCQTGKMHILPLVGGKIEEVKRRQYSLAFSSAGAQAQTYHVSFETLAEYQRWQRQ
ASKVVSQRISTVDLSCYSLEEVPEHLFYSQDITYLNLRHNFMQLERPGGLDTLYKFSQLKG
LNLSHNKLGLFPILLCEISTLTELNLSCNGFHDLPSQIGNLLNLQTLCLDGNFLTTLPEELGN
LQQLSSLGISFNNFSQIPEVYEKLTMLDRVVMAGNCLEVLNLGVLNRMNHIKHVDLRMN
HLKTMVIENLEGNKHITHVDLRDNRLTDLDLSSLCSLEQLHCGRNQLRELTLSGFSLRTLY
ASSNRLTAVNVYPVPSLLTFLDLSRNLLECVPDWACEAKKIEVLDVSYNLLTEVPVRILSSL
SLRKLMLGHNHVQNLPTLVEHIPLEVLDLQHNALTRLPDTLFSKALNLRYLNASANSLESL
PSACTGEESLSMLQLLYLTNNLLTDQCIPVLVGHLHLRILHLANNQLQTFPASKLNKLEQLE

ELNLSGNKLKTIPTTIANCKRLHTLVAHSNNISIFPEILQLPQIQFVDLSCNDLTEILIPEALPA
TLQDLDLTGNTNLVLEHKTLDIFSHITTLKIDQKPLPTTDSTVTSTFWSHGLAEMAGQRNK
LCVSALAMDSFAEGVGAVYGMFDGDRNEELPRLLQCTMADVLLEEVQQSTNDTVFMAN
TFLVSHRKLGMAGQKLGSSALLCYIRPDTADPASSFSLTVANVGTCQAVLCRGGKPVPLSK
VFSLEQDPEEAQRVKDQKAIITEDNKVNGVTCCTRMLGCTYLYPWILPKPHISSTPLTIQDE
LLILGNKALWEHLSYTEAVNAVRHVQDPLAAAKKLCTLAQSYGCQDNVGAMVVYLNIG
EEGCTCEMNGLTLPGPVGFASTTTIKDAPKPATPSSSSGIASEFSSEMSTSEVSSEVGSTASD
EHNAGGLDTALLPRPERRCSLHPTPTSGLFQRQPSSATFSSNQSDNGLDSDDDQPVEGVIT
NGSKVEVEVDIHCCRGRDLENSPPLIESSPTLCSEEHARGSCFGIRRQNSVNSGMLLPMSK
DRMELQKSPSTSCLYGKKLSNGSIVPLEDSLNLIEVATEVPKRKTGYFAAPTQMEPEDQFV
VPHDLEEEVKEQMKQHQDSRLEPEPHEEDRTEPPEEFDTAL

>Q86YT9 single-pass type I membrane protein
MFCPLKLILLPVLLDYSLGLNDLNVSPPELTVHVGDSALMGCVFQSTEDKCIFKIDWTLSP
GEHAKDEYVLYYYSNLSVPIGRFQNRVHLMGDILCNDGSLLLQDVQEADQGTYICEIRLK
GESQVFKKAVVLHVLPEEPKELMVHVGGLIQMGCVFQSTEVKHVTKVEWIFSGRRAKEEI
VFRYYHKLRMSVEYSQSWGHFQNRVNLVGDIFRNDGSIMLQGVRESDGGNYTCSIHLGN
LVFKKTIVLHVSPEEPRTLVTPAALRPLVLGGNQLVIIVGIVCATILLLPVLILIVKKTCGNKS
SVNSTVLVKNTKKTNPEIKEKPCHFERCEGEKHIYSPIIVREVIEEEEPSEKSEATYMTMHP
VWPSLRSDRNNSLEKKSGGGMPKTQQAF

>Q7Z698 peripheral membrane protein
MTEETHPDDDSYIVRVKAVVMTRDDSSGGWFPQEGGGISRVGVCKVMHPEGNGRSGFLI
HGERQKDKLVVLECYVRKDLVYTKANPTFHHWKVDNRKFGLTFQSPADARAFDRGVRK
AIEDLIEGSTTSSSTIHNEAELGDDDVFTTATDSSSNSSQKREQPTRTISSPTSCEHRRIYTLG
HLHDSYPTDHYHLDQPMPRPYRQVSFPDDDEEIVRINPREKIWMTGYEDYRHAPVRGKY
PDPSEDADSSYVRFAKGEVPKHDYNYPYVDSSDFGLGEDPKGRGGSVIKTQPSRGKSRRR
KEDGERSRCVYCRDMFNHEENRRGHCQDAPDSVRTCIRRVSCMWCADSMLYHCMSDPE
GDYTDPCSCDTSDEKFCLRWMALIALSFLAPCMCCYLPLRACYHCGVMCRCCGGKHKA
AA

>P48449 peripheral membrane protein
MTEGTCLRRRGGPYKTEPATDLGRWRLNCERGRQTWTYLQDERAGREQTGLEAYALGL
DTKNYFKDLPKAHTAFEGALNGMTFYVGLQAEDGHWTGDYGGPLFLLPGLLITCHVARI
PLPAGYREEIVRYLRSVQLPDGGWGLHIEDKSTVFGTALNYVSLRILGVGPDDPDLVRARN
ILHKKGGAVAIPSWGKFWLAVLNVYSWEGLNTLFPEMWLFPDWAPAHPSTLWCHCRQVY
LPMSYCYAVRLSAAEDPLVQSLRQELYVEDFASIDWLAQRNNVAPDELYTPHSWLLRVVY
ALLNLYEHHHSAHLRQRAVQKLYEHIVADDRFTKSISIGPISKTINMLVRWYVDGPASTAFQ
EHVSRIPDYLWMGLDGMKMQGTNGSQIWDTAFAIQALLEAGGHHRPEFSSCLQKAHEFL
RLSQVPDNPPDYQKYYRQMRKGGFSFSTLDCGWIVSDCTAEALKAVLLLQEKCPHVTEHI
PRERLCDAVAVLLNMRNPDGGFATYETKRGGHLLELLNPSEVFGDIMIDYTYVECTSAVM
QALKYFHKRFPEHRAAEIRETLTQGLEFCRRQQRADGSWEGSWGVCFTYGTWFGLEAFA
CMGQTYRDGTACAEVSRACDFLLSRQMADGGWGEDFESCEERRYLQSAQSQIHNTCWA
MMGLMAVRHPDIEAQERGVRCLLEKQLPNGDWPQENIAGVFNKSCAISYTSYRNIFPIWA

LGRFSQLYPERALAGHP

>O00562	peripheral membrane protein
MLIKEYHILLPMSLDEYQVAQLYMIQKKSREESSGEGSGVEILANRPYTDGPGGSGQYTH
KVYHVGSHIPGWFRALLPKAALQVEEESWNAYPYTRTRYTCPFVEKFSIEIETYYLPDGGQ
QPNVFNLSGAERRQRILDTIDIVRDAVAPGEYKAEEDPRLYHSVKTGRGPLSDDWARTAA
QTGPLMCAYKLCKVEFRYWGMQAKIEQFIHDVGLRRVMLRAHRQAWCWQDEWTELSM
ADIRALEEETARMLAQRMAKCNTGSEGSEAQPPGKPSTEARSAASNTGTPDGPEAPPGPD
ASPDASFGKQWSSSSRSSYSSQHGGAVSPQSLSEWRMQNIARDSENSSEEEFFDAHEGFSD
SEEVFPKEMTKWNSNDFIDAFASPVEAEGTPEPGAEAAKGIEDGAQAPRDSEGLDGAGEL
GAEACAVHALFLILHSGNILDSGPGDANSKQADVQTLSSAFEAVTRIHFPEALGHVALRLV
PCPPICAAAYALVSNLSPYSHDGDSLSRSQDHIPLAALPLLATSSSRYQGAVATVIARTNQAY
SAFLRSPEGAGFCGQVALIGDGVGGILGFDALCHSANAGTGSRGSSRRGSMNNELLSPEFG
PVRDPLADGVEGLGRGSPEPSALPPQRIPSDMASPEPEGSQNSLQAAPATTSSWEPRRAST
AFCPPAASSEAPDGPSSTARLDFKVSGFFLFGSPLGLVLALRKTVMPALEAAQMRPACEQI
YNLFHAADPCASRLEPLLAPKFQAIAPLTVPRYQKFPLGDGSSLLLADTLQTHSSLFLEELE
MLVPSTPTSTSGAFWKGSELATDPPAQPAAPSTTSEVVKILERWWGTKRIDYSLYCPEALT
AFPTVTLPHLFHASYWESADVVAFILRQVIEKERPQLAECEEPSIYSPAFPREKWQRKRTQ
VKIRNVTSNHRASDTVVCEGRPQVLSGRFMYGPLDVVTLTGEKVDVYIMTQPLSGKWIH
FGTEVTNSSGRLTFPVPPERALGIGVYPVRMVVRGDHTYAECCLTVVARGTEAVVFSIDGS
FTASVSIMGSDPKVRAGAVDVVRHWQDSGYLIVYVTGRPDMQKHRVVAWLSQHNFPHG
VVSFCDGLTHDPLRQKAMFLQSLVQEVELNIVAGYGSPKDVAVYAALGLSPSQTYIVGRA
VRKLQAQCQFLSDGYVAHLGQLEAGSHSHASSGPPRAALGKSSYGVAAPVDFLRKQSQL
LRSRGPSQAEREGPGTPPTTLARGKARSISLKLDSEE

>P62834	lipid-anchor
MREYKLVVLGSGGVGKSALTVQFVQGIFVEKYDPTIEDSYRKQVEVDCQQCMLEILDTA
GTEQFTAMRDLYMKNGQGFALVYSITAQSTFNDLQDLREQILRVKDTEDVPMILVGNKCD
LEDERVVGKEQGQNLARQWCNCAFLESSAKSKINVNEIFYDLVRQINRKTPVEKKKPKKK
SCLLL

>P23468	single-pass type I membrane protein
MVHVARLLLLLLTFFLRTDAETPPRFTRTPVDQTGVSGGVASFICQATGDPRPKIVWNKKG
KKVSNQRFEVIEFDDGSGSVLRIQPLRTPRDEAIYECVASNNVGEISVSTRLTVLREDQIPR
GFPTIDMGPQLKVVERTRTATMLCAASGNPDPEITWFKDFLPVDTSNNNGRIKQLRSESIG
GTPIRGALQIEQSEESDQGKYECVATNSAGTRYSAPANLYVRELREVRRVPPRFSIPPTNHEI
MPGGSVNITCVAVGSPMPYVKWMLGAEDLTPEDDMPIGRNVLELNDVRQSANYTCVAM
STLGVIEAIAQITVKALPKPPGTPVVTESTATSITLTWDSGNPEPVSYYIIQHKPKNSEELYK
EIDGVATTRYSVAGLSPYSDYEFRVVAVNNIGRGPPSEPVLTQTSEQAPSSAPRDVQARMLS
STTILVQWKEPEEPNGQIQGYRVYYTMDPTQHVNNWMKHNVADSQITTIGNLVPQKTYS
VKVLAFTSIGDGPLSSDIQVITQTGVPGQPLNFKAEPESETSILLSWTPPRSDTIANYELVYK
DGEHGEEQRITIEPGTSYRLQGLKPNSLYYFRLAARSPQGLGASTAEISARTMQSKPSAPPQ
DISCTSPSSTSILVSWQPPPVEKQNGIITEYSIKYTAVDGEDDKPHEILGIPSDTTKYLLEQLE
KWTEYRITVTAHTDVGPGPESLSVLIRTNEDVPSGPPRKVEVEAVNSTSVKVSWRSPVPNK

QHGQIRGYQVHYVRMENGEPKGQPMLKDVMLADAQWEFDDTTEHDMIISGLQPETSYS
LTVTAYTTKGDGARSKPKLVSTTGAVPGKPRLVINHTQMNTALIQWHPPVDTFGPLQGYR
LKFGRKDMEPLTTLEFSEKEDHFTATDIHKGASYVFRLSARNKVGFGEEMVKEISIPEEVPT
GFPQNLHSEGTTSTSVQLSWQPPVLAERNGIITKYTLLYRDINIPLLPMEQLIVPADTTMTLT
GLKPDTTYDVKVRAHTSKGPGPYSPSVQFRTLPVDQVFAKNFHVKAVMKTSVLLSWEIPE
NYNSAMPFKILYDDGKMVEEVDGRATQKLIVNLKPEKSYSFVLTNRGNSAGGLQHRVTA
KTAPDVLRTKPAFIGKTNLDGMITVQLPEVPANENIKGYYIIIVPLKKSRGKFIKPWESPDE
MELDELLKEISRKRRSIRYGREVELKPYIAAHFDVLPTEFTLGDDKHYGGFTNKQLQSGQE
YVFFVLAVMEHAESKMYATSPYSDPVVSMDLDPQPITDEEEGLIWVVGPVLAVVFIICIVIA
ILLYKRKRAESDSRKSSIPNNKEIPSHHPTDPVELRRLNFQTPGMASHPPIPILELADHIERL
KANDNLKFSQEYESIDPGQQFTWEHSNLEVNKPKNRYANVIAYDHSRVLLSAIEGIPGSDY
VNANYIDGYRKQNAYIATQGSLPETFGDFWRMIWEQRSATVVMMTKLEERSRVKCDQY
WPSRGTETHGLVQVTLLDTVELATYCVRTFALYKNGSSEKREVRQFQFTAWPDHGVPEHP
TPFLAFLRRVKTCNPPDAGPMVVHCSAGVGRTGCFIVIDAMLERIKHEKTVDIYGHVTLM
RAQRNYMVQTEDQYIFIHDALLEAVTCGNTEVPARNLYAYIQKLTQIETGENVTGMELEFK
RLASSKAHTSRFISANLPCNKFKNRLVNIMPYESTRVCLQPIRGVEGSDYINASFIDGYRQQ
KAYIATQGPLAETTEDFWRMLWEHNSTIVVMLTKLREMGREKCHQYWPAERSARYQYFV
VDPMAEYNMPQYILREFKVTDARDGQSRTVRQFQFTDWPEQGVPKSGEGFIDFIGQVHK
TKEQFGQDGPISVHCSAGVGRTGVFITLSIVLERMRYEGVVDIFQTVKMLRTQRPAMVQT
EDQYQFSYRAALEYLGSFDHYAT

>Q9UJZ1 peripheral membrane protein
MLARAARGTGALLLRGSLLASGRAPRRASSGLPRNTVVLFVPQQEAWVVERMGRFHRIL
EPGLNILIPVLDRIRYVQSLKEIVINVPEQSAVTLDNVTLQIDGVLYLRIMDPYKASYGVED
PEYAVTQLAQTTMRSELGKLSLDKVFRERESLNASIVDAINQAADCWGIRCLRYEIKDIHV
PPRVKESMQMQVEAERRKRATVLESEGTRESAINVAEGKKQAQILASEAEKAEQINQAAG
EASAVLAKAKAKAEAIRILAAALTQHNGDAAASLTVAEQYVSAFSKLAKDSNTILLPSNPG
DVTSMVAQAMGVYGALTKAPVPGTPDSLSSGSSRDVQGTDASLDEELDRVKMS

>P01833 single-pass type I membrane protein
MLLFVLTCLLAVFPAISTKSPIFGPEEVNSVEGNSVSITCYYPPTSVNRHTRKYWCRQGARG
GCITLISSEGYVSSKYAGRANLTNFPENGTFVVNIAQLSQDDSGRYKCGLGINSRGLSFDVS
LEVSQGPGLLNDTKVYTVDLGRTVTINCPFKTENAQKRKSLYKQIGLYPVLVIDSSGYVNP
NYTGRIRLDIQGTGQLLFSVVINQLRLSDAGQYLCQAGDDSNSNKKNADLQVLKPEPELV
YEDLRGSVTFHCALGPEVANVAKFLCRQSSGENCDVVVNTLGKRAPAFEGRILLNPQDKD
GSFSVVITGLRKEDAGRYLCGAHSDGQLQEGSPIQAWQLFVNEESTIPRSPTVVKGVAGGS
VAVLCPYNRKESKSIKYWCLWEGAQNGRCPLLVDSEGWVKAQYEGRLSLLEEPGNGTFT
VILNQLTSRDAGFYWCLTNGDTLWRTTVEIKIIEGEPNLKVPGNVTAVLGETLKVPCHFPC
KFSSYEKYWCKWNNTGCQALPSQDEGPSKAFVNCDENSRLVSLTLNLVTRADEGWYWC
GVKQGHFYGETAAVYVAVEERKAAGSRDVSLAKADAAPDEKVLDSGFREIENKAIQDPR
LFAEEKAVADTRDQADGSRASVDSGSSEEQGGSSRALVSTLVPLGLVLAVGAVAVGVARAR
HRKNVDRVSIRSYRTDISMSDFENSREFGANDNMGASSITQETSLGGKEEFVATTESTTETK
EPKKAKRSSKEEAEMAYKDFLLQSSTVAAEAQDGPQEA

>Q8IV08 single-pass type II membrane protein
MKPKLMYQELKVPAEEPANELPMNEIEAWKAAEKKARWVLLVLILAVVGFGALMTQLFL
WEYGDLHLFGPNQRPAPCYDPCEAVLVESIPEGLDFPNASTGNPSTSQAWLGLLAGAHSSL
DIASFYWTLTNNDTHTQEPSAQQGEEVLRQLQTLAPKGVNVRIAVSKPSGPQPQADLQAL
LQSGAQVRMVDMQKLTHGVLHTKFWVVDQTHFYLGSANMDWRSLTQVKELGVVMYN
CSCLARDLTKIFEAYWFLGQAGSSIPSTWPRFYDTRYNQETPMEICLNGTPALAYLASAPPP
LCPSGRTPDLKALLNVVDNARSFIYVAVMNYLPTLEFSHPHRFWPAIDDGLRRATYERGV
KVRLLISCWGHSEPSMRAFLLSLAALRDNHTHSDIQVKLFVVPADEAQARIPYARVNHNK
YMVTERATYIGTSNWSGNYFTETAGTSLLVTQNGRGGLRSQLEAIFLRDWDSPYSHDLDT
SADSVGNACRLL

>Q9BZ11 single-pass type I membrane protein
MGWRPRRARGTPLLLLLLLLLLLWPVPGAGVLQGHIPGQPVTPHWVLDGQPWRTVSLEEP
VSKPDMGLVALEAEGQELLLELEKNHRLLAPGYIETHYGPDGQPVVLAPNHTDHCHYQG
RVRGFPDSWVVLCTCSGMSGLITLSRNASYYLRPWPPRGSKDFSTHEIFRMEQLLTWKGT
CGHRDPGNKAGMTSLPGGPQSRGRREARRTRKYLELYIVADHTLFLTRHRNLNHTKQRLL
EVANYVDQLLRTLDIQVALTGLEVWTERDRSRVTQDANATLWAFLQWRRGLWAQRPHDS
AQLLTGRAFQGATVGLAPVEGMCRAESSGGVSTDHSELPIGAAATMAHEIGHSLGLSHDP
DGCCVEAAAESGGCVMAAATGHPFPRVFSACSRRQLRAFFRKGGGACLSNAPDPGLPVPP
ALCGNGFVEAGEECDCGPGQECRDLCCFAHNCSLRPGAQCAHGDCCVRCLLKPAGALCR
QAMGDCDLPEFCTGTSSHCPPDVYLLDGSPCARGSGYCWDGACPTLEQQCQQLWGPGS
HPAPEACFQVVNSAGDAHGNCGQDSEGHFLPCAGRDALCGKLQCQGGKPSLLAPHMVP
VDSTVHLDGQEVTCRGALALPSAQLDLLGLGLVEPGTQCGPRMVCQSRRCRKNAFQELQ
RCLTACHSHGVCNSNHNCHCAPGWAPPFCDKPGFGGSMDSGPVQAENHDTFLLAMLLSV
LLPLLPGAGLAWCCYRLPGAHLQRCSWGCRRDPACSGPKDGPHRDHPLGGVHPMELGPT
ATGQPWPLDPENSHEPSSHPEKPLPAVSPDPQADQVQMPRSCLW

>O60476 single-pass type II membrane protein
MTTPALLPLSGRRIPPLNLGPPSFPHHRATLRLSEKFILLLILSAFITLCFGAFFFLPDSSKHKR
FDLGLEDVLIPHVDAGKGAKNPGVFLIHGPDEHRHREEEERLRNKIRADHEKALEEAKEK
LRKSREEIRAEIQTEKNKVVQEMKIKENKPLPPVPIPNLVGIRGGDPEDNDIREKREKIKEM
MKHAWDNYRTYGWGHNELRPIARKGHSPNIFGSSQMGATIVDALDTLYIMGLHDEFLDG
QRWIEDNLDFSVNSEVSVFEVNIRFIGGLLAAYYLSGEEIFKIKAVQLAEKLLPAFNTPTGIP
WAMVNLKSGVGRNWGWASAGSSILAEFGTLHMEFIHLSYLTGDLTYYKKVMHIRKLLQK
MDRPNGLYPNYLNPRTGRWGQYHTSVGGLGDSFYEYLLKAWLMSDKTDHEARKMYDD
AIEAIEKHLIKKSRGGLTFIGEWKNGHLEKKMGHLACFAGGMFALGADGSRADKAGHYL
ELGAEIARTCHESYDRTALKLGPESFKFDGAVEAVAVRQAEKYYILRPEVIETYWYLWRFT
HDPRYRQWGWEAALAIEKYCRVNGGFSGVKDVYSSTPTHDDVQQSFFLAETLKYLYLLF
SGDDLLPLDHWVFNTEAHPLPVLHLANTTLSGNPAVR

>O75381 peripheral membrane protein
MASSEQAEQPSQPSSTPGSENVLPREPLIATAVKFLQNSRVRQSPLATRRAFLKKKGLTDEE
IDMAFQQSGTAADEPSSLGPATQVVPVQPPHLISQPYSPAGSRWRDYGALAIIMAGIAFGFH
QLYKKYLLPLILGGREDRKQLERMEAGLSELSGSVAQTVTQLQTTLASVQELLIQQQQKIQ

ELAHELAAAKATTSTNWILESQNINELKSEINSLKGLLLNRRQFPPSPSAPKIPSWQIPVKSP
SPSSPAAVNHHSSSDISPVSNESTSSSPGKEGHSPEGSTVTYHLLGPQEEGEGVVDVKGQV
RMEVQGEEEKREDKEDEEDEEDDDVSHVDEEDCLGVQREDRRGGDGQINEQVEKLRRP
EGASNESERD

>Q96J66 multipass membrane protein
MTRKRTYWVPNSSGGLVNRGIDIGDDMVSGLIYKTYTLQDGPWSQQERNPEAPGRAAVP
PWGKYDAALRTMIPFRPKPRFPAPQPLDNAGLFSYLTVSWLTPLMIQSLRSRLDENTIPPLS
VHDASDKNVQRLHRLWEEEVSRRGIEKASVLLVMLRFQRTRLIFDALLGICFCIASVLGPIL
IIPKILEYSEEQLGNVVHGVGLCFALFLSECVKSLSFSSSWIINQRTAIRFRAAVSSFAFEKLI
QFKSVIHITSGEAISFFTGDVNYLFEGVCYGPLVLITCASLVICSISSYFIIGYTAFIAILCYLLV
FPLAVFMTRMAVKAQHHTSEVSDQRIRVTSEVLTCIKLIKMYTWEKPFAKIIEDLRRKERK
LLEKCGLVQSLTSITLFIIPTVATAVWVLIHTSLKLKLTASMAFSMLASLNLLRLSVFFVPIAV
KGLTNSKSAVMRFKKFFLQESPVFYVQTLQDPSKALVFEEATLSWQQTCPGIVNGALELER
NGHASEGMTRPRDALGPEEEGNSLGPELHKINLVVSKGMMLGVCGNTGSGKSSLLSAILE
EMHLLEGSVGVQGSLAYVPQQAWIVSGNIRENILMGGAYDKARYLQVLHCCSLNRDLEL
LPFGDMTEIGERGLNLSGGQKQRISLARAVYSDRQIYLLDDPLSAVDAHVGKHIFEECIKK
TLRGKTVVLVTHQLQYLEFCGQIILLENGKICENGTHSELMQKKGKYAQLIQKMHKEATS
DMLQDTAKIAEKPKVESQALATSLEESLNGNAVPEHQLTQEEEMEEGSLSWRVYHHYIQA
AGGYMVSCIIFFFVVLIVFLTIFSFWWLSYWLEQGSGTNSSRESNGTMADLGNIADNPQLS
FYQLVYGLNALLLICVGVCSSGIFTKVTRKASTALHNKLFNKVFRCPMSFFDTIPIGRLLNC
FAGDLEQLDQLLPIFSEQFLVLSLMVIAVLLIVSVLSPYILLMGAIIMVICFIYYMMFKKAIG
VFKRLENYSRSPLFSHILNSLQGLSSIHVYGKTEDFISQFKRLTDAQNNYLLLFLSSTRWMA
LRLEIMTNLVTLAVALFVAFGISSTPYSFKVMAVNIVLQLASSFQATARIGLETEAQFTAVER
ILQYMKMCVSEAPLHMEGTSCPQGWPQHGEIIFQDYHMKYRDNTPTVLHGINLTIRGHEV
VGIVGRTGSGKSSLGMALFRLVEPMAGRILIDGVDICSIGLEDLRSKLSVIPQDPVLLSGTIR
FNLDPFDRHTDQQIWDALERTFLTKAISKFPKKLHTDVVENGGNFSVGERQLLCIARAVLR
NSKIILIDEATASIDMETDTLIQRTIREAFQGCTVLVIAHRVTTVLNCDHILVMGNGKVVEFD
RPEVLRKKPGSLFAALMATATSSLR

>Q96D96 multipass membrane protein
MATWDEKAVTRRAKVAPAERMSKFLRHFTVVGDDYHAWNINYKKWENEEEEEEEEQPP
PTPVSGEEGRAAAPDVAPAPGPAPRAPLDFRGMLRKLFSSHRFQVIIICLVVLDALLVLAELI
LDLKIIQPDKNNYAAMVFHYMSITILVFFMMEIIFKLFVFRLEFFHHKFEILDAVVVVVSFIL
DIVLLFQEHQFEALGLLILLRLWRVARIINGIIISVKTRSERQLLRLKQMNVQLAAKIQHLEF
SCSEKEQEIERLNKLLRQHGLLGEVN

>Q9BUF7 single-pass type I membrane protein
MANPGLGLLLALGLPFLLARWGRAWGQIQTTSANENSTVLPSSTSSSSDGNLRPEAITAIIV
VFSLLAALLLAVGLALLVRKLREKRQTEGTYRPSSEEQVGARVPPTPNLKLPPEERLI

>P20701 single-pass type I membrane protein
MKDSCITVMAMALLSGFFFFAPASSYNLDVRGARSFSPPRAGRHFGYRVLQVGNGVIVGA
PGEGNSTGSLYQCQSGTGHCLPVTLRGSNYTSKYLGMTLATDPTDGSILACDPGLSRTCD

QNTYLSGLCYLFRQNLQGPMLQGRPGFQECIKGNVDLVFLFDGSMSLQPDEFQKILDFMK
DVMKKLSNTSYQFAAVQFSTSYKTEFDFSDYVKRKDPDALLKHVKHMLLLTNTFGAINY
VATEVFREELGARPDATKVLIIITDGEATDSGNIDAAKDIIRYIIGIGKHFQTKESQETLHKFA
SKPASEFVKILDTFEKLKDLFTELQKKIYVIEGTSKQDLTSFNMELSSSGISADLSRGHAVV
GAVGAKDWAGGFLDLKADLQDDTFIGNEPLTPEVRAGYLGYTVTWLPSRQKTSLLASGA
PRYQHMGRVLLFQEPQGGGHWSQVQTIHGTQIGSYFGGELCGVDVDQDGETELLLIGAPL
FYGEQRGGRVFIYQRRQLGFEEVSELQGDPGYPLGRFGEAITALTDINGDGLVDVAVGAPL
EEQGAVYIFNGRHGGLSPQPSQRIEGTQVLSGIQWFGRSIHGVKDLEGDGLADVAVGAES
QMIVLSSRPVVDMVTLMSFSPAEIPVHEVECSYSTSNKMKEGVNITICFQIKSLIPQFQGRL
VANLTYTLQLDGHRTRRRGLFPGGRHELRRNIAVTTSMSCTDFSFHFPVCVQDLISPINVSL
NFSLWEEEGTPRDQRAQGKDIPPILRPSLHSETWEIPFEKNCGEDKKCEANLRVSFSPARSR
ALRLTAFASLSVELSLSNLEEDAYWVQLDLHFPPGLSFRKVEMLKPHSQIPVSCEELPEESR
LLSRALSCNVSSPIFKAGHSVALQMMFNTLVNSSWGDSVELHANVTCNNEDSDLLEDNSA
TTIIPILYPINILIQDQEDSTLYVSFTPKGPKIHQVKHMYQVRIQPSIHDHNIPTLEAVVGVPQ
PPSEGPITHQWSVQMEPPVPCHYEDLERLPDAAEPCLPGALFRCPVVFRQEILVQVIGTLEL
VGEIEASSMFSLCSSLSISFNSSKHFHLYGSNASLAQVVMKVDVVYEKQMLYLYVLSGIGG
LLLLLLIFIVLYKVGFFKRNLKEKMEAGRGVPNGIPAEDSEQLASGQEAGDPGCLKPLHEK
DSESGGGKD

>Q01082 peripheral membrane protein
MTTTVATDYDNIEIQQQYSDVNNRWDVDDWDNENSSARLFERSRIKALADEREAVQKKT
FTKWVNSHLARVSCRITDLYTDLRDGRMLIKLLEVLSGERLPKPTKGRMRIHCLENVDKA
LQFLKEQRVHLENMGSHDIVDGNHRLTLGLIWTIILRFQIQDISVETEDNKEKKSAKDALL
LWCQMKTAGYPNVNIHNFTTSWRDGMAFNALIHKHRPDLIDFDKLKKSNAHYNLQNAF
NLAEQHLGLTKLLDPEDISVDHPDEKSIITYVVTYYHYFSKMKALAVEGKRIGKVLDNAIE
TEKMIEKYESLASDLLEWIEQTIIILNNRKFANSLVGVQQQLQAFNTYRTVEKPPKFTEKGN
LEVLLFTIQSKMRANNQKVYMPREGKLISDINKAWERLEKAEHERELALRNELIRQEKLE
QLARRFDRKAAMRETWLSENQRLVSQDNFGFDLPAVEAATKKHEAIETDIAAYEERVQAV
VAVARELEAENYHDIKRITARKDNVIRLWEYLLELLRARRQRLEMNLGLQKIFQEMLYIM
DWMDEMKVLVLSQDYGKHLLGVEDLLQKHTLVEADIGIQAERVRGVNASAQKFATDGE
GYKPCDPQVIRDRVAHMEFCYQELCQLAAERRARLEESRRLWKFFWEMAEEEGWIREKE
KILSSDDYGKDLTSVMRLLSKHRAFEDEMSGRSGHFEQAIKEGEDMIAEEHFGSEKIRERII
YIREQWANLEQLSAIRKKRLEEASLLHQFQADADDIDAWMLDILKIVSSSDVGHDEYSTQ
SLVKKHKDVAEEIANYRPTLDTLHEQASALPQEHAESPDVRGRLSGIEERYKEVAELTRLR
KQALQDTLALYKMFSEADACELWIDEKEQWLNNMQIPEKLEDLEVIQHRFESLEPEMNN
QASRVAVVNQIARQLMHSGHPSEKEIKAQQDKLNTRWSQFRELVDRKKDALLSALSIQNY
HLECNETKSWIREKTKVIESTQDLGNDLAGVMALQRKLTGMERDLVAIEAKLSDLQKEAE
KLESEHPDQAQAILSRLAEISDVWEEMKTTLKNREASLGEASKLQQFLRDLDDFQSWLSR
TQTAIASEDMPNTLTEAEKLLTQHENIKNEIDNYEEDYQKMRDMGEMVTQGQTDAQYMF
LRQRLQALDTGWNELHKMWENRQNLLSQSHAYQQFLRDTKQAEAFLNNQEYVLAHTE
MPTTLEGAEAAIKKQEDFMTTMDANEEKINAVVETGRRLVSDGNINSDRIQEKVDSIDDR
HRKNRETASELLMRLKDNRDLQKFLQDCQELSLWINEKMLTAQDMSYDEARNLHSKWL
KHQAFMAELASNKEWLDKIEKEGMQLISEKPETEAVVKEKLTGLHKMWEVLESTTQTKA
QRLFDANKAELFTQSCADLDKWLHGLESQIQSDDYGKDLTSVNILLKKQQMLENQMEVR

KKEIEELQSQAQALSQEGKSTDEVDSKRLTVQTKFMELLEPLNERKHNLLASKEIHQFNR
DVEDEILWVGERMPLATSTDHGHNLQTVQLLIKKNQTLQKEIQGHQPRIDDIFERSQNIVT
DSSSLSAEAIRQRLADLKQLWGLLIEETEKRHRRLEEAHRAQQYYFDAAEAEAWMSEQEL
YMMSEEKAKDEQSAVSMLKKHQILEQAVEDYAETVHQLSKTSRALVADSHPESERISMRQ
SKVDKLYAGLKDLAEERRGKLDERHRLFQLNREVDDLEQWIAEREVVAGSHELGQDYEH
VTMLQERFREFARDTGNIGQERVDTVNHLADELINSGHSDAATIAEWKDGLNEAWADLLE
LIDTRTQILAASYELHKFYHDAKEIFGRIQDKHKKLPEELGRDQNTVETLQRMHTTFEHDI
QALGTQVRQLQEDAARLQAAYAGDKADDIQKRENEVLEAWKSLLDACESRRVRLVDTG
DKFRFFSMVRDLMLWMEDVIRQIEAQEKPRDVSSVELLMNNHQGIKAEIDARNDSFTTCI
ELGKSLLARKHYASEEIKEKLLQLTEKRKEMIDKWEDRWEWLRLILEVHQFSRDASVAEA
WLLGQEPYLSSREIGQSVDEVEKLIKRHEAFEKSAATWDERFSALERLTTLELLEVRRQQE
EEERKRRPPSPEPSTKVSEEAESQQQWDTSKGEQVSQNGLPAEQGSPRMAETVDTSEMVN
GATEQRTSSKESSPIPSPTSDRKAKTALPAQSAATLPARTQETPSAQMEGFLNRKHEWEAH
NKKASSRSWHNVYCVINNQEMGFYKDAKTAASGIPYHSEVPVSLKEAVCEVALDYKKKK
HVFKLRLNDGNEYLFQAKDDEEMNTWIQAISSAISSDKHEVSASTQSTPASSRAQTLPTSV
VTITSESSPGKREKDKEKDKEKRFSLFGKKK

>Q02297 single-pass type I membrane protein
MSERKEGRGKGKGKKKERGSGKKPESAAGSQSPALPPRLKEMKSQESAAGSKLVLRCET
SSEYSSLRFKWFKNGNELNRKNKPQNIKIQKKPGKSELRINKASLADSGEYMCKVISKLG
NDSASANITIVESNEIITGMPASTEGAYVSSESPIRISVSTEGANTSSSTSTSTTGTSHLVKCA
EKEKTFCVNGGECFMVKDLSNPSRYLCKCQPGFTGARCTENVPMKVQNQEKAEELYQKR
VLTITGICIALLVVGIMCVVAYCKTKKQRKKLHDRLRQSLRSERNNMMNIANGPHHPNPPP
ENVQLVNQYVSKNVISSEHIVEREAETSFSTSHYTSTAHHSTTVTQTPSHSWSNGHTESILS
ESHSVIVMSSVENSRHSSPTGGPRGRLNGTGGPRECNSFLRHARETPDSYRDSPHSERYVS
AMTTPARMSPVDFHTPSSPKSPPSEMSPPVSSMTVSMPSMAVSPFMEEERPLLLVTPPRLRE
KKFDHHPQQFSSFHHNPAHDSNSLPASPLRIVEDEEYETTQEYEPAQEPVKKLANSRRAKR
TKPNGHIANRLEVDSNTSSQSSNSESETEDERVGEDTPFLGIQNPLAASLEATPAFRLADSR
TNPAGRFSTQEEIQARLSSVIANQDPIAV

>O60909 single-pass type II membrane protein
MSRLLGGTLERVCKAVLLLCLLHFLVAVILYFDVYAQHLAFFSRFSARGPAHALHPAASSSS
SSSNCSRPNATASSSGLPEVPSALPGPTAPTLPPCPDSPPGLVGRLLIEFTSPMPLERVQRENP
GVLMGGRYTPPDCTPAQTVAVIIPFRHREHHLRYWLHYLHPILRRQRLRYGVYVINQHGE
DTFNRAKLLNVGFLEALKEDAAYDCFIFSDVDLVPMDDRNLYRCGDQPRHFAIAMDKFGF
RLPYAGYFGGVSGLSKAQFLRINGFPNEYWGWGGEDDDIFNRISLTGMKISRPDIRIGRYR
MIKHDRDKHNEPNPQRFTKIQNTKLTMKRDGIGSVRYQVLEVSRQPLFTNITVDIGRPPSW
PPRG

>Q8WY64 peripheral membrane protein
MLCYVTRPDAVLMEVEVEAKANGEDCLNQVCRRLGIIEVDYFGLQFTGSKGESLWLNLR
NRISQQMDGLAPYRLKLRVKFFVEPHLILQEQTRHIFFLHIKEALLAGHLLCSPEQAVELSA
LLAQTKFGDYNQNTAKYNYEELCAKELSSATLNSIVAKHKELEGTSQASAEYQVLQIVSA
MENYGIEWHSVRDSEGQKLLIGVGPEGISICKDDFSPINRIAYPVVQMATQSGKNVYLTVT

KESGNSIVLLFKMISTRAASGLYRAITETHAFYRCDTVTSAVMMQYSRDLKGHLASLFLNE
NINLGKKYVFDIKRTSKEVYDHARRALYNAGVVDLVSRNNQSPSHSPLKSSESSMNCSSC
EGLSCQQTRVLQEKLRKLKEAMLCMVCCEEEINSTFCPCGHTVCCESCAAQLQSCPVCRS
RVEHVQHVYLPTHTSLLNLTVI

>O60312 multipass membrane protein
MEREPAGTEEPGPPGRRRRREGRTRTVRSNLLPPPGAEDPAAGAAKGERRRRRGCAQHLA
DNRLKTTKYTLLSFLPKNLFEQFHRPANVYFVFIALLNFVPAVNAFQPGLALAPVLFILAIT
AFRDLWEDYSRHRSDHKINHLGCLVFSREEKKYVNRFWKEIHVGDFVRLRCNEIFPADILL
LSSSDPDGLCHIETANLDGETNLKRRQVVRGFSELVSEFNPLTFTSVIECEKPNNDLSRFRG
CIIHDNGKKAGLYKENLLLRGCTLRNTDAVVGIVIYAGHETKALLNNSGPRYKRSKLERQ
MNCDVLWCVLLLVCMSLFSAVGHGLWIWRYQEKKSLFYVPKSDGSSLSPVTAAVYSFLT
MIIVLQVLIPISLYVSIEIVKACQVYFINQDMQLYDEETDSQLQCRALNITEDLGQIQYIFSD
KTGTLTENKMVFRRCTVSGVEYSHDANAQRLARYQEADSEEEEVVPRGGSVSQRGSIGS
HQSVRVVHRTQSTKSHRRTGSRAEAKRASMLSKHTAFSSPMEKDITPDPKLLEKVSECDK
SLAVARHQEHLLAHLSPELSDVFDFFIALTICNTVVVTSPDQPRTKVRVRFELKSPVKTIEDF
LRRFTPSCLTSGCSSIGSLAANKSSHKLGSSFPSTPSSDGMLLRLEERLGQPTSAIASNGYSS
QADNWASELAQEQESERELRYEAESPDEAALVYAARAYNCVLVERLHDQVSVELPHLGR
LTFELLHTLGFDSVRKRMSVVIRHPLTDEINVYTKGADSVVMDLLQPCSSVDARGRHQKK
IRSKTQNYLNVYAAEGLRTLCIAKRVLSKEEYACWLQSHLEAESSLENSEELLFQSAIRLET
NLHLLGATGIEDRLQDGVPETISKLRQAGLQIWVLTGDKQETAVNIAYACKLLDHDEEVIT
LNATSQEACAALLDQCLCYVQSRGLQRAPEKTKGKVSMRFSSLCPPSTSTASGRRPSLVID
GRSLAYALEKNLEDKFLFLAKQCRSVLCCRSTPLQKSMVVKLVRSKLKAMTLAIGDGAN
DVSMIQVADVGVGISGQEGMQAVMASDFAVPKFRYLERLLILHGHWCYSRLANMVLYFF
YKNTMFVGLLFWFQFFCGFSASTMIDQWYLIFFNLLFSSLPPLVTGVLDRDVPANVLLTNP
QLYKSGQNMEEYRPRTFWFNMADAAFQSLVCFSIPYLAYYDSNVDLFTWGTPIVTIALLTF
LLHLGIETKTWTWLNWITCGFSVLLFFTVALIYNASCATCYPPSNPYWTMQALLGDPVFY
LTCLMTPVAALLPRLFFRSLQGRVFPTQLQLARQLTRKSPRRCSAPKETFAQGRLPKDSGT
EHSSGRTVKTSVPLSQPSWHTQQPVCSLEASGEPSTVDMSMPVREHTLLEGLSAPAPMSS
APGEAVLRSPGGCPEESKVRAASTGRVTPLSSLFSLPTFSLLNWISSWSLVSRLGSVLQFSR
TEQLADGQAGRGLPVQPHSGRSGLQGPDHRLLIGASSRRSQ

>O14678 multipass membrane protein
MAVAGPAPGAGARPRLDLQFLQRFLQILKVLFPSWSSQNALMFLTLLCLTLLLEQFVIYQVG
LIPSQYYGVLGNKDLEGFKTLTFLAVMLIVLNSTLKSFDQFTCNLLYVSWRKDLTEHLHRL
YFRGRAYYTLNVLRDDIDNPDQRISQDVERFCRQLSSMASKLIISPFTLVYYTYQCFQSTG
WLGPVSIFGYFILGTVVNKTLMGPIVMKLVHQEKLEGDFRFKHMQIRVNAEPAAFYRAGH
VEHMRTDRRLQRLLQTQRELMSKELWLYIGINTFDYLGSILSYVVIAIPIFSGVYGDLSPAE
LSTLVSKNAFVCIYLISCFTQLIDLSTTLSDVAGYTHRIGQLRETLLDMSLKSQDCEILGESE
WGLDTPPGWPAAEPADTAFLLERVSISAPSSDKPLIKDLSLKISEGQSLLITGNTGTGKTSLL
RVLGGLWTSTRGSVQMLTDFGPHGVLFLPQKPFFTDGTLREQVIYPLKEVYPDSGSADDE
RILRFLELAGLSNLVARTEGLDQQVDWNWYDVLSPGEMQRLSFARLFYLQPKYAVLDEAT
SALTEEVESELYRIGQQLGMTFISVGHRQSLEKFHSLVLKLCGGGRWELMRIKVE

>Q9NP72 lipid-anchor
MDEDVLTTLKILIIGESGVGKSSLLLRFTDDTFDPELAATIGVDFKVKTISVDGNKAKLAIW
DTAGQERFRTLTPSYYRGAQGVILVYDVTRRDTFVKLDNWLNELETYCTRNDIVNMLVG
NKIDKENREVDRNEGLKFARKHSMLFIEASAKTCDGVQCAFEELVEKIIQTPGLWESENQN
KGVKLSHREEGQGGGACGGYCSVL

>Q13444 single-pass type I membrane protein
MRLALLWALGLLGAGSPLPSWPLPNIGGTEEQQAESEKAPREPLEPQVLQDDLPISLKKVL
QTSLPEPLRIKLELDGDSHILELLQNRELVPGRPTLVWYQPDGTRVVSEGHTLENCCYQGR
VRGYAGSWVSICTCSGLRGLVVLTPERSYTLEQGPGDLQGPPIISRIQDLHLPGHTCALSWR
ESVHTQKPPEHPLGQRHIRRRRDVVTETKTVELVIVADHSEAQKYRDFQHLLNRTLEVALL
LDTFFRPLNVRVALVGLEAWTQRDLVEISPNPAVTLENFLHWRRAHLLPRLPHDSAQLVTG
TSFSGPTVGMAIQNSICSPDFSGGVNMDHSTSILGVASSIAHELGHSLGLDHDLPGNSCPCP
GPAPAKTCIMEASTDFLPGLNFSNCSRRALEKALLDGMGSCLFERLPSLPPMAAFCGNMF
VEPGEQCDCGFLDDCVDPCCDSLTCQLRPGAQCASDGPCCQNCQLRPSGWQCRPTRGDC
DLPEFCPGDSSQCPPDVSLGDGEPCAGGQAVCMHGRCASYAQQCQSLWGPGAQPAAPLC
LQTANTRGNAFGSCGRNPSGSYVSCTPRDAICGQLQCQTGRTQPLLGSIRDLLWETIDVNG
TELNCSWVHLDLGSDVAQPLLTLPGTACGPGLVCIDHRCQRVDLLGAQECRSKCHGHGV
CDSNRHCYCEEGWAPPDCTTQLKATSSLTTGLLLSLLVLLVLVMLGASYWYRARLHQRLC
QLKGPTCQYRAAQSGPSERPGPPQRALLARGTKQASALSFPAPPSRPLPPDPVSKRLQAEL
ADRPNPPTRPLPADPVVRSPKSQGPAKPPPPRKPLPADPQGRCPSGDLPGPGAGIPPLVVPS
RPAPPPPTVSSLYL

>P11717 single-pass type I membrane protein
MGAAAGRSPHLGPAPARRPQRSLLLLQLLLLVAAPGSTQAQAAPFPELCSYTWEAVDTKN
NVLYKINICGSVDIVQCGPSSAVCMHDLKTRTYHSVGDSVLRSATRSLLEFNTTVSCDQQG
TNHRVQSSIAFLCGKTLGTPEFVTATECVHYFEWRTTAACKKDIFKANKEVPCYVFDEELR
KHDLNPLIKLSGAYLVDDSDPDTSLFINVCRDIDTLRDPGSQLRACPPGTAACLVRGHQAF
DVGQPRDGLKLVRKDRLVLSYVREEAGKLDFCDGHSPAVTITFVCPSERREGTIPKLTAKS
NCRYEIEWITEYACHRDYLESKTCSLSGEQQDVSIDLTPLAQSGGSSYISDGKEYLFYLNVC
GETEIQFCNKKQAAVCQVKKSDTSQVKAAGRYHNQTLRYSDGDLTLIYFGGDECSSGFQR
MSVINFECNKTAGNDGKGTPVFTGEVDCTYFFTWDTEYACVKEKEDLLCGATDGKKRYD
LSALVRHAEPEQNWEAVDGSQTETEKKHFFINICHRVLQEGKARGCPEDAAVCAVDKNGS
KNLGKFISSPMKEKGNIQLSYSDGDDCGHGKKIKTNITLVCKPGDLESAPVLRTSGEGGCF
YEFEWHTAAACVLSKTEGENCTVFDSQAGFSFDLSPLTKKNGAYKVETKKYDFYINVCGP
VSVSPCQPDSGACQVAKSDEKTWNLGLSNAKLSYYDGMIQLNYRGGTPYNNERHTPRAT
LITFLCDRDAGVGFPEYQEEDNSTYNFRWYTSYACPEEPLECVVTDPSTLEQYDLSSLAKS
EGGLGGNWYAMDNSGEHVTWRKYYINVCRPLNPVPGCNRYASACQMKYEKDQGSFTE
VVSISNLGMAKTGPVVEDSGSLLLEYVNGSACTTSDGRQTTYTTRIHLVCSRGRLNSHPIF
SLNWECVVSFLWNTEAACPIQTTTDTDQACSIRDPNSGFVFNLNPLNSSQGYNVSGIGKIF
MFNVCGTMPVCGTILGKPASGCEAETQTEELKNWKPARPVGIEKSLQLSTEGFITLTYKGP
LSAKGTADAFIVRFVCNDDVYSGPLKFLHQDIDSGQGIRNTYFEFETALACVPSPVDCQVT
DLAGNEYDLTGLSTVRKPWTAVDTSVDGRKRTFYLSVCNPLPYIPGCQGSAVGSCLVSEG
NSWNLGVVQMSPQAAANGSLSIMYVNGDKCGNQRFSTRITFECAQISGSPAFQLQDGCE

YVFIWRTVEACPVVRVEGDNCEVKDPRHGNLYDLKPLGLNDTIVSAGEYTYYFRVCGKL
SSDVCPTSDKSKVVSSCQEKREPQGFHKVAGLLTQKLTYENGLLKMNFTGGDTCHKVYQ
RSTAIFFYCDRGTQRPVFLKETSDCSYLFEWRTQYACPPFDLTECSFKDGAGNSFDLSSLSR
YSDNWEAITGTGDPEHYLINVCKSLAPQAGTEPCPPEAAACLLGGSKPVNLGRVRDGPQ
WRDGIIVLKYVDGDLCPDGIRKKSTTIRFTCSESQVNSRPMFISAVEDCEYTFAWPTATACP
MKSNEHDDCQVTNPSTGHLFDLSSLSGRAGFTAAYSEKGLVYMSICGENENCPPGVGACF
GQTRISVGKANKRLRYVDQVLQLVYKDGSPCPSKSGLSYKSVISFVCRPEARPTNRPMLIS
LDKQTCTLFFSWHTPLACEQATECSVRNGSSIVDLSPLIHRTGGYEAYDESEDDASDTNPD
FYINICQPLNPMHGVPCPAGAAVCKVPIDGPPIDIGRVAGPPILNPIANEIYLNFESSTPCLAD
KHFNYTSLIAFHCKRGVSMGTPKLLRTSECDFVFEWETPVVCPDEVRMDGCTLTDEQLLY
SFNLSSLSTSTFKVTRDSRTYSVGVCTFAVGPEQGGCKDGGVCLLSGTKGASFGRLQSMK
LDYRHQDEAVVLSYVNGDRCPPETDDGVPCVFPFIFNGKSYEECIIESRAKLWCSTTADYD
RDHEWGFCRHSNSYRTSSIIFKCDEDEDIGRPQVFSEVRGCDVTFEWKTKVVCPPKKLEC
KFVQKHKTYDLRLLSSLTGSWSLVHNGVSYYINLCQKIYKGPLGCSERASICRRTTTGDVQ
VLGLVHTQKLGVIGDKVVVTYSKGYPCGGNKTASSVIELTCTKTVGRPAFKRFDIDSCTY
YFSWDSRAACAVKPQEVQMVNGTITNPINGKSFSLGDIYFKLFRASGDMRTNGDNYLYEI
QLSSITSSRNPACSGANICQVKPNDQHFSRKVGTSDKTKYYLQDGDLDVVFASSSKCGKD
KTKSVSSTIFFHCDPLVEDGIPEFSHETADCQYLFSWYTSAVCPLGVGFDSENPGDDGQMH
KGLSERSQAVGAVLSLLLVALTCCLLALLLYKKERRETVISKLTTCCRRSSNVSYKYSKVN
KEEETDENETEWLMEEIQLPPPRQGKEGQENGHITTKSVKALSSLHGDDQDSEDEVLTIPE
VKVHSGRGAGAESSHPVRNAQSNALQEREDDRVGLVRGEKARKGKSSSAQQKTVSSTKL
VSFHDDSDEDLLHI

>Q96E93 single-pass type II membrane protein
MTDSVIYSMLELPTATQAQNDYGPQQKSSSSRPSCSCLVAIALGLLTAVLLSVLLYQWILCQ
GSNYSTCASCPSCPDRWMKYGNHCYYFSVEEKDWNSSLEFCLARDSHLLVITDNQEMSL
LQVFLSEAFCWIGLRNNSGWRWEDGSPLNFSRISSNSFVQTCGAINKNGLQASSCEVPLH
WVCKKCPFADQALF

>Q9BXC0 multipass membrane protein
MYNGSCCRIEGDTISQVMPPLLIVAFVLGALGNGVALCGFCFHMKTWKPSTVYLFNLAVA
DFLLMICLPFRTDYYLRRRHWAFGDIPCRVGLFTLAMNRAGSIVFLTVVAADRYFKVVHP
HHAVNTISTRVAAGIVCTLWALVILGTVYLLLENHLCVQETAVSCESFIMESANGWHDIMF
QLEFFMPLGIILFCSFKIVWSLRRRQQLARQARMKKATRFIMVVAIVFITCYLPSVSARLYF
LWTVPSSACDPSVHGALHITLSFTYMNSMLDPLVYYFSSPSFPKFYNKLKICSLKPKQPGH
SKTQRPEEMPISNLGRRSCISVANSFQSQSDGQWDPHIVEWH

>Q14160 peripheral membrane protein
MLKCIPLWRCNRHVESVDKRHCSLQAVPEEIYRYSRSLEELLLDANQLRELPKPFFRLLNL
RKLGLSDNEIQRLPPEVANFMQLVELDVSRNDIPEIPESIKFCKALEIADFSGNPLSRLPDGF
TQLRSLAHLALNDVSLQALPGDVGNLANLVTLELRENLLKSLPASLSFLVKLEQLDLGGN
DLEVLPDTLGALPNLRELWLDRNQLSALPPELGNLRRLVCLDVSENRLEELPAELGGLVLL
TDLLLSQNLLRRLPDGIGQLKQLSILKVDQNRLCEVTEAIGDCENLSELILTENLLMALPRS
LGKLTKLTNLNVDRNHLEALPPEIGGCVALSVLSLRDNRLAVLPPELAHTTELHVLDVAGN

RLQSLPFALTHLNLKALWLAENQAQPMLRFQTEDDARTGEKVLTCYLLPQQPPPSLEDAG
QQGSLSETWSDAPPSRVSVIQFLEAPIGDEDAEEAAAEKRGLQRRATPHPSELKVMKRSIE
GRRSEACPCQPDSGSPLPAEEEKRLSAESGLSEDSRPSASTVSEAEPEGPSAEAQGGSQQEA
TTAGGEEDAEEDYQEPTVHFAEDALLPGDDREIEEGQPEAPWTLPGGRQRLIRKDTPHYK
KHFKISKLPQPEAVVALLQGMQPDGEGPVAPGGWHNGPHAPWAPRAQKEEEEEEEGSPQ
EEEVEEEEENRAEEEEASTEEEDKEGAVVSAPSVKGVSFDQANNLLIEPARIEEEELTLTILR
QTGGLGISIAGGKGSTPYKGDDEGIFISRVSEEGPAARAGVRVGDKLLEVNGVALQGAEH
HEAVEALRGAGTAVQMRVWRERMVEPENAVTITPLRPEDDYSPRERRGGGLRLPLLPPES
PGPLRQRHVACLARSERGLGFSIAGGKGSTPYRAGDAGIFVSRIAEGGAAHRAGTLQVGD
RVLSINGVDVTEARHDHAVSLLTAASPTIALLLEREAGGPLPPSPLPHSSPPTAAVATTSITTA
TPGVPGLPSLAPSLLAAALEGPYPVEEIRLPRAGGPLGLSIVGGSDHSSHPFGVQEPGVFIS
KVLPRGLAARSGLRVGDRILAVNGQDVRDATHQEAVSALLRPCLELSLLVRRDPAPPGLRE
LCIQKAPGERLGISIRGGARGHAGNPRDPTDEGIFISKVSPTGAAGRDGRLRVGLRLLEVN
QQSLLGLTHGEAVQLLRSVGDTLTVLVCDGFEASTDAALEVSPGVIANPFAAGIGHRNSLE
SISSIDRELSPEGPGKEKELPGQTLHWGPEATEAAGRGLQPLKLDYRALAAVPSAGSVQRV
PSGAAGGKMAESPCSPSGQQPPSPPSPDELPANVKQAYRAFAAVPTSHPPEDAPAQPPTPGP
AASPEQLSFRERQKYFELEVRVPQAEGPPKRVSLVGADDLRKMQEEEARKLQQKRAQML
REAAEAGAEARLALDGETLGEEEQEDEQPPWASPSPTSRQSPASPPPLGGGAPVRTAKAER
RHQERLRVQSPEPPAPERALSPAELRALEAEKRALWRAARMKSLEQDALRAQMVLSRSQ
EGRGTRGPLERLAEAPSPAPTPSPTPVEDLGPQTSTSPGRLSPDFAEELRSLEPSPSPGPQEE
DGEVALVLLGRPSPGAVGPEDVALCSSRRPVRPGRRGLGPVPS

>P28328 multipass membrane protein
MASRKENAKSANRVLRISQLDALELNKALEQLVWSQFTQCFHGFKPGLLARFEPEVKACL
WVFLWRFTIYSKNATVGQSVLNIKYKNDFSPNLRYQPPSKNQKIWYAVCTIGGRWLEERC
YDLFRNHHLASFGKVKQCVNFVIGLLKLGGLINFLIFLQRGKFATLTERLLGIHSVFCKPQN
ICEVGFEYMNRELLWHGFAEFLIFLLPLINVQKLKAKLSSWCIPLTGAPNSDNTLATSGKEC
ALCGEWPTMPHTIGCEHIFCYFCAKSSFLFDVYFTCPKCGTEVHSLQPLKSGIEMSEVNAL

>P61018 lipid-anchor
MAETYDFLFKFLVIGSAGTGKSCLLHQFIENKFKQDSNHTIGVEFGSRVVNVGGKTVKLQI
WDTAGQERFRSVTRSYYRGAAGALLVYDITSRETYNSLAAWLTDARTLASPNIVVILCGN
KKDLDPEREVTFLEASRFAQENELMFLETSALTGENVEEAFLKCARTILNKIDSGELDPER
MGSGIQYGDASLRQLRQPRSAQAVAPQPCGC

>Q86V24 multipass membrane protein
MNEPTENRLGCSRTPEPDIRLRKGHQLDGTRRGDNDSHQGDLEPILEASVLSSHHKKSSEE
HEYSDEAPQEDEGFMGMSPLLQAHHAMEKMEEFVCKVWEGRWRVIPHDVLPDWLKDN
DFLLHGHRPPMPSFRACFKSIFRIHTETGNIWTHLLGCVFFLCLGIFYMFRPNISFVAPLQEK
VVFGLFFLGAILCLSFSWLFHTVYCHSEGVSRLFSKLDYSGIALLIMGSFVPWLYYSFYCN
PQPCFIYLIVICVLGIAAIIVSQWDMFATPQYRGVRAGVFLGLGLSGIIPTLHYVISEGFLKA
ATIGQIGWLMLMASLYITGAALYAARIPERFFPGKCDIWFHSHQLFHIFVVAGAFVHFHGV
SNLQEFRFMIGGGCSEEDAL

>Q9Y397 multipass membrane protein
MSVMVVRKKVTRKWEKLPGRNTFCCDGRVMMARQKGIFYLTLFLILGTCTLFFAFECRY
LAVQLSPAIPVFAAMLFLFSMATLLRTSFSDPGVIPRALPDEAAFIEMEIEATNGAVPQGQRP
PPRIKNFQINNQIVKLKYCYTCKIFRPPRASHCSICDNCVERFDHHCPWVGNCVGKRNYR
YFYLFILSLSLLTIYVFAFNIVYVALKSLKIGFLETLKETPGTVLEVLICFFTLWSVVGLTGFH
TFLVALNQTTNEDIKGSWTGKNRVQNPYSHGNIVKNCCEVLCGPLPPSVLDRRGILPLEES
GSRPPSTQETSSSLLPQSPAPTEHLNSNEMPEDSSTPEEMPPPEPPEPPQEAAEAEK

>Q8TD07 single-pass type I membrane protein
MRRISLTSSPVRLLLFLLLLLIALEIMVGGHSLCFNFTIKSLSRPGQPWCEAQVFLNKNLFL
QYNSDNNMVKPLGLLGKKVYATSTWGELTQTLGEVGRDLRMLLCDIKPQIKTSDPSTLQ
VEMFCQREAERCTGASWQFATNGEKSLLFDAMNMTWTVINHEASKIKETWKKDRGLEK
YFRKLSKGDCDHWLREFLGHWEAMPEPTVSPVNASDIHWSSSSLPDRWIILGAFILLVLM
GIVLICVWWQNGEWQAGLWPLRTS

>Q8NGY9 multipass membrane protein
MENYNQTSTDFILLGLFPPSRIDLFFFILIVFIFLMALIGNLSMILLIFLDTHLHTPMYFLLSQ
LSLIDLNYISTIVPKMASDFLHGNKSISFTGCGIQSFFFLALGGAEALLLASMAYDRYIAICF
PLHYLIRMSKRVCVLMITGSWIIGSINACAHTVYVLHIPYCRSRAINHFFCDVPAMVTLAC
MDTWVYEGTVFLSATIFLVFPFIGISCSYGQVLFAVYHMKSAEGRKKAYLTCSTHLTVVTF
YYAPFVYTYLRPRSLRSPTEDKVLAVFYTILTPMLNPIIYSLRNKEVMGALTRVSQRICSVK
M

>P61077 peripheral membrane protein
MALKRINKELSDLARDPPAQCSAGPVGDDMFHWQATIMGPNDSPYQGGVFFLTIHFPTDY
PFKPPKVAFTTRIYHPNINSNGSICLDILRSQWSPALTISKVLLSICSLLCDPNPDDPLVPEIAR
IYKTDRDKYNRISREWTQKYAM

>Q7L591 peripheral membrane protein
MTRGARLRSDARAQLNQLSLDGGTGSGQKGKCEEFPSSLSSVSPGLEAAALLLAVTMDPL
ETPIKDGILYQQHVKFGKKCWRKVWALLYAGGPSGVARLESWEVRDGGLGAAGDRSAGP
GRRGERRVIRLADCVSVLPADGESCPRDTGAFLLTTTERSHLLAAQHRQAWMGPICQLAF
PGTGEASSGSTDAQSPKRGLVPMEENSIYSSWQEVGEFPVVVQRTEAATRCQLKGPALLV
LGPDAIQLREAKGTQALYSWPYHFLRKFGSDKGVFSFEAGRRCHSGEGLFAFSTPCAPDL
CRAVAGAIARQRERLPELTRPQPCPLPRATSLPSLDTPGELREMPPGPEPPTSRKMHLAEPG
PQSLPLLLGPEPNDLASGLYASVCKRASGPPGNEHLYENLCVLEASPTLHGGEPEPHEGPG
SRSPTTSPIYHNGQDLSWPGPANDSTLEAQYRRLLELDQVEGTGRPDPQAGFKAKLVTLLS
RERRKGPAPCDRP

>Q9BXW4 lipid-anchor
MPPPQKIPSVRPFKQRKSLAIRQEEVAGIRAKFPNKIPVVVERYPRETFLPPLDKTKFLVPQE
LTMTQFLSIIRSRMVLRATEAFYLLVNNKSLVSMSATMAEIYRDYKDEDGFVYMTYASQE
TFGCLESAAPRDGSSLEDRPCNPL

>Q8NGC1 multipass membrane protein
MHFLSQNDLNINLIPHLCLHRHSVIAGAFTIHRHMKIFNSPSNSSTFTGFILLGFPCPREGQI
LLFVLFTVVYLLTLMGNGSIICAVHWDQRLHAPMYILLANFSFLEICYVTSTVPSMLANFL
SDTKIISFSGCFLQFYFFFSLGSTECFFLAVMAFDRYLAICRPLRYPTIMTRRLCTNLVVNCW
VLGFIWFLIPIVNISQMSFCGSRIIDHFLCDPAPLLTLTCKKGPVIELVFSVLSPLPVFMLFLFI
VGSYALVVRAVLRVPSAAGRRKAFSTCGSHLAVVSLFYGSVLVMYGSPPSKNEAGKQKTV
TLFYSVVTPLLNPVIYSLRNKDMRKALKKFWGT

>Q13332 single-pass type I membrane protein
MAPTWGPGMVSVVGPMGLLVVLLVGGCAAEEPPRFIKEPKDQIGVSGGVASFVCQATGD
PKPRVTWNKKGKKVNSQRFETIEFDESAGAVLRIQPLRTPRDENVYECVAQNSVGEITVHA
KLTVLREDQLPSGFPNIDMGPQLKVVERTRTATMLCAASGNPDPEITWFKDFLPVDPSASN
GRIKQLRSETFESTPIRGALQIESSEETDQGKYECVATNSAGVRYSSPANLYVRELREVRRV
APRFSILPMSHEIMPGGNVNITCVAVGSPMPYVKWMQGAEDLTPEDDMPVGRNVLELTD
VKDSANYTCVAMSSLGVIEAVAQITVKSLPKAPGTPMVTENTATSITITWDSGNPDPVSYY
VIEYKSKSQDGPYQIKEDITTTRYSIGGLSPNSEYEIWVSAVNSIGQGPPSESVVTRTGEQAP
ASAPRNVQARMLSATTMIVQWEEPVEPNGLIRGYRVYYTMEPEHPVGNWQKHNVDDSL
LTTVGSLLEDETYTVRVLAFTSVGDGPLSDPIQVKTQQGVPGQPMNLRAEARSETSITLSW
SPPRQESIIKYELLFREGDHGREVGRTFDPTTSYVVEDLKPNTEYAFRLAARSPQGLGAFTP
VVRQRTLQSKPSAPPQDVKCVSVRSTAILVSWRPPPPETHNGALVGYSVRYRPLGSEDPEP
KEVNGIPPTTTQILLEALEKWTQYRITTVAHTEVGPGPESSPVVVRTDEDVPSAPPRKVEAE
ALNATAIRVLWRSPAPGRQHGQIRGYQVHYVRMEGAEARGPPRIKDVMLADAQWETDDT
AEYEMVITNLQPETAYSITVAAYTMKGDGARSKPKVVVTKGAVLGRPTLSVQQTPEGSLL
ARWEPPAGTAEDQVLGYRLQFGREDSTPLATLEFPPSEDRYTASGVHKGATYVFRLAARS
RGGLGEEAAEVLSIPEDTPRGHPQILEAAGNASAGTVLLRWLPPVPAERNGAIVKYTVAVR
EAGALGPARETELPAAAEPGAENALTLQGLKPDTAYDLQVRAHTRRGPGPFSPPVRYRTFL
RDQVSPKNFKVKMIMKTSVLLSWEFPDNYNSPTPYKIQYNGLTLDVDGRTTKKLITHLKP
HTFYNFVLTNRGSSLGGLQQTVTAWTAFNLLNGKPSVAPKPDADGFIMVYLPDGQSPVPV
QSYFIVMVPLRKSRGGQFLTPLGSPEDMDLEELIQDISRLQRRSLRHSRQLEVPRPYIAARF
SVLPPTFHPGDQKQYGGFDNRGLEPGHRYVLFVLAVLQKSEPTFAASPFSDPFQLDNPDPQ
PIVDGEEGLIWVIGPVLAVVFIICIVIAILLYKNKPDSKRKDSEPRTKCLLNNADLAPHHPKD
PVEMRRINFQTPDSGLRSPLREPGFHFESMLSHPPIPIADMAEHTERLKANDSLKLSQEYES
IDPGQQFTWEHSNLEVNKPKNRYANVIAYDHSRVILQPIEGIMGSDYINANYVDGYRCQN
AYIATQGPLPETFGDFWRMVWEQRSATIVMMTRLEEKSRIKCDQYWPNRGTETYGFIQVT
LLDTIELATFCVRTFSLHKNGSSEKREVRQFQFTAWPDHGVPEYPTPFLAFLRRVKTCNPPD
AGPIVVHCSAGVGRTGCFIVIDAMLERIKPEKTVDVYGHVTLMRSQRNYMVQTEDQYSFI
HEALLEAVGCGNTEVPARSLYAYIQKLAQVEPGEHVTGMELEFKRLANSKAHTSRFISANL
PCNKFKNRLVNIMPYESTRVCLQPIRGVEGSDYINASFIDGYRQQKAYIATQGPLAETTEDF
WRMLWENNSTIVVMLTKLREMGREKCHQYWPAERSARYQYFVVDPMAEYNMPQYILR
EFKVTDARDGQSRTVRQFQFTDWPEQGVPKSGEGFIDFIGQVHKTKEQFGQDGPISVHCS
AGVGRTGVFITLSIVLERMRYEGVVDIFQTVKMLRTQRPAMVQTEDEYQFCYQAALEYL
GSFDHYAT

>Q8NGF7 multipass membrane protein

MENNTEVSEFILLGLTNAPELQVPLFIMFTLIYLITLTGNLGMIILILLDSHLHTPMYFFLSNL
SLAGIGYSSAVTPKVLTGLLIEDKAISYSACAAQMFFCAVFATVENYLLSSMAYDRYAAVC
NPLHYTTTMTTRVCACLAIGCYVIGFLNASIQIGDTFRLSFCMSNVIHHFFCDKPAVITLTCS
EKHISELILVLISSFNVFFALLVTLISYLFILITILKRHTGKGYQKPLSTCGSHLIAIFLFYITVII
MYIRPSSSHSMDTDKIASVFYTMIIPMLSPIVYTLRNKDVKNAFMKVVEKAKYSLDSVF

>O00421 multipass membrane protein
MANYTLAPEDEYDVLIEGELESDEAEQCDKYDAQALSAQLVPSLCSAVFVIGVLDNLLVV
LILVKYKGLKRVENIYLLNLAVSNLCFLLTLPFWAHAGGDPMCKILIGLYFVGLYSETFFNC
LLTVQRYLVFLHKGNFFSARRRVPCGIITSVLAWVTAILATLPEFVVYKPQMEDQKYKCAF
SRTPFLPADETFWKHFLTLKMNISVLVLPLFIFTFLYVQMRKTLRFREQRYSLFKLVFAIMV
VFLLMWAPYNIAFFLSTFKEHFSLSDCKSSYNLDKSVHITKLIATTHCCINPLLYAFLDGTFS
KYLCRCFHLRSNTPLQPRGQSAQGTSREEPDHSTEV

>Q9Y282 multipass membrane protein
MEALGKLKQFDAYPKTLEDFRVKTCGGATVTIVSGLLMLLLFLSELQYYLTTEVHPELYV
DKSRGDKLKINIDVLFPHMPCAYLSIDAMDVAGEQQLDVEHNLFKQRLDKDGIPVSSEAE
RHELGKVEVTVFDPDSLDPDRCESCYGAEAEDIKCCNTCEDVREAYRRRGWAFKNPDTIE
QCRREGFSQKMQEQKNEGCQVYGFLEVNKVAGNFHFAPGKSFQQSHVHVHDLQSFGLD
NINMTHYIQHLSFGEDYPGIVNPLDHTNVTAPQASMMFQYFVKVVPTVYMKVDGEVLRT
NQFSVTRHEKVANGLLGDQGLPGVFVLYELSPMMVKLTEKHRSFTHFLTGVCAIIGGMFT
VAGLIDSLIYHSARAIQKKIDLGKTT

>P40198 single-pass type I membrane protein
MGPPSASPHRECIPWQGLLLTASLLNFWNPPTTAKLTIESMPLSVAEGKEVLLLVHNLPQHL
FGYSWYKGERVDGNSLIVGYVIGTQQATPGAAYSGRETIYTNASLLIQNVTQNDIGFYTLQ
VIKSDLVNEEATGQFHVYQENAPGLPVGAVAGIVTGVLVGVALVAALVCFLLLAKTGRTSI
QRDLKEQQPQALAPGRGPSHSSAFSMSPLSTAQAPLPNPRTAASIYEELLKHDTNIYCRMD
HKAEVAS

>P51677 multipass membrane protein
MTTSLDTVETFGTTSYYDDVGLLCEKADTRALMAQFVPPLYSLVFTVGLLGNVVVVMILI
KYRRLRIMTNIYLLNLAISDLLFLVTLPFWIHYVRGHNWVFGHGMCKLLSGFYHTGLYSEI
FFIILLTIDRYLAIVHAVFALRARTVTFGVITSIVTWGLAVLAALPEFIFYETEELFEETLCSAL
YPEDTVYSWRHFHTLRMTIFCLVLPLLVMAICYTGIIKTLLRCPSKKKYKAIRLIFVIMAVF
FIFWTPYNVAILLSSYQSILFGNDCERSKHLDLVMLVTEVIAYSHCCMNPVIYAFVGERFRK
YLRHFFHRHLLMHLGRYIPFLPSEKLERTSSVSPSTAEPELSIVF

>Q9GZM6 multipass membrane protein
MATSNHSSGAEFILAGLTQRPELQLPLFLLFLGIYVVTVVGNLGMIFLIALSSQLYPPVYYF
LSHLSFIDLCYSSVITPKMLVNFVPEENIISFLECITQLYFFLIFVIAEGYLLTAMEYDRYVAIC
RPLLYNIVMSHRVCSIMMAVVYSLGFLWATVHTTRMSVLSFCRSHTVSHYFCDILPLLTLS
CSSTHINEILLFIIGGVNTLATTLAVLISYAFIFSSILGIHSTEGQSKAFGTCSSHLLAVGIFFGSI
TFMYFKPPSSTTMEKEKVSSVFYITIIPMLNPLIYSLRNKDVKNALKKMTRGRQSS

>Q14542 multipass membrane protein
MARGDAPRDSYHLVGISFFILGLGTLLPWNFFITAIPYFQARLAGAGNSTARILSTNHTGPE
DAFNFNNWVTLLSQLPLLLFTLLNSFLYQCVPETVRILGSLLAILLLFALTAALVKVDMSPG
PFFSITMASVCFINSFSAVLQGSLFGQLGTMPSTYSTLFLSGQGLAGIFAALAMLLSMASGV
DAETSALGYFITPCVGILMSIVCYLSLPHLKFARYYLANKSSQAQAQELETKAELLQSDEN
GIPSSPQKVALTLDLDLEKEPESEPDEPQKPGKPSVFTVFQKIWLTALCLVLVFTVTLSVFPA
ITAMVTSSTSPGKWSQFFNPICCFLLFNIMDWLGRSLTSYFLWPDEDSRLLPLLVCLRFLFV
PLFMLCHVPQRSRLPILFPQDAYFITFMLLFAVSNGYLVSLTMCLAPRQVLPHEREVAGAL
MTFFLALGLSCGASLSFLFKALL

>P30874 multipass membrane protein
MDMADEPLNGSHTWLSIPFDLNGSVVSTNTSNQTEPYYDLTSNAVLTFIYFVVCIIGLCGN
TLVIYVILRYAKMKTITNIYILNLAIADELFMLGLPFLAMQVALVHWPFGKAICRVVMTVD
GINQFTSIFCLTVMSIDRYLAVVHPIKSAKWRRPRTAKMITMAVWGVSLLVILPIMIYAGLR
SNQWGRSSCTINWPGESGAWYTGFIIYTFILGFLVPLTIICLCYLFIIIKVKSSGIRVGSSKRK
KSEKKVTRMVSIVVAVFIFCWLPFYIFNVSSVSMAISPTPALKGMFDFVVVLTYANSCANPI
LYAFLSDNFKKSFQNVLCLVKVSGTDDGERSDSKQDKSRLNETTETQRTLLNGDLQTSI

>Q10567 peripheral membrane protein
MTDSKYFTTTKKGEIFELKAELNSDKKEKKKEAVKKVIASMTVGKDVSALFPDVVNCMQ
TDNLELKKLVYLYLMNYAKSQPDMAIMAVNTFVKDCEDPNPLIRALAVRTMGCIRVDKIT
EYLCEPLRKCLKDEDPYVRKTAAVCVAKLHDINAQLVEDQGFLDTLKDLISDSNPMVVAN
AVAALSEIAESHPSSNLLDLNPQSINKLLTALNECTEWGQIFILDCLANYMPKDDREAQSIC
ERVTPRLSHANSAVVLSAVKVLMKFMEMLSKDLDYYGTLLKKLAPPLVTLLSAEPELQYV
ALRNINLIVQKRPEILKHEMKVFFVKYNDPIYVKLEKLDIMIRLASQANIAQVLAELKEYA
TEVDVDFVRKAVRAIGRCAIKVEQSAERCVSTLLDLIQTKVNYVVQEAIVVIKDIFRKYPN
KYESVIATLCENLDSLDEPEARAAMIWIVGEYAERIDNADELLESFLEGFHDESTQVQLQL
LTAIVKLFLKKPTETQELVQQVLSLATQDSDNPDLRDRGYIYWRLLSTDPVAAKEVVLAEK
PLISEETDLIEPTLLDELICYIGTLASVYHKPPSAFVEGGRGVVHKSLPPRTASSESAESPETA
PTGAPPGEQPDVIPAQGDLLGDLLNLDLGPPVSGPPLATSSVQMGAVDLLGGGLDSLMGD
EPEGIGGTNFVAPPTAAVPANLGAPIGSGLSDLFDLTSGVGTLSGSYVAPKAVWLPAMKAK
GLEISGTFTRQVGSISMDLQLTNKALQVMTDFAIQFNRNSFGLAPATPLQVHAPLSPNQTV
EISLPLSTVGSVMKMEPLNNLQVAVKNNIDVFYFSTLYPLHILFVEDGKMDRQMFLATWK
DIPNENEAQFQIRDCPLNAEAASSKLQSSNIFTVAKRNVEGQDMLYQSLKLTNGIWVLAEL
RIQPGNPSCTDLELSLKCRAPEVSQHVYQAYETILKN

>Q8NGF3 multipass membrane protein
MQKPQLLVPIIATSNGNLVHAAYFLLVGIPGLGPTIHFWLAFPLCFMYALATLGNLTIVLIIR
VERRLHEPMYLFLAMLSTIDLVLSSITMPKMASLFLMGIQEIEFNICLAQMFLIHALSAVES
AVLLAMAFDRFVAICHPLRHASVLTGCTVAKIGLSALTRGFVFFFPLPFILKWLSYCQTHTV
THSFCLHQDIMKLSCTDTRVNVVYGLFIILSVMGVDSLFIGFSYILILWAVLELSSRRAALK
AFNTCISHLCAVLVFYVPLIGLSVVHRLGGPTSLLHVVMANTYLLLPPVVNPLVYGAKTKE
ICSRVLCMFSQGGK

>Q8NHC5 multipass membrane protein
MANLTIVTEFILMGFSTNKNMCILHSILFLLIYLCALMGNVLIIMITTLDHHLHTPVYFFLK
NLSFLDLCLISVTAPKSIANSLIHNNSISFLGCVSQVFLLLSSASAELLLLTVMSFDRYTAICH
PLHYDVIMDRSTCVQRATVSWLYGGLIAVMHTAGTFSLSYCGSNMVHQFFCDIPQLLAISC
SENLIREIALILINVVLDFCCFIVIIITYVHVFSTVKKIPSTEGQSKAYSICLPHLLVVLFLSTGF
IAYLKPASESPSILDAVISVFYTMLPPTFNPIIYSLRNKAIKVALGMLIKGKLTKK

>P51686 multipass membrane protein
MTPTDFTSPIPNMADDYGSESTSSMEDYVNFNFTDFYCEKNNVRQFASHFLPPLYWLVFIV
GALGNSLVILVYWYCTRVKTMTDMFLLNLAIADLLFLVTLPFWAIAAADQWKFQTFMCK
VVNSMYKMNFYSCVLLIMCISVDRYIAIAQAMRAHTWREKRLLYSKMVCFTIWVLAAAL
CIPEILYSQIKEESGIAICTMVYPSDESTKLKSAVLTLKVILGFFLPFVVMACCYTIIIHTLIQA
KKSSKHKALKVTITVLTVFVLSQFPYNCILLVQTIDAYAMFISNCAVSTNIDICFQVTQTIAF
FHSCLNPVLYVFVGERFRRDLVKTLKNLGCISQAQWVSFTRREGSLKLSSMLLETTSGALS
L

>Q8NEW0 multipass membrane protein
MLPLSIKDDEYKPPKFNLFGKISGWFRSILSDKTSRNLFFFLCLNLSFAFVELLYGIWSNCL
GLISDSFHMFFDSTAILAGLAASVISKWRDNDAFSYGYVRAEVLAGFVNGLFLIFTAFFIFS
EGVERALAPPDVHHERLLLVSILGFVVNLIGIFVFKHGGHGHSHGSGHGHSHSLFNGALD
QAHGHVDHCHSHEVKHGAAHSHDHAHGHGHFHSHDGPSLKETTGPSRQILQGVFLHILA
DTLGSIGVIASAIMMQNFGLMIADPICSILIAILIVVSVIPLLRESVGILMQRTPPLLENSLPQC
YQRVQQLQGVYSLQEQHFWTLCSDVYVGTLKLIVAPDADARWILSQTHNIFTQAGVRQL
YVQIDFAAM

>Q14831 multipass membrane protein
MVQLRKLLRVLTLMKFPCCVLEVLLCALAAAARGQEMYAPHSIRIEGDVTLGGLFPVHA
KGPSGVPCGDIKRENGIHRLEAMLYALDQINSDPNLLPNVTLGARILDTCSRDTYALEQSLT
FVQALIQKDTSDVRCTNGEPPVFVKPEKVVGVIGASGSSVSIMVANILRLFQIPQISYASTAP
ELSDDRRYDFFSRVVPPDSFQAQAMVDIVKALGWNYVSTLASEGSYGEKGVESFTQISKE
AGGLCIAQSVRIPQERKDRTIDFDRIIKQLLDTPNSRAVVIFANDEDIKQILAAAKRADQVG
HFLWVGSDSWGSKINPLHQHEDIAEGAITIQPKRATVEGFDAYFTSRTLENNRRNVWFAEY
WEENFNCKLTISGSKKEDTDRKCTGQERIGKDSNYEQEGKVQFVIDAVYAMAHALHHMN
KDLCADYRGVCPEMEQAGGKKLLKYIRNVNFNGSAGTPVMFNKNGDAPGRYDIFQYQT
TNTSNPGYRLIGQWTDELQLNIEDMQWGKGVREIPASVCTLPCKPGQRKKTQKGTPCCW
TCEPCDGYQYQFDEMTCQHCPYDQRPNENRTGCQDIPIIKLEWHSPWAVIPVFLAMLGIIA
TIFVMATFIRYNDTPIVRASGRELSYVLLTGIFLCYIITFLMIAKPDVAVCSFRRVFLGLGMCI
SYAALLTKTNRIYRIFEQGKKSVTAPRLISPTSQLAITSSLISVQLLGVFIWFGVDPPNIIIDYD
EHKTMNPEQARGVLKCDITDLQIICSLGYSILLMVTCTVYAIKTRGVPENFNEAKPIGFTM
YTTCIVWLAFIPIFFGTAQSAEKLYIQTTTLTISMNLSASVALGMLYMPKVYIIIFHPELNVQ
KRKRSFKAVVTAATMSSRLSHKPSDRPNGEAKTELCENVDPNSPAAKKKYVSYNNLVI

>Q12891 GPI-anchor, lipid-anchor

MRAGPGPTVTLALVLAVSWAMELKPTAPPIFTGRPFVVAWDVPTQDCGPRLKVPLDLNAF
DVQASPNEGFVNQNITIFYRDRLGLYPRFDSAGRSVHGGVPQNVSLWAHRKMLQKRVEH
YIRTQESAGLAVIDWEDWRPVWVRNWQDKDVYRRLSRQLVASRHPDWPPDRIVKQAQY
EFEFAAQQFMLETLRYVKAVRPRHLWGFYLFPDCYNHDYVQNWESYTGRCPDVEVARN
DQLAWLWAESTALFPSVYLDETLASSRHGRNFVSFRVQEALRVARTHHANHALPVYVFTR
PTYSRRLTGLSEMDLISTIGESAALGAAGVILWGDAGYTTSTETCQYLKDYLTRLLVPYVV
NVSWATQYCSRAQCHGHGRCVRRNPSASTFLHLSTNSFRLVPGHAPGEPQLRPVGELSWA
DIDHLQTHFRCQCYLGWSGEQCQWDHRQAAGGASEAWAGSHLTSLLALAALAFTWTL

>Q53HI1 multipass membrane protein
MLPSTSVNSLVQGNGVLNSRDAARHTAGAKRYKYLRRLFRFRQMDFEFAAWQMLYLFTS
PQRVYRNFHYRKQTKDQWARDDPAFLVLLSIWLCVSTIGFGFVLDMGFFETIKLLLWVVLI
DCVGVGLLIATLMWFISNKYLVKRQSRDYDVEWGYAFDVHLNAFYPLLVILHFIQLFFINH
VILTDTFIGYLVGNTLWLVAVGYYIYVTFLGYSALPFLKNTVILLYPFAPLILLYGLSLALGW
NFTHTLCSFYKYRVK

>Q8N7J2 peripheral membrane protein
METSRSRGGGGAVSERGGAGASVGVCRRKAEAGAGTGTLAADMDLHCDCAAETPAAEP
PSGKINKAAFKLFKKRKSGGTMPSIFGVKNKGDGKSSGPTGLVRSRTHDGLAEVLVLESG
RKEEPRGGGDSGGGGGGRPNPGPPRAAGPGGGSLASSSVAKSHSFFSLLKKNGRSENGKG
EPVDASKAGGKQKRGLRGLFSGMRWHRKDKRAKAEAAEGRAPGGGLILPGSLTASLECV
KEETPRAAREPEEPSQDAPRDPAGEPAGGEEVPAPADRAPARSCREAEGLAHPGDTGARG
EDAAGHRRAEPGPGEVRTAEDASRTGAVPVKTVPLVDSEGGSGRAPAAPDPASVDPPSDPS
ADRICLMFSDVTSLKSFDSLTGCGDIIADQEEEAGPSCDKHVPGPGKPALSKKNPGVVAYQ
GGGEEMASPDEVDDTYLQEFWDMLSQTEEQGPEPQEGAAKVAAALETKVVPETPKDTR
CVEAAKDASSVKRRRLNRIPIEPHPKEEPKHPEKEQQEGVPNSDEGYWDSTTPGPEEDSSS
SGKKAGIPRDSYSGDALYDLYADPDGSPATLPGGKDNEETSSLSRLKPVSPGTITCPLRTPG
SLLKDSKIPISIKHLTNLPSSHPVVHQQPSRSEMPRTKIPVSKVLVRRVSNRGLAGTTIRATA
CHDSAKKL

>Q9BY07 multipass membrane protein
MKVKEEKAGVGKLDHTNHRRRFPDQKECPPIHIGLPVPTYPQRKTDQKGHLSGLQKVHW
GLRPDQPQQELTGPGSGASSQDSSMDLISRTRSPAAEQLQDILGEEDEAPNPTLFTEMDTL
QHDGDQMEWKESARWIKFEEKVEEGGERWSKPHVSTLSLHSLFELRTCLQTGTVLLDLD
SGSLPQIIDDVIEKQIEDGLLRPELRERVSYVLLRRHRHQTKKPIHRSLADIGKSVSTTNRSP
ARSPGAGPSLHHSTEDLRMRQSANYGRLCHAQSRSMNDISLTPNTDQRKNKFMKKIPKDS
EASNVLVGEVDFLDQPFIAFVRLIQSAMLGGVTEVPVPTRFLFILLGPSGRAKSYNEIGRAI
ATLMVDDLFSDVAYKARNREDLIAGIDEFLDEVIVLPPGEWDPNIRIEPPKKVPSADKRKS
VFSLAELGQMNGSVGGGGGAPGGGNGGGGGGGSGGGAGSGGAGGTSSGDDGEMPAMH
EIGEELIWTGRFFGGLCLDIKRKLPWFPSDFYDGFHIQSISAILFIYLGCITNAITFGGLLGDA
TDNYQGVMESFLGTAMAGSLFCLFSGQPLIILSSTGPILIFEKLLFDFSKGNGLDYMEFRLW
IGLHSAVQCLILVATDASFIIKYITRFTEEGFSTLISFIFIYDAIKKMIGAFKYYPINMDFKPNFI
TTYKCECVAPDTVNTTVFNASAPLAPDTNASLYNLLNLTALDWSLLSKKECLSYGGRLLG
NSCKFIPDLALMSFILFFGTYSMTLTLKKFKFSRYFPTKVRALVADFSIVFSILMFCGIDACF

GLETPKLHVPSVIKPTRPDRGWFVAPFGKNPWWVYPASILPALLVTILIFMDQQITAVIVNR
KENKLKKAAGYHLDLFWVGILMALCSFMGLPWYVAATVISIAHIDSLKMETETSAPGEQP
QFLGVREQRVTGIIVFILTGISVFLAPILKCIPLPVLYGVFLYMGVASLNGIQMGTGGSEFKIQ
KKLTPFWERCKLFLMPAKHQPDHAFLRHVPLRRIHLFTLVQILCLAVLWILKSTVAAIIFPV
MILGLIIVRRLLDFIFSQHDLAWIDNILPEKEKKETDKKRKRKKGAHEDCDEEPQFPPPSVI
KIPMESVQSDPQNGIHCIARKRSSSWSYSL

>Q6Q4G3 single-pass type II membrane protein
MGPPSSSGFYVSRAVALLLAGLVAALLLALAVLAALYGHCERVPPSELPGLRDLEAESSPPL
RQKPTPTPKPSSARELAVTTTPSNWRPPGPWDQLRLPPWLVPLHYDLELWPQLRPDELPA
GSLPFTGRVNITVRCTVATSRLLLHSLFQDCERAEVRGPLSPGTGNATVGRVPVDDVWFAL
DTEYMVLELSEPLKPGSSYELQLSFSGLVKEDLREGLFLNVYTDQGERRALLASQLEPTFA
RYVFPCFDEPALKATFNITMIHHPSYVALSNMPKLGQSEKEDVNGSKWTVTTFSTTPHMPT
YLVAFVICDYDHVNRTERGKEIRIWARKDAIANGSADFALNITGPIFSFLEDLFNISYSLPKT
DIIALPSFDNHAMENWGLMIFDESGLLLEPKDQLTEKKTLISYVVSHEIGHQWFGNLVTM
NWWNNIWLNEGFASYFEFEVINYFNPKLPRNEIFFSNILHNILREDHALVTRAVAMKVENF
KTSEIQELFDIFTYSKGASMARMLSCFLNEHLFVSALKSYLKTFSYSNAEQDDLWRHFQM
AIDDQSTVILPATIKNIMDSWTHQSGFPVITLNVSTGVMKQEPFYLENIKNRTLLTSNDTWI
VPILWIKNGTTQPLVWLDQSSKVFPEMQVSDSDHDWVILNLNMTGYYRVNYDKLGWKK
LNQQLEKDPKAIPVIHRLQLIDDAFSLSKNNYIEIETALELTKYLAEEDEIIVWHTVLVNLVT
RDLVSEVNIYDIYSLLKRYLLKRLNLIWNIYSTIIRENVLALQDDYLALISLEKLFVTACWL
GLEDCLQLSKELFAKWVDHPENEIPYPIKDVVLCYGIALGSDKEWDILLNTYTNTTNKEE
KIQLAYAMSCSKDPWILNRYMEYAISTSPFTSNETNIIEVVASSEVGRYVAKDFLVNNWQA
VSKRYGTQSLINLIYTIGRTVTTDLQIVELQQFFSNMLEEHQRIRVHANLQTIKNENLKNKK
LSARIAAWLRRNT

>O95754 single-pass type I membrane protein
MPASAARPRPGPGQPTASPFPLLLLAVLSGPVSGRVPRSVPRTSLPISEADSCLTRFAVPHTY
NYSVLLVDPASHTLYVGARDTIFALSLPFSGERPRRIDWMVPEAHRQNCRKKGKKEDECH
NFVQILAIANASHLLTCGTFAFDPKCGVIDVSRFQQVERLESGRGKCPFEPAQRSAAVMAG
GVLYAATVKNYLGTEPIITRAVGRAEDWIRTDTLPSWLNAPAFVAAVALSPAEWGDEDGD
DEIYFFFTETSRAFDSYERIKVPRVARVCAGDLGGRKTLQQRWTTFLKADLLCPGPEHGRA
SSVLQDVAVLRPELGAGTPIFYGIFSSQWEGATISAVCAFRPQDIRTVLNGPFRELKHDCNR
GLPVVDNDVPQPRPGECITNNMKLRHFGSSLSLPDRVLTFIRDHPLMDRPVFPADGHPLLV
TTDTAYLRVVAHRVTSLSGKEYDVLYLGTEDGHLHRAVRIGAQLSVLEDLALFPEPQPVEN
MKLYHSWLLVGSRTEVTQVNTTNCGRLQSCSECILAQDPVCAWSFRLDECVAHAGEHRG
LVQDIESADVSSLCPKEPGERPVVFEVPVATAAHVVLPCSPSSAWASCVWHQPSGVTALTP
RRDGLEVVVTPGAMGAYACECQEGGAAHVVAAYSLVWGSQRDAPSRAHTVGAGLAGFF
LGILAASLTLILIGRRQQRRRQRELLARDKVGLDLGAPPSGTTSYSQDPPSPSPEDERLPLA
LAKRGSGFGGFSPPFLLDPCPSPAHIRLTGAPLATCDETSI

>P08235 peripheral membrane protein
METKGYHSLPEGLDMERRWGQVSQAVERSSLGPTERTDENNYMEIVNVSCVSGAIPNNST
QGSSKEKQELLPCLQQDNNRPGILTSDIKTELESKELSATVAESMGLYMDSVRDADYSYEQ

QNQQGSMSPAKIYQNVEQLVKFYKGNGHRPSTLSCVNTPLRSFMSDSGSSVNGGVMRAI
VKSPIMCHEKSPSVCSPLNMTSSVCSPAGINSVSSTTASFGSFPVHSPITQGTPLTCSPNAEN
RGSRSHSPAHASNVGSPLSSPLSSMKSSISSPPSHCSVKSPVSSPNNVTLRSSVSSPANINNS
RCSVSSPSNTNNRSTLSSPAASTVGSICSPVNNAFSYTASGTSAGSSTLRDVVPSPDTQEKG
AQEVPFPKTEEVESAISNGVTGQLNIVQYIKPEPDGAFSSSCLGGNSKINSDSSFSVPIKQES
TKHSCSGTSFKGNPTVNPFPFMDGSYFSFMDDKDYYSLSGILGPPVPGFDGNCEGSGFPV
GIKQEPDDGSYYPEASIPSSAIVGVNSGGQSFHYRIGAQGTISLSRSARDQSFQHLSSFPPVN
TLVESWKSHGDLSSRRSDGYPVLEYIPENVSSSTLRSVSTGSSRPSKICLVCGDEASGCHYG
VVTCGSCKVFFKRAVEGQHNYLCAGRNDCIIDKIRRKNCPACRLQKCLQAGMNLGARKS
KKLGKLKGIHEEQPQQQQPPPPPPPPQSPEEGTTYIAPAKEPSVNTALVPQLSTISRALTPSP
VMVLENIEPEIVYAGYDSSKPDTAENLLSTLNRLAGKQMIQVVKWAKVLPGFKNLPLEDQ
ITLIQYSWMCLSSFALSWRSYKHTNSQFLYFAPDLVFNEEKMHQSAMYELCQGMHQISLQ
FVRLQLTFEEYTIMKVLLLLSTIPKDGLKSQAAFEEMRTNYIKELRKMVTKCPNNSGQSW
QRFYQLTKLLDSMHDLVSDLLEFCFYTFRESHALKVEFPAMLVEIISDQLPKVESGNAKPL
YFHRK

>Q8N349 multipass membrane protein
MEKWNHTSNDFILLGLLPPNQTGIFLLCLIILIFFLASVGNSAMIHLIHVDPRLHTPMYFLLS
QLSLMDLMYISTTVPKMAYNFLSGQKGISFLGCGVQSFFFLTMACSEGLLLTSMAYDRYL
AICHSLYYPIRMSKMMCVKMIGGSWTLGSINSLAHTVFALHIPYCRSRAIDHFFCDVPAML
LLACTDTWVYEYMVFVSTSLFLLFPFIGITSSCGRVLFAVYHMHSKEGRKKAFTTISTHLTV
VIFYYAPFVYTYLRPRNLRSPAEDKILAVFYTILTPMLNPIIYSLRNKEVLGAMRRVFGIFSF
LKE

>Q99500 multipass membrane protein
MATALPPRLQPVRGNETLREHYQYVGKLAGRLKEASEGSTLTTVLFLVICSFIVLENLMVL
IAIWKNNKFHNRMYFFIGNLALCDLLAGIAYKVNILMSGKKTFSLSPTVWFLREGSMFVA
LGASTCSLLAIAIERHLTMIKMRPYDANKRHRVFLLIGMCWLIAFTLGALPILGWNCLHNL
PDCSTILPLYSKKYIAFCISIFTAILVTIVILYARIYFLVKSSSRKVANHNNSERSMALLRTVVI
VVSVFIACWSPLFILFLIDVACRVQACPILFKAQWFIVLAVLNSAMNPVIYTLASKEMRRAF
FRLVCNCLVRGRGARASPIQPALDPSRSKSSSSNNSSHSPKVKEDLPHTAPSSCIMDKNAAL
QNGIFCN

>P54760 single-pass type I membrane protein
MELRVLLCWASLAAALEETLLNTKLETADLKWVTFPQVDGQWEELSGLDEEQHSVRTYE
VCDVQRAPGQAHWLRTGWVPRRGAVHVYATLRFTMLECLSLPRAGRSCKETFTVFYYES
DADTATALTPAWMENPYIKVDTVAAEHLTRKRPGAEATGKVNVKTLRLGPLSKAGFYLAF
QDQGACMALLSLHLFYKKCAQLTVNLTRFPETVPRELVVPVAGSCVVDAVPAPGPSPSLY
CREDGQWAEQPVTGCSCAPGFEAAEGNTKCRACAQGTFKPLSGEGSCQPCPANSHSNTIG
SAVCQCRVGYFRARTDPRGAPCTTPPSAPRSVVSRLNGSSLHLEWSAPLESGGREDLTYAL
RCRECRPGGSCAPCGGDLTFDPGPRDLVEPWVVVRGLRPDFTYTFEVTALNGVSSLATGP
VPFEPVNVTTDREVPPAVSDIRVTRSSPSSLSLAWAVPRAPSGAVLDYEVKYHEKGAEGPSS
VRFLKTSENRAELRGLKRGASYLVQVRARSEAGYGPFGQEHHSQTQLDESEGWREQLAL
IAGTAVVGVVLVLVVIVVAVLCLRKQSNGREAEYSDKHGQYLIGHGTKVYIDPFTYEDPNE

AVREFAKEIDVSYVKIEEVIGAGEFGEVCRGRLKAPGKKESCVAIKTLKGGYTERQRREFL
SEASIMGQFEHPNIIRLEGVVTNSMPVMILTEFMENGALDSFLRLNDGQFTVIQLVGMLRG
IASGMRYLAEMSYVHRDLAARNILVNSNLVCKVSDFGLSRFLEENSSDPTYTSSLGGKIPIR
WTAPEAIAFRKFTSASDAWSYGIVMWEVMSFGERPYWDMSNQDVINAIEQDYRLPPPPD
CPTSLHQLMLDCWQKDRNARPRFPQVVSALDKMIRNPASLKIVARENGGASHPLLDQRQ
PHYSAFGSVGEWLRAIKMGRYEESFAAAGFGSFELVSQISAEDLLRIGVTLAGHQKKILAS
VQHMKSQAKPGTPGGTGGPAPQY

>Q9HC24 multipass membrane protein
MADPDPRYPRSSIEDDFNYGSSVASATVHIRMAFLRKVYSILSLQVLLTTVTSTVFLYFESV
RTFVHESPALILLFALGSLGLIFALILNRHKYPLNLYLLFGFTLLEALTVAVVVTFYDVYIILQ
AFILTTTVFFGLTVYTLQSKKDFSKFGAGLFALLWILCLSGFLKFFFYSEIMELVLAAAGAL
LFCGFIIYDTHSLMHKLSPEEYVLAAISLYLDIINLFLHLLRFLEAVNKK

>Q9Y691 multipass membrane protein
MFIWTSGRTSSSYRHDEKRNIYQKIRDHDLLDKRKTVTALKAGEDRAILLGLAMMVCSIM
MYFLLGITLLRSYMQSVWTEESQCTLLNASITETFNCSFSCGPDCWKLSQYPCLQVYVNL
TSSGEKLLLYHTEETIKINQKCSYIPKCGKNFEESMSLVNVVMENFRKYQHFSCYSDPEGN
QKSVILTKLYSSNVLFHSLFWPTCMMAGGVAIVAMVKLTQYLSLLCERIQRINR

>Q8WXF7 multipass membrane protein
MAKNRRDRNSWGGFSEKTYEWSSEEEEPVKKAGPVQVLIVKDDHSFELDETALNRILLSE
AVRDKEVVAVSVAGAFRKGKSFLMDFMLRYMYNQESVDWVGDYNEPLTGFSWRGGSER
ETTGIQIWSEIFLINKPDGKKVAVLLMDTQGTFDSQSTLRDSATVFALSTMISSIQVYNLSQ
NVQEDDLQHLQLFTEYGRLAMEETFLKPFQSLIFLVRDWSFPYEFSYGADGGAKFLEKRL
KVSGNQHEELQNVRKHIHSCFTNISCFLLPHPGLKVATNPNFDGKLKEIDDEFIKNLKILIP
WLLSPESLDIKEINGNKITCRGLVEYFKAYIKIYQGEELPHPKSMLQATAEANNLAAVATAK
DTYNKKMEEICGGDKPFLAPNDLQTKHLQLKEESVKLFRGVKKMGGEEFSRRYLQQLES
EIDELYIQYIKHNDSKNIFHAARTPATLFVVIFITYVIAGVTGFIGLDIIASLCNMIMGLTLITL
CTWAYIRYSGEYRELGAVIDQVAAALWDQGSTNEALYKLYSAAATHRHLYHQAFPTPKSE
STEQSEKKKM

>P29317 single-pass type I membrane protein
MELQAARACFALLWGCALAAAAAAQGKEVVLLDFAAAGGELGWLTHPYGKGWDLMQ
NIMNDMPIYMYSVCNVMSGDQDNWLRTNWVYRGEAERIFIELKFTVRDCNSFPGGASSC
KETFNLYYAESDLDYGTNFQKRLFTKIDTIAPDEITVSSDFEARHVKLNVEERSVGPLTRKG
FYLAFQDIGACVALLSVRVYYKKCPELLQGLAHFPETIAGSDAPSLATVAGTCVDHAVVPP
GGEEPRMHCAVDGEWLVPIGQCLCQAGYEKVEDACQACSPGFFKFEASESPCLECPEHTL
PSPEGATSCECEEGFFRAPQDPASMPCTRPPSAPHYLTAVGMGAKVELRWTPPQDSGGRED
IVYSVTCEQCWPESGECGPCEASVRYSEPPHGLTRTSVTVSDLEPHMNYTFTVEARNGVS
GLVTSRSFRTASVSINQTEPPKVRLEGRSTTSLSVSWSIPPPQQSRVWKYEVTYRKKGDSNS
YNVRRTEGFSVTLDDLAPDTTYLVQVQALTQEGQGAGSKVHEFQTLSPEGSGNLAVIGGV
AVGVVLLLVLAGVGFFIHRRRKNQRARQSPEDVYFSKSEQLKPLKTYVDPHTYEDPNQAV
LKFTTEIHPSCVTRQKVIGAGEFGEVYKGMLKTSSGKKEVPVAIKTLKAGYTEKQRVDFL

GEAGIMGQFSHHNIIRLEGVISKYKPMMIITEYMENGALDKFLREKDGEFSVLQLVGMLR
GIAAGMKYLANMNYVHRDLAARNILVNSNLVCKVSDFGLSRVLEDDPEATYTTSGGKIPI
RWTAPEAISYRKFTSASDVWSFGIVMWEVMTYGERPYWELSNHEVMKAINDGFRLPTPM
DCPSAIYQLMMQCWQQERARRPKFADIVSILDKLIRAPDSLKTLADFDPRVSIRLPSTSGSE
GVPFRTVSEWLESIKMQQYTEHFMAAGYTAIEKVVQMTNDDIKRIGVRLPGHQKRIAYSL
LGLKDQVNTVGIPI

>A6NF89 multipass membrane protein
MKNKSMEIEFILLGLTDDPQLQIVIFLFLFLNYTLSLMGNLIIIILTLLDPRLKTPMYFFLRNF
SFLEVIFTTVCIPRFLITIVTRDKTISYNNCATQLFFILLPGVTEFYLLAAMSYDRYVAICKPL
HYPIIMSSKVCYQLVLSSWVTGFLIIFPPLVMGLKLDFCASKTIDHFMCETSPILQISCTDTH
VLELMSFTLAVVTLVVTLVLVILSYTCIIKTILKFSSAQQRNKAFSTCTSHMIVVSMTYGSCI
FMYIKPSAKERVTVSKGVALLYTSIAPLLNPFIYTLRNQQVKEVFWDVLQKNLCFSKRPF

>P15421 single-pass type I membrane protein
MYGKIIFVLLLSGIVSISASSTTGVAMHTSTSSSVTKSYISSQTNGITLINWWAMARVIFEVM
LVVVGMIILISYCIR

>Q9NZP5 multipass membrane protein
MDISEGNKTLVTEFVLTGLTDRPWLHVLFFVVFLVVYLITMVGNLGLIVLIWNDPHLHMP
MYLFLGGLAFSDACTSTSITPRMLVNFLDKTAMISLAECITQFYFFASSATTECFLLVMMAY
DRYVAICNPLLYPVMMSNKLSAQLLSISYVIGFLHPLVHVSLLLRLTFCRFNIIHYFYCEILQ
LFKISCNGPSINALMIFIFGAFIQIPTLMTIIISYTRVLFDILKKKSEKGRSKAFSTCGAHLLSV
SLYYGTLIFMYVRPASGLAEDQDKVYSLFYTIIIPLLNPFIYSLRNKKVMHALRRVIRK

>Q9UQQ1 single-pass type II membrane protein
MQWTKVLGLGLGAAALLGLGIILGHFAIPKKANSLAPQDLDLEILETVMGQLDAHRIREN
LRELSREPHLASSPRDEDLVQLLLQRWKDPESGLDSAEASTYEVLLSFPSQEQPNVVDIVG
PTGGIIHSCHRTEENVTGEQGGPDVVQPYAAYAPSGTPQGLLVYANRGAEEDFKELQTQGI
KLEGTIALTRYGGVGRGAKAVNAAKHGVAGVLVYTDPADINDGLSSPDETFPNSWYLPPS
GVERGSYYEYFGDPLTPYLPAVPSSFRVDLANVSGFPPIPTQPIGFQDARDLLCNLNGTLAP
ATWQGALGCHYRLGPGFRPDGDFPADSQVNVSVYNRLELRNSSNVLGIIRGAVEPDRYVL
YGNHRDSWVHGAVDPSSGTAVLLELSRVLGTLLKKGTWRPRRSIVFASWGAEEFGLIGST
EFTEEFFNKLQERTVAYINVDISVFANATLRVQGTPPVQSVVFSATKEIRSPGPGDLSIYDN
WIRYFNRSSPVYGLVPSLGSLGAGSDYAPFVHFLGISSMDIAYTYDRSKTSARIYPTYHTAF
DTFDYVDKFLDPGFSSHQAVARTAGSVILRLSDSFFLPLKVSDYSETLRSFLQAAQQDLGA
LLEQHSISLGPLVTAVEKFEAEAAALGQRISTLQKGSPDPLQVRMLNDQLMLLERTFLNPR
AFPEERYYSHVLWAPRTGSVVTFPGLSNACSRARDTASGSEAWAEVQRQLSIVVTALEGA
AATLRPVADL

>P04629 single-pass type I membrane protein
MLRGGRRGQLGWHSWAAGPGSLLAWLILASAGAAPCPDACCPHGSSGLRCTRDGALDS
LHHLPGAENLTELYIENQQHLQHLELRDLRGLGELRNLTIVKSGLRFVAPDAFHFTPRLSRL
NLSFNALESLSWKTVQGLSLQELVLSGNPLHCSCALRWLQRWEEEGLGGVPEQKLQCHG

QGPLAHMPNASCGVPTLKVQVPNASVDVGDDVLLRCQVEGRGLEQAGWILTELEQSATV
MKSGGLPSLGLTLANVTSDLNRKNVTCWAENDVGRAEVSVQVNVSFPASVQLHTAVEM
HHWCIPFSVDGQPAPSLRWLFNGSVLNETSFIFTEFLEPAANETVRHGCLRLNQPTHVNNG
NYTLLAANPFGQASASIMAAFMDNPFEFNPEDPIPVSFSPVDTNSTSGDPVEKKDETPFGV
SVAVGLAVFACLFLSTLLLVLNKCGRRNKFGINRPAVLAPEDGLAMSLHFMTLGGSSLSPT
EGKGSGLQGHIIENPQYFSDACVHHIKRRDIVLKWELGEGAFGKVFLAECHNLLPEQDKM
LVAVKALKEASESARQDFQREAELLTMLQHQHIVRFFGVCTEGRPLLMVFEYMRHGDLN
RFLRSHGPDAKLLAGGEDVAPGPLGLGQLLAVASQVAAGMVYLAGLHFVHRDLATRNCL
VGQGLVVKIGDFGMSRDIYSTDYYRVGGRTMLPIRWMPPESILYRKFTTESDVWSFGVVL
WEIFTYGKQPWYQLSNTEAIDCITQGRELERPRACPPEVYAIMRGCWQREPQQRHSIKDV
HARLQALAQAPPVYLDVLG

>P31994 single-pass type I membrane protein
MGILSFLPVLATESDWADCKSPQPWGHMLLWTAVLFLAPVAGTPAAPPKAVLKLEPQWIN
VLQEDSVTLTCRGTHSPESDSIQWFHNGNLIPTHTQPSYRFKANNNDSGEYTCQTGQTSLS
DPVHLTVLSEWLVLQTPHLEFQEGETIVLRCHSWKDKPLVKVTFFQNGKSKKFSRSDPNFS
IPQANHSHSGDYHCTGNIGYTLYSSKPVTITVQAPSSSPMGIIVAVVTGIAVAAIVAAVVALI
YCRKKRISALPGYPECREMGETLPEKPANPTNPDEADKVGAENTITYSLLMHPDALEEPD
DQNRI

>P22083 single-pass type II membrane protein
MRRLWGAARKPSGAGWEKEWAEAPQEAPGAWSGRLGPGRSGRKGRAVPGWASWPAHL
ALAARPARHLGGAGQGPRPLHSGTAPFHSRASGERQRRLEPQLQHESRCRSSTPADAWRA
EAALPVRAMGAPWGSPTAAAGGRRGWRRGRGLPWTVCVLAAAGLTCTALITYACWGQL
PPLPWASPTPSRPVGVLLWWEPFGGRDSAPRPPPDCRLRFNISGCRLLTDRASYGEAQAVL
FHHRDLVKGPPDWPPPWGIQAHTAEEVDLRVLDYEEAAAAAEALATSSPRPPGQRWVW
MNFESPSHSPGLRSLASNLFNWTLSYRADSDVFVPYGYLYPRSHPGDPPSGLAPPLSRKQG
LVAWVVSHWDERQARVRYYHQLSQHVTVDVFGRGGPGQPVPEIGLLHTVARYKFYLAFE
NSQHLDYITEKLWRNALLAGAVPVVLGPDRANYERFVPRGAFIHVDDFPSASSLASYLLF
LDRNPAVYRRYFHWRRSYAVHITSFWDEPWCRVCQAVQRAGDRPKSIRNLASWFER

>O60353 multipass membrane protein
MEMFTFLLTCIFLPLLRGHSLFTCEPITVPRCMKMAYNMTFFPNLMGHYDQSIAAVEMEHF
LPLANLECSPNIETFLCKAFVPTCIEQIHVVPPCRKLCEKVYSDCKKLIDTFGIRWPEELECD
RLQYCDETVPVTFDPHTEFLGPQKKTEQVQRDIGFWCPRHLKTSGGQGYKFLGIDQCAPP
CPNMYFKSDELEFAKSFIGTVSIFCLCATLFTFLTFLIDVRRFRYPERPIIYYSVCYSIVSLMY
FIGFLLGDSTACNKADEKLELGDTVVLGSQNKACTVLFMLLYFFTMAGTVWWVILTITWF
LAAGRKWSCEAIEQKAVWFHAVAWGTPGFLTVMLLAMNKVEGDNISGVCFVGLYDLDA
SRYFVLLPLCLCVFVGLSLLLAGIISLNHVRQVIQHDGRNQEKLKKFMIRIGVFSGLYLVPL
VTLLGCYVYEQVNRITWEITWVSDHCRQYHIPCPYQAKAKARPELALFMIKYLMTLIVGI
SAVFWVGSKKTCTEWAGFFKRNRKRDPISESRRVLQESCEFFLKHNSKVKHKKKHYKPSS
HKLKVISKSMGTSTGATANHGTSAVAITSHDYLGQETLTEIQTSPETSMREVKADGASTPR
LREQDCGEPASPAASISRLSGEQVDGKGQAGSVSESARSEGRISPKSDITDTGLAQSNNLQ
VPSSSEPSSLKGSTSLLVHPVSGVRKEQGGGCHSDT

>O14994 peripheral membrane protein
MNFLRRRLSDSSFMANLPNGYMTDLQRPDSSTSSPASPAMERRHPQPLAASFSSPGSSLFS
SLSSAMKQAPQATSGLMEPPGPSTPIVQRPRILLVIDDAHTDWSKYFHGKKVNGEIEIRVE
QAEFSELNLAAYVTGGCMVDMQVVRNGTKVVSRSFKPDFILVRQHAYSMALGEDYRSLV
IGLQYGGLPAVNSLYSVYNFCSKPWVFSQLIKIFHSLGPEKFPLVEQTFFPNHKPMVTAPHF
PVVVKLGHAHAGMGKIKVENQLDFQDITSVVAMAKTYATTEAFIDSKYDIRIQKIGSNYK
AYMRTSISGNWKANTGSAMLEQVAMTERYRLWVDSCSEMFGGLDICAVKAVHSKDGRD
YIIEVMDSSMPLIGEHVEEDRQLMADLVVSKMSQLPMPGGTAPSPLRPWAPQIKSAKSPG
QAQLGPQLGQPQPRPPPQGGPRQAQSPQPQRSGSPSQQRLSPQGQQPLSPQSGSPQQQRSP
GSPQLSRASSGSSPNQASKPGATLASQPRPPVQGRSTSQQGEESKKPAPPHPHLNKSQSLTN
SLSTSDTSQRGTPSEDEAKAETIRNLRKSFASLFSD

>P30622 peripheral membrane protein
MSMLKPSGLKAPTKILKPGSTALKTPTAVVAPVEKTISSEKASSTPSSETQEEFVDDFRVGE
RVWVNGNKPGFIQFLGETQFAPGQWAGIVLDEPIGKNDGSVAGVRYFQCEPLKGIFTRPSK
LTRKVQAEDEANGLQTTPASRATSPLCTSTASMVSSSPSTPSNIPQKPSQPAAKEPSATPPIS
NLTKTASESISNLSEAGSIKKGERELKIGDRVLVGGTKAGVVRFLGETDFAKGEWCGVELD
EPLGKNDGAVAGTRYFQCQPKYGLFAPVHKVTKIGFPSTTPAKAKANAVRRVMATTSASL
KRSPSASSLSSMSSVASSVSSRPSRTGLLTETSSRYARKISGTTALQEALKEKQQHIEQLLAE
RDLERAEVAKATSHVGEIEQELALARDGHDQHVLELEAKMDQLRTMVEAADREKVELL
NQLEEEKRKVEDLQFRVEEESITKGDLEQKSQISEDPENTQTKLEHARIKELEQSLLFEKTK
ADKLQRELEDTRVATVSEKSRIMELEKDLALRVQEVAELRRRLESNKPAGDVDMSLSLLQ
EISSLQEKLEVTRTDHQREITSLKEHFGAREETHQKEIKALYTATEKLSKENESLKSKLEHA
NKENSDVIALWKSKLETAIASHQQAMEELKVSFSKGLGTETAEFAELKTQIEKMRLDYQH
EIENLQNQQDSERAAHAKEMEALRAKLMKVIKEKENSLEAIRSKLDKAEDQHLVEMEDT
LNKLQEAEIKVKELEVLQAKCNEQTKVIDNFTSQLKATEEKLLDLDALRKASSEGKSEMK
KLRQQLEAAEKQIKHLEIEKNAESSKASSITRELQGRELKLTNLQENLSEVSQVKETLEKE
LQILKEKFAEASEEAVSVQRSMQETVNKLHQKEEQFNMLSSDLEKLRENLADMEAKFRE
KDEREEQLIKAKEKLENDIAEIMKMSGDNSSQLTKMNDELRLKERDVEELQLKLTKANEN
ASFLQKSIEDMTVKAEQSQQEAAKKHEEEKKELERKLSDLEKKMETSHNQCQELKARYE
RATSETKTKHEEILQNLQKTLLDTEDKLKGAREENSGLLQELEELRKQADKAKAAQTAED
AMQIMEQMTKEKTETLASLEDTKQTNAKLQNELDTLKENNLKNVEELNKSKELLTVENQ
KMEEFRKEIETLKQAAAQKSQQLSALQEENVKLAEELGRSRDEVTSHQKLEEERSVLNN
QLLEMKKRESKFIKDADEEKASLQKSISITSALLTEKDAELEKLRNEVTVLRGENASAKSL
HSVVQTLESDKVKLELKVKNLELQLKENKRQLSSSSGNTDTQADEDERAQESQIDFLNSV
IVDLQRKNQDLKMKVEMMSEAALNGNGDDLNNYDSDDQEKQSKKKPRLFCDICDCFDL
HDTEDCPTQAQMSEDPPHSTHHGSRGEERPYCEICEMFGHWATNCNDDETF

>Q5SRI9 single-pass type II membrane protein
MAKFRRRTCIILALFILFIFSLMMGLKMLRPNTATFGAPFGLDLLPELHQRTIHLGKNFDFQ
KSDRINSETNTKNLKSVEITMKPSKASELNLDELPPLNNYLHVFYYSWYGNPQFDGKYIH
WNHPVLEHWDPRIAKNYPQGRHNPPDDIGSSFYPELGSYSSRDPSVIETHMRQMRSASIG
VLALSWYPPDVNDENGEPTDNLVPTILDKAHKYNLKVTFHIEPYSNRDDQNMYKNVKYII

DKYGNHPAFYRYKTKTGNALPMFYVYDSYITKPEKWANLLTTSGSRSIRNSPYDGLFIALL
VEEKHKYDILQSGFDGIYTYFATNGFTYGSSHQNWASLKLFCDKYNLIFIPSVGPGYIDTSI
RPWNTQNTRNRINGKYYEIGLSAALQTRPSLISITSFNEWHEGTQIEKAVPKRTSNTVYLD
YRPHKPGLYLELTRKWSEKYSKERATYALDRQLPVS

>Q9NUT2 multipass membrane protein
MLVHLFRVGIRGGPFPGRLLPPLRFQTFSAVRNTWRNGKTGQLHKAEGEYSDGYRSSSLL
RAVAHLRSQLWAHLPRAPLAPRWSPSAWCWVGGALLGPMVLSKHPHLCLVALCEAEEAP
PASSTPHVVGSRFNWKLFWQFLHPHLLVLGVAVVLALGAALVNVQIPLLLGQLVEVVAKY
TRDHVGSFMTESQNLSTHLLILYGVQGLLTFGYLVLLSHVGERMAVDMRRALFSSLLRQD
ITFFDANKTGQLVSRLTTDVQEFKSSFKLVISQGLRSCTQVAGCLVSLSMLSTRLTLLLMVA
TPALMGVGTLMGSGLRKLSRQCQEQIARAMGVADEALGNVRTVRAFAMEQREERYGA
ELEACRCRAEELGRGIALFQGLSNIAFNCMVLGTLFIGGSLVAGQQLTGGDLMSFLVASQT
VQRSMANLSVLFGQVVRGLSAGARVFEYMALNPCIPLSGGCCVPKEQLRGSVTFQNVCF
SYPCRPGFEVLKDFTLTLPPGKIVALVGQSGGGKTTVASLLERFYDPTAGVVMLDGRDLRT
LDPSWLRGQVVGFISQEPVLFGTTIMENIRFGKLEASDEEVYTAAREANAHEFITSFPEGY
NTVVGERGTTLSGGQKQRLAIARALIKQPTVLILDEATSALDAESERVVQEALDRASAGRT
VLVIAHRLSTVRGAHCIVVMADGRVWEAGTHEELLKKGGLYAELIRRQALDAPRTAAPPP
KKPEGPRSHQHKS

>Q92633 multipass membrane protein
MAAISTSIPVISQPQFTAMNEPQCFYNESIAFFYNRSGKHLATEWNTVSKLVMGLGITVCIFI
MLANLLVMVAIYVNRRFHFPIYYLMANLAAADFFAGLAYFYLMFNTGPNTRRLTVSTWL
LRQGLIDTSLTASVANLLAIAIERHITVFRMQLHTRMSNRRVVVVIVVIWTMAIVMGAIPSV
GWNCICDIENCSNMAPLYSDSYLVFWAIFNLVTFVVMVVLYAHIFGYVRQRTMRMSRHSS
GPRRNRDTMMSLLKTVVIVLGAFIICWTPGLVLLLLDVCCPQCDVLAYEKFFLLLAEFNSA
MNPIIYSYRDKEMSATFRQILCCQRSENPTGPTEGSDRSASSLNHTILAGVHSNDHSVV

>Q9NX47 multipass membrane protein
MPDQALQQMLDRSCWVCFATDEDDRTAEWVRPCRCRGSTKWVHQACLQRWVDEKQRG
NSTARVACPQCNAEYLIVFPKLGPVVYVLDLADRLISKACPFAAAGIMVGSIYWTAVTYGA
VTVMQVVGHKEGLDVMERADPLFLLIGLPTIPVMLILGKMIRWEDYVLRLWRKYSNKLQ
ILNSIFPGIGCPVPRIPAEANPLADHVSATRILCGALVFPTIATIVGKLMFSSVNSNLQRTILG
GIAFVAIKGAFKVYFKQQQYLRQAHRKILNYPEQEEA

>P37173 single-pass type I membrane protein
MGRGLLRGLWPLHIVLWTRIASTIPPHVQKSVNNDMIVTDNNGAVKFPQLCKFCDVRFST
CDNQKSCMSNCSITSICEKPQEVCVAVWRKNDENITLETVCHDPKLPYHDFILEDAASPKC
IMKEKKKPGETFFMCSCSSDECNDNIIFSEEYNTSNPDLLLVIFQVTGISLLPPLGVAISVIIIF
YCYRVNRQQKLSSTWETGKTRKLMEFSEHCAIILEDDRSDISSTCANNINHNTELLPIELDT
LVGKGRFAEVYKAKLKQNTSEQFETVAVKIFPYEEYASWKTEKDIFSDINLKHENILQFLTA
EERKTELGKQYWLITAFHAKGNLQEYLTRHVISWEDLRKLGSSLARGIAHLHSDHTPCGR
PKMPIVHRDLKSSNILVKNDLTCCLCDFGLSLRLDPTLSVDDLANSGQVGTARYMAPEVL
ESRMNLENVESFKQTDVYSMALVLWEMTSRCNAVGEVKDYEPPFGSKVREHPCVESMK

DNVLRDRGRPEIPSFWLNHQGIQMVCETLTECWDHDPEARLTAQCVAERFSELEHLDRLS
GRSCSEEKIPEDGSLNTTK

>O95782 peripheral membrane protein
MPAVSKGDGMRGLAVFISDIRNCKSKEAEIKRINKELANIRSKFKGDKALDGYSKKKYVC
KLLFIFLLGHDIDFGHMEAVNLLSSNKYTEKQIGYLFISVLVNSNSELIRLINNAIKNDLASR
NPTFMCLALHCIANVGSREMGEAFAADIPRILVAGDSMDSVKQSAALCLLRLYKASPDLVP
MGEWTARVVHLLNDQHMGVVTAAVSLITCLCKKNPDDFKTCVSLAVSRLSRIVSSASTDL
QDYTYYFVPAPWLSVKLLRLLQCYPPPEDAAVKGRLVECLETVLNKAQEPPKSKKVQHS
NAKNAILFETISLIIHYDSEPNLLVRACNQLGQFLQHRETNLRYLALESMCTLASSEFSHEA
VKTHIDTVINALKTERDVSVRQRAADLLYAMCDRSNAKQIVSEMLRYLETADYAIREEIVL
KVAILAEKYAVDYSWYVDTILNLIRIAGDYVSEEVWYRVLQIVTNRDDVQGYAAKTVFEA
LQAPACHENMVKVGGYILGEFGNLIAGDPRSSPPVQFSLLHSKFHLCSVATRALLLSTYIKF
INLFPETKATIQGVLRAGSQLRNADVELQQRAVEYLTLSSVASTDVLATVLEEMPPFPERES
SILAKLKRKKGPGAGSALDDGRRDPSSNDINGGMEPTPSTVSTPSPSADLLGLRAAPPPAA
PPASAGAGNLLVDVFDGPAAQPSLGPTPEEAFLSELEPPAPESPMALLADPAPAADPGPEDI
GPPIPEADELLNKFVCKNNGVLFENQLLQIGVKSEFRQNLGRMYLFYGNKTSVQFQNFSP
TVVHPGDLQTQLAVQTKRVAAQVDGGAQVQQVLNIECLRDFLTPPLLSVRFRYGGAPQAL
TLKLPVTINKFFQPTEMAAQDFFQRWKQLSLPQQEAQKIFKANHPMDAEVTKAKLLGFG
SALLDNVDPNPENFVGAGIIQTKALQVGCLLRLEPNAQAQMYRLTLRTSKEPVSRHLCEL
LAQQF

>Q8NHU3 multipass membrane protein
MDIIETAKLEEHLENQPSDPTNTYARPAEPVEEENKNGNGKPKSLSSGLRKGTKKYPDYIQI
AMPTESRNKFPLEWWKTGIAFIYAVFNLVLTTVMITVVHERVPPKELSPPLPDKFFDYIDRV
KWAFSVSEINGIILVGLWITQWLFLRYKSIVGRRFCFIIGTLYLYRCITMYVTTLPVPGMHFQ
CAPKLNGDSQAKVQRILRLISGGGLSITGSHILCGDFLFSGHTVTLTLTYLFIKEYSPRHFW
WYHLICWLLSAAGIICILVAHEHYTIDVIIAYYITTRLFWWYHSMANEKNLKVSSQTNFLS
RAWWFPIFYFFEKNVQGSIPCCFSWPLSWPPGCFKSSCKKYSRVQKIGEDNEKST

>O43497 multipass membrane protein
MDEEEDGAGAEESGQPRSFMRLNDLSGAGGRPGPGSAEKDPGSADSEAEGLPYPALAPV
VFFYLSQDSRPRSWCLRTVCNPWFERISMLVILLNCVTLGMFRPCEDIACDSQRCRILQAF
DDFIFAFFAVEMVVKMVALGIFGKKCYLGDTWNRLDFFIVIAGMLEYSLDLQNVSFSAVRT
VRVLRPLRAINRVPSMRILVTLLLDTLPMLGNVLLLCFFVFFIFGIVGVQLWAGLLRNRCFL
PENFSLPLSVDLERYYQTENEDESPFICSQPRENGMRSCRSVPTLRGDGGGGPPCGLDYEA
YNSSSNTTCVNWNQYYTNCSAGEHNPFKGAINFDNIGYAWIAIFQVITLEGWVDIMYFVM
DAHSFYNFIYFILLIIVGSFFMINLCLVVIATQFSETKQRESQLMREQRVRFLSNASTLASFSE
PGSCYEELLKYLVYILRKAARRLAQVSRAAGVRVGLLSSPAPLGGQETQPSSSCSRSHRRL
SVHHLVHHHHHHHHHYHLGNGTLRAPRASPEIQDRDANGSRRLMLPPPSTPALSGAPPGG
AESVHSFYHADCHLEPVRCQAPPPRSPSEASGRTVGSGKVYPTVHTSPPPETLKEKALVEV
AASSGPPTLTSLNIPPGPYSSMHKLLETQSTGACQSSCKISSPCLKADSGACGPDSCPYCAR
AGAGEVELADREMPDSDSEAVYEFTQDAQHSDLRDPHSRRQRSLGPDAEPSSVLAFWRLI
CDTFRKIVDSKYFGRGIMIAILVNTLSMGIEYHEQPEELTNALEISNIVFTSLFALEMLLKLL

VYGPFGYIKNPYNIFDGVIVVISVWEIVGQQGGGLSVLRTFRLMRVLKLVRFLPALQRQLV
VLMKTMDNVATFCMLLMLFIFIFSILGMHLFGCKFASERDGDTLPDRKNFDSLLWAIVTVF
QILTQEDWNKVLYNGMASTSSWAALYFIALMTFGNYVLFNLLVAILVEGFQAEEISKREDA
SGQLSCIQLPVDSQGGDANKSESEPDFFSPSLDGDGDRKKCLALVSLGEHPELRKSLLPPLI
IHTAATPMSLPKSTSTGLGEALGPASRRTSSSGSAEPGAAHEMKSPPSARSSPHSPWSAASS
WTSRRSSRNSLGRAPSLKRRSPSGERRSLLSGEGQESQDEEESSEEERASPAGSDHRHRGS
LEREAKSSFDLPDTLQVPGLHRTASGRGSASEHQDCNGKSASGRLARALRPDDPPLDGDD
ADDEGNLSKGERVRAWIRARLPACCLERDSWSAYIFPPQSRFRLLCHRIITHKMFDHVVLV
IIFLNCITIAMERPKIDPHSAERIFLTLSNYIFTAVFLAEMTVKVVALGWCFGEQAYLRSSWN
VLDGLLVLISVIDILVSMVSDSGTKILGMLRVLRLLRTLRPLRVISRAQGLKLVVETLMSSL
KPIGNIVVICCAFFIIFGILGVQLFKGKFFVCQGEDTRNITNKSDCAEASYRWVRHKYNFDN
LGQALMSLFVLASKDGWVDIMYDGLDAVGVDQQPIMNHNPWMLLYFISFLLIVAFFVLN
MFVGVVVENFHKCRQHQEEEEARRREEKRLRRLEKKRRNLMLDDVIASGSSASAASEAQ
CKPYYSDYSRFRLLVHHLCTSHYLDLFITGVIGLNVVTMAMEHYQQPQILDEALKICNYIF
TVIFVLESVFKLVAFGFRRFFQDRWNQLDLAIVLLSIMGITLEEIEVNASLPINPTIIRIMRVL
RIARVLKLLKMAVGMRALLDTVMQALPQVGNLGLLFMLLFFIFAALGVELFGDLECDET
HPCEGLGRHATFRNFGMAFLTLFRVSTGDNWNGIMKDTLRDCDQESTCYNTVISPIYFVSF
VLTAQFVLVNVVIAVLMKHLEESNKEAKEEAELEAELELEMKTLSPQPHSPLGSPFLWPGV
EGPDSPDSPKPGALHPAAHARSASHFSLEHPTDRQLFDTISLLIQGSLEWELKLMDELAGP
GGQPSAFPSAPSLGGSDPQIPLAEMEALSLTSEIVSEPSCSLALTDDSLPDDMHTLLLSALES
NMQPHPTELPGPDLLTVRKSGVSRTHSLPNDSYMCRHGSTAEGPLGHRGWGLPKAQSGS
VLSVHSQPADTSYILQLPKDAPHLLQPHSAPTWGTIPKLPPPGRSPLAQRPLRRQAAIRTDS
LDVQGLGSREDLLAEVSGPSPPLARAYSFWGQSSTQAQQHSRSHSKISKHMTPPAPCPGPE
PNWGKGPPETRSSLELDTELSWISGDLLPPGGQEEPPSPRDLKKCYSVEAQSCQRRPTSWL
DEQRRHSIAVSCLDSGSQPHLGTDPSNLGGQPLGGPGSRPKKKLSPPSITIDPPESQGPRTPP
SPGICLRRRAPSSDSKDPLASGPPDSMAASPSPKKDVLSLSGLSSDPADLDP

>O43526 multipass membrane protein
MVQKSRNGGVYPGPSGEKKLKVGFVGLDPGAPDSTRDGALLIAGSEAPKRGSILSKPRAG
GAGAGKPPKRNAFYRKLQNFLYNVLERPRGWAFIYHAYVFLLVFSCLVLSVFSTIKEYEKS
SEGALYILEIVTIVVFGVEYFVRIWAAGCCCRYRGWRGRLKFARKPFCVIDIMVLIASIAVL
AAGSQGNVFATSALRSLRFLQILRMIRMDRRGGTWKLLGSVVYAHSKELVTAWYIGFLCL
ILASFLVYLAEKGENDHFDTYADALWWGLITLTTIGYGDKYPQTWNGRLLAATFTLIGVSF
FALPAGILGSGFALKVQEQHRQKHFEKRRNPAAGLIQSAWRFYATNLSRTDLHSTWQYYE
RTVTVPMYSSQTQTYGASRLIPPLNQLELLRNLKSKSGLAFRKDPPPEPSPSKGSPCRGPLC
GCCPGRSSQKVSLKDRVFSSPRGVAAKGKGSPQAQTVRRSPSADQSLEDSPSKVPKSWSF
GDRSRARQAFRIKGAASRQNSEEASLPGEDIVDDKSCPCEFVTEDLTPGLKVSIRAVCVMR
FLVSKRKFKESLRPYDVMDVIEQYSAGHLDMLSRIKSLQSRVDQIVGRGPAITDKDRTKGP
AEAELPEDPSMMGRLGKVEKQVLSMEKKLDFLVNIYMQRMGIPPTETEAYFGAKEPEPAP
PYHSPEDSREHVDRHGCIVKIVRSSSSTGQKNFSAPPAAPPVQCPPSTSWQPQSHPRQGHG
TSPVGDHGSLVRIPPPPAHERSLSAYGGGNRASMEFLRQEDTPGCRPPEGNLRDSDTSISIPS
VDHEELERSFSGFSISQSKENLDALNSCYAAVAPCAKVRPYIAEGESDTDSDLCTPCGPPPR
SATGEGPFGDVGWAGPRK

>P26715 single-pass type II membrane protein
MDNQGVIYSDLNLPPNPKRQQRKPKGNKNSILATEQEITYAELNLQKASQDFQGNDKTYH
CKDLPSAPEKLIVGILGIICLILMASVVTIVVIPSTLIQRHNNSSLNTRTQKARHCGHCPEEW
ITYSNSCYYIGKERRTWEESLLACTSKNSSLLSIDNEEEMKFLSIISPSSWIGVFRNSSHHPW
VTMNGLAFKHEIKDSDNAELNCAVLQVNRLKSAQCGSSIIYHCKHKL

>Q86VB7 single-pass type I membrane protein
MSKLRMVLLEDSGSADFRRHFVNLSPFTITVVLLLSACFVTSSLGGTDKELRLVDGENKCS
GRVEVKVQEEWGTVCNNGWSMEAVSVICNQLGCPTAIKAPGWANSSAGSGRIWMDHVS
CRGNESALWDCKHDGWGKHSNCTHQQDAGVTCSDGSNLEMRLTRGGNMCSGRIEIKFQ
GRWGTVCDDNFNIDHASVICRQLECGSAVSFSGSSNFGEGSGPIWFDDLICNGNESALWN
CKHQGWGKHNCDHAEDAGVICSKGADLSLRLVDGVTECSGRLEVRFQGEWGTICDDGW
DSYDAAVACKQLGCPTAVTAIGRVNASKGFGHIWLDSVSCQGHEPAIWQCKHHEWGKHY
CNHNEDAGVTCSDGSDLELRLRGGGSRCAGTVEVEIQRLLGKVCDRGWGLKEADVVCR
QLGCGSALKTSYQVYSKIQATNTWLFLSSCNGNETSLWDCKNWQWGGLTCDHYEEAKIT
CSAHREPRLVGGDIPCSGRVEVKHGDTWGSICDSDFSLEAASVLCRELQCGTVVSILGGA
HFGEGNGQIWAEEFQCEGHESHLSLCPVAPRPEGTCSHSRDVGVVCSRYTEIRLVNGKTPC
EGRVELKTLGAWGSLCNSHWDIEDAHVLCQQLKCGVALSTPGGARFGKGNGQIWRHMF
HCTGTEQHMGDCPVTALGASLCPSEQVASVICSGNQSQTLSSCNSSSLGPTRPTIPEESAVA
CIESGQLRLVNGGGRCAGRVEIYHEGSWGTICDDSWDLSDAHVVCRQLGCGEAINATGSA
HFGEGTGPIWLDEMKCNGKESRIWQCHSHGWGQQNCRHKEDAGVICSEFMSLRLTSEAS
REACAGRLEVFYNGAWGTVGKSSMSETTVGVVCRQLGCADKGKINPASLDKAMSIPMW
VDNVQCPKGPDTLWQCPSSPWEKRLASPSEETWITCDNKIRLQEGPTSCSGRVEIWHGGS
WGTVCDDSWDLDDAQVVCQQLGCGPALKAFKEAEFGQGTGPIWLNEVKCKGNESSLW
DCPARRWGHSECGHKEDAAVNCTDISVQKTPQKATTGRSSRQSSFIAVGILGVVLLAIFVA
LFFLTKKRRQRQRLAVSSRGENLVHQIQYREMNSCLNADDLDLMNSSENSHESADFSAAE
LISVSKFLPISGMEKEAILSHTEKENGNL

>O60513 single-pass type II membrane protein
MGFNLTFHLSYKFRLLLLLTLCLTVVGWATSNYFVGAIQEIPKAKEFMANFHKTLILGKGK
TLTNEASTKKVELDNCPSVSPYLRGQSKLIFKPDLTLEEVQAENPKVSRGRYRPQECKALQ
RVAILVPHRNREKHLMYLLEHLHPFLQRQQLDYGIYVIHQAEGKKFNRAKLLNVGYLEAL
KEENWDCFIFHDVDLVPENDFNLYKCEEHPKHLVVGRNSTGYRLRYSGYFGGVTALSREQ
FFKVNGFSNNYWGWGGEDDDLRLRVELQRMKISRPLPEVGKYTMVFHTRDKGNEVNAE
RMKLLHQVSRVWRTDGLSSCSYKLVSVEHNPLYINITVDFWFGA

>P53779 lipid-anchor
MSLHFLYYCSEPTLDVKIAFCQGFDKQVDVSYIAKHYNMSKSKVDNQFYSVEVGDSTFT
VLKRYQNLKPIGSGAQGIVCAAYDAVLDRNVAIKKLSRPFQNQTHAKRAYRELVLMKCV
NHKNIISLLNVFTPQKTLEEFQDVYLVMELMDANLCQVIQMELDHERMSYLLYQMLCGIK
HLHSAGIIHRDLKPSNIVVKSDCTLKILDFGLARTAGTSFMMTPYVVTRYYRAPEVILGMG
YKENVDIWSVGCIMGEMVRHKILFPGRDYIDQWNKVIEQLGTPCPEFMKKLQPTVRNYV
ENRPKYAGLTFPKLFPDSLFPADSEHNKLKASQARDLLSKMLVIDPAKRISVDDALQHPYI
NVWYDPAEVEAPPPQIYDKQLDEREHTIEEWKELIYKEVMNSEEKTKNGVVKGQPSPSG

AAVNSSESLPPSSSVNDISSMSTDQTLASDTDSSLEASAGPLGCCR

>Q9UKF5 single-pass type I membrane protein
MKMLLLLHCLGVFLSCSGHIQDEHPQYHSPPDVVIPVRITGTTRGMTPPGWLSYILPFGGQ
KHIIHIKVKKLLFSKHLPVFTYTDQGAILEDQPFVQNNCYYHGYVEGDPESLVSLSTCFGG
FQGILQINDFAYEIKPLAFSTTFEHLVYKMDSEEKQFSTMRSGFMQNEITCRMEFEEIDNST
QKQSSYVGWWIHFRIVEIVVVIDNYLYIRYERNDSKLLEDLYVIVNIVDSILDVIGVKVLLF
GLEIWTNKNLIVVDDVRKSVHLYCKWKSENITPRMQHDTSHLFTTLGLRGLSGIGAFRGM
CTPHRSCAIVTFMNKTLGTFSIAVAHHLGHNLGMNHDEDTCRCSQPRCIMHEGNPPITKFS
NCSYGDFWEYTVERTKCLLETVHTKDIFNVKRCGNGVVEEGEECDCGPLKHCAKDPCCL
SNCTLTDGSTCAFGLCCKDCKFLPSGKVCRKEVNECDLPEWCNGTSHKCPDDFYVEDGIP
CKERGYCYEKSCHDRNEQCRRIFGAGANTASETCYKELNTLGDRVGHCGIKNATYIKCNI
SDVQCGRIQCENVTEIPNMSDHTTVHWARFNDIMCWSTDYHLGMKGPDIGEVKDGTECG
IDHICIHRHCVHITILNSNCSPAFCNKRGICNNKHHCHCNYLWDPPNCLIKGYGGSVDSGPP
PKRKKKKKFCYLCILLLIVLFILLCCLYRLCKKSKPIKKQQDVQTPSAKEEEKIQRRPHELP
PQSQPWVMPSQSQPPVTPSQSHPQVMPSQSQPPVTPSQSQPRVMPSQSQPPVMPSQSHPQL
TPSQSQPPVTPSQRQPQLMPSQSQPPVTPS

>Q9NZM1 single-pass type II membrane protein
MLRVIVESASNIPKTKFGKPDPIVSVIFKDEKKKTKKVDNELNPVWNEILEFDLRGIPLDFS
SSLGIIVKDFETIGQNKLIGTATVALKDLTGDQSRSLPYKLISLLNEKGQDTGATIDLVIGYD
PPSAPHPNDLSGPSVPGMGGDGEEDEGDEDRLDNAVRGPGPKGPVGTVSEAQLARRLTK
VKNSRRMLSNKPQDFQIRVRVIEGRQLSGNNIRPVVKVHVCGQTHRTRIKRGNNPFFDELF
FYNVNMTPSELMDEIISIRVYNSHSLRADCLMGEFKIDVGFVYDEPGHAVMRKWLLLNDP
EDTSSGSKGYMKVSMFVLGTGDEPPPERRDRDNDSDDVESNLLLPAGIALRWVTFLLKIY
RAEDIPQMDDAFSQTVKEIFGGNADKKNLVDPFVEVSFAGKKVCTNIIEKNANPEWNQVV
NLQIKFPSVCEKIKLTIYDWDRLTKNDVVGTTYLHLSKIAASGGEVEDFSSSGTGAASYTV
NTGETEVGFVPTFGPCYLNLYGSPREYTGFPDPYDELNTGKGEGVAYRGRILVELATFLEK
TPPDKKLEPISNDDLLVVEKYQRRRKYSLSAVFHSATMLQDVGEAIQFEVSIGNYGNKFDT
TCKPLASTTQYSRAVFDGNYYYYLPWAHTKPVVTLTSYWEDISHRLDAVNTLLAMAERL
QTNIEALKSGIQGKIPANQLAELWLKLIDEVIEDTRYTLPLTEGKANVTVLDTQIRKLRSRS
LSQIHEAAVRMRSEATDVKSTLAEIEDWLDKLMQLTEEPQNSMPDIIIWMIRGEKRLAYAR
IPAHQVLYSTSGENASGKYCGKTQTIFLKYPQEKNNGPKVPVELRVNIWLGLSAVEKKFNS
FAEGTFTVFAEMYENQALMFGKWGTSGLVGRHKFSDVTGKIKLKREFFLPPKGWEWEGE
WIVDPERSLLTEADAGHTEFTDEVYQNESRYPGGDWKPAEDTYTDANGDKAASPSELTCP
PGWEWEDDAWSYDINRAVDEKGWEYGITIPPDHKPKSWVAAEKMYHTHRRRRLVRKRK
KDLTQTASSTARAMEELQDQEGWEYASLIGWKFHWKQRSSDTFRRRRWRRKMAPSETH
GAAAIFKLEGALGADTTEDGDEKSLEKQKHSATTVFGANTPIVSCNFDRVYIYHLRCYVY
QARNLLALDKDSFSDPYAHICFLHRSKTTEIIHSTLNPTWDQTIIFDEVEIYGEPQTVLQNPP
KVIMELFDNDQVGKDEFLGRSIFSPVVKLNSEMDITPKLLWHPVMNGDKACGDVLVTAE
LILRGKDGSNLPILPPQRAPNLYMVPQGIRPVVQLTAIEILAWGLRNMKNFQMASITSPSLV
VECGGERVESVVIKNLKKTPNFPSSVLFMKVFLPKEELYMPPLVIKVIDHRQFGRKPVVGQ
CTIERLDRFRCDPYAGKEDIVPQLKASLLSAPPCRDIVIEMEDTKPLLASKLTEKEEEIVDW
WSKFYASSGEHEKCGQYIQKGYSKLKIYNCELENVAEFEGLTDFSDTFKLYRGKSDENEDP

SVVGEFKGSFRIYPLPDDPSVPAPPRQFRELPDSVPQECTVRIYIVRGLELQPQDNNGLCDP
YIKITLGKKVIEDRDHYIPNTLNPVFGRMYELSCYLPQEKDLKISVYDYDTFTRDEKVGET
IIDLENRFLSRFGSHCGIPEEYCVSGVNTWRDQLRPTQLLQNVARFKGFPQPILSEDGSRIR
YGGRDYSLDEFEANKILHQHLGAPEERLALHILRTQGLVPEHVETRTLHSTFQPNISQGKL
QMWVDVFPKSLGPPGPPFNITPRKAKKYYLRVIIWNTKDVILDEKSITGEEMSDIYVKGWI
PGNEENKQKTDVHYRSLDGEGNFNWRFVFPFDYLPAEQLCIVAKKEHFWSIDQTEFRIPPR
LIIQIWDNDKFSLDDYLGFLELDLRHTIIPAKSPEKCRLDMIPDLKAMNPLKAKTASLFEQK
SMKGWWPCYAEKDGARVMAGKVEMTLEILNEKEADERPAGKGRDEPNMNPKLDLPNRP
ETSFLWFTNPCKTMKFIVWRRFKWVIIGLLFLLILLLFVAVLLYSLPNYLSMKIVKPNV

>O95528 multipass membrane protein
MGHSPPVLPLCASVSLLGGLTFGYELAVISGALLPLQLDFGLSCLEQEFLVGSLLLGALLAS
LVGGFLIDCYGRKQAILGSNLVLLAGSLTLGLAGSLAWLVLGRAVVGFAISLSSMACCIYV
SELVGPRQRGVLVSLYEAGITVGILLSYALNYALAGTPWGWRHMFGWATAPAVLQSLSLLF
LPAGTDETATHKDLIPLQGGEAPKLGPGRPRYSFLDLFRARDNMRGRTTVGLGLVLFQQLT
GQPNVLCYASTIFSSVGFHGGSSAVLASVGLGAVKVAATLTAMGLVDRAGRRALLLAGCA
LMALSVSGIGLVSFAVPMDSGPSCLAVPNATGQTGLPGDSGLLQDSSLPPIPRTNEDQREPIL
STAKKTKPHPRSGDPSAPPRLALSSALPGPPLPARGHALLRWTALLCLMVFVSAFSFGFGP
VTWLVLSEIYPVEIRGRAFAFCNSFNWAANLFISLSFLDLIGTIGLSWTFLLYGLTAVLGLGFI
YLFVPETKGQSLAEIDQQFQKRRFTLSFGHRQNSTGIPYSRIEISAAS

>Q96DZ9 multipass membrane protein
MLSARDRRDRHPEEGVVAELQGFAVDKAFLTSHKGILLETELALTLIIFICFTASISAYMAAA
LLEFFITLAFLFLYATQYYQRFDRINWPCLLQGHGQSGGPHPLDLLSHSAKVQPQPWPGLT
PPGWHTPAAVPWVPAPAPGFWSWLLWFICFHSLGSSDFLRCVSAIIIFLVVSFAAVTSRDGA
AIAAFVFGIILVSIFAYDAFKIYRTEMAPGASQGDQQ

>O15031 single-pass type I membrane protein
MALQLWALTLLGLLGAGASLRPRKLDFFRSEKELNHLAVDEASGVVYLGAVNALYQLDA
KLQLEQQVATGPALDNKKCTPPIEASQCHEAEMTDNVNQLLLLDPPRKRLVECGSLFKGIC
ALRALSNISLRLFYEDGSGEKSFVASNDEGVATVGLVSSTGPGGDRVLFVGKGNGPHDNGI
IVSTRLLDRTDSREAFEAYTDHATYKAGYLSTNTQQFVAAFEDGPYVFFVFNQQDKHPAR
NRTLLARMCREDPNYYSYLEMDLQCRDPDIHAAAFGTCLAASVAAPGSGRVLYAVFSRDS
RSSGGPGAGLCLFPLDKVHAKMEANRNACYTGTREARDIFYKPFHGDIQCGGHAPGSSK
SFPCGSEHLPYPLGSRDGLRGTAVLQRGGLNLTAVTVAAENNHTVAFLGTSDGRILKVYLT
PDGTSSEYDSILVEINKRVKRDLVLSGDLGSLYAMTQDKVFRLPVQECLSYPTCTQCRDSQ
DPYCGWCVVEGRCTRKAECPRAEEASHWLWSRSKSCVAVTSAQPQNMSRRAQGEVQLT
VSPLPALSEEDELLCLFGESPPHPARVEGEAVICNSPSSIPVTPPGQDHVAVTIQLLLRRGNIF
LTSYQYPFYDCRQAMSLEENLPCISCVSNRWTCQWDLRYHECREASPNPEDGIVRAHME
DSCPQFLGPSPLVIPMNHETDVNFQGKNLDTVKGSSLHVGSDLLKFMEPVTMQESGTFAF
RTPKLSHDANETLPLHLYVKSYGKNIDSKLHVTLYNCSFGRSDCSLCRAANPDYRCAWCG
GQSRCVYEALCNTTSECPPPVITRIQPETGPLGGGIRITILGSNLGVQAGDIQRISVAGRNCS
FQPERYSVSTRIVCVIEAAETPFTGGVEVDVFGKLGRSPPNVQFTFQQPKPLSVEPQQGPQ
AGGTTLTIHGTHLDTGSQEDVRVTLNGVPCKVTKFGAQLQCVTGPQATRGQMLLEVSYG

GSPVPNPGIFFTYRENPVLRAFEPLRSFASGGRSINVTGQGFSLIQRFAMVVIAEPLQSWQP
PREAESLQPMTVVGTDYVFHNDTKVVFLSPAVPEEPEAYNLTVLIEMDGHRALLRTEAGA
FEYVPDPTFENFTGGVKKQVNKLIHARGTNLNKAMTLQEAEAFVGAERCTMKTLTETDL
YCEPPEVQPPPKRRQKRDTTHNLPEFIVKFGSREWVLGRVEYDTRVSDVPLSLILPLVIVPM
VVVIAVSVYCYWRKSQQAEREYEKIKSQLEGLEESVRDRCKKEFTDLMIEMEDQTNDVH
EAGIPVLDYKTYTDRVFFLPSKDGDKDVMITGKLDIPEPRRPVVEQALYQFSNLLNSKSFLI
NFIHTLENQREFSARAKVYFASLLTVALHGKLEYYTDIMHTLFLELLEQYVVAKNPKLML
RRSETVVERMLSNWMSICLYQYLKDSAGEPLYKLFKAIKHQVEKGPVDAVQKKAKYTLN
DTGLLGDDVEYAPLTVSVIVQDEGVDAIPVKVLNCDTISQVKEKIIDQVYRGQPCSCWPRP
DSVVLEWRPGSTAQILSDLDLTSQREGRWKRVNTLMHYNVRDGATLILSKVGVSQQPEDS
QQDLPGERHALLEEENRVWHLVRPTDEVDEGKSKRGSVKEKERTKAITEIYLTRLLSVKG
TLQQFVDNFFQSVLAPGHAVPPAVKYFFDFLDEQAEKHNIQDEDTIHIWKTNSLPLRFWVN
ILKNPHFIFDVHVHEVVDASLSVIAQTFMDACTRTEHKLSRDSPSNKLLYAKEISTYKKMV
EDYYKGIRQMVQVSDQDMNTHLAEISRAHTDSLNTLVALHQLYQYTQKYYDEIINALEED
PAAQKMQLAFRLQQIAAALENKVTDL

>Q8NH64 multipass membrane protein
MSVLNNSEVKLFLLIGIPGLEHAHIWFSIPICLMYLLAIMGNCTILFIIKTEPSLHEPMYYFL
AMLAVSDMGLSLSSLPTMLRVFLFNAMGISPNACFAQEFFIHGFTVMESSVLLIMSLDRFL
AIHNPLRYSSILTSNRVAKMGLILAIRSILLVIPFPFTLRRLKYCQKNLLSHSYCLHQDTMKL
ACSDNKTNVIYGFFIALCTMLDLALIVLSYVLILKTILSIASLAERLKALNTCVSHICAVLTF
YVPIITLAAMHHFAKHKSPLVVILIADMFLLVPPLMNPIVYCVKTRQIWEKILGKLLNVCG
R

>P47804 multipass membrane protein
MAETSALPTGFGELEVLAVGMVLLVEALSGLSLNTLTIFSFCKTPELRTPCHLLVLSLALAD
SGISLNALVAATSSLLRRWPYGSDGCQAHGFQGFVTALASICSSAAIAWGRYHHYCTRSQL
AWNSAVSLVLFVWLSSAFWAALPLLGWGHYDYEPLGTCCTLDYSKGDRNFTSFLFTMSF
FNFAMPLFITITSYSLMEQKLGKSGHLQVNTTLPARTLLLGWGPYAILYLYAVIADVTSISPK
LQMVPALIAKMVPTINAINYALGNEMVCRGIWQCLSPQKREKDRTK

>P54851 multipass membrane protein
MLVLLAFIIAFHITSAALLFIATVDNAWWVGDEFFADVWRICTNNTNCTVINDSFQEYSTL
QAVQATMILSTILCCIAFFIFVLQLFRLKQGERFVLTSIIQLMSCLCVMIAASIYTDRREDIHD
KNAKFYPVTREGSYGYSYILAWVAFACTFISGMMYLILRKRK

>Q92835 peripheral membrane protein
MVPCWNHGNITRSKAEELLSRTGKDGSFLVRASESISRAYALCVLYRNCVYTYRILPNEDD
KFTVQASEGVSMRFFTKLDQLIEFYKKENMGLVTHLQYPVPLEEEDTGDDPEEDTVESVV
SPPELPPRNIPLTASSCEAKEVPFSNENPRATETSRPSLSETLFQRLQSMDTSGLPEEHLKAIQ
DYLSTQLAQDSEFVKTGSSSLPHLKKLTTLLCKELYGEVIRTLPSLESLQRLFDQQLSPGLR
PRPQVPGEANPINMVSKLSQLTSLLSSIEDKVKALLHEGPESPHRPSLIPPVTFEVKAESLGI
PQKMQLKVDVESGKLIIKKSKDGSEDKFYSHKKILQLIKSQKFLNKLVILVETEKEKILRKE
YVFADSKKREGFCQLLQQMKNKHSEQPEPDMITIFIGTWNMGNAPPPKKITSWFLSKGQG

KTRDDSADYIPHDIYVIGTQEDPLSEKEWLEILKHSLQEITSVTFKTVAIHTLWNIRIVVLAK
PEHENRISHICTDNVKTGIANTLGNKGAVGVSFMFNGTSLGFVNSHLTSGSEKKLRRNQN
YMNILRFLALGDKKLSPFNITHRFTHLFWFGDLNYRVDLPTWEAETIIQKIKQQQYADLLS
HDQLLTERREQKVFLHFEEEEITFAPTYRFERLTRDKYAYTKQKATGMKYNLPSWCDRVL
WKSYPLVHVVCQSYGSTSDIMTSDHSPVFATFEAGVTSQFVSKNGPGTVDSQGQIEFLRCY
ATLKTKSQTKFYLEFHSSCLESFVKSQEGENEEGSEGELVVKFGETLPKLKPIISDPEYLLD
QHILISIKSSDSDESYGEGCIALRLEATETQLPIYTPLTHHGELTGHFQGEIKLQTSQGKTRE
KLYDFVKTERDESSGPKTLKSLTSHDPMKQWEVTSRAPPCSGSSITEIINPNYMGVGPFGPP
MPLHVKQTLSPDQQPTAWSYDQPPKDSPLGPCRGESPPTPPGQPPISPKKFLPSTANRGLPP
RTQESRPSDLGKNAGDTLPQEDLPLTKPEMFENPLYGSLSSFPKPAPRKDQESPKMPRKEP
PPCPEPGILSPSIVLTKAQEADRGEGPGKQVPAPRLRSFTCSSSAEGRAAGGDKSQGKPKTP
VSSQAPVPAKRPIKPSRSEINQQTPPTPTPRPPLPVKSPAVLHLQHSKGRDYRDNTELPHHG
KHRPEEGPPGPLGRTAMQ

>P21817 multipass membrane protein
MGDAEGEDEVQFLRTDDEVVLQCSATVLKEQLKLCLAAEGFGNRLCFLEPTSNAQNVPP
DLAICCFVLEQSLSVRALQEMLANTVEAGVESSQGGGHRTLLYGHAILLRHAHSRMYLSC
LTTSRSMTDKLAFDVGLQEDATGEACWWTMHPASKQRSEGEKVRVGDDIILVSVSSERYL
HLSTASGELQVDASFMQTLWNMNPICSRCEEGFVTGGHVLRLFHGHMDECLTISPADSDD
QRRLVYYEGGAVCTHARSLWRLEPLRISWSGSHLRWGQPLRVRHVTTGQYLALTEDQGL
VVVDASKAHTKATSFCFRISKEKLDVAPKRDVEGMGPPEIKYGESLCFVQHVASGLWLTY
AAPDPKALRLGVLKKKAMLHQEGHMDDALSLTRCQQEESQAARMIHSTNGLYNQFIKSL
DSFSGKPRGSGPPAGTALPIEGVILSLQDLIIYFEPPSEDLQHEEKQSKLRSLRNRQSLFQEE
GMLSMVLNCIDRLNVYTTAAHFAEFAGEEAAESWKEIVNLLYELLASLIRGNRSNCALFST
NLDWLVSKLDRLEASSGILEVLYCVLIESPEVLNIIQENHIKSIISLLDKHGRNHKVLDVLCS
LCVCNGVAVRSNQDLITENLLPGRELLLQTNLINYVTSIRPNIFVGRAEGTTQYSKWYFEV
MVDEVTPFLTAQATHLRVGWALTEGYTPYPGAGEGWGGNGVGDDLYSYGFDGLHLWTG
HVARPVTSPGQHLLAPEDVISCCLDLSVPSISFRINGCPVQGVFESFNLDGLFFPVVSFSAG
VKVRFLLGGRHGEFKFLPPPGYAPCHEAVLPRERLHLEPIKEYRREGPRGPHLVGPSRCLS
HTDFVPCPVDTVQIVLPPHLERIREKLAENIHELWALTRIEQGWTYGPVRDDNKRLHPCLV
DFHSLPEPERNYNLQMSGETLKTLLALGCHVGMADEKAEDNLKKTKLPKTYMMSNGYK
PAPLDLSHVRLTPAQTTLVDRLAENGHNVWARDRVGQGWSYSAVQDIPARRNPRLVPYRL
LDEATKRSNRDSLCQAVRTLLGYGYNIEPPDQEPSQVENQSRCDRVRIFRAEKSYTVQSGR
WYFEFEAVTTGEMRVGWARPELRPDVELGADELAYVFNGHRGQRWHLGSEPFGRPWQP
GDVVGCMIDLTENTIIFTLNGEVLMSDSGSETAFREIEIGDGFLPVCSLGPGQVGHLNLGQD
VSSLRFFAICGLQEGFEPFAINMQRPVTTWFSKGLPQFEPVPLEHPHYEVSRVDGTVDTPPC
LRLTHRTWGSQNSLVEMLFLRLSLPVQFHQHFRCTAGATPLAPPGLQPPAEDEARAAEPDP
DYENLRRSAGGWSEAENGKEGTAKEGAPGGTPQAGGEAQPARAENEKDATTEKNKKRG
FLFKAKKVAMMTQPPATPTLPRLPHDVVPADNRDDPEIILNTTTYYYSVRVFAGQEPSCVW
AGWVTPDYHQHDMSFDLSKVRVVTVTMGDEQGNVHSSLKCSNCYMVWGGDFVSPGQQ
GRISHTDLVIGCLVDLATGLMTFTANGKESNTFFQVEPNTKLFPAVFVLPTHQNVIQFELGK
QKNIMPLSAAMFQSERKNPAPQCPPRLEMQMLMPVSWSRMPNHFLQVETRRAGERLGW
AVQCQEPLTMMALHIPEENRCMDILELSERLDLQRFHSHTLRLYRAVCALGNNRVAHALC
SHVDQAQLLHALEDAHLPGPLRAGYYDLLISIHLESACRSRRSMLSEYIVPLTPETRAITLF

PPGRSTENGHPRHGLPGVGVTTSLRPPHHFSPPCFVAALPAAGAAEAPARLSPAIPLEALRD
KALRMLGEAVRDGGQHARDPVGGSVEFQFVPVLKLVSTLLVMGIFGDEDVKQILKMIEPE
VFTEEEEEEDEEEEGEEEDEEEKEEDEEETAQEKEDEEKEEEEAAEGEKEEGLEEGLLQMK
LPESVKLQMCHLLEYFCDQELQHRVESLAAFAERYVDKLQANQRSRYGLLIKAFSMTAAE
TARRTREFRSPPQEQINMLLQFKDGTDEEDCPLPEEIRQDLLDFHQDLLAHCGIQLDGEEE
EPEEETTLGSRLMSLLEKVRLVKKKEEKPEEERSAEESKPRSLQELVSHMVVRWAQEDFV
QSPELVRAMFSLLHRQYDGLGELLRALPRAYTISPSSVEDTMSLLECLGQIRSLLIVQMGP
QEENLMIQSIGNIMNNKVFYQHPNLMRALGMHETVMEVMVNVLGGGESKEIRFPKMVT
SCCRFLCYFCRISRQNQRSMFDHLSYLLENSGIGLGMQGSTPLDVAAASVIDNNELALALQ
EQDLEKVVSYLAGCGLQSCPMLVAKGYPDIGWNPCGGERYLDFLRFAVFVNGESVEENA
NVVVRLLIRKPECFGPALRGEGGSGLLAAIEEAIRISEDPARDGPGIRRDRRREHFGEEPPEE
NRVHLGHAIMSFYAALIDLLGRCAPEMHLIQAGKGEALRIRAILRSLVPLEDLVGIISLPLQI
PTLGKDGALVQPKMSASFVPDHKASMVLFLDRVYGIENQDFLLHVLDVGFLPDMRAAAS
LDTATFSTTEMALALNRYLCLAVLPLITKCAPLFAGTEHRAIMVDSMLHTVYRLSRGRSLT
KAQRDVIEDCLMSLCRYIRPSMLQHLLRRLVFDVPILNEFAKMPLKLLTNHYERCWKYYC
LPTGWANFGVTSEEELHLTRKLFWGIFDSLAHKKYDPELYRMAMPCLCAIAGALPPDYVD
ASYSSKAEKKATVDAEGNFDPRPVETLNVIIPEKLDSFINKFAEYTHEKWAFDKIQNNWSY
GENIDEELKTHPMLRPYKTFSEKDKEIYRWPIKESLKAMIAWEWTIEKAREGEEEKTEKK
KTRKISQSAQTYDPREGYNPQPPDLSAVTLSRELQAMAEQLAENYHNTWGRKKKQELEA
KGGGTHPLLVPYDTLTAKEKARDREKAQELLKFLQMNGYAVTRGLKDMELDSSSIEKRFA
FGFLQQLLRWMDISQEFIAHLEAVVSSGRVEKSPHEQEIKFFAKILLPLINQYFTNHCLYFLS
TPAKVLGSGGHASNKEKEMITSLFCKLAALVRHRVSLFGTDAPAVVNCLHILARSLDARTV
MKSGPEIVKAGLRSFFESASEDIEKMVENLRLGKVSQARTQVKGVGQNLTYTTVALLPVL
TTLFQHIAQHQFGDDVILDDVQVSCYRTLCSIYSLGTTKNTYVEKLRPALGECLARLAAA
MPVAFLEPQLNEYNACSVYTTKSPRERAILGLPNSVEEMCPDIPVLERLMADIGGLAESGA
RYTEMPHVIEITLPMLCSYLPRWWERGPEAPPSALPAGAPPPCTAVTSDHLNSLLGNILRIIV
NNLGIDEASWMKRLAVFAQPIVSRARPELLQSHFIPTIGRLRKRAGKVVSEEEQLRLEAKA
EAQEGELLVRDEFSVLCRDLYALYPLLIRYVDNNRAQWLTEPNPSAEELFRMVGEIFIYWS
KSHNFKREEQNFVVQNEINNMSFLTADNKSKMAKAGDIQSGGSDQERTKKKRRGDRYSV
QTSLIVATLKKMLPIGLNMCAPTDQDLITLAKTRYALKDTDEEVREFLHNNLHLQGKVEG
SPSLRWQMALYRGVPGREEDADDPEKIVRRVQEVSAVLYYLDQTEHPYKSKKAVWHKLL
SKQRRRAVVACFRMTPLYNLPTHRACNMFLESYKAAWILTEDHSFEDRMIDDLSKAGEQE
EEEEEVEEKKPDPLHQLVLHFSRTALTEKSKLDEDYLYMAYADIMAKSCHLEEGGENGEA
EEEVEVSFEEKQMEKQRLLYQQARLHTRGAAEMVLQMISACKGETGAMVSSTLKLGISIL
NGGNAEVQQKMLDYLKDKKEVGFFQSIQALMQTCSVLDLNAFERQNKAEGLGMVNED
GTVINRQNGEKVMADDEFTQDLFRFLQLLCEGHNNDFQNYLRTQTGNTTTINIIICTVDYL
LRLQESISDFYWYYSGKDVIEEQGKRNFSKAMSVAKQVFNSLTEYIQGPCTGNQQSLAHS
RLWDAVVGFLHVFAHMMMKLAQDSSQIELLKELLDLQKDMVVMLLSLLEGNVVNGMIA
RQMVDMLVESSSNVEMILKFFDMFLKLKDIVGSEAFQDYVTDPRGLISKKDFQKAMDSQ
KQFSGPEIQFLLSCSEADENEMINCEEFANRFQEPARDIGFNVAVLLTNLSEHVPHDPRLHN
FLELAESILEYFRPYLGRIEIMGASRRIERIYFEISETNRAQWEMPQVKESKRQFIFDVVNEG
GEAEKMELFVSFCEDTIFEMQIAAQISEPEGEPETDEDEGAGAAEAGAEGAEEGAAGLEG
TAATAAAGATARVVAAAGRALRGLSYRSLRRRVRRLRRLTAREAATAVAALLWAAVTRAG
AAGAGAAAGALGLLWGSLFGGGLVEGAKKVTVTELLAGMPDPTSDEVHGEQPAGPGGD

ADGEGASEGAGDAAEGAGDEEEAVHEAGPGGADGAVAVTDGGPFRPEGAGGLGDMGDT
TPAEPPTPEGSPILKRKLGVDGVEEELPPEPEPEPEPELEPEKADAENGEKEEVPEPTPEPPK
KQAPPSPPPKKEEAGGEFWGELEVQRVKFLNYLSRNFYTLRFLALFLAFAINFILLFYKVS
DSPPGEDDMEGSAAGDVSGAGSGGSSGWGLGAGEEAEGDEDENMVYYFLEESTGYMEP
ALRCLSLLHTLVAFLCIIGYNCLKVPLVIFKREKELARKLEFDGLYITEQPEDDDVKGQWD
RLVLNTPSFPSNYWDKFVKRKVLDKHGDIYGRERIAELLGMDLATLEITAHNERKPNPPPG
LLTWLMSIDVKYQIWKFGVIFTDNSFLYLGWYMVMSLLGHYNNFFFAAHLLDIAMGVKT
LRTILSSVTHNGKQLVMTVGLLAVVVYLYTVVAFNFFRKFYNKSEDEDEPDMKCDDMMT
CYLFHMYVGVRAGGGIGDEIEDPAGDEYELYRVVFDITFFFFVIVILLAIIQGLIIDAFGELR
DQQEQVKEDMETKCFICGIGSDYFDTTPHGFETHTLEEHNLANYMFFLMYLINKDETEHT
GQESYVWKMYQERCWDFFPAGDCFRKQYEDQLS

>Q8NGY2 multipass membrane protein
MESPNRTTIQEFIFSAFPYSWVKSVVCFVPLLFIYAFIVVGNLVIITVVQLNTHLHTPMYTFI
SALSFLEIWYTTATIPKMLSSLLSERSISFNGCLLQMYFFHSTGICEVCLLTVMAFDHYLAIC
SPLHYPSIMTPKLCTQLTLSCCVCGFITPLPEIAWISTLPFCGSNHLEHIFCDFLPVLRLACTD
TRAIVMIQVVDVIHAVEIITAVMLIFMSYDGIVAVILRIHSAGGRRTAFSTCVSHFIVFSLFFG
SVTLMYLRFSATYSLFWDIAIALAFAVLSPFFNPIIYSLRNKEIKEAIKKHIGQAKIFFSVRPG
TSSKIF

>P98194 multipass membrane protein
MKVARFQKIPNGENETMIPVLTSKKASELPVSEVASILQADLQNGLNKCEVSHRRAFHGW
NEFDISEDEPLWKKYISQFKNPLIMLLLASAVISVLMHQFDDAVSITVAILIVVTVAFVQEYR
SEKSLEELSKLVPPECHCVREGKLEHTLARDLVPGDTVCLSVGDRVPADLRLFEAVDLSID
ESSLTGETTPCSKVTAPQPAATNGDLASRSNIAFMGTLVRCGKAKGVVIGTGENSEFGEVF
KMMQAEEAPKTPLQKSMDLLGKQLSFYSFGIIGIIMLVGWLLGKDILEMFTISVSLAVAAIP
EGLPIVVTVTLALGVMRMVKKRAIVKKLPIVETLGCCNVICSDKTGTLTKNEMTVTHIFTS
DGLHAEVTGVGYNQFGEVIVDGDVVHGFYNPAVSRIVEAGCVCNDAVIRNNTLMGKPTE
GALIALAMKMGLDGLQQDYIRKAEYPFSSEQKWMAVKCVHRTQQDRPEICFMKGAYEQ
VIKYCTTYQSKGQTLTLTQQQRDVYQQEKARMGSAGLRVLALASGPELGQLTFLGLVGII
DPPRTGVKEAVTTLIASGVSIKMITGDSQETAVAIASRLGLYSKTSQSVSGEEIDAMDVQQL
SQIVPKVAVFYRASPRHKMKIIKSLQKNGSVVAMTGDGVNDAVALKAADIGVAMGQTGT
DVCKEAADMILVDDDFQTIMSAIEEGKGIYNNIKNFVRFQLSTSIAALTLISLATLMNFPNP
LNAMQILWINIIMDGPPAQSLGVEPVDKDVIRKPPRNWKDSILTKNLILKILVSSIIIVCGTLF
VFWRELRDNVITPRDTTMTFTCFVFFDMFNALSSRSQTKSVFEIGLCSNRMFCYAVLGSIM
GQLLVIYFPPLQKVFQTESLSILDLLFLLGLTSSVCIVAEIIKKVERSREKIQKHVSSTSSSFLE
V

>P48065 multipass membrane protein
MDGKVAVQECGPPAVSWVPEEGEKLDQEDEDQVKDRGQWTNKMEFVLSVAGEIIGLGN
VWRFPYLCYKNGGGAFFIPYFIFFFVCGIPVFFLEVALGQYTSQGSVTAWRKICPLFQGIGL
ASVVIESYLNVYYIIILAWALFYLFSSFTSELPWTTCNNFWNTEHCTDFLNHSGAGTVTPFE
NFTSPVMEFWERRVLGITSGIHDLGSLRWELALCLLLAWVICYFCIWKGVKSTGKVVYFT
ATFPYLMLVILLIRGVTLPGAYQGIIYYLKPDLFRLKDPQVWMDAGTQIFFSFAICQGCLTA

LGSYNKYHNNCYKDCIALCFLNSATSFVAGFVVFSILGFMSQEQGVPISEVAESGPGLAFIA
FPKAVTMMPLSQLWSCLFFIMLIFLGLDSQFVCVECLVTASIDMFPRQLRKSGRRELLILTIA
VMCYLIGLFLVTEGGMYIFQLFDYYASSGICLLFLSLFEVVCISWVYGADRFYDNIEDMIG
YRPWPLVKISWLFLTPGLCLATFLFSLSKYTPLKYNNVYVYPPWGYSIGWFLALSSMVCV
PLFVVITLLKTRGPFRKRLRQLITPDSSLPQPKQHPCLDGSAGRNFGPSPTREGLIAGEKET
HL

>Q96R28 multipass membrane protein
MAWENQTFNSDFILLGIFNHSPPHTFLFFLVLGIFLVAFMGNSVMVLLIYLDTQLHTPMYFL
LSQLSLMDLMLICTTVPKMAFNYLSGSKSISMAGCVTQIFFYISLSGSECFLLAVMAYDRYI
AICHPLRYTNLMNPKICGLMATFSWILGSTDGIIDAVATFSFSFCGSREIAHFFCEFPSLLILS
CNDTSIFEEVIFICCIVMLVFPVAIIIASYARVILAVIHMGSGEGRCKAFTTCSSHLMVVGMY
YGAALFMYIRPTSDHSPTQDKMVSVFYTILTPMLNPLIYSLRNKEVTRAFMKILGKGKSES
ELPHKLYVLLFAKFFFLISIFFYDVKILALIMYIA

>Q8NGZ3 multipass membrane protein
MNHSVVTEFIILGLTKKPELQGIIFLFFLIVYLVAFLGNMLIIIAKIYNNTLHTPMYVFLLTLA
VVDIICTTSIIPKMLGTMLTSENTISYAGCMSQLFLFTWSLGAEMVLFTTMAYDRYVAICFP
LHYSTIMNHHMCVALLSMVMAIAVTNSWVHTALIMRLTFCGPNTIDHFFCEIPPLLALSCS
PVRINEVMVYVADITLAIGDFILTCISYGFIIVAILRIRTVEGKRKAFSTCSSHLTVVTLYYSP
VIYTYIRPASSYTFERDKVVAALYTLVTPTLNPMVYSFQNREMQAGIRKVFAFLKH

>Q6W5P4 multipass membrane protein
MPANFTEGSFDSSGTGQTLDSSPVACTETVTFTEVVEGKEWGSFYYSFKTEQLITLWVLFV
FTIVGNSVVLFSTWRRKKKSRMTFFVTQLAITDSFTGLVNILTDINWRFTGDFTAPDLVCRV
VRYLQVVLLYASTYVLVSLSIDRYHAIVYPMKFLQGEKQARVLIVIAWSLSFLFSIPTLIIFG
KRTLSNGEVQCWALWPDDSYWTPYMTIVAFLVYFIPLTIISIMYGIVIRTIWIKSKTYETVIS
NCSDGKLCSSYNRGLISKAKIKAIKYSIIIILAFICCWSPYFLFDILDNFNLLPDTQERFYASVI
IQNLPALNSAINPLIYCVFSSSISFPCREQRSQDSRMTFRERTERHEMQILSKPEFI

>Q93084 multipass membrane protein
MEAAHLLPAADVLRHFSVTAEGGLSPAQVTGARERYGPNELPSEEGKSLWELVLEQFEDL
LVRILLLAALVSFVLAWFEEGEETTTAFVEPLVIMLILVANAIVGVWQERNAESAIEALKEY
EPEMGKVIRSDRKGVQRIRARDIVPGDIVEVAVGDKVPADLRLIEIKSTTLRVDQSILTGESV
SVTKHTEAIPDPRAVNQDKKNMLFSGTNITSGKAVGVAVATGLHTELGKIRSQMAAVEPER
TPLQRKLDEFGRQLSHAISVICVAVWVINIGHFADPAHGGSWLRGAVYYFKIAVALAVAAIP
EGLPAVITTCLALGTRRMARKNAIVRSLPSVETLGCTSVICSDKTGTLTTNQMSVCRMFVV
AEADAGSCLLHEFTISGTTYTPEGEVRQGDQPVRCGQFDGLVELATICALCNDSALDYNE
AKGVYEKVGEATETALTCLVEKMNVFDTDLQALSRVERAGACNTVIKQLMRKEFTLEFSR
DRKSMSVYCTPTRPHPTGQGSKMFVKGAPESVIERCSSVRVGSRTAPLTPTSREQILAKIRD
WGSGSDTLRCLALATRDAPPRKEDMELDDCSKFVQYETDLTFVGCVGMLDPPRPEVAACI
TRCYQAGIRVVMITGDNKGTAVAICRRLGIFGDTEDVAGKAYTGREFDDLSPEQQRQACRT
ARCFARVEPAHKSRIVENLQSFNEITAMTGDGVNDAPALKKAEIGIAMGSGTAVAKSAAE
MVLSDDNFASIVAAVEEGRAIYSNMKQFIRYLISSNVGEVVCIFLTAILGLPEALIPVQLLWV

NLVTDGLPATALGFNPPDLDIMEKLPRSPREALISGWLFFRYLAIGVYVGLATVAAATWWF
VYDAEGPHINFYQLRNFLKCSEDNPLFAGIDCEVFESRFPTTMALSVLVTIEMCNALNSVS
ENQSLLRMPPWMNPWLLVAVAMSMALHFLILLVPPLPLIFQVTPLSGRQWVVVLQISLPVI
LLDEALKYLSRNHMHACLYPGLLRTVSQAWSRQPLTTSWTPDHTGRNEPEVSAGNRVESP
VCTSD

>Q13286 multipass membrane protein
MGGCAGSRRRFSDSEGEETVPEPRLPLLDHQGAHWKNAVGFWLLGLCNNFSYVVMLSA
AHDILSHKRTSGNQSHVDPGPTPIPHNSSSRFDCNSVSTAAVLLADILPTLVIKLLAPLGLHL
LPYSPRVLVSGICAAGSFVLVAFSHSVGTSLCGVVFASISSGLGEVTFLSLTAFYPRAVISWW
SSGTGGAGLLGALSYLGLTQAGLSPQQTLLSMLGIPALLLASYFLLLTSPEAQDPGGEEEA
ESAARQPLIRTEAPESKPGSSSSLSLRERWTVFKGLLWYIVPLVVVYFAEYFINQGLFELLFF
WNTSLSHAQQYRWYQMLYQAGVFASRSSLRCCRIRFTWALALLQCLNLVFLLADVWFGF
LPSIYLVFLIILYEGLLGGAAYVNTFHNIALETSDEHREFAMAATCISDTLGISLSGLLALPLH
DFLCQLS

>Q86SQ9 peripheral membrane protein
MSWIKEGELSLWERFCANIIKAGPMPKHIAFIMDGNRRYAKKCQVERQEGHSQGFNKLAE
TLRWCLNLGILEVTVYAFSIENFKRSKSEVDGLMDLARQKFSRLMEEKEKLQKHGVCIRV
LGDLHLLPLDLQELIAQAVQATKNYNKCFLNVCFAYTSRHEISNAVREMAWGVEQGLLDP
SDISESLLDKCLYTNRSPHPDILIRTSGEVRLSDFLLWQTSHSCLVFQPVLWPEYTFWNLFE
AILQFQMNHSVLQKARDMYAEERKRQQLERDQATVTEQLLREGLQASGDAQLRRTRLHK
LSARREERVQGFLQALELKRADWLARLGTASA

>O94856 single-pass type I membrane protein
MARQPPPPWVHAAFLLCLLSLGGAIEIPMDPSIQNELTQPPTITKQSAKDHIVDPRDNILIEC
EAKGNPAPSFHWTRNSRFFNIAKDPRVSMRRRSGTLVIDFRSGGRPEEYEGEYQCFARNKF
GTALSNRIRLQVSKSPLWPKENLDPVVVQEGAPLTLQCNPPPGLPSPVIFWMSSSMEPITQD
KRVSQGHNGDLYFSNVMLQDMQTDYSCNARFHFTHTIQQKNPFTLKVLTTRGVAERTPSF
MYPQGTASSQMVLRGMDLLLECIASGVPTPDIAWYKKGGDLPSDKAKFENFNKALRITN
VSEEDSGEYFCLASNKMGSIRHTISVRVKAAPYWLDEPKNLILAPGEDGRLVCRANGNPK
PTVQWMVNGEPLQSAPPNPNREVAGDTIIFRDTQISSRAVYQCNTSNEHGYLLANAFVSV
LDVPPRMLSPRNQLIRVILYNRTRLDCPFFGSPIPTLRWFKNGQGSNLDGGNYHVYENGSL
EIKMIRKEDQGIYTCVATNILGKAENQVRLEVKDPTRIYRMPEDQVARRGTTVQLECRVK
HDPSLKLTVSWLKDDEPLYIGNRMKKEDDSLTIFGVAERDQGSYTCVASTELDQDLAKAY
LTVLADQATPTNRLAALPKGRPDRPRDLELTDLAERSVRLTWIPGDANNSPITDYVVQFEE
DQFQPGVWHDHSKYPGSVNSAVLRLSPYVNYQFRVIAINEVGSSHPSLPSERYRTSGAPPE
SNPGDVKGEGTRKNNMEITWTPMNATSAFGPNLRYIVKWRRRETREAWNNVTVWGSRY
VVGQTPVYVPYEIRVQAENDFGKGPEPESVIGYSGEDYPRAAPTEVKVRVMNSTAISLQW
NRVYSDTVQGQLREYRAYYWRESSLLKNLWVSQKRQQASFPGDRLRGVVSRLFPYSNYK
LEMVVVNGRGDGPRSETKEFTTPEGVPSAPRRFRVRQPNLETINLEWDHPEHPNGIMIGYT
LKYVAFNGTKVGKQIVENFSPNQTKFTVQRTDPVSRYRFTLSARTQVGSGEAVTEESPAPP
NEATPTAAPPTLPPTTVGATGAVSSTDATAIAATTEATTVPIIPTVAPTTIATTTTVATTTTTTA
AATTTTESPPTTTSGTKIHESAPDEQSIWNVTVLPNSKWANITWKHNFGPGTDFVVEYIDS

NHTKKTVPVKAQAQPIQLTDLYPGMTYTLRVYSRDNEGISSTVITFMTSTAYTNNQADIAT
QGWFIGLMCAIALLVLILLIVCFIKRSRGGKYPVREKKDVPLGPEDPKEEDGSFDYSDEDN
KPLQGSQTSLDGTIKQQESDDSLVDYGEGGEGQFNEDGSFIGQYTVKKDKEETEGNESSE
ATSPVNAIYSLA

>Q96MX0 multipass membrane protein
MWPPDPDPDPDPEPAGGSRPGPAVPGLRALLPARAFLCSLKGRLLLAESGLSFITFICYVAS
SASAFLTAPLLEFLLALYFLFADAMQLNDKWQGLCWPMMDFLRCVTAALIYFAISITAIAK
YSDGASKAAGVFGFFATIVFATDFYLIFNDVAKFLKQGDSADETTAHKTEEENSDSDSD

>P02786 single-pass type II membrane protein
MMDQARSAFSNLFGGEPLSYTRFSLARQVDGDNSHVEMKLAVDEEENADNNTKANVTK
PKRCSGSICYGTIAVIVFFLIGFMIGYLGYCKGVEPKTECERLAGTESPVREEPGEDFPAAR
RLYWDDLKRKLSEKLDSTDFTGTIKLLNENSYVPREAGSQKDENLALYVENQFREFKLSK
VWRDQHFVKIQVKDSAQNSVIIVDKNGRLVYLVENPGGYVAYSKAATVTGKLVHANFGT
KKDFEDLYTPVNGSIVIVRAGKITFAEKVANAESLNAIGVLIYMDQTKFPIVNAELSFFGHA
HLGTGDPYTPGFPSFNHTQFPPSRSSGLPNIPVQTISRAAAEKLFGNMEGDCPSDWKTDST
CRMVTSESKNVKLTVSNVLKEIKILNIFGVIKGFVEPDHYVVVGAQRDAWGPGAAKSGV
GTALLLKLAQMFSDMVLKDGFQPSRSIIFASWSAGDFGSVGATEWLEGYLSSLHLKAFTYI
NLDKAVLGTSNFKVSASPLLYTLIEKTMQNVKHPVTGQFLYQDSNWASKVEKLTLDNAAF
PFLAYSGIPAVSFCFCEDTDYPYLGTTMDTYKELIERIPELNKVARAAAEVAGQFVIKLTHD
VELNLDYERYNSQLLSFVRDLNQYRADIKEMGLSLQWLYSARGDFFRATSRLTTDFGNAE
KTDRFVMKKLNDRVMRVEYHFLSPYVSPKESPFRHVFWGSGSHTLPALLENLKLRKQNN
GAFNETLFRNQLALATWTIQGAANALSGDVWDIDNEF

>P16150 single-pass type I membrane protein
MATLLLLLGVLVVSPDALGSTTAVQTPTSGEPLVSTSEPLSSKMYTTSITSDPKADSTGDQT
SALPPSTSINEGSPLWTSIGASTGSPLPEPTTYQEVSIKMSSVPQETPHATSHPAVPITANSLG
SHTVTGGTITTNSPETSSRTSGAPVTTAASSLETSRGTSGPPLTMATVSLETSKGTSGPPVTM
ATDSLETSTGTTGPPVTMTTGSLEPSSGASGPQVSSVKLSTMMSPTTSTNASTVPFRNPDE
NSRGMLPVAVLVALLAVIVLVALLLLWRRRQKRRTGALVLSRGGKRNGVVDAWAGPAQV
PEEGAVTVTVGGSGGDKGSGFPDGEGSSRRPTLTTFFGRRKSRQGSLAMEELKSGSGPSLK
GEEEPLVASEDGAVDAPAPDEPEGGDGAAP

>Q96RQ1 multipass membrane protein
MRRLNRKKTLSLVKELDAFPKVPESYVETSASGGTVSLIAFTTMALLTIMEFSVYQDTWM
KYEYEVDKDFSSKLRINIDITVAMKCQYVGADVLDLAETMVASADGLVYEPTVFDLSPQQ
KEWQRMLQLIQSRLQEEHSLQDVIFKSAFKSTSTALPPREDDSSQSPNACRIHGHLYVNKV
AGNFHITVGKAIPHPRGHAHLAALVNHESYNFSHRIDHLSFGELVPAIINPLDGTEKIAIDH
NQMFQYFITVVPTKLHTYKISADTHQFSVTERERIINHAAGSHGVSGIFMKYDLSSLMVTV
TEEHMPFWQFFVRLCGIVGGIFSTTGMLHGIGKFIVEIICCRFRLGSYKPVNSVPFEDGHTD
NHLPLLENNTH

>Q8NGK5 multipass membrane protein

MLTFHNVCSVPSSFWLTGIPGLESLHVWLSIPFGSMYLVAVVGNVTILAVVKIERSLHQPM
YFFLCMLAAIDLVLSTSTIPKLLGIFWFGACDIGLDACLGQMFLIHCFATVESGIFLAMAFD
RYVAICNPLRHSMVLTYTVVGRLGLVSLLRGVLYIGPLPLMIRLRLPLYKTHVISHSYCEH
MAVVALTCGDSRVNNVYGLSIGFLVLILDSVAIAASYVMIFRAVMGLATPEARLKTLGTCA
SHLCAILIFYVPIAVSSLIHRFGQCVPPPVHTLLANFYLLIPPILNPIVYAVRTKQIRESLLQIPR
IEMKIR

>O00555 multipass membrane protein
MARFGDEMPARYGGGGSGAAAGVVVGSGGGRGAGGSRQGGQPGAQRMYKQSMAQRA
RTMALYNPIPVRQNCLTVNRSLFLFSEDNVVRKYAKKITEWPPFEYMILATIIANCIVLALE
QHLPDDDKTPMSERLDDTEPYFIGIFCFEAGIKIIALGFAFHKGSYLRNGWNVMDFVVVLT
GILATVGTEFDLRTLRAVRVLRPLKLVSGIPSLQVVLKSIMKAMIPLLQIGLLLFFAILIFAIIG
LEFYMGKFHTTCFEEGTDDIQGESPAPCGTEEPARTCPNGTKCQPYWEGPNNGITQFDNIL
FAVLTVFQCITMEGWTDLLYNSNDASGNTWNWLYFIPLIIIGSFFMLNLVLGVLSGEFAKER
ERVENRRAFLKLRRQQQIERELNGYMEWISKAEEVILAEDETDGEQRHPFDGALRRTTIK
KSKTDLLNPEEAEDQLADIASVGSPFARASIKSAKLENSTFFHKKERRMRFYIRRMVKTQA
FYWTVLSLVALNTLCVAIVHYNQPEWLSDFLYYAEFIFLGLFMSEMFIKMYGLGTRPYFHS
SFNCFDCGVIIGSIFEVIWAVIKPGTSFGISVLRALRLLRIFKVTKYWASLRNLVVSLLNSMK
SIISLLFLLFLFIVVFALLGMQLFGGQFNFDEGTPPTNFDTFPAAIMTVFQILTGEDWNEVM
YDGIKSQGGVQGGMVFSIYFIVLTLFGNYTLLNVFLAIAVDNLANAQELTKDEQEEEEAA
NQKLALQKAKEVAEVSPLSAANMSIAVKEQQKNQKPAKSVWEQRTSEMRKQNLLASREA
LYNEMDPDERWKAAYTRHLRPDMKTHLDRPLVVDPQENRNNNTNKSRAAEPTVDQRLG
QQRAEDFLRKQARYHDRARDPSGSAGLDARRPWAGSQEAELSREGPYGRESDHHAREGS
LEQPGFWEGEAERGKAGDPHRRHVHRQGGSRESRSGSPRTGADGEHRRHRAHRRPGEEG
PEDKAERRARHREGSRPARGGEGEGEGPDGGERRRRHRHGAPATYEGDARREDKERRHR
RRKENQGSGVPVSGPNLSTTRPIQQDLGRQDPPLAEDIDNMKNNKLATAESAAPHGSLGH
AGLPQSPAKMGNSTDPGPMLAIPAMATNPQNAASRRTPNNPGNPSNPGPPKTPENSLIVTN
PSGTQTNSAKTARKPDHTTVDIPPACPPPLNHTVVQVNKNANPDPLPKKEEEKKEEEEDD
RGEDGPKPMPPYSSMFILSTTNPLRRLCHYILNLRYFEMCILMVIAMSSIALAAEDPVQPN
APRNNVLRYFDYVFTGVFTFEMVIKMIDLGLVLHQGAYFRDLWNILDFIVVSGALVAFAFT
GNSKGKDINTIKSLRVLRVLRPLKTIKRLPKLKAVFDCVVNSLKNVFNILIVYMLFMFIFAV
VAVQLFKGKFFHCTDESKEFEKDCRGKYLLYEKNEVKARDREWKKYEFHYDNVLWALLT
LFTVSTGEGWPQVLKHSVDATFENQGPSPGYRMEMSIFYVVYFVVFPFFFVNIFVALIIITF
QEQGDKMMEEYSLEKNERACIDFAISAKPLTRHMPQNKQSFQYRMWQFVVSPPFEYTIM
AMIALNTIVLMMKFYGASVAYENALRVFNIVFTSLFSLECVLKVMAFGILNYFRDAWNIF
DFVTVLGSITDILVTEFGNNFINLSFLRLFRAARLIKLLRQGYTIRILLWTFVQSFKALPYVC
LLIAMLFFIYAIIGMQVFGNIGIDVEDEDSDEDEFQITEHNNFRTFFQALMLLFRSATGEAW
HNIMLSCLSGKPCDKNSGILTRECGNEFAYFYFVSFIFLCSFLMLNLFVAVIMDNFEYLTRD
SSILGPHHLDEYVRVWAEYDPAAWGRMPYLDMYQMLRHMSPPLGLGKKCPARVAYKRL
LRMDLPVADDNTVHFNSTLMALIRTALDIKIAKGGADKQQMDAELRKEMMAIWPNLSQK
TLDLLVTPHKSTDLTVGKIYAAMMIMEYYRQSKAKKLQAMREEQDRTPLMFQRMEPPSP
TQEGGPGQNALPSTQLDPGGALMAHESGLKESPSWVTQRAQEMFQKTGTWSPEQGPPTD
MPNSQPNSQSVEMREMGRDGYSDSEHYLPMEGQGRAASMPRLPAENQRRRGRPRGNNL
STISDTSPMKRSASVLGPKARRLDDYSLERVPPEENQRHHQRRRDRSHRASERSLGRYTD

VDTGLGTDLSMTTQSGDLPSKERDQERGRPKDRKHRQHHHHHHHHHHPPPPDKDRYAQ
ERPDHGRARARDQRWSRSPSEGREHMAHRQGSSSVSGSPAPSTSGTSTPRRGRRQLPQTP
STPRPHVSYSPVIRKAGGSGPPQQQQQQQQQQQAVARPGRAATSGPRRYPGPTAEPLAGD
RPPTGGHSSGRSPRMERRVPGPARSESPRACRHGGARWPASGPHVSEGPPGPRHHGYYRG
SDYDEADGPGSGGGEEAMAGAYDAPPPVRHASSGATGRSPRTPRASGPACASPSRHGRRL
PNGYYPAHGLARPRGPGSRKGLHEPYSESDDDWC

>O95471 multipass membrane protein
MANSGLQLLGFSMALLGWVGLVACTAIPQWQMSSYAGDNIITAQAMYKGLWMDCVTQS
TGMMSCKMYDSVLALSAALQATRALMVVSLVLGFLAMFVATMGMKCTRCGGDDKVKK
ARIAMGGGIIFIVAGLAALVACSWYGHQIVTDFYNPLIPTNIKYEFGPAIFIGWAGSALVILG
GALLSCSCPGNESKAGYRVPRSYPKSNSSKEYV

>Q7Z3T1 multipass membrane protein
MDGTNGSTQTHFILLGFSDRPHLERILFVVILIAYLLTLVGNTTIILVSRLDPHLHTPMYFFL
AHLSFLDLSFTTSSIPQLLYNLNGCDKTISYMGCAIQLFLFLGLGGVECLLLAVMAYDRCV
AICKPLHYMVIMNPRLCRGLVSVTWGCGVANSLAMSPVTLRLPRCGHHEVDHFLREMPA
LIRMACVSTVAIEGTVFVLAVGVVLSPLVFILLSYSYIVRAVLQIRSASGRQKAFGTCGSHLT
VVSLFYGNIIYMYMQPGASSSQDQGMFLMLFYNIVTPLLNPLIYTLRNREVKGALGRLLL
GKRELGKE

>O60462 single-pass type I membrane protein
MDMFPLTWVFLALYFSRHQVRGQPDPPCGGRLNSKDAGYITSPGYPQDYPSHQNCEWIV
YAPEPNQKIVLNFNPHFEIEKHDCKYDFIEIRDGDSESADLLGKHCGNIAPPTIISSGSMLYI
RFTSDYARQGAGFSLRYEIFKTGSEDCSKNFTSPNGTIESPGFPEKYPHNLDCTFTILAKPK
MEIILQFLIFDLEHDPLQVGEGDCKYDWLDIWDGIPHVGPLIGKYCGTKTPSELRSSTGILS
LTFHTDMAVAKDGFSARYYLVHQEPLENFQCNVPLGMESGRIANEQISASSTYSDGRWTP
QQSRLHGDDNGWTPNLDSNKEYLQVDLRFLTMLTAIATQGAISRETQNGYYVKSYKLEV
STNGEDWMVYRHGKNHKVFQANNDATEVVLNKLHAPLLTRFVRIRPQTWHSGIALRLEL
FGCRVTDAPCSNMLGMLSGLIADSQISASSTQEYLWSPSAARLVSSRSGWFPRIPQAQPGE
EWLQVDLGTPKTVKGVIIQGARGGDSITAVEARAFVRKFKVSYSLNGKDWEYIQDPRTQQ
PKLFEGNMHYDTPDIRRFDPIPAQYVRVYPERWSPAGIGMRLEVLGCDWTDSKPTVETLG
PTVKSEETTTPYPTEEEATECGENCSFEDDKDLQLPSGFNCNFDFLEEPCGWMYDHAKWL
RTTWASSSSPNDRTFPDDRNFLRLQSDSQREGQYARLISPPVHLPRSPVCMEFQYQATGGR
GVALQVVREASQESKLLWVIREDQGGEWKHGRIILPSYDMEYQIVFEGVIGKGRSGEIAID
DIRISTDVPLENCMEPISAFAGENFKVDIPEIHEREGYEDEIDDEYEVDWSNSSSATSGSGAP
STDKEKSWLYTLDPILITIIAMSSLGVLLGATCAGLLLYCTCSYSGLSSRSCTTLENYNFELY
DGLKHKVKMNHQKCCSEA

>Q14574 single-pass type I membrane protein
MAAAGPRRSVRGAVCLHLLLTLVIFSRAGEACKKVILNVPSKLEADKIIGRVNLEECFRSA
DLIRSSDPDFRVLNDGSVYTARAVALSDKKRSFTIWLSDKRKQTQKEVTVLLEHQKKVSK
TRHTRETVLRRAKRRWAPIPCSMQENSLGPFPLFLQQVESDAAQNYTVFYSISGRGVDKE
PLNLFYIERDTGNLFCTRPVDREEYDVFDLIAYASTADGYSADLPLPLPIRVEDENDNHPVF

TEAIYNFEVLESSRPGTTVGVVCATDRDEPDTMHTRLKYSILQQTPRSPGLFSVHPSTGVIT
TVSHYLDREVVDKYSLIMKVQDMDGQFFGLIGTSTCIITVTDSNDNAPTFRQNAYEAFVE
ENAFNVEILRIPIEDKDLINTANWRVNFTILKGNENGHFKISTDKETNEGVLSVVKPLNYEE
NRQVNLEIGVNNEAPFARDIPRVTALNRALVTVHVRDLDEGPECTPAAQYVRIKENLAVGS
KINGYKAYDPENRNGNGLRYKKLHDPKGWITIDEISGSIITSKILDREVETPKNELYNITVL
AIDKDDRSCTGTLAVNIEDVNDNPPEILQEYVVICKPKMGYTDILAVDPDEPVHGAPFYFS
LPNTSPEISRLWSLTKVNDTAARLSYQKNAGFQEYTIPITVKDRAGQAATKLLRVNLCECT
HPTQCRATSRSTGVILGKWAILAILLGIALLFSVLLTLVCGVFGATKGKRFPEDLAQQNLIIS
NTEAPGDDRVCSANGFMTQTTNNSSQGFCGTMGSGMKNGGQETIEMMKGGNQTLESCR
GAGHHHTLDSCRGGHTEVDNCRYTYSEWHSFTQPRLGEKLHRCNQNEDRMPSQDYVLT
YNYEGRGSPAGSVGCCSEKQEEDGLDFLNNLEPKFITLAEACTKR

>P25189 single-pass type I membrane protein
MAPGAPSSSPSPILAVLLFSSLVLSPAQAIVVYTDREVHGAVGSRVTLHCSFWSSEWVSDDI
SFTWRYQPEGGRDAISIFHYAKGQPYIDEVGTFKERIQWVGDPRWKDGSIVIHNLDYSDN
GTFTCDVKNPPDIVGKTSQVTLYVFEKVPTRYGVVLGAVIGGVLGVVLLLLLLFYVVRYC
WLRRQAALQRRLSAMEKGKLHKPGKDASKRGRQTPVLYAMLDHSRSTKAVSEKKAKGL
GESRKDKK

>P55283 single-pass type I membrane protein
MTAGAGVLLLLLLSLSGALRAHNEDLTTRETCKAGFSEDDYTALISQNILEGEKLLQVKFSS
CVGTKGTQYETNSMDFKVGADGTVFATRELQVPSEQVAFTVTAWDSQTAEKWDAVVRLL
VAQTSSPHSGHKPQKGKKVVALDPSPPPKDTLLPWPQHQNANGLRRRKRDWVIPPINVPE
NSRGPFPQQLVRIRSDKDNDIPIRYSITGVGADQPPMEVFSIDSMSGRMYVTRPMDREEHA
SYHLRAHAVDMNGNKVENPIDLYIYVIDMNDNRPEFINQVYNGSVDEGSKPGTYVMTVT
ANDADDSTTANGMVRYRIVTQTPQSPSQNMFTINSETGDIVTVAAGLDREKVQQYTVIVQ
ATDMEGNLNYGLSNTATAIITVTDVNDNPPEFTASTFAGEVPENRVETVVANLTVMDRDQP
HSPNWNAVYRIISGDPSGHFSVRTDPVTNEGMVTVVKAVDYELNRAFMLTVMVSNQAPL
ASGIQMSFQSTAGVTISIMDINEAPYFPSNHKLIRLEEGVPPGTVLTTFSAVDPDRFMQQAV
RYSKLSDPASWLHINATNGQITTAAVLDRESLYTKNNVYEATFLAADNGIPPASGTGTLQIY
LIDINDNAPELLPKEAQICEKPNLNAINITAADADVDPNIGPYVFELPFVPAAVRKNWTITR
LNGDYAQLSLRILYLEAGMYDVPIIVTDSGNPPLSNTSIIKVKVCPCDDNGDCTTIGAVAAA
GLGTGAIVAILICILILLTMVLLFVMWMKRREKERHTKQLLIDPEDDVRDNILKYDEEGGG
EEDQDYDLSQLQQPEAMGHVPSKAPGVRRVDERPVGAEPQYPIRPMVPHPGDIGDFINEG
LRAADNDPTAPPYDSLLVFDYEGSGSTAGSVSSLNSSSSGDQDYDYLNDWGPRFKKLAD
MYGGGEED

>O00624 multipass membrane protein
MDGKPATRKGPDFCSLRYGLALIMHFSNFTMITQRVSLSIAIIAMVNTTQQQGLSNASTEG
PVADAFNNSSISIKEFDTKASVYQWSPETQGIIFSSINYGIILTLIPSGYLAGIFGAKKMLGAG
LLISSLLTLFTPLAADFGVILVIMVRTVQGMAQGMAWTGQFTIWAKWAPPLERSKLTTIAG
SGSAFGSFIILCVGGLISQALSWPFIFYIFGSTGCVCCLLWFTVIYDDPMHHPCISVREKEHIL
SSLAQQPSSPGRAVPIKAMVTCLPLWAIFLGFFSHFWLCTIILTYLPTYISTLLHVNIRDSGVL
SSLPFIAAASCTILGGQLADFLLSRNLLRLITVRKLFSSLGLLLPSICAVALPFVASSYVITIILL

ILIPGTSNLCDSGFIINTLDIAPRYASFLMGISRGFGLIAGIISSTATGFLISQVGPVY

>P08473 single-pass type II membrane protein
MGKSESQMDITDINTPKPKKKQRWTPLEISLSVLVLLLTIIAVTMIALYATYDDGICKSSDCI
KSAARLIQNMDATTEPCTDFFKYACGGWLKRNVIPETSSRYGNFDILRDELEVVLKDVLQ
EPKTEDIVAVQKAKALYRSCINESAIDSRGGEPLLKLLPDIYGWPVATENWEQKYGASWTA
EKAIAQLNSKYGKKVLINLFVGTDDKNSVNHVIHIDQPRLGLPSRDYYECTGIYKEACTAY
VDFMISVARLIRQEERLPIDENQLALEMNKVMELEKEIANATAKPEDRNDPMLLYNKMTL
AQIQNNFSLEINGKPFSWLNFTNEIMSTVNISITNEEDVVVYAPEYLTKLKPILTKYSARDL
QNLMSWRFIMDLVSSLSRTYKESRNAFRKALYGTTSETATWRRCANYVNGNMENAVGRL
YVEAAFAGESKHVVEDLIAQIREVFIQTLDDLTWMDAETKKRAEEKALAIKERIGYPDDIV
SNDNKLNNEYLELNYKEDEYFENIIQNLKFSQSKQLKKLREKVDKDEWISGAAVVNAFYS
SGRNQIVFPAGILQPPFFSAQQSNSLNYGGIGMVIGHEITHGFDDNGRNFNKDGDLVDWW
TQQSASNFKEQSQCMVYQYGNFSWDLAGGQHLNGINTLGENIADNGGLGQAYRAYQNY
IKKNGEEKLLPGLDLNHKQLFFLNFAQVWCGTYRPEYAVNSIKTDVHSPGNFRIIGTLQNS
AEFSEAFHCRKNSYMNPEKKCRVW

>Q9NRR3 lipid-anchor
MSEFWLCFNCCIAEQPQPKRRRRIDRSMIGEPTNFVHTAHVGSGDLFSGMNSVSSIQNQM
QSKGGYGGGMPANVQMQLVDTKAG

>P27487 single-pass type II membrane protein
MKTPWKVLLGLLGAAALVTIITVPVVLLNKGTDDATADSRKTYTLTDYLKNTYRLKLYSL
RWISDHEYLYKQENNILVFNAEYGNSSVFLENSTFDEFGHSINDYSISPDGQFILLEYNYVK
QWRHSYTASYDIYDLNKRQLITEERIPNNTQWVTWSPVGHKLAYVWNNDIYVKIEPNLPS
YRITWTGKEDIIYNGITDWVYEEEVFSAYSALWWSPNGTFLAYAQFNDTEVPLIEYSFYSD
ESLQYPKTVRVPYPKAGAVNPTVKFFVVNTDSLSSVTNATSIQITAPASMLIGDHYLCDVT
WATQERISLQWLRRIQNYSVMDICDYDESSGRWNCLVARQHIEMSTTGWVGRFRPSEPHF
TLDGNSFYKIISNEEGYRHICYFQIDKKDCTFITKGTWEVIGIEALTSDYLYYISNEYKGMP
GGRNLYKIQLSDYTKVTCLSCELNPERCQYYSVSFSKEAKYYQLRCSGPGLPLYTLHSSVN
DKGLRVLEDNSALDKMLQNVQMPSKKLDFIILNETKFWYQMILPPHFDKSKKYPLLLDVY
AGPCSQKADTVFRLNWATYLASTENIIVASFDGRGSGYQGDKIMHAINRRLGTFEVEDQIE
AARQFSKMGFVDNKRIAIWGWSYGGYVTSMVLGSGSGVFKCGIAVAPVSRWEYYDSVY
TERYMGLPTPEDNLDHYRNSTVMSRAENFKQVEYLLIHGTADDNVHFQQSAQISKALVD
VGVDFQAMWYTDEDHGIASSTAHQHIYTHMSHFIKQCFSLP

>Q9ULZ9 GPI-anchor, lipid-anchor
MRRRAARGPGPPPPGPGLSRLPLPLLLLLALGTRGGCAAPAPAPRAEDLSLGVEWLSRFG
YLPPADPTTGQLQTQEELSKAITAMQQFGGLEATGILDEATLALMKTPRCSLPDLPVLTQA
RRRRQAPAPTKWNKRNLSWRVRTFPRDSPLGHDTVRALMYYALKVWSDIAPLNFHEVAG
SAADIQIDFSKADHNDGYPFDGPGGTVAHAFFPGHHHTAGDTHFDDDEAWTFRSSDAHG
MDLFAVAVHEFGHAIGLSHVAAAHSIMRPYYQGPVGDPLRYGLPYEDKVRVWQLYGVRE
SVSPTAQPEEPPLLPEPPDNRSSAPPRKDVPHRCSTHFDAVAQIRGEAFFFKGKYFWRLTRD
RHLVSLQPAQMHRFWRGLPLHLDSVDAVYERTSDHKIVFFKGDRYWVFKDNNVEEGYPR

PVSDFSLPPGGIDAAFSWAHNDRTYFFKDQLYWRYDDHTRHMDPGYPAQSPLWRGVPST
LDDAMRWSDGASYFFRGQEYWKVLDGELEVAPGYPQSTARDWLVCGDSQADGSVAAG
VDAAEGPRAPPGQHDQSRSEDGYEVCSCTSGASSPPGAPGPLVAATMLLLLPPLSPGALWT
AAQALTL

>P10635 peripheral membrane protein
MGLEALVPLAVIVAIFLLLVDLMHRRQRWAARYPPGPLPLPGLGNLLHVDFQNTPYCFDQ
LRRRFGDVFSLQLAWTPVVVLNGLAAVREALVTHGEDTADRPPVPITQILGFGPRSQGVFL
ARYGPAWREQRRFSVSTLRNLGLGKKSLEQWVTEEAACLCAAFANHSGRPFRPNGLLDK
AVSNVIASLTCGRRFEYDDPRFLRLLDLAQEGLKEESGFLREVLNAVPVLLHIPALAGKVL
RFQKAFLTQLDELLTEHRMTWDPAQPPRDLTEAFLAEMEKAKGNPESSFNDENLRIVVAD
LFSAGMVTTSTTLAWGLLLMILHPDVQRRVQQEIDDVIGQVRRPEMGDQAHMPYTTAVIH
EVQRFGDIVPLGVTHMTSRDIEVQGFRIPKGTTLITNLSSVLKDEAVWEKPFRFHPEHFLD
AQGHFVKPEAFLPFSAGRRACLGEPLARMELFLFFTSLLQHFSFSVPTGQPRPSHHGVFAF
LVSPSPYELCAVPR

>Q13639 multipass membrane protein
MDKLDANVSSEEGFGSVEKVVLLTFLSTVILMAILGNLLVMVAVCWDRQLRKIKTNYFIV
SLAFADLLVSVLVMPFGAIELVQDIWIYGEVFCLVRTSLDVLLTTASIFHLCCISLDRYYAIC
CQPLVYRNKMTPLRIALMLGGCWVIPTFISFLPIMQGWNNIGIIDLIEKRKFNQNSNSTYCV
FMVNKPYAITCSVVAFYIPFLLMVLAYYRIYVTAKEHAHQIQMLQRAGASSESRPQSADQ
HSTHRMRTETKAAKTLCIIMGCFCLCWAPFFVTNIVDPFIDYTVPGQVWTAFLWLGYINSG
LNPFLYAFLNKSFRRAFLIILCCDDERYRRPSILGQTVPCSTTTINGSTHVLRDAVECGGQW
ESQCHPPATSPLVAAQPSDT

>P40967 single-pass type I membrane protein
MDLVLKRCLLHLAVIGALLAVGATKVPRNQDWLGVSRQLRTKAWNRQLYPEWTEAQRL
DCWRGGQVSLKVSNDGPTLIGANASFSIALNFPGSQKVLPDGQVIWVNNTIINGSQVWGG
QPVYPQETDDACIFPDGGPCPSGSWSQKRSFVYVWKTWGQYWQVLGGPVSGLSIGTGRA
MLGTHTMEVTVYHRRGSRSYVPLAHSSSAFTITDQVPFSVSVSQLRALDGGNKHFLRNQP
LTFALQLHDPSGYLAEADLSYTWDFGDSSGTLISRALVVTHTYLEPGPVTAQVVLQAAIPL
TSCGSSPVPGTTDGHRPTAEAPNTTAGQVPTTEVVGTTPGQAPTAEPSGTTSVQVPTTEVIS
TAPVQMPTAESTGMTPEKVPVSEVMGTTLAEMSTPEATGMTPAEVSIVVLSGTTAAQVTT
TEWVETTARELPIPEPEGPDASSIMSTESITGSLGPLLDGTATLRLVKRQVPLDCVLYRYGSF
SVTLDIVQGIESAEILQAVPSGEGDAFELTVSCQGGLPKEACMEISSPGCQPPAQRLCQPVL
PSPACQLVLHQILKGGSGTYCLNVSLADTNSLAVVSTQLIMPGQEAGLGQVPLIVGILLVL
MAVVLASLIYRRRLMKQDFSVPQLPHSSSHWLRLPRIFCSCPIGENSPLLSGQQV

>P02724 single-pass type I membrane protein
MYGKIIFVLLLSEIVSISASSTTGVAMHTSTSSSVTKSYISSQTNDTHKRDTYAATPRAHEVS
EISVRTVYPPEEETGERVQLAHHFSEPEITLIIFGVMAGVIGTILLISYGIRRLIKKSPSDVKPL
PSPDTDVPLSSVEIENPETSDQ

>Q9BX84 multipass membrane protein

MKEQPVLERLQSQKSWIKGVFDKRECSTIIPSSKNPHRCTPVCQVCQNLIRCYCGRLIGDH
AGIDYSWTISAAKGKESEQWSVEKHTTKSPTDTFGTINFQDGEHTHHAKYIRTSYDTKLD
HLLHLMLKEWKMELPKLVISVHGGIQNFTMPSKFKEIFSQGLVKAAETTGAWIITEGINTG
VSKHVGDALKSHSSHSLRKIWTVGIPPWGVIENQRDLIGKDVVCLYQTLDNPLSKLTTLNS
MHSHFILSDDGTVGKYGNEMKLRRNLEKYLSLQKIHCRSRQGVPVVGLVVEGGPNVILS
VWETVKDKDPVVVCEGTGRAADLLAFTHKHLADEGMLRPQVKEEIICMIQNTFNFSLKQ
SKHLFQILMECMVHRDCITIFDADSEEQQDLDLAILTALLKGTNLSASEQLNLAMAWDRV
DIAKKHILIYEQHWKPDALEQAMSDALVMDRVDFVKLLIEYGVNLHRFLTIPRLEELYNTK
QGPTNTLLHHLVQDVKQHTLLSGYRITLIDIGLVVEYLIGRAYRSNYTRKHFRALYNNLYR
KYKHQRHSSGNRNESAESTLHSQFIRTAQPYKFKEKSIVLHKSRKKSKEQNVSDDPESTGF
LYPYNDLLVWAVLMKRQKMAMFFWQHGEEATVKAVIACILYRAMAHEAKESHMVDDAS
EELKNYSKQFGQLALDLLEKAFKQNERMAMTLLTYELRNWSNSTCLKLAVSGGLRPFVS
HTCTQMLLTDMWMGRLKMRKNSWLKIIISIILPPTILTLEFKSKAEMSHVPQSQDFQFMW
YYSDQNASSSKESASVKEYDLERGHDEKLDENQHFGLESGHQHLPWTRKVYEFYSAPIV
KFWFYTMAYLAFLMLFTYTVLVEMQPQPSVQEWLVSIYIFTNAIEVVREICISEPGKFTQK
VKVWISEYWNLTETVAIGLFSAGFVLRWGDPPFHTAGRLIYCIDIIFWFSRLLDFFAVNQHA
GPYVTMIAKMTANMFYIVIIMAIVLLSFGVARKAILSPKEPPSWSLARDIVFEPYWMIYGE
VYAGEIDVCSSQPSCPPGSFLTPFLQAVYLFVQYIIMVNLLIAFFNNVYLDMESISNNLWKY
NRYRYIMTYHEKPWLPPPLILLSHVGLLLRRLCCHRAPHDQEEGDVGLKLYLSKEDLKKL
HDFEEQCVEKYFHEKMEDVNCSCEERIRVTSERVTEMYFQLKEMNEKVSFIKDSLLSLDS
QVGHLQDLSALTVDTLKVLSAVDTLQEDEALLAKRKHSTCKKLPHSWSNVICAEVLGSM
EIAGEKKYQYYSMPSSLLRSLAGGRHPPRVQRGALLEITNSKREATNVRNDQERQETQSSI
VVSGVSPNRQAHSKYGQFLLVPSNLKRVPFSAETVLPLSRPSVPDVLATEQDIQTEVLVHL
TGQTPVVSDWASVDEPKEKHEPIAHLLDGQDKAEQVLPTLSCTPEPMTMSSPLSQAKIMQ
TGGGYVNWAFSEGDETGVFSIKKKWQTCLPSTCDSDSSRSEQHQKQAQDSSLSDNSTRSA
QSSECSEVGPWLQPNTSFWINPLRRYRPFARSHSFRFHKEEKLMKICKIKNLSGSSEIGQGA
WVKAKMLTKDRRLSKKKKNTQGLQVPIITVNACSQSDQLNPEPGENSISEEEYSKNWFTV
SKFSHTGVEPYIHQKMKTKEIGQCAIQISDYLKQSQEDLSKNSLWNSRSTNLNRNSLLKSSI
GVDKISASLKSPQEPHHHYSAIERNNLMRLSQTIPFTPVQLFAGEEITVYRLEESSPLNLDK
SMSSWSQRGRAAMIQVLSREEMDGGLRKAMRVVSTWSEDDILKPGQVFIVKSFLPEVVR
TWHKIFQESTVLHLCLREIQQQRAAQKLIYTFNQVKPQTIPYTPRFLEVFLIYCHSANQWL
TIEKYMTGEFRKYNNNNGDEITPTNTLEELMLAFSHWTYEYTRGELLVLDLQGVGENLTD
PSVIKPEVKQSRGMVFGPANLGEDAIRNFIAKHHCNSCCRKLKLPDLKRNDYSPERINSTF
GLEIKIESAEEPPARETGRNSPEDDMQL

>P49447 multipass membrane protein
MEGGAAAATPTALPYYVAFSQLLGLTLVAMTGAWLGLYRGGIAWESDLQFNAHPLCMVI
GLIFLQGNALLVYRVFRNEAKRTTKVLHGLLHIFALVIALVGLVAVFDYHRKKGYADLYSL
HSWCGILVFVLYFVQWLVGFSFFLFPGASFSLRSRYRPQHIFFGATIFLLSVGTALLGLKEAL
LFNLGGKYSAFEPEGVLANVLGLLLACFGGAVLYILTRADWKRPSQAEEQALSMDFKTLT
EGDSPGSQ

>Q8IWY4 peripheral membrane protein
MGAAAVRWHLCVLLALGTRGRLAGGSGLPGSVDVDECSEGTDDCHIDAICQNTPKSYKC

LCKPGYKGEGKQCEDIDECENDYYNGGCVHECINIPGNYRCTCFDGFMLAHDGHNCLDV
DECQDNNGGCQQICVNAMGSYECQCHSGFFLSDNQHTCIHRSNEGMNCMNKDHGCAHI
CRETPKGGVACDCRPGFDLAQNQKDCTLTCNYGNGGCQHSCEDTDTGPTCGCHQKYAL
HSDGRTCIETCAVNNGGCDRTCKDTATGVRCSCPVGFTLQPDGKTCKDINECLVNNGGCD
HFCRNTVGSFECGCRKGYKLLTDERTCQDIDECSFERTCDHICINSPGSFQCLCHRGYILYG
TTHCGDVDECSMSNGSCDQGCVNTKGSYECVCPPGRRLHWNGKDCVETGKCLSRAKTS
PRAQLSCSKAGGVESCFLSCPAHTLFVPDSENSYVLSCGVPGPQGKALQKRNGTSSGLGPS
CSDAPTTPIKQKARFKIRDAKCHLRPHSQARAKETARQPLLDHCHVTFVTLKCDSSKKRR
RGRKSPSKEVSHITAEFEIETKMEEASDTCEADCLRKRAEQSLQAAIKTLRKSIGRQQFYV
QVSGTEYEVAQRPAKALEGQGACGAGQVLQDSKCVACGPGTHFGGELGQCVSCMPGTY
QDMEGQLSCTPCPSSDGLGLPGARNVSECGGQCSPGFFSADGFKPCQACPVGTYQPEPGR
TGCFPCGGGLLTKHEGTTSFQDCEAKVHCSPGHHYNTTTHRCIRCPVGTYQPEFGQNHCI
TCPGNTSTDFDGSTNVTHCKNQHCGGELGDYTGYIESPNYPGDYPANAECVWHIAPPPKR
RILIVVPEIFLPIEDECGDVLVMRKSASPTSITTYETCQTYERPIAFTSRSRKLWIQFKSNEGN
SGKGFQVPYVTYDEDYQQLIEDIVRDGRLYASENHQEILKDKKLIKALFDVLAHPQNYFK
YTAQESKEMFPRSFIKLLRSKVSRFLRPYK

>P31641 multipass membrane protein
MATKEKLQCLKDFHKDILKPSPGKSPGTRPEDEAEGKPPQREKWSSKIDFVLSVAGGFVG
LGNVWRFPYLCYKNGGGAFLIPYFIFLFGSGLPVFFLEIIIGQYTSEGGITCWEKICPLFSGIG
YASVVIVSLLNVYYIVILAWATYYLFQSFQKELPWAHCNHSWNTPHCMEDTMRKNKSVW
ITISSTNFTSPVIEFWERNVLSLSPGIDHPGSLKWDLALCLLLVWLVCFFCIWKGVRSTGKV
VYFTATFPFAMLLVLLVRGLTLPGAGAGIKFYLYPDITRLEDPQVWIDAGTQIFFSYAICLGA
MTSLGSYNKYKYNSYRDCMLLGCLNSGTSFVSGFAIFSILGFMAQEQGVDIADVAESGPG
LAFIAYPKAVTMMPLPTFWSILFFIMLLLLGLDSQFVEVEGQITSLVDLYPSFLRKGYRREIF
IAFVCSISYLLGLTMVTEGGMYVFQLFDYYAASGVCLLWVAFFECFVIAWIYGGDNLYDGI
EDMIGYRPGPWMKYSWAVITPVLCVGCFIFSLVKYVPLTYNKTYVYPNWAIGLGWSLALS
SMLCVPLVIVIRLCQTEGPFLVRVKYLLTPREPNRWAVEREGATPYNSRTVMNGALVKPTHI
IVETMM

>Q16288 single-pass type I membrane protein
MDVSLCPAKCSFWRIFLLGSVWLDYVGSVLACPANCVCSKTEINCRRPDDGNLFPLLEGQ
DSGNSNGNASINITDISRNITSIHIENWRSLHTLNAVDMELYTGLQKLTIKNSGLRSIQPRAF
AKNPHLRYINLSSNRLTTLSWQLFQTLSLRELQLEQNFFNCSCDIRWMQLWQEQGEAKLN
SQNLYCINADGSQLPLFRMNISQCDLPEISVSHVNLTVREGDNAVITCNGSGSPLPDVDWIV
TGLQSINTHQTNLNWTNVHAINLTLVNVTSEDNGFTLTCIAENVVGMSNASVALTVYYPPR
VVSLEEPELRLEHCIEFVVRGNPPPTLHWLHNGQPLRESKIIHVEYYQEGEISEGCLLFNKP
THYNNGNYTLIAKNPLGTANQTINGHFLKEPFPESTDNFILFDEVSPTPPITVTHKPEEDTFG
VSIAVGLAAFACVLLVVLFVMINKYGRRSKFGMKGPVAVISGEEDSASPLHHINHGITTPSS
LDAGPDTVVIGMTRIPVIENPQYFRQGHNCHKPDTYVQHIKRRDIVLKRELGEGAFGKVF
LAECYNLSPTKDKMLVAVKALKDPTLAARKDFQREAELLTNLQHEHIVKFYGVCGDGDP
LIMVFEYMKHGDLNKFLRAHGPDAMILVDGQPRQAKGELGLSQMLHIASQIASGMVYLA
SQHFVHRDLATRNCLVGANLLVKIGDFGMSRDVYSTDYYRLFNPSGNDFCIWCEVGGHT
MLPIRWMPPESIMYRKFTTESDVWSFGVILWEIFTYGKQPWFQLSNTEVIECITQGRVLERP

RVCPKEVYDVMLGCWQREPQQRLNIKEIYKILHALGKATPIYLDILG

>Q9Y5U8 multipass membrane protein
MAGALVRKAADYVRSKDFRDYLMSTHFWGPVANWGLPIAAINDMKKSPEIISGRMTFAL
CCYSLTFMRFAYKVQPRNWLLFACHATNEVAQLIQGGRLIKHEMTKTASA

>P22413 single-pass type II membrane protein
MERDGCAGGGSRGGEGGRAPREGPAGNGRDRGRSHAAEAPGDPQAAASLLAPMDVGEE
PLEKAARARTAKDPNTYKVLSLVLSVCVLTTILGCIFGLKPSCAKEVKSCKGRCFERTFGN
CRCDAACVELGNCCLDYQETCIEPEHIWTCNKFRCGEKRLTRSLCACSDDCKDKGDCCIN
YSSVCQGEKSWVEEPCESINEPQCPAGFETPPTLLFSLDGFRAEYLHTWGGLLPVISKLKK
CGTYTKNMRPVYPTKTFPNHYSIVTGLYPESHGIIDNKMYDPKMNASFSLKSKEKFNPEW
YKGEPIWVTAKYQGLKSGTFFWPGSDVEINGIFPDIYKMYNGSVPFEERILAVLQWLQLPK
DERPHFYTLYLEEPDSSGHSYGPVSSEVIKALQRVDGMVGMLMDGLKELNLHRCLNLILIS
DHGMEQGSCKKYIYLNKYLGDVKNIKVIYGPAARLRPSDVPDKYYSFNYEGIARNLSCRE
PNQHFKPYLKHFLPKRLHFAKSDRIEPLTFYLDPQWQLALNPSERKYCGSGFHGSDNVFS
NMQALFVGYGPGFKHGIEADTFENIEVYNLMCDLLNLTPAPNNGTHGSLNHLLKNPVYTP
KHPKEVHPLVQCPFTRNPRDNLGCSCNPSILPIEDFQTQFNLTVAEEKIIKHETLPYGRPRVL
QKENTICLLSQHQFMSGYSQDILMPLWTSYTVDRNDSFSTEDFSNCLYQDFRIPLSPVHKC
SFYKNNTKVSYGFLSPPQLNKNSSGIYSEALLTTNIVPMYQSFQVIWRYFHDTLLRKYAEE
RNGVNVVSGPVFDFDYDGRCDSLENLRQKRRVIRNQEILIPTHFFIVLTSCKDTSQTPLHCE
NLDTLAFILPHRTDNSESCVHGKHDSSWVEELLMLHRARITDVEHITGLSFYQQRKEPVSD
ILKLKTHLPTFSQED

>P57735 lipid-anchor
MGNGTEEDYNFVFKVVLIGESGVGKTNLLSRFTRNEFSHDSRTTIGVEFSTRTVMLGTAAV
KAQIWDTAGLERYRAITSAYYRGAVGALLVFDLTKHQTYAVVERWLKELYDHAEATIVVM
LVGNKSDLSQAREVPTEEARMFAENNGLLFLETSALDSTNVELAFETVLKEIFAKVSKQRQ
NSIRTNAITLGSAQAGQEPGPGEKRACCISL

>P07357 multipass membrane protein
MFAVVFFILSLMTCQPGVTAQEKVNQRVRRAATPAAVTCQLSNWSEWTDCFPCQDKKYR
HRSLLQPNKFGGTICSGDIWDQASCSSSTTCVRQAQCGQDFQCKETGRCLKRHLVCNGD
QDCLDGSDEDDCEDVRAIDEDCSQYEPIPGSQKAALGYNILTQEDAQSVYDASYYGGQCE
TVYNGEWRELRYDSTCERLYYGDDEKYFRKPYNFLKYHFEALADTGISSEFYDNANDLL
SKVKKDKSDSFGVTIGIGPAGSPLLVGVGVSHSQDTSFLNELNKYNEKKFIFTRIFTKVQTA
HFKMRKDDIMLDEGMLQSLMELPDQYNYGMYAKFINDYGTHYITSGSMGGIYEYILVID
KAKMESLGITSRDITTCFGGSLGIQYEDKINVGGGLSGDHCKKFGGGKTERARKAMAVED
IISRVRGGSSGWSGGLAQNRSTITYRSWGRSLKYNPVVIDFEMQPIHEVLRHTSLGPLEAK
RQNLRRALDQYLMEFNACRCGPCFNNGVPILEGTSCRCQCRLGSLGAACEQTQTEGAKA
DGSWSCWSSWSVCRAGIQERRRECDNPAPQNGGASCPGRKVQTQAC

>Q12772 multipass membrane protein
MDDSGELGGLETMETLTELGDELTLGDIDEMLQFVSNQVGEFPDLFSEQLCSSFPGSGGSG

SSSGSSGSSSSSSNGRGSSSGAVDPSVQRSFTQVTLPSFSPSAASPQAPTLQVKVSPTSVPTT
PRATPILQPRPQPQPQPQTQLQQQTVMITPTFSTTPQTRIIQQPLIYQNAATSFQVLQPQVQS
LVTSSQVQPVTIQQQVQTVQAQRVLTQTANGTLQTLAPATVQTVAAPQVQQVPVLVQPQII
KTDSLVLTTLKTDGSPVMAAVQNPALTALTTPIQTAALQVPTLVGSSGTILTTMPVMMGQE
KVPIKQVPGGVKQLEPPKEGERRTTHNIIEKRYRSSINDKIIELKDLVMGTDAKMHKSGVL
RKAIDYIKYLQQVNHKLRQENMVLKLANQKNKLLKGIDLGSLVDNEVDLKIEDFNQNVL
LMSPPASDSGSQAGFSPYSIDSEPGSPLLDDAKVKDEPDSPPVALGMVDRSRILLCVLTFLC
LSFNPLTSLLQWGGAHDSDQHPHSGSGRSVLSFESGSGGWFDWMMPTLLLWLVNGVIVL
SVFVKLLVHGEPVIRPHSRSSVTFWRHRKQADLDLARGDFAAAAGNLQTCLAVLGRALPT
SRLDLACSLSWNVIRYSLQKLRLVRWLLKKVFQCRRATPATEAGFEDEAKTSARDAALAY
HRLHQLHITGKLPAGSACSDVHMALCAVNLAECAEEKIPPSTLVEIHLTAAMGLKTRCGG
KLGFLASYFLSRAQSLCGPEHSAVPDSLRWLCHPLGQKFFMERSWSVKSAAKESLYCAQR
NPADPIAQVHQAFCKNLLERAIESLVKPQAKKKAGDQEEESCEFSSALEYLKLLHSFVDSV
GVMSPPLSRSSVLKSALGPDIICRWWTSAITVAISWLQGDDAAVRSHFTKVERIPKALEVT
ESPLVKAIFHACRAMHASLPGKADGQQSSFCHCERASGHLWSSLNVSGATSDPALNHVVQ
LLTCDLLLSLRTALWQKQASASQAVGETYHASGAELAGFQRDLGSLRRLAHSFRPAYRKV
FLHEATVRLMAGASPTRTHQLLEHSLRRRTTQSTKHGEVDAWPGQRERATAILLACRHLP
LSFLSSPGQRAVLLAEAARTLEKVGDRRSCNDCQQMIVKLGGGTAIAAS

>Q13439 peripheral membrane protein
MFKKLKQKISEEQQQLQQALAPAQASSNSSTPTRMRSRTSSFTEQLDEGTPNRESGDTQSF
AQKLQLRVPSVESLFRSPIKESLFRSSSKESLVRTSSRESLNRLDLDSSTASFDPPSDMDSEA
EDLVGNSDSLNKEQLIQRLRRMERSLSSYRGKYSELVTAYQMLQREKKKLQGILSQSQDK
SLRRIAELREELQMDQQAKKHLQEEFDASLEEKDQYISVLQTQVSLLKQRLRNGPMNVD
VLKPLPQLEPQAEVFTKEENPESDGEPVVEDGTSVKTLETLQQRVKRQENLLKRCKETIQS
HKEQCTLLTSEKEALQEQLDERLQELEKIKDLHMAEKTKLITQLRDAKNLIEQLEQDKGM
VIAETKRQMHETLEMKEEEIAQLRSRIKQMTTQGEELREQKEKSERAAFEELEKALSTAQ
KTEEARRKLKAEMDEQIKTIEKTSEEERISLQQELSRVKQEVVDVMKKSSEEQIAKLQKLH
EKELARKEQELTKKLQTREREFQEQMKVALEKSQSEYLKISQEKEQQESLALEELELQKK
AILTESENKLRDLQQEAETYRTRILELESSLEKSLQENKNQSKDLAVHLEAEKNKHNKEIT
VMVEKHKTELESLKHQQDALWTEKLQVLKQQYQTEMEKLREKCEQEKETLLKDKEIIFQ
AHIEEMNEKTLEKLDVKQTELESLSSELSEVLKARHKLEEELSVLKDQTDKMKQELEAK
MDEQKNHHQQQVDSIIKEHEVSIQRTEKALKDQINQLELLLKERDKHLKEHQAHVENLEA
DIKRSEGELQQASAKLDVFQSYQSATHEQTKAYEEQLAQLQQKLLDLETERILLTKQVAE
VEAQKKDVCTELDAHKIQVQDLMQQLEKQNSEMEQKVKSLTQVYESKLEDGNKEQEQT
KQILVEKENMILQMREGQKKEIEILTQKLSAKEDSIHILNEEYETKFKNQEKKMEKVKQKA
KEMQETLKKKLLDQEAKLKKELENTALELSQKEKQFNAKMLEMAQANSAGISDAVSRLE
TNQKEQIESLTEVHRRELNDVISIWEKKLNQQAEELQEIHEIQLQEKEQEVAELKQKILLFG
CEKEEMNKEITWLKEEGVKQDTTLNELQEQLKQKSAHVNSLAQDETKLKAHLEKLEVDL
NKSLKENTFLQEQLVELKMLAEEDKRKVSELTSKLKTTDEEFQSLKSSHEKSNKSLEDKSL
EFKKLSEELAIQLDICCKKTEALLEAKTNELINISSSKTNAILSRISHCQHRTTKVKEALLIKT
CTVSELEAQLRQLTEEQNTLNISFQQATHQLEEKENQIKSMKADIESLVTEKEALQKEGGN
QQQAASEKESCITQLKKELSENINAVTLMKEELKEKKVEISSLSKQLTDLNVQLQNSISLSE
KEAAISSLRKQYDEEKCELLDQVQDLSFKVDTLSKEKISALEQVDDWSNKFSEWKKKAQ

SRFTQHQNTVKELQIQLELKSKEAYEKDEQINLLKEELDQQNKRFDCLKGEMEDDKSKM
EKKESNLETELKSQTARIMELEDHITQKTIEIESLNEVLKNYNQQKDIEHKELVQKLQHFQE
LGEEKDNRVKEAEEKILTLENQVYSMKAELETKKKELEHVNLSVKSKEEELKALEDRLES
ESAAKLAELKRKAEQKIAAIKKQLLSQMEEKEEQYKKGTESHLSELNTKLQEREREVHIL
EEKLKSVESSQSETLIVPRSAKNVAAYTEQEEADSQGCVQKTYEEKISVLQRNLTEKEKLL
QRVGQEKEETVSSHFEMRCQYQERLIKLEHAEAKQHEDQSMIGHLQEELEEKNKKYSLIV
AQHVEKEGGKNNIQAKQNLENVFDDVQKTLQEKELTCQILEQKIKELDSCLVRQKEVHR
VEMEELTSKYEKLQALQQMDGRNKPTELLEENTEEKSKSHLVQPKLLSNMEAQHNDLEF
KLAGAEREKQKLGKEIVRLQKDLRMLRKEHQQELEILKKEYDQEREEKIKQEQEDLELK
HNSTLKQLMREFNTQLAQKEQELEMTIKETINKAQEVEAELLESHQEETNQLLKKIAEKD
DDLKRTAKRYEEILDAREEEMTAKVRDLQTQLEELQKKYQQKLEQEENPGNDNVTIMEL
QTQLAQKTTLISDSKLKEQEFREQIHNLEDRLKKYEKNVYATTVGTPYKGGNLYHTDVSL
FGEPTEFEYLRKVLFEYMMGRETKTMAKVITTVLKFPDDQTQKILEREDARLMFTSPRSGI
F

>P00395 multipass membrane protein
MFADRWLFSTNHKDIGTLYLLFGAWAGVLGTALSLLIRAELGQPGNLLGNDHIYNVIVTA
HAFVMIFFMVMPIMIGGFGNWLVPLMIGAPDMAFPRMNNMSFWLLPPSLLLLLASAMVE
AGAGTGWTVYPPLAGNYSHPGASVDLTIFSLHLAGVSSILGAINFITTIINMKPPAMTQYQT
PLFVWSVLITAVLLLLSLPVLAAGITMLLTDRNLNTTFFDPAGGGDPILYQHLFWFFGHPEV
YILILPGFGMISHIVTYYSGKKEPFGYMGMVWAMMSIGFLGFIVWAHHMFTVGMDVDTR
AYFTSATMIIAIPTGVKVFSWLATLHGSNMKWSAAVLWALGFIFLFTVGGLTGIVLANSSL
DIVLHDTYYVVAHFHYVLSMGAVFAIMGGFIHWFPLFSGYTLDQTYAKIHFTIMFIGVNLT
FFPQHFLGLSGMPRRYSDYPDAYTTWNILSSVGSFISLTAVMLMIFMIWEAFASKRKVLMV
EEPSMNLEWLYGCPPPYHTFEEPVYMKS

>P21964 single-pass type II membrane protein
MPEAPPLLLAAVLLGLVLLVVLLLLLRHWGWGLCLIGWNEFILQPIHNLLMGDTKEQRIL
NHVLQHAEPGNAQSVLEAIDTYCEQKEWAMNVGDKKGKIVDAVIQEHQPSVLLELGAYC
GYSAVRMARLLSPGARLITIEINPDCAAITQRMVDFAGVKDKVTLVVGASQDIIPQLKKKY
DVDTLDMVFLDHWKDRYLPDTLLLEECGLLRKGTVLLADNVICPGAPDFLAHVRGSSCF
ECTHYQSFLEYREVVDGLEKAIYKGPGSEAGP

>Q8WWB7 single-pass type I membrane protein
MRGSVECTWGWGHCAPSPLLLWTLLLFAAPFGLLGEKTRQVSLEVIPNWLGPLQNLLHIR
AVGTNSTLHYVWSSLGPLAVVMVATNTPHSTLSVNWSLLLSPEPDGGLMVLPKDSIQFSS
ALVFTRLLEFDSTNVSDTAAKPLGRPYPPYSLADFSWNNITDSLDPATLSATFQGHPMNDP
TRTFANGSLAFRVQAFSRSSRPAQPPRLLHTADTCQLEVALIGASPRGNRSLFGLEVATLGQ
GPDCPSMQEQHSIDDEYAPAVFQLDQLLWGSLPSGFAQWRPVAYSQKPGGRESALPCQAS
PLHPALAYSLPQSPIVRAFFGSQNNFCAFNLTFGASTGPGYWDQHYLSWSMLLGVGFPPV
DGLSPLVLGIMAVALGAPGLMLLGGGLVLLLHHKKYSEYQSIN

>P42345 peripheral membrane protein
MLGTGPAAATTAATTSSNVSVLQQFASGLKSRNEETRAKAAKELQHYVTMELREMSQEE

STRFYDQLNHHIFELVSSSDANERKGGILAIASLIGVEGGNATRIGRFANYLRNLLPSNDPV
VMEMASKAIGRLAMAGDTFTAEYVEFEVKRALEWLGADRNEGRRHAAVLVLRELAISVP
TFFFQQVQPFFDNIFVAVWDPKQAIREGAVAALRACLILTTQREPKEMQKPQWYRHTFEEA
EKGFDETLAKEKGMNRDDRIHGALLILNELVRISSMEGERLREEMEEITQQQLVHDKYCK
DLMGFGTKPRHITPFTSFQAVQPQQSNALVGLLGYSSHQGLMGFGTSPSPAKSTLVESRCC
RDLMEEKFDQVCQWVLKCRNSKNSLIQMTILNLLPRLAAFRPSAFTDTQYLQDTMNHVL
SCVKKEKERTAAFQALGLLSVAVRSEFKVYLPRVLDIIRAALPPKDFAHKRQKAMQVDAT
VFTCISMLARAMGPGIQQDIKELLEPMLAVGLSPALTAVLYDLSRQIPQLKKDIQDGLLKM
LSLVLMHKPLRHPGMPKGLAHQLASPGLTTLPEASDVGSITLALRTLGSFEFEGHSLTQFV
RHCADHFLNSEHKEIRMEAARTCSRLLTPSIHLISGHAHVVSQTAVQVVADVLSKLLVVGI
TDPDPDIRYCVLASLDERFDAHLAQAENLQALFVALNDQVFEIRELAICTVGRLSSMNPAF
VMPFLRKMLIQILTELEHSGIGRIKEQSARMLGHLVSNAPRLIRPYMEPILKALILKLKDPDP
DPNPGVINNVLATIGELAQVSGLEMRKWVDELFIIIMDMLQDSSLLAKRQVALWTLGQLV
ASTGYVVEPYRKYPTLLEVLLNFLKTEQNQGTRREAIRVLGLLGALDPYKHKVNIGMIDQ
SRDASAVSLSESKSSQDSSDYSTSEMLVNMGNLPLDEFYPAVSMVALMRIFRDQSLSHHHT
MVVQAITFIFKSLGLKCVQFLPQVMPTFLNVIRVCDGAIREFLFQQLGMLVSFVKSHIRPY
MDEIVTLMREFWVMNTSIQSTIILLIEQIVVALGGEFKLYLPQLIPHMLRVFMHDNSPGRIV
SIKLLAAIQLFGANLDDYLHLLLPPIVKLFDAPEAPLPSRKAALETVDRLTESLDFTDYASRI
IHPIVRTLDQSPELRSTAMDTLSSLVFQLGKKYQIFIPMVNKVLVRHRINHQRYDVLICRIV
KGYTLADEEEDPLIYQHRMLRSGQGDALASGPVETGPMKKLHVSTINLQKAWGAARRVS
KDDWLEWLRRLSLELLKDSSSPSLRSCWALAQAYNPMARDLFNAAFVSCWSELNEDQQ
DELIRSIELALTSQDIAEVTQTLLNLAEFMEHSDKGPLPLRDDNGIVLLGERAAKCRAYAK
ALHYKELEFQKGPTPAILESLISINNKLQQPEAAAGVLEYAMKHFGELEIQATWYEKLHEW
EDALVAYDKKMDTNKDDPELMLGRMRCLEALGEWGQLHQQCCEKWTLVNDETQAKM
ARMAAAAAWGLGQWDSMEEYTCMIPRDTHDGAFYRAVLALHQDLFSLAQQCIDKARD
LLDAELTAMAGESYSRAYGAMVSCHMLSELEEVIQYKLVPERREIIRQIWWERLQGCQRI
VEDWQKILMVRSLVVSPHEDMRTWLKYASLCGKSGRLALAHKTLVLLLGVDPSRQLDHP
LPTVHPQVTYAYMKNMWKSARKIDAFQHMQHFVQTMQQQAQHAIATEDQQHKQELHK
LMARCFLKLGEWQLNLQGINESTIPKVLQYYSAATEHDRSWYKAWHAWAVMNFEAVLH
YKHQNQARDEKKKLRHASGANITNATTAATTAATATTTASTEGSNSESEAESTENSPTPSPL
QKKVTEDLSKTLLMYTVPAVQGFFRSISLSRGNNLQDTLRVLTLWFDYGHWPDVNEALVE
GVKAIQIDTWLQVIPQLIARIDTPRPLVGRLIHQLLTDIGRYHPQALIYPLTVASKSTTTARH
NAANKILKNMCEHSNTLVQQAMMVSEELIRVAILWHEMWHEGLEEASRLYFGERNVKG
MFEVLEPLHAMMERGPQTLKETSFNQAYGRDLMEAQEWCRKYMKSGNVKDLTQAWDL
YYHVFRRISKQLPQLTSLELQYVSPKLLMCRDLELAVPGTYDPNQPIIRIQSIAPSLQVITSK
QRPRKLTLMGSNGHEFVFLLKGHEDLRQDERVMQLFGLVNTLLANDPTSLRKNLSIQRYA
VIPLSTNSGLIGWVPHCDTLHALIRDYREKKKILLNIEHRIMLRMAPDYDHLTLMQKVEVF
EHAVNNTAGDDLAKLLWLKSPSSEVWFDRRTNYTRSLAVMSMVGYILGLGDRHPSNLML
DRLSGKILHIDFGDCFEVAMTREKFPEKIPFRLTRMLTNAMEVTGLDGNYRITCHTVMEVL
REHKDSVMAVLEAFVYDPLLNWRLMDTNTKGNKRSRTRTDSYSAGQSVEILDGVELGEP
AHKKTGTTVPESIHSFIGDGLVKPEALNKKAIQIINRVRDKLTGRDFSHDDTLDVPTQVELL
IKQATSHENLCQCYIGWCPFW

>P07766 single-pass type I membrane protein

MQSGTHWRVLGLCLLSVGVWGQDGNEEMGGITQTPYKVSISGTTVILTCPQYPGSEILWQ
HNDKNIGGDEDDKNIGSDEDHLSLKEFSELEQSGYYVCYPRGSKPEDANFYLYLRARVCE
NCMEMDVMSVATIVIVDICITGGLLLLVYYWSKNRKAKAKPVTRGAGAGGRQRGQNKE
RPPPVPNPDYEPIRKGQRDLYSGLNQRRI

>P23219 peripheral membrane protein
MSRSLLLWFLLFLLLLPPLPVLLADPGAPTPVNPCCYYPCQHQGICVRFGLDRYQCDCTRT
GYSGPNCTIPGLWTWLRNSLRPSPSFTHFLLTHGRWFWEFVNATFIREMLMRLVLTVRSNL
IPSPPTYNSAHDYISWESFSNVSYYTRILPSVPKDCPTPMGTKGKKQLPDAQLLARRFLLR
RKFIPDPQGTNLMFAFFAQHFTHQFFKTSGKMGPGFTKALGHGVDLGHIYGDNLERQYQL
RLFKDGKLKYQVLDGEMYPPSVEEAPVLMHYPRGIPPQSQMAVGQEVFGLLPGLMLYAT
LWLREHNRVCDLLKAEHPTWGDEQLFQTTRLILIGETIKIVIEEYVQQLSGYFLQLKFDPEL
LFGVQFQYRNRIAMEFNHLYHWHPLMPDSFKVGSQEYSYEQFLFNTSMLVDYGVEALVD
AFSRQIAGRIGGGRNMDHHILHVAVDVIRESREMRLQPFNEYRKRFGMKPYTSFQELVGE
KEMAAELEELYGDIDALEFYPGLLLEKCHPNSIFGESMIEIGAPFSLKGLLGNPICSPEYWK
PSTFGGEVGFNIVKTATLKKLVCLNTKTCPYVSFRVPDASQDDGPAVERPSTEL

>Q9HCE7 peripheral membrane protein
MSNPGTRRNGSSIKIRLTVLCAKNLAKKDFFRLPDPFAKIVVDGSGQCHSTDTVKNTLDPK
WNQHYDLYVGKTDSITISVWNHKKIHKKQGAGFLGCVRLLSNAISRLKDTGYQRLDLCK
LNPSDTDAVRGQIVVSLQTRDRIGTGGSVVDCRGLLENEGTVYEDSGPGRPLSCFMEEPAP
YTDSTGAAAGGGNCRFVESPSQDQRLQAQRLRNPDVRGSLQTPQNRPHGHQSPELPEGY
EQRTTVQGQVYFLHTQTGVSTWHDPRIPSPSGTIPGGDAAFLYEFLLQGHTSEPRDLNSVN
CDELGPLPPGWEVRSTVSGRIYFVDHNNRTTQFTDPRLHHIMNHQCQLKEPSQPLPLPSEG
SLEDEELPAQRYERDLVQKLKVLRHELSLQQPQAGHCRIEVSREEIFEESYRQIMKMRPKD
LKKRLMVKFRGEEGLDYGGVAREWLYLLCHEMLNPYYGLFQYSTDNIYMLQINPDSSINP
DHLSYFHFVGRIMGLAVFHGHYINGGFTVPFYKQLLGKPIQLSDLESVDPELHKSLVWILE
NDITPVLDHTFCVEHNAFGRILQHELKPNGRNVPVTEENKKEYVRLYVNWRFMRGIEAQF
LALQKGFNELIPQHLLKPFDQKELELIIGGLDKIDLNDWKSNTRLKHCVADSNIVRWFWQ
AVETFDEERRARLLQFVTGSTRVPLQGFKALQGSTGAAGPRLFTIHLIDANTDNLPKAHTC
FNRIDIPPYESYEKLYEKLLTAVEETCGFAVE

>P00846 multipass membrane protein
MNENLFASFIAPTILGLPAAVLIILFPPLLIPTSKYLINNRLITTQQWLIKLTSKQMMTMHNT
KGRTWSLMLVSLIIFIATTNLLGLLPHSFTPTTQLSMNLAMAIPLWAGTVIMGFRSKIKNAL
AHFLPQGTPTPLIPMLVIIETISLLIQPMALAVRLTANITAGHLLMHLIGSATLAMSTINLPST
LIIFTILILLTILEIAVALIQAYVFTLLVSLYLHDNT

>Q9H0U3 multipass membrane protein
MAARWRFWCVSVTMVVALLIVCDVPSASAQRKKEMVLSEKVSQLMEWTNKRPVIRMN
GDKFRRLVKAPPRNYSVIVMFTALQLHRQCVVCKQADEEFQILANSWRYSSAFTNRIFFA
MVDFDEGSDVFQMLNMNSAPTFINFPAKGKPKRGDTYELQVRGFSAEQIARWIADRTDV
NIRVIRPPNYAGPLMLGLLLAVIGGLVYLRRSNMEFLFNKTGWAFAALCFVLAMTSGQMW
NHIRGPPYAHKNPHTGHVNYIHGSSQAQFVAETHIVLLFNGGVTLGMVLLCEAATSDMDI

GKRKIMCVAGIGLVVLFFSWMLSIFRSKYHGYPYSFLMS

>P25929 multipass membrane protein
MNSTLFSQVENHSVHSNFSEKNAQLLAFENDDCHLPLAMIFTLALAYGAVIILGVSGNLAL
IIIILKQKEMRNVTNILIVNLSFSDLLVAIMCLPFTFVYTLMDHWVFGEAMCKLNPFVQCVS
ITVSIFSLVLIAVERHQLIINPRGWRPNNRHAYVGIAVIWVLAVASSLPFLIYQVMTDEPFQN
VTLDAYKDKYVCFDQFPSDSHRLSYTTLLLVLQYFGPLCFIFICYFKIYIRLKRRNNMMDK
MRDNKYRSSETKRINIMLLSIVVAFAVCWLPLTIFNTVFDWNHQIIATCNHNLLFLLCHLTA
MISTCVNPIFYGFLNKNFQRDLQFFFNFCDFRSRDDDYETIAMSTMHTDVSKTSLKQASPV
AFKKINNNDDNEKI

>Q6P0Q8 peripheral membrane protein
MKRSRCRDRPQPPPPDRREDGVQRAAELSQSLPPRRRAPPGRQRLEERTGPAGPEGKEQD
VVTGVSPLLFRKLSNPDIFSSTGKVKLQRQLSQDDCKLWRGNLASSLSGKQLLPLSSSVHS
SVGQVTWQSSGEASNLVRMRNQSLGQSAPSLTAGLKELSLPRRGSFCRTSNRKSLIVTSST
SPTLPRPHSPLHGHTGNSPLDSPRNFSPNAPAHFSFVPARRTDGRRWSLASLPSSGYGTNTP
SSTVSSSCSSQEKLHQLPFQPTADELHFLTKHFSTESVPDEEGRQSPAMRPRSRSLSPGRSPV
SFDSEIIMMNHVYKERFPKATAQMEERLAEFISSNTPDSVLPLADGALSFIHHQVIEMARD
CLDKSRSGLITSQYFYELQDNLEKLLQDAHERSESSEVAFVMQLVKKLMIIIARPARLLECL
EFDPEEFYHLLEAAEGHAKEGQGIKCDIPRYIVSQLGLTRDPLEEMAQLSSCDSPDTPETD
DSIEGHGASLPSKKTPSEEDFETIKLISNGAYGAVFLVRHKSTRQRFAMKKINKQNLILRNQ
IQQAFVERDILTFAENPFVVSMFCSFDTKRHLCMVMEYVEGGDCATLLKNIGALPVDMVR
LYFAETVLALEYLHNYGIVHRDLKPDNLLITSMGHIKLTDFGLSKIGLMSLTTNLYEGHIEK
DAREFLDKQVCGTPEYIAPEVILRQGYGKPVDWWAMGIILYEFLVGCVPFFGDTPEELFGQ
VISDEIVWPEGDEALPPDAQDLTSKLLHQNPLERLGTGSAYEVKQHPFFTGLDWTGLLRQ
KAEFIPQLESEDDTSYFDTRSERYHHMDSEDEEEVSEDGCLEIRQFSSCSPRFNKVYSSME
RLSLLEERRTPPPTKRSLSEEKEDHSDGLAGLKGRDRSWVIGSPEILRKRLSVSESSHTESD
SSPPMTVRRRCSGLLDAPRFPEGPEEASSTLRRQPQEGIWVLTPPSGEGVSGPVTEHSGEQ
RPKLDEEAVGRSSGSSPAMETRGRGTSQLAEGATAKAISDLAVRRARHRLLSGDSTEKRTA
RPVNKVIKSASATALSLLIPSEHHTCSPLASPMSPHSQSSNPSSRDSSPSRDFLPALGSMRPPI
IIHRAGKKYGFTLRAIRVYMGDSDVYTVHHMVWHVEDGGPASEAGLRQGDLITHVNGEP
VHGLVHTEVVELILKSGNKVAISTTPLENTSIKVGPARKGSYKAKMARRSKRSRGKDGQE
SRKRSSLFRKITKQASLLHTSRSLSSLNRSLSSGESGPGSPTHSHSLSPRSPTQGYRVTPDAV
HSVGGNSSQSSSPSSSVPSSPAGSGHTRPSSLHGLAPKLQRQYRSPRRKSAGSIPLSPLAHTP
SPPPPTASPQRSPSPLSGHVAQAFPTKLHLSPPLGRQLSRPKSAEPPRSPLLKRVQSAEKLAA
ALAASEKKLATSRKHSLDLPHSELKKELPPREVSPLEVVGARSVLSGKGALPGKGVLQPA
PSRALGTLRQDRAERRESLQKQEAIREVDSSEDDTEEGPENSQGAQELSLAPHPEVSQSVA
PKGAGESGEEDPFPSRDPRSLGPMVPSLLTGITLGPPRMESPSGPHRRLGSPQAIEEAASSSS
AGPNLGQSGATDPIPPEGCWKAQHLHTQALTALSPSTSGLTPTSSCSPPSSTSGKLSMWSW
KSLIEGPDRASPSRKATMAGGLANLQDLENTTPAQPKNLSPREQGKTQPPSAPRLAHPSYE
DPSQGWLWESECAQAVKEDPALSITQVPDASGDRRQDVPCRGCPLTQKSEPSLRRGQEPG
GHQKHRDLALVPDELLKQT

>Q96H72 multipass membrane protein

MPGCPCPGCGMAGPRLLFLTALALELLERAGGSQPALRSRGTATACRLDNKESESWGALL
SGERLDTWICSLLGSLMVGLSGVFPLLVIPLEMGTMLRSEAGAWRLKQLLSFALGGLLGN
VFLHLLPEAWAYTCSASPGGEGQSLQQQQQLGLWVIAGILTFLALEKMFLDSKEEGTSQAP
NKDPTAAAAALNGGHCLAQPAAEPGLGAVVRSIKVSGYLNLLANTIDNFTHGLAVAASFL
VSKKIGLLTTMAILLHEIPHEVGDFAILLRAGFDRWSAAKLQLSTALGGLLGAGFAICTQSP
KGVVGCSPAAEETAAWVLPFTSGGFLYIALVNVLPDLLEEEDPWRSLQQLLLLCAGIVVM
VLFSLFVD

>Q96RD9 single-pass type I membrane protein
MLLWVILLVLAPVSGQFARTPRPIIFLQPPWTTVFQGERVTLTCKGFRFYSPQKTKWYHRY
LGKEILRETPDNILEVQESGEYRCQAQGSPLSSPVHLDFSSASLILQAPLSVFEGDSVVLRC
RAKAEVTLNNTIYKNDNVLAFLNKRTDFHIPHACLKDNGAYRCTGYKESCCPVSSNTVKI
QVQEPFTRPVLRASSFQPISGNPVTLTCETQLSLERSDVPLRFRFFRDDQTLGLGWSLSPNF
QITAMWSKDSGFYWCKAATMPYSVISDSPRSWIQVQIPASHPVLTLSPEKALNFEGTKVTL
HCETQEDSLRTLYRFYHEGVPLRHKSVRCERGASISFSLTTENSGNYYCTADNGLGAKPSK
AVSLSVTVPVSHPVLNLSSPEDLIFEGAKVTLHCEAQRGSLPILYQFHHEGAALERRSANSA
GGVAISFSLTAEHSGNYYCTADNGFGPQRSKAVSLSVTVPVSHPVLTLSSAEALTFEGATVT
LHCEVQRGSPQILYQFYHEDMPLWSSSTPSVGRVSFSFSLTEGHSGNYYCTADNGFGPQRS
EVVSLFVTVPVSRPILTLRVPRAQAVVGDLLELHCEAPRGSPPILYWFYHEDVTLGSSSAPS
GGEASFNLSLTAEHSGNYSCEANNGLVAQHSDTISLSVIVPVSRPILTFRAPRAQAVVGDLL
ELHCEALRGSSPILYWFYHEDVTLGKISAPSGGGASFNLSLTTEHSGIYSCEADNGLEAQRS
EMVTLKVAVPVSRPVLTLRAPGTHAAVGDLLELHCEALRGSPLILYRFFHEDVTLGNRSSP
SGGASLNLSLTAEHSGNYSCEADNGLGAQRSETVTLYITGLTANRSGPFATGVAGGLLSIAG
LAAGALLLYCWLSRKAGRKPASDPARSPSDSDSQEPTYHNVPAWEELQPVYTNANPRGEN
VVYSEVRIIQEKKKHAVASDPRHLRNKGSPIIYSEVKVASTPVSGSLFLASSAPHR

>Q8NGY1 multipass membrane protein
MGQTNVTSWRDFVFLGFSSSGELQLLLFALFLSLYLVTLTSNVFIIIAIRLDSHLHTPMYLFL
SFLSFSETCYTLGIIPRMLSGLAGGDQAISYVGCAAQMFFSASWACTNCFLLAAMGFDRYV
AICAPLHYASHMNPTLCAQLVITSFLTGYLFGLGMTLVIFHLSFCSSHEIQHFFCDTPPVLSL
ACGDTGPSELRIFILSLLVLLVSFFFITISYAYILAAILRIPSAEGQKKAFSTCASHLTVVIIHYG
CASFVYLRPKASYSLERDQLIAMTYTVVTPLLNPIVYSLRNRAIQTALRNAFRGRLLGKG

>O43164 peripheral membrane protein
MSQYTEKEPAAMDQESGKAVWPKPAGGYQTITGRRYGRRHAYVSFKPCMTRHERSLGRA
GDDYEVLELDDVPKENSSGSSPLDQVDSSLPSEPIFEKSETEIPTCGSALNQTTESSQSFVAV
HHSEEGRDTLGSSTNLHNHSEGEYIPGACSASSVQNGIALVHTDSYDPDGKHGEDNDHLQ
LSAEVVEGSRYQESLGNTVFELENREAEAYTGLSPPVPSFNCEVRDEFEELDSVPLVKSSA
GDTEFVHQNSQEIQRSSQDEMVSTKQQNNTSQERQTEHSPEDAACGPGHICSEQNTNDRE
KNHGSSPEQVVRPKVRKLISSSQVDQETGFNRHEAKQRSVQRWREALEVEESGSDDLLIK
CEEYDGEHDCMFLDPPYSRVITQRETENNQMTSESGATAGRQEVDNTFWNGCGDYYQLY
DKDEDSSECSDGEWSASLPHRFSGTEKDQSSSDESWETLPGKDENEPELQSDSSGPEEENQ
ELSLQEGEQTSLEEGEIPWLQYNEVNESSSDEGNEPANEFAQPAFMLDGNNNLEDDSSVSE
DLDVDWSLFDGFADGLGVAEAISYVDPQFLTYMALEERLAQAMETALAHLESLAVDVEV

ANPPASKESIDGLPETLVLEDHTAIGQEQCCPICCSEYIKDDIATELPCHHFFHKPCVSIWLQ
KSGTCPVCRRHFPPAVIEASAAPSSEPDPDAPPSNDSIAEAP

>P20963 single-pass type I membrane protein
MKWKALFTAAILQAQLPITEAQSFGLLDPKLCYLLDGILFIYGVILTALFLRVKFSRSADAP
AYQQGQNQLYNELNLGRREEYDVLDKRRGRDPEMGGKPQRRKNPQEGLYNELQKDKM
AEAYSEIGMKGERRRGKGHDGLYQGLSTATKDTYDALHMQALPPR

>Q8NGL4 multipass membrane protein
MMASERNQSSTPTFILLGFSEYPEIQVPLFLVFLFVYTVTVVGNLGMIIIIRLNSKLHTIMCF
FLSHLSLTDFCFSTVVTPKLLENLVVEYRTISFSGCIMQFCFACIFGVTETFMLAAMAYDRF
VAVCKPLLYTTIMSQKLCALLVAGSYTWGIVCSLILTYFLLDLSFCESTFINNFICDHSVIVS
ASYSDPYISQRLCFIIAIFNEVSSLIIILTSYMLIFTTIMKMRSASGRQKTFSTCASHLTAITIFH
GTILFLYCVPNPKTSSLIVTVASVFYTVAIPMLNPLIYSLRNKDINNMFEKLVVTKLIYH

>P14416 multipass membrane protein
MDPLNLSWYDDDLERQNWSRPFNGSDGKADRPHYNYYATLLTLLIAVIVFGNVLVCMAV
SREKALQTTTNYLIVSLAVADLLVATLVMPWVVYLEVVGEWKFSRIHCDIFVTLDVMMCT
ASILNLCAISIDRYTAVAMPMLYNTRYSSKRRVTVMISIVWVLSFTISCPLLFGLNNADQNE
CIIANPAFVVYSSIVSFYVPFIVTLLVYIKIYIVLRRRRKRVNTKRSSRAFRAHLRAPLKGNC
THPEDMKLCTVIMKSNGSFPVNRRRVEAARRAQELEMEMLSSTSPPERTRYSPIPPSHHQL
TLPDPSHHGLHSTPDSPAKPEKNGHAKDHPKIAKIFEIQTMPNGKTRTSLKTMSRRKLSQQ
KEKKATQMLAIVLGVFIICWLPFFITHILNIHCDCNIPPVLYSAFTWLGYVNSAVNPIIYTTF
NIEFRKAFLKILHC

>P00403 multipass membrane protein
MAHAAQVGLQDATSPIMEELITFHDHALMIIFLICFLVLYALFLTLTTKLTNTNISDAQEMET
VWTILPAIILVLIALPSLRILYMTDEVNDPSLTIKSIGHQWYWTYEYTDYGGLIFNSYMLPPL
FLEPGDLRLLDVDNRVVLPIEAPIRMMITSQDVLHSWAVPTLGLKTDAIPGRLNQTTFTATR
PGVYYGQCSEICGANHSFMPIVLELIPLKIFEMGPVFTL

>Q8IYT8 peripheral membrane protein
MEVVGDFEYSKRDLVGHGAFAVVFRGRHRQKTDWEVAIKSINKKNLSKSQILLGKEIKIL
KELQHENIVALYDVQELPNSVFLVMEYCNGGDLADYLQAKGTLSEDTIRVFLHQIAAAMR
ILHSKGIIHRDLKPQNILLSYANRRKSSVSGIRIKIADFGFARYLHSNMMAATLCGSPMYMA
PEVIMSQHYDAKADLWSIGTVIYQCLVGKPPFQANSPQDLRMFYEKNRSLMPSIPRETSPY
LANLLLGLLQRNQKDRMDFEAFFSHPFLEQGPVKKSCPVPVPMYSGSVSGSSCGSSPSCRF
ASPPSLPDMQHIQEENLSSPPLGPPNYLQVSKDSASTSSKNSSCDTDDFVLVPHNISSDHSC
DMPVGTAGRRASNEFLVCGGQCQPTVSPHSETAPIPVPTQIRNYQRIEQNLTSTASSGTNVH
GSPRSAVVRRSNTSPMGFLRPGSCSPVPADTAQTVGRRLSTGSSRPYSPSPLVGTIPEQFSQ
CCCGHPQGHDSRSRNSSGSPVPQAQSPQSLLSGARLQSAPTLTDIYQNKQKLRKQHSDPV
CPSHTGAGYSYSPQPSRPGSLGTSPTKHLGSSPRSSDWFFKTPLPTIIGSPTKTTAPFKIPKTQ
ASSNLLALVTRHGPAEEQSKDGNEPRECAHCLLVQGSERQRAEQQSKAVFGRSVSTGKLS
DQQGKTPICRHQGSTDSLNTERPMDIAPAGACGGVLAPPAGTAASSKAVLFTVGSPPHSAA

APTCTHMFLRTRTTSVGPSNSGGSLCAMSGRVCVGSPPGPGFGSSPPGAEAAPSLRYVPYG
ASPPSLEGLITFEAPELPEETLMEREHTDTLRHLNVMLMFTECVLDLTAMRGGNPELCTSA
VSLYQIQESVVVDQISQLSKDWGRVEQLVLYMKAAQLLAASLHLAKAQIKSGKLSPSTAV
KQVVKNLNERYKFCITMCKKLTEKLNRFFSDKQRFIDEINSVTAEKLIYNCAVEMVQSAAL
DEMFQQTEDIVYRYHKAALLLEGLSRILQDPADIENVHKYKCSIERRLSALCHSTATV

>Q6Q0C1 multipass membrane protein
MDFVAGAIGGVCGVAVGYPLDTVKVRIQTEPKYTGIWHCVRDTYHRERVWGFYRGLSLP
VCTVSLVSSVSFGTYRHCLAHICRLRYGNPDAKPTKADITLSGCASGLVRVFLTSPTEVAK
VRLQTQTQAQKQQRRLSASGPLAVPPMCPVPPACPEPKYRGPLHCLATVAREEGLCGLYK
GSSALVLRDGHSFATYFLSYAVLCEWLSPAGHSRPDVPGVLVAGGCAGVLAWAVATPMDV
IKSRLQADGQGQRRYRGLLHCMVTSVREEGPRVLFKGLVLNCCRAFPVNMVVFVAYEAV
LRLARGLLT

>P08034 multipass membrane protein
MNWTGLYTLLSGVNRHSTAIGRVWLSVIFIFRIMVLVVAAESVWGDEKSSFICNTLQPGCN
SVCYDQFFPISHVRLWSLQLILVSTPALLVAMHVAHQQHIEKKMLRLEGHGDPLHLEEVKR
HKVHISGTLWWTYVISVVFRLLFEAVFMYVFYLLYPGYAMVRLVKCDVYPCPNTVDCFV
SRPTEKTVFTVFMLAASGICIILNVAEVVYLIIRACARRAQRRSNPPSRKGSGFGHRLSPEY
KQNEINKLLSEQDGSLKDILRRSPGTGAGLAEKSDRCSAC

>Q14D04 peripheral membrane protein
MHQLFRLVLGQKDLSRAGDLFSLDDSEIEDSLTEALEQIKIISSSSDYQTNNNDQAVVEICIT
RITTAIRETESIEKHAKALVGLWDSCLEHNLRPFGKDEDTPHAKIASDIMSCILQNYNRPPV
MALAIPIAVKFLHRGNKELCRNMSNYLSLAAITKADLLADHTEVIVKSILQGNTMLLRVLP
AVYEKQPQPINRHLTELLALMSQLEQPEQYHLLRLLHVAAKKKQLEVVQKCIPFLIGHLKD
STHNDIILNILIEIAVYEPVALNSFLPMLKEIGERFPYLTGQMARIYGAVGHVDEERARSCLT
YLVSQLANMEHSFHHILLLEIKSITDTFSSILGPQSRDIFRMSNSFTAIAKLLTRQLENTKAG
SGRRKISTEIEFPEKLEETKLIVTENEDHEKLQVKIQAFEDKINAGSNTPGSIRRYSLGQVSK
EERKNIRFNRSKSLAFHTMLTKGVGSDDGEDENRGDIPASISLSEIDPLGQGNDKLPFKTDT
ERSQLGESSVSYPNIIHIDSENLSETVKENSQEETPETTASPIEYQDKLYLHLKKNLSKVKAY
AMEIGKKIPVPDQCTIEDTVRSCVAKLFFTCSLKGHYCLYSKSSFILISQEPQPWIQIMFLFQ
QSLFPEPLSIQSHSVQFLRALWEKTQAGGAHSFETAMMESTFPQQKDLDQVQLHLEEVRF
FDVFGFSETAGAWQCFMCNNPEKATVVNQDGQPLIEGKLKEKQVRWKFIKRWKTRYFTL
AGNQLLFQKGKSKDDPDDCPIELSKVQSVKAVAKKRRDRSLPRAFEIFTDNKTYVFKAKD
EKNAEEWLQCINVAVAQAKERESREVTTYL

>P11532 peripheral membrane protein
MLWWEEVEDCYEREDVQKKTFTKWVNAQFSKFGKQHIENLFSDLQDGRRLLDLLEGLT
GQKLPKEKGSTRVHALNNVNKALRVLQNNNVDLVNIGSTDIVDGNHKLTLGLIWNIILHW
QVKNVMKNIMAGLQQTNSEKILLSWVRQSTRNYPQVNVINFTTSWSDGLALNALIHSHR
PDLFDWNSVVCQQSATQRLEHAFNIARYQLGIEKLLDPEDVDTTYPDKKSILMYITSLFQV
LPQQVSIEAIQEVEMLPRPPKVTKEEHFQLHHQMHYSQQITVSLAQGYERTSSPKPRFKSY
AYTQAAYVTTSDPTRSPFPSQHLEAPEDKSFGSSLMESEVNLDRYQTALEEVLSWLLSAED

TLQAQGEISNDVEVVKDQFHTHEGYMMDLTAHQGRVGNILQLGSKLIGTGKLSEDEETEV
QEQMNLLNSRWECLRVASMEKQSNLHRVLMDLQNQKLKELNDWLTKTEERTRKMEEEP
LGPDLEDLKRQVQQHKVLQEDLEQEQVRVNSLTHMVVVVDESSGDHATAALEEQLKVL
GDRWANICRWTEDRWVLLQDILLKWQRLTEEQCLFSAWLSEKEDAVNKIHTTGFKDQNE
MLSSLQKLAVLKADLEKKKQSMGKLYSLKQDLLSTLKNKSVTQKTEAWLDNFARCWDN
LVQKLEKSTAQISQAVTTTQPSLTQTTVMETVTTVTTREQILVKHAQEELPPPPPQKKRQIT
VDSEIRKRLDVDITELHSWITRSEAVLQSPEFAIFRKEGNFSDLKEKVNAIEREKAEKFRKL
QDASRSAQALVEQMVNEGVNADSIKQASEQLNSRWIEFCQLLSERLNWLEYQNNIIAFYN
QLQQLEQMTTTAENWLKIQPTTPSEPTAIKSQLKICKDEVNRLSDLQPQIERLKIQSIALKE
KGQGPMFLDADFVAFTNHFKQVFSDVQAREKELQTIFDTLPPMRYQETMSAIRTWVQQSE
TKLSIPQLSVTDYEIMEQRLGELQALQSSLQEQQSGLYYLSTTVKEMSKKAPSEISRKYQS
EFEEIEGRWKKLSSQLVEHCQKLEEQMNKLRKIQNHIQTLKKWMAEVDVFLKEEWPALG
DSEILKKQLKQCRLLVSDIQTIQPSLNSVNEGGQKIKNEAEPEFASRLETELKELNTQWDH
MCQQVYARKEALKGGLEKTVSLQKDLSEMHEWMTQAEEEYLERDFEYKTPDELQKAVE
EMKRAKEEAQQKEAKVKLLTESVNSVIAQAPPVAQEALKKELETLTTNYQWLCTRLNGK
CKTLEEVWACWHELLSYLEKANKWLNEVEFKLKTTENIPGGAEEISEVLDSLENLMRHSE
DNPNQIRILAQTLTDGGVMDELINEELETFNSRWRELHEEAVRRQKLLEQSIQSAQETEKS
LHLIQESLTFIDKQLAAYIADKVDAAQMPQEAQKIQSDLTSHEISLEEMKKHNQGKEAAQR
VLSQIDVAQKKLQDVSMKFRLFQKPANFEQRLQESKMILDEVKMHLPALETKSVEQEVV
QSQLNHCVNLYKSLSEVKSEVEMVIKTGRQIVQKKQTENPKELDERVTALKLHYNELGA
KVTERKQQLEKCLKLSRKMRKEMNVLTEWLAATDMELTKRSAVEGMPSNLDSEVAWGK
ATQKEIEKQKVHLKSITEVGEALKTVLGKKETLVEDKLSLLNSNWIAVTSRAEEWLNLLLE
YQKHMETFDQNVDHITKWIIQADTLLDESEKKKPQQKEDVLKRLKAELNDIRPKVDSTR
DQAANLMANRGDHCRKLVEPQISELNHRFAAISHRIKTGKASIPLKELEQFNSDIQKLLEPL
EAEIQQGVNLKEEDFNKDMNEDNEGTVKELLQRGDNLQQRITDERKREEIKIKQQLLQTK
HNALKDLRSQRRKKALEISHQWYQYKRQADDLLKCLDDIEKKLASLPEPRDERKIKEIDR
ELQKKKEELNAVRRQAEGLSEDGAAMAVEPTQIQLSKRWREIESKFAQFRRLNFAQIHTV
REETMMVMTEDMPLEISYVPSTYLTEITHVSQALLEVEQLLNAPDLCAKDFEDLFKQEES
LKNIKDSLQQSSGRIDIIHSKKTAALQSATPVERVKLQEALSQLDFQWEKVNKMYKDRQG
RFDRSVEKWRRFHYDIKIFNQWLTEAEQFLRKTQIPENWEHAKYKWYLKELQDGIGQRQ
TVVRTLNATGEEIIQQSSKTDASILQEKLGSLNLRWQEVCKQLSDRKKRLEEQKNILSEFQ
RDLNEFVLWLEEADNIASIPLEPGKEQQLKEKLEQVKLLVEELPLRQGILKQLNETGGPVL
VSAPISPEEQDKLENKLKQTNLQWIKVSRALPEKQGEIEAQIKDLGQLEKKLEDLEEQLNH
LLLWLSPIRNQLEIYNQPNQEGPFDVKETEIAVQAKQPDVEEILSKGQHLYKEKPATQPVKR
KLEDLSSEWKAVNRLLQELRAKQPDLAPGLTTIGASPTQTVTLVTQPVVTKETAISKLEMP
SSLMLEVPALADFNRAWTELTDWLSLLDQVIKSQRVMVGDLEDINEMIIKQKATMQDLEQ
RRPQLEELITAAQNLKNKTSNQEARTIITDRIERIQNQWDEVQEHLQNRRQQLNEMLKDST
QWLEAKEEAEQVLGQARAKLESWKEGPYTVDAIQKKITETKQLAKDLRQWQTNVDVAN
DLALKLLRDYSADDTRKVHMITENINASWRSIHKRVSEREAALEETHRLLQQFPLDLEKF
LAWLTEAETTANVLQDATRKERLLEDSKGVKELMKQWQDLQGEIEAHTDVYHNLDENS
QKILRSLEGSDDAVLLQRRLDNMNFKWSELRKKSLNIRSHLEASSDQWKRLHLSLQELLV
WLQLKDDELSRQAPIGGDFPAVQKQNDVHRAFKRELKTKEPVIMSTLETVRIFLTEQPLEG
LEKLYQEPRELPPEERAQNVTRLLRKQAEEVNTEWEKLNLHSADWQRKIDETLERLQELQ
EATDELDLKLRQAEVIKGSWQPVGDLLIDSLQDHLEKVKALRGEIAPLKENVSHVNDLAR

QLTTLGIQLSPYNLSTLEDLNTRWKLLQVAVEDRVRQLHEAHRDFGPASQHFLSTSVQGP
WERAISPNKVPYYINHETQTTCWDHPKMTELYQSLADLNNVRFSAYRTAMKLRRLQKAL
CLDLLSLSAACDALDQHNLKQNDQPMDILQIINCLTTIYDRLEQEHNNLVNVPLCVDMCL
NWLLNVYDTGRTGRIRVLSFKTGIISLCKAHLEDKYRYLFKQVASSTGFCDQRRLGLLLHD
SIQIPRQLGEVASFGGSNIEPSVRSCFQFANNKPEIEAALFLDWMRLEPQSMVWLPVLHRV
AAAETAKHQAKCNICKECPIIGFRYRSLKHFNYDICQSCFFSGRVAKGHKMHYPMVEYCT
PTTSGEDVRDFAKVLKNKFRTKRYFAKHPRMGYLPVQTVLEGDNMETPVTLINFWPVDS
APASSPQLSHDDTHSRIEHYASRLAEMENSNGSYLNDSISPNESIDDEHLLIQHYCQSLNQD
SPLSQPRSPAQILISLESEERGELERILADLEEENRNLQAEYDRLKQQHEHKGLSPLPSPPEM
MPTSPQSPRDAELIAEAKLLRQHKGRLEARMQILEDHNKQLESQLHRLRQLLEQPQAEAK
VNGTTVSSPSTSLQRSDSSQPMLLRVVGSQTSDSMGEEDLLSPPQDTSTGLEEVMEQLNNS
FPSSRGRNTPGKPMREDTM

>Q9Y2E8 multipass membrane protein
MGEKMAEEERFPNTTHEGFNVTLHTTLVVTTKLVLPTPGKPILPVQTGEQAQQEEQSSGM
TIFFSLLVLAICIILVHLLIRYRLHFLPESVAVVSLGILMGAVIKIIEFKKLANWKEEEMFRPN
MFFLLLLPPIIFESGYSLHKGNFFQNIGSITLFAVFGTAISAFVVGGGIYFLGQADVISKLNM
TDSFAFGSLISAVDPVATIAIFNALHVDPVLNMLVFGESILNDAVSIVLTNTAEGLTRKNMSD
VSGWQTFLQALDYFLKMFFGSAALGTLTGLISALVLKHIDLRKTPSLEFGMMIIFAYLPYG
LAEGISLSGIMAILFSGIVMSHYTHHNLSPVTQILMQQTLRTVAFLCETCVFAFLGLSIFSFP
HKFEISFVIWCIVLVLFGRAVNIFPLSYLLNFFRDHKITPKMMFIMWFSGLRGAIPYALSLHL
DLEPMEKRQLIGTTTIVIVLFTILLLGGSTMPLIRLMDIEDAKAHRRNKKDVNLSKTEKMG
NTVESEHLSELTEEEYEAHYIRRQDLKGFVWLDAKYLNPFFTRRLTQEDLHHGRIQMKTL
TNKWYEEVRQGPSGSEDDEQELL

>P45844 multipass membrane protein
MACLMAAFSVGTAMNASSYSAEMTEPKSVCVSVDEVVSSNMEATETDLLNGHLKKVDN
NLTEAQRFSSLPRRAAVNIEFRDLSYSVPEGPWWRKKGYKTLLKGISGKFNSGELVAIMGP
SGAGKSTLMNILAGYRETGMKGAVLINGLPRDLRCFRKVSCYIMQDDMLLPHLTVQEAM
MVSAHLKLQEKDEGRREMVKEILTALGLLSCANTRTGSLSGGQRKRLAIALELVNNPPVM
FFDEPTSGLDSASCFQVVSLMKGLAQGGRSIICTIHQPSAKLFELFDQLYVLSQGQCVYRG
KVCNLVPYLRDLGLNCPTYHNPADFVMEVASGEYGDQNSRLVRAVREGMCDSDHKRDL
GGDAEVNPFLWHRPSEEVKQTKRLKGLRKDSSSMEGCHSFSASCLTQFCILFKRTFLSIMR
DSVLTHLRITSHIGIGLLIGLLYLGIGNEAKKVLSNSGFLFFSMLFLMFAALMPTVLTFPLEM
GVFLREHLNYWYSLKAYYLAKTMADVPFQIMFPVAYCSIVYWMTSQPSDAVRFVLFAAL
GTMTSLVAQSLGLLIGAASTSLQVATFVGPVTAIPVLLFSGFFVSFDTIPTYLQWMSYISYVR
YGFEGVILSIYGLDREDLHCDIDETCHFQKSEAILRELDVENAKLYLDFIVLGIFFISLRLIAY
FVLRYKIRAER

>Q9BXA5 multipass membrane protein
MLGIMAWNATCKNWLAAEAALEKYYLSIFYGIEFVVGVLGNTIVVYGYIFSLKNWNSSNI
YLFNLSVSDLAFLCTLPMLIRSYANGNWIYGDVLCISNRYVLHANLYTSILFLTFISIDRYLII
KYPFREHLLQKKEFAILISLAIWVLVTLELLPILPLINPVITDNGTTCNDFASSGDPNYNLIYS
MCLTLLGFLIPLFVMCFFYYKIALFLKQRNRQVATALPLEKPLNLVIMAVVIFSVLFTPYHV

MRNVRIASRLGSWKQYQCTQVVINSFYIVTRPLAFLNSVINPVFYFLLGDHFRDMLMNQL
RHNFKSLTSFSRWAHELLLSFREK

>Q96LA5 single-pass type I membrane protein
MLLWSLLVIFDAVTEQADSLTLVAPSSVFEGDSIVLKCQGEQNWKIQKMAYHKDNKELSV
FKKFSDFLIQSAVLSDSGNYFCSTKGQLFLWDKTSNIVKIKVQELFQRPVLTASSFQPIEGGP
VSLKCETRLSPQRLDVQLQFCFFRENQVLGSGWSSSPELQISAVWSEDTGSYWCKAETVT
HRIRKQSLQSQIHVQRIPISNVSLEIRAPGGQVTEGQKLILLCSVAGGTGNVTFSWYREATG
TSMGKKTQRSLSAELEIPAVKESDAGKYYCRADNGHVPIQSKVVNIPVRIPVSRPVLTLRSP
GAQAAVGDLLELHCEALRGSPPILYQFYHEDVTLGNSSAPSGGGASFNLSLTAEHSGNYSC
EANNGLGAQCSEAVPVSISGPDGYRRDLMTAGVLWGLFGVLGFTGVALLLYALFHKISGE
SSATNEPRGASRPNPQEFTYSSPTPDMEELQPVYVNVGSVDVDVVYSQVWSMQQPESSA
NIRTLLENKDSQVIYSSVKKS

>Q7Z3C6 multipass membrane protein
MAQFDTEYQRLEASYSDSPPGEEDLLVHVAEGSKSPWHHIENLDLFFSRVYNLHQKNGFT
CMLIGEIFELMQFLFVVAFTTFLVSCVDYDILFANKMVNHSLHPTEPVKVTLPDAFLPAQV
CSARIQENGSLITILVIAGVFWIHRLIKFIYNICCYWEIHSFYLHALRIPMSALPYCTWQEVQ
ARIVQTQKEHQICIHKRELTELDIYHRILRFQNYMVALVNKSLLPLRFRLPGLGEAVFFTRG
LKYNFELILFWGPGSLFLNEWSLKAEYKRGGQRLELAQRLSNRILWIGIANFLLCPLILIWQ
ILYAFFSYAEVLKREPGALGARCWSLYGRCYLRHFNELEHELQSRLNRGYKPASKYMNCF
LSPLLTLLAKNGAFFAGSILAVLIALTIYDEDVLAVEHVLTTVTLLGVTVTVCRSFIPDQHM
VFCPEQLLRVILAHIHYMPDHWQGNAHRSQTRDEFAQLFQYKAVFILEELLSPIVTPLILIFC
LRPRALEIIDFFRNFTVEVVGVGDTCSFAQMDVRQHGHPQWLSAGQTEASVYQQAEDGK
TELSLMHFAITNPGWQPPRESTAFLGFLKEQVQRDGAAASLAQGGLLPENALFTSIQSLQS
ESEPLSLIANVVAGSSCRGPPLPRDLQGSRHRAEVASALRSFSPLQPGQAPTGRAHSTMTG
SGVDARTASSGSSVWEGQLQSLVLSEYASTEMSLHALYMHQLHKQQAQAEPERHVWHR
RESDESGESAPDEGGEGARAPQSIPRSASYPCAAPRPGAPETTALHGGFQRRYGGITDPGT
VPRVPSHFSRLPLGGWAEDGQSASRHPEPVPEEGSEDELPPQVHKV

>Q14494 single-pass type II membrane protein
MLSLKKYLTEGLLQFTILLSLIGVRVDVDTYLTSQLPPLREIILGPSSAYTQTQFHNLRNTLD
GYGIHPKSIDLDNYFTARRLLSQVRALDRFQVPTTEVNAWLVHRDPEGSVSGSQPNSGLA
LESSSGLQDVTGPDNGVRESETEQGFGEDLEDLGAVAPPVSGDLTKEDIDLIDILWRQDIDL
GAGREVFDYSHRQKEQDVEKELRDGGEQDTWAGEGAEALARNLLVDGETGESFPAQVPS
GEDQTALSLEECLRLLEATCPFGENAEFPADISSITEAVPSESEPPALQNNLLSPLLTGTESPF
DLEQQWQDLMSIMEMQAMEVNTSASEILYSAPPGDPLSTNYSLAPNTPINQNVSLHQASL
GGCSQDFLLFSPEVESLPVASSSTLLPLAPSNSTSLNSTFGSTNLTGLFFPPQLNGTANDTAG
PELPDPLGGLLDEAMLDEISLMDLAIEEGFNPVQASQLEEEFDSDSGLSLDSSHSPSSLSSSE
GSSSSSSSSSSSSSSASSSASSSFSEEGAVGYSSDSETLDLEEAEGAVGYQPEYSKFCRMSYQ
DPAQLSCLPYLEHVGHNHTYNMAPSALDSADLPPPSALKKGSKEKQADFLDKQMSRDEH
RARAMKIPFTNDKIINLPVEEFNELLSKYQLSEAQLSLIRDIRRRGKNKMAAQNCRKRKLD
TILNLERDVEDLQRDKARLLREKVEFLRSLRQMKQKVQSLYQEVFGRLRDENGRPYSPSQ
YALQYAGDGSVLLIPRTMADQQARRQERKPKDRRK

>Q13698	multipass membrane protein
MEPSSPQDEGLRKKQPKKPVPEILPRPPRALFCLTLENPLRKACISIVEWKPFETIILLTIFAN
CVALAVYLPMPEDDNNSLNLGLEKLEYFFLIVFSIEAAMKIIAYGFLFHQDAYLRSGWNVL
DFTIVFLGVFTVILEQVNVIQSHTAPMSSKGAGLDVKALRAFRVLRPLRLVSGVPSLQVVL
NSIFKAMLPLFHIALLVLFMVIIYAIIGLELFKGKMHKTCYFIGTDIVATVENEEPSPCARTGS
GRRCTINGSECRGGWPGPNHGITHFDNFGFSMLTVYQCITMEGWTDVLYWVNDAIGNEW
PWIYFVTLILLGSFFILNLVLGVLSGEFTKEREKAKSRGTFQKLREKQQLDEDLRGYMSWI
TQGEVMDVEDFREGKLSLDEGGSDTESLYEIAGLNKIIQFIRHWRQWNRIFRWKCHDIVK
SKVFYWLVILIVALNTLSIASEHHNQPLWLTRLQDIANRVLLSLFTTEMLMKMYGLGLRQY
FMSIFNRFDCFVVCSGILEILLVESGAMTPLGISVLRCIRLLRIFKITKYWTSLSNLVASLLNS
IRSIASLLLLLFLFIVIFALLGMQLFGGRYDFEDTEVRRSNFDNFPQALISVFQVLTGEDWTS
MMYNGIMAYGGPSYPGMLVCIYFIILFVCGNYILLNVFLAIAVDNLAEAESLTSAQKAKAE
EKKRRKMSKGLPDKSEEEKSTMAKKLEQKPKGEGIPTTAKLKIDEFESNVNEVKDPYPSA
DFPGDDEEDEPEIPLSPRPRPLAELQLKEKAVPIPEASSFFIFSPTNKIRVLCHRIVNATWFTN
FILLFILLSSAALAAEDPIRADSMRNQILKHFDIGFTSVFTVEIVLKMTTYGAFLHKGSFCR
NYFNMLDLLVVAVSLISMGLESSAISVVKILRVLRVLRPLRAINRAKGLKHVVQCMFVAIST
IGNIVLVTTLLQFMFACIGVQLFKGKFFRCTDLSKMTEEECRGYYYVYKDGDPMQIELRH
REWVHSDFHFDNVLSAMMSLFTVSTFEGWPQLLYKAIDSNAEDVGPIYNNRVEMAIFFIIY
IILIAFFMMNIFVGFVIVTFQEQGETEYKNCELDKNQRQCVQYALKARPLRCYIPKNPYQY
QVWYIVTSSYFEYLMFALIMLNTICLGMQHYNQSEQMNHISDILNVAFTIIFTLEMILKLM
AFKARGYFGDPWNVFDFLIVIGSIIDVILSEIDTFLASSGGLYCLGGGCGNVDPDESARISSA
FFRLFRVMRLIKLLSRAEGVRTLLWTFIKSFQALPYVALLIVMLFFIYAVIGMQMFGKIALV
DGTQINRNNNFQTFPQAVLLLFRCATGEAWQEILLACSYGKLCDPESDYAPGEEYTCGTNF
AYYYFISFYMLCAFLVINLFVAVIMDNFDYLTRDWSILGPHHLDEFKAIWAEYDPEAKGRI
KHLDVVTLLRRIQPPLGFGKFCPHRVACKRLVGMNMPLNSDGTVTFNATLFALVRTALKIK
TEGNFEQANEELRAIIKKIWKRTSMKLLDQVIPPIGDDEVTVGKFYATFLIQEHFRKFMKR
QEEYYGYRPKKDIVQIQAGLRTIEEEAAPEICRTVSGDLAAEEELERAMVEAAMEEGIFRR
TGGLFGQVDNFLERTNSLPPVMANQRPLQFAEIEMEEMESPVFLEDFPQDPRTNPLARANT
NNANANVAYGNSNHSNSHVFSSVHYEREFPEETETPATRGRALGQPCRVLGPHSKPCVEM
LKGLLTQRAMPRGQAPPAPCQCPRVESSMPEDRKSSTPGSLHEETPHSRSTRENTSRCSAPA
TALLIQKALVRGGLGTLAADANFIMATGQALADACQMEPEEVEIMATELLKGREAPEGM
ASSLGCLNLGSSLGSLDQHQGSQETLIPPRL

>P56180	multipass membrane protein
MNESPDPTDLAGVIIELGPNDSPQTSEFKGATEEAPAKESPHTSEFKGAARVSPISESVLARL
SKFEVEDAENVASYDSKIKKIVHSIVSSFAFGLFGVFLVLLDVTLILADLIFTDSKLYIPLEYR
SISLAIALFFLMDVLLRVFVERRQQYFSDLFNILDTAIIVILLLVDVVYIFFDIKLLRNIPRWT
HLLRLLRLIILLRIFHLFHQKRQLEKLIRRRVSENKRRYTRDGFDLDLTYVTERIIAMSFPSS
GRQSFYRNPIKEVVRFLDKKHRNHYRVYNLCSERAYDPKHFHNRVVRIMIDDHNVPTLH
QMVVFTKEVNEWMAQDLENIVAIHCKGGTDRTGTMVCAFLIASEICSTAKESLYYFGERR
TDKTHSEKFQGVKTPSQKRYVAYFAQVKHLYNWNLPPRRILFIKHFIIYSIPRYVRDLKIQIE
MEKKVVFSTISLGKCSVLDNITTDKILIDVFDGLPLYDDVKVQFFYSNLPTYYDNCSFYFW
LHTSFIENNRLYLPKNELDNLHKQKARRIYPSDFAVEILFGEKMTSSDVVAGSD

>Q9UNH7 peripheral membrane protein
MMEGLDDGPDFLSEEDRGLKAINVDLQSDAALQVDISDALSERDKVKFTVHTKSSLPNFK
QNEFSVVRQHEEFIWLHDSFVENEDYAGYIIPPAPPRPDFDASREKLQKLGEGEGSMTKEE
FTKMKQELEAEYLAIFKKTVAMHEVFLCRVAAHPILRRDLNFHVFLEYNQDLSVRGKNKK
EKLEDFFKNMVKSADGVIVSGVKDVDDFFEHERTFLLEYHNRVKDASAKSDRMTRSHKS
AADDYNRIGSSLYALGTQDSTDICKFFLKVSELFDKTRKIEARVSADEDLKLSDLLKYYLR
ESQAAKDLLYRRSRSLVDYENANKALDKARAKNKDVLQAETSQQLCCQKFEKISESAKQ
ELIDFKTRRVAAFRKNLVELAELELKHAKGNLQLLQNCLAVLNGDT

>Q14457 peripheral membrane protein
MEGSKTSNNSTMQVSFVCQRCSQPLKLDTSFKILDRVTIQELTAPLLTTAQAKPGETQEEET
NSGEEPFIETPRQDGVSRRFIPPARMMSTESANSFTLIGEASDGGTMENLSRRLKVTGDLFD
IMSGQTDVDHPLCEECTDTLLDQLDTQLNVTENECQNYKRCLEILEQMNEDDSEQLQME
LKELALEEERLIQELEDVEKNRKIVAENLEKVQAEAERLDQEEAQYQREYSEFKRQQLEL
DDELKSVENQMRYAQTQLDKLKKTNVFNATFHIWHSGQFGTINNFRLGRLPSVPVEWNEI
NAAWGQTVLLLHALANKMGLKFQRYRLVPYGNHSYLESLTDKSKELPLYCSGGLRFFWD
NKFDHAMVAFLDCVQQFKEEVEKGETRFCLPYRMDVEKGKIEDTGGSGGSYSIKTQFNSE
EQWTKALKFMLTNLKWGLAWVSSQFYNK

>Q9Y5S2 peripheral membrane protein
MSAKVRLKKLEQLLLDGPWRNESALSVETLLDVLVCLYTECSHSALRRDKYVAEFLEWA
KPFTQLVKEMQLHREDFEIIKVIGRGAFGEVAVVKMKNTERIYAMKILNKWEMLKRAETA
CFREERDVLVNGDCQWITALHYAFQDENHLYLVMDYYVGGDLLTLLSKFEDKLPEDMAR
FYIGEMVLAIDSIHQLHYVHRDIKPDNVLLDVNGHIRLADFGSCLKMNDDGTVQSSVAVG
TPDYISPEILQAMEDGMGKYGPECDWWSLGVCMYEMLYGETPFYAESLVETYGKIMNHE
ERFQFPSHVTDVSEEAKDLIQRLICSRERRLGQNGIEDFKKHAFFEGLNWENIRNLEAPYIP
DVSSPSDTSNFDVDDDVLRNTEILPPGSHTGFSGLHLPFIGFTFTTESCFSDRGSLKSIMQSN
TLTKDEDVQRDLEHSLQMEAYERRIRRLEQEKLELSRKLQESTQTVQSLHGSSRALSNSNR
DKEIKKLNEEIERLKNKIADSNRLERQLEDTVALRQEREDSTQRLRGLEKQHRVVRQEKEE
LHKQLVEASERLKSQAKELKDAHQQRKLALQEFSELNERMAELRAQKQKVSRQLRDKEE
EMEVATQKVDAMRQEMRRAEKLRKELEAQLDDAVAEASKERKLREHSENFCKQMESEL
EALKVKQGGRGAGATLEHQQEISKIKSELEKKVLFYEEELVRREASHVLEVKNVKKEVHD
SESHQLALQKEILMLKDKLEKSKRERHNEMEEAVGTIKDKYERERAMLFDENKKLTAENE
KLCSFVDKLTAQNRQLEDELQDLAAKKESVAHWEAQIAEIIQWVSDEKDARGYLQALAS
KMTEELEALRSSSLGSRTLDPLWKVRRSQKLDMSARLELQSALEAEIRAKQLVQEELRKV
KDANLTLESKLKDSEAKNRELLEEMEILKKKMEEKFRADTGLKLPDFQDSIFEYFNTAPLA
HDLTFRTSSASEQETQAPKPEASPSMSVAASEQQEDMARPPQRPSAVPLPTTQALALAGPK
PKAHQFSIKSFSSPTQCSHCTSLMVGLIRQGYACEVCSFACHVSCKDGAPQVCPIPPEQSKR
PLGVDVQRGIGTAYKGHVKVPKPTGVKKGWQRAYAVVCDCKLFLYDLPEGKSTQPGVIA
SQVLDLRDDEFSVSSVLASDVIHATRRDIPCIFRVTASLLGAPSKTSSLLILTENENEKRKWV
GILEGLQSILHKNRLRNQVVHVPLEAYDSSLPLIKAILTAAIVDADRIAVGLEEGLYVIEVTR
DVIVRAADCKKVHQIELAPREKIVILLCGRNHHVHLYPWSSLDGAEGSFDIKLPETKGCQL
MATATLKRNSGTCLFVAVKRLILCYEIQRTKPFHRKFNEIVAPGSVQCLAVLRDRLCVGYPS

GFCLLSIQGDGQPLNLVNPNDPSLAFLSQQSFDALCAVELESEEYLLCFSHMGLYVDPQGR
RARAQELMWPAAPVACSCSPTHVTVYSEYGVDVFDVRTMEWVQTIGLRRIRPLNSEGTL
NLLNCEPPRLIYFKSKFSGAVLNVPDTSDNSKKQMLRTRSKRRFVFKVPEEERLQQRREM
LRDPELRSKMISNPTNFNHVAHMGPGDGMQVLMDLPLSAVPPSQEERPGPAPTNLARQPP
SRNKPYISWPSSGGSEPSVTVPLRSMSDPDQDFDKEPDSDSTKHSTPSNSSNPSGPPSPNSP
HRSQLPLEGLEQPACDT

>B0YJ81 multipass membrane protein
MGRLTEAAAAGSGSRAAGWAGSPPTLLPLSPTSPRCAATMASSDEDGTNGGASEAGEDR
EAPGERRRLGVLATAWLTFYDIAMTAGWLVLAIAMVRFYMEKGTHRGLYKSIQKTLKFFQ
TFALLEIVHCLIGIVPTSVIVTGVQVSSRIFMVWLITHSIKPIQNEESVVLFLVAWTVTEITRY
SFYTFSLLDHLPYFIKWARYNFFIILYPVGVAGELLTIYAALPHVKKTGMFSIRLPNKYNVSF
DYYYFLLITMASYIPLFPQLYFHMLRQRRKVLHGEVIVEKDD

>Q14832 multipass membrane protein
MKMLTRLQVLTLALFSKGFLLSLGDHNFLRREIKIEGDLVLGGLFPINEKGTGTEECGRINE
DRGIQRLEAMLFAIDEINKDDYLLPGVKLGVHILDTCSRDTYALEQSLEFVRASLTKVDEA
EYMCPDGSYAIQENIPLLIAGVIGGSYSSVSIQVANLLRLFQIPQISYASTSAKLSDKSRYDYF
ARTVPPDFYQAKAMAEILRFFNWTYVSTVASEGDYGETGIEAFEQEARLRNICIATAEKVG
RSNIRKSYDSVIRELLQKPNARVVVLFMRSDDSRELIAAASRANASFTWVASDGWGAQES
IIKGSEHVAYGAITLELASQPVRQFDRYFQSLNPYNNHRNPWFRDFWEQKFQCSLQNKRN
HRRVCDKHLAIDSSNYEQESKIMFVVNAVYAMAHALHKMQRTLCPNTTKLCDAMKILDG
KKLYKDYLLKINFTAPFNPNKDADSIVKFDTFGDGMGRYNVFNFQNVGGKYSYLKVGHW
AETLSLDVNSIHWSRNSVPTSQCSDPCAPNEMKNMQPGDVCCWICIPCEPYEYLADEFTC
MDCGSGQWPTADLTGCYDLPEDYIRWEDAWAIGPVTIACLGFMCTCMVVTVFIKHNNTP
LVKASGRELCYILLFGVGLSYCMTFFFIAKPSPVICALRRLGLGSSFAICYSALLTKTNCIAR
IFDGVKNGAQRPKFISPSSQVFICLGLILVQIVMVSVWLILEAPGTRRYTLAEKRETVILKC
NVKDSSMLISLTYDVILVILCTVYAFKTRKCPENFNEAKFIGFTMYTTCIIWLAFLPIFYVTS
SDYRVQTTTMCISVSLSGFVVLGCLFAPKVHIILFQPQKNVVTHRLHLNRFSVSGTGTTYS
QSSASTYVPTVCNGREVLDSTTSSL

>Q8N130 multipass membrane protein
MPSSLPGSQVPHPTLDAVDLVEKTLRNEGTSSSAPVLEEGDTDPWTLPQLKDTSQPWKEL
RVAGRLRRVAGSVLKACGLLGSLYFFICSLDVLSSAFQLLGSKVAGDIFKDNVVLSNPVAG
LVIGVLVTALVQSSSTSSSIVVSMVAAKLLTVRVSVPIIMGVNVGTSITSTLVSMAQSGDRD
EFQRAFSGSAVHGIFNWLTVLVLLPLESATALLERLSELALGAASLTPRAQAPDILKVLTKP
LTHLIVQLDSDMIMSSATGNATNSSLIKHWCGTTGQPTQENSSCGAFGPCTEKNSTAPADR
LPCRHLFAGTELTDLAVGCILLAGSLLVLCGCLVLIVKLLNSVLRGRVAQVVRTVINADFPF
PLGWLGGYLAVLAGAGLTFALQSSSVFTAAVVPLMGVGVISLDRAYPLLLGSNIGTTTTAL
LAALASPADRMLSALQVALIHFFFNLAGILLWYLVPALRLPIPLARHFGVVTARYRWVAGV
YLLLGFLLLPLAAFGLSLAGGMELAAVGGPLVGLVLLVILVTVLQRRRPAWLPVRLRSWA
WLPVWLHSLEPWDRLVTRCCPCNVCSPPKATTKEAYCYENPEILASQQL

>Q08722 multipass membrane protein

MWPLVAALLLGSACCGSAQLLFNKTKSVEFTFCNDTVVIPCFVTNMEAQNTTEVYVKWK
FKGRDIYTFDGALNKSTVPTDFSSAKIEVSQLLKGDASLKMDKSDAVSHTGNYTCEVTEL
TREGETIIELKYRVVSWFSPNENILIVIFPIFAILLFWGQFGIKTLKYRSGGMDEKTIALLVAG
LVITVIVIVGAILFVPGEYSLKNATGLGLIVTSTGILILLHYYVFSTAIGLTSFVIAILVIQVIAYI
LAVVGLSLCIAACIPMHGPLLISGLSILALAQLLGLVYMKFVASNQKTIQPPRKAVEEPLNA
FKESKGMMNDE

>Q9ULL4 single-pass type I membrane protein
MCHAAQETPLLHHFMAPVMARWPPFGLCLLLLLLLSPPPLPLTGAHRFSAPNTTLNHLALA
PGRGTLYVGAVNRLFQLSPELQLEAVAVTGPVIDSPDCVPFRDPAECPQAQLTDNANQLLL
VSSRAQELVACGQVRQGVCETRRLGDVAEVLYQAEDPGDGQFVAANTPGVATVGLVVPL
PGRDLLLVARGLAGKLSAGVPPLAIRQLAGSQPFSSEGLGRLVVGDFSDYNNSYVGAFAD
ARSAYFVFRRRGARAQAEYRSYVARVCLGDTNLYSYVEVPLACQGQGLIQAAFLAPGTLL
GVFAAGPRGTQAALCAFPMVELGASMEQARRLCYTAGGRGPSGAEEATVEYGVTSRCVT
LPLDSPESYPCGDEHTPSPIAGRQPLEVQPLLKLGQPVSAVAALQADGHMIAFLGDTQGQL
YKVFLHGSQGQVYHSQQVGPPGSAISPDLLLDSSGSHLYVLTAHQVDRIPVAACPQFPDCA
SCLQAQDPLCGWCVLQGRCTRKGQCGRAGQLNQWLWSYEEDSHCLHIQSLLPGHHPRQ
EQGQVTLSVPRLPILDADEYFHCAFGDYDSLAHVEGPHVACVTPPQDQVPLNPPGTDHVT
VPLALMFEDVTVAATNFSFYDCSAVQALEAAAPCRACVGSIWRCHWCPQSSHCVYGEHC
PEGERTIYSAQEVDIQVRGPGACPQVEGLAGPHLVPVGWESHLALRVRNLQHFRGLPASF
HCWLELPGELRGLPATLEETAGDSGLIHCQAHQFYPSMSQRELPVPIYVTQGEAQRLDNT
HALYVILYDCAMGHPDCSHCQAANRSLGCLWCADGQPACRYGPLCPPGAVELLCPAPSID
AVEPLTGPPEGGLALTILGSNLGRAFADVQYAVSVASRPCNPEPSLYRTSARIVCVTSPAPNG
TTGPVRVAIKSQPPGISSQHFTYQDPVLLSLSPRWGPQAGGTQLTIRGQHLQTGGNTSAFV
GGQPCPILEPVCPEAIVCRTRPQAAPGEAAVLVVFGHAQRTLLASPFRYTANPQLVAAEPSA
SFRGGGRLIRVRGTGLDVVQRPLLSVWLEADAEVQASRAQPQDPQPRRSCGAPAADPQA
CIQLGGGLLQCSTVCSVNSSSLLLCRSPAVPDRAHPQRVFFTLDNVQVDFASASGGQGFLY
QPNPRLAPLSREGPARPYRLKPGHVLDVEGEGLNLGISKEEVRVHIGRGECLVKTLTRTHL
YCEPPAHAPQPANGSGLPQFVVQMGNVQLALGPVQYEAEPPLSAFPVEAQAGVGMGAAV
LIAAVLLLTLMYRHKSKQALRDYQKVLVQLESLETGVGDQCRKEFTDLMTEMTDLSSDL
EGSGIPFLDYRTYAERAFFPGHGGCPLQPKPEGPGEDGHCATVRQGLTQLSNLLNSKLFLL
TLIHTLEEQPSFSQRDRCHVASLLSLALHGKLEYLTDIMRTLLGDLAAHYVHRNPKLMLRR
TETMVEKLLTNWLSICLYAFLREVAGEPLYMLFRAIQYQVDKGPVDAVTGKAKRTLNDSR
LLREDVEFQPLTLMVLVGPGAGGAAGSSEMQRVPARVLDTDTITQVKEKVLDQVYKGTP
FSQRPSVHALDLEWRSGLAGHLTLSDEDLTSVTQNHWKRLNTLQHYKVPDGATVGLVPQ
LHRGSTISQSLAQRCPLGENIPTLEDGEEGGVCLWHLVKATEEPEGAKVRCSSLREREPAR
AKAIPEIYLTRLLSMKGTLQKFVDDTFQAILSVNRPIPIAVKYLFDLLDELAEKHGIEDPGTL
HIWKTNSLLLRFWVNALKNPQLIFDVRVSDNVDAILAVIAQTFIDSCTTSEHKVGRDSPVN
KLLYAREIPRYKQMVERYYADIRQSSPASYQEMNSALAELSGNYTSAPHCLEALQELYNHI
HRYYDQIISALEEDPVGQKLQLACRLQQVAALVENKVTDL

>Q86WA9 multipass membrane protein
MPSSVTALGQARSSGPGMAPSACCCSPAALQRRLPILAWLPSYSLQWLKMDFVAGLSVGL
TAIPQALAYAEVAGLPPQYGLYSAFMGCFVYFFLGTSRDVTLGPTAIMSLLVSFYTFHEPAY

AVLLAFLSGCIQLAMGVLRLGFLLDFISYPVIKGFTSAAAVTIGFGQIKNLLGLQNIPRPFFL
QVYHTFLRIAETRVGDAVLGLVCMLLLLVLKLMRDHVPPVHPEMPPGVRLSRGLVWAATT
ARNALVVSFAALVAYSFEVTGYQPFILTGETAEGLPPVRIPPFSVTTANGTISFTEMVQDMG
AGLAVVPLMGLLESIAVAKAFASQNNYRIDANQELLAIGLTNMLGSLVSSYPVTGSFGRTA
VNAQSGVCTPAGGLVTGVLVLLSLDYLTSLFYYIPKSALAAVIIMAVAPLFDTKIFRTLWRV
KRLDLLPLCVTFLLCFWEVQYGILAGALVSLLMLLHSAARPETKVSEGPVLVLQPASGLSF
PAMEALREEILSRALEVSPPRCLVLECTHVCSIDYTVVLGLGELLQDFQKQGVALAFVGLQ
VPVLRVLLSADLKGFQYFSTLEEAEKHLRQEPGTQPYNIREDSILDQKVALLKA

>O94898 single-pass type I membrane protein
MAPAPLGVPEEQLLGCRSRVLSRLLFIAQTALLLLPAAGAGLCPAPCSCRIPLLDCSRRKLP
APSWRALSGLLPPDTAILDFSHNRLSNWNISLESQTLQEVKMNYNELTEIPYFGEPTSNITL
LSLVHNIIPEINAQALQFYPALESLDLSSNIISEIKTSSFPRMQLKYLNLSNNRITTLEAGCFD
NLSSSLLVVKLNRNRMSMIPPKIFKLPHLQFLELKRNRIKIVEGLTFQGLDSLRSLKMQRNG
ISKLKDGAFFGLNNMEELELEHNNLTRVNKGWLYGLRMLQQLYVSQNAIERISPDAWEFC
QRLSELDLSYNQLTRLDESAFVGLSLLERLNLGDNRVTHIADGVFRFLSNLQTLDLRNNEI
SWAIEDASEAFAGLTSLTKLILQGNQIKSITKKAFIGLESLEHLDLNNNAIMSIQENAFSQTH
LKELILNTSSLLCDCHLKWLLQWLVDNNFQHSVNVSCAHPEWLAGQSILNVDLKDFVCD
DFLKPQIRTHPETIIALRGMNVTLTCTAVSSSDSPMSTVWRKDSEILYDVDTENFVRYWQQ
AGEALEYTSILHLFNVNFTDEGKYQCIVTNHFGSNYSQKAKLTVNEMPSFLKTPMDLTIRT
GAMARLECAAEGHPAPQISWQKDGGTDFPAARERRMHVMPEDDVFFIANVKIEDMGIYS
CMAQNTAGGLSANASLTVLETPSFIRPLEDKTVTRGETAVLQCIAGGSPAPRLNWTKDDGP
LLVTERHFFAAANQLLIIVDAGLEDAGKYTCIMSNTLGTERGHIYLNVISSPNCDSSQSSIG
HEDDGWTTVGIVIIVVVCCVVGTSLIWVIVIYHMRRKNEDYSITNTEELNLPADIPSYLSSQ
GTLSEPQEGYSNSEAGSHQQLMPPANGYIHKGTDGGTGTRVICSDCYDNANIYSRTREYC
PYTYIAEEDVLDQTLSSLMVQMPKETYLVHPPQDTTALESLIPSANREPSAFPTNHERISEK
KLPSTQMSGETLQRPVWNINRELGLPHPPFSQQPVHESPQLHQNEGLAGREPDCSASSMS
CHRLQDHAFDFSRTRNIQDGSEGT

>P26232 peripheral membrane protein
MTSATSPIILKWDPKSLEIRTLTVERLLEPLVTQVTTLVNTSNKGPSGKKKGRSKKAHVLA
ASVEQATQNFLEKGEQIAKESQDLKEELVAAVEDVRKQGETMRIASSEFADDPCSSVKRGT
MVRAARALLSAVTRLLILADMADVMRLLSHLKIVEEALEAVKNATNEQDLANRFKEFGK
EMVKLNYVAARRQQELKDPHCRDEMAAARGALKKNATMLYTASQAFLRHPDVAATRAN
RDYVFKQVQEAIAGISNAAQATSPTDEAKGHTGIGELAAALNEFDNKIILDPMTFSEARFR
PSLEERLESIISGAALMADSSCTRDDRRERIVAECNAVRQALQDLLSEYMNNTGRKEKGDP
LNIAIDKMTKKTRDLRRQLRKAVMDHISDSFLETNVPLLVLIEAAKSGNEKEVKEYAQVF
REHANKLVEVANLACSISNNEEGVKLVRMAATQIDSLCPQVINAALTLAARPQSKVAQDN
MDVFKDQWEKQVRVLTEAVDDITSVDDFLSVSENHILEDVNKCVIALQEGDVDTLDRTA
GAIRGRAARVIHIINAEMENYEAGVYTEKVLEATKLLSETVMPRFAEQVEVAIEALSANVP
QPFEENEFIDASRLVYDGVRDIRKAVLMIRTPEELEDDSDFEQEDYDVRSRTSVQTEDDQLI
AGQSARAIMAQLPQEEKAKIAEQVEIFHQEKSKLDAEVAKWDDSGNDIIVLAKQMCMIM
MEMTDFTRGKGPLKNTSDVINAAKKIAEAGSRMDKLARAVADQCPDSACKQDLLAYLQR
IALYCHQLNICSKVKAEVQNLGGELIVSGTGVQSTFTTFYEVDCDVIDGGRASQLSTHLPT

CAEGAPIGSGSSDSSMLDSATSLIQAAKNLMNAVVLTVKASYVASTKYQKVYGTAAVNSP
VVSWKMKAPEKKPLVKREKPEEFQTRVRRGSQKKHISPVQALSEFKAMDSF

>O43869 multipass membrane protein
MWQEYYFLNVFFPLLKVCCLTINSHVVILLPWECYHLIWKILPYIGTTVGSMEEYNTSSTD
FTFMGLFNRKETSGLIFAIISIIFFTALMANGVMIFLIQTDLRLHTPMYFLLSHLSLIDMMYIS
TIVPKMLVNYLLDQRTISFVGCTAQHFLYLTLVGAEFFLLGLMAYDRYVAICNPLRYPVLM
SRRVCWMIIAGSWFGGSLDGFLLTPITMSFPFCNSREINHFFCEAPAVLKLACADTALYETV
MYVCCVLMLLIPFSVVLASYARILTTVQCMSSVEGRKKAFATCSSHMTVVSLFYGAAMYT
YMLPHSYHKPAQDKVLSVFYTILTPMLNPLIYSLRNKDVTGALKRALGRFKGPQRVSGGV
F

>Q8NH00 multipass membrane protein
MDNITWMASHTGWSDFILMGLFRQSKHPMANITWMANHTGWSDFILLGLFRQSKHPALL
CVVIFVVFLMALSGNAVLILLIHCDAHLHTPMYFFISQLSLMDMAYISVTVPKMLLDQVM
GVNKISAPECGMQMFFYVTLAGSEFFLLATMAYDRYVAICHPLRYPVLMNHRVCLFLSSG
CWFLGSVDGFTFTPITMTFPFRGSREIHHFFCEVPAVLNLSCSDTSLYEIFMYLCCVLMLLIP
VVIISSSYLLILLTIHGMNSAEGRKKAFATCSSHLTVVILFYGAAIYTYMLPSSYHTPEKDM
MVSVFYTILTPVVNPLIYSLRNKDVMGALKKMLTVEPAFQKAME

>Q8NG84 multipass membrane protein
MNISDVISFDILVSAMKTGNQSFGTDFLLVGLFQYGWINSLLFVVIATLFTVALTGNIMLIHL
IRLNTRLHTPMYFLLSQLSIVDLMYISTTVPKMAVSFLSQSKTIRFLGCEIQTYVFLALGGT
EALLLGFMSYDRYVAICHPLHYPMLMSKKICCLMVACAWASGSINAFIHTLYVFQLPFCRS
RLINHFFCEVPALLSLVCQDTSQYEYTVLLSGLIILLLPFLAILASYARVLIVVFQMSSGKGQ
AKAVSTCSSHLIVASLFYATTLFTYTRPHSLRSPSRDKAVAVFYTIVTPLLNPFIYSLRNKEVT
GAVRRLLGYWICCRKYDFRSLY

>Q96R54 multipass membrane protein
MANVTLVTGFLLMGFSNIQKLRILYGVLFLLIYLAALMSNLLIITLITLDVKLQTPMYFFLK
NLSFLDVFLVSVPIPKFIVNNLTHNNSISILGCAFQLLLMTSFSAGEIFILTAMSYDRYVAICC
PLNYEVIMNTGVCVLMASVSWAIGGLFGTAYTAGTFSMPFCGSSVIPQFFCDVPSLLRISCS
ETLMVIYAGIGVGACLSISCFICIVISYIYIFSTVLKIPTTKGQSKAFSTCFPHLTVFTVFIITAY
FVYLKPPSNSPSVIDRLLSVIYTVMPPVFNPVTYSLRNNDMKCALIRLLQKTYGQEAYFI

>Q8N628 multipass membrane protein
MMEIANVSSPEVFVLLGFSTRPSLETVLFIVVLSFYMVSILGNGIIILVSHTDVHLHTPMYFF
LANLPFLDMSFTTSIVPQLLANLWGPQKTISYGGCVVQFYISHWLGATECVLLATMSYDRY
AAICRPLHYTVIMHPQLCLGLALASWLGGLTTSMVGSTLTMLLPLCGNNCIDHFFCEMPLI
MQLACVDTSLNEMEMYLASFVFVVLPLGLILVSYGHIARAVLKIRSAEGRRKAFNTCSSH
VAVVSLFYGSIIFMYLQPAKSTSHEQGKFIALFYTVVTPALNPLIYTLRNTEVKSALRHMVL
ENCCGSAGKLAQI

>Q9H1Y3 multipass membrane protein

MYSGNRSGGHGYWDGGGAAGAEGPAPAGTLSPAPLFSPGTYERLALLLGSIGLLGVGNN
LLVLVLYYKFQRLRTPTHLLLVNISLSDLLVSLFGVTFTFVSCLRNGWVWDTVGCVWDGF
SGSLFGIVSIATLTVLAYERYIRVVHARVINFSWAWRAITYIWLYSLAWAGAPLLGWNRYIL
DVHGLGCTVDWKSKDANDSSFVLFLFLGCLVVPLGVIAHCYGHILYSIRMLRCVEDLQTI
QVIKILKYEKKLAKMCFLMIFTFLVCWMPYIVICFLVVNGHGHLVTPTISIVSYLFAKSNTV
YNPVIYVFMIRKFRRSLLQLLCLRLLRCQRPAKDLPAAGSEMQIRPIVMSQKDGDRPKKK
VTFNSSSIIFIITSDESLSVDDSDKTNGSKVDVIQVRPL

>O15229 multipass membrane protein
MDSSVIQRKKVAVIGGGLVGSLQACFLAKRNFQIDVYEAREDTRVATFTRGRSINLALSHR
GRQALKAVGLEDQIVSQGIPMRARMIHSLSGKKSAIPYGTKSQYILSVSRENLNKDLLTAA
EKYPNVKMHFNHRLLKCNPEEGMITVLGSDKVPKDVTCDLIVGCDGAYSTVRSHLMKKP
RFDYSQQYIPHGYMELTIPPKNGDYAMEPNYLHIWPRNTFMMIALPNMNKSFTCTLFMPF
EEFEKLLTSNDVVDFFQKYFPDAIPLIGEKLLVQDFFLLPAQPMISVKCSSFHFKSHCVLLG
DAAHAIVPFFGQGMNAGFEDCLVFDELMDKFSNDLSLCLPVFSRLRIPDDHAISDLSMYN
YIEMRAHVNSSWFIFQKNMERFLHAIMPSTFIPLYTMVTFSRIRYHEAVQRWHWQKKVIN
KGLFFLGSLIAISSTYLLIHYMSPRSFLRLRRPWNWIAHFRNTTCFPAKAVDSLEQISNLISR

>Q12836 single-pass type I membrane protein
MWLLRCVLLCVSLSLAVSGQHKPEAPDYSSVLHCGPWSFQFAVNLNQEATSPPVLIAWDN
QGLLHELQNDSDCGTWIRKGPGSSVVLEATYSSCYVTEWDSHYIMPVGVEGAGAAEHKV
VTERKLLKCPMDLLARDAPDTDWCDSIPARDRLPCAPSPISRGDCEGLGCCYSSEEVNSC
YYGNTVTLHCTREGHFSIAVSRNVTSPPLLLDSVRLALRNDSACNPVMATQAFVLFQFPFT
SCGTTRQITGDRAVYENELVATRDVKNGSRGSVTRDSIFRLHVSCSYSVSSNSLPINVQVFT
LPPPFPETQPGPLTLELQIAKDKNYGSYYGVGDYPVVKLLRDPIYVEVSILHRTDPYLGLLL
QQCWATPSTDPLSQPQWPILVKGCPYIGDNYQTQLIPVQKALDLPFPSHHQRFSIFTFSFVN
PTVEKQALRGPVHLHCSVSVCQPAETPSCVVTCPDLSRRRNFDNSSQNTTASVSSKGPMIL
LQATKDPPEKLRVPVDSKVLWVAGLSGTLILGALLVSYLAVKKQKSCPDQMCQ

>Q92736 multipass membrane protein
MADGGEGEDEIQFLRTDDEVVLQCTATIHKEQQKLCLAAEGFGNRLCFLESTSNSKNVPP
DLSICTFVLEQSLSVRALQEMLANTVEKSEGQVDVEKWKFMMKTAQGGGHRTLLYGHAI
LLRHSYSGMYLCCLSTSRSSTDKLAFDVGLQEDTTGEACWWTIHPASKQRSEGEKVRVG
DDLILVSVSSERYLHLSYGNGSLHVDAAFQQTLWSVAPISSGSEAAQGYLIGGDVLRLLHG
HMDECLTVPSGEHGEEQRRTVHYEGGAVSVHARSLWRLETLRVAWSGSHIRWGQPFRLR
HVTTGKYLSLMEDKNLLLMDKEKADVKSTAFTFRSSKEKLDVGVRKEVDGMGTSEIKY
GDSVCYIQHVDTGLWLTYQSVDVKSVRMGSIQRKAIMHHEGHMDDGISLSRSQHEESRT
ARVIRSTVFLFNRFIRGLDALSKKAKASTVDLPIESVSLSLQDLIGYFHPPDEHLEHEDKQN
RLRALKNRQNLFQEEGMINLVLECIDRLHVYSSAAHFADVAGREAGESWKSILNSLYELL
AALIRGNRKNCAQFSGSLDWLISRLERLEASSGILEVLHCVLVESPEALNIIKEGHIKSIISLL
DKHGRNHKVLDVLCSLCVCHGVAVRSNQHLICDNLLPGRDLLLQTRLVNHVSSMRPNIFL
GVSEGSAQYKKWYYELMVDHTEPFVTAEATHLRVGWASTEGYSPYPGGGEEWGGNGVG
DDLFSYGFDGLHLWSGCIARTVSSPNQHLLRTDDVISCCLDLSAPSISFRINGQPVQGMFEN
FNIDGLFFPVVSFSAGIKVRFLLGGRHGEFKFLPPPGYAPCYEAVLPKEKLKVEHSREYKQE

RTYTRDLLGPTVSLTQAAFTPIPVDTSQIVLPPHLERIREKLAENIHELWVMNKIELGWQYG
PVRDDNKRQHPCLVEFSKLPEQERNYNLQMSLETLKTLLALGCHVGISDEHAEDKVKKM
KLPKNYQLTSGYKPAPMDLSFIKLTPSQEAMVDKLAENAHNVWARDRIRQGWTYGIQQD
VKNRRNPRLVPYTLLDDRTKKSNKDSLREAVRTLLGYGYNLEAPDQDHAARAEVCSGTG
ERFRIFRAEKTYAVKAGRWYFEFETVTAGDMRVGWSRPGCQPDQELGSDERAFAFDGFK
AQRWHQGNEHYGRSWQAGDVVGCMVDMNEHTMMFTLNGEILLDDSGSELAFKDFDV
GDGFIPVCSLGVAQVGRMNFGKDVSTLKYFTICGLQEGYEPFAVNTNRDITMWLSKRLPQ
FLQVPSNHEHIEVTRIDGTIDSSPCLKVTQKSFGSQNSNTDIMFYRLSMPIECAEVFSKTVA
GGLPGAGLFGPKNDLEDYDADSDFEVLMKTAHGHLVPDRVDKDKEATKPEFNNHKDYA
QEKPSRLKQRFLLRRTKPDYSTSHSARLTEDVLADDRDDYDFLMQTSTYYYSVRIFPGQEP
ANVWVGWITSDFHQYDTGFDLDRVRTVTVTLGDEKGKVHESIKRSNCYMVCAGESMSP
GQGRNNNGLEIGCVVDAASGLLTFIANGKELSTYYQVEPSTKLFPAVFAQATSPNVFQFEL
GRIKNVMPLSAGLFKSEHKNPVPQCPPRLHVQFLSHVLWSRMPNQFLKVDVSRISERQGW
LVQCLDPLQFMSLHIPEENRSVDILELTEQEELLKFHYHTLRLYSAVCALGNHRVAHALCS
HVDEPQLLYAIENKYMPGLLRAGYYDLLIDIHLSSYATARLMMNNEYIVPMTEETKSITLFP
DENKKHGLPGIGLSTSLRPRMQFSSPSFVSISNECYQYSPEFPLDILKSKTIQMLTEAVKEGS
LHARDPVGGTTEFLFVPLIKLFYTLLIMGIFHNEDLKHILQLIEPSVFKEAATPEEESDTLEK
ELSVDDAKLQGAGEEEAKGGKRPKEGLLQMKLPEPVKLQMCLLLQYLCDCQVRHRIEAI
VAFSDDFVAKLQDNQRFRYNEVMQALNMSAALTARKTKEFRSPPQEQINMLLNFKDDKS
ECPCPEEIRDQLLDFHEDLMTHCGIELDEDGSLDGNSDLTIRGRLLSLVEKVTYLKKKQAE
KPVESDSKKSSTLQQLISETMVRWAQESVIEDPELVRAMFVLLHRQYDGIGGLVRALPKTY
TINGVSVEDTINLLASLGQIRSLLSVRMGKEEEKLMIRGLGDIMNNKVFYQHPNLMRALG
MHETVMEVMVNVLGGGESKEITFPKMVANCCRFLCYFCRISRQNQKAMFDHLSYLLENS
SVGLASPAMRGSTPLDVAAASVMDNNELALALREPDLEKVVRYLAGCGLQSCQMLVSKG
YPDIGWNPVEGERYLDFLRFAVFCNGESVEENANVVVRLLIRRPECFGPALRGEGGNGLL
AAMEEAIKIAEDPSRDGPSPNSGSSKTLDTEEEEDDTIHMGNAIMTFYSALIDLLGRCAPE
MHLIHAGKGEAIRIRSILRSLIPLGDLVGVISIAFQMPTIAKDGNVVEPDMSAGFCPDHKAA
MVLFLDRVYGIEVQDFLLHLLEVGFLPDLRAAASLDTAALSATDMALALNRYLCTAVLPL
LTRCAPLFAGTEHHASLIDSLLHTVYRLSKGCSLTKAQRDSIEVCLLSICGQLRPSMMQHL
LRRLVFDVPLLNEHAKMPLKLLTNHYERCWKYYCLPGGWGNFGAASEEELHLSRKLFW
GIFDALSQKKYEQELFKLALPCLSAVAGALPPDYMESNYVSMMEKQSSMDSEGNFNPQP
VDTSNITIPEKLEYFINKYAEHSHDKWSMDKLANGWIYGEIYSDSSKVQPLMKPYKLLSE
KEKEIYRWPIKESLKTMLAWGWRIERTREGDSMALYNRTRRISQTSQVSVDAAHGYSPRA
IDMSNVTLSRDLHAMAEMMAENYHNIWAKKKKMELESKGGGNHPLLVPYDTLTAKEKA
KDREKAQDILKFLQINGYAVSRGFKDLELDTPSIEKRFAYSFLQQLIRYVDEAHQYILEFDG
GSRGKGEHFPYEQEIKFFAKVVLPLIDQYFKNHRLYFLSAASRPLCSGGHASNKEKEMVTS
LFCKLGVLVRHRISLFGNDATSIVNCLHILGQTLDARTVMKTGLESVKSALRAFLDNAAED
LEKTMENLKQGQFTHTRNQPKGVTQIINYTTVALLPMLSSLFEHIGQHQFGEDLILEDVQV
SCYRILTSLYALGTSKSIYVERQRSALGECLAAFAGAFPVAFLETHLDKHNIYSIYNTKSSRE
RAALSLPTNVEDVCPNIPSLEKLMEEIVELAESGIRYTQMPHVMEVILPMLCSYMSRWWE
HGPENNPERAEMCCTALNSEHMNTLLGNILKIIYNNLGIDEGAWMKRLAVFSQPIINKVKP
QLLKTHFLPLMEKLKKKAATVVSEEDHLKAEARGDMSEAELLILDEFTTLARDLYAFYPL
LIRFVDYNRAKWLKEPNPEAEELFRMVAEVFIYWSKSHNFKREEQNFVVQNEINNMSFLI
TDTKSKMSKAAVSDQERKKMKRKGDRYSMQTSLIVAALKRLLPIGLNICAPGDQELIALA

KNRFSLKDTEDEVRDIIRSNIHLQGKLEDPAIRWQMALYKDLPNRTDDTSDPEKTVERVLD
IANVLFHLEQKSKRVGRRHYCLVEHPQRSKKAVWHKLLSKQRKRAVVACFRMAPLYNLP
RHRAVNLFLQGYEKSWIETEEHYFEDKLIEDLAKPGAEPPEEDEGTKRVDPLHQLILLFSRT
ALTEKCKLEEDFLYMAYADIMAKSCHDEEDDDGEEEVKSFEEKEMEKQKLLYQQARLHD
RGAAEMVLQTISASKGETGPMVAATLKLGIAILNGGNSTVQQKMLDYLKEKKDVGFFQS
LAGLMQSCSVLDLNAFERQNKAEGLGMVTEEGSGEKVLQDDEFTCDLFRFLQLLCEGHN
SDFQNYLRTQTGNNTTVNIIISTVDYLLRVQESISDFYWYYSGKDVIDEQGQRNFSKAIQV
AKQVFNTLTEYIQGPCTGNQQSLAHSRLWDAVVGFLHVFAHMQMKLSQDSSQIELLKEL
MDLQKDMVVMLLSMLEGNVVNGTIGKQMVDMLVESSNNVEMILKFFDMFLKLKDLTSS
DTFKEYDPDGKGVISKRDFHKAMESHKHYTQSETEFLLSCAETDENETLDYEEFVKRFHE
PAKDIGFNVAVLLTNLSEHMPNDTRLQTFLELAESVLNYFQPFLGRIEIMGSAKRIERVYFEI
SESSRTQWEKPQVKESKRQFIFDVVNEGGEKEKMELFVNFCEDTIFEMQLAAQISESDLNE
RSANKEESEKERPEEQGPRMAFFSILTVRSALFALRYNILTLMRMLSLKSLKKQMKKVKK
MTVKDMVTAFFSSYWSIFMTLLHFVASVFRGFFRIICSLLLGGSLVEGAKKIKVAELLANM
PDPTQDEVRGDGEEGERKPLEAALPSEDLTDLKELTEESDLLSDIFGLDLKREGGQYKLIP
HNPNAGLSDLMSNPVPMPEVQEKFQEQKAKEEEKEEKEETKSEPEKAEGEDGEKEEKAK
EDKGKQKLRQLHTHRYGEPEVPESAFWKKIIAYQQKLLNYFARNFYNMRMLALFVAFAIN
FILLFYKVSTSSVVEGKELPTRSSSENAKVTSLDSSSHRIIAVHYVLEESSGYMEPTLRILAIL
HTVISFFCIIGYYCLKVPLVIFKREKEVARKLEFDGLYITEQPSEDDIKGQWDRLVINTQSFP
NNYWDKFVKRKVMDKYGEFYGRDRISELLGMDKAALDFSDAREKKKPKKDSSLSAVLN
SIDVKYQMWKLGVVFTDNSFLYLAWYMTMSVLGHYNNFFFAAHLLDIAMGFKTLRTILS
SVTHNGKQLVLTVGLLAVVVYLYTVVAFNFFRKFYNKSEDGDTPDMKCDDMLTCYMFH
MYVGVRAGGGIGDEIEDPAGDEYEIYRIIFDITFFFFVIVILLAIIQGLIIDAFGELRDQQEQV
KEDMETKCFICGIGNDYFDTVPHGFETHTLQEHNLANYLFFLMYLINKDETEHTGQESYV
WKMYQERCWEFFPAGDCFRKQYEDQLN

>P50150 lipid-anchor
MKEGMSNNSTTSISQARKAVEQLKMEACMDRVKVSQAAADLLAYCEAHVREDPLIIPVP
ASENPFREKKFFCTIL

>O15228 peripheral membrane protein
MESSSSSNSYFSVGPTSPSAVVLLYSKELKKWDEFEDILEERRHVSDLKFAMKCYTPLVYK
GITPCKPIDIKCSVLNSEEIHYVIKQLSKESLQSVDVLREEVSEILDEMSHKLRLGAIRFCAF
TLSKVFKQIFSKVCVNEEGIQKLQRAIQEHPVVLLPSHRSYIDFLMLSFLLYNYDLPVPVIA
AGMDFLGMKMVGELLRMSGAFFMRRTFGGNKLYWAVFSEYVKTMLRNGYAPVEFFLEG
TRSRSAKTLTPKFGLLNIVMEPFFKREVFDTYLVPISISYDKILEETLYVYELLGVPKPKEST
TGLLKARKILSENFGSIHVYFGDPVSLRSLAAGRMSRSSYNLVPRYIPQKQSEDMHAFVTE
VAYKMELLQIENMVLSPWTLIVAVLLQNRPSMDFDALVEKTLWLKGLTQAFGGFLIWPDN
KPAEEVVPASILLHSNIASLVKDQVILKVDSGDSEVVDGLMLQHITLLMCSAYRNQLLNIF
VRPSLVAVALQMTPGFRKEDVYSCFRFLRDVFADEFIFLPGNTLKDFEEGCYLLCKSEAIQV
TTKDILVTEKGNTVLEFLVGLFKPFVESYQIICKYLLSEEEDHFSEEQYLAAVRKFTSQLLD
QGTSQCYDVLSSDVQKNALAACVRLGVVEKKKINNNCIFNVNEPATTKLEEMLGCKTPIG
KPATAKL

>Q14746	peripheral membrane protein
MEKSRMNLPKGPDTLCFDKDEFMKEDFDVDHFVSDCRKRVQLEELRDDLELYYKLLKTA
MVELINKDYADFVNLSTNLVGMDKALNQLSVPLGQLREEVLSLRSSVSEGIRAVDERMSK
QEDIRKKKMCVLRLIQVIRSVEKIEKILNSQSSKETSALEASSPLLTGQILERIATEFNQLQFH
AVQSKGMPLLDKVRPRIAGITAMLQQSLEGLLLEGLQTSDVDIIRHCLRTYATIDKTRDAE
ALVGQVLVKPYIDEVIIEQFVESHPNGLQVMYNKLLEFVPHHCRLLREVTGGAISSEKGNT
VPGYDFLVNSVWPQIVQGLEEKLPSLFNPGNPDAFHEKYTISMDFVRRLERQCGSQASVK
RLRAHPAYHSFNKKWNLPVYFQIRFREIAGSLEAALTDVLEDAPAESPYCLLASHRTWSSL
RRCWSDEMFLPLLVHRLWRLTLQILARYSVFVNELSLRPISNESPKEIKKPLVTGSKEPSITQ
GNTEDQGSGPSETKPVVSISRTQLVYVVADLDKLQEQLPELLEIIKPKLEMIGFKNFSSISAA
LEDSQSSFSACVPSLSSKIIQDLSDSCFGFLKSALEVPRLYRRTNKEVPTTASSYVDSALKPL
FQLQSGHKDKLKQAIIQQWLEGTLSESTHKYYETVSDVLNSVKKMEESLKRLKQARKTTP
ANPVGPSGGMSDDDKIRLQLALDVEYLGEQIQKLGLQASDIKSFSALAELVAAAKDQATA
EQP

>Q10471	single-pass type II membrane protein
MRRRSRMLLCFAFLWVLGIAYYMYSGGGSALAGGAGGGAGRKEDWNEIDPIKKKDLHH
SNGEEKAQSMETLPPGKVRWPDFNQEAYVGGTMVRSGQDPYARNKFNQVESDKLRMDR
AIPDTRHDQCQRKQWRVDLPATSVVITFHNEARSALLRTVVSVLKKSPPHLIKEIILVDDYS
NDPEDGALLGKIEKVRVLRNDRREGLMRSRVRGADAAQAKVLTFLDSHCECNEHWLEPL
LERVAEDRTRVVSPIIDVINMDNFQYVGASADLKGGFDWNLVFKWDYMTPEQRRSRQGN
PVAPIKTPMIAGGLFVMDKFYFEELGKYDMMMDVWGGENLEISFRVWQCGGSLEIIPCSR
VGHVFRKQHPYTFPGGSGTVFARNTRRAAEVWMDEYKNFYYAAVPSARNVPYGNIQSRL
ELRKKLSCKPFKWYLENVYPELRVPDHQDIAFGALQQGTNCLDTLGHFADGVVGVYECH
NAGGNQEWALTKEKSVKHMDLCLTVVDRAPGSLIKLQGCRENDSRQKWEQIEGNSKLRH
VGSNLCLDSRTAKSGGLSVEVCGPALSQQWKFTLNLQQ

>Q9NRK6	multipass membrane protein
MRGPPAWPLRLLEPPSPAEPGRLLPVACVWAAASRVPGSLSPFTGLRPARLWGAGPALLWG
VGAARRWRSGCRGGGPGASRGVLGLARLLGLWARGPGSCRCGAFAGPGAPRLPRARFPG
GPAAAAWAGDEAWRRGPAAPPGDKGRLRPAAAGLPEARKLLGLAYPERRRLAAAVGFLT
MSSVISMSAPFFLGKIIDVIYTNPTVDYSDNLTRLCLGLSAVFLCGAAANAIRVYLMQTSG
QRIVNRLRTSLFSSILRQEVAFFDKTRTGELINRLSSDTALLGRSVTENLSDGLRAGAQASV
GISMMFFVSPNLATFVLSVVPPVSIIAVIYGRYLRKLTKVTQDSLAQATQLAEERIGNVRTV
RAFGKEMTEIEKYASKVDHVMQLARKEAFARAGFFGATGLSGNLIVLSVLYKGGLLMGS
AHMTVGELSSFLMYAFWVGISIGGLSSFYSELMKGLGAGGRLWELLEREPKLPFNEGVIL
NEKSFQGALEFKNVHFAYPARPEVPIFQDFSLSIPSGSVTALVGPSGSGKSTVLSLLLRLYDP
ASGTISLDGHDIRQLNPVWLRSKIGTVSQEPILFSCSIAENIAYGADDPSSVTAEEIQRVAEV
ANAVAFIRNFPQGFNTVVGEKGVLLSGGQKQRIAIARALLKNPKILLLDEATSALDAENEY
LVQEALDRLMDGRTVLVIAHRLSTIKNANMVAVLDQGKITEYGKHEELLSKPNGIYRKLM
NKQSFISA

>Q7L0Q8	lipid-anchor
MPPQQGDPAFPDRCEAPPVPPRRERGGRGGRGPGEPGGRGRAGGAEGRGVKCVLVGDGA

VGKTSLVVSYTTNGYPTEYIPTAFDNFSAVVSVDGRPVRLQLCDTAGQDEFDKLRPLCYT
NTDIFLLCFSVVSPSSFQNVSEKWVPEIRCHCPKAPIILVGTQSDLREDVKVLIELDKCKEK
PVPEEAAKLCAEEIKAASYIECSALTQKNLKEVFDAAIVAGIQYSDTQQQPKKSKSRTPDK
MKNLSKSWWKKYCCFV

>Q5T442 multipass membrane protein
MTNMSWSFLTRLLEEIHNHSTFVGKVWLTVLVVFRIVLTAVGGEAIYSDEQAKFTCNTRQP
GCDNVCYDAFAPLSHVRFWVFQIVVISTPSVMYLGYAVHRLARASEQERRRALRRRPGPR
RAPRAHLPPPHAGWPEPADLGEEEPMLGLGEEEEEEETGAAEGAGEEAEEAGAEEACTKA
VGADGKAAGTPGPTGQHDGRRRIQREGLMRVYVAQLVARAAFEVAFLVGQYLLYGFEVR
PFFPCSRQPCPHVVDCFVSRPTEKTVFLLVMYVVSCLCLLLNLCEMAHLGLGSAQDAVRG
RRGPPASAPAPAPRPPPCAFPAAAAGLACPPDYSLVVRAAERARAHDQNLANLALQALRD
GAAAGDRDRDSSPCVGLPAASRGPPRAGAPASRTGSATSAGTVGEQGRPGTHERPGAKPR
AGSEKGSASSRDGKTTVWI

>O00548 single-pass type I membrane protein
MGSRCALALAVLSALLCQVWSSGVFELKLQEFVNKKGLLGNRNCCRGGAGPPPCACRTF
FRVCLKHYQASVSPEPPCTYGSAVTPVLGVDSFSLPDGGGADSAFSNPIRFPFGFTWPGTFS
LIIEALHTDSPDDLATENPERLISRLATQRHLTVGEEWSQDLHSSGRTDLKYSYRFVCDEHY
YGEGCSVFCRPRDDAFGHFTCGERGEKVCNPGWKGPYCTEPICLPGCDEQHGFCDKPGE
CKCRVGWQGRYCDECIRYPGCLHGTCQQPWQCNCQEGWGGLFCNQDLNYCTHHKPCK
NGATCTNTGQGSYTCSCRPGYTGATCELGIDECDPSPCKNGGSCTDLENSYSCTCPPGFYG
KICELSAMTCADGPCFNGGRCSDSPDGGYSCRCPVGYSGFNCEKKIDYCSSSPCSNGAKC
VDLGDAYLCRCQAGFSGRHCDDNVDDCASSPCANGGTCRDGVNDFSCTCPPGYTGRNC
SAPVSRCEHAPCHNGATCHERGHRYVCECARGYGGPNCQFLLPELPPGPAVVDLTEKLEG
QGGPFPWVAVCAGVILVLMLLLGCAAVVVCVRLRLQKHRPPADPCRGETETMNNLANCQ
REKDISVSIIGATQIKNTNKKADFHGDHSADKNGFKARYPAVDYNLVQDLKGDDTAVRDA
HSKRDTKCQPQGSSGEEKGTPTTLRGGEASERKRPDSGCSTSKDTKYQSVYVISEEKDEC
VIATEV

>P49810 multipass membrane protein
MLTFMASDSEEEVCDERTSLMSAESPTPRSCQEGRQGPEDGENTAQWRSQENEEDGEEDP
DRYVCSGVPGRPPGLEEELTLKYGAKHVIMLFVPVTLCMIVVVATIKSVRFYTEKNGQLIY
TPFTEDTPSVGQRLLNSVLNTLIMISVIVVMTIFLVVLYKYRCYKFIHGWLIMSSLMLLFLF
TYIYLGEVLKTYNVAMDYPTLLLTVWNFGAVGMVCIHWKGPLVLQQAYLIMISALMALV
FIKYLPEWSAWVILGAISVYDLVAVLCPKGPLRMLVETAQERNEPIFPALIYSSAMVWTVG
MAKLDPSSQGALQLPYDPEMEEDSYDSFGEPSYPEVFEPPLTGYPGEELEEEEERGVKLGL
GDFIFYSVLVGKAAATGSGDWNTTLACFVAILIGLCLTLLLLAVFKKALPALPISITFGLIFYF
STDNLVRPFMDTLASHQLYI

>Q9H3P7 peripheral membrane protein
MAAVLNAERLEVSVDGLTLSPDPEERPGAEGAPLLPPPLPPPSPPGSGRGPGASGEQPEPGE
AAAGGAAEEARRLEQRWGFGLEELYGLALRFFKEKDGKAFHPTYEEKLKLVALHKQVL
MGPYNPDTCPEVGFFDVLGNDRRREWAALGNMSKEDAMVEFVKLLNRCCHLFSTYVAS

HKIEKEEQEKKRKEEEERRRREEEERERLQKEEEKRRREEEERLRREEEERRRIEEERLRLE
QQKQQIMAALNSQTAVQFQQYAAQQYPGNYEQQQILIRQLQEQHYQQYMQQLYQVQLA
QQQAALQKQQEVVVAGSSLPTSSKVNATVPSNMMSVNGQAKTHTDSSEKELEPEAAEEA
LENGPKESLPVIAAPSMWTRPQIKDFKEKIQQDADSVITVGRGEVVTVRVPTHEEGSYLF
WEFATDNYDIGFGVYFEWTDSPNTAVSVHVSESSDDDEEEEENIGCEEKAKKNANKPLLD
EIVPVYRRDCHEEVYAGSHQYPGRGVYLLKFDNSYSLWRSKSVYYRVYYTR

>O00270 multipass membrane protein
MPFPNCSAPSTVVATAVGVLLGLECGLGLLGNAVALWTFLFRVRVWKPYAVYLLNLALAD
LLLAACLPFLAAFYLSLQAWHLGRVGCWALHFLLDLSRSVGMAFLAAVALDRYLRVVHP
RLKVNLLSPQAALGVSGLVWLLMVALTCPGLLISEAAQNSTRCHSFYSRADGSFSIIWQEA
LSCLQFVLPFGLIVFCNAGIIRALQKRLREPEKQPKLQRAQALVTLVVVLFALCFLPCFLAR
VLMHIFQNLGSCRALCAVAHTSDVTGSLTYLHSVLNPVVYCFSSPTFRSSYRRVFHTLRGK
GQAAEPPDFNPRDSYS

>Q969Z3 peripheral membrane protein
MGASSSSALARLGLPARPWPRWLGVAALGLAAVALGTVAWRRAWPRRRRRLQQVGTVA
KLWIYPVKSCKGVPVSEAECTAMGLRSGNLRDRFWLVIKEDGHMVTARQEPRLVLISIIYE
NNCLIFRAPDMDQLVLPSKQPSSNKLHNCRIFGLDIKGRDCGNEAAKWFTNFLKTEAYRL
VQFETNMKGRTSRKLLPTLDQNFQVAYPDYCPLLIMTDASLVDLNTRMEKKMKMENFRP
NIVVTGCDAFEEDTWDELLIGSVEVKKVMACPRCILTTVDPDTGVIDRKQPLDTLKSYRL
CDPSERELYKLSPLFGIYYSVEKIGSLRVGDPVYRMV

>Q9P0L2 peripheral membrane protein
MSARTPLPTVNERDTENHTSVDGYTEPHIQPTKSSSRQNIPRCRNSITSATDEQPHIGNYRL
QKTIGKGNFAKVKLARHVLTGREVAVKIIDKTQLNPTSLQKLFREVRIMKILNHPNIVKLFE
VIETEKTLYLVMEYASGGEVFDYLVAHGRMKEKEARAKFRQIVSAVQYCHQKYIVHRDL
KAENLLLDGDMNIKIADFGFSNEFTVGNKLDTFCGSPPYAAPELFQGKKYDGPEVDVWSL
GVILYTLVSGSLPFDGQNLKELRERVLRGKYRIPFYMSTDCENLLKKLLVLNPIKRGSLEQI
MKDRWMNVGHEEEELKPYTEPDPDFNDTKRIDIMVTMGFARDEINDALINQKYDEVMAT
YILLGRKPPEFEGGESLSSGNLCQRSRPSSDLNNSTLQSPAHLKVQRSISANQKQRRFSDHA
GPSIPPAVSYTKRPQANSVESEQKEEWDKDVARKLGSTTVGSKSEMTASPLVGPERKKSST
IPSNNVYSGGSMARRNTYVCERTTDRYVALQNGKDSSLTEMSVSSISSAGSSVASAVPSAR
PRHQKSMSTSGHPIKVTLPTIKDGSEAYRPGTTQRVPAASPSAHSISTATPDRTRFPRGSSSR
STFHGEQLRERRSVAYNGPPASPSHETGAFAHARRGTSTGIISKITSKFVRRDPSEGEASGRT
DTSRSTSGEPKERDKEEGKDSKPRSLRFTWSMKTTSSMDPNDMMREIRKVLDANNCDYE
QKERFLLFCVHGDARQDSLVQWEMEVCKLPRLSLNGVRFKRISGTSIAFKNIASKIANELK
L

>O15245 multipass membrane protein
MPTVDDILEQVGESGWFQKQAFLILCLLSAAFAPICVGIVFLGFTPDHHCQSPGVAELSQR
CGWSPAEELNYTVPGLGPAGEAFLGQCRRYEVDWNQSALSCVDPLASLATNRSHLPLGPC
QDGWVYDTPGSSIVTEFNLVCADSWKLDLFQSCLNAGFLFGSLGVGYFADRFGRKLCLL
GTVLVNAVSGVLMAFSPNYMSMLLFRLLQGLVSKGNWMAGYTLITEFVGSGSRRTVAIM

YQMAFTVGLVALTGLAYALPHWRWLQLAVSLPTFLFLLYYWCVPESPRWLLSQKRNTEAI
KIMDHIAQKNGKLPPADLKMLSLEEDVTEKLSPSFADLFRTPRLRKRTFILMYLWFTDSVL
YQGLILHMGATSGNLYLDFLYSALVEIPGAFIALITIDRVGRIYPMAMSNLLAGAACLVMIFI
SPDLHWLNIIIMCVGRMGITIAIQMICLVNAELYPTFVRNLGVMVCSSLCDIGGIITPFIVFRL
REVWQALPLILFAVLGLLAAGVTLLLPETKGVALPETMKDAENLGRKAKPKENTIYLKVQ
TSEPSGT

>Q9NZJ0 peripheral membrane protein
MLFNSVLRQPQLGVLRNGWSSQYPLQSLLTGYQCSGNDEHTSYGETGVPVPPFGCTFSSA
PNMEHVLAVANEEGFVRLYNTESQSFRKKCFKEWMAHWNAVFDLAWVPGELKLVTAAG
DQTAKFWDVKAGELIGTCKGHQCSLKSVAFSKFEKAVFCTGGRDGNIMVWDTRCNKKD
GFYRQVNQISGAHNTSDKQTPSKPKKKQNSKGLAPSVDFQQSVTVVLFQDENTLVSAGA
VDGIIKVWDLRKNYTAYRQEPIASKSFLYPGSSTRKLGYSSLILDSTGSTLFANCTDDNIYM
FNMTGLKTSPVAIFNGHQNSTFYVKSSLSPDDQFLVSGSSDEAAYIWKVSTPWQPPTVLLG
HSQEVTSVCWCPSDFTKIATCSDDNTLKIWRLNRGLEEKPGGDKLSTVGWASQKKKESRP
GLVTVTSSQSTPAKAPRAKCNPSNSSPSSAACAPSCAGDLPLPSNTPTFSIKTSPAKARSPIN
RRGSVSSVSPKPPSSFKMSIRNWVTRTPSSSPPITPPASETKIMSPRKALIPVSQKSSQAEACS
ESRNRVKRRLDSSCLESVKQKCVKSCNCVTELDGQVENLHLDLCCLAGNQEDLSKDSLG
PTKSSKIEGAGTSISEPPSPISPYASESCGTLPLPLRPCGEGSEMVGKENSSPENKNWLLAM
AAKRKAENPSPRSPSSQTPNSRRQSGKKLPSPVTITPSSMRKICTYFHRKSQEDFCGPEHST
EL

>Q92604 multipass membrane protein
MAITLEEAPWLGWLLVKALMRFAFMVVNNLVAIPSYICYVIILQPLRVLDSKRFWYIEGIM
YKWLLGMVASWGWYAGYTVMEWGEDIKAVSKDEAVMLVNHQATGDVCTLMMCLQDK
GLVVAQMMWLMDHIFKYTNFGIVSLVHGDFFIRQGRSYRDQQLLLLKKHLENNYRSRDR
KWIVLFPEGGFLRKRRETSQAFAKKNNLPFLTNVTLPRSGATKIILNALVAQQKNGSPAGG
DAKELDSKSKGLQWIIDTTIAYPKAEPIDIQTWILGYRKPTVTHVHYRIFPIKDVPLETDDLT
TWLYQRFVEKEDLLSHFYETGAFPPSKGHKEAVSREMTLSNLWIFLIQSFAFLSGYMWYNI
IQYFYHCLF

>P20023 single-pass type I membrane protein
MGAAGLLGVFLALVAPGVLGISCGSPPPILNGRISYYSTPIAVGTVIRYSCSGTFRLIGEKSL
LCITKDKVDGTWDKPAPKCEYFNKYSSCPEPIVPGGYKIRGSTPYRHGDSVTFACKTNFS
MNGNKSVWCQANNMWGPTRLPTCVSVFPLECPALPMIHNGHHTSENVGSIAPGLSVTYS
CESGYLLVGEKIINCLSSGKWSAVPPTCEEARCKSLGRFPNGKVKEPPILRVGVTANFFCDE
GYRLQGPPSSRCVIAGQGVAWTKMPVCEEIFCPSPPPILNGRHIGNSLANVSYGSIVTYTCD
PDPEEGVNFILIGESTLRCTVDSQKTGTWSGPAPRCELSTSAVQCPHPQILRGRMVSGQKD
RYTYNDTVIFACMFGFTLKGSKQIRCNAQGTWEPSAPVCEKECQAPPNILNGQKEDRHM
VRFDPGTSIKYSCNPGYVLVGEESIQCTSEGVWTPPVPQCKVAACEATGRQLLTKPQHQFV
RPDVNSSCGEGYKLSGSVYQECQGTIPWFMEIRLCKEITCPPPPVIYNGAHTGSSLEDFPY
GTTVTYTCNPGPERGVEFSLIGESTIRCTSNDQERGTWSGPAPLCKLSLLAVQCSHVHIAN
GYKISGKEAPYFYNDTVTFKCYSGFTLKGSSQIRCKADNTWDPEIPVCEKETCQHVRQSL
QELPAGSRVELVNTSCQDGYQLTGHAYQMCQDAENGIWFKKIPLCKVIHCHPPPVIVNGK

HTGMMAENFLYGNEVSYECDQGFYLLGEKKLQCRSDSKGHGSWSGPSPQCLRSPPVTRC
PNPEVKHGYKLNKTHSAYSHNDIVYVDCNPGFIMNGSRVIRCHTDNTWVPGVPTCIKKAF
IGCPPPPKTPNGNHTGGNIARFSPGMSILYSCDQGYLLVGEALLLCTHEGTWSQPAPHCKE
VNCSSPADMDGIQKGLEPRKMYQYGAVVTLECEDGYMLEGSPQSQCQSDHQWNPPLAV
CRSRSLAPVLCGIAAGLILLTFLIVITLYVISKHRARNYYTDTSQKEAFHLEAREVYSVDPY
NPAS

>P47901 multipass membrane protein
MDSGPLWDANPTPRGTLSAPNATTPWLGRDEELAKVEIGVLATVLVLATGGNLAVLLTLG
QLGRKRSRMHLFVLHLALTDLAVALFQVLPQLLWDITYRFQGPDLLCRAVKYLQVLSMFA
STYMLLAMTLDRYLAVCHPLRSLQQPGQSTYLLIAAPWLLAAIFSLPQVFIFSLREVIQGSG
VLDCWADFGFPWGPRAYLTWTTLAIFVLPVTMLTACYSLICHEICKNLKVKTQAWRVGGG
GWRTWDRPSPSTLAATTRGLPSRVSSINTISRAKIRTVKMTFVIVLAYIACWAPFFSVQMWS
VWDKNAPDEDSTNVAFTISMLLGNLNSCCNPWIYMGFNSHLLPRPLRHLACCGGPQPRM
RRRLSDGSLSSRHTTLLTRSSCPATLSLSLSLTLSGRPRPEESPRDLELADGEGTAETIIF

>Q8IVJ1 multipass membrane protein
MSSKPEPKDVHQLNGTGPSASPCSSDGPGREPLAGTSEFLGPDGAGVEVVIESRANAKGV
REEDALLENGSQSNESDDVSTDRGPAPPSPLKETSFSIGLQVLFPFLLAGFGTVAAGMVLDI
VQHWEVFQKVTEVFILVPALLGLKGNLEMTLASRLSTAANIGHMDTPKELWRMITGNMA
LIQVQATVVGFLASIAAVVFGWIPDGHFSIPHAFLLCASSVATAFIASLVLGMIMIGVIIGSRK
IGINPDNVATPIAASLGDLITLALLSGISWGLYLELNHWRYIYPLVCAFFVALLPVWVVLAR
RSPATREVLYSGWEPVIIAMAISSVGGLILDKTVSDPNFAGMAVFTPVINGVGGNLVAVQAS
RISTFLHMNGMPGENSEQAPRRCPSPCTTFFSPDVNSRSARVLFLLVVPGHLVFLYTISCMQ
GGHTTLTLIFIIFYMTAALLQVLILLYIADWMVHWMWGRGLDPDNFSIPYLTALGDLLGTG
LLALSFHVLWLIGDRDTDVGD

>P56880 multipass membrane protein
MASAGLQLLAFILALSGVSGVLTATLLPNWKVNVDVDSNIITAIVQLHGLWMDCTWYSTG
MFSCALKHSILSLPIHVQAARATMVLACVLSALGICTSTVGMKCTRLGGDRETKSHASFA
GGVCFMSAGISSLISTVWYTKEIIANFLDLTVPESNKHEPGGAIYIGFISAMLLFISGMIFCTS
CIKRNPEARLDPPTQQPISNTQLENNSTHNLKDYV

>Q9HBX8 multipass membrane protein
MPSPPGLRALWLCAALCASRRAGGAPQPGPGPTACPAPCHCQEDGIMLSADCSELGLSAV
PGDLDPLTAYLDLSMNNLTELQPGLFHHLRFLEELRLSGNHLSHIPGQAFSGLYSLKILMLQ
NNQLGGIPAEALWELPSLQSLRLDANLISLVPERSFEGLSSLRHLWLDDNALTEIPVRALNN
LPALQAMTLALNRISHIPDYAFQNLTSLVVLHLHNNRIQHLGTHSFEGLHNLETLDLNYNK
LQEFPVAIRTLGRLQELGFHNNNIKAIPEKAFMGNPLLQTIHFYDNPIQFVGRSAFQYLPKL
HTLSLNGAMDIQEFPDLKGTTSLEILTLTRAGIRLLPSGMCQQLPRLRVLELSHNQIEELPSL
HRCQKLEEIGLQHNRIWEIGADTFSQLSSLQALDLSWNAIRSIHPEAFSTLHSLVKLDLTDN
QLTTLPLAGLGGLMHLKLKGNLALSQAFSKDSFPKLRILEVPYAYQCCPYGMCASFFKAS
GQWEAEDLHLDDEESSKRPLGLLARQAENHYDQDLDELQLEMEDSKPHPSVQCSPTPGP
FKPCEYLFESWGIRLAVWAIVLLSVLCNGLVLLTVFAGGPVPLPPVKFVVGAIAGANTLTGI

SCGLLASVDALTFGQFSEYGARWETGLGCRATGFLAVLGSEASVLLLTLAAVQCSVSVSC
VRAYGKSPSLGSVRAGVLGCLALAGLAAALPLASVGEYGASPLCLPYAPPEGQPAALGFT
VALVMMNSFCFLVVAGAYIKLYCDLPRGDFEAVWDCAMVRHVAWLIFADGLLYCPVAFLS
FASMLGLFPVTPEAVKSVLLVVLPLPACLNPLLYLLFNPHFRDDLRRLRPRAGDSGPLAYA
AAGELEKSSCDSTQALVAFSDVDLILEASEAGRPPGLETYGFPSVTLISCQQPGAPRLEGSH
CVEPEGNHFGNPQPSMDGELLLRAEGSTPAGGGLSGGGGFQPSGLAFASHV

>O60883 multipass membrane protein
MRWLWPLAVSLAVILAVGLSRVSGGAPLHLGRHRAETQEQQSRSKRGTEDEEAKGVQQY
VPEEWAEYPRPIHPAGLQPTKPLVATSPNPGKDGGTPDSGQELRGNLTGAPGQRLQIQNPL
YPVTESSYSAYAIMLLALVVFAVGIVGNLSVMCIVWHSYYLKSAWNSILASLALWDFLVLF
FCLPIVIFNEITKQRLLGDVSCRAVPFMEVSSLGVTTFSLCALGIDRFHVATSTLPKVRPIER
CQSILAKLAVIWVGSMTLAVPELLLWQLAQEPAPTMGTLDSCIMKPSASLPESLYSLVMTY
QNARMWWYFGCYFCLPILFTVTCQLVTWRVRGPPGRKSECRASKHEQCESQLNSTVVGL
TVVYAFCTLPENVCNIVVAYLSTELTRQTLDLLGLINQFSTFFKGAITPVLLLCICRPLGQAF
LDCCCCCCCEECGGASEASAANGSDNKLKTEVSSSIYFHKPRESPPLLPLGTPC

>Q9Y5J5 peripheral membrane protein
MTAAATATVLKEGVLEKRSGGLLQLWKRKRCVLTERGLQLFEAKGTGGRPKELSFARIKA
VECVESTGRHIYFTLVTEGGGEIDFRCPLEDPGWNAQITLGLVKFKNQQAIQTVRARQSLG
TGTLVS

>Q9P0T7 single-pass type I membrane protein
MKLLSLVAVVGCLLVPPAEANKSSEDIRCKCICPPYRNISGHIYNQNVSQKDCNCLHVVEP
MPVPGHDVEAYCLLCECRYEERSTTTIKVIIVIYLSVVGALLLYMAFLMLVDPLIRKPDAYT
EQLHNEEENEDARSMAAAAASLGGPRANTVLERVEGAQQRWKLQVQEQRKTVFDRHK
MLS

>Q6H3X3 single-pass type I membrane protein
MAAAASPAFLLRLPLLLLLSSWCRTGLADPHSLCYDITVIPKFRPGPRWCAVQGQVDEKTF
LHYDCGSKTVTPVSPLGKKLNVTTAWKAQNPVLREVVDILTEQLLDIQLENYIPKEPLTLQ
ARMSCEQKAEGHGSGSWQLSFDGQIFLLFDSENRMWTTVHPGARKMKEKWENDKDMT
MSFHYISMGDCTGWLEDFLMGMDSTLEPSAGAPPTMSSGTAQPRATATTLILCCLLIMCLL
ICSRHSLTQSHGHHPQSLQPPPHPPLLHPTWLLRRVLWSDSYQIAKRPLSGGHVTRVTLPII
GDDSHSLPCPLALYTINNGAARYSEPLQVSIS

>P08575 single-pass type I membrane protein
MYLWLKLLAFGFAFLDTEVFVTGQSPTPSPTGLTTAKMPSVPLSSDPLPTHTTAFSPASTFE
RENDFSETTTSLSPDNTSTQVSPDSLDNASAFNTTGVSSVQTPHLPTHADSQTPSAGTDTQ
TFSGSAANAKLNPTPGSNAISDVPGERSTASTFPTDPVSPLTTTLSLAHHSSAALPARTSNTT
ITANTSDAYLNASETTTLSPSGSAVISTTTIATTPSKPTCDEKYANITVDYLYNKETKLFTAK
LNVNENVECGNNTCTNNEVHNLTECKNASVSISHNSCTAPDKTLILDVPPGVEKFQLHDC
TQVEKADTTICLKWKNIETFTCDTQNITYRFQCGNMIFDNKEIKLENLEPEHEYKCDSEILY
NNHKFTNASKIIKTDFGSPGEPQIIFCRSEAAHQGVITWNPPQRSFHNFTLCYIKETEKDCL

NLDKNLIKYDLQNLKPYTKYVLSLHAYIIAKVQRNGSAAMCHFTTKSAPPSQVWNMTVS
MTSDNSMHVKCRPPRDRNGPHERYHLEVEAGNTLVRNESHKNCDFRVKDLQYSTDYTF
KAYFHNGDYPGEPFILHHSTSYNSKALIAFLAFLIIVTSIALLVVLYKIYDLHKKRSCNLDEQ
QELVERDDEKQLMNVEPIHADILLETYKRKIADEGRLFLAEFQSIPRVFSKFPIKEARKPFN
QNKNRYVDILPYDYNRVELSEINGDAGSNYINASYIDGFKEPRKYIAAQGPRDETVDDFW
RMIWEQKATVIVMVTRCEEGNRNKCAEYWPSMEEGTRAFGDVVVKINQHKRCPDYIIQK
LNIVNKKEKATGREVTHIQFTSWPDHGVPEDPHLLLKLRRRVNAFSNFFSGPIVVHCSAGV
GRTGTYIGIDAMLEGLEAENKVDVYGYVVKLRRQRCLMVQVEAQYILIHQALVEYNQFG
ETEVNLSELHPYLHNMKKRDPPSEPSPLEAEFQRLPSYRSWRTQHIGNQEENKSKNRNSN
VIPYDYNRVPLKHELEMSKESEHDSDESSDDDSDSEEPSKYINASFIMSYWKPEVMIAAQG
PLKETIGDFWQMIFQRKVKVIVMLTELKHGDQEICAQYWGEGKQTYGDIEVDLKDTDKS
STYTLRVFELRHSKRKDSRTVYQYQYTNWSVEQLPAEPKELISMIQVVKQKLPQKNSSEG
NKHHKSTPLLIHCRDGSQQTGIFCALLNLLESAETEEVVDIFQVVKALRKARPGMVSTFEQ
YQFLYDVIASTYPAQNGQVKKNNHQEDKIEFDNEVDKVKQDANCVNPLGAPEKLPEAKE
QAEGSEPTSGTEGPEHSVNGPASPALNQGS

>P82279 single-pass type I membrane protein
MALKNINYLLIFYLSFSLLIYIKNSFCNKNNTRCLSNSCQNNSTCKDFSKDNDCSCSDTAN
NLDKDCDNMKDPCFSNPCQGSATCVNTPGERSFLCKCPPGYSGTICETTIGSCGKNSCQH
GGICHQDPIYPVCICPAGYAGRFCEIDHDECASSPCQNGAVCQDGIDGYSCFCVPGYQGRH
CDLEVDECASDPCKNEATCLNEIGRYTCICPHNYSGVNCELEIDECWSQPCLNGATCQDA
LGAYFCDCAPGFLGDHCELNTDECASQPCLHGGLCVDGENRYSCNCTGSGFTGTHCETL
MPLCWSKPCHNNATCEDSVDNYTCHCWPGYTGAQCEIDLNECNSNPCQSNGECVELSSE
KQYGRITGLPSSFSYHEASGYVCICQPGFTGIHCEEDVNECSSNPCQNGGTCENLPGNYTC
HCPFDNLSRTFYGGRDCSDILLGCTHQQCLNNGTCIPHFQDGQHGFSCLCPSGYTGSLCEI
ATTLSFEGDGFLWVKSGSVTTKGSVCNIALRFQTVQPMALLLFRSNRDVFVKLELLSGYIH
LSIQVNNQSKVLLFISHNTSDGEWHFVEVIFAEAVTLTLIDDSCKEKCIAKAPTPLESDQSIC
AFQNSFLGGLPVGMTSNGVALLNFYNMPSTPSFVGCLQDIKIDWNHITLENISSGSSLNVK
AGCVRKDWCESQPCQSRGRCINLWLSYQCDCHRPYEGPNCLREYVAGRFGQDDSTGYVI
FTLDESYGDTISLSMFVRTLQPSGLLLALENSTYQYIRVWLERGRLAMLTPNSPKLVVKFV
LNDGNVHLISLKIKPYKIELYQSSQNLGFISASTWKIEKGDVIYIGGLPDKQETELNGGFFK
GCIQDVRLNNQNLEFFPNPTNNASLNPVLVNVTQGCAGDNSCKSNPCHNGGVCHSRWDD
FSCSCPALTSGKACEEVQWCGFSPCPHGAQCQPVLQGFECIANAVFNGQSGQILFRSNGNI
TRELTNITFGFRTRDANVIILHAEKEPEFLNISIQDSRLFFQLQSGNSFYMLSLTSLQSVNDG
TWHEVTLSMTDPLSQTSRWQMEVDNETPFVTSTIATGSLNFLKDNTDIYVGDRAIDNIKG
LQGCLSTIEIGGIYLSYFENVHGFINKPQEEQFLKISTNSVVTGCLQLNVCNSNPCLHGGNC
EDIYSSYHCSCPLGWSGKHCELNIDECFSNPCIHGNCSDRVAAYHCTCEPGYTGVNCEVDI
DNCQSHQCANGATCISHTNGYSCLCFGNFTGKFCRQSRLPSTVCGNEKTNLTCYNGGNCT
EFQTELKCMCRPGFTGEWCEKDIDECASDPCVNGGLCQDLLNKFQCLCDVAFAGERCEV
DLADDLISDIFTTIGSVTVALLLILLLAIVASVVTSNKRATQGTYSPSRQEKEGSRVEMWNL
MPPPAMERLI

>P35354 peripheral membrane protein
MLARALLLCAVLALSHTANPCCSHPCQNRGVCMSVGFDQYKCDCTRTGFYGENCSTPEF

LTRIKLFLKPTPNTVHYILTHFKGFWNVVNNIPFLRNAIMSYVLTSRSHLIDSPPTYNADYG
YKSWEAFSNLSYYTRALPPVPDDCPTPLGVKGKKQLPDSNEIVEKLLLRRKFIPDPQGSN
MMFAFFAQHFTHQFFKTDHKRGPAFTNGLGHGVDLNHIYGETLARQRKLRLFKDGKMK
YQIIDGEMYPPTVKDTQAEMIYPPQVPEHLRFAVGQEVFGLVPGLMMYATIWLREHNRVC
DVLKQEHPEWGDEQLFQTSRLILIGETIKIVIEDYVQHLSGYHFKLKFDPELLFNKQFQYQ
NRIAAEFNTLYHWHPLLPDTFQIHDQKYNYQQFIYNNSILLEHGITQFVESFTRQIAGRVAG
GRNVPPAVQKVSQASIDQSRQMKYQSFNEYRKRFMLKPYESFEELTGEKEMSAELEALYG
DIDAVELYPALLVEKPRPDAIFGETMVEVGAPFSLKGLMGNVICSPAYWKPSTFGGEVGFQ
IINTASIQSLICNNVKGCPFTSFSVPDPELIKTVTINASSSRSGLDDINPTVLLKERSTEL

>P12270 peripheral membrane protein
MAAVLQQVLERTELNKLPKSVQNKLEKFLADQQSEIDGLKGRHEKFKVESEQQYFEIEKR
LSHSQERLVNETRECQSLRLELEKLNNQLKALTEKNKELEIAQDRNIAIQSQFTRTKEELEA
EKRDLIRTNERLSQELEYLTEDVKRLNEKLKESNTTKGELQLKLDELQASDVSVKYREKR
LEQEKELLHSQNTWLNTELKTKTDELLALGREKGNEILELKCNLENKKEEVSRLEEQMN
GLKTSNEHLQKHVEDLLTKLKEAKEQQASMEEKFHNELNAHIKLSNLYKSAADDSEAKS
NELTRAVEELHKLLKEAGEANKAIQDHLLEVEQSKDQMEKEMLEKIGRLEKELENANDL
LSATKRKGAILSEEELAAMSPTAAAVAKIVKPGMKLTELYNAYVETQDQLLLEKLENKRIN
KYLDEIVKEVEAKAPILKRQREEYERAQKAVASLSVKLEQAMKEIQRLQEDTDKANKQSS
VLERDNRRMEIQVKDLSQQIRVLLMELEEARGNHVIRDEEVSSADISSSSEVISQHLVSYRN
IEELQQQNQRLLVALRELGETREREEQETTSSKITELQLKLESALTELEQLRKSRQHQMQLV
DSIVRQRDMYRILLSQTTGVAIPLHASSLDDVSLASTPKRPSTSQTVSTPAPVPVIESTEAIE
AKAALKQLQEIFENYKKEKAENEKIQNEQLEKLQEQVTDLRSQNTKISTQLDFASKRYEM
LQDNVEGYRREITSLHERNQKLTATTQKQEQIINTMTQDLRGANEKLAVAEVRAENLKKE
KEMLKLSEVRLSQQRESLLAEQRGQNLLLTNLQTIQGILERSETETKQRLSSQIEKLEHEIS
HLKKKLENEVEQRHTLTRNLDVQLLDTKRQLDTETNLHLNTKELLKNAQKEIATLKQHLS
NMEVQVASQSSQRTGKGQPSNKEDVDDLVSQLRQTEEQVNDLKERLKTSTSNVEQYQA
MVTSLEESLNKEKQVTEEVRKNIEVRLKESAEFQTQLEKKLMEVEKEKQELQDDKRRAIE
SMEQQLSELKKTLSSVQNEVQEALQRASTALSNEQQARRDCQEQAKIAVEAQNKYEREL
MLHAADVEALQAAKEQVSKMASVRQHLEETTQKAESQLLECKASWEERERMLKDEVS
KCVCRCEDLEKQNRLLHDQIEKLSDKVVASVKEGVQGPLNVSLSEEGKSQEQILEILRFIR
REKEIAETRFEVAQVESLRYRQRVELLERELQELQDSLNAEREKVQVTAKTMAQHEELMK
KTETMNVVMETNKMLREEKERLEQDLQQMQAKVRKLELDILPLQEANAELSEKSGMLQ
AEKKLLEEDVKRWKARNQHLVSQQKDPDTEEYRKLLSEKEVHTKRIQQLTEEIGRLKAEI
ARSNASLTNNQNLIQSLKEDLNKVRTEKETIQKDLDAKIIDIQEKVKTITQVKKIGRRYKTQ
YEELKAQQDKVMETSAQSSGDHQEQHVSVQEMQELKETLNQAETKSKSLESQVENLQK
TLSEKETEARNLQEQTVQLQSELSRLRQDLQDRTTQEEQLRQQITEKEEKTRKAIVAAKSK
IAHLAGVKDQLTKENEELKQRNGALDQQKDELDVRITALKSQYEGRISRLERELREHQER
HLEQRDEPQEPSNKVPEQQRQITLKTTPASGERGIASTSDPPTANIKPTPVVSTPSKVTAAA
MAGNKSTPRASIRPMVTPATVTNPTTTPTATVMPTTQVESQEAMQSEGPVEHVPVFGSTS
GSVRSTSPNVQPSISQPILTVQQQTQATAFVQPTQQSHPQIEPANQELSSNIVEVVQSSPVER
PSTSTAVFGTVSATPSSSLPKRTREEEEDSTIEASDQVSDDTVEMPLPKKLKSVTPVGTEEE
VMAEESTDGEVETQVYNQDSQDSIGEGVTQGDYTPMEDSEETSQSLQIDLGPLQSDQQTT
TSSQDGQGKGDDVIVIDSDDEEEDDDENDGEHEDYEEDEEDDDDDEDDTGMGDEGEDS

NEGTGSADGNDGYEADDAEGGDGTDPGTETEESMGGGEGNHRAADSQNSGEGNTGAA
ESSFSQEVSREQQPSSASERQAPRAPQSPRRPPHPLPPRLTIHAPPQELGPPVQRIQMTRRQS
VGRGLQLTPGIGGMQQHFFDDEDRTVPSTPTLVVPHRTDGFAEAIHSPQVAGVPRFRFGPP
EDMPQTSSSHSDLGQLASQGGLGMYETPLFLAHEEESGGRSVPTTPLQVAAPVTVFTESTT
SDASEHASQSVPMVTTSTGTLSTTNETATGDDGDEVFVEAESEGISSEAGLEIDSQQEEEPV
QASDESDLPSTSQDPPSSSSVDTSSSQPKPFRRVRLQTTLRQGVRGRQFNRQRGVSHAMG
GRGGINRGNIN

>P46939 peripheral membrane protein
MAKYGEHEASPDNGQNEFSDIIKSRSDEHNDVQKKTFTKWINARFSKSGKPPINDMFTDL
KDGRKLLDLLEGLTGTSLPKERGSTRVHALNNVNRVLQVLHQNNVELVNIGGTDIVDGN
HKLTLGLLWSIILHWQVKDVMKDVMSDLQQTNSEKILLSWVRQTTRPYSQVNVLNFTTS
WTDGLAFNAVLHRHKPDLFSWDKVVKMSPIERLEHAFSKAQTYLGIEKLLDPEDVAVQLP
DKKSIIMYLTSLFEVLPQQVTIDAIREVETLPRKYKKECEEEAINIQSTAPEEEHESPRAETPS
TVTEVDMDLDSYQIALEEVLTWLLSAEDTFQEQDDISDDVEEVKDQFATHEAFMMELTAH
QSSVGSVLQAGNQLITQGTLSDEEEFEIQEQMTLLNARWEALRVESMDRQSRLHDVLMEL
QKKQLQQLSAWLTLTEERIQKMETCPLDDDVKSLQKLLEEHKSLQSDLEAEQVKVNSLTH
MVVIVDENSGESATAILEDQLQKLGERWTAVCRWTEERWNRLQEINILWQELLEEQCLLK
AWLTEKEEALNKVQTSNFKDQKELSVSVRRLAILKEDMEMKRQTLDQLSEIGQDVGQLL
DNSKASKKINSDSEELTQRWDSLVQRLEDSSNQVTQAVAKLGMSQIPQKDLLETVRVREQ
AITKKSKQELPPPPPPKKRQIHVDIEAKKKFDAISAELLNWILKWKTAIQTTEIKEYMKMQ
DTSEMKKKLKALEKEQRERIPRADELNQTGQILVEQMGKEGLPTEEIKNVLEKVSSEWKN
VSQHLEDLERKIQLQEDINAYFKQLDELEKVIKTKEEWVKHTSISESSRQSLPSLKDSCQRE
LTNLLGLHPKIEMARASCSALMSQPSAPDFVQRGFDSFLGRYQAVQEAVEDRQQHLENEL
KGQPGHAYLETLKTLKDVLNDSENKAQVSLNVLNDLAKVEKALQEKKTLDEILENQKPA
LHKLAEETKALEKNVHPDVEKLYKQEFDDVQGKWNKLKVLVSKDLHLLEEIALTLRAFE
ADSTVIEKWMDGVKDFLMKQQAAQGDDAGLQRQLDQCSAFVNEIETIESSLKNMKEIET
NLRSGPVAGIKTWVQTRLGDYQTQLEKLSKEIATQKSRLSESQEKAANLKKDLAEMQEW
MTQAEEEYLERDFEYKSPEELESAVEEMKRAKEDVLQKEVRVKILKDNIKLLAAKVPSGG
QELTSELNVVLENYQLLCNRIRGKCHTLEEVWSCWIELLHYLDLETTWLNTLEERMKSTE
VLPEKTDAVNEALESLESVLRHPADNRTQIRELGQTLIDGGILDDIISEKLEAFNSRYEDLSH
LAESKQISLEKQLQVLRETDQMLQVLQESLGELDKQLTTYLTDRIDAFQVPQEAQKIQAEI
SAHELTLEELRRNMRSQPLTSPESRTARGGSQMDVLQRKLREVSTKFQLFQKPANFEQRM
LDCKRVLDGVKAELHVLDVKDVDPDVIQTHLDKCMKLYKTLSEVKLEVETVIKTGRHIV
QKQQTDNPKGMDEQLTSLKVLYNDLGAQVTEGKQDLERASQLARKMKKEAASLSEWLS
ATETELVQKSTSEGLLGDLDTEISWAKNVLKDLEKRKADLNTITESSAALQNLIEGSEPILE
ERLCVLNAGWSRVRTWTEDWCNTLMNHQNQLEIFDGNVAHISTWLYQAEALLDEIEKKP
TSKQEEIVKRLVSELDDANLQVENVRDQALILMNARGSSSRELVEPKLAELNRNFEKVSQ
HIKSAKLLIAQEPLYQCLVTTETFETGVPFSDLEKLENDIENMLKFVEKHLESSDEDEKMD
EESAQIEEVLQRGEEMLHQPMEDNKKEKIRLQLLLLHTRYNKIKAIPIQQRKMGQLASGIR
SSLLPTDYLVEINKILLCMDDVELSLNVPELNTAIYEDFSFQEDSLKNIKDQLDKLGEQIAVI
HEKQPDVILEASGPEAIQIRDTLTQLNAKWDRINRMYSDRKGCFDRAMEEWRQFHCDLN
DLTQWITEAEELLVDTCAPGGSLDLEKARIHQQELEVGISSHQPSFAALNRTGDGIVQKLS
QADGSFLKEKLAGLNQRWDAIVAEVKDRQPRLKGESKQVMKYRHQLDEIICWLTKAEHA

MQKRSTTELGENLQELRDLTQEMEVHAEKLKWLNRTELEMLSDKSLSLPERDKISESLRT
VNMTWNKICREVPTTLKECIQEPSSVSQTRIAAHPNVQKVVLVSSASDIPVQSHRTSEISIPA
DLDKTITELADWLVLIDQMLKSNIVTVGDVEEINKTVSRMKITKADLEQRHPQLDYVFTL
AQNLKNKASSSDMRTAITEKLERVKNQWDGTQHGVELRQQQLEDMIIDSLQWDDHREET
EELMRKYEARLYILQQARRDPLTKQISDNQILLQELGPGDGIVMAFDNVLQKLLEEYGSD
DTRNVKETTEYLKTSWINLKQSIADRQNALEAEWRTVQASRRDLENFLKWIQEAETTVN
VLVDASHRENALQDSILARELKQQMQDIQAEIDAHNDIFKSIDGNRQKMVKALGNSEEAT
MLQHRLDDMNQRWNDLKAKSASIRAHLEASAEKWNRLLMSLEELIKWLNMKDEELKK
QMPIGGDVPALQLQYDHCKALRRELKEKEYSVLNAVDQARVFLADQPIEAPEEPRRNLQS
KTELTPEERAQKIAKAMRKQSSEVKEKWESLNAVTSNWQKQVDKALEKLRDLQGAMDD
LDADMKEAESVRNGWKPVGDLLIDSLQDHIEKIMAFREEIAPINFKVKTVNDLSSQLSPLD
LHPSLKMSRQLDDLNMRWKLLQVSVDDRLKQLQEAHRDFGPSSQHFLSTSVQLPWQRSI
SHNKVPYYINHQTQTTCWDHPKMTELFQSLADLNNVRFSAYRTAIKIRRLQKALCLDLLE
LSTTNEIFKQHKLNQNDQLLSVPDVINCLTTTYDGLEQMHKDLVNVPLCVDMCLNWLLN
VYDTGRTGKIRVQSLKIGLMSLSKGLLEEKYRYLFKEVAGPTEMCDQRQLGLLLHDAIQIP
RQLGEVAAFGGSNIEPSVRSCFQQNNNKPEISVKEFIDWMHLEPQSMVWLPVLHRVAAAE
TAKHQAKCNICKECPIVGFRYRSLKHFNYDVCQSCFFSGRTAKGHKLHYPMVEYCIPTTS
GEDVRDFTKVLKNKFRSKKYFAKHPRLGYLPVQTVLEGDNLETPITLISMWPEHYDPSQS
PQLFHDDTHSRIEQYATRLAQMERTNGSFLTDSSSTTGSVEDEHALIQQYCQTLGGESPVS
QPQSPAQILKSVEREERGELERIIADLEEEQRNLQVEYEQLKDQHLRRGLPVGSPPESIISPH
HTSEDSELIAEAKLLRQHKGRLEARMQILEDHNKQLESQLHRLRQLLEQPESDSRINGVSP
WASPQHSALSYSLDPDASGPQFHQAAGEDLLAPPHDTSTDLTEVMEQIHSTFPSCCPNVPS
RPQAM

>Q15878 multipass membrane protein
MARFGEAVVARPGSGDGDSDQSRNRQGTPVPASGQAAAYKQTKAQRARTMALYNPIPVR
QNCFTVNRSLFIFGEDNIVRKYAKKLIDWPPFEYMILATIIANCIVLALEQHLPEDDKTPMS
RRLEKTEPYFIGIFCFEAGIKIVALGFIFHKGSYLRNGWNVMDFIVVLSGILATAGTHFNTH
VDLRTLRAVRVLRPLKLVSGIPSLQIVLKSIMKAMVPLLQIGLLLFFAILMFAIIGLEFYSGKL
HRACFMNNSGILEGFDPPHPCGVQGCPAGYECKDWIGPNDGITQFDNILFAVLTVFQCITM
EGWTTVLYNTNDALGATWNWLYFIPLIIIGSFFVLNLVLGVLSGEFAKERERVENRRAFMK
LRRQQQIERELNGYRAWIDKAEEVMLAEENKNAGTSALEVLRRATIKRSRTEAMTRDSSD
EHCVDISSVGTPLARASIKSAKVDGVSYFRHKERLLRISIRHMVKSQVFYWIVLSLVALNT
ACVAIVHHNQPQWLTHLLYYAEFLFLGLFLLEMSLKMYGMGPRLYFHSSFNCFDFGVTVG
SIFEVVWAIFRPGTSFGISVLRALRLLRIFKITKYWASLRNLVVSLMSSMKSIISLLFLLFLFIV
VFALLGMQLFGGRFNFNDGTPSANFDTFPAAIMTVFQILTGEDWNEVMYNGIRSQGGVSS
GMWSAIYFIVLTLFGNYTLLNVFLAIAVDNLANAQELTKDEQEEEEAFNQKHALQKAKEV
SPMSAPNMPSIERDRRRRHHMSMWEPRSSHLRERRRRHHMSVWEQRTSQLRKHMQMSS
QEALNREEAPTMNPLNPLNPLSSLNPLNAHPSLYRRPRAIEGLALGLALEKFEEERISRGGS
LKGDGGDRSSALDNQRTPLSLGQREPPWLARPCHGNCDPTQQEAGGGEAVVTFEDRARH
RQSQRRSRHRRVRTEGKESSSASRSRSASQERSLDEAMPTEGEKDHELRGNHGAKEPTIQE
ERAQDLRRTNSLMVSRGSGLAGGLDEADTPLVLPHPELEVGKHVVLTEQEPEGSSEQALL
GNVQLDMGRVISQSEPDLSCITANTDKATTESTSVTVAIPDVDPLVDSTVVHISNKTDGEAS
PLKEAEIREDEEEVEKKKQKKEKRETGKAMVPHSSMFIFSTTNPIRRACHYIVNLRYFEMC

ILLVIAASSIALAAEDPVLTNSERNKVLRYFDYVFTGVFTFEMVIKMIDQGLILQDGSYFRD
LWNILDFVVVVGALVAFALANALGTNKGRDIKTIKSLRVLRVLRPLKTIKRLPKLKAVFDC
VVTSLKNVFNILIVYKLFMFIFAVIAVQLFKGKFFYCTDSSKDTEKECIGNYVDHEKNKME
VKGREWKRHEFHYDNIIWALLTLFTVSTGEGWPQVLQHSVDVTEEDRGPSRSNRMEMSIF
YVVYFVVFPFFFVNIFVALIIITFQEQGDKMMEECSLEKNERACIDFAISAKPLTRYMPQNR
HTFQYRVWHFVVSPSFEYTIMAMIALNTVVLMMKYYSAPCTYELALKYLNIAFTMVFSL
ECVLKVIAFGFLNYFRDTWNIFDFITVIGSITEIILTDSKLVNTSGFNMSFLKLFRAARLIKLL
RQGYTIRILLWTFVQSFKALPYVCLLIAMLFFIYAIIGMQVFGNIKLDEESHINRHNNFRSFF
GSLMLLFRSATGEAWQEIMLSCLGEKGCEPDTTAPSGQNENERCGTDLAYVYFVSFIFFCS
FLMLNLFVAVIMDNFEYLTRDSSILGPHHLDEFVRVWAEYDRAACGRIHYTEMYEMLTLM
SPPLGLGKRCPSKVAYKRLVLMNMPVAEDMTVHFTSTLMALIRTALDIKIAKGGADRQQL
DSELQKETLAIWPHLSQKMLDLLVPMPKASDLTVGKIYAAMMIMDYYKQSKVKKQRQQ
LEEQKNAPMFQRMEPSSLPQEIIANAKALPYLQQDPVSGLSGRSGYPSMSPLSPQDIFQLA
CMDPADDGQFQERQSLEPEVSELKSVQPSNHGIYLPSDTQEHAGSGRASSMPRLTVDPQV
VTDPSSMRRSFSTIRDKRSNSSWLEEFSMERSSENTYKSRRRSYHSSLRLSAHRLNSDSGH
KSDTHRSGGRERGRSKERKHLLSPDVSRCNSEERGTQADWESPERRQSRSPSEGRSQTPN
RQGTGSLSESSIPSVSDTSTPRRSRRQLPPVPPKPRPLLSYSSLIRHAGSISPPADGSEEGSPLT
SQALESNNACLTESSNSPHPQQSQHASPQRYISEPYLALHEDSHASDCGEEETLTFEAAVAT
SLGRSNTIGSAPPLRHSWQMPNGHYRRRRRGGPGPGMMCGAVNNLLSDTEEDDKC

>P56589 multipass membrane protein
MLRSVWNFLKRHKKKCIFLGTVLGGVYILGKYGQKKIREIQEREAAEYIAQARRQYHFES
NQRTCNMTVLSMLPTLREALMQQLNSESLTALLKNRPSNKLEIWEDLKIISFTRSTVAVYS
TCMLVVLLRVQLNIIGGYIYLDNAAVGKNGTTILAPPDVQQQYLSSIQHLLGDGLTELITVI
KQAVQKVLGSVSLKHSLSLLDLEQKLKEIRNLVEQHKSSSWINKDGSKPLLCHYMMPDEE
TPLAVQACGLSPRDITTIKLLNETRDMLESPDFSTVLNTCLNRGFSRLLDNMAEFFRPTEQ
DLQHGNSMNSLSSVSLPLAKIIPIVNGQIHSVCSETPSHFVQDLLTMEQVKDFAANVYEAF
STPQQLEK

>Q86SQ4 multipass membrane protein
MMFRSDRMWSCHWKWKPSPLLFLFALYIMCVPHSVWGCANCRVVLSNPSGTFTSPCYPN
DYPNSQACMWTLRAPTGYIIQITFNDFDIEEAPNCIYDSLSLDNGESQTKFCGATAKGLSFN
SSANEMHVSFSSDFSIQKKGFNASYIRVAVSLRNQKVILPQTSDAYQVSVAKSISIPELSAFT
LCFEATKVGHEDSDWTAFSYSNASFTQLLSFGKAKSGYFLSISDSKCLLNNALPVKEKEDI
FAESFEQLCLVWNNSLGSIGVNFKRNYETVPCDSTISKVIPGNGKLLLGSNQNEIVSLKGDI
YNFRLWNFTMNAKILSNLSCNVKGNVVDWQNDFWNIPNLALKAESNLSCGSYLIPLPAA
ELASCADLGTLCQATVNSPSTTPPTVTTNMPVTNRIDKQRNDGIIYRISVVIQNILRHPEVK
VQSKVAEWLNSTFQNWNYTVYVVNISFHLSAGEDKIKVKRSLEDEPRLVLWALLVYNAT
NNTNLEGKIIQQKLLKNNESLDEGLRLHTVNVRQLGHCLAMEEPKGYYWPSIQPSEYVLP
CPDKPGFSASRICFYNATNPLVTYWGPVDISNCLKEANEVANQILNLTADGQNLTSANITNI
VEQVKRIVNKEENIDITLGSTLMNIFSNILSSSDSDLLESSSEALKTIDELAFKIDLNSTSHVN
ITTRNLALSVSSLLPGTNAISNFSIGLPSNNESYFQMDFESGQVDPLASVILPPNLLENLSPE
DSVLVRRAQFTFFNKTGLFQDVGPQRKTLVSYVMACSIGNITIQNLKDPVQIKIKHTRTQE
VHHPICAFWDLNKNKSFGGWNTSGCVAHRDSDASETVCLCNHFTHFGVLMDLPRSASQL

DARNTKVLTFISYIGCGISAIFSAATLLTYVAFEKLRRDYPSKILMNLSTALLFLNLLFLLDG
WITSFNVDGLCIAVAVLLHFFLLATFTWMGLEAIHMYIALVKVFNTYIRRYILKFCIIGWGLP
ALVVSVVLASRNNNEVYGKESYGKEKGDEFCWIQDPVIFYVTCAGYFGVMFFLNIAMFIV
VMVQICGRNGKRSNRTLREEVLRNLRSVVSLTFLLGMTWGFAFFAWGPLNIPFMYLFSIFN
SLQGLFIFIFHCAMKENVQKQWRQHLCCGRFRLADNSDWSKTATNIIKKSSDNLGKSLSSS
SIGSNSTYLTSKSKSSSTTYFKRNSHTDNVSYEHSFNKSGSLRQCFHGQVLVKTGPC

>P35610 multipass membrane protein
MVGEEKMSLRNRLSKSRENPEEDEDQRNPAKESLETPSNGRIDIKQLIAKKIKLTAEAEEL
KPFFMKEVGSHFDDFVTNLIEKSASLDNGGCALTTFSVLEGEKNNHRAKDLRAPPEQGKI
FIARRSLLDELLEVDHIRTIYHMFIALLILFILSTLVVDYIDEGRLVLEFSLLSYAFGKFPTVV
WTWWIMFLSTFSVPYFLFQHWATGYSKSSHPLIRSLFHGFLFMIFQIGVLGFGPTYVVLAY
TLPPASRFIIIFEQIRFVMKAHSFVRENVPRVLNSAKEKSSTVPIPTVNQYLYFLFAPTLIYRD
SYPRNPTVRWGYVAMKFAQVFGCFFYVYYIFERLCAPLFRNIKQEPFSARVLVLCVFNSIL
PGVLILFLTFFAFLHCWLNAFAEMLRFGDRMFYKDWWNSTSYSNYYRTWNVVVHDWLY
YYAYKDFLWFFSKRFKSAAMLAVFAVSAVVHEYALAVCLSFFYPVLFVLFMFFGMAFNFIV
NDSRKKPIWNVLMWTSLFLGNGVLLCFYSQEWYARQHCPLKNPTFLDYVRPRSWTCRY
VF

>Q9Y2T5 multipass membrane protein
MNESRWTEWRILNMSSGIVNVSERHSCPLGFGHYSVVDVCIFETVVIVLLTFLIIAGNLTVI
FVFHCAPLLHHYTTSYFIQTMAYADLFVGVSCLVPTLSLLHYSTGVHESLTCQVFGYIISVL
KSVSMACLACISVDRYLAITKPLSYNQLVTPCRLRICIILIWIYSCLIFLPSFFGWGKPGYHG
DIFEWCATSWLTSAYFTGFIVCLLYAPAAFVVCFTYFHIFKICRQHTKEINDRRARFPSHEV
DSSRETGHSPDRRYAMVLFRITSVFYMLWLPYIIYFLLESSRVLDNPTLSFLTTWLAISNSFC
NCVIYSLSNSVFRLGLRRLSETMCTSCMCVKDQEAQEPKPRKRANSCSI

>Q5TAH2 multipass membrane protein
MSSYFWAQNESNRPDLLCGQPADYLVEEKHFTTLVCFIVVLGGLLKMCLKNCEVIVLTILS
LSGFVIGHMAYNSVEVHQIVYPLLRTSSFSLYSYFSPLIIFMVALDVEFYTLKKMFWQVLLT
GLISFSTASIIIGYVVIKFNKDSWDLQSCLLFSITLGIIDPLRSVNSLKTIGISKIYIDLIRGESLII
CSIASIFFGNFRGNRIHFSIFRDLHVGIELSYDILGSIIFGYWCAKIIQCILADVFSNMLTNIILC
FSMVYMTFYIVEFLGMSGTLALAAVGLNLDSLTFKPKIELVITKFLRIFSSVYEHLIYAFFGI
VIGCGELSHYEFHTIPFIFILFTTVNLVRLLTILLVSPILMHSNYEYNWRWGVVITWSGIKGV
FNLLWAPDVYNLAERKVEVPQMFILYVQVISLLTMGINSYVMTQSARKLDLCVLSLPRQM
ILQNATQHIQEIVQNTITLFKTEKILTNVNWTLVEDKTRIEYIPFSHVSHNDMKTESTTDEAL
MEEARLHVAAIQMSSFEKQRNNGILEIEAARILIGAAKCYYSIQGKFMSIYDVSTYMRTRS
WLIKFKNVLTFLEYCIEKIHFIPPESNTFLTFIFHIVFSEEFEYTGQIINLIYIYPMIIHLWPMAR
GLNVSALISINYYFMFLYVLESTLKIIILKRKYFQQCWNTLEFFILVIGIIDIFCVYFVKLRPD
NLALIQLTVIMGYLRIIRFLPLFKIIVPILIRIADVQIKKRLSLMYSITKGYIKSQEDAKLLIKQI
AVCESIYQKLCEILETNKQDAVKELVLMEHEGRDVVIALKTKQAIRNVIAKALKNLTFLCS
RGIIDKHEVIEINKVLLKKLKALNNFPKAIPPPTPDIYLHNIIWLEGKDVLIDFFKERAKLAC
FDSGDTICKGGEMPQGIYLIISGMAILHSLSPTFGIESNQRCDRGSRDMFTEFCTTGDIIGEL
SCLLKREIEYTVICETSLQACFISLEDLYEGFDAFWPSLEYKIWLKLALSTAYQYFESSLIDE

DLRFQNCVMFNQAYVETLSSYSDMIIDNMTMKFVIIVYGSVIDTKTEEPYFAPCIIPTTCEQ
VQGTSDLSKLLIIQASELTQRNSNTNVMASVNTVFEQPGKNINGRQKMS

>P48023 single-pass type II membrane protein
MQQPFNYPYPQIYWVDSSASSPWAPPGTVLPCPTSVPRRPGQRRPPPPPPPPPLPPPPPPPL
PPLPLPPLKKRGNHSTGLCLLVMFFMVLVALVGLGLGMFQLFHLQKELAELRESTSQMHT
ASSLEKQIGHPSPPPEKKELRKVAHLTGKSNSRSMPLEWEDTYGIVLLSGVKYKKGGLVIN
ETGLYFVYSKVYFRGQSCNNLPLSHKVYMRNSKYPQDLVMMEGKMMSYCTTGQMWAR
SSYLGAVFNLTSADHLYVNVSELSLVNFEESQTFFGLYKL

>P15260 single-pass type I membrane protein
MALLFLLPLVMQGVSRAEMGTADLGPSSVPTPTNVTIESYNMNPIVYWEYQIMPQVPVFT
VEVKNYGVKNSEWIDACINISHHYCNISDHVGDPSNSLWVRVKARVGQKESAYAKSEEFA
VCRDGKIGPPKLDIRKEEKQIMIDIFHPSVFVNGDEQEVDYDPETTCYIRVYNVYVRMNGS
EIQYKILTQKEDDCDEIQCQLAIPVSSLNSQYCVSAEGVLHVWGVTTEKSKEVCITIFNSSI
KGSLWIPVVAALLLFLVLSLVFICFYIKKINPLKEKSIILPKSLISVVRSATLETKPESKYVSLI
TSYQPFSLEKEVVCEEPLSPATVPGMHTEDNPGKVEHTEELSSITEVVTTEENIPDVVPGSH
LTPIERESSSPLSSNQSEPGSIALNSYHSRNCSESDHSRNGFDTDSSCLESHSSLSDSEFPPNN
KGEIKTEGQELITVIKAPTSFGYDKPHVLVDLLVDDSGKESLIGYRPTEDSKEFS

>P05026 single-pass type II membrane protein
MARGKAKEEGSWKKFIWNSEKKEFLGRTGGSWFKILLFYVIFYGCLAGIFIGTIQVMLLTI
SEFKPTYQDRVAPPGLTQIPQIQKTEISFRPNDPKSYEAYVLNIVRFLEKYKDSAQRDDMIF
EDCGDVPSEPKERGDFNHERGERKVCRFKLEWLGNCSGLNDETYGYKEGKPCIIIKLNRV
LGFKPKPPKNESLETYPVMKYNPNVLPVQCTGKRDEDKDKVGNVEYFGLGNSPGFPLQY
YPYYGKLLQPKYLQPLLAVQFTNLTMDTEIRIECKAYGENIGYSEKDRFQGRFDVKIEVKS

>Q96PN6 peripheral membrane protein
MNTPKEEFQDWPIVRIAAHLPDLIVYGHFSPERPFMDYFDGVLMFVDISGFTAMTEKFSSA
MYMDRGAEQLVEILNYHISAIVEKVLIFGGDILKFAGDALLALWRVERKQLKNIITVVIKC
SLEIHGLFETQEWEEGLDIRVKIGLAAGHISMLVFGDETHSHFLVIGQAVDDVRLAQNMAQ
MNDVILSPNCWQLCDRSMIEIESVPDQRAVKVNFLKPPPNFNFDEFFTKCTTFMHYYPSGE
HKNLLRLACTLKPDPELEMSLQKYVMESILKQIDNKQLQGYLSELRPVTIVFVNLMFEDQ
DKAEEIGPAIQDAYMHITSVLKIFQGQINKVFMFDKGCSFLCVFGFPGEKVPDELTHALEC
AMDIFDFCSQVHKIQTVSIGVASGIVFCGIVGHTVRHEYTVIGQKVNLAARMMMYYPGIV
TCDSVTYNGSNLPAYFFKELPKKVMKGVADSGPLYQYWGRTEKVMFGMACLICNRKED
YPLLGRNKEINYFMYTMKKFLISNSSQVLMYEGLPGYGKSQILMKIEYLAQGKNHRIIAIS
LNKISFHQTFYTIQMFMANVLGLDTCKHYKERQTNLRNKVMTLLDEKFYCLLNDIFHVQ
FPISREISRMSTLKKQKQLEILFMKILKLIVKEERIIFIIDEAQFVDSTSWRFMEKLIRTLPIFII
MSLCPFVNIPCAAARAVIKNRNTTYIVIGAVQPNDISNKICLDLNVSCISKELDSYLGEGSC
GIPFYCEELLKNLEHHEVLVFQQTESEEKTNRTWNNLFKYSIKLTEKLNMVTLHSDKESEE
VCHLTSGVRLKNLSPPTSLKEISLIQLDSMRLSHQMLVRCAAIIGLTFTTELLFEILPCWNM
KMMIKTLATLVESNIFYCFRNGKELQKALKQNDPSFEVHYRSLSLKPSEGMDHGEEEQLR
ELENEVIECHRIRFCNPMMQKTAYELWLKDQRKAMHLKCARFLEEDAHRCDHCRGRDFI

PYHHFTVNIRLNALDMDAIKKMAMSHGFKTEEKLILSNSEIPETSAFFPENRSPEEIREKIL
NFFDHVLTKMKTSDEDIIPLESCQCEEILEIVILPLAHHFLALGENDKALYYFLEIASAYLIFC
DNYMAYMYLNEGQKLLKTLKKDKSWSQTFESATFYSLKGEVCFNMGQIVLAKKMLRKA
LKLLNRIFPYNLISLFLHIHVEKNRHFHYVNRQAQESPPPGKKRLAQLYRQTVCLSLLWRI
YSYSYLFHCKYYAHLAVMMQMNTALETQNCFQIIKAYLDYSLYHHLAGYKGVWFKYEV
MAMEHIFNLPLKGEGIEIVAYVAETLVFNKLIMGHLDLAIELGSRALQMWALLQNPNRHY
QSLCRLSRCLLLNSRYPQLIQVLGRLWELSVTQEHIFSKAFFYFVCLDILLYSGFVYRTFEE
CLEFIHQYENNRILKFHSGLLLGLYSSVAIWYARLQEWDNFYKFSNRAKNLLPRRTMTLTY
YDGISRYMEGQVLHLQKQIKEQSENAQASGEELLKNLENLVAQNTTGPVFCPRLYHLMAY
VCILMGDGQKCGLFLNTALRLSETQGNILEKCWLNMNKESWYSTSELKEDQWLQTILSLP
SWEKIVAGRVNIQDLQKNKFLMRANTVDNHF

>Q99795 single-pass type I membrane protein
MVGKMWPVLWTLCAVRVTVDAISVETPQDVLRASQGKSVTLPCTYHTSTSSREGLIQWD
KLLLTHTERVVIWPFSNKNYIHGELYKNRVSISNNAEQSDASITIDQLTMADNGTYECSVSL
MSDLEGNTKSRVRLLVLVPPSKPECGIEGETIIGNNIQLTCQSKEGSPTPQYSWKRYNILNQ
EQPLAQPASGQPVSLKNISTDTSGYYICTSSNEEGTQFCNITVAVRSPSMNVALYVGIAVGV
VAALIIIGIIIYCCCCRGKDDNTEDKEDARPNREAYEEPPEQLRELSREREEEDDYRQEEQR
STGRESPDHLDQ

>Q9UM00 multipass membrane protein
MSTMFADTLLIVFISVCTALLAEGITWVLVYRTDKYKRLKAEVEKQSKKLEKKKETITESA
GRQQKKKIERQEEKLKNNNRDLSMVRMKSMFAIGFCFTALMGMFNSIFDGRVVAKLPFTP
LSYIQGLSHRNLLGDDTTDCSFIFLYILCTMSIRQNIQKILGLAPSRAATKQAGGFLGPPPPS
GKFS

>Q16832 single-pass type I membrane protein
MILIPRMLLVLFLLLPILSSAKAQVNPAICRYPLGMSGGQIPDEDITASSQWSESTAAKYGRL
DSEEGDGAWCPEIPVEPDDLKEFLQIDLHTLHFITLVGTQGRHAGGHGIEFAPMYKINYSR
DGTRWISWRNRHGKQVLDGNSNPYDIFLKDLEPPIVARFVRFIPVTDHSMNVCMRVELYG
CVWLDGLVSYNAPAGQQFVLPGGSIIYLNDSVYDGAVGYSMTEGLGQLTDGVSGLDDFT
QTHEYHVWPGYDYVGWRNESATNGYIEIMFEFDRIRNFTTMKVHCNNMFAKGVKIFKEV
QCYFRSEASEWEPNAISFPLVLDDVNPSARFVTVPLHHRMASAIKCQYHFADTWMMFSEI
TFQSDAAMYNNSEALPTSPMAPTTYDPMLKVDDSNTRILIGCLVAIIFILLAIIVIILWRQFW
QKMLEKASRRMLDDEMTVSLSLPSDSSMFNNNRSSSPSEQGSNSTYDRIFPLRPDYQEPSR
LIRKLPEFAPGEEESGCSGVVKPVQPSGPEGVPHYAEADIVNLQGVTGGNTYSVPAVTMDL
LSGKDVAVEEFPRKLLTFKEKLGEGQFGEVHLCEVEGMEKFKDKDFALDVSANQPVLVAV
KMLRADANKNARNDFLKEIKIMSRLKDPNIIHLLAVCITDDPLCMITEYMENGDLNQFLSR
HEPPNSSSSDVRTVSYTNLKFMATQIASGMKYLSSLNFVHRDLATRNCLVGKNYTIKIADF
GMSRNLYSGDYYRIQGRAVLPIRWMSWESILLGKFTTASDVWAFGVTLWETFTFCQEQPY
SQLSDEQVIENTGEFFRDQGRQTYLPQPAICPDSVYKLMLSCWRRDTKNRPSFQEIHLLLL
QQGDE

>Q9P1P5 multipass membrane protein

MAVSSEQHELSHFKRTQTKKEKFNCSEYGNRSCPENERSLGVRVAMYSFMAGSIFITIFGN
LAMIISISYFKQLHTPTNFLILSMAITDFLLGFTIMPYSMIRSVENCWYFGLTFCKIYYSFDL
MLSITSIFHLCSVAIDRFYAICYPLLYSTKITIPVIKRLLLLCWSVPGAFAFGVVFSEAYADGIE
GYDILVACSSSCPVMFNKLWGTTLFMAGFFTPGSMMVGIYGKIFAVSRKHAHAINNLREN
QNNQVKKDKKAAKTLGIVIGVFLLCWFPCFFTILLDPFLNFSTPVVLFDALTWFGYFNSTC
NPLIYGFFYPWFRRALKYILLGKIFSSCFHNTILCMQKESE

>P18850 single-pass type II membrane protein
MGEPAGVAGTMESPFSPGLFHRLDEDWDSALFAELGYFTDTDELQLEAANETYENNFDN
LDFDLDLMPWESDIWDINNQICTVKDIKAEPQPLSPASSSYSVSSPRSVDSYSSTQHVPEEL
DLSSSSQMSPLSLYGENSNSLSSAEPLKEDKPVTGPRNKTENGLTPKKKIQVNSKPSIQPKP
LLLPAAPKTQTNSSVPAKTIIIQTVPTLMPLAKQQPIISLQPAPTKGQTVLLSQPTVVQLQAP
GVLPSAQPVLAVAGGVTQLPNHVVNVVPAPSANSPVNGKLSVTKPVLQSTMRNVGSDIAV
LRRQQRMIKNRESACQSRKKKKEYMLGLEARLKAALSENEQLKKENGTLKRQLDEVVS
ENQRLKVPSPKRRVVCVMIVLAFIILNYGPMSMLEQDSRRMNPSVSPANQRRHLLGFSAK
EAQDTSDGIIQKNSYRYDHSVSNDKALMVLTEEPLLYIPPPPCQPLINTTESLRLNHELRGW
VHRHEVERTKSRRMTNNQQKTRILQGALEQGSNSQLMAVQYTETTSSISRNSGSELQVYY
ASPRSYQDFFEAIRRRGDTFYVVSFRRDHLLLPATTHNKTTRPKMSIVLPAININENVINGQ
DYEVMMQIDCQVMDTRILHIKSSSVPPYLRDQQRNQTNTFFGSPPAATEATHVVSTIPESL
Q

>Q99643 multipass membrane protein
MAALLLRHVGRHCLRAHFSPQLCIRNAVPLGTTAKEEMERFWNKNIGSNRPLSPHITIYSW
SLPMAMSICHRGTGIALSAGVSLFGMSALLLPGNFESYLELVKSLCLGPALIHTAKFALVFP
LMYHTWNGIRHLMWDLGKGLKIPQLYQSGVVVLVLTVLSSMGLAAM

>O75306 peripheral membrane protein
MAALRALCGFRGVAAQVLRPGAGVRLPIQPSRGVRQWQPDVEWAQQFGGAVMYPSKET
AHWKPPPWNDVDPPKDTIVKNITLNFGPQHPAAHGVLRLVMELSGEMVRKCDPHIGLLH
RGTEKLIEYKTYLQALPYFDRLDYVSMMCNEQAYSLAVEKLLNIRPPPRAQWIRVLFGEIT
RLLNHIMAVTTHALDLGAMTPFFWLFEEREKMFEFYERVSGARMHAAYIRPGGVHQDLP
LGLMDDIYQFSKNFSLRLDELEELLTNNRIWRNRTIDIGVVTAEEALNYGFSGVMLRGSGI
QWDLRKTQPYDVYDQVEFDVPVGSRGDCYDRYLCRVEEMRQSLRIIAQCLNKMPPGEIK
VDDAKVSPPKRAEMKTSMESLIHHFKLYTEGYQVPPGATYTAIEAPKGEFGVYLVSDGSS
RPYRCKIKAPGFAHLAGLDKMSKGHMLADVVAIIGTQDIVFGEVDR

>Q9HBG7 single-pass type I membrane protein
MVAPKSHTDDWAPGPFSSKPQRSQLQIFSSVLQTSLLFLLMGLRASGKDSAPTVVSGILGG
SVTLPLNISVDTEIENVIWIGPKNALAFARPKENVTIMVKSYLGRLDITKWSYSLCISNLTL
NDAGSYKAQINQRNFEVTTEEEFTLFVYEQLQEPQVTMKSVKVSENFSCNITLMCSVKGA
EKSVLYSWTPREPHASESNGGSILTVSRTPCDPDLPYICTAQNPVSQRSSLPVHVGQFCTDP
GASRGGTTGETVVGVLGEPVTLPLALPACRDTEKVVWLFNTSIISKEREEAATADPLIKSR
DPYKNRVWVSSQDCSLKISQLKIEDAGPYHAYVCSEASSVTSMTHVTLLIYRRLRKPKITW
SLRHSEDGICRISLTCSVEDGGNTVMYTWTPLQKEAVVSQGESHLNVSWRSSENHPNLTC

TASNPVSRSSHQFLSENICSGPERNTKLWIGLFLMVCLLCVGIFSWCIWKRKGRCSVPAFCS
SQAEAPADTPEPTAGHTLYSVLSQGYEKLDTPLRPARQQPTPTSDSSSDSNLTTEEDEDRPE
VHKPISGRYEVFDQVTQEGAGHDPAPEGQADYDPVTPYVTEVESVVGENTMYAQVFNLQ
GKTPVSQKEESSATIYCSIRKPQVVPPPQQNDLEIPESPTYENFT

>Q9NQ25 single-pass type I membrane protein
MAGSPTCLTLIYILWQLTGSAASGPVKELVGSVGGAVTFPLKSKVKQVDSIVWTFNTTPLV
TIQPEGGTIIVTQNRNRERVDFPDGGYSLKLSKLKKNDSGIYYVGIYSSSLQQPSTQEYVLH
VYEHLSKPKVTMGLQSNKNGTCVTNLTCCMEHGEEDVIYTWKALGQAANESHNGSILPI
SWRWGESDMTFICVARNPVSRNFSSPILARKLCEGAADDPDSSMVLLCLLLVPLLLSLFVL
GLFLWFLKRERQEEYIEEKKRVDICRETPNICPHSGENTEYDTIPHTNRTILKEDPANTVYST
VEIPKKMENPHSLLTMPDTPRLFAYENVI

>P09326 GPI-anchor, lipid-anchor
MCSRGWDSCLALELLLLPLSLLVTSIQGHLVHMTVVSGSNVTLNISESLPENYKQLTWFYT
FDQKIVEWDSRKSKYFESKFKGRVRLDPQSGALYISKVQKEDNSTYIMRVLKKTGNEQEW
KIKLQVLDPVPKPVIKIEKIEDMDDNCYLKLSCVIPGESVNYTWYGDKRPFPKELQNSVLE
TTLMPHNYSRCYTCQVSNSVSSKNGTVCLSPPCTLARSFGVEWIASWLVVTVPTILGLLLT

>Q9UIB8 single-pass type I membrane protein
MAQHHLWILLLCLQTWPEAAGKDSEIFTVNGILGESVTFPVNIQEPRQVKIIAWTSKTSVA
YVTPGDSETAPVVTVTHRNYYERIHALGPNYNLVISDLRMEDAGDYKADINTQADPYTTT
KRYNLQIYRRLGKPKITQSLMASVNSTCNVTLTCSVEKEEKNVTYNWSPLGEEGNVLQIF
QTPEDQELTYTCTAQNPVSNNSDSISARQLCADIAMGFRTHHTGLLSVLAMFFLLVLILSSV
FLFRLFKRRQGRIFPEGSCLNTFTKNPYAASKKTIYTYIMASRNTQPAESRIYDEILQSKVLP
SKEEPVNTVYSEVQFADKMGKASTQDSKPPGTSSYEIVI

>P53621 peripheral membrane protein
MLTKFETKSARVKGLSFHPKRPWILTSLHNGVIQLWDYRMCTLIDKFDEHDGPVRGIDFH
KQQPLFVSGGDDYKIKVWNYKLRRCLFTLLGHLDYIRTTFFHHEYPWILSASDDQTIRVW
NWQSRTCVCVLTGHNHYVMCAQFHPTEDLVVSASLDQTVRVWDISGLRKKNLSPGAVES
DVRGITGVDLFGTTDAVVKHVLEGHDRGVNWAAFHPTMPLIVSGADDRQVKIWRMNES
KAWEVDTCRGHYNNVSCAVFHPRQELILSNSEDKSIRVWDMSKRTGVQTFRRDHDRFWV
LAAHPNLNLFAAGHDGGMIVFKLERERPAYAVHGNMLHYVKDRFLRQLDFNSSKDVAVM
QLRSGSKFPVFNMSYNPAENAVLLCTRASNLENSTYDLYTIPKDADSQNPDAPEGKRSSGL
TAVWVARNRFAVLDRMHSLLIKNLKNEITKKVQVPNCDEIFYAGTGNLLLRDADSITLFDV
QQKRTLASVKISKVKYVIWSADMSHVALLAKHAIVICNRKLDALCNIHENIRVKSGAWDE
SGVFIYTTSNHIKYAVTTGDHGIIRTLDLPIYVTRVKGNNVYCLDRECRPRVLTIDPTEFKFK
LALINRKYDEVLHMVRNAKLVGQSIIAYLQKKGYPEVALHFVKDEKTRFSLALECGNIEIA
LEAAKALDDKNCWEKLGEVALLQGNHQIVEMCYQRTKNFDKLSFLYLITGNLEKLRKM
MKIAEIRKDMSGHYQNALYLGDVSERVRILKNCGQKSLAYLTAATHGLDEEAESLKETFD
PEKETIPDIDPNAKLLQPPAPIMPLDTNWPLLTVSKGFFEGTIASKGKGGALAADIDIDTVG
TEGWGEDAELQLDEDGFVEATEGLGDDALGKGQEEGGGWDVEEDLELPPELDISPGAAG
GAEDGFFVPPTKGTSPTQIWCNNSQLPVDHILAGSFETAMRLLHDQVGVIQFGPYKQLFL

QTYARGRTTYQALPCLPSMYGYPNRNWKDAGLKNGVPAVGLKLNDLIQRLQLCYQLTTV
GKFEEAVEKFRSILLSVPLLVVDNKQEIAEAQQLITICREYIVGLSVETERKKLPKETLEQQ
KRICEMAAYFTHSNLQPVHMILVLRTALNLFFKLKNFKTAATFARRLLELGPKPEVAQQTR
KILSACEKNPTDAYQLNYDMHNPFDICAASYRPIYRGKPVEKCPLSGACYSPEFKGQICRV
TTVTEIGKDVIGLRISPLQFR

>P40855 lipid-anchor
MAAAEEGCSVGAEADRELEELLESALDDFDKAKPSPAPPSTTTAPDASGPQKRSPGDTAK
DALFASQEKFFQELFDSELASQATAEFEKAMKELAEEEPHLVEQFQKLSEAAGRVGSDMT
SQQEFTSCLKETLSGLAKNATDLQNSSMSEEELTKAMEGLGMDEGDGEGNILPIMQSIMQ
NLLSKDVLYPSLKEITEKYPEWLQSHRESLPPEQFEKYQEQHSVMCKICEQFEAETPTDSE
TTQKARFEMVLDLMQQLQDLGHPPKELAGEMPPGLNFDLDALNLSGPPGASGEQCLIM

>Q13733 multipass membrane protein
MGLWGKKGTVAPHDQSPRRRPKKGLIKKKMVKREKQKRNMEELKKEVVMDDHKLTLE
ELSTKYSVDLTKGHSHQRAKEILTRGGPNTVTPPPTTPEWVKFCKQLFGGFSLLLWTGAIL
CFVAYSIQIYFNEEPTKDNLYLSIVLSVVVIVTGCFSYYQEAKSSKIMESFKNMVPQQALVI
RGGEKMQINVQEVVLGDLVEIKGGDRVPADLRLISAQGCKVDNSSLTGESEPQSRSPDFTH
ENPLETRNICFFSTNCVEGTARGIVIATGDSTVMGRIASLTSGLAVGQTPIAAEIEHFIHLITV
VAVFLGVTFFALSLLLGYGWLEAIIFLIGIIVANVPEGLLATVTVCLTLTAKRMARKNCLVK
NLEAVETLGSTSTICSDKTGTLTQNRMTVAHMWFDMTVYEADTTEEQTGKTFTKSSDTW
FMLARIAGLCNRADFKANQEILPIAKRATTGDASESALLKFIEQSYSSVAEMREKNPKVAEI
PFNSTNKYQMSIHLREDSSQTHVLMMKGAPERILEFCSTFLLNGQEYSMNDEMKEAFQN
AYLELGGLGERVLGFCFLNLPSSFSKGFPFNTDEINFPMDNLCFVGLISMIDPPRAAVPDAV
SKCRSAGIKVIMVTGDHPITAKAIAKGVGIISEGTETAEEVAARLKIPISKVDASAAKAIVVH
GAELKDIQSKQLDQILQNHPEIVFARTSPQQKLIIVEGCQRLGAVVAVTGDGVNDSPALKK
ADIGIAMGISGSDVSKQAADMILLDDNFASIVTGVEEGRLIFDNLKKSIMYTLTSNIPEITPF
LMFIILGIPLPLGTITILCIDLGTDMVPAISLAYESAESDIMKRLPRNPKTDNLVNHRLIGMAY
GQIGMIQALAGFFTYFVILAENGFRPVDLLGIRLHWEDKYLNDLEDSYGQQWTYEQRKV
VEFTCQTAFFVTIVVVQWADLIISKTRRNSLFQQGMRNKVLIFGILEETLLAAFLSYTPGMD
VALRMYPLKITWWLCAIPYSILIFVYDEIRKLLIRQHPDGWVERETYY

>Q92806 multipass membrane protein
MAQENAAFSPGQEEPPRRRGRQRYVEKDGRCNVQQGNVRETYRYLTDLFTTLVDLQWR
LSLLFFVLAYALTWLFFGAIWWLIAYGRGDLEHLEDTAWTPCVNNLNGFVAAFLFSIETET
TIGYGHRVITDQCPEGIVLLLLQAILGSMVNAFMVGCMFVKISQPNKRAATLVFSSHAVVS
LRDGRLCLMFRVGDLRSSHIVEASIRAKLIRSRQTLEGEFIPLHQTDLSVGFDTGDDRLFLV
SPLVISHEIDAASPFWEASRRALERDDFEIVVILEGMVEATGMTCQARSSYLVDEVLWGHR
FTSVLTLEDGFYEVDYASFHETFEVPTPSCSARELAEAAARLDAHLYWSIPSRLDEKVEEE
GAGEGAGGEAGADKEQNGCLPPPESESKV

>P78508 multipass membrane protein
MTSVAKVYYSQTTQTESRPLMGPGIRRRRVLTKDGRSNVRMEHIADKRFLYLKDLWTTFI
DMQWRYKLLLFSATFAGTWFLFGVVWYLVAVAHGDLLELDPPANHTPCVVQVHTLTGAF

LFSLESQTTIGYGFRYISEECPLAIVLLIAQLVLTTILEIFITGTFLAKIARPKKRAETIRFSQHA
VVASHNGKPCLMIRVANMRKSLLIGCQVTGKLLQTHQTKEGENIRLNQVNVTFQVDTASD
SPFLILPLTFYHVVDETSPLKDLPLRSGEGDFELVLILSGTVESTSATCQVRTSYLPEEILWG
YEFTPAISLSASGKYIADFSLFDQVVKVASPSGLRDSTVRYGDPEKLKLEESLREQAEKEGS
ALSVRISNV

>Q9H3S5 multipass membrane protein
MGSTKHWGEWLLNLKVAPAGVFGVAFLARVALVFYGVFQDRTLHVRYTDIDYQVFTDAA
RFVTEGRSPYLRATYRYTPLLGWLLTPNIYLSELFGKFLFISCDLLTAFLLYRLLLLKGLGRR
QACGYCVFWLLNPLPMAVSSRGNADSIVASLVLMVLYLIKKRLVACAAVFYGFAVHMKIY
PVTYILPITLHLLPDRDNDKSLRQFRYTFQACLYELLKRLCNRAVLLFVAVAGLTFFALSFGF
YYEYGWEFLEHTYFYHLTRRDIRHNFSPYFYMLYLTAESKWSFSLGIAAFLPQLILLSAVSF
AYYRDLVFCCFLHTSIFVTFNKVCTSQYFLWYLCLLPLVMPLVRMPWKRAVVLLMLWFIG
QAMWLAPAYVLEFQGKNTFLFIWLAGLFFLLINCSILIQIISHYKEEPLTERIKYD

>Q96A28 single-pass type I membrane protein
MCAFPWLLLLLLLQEGSQRRLWRWCGSEEVVAVLQESISLPLEIPPDEEVENIIWSSHKSLA
TVVPGKEGHPATIMVTNPHYQGQVSFLDPSYSLHISNLSWEDSGLYQAQVNLRTSQISTMQ
QYNICVYRWLSEPQITVNFESSGEGACSMSLVCSVEKAGMDMTYSWLSRGDSTYTFHEG
PVLSTSWRPGDSALSYTCRANNPISNVSSCPIPDGPFYADPNYASEKPSTAFCLLAKGLLIFL
LLVILAMGLWVIRVQKRHKMPRMKKLMRNRMKLRKEAKPGSSPA

>Q16570 multipass membrane protein
MGNCLHRAELSPSTENSSQLDFEDVWNSSYGVNDSFPDGDYGANLEAAAPCHSCNLLDD
SALPFFILTSVLGILASSTVLFMLFRPLFRWQLCPGWPVLAQLAVGSALFSIVVPVLAPGLG
STRSSALCSLGYCVWYGSAFAQALLLGCHASLGHRLGAGQVPGLTLGLTVGIWGVAALLT
LPVTLASGASGGLCTLIYSTELKALQATHTVACLAIFVLLPLGLFGAKGLKKALGMGPGP
WMNILWAWFIFWWPHGVVLGLDFLVRSKLLLLSTCLAQQALDLLLNLAEALAILHCVATP
LLLALFCHQATRTLLPSLPLPEGWSSHLDTLGSKS

>Q8NGW6 multipass membrane protein
MKQYSVGNQHSNYRSLLFPFLCSQMTQLTASGNQTMVTEFLFSMFPHAHRGGLLFFIPLL
LIYGFILTGNLIMFIVIQVGMALHTPLYFFISVLSFLEICYTTTTIPKMLSCLISEQKSISVAGC
LLQMYFFHSLGITESCVLTAMAIDRYIAICNPLRYPTIMIPKLCIQLTVGSCFCGFLLVLPEIA
WISTLPFCGSNQIHQIFCDFTPVLSLACTDTFLVVIVDAIHAAEIVASFLVIALSYIRIIIVILGM
HSAEGHHKAFSTCAAHLAVFLLFFGSVAVMYLRFSATYSVFWDTAIAVTFVILAPFFNPIIY
SLKNKDMKEAIGRLFHYQKRAGWAGK

>Q8NGY3 multipass membrane protein
MCWTMPSPFTGSSTRNMESGNQSTVTEFIFTGFPQLQDGSLLYFFPLLFIYTFIIIDNLLIFSA
VRLDTHLHNPMYNFISIFSFLEIWYTTATIPKMLSNLISEKKAISMTGCILQMYFFHSLENSE
GILLTTMAIDRYVAICNPLRYQMIMTPRLCAQLSAGSCLFGFLILLPEIVMISTLPFCGPNQI
HQIFCDLVPVLSLACTDTSMILIEDVIHAVTIIITFLIIALSYVRIVTVILRIPSSEGRQKAFSTC
AGHLMVFPIFFGSVSLMYLRFSDTYPPVLDTAIALMFTVLAPFFNPIIYSLRNKDMNNAIKK

LFCLQKVLNKPGG

>Q8NGY0 multipass membrane protein
MVLNVYCCFFQISDIQTMKINQTILKEFILVGFSVYPHVQTFLFVVFFCLYLLTLAGNLIIMG
LTWVDRSLHTPMYLFLSALSFSETCYTLTIVPKMLEDLLAKDRSISVTGCSLQMCFFLGLG
GTNCIILTLMGYDRFLAICNPLRYPLLMTNIVCGQLVASACTAGFFISLTETALIFRDSFCRPN
LVKHFFCHMLAVIRLSCIDSNHTEFIITLISVSGLLGTLLLIILTDVFIISTVLRIPSAEGKQKAF
TTCASHLTVVIIHFGFASIVYLKPEASGDDTLIAVPYTVITPFLSPIIFSLRNKDMKNAFRRM
MGNTVALKK

>Q8NGX6 multipass membrane protein
MPQILIFTYLNMFYFFPPLQILAENLTMVTEFLLLGFSSLGEIQLALFVVFLFLYLVILSGNVT
IISVIHLDKSLHTPMYFFLGILSTSETFYTFVILPKMLINLLSVARTISFNCCALQMFFFLGFAI
TNCLLLGVMGYDRYAAICHPLHYPTLMSWQVCGKLAAACAIGGFLASLTVVNLVFSLPFC
SANKVNHYFCDISAVILLACTNTDVNEFVIFICGVLVLVVPFLFICVSYLCILRTILKIPSAEG
RRKAFSTCASHLSVVIVHYGCASFIYLRPTANYVSNKDRLVTVTYTIVTPLLNPMVYSLRN
KDVQLAIRKVLGKKGSLKLYN

>P15812 single-pass type I membrane protein
MLLLFLLFEGLCCPGENTAAPQALQSYHLAAEEQLSFRMLQTSSFANHSWAHSEGSGWLG
DLQTHGWDTVLGTIRFLKPWSHGNFSKQELKNLQSLFQLYFHSFIQIVQASAGQFQLEYPF
EIQILAGCRMNAPQIFLNMAYQGSDFLSFQGISWEPSPGAGIRAQNICKVLNRYLDIKEILQ
SLLGHTCPRFLAGLMEAGESELKRKVKPEAWLSCGPSPGPGRLQLVCHVSGFYPKPVWV
MWMRGEQEQRGTQRGDVLPNADETWYLRATLDVAAGEAAGLSCRVKHSSLGGHDLIIH
WGGYSIFLILICLTVIVTLVILVVVDSRLKKQSSNKNILSPHTPSPVFLMGANTQDTKNSRH
QFCLAQVSWIKNRVLKKWKTRLNQLW

>P29016 single-pass type I membrane protein
MLLLPFQLLAVLFPGGNSEHAFQGPTSFHVIQTSSFTNSTWAQTQGSGWLDDLQIHGWDS
DSGTAIFLKPWSKGNFSDKEVAELEEIFRVYIFGFAREVQDFAGDFQMKYPFEIQGIAGCEL
HSGGAIVSFLRGALGGLDFLSVKNASCVPSPEGGSRAQKFCALIIQYQGIMETVRILLYETC
PRYLLGVLNAGKADLQRQVKPEAWLSSGPSPGPGRLQLVCHVSGFYPKPVWVMWMRGE
QEQQGTQLGDILPNANWTWYLRATLDVADGEAAGLSCRVKHSSLEGQDIILYWRNPTSIG
SIVLAIIVPSLLLLLCLALWYMRRRSYQNIP

>P29017 single-pass type I membrane protein
MLFLQFLLLALLLPGGDNADASQEHVSFHVIQIFSFVNQSWARGQGSGWLDELQTHGWD
SESGTIIFLHNWSKGNFSNEELSDLELLFRFYLFGLTREIQDHASQDYSKYPFEVQVKAGCE
LHSGKSPEGFFQVAFNGLDLLSFQNTTWVPSPGCGSLAQSVCHLLNHQYEGVTETVYNLI
RSTCPRFLLGLLDAGKMYVHRQVRPEAWLSSRPSLGSGQLLLVCHASGFYPKPVWVTW
MRNEQEQLGTKHGDILPNADGTWYLQVILEVASEEPAGLSCRVRHSSLGGQDIILYWGHH
FSMNWIALVVIVPLVILIVLVLWFKKHCSYQDIL

>P15813 single-pass type I membrane protein

MGCLLFLLLWALLQAWGSAEVPQRLFPLRCLQISSFANSSWTRTDGLAWLGELQTHSWSN
DSDTVRSLKPWSQGTFSDQQWETLQHIFRVYRSSFTRDVKEFAKMLRLSYPLELQVSAGC
EVHPGNASNNFFHVAFQGKDILSFQGTSWEPTQEAPLWVNLAIQVLNQDKWTRETVQWL
LNGTCPQFVSGLLESGKSELKKQVKPKAWLSRGPSPGPGRLLLVCHVSGFYPKPVWVKW
MRGEQEQQGTQPGDILPNADETWYLRATLDVVAGEAAGLSCRVKHSSLEGQDIVLYWGG
SYTSMGLIALAVLACLLFLLIVGFTSRFKRQTSYQGVL

>Q96LA6 single-pass type I membrane protein
MLPRLLLLICAPLCEPAELFLIASPSHPTEGSPVTLTCKMPFLQSSDAQFQFCFFRDTRALGP
GWSSSPKLQIAAMWKEDTGSYWCEAQTMASKVLRSRRSQINVHRVPVADVSLETQPPGG
QVMEGDRLVLICSVAMGTGDITFLWYKGAVGLNLQSKTQRSLTAEYEIPSVRESDAEQYY
CVAENGYGPSPSGLVSITVRIPVSRPILMLRAPRAQAAVEDVLELHCEALRGSPPILYWFYH
EDITLGSRSAPSGGGASFNLSLTEEHSGNYSCEANNGLGAQRSEAVTLNFTVPTGARSNHL
TSGVIEGLLSTLGPATVALLFCYGLKRKIGRRSARDPLRSLPSPLPQEFTYLNSPTPGQLQPI
YENVNVVSGDEVYSLAYYNQPEQESVAAETLGTHMEDKVSLDIYSRLRKANITDVDYED
AM

>Q96P31 single-pass type I membrane protein
MLLWLLLLILTPGREQSGVAPKAVLLLNPPWSTAFKGEKVALICSSISHSLAQGDTYWYHD
EKLLKIKHDKIQITEPGNYQCKTRGSSLSDAVHVEFSPDWLILQALHPVFEGDNVILRCQG
KDNKNTHQKVYYKDGKQLPNSYNLEKITVNSVSRDNSKYHCTAYRKFYILDIEVTSKPLN
IQVQELFLHPVLRASSSTPIEGSPMTLTCETQLSPQRPDVQLQFSLFRDSQTLGLGWSRSPR
LQIPAMWTEDSGSYWCEVETVTHSIKKRSLRSQIRVQRVPVSNVNLEIRPTGGQLIEGENM
VLICSVAQGSGTVTFSWHKEGRVRSLGRKTQRSLLAELHVLTVKESDAGRYYCAADNVH
SPILSTWIRVTVRIPVSHPVLTFRAPRAHTVVGDLLELHCESLRGSPPILYRFYHEDVTLGNS
SAPSGGGASFNLSLTAEHSGNYSCDADNGLGAQHSHGVSLRVTVPVSRPVLTLRAPGAQA
VVGDLLELHCESLRGSFPILYWFYHEDDTLGNISAHSGGGASFNLSLTTEHSGNYSCEADN
GLGAQHSKVVTLNVTGTSRNRTGLTAAGITGLVLSILVLAAAAALLHYARARRKPGGLSA
TGTSSHSPSECQEPSSSRPSRIDPQEPTHSKPLAPMELEPMYSNVNPGDSNPIYSQIWSIQHT
KENSANCPMMHQEHEELTVLYSELKKTHPDDSAGEASSRGRAHEEDDEENYENVPRVLL
ASDH

>P14616 single-pass type I membrane protein
MAVPSLWPWGACLPVIFLSLGFGLDTVEVCPSLDIRSEVAELRQLENCSVVEGHLQILLMF
TATGEDFRGLSFPRLTQVTDYLLLFRVYGLESLRDLFPNLAVIRGTRLFLGYALVIFEMPHL
RDVALPALGAVLRGAVRVEKNQELCHLSTIDWGLLQPAPGANHIVGNKLGEECADVCPGV
LGAAGEPCAKTTFSGHTDYRCWTSSHCQRVCPCPHGMACTARGECCHTECLGGCSQPED
PRACVACRHLYFQGACLWACPPGTYQYESWRCVTAERCASLHSVPGRASTFGIHQGSCLA
QCPSGFTRNSSSIFCHKCEGLCPKECKVGTKTIDSIQAAQDLVGCTHVEGSLILNLRQGYN
LEPQLQHSLGLVETITGFLKIKHSFALVSLGFFKNLKLIRGDAMVDGNYTLYVLDNQNLQQ
LGSWVAAGLTIPVGKIYFAFNPRLCLEHIYRLEEVTGTRGRQNKAEINPRTNGDRAACQTR
TLRFVSNVTEADRILLRWERYEPLEARDLLSFIVYYKESPFQNATEHVGPDACGTQSWNLL
DVELPLSRTQEPGVTLASLKPWTQYAVFVRAITLTTEEDSPHQGAQSPIVYLRTLPAAPTVP
QDVISTSNSSSHLLVRWKPPTQRNGNLTYYLVLWQRLAEDGDLYLNDYCHRGLRLPTSNN

DPRFDGEDGDPEAEMESDCCPCQHPPPGQVLPPLEAQEASFQKKFENFLHNAITIPISPWK
VTSINKSPQRDSGRHRRAAGPLRLGGNSSDFEIQEDKVPRERAVLSGLRHFTEYRIDIHAC
NHAAHTVGCSAATFVFARTMPHREADGIPGKVAWEASSKNSVLLRWLEPPDPNGLILKYE
IKYRRLGEEATVLCVSRLRYAKFGGVHLALLPPGNYSARVRATSLAGNGSWTDSVAFYIL
GPEEEDAGGLHVLLTATPVGLTLLIVLAALGFFYGKKRNRTLYASVNPEYFSASDMYVPDE
WEVPREQISIIRELGQGSFGMVYEGLARGLEAGEESTPVALKTVNELASPRECIEFLKEASV
MKAFKCHHVVRLLGVVSQGQPTLVIMELMTRGDLKSHLRSLRPEAENNPGLPQPALGEM
IQMAGEIADGMAYLAANKFVHRDLAARNCMVSQDFTVKIGDFGMTRDVYETDYYRKG
GKGLLPVRWMAPESLKDGIFTTHSDVWSFGVVLWEIVTLAEQPYQGLSNEQVLKFVMDG
GVLEELEGCPLQLQELMSRCWQPNPRLRPSFTHILDSIQEELRPSFRLLSFYYSPECRGARG
SLPTTDAEPDSSPTPRDCSPQNGGPGH

>Q15262 single-pass type I membrane protein
MDTTAAAALPAFVALLLLSPWPLLGSAQGQFSAGGCTFDDGPGACDYHQDLYDDFEWVH
VSAQEPHYLPPEMPQGSYMIVDSSDHDPGEKARLQLPTMKENDTHCIDFSYLLYSQKGLN
PGTLNILVRVNKGPLANPIWNVTGFTGRDWLRAELAVSTFWPNEYQVIFEAEVSGGRSGYI
AIDDIQVLSYPCDKSPHFLRLGDVEVNAGQNATFQCIATGRDAVHNKLWLQRRNGEDIPV
AQTKNINHRRFAASFRLQEVTKTDQDLYRCVTQSERGSGVSNFAQLIVREPPRPIAPPQLL
GVGPTYLLIQLNANSIIGDGPIILKEVEYRMTSGSWTETHAVNAPTYKLWHLDPDTEYEIR
VLLTRPGEGGTGLPGPPLITRTKCAEPMRTPKTLKIAEIQARRIAVDWESLGYNITRCHTFN
VTICYHYFRGHNESKADCLDMDPKAPQHVVNHLPPYTNVSLKMILTNPEGRKESEETIIQT
DEDVPGPVPVKSLQGTSFENKIFLNWKEPLDPNGIITQYEISYSSIRSFDPAVPVAGPPQTVS
NLWNSTHHVFMHLHPGTTYQFFIRASTVKGFGPATAINVTTNISAPTLPDYEGVDASLNET
ATTITVLLRPAQAKGAPISAYQIVVEELHPHRTKREAGAMECYQVPVTYQNAMSGGAPYY
FAAELPPGNLPEPAPFTVGDNRTYQGFWNPPLAPRKGYNIYFQAMSSVEKETKTQCVRIAT
KAATEEPEVIPDPAKQTDRVVKIAGISAGILVFILLLLVVILIVKKSKLAKKRKDAMGNTRQ
EMTHMVNAMDRSYADQSTLHAEDPLSITFMDQHNFSPRYENHSATAESSRLLDVPRYLCE
GTESPYQTGQLHPAIRVADLLQHINLMKTSDSYGFKEEYESFFEGQSASWDVAKKDQNRA
KNRYGNIIAYDHSRVILQPVEDDPSSDYINANYIDGYQRPSHYIATQGPVHETVYDFWRMI
WQEQSACIVMVTNLVEVGRVKCYKYWPDDTEVYGDFKVTCVEMEPLAEYVVRTFTLER
RGYNEIREVKQFHFTGWPDHGVPYHATGLLSFIRRVKLSNPPSAGPIVVHCSAGAGRTGC
YIVIDIMLDMAEREGVVDIYNCVKALRSRRINMVQTEEQYIFIHDAILEACLCGETAIPVCE
FKAAYFDMIRIDSQTNSSHLKDEFQTLNSVTPRLQAEDCSIACLPRNHDKNRFMDMLPPD
RCLPFLITIDGESSNYINAALMDSYRQPAAFIVTQYPLPNTVKDFWRLVYDYGCTSIVMLN
EVDLSQGCPQYWPEEGMLRYGPIQVECMSCSMDCDVINRIFRICNLTRPQEGYLMVQQFQ
YLGWASHREVPGSKRSFLKLILQVEKWQEECEEGEGRTIIHCLNGGGRSGMFCAIGIVVE
MVKRQNVVDVFHAVKTLRNSKPNMVEAPEQYRFCYDVALEYLESS

>Q9Y2Q5 peripheral membrane protein
MLRPKALTQVLSQANTGGVQSTLLLNNEGSLLAYSGYGDTDARVTAAIASNIWAAYDRN
GNQAFNEDNLKFILMDCMEGRVAITRVANLLLCMYAKETVGFGMLKAKAQALVQYLEEP
LTQVAAS

>Q8TDU9 multipass membrane protein

MPTLNTSASPPTFFWANASGGSVLSADDAPMPVKFLALRLMVALAYGLVGAIGLLGNLAV
LWVLSNCARRAPGPPSDTFVFNLALADLGLALTLPFWAAESALDFHWPFGGALCKMVLT
ATVLNVYASIFLITALSVARYWVVAMAAGPGTHLSLFWARIATLAVWAAAALVTVPTAVFG
VEGEVCGVRLCLLRFPSRYWLGAYQLQRVVLAFMVPLGVITTSYLLLLAFLQRRQRRRQD
SRVVARSVRILVASFFLCWFPNHVVTLWGVLVKFDLVPWNSTFYTIQTYVFPVTTCLAHSN
SCLNPVLYCLLRREPRQALAGTFRDLRLRLWPQGGGWVQQVALKQVGRRWVASNPRESR
PSTLLTNLDRGTPG

>Q9P1Z3 multipass membrane protein
MEAEQRPAAGASEGATPGLEAVPPVAPPPATAASGPIPKSGPEPKRRHLGTLLQPTVNKFSL
RVFGSHKAVEIEQERVKSAGAWIIHPYSDFRFYWDLIMLLLMVGNLIVLPVGITFFKEENSP
PWIVFNVLSDTFFLLDLVLNFRTGIVVEEGAEILLAPRAIRTRYLRTWFLVDLISSIPVDYIFL
VVELEPRLDAEVYKTARALRIVRFTKILSLLRLLRLSRLIRYIHQWEEIFHMTYDLASAVVR
IFNLIGMMLLLCHWDGCLQFLVPMLQDFPPDCWVSINHMVNHSWGRQYSHALFKAMSH
MLCIGYGQQAPVGMPDVWLTMLSMIVGATCYAMFIGHATALIQSLDSSRRQYQEKYKQV
EQYMSFHKLPADTRQRIHEYYEHRYQGKMFDEESILGELSEPLREEIINFTCRGLVAHMPLF
AHADPSFVTAVLTKLRFEVFQPGDLVVREGSVGRKMYFIQHGLLSVLARGARDTRLTDGS
YFGEICLLTRGRRTASVRADTYCRLYSLSVDHFNAVLEEFPMMRRAFETVAMDRLLRIGK
KNSILQRKRSEPSPGSSGGIMEQHLVQHDRDMARGVRGRAPSTGAQLSGKPVLWEPLVHA
PLQAAAVTSNVAIALTHQRGPLPLSPDSPATLLARSAWRSAGSPASPLVPVRAGPWASTSRL
PAPPARTLHASLSRAGRSQVSLLGPPPGGGGRRLGPRGRPLSASQPSLPQRATGDGSPGRK
GSGSERLPPSGLLAKPPRTAQPPRPPVPEPATPRGLQLSANM

>Q9P2X0 multipass membrane protein
MTKLAQWLWGLAILGSTWVALTTGALGLELPLSCQEVLWPLPAYLLVSAGCYALGTVGY
RVATFHDCEDAARELQSQIQEARADLARRGLRF

>Q9BRV3 multipass membrane protein
MEAGGFLDSLIYGACVVFTLGMFSAGLSDLRHMRMTRSVDNVQFLPFLTTEVNNLGWLS
YGALKGDGILIVVNTVGAALQTLYILAYLHYCPRKRVVLLQTATLLGVLLLGYGYFWLLV
PNPEARLQQLGLFCSVFTISMYLSPLADLAKVIQTKSTQCLSYPLTIATLLTSASWCLYGFR
LRDPYIMVSNFPGIVTSFIRFWLFWKYPQEQDRNYWLLQT

>P20827 GPI-anchor, lipid-anchor
MEFLWAPLLGLCCSLAAADRHTVFWNSSNPKFRNEDYTIHVQLNDYVDIICPHYEDHSVA
DAAMEQYILYLVEHEEYQLCQPQSKDQVRWQCNRPSAKHGPEKLSEKFQRFTPFTLGKEF
KEGHSYYYISKPIHQHEDRCLRLKVTVSGKITHSPQAHDNPQEKRLAADDPEVRVLHSIGH
SAAPRLFPLAWTVLLLPLLLLQTP

>P52797 GPI-anchor, lipid-anchor
MAAAPLLLLLLLVPVPLLPLLAQGPGGALGNRHAVYWNSSNQHLRREGYTVQVNVNDY
LDIYCPHYNSSGVGPGAGPGPGGGAEQYVLYMVSRNGYRTCNASQGFKRWECNRPHAP
HSPIKFSEKFQRYSAFSLGYEFHAGHEYYYISTPTHNLHWKCLRMKVFVCCASTSHSGEK
PVPTLPQFTMGPNVKINVLEDFEGENPQVPKLEKSISGTSPKREHLPLAVGIAFFLMTFLAS

>P33908 single-pass type II membrane protein
MPVGGLLPLFSSPAGGVLGGGLGGGGGRKGSGPAALRLTEKFVLLLVFSAFITLCFGAIFFL
PDSSKLLSGVLFHSSPALQPAADHKPGPGARAEDAAEGRARRREEGAPGDPEAALEDNLA
RIRENHERALREAKETLQKLPEEIQRDILLEKKKVAQDQLRDKAPFRGLPPVDFVPPIGVES
REPADAAIREKRAKIKEMMKHAWNNYKGYAWGLNELKPISKGGHSSSLFGNIKGATIVDA
LDTLFIMEMKHEFEEAKSWVEENLDFNVNAEISVFEVNIRFVGGLLSAYYLSGEEIFRKKA
VELGVKLLPAFHTPSGIPWALLNMKSGIGRNWPWASGGSSILAEFGTLHLEFMHLSHLSGN
PIFAEKVMNIRTVLNKLEKPQGLYPNYLNPSSGQWGQHHVSVGGLGDSFYEYLLKAWLM
SDKTDLEAKKMYFDAVQAIETHLIRKSSSGLTYIAEWKGGLLEHKMGHLTCFAGGMFALG
ADAAPEGMAQHYLELGAEIARTCHESYNRTFMKLGPEAFRFDGGVEAIATRQNEKYYILR
PEVMETYMYMWRLTHDPKYRKWAWEAVEALENHCRVNGGYSGLRDVYLLHESYDDVQ
QSFFLAETLKYLYLIFSDDDLLPLEHWIFNSEAHLLPILPKDKKEVEIREE

>P17787 multipass membrane protein
MARRCGPVALLLGFGLLRLCSGVWGTDTEERLVEHLLDPSRYNKLIRPATNGSELVTVQL
MVSLAQLISVHEREQIMTTNVWLTQEWEDYRLTWKPEEFDNMKKVRLPSKHIWLPDVVL
YNNADGMYEVSFYSNAVVSYDGSIFWLPPAIYKSACKIEVKHFPFDQQNCTMKFRSWTY
DRTEIDLVLKSEVASLDDFTPSGEWDIVALPGRRNENPDDSTYVDITYDFIIRRKPLFYTINLI
IPCVLITSLAILVFYLPSDCGEKMTLCISVLLALTVFLLLISKIVPPTSLDVPLVGKYLMFTM
VLVTFSIVTSVCVLNVHHRSPTTHTMAPWVKVVFLEKLPALLFMQQPRHHCARQRLRLR
RRQREREGAGALFFREAPGADSCTCFVNRASVQGLAGAFGAEPAPVAGPGRSGEPCGCGL
REAVDGVRFIADHMRSEDDDQSVSEDWKYVAMVIDRLFLWIFVFVCVFGTIGMFLQPLFQ
NYTTTTFLHSDHSAPSSK

>P08887 single-pass type I membrane protein
MLAVGCALLAALLAAPGAALAPRRCPAQEVARGVLTSLPGDSVTLTCPGVEPEDNATVH
WVLRKPAAGSHPSRWAGMGRRLLLRSVQLHDSGNYSCYRAGRPAGTVHLLVDVPPEEPQ
LSCFRKSPLSNVVCEWGPRSTPSLTTKAVLLVRKFQNSPAEDFQEPCQYSQESQKFSCQLA
VPEGDSSFYIVSMCVASSVGSKFSKTQTFQGCGILQPDPPANITVTAVARNPRWLSVTWQD
PHSWNSSFYRLRFELRYRAERSKTFTTWMVKDLQHHCVIHDAWSGLRHVVQLRAQEEFG
QGEWSEWSPEAMGTPWTESRSPPAENEVSTPMQALTTNKDDDNILFRDSANATSLPVQDS
SSVPLPTFLVAGGSLAFGTLLCIAIVLRFKKTWKLRALKEGKTSMHPPYSLGQLVPERPRPT
PVLVPLISPPVSPSSLGSDNTSSHNRPDARDPRSPYDISNTDYFFPR

>P98198 multipass membrane protein
MTVPKEMPEKWARAQAPPSWSRKKPSWGTEEERRARANDREYNEKFQYASNCIKTSKY
NILTFLPVNLFEQFQEVANTYFLFLLILQLIPQISSLSWFTTIVPLVLVLTITAVKDATDDYFRH
KSDNQVNNRQSQVLINGILQQEQWMNVCVGDIIKLENNQFVAADLLLLSSSEPHGLCYIET
AELDGETNMKVRQAIPVTSELGDISKLAKFDGEVICEPPNNKLDKFSGTLYWKENKFPLS
NQNMLLRGCVLRNTEWCFGLVIFAGPDTKLMQNSGRTKFKRTSIDRLMNTLVLWIFGFLV
CMGVILAIGNAIWEHEVGMRFQVYLPWDEAVDSAFFSGFLSFWSYIIILNTVVPISLYVSVE
VIRLGHSYFINWDKKMFCMKKRTPAEARTTTLNEELGQVEYIFSDKTGTLTQNIMVFNKC
SINGHSYGDVFDVLGHKAELGERPEPVDFSFNPLADKKFLFWDPSLLEAVKIGDPHTHEFF

RLLSLCHTVMSEEKNEGELYYKAQSPDEGALVTAARNFGFVFRSRTPKTITVHEMGTAITY
QLLAILDFNNIRKRMSVIVRNPEGKIRLYCKGADTILLDRLHHSTQELLNTTMDHLNEYAG
EGLRTLVLAYKDLDEEYYEEWAERRLQASLAQDSREDRLASIYEEVENNMMLLGATAIED
KLQQGVPETIALLTLANIKIWVLTGDKQETAVNIGYSCKMLTDDMTEVFIVTGHTVLEVRE
ELRKAREKMMDSSRSVGNGFTYQDKLSSSKLTSVLEAVAGEYALVINGHSLAHALEADM
ELEFLETACACKAVICCRVTPLQKAQVVELVKKYKKAVTLAIGDGANDVSMIKTAHIGVGI
SGQEGIQAVLASDYSFSQFKFLQRLLLVHGRWSYLRMCKFLCYFFYKNFAFTMVHFWFGF
FCGFSAQTVYDQYFITLYNIVYTSLPVLAMGVFDQDVPEQRSMEYPKLYEPGQLNLLFNK
REFFICIAQGIYTSVLMFFIPYGVFADATRDDGTQLADYQSFAVTVATSLVIVVSVQIGLDTG
YWTAINHFFIWGSLAVYFAILFAMHSNGLFDMFPNQFRFVGNAQNTLAQPTVWLTIVLTT
VVCIMPVVAFRFLRLNLKPDLSDTVRYTQLVRKKQKAQHRCMRRVGRTGSRRSGYAFSH
QEGFGELIMSGKNMRLSSLALSSFTTRSSSSWIESLRRKKSDSASSPSGGADKPLKG

>Q96E22 single-pass type I membrane protein
MTGLYELVWRVLHALLCLHRTLTSWLRVRFGTWNWIWRRCCRAASAAVLAPLGFTLRKP
PAVGRNRRHHRHPRGGSCLAAAHHRMRWRADGRSLEKLPVHMGLVITEVEQEPSFSDIAS
LVVWCMAVGISYISVYDHQGIFKRNNSRLMDEILKQQQELLGLDCSKYSPEFANSNDKDD
QVLNCHLAVKVLSPEDGKADIVRAAQDFCQLVAQKQKRPTDLDVDTLASLLSSNGCPDP
DLVLKFGPVDSTLGFLPWHIRLTEIVSLPSHLNISYEDFFSALRQYAACEQRLGK

>Q9HD26 peripheral membrane protein
MSAGGPCPAAAGGGPGGASCSVGAPGGVSMFRWLEVLEKEFDKAFVDVDLLLGEIDPDQ
ADITYEGRQKMTSLSSCFAQLCHKAQSVSQINHKLEAQLVDLKSELTETQAEKVVLEKEV
HDQLLQLHSIQLQLHAKTGQSADSGTIKAKLSGPSVEELERELEANKKEKMKEAQLEAEV
KLLRKENEALRRHIAVLQAEVYGARLAAKYLDKELAGRVQQIQLLGRDMKGPAHDKLW
NQLEAEIHLHRHKTVIRACRGRNDLKRPMQAPPGHDQDSLKKSQGVGPIRKVLLLKEDH
EGLGISITGGKEHGVPILISEIHPGQPADRCGGLHVGDAILAVNGVNLRDTKHKEAVTILSQ
QRGEIEFEVVYVAPEVDSDDENVEYEDESGHRYRLYLDELEGGGNPGASCKDTSGEIKVL
QGFNKKAVTDTHENGDLGTASETPLDDGASKLDDLHTLYHKKSY

>P51153 lipid-anchor
MAKAYDHLFKLLLIGDSGVGKTCLIIRFAEDNFNNTYISTIGIDFKIRTVDIEGKKIKLQVW
DTAGQERFKTITTAYYRGAMGIILVYDITDEKSFENIQNWMKSIKENASAGVERLLLGNKC
DMEAKRKVQKEQADKLAREHGIRFFETSAKSSMNVDEAFSSLARDILLKSGGRRSGNGN
KPPSTDLKTCDKKNTNKCSLG

>Q14642 lipid-anchor
MAGKAAAPGTAVLLVTANVGSLFDDPENLQKNWLREFYQVVHTHKPHFMALHCQEFGG
KNYEASMSHVDKFVKELLSSDAMKEYNRARVYLDENYKSQEHFTALGSFYFLHESLKNI
YQFDFKAKKYRKVAGKEIYSDTLESTPMLEKEKFPQDYFPECKWSRKGFIRTRWCIADCA
FDLVNIHLFHDASNLVAWETSPSVYSGIRHKALGYVLDRIIDQRFEKVSYFVFGDFNFRLDS
KSVVETLCTKATMQTVRAADTNEVVKLIFRESDNDRKVMLQLEKKLFDYFNQEVFRDNN
GTALLEFDKELSVFKDRLYELDISFPPSYPYSEDARQGEQYMNTRCPAWCDRILMSPSAKE
LVLRSESEEKVVTYDHIGPNVCMGDHKPVFLAFRIMPGAGKPHAHVHKCCVVQ

>O43184 single-pass type I membrane protein
MAARPLPVSPARALLLALAGALLAPCEARGVSLWNQGRADEVVSASVGSGDLWIPVKSF
DSKNHPEVLNIRLQRESKELIINLERNEGLIASSFTETHYLQDGTDVSLARNYTVILGHCYY
HGHVRGYSDSAVSLSTCSGLRGLIVFENESYVLEPMKSATNRYKLFPAKKLKSVRGSCGS
HHNTPNLAAKNVFPPPSQTWARRHKRETLKATKYVELVIVADNREFQRQGKDLEKVKQR
LIEIANHVDKFYRPLNIRIVLVGVEVWNDMDKCSVSQDPFTSLHEFLDWRKMKLLPRKSH
DNAQLVSGVYFQGTTIGMAPIMSMCTADQSGGIVMDHSDNPLGAAVTLAHELGHNFGM
NHDTLDRGCSCQMAVEKGGCIMNASTGYPFPMVFSSCSRKDLETSLEKGMGVCLFNLPE
VRESFGGQKCGNRFVEEGEECDCGEPEECMNRCCNATTCTLKPDAVCAHGLCCEDCQLK
PAGTACRDSSNSCDLPEFCTGASPHCPANVYLHDGHSCQDVDGYCYNGICQTHEQQCVTL
WGPGAKPAPGICFERVNSAGDPYGNCGKVSKSSFAKCEMRDAKCGKIQCQGGASRPVIGT
NAVSIETNIPLQQGGRILCRGTHVYLGDDMPDPGLVLAGTKCADGKICLNRQCQNISVFG
VHECAMQCHGRGVCNNRKNCHCEAHWAPPFCDKFGFGGSTDSGPIRQADNQGLTIGILV
TILCLLAAGFVVYLKRKTLIRLLFTNKKTTIEKLRCVRPSRPPRGFQPCQAHLGHLGKGLM
RKPPDSYPPKDNPRRLLQCQNVDISRPLNGLNVPQPQSTQRVLPPLHRAPRAPSVPARPLP
AKPALRQAQGTCKPNPPQKPLPADPLARTTRLTHALARTPGQWETGLRLAPLRPAPQYPH
QVPRSTHTAYIK

>P16066 single-pass type I membrane protein
MPGPRRPAGSRLRLLLLLLLPPLLLLLRGSHAGNLTVAVVLPLANTSYPWSWARVGPAVEL
ALAQVKARPDLLPGWTVRTVLGSSENALGVCSDTAAPLAAVDLKWEHNPAVFLGPGCVY
AAAPVGRFTAHWRVPLLTAGAPALGFGVKDEYALTTRAGPSYAKLGDFVAALHRRLGWE
RQALMLYAYRPGDEEHCFFLVEGLFMRVRDRLNITVDHLEFAEDDLSHYTRLLRTMPRKG
RVIYICSSPDAFRTLMLLALEAGLCGEDYVFFHLDIFGQSLQGGQGPAPRRPWERGDGQD
VSARQAFQAAKIITYKDPDNPEYLEFLKQLKHLAYEQFNFTMEDGLVNTIPASFHDGLLLY
IQAVTETLAHGGTVTDGENITQRMWNRSFQGVTGYLKIDSSGDRETDFSLWDMDPENGA
FRVVLNYNGTSQELVAVSGRKLNWPLGYPPPDIPKCGFDNEDPACNQDHLSTLEVLALVG
SLSLLGILIVSFFIYRKMQLEKELASELWRVRWEDVEPSSLERHLRSAGSRLTLSGRGSNYG
SLLTTEGQFQVFAKTAYYKGNLVAVKRVNRKRIELTRKVLFELKHMRDVQNEHLTRFVGA
CTDPPNICILTEYCPRGSLQDILENESITLDWMFRYSLTNDIVKGMLFLHNGAICSHGNLKS
SNCVVDGRFVLKITDYGLESFRDLDPEQGHTVYAKKLWTAPELLRMASPPVRGSQAGDV
YSFGIILQEIALRSGVFHVEGLDLSPKEIIERVTRGEQPPFRPSLALQSHLEELGLLMQRCWA
EDPQERPPFQQIRLTLRKFNRENSSNILDNLLSRMEQYANNLEELVEERTQAYLEEKRKAE
ALLYQILPHSVAEQLKRGETVQAEAFDSVTIYFSDIVGFTALSAESTPMQVVTLLNDLYTCF
DAVIDNFDVYKVETIGDAYMVVSGLPVRNGRLHACEVARMALALLDAVRSFRIRHRPQE
QLRLRIGIHTGPVCAGVVGLKMPRYCLFGDTVNTASRMESNGEALKIHLSSETKAVLEEFG
GFELELRGDVEMKGKGKVRTYWLLGERGSSTRG

>O95295 peripheral membrane protein
MAGAGSAAVSGAGTPVAGPTGRDLFAEGLLEFLRPAVQQLDSHVHAVRESQVELREQIDN
LATELCRINEDQKVALDLDPYVKKLLNARRRVVLVNNILQNAQERLRRLNHSVAKETARR
RAMLDSGIYPPGSPGK

>P06703 peripheral membrane protein
MACPLDQAIGLLVAIFHKYSGREGDKHTLSKKELKELIQKELTIGSKLQDAEIARLMEDLD
RNKDQEVNFQEYVTFLGALALIYNEALKG

>P05109 peripheral membrane protein
MLTELEKALNSIIDVYHKYSLIKGNFHAVYRDDLKKLLETECPQYIRKKGADVWFKELDI
NTDGAVNFQEFLILVIKMGVAAHKKSHEESHKE

>P06702 peripheral membrane protein
MTCKMSQLERNIETIINTFHQYSVKLGHPDTLNQGEFKELVRKDLQNFLKKENKNEKVIE
HIMEDLDTNADKQLSFEEFIMLMARLTWASHEKMHEGDEGPGHHHKPGLGEGTP

>P49901 peripheral membrane protein
MCDQTKHSKCCPAKGNQCCPPQQNQCCQSKGNQCCPPKQNQCCQPKGSQCCPPKHNHC
CQPKPPCCIQARCCGLETKPEVSPLNMESEPNSPQTQDKGCQTQQQPHSPQNESRPSK

>Q9HB21 peripheral membrane protein
MPYVDRQNRICGFLDIEENENSGKFLRRYFILDTREDSFVWYMDNPQNLPSGSSRVGAIKL
TYISKVSDATKLRPKAEFCFVMNAGMRKYFLQANDQQDLVEWVNVLNKAIKITVPKQSD
SQPNSDNLSRHGECGKKQVSYRTDIVGGVPIITPTQKEEVNECGESIDRNNLKRSQSHLPY
FTPKPPQDSAVIKAGYCVKQGAVMKNWKRRYFQLDENTIGYFKSELEKEPLRVIPLKEVH
KVQECKQSDIMMRDNLFEIVTTSRTFYVQADSPEEMHSWIKAVSGAIVAQRGPGRSASSE
HPPGPSESKHAFRPTNAATATSHSTASRSNSLVSTFTMEKRGFYESLAKVKPGNFKVQTVS
PREPASKVTEQALLRPQSKNGPQEKDCDLVDLDDASLPVSDV

>Q9UGP8 multipass membrane protein
MAGQQFQYDDSGNTFFYFLTSFVGLIVIPATYYLWPRDQNAEQIRLKNIRKVYGRCMWYR
LRLLKPQPNIIPTVKKIVLLAGWALFLFLAYKVSKTDREYQEYNPYEVLNLDPGATVAEIK
KQYRLLSLKYHPDKGGDEVMFMRIAKAYAALTDEESRKNWEEFGNPDGPQATSFGIALPA
WIVDQKNSILVLLVYGLAFMVILPVVVGSWWYRSIRYSGDQILIRTTQIYTYFVYKTRNMD
MKRLIMVLAGASEFDPQYNKDATSRPTDNILIPQLIREIGSINLKKNEPPLTCPYSLKARVLL
LSHLARMKIPETLEEDQQFMLKKCPALLQEMVNVICQLIVMARNREEREFRAPTLASLEN
CMKLSQMAVQGLQQFKSPLLQLPHIEEDNLRRVSNHKKYKIKTIQDLVSLKESDRHTLLHF
LEDEKYEEVMAVLGSFPYVTMDIKSQVLDDEDSNNITVGSLVTVLVKLTRQTMAEVFEKE
QSICAAEEQPAEDGQGETNKNRTKGGWQQKSKGPKKTAKSKKKKPLKKKPTPVLLPQSK
QQKQKQANGVVGNEAAVKEDEEEVSDKGSDSEEEETNRDSQSEKDDGSDRDSDREQDE
KQNKDDEAEWQELQQSIQRKERALLETKSKITHPVYSLYFPEEKQEWWWLYIADRKEQT
LISMPYHVCTLKDTEEVELKFPAPGKPGNYQYTVFLRSDSYMGLDQIKPLKLEVHEAKPV
PENHPQWDTAIEGDEDQEDSEGFEDSFEEEEEEEEDDD

>Q9Y6Y8 peripheral membrane protein
MAERKPNGGSGGASTSSSGTNLLFSSSATEFSFNVPFIPVTQASASPASLLLPGEDSTDVGE
EDSFLGQTSIHTSAPQTFSYFSQVSSSSDPFGNIGQSPLTTAATSVGQSGFPKPLTALPFTTGS
QDVSNAFSPSISKAQPGAPPSSLMGINSYLPSQPSSLPPSYFGNQPQGIPQPGYNPYRHTPGS

SRANPYIAPPQLQQCQTPGPPAHPPPSGPPVQMYQMPPGSLPPVPSSVQSPAQQQVPARPG
APSVQVPSPFLLQNQYEPVQPHWFYCKEVEYKQLWMPFSVFDSLNLEEIYNSVQPDPESV
VLGTDGGRYDVYLYDRIRKAAYWEEEPAEVRRCTWFYKGDTDSRFIPYTEEFSEKLEAEY
KKAVTTNQWHRRLEFPSGETIVMHNPKVIVQFQPSSVPDEWGTTQDGQTRPRVVKRGIDD
NLDEIPDGEMPQVDHLVFVVHGIGPVCDLRFRSIIECVDDFRVVSLKLLRTHFKKSLDDGK
VSRVEFLPVHWHSSLGGDATGVDRNIKKITLPSIGRFRHFTNETLLDILFYNSPTYCQTIVE
KVGMEINHLHALFMSRNPDFKGGVSVAGHSLGSLILFDILSNQKDLNLSKCPGPLAVANG
VVKQLHFQEKQMPEEPKLTLDESYDLVVENKEVLTLQETLEALSLSEYFSTFEKEKIDMES
LLMCTVDDLKEMGIPLGPRKKIANFVEHKAAKLKKAASEKKAVAATSTKGQEQSAQKTK
DMASLPSESNEPKRKLPVGACVSSVCVNYESFEVGAGQVSVAYNSLDFEPEIFFALGSPIA
MFLTIRGVDRIDENYSLPTCKGFFNIYHPLDPVAYRLEPMIVPDLDLKAVLIPHHKGRKRLH
LELKESLSRMGSDLKQGFISSLKSAWQTLNEFARAHTSSTQLQEELEKVANQIKEEEEKQV
VEAEKVVESPDFSKDEDYLGKVGMLNGGRRIDYVLQEKPIESFNEYLFALQSHLCYWESE
DTALLLLKEIYRTMNISPEQPQH

>Q96BI3 multipass membrane protein
MGAAVFFGCTFVAFGPAFALFLITVAGDPLRVIILVAGAFFWLVSLLLASVVWFILVHVTDR
SDARLQYGLLIFGAAVSVLLQEVFRFAYYKLLKKADEGLASLSEDGRSPISIRQMAYVSGL
SFGIISGVFSVINILADALGPGVVGIHGDSPYYFLTSAFLTAAIILLHTFWGVVFFDACERRR
YWALGLVVGSHLLTSGLTFLNPWYEASLLPIYAVTVSMGLWAFITAGGSLRSIQRSLLCRR
QEDSRVMVYSALRIPPED

>P12314 single-pass type I membrane protein
MWFLTTLLLWVPVDGQVDTTKAVITLQPPWVSVFQEETVTLHCEVLHLPGSSSTQWFLN
GTATQTSTPSYRITSASVNDSGEYRCQRGLSGRSDPIQLEIHRGWLLLQVSSRVFTEGEPLA
LRCHAWKDKLVYNVLYYRNGKAFKFFHWNSNLTILKTNISHNGTYHCSGMGKHRYTSA
GISVTVKELFPAPVLNASVTSPLLEGNLVTLSCETKLLLQRPGLQLYFSFYMGSKTLRGRNT
SSEYQILTARREDSGLYWCEAATEDGNVLKRSPELELQVLGLQLPTPVWFHVLFYLAVGIM
FLVNTVLWVTIRKELKRKKKWDLEISLDSGHEKKVISSLQEDRHLEEELKCQEQKEEQLQ
EGVHRKEPQGAT

>P08588 multipass membrane protein
MGAGVLVLGASEPGNLSSAAPLPDGAATAARLLVPASPPASLLPPASESPEPLSQQWTAGM
GLLMALIVLLIVAGNVLVIVAIAKTPRLQTLTNLFIMSLASADLVMGLLVVPFGATIVVWGR
WEYGSFFCELWTSVDVLCVTASIETLCVIALDRYLAITSPFRYQSLLTRARARGLVCTVWAI
SALVSFLPILMHWWRAESDEARRCYNDPKCCDFVTNRAYAIASSVVSFYVPLCIMAFVYL
RVFREAQKQVKKIDSCERRFLGGPARPPSPSPSPVPAPAPPPGPPRPAAAAATAPLANGRAG
KRRPSRLVALREQKALKTLGIIMGVFTLCWLPFFLANVVKAFHRELVPDRLFVFFNWLGY
ANSAFNPIIYCRSPDFRKAFQRLLCCARRAARRRHATHGDRPRASGCLARPGPPPSPGAAS
DDDDDDVVGATPPARLLEPWAGCNGGAAADSDSSLDEPCRPGFASESKV

>O75578 single-pass type I membrane protein
MELPFVTHLFLPLVFLTGLCSPFNLDEHHPRLFPGPPEAEFGYSVLQHVGGGQRWMLVGA
PWDGPSGDRRGDVYRCPVGGAHNAPCAKGHLGDYQLGNSSHPAVNMHLGMSLLETDG

DGGFMACAPLWSRACGSSVFSSGICARVDASFQPQGSLAPTAQRCPTYMDVVIVLDGSNS
IYPWSEVQTFLRRLVGKLFIDPEQIQVGLVQYGESPVHEWSLGDFRTKEEVVRAAKNLSRR
EGRETKTAQAIMVACTEGFSQSHGGRPEAARLLVVVTDGESHDGEELPAALKACEAGRVT
RYGIAVLGHYLRRQRDPSSFLREIRTIASDPDERFFFNVTDEAALTDIVDALGDRIFGLEGSH
AENESSFGLEMSQIGFSTHRLKDGILFGMVGAYDWGGSVLWLEGGHRLFPPRMALEDEFP
PALQNHAAYLGYSVSSMLLRGGRRLFLSGAPRFRHRGKVIAFQLKKDGAVRVAQSLQGEQ
IGSYFGSELCPLDTDRDGTTDVLLVAAPMFLGPQNKETGRVYVYLVGQQSLLTLQGTLQP
EPPQDARFGFAMGALPDLNQDGFADVAVGAPLEDGHQGALYLYHGTQSGVRPHPAQRIA
AASMPHALSYFGRSVDGRLDLDGDDLVDVAVGAQGAAILLSSRPIVHLTPSLEVTPQAISV
VQRDCRRRGQEAVCLTAALCFQVTSRTPGRWDHQFYMRFTASLDEWTAGARAAFDGSG
QRLSPRRLRLSVGNVTCEQLHFHVLDTSDYLRPVALTVTFALDNTTKPGPVLNEGSPTSIQ
KLVPFSKDCGPDNECVTDLVLQVNMDIRGSRKAPFVVRGGRRKVLVSTTLENRKENAYN
TSLSLIFSRNLHLASLTPQRESPIKVECAAPSAHARLCSVGHPVFQTGAKVTFLLEFEFSCSS
LLSQVFVKLTASSDSLERNGTLQDNTAQTSAYIQYEPHLLFSSESTLHRYEVHPYGTLPVGP
GPEFKTTLRVQNLGCYVVSGLIISALLPAVAHGGNYFLSLSQVITNNASCIVQNLTEPPGPPV
HPEELQHTNRLNGSNTQCQVVRCHLGQLAKGTEVSVGLLRLVHNEFFRRAKFKSLTVVST
FELGTEEGSVLQLTEASRWSESLLEVVQTRPILISLWILIGSVLGGLLLLALLVFCLWKLGFF
AHKKIPEEEKREEKLEQ

>O43318 peripheral membrane protein
MSTASAASSSSSSSAGEMIEAPSQVLNFEEIDYKEIEVEEVVGRGAFGVVCKAKWRAKDV
AIKQIESESERKAFIVELRQLSRVNHPNIVKLYGACLNPVCLVMEYAEGGSLYNVLHGAEP
LPYYTAAHAMSWCLQCSQGVAYLHSMQPKALIHRDLKPPNLLLVAGGTVLKICDFGTAC
DIQTHMTNNKGSAAWMAPEVFEGSNYSEKCDVFSWGIILWEVITRRKPFDEIGGPAFRIM
WAVHNGTRPPLIKNLPKPIESLMTRCWSKDPSQRPSMEEIVKIMTHLMRYFPGADEPLQYP
CQYSDEGQSNSATSTGSFMDIASTNTSNKSDTNMEQVPATNDTIKRLESKLLKNQAKQQS
ESGRLSLGASRGSSVESLPPTSEGKRMSADMSEIEARIAATTAYSKPKRGHRKTASFGNILD
VPEIVISGNGQPRRRSIQDLTVTGTEPGQVSSRSSSPSVRMITTSGPTSEKPTRSHPWTPDDS
TDTNGSDNSIPMAYLTLDHQLQPLAPCPNSKESMAVFEQHCKMAQEYMKVQTEIALLLQ
RKQELVAELDQDEKDQQNTSRLVQEHKKLLDENKSLSTYYQQCKKQLEVIRSQQQKRQG
TS

>Q92637 single-pass type I membrane protein
MWFLTTLLLWVPVDGQVDTTKAVITLQPPWVSVFQEETVTLHCEVLHLPGSSSTQWFLN
GTATQTSTPSYRITSASVNDSGEYRCQRGLSGRSDPIQLEIHRGWLLLQVSSRVFMEGEPLA
LRCHAWKDKLVYNVLYYRNGKAFKFFHWNSNLTILKTNISHNGTYHCSGMGKHRYTSA
GISQYTVKGLQLPTPVWFHVLFYLAVGIMFLVNTVLWVTIRKELKRKKKWNLEISLDSGH
EKKVISSLQEDRHLEEELKCQEQKEEQLQEGVHRKEPQGAT

>Q9UKF2 single-pass type I membrane protein
MRSVQIFLSQCRLLLLLVPTMLLKSLGEDVIFHPEGEFDSYEVTIPEKLSFRGEVQGVVSPV
SYLLQLKGKKHVLHLWPKRLLLPRHLRVFSFTEHGELLEDHPYIPKDCNYMGSVKESLDS
KATISTCMGGLRGVFNIDAKHYQIEPLKASPSFEHVVYLLKKEQFGNQVCGLSDDEIEWQ
MAPYENKARLRDFPGSYKHPKYLELILLFDQSRYRFVNNNLSQVIHDAILLTGIMDTYFQD

VRMRIHLKALEVWTDFNKIRVGYPELAEVLGRFVIYKKSVLNARLSSDWAHLYLQRKYN
DALAWSFGKVCSLEYAGSVSTLLDTNILAPATWSAHELGHAVGMSHDEQYCQCRGRLNCI
MGSGRTGFSNCSYISFFKHISSGATCLNNIPGLGYVLKRCGNKIVEDNEECDCGSTEECQK
DRCCQSNCKLQPGANCSIGLCCHDCRFRPSGYVCRQEGNECDLAEYCDGNSSSCPNDVY
KQDGTPCKYEGRCFRKGCRSRYMQCQSIFGPDAMEAPSECYDAVNLIGDQFGNCEITGIR
NFKKCESANSICGRLQCINVETIPDLPEHTTIISTHLQAENLMCWGTGYHLSMKPMGIPDL
GMINDGTSCGEGRVCFKKNCVNSSVLQFDCLPEKCNTRGVCNNRKNCHCMYGWAPPFC
EEVGYGGSIDSGPPGLLRGAIPSSIWVVSIIMFRLILLILSVVFVFFRQVIGNHLKPKQEKMP
LSKAKTEQEESKTKTVQEESKTKTGQEESEAKTGQEESKAKTGQEESKANIESKRPKAKS
VKKQKK

>Q9H6R6 multipass membrane protein
MGTFCSVIKFENLQELKRLCHWGPIIALGVIAICSTMAMIDSVLWYWPLHTTGGSVNFIML
INWTVMILYNYFNAMFVGPGFVPLGWKPEISQDTMYLQYCKVCQAYKAPRSHHCRKCN
RCVMKMDHHCPWINNCCGYQNHASFTLFLLLAPLGCIHAAFIFVMTMYTQLYHRLSFGW
NTVKIDMSAARRDPLPIVPFGLAAFATTLFALGLALGTTIAVGMLFFIQMKIILRNKTSIESW
IEEKAKDRIQYYQLDEVFVFPYDMGSRWRNFKQVFTWSGVPEGDGLEWPVREGCHQYSL
TIEQLKQKADKRVRSVRYKVIEDYSGACCPLNKGIKTFFTSPCTEEPRIQLQKGEFILATRG
LRYWLYGDKILDDSFIEGVSRIRGWFPRKCVEKCPCDAETDQAPEGEKKNR

>Q96PL2 GPI-anchor, lipid-anchor
MVTKAFVLLAIFAEASAKSCAPNKADVILVFCYPKTIITKIPECPYGWEVHQLALGGLCYN
GVHEGGYYQFVIPDLSPKNKSYCGTQSEYKPPIYHFYSHIVSNDTTVIVKNQPVNYSFSCT
YHSTYLVNQAAFDQRVATVHVKNGSMGTFESQLSLNFYTNAKFSIKKEAPFVLEASEIGSD
LFAGVEAKGLSIRFKVVLNSCWATPSADFMYPLQWQLINKGCPTDETVLVHENGRDHRAT
FQFNAFRFQNIPKLSKVWLHCETFICDSEKLSCPVTCDKRKRLLRDQTGGVLVVELSLRSR
GFSSLYSFSDVLHHLIMMLGICAVL

>P06729 single-pass type I membrane protein
MSFPCKFVASFLLIFNVSSKGAVSKEITNALETWGALGQDINLDIPSFQMSDDIDDIKWEKT
SDKKKIAQFRKEKETFKEKDTYKLFKNGTLKIKHLKTDDQDIYKVSIYDTKGKNVLEKIF
DLKIQERVSKPKISWTCINTTLTCEVMNGTDPELNLYQDGKHLKLSQRVITHKWTTSLSAK
FKCTAGNKVSKESSVEPVSCPEKGLDIYLIIGICGGGSLLMVFVALLVFYITKRKKQRSRRN
DEELETRAHRVATEERGRKPHQIPASTPQNPATSQHPPPPPGHRSQAPSHRPPPPGHRVQHQ
PQKRPPAPSGTQVHQQKGPPLPRPRVQPKPPHGAAENSLSPSSN

>P19256 GPI-anchor, lipid-anchor, single-pass type I membrane protein
MVAGSDAGRALGVLSVVCLLHCFGFISCFSQQIYGVVYGNVTFHVPSNVPLKEVLWKKQ
KDKVAELENSEFRAFSSFKNRVYLDTVSGSLTIYNLTSSDEDEYEMESPNITDTMKFFLYVL
ESLPSPTLTCALTNGSIEVQCMIPEHYNSHRGLIMYSWDCPMEQCKRNSTSIYFKMENDLP
QKIQCTLSNPLFNTTSSIILTTCIPSSGHSRHRYALIPIPLAVITTCIVLYMNGILKCDRKPDRT
NSN

>P21554 multipass membrane protein

MKSILDGLADTTFRTITTDLLYVGSNDIQYEDIKGDMASKLGYFPQKFPLTSFRGSPFQEK
MTAGDNPQLVPADQVNITEFYNKSLSSFKENEENIQCGENFMDIECFMVLNPSQQLAIAVL
SLTLGTFTVLENLLVLCVILHSRSLRCRPSYHFIGSLAVADLLGSVIFVYSFIDFHVFHRKDS
RNVFLFKLGGVTASFTASVGSLFLTAIDRYISIHRPLAYKRIVTRPKAVVAFCLMWTIAIVIA
VLPLLGWNCEKLQSVCSDIFPHIDETYLMFWIGVTSVLLLFIVYAYMYILWKAHSHAVRMI
QRGTQKSIIIHTSEDGKVQVTRPDQARMDIRLAKTLVLILVVLIICWGPLLAIMVYDVFGK
MNKLIKTVFAFCSMLCLLNSTVNPIIYALRSKDLRHAFRSMFPSCEGTAQPLDNSMGDSDC
LHKHANNAASVHRAAESCIKSTVKIAKVTMSVSTDTSAEAL

>Q00013 lipid-anchor
MTLKASEGESGGSMHTALSDLYLEHLLQKRSRPEAVSHPLNTVTEDMYTNGSPAPGSPAQ
VKGQEVRKVRLIQFEKVTEEPMGITLKLNEKQSCTVARILHGGMIHRQGSLHVGDEILEIN
GTNVTNHSVDQLQKAMKETKGMISLKVIPNQQSRLPALQMFMRAQFDYDPKKDNLIPCK
EAGLKFATGDIIQIINKDDSNWWQGRVEGSSKESAGLIPSPELQEWRVASMAQSAPSEAPS
CSPFGKKKKYKDKYLAKHSSIFDQLDVVSYEEVVRLPAFKRKTLVLIGASGVGRSHIKNA
LLSQNPEKFVYPVPYTTRPPRKSEEDGKEYHFISTEEMTRNISANEFLEFGSYQGNMFGTK
FETVHQIHKQNKIAILDIEPQTLKIVRTAELSPFIVFIAPTDQGTQTEALQQLQKDSEAIRSQY
AHYFDLSLVNNGVDETLKKLQEAFDQACSSPQWVPVSWVY

>P78382 multipass membrane protein
MAAPRDNVTLLFKLYCLAVMTLMAAVYTIALRYTRTSDKELYFSTTAVCITEVIKLLLSVGI
LAKETGSLGRFKASLRENVLGSPKELLKLSVPSLVYAVQNNMAFLALSNLDAAVYQVTYQ
LKIPCTALCTVLMLNRTLSKLQWVSVFMLCAGVTLVQWKPAQATKVVVEQNPLLGFGAI
AIAVLCSGFAGVYFEKVLKSSDTSLWVRNIQMYLSGIIVTLAGVYLSDGAEIKEKGFFYGY
TYYVWFVIFLASVGGLYTSVVVKYTDNIMKGFSAAAAIVLSTIASVMLFGLQITLTFALGT
LLVCVSIYLYGLPRQDTTSIQQGETASKERVIGV

>P53985 multipass membrane protein
MPPAVGGPVGYTPPDGGWGWAVVIGAFISIGFSYAFPKSITVFFKEIEGIFHATTSEVSWISSI
MLAVMYGGGPISSILVNKYGSRIVMIVGGCLSGCGLIAASFCNTVQQLYVCIGVIGGLGLA
FNLNPALTMIGKYFYKRRPLANGLAMAGSPVFLCTLAPLNQVFFGIFGWRGSFLILGGLLL
NCCVAGALMRPIGPKPTKAGKDKSKASLEKAGKSGVKKDLHDANTDLIGRHPKQEKRSV
FQTINQFLDLTLFTHRGFLLYLSGNVIMFFGLFAPLVFLSSYGKSQHYSSEKSAFLLSILAFV
DMVARPSMGLVANTKPIRPRIQYFFAASVVANGVCHMLAPLSTTYVGFCVYAGFFGFAFG
WLSSVLFETLMDLVGPQRFSSAVGLVTIVECCPVLLGPPLLGRLNDMYGDYKYTYWACG
VVLIISGIYLFIGMGINYRLLAKEQKANEQKKESKEEETSIDVAGKPNEVTKAAESPDQKD
TDGGPKEEESPV

>Q9UPU3 single-pass type I membrane protein
MEAARTERPAGRPGAPLVRTGLLLLSTWVLAGAEITWDATGGPGRPAAPASRPPALSPLSP
RAVASQWPEELASARRAAVLGRRAGPELLPQQGGGRGGEMQVEAGGTSPAGERRGRGIP
APAKLGGARRSRRAQPPITQERGDAWATAPADGSRGSRPLAKGSREEVKAPRAGGSAAED
LRLPSTSFALTGDSAHNQAMVHWSGHNSSVILILTKLYDFNLGSVTESSLWRSTDYGTTYE
KLNDKVGLKTVLSYLYVNPTNKRKIMLLSDPEMESSILISSDEGATYQKYRLTFYIQSLLFH

PKQEDWVLAYSLDQKLYSSMDFGRRWQLMHERITPNRFYWSVAGLDKEADLVHMEVRT
TDGYAHYLTCRIQECAETTRSGPFARSIDISSLVVQDEYIFIQVTTSGRASYYVSYRREAFAQ
IKLPKYSLPKDMHIISTDENQVFAAVQEWNQNDTYNLYISDTRGIYFTLAMENIKSSRGLM
GNIIIELYEVAGIKGIFLANKKVDDQVKTYITYNKGRDWRLLQAPDVDLRGSPVHCLLPFC
SLHLHLQLSENPYSSGRISSKETAPGLVVATGNIGPELSYTDIGVFISSDGGNTWRQIFDEEY
NVWFLDWGGALVAMKHTPLPVRHLWVSFDEGHSWDKYGFTSVPLFVDGALVEAGMET
HIMTVFGHFSLRSEWQLVKVDYKSIFSRHCTKEDYQTWHLLNQGEPCVMGERKIFKKRK
PGAQCALGRDHSGSVVSEPCVCANWDFECDYGYERHGESQCVPAFWYNPASPSKDCSLG
QSYLNSTGYRRIVSNNCTDGLREKYTAKAQMCPGKAPRGLHVVTTDGRLVAEQGHNATF
IILMEEGDLQRTNIQLDFGDGIAVSYANFSPIEDGIKHVYKSAGIFQVTAYAENNLGSDTAVL
FLHVVCPVEHVHLRVPFVAIRNKEVNISAVVWPSQLGTLTYFWWFGNSTKPLITLDSSISFT
FLAEGTDTITVQVAAGNALIQDTKEIAVHEYFQSQLLSFSPNLDYHNPDIPEWRKDIGNVIK
RALVKVTSVPEDQILIAVFPGLPTSAELFILPPKNLTERRKGNEGDLEQIVETLFNALNQNLV
QFELKPGVQVIVYVTQLTLAPLVDSSAGHSSSAMLMLLSVVFVGLAVFLIYKFKRKIPWINI
YAQVQHDKEQEMIGSVSQSENAPKITLSDFTEPEELLDKELDTRVIGGIATIANSESTKEIPN
CTSV

>P33765 multipass membrane protein
MPNNSTALSLANVTYITMEIFIGLCAIVGNVLVICVVKLNPSLQTTTFYFIVSLALADIAVGV
LVMPLAIVVSLGITIHFYSCLFMTCLLLIFTHASIMSLLAIAVDRYLRVKLTVRYKRVTTHRR
IWLALGLCWLVSFLVGLTPMFGWNMKLTSEYHRNVTFLSCQFVSVMRMDYMVYFSFLT
WIFIPLVVMCAIYLDIFYIIRNKLSLNLSNSKETGAFYGREFKTAKSLFLVLFLFALSWLPLSI
INCIIYFNGEVPQLVLYMGILLSHANSMMNPIVYAYKIKKFKETYLLILKACVVCHPSDSLD
TSIEKNSE

>P48146 multipass membrane protein
MQAAGHPEPLDSRGSFSLPTMGANVSQDNGTGHNATFSEPLPFLYVLLPAVYSGICAVGLT
GNTAVILVILRAPKMKTVTNVFILNLAVADGLFTLVLPVNIAEHLLQYWPFGELLCKLVLAV
DHYNIFSSIYFLAVMSVDRYLVVLATVRSRHMPWRTYRGAKVASLCVWLGVTVLVLPFFS
FAGVYSNELQVPSCGLSFPWPEQVWFKASRVYTLVLGFVLPVCTICVLYTDLLRRLRAVRL
RSGAKALGKARRKVTVLVLVVLAVCLLCWTPFHLASVVALTTDLPQTPLVISMSYVITSLS
YANSCLNPFLYAFLDDNFRKNFRSILRC

>P22001 multipass membrane protein
MDERLSLLRSPPPPSARHRAHPPQRPASSGGAHTLVNHGYAEPAAGRELPPDMTVVPGDH
LLEPEVADGGGAPPQGGCGGGGCDRYEPLPPSLPAAGEQDCCGERVVINISGLRFETQLKT
LCQFPETLLGDPKRRMRYFDPLRNEYFFDRNRPSFDAILYYYQSGGRIRRPVNVPIDIFSEEI
RFYQLGEEAMEKFREDEGFLREEERPLPRRDFQRQVWLLFEYPESSGPARGIAIVSVLVILI
SIVIFCLETLPEFRDEKDYPASTSQDSFEAAGNSTSGSRAGASSFSDPFFVVETLCIIWFSFEL
LVRFFACPSKATFSRNIMNLIDIVAIIPYFITLGTELAERQGNGQQAMSLAILRVIRLVRVFRIF
KLSRHSKGLQILGQTLKASMRELGLLIFFLFIGVILFSSAVYFAEADDPTSGFSSIPDAFWWA
VVTMTTVGYGDMHPVTIGGKIVGSLCAIAGVLTIALPVPVIVSNFNYFYHRETEGEEQSQY
MHVGSCQHLSSSAEELRKARSNSTLSKSEYMVIEEGGMNHSAFPQTPFKTGNSTATCTTN
NNPNSCVNIKKIFTDV

>Q16322 multipass membrane protein
MDVCGWKEMEVALVNFDNSDEIQEEPGYATDFDSTSPKGRPGGSSFSNGKILISESTNHET
AFSKLPGDYADPPGPEPVVLNEGNQRVIINIAGLRFETQLRTLSQFPETLLGDREKRMQFFD
SMRNEYFFDRNRPSFDGILYYYQSGGKIRRPANVPIDIFADEISFYELGSEAMDQFREDEGFI
KDPETLLPTNDIHRQFWLLFEYPESSSAARAVAVVSVLVVVISITIFCLETLPEFREDRELKV
VRDPNLNMSKTVLSQTMFTDPFFMVESTCIVWFTFELVLRFVVCPSKTDFFRNIMNIIDIISI
IPYFATLITELVQETEPSAQQNMSLAILRIIRLVRVFRIFKLSRHSKGLQILGQTLKASMRELG
LLIFFLFIGVILFSSAVYFAEVDEPESHFSSIPDGFWWAVVTMTTVGYGDMCPTTPGGKIVG
TLCAIAGVLTIALPVPVIVSNFNYFYHRETENEEKQNIPGEIERILNSVGSRMGSTDSLNKT
NGGCSTEKSRK

>O15374 multipass membrane protein
MLKREGKVQPYTKTLDGGWGWMIVIHFFLVNVFVMGMTKTFAIFFVVFQEEFEGTSEQI
GWIGSIMSSLRFCAGPLVAIICDILGEKTTSILGAFVVTGGYLISSWATSIPFLCVTMGLLPGL
GSAFLYQVAAVVTTKYFKKRLALSTAIARSGMGLTFLLAPFTKFLIDLYDWTGALILFGAIA
LNLVPSSMLLRPIHIKSENNSGIKDKGSSLSAHGPEAHATETHCHETEESTIKDSTTQKAGL
PSKNLTVSQNQSEEFYNGPNRNRLLLKSDEESDKVISWSCKQLFDISLFRNPFFYIFTWSFL
LSQLAYFIPTFHLVARAKTLGIDIMDASYLVSVAGILETVSQIISGWVADQNWIKKYHYHKS
YLILCGITNLLAPLATTFPLLMTYTICFAIFAGGYLALILPVLVDLCRNSTVNRFLGLASFFA
GMAVLSGPPIAGWLYDYTQTYNGSFYFSGICYLLSSVSFFFVPLAERWKNSLT

>Q03721 multipass membrane protein
MISSVCVSSYRGRKSGNKPPSKTCLKEEMAKGEASEKIIINVGGTRHETYRSTLRTLPGTR
LAWLADPDGGGRPETDGGGVGSSGSSGGGGCEFFFDRHPGVFAYVLNYYRTGKLHCPAD
VCGPLFEEELTFWGIDETDVEPCCWMTYRQHRDAEEALDIFESPDGGGSGAGPSDEAGDD
ERELALQRLGPHEGGAGHGAGSGGCRGWQPRMWALFEDPYSSRAARVVAFASLFFILVSI
TTFCLETHEAFNIDRNVTEILRVGNITSVHFRREVETEPILTYIEGVCVLWFTLEFLVRIVCCP
DTLDFVKNLLNIIDFVAILPFYLEVGLSGLSSKAARDVLGFLRVVRFVRILRIFKLTRHFVGL
RVLGHTLRASTNEFLLLIIFLALGVLIFATMIYYAERIGARPSDPRGNDHTDFKNIPIGFWWA
VVTMTTLGYGDMYPKTWSGMLVGALCALAGVLTIAMPVPVIVNNFGMYYSLAMAKQK
LPKKRKKHVPRPAQLESPMYCKSEETSPRDSTCSDTSPPAREEGMIERKRADSKQNGDAN
AVLSDEEGAGLTQPLASSPTPEERRALRRSTTRDRNKKAAACFLLSTGDYACADGSVRKG
TFVLRDLPLQHSPEAACPPTAGTLFLPH

>Q9GZR5 multipass membrane protein
MGLLDSEPGSVLNVVSTALNDTVEFYRWTWSIADKRVENWPLMQSPWPTLSISTLYLLFV
WLGPKWMKDREPFQMRLVLIIYNFGMVLLNLFIFRELFMGSYNAGYSYICQSVDYSNNV
HEVRIAAALWWYFVSKGVEYLDTVFFILRKKNNQVSFLHVYHHCTMFTLWWIGIKWVA
GGQAFFGAQLNSFIHVIMYSYYGLTAFGPWIQKYLWWKRYLTMLQLIQFHVTIGHTALSL
YTDCPFPKWMHWALIAYAISFIFLFLNFYIRTYKEPKKPKAGKTAMNGISANGVSKSEKQL
MIENGKKQKNGKAKGD

>P04001 multipass membrane protein

MAQQWSLQRLAGRHPQDSYEDSTQSSIFTYTNSNSTRGPFEGPNYHIAPRWVYHLTSVW
MIFVVIASVFTNGLVLAATMKFKKLRHPLNWILVNLAVADLAETVIASTISVVNQVYGYFV
LGHPMCVLEGYTVSLCGITGLWSLAIISWERWMVVCKPFGNVRFDAKLAIVGIAFSWIWA
AVWTAPPIFGWSRYWPHGLKTSCGPDVFSGSSYPGVQSYMIVLMVTCCITPLSIIVLCYLQ
VWLAIRAVAKQQKESESTQKAEKEVTRMVVVMVLAFCFCWGPYAFFACFAAANPGYPFH
PLMAALPAFFAKSATIYNPVIYVFMNRQFRNCILQLFGKKVDDGSELSSASKTEVSSVSSVS
PA

>P28222 multipass membrane protein
MEEPGAQCAPPPPAGSETWVPQANLSSAPSQNCSAKDYIYQDSISLPWKVLLVMLLALITL
ATTLSNAFVIATVYRTRKLHTPANYLIASLAVTDLLVSILVMPISTMYTVTGRWTLGQVVC
DFWLSSDITCCTASILHLCVIALDRYWAITDAVEYSAKRTPKRAAVMIALVWVFSISISLPPF
FWRQAKAEEEVSECVVNTDHILYTVYSTVGAFYFPTLLLIALYGRIYVEARSRILKQTPNR
TGKRLTRAQLITDSPGSTSSVTSINSRVPDVPSESGSPVYVNQVKVRVSDALLEKKKLMAA
RERKATKTLGIILGAFIVCWLPFFIISLVMPICKDACWFHLAIFDFFTWLGYLNSLINPIIYTM
SNEDFKQAFHKLIRFKCTS

>Q9UM54 peripheral membrane protein
MEDGKPVWAPHPTDGFQMGNIVDIGPDSLTIEPLNQKGKTFLALINQVFPAEEDSKKDVE
DNCSLMYLNEATLLHNIKVRYSKDRIYTYVANILIAVNPYFDIPKIYSSEAIKSYQGKSLGT
RPPHVFAIADKAFRDMKVLKMSQSIIVSGESGAGKTENTKFVLRYLTESYGTGQDIDDRIV
EANPLLEAFGNAKTVRNNNSSRFGKFVEIHFNEKSSVVGGFVSHYLLEKSRICVQGKEER
NYHIFYRLCAGASEDIREKLHLSSPDNFRYLNRGCTRYFANKETDKQILQNRKSPEYLKAG
SMKDPLLDDHGDFIRMCTAMKKIGLDDEEKLDLFRVVAGVLHLGNIDFEEAGSTSGGCNL
KNKSAQSLEYCAELLGLDQDDLRVSLTTRVMLTTAGGTKGTVIKVPLKVEQANNARDAL
AKTVYSHLFDHVVNRVNQCFPFETSSYFIGVLDIAGFEYFEHNSFEQFCINYCNEKLQQFF
NERILKEEQELYQKEGLGVNEVHYVDNQDCIDLIEAKLVGILDILDEENRLPQPSDQHFTSA
VHQKHKDHFRLTIPRKSKLAVHRNIRDDEGFIIRHFAGAVCYETTQFVEKNNDALHMSLES
LICESRDKFIRELFESSTNNNKDTKQKAGKLSFISVGNKFKTQLNLLLDKLRSTGASFIRCI
KPNLKMTSHHFEGAQILSQLQCSGMVSVLDLMQGGYPSRASFHELYNMYKKYMPDKLA
RLDPRLFCKALFKALGLNENDYKFGLTKVFFRPGKFAEFDQIMKSDPDHLAELVKRVNH
WLTCSRWKKVQWCSLSVIKLKNKIKYRAEACIKMQKTIRMWLCKRRHKPRIDGLVKVGT
LKKRLDKFNEVVSVLKDGKPEMNKQIKNLEISIDTLMAKIKSTMMTQEQIQKEYDALVKS
SEELLSALQKKKQQEEEAERLRRIQEEMEKERKRREEDEKRRRKEEEERRMKLEMEAKR
KQEEEERKKREDDEKRIQAEVEAQLARQKEEESQQQAVLEQERRDRELALRIAQSEAELIS
DEAQADLALRRSLDSYPVSKNDGTRPKMTPEQMAKEMSEFLSRGPAVLATKAAAGTKKY
DLSKWKYAELRDTINTSCDIELLAACREEFHRRLKVYHAWKSKNKKRNTETEQRAPKSV
TDYDFAPFLNNSPQQNPAAQIPARQREIEMNRQQRFFRIPFIRPADQYKDPQSKKKGWWYA
HFDGPWIARQMELHPDKPPILLVAGKDDMEMCELNLEETGLTRKRGAEILPRQFEEIWER
CGGIQYLQNAIESRQARPTYATAMLQSLLK

>Q9HB03 multipass membrane protein
MVTAMNVSHEVNQLFQPYNFELSKDMRPFFEEYWATSFPIALIYLVLIAVGQNYMKERKG
FNLQGPLILWSFCLAIFSILGAVRMWGIMGTVLLTGGLKQTVCFINFIDNSTVKFWSWVFL

LSKVIELGDTAFIILRKRPLIFIHWYHHSTVLVYTSFGYKNKVPAGGWFVTMNFGVHAIMY
TYYTLKAANVKPPKMLPMLITSLQILQMFVGAIVSILTYIWRQDQGCHTTMEHLFWSFILY
MTYFILFAHFFCQTYIRPKVKAKTKSQ

>P32004 single-pass type I membrane protein
MVVALRYVWPLLLCSPCLLIQIPEEYEGHHVMEPPVITEQSPRRLVVFPTDDISLKCEASGK
PEVQFRWTRDGVHFKPKEELGVTVYQSPHSGSFTITGNNSNFAQRFQGIYRCFASNKLGTA
MSHEIRLMAEGAPKWPKETVKPVEVEEGESVVLPCNPPPSAEPLRIYWMNSKILHIKQDE
RVTMGQNGNLYFANVLTSDNHSDYICHAHFPGTRTIIQKEPIDLRVKATNSMIDRKPRLLFP
TNSSSHLVALQGQPLVLECIAEGFPTPTIKWLRPSGPMPADRVTYQNHNKTLQLLKVGEED
DGEYRCLAENSLGSARHAYYVTVEAAPYWLHKPQSHLYGPGETARLDCQVQGRPQPEVT
WRINGIPVEELAKDQKYRIQRGALILSNVQPSDTMVTQCEARNRHGLLLANAYIYVVQLP
AKILTADNQTYMAVQGSTAYLLCKAFGAPVPSVQWLDEDGTTVLQDERFFPYANGTLGIR
DLQANDTGRYFCLAANDQNNVTIMANLKVKDATQITQGPRSTIEKKGSRVTFTCQASFDP
SLQPSITWRGDGRDLQELGDSDKYFIEDGRLVIHSLDYSDQGNYSCVASTELDVVESRAQL
LVVGSPGPVPRLVLSDLHLLTQSQVRVSWSPAEDHNAPIEKYDIEFEDKEMAPEKWYSLG
KVPGNQTSTTLKLSPYVHYTFRVTAINKYGPGEPSPVSETVVTPEAAPEKNPVDVKGEGN
ETTNMVITWKPLRWMDWNAPQVQYRVQWRPQGTRGPWQEQIVSDPFLVVSNTSTFVPY
EIKVQAVNSQGKGPEPQVTIGYSGEDYPQAIPELEGIEILNSSAVLVKWRPVDLAQVKGHL
RGYNVTYWREGSQRKHSKRHIHKDHVVVPANTTSVILSGLRPYSSYHLEVQAFNGRGSGP
ASEFTFSTPEGVPGHPEALHLECQSNTSLLLRWQPPLSHNGVLTGYVLSYHPLDEGGKGQ
LSFNLRDPELRTHNLTDLSPHLRYRFQLQATTKEGPGEAIVREGGTMALSGISDFGNISATA
GENYSVVSWVPKEGQCNFRFHILFKALGEEKGGASLSPQYVSYNQSSYTQWDLQPDTDY
EIHLFKERMFRHQMAVKTNGTGRVRLPPAGFATEGWFIGFVSAIILLLLVLLILCFIKRSKGG
KYSVKDKEDTQVDSEARPMKDETFGEYRSLESDNEEKAFGSSQPSLNGDIKPLGSDDSLA
DYGGSVDVQFNEDGSFIGQYSGKKEKEAAGGNDSSGATSPINPAVALE

>Q9Y2D2 multipass membrane protein
MFANLKYVSLGILVFQTTSLVLTMRYSRTLKEEGPRYLSSTAVVVAELLKIMACILLVYKDS
KCSLRALNRVLHDEILNKPMETLKLAIPSGIYTLQNNLLYVALSNLDAATYQVTYQLKILTT
ALFSVSMLSKKLGVYQWLSLVILMTGVAFVQWPSDSQLDSKELSAGSQFVGLMAVLTACF
SSGFAGVYFEKILKETKQSVWIRNIQLGFFGSIFGLMGVYIYDGELVSKNGFFQGYNRLTW
IVVVLQALGGLVIAAVIKYADNILKGFATSLSIILSTLISYFWLQDFVPTSVFFLGAILVITATF
LYGYDPKPAGNPTKA

>P28288 multipass membrane protein
MAAFSKYLTARNSSLAGAAFLLLCLLHKRRRALGLHGKKSGKPPLQNNEKEGKKERAVV
DKVFFSRLIQILKIMVPRTFCKETGYLVLIAVMLVSRTYCDVWMIQNGTLIESGIIGRSRKDF
KRYLLNFIAAMPLISLVNNFLKYGLNELKLCFRVRLTKYLYEEYLQAFTYYKMGNLDNRI
ANPDQLLTQDVEKFCNSVVDLYSNLSKPFLDIVLYIFKLTSAIGAQGPASMMAYLVVSGLFL
TRLRRPIGKMTITEQKYEGEYRYVNSRLITNSEEIAFYNGNKREKQTVHSVFRKLVEHLHN
FILFRFSMGFIDSIIAKYLATVVGYLVVSRPFLDLSHPRHLKSTHSELLEDYYQSGRMLLRM
SQALGRIVLAGREMTRLAGFTARITELMQVLKDLNHGKYERTMVSQQEKGIEGVQVIPLI
PGAGEIIIADNIIKFDHVPLATPNGDVLIRDLNFEVRSGANVLICGPNGCGKSSLFRVLGEL

WPLFGGRLTKPERGKLFYVPQRPYMTLGTLRDQVIYPDGREDQKRKGISDLVLKEYLDNV
QLGHILEREGGWDSVQDWMDVLSGGEKQRMAMARLFYHKPQFAILDECTSAVSVDVEG
YIYSHCRKVGITLFTVSHRKSLWKHHEYYLHMDGRGNYEFKQITEDTVEFGS

>P78363 multipass membrane protein
MGFVRQIQLLLWKNWTLRKRQKIRFVVELVWPLSLFLVLIWLRNANPLYSHHECHFPNKA
MPSAGMLPWLQGIFCNVNNPCFQSPTPGESPGIVSNYNNSILARVYRDFQELLMNAPESQ
HLGRIWTELHILSQFMDTLRTHPERIAGRGIRIRDILKDEETLTLFLIKNIGLSDSVVYLLINS
QVRPEQFAHGVPDLALKDIACSEALLERFIIFSQRRGAKTVRYALCSLSQGTLQWIEDTLYA
NVDFFKLFRVLPTLLDSRSQGINLRSWGGILSDMSPRIQEFIHRPSMQDLLWVTRPLMQNG
GPETFTKLMGILSDLLCGYPEGGGSRVLSFNWYEDNNYKAFLGIDSTRKDPIYSYDRRTTS
FCNALIQSLESNPLTKIAWRAAKPLLMGKILYTPDSPAARRILKNANSTFEELEHVRKLVKA
WEEVGPQIWYFFDNSTQMNMIRDTLGNPTVKDFLNRQLGEEGITAEAILNFLYKGPRESQ
ADDMANFDWRDIFNITDRTLRLVNQYLECLVLDKFESYNDETQLTQRALSLLEENMFWA
GVVFPDMYPWTSSLPPHVKYKIRMDIDVVEKTNKIKDRYWDSGPRADPVEDFRYIWGGF
AYLQDMVEQGITRSQVQAEAPVGIYLQQMPYPCFVDDSFMIILNRCFPIFMVLAWIYSVS
MTVKSIVLEKELRLKETLKNQGVSNAVIWCTWFLDSFSIMSMSIFLLTIFIMHGRILHYSDP
FILFLFLLAFSTATIMLCFLLSTFFSKASLAAACSGVIYFTLYLPHILCFAWQDRMTAELKKA
VSLLSPVAFGFGTEYLVRFEEQGLGLQWSNIGNSPTEGDEFSFLLSMQMMLLDAAVYGLL
AWYLDQVFPGDYGTPLPWYFLLQESYWLGGEGCSTREERALEKTEPLTEETEDPEHPEGI
HDSFFEREHPGWVPGVCVKNLVKIFEPCGRPAVDRLNITFYENQITAFLGHNGAGKTTTLSI
LTGLLPPTSGTVLVGGRDIETSLDAVRQSLGMCPQHNILFHHLTVAEHMLFYAQLKGKSQE
EAQLEMEAMLEDTGLHHKRNEEAQDLSGGMQRKLSVAIAFVGDAKVVILDEPTSGVDPY
SRRSIWDLLLKYRSGRTIIMSTHHMDEADLLGDRIAIIAQGRLYCSGTPLFLKNCFGTGLYL
TLVRKMKNIQSQRKGSEGTCSCSSKGFSTTCPAHVDDLTPEQVLDGDVNELMDVVLHHV
PEAKLVECIGQELIFLLPNKNFKHRAYASLFRELEETLADLGLSSFGISDTPLEEIFLKVTEDS
DSGPLFAGGAQQKRENVNPRHPCLGPREKAGQTPQDSNVCSPGAPAAHPEGQPPPEPECP
GPQLNTGTQLVLQHVQALLVKRFQHTIRSHKDFLAQIVLPATFVFLALMLSIVIPPFGEYPA
LTLHPWIYGQQYTFFSMDEPGSEQFTVLADVLLNKPGFGNRCLKEGWLPEYPCGNSTPW
KTPSVSPNITQLFQKQKWTQVNPSPSCRCSTREKLTMLPECPEGAGGLPPPQRTQRSTEILQ
DLTDRNISDFLVKTYPALIRSSLKSKFWVNEQRYGGISIGGKLPVVPITGEALVGFLSDLGRI
MNVSGGPITREASKEIPDFLKHLETEDNIKVWFNNKGWHALVSFLNVAHNAILRASLPKD
RSPEEYGITVISQPLNLTKEQLSEITVLTTSVDAVVAICVIFSMSFVPASFVLYLIQERVNKSK
HLQFISGVSPTTYWVTNFLWDIMNYSVSAGLVVGIFIGFQKKAYTSPENLPALVALLLLYG
WAVIPMMYPASFLFDVPSTAYVALSCANLFIGINSSAITFILELFENNRTLLRFNAVLRKLLIV
FPHFCLGRGLIDLALSQAVTDVYARFGEEHSANPFHWDLIGKNLFAMVVEGVVYFLLTLL
VQRHFFLSQWIAEPTKEPIVDEDDDVAEERQRIITGGNKTDILRLHELTKIYPGTSSPAVDRL
CVGVRPGECFGLLGVNGAGKTTTFKMLTGDTTVTSGDATVAGKSILTNISEVHQNMGYCP
QFDAIDELLTGREHLYLYARLRGVPAEEIEKVANWSIKSLGLTVYADCLAGTYSGGNKRKL
STAIALIGCPPLVLLDEPTTGMDPQARRMLWNVIVSIIREGRAVVLTSHSMEECEALCTRLA
IMVKGAFRCMGTIQHLKSKFGDGYIVTMKIKSPKDDLLPDLNPVEQFFQGNFPGSVQRER
HYNMLQFQVSSSSLARIFQLLLSHKDSLLIEEYSVTQTTLDQVFVNFAKQQTESHDLPLHP
RAAGASRQAQD

>P43681 multipass membrane protein
MELGGPGAPRLLPPLLLLLGTGLLRASSHVETRAHAEERLLKKLFSGYNKWSRPVANISD
VVLVRFGLSIAQLIDVDEKNQMMTTNVWVKQEWHDYKLRWDPADYENVTSIRIPSELIW
RPDIVLYNNADGDFAVTHLTKAHLFHDGRVQWTPPAIYKSSCSIDVTFFPFDQQNCTMKFG
SWTYDKAKIDLVNMHSRVDQLDFWESGEWVIVDAVGTYNTRKYECCAEIYPDITYAFVIR
RLPLFYTINLIIPCLLISCLTVLVFYLPSECGEKITLCISVLLSLTVFLLLITEIIPSTSLVIPLIGE
YLLFTMIFVTLSIVITVFVLNVHHRSPRTHTMPTWVRRVFLDIVPRLLLMKRPSVVKDNCR
RLIESMHKMASAPRFWPEPEGEPPATSGTQSLHPPSPSFCVPLDVPAEPGPSCKSPSDQLPP
QQPLEAEKASPHPSPGPCRPPHGTQAPGLAKARSLSVQHMSSPGEAVEGGVRCRSRSIQYC
VPRDDAAPEADGQAAGALASRNTHSAELPPPDQPSPCKCTCKKEPSSVSPSATVKTRSTK
APPPHLPLSPALTRAVEGVQYIADHLKAEDTDFSVKEDWKYVAMVIDRIFLWMFIIVCLLG
TVGLFLPPWLAGMI

>Q9Y3A6 single-pass type I membrane protein
MGDKIWLPFPVLLLAALPPVLLPGAAGFTPSLDSDFTFTLPAGQKECFYQPMPLKASLEIE
YQVLDGAGLDIDFHLASPEGKTLVFEQRKSDGVHTVETEVGDYMFCFDNTFSTISEKVIFF
ELILDNMGEQAQEQEDWKKYITGTDILDMKLEDILESINSIKSRLSKSGHIQTLLRAFEARD
RNIQESNFDRVNFWSMVNLVVMVVVSAIQVYMLKSLFEDKRKSRT

>Q9UN88 multipass membrane protein
MGIRGMLRAAVILLLIRTWLAEGNYPSPIPKFHFEFSSAVPEVVLNLFNCKNCANEAVVQKI
LDRVLSRYDVRLRPNFGGAPVPVRISIYVTSIEQISEMNMDYTITMFFHQTWKDSRLAYYE
TTLNLTLDYRMHEKLWVPDCYFLNSKDAFVHDVTVENRVFQLHPDGTVRYGIRLTTTAA
CSLDLHKFPMDKQACNLVVESYGYTVEDIILFWDDNGNAIHMTEELHIPQFTFLGRTITSK
EVYFYTGSYIRLILKFQVQREVNSYLVQVYWPTVLTTITSWISFWMNYDSSAARVTIGLTS
MLILTTIDSHLRDKLPNISCIKAIDIYILVCLFFVFLSLLEYVYINYLFYSRGPRRQPRRHRRP
RRVIARYRYQQVVVGNVQDGLINVEDGVSSLPITPAQAPLASPESLGSLTSTSEQAQLATSE
SLSPLTSLSGQAPLATGESLSDLPSTSEQARHSYGVRFNGFQADDSIFPTEIRNRVEAHGHG
VTHDHEDSNESLSSDERHGHGPSGKPMLHHGEKGVQEAGWDLDDNNDKSDCLAIKEQF
KCDTNSTWGLNDDELMAHGQEKDSSSESEDSCPPSPGCSFTEGFSFDLFNPDYVPKVDK
WSRFLFPLAFGLFNIVYWVYHMY

>P34903 multipass membrane protein
MIITQTSHCYMTSLGILFLINILPGTTGQGESRRQEPGDFVKQDIGGLSPKHAPDIPDDSTDN
ITIFTRILDRLLDGYDNRLRPGLGDAVTEVKTDIYVTSFGPVSDTDMEYTIDVFFRQTWHD
ERLKFDGPMKILPLNNLLASKIWTPDTFFHNGKKSVAHNMTTPNKLLRLVDNGTLLYTMR
LTIHAECPMHLEDFPMDVHACPLKFGSYAYTTAEVVYSWTLGKNKSVEVAQDGSRLNQY
DLLGHVVGTEIIRSSTGEYVVMTTHFHLKRKIGYFVIQTYLPCIMTVILSQVSFWLNRESVP
ARTVFGVTTVLTMTTLSISARNSLPKVAYATAMDWFIAVCYAFVFSALIEFATVNYFTKRSW
AWEGKKVPEALEMKKKTPAAPAKKTSTTFNIVGTTYPINLAKDTEFSTISKGAAPSASSTP
TIIASPKATYVQDSPTETKTYNSVSKVDKISRIIFPVLFAIFNLVYWATYVNRESAIKGMIRK
Q

>P78334 multipass membrane protein

MLSKVLPVLLGILLILQSRVEGPQTESKNEASSRDVVYGPQPQPLENQLLSEETKSTETETG
SRVGKLPEASRILNTILSNYDHKLRPGIGEKPTVVTVEISVNSLGPLSILDMEYTIDIIFSQTW
YDERLCYNDTFESLVLNGNVVSQLWIPDTFFRNSKRTHEHEITMPNQMVRIYKDGKVLYT
IRMTIDAGCSLHMLRFPMDSHSCPLSFSSFSYPENEMIYKWENFKLEINEKNSWKLFQFDF
TGVSNKTEIITTPVGDFMVMTIFFNVSRRFGYVAFQNYVPSSVTTMLSWVSFWIKTESAPA
RTSLGITSVLTMTTLGTFSRKNFPRVSYITALDFYIAICFVFCFCALLEFAVLNFLIYNQTKAH
ASPKLRHPRINSRAHARTRARSRACARQHQEAFVCQIVTTEGSDGEERPSCSAQQPPSPGS
PEGPRSLCSKLACCEWCKRFKKYFCMVPDCEGSTWQQGRLCIHVYRLDNYSRVVFPVTF
FFFNVLYWLVCLNL

>Q9NQW1 peripheral membrane protein
MKLKELERPAVQAWSPASQYPLYLATGTSAQQLDSSFSTNGTLEIFEVDFRDPSLDLKHRG
VLSALSRFHKLVWGSFGSGLLESSGVIVGGGDNGMLILYNVTHILSSGKEPVIAQKQKHTG
AVRALDLNPFQGNLLASGASDSEIFIWDLNNLNVPMTLGSKSQQPPEDIKALSWNRQAQH
ILSSAHPSGKAVVWDLRKNEPIIKVSDHSNRMHCSGLAWHPDIATQLVLCSEDDRLPVIQL
WDLRFASSPLKVLESHSRGILSVSWSQADAELLLTSAKDSQILCRNLGSSEVVYKLPTQSS
WCFDVQWCPRDPSVFSAASFNGWISLYSVMGRSWEVQHMRQADKISSSFSKGQPLPPLQ
VPEQVAQAPLIPPLKKPPKWIRRPTGVSFAFGGKLVTFGLPSTPAHLVPQPCPRLVFISQVTT
ESEFLMRSAELQEALGSGNLLNYCQNKSQQALLQSEKMLWQFLKVTLEQDSRMKFLKLL
GYSKDELQKKVATWLKSDVGLGESPQPKGNDLNSDRQQAFCSQASKHTTKEASASSAFF
DELVPQNMTPWEIPITKDIDGLLSQALLLGELGPAVELCLKEERFADAIILAQAGGTDLLKQ
TQERYLAKKKTKISSLLACVVQKNWKDVVCTCSLKNWREALALLLTYSGTEKFPELCDM
LGTRMEQEGSRALTSEARLCYVCSGSVERLVECWAKCHQALSPMALQDLMEKVMVLNR
SLEQLRGPHGVSPGPATTYRVTQYANLLAAQGSLATAMSFLPRDCAQPPVQQLRDRLFHA
QGSAVLGQQSPPFPFPRIVVGATLHSKETSSYRLGSQPSHQVPTPSPRPRVFTPQSSPAMPLA
PSHPSPYQGPRTQNISDYRAPGPQAIQPLPLSPGVRPASSQPQLLGGQRVQVPNPVGFPGT
WPLPGSPLPMACPGIMRPGSTSLPETPRLFPLLPLRPLGPGRMVSHTPAPPASFPVPYLPGD
PGAPCSSVLPTTGILTPHPGPQDSWKEAPAPRGNLQRNKLPETFMPPAPITAPVMSLTPELQ
GILPSQPPVSSVSHAPPGVPGELSLQLQHLPPEKMERKELPPEHQSLKSSFEALLQRCSLSA
TDLKTKRKLEEAAQRLEYLYEKLCEGTLSPHVVAGLHEVARCVDAGSFEQGLAVHAQVA
GCSSFSEVSSFMPILKAVLIIAHKLLV

>Q9BZD7 single-pass type I membrane protein
MAVFLEAKDAHSVLKRFPRANEFLEELRQGTIERECMEEICSYEEVKEVFENKEKTMEFW
KGYPNAVYSVRDPSQSSDAMYVVVPLLGVALLIVIALFIIWRCQLQKATRHHPSYAQNRYL
ASRAGHTLPRVMVYRGTVHSQGEPSGHREAANSPQVVLGPSRGGRTTVRLESTLYLPELS
LSRLSSTTPPPSYEEVTAPQESSSEEASVSYSDPPPKYEEIVAANPGADK

>Q8TCZ2 single-pass type I membrane protein
MVAWRSAFLVCLAFSLATLVQRGSGDFDDFNLEDAVKETSSVKQPWDHTTTTTTNRPGTT
RAPAKPPGSGLDLADALDDQDDGRRKPGIGGRERWNHVTTTTKRPVTTRAPANTLGNDF
DLADALDDRNDRDDGRRKPIAGGGGFSDKDLEDIVGGGEYKPDKGKGDGRYGSNDDPG
SGMVAEPGTIAGVASALAMALIGAVSSYISYQQKKFCFSIQQGLNADYVKGENLEAVVCE
EPQVKYSTLHTQSAEPPPPPEPARI

>Q13613 peripheral membrane protein
MDRPAAAAAAGCEGGGGPNPGPAGGRRPPRAAGGATAGSRQPSVETLDSPTGSHVEWCK
QLIAATISSQISGSVTSENVSRDYKALRDGNKLAQMEEAPLFPGESIKAIVKDVMYICPFM
GAVSGTLTVTDFKLYFKNVERDPHFILDVPLGVISRVEKIGAQSHGDNSCGIEIVCKDMRN
LRLAYKQEEQSKLGIFENLNKHAFPLSNGQALFAFSYKEKFPINGWKVYDPVSEYKRQGL
PNESWKISKINSNYEFCDTYPAIIVVPTSVKDDDLSKVAAFRAKGRVPVLSWIHPESQATIT
RCSQPLVGPNDKRCKEDEKYLQTIMDANAQSHKLIIFDARQNSVADTNKTKGGGYESESA
YPNAELVFLEIHNIHVMRESLRKLKEIVYPSIDEARWLSNVDGTHWLEYIRMLLAGAVRIA
DKIESGKTSVVVHCSDGWDRTAQLTSLAMLMLDSYYRTIKGFETLVEKEWISFGHRFALR
VGHGNDNHADADRSPIFLQFVDCVWQMTRQFPSAFEFNELFLITILDHLYSCLFGTFLCNC
EQQRFKEDVYTKTISLWSYINSQLDEFSNPFFVNYENHVLYPVASLSHLELWVNYYVRWN
PRMRPQMPIHQNLKELLAVRAELQKRVEGLQREVATRAVSSSSERGSSPSHSATSVHTSV

>Q13496 peripheral membrane protein
MASASTSKYNSHSLENESIKRTSRDGVNRDLTEAVPRLPGETLITDKEVIYICPFNGPIKGRV
YITNYRLYLRSLETDSSLILDVPLGVISRIEKMGGATSRGENSYGLDITCKDMRNLRFALKQ
EGHSRRDMFEILTRYAFPLAHSLPLFAFLNEEKFNVDGWTVYNPVEEYRRQGLPNHHWRI
TFINKCYELCDTYPALLVVPYRASDDDLRRVATFRSRNRIPVLSWIHPENKTVIVRCSQPLV
GMSGKRNKDDEKYLDVIRETNKQISKLTIYDARPSVNAVANKATGGGYESDDAYHNAELF
FLDIHNIHVMRESLKKVKDIVYPNVEESHWLSSLESTHWLEHIKLVLTGAIQVADKVSSGK
SSVLVHCSDGWDRTAQLTSLAMLMLDSFYRSIEGFEILVQKEWISFGHKFASRIGHGDKNH
TDADRSPIFLQFIDCVWQMSKQFPTAFEFNEQFLIIILDHLYSCRFGTFLFNCESARERQKVT
ERTVSLWSLINSNKEKFKNPFYTKEINRVLYPVASMRHLELWVNYYIRWNPRIKQQQPNPV
EQRYMELLALRDEYIKRLEELQLANSAKLSDPPTSPSSPSQMMPHVQTHF

>Q9NR82 multipass membrane protein
MPRHHAGGEEGGAAGLWVKSGAAAAAAGGGRLGSGMKDVESGRGRVLLNSAAARGD
GLLLLGTRAATLGGGGGGLRESRRGKQGARMSLLGKPLSYTSSQSCRRNVKYRRVQNYL
YNVLERPRGWAFIYHAFVFLLVFGCLILSVFSTIPEHTKLASSCLLILEFVMIVVFGLEFIIRI
WSAGCCCRYRGWQGRLRFARKPFCVIDTIVLIASIAVVSAKTQGNIFATSALRSLRFLQILR
MVRMDRRGGTWKLLGSVVYAHSKELITAWYIGFLVLIFSSFLVYLVEKDANKEFSTYADA
LWWGTITLTTIGYGDKTPLTWLGRLLSAGFALLGISFFALPAGILGSGFALKVQEQHRQKHF
EKRRNPAANLIQCVWRSYAADEKSVSIATWKPHLKALHTCSPTKKEQGEASSSQKLSFKE
RVRMASPRGQSIKSRQASVGDRRSPSTDITAEGSPTKVQKSWSFNDRTRFRPSLRLKSSQP
KPVIDADTALGTDDVYDEKGCQCDVSVEDLTPPLKTVIRAIRIMKFHVAKRKFKETLRPYD
VKDVIEQYSAGHLDMLCRIKSLQTRVDQILGKGQITSDKKSREKITAEHETTDDLSMLGRV
VKVEKQVQSIESKLDCLLDIYQQVLRKGSASALALASFQIPPFECEQTSDYQSPVDSKDLS
GSAQNSGCLSRSTSANISRGLQFILTPNEFSAQTFYALSPTMHSQATQVPISQSDGSAVAATN
TIANQINTAPKPAAPTTLQIPPPLPAIKHLPRPETLHPNPAGLQESISDVTTCLVASKENVQVA
QSNLTKDRSMRKSFDMGGETLLSVCPMVPKDLGKSLSVQNLIRSTEELNIQLSGSESSGSR
GSQDFYPKWRESKLFITDEEVGPEETETDTFDAAPQPAREAAFASDSLRTGRSRSSQSICKA
GESTDALSLPHVKLK

>Q92887 multipass membrane protein
MLEKFCNSTFWNSSFLDSPEADLPLCFEQTVLVWIPLGYLWLLAPWQLLHVYKSRTKRSS
TTKLYLAKQVFVGFLLILAAIELALVLTEDSGQATVPAVRYTNPSLYLGTWLLVLLIQYSRQ
WCVQKNSWFLSLFWILSILCGTFQFQTLIRTLLQGDNSNLAYSCLFFISYGFQILILIFSAFSE
NNESSNNPSSIASFLSSITYSWYDSIILKGYKRPLTLEDVWEVDEEMKTKTLVSKFETHMK
RELQKARRALQRRQEKSSQQNSGARLPGLNKNQSQSQDALVLEDVEKKKKKSGTKKDV
PKSWLMKALFKTFYMVLLKSFLLKLVNDIFTFVSPQLLKLLISFASDRDTYLWIGYLCAILL
FTAALIQSFCLQCYFQLCFKLGVKVRTAIMASVYKKALTLSNLARKEYTVGETVNLMSVD
AQKLMDVTNFMHMLWSSVLQIVLSIFFLWRELGPSVLAGVGVMVLVIPINAILSTKSKTIQ
VKNMKNKDKRLKIMNEILSGIKILKYFAWEPSFRDQVQNLRKKELKNLLAFSQLQCVVIF
VFQLTPVLVSVVTFSVYVLVDSNNILDAQKAFTSITLFNILRFPLSMLPMMISSMLQASVST
ERLEKYLGGDDLDTSAIRHDCNFDKAMQFSEASFTWEHDSEATVRDVNLDIMAGQLVAVI
GPVGSGKSSLISAMLGEMENVHGHITIKGTTAYVPQQSWIQNGTIKDNILFGTEFNEKRYQ
QVLEACALLPDLEMLPGGDLAEIGEKGINLSGGQKQRISLARATYQNLDIYLLDDPLSAVD
AHVGKHIFNKVLGPNGLLKGKTRLLVTHSMHFLPQVDEIVVLGNGTIVEKGSYSALLAKK
GEFAKNLKTFLRHTGPEEEATVHDGSEEEDDDYGLISSVEEIPEDAASITMRRENSFRRTLS
RSSRSNGRHLKSLRNSLKTRNVNSLKEDEELVKGQKLIKKEFIETGKVKFSIYLEYLQAIGL
FSIFFIILAFVMNSVAFIGSNLWLSAWTSDSKIFNSTDYPASQRDMRVGVYGALGLAQGIFV
FIAHFWSAFGFVHASNILHKQLLNNILRAPMRFFDTTPTGRIVNRFAGDISTVDDTLPQSLR
SWITCFLGIISTLVMICMATPVFTIIVIPLGIIYVSVQMFYVSTSRQLRRLDSVTRSPIYSHFSE
TVSGLPVIRAFEHQQRFLKHNEVRIDTNQKCVFSWITSNRWLAIRLELVGNLTVFFSALMM
VIYRDTLSGDTVGFVLSNALNITQTLNWLVRMTSEIETNIVAVERITEYTKVENEAPWVTD
KRPPPDWPSKGKIQFNNYQVRYRPELDLVLRGITCDIGSMEKIGVVGRTGAGKSSLTNCLF
RILEAAGGQIIIDGVDIASIGLHDLREKLTIIPQDPILFSGSLRMNLDPFNNYSDEEIWKALEL
AHLKSFVASLQLGLSHEVTEAGGNLSIGQRQLLCLGRALLRKSKILVLDEATAAVDLETDN
LIQTTIQNEFAHCTVITIAHRLHTIMDSDKVMVLDNGKIIECGSPEELLQIPGPFYFMAKEA
GIENVNSTKF

>Q8IYB5 peripheral membrane protein
MATRSCREKAQKLNEQHQLILSKLLREEDNKYCADCEAKGPRWASWNIGVFICIRCAGIH
RNLGVHISRVKSVNLDQWTAEQIQCMQDMGNTKARLLYEANLPENFRRPQTDQAVEFFIR
DKYEKKKYYDKNAIAITNISSSDAPLQPLVSSPSLQAAVDKNKLEKEKEKKKEEKKREKEP
EKPAKPLTAEKLQKKDQQLEPKKSTSPKKAAEPTVDLLGLDGPAVAPVTNGNTTVPPLND
DLDIFGPMISNPLPATVMPPAQGTPSAPAAATLSTVTSGDLDLFTEQTTKSEEVAKKQLSKD
SILSLYGTGTIQQQSTPGVFMGPTNIPFTSQAPAAFQGFPSMGVPVPAAPGLIGNVMGQSPS
MMVGMPMPNGFMGNAQTGVMPLPQNVVGPQGGMVGQMGAPQSKFGLPQAQQPQWS
LSQMNQQMAGMSISSATPTAGFGQPSSTTAGWSGSSSGQTLSTQLWK

>P32455 lipid-anchor
MASEIHMTGPMCLIENTNGRLMANPEALKILSAITQPMVVVAIVGLYRTGKSYLMNKLAG
KKKGFSLGSTVQSHTKGIWMWCVPHPKKPGHILVLLDTEGLGDVEKGDNQNDSWIFALA
VLLSSTFVYNSIGTINQQAMDQLYYVTELTHRIRSKSSPDENENEVEDSADFVSFFPDFVW
TLRDFSLDLEADGQPLTPDEYLTYSLKLKKGTSQKDETFNLPRLCIRKFFPKKKCFVFDRP
VHRRKLAQLEKLQDEELDPEFVQQVADFCSYIFSNSKTKTLSGGIQVNGPRLESLVLTYVN

AISSGDLPCMENAVLALAQIENSAAVQKAIAHYEQQMGQKVQLPTETLQELLDLHRDSER
EAIEVFIRSSFKDVDHLFQKELAAQLEKKRDDFCKQNQEASSDRCSALLQVIFSPLEEEVK
AGIYSKPGGYRLFVQKLQDLKKKYYEEPRKGIQAEEILQTYLKSKESMTDAILQTDQTLTE
KEKEIEVERVKAESAQASAKMLQEMQRKNEQMMEQKERSYQEHLKQLTEKMENDRVQL
LKEQERTLALKLQEQEQLLKEGFQKESRIMKNEIQDLQTKMRRRKACTIS

>Q96A46 multipass membrane protein
MELEGRGAGGVAGGPAAGPGRSPGESALLDGWLQRGVGRGAGGGEAGACRPPVRQDPD
SGPDYEALPAGATVTTHMVAGAVAGILEHCVMYPIDCVKTRMQSLQPDPAARYRNVLEAL
WRIIRTEGLWRPMRGLNVTATGAGPAHALYFACYEKLKKTLSDVIHPGGNSHIANGAAGC
VATLLHDAAMNPAEVVKQRMQMYNSPYHRVTDCVRAVWQNEGAGAFYRSYTTQLTMN
VPFQAIHFMTYEFLQEHFNPQRRYNPSSHVLSGACAGAVAAAATTPLDVCKTLLNTQESL
ALNSHITGHITGMASAFRTVYQVGGVTAYFRGVQARVIYQIPSTAIAWSVYEFFKYLITKR
QEEWRAGK

>P30989 multipass membrane protein
MRLNSSAPGTPGTPAADPFQRAQAGLEEALLAPGFGNASGNASERVLAAPSSELDVNTDI
YSKVLVTAVYLALFVVGTVGNTVTAFTLARKKSLQSLQSTVHYHLGSLALSDLLTLLLAM
PVELYNFIWVHHPWAFGDAGCRGYYFLRDACTYATALNVASLSVERYLAICHPFKAKTLM
SRSRTKKFISAIWLASALLAVPMLFTMGEQNRSADGQHAGGLVCTPTIHTATVKVVIQVNT
FMSFIFPMVVISVLNTIIANKLTVMVRQAAEQGQVCTVGGEHSTFSMAIEPGRVQALRHG
VRVLRAVVIAFVVCWLPYHVRRLMFCYISDEQWTPFLYDFYHYFYMVTNALFYVSSTINP
ILYNLVSANFRHIFLATLACLCPVWRRRRKRPAFSRKADSVSSNHTLSSNATRETLY

>Q9UQC9 single-pass type I membrane protein
MTQRSIAGPICNLKFVTLLVALSSELPFLGAGVQLQDNGYNGLLIAINPQVPENQNLISNIK
EMITEASFYLFNATKRRVFFRNIKILIPATWKANNNSKIKQESYEKANVIVTDWYGAHGDD
PYTLQYRGCGKEGKYIHFTPNFLLNDNLTAGYGSRGRVFVHEWAHLRWGVFDEYNNDKP
FYINGQNQIKVTRCSSDITGIFVCEKGPCPQENCIISKLFKEGCTFIYNSTQNATASIMFMQS
LSSVVEFCNASTHNQEAPNLQNQMCSLRSAWDVITDSADFHHSFPMNGTELPPPPTFSLV
QAGDKVVCLVLDVSSKMAEADRLLQLQQAAEFYLMQIVEIHTFVGIASFDSKGEIRAQLH
QINSNDDRKLLVSYLPTTVSAKTDISICSGLKKGFEVVEKLNGKAYGSVMILVTSGDDKLL
GNCLPTVLSSGSTIHSIALGSSAAPNLEELSRLTGGLKFFVPDISNSNSMIDAFSRISSGTGDI
FQQHIQLESTGENVKPHHQLKNTVTVDNTVGNDTMFLVTWQASGPPEIILFDPDGRKYYT
NNFITNLTFRTASLWIPGTAKPGHWTYTLNNTHHSLQALKVTVTSRASNSAVPPATVEAFV
ERDSLHFPHPVMIYANVKQGFYPILNATVTATVEPETGDPVTLRLLDDGAGADVIKNDGIY
SRYFFSFAANGRYSLKVHVNHSPSISTPAHSIPGSHAMYVPGYTANGNIQMNAPRKSVGRN
EEERKWGFSRVSSGGSFSVLGVPAGPHPDVFPPCKIIDLEAVKVEEELTLSWTAPGEDFDQ
GQATSYEIRMSKSLQNIQDDFNNAILVNTSKRNPQQAGIREIFTFSPQISTNGPEHQPNGET
HESHRIYVAIRAMDRNSLQSAVSNIAQAPLFIPPNSDPVPARDYLILKGVLTAMGLIGIICLII
VVTHHTLSRKKRADKKENGTKLL

>Q9NUN5 multipass membrane protein
MATSGAASAELVIGWCIFGLLLLAILAFCWIYVRKYQSRRESEVVSTITAIFSLAIALITSAL

LPVDIFLVSYMKNQNGTFKDWANANVSRQIEDTVLYGYYTLYSVILFCVFFWIPFVYFYYE
EKDDDDTSKCTQIKTALKYTLGFVVICALLLLVGAFVPLNVPNNKNSTEWEKVKSLFEEL
GSSHGLAALSFSISSLTLIGMLAAITYTAYGMSALPLNLIKGTRSAAYERLENTEDIEEVEQH
IQTIKSKSKDGRPLPARDKRALKQFEERLRTLKKRERHLEFIENSWWTKFCGALRPLKIVW
GIFFILVALLFVISLFLSNLDKALHSAGIDSGFIIFGANLSNPLNMLLPLLQTVFPLDYILITIII
MYFIFTSMAGIRNIGIWFFWIRLYKIRRGRTRPQALLFLCMILLLIVLHTSYMIYSLAPQYV
MYGSQNYLIETNITSDNHKGNSTLSVPKRCDADAPEDQCTVTRTYLFLHKFWFFSAAYYF
GNWAFLGVFLIGLIVSCCKGKKSVIEGVDEDSDISDDEPSVYSA

>Q9UBY5 multipass membrane protein
MNECHYDKHMDFFYNRSNTDTVDDWTGTKLVIVLCVGTFFCLFIFFSNSLVIAAVIKNRKF
HFPFYYLLANLAAADFFAGIAYVFLMFNTGPVSKTLTVNRWFLRQGLLDSSLTASLTNLLV
IAVERHMSIMRMRVHSNLTKKRVTLLILLVWAIAIFMGAVPTLGWNCLCNISACSSLAPIYS
RSYLVFWTVSNLMAFLIMVVVYLRIYVYVKRKTNVLSPHTSGSISRRRTPMKLMKTVMT
VLGAFVVCWTPGLVVLLLDGLNCRQCGVQHVKRWFLLLALLNSVVNPIIYSYKDEDMY
GTMKKMICCFSQENPERRPSRIPSTVLSRSDTGSQYIEDSISQGAVCNKSTS

>P29965 single-pass type II membrane protein
MIETYNQTSPRSAATGLPISMKIFMYLLTVFLITQMIGSALFAVYLHRRLDKIEDERNLHED
FVFMKTIQRCNTGERSLSLLNCEEIKSQFEGFVKDIMLNKEETKKENSFEMQKGDQNPQIA
AHVISEASSKTTSVLQWAEKGYYTMSNNLVTLENGKQLTVKRQGLYYIYAQVTFCSNREA
SSQAPFIASLCLKSPGRFERILLRAANTHSSAKPCGQQSIHLGGVFELQPGASVFVNVTDPS
QVSHGTGFTSFGLLKL

>P63218 lipid-anchor
MSGSSSVAAMKKVVQQLRLEAGLNRVKVSQAAADLKQFCLQNAQHDPLLTGVSSSTNPF
RPQKVCSFL

>P32247 multipass membrane protein
MAQRQPHSPNQTLISITNDTESSSSVVSNDNTNKGWSGDNSPGIEALCAIYITYAVIISVGIL
GNAILIKVFFKTKSMQTVPNIFITSLAFGDLLLLLTCVPVDATHYLAEGWLFGRIGCKVLSF
IRLTSVGVSVFTLTILSADRYKAVVKPLERQPSNAILKTCVKAGCVWIVSMIFALPEAIFSNV
YTFRDPNKNMTFESCTSYPVSKKLLQEIHSLLCFLVFYIIPLSIISVYYSLIARTLYKSTLNIPT
EEQSHARKQIESRKRIARTVLVLVALFALCWLPNHLLYLYHSFTSQTYVDPSAMHFIFTIFSR
VLAFSNSCVNPFALYWLSKSFQKHFKAQLFCCKAERPEPPVADTSLTTLAVMGTVPGTGSI
QMSEISVTSFTGCSVKQAEDRF

>Q8IZF6 multipass membrane protein
MKEHIIYQKLYGLILMSSFIFLSDTLSLKGKKLDFFGRGDTYVSLIDTIPELSRFTACIDLVF
MDDNSRYWMAFSYITNNALLGREDIDLGLAGDHQQLILYRLGKTFSIRHHLASFQWHTIC
LIWDGVKGKLELFLNKERILEVTDQPHNLTPHGTLFLGHFLKNESSEVKSMMRSFPGSLYY
FQLWDHILENEEFMKCLDGNIVSWEEDVWLVNKIIPTVDRTLRCFVPENMTIQEKSTTVSQ
QIDMTTPSQITGVKPQNTAHSSTLLSQSIPIFATDYTTISYSNTTSPPLETMTAQKILKTLVDE
TATFAVDVLSTSSAISLPTQSISIDNTTNSMKKTKSPSSESTKTTKMVEAMATEIFQPPTPSNF

LSTSRFTKNSVVSTTSAIKSQSAVTKTTSLFSTIESTSMSTTPCLKQKSTNTGALPISTAGQEF
IESTAAGTVPWFTVEKTSPASTHVGTASSFPPEPVLISTAAPVDSVFPRNQTAFPLATTDMKI
AFTVHSLTLPTRLIETTPAPRTAETELTSTNFQDVSLPRVEDAMSTSMSKETSSKTFSFLTSFS
FTGTESVQTVIDAEATRTALTPEITLASTVAETMLSSTITGRVYTQNTPTADGHLLTLMSTRS
ASTSKAPESGPTSTTDEAAHLFSSNETIWTSRPDQALLASMNTTTILTFVPNENFTSAFHEN
TTYTEYLSATTNITPLKASPEGKGTTANDATTARYTTAVSKLTSPWFANFSIVSGTTSITNMP
EFKLTTLLLKTIPMSTKPANELPLTPRETVVPSVDIISTLACIQPNFSTEESASETTQTEINGAI
VFGGTTTPVPKSATTQRLNATVTRKEATSHYLMRKSTIAAVAEVSPFSTMLEVTDESAQRV
TASVTVSSFPDIEKLSTPLDNKTATTEVRESWLLTKLVKTTPRSSYNEMTEMFNFNHTYVA
HWTSETSEGISAGSPTSGSTHIFGEPLGASTTRISETSFSTTPTDRTATSLSDGILPPQPTAAHS
SATPVPVTHMFSLPVNGSSVVAEETEVTMSEPSTLARAFSTSVLSDVSNLSSTTMTTALVPP
LDQTASTTIVIVPTHGDLIRTTSEATVISVRKTSMAVPSLTETPFHSLRLSTPVTAKAETTLFS
TSVDTVTPSTHTLVCSKPPPDNIPPASSTHVISTTSTPEATQPISQVEETSTYALSFPYTFSGG
GVVASLATGTTETSVVDETTPSHISANKLTTSVNSHISSSATYRVHTPVSIQLVTSTSVLSSD
KDQMTISLGKTPRTMEVTEMSPSKNSFISYSRGTPSLEMTDTGFPETTKISSHQTHSPSEIPL
GTPSDGNLASSPTSGSTQITPTLTSSNTVGVHIPEMSTSLGKTALPSQALTITTFLCPEKESTS
ALPAYTPRTVEMIVNSTYVTHSVSYGQDTSFVDTTTSSSTRISNPMDINTTFSHLHSLRTQP
EVTSVASFISESTQTFPESLSLSTAGLYNDGFTVLSDRITTAFSVPNVPTMLPRESSMATSTPI
YQMSSLPVNVTAFTSKKVSDTPPIVITKSSKTMHPGCLKSPCTATSGPMSEMSSIPVNNSAF
TPATVSSDTSTRVGLFSTLLSSVTPRTTMTMQTSTLDVTPVIYAGATSKNKMVSSAFTTEMI
EAPSRITPTTFLSPTEPTLPFVKTVPTTIMAGIVTPFVGTTAFSPLSSKSTGAISSIPKTTFSPFL
SATQQSSQADEATTLGILSGITNRSLSTVNSGTGVALTDTYSRITVPENMLSPTHADSLHTSF
NIQVSPSLTSFKSASGPTKNVKTTTNCFSSNTRKMTSLLEKTSLTNYATSLNTPVSYPPWTP
SSATLPSLTSFVYSPHSTEAEISTPKTSPPPTSQMVEFPVLGTRMTSSNTQPLLMTSWNIPTA
EGSQFPISTTINVPTSNEMETETLHLVPGPLSTFTASQTGLVSKDVMAMSSIPMSGILPNHGL
SENPSLSTSLRAITSTLADVKHTFEKMTTSVTPGTTLPSILSGATSGSVISKSPILTWLLSSLP
SGSPPATVSNAPHVMTSSTVEVSKSTFLTSDMISAHPFTNLTTLPSATMSTILTRTIPTPTLGG
ITTGFPTSLPMSINVTDDIVYISTHPEASSRTTITANPRTVSHPSSFSRKTMSPSTTDHTLSVG
AMPLPSSTITSSWNRIPTASSPSTLIIPKPTLDSLLNIMTTTSTVPGASFPLISTGVTYPFTATV
SSPISSFFETTWLDSTPSFLSTEASTSPTATKSTVSFYNVEMSFSVFVEEPRIPITSVINEFTEN
SLNSIFQNSEFSLATLETQIKSRDISEEEMVMDRAILEQREGQEMATISYVPYSCVCQVIIKA
SSSLASSELMRKIKSKIHGNFTHGNFTQDQLTLLVNCEHVAVKKLEPGNCKADETASKYKG
TYKWLLTNPTETAQTRCIKNEDGNATRFCSISINTGKSQWEKPKFKQCKLLQELPDKIVDL
ANITISDENAEDVAEHILNLINESPALGKEETKIIVSKISDISQCDEISMNLTHVMLQIINVVL
EKQNNSASDLHEISNEILRIIERTGHKMEFSGQIANLTVAGLALAVLRGDHTFDGMAFSIHS
YEEGTDPEIFLGNVPVGGILASIYLPKSLTERIPLSNLQTILFNFFGQTSLFKTKNVTKALTTY
VVSASISDDMFIQNLADPVVITLQHIGGNQNYGQVHCAFWDFENNNGLGGWNSSGCKVK
ETNVNYTICQCDHLTHFGVLMDLSRSTVDSVNEQILALITYTGCGISSIFLGVAVVTYIAFH
KLRKDYPAKILINLCTALLMLNLVFLINSWLSSFQKVGVCITAAVALHYFLLVSFTWMGLE
AVHMYLALVKVFNIYIPNYILKFCLVGWGIPAIMVAITVSVKKDLYGTLSPTTPFCWIKDDS
IFYISVVAYFCLIFLMNLSMFCTVLVQLNSVKSQIQKTRRKMILHDLKGTMSLTFLLGLTW
GFAFFAWGPMRNFFLYLFAIFNTLQGFFIFVFHCVMKESVREQWQIHLCCGWLRLDNSSD
GSSRCQIKVGYKQEGLKKIFEHKLLTPSLKSTATSSTFKSLGSAQGTPSEISFPNDDFDKDPY
CSSP

>Q92581 multipass membrane protein
MARRGWRRAPLRRGVGSSPRARRLMRPLWLLLAVGVFDWAGASDGGGGEARAMDEEI
VSEKQAEESHRQDSANLLIFILLLTLTILTIWLFKHRRARFLHETGLAMIYGLLVGLVLRYGI
HVPSDVNNVTLSCEVQSSPTTLLVTFDPEVFFNILLPPIIFYAGYSLKRRHFFRNLGSILAYAF
LGTAISCFVIGSIMYGCVTLMKVTGQLAGDFYFTDCLLFGAIVSATDPVTVLAIFHELQVD
VELYALLFGESVLNDAVAIVLSSSIVAYQPAGDNSHTFDVTAMFKSIGIFLGIFSGSFAMGAA
TGVVTALVTKFTKLREFQLLETGLFFLMSWSTFLLAEAWGFTGVVAVLFCGITQAHYTYN
NLSTESQHRTKQLFELLNFLAENFIFSYMGLTLFTFQNHVFNPTFVVGAFVAIFLGRAANIY
PLSLLLNLGRRSKIGSNFQHMMMFAGLRGAMAFALAIRDTATYARQMMFSTTLLIVFFTV
WVFGGGTTAMLSCLHIRVGVDSDQEHLGVPENERRTTKAESAWLFRMWYNFDHNYLKP
LLTHSGPPLTTTLPACCGPIARCLTSPQAYENQEQLKDDDSDLILNDGDISLTYGDSTVNTEP
ATSSAPRRFMGNSSEDALDRELAFGDHELVIRGTRLVLPMDDSEPPLNLLDNTRHGPA

>Q9HBW9 multipass membrane protein
MKRLPLLVVFSTLLNCSYTQNCTKTPCLPNAKCEIRNGIEACYCNMGFSGNGVTICEDDN
ECGNLTQSCGENANCTNTEGSYYCMCVPGFRSSSNQDRFITNDGTVCIENVNANCHLDN
VCIAANINKTLTKIRSIKEPVALLQEVYRNSVTDLSPTDIITYIEILAESSSLLGYKNNTISAK
DTLSNSTLTEFVKTVNNFVQRDTFVVWDKLSVNHRRTHLTKLMHTVEQATLRISQSFQKT
TEFDTNSTDIALKVFFFDSYNMKHIHPHMNMDGDYINIFPKRKAAYDSNGNVAVAFVYYK
SIGPLLSSSDNFLLKPQNYDNSEEEERVISSVISVSMSSNPPTLYELEKITFTLSHRKVTDRYR
SLCAFWNYSPDTMNGSWSSEGCELTYSNETHTSCRCNHLTHFAILMSSGPSIGIKDYNILTR
ITQLGIIISLICLAICIFTFWFFSEIQSTRTTIHKNLCCSLFLAELVFLVGINTNTNKLFCSIIAGL
LHYFFLAAFAWMCIEGIHLYLIVVGVIYNKGFLHKNFYIFGYLSPAVVVGFSAALGYRYYG
TTKVCWLSTENNFIWSFIGPACLIILVNLLAFGVIIYKVFRHTAGLKPEVSCFENIRSCARGA
LALLFLLGTTWIFGVLHVVHASVVTAYLFTVSNAFQGMFIFLFLCVLSRKIQEEYYRLFKN
VPCCFGCLR

>P43088 multipass membrane protein
MSMNNSKQLVSPAAALLSNTTCQTENRLSVFFSVIFMTVGILSNSLAIAILMKAYQRFRQK
SKASFLLLASGLVITDFFGHLINGAIAVFVYASDKEWIRFDQSNVLCSIFGICMVFSGLCPLL
LGSVMAIERCIGVTKPIFHSTKITSKHVKMMLSGVCLFAVFIALLPILGHRDYKIQASRTWC
FYNTEDIKDWEDRFYLLLFSFLGLLALGVSLLCNAITGITLLRVKFKSQQHRQGRSHHLEM
VIQLLAIMCVSCICWSPFLVTMANIGINGNHSLETCETTLFALRMATWNQILDPWVYILLR
KAVLKNLYKLASQCCGVHVISLHIWELSSIKNSLKVAAISESPVAEKSAST

>Q92643 single-pass type I membrane protein
MAVTDSLSRAATVLATVLLLSFGSVAASHIEDQAEQFFRSGHTNNWAVLVCTSRFWFNYR
HVANTLSVYRSVKRLGIPDSHIVLMLADDMACNPRNPKPATVFSHKNMELNVYGDDVEV
DYRSYEVTVENFLRVLTGRIPPSTPRSKRLLSDDRSNILIYMTGHGGNGFLKFQDSEEITNIE
LADAFEQMWQKRRYNELLFIIDTCQGASMYERFYSPNIMALASSQVGEDSLSHQPDPAIG
VHLMDRYTFYVLEFLEEINPASQTNMNDLFQVCPKSLCVSTPGHRTDLFQRDPKNVLITDF
FGSVRKVEITTETIKLQQDSEIMESSYKEDQMDEKLMEPLKYAEQLPVAQIIHQKPKLKDW
HPPGGFILGLWALIIMVFFKTYGIKHMKFIF

>P51654	GPI-anchor, lipid-anchor
MAGTVRTACLVVAMLLSLDFPGQAQPPPPPPDATCHQVRSFFQRLQPGLKWVPETPVPGS
DLQVCLPKGPTCCSRKMEEKYQLTARLNMEQLLQSASMELKFLIIQNAAVFQEAFEIVVR
HAKNYTNAMFKNNYPSLTPQAFEFVGEFFTDVSLYILGSDINVDDMVNELFDSLFPVIYTQ
LMNPGLPDSALDINECLRGARRDLKVFGNFPKLIMTQVSKSLQVTRIFLQALNLGIEVINT
TDHLKFSKDCGRMLTRMWYCSYCQGLMMVKPCGGYCNVVMQGCMAGVVEIDKYWRE
YILSLEELVNGMYRIYDMENVLLGLFSTIHDSIQYVQKNAGKLTTTIGKLCAHSQQRQYRS
AYYPEDLFIDKKVLKVAHVEHEETLSSRRRELIQKLKSFISFYSALPGYICSHSPVAENDTLC
WNGQELVERYSQKAARNGMKNQFNLHELKMKGPEPVVSQIIDKLKHINQLLRTMSMPK
GRVLDKNLDEEGFESGDCGDDEDECIGGSGDGMIKVKNQLRFLAELAYDLDVDDAPGNS
QQATPKDNEISTFHNLGNVHSPLKLLTSMAISVVCFFFLVH

>O75487	GPI-anchor, lipid-anchor
MARFGLPALLCTLAVLSAALLAAELKSKSCSEVRRLYVSKGFNKNDAPLHEINGDHLKICP
QGSTCCSQEMEEKYSLQSKDDFKSVVSEQCNHLQAVFASRYKKFDEFFKELLENAEKSLN
DMFVKTYGHLYMQNSELFKDLFVELKRYYVVGNVNLEEMLNDFWARLLERMFRLVNSQ
YHFTDEYLECVSKYTEQLKPFGDVPRKLKLQVTRAFVAARTFAQGLAVAGDVVSKVSVV
NPTAQCTHALLKMIYCSHCRGLVTVKPCYNYCSNIMRGCLANQGDLDFEWNNFIDAML
MVAERLEGPFNIESVMDPIDVKISDAIMNMQDNSVQVSQKVFQGCGPPKPLPAGRISRSISE
SAFSARFRPHHPEERPTTAAGTSLDRLVTDVKEKLKQAKKFWSSLPSNVCNDERMAAGN
GNEDDCWNGKGKSRYLFAVTGNGLANQGNNPEVQVDTSKPDILILRQIMALRVMTSKMK
NAYNGNDVDFFDISDESSGEGSGSGCEYQQCPSEFDYNATDHAGKSANEKADSAGVRPG
AQAYLLTVFCILFLVMQREWR

>O43614	multipass membrane protein
MSGTKLEDSPPCRNWSSASELNETQEPFLNPTDYDDEEFLRYLWREYLHPKEYEWVLIAG
YIIVFVVALIGNVLVCVAVWKNHHMRTVTNYFIVNLSLADVLVTITCLPATLVVDITETWFF
GQSLCKVIPYLQTVSVSVSVLTLSCIALDRWYAICHPLMFKSTAKRARNSIVIIWIVSCIIMIP
QAIVMECSTVFPGLANKTTLFTVCDERWGGEIYPKMYHICFFLVTYMAPLCLMVLAYLQI
FRKLWCRQIPGTSSVVQRKWKPLQPVSQPRGPGQPTKSRMSAVAAEIKQIRARRKTARML
MIVLLVFAICYLPISILNVLKRVFGMFAHTEDRETVYAWFTFSHWLVYANSAANPIIYNFLS
GKFREEFKAAFSCCCLGVHHRQEDRLTRGRTSTESRKSLTTQISNFDNISKLSEQVVLTSIST
LPAANGAGPLQNW

>Q9BTT6	peripheral membrane protein
MFHCIPLWRCNRHVESIDKRHCSLVYVPEEIYRYARSLEELLLDANQLRELPEQFFQLVKL
RKLGLSDNEIQRLPPEIANFMQLVELDVSRNEIPEIPESISFCKALQVADFSGNPLTRLPESFP
ELQNLTCLSVNDISLQSLPENIGNLYNLASLELRENLLTYLPDSLTQLRRLEELDLGNNEIY
NLPESIGALLHLKDLWLDGNQLSELPQEIGNLKNLLCLDVSENRLERLPEEISGLTSLTDLVI
SQNLLETIPDGIGKLKKLSILKVDQNRLTQLPEAVGECESLTELVLTENQLLTLPKSIGKLKK
LSNLNADRNKLVSLPKEIGGCCSLTVFCVRDNRLTRIPAEVSQATELHVLDVAGNRLLHLP
LSLTALKLKALWLSDNQSQPLLTFQTDTDYTTGEKILTCVLLPQLPSEPTCQENLPRCGALE
NLVNDVSDEAWNERAVNRVSAIRFVEDEKDEEDNETRTLLRRATPHPGELKHMKKTVEN

LRNDMNAAKGLDSNKNEVNHAIDRVTTSV

>O95661 lipid-anchor
MGNASFGSKEQKLLKRLRLLPALLILRAFKPHRKIRDYRVVVVGTAGVGKSTLLHKWASG
NFRHEYLPTIENTYCQLLGCSHGVLSLHITDSKSGDGNRALQRHVIARGHAFVLVYSVTK
KETLEELKAFYELICKIKGNNLHKFPIVLVGNKSDDTHREVALNDGATCAMEWNCAFMEI
SAKTDVNVQELFHMLLNYKKKPTTGLQEPEKKSQMPNTTEKLLDKCIIM

>Q9UBI6 lipid-anchor
MSSKTASTNNIAQARRTVQQLRLEASIERIKVSKASADLMSYCEEHARSDPLLIGIPTSENP
FKDKKTCIIL

>Q9BQI5 peripheral membrane protein
MMEGLKKRTRKAFGIRKKEKDTDSTGSPDRDGIQPSPHEPPYNSKAECAREGGKKVSKKS
NGAPNGFYAEIDWERYNSPELDEEGYSIRPEEPGSTKGKHFYSSSESEEEEESHKKFNIKIK
PLQSKDILKNAATVDELKASIGNIALSPSPVRKSPRRSPGAIKRNLSSEEVARPRRSTPTPELI
SKKPPDDTTALAPLFGPPLESAFDEQKTEVLLDQPEIWGSGQPINPSMESPKLTRPFPTGTPP
PLPPKNVPATPPRTGSPLTIGPGNDQSATEVKIEKLPSINDLDSIFGPVLSPKSVAVNAEEKW
VHFSDTSPEHVTPELTPREKVVSPPATPDNPADSPAPGPLGPPGPTGPPGPPGPPRNVLSPLN
LEEVQKKVAEQTFIKDDYLETISSPKDFGLGQRATPPPPPPPTYRTVVSSPGPGSGPGPGTTS
GASSPARPATPLVPCRSTTPPPPPPRPPSRPKLPPGKPGVGDVSRPFSPPIHSSSPPPIAPLARA
ESTSSISSTNSLSAATTPTVENEQPSLVWFDRGKFYLTFEGSSRGPSPLTMGAQDTLPVAAA
FTETVNAYFKGADPSKCIVKITGEMVLSFPAGITRHFANNPSPAALTFRVINFSRLEHVLPNP
QLLCCDNTQNDANTKEFWVNMPNLMTHLKKVSEQKPQATYYNVDMLKYQVSAQGIQS
TPLNLAVNWRCEPSSTDLRIDYKYNTDAMTTAVALNNVQFLVPIDGGVTKLQAVLPPAVW
NAEQQRILWKIPDISQKSENGGVGSLLARFQLSEGPSKPSPLVVQFTSEGSTLSGCDIELVG
AGYRFSLIKKRFAAGKYLADN

>Q01973 single-pass type I membrane protein
MHRPRRRGTRPPLLALLAALLLAARGAAAQETELSVSAELVPTSSWNISSELNKDSYLTLD
EPMNNITTSLGQTAELHCKVSGNPPPTIRWFKNDAPVVQEPRRLSFRSTIYGSRLRIRNLDT
TDTGYFQCVATNGKEVVSSTGVLFVKFGPPPTASPGYSDEYEEDGFCQPYRGIACARFIGN
RTVYMESLHMQGEIENQITAAFTMIGTSSHLSDKCSQFAIPSLCHYAFPYCDETSSVPKPRD
LCRDECEILENVLCQTEYIFARSNPMILMRLKLPNCEDLPQPESPEAANCIRIGIPMADPINK
NHKCYNSTGVDYRGTVSVTKSGRQCQPWNSQYPHTHTFTALRFPELNGGHSYCRNPGNQ
KEAPWCFTLDENFKSDLCDIPACDSKDSKEKNKMEILYILVPSVAIPLAIALLFFFICVCRNN
QKSSSAPVQRQPKHVRGQNVEMSMLNAYKPKSKAKELPLSAVRFMEELGECAFGKIYKG
HLYLPGMDHAQLVAIKTLKDYNNPQQWTEFQQEASLMAELHHPNIVCLLGAVTQEQPVC
MLFEYINQGDLHEFLIMRSPHSDVGCSSDEDGTVKSSLDHGDFLHIAIQIAAGMEYLSSHF
FVHKDLAARNILIGEQLHVKISDLGLSREIYSADYYRVQSKSLLPIRWMPPEAIMYGKFSSD
SDIWSFGVVLWEIFSFGLQPYYGFSNQEVIEMVRKRQLLPCSEDCPPRMYSLMTECWNEIP
SRRPRFKDIHVRLRSWEGLSSHTSSTTPSGGNATTQTTSLSASPVSNLSNPRYPNYMFPSQG
ITPQGQIAGFIGPPIPQNQRFIPINGYPIPPGYAAFPAAHYQPTGPPRVIQHCPPPKSRSPSSAS
GSTSTGHVTSLPSSGSNQEANIPLLPHMSIPNHPGGMGITVFGNKSQKPYKIDSKQASLLG

DANIHGHTESMISAEL

>O43895 GPI-anchor, lipid-anchor
MARAHWGCCPWLVLLCACAWGHTKPVDLGGQDVRNCSTNPPYLPVTVVNTTMSLTALR
QQMQTQNLSAYIIPGTDAHMNEYIGQHDERRAWITGFTGSAGTAVVTMKKAAVWTDSRY
WTQAERQMDCNWELHKEVGTTPIVTWLLTEIPAGGRVGFDPFLLSIDTWESYDLALQGSN
RQLVSITTNLVDLVWGSERPPVPNQPIYALQEAFTGSTWQEKVSGVRSQMQKHQKVPTAV
LLSALEETAWLFNLRASDIPYNPFFYSYTLLTDSSIRLFANKSRFSSETLSYLNSSCTGPMCV
QIEDYSQVRDSIQAYSLGDVRIWIGTSYTMYGIYEMIPKEKLVTDTYSPVMMTKAVKNSK
EQALLKASHVRDAVAVIRYLVWLEKNVPKGTVDEFSGAEIVDKFRGEEQFSSGPSFETISAS
GLNAALAHYSPTKELNRKLSSDEMYLLDSGGQYWDGTTDITRTVHWGTPSAFQKEAYTR
VLIGNIDLSRLIFPAATSGRMVEAFARRALWDAGLNYGHGTGHGIGNFLCVHEWPVGFQS
NNIAMAKGMFTSIEPGYYKDGEFGIRLEDVALVVEAKTKYPGSYLTFEVVSFVPYDRNLID
VSLLSPEHLQYLNRYYQTIREKVGPELQRRQLLEEFEWLQQHTEPLAARAPDTASWASVL
VVSTLAILGWSV

>Q96PE5 single-pass type I membrane protein
MSFSLNFTLPANTTSSPVTGGKETDCGPSLGLAAGIPLLVATALLVALLFTLIHRRRSSIEAM
EESDRPCEISEIDDNPKISENPRRSPTHEKNTMGAQEAHIYVKTVAGSEEPVHDRYRPTIEM
ERRRGLWWLVPRLSLE

>Q02094 multipass membrane protein
MRFTFPLMAIVLEIAMIVLFGLFVEYETDQTVLEQLNITKPTDMGIFFELYPLFQDVHVMIF
VGFGFLMTFLKKYGFSSVGINLLVAALGLQWGTIVQGILQSQGQKFNIGIKNMINADFSAA
TVLISFGAVLGKTSPTQMLIMTILEIVFFAHNEYLVSEIFKASDIGASMTIHAFGAYFGLAVA
GILYRSGLRKGHENEESAYYSDLFAMIGTLFLWMFWPSFNSAIAEPGDKQCRAIVNTYFSL
AACVLTAFAFSSLVEHRGKLNMVHIQNATLAGGVAVGTCADMAIHPFGSMIIGSIAGMVSV
LGYKFLTPLFTTKLRIHDTCGVHNLHGLPGVVGGLAGIVAVAMGASNTSMAMQAAALGS
SIGTAVVGGLMTGLILKLPLWGQPSDQNCYDDSVYWKVPKTR

>Q9BX74 multipass membrane protein
MAAAWPSGPSAPEAVTARLVGVLWFVSVTTGPWGAVATSAGGEESLKCEDLKVGQYICK
DPKINDATQEPVNCTNYTAHVSCFPAPNITCKDSSGNETHFTGNEVGFFKPISCRNVNGYS
YKVAVALSLFLGWLGADRFYLGYPALGLLKFCTVGFCGIGSLIDFILISMQIVGPSDGSSYII
DYYGTRLTRLSITNETFRKTQLYP

>P51589 peripheral membrane protein
MLAAMGSLAAALWAVVHPRTLLLGTVAFLLAADFLKRRRPKNYPPGPWRLPFLGNFFLV
DFEQSHLEVQLFVKKYGNLFSLELGDISAVLITGLPLIKEALIHMDQNFGNRPVTPMREHIF
KKNGLIMSSGQAWKEQRRFTLTALRNFGLGKKSLEERIQEEAQHLTEAIKEENGQPFDPHF
KINNAVSNIICSITFGERFEYQDSWFQQLLKLLDEVTYLEASKTCQLYNVFPWIMKFLPGPH
QTLFSNWKKLKLFVSHMIDKHRKDWNPAETRDFIDAYLKEMSKHTGNPTSSFHEENLICS
TLDLFFAGTETTSTTLRWALLYMALYPEIQEKVQAEIDRVIGQGQQPSTAARESMPYTNAVI
HEVQRMGNIIPLNVPREVTVDTTLAGYHLPKGTMILTNLTALHRDPTEWATPDTFNPDHFL

ENGQFKKREAFMPFSIGKRACLGEQLARTELFIFFTSLMQKFTFRPPNNEKLSLKFRMGITI
SPVSHRLCAVPQV

>P09758 single-pass type I membrane protein
MARGPGLAPPPLRLPLLLLVLAAVTGHTAAQDNCTCPTNKMTVCSPDGPGGRCQCRALG
SGMAVDCSTLTSKCLLLKARMSAPKNARTLVRPSEHALVDNDGLYDPDCDPEGRFKARQ
CNQTSVCWCVNSVGVRRTDKGDLSLRCDELVRTHHILIDLRHRPTAGAFNHSDLDAELRR
LFRERYRLHPKFVAAVHYEQPTIQIELRQNTSQKAAGDVDIGDAAYYFERDIKGESLFQGR
GGLDLRVRGEPLQVERTLIYYLDEIPPKFSMKRLTAGLIAVIVVVVVALVAGMAVLVITNRR
KSGKYKKVEIKELGELRKEPSL

>Q5T601 multipass membrane protein
MKVGVLWLISFFTFTDGHGGFLGKNDGIKTKKELIVNKKKHLGPVEEYQLLLQVTYRDS
KEKRDLRNFLKLLKPPLLWSHGLIRIIRAKATTDCNSLNGVLQCTCEDSYTWFPPSCLDPQ
NCYLHTAGALPSCECHLNNLSQSVNFCERTKIWGTFKINERFTNDLLNSSSAIYSKYANGIE
IQLKKAYERIQGFESVQVTQFRNGSIVAGYEVVGSSSASELLSAIEHVAEKAKTALHKLFPL
EDGSFRVFGKAQCNDIVFGFGSKDDEYTLPCSSGYRGNITAKCESSGWQVIRETCVLSLLE
ELNKNFSMIVGNATEAAVSSFVQNLSVIIRQNPSTTVGNLASVVSILSNISSLSLASHFRVSN
STMEDVISIADNILNSASVTNWTVLLREEKYASSRLLETLENISTLVPPTALPLNFSRKFIDW
KGIPVNKSQLKRGYSYQIKMCPQNTSIPIRGRVLIGSDQFQRSLPETIISMASLTLGNILPVS
KNGNAQVNGPVISTVIQNYSINEVFLFFSKIESNLSQPHCVFWDFSHLQWNDAGCHLVNE
TQDIVTCQCTHLTSFSILMSPFVPSTIFPVVKWITYVGLGISIGSLILCLIIEALFWKQIKKSQT
SHTRRICMVNIALSLLIADVWFIVGATVDTTVNPSGVCTAAVFFTHFFYLSLFFWMLMLGI
LLAYRIILVFHHMAQHLMMAVGFCLGYGCPLIISVITIAVTQPSNTYKRKDVCWLNWSNGS
KPLLAFVVPALAIVAVNFVVVLLVLTKLWRPTVGERLSRDDKATIIRVGKSLLILTPLLGLT
WGFGIGTIVDSQNLAWHVIFALLNAFQGFFILCFGILLDSKLRQLLFNKLSALSSWKQTEK
QNSSDLSAKPKFSKPFNPLQNKGHYAFSHTGDSSDNIMLTQFVSNE

>O95847 multipass membrane protein
MSVPEEEERLLPLTQRWPRASKFLLSGCAATVAELATFPLDLTKTRLQMQGEAALARLGD
GARESAPYRGMVRTALGIIEEEGFLKLWQGVTPAIYRHVVYSGGRMVTYEHLREVVFGKS
EDEHYPLWKSVIGGMMAGVIGQFLANPTDLVKVQMQMEGKRKLEGKPLRFRGVHHAFA
KILAEGGIRGLWAGWVPNIQRAALVNMGDLTTYDTVKHYLVLNTPLEDNIMTHGLSSLCS
GLVASILGTPADVIKSRIMNQPRDKQGRGLLYKSSTDCLIQAVQGEGFMSLYKGFLPSWLR
MTPWSMVFWLTYEKIREMSGVSPF

>Q9BTX1 multipass membrane protein
MATAVSRPCAGRSRDILWRVLGWRIVASIVWSVLFLPICTTVFIIFSRIDLFHPIQWLSDSFSD
LYSSYVIFYFLLLSVVIIIISIFNVEFYAVVPSIPCSRLALIGKIIHPQQLMHSFIHAAMGMVMA
WCAAVITQGQYSFLVVPCTGTNSFGSPAAQTCLNEYHLFFLLTGAFMGYSYSLLYFVNNM
NYLPFPIIQQYKFLRFRRSLLLLVKHSCVESLFLVRNFCILYYFLGYIPKAWISTAMNLHIDE
QVHRPLDTVSGLLNLSLLYHVWLCGVFLLTTWYVSWILFKIYATEAHVFPVQPPFAEGSDE
CLPKVLNSNPPPIIKYLALQDLMLLSQYSPSRRQEVFSLSQPGGHPHNWTAISRECLNLLN
GMTQKLILYQEAAATNGRVSSSYPVEPKKLNSPEETAFQTPKSSQMPRPSVPPLVKTSLFSS

KLSTPDVVSPFGTPFGSSVMNRMAGIFDVNTCYGSPQSPQLIRRGPRLWTSASDQQMTEFS
NPSPSTSISAEGKTMRQPSVIYSWIQNKREQIKNFLSKRVLIMYFFSKHPEASIQAVFSDAQ
MHIWALEGLSHLVAASFTEDRFGVVQTTLPAILNTLLTLQEAVDKYFKLPHASSKPPRISGS
LVDTSYKTLRFAFRASLKTAIYRITTTFGEHLNAVQASAEHQKRLQQFLEFKE

>P51160 lipid-anchor
MGEINQVAVEKYLEENPQFAKEYFDRKLRVEVLGEIFKNSQVPVQSSMSFSELTQVEESAL
CLELLWTVQEEGGTPEQGVHRALQRLAHLLQADRCSMFLCRSRNGIPEVASRLLDVTPTS
KFEDNLVGPDKEVVFPLDIGIVGWAAHTKKTHNVPDVKKNSHFSDFMDKQTGYVTKNLL
ATPIVVGKEVLAVIMAVNKVNASEFSKQDEEVFSKYLNFVSIILRLHHTSYMYNIESRRSQI
LMWSANKVFEELTDVERQFHKALYTVRSYLNCERYSIGLLDMTKEKEFYDEWPIKLGEV
EPYKGPKTPDGREVNFYKIIDYILHGKEEIKVIPTPPADHWTLISGLPTYVAENGFICNMMN
APADEYFTFQKGPVDETGWVIKNVLSLPIVNKKEDIVGVATFYNRKDGKPFDEHDEYITET
LTQFLGWSLLNTDTYDKMNKLENRKDIAQEMLMNQTKATPEEIKSILKFQEKLNVDVIDD
CEEKQLVAILKEDLPDPRSAELYEFRFSDFPLTEHGLIKCGIRLFFEINVVEKFKVPVEVLTR
WMYTVRKGYRAVTYHNWRHGFNVGQTMFTLLMTGRLKKYYTDLEAFAMLAAAFCHDI
DHRGTNNLYQMKSTSPLARLHGSSILERHHLEYSKTLLQDESLNIFQNLNKRQFETVIHLF
EVAIIATDLALYFKKRTMFQKIVDACEQMQTEEEAIKYVTVDPTKKEIIMAMMMTACDLS
AITKPWEVQSQVALMVANEFWEQGDLERTVLQQQPIPMMDRNKRDELPKLQVGFIDFVC
TFVYKEFSRFHKEITPMLSGLQNNRVEWKSLADEYDAKMKVIEEEAKKQEGGAEKAAED
SGGGDDKKSKTCLML

>Q6X4W1 peripheral membrane protein
MGAAASRRRALRSEAMSSVAAKVRAARAFGEYLSQSHPENRNGADHLLADAYSGHDGS
PEMQPAPQNKRRLSLVSNGCYEGSLSEEPSIRKPAGEGPQPRVYTISGEPALLPSPEAEAIEL
AVVKGRRQRHPHHHSQPLRASPGGSREDVSRPCQSWAGSRQGSKECPGCAQLAPGPTPR
AFGLDQPPLPETSGRRKKLERMYSVDRVSDDIPIRTWFPKENLFSFQTATTTMQAISVFRGY
AERKRRKRENDSASVIQRNFRKHLRMVGSRRVKAQTFAERRERSFSRSWSDPTPMKADT
SHDSRDSSDLQSSHCTLDEAFEDLDWDTEKGLEAVACDTEGFVPPKVMLISSKVPKAEYIP
TIIRRDDPSIIPILYDHEHATFEDILEEIERKLNVYHKGAKIWKMLIFCQGGPGHLYLLKNKV
ATFAKVEKEEDMIHFWKRLSRLMSKVNPEPNVIHIMGCYILGNPNGEKLFQNLRTLMTPY
RVTFESPLELSAQGKQMIETYFDFRLYRLWKSRQHSKLLDFDDVL

>P23786 peripheral membrane protein
MVPRLLLRAWPRGPAVGPGAPSRPLSAGSGPGQYLQRSIVPTMHYQDSLPRLPIPKLEDTI
RRYLSAQKPLLNDGQFRKTEQFCKSFENGIGKELHEQLVALDKQNKHTSYISGPWFDMYL
SARDSVVLNFNPFMAFNPDPKSEYNDQLTRATNMTVSAIRFLKTLRAGLLEPEVFHLNPA
KSDTITFKRLIRFVPSSLSWYGAYLVNAYPLDMSQYFRLFNSTRLPKPSRDELFTDDKARH
LLVLRKGNFYIFDVLDQDGNIVSPSEIQAHLKYILSDSSPAPEFPLAYLTSENRDIWAELRQK
LMSSGNEESLRKVDSAVFCLCLDDFPIKDLVHLSHNMLHGDGTNRWFDKSFNLIIAKDGST
AVHFEHSWGDGVAVLRFFNEVFKDSTQTPAVTPQSQPATTDSTVTVQKLNFELTDALKTGI
TAAKEKFDATMKTLTIDCVQFQRGGKEFLKKQKLSPDAVAQLAFQMAFLRQYGQTVATY
ESCSTAAFKHGRTETIRPASVYTKRCSEAFVREPSRHSAGELQQMMVECSKYHGQLTKEA
AMGQGFDRHLFALRHLAAAKGIILPELYLDPAYGQINHNVLSTSTLSSPAVNLGGFAPVVS

DGFGVGYAVHDNWIGCNVSSYPGRNAREFLQCVEKALEDMFDALEGKSIKS

>O00341 multipass membrane protein
MVPHAILARGRDVCRRNGLLILSVLSVIVGCLLGFFLRTRRLSPQEISYFQFPGELLMRML
KMMILPLVVSSLMSGLASLDAKTSSRLGVLTVAYYLWTTFMAVIVGIFMVSIIHPGSAAQK
ETTEQSGKPIMSSADALLDLIRNMFPANLVEATFKQYRTKTTPVVKSPKVAPEEAPPRRILI
YGVQEENGSHVQNFALDLTPPPEVVYKSEPGTSDGMNVLGIVFFSATMGIMLGRMGDSG
APLVSFCQCLNESVMKIVAVAVWYFPFGIVFLIAGKILEMDDPRAVGKKLGFYSVTVVCGL
VLHGLFILPLLYFFITKKNPIVFIRGILQALLIALATSSSSATLPITFKCLLENNHIDRRIARFVL
PVGATINMDGTALYEAVAAIFIAQVNNYELDFGQIITISITATAASIGAAGIPQAGLVTMVIVL
TSVGLPTDDITLIIAVDWALDRFRTMINVLGDALAAGIMAHICRKDFARDTGTEKLLPCET
KPVSLQEIVAAQQNGCVKSVAEASELTLGPTCPHHVPVQVEQDEELPAASLNHCTIQISELE
TNV

>Q2I0M4 single-pass type I membrane protein
MRGPSWSRPRPLLLLLLLLSPWPVWAQVSATASPSGSLGAPDCPEVCTCVPGGLASCSALS
LPAVPPGLSLRLRALLLDHNRVRALPPGAFAGAGALQRLDLRENGLHSVHVRAFWGLGA
LQLLDLSANQLEALAPGTFAPLRALRNLSLAGNRLARLEPAALGALPLLRSLSLQDNELA
ALAPGLLGRLPALDALHLRGNPWGCGCALRPLCAWLRRHPLPASEAETVLCVWPGRLTL
SPLTAFSDAAFSHCAQPLALRDLAVVYTLGPASFLVSLASCLALGSGLTACRARRRRLRTA
ALRPPRPPDPNPDPDPHGCASPADPGSPAAAAQA

>Q9UIX4 multipass membrane protein
MTLLPGDNSDYDYSALSCTSDASFHPAFLPQRQAIKGAFYRRAQRLRPQDEPRQGCQPED
RRRRIIINVGGIKYSLPWTTLDEFPLTRLGQLKACTNFDDILNVCDDYDVTCNEFFFDRNPG
AFGTILTFLRAGKLRLLREMCALSFQEELLYWGIAEDHLDGCCKRRYLQKIEEFAEMVERE
EEDDALDSEGRDSEGPAEGEGRLGRCMRRLRDMVERPHSGLPGKVFACLSVLFVTVTAV
NLSVSTLPSLREEEEQGHCSQMCHNVFIVESVCVGWFSLEFLLRLIQAPSKFAFLRSPLTLI
DLVAILPYYITLLVDGAAAGRRKPGAGNSYLDKVGLVLRVLRALRILYVMRLARHSLGLQ
TLGLTARRCTREFGLLLLFLCVAIALFAPLLYVIENEMADSPEFTSIPACYWWAVITMTTVG
YGDMVPRSTPGQVVALSSILSGILLMAFPVTSIFHTFSRSYLELKQEQERVMFRRAQFLIKT
KSQLSVSQDSDILFGSASSDTRDNN

>Q9UKM7 single-pass type II membrane protein
MAACEGRRSGALGSSQSDFLTPPVGGAPWAVATTVVMYPPPPPPPHRDFISVTLSFGENYD
NSKSWRRRSCWRKWKQLSRLQRNMILFLLAFLLFCGLLFYINLADHWKALAFRLEEEQK
MRPEIAGLKPANPPVLPAPQKADTDPENLPEISSQKTQRHIQRGPPHLQIRPPSQDLKDGTQ
EEATKRQEAPVDPRPEGDPQRTVISWRGAVIEPEQGTELPSRRAEVPTKPPLPPARTQGTPV
HLNYRQKGVIDVFLHAWKGYRKFAWGHDELKPVSRSFSEWFGLGLTLIDALDTMWILGL
RKEFEEARKWVSKKLHFEKDVDVNLFESTIRILGGLLSAYHLSGDSLFLRKAEDFGNRLM
PAFRTPSKIPYSDVNIGTGVAHPPRWTSDSTVAEVTSIQLEFRELSRLTGDKKFQEAVEKVT
QHIHGLSGKKDGLVPMFINTHSGLFTHLGVFTLGARADSYYEYLLKQWIQGGKQETQLLE
DYVEAIEGVRTHLLRHSEPSKLTFVGELAHGRFSAKMDHLVCFLPGTLALGVYHGLPASH
MELAQELMETCYQMNRQMETGLSPEIVHFNLYPQPGRRDVEVKPADRHNLLRPETVESL

FYLYRVTGDRKYQDWGWEILQSFSRFTRVPSGGYSSINNVQDPQKPEPRDKMESFFLGET
LKYLFLLFSDDPNLLSLDAYVFNTEAHPLPIWTPA

>P18031 peripheral membrane protein
MEMEKEFEQIDKSGSWAAIYQDIRHEASDFPCRVAKLPKNKNRNRYRDVSPFDHSRIKLH
QEDNDYINASLIKMEEAQRSYILTQGPLPNTCGHFWEMVWEQKSRGVVMLNRVMEKGSL
KCAQYWPQKEEKEMIFEDTNLKLTLISEDIKSYYTVRQLELENLTTQETREILHFHYTTWP
DFGVPESPASFLNFLFKVRESGSLSPEHGPVVVHCSAGIGRSGTFCLADTCLLLMDKRKDP
SSVDIKKVLLEMRKFRMGLIQTADQLRFSYLAVIEGAKFIMGDSSVQDQWKELSHEDLEP
PPEHIPPPPRPPKRILEPHNGKCREFFPNHQWVKEETQEDKDCPIKEEKGSPLNAAPYGIES
MSQDTEVRSRVVGGSLRGAQAASPAKGEPSLPEKDEDHALSYWKPFLVNMCVATVLTAG
AYLCYRFLFNSNT

>A5PLL7 multipass membrane protein
MAGAENWPGQQLELDEDEASCCRWGAQHAGARELAALYSPGKRLQEWCSVILCFSLIAH
NLVHLLLLARWEDTPLVILGVVAGALIADFLSGLVHWGADTWGSVELPIVGKAFIRPFREH
HIDPTAITRHDFIETNGDNCLVTLLPLLNMAYKFRTHSPEALEQLYPWECFVFCLIIFGTFTN
QIHKWSHTYFGLPRWVTLLQDWHVILPRKHHRIHHVSPHETYFCITTGWLNYPLEKIGFW
RRLEDLIQGLTGEKPRADDMKWAQKIK

>P78552 single-pass type I membrane protein
MEWPARLCGLWALLLCAGGGGGGGGAAPTETQPPVTNLSVSVENLCTVIWTWNPPEGAS
SNCSLWYFSHFGDKQDKKIAPETRRSIEVPLNERICLQVGSQCSTNESEKPSILVEKCISPPE
GDPESAVTELQCIWHNLSYMKCSWLPGRNTSPDTNYTLYYWHRSLEKIHQCENIFREGQY
FGCSFDLTKVKDSSFEQHSVQIMVKDNAGKIKPSFNIVPLTSRVKPDPPHIKNLSFHNDDLY
VQWENPQNFISRCLFYEVEVNNSQTETHNVFYVQEAKCENPEFERNVENTSCFMVPGVLP
DTLNTVRIRVKTNKLCYEDDKLWSNWSQEMSIGKKRNSTLYITMLLIVPVIVAGAIIVLLLY
LKRLKIIIFPPIPDPGKIFKEMFGDQNDDTLHWKKYDIYEKQTKEETDSVVLIENLKKASQ

>Q99808 multipass membrane protein
MTTSHQPQDRYKAVWLIFFMLGLGTLLPWNFFMTATQYFTNRLDMSQNVSLVTAELSKD
AQASAAPAAPLPERNSLSAIFNNVMTLCAMLPLLLFTYLNSFLHQRIPQSVRILGSLVAILLV
FLITAILVKVQLDALPFFVITMIKIVLINSFGAILQGSLFGLAGLLPASYTAPIMSGQGLAGFF
ASVAMICAIASGSELSESAFGYFITACAVIILTIICYLGLPRLEFYRYYQQLKLEGPGEQETKL
DLISKGEEPRAGKEESGVSVSNSQPTNESHSIKAILKNISVLAFSVCFIFTITIGMFPAVTVEV
KSSIAGSSTWERYFIPVSCFLTFNIFDWLGRSLTAVFMWPGKDSRWLPSLVLARLVFVPLLL
LCNIKPRRYLTVVFEHDAWFIFFMAAFAFSNGYLASLCMCFGPKKVKPAEAETAGAIMAFF
LCLGLALGAVFSFLFRAIV

>O43286 single-pass type II membrane protein
MRARRGLLRLPRRSLLAALFFFSLSSSLLYFVYVAPGIVNTYLFMMQAQGILIRDNVRTIGA
QVYEQVLRSAYAKRNSSVNDSDYPLDLNHSETFLQTTTFLPEDFTYFANHTCPERLPSMK
GPIDINMSEIGMDYIHELFSKDPTIKLGGHWKPSDCMPRWKVAILIPFRNRHEHLPVLFRHL
LPMLQRQRLQFAFYVVEQVGTQPFNRAMLFNVGFQEAMKDLDWDCLIFHDVDHIPESDR

NYYGCGQMPRHFATKLDKYMYLLPYTEFFGGVSGLTVEQFRKINGFPNAFWGWGGEDD
DLWNRVQNAGYSVSRPEGDTGKYKSIPHHHRGEVQFLGRYALLRKSKERQGLDGLNNLN
YFANITYDALYKNITVNLTPELAQVNEY

>Q9NRR6 peripheral membrane protein
MPSKAENLRPSEPAPQPPEGRTLQGQLPGAPPAQRAGSPPDAPGSESPALACSTPATPSGED
PPARAAPIAPRPPARPRLERALSLDDKGWRRRRFRGSQEDLEARNGTSPSRGSVQSEGPGA
PAHSCSPPCLSTSLQEIPKSRGVLSSERGSPSSGGNPLSGVASSSPNLPHRDAAVAGSSPRLPS
LLPPRPPPALSLDIASDSLRTANKVDSDLADYKLRAQPLLVRAHSSLGPGRPRSPLACDDCS
LRSAKSSFSLLAPIRSKDVRSRSYLEGSLLASGALLGADELARYFPDRNVALFVATWNMQG
QKELPPSLDEFLLPAEADYAQDLYVIGVQEGCSDRREWETRLQETLGPHYVLLSSAAHGV
LYMSLFIRRDLIWFCSEVECSTVTTRIVSQIKTKGALGISFTFFGTSFLFITSHFTSGDGKVAE
RLLDYTRTVQALVLPRNVPDTNPYRSSAADVTTRFDEVFWFGDFNFRLSGGRTVVDALLC
QGLVVDVPALLQHDQLIREMRKGSIFKGFQEPDIHFLPSYKFDIGKDTYDSTSKQRTPSYT
DRVLYRSRHKGDICPVSYSSCPGIKTSDHRPVYGLFRVKVRPGRDNIPLAAGKFDRELYLL
GIKRRISKEIQRQQALQSQNSSTICSVS

>P42566 peripheral membrane protein
MAAAAQLSLTQLSSGNPVYEKYYRQVDTGNTGRVLASDAAAFLKKSGLPDLILGKIWDL
ADTDGKGILNKQEFFVALRLVACAQNGLEVSLSSLNLAVPPPRFHDTSSPLLISGTSAAELP
WAVKPEDKAKYDAIFDSLSPVNGFLSGDKVKPVLLNSKLPVDILGRVWELSDIDHDGMLD
RDEFAVAMFLVYCALEKEPVPMSLPPALVPPSKRKTWVVSPAEKAKYDEIFLKTDKDMDG
FVSGLEVREIFLKTGLPSTLLAHIWSLCDTKDCGKLSKDQFALAFHLISQKLIKGIDPPHVL
TPEMIPPSDRASLQKNIIGSSPVADFSAIKELDTLNNEIVDLQREKNNVEQDLKEKEDTIKQ
RTSEVQDLQDEVQRENTNLQKLQAQKQQVQELLDELDEQKAQLEEQLKEVRKKCAEEA
QLISSLKAELTSQESQISTYEEELAKAREELSRLQQETAELEESVESGKAQLEPLQQHLQDS
QQEISSMQMKLMEMKDLENHNSQLNWCSSPHSILVNGATDYCSLSTSSSETANLNEHVEG
QSNLESEPIHQESPARSSPELLPSGVTDENEVTTAVTEKVCSELDNNRHSKEEDPFNVDSSS
LTGPVADTNLDFFQSDPFVGSDPFKDDPFGKIDPFGGDPFKGSDPFASDCFFRQSTDPFATSS
TDPFSAANNSSITSVETLKHNDPFAPGGTVVAASDSATDPFASVFGNESFGGGFADFSTLSK
VNNEDPFRSATSSSVSNVVITKNVFEETSVKSEDEPPALPPKIGTPTRPCPLPPGKRSINKLD
SPDPFKLNDPFQPFPGNDSPKEKDPEIFCDPFTSATTTTNKEADPSNFANFSAYPSEEDMIEW
AKRESEREEEQRLARLNQQEQEDLELAIALSKSEISEA

>Q14721 multipass membrane protein
MPAGMTKHGSRSTSSLPPEPMEIVRSKACSRRVRLNVGGLAHEVLWRTLDRLPRTRLGKL
RDCNTHDSLLEVCDDYSLDDNEYFFDRHPGAFTSILNFYRTGRLHMMEEMCALSFSQELD
YWGIDEIYLESCCQARYHQKKEQMNEELKREAETLREREGEEFDNTCCAEKRKKLWDLL
EKPNSSVAAKILAIISIMFIVLSTIALSLNTLPELQSLDEFGQSTDNPQLAHVEAVCIAWFTM
EYLLRFLSSPKKWKFFKGPLNAIDLLAILPYYVTIFLTESNKSVLQFQNVRRVVQIFRIMRIL
RILKLARHSTGLQSLGFTLRRSYNELGLLILFLAMGIMIFSSLVFFAEKDEDDTKFKSIPASF
WWATITMTTVGYGDIYPKTLLGKIVGGLCCIAGVLVIALPIPIIVNNFSEFYKEQKRQEKAI
KRREALERAKRNGSIVSMNMKDAFARSIEMMDIVVEKNGENMGKKDKVQDNHLSPNK
WKWTKRTLSETSSSKSFETKEQGSPEKARSSSSPQHLNVQQLEDMYNKMAKTQSQPILNT

KESAAQSKPKEELEMESIPSPVAPLPTRTEGVIDMRSMSSIDSFISCATDFPEATRFSHSPLTS
LPSKTGGSTAPEVGWRGALGASGGRFVEANPSPDASQHSSFFIESPKSSMKTNNPLKLRAL
KVNFMEGDPSPLLPVLGMYHDPLRNRGSAAAAVAGLECATLLDKAVLSPESSIYTTASAK
TPPRSPEKHTAIAFNFEAGVHQYIDADTDDEGQLLYSVDSSPPKSLPGSTSPKFSTGTRSEK
NHFESSPLPTSPKFLRQNCIYSTEALTGKGPSGQEKCKLENHISPDVRVLPGGGAHGSTRD
QSI

>Q5H943 single-pass type II membrane protein
MNFYLLLASSILCALIVFWKYRRFQRNTGEMSSNSTALALVRPSSSGLINSNTDNNLAVYD
LSRDILNNFPHSIARQKRILVNLSMVENKLVELEHTLLSKGFRGASPHRKST

>Q9UN76 multipass membrane protein
MDKLKCPSFFKCREKEKVSASSENFHVGENDENQDRGNWSKKSDYLLSMIGYAVGLGNV
WRFPYLTYSNGGGAFLIPYAIMLALAGLPLFFLECSLGQFASLGPVSVWRILPLFQGVGITM
VLISIFVTIYYNVIIAYSLYYMFASFQSELPWKNCSSWSDKNCSRSPIVTHCNVSTVNKGIQE
IIQMNKSWVDINNFTCINGSEIYQPGQLPSEQYWNKVALQRSSGMNETGVIVWYLALCLL
LAWLIVGAALFKGIKSSGKVVYFTALFPYVVLLILLVRGATLEGASKGISYYIGAQSNFTKL
KEAEVWKDAATQIFYSLSVAWGGLVALSSYNKFKNNCFSDAIVVCLTNCLTSVFAGFAIFSI
LGHMAHISGKEVSQVVKSGFDLAFIAYPEALAQLPGGPFWSILFFFMLLTLGLDSQFASIET
ITTTIQDLFPKVMKKMRVPITLGCCLVLFLLGLVCVTQAGIYWVHLIDHFCAGWGILIAAIL
ELVGIIWIYGGNRFIEDTEMMIGAKRWIFWLWWRACWFVITPILLIAIFIWSLVQFHRPNYG
AIPYPDWGVALGWCMIVFCIIWIPIMAIIKIIQAKGNIFQRLISCCRPASNWGPYLEQHRGER
YKDMVDPKKEADHEIPTVSGSRKPE

>P50052 multipass membrane protein
MKGNSTLATTSKNITSGLHFGLVNISGNNESTLNCSQKPSDKHLDAIPILYYIIFVIGFLVNIV
VVTLFCCQKGPKKVSSIYIFNLAVADLLLLATLPLWATYYSYRYDWLFGPVMCKVFGSFLT
LNMFASIFFITCMSVDRYQSVIYPFLSQRRNPWQASYIVPLVWCMACLSSLPTFYFRDVRTI
EYLGVNACIMAFPPEKYAQWSAGIALMKNILGFIIPLIFIATCYFGIRKHLLKTNSYGKNRIT
RDQVLKMAAAVVLAFIICWLPFHVLTFLDALAWMGVINSCEVIAVIDLALPFAILLGFTNSC
VNPFLYCFVGNRFQQKLRSVFRVPITWLQGKRESMSCRKSSSLREMETFVS

>P13584 peripheral membrane protein
MVPSFLSLSFSSLGLWASGLILVLGFLKLIHLLLRRQTLAKAMDKFPGPPTHWLFGHALEIQ
ETGSLDKVVSWAHQFPYAHPLWFGQFIGFLNIYEPDYAKAVYSRGDPKAPDVYDFFLQWI
GRGLLVLEGPKWLQHRKLLTPGFHYDVLKPYVAVFTESTRIMLDKWEEKAREGKSFDIFC
DVGHMALNTLMKCTFGRGDTGLGHRDSSYYLAVSDLTLLMQQRLVSFQYHNDFIYWLTP
HGRRFLRACQVAHDHTDQVIRERKAALQDEKVRKKIQNRRHLDFLDILLGARDEDDIKLS
DADLRAEVDTFMFEGHDTTTSGISWFLYCMALYPEHQHRCREEVREILGDQDFFQWDDL
GKMTYLTMCIKESFRLYPPVPQVYRQLSKPVTFVDGRSLPAGSLISMHIYALHRNSAVWPD
PEVFDSLRFSTENASKRHPFAFMPFSAGPRNCIGQQFAMSEMKVVTAMCLLRFEFSLDPSR
LPIKMPQLVLRSKNGFHLHLKPLGPGSGK

>O15260 multipass membrane protein

MGQNDLMGTAEDFADQFLRVTKQYLPHVARLCLISTFLEDGIRMWFQWSEQRDYIDTTW
NCGYLLASSFVFLNLLGQLTGCVLVLSRNFVQYACFGLFGIIALQTIAYSILWDLKFLMRNL
ALGGGLLLLLAESRSEGKSMFAGVPTMRESSPKQYMQLGGRVLLVLMFMTLLHFDASFFS
IVQNIVGTALMILVAIGFKTKLAALTLVVWLFAINVYFNAFWTIPVYKPMHDFLKYDFFQT
MSVIGGLLLVVALGPGGVSMDEKKKEW

>Q9UHM6 multipass membrane protein
MNPPSGPRVPPSPTQEPSCMATPAPPSWWDSSQSSISSLGRLPSISPTAPGTWAAAWVPLPT
VDVPDHAHYTLGTVILLVGLTGMLGNLTVIYTFCRSRSLRTPANMFIINLAVSDFLMSFTQA
PVFFTSSLYKQWLFGETGCEFYAFCGALFGISSMITLTAIALDRYLVITRPLATFGVASKRRA
AFVLLGVWLYALAWSLPPFFGWSAYVPEGLLTSCSWDYMSFTPAVRAYTMLLCCFVFFLP
LLIIIYCYIFIFRAIRETGRALQTFGACKGNGESLWQRQRLQSECKMAKIMLLVILLFVLSW
APYSAVALVAFAGYAHVLTPYMSSVPAVIAKASAIHNPIIYAITHPKYRVAIAQHLPCLGVLL
GVSRRHSRPYPSYRSTHRSTLTSHTSNLSWISIRRRQESLGSESEVGWTHMEAAAVWGAA
QQANGRSLYGQGLEDLEAKAPPRPQGHEAETPGKTKGLIPSQDPRM

>Q9UJ90 single-pass type I membrane protein
MNCSESQRLRTLLSRLLLELHHRGNASGLGAGPRPSMGMGVVPDPFVGREVTSAKGDDA
YLYILLIMIFYACLAGGLILAYTRSRKLVEAKDEPSQACAEHEWAPGGALTADAEAAAGSQ
AEGRRQLASEGLPALAQGAERV

>O14654 peripheral membrane protein
MASCSFTRDQATRRLRGAAAAAAAALAAVVTTPLLSSGTPTALIGTGSSCPGAMWLSTAT
GSRSDSESEEEDLPVGEEVCKRGYLRKQKHGHRRYFVLKLETADAPARLEYYENARKFR
HSVRAAAAAAAAAASGAAIPPLIPPRRVITLYQCFSVSQRADARYRHLIALFTQDEYFAMV
AENESEQESWYLLLSRLILESKRRRCGTLGAQPDGEPAALAAAAAAEPPFYKDVWQVIVK
PRGLGHRKELSGVFRLCLTDEEVVFVRLNTEVASVVVQLLSIRRCGHSEQYFFLEVGRSTV
IGPGELWMQVDDCVVAQNMHELFLEKMRALCADEYRARCRSYSISIGAHLLTLLSARRHL
GLVPLEPGGWLRRSRFEQFCHLRAIGDGEDEMLFTRRFVTPSEPVAHSRRGRLHLPRGRRS
RRAVSVPASFFRRLAPSPARPRHPAEAPNNGARLSSEVSGSGSGNFGEEGNPQGKEDQEGS
GGDYMPMNNWGSGNGRGSGGGQGSNGQGSSSHSSGGNQCSGEGQGSRGGQGSNGQGS
GGNQCSRDGQGTAGGHGSGGGQRPGGGHGSGGGQGPGDGHGSGGGKNSGGGKGSGSG
KGSDGDGERGKSLKKRSYFGKLTQSKQQQMPPPPPPPPPPPPAGGTGGKGKSGGRFRLYF
CVDRGATKECKEAKEVKDAEIPEGAARGPHRARAFDEDEDDPYVPMRPGVATPLVSSSDY
MPMAPQNVSASKKRHSRSPFEDSRGYMMMFPRVSPPPAPSPPKAPDTNKEDDSKDNDSES
DYMFMAPGAGAIPKNPRNPQGGSSSKSWSSYFSLPNPFRSSPLGQNDNSEYVPMLPGKFL
GRGLDKEVSYNWDPKDAASKPSGEGSFSKPGDGGSPSKPSDHEPPKNKAKRPNRLSFITK
GYKIKPKPQKPTHEQREADSSSDYVNMDFTKRESNTPAPSTQGLPDSWGIIAEPRQSAFSN
YVNVEFGVPFPNPANDLSDLLRAIPRANPLSLDSARWPLPPLPLSATGSNAIEEEGDYIEVIF
NSAMTPAMALADSAIRYDAETGRIYVVDPFSECCMDISLSPSRCSEPPPVARLLQEEEQER
RRPQSRSQSFFAAARAAVSAFPTDSLERDLSPSSAPAVASAAEPTLALSQVVAAASALAAAP
GIGAAAAAAGFDSASARWFQPVANAADAEAVRGAQDVAGGSNPGAHNPSANLARGDNQ
AGGAAAAAAAAPEPPPRSRRVPRPPEREDSDNDDDTHVRMDFARRDNQFDSPKRGR

>Q9H3K2 multipass membrane protein
MLAARLVCLRTLPSRVFHPAFTKASPVVKNSITKNQWLLTPSREYATKTRIGIRRGRTGQEL
KEAALEPSMEKIFKIDQMGRWFVAGGAAVGLGALCYYGLGLSNEIGAIEKAVIWPQYVKD
RIHSTYMYLAGSIGLTALSAIAISRTPVLMNFMMRGSWVTIGVTFAAMVGAGMLVRSIPYD
QSPGPKHLAWLLHSGVMGAVVAPLTILGGPLLIRAAWYTAGIVGGLSTVAMCAPSEKFLN
MGAPLGVGLGLVFVSSLGSMFLPPTTVAGATLYSVAMYGGLVLFSMFLLYDTQKVIKRAE
VSPMYGVQKYDPINSMLSIYMDTLNIFMRVATMLATGGNRKK

>P56975 single-pass type I membrane protein
MSEGAAAASPPGAASAAAASAEEGTAAAAAAAAAGGGPDGGGEGAAEPPRELRCSDCIV
WNRQQTWLCVVPLFIGFIGLGLSLMLLKWIVVGSVKEYVPTDLVDSKGMGQDPFFLSKPS
SFPKAMETTTTTTTSTTSPATPSAGGAASSRTPNRISTRLTTITRAPTRFPGHRVPIRASPRSTT
ARNTAAPATVPSTTAPFFSSSTLGSRPPVPGTPSTQAMPSWPTAAYATSSYLHDSTPSWTLSP
FQDAASSSSSSSSSSATTTTPETSTSPKFHTTTYSTERSEHFKPCRDKDLAYCLNDGECFVIET
LTGSHKHCRCKEGYQGVRCDQFLPKTDSILSDPTDHLGIEFMESEEVYQRQVLSISCIIFGI
VIVGMFCAAFYFKSKKQAKQIQEQLKVPQNGKSYSLKASSTMAKSENLVKSHVQLQNYS
KVERHPVTALEKMMESSFVGPQSFPEVPSPDRGSQSVKHHRSLSSCCSPGQRSGMLHRNA
FRRTPPSPRSRLGGIVGPAYQQLEESRIPDQDTIPCQGIEVRKTISHLPIQLWCVERPLDLKYS
SSGLKTQRNTSINMQLPSRETNPYFNSLEQKDLVGYSSTRASSVPIIPSVGLEETCLQMPGIS
EVKSIKWCKNSYSADVVNVSIPVSDCLIAEQQEVKILLETVQEQIRILTDARRSEDYELASV
ETEDSASENTAFLPLSPTAKSEREAQFVLRNEIQRDSALTK

>Q9Y6C5 multipass membrane protein
MTRSPPLRELPPSYTPPARTAAPQILAGSLKAPLWLRAYFQGLLFSLGCGIQRHCGKVLFLG
LLAFGALALGLRMAIIETNLEQLWVEVGSRVSQELHYTKEKLGEEAAYTSQMLIQTARQE
GENILTPEALGLHLQAALTASKVQVSLYGKSWDLNKICYKSGVPLIENGMIERMIEKLFPC
VILTPLDCFWEGAKLQGGSAYLPGRPDIQWTNLDPEQLLEELGPFASLEGFRELLDKAQVG
QAYVGRPCLHPDDLHCPPSAPNHHSRQAPNVAHELSGGCHGFSHKFMHWQEELLLGGM
ARDPQGELLRAEALQSTFLLMSPRQLYEHFRGDYQTHDIGWSEEQASTVLQAWQRRFVQ
LAQEALPENASQQIHAFSSTTLDDILHAFSEVSAARVVGGYLLMLAYACVTMLRWDCAQS
QGSVGLAGVLLVALAVASGLGLCALLGITFNAATTQVLPFLALGIGVDDVFLLAHAFTEAL
PGTPLQERMGECLQRTGTSVVLTSINNMAAFLMAALVPIPALRAFSLQAAIVVGCTFVAVM
LVFPAILSLDLRRRHCQRLDVLCCFSSPCSAQVIQILPQELGDGTVPVGIAHLTATVQAFTH
CEASSQHVVTILPPQAHLVPPPSDPLGSELFSPGGSTRDLLGQEEETRQKAACKSLPCARW
NLAHFARYQFAPLLLQSHAKAIVLVLFGALLGLSLYGATLVQDGLALTDVVPRGTKEHAFL
SAQLRYFSLYEVALVTQGGFDYAHSQRALFDLHQRFSSLKAVLPPPATQAPRTWLHYYRN
WLQGIQAAFDQDWASGRITRHSYRNGSEDGALAYKLLIQTGDAQEPLDFSQLTTRKLVDR
EGLIPPELFYMGLTVWVSSDPLGLAASQANFYPPPPEWLHDKYDTTGENLRIPPAQPLEFA
QFPFLLRGLQKTADFVEAIEGARAACAEAGQAGVHAYPSGSPFLFWEQYLGLRRCFLLAV
CILLVCTFLVCALLLLNPWTAGLIVLVLAMMTVELFGIMGFLGIKLSAIPVVILVASVGIGVE
FTVHVALGFLTTQGSRNLRAAHALEHTFAPVTDGAISTLLGLLMLAGSHFDFIVRYFFAAL
TVLTLLGLLHGLVLLPVLLSILGPPPEVIQMYKESPEILSPPAPQGGGLRWGASSSLPQSFAR
VTTSMTVAIHPPPLPGAYIHPAPDEPPWSPAATSSGNLSSRGPGPATG

>Q8NFU0 multipass membrane protein
MTVSYTLKVAEARFGGFSGLLLRWRGSIYKLLYKEFLLFGALYAVLSITYRLLLTQEQRYV
YAQVARYCNRSADLIPLSFVLGFYVTLVVNRWWSQYTSIPLPDQLMCVISASVHGVDQRG
RLLRRTLIRYANLASVLVLRSVSTRVLKRFPTMEHVVDAGFMSQEERKKFESLKSDFNKY
WVPCVWFTNLAAQARRDGRIRDDIALCLLLEELNKYRAKCSMLFHYDWISIPLVYTQVV
TIAVYSFFALSLVGRQFVEPEAGAAKPQKLLKPGQEPAPALGDPDMYVPLTTLLQFFFYAG
WLKVAEQIINPFGEDDDDFETNQLIDRNLQVSLLSVDEMYQNLPPAEKDQYWDEDQPQPP
YTVATAAESLRPSFLGSTFNLRMSDDPEQSLQVEASPGSGRPAPAAQTPLLGRFLGVGAPSP
AISLRNFGRVRGTPRPPHLLRFRAEEGGDPEAAARIEEESAESGDEALEP

>Q9Y6A1 multipass membrane protein
MWGFLKRPVVVTADINLSLVALTGMGLLSRLWRLTYPRAVVFDEVYYGQYISFYMKQIFF
LDDSGPPFGHMVLALGGYLGGFDGNFLWNRIGAEYSSNVPVWSLRLLPALAGALSVPMA
YQIVLELHFSHCAAMGAALLMLIENALITQSRLMLLESVLIFFNLLAVLSYLKFFNCQKHS
PFSLSWWFWLTLTGVACSCAVGIKYMGVFTYVLVLGVAAVHAWHLLGDQTLSNVGADV
QCCMRPACMGQMQMSQGVCVFCHLLARAVALLVIPVVLYLLFFYVHLILVFRSGPHDQIM
SSAFQASLEGGLARITQGQPLEVAFGSQVTLRNVFGKPVPCWLHSHQDTYPMIYENGRGS
SHQQQVTCYPFKDVNNWWIVKDPRRHQLVVSSPPRPVRHGDMVQLVHGMTTRSLNTHD
VAAPLSPHSQEVSCYIDYNISMPAQNLWRLEIVNRGSDTDVWKTILSEVRFVHVNTSAVLK
LSGAHLPDWGYRQLEIVGEKLSRGYHGSTVWNVEEHRYGASQEQREREREL HSPAQVDV
SRNLSFMARFSELQWRMLALRSDDSEHKYSSSPLEWVTLDTNIAYWLHPRTSAQIHLLGN
IVIWVSGSLALAIYALLSLWYLLRRRRNVHDLPQDAWLRWVLAGALCAGGWAVNYLPFF
LMEKTLFLYHYLPALTFQILLLPVVLQHISDHLCRSQLQRSIFSALVVAWYSSACHVSNTLR
PLTYGDKSLSPHELKALRWKDSWDILIRKH

>Q8NBV4 multipass membrane protein
MPASQSRARARDRNNVLNRAEFLSLNQPPKGGPEPRSSGRKASGPSAQPPPAGDGARERR
QSQQLPEEDCMQLNPSFKGIAFNSLLAIDICMSKRLGVCAGRAASWASARSMVKLIGITG
HGIPWIGGTILCLVKSSTLAGQEVLMNLLLALLLDIMTVAGVQKLIKRRGPYETSPSLLDYL
TMDIYAFPAGHASRAAMVSKFFLSHLVLAVPLRVLLVLWALCVGLSRVMIGRHHVTDVLS
GFVIGYLQFRLVELVWMPSSTCQMLISAW

>P25942 single-pass type I membrane protein
MVRLPLQCVLWGCLLTAVHPEPPTACREKQYLINSQCCSLCQPGQKLVSDCTEFTETECLP
CGESEFLDTWNRETHCHQHKYCDPNLGLRVQQKGTSETDTICTCEEGWHCTSEACESCV
LHRSCSPGFGVKQIATGVSDTICEPCPVGFFSNVSSAFEKCHPWTSCETKDLVVQQAGTNK
TDVVCGPQDRLRALVVIPIIFGILFAILLVLVFIKKVAKKPTNKAPHPKQEPQEINFPDDLPGS
NTAAPVQETLHGCQPVTQEDGKESRISVQERQ

>P48067 multipass membrane protein
MSGGDTRAAIARPRMAAAHGPVAPSSPEQVTLLPVQRSFFLPPFSGATPSTSLAESVLKVW
HGAYNSGLLPQLMAQHSLAMAQNGAVPSEATKRDQNLKRGNWGNQIEFVLTSVGYAVG
LGNVWRFPYLCYRNGGGAFMFPYFIMLIFCGIPLFFMELSFGQFASQGCLGVWRISPMFK
GVGYGMMVVSTYIGIYYNVVICIAFYYFFSSMTHVLPWAYCNNPWNTHDCAGVLDASNL

TNGSRPAALPSNLSHLLNHSLQRTSPSEEYWRLYVLKLSDDIGNFGEVRLPLLGCLGVSWL
VVFLCLIRGVKSSGKVVYFTATFPYVVLTILFVRGVTLEGAFDGIMYYLTPQWDKILEAKV
WGDAASQIFYSLGCAWGGLITMASYNKFHNNCYRDSVIISITNCATSVYAGFVIFSILGFM
ANHLGVDVSRVADHGPGLAFVAYPEALTLLPISPLWSLLFFFMLILLGLGTQFCLLETLVTAI
VDEVGNEWILQKKTYVTLGVAVAGFLLGIPLTSQAGIYWLLLMDNYAASFSLVVISCIMCV
AIMYIYGHRNYFQDIQMMLGFPPPLFFQICWRFVSPAIIFFILVFTVIQYQPITYNHYQYPGW
AVAIGFLMALSSVLCIPLYAMFRLCRTDGDTLLQRLKNATKPSRDWGPALLEHRTGRYAPTI
APSPEDGFEVQPLHPDKAQIPIVGSNGSSRLQDSRI

>P00519 lipid-anchor
MLEICLKLVGCKSKKGLSSSSSCYLEEALQRPVASDFEPQGLSEAARWNSKENLLAGPSEN
DPNLFVALYDFVASGDNTLSITKGEKLRVLGYNHNGEWCEAQTKNGQGWVPSNYITPVN
SLEKHSWYHGPVSRNAAEYLLSSGINGSFLVRESESSPGQRSISLRYEGRVYHYRINTASDG
KLYVSSESRFNTLAELVHHHSTVADGLITTLHYPAPKRNKPTVYGVSPNYDKWEMERTDI
TMKHKLGGGQYGEVYEGVWKKYSLTVAVKTLKEDTMEVEEFLKEAAVMKEIKHPNLVQ
LLGVCTREPPFYIITEFMTYGNLLDYLRECNRQEVNAVVLLYMATQISSAMEYLEKKNFIH
RDLAARNCLVGENHLVKVADFGLSRLMTGDTYTAHAGAKFPIKWTAPESLAYNKFSIKSD
VWAFGVLLWEIATYGMSPYPGIDLSQVYELLEKDYRMERPEGCPEKVYELMRACWQWN
PSDRPSFAEIHQAFETMFQESSISDEVEKELGKQGVRGAVSTLLQAPELPTKTRTSRRAAEH
RDTTDVPEMPHSKGQGESDPLDHEPAVSPLLPRKERGPPEGGLNEDERLLPKDKKTNLFS
ALIKKKKKTAPTPPKRSSSFREMDGQPERRGAGEEEGRDISNGALAFTPLDTADPAKSPKP
SNGAGVPNGALRESGGSGFRSPHLWKKSSTLTSSRLATGEEEGGGSSSKRFLRSCSASCVP
HGAKDTEWRSVTLPRDLQSTGRQFDSSTFGGHKSEKPALPRKRAGENRSDQVTRGTVTPP
PRLVKKNEEAADEVFKDIMESSPGSSPPNLTPKPLRRQVTVAPASGLPHKEEAGKGSALGT
PAAAEPVTPTSKAGSGAPGGTSKGPAEESRVRRHKHSSESPGRDKGKLSRLKPAPPPPPAA
SAGKAGGKPSQSPSQEAAGEAVLGAKTKATSLVDAVNSDAAKPSQPGEGLKKPVLPATPK
PQSAKPSGTPISPAPVPSTLPSASSALAGDQPSSTAFIPLISTRVSLRKTRQPPERIASGAITKG
VVLDSTEALCLAISRNSEQMASHSAVLEAGKNLYTFCVSYVDSIQQMRNKFAFREAINKLE
NNLRELQICPATAGSGPAATQDFSKLLSSVKEISDIVQR

>Q8TDM6 peripheral membrane protein
MEPQRRELLAQCQQSLAQAMTEVEAVLGLLEAAGALSPGERRQLDEEAGGAKAELLLKL
LLAKERDHFQDLRAALEKTQPHLLPILYLNGVVGPPQPAEGAGSTYSVLSTMPSDSESSSS
LSSVGTTGKAPSPPPLLTDQQVNEKVENLSIQLRLMTRERNELRKRLAFATHGTAFDKRPY
HRLNPDYERLKIQCVRAMSDLQSLQNQHTNALKRCEEVAKETDFYHTLHSRLLSDQTRL
KDDVDMLRRENGQLLRERNLLQQSWEDMKRLHEEDQKEIGDLRAQQQQVLKHNGSSEI
LNKLYDTAMDKLEVVKKDYDALRKRYSEKVAIHNADLSRLEQLGEENQRLLKQTEMLTQ
QRDTAIQLQHQCALSLRRFEAIHHELNKATAQNKDLQWEMELLQSELTELRTTQVKTAKE
SEKYREERDAVYSEYKLIMSERDQVISELDKLQTEVELAESKLKSSTSEKKAANEEMEAL
RQIKDTVTMDAGRANKEVEILRKQCKALCQELKEALQEADVAKCRRDWAFQERDKIVAE
RDSIRTLCDNLRRERDRAVSELAEALRSLDDTRKQKNDVSRELKELKEQMESQLEKEARF
RQLMAHSSHDSAIDTDSMEWETEVVEFERETEDIDLKALGFDMAEGVNEPCFPGDCGIFV
TKVDKGSIADGRLRVNDWLLRINDVDLINKDKKQAIKALLNGEGAINMVVRRRKSLGGK
VVTPLHINLSGQKDSGISLENGVYAAAVLPGSPAAKEGSLAVGDRIVAINGIALDNKSLNEC

ESLLRSCQDSLTLSLLKVFPQSSSWSGQNIFENIKDSDKMLSFRAHGPEVQAHNKRNLIQH
NNSTQTDIFYTDRLEDRKEPGPPGGSSSFLHKPFPGGPLQVCPQACPSASERSLSSFRSDAS
GDRGFGLVDVRGRRPLLPFETEVGPCGVGEASLDKADSEGSNSGGTWPKAMLSSTAVPEK
LSVYKKPKQRKSIFDPNTFKRPQTPPKIDYLLPGPGPAHSPQPSKRAGPLTPPKPPRRSDSIK
FQHRLETSSESEATLVGSSPSTSPPSALPPDVDPGEPMHASPPRKARVRIASSYYPEGDGDS
SHLPAKKSCDEDLTSQKVDELGQKRRRPKSAPSFRPKLAPVVIPAQFLEEQKCVPASGELSP
ELQEWAPYSPGHSSRHSNPPLYPSRPSVGTVPRSLTPSTTVSSILRNPIYTVRSHRVGPCSSPP
AARDAGPQGLHPSVQHQGRLSLDLSHRTCSDYSEMRATHGSNSLPSSARLGSSSNLQFKA
ERIKIPSTPRYPRSVVGSERGSVSHSECSTPPQSPLNIDTLSSCSQSQTSASTLPRIAVNPASL
GERRKDRPYVEEPRHVKVQKGSEPLGISIVSGEKGGIYVSKVTVGSIAHQAGLEYGDQLL
EFNGINLRSATEQQARLIIGQQCDTITILAQYNPHVHQLSSHSRSSSHLDPAGTHSTLQGSG
TTTPEHPSVIDPLMEQDEGPSTPPAKQSSSRIAGDANKKTLEPRVVFIKKSQLELGVHLCGG
NLHGVFVAEVEDDSPAKGPDGLVPGDLILEYGSLDVRNKTVEEVYVEMLKPRDGVRLKV
QYRPEEFTKAKGLPGDSFYIRALYDRLADVEQELSFKKDDILYVDDTLPQGTFGSWMAW
QLDENAQKIQRGQIPSKYVMDQEFSRRLSMSEVKDDNSATKTLSAAARRSFFRRKHKHK
RSGSKDGKDLLALDAFSSDSIPLFEDSVSLAYQRVQKVDCTALRPVLILGPLLDVVKEMLV
NEAPGKFCRCPLEVMKASQQAIERGVKDCLFVDYKRRSGHFDVTTVASIKEITEKNRHCL
LDIAPHAIERLHHMHIYPIVIFIHYKSAKHIKEQRDPIYLRDKVTQRHSKEQFEAAQKLEQE
YSRYFTGVIQGGALSSICTQILAMVNQEQNKVLWIPACPL

>P62166 peripheral membrane protein
MGKSNSKLKPEVVEELTRKTYFTEKEVQQWYKGFIKDCPSGQLDAAGFQKIYKQFFPFGD
PTKFATFVFNVFDENKDGRIEFSEFIQALSVTSRGTLDEKLRWAFKLYDLDNDGYITRNEM
LDIVDAIYQMVGNTVELPEEENTPEKRVDRIFAMMDKNADGKLTLQEFQEGSKADPSIVQ
ALSLYDGLV

>Q5VW38 multipass membrane protein
MAALAPVGSPASRGPRLAAGLRLLPMLGLLQLLAEPGLGRVHHLALKDDVRHKVHLNTF
GFFKDGYMVVNVSSLSLNEPEDKDVTIGFSLDRTKNDGFSSYLDEDVNYCILKKQSVSVT
LLILDISRSEVRVKSPPEAGTQLPKIIFSRDEKVLGQSQEPNVNPASAGNQTQKTQDGGKSK
RSTVDSKAMGEKSFSVHNNGGAVSFQFFFNISTDDQEGLYSLYFHKCLGKELPSDKFTFSL
DIEITEKNPDSYLSAGEIPLPKLYISMAFFFFLSGTIWIHILRKRRNDVFKIHWLMAALPFTK
SLSLVFHAIDYHYISSQGFPIEGWAVVYYITHLLKGALLFITIALIGTGWAFIKHILSDKDKKI
FMIVIPLQVLANVAYIIIESTEEGTTEYGLWKDSLFLVDLLCCGAILFPVVWSIRHLQEASAT
DGKGDSMGPLQQRANLRAGSRIESHHFAQADLELLASSCPPASVSQRAGITAAINLAKLKL
FRHYYVLIVCYIYFTRIIAFLLKLAVPFQWKWLYQLLDETATLVFFVLTGYKFRPASDNPYL
QLSQEEEDLEMESVVTTSGVMESMKKVKKVTNGSVEPQGEWEGAV

>Q9BW60 multipass membrane protein
MEAVVNLYQEVMKHADPRIQGYPLMGSPLLMTSILLTYVYFVLSLGPRIMANRKPFQLRG
FMIVYNFSLVALSLYIVYEFLMSGWLSTYTWRCDPVDYSNSPEALRMVRVAWLFLFSKFIE
LMDTVIFILRKKDGQVTFLHVFHHSVLPWSWWWGVKIAPGGMGSFHAMINSSVHVIMYL
YYGLSAFGPVAQPYLWWKKHMTAIQLIQFVLVSLHISQYYFMSSCNYQYPVIIHLIWMYG
TIFFMLFSNFWYHSYTKGKRLPRALQQNGAPGIAKVKAN

>P40238 single-pass type I membrane protein
MPSWALFMVTSCLLLAPQNLAQVSSQDVSLLASDSEPLKCFSRTFEDLTCFWDEEEAAPS
GTYQLLYAYPREKPRACPLSSQSMPHFGTRYVCQFPDQEEVRLFFPLHLWVKNVFLNQTR
TQRVLFVDSVGLPAPPSIIKAMGGSQPGELQISWEEPAPEISDFLRYELRYGPRDPKNSTGPT
VIQLIATETCCPALQRPHSASALDQSPCAQPTMPWQDGPKQTSPSREASALTAEGGSCLISG
LQPGNSYWLQLRSEPDGISLGGSWGSWSLPVTVDLPGDAVALGLQCFTLDLKNVTCQWQ
QQDHASSQGFFYHSRARCCPRDRYPIWENCEEEEKTNPGLQTPQFSRCHFKSRNDSIIHILV
EVTTAPGTVHSYLGSPFWIHQAVRLPTPNLHWREISSGHLELEWQHPSSWAAQETCYQLR
YTGEGHQDWKVLEPPLGARGGTLELRPRSRYRLQLRARLNGPTYQGPWSSWSDPTRVET
ATETAWISLVTALHLVLGLSAVLGLLLLRWQFPAHYRRLRHALWPSLPDLHRVLGQYLRDT
AALSPPKATVSDTCEEVEPSLLEILPKSSERTPLPLCSSQAQMDYRRLQPSCLGTMPLSVCP
PMAESGSCCTTHIANHSYLPLSYWQQP

>P35590 single-pass type I membrane protein
MVWRVPPFLLPILFLASHVGAAVDLTLLANLRLTDPQRFFLTCVSGEAGAGRGSDAWGPP
LLLEKDDRIVRTPPGPPLRLARNGSHQVTLRGFSKPSDLVGVFSCVGGAGARRTRVIYVHN
SPGAHLLPDKVTHTVNKGDTAVLSARVHKEKQTDVIWKSNGSYFYTLDWHEAQDGRFLL
QLPNVQPPSSGIYSATYLEASPLGSAFFRLIVRGCGAGRWGPGCTKECPGCLHGGVCHDH
DGECVCPPGFTGTRCEQACREGRFGQSCQEQCPGISGCRGLTFCLPDPYGCSCGSGWRGS
QCQEACAPGHFGADCRLQCQCQNGGTCDRFSGCVCPSGWHGVHCEKSDRIPQILNMASE
LEFNLETMPRINCAAAGNPFPVRGSIELRKPDGTVLLSTKAIVEPEKTTAEFEVPRLVLADS
GFWECRVSTSGGQDSRRFKVNVKVPPVPLAAPRLLTKQSRQLVVSPLVSFSGDGPISTVRL
HYRPQDSTMDWSTIVVDPSENVTLMNLRPKTGYSVRVQLSRPGEGGEGAWGPPTLMTTD
CPEPLLQPWLEGWHVEGTDRLRVSWSLPLVPGPLVGDGFLLRLWDGTRGQERRENVSSP
QARTALLTGLTPGTHYQLDVQLYHCTLLGPASPPAHVLLPPSGPPAPRHLHAQALSDSEIQL
TWKHPEALPGPISKYVVEVQVAGGAGDPLWIDVDRPEETSTIIRGLNASTRYLFRMRASIQ
GLGDWSNTVEESTLGNGLQAEGPVQESRAAEEGLDQQLILAVVGSVSATCLTILAALLTLV
CIRRSCLHRRRTFTYQSGSGEETILQFSSGTLTLTRRPKLQPEPLSYPVLEWEDITFEDLIGEG
NFGQVIRAMIKKDGLKMNAAIKMLKEYASENDHRDFAGELEVLCKLGHHPNIINLLGAC
KNRGYLYIAIEYAPYGNLLDFLRKSRVLETDPAFAREHGTASTLSSRQLLRFASDAANGMQ
YLSEKQFIHRDLAARNVLVGENLASKIADFGLSRGEEVYVKKTMGRLPVRWMAIESLNYS
VYTTKSDVWSFGVLLWEIVSLGGTPYCGMTCAELYEKLPQGYRMEQPRNCDDEVYELM
RQCWRDRPYERPPFAQIALQLGRMLEARKAYVNMSLFENFTYAGIDATAEEA

>Q96PL5 single-pass type I membrane protein
MEMASSAGSWLSGCLIPLVFLRLSVHVSGHAGDAGKFHVALLGGTAELLCPLSLWPGTVP
KEVRWLRSPFPQRSQAVHIFRDGKDQDEDLMPEYKGRTVLVRDAQEGSVTLQILDVRLED
QGSYRCLIQVGNLSKEDTVILQVAAPSVGSLSPSAVALAVILPVLVLLIMVCLCLIWKQRRA
KEKLLYEHVTEVDNLLSDHAKEKGKLHKAVKKLRSELKLKRAAANSGWRRARLHFVAV
TLDPDTAHPKLILSEDQRCVRLGDRRQPVPDNPQRFDFVVSILGSEYFTTGCHYWEVYVG
DKTKWILGVCSESVSRKGKVTASPANGHWLLRQSRGNEYEALTSPQTSFRLKEPPRCVGIF
LDYEAGVISFYNVTNKSHIFTFTHNFSGPLRPFFEPCLHDGGKNTAPLVICSELHKSEESIVP
RPEGKGHANGDVSLKVNSSLLPPKAPELKDIILSLPPDLGPALQELKAPSF

>Q9Y694 multipass membrane protein
MGFEELLEQVGGFGPFQLRNVALLALPRVLLPLHFLLPIFLAAVPAHRCALPGAPANFSHQ
DVWLEAHLPREPDGTLSSCLRFAYPQALPNTTLGEERQSRGELEDEPATVPCSQGWEYDH
SEFSSTIATESQWDLVCEQKGLNRAASTFFFAGVLVGAVAFGYLSDRFGRRRLLLVAYVSTL
VLGLASAASVSYVMFAITRTLTGSALAGFTIIVMPLELEWLDVEHRTVAGVLSSTFWTGGV
MLLALVGYLIRDWRWLLLAVTLPCAPGILSLWWVPESARWLLTQGHVKEAHRYLLHCAR
LNGRPVCEDSFSQEAVSKVAAGERVVRRPSYLDLFRTPRLRHISLCCVVVWFGVNFSYYG
LSLDVSGLGLNVYQTQLLFGAVELPSKLLVYLSVRYAGRRLTQAGTLLGTALAFGTRLLVS
SDMKSWSTVLAVMGKAFSEAAFTTAYLFTSELYPTVLRQTGMGLTALVGRLGGSLAPLAA
LLDGVWLSLPKLTYGGIALLAAGTALLLPETRQAQLPETIQDVERKSAPTSLQEEEMPMK
QVQN

>P31431 single-pass type I membrane protein
MAPARLFALLLFFVGGVAESIRETEVIDPQDLLEGRYFSGALPDDEDVVGPGQESDDFELS
GSGDLDDLEDSMIGPEVVHPLVPLDNHIPERAGSGSQVPTEPKKLEENEVIPKRISPVEESE
DVSNKVSMSSTVQGSNIFERTEVLAALIVGGIVGILFAVFLILLLMYRMKKKDEGSYDLGK
KPIYKKAPTNEFYA

>O75844 multipass membrane protein
MGMWASLDALWEMPAEKRIFGAVLLFSWTVYLWETFLAQRQRRIYKTTTHVPPELGQIM
DSETFEKSRLYQLDKSTFSFWSGLYSETEGTLILLFGGIPYLWRLSGRFCGYAGFGPEYEIT
QSLVFLLLATLFSALTGLPWSLYNTFVIEEKHGFNQQTLGFFMKDAIKKFVVTQCILLPVSS
LLLYIIKIGGDYFFIYAWLFTLVVSLVLVTIYADYIAPLFDKFTPLPEGKLKEEIEVMAKSIDF
PLTKVYVVEGSKRSSHSNAYFYGFFKNKRIVLFDTLLEEYSVLNKDIQEDSGMEPRNEEEG
NSEEIKAKVKNKKQGCKNEEVLAVLGHELGHWKLGHTVKNIIISQMNSFLCFFLFAVLIGR
KELFAAFGFYDSQPTLIGLLIIFQFIFSPYNEVLSFCLTVLSRRFEFQADAFAKKLGKAKDLY
SALIKLNKDNLGFPVSDWLFSMWHYSHPPLLERLQALKTMKQH

>Q15785 peripheral membrane protein
MAPKFPDSVEELRAAGNESFRNGQYAEASALYGRALRVLQAQGSSDPEEESVLYSNRAAC
HLKDGNCRDCIKDCTSALALVPFSIKPLLRRASAYEALEKYPMAYVDYKTVLQIDDNVTS
AVEGINRMTRALMDSLGPEWRLKLPSIPLVPVSAQKRWNSLPSENHKEMAKSKSKETTAT
KNRVPSAGDVEKARVLKEEGNELVKKGNHKKAIEKYSESLLCSNLESATYSNRALCYLVL
KQYTEAVKDCTEALKLDGKNVKAFYRRAQAHKALKDYKSSFADISNLLQIEPRNGPAQK
LRQEVKQNLH

>Q9H427 multipass membrane protein
MRRPSVRAAGLVLCTLCYLLVGAAVFDALESEAESGRQRLLVQKRGALRRKFGFSAEDY
RELERLALQAEPHRAGRQWKFPGSFYFAITVITTIGYGHAAPGTDSGKVFCMFYALLGIPL
TLVTFQSLGERLNAVVRRLLLAAKCCLGLRWTCVSTENLVVAGLLACAATLALGAVAFSH
FEGWTFFHAYYYCFITLTTIGFGDFVALQSGEALQRKLPYVAFSFLYILLGLTVIGAFLNLVV
LRFLVASADWPERAARPPSPRPPGAPESRGLWLPRRPARSVGSASVFCHVHKLERCARDN
LGFSPPSSPGVVRGGQAPRPGARWKSI

>P00813 peripheral membrane protein
MAQTPAFDKPKVELHVHLDGSIKPETILYYGRRRGIALPANTAEGLLNVIGMDKPLTLPDF
LAKFDYYMPAIAGCREAIKRIAYEFVEMKAKEGVVYVEVRYSPHLLANSKVEPIPWNQAE
GDLTPDEVVALVGQGLQEGERDFGVKARSILCCMRHQPNWSPKVVELCKKYQQQTVVAI
DLAGDETIPGSSLLPGHVQAYQEAVKSGIHRTVHAGEVGSAEVVKEAVDILKTERLGHGY
HTLEDQALYNRLRQENMHFEICPWSSYLTGAWKPDTEHAVIRLKNDQANYSLNTDDPLIF
KSTLDTDYQMTKRDMGFTEEEFKRLNINAAKSSFLPEDEKRELLDLLYKAYGMPPSASAG
QNL

>O60220 peripheral membrane protein
MDSSSSSSAAGLGAVDPQLQHFIEVETQKQRFQQLVHQMTELCWEKCMDKPGPKLDSRA
EACFVNCVERFIDTSQFILNRLEQTQKSKPVFSESLSD

>Q9Y5S8 multipass membrane protein
MGNWVVNHWFSVLFLVVWLGLNVFLFVDAFLKYEKADKYYYTRKILGSTLACARASAL
CLNFNSTLILLPVCRNLLSFLRGTCSFCSRTLRKQLDHNLTFHKLVAYMICLHTAIHIIAHLF
NFDCYSRSRQATDGSLASILSSLSHDEKKGGSWLNPIQSRNTTVEYVTFTSIAGLTGVIMTI
ALILMVTSATEFIRRSYFEVFWYTHHLFIFYILGLGIHGIGGIVRGQTEESMNESHPRKCAES
FEMWDDRDSHCRRPKFEGHPPESWKWILAPVILYICERILRFYRSQQKVVITKVVMHPSK
VLELQMNKRGFSMEVGQYIFVNCPSISLLEWHPFTLTSAPEEDFFSIHIRAAGDWTENLIRA
FEQQYSPIPRIEVDGPFGTASEDVFQYEVAVLVGAGIGVTPFASILKSIWYKFQCADHNLKT
KKIYFYWICRETGAFSWFNNLLTSLEQEMEELGKVGFLNYRLFLTGWDSNIVGHAALNFD
KATDIVTGLKQKTSFGRPMWDNEFSTIATSHPKSVVGVFLCGPRTLAKSLRKCCHRYSSLD
PRKVQFYFNKENF

>O43657 multipass membrane protein
MASPSRRLQTKPVITCFKSVLLIYTFIFWITGVILLAVGIWGKVSLENYFSLLNEKATNVPFV
LIATGTVIILLGTFGCFATCRASAWMLKLYAMFLTLVFLVELVAAIVGFVFRHEIKNSFKNNY
EKALKQYNSTGDYRSHAVDKIQNTLHCCGVTDYRDWTDTNYYSEKGFPKSCCKLEDCTP
QRDADKVNNEGCFIKVMTIIESEMGVVAGISFGVACFQLIGIFLAYCLSRAITNNQYEIV

>P32019 peripheral membrane protein
MDQSVAIQETLAEGEYCVIAVQGVLCEGDSRQSRLLGLVRYRLEHGGQEHALFLYTHRRM
AITGDDVSLDQIVPVSRDFTLEEVSPDGELYILGSDVTVQLDTAELSLVFQLPFGSQTRMFL
HEVARACPGFDSATRDPEFLWLSRYRCAELELEMPTPRGCNSALVTWPGYATIGGGRYPSR
KKRWGLEEARPQGAGSVLFWGGAMEKTGFRLMERAHGGGFVWGRSARDGRRDEELEE
AGREMSAAAGSRERNTAGGSNFDGLRPNGKGVPMDQSSRGQDKPESLQPRQNKSKSEIT
DMVRSSTITVSDKAHILSMQKFGLRDTIVKSHLLQKEEDYTYIQNFRFFAGTYNVNGQSPK
ECLRLWLSNGIQAPDVYCVGFQELDLSKEAFFFHDTPKEEEWFKAVSEGLHPDAKYAKVK
LIRLVGIMLLLYVKQEHAAYISEVEAETVGTGIMGRMGNKGGVAIRFQFHNTSICVVNSHL
AAHIEEYERRNQDYKDICSRMQFCQPDPSLPPLTISNHDVILWLGDLNYRIEELDVEKVKK
LIEEKDFQMLYAYDQLKIQVAAKTVFEGFTEGELTFQPTYKYDTGSDDWDTSEKCRAPAW
CDRILWKGKNITQLSYQSHMALKTSDHKPVSSVFDIGVRVVNDELYRKTLEEIVRSLDKM

ENANIPSVSLSKREFCFQNVKYMQLKVESFTIHNGQVPCHFEFINKPDEESYCKQWLNAN
PSRGFLLPDSDVEIDLELFVNKMTATKLNSGEDKIEDILVLHLDRGKDYFLSVSGNYLPSCF
GSPIHTLCYMREPILDLPLETISELTLMPVWTGDDGSQLDSPMEIPKELWMMVDYLYRNAV
QQEDLFQQPGLRSEFEHIRDCLDTGMIDNLSASNHSVAEALLLFLESLPEPVICYSTYHNCL
ECSGNYTASKQVISTLPIFHKNVFHYLMAFLRELLKNSAKNHLDENILASIFGSLLLRNPAG
HQKLDMTEKKKAQEFIHQFLCNPL

>Q86U90 peripheral membrane protein
MSPARRCRGMRAAVAASVGLSEGPAGSRSGRLFRPPSPAPAAPGARLLRLPGSGAVQAASP
ERAGWTEALRAAVAELRAGAVVAVPTDTLYGLACAASCSAALRAVYRLKGRSEAKPLAV
CLGRVADVYRYCRVRVPEGLLKDLLPGPVTLVMERSEELNKDLNPFTPLVGIRIPDHAFMQ
DLAQMFEGPLALTSANLSSQASSLNVEEFQDLWPQLSLVIDGGQIGDGQSPECRLGSTVVD
LSVPGKFGIIRPGCALESTTAILQQKYGLLPSHASYL

>Q8NE86 multipass membrane protein
MAAAAGRSLLLLLSSRGGGGGGAGGCGALTAGCFPGLGVSRHRQQQHHRTVHQRIASW
QNLGAVYCSTVVPSDDVTVVYQNGLPVISVRLPSRRERCQFTLKPISDSVGVFLRQLQEED
RGIDRVAIYSPDGVRVAASTGIDLLLLDDFKLVINDLTYHVRPPKRDLLSHENAATLNDVKT
LVQQLYTTLCIEQHQLNKERELIERLEDLKEQLAPLEKVRIEISRKAEKRTTLVLWGGLAY
MATQFGILARLTWWEYSWDIMEPVTYFITYGSAMAMYAYFVMTRQEYVYPEARDRQYL
LFFHKGAKKSRFDLEKYNQLKDAIAQAEMDLKRLRDPLQVHLPLRQIGEKD

>Q6KCM7 multipass membrane protein
MLCLCLYVPVIGEAQTEFQYFESKGLPAELKSIFKLSVFIPSQEFSTYRQWKQKIVQAGDK
DLDGQLDFEEFVHYLQDHEKKLRLVFKSLDKKNDGRIDAQEIMQSLRDLGVKISEQQAEK
ILKSMDKNGTMTIDWNEWRDYHLLHPVENIPEIILYWKHSTIFDVGENLTVPDEFTVEERQ
TGMWWRHLVAGGGAGAVSRTCTAPLDRLKVLMQVHASRSNNMGIVGGFTQMIREGGAR
SLWRGNGINVLKIAPESAIKFMAYEQIKRLVGSDQETLRIHERLVAGSLAGAIAQSSIYPME
VLKTRMALRKTGQYSGMLDCARRILAREGVAAFYKGYVPNMLGIIPYAGIDLAVYETLK
NAWLQHYAVNSADPGVFVLLACGTMSSTCGQLASYPLALVRTRMQAQASIEGAPEVTMS
SLFKHILRTEGAFGLYRGLAPNFMKVIPAVSISYVVYENLKITLGVQSR

>Q13003 multipass membrane protein
MTAPWRRLRSLVWEYWAGLLVCAFWIPDSRGMPHVIRIGGIFEYADGPNAQVMNAEEHA
FRFSANIINRNRTLLPNTTLTYDIQRIHFHDSFEATKKACDQLALGVVAIFGPSQGSCTNAVQ
SICNALEVPHIQLRWKHHPLDNKDTFYVNLYPDYASLSHAILDLVQYLKWRSATVVYDDS
TGLIRLQELIMAPSRYNIRLKIRQLPIDSDDSRPLLKEMKRGREFRIIFDCSHTMAAQILKQA
MAMGMMTEYYHFIFTTLDLYALDLEPYRYSGVNLTGFRILNVDNPHVSAIVEKWSMERL
QAAPRSESGLLDGVMMTDAALLYDAVHIVSVCYQRAPQMTVNSLQCHRHKAWRFGGRF
MNFIKEAQWEGLTGRIVFNKTSGLRTDFDLDIISLKEDGLEKVGVWSPADGLNITEVAKGR
GPNVTDSLTNRSLIVTTVLEEPFVMFRKSDRTLYGNDRFEGYCIDLLKELAHILGFSYEIRL
VEDGKYGAQDDKGQWNGMVKELIDHKADLAVAPLTITHVREKAIDFSKPFMTLGVSILYR
KPNGTNPSVFSFLNPLSPDIWMYVLLAYLGVSCVLFVIARFSPYEWYDAHPCNPGSEVVE
NNFTLLNSFWFGMGSLMQQGSELMPKALSTRIIGGIWWFFTLIIISSYTANLAAFLTVERME

SPIDSADDLAKQTKIEYGAVKDGATMTFFKKSKISTFEKMWAFMSSKPSALVKNNEEGIQR
ALTADYALLMESTTIEYVTQRNCNLTQIGGLIDSKGYGIGTPMGSPYRDKITIAILQLQEED
KLHIMKEKWWRGSGCPEEENKEASALGIQKIGGIFIVLAAGLVLSVLVAVGEFVYKLRKTA
EREQRSFCSTVADEIRFSLTCQRRVKHKPQPPMMVKTDAVINMHTFNDRRLPGKDSMACS
TSLAPVFP

>Q86YW5 single-pass type I membrane protein
MGLTLLLLLLLGLEGQGIVGSLPEVLQAPVGSSILVQCHYRLQDVKAQKVWCRFLPEGCQ
PLVSSAVDRRAPAGRRTFLTDLGGGLLQVEMVTLQEEDAGEYGCMVDGARGPQILHRVSL
NILPPEEEEETHKIGSLAENAFSDPAGSANPLEPSQDEKSIPLIWGAVLLVGLLVAAVVLFAV
MAKRKQGNRLGVCGRFLSSRVSGMNPSSVVHHVSDSGPAAELPLDVPHIRLDSPPSFDNT
TYTSLPLDSPSGKPSLPAPSSLPPLPPKVLVCSKPVTYATVIFPGGNKGGGTSCGPAQNPPNN
QTPSS

>Q5TGU0 multipass membrane protein
MRLQGAIFVLLPHLGPILVWLFTRDHMSGWCEGPRMLSWCPFYKVLLLVQTAIYSVVGY
ASYLVWKDLGGGLGWPLALPLGLYAVQLTISWTVLVLFFTVHNPGLALLHLLLLYGLVVS
TALIWHPINKLAALLLLPYLAWLTVTSALTYHLWRDSLCPVHQPQPTEKSD

>O14522 single-pass type I membrane protein
MASLAALALSLLLRLQLPPLPGARAQSAAGGCSFDEHYSNCGYSVALGTNGFTWEQINT
WEKPMLDQAVPTGSFMMVNSSGRASGQKAHLLLPTLKENDTHCIDFHYYFSSRDRSSPG
ALNVYVKVNGGPQGNPVWNVSGVVTEGWVKAELAISTFWPHFYQVIFESVSLKGHPGYI
AVDEVRVLAHPCRKAPHFLRLQNVEVNVGQNATFQCIAGGKWSQHDKLWLQQWNGRDT
ALMVTRVVNHRRFSATVSVADTAQRSVSKYRCVIRSDGGSGVSNYAELIVKEPPTPIAPPE
LLAVGATYLWIKPNANSIIGDGPIILKEVEYRTTTGTWAETHIVDSPNYKLWHLDPDVEYEI
RVLLTRPGEGGTGPPGPPLTTRTKCADPVHGPQNVEIVDIRARQLTLQWEPFGYAVTRCHS
YNLTVQYQYVFNQQQYEAEEVIQTSSHYTLRGLRPFMTIRLRLLLSNPEGRMESEELVVQ
TEEDVPGAVPLESIQGGPFEEKIYIQWKPPNETNGVITLYEINYKAVGSLDPSADLSSQRGK
VFKLRNETHHLFVGLYPGTTYSFTIKASTAKGFGPPVTTRIATKISAPSMPEYDTDTPLNET
DTTITVMLKPAQSRGAPVSVYQLVVKEERLQKSRRAADIIECFSVPVSYRNASSLDSLHYF
AAELKPANLPVTQPFTVGDNKTYNGYWNPPLSPLKSYSIYFQALSKANGETKINCVRLAT
KGASTQNSNTVEPEKQVDNTVKMAGVIAGLLMFIIILLGVMLTIKRRRNAYSYSYYLKLA
KKQKETQSGAQREMGPVASADKPTTKLSASRNDEGFSSSSQDVNGFTDGSRGELSQPTLTI
QTHPYRTCDPVEMSYPRDQFQPAIRVADLLQHITQMKRGQGYGFKEEYEALPEGQTASW
DTAKEDENRNKNRYGNIISYDHSRVRLLVLDGDPHSDYINANYIDGYHRPRHYIATQGPM
QETVKDFWRMIWQENSASIVMVTNLVEVGRVKCVRYWPDDTEVYGDIKVTLIETEPLAE
YVIRTFTVQKKGYHEIRELRLFHFTSWPDHGVPCYATGLLGFVRQVKFLNPPEAGPIVVHC
SAGAGRTGCFIAIDTMLDMAENEGVVDIFNCVRELRAQRVNLVQTEEQYVFVHDAILEAC
LCGNTAIPVCEFRSLYYNISRLDPQTNSSQIKDEFQTLNIVTPRVRPEDCSIGLLPRNHDKNR
SMDVLPLDRCLPFLISVDGESSNYINAALMDSHKQPAAFVVTQHPLPNTVADFWRLVFDY
NCSSVVMLNEMDTAQFCMQYWPEKTSGCYGPIQVEFVSADIDEDIIHRIFRICNMARPQD
GYRIVQHLQYIGWPAYRDTPPSKRSLLKVVRRLEKWQEQYDGREGRTVVHCLNGGGRSG
TFCAICSVCEMIQQQNIIDVFHIVKTLRNNKSNMVETLEQYKFVYEVALEYLSSF

>Q9BZD2 multipass membrane protein
MAVVSEDDFQHSSNSTYRTTSSSLRADQEALLEKLLDRPPPGLQRPEDRFCGTYIIFFSLGI
GSLLPWNFFITAKEYWMFKLRNSSSPATGEDPEGSDILNYFESYLAVASTVPSMLCLVANFL
LVNRVAVHIRVLASLTVILAIFMVITALVKVDTSSWTRGFFAVTIVCMVILSGASTVFSSSIYG
MTGSFPMRNSQALISGGAMGGTVSAVASLVDLAASSDVRNSALAFFLTATVFLVLCMGLY
LLLSRLEYARYYMRPVLAAHVFSGEEELPQDSLSAPSVASRFIDSHTPPLRPILKKTASLGF
CVTYVFFITSLIYPAICTNIESLNKGSGSLWTTKFFIPLTTFLLYNFADLCGRQLTAWIQVPGP
NSKALPGFVLLRTCLIPLFVLCNYQPRVHLKTVVFQSDVYPALLSSLLGLSNGYLSTLALL
YGPKIVPRELAEATGVVMSFYVCLGLTLGSACSTLLVHLI

>P17813 single-pass type I membrane protein
MDRGTLPLAVALLLASCSLSPTSLAETVHCDLQPVGPERGEVTYTTSQVSKGCVAQAPNAI
LEVHVLFLEFPTGPSQLELTLQASKQNGTWPREVLLVLSVNSSVFLHLQALGIPLHLAYNS
SLVTFQEPPGVNTTELPSFPKTQILEWAAERGPITSAAELNDPQSILLRLGQAQGSLSFCML
EASQDMGRTLEWRPRTPALVRGCHLEGVAGHKEAHILRVLPGHSAGPRTVTVKVELSCAP
GDLDAVLILQGPPYVSWLIDANHNMQIWTTGEYSFKIFPEKNIRGFKLPDTPQGLLGEARM
LNASIVASFVELPLASIVSLHASSCGGRLQTSPAPIQTTPPKDTCSPELLMSLIQTKCADDAM
TLVLKKELVAHLKCTITGLTFWDPSCEAEDRGDKFVLRSAYSSCGMQVSASMISNEAVVNI
LSSSSPQRKKVHCLNMDSLSFQLGLYLSPHFLQASNTIEPGQQSFVQVRVSPSVSEFLLQLD
SCHLDLGPEGGTVELIQGRAAKGNCVSLLSPSPEGDPRFSFLLHFYTVPIPKTGTLSCTVAL
RPKTGSQDQEVHRTVFMRLNIISPDLSGCTSKGLVLPAVLGITFGAFLIGALLTAALWYIYS
HTRSPSKREPVVAVAAPASSESSSTNHSIGSTQSTPCSTSSMA

>Q96T54 multipass membrane protein
MYRPRARAAPEGRVRGCAVPSTVLLLLAYLAYLALGTGVFWTLEGRAAQDSSRSFQRDK
WELLQNFTCLDRPALDSLIRDVVQAYKNGASLLSNTTSMGRWELVGSFFFSVSTITTIGYG
NLSPNTMAARLFCIFFALVGIPLNLVVLNRLGHLMQQGVNHWASRLGGTWQDPDKARWL
AGSGALLSGLLLFLLLPPLLFSHMEGWSYTEGFYFAFITLSTVGFGDYVIGMNPSQRYPLW
YKNMVSLWILFGMAWLALIIKLILSQLETPGRVCSCCHHSSKEDFKSQSWRQGPDREPESH
SPQQGCYPEGPMGIIQHLEPSAHAAGCGKDS

>P43220 multipass membrane protein
MAGAPGPLRLALLLLGMVGRAGPRPQGATVSLWETVQKWREYRRQCQRSLTEDPPPATD
LFCNRTFDEYACWPDGEPGSFVNVSCPWYLPWASSVPQGHVYRFCTAEGLWLQKDNSSL
PWRDLSECEESKRGERSSPEEQLLFLYIIYTVGYALSFSALVIASAILLGFRHLHCTRNYIHL
NLFASFILRALSVFIKDAALKWMYSTAAQQHQWDGLLSYQDSLSCRLVFLLMQYCVAAN
YYWLLVEGVYLYTLLAFSVLSEQWIFRLYVSIGWGVPLLFVVPWGIVKYLYEDEGCWTR
NSNMNYWLIIRLPILFAIGVNFLIFVRVICIVVSKLKANLMCKTDIKCRLAKSTLTLIPLLGT
HEVIFAFVMDEHARGTLRFIKLFTELSFTSFQGLMVAILYCFVNNEVQLEFRKSWERWRLE
HLHIQRDSSMKPLKCPTSSLSSGATAGSSMYTATCQASCS

>Q99677 multipass membrane protein
MGDRRFIDFQFQDSNSSLRPRLGNATANNTCIVDDSFKYNLNGAVYSVVFILGLITNSVSLF

VFCFRMKMRSETAIFITNLAVSDLLFVCTLPFKIFYNFNRHWPFGDTLCKISGTAFLTNIYGS
MLFLTCISVDRFLAIVYPFRSRTIRTRRNSAIVCAGVWILVLSGGISASLFSTTNVNNATTTC
FEGFSKRVWKTYLSKITIFIEVVGFIIPLILNVSCSSVVLRTLRKPATLSQIGTNKKKVLKMIT
VHMAVFVVCFVPYNSVLFLYALVRSQAITNCFLERFAKIMYPITLCLATLNCCFDPFIYYFT
LESFQKSFYINAHIRMESLFKTETPLTTKPSLPAIQEEVSDQTTNNGGELMLESTF

>P61764 peripheral membrane protein
MAPIGLKAVVGEKIMHDVIKKVKKKGEWKVLVVDQLSMRMLSSCCKMTDIMTEGITIVE
DINKRREPLPSLEAVYLITPSEKSVHSLISDFKDPPTAKYRAAHVFFTDSCPDALFNELVKSR
AAKVIKTLTEINIAFLPYESQVYSLDSADSFQSFYSPHKAQMKNPILERLAEQIATLCATLKE
YPAVRYRGEYKDNALLAQLIQDKLDAYKADDPTMGEGPDKARSQLLILDRGFDPSSPVLH
ELTFQAMSYDLLPIENDVYKYETSGIGEARVKEVLLDEDDDLWIALRHKHIAEVSQEVTRS
LKDFSSSKRMNTGEKTTMRDLSQMLKKMPQYQKELSKYSTHLHLAEDCMKHYQGTVD
KLCRVEQDLAMGTDAEGEKIKDPMRAIVPILLDANVSTYDKIRIILLYIFLKNGITEENLNK
LIQHAQIPPEDSEIITNMAHLGVPIVTDSTLRRRSKPERKERISEQTYQLSRWTPIIKDIMEDT
IEDKLDTKHYPYISTRSSASFSTTAVSARYGHWHKNKAPGEYRSGPRLIIFILGGVSLNEMR
CAYEVTQANGKWEVLIGSTHILTPQKLLDTLKKLNKTDEEISS

>Q9Y271 multipass membrane protein
MDETGNLTVSSATCHDTIDDFRNQVYSTLYSMISVVGFFGNGFVLYVLIKTYHKKSAFQV
YMINLAVADLLCVCTLPLRVVYYVHKGIWLFGDFLCRLSTYALYVNLYCSIFFMTAMSFFR
CIAIVFPVQNINLVTQKKARFVCVGIWIFVILTSSPFLMAKPQKDEKNNTKCFEPPQDNQTK
NHVLVLHYVSLFVGFIIPFVIIIVCYTMIILTLLKKSMKKNLSSHKKAIGMIMVVTAAFLVSF
MPYHIQRTIHLHFLHNETKPCDSVLRMQKSVVITLSLAASNCCFDPLLYFFSGGNFRKRLS
TFRKHSLSSVTYVPRKKASLPEKGEEICKV

>P21452 multipass membrane protein
MGTCDIVTEANISSGPESNTTGITAFSMPSWQLALWATAYLALVLVAVTGNAIVIWIILAHR
RMRTVTNYFIVNLALADLCMAAFNAAFNFVYASHNIWYFGRAFCYFQNLFPITAMFVSIY
SMTAIAADRYMAIVHPFQPRLSAPSTKAVIAGIWLVALALASPQCFYSTVTMDQGATKCVV
AWPEDSGGKTLLLYHLVVIALIYFLPLAVMFVAYSVIGLTLWRRAVPGHQAHGANLRHLQ
AMKKFVKTMVLVVLTFAICWLPYHLYFILGSFQEDIYCHKFIQQVYLALFWLAMSSTMYN
PIIYCCLNHRFRSGFRLAFRCCPWVTPTKEDKLELTPTTSLSTRVNRCHTKETLFMAGDTAP
SEATSGEAGRPQDGSGLWFGYGLLAPTKTHVEI

>O75712 multipass membrane protein
MDWKTLQALLSGVNKYSTAFGRIWLSVVFVFRVLVYVVAAERVWGDEQKDFDCNTKQP
GCTNVCYDNYFPISNIRLWALQLIFVTCPSLLVILHVAYREERERRHRQKHGDQCAKLYDN
AGKKHGGLWWTYLFSLIFKLIIEFLFLYLLHTLWHGFNMPRLVQCANVAPCPNIVDCYIAR
PTEKKIFTYFMVGASAVCIVLTICELCYLICHRVLRGLHKDKPRGGCSPSSSASRASTCRCH
HKLVEAGEVDPDPGNNKLQASAPNLTPI

>Q6ZMZ0 multipass membrane protein
MGSEKDSESPRSTSLHAAAPDPKCRSGGRRRRLTLHSVFSASARGRRARAKPQAEPPPPAA

QPPPAPAPAAAQGPPPEALPAEPAAEAEAEAAAAAAEPGFDDEEAAEGGGPGAEEVECPL
CLVRLPPERAPRLLSCPHRSCRDCLRHYLRLEISESRVPISCPECSERLNPHDIRLLLADPPL
MHKYEEFMLRRYLASDPDCRWCPAPDCGYAVIAYGCASCPKLTCEREGCQTEFCYHCKQI
WHPNQTCDMARQQRAQTLRVRTKHTSGLSYGQESGPADDIKPCPRCSAYIIKMNDGSCN
HMTCAVCGCEFCWLCMKEISDLHYLSPSGCTFWGKKPWSRKKKILWQLGTLIGAPVGISL
IAGIAIPAMVIGIPVYVGRKIHSRYEGRKTSKHKRNLAITGGVTLSVIASPVIAAVSVGIGVPI
MLAYVYGVVPISLCRGGGCGVSTANGKGVKIEFDEDDGPITVADAWRALKNPSIGESSIEG
LTSVLSTSGSPTDGLSVMQGPYSETASFAALSGGTLSGGILSSGKGKYSRLEVQADVQKEIF
PKDTASLGAISDNASTRAMAGSIISSYNPQDRECNNMEIQVDIEAKPSHYQLVSGSSTEDSL
HVHAQMAENEEEGSGGGGSEEDPPCRHQSCEQKDCLASKPWDISLAQPESIRSDLESSDA
QSDDVPDITSDECGSPRSHTAACPSTPRAQGAPSPSAHMNLSALAEGQTVLKPEGGEARV

>Q8NAU1 single-pass type I membrane protein
MHPGSPSAWPPRARAALRLWLGCVCFALVQADSPSAPVNVTVRHLKANSAVVSWDVLE
DEVVIGFAISQQKKDVRMLRFIQEVNTTTRSCALWDLEEDTEYIVHVQAISIQGQSPASEPV
LFKTPREAEKMASKNKDEVTMKEMGRNQQLRTGEVLIIVVVLFMWAGVIALFCRQYDIIK
DNEPNNNKEKTKSASETSTPEHQGGGLLRSKI

>P46020 lipid-anchor
MRSRSNSGVRLDGYARLVQQTILCHQNPVTGLLPASYDQKDAWVRDNVYSILAVWGLGL
AYRKNADRDEDKAKAYELEQSVVKLMRGLLHCMIRQVDKVESFKYSQSTKDSLHAKYN
TKTCATVVGDDQWGHLQLDATSVYLLFLAQMTASGLHIIHSLDEVNFIQNLVFYIEAAYKT
ADFGIWERGDKTNQGISELNASSVGMAKAALEALDELDLFGVKGGPQSVIHVLADEVQH
CQSILNSLLPRASTSKEVDASLLSVVSFPAFAVEDSQLVELTKQEIITKLQGRYGCCRFLRDG
YKTPKEDPNRLYYEPAELKLFENIECEWPLFWTYFILDGVFSGNAEQVQEYKEALEAVLIK
GKNGVPLLPELYSVPPDRVDEEYQNPHTVDRVPMGKLPHMWGQSLYILGSLMAEGFLAP
GEIDPLNRRFSTVPKPDVVVQVSILAETEEIKTILKDKGIYVETIAEVYPIRVQPARILSHIYS
SLGCNNRMKLSGRPYRHMGVLGTSKLYDIRKTIFTFTPQFIDQQQFYLALDNKMIVEMLR
TDLSYLCSRWRMTGQPTITFPISHSMLDEDGTSLNSSILAALRKMQDGYFGGARVQTGKL
SEFLTTSCCTHLSFMDPGPEGKLYSEDYDDNYDYLESGNWMNDYDSTSHARCGDEVARY
LDHLLAHTAPHPKLAPTSQKGGLDRFQAAVQTTCDLMSLVTKAKELHVQNVHMYLPTKL
FQASRPSFNLLDSPHPRQENQVPSVRVEIHLPRDQSGEVDFKALVLQLKETSSLQEQADILY
MLYTMKGPDWNTELYNERSATVRELLTELYGKVGEIRHWGLIRYISGILRKKVEALDEAC
TDLLSHQKHLTVGLPPEPREKTISAPLPYEALTQLIDEASEGDMSISILTQEIMVYLAMYMR
TQPGLFAEMFRLRIGLIIQVMATELAHSLRCSAEEATEGLMNLSPSAMKNLLHHILSGKEF
GVERSVRPTDSNVSPAISIHEIGAVGATKTERTGIMQLKSEIKQVEFRRLSISAESQSPGTSM
TPSSGSFPSAYDQQSSKDSRQGQWQRRRRLDGALNRVPVGFYQKVWKVLQKCHGLSVE
GFVLPSSTTREMTPGEIKFSVHVESVLNRVPQPEYRQLLVEAILVLTMLADIEIHSIGSIIAVE
KIVHIANDLFLQEQKTLGADDTMLAKDPASGICTLLYDSAPSGRFGTMTYLSKAAATYVQ
EFLPHSICAMQ

>Q92805 peripheral membrane protein
MFAKLKKKIAEETAVAQRPGGATRIPRSVSKESVASMGADSGDDFASDGSSSREDLSSQLL
RRNEQIRKLEARLSDYAEQVRNLQKIKEKLEIALEKHQDSSMRKFQEQNETFQANRAKM

AEGLALALARKDQEWSEKMDQLEKEKNILTAQLQEMKNQSMNLFQRRDEMDELEGFQQ
QELSKIKHMLLKKEESLGKMEQELEARTRELSRTQEELMNSNQMSSDLSQKLEELQRHYS
TLEEQRDHVIASKTGAESKITALEQKEQELQALIQQLSIDLQKVTAETQEKEDVITHLQEKV
ASLEKRLEQNLSGEEHLQELLKEKTLAEQNLEDTRQQLLAARSSQAKAINTLETRVRELE
QTLQASEEQLQQSKGIVAAQETQIQELAAANQESSHVQQQALALEQQFLERTQALEAQIV
ALERTRAADQTTAEQGMRQLEQENAALKECRNEYERSLQNHQFELKKLKEEWSQREIVS
VAMAQALEEVRKQREEFQQQAANLTAIIDEKEQNLREKTEVLLQKEQEILQLERGHNSAL
LQIHQLQAELEALRTLKAEEAAVVAEQEDLLRLRGPLQAEALSVNESHVTSRAMQDPVFQ
LPTAGRTPNGEVGAMDLTQLQKEKQDLEQQLLEKNKTIKQMQQRMLELRKTLQKELKIR
PDNELFEVREKPGPEMANMAPSVTNNTDLTDAREINFEYLKHVVLKFMSCRESEAFHLIK
AVSVLLNFSQEEENMLKETLEYKMSWFGSKPAPKGSIRPSISNPRIPWS

>Q5IJ48 single-pass type I membrane protein
MALARPGTPDPQALASVLLLLLWAPALSLLAGTVPSEPPSACASDPCAPGTECQATESGGY
TCGPMEPRGCATQPCHHGALCVPQGPDPTGFRCYCVPGFQGPRCELDIDECASRPCHHGA
TCRNLADRYECHCPLGYAGVTCEMEVDECASAPCLHGGSCLDGVGSFRCVCAPGYGGTR
CQLDLDECQSQPCAHGGTCHDLVNGFRCDCAGTGYEGTHCEREVLECASAPCEHNASCL
EGLGSFRCLCWPGYSGELCEVDEDECASSPCQHGGRCLQRSDPALYGGVQAAFPGAFSFR
HAAGFLCHCPPGFEGADCGVEVDECASRPCLNGGHCQDLPNGFQCHCPDGYAGPTCEED
VDECLSDPCLHGGTCSDTVAGYICRCPETWGGRDCSVQLTGCQGHTCPLAATCIPIFESGV
HSYVCHCPPGTHGPFCGQNTTFSVMAGSPIQASVPAGGPLGLALRFRTTLPAGTLATRNDT
KESLELALVAATLQATLWSYSTTVLVLRLPDLALNDGHWHQVEVVLHLATLELRLWHEG
CPARLCVASGPVALASTASATPLPAGISSAQLGDATFAGCLQDVRVDGHLLLPEDLGENVL
LGCERREQCRPLPCVHGGSCVDLWTHFRCDCARPHRGPTCADEIPAATFGLGGAPSSASFL
LQELPGPNLTVSFLLRTRESAGLLLQFANDSAAGLTVFLSEGRIRAEVPGSPAVVLPGRWD
DGLRHLVMLSFGPDQLQDLGQHVHVGGRLLAADSQPWGGPFRGCLQDLRLDGCHLPFFP
LPLDNSSQPSELGGRQSWNLTAGCVSEDMCSPDPCFNGGTCLVTWNDFHCTCPANFTGPT
CAQQLWCPGQPCLPPATCEEVPDGFVCVAEATFREGPPAAFSGHNASSGRLLGGLSLAFRT
RDSEAWLLRAAAGALEGVWLAVRNGSLAGGVRGGHGLPGAVLPIPGPRVADGAWHRVR
LAMERPAATTSRWLLWLDGAATPVALRGLASDLGFLQGPGAVRILLAENFTGCLGRVALG
GLPLPLARPRPGAAPGAREHFASWPGTPAPILGCRGAPVCAPSPCLHDGACRDLFDAFACA
CGPGWEGPRCEAHVDPCHSAPCARGRCHTHPDGRFECRCPPGFGGPRCRLPVPSKECSLN
VTCLDGSPCEGGSPAANCSCLEGLAGQRCQVPTLPCEANPCLNGGTCRAAGGVSECICNA
RFSGQFCEVAKGLPLPLPFPLLEVAVPAACACLLLLLLLGLLSGILAARKRRQSEGTYSPSQQ
EVAGARLEMDSVLKVPPEERLI

>Q99679 multipass membrane protein
MNSTLDGNQSSHPFCLLAFGYLETVNFCLLEVLIIVFLTVLIISGNIIVIFVFHCAPLLNHHTT
SYFIQTMAYADLFVGVSCVVPSLSLLHHPLPVEESLTCQIFGFVVSVLKSVSMASLACISIDR
YIAITKPLTYNTLVTPWRLRLCIFLIWLYSTLVFLPSFFHWGKPGYHGDVFQWCAESWHTD
SYFTLFIVMMLYAPAALIVCFTYFNIFRICQQHTKDISERQARFSSQSGETGEVQACPDKRY
AMVLFRITSVFYILWLPYIIYFLLESSTGHSNRFASFLTTWLAISNSFCNCVIYSLSNSVFQRG
LKRLSGAMCTSCASQTTANDPYTVRSKGPLNGCHI

>O60241 multipass membrane protein
MENTGWMGKGHRMTPACPLLLSVILSLRLATAFDPAPSACSALASGVLYGAFSLQDLFPTI
ASGCSWTLENPDPTKYSLYLRFNRQEQVCAHFAPRLLPLDHYLVNFTCLRPSPEEAVAQAE
SEVGRPEEEEAEAAAGLELCSGSGPFTFLHFDKNFVQLCLSAEPSEAPRLLAPAALAFRFV
EVLLINNNNSSQFTCGVLCRWSEECGRAAGRACGFAQPGCSCPGEAGAGSTTTTSPGPPA
AHTLSNALVPGGPAPPAEADLHSGSSNDLFTTEMRYGEEPEEEPKVKTQWPRSADEPGLY
MAQTGDPAAEEWSPWSVCSLTCGQGLQVRTRSCVSSPYGTLCSGPLRETRPCNNSATCPV
HGVWEEWGSWSLCSRSCGRGSRSRMRTCVPPQHGGKACEGPELQTKLCSMAACPVEGQ
WLEWGPWGPCSTSCANGTQQRSRKCSVAGPAWATCTGALTDTRECSNLECPATDSKWGP
WNAWSLCSKTCDTGWQRRFRMCQATGTQGYPCEGTGEEVKPCSEKRCPAFHEMCRDEY
VMLMTWKKAAAGEIIYNKCPPNASGSASRRCLLSAQGVAYWGLPSFARCISHEYRYLYLS
LREHLAKGQRMLAGEGMSQVVRSLQELLARRTYYSGDLLFSVDILRNVTDTFKRATYVP
SADDVQRFFQVVSFMVDAENKEKWDDAQQVSPGSVHLLRVVEDFIHLVGDALKAFQSSL
IVTDNLVISIQREPVSAVSSDITFPMRGRRGMKDWVRHSEDRLFLPKEVLSLSSPGKPATSG
AAGSPGRGRGPGTVPPGPGHSHQRLLPADPDESSYFVIGAVLYRTLGLILPPPRPPLAVTSR
VMTVTVRPPTQPPAEPLITVELSYIINGTTDPHCASWDYSRADASSGDWDTENCQTLETQ
AAHTRCQCQHLSTFAVLAQPPKDLTLELAGSPSVPLVIGCAVSCMALLTLLAIYAAFWRFIK
SERSIILLNFCLSILASNILILVGQSRVLSKGVCTMTAAFLHFFFLSSFCWVLTEAWQSYLAVI
GRMRTRLVRKRFLCLGWGLPALVVAVSVGFTRTKGYGTSSYCWLSLEGGLLYAFVGPAAV
IVLVNMLIGIIVFNKLMARDGISDKSKKQRAGSERCPWASLLLPCSACGAVPSPLLSSASAR
NAMASLWSSCVVLPLLALTWMSAVLAMTDRRSVLFQALFAVFNSAQGFVITAVHCFLRRE
VQDVVKCQMGVCRADESEDSPDSCKNGQLQILSDFEKDVDLACQTVLFKEVNTCNPSTI
TGTLSRLSLDEDEEPKSCLVGPEGSLSFSPLPGNILVPMAASPGLGEPPPPQEANPVYMCGE
GGLRQLDLTWLRPTEPGSEGDYMVLPRRTLSLQPGGGGGGGEDAPRARPEGTPRRAAKT
VAHTEGYPSFLSVDHSGLGLGPAYGSLQNPYGMTFQPPPPTPSARQVPEPGERSRTMPRTV
PGSTMKMGSLERKKLRYSDLDFEKVMHTRKRHSELYHELNQKFHTFDRYRSQSTAKREK
RWSVSSGGAAERSVCTDKPSPGERPSLSQHRRHQSWSTFKSMTLGSLPPKPRERLTLHRA
AAWEPTEPPDGDFQTEV

>Q8NGR4 multipass membrane protein
MNSENLTRAAVAPAEFVLLGITNRWDLRVALFLTCLPVYLVSLLGNMGMALLIRMDARLH
TPMYFFLANLSLLDACYSSAIGPKMLVDLLLPRATIPYTACALQMFVFAGLADTECCLLAA
MAYDRYVAIRNPLLYTTAMSQRLCLALLGASGLGGAVSAFVHTTLTFRLSFCRSRKINSFF
CDIPPLLAISCSDTSLNELLLFAICGFIQTATVLAITVSYGFIAGAVIHMRSVEGSRRAASTGG
SHLTAVAMMYGTLIFMYLRPSSSYALDTDKMASVFYTLVIPSLNPLIYSLRNKEVKEALRQ
TWSRFHCPGQGSQ

>Q8NGR2 multipass membrane protein
MSYFYRLKLMKEAVLVKLPFTSLPLLLQTLSRKSRDMEIKNYSSSTSGFILLGLSSNPQLQK
PLFAIFLIMYLLAAVGNVLIIPAIYSDPRLHTPMYFFLSNLSFMDICFTTVIVPKMLVNFLSET
KVISYVGCLAQMYFFMAFGNTDSYLLASMAIDRLVAICNPLHYDVVMKPRHCLLMLLGS
CSISHLHSLFRVLLMSRLSFCASHIIKHFFCDTQPVLKLSCSDTSSSQMVVMTETLAVIVTPF
LCIIFSYLRIMVTVLRIPSAAGKWKAFSTCGSHLTAVALFYGSIIYVYFRPLSMYSVVRDRVA
TVMYTVVTPMLNPFIYSLRNKDMKRGLKKLQDRIYR

>Q8NH94 multipass membrane protein
MERNHNPDNCNVLNFFFADKKNKRRNFGQIVSDVGRICYSVSLSLGEPTTMGRNNLTRPS
EFILLGLSSRPEDQKPLFAVFLPIYLITVIGNLLIILAIRSDTRLQTPMYFFLSILSFVDICYVTV
IIPKMLVNFLSETKTISYSECLTQMYFFLAFGNTDSYLLAAMAIDRYVAICNPFHYITIMSHR
CCVLLLVLSFCIPHFHSLLHILLTNQLIFCASNVIHHFFCDDQPVLKLSCSSHFVKEITVMTE
GLAVIMTPFSCIIISYLRILITVLKIPSAAGKRKAFSTCGSHLTVVTLFYGSISYLYFQPLSNYT
VKDQIATIIYTVLTPMLNPFIYSLRNKDMKQGLAKLMHRMKCQ

>Q8NGR9 multipass membrane protein
MEGFYLRRSHELQGMGKPGRVNQTTVSDFLLLGLSEWPEEQPLLFGIFLGMYLVTMVGN
LLIILAISSDPHLHTPMYFFLANLSLTDACFTSASIPKMLANIHTQSQIISYSGCLAQLYFLLM
FGGLDNCLLAVMAYDRYVAICQPLHYSTSMSPQLCALMLGVCWVLTNCPALMHTLLLTR
VAFCAQKAIPHFYCDPSALLKLACSDTHVNELMIITMGLLFLTVPLLLIVFSYVRIFWAVFVI
SSPGGRWKAFSTCGSHLTVVLLFYGSLMGVYLLPPSTYSTERESRAAVLYMVIIPTLNPFIY
SLRNRDMKEALGKLFVSGKTFFL

>P49682 multipass membrane protein
MVLEVSDHQVLNDAEVAALLENFSSSYDYGENESDSCCTSPPCPQDFSLNFDRAFLPALYS
LLFLLGLLGNGAVAAVLLSRRTALSSTDTFLLHLAVADTLLVLTLPLWAVDAAVQWVFGSG
LCKVAGALFNINFYAGALLLACISFDRYLNIVHATQLYRRGPPARVTLTCLAVWGLCLLFAL
PDFIFLSAHHDERLNATHCQYNFPQVGRTALRVLQLVAGFLLPLLVMAYCYAHILAVLLVS
RGQRRLRAMRLVVVVVVAFALCWTPYHLVVLVDILMDLGALARNCGRESRVDVAKSVTS
GLGYMHCCLNPLLYAFVGVKFRERMWMLLLRLGCPNQRGLQRQPSSSRRDSSWSETSEA
SYSGL

>Q9UBV8 peripheral membrane protein
MASYPYRQGCPGAAGQAPGAPPGSYYPGPPNSGGQYGSGLPPGGGYGGPAPGGPYGPPA
GGGPYGHPNPGMFPSGTPGGPYGGAAPGGPYGQPPPSSYGAQQPGLYGQGGAPPNVDPE
AYSWFQSVDSDHSGYISMKELKQALVNCNWSSFNDETCLMMINMFDKTKSGRIDVYGFS
ALWKFIQQWKNLFQQYDRDRSGSISYTELQQALSQMGYNLSPQFTQLLVSRYCPRSANPA
MQLDRFIQVCTQLQVLTEAFREKDTAVQGNIRLSFEDFVTMTASRML

>O43613 multipass membrane protein
MEPSATPGAQMGVPPGSREPSPVPPDYEDEFLRYLWRDYLYPKQYEWVLIAAYVAVFVVA
LVGNTLVCLAVWRNHHMRTVTNYFIVNLSLADVLVTAICLPASLLVDITESWLFGHALCK
VIPYLQAVSVSVAVLTLSFIALDRWYAICHPLLFKSTARRARGSILGIWAVSLAIMVPQAAV
MECSSVLPELANRTRLFSVCDERWADDLYPKIYHSCFFIVTYLAPLGLMAMAYFQIFRKLW
GRQIPGTTSALVRNWKRPSDQLGDLEQGLSGEPQPRARAFLAEVKQMRARRKTAKMLM
VVLLVFALCYLPISVLNVLKRVFGMFRQASDREAVYACFTFSHWLVYANSAANPIIYNFLS
GKFREQFKAAFSCCLPGLGPCGSLKAPSPRSSASHKSLSLQSRCSISKISEHVVLTSVTTVLP

>P61106 lipid-anchor
MATAPYNYSYIFKYIIIGDMGVGKSCLLHQFTEKKFMADCPHTIGVEFGTRIIEVSGQKIKL

QIWDTAGQERFRAVTRSYYRGAAGALMVYDITRRSTYNHLSSWLTDARNLTNPNTVIILIG
NKADLEAQRDVTYEEAKQFAEENGLLFLEASAKTGENVEDAFLEAAKKIYQNIQDGSLD
LNAAESGVQHKPSAPQGGRLTSEPQPQREGCGC

>O00206 single-pass type I membrane protein
MMSASRLAGTLIPAMAFLSCVRPESWEPCVEVVPNITYQCMELNFYKIPDNLPFSTKNLDL
SFNPLRHLGSYSFFSFPELQVLDLSRCEIQTIEDGAYQSLSHLSTLILTGNPIQSLALGAFSGL
SSLQKLVAVETNLASLENFPIGHLKTLKELNVAHNLIQSFKLPEYFSNLTNLEHLDLSSNKIQ
SIYCTDLRVLHQMPLLNLSLDLSLNPMNFIQPGAFKEIRLHKLTLRNNFDSLNVMKTCIQG
LAGLEVHRLVLGEFRNEGNLEKFDKSALEGLCNLTIEEFRLAYLDYYLDDIIDLFNCLTNVS
SFSLVSVTIERVKDFSYNFGWQHLELVNCKFGQFPTLKLKSLKRLTFTSNKGGNAFSEVDL
PSLEFLDLSRNGLSFKGCCSQSDFGTTSLKYLDLSFNGVITMSSNFLGLEQLEHLDFQHSN
LKQMSEFSVFLSLRNLIYLDISHTHTRVAFNGIFNGLSSLEVLKMAGNSFQENFLPDIFTELR
NLTFLDLSQCQLEQLSPTAFNSLSSLQVLNMSHNNFFSLDTFPYKCLNSLQVLDYSLNHIM
TSKKQELQHFPSSLAFLNLTQNDFACTCEHQSFLQWIKDQRQLLVEVERMECATPSDKQG
MPVLSLNITCQMNKTIIGVSVLSVLVVSVVAVLVYKFYFHLMLLAGCIKYGRGENIYDAFV
IYSSQDEDWVRNELVKNLEEGVPPFQLCLHYRDFIPGVAIAANIIHEGFHKSRKVIVVVSQ
HFIQSRWCIFEYEIAQTWQFLSSRAGIIFIVLQKVEKTLLRQQVELYRLLSRNTYLEWEDSV
LGRHIFWRRLRKALLDGKSWNPEGTVGTGCNWQEATSI

>P09769 lipid-anchor, peripheral membrane protein
MGCVFCKKLEPVATAKEDAGLEGDFRSYGAADHYGPDPTKARPASSFAHIPNYSNFSSQAI
NPGFLDSGTIRGVSGIGVTLFIALYDYEARTEDDLTFTKGEKFHILNNTEGDWWEARSLSS
GKTGCIPSNYVAPVDSIQAEEWYFGKIGRKDAERQLLSPGNPQGAFLIRESETTKGAYSLSI
RDWDQTRGDHVKHYKIRKLDMGGYYITTRVQFNSVQELVQHYMEVNDGLCNLLIAPCTI
MKPQTLGLAKDAWEISRSSITLERRLGTGCFGDVWLGTWNGSTKVAVKTLKPGTMSPKA
FLEEAQVMKLLRHDKLVQLYAVVSEEPIYIVTEFMCHGSLLDFLKNPEGQDLRLPQLVDM
AAQVAEGMAYMERMNYIHRDLRAANILVGERLACKIADFGLARLIKDDEYNPCQGSKFPI
KWTAPEAALFGRFTIKSDVWSFGILLTELITKGRIPYPGMNKREVLEQVEQGYHMPCPPGC
PASLYEAMEQTWRLDPEERPTFEYLQSFLEDYFTSAEPQYQPGDQT

>P46089 multipass membrane protein
MMWGAGSPLAWLSAGSGNVNVSSVGPAEGPTGPAAPLPSPKAWDVVLCISGTLVSCENA
LVVAIIVGTPAFRAPMFLLVGSLAVADLLAGLGLVLHFAAVFCIGSAEMSLVLVGVLAMAFT
ASIGSLLAITVDRYLSLYNALTYYSETTVTRTYVMLALVWGGALGLGLLPVLAWNCLDGL
TTCGVVYPLSKNHLVVLAIAFFMVFGIMLQLYAQICRIVCRHAQQIALQRHLLPASHYVAT
RKGIATLAVVLGAFAACWLPFTVYCLLGDAHSPPLYTYLTLLPATYNSMINPIIYAFRNQDV
QKVLWAVCCCCSSSKIPFRSRSPSDV

>Q16572 multipass membrane protein
MESAEPAGQARAAATKLSEAVGAALQEPRRQRRLVLVIVCVALLLDNMLYMVIVPIVPDYI
AHMRGGGEGPTRTPEVWEPTLPLPTPANASAYTANTSASPTAAWPAGSALRPRYPTESEDV
KIGVLFASKAILQLLVNPLSGPFIDRMSYDVPLLIGLGVMFASTVLFAFAEDYATLFAARSL
QGLGSAFADTSGIAMIADKYPEEPERSRALGVALAFISFGSLVAPPFGGILYEFAGKRVPFLV

LAAVSLFDALLLLAVAKPFSAAARARANLPVGTPIHRLMLDPYIAVVAGALTTCNIPLAFLE
PTIATWMKHTMAASEWEMGMAWLPAFVPHVLGVYLTVRLAARYPHLQWLYGALGLAVI
GASSCIVPACRSFAPLVVSLCGLCFGIALVDTALLPTLAFLVDVRHVSVYGSVYAIADISYSV
AYALGPIVAGHIVHSLGFEQLSLGMGLANLLYAPVLLLLRNVGLLTRSRSERDVLLDEPPQ
GLYDAVRLRERPVSGQDGEPRSPPGPFDACEDDYNYYYTRS

>Q14833 multipass membrane protein
MPGKRGLGWWWARLPLCLLLSLYGPWMPSSLGKPKGHPHMNSIRIDGDITLGGLFPVHG
RGSEGKPCGELKKEKGIHRLEAMLFALDRINNDPDLLPNITLGARILDTCSRDTHALEQSL
TFVQALIEKDGTEVRCGSGGPPIITKPERVVGVIGASGSSVSIMVANILRLFKIPQISYASTAP
DLSDNSRYDFFSRVVPSDTYQAQAMVDIVRALKWNYVSTVASEGSYGESGVEAFIQKSRE
DGGVCIAQSVKIPREPKAGEFDKIIRRLLETSNARAVIIFANEDDIRRVLEAARRANQTGHFF
WMGSDSWGSKIAPVLHLEEVAEGAVTILPKRMSVRGFDRYFSSRTLDNNRRNIWFAEFWE
DNFHCKLSRHALKKGSHVKKCTNRERIGQDSAYEQEGKVQFVIDAVYAMGHALHAMHR
DLCPGRVGLCPRMDPVDGTQLLKYIRNVNFSGIAGNPVTFNENGDAPGRYDIYQYQLRN
DSAEYKVIGSWTDHLHLRIERMHWPGSGQQLPRSICSLPCQPGERKKTVKGMPCCWHCE
PCTGYQYQVDRYTCKTCPYDMRPTENRTGCRPIPIIKLEWGSPWAVLPLFLAVVGIAATLF
VVITFVRYNDTPIVKASGRELSYVLLAGIFLCYATTFLMIAEPDLGTCSLRRIFLGLGMSISY
AALLTKTNRIYRIFEQGKRSVSAPRFISPASQLAITFSLISLQLLGICVWFVVDPSHSVVDFQ
DQRTLDPRFARGVLKCDISDLSLICLLGYSMLLMVTCTVYAIKTRGVPETFNEAKPIGFTM
YTTCIVWLAFIPIFFGTSQSADKLYIQTTTLTVSVSLSASVSLGMLYMPKVYIILFHPEQNVP
KRKRSLKAVVTAATMSNKFTQKGNFRPNGEAKSELCENLEAPALATKQTYVTYTNHAI

>O15431 multipass membrane protein
MDHSHHMGMSYMDSNSTMQPSHHHPTTSASHSHGGGDSSMMMMPMTFYFGFKNVELL
FSGLVINTAGEMAGAFVAVFLLAMFYEGLKIARESLLRKSQVSIRYNSMPVPGPNGTILME
THKTVGQQMLSFPHLLQTVLHIIQVVISYFLMLIFMTYNGYLCIAVAAGAGTGYFLFSWK
KAVVVDITEHCH

>P31358 GPI-anchor, lipid-anchor
MKRFLFLLLTISLLVMVQIQTGLSGQNDTSQTSSPSASSNISGGIFLFFVANAIIHLFCFS

>Q9UMY4 peripheral membrane protein
MSDTAVADTRRLNSKPQDLTDAYGPPSNFLEIDIFNPQTVGVGRARFTTYEVRMRTNLPIF
KLKESCVRRRYSDFEWLKNELERDSKIVVPPLPGKALKRQLPFRGDEGIFEESFIEERRQGL
EQFINKIAGHPLAQNERCLHMFLQEEAIDRNYVPGKSLAVSCPGWSAVA

>Q9BRI3 multipass membrane protein
MEAKEKQHLLDARPAIRSYTGSLWQEGAGWIPLPRPGLDLQAIELAAQSNHHCHAQKGP
DSHCDPKKGKAQRQLYVASAICLLFMIGEVVEILGALVSVLSIWVVTGVLVYLAVERLISG
DYEIDGGTMLITSGCAVAVNIIMGLTLHQSGHGHSHGTTNQQEENPSVRAAFIHVIGDFMQ
SMGVLVAAYILYFKPEYKYVDPICTFVFSILVLGTTLTILRDVILVLMEGTPKGVDFTAVRDL
LLSVEGVEALHSLHIWALTVAQPVLSVHIAIAQNTDAQAVLKTASSRLQGKFHFHTVTIQIE
DYSEDMKDCQACQGPSD

>P50151 lipid-anchor
MSSGASASALQRLVEQLKLEAGVERIKVSQAAAELQQYCMQNACKDALLVGVPAGSNPF
REPRSCALL

>P50391 multipass membrane protein
MNTSHLLALLLPKSPQGENRSKPLGTPYNFSEHCQDSVDVMVFIVTSYSIETVVGVLGNL
CLMCVTVRQKEKANVTNLLIANLAFSDFLMCLLCQPLTAVYTIMDYWIFGETLCKMSAFI
QCMSVTVSILSLVLVALERHQLIINPTGWKPSISQAYLGIVLIWVIACVLSLPFLANSILENVF
HKNHSKALEFLADKVVCTESWPLAHHRTIYTTFLLLFQYCLPLGFILVCYARIYRRLQRQG
RVFHKGTYSLRAGHMKQVNVVLVVMVVAFAVLWLPLHVFNSLEDWHHEAIPICHGNLIFL
VCHLLAMASTCVNPFIYGFLNTNFKKEIKALVLTCQQSAPLEESEHLPLSTVHTEVSKGSL
RLSGRSNPI

>Q14573 multipass membrane protein
MSEMSSFLHIGDIVSLYAEGSVNGFISTLGLVDDRCVVEPAAGDLDNPPKKFRDCLFKVCP
MNRYSAQKQYWKAKQTKQDKEKIADVVLLQKLQHAAQMEQKQNDTENKKVHGDVVK
YGSVIQLLHMKSNKYLTVNKRLPALLEKNAMRVTLDATGNEGSWLFIQPFWKLRSNGDN
VVVGDKVILNPVNAGQPLHASNYELSDNAGCKEVNSVNCNTSWKINLFMQFRDHLEEVL
KGGDVVRLFHAEQEKFLTCDEYKGKLQVFLRTTLRQSATSATSSNALWEVEVVHHDPCR
GGAGHWNGLYRFKHLATGNYLAAEENPSYKGDASDPKAAGMGAQGRTGRRNAGEKIK
YCLVAVPHGNDIASLFELDPTTLQKTDSFVPRNSYVRLRHLCTNTWIQSTNVPIDIEEERPIR
LMLGTCPTKEDKEAFAIVSVPVSEIRDLDFANDASSMLASAVEKLNEGFISQNDRRFVIQLL
EDLVFFVSDVPNNGQNVLDIMVTKPNRERQKLMREQNILKQVFGILKAPFREKGGEGPLV
RLEELSDQKNAPYQHMFRLCYRVLRHSQEDYRKNQEHIAKQFGMMQSQIGYDILAEDTIT
ALLHNNRKLLEKHITKTEVETFVSLVRKNREPRFLDYLSDLCVSNHIAIPVTQELICKCVLD
PKNSDILIRTELRPVKEMAQSHEYLSIEYSEEEVWLTWTDKNNEHHEKSVRQLAQEARAG
NAHDENVLSYYRYQLKLFARMCLDRQYLAIDEISQQLGVDLIFLCMADEMLPFDLRASFC
HLMLHVHVDRDPQELVTPVKFARLWTEIPTAITIKDYDSNLNASRDDKKNKFANTMEFVE
DYLNNVVSEAVPFANEEKNKLTFEVVSLAHNLIYFGFYSFSELLRLTRTLLGIIDCVQGPPA
MLQAYEDPGGKNVRRSIQGVGHMMSTMVLSRKQSVFSAPSLSAGASAAEPLDRSKFEEN
EDIVVMETKLKILEILQFILNVRLDYRISYLLSVFKKEFVEVFPMQDSGADGTAPAFDSTTA
NMNLDRIGEQAEAMFGVGKTSSMLEVDDEGGRMFLRVLIHLTMHDYAPLVSGALQLLFK
HFSQRQEAMHTFKQVQLLISAQDVENYKVIKSELDRLRTMVEKSELWVDKKGSGKGEEV
EAGAAKDKKERPTDEEGFLHPPGEKSSENYQIVKGILERLNKMCGVGEQMRKKQQRLLK
NMDAHKVMLDLLQIPYDKGDAKMMEILRYTHQFLQKFCAGNPGNQALLHKHLHLFLTP
GLLEAETMQHIFLNNYQLCSEISEPVLQHFVHLLATHGRHVQYLDFLHTVIKAEGKYVKK
CQDMIMTELTNAGDDVVVFYNDKASLAHLLDMMKAARDGVEDHSPLMYHISLVDLLAA
CAEGKNVYTEIKCTSLLPLEDVVSVVTHEDCITEVKMAYVNFVNHCYVDTEVEMKEIYTS
NHIWTLFENFTLDMARVCSKREKRVADPTLEKYVLSVVLDTINAFFSSPFSENSTSLQTHQ
TIVVQLLQSTTRLLECPWLQQQHKGSVEACIRTLAMVAKGRAILLPMDLDAHISSMLSSG
ASCAAAAQRNASSYKATTRAFPRVTPTANQWDYKNIIEKLQDIITALEERLKPLVQAELSV
LVDVLHWPELLFLEGSEAYQRCESGGFLSKLIQHTKDLMESEEKLCIKVLRTLQQMLLKK
TKYGDRGNQLRKMLLQNYLQNRKSTSRGDLPDPIGTGLDPDWSAIAATQCRLDKEGATK

LVCDLITSTKNEKIFQESIGLAIHLLDGGNTEIQKSFHNLMMSDKKSERFFKVLHDRMKRA
QQETKSTVAVNMNDLGSQPHEDREPVDPTTKGRVASFSIPGSSSRYSLGPSLRRGHEVSER
VQSSEMGTSVLIMQPILRFLQLLCENHNRDLQNFLRCQNNKTNYNLVCETLQFLDIMCGS
TTGGLGLLGLYINEDNVGLVIQTLETLTEYCQGPCHENQTCIVTHESNGIDIITALILNDISPL
CKYRMDLVLQLKDNASKLLLALMESRHDSENAERILISLRPQELVDVIKKAYLQEEERENS
EVSPREVGHNIYILALQLSRHNKQLQHLLKPVKRIQEEEAEGISSMLSLNNKQLSQMLKSS
APAQEEEEDPLAYYENHTSQIEIVRQDRSMEQIVFPVPGICQFLTEETKHRLFTTTEQDEQG
SKVSDFFDQSSFLHNEMEWQRKLRSMPLIYWFSRRMTLWGSISFNLAVFINIIIAFFYPYME
GASTGVLDSPLISLLFWILICFSIAALFTKRYSIRPLIVALILRSIYYLGIGPTLNILGALNLTNK
IVFVVSFVGNRGTFIRGYKAMVMDMEFLYHVGYILTSVLGLFAHELFYSILLFDLIYREETL
FNVIKSVTRNGRSILLTALLALILVYLFSIVGFLFLKDDFILEVDRLPNNHSTASPLGMPHGA
AAFVDTCSGDKMDCVSGLSVPEVLEEDRELDSTERACDTLLMCIVTVMNHGLRNGGGV
GDILRKPSKDESLFPARVVYDLLFFFIVIIIVLNLIFGVIIDTFADLRSEKQKKEEILKTTCFIC
GLERDKFDNKTVSFEEHIKLEHNMWNYLYFIVLVRVKNKTDYTGPESYVAQMIKNKNLD
WFPRMRAMSLVSNEGEGEQNEIRILQDKLNSTMKLVSHLTAQLNELKEQMTEQRKRRQR
LGFVDVQNCISR

>Q9NR34 single-pass type II membrane protein
MLMRKVPGFVPASPWGLRLPQKFLFLLFLSGLVTLCFGALFLLPHSSRLKRLFLAPRTQQP
GLEVVAEIAGHAPAREQEPPPNPAPAAPAPGEDDPSSWASPRRRKGGLRRTRPTGPREEATA
ARGNSIPASRPGDEGVPFRFDFNAFRSRLRHPVLGTRADESQEPQSQVRAQREKIKEMMQ
FAWQSYKRYAMGKNELRPLTKDGYEGNMFGGLSGATVIDSLDTLYLMELKEEFQEAKAW
VGESFHLNVSGEASLFEVNIRYIGGLLSAFYLTGEEVFRIKAIRLGEKLLPAFNTPTGIPKGV
VSFKSGNWGWATAGSSSILAEFGSLHLEFLHLTELSGNQVFAEKVRNIRKVLRKIEKPFGLY
PNFLSPVSGNWVQHHVSVGGLGDSFYEYLIKSWLMSGKTDMEAKNMYYEALEAIETYL
LNVSPGGLTYIAEWRGGILDHKMGHLACFSGGMIALGAEDAKEEKRAHYRELAAQITKT
CHESYARSDTKLGPEAFWFNSGREAVATQLSESYYILRPEVVESYMYLWRQTHNPIYREW
GWEVVLALEKYCRTEAGFSGIQDVYSSTPNHDNKQQSFFLAETLKYLYLLFSEDDLLSLE
DWVFNTEAHPLPVNHSDSSGRAWGRH

>Q9HCC8 multipass membrane protein
MAESPGCCSVWARCLHCLYSCHWRKCPRERMQTSKCDCIWFGLLFLTFLLSLSWLYIGLV
LLNDLHNFNEFLFRRWGHWMDWSLAFLLVISLLVTYASLLLVLALLLRLCRQPLHLHSLH
KVLLLLIMLLVAAGLVGLDIQWQQEWHSLRVSLQATAPFLHIGAAAGIALLAWPVADTFY
RIHRRGPKILLLLLLFFGVVLVIYLAPLCISSPCIMEPRDLPPKPGLVGHRGAPMLAPENTLMS
LRKTAECGATVFETDVMVSSDGVPFLMHDEHLSRTTNVASVFPTRITAHSSDFSWTELKRL
NAGSWFLERRPFWGAKPLAGPDQKEAESQTVPALEELLEEAAALNLSIMFDLRRPPQNHT
YYDTFVIQTLETVLNARVPQAMVFWLPDEDRANVQRRAPGMRQIYGRQGGNRTERPQFL
NLPYQDLPLLDIKALHKDNVSVNLFVVNKPWLFSLLWCAGVDSVTTNDCQLLQQMRYPI
WLITPQTYLIIWVITNCVSTMLLLWTFLLQRRFVKKRGKTGLETAVLLTRINNFMME

>P09917 peripheral membrane protein
MPSYTVTVATGSQWFAGTDDYIYLSLVGSAGCSEKHLLDKPFYNDFERGAVDSYDVTVDE
ELGEIQLVRIEKRKYWLNDDWYLKYITLKTPHGDYIEFPCYRWITGDVEVVLRDGRAKLA

RDDQIHILKQHRRKELETRQKQYRWMEWNPGFPLSIDAKCHKDLPRDIQFDSEKGVDFVL
NYSKAMENLFINRFMHMFQSSWNDFADFEKIFVKISNTISERVMNHWQEDLMFGYQFLN
GCNPVLIRRCTELPEKLPVTTEMVECSLERQLSLEQEVQQGNIFIVDFELLDGIDANKTDPC
TLQFLAAPICLLYKNLANKIVPIAIQLNQIPGDENPIFLPSDAKYDWLLAKIWVRSSDFHVH
QTITHLLRTHLVSEVFGIAMYRQLPAVHPIFKLLVAHVRFTIAINTKAREQLICECGLFDKAN
ATGGGGHVQMVQRAMKDLTYASLCFPEAIKARGMESKEDIPYYFYRDDGLLVWEAIRTF
TAEVVDIYYEGDQVVEEDPELQDFVNDVYVYGMRGRKSSGFPKSVKSREQLSEYLTVVIF
TASAQHAAVNFGQYDWCSWIPNAPPTMRAPPPTAKGVVTIEQIVDTLPDRGRSCWHLGA
VWALSQFQENELFLGMYPEEHFIEKPVKEAMARFRKNLEAIVSVIAERNKKKQLPYYYLS
PDRIPNSVAI

>Q5YKI7 peripheral membrane protein
MKEEDSSFKLCVPGIVALQSPPNKAFRSTDTVGFLESELKKLLGMQQESRLWKLGSQEGR
ELLTRPEITVVEGEGYEVQRRLRHLPSPISVAQCLLLEEKGEMGNWPPE

>P34972 multipass membrane protein
MEECWVTEIANGSKDGLDSNPMKDYMILSGPQKTAVAVLCTLLGLLSALENVAVLYLILSS
HQLRRKPSYLFIGSLAGADFLASVVFACSFVNFHVFHGVDSKAVFLLKIGSVTMTFTASVG
SLLLTAIDRYLCLRYPPSYKALLTRGRALVTLGIMWVLSALVSYLPLMGWTCCPRPCSELF
PLIPNDYLLSWLLFIAFLFSGIIYTYGHVLWKAHQHVASLSGHQDRQVPGMARMRLDVRL
AKTLGLVLAVLLICWFPVLALMAHSLATTLSDQVKKAFAFCSMLCLINSMVNPVIYALRSG
EIRSSAHHCLAHWKKCVRGLGSEAKEEAPRSSVTETEADGKITPWPDSRDLDLSDC

>P51582 multipass membrane protein
MASTESSLLRSLGLSPGPGSSEVELDCWFDEDFKFILLPVSYAVVFVLGLGLNAPTLWLFIF
RLRPWDATATYMFHLALSDTLYVLSLPTLIYYYAAHNHWPFGTEICKFVRFLFYWNLYCS
VLFLTCISVHRYLGICHPLRALRWGRPRLAGLLCLAVWLVVAGCLVPNLFFVTTSNKGTTV
LCHDTTRPEEFDHYVHFSSAVMGLLFGVPCLVTLVCYGLMARRLYQPLPGSAQSSSRLRSL
RTIAVVLTVFAVCFVPFHITRTIYYLARLLEADCRVLNIVNVVYKVTRPLASANSCLDPVLY
LLTGDKYRRQLRQLCGGGKPQPRTAASSLALVSLPEDSSCRWAATPQDSSCSTPRADRL

>Q92838 single-pass type II membrane protein
MGYPEVERRELLPAAAPRERGSQGCGCGGAPARAGEGNSCLLFLGFFGLSLALHLLTLCC
YLELRSELRRERGAESRLGGSGTPGTSGTLSSLGGLDPDSPITSHLGQPSPKQQPLEPGEAA
LHSDSQDGHQMALLNFFFPDEKPYSEEESRRVRRNKRSKSNEGADGPVKNKKKGKKAGP
PGPNGPPGPPGPPGPQGPPGIPGIPGIPGTTVMGPPGPPGPPGPQGPPGLQGPSGAADKAGT
RENQPAVVHLQGQGSAIQVKNDLSGGVLNDWSRITMNPKVFKLHPRSGELEVLVDGTYFI
YSQVEVYYINFTDFASYEVVVDEKPFLQCTRSIETGKTNYNTCYTAGVCLLKARQKIAVK
MVHADISINMSKHTTFFGAIRLGEAPAS

>Q13873 single-pass type I membrane protein
MTSSLQRPWRVPWLPWTILLVSTAAASQNQERLCAFKDPYQQDLGIGESRISHENGTILCS
KGSTCYGLWEKSKGDINLVKQGCWSHIGDPQECHYEECVVTTTPPSIQNGTYRFCCCSTD
LCNVNFTENFPPPDTTPLSPPHSFNRDETIIIALASVSVLAVLIVALCFGYRMLTGDRKQGLH

SMNMMEAAASEPSLDLDNLKLLELIGRGRYGAVYKGSLDERPVAVKVFSFANRQNFINEK
NIYRVPLMEHDNIARFIVGDERVTADGRMEYLLVMEYYPNGSLCKYLSLHTSDWVSSCRL
AHSVTRGLAYLHTELPRGDHYKPAISHRDLNSRNVLVKNDGTCVISDFGLSMRLTGNRLV
RPGEEDNAAISEVGTIRYMAPEVLEGAVNLRDCESALKQVDMYALGLIYWEIFMRCTDLF
PGESVPEYQMAFQTEVGNHPTFEDMQVLVSREKQRPKFPEAWKENSLAVRSLKETIEDCW
DQDAEARLTAQCAEERMAELMMIWERNKSVSPTVNPMSTAMQNERNLSHNRRVPKIGPY
PDYSSSSYIEDSIHHTDSIVKNISSEHSMSSTPLTIGEKNRNSINYERQQAQARIPSPETSVTS
LSTNTTTTNTTGLTPSTGMTTISEMPYPDETNLHTTNVAQSIGPTPVCLQLTEEDLETNKLD
PKEVDKNLKESSDENLMEHSLKQFSGPDPLSSTSSSLLYPLIKLAVEATGQQDFTQTANGQ
ACLIPDVLPTQIYPLPKQQNLPKRPTSLPLNTKNSTKEPRLKFGSKHKSNLKQVETGVAKM
NTINAAEPHVVTVTMNGVAGRNHSVNSHAATTQYANGTVLSGQTTNIVTHRAQEMLQN
QFIGEDTRLNINSSPDEHEPLLRREQQAGHDEGVLDRLVDRRERPLEGGRTNSNNNNSNPC
SEQDVLAQGVPSTAADPGPSKPRRAQRPNSLDLSATNVLDGSSIQIGESTQDGKSGSGEKI
KKRVKTPYSLKRWRPSTWVISTESLDCEVNNNGSNRAVHSKSSTAVYLAEGGTATTMVSK
DIGMNCL

>P29323 single-pass type I membrane protein
MALRRLGAALLLLPLLAAVEETLMDSTTATAELGWMVHPPSGWEEVSGYDENMNTIRTY
QVCNVFESSQNNWLRTKFIRRRGAHRIHVEMKFSVRDCSSIPSVPGSCKETFNLYYYEADF
DSATKTFPNWMENPWVKVDTIAADESFSQVDLGGRVMKINTEVRSFGPVSRSGFYLAFQ
DYGGCMSLIAVRVFYRKCPRIIQNGAIFQETLSGAESTSLVAARGSCIANAEEVDVPIKLYC
NGDGEWLVPIGRCMCKAGFEAVENGTVCRGCPSGTFKANQGDEACTHCPINSRTTSEGAT
NCVCRNGYYRADLDPLDMPCTTIPSAPQAVISSVNETSLMLEWTPPRDSGGREDLVYNIIC
KSCGSGRGACTRCGDNVQYAPRQLGLTEPRIYISDLLAHTQYTFEIQAVNGVTDQSPFSPQ
FASVNITTNQAAPSAVSIMHQVSRTVDSITLSWSQPDQPNGVILDYELQYYEKELSEYNAT
AIKSPTNTVTVQGLKAGAIYVFQVRARTVAGYGRYSGKMYFQTMTEAEYQTSIQEKLPLII
GSSAAGLVFLIAVVVIAIVCNRRGFERADSEYTDKLQHYTSGHMTPGMKIYIDPFTYEDPN
EAVREFAKEIDISCVKIEQVIGAGEFGEVCSGHLKLPGKREIFVAIKTLKSGYTEKQRRDFLS
EASIMGQFDHPNVIHLEGVVTKSTPVMIITEFMENGSLDSFLRQNDGQFTVIQLVGMLRGI
AAGMKYLADMNYVHRDLAARNILVNSNLVCKVSDFGLSRFLEDDTSDPTYTSALGGKIPI
RWTAPEAIQYRKFTSASDVWSYGIVMWEVMSYGERPYWDMTNQDVINAIEQDYRLPPP
MDCPSALHQLMLDCWQKDRNHRPKFGQIVNTLDKMIRNPNSLKAMAPLSSGINLPLLDR
TIPDYTSFNTVDEWLEAIKMGQYKESFANAGFTSFDVVSQMMMEDILRVGVTLAGHQKK
ILNSIQVMRAQMNQIQSVEGQPLARRPRATGRTKRCQPRDVTKKTCNSNDGKKKGMGKK
KTDPGRGREIQGIFFKEDSHKESNDCSCGG

>Q9H461 multipass membrane protein
MEWGYLLEVTSLLAALALLQRSSGAAAASAKELACQEITVPLCKGIGYNYTYMPNQFNH
DTQDEAGLEVHQFWPLVEIQCSPDLKFFLCSMYTPICLEDYKKPLPPCRSVCERAKAGCAP
LMRQYGFAWPDRMRCDRLPEQGNPDTLCMDYNRTDLTTAAPSPPRRLPPPPPGEQPPSGS
GHGRPPGARPPHRGGGRGGGGGDAAAPPARGGGGGGKARPPGGGAAPCEPGCQCRAPM
VSVSSERHPLYNRVKTGQIANCALPCHNPFFSQDERAFTVFWIGLWSVLCFVSTFATVSTFL
IDMERFKYPERPIIFLSACYLFVSVGYLVRLVAGHEKVACSGGAPGAGGAGGAGGAAAGA
GAAGAGAGGPGGRGEYEELGAVEQHVRYETTGPALCTVVFLLVYFFGMASSIWWVILSLT

WFLAAGMKWGNEAIAGYSQYFHLAAWLVPSVKSIAVLALSSVDGDPVAGICYVGNQSLD
NLRGFVLAPLVIYLFIGTMFLLAGFVSLFRIRSVIKQQDGPTKTHKLEKLMIRLGLFTVLYT
VPAAVVVACLFYEQHNRPRWEATHNCPCLRDLQPDQARRPDYAVFMLKYFMCLVVGITS
GVWVWSGKTLESWRSLCTRCCWASKGAAVGGGAGATAAGGGGGPGGGGGGGPGGGG
GPGGGGGSLYSDVSTGLTWRSGTASSVSYPKQMPLSQV

>Q8ND76 lipid-anchor
MGNTTSCCVSSSPKLRRNAHSRLESYRPDTDLSREDTGCNLQHISDRENIDDLNMEFNPSD
HPRASTIFLSKSQTDVREKRKSLFINHHPPGQIARKYSSCSTIFLDDSTVSQPNLKYTIKCVA
LAIYYHIKNRDPDGRMLLDIFDENLHPLSKSEVPPDYDKHNPEQKQIYRFVRTLFSAAQLT
AECAIVTLVYLERLLTYAEIDICPANWKRIVLGAILLASKVWDDQAVWNVDYCQILKDITV
EDMNELERQFLELLQFNINVPSSVYAKYYFDLRSLAEANNLSFPLEPLSRERAHKLEAISRL
CEDKYKDLRRSARKRSASADNLTLPRWSPAIIS

>Q8WWI5 multipass membrane protein
MGCCSSASSAAQSSKREWKPLEDRSCTDIPWLLLFILFCIGMGFICGFSIATGAAARLVSGY
DSYGNICGQKNTKLEAIPNSGMDHTQRKYVFFLDPCNLDLINRKIKSVALCVAACPRQEL
KTLSDVQKFAEINGSALCSYNLKPSEYTTSPKSSVLCPKLPVPASAPIPFFHRCAPVNISCYA
KFAEALITFVSDNSVLHRLISGVMTSKEIILGLCLLSLVLSMILMVIIRYISRVLVWILTILVIL
GSLGGTGVLWWLYAKQRRSPKETVTPEQLQIAEDNLRALLIYAISATVFTVILFLIMLVMR
KRVALTIALFHVAGKVFIHLPLLVFQPFWTFFALVLFWVYWIMTLLFLGTTGSPVQNEQGF
VEFKISGPLQYMWWYHVVGLIWISEFILACQQMTVAGAVVTYYFTRDKRNLPFTPILASV
NRLIRYHLGTVAKGSFIITLVKIPRMILMYIHSQLKGKENACARCVLKSCICCLWCLEKCLN
YLNQNAYTATAINSTNFCTSAKDAFVILVENALRVATINTVGDFMLFLGKVLIVCSTGLAGI
MLLNYQQDYTVWVLPLIIVCLFAFLVAHCFLSIYEMVVDVLFLCFAIDTKYNDGSPGREFY
MDKVLMEFVENSRKAMKEAGKGGVADSRELKPMASGASSA

>O95477 multipass membrane protein
MACWPQLRLLLWKNLTFRRRQTCQLLLEVAWPLFIFLILISVRLSYPPYEQHECHFPNKAM
PSAGTLPWVQGIICNANNPCFRYPTPGEAPGVVGNFNKSIVARLFSDARRLLLYSQKDTSM
KDMRKVLRTLQQIKKSSSNLKLQDFLVDNETFSGFLYHNLSLPKSTVDKMLRADVILHKV
FLQGYQLHLTSLCNGSKSEEMIQLGDQEVSELCGLPREKLAAAERVLRSNMDILKPILRTL
NSTSPFPSKELAEATKTLLHSLGTLAQELFSMRSWSDMRQEVMFLTNVNSSSSSTQIYQAV
SRIVCGHPEGGGLKIKSLNWYEDNNYKALFGGNGTEEDAETFYDNSTTPYCNDLMKNLE
SSPLSRIIWKALKPLLVGKILYTPDTPATRQVMAEVNKTFQELAVFHDLEGMWEELSPKIW
TFMENSQEMDLVRMLLDSRDNDHFWEQQLDGLDWTAQDIVAFLAKHPEDVQSSNGSVY
TWREAFNETNQAIRTISRFMECVNLNKLEPIATEVWLINKSMELLDERKFWAGIVFTGITPG
SIELPHHVKYKIRMDIDNVERTNKIKDGYWDPGPRADPFEDMRYVWGGFAYLQDVVEQA
IIRVLTGTEKKTGVYMQQMPYPCYVDDIFLRVMSRSMPLFMTLAWIYSVAVIIKGIVYEKE
ARLKETMRIMGLDNSILWFSWFISSLIPLLVSAGLLVVILKLGNLLPYSDPSVVFVFLSVFAV
VTILQCFLISTLFSRANLAAACGGIIYFTLYLPYVLCVAWQDYVGFTLKIFASLLSPVAFGFG
CEYFALFEEQGIGVQWDNLFESPVEEDGFNLTTSVSMMLFDTFLYGVMTWYIEAVFPGQY
GIPRPWYFPCTKSYWFGEESDEKSHPGSNQKRISEICMEEEPTHLKLGVSIQNLVKVYRDG
MKVAVDGLALNFYEGQITSFLGHNGAGKTTTMSILTGLFPPTSGTAYILGKDIRSEMSTIRQ

NLGVCPQHNVLFDMLTVEEHIWFYARLKGLSEKHVKAEMEQMALDVGLPSSKLKSKTSQ
LSGGMQRKLSVALAFVGGSKVVILDEPTAGVDPYSRRGIWELLLKYRQGRTIILSTHHMD
EADVLGDRIAIISHGKLCCVGSSLFLKNQLGTGYYLTLVKKDVESSLSSCRNSSSTVSYLK
KEDSVSQSSSDAGLGSDHESDTLTIDVSAISNLIRKHVSEARLVEDIGHELTYVLPYEAAKE
GAFVELFHEIDDRLSDLGISSYGISETTLEEIFLKVAEESGVDAETSDGTLPARRNRRAFGD
KQSCLRPFTEDDAADPNDSDIDPESRETDLLSGMDGKGSYQVKGWKLTQQQFVALLWKR
LLIARRSRKGFFAQIVLPAVFVCIALVFSLIVPPFGKYPSLELQPWMYNEQYTFVSNDAPED
TGTLELLNALTKDPGFGTRCMEGNPIPDTPCQAGEEEWTTAPVPQTIMDLFQNGNWTMQ
NPSPACQCSSDKIKKMLPVCPPGAGGLPPPQRKQNTADILQDLTGRNISDYLVKTYVQIIAK
SLKNKIWVNEFRYGGFSLGVSNTQALPPSQEVNDAIKQMKKHLKLAKDSSADRFLNSLG
RFMTGLDTKNNVKVWFNNKGWHAISSFLNVINNAILRANLQKGENPSHYGITAFNHPLNL
TKQQLSEVALMTTSVDVLVSICVIFAMSFVPASFVVFLIQERVSKAKHLQFISGVKPVIYWL
SNFVWDMCNYVVPATLVIIIFICFQQKSYVSSTNLPVLALLLLLYGWSITPLMYPASFVFKIP
STAYVVLTSVNLFIGINGSVATFVLELFTDNKLNNINDILKSVFLIFPHFCLGRGLIDMVKNQ
AMADALERFGENRFVSPLSWDLVGRNLFAMAVEGVVFFLITVLIQYRFFIRPRPVNAKLSP
LNDEDEDVRRERQRILDGGGQNDILEIKELTKIYRRKRKPAVDRICVGIPPGECFGLLGVNG
AGKSSTFKMLTGDTTVTRGDAFLNKNSILSNIHEVHQNMGYCPQFDAITELLTGREHVEFF
ALLRGVPEKEVGKVGEWAIRKLGLVKYGEKYAGNYSGGNKRKLSTAMALIGGPPVVFLD
EPTTGMDPKARRFLWNCALSVVKEGRSVVLTSHSMEECEALCTRMAIMVNGRFRCLGSV
QHLKNRFGDGYTIVVRIAGSNPDLKPVQDFFGLAFPGSVLKEKHRNMLQYQLPSSLSSLA
RIFSILSQSKKRLHIEDYSVSQTTLDQVFVNFAKDQSDDDHLKDLSLHKNQTVVDVAVLTS
FLQDEKVKESYV

>P47736 peripheral membrane protein
MIEKMQGSRMDEQRCSFPPPLKTEEDYIPYPSVHEVLGREGPFPLILLPQFGGYWIEGTNH
EITSIPETEPLQSPTTKVKLECNPTARIYRKHFLGKEHFNYYSLDAALGHLVFSLKYDVIGD
QEHLRLLLRTKCRTYHDVIPISCLTEFPNVVQMAKLVCEDVNVDRFYPVLYPKASRLIVTF
DEHVISNNFKFGVIYQKLGQTSEEELFSTNEESPAFVEFLEFLGQKVKLQDFKGFRGGLDV
THGQTGTESVYCNFRNKEIMFHVSTKLPYTEGDAQQLQRKRHIGNDIVAVVFQDENTPFV
PDMIASNFLHAYVVVQAEGGGPDGPLYKVSVTARDDVPFFGPPLPDPAVFRKGPEFQEFLL
TKLINAEYACYKAEKFAKLEERTRAALLETLYEELHIHSQSMMGLGGDEDKMENGSGGG
GFFESFKRVIRSRSQSMDAMGLSNKKPNTVSTSHSGSFAPNNPDLAKAAGISLIVPGKSPTR
KKSGPFGSRRSSAIGIENIQEVQEKRESPPAGQKTPDSGHVSQEPKSENSSTQSSPEMPTTK
NRAETAAQRAEALKDFSRSSSSASSFASVVEETEGVDGEDTGLESVSSSGTPHKRDSFIYST
WLEDSVSTTSGGSSPGPSRSPHPDAGKLGDPACPEIKIQLEASEQHMPQLGC

>Q8NGS6 multipass membrane protein
MIVQLICTVCFLAVNTFHVRSSFDFLKADDMGEINQTLVSEFLLLGLSGYPKIEIVYFALILV
MYLVILIGNGVLIIASIFDSHFHTPMYFFLGNLSFLDICYTSSSVPSTLVSLISKKRNISFSGCA
VQMFFGFAMGSTECLLLGMMAFDRYVAICNPLRYPIILSKVAYVLMASVSWLSGGINSAV
QTLLAMRLPFCGNNIINHFACEILAVLKLACADISLNIITMVISNMAFLVLPLMVIFFSYMFIL
YTILQMNSATGRRKAFSTCSAHLTVVIIFYGTIFFMYAKPKSQDLIGEEKLQALDKLISLFY
GVVTPMLNPILYSLRNKDVKAAVKYLLNKKPIH

>P05186 GPI-anchor, lipid-anchor
MISPFLVLAIGTCLTNSLVPEKEKDPKYWRDQAQETLKYALELQKLNTNVAKNVIMFLGD
GMGVSTVTAARILKGQLHHNPGEETRLEMDKFPFVALSKTYNTNAQVPDSAGTATAYLCG
VKANEGTVGVSAATERSRCNTTQGNEVTSILRWAKDAGKSVGIVTTTRVNHATPSAAYAH
SADRDWYSDNEMPPEALSQGCKDIAYQLMHNIRDIDVIMGGGRKYMYPKNKTDVEYES
DEKARGTRLDGLDLVDTWKSFKPRYKHSHFIWNRTELLTLDPHNVDYLLGLFEPGDMQY
ELNRNNVTDPSLSEMVVVAIQILRKNPKGFFLLVEGGRIDHGHHEGKAKQALHEAVEMDR
AIGQAGSLTSSEDTLTVVTADHSHVFTFGGYTPRGNSIFGLAPMLSDTDKKPFTAILYGNGP
GYKVVGGERENVSMVDYAHNNYQAQSAVPLRHETHGGEDVAVFSKGPMAHLLHGVHE
QNYVPHVMAYAACIGANLGHCAPASSAGSLAAGPLLLALALYPLSVLF

>P28067 single-pass type I membrane protein
MGHEQNQGAALLQMLPLLWLLPHSWAVPEAPTPMWPDDLQNHTFLHTVYCQDGSPSVG
LSEAYDEDQLFFFDFSQNTRVPRLPEFADWAQEQGDAPAILFDKEFCEWMIQQIGPKLDGK
IPVSRGFPIAEVFTLKPLEFGKPNTLVCFVSNLFPPMLTVNWHDHSVPVEGFGPTFVSAVDG
LSFQAFSYLNFTPEPSDIFSCIVTHEIDRYTAIAYWVPRNALPSDLLENVLCGVAFGLGVLGI
IVGIVLIIYFRKPCSGD

>Q8IYR6 single-pass type I membrane protein
MGAAAAEAPLRLPAAPPLAFCCYTSVLLLFAFSLPGSRASNQPPGGGGGSGGDCPGGKGK
SINCSELNVRESDVRVCDESSCKYGGVCKEDGDGLKCACQFQCHTNYIPVCGSNGDTYQ
NECFLRRAACKHQKEITVIARGPCYSDNGSGSGEGEEEGSGAEVHRKHSKCGPCKYKAE
CDEDAENVGCVCNIDCSGYSFNPVCASDGSSYNNPCFVREASCIKQEQIDIRHLGHCTDTD
DTSLLGKKDDGLQYRPDVKDASDQREDVYIGNHMPCPENLNGYCIHGKCEFIYSTQKAS
CRCESGYTGQHCEKTDFSILYVVPSRQKLTHVLIAAIIGAVQIAIIVAIVMCITRKCPKNNRG
RRQKQNLGHFTSDTSSRMV

>P42892 single-pass type II membrane protein
MRGVWPPPVSALLSALGMSTYKRATLDEEDLVDSLSEGDAYPNGLQVNFHSPRSGQRCW
AARTQVEKRLVVLVVLLAAGLVACLAALGIQYQTRSPSVCLSEACVSVTSSILSSMDPTVD
PCHDFFSYACGGWIKANPVPDGHSRWGTFSNLWEHNQAIIKHLLENSTASVSEAERKAQV
YYRACMNETRIEELRAKPLMELIERLGGWNITGPWAKDNFQDTLQVVTAHYRTSPFFSVY
VSADSKNSNSNVIQVDQSGLGLPSRDYYLNKTENEKVLTGYLNYMVQLGKLLGGGDEEA
IRPQMQQILDFETALANITIPQEKRRDEELIYHKVTAAELQTLAPAINWLPFLNTIFYPVEIN
ESEPIVVYDKEYLEQISTLINTTDRCLLNNYMIWNLVRKTSSFLDQRFQDADEKFMEVMY
GTKKTCLPRWKFCVSDTENNLGFALGPMFVKATFAEDSKSIATEIILEIKKAFEESLSTLKW
MDEETRKSAKEKADAIYNMIGYPNFIMDPKELDKVFNDYTAVPDLYFENAMRFFNFSWR
VTADQLRKAPNRDQWSMTPPMVNAYYSPTKNEIVFPAGILQAPFYTRSSPKALNFGGIGV
VVGHELTHAFDDQGREYDKDGNLRPWWKNSSVEAFKRQTECMVEQYSNYSVNGEPVN
GRHTLGENIADNGGLKAAYRAYQNWVKKNGAEHSLPTLGLTNNQLFFLGFAQVWCSVR
TPESSHEGLITDPHSPSRFRVIGSLSNSKEFSEHFRCPPGSPMNPPHKCEVW

>Q9NXF1 multipass membrane protein
MTKKRKRQHDFQKVKLKVGKKKPKLQNATPTNFKTKTIHLPEQLKEDGTLPTNNRKLNI

KDLLSQMHHYNAGVKQSALLGLKDLLSQYPFIIDAHLSNILSEVTAVFTDKDANVRLAAV
QLLQFLAPKIRAEQISPFFPLVSAHLSSAMTHITEGIQEDSLKVLDILLEQYPALITGRSSILL
KNFVELISHQQLSKGLINRDRSQSWILSVNPNRRLTSQQWRLKVLVRLSKFLQALADGSSR
LRESEGLQEQKENPHATSNSIFINWKEHANDQQHIQVYENGGSQPNVSSQFRLRYLVGGLS
GVDEGLSSTENLKGFIEIIIPLLIECWVEAVPPQLATPVGNGIEREPLQVMQQVLNIISLLWK
LSKQQDETHKLESWLRKNYLIDFKHHFMSRFPYVLKEITKHKRKEPNKSIKHCTVLSNNI
DHLLLNLTLSDIMVSLANASTLQKDCSWIEMIRKFVTETLEDGSRLNSKQLNRLLGVSWR
LMQIQPNREDTETLIKAVYTLYQQRGLILPVRTLLLKFFSKIYQTEELRSCRFRYRSKVLSR
WLAGLPLQLAHLGSRNPELSTQLIDIIHTAAARANKELLKSLQATALRIYDPQEGAVVVLP
ADSQQRLVQLVYFLPSLPADLLSRLSRCCIMGRLSSSLAAMLIGILHMRSSFSGWKYSAKD
WLMSDVDYFSFLFSTLTGFSKEELTWLQSLRGVPHVIQTQLSPVLLYLTDLDQFLHHWDV
TEAVFHSLLVIPARSQNFDILQSAISKHLVGLTVIPDSTAGCVFGVICKLLDHTCVVSETLLPF
LASCCYSLLYFLLTIEKGEAEHLRKRDKLWGVCVSILALLPRVLRLMLQSLRVNRVGPEEL
PVVGQLLRLLLQHAPLRTHMLTNAILVQQIIKNITTLKSGSVQEQWLTDLHYCFNVYITGH
PQGPSALATVY

>P01906 single-pass type I membrane protein
MILNKALLLGALALTAVMSPCGGEDIVADHVASYGVNFYQSHGPSGQYTHEFDGDEEFYV
DLETKETVWQLPMFSKFISFDPQSALRNMAVGKHTLEFMMRQSNSTAATNEVPEVTVFSK
FPVTLGQPNTLICLVDNIFPPVVNITWLSNGHSVTEGVSETSFLSKSDHSFFKISYLTFLPSA
DEIYDCKVEHWGLDEPLLKHWEPEIPAPMSELTETLVCALGLSVGLMGIVVGTVFIIQGLR
SVGASRHQGLL

>P05556 single-pass type I membrane protein
MNLQPIFWIGLISSVCCVFAQTDENRCLKANAKSCGECIQAGPNCGWCTNSTFLQEGMPT
SARCDDLEALKKKGCPPDDIENPRGSKDIKKNKNVTNRSKGTAEKLKPEDITQIQPQQLVL
RLRSGEPQTFTLKFKRAEDYPIDLYYLMDLSYSMKDDLENVKSLGTDLMNEMRRITSDFR
IGFGSFVEKTVMPYISTTPAKLRNPCTSEQNCTSPFSYKNVLSLTNKGEVFNELVGKQRISG
NLDSPEGGFDAIMQVAVCGSLIGWRNVTRLLVFSTDAGFHFAGDGKLGGIVLPNDGQCHL
ENNMYTMSHYYDYPSIAHLVQKLSENNIQTIFAVTEEFQPVYKELKNLIPKSAVGTLSANS
SNVIQLIIDAYNSLSSEVILENGKLSEGVTISYKSYCKNGVNGTGENGRKCSNISIGDEVQFE
ISITSNKCPKKDSDSFKIRPLGFTEEVEVILQYICECECQSEGIPESPKCHEGNGTFECGACR
CNEGRVGRHCECSTDEVNSEDMDAYCRKENSSEICSNNGECVCGQCVCRKRDNTNEIYS
GKFCECDNFNCDRSNGLICGGNGVCKCRVCECNPNYTGSACDCSLDTSTCEASNGQICNG
RGICECGVCKCTDPKFQGQTCEMCQTCLGVCAEHKECVQCRAFNKGEKKDTCTQECSYF
NITKVESRDKLPQPVQPDPVSHCKEKDVDDCWFYFTYSVNGNNEVMVHVVENPECPTGP
DIIPIVAGVVAGIVLIGLALLLIWKLLMIIHDRREFAKFEKEKMNAKWDTGENPIYKSAVTT
VVNPKYEGK

>P36897 single-pass type I membrane protein
MEAAVAAPRPRLLLLVLAAAAAAAAALLPGATALQCFCHLCTKDNFTCVTDGLCFVSVTE
TTDKVIHNSMCIAEIDLIPRDRPFVCAPSSKTGSVTTTYCCNQDHCNKIELPTTVKSSPGLG
PVELAAVIAGPVCFVCISLMLMVYICHNRTVIHHRVPNEEDPSLDRPFISEGTTLKDLIYDM
TTSGSGSGLPLLVQRTIARTIVLQESIGKGRFGEVWRGKWRGEEVAVKIFSSREERSWFREA

EIYQTVMLRHENILGFIAADNKDNGTWTQLWLVSDYHEHGSLFDYLNRYTVTVEGMIKL
ALSTASGLAHLHMEIVGTQGKPAIAHRDLKSKNILVKKNGTCCIADLGLAVRHDSATDTID
IAPNHRVGTKRYMAPEVLDDSINMKHFESFKRADIYAMGLVFWEIARRCSIGGIHEDYQLP
YYDLVPSDPSVEEMRKVVCEQKLRPNIPNRWQSCEALRVMAKIMRECWYANGAARLTAL
RIKKTLSQLSQQEGIKM

>P39656 single-pass type I membrane protein
MGYFRCARAGSFGRRRKMEPSTAARAWALFWLLLPLLGAVCASGPRTLVLLDNLNVRET
HSLFFRSLKDRGFELTFKTADDPSLSLIKYGEFLYDNLIIFSPSVEDFGGNINVETISAFIDGG
GSVLVAASSDIGDPLRELGSECGIEFDEEKTAVIDHHNYDISDLGQHTLIVADTENLLKAPTI
VGKSSLNPILFRGVGMVADPDNPLVLDILTGSSTSYSFFPDKPITQYPHAVGKNTLLIAGLQ
ARNNARVIFSGSLDFFSDSFFNSAVQKAAPGSQRYSQTGNYELAVALSRWVFKEEGVLRV
GPVSHHRVGETAPPNAYTVTDLVEYSIVIQQLSNGKWVPFDGDDIQLEFVRIDPFVRTFLK
KKGGKYSVQFKLPDVYGVFQFKVDYNRLGYTHLYSSTQVSVRPLQHTQYERFIPSAYPYY
ASAFSMMLGLFIFSIVFLHMKEKEKSD

>Q15109 single-pass type I membrane protein
MAAGTAVGAWVLVLSLWGAVVGAQNITARIGEPLVLKCKGAPKKPPQRLEWKLNTGRTE
AWKVLSPQGGGPWDSVARVLPNGSLFLPAVGIQDEGIFRCQAMNRNGKETKSNYRVRVY
QIPGKPEIVDSASELTAGVPNKVGTCVSEGSYPAGTLSWHLDGKPLVPNEKGVSVKEQTRR
HPETGLFTLQSELMVTPARGGDPRPTFSCSFSPGLPRHRALRTAPIQPRVWEPVPLEEVQLV
VEPEGGAVAPGGTVTLTCEVPAQPSPQIHWMKDGVPLPLPPSPVLILPEIGPQDQGTYSCVA
THSSHGPQESRAVSISIIEPGEEGPTAGSVGGSGLGTLALALGILGGLGTAALLIGVILWQRR
QRRGEERKAPENQEEEEERAELNQSEEPEAGESSTGGP

>Q99942 multipass membrane protein
MAAAEEEDGGPEGPNRERGGAGATFECNICLETAREAVVSVCGHLYCWPCLHQWLETRP
ERQECPVCKAGISREKVVPLYGRGSQKPQDPRLKTPPRPQGQRPAPESRGGFQPFGDTGGF
HFSFGVGAFPFGFFTTVFNAHEPFRRGTGVDLGQGHPASSWQDSLFLFLAIFFFFWLLSI

>P14555 peripheral membrane protein
MKTLLLLAVIMIFGLLQAHGNLVNFHRMIKLTTGKEAALSYGFYGCHCGVGGRGSPKDAT
DRCCVTHDCCYKRLEKRGCGTKFLSYKFSNSGSRITCAKQDSCRSQLCECDKAAATCFAR
NKTTYNKKYQYYSNKHCRGSTPRC

>Q6ZP29 multipass membrane protein
MVWKKLGSRNFSSCPSGSIQWIWDVLGECAQDGWDEASVGLGLISILCFAASTFPQFIKA
YKTGNMDQALSLWFLLGWIGGDSCNLIGSFLADQLPLQTYTAVYYVLADLVMLTLYFYY
KFRTRPSLLSAPINSVLLFLMGMACATPLLSAAGPVAAPREAFRGRALLSVESGSKPFTRQE
VIGFVIGSISSVLYLLSRLPQIRTNFLRKSTQGISYSLFALVMLGNTLYGLSVLLKNPEEGQSE
GSYLLHHLPWLVGSLGVLLLDTIISIQFLVYRRSTAASELEPLLPS

>P21731 multipass membrane protein
MWPNGSSLGPCFRPTNITLEERRLIASPWFAASFCVVGLASNLLALSVLAGARQGGSHTRS

SFLTFLCGLVLTDFLGLLVTGTIVVSQHAALFEWHAVDPGCRLCRFMGVVMIFFGLSPLLL
GAAMASERYLGITRPFSRPAVASQRRAWATVGLVWAAALALGLLPLLGVGRYTVQYPGS
WCFLTLGAESGDVAFGLLFSMLGGLSVGLSFLLNTVSVATLCHVYHGQEAAQQRPRDSEV
EMMAQLLGIMVVASVCWLPLLVFIAQTVLRNPPAMSPAGQLSRTTEKELLIYLRVATWNQI
LDPWVYILFRRAVLRRLQPRLSTRPRSLSLQPQLTQRSGLQ

>Q9NQR9 multipass membrane protein
MDFLHRNGVLIIQHLQKDYRAYYTFLNFMSNVGDPRNIFFIYFPLCFQFNQTVGTKMIWVA
VIGDWLNLIFKWILFGHRPYWWVQETQIYPNHSSPCLEQFPTTCETGPGSPSGHAMGASC
VWYVMVTAALSHTVCGMDKFSITLHRLTWSFLWSVFWLIQISVCISRVFIATHFPHQVILG
VIGGMLVAEAFEHTPGIQTASLGTYLKTNLFLFLFAVGFYLLLRVLNIDLLWSVPIAKKWC
ANPDWIHIDTTPFAGLVRNLGVLFGLGFAINSEMFLLSCRGGNNYTLSFRLLCALTSLTILQ
LYHFLQIPTHEEHLFYVLSFCKSASIPLTVVAFIPYSVHMLMKQSGKKSQ

>Q8TE23 multipass membrane protein
MGPRAKTISSLFFLLWVLAEPAENSDFYLPGDYLLGGLFSLHANMKGIVHLNFLQVPMCK
EYEVKVIGYNLMQAMRFAVEEINNDSSLLPGVLLGYEIVDVCYISNNVQPVLYFLAHEDN
LLPIQEDYSNYISRVVAVIGPDNSESVMTVANFLSLFLLPQITYSAISDELRDKVRFPALLRTT
PSADHHIEAMVQLMLHFRWNWIIVLVSSDTYGRDNGQLLGERVARRDICIAFQETLPTLQP
NQNMTSEERQRLVTIVDKLQQSTARVVVVFSPDLTLYHFFNEVLRQNFTGAVWIASESWAI
DPVLHNLTELRHLGTFLGITIQSVPIPGFSEFREWGPQAGPPPLSRTSQSYTCNQECDNCLN
ATLSFNTILRLSGERVVYSVYSAVYAVAHALHSLLGCDKSTCTKRVVYPWQLLEEIWKVNF
TLLDHQIFFDPQGDVALHLEIVQWQWDRSQNPFQSVASYYPLQRQLKNIQDISWHTINNTI
PMSMCSKRCQSGQKKKPVGIHVCCFECIDCLPGTFLNHTEDEYECQACPNNEWSYQSETS
CFKRQLVFLEWHEAPTIAVALLAALGFLSTLAILVIFWRHFQTPIVRSAGGPMCFLMLTLLL
VAYMVVPVYVGPPKVSTCLCRQALFPLCFTICISCIAVRSFQIVCAFKMASRFPRAYSYWV
RYQGPYVSMAFITVLKMVIVVIGMLATGLSPTTRTDPDDPKITIVSCNPNYRNSLLFNTSLD
LLLSVVGFSFAYMGKELPTNYNEAKFITLSMTFYFTSSVSLCTFMSAYSGVLVTIVDLLVTV
LNLLAISLGYFGPKCYMILFYPERNTPAYFNSMIQGYTMRRD

>P35498 multipass membrane protein
MEQTVLVPPGPDSFNFFTRESLAAIERRIAEEKAKNPKPDKKDDDENGPKPNSDLEAGKN
LPFIYGDIPPEMVSEPLEDLDPYYINKKTFIVLNKGKAIFRFSATSALYILTPFNPLRKIAIKIL
VHSLFSMLIMCTILTNCVFMTMSNPPDWTKNVEYTFTGIYTFESLIKIIARGFCLEDFTFLR
DPWNWLDFTVITFAYVTEFVDLGNVSALRTFRVLRALKTISVIPGLKTIVGALIQSVKKLSD
VMILTVFCLSVFALIGLQLFMGNLRNKCIQWPPTNASLEEHSIEKNITVNYNGTLINETVFE
FDWKSYIQDSRYHYFLEGFLDALLCGNSSDAGQCPEGYMCVKAGRNPNYGYTSFDTFSW
AFLSLFRLMTQDFWENLYQLTLRAAGKTYMIFFVLVIFLGSFYLINLILAVVAMAYEEQNQ
ATLEEAEQKEAEFQQMIEQLKKQQEAAQQAATATASEHSREPSAAGRLSDSSSEASKLSSK
SAKERRNRRKKRKQKEQSGGEEKDEDEFQKSESEDSIRRKGFRFSIEGNRLTYEKRYSSPH
QSLLSIRGSLFSPRRNSRTSLFSFRGRAKDVGSENDFADDEHSTFEDNESRRDSLFVPRRHG
ERRNSNLSQTSRSSRMLAVFPANGKMHSTVDCNGVVSLVGGPSVPTSPVGQLLPEVIIDKP
ATDDNGTTTETEMRKRRSSSFHVSMDFLEDPSQRQRAMSIASILTNTVEELEESRQKCPPC
WYKFSNIFLIWDCSPYWLKVKHVVNLVVMDPFVDLAITICIVLNTLFMAMEHYPMTDHF

NNVLTVGNLVFTGIFTAEMFLKIIAMDPYYYFQEGWNIFDGFIVTLSLVELGLANVEGLSV
LRSFRLLRVFKLAKSWPTLNMLIKIIGNSVGALGNLTLVLAIIVFIFAVVGMQLFGKSYKDC
VCKIASDCQLPRWHMNDFFHSFLIVFRVLCGEWIETMWDCMEVAGQAMCLTVFMMVMV
IGNLVVLNLFLALLLSSFSADNLAATDDDNEMNNLQIAVDRMHKGVAYVKRKIYEFIQQSF
IRKQKILDEIKPLDDLNNKKDSCMSNHTAEIGKDLDYLKDVNGTTSGIGTGSSVEKYIIDES
DYMSFINNPSLTVTVPIAVGESDFENLNTEDFSSESDLEESKEKLNESSSSSEGSTVDIGAPV
EEQPVVEPEETLEPEACFTEGCVQRFKCCQINVEEGRGKQWWNLRRTCFRIVEHNWFETF
IVFMILLSSGALAFEDIYIDQRKTIKTMLEYADKVFTYIFILEMLLKWVAYGYQTYFTNAW
CWLDFLIVDVSLVSLTANALGYSELGAIKSLRTLRALRPLRALSRFEGMRVVVNALLGAIP
SIMNVLLVCLIFWLIFSIMGVNLFAGKFYHCINTTTGDRFDIEDVNNHTDCLKLIERNETAR
WKNVKVNFDNVGFGYLSLLQVATFKGWMDIMYAAVDSRNVELQPKYEESLYMYLYFVIF
IIFGSFFTLNLFIGVIIDNFNQQKKKFGGQDIFMTEEQKKYYNAMKKLGSKKPQKPIPRPGN
KFQGMVFDFVTRQVFDISIMILICLNMVTMMVETDDQSEYVTTILSRINLVFIVLFTGECVL
KLISLRHYYFTIGWNIFDFVVVILSIVGMFLAELIEKYFVSPTLFRVIRLARIGRILRLIKGAK
GIRTLLFALMMSLPALFNIGLLLFLVMFIYAIFGMSNFAYVKREVGIDDMFNFETFGNSMIC
LFQITTSAGWDGLLAPILNSKPPDCDPNKVNPGSSVKGDCGNPSVGIFFFVSYIIISFLVVVN
MYIAVILENFSVATEESAEPLSEDDFEMFYEVWEKFDPDATQFMEFEKLSQFAAALEPPLN
LPQPNKLQLIAMDLPMVSGDRIHCLDILFAFTKRVLGESGEMDALRIQMEERFMASNPSK
VSYQPITTTLKRKQEEVSAVIIQRAYRRHLLKRTVKQASFTYNKNKIKGGANLLIKEDMIID
RINENSITEKTDLTMSTAACPPSYDRVTKPIVEKHEQEGKDEKAKGK

>Q92982 multipass membrane protein
MDSGTEEYELNGGLPPGTPGSPDASPARWGWRHGPINVNHYASKKSAAESMLDIALLMA
NASQLKAVVEQGPSFAFYVPLVVLISISLVLQIGVGVLLIFLVKYDLNNPAKHAKLDFLNNL
ATGLVFIIVVVNIFITAFGVQKPLMDMAPQQ

>P21912 peripheral membrane protein
MAAVVALSLRRRLPATTLGGACLQASRGAQTAAATAPRIKKFAIYRWDPDKAGDKPHMQ
TYEVDLNKCGPMVLDALIKIKNEVDSTLTFRRSCREGICGSCAMNINGGNTLACTRRIDTN
LNKVSKIYPLPHMYVIKDLVPDLSNFYAQYKSIEPYLKKKDESQEGKQQYLQSIEEREKLD
GLYECILCACCSTSCPSYWWNGDKYLGPAVLMQAYRWMIDSRDDFTEERLAKLQDPFSLY
RCHTIMNCTRTCPKGLNPGKAIAEIKKMMATYKEKKASV

>O14521 multipass membrane protein
MAVLWRLSAVCGALGGRALLLRTPVVRPAHISAFLQDRPIPEWCGVQHIHLSPSHHSGSKA
ASLHWTSERVVSVLLLGLLPAAYLNPCSAMDYSLAAALTLHGHWGLGQVVTDYVHGDA
LQKAAKAGLLALSALTFAGLCYFNYHDVGICKAVAMLWKL

>Q01974 single-pass type I membrane protein
MARGSALPRRPLLCIPAVWAAAALLLSVSRTSGEVEVLDPNDPLGPLDGQDGPIPTLKGYF
LNFLEPVNNITIVQGQTAILHCKVAGNPPPNVRWLKNDAPVVQEPRRIIIRKTEYGSRLRIQ
DLDTTDTGYYQCVATNGMKTITATGVLFVRLGPTHSPNHNFQDDYHEDGFCQPYRGIACA
RFIGNRTIYVDSLQMQGEIENRITAAFTMIGTSTHLSDQCSQFAIPSFCHFVFPLCDARSRTP
KPRELCRDECEVLESDLCRQEYTIARSNPLILMRLQLPKCEALPMPESPDAANCMRIGIPAE

RLGRYHQCYNGSGMDYRGTASTTKSGHQCQPWALQHPHSHHLSSTDFPELGGGHAYCR
NPGGQMEGPWCFTQNKNVRMELCDVPSCSPRDSSKMGILYILVPSIAIPLVIACLFFLVCM
CRNKQKASASTPQRRQLMASPSQDMEMPLINQHKQAKLKEISLSAVRFMEELGEDRFGK
VYKGHLFGPAPGEQTQAVAIKTLKDKAEGPLREEFRHEAMLRARLQHPNVVCLLGVVTK
DQPLSMIFSYCSHGDLHEFLVMRSPHSDVGSTDDDRTVKSALEPPDFVHLVAQIAAGMEY
LSSHHVVHKDLATRNVLVYDKLNVKISDLGLFREVYAADYYKLLGNSLLPIRWMAPEAI
MYGKFSIDSDIWSYGVVLWEVFSYGLQPYCGYSNQDVVEMIRNRQVLPCPDDCPAWVYA
LMIECWNEFPSRRPRFKDIHSRLRAWGNLSNYNSSAQTSGASNTTQTSSLSTSPVSNVSNA
RYVGPKQKAPPFPQPQFIPMKGQIRPMVPPPQLYVPVNGYQPVPAYGAYLPNFYPVQIPMQ
MAPQQVPPQMVPKPSSHHSGSGSTSTGYVTTAPSNTSMADRAALLSEGADDTQNAPEDG
AQSTVQEAEEEEEGSVPETELLGDCDTLQVDEAQVQLEA

>Q96HU8 lipid-anchor
MPEQSNDYRVAVFGAGGVGKSSLVLRFVKGTFRESYIPTVEDTYRQVISCDKSICTLQITDT
TGSHQFPAMQRLSISKGHAFILVYSITSRQSLEELKPIYEQICEIKGDVESIPIMLVGNKCDES
PSREVQSSEAEALARTWKCAFMETSAKLNHNVKELFQELLNLEKRRTVSLQIDGKKSKQQ
KRKEKLKGKCVIM

>P08631 lipid-anchor
MGGRSSCEDPGCPRDEERAPRMGCMKSKFLQVGGNTFSKTETSASPHCPVYVPDPTSTIK
PGPNSHNSNTPGIREAGSEDIIVVALYDYEAIHHEDLSFQKGDQMVVLEESGEWWKARSL
ATRKEGYIPSNYVARVDSLETEEWFFKGISRKDAERQLLAPGNMLGSFMIRDSETTKGSYS
LSVRDYDPRQGDTVKHYKIRTLDNGGFYISPRSTFSTLQELVDHYKKGNDGLCQKLSVPC
MSSKPQKPWEKDAWEIPRESLKLEKKLGAGQFGEVWMATYNKHTKVAVKTMKPGSMSV
EAFLAEANVMKTLQHDKLVKLHAVVTKEPIYIITEFMAKGSLLDFLKSDEGSKQPLPKLID
FSAQIAEGMAFIEQRNYIHRDLRAANILVSASLVCKIADFGLARVIEDNEYTAREGAKFPIK
WTAPEAINFGSFTIKSDVWSFGILLMEIVTYGRIPYPGMSNPEVIRALERGYRMPRPENCPE
ELYNIMMRCWKNRPEERPTFEYIQSVLDDFYTATESQYQQQP

>Q9Y275 single-pass type II membrane protein
MDDSTEREQSRLTSCLKKREEMKLKECVSILPRKESPSVRSSKDGKLLAATLLLALLSCCL
TVVSFYQVAALQGDLASLRAELQGHHAEKLPAGAGAPKAGLEEAPAVTAGLKIFEPPAPG
EGNSSQNSRNKRAVQGPEETVTQDCLQLIADSETPTIQKGSYTFVPWLLSFKRGSALEEKE
NKILVKETGYFFIYGQVLYTDKTYAMGHLIQRKKVHVFGDELSLVTLFRCIQNMPETLPNN
SCYSAGIAKLEEGDELQLAIPRENAQISLDGDVTFFGALKLL

>P55008 peripheral membrane protein
MSQTRDLQGGKAFGLLKAQQEERLDEINKQFLDDPKYSSDEDLPSKLEGFKEKYMEFDL
NGNGDIDIMSLKRMLEKLGVPKTHLELKKLIGEVSSGSGETFSYPDFLRMMLGKRSAILK
MILMYEEKAREKEKPTGPPAKKAISELP

>P10321 single-pass type I membrane protein
MRVMAPRALLLLLSGGLALTETWACSHSMRYFDTAVSRPGRGEPRFISVGYVDDTQFVRF
DSDAASPRGEPRAPWVEQEGPEYWDRETQKYKRQAQADRVSLRNLRGYYNQSEDGSHT

LQRMSGCDLGPDGRLLRGYDQSAYDGKDYIALNEDLRSWTAADTAAQITQRKLEAARA
AEQLRAYLEGTCVEWLRRYLENGKETLQRAEPPKTHVTHHPLSDHEATLRCWALGFYPA
EITLTWQRDGEDQTQDTELVETRPAGDGTFQKWAAVVVPSGQEQRYTCHMQHEGLQEPL
TLSWEPSSQPTIPIMGIVAGLAVLVVLAVLGAVVTAMMCRRKSSGGKGGSCSQAACSNSA
QGSDESLITCKA

>P20333 single-pass type I membrane protein
MAPVAVWAALAVGLELWAAAHALPAQVAFTPYAPEPGSTCRLREYYDQTAQMCCSKCSP
GQHAKVFCTKTSDTVCDSCEDSTYTQLWNWVPECLSCGSRCSSDQVETQACTREQNRIC
TCRPGWYCALSKQEGCRLCAPLRKCRPGFGVARPGTETSDVVCKPCAPGTFSNTTSSTDIC
RPHQICNVVAIPGNASMDAVCTSTSPTRSMAPGAVHLPQPVSTRSQHTQPTPEPSTAPSTSF
LLPMGPSPPAEGSTGDFALPVGLIVGVTALGLLIIGVVNCVIMTQVKKKPLCLQREAKVPH
LPADKARGTQGPEQQHLLITAPSSSSSSLESSASALDRRAPTRNQPQAPGVEASGAGEARA
STGSSDSSPGGHGTQVNVTCIVNVCSSSDHSSQCSSQASSTMGDTDSSPSESPKDEQVPFS
KEECAFRSQLETPETLLGSTEEKPLPLGVPDAGMKPS

>O60840 multipass membrane protein
MSESEGGKDTTPEPSPANGAGPGPEWGLCPGPPAVEGESSGASGLGTPKRRNQHSKHKTV
AVASAQRSPRALFCLTLANPLRRSCISIVEWKPFDILILLTIFANCVALGVYIPFPEDDSNTAN
HNLEQVEYVFLVIFTVETVLKIVAYGLVLHPSAYIRNGWNLLDFIIVVVGLFSVLLEQGPGR
PGDAPHTGGKPGGFDVKALRAFRVLRPLRLVSGVPSLHIVLNSIMKALVPLLHIALLVLFVI
IIYAIIGLELFLGRMHKTCYFLGSDMEAEEDPSPCASSGSGRACTLNQTECRGRWPGPNGGI
TNFDNFFFAMLTVFQCVTMEGWTDVLYWMQDAMGYELPWVYFVSLVIFGSFFVLNLVL
GVLSGEFSKEREKAKARGDFQKQREKQQMEEDLRGYLDWITQAEELDMEDPSADDNLG
SMAEEGRAGHRPQLAELTNRRRGRLRWFSHSTRSTHSTSSHASLPASDTGSMTETQGDED
EEEGALASCTRCLNKIMKTRVCRRLRRANRVLRARCRRAVKSNACYWAVLLLVFLNTLTI
ASEHHGQPVWLTQIQEYANKVLLCLFTVEMLLKLYGLGPSAYVSSFFNRFDCFVVCGGILE
TTLVEVGAMQPLGISVLRCVRLLRIFKVTRHWASLSNLVASLLNSMKSIASLLLLLFLFIIIFS
LLGMQLFGGKFNFDQTHTKRSTFDTFPQALLTVFQILTGEDWNVVMYDGIMAYGGPFFPG
MLVCIYFIILFICGNYILLNVFLAIAVDNLASGDAGTAKDKGGEKSNEKDLPQENEGLVPGV
EKEEEEGARREGADMEEEEEEEEEEEEEEEEEGAGGVELLQEVVPKEKVVPIPEGSAFFCL
SQTNPLRKGCHTLIHHHVFTNLILVFIILSSVSLAAEDPIRAHSFRNHILGYFDYAFTSIFTVEI
LLKMTVFGAFLHRGSFCRSWFNMLDLLVVSVSLISFGIHSSAISVVKILRVLRVLRPLRAIN
RAKGLKHVVQCVFVAIRTIGNIMIVTTLLQFMFACIGVQLFKGKFYTCTDEAKHTPQECKG
SFLVYPDGDVSRPLVRERLWVNSDFNFDNVLSAMMALFTVSTFEGWPALLYKAIDAYAED
HGPIYNYRVEISVFFIVYIIIIAFFMMNIFVGFVIITFRAQGEQEYQNCELDKNQRQCVEYAL
KAQPLRRYIPKNPHQYRVWATVNSAAFEYLMFLLILLNTVALAMQHYEQTAPFNYAMDIL
NMVFTGLFTIEMVLKIIAFKPKHYFTDAWNTFDALIVVGSIVDIAVTEVNNGGHLGESSED
SSRISITFFRLFRVMRLVKLLSKGEGIRTLLWTFIKSFQALPYVALLIAMIFFIYAVIGMQMFG
KVALQDGTQINRNNNFQTFPQAVLLLFRCATGEAWQEIMLASLPGNRCDPESDFGPGEEFT
CGSNFAIAYFISFFMLCAFLIINLFVAVIMDNFDYLTRDWSILGPHHLDEFKRIWSEYDPGAK
GRIKHLDVVALLRRIQPPLGFGKLCPHRVACKRLVAMNMPLNSDGTVTFNATLFALVRTSL
KIKTEGNLEQANQELRIVIKKIWKRMKQKLLDEVIPPPDEEEVTVGKFYATFLIQDYFRKF
RRRKEKGLLGNDAAPSTSSALQAGLRSLQDLGPEMRQALTCDTEEEEEEGQEGVEEEDEK

DLETNKATMVSQPSARRGSGISVSLPVGDRLPDSLSFGPSDDDRGTPTSSQPSVPQAGSNT
HRRGSGALIFTIPEEGNSQPKGTKGQNKQDEDEEVPDRLSYLDEQAGTPPCSVLLPPHRAQ
RYMDGHLVPRRRLLPPTPAGRKPSFTIQCLQRQGSCEDLPIPGTYHRGRNSGPNRAQGSWA
TPPQRGRLLYAPLLLVEEGAAGEGYLGRSSGPLRTFTCLHVPGTHSDPSHGKRGSADSLVE
AVLISEGLGLFARDPRFVALAKQEIADACRLTLDEMDNAASDLLAQGTSSLYSDEESILSRF
DEEDLGDEMACVHAL

>Q15615 multipass membrane protein
MEPQNTTQVSMFVLLGFSQTQELQKFLFLLFLLVYVTTIVGNLLIMVTVTFDCRLHTPMY
FLLRNLALIDLCYSTVTSPKMLVDFLHETKTISYQGCMAQIFFFHLLGGGTVFFLSVMAYD
RYIAISQPLRYVTIMNTQLCVGLVVAAWVGGFVHSIVQLALILPLPFCGPNILDNFYCDVPQ
VLRLACTDTSLLEFLMISNSGLLVIIWFLLLLISYTVILVMLRSHSGKARRKAASTCTTHIIV
VSMIFIPCIYIYTWPFTPFLMDKAVSISYTVMTPMLNPMIYTLRNQDMKAAMRRLGKCLVI
CRE

>Q5SSG8 single-pass type I membrane protein
MKMQKGNVLLMFGLLLHLEAATNSNETSTSANTGSSVISSGASTATNSGSSVTSSGVSTAT
ISGSSVTSNGVSIVTNSEFHTTSSGISTATNSEFSTVSSGISIATNSESSTTSSGASTATNSESST
PSSGASTATNSDSSTTSSGASTATNSDSSTTSSEASTATNSESSTTSSGASTATNSESSTVSSR
ASTATNSESSTTSSGASTATNSESRTTSNGAGTATNSESSTTSSGASTATNSESSTPSSGAGTA
TNSESSTTSSGAGTATNSESSTVSSGISTVTNSESSTPSSGANTATNSESSTTSSGANTATNSD
SSTTSSGASTATNSESSTTSSGASTATNSESSTTSSGASTATNSGSSTTSSGTSTATNSESSTVS
SGASTATTSESSTTSSGASTATNSESSTVSSGASTATNSESSTTSSGANTATNSGSSVTSAGSG
TAALTGMHTTSHSASTAVSEAKPGGSLVPWEIFLITLVSVVAAVGLFAGLFFCVRNSLSLRN
TFNTAVYHPHGLNHGLGPGPGGNHGAPHRPRWSPNWFWRRPVSSIAMEMSGRNSGP

>Q04941 multipass membrane protein
MADSERLSAPGCWAACTNFSRTRKGILLFAEIILCLVILICFSASTPGYSSLSVIEMILAAIFF
VVYMCDLHTKIPFINWPWSDFFRTLIAAILYLITSIVVLVERGNHSKIVAGVLGLIATCLFGY
DAYVTFPVRQPRHTAAPTDPADGPV

>Q5T848 multipass membrane protein
MGAMAYPLLLCLLLAQLGLGAVGASRDPQGRPDSPRERTPKGKPHAQQPGRASASDSSA
PWSRSTDGTILAQKLAEEVPMDVASYLYTGDSHQLKRANCSGRYELAGLPGKWPALASA
HPSLHRALDTLTHATNFLNVMLQSNKSREQNLQDDLDWYQALVWSLLEGEPSISRAAITF
STDSLSAPAPQVFLQATREESRILLQDLSSSAPHLANATLETEWFHGLRRKWRPHLHRRGP
NQGPRGLGHSWRRKDGLGGDKSHFKWSPPYLECENGSYKPGWLVTLSSAIYGLQPNLVP
EFRGVMKVDINLQKVDIDQCSSDGWFSGTHKCHLNNSECMPIKGLGFVLGAYECICKAGF
YHPGVLPVNNFRRRGPDQHISGSTKDVSEEAYVCLPCREGCPFCADDSPCFVQEDKYLRL
AIISFQALCMLLDFVSMLVVYHFRKAKSIRASGLILLETILFGSLLLYFPVVILYFEPSTFRCI
LLRWARLLGFATVYGTVTLKLHRVLKVFLSRTAQRIPYMTGGRVMRMLAVILLVVFWFLI
GWTSSVCQNLEKQISLIGQGKTSDHLIFNMCLIDRWDYMTAVAEFLFLLWGVYLCYAVRT
VPSAFHEPRYMAVAVHNELIISAIFHTIRFVLASRLQSDWMLMLYFAHTHLTVTVTIGLLLIP
KFSHSSNNPRDDIATEAYEDELDMGRSGSYLNSSINSAWSEHSLDPEDIRDELKKLYAQLEI

YKRKKMITNNPHLQKKRCSKKGLGRSIMRRITEIPETVSRQCSKEDKEGADHGTAKGTALI
RKNPPESSGNTGKSKEETLKNRVFSLKKSHSTYDHVRDQTEESSSLPTESQEEETTENSTLE
SLSGKKLTQKLKEDSEAESTESVPLVCKSASAHNLSSEKKTGHPRTSMLQKSLSVIASAKE
KTLGLAGKTQTAGVEERTKSQKPLPKDKETNRNHSNSDNTETKDPAPQNSNPAEEPRKPQ
KSGIMKQQRVNPTTANSDLNPGTTQMKDNFDIGEVCPWEVYDLTPGPVPSESKVQKHVSI
VASEMEKNPTFSLKEKSHHKPKAAEVCQQSNQKRIDKAEVCLWESQGQSILEDEKLLISK
TPVLPERAKEENGGQPRAANVCAGQSEELPPKAVASKTENENLNQIGHQEKKTSSSEENV
RGSYNSSNNFQQPLTSRAEVCPWEFETPAQPNAGRSVALPASSALSANKIAGPRKEEIWDS
FKV

>P32249 multipass membrane protein
MDIQMANNFTPPSATPQGNDCDLYAHHSTARIVMPLHYSLVFIIGLVGNLLALVVIVQNRK
KINSTTLYSTNLVISDILFTTALPTRIAYYAMGFDWRIGDALCRITALVFYINTYAGVNFMTC
LSIDRFIAVVHPLRYNKIKRIEHAKGVCIFVWILVFAQTLPLLINPMSKQEAERITCMEYPNF
EETKSLPWILLGACFIGYVLPLIIILICYSQICCKLFRTAKQNPLTEKSGVNKKALNTIILIIVV
FVLCFTPYHVAIIQHMIKKLRFSNFLECSQRHSFQISLHFTVCLMNFNCCMDPFIYFFACKG
YKRKVMRMLKRQVSVSISSAVKSAPEENSREMTETQMMIHSKSSNGK

>P46059 multipass membrane protein
MGMSKSHSFFGYPLSIFFIVVNEFCERFSYYGMRAILILYFTNFISWDDNLSTAIYHTFVALC
YLTPILGALIADSWLGKFKTIVSLSIVYTIGQAVTSVSSINDLTDHNHDGTPDSLPVHVVLSL
IGLALIALGTGGIKPCVSAFGGDQFEEGQEKQRNRFFSIFYLAINAGSLLSTIITPMLRVQQC
GIHSKQACYPLAFGVPAALMAVALIVFVLGSGMYKKFKPQGNIMGKVAKCIGFAIKNRFR
HRSKAFPKREHWLDWAKEKYDERLISQIKMVTRVMFLYIPLPMFWALFDQQGSRWTLQA
TTMSGKIGALEIQPDQMQTVNAILIVIMVPIFDAVLYPLIAKCGFNFTSLKKMAVGMVLAS
MAFVVAAIVQVEIDKTLPVFPKGNEVQIKVLNIGNNTMNISLPGEMVTLGPMSQTNAFMT
FDVNKLTRINISSPGSPVTAVTDDFKQGQRHTLLVWAPNHYQVVKDGLNQKPEKGENGIR
FVNTFNELITITMSGKVYANISSYNASTYQFFPSGIKGFTISSTEIPPQCQPNFNTFYLEFGSA
YTYIVQRKNDSCPEVKVFEDISANTVNMALQIPQYFLLTCGEVVFSVTGLEFSYSQAPSNM
KSVLQAGWLLTVAVGNIIVLIVAGAGQFSKQWAEYILFAALLLVVCVIFAIMARFYTYINPA
EIEAQFDEDEKKNRLEKSNPYFMSGANSQKQM

>O60830 multipass membrane protein
MEEYAREPCPWRIVDDCGGAFTMGVIGGGVFQAIKGFRNAPVGIRHRLRGSANAVRIRAP
QIGGSFAVWGGLFSTIDCGLVRLRGKEDPWNSITSGALTGAVLAARSGPLAMVGSAMMG
GILLALIEGVGILLTRYTAQQFRNAPPFLEDPSQLPPKDGTPAPGYPSYQQYH

>O75354 single-pass type II membrane protein
MKKGIRYETSRKTSYIFQQPQHGPWQTRMRKISNHGSLRVAKVAYPLGLCVGVFIYVAYIK
WHRATATQAFFSITRAAPGARWGQQAHSPLGTAADGHEVFYGIMFDAGSTGTRVHVFQF
TRPPRETPTLTHETFKALKPGLSAYADDVEKSAQGIRELLDVAKQDIPFDFWKATPLVLKAT
AGLRLLPGEKAQKLLQKVKKVFKASPFLVGDDCVSIMNGTDEGVSAWITINFLTGSLKTP
GGSSVGMLDLGGGSTQIAFLPRVEGTLQASPPGYLTALRMFNRTYKLYSYSYLGLGLMSA
RLAILGGVEGQPAKDGKELVSPCLSPSFKGEWEHAEVTYRVSGQKAAASLHELCAARVSE

VLQNRVHRTEEVKHVDFYAFSYYYDLAAGVGLIDAEKGGSLVVGDFEIAAKYVCRTLET
QPQSSPFSCMDLTYVSLLLQEFGFPRSKVLKLTRKIDNVETSWALGAIFHYIDSLNRQKSPA
S

>Q02742 single-pass type II membrane protein
MLRTLLRRRLFSYPTKYYFMVLVLSLITFSVLRIHQKPEFVSVRHLELAGENPSSDINCTKV
LQGDVNEIQKVKLEILTVKFKKRPRWTPDDYINMTSDCSSFIKRRKYIVEPLSKEEAEFPIA
YSIVVHHKIEMLDRLLRAIYMPQNFYCIHVDTKSEDSYLAAVMGIASCFSNVFVASRLESV
VYASWSRVQADLNCMKDLYAMSANWKYLINLCGMDFPIKTNLEIVRKLKLLMGENNLET
ERMPSHKEERWKKRYEVVNGKLTNTGTVKMLPPLETPLFSGSAYFVVSREYVGYVLQNE
KIQKLMEWAQDTYSPDEYLWATIQRIPEVPGSLPASHKYDLSDMQAVARFVKWQYFEGDV
SKGAPYPPCDGVHVRSVCIFGAGDLNWMLRKHHLFANKFDVDVDLFAIQCLDEHLRHKA
LETLKH

>Q92824 single-pass type I membrane protein
MGWGSRCCCPGRLDLLCVLALLGGCLLPVCRTRVYTNHWAVKIAGGFPEANRIASKYGFI
NIGQIGALKDYYHFYHSRTIKRSVISSRGTHSFISMEPKVEWIQQQVVKKRTKRDYDFSRA
QSTYFNDPKWPSMWYMHCSDNTHPCQSDMNIEGAWKRGYTGKNIVVTILDDGIERTHPD
LMQNYDALASCDVNGNDLDPMPRYDASNENKHGTRCAGEVAAAANNSHCTVGIAFNA
KIGGVRMLDGDVTDMVEAKSVSFNPQHVHIYSASWGPDDDGKTVDGPAPLTRQAFENG
VRMGRRGLGSVFVWASGNGGRSKDHCSCDGYTNSIYTISISSTAESGKKPWYLEECSSTLA
TTYSSGESYDKKIITTDLRQRCTDNHTGTSASAPMAAGIIALALEANPFLTWRDVQHVIVR
TSRAGHLNANDWKTNAAGFKVSHLYGFGLMDAEAMVMEAEKWTTVPRQHVCVESTDR
QIKTIRPNSAVRSIYKASGCSDNPNRHVNYLEHVVVRITITHPRRGDLAIYLTSPSGTRSQLL
ANRLFDHSMEGFKNWEFMTIHCWGERAAGDWVLEVYDTPSQLRNFKTPGKLKEWSLVL
YGTSVQPYSPTNEFPKVERFRYSRVEDPTDDYGTEDYAGPCDPECSEVGCDGPGPDHCND
CLHYYYKLKNNTRICVSSCPPGHYHADKKRCRKCAPNCESCFGSHGDQCMSCKYGYFLN
EETNSCVTHCPDGSYQDTKKNLCRKCSENCKTCTEFHNCTECRDGLSLQGSRCSVSCEDG
RYFNGQDCQPCHRFCATCAGAGADGCINCTEGYFMEDGRCVQSCSISYYFDHSSENGYK
SCKKCDISCLTCNGPGFKNCTSCPSGYLLDLGMCQMGAICKDGEYVDEHGHCQTCEASC
AKCQGPTQEDCTTCPMTRIFDDGRCVSNCPSWKFEFENQCHPCHHTCQRCQGSGPTHCTS
CGADNYGREHFLYQGECGDSCPEGHYATEGNTCLPCPDNCELCHSVHVCTRCMKGYFIA
PTNHTCQKLECGQGEVQDPDYEECVPCEEGCLGCSLDDPGTCTSCAMGYYRFDHHCYKT
CPEKTYSEEVECKACDSNCGSCDQNGCYWCEEGFFLLGGSCVRKCGPGFYGDQEMGEC
ESCHRACETCTGPGHDECSSCQEGLQLLRGMCVHATKTQEEGKFWNDILRKLQPCHSSC
KTCNGSATLCTSCPKGAYLLAQACVSSCPQGTWPSVRSGSCENCTEACAICSGADLCKKC
QMQPGHPLFLHEGRCYSKCPEGSYAEDGICERCSSPCRTCEGNATNCHSCEGGHVLHHGV
CQENCPERHVAVKGVCKHCPEMCQDCIHEKTCKECTPEFFLHDDMCHQSCPRGFYADSR
HCVPCHKDCLECSGPKADDCELCLESSWVLYDGLCLEECPAGTYYEKETKECRDCHKSCL
TCSSSGTCTTCQKGLIMNPRGSCMANEKCSPSEYWDEDAPGCKPCHVKCFHCMGPAEDQ
CQTCPMNSLLLNTTCVKDCPEGYYADEDSNRCAHCHSSCRTCEGRHSRQCHSCRPGWFQ
LGKECLLQCREGYYADNSTGRCERCNRSCKGCQGPRPTDCLSCDRFFFLLRSKGECHRSC
PDHYYVEQSTQTCERCHPTCDQCKGKGALNCLSCVWSYHLMGGICTSDCLVGEYRVGEG
EKFNCEKCHESCMECKGPGAKNCTLCPANLVLHMDDSHCLHCCNTSDPPSAQECCDCQD

TTDECILRTSKVRPATEHFKTALFITSSMMLVLLLGAAVVVWKKSRGRVQPAAKAGYEKL
ADPNKSYSSYKSSYRESTSFEEDQVIEYRDRDYDEDDDDDIVYMGQDGTVYRKFKYGLL
DDDDIDELEYDDESYSYYQ

>Q9Y5Z9 multipass membrane protein
MAASQVLGEKINILSGETVKAGDRDPLGNDCPEQDRLPQRSWRQKCASYVLALRPWSFS
ASLTPVALGSALAYRSHGVLDPRLLVGCAVAVLAVHGAGNLVNTYYDFSKGIDHKKSDDR
TLVDRILEPQDVVRFGVFLYTLGCVCAACLYYLSPLKLEHLALIYFGGLSGSFLYTGGIGFK
YVALGDLIILITFGPLAVMFAYAIQVGSLAIFPLVYAIPLALSTEAILHSNNTRDMESDREAGI
VTLAILIGPTFSYILYNTLLFLPYLVFSILATHCTISLALPLLTIPMAFSLERQFRSQAFNKLPQ
RTAKLNLLLGLFYVFGIILAPAGSLPKI

>P63244 peripheral membrane protein
MTEQMTLRGTLKGHNGWVTQIATTPQFPDMILSASRDKTIIMWKLTRDETNYGIPQRALR
GHSHFVSDVVISSDGQFALSGSWDGTLRLWDLTTGTTTRRFVGHTKDVLSVAFSSDNRQI
VSGSRDKTIKLWNTLGVCKYTVQDESHSEWVSCVRFSPNSSNPIIVSCGWDKLVKVWNL
ANCKLKTNHIGHTGYLNTVTVSPDGSLCASGGKDGQAMLWDLNEGKHLYTLDGGDIINA
LCFSPNRYWLCAATGPSIKIWDLEGKIIVDELKQEVISTSSKAEPPQCTSLAWSADGQTLFA
GYTDNLVRVWQVTIGTR

>O15439 multipass membrane protein
MLPVYQEVKPNPLQDANLCSRVFFWWLNPLFKIGHKRRLEEDDMYSVLPEDRSQHLGEE
LQGFWDKEVLRAENDAQKPSLTRAIIKCYWKSYLVLGIFTLIEESAKVIQPIFLGKIINYFEN
YDPMDSVALNTAYAYATVLTFCTLILAILHHLYFYHVQCAGMRLRVAMCHMIYRKALRLS
NMAMGKTTTGQIVNLLSNDVNKFDQVTVFLHFLWAGPLQAIAVTALLWMEIGISCLAGM
AVLIILLPLQSCFGKLFSSLRSKTATFTDARIRTMNEVITGIRIIKMYAWEKSFSNLITNLRKK
EISKILRSSCLRGMNLASFFSASKIIVFVTFTTYVLLGSVITASRVFVAVTLYGAVRLTVTLFF
PSAIERVSEAIVSIRRIQTFLLLDEISQRNRQLPSDGKKMVHVQDFTAFWDKASETPTLQGL
SFTVRPGELLAVVGPVGAGKSSLLSAVLGELAPSHGLVSVHGRIAYVSQQPWVFSGTLRSN
ILFGKKYEKERYEKVIKACALKKDLQLLEDGDLTVIGDRGTTLSGGQKARVNLARAVYQD
ADIYLLDDPLSAVDAEVSRHLFELCICQILHEKITILVTHQLQYLKAASQILILKDGKMVQK
GTYTEFLKSGIDFGSLLKKDNEESEQPPVPGTPTLRNRTFSESSVWSQQSSRPSLKDGALES
QDTENVPVTLSEENRSEGKVGFQAYKNYFRAGAHWIVFIFLILLNTAAQVAYVLQDWWLS
YWANKQSMLNVTVNGGGNVTEKLDLNWYLGIYSGLTVATVLFGIARSLLVFYVLVNSSQ
TLHNKMFESILKAPVLFFDRNPIGRILNRFSKDIGHLDDLLPLTFLDFIQTLLQVVGVVSVAV
AVIPWIAIPLVPLGIIFIFLRRYFLETSRDVKRLESTTRSPVFSHLSSSLQGLWTIRAYKAEERC
QELFDAHQDLHSEAWFLFLTTSRWFAVRLDAICAMFVIIVAFGSLILAKTLDAGQVGLALS
YALTLMGMFQWCVRQSAEVENMMISVERVIEYTDLEKEAPWEYQKRPPPAWPHEGVIIFD
NVNFMYSPGGPLVLKHLTALIKSQEKVGIVGRTGAGKSSLISALFRLSEPEGKIWIDKILTTE
IGLHDLRKKMSIIPQEPVLFTGTMRKNLDPFNEHTDEELWNALQEVQLKETIEDLPGKMD
TELAESGSNFSVGQRQLVCLARAILRKNQILIIDEATANVDPRTDELIQKKIREKFAHCTVLT
IAHRLNTIIDSDKIMVLDSGRLKEYDEPYVLLQNKESLFYKMVQQLGKAEAAALTETAKQ
VYFKRNYPHIGHTDHMVTNTSNGQPSTLTIFETAL

>O95070 multipass membrane protein
MAYHSGYGAHGSKHRARAAPDPPPLFDDTSGGYSSQPGGYPATGADVAFSVNHLLGDPM
ANVAMAYGSSIASHGKDMVHKELHRFVSVSKLKYFFAVDTAYVAKKLGLLVFPYTHQNW
EVQYSRDAPLPPRQDLNAPDLYIPTMAFITYVLLAGMALGIQKRFSPEVLGLCASTALVW
VVMEVLALLLGLYLATVRSDLSTFHLLAYSGYKYVGMILSVLTGLLFGSDGYYVALAWTS
SALMYFIVRSLRTAALGPDSMGGPVPRQRLQLYLTLGAAAFQPLIIYWLTFHLVR

>Q96KC8 single-pass type I membrane protein
MTAPCSQPAQLPGRRQLGLVPFPPPPPRTPLLWLLLLLLAAVAPARGWESGDLELFDLVEE
VQLNFYQFLGVQQDASSADIRKAYRKLSLTLHPDKNKDENAETQFRQLVAIYEVLKDDER
RQRYDDILINGLPDWRQPVFYYRRVRKMSNAELALLLFIILTVGHYAVVWSIYLEKQLDEL
LSRKKREKKKKTGSKSVDVSKLGASEKNERLLMKPQWHDLLPCKLGIWFCLTLKALPHLI
QDAGQFYAKYKETRLKEKEDALTRTELETLQKQKKVKKPKPEFPVYTPLETTYIQSYDHG
TSIEEIEEQMDDWLENRNRTQKKQAPEWTEEDLSQLTRSMVKFPGGTPGRWEKIAHELGR
SVTDVTTKAKQLKDSVTCSPGMVRLSELKSTVQNSRPIKTATTLPDDMITQREDAEGVAA
EEEQEGDSGEQETGATDARPRRRKPARLLEATAKPEPEEKSRAKRQKDFDIAEQNESSDEE
SLRKERARSAEEPWTQNQQKLLELALQQYPRGSSDRWDKIARCVPSKSKEDCIARYKLLV
ELVQKKKQAKS

>Q9UBS5 multipass membrane protein
MLLLLLLAPLFLRPPGAGGAQTPNATSEGCQIIHPPWEGGIRYRGLTRDQVKAINFLPVDY
EIEYVCRGEREVVGPKVRKCLANGSWTDMDTPSRCVRICSKSYLTLENGKVFLTGGDLPA
LDGARVDFRCDPDFHLVGSSRSICSQGQWSTPKPHCQVNRTPHSERRAVYIGALFPMSGG
WPGGQACQPAVEMALEDVNSRRDILPDYELKLIHHDSKCDPGQATKYLYELLYNDPIKIIL
MPGCSSVSTLVAEAARMWNLIVLSYGSSSPALSNRQRFPTFFRTHPSATLHNPTRVKLFEK
WGWKKIATIQQTTEVFTSTLDDLEERVKEAGIEITFRQSFFSDPAVPVKNLKRQDARIIVGL
FYETEARKVFCEVYKERLFGKKYVWFLIGWYADNWFKIYDPSINCTVDEMTEAVEGHITT
EIVMLNPANTRSISNMTSQEFVEKLTKRLKRHPEETGGFQEAPLAYDAIWALALALNKTSG
GGGRSGVRLEDFNYNNQTITDQIYRAMNSSSFEGVSGHVVFDASGSRMAWTLIEQLQGG
SYKKIGYYDSTKDDLSWSKTDKWIGGSPPADQTLVIKTFRFLSQKLFISVSVLSSLGIVLAV
VCLSFNIYNSHVRYIQNSQPNLNNLTAVGCSLALAAVFPLGLDGYHIGRNQFPFVCQARLW
LLGLGFSLGYGSMFTKIWWVHTVFTKKEEKKEWRKTLEPWKLYATVGLLVGMDVLTLAI
WQIVDPLHRTIETFAKEEPKEDIDVSILPQLEHCSSRKMNTWLGIFYGYKGLLLLLGIFLAY
ETKSVSTEKINDHRAVGMAIYNVAVLCLITAPVTMILSSQQDAAFAFASLAIVFSSYITLVVL
FVPKMRRLITRGEWQSEAQDTMKTGSSTNNNEEEKSRLLEKENRELEKIIAEKEERVSELR
HQLQSRQQLRSRRHPPTPPEPSGGLPRGPPEPPDRLSCDGSRVHLLYK

>Q9Y625 GPI-anchor, lipid-anchor
MPSWIGAVILPLLGLLLSLPAGADVKARSCGEVRQAYGAKGFSLADIPYQEIAGEHLRICP
QEYTCCTTEMEDKLSQQSKLEFENLVEETSHFVRTTFVSRHKKFDEFFRELLENAEKSLND
MFVRTYGMLYMQNSEVFQDLFTELKRYYTGGNVNLEEMLNDFWARLLERMFQLINPQY
HFSEDYLECVSKYTDQLKPFGDVPRKLKIQVTRAFIAARTFVQGLTVGREVANRVSKVSPT
PGCIRALMKMLYCPYCRGLPTVRPCNNYCLNVMKGCLANQADLDTEWNLFIDAMLLVA
ERLEGPFNIESVMDPIDVKISEAIMNMQENSMQVSAKVFQGCGQPKPAPALRSARSAPENF

NTRFRPYNPEERPTTAAGTSLDRLVTDIKEKLKLSKKVWSALPYTICKDESVTAGTSNEEE
CWNGHSKARYLPEIMNDGLTNQINNPEVDVDITRPDTFIRQQIMALRVMTNKLKNAYNG
NDVNFQDTSDESSGSGSGSGCMDDVCPTEFEFVTTEAPAVDPDRREVDSSAAQRGHSLLS
WSLTCIVLALQRLCR

>P78333 GPI-anchor, lipid-anchor
MDAQTWPVGFRCLLLLALVGSARSEGVQTCEEVRKLFQWRLLGAVRGLPDSPRAGPDLQ
VCISKKPTCCTRKMEERYQIAARQDMQQFLQTSSSTLKFLISRNAAAFQETLETLIKQAEN
YTSILFCSTYRNMALEAAASVQEFFTDVGLYLFGADVNPEEFVNRFFDSLFPLVYNHLINP
GVTDSSLEYSECIRMARRDVSPFGNIPQRVMGQMGRSLLPSRTFLQALNLGIEVINTTDYL
HFSKECSRALLKMQYCPHCQGLALTKPCMGYCLNVMRGCLAHMAELNPHWHAYIRSLE
ELSDAMHGTYDIGHVLLNFHLLVNDAVLQAHLNGQKLLEQVNRICGRPVRTPTQSPRCSF
DQSKEKHGMKTTTRNSEETLANRRKEFINSLRLYRSFYGGLADQLCANELAAADGLPCW
NGEDIVKSYTQRVVGNGIKAQSGNPEVKVKGIDPVINQIIDKLKHVVQLLQGRSPKPDKW
ELLQLGSGGGMVEQVSGDCDDEDGCGGSGSGEVKRTLKITDWMPDDMNFSDVKQIHQT
DTGSTLDTTGAGCAVATESMTFTLISVVMLLPGIW

>Q6P4Q7 multipass membrane protein
MAPVGGGGRPVGGPARGRLLLAAPVLLVLLWALGARGQGSPQQGTIVGMRLASCNKSC
GTNPDGIIFVSEGSTVNLRLYGYSLGNISSNLISFTEVDDAETLHKSTSCLELTKDLVVQQLV
NVSRGNTSGVLVVLTKFLRRSESMKLYALCTRAQPDGPWLKWTDKDSLLFMVEEPGRFL
PLWLHILLITVLLVLSGIFSGLNLGLMALDPMELRIVQNCGTEKERRYARKIEPIRRKGNYL
LCSLLLGNVLVNTSLTILLDNLIGSGLMAVASSTIGIVIFGEILPQALCSRHGLAVGANTILLT
KFFMLLTFPLSFPISKLLDFFLGQEIRTVYNREKLMEMLKVTEPYNDLVKEELNMIQGALE
LRTKTVEDIMTQLQDCFMIRSDAILDFNTMSEIMESGYTRIPVFEDEQSNIVDILYVKDLAF
VDPDDCTPLKTITRFYNHPVHFVFHDTKLDAMLEEFKKGKSHLAIVQKVNNEGEGDPFYE
VLGLVTLEDVIEEIIKSEILDESDMYTDNRSRKRVSEKNKRDFSAFKDADNELKVKISPQLL
LAAHRFLATEVSQFSPSLISEKILLRLLKYPDVIQELKFDEHNKYYARHYLYTRNKPADYFI
LILQGKVEVEAGKENMKFETGAFSYYGTMALTSVPSDRSPAHPTPLSRSASLSYPDRTDVS
TAATLAGSSNQFGSSVLGQYISDFSVRALVDLQYIKITRQQYQNGLLASRMENSPQFPIDG
CTTHMENLAEKSELPVVDETTTLLNERNSLLHKASHENAI

>Q9H0V9 single-pass type I membrane protein
MAATLGPLGSWQQWRRCLSARDGSRMLLLLLLLGSGQGPQQVGAGQTFEYLKREHSLS
KPYQGVGTGSSSLWNLMGNAMVMTQYIRLTPDMQSKQGALWNRVPCFLRDWELQVHF
KIHGQGKKNLHGDGLAIWYTKDRMQPGPVFGNMDKFVGLGVFVDTYPNEEKQQERVFP
YISAMVNNGSLSYDHERDGRPTELGGCTAIVRNLHYDTFLVIRYVKRHLTIMMDIDGKHE
WRDCIEVPGVRLPRGYYFGTSSITGDLSDNHDVISLKLFELTVERTPEEEKLHRDVFLPSVD
NMKLPEMTAPLPPLSGLALFLIVFFSLVFSVFAIVIGIILYNKWQEQSRKRFY

>Q9BZJ4 multipass membrane protein
MADQDPAGISPLQQMVASGTGAVVTSLFMTPLDVVKVRLQSQRPSMASELMPSSRLWSLS
YTKLPSSLQSTGKCLLYCNGVLEPLYLCPNGARCATWFQDPTRFTGTMDAFVKIVRHEGT
RTLWSGLPATLVMTVPATAIYFTAYDQLKAFLCGRALTSDLYAPMVAGALARLGTVTVISPL

ELMRTKLQAQHVSYRELGACVRTAVAQGGWRSLWLGWGPTALRDVPFSALYWFNYELV
KSWLNGFRPKDQTSVGMSFVAGGISGTVAAVLTLPFDVVKTQRQVALGAMEAVRVNPLH
VDSTWLLLRRIRAESGTKGLFAGFLPRIIKAAPSCAIMISTYEFGKSFFQRLNQDRLLGG

>P07204 single-pass type I membrane protein
MLGVLVLGALALAGLGFPAPAEPQPGGSQCVEHDCFALYPGPATFLNASQICDGLRGHLM
TVRSSVAADVISLLLNGDGGVGRRRLWIGLQLPPGCGDPKRLGPLRGFQWVTGDNNTSYS
RWARLDLNGAPLCGPLCVAVSAAEATVPSEPIWEEQQCEVKADGFLCEFHFPATCRPLAVE
PGAAAAAVSITYGTPFAARGADFQALPVGSSAAVAPLGLQLMCTAPPGAVQGHWAREAPG
AWDCSVENGGCEHACNAIPGAPRCQCPAGAALQADGRSCTASATQSCNDLCEHFCVPNP
DQPGSYSCMCETGYRLAADQHRCEDVDDCILEPSPCPQRCVNTQGGFECHCYPNYDLVD
GECVEPVDPCFRANCEYQCQPLNQTSYLCVCAEGFAPIPHEPHRCQMFCNQTACPADCDP
NTQASCECPEGYILDDGFICTDIDECENGGFCSGVCHNLPGTFECICGPDSALARHIGTDCD
SGKVDGGDSGSGEPPPSPTPGSTLTPPAVGLVHSGLLIGISIASLCLVVALLALLCHLRKKQG
AARAKMEYKCAAPSKEVVLQHVRTERTPQRL

>Q9HCF6 multipass membrane protein
MPEPWGTVYFLGIAQVFSFLFSWWNLEGVMNQADAPRPLNWTIRKLCHAAFLPSVRLLK
AQKSWIERAFYKRECVHIIPSTKDPHRCCCGRLIGQHVGLTPSISVLQNEKNESRLSRNDIQ
SEKWSISKHTQLSPTDAFGTIEFQGGGHSNKAMYVRVSFDTKPDLLLHLMTKEWQLELPK
LLISVHGGLQNFELQPKLKQVFGKGLIKAAMTTGAWIFTGGVNTGVIRHVGDALKDHAS
KSRGKICTIGIAPWGIVENQEDLIGRDVVRPYQTMSNPMSKLTVLNSMHSHFILADNGTTG
KYGAEVKLRRQLEKHISLQKINTRCLPFFSLDSRLFYSFWGSCQLDSVGIGQGVPVVALIV
EGGPNVISIVLEYLRDTPPVPVVVCDGSGRASDILAFGHKYSEEGGLINESLRDQLLVTIQK
TFTYTRTQAQHLFIILMECMKKKELITVFRMGSEGHQDIDLAILTALLKGANASAPDQLSL
ALAWNRVDIARSQIFIYGQQWPVGSLEQAMLDALVLDRVDFVKLLIENGVSMHRFLTISRL
EELYNTRHGPSNTLYHLVRDVKKGNLPPDYRISLIDIGLVIEYLMGGAYRCNYTRKRFRTL
YHNLFGPKRPKALKLLGMEDDIPLRRGRKTTKKREEEVDIDLDDPEINHFPFPFHELMVW
AVLMKRQKMALFFWQHGEEAMAKALVACKLCKAMAHEASENDMVDDISQELNHNSRD
FGQLAVELLDQSYKQDEQLAMKLLTYELKNWSNATCLQLAVAAKHRDFIAHTCSQMLLT
DMWMGRLRMRKNSGLKVILGILLPPSILSLEFKNKDDMPYMSQAQEIHLQEKEAEEPEKP
TKEKEEEDMELTAMLGRNNGESSRKKDEEEVQSKHRLIPLGRKIYEFYNAPIVKFWFYTL
AYIGYLMLFNYIVLVKMERWPSTQEWIVISYIFTLGIEKMREILMSEPGKLLQKVKVWLQE
YWNVTDLIAILLFSVGMILRLQDQPFRSDGRVIYCVNIIYWYIRLLDIFGVNKYLGPYVMM
IGKMMIDMMYFVIIMLVVLMSFGVARQAILFPNEEPSWKLAKNIFYMPYWMIYGEVFAD
QIDPPCGQNETREDGKIIQLPPCKTGAWIVPAIMACYLLVANILLVNLLIAVFNNTFFEVKSI
SNQVWKFQRYQLIMTFHERPVLPPPLIIFSHMTMIFQHLCCRWRKHESDPDERDYGLKLFI
TDDELKKVHDFEEQCIEEYFREKDDRFNSSNDERIRVTSERVENMSMRLEEVNEREHSMK
ASLQTVDIRLAQLEDLIGRMATALERLTGLERAESNKIRSRTSSDCTDAAYIVRQSSFNSQE
GNTFKLQESIDPAGEETMSPTSPTLMPRMRSHSFYSVNMKDKGGIEKLESIFKERSLSLHRA
TSSHSVAKEPKAPAAPANTLAIVPDSRRPSSCIDIYVSAMDELHCDIDPLDNSVNILGLGEPS
FSTPVPSTAPSSSAYATLAPTDRPPSRSIDFEDITSMDTRSFSSDYTHLPECQNPWDSEPPMY
HTIERSKSSRYLATTPFLLEEAPIVKSHSFMFSPSRSYYANFGVPVKTAEYTSITDCIDTRCV
NAPQAIADRAAFPGGLGDKVEDLTCCHPEREAELSHPSSDSEENEAKGRRATIAISSQEGD

NSERTLSNNITVPKIERANSYSAEEPSAPYAHTRKSFSISDKLDRQRNTASLRNPFQRSKSSK
PEGRGDSLSMRRLSRTSAFQSFESKHN

>Q86TM6 multipass membrane protein
MFRTAVMMAASLALTGAVVAHAYYLKHQFYPTVVYLTKSSPSMAVLYIQAFVLVFLLGKV
MGKVFFGQLRAAEMEHLLERSWYAVTETCLAFTVFRDDFSPRFVALFTLLLFLKCFHWLA
EDRVDFMERSPNISWLFHCRIVSLMFLLGILDFLFVSHAYHSILTRGASVQLVFGFEYAILM
TMVLTIFIKYVLHSVDLQSENPWDNKAVYMLYTELFTGFIKVLLYMAFMTIMIKVHTFPLF
AIRPMYLAMRQFKKAVTDAIMSRRAIRNMNTLYPDATPEELQAMDNVCIICREEMVTGAK
RLPCNHIFHTSCLRSWFQRQQTCPTCRMDVLRASLPAQSPPPPEPADQGPPPAPHPPPLLPQ
PPNFPQGLLPPFPPGMFPLWPPMGPFPPVPPPPSSGEAVAPPSTSAAALSRPSGAATTTAAGT
SATAASATASGPGSGSAPEAGPAPGFPFPPPWMGMPLPPPFAFPPMPVPPAGFAGLTPEELR
ALEGHERQHLEARLQSLRNIHTLLDAAMLQINQYLTVLASLGPPRPATSVNSTEETATTVV
AAASSTSIPSSEATTPTPGASPPAPEMERPPAPESVGTEEMPEDGEPDAAELRRRRLQKLESP
VAH

>Q9UDY2 peripheral membrane protein
MPVRGDRGFPPRRELSGWLRAPGMEELIWEQYTVTLQKDSKRGFGIAVSGGRDNPHFEN
GETSIVISDVLPGGPADGLLQENDRVVMVNGTPMEDVLHSFAVQQLRKSGKVAAIVVKRP
RKVQVAALQASPPLDQDDRAFEVMDEFDGRSFRSGYSERSRLNSHGGRSRSWEDSPERG
RPHERARSRERDLSRDRSRGRSLERGLDQDHARTRDRSRGRSLERGLDHDFGPSRDRDRD
RSRGRSIDQDYERAYHRAYDPDYERAYSPEYRRGARHDARSRGPRSRSREHPHSRSPSPEP
RGRPGPIGVLLMKSRANEEYGLRLGSQIFVKEMTRTGLATKDGNLHEGDIILKINGTVTEN
MSLTDARKLIEKSRGKLQLVVLRDSQQTLINIPSLNDSDSEIEDISEIESNRSFSPEERRHQYS
DYDYHSSSEKLKERPSSREDTPSRLSRMGATPTPFKSTGDIAGTVVPETNKEPRYQEDPPAP
QPKAAPRTFLRPSPEDEAIYGPNTKMVRFKKGDSVGLRLAGGNDVGIFVAGIQEGTSAEQ
EGLQEGDQILKVNTQDFRGLVREDAVLYLLEIPKGEMVTILAQSRADVYRDILACGRGDSF
FIRSHFECEKETPQSLAFTRGEVFRVVDTLYDGKLGNWLAVRIGNELEKGLIPNKSRAEQM
ASVQNAQRDNAGDRADFWRMRGQRSGVKKNLRKSREDLTAVVSVSTKFPAYERVLLRE
AGFKRPVVLFGPIADIAMEKLANELPDWFQTAKTEPKDAGSEKSTGVVRLNTVRQIIEQD
KHALLDVTPKAVDLLNYTQWFPIVIFFNPDSRQGVKTMRQRLNPTSNKSSRKLFDQANKL
KKTCAHLFTATINLNSANDSWFGSLKDTIQHQQGEAVWVSEGKMEGMDDDPEDRMSYLT
AMGADYLSCDSRLISDFEDTDGEGGAYTDNELDEPAEEPLVSSITRSSEPVQHEESIRKPSP
EPRAQMRRAASSDQLRDNSPPPAFKPEPPKAKTQNKEESYDFSKSYEYKSNPSAVAGNETP
GASTKGYPPPVAAKPTFGRSILKPSTPIPPQEGEEVGESSEEQDNAPKSVLGKVKIFEKMDH
KARLQRMQELQEAQNARIEIAQKHPDIYAVPIKTHKPDPGTPQHTSSRPPEPQKAPSRPYQ
DTRGSYGSDAEEEEYRQQLSEHSKRGYYGQSARYRDTEL

>P25021 multipass membrane protein
MAPNGTASSFCLDSTACKITITVVLAVLILITVAGNVVVCLAVGLNRRLRNLTNCFIVSLAIT
DLLLGLLVLPFSAIYQLSCKWSFGKVFCNIYTSLDVMLCTASILNLFMISLDRYCAVMDPLR
YPVLVTPVRVAISLVLIWVISITLSFLSIHLGWNSRNETSKGNHTTSKCKVQVNEVYGLVDG
LVTFYLPLLIMCITYYRIFKVARDQAKRINHISSWKAATIREHKATVTLAAVMGAFIICWFP
YFTAFVYRGLRGDDAINEVLEAIVLWLGYANSALNPILYAALNRDFRTGYQQLFCCRLAN

RNSHKTSLRSNASQLSRTQSREPRQQEEKPLKLQVWSGTEVTAPQGATDR

>O94985 single-pass type I membrane protein
MLRRPAPALAPAARLLLAGLLCGGGVWAARVNKHKPWLEPTYHGIVTENDNTVLLDPPLI
ALDKDAPLRFAESFEVTVTKEGEICGFKIHGQNVPFDAVVVDKSTGEGVIRSKEKLDCELQ
KDYSFTIQAYDCGKGPDGTNVKKSHKATVHIQVNDVNEYAPVFKEKSYKATVIEGKQYD
SILRVEAVDADCSPQFSQICSYEIITPDVPFTVDKDGYIKNTEKLNYGKEHQYKLTVTAYDC
GKKRATEDVLVKISIKPTCTPGWQGWNNRIEYEPGTGALAVFPNIHLETCDEPVASVQATV
ELETSHIGKGCDRDTYSEKSLHRLCGAAAGTAELLPSPSGSLNWTMGLPTDNGHDSDQVF
EFNGTQAVRIPDGVVSVSPKEPFTISVWMRHGPFGRKKETILCSSDKTDMNRHHYSLYVH
GCRLIFLFRQDPSEEKKYRPAEFHWKLNQVCDEEWHHYVLNVEFPSVTLYVDGTSHEPFS
VTEDYPLHPSKIETQLVVGACWQEFSGVENDNETEPVTVASAGGDLHMTQFFRGNLAGLT
LRSGKLADKKVIDCLYTCKEGLDLQVLEDSGRGVQIQAHPSQLVLTLEGEDLGELDKAMQ
HISYLNSRQFPTPGIRRLKITSTIKCFNEATCISVPPVDGYVMVLQPEEPKISLSGVHHFARA
ASEFESSEGVFLFPELRIISTITREVEPEGDGAEDPTVQESLVSEEIVHDLDTCEVTVEGEEL
NHEQESLEVDMARLQQKGIEVSSSELGMTFTGVDTMASYEEVLHLLRYRNWHARSLLDR
KFKLICSELNGRYISNEFKVEVNVIHTANPMEHANHMAAQPQFVHPEHRSFVDLSGHNLA
NPHPFAVVPSTATVVIVVCVSFLVFMIILGVFRIRAAHRRTMRDQDTGKENEMDWDDSALT
ITVNPMETYEDQHSSEEEEEEEEEEEESEDGEEEDDITSAESESSEEEEGEQGDPQNATRQQQ
LEWDDSTLSY

>Q9UK41 peripheral membrane protein
MFHGIPATPGIGAPGNKPELYEEVKLYKNAREREKYDNMAELFAVVKTMQALEKAYIKDC
VSPSEYTAACSRLLVQYKAAFRQVQGSEISSIDEFCRKFRLDCPLAMERIKEDRPITIKDDK
GNLNRCIADVVSLFITVMDKLRLEIRAMDEIQPDLRELMETMHRMSHLPPDFEGRQTVSQ
WLQTLSGMSASDELDDSQVRQMLFDLESAYNAFNRFLHA

>O43493 single-pass type I membrane protein
MRFVVALVLLNVAAAGAVPLLATESVKQEEAGVRPSAGNVSTHPSLSQRPGGSTKSHPEP
QTPKDSPSKSSAEAQTPEDTPNKSGAEAKTQKDSSNKSGAEAKTQKGSTSKSGSEAQTTK
DSTSKSHPELQTPKDSTGKSGAEAQTPEDSPNRSGAEAKTQKDSPSKSGSEAQTTKDVPN
KSGADGQTPKDGSSKSGAEDQTPKDVPNKSGAEKQTPKDGSNKSGAEEQGPIDGPSKSG
AEEQTSKDSPNKVVPEQPSRKDHSKPISNPSDNKELPKADTNQLADKGKLSPHAFKTESG
EETDLISPPQEEVKSSEPTEDVEPKEAEDDDTGPEEGSPPKEEKEKMSGSASSENREGTLSD
STGSEKDDLYPNGSGNGSAESSHFFAYLVTAAILVAVLYIAHHNKRKIIAFVLEGKRSKVTR
RPKASDYQRLDQKYVLILNVFPAPPKRSFLPQVLTEWYIPLEKDERHQWIVLLSFQL

>Q5UAW9 multipass membrane protein
MQPSPPPTELVPSERAVVLLSCALSALGSGLLVATHALWPDLRSRARRLLLFLSLADLLSAA
SYFYGVLQNFAGPSWDCVLQGALSTFANTSSFFWTVAIALYLYLSIVRAARGPRTDRLLWA
FHVVSWGVPLVITVAAVALKKIGYDASDVSVGWCWIDLEAKDHVLWMLLTGKLWEMLA
YVLLPLLYLLVRKHINRAHTALSEYRPILSQEHRLLRHSSMADKKLVLIPLIFIGLRVWSTV
RFVLTLCGSPAVQTPVLVVLHGIGNTFQGGANCIMFVLCTRAVRTRLFSLCCCCCSSQPPTK
SPAGTPKAPAPSKPGESQESQGTPGELPST

>P22732 multipass membrane protein
MEQQDQSMKEGRLTLVLALATLIAAFGSSFQYGYNVAAVNSPALLMQQFYNETYYGRTG
EFMEDFPLTLLWSVTVSMFPFGGFIGSLLVGPLVNKFGRKGALLFNNIFSIVPAILMGCSRVA
TSFELIIISRLLVGICAGVSSNVVPMYLGELAPKNLRGALGVVPQLFITVGILVAQIFGLRNL
LANVDGWPILLGLTGVPAALQLLLLPFFPESPRYLLIQKKDEAAAKKALQTLRGWDSVDR
EVAEIRQEDEAEKAAGFISVLKLFRMRSLRWQLLSIIVLMGGQQLSGVNAIYYYADQIYLS
AGVPEEHVQYVTAGTGAVNVVMTFCAVFVVELLGRRLLLLLGFSICLIACCVLTAALALQ
DTVSWMPYISIVCVISYVIGHALGPSPIPALLITEIFLQSSRPSAFMVGGSVHWLSNFTVGLI
FPFIQEGLGPYSFIVFAVICLLTTIYIFLIVPETKAKTFIEINQIFTKMNKVSEVYPEKEELKELP
PVTSEQ

>Q9UJM3 peripheral membrane protein
MSIAGVAAQEIRVPLKTGFLHNGRAMGNMRKTYWSSRSEFKNNFLNIDPITMAYSLNSSA
QERLIPLGHASKSAPMNGHCFAENGPSQKSSLPPLLIPPSENLGPHEEDQVVCGFKKLTVN
GVCASTPPLTPIKNSPSLFPCAPLCERGSRPLPPLPISEALSLDDTDCEVEFLTSSDTDFLLED
STLSDFKYDVPGRRSFRGCGQINYAYFDTPAVSAADLSYVSDQNGGVPDPNPPPPQTHRRL
RRSHSGPAGSFNKPAIRISNCCIHRASPNSDEDKPEVPPRVPIPPRPVKPDYRRWSAEVTSST
YSDEDRPPKVPPREPLSPSNSRTPSPKSLPSYLNGVMPPTQSFAPDPKYVSSKALQRQNSEG
SASKVPCILPIIENGKKVSSTHYYLLPERPPYLDKYEKFFREAEETNGGAQIQPLPADCGISS
ATEKPDSKTKMDLGGHVKRKHLSYVVSP

>Q07011 single-pass type I membrane protein
MGNSCYNIVATLLLVLNFERTRSLQDPCSNCPAGTFCDNNRNQICSPCPPNSFSSAGGQRTC
DICRQCKGVFRTRKECSSTSNAECDCTPGFHCLGAGCSMCEQDCKQGQELTKKGCKDCC
FGTFNDQKRGICRPWTNCSLDGKSVLVNGTKERDVVCGPSPADLSPGASSVTPPAPAREPG
HSPQIISFFLALTSTALLFLLFFLTLRFSVVKRGRKKLLYIFKQPFMRPVQTTQEEDGCSCRF
PEEEEGGCEL

>Q92783 peripheral membrane protein
MPLFATNPFDQDVEKATSEMNTAEDWGLILDICDKVGQSRTGPKDCLRSIMRRVNHKDPH
VAMQALTLLGACVSNCGKIFHLEVCSRDFASEVSNVLNKGHPKVCEKLKALMVEWTDEF
KNDPQLSLISAMIKNLKEQGVTFPAIGSQAAEQAKASPALVAKDPGTVANKKEEEDLAKAI
ELSLKEQRQQSTTLSTLYPSTSSLLTNHQHEGRKVRAIYDFEAAEDNELTFKAGEIITVLDD
SDPNWWKGETHQGIGLFPSNFVTADLTAEPEMIKTEKKTVQFSDDVQVETIEPEPEPAFIDE
DKMDQLLQMLQSTDPSDDQPDLPELLHLEAMCHQMGPLIDEKLEDIDRKHSELSELNVK
VMEALSLYTKLMNEDPMYSMYAKLQNQPYYMQSSGVSGSQVYAGPPPSGAYLVAGNAQ
MSHLQSYSLPPEQLSSLSQAVVPPSANPALPSQQTQAAYPNTMVSSVQGNTYPSQAPVYSP
PPAATAAAATADVTLYQNAGPNMPQVPNYNLTSSTLPQPGGSQQPPQPQQPYSQKALL

>Q96S37 multipass membrane protein
MAFSELLDLVGGLGRFQVLQTMALMVSIMWLCTQSMLENFSAAVPSHRCWAPLLDNSTA
QASILGSLSPEALLAISIPPGPNQRPHQCRRFRQPQWQLLDPNATATSWSEADTEPCVDGW
VYDRSIFTSTIVAKWNLVCDSHALKPMAQSIYLAGILVGAAACGPASDRFGRRLVLTWSYL

QMAVMGTAAAFAPAFPVYCLFRFLLAFAVAGVMMNTGTLLMEWTAARARPLVMTLNSL
GFSFGHGLTAAVAYGVRDWTLLQLVVSVPFFLCFLYSWWLAESARWLLTTGRLDWGLQE
LWRVAAINGKGAVQDTLTPEVLLSAMREELSMGQPPASLGTLLRMPGLRFRTCISTLCWFA
FGFTFFGLALDLQALGSNIFLLQMFIGVVDIPAKMGALLLLSHLGRRPTLAASLLLAGLCIL
ANTLVPHEMGALRSALAVLGLGGVGAAFTCITIYSSELFPTVLRMTAVGLGQMAARGGAI
LGPLVRLLGVHGPWLPLLVYGTVPVLSGLAALLLPETQSLPLPDTIQDVQNQAVKKATHG
TLGNSVLKSTQF

>Q15464 peripheral membrane protein
MAKWLNKYFSLGNSKTKSPPQPPRPDYREQRRRGERPSQPPQAVPQASSAASASCGPATA
SCFSASSGSLPDDSGSTSDLIRAYRAQKERDFEDPYNGPGSSLRKLRAMCRLDYCGGSGEP
GGVQRAFSASSASGAAGCCCASSGAGAAASSSSSGSPHLYRSSSERRPATPAEVRYISPKH
RLIKVESAAGGGAGDPLGGACAGGRTWSPTACGGKKLLNKCAASAAEESGAGKKDKVTI
ADDYSDPFDAKNDLKSKAGKGESAGYMEPYEAQRIMTEFQRQESVRSQHKGIQLYDTPY
EPEGQSVDSDSESTVSPRLRESKLPQDDDRPADEYDQPWEWNRVTIPALAAQFNGNEKRQ
SSPSPSRDRRRQLRAPGGGFKPIKHGSPEFCGILGERVDPAVPLEKQIWYHGAISRGDAENL
LRLCKECSYLVRNSQTSKHDYSLSLRSNQGFMHMKLAKTKEKYVLGQNSPPFDSVPEVIH
YYTTRKLPIKGAEHLSLLYPVAVRTL

>Q99678 multipass membrane protein
MPSVSPAGPSAGAVPNATAVTTVRTNASGLEVPLFHLFARLDEELHGTFPGLWLALMAVH
GAIFLAGLVLNGLALYVFCCRTRAKTPSVIYTINLVVTDLLVGLSLPTRFAVYYGARGCLRC
AFPHVLGYFLNMHCSILFLTCICVDRYLAIVRPEGSRRCRQPACARAVCAFVWLAAGAVTL
SVLGVTGSRPCCRVFALTVLEFLLPLLVISVFTGRIMCALSRPGLLHQGRQRRVRAMQLLLT
VLIIFLVCFTPFHARQVAVALWPDMPHHTSLVVYHVAVTLSSLNSCMDPIVYCFVTSGFQAT
VRGLFGQHGEREPSSGDVVSMHRSSKGSGRHHILSAGPHALTQALANGPEA

>Q9Y5X3 peripheral membrane protein
MAAVPELLQQQEEDRSKLRSVSVDLNVDPSLQIDIPDALSERDKVKFTVHTKTTLPTFQSP
EFSVTRQHEDFVWLHDTLIETTDYAGLIIPPAPTKPDFDGPREKMQKLGEGEGSMTKEEFA
KMKQELEAEYLAVFKKTVSSHEVFLQRLSSHPVLSKDRNFHVFLEYDQDLSVRRKNTKE
MFGGFFKSVVKSADEVLFTGVKEVDDFFEQEKNFLINYYNRIKDSCVKADKMTRSHKNV
ADDYIHTAACLHSLALEEPTVIKKYLLKVAELFEKLRKVEGRVSSDEDLKLTELLRYYMLN
IEAAKDLLYRRTKALIDYENSNKALDKARLKSKDVKLAEAHQQECCQKFEQLSESAKEEL
INFKRKRVAAFRKNLIEMSELEIKHARNNVSLLQSCIDLFKNN

>Q5SYB0 peripheral membrane protein
MEELETSLFQTRKAHRIEQMVARWLRRSRDSSARAKVAAADGPARNPTQTLIPVRHTVKI
DKDTLLQDYGFHISESLPLTVVAVTAGGSAHGKLFPGDQILQMNNEPAEDLSWERAVDILR
EAEDSLSITVVRCTSGVPKSSFLTEEKRARLKTNPVKVHFAEEVLISGHSQGNSLLCMPNV
LKLYLENGQTKAFKFEANTTVKDIILTVKEKLSIRSIEYFALALEEQYSISRLHLLHEEELIQ
QVVEREESHDYRCLFRVCFVPKDPLDLLKEDPVAFEYLYLQSCSDVLQERFAVEMKCSSA
LRLAALHIQERIYACAQPQKISLKYIEKDWGIENFISPTLLRNMKGKDIKKAISFHMKRNQ
NLLEPRQKQLISAAQLRLNYLQILGELKTYGGRIFNATLMLQDRESYIALLVGAKYGISQVI

NSKLNIMSTLAEFANISRVELTEESEKVSVVKVYLQDVKVLTLLLESNSAKDLACLIAGYY
RLLVDPVTSIFLWPGNKQQAHRVSAEEGYESRACSDSEESSEVDCVLEPLSDRRLVKLAPC
RSLIKEEQPPGNSPTPEVARRGPSTCGASSTTDSAESEASDSANTESRGYRTSGSSESMDAL
EEDDLDTCSSSRSTFFHFGSPGLAESIDSDSQEERSGIETSGFLCLLDLAQRANPQCQKTEFS
ESAALETFGWAPELSTVRLDPRLYEGSHADYYSLCSSVSPASYLSDSSESTASRQGGAPPA
WGQQGWTEAQPSSMLEPLALHPPLAFEDGSSDEEYYDAADKLTPPGPPSGPRDVSTAEPS
ATSLQNKASTSSPENSLPCGPDGRQPSRRGGVKKYAKTLRKRRSFLQTDYTSQVSFPLVPS
ASLESVDDVCYYDREPYLALGAPSPTVSSLQDMQGEPGLLETKALGLLAPLRETKSTNPA
SRVMEMEPETMETKSVIDSRVSSISAIRFRIDPNNKENSGVVPAASSSASTPHCSNPGSSGPD
TAQARPSQILPLSQDLDGIAPKEPTIEHGDSSFSLSSGDPNPDRACLASNPGLNNVSQGDTL
ELQLEPHVQLEMGLESFCTNHIQETAPKYTEPLLSPRDEPRSDECGINPGEKIASIPTKEEPQ
GQLSLERDREVTNKNGTNVFQEESRKDSGDSPGDVSNNVSQTLDISSPAGKIVTSLSLDAP
VTGTEQIPPHPPRDPQGQSREPPGQGCQAQEQKLFVELDLDPDFFLGKQTVSPAVPPEGIK
AEAPNHVTGQDIAPRDSPEWVCFNPEPSLPEPLPCPQEDPHLETSNHCLLSEGKSDSSSICL
SAEKSFLCFAPESHPEVSASLRVATSLGFAGMNEMVAPRIGMDQCSCQFSYATCFRGPQPET
EEEDRDLEAHPMAPLTSPPSAGSPVVLPWRPARAHSCTTAPLSRKSHIWPEYCSRALRQLK
ATPASTPEGFIQLMESLLELQDILETSWGVGNKHPPEKCTWHFTESRSRLCMGSQKLLSSC
RHVIRMDQSPEEMQGAVRDTFQHLVQLAGLCFQFTDCSRCSARHREAAGNLRDVVYTYH
QFIEAAKSTCERGYHDLSVKLLARQCTALTAAVFCLTQKFRASTAL

>Q93038 single-pass type I membrane protein
MEQRPRGCAAVAAALLLVLLGARAQGGTRSPRCDCAGDFHKKIGLFCCRGCPAGHYLKA
PCTEPCGNSTCLVCPQDTFLAWENHHNSECARCQACDEQASQVALENCSAVADTRCGCKP
GWFVECQVSQCVSSSPFYCQPCLDCGALHRHTRLLCSRRDTDCGTCLPGFYEHGDGCVS
CPTSTLGSCPERCAAVCGWRQMFWVQVLLAGLVVPLLLGATLTYTYRHCWPHKPLVTAD
EAGMEALTPPPATHLSPLDSAHTLLAPPDSSEKICTVQLVGNSWTPGYPETQEALCPQVTW
SWDQLPSRALGPAAAPTLSPESPAGSPAMMLQPGPQLYDVMDAVPARRWKEFVRTLGLRE
AEIEAVEVEIGRFRDQQYEMLKRWRQQQPAGLGAVYAALERMGLDGCVEDLRSRLQRGP

>P08195 single-pass type II membrane protein
MELQPPEASIAVVSIPRQLPGSHSEAGVQGLSAGDDSELGSHCVAQTGLELLASGDPLPSA
SQNAEMIETGSDCVTQAGLQLLASSDPPALASKNAEVTGTMSQDTEVDMKEVELNELEP
EKQPMNAASGAAMSLAGAEKNGLVKIKVAEDEAEAAAAAKFTGLSKEELLKVAGSPGW
VRTRWALLLLFWLGWLGMLAGAVVIIVRAPRCRELPAQKWWHTGALYRIGDLQAFQGH
GAGNLAGLKGRLDYLSSLKVKGLVLGPIHKNQKDDVAQTDLLQIDPNFGSKEDFDSLLQS
AKKKSIRVILDLTPNYRGENSWFSTQVDTVATKVKDALEFWLQAGVDGFQVRDIENLKDA
SSFLAEWQNITKGFSEDRLLIAGTNSSDLQQILSLLESNKDLLLTSSYLSDSGSTGEHTKSLV
TQYLNATGNRWCSWSLSQARLLTSFLPAQLLRLYQLMLFTLPGTPVFSYGDEIGLDAAALP
GQPMEAPVMLWDESSFPDIPGAVSANMTVKGQSEDPGSLLSLFRRLSDQRSKERSLLHGD
FHAFSAGPGLFSYIRHWDQNERFLVVLNFGDVGLSAGLQASDLPASASLPAKADLLLSTQP
GREEGSPLELERLKLEPHEGLLLRFPYAA

>Q6NV75 multipass membrane protein
MSDERRLPGSAVGWLVCGGLSLLANAWGILSVGAKQKKWKPLEFLLCTLAATHMLNVAV

PIATYSVVQLRRQRPDFEWNEGLCKVFVSTFYTLTLATCFSVTSLSYHRMWMVCWPVNY
RLSNAKKQAVHTVMGIWMVSFILSALPAVGWHDTSERFYTHGCRFIVAEIGLGFGVCFLLL
VGGSVAMGVICTAIALFQTLAVQVGRQADRRAFTVPTIVVEDAQGKRRSSIDGSEPAKTSL
QTTGLVTTIVFIYDCLMGFPVLVVSFSSLRADASAPWMALCVLWCSVAQALLLPVFLWAC
DRYRADLKAVREKCMALMANDEESDDETSLEGGISPDLVLERSLDYGYGGDFVALDRMA
KYEISALEGGLPQLYPLRPLQEDKMQYLQVPPTRRFSHDDADVWAAVPLPAFLPRWGSGE
DLAALAHLVLPAGPERRRASLLAFAEDAPPSRARRRSAESLLSLRPSALDSGPRGARDSPP
GSPRRRPGPGPRSASASLLPDAFALTAFECEPQALRRPPGPFPAAPAAPDGADPGEAPTPPSS
AQRSPGPRPSAHSHAGSLRPGLSASWGEPGGLRAAGGGGSTSSFLSSPSESSGYATLHSDS
LGSAS

>Q9H4G4 lipid-anchor
MGKSASKQFHNEVLKAHNEYRQKHGVPPLKLCKNLNREAQQYSEALASTRILKHSPESS
RGQCGENLAWASYDQTGKEVADRWYSEIKNYNFQQPGFTSGTGHFTAMVWKNTKKMG
VGKASASDGSSFVVARYFPAGNVVNEGFFEENVLPPKK

>O76038 peripheral membrane protein
MDSSREPTLGRLDAAGFWQVWQRFDADEKGYIEEKELDAFFLHMLMKLGTDDTVMKA
NLHKVKQQFMTTQDASKDGRIRMKELAGMFLSEDENFLLLFRRENPLDSSVEFMQIWRK
YDADSSGFISAAELRNFLRDLFLHHKKAISEAKLEEYTGTMMKIFDRNKDGRLDLNDLAR
ILALQENFLLQFKMDACSTEERKRDFEKIFAYYDVSKTGALEGPEVDGFVKDMMELVQPS
ISGVDLDKFREILLRHCDVNKDGKIQKSELALCLGLKINP

>O95980 GPI-anchor, lipid-anchor
MATVRASLRGALLLLLLAVAGVAEVAGGLAPGSAGALCCNHSKDNQMCRDVCEQIFSSKS
ESRLKHLLQRAPDYCPETMVEIWNCMNSSLPGVFKKSDGWVGLGCCELAIALECRQACK
QASSKNDISKVCRKEYENALFSCISRNEMGSVCCSYAGHHTNCREYCQAIFRTDSSPGPSQ
IKAVENYCASISPQLIHCVNNYTQSYPMRNPTDSLYCCDRAEDHACQNACKRILMSKKTE
MEIVDGLIEGCKTQPLPQDPLWQCFLESSQSVHPGVTVHPPPSTGLDGAKLHCCSKANTST
CRELCTKLYSMSWGNTQSWQEFDRFCEYNPVEVSMLTCLADVREPCQLGCRNLTYCTNF
NNRPTELFRSCNAQSDQGAMNDMKLWEKGSIKMPFINIPVLDIKKCQPEMWKAIACSLQI
KPCHSKSRGSIICKSDCVEILKKCGDQNKFPEDHTAESICELLSPTDDLKNCIPLDTYLRPST
LGNIVEEVTHPCNPNPCPANELCEVNRKGCPSGDPCLPYFCVQGCKLGEASDFIVRQGTLI
QVPSSAGEVGCYKICSCGQSGLLENCMEMHCIDLQKSCIVGGKRKSHGTSFSIDCNVCSCF
AGNLVCSTRLCLSEHSSEDDRRTFTGLPCNCADQFVPVCGQNGRTYPSACIARCVGLQDH
QFEFGSCMSKDPCNPNPCQKNQRCIPKPQVCLTTFDKFGCSQYECVPRQLACDQVQDPVC
DTDHMEHNNLCTLYQRGKSLSYKGPCQPFCRATEPVCGHNGETYSSVCAAYSDRVAVDY
YGDCQAVGVLSEHSSVAECASVKCPSLLAAGCKPIIPPGACCPLCAGMLRVLFDKEKLDTI
AKVTNKKPITVLEILQKIRMHVSVPQCDVFGYFSIESEIVILIIPVDHYPKALQIEACNKEAE
KIESLINSDSPTLASHVPLSALIISQVQVSSSVPSAGVRARPSCHSLLLPLSLGLALHLLWTY
N

>Q8NGT2 multipass membrane protein
MEPLNRTEVSEFFLKGFSGYPALEHLLFPLCSAMYLVTLLGNTAIMAVSVLDIHLHTPVYFF

LGNLSTLDICYTPTFVPLMLVHLLSSRKTISFAVCAIQMCLSLSTGSTECLLLAITAYDRYLA
ICQPLRYHVLMSHRLCVLLMGAAWVLCLLKSVTEMVISMRLPFCGHHVVSHFTCKILAVL
KLACGNTSVSEDFLLAGSILLLPVPLAFICLSYLLILATILRVPSAARCCKAFSTCLAHLAVV
LLFYGTIIFMYLKPKSKEAHISDEVFTVLYAMVTTMLNPTIYSLRNKEVKEAARKVWGRS
RASR

>Q07065 single-pass type II membrane protein
MPSAKQRGSKGGHGAASPSEKGAHPSGGADDVAKKPPPAPQQPPPPPAPHPQQHPQQHPQ
NQAHGKGGHRGGGGGGGKSSSSSSASAAAAAAAASSSASCSRRLGRALNFLFYLALVAA
AAFSGWCVHHVLEEVQQVRRSHQDFSRQREELGQGLQGVEQKVQSLQATFGTFESILRSS
QHKQDLTEKAVKQGESEVSRISEVLQKLQNEILKDLSDGIHVVKDARERDFTSLENTVEER
LTELTKSINDNIAIFTEVQKRSQKEINDMKAKVASLEESEGNKQDLKALKEAVKEIQTSAKS
REWDMEALRSTLQTMESDIYTEVRELVSLKQEQQAFKEAADTERLALQALTEKLLRSEES
VSRLPEEIRRLEEELRQLKSDSHGPKEDGGFRHSEAFEALQQKSQGLDSRLQHVEDGVLS
MQVASARQTESLESLLSKSQEHEQRLAALQGRLEGLGSSEADQDGLASTVRSLGETQLVL
YGDVEELKRSVGELPSTVESLQKVQEQVHTLLSQDQAQAARLPPQDFLDRLSSLDNLKAS
VSQVEADLKMLRTAVDSLVAYSVKIETNENNLESAKGLLDDLRNDLDRLFVKVEKIHEKV

>Q8IVP5 multipass membrane protein
MATRNPPPQDYESDDDSYEVLDLTEYARRHQWWNRVFGHSSGPMVEKYSVATQIVMGG
VTGWCAGFLFQKVGKLAATAVGGGFLLLQIASHSGYVQIDWKRVEKDVNKAKRQIKKRA
NKAAPEINNLIEEATEFIKQNIVISSGFVGGFLLGLAS

>P53708 single-pass type I membrane protein
MSPGASRGPRGSQAPLIAPLCCAAAALGMLLWSPACQAFNLDVEKLTVYSGPKGSYFGYA
VDFHIPDARTASVLVGAPKANTSQPDIVEGGAVYYCPWPAEGSAQCRQIPFDTTNNRKIRV
NGTKEPIEFKSNQWFGATVKAHKGKVVACAPLYHWRTLKPTPEKDPVGTCYVAIQNFSAY
AEFSPCRNSNADPEGQGYCQAGFSLDFYKNGDLIVGGPGSFYWQGQVITASVADIIANYSF
KDILRKLAGEKQTEVAPASYDDSYLGYSVAAGEFTGDSQQELVAGIPRGAQNFGYVSIINS
TDMTFIQNFTGEQMASYFGYTVVVSDVNSDGLDDVLVGAPLFMEREFESNPREVGQIYLY
LQVSSLLFRDPQILTGTETFGRFGSAMAHLGDLNQDGYNDIAIGVPFAGKDQRGKVLIYN
GNKDGLNTKPSQVLQGVWASHAVPSGFGFTLRGDSDIDKNDYPDLIVGAFGTGKVAVYR
ARPVVTVDAQLLLHPMIINLENKTCQVPDSMTSAACFSLRVCASVTGQSIANTIVLMAEV
QLDSLKQKGAIKRTLFLDNHQAHRVFPLVIKRQKSHQCQDFIVYLRDETEFRDKLSPINISL
NYSLDESTFKEGLEVKPILNYYRENIVSEQAHILVDCGEDNLCVPDLKLSARPDKHQVIIG
DENHLMLIINARNEGEGAYEAELFVMIPEEADYVGIERNNKGFRPLSCEYKMENVTRMV
VCDLGNPMVSGTNYSLGLRFAVPRLEKTNMSINFDLQIRSSNKDNPDSNFVSLQINITAVAQ
VEIRGVSHPPQIVLPIHNWEPEEEPHKEEEVGPLVEHIYELHNIGPSTISDTILEVGWPFSAR
DEFLLYIFHIQTLGPLQCQPNPNINPQDIKPAASPEDTPELSAFLRNSTIPHLVRKRDVHVVE
FHRQSPAKILNCTNIECLQISCAVGRLEGGESAVLKVRSRLWAHTFLQRKNDPYALASLVSF
EVKKMPYTDQPAKLPEGSIVIKTSVIWATPNVSFSIPLWVIILAILLGLLVLAILTLALWKCGF
FDRARPPQEDMTDREQLTNDKTPEA

>Q16394 single-pass type II membrane protein

MQAKKRYFILLSAGSCLALLFYFGGLQFRASRSHSRREEHSGRNGLHHPSPDHFWPRFPD
ALRPFVPWDQLENEDSSVHISPRQKRDANSSIYKGKKCRMESCFDFTLCKKNGFKVYVYP
QQKGEKIAESYQNILAAIEGSRFYTSDPSQACLFVLSLDTLDRDQLSPQYVHNLRSKVQSL
HLWNNGRNHLIFNLYSGTWPDYTEDVGFDIGQAMLAKASISTENFRPNFDVSIPLFSKDHP
RTGGERGFLKFNTIPPLRKYMLVFKGKRYLTGIGSDTRNALYHVHNGEDVVLLTTCKHGK
DWQKHKDSRCDRDNTEYEKYDYREMLHNATFCLVPRGRRLGSFRFLEALQAACVPVML
SNGWELPFSEVINWNQAAVIGDERLLLQIPSTIRSIHQDKILALRQQTQFLWEAYFSSVEKI
VLTTLEIIQDRIFKHISRNSLIWNKHPGGLFVLPQYSSYLGDFPYYYANLGLKPPSKFTAVIH
AVTPLVSQSQPVLKLLVAAAKSQYCAQIIVLWNCDKPLPAKHRWPATAVPVVVIEGESKVM
SSRFLPYDNIITDAVLSLDEDTVLSTTEVDFAFTVWQSFPERIVGYPARSHFWDNSKERWG
YTSKWTNDYSMVLTGAAIYHKYYHYLYSHYLPASLKNMVDQLANCEDILMNFLVSAVTK
LPPIKVTQKKQYKETMMGQTSRASRWADPDHFAQRQSCMNTFASWFGYMPLIHSQMRL
DPVLFKDQVSILRKKYRDIERL

>Q5VV43 single-pass type I membrane protein
MAPPTGVLSSLLLLVTIAGCARKQCSEGRTYSNAVISPNLETTRIMRVSHTFPVVDCTAACC
DLSSCDLAWWFEGRCYLVSCPHKENCEPKKMGPIRSYLTFVLRPVQRPAQLLDYGDMML
NRGSPSGIWGDSPEDIRKDLTFLGKDWGLEEMSEYSDDYRELEKDLLQPSGKQEPRGSAE
YTDWGLLPGSEGAFNSSVGDSPAVPAETQQDPELHYLNESASTPAPKLPERSVLLPLPTTPS
SGEVLEKEKASQLQEQSSNSSGKEVLMPSHSLPPASLELSSVTVEKSPVLTVTPGSTEHSIPT
PPTSAAPSESTPSELPISPTTAPRTVKELTVSAGDNLIITLPDNEVELKAFVAPAPPVETTYNY
EWNLISHPTDYQGEIKQGHKQTLNLSQLSVGLYVFKVTVSSENAFGEGFVNVTVKPARRV
NLPPVAVVSPQLQELTLPLTSALIDGSQSTDDTEIVSYHWEEINGPFIEEKTSVDSPVLRLSN
LDPGNYSFRLTVTDSDGATNSTTAALIVNNAVDYPPVANAGPNHTITLPQNSITLNGNQSS
DDHQIVLYEWSLGPGSEGKHVVMQGVQTPYLHLSAMQEGDYTFQLKVTDSSRQQSTAV
VTVIVQPENNRPPVAVAGPDKELIFPVESATLDGSSSSDDHGIVFYHWEHVRGPSAVEMENI
DKAIATVTGLQVGTYHFRLTVKDQQGLSSTSTLTVAVKKENNSPPRARAGGRHVLVLPNN
SITLDGSRSTDDQRIVSYLWIRDGQSPAAGDVIDGSDHSVALQLTNLVEGVYTFHLRVTDS
QGASDTDTATVEVQPDPRKSGLVELTLQVGVGQLTEQRKDTLVRQLAVLLNVLDSDIKVQ
KIRAHSDLSTVIVFYVQSRPPFKVLKAAEVARNLHMRLSKEKADFLLFKVLRVDTAGCLL
KCSGHGHCDPLTKRCICSHLWMENLIQRYIWDGESNCEWSIFYVTVLAFTLIVLTGGFTWL
CICCCKRQKRTKIRKKTKYTILDNMDEQERMELRPKYGIKHRSTEHNSSLMVSESEFDSD
QDTIFSREKMERGNPKVSMNGSIRNGASFSYCSKDR

>Q16790 single-pass type I membrane protein
MAPLCPSPWLPLLIPAPAPGLTVQLLLSLLLLVPVHPQRLPRMQEDSPLGGGSSGEDDPLGE
EDLPSEEDSPREEDPPGEEDLPGEEDLPGEEDLPEVKPKSEEEGSLKLEDLPTVEAPGDPQE
PQNNAHRDKEGDDQSHWRYGGDPPWPRVSPACAGRFQSPVDIRPQLAAFCPALRPLELLG
FQLPPLPELRLRNNGHSVQLTLPPGLEMALGPGREYRALQLHLHWGAAGRPGSEHTVEG
HRFPAEIHVVHLSTAFARVDEALGRPGGLAVLAAFLEEGPEENSAYEQLLSRLEEIAEEGSE
TQVPGLDISALLPSDFSRYFQYEGSLTTPPCAQGVIWTVFNQTVMLSAKQLHTLSDTLWGP
GDSRLQLNFRATQPLNGRVIEASFPAGVDSSPRAAEPVQLNSCLAAGDILALVFGLLFAVTS
VAFLVQMRRQHRRGTKGGVSYRPAEVAETGA

>Q8NGK9 multipass membrane protein
MFLTERNTTSEATFTLLGFSDYLELQIPLFFVFLAVYGFSVVGNLGMIVIIKINPKLHTPMYF
FLNHLSFVDFCYSSIIAPMMLVNLVVEDRTISFSGCLVQFFFFCTFVVTELILFAVMAYDHFV
AICNPLLYTVAISQKLCAMLVVVLYAWGVACSLTLACSALKLSFHGFNTINHFFCELSSLISL
SYPDSYLSQLLLFTVATFNEISTLLIILTSYAFIIVTTLKMPSASGHRKVFSTCASHLTAITIFH
GTILFLYCVPNSKNSRHTVKVASVFYTVVIPLLNPLIYSLRNKDVKDAIRKIINTKYFHIKHR
HWYPFNFVIEQ

>P41732 multipass membrane protein
MASRRMETKPVITCLKTLLIIYSFVFWITGVILLAVGVWGKLTLGTYISLIAENSTNAPYVLI
GTGTTIVVFGLFGCFATCRGSPWMLKLYAMFLSLVFLAELVAGISGFVFRHEIKDTFLRTYT
DAMQTYNGNDERSRAVDHVQRSLSCCGVQNYTNWSTSPYFLEHGIPPSCCMNETDCNPQ
DLHNLTVAATKVNQKGCYDLVTSFMETNMGIIAGVAFGIAFSQLIGMLLACCLSRFITANQ
YEMV

>Q969S0 multipass membrane protein
MRPALAVGLVFAGCCSNVIFLELLARKHPGCGNIVTFAQFLFIAVEGFLFEADLGRKPPAIPI
RYYAIMVTMFFTVSVVNNYALNLNIAMPLHMIFRSGSLIANMILGIIILKKRYSIFKYTSIAL
VSVGIFICTFMSAKQVTSQSSLSENDGFQAFVWWLLGIGALTFALLMSARMGIFQETLYKR
FGKHSKEALFYNHALPLPGFVFLASDIYDHAVLFNKSELYEIPVIGVTLPIMWFYLLMNIIT
QYVCIRGVFILTTECASLTVTLVVTLRKFVSLIFSILYFQNPFTLWHWLGTLFVFIGTLMYTE
VWNNLGTTKSEPQKDSKKN

>Q9Y287 single-pass type II membrane protein
MVKVTFNSALAQKEAKKDEPKSGEEALIIPPDAVAVDCKDPDDVVPVGQRRAWCWCMCF
GLAFMLAGVILGGAYLYKYFALQPDDVYYCGIKYIKDDVILNEPSADAPAALYQTIEENIKI
FEEEEVEFISVPVPEFADSDPANIVHDFNKKLTAYLDLNLDKCYVIPLNTSIVMPPRNLLELL
INIKAGTYLPQSYLIHEHMVITDRIENIDHLGFFIYRLCHDKETYKLQRRETIKGIQKREASN
CFAIRHFENKFAVETLICS

>O14764 multipass membrane protein
MDAPARLLAPLLLLCAQQLRGTRAMNDIGDYVGSNLEISWLPNLDGLIAGYARNFRPGIG
GPPVNVALALEVASIDHISEANMEYTMTVFLHQSWRDSRLSYNHTNETLGLDSRFVDKLW
LPDTFIVNAKSAWFHDVTVENKLIRLQPDGVILYSIRITSTVACDMDLAKYPMDEQECMLD
LESYGYSSEDIVYYWSESQEHIHGLDKLQLAQFTITSYRFTTELMNFKSAGQFPRLSLHFH
LRRNRGVYIIQSYMPSVLLVAMSWVSFWISQAAVPARVSLGITTVLTMTTLMVSARSSLPR
ASAIKALDVYFWICYVFVFAALVEYAFAHFNADYRKKQKAKVKVSRPRAEMDVRNAIVL
FSLSAAGVTQELAISRRQRRVPGNLMGSYRSVGVETGETKKEGAARSGGQGGIRARLRPI
DADTIDIYARAVFPAAFAAVNVIYWAAYAM

>P04839 multipass membrane protein
MGNWAVNEGLSIFVILVWLGLNVFLFVWYYRVYDIPPKFFYTRKLLGSALALARAPAACL
NFNCMLILLPVCRNLLSFLRGSSACCSTRVRRQLDRNLTFHKMVAWMIALHSAIHTIAHLF
NVEWCVNARVNNSDPYSVALSELGDRQNESYLNFARKRIKNPEGGLYLAVTLLAGITGVV

ITLCLILIITSSTKTIRRSYFEVFWYTHHLFVIFFIGLAIHGAERIVRGQTAESLAVHNITVCEQ
KISEWGKIKECPIPQFAGNPPMTWKWIVGPMFLYLCERLVRFWRSQQKVVITKVVTHPFK
TIELQMKKKGFKMEVGQYIFVKCPKVSKLEWHPFTLTSAPEEDFFSIHIRIVGDWTEGLFN
ACGCDKQEFQDAWKLPKIAVDGPFGTASEDVFSYEVVMLVGAGIGVTPFASILKSVWYKY
CNNATNLKLKKIYFYWLCRDTHAFEWFADLLQLLESQMQERNNAGFLSYNIYLTGWDES
QANHFAVHHDEEKDVITGLKQKTLYGRPNWDNEFKTIASQHPNTRIGVFLCGPEALAETL
SKQSISNSESGPRGVHFIFNKENF

>Q8TDS5 multipass membrane protein
MLCHRGGQLIVPIIPLCPEHSCRGRRLQNLLSGPWPKQPMELHNLSSPSPSLSSSVLPPSFSP
SPSSAPSAFTTVGGSSGGPCHPTSSSLVSAFLAPILALEFVLGLVGNSLALFIFCIHTRPWTSN
TVFLVSLVAADFLLISNLPLRVDYYLLHETWRFGAAACKVNLFMLSTNRTASVVFLTAIAL
NRYLKVVQPHHVLSRASVGAAARVAGGLWVGILLLNGHLLLSTFSGPSCLSYRVGTKPSA
SLRWHQALYLLEFFLPLALILFAIVSIGLTIRNRGLGGQAGPQRAMRVLAMVVAVYTICFLP
SIIFGMASMVAFWLSACRSLDLCTQLFHGSLAFTYLNSVLDPVLYCFSSPNFLHQSRALLG
LTRGRQGPVSDESSYQPSRQWRYREASRKAEAIGKLKVQGEVSLEKEGSSQG

>Q9UJA2 multipass membrane protein
MLALRVARGSWGALRGAAWAPGTRPSKRRACWALLPPVPCCLGCLAERWRLRPAALGL
RLPGIGQRNHCSGAGKAAPRPAAGAGAAAEAPGGQWGPASTPSLYENPWTIPNMLSMTRI
GLAPVLGYLIIEEDFNIALGVFALAGLTDLLDGFIARNWANQRSALGSALDPLADKILISILY
VSLTYADLIPVPLTYMIISRDVMLIAAVFYVRYRTLPTPRTLAKYFNPCYATARLKPTFISKV
NTAVQLILVAASLAAPVFNYADSIYLQILWCFTAFTTAASAYSYYHYGRKTVQVIKD

>O14640 peripheral membrane protein
MAETKIIYHMDEEETPYLVKLPVAPERVTLADFKNVLSNRPVHAYKFFFKSMDQDFGVVK
EEIFDDNAKLPCFNGRVVSWLVLAEGAHSDAGSQGTDSHTDLPPPLERTGGIGDSRPPSFH
PNVASSRDGMDNETGTESMVSHRRERARRRNREEAARTNGHPRGDRRRDVGLPPDSAST
ALSSELESSSFVDSDEDGSTSRLSSSTEQSTSSRLIRKHKRRRRKQRLRQADRASSFSSITDS
TMSLNIVTVTLNMERHHFLGISIVGQSNDRGDGGIYIGSIMKGGAVAADGRIEPGDMLLQV
NDVNFENMSNDDAVRVLREIVSQTGPISLTVAKCWDPTPRSYFTVPRADPVRPIDPAAWLS
HTAALTGALPRYGTSPCSSAVTRTSSSSLTSSVPGAPQLEEAPLTVKSDMSAVVRVMQLPDS
GLEIRDRMWLKITIANAVIGADVVDWLYTHVEGFKERREARKYASSLLKHGFLRHTVNKI
TFSEQCYYVFGDLCSNLATLNLNSGSSGTSDQDTLAPLPHPAAPWPLGQGYPYQYPGPPP
CFPPAYQDPGFSYGSGSTGSQQSEGSKSSGSTRSSRRAPGREKERRAAGAGGSGSESDHTA
PSGVGSSWRERPAGQLSRGSSPRSQASATAPGLPPPHPTTKAYTVVGGPPGGPPVRELAAV
PPELTGSRQSFQKAMGNPCEFFVDIM

>P32189 peripheral membrane protein
MAASKKAVLGPLVGAVDQGTSSTRFLVFNSKTAELLSHHQVEIKQEFPREGWVEQDPKEIL
HSVYECIEKTCEKLGQLNIDISNIKAIGVSNQRETTVVWDKITGEPLYNAVVWLDLRTQST
VESLSKRIPGNNNFVKSKTGLPLSTYFSAVKLRWLLDNVRKVQKAVEEKRALFGTIDSWLI
WSLTGGVNGGVHCTDVTNASRTMLFNIHSLEWDKQLCEFFGIPMEILPNVRSSSEIYGLM
KISHSVKAGALEGVPISGCLGDQSAALVGQMCFQIGQAKNTYGTGCFLLCNTGHKCVFSD

HGLLTTVAYKLGRDKPVYYALEGSVAIAGAVIRWLRDNLGIIKTSEEIEKLAKEVGTSYGC
YFVPAFSGLYAPYWEPSARGIICGLTQFTNKCHIAFAALEAVCFQTREILDAMNRDCGIPLS
HLQVDGGMTSNKILMQLQADILYIPVVKPSMPETTALGAAMAAGAAEGVGVWSLEPEDL
SAVTMERFEPQINAEESEIRYSTWKKAVMKSMGWVTTQSPESGDPSIFCSLPLGFFIVSSMV
MLIGARYISGIP

>Q8NHH9 multipass membrane protein
MAEGDEAARGQQPHQGLWRRRRTSDPSAAVNHVSSTTSLGENYEDDDLVNSDEVMKKP
CPVQIVLAHEDDHNFELDEEALEQILLQEHIRDLNIVVVSVAGAFRKGKSFLLDFMLRYM
YNKDSQSWIGGNNEPLTGFTWRGGCERETTGIQVWNEVFVIDRPNGTKVAVLLMDTQGA
FDSQSTIKDCATVFALSTMTSSVQVYNLSQNIQEDDLQHLQLFTEYGRLAMEEIYQKPFQT
LMFLIRDWSYPYEHSYGLEGGKQFLEKRLQVKQNQHEELQNVRKHIHNCFSNLGCFLLP
HPGLKVATNPSFDGRLKDIDEDFKRELRNLVPLLLAPENLVEKEISGSKVTCRDLVEYFKA
YIKIYQGEELPHPKSMLQATAEANNLAAVAGARDTYCKSMEQVCGGDKPYIAPSDLERKH
LDLKEVAIKQFRSVKKMGGDEFCRRYQDQLEAEIEETYANFIKHNDGKNIFYAARTPATLF
AVMFAMYIISGLTGFIGLNSIAVLCNLVMGLALIFLCTWAYVKYSGEFREIGTVIDQIAETLW
EQVLKPLGDNLMEENIRQSVTNSIKAGLTDQVSHHARLKTD

>Q01151 single-pass type I membrane protein
MSRGLQLLLLSCAYSLAPATPEVKVACSEDVDLPCTAPWDPQVPYTVSWVKLLEGGEER
METPQEDHLRGQHYHQKGQNGSFDAPNERPYSLKIRNTTSCNSGTYRCTLQDPDGQRNL
SGKVILRVTGCPAQRKEETFKKYRAEIVLLLALVIFYLTLIIFTCKFARLQSIFPDFSKAGME
RAFLPVTSPNKHLGLVTPHKTELV

>Q96L58 single-pass type II membrane protein
MKLLRRAWRRRAALGLGTLALCGAALLYLARCAAEPGDPRAMSGRSPPPPAPARAAAFL
AVLVASAPRAAERRSVIRSTWLARRGAPGDVWARFAVGTAGLGAEERRALEREQARHGD
LLLLPALRDAYENLTAKVLAMLAWLDEHVAFEFVLKADDDSFARLDALLAELRAREPARR
RRLYWGFFSGRGRVKPGGRWREAAWQLCDYYLPYALGGGYVLSADLVHYLRLSRDYLR
AWHSEDVSLGAWLAPVDVQREHDPRFDTEYRSRGCSNQYLVTHKQSLEDMLEKHATLA
REGRLCKREVQLRLSYVYDWSAPPSQCCQRREGIP

>Q9BXB1 multipass membrane protein
MPGPLGLLCFLALGLLGSAGPSGAAPPLCAAPCSCDGDRRVDCSGKGLTAVPEGLSAFTQ
ALDISMNNITQLPEDAFKNFPFLEELQLAGNDLSFIHPKALSGLKELKVLTLQNNQLKTVP
SEAIRGLSALQSLRLDANHITSVPEDSFEGLVQLRHLWLDDNSLTEVPVHPLSNLPTLQALT
LALNKISSIPDFAFTNLSSLVVLHLHNNKIRSLSQHCFDGLDNLETLDLNYNNLGEFPQAIK
ALPSLKELGFHSNSISVIPDGAFDGNPLLRTIHLYDNPLSFVGNSAFHNLSDLHSLVIRGAS
MVQQFPNLTGTVHLESLTLTGTKISSIPNNLCQEQKMLRTLDLSYNNIRDLPSFNGCHALEE
ISLQRNQIYQIKEGTFQGLISLRILDLSRNLIHEIHSRAFATLGPITNLDVSFNELTSFPTEGLN
GLNQLKLVGNFKLKEALAAKDFVNLRSLSVPYAYQCCAFWGCDSYANLNTEDNSLQDHS
VAQEKGTADAANVTSTLENEEHSQIIIHCTPSTGAFKPCEYLLGSWMIRLTVWFIFLVALFF
NLLVILTTFASCTSLPSSKLFIGLISVSNLFMGIYTGILTFLDAVSWGRFAEFGIWWETGSGC
KVAGFLAVFSSESAIFLLMLATVERSLSAKDIMKNGKSNHLKQFRVAALLAFLGATVAGCF

PLFHRGEYSASPLCLPFPTGETPSLGFTVTLVLLNSLAFLLMAVIYTKLYCNLEKEDLSENS
QSSMIKHVAWLIFTNCIFFCPVAFFSFAPLITAISISPEIMKSVTLIFFPLPACLNPVLYVFFNPK
FKEDWKLLKRRVTKKSGSVSVSISSQGGCLEQDFYYDCGMYSHLQGNLTVCDCCESFLLT
KPVSCKHLIKSHSCPALAVASCQRPEGYWSDCGTQSAHSDYADEEDSFVSDSSDQVQACG
RACFYQSRGFPLVRYAYNLPRVKD

>P43489 single-pass type I membrane protein
MCVGARRLGRGPCAALLLLGLGLSTVTGLHCVGDTYPSNDRCCHECRPGNGMVSRCSRS
QNTVCRPCGPGFYNDVVSSKPCKPCTWCNLRSGSERKQLCTATQDTVCRCRAGTQPLDS
YKPGVDCAPCPPGHFSPGDNQACKPWTNCTLAGKHTLQPASNSSDAICEDRDPPATQPQE
TQGPPARPITVQPTEAWPRTSQGPSTRPVEVPGGRAVAAILGLGLVLGLLGPLAILLALYLL
RRDQRLPPDAHKPPGGGSFRTPIQEEQADAHSTLAKI

>Q96NR3 multipass membrane protein
MLRQVLHRGLRTCFSRLGHFIASHPVFFASAPVLISILLGASFSRYQVEESVEHLLAPQHSL
AKIERNLVNSLFPVNRSKHRLYSDLQTPGRYGRVIVTSFQKANMLDQHHTDLILKLHAAVT
KIQVPRPGFNYTFAHICILNNDKTCIVDDIVHVLEELKNARATNRTNFAITYPITHLKDGRA
VYNGHQLGGVTVHSKDRVKSAEAIQLTYYLQSINSLNDMVAERWESSFCDTVRLFQKSNS
KVKMYPYTSSSLREDFQKTSRVSERYLVTSLILVVTMAILCCSMQDCVRSKPWLGLLGLV
TISLATLTAAGIINLTGGKYNSTFLGVPFVMLGHGLYGTFEMLSSWRKTREDQHVKERTAA
VYADSMLSFSLTTAMYLVTFGIGASPFTNIEAARIFCCNSCIAIFFNYLYVLSFYGSSLVFTG
YIENNYQHSIFCRKVPKPEALQEKPAWYRFLLTARFSEDTAEGEEANTYESHLLVCFLKRY
YCDWITNTYVKPFVVLFYLIYISFALMGYLQVSEGSDLSNIVATATQTIEYTTAQQKYFSNY
SPVIGFYIYESIEYWNTSVQEDVLEYTKGFVRISWFESYLNYLRKLNVSTGLPKKNFTDML
RNSFLKAPQFSHFQEDIIFSKKYNDEVDVVASRMFLVAKTMETNREELYDLLETLRRLSVT
SKVKFIVFNPSFVYMDRYASSLGAPLHNSCISALFLLFFSAFLVADSLINVWITLTVVSVEFG
VIGFMTLWKVELDCISVLCLIYGINYTIDNCAPMLSTFVLGKDFTRTKWVKNALEVHGVAI
LQSYLCYIVGLIPLAAVPSNLTCTLFRCLFLIAFVTFFHCFAILPVILTFLPPSKKKRKEKKNP
ENREEIECVEMVDIDSTRVVDQITTV

>P25100 multipass membrane protein
MTFRDLLSVSFEGPRPDSSAGGSSAGGGGGSAGGAAPSEGPAVGGVPGGAGGGGGVVGA
GSGEDNRSSAGEPGSAGAGGDVNGTAAVGGLVVSAQGVGVGVFLAAFILMAVAGNLLVI
LSVACNRHLQTVTNYFIVNLAVADLLLSATVLPFSATMEVLGFWAFGRAFCDVWAAVDVL
CCTASILSLCTISVDRYVGVRHSLKYPAIMTERKAAAILALLWVVALVVSVGPLLGWKEPV
PPDERFCGITEEAGYAVFSSVCSFYLPMAVIVVMYCRVYVVARSTTRSLEAGVKRERGKAS
EVVLRIHCRGAATGADGAHGMRSAKGHTFRSSLSVRLLKFSREKKAAKTLAIVVGVFVL
CWFPFFFVLPLGSLFPQLKPSEGVFKVIFWLGYFNSCVNPLIYPCSSREFKRAFLRLLRCQC
RRRRRRRPLWRVYGHHWRASTSGLRQDCAPSSGDAPPGAPLALTALPDPDPEPPGTPEMQ
APVASRRKPPSAFREWRLLGPFRRPTTQLRAKVSSLSHKIRAGGAQRAEAACAQRSEVEA
VSLGVPHEVAEGATCQAYELADYSNLRETDI

>Q12797 single-pass type II membrane protein
MAQRKNAKSSGNSSSSGSGSGSTSAGSSSPGARRETKHGGHKNGRKGGLSGTSFFTWFM

VIALLGVWTSVAVVWFDLVDYEEVLGKLGIYDADGDGDFDVDDAKVLLGLKERSTSEPA
VPPEEAEPHTEPEEQVPVEAEPQNIEDEAKEQIQSLLHEMVHAEHVEGEDLQQEDGPTGEP
QQEDDEFLMATDVDDRFETLEPEVSHEETEHSYHVEETVSQDCNQDMEEMMSEQENPDS
SEPVVEDERLHHDTDDVTYQVYEEQAVYEPLENEGIEITEVTAPPEDNPVEDSQVIVEEVSI
FPVEEQQEVPPETNRKTDDPEQKAKVKKKKPKLLNKFDKTIKAELDAAEKLRKRGKIEEA
VNAFKELVRKYPQSPRARYGKAQCEDDLAEKRRSNEVLRGAIETYQEVASLPDVPADLLK
LSLKRRSDRQQFLGHMRGSLLTLQRLVQLFPNDTSLKNDLGVGYLLIGDNDNAKKVYEE
VLSVTPNDGFAKVHYGFILKAQNKIAESIPYLKEGIESGDPGTDDGRFYFHLGDAMQRVG
NKEAYKWYELGHKRGHFASVWQRSLYNVNGLKAQPWWTPKETGYTELVKSLERNWKLI
RDEGLAVMDKAKGLFLPEDENLREKGDWSQFTLWQQGRRNENACKGAPKTCTLLEKFP
ETTGCRRGQIKYSIMHPGTHVWPHTGPTNCRLRMHLGLVIPKEGCKIRCANETKTWEEGK
VLIFDDSFEHEVWQDASSFRLIFIVDVWHPELTPQQRRSLPAI

>Q8IZ96 multipass membrane protein
MDPEHAKPESSEAPSGNLKQPETAAALSLILGALACFIITQANESFITITSLEICIVVFFILIYV
LTLHHLLTYLHWPLLDLTNSIITAVFLSVVAILAMQEKKRRHLLYVGGSLCLTAVIVCCIDAF
VVTTKMRTNLKRFLGVEVERKLSPAKDAYPETGPDAPQRPA

>Q8WXI2 peripheral membrane protein
MALIMEPVSKWSPSQVVDWMKGLDDCLQQYIKNFEREKISGDQLLRITHQELEDLGVSRI
GHQELILEAVDLLCALNYGLETENLKTLSHKLNASAKNLQNFITGRRRSGHYDGRTSRKL
PNDFLTSVVDLIGAAKSLLAWLDRSPFAAVTDYSVTRNNVIQLCLELTTIVQQDCTVYETE
NKILHVCKTLSGVCDHIISLSSDPLVSQSAHLEVIQLANIKPSEGLGMYIKSTYDGLHVITGT
TENSPADRCKKIHAGDEVIQVNHQTVVGWQLKNLVNALREDPSGVILTLKKRPQSMLTSA
PALLKNMRWKPLALQPLIPRSPTSSVATPSSTISTPTKRDSSALQDLYIPPPPAEPYIPRDEKG
NLPCEDLRGHMVGKPVHKGSESPNSFLDQEYRKRFNIVEEDTVLYCYEYEKGRSSSQGRR
ESTPTYGKLRPISMPVEYNWVGDYEDPNKMKRDSRRENSLLRYMSNEKIAQEEYMFQRN
SKKDTGKKSKKKGDKSNSPTHYSLLPSLQMDALRQDIMGTPVPETTLYHTFQQSSLQHKS
KKKNKGPIAGKSKRRISCKDLGRGDCEGWLWKKKDAKSYFSQKWKKYWFVLKDASLY
WYINEEDEKAEGFISLPEFKIDRASECRKKYAFKACHPKIKSFYFAAEHLDDMNRWLNRIN
MLTAGYAERERIKQEQDYWSESDKEEADTPSTPKQDSPPPPYDTYPRPPSMSCASPYVEA
KHSRLSSTETSQSQSSHEEFRQEVTGSSAVSPIRKTASQRRSWQDLIETPLTSSGLHYLQTLP
LEDSVFSDSAAISPEHRRQSTLPTQKCHLQDHYGPYPLAESERMQVLNGNGGKPRSFTLP
RDSGFNHCCLNAPVSACDPQDDVQPPEVEEEEEEEEEEGEAAGENIGEKSESREEKLGDSL
QDLYRALEQASLSPLGEHRISTKMEYKLSFIKRCNDPVMNEKLHRLRILKSTLKAREGEVA
IIDKVLDNPDLTSKEFQQWKQMYLDLFLDICQNTTSNDPLSISSEVDVITSSLAHTHSYIET
HV

>O60749 peripheral membrane protein
MAAEREPPPLGDGKPTDFEDLEDGEDLFTSTVSTLESSPSSPEPASLPAEDISANSNGPKPTE
VVLDDDREDLFAEATEEVSLDSPEREPILSSEPSPAVTPVTPTTLIAPRIESKSMSAPVIFDRS
REEIEEEANGDIFDIEIGVSDPEKVGDGMNAYMAYRVTTKTSLSMFSKSEFSVKRRFSDFL
GLHSKLASKYLHVGYIVPPAPEKSIVGMTKVKVGKEDSSSTEFVEKRRAALERYLQRTVK
HPTLLQDPDLRQFLESSELPRAVNTQALSGAGILRMVNKAADAVNKMTIKMNESDAWFE

EKQQQFENLDQQLRKLHVSVEALVCHRKELSANTAAFAKSAAMLGNSEDHTALSRALSQ
LAEVEEKIDQLHQEQAFADFYMFSELLSDYIRLIAAVKGVFDHRMKCWQKWEDAQITLL
KKREAEAKMMVANKPDKIQQAKNEIREWEAKVQQGERDFEQISKTIRKEVGRFEKERVK
DFKTVIIKYLESLVQTQQQLIKYWEAFLPEAKAIA

>Q68CP4 multipass membrane protein
MTGARASAAEQRRAGRSGQARAAERAAGMSGAGRALAALLLAASVLSAALLAPGGSSG
RDAQAAPPRDLDKKRHAELKMDQALLLIHNELLWTNLTVYWKSECCYHCLFQVLVNVP
QSPKAGKPSAAAASVSTQHGSILQLNDTLEEKEVCRLEYRFGEFGNYSLLVKNIHNGVSEI
ACDLAVNEDPVDSNLPVSIAFLIGLAVIIVISFLRLLLSLDDFNNWISKAISSRETDRLINSEL
GSPSRTDPLDGDVQPATWRLSALPPRLRSVDTFRGIALILMVFVNYGGGKYWYFKHASW
NGLTVADLVFPWFVFIMGSSIFLSMTSILQRGCSKFRLLGKIAWRSFLLICIGIIIVNPNYCLG
PLSWDKVRIPGVLQRLGVTYFVVAVLELLFAKPVPEHCASERSCLSLRDITSSWPQWLLIL
VLEGLWLGLTFLLPVPGCPTGYLGPGGIGDFGKYPNCTGGAAGYIDRLLLGDDHLYQHPS
SAVLYHTEVAYDPEGILGTINSIVMAFLGVQAGKILLYYKARTKDILIRFTAWCCILGLISVA
LTKVSENEGFIPVNKNLWSLSYVTTLSSFAFFILLVLYPVVDVKGLWTGTPFFYPGMNSILV
YVGHEVFENYFPFQWKLKDNQSHKEHLTQNIVATALWVLIAYILYRKKIFWKI

>Q13507 multipass membrane protein
MREKGRRQAVRGPAFMFNDRGTSLTAEEERFLDAAEYGNIPVVRKMLEESKTLNVNCVD
YMGQNALQLAVGNEHLEVTELLLKKENLARIGDALLLAISKGYVRIVEAILNHPGFAASK
RLTLSPCEQELQDDDFYAYDEDGTRFSPDITPIILAAHCQKYEVVHMLLMKGARIERPHDY
FCKCGDCMEKQRHDSFSHSRSRINAYKGLASPAYLSLSSEDPVLTALELSNELAKLANIEK
EFKNDYRKLSMQCKDFVVGVLDLCRDSEEVEAILNGDLESAEPLEVHRHKASLSRVKLAI
KYEVKKFVAHPNCQQQLLTIWYENLSGLREQTIAIKCLVVLVVALGLPFLAIGYWIAPCSR
LGKILRSPFMKFVAHAASFIIFLGLLVFNASDRFEGITTLPNITVTDYPKQIFRVKTTQFTWT
EMLIMVWVLGMMWSECKELWLEGPREYILQLWNVLDFGMLSIFIAAFTARFLAFLQATK
AQQYVDSYVQESDLSEVTLPPEIQYFTYARDKWLPSDPQIISEGLYAIAVVLSFSRIAYILPA
NESFGPLQISLGRTVKDIFKFMVLFIMVFFAFMIGMFILYSYYLGAKVNAAFTTVEESFKTL
FWSIFGLSEVTSVVLKYDHKFIENIGYVLYGIYNVTMVVVLLNMLIAMINSSYQEIEDDSD
VEWKFARSKLWLSYFDDGKTLPPPFSLVPSPKSFVYFIMRIVNFPKCRRRRLQKDIEMGMG
NSKSRLNLFTQSNSRVFESHSFNSILNQPTRYQQIMKRLIKRYVLKAQVDKENDEVNEGEL
KEIKQDISSLRYELLEDKSQATEELAILIHKLSEKLNPSMLRCE

>Q9H1N7 multipass membrane protein
MDLTQQAKDIQNITVQETNKNNSESIECSKITMDLKFNNSRKYISITVPSKTQTMSPHIKSV
DDVVVLGMNLSKFNKLTQFFICVAGVFVFYLIYGYLQELIFSVEGFKSCGWYLTLVQFAFY
SIFGLIELQLIQDKRRRIPGKTYMIIAFLTVGTMGLSNTSLGYLNYPTQVIFKCCKLIPVMLG
GVFIQGKRYNVADVSAAICMSLGLIWFTLADSTTAPNFNLTGVVLISLALCADAVIGNVQE
KAMKLHNASNSEMVLYSYSIGFVYILLGLTCTSGLGPAVTFCAKNPVRTYGYAFLFSLTGY
FGISFVLALIKIFGALIAVTVTTGRKAMTIVLSFIFFAKPFTFQYVWSGLLVVLGIFLNVYSK
NMDKIRLPSLYDLINKSVEARKSRTLAQTV

>Q96RD1 multipass membrane protein

MRNHTEITEFILLGLTDDPNFQVVIFVFLLITYMLSITGNLTLITITLLDSHLQTPMYFFLRNF
SILEISFTTVSIPKFLGNIISGDKTISFNNCIVQLFFFILLGVTEFYLLAAMSYDRYVAICKPLH
CLSIMNRRVCTLLVFTSWLVSFLIIFPALMLLLKLHYCRSNIIDHFTCDYFPLLQLACSDTKF
LEVMGFSCAAFTLMFTLALIFLSYIYIIRTILRIPSTSQRTKAFSTCSSHMVVVSISYGSCIFM
YIKPSAKDRVSLSKGVAILNTSVAPMMNPFIYSLRNQQVKQAFINMARKTVFFTST

>Q9UBN4 multipass membrane protein
MAQFYYKRNVNAPYRDRIPLRIVRAESELSPSEKAYLNAVEKGDYASVKKSLEEAEIYFKI
NINCIDPLGRTALLIAIENENLELIELLLSFNVYVGDALLHAIRKEVVGAVELLLNHKKPSG
EKQVPPILLDKQFSEFTPDITPIILAAHTNNYEIIKLLVQKGVSVPRPHEVRCNCVECVSSSD
VDSLRHSRSRLNIYKALASPSLIALSSEDPFLTAFQLSWELQELSKVENEFKSEYEELSRQC
KQFAKDLLDQTRSSRELEIILNYRDDNSLIEEQSGNDLARLKLAIKYRQKEFVAQPNCQQL
LASRWYDEFPGWRRRHWAVKMVTCFIIGLLFPVFSVCYLIAPKSPLGLFIRKPFIKFICHTAS
YLTFLFLLLLASQHIDRSDLNRQGPPPTIVEWMILPWVLGFIWGEIKQMWDGGLQDYIHD
WWNLMDFVMNSLYLATISLKIVAFVKYSALNPRESWDMWHPTLVAEALFAIANIFSSLRLI
SLFTANSHLGPLQISLGRMLLDILKFLFIYCLVLLAFANGLNQLYFYYEETKGLTCKGIRCE
KQNNAFSTLFETLQSLFWSIFGLINLYVTNVKAQHEFTEFVGATMFGTYNVISLVVLLNML
IAMMNNSYQLIADHADIEWKFARTKLWMSYFEEGGTLPTPFNVIPSPKSLWYLIKWIWTH
LCKKKMRRKPESFGTIGRRAADNLRRHHQYQEVMRNLVKRYVAAMIRDAKTEEGLTEEN
FKELKQDISSFRFEVLGLLRGSKLSTIQSANASKESSNSADSDEKSDSEGNSKDKKKNFSLF
DLTTLIHPRSAAIASERHNISNGSALVVQEPPREKQRKVNFVTDIKNFGLFHRRSKQNAAE
QNANQIFSVSEEVARQQAAGPLERNIQLESRGLASRGDLSIPGLSEQCVLVDHRERNTDTL
GLQVGKRVCPFKSEKVVVEDTVPIIPKEKHAKEEDSSIDYDLNLPDTVTHEDYVTTRL

>Q9Y653 multipass membrane protein
MTPQSLLQTTLFLLSLLFLVQGAHGRGHREDFRFCSQRNQTHRSSLHYKPTPDLRISIENSE
EALTVHAPFPAAHPASRSFPDPRGLYHFCLYWNRHAGRLHLLYGKRDFLLSDKASSLLCFQ
HQEESLAQGPPLLATSVTSWWSPQNISLPSAASFTFSFHSPPHTAAHNASVDMCELKRDLQ
LLSQFLKHPQKASRRPSAAPASQQLQSLESKLTSVRFMGDMVSFEEDRINATVWKLQPTA
GLQDLHIHSRQEEEQSEIMEYSVLLPRTLFQRTKGRSGEAEKRLLLVDFSSQALFQDKNSS
QVLGEKVLGIVVQNTKVANLTEPVVLTFQHQLQPKNVTLQCVFWVEDPTLSSPGHWSSA
GCETVRRETQTSCFCNHLTYFAVLMVSSVEVDAVHKHYLSLLSYVGCVVSALACLVTIAA
YLCSRVPLPCRRKPRDYTIKVHMNLLLAVFLLDTSFLLSEPVALTGSEAGCRASAIFLHFSL
LTCLSWMGLEGYNLYRLVVEVFGTYVPGYLLKLSAMGWGFPIFLVTLVALVDVDNYGPII
LAVHRTPEGVIYPSMCWIRDSLVSYITNLGLFSLVFLFNMAMLATMVVQILRLRPHTQKWS
HVLTLLGLSLVLGLPWALIFFSFASGTFQLVVLYLFSIITSFQGFLIFIWYWSMRLQARGGPS
PLKSNSDSARLPISSGSTSSSRI

>P15291 single-pass type II membrane protein
MRLREPLLSGSAAMPGASLQRACRLLVAVCALHLGVTLVYYLAGRDLSRLPQLVGVSTPL
QGGSNSAAAIGQSSGELRTGGARPPPPLGASSQPRPGGDSSPVVDSGPGPASNLTSVPVPHT
TALSLPACPEESPLLVGPMLIEFNMPVDLELVAKQNPNVKMGGRYAPRDCVSPHKVAIIIPF
RNRQEHLKYWLYYLHPVLQRQQLDYGIYVINQAGDTIFNRAKLLNVGFQEALKDYDYTC
FVFSDVDLIPMNDHNAYRCFSQPRHISVAMDKFGFSLPYVQYFGGVSALSKQQFLTINGFP

NNYWGWGGEDDDIFNRLVFRGMSISRPNAVVGRCRMIRHSRDKKNEPNPQRFDRIAHTK
ETMLSDGLNSLTYQVLDVQRYPLYTQITVDIGTPS

>Q86UG4 multipass membrane protein
MFVGVARHSGSQDEVSRGVEPLEAARAQPAKDRRAKGTPKSSKPGKKHRYLRLLPEALIR
FGGFRKRKKAKSSVSKKPGEVDDSLEQPCGLGCLVSTCCECCNNIRCFMIFYCILLICQGV
VFGLIDVSIGDFQKEYQLKTIEKLALEKSYDISSGLVAIFIAFYGDRKKVIWFVASSFLIGLGS
LLCAFPSINEENKQSKVGIEDICEEIKVVSGCQSSGISFQSKYLSFFILGQTVQGIAGMPLYIL
GITFIDENVATHSAGIYLGIAECTSMIGYALGYVLGAPLVKVPENTTSATNTTVNNGSPEWL
WTWWINFLFAAVVAWCTLIPLSCFPNNMPGSTRIKARKRKQLHFFDSRLKDLKLGTNIKD
LCAALWILMKNPVLICLALSKATEYLVIIGASEFLPIYLENQFILTPTVATTLAGLVLIPGGAL
GQLLGGVIVSTLEMSCKALMRFIMVTSVISLILLVFIIFVRCNPVQFAGINEDYDGTGKLGN
LTAPCNEKCRCSSSIYSSICGRDDIEYFSPCFAGCTYSKAQNQKKMYYNCSCIKEGLITADA
EGDFIDARPGKCDAKCYKLPLFIAFIFSTLIFSGFSGVPIVLAMTRVVPDKLRSLALGVSYVI
LRIFGTIPGPSIFKMSGETSCILRDVNKCGHTGRCWIYNKTKMAFLLVGICFLCKLCTIIFTTI
AFFIYKRRLNENTDFPDVTVKNPKVKKKEETDL

>Q8IZP9 multipass membrane protein
MVFSVRQCGHVGRTEEVLLTFKIFLVIICLHVVLVTSLEEDTDNSSLSPPPAKLSVVSFAPSS
NGTPEVETTSLNDVTLSLLPSNETEKTKITIVKTFNASGVKPQRNICNLSSICNDSAFFRGEI
MFQYDKESTVPQNQHITNGTLTGVLSLSELKRSELNKTLQTLSETYFIMCATAEAQSTLNC
TFTIKLNNTMNACAVIAALERVKIRPMEHCCCSVRIPCPSSPEELEKLQCDLQDPIVCLADH
PRGPPFSSSQSIPVVPRATVLSQVPKATSFAEPPDYSPVTHNVPSPIGEIQPLSPQPSAPIASSP
AIDMPPQSETISSPMPQTHVSGTPPPVKASFSSPTVSAPANVNTTSAPPVQTDIVNTSSISDL
ENQVLQMEKALSLGSLEPNLAGEMINQVSRLLHSPPDMLAPLAQRLLKVVDDIGLQLNFS
NTTISLTSPSLALAVIRVNASSFNTTTFVAQDPANLQVSLETQAPENSIGTITLPSSLMNNLPA
HDMELASRVQFNFFETPALFQDPSLENLSLISYVISSSVANLTVRNLTRNVTVTLKHINPSQ
DELTVRCVFWDLGRNGGRGGWSDNGCSVKDRRLNETICTCSHLTSFGVLLDLSRTSVLPA
QMMALTFITYIGCGLSSIFLSVTLVTYIAFEKIRRDYPSKILIQLCAALLLLNLVFLLDSWIAL
YKMQGLCISVAVFLHYFLLVSFTWMGLEAFHMYLALVKVFNTYIRKYILKFCIVGWGVPA
VVVTIILTISPDNYGLGSYGKFPNGSPDDFCWINNNAVFYITVVGYFCVIFLLNVSMFIVVL
VQLCRIKKKKQLGAQRKTSIQDLRSIAGLTFLLGITWGFAFFAWGPVNVTFMYLFAIFNTL
QGFFIFIFYCVAKENVRKQWRRYLCCGKLRLAENSDWSKTATNGLKKQTVNQGVSSSSNS
LQSSSNSTNSTTLLVNNDCSVHASGNGNASTERNGVSFSVQNGDVCLHDFTGKQHMFNE
KEDSCNGKGRMALRRTSKRGSLHFIEQM

>P23975 multipass membrane protein
MLLARMNPQVQPENNGADTGPEQPLRARKTAELLVVKERNGVQCLLAPRDGDAQPRET
WGKKIDFLLSVVGFAVDLANVWRFPYLCYKNGGGAFLIPYTLFLIIAGMPLFYMELALGQ
YNREGAATVWKICPFFKGVGYAVILIALYVGFYYNVIIAWSLYYLFSSFTLNLPWTDCGHT
WNSPNCTDPKLLNGSVLGNHTKYSKYKFTPAAEFYERGVLHLHESSGIHDIGLPQWQLLL
CLMVVVIVLYFSLWKGVKTSGKVVWITATLPYFVLFVLLVHGVTLPGASNGINAYLHIDF
YRLKEATVWIDAATQIFFSLGAGFGVLIAFASYNKFDNNCYRDALLTSSINCITSFVSGFAIF
SILGYMAHEHKVNIEDVATEGAGLVFILYPEAISTLSGSTFWAVVFFVMLLALGLDSSMGG

MEAVITGLADDFQVLKRHRKLFTFGVTFSTFLLALFCITKGGIYVLTLLDTFAAGTSILFAV
LMEAIGVSWFYGVDRFSNDIQQMMGFRPGLYWRLCWKFVSPAFLLFVVVVSIINFKPLTY
DDYIFPPWANWVGWGIALSSMVLVPIYVIYKFLSTQGSLWERLAYGITPENEHHLVAQRDI
RQFQLQHWLAI

>P46019 lipid-anchor
MRSRSNSGVRLDGYARLVQQTILCYQNPVTGLLSASHEQKDAWVRDNIYSILAVWGLGM
AYRKNADRDEDKAKAYELEQNVVKLMRGLLQCMMRQVAKVEKFKHTQSTKDSLHAKY
NTATCGTVVGDDQWGHLQVDATSLFLLFLAQMTASGLRIIFTLDEVAFIQNLVFYIEAAYK
VADYGMWERGDKTNQGIPELNASSVGMAKAALEAIDELDLFGAHGGRKSVIHVLPDEVE
HCQSILFSMLPRASTSKEIDAGLLSIISFPAFAVEDVNLVNVTKNEIISKLQGRYGCCRFLRD
GYKTPREDPNRLHYDPAELKLFENIECEWPVFWTYFIIDGVFSGDAVQVQEYREALEGILI
RGKNGIRLVPELYAVPPNKVDEEYKNPHTVDRVPMGKVPHLWGQSLYILSSLLAEGFLAA
GEIDPLNRRFSTSVKPDVVVQVTVLAENNHIKDLLRKHGVNVQSIADIHPIQVQPGRILSHI
YAKLGRNKNMNLSGRPYRHIGVLGTSKLYVIRNQIFTFTPQFTDQHHFYLALDNEMIVEM
LRIELAYLCTCWRMTGRPTLTFPISRTMLTNDGSDIHSAVLSTIRKLEDGYFGGARVKLGNL
SEFLTTSFYTYLTFLDPDCDEKLFDNASEGTFSPDSDSDLVGYLEDTCNQESQDELDHYIN
HLLQSTSLRSYLPPLCKNTEDRHVFSAIHSTRDILSVMAKAKGLEVPFVPMTLPTKVLSAH
RKSLNLVDSPQPLLEKVPESDFQWPRDDHGDVDCEKLVEQLKDCSNLQDQADILYILYVI
KGPSWDTNLSGQHGVTVQNLLGELYGKAGLNQEWGLIRYISGLLRKKVEVLAEACTDLL
SHQKQLTVGLPPEPREKIISAPLPPEELTKLIYEASGQDISIAVLTQEIVVYLAMYVRAQPSLF
VEMLRLRIGLIIQVMATELARSLNCSGEEASESLMNLSPFDMKNLLHHILSGKEFGVERSV
RPIHSSTSSPTISIHEVGHTGVTKTERSGINRLRSEMKQMTRRFSADEQFFSVGQAASSSAH
SSKSARSSTPSSPTGTSSSDSGGHHIGWGERQGQWLRRRRLDGAINRVPVGFYQRVWKIL
QKCHGLSIDGYVLPSSTTREMTPHEIKFAVHVESVLNRVPQPEYRQLLVEAIMVLTLLSDTE
MTSIGGIIHVDQIVQMASQLFLQDQVSIGAMDTLEKDQATGICHFFYDSAPSGAYGTMTYL
TRAVASYLQELLPNSGCQMQ

>P01589 single-pass type I membrane protein
MDSYLLMWGLLTFIMVPGCQAELCDDDPPEIPHATFKAMAYKEGTMLNCECKRGFRRIK
SGSLYMLCTGNSSHSSWDNQCQCTSSATRNTTKQVTPQPEEQKERKTTEMQSPMQPVDQ
ASLPGHCREPPPWENEATERIYHFVVGQMVYYQCVQGYRALHRGPAESVCKMTHGKTR
WTQPQLICTGEMETSQFPGEEKPQASPEGRPESETSCLVTTTDFQIQTEMAATMETSIFTTE
YQVAVAGCVFLLISVLLLSGLTWQRRQRKSRRTI

>Q13261 single-pass type I membrane protein
MAPRRARGCRTLGLPALLLLLLLLRPPATRGITCPPPMSVEHADIWVKSYSLYSRERYICNSG
FKRKAGTSSLTECVLNKATNVAHWTTPSLKCIRDPALVHQRPAPPSTVTTAGVTPQPESLSP
SGKEPAASSPSSNNTAATTAAIVPGSQLMPSKSPSTGTTEISSHESSHGTPSQTTAKNWELTA
SASHQPPGVYPQGHSDTTVAISTSTVLLCGLSAVSLLACYLKSRQTPPLASVEMEAMEALP
VTWGTSSRDEDLENCSHHL

>Q8NGU9 multipass membrane protein
MEDLFSPSILPPAPNISVPILLGWGLNLTLGQGAPASGPPSRRVRLVFLGVILVVAVAGNTTV

LCRLCGGGGPWAGPKRRKMDFLLVQLALADLYACGGTALSQLAWELLGEPRAATGDLAC
RFLQLLQASGRGASAHLVVLIALERRRAVRLPHGRPLPARALAALGWLLALLLALPPAFV
VRGDSPSPLPPPPPPTSLQPGAPPAARAWPGERRCHGIFAPLPRWHLQVYAFYEAVAGFVAP
VTVLGVACGHLLSVWWRHRPQAPAAAAPWSASPGRAPAPSALPRAKVQSLKMSLLLALL
FVGCELPYFAARLAAAWSSGPAGDWEGEGLSAALRVVAMANSALNPFVYLFFQAGDCRL
RRQLRKRLGSLCCAPQGGAEDEEGPRGHQALYRQRWPHPHYHHARREPLDEGGLRPPPP
RPRPLPCSCESAF

>Q8NBS3 multipass membrane protein
MSQVGGRGDRCTQEVQGLVHGAGDLSASLAENSPTMSQNGYFEDSSYYKCDTDDTFEA
REEILGDEAFDTANSSIVSGESIRFFVNVNLEMQATNTENEATSGGCVLLHTSRKYLKLKN
FKEEIRAHRDLDGFLAQASIVLNETATSLDNVLRTMLRRFARDPDNNEPNCNLDLLMAML
FTDAGAPMRGKVHLLSDTIQGVTATVTGVRYQQSWLCIICTMKALQKRHVCISRLVRPQN
WGENSCEVRFVILVLAPPKMKSTKTAMEVARTFATMFSDIAFRQKLLETRTEEEFKEALVH
QRQLLTMVSHGPVAPRTKERSTVSLPAHRHPEPPKCKDFVPFGKGIREDIARRFPLYPLDFT
DGIIGKNKAVGKYITTTLFLYFACLLPTIAFGSLNDENTDGAIDVQKTIAGQSIGGLLYALFS
GQPLVILLTTAPLALYIQVIRVICDDYDLDFNSFYAWTGLWNSFFLALYAFFNLSLVMSLFKR
STEEIIALFISITFVLDAVKGTVKIFWKYYYGHYLDDYHTKRTSSLVSLSGLGASLNASLHT
ALNASFLASPTELPSATHSGQATAVLSLLIMLGTLWLGYTLYQFKKSPYLHPCVREILSDCA
LPIAVLAFSLISSHGFREIEMSKFRYNPSESPFAMAQIQSLSLRAVSGAMGLGFLLSMLFFIE
QNLVAALVNAPENRLVKGTAYHWDLLLLAIINTGLSLFGLPWIHAAYPHSPLHVRALALVE
ERVENGHIYDTIVNVKETRLTSLGASVLVGLSLLLLPVPLQWIPKPVLYGLFLYIALTSLDG
NQLVQRVALLLKEQTAYPPTHYIRRVPQRKIHYFTGLQVLQLLLLCAFGMSSLPYMKMIFP
LIMIAMIPIRYILLPRIIEAKYLDVMDAEHRP

>Q687X5 multipass membrane protein
MEKTCIDALPLTMNSSEKQETVCIFGTGDFGRSLGLKMLQCGYSVVFGSRNPQKTTLLPS
GAEVLSYSEAAKKSGIIIIAIHREHYDFLTELTEVLNGKILVDISNNLKINQYPESNAEYLAH
LVPGAHVVKAFNTISAWALQSGALDASRQVFVCGNDSKAKQRVMDIVRNLGLTPMDQGS
LMAAKEIEKYPLQLFPMWRFPFYLSAVLCVFLFFYCVIRDVIYPYVYEKKDNTFRMAISIP
NRIFPITALTLLALVYLPGVIAAILQLYRGTKYRRFPDWLDHWMLCRKQLGLVALGFAFLH
VLYTLVIPIRYYVRWRLGNLTVTQAILKKENPFSTSSAWLSDSYVALGILGFFLFVLLGITSL
PSVSNAVNWREFRFVQSKLGYLTLILCTAHTLVYGGKRFLSPSNLRWYLPAAYVLGLIIPCT
VLVIKFVLIMPCVDNTLTRIRQGWERNSKH

>Q9UEF7 single-pass type I membrane protein
MPASAPPRRPRPPPPSLSLLLVLLGLGGRRLRAEPGDGAQTWARFSRPPAPEAAGLFQGTF
PDGFLWAVGSAAYQTEGGWQQHGKGASIWDTFTHHPLAPPGDSRNASLPLGAPSPLQPAT
GDVASDSYNNVFRDTEALRELGVTHYRFSISWARVLPNGSAGVPNREGLRYYRRLLERLR
ELGVQPVVTLYHWDLPQRLQDAYGGWANRALADHFRDYAELCFRHFGGQVKYWITIDN
PYVVAWHGYATGRLAPGIRGSPRLGYLVAHNLLLAHAKVWHLYNTSFRPTQGGQVSIALS
SHWINPRRMTDHSIKECQKSLDFVLGWFAKPVFIDGDYPESMKNNLSSILPDFTESEKKFI
KGTADFFALCFGPTLSFQLLDPHMKFRQLESPNLRQLLSWIDLEFNHPQIFIVENGWFVSG
TTKRDDAKYMYYLKKFIMETLKAIKLDGVDVIGYTAWSLMDGFEWHRGYSIRRGLFYV

DFLSQDKMLLPKSSALFYQKLIEKNGFPPLPENQPLEGTFPCDFAWGVVDNYIQVDTTLSQ
FTDLNVYLWDVHHSKRLIKVDGVVTKKRKSYCVDFAAIQPQIALLQEMHVTHFRFSLDW
ALILPLGNQSQVNHTILQYYRCMASELVRVNITPVVALWQPMAPNQGLPRLLARQGAWE
NPYTALAFAEYARLCFQELGHHVKLWITMNEPYTRNMTYSAGHNLLKAHALAWHVYNE
KFRHAQNGKISIALQADWIEPACPFSQKDKEVAERVLEFDIGWLAEPIFGSGDYPWVMRD
WLNQRNNFLLPYFTEDEKKLIQGTFDFLALSHYTTILVDSEKEDPIKYNDYLEVQEMTDIT
WLNSPSQVAVVPWGLRKVLNWLKFKYGDLPMYIISNGIDDGLHAEDDQLRVYYMQNYIN
EALKAHILDGINLCGYFAYSFNDRTAPRFGLYRYAADQFEPKASMKHYRKIIDSNGFPGPET
LERFCPEEFTVCTECSFFHTRKSLLAFIAFLFFASIISLSLIFYYSKKGRRSYK

>Q9H343 multipass membrane protein
MLGLNGTPFQPATLQLTGIPGIQTGLTWVALIFCILYMISIVGNLSILTLVFWEPALHQPMYY
FLSMLALNDLGVSFSTLPTVISTFCFNYNHVAFNACLVQMFFIHTFSFMESGILLAMSLDRF
VAICYPLRYVTVLTHNRILAMGLGILTKSFTTLFPFPFVVKRLPFCKGNVLHHSYCLHPDL
MKVACGDIHVNNIYGLLVIIFTYGMDSTFILLSYALILRAMLVIISQEQRLKALNTCMSHICA
VLAFYVPIIAVSMIHRFWKSAPPVVHVMMSNVYLFVPPMLNPIIYSVKTKEIRKGILKFFHK
SQA

>Q9H340 multipass membrane protein
MGLNKSASTFQLTGFPGMEKAHHWIFIPLLAAYISILLGNGTLLFLIRNDHNLHEPMYYFL
AMLAATDLGVTLTTMPTVLGVLWLDHREIGHGACFSQAYFIHTLSVMESGVLLAMAYDC
FITIRSPLRYTSILTNTQVMKIGVRVLTRAGLSIMPIVVRLHWFPYCRSHVLSHAFCLHQDVI
KLACADITFNRLYPVVVLFAMVLLDFLIIFFSYILILKTVMGIGSGGERAKALNTCVSHICCI
LVFYVTVVCLTFIHRFGKHVPHVVHITMSYIHFLFPPFMNPFIYSIKTKQIQSGILRLFSLPHS
RA

>Q9Y5P0 multipass membrane protein
MWYNNSAGPFLLTGFLGSEAVHYRISMSFFVIYFSVLFGNGTLLVLIWNDHSLHEPMYYFL
AMLADTDLGMTFTTMPTVLGVLLLDQREIAHAACFTQSFIHSLAIVESGILLVLAYDCFIAI
RTPLRYNCILTNSRVMNIGLGVLMRGFMSILPIILSLYCYPYCGSRALLHTFCLHQDVIKLA
CADITFNHIYPIIQTSLTVFLDALIIIFSYILILKTVMGIASGQEEAKSLNTCVSHISCVLVFHIT
VMGLSFIHRFGKHAPHVVPITMSYVHFLFPPFVNPIIYSIKTKQIQRSIIRLFSGQSRA

>Q13596 peripheral membrane protein
MASGGGGCSASERLPPPFPGLEPESEGAAGGSEPEAGDSDTEGEDIFTGAAVVSKHQSPKI
TTSLLPINNGSKENGIHEEQDQEPQDLFADATVELSLDSTQNNQKKVLAKTLISLPPQEATN
SSKPQPTYEELEEEEQEDQFDLTVGITDPEKIGDGMNAYVAYKVTTQTSLPLFRSKQFAVK
RRFSDFLGLYEKLSEKHSQNGFIVPPPPEKSLIGMTKVKVGKEDSSSAEFLEKRRAALERY
LQRIVNHPTMLQDPDVREFLEKEELPRAVGTQTLSGAGLLKMFNKATDAVSKMTIKMNES
DIWFEEKLQEVECEEQRLRKLHAVVETLVNHRKELALNTAQFAKSLAMLGSSEDNTALSR
ALSQLAEVEEKIEQLHQEQANNDFFLLAELLSDYIRLLAIVRAAFDQRMKTWQRWQDAQ
ATLQKKREAEARLLWANKPDKLQQAKDEILEWESRVTQYERDFERISTVVRKEVIRFEKE
KSKDFKNHVIKYLETLLYSQQQLAKYWEAFLPEAKAIS

>P30550 multipass membrane protein
MALNDCFLLNLEVDHFMHCNISSHSADLPVNDDWSHPGILYVIPAVYGVIILIGLIGNITLIK
IFCTVKSMRNVPNLFISSLALGDLLLLITCAPVDASRYLADRWLFGRIGCKLIPFIQLTSVGV
SVFTLTALSADRYKAIVRPMDIQASHALMKICLKAAFIWIISMLLAIPEAVFSDLHPFHEEST
NQTFISCAPYPHSNELHPKIHSMASFLVFYVIPLSIISVYYYFIAKNLIQSAYNLPVEGNIHVK
KQIESRKRLAKTVLVFVGLFAFCWLPNHVIYLYRSYHYSEVDTSMLHFVTSICARLLAFTN
SCVNPFALYLLSKSFRKQFNTQLLCCQPGLIIRSHSTGRSTTCMTSLKSTNPSVATFSLINGNI
CHERYV

>Q8NH60 multipass membrane protein
MFYHNKSIFHPVTFFLIGIPGLEDFHMWISGPFCSVYLVALLGNATILLVIKVEQTLREPMFY
FLAILSTIDLALSTTSVPRMLGIFWFDAHEINYGACVAQMFLIHAFTGMEAEVLLAMAFDR
YVAVCAPLHYATILTSQVLVGISMCIVIRPVLLTLPMVYLIYRLPFCQAHIIAHSYCEHMGIA
KLSCGNIRINGIYGLFVVSFFVLNLVLIGISYVYILRAVFRLPSHDAQLKALSTCGAHVGVIC
VFYIPSVFSFLTHRFGHQIPGYIHILVANLYLIIPPSLNPIIYGVRTKQIRERVLYVFTKK

>Q8NGJ7 multipass membrane protein
MSIINTSYVEITTFFLVGMPGLEYAHIWISIPICSMYLIAILGNGTILFIIKTEPSLHGPMYYFL
SMLAMSDLGLSLSSLPTVLSIFLFNAPETSSSACFAQEFFIHGFSVLESSVLLIMSFDRFLAIH
NPLRYTSILTTVRVAQIGIVFSFKSMLLVLPFPFTLRSLRYCKKNQLSHSYCLHQDVMKLAC
SDNRIDVIYGFFGALCLMVDFILIAVSYTLILKTVPGIASKKEELKALNTCVSHICAVIIFYLP
IINLAVVHRFAGHVSPLINVLMANVLLLVPPLMKPIVYCVKTKQIRVRVVAKLCQWKI

>Q8NGJ9 multipass membrane protein
MAIFNNTTSSSSNFLLTAFPGLECAHVWISIPVCCLYTIALLGNSMIFLVIITKRRLHKPMYY
FLSMLAAVDLCLTITTLPTVLGVLWFHAREISFKACFIQMFFVHAFSLLESSVLVAMAFDRF
VAICNPLNYATILTDRMVLVIGLVICIRPAVFLLPLLVAINTVSFHGGHELSHPFCYHPEVIKY
TYSKPWISSFWGLFLQLYLNGTDVLFILFSYVLILRTVLGIVARKKQQKALSTCVCHICAVT
IFYVPLISLSLAHRLFHSTPRVLCSTLANIYLLLPPVLNPIIYSLKTKTIRQAMFQLLQSKGS
WGFNVRGLRGRWD

>Q8NGF1 multipass membrane protein
MVLASGNSSSHPVSFILLGIPGLESFQLWIAFPFCATYAVAVVGNITLLHVIRIDHTLHEPMY
LFLAMLAITDLVLSSSTQPKMLAIFWFHAHEIQYHACLIQVFFIHAFSSVESGVLMAMALD
CYVAICFPLRHSSILTPSVVIKLGTIVMLRGLLWVSPFCFMVSRMPFCQHQAIPQSYCEHMA
VLKLVCADTSISRGNGLFVAFSVAGFDMIVIGMSYVMILRAVLQLPSGEARLKAFSTRSSHI
CVILALYIPALFSFLTYRFGHDVPRVVHILFANLYLLIPPMLNPIIYGVRTKQIGDRVIQGCCG
NIP

>P18433 single-pass type I membrane protein
MDSWFILVLLGSGLICVSANNATTVAPSVGITRLINSSTAEPVKEEAKTSNPTSSLTSLSVAP
TFSPNITLGPTYLTTVNSSDSDNGTTRTASTNSIGITISPNGTWLPDNQFTDARTEPWEGNSS
TAATTPETFPPSDETPIIAVMVALSSLLVIVFIIIVLYMLRFKKYKQAGSHSNSKQAGSHSNSF
RLSNGRTEDVEPQSVPLLARSPSTNRKYPPLPVDKLEEEINRRMADDNKLFREEFNALPAC

PIQATCEAASKEENKEKNRYVNILPYDHSRVHLTPVEGVPDSDYINASFINGYQEKNKFIAA
QGPKEETVNDFWRMIWEQNTATIVMVTNLKERKECKCAQYWPDQGCWTYGNIRVSVED
VTVLVDYTVRKFCIQQVGDMTNRKPQRLITQFHFTSWPDFGVPFTPIGMLKFLKKVKACN
PQYAGAIVVHCSAGVGRTGTFVVIDAMLDMMHTERKVDVYGFVSRIRAQRCQMVQTDM
QYVFIYQALLEHYLYGDTELEVTSLETHLQKIYNKIPGTSNNGLEEEFKKLTSIKIQNDKMR
TGNLPANMKKNRVLQIIPYEFNRVIIPVKRGEENTDYVNASFIDGYRQKDSYIASQGPLLHT
IEDFWRMIWEWKSCSIVMLTELEERGQEKCAQYWPSDGLVSYGDITVELKKEEECESYTV
RDLLVTNTRENKSRQIRQFHFHGWPEVGIPSDGKGMISIIAAVQKQQQQSGNHPITVHCSA
GAGRTGTFCALSTVLERVKAEGILDVFQTVKSLRLQRPHMVQTLEQYEFCYKVVQEYIDA
FSDYANFK

>Q9H269 peripheral membrane protein
MDCYTANWNPLGDSAFYRKYELYSMDWDLKEELRDCLVAAAPYGGPIALLRNPWRKEK
AASVRPVLDIYSASGMPLASLLWKSGPVVSLGWSAEEELLCVQEDGAVLVYGLHGDFRR
HFSMGNEVLQNRVLDARIFHTEFGSGVAILTGAHRFTLSANVGDLKLRRMPEVPGLQSAP
SCWTVLCQDRVAHILLAVGPDLYLLDHAACSAVTPPGLAPGVSSFLQMAVSFTYRHLALFT
DTGYIWMGTASLKEKLCEFNCNIRAPPKQMVWCSRPRSKERAVVVAWERRLMVVGDAP
ESIQFVLDEDSYLVPELDGVRIFSRSTHEFLHEVPAASEEIFKIASMAPGALLLEAQKEYEKE
SQKADEYLREIQELGQLTQAVQQCIEAAGHEHQPDMQKSLLRAASFGKCFLDRFPPDSFV
HMCQDLRVLNAVRDYHIGIPLTYSQYKQLTIQVLLDRLVLRRLYPLAIQICEYLRLPEVQGV
SRILAHWACYKVQQKDVSDEDVARAINQKLGDTPGVSYSDIAARAYGCGRTELAIKLLEY
EPRSGEQVPLLLKMKRSKLALSKAIESGDTDLVFTVLLHLKNELNRGDFFMTLRNQPMAL
SLYRQFCKHQELETLKDLYNQDDNHQELGSFHIRASYAAEERIEGRVAALQTAADAFYKA
KNEFAAKATEDQMRLLRLQRRLEDELGGQFLDLSLHDTVTTLILGGHNKRAEQLARDFRI
PDKRLWWLKLTALADLEDWEELEKFSKSKKSPIGYLPFVEICMKQHNKYEAKKYASRVG
PEQKVKALLLVGDVAQAADVAIEHRNEAELSLVLSHCTGATDGATADKIQRARAQAQKK

>P20292 multipass membrane protein
MDQETVGNVVLLAIVTLISVVQNGFFAHKVEHESRTQNGRSFQRTGTLAFERVYTANQNC
VDAYPTFLAVLWSAGLLCSQVPAAFAGLMYLFVRQKYFVGYLGERTQSTPGYIFGKRIILF
LFLMSVAGIFNYYLIFFFGSDFENYIKTISTTISPLLLIP

>P35348 multipass membrane protein
MVFLSGNASDSSNCTQPPAPVNISKAILLGVILGGLILFGVLGNILVILSVACHRHLHSVTHY
YIVNLAVADLLLTSTVLPFSAIFEVLGYWAFGRVFCNIWAAVDVLCCTASIMGLCIISIDRYI
GVSYPLRYPTIVTQRRGLMALLCVWALSLVISIGPLFGWRQPAPEDETICQINEEPGYVLFS
ALGSFYLPLAIILVMYCRVYVVAKRESRGLKSGLKTDKSDSEQVTLRIHRKNAPAGGSGM
ASAKTKTHFSVRLLKFSREKKAAKTLGIVVGCFVLCWLPFFLVMPIGSFFPDFKPSETVFKI
VFWLGYLNSCINPIIYPCSSQEFKKAFQNVLRIQCLCRKQSSKHALGYTLHPPSQAVEGQH
KDMVRIPVGSRETFYRISKTDGVCEWKFFSSMPRGSARITVSKDQSSCTTARVRSKSFLQV
CCCVGPSTPSLDKNHQVPTIKVHTISLSENGEEV

>Q99962 peripheral membrane protein
MSVAGLKKQFHKATQKVSEKVGGAEGTKLDDDFKEMERKVDVTSRAVMEIMTKTIEYL

QPNPASRAKLSMINTMSKIRGQEKGPGYPQAEALLAEAMLKFGRELGDDCNFGPALGEV
GEAMRELSEVKDSLDIEVKQNFIDPLQNLHDKDLREIQHHLKKLEGRRLDFDYKKKRQG
KIPDEELRQALEKFDESKEIAESSMFNLLEMDIEQVSQLSALVQAQLEYHKQAVQILQQVT
VRLEERIRQASSQPRREYQPKPRMSLEFPTGDSTQPNGGLSHTGTPKPSGVQMDQPCCRA
LYDFEPENEGELGFKEGDIITLTNQIDENWYEGMLHGHSGFFPINYVEILVALPH

>Q6TCH7 multipass membrane protein
MHQKLLKSAHYIELGSYQYWPVLVPRGIRLYTYEQIPGSLKDNPYITDGYRAYLPSRLCIK
SLFILSNETVNIWSHLLGFFLFFTLGIYDMTSVLPSASASREDFVICSICLFCFQVCMLCSVG
YHLFSCHRSEKTCRRWMALDYAGISIGILGCYVSGVFYAFYCNNYWRQVYLITVLAMILA
VFFAQIHPNYLTQQWQRLRSIIFCSVSGYGVIPTLHWVWLNGGIGAPIVQDFAPRVIVMYM
IALLAFLFYISKVPERYFPGQLNYLGSSHQIWHILAVVMLYWWHQSTVYYVMQYRHSKPCP
DYVSHL

>P30825 multipass membrane protein
MGCKVLLNIGQQMLRRKVVDCSREETRLSRCLNTFDLVALGVGSTLGAGVYVLAGAVAR
ENAGPAIVISFLIAALASVLAGLCYGEFGARVPKTGSAYLYSYVTVGELWAFITGWNLILSY
IIGTSSVARAWSATFDELIGRPIGEFSRTHMTLNAPGVLAENPDIFAVIIILILTGLLTLGVKES
AMVNKIFTCINVLVLGFIMVSGFVKGSVKNWQLTEEDFGNTSGRLCLNNDTKEGKPGVG
GFMPFGFSGVLSGAATCFYAFVGFDCIATTGEEVKNPQKAIPVGIVASLLICFIAYFGVSAAL
TLMMPYFCLDNNSPLPDAFKHVGWEGAKYAVAVGSLCALSASLLGSMFPMPRVIYAMAE
DGLLFKFLANVNDRTKTPIIATLASGAVAAVMAFLFDLKDLVDLMSIGTLLAYSLVAACVL
VLRYQPEQPNLVYQMASTSDELDPADQNELASTNDSQLGFLPEAEMFSLKTILSPKNMEPS
KISGLIVNISTSLIAVLIITFCIVTVLGREALTKGALWAVFLLAGSALLCAVVTGVIWRQPESK
TKLSFKVPFLPVLPILSIFVNVYLMMQLDQGTWVRFAVWMLIGFIIYFGYGLWHSEEASLD
ADQARTPDGNLDQCK

>Q6UWI2 single-pass type I membrane protein
MVYKTLFALCILTAGWRVQSLPTSAPLSVSLPTNIVPPTTIWTSSPQNTDADTASPSNGTHN
NSVLPVTASAPTSLLPKNISIESREEEITSPGSNWEGTNTDPSPSGFSSTSGGVHLTTTLEEHS
SGTPEAGVAATLSQSAAEPPTLISPQAPASSPSSLSTSPPEVFSASVTTNHSSTVTSTQPTGAP
TAPESPTEESSSDHTPTSHATAEPVPQEKTPPTTVSGKVMCELIDMETTTTFPRVIMQEVEH
ALSSGSIAAITVTVIAVVLLVFGVAAYLKIRHSSYGRLLDDHDYGSWGNYNNPLYDDS

>P51810 multipass membrane protein
MASPRLGTFCCPTRDAATQLVLSFQPRAFHALCLGSGGLRLALGLLQLLPGRRPAGPGSPA
TSPPASVRILRAAAACDLLGCLGMVIRSTVWLGFPNFVDSVSDMNHTEIWPAAFCVGSAM
WIQLLYSACFWWLFCYAVDAYLVIRRSAGLSTILLYHIMAWGLATLLCVEGAAMLYYPSV
SRCERGLDHAIPHYVTMYLPLLLVLVANPILFQKTVTAVASLLKGRQGIYTENERRMGAVI
KIRFFKIMLVLIICWLSNIINESLLFYLEMQTDINGGSLKPVRTAAKTTWFIMGILNPAQGFL
LSLAFYGWTGCSLGFQSPRKEIQWESLTTSAAEGAHPSPLMPHENPASGKVSQVGGQTSD
EALSMLSEGSDASTIEIHTASESCNKNEGDPALPTHGDL

>Q13621 multipass membrane protein

MSLNNSSNVFLDSVPSNTNRFQVSVINENHESSAAADDNTDPPHYEETSFGDEAQKRLRIS
FRPGNQECYDNFLQSGETAKTDASFHAYDSHTNTYYLQTFGHNTMDAVPKIEYYRNTGSI
SGPKVNRPSLLEIHEQLAKNVAVTPSSADRVANGDGIPGDEQAENKEDDQAGVVKFGWV
KGVLVRCMLNIWGVMLFIRLSWIVGEAGIGLGVLIILLSTMVTSITGLSTSAIATNGFVRGG
GAYYLISRSLGPEFGGSIGLIFAFANAVAVAMYVVGFAETVVDLLKESDSMMVDPTNDIRII
GSITVVILLGISVAGMEWEAKAQVILLVILLIAIANFFIGTVIPSNNEKKSRGFFNYQASIFAE
NFGPRFTKGEGFFSVFAIFFPAATGILAGANISGDLEDPQDAIPRGTMLAIFITTVAYLGVAIC
VGACVVRDATGNMNDTIISGMNCNGSAACGLGYDFSRCRHEPCQYGLMNNFQVMSMVS
GFGPLITAGIFSATLSSALASLVSAPKVFQALCKDNIYKALQFFAKGYGKNNEPLRGYILTF
LIAMAFILIAELNTIAPIISNFFLASYALINFSCFHASYAKSPGWRPAYGIYNMWVSLFGAVL
CCAVMFVINWWAAVITYVIEFFLYVYVTCKKPDVNWGSSTQALSYVSALDNALELTTVED
HVKNFRPQCIVLTGGPMTRPALLDITHAFTKNSGLCICCEVFVGPRKLCVKEMNSGMAKK
QAWLIKNKIKAFYAAVAADCFRDGVRSLLQASGLGRMKPNTLVIGYKKNWRKAPLTEIEN
YVGIIHDAFDFEIGVVIVRISQGFDISQVLQVQEELERLEQERLALEATIKDNECEEESGGIR
GLFKKAGKLNITKTTPKKDGSINTSQSMHVGEFNQKLVEASTQFKKKQEKGTIDVWWLF
DDGGLTLLIPYILTLRKKWKDCKLRIYVGGKINRIEEEKIVMASLLSKFRIKFADIHIIGDINI
RPNKESWKVFEEMIEPYRLHESCKDLTTAEKLKRETPWKITDAELEAVKEKSYRQVRLNE
LLQEHSRAANLIVLSLPVARKGSISDLLYMAWLEILTKNLPPVLLVRGNHKNVLTFYS

>O75970 peripheral membrane protein
MLEAIDKNRALHAAERLQTKLRERGDVANEDKLSLLKSVLQSPLFSQILSLQTSVQQLKD
QVNIATSATSNIEYAHVPHLSPAVIPTLQNESFLLSPNNGNLEALTGPGIPHINGKPACDEFD
QLIKNMAQGRHVEVFELLKPPSGGLGFSVVGLRSENRGELGIFVQEIQEGSVAHRDGRLK
ETDQILAINGQALDQTITHQQAISILQKAKDTVQLVIARGSLPQLVSPIVSRSPSAASTISAHS
NPVHWQHMETIELVNDGSGLGFGIIGGKATGVIVKTILPGGVADQHGRLCSGDHILKIGDT
DLAGMSSEQVAQVLRQCGNRVKLMIARGAIEERTAPTALGITLSSSPTSTPELRVDASTQK
GEESETFDVELTKNVQGLGITIAGYIGDKKLEPSGIFVKSITKSSAVEHDGRIQIGDQIIAVDG
TNLQGFTNQQAVEVLRHTGQTVLLTLMRRGMKQEAELMSREDVTKDADLSPVNASIIKE
NYEKDEDFLSSTRNTNILPTEEEGYPLLSAEIEEIEDAQKQEAALLTKWQRIMGINYEIVVA
HVSKFSENSGLGISLEATVGHHFIRSVLPEGPVGHSGKLFSGDELLEVNGITLLGENHQDV
VNILKELPIEVTMVCCRRTVPPTTQSELDSLDLCDIELTEKPHVDLGEFIGSSETEDPVLAM
TDAGQSTEEVQAPLAMWEAGIQHIELEKGSKGLGFSILDYQDPIDPASTVIIIRSLVPGGIAE
KDGRLLPGDRLMFVNDVNLENSSLEEAVEALKGAPSGTVRIGVAKPLPLSPEEGYVSAKE
DSFLYPPHSCEEAGLADKPLFRADLALVGTNDADLVDESTFESPYSPENDSIYSTQASILSL
HGSSCGDGLNYGSSLPSSPPKDVIENSCDPVLDLHMSLEELYTQNLLQRQDENTPSVDISM
GPASGFTINDYTPANAIEQQYECENTIVWTESHLPSEVISSAELPSVLPDSAGKGSEYLLEQS
SLACNAECVMLQNVSKESFERTINIAKGNSSLGMTVSANKDGLGMIVRSIIHGGAISRDGR
IAIGDCILSINEESTISVTNAQARAMLRRHSLIGPDIKITYVPAEHLEEFKISLGQQSGRVMAL
DIFSSYTGRDIPELPEREEGEGEESELQNTAYSNWNQPRRVELWREPSKSLGISIVGGRGMG
SRLSNGEVMRGIFIKHVLEDSPAGKNGTLKPGDRIVEVDGMDLRDASHEQAVEAIRKAGN
PVVFMVQSIINRPRKSPLPSLLHNLYPKYNFSSTNPFADSLQINADKAPSQSESEPEKAPLCS
VPPPPPSAFAEMGSDHTQSSASKISQDVDKEDEFGYSWKNIRERYGTLTGELHMIELEKGH
SGLGLSLAGNKDRSRMSVFIVGIDPNGAAGKDGRLQIADELLEINGQILYGRSHQNASSIIK
CAPSKVKIIFIRNKDAVNQMAVCPGNAVEPLPSNSENLQNKETEPTVTTSDAAVDLSSFKN

VQHLELPKDQGGLGIAISEEDTLSGVIIKSLTEHGVAATDGRLKVGDQILAVDDEIVVGYPI
EKFISLLKTAKMTVKLTIHAENPDSQAVPSAAGAASGEKKNSSQSLMVPQSGSPEPESIRNT
SRSSTPAIFASDPATCPIIPGCETTIEISKGRTGLGLSIVGGSDTLLGAIIIHEVYEEGAACKDG
RLWAGDQILEVNGIDLRKATHDEAINVLRQTPQRVRLTLYRDEAPYKEEEVCDTLTIELQK
KPGKGLGLSIVGKRNDTGVFVSDIVKGGIADADGRLMQGDQILMVNGEDVRNATQEAVA
ALLKCSLGTVTLEVGRIKAGPFHSERRPSQSSQVSEGSLSSFTFPLSGSSTSESLESSSKKNA
LASEIQGLRTVEMKKGPTDSLGISIAGGVGSPLGDVPIFIAMMHPTGVAAQTQKLRVGDRI
VTICGTSTEGMTHTQAVNLLKNASGSIEMQVVAGGDVSVVTGHQQEPASSSLSFTGLTSSS
IFQDDLGPPQCKSITLERGPDGLGFSIVGGYGSPHGDLPIYVKTVFAKGAASEDGRLKRGD
QIIAVNGQSLEGVTHEEAVAILKRTKGTVTLMVLS

>P53794 multipass membrane protein
MRAVLDTADIAIVALYFILVMCIGFFAMWKSNRSTVSGYFLAGRSMTWVTIGASLFVSNIG
SEHFIGLAGSGAASGFAVGAWEFNALLLLQLLGWVFIPIYIRSGVYTMPEYLSKRFGGHRI
QVYFAALSLILYIFTKLSVDLYSGALFIQESLGWNLYVSVILLIGMTALLTVTGGLVAVIYTD
TLQALLMIIGALTLMIISIMEIGGFEEVKRRYMLASPDVTSILLTYNLSNTNSCNVSPKKEAL
KMLRNPTDEDVPWPGFILGQTPASVWYWCADQVIVQRVLAAKNIAHAKGSTLMAGFLK
LLPMFIIVVPGMISRILFTDDIACINPEHCMLVCGSRAGCSNIAYPRLVMKLVPVGLRGLMM
AVMIAALMSDLDSIFNSASTIFTLDVYKLIRKSASSRELMIVGRIFVAFMVVISIAWVPIIVE
MQGGQMYLYIQEVADYLTPPVAALFLLAIFWKRCNEQGAFYGGMAGFVLGAVRLILAFA
YRAPECDQPDNRPGFIKDIHYMYVATGLFWVTGLITVIVSLLTPPPTKEQIRTTTFWSKKNL
VVKENCSPKEEPYQMQEKSILRCSENNETINHIIPNGKSEDSIKGLQPEDVNLLVTCREEGN
PVASLGHSEAETPVDAYSNGQAALMGEKERKKETDDGGRYWKFIDWFCGFKSKSLSKRS
LRDLMEEEAVCLQMLEETRQVKVILNIGLFAVCSLGIFMFVVYFSL

>Q7Z6B0 peripheral membrane protein
MDDDDFGGFEAAETFDGGSGETQTTSPAIPWAAFPAVSGVHLSPSSPEIVLDRDHSSSIGCL
SSDAIISSPENTHAANSIVSQTIPKAQIQQSTHTHLDISLFPLGLTDEKSNGTIALVDDSEDPG
ANVSNIQLQQKISSLEIKLKVSEEEKQRIKQDVESLMEKHNVLEKGFLKEKEQEAISFQDR
YKELQEKHKQELEDMRKAGHEALSIIVDEYKALLQSSVKQQVEAIEKQYISAIEKQAHKC
EELLNAQHQRLLEMLDTEKELLKEKIKEALIQQSQEQKEILEKCLEEERQRNKEALVSAAK
LEKEAVKDAVLKVVEEERKNLEKAHAEERELWKTEHAKDQEKVSQEIQKAIQEQRKISQE
TVKAAIIEEQKRSEKAVEEAVKRTRDELIEYIKEQKRLDQVIRQRSLSSLELFLSCAQKQLS
ALIATEPVDIE

>P57103 multipass membrane protein
MAWLRLQPLTSAFLHFGLVTFVLFLNGLRAEAGGSGDVPSTGQNNESCSGSSDCKEGVIL
PIWYPENPSLGDKIARVIVYFVALIYMFLGVSIIADRFMASIEVITSQEREVTIKKPNGETSTT
TIRVWNETVSNLTLMALGSSAPEILLSLIEVCGHGFIAGDLGPSTIVGSAAFNMFIIIGICVYV
IPDGETRKIKHLRVFFITAAWSIFAYIWLYMILAVFSPGVVQVWEGLLTLFFFPVCVLLAWV
ADKRLLFYKYMHKKYRTDKHRGIIIETEGDHPKGIEMDGKMMNSHFLDGNLVPLEGKEV
DESRREMIRILKDLKQKHPEKDLDQLVEMANYYALSHQQKSRAFYRIQATRMMTGAGNI
LKKHAAEQAKKASSMSEVHTDEPEDFISKVFFDPCSYQCLENCGAVLLTVVRKGGDMSK
TMYVDYKTEDGSANAGADYEFTEGTVVLKPGETQKEFSVGIIDDDIFEEDEHFFVRLSNV

RIEEEQPEEGMPPAIFNSLPLPRAVLASPCVATVTILDDDHAGIFTFECDTIHVSESIGVMEV
KVLRTSGARGTVIVPFRTVEGTAKGGGEDFEDTYGELEFKNDETVKTIRVKIVDEEEYERQ
ENFFIALGEPKWMERGISALLLSPDVTDRKLTMEEEEAKRIAEMGKPVLGEHPKLEVIIEES
YEFKTTVDKLIKKTNLALVVGTHSWRDQFMEAITVSAAGDEDEDESGEERLPSCFDYVM
HFLTVFWKVLFACVPPTEYCHGWACFAVSILIIGMLTAIIGDLASHFGCTIGLKDSVTAVVFV
AFGTSVPDTFASKAAALQDVYADASIGNVTGSNAVNVFLGIGLAWSVAAIYWALQGQEFH
VSAGTLAFSVTLFTIFAFVCISVLLYRRRPHLGGELGGPRGCKLATTWLFVSLWLLYILFAT
LEAYCYIKGF

>Q86VZ1 multipass membrane protein
MQVPNSTGPDNATLQMLRNPAIAVALPVVYSLVAAVSIPGNLFSLWVLCRRMGPRSPSVIF
MINLSVTDLMLASVLPFQIYYHCNRHHWVFGVLLCNVVTVAFYANMYSSILTMTCISVER
FLGVLYPLSSKRWRRRRYAVAACAGTWLLLLTALSPLARTDLTYPVHALGIITCFDVLKWT
MLPSVAMWAVFLFTIFILLFLIPFVITVACYTATILKLLRTEEAHGREQRRRAVGLAAVVLLA
FVTCFAPNNFVLLAHIVSRLFYGKSYYHVYKLTLCLSCLNNCLDPFVYYFASREFQLRLRE
YLGCRRVPRDTLDTRRESLFSARTTSVRSEAGAHPEGMEGATRPGLQRQESVF

>Q14571 multipass membrane protein
MTEKMSSFLYIGDIVSLYAEGSVNGFISTLGLVDDRCVVHPEAGDLANPPKKFRDCLFKVC
PMNRYSAQKQYWKAKQAKQGNHTEAALLKKLQHAAELEQKQNESENKKLLGEIVKYS
NVIQLLHIKSNKYLTVNKRLPALLEKNAMRVSLDAAGNEGSWFYIHPFWKLRSEGDNIVV
GDKVVLMPVNAGQPLHASNIELLDNPGCKEVNAVNCNTSWKITLFMKYSSYREDVLKGG
DVVRLFHAEQEKFLTCDEYEKKQHIFLRTTLRQSATSATSSKALWEIEVVHHDPCRGGAG
QWNSLFRFKHLATGNYLAAELNPDYRDAQNEGKNVRDGVPPTSKKKRQAGEKIMYTLV
SVPHGNDIASLFELDATTLQRADCLVPRNSYVRLRHLCTNTWVTSTSIPIDTDEERPVMLKI
GTCQTKEDKEAFAIVSVPLSEVRDLDFANDANKVLATTVKKLENGTITQNERRFVTKLLE
DLIFFVADVPNNGQEVLDVVITKPNRERQKLMREQNILAQVFGILKAPFKEKAGEGSMLR
LEDLGDQRYAPYKYMLRLCYRVLRHSQQDYRKNQEYIAKNFCVMQSQIGYDILAEDTITA
LLHNNRKLLEKHITAKEIETFVSLLRRNREPRFLDYLSDLCVSNTTAIPVTQELICKFMLSP
GNADILIQTKVVSMQADNPMESSILSDDIDDEEVWLYWIDSNKEPHGKAIRHLAQEAKEG
TKADLEVLTYYRYQLNLFARMCLDRQYLAINQISTQLSVDLILRCVSDESLPFDLRASFCR
LMLHMHVDRDPQESVVPVRYARLWTEIPTKITIHEYDSITDSSRNDMKRKFALTMEFVEEY
LKEVVNQPFPFGDKEKNKLTFEVVHLARNLIYFGFYSFSELLRLTRTLLAILDIVQAPMSSY
FERLSKFQDGGNNVMRTIHGVGEMMTQMVLSRGSIFPMSVPDVPPSIHPSKQGSPTEHED
VTVMDTKLKIIEILQFILSVRLDYRISYMLSIYKKEFGEDNDNAETSASGSPDTLLPSAIVPD
IDEIAAQAETMFAGRKEKNPVQLDDEGGRTFLRVLIHLIMHDYPPLLSGALQLLFKHFSQR
AEVLQAFKQVQLLVSNQDVDNYKQIKADLDQLRLTVEKSELWVEKSSNYENGEIGESQV
KGGEEPIEESNILSPVQDGTKKPQIDSNKSNNYRIVKEILIRLSKLCVQNKKCRNQHQRLLK
NMGAHSVVLDLLQIPYEKNDEKMNEVMNLAHTFLQNFCRGNPQNQVLLHKHLNLFLTP
GLLEAETMRHIFMNNYHLCNEISERVVQHFVHCIETHGRHVEYLRFLQTIVKADGKYVKK
CQDMVMTELINGGEDVLIFYNDRASFPILLHMMCSERDRGDESGPLAYHITLVELLAACT
EGKNVYTEIKCNSLLPLDDIVRVVTHDDCIPEVKIAYVNFVNHCYVDTEVEMKEIYTSNHI
WKLFENFLVDMARVCNTTTDRKHADIFLEKCVTESIMNIVSGFFNSPFSDNSTSLQTHQPV
FIQLLQSAFRIYNCTWPNPAQKASVESCIRTLAEVAKNRGIAIPVDLDSQVNTLFMKSHSN

MVQRAAMGWRLSARSGPRFKEALGGPAWDYRNIIEKLQDVVASLEHQFSPMMQAEFSVL
VDVLYSPELLFPEGSDARIRCGAFMSKLINHTKKLMEKEEKLCIKILQTLREMLEKKDSFV
EEGNTLRKILLNRYFKGDYSIGVNGHLSGAYSKTAQVGGSFSGQDSDKMGISMSDIQCLL
DKEGASELVIDVIVNTKNDRIFSEGIFLGIALLEGGNTQTQYSFYQQLHEQKKSEKFFKVLY
DRMKAAQKEIRSTVTVNTIDLGNKKRDDDNELMTSGPRMRVRDSTLHLKEGMKGQLTE
ASSATSKAYCVYRREMDPEIDIMCTGPEAGNTEEKSAEEVTMSPAIAIMQPILRFLQLLCEN
HNRELQNFLRNQNNKTNYNLVCETLQFLDCICGSTTGGLGLLGLYINEKNVALVNQNLES
LTEYCQGPCHENQTCIATHESNGIDIIIALILNDINPLGKYRMDLVLQLKNNASKLLLAIMES
RHDSENAERILFNMRPRELVDVMKNAYNQGLECDHGDDEGGDDGVSPKDVGHNIYILAH
QLARHNKLLQQMLKPGSDPDEGDEALKYYANHTAQIEIVRHDRTMEQIVFPVPNICEYLT
RESKCRVFNTTERDEQGSKVNDFFQQTEDLYNEMKWQKKIRNNPALFWFSRHISLWGSIS
FNLAVFINLAVALFYPFGDDGDEGTLSPLFSVLLWIAVAICTSMLFFFSKPVGIRPFLVSIMLR
SIYTIGLGPTLILLGAANLCNKIVFLVSFVGNRGTFTRGYRAVILDMAFLYHVAYVLVCMLG
LFVHEFFYSFLLFDLVYREETLLNVIKSVTRNGRSIILTAVLALILVYLFSIIGFLFLKDDFTM
EVDRLKNRTPVTGSHQVPTMTLTTMMEACAKENCSPTIPASNTADEEYEDGIERTCDTLL
MCIVTVLNQGLRNGGGVGDVLRRPSKDEPLFAARVVYDLLFYFIVIIIVLNLIFGVIIDTFAD
LRSEKQKKEEILKTTCFICGLERDKFDNKTVSFEEHIKSEHNMWHYLYFIVLVKVKDPTEY
TGPESYVAQMIVEKNLDWFPRMRAMSLVSNEGDSEQNEIRSLQEKLESTMSLVKQLSGQL
AELKEQMTEQRKNKQRLGFLGSNTPHVNHHMPPH

>Q8TC12 single-pass type II membrane protein
MVELMFPLLLLLLPFLLYMAAPQIRKMLSSGVCTSTVQLPGKVVVVTGANTGIGKETAKE
LAQRGARVYLACRDVEKGELVAKEIQTTTGNQQVLVRKLDLSDTKSIRAFAKGFLAEEKH
LHVLINNAGVMMCPYSKTADGFEMHIGVNHLGHFLLTHLLLEKLKESAPSRIVNVSSLAH
HLGRIHFHNLQGEKFYNAGLAYCHSKLANILFTQELARRLKGSGVTTYSVHPGTVQSELV
RHSSFMRWMWWLFSFFIKTPQQGAQTSLHCALTEGLEILSGNHFSDCHVAWVSAQARNE
TIARRLWDVSCDLLGLPID

>Q6DWJ6 multipass membrane protein
MEHTHAHLAANSSLSWWSPGSACGLGFVPVVYYSLLLCLGLPANILTVIILSQLVARRQKS
SYNYLLALAAADILVLFFIVFVDFLLEDFILNMQMPQVPDKIIEVLEFSSIHTSIWITVPLTID
RYIAVCHPLKYHTVSYPARTRKVIVSVYITCFLTSIPYYWWPNIWTEDYISTSVHHVLIWIH
CFTVYLVPCSIFFILNSIIVYKLRRKSNFRLRGYSTGKTTAILFTITSIFATLWAPRIIMILYHLY
GAPIQNRWLVHIMSDIANMLALLNTAINFFLYCFISKRFRTMAAATLKAFFKCQKQPVQFY
TNHNFSITSSPWISPANSHCIKMLVYQYDKNGKPIKVSP

>P42680 peripheral membrane protein
MNFNTILEEILIKRSQQKKKTSPLNYKERLFVLTKSMLTYYEGRAEKKYRKGFIDVSKIKC
VEIVKNDDGVIPCQNKYPFQVVHDANTLYIFAPSPQSRDLWVKKLKEEIKNNNNIMIKYHP
KFWTDGSYQCCRQTEKLAPGCEKYNLFESSIRKALPPAPETKKRRPPPPIPLEEEDNSEEIV
VAMYDFQAAEGHDLRLERGQEYLILEKNDVHWWRARDKYGNEGYIPSNYVTGKKSNNL
DQYEWYCRNMNRSKAEQLLRSEDKEGGFMVRDSSQPGLYTVSLYTKFGGEGSSGFRHY
HIKETTTSPKKYYLAEKHAFGSIPEIIEYHKHNAAGLVTRLRYPVSVKGKNAPTTAGFSYE
KWEINPSELTFMRELGSGLFGVVRLGKWRAQYKVAIKAIREGAMCEEDFIEEAKVMMKL

THPKLVQLYGVCTQQKPIYIVTEFMERGCLLNFLRQRQGHFSRDVLLSMCQDVCEGMEY
LERNSFIHRDLAARNCLVSEAGVVKVSDFGMARYVLDDQYTSSSGAKFPVKWCPPEVFN
YSRFSSKSDVWSFGVLMWEVFTEGRMPFEKYTNYEVVTMVTRGHRLYQPKLASNYVYE
VMLRCWQEKPEGRPSFEDLLRTIDELVECEETFGR

>P15509 single-pass type I membrane protein
MLLLVTSLLLCELPHPAFLLIPEKSDLRTVAPASSLNVRFDSRTMNLSWDCQENTTFSKCFL
TDKKNRVVEPRLSNNECSCTFREICLHEGVTFEVHVNTSQRGFQQKLLYPNSGREGTAAQ
NFSCFIYNADLMNCTWARGPTAPRDVQYFLYIRNSKRRREIRCPYYIQDSGTHVGCHLDN
LSGLTSRNYFLVNGTSREIGIQFFDSLLDTKKIERFNPPSNVTVRCNTTHCLVRWKQPRTYQ
KLSYLDFQYQLDVHRKNTQPGTENLLINVSGDLENRYNFPSSEPRAKHSVKIRAADVRIL
NWSSWSEAIEFGSDDGNLGSVYIYVLLIVGTLVCGIVLGFLFKRFLRIQRLFPPVPQIKDKL
NDNHEVEDEIIWEEFTPEEGKGYREEVLTVKEIT

>Q9NZQ7 single-pass type I membrane protein
MRIFAVFIFMTYWHLLNAFTVTVPKDLYVVEYGSNMTIECKFPVEKQLDLAALIVYWEME
DKNIIQFVHGEEDLKVQHSSYRQRARLLKDQLSLGNAALQITDVKLQDAGVYRCMISYG
GADYKRITVKVNAPYNKINQRILVVDPVTSEHELTCQAEGYPKAEVIWTSSDHQVLSGKT
TTTNSKREEKLFNVTSTLRINTTTNEIFYCTFRRLDPEENHTAELVIPELPLAHPPNERTHLVI
LGAILLCLGVALTFIFRLRKGRMMDVKKCGIQDTNSKKQSDTHLEET

>Q9NTI2 multipass membrane protein
MSRATSVGDQLEAPARTIYLNQPHLNKFRDNQISTAKYSVLTFLPRFLYEQIRRAANAFFLF
IALLQQIPDVSPTGRYTTLVPLIIILTIAGIKEIVEDFKRHKADNAVNKKKTIVLRNGMWHTI
MWKEVAVGDIVKVVNGQYLPADVVLLSSSEPQAMCYVETANLDGETNLKIRQGLSHTAD
MQTREVLMKLSGTIECEGPNRHLYDFTGNLNLDGKSLVALGPDQILLRGTQLRNTQWVFG
IVVYTGHDTKLMQNSTKAPLKRSNVEKVTNVQILVLFGILLVMALVSSAGALYWNRSHGE
KNWYIKKMDTTSDNFGYNLLTFIILYNNLIPISLLVTLEVVKYTQALFINWDTDMYYIGND
TPAMARTSNLNEELGQVKYLFSDKTGTLTCNIMNFKKCSIAGVTYGHFPELAREPSSDDFC
RMPPPCSDSCDFDDPRLLKNIEDRHPTAPCIQEFLTLLAVCHTVVPEKDGDNIIYQASSPDE
AALVKGAKKLGFVFTARTPFSVIIEAMGQEQTFGILNVLEFSSDRKRMSVIVRTPSGRLRLY
CKGADNVIFERLSKDSKYMEETLCHLEYFATEGLRTLCVAYADLSENEYEEWLKVYQEAS
TILKDRAQRLEECYEIIEKNLLLLGATAIEDRLQAGVPETIATLLKAEIKIWVLTGDKQETAI
NIGYSCRLVSQNMALILLKEDSLDATRAAITQHCTDLGNLLGKENDVALIIDGHTLKYALS
FEVRRSFLDLALSCKAVICCRVSPLQKSEIVDVVKKRVKAITLAIGDGANDVGMIQTAHVG
VGISGNEGMQATNNSDYAIAQFSYLEKLLLVHGAWSYNRVTKCILYCFYKNVVLYIIELWF
AFVNGFSGQILFERWCIGLYNVIFTALPPFTLGIFERSCTQESMLRFPQLYKITQNGEGFNTK
VFWGHCINALVHSLILFWFPMKALEHDTVLTSGHATDYLFVGNIVYTYVVVTVCLKAGLE
TTAWTKFSHLAVWGSMLTWLVFFGIYSTIWPTIPIAPDMRGQATMVLSSAHFWLGLFLVPT
ACLIEDVAWRAAKHTCKKTLLEEVQELETKSRVLGKAVLRDSNGKRLNERDRLIKRLGRK
TPPTLFRGSSLQQGVPHGYAFSQEEHGAVSQEEVIRAYDTTKKKSRKK

>Q6UXY1 peripheral membrane protein
MAPEMDQFYRSTMAIYKSIMEQFNPALENLVYLGNNYLRAFHALSEAAEVYFSAIQKIGE

RALQSPTSQILGEILVQMSDTQRHLNSDLEVVVQTFHGGLLQHMEKNTKLDMQFIKDSRQ
HYELEYRHRAANLEKCMSELWRMERKRDKNVREMKESVNRLHAQMQAFVSESQRAAE
LEEKRRYRFLAEKHLLLSNTFLQFFGRARGMLQNRVLLWKEQSEASRSPSRAHSPGLLGP
ALGPPYPSGRLTPTCLDMPPRPLGEFSSPRSRHGSGSYGTEPDARPASQLEPDRRSLPRTPS
ASSLYSGSAQSSRSNSFGERPGGGGGARRVRALVSHSEGANHTLLRFSAGDVVEVLVPEA
QNGWLYGKLEGSSASGWFPEAYVKALEEGPVNPMTPVTPMTSMTSMSPMTPMNPGNEL
PSRSYPLRGSHSLDDLLDRPGNSIAPSEYWDGQSRSRTPSRVPSRAPSPAPPPLPSSRRSSMG
STAVATDVKKLMSSEQYPPQELFPRGTNPFATVKLRPTITNDRSAPLIR

>Q9BVL2 peripheral membrane protein
MSTGFSFGSGTLGSTTVAAGGTSTGGVFSFGTGASSNPSVGLNFGNLGSTSTPATTSAPSSG
FGTGLFGSKPATGFTLGGTNTGIATTITTGLTLGTPATTSAATTGFSLGFNKPAASATPFALPI
TSTSASGLTLSSALTSTPAASTGFTLNNLGGTTATTTTASTGLSLGGALAGLGGSLFQSTNT
GTSGLGQNALGLTLGTTAATSTAGNEGLGGIDFSSSSDKKSDKTGTRPEDSKALKDENLPP
VICQDVENLQKFVKEQKQVQEEISRMSSKAMLKVQEDIKALKQLLSLAANGIQRNTLNID
KLKIETAQELKNAEIALRTQKTPPGLQHEYAAPADYFRILVQQFEVQLQQYRQQIEELENH
LATQANNSHITPQDLSMAMQKIYQTFVALAAQLQSIHENVKVLKEQYLGYRKMFLGDAV
DVFETRRAEAKKWQNTPRVTTGPTPFSTMPNAAAVAMAATLTQQQQPATGPQPSLGVSFG
TPFGSGIGTGLQSSGLGSSNLGGFGTSSGFGCSTTGASTFGFGTTNKPSGSLSAGFGSSSTS
GFNFSNPGITASAGLTFGVSNPASAGFGTGGQLLQLKKPPAGNKRGKR

>Q9NPI0 multipass membrane protein
MLQTSNYSLVLSLQFLLLSYDLFVNSFSELLQKTPVIQLVLFIIQDIAVLFNIIIIFLMFFNTFV
FQAGLVNLLFHKFKGTIILTAVYFALSISLHVWVMNLRWKNSNSFIWTDGLQMLFVFQRL
AAVLYCYFYKRTAVRLGDPHFYQDSLWLRKEFMQVRR

>Q15669 lipid-anchor
MLSSIKCVLVGDSAVGKTSLLVRFTSETFPEAYKPTVYENTGVDVFMDGIQISLGLWDTAG
NDAFRSIRPLSYQQADVVLMCYSVANHNSFLNLKNKWIGEIRSNLPCTPVLVVATQTDQR
EMGPHRASCVNAMEGKKLAQDVRAKGYLECSALSNRGVQQVFECAVRTAVNQARRRNR
RRLFSINECKIF

>Q717R9 lipid-anchor
MGSGSSRSSRTLRRRRSPESLPAGPGAAALEGGTRRRVPVAAAEVPGAAAEEAPGRDPSPV
APPDGRDETLRLLDELLAESAAWGPPEPAPRRPARLRPTAVAGSAVCAEQSTEGHPGSGNV
SEAPGSGRKKPERPAAISYDHSEEGLMASIEREYCR

>O60931 multipass membrane protein
MIRNWLTIFILFPLKLVEKCESSVSLTVPPVVKLENGSSTNVSLTLRPPLNATLVITFEITFRS
KNITILELPDEVVVPPGVTNSSFQVTSQNVGQLTVYLHGNHSNQTGPRIRFLVIRSSAISIIN
QVIGWIYFVAWSISFYPQVIMNWRRKSVIGLSFDFVALNLTGFVAYSVFNIGLLWVPYIKEQ
FLLKYPNGVNPVNSNDVFFSLHAVVLTLIIIVQCCLYERGGQRVSWPAIGFLVLAWLFAFVT
MIVAAVGVTTWLQFLFCFSYIKLAVTLVKYFPQAYMNFYYKSTEGWSIGNVLLDFTGGSFS
LLQMFLQSYNNDQWTLIFGDPTKFGLGVFSIVFDVVFFIQHFCLYRKRPGYDQLN

>Q07444 single-pass type II membrane protein
MSKQRGTFSEVSLAQDPKWQQRKPKGNKSSISGTEQEIFQVELNLQNASLNHQGIDKIYD
CQGLLPPPEKLTAEVLGIICIVLMATVLKTIVLIPFLEQNNSSPNARTQKARHCGHCPEEWIT
YSNSCYYIGKERRTWEESLQACASKNSSSLLCIDNEEEMKFLASILPSSWIGVFRNSSHHP
WVTINGLAFKHEIKDSDHAERNCAMLHVRGLISDQCGSSRIIRRGFIMLTRLVLNS

>Q07912 peripheral membrane protein
MQPEEGTGWLLELLSEVQLQQYFLRLRDDLNVTRLSHFEYVKNEDLEKIGMGRPGQRRL
WEAVKRRKALCKRKSWMSKVFSGKRLEAEFPPHHSQSTFRKTSPAPGGPAGEGPLQSLTC
LIGEKDLRLLEKLGDGSFGVVRRGEWDAPSGKTVSVAVKCLKPDVLSQPEAMDDFIREVN
AMHSLDHRNLIRLYGVVLTPPMKMVTELAPLGSLLDRLRKHQGHFLLGTLSRYAVQVAEG
MGYLESKRFIHRDLAARNLLLATRDLVKIGDFGLMRALPQNDDHYVMQEHRKVPFAWC
APESLKTRTFSHASDTWMFGVTLWEMFTYGQEPWIGLNGSQILHKIDKEGERLPRPEDCP
QDIYNVMVQCWAHKPEDRPTFVALRDFLLEAQPTDMRALQDFEEPDKLHIQMNDVITVIE
GRAENYWWRGQNTRTLCVGPFPRNVVTSVAGLSAQDISQPLQNSFIHTGHGDSDPRHCW
GFPDRIDELYLGNPMDPPDLLSVELSTSRPPQHLGGVKKPTYDPVSEDQDPLSSDFKRLGL
RKPGLPRGLWLAKPSARVPGTKASRGSGAEVTLIDFGEEPVVPALRPCAPSLAQLAMDAC
SLLDETPPQSPTRALPRPLHPTPVVDWDARPLPPPPAYDDVAQDEDDFEICSINSTLVGAGV
PAGPSQGQTNYAFVPEQARPPPPLEDNLFLPPQGGGKPPSSAQTAEIFQALQQECMRQLQA
PAGSPAPSPSPGGDDKPQVPPRVPIPPRPTRPHVQLSPAPPGEEETSQWPGPASPPRVPPREPL
SPQGSRTPSPLVPPGSSPLPPRLSSSPGKTMPTTQSFASDPKYATPQVIQAPGPRAGPCILPIV
RDGKKVSSTHYYLLPERPSYLERYQRFLREAQSPEEPTPLPVPLLLPPPSTPAPAAPTATVRP
MPQAALDPKANFSTNNSNPGARPPPPRATARLPQRGCPGDGPEAGRPADKIQMAMVHGV
TTEECQAALQCHGWSVQRAAQYLKVEQLFGLGLRPRGECHKVLEMFDWNLEQAGCHL
LGSWGPAHHKR

>P54707 multipass membrane protein
MHQKTPEIYSVELSGTKDIVKTDKGDGKEKYRGLKNNCLELKKKNHKEEFQKELHLDDH
KLSNRELEEKYGTDIIMGLSSTRAAELLARDGPNSLTPPKQTPEIVKFLKQMVGGFSILLW
VGAFLCWIAYGIQYSSDKSASLNNVYLGCVLGLVVILTGIFAYYQEAKSTNIMSSFNKMIP
QQALVIRDSEKKTIPSEQLVVGDIVEVKGGDQIPADIRVLSSQGCRVDNSSLTGESEPQPRSS
EFTHENPLETKNICFYSTTCLEGTVTGMVINTGDRTIIGHIASLASGVGNEKTPIAIEIEHFV
HIVAGVAVSIGILFFIIAVSLKYQVLDSIIFLIGIIVANVPEGLLATVTVTLSLTAKRMAKKNCL
VKNLEAVETLGSTSIICSDKTGTLTQNRMTVAHLWFDNQIFVADTSEDHSNQVFDQSSRTW
ASLSKIITLCNRAEFKPGQENVPIMKKAVIGDASETALLKFSEVILGDVMEIRKRNRKVAEIP
FNSTNKFQLSIHEMDDPHGKRFLMVMKGAPERILEKCSTIMINGEEHPLDKSTAKTFHTAY
MELGGLGERVLGFCHLYLPADEFPETYSFDIDAMNFPTSNLCFVGLLSMIDPPRSTVPDAV
TKCRSAGIKVIMVTGDHPITAKAIAKSVGIISANSETVEDIAHRLNIAVEQVNKRDAKAAVV
TGMELKDMSSEQLDEILANYQEIVFARTSPQQKLIIVEGCQRQDAVVAVTGDGVNDSPALK
KADIGIAMGIAGSDAAKNAADMVLLDDNFASIVTGVEEGRLIFDNLKKTIAYSLTKNIAEL
CPFLIYIIVGLPLPIGTITILFIDLGTDIIPSIALAYEKAESDIMNRKPRHKNKDRLVNQPLAVY
SYLHIGLMQALGAFLVYFTVYAQEGFLPRTLINLRVEWEKDYVNDLKDSYGQEWTRYQR
EYLEWTGYTAFFVGILVQQIADLIIRKTRRNSIFQQGLFRNKVIWVGITSQIIIGLILSYGLGS

VTALSFTMLRAQYWFVAVPHAILIWVYDEVRKLFIRLYPGSWWDKNMYY

>P16471 single-pass type I membrane protein
MKENVASATVFTLLLFLNTCLLNGQLPPGKPEIFKCRSPNKETFTCWWRPGTDGGLPTNY
SLTYHREGETLMHECPDYITGGPNSCHFGKQYTSMWRTYIMMVNATNQMGSSFSDELYV
DVTYIVQPDPPLELAVEVKQPEDRKPYLWIKWSPPTLIDLKTGWFTLLYEIRLKPEKAAEW
EIHFAGQQTEFKILSLHPGQKYLVQVRCKPDHGYWSAWSPATFIQIPSDFTMNDTTVWISV
AVLSAVICLIIVWAVALKGYSMVTCIFPPVPGPKIKGFDAHLLEKGKSEELLSALGCQDFPPT
SDYEDLLVEYLEVDDSEDQHLMSVHSKEHPSQGMKPTYLDPDTDSGRGSCDSPSLLSEKC
EEPQANPSTFYDPEVIEKPENPETTHTWDPQCISMEGKIPYFHAGGSKCSTWPLPQPSQHN
PRSSYHNITDVCELAVGPAGAPATLLNEAGKDALKSSQTIKSREEGKATQQREVESFHSET
DQDTPWLLPQEKTPFGSAKPLDYVEIHKVNKDGALSLLPKQRENSGKPKKPGTPENNKEY
AKVSGVMDNNILVLVPDPHAKNVACFEESAKEAPPSLEQNQAEKALANFTATSSKCRLQL
GGLDYLDPACFTHSFH

>Q8WXG1 peripheral membrane protein
MWVLTPAAFAGKLLSVFRQPLSSLWRSLVPLFCWLRATFWLLATKRRKQQLVLRGPDETK
EEEEDPPLPTTPTSVNYHFTRQCNYKCGFCFHTAKTSFVLPLEEAKRGLLLLKEAGMEKIN
FSGGEPFLQDRGEYLGKLVRFCKVELRLPSVSIVSNGSLIRERWFQNYGEYLDILAISCDSF
DEEVNVLIGRGQGKKNHVENLQKLRRWCRDYRVAFKINSVINRFNVEEDMTEQIKALNP
VRWKVFQCLLIEGENCGEDALREAERFVIGDEEFERFLERHKEVSCLVPESNQKMKDSYLI
LDEYMRFLNCRKGRKDPSKSILDVGVEEAIKFSGFDEKMFLKRGGKYIWSKADLKLDW

>O95436 multipass membrane protein
MAPWPELGDAQPNPDKYLEGAAGQQPTAPDKSKETNKTDNTEAPVTKIELLPSYSTATLI
DEPTEVDDPWNLPTLQDSGIKWSERDTKGKILCFFQGIGRLILLLGFLYFFVCSLDILSSAF
QLVGGKMAGQFFSNSSIMSNPLLGLVIGVLVTVLVQSSSTSTSIVVSMVSSSLLTVRAAIPII
MGANIGTSITNTIVALMQVGDRSEFRRAFAGATVHDFFNWLSVLVLLPVEVATHYLEIITQL
IVESFHFKNGEDAPDLLKVITKPFTKLIVQLDKKVISQIAMNDEKAKNKSLVKIWCKTFTN
KTQINVTVPSTANCTSPSLCWTDGIQNWTMKNVTYKENIAKCQHIFVNFHLPDLAVGTILL
ILSLLVLCGCLIMIVKILGSVLKGQVATVIKKTINTDFPFPFAWLTGYLAILVGAGMTFIVQSS
SVFTSALTPLIGIGVITIERAYPLTLGSNIGTTTTAILAALASPGNALRSSLQIALCHFFFNISGI
LLWYPIPFTRLPIRMAKGLGNISAKYRWFAVFYLIIFFFLIPLTVFGLSLAGWRVLVGVGVPV
VFIIILVLCLRLLQSRCPRVLPKKLQNWNFLPLWMRSLKPWDAVVSKFTGCFQMRCCCCC
RVCCRACCLLCDCPKCCRCSKCCEDLEEAQEGQDVPVKAPETFDNITISREAQGEVPASDS
KTECTAL

>Q8TDN2 multipass membrane protein
MLKQSERRRSWSYRPWNTTENEGSQHRRSICSLGARSGSQASIHGWTEGNYNYYIEEDE
DGEEEDQWKDDLAEEDQQAGEVTTAKPEGPSDPPALLSTLNVNVGGHSYQLDYCELAGF
PKTRLGRLATSTSRSRQLSLCDDYEEQTDEYFFDRDPAVFQLVYNFYLSGVLLVLDGLCPR
RFLEELGYWGVRLKYTPRCCRICFEERRDELSERLKIQHELRAQAQVEEAEELFRDMRFY
GPQRRRLWNLMEKPFSSVAAKAIGVASSTFVLVSVVALALNTVEEMQQHSGQGEGGPDL
RPILEHVEMLCMGFFTLEYLLRLASTPDLRRFARSALNLVDLVAILPLYLQLLLECFTGEGH

QRGQTVGSVGKVGQVLRVMRLMRIFRILKLARHSTGLRAFGFTLRQCYQQVGCLLLFIA
MGIFTFSAAVYSVEHDVPSTNFTTIPHSWWWAAVSISTVGYGDMYPETHLGRFFAFLCIAF
GIILNGMPISILYNKFSDYYSKLKAYEYTTIRRERGEVNFMQRARKKIAECLLGSNPQLTPR
QEN

>P98155 single-pass type I membrane protein
MGTSALWALWLLLALCWAPRESGATGTGRKAKCEPSQFQCTNGRCITLLWKCDGDEDCV
DGSDEKNCVKKTCAESDFVCNNGQCVPSRWKCDGDPDCEDGSDESPEQCHMRTCRIHEI
SCGAHSTQCIPVSWRCDGENDCDSGEDEENCGNITCSPDEFTCSSGRCISRNFVCNGQDDC
SDGSDELDCAPPTCGAHEFQCSTSSCIPISWVCDDDADCSDQSDESLEQCGRQPVIHTKCP
ASEIQCGSGECIHKKWRCDGDPDCKDGSDEVNCPSRTCRPDQFECEDGSCIHGSRQCNGI
RDCVDGSDEVNCKNVNQCLGPGKFKCRSGECIDISKVCNQEQDCRDWSDEPLKECHINE
CLVNNGGCSHICKDLVIGYECDCAAGFELIDRKTCGDIDECQNPGICSQICINLKGGYKCEC
SRGYQMDLATGVCKAVGKEPSLIFTNRRDIRKIGLERKEYIQLVEQLRNTVALDADIAAQK
LFWADLSQKAIFSASIDDKVGRHVKMIDNVYNPAAIAVDWVYKTIYWTDAASKTISVATL
DGTKRKFLFNSDLREPASIAVDPLSGFVYWSDWGEPAKIEKAGMNGFDRRPLVTADIQWP
NGITLDLIKSRLYWLDSKLHMLSSVDLNGQDRRIVLKSLEFLAHPLALTIFEDRVYWIDGE
NEAVYGANKFTGSELATLVNNLNDAQDIIVYHELVQPSGKNWCEEDMENGGCEYLCLPA
PQINDHSPKYTCSCPSGYNVEENGRDCQSTATTVTYSETKDTNTTEISATSGLVPGGINVTT
AVSEVSVPPKGTSAAWAILPLLLLVMAAVGGYLMWRNWQHKNMKSMNFDNPVYLKTTE
EDLSIDIGRHSASVGHTYPAISVVSTDDDLA

>Q68DD2 peripheral membrane protein
MLWALWPRWLADKMLPLLGAVLLQKREKRGPLWRHWRRETYPYYDLQVKVLRATNIRG
TDLLSKADCYVQLWLPTASPSPAQTRIVANCSDPEWNETFHYQIHGAVKNVLELTLYDKDI
LGSDQLSLLLFDLRSLKCGQPHKHTFPLNHQDSQELQVEFVLEKSQVPASEVITNGVLVAH
PCLRIQGTLRGDGTAPREEYGSRQLQLAVPGAYEKPQLLPLQPPTEPGLPPTFTFHVNPVLS
SRLHVELMELLAAVQSGPSAELEAQTSKLGEGGILLSSLPLGQEEQCSVALGEGQEVALSM
KVEMSSGDLDLRLGFDLSDGEQEFLDRRKQVVSKALQQVLGLSEALDSGQVPVVAVLGS
GGGTRAMSSLYGSLAGLQELGLLDTVTYLSGVSGSTWCISTLYRDPAWSQVALQGPIERA
QVHVCSSKMGALSTERLQYYTQELGVRERSGHSVSLIDLWGLLVEYLLYQEENPAKLSDQ
QEAVRQGQNPYPIYTSVNVRTNLSGEDFAEWCEFTPYEVGFPKYGAYVPTELFGSELFMG
RLLQLQPEPRICYLQGMWGSAFATSLDEIFLKTAGSGLSFLEWYRGSVNITDDCQKPQLHN
PSRLRTRLLTPQGPFSQAVLDIFTSRFTSAQSFNFTRGLCLHKDYVAGREFVAWKDTHPDAF
PNQLTPMRDCLYLVDGGFAINSPFPLALLPQRAVDLILSFDYSLEAPFEVLKMTEKYCLDR
GIPFPSIEVGPEDMEEARECYLFAKAEDPRSPIVLHFPLVNRTFRTHLAPGVERQTAEEKAF
GDFVINRPDTPYGMMNFTYEPQDFYRLVALSRYNVLNNVETLKCALQLALDRHQARERA
GA

>P0C869 peripheral membrane protein
MAVAEVSRTCLLTVRVLQAHRLPSKDLVTPSDCYVTLWLPTACSHRLQTRTVKNSSSPVW
NQSFHFRIHRQLKNVMELKVFDQDLVTGDDPVLSVLFDAGTLRAGEFRRESFSLSPQGEG
RLEVEFRLQSLADRGEWLVSNGVLVARELSCLHVQLEETGDQKSSEHRVQLVVPGSCEGP
QEASVGTGTFRFHCPACWEQELSIRLQDAPEEQLKAPLSALPSGQVVRLVFPTSQEPLMRV

ELKKEAGLRELAVRLGFGPCAEEQAFLSRRKQVVAAALRQALQLDGDLQEDEIPVVAIMA
TGGGIRAMTSLYGQLAGLKELGLLDCVSYITGASGSTWALANLYEDPEWSQKDLAGPTEL
LKTQVTKNKLGVLAPSQLQRYRQELAERARLGYPSCFTNLWALINEALLHDEPHDHKLSD
QREALSHGQNPLPIYCALNTKGQSLTTFEFGEWCEFSPYEVGFPKYGAFIPSELFGSEFFMG
QLMKRLPESRICFLEGIWSNLYAANLQDSLYWASEPSQFWDRWVRNQANLDKEQVPLLKI
EEPPSTAGRIAEFFTDLLTWRPLAQATHNFLRGLHFHKDYFQHPHFSTWKATTLDGLPNQL
TPSEPHLCLLDVGYLINTSCLPLLQPTRDVDLILSLDYNLHGAFQQLQLLGRFCQEQGIPFP
PISPSPEEQLQPRECHTFSDPTCPGAPAVLHFPLVSDSFREYSAPGVRRTPEEAAAGEVNLSS
SDSPYHYTKVTYSQEDVDKLLHLTHYNVCNNQEQLLEALRQAVQRRRQRRPH

>Q13017 peripheral membrane protein
MMAKNKEPRPPSYTISIVGLSGTEKDKGNCGVGKSCLCNRFVRSKADEYYPEHTSVLSTI
DFGGRVVNNDHFLYWGDIIQNSEDGVECKIHVIEQTEFIDDQTFLPHRSTNLQPYIKRAAA
SKLQSAEKLMYICTDQLGLEQDFEQKQMPEGKLNVDGFLLCIDVSQGCNRKFDDQLKFV
NNLFVQLSKSKKPVIIAATKCDECVDHYLREVQAFASNKKNLLVVETSARFNVNIETCFTA
LVQMLDKTRSKPKIIPYLDAYKTQRQLVVTATDKFEKLVQTVRDYHATWKTVSNKLKNHP
DYEEYINLEGTRKARNTFSKHIEQLKQEHIRKRREEYINTLPRAFNTLLPNLEEIEHLNWSE
ALKLMEKRADFQLCFVVLEKTPWDETDHIDKINDRRIPFDLLSTLEAEKVYQNHVQHLIS
EKRRVEMKEKFKKTLEKIQFISPGQPWEEVMCFVMEDEAYKYITEADSKEVYGRHQREIV
EKAKEEFQEMLFEHSELFYDLDLNATPSSDKMSEIHTVLSEEPRYKALQKLAPDRESLLLK
HIGFVYHPTKETCLSGQNCTDIKVEQLLASSLLQLDHGRLRLYHDSTNIDKVNLFILGKDG
LAQELANEIRTQSTDDEYALDGKIYELDLRPVDAKSPYFLSQLWTAAFKPHGCFCVFNSIE
SLSFIGEFIGKIRTEASQIRKDKYMANLPFTLILANQRDSISKNLPILRHQGQQLANKLQCPF
VDVPAGTYPRKFNETQIKQALRGVLESVKHNLDVVSPIPANKDLSEADLRIVMCAMCGDP
FSVDLILSPFLDSHSCSAAQAGQNNSLMLDKIIGEKRRRIQITILSYHSSIGVRKDELVHGYI
LVYSAKRKASMGMLRAFLSEVQDTIPVQLVAVTDSQADFFENEAIKELMTEGEHIATEITA
KFTALYSLSQYHRQTEVFTLFFSDVLEKKNMIENSYLSDNTRESTHQSEDVFLPSPRDCFPY
NNYPDSDDDTEAPPPYSPIGDDVQLLPTPSDRSRYRLDLEGNEYPIHSTPNCHDHERNHKV
PPPIKPKPVVPKTNVKKLDPNLLKTIEAGIGKNPRKQTSRVPLAHPEDMDPSDNYAEPIDTI
FKQKGYSDEIYVVPDDSQNRIKIRNSFVNNTQGDEENGFSDRTSKSHGERRPSKYKYKSK
TLFSKAKSYYRRTHSDASDDEAFTTSKTKRKGRHRGSEEDPLLSPVETWKGGIDNPAITSD
QELDDKKMKKKTHKVKEDKKQKKKTKNFNPPTRRNWESNYFGMPLQDLVTAEKPIPLF
VEKCVEFIEDTGLCTEGLYRVSGNKTDQDNIQKQFDQDHNINLVSMEVTVNAVAGALKAF
FADLPDPLIPYSLHPELLEAAKIPDKTERLHALKEIVKKFHPVNYDVFRYVITHLNRVSQQH
KINLMTADNLSICFWPTLMRPDFENREFLSTTKIHQSVVETFIQQCQFFFYNGEIVETTNIVA
PPPPSNPGQLVEPMVPLQLPPPLQPQLIQPQLQTDPLGII

>Q96P69 multipass membrane protein
MGPGEALLAGLLVMVLAVALLSNALVLLCCAYSAELRTRASGVLLVNLSLGHLLLAALD
MPFTLLGVMRGRTPSAPGACQVIGFLDTFLASNAALSVAALSADQWLAVGFPLRYAGRLR
PRYAGLLLGCAWGQSLAFSGAALGCSWLGYSSAFASCSLRLPPEPERPRFAAFTATLHAVG
FVLPLAVLCLTSLQVHRVARRHCQRMDTVTMKALALLADLHPSVRQRCLIQQKRRRHRA
TRKIGIAIATFLICFAPYVMTRLAELVPFVTVNAQWGILSKCLTYSKAVADPFTYSLLRRPFR
QVLAGMVHRLLKRTPRPASTHDSSLDVAGMVHQLLKRTPRPASTHNGSVDTENDSCLQQ

TH

>Q9NYW7 multipass membrane protein
MLESHLIIYFLLAVIQFLLGIFTNGIIVVVNGIDLIKHRKMAPLDLLLSCLAVSRIFLQLFIFYV
NVIVIFFIEFIMCSANCAILLFINELELWLATWLGVFYCAKVASVRHPLFIWLKMRISKLVP
WMILGSLLYVSMICVFHSKYAGFMVPYFLRKFFSQNATIQKEDTLAIQIFSFVAEFSVPLLIF
LFAVLLLIFSLGRHTRQMRNTVAGSRVPGRGAPISALLSILSFLILYFSHCMIKVFLSSLKFHI
RRFIFLFFILVIGIYPSGHSLILILGNPKLKQNAKKFLLHSKCCQ

>Q13591 single-pass type I membrane protein
MKGTCVIAWLFSSLGLWRLAHPEAQGTTQCQRTEHPVISYKEIGPWLREFRAKNAVDFSQ
LTFDPGQKELVVGARNYLFRLQLEDLSLIQAVEWECDEATKKACYSKGKSKEECQNYIRV
LLVGGDRLFTCGTNAFTPVCTNRSLSNLTEIHDQISGMARCPYSPQHNSTALLTAGGELYAA
TAMDFPGRDPAIYRSLGILPPLRTAQYNSKWLNEPNFVSSYDIGNFTYFFFRENAVEHDCG
KTVFSRAARVCKNDIGGRFLLEDTWTTFMKARLNCSRPGEVPFYYNELQSTFFLPELDLIY
GIFTTNVNSIAASAVCVFNLSAIAQAFSGPFKYQENSRSAWLPYPNPNPHFQCGTVDQGLY
VNLTERNLQDAQKFILMHEVVQPVTTVPSFMEDNSRFSHVAVDVVQGREALVHIIYLATD
YGTIKKVRVPLNQTSSSCLLEEIELFPERRREPIRSLQILHSQSVLFVGLREHVVKIPLKRCQ
FYRTRSTCIGAQDPYCGWDVVMKKCTSLEESLSMTQWEQSISACPTRNLTVDGHFGVWS
PWTPCTHTDGSAVGSCLCRTRSCDSPAPQCGGWQCEGPGMEIANCSRNGGWTPWTSWSP
CSTTCGIGFQVRQRSCSNPTPRHGGRVCVGQNREERYCNEHLLCPPHMFWTGWGPWERC
TAQCGGGIQARRRICENGPDCAGCNVEYQSCNTNPCPELKKTTPWTPWTPVNISDNGGH
YEQRFRYTCKARLADPNLLEVGRQRIEMRYCSSDGTSGCSTDGLSGDFLRAGRYSAHTVN
GAWSAWTSWSQCSRDCSRGIRNRKRVCNNPEPKYGGMPCLGPSLEYQECNILPCPVDGV
WSCWSPWTKCSATCGGGHYMRTRSCSNPAPAYGGDICLGLHTEEALCNTQPCPESWSEW
SDWSECEASGVQVRARQCILLFPMGSQCSGNTTESRPCVFDSNFIPEVSVARSSSVEEKRC
GEFNMFHMIAVGLSSSILGCLLTLLVYTYCQRYQQQSHDATVIHPVSPAPLNTSITNHINKL
DKYDSVEAIKAFNKNNLILEERNKYFNPHLTGKTYSNAYFTDLNNYDEY

>Q9NR22 lipid-anchor
MGMKHSSRCLLLRRKMAENAAESTEVNSPPSQPPQPVVPAKPVQCVHHVSTQPSCPGRG
KMSKLLNPEEMTSRDYYFDSYAHFGIHEEMLKDEVRTLTYRNSMYHNKHVFKDKVVLD
VGSGTGILSMFAAKAGAKKVFGIECSSISDYSEKIIKANHLDNIITIFKGKVEEVELPVEKVD
IIISEWMGYCLFYESMLNTVIFARDKWLKPGGLMFPDRAALYVVAIEDRQYKDFKIHWWE
NVYGFDMTCIRDVAMKEPLVDIVDPKQVVTNACLIKEVDIYTVKTEELSFTSAFCLQIQRN
DYVHALVTYFNIEFTKCHKKMGFSTAPDAPYTHWKQTVFYLEDYLTVRRGEEIYGTISMK
PNAKNVRDLDFTVDLDFKGQLCETSVSNDYKMR

>Q13421 GPI-anchor, lipid-anchor
MALPTARPLLGSCGTPALGSLLFLLFSLGWVQPSRTLAGETGQEAAPLDGVLANPPNISSL
SPRQLLGFPCAEVSGLSTERVRELAVALAQKNVKLSTEQLRCLAHRLSEPPEDLDALPLDL
LLFLNPDAFSGPQACTRFFSRITKANVDLLPRGAPERQRLLPAALACWGVRGSLLSEADV
RALGGLACDLPGRFVAESAEVLLPRLVSCPGPLDQDQQEAARAALQGGGPPYGPPSTWSV
STMDALRGLLPVLGQPIIRSIPQGIVAAWRQRSSRDPSWRQPERTILRPRFRREVEKTACPS

GKKAREIDESLIFYKKWELEACVDAALLATQMDRVNAIPFTYEQLDVLKHKLDELYPQGY
PESVIQHLGYLFLKMSPEDIRKWNVTSLETLKALLEVNKGHEMSPQAPRRPLPQVATLIDR
FVKGRGQLDKDTLDTLTAFYPGYLCSLSPEELSSVPPSSIWAVRPQDLDTCDPRQLDVLYP
KARLAFQNMNGSEYFVKIQSFLGGAPTEDLKALSQQNVSMDLATFMKLRTDAVLPLTVAE
VQKLLGPHVEGLKAEERHRPVRDWILRQRQDDLDTLGLGLQGGIPNGYLVLDLSMQEAL
SGTPCLLGPGPVLTVLALLLASTLA

>P22830 peripheral membrane protein
MRSLGANMAAALRAAGVLLRDPLASSSWRVCQPWRWKSGAAAAAVTTETAQHAQGAK
PQVQPQKRKPKTGILMLNMGGPETLGDVHDFLLRLFLDRDLMTLPIQNKLAPFIAKRRTP
KIQEQYRRIGGGSPIKIWTSKQGEGMVKLLDELSPNTAPHKYYIGFRYVHPLTEEAIEEMER
DGLERAIAFTQYPQYSCSTTGSSLNAIYRYYNQVGRKPTMKWSTIDRWPTHHLLIQCFAD
HILKELDHFPLEKRSEVVILFSAHSLPMSVVNRGDPYPQEVSATVQKVMERLEYCNPYRL
VWQSKVGPMPWLGPQTDESIKGLCERGRKNILLVPIAFTSDHIETLYELDIEYSQVLAKEC
GVENIRRAESLNGNPLFSKALADLVHSHIQSNELCSKQLTLSCPLCVNPVCRETKSFFTSQQ
L

>Q03519 multipass membrane protein
MRLPDLRPWTSLLLVDAALLWLLQGPLGTLLPQGLPGLWLEGTLRLGGLWGLLKLRGLL
GFVGTLLLPLCLATPLTVSLRALVAGASRAPPARVASAPWSWLLVGYGAAGLSWSLWAVL
SPPGAQEKEQDQVNNKVLMWRLLKLSRPDLPLLVAAFFFLVLAVLGETLIPHYSGRVIDIL
GGDFDPHAFASAIFFMCLFSFGSSLSAGCRGGCFTYTMSRINLRIREQLFSSLLRQDLGFFQ
ETKTGELNSRLSSDTTLMSNWLPLNANVLLRSLVKVVGLYGFMLSISPRLTLLSLLHMPFTI
AAEKVYNTRHQEVLREIQDAVARAGQVVREAVGGLQTVRSFGAEEHEVCRYKEALEQCR
QLYWRRDLERALYLLVRRVLHLGVQMLMLSCGLQQMQDGELTQGSLLSFMIYQESVGSY
VQTLVYIYGDMLSNVGAAEKVFSYMDRQPNLPSPGTLAPTTLQGVVKFQDVSFAYPNRP
DRPVLKGLTFTLRPGEVTALVGPNGSGKSTVAALLQNLYQPTGGQVLLDEKPISQYEHCYL
HSQVVSVGQEPVLFSGSVRNNIAYGLQSCEDDKVMAAAQAAHADDFIQEMEHGIYTDVG
EKGSQLAAGQKQRLAIARALVRDPRVLILDEATSALDVQCEQALQDWNSRGDRTVLVIAH
RLQTVQRAHQILVLQEGKLQKLAQL

>P55087 multipass membrane protein
MSDRPTARRWGKCGPLCTRENIMVAFKGVWTQAFWKAVTAEFLAMLIFVLLSLGSTINW
GGTEKPLPVDMVLISLCFGLSIATMVQCFGHISGGHINPAVTVAMVCTRKISIAKSVFYIAA
QCLGAIIGAGILYLVTPPSVVGGLGVTMVHGNLTAGHGLLVELIITFQLVFTIFASCDSKRTD
VTGSIALAIGFSVAIGHLFAINYTGASMNPARSFGPAVIMGNWENHWIYWVGPIIGAVLAG
GLYEYVFCPDVEFKRRFKEAFSKAAQQTKGSYMEVEDNRSQVETDDLILKPGVVHVIDV
DRGEEKKGKDQSGEVLSSV

>Q9Y3N9 multipass membrane protein
MDQSNYSSLHGFILLGFSNHPKMEMILSGVVAIFYLITLVGNTAIILASLLDSQLHTPMYFFL
RNLSFLDLCFTTSIIPQMLVNLWGPDKTISYVGCIIQLYVYMWLGSVECLLLAVMSYDRFT
AICKPLHYFVVMNPHLCLKMIIMIWSISLANSVVLCTLTLNLPTCGNNILDHFLCELPALVK
IACVDTTTVEMSVFALGIIIVLTPLILILISYGYIAKAVLRTKSKASQRKAMNTCGSHLTVVS

MFYGTIIYMYLQPGNRASKDQGKFLTLFYTVITPSLNPLIYTLRNKDMKDALKKLMRFHH
KSTKIKRNCKS

>Q6Y1H2 multipass membrane protein
MAAVAATAAAKGNGGGGGRAGAGDASGTRKKKGPGPLATAYLVIYNVVMTAGWLVIAV
GLVRAYLAKGSYHSLYYSIEKPLKFFQTGALLEILHCAIGIVPSSVVLTSFQVMSRVFLIWAV
THSVKEVQSEDSVLLFVIAWTITEIIRYSFYTFSLLNHLPYLIKWARYTLFIVLYPMGVSGEL
LTIYAALPFVRQAGLYSISLPNKYNFSFDYYAFLILIMISYIPIFPQLYFHMIHQRRKILSHTEE
HKKFE

>Q495A1 single-pass type I membrane protein
MRWCLLLIWAQGLRQAPLASGMMTGTIETTGNISAEKGGSIILQCHLSSTTAQVTQVNWE
QQDQLLAICNADLGWHISPSFKDRVAPGPGLGLTLQSLTVNDTGEYFCIYHTYPDGTYTGR
IFLEVLESSVAEHGARFQIPLLGAMAATLVVICTAVIVVVALTRKKKALRIHSVEGDLRRKS
AGQEEWSPSAPSPPGSCVQAEAAPAGLCGEQRGEDCAELHDYFNVLSYRSLGNCSFFTET
G

>P35462 multipass membrane protein
MASLSQLSSHLNYTCGAENSTGASQARPHAYYALSYCALILAIVFGNGLVCMAVLKERAL
QTTTNYLVVSLAVADLLVATLVMPWVVYLEVTGGVWNFSRICCDVFVTLDVMMCTASIL
NLCAISIDRYTAVVMPVHYQHGTGQSSCRRVALMITAVWVLAFAVSCPLLFGFNTTGDPTV
CSISNPDFVIYSSVVSFYLPFGVTVLVYARIYVVLKQRRRKRILTRQNSQCNSVRPGFPQQT
LSPDPAHLELKRYYSICQDTALGGPGFQERGGELKREEKTRNSLSPTIAPKLSLEVRKLSNG
RLSTSLKLGPLQPRGVPLREKKATQMVAIVLGAFIVCWLPFFLTHVLNTHCQTCHVSPELY
SATTWLGYVNSALNPVIYTTFNIEFRKAFLKILSC

>Q8NGV6 multipass membrane protein
MFLYLCFIFQRTCSEEMEEENATLLTEFVLTGFLHQPDCKIPLFLAFLVIYLITIMGNLGLIVL
IWKDPHLHIPMYLFLGSLAFVDASLSSTVTPKMLINFLAKSKMISLSECMVQFFSLVTTVTT
ECFLLATMAYDRYVAICKALLYPVIMTNELCIQLLVLSFIGGLLHALIHEAFSFRLTFCNSNII
QHFYCDIIPLLKISCTDSSINFLMVFIFAGSVQVFTIGTILISYTIILFTILEKKSIKGIRKAVSTC
GAHLLSVSLYYGPLTFKYLGSASPQADDQDMMESLFYTVIVPLLNPMIYSLRNKQVIASFT
KMFKSNV

>Q9ULU8 peripheral membrane protein
MLDPSSSEEESDEIVEEESGKEVLGSAPSGARLSPSRTSEGSAGSAGLGGGGAGAGAGVG
AGGGGGSGASSGGGAGGLQPSSRAGGGRPSSPSPSVVSEKEKEELERLQKEEEERKKRLQ
LYVFVMRCIAYPFNAKQPTDMARRQQKISKQQLQTVKDRFQAFLNGETQIMADEAFMNA
VQSYYEVFLKSDRVARMVQSGGCSANDSREVFKKHIEKRVRSLPEIDGLSKETVLSSWMA
KFDAIYRGEEDPRKQQARMTASAASELILSKEQLYEMFQNILGIKKFEHQLLYNACQLDNP
DEQAAQIRRELDGRLQMADQIARERKFPKFVSKEMENMYIEELKSSVNLLMANLESMPV
SKGGEFKLQKLKRSHNASIIDMGEESENQLSKSDVVLSFSLEVVIMEVQGLKSLAPNRIVY
CTMEVEGGEKLQTDQAEASKPTWGTQGDFSTTHALPAVKVKLFTESTGVLALEDKELGR
VILHPTPNSPKQSEWHKMTVSKNCPDQDLKIKLAVRMDKPQNMKHSGYLWAIGKNVWK

RWKKRFFVLVQVSQYTFAMCSYREKKAEPQELLQLDGYTVDYTDPQPGLEGGRAFFNAV
KEGDTVIFASDDEQDRILWVQAMYRATGQSHKPVPPTQVQKLNAKGGNVPQLDAPISQFY
ADRAQKHGMDEFISSNPCNFDHASLFEMVQRLTLDHRLNDSYSCLGWFSPGQVFVLDEY
CARNGVRGCHRHLCYLRDLLERAENGAMIDPTLLHYSFAFCASHVHGNRPDGIGTVTVE
EKERFEEIKERLRVLLENQITHFRYCFPFGRPEGALKATLSLLERVLMKDIVTPVPQEEVKT
VIRKCLEQAALVNYSRLSEYAKIEENQKDAENVGRLITPAKKLEDTIRLAELVIEVLQQNEE
HHAEPHVDKGEAFAWWSDLMVEHAETFLSLFAVDMDAALEVQPPDTWDSFPLFQLLNDF
LRTDYNLCNGKFHKHLQDLFAPLVVRYVDLMESSIAQSIHRGFERESWEPVKSLTSNLPNV
NLPNVNLPKVPNLPVNIPLGIPQMPTFSAPSWMAAIYDADNGSGTSEDLFWKLDALQTFIR
DLHWPEEEFGKHLEQRLKLMASDMIESCVKRTRIAFEVKLQKTSRSTDFRVPQSICTMFN
VMVDAKAQSTKLCSMEMGQEHQYHSKIDELIEETVKEMITLLVAKFVTILEGVLAKLSRY
DEGTLFSSFLSFTVKAASKYVDVPKPGMDVADAYVTFVRHSQDVLRDKVNEEMYIERLF
DQWYNSSMNVICTWLTDRMDLQLHIYQLKTLIRMVKKTYRDFRLQGVLDSTLNSKTYET
IRNRLTVEEATASVSEGGGLQGISMKDSDEEDEEDD

>Q8NBJ4 single-pass type II membrane protein
MMGLGNGRRSMKSPPLVLAALVACIIVLGFNYWIASSRSVDLQTRIMELEGRVRRAAAER
GAVELKKNEFQGELEKQREQLDKIQSSHNFQLESVNKLYQDEKAVLVNNITTGERLIRVLQ
DQLKTLQRNYGRLQQDVLQFQKNQTNLERKFSYDLSQCINQMKEVKEQCEERIEEVTKK
GNEAVASRDLSENNDQRQQLQALSEPQPRLQAAGLPHTEVPQGKGNVLGNSKSQTPAPSS
EVVLDSKRQVEKEETNEIQVVNEEPQRDRLPQEPGREQVVEDRPVGGRGFGGAGELGQT
PQVQAALSVSQENPEMEGPERDQLVIPDGQEEEQEAAGEGRNQQKLRGEDDYNMDENE
AESETDKQAALAGNDRNIDVFNVEDQKRDTINLLDQREKRNHTL

>Q8WWQ8 single-pass type I membrane protein
MMLQHLVIFCLGLVVQNFCSPAETTGQARRCDRKSLLTIRTECRSCALNLGVKCPDGYTMI
TSGSVGVRDCRYTFEVRTYSLSLPGCRHICRKDYLQPRCCPGRWGPDCIECPGGAGSPCN
GRGSCAEGMEGNGTCSCQEGFGGTACETCADDNLFGPSCSSVCNCVHGVCNSGLDGDGT
CECYSAYTGPKCDKPIPECAALLCPENSRCSPSTEDENKLECKCLPNYRGDGKYCDPINPC
LRKICHPHAHCTYLGPNRHSCTCQEGYRGDGQVCLPVDPCQINFGNCPTKSTVCKYDGP
GQSHCECKEHYQNFVPGVGCSMTDICKSDNPCHRNANCTTVAPGRTECICQKGYVGDGL
TCYGNIMERLRELNTEPRGKWQGRLTSFISLLDKAYAWPLSKLGPFTVLLPTDKGLKGFN
VNELLVDNKAAQYFVKLHIIAGQMNIEYMNNTDMFYTLTGKSGEIFNSDKDNQIKLKLHG
GKKKVKIIQGDIIASNGLLHILDRAMDKLEPTFESNNEQTIMTMLQPRYSKFRSLLEETNLG
HALDEDGVGGPYTIFVPNNEALNNMKDGTLDYLLSPEGSRKLLELVRYHIVPFTQLEVATL
ISTPHIRSMANQLIQFNTTDNGQILANDVAMEEIEITAKNGRIYTLTGVLIPPSIVPILPHRCD
ETKREMKLGTCVSCSLVYWSRCPANSEPTALFTHRCVYSGRFGSLKSGCARYCNATVKIP
KCCKGFYGPDCNQCPGGFSNPCSGNGQCADSLGGNGTCICEEGFQGSQCQFCSDPNKYG
PRCNKKCLCVHGTCNNRIDSDGACLTGTCRDGSAGRLCDKQTSACGPYVQFCHIHATCE
YSNGTASCICKAGYEGDGTLCSEMDPCTGLTPGGCSRNAECIKTGTGTHTCVCQQGWTG
NGRDCSEINNCLLPSAGGCHDNASCLYVGPGQNECECKKGFRGNGIDCEPITSCLEQTGK
CHPLASCQSTSSGVWSCVCQEGYEGDGFLCYGNAAVELSFLSEAAIFNRWINNASLQPTLS
ATSNLTVLVPSQQATEDMDQDEKSFWLSQSNIPALIKYHMLLGTYRVADLQTLSSSDMLAT
SLQGNFLHLAKVDGNITIEGASIVDGDNAATNGVIHIINKVLVPQRRLTGSLPNLLMRLEQ

MPDYSIFRGYIIQYNLANAIEAADAYTVFAPNNNAIENYIREKKVLSLEEDVLRYHVVLEE
KLLKNDLHNGMHRETMLGFSYFLSFFLHNDQLYVNEAPINYTNVATDKGVIHGLGKVLEI
QKNRCDNNDTTIIRGRCRTCSSELTCPFGTKSLGNEKRRCIYTSYFMGRRTLFIGCQPKCVR
TVITRECCAGFFGPQCQPCPGNAQNVCFGNGICLDGVNGTGVCECGEGFSGTACETCTEG
KYGIHCDQACSCVHGRCNQGPLGDGSCDCDVGWRGVHCDNATTEDNCNGTCHTSANCL
TNSDGTASCKCAAGFQGNGTICTAINACEISNGGCSAKADCKRTTPGRRVCTCKAGYTGD
GIVCLEINPCLENHGGCDKNAECTQTGPNQAACNCLPAYTGDGKVCTLINVCLTKNGGCS
EFAICNHTGQVERTCTCKPNYIGDGFTCRGSIYQELPKNPKTSQYFFQLQEHFVKDLVGPG
PFTVFAPLSAAFDEEARVKDWDKYGLMPQVLRYHVVACHQLLLENLKLISNATSLQGEPI
VISVSQSTVYINNKAKIISSDIISTNGIVHIIDKLLSPKNLLITPKDNSGRILQNLTTLATNNGY
IKFSNLIQDSGLLSVITDPIHTPVTLFWPTDQALHALPAEQQDFLFNQDNKDKLKEYLKFH
VIRDAKVLAVDLPTSTAWKTLQGSELSVKCGAGRDIGDLFLNGQTCRIVQRELLFDLGVA
YGIDCLLIDPTLGGRCDTFTTFDASGECGSCVNTPSCPRWSKPKGVKQKCLYNLPFKRNLE
GCRERCSLVIQIPRCCKGYFGRDCQACPGGPDAPCNNRGVCLDQYSATGECKCNTGFNGT
ACEMCWPGRFGPDCLPCGCSDHGQCDDGITGSGQCLCETGWTGPSCDTQAVLPAVCTPP
CSAHATCKENNTCECNLDYEGDGITCTVVDFCKQDNGGCAKVARCSQKGTKVSCSCQKG
YKGDGHSCTEIDPCADGLNGGCHEHATCKMTGPGKHKCECKSHYVGDGLNCEPEQLPID
RCLQDNGQCHADAKCVDLHFQDTTVGVFHLRSPLGQYKLTFDKAREACANEAATMATY
NQLSYAQKAKYHLCSAGWLETGRVAYPTAFASQNCGSGVVGIVDYGPRPNKSEMWDVFC
YRMKDVNCTCKVGYVGDGFSCSGNLLQVLMSFPSLTNFLTEVLAYSNSSARGRAFLEHLT
DLSIRGTLFVPQNSGLGENETLSGRDIEHHLANVSMFFYNDLVNGTTLQTRLGSKLLITAS
QDPLQPTETRFVDGRAILQWDIFASNGIIHVISRPLKAPPAPVTLTHTGLGAGIFFAIILVTGA
VALAAYSYFRINRRTIGFQHFESEEDINVAALGKQQPENISNPLYESTTSAPPEPSYDPFTDS
EERQLEGNDPLRTL

>Q14242 single-pass type I membrane protein
MPLQLLLLLILLGPGNSLQLWDTWADEAEKALGPLLARDRRQATEYEYLDYDFLPETEPP
EMLRNSTDTTPLTGPGTPESTTVEPAARRSTGLDAGGAVTELTTELANMGNLSTDSAAMEI
QTTQPAATEAQTTQPVPTEAQTTPLAATEAQTTRLTATEAQTTPLAATEAQTTPPAATEAQT
TQPTGLEAQTTAPAAMEAQTTAPAAMEAQTTPPAAMEAQTTQTTAMEAQTTAPEATEAQ
TTQPTATEAQTTPLAAMEALSTEPSATEALSMEPTTKRGLFIPFSVSSVTHKGIPMAASNLS
VNYPVGAPDHISVKQCLLAILILALVATIFFVCTVVLAVRLSRKGHMYPVRNYSPTEMVCIS
SLLPDGGEGPSATANGGLSKAKSPGLTPEPREDREGDDLTLHSFLP

>O43525 multipass membrane protein
MGLKARRAAGAAGGGGDGGGGGGGAANPAGGDAAAAGDEERKVGLAPGDVEQVTLA
LGAGADKDGTLLLEGGGRDEGQRRTPQGIGLLAKTPLSRPVKRNNAKYRRIQTLIYDALE
RPRGWALLYHALVFLIVLGCLILAVLTTFKEYETVSGDWLLLLETFAIFIFGAEFALRIWAAG
CCCRYKGWRGRLKFARKPLCMLDIFVLIASVPVVAVGNQGNVLATSLRSLRFLQILRMLR
MDRRGGTWKLLGSAICAHSKELITAWYIGFLTLILSSFLVYLVEKDVPEVDAQGEEMKEEF
ETYADALWWGLITLATIGYGDKTPKTWEGRLIAATFSLIGVSFFALPAGILGSGLALKVQEQ
HRQKHFEKRRKPAAELIQAAWRYYATNPNRIDLVATWRFYESVVSFPFFRKEQLEAASSQK
LGLLDRVRLSNPRGSNTKGKLFTPLNVDAIEESPSKEPKPVGLNNKERFRTAFRMKAYAF
WQSSEDAGTGDPMAEDRGYGNDFPIEDMIPTLKAAIRAVRILQFRLYKKKFKETLRPYDV

KDVIEQYSAGHLDMLSRIKYLQTRIDMIFTPGPPSTPKHKKSQKGSAFTFPSQQSPRNEPYV
ARPSTSEIEDQSMMGKFVKVERQVQDMGKKLDFLVDMHMQHMERLQVQVTEYYPTKG
TSSPAEAEKKEDNRYSDLKTIICNYSETGPPEPPYSFHQVTIDKVSPYGFFAHDPVNLPRGG
PSSGKVQATPPSSATTYVERPTVLPILTLLDSRVSCHSQADLQGPYSDRISPRQRRSITRDSD
TPLSLMSVNHEELERSPSGFSISQDRDDYVFGPNGGSSWMREKRYLAEGETDTDTDPFTPS
GSMPLSSTGDGISDSVWTPSNKPI

>Q9UM73 single-pass type I membrane protein
MGAIGLLWLLPLLLSTAAVGSGMGTGQRAGSPAAGPPLQPREPLSYSRLQRKSLAVDFVV
PSLFRVYARDLLLPPSSSELKAGRPEARGSLALDCAPLLRLLGPAPGVSWTAGSPAPAEART
LSRVLKGGSVRKLRRAKQLVLELGEEAILEGCVGPPGEAAVGLLQFNLSELFSWWIRQGE
GRLRIRLMPEKKASEVGREGRLSAAIRASQPRLLFQIFGTGHSSLESPTNMPSPSPDYFTWN
LTWIMKDSFPFLSHRSRYGLECSFDFPCELEYSPPLHDLRNQSWSWRRIPSEEASQMDLLD
GPGAERSKEMPRGSFLLLNTSADSKHTILSPWMRSSSEHCTLAVSVHRHLQPSGRYIAQLL
PHNEAAREILLMPTPGKHGWTVLQGRIGRPDNPFRVALEYISSGNRSLSAVDFFALKNCSE
GTSPGSKMALQSSFTCWNGTVLQLGQACDFHQDCAQGEDESQMCRKLPVGFYCNFEDG
FCGWTQGTLSPHTPQWQVRTLKDARFQDHQDHALLLSTTDVPASESATVTSATFPAPIKSS
PCELRMSWLIRGVLRGNVSLVLVENKTGKEQGRMVWHVAAYEGLSLWQWMVLPLLDVS
DRFWLQMVAWWGQGSRAIVAFDNISISLDCYLTISGEDKILQNTAPKSRNLFERNPNKELK
PGENSPRQTPIFDPTVHWLFTTCGASGPHGPTQAQCNNAYQNSNLSVEVGSEGPLKGIQIW
KVPATDTYSISGYGAAGGKGGKNTMMRSHGVSVLGIFNLEKDDMLYILVGQQGEDACPS
TNQLIQKVCIGENNVIEEEIRVNRSVHEWAGGGGGGGGATYVFKMKDGVPVPLIIAAGGG
GRAYGAKTDTFHPERLENNSSVLGLNGNSGAAGGGGGWNDNTSLLWAGKSLQEGATGG
HSCPQAMKKWGWETRGGFGGGGGGCSSGGGGGGYIGGNAASNNDPEMDGEDGVSFISP
LGILYTPALKVMEGHGEVNIKHYLNCSHCEVDECHMDPESHKVICFCDHGTVLAEDGVSC
IVSPTPEPHLPLSLILSVVTSALVAALVLAFSGIMIVYRRKHQELQAMQMELQSPEYKLSKL
RTSTIMTDYNPNYCFAGKTSSISDLKEVPRKNITLIRGLGHGAFGEVYEGQVSGMPNDPSP
LQVAVKTLPEVCSEQDELDFLMEALIISKFNHQNIVRCIGVSLQSLPRFILLELMAGGDLKS
FLRETRPRPSQPSSLAMLDLLHVARDIACGCQYLEENHFIHRDIAARNCLLTCPGPGRVAKI
GDFGMARDIYRASYYRKGGCAMLPVKWMPPEAFMEGIFTSKTDTWSFGVLLWEIFSLGY
MPYPSKSNQEVLEFVTSGGRMDPPKNCPGPVYRIMTQCWQHQPEDRPNFAIILERIEYCTQ
DPDVINTALPIEYGPLVEEEEKVPVRPKDPEGVPPLLVSQQAKREEERSPAAPPPLPTTSSGK
AAKKPTAAEISVRVPRGPAVEGGHVNMAFSQSNPPSELHKVHGSRNKPTSLWNPTYGSWF
TEKPTKKNNPIAKKEPHDRGNLGLEGSCTVPPNVATGRLPGASLLLEPSSLTANMKEVPLF
RLRHFPCGNVNYGYQQQGLPLEAATAPGAGHYEDTILKSKNSMNQPGP

>Q13349 single-pass type I membrane protein
MTFGTVLLLSVLASYHGFNLDVEEPTIFQEDAGGFGQSVVQFGGSRLVVGAPLEVVAANQ
TGRLYDCAAATGMCQPIPLHIRPEAVNMSLGLTLAASTNGSRLLACGPTLHRVCGENSYSK
GSCLLLGSRWEIIQTVPDATPECPHQEMDIVFLIDGSGSIDQNDFNQMKGFVQAVMGQFEG
TDTLFALMQYSNLLKIHFTFTQFRTSPSQQSLVDPIVQLKGLTFTATGILTVVTQLFHHKNG
ARKSAKKILIVITDGQKYKDPLEYSDVIPQAEKAGIIRYAIGVGHAFQGPTARQELNTISSAP
PQDHVFKVDNFAALGSIQKQLQEKIYAVEGTQSRASSSFQHEMSQEGFSTALTMDGLFLGA
VGSFSWSGGAFLYPPNMSPTFINMSQENVDMRDSYLGYSTELALWKGVQNLVLGAPRYQ

HTGKAVIFTQVSRQWRKKAEVTGTQIGSYFGASLCSVDVDSDGSTDLILIGAPHYYEQTR
GGQVSVCPLPRGRVQWQCDAVLRGEQGHPWGRFGAALTVLGDVNEDKLIDVAIGAPGEQ
ENRGAVYLFHGASESGISPSHSQRIASSQLSPRLQYFGQALSGGQDLTQDGLMDLAVGAR
GQVLLLRSLPVLKVGVAMRFSPVEVAKAVYRCWEEKPSALEAGDATVCLTIQKSSLDQLG
DIQSSVRFDLALDPGRLTSRAIFNETKNPTLTRRKTLGLGIHCETLKLLLPDCVEDVVSPIIL
HLNFSLVREPIPSPQNLRPVLAVGSQDLFTASLPFEKNCGQDGLCEGDLGVTLSFSGLQTLT
VGSSLELNVIVTVWNAGEDSYGTVVSLYYPAGLSHRRVSGAQKQPHQSALRLACETVPTE
DEGLRSSRCSVNHPIFHEGSNGTFIVTFDVSYKATLGDRMLMRASASSENNKASSSKATFQ
LELPVKYAVYTMISRQEESTKYFNFATSDEKKMKEAEHRYRVNNLSQRDLAISINFWVPVL
LNGVAVWDVVMEAPSQSLPCVSERKPPQHSDFLTQISRSPMLDCSIADCLQFRCDVPSFSV
QEELDFTLKGNLSFGWVRETLQKKVLVVSVAEITFDTSVYSQLPGQEAFMRAQMEMVLE
EDEVYNAIPIIMGSSVGALLLLALITATLYKLGFFKRHYKEMLEDKPEDTATFSGDDFSCVA
PNVPLS

>Q6ZMC9 single-pass type I membrane protein
MEKSIWLLACLAWVLPTGSFVRTKIDTTENLLNTEVHSSPAQRWSMQVPPEVSAEAGDAA
VLPCTFTHPHRHYDGPLTAIWRAGEPYAGPQVFRCAAARGSELCQTALSLHGRFRLLGNP
RRNDLSLRVERLALADDRRYFCRVEFAGDVHDRYESRHGVRLHVTAAPRIVNISVLPSPAH
AFRALCTAEGEPPPALAWSGPALGNSLAAVRSPREGHGHLVTAELPALTHDGRYTCTAANS
LGRSEASVYLFRFHGASGASTVALLLGALGFKALLLLGVLAARAARRRPEHLDTPDTPPR
SQAQESNYENLSQMNPRSPPATMCSP

>P07510 multipass membrane protein
MHGGQGPLLLLLLLAVCLGAQGRNQEERLLADLMQNYDPNLRPAERDSDVVNVSLKLTL
TNLISLNEREEALTTNVWIEMQWCDYRLRWDPRDYEGLWVLRVPSTMVWRPDIVLENNV
DGVFEVALYCNVLVSPDGCIYWLPPAIFRSACSISVTYFPFDWQNCSLIFQSQTYSTNEIDLQ
LSQEDGQTIEWIFIDPEAFTENGEWAIQHRPAKMLLDPAAPAQEAGHQKVVFYLLIQRKPL
FYVINIIAPCVLISSVAILIHFLPAKAGGQKCTVAINVLLAQTVFLFLVAKKVPETSQAVPLIS
KYLTFLLVVTILIVVNAVVVLNVSLRSPHTHSMARGVRKVFLRLLPQLLRMHVRPLAPAA
VQDTQSRLQNGSSGWSITTGEEVALCLPRSELLFQQWQRQGLVAAALEKLEKGPELGLSQ
FCGSLKQAAPAIQACVEACNLIACARHQQSHFDNGNEEWFLVGRVLDRVCFLAMLSLFIC
GTAGIFLMAHYNRVPALPFPGDPRPYLPSPD

>Q86SP6 multipass membrane protein
MSLFLSNLSTNDSSLWKENHNSTDLLNPPGTLNIYLFCLTCLMTFAALVGSIYSLISLLKMQ
NRTVVSMLVASWSVDDLMSVLSVTIFMFLQWPNEVPGYFQFLCTTSALMYLCQGLSSNL
KATLLVSYNFYTMHRGVGSQTASRRSGQVLGVVLTVWAASLLLSALPLCGWGAFVRTPW
GCLVDCSSSYVLFLSIVYALAFGLLVGLSVPLTHRLLCSEEPPRLHSNYQEISRGASIPGTPPT
AGRVVSLSPEDAPGPSLRRSGGCSPSSDTVFGPGAPAAAGAEACRRENRGTLYGTRSFTVS
VAQKRFALILALTKVVLWLPMMMHMVVQNVVGFQSLPLETFSFLLTLLATTVTPVFVLSK
RWTHLPCGCIINCRQNAYAVASDGKKIKRKGFEFNLSFQKSYGIYKIAHEDYYDDDENSIF
YHNLMNSECETTKDPQRDNRNIFNAIKVEISTTPSLDSSTQRGINKCTNTDITEAKQDSNN
KKDAFSDKTGGDINYEETTFSEGPERRLSHEESQKPDLSDWEWCRSKSERTPRQRSGYAL
AIPLCAFQGTVSLHAPTGKTLSLSTYEVSAEGQKITPASKKIEVYRSKSVGHEPNSEDSSST

FVDTSVKIHLEVLEICDNEEALDTVSIISNISQSSTQVRSPSLRYSRKENRFVSCDLGETASY
SLFLPTSNPDGDINISIPDTVEAHRQNSKRQHQERDGYQEEIQLLNKAYRKREEESKGS

>Q99569 peripheral membrane protein
MPAPEQASLVEEGQPQTRQEAASTGPGMEPETTATTILASVKEQELQFQRLTRELEVERQIV
ASQLERCRLGAESPSIASTSSTEKSFPWRSTDVPNTGVSKPRVSDAVQPNNYLIRTEPEQGT
LYSPEQTSLHESEGSLGNSRSSTQMNSYSDSGYQEAGSFHNSQNVSKADNRQQHSFIGSTN
NHVVRNSRAEGQTLVQPSVANRAMRRVSSVPSRAQSPSYVISTGVSPSRGSLRTSLGSGFG
SPSVTDPRPLNPSAYSSTTLPAARAASPYSQRPASPTAIRRIGSVTSRQTSNPNGPTPQYQTT
ARVGSPLTLTDAQTRVASPSQGQVGSSSPKRSGMTAVPQHLGPSLQRTVHDMEQFGQQQY
DIYERMVPPRPDSLTGLRSSYASQHSQLGQDLRSAVSPDLHITPIYEGRTYYSPVYRSPNHG
TVELQGSQTALYRTGSVGIGNLQRTSSQRSTLTYQRNNYALNTTATYAEPYRPIQYRVQEC
NYNRLQHAVPADDGTTRSPSIDSIQKDPREFAWRDPELPEVIHMLQHQFPSVQANAAAYL
QHLCFGDNKVKMEVCRLGGIKHLVDLLDHRVLEVQKNACGALRNLVFGKSTDENKIAM
KNVGGIPALLRLLRKSIDAEVRELVTGVLWNLSSCDAVKMTIIRDALSTLTNTVIVPHSGW
NNSSFDDDHKIKFQTSLVLRNTTGCLRNLSSAGEEARKQMRSCEGLVDSLLYVIHTCVNTS
DYDSKTVENCVCTLRNLSYRLELEVPQARLLGLNELDDLLGKESPSKDSEPSCWGKKKK
KKKRTPQEDQWDGVGPIPGLSKSPKGVEMLWHPSVVKPYLTLLAESSNPATLEGSAGSLQ
NLSAGNWKFAAYIRAAVRKEKGLPILVELLRMDNDRVVSSVATALRNMALDVRNKELIGK
YAMRDLVNRLPGGNGPSVLSDETMAAICCALHEVTSKNMENAKALADSGGIEKLVNITK
GRGDRSSLKVVKAAAQVLNTLWQYRDLRSIYKKDGWNQNHFITPVSTLERDRFKSHPSL
STTNQQMSPIIQSVGSTSSSPALLGIRDPRSEYDRTQPPMQYYNSQGDATHKGLYPGSSKPS
PIYISSYSSPAREQNRRLQHQQLYYSQDDSNRKNFDAYRLYLQSPHSYEDPYFDDRVHFPA
STDYSTQYGLKSTTNYVDFYSTKRPSYRAEQYPGSPDSWV

>Q09428 multipass membrane protein
MPLAFCGSENHSAAYRVDQGVLNNGCFVDALNVVPHVFLLFITFPILFIGWGSQSSKVHIH
HSTWLHFPGHNLRWILTFMLLFVLVCEIAEGILSDGVTESHHLHLYMPAGMAFMAAVTSV
VYYHNIETSNFPKLLIALLVYWTLAFITKTIKFVKFLDHAIGFSQLRFCLTGLLVILYGMLLL
VEVNVIRVRRYIFFKTPREVKPPEDLQDLGVRFLQPFVNLLSKGTYWWMNAFIKTAHKKP
IDLRAIGKLPIAMRALTNYQRLCEAFDAQVRKDIQGTQGARAIWQALSHAFGRRLVLSSTF
RILADLLGFAGPLCIFGIVDHLGKENDVFQPKTQFLGVYFVSSQEFLANAYVLAVLLFLAL
LLQRTFLQASYYVAIETGINLRGAIQTKIYNKIMHLSTSNLSMGEMTAGQICNLVAIDTNQL
MWFFFLCPNLWAMPVQIIVGVILLYYILGVSALIGAAVIILLAPVQYFVATKLSQAQRSTLE
YSNERLKQTNEMLRGIKLLKLYAWENIFRTRVETTRRKEMTSLRAFAIYTSISIFMNTAIPIA
AVLITFVGHVSFFKEADFSPSVAFASLSLFHILVTPLFLLSSVVRSTVKALVSVQKLSEFLSSA
EIREEQCAPHEPTPQGPASKYQAVPLRVVNRKRPAREDCRGLTGPLQSLVPSADGDADNCC
VQIMGGYFTWTPDGIPTLSNITIRIPRGQLTMIVGQVGCGKSSLLLAALGEMQKVSGAVFW
SSLPDSEIGEDPSPERETATDLDIRKRGPVAYASQKPWLLNATVEENIIFESPFNKQRYKMVI
EACSLQPDIDILPHGDQTQIGERGINLSGGQRQRISVARALYQHANVVFLDDPFSALDIHLS
DHLMQAGILELLRDDKRTVVLVTHKLQYLPHADWIIAMKDGTIQREGTLKDFQRSECQLF
EHWKTLMNRQDQELEKETVTERKATEPPQGLSRAMSSRDGLLQDEEEEEEEAAESEEDD
NLSSMLHQRAEIPWRACAKYLSSAGILLLSLLVFSQLLKHMVLVAIDYWLAKWTDSALTL
TPAARNCSLSQECTLDQTVYAMVFTVLCSLGIVLCLVTSVTVEWTGLKVAKRLHRSLLNRI

ILAPMRFFETTPLGSILNRFSSDCNTIDQHIPSTLECLSRSTLLCVSALAVISYVTPVFLVALLP
LAIVCYFIQKYFRVASRDLQQLDDTTQLPLLSHFAETVEGLTTIRAFRYEARFQQKLLEYTD
SNNIASLFLTAANRWLEVRMEYIGACVVLIAAVTSISNSLHRELSAGLVGLGLTYALMVSN
YLNWMVRNLADMELQLGAVKRIHGLLKTEAESYEGLLAPSLIPKNWPDQGKIQIQNLSVR
YDSSLKPVLKHVNALIAPGQKIGICGRTGSGKSSFSLAFFRMVDTFEGHIIIDGIDIAKLPLH
TLRSRLSIILQDPVLFSGTIRFNLDPERKCSDSTLWEALEIAQLKLVVKALPGGLDAIITEGG
ENFSQGQRQLFCLARAFVRKTSIFIMDEATASIDMATENILQKVVMTAFADRTVVTIAHRV
HTILSADLVIVLKRGAILEFDKPEKLLSRKDSVFASFVRADK

>Q8TDS4 multipass membrane protein
MNRHHLQDHFLEIDKKNCCVFRDDFIVKVLPPVLGLEFIFGLLGNGLALWIFCFHLKSWK
SSRIFLFNLAVADFLLIICLPFLMDNYVRRWDWKFGDIPCRLMLFMLAMNRQGSIIFLTVVA
VDRYFRVVHPHHALNKISNRTAAIISCLLWGITIGLTVHLLKKKMPIQNGGANLCSSFSICH
TFQWHEAMFLLEFFLPLGIILFCSARIIWSLRQRQMDRHAKIKRAITFIMVVAIVFVICFLPS
VVVRIRIFWLLHTSGTQNCEVYRSVDLAFFITLSFTYMNSMLDPVVYYFSSPSFPNFFSTLI
NRCLQRKMTGEPDNNRSTSVELTGDPNKTRGAPEALMANSGEPWSPSYLGPTSP

>Q86Y22 single-pass type II membrane protein
MGPGERAGGGGDAGKGNAAGGGGGGRSATTAGSRAVSALCLLLSVGSAAACLLLGVQA
AALQGRVAALEEERELLRRAGPPGALDAWAEPHLERLLREKLDGLAKIRTAREAPSECVC
PPGPPGRRGKPGRRGDPGPPGQSGRDGYPGPLGLDGKPGLPGPKGEKGAPGDFGPRGDQ
GQDGAAGPPGPPGPPGARGPPGDTGKDGPRGAQGPAGPKGEPGQDGEMGPKGPPGPKGE
PGVPGKKGDDGTPSQPGPPGPKGEPGSMGPRGENGVDGAPGPKGEPGHRGTDGAAGPR
GAPGLKGEQGDTVVIDYDGRILDALKGPPGPQGPPGPPGIPGAKGELGLPGAPGIDGEKGP
KGQKGDPGEPGPAGLKGEAGEMGLSGLPGADGLKGEKGESASDSLQESLAQLIVEPGPPG
PPGPPGPMGLQGIQGPKGLDGAKGEKGASGERGPSGLPGPVGPPGLIGLPGTKGEKGRPG
EPGLDGFPGPRGEKGDRSERGEKGERGVPGRKGVKGQKGEPGPPGLDQPCPVGPDGLPV
PGCWHK

>P59542 multipass membrane protein
MMCFLLIISSILVVFAFVLGNVANGFIALVNVIDWVNTRKISSAEQILTALVVSRIGLLWVML
FLWYATVFNSALYGLEVRIVASNAWAVTNHFSMWLAASLSIFCLLKIANFSNLISLHLKKRI
KSVVLVILLGPLVFLICNLAVITMDERVWTKEYEGNVTWKIKLRNAIHLSSLTVTTLANLIP
FTLSLICFLLLICSLCKHLKKMRLHSKGSQDPSTKVHIKALQTVTSFLMLFAIYFLCIITSTW
NLRTQQSKLVLLLCQTVAIMYPSFHSFILIMGSRKLKQTFLSVLWQMTR

>P59538 multipass membrane protein
MTTFIPIIFSSVVVVLFVIGNFANGFIALVNSIERVKRQKISFADQILTALAVSRVGLLWVLLL
NWYSTVFNPAFYSVEVRTTAYNVWAVTGHFSNWLATSLSIFYLLKIANFSNLIFLHLKRRV
KSVILVMLLGPLLFLACQLFVINMKEIVRTKEYEGNLTWKIKLRSAVYLSDATVTTLGNLV
PFTLTLLCFLLLICSLCKHLKKMQLHGKGSQDPSTKVHIKALQTVIFFLLLCAVYFLSIMISV
WSFGSLENKPVFMFCKAIRFSYPSIHPFILIWGNKKLKQTFLSVLRQVRYWVKGEKPSSP

>Q9NYV9 multipass membrane protein

MESALPSIFTLVIIAEFIIGNLSNGFIVLINCIDWVSKRELSSVDKLLIILAISRIGLIWEILVSW
FLALHYLAIFVSGTGLRIMIFSWIVSNHFNLWLATIFSIFYLLKIASFSSPAFLYLKWRVNKVI
LMILLGTLVFLFLNLIQINMHIKDWLDRYERNTTWNFSMSDFETFSVSVKFTMTMFSLTPF
TVAFISFLLLIFSLQKHLQKMQLNYKGHRDPRTKVHTNALKIVISFLLFYASFFLCVLISWIS
ELYQNTVIYMLCETIGVFSPSSHSFLLILGNAKLRQAFLLVAAKVWAKR

>Q6GTX8 single-pass type I membrane protein
MSPHPTALLGLVLCLAQTIHTQEEDLPRPSISAEPGTVIPLGSHVTFVCRGPVGVQTFRLER
ESRSTYNDTEDVSQASPSESEARFRIDSVSEGNAGPYRCIYYKPPKWSEQSDYLELLVKET
SGGPDSPDTEPGSSAGPTQRPSDNSHNEHAPASQGLKAEHLYILIGVSVVFLFCLLLLVLFC
LHRQNQIKQGPPRSKDEEQKPQQRPDLAVDVLERTADKATVNGLPEKDRETDTSALAAGS
SQEVTYAQLDHWALTQRTARAVSPQSTKPMAESITYAAVARH

>Q10981 single-pass type II membrane protein
MLVVQMPFSFPMAHFILFVFTVSTIFHVQQRLAKIQAMWELPVQIPVLASTSKALGPSQLR
GMWTINAIGRLGNQMGEYATLYALAKMNGRPAFIPAQMHSTLAPIFRITLPVLHSATASRIP
WQNYHLNDWMEEEYRHIPGEYVRFTGYPCSWTFYHHLRQEILQEFTLHDHVREEAQKFL
RGLQVNGSRPGTFVGVHVRRGDYVHVMPKVWKGVVADRRYLQQALDWFRARYSSLIFV
VTSNGMAWCRENIDTSHGDVVFAGDGIEGSPAKDFALLTQCNHTIMTIGTFGIWAAYLTGG
DTIYLANYTLPDSPFLKIFKPEAAFLPEWTGIAADLSPLLKH

>P05187 GPI-anchor, lipid-anchor
MLGPCMLLLLLLLGLRLQLSLGIIPVEEENPDFWNREAAEALGAAKKLQPAQTAAKNLIIF
LGDGMGVSTVTAARILKGQKKDKLGPEIPLAMDRFPYVALSKTYNVDKHVPDSGATATAY
LCGVKGNFQTIGLSAAARFNQCNTTRGNEVISVMNRAKKAGKSVGVVTTTRVQHASPAG
TYAHTVNRNWYSDADVPASARQEGCQDIATQLISNMDIDVILGGGRKYMFRMGTPDPEY
PDDYSQGGTRLDGKNLVQEWLAKRQGARYVWNRTELMQASLDPSVTHLMGLFEPGDM
KYEIHRDSTLDPSLMEMTEAALRLLSRNPRGFFLFVEGGRIDHGHHESRAYRALTETIMFD
DAIERAGQLTSEEDTLSLVTADHSHVFSFGGYPLRGSSIFGLAPGKARDRKAYTVLLYGNG
PGYVLKDGARPDVTESESGSPEYRQQSAVPLDEETHAGEDVAVFARGPQAHLVHGVQEQT
FIAHVMAFAACLEPYTACDLAPPAGTTDAAHPGRSVVPALLPLLAGTLLLLETATAP

>P31751 peripheral membrane protein
MNEVSVIKEGWLHKRGEYIKTWRPRYFLLKSDGSFIGYKERPEAPDQTLPPLNNFSVAEC
QLMKTERPRPNTFVIRCLQWTTVIERTFHVDSPDEREEWMRAIQMVANSLKQRAPGEDP
MDYKCGSPSDSSTTEEMEVAVSKARAKVTMNDFDYLKLLGKGTFGKVILVREKATGRYY
AMKILRKEVIIAKDEVAHTVTESRVLQNTRHPFLTALKYAFQTHDRLCFVMEYANGGELFF
HLSRERVFTEERARFYGAEIVSALEYLHSRDVVYRDIKLENLMLDKDGHIKITDFGLCKEG
ISDGATMKTFCGTPEYLAPEVLEDNDYGRAVDWWGLGVVMYEMMCGRLPFYNQDHER
LFELILMEEIRFPRTLSPEAKSLLAGLLKKDPKQRLGGGPSDAKEVMEHRFFLSINWQDVV
QKKLLPPFKPQVTSEVDTRYFDDEFTAQSITITPPDRYDSLGLLELDQRTHFPQFSYSASIRE

>Q9Y2T6 multipass membrane protein
MSQQNTSGDCLFDGVNELMKTLQFAVHIPTFVLGLLLNLLAIHGFSTFLKNRWPDYAATSI

YMINLAVFDLLLVLSLPFKMVLSQVQSPFPSLCTLVECLYFVSMYGSVFTICFISMDRFLAIR
YPLLVSHLRSPRKIFGICCTIWVLVWTGSIPIYSFHGKVEKYMCFHNMSDDTWSAKVFFPL
EVFGFLLPMGIMGFCCSRSIHILLGRRDHTQDWVQQKACIYSIAASLAVFVVSFLPVHLGF
FLQFLVRNSFIVECRAKQSISFFLQLSMCFSNVNCCLDVFCYYFVIKEFRMNIRAHRPSRVQ
LVLQDTTISRG

>Q9Y5X1 peripheral membrane protein
MATKARVMYDFAAEPGNNELTVNEGEIITITNPDVGGGWLEGRNIKGERGLVPTDYVEILP
SDGKDQFSCGNSVADQAFLDSLSASTAQASSSAASNNHQVGSGNDPWSAWSASKSGNWE
SSEGWGAQPEGAGAQRNTNTPNNWDTAFGHPQAYQGPATGDDDDWDEDWDGPKSSSY
FKDSESADAGGAQRGNSRASSSSMKIPLNKFPGFAKPGTEQYLLAKQLAKPKEKIPIIVGD
YGPMWVYPTSTFDCVVADPRKGSKMYGLKSYIEYQLTPTNTNRSVNHRYKHFDWLYERL
LVKFGSAIPIPSLPDKQVTGRFEEEFIKMRMERLQAWMTRMCRHPVISESEVFQQFLNFRD
EKEWKTGKRKAERDELAGVMIFSTMEPEAPDLDLVEIEQKCEAVGKFTKAMDDGVKELL
TVGQEHWKRCTGPLPKEYQKIGKALQSLATVFSSSGYQGETDLNDAITEAGKTYEEIASLV
AEQPKKDLHFLMECNHEYKGFLGCFPDIIGTHKGAIEKVKESDKLVATSKITLQDKQNMV
KRVSIMSYALQAEMNHFHSNRIYDYNSVIRLYLEQQVQFYETIAEKLRQALSRFPVM

>O15427 multipass membrane protein
MGGAVVDEGPTGVKAPDGGWGWAVLFGCFVITGFSYAFPKAVSVFFKELIQEFGIGYSDT
AWISSILLAMLYGTGPLCSVCVNRFGCRPVMLVGGLFASLGMVAASFCRSIIQVYLTTGVIT
GLGLALNFQPSLIMLNRYFSKRRPMANGLAAAGSPVFLCALSPLGQLLQDRYGWRGGFLI
LGGLLLNCCVCAALMRPLVVTAQPGSGPPRPSRRLLDLSVFRDRGFVLYAVAASVMVLGL
FVPPVFVVSYAKDLGVPDTKAAFLLTILGFIDIFARPAAGFVAGLGKVRPYSVYLFSFSMFF
NGLADLAGSTAGDYGGLVVFCIFFGISYGMVGALQFEVLMAIVGTHKFSSAIGLVLLMEA
VAVLVGPPSGGKLLDATHVYMYVFILAGAEVLTSSLILLLGNFFCIRKKPKEPQPEVAAAEE
EKLHKPPADSGVDLREVEHFLKAEPEKNGEVVHTPETSV

>Q16602 multipass membrane protein
MEKKCTLNFLVLLPFFMILVTAELEESPEDSIQLGVTRNKIMTAQYECYQKIMQDPIQQAE
GVYCNRTWDGWLCWNDVAAGTESMQLCPDYFQDFDPSEKVTKICDQDGNWFRHPASNR
TWTNYTQCNVNTHEKVKTALNLFYLTIIGHGLSIASLLISLGIFFYFKSLSCQRITLHKNLFF
SFVCNSVVTIIHLTAVANNQALVATNPVSCKVSQFIHLYLMGCNYFWMLCEGIYLHTLIVVA
VFAEKQHLMWYYFLGWGFPLIPACIHAIARSLYYNDNCWISSDTHLLYIIHGPICAALLVNL
FFLLNIVRVLITKLKVTHQAESNLYMKAVRATLILVPLLGIEFVLIPWRPEGKIAEEVYDYIM
HILMHFQGLLVSTIFCFFNGEVQAILRRNWNQYKIQFGNSFSNSEALRSASYTVSTISDGPG
YSHDCPSEHLNGKSIHDIENVLLKPENLYN

>Q9H2K8 peripheral membrane protein
MRKGVLKDPEIADLFYKDDPEELFIGLHEIGHGSFGAVYFATNAHTSEVVAIKKMSYSGKQ
THEKWQDILKEVKFLRQLKHPNTIEYKGCYLKEHTAWLVMEYCLGSASDLLEVHKKPLQ
EVEIAAITHGALHGLAYLHSHALIHRDIKAGNILLTEPGQVKLADFGSASMASPANSFVGT
PYWMAPEVILAMDEGQYDGKVDIWSLGITCIELAERKPPLFNMNAMSALYHIAQNDSPTL
QSNEWTDSFRRFVDYCLQKIPQERPTSAELLRHDFVRRDRPLRVLIDLIQRTKDAVRELDN

LQYRKMKKILFQETRNGPLNESQEDEEDSEHGTSLNREMDSLGSNHSIPSMSVSTGSQSSS
VNSMQEVMDESSSELVMMHDDESTINSSSSVVHKKDHVFIRDEAGHGDPRPEPRPTQSVQ
SQALHYRNRERFATIKSASLVTRQIHEHEQENELREQMSGYKRMRRQHQKQLIALENKLK
AEMDEHRLKLQKEVETHANNSSIELEKLAKKQVAIIEKEAKVAAADEKKFQQQILAQQKK
DLTTFLESQKKQYKICKEKIKEEMNEDHSTPKKEKQERISKHKENLQHTQAEEEAHLLTQ
QRLYYDKNCRFFKRKIMIKRHEVEQQNIREELNKKRTQKEMEHAMLIRHDESTRELEYRQ
LHTLQKLRMDLIRLQHQTELENQLEYNKRRERELHRKHVMELRQQPKNLKAMEMQIKK
QFQDTCKVQTKQYKALKNHQLEVTPKNEHKTILKTLKDEQTRKLAILAEQYEQSINEMM
ASQALRLDEAQEAECQALRLQLQQEMELLNAYQSKIKMQTEAQHERELQKLEQRVSLRR
AHLEQKIEEELAALQKERSERIKNLLERQEREIETFDMESLRMGFGNLVTLDFPKEDYR

>Q9ULQ1 multipass membrane protein
MAVSLDDDVPLILTLDEGGSAPLAPSNGLGQEELPSKNGGSYAIHDSQAPSLSSGGESSPSS
PAHNWEMNYQEAAIYLQEGENNDKFFTHPKDAKALAAYLFAHNHLFYLMELATALLLLL
LSLCEAPAVPALRLGIYVHATLELFALMVVVFELCMKLRWLGLHTFIRHKRTMVKTSVLV
VQFVEAIVVLVRQMSHVRVTRALRCIFLVDCRYCGGVRRNLRQIFQSLPPFMDILLLLLFF
MIIFAILGFYLFSPNPSDPYFSTLENSIVSLFVLLTTANFPDVMMPSYSRNPWSCVFFIVYLSI
ELYFIMNLLLAVVFDTFNDIEKRKFKSLLLHKRTAIQHAYRLLISQRRPAGISYRQFEGLMR
FYKPRMSARERYLTFKALNQNNTPLLSLKDFYDIYEVAALKWKAKKNREHWFDELPRTA
LLIFKGINILVKSKAFQYFMYLVVAVNGVWILVETFMLKGGNFFSKHVPWSYLVFLTIYGV
ELFLKVAGLGPVEYLSSGWNLFDFSVTVFAFLGLLALALNMEPFYFIVVLRPLQLLRLFKL
KERYRNVLDTMFELLPRMASLGLTLLIFYYSFAIVGMEFFCGIVFPNCCNTSTVADAYRWR
NHTVGNRTVVEEGYYYLNNFDNILNSFVTLFELTVVNNWYIIMEGVTSQTSHWSRLYFMT
FYIVTMVVMTIIVAFILEAFVFRMNYSRKNQDSEVDGGITLEKEISKEELVAVLELYREARG
ASSDVTRLLETLSQMERYQQHSMVFLGRRSRTKSDLSLKMYQEEIQEWYEEHAREQEQQ
RQLSSSAAPAAQQPPGSRQRSQTVT

>Q86SQ6 multipass membrane protein
MDLKTVLSLPRYPGEFLHPVVYACTAVMLLCLLASFVTYIVHQSAIRISRKGRHTLLNFCF
HAALTFTVFAGGINRTKYPILCQAVGIVLHYSTLSTMLWIGVTARNIYKQVTKKAPLCLDT
DQPPYPRQPLLRFYLVSGGVPFIICGVTAATNIRNYGTEDEDTAYCWMAWEPSLGAFYGPA
AIITLVTCVYFLGTYVQLRRHPGRRYELRTQPEEQRRLATPEGGRGIRPGTPPAHDAPGASV
LQNEHSFQAQLRAAAFTLFLFTATWAFGALAVSQGHFLDMVFSCLYGAFCVTLGLFVLIH
HCAKREDVWQCWWACCPPRKDAHPALDANGAALGRAACLHSPGLGQPRGFAHPPGPCK
MTNLQAAQGHASCLSPATPCCAKMHCEPLTADEAHVHLQEEGAFGHDPHLHGCLQGRTK
PPYFSRHPAEEPEYAYHIPSSLDGSPRSSRTDSPPSSLDGPAGTHTLACCTQGDPFPMVTQPE
GSDGSPALYSCPTQPGREAALGPGHLEMLRRTQSLPFGGPSQNGLPKGKLLEGLPFGTDGT
GNIRTGPWKNETTV

>Q14435 single-pass type II membrane protein
MAHLKRLVKLHIKRHYHKKFWKLGAVIFFFIIVLVLMQREVSVQYSKEESRMERNMKNK
NKMLDLMLEAVNNIKDAMPKMQIGAPVRQNIDAGERPCLQGYYTAAELKPVLDRPPQDS
NAPGASGKAFKTTNLSVEEQKEKERGEAKHCFNAFASDRISLHRDLGPDTRPPECIEQKFK
RCPPLPTTSVIIVFHNEAWSTLLRTVHSVLYSSPAILLKEIILVDDASVDEYLHDKLDEYVKQ

FSIVKIVRQRERKGLITARLLGATVATAETLTFLDAHCECFYGWLEPLLARIAENYTAVVSP
DIASIDLNTFEFNKPSPYGSNHNRGNFDWSLSFGWESLPDHEKQRRKDETYPIKTPTFAGG
LFSISKEYFEYIGSYDEEMEIWGGENIEMSFRVWQCGGQLEIMPCSVVGHVFRSKSPHSFP
KGTQVIARNQVRLAEVWMDEYKEIFYRRNTDAAKIVKQKAFGDLSKRFEIKHRLQCKNF
TWYLNNIYPEVYVPDLNPVISGYIKSVGQPLCLDVGENNQGGKPLIMYTCHGLGGNQYFE
YSAQHEIRHNIQKELCLHAAQGLVQLKACTYKGHKTVVTGEQIWEIQKDQLLYNPFLKM
CLSANGEHPSLVSCNPSDPLQKWILSQND

>P46977 multipass membrane protein
MTKFGFLRLSYEKQDTLLKLLILSMAAVLSFSTRLFAVLRFESVIHEFDPYFNYRTTRFLAE
EGFYKFHNWFDDRAWYPLGRIIGGTIYPGLMITSAAIYHVLHFFHITIDIRNVCVFLAPLFSS
FTTIVTYHLTKELKDAGAGLLAAAMIAVVPGYISRSVAGSYDNEGIAIFCMLLTYYMWIKA
VKTGSICWAAKCALAYFYMVSSWGGYVFLINLIPLHVLVLMLTGRFSHRIYVAYCTVYCL
GTILSMQISFVGFQPVLSSEHMAAFGVFGLCQIHAFVDYLRSKLNPQQFEVLFRSVISLVGF
VLLTVGALLMLTGKISPWTGRFYSLLDPSYAKNNIPIIASVSEHQPTTWSSYYFDLQLLVFM
FPVGLYYCFSNLSDARIFIIMYGVTSMYFSAVMVRLMLVLAPVMCILSGIGVSQVLSTYMK
NLDISRPDKKSKKQQDSTYPIKNEVASGMILVMAFFLITYTFHSTWVTSEAYSSPSIVLSAR
GGDGSRIIFDDFREAYYWLRHNTPEDAKVMSWWDYGYQITAMANRTILVDNNTWNNTHI
SRVGQAMASTEEKAYEIMRELDVSYVLVIFGGLTGYSSDDINKFLWMVRIGGSTDTGKHI
KENDYYTPTGEFRVDREGSPVLLNCLMYKMCYYRFGQVYTEAKRPPGFDRVRNAEIGNK
DFELDVLEEAYTTEHWLVRIYKVKDLDNRGLSRT

>P40259 single-pass type I membrane protein
MARLALSPVPSHWMVALLLLLSAEPVPAARSEDRYRNPKGSACSRIWQSPRFIARKRGFT
VKMHCYMNSASGNVSWLWKQEMDENPQQLKLEKGRMEESQNESLATLTIQGIRFEDNGI
YFCQQKCNNTSEVYQGCGTELRVMGFSTLAQLKQRNTLKDGIIMIQTLLIILFIIVPIFLLLD
KDDSKAGMEEDHTYEGLDIDQTATYEDIVTLRTGEVKWSVGEHPGQE

>P34947 peripheral membrane protein
MELENIVANTVLLKAREGGGGKRKGKSKKWKEILKFPHISQCEDLRRTIDRDYCSLCDKQ
PIGRLLFRQFCETRPGLECYIQFLDSVAEYEVTPDEKLGEKGKEIMTKYLTPKSPVFIAQVG
QDLVSQTEEKLLQKPCKELFSACAQSVHEYLRGEPFHEYLDSMFFDRFLQWKWLERQPV
TKNTFRQYRVLGKGGFGEVCACQVRATGKMYACKRLEKKRIKKRKGESMALNEKQILEK
VNSQFVVNLAYAYETKDALCLVLTIMNGGDLKFHIYNMGNPGFEEERALFYAAEILCGLE
DLHRENTVYRDLKPENILLDDYGHIRISDLGLAVKIPEGDLIRGRVGTVGYMAPEVLNNQR
YGLSPDYWGLGCLIYEMIEGQSPFRGRKEKVKREEVDRRVLETEEVYSHKFSEEAKSICK
MLLTKDAKQRLGCQEEGAAEVKRHPFFRNMNFKRLEAGMLDPPFVPDPRAVYCKDVLDI
EQFSTVKGVNLDHTDDDFYSKFSTGSVSIPWQNEMIETECFKELNVFGPNGTLPPDLNRN
HPPEPPKKGLLQRLFKRQHQNNSKSSPSSKTSFNHHINSNHVSSNSTGSS

>Q13607 multipass membrane protein
MGTDNQTWVSEFILLGLSSDWDTRVSLFVLFLVMYVVTVLGNCLIVLLIRLDSRLHTPMY
FFLTNLSLVDVSYATSVVPQLLAHFLAEHKAIPFQSCAAQLFFSLALGGIEFVLLAVMAYDR
YVAVCDALRYSAIMHGGLCARLAITSWVSGFISSPVQTAITFQLPMCRNKFIDHISCELLAV

VRLACVDTSSNEVTIMVSSIVLLMTPFCLVLLSYIQIISTILKIQSREGRKKAFHTCASHLTV
VALCYGVAIFTYIQPHSSPSVLQEKLFSVFYAILTPMLNPMIYSLRNKEVKGAWQKLLWKF
SGLTSKLAT

>O15197 single-pass type I membrane protein
MATEGAAQLGNRVAGMVCSLWVLLLVSSVLALEEVLLDTTGETSEIGWLTYPPGGWDEV
SVLDDQRRLTRTFEACHVAGAPPGTGQDNWLQTHFVERRGAQRAHIRLHFSVRACSSLG
VSGGTCRETFTLYYRQAEEPDSPDSVSSWHLKRWTKVDTIAADESFPSSSSSSSSSSAAWA
VGPHGAGQRAGLQLNVKERSFGPLTQRGFYVAFQDTGACLALVAVRLFSYTCPAVLRSFA
SFPETQASGAGGASLVAAVGTCVAHAEPEEDGVGGQAGGSPPRLHCNGEGKWMVAVGGC
RCQPGYQPARGDKACQACPRGLYKSSAGNAPCSPCPARSHAPNPAAPVCPCLEGFYRASS
DPPEAPCTGPPSAPQELWFEVQGSALMLHWRLPRELGGRGDLLFNVVCKECEGRQEPAS
GGGGTCHRCRDEVHFDPRQRGLTESRVLVGGLRAHVPYILEVQAVNGVSELSPDPPQAAA
INVSTSHEVPSAVPVVHQVSRASNSITVSWPQPDQTNGNILDYQLRYYDQAEDESHSFTLT
SETNTATVTQLSPGHIYGFQVRARTAAGHGPYGGKVYFQTLPQGELSSQLPERLSLVIGSIL
GALAFLLLAAITVLAVVFQRKRRGTGYTEQLQQYSSPGLGVKYYIDPSTYEDPCQAIRELA
REVDPAYIKIEEVIGTGSFGEVRQGRLQPRGRREQTVAIQALWAGGAESLQMTFLGRAAVL
GQFQHPNILRLEGVVTKSRPLMVLTEFMELGPLDSFLRQREGQFSSLQLVAMQRGVAAAM
QYLSSFAFVHRSLSAHSVLVNSHLVCKVARLGHSPQGPSCLLRWAAPEVIAHGKHTTSSDV
WSFGILMWEVMSYGERPYWDMSEQEVLNAIEQEFRLPPPPGCPPGLHLLMLDTWQKDR
ARRPHFDQLVAAFDKMIRKPDTLQAGGDPGERPSQALLTPVALDFPCLDSPQAWLSAIGLE
CYQDNFSKFGLCTFSDVAQLSLEDLPALGITLAGHQKKLLHHIQLLQQHLRQQGSVEV

>P09172 single-pass type II membrane protein
MPALSRWASLPGPSMREAAFMYSTAVAIFLVILVAALQGSAPRESPLPYHIPLDPEGSLELS
WNVSYTQEAIHFQLLVRRLKAGVLFGMSDRGELENADLVVLWTDGDTAYFADAWSDQK
GQIHLDPQQDYQLLQVQRTPEGLTLLFKRPFGTCDPKDYLIEDGTVHLVYGILEEPFRSLEA
INGSGLQMGLQRVQLLKPNIPEPELPSDACTMEVQAPNIQIPSQETTYWCYIKELPKGFSRH
HIIKYEPIVTKGNEALVHHMEVFQCAPEMDSVPHFSGPCDSKMKPDRLNYCRHVLAAWA
LGAKAFYYPEEAGLAFGGPGSSRYLRLEVHYHNPLVIEGRNDSSGIRLYYTAKLRRFNAGI
MELGLVYTPVMAIPPRETAFILTGYCTDKCTQLALPPSGIHIFASQLHTHLTGRKVVTVLVR
DGREWEIVNQDNHYSPHFQEIRMLKKVVSVHPGDVLITSCTYNTEDRELATVGGFGILEE
MCVNYVHYYPQTQLELCKSAVDAGFLQKYFHLINRFNNEDVCTCPQASVSQQFTSVPWN
SFNRDVLKALYSFAPISMHCNKSSAVRFQGEWNLQPLPKVISTLEEPTPQCPTSQGRSPAGP
TVVSIGGGKG

>Q96PR1 multipass membrane protein
MGKIENNERVILNVGGTRHETYRSTLKTLPGTRLALLASSEPPGDCLTTAGDKLQPSPPPLS
PPPRAPPLSPGPGGCFEGGAGNCSSRGGRASDHPGGGREFFFDRHPGVFAYVLNYYRTGK
LHCPADVCGPLFEEELAFWGIDETDVEPCCWMTYRQHRDAEEALDIFETPDLIGGDPGDD
EDLAAKRLGIEDAAGLGGPDGKSGRWRRLQPRMWALFEDPYSSRAARFIAFASLFFILVSI
TTFCLETHEAFNIVKNKTEPVINGTSVVLQYEIETDPALTYVEGVCVVWFTFEFLVRIVFSP
NKLEFIKNLLNIIDFVAILPFYLEVGLSGLSSKAAKDVLGFLRVVRFVRILRIFKLTRHFVGL
RVLGHTLRASTNEFLLLIIFLALGVLIFATMIYYAERVGAQPNDPSASEHTQFKNIPIGFWWA

VVTMTTLGYGDMYPQTWSGMLVGALCALAGVLTIAMPVPVIVNNFGMYYSLAMAKQK
LPRKRKKHIPPAPQASSPTFCKTELNMACNSTQSDTCLGKDNRLLEHNRSVLSGDDSTGSE
PPLSPPERLPIRRSSTRDKNRRGETCFLLTTGDYTCASDGGIRKGYEKSRSLNNIAGLAGNA
LRLSPVTSPYNSPCPLRRSRSPIPSIL

>P23471 single-pass type I membrane protein
MRILKRFLACIQLLCVCRLDWANGYYRQQRKLVEEIGWSYTGALNQKNWGKKYPTCNSP
KQSPINIDEDLTQVNVNLKKLKFQGWDKTSLENTFIHNTGKTVEINLTNDYRVSGGVSEM
VFKASKITFHWGKCNMSSDGSEHSLEGQKFPLEMQIYCFDADRFSSFEEAVKGKGKLRAL
SILFEVGTEENLDFKAIIDGVESVSRFGKQAALDPFILLNLLPNSTDKYYIYNGSLTSPPCTD
TVDWIVFKDTVSISESQLAVFCEVLTMQQSGYVMLMDYLQNNFREQQYKFSRQVFSSYT
GKEEIHEAVCSSEPENVQADPENYTSLLVTWERPRVVYDTMIEKFAVLYQQLDGEDQTKH
EFLTDGYQDLGAILNNLLPNMSYVLQIVAICTNGLYGKYSDQLIVDMPTDNPELDLFPELI
GTEEIIKEEEEGKDIEEGAIVNPGRDSATNQIRKKEPQISTTTHYNRIGTKYNEAKTNRSPTR
GSEFSGKGDVPNTSLNSTSQPVTKLATEKDISLTSQTVTELPPHTVEGTSASLNDGSKTVLR
SPHMNLSGTAESLNTVSITEYEEESLLTSFKLDTGAEDSSGSSPATSAIPFISENISQGYIFSSE
NPETITYDVLIPESARNASEDSTSSGSEESLKDPSMEGNVWFPSSTDITAQPDVGSGRESFL
QTNYTEIRVDESEKTTKSFSAGPVMSQGPSVTDLEMPHYSTFAYFPTEVTPHAFTPSSRQQ
DLVSTVNVVYSQTTQPVYNGETPLQPSYSSEVFPLVTPLLLDNQILNTTPAASSSDSALHAT
PVFPSVDVSFESILSSYDGAPLLPFSSASFSSELFRHLHTVSQILPQVTSATESDKVPLHASLP
VAGGDLLLEPSLAQYSDVLSTTHAASETLEFGSESGVLYKTLMFSQVEPPSSDAMMHARS
SGPEPSYALSDNEGSQHIFTVSYSSAIPVHDSVGVTYQGSLFSGPSHIPIPKSSLITPTASLLQ
PTHALSGDGEWSGASSDSEFLLPDTDGLTALNISSPVSVAEFTYTTSVFGDDNKALSKSEII
YGNETELQIPSFNEMVYPSESTVMPNMYDNVNKLNASLQETSVSISSTKGMFPGSLAHTT
TKVFDHEISQVPENNFSVQPTHTVSQASGDTSLKPVLSANSEPASSDPASSEMLSPSTQLLF
YETSASFSTEVLLQPSFQASDVDTLLKTVLPAVPSDPILVETPKVDKISSTMLHLIVSNSASS
ENMLHSTSVPVFDVSPTSHMHSASLQGLTISYASEKYEPVLLKSESSHQVVPSLYSNDELF
QTANLEINQAHPPKGRHVFATPVLSIDEPLNTLINKLIHSDEILTSTKSSVTGKVFAGIPTVAS
DTFVSTDHSVPIGNGHVAITAVSPHRDGSVTSTKLLFPSKATSELSHSAKSDAGLVGGGED
GDTDDDGDDDDDDRGSDGLSIHKCMSCSSYRESQEKVMNDSDTHENSLMDQNNPISYSL
SENSEEDNRVTSVSSDSQTGMDRSPGKSPSANGLSQKHNDGKEENDIQTGSALLPLSPESK
AWAVLTSDEESGSGQGTSDSLNENETSTDFSFADTNEKDADGILAAGDSEITPGFPQSPTSS
VTSENSEVFHVSEAEASNSSHESRIGLAEGLESEKKAVIPLVIVSALTFICLVVLVGILIYWR
KCFQTAHFYLEDSTSPRVISTPPTPIFPISDDVGAIPIKHFPKHVADLHASSGFTEEFETLKEF
YQEVQSCTVDLGITADSSNHPDNKHKNRYINIVAYDHSRVKLAQLAEKDGKLTDYINANY
VDGYNRPKAYIAAQGPLKSTAEDFWRMIWEHNVEVIVMITNLVEKGRRKCDQYWPADGS
EEYGNFLVTQKSVQVLAYYTVRNFTLRNTKIKKGSQKGRPSGRVVTQYHYTQWPDMGV
PEYSLPVLTFVRKAAYAKRHAVGPVVVHCSAGVGRTGTYIVLDSMLQQIQHEGTVNIFGF
LKHIRSQRNYLVQTEEQYVFIHDTLVEAILSKETEVLDSHIHAYVNALLIPGPAGKTKLEKQ
FQLLSQSNIQQSDYSAALKQCNREKNRTSSIIPVERSRVGISSLSGEGTDYINASYIMGYYQ
SNEFIITQHPLLHTIKDFWRMIWDHNAQLVVMIPDGQNMAEDEFVYWPNKDEPINCESFK
VTLMAEEHKCLSNEEKLIIQDFILEATQDDYVLEVRHFQCPKWPNPDSPISKTFELISVIKEE
AANRDGPMIVHDEHGGVTAGTFCALTTLMHQLEKENSVDVYQVAKMINLMRPGVFADIE
QYQFLYKVILSLVSTRQEENPSTSLDSNGAALPDGNIAESLESLV

>Q96T51 peripheral membrane protein
MADREGGCAAGRGRELEPELEPGPGPGSALEPGEEFEIVDRSQLPGPGDLRSATRPRAAEG
WSAPILTLARRATGNLSASCGSALRAAAGLGGGDSGDGTARAASKCQMMEERANLMHM
MKLSIKVLLQSALSLGRSLDADHAPLQQFFVVMEHCLKHGLKVKKSFIGQNKSFFGPLEL
VEKLCPEASDIATSVRNLPELKTAVGRGRAWLYLALMQKKLADYLKVLIDNKHLLSEFYE
PEALMMEEEGMVIVGLLVGLNVLDANLCLKGEDLDSQVGVIDFSLYLKDVQDLDGGKEH
ERITDVLDQKNYVEELNRHLSCTVGDLQTKIDGLEKTNSKLQEELSAATDRICSLQEEQQQ
LREQNELIRERSEKSVEITKQDTKVELETYKQTRQGLDEMYSDVWKQLKEEKKVRLELE
KELELQIGMKTEMEIAMKLLEKDTHEKQDTLVALRQQLEEVKAINLQMFHKAQNAESSL
QQKNEAITSFEGKTNQVMSSMKQMEERLQHSERARQGAEERSHKLQQELGGRIGALQLQ
LSQLHEQCSSLEKELKSEKEQRQALQRELQHEKDTSSLLRMELQQVEGLKKELRELQDEK
AELQKICEEQEQALQEMGLHLSQSKLKMEDIKEVNQALKGHAWLKDDEATHCRQCEKEF
SISRRKHHCRNCGHIFCNTCSSNELALPSYPKPVRVCDSCHTLLLQRCSSTAS

>O43747 peripheral membrane protein
MPAPIRLRELIRTIRTARTQAEEREMIQKECAAIRSSFREEDNTYRCRNVAKLLYMHMLGYP
AHFGQLECLKLIASQKFTDKRIGYLGAMLLLDERQDVHLLMTNCIKNDLNHSTQFVQGL
ALCTLGCMGSSEMCRDLAGEVEKLLKTSNSYLRKKAALCAVHVIRKVPELMEMFLPATK
NLLNEKNHGVLHTSVVLLTEMCERSPDMLAHFRKLVPQLVRILKNLIMSGYSPEHDVSGIS
DPFLQVRILRLLRILGRNDDDSSEAMNDILAQVATNTETSKNVGNAILYETVLTIMDIKSES
GLRVLAINILGRFLLNNDKNIRYVALTSLLKTVQTDHNAVQRHRSTIVDCLKDLDVSIKRR
AMELSFALVNGNNIRGMMKELLYFLDSCEPEFKADCASGIFLAAEKYAPSKRWHIDTIMR
VLTTAGSYVRDDAVPNLIQLITNSVEMHAYTVQRLYKAILGDYSQQPLVQVAAWCIGEYG
DLLVSGQCEEEEPIQVTEDEVLDILESVLISNMSTSVTRGYALTAIMKLSTRFTCTVNRIKK
VVSIYGSSIDVELQQRAVEYNALFKKYDHMRSALLERMPVMEKVTTNGPTEIVQTNGETE
PAPLETKPPPSGPQPTSQANDLLDLLGGNDITPVIPTAPTSKPSSAGGELLDLLGDINLTGAP
AAAPAPASVPQISQPPFLLDGLSSQPLFNDIAAGIPSITAYSKNGLKIEFTFERSNTNPSVTVIT
IQASNSTELDMTDFVFQAAVPKTFQLQLLSPSSSIVPAFNTGTITQVIKVLNPQKQQLRMRI
KLTYNHKGSAMQDLAEVNNFPPQSWQ

>Q96P48 peripheral membrane protein
MAEAGDAALSVAEWLRALHLEQYTGLFEQHGLVWATECQGLSDTRLMDMGMLLPGHR
RRILAGLLRAHTSPAPAPRPTPRPVPMKRHIFRSPPVPATPPEPLPTTTEDEGLPAAPPIPPRR
SCLPPTCFTTPSTAAPDPVLPPLPAKRHLAELSVPPVPPRTGPPRLLVSLPTKEEESLLPSLSS
PPQPQSEEPLSTLPQGPPQPPSPPPCPPEIPPKPVRLFPEFDDSDYDEVPEEGPGAPARVMTK
KEEPPPSRVPRAVRVASLLSEGEELSGDDQGDEEEDDHAYEGVPNGGWHTSSLSLSLPSTIA
APHPMDGPPGGSTPVTPVIKAGWLDKNPPQGSYIYQKRWVRLDTDHLRYFDSNKDAYSK
RFISVACISHVAAIGDQKFEVITNNRTFAFRAESDVERKEWMQALQQAMAEQRARARLSS
AYLLGVPGSEQPDRAGSLELRGFKNKLYVAVVGDKVQLYKNLEEYHLGIGITFIDMSVGN
VKEVDRRSFDLTTPYRIFSFSADSELEKEQWLEAMQGAIAEALSTSEVAERIWAAAPNRFC
ADCGAPQPDWASINLCVVICKRCAGEHRGLGAGVSKVRSLKMDRKVWTETLIELFLQLG
NGAGNRFWAANVPPSEALQPSSSPSTRRCHLEAKYREGKYRRYHPLFGNQEELDKALCA
AVTTTDLAETQALLGCGAGINCFSGDPEAPTPLALAEQAGQTLQMEFLRNNRTTEVPRLD

SMKPLEKHYSVVLPTVSHSGFLYKTASAGKLLQDRRAREEFSRRWCVLGDGVLSYFENER
AVTPNGEIRASEIVCLAVPPPDTHGFEHTFEVYTEGERLYLFGLESAEQAHEWVKCIAKAF
VPPLAEDLLARDFERLGRLPYKAGLSLQRAQEGWFSLSGSELRAVFPEGPCEEPLQLRKLQ
ELSIQGDSENQVLVLVERRRTLYIQGERRLDFMGWLGAIQKAAASMGDTLSEQQLGDSDI
PVIVYRCVDYITQCGLTSEGIYRKCGQTSKTQRLLESLRQDARSVHLKEGEQHVDDVSSA
LKRFLRDLPDGLFTRAQRLTWLEASEIEDEEEKVSRYRELLVRLPPVNRATVKALISHLYC
VQCFSDTNQMNVHNLAIVFGPTLFQTDGQDYKAGRVVEDLINHYVVVFSVDEEELRKQR
EEITAIVKMRVAGTASGTQHAGDFICTVYLEEKKAETEQHIKVPASMTAEELTLEILDRRNV
GIREKDYWTCFEVNEREEAERPLHFAEKVLPILHGLGTDSHLVVKKHQAMEAMLLYLASR
VGDTKHGMMKFREDRSLLGLGLPSGGFHDRYFILNSSCLRLYKEVRSQRPWSGAPETSHR
PEKEWPIKSLKVYLGVKKKLRPPTCWGFTVVHETEKHEKQQWYLCCDTQMELREWFAT
FLFVQHDGLVWPSEPSRVSRAVPEVRLGSVSLIPLRGSENEMRRSVAAFTADPLSLLRNV

>P17677 peripheral membrane protein
MLCCMRRTKQVEKNDDDQKIEQDGIKPEDKAHKAATKIQASFRGHITRKKLKGEKKDDV
QAAEAEANKKDEAPVADGVEKKGEGTTTAEAAPATGSKPDEPGKAGETPSEEKKGEGDA
ATEQAAPQAPASSEEKAGSAETESATKASTDNSPSSKAEDAPAKEEPKQADVPAAVTAAAA
TTPAAEDAAAKATAQPPTETGESSQAEENIEAVDETKPKESARQDEGKEEEPEADQEHA

>Q969X5 multipass membrane protein
MPFDFRRFDIYRKVPKDLTQPTYTGAIISICCCLFILFLFLSELTGFITTEVVNELYVDDPDKD
SGGKIDVSLNISLPNLHCELVGLDIQDEMGRHEVGHIDNSMKIPLNNGAGCRFEGQFSINK
VPGNFHVSTHSATAQPQNPDMTHVIHKLSFGDTLQVQNIHGAFNALGGADRLTSNPLASH
DYILKIVPTVYEDKSGKQRYSYQYTVANKEYVAYSHTGRIIPAIWFRYDLSPITVKYTERRQ
PLYRFITTICAIIGGTFTVAGILDSCIFTASEAWKKIQLGKMH

>Q8TB61 multipass membrane protein
MDARWWAVVVLAAFPSLGAGGETPEAPPESWTQLWFFRFVVNAAGYASFMVPGYLLVQ
YFRRKNYLETGRGLCFPLVKACVFGNEPKASDEVPLAPRTEAAETTPMWQALKLLFCATG
LQVSYLTWGVLQERVMTRSYGATATSPGERFTDSQFLVLMNRVLALIVAGLSCVLCKQPR
HGAPMYRYSFASLSNVLSSWCQYEALKFVSFPTQVLAKASKVIPVMLMGKLVSRRSYEH
WEYLTATLISIGVSMFLLSSGPEPRSSPATTLSGLILLAGYIAFDSFTSNWQDALFAYKMSSV
QMMFGVNFFSCLFTVGSLLEQGALLEGTRFMGRHSEFAAHALLLSICSACGQLFIFYTIGQ
FGAAVFTIIMTLRQAFAILLSCLLYGHTVTVVGGLGVAVVFAALLLRVYARGRLKQRGKKA
VPVESPVQKV

>P09543 lipid-anchor
MNRGFSRKSHTFLPKIFFRKMSSSGAKDKPELQFPFLQDEDTVATLLECKTLFILRGLPGSG
KSTLARVIVDKYRDGTKMVSADAYKITPGARGAFSEEYKRLDEDLAAYCRRRDIRILVLD
DTNHERERLEQLFEMADQYQYQVVLVEPKTAWRLDCAQLKEKNQWQLSADDLKKLKP
GLEKDFLPLYFGWFLTKKSSETLRKAGQVFLEELGNHKAFKKELRQFVPGDEPREKMDLV
TYFGKRPPGVLHCTTKFCDYGKAPGAEEYAQQDVLKKSYSKAFTLTISALFVTPKTTGAR
VELSEQQLQLWPSDVDKLSPTDNLPRGSRAHITLGCAADVEAVQTGLDLLEILRQEKGGS
RGEEVGELSRGKLYSLGNGRWMLTLAKNMEVRAIFTGYYGKGKPVPTQGSRKGGALQS

CTII

>Q86SQ0 peripheral membrane protein
MEEHSYIQKELDLQNGSLEEDSVVHSVENDSQNMMESLSPKKYSSSLRFKANGDYSGSYL
TLSQPVPAKRSPSPLGTSVRSSPSLAKIQGSKQFSYDGTDKNIPMKPPTPLLNTTSSLSGYPL
GRADFDHYTGRDSERALRLSEKPPYSKYSSRHKSHDNVYSLGGLEGRKASGSLLAMWN
GSSLSDAGPPPISRSGAASMPSSPKQARKMSIQDSLALQPKLTRHKELASENINLRTRKYSS
SSLSHMGAYSRSLPRLYRATENQLTPLSLPPRNSLGNSKRTKLGEKDLPHSVIDNDNYLNFS
SLSSGALPYKTSASEGNPYVSSTLSVPASPRVARKMLLASTSSCASDDFDQASYVGTNPSH
SLLAGESDRVFATRRNFSCGSVEFDEADLESLRQASGTPQPALRERKSSISISGRDDLMDY
HRRQREERLREQEMERLERQRLETILSLCAEYTKPDSRLSTGTTVEDVQKINKELEKLQLS
DEESVFEEALMSPDTRYRCHRKDSLPDADLASCGSLSQSSASFFTPRSTRNDELLSDLTRTP
PPPSSTFPKASSESSYLSILPKTPEGISEEQRSQELAAMEETRIVILNNLEELKQKIKDINDQM
DESFRELDMECALLDGEQKSETTELMKEKEILDHLNRKIAELEKNIVGEKTKEKVKLDAE
REKLERLQELYSEQKTQLDNCPESMREQLQQQLKRDADLLDVESKHFEDLEFQQLEHESR
LDEEKENLTQQLLREVAEYQRNIVSRKEKISALKKQANHIVQQAQREQDHFVKEKNNLIM
MLQREKENLCNLEKKYSSLSGGKGFPVNPNTLKEGYISVNEINEPCGNSTNLSPSTQFPAD
ADAVATEPATAVLASQPQSKEHFRSLEERKKQHKEGLYLSDTLPRKKTTSSISPHFSSATMG
RSITPKAHLPLGQSNSCGSVLPPSLAAMAKDSESRRMLRGYNHQQMSEGHRQKSEFYNRT
ASESNVYLNSFHYPDHSYKDQAFDTLSLDSSDSMETSISACSPDNISSASTSNIARIEEMER
LLKQAHAEKTRLLESREREMEAKKRALEEEKRRREILEKRLQEETSQRQKLIEKEVKIRER
QRAQARPLTRYLPVRKEDFDLRSHVETAGHNIDTCYHVSITEKTCRGFLIKMGGKIKTWK
KRWFVFDRNKRTFSYYADKHETKLKGVIYFQAIEEVYYDHLKNANKSPNPLLTFSVKTHD
RIYYMVAPSPEAMRIWMDVIVTGAEGYTHFLL

>Q14C86 peripheral membrane protein
MVKLDIHTLAHHLKQERLYVNSEKQLIQRLNADVLKTAEKLYRTAWIAKQQRINLDRLIIT
SAEASPAECCQHAKILEDTQFVDGYKQLGFQETAYGEFLSRLRENPRLIASSLVAGEKLNQ
ENTQSVIYTVFTSLYGNCIMQEDESYLLQVLRYLIEFELKESDNPRRLLRRGTCAFSILFKLF
SEGLFSAKLFLTATLHEPIMQLLVEDEDHLETDPNKLIERFSPSQQEKLFGEKGSDRFRQKV
QEMVESNEAKLVALVNKFIGYLKQNTYCFPHSLRWIVSQMYKTLSCVDRLEVGEVRAMC
TDLLLACFICPAVVNPEQYGIISDAPINEVARFNLMQVGRLLQQLAMTGSEEGDPRTKSSLG
KFDKSCVAAFLDVVIGGRAVETPPLSSVNLLEGLSRTVVYITYSQLITLVNFMKSVMSGDQ
LREDRMALDNLLANLPPAKPGKSSSLEMTPYNTPQLSPATTPANKKNRLPIATRSRSRTNM
LMDLHMDHEGSSQETIQEVQPEEVLVISLGTGPQLTPGMMSENEVLNMQLSDGGQGDVP
VDENKLHGKPDKTLRFSLCSDNLEGISEGPSNRSNSVSSLDLEGESVSELGAGPSGSNGVE
ALQLLEHEQATTQDNLDDKLRKFEIRDMMGLTDDRDISETVSETWSTDVLGSDFDPNIDE
DRLQEIAGAAAENMLGSLLCLPGSGSVLLDPCTGSTISETTSEAWSVEVLPSDSEAPDLKQ
EERLQELESCSGLGSTSDDTDVREVSSRPSTPGLSVVSGISATSEDIPNKIEDLRSECSSDFG
GKDSVTSPDMDEITHGAHQLTSPPSQSESLLAMFDPLSSHEGASAVVRPKVHYARPSHPPP
DPPILEGAVGGNEARLPNFGSHVLTPAEMEAFKQRHSYPERLVRSRSSDIVSSVRRPMSDPS
WNRRPGNEERELPPAAAIGATSLVAAPHSSSSSPSKDSSRGETEERKDSDDEKSDRNRPWW
RKRFVSAMPKAPIPFRKKEKQEKDKDDLGPDRFSTLTDDPSPRLSAQAQVAEDILDKYRN
AIKRTSPSDGAMANYESTGDNHDRDLSSKLLYHSDKEVMGDGESAHDSPRDEALQNISA

DDLPDSASQAAHPQDSAFSYRDAKKKLRLALCSADSVAFPVLTHSTRNGLPDHTDPEDNE
IVCFLKVQIAEAINLQDKNLMAQLQETMRCVCRFDNRTCRKLLASIAEDYRKRAPYIAYLT
RCRQGLQTTQAHLERLLQRVLRDKEVANRYFTTVCVRLLLESKEKKIREFIQDFQKLTAAD
DKTAQVEDFLQFLYGAMAQDVIWQNASEEQLQDAQLAIERSVMNRIFKLAFYPNQDGDI
LRDQVLHEHIQRLSKVVTANHRALQIPEVYLREAPWPSAQSEIRTISAYKTPRDKVQCILR
MCSTIMNLLSLANEDSVPGADDFVPVLVFVLIKANPPCLLSTVQYISSFYASCLSGEESYW
WMQFTAAVEFIKTIDDRK

>Q9BRT3 lipid-anchor
MSGEPGQTSVAPPPEEVEPGSGVRIVVEYCEPCGFEATYLELASAVKEQYPGIEIESRLGGT
GAFEIEINGQLVFSKLENGGFPYEKDLIEAIRRASNGETLEKITNSRPPCVIL

>Q02641 peripheral membrane protein
MVQKTSMSRGPYPPSQEIPMEVFDPSPQGKYSKRKGRFKRSDGSTSSDTTSNSFVRQGSA
ESYTSRPSDSDVSLEEDREALRKEAERQALAQLEKAKTKPVAFAVRTNVGYNPSPGDEVP
VQGVAITFEPKDFLHIKEKYNNDWWIGRLVKEGCEVGFIPSPVKLDSLRLLQEQKLRQNR
LGSSKSGDNSSSSLGDVVTGTRRPTPPASAKQKQKSTEHVPPYDVVPSMRPIILVGPSLKG
YEVTDMMQKALFDFLKHRFDGRISITRVTADISLAKRSVLNNPSKHIIIERSNTRSSLAEVQ
SEIERIFELARTLQLVALDADTINHPAQLSKTSLAPIIVYIKITSPKVLQRLIKSRGKSQSKHL
NVQIAASEKLAQCPPEMFDIILDENQLEDACEHLAEYLEAYWKATHPPSSTPPNPLLNRTM
ATAALAASPAPVSNLQGPYLASGDQPLERATGEHASMHEYPGELGQPPGLYPSSHPPGRA
GTLRALSRQDTFDADTPGSRNSAYTELGDSCVDMETDPSEGPGLGDPAGGGTPPARQGS
WEDEEEDYEEELTDNRNRGRNKARYCAEGGGPVLGRNKNELEGWGRGVYIR

>Q9P289 peripheral membrane protein
MAHSPVAVQVPGMQNNIADPEELFTKLERIGKGSFGEVFKGIDNRTQQVVAIKIIDLEEAED
EIEDIQQEITVLSQCDSSYVTKYYGSYLKGSKLWIIMEYLGGGSALDLLRAGPFDEFQIATM
LKEILKGLDYLHSEKKIHRDIKAANVLLSEQGDVKLADFGVAGQLTDTQIKRNTFVGTPF
WMAPEVIQQSAYDSKADIWSLGITAIELAKGEPPNSDMHPMRVLFLIPKNNPPTLVGDFTK
SFKEFIDACLNKDPSFRPTAKELLKHKFIVKNSKKTSYLTELIDRFKRWKAEGHSDDESDSE
GSDSESTSRENNTHPEWSFTTVRKKPDPKKVQNGAEQDLVQTLSCLSMIITPAFAELKQQD
ENNASRNQAIEELEKSIAVAEAACPGITDKMVKKLIEKFQKCSADESP

>A8MVW5 single-pass type I membrane protein
MGQDAFMEPFGDTLGVFQCKIYLLLFGACSGLKVTVPSHTVHGVRGQALYLPVHYGFHT
PASDIQIIWLFERPHTMPKYLLGSVNKSVVPDLEYQHKFTMMPPNASLLINPLQFPDEGNY
IVKVNIQGNGTLSASQKIQVTVDDPVTKPVVQIHPPSGAVEYVGNMTLTCHVEGGTRLAY
QWLKNGRPVHTSSTYSFSPQNNTLHIAPVTKEDIGNYSCLVRNPVSEMESDIIMPIIYYGPY
GLQVNSDKGLKVGEVFTVDLGEAILFDCSADSHPPNTYSWIRRTDNTTYIIKHGPRLEVAS
EKVAQKTMDYVCCAYNNITGRQDETHFTVIITSVGLEKLAQKGKSLSPLASITGISLFLIIS
MCLLFLWKKYQPYKVIKQKLEGRPETEYRKAQTFSGHEDALDDFGIYEFVAFPDVSGVSR
IPSRSVPASDCVSGQDLHSTVYEVIQHIPAQQQDHPE

>O95800 multipass membrane protein

MNSTGHLQDAPNATSLHVPHSQEGNSTSLQEGLQDLIHTATLVTCTFLLAVIFCLGSYGNFI
VFLSFFDPAFRKFRTNFDFMILNLSFCDLFICGVTAPMFTFVLFFSSASSIPDAFCFTFHLTSS
GFIIMSLKTVAVIALHRLRMVLGKQPNRTASFPCTVLLTLLLWATSFTLATLATLKTSKSHLC
LPMSSLIAGKGKAILSLYVVDFTFCVAVVSVSYIMIAQTLRKNAQVRKCPPVITVDASRPQP
FMGVPVQGGGDPIQCAMPALYRNQNYNKLQHVQTRGYTKSPNQLVTPAASRLQLVSAIN
LSTAKDSKAVVTCVIIVLSVLVCCLPLGISLVQVVLSSNGSFILYQFELFGFTLIFFKSGLNPFI
YSRNSAGLRRKVLWCLQYIGLGFFCCKQKTRLRAMGKGNLEVNRNKSSHHETNSAYML
SPKPQKKFVDQACGPSHSKESMVSPKISAGHQHCGQSSSTPINTRIEPYYSIYNSSPSQEESS
PCNLQPVNSFGFANSYIAMHYHTTNDLVQEYDSTSAKQIPVPSV

>P21754 single-pass type I membrane protein
MELSYRLFICLLLWGSTELCYPQPLWLLQGGASHPETSVQPVLVECQEATLMVMVSKDLF
GTGKLIRAADLTLGPEACEPLVSMDTEDVVRFEVGLHECGNSMQVTDDALVYSTFLLHDP
RPVGNLSIVRTNRAEIPIECRYPRQGNVSSQAILPTWLPFRTTVFSEEKLTFSLRLMEENWN
AEKRSPTFHLGDAAHLQAEIHTGSHVPLRLFVDHCVATPTPDQNASPYHTIVDFHGCLVD
GLTDASSAFKVPRPGPDTLQFTVDVFHFANDSRNMIYITCHLKVTLAEQDPDELNKACSFS
KPSNSWFPVEGSADICQCCNKGDCGTPSHSRRQPHVMSQWSRSASRNRRHVTEEADVTV
GPLIFLDRRGDHEVEQWALPSDTSVVLLGVGLAVVVSLTLTAVILVLTRRCRTASHPVSASE

>Q14254 peripheral membrane protein
MGNCHTVGPNEALVVSGGCCGSDYKQYVFGGWAWAWWCISDTQRISLEIMTLQPRCED
VETAEGVALTVTGVAQVKIMTEKELLAVACEQFLGKNVQDIKNVVLQTLEGHLRSILGTLT
VEQIYQDRDQFAKLVREVAAPDVGRMGIEILSFTIKDVYDKVDYLSSLGKTQTAVVQRDA
DIGVAEAERDAGIREAECKKEMLDVKFMADTKIADSKRAFELQKSAFSEEVNIKTAEAQL
AYELQGAREQQKIRQEEIEIEVVQRKKQIAVEAQEILRTDKELIATVRRPAEAEAHRIQQIAE
GEKVKQVLLAQAEAEKIRKIGEAEAAVIEAMGKAEAERMKLKAEAYQKYGDAAKMALV
LEALPQIAAKIAAPLTKVDEIVVLSGDNSKVTSEVNRLLAELPASVHALTGVDLSKIPLIKK
ATGVQV

>Q9BQB6 multipass membrane protein
MGSTWGSPGWVRLALCLTGLVLSLYALHVKAARARDRDYRALCDVGTAISCSRVFSSRW
GRGFGLVEHVLGQDSILNQSNSIFGCIFYTLQLLLGCLRTRWASVLMLLSSLVSLAGSVYLA
WILFFVLYDFCIVCITTYAINVSLMWLSFRKVQEPQGKAKRH

>Q8WVH0 lipid-anchor
MAFMVKTMVGGQLKNLTGSLGGGEDKGDGDKSAAEAQGMSREEYEEYQKQLVEEKME
RDAQFTQRKAERATLRSHFRDKYRLPKNETDESQIQMAGGDVELPRELAKMIEEDTEEEE
EKASVLGQLASLPGLNLGSLKDKAQATLGDLKQSAEKCHVM

>Q14416 multipass membrane protein
MGSLLALLALLLLWGAVAEGPAKKVLTLEGDLVLGGLFPVHQKGGPAEDCGPVNEHRGIQ
RLEAMLFALDRINRDPHLLPGVRLGAHILDSCSKDTHALEQALDFVRASLSRGADGSRHIC
PDGSYATHGDAPTAITGVIGGSYSDVSIQVANLLRLFQIPQISYASTSAKLSDKSRYDYFART
VPPDFFQAKAMAEILRFFNWTYVSTVASEGDYGETGIEAFELEARARNICVATSEKVGRA

MSRAAFEGVVRALLQKPSARVAVLFTRSEDARELLAASQRLNASFTWVASDGWGALESV
VAGSEGAAEGAITIELASYPISDFASYFQSLDPWNNSRNPWFREFWEQRFRCSFRQRDCAA
HSLRAVPFEQESKIMFVVNAVYAMAHALHNMHRALCPNTTRLCDAMRPVNGRRLYKDF
VLNVKFDAPFRPADTHNEVRFDRFGDGIGRYNIFTYLRAGSGRYRYQKVGYWAEGLTLDT
SLIPWASPSAGPLPASRCSEPCLQNEVKSVQPGEVCCWLCIPCQPYEYRLDEFTCADCGLG
YWPNASLTGCFELPQEYIRWGDAWAVGPVTIACLGALATLFVLGVFVRHNATPVVKASGR
ELCYILLGGVFLCYCMTFIFIAKPSTAVCTLRRLGLGTAFSVCYSALLTKTNRIARIFGGARE
GAQRPRFISPASQVAICLALISGQLLIVVAWLVVEAPGTGKETAPERREVVTLRCNHRDAS
MLGSLAYNVLLIALCTLYAFKTRKCPENFNEAKFIGFTMYTTCIIWLAFLPIFYVTSSDYRV
QTTTMCVSVSLSGSVVLGCLFAPKLHIILFQPQKNVVSHRAPTSRFGSAAARASSSLGQGS
GSQFVPTVCNGREVVDSTTSSL

>Q8NGF6 multipass membrane protein
MEFVFLAYPSCPELHILSFLGVSLVYGLIITGNILIVVSIHTETCLCTSMYYFLGSLSGIEICYT
AVVVPHILANTLQSEKTITLLGCATQMAFFIALGSADCFLLAAMAYDRYVAICHPLQYPLL
MTLTLCVHLVVASVISGLFLSLQLVAFIFSLPFCQAQGIEHFFCDVPPVMHVVCAQSHIHEQ
SVLVAAILAIAVPFFLITTSYTFIVAALLKIHSAAGRHRAFSTCSSHLTVVLLQYGCCAFMYL
CPSSSYNPKQDRFISLVYTLGTPLLNPLIYALRNSEMKGAVGRVLTRNCLSQNS

>O75575 peripheral membrane protein
MEVKDANSALLSNYEVFQLLTDLKEQRKESGKNKHSSGQQNLNTITYETLKYISKTPCRH
QSPEIVREFLTALKSHKLTKAEKLQLLNHRPVTAVEIQLMVEESEERLTEEQIEALLHTVTSI
LPAEPEAEQKKNTNSNVAMDEEDPA

>Q9NR71 single-pass type II membrane protein
MAKRTFSNLETFLIFLLVMMSAITVALLSLLFITSGTIENHKDLGGHFFSTTQSPPATQGSTA
AQRSTATQHSTATQSSTATQTSPVPLTPESPLFQNFSGYHIGVGRADCTGQVADINLMGYG
KSGQNAQGILTRLYSRAFIMAEPDGSNRTVFVSIDIGMVSQRLRLEVLNRLQSKYGSLYRR
DNVILSGTHTHSGPAGYFQYTVFVIASEGFSNQTFQHMVTGILKSIDIAHTNMKPGKIFINK
GNVDGVQINRSPYSYLQNPQSERARYSSNTDKEMIVLKMVDLNGDDLGLISWFAIHPVSM
NNSNHLVNSDNVGYASYLLEQEKNKGYLPGQGPFVAAFASSNLGDVSPNILGPRCINTGES
CDNANSTCPIGGPSMCIAKGPGQDMFDSTQIIGRAMYQRAKELYASASQEVTGPLASAHQ
WVDMTDVTVWLNSTHASKTCKPALGYSFAAGTIDGVGGLNFTQGKTEGDPFWDTIRDQI
LGKPSEEIKECHKPKPILLHTGELSKPHPWHPDIVDVQIITLGSLAITAIPGEFTTMSGRRLR
EAVQAEFASHGMQNMTVVISGLCNVYTHYITTYEEYQAQRYEAASTIYGPHTLSAYIQLF
RNLAKAIATDTVANLSRGPEPPFFKQLIVPLIPSIVDRAPKGRTFGDVLQPAKPEYRVGEVA
EVIFVGANPKNSVQNQTHQTFLTVEKYEATSTSWQIVCNDASWETRFYWHKGLLGLSNA
TVEWHIPDTAQPGIYRIRYFGHNRKQDILKPAVILSFEGTSPAFEVVTI

>Q9BXS9 multipass membrane protein
MGLADASGPRDTQALLSATQAMDLRRRDYHMERPLLNQEHLEELGRWGSAPRTHQWRT
WLQCSRARAYALLLQHLPVLVWLPRYPVRDWLLGDLLSGLSVAIMQLPQGLAYALLAGL
PPVFGLYSSFYPVFIYFLFGTSRHISVGTFAVMSVMVGSVTESLAPQALNDSMINETARDAA
RVQVASTLSVLVGLFQVGLGLIHFGFVVTYLSEPLVRGYTTAAAVQVFVSQLKYVFGLHLS

SHSGPLSLIYTVLEVCWKLPQSKVGTVVTAAVAGVVLVVVKLLNDKLQQQLPMPIPGELL
TLIGATGISYGMGLKHRFEVDVVGNIPAGLVPPVAPNTQLFSKLVGSAFTIAVVGFAIAISLG
KIFALRHGYRVDSNQELVALGLSNLIGGIFQCFPVSCSMSRSLVQESTGGNSQVAGAISSLFI
LLIIVKLGELFHDLPKAVLAAIIIVNLKGMLRQLSDMRSLWKANRADLLIWLVTFTATILLN
LDLGLVVAVIFSLLLVVVRTQMPHYSVLGQVPDTDIYRDVAEYSEAKEVRGVKVFRSSATV
YFANAEFYSDALKQRCGVDVDFLISQKKKLLKKQEQLKLKQLQKEEKLRKQAASPKGAS
VSINVNTSLEDMRSNNVEDCKMMQVSSGDKMEDATANGQEDSKAPDGSTLKALGLPQP
DFHSLILDLGALSFVDTVCLKSLKNIFHDFREIEVEVYMAACHSPVVSQLEAGHFFDASIT
KKHLFASVHDAVTFALQHPRPVPDSPVSVTRL

>Q13884 peripheral membrane protein
MAVAAAAAAAGPAGAGGGRAQRSGLLEVLVRDRWHKVLVNLSEDALVLSSEEGAAAYN
GIGTATNGSFCRGAGAGHPGAGGAQPPDSPAGVRTAFTDLPEQVPESISNQKRGVKVLKQ
ELGGLGISIKGGKENKMPILISKIFKGLAADQTQALYVGDAILSVNGADLRDATHDEAVQA
LKRAGKEVLLEVKYMREATPYVKKGSPVSEIGWETPPPESPRLGGSTSDPPSSQSFSFHRD
RKSIPLKMCYVTRSMALADPENRQLEIHSPDAKHTVILRSKDSATAQAWFSAIHSNVNDLL
TRVIAEVREQLGKTGIAGSREIRHLGWLAEKVPGESKKQWKPALVVLTEKDLLIYDSMPR
RKEAWFSPVHTYPLLATRLVHSGPGKGSPQAGVDLSFATRTGTRQGIETHLFRAETSRDLS
HWTRSIVQGCHNSAELIAEISTACTYKNQECRLTIHYENGFSITTEPQEGAFPKTIIQSPYEK
LKMSSDDGIRMLYLDFGGKDGEIQLDLHSCPKPIVFIIHSFLSAKITRLGLVA

>Q96HE7 peripheral membrane protein
MGRGWGFLFGLLGAVWLLSSGHGEEQPPETAAQRCFCQVSGYLDDCTCDVETIDRFNNY
RLFPRLQKLLESDYFRYYKVNLKRPCPFWNDISQCGRRDCAVKPCQSDEVPDGIKSASYK
YSEEANNLIEECEQAERLGAVDESLSEETQKAVLQWTKHDDSSDNFCEADDIQSPEAEYV
DLLLNPERYTGYKGPDAWKIWNVIYEENCFKPQTIKRPLNPLASGQGTSEENTFYSWLEG
LCVEKRAFYRLISGLHASINVHLSARYLLQETWLEKKWGHNITEFQQRFDGILTEGEGPRR
LKNLYFLYLIELRALSKVLPFFERPDFQLFTGNKIQDEENKMLLLEILHEIKSFPLHFDENSF
FAGDKKEAHKLKEDFRLHFRNISRIMDCVGCFKCRLWGKLQTQGLGTALKILFSEKLIAN
MPESGPSYEFHLTRQEIVSLFNAFGRISTSVKELENFRNLLQNIH

>Q9H903 peripheral membrane protein
MTVPVRGFSLLRGRLGRAPALGRSTAPSVRAPGEPGSAFRGFRSSGVRHEAIIISGTEMAK
HIQKEIQRGVESWVSLGNRRPHLSIILVGDNPASHTYVRNKIRAASAVGICSELILKPKDVS
QEELLDVTDQLNMDPRVSGILVQLPLPDHVDERTICNGIAPEKDVDGFHIINIGRLCLDQHS
LIPATASAVWEIIKRTGIQTFGKNVVVAGRSKNVGMPIAMLLHTDGEHERPGGDATVTIAH
RYTPKEQLKIHTQLADIIIVAAGIPKLITSDMVKEGAAVIDVGINYVHDPVTGKTKLVGDVD
FEAVKKKAGFITPVPGGVGPMTVAMLLKNTLLAAKKIIY

>Q1EHB4 multipass membrane protein
MEVKNFAVWDYVVFAALFFISSGIGVFFAIKERKKATSREFLVGGRQMSFGPVGLSLTASF
MSAVTVLGTPSEVYRFGASFLVFFIAYLFVILLTSELFLPVFYRSGITSTYEYLQLRFNKPVR
YAATVIYIVQTILYTGVVVYAPALALNQVTGFDLWGSVFATGIVCTFYCTLGGLKAVVWTD
AFQMVVMIVGFLTVLIQGSTHAGGFHNVLEQSTNGSRLHIFDFDVDPLRRHTFWTITVGG

TFTWLGIYGVNQSTIQRCISCKTEKHAKLALYFNLLGLWIILVCAVFSGLIMYSHFKDCDP
WTSGIISAPDQLMPYFVMEIFATMPGLPGLFVACAFSGTLSTVASSINALATVTFEDFVKSC
FPHLSDKLSTWISKGLCLLFGVMCTSMAVAASVMGGVVQASLSIHGMCGGPMLGLFSLGI
VFPFVNWKGALGGLLTGITLSFWVAIGAFIYPAPASKTWPLPLSTDQCIKSNVTATGPPVLS
SRPGIADTWYSISYLYYSAVGCLGCIVAGVIISLITGRQRGEDIQPLLIRPVCNLFCFWSKKY
KTLCWCGVQHDSGTEQENLENGSARKQGAESVLQNGLRRESLVHVPGYDPKDKSYNNM
AFETTHF

>Q2QL34 multipass membrane protein
MAGWWPALSRAARRHPWPTNVLLYGSLVSAGDALQQRLQGREANWRQTRRVATLVVTF
HANFNYVWLRLLERALPGRAPHALLAKLLCDQVVGAPIAVSAFYVGMSILQGKDDIFLDL
KQKFWNTYLSGLMYWPFVQLTNFSLVPVQWRTAYAGVCGFLWATFICFSQQSGDGTFKS
AFTILYTKGTSATEGYPKK

>Q5T5U3 peripheral membrane protein
MATRRTGLSEGDGDKLKACEVSKNKDGKEQSETVSLSEDETFSWPGPKTVTLKRTSQGFG
FTLRHFIVYPPESAIQFSYKDEENGNRGGKQRNRLEPMDTIFVKQVKEGGPAFEAGLCTGD
RIIKVNGESVIGKTYSQVIALIQNSDTTLELSVMPKDEDILQVLQFTKDVTALAYSQDAYLK
GNEAYSGNARNIPEPPPICYPWLPSAPSAMAQPVEISPPDSSLSKQQTSTPVLTQPGRAYRM
EIQVPPSPTDVAKSNTAVCVCNESVRTVIVPSEKVVDLLSNRNNHTGPSHRTEEVRYGVSE
QTSLKTVSRTTSPPLSIPTTHLIHQPAGSRSLEPSGILLKSGNYSGHSDGISSSRSQAVEAPSV
SVNHYSPNSHQHIDWKNYKTYKEYIDNRRLHIGCRTIQERLDSLRAASQSTTDYNQVVPN
RTTLQGRRRSTSHDRVPQSVQIRQRSVSQERLEDSVLMKYCPRSASQGALTSPSVSFSNHR
TRSWDYIEGQDETLENVNSGTPIPDSNGEKKQTYKWSGFTEQDDRRGICERPRQQEIHKSF
RGSNFTVAPSVVNSDNRRMSGRGVGSVSQFKKIPPDLKTLQSNRNFQTTCGMSLPRGISQ
DRSPLVKVRSNSLKAPSTHVTKPSFSQKSFVSIKDQRPVNHLHQNSLLNQQTWVRTDSAP
DQQVETGKSPSLSGASAKPAPQSSENAGTSDLELPVSQRNQDLSLQEAETEQSDTLDNKE
AVILREKPPSGRQTPQPLRHQSYILAVNDQETGSDTTCWLPNDARREVHIKRMEERKASST
SPPGDSLASIPFIDEPTSPSIDHDIAHIPASAVISASTSQVPSIATVPPCLTTSAPLIRRQLSHDH
ESVGPPSLDAQPNSKTERSKSYDEGLDDYREDAKLSFKHVSSLKGIKIADSQKSSEDSGSR
KDSSSEVFSDAAKEGWLHFRPLVTDKGKRVGGSIRPWKQMYVVLRGHSLYLYKDKREQT
TPSEEEQPISVNACLIDISYSETKRKNVFRLTTSDCECLFQAEDRDDMLAWIKTIQESSNLN
EEDTGVTNRDLISRRIKEYNNLMSKAEQLPKTPRQSLSIRQTLLGAKSEPKTQSPHSPKEES
ERKLLSKDDTSPPKDKGTWRKGIPSIMRKTFEKKPTATGTFGVRLDDCPPAHTNRYIPLIVD
ICCKLVEERGLEYTGIYRVPGNNAAISSMQEELNKGMADIDIQDDKWRDLNVISSLLKSFF
RKLPEPLFTNDKYADFIEANRKEDPLDRLKTLKRLIHDLPEHHYETLKFLSAHLKTVAENS
EKNKMEPRNLAIVFGPTLVRTSEDNMTHMVTHMPDQYKIVETLIQHHDWFFTEEGAEEPL
TTVQEESTVDSQPVPNIDHLLTNIGRTGVSPGDVSDSATSDSTKSKGSWGSGKDQYSRELL
VSSIFAAASRKRKKPKEKAQPSSSEDELDNVFFKKENVEQCHNDTKEESKKESETLGRKQ
KIIIAKENSTRKDPSTTKDEKISLGKESTPSEEPSPPHNSKHNKSPTLSCRFAILKESPRSLLA
QKSSHLEETGSDSGTLLSTSSQASLARFSMKKSTSPETKHSEFLANVSTITSDYSTTSSATYL
TSLDSSRLSPEVQSVAESKGDEADDERSELISEGRPVETDSESEFPVFPTALTSERLFRGKLQ
EVTKSSRRNSEGSELSCTEGSLTSSLDSRRQLFSSHKLIECDTLSRKKSARFKSDSGSLGDA
KNEKEAPSLTKVFDVMKKGKSTGSLLTPTRGESEKQEPTWKTKIADRLKLRPRAPADDMF

GVGNHKVNAETAKRKSIRRRHTLGGHRDATEISVLNFWKVHEQSGERESELSAVNRLKPK
CSAQDLSISDWLARERLRTSTSDLSRGEIGDPQTENPSTREIATTDTPLSLHCNTGSSSSTLA
STNRPLLSIPPQSPDQINGESFQNVSKNASSAANAQPHKLSETPGSKAEFHPCL

>Q8TAD4 multipass membrane protein
MEEKYGGDVLAGPGGGGGLGPVDVPSARLTKYIVLLCFTKFLKAVGLFESYDLLKAVHIV
QFIFILKLGTAFFMVLFQKPFSSGKTITKHQWIKIFKHAVAGCIISLLWFFGLTLCGPLRTLLL
FEHSDIVVISLLSVLFTSSGGGPAKTRGAAFFIIAVICLLLFDNDDLMAKMAEHPEGHHDSA
LTHMLYTAIAFLGVADHKGGVLLLVLALCCKVGFHTASRKLSVDVGGAKRLQALSHLVS
VLLLCPWVIVLSVTTESKVESWFSLIMPFATVIFFVMILDFYVDSICSVKMEVSKCARYGSF
PIFISALLFGNFWTHPITDQLRAMNKAAHQESTEHVLSGGVVVSAIFFILSANILSSPSKRG
QKGTLIGYSPEGTPLYNFMGDAFQHSSQSIPRFIKESLKQILEESDSRQIFYFLCLNLLFTFV
ELFYGVLTNSLGLISDGFHMLFDCSALVMGLFAALMSRWKATRIFSYGYGRIEILSGFINGL
FLIVIAFFVFMESVARLIDPPELDTHMLTPVSVGGLIVNLIGICAFSHAHSHAHGASQGSCHS
SDHSHSHHMHGHSDHGHGHSHGSAGGGMNANMRGVFLHVLADTLGSIGVIVSTVLIEQF
GWFIADPLCSLFIAILIFLSVVPLIKDACQVLLLRLPPEYEKELHIALEKIQKIEGLISYRDPH
FWRHSASIVAGTIHIQVTSDVLEQRIVQQVTGILKDAGVNNLTIQVEKEAYFQHMSGLSTG
FHDVLAMTKQMESMKYCKDGTYIM

>Q96PC3 peripheral membrane protein
MIHFILLFSRQGKLRLQKWYITLPDKERKKITREIVQIILSRGHRTSSFVDWKELKLVYKRY
ASLYFCCAIENQDNELLTLEIVHRYVELLDKYFGNVCELDIIFNFEKAYFILDEFIIGGEIQET
SKKIAVKAIEDSDMLQEVSTVSQTMGER

>P50440 peripheral membrane protein
MLRVRCLRGGSRGAEAVHYIGSRLGRTLTGWVQRTFQSTQAATASSRNSCAADDKATEPL
PKDCPVSSYNEWDPLEEVIVGRAENACVPPFTIEVKANTYEKYWPFYQKQGGHYFPKDH
LKKAVAEIEEMCNILKTEGVTVRRPDPIDWSLKYKTPDFESTGLYSAMPRDILIVVGNEIIE
APMAWRSRFFEYRAYRSIIKDYFHRGAKWTTAPKPTMADELYNQDYPIHSVEDRHKLAA
QGKFVTTEFEPCFDAADFIRAGRDIFAQRSQVTNYLGIEWMRRHLAPDYRVHIISFKDPNP
MHIDATFNIIGPGIVLSNPDRPCHQIDLFKKAGWTIITPPTPIIPDDHPLWMSSKWLSMNVL
MLDEKRVMVDANEVPIQKMFEKLGITTIKVNIRNANSLGGGFHCWTCDVRRRGTLQSYL
D

>Q8N6M3 multipass membrane protein
MEHLERCEWLLRGTLVRAAVRRYLPWALVASMLAGSLLKELSPLPESYLSNKRNVLNVYF
VKVAWAWTFCLLLPFIALTNYHLTGKAGLVLRRLSTLLVGTAIWYICTSIFSNIEHYTGSCY
QSPALEGVRKEHQSKQQCHQEGGFWHGFDISGHSFLLTFCALMIVEEMSVLHEVKTDRSH
CLHTAITTLVVALGILTFIWVLMFLCTAVYFHNLSQKVFGTLFGLLSWYGTYGFWYPKAFS
PGLPPQSCSLNLKQDSYKK

>Q9H255 multipass membrane protein
MSSCNFTHATFVLIGIPGLEKAHFWVGFPLLSMYVVAMFGNCIVVFIVRTERSLHAPMYLF
LCMLAAIDLALSTSTMPKILALFWFDSREISFEACLTQMFFIHALSAIESTILLAMAFDRYVA

ICHPLRHAAVLNNTVTAQIGIVAVVRGSLFFFPLPLLIKRLAFCHSNVLSHSYCVHQDVMKL
AYADTLPNVVYGLTAILLVMGVDVMFISLSYFLIIRTVLQLPSKSERAKAFGTCVSHIGVVL
AFYVPLIGLSVVHRFGNSLHPIVRVVMGDIYLLLPPVINPIIYGAKTKQIRTRVLAMFKISCD
KDLQAVGGK

>Q8TCB6 multipass membrane protein
MVDPNGNESSATYFILIGLPGLEEAQFWLAFPLCSLYLIAVLGNLTIIYIVRTEHSLHEPMYIF
LCMLSGIDILISTSSMPKMLAIFWFNSTTIQFDACLLQMFAIHSLSGMESTVLLAMAFDRYV
AICHPLRHATVLTLPRVTKIGVAAVVRGAALMAPLPVFIKQLPFCRSNILSHSYCLHQDVM
KLACDDIRVNVVYGLIVIISAIGLDSLLISFSYLLILKTVLGLTREAQAKAFGTCVSHVCAVF
IFYVPFIGLSMVHRFSKRRDSPLPVILANIYLLVPPVLNPIVYGVKTKEIRQRILRLFHVATH
ASEP

>Q86UL3 multipass membrane protein
MFLLLPFDSLIVNLLGISLTVLFTLLLVFIIVPAIFGVSFGIRKLYMKSLLKIFAWATLRMERG
AKEKNHQLYKPYTNGIIAKDPTSLEEEIKEIRRSGSSKALDNTPEFELSDIFYFCRKGMETI
MDDEVTKRFSAEELESWNLLSRTNYNFQYISLRLTVLWGLGVLIRYCFLLPLRIALAFTGIS
LLVVGTTVVGYLPNGRFKEFMSKHVHLMCYRICVRALTAIITYHDRENRPRNGGICVANH
TSPIDVIILASDGYYAMVGQVHGGLMGVIQRAMVKACPHVWFERSEVKDRHLVAKRLTE
HVQDKSKLPILIFPEGTCINNTSVMMFKKGSFEIGATVYPVAIKYDPQFGDAFWNSSKYGM
VTYLLRMMTSWAIVCSVWYLPPMTREADEDAVQFANRVKSAIARQGGLVDLLWDGGLK
REKVKDTFKEEQQKLYSKMIVGNHKDRSRS

>P13612 single-pass type I membrane protein
MAWEARREPGPRRAAVRETVMLLLCLGVPTGRPYNVDTESALLYQGPHNTLFGYSVVLH
SHGANRWLLVGAPTANWLANASVINPGAIYRCRIGKNPGQTCEQLQLGSPNGEPCGKTCL
EERDNQWLGVTLSRQPGENGSIVTCGHRWKNIFYIKNENKLPTGGCYGVPPDLRTELSKRI
APCYQDYVKKFGENFASCQAGISSFYTKDLIVMGAPGSSYWTGSLFVYNITTNKYKAFLD
KQNQVKFGSYLGYSVGAGHFRSQHTTEVVGGAPQHEQIGKAYIFSIDEKELNILHEMKGK
KLGSYFGASVCAVDLNADGFSDLLVGAPMQSTIREEGRVFVYINSGSGAVMNAMETNLV
GSDKYAARFGESIVNLGDIDNDGFEDVAIGAPQEDDLQGAIYIYNGRADGISSTFSQRIEGL
QISKSLSMFGQSISGQIDADNNGYVDVAVGAFRSDSAVLLRTRPVVIVDASLSHPESVNRT
KFDCVENGWPSVCIDLTLCFSYKGKEVPGYIVLFYNMSLDVNRKAESPPRFYFSSNGTSD
VITGSIQVSSREANCRTHQAFMRKDVRDILTPIQIEAAYHLGPHVISKRSTEEFPPLQPILQQ
KKEKDIMKKTINFARFCAHENCSADLQVSAKIGFLKPHENKTYLAVGSMKTLMLNVSLFN
AGDDAYETTLHVKLPVGLYFIKILELEEKQINCEVTDNSGVVQLDCSIGYIYVDHLSRIDIS
FLLDVSSLSRAEEDLSITVHATCENEEEMDNLKHSRVTVAIPLKYEVKLTVHGFVNPTSFV
YGSNDENEPETCMVEKMNLTFHVINTGNSMAPNVSVEIMVPNSFSPQTDKLFNILDVQTT
TGECHFENYQRVCALEQQKSAMQTLKGIVRFLSKTDKRLLYCIKADPHCLNFLCNFGKME
SGKEASVHIQLEGRPSILEMDETSALKFEIRATGFPEPNPRVIELNKDENVAHVLLEGLHHQ
RPKRYFTIVIISSSLLLGLIVLLLISYVMWKAGFFKRQYKSILQEENRRDSWSYINSKSNDD

>O00476 multipass membrane protein
MATKTELSPTARESKNAQDMQVDETLIPRKVPSLCSARYGIALVLHFCNFTTIAQNVIMNIT

MVAMVNSTSPQSQLNDSSEVLPVDSFGGLSKAPKSLPAKSSILGGQFAIWEKWGPPQERSR
LCSIALSGMLLGCFTAILIGGFISETLGWPFVFYIFGGVGCVCCLLWFVVIYDDPVSYPWIST
SEKEYIISSLKQQVGSSKQPLPIKAMLRSLPIWSICLGCFSHQWLVSTMVVYIPTYISSVYHV
NIRDNGLLSALPFIVAWVIGMVGGYLADFLLTKKFRLITVRKIATILGSLPSSALIVSLPYLN
SGYITATALLTLSCGLSTLCQSGIYINVLDIAPRYSSFLMGASRGFSSIAPVIVPTVSGFLLSQ
DPEFGWRNVFFLLFAVNLLGLLFYLIFGEADVQEWAKERKLTRL

>P11362 single-pass type I membrane protein
MWSWKCLLFWAVLVTATLCTARPSPTLPEQAQPWGAPVEVESFLVHPGDLLQLRCRLRD
DVQSINWLRDGVQLAESNRTRITGEEVEVQDSVPADSGLYACVTSSPSGSDTTYFSVNVSD
ALPSSEDDDDDDDSSSEEKETDNTKPNRMPVAPYWTSPEKMEKKLHAVPAAKTVKFKCP
SSGTPNPTLRWLKNGKEFKPDHRIGGYKVRYATWSIIMDSVVPSDKGNYTCIVENEYGSIN
HTYQLDVVERSPHRPILQAGLPANKTVALGSNVEFMCKVYSDPQPHIQWLKHIEVNGSKI
GPDNLPYVQILKTAGVNTTDKEMEVLHLRNVSFEDAGEYTCLAGNSIGLSHHSAWLTVLE
ALEERPAVMTSPLYLEIIIYCTGAFLISCMVGSVIVYKMKSGTKKSDFHSQMAVHKLAKSIP
LRRQVTVSADSSASMNSGVLLVRPSRLSSSGTPMLAGVSEYELPEDPRWELPRDRLVLGK
PLGEGCFGQVVLAEAIGLDKDKPNRVTKVAVKMLKSDATEKDLSDLISEMEMMKMIGKH
KNIINLLGACTQDGPLYVIVEYASKGNLREYLQARRPPGLEYCYNPSHNPEEQLSSKDLVS
CAYQVARGMEYLASKKCIHRDLAARNVLVTEDNVMKIADFGLARDIHHIDYYKKTTNGR
LPVKWMAPEALFDRIYTHQSDVWSFGVLLWEIFTLGGSPYPGVPVEELFKLLKEGHRMD
KPSNCTNELYMMMRDCWHAVPSQRPTFKQLVEDLDRIVALTSNQEYLDLSMPLDQYSPSF
PDTRSSTCSSGEDSVFSHEPLPEEPCLPRHPAQLANGGLKRR

>Q8NFA2 peripheral membrane protein
MAGPRYPVSVQGAALVQIKRLQTFAFSVRWSDGSDTFVRRSWDEFRQLKKTLKETFPVE
AGLLRRSDRVLPKLLGQASLDAPLLGRVGRTSRGLARLQLLETYSRRLLATAERVARSPTIT
GFFAPQPLDLEPALPPGSRVILPTPEEQPLSRAAGRLSIHSLEAQSLRCLQPFCTQDTRDRPF
QAQAQESLDVLLRHPSGWWLVENEDRQTAWFPAPYLEEAAPGQGREGGPSLGSSGPQFC
ASRAYESSRADELSVPAGARVRVLETSDRGWWLCRYGDRAGLLPAVLLRPEGLGALLSGT
GFRGGDDPAGEARGFPEPSQATAPPPTVPTRPSPGAIQSRCCTVTRRALERRPRRQGRPRG
CVDSVPHPTTEQ

>Q8IWV2 GPI-anchor, lipid-anchor
MRLPWELLVLQSFILCLADDSTLHGPIFIQEPSPVMFPLDSEEKKVKLNCEVKGNPKPHIR
WKLNGTDVDTGMDFRYSVVEGSLLINNPNKTQDAGTYQCTATNSFGTIVSREAKLQFAYL
DNFKTRTRSTVSVRRGQGMVLLCGPPPHSGELSYAWIFNEYPSYQDNRRFVSQETGNLYIA
KVEKSDVGNYTCVVTNTVTNHKVLGPPTPLILRNDGVMGEYEPKIEVQFPETVPTAKGAT
VKLECFALGNPVPTIIWRRADGKPIARKARRHKSNGILEIPNFQQEDAGLYECVAENSRGK
NVARGQLTFYAQPNWIQKINDIHVAMEENVFWECKANGRPKPTYKWLKNGEPLLTRDRI
QIEQGTLNITIVNLSDAGMYQCLAENKHGVIFSNAELSVIAVGPDFSRTLLKRVTLVKVGG
EVVIECKPKASPKPVYTWKKGRDILKENERITISEDGNLRIINVTKSDAGSYTCIATNHFGT
ASSTGNLVVKDPTRVMVPPSSMDVTVGESIVLPCQVTHDHSLDIVFTWSFNGHLIDFDRD
GDHFERVGGQDSAGDLMIRNIQLKHAGKYVCMVQTSVDRLSAAADLIVRGPPGPPEAVTI
DEITDTTAQLSWRPGPDNHSPITMYVIQARTPFSVGWQAVSTVPELIDGKTFTATVVGLNP

WVEYEFRTVAANVIGIGEPSRPSEKRRTEEALPEVTPANVSGGGGSKSELVITWETVPEELQ
NGRGFGYVVAFRPYGKMIWMLTVLASADASRYVFRNESVHPFSPFEVKVGVFNNKGEGP
FSPTTVVYSAEEEPTKPPASIFARSLSATDIEVFWASPLEKNRGRIQGYEVKYWRHEDKEEN
ARKIRTVGNQTSTKITNLKGSVLYHLAVKAYNSAGTGPSSATVNVTTRKPPPSQPPGNIIW
NSSDSKIILNWDQVKALDNESEVKGYKVLYRWNRQSSTSVIETNKTSVELSLPFDEDYIIEI
KPFSDGGDGSSSEQIRIPKISNAYARGSGASTSNACTLSAISTIMISLTARSSL

>Q9UBQ0 peripheral membrane protein
MLVLVLGDLHIPHRCNSLPAKFKKLLVPGKIQHILCTGNLCTKESYDYLKTLAGDVHIVRG
DFDENLNYPEQKVVTVGQFKIGLIHGHQVIPWGDMASLALLQRQFDVDILISGHTHKFEA
FEHENKFYINPGSATGAYNALETNIIPSFVLMDIQASTVVTYVYQLIGDDVKVERIEYKKP

>Q9BQ51 single-pass type I membrane protein
MIFLLLMLSLELQLHQIAALFTVTVPKELYIIEHGSNVTLECNFDTGSHVNLGAITASLQKV
ENDTSPHRERATLLEEQLPLGKASFHIPQVQVRDEGQYQCIIIYGVAWDYKYLTLKVKASY
RKINTHILKVPETDEVELTCQATGYPLAEVSWPNVSVPANTSHSRTPEGLYQVTSVLRLKPP
PGRNFSCVFWNTHVRELTLASIDLQSQMEPRTHPTWLLHIFIPFCIIAFIFIATVIALRKQLCQ
KLYSSKDTTKRPVTTTKREVNSAI

>Q96B97 peripheral membrane protein
MVEAIVEFDYQAQHDDELTISVGEIITNIRKEDGGWWEGQINGRRGLFPDNFVREIKKEM
KKDPLTNKAPEKPLHEVPSGNSLLSSETILRTNKRGERRRRRCQVAFSYLPQNDDELELKV
GDIIEVVGEVEEGWWEGVLNGKTGMFPSNFIKELSGESDELGISQDEQLSKSSLRETTGSE
SDGGDSSSTKSEGANGTVATAAIQPKKVKGVGFGDIFKDKPIKLRPRSIEVENDFLPVEKTI
GKKLPATTATPDSSKTEMDSRTKSKDYCKVIFPYEAQNDDELTIKEGDIVTLINKDCIDVG
WWEGELNGRRGVFPDNFVKLLPPDFEKEGNRPKKPPPPSAPVIKQGAGTTERKHEIKKIPP
ERPEMLPNRTEEKERPEREPKLDLQKPSVPAIPPKKPRPPKTNSLSRPGALPPRRPERPVGPL
THTRGDSPKIDLAGSSLSGILDKDLSDRSNDIDLEGFDSVVSSTEKLSHPTTSRPKATGRRP
PSQSLTSSSLSSPDIFDSPSPEEDKEEHISLAHRGVDASKKTSKTVTISQVSDNKASLPPKPG
TMAAGGGGPAPLSSAAPSPLSSSLGTAGHRANSPSLFGTEGKPKMEPAASSQAAVEELRTQ
VRELRSIIETMKDQQKREIKQLLSELDEEKKIRLRLQMEVNDIKKALQSK

>Q86T03 multipass membrane protein
MAADGERSPLLSEPIDGGAGGNGLVGPGGSGAGPGGGLTPSAPPYGAAFPPFPEGHPAVLP
GEDPPPYSPLTSPDSGSAPMITCRVCQSLINVEGKMHQHVVKCGVCNEATPIKNAPPGKKY
VRCPCNCLLICKVTSQRIACPRPYCKRIINLGPVHPGPLSPEPQPMGVRVICGHCKNTFLWT
EFTDRTLARCPHCRKVSSIGRRYPRKRCICCFLLGLLLAVTATGLAFGTWKHARRYGGIYA
AWAFVILLAVLCLGRALYWACMKVSHPVQNFS

>P52569 multipass membrane protein
MIPCRAALTFARCLIRRKIVTLDSLEDTKLCRCLSTMDLIALGVGSTLGAGVYVLAGEVAK
ADSGPSIVVSFLIAALASVMAGLCYAEFGARVPKTGSAYLYTYVTVGELWAFITGWNLILS
YVIGTSSVARAWSGTFDELLSKQIGQFLRTYFRMNYTGLAEYPDFFAVCLILLLAGLLSFGV
KESAWVNKVFTAVNILVLLFVMVAGFVKGNVANWKISEEFLKNISASAREPPSENGTSIYG

AGGFMPYGFTGTLAGAATCFYAFVGFDCIATTGEEVRNPQKAIPIGIVTSLLVCFMAYFGV
SAALTLMMPYYLLDEKSPLPVAFEYVGWGPAKYVVAAGSLCALSTSLLGSIFPMPRVIYA
MAEDGLLFKCLAQINSKTKTPIIATLSSGAVAALMAFLFDLKALVDMMSIGTLMAYSLVAA
CVLILRYQPGLSYDQPKCSPEKDGLGSSPRVTSKSESQVTMLQRQGFSMRTLFCPSLLPTQ
QSASLVSFLVGFLAFLVLGLSVLTTYGVHAITRLEAWSLALLALFLVLFVAIVLTIWRQPQN
QQKVAFMVPFLPFLPAFSILVNIYLMVQLSADTWVRFSIWMAIGFLIYFSYGIRHSLEGHLR
DENNEEDAYPDNVHAAAEEKSAIQANDHHPRNLSSPFIFHEKTSEF

>P34998 multipass membrane protein
MGGHPQLRLVKALLLLGLNPVSASLQDQHCESLSLASNISGLQCNASVDLIGTCWPRSPA
GQLVVRPCPAFFYGVRYNTTNNGYRECLANGSWAARVNYSECQEILNEEKKSKVHYHVA
VIINYLGHCISLVALLVAFVLFLRLRPGCTHWGDQADGALEVGAPWSGAPFQVRRSIRCLR
NIIHWNLISAFILRNATWFVVQLTMSPEVHQSNVGWCRLVTAAYNYFHVTNFFWMFGEGC
YLHTAIVLTYSTDRLRKWMFICIGWGVPFPIIVAWAIGKLYYDNEKCWFGKRPGVYTDYIY
QGPMILVLLINFIFLFNIVRILMTKLRASTTSETIQYRKAVKATLVLLPLLGITYMLFFVNPGE
DEVSRVVFIYFNSFLESFQGFFVSVFYCFLNSEVRSAIRKRWHRWQDKHSIRARVARAMSI
PTSPTRVSFHSIKQSTAV

>P20591 peripheral membrane protein
MVVSEVDIAKADPAAASHPLLLNGDATVAQKNPGSVAENNLCSQYEEKVRPCIDLIDSLR
ALGVEQDLALPAIAVIGDQSSGKSSVLEALSGVALPRGSGIVTRCPLVLKLKKLVNEDKWR
GKVSYQDYEIEISDASEVEKEINKAQNAIAGEGMGISHELITLEISSRDVPDLTLIDLPGITRV
AVGNQPADIGYKIKTLIKKYIQRQETISLVVVPSNVDIATTEALSMAQEVDPEGDRTIGILTK
PDLVDKGTEDKVVDVVRNLVFHLKKGYMIVKCRGQQEIQDQLSLSEALQREKIFFENHPY
FRDLLEEGKATVPCLAEKLTSELITHICKSLPLLENQIKETHQRITEELQKYGVDIPEDENEK
MFFLIDKVNAFNQDITALMQGEETVGEEDIRLFTRLRHEFHKWSTIIENNFQEGHKILSRKI
QKFENQYRGRELPGFVNYRTFETIVKQQIKALEEPAVDMLHTVTDMVRLAFTDVSIKNFE
EFFNLHRTAKSKIEDIRAEQEREGEKLIRLHFQMEQIVYCQDQVYRGALQKVREKELEEEK
KKKSWDFGAFQSSSATDSSMEEIFQHLMAYHQEASKRISSHIPLIIQFFMLQTYGQQLQKA
MLQLLQDKDTYSWLLKERSDTSDKRKFLKERLARLTQARRRLAQFPG

>Q9H2H9 multipass membrane protein
MMHFKSGLELTELQNMTVPEDDNISNDSNDFTEVENGQINSKFISDRESRRSLTNSHLEKK
KCDEYIPGTTSLGMSVFNLSNAIMGSGILGLAFALANTGILLFLVLLTSVTLLSIYSINLLLIC
SKETGCMVYEKLGEQVFGTTGKFVIFGATSLQNTGAMLSYLFIVKNELPSAIKFLMGKEE
TFSAWYVDGRVLVVIVTFGIILPLCLLKNLGYLGYTSGFSLSCMVFFLIVVIYKKFQIPCIVP
ELNSTISANSTNADTCTPKYVTFNSKTVYALPTIAFAFVCHPSVLPIYSELKDRSQKKMQM
VSNISFFAMFVMYFLTAIFGYLTFYDNVQSDLLHKYQSKDDILILTVRLAVIVAVILTVPVLF
FTVRSSLFELAKKTKFNLCRHTVVTCILLVVINLLVIFIPSMKDIFGVVGVTSANMLIFILPSS
LYLKITDQDGDKGTQRIWAALFLGLGVLFSLVSIPLVIYDWACSSSSDEGH

>Q8TB92 peripheral membrane protein
MGNVPSAVKHCLSYQQLLREHLWIGDSVAGALDPAQTSLLTNLHCFQPDVSGFSVSLAGT
VACIHWETSQLSGLPEFVKIVEVGPRDGLQNEKVIVPTDIKIEFINRLSQTGLSVIEVTSFVS

SRWVPQMADHTEVMKGIHQYPGVRYPVLTPNLQGFHHAVAAGATEISVFGAASESFSKKN
INCSIEESMGKFEEVVKSARHMNIPARGYVSCALGCPYEGSITPQKVTEVSKRLYGMGCYE
ISLGDTIGVGTPGSMKRMLESVMKEIPPGALAVHCHDTYGQALANILTALQMGINVVDSA
VSGLGGCPYAKGASGNVATEDLIYMLNGLGLNTGVNLYKVMEAGDFICKAVNKTTNSKV
AQASFNA

>Q8IZF7 multipass membrane protein
MGLTAYGNRRVQPGELPFGANLTLIHTRAQPVICSKLLLTKRVSPISFFLSKFQNSWGEDG
WVQLDQLPSPNAVSSDQVHCSAGCTHRKCGWAASKSKEKVPARPHGVCDGVCTDYSQC
TQPCPPDTQGNMGFSCRQKTWHKITDTCQTLNALNIFEEDSRLVQPFEDNIKISVYTGKSE
TITDMLLQKCPTDLSCVIRNIQQSPWIPGNIAVIVQLLHNISTAIWTGVDEAKMQSYSTIAN
HILNSKSISNWTFIPDRNSSYILLHSVNSFARRLFIDKHPVDISDVFIHTMGTTISGDNIGKNF
TFSMRINDTSNEVTGRVLISRDELRKVPSPSQVISIAFPTIGAILEASLLENVTVNGLVLSAIL
PKELKRISLIFEKISKSEERRTQCVGWHSVENRWDQQACKMIQENSQQAVCKCRPSKLFTS
FSILMSPHILESLILTYITYVGLGISICSLILCLSIEVLVWSQVTKTEITYLRHVCIVNIAATLL
MADVWFIVASFLSGPITHHKGCVAATFFVHFFYLSVFFWMLAKALLILYGIMIVFHTLPKS
VLVASLFSVGYGCPLAIAAITVAATEPGKGYLRPEICWLNWDMTKALLAFVIPALAIVVVN
LITVTLVIVKTQRAAIGNSMFQEVRAIVRISKNIAILTPLLGLTWGFGVATVIDDRSLAFHIIF
SLLNAFQVSPDASDQVQSERIHEDVL

>Q6DD88 multipass membrane protein
MLSPQRVAAAASRGADDAMESSKPGPVQVVLVQKDQHSFELDEKALASILLQDHIRDLD
VVVVSVAGAFRKGKSFILDFMLRYLYSQKESGHSNWLGDPEEPLTGFSWRGGSDPETTGI
QIWSEVFTVEKPGGKKVAVVLMDTQGAFDSQSTVKDCATIFALSTMTSSVQIYNLSQNIQE
DDLQQLQLFTEYGRLAMDEIFQKPFQTLMFLVRDWSFPYEYSYGLQGGMAFLDKRLQVK
EHQHEEIQNVRNHIHSCFSDVTCFLLPHPGLQVATSPDFDGKLKDIAGEFKEQLQALIPYVL
NPSKLMEKEINGSKVTCRGLLEYFKAYIKIYQGEDLPHPKSMLQATAEANNLAAAASAKD
IYYNNMEEVCGGEKPYLSPDILEEKHCEFKQLALDHFKKTKKMGGKDFSFRYQQELEEEI
KELYENFCKHNGSKNVFSTFRTPAVLFTGIVALYIASGLTGFIGLEVVAQLFNCMVGLLLIA
LLTWGYIRYSGQYRELGGAIDFGAAYVLEQASSHIGNSTQATVRDAVVGRPSMDKKAQ

>P49238 multipass membrane protein
MDQFPESVTENFEYDDLAEACYIGDIVVFGTVFLSIFYSVIFAIGLVGNLLVVFALTNSKKP
KSVTDIYLLNLALSDLLFVATLPFWTHYLINEKGLHNAMCKFTTAFFFIGFFGSIFFITVISID
RYLAIVLAANSMNNRTVQHGVTISLGVWAAAILVAAPQFMFTKQKENECLGDYPEVLQEI
WPVLRNVETNFLGFLLPLLIMSYCYFRIIQTLFSCKNHKKAKAIKLILLVVIVFFLFWTPYN
VMIFLETLKLYDFFPSCDMRKDLRLALSVTETVAFSHCCLNPLIYAFAGEKFRRYLYHLYG
KCLAVLCGRSVHVDFSSSESQRSRHGSVLSSNFTYHTSDGDALLLL

>Q6ZRI0 peripheral membrane protein
MGVLASALCWLLCVWLPWGEQAAESLRVQRLGERVVDSGRSGARGMRNVKGMRNGPA
QTRVSSSSSHQEATLAMGDKATVVGGQQAEAPDSVAMSSWERRLHRAKCAPSYLFSCFN
GGECVHPAFCDCRRFNATGPRCQMVYNAGPERDSICRAWGQHHVETFDGLYYYLSGKGS
YTLVGRHEPEGQSFSIQVHNDPQCGSSPYTCSRAVSLFFVGEQEIHLAKEVTHGGMRVQLP

HVMGSARLQQLAGYVIVRHQSAFTLAWDGASAVYIKMSPELLGWTHGLCGNNNADPKD
DLVTSSGKLTDDVVEFVHSWQEQAPNQPPGPTTSSLPRPPCLQQNPGTMQGVYEQCEALL
RPPFDACHAYVSPLPFTASCTSDLCQSMGDVATWCRALAEYARACAQAGRPLQGWRTQL
RQCTVHCKEKAFTYNECIACCPASCHPRASCVDSEIACVDGCYCPNGLIFEDGGCVAPAEC
PCEFHGTLYPPGSVVKEDCNTCTCTSGKWECSTAVCPAECSVTGDIHFTTFDGRRYTFPAT
CQYILAKSRSSGTFTVTLQNAPCGLNQDGACVQSVSVILHQDPRRQVTLTQAGDVLLFDQ
YKIIPPYTDDAFEIRRLSSVFLRVRTNVGVRVLYDREGLRLYLQVDQRWVEDTVGLCGTFN
GNTQDDFLSPVGVPESTPQLFGNSWKTLSACSPLVSGSPLDPCDVHLQAASYSVQACSVL
TGEMFAPCSAFLSPVPYFEQCRRDACRCGQPCLCATLAHYAHLCRRHGLPVDFRARLPAC
ALSCEASKEYSPCVAPCGRTCQDLASPEACGVDGGDDLSRDECVEGCACPPDTYLDTQA
DLCVPRNQCSCHFQGVDYPPGDSDIPSLGHCHCKDGVMSCDSRAPAAACPAGQVFVNCS
DLHTDLELSRERTCEQQLLNLSVSARGPCLSGCACPQGLLRHGDACFLPEECPCTWKGKE
YFPGDQVMSPCHTCVCQRGSFQCTLHPCASTCTAYGDRHYRTFDGLPFDFVGACKVHLV
KSTSDVSFSVIVENVNCYSSGMICRKFISINVGNSLIVFDDDSGNPSPESFLDDKQEVHTWR
VGFFTLVHFPQEHITLLWDQRTTVHVQAGPQWQGQLAGLCGNFDLKTINEMRTPENLELT
NPQEFGSSWAAVECPDTLDPRDMCVLNPLREPFAKKECSILLSEVFEICHPVVDVTWFYSN
CLTDTCGCSQGGDCECFCASVSAYAHQCCQHGVAVDWRTPRLCPYDCDFFNKVLGKGPY
QLSSLAAGGALVGMKAVGDDIVLVRTEDVAPADIVSFLLTAALYKAKAHDPDVVSLEAAD
RPNFFLHVTANGSLELAKWQGRDTFQQHASFLLHRGTRQAGLVALESLAKPSSFLYVSGA
VLALRLYEHTEVFRRGTLFRLLDAKPSGAAYPICEWRYDACASPCFQTCRDPRAASCRDV
PRVEGCVPVCPTPQVLDEVTQRCVYLEDCVEPAVWVPTEALGNETLPPSQGLPTPSDEEPQ
LSQESPRTPTHRPALTPAAPLTTALNPPVTATEEPVVSPGPTQTTLQQPLELTASQLPAGPTES
PASKGVTASLLAIPHTPESSSLPVALQTPTPGMVSGAMETTRVTVIFAGSPNITVSSRSPPAP
RFPLMTKAVTVRGHGSLPVRTTPPQPSLTASPSSRPVASPGAISRSPTSSGSHKAVLTPAVTK
VISRTGVPQPTQAQSASSPSTPLTVAGTAAEQVPVSPLATRSLEIVLSTEKGEAGHSQPMGS
PASPQPHPLPSAPPRPAQHTTMATRSPALPPETPAAASLSTATDGLAATPFMSLESTRPSQLL
SGLPPDTSLPLAKVGTSAPVATPGPKASVITTPLQPQATTLPAQTLSPVLPFTPAAMTQAHPP
THIAPPAAGTAPGLLLGATLPTSGVLPVAEGTASMVSVVPRKSTTGKVAILSKQVSLPTSMY
GSAEGGPTELTPATSHPLTPLVAEPEGAQAGTALPVPTSYALSRVSARTAPQDSMLVLLPQL
AEAHGTSAGPHLAAEPVDEATTEPSGRSAPALSIVEGLAEALATTTEANTSTTCVPIAEQDC
VRHICLEGQLIRVNQSQHCPQGAAPPRCGILGLAVRVGGDRCCPLWECACRCSIFPDLSFV
TFDGSHVALFKEAIYILSQSPDEMLTVHVLDCKSANLGHLNWPPFCLVMLNMTHLAHQVT
IDRFNRKVTVDLQPVWPPVSRYGFRIEDTGHMYMILTPSDIQIQWLHSSGLMIVEASKTSK
AQGHGLCGICDGDAANDLTLKDGSVVGGAEDPAPFLDSWQVPSSLTSVGQTRFRPDSCAT
TDCSPCLRMVSNRTFSACHRFVPPESFCELWIRDTKYVQQPCVALTVYVAMCHKFHVCIE
WRRSDYCPFLCSSDSTYQACVTACEPPKTCQDGILGPLDPEHCQVLGEGCVCSEGTILHRR
HSALCIPEAKCACTDSMGVPRALGETWNSSLSGCCQHQCQAPDTIVPVDLGCPSPRPESC
LRFGEVALLLPTKDPCCLGTVCVCNQTLCEGLAPTCRPGHRLLTHFQEDSCCPSYSCECDP
DLCEAELVPSCRQDQILITGRLGDSCCTSYFCACGDCPDSIPECQEGEALTVHRNTTELCCP
LYQCVCENFRCPQVQCGLGTALVEVWSPDRCCPYKSCECDCDTIPVPRCHLWEKSQLDEE
FMHSVENVCGCAKYECVKAPVCLSRELGVMQPGQTVVELSADGVCHTSRCTTVLDPLTN
FYQINTTSVLCDIHCEANQEYEHPRDLAACCGSCRNVSCLFTFPNGTTSLFLPGASWIADC
ARHHCSSTPLGAVLVRSPISCPPLNETECAKVGGSVVPSLEGCCRTCKEDGRSCKKVTIRM
TIRKNECRSSTPVNLVSCDGRCPSASIYNYNINTYARFCKCCREVGLQRRSVQLFCATNAT

WVPYTVQEPTDCACQWS

>P33527 multipass membrane protein
MALRGFCSADGSDPLWDWNVTWNTSNPDFTKCFQNTVLVWVPCFYLWACFPFYFLYLSR
HDRGYIQMTPLNKTKTALGFLLWIVCWADLFYSFWERSRGIFLAPVFLVSPTLLGITMLLA
TFLIQLERRKGVQSSGIMLTFWLVALVCALAILRSKIMTALKEDAQVDLFRDITFYVYFSLL
LIQLVLSCFSDRSPLFSETIHDPNPCPESSASFLSRITFWWITGLIVRGYRQPLEGSDLWSLN
KEDTSEQVVPVLVKNWKKECAKTRKQPVKVVYSSKDPAQPKESSKVDANEEVEALIVKS
PQKEWNPSLFKVLYKTFGPYFLMSFFFKAIHDLMMFSGPQILKLLIKFVNDTKAPDWQGY
FYTVLLFVTACLQTLVLHQYFHICFVSGMRIKTAVIGAVYRKALVITNSARKSSTVGEIVNL
MSVDAQRFMDLATYINMIWSAPLQVILALYLLWLNLGPSVLAGVAVMVLMVPVNAVMA
MKTKTYQVAHMKSKDNRIKLMNEILNGIKVLKLYAWELAFKDKVLAIRQEELKVLKKSA
YLSAVGTFTWVCTPFLVALCTFAVYVTIDENNILDAQTAFVSLALFNILRFPLNILPMVISSIV
QASVSLKRLRIFLSHEELEPDSIERRPVKDGGGTNSITVRNATFTWARSDPPTLNGITFSIPE
GALVAVVGQVGCGKSSLLSALLAEMDKVEGHVAIKGSVAYVPQQAWIQNDSLRENILFGC
QLEEPYYRSVIQACALLPDLEILPSGDRTEIGEKGVNLSGGQKQRVSLARAVYSNADIYLF
DDPLSAVDAHVGKHIFENVIGPKGMLKNKTRILVTHSMSYLPQVDVIIVMSGGKISEMGS
YQELLARDGAFAEFLRTYASTEQEQDAEENGVTGVSGPGKEAKQMENGMLVTDSAGKQ
LQRQLSSSSSYSGDISRHHNSTAELQKAEAKKEETWKLMEADKAQTGQVKLSVYWDYM
KAIGLFISFLSIFLFMCNHVSALASNYWLSLWTDDPIVNGTQEHTKVRLSVYGALGISQGIA
VFGYSMAVSIGGILASRCLHVDLLHSILRSPMSFFERTPSGNLVNRFSKELDTVDSMIPEVIK
MFMGSLFNVIGACIVILLATPIAAIIIPPLGLIYFFVQRFYVASSRQLKRLESVSRSPVYSHFN
ETLLGVSVIRAFEEQERFIHQSDLKVDENQKAYYPSIVANRWLAVRLECVGNCIVLFAALFA
VISRHSLSAGLVGLSVSYSLQVTTYLNWLVRMSSEMETNIVAVERLKEYSETEKEAPWQIQ
ETAPPSSWPQVGRVEFRNYCLRYREDLDFVLRHINVTINGGEKVGIVGRTGAGKSSLTLGL
FRINESAEGEIIIDGINIAKIGLHDLRFKITIIPQDPVLFSGSLRMNLDPFSQYSDEEVWTSLEL
AHLKDFVSALPDKLDHECAEGGENLSVGQRQLVCLARALLRKTKILVLDEATAAVDLETD
DLIQSTIRTQFEDCTVLTIAHRLNTIMDYTRVIVLDKGEIQEYGAPSDLLQQRGLFYSMAKD
AGLV

>Q13464 peripheral membrane protein
MSTGDSFETRFEKMDNLLRDPKSEVNSDCLLDGLDALVYDLDFPALRKNKNIDNFLSRYK
DTINKIRDLRMKAEDYEVVKVIGRGAFGEVQLVRHKSTRKVYAMKLLSKFEMIKRSDSAF
FWEERDIMAFANSPWVVQLFYAFQDDRYLYMVMEYMPGGDLVNLMSNYDVPEKWARF
YTAEVVLALDAIHSMGFIHRDVKPDNMLLDKSGHLKLADFGTCMKMNKEGMVRCDTAV
GTPDYISPEVLKSQGGDGYYGRECDWWSVGVFLYEMLVGDTPFYADSLVGTYSKIMNHK
NSLTFPDDNDISKEAKNLICAFLTDREVRLGRNGVEEIKRHLFFKNDQWAWETLRDTVAPV
VPDLSSDIDTSNFDDLEEDKGEEETFPIPKAFVGNQLPFVGFTYYSNRRYLSSANPNDNRTS
SNADKSLQESLQKTIYKLEEQLHNEMQLKDEMEQKCRTSNIKLDKIMKELDEEGNQRRN
LESTVSQIEKEKMLLQHRINEYQRKAEQENEKRRNVENEVSTLKDQLEDLKKVSQNSQL
ANEKLSQLQKQLEEANDLLRTESDTAVRLRKSHTEMSKSISQLESLNRELQERNRILENSK
SQTDKDYYQLQAILEAERRDRGHDSEMIGDLQARITSLQEEVKHLKHNLEKVEGERKEA
QDMLNHSEKEKNNLEIDLNYKLKSLQQRLEQEVNEHKVTKARLTDKHQSIEEAKSVAMC
EMEKKLKEEREAREKAENRVVQIEKQCSMLDVDLKQSQQKLEHLTGNKERMEDEVKNL

TLQLEQESNKRLLLQNELKTQAFEADNLKGLEKQMKQEINTLLEAKRLLEFELAQLTKQY
RGNEGQMRELQDQLEAEQYFSTLYKTQVKELKEEIEEKNRENLKKIQELQNEKETLATQL
DLAETKAESEQLARGLLEEQYFELTQESKKAASRNRQEITDKDHTVSRLEEANSMLTKDIE
ILRRENEELTEKMKKAEEEYKLEKEEEISNLKAAFEKNINTERTLKTQAVNKLAEIMNRKD
FKIDRKKANTQDLRKKEKENRKLQLELNQEREKFNQMVVKHQKELNDMQAQLVEECAH
RNELQMQLASKESDIEQLRAKLLDLSDSTSVASFPSADETDGNLPESRIEGWLSVPNRGNI
KRYGWKKQYVVVSSKKILFYNDEQDKEQSNPSMVLDIDKLFHVRPVTQGDVYRAETEEI
PKIFQILYANEGECRKDVEMEPVQQAEKTNFQNHKGHEFIPTLYHFPANCDACAKPLWHV
FKPPPALECRRCHVKCHRDHLDKKEDLICPCKVSYDVTSARDMLLLACSQDEQKKWVTH
LVKKIPKNPPSGFVRASPRTLSTRSTANQSFRKVVKNTSGKTS

>Q01628 single-pass type II membrane protein
MNHTVQTFFSPVNSGQPPNYEMLKEEHEVAVLGAPHNPAPPTSTVIHIRSETSVPDHVVWS
LFNTLFMNPCCLGFIAFAYSVKSRDRKMVGDVTGAQAYASTAKCLNIWALILGILMTILLIV
IPVLIFQAYG

>Q5KU26 single-pass type II membrane protein
MKDDFAEEEEVQSFGYKRFGIQEGTQCTKCKNNWALKFSIILLYILCALLTITVAILGYKVV
EKMDNVTGGMETSRQTYDDKLTAVESDLKKLGDQTGKKAISTNSELSTFRSDILDLRQQL
REITEKTSKNKDTLEKLQASGDALVDRQSQLKETLENNSFLITTVNKTLQAYNGYVTNLQ
QDTSVLQGNLQNQMYSHNVVIMNLNNLNLTQVQQRNLITNLQRSVDDTSQAIQRIKNDF
QNLQQVFLQAKKDTDWLKEKVQSLQTLAANNSALAKANNDTLEDMNSQLNSFTGQME
NITTISQANEQNLKDLQDLHKDAENRTAIKFNQLEERFQLFETDIVNIISNISYTAHHLRTLT
SNLNEVRTTCTDTLTKHTDDLTSLNNTLANIRLDSVSLRMQQDLMRSRLDTEVANLSVIM
EEMKLVDSKHGQLIKNFTILQGPPGPRGPRGDRGSQGPPGPTGNKGQKGEKGEPGPPGPA
GERGPIGPAGPPGERGGKGSKGSQGPKGSRGSPGKPGPQGSSGDPGPPGPPGKEGLPGPQG
PPGFQGLQGTVGEPGVPGPRGLPGLPGVPGMPGPKGPPGPPGPSGAVVPLALQNEPTPAPE
DNGCPPHWKNFTDKCYYFSVEKEIFEDAKLFCEDKSSHLVFINTREEQQWIKKQMVGRES
HWIGLTDSERENEWKWLDGTSPDYKNWKAGQPDNWGHGHGPGEDCAGLIYAGQWNDF
QCEDVNNFICEKDRETVLSSAL

>P48051 multipass membrane protein
MAKLTESMTNVLEGDSMDQDVESPVAIHQPKLPKQARDDLPRHISRDRTKRKIQRYVRKD
GKCNVHHGNVRETYRYLTDIFTTLVDLKWRFNLLIFVMVYTVTWLFFGMIWWLIAYIRG
DMDHIEDPSWTPCVTNLNGFVSAFLFSIETETTIGYGYRVITDKCPEGIILLLIQSVLGSIVN
AFMVGCMFVKISQPKKRAETLVFSTHAVISMRDGKLCLMFRVGDLRNSHIVEASIRAKLIK
SKQTSEGEFIPLNQTDINVGYYTGDDRLFLVSPLIISHEINQQSPFWEISKAQLPKEELEIVVI
LEGMVEATGMTCQARSSYITSEILWGYRFTPVLTLEDGFYEVDYNSFHETYETSTPSLSAK
ELAELASRAELPLSWSVSSKLNQHAELETEEEEKNLEEQTERNGDVANLENESKV

>P13224 single-pass type I membrane protein
MGSGPRGALSLLLLLLLAPPSRPAAGCPAPCSCAGTLVDCGRRGLTWASLPTAFPVDTTELV
LTGNNLTALPPGLLDALPALRTAHLGANPWRCDCRLVPLRAWLAGRPERAPYRDLRCVAP
PALRGRLLPYLAEDELRAACAPGPLCWGALAAQLALLGLGLLHALLLVLLLCRLRRLRAR

ARARAAARLSLTDPLVAERAGTDES

>O15394 single-pass type I membrane protein
MSLLLSFYLLGLLVSSGQALLQVTISLSKVELSVGESKFFTCTAIGEPESIDWYNPQGEKIIS
TQRVVVQKEGVRSRLTIYNANIEDAGIYRCQATDAKGQTQEATVVLEIYQKLTFREVVSPQ
EFKQGEDAEVVCRVSSSPAPAVSWLYHNEEVTTISDNRFAMLANNNLQILNINKSDEGIYR
CEGRVEARGEIDFRDIIVIVNVPPAISMPQKSFNATAERGEEMTFSCRASGSPEPAISWFRNG
KLIEENEKYILKGSNTELTVRNIINSDGGPYVCRATNKAGEDEKQAFLQVFVQPHIIQLKNE
TTYENGQVTLVCDAEGEPIPEITWKRAVDGFTFTEGDKSLDGRIEVKGQHGSSSLHIKDVK
LSDSGRYDCEAASRIGGHQKSMYLDIEYAPKFISNQTIYYSWEGNPINISCDVKSNPPASIH
WRRDKLVLPAKNTTNLKTYSTGRKMILEIAPTSDNDFGRYNCTATNHIGTRFQEYILALAD
VPSSPYGVKIIELSQTTAKVSFNKPDSHGGVPIHHYQVDVKEVASEIWKIVRSHGVQTMVV
LNNLEPNTTYEIRVAAVNGKGQGDYSKIEIFQTLPVREPSPPSIHGQPSSGKSFKLSITKQDD
GGAPILEYIVKYRSKDKEDQWLEKKVQGNKDHIILEHLQWTMGYEVQITAANRLGYSEP
TVYEFSMPPKPNIIKDTLFNGLGLGAVIGLGVAALLLILVVTDVSCFFIRQCGLLMCITRRM
CGKKSGSSGKSKELEEGKAAYLKDGSKEPIVEMRTEDERVTNHEDGSPVNEPNETTPLTEP
EKLPLKEEDGKEALNPETIEIKVSNDIIQSKEDDSKA

>Q9GZK7 multipass membrane protein
MEIVSTGNETITEFVLLGFYDIPELHFLFFIVFTAVYVFIIIGNMLIIVAVVSSQRLHKPMYIFL
ANLSFLDILYTSAVMPKMLEGFLQEATISVAGCLLQFFIFGSLATAECLLLAVMAYDRYLAI
CYPLHYPLLMGPRRYMGLVVTTWLSGFVVDGLVVALVAQLRFCGPNHIDQFYCDFMLFV
GLACSDPRVAQVTTLILSVFCLTIPFGLILTSYARIVVAVLRVPAGASRRRAFSTCSSHLAVVT
TFYGTLMIFYVAPSAVHSQLLSKVFSLLYTVVTPLFNPVIYTMRNKEVHQALRKILCIKQTE
TLD

>P47871 multipass membrane protein
MPPCQPQRPLLLLLLLLACQPQVPSAQVMDFLFEKWKLYGDQCHHNLSLLPPPTELVCNR
TFDKYSCWPDTPANTTANISCPWYLPWHHKVQHRFVFKRCGPDGQWVRGPRGQPWRDA
SQCQMDGEEIEVQKEVAKMYSSFQVMYTVGYSLSLGALLLALAILGGLSKLHCTRNAIHA
NLFASFVLKASSVLVIDGLLRTRYSQKIGDDLSVSTWLSDGAVAGCRVAAVFMQYGIVANY
CWLLVEGLYLHNLLGLATLPERSFFSLYLGIGWGAPMLFVVPWAVVKCLFENVQCWTSN
DNMGFWWILRFPVFLAILINFFIFVRIVQLLVAKLRARQMHHTDYKFRLAKSTLTLIPLLGV
HEVVFAFVTDEHAQGTLRSAKLFFDLFLSSFQGLLVAVLYCFLNKEVQSELRRRWHRWRL
GKVLWEERNTSNHRASSSPGHGPPSKELQFGRGGGSQDSSAETPLAGGLPRLAESPF

>Q6PXP3 multipass membrane protein
MENKEAGTPPPIPSREGRLQPTLLLATLSAAFGSAFQYGYNLSVVNTPHKVFKSFYNETYF
ERHATFMDGKLMLLLWSCTVSMFPLGGLLGSLLVGLLVDSCGRKGTLLINNIFAIIPAILMG
VSKVAKAFELIVFSRVVLGVCAGISYSALPMYLGELAPKNLRGMVGTMTEVFVIVGVFLA
QIFSLQAILGNPAGWPVLLALTGVPALLQLLTLPFFPESPRYSLIQKGDEATARQALRRLRG
HTDMEAELEDMRAEARAERAEGHLSVLHLCALRSLRWQLLSIIVLMAGQQLSGINAINYY
ADTIYTSAGVEAAHSQYVTVGSGVVNIVMTITSAVLVERLGRRHLLLAGYGICGSACLVLT
VVLLFQNRVPELSYLGIICVFAYIAGHSIGPSPVPSVVRTEIFLQSSRRAAFMVDGAVHWLT

NFIIGFLFPSIQEAIGAYSFIIFAGICLLTAIYIYVVIPETKGKTFVEINRIFAKRNRVKLPEEKEE
TIDAGPPTASPAKETSF

>P49758 peripheral membrane protein
MAQGSGDQRAVGVADPEESSPNMIVYCKIEDIITKMQDDKTGGVPIRTVKSFLSKIPSVVT
GTDIVQWLMKNLSIEDPVEAIHLGSLIAAQGYIFPISDHVLTMKDDGTFYRFQAPYFWPSN
CWEPENTDYAIYLCKRTMQNKARLELADYEAENLARLQRAFARKWEFIFMQAEAQVKID
RKKDKTERKILDSQERAFWDVHRPVPGCVNTTEMDIRKCRRLKNPQKVKKSVYGVTEES
QAQSPVHVLSQPIRKTTKEDIRKQITFLNAQIDRHCLKMSKVAESLIAYTEQYVEYDPLITP
AEPSNPWISDDVALWDIEMSKEPSQQRVKRWGFSFDEILKDQVGRDQFLRFLESEFSSENL
RFWLAVQDLKKQPLQDVAKRVEEIWQEFLAPGAPSAINLDSHSYEITSQNVKDGGRYTFE
DAQEHIYKLMKSDSYARFLRSNAYQDLLLAKKKGKSLAGKRLTGLMQSS

>Q02763 single-pass type I membrane protein
MDSLASLVLCGVSLLLSGTVEGAMDLILINSLPLVSDAETSLTCIASGWRPHEPITIGRDFEA
LMNQHQDPLEVTQDVTREWAKKVVWKREKASKINGAYFCEGRVRGEAIRIRTMKMRQQ
ASFLPATLTMTVDKGDNVNISFKKVLIKEEDAVIYKNGSFIHSVPRHEVPDILEVHLPHAQP
QDAGVYSARYIGGNLFTSAFTRLIVRRCEAQKWGPECNHLCTACMNNGVCHEDTGECIC
PPGFMGRTCEKACELHTFGRTCKERCSGQEGCKSYVFCLPDPYGCSCATGWKGLQCNEA
CHPGFYGPDCKLRCSCNNGEMCDRFQGCLCSPGWQGLQCEREGIQRMTPKIVDLPDHIE
VNSGKFNPICKASGWPLPTNEEMTLVKPDGTVLHPKDFNHTDHFSVAIFTIHRILPPDSGV
WVCSVNTVAGMVEKPFNISVKVLPKPLNAPNVIDTGHNFAVINISSEPYFGDGPIKSKKLLY
KPVNHYEAWQHIQVTNEIVTLNYLEPRTEYELCVQLVRRGEGGEGHPGPVRRFTTASIGLP
PPRGLNLLPKSQTTLNLTWQPIFPSSEDDFYVEVERRSVQKSDQQNIKVPGNLTSVLLNNL
HPREQYVVRARVNTKAQGEWSEDLTAWTLSDILPPQPENIKISNITHSSAVISWTILDGYSIS
SITIRYKVQGKNEDQHVDVKIKNATITQYQLKGLEPETAYQVDIFAENNIGSSNPAFSHELV
TLPESQAPADLGGGKMLLIAILGSAGMTCLTVLLAFLIILQLKRANVQRRMAQAFQNVRE
EPAVQFNSGTLALNRKVKNNPDPTIYPVLDWNDIKFQDVIGEGNFGQVLKARIKKDGLRM
DAAIKRMKEYASKDDHRDFAGELEVLCKLGHHPNIINLLGACEHRGYLYLAIEYAPHGNL
LDFLRKSRVLETDPAFAIANSTASTLSSQQLLHFAADVARGMDYLSQKQFIHRDLAARNIL
VGENYVAKIADFGLSRGQEVYVKKTMGRLPVRWMAIESLNYSVYTTNSDVWSYGVLLW
EIVSLGGTPYCGMTCAELYEKLPQGYRLEKPLNCDDEVYDLMRQCWREKPYERPSFAQIL
VSLNRMLEERKTYVNTTLYEKFTYAGIDCSAEEAA

>P32927 single-pass type I membrane protein
MVLAQGLLSMALLALCWERSLAGAEETIPLQTLRCYNDYTSHITCRWADTQDAQRLVNV
TLIRRVNEDLLEPVSCDLSDDMPWSACPHPRCVPRRCVIPCQSFVVTDVDYFSFQPDRPLG
TRLTVTLTQHVQPPEPRDLQISTDQDHFLLTWSVALGSPQSHWLSPGDLEFEVVYKRLQDS
WEDAAILLSNTSQATLGPEHLMPSSTYVARVRTRLAPGSRLSGRPSKWSPEVCWDSQPGD
EAQPQNLECFFDGAAVLSCSWEVRKEVASSVSFGLFYKPSPDAGEEECSPVLREGLGSLHT
RHHCQIPVPDPATHGQYIVSVQPRRAEKHIKSSVNIQMAPPSLNVTKDGDSYSLRWETMK
MRYEHIDHTFEIQYRKDTATWKDSKTETLQNAHSMALPALEPSTRYWARVRVRTSRTGYN
GIWSEWSEARSWDTESVLPMWVLALIVIFLTIAVLLALRFCGIYGYRLRRKWEEKIPNPSK
SHLFQNGSAELWPPGSMSAFTSGSPPHQGPWGSRFPELEGVFPVGFGDSEVSPLTIEDPKH

VCDPPSGPDTTPAASDLPTEQPPSPQPGPPAASHTPEKQASSFDFNGPYLGPPHSRSLPDILG
QPEPPQEGGSQKSPPPGSLEYLCLPAGGQVQLVPLAQAMGPGQAVEVERRPSQGAAGSPS
LESGGGPAPPALGPRVGGQDQKDSPVAIPMSSGDTEDPGVASGYVSSADLVFTPNSGASSV
SLVPSLGLPSDQTPSLCPGLASGPPGAPGPVKSGFEGYVELPPIEGRSPRSPRNNPVPPEAKS
PVLNPGERPADVSPTSPQPEGLLVLQQVGDYCFLPGLGPGPLSLRSKPSSPGPGPEIKNLDQ
AFQVKKPPGQAVPQVPVIQLFKALKQQDYLSLPPWEVNKPGEVC

>Q8N3J6 single-pass type I membrane protein
MIWKRSAVLRFYSVCGLLLQGSQGQFPLTQNVTVVEGGTAILTCRVDQNDNTSLQWSNPA
QQTLYFDDKKALRDNRIELVRASWHELSISVSDVSLSDEGQYTCSLFTMPVKTSKAYLTVL
GVPEKPQISGFSSPVMEGDLMQLTCKTSGSKPAADIRWFKNDKEIKDVKYLKEEDANRKT
FTVSSTLDFRVDRSDDGVAVICRVDHESLNATPQVAMQVLEIHYTPSVKIIPSTPFPQEGQPL
ILTCESKGKPLPEPVLWTKDGGELPDPDRMVVSGRELNILFLNKTDNGTYRCEATNTIGQS
SAEYVLIVHDVPNTLLPTTIIPSLTTATVTTTVAITTSPTTSATTSSIRDPNALAGQNGPDHAL
IGGIVAVVVFVTLCSIFLLGRYLARHKGTYLTNEAKGAEDAPDADTAIINAEGSQVNAEEK
KEYFI

>P29973 multipass membrane protein
MKLSMKNNIINTQQSFVTMPNVIVPDIEKEIRRMENGACSSFSEDDDSASTSEESENENPH
ARGSFSYKSLRKGGPSQREQYLPGAIALFNVNNSSNKDQEPEEKKKKKKEKKSKSDDKN
ENKNDPEKKKKKKDKEKKKKEEKSKDKKEEEKKEVVVIDPSGNTYYNWLFCITLPVMY
NWTMVIARACFDELQSDYLEYWLILDYVSDIVYLIDMFVRTRTGYLEQGLLVKEELKLIN
KYKSNLQFKLDVLSLIPTDLLYFKLGWNYPEIRLNRLLRFSRMFEFFQRTETRTNYPNIFRIS
NLVMYIVIIIHWNACVFYSISKAIGFGNDTWVYPDINDPEFGRLARKYVYSLYWSTLTLTTI
GETPPPVRDSEYVFVVVDFLIGVLIFATIVGNIGSMISNMNAARAEFQARIDAIKQYMHFRN
VSKDMEKRVIKWFDYLWTNKKTVDEKEVLKYLPDKLRAEIAINVHLDTLKKVRIFADCE
AGLLVELVLKLQPQVYSPGDYICKKGDIGREMYIIKEGKLAVVADDGVTQFVVLSDGSYF
GEISILNIKGSKAGNRRTANIKSIGYSDLFCLSKDDLMEALTEYPDAKTMLEEKGKQILMK
DGLLDLNIANAGSDPKDLEEKVTRMEGSVDLLQTRFARILAEYESMQQKLKQRLTKVEKF
LKPLIDTEFSSIEGPGAESGPIDST

>O00501 multipass membrane protein
MGSAALEILGLVLCLVGWGGLILACGLPMWQVTAFLDHNIVTAQTTWKGLWMSCVVQS
TGHMQCKVYDSVLALSTEVQAARALTVSAVLLAFVALFVTLAGAQCTTCVAPGPAKARV
ALTGGVLYLFCGLLALVPLCWFANIVVREFYDPSVPVSQKYELGAALYIGWAATALLMVG
GCLLCCGAWVCTGRPDLSFPVKYSAPRRPTATGDYDKKNYV

>Q8WXG9 multipass membrane protein
MSVFLGPGMPSASLLVNLLSALLILFVFGETEIRFTGQTEFVVNETSTTVIRLIIERIGEPANV
TAIVSLYGEDAGDFFDTYAAAFIPAGETNRTVYIAVCDDDLPEPDETFIFHLTLQKPSANVK
LGWPRTVTVTILSNDNAFGIISFNMLPSIAVSEPKGRNESMPLTLIREKGTYGMVMVTFEVE
GGPNPPDEDLSPVKGNITFPPGRATVIYNLTVLDDEVPENDEIFLIQLKSVEGGAEINTSRNS
IEIIIKKNDSPVRFLQSIYLVPEEDHILIIPVVRGKDNNGNLIGSDEYEVSISYAVTTGNSTAHA
QQNLDFIDLQPNTTVVFPPFIHESHLKFQIVDDTIPEIAESFHIMLLKDTLQGDAVLISPSVV

QVTIKPNDKPYGVLSFNSVLFERTVIIDEDRISRYEEITVVRNGGTHGNVSANWVLTRNST
DPSPVTADIRPSSGVLHFAQGQMLATIPLTVVDDDLPEEAEAYLLQILPHTIRGGAEVSEPA
ELLFYIQDSDDVYGLITFFPMENQKIESSPGERYLSLSFTRLGGTKGDVRLLYSVLYIPAGAV
DPLQAKEGILNISRRNDLIFPEQKTQVTTKLPIRNDAFLQNGAHFLVQLETVELLNIIPLIPPI
SPRFGEICNISLLVTPAIANGEIGFLSNLPIILHEPEDFAAEVVYIPLHRDGTDGQATVYWSLK
PSGFNSKAVTPDDIGPFNGSVLFLSGQSDTTINITIKGDDIPEMNETVTLSLDRVNVENQVL
KSGYTSRDLIILENDDPGGVFEFSPASRGPYVIKEGESVELHIIRSRGSLVKQFLHYRVEPRD
SNEFYGNTGVLEFKPGEREIVITLLARLDGIPELDEHYWVVLSSHGERESKLGSATIVNITIL
KNDDPHGIIEFVSDGLIVMINESKGDAIYSAVYDVVRNRGNFGDVSVSWVVSPDFTQDVF
PVQGTVVFGDQEFSKNITIYSLPDEIPEEMEEFTVILLNGTGGAKVGNRTTATLRIRRNDDPI
YFAEPRVVRVQEGETANFTVLRNGSVDVTCMVQYATKDGKATARERDFIPVEKGETLIFEV
GSRQQSISIFVNEDGIPETDEPFYIILLNSTGDTVVYQYGVATVIIEANDDPNGIFSLEPIDKA
VEEGKTNAFWILRHRGYFGSVSVSWQLFQNDSALQPGQEFYETSGTVNFMDGEEAKPIIL
HAFPDKIPEFNEFYFLKLVNISGGSPGPGGQLAETNLQVTVMVPFNDDPFGVFILDPECLER
EVAEDVLSEDDMSYITNFTILRQQGVFGDVQLGWEILSSEFPAGLPPMIDFLLVGIFPTTVH
LQQHMRRHHSGTDALYFTGLEGAFGTVNPKYHPSRNNTIANFTFSAWVMPNANTNGFIIA
KDDGNGSIYYGVKIQTNESHVTLSLHYKTLGSNATYIAKTTVMKYLEESVWLHLLIILEDG
IIEFYLDGNAMPRGIKSLKGEAITDGPGILRIGAGINGNDRFTGLMQDVRSYERKLTLEEIY
ELHAMPAKSDLHPISGYLEFRQGETNKSFIISARDDNDEEGEELFILKLVSVYGGARISEEN
TTARLTIQKSDNANGLFGFTGACIPEIAEEGSTISCVVERTRGALDYVHVFYTISQIETDGIN
YLVDDFANASGTITFLPWQRSEVLNIYVLDDDIPELNEYFRVTLVSAIPGDGKLGSTPTSGA
SIDPEKETTDITIKASDHPYGLLQFSTGLPPQPKDAMTLPASSVPHITVEEEDGEIRLLVIRA
QGLLGRVTAEFRTVSLTAFSPEDYQNVAGTLEFQPGERYKYIFINITDNSIPELEKSFKVELL
NLEGGVAELFRVDGSGSGDGDMEFFLPTIHKRASLGVASQILVTIAASDHAHGVFEFSPESL
FVSGTEPEDGYSTVTLNVIRHHGTLSPVTLHWNIDSDPDGDLAFTSGNITFEIGQTSANITV
EILPDEDPELDKAFSVSVLSVSSGSLGAHINATLTVLASDDPYGIFIFSEKNRPVKVEEATQN
ITLSIIRLKGLMGKVLVSYATLDDMEKPPYFPPNLARATQGRDYIPASGFALFGANQSEATI
AISILDDDEPERSESVFIELLNSTLVAKVQSRSIPNSPRLGPKVETIAQLIIIANDDAFGTLQLS
APIVRVAENHVGPIINVTRTGGAFADVSVKFKAVPITAIAGEDYSIASSDVVLLEGETSKAVP
IYVINDIYPELEESFLVQLMNETTGGARLGALTEAVIIIEASDDPYGLFGFQITKLIVEEPEFN
SVKVNLPIIRNSGTLGNVTVQWVATINGQLATGDLRVVSGNVTFAPGETIQTLLLEVLADD
VPEIEEVIQVQLTDASGGGTIGLDRIANIIIPANDDPYGTVAFAQMVYRVQEPLERSSCANIT
VRRSGGHFGRLLLFYSTSDIDVVALAMEEGQDLLSYYESPIQGVPDPLWRTWMNVSAVGE
PLYTCATLCLKEQACSAFSFFSASEGPQCFWMTSWISPAVNNSDFWTYRKNMTRVASLFSG
QAVAGSDYEPVTRQWAIMQEGDEFANLTVSILPDDFPEMDESFLISLLEVHLMNISASLKN
QPTIGQPNISTVVIALNGDAFGVFVIYNISPNTSEDGLFVEVQEQPQTLVELMIHRTGGSLG
QVAVEWRVVGGTATEGLDFIGAGEILTFAEGETKKTVILTILDDSEPEDDESIIVSLVYTEGG
SRILPSSDTVRVNILANDNVAGIVSFQTASRSVIGHEGEILQFHVIRTFPGRGNVTVNWKIIG
QNLELNFANFSGQLFFPEGSLNTTLFVHLLDDNIPEEKEVYQVILYDVRTQGVPPAGIALLD
AQGYAAVLTVEASDEPHGVLNFALSSRFVLLQEANITIQLFINREFGSLGAINVTYTTVPGM
LSLKNQTVGNLAEPEVDFVPIIGFLILEEGETAAAINITILEDDVPELEEYFLVNLTYVGLTM
AASTSFPPRLDSEGLTAQVIIDANDGARGVIEWQQSRFEVNETHGSLTLVAQRSREPLGHVS
LFVYAQNLEAQVGLDYIFTPMILHFADGERYKNVNIMILDDDIPEGDEKFQLILTNPSPGLE
LGKNTIALIIVLANDDGPGVLSFNNSEHFFLREPTALYVQESVAVLYIVREPAQGLFGTVTV

QFIVTEVNSSNESKDLTPSKGYIVLEEGVRFKALQISAILDTEPEMDEYFVCTLFNPTGGAR
LGVHVQTLITVLQNQAPLGLFSISAVENRATSIDIEEANRTVYLNVSRTNGIDLAVSVQWET
VSETAFGMRGMDVVFSVFQSFLDESASGWCFFTLENLIYGIMLRKSSVTVYRWQGIFIPVE
DLNIENPKTCEAFNIGFSPYFVITHEERNEEKPSLNSVFTFTSGFKLFLVQTIIILESSQVRYFT
SDSQDYLIIASQRDDSELTQVFRWNGGSFVLHQKLPVRGVLTVALFNKGGSVFLAISQANA
RLNSLLFRWSGSGFINFQEVPVSGTTEVEALSSANDIYLIFAENVFLGDQNSIDIFIWEMGQ
SSFRYFQSVDFAAVNRIHSFTPASGIAHILLIGQDMSALYCWNSERNQFSFVLEVPSAYDVA
SVTVKSLNSSKNLIALVGAHSHIYELAYISSHSDFIPSSGELIFEPGEREATIAVNILDDTVPE
KEESFKVQLKNPKGGAEIGINDSVTITILSNDDAYGIVAFAQNSLYKQVEEMEQDSLVTLN
VERLKGTYGRITIAWEADGSISDIFPTSGVILFTEGQVLSTITLTILADNIPELSEVVIVTLTRI
TTEGVEDSYKGATIDQDRSKSVITTLPNDSPFGLVGWRAASVFIRVAEPKENTTTLQLQIAR
DKGLLGDIAIHLRAQPNFLLHVDNQATENEDYVLQETIIIMKENIKEAHAEVSILPDDLPEL
EEGFIVTITEVNLVNSDFSTGQPSVRRPGMEIAEIMIEENDDPRGIFMFHVTRGAGEVITAYE
VPPPLNVLQVPVVRLAGSFGAVNVYWKASPDSAGLEDFKPSHGILEFADKQVTAMIEITII
DDAEFELTETFNISLISVAGGGRLGDDVVVTVVIPQNDSPFGVFGFEEKTVMIDESLSSDDP
DSYVTLTVVRSPGGKGTVRLEWTIDEKAKHNLSPLNGTLHFDETESQKTIVLHTLQDTVL
EEDRRFTIQLISIDEVEISPVKGSASIIIRGDKRASGEVGIAPSSRHILIGEPSAKYNGTAIISLV
RGPGILGEVTVFWRIFPPSVGEFAETSGKLTMRDEQSAVIVVIQALNDDIPEEKSFYEFQLTA
VSEGGVLSESSSTANITVVASDSPYGRFAFSHEQLRVSEAQRVNITIIRSSGDFGHVRLWYK
TMSGTAEAGLDFVPAAGELLFEAGEMRKSLHVEILDDDYPEGPEEFSLTITKVELQGRGYD
FTIQENGLQIDQPPEIGNISIVRIIIMKNDNAEGIIEFDPKYTAFEVEEDVGLIMIPVVRLHGT
YGYVTADFISQSSSASPGGVDYILHGSTVTFQHGQNLSFINISIIDDNESEFEEPIEILLTGATG
GAVLGRHLVSRIIIAKSDSPFGVIRFLNQSKISIANPNSTMILSLVLERTGGLLGEIQVNWETV
GPNSQEALLPQNRDIADPVSGLFYFGEGEGGVRTIILTIYPHEEIEVEETFIIKLHLVKGEAKL
DSRAKDVTLTIQEFGDPNGVVQFAPETLSKKTYSEPLALEGPLLITFFVRRVKGTFGEIMVY
WELSSEFDITEDFLSTSGFFTIADGESEASFDVHLLPDEVPEIEEDYVIQLVSVEGGAELDLE
KSITWFSVYANDDPHGVFALYSDRQSILIGQNLIRSIQINITRLAGTFGDVAVGLRISSDHKE
QPIVTENAERQLVVKDGATYKVDVVPIKNQVFLSLGSNFTLQLVTVMLVGGRFYGMPTIL
QEAKSAVLPVSEKAANSQVGFESTAFQLMNITAGTSHVMISRRGTYGALSVAWTTGYAPG
LEIPEFIVVGNMTPTLGSLSFSHGEQRKGVFLWTFPSPGWPEAFVLHLSGVQSSAPGGAQL
RSGFIVAEIEPMGVFQFSTSSRNIIVSEDTQMIRLHVQRLFGFHSDLIKVSYQTTAGSAKPLE
DFEPVQNGELFFQKFQTEVDFEITIINDQLSEIEEFFYINLTSVEIRGLQKFDVNWSPRLNLD
FSVAVITILDNDDLAGMDISFPETTVAVAVDTTLIPVETESTTYLSTSKTTTILQPTNVVAIVT
EATGVSAIPEKLVTLHGTPAVSEKPDVATVTANVSIHGTFSLGPSIVYIEEEMKNGTFNTAEV
LIRRTGGFTGNVSITVKTFGERCAQMEPNALPFRGIYGISNLTWAVEEEDFEEQTLTLIFLDG
ERERKVSVQILDDDEPEGQEFFYVFLTNPQGGAQIVEEKDDTGFAAFAMVIITGSDLHNGII
GFSEESQSGLELREGAVMRRLHLIVTRQPNRAFEDVKVFWRVTLNKTVVVLQKDGVNLV
EELQSVSGTTTCTMGQTKCFISIELKPEKVPQVEVYFFVELYEATAGAAINNSARFAQIKILE
SDESQSLVYFSVGSRLAVAHKKATLISLQVARDSGTGLMMSVNFSTQELRSAETIGRTIISPA
ISGKDFVITEGTLVFEPGQRSTVLDVILTPETGSLNSFPKRFQIVLFDPKGGARIDKVYGTAN
ITLVSDADSQAIWGLADQLHQPVNDDILNRVLHTISMKVATENTDEQLSAMMHLIEKITTE
GKIQAFSVASRTLFYEILCSLINPKRKDTRGFSHFAEVTENFAFSLLTNVTCGSPGEKSKTIL
DSCPYLSILALHWYPQQINGHKFEGKEGDYIRIPERLLDVQDAEIMAGKSTCKLVQFTEYS
SQQWFISGNNLPTLKNKVLSLSVKGQSSQLLTNDNEVLYRIYAAEPRIIPQTSLCLLWNQA

AASWLSDSQFCKVVEETADYVECACSHMSVYAVYARTDNLSSYNEAFFTSGFICISGLCLA
VLSHIFCARYSMFAAKLLTHMMAASLGTQILFLASAYASPQLAEESCSAMAAVTHYLYLC
QFSWMLIQSVNFWYVLVMNDEHTERRYLLFFLLSWGLPAFVVILLIVILKGIYHQSMSQIY
GLIHGDLCFIPNVYAALFTAALVPLTCLVVVFVVFIHAYQVKPQWKAYDDVFRGRTNAAEI
PLILYLFALISVTWLWGGLHMAYRHFWMLVLFVIFNSLQGLYVFMVYFILHNQMCCPMK
ASYTVEMNGHPGPSTAFFTPGSGMPPAGGEISKSTQNLIGAMEEVPPDWERASFQQGSQA
SPDLKPSPQNGATFPSSGGYGQGSLIADEESQEFDDLIFALKTGAGLSVSDNESGQGSQEG
GTLTDSQIVELRRIPIADTHL

>Q13615 peripheral membrane protein
MDEETRHSLECIQANQIFPRKQLIREDENLQVPFLELHGESTEFVGRAEDAIIALSNYRLHI
KFKESLVNVPLQLIESVECRDIFQLHLTCKDCKVIRCQFSTFEQCQEWLKRLNNAIRPPAKI
EDLFSFAYHAWCMEVYASEKEQHGDLCRPGEHVTSRFKNEVERMGFDMNNAWRISNINE
KYKLCGSYPQELIVPAWITDKELESVSSFRSWKRIPAVIYRHQSNGAVIARCGQPEVSWWG
WRNADDEHLVQSVAKACASDSRSSGSKLSTRNTSRDFPNGGDLSDVEFDSSLSNASGAES
LAIQPQKLLILDARSYAAAVANRAKGGGCECPEYYPNCEVVFMGMANIHSIRRSFQSLRLL
CTQMPDPGNWLSALESTKWLHHLSVLLKSALLVVHAVDQDQRPVLVHCSDGWDRTPQIV
ALAKLLLDPYYRTIEGFQVLVEMEWLDFGHKFADRCGHGENSDDLNERCPVFLQWLDCV
HQLQRQFPCSFEFNEAFLVKLVQHTYSCLFGTFLCNNAKERGEKHTQERTCSVWSLLRAG
NKAFKNLLYSSQSEAVLYPVCHVRNLMLWSAVYLPCPSPTTPVDDSCAPYPAPGTSPDDPP
LSRLPKTRSYDNLTTACDNTVPLASRRCSDPSLNEKWQEHRRSLELSSLAGPGEDPLSADS
LGKPTRVPGGAELSVAAGVAEGQMENILQEATKEESGVEEPAHRAGIEIQEGKEDPLLEKE
SRRKTPEASAIGLHQDPELGDAALRSHLDMSWPLFSQGISEQQSGLSVLLSSLQVPPRGED
SLEVPVEQFRIEEIAEGREEAVLPIPVDAKVGYGTSQSCSLLPSQVPFETRGPNVDSSTDML
VEDKVKSVSGPQGHHRSCLVNSGKDRLPQTMEPSPSETSLVERPQVGSVVHRTSLGSTLSL
TRSPCALPLAECKEGLVCNGAPETENRASEQPPGLSTLQMYPTPNGHCANGEAGRSKDSL
SRQLSAMSCSSAHLHSRNLHHKWLHSHSGRPSATSSPDQPSRSHLDDDGMSVYTDTIQQR
LRQIESGHQQEVETLKKQVQELKSRLESQYLTSSLHFNGDFGDEVTSIPDSESNLDQNCLS
RCSTEIFSEASWEQVDKQDTEMTRWLPDHLAAHCYACDSAFWLASRKHHCRNCGNVFC
SSCCNQKVPVPSQQLFEPSRVCKSCYSSLHPTSSSIDLELDKPIAATSN

>Q9HBW0 multipass membrane protein
MVIMGQCYYNETIGFFYNNSGKELSSHWRPKDVVVVALGLTVSVLVLLTNLLVIAAIASN
RRFHQPIYYLLGNLAAADLFAGVAYLFLMFHTGPRTARLSLEGWFLRQGLLDTSLTASVAT
LLAIAVERHRSVMAVQLHSRLPRGRVVMLIVGVWVAALGLGLLPAHSWHCLCALDRCSR
MAPLLSRSYLAVWALSSLLVFLLMVAVYTRIFFYVRRRVQRMAEHVSCHPRYRETTLSLV
KTVVIILGAFVVCWTPGQVVLLLDGLGCESCNVLAVEKYFLLLAEANSLVNAAVYSCRDA
EMRRTFRRLLCCACLRQSTRESVHYTSSAQGGASTRIMLPENGHPLMDSTL

>Q8IY49 multipass membrane protein
MFAPRLLDFQKTKYARFMNHRVPAHKRYQPTEYEHAANCATHAFWIIPSILGSSNLYFLSD
DDWETISAWIYGLGLCGLFVVSTVFHTISWKKSHLRMVEHCLHMFDRMVIYFFIAASYAP
WLNLRELGPWASHMRWLVWIMASVGTIYVFFFHERTGSCVQFLRGEACPKAGTACLPAR
YKLVELLCYVVMGFFPALVILSMPNTEGIWELVTGGVFYCLGMVFFKSDGRIPFAHAIWH

LFVAFGAGTHYYAIWRYLYLPSTLQTKVSK

>P23945 multipass membrane protein
MALLLVSLLAFLSLGSGCHHRICHCSNRVFLCQESKVTEIPSDLPRNAIELRFVLTKLRVIQK
GAFSGFGDLEKIEISQNDVLEVIEADVFSNLPKLHEIRIEKANNLLYINPEAFQNLPNLQYLL
ISNTGIKHLPDVHKIHSLQKVLLDIQDNINIHTIERNSFVGLSFESVILWLNKNGIQEIHNCAF
NGTQLDELNLSDNNNLEELPNDVFHGASGPVILDISRTRIHSLPSYGLENLKKLRARSTYN
LKKLPTLEKLVALMEASLTYPSHCCAFANWRRQISELHPICNKSILRQEVDYMTQARGQRS
SLAEDNESSYSRGFDMTYTEFDYDLCNEVVDVTCSPKPDAFNPCEDIMGYNILRVLIWFISI
LAITGNIIVLVILTTSQYKLTVPRFLMCNLAFADLCIGIYLLLIASVDIHTKSQYHNYAIDWQ
TGAGCDAAGFFTVFASELSVYTLTAITLERWHTITHAMQLDCKVQLRHAASVMVMGWIF
AFAAALFPIFGISSYMKVSICLPMDIDSPLSQLYVMSLLVLNVLAFVVICGCYIHIYLTVRNP
NIVSSSSDTRIAKRMAMLIFTDFLCMAPISFFAISASLKVPLITVSKAKILLVLFHPINSCANP
FLYAIFTKNFRRDFFILLSKCGCYEMQAQIYRTETSSTVHNTHPRNGHCSSAPRVTNGSTYI
LVPLSHLAQN

>O75477 single-pass type II membrane protein
MTQARVLVAAVVGLVAVLLYASIHKIEEGHLAVYYRGGALLTSPSGPGYHIMLPFITTFRSV
QTTLQTDEVKNVPCGTSGGVMIYIDRIEVVNMLAPYAVFDIVRNYTADYDKTLIFNKIHHE
LNQFCSAHTLQEVYIELFDQIDENLKQALQKDLNLMAPGLTIQAVRVTKPKIPEAIRRNFEL
MEAEKTKLLIAAQKQKVVEKEAETERKKAVIEAEKIAQVAKIRFQQKVMEKETEKRISEIE
DAAFLAREKAKADAEYYAAHKYATSNKHKLTPEYLELKKYQAIASNSKIYFGSNIPNMFV
DSSCALKYSDIRTGRESSLPSKEALEPSGENVIQNKESTG

>Q4LDE5 peripheral membrane protein
MWPRLAFCCWGLALVSGWATFQQMSPSRNFSFRLFPETAPGAPGSIPAPPAPGDEAAGSRV
ERLGQAFRRRVRLLRELSERLELVFLVDDSSSVGEVNFRSELMFVRKLLSDFPVVPTATRV
AIVTFSSKNYVVPRVDYISTRRARQHKCALLLQEIPAISYRGGGTYTKGAFQQAAQILLHA
RENSTKVVFLITDGYSNGGDPRPIAASLRDSGVEIFTFGIWQGNIRELNDMASTPKEEHCY
LLHSFEEFEALARRALHEDLPSGSFIQDDMVHCSYLCDEGKDCCDRMGSCKCGTHTGHF
ECICEKGYYGKGLQYECTACPSGTYKPEGSPGGISSCIPCPDENHTSPPGSTSPEDCVCREG
YRASGQTCELVHCPALKPPENGYFIQNTCNNHFNAACGVRCHPGFDLVGSSIILCLPNGLW
SGSESYCRVRTCPHLRQPKHGHISCSTREMLYKTTCLVACDEGYRLEGSDKLTCQGNSQW
DGPEPRCVERHCSTFQMPKDVIISPHNCGKQPAKFGTICYVSCRQGFILSGVKEMLRCTTS
GKWNVGVQAAVCKDVEAPQINCPKDIEAKTLEQQDSANVTWQIPTAKDNSGEKVSVHV
HPAFTPPYLFPIGDVAIVYTATDLSGNQASCIFHIKVIDAEPPVIDWCRSPPPVQVSEKVHAA
SWDEPQFSDNSGAELVITRSHTQGDLFPQGETIVQYTATDPSGNNRTCDIHIVIKGSPCEIPF
TPVNGDFICTPDNTGVNCTLTCLEGYDFTEGSTDKYYCAYEDGVWKPTYTTEWPDCAKK
RFANHGFKSFEMFYKAARCDDTDLMKKFSEAFETTLGKMVPSFCSDAEDIDCRLEENLTK
KYCLEYNYDYENGFAIGPGGWGAANRLDYSYDDFLDTVQETATSIGNAKSSRIKRSAPLS
DYKIKLIFNITASVPLPDERNDTLEWENQQRLLQTLETITNKLKRTLNKDPMYSFQLASEIL
IADSNSLETKKASPFCRPGSVLRGRMCVNCPLGTYYNLEHFTCESCRIGSYQDEEGQLECK
LCPSGMYTEYIHSRNISDCKAQCKQGTYSYSGLETCESCPLGTYQPKFGSRSCLSCPENTS
TVKRGAVNISACGVPCPEGKFSRSGLMPCHPCPRDYYQPNAGKAFCLACPFYGTTPFAGS

RSITECSSFSSTFSAAEESVVPPASLGHIKKRHEISSQVFHECFFNPCHNSGTCQQLGRGYVC
LCPLGYTGLKCETDIDECSPLPCLNNGVCKDLVGEFICECPSGYTGQRCEENINECSSSPCL
NKGICVDGVAGYRCTCVKGFVGLHCETEVNECQSNPCLNNAVCEDQVGGFLCKCPPGFL
GTRCGKNVDECLSQPCKNGATCKDGANSFRCLCAAGFTGSHCELNINECQSNPCRNQAT
CVDELNSYSCKCQPGFSGKRCETEQSTGFNLDFEVSGIYGYVMLDGMLPSLHALTCTFW
MKSSDDMNYGTPISYAVDNGSDNTLLLTDYNGWVLYVNGREKITNCPSVNDGRWHHIAI
TWTSANGIWKVYIDGKLSDGGAGLSVGLPIPGGGALVLGQEQDKKGEGFSPAESFVGSIS
QLNLWDYVLSPQQVKSLATSCPEELSKGNVLAWPDFLSGIVGKVKIDSKSIFCSDCPRLGG
SVPHLRTASEDLKPGSKVNLFCDPGFQLVGNPVQYCLNQGQWTQPLPHCERISCGVPPPLE
NGFHSADDFYAGSTVTYQCNNGYYLLGDSRMFCTDNGSWNGVSPSCLDVDECAVGSDC
SEHASCLNVDGSYICSCVPPYTGDGKNCAEPIKCKAPGNPENGHSSGEIYTVGAEVTFSCQ
EGYQLMGVTKITCLESGEWNHLIPYCKAVSCGKPAIPENGCIEELAFTFGSKVTYRCNKGY
TLAGDKESSCLANSSWSHSPPVCEPVKCSSPENINNGKYILSGLTYLSTASYSCDTGYSLQ
GPSIIECTASGIWDRAPPACHLVFCGEPPAIKDAVITGNNFTFRNTVTYTCKEGYTLAGLDTI
ECLADGKWSRSDQQCLAVSCDEPPIVDHASPETAHRLFGDIAFYYCSDGYSLADNSQLLC
NAQGKWVPPEGQDMPRCIAHFCEKPPSVSYSILESVSKAKFAAGSVVSFKCMEGFVLNTS
AKIECMRGGQWNPSPMSIQCIPVRCGEPPSIMNGYASGSNYSFGAMVAYSCNKGFYIKGE
KKSTCEATGQWSSPIPTCHPVSCGEPPKVENGFLEHTTGRIFESEVRYQCNPGYKSVGSPV
FVCQANRHWHSESPLMCVPLDCGKPPPIQNGFMKGENFEVGSKVQFFCNEGYELVGDSS
WTCQKSGKWNKKSNPKCMPAKCPEPPLLENQLVLKELTTEVGVVTFSCKEGHVLQGPSV
LKCLPSQQWNDSFPVCKIVLCTPPPLISFGVPIPSSALHFGSTVKYSCVGGFFLRGNSTTLC
QPDGTWSSPLPECVPVECPQPEEIPNGIIDVQGLAYLSTALYTCKPGFELVGNTTTLCGENG
HWLGGKPTCKAIECLKPKEILNGKFSYTDLHYGQTVTYSCNRGFRLEGPSALTCLETGDW
DVDAPSCNAIHCDSPQPIENGFVEGADYSYGAIIIYSCFPGFQVAGHAMQTCEESGWSSSIP
TCMPIDCGLPPHIDFGDCTKLKDDQGYFEQEDDMMEVPYVTPHPPYHLGAVAKTWENTK
ESPATHSSNFLYGTMVSYTCNPGYELLGNPVLICQEDGTWNGSAPSCISIECDLPTAPENGF
LRFTETSMGSAVQYSCKPGHILAGSDLRLCLENRKWSGASPRCEAISCKKPNPVMNGSIK
GSNYTYLSTLYYECDPGYVLNGTERRTCQDDKNWDEDEPICIPVDCSSPPVSANGQVRGD
EYTFQKEIEYTCNEGFLLEGARSRVCLANGSWSGATPDCVPVRCATPPQLANGVTEGLDY
GFMKEVTFHCHEGYILHGAPKLTCQSDGNWDAEIPLCKPVNCGPPEDLAHGFPNGFSFIH
GGHIQYQCFPGYKLHGNSSRRCLSNGSWSGSSPSCLPCRCSTPVIEYGTVNGTDFDCGKA
ARIQCFKGFKLLGLSEITCEADGQWSSGFPHCEHTSCGSLPMIPNAFISETSSWKENVITYS
CRSGYVIQGSSDLICTEKGVWSQPYPVCEPLSCGSPPSVANAVATGEAHTYESEVKLRCLE
GYTMDTDTDTFTCQKDGRWFPERISCSPKKCPLPENITHILVHGDDFSVNRQVSVSCAEGY
TFEGVNISVCQLDGTWEPPFSDESCSPVSCGKPESPEHGFVVGSKYTFESTIIYQCEPGYEL
EGNRERVCQENRQWSGGVAICKETRCETPLEFLNGKADIENRTTGPNVVYSCNRGYSLEG
PSEAHCTENGTWSHPVPLCKPNPCPVPFVIPENALLSEKEFYVDQNVSIKCREGFLLQGHG
IITCNPDETWTQTSAKCEKISCGPPAHVENAIARGVHYQYGDMITYSCYSGYMLEGFLRSV
CLENGTWTSPPICRAVCRFPCQNGGICQRPNACSCPEGWMGRLCEEPICILPCLNGGRCVA
PYQCDCPPGWTGSRCHTAVCQSPCLNGGKCVRPNRCHCLSSWTGHNCSRKRRTGF

>Q9P0X4 multipass membrane protein
MAESASPPSSSAAAPAAEPGVTTEQPGPRSPPSSPPGLEEPLDGADPHVPHPDLAPIAFFCL
RQTTSPRNWCIKMVCNPWFECVSMLVILLNCVTLGMYQPCDDMDCLSDRCKILQVFDDF

IFIFFAMEMVLKMVALGIFGKKCYLGDTWNRLDFFIVMAGMVEYSLDLQNINLSAIRTVR
VLRPLKAINRVPSMRILVNLLLDTLPMLGNVLLLCFFVFFIFGIIGVQLWAGLLRNRCFLEE
NFTIQGDVALPPYYQPEEDDEMPFICSLSGDNGIMGCHEIPPLKEQGRECCLSKDDVYDFG
AGRQDLNASGLCVNWNRYYNVCRTGSANPHKGAINFDNIGYAWIVIFQVITLEGWVEIM
YYVMDAHSFYNFIYFILLIIVGSFFMINLCLVVIATQFSETKQREHRLMLEQRQRYLSSSTV
ASYAEPGDCYEEIFQYVCHILRKAKRRALGLYQALQSRRQALGPEAPAPAKPGPHAKEPR
HYHGKTKGQGDEGRHLGSRHCQTLHGPASPGNDHSGRELCPQHSPLDATPHTLVQPIPAT
LASDPASCPCCQHEDGRRPSGLGSTDSGQEGSGSGSSAGGEDEADGDGARSSEDGASSEL
GKEEEEEEQADGAVWLCGDVWRETRAKLRGIVDSKYFNRGIMMAILVNTVSMGIEHHEQ
PEELTNILEICNVVFTSMFALEMILKLAAFGLFDYLRNPYNIFDSIIVIISIWEIVGQADGGLS
VLRTFRLLRVLKLVRFMPALRRQLVVLMKTMDNVATFCMLLMLFIFIFSILGMHIFGCKFSL
RTDTGDTVPDRKNFDSLLWAIVTVFQILTQEDWNVVLYNGMASTSPWASLYFVALMTFGN
YVLFNLLVAILVEGFQAEGDANRSYSDEDQSSSNIEEFDKLQEGLDSSGDPKLCPIPMTPNG
HLDPSLPLGGHLGPAGAAGPAPRLSLQPDPMLVALGSRKSSVMSLGRMSYDQRSLSSSRSS
YYGPWGRSAAWASRRSSWNSLKHKPPSAEHESLLSAERGGGARVCEVAADEGPPRAAPL
HTPHAHHIHHGPHLAHRHRHHRRTLSLDNRDSVDLAELVPAVGAHPRAAWRAAGPAPGH
EDCNGRMPSIAKDVFTKMGDRGDRGEDEEEIDYTLCFRVRKMIDVYKPDWCEVREDWS
VVYLFSPENRFRVLCQTIIAHKLFDYVVLAFIFLNCITIALERPQIEAGSTERIFLTVSNYIFTAI
FVGEMTLKVVSLGLYFGEQAYLRSSWNVLDGFLVFVSIIDIVVSLASAGGAKILGVLRVLR
LLRTLRPLRVISRAPGLKLVVETLISSLKPIGNIVLICCAFFIIFGILGVQLFKGKFYHCLGVD
TRNITNRSDCMAANYRWVHHKYNFDNLGQALMSLFVLASKDGWVNIMYNGLDAVAVD
QQPVTNHNPWMLLYFISFLLIVSFFVLNMFVGVVVENFHKCRQHQEAEEARRREEKRLRR
LEKKRRKAQRLPYYATYCHTRLLIHSMCTSHYLDIFITFIICLNVVTMSLEHYNQPTSLETA
LKYCNYMFTTVFVLEAVLKLVAFGLRRFFKDRWNQLDLAIVLLSVMGITLEEIEINAALPI
NPTIIRIMRVLRIARVLKLLKMATGMRALLDTVVQALPQVGNLGLLFMLLFFIYAALGVEL
FGKLVCNDENPCEGMSRHATFENFGMAFLTLFQVSTGDNWNGIMKDTLRDCTHDERSCL
SSLQFVSPLYFVSFVLTAQFVLINVVVAVLMKHLDDSNKEAQEDAEMDAELELEMAHGLG
PGPRLPTGSPGAPGRGPGGAGGGGDTEGGLCRRCYSPAQENLWLDSVSLIIKDSLEGELTII
DNLSGSIFHHYSSPAGCKKCHHDKQEVQLAETEAFSLNSDRSSSILLGDDLSLEDPTACPPG
RKDSKGELDPPEPMRVGDLGECFFPLSSTAVSPDPENFLCEMEEIPFNPVRSWLKHDSSQA
PPSPFSPDASSPLLPMPAEFFHPAVSASQKGPEKGTGTGTLPKIALQGSWASLRSPRVNCTL
LRQATGSDTSLDASPSSSAGSLQTTLEDSLTLSDSPRRALGPPAPAPGPRAGLSPAARRRLSL
RGRGLFSLRGLRAHQRSHSSGGSTSPGCTHHDSMDPSDEEGRGGAGGGGAGSEHSETLSS
LSLTSLFCPPPPPPAPGLTPARKFSSTSSLAAPGRPHAAALAHGLARSPSWAADRSKDPPGR
APLPMGLGPLAPPPQPLPGELEPGDAASKRKR

>Q06136 multipass membrane protein
MLLLAAAFLVAFVLLLYMVSPLISPKPLALPGAHVVVTGGSSGIGKCIAIECYKQGAFITLV
ARNEDKLLQAKKEIEMHSINDKQVVLCISVDVSQDYNQVENVIKQAQEKLGPVDMLVNC
AGMAVSGKFEDLEVSTFERLMSINYLGSVYPSRAVITTMKERRVGRIVFVSSQAGQLGLFG
FTAYSASKFAIRGLAEALQMEVKPYNVYITVAYPPDTDTPGFAEENRTKPLETRLISETTSV
CKPEQVAKQIVKDAIQGNFNSSLGSDGYMLSALTCGMAPVTSITEGLQQVVTMGLFRTIA
LFYLGSFDSIVRRCMMQREKSENADKTA

>Q8WWR8 peripheral membrane protein
MGVPRTPSRTVLFERERTGLTYRVPSLLPVPPGPTLLAFVEQRLSPDDSHAHRLVLRRGTL
AGGSVRWGALHVLGTAALAEHRSMNPCPVHDAGTGTVFLFFIAVLGHTPEAVQIATGRNA
ARLCCVASRDAGLSWGSARDLTEEAIGGAVQDWATFAVGPGHGVQLPSGRLLVPAYTYRV
DRRECFGKICRTSPHSFAFYSDDHGRTWRCGGLVPNLRSGECQLAAVDGGQAGSFLYCNA
RSPLGSRVQALSTDEGTSFLPAERVASLPETAWGCQGSIVGFPAPAPNRPRDDSWSVGPGSP
LQPPLLGPGVHEPPEEAAVDPRGGQVPGGPFSRLQPRGDGPRQPGPRPGVSGDVGSWTLA
LPMPFAAPPQSPTWLLYSHPVGRRARLHMGIRLSQSPLDPRSWTEPWVIYEGPSGYSDLAS
IGPAPEGGLVFACLYESGARTSYDEISFCTFSLREVLENVPASPKPPNLGDKPRGCCWPS

>Q68DV7 single-pass type I membrane protein
MSGGHQLQLAALWPWLLMATLQAGFGRTGLVLAAAVESERSAEQKAIIRVIPLKMDPTG
KLNLTLEGVFAGVAEITPAEGKLMQSHPLYLCNASDDDNLEPGFISIVKLESPRRAPRPCLS
LASKARMAGERGASAVLFDITEDRAAAEQLQQPLGLTWPVVLIWGNDAEKLMEFVYKN
QKAHVRIELKEPPAWPDYDVWILMTVVGTIFVIILASVLRIRCRPRHSRPDPLQQRTAWAIS
QLATRRYQASCRQARGEWPDSGSSCSSAPVCAICLEEFSEGQELRVISCLHEFHRNCVDPW
LHQHRTCPLCMFNITEGDSFSQSLGPSRSYQEPGRRLHLIRQHPGHAHYHLPAAYLLGPSR
SAVARPPRPGPFLPSQEPGMGPRHHRFPRAAHPRAPGEQQRLAGAQHPYAQGWGLSHLQS
TSQHPAACPVPLRRARPPDSSGSGESYCTERSGYLADGPASDSSSGPCHGSSSDSVVNCTDI
SLQGVHGSSSTFCSSLSSDFDPLVYCSPKGDPQRVDMQPSVTSRPRSLDSVVPTGETQVSS
HVHYHRHRHHHYKKRFQWHGRKPGPETGVPQSRPPIPRTQPQPEPPSPDQQVTRSNSAAP
SGRLSNPQCPRALPEPAPGPVDASSICPSTSSLFNLQKSSLSARHPQRKRRGGPSEPTPGSRP
QDATVHPACQIFPHYTPSVAYPWSPEAHPLICGPPGLDKRLLPETPGPCYSNSQPVWLCLTP
RQPLEPHPPGEGPSEWSSDTAEGRPCPYPHCQVLSAQPGSEEELEELCEQAV

>Q96QZ7 peripheral membrane protein
MSKVIQKKNHWTSRVHECTVKRGPQGELGVTVLGGAEHGEFPYVGAVAAVEAAGLPGG
GEGPRLGEGELLLEVQGVRVSGLPRYDVLGVIDSCKEAVTFKAVRQGGRLNKDLRHFLNQ
RFQKGSPDHELQQTIRDNLYRHAVPCTTRSPREGEVPGVDYNFLTVKEFLDLEQSGTLLEV
GTYEGNYYGTPKPPSQPVSGKVITTDALHSLQSGSKQSTPKRTKSYNDMQNAGIVHAENE
EEDDVPEMNSSFTADSGEQEEHTLQETALPPVNSSIIAAPITDPSQKFPQYLPLSAEDNLGPL
PENWEMAYTENGEVYFIDHNTKTTSWLDPRCLNKQQKPLEECEDDEGVHTEELDSELELP
AGWEKIEDPVYGIYYVDHINRKTQYENPVLEAKRKKQLEQQQQQQQQQQQQQQQQQQ
QQTEEWTEDHSALVPPVIPNHPPSNPEPAREVPLQGKPFFTRNPSELKGKFIHTKLRKSSRG
FGFTVVGGDEPDEFLQIKSLVLDGPAALDGKMETGDVIVSVNDTCVLGHTHAQVVKIFQS
IPIGASVDLELCRGYPLPFDPDDPNTSLVTSVAILDKEPIIVNGQETYDSPASHSSKTGKVNG
MKDARPSSPADVASNSSHGYPNDTVSLASSIATQPELITVHIVKGPMGFGFTIADSPGGGG
QRVKQIVDSPRCRGLKEGDLIVEVNKKNVQALTHNQVVDMLVECPKGSEVTLLVQRGGL
PVPKKSPKSQPLERKDSQNSSQHSVSSHRSLHTASPSHSTQVLPEFPPAEAQAPDQTDSSGQ
KKPDPFKIWAQSRSMYENRPMSPSPASGLSKGEREREINSTNFGECPIPDYQEQDIFLWRKE
TGFGFRILGGNEPGEPIYIGHIVPLGAADTDGRLRSGDELICVDGTPVIGKSHQLVVQLMQ
QAAKQGHVNLTVRRKVVFAVPKTENEVPSPASSHHSSNQPASLTEEKRTPQGSQNSLNTVS
SGSGSTSGIGSGGGGGSGVVSTVVQPYDVEIRRGENEGFGFVIVSSVSRPEAGTTFAGNAC
VAMPHKIGRIIEGSPADRCGKLKVGDRILAVNGCSITNKSHSDIVNLIKEAGNTVTLRIIPGD

ESSNATLLTNAEKIATITTTHTPSQQGTQETRNTTKPKQESQFEFKAPQATQEQDFYTVELE
RGAKGFGFSLRGGREYNMDLYVLRLAEDGPAERCGKMRIGDEILEINGETTKNMKHSRAI
ELIKNGGRRVRLFLKRGDGSVPEYDPSSDRHGPATGPQGVPEVRAGPDRRQHPSLESSYPP
DLHKSSPHGEKRAHARDPKGSREYSRQPNEHHTWNGTSRKPDSGACRPKDRAPEGRRDA
QAERAAAANGPKRRSPEKRREGTRSADNTLERREKHEKRRDVSPERRRERSPTRRRDGSP
SRRRRSLERLLEQRRSPERRRGGSPERRAKSTDRRRARSPERRRERSLDKRNREDRASHRE
REEANLKQDAGRSSRHPPEQRRRPYKECSTDLSI

>Q9UNG2 single-pass type II membrane protein
MTLHPSPITCEFLFSTALISPKMCLSHLENMPLSHSRTQGAQRSSWKLWLFCSIVMLLFLCS
FSWLIFIFLQLETAKEPCMAKFGPLPSKWQMASSEPPCVNKVSDWKLEILQNGLYLIYGQV
APNANYNDVAPFEVRLYKNKDMIQTLTNKSKIQNVGGTYELHVGDTIDLIFNSEHQVLKN
NTYWGIILLANPQFIS

>Q96CS7 peripheral membrane protein
MAFVKSGWLLRQSTILKRWKKNWFDLWSDGHLIYYDDQTRQNIEDKVHMPMDCINIRT
GQECRDTQPPDGKSKDCMLQIVCRDGKTISLCAESTDDCLAWKFTLQDSRTNTAYVGSAV
MTDETSVVSSPPPYTAYAAPAPEQAYGYGPYGGAYPPGTQVVYAANGQAYAVPYQYPYA
GLYGQQPANQVIIRERYRDNDSDLALGMLAGAATGMALGSLFWVF

>P28476 multipass membrane protein
MVKPGGICSATGYWKAAFCLTDVHKMPYFTRLILFLFCLMVLVESRKPKRKRWTGQVEM
PKPSHLYKKNLDVTKIRKGKPQQLLRVDEHDFSMRPAFGGPAIPVGVDVQVESLDSISEVD
MDFTMTLYLRHYWKDERLAFSSASNKSMTFDGRLVKKIWVPDVFFVHSKRSFTHDTTTD
NIMLRVFPDGHVLYSMRITVTAMCNMDFSHFPLDSQTCSLELESYAYTDEDLMLYWKNG
DESLKTDEKISLSQFLIQKFHTTSRLAFYSSTGWYNRLYINFTLRRHIFFFLLQTYFPATLMV
MLSWVSFWIDRRAVPARVSLGITTVLTMTTIITGVNASMPRVSYVKAVDIYLWVSFVFVFL
SVLEYAAVNYLTTVQERKERKLREKFPCMCGMLHSKTMMLDGSYSESEANSLAGYPRSH
ILTEEERQDKIVVHLGLSGEANAARKKGLLKGQTGFRIFQNTHAIDKYSRLIFPASYIFFNLI
YWSVFS

>P18825 multipass membrane protein
MASPALAAALAVAAAAGPNASGAGERGSGGVANASGASWGPPRGQYSAGAVAGLAAVV
GFLIVFTVVGNVLVVIAVLTSRALRAPQNLFLVSLASADILVATLVMPFSLANELMAYWYF
GQVWCGVYLALDVLFCTSSIVHLCAISLDRYWSVTQAVEYNLKRTPRRVKATIVAVWLISA
VISFPPLVSLYRQPDGAAYPQCGLNDETWYILSSCIGSFFAPCLIMGLVYARIYRVAKLRTRT
LSEKRAPVGPDGASPTTENGLGAAAGAGENGHCAPPPADVEPDESSAAAERRRRRGALR
RGGRRRAGAEGGAGGADGQGAGPGAAESGALTASRSPGPGGRLSRASSRSVEFFLSRRR
RARSSVCRRKVAQAREKRFTFVLAVVMGVFVLCWFPFFFSYSLYGICREACQVPGPLFKFF
FWIGYCNSSLNPVIYTVFNQDFRRSFKHILFRRRRRGFRQ

>Q5DID0 single-pass type I membrane protein
MLRTSGLALLALVSAVGPSQASGFTEKGLSLLGYQLCSHRVTHTVQKVEAVQTSYTSYVS
CGGWIPWRRCPKMVYRTQYLVVEVPESRNVTDCCEGYEQLGLYCVLPLNQSGQFTSRPG

ACPAEGPEPSTSPCSLDIDCPGLEKCCPWSGGRYCMAPAPQAPERDPVGSWYNVTILVKM
DFKELQQVDPRLLNHMRLLHSLVTSALQPMASTVHHLHSAPGNASTTVSRLLLGLPRPLP
VADVSTLLGDIAKRVYEVISVQVQDVNECFYEELNACSGRELCANLEGSYWCVCHQEAPA
TSPRKLNLEWEDCPPVSDYVVLNVTSDSFQVSWRLNSTQNHTFHVRVYRGMELLRSART
QSQALAVAGLEAGVLYRVKTSYQGCGADVSTTLTIKTNAQVFEVTIKIVNHNLTEKLLNRS
SVEYQDFSRQLLHEVESSFPPVVSDLYRSGKLRMQIVSLQAGSVVVRLKLTVQDPGFPMGI
STLAPILQPLLASTVFQIDRQGTRVQDWDECVDSAEHDCSPAAWCINLEGSYTCQCRTTRD
ATPSRAGRACEGDLVSPMGGGLSAATGVTVPGLGTGTAALGLENFTLSPSPGYPQGTPAA
GQAWTPEPSPRRGGSNVVGYDRNNTGKGVEQELQGNSIMEPPSWPSPTEDPTGHFLWHA
TRSTRETLLNPTWLRNEDSGPSGSVDLPLTSTLTALKTPACVPVSIGRIMVSNVTSTGFHLA
WEADLAMDSTFQLTLTSMWSPAVVLETWNTSVTLSGLEPGVLHLVEIMAKACGKEGARA
HLKVRTAARKLIGKVRIKNVRYSESFRNASSQEYRDFLELFFRMVRGSLPATMCQHMDAG
GVRMEVVSVTNGSIVVEFHLLIIADVDVQEVSAAFLTAFQTVPLLEVIRGDTFIQDYDECE
RKEDDCVPGTSCRNTLGSFTCSCEGGAPDFPVEYSERPCEGDSPGNETWATSPERPLTTAG
TKAAFVQGTSPTPQGLPQRLNLTGAVRVLCEIEKVVVAIQKRFLQQESIPESSLYLSHPSCN
VSHSNGTHVLLEAGWSECGTLMQSNMTNTVVRTTLRNDLSQEGIIHHLKILSPIYCAFQN
DLLTSSGFTLEWGVYTIIEDLHGAGNFVTEMQLFIGDSPIPQNYSVSASDDVRIEVGLYRQK
SNLKVVLTECWATPSSNARDPITFSFINNSCPVPNTYTNVIENGNSNKAQFKLRIFSFINDSI
VYLHCKLRVCMESPGATCKINCNNFRLLQNSETSATHQMSWGPLIRSEGEPPHAEAGLGA
GYVVLIVVAIFVLVAGTATLLIVRYQRMNGRYNFKIQSNNFSYQVFYE

>Q15619 multipass membrane protein
MEKRNLTVVREFVLLGLPSSAEQQHLLSVLFLCMYLATTLGNMLIIATIGFDSHLHSPMYF
FLSNLAFVDICFTSTTVPQMVVNILTGTKTISFAGCLTQLFFFVSFVNMDSLLLCVMAYDRY
VAICHPLHYTARMNLCLCVQLVAGLWLVTYLHALLHTVLIAQLSFCASNIIHHFFCDLNPL
LQLSCSDVSFNVMIIFAVGGLLALTPLVCILVSYGLIFSTVLKITSTQGKQRAVSTCSCHLSV
VVLFYGTAIAVYFSPSSPHMPESDTLSTIMYSMVAPMLNPFIYTLRNRDMKRGLQKMLLK
CTVFQQQ

>O95007 multipass membrane protein
MELENQTRVTKFILVGFPGSLSMRAAMFLIFLVAYILTVAENVIIILLVLQNRPLHKPMYFFL
ANLSFLETWYISVTVPKLLFSFWSVNNSISFTLCMIQLYFFIALMCTECVLLAAMAYDRYV
AICRPLHYPTIMSHGLCFRLALGSWAIGFGISLAKIYFISCLSFCGPNVINHFFCDISPVLNLS
CTDMSITELVDFILALVIFLFPLFITVLSYGCILATILCMPTGKQKAFSTCASHLVVVTIFYSAI
IFMYARPRVIHAFNMNKIISIFYAIVTPSLNPFIYCLRNREVKEALKKLAYCQASRSD

>Q8NGK2 multipass membrane protein
MPTVNHSGTSHTVFHLLGIPGLQDQHMWISIPFFISYVTALLGNSLLIFIILTKRSLHEPMYL
FLCMLAGADIVLSTCTIPQALAIFWFRAGDISLDRCITQLFFIHSTFISESGILLVMAFDHYIA
ICYPLRYTTILTNALIKKICVTVSLRSYGTIFPIIFLLKRLTFCQNNIIPHTFCEHIGLAKYACN
DIRINIWYGFSILMSTVVLDVVLIFISYMLILHAVFHMPSPDACHKALNTFGSHVCIIILFYG
SGIFTILTQRFGRHIPPCIHIPLANVCILAPPMLNPIIYGIKTKQIQEQVVQFLFIKQK

>Q8NGC2 multipass membrane protein

MDSLNQTRVTEFVFLGLTDNRVLEMLFFMAFSAIYMLTLSGNILIIIATVFTPSLHTPMYFFL
SNLSFIDICHSSVTVPKMLEGLLLERKTISFDNCITQLFFLHLFACAEIFLLIIVAYDRYVAICT
PLHYPNVMNMRVCIQLVFALWLGGTVHSLGQTFLTIRLPYCGPNIIDSYFCDVPLVIKLACT
DTYLTGILIVTNSGTISLSCFLAVVTSYMVILVSLRKHSAEGRQKALSTCSAHFMVVALFFG
PCIFIYTRPDTSFSIDKVVSVFYTVVTPLLNPFIYTLRNEEVKSAMKQLRQRQVFFTKSYT

>P59536 multipass membrane protein
MQAALTAFFVLLFSLLSLLGIAANGFIVLVLGREWLRYGRLLPLDMILISLGASRFCLQLVG
TVHNFYYSAQKVEYSGGLGRQFFHLHWHFLNSATFWFCSWLSVLFCVKIANITHSTFLWL
KWRFPGWVPWLLLGSVLISFIITLLFFWVNYPVYQEFLIRKFSGNMTYKWNTRIETYYFPS
LKLVIWSIPFSVFLVSIMLLINSLRRHTQRMQHNGHSLQDPSTQAHTRALKSLISFLILYALS
FLSLIIDAAKFISMQNDFYWPWQIAVYLCISVHPFILIFSNLKLRSVFSQLLLLARGFWVA

>Q6IF42 multipass membrane protein
MEGNQTWITDITLLGFQVGPALAILLCGLFSVFYTLTLLGNGVIFGIICLDSKLHTPMYFFLS
HLAIIDMSYASNNVPKMLANLMNQKRTISFVPCIMQTFLYLAFAVTECLILVVMSYDRYVA
ICHPFQYTVIMSWRVCTILVLTSWSCGFALSLVHEILLLRLPFCGPRDVNHLFCEILSVLKLA
CADTWVNQVVIFATCVFVLVGPLSLILVSYMHILGAILKIQTKEGRIKAFSTCSSHLCVVGL
FFGIAMVVYMVPDSNQREEQEKMLSLFHSVFNPMLNPLIYSLRNAQLKGALHRALQRKR
SMRTVYGLCL

>P59535 multipass membrane protein
MATVNTDATDKDISKFKVTFTLVVSGIECITGILGSGFITAIYGAEWARGKTLPTGDRIMLM
LSFSRLLLQIWMMLENIFSLLFRIVYNQNSVYILFKVITVFLNHSNLWFAAWLKVFYCLRIA
NFNHPLFFLMKRKIIVLMPWLLRLSVLVSLSFSFPLSRDVFNVYVNSSIPIPSSNSTEKKYFS
ETNMVNLVFFYNMGIFVPLIMFILAATLLILSLKRHTLHMGSNATGSRDPSMKAHIGAIKAT
SYFLILYIFNAIALFLSTSNIFDTYSSWNILCKIIMAAYPAGHSVQLILGNPGLRRAWKRFQH
QVPLYLKGQTL

>Q15858 multipass membrane protein
MAMLPPPGPQSFVHFTKQSLALIEQRIAERKSKEPKEEKKDDDEEAPKPSSDLEAGKQLPF
IYGDIPPGMVSEPLEDLDPYYADKKTFIVLNKGKTIFRFNATPALYMLSPFSPLRRISIKILVH
SLFSMLIMCTILTNCIFMTMNNPPDWTKNVEYTFTGIYTFESLVKILARGFCVGEFTFLRDP
WNWLDFVVIVFAYLTEFVNLGNVSALRTFRVLRALKTISVIPGLKTIVGALIQSVKKLSDV
MILTVFCLSVFALIGLQLFMGNLKHKCFRNSLENNETLESIMNTLESEEDFRKYFYYLEGS
KDALLCGFSTDSGQCPEGYTCVKIGRNPDYGYTSFDTFSWAFLALFRLMTQDYWENLYQ
QTLRAAGKTYMIFFVVVIFLGSFYLINLILAVVAMAYEEQNQANIEEAKQKELEFQQMLDR
LKKEQEEAEAIAAAAAEYTSIRRSRIMGLSESSSETSKLSSKSAKERRNRRKKKNQKKLSS
GEEKGDAEKLSKSESEDSIRRKSFHLGVEGHRRAHEKRLSTPNQSPLSIRGSLFSARRSSRT
SLFSFKGRGRDIGSETEFADDEHSIFGDNESRRGSLFVPHRPQERRSSNISQASRSPPMLPVN
GKMHSAVDCNGVVSLVDGRSALMLPNGQLLPEVIIDKATSDDSGTTNQIHKKRRCSSYLL
SEDMLNDPNLRQRAMSRASILTNTVEELEESRQKCPPWWYRFAHKFLIWNCSPYWIKFKK
CIYFIVMDPFVDLAITICIVLNTLFMAMEHHPMTEEFKNVLAIGNLVFTGIFAAEMVLKLIA
MDPYEYFQVGWNIFDSLIVTLSLVELFLADVEGLSVLRSFRLLRVFKLAKSWPTLNMLIKII

GNSVGALGNLTLVLAIIVFIFAVVGMQLFGKSYKECVCKINDDCTLPRWHMNDFFHSFLIV
FRVLCGEWIETMWDCMEVAGQAMCLIVYMMVMVIGNLVVLNLFLALLLSSFSSDNLTAI
EEDPDANNLQIAVTRIKKGINYVKQTLREFILKAFSKKPKISREIRQAEDLNTKKENYISNH
TLAEMSKGHNFLKEKDKISGFGSSVDKHLMEDSDGQSFIHNPSLTVTVPIAPGESDLENMN
AEELSSDSDSEYSKVRLNRSSSSECSTVDNPLPGEGEEAEAEPMNSDEPEACFTDGCVWRF
SCCQVNIESGKGKIWWNIRKTCYKIVEHSWFESFIVLMILLSSGALAFEDIYIERKKTIKIIL
EYADKIFTYIFILEMLLKWIAYGYKTYFTNAWCWLDFLIVDVSLVTLVANTLGYSDLGPIKS
LRTLRALRPLRALSRFEGMRVVVNALIGAIPSIMNVLLVCLIFWLIFSIMGVNLFAGKFYECI
NTTDGSRFPASQVPNRSECFALMNVSQNVRWKNLKVNFDNVGLGYLSLLQVATFKGWTII
MYAAVDSVNVDKQPKYEYSLYMYIYFVVFIIFGSFFTLNLFIGVIIDNFNQQKKKLGGQDIF
MTEEQKKYYNAMKKLGSKKPQKPIPRPGNKIQGCIFDLVTNQAFDISIMVLICLNMVTMM
VEKEGQSQHMTEVLYWINVVFIILFTGECVLKLISLRHYYFTVGWNIFDFVVVIISIVGMFL
ADLIETYFVSPTLFRVIRLARIGRILRLVKGAKGIRTLLFALMMSLPALFNIGLLLFLVMFIYA
IFGMSNFAYVKKEDGINDMFNFETFGNSMICLFQITTSAGWDGLLAPILNSKPPDCDPKKV
HPGSSVEGDCGNPSVGIFYFVSYIIISFLVVVNMYIAVILENFSVATEESTEPLSEDDFEMFYE
VWEKFDPDATQFIEFSKLSDFAAALDPPLLIAKPNKVQLIAMDLPMVSGDRIHCLDILFAFT
KRVLGESGEMDSLRSQMEERFMSANPSKVSYEPITTTLKRKQEDVSATVIQRAYRRYRLR
QNVKNISSIYIKDGDRDDDLLNKKDMAFDNVNENSSPEKTDATSSTTSPPSYDSVTKPDKE
KYEQDRTEKEDKGKDSKESKK

>Q9UJ71 single-pass type II membrane protein
MTVEKEAPDAHFTVDKQNISLWPREPPPKSGPSLVPGKTPTVRAALICLTLVLVASVLLQA
VLYPRFMGTISDVKTNVQLLKGRVDNISTLDSEIKKNSDGMEAAGVQIQMVNESLGYVRS
QFLKLKTSVEKANAQIQILTRSWEEVSTLNAQIPELKSDLEKASALNTKIRALQGSLENMS
KLLKRQNDILQVVSQGWKYFKGNFYYFSLIPKTWYSAEQFCVSRNSHLTSVTSESEQEFL
YKTAGGLIYWIGLTKAGMEGDWSWVDDTPFNKVQSVRFWIPGEPNNAGNNEHCGNIKA
PSLQAWNDAPCDKTFLFICKRPYVPSEP

>Q8N608 single-pass type II membrane protein
MNQTASVSHHIKCQPSKTIKELGSNSPPQRNWKGIAIALLVILVVCSLITMSVILLTPDELTN
SSETRLSLEDLFRKDFVLHDPEARWINDTDVVYKSENGHVIKLNIETNATTLLLENTTFVT
FKASRHSVSPDLKYVLLAYDVKQIFHYSYTASYVIYNIHTREVWELNPPEVEDSVLQYAA
WGVQGQQLIYIFENNIYYQPDIKSSSLRLTSSGKEEIIFNGIADWLYEEELLHSHIAHWWSP
DGERLAFLMINDSLVPTMVIPRFTGALYPKGKQYPYPKAGQVNPTIKLYVVVNLYGPTHTLE
LMPPDSFKSREYYITMVKWVSNTKTVVRWLNRAQNISILTVCETTTGACSKKYEMTSDT
WLSQQNEEPVFSRDGSKFFMTVPVKQGGRGEFHHVAMFLIQSKSEQITVRHLTSGNWEVI
KILAYDETTQKIYFLSTESSPRGRQLYSASTEGLLNRQCISCNFMKEQCTYFDASFSPMNQH
FLLFCEGPRVPVVSLHSTDNPAKYFILESNSMLKEAILKKKIGKPEIKILHIDDYELPLQLSLP
KDFMDRNQYALLLIMDEEPGGQLVTDKFHIDWDSVLIDMDNVIVARFDGRGSGFQGLKIL
QEIHRRLGSVEVKDQITAVKFLLKLPYIDSKRLSIFGKGYGGYIASMILKSDEKLFKCGSVV
APITDLKLYASAFSERYLGMPSKEESTYQAASVLHNVHGLKEENILIIHGTADTKVHFQHS
AELIKHLIKAGVNYTMQVYPDEGHNVSEKSKYHLYSTILKFFSDCLKEEISVLPQEPEEDE

>Q96N16 peripheral membrane protein

MSKKGRSKGEKPEMETDAVQMANEELRAKLTSIQIEFQQEKSKVGKLRERLQEAKLERE
QEQRRHTAYISELKAKLHEEKTKELQALREGLIRQHEQEAARTAKIKEGELQRLQATLNVL
RDGAADKVKTALLTEAREEARRAFDGERLRLQQEILELKAARKQAEEALSNCMQADKTK
AADLRAAYQAHQDEVHRIKRECERDIRRLMDEIKGKDRVILALEKELGVQAGQTQKLLL
QKEALDEQLVQVKEAERHHSSPKRELPPGIGDMVELMGVQDQHMDERDVRRFQLKIAEL
NSVIRKLEDRNTLLADERNELLKRSRETEVQLKPLVEKNKRMNKKNEDLLQSIQRMEEKI
KNLTRENVEMKEKLSAQASLKRHTSLNDLSLTRDEQEIEFLRLQVLEQQHVIDDLSLERER
LLRSKRHRGKSLKPPKKHVVETFFGFDEESVDSETLSETSYNTDRTDRTPATPEEDLDDAT
AREEADLRFCQLTREYQALQRAYALLQEQVGGTLDAEREARTREQLQADLLRCQAKIEDL
EKLLVEKGQDSKWVEEKQLLIRTNQDLLEKIYRLEMEENQLKNEMQDAKDQNELLEFRV
LELEVRDSICCKLSNGADILFEPKLKFM

>Q01118 multipass membrane protein
MLASPEPKGLVPFTKESFELIKQHIAKTHNEDHEEEDLKPTPDLEVGKKLPFIYGNLSQGM
VSEPLEDVDPYYYKKKNTFIVLNKNRTIFRFNAASILCTLSPFNCIRRTTIKVLVHPFFQLFIL
ISVLIDCVFMSLTNLPKWRPVLENTLLGIYTFEILVKLFARGVWAGSFSFLGDPWNWLDFS
VTVFEVIIRYSPLDFIPTLQTARTLRILKIIPLNQGLKSLVGVLIHCLKQLIGVIILTLFFLSIFSL
IGMGLFMGNLKHKCFRWPQENENETLHNRTGNPYYIRETENFYYLEGERYALLCGNRTD
AGQCPEGYVCVKAGINPDQGFTNFDSFGWALFALFRLMAQDYPEVLYHQILYASGKVYMI
FFVVVSFLFSFYMASLFLGILAMAYEEEKQRVGEISKKIEPKFQQTGKELQEGNETDEAKTI
QIEMKKRSPISTDTSLDVLEDATLRHKEELEKSKKICPLYWYKFAKTFLIWNCSPCWLKLK
EFVHRIIMAPFTDLFLIICIILNVCFLTLEHYPMSKQTNTLLNIGNLVFIGIFTAEMIFKIIAMH
PYGYFQVGWNIFDSMIVFHGLIELCLANVAGMALLRLFRMLRIFKLGKYWPTFQILMWSL
SNSWVALKDLVLLLFTFIFFSAAFGMKLFGKNYEEFVCHIDKDCQLPRWHMHDFFHSFLN
VFRILCGEWVETLWDCMEVAGQSWCIPFYLMVILIGNLLVLYLFLALVSSFSSCKDVTAEE
NNEAKNLQLAVARIKKGINYVLLKILCKTQNVPKDTMDHVNEVYVKEDISDHTLSELSNT
QDFLKDKEKSSGTEKNATENESQSLIPSPSVSETVPIASGESDIENLDNKEIQSKSGDGGSKE
KIKQSSSSECSTVDIAISEEEEMFYGGERSKHLKNGCRRGSSLGQISGASKKGKIWQNIRKT
CCKIVENNWFKCFIGLVTLLSTGTLAFEDIYMDQRKTIKILLEYADMIFTYIFILEMLLKWM
AYGFKAYFSNGWYRLDFVVVIVFCLSLIGKTREELKPLISMKFLRPLRVLSQFERMKVVVR
ALIKTTLPTLNVFLVCLMIWLIFSIMGVDLFAGRFYECIDPTSGERFPSSEVMNKSRCESLLF
NESMLWENAKMNFDNVGNGFLSLLQVATFNGWITIMNSAIDSVAVNIQPHFEVNIYMYCY
FINFIIFGVFLPLSMLITVIIDNFNKHKIKLGGSNIFITVKQRKQYRRLKKLMYEDSQRPVPR
PLNKLQGFIFDVVTSQAFNVIVMVLICFQAIAMMIDTDVQSLQMSIALYWINSIFVMLYTM
ECILKLIAFRCFYFTIAWNIFDFMVVIFSITGLCLPMTVGSYLVPPSLVQLILLSRIIHMLRLG
KGPKVFHNLMLPLMLSLPALLNIILLIFLVMFIYAVFGMYNFAYVKKEAGINDVSNFETFGN
SMLCLFQVAIFAGWDGMLDAIFNSKWSDCDPDKINPGTQVRGDCGNPSVGIFYFVSYILIS
WLIIVNMYIVVVMEFLNIASKKKNKTLSEDDFRKFFQVWKRFDPDRTQYIDSSKLSDFAA
ALDPPLFMAKPNKGQLIALDLPMAVGDRIHCLDILLAFTKRVMGQDVRMEKVVSEIESGF
LLANPFKITCEPITTTLKRKQEAVSATIIQRAYKNYRLRRNDKNTSDIHMIDGDRDVHATKE
GAYFDKAKEKSPIQSQI

>O75923 single-pass type II membrane protein
MLRVFILYAENVHTPDTDISDAYCSAVFAGVKKRTKVIKNSVNPVWNEGFEWDLKGIPLD

QGSELHVVVKDHETMGRNRFLGEAKVPLREVLATPSLSASFNAPLLDTKKQPTGASLVLQ
VSYTPLPGAVPLFPPPTPLEPSPTLPDLDVVADTGGEEDTEDQGLTGDEAEPFLDQSGGPGA
PTTPRKLPSRPPPHYPGIKRKRSAPTSRKLLSDKPQDFQIRVQVIEGRQLPGVNIKPVVKVT
AAGQTKRTRIHKGNSPLFNETLFFNLFDSPGELFDEPIFITVVDSRSLRTDALLGEFRMDVG
TIYREPRHAYLRKWLLLSDPDDFSAGARGYLKTSLCVLGPGDEAPLERKDPSEDKEDIESN
LLRPTGVALRGAHFCLKVFRAEDLPQMDDAVMDNVKQIFGFESNKKNLVDPFVEVSFAG
KMLCSKILEKTANPQWNQNITLPAMFPSMCEKMRIRIIDWDRLTHNDIVATTYLSMSKISA
PGGEIEEEPAGAVKPSKASDLDDYLGFLPTFGPCYINLYGSPREFTGFPDPYTELNTGKGEG
VAYRGRLLLSLETKLVEHSEQKVEDLPADDILRVEKYLRRRKYSLFAAFYSATMLQDVDD
AIQFEVSIGNYGNKFDMTCLPLASTTQYSRAVFDGCHYYYLPWGNVKPVVVLSSYWEDIS
HRIETQNQLLGIADRLEAGLEQVHLALKAQCSTEDVDSLVAQLTDELIAGCSQPLGDIHET
PSATHLDQYLYQLRTHHLSQITEAALALKLGHSELPAALEQAEDWLLRLRALAEEPQNSLP
DIVIWMLQGDKRVAYQRVPAHQVLFSRRGANYCGKNCGKLQTIFLKYPMEKVPGARMPV
QIRVKLWFGLSVDEKEFNQFAEGKLSVFAETYENETKLALVGNWGTTGLTYPKFSDVTGK
IKLPKDSFRPSAGWTWAGDWFVCPEKTLLHDMDAGHLSFVEEVFENQTRLPGGQWIYMS
DNYTDVNGEKVLPKDDIECPLGWKWEDEEWSTDLNRAVDEQGWEYSITIPPERKPKHWV
PAEKMYYTHRRRRWVRLRRRDLSQMEALKRHRQAEAEGEGWEYASLFGWKFHLEYRK
TDAFRRRRWRRRMEPLEKTGPAAVFALEGALGGVMDDKSEDSMSVSTLSFGVNRPTISCI
FDYGNRYHLRCYMYQARDLAAMDKDSFSDPYAIVSFLHQSQKTVVVKNTLNPTWDQTLI
FYEIEIFGEPATVAEQPPSIVVELYDHDTYGADEFMGRCICQPSLERMPRLAWFPLTRGSQPS
GELLASFELIQREKPAIHHIPGFEVQETSRILDESEDTDLPYPPPQREANIYMVPQNIKPALQ
RTAIEILAWGLRNMKSYQLANISSPSLVVECGGQTVQSCVIRNLRKNPNFDICTLFMEVML
PREELYCPPITVKVIDNRQFGRRPVVGQCTIRSLESFLCDPYSAESPSPQGGPDDVSLLSPGE
DVLIDIDDKEPLIPIQEEEFIDWWSKFFASIGEREKCGSYLEKDFDTLKVYDTQLENVEAFE
GLSDFCNTFKLYRGKTQEETEDPSVIGEFKGLFKIYPLPEDPAIPMPPRQFHQLAAQGPQEC
LVRIYIVRAFGLQPKDPNGKCDPYIKISIGKKSVSDQDNYIPCTLEPVFGKMFELTCTLPLEK
DLKITLYDYDLLSKDEKIGETVVDLENRLLSKFGARCGLPQTYCVSGPNQWRDQLRPSQL
LHLFCQQHRVKAPVYRTDRVMFQDKEYSIEEIEAGRIPNPHLGPVEERLALHVLQQQGLVP
EHVESRPLYSPLQPDIEQGKLQMWVDLFPKALGRPGPPFNITPRRARRFFLRCIIWNTRDVI
LDDLSLTGEKMSDIYVKGWMIGFEEHKQKTDVHYRSLGGEGNFNWRFIFPFDYLPAEQV
CTIAKKDAFWRLDKTESKIPARVVFQIWDNDKFSFDDFLGSLQLDLNRMPKPAKTAKKCS
LDQLDDAFHPEWFVSLFEQKTVKGWWPCVAEEGEKKILAGKLEMTLEIVAESEHEERPAG
QGRDEPNMNPKLEDPRRPDTSFLWFTSPYKTMKFILWRRFRWAIILFIILFILLLFLAIFIYAF
PNYAAMKLVKPFS

>P61073 multipass membrane protein
MEGISIYTSDNYTEEMGSGDYDSMKEPCFREENANFNKIFLPTIYSIIFLTGIVGNGLVILVM
GYQKKLRSMTDKYRLHLSVADLLFVITLPFWAVDAVANWYFGNFLCKAVHVIYTVNLYSS
VLILAFISLDRYLAIVHATNSQRPRKLLAEKVVYVGVWIPALLLTIPDFIFANVSEADDRYIC
DRFYPNDLWVVVFQFQHIMVGLILPGIVILSCYCIIISKLSHSKGHQKRKALKTTVILILAFF
ACWLPYYIGISIDSFILLEIIKQGCEFENTVHKWISITEALAFFHCCLNPILYAFLGAKFKTSA
QHALTSVSRGSSLKILSKGKRGGHSSVSTESESSSFHSS

>A6BM72 single-pass type I membrane protein

MVLSLTGLIAFSFLQATLALNPEDPNVCSHWESYAVTVQESYAHPFDQIYYTRCTDILNWF
KCTRHRISYKTAYRRGLRTMYRRRSQCCPGYYESGDFCIPLCTEECVHGRCVSPDTCHCEP
GWGGPDCSSGCDSDHWGPHCSNRCQCQNGALCNPITGACVCAAGFRGWRCEELCAPGT
HGKGCQLPCQCRHGASCDPRAGECLCAPGYTGVYCEELCPPGSHGAHCELRCPCQNGGT
CHHITGECACPPGWTGAVCAQPCPPGTFGQNCSQDCPCHHGGQCDHVTGQCHCTAGYM
GDRCQEECPFGSFGFQCSQHCDCHNGGQCSPTTGACECEPGYKGPRCQERLCPEGLHGPG
CTLPCPCDADNTISCHPVTGACTCQPGWSGHHCNESCPVGYYGDGCQLPCTCQNGADCH
SITGGCTCAPGFMGEVCAVSCAAGTYGPNCSSICSCNNGGTCSPVDGSCTCKEGWQGLDC
TLPCPSGTWGLNCNESCTCANGAACSPIDGSCSCTPGWLGDTCELPCPDGTFGLNCSEHC
DCSHADGCDPVTGHCCCLAGWTGIRCDSTCPPGRWGPNCSVSCSCENGGSCSPEDGSCE
CAPGFRGPLCQRICPPGFYGHGCAQPCPLCVHSSRPCHHISGICECLPGFSGALCNQVCAG
GYFGQDCAQLCSCANNGTCSPIDGSCQCFPGWIGKDCSQACPPGFWGPACFHACSCHNG
ASCSAEDGACHCTPGWTGLFCTQRCPAAFFGKDCGRVCQCQNGASCDHISGKCTCRTGF
TGQHCEQRCAPGTFGYGCQQLCECMNNSTCDHVTGTCYCSPGFKGIRCDQAALMMEEL
NPYTKISPALGAERHSVGAVTGIMLLLFLIVVLLGLFAWHRRRQKEKGRDLAPRVSYTPAM
RMTSTDYSLSGACGMDRRQNTYIMDKGFKDYMKESVCSSSTCSLNSSENPYATIKDPPILT
CKLPESSYVEMKSPVHMGSPYTDVPSLSTSNKNIYEVEPTVSVVQEGCGHNSSYIQNAYD
LPRNSHIPGHYDLLPVRQSPANGPSQDKQS

>O43731 multipass membrane protein
MNVFRILGDLSHLLAMILLLGKIWRSKCCKGISGKSQILFALVFTTRYLDLFTNFISIYNTV
MKVVFLLCAYVTVYMIYGKFRKTFDSENDTFRLEFLLVPVIGLSFLENYSFTLLEILWTFSI
YLESVAILPQLFMISKTGEAETITTHYLFFLGLYRALYLANWIRRYQTENFYDQIAVVSGVV
QTIFYCDFFYLYVTKVLKGKKLSLPMPI

>P18089 multipass membrane protein
MDHQDPYSVQATAAIAAAITFLILFTIFGNALVILAVLTSRSLRAPQNLFLVSLAAADILVAT
LIIPFSLANELLGYWYFRRTWCEVYLALDVLFCTSSIVHLCAISLDRYWAVSRALEYNSKR
TPRRIKCIILTVWLIAAVISLPPLIYKGDQGPQPRGRPQCKLNQEAWYILASSIGSFFAPCLIM
ILVYLRIYLIAKRSNRRGPRAKGGPGQGESKQPRPDHGGALASAKLPALASVASAREVNG
HSKSTGEKEEGETPEDTGTRALPPSWAALPNSGQGQKEGVCGASPEDEAEEEEEEEEEEE
CEPQAVPVSPASACSPPLQQPQGSRVLATLRGQVLLGRGVGAIGGQWWRRRAQLTREKRF
TFVLAVVIGVFVLCWFPFFFSYSLGAICPKHCKVPHGLFQFFFWIGYCNSSLNPVIYTIFNQ
DFRRAFRRILCRPWTQTAW

>Q6IFN5 multipass membrane protein
MSYFPILFFFFLKRCPSYTEPQNLTGVSEFLLLGLSEDPELQPVLAGLFLSMYLVTVLGNLLI
ILAVSSDSHLHTPMYFFLSNLSLADIGFTSTTVPKMIVDMQTHSRVISYEGCLTQMSFFVLF
ACMDDMLLSVMAYDRFVAICHPLHYRIIMNPRLCGFLILLSFFISLLDSQLHNLIMLQLTCF
KDVDISNFFCDPSQLLHLRCSDTFINEMVIYFMGAIFGCLPISGILFSYYKIVSPILRVPTSDG
KYKAFSTCGSHLAVVCLFYGTGLVGYLSSAVLPSPRKSMVASVMYTVVTPMLNPFIYSLR
NKDIQSALCRLHGRIIKSHHLHPFCYMG

>Q9H2X9 multipass membrane protein

MSRRFTVTSLPPAGPARSPDPESRRHSVADPRHLPGEDVKGDGNPKESSPFINSTDTEKGKE
YDGKNMALFEEEMDTSPMVSSLLSGLANYTNLPQGSREHEEAENNEGGKKKPVQAPRM
GTFMGVYLPCLQNIFGVILFLRLTWVVGIAGIMESFCMVFICCSCTMLTAISMSAIATNGVV
PAGGSYYMISRSLGPEFGGAVGLCFYLGTTFAGAMYILGTIEILLAYLFPAMAIFKAEDASG
EAAAMLNNMRVYGTCVLTCMATVVFVGVKYVNKFALVFLGCVILSILAIYAGVIKSAFDP
PNFPICLLGNRTLSRHGFDVCAKLAWEGNETVTTRLWGLFCSSRFLNATCDEYFTRNNVT
EIQGIPGAASGLIKENLWSSYLTKGVIVERSGMTSVGLADGTPIDMDHPYVFSDMTSYFTL
LVGIYFPSVTGIMAGSNRSGDLRDAQKSIPTGTILAIATTSAVYISSVVLFGACIEGVVLRDK
FGEAVNGNLVVGTLAWPSPWVIVIGSFFSTCGAGLQSLTGAPRLLQAISRDGIVPFLQVFG
HGKANGEPTWALLLTACICEIGILIASLDEVAPILSMFFLMCYMFVNLACAVQTLLRTPNW
RPRFRYYHWTLSFLGMSLCLALMFICSWYYALVAMLIAGLIYKYIEYRGAEKEWGDGIRG
LSLSAARYALLRLEEGPPHTKNWRPQLLVLVRVDQDQNVVHPQLLSLTSQLKAGKGLTIV
GSVLEGTFLENHPQAQRAEESIRRLMEAEKVKGFCQVVISSNLRDGVSHLIQSGGLGGLQ
HNTVLVGWPRNWRQKEDHQTWRNFIELVRETTAGHLALLVTKNVSMFPGNPERFSEGSI
DVWWIVHDGGMLMLLPFLLRHHKVWRKCKMRIFTVAQMDDNSIQMKKDLTTFLYHLRI
TAEVEVVEMHESDISAYTYEKTLVMEQRSQILKQMHLTKNEREREIQSITDESRGSIRRKNP
ANTRLRLNVPEETAGDSEEKPEEEVQLIHDQSAPSCPSSSPSPGEEPEGEGETDPEKVHLTW
TKDKSVAEKNKGPSPVSSEGIKDFFSMKPEWENLNQSNVRRMHTAVRLNEVIVKKSRDA
KLVLLNMPGPPRNRNGDENYMEFLEVLTEHLDRVMLVRGGGREVITIYS

>Q14677 peripheral membrane protein
MLNMWKVRELVDKATNVVMNYSEIESKVREATNDDPWGPSGQLMGEIAKATFMYEQFP
ELMNMLWSRMLKDNKKNWRRVYKSLLLLAYLIRNGSERVVTSAREHIYDLRSLENYHFV
DEHGKDQGINIRQKVKELVEFAQDDDRLREERKKAKKNKDKYVGVSSDSVGGFRYSERY
DPEPKSKWDEEWDKNKSAFPFSDKLGELSDKIGSTIDDTISKFRRKDREDSPERCSDSDEE
KKARRGRSPKGEFKDEEETVTTKHIHITQATETTTTRHKRTANPSKTIDLGAAAHYTGDKA
SPDQNASTHTPQSSVKTSVPSSKSSGDLVDLFDGTSQSTGGSADLFGGFADFGSAAASGSF
PSQVTATSGNGDFGDWSAFNQAPSGPVASSGEFFGSASQPAVELVSGSQSALGPPPAASNSS
DLFDLMGSSQATMTSSQSMNFSMMSTNTVGLGLPMSRSQNTDMVQKSVSKTLPSTWSDP
SVNISLDNLLPGMQPSKPQQPSLNTMIQQQNMQQPMNVMTQSFGAVNLSSPSNMLPVRP
QTNALIGGPMPMSMPNVMTGTMGMAPLGNTPMMNQSMMGMNMNIGMSAAGMGLTG
TMGMGMPNIAMTSGTVQPKQDAFANFANFSK

>O75746 multipass membrane protein
MAVKVQTTKRGDPHELRNIFLQYASTEVDGERYMTPEDFVQRYLGLYNDPNSNPKIVQLL
AGVADQTKDGLISYQEFLAFESVLCAPDSMFIVAFQLFDKSGNGEVTFENVKEIFGQTIIHH
HIPFNWDCEFIRLHFGHNRKKHLNYTEFTQFLQELQLEHARQAFALKDKSKSGMISGLDFS
DIMVTIRSHMLTPFVEENLVSAAGGSISHQVSFSYFNAFNSLLNNMELVRKIYSTLAGTRK
DVEVTKEEFAQSAIRYGQVTPLEIDILYQLADLYNASGRLTLADIERIAPLAEGALPYNLAE
LQRQQSPGLGRPIWLQIAESAYRFTLGSVAGAVGATAVYPIDLVKTRMQNQRGSGSVVGEL
MYKNSFDCFKKVLRYEGFFGLYRGLIPQLIGVAPEKAIKLTVNDFVRDKFTRRDGSVPLPA
EVLAGGCAGGSQVIFTNPLEIVKIRLQVAGEITTGPRVSALNVLRDLGIFGLYKGAKACFLR
DIPFSAIYFPVYAHCKLLLADENGHVGGLNLLAAGAMAGVPAASLVTPADVIKTRLQVAA
RAGQTTYSGVIDCFRKILREEGPSAFWKGTAARVFRSSPQFGVTLVTYELLQRWFYIDFGG

LKPAGSEPTPKSRIADLPPANPDHIGGYRLATATFAGIENKFGLYLPKFKSPSVAVVQPKAAV
AATQ

>Q8N6Q3 GPI-anchor, lipid-anchor
MSAVLLLALLGFILPLPGVQALLCQFGTVQHVWKVSDLPRQWTPKNTSCDSGLGCQDTL
MLIESGPQVSLVLSKGCTEAKDQEPRVTEHRMGPGLSLISYTFVCRQEDFCNNLVNSLPLW
APQPPADPGSLRCPVCLSMEGCLEGTTEEICPKGTTHCYDGLLRLRGGGIFSNLRVQGCMP
QPGCNLLNGTQEIGPVGMTENCNRKDFLTCHRGTTIMTHGNLAQEPTDWTTSNTEMCEV
GQVCQETLLLLDVGLTSTLVGTKGCSTVGAQNSQKTTIHSAPPGVLVASYTHFCSSDLCNS
ASSSSVLLNSLPPQAAPVPGDRQCPTCVQPLGTCSSGSPRMTCPRGATHCYDGYIHLSGGG
LSTKMSIQGCVAQPSSFLLNHTRQIGIFSAREKRDVQPPASQHEGGGAEGLESLTWGVGLA
LAPALWWGVVCPSC

>O75955 peripheral membrane protein
MFFTCGPNEAMVVSGFCRSPPVMVAGGRVFVLPCIQQIQRISLNTLTLNVKSEKVYTRHG
VPISVTGIAQVKIQGQNKEMLAAACQMFLGKTEAEIAHIALETLEGHQRAIMAHMTVEEI
YKDRQKFSEQVFKVASSDLVNMGISVVSYTLKDIHDDQDYLHSLGKARTAQVQKDARIG
EAEAKRDAGIREAKAKQEKVSAQYLSEIEMAKAQRDYELKKAAYDIEVNTRRAQADLAY
QLQVAKTKQQIEEQRVQVQVVERAQQVAVQEQEIARREKELEARVRKPAEAERYKLERLA
EAEKSQLIMQAEAEAASVRMRGEAEAFAIGARARAEAEQMAKKAEAFQLYQEAAQLDM
LLEKLPQVAEEISGPLTSANKITLVSSGSGTMGAAKVTGEVLDILTRLPESVERLTGVSISQV
NHKPLRTA

>P43146 single-pass type I membrane protein
MENSLRCVWVPKLAFVLFGASLFSAHLQVTGFQIKAFTALRFLSEPSDAVTMRGGNVLLD
CSAESDRGVPVIKWKKDGIHLALGMDERKQQLSNGSLLIQNILHSRHHKPDEGLYQCEAS
LGDSGSIISRTAKVAVAGPLRFLSQTESVTAFMGDTVLLKCEVIGEPMPTIHWQKNQQDLTP
IPGDSRVVVLPSGALQISRLQPGDIGIYRCSARNPASSRTGNEAEVRILSDPGLHRQLYFLQR
PSNVVAIEGKDAVLECCVSGYPPPSFTWLRGEEVIQLRSKKYSLLGGSNLLISNVTDDDSG
MYTCVVTYKNENISASAELTVLVPPWFLNHPSNLYAYESMDIEFECTVSGKPVPTVNWMK
NGDVVIPSDYFQIVGGSNLRILGVVKSDEGFYQCVAENEAGNAQTSAQLIVPKPAIPSSSVL
PSAPRDVVPVLVSSRFVRLSWRPPAEAKGNIQTFTVFFSREGDNRERALNTTQPGSLQLTV
GNLKPEAMYTFRVVAYNEWGPGESSQPIKVATQPELQVPGPVENLQAVSTSPTSILITWEPP
AYANGPVQGYRLFCTEVSTGKEQNIEVDGLSYKLEGLKKFTEYSLRFLAYNRYGPGVSTD
DITVVTLSDVPSAPPQNVSLEVVNSRSIKVSWLPPPSGTQNGFITGYKIRHRKTTRRGEME
TLEPNNLWYLFTGLEKGSQYSFQVSAMTVNGTGPPSNWYTAETPENDLDESQVPDQPSSL
HVRPQTNCIIMSWTPPLNPNIVVRGYIIGYGVGSPYAETVRVDSKQRYYSIERLESSSHYVIS
LKAFNNAGEGVPLYESATTRSITDPTDPVDYYPLLDDFPTSVPDLSTPMLPPVGVQAVALT
HDAVRVSWADNSVPKNQKTSEVRLYTVRWRTSFSASAKYKSEDTTSLSYTATGLKPNTM
YEFSVMVTKNRRSSTWSMTAHATTYEAAPTSAPKDLTVITREGKPRAVIVSWQPPLEANG
KITAYILFYTLDKNIPIDDWIMETISGDRLTHQIMDLNLDTMYYFRIQARNSKGVGPLSDPIL
FRTLKVEHPDKMANDQGRHGDGGYWPVDTNLIDRSTLNEPPIGQMHPPHGSVTPQKNSN
LLVIIVVTVGVITVLVVVIVAVICTRRSSAQQRKKRATHSAGKRKGSQKDLRPPDLWIHHEE
MEMKNIEKPSGTDPAGRDSPIQSCQDLTPVSHSQSETQLGSKSTSHSGQDTEEAGSSMSTL

ERSLAARRAPRAKLMIPMDAQSNNPAVVSAIPVPTLESAQYPGILPSPTCGYPHPQFTLRPV
PFPTLSVDRGFGAGRSQSVSEGPTTQQPPMLPPSQPEHSSSEEAPSRTIPTACVRPTHPLRSF
ANPLLPPPMSAIEPKVPYTPLLSQPGPTLPKTHVKTASLGLAGKARSPLLPVSVPTAPEVSE
ESHKPTEDSANVYEQDDLSEQMASLEGLMKQLNAITGSAF

>Q7KZI7 peripheral membrane protein
MSSARTPLPTLNERDTEQPTLGHLDSKPSSKSNMIRGRNSATSADEQPHIGNYRLLKTIGK
GNFAKVKLARHILTGKEVAVKIIDKTQLNSSSLQKLFREVRIMKVLNHPNIVKLFEVIETEK
TLYLVMEYASGGEVFDYLVAHGRMKEKEARAKFRQIVSAVQYCHQKFIVHRDLKAENLL
LDADMNIKIADFGFSNEFTFGNKLDTFCGSPPYAAPELFQGKKYDGPEVDVWSLGVILYTL
VSGSLPFDGQNLKELRERVLRGKYRIPFYMSTDCENLLKKFLILNPSKRGTLEQIMKDRW
MNVGHEDDELKPYVEPLPDYKDPRRTELMVSMGYTREEIQDSLVGQRYNEVMATYLLLG
YKSSELEGDTITLKPRPSADLTNSSAPSPSHKVQRSVSANPKQRRFSDQAAGPAIPTSNSYS
KKTQSNNAENKRPEEDRESGRKASSTAKVPASPLPGLERKKTTPTPSTNSVLSTSTNRSRN
SPLLERASLGQASIQNGKDSLTMPGSRASTASASAAVSAARPRQHQKSMSASVHPNKASG
LPPTESNCEVPRPSTAPQRVPVASPSAHNISSSGGAPDRTNFPRGVSSRSTFHAGQLRQVRD
QQNLPYGVTPASPSGHSQGRRGASGSIFSKFTSKFVRRNLSFRFARRNLNEPESKDRVETLR
PHVVGSGGNDKEKEEFREAKPRSLRFTWSMKTTSSMEPNEMMREIRKVLDANSCQSELH
EKYMLLCMHGTPGHEDFVQWEMEVCKLPRLSLNGVRFKRISGTSMAFKNIASKIANELK
L

>P55061 multipass membrane protein
MNIFDRKINFDALLKFSHITPSTQQHLKKVYASFALCMFVAAAGAYVHMVTHFIQAGLLS
ALGSLILMIWLMATPHSHETEQKRLGLLAGFAFLTGVGLGPALEFCIAVNPSILPTAFMGTA
MIFTCFTLSALYARRRSYLFLGGILMSALSLLLLSSLGNVFFGSIWLFQANLYVGLVVMCGF
VLFDTQLIIEKAEHGDQDYIWHCIDLFLDFITVFRKLMMILAMNEKDKKKEKK

>P04440 single-pass type I membrane protein
MMVLQVSAAPRTVALTALLMVLLTSVVQGRATPENYLFQGRQECYAFNGTQRFLERYIYN
REEFARFDSDVGEFRAVTELGRPAAEYWNSQKDILEEKRAVPDRMCRHNYELGGPMTLQ
RRVQPRVNVSPSKKGPLQHHNLLVCHVTDFYPGSIQVRWFLNGQEETAGVVSTNLIRNGD
WTFQILVMLEMTPQQGDVYTCQVEHTSLDSPVTVEWKAQSDSARSKTLTGAGGFVLGLII
CGVGIFMHRRSKKVQRGSA

>Q5HYA8 multipass membrane protein
MATRGGAGVAMAVWSLLSARAVTAFLLLFLPRFLQAQTFSFPFQQPEKCDNNQYFDISAL
SCVPCGANQRQDARGTSCVCLPGFQMISNNGGPAIICKKCPENMKGVTEDGWNCISCPSD
LTAEGKCHCPIGHILVERDINGTLLSQATCELCDGNENSFMVVNALGDRCVRCEPTFVNTS
RSCACSEPNILTGGLCFSSTGNFPLRRISAARYGEVGMSLTSEWFAKYLQSSAAACWVYAN
LTSCQALGNMCVMNMNSYDFATFDACGLFQFIFENTAGLSTVHSISFWRQNLPWLFYGDQ
LGLAPQVLSSTSLPTNFSFKGENQNTKLKFVAASYDIRGNFLKWQTLEGGVLQLCPDTET
RLNAAYSFGTTYQQNCEIPISKILIDFPTPIFYDVYLEYTDENQHQYILAVPVLNLNLQHNKI
FVNQDSNSGKWLLTRRIFLVDAVSGRENDLGTQPRVIRVATQISLSVHLVPNTINGNIYPPLI
TIAYSDIDIKDANSQSVKVSFSVTYEMDHGEAHVQTDIALGVLGGLAVLASLLKTAGWKR

RIGSPMIDLQTVVKFLVYYAGDLANVFFIITVGTGLYWLIFFKAQKSVSVLLPMPIQEERFV
TYVGCAFALKALQFLHKLISQITIDVFFIDWERPKGKVLKAVEGEGGVRSATVPVSIWRTY
FVANEWNEIQTVRKINSLFQVLTVLFFLEVVGFKNLALMDSSSSLSRNPPSYIAPYSCILRYA
VSAALWLAIGIIQVVFFAVFYERFIEDKIRQFVDLCSMSNISVFLLSHKCFGYYIHGRSVHG
HADTNMEEMNMNLKREAENLCSQRGLVPNTDGQTFEIAISNQMRQHYDRIHETLIRKNGP
ARLLSSSASTFEQSIKAYHMMNKFLGSFIDHVHKEMDYFIKDKLLLERILGMEFMEPMEK
SIFYNDEGYSFSSVLYYGNEATLLIFDLLFFCVVDLACQNFILASFLTYLQQEIFRYIRNTVG
QKNLASKTLVDQRFLI

>P13765 single-pass type I membrane protein
MGSGWVPWVVALLVNLTRLDSSMTQGTDSPEDFVIQAKADCYFTNGTEKVQFVVRFIFN
LEEYVRFDSDVGMFVALTKLGQPDAEQWNSRLDLLERSRQAVDGVCRHNYRLGAPFTVG
RKVQPEVTVYPERTPLLHQHNLLHCSVTGFYPGDIKIKWFLNGQEERAGVMSTGPIRNGD
WTFQTVVMLEMTPELGHVYTCLVDHSSLLSPVSVEWRAQSEYSWRKMLSGIAAFLLGLI
FLLVGIVIQLRAQKGYVRTQMSGNEVSRAVLLPQSC

>Q15751 peripheral membrane protein
MATMIPPVKLKWLEHLNSSWITEDSESIATREGVAVLYSKLVSNKEVVPLPQQVLCLKGPQ
LPDFERESLSSDEQDHYLDALLSSQLALAKMVCSDSPFAGALRKRLLVLQRVFYALSNKY
HDKGKVKQQQHSPESSSGSADVHSVSERPRSSTDALIEMGVRTGLSLLFALLRQSWMMP
VSGPGLSLCNDVIHTAIEVVSSLPPLSLANESKIPPMGLDCLSQVTTFLKGVTIPNSGADTL
GRRLASELLLGLAAQRGSLRYLLEWIEMALGASAVVHTMEKGKLLSSQEGMISFDCFMTI
LMQMRRSLGSSADRSQWREPTRTSDGLCSLYEAALCLFEEVCRMASDYSRTCASPDSIQT
GDAPIVSETCEVYVWGSNSSHQLVEGTQEKILQPKLAPSFSDAQTIEAGQYCTFVISTDGS
VRACGKGSYGRLGLGDSNNQSTLKKLTFEPHRSIKKVSSSKGSDGHTLAFTTEGEVFSWG
DGDYGKLGHGNSSTQKYPKLIQGPLQGKVVVCVSAGYRHSAAVTEDGELYTWGEGDFG
RLGHGDSNSRNIPTLVKDISNVGEVSCGSSHTIALSKDGRTVWSFGGGDNGKLGHGDTNR
VYKPKVIEALQGMFIRKVCAGSQSSLALTSTGQVYAWGCGACLGCGSSEATALRPKLIEEL
AATRIVDVSIGDSHCLALSHDNEVYAWGNNSMGQCGQGNSTGPITKPKKVSGLDGIAIQQI
SAGTSHSLAWTALPRDRQVVAWHRPYCVDLEESTFSHLRSFLERYCDKINSEIPPLPFPSSR
EHHSFLKLCLKLLSNHLALALAGGVATSILGRQAGPLRNLLFRLMDSTVPDEIQEVVIETLS
VGATMLLPPLRERMELLHSLLPQGPDRWESLSKGQRMQLDIILTSLQDHTHVASLLGYSSP
SDAADLSSVCTGYGNLSDQPYGTQSCHPDTHLAEILMKTLLRNLGFYTDQAFGELEKNSD
KFLLGTSSSENSQPAHLHELLCSLQKQLLAFCHINNISENSSSVALLHKHLQLLLPHATDIYS
RSANLLKESPWNGSVGEKLRDVIYVSAAGSMLCQIVNSLLLLPVSVARPLLSYLLDLLPPL
DCLNRLLPAADLLEDQELQWPLHGGPELIDPAGLPLPQPAQSWVWLVDLERTIALLIGRCL
GGMLQGSPVSPEEQDTAYWMKTPLFSDGVEMDTPQLDKCMSCLLEVALSGNEEQKPFDY
KLRPEIAVYVDLALGCSKEPARSLWISMQDYAVSKDWDSATLSNESLLDTVSRFVLAALLK
HTNLLSQACGESRYQPGKHLSEVYRCVYKVRSRLLACKNLELIQTRSSSRDRWISENQDS
ADVDPQEHSFTRTIDEEAEMEEQAERDREEGHPEPEDEEEEREHEVMTAGKIFQCFLSARE
VARSRDRDRMNSGAGSGARADDPPPQSQQERRVSTDLPEGQDVYTAACNSVIHRCALLIL
GVSPVIDELQKRREEGQLQQPSTSASEGGGLMTRSESLTAESRLVHTSPNYRLIKSRSESDL
SQPESDEEGYALSGRRNVDLDLAASHRKRGPMHSQLESLSDSWARLKHSRDWLCNSSYS
FESDFDLTKSLGVHTLIENVVSFVSGDVGNAPGFKEPEESMSTSPQASIIAMEQQQLRAEL

RLEALHQILVLLSGMEEKGSISLAGSRLSSGFQSSTLLTSVRLQFLAGCFGLGTVGHTGGK
GESGRLHHYQDGIRAAKRNIQIEIQVAVHKIYQQLSATLERALQANKHHIEAQQRLLLVTV
FALSVHYQPVDVSLAISTGLLNVLSQLCGTDTMLGQPLQLLPKTGVSQLSTALKVASTRLL
QILAITTGTYADKLSPKVVQSLLDLLCSQLKNLLSQTGVLHMASFGEGEQEDGEEEEKKV
DSSGETEKKDFRAALRKQHAAELHLGDFLVFLRRVVSSKAIQSKMASPKWTEVLLNIASQ
KCSSGIPLVGNLRTRLLALHVLEAVLPACESGVEDDQMAQIVERLFSLLSDCMWETPIAQA
KHAIQIKEKEQEIKLQKQGELEEEDENLPIQEVSFDPEKAQCCLVENGQILTHGSGGKGYG
LASTGVTSGCYQWKFYIVKENRGNEGTCVGVSRWPVHDFNHRTTSDMWLYRAYSGNLY
HNGEQTLTLSSFTQGDFITCVLDMEARTISFGKNGEEPKLAFEDVDAAELYPCVMFYSSNP
GEKVKICDMQMRGTPRDLLPGDPICSPVAAVLAEATIQLIRILHRTDRWTYCINKKMMERL
HKIKICIKESGQKLKKSRSVQSREENEMREEKESKEEEKGKHTRHGLADLSELQLRTLCIE
VWPVLAVIGGVDAGLRVGGRCVHKQTGRHATLLGVVKEGSTSAKVQWDEAEITISFPTF
WSPSDTPLYNLEPCEPLPFDVARFRGLTASVLLDLTYLTGVHEDMGKQSTKRHEKKHRHE
SEEKGDVEQKPESESALDMRTGLTSDDVKSQSTTSSKSENEIASFSLDPTLPSVESQHQITE
GKRKNHEHMSKNHDVAQSEIRAVQLSYLYLGAMKSLSALLGCSKYAELLLIPKVLAENGH
NSDCASSPVVHEDVEMRAALQFLMRHMVKRAVMRSPIKRALGLADLERAQAMIYKLVV
HGLLEDQFGGKIKQEIDQQAEESDPAQQAQTPVTTSPSASSTTSFMSSSLEDTTTATTPVTD
TETVPASESPGVMPLSLLRQMFSSYPTTTVLPTRRAQTPPISSLPTSPSDEVGRRQSLTSPDS
QSARPANRTALSDPSSRLSTSPPPPAIAVPLLEMGFSLRQIAKAMEATGARGEADAQNITVL
AMWMIEHPGHEDEEEPQSGSTADSRPGAAVLGSGGKSNDPCYLQSPGDIPSADAAEMEE
GFSESPDNLDHTENAASGSGPSARGRSAVTRRHKFDLAARTLLARAAGLYRSVQAHRNQS
RREGISLQQDPGALYDFNLDEELEIDLDDEAMEAMFGQDLTSDNDILGMWIPEVLDWPT
WHVCESEDREEVVVCELCECSVVSFNQHMKRNHPGCGRSANRQGYRSNGSYVDGWFG
GECGSGNPYYLLCGTCREKYLAMKTKSKSTSSERYKGQAPDLIGKQDSVYEEDWDMLD
VDEDEKLTGEEEFELLAGPLGLNDRRIVPEPVQFPDSDPLGASVAMVTATNSMEETLMQIG
CHGSVEKSSSGRITLGEQAAALANPHDRVVALRRVTAAAQVLLARTMVMRALSLLSVSGS
SCSLAAGLESLGLTDIRTLVRLMCLAAAGRAGLSTSPSAMASTSERSRGGHSKANKPISCL
AYLSTAVGCLASNAPSAAKLLVQLCTQNLISAATGVNLTTVDDSIQRKFLPSFLRGIAEENK
LVTSPNFVVTQALVALLADKGAKLRPNYDKSEVEKKGPLELANALAACCLSSRLSSQHRQ
WAAQQLVRTLAAHDRDNQTTLQTLADMGGDLRKCSFIKLEAHQNRVMTCVWCNKKGL
LATSGNDGTIRVWNVTKKQYSLQQTCVFNRLEGDAEESLGSPSDPSFSPVSWSISGKYLAG
ALEKMVNIWQVNGGKGLVDIQPHWVSALAWPEEGPATAWSGESPELLLVGRMDGSLGLI
EVVDVSTMHRRELEHCYRKDVSVTCIAWFSEDRPFAVGYFDGKLLLGTKEPLEKGGIVLI
DAHKDTLISMKWDPTGHILMTCAKEDSVKLWGSISGCWCCLHSLCHPSIVNGIAWCRLPG
KGSKLQLLMATGCQSGLVCVWRIPQDTTQTNVTSAEGWWEQESNCQDGYRKSSGAKCV
YQLRGHITPVRTVAFSSDGLALVSGGLGGLMNIWSLRDGSVLQTVVIGSGAIQTTVWIPEV
GVAACSNRSKDVLVVNCTAEWAAANHVLATCRTALKQQGVLGLNMAPCMRAFLERLPM
MLQEQYAYEKPHVVCGDQLVHSPYMQCLASLAVGLHLDQLLCNPPVPPHHQNCLPDPAS
WNPNEWAWLECFSTTIKAAEALTNGAQFPESFTVPDLEPVPEDELVFLMDNSKWINGMDE
QIMSWATSRPEDWHLGGKCDVYLWGAGRHGQLAEAGRNVMVPAAAPSFSQAQQVICGQ
NCTFVIQANGTVLACGEGSYGRLGQGNSDDLHVLTVISALQGFVVTQLVTSCGSDGHSM
ALTESGEVFSWGDGDYGKLGHGNSDRQRRPRQIEALQGEEVVQMSCGFKHSAVVTSDGK
LFTFGNGDYGRLGLGNTSNKKLPERVTALEGYQIGQVACGLNHTLAVSADGSMVWAFGD
GDYGKLGLGNSTAKSSPQKIDVLCGIGIKKVACGTQFSVALTKDGHVYTFGQDRLIGLPEG

RARNHNRPQQIPVLAGVIIEDVAVGAEHTLALASNGDVYAWGSNSEGQLGLGHTNHVREP
TLVTGLQGKNVRQISAGRCHSAAWTAPPVPPRAPGVSVPLQLGLPDTVPPQYGALREVSI
HTVRARLRLLYHFSDLMYSSWRLLNLSPNNQNSTSHYNAGTWGIVQGQLRPLLAPRVYT
LPMVRSIGKTMVQGKNYGPQITVKRISTRGRKCKPIFVQIARQVVKLNASDLRLPSRAWK
VKLVGEGADDAGGVFDDTITEMCQELETGIVDLLIPSPNATAEVGYNRDRFLFNPSACLDE
HLMQFKFLGILMGVAIRTKKPLDLHLAPLVWKQLCCVPLTLEDLEEVDLLYVQTLNSILHI
EDSGITEESFHEMIPLDSFVGQSADGKMVPIIPGGNSIPLTFSNRKEYVERAIEYRLHEMDR
QVAAVREGMSWIVPVPLLSLLTAKQLEQMVCGMPEISVEVLKKVVRYREVDEQHQLVQW
FWHTLEEFSNEERVLFMRFVSGRSRLPANTADISQRFQIMKVDRPYDSLPTSQTCFFQLRLP
PYSSQLVMAERLRYAINNCRSIDMDNYMLSRNVDNAEGSDTDY

>O94850 peripheral membrane protein
MLDGPLFSEGPDSPRELQDEESGSCLWVQKSKLLVIEVKTISCHYSRRAPSRQPMDFQASH
WARGFQNRTCGPRPGSPQPPPRRPWASRVLQEATNWRAGPLAEVRAREQEKRKAASQER
EAKETERKRRKAGGARRSPPGRPRPEPRNAPRVAQLAGLPAPLRPERLAPVGRAPRPSAQP
QSDPGSAWAGPWGGRRPGPPSYEAHLLLRGSAGTAPRRRWDRPPPYVAPPSYEGPHRTLG
TKRGPGNSQVPTSSAPAATPARTDGGRTKKRLDPRIYRDVLGAWGLRQGQGLLGGSPGCG
AARARPEPGKGVVEKSLGLAAADLNSGSDSHPQAKATGSAGTEIAPAGSATAAPCAPHPA
PRSRHHLKGSREGKEGEQIWFPKCWIPSPKKQPPRHSQTLPRPWAPGGTGWRESLGLGEG
AGPETLEGWKATRRAHTLPRSSQGLSRGEGVFVIDATCVVIRSQYVPTPRTQQVQLLPSGV
TRVVGDSPSQSKPGKEEGEGATVFPSPCQKRLSSSRLLHQPGGGRGGEAEGGRPGDSTLEE
RTFRILGLPAPEVNLRDAPTQPGSPEHQALGPAASGAQGRAEGSEVAVVQRRAGRGWART
PGPYAGALREAVSRIRRHTAPDSDTDEAEELSVHSGSSDGSDTEAPGASWRNERTLPEVGN
SSPEEDGKTAELSDSVGEILDVISQTEEVLFGVRDIRGTQQGNRKRQ

>O43808 multipass membrane protein
MASVLSYESLVHAVAGAVGSVTAMTVFFPLDTARLRLQVDEKRKSKTTHMVLLEIIKEEG
LLAPYRGWFPVISSLCCSNFVYFYTFNSLKALWVKGQHSTTGKDLVVGFVAGVVNVLLTT
PLWVVNTRLKLQGAKFRNEDIVPTNYKGIIDAFHQIIRDEGISALWNGTFPSLLLVFNPAIQ
FMFYEGLKRQLLKKRMKLSSLDVFIIGAVAKAIATTVTYPLQTVQSILRFGRHRLNPENRTL
GSLRNILYLLHQRVRRFGIMGLYKGLEAKLLQTVLTAALMFLVYEKLTAATFTVMGLKRA
HQH

>Q96T55 multipass membrane protein
MPSAGLCSCWGGRVLPLLLAYVCYLLLGATIFQLLERQAEAQSRDQFQLEKLRFLENYTC
LDQWAMEQFVQVIMEAWVKGVNPKGNSTNPSNWDFGSSFFFAGTVVTTIGYGNLAPSTE
AGQVFCVFYALLGIPLNVIFLNHLGTGLRAHLAAIERWEDRPRRSQVLQVLGLALFLTLGT
LVILIFPPMVFSHVEGWSFSEGFYFAFITLSTIGFGDYVVGTDPSKHYISVYRSLAAIWILLG
LAWLALILPLGPLLLHRCCQLWLLSLRQGCGAKAAPGRRPRRGSTAARGVQVTPQDFPIS
KKGLGS

>P35410 multipass membrane protein
MVWGKICWFSQRAGWTVFAESQISLSCSLCLHSGDQEAQNPNLVSQLCGVFLQNETNETI
HMQMSMAVGQQALPLNIIAPKAVLVSLCGVLLNGTVFWLLCCGATNPYMVYILHLVAAD

VIYLCCSAVGFLQVTLLTYHGVVFFIPDFLAILSPFSFEVCLCLLVAISTERCVCVLFPIWYR
CHRPKYTSNVVCTLIWGLPFCINIVKSLFLTYWKHVKACVIFLKLSGLFHAILSLVMCVSSL
TLLIRFLCCSQQQKATRVYAVVQISAPMFLLWALPLSVAPLITDFKMFVTTSYLISLFLIINSS
ANPIIYFFVGSLRKKRLKESLRVILQRALADKPEVGRNKKAAGIDPMEQPHSTQHVENLLP
REHRVDVET

>P51572 multipass membrane protein
MSLQWTAVATFLYAEVFVVLLLCIPFISPKRWQKIFKSRLVELLVSYGNTFFVVLIVILVLLVI
DAVREIRKYDDVTEKVNLQNNPGAMEHFHMKLFRAQRNLYIAGFSLLLSFLLRRLVTLIS
QQATLLASNEAFKKQAESASEAAKKYMEENDQLKKGAAVDGGKLDVGNAEVKLEEENR
SLKADLQKLKDELASTKQKLEKAENQVLAMRKQSEGLTKEYDRLLEEHAKLQAAVDGP
MDKKEE

>P40126 single-pass type I membrane protein
MSPLWWGFLLSCLGCKILPGAQGQFPRVCMTVDSLVNKECCPRLGAESANVCGSQQGRG
QCTEVRADTRPWSGPYILRNQDDRELWPRKFFHRTCKCTGNFAGYNCGDCKFGWTGPNC
ERKKPPVIRQNIHSLSPQEREQFLGALDLAKKRVHPDYVITTQHWLGLLGPNGTQPQFAN
CSVYDFFVWLHYYSVRDTLLGPGRPYRAIDFSHQGPAFVTWHRYHLLCLERDLQRLIGNE
SFALPYWNFATGRNECDVCTDQLFGAARPDDPTLISRNSRFSSWETVCDSLDDYNHLVTL
CNGTYEGLLRRNQMGRNSMKLPTLKDIRDCLSLQKFDNPPFFQNSTFSFRNALEGFDKAD
GTLDSQVMSLHNLVHSFLNGTNALPHSAANDPIFVVLHSFTDAIFDEWMKRFNPPADAWP
QELAPIGHNRMYNMVPFFPPVTNEELFLTSDQLGYSYAIDLPVSVEETPGWPTTLLVVMGT
LVALVGLFVLLAFLQYRRLRKGYTPLMETHLSSKRYTEEA

>Q86YR5 peripheral membrane protein
MAGPAPPVADELPGPAARRLYSRMEASCLELALEGERLCKAGDFKTGVAFFEAAVQVGTE
DLKTLSAIYSQLGNAYFYLKEHGRALEYHKHDLLLARTIGDRMGEAKASGNLGNTLKVL
GRFDEAAVCCQRHLSIAQEQGDKVGEARALYNIGNVYHAKGKQLSWNAANATQDPGHL
PPDVRETLCKASEFYERNLSLVKELGDRAAQGRAYGNLGNTHYLLGNFTEATTFHKERLA
IAKEFGDKAAERRAYSNLGNAHVFLGRFDVAAEYYKKTLQLSRQLRDQAVEAQACYSLG
NTYTLLQDYERAAEYHLRHLLIAQELADRVGEGRACWSLGNAYVSMGRPAQALTFAKKH
LQISQEIGDRHGELTARMNVAQLQLVLGRLTSPAASEKPDLAGYEAQGARPKRTQRLSAET
WDLLRLPLEREQNGDSHHSGDWRGPSRDSLPLPVRSRKYQEGPDAERRPREGSHSPLDSA
DVRVHVPRTSIPRAPSSDEECFFDLLTKFQSSRMDDQRCPLDDGQAGAAEATAAPTLEDRI
AQPSMTASPQTEEFFDLIASSQSRRLDDQRASVGSLPGLRITHSNAGHLRGHGEPQEPGDD
FFNMLIKYQSSRIDDQRCPPPDVLPRGPTMPDEDFFSLIQRVQAKRMDEQRVDLAGGPEQ
GAGGPPEPQQQCQPGAS

>Q86SM8 multipass membrane protein
MEPREAGQHVGAANGAQEDVAFNLIILSLTEGLGLGGLLGNGAVLWLLSSNVYRNPFAIY
LLDVACADLIFLGCHMVAIVPDLLQGRLDFPGFVQTSLATLRFFCYIVGLSLLAAVSVEQCL
AALFPAWYSCRRPRHLTTCVCALTWALCLLLHLLLSGACTQFFGEPSRHLCRTLWLVAAVL
LALLCCTMCGASLMLLLRVERGPQRPPPRGFPGLILLTVLLFLFCGLPFGIYWLSRNLLWYI
PHYFYHFSFLMAAVHCAAKPVVYFCLGSAQGRRLPLRLVLQRALGDEAELGAVRETSRR

GLVDIAA

>Q9Y6R1 multipass membrane protein
MEDEAVLDRGASFLKHVCDEEEVEGHHTIYIGVHVPKSYRRRRRHKRKTGHKEKKEKER
ISENYSDKSDIENADESSSSILKPLISPAAERIRFILGEEDDSPAPPQLFTELDELLAVDGQEM
EWKETARWIKFEEKVEQGGERWSKPHVATLSLHSLFELRTCMEKGSIMLDREASSLPQLVE
MIVDHQIETGLLKPELKDKVTYTLLRKHRHQTKKSNLRSLADIGKTVSSASRMFTNPDNG
SPAMTHRNLTSSSLNDISDKPEKDQLKNKFMKKLPRDAEASNVLVGEVDFLDTPFIAFVRL
QQAVMLGALTEVPVPTRFLFILLGPKGKAKSYHEIGRAIATLMSDEVFHDIAYKAKDRHDL
IAGIDEFLDEVIVLPPGEWDPAIRIEPPKSLPSSDKRKNMYSGGENVQMNGDTPHDGGHGG
GGHGDCEELQRTGRFCGGLIKDIKRKAPFFASDFYDALNIQALSAILFIYLATVTNAITFGG
LLGDATDNMQGVLESFLGTAVSGAIFCLFAGQPLTILSSTGPVLVFERLLFNFSKDNNFDYL
EFRLWIGLWSAFLCLILVATDASFLVQYFTRFTEEGFSSLISFIFIYDAFKKMIKLADYYPINS
NFKVGYNTLFSCTCVPPDPANISISNDTTLAPEYLPTMSSTDMYHNTTFDWAFLSKKECSK
YGGNLVGNNCNFVPDITLMSFILFLGTYTSSMALKKFKTSPYFPTTARKLISDFAIILSILIFC
VIDALVGVDTPKLIVPSEFKPTSPNRGWFVPPFGENPWWVCLAAAIPALLVTILIFMDQQIT
AVIVNRKEHKLKKGAGYHLDLFWVAILMVICSLMALPWYVAATVISIAHIDSLKMETETS
APGEQPKFLGVREQRVTGTLVFILTGLSVFMAPILKFIPMPVLYGVFLYMGVASLNGVQFM
DRLKLLLMPLKHQPDFIYLRHVPLRRVHLFTFLQVLCLALLWILKSTVAAIIFPVMILALVA
VRKGMDYLFSQHDLSFLDDVIPEKDKKKKEDEKKKKKKKGSLDSDNDDSDCPYSEKVPS
IKIPMDIMEQQPFLSDSKPSDRERSPTFLERHTSC

>Q0JRZ9 peripheral membrane protein
MVMAYFVENFWGEKNSGFDVLYHNMKHGQISTKELADFVRERATIEEAYSRSMTKLAKS
ASNYSQLGTFAPVWDVFKTSTEKLANCHLDLVRKLQELIKEVQKYGEEQVKSHKKTKEE
VAGTLEAVQTIQSITQALQKSKENYNAKCVEQERLKKEGATQREIEKAAVKSKKATDTYK
LYVEKYALAKADFEQKMTETAQKFQDIEETHLIHIKEIIGSLSNAIKEIHLQIGQVHEEFINN
MANTTVESLIQKFAESKGTGKERPGLIEFEECDTASAVEGIKPRKRKTFALPGIIKKEKDAE
SVECPDADSLNIPDVDEEGYSIKPETNQNDTKENHFYSSSDSDSEDEEPKKYRIEIKPMHPN
NSHHTMASLDELKVSIGNITLSPAISRHSPVQMNRNLSNEELTKSKPSAPPNEKGTSDLLA
WDPLFGPSLDSSSSSSLTSSSSARPTTPLSVGTIVPPPRPASRPKLTSGKLSGINEIPRPFSPPV
TSNTSPPPAAPLARAESSSSISSSASLSAANTPTVGVSRGPSPVSLGNQDTLPVAVALTESVN
AYFKGADPTKCIVKITGDMTMSFPSGIIKVFTSNPTPAVLCFRVKNISRLEQILPNAQLVFSD
PSQCDSNTKDFWMNMQAVTVYLKKLSEQNPAASYYNVDVLKYQVSSNGIQSTPLNLATY
WKCSASTTDLRVDYKYNPEAMVAPSVLSNIQVVVPVDGGVTNMQSLPPAIWNAEQMKA
FWKLSSISEKSENGGSGSLRAKFDLSEGPSKPTTLAVQFLSEGSTLSGVDFELVGTGYRLSL
IKKRFATGRYLADC

>Q8NHB8 multipass membrane protein
MVEENHTMKNEFILTGFTDHPELKTLLFVVFFAIYLITVVGNISLVALIFTHCRLHTPMYIFL
GNLALVDSCCACAITPKMLENFFSEGKRISLYECAVQFYFLCTVETADCFLLAAVAYDRYV
AICNPLQYHIMMSKKLCIQMTTGAFIAGNLHSMIHVGLVFRLVFCGLNHINHFYCDTLPLY
RLSCVDPFINELVLFIFSGSVQVFTIGSVLISYLYILLTIFRMKSKEGRAKAFSTCASHFSSVS
LFYGSIFFLYIRPNLLEEGGNDIPAAILFTIVVPLLNPFIYSLRNKEVISVLRKILLKIKSQGSV

NK

>Q8TCS8 peripheral membrane protein
MAACRYCCSCLRLRPLSDGPFLLPRRDRALTQLQVRALWSSAGSRAVAVDLGNRKLEISS
GKLARFADGSAVVQSGDTAVMVTAVSKTKPSPSQFMPLVVDYRQKAAAAGRIPTNYLRR
EIGTSDKEILTSRIIDRSIRPLFPAGYFYDTQVLCNLLAVDGVNEPDVLAINGASVALSLSDIP
WNGPVGAVRIGIIDGEYVVNPTRKEMSSSTLNLVVAGAPKSQIVMLEASAENILQQDFCHA
IKVGVKYTQQIIQGIQQLVKETGVTKRTPQKLFTPSPEIVKYTHKLAMERLYAVFTDYEHD
KVSRDEAVNKIRLDTEEQLKEKFPEADPYEIIESFNVVAKEVFRSIVLNEYKRCDGRDLTSL
RNVSCEVDMFKTLHGSALFQRGQTQVLCTVTFDSLESGIKSDQVITAINGIKDKNFMLHY
EFPPYATNEIGKVTGLNRRELGHGALAEKALYPVIPRDFPFTIRVTSEVLESNGSSSMASAC
GGSLALMDSGVPISSAVAGVAIGLVTKTDPEKGEIEDYRLLTDILGIEDYNGDMDFKIAGTN
KGITALQADIKLPGIPIKIVMEAIQQASVAKKEILQIMNKTISKPRASRKENGPVVETVQVPL
SKRAKFVGPGGYNLKKLQAETGVTISQVDEETFSVFAPTPSAMHEARDFITEICKDDQEQQ
LEFGAVYTATITEIRDTGVMVKLYPNMTAVLLHNTQLDQRKIKHPTALGLEVGQEIQVKYF
GRDPADGRMRLSRKVLQSPATTVVRTLNDRSSIVMGEPISQSSSNSQ

>Q6IEY1 multipass membrane protein
MDGENHSVVSEFLFLGLTHSWEIQLLLLVFSSVLYVASITGNILIVFSVTTDPHLHSPMYFLL
ASLSFIDLGACSVTSPKMIYDLFRKRKVISFGGCIAQIFFIHVVGGVEMVLLIAMAFDRYVA
LCKPLHYLTIMSPRMCLSFLAVAWTLGVSHSLFQLAFLVNLAFCGPNVLDSFYCDLPRLLR
LACTDTYRLQFMVTVNSGFICVGTFFILLISYVFILFTVWKHSSGGSSKALSTLSAHSTVVL
LFFGPPMFVYTRPHPNSQMDKFLAIFDAVLTPFLNPVVYTFRNKEMKAAIKRVCKQLVIYK
RIS

>P35372 multipass membrane protein
MDSSAAPTNASNCTDALAYSSCSPAPSPGSWVNLSHLDGNLSDPCGPNRTDLGGRDSLCP
PTGSPSMITAITIMALYSIVCVVGLFGNFLVMYVIVRYTKMKTATNIYIFNLALADALATSTL
PFQSVNYLMGTWPFGTILCKIVISIDYYNMFTSIFTLCTMSVDRYIAVCHPVKALDFRTPRN
AKIINVCNWILSSAIGLPVMFMATTKYRQGSIDCTLTFSHPTWYWENLLKICVFIFAFIMPV
LIITVCYGLMILRLKSVRMLSGSKEKDRNLRRITRMVLVVVAVFIVCWTPIHIYVIIKALVTI
PETTFQTVSWHFCIALGYTNSCLNPVLYAFLDENFKRCFREFCIPTSSNIEQQNSTRIRQNTR
DHPSTANTVDRTNHQLENLEAETAPLP

>P28676 peripheral membrane protein
MAYPGYGGGFGNFSIQVPGMQMGQPVPETGPAILLDGYSGPAYSDTYSSAGDSVYTYFSA
VAGQDGEVDAEELQRCLTQSGINGTYSPFSLETCRIMIAMLDRDHTGKMGFNAFKELWAA
LNAWKENFMTVDQDGSGTVEHHELRQAIGLMGYRLSPQTLTTIVKRYSKNGRIFFDDYVA
CCVKLRALTDFFRKRDHLQQGSANFIYDDFLQGTMAI

>Q96NT5 multipass membrane protein
MEGSASPPEKPRARPAAAVLCRGPVEPLVFLANFALVLQGPLTTQYLWHRFSADLGYNGT
RQRGGCSNRSADPTMQEVETLTSHWTLYMNVGGFLVGLFSSTLLGAWSDSVGRRPLLVL
ASLGLLLQALVSVFVVQLQLHVGYFVLGRILCALLGDFGGLLAASFASVADVSSSRSRTFR

MALLEASIGVAGMLASLLGGHWLRAQGYANPFWLALALLIAMTLYAAFCFGETLKEPKS
TRLFTFRHHRSIVQLYVAPAPEKSRKHLALYSLAIFVVITVHFGAQDILTLYELSTPLCWDSK
LIGYGSAAQHLPYLTSLLALKLLQYCLADAWVAEIGLAFNILGMVVFAFATITPLMFTGYG
LLFLSLVITPVIRAKLSKLVRETEQGALFSAVACVNSLAMLTASGIFNSLYPATLNFMKGFPF
LLGAGLLLIPAVLIGMLEKADPHLEFQQFPQSP

>Q6U841 multipass membrane protein
MEIKDQGAQMEPLLPTRNDEEAVVDRGGTRSILKTHFEKEDLEGHRTLFIGVHVPLGGRK
SHRRHRHRGHKHRKRDRERDSGLEDGRESPSFDTPSQRVQFILGTEDDDEEHIPHDLFTEL
DEICWREGEDAEWRETARWLKFEEDVEDGGERWSKPYVATLSLHSLFELRSCILNGTVLL
DMHANTLEEIADMVLDQQVSSGQLNEDVRHRVHEALMKQHHHQNQKKLTNRIPIVRSFA
DIGKKQSEPNSMDKNAGQVVSPQSAPACVENKNDVSRENSTVDFSKGLGGQQKGHTSPC
GMKQRHEKGPPHQQEREVDLHFMKKIPPGAEASNILVGELEFLDRTVVAFVRLSPAVLLQ
GLAEVPIPTRFLFILLGPLGKGQQYHEIGRSIATLMTDEVFHDVAYKAKDRNDLVSGIDEFL
DQVTVLPPGEWDPSIRIEPPKNVPSQEKRKIPAVPNGTAAHGEAEPHGGHSGPELQRTGRIF
GGLILDIKRKAPYFWSDFRDAFSLQCLASFLFLYCACMSPVITFGGLLGEATEGRISAIESLF
GASMTGIAYSLFGGQPLTILGSTGPVLVFEKILFKFCKEYGLSYLSLRASIGLWTATLCIILVA
TDASSLVCYITRFTEEAFASLICIIFIYEALEKLFELSEAYPINMHNDLELLTQYSCNCVEPHN
PSNGTLKEWRESNISASDIIWENLTVSECKSLHGEYVGRACGHDHPYVPDVLFWSVILFFS
TVTLSATLKQFKTSRYFPTKVRSIVSDFAVFLTILCMVLIDYAIGIPSPKLQVPSVFKPTRDDR
GWFVTPLGPNPWWTVIAAIIPALLCTILIFMDQQITAVIINRKEHKLKKGCGYHLDLLMVAV
MLGVCSIMGLPWFVAATVLSITHVNSLKLESECSAPGEQPKFLGIREQRVTGLMIFILMGSS
VFMTSILKFIPMPVLYGVFLYMGASSLKGIQFFDRIKLFWMPAKHQPDFIYLRHVPLRKVH
LFTIIQMSCLGLLWIIKVSRAAIVFPMMVLALVFVRKLMDLLFTKRELSWLDDLMPESKK
KKLEDAEKEEEQSMLAMEDEGTVQLPLEGHYRDDPSVINISDEMSKTALWRNLLITADNS
KDKESSFPSKSSPS

>Q8N148 multipass membrane protein
MANLSQPSEFVLLGFSSFGELQALLYGPFLMLYLLAFMGNTIIIVMVIADTHLHTPMYFFL
GNFSLLEILVTMTAVPRMLSDLLVPHKVITFTGCMVQFYFHFSLGSTSFLILTDMALDRFVA
ICHPLRYGTLMSRAMCVQLAGAAWAAPFLAMVPTVLSRAHLDYCHGDVINHFFCDNEPL
LQLSCSDTRLLEFWDFLMALTFVLSSFLVTLISYGYIVTTVLRIPSASSCQKAFSTCGSHLTL
VFIGYSSTIFLYVRPGKAHSVQVRKVVALVTSVLTPFLNPFILTFCNQTVKTVLQGQMQRL
KGLCKAQ

>Q6UVW9 single-pass type II membrane protein
MINPELRDGRADGFIHRIVPKLIQNWKIGLMCFLSIIITTVCIIMIATWSKHAKPVACSGDWL
GVRDKCFYFSDDTRNWTASKIFCSLQKAELAQIDTQEDMEFLKRYAGTDMHWIGLSRKQ
GDSWKWTNGTTFNGWFEIIGNGSFAFLSADGVHSSRGFIDIKWICSKPKYFL

>P35499 multipass membrane protein
MARPSLCTLVPLGPECLRPFTRESLAAIEQRAVEEEARLQRNKQMEIEEPERKPRSDLEAG
KNLPMIYGDPPPEVIGIPLEDLDPYYSNKKTFIVLNKGKAIFRFSATPALYLLSPFSVVRRGA
IKVLIHALFSMFIMITILTNCVFMTMSDPPPWSKNVEYTFTGIYTFESLIKILARGFCVDDFT

FLRDPWNWLDFSVIMMAYLTEFVDLGNISALRTFRVLRALKTITVIPGLKTIVGALIQSVKK
LSDVMILTVFCLSVFALVGLQLFMGNLRQKCVRWPPPFNDTNTTWYSNDTWYGNDTWY
GNEMWYGNDSWYANDTWNSHASWATNDTFDWDAYISDEGNFYFLEGSNDALLCGNSS
DAGHCPEGYECIKTGRNPNYGYTSYDTFSWAFLALFRLMTQDYWENLFQLTLRAAGKTY
MIFFVVIIFLGSFYLINLILAVVAMAYAEQNEATLAEDKEKEEEFQQMLEKFKKHQEELEKA
KAAQALEGGEADGDPAHGKDCNGSLDTSQGEKGAPRQSSSGDSGISDAMEELEEAHQKC
PPWWYKCAHKVLIWNCCAPWLKFKNIIHLIVMDPFVDLGITICIVLNTLFMAMEHYPMTE
HFDNVLTVGNLVFTGIFTAEMVLKLIAMDPYEYFQQGWNIFDSIIVTLSLVELGLANVQGL
SVLRSFRLLRVFKLAKSWPTLNMLIKIIGNSVGALGNLTLVLAIIVFIFAVVGMQLFGKSYK
ECVCKIALDCNLPRWHMHDFFHSFLIVFRILCGEWIETMWDCMEVAGQAMCLTVFLMVM
VIGNLVVLNLFLALLLSSFSADSLAASDEDGEMNNLQIAIGRIKLGIGFAKAFLLGLLHGKI
LSPKDIMLSLGEADGAGEAGEAGETAPEDEKKEPPEEDLKKDNHILNHMGLADGPPSSLE
LDHLNFINNPYLTIQVPIASEESDLEMPTEEETDTFSEPEDSKKPPQPLYDGNSSVCSTADYK
PPEEDPEEQAEENPEGEQPEECFTEACVQRWPCLYVDISQGRGKKWWTLRRACFKIVEHN
WFETFIVFMILLSSGALAFEDIYIEQRRVIRTILEYADKVFTYIFIMEMLLKWVAYGFKVYFT
NAWCWLDFLIVDVSIISLVANWLGYSELGPIKSLRTLRALRPLRALSRFEGMRVVVNALLG
AIPSIMNVLLVCLIFWLIFSIMGVNLFAGKFYYCINTTTSERFDISEVNNKSECESLMHTGQ
VRWLNVKVNYDNVGLGYLSLLQVATFKGWMDIMYAAVDSREKEEQPQYEVNLYMYLYF
VIFIIFGSFFTLNLFIGVIIDNFNQQKKKLGGKDIFMTEEQKKYYNAMKKLGSKKPQKPIPR
PQNKIQGMVYDLVTKQAFDITIMILICLNMVTMMVETDNQSQLKVDILYNINMIFIIIFTGE
CVLKMLALRQYYFTVGWNIFDFVVVILSIVGLALSDLIQKYFVSPTLFRVIRLARIGRVLRL
IRGAKGIRTLLFALMMSLPALFNIGLLLFLVMFIYSIFGMSNFAYVKKESGIDDMFNFETFG
NSIICLFEITTSAGWDGLLNPILNSGPPDCDPNLENPGTSVKGDCGNPSIGICFFCSYIIISFLI
VVNMYIAIILENFNVATEESSEPLGEDDFEMFYETWEKFDPDATQFIAYSRLSDFVDTLQEP
LRIAKPNKIKLITLDLPMVPGDKIHCLDILFALTKEVLGDSGEMDALKQTMEEKFMAANPS
KVSYEPITTTLKRKHEEVCAIKIQRAYRRHLLQRSMKQASYMYRHSHDGSGDDAPEKEGL
LANTMSKMYGHENGNSSSPSPEEKGEAGDAGPTMGLMPISPSDTAWPPAPPPGQTVRPGV
KESLV

>Q07699 single-pass type I membrane protein
MGRLLALVVGAALVSSACGGCVEVDSETEAVYGMTFKILCISCKRRSETNAETFTEWTFR
QKGTEEFVKILRYENEVLQLEEDERFEGRVVWNGSRGTKDLQDLSIFITNVTYNHSGDYE
CHVYRLLFFENYEHNTSVVKKIHIEVVDKANRDMASIVSEIMMYVLIVVLTIWLVAEMIYC
YKKIAAATETAAQENASEYLAITSESKENCTGVQVAE

>P20036 single-pass type I membrane protein
MRPEDRMFHIRAVILRALSLAFLLSLRGAGAIKADHVSTYAAFVQTHRPTGEFMFEFDEDE
MFYVDLDKKETVWHLEEFGQAFSFEAQGGLANIAILNNNLNTLIQRSNHTQATNDPPEVT
VFPKEPVELGQPNTLICHIDKFFPPVLNVTWLCNGELVTEGVAESLFLPRTDYSFHKFHYLT
FVPSAEDFYDCRVEHWGLDQPLLKHWEAQEPIQMPETTETVLCALGLVLGLVGIIVGTVLI
IKSLRSGHDPRAQGTL

>P17342 single-pass type I membrane protein
MPSLLVLTFSPCVLLGWALLAGGTGGGGVGGGGGGAGIGGGRQEREALPPQKIEVLVLLP

QDDSYLFSLTRVRPAIEYALRSVEGNGTGRRLLPPGTRFQVAYEDSDCGNRALFSLVDRVA
AARGAKPDLILGPVCEYAAAPVARLASHWDLPMLSAGALAAGFQHKDSEYSHLTRVAPAY
AKMGEMMLALFRHHHWSRAALVYSDDKLERNCYFTLEGVHEVFQEEGLHTSIYSFDETK
DLDLEDIVRNIQASERVVIMCASSDTIRSIMLVAHRHGMTSGDYAFFNIELFNSSSYGDGSW
KRGDKHDFEAKQAYSSLQTVTLLRTVKPEFEKFSMEVKSSVEKQGLNMEDYVNMFVEGF
HDAILLYVLALHEVLRAGYSKKDGGKIIQQTWNRTFEGIAGQVSIDANGDRYGDFSVIAM
TDVEAGTQEVIGDYFGKEGRFEMRPNVKYPWGPLKLRIDENRIVEHTNSSPCKSSGGLEE
SAVTGIVVGALLGAGLLMAFYFFRKKYRITIERRTQQEESNLGKHRELREDSIRSHFSVA

>O96024 single-pass type II membrane protein
MQLRLFRRLLLAALLLVIVWTLFGPSGLGEELLSLSLASLLPAPASPGPPLALPRLLIPNQEA
CSGPGAPPFLLILVCTAPENLNQRNAIRASWGGLREARGLRVQTLFLLGEPNAQHPVWGS
QGSDLASESAAQGDILQAAFQDSYRNLTLKTLSGLNWAEKHCPMARYVLKTDDDVYVN
VPELVSELVLRGGRWGQWERSTEPQREAEQEGGQVLHSEEVPLLYLGRVHWRVNPSRTPG
GRHRVSEEQWPHTWGPFPPYASGTGYVLSASAVQLILKVASRAPLLPLEDVFVGVSARRG
GLAPTQCVKLAGATHYPLDRCCYGKFLLTSHRLDPWKMQEAWKLVGGSDGERTAPFCS
WFQGVLGILRCRAIAWLQS

>Q9Y5W8 peripheral membrane protein
MLTEASLSIWGWGSLGIVLFLITFGPFVIFYLTFYILCFVGGGLVVTLLFGKTNSEKYLEQC
EHSFLPPTSPGVPKCLEEMKREARTIKIDRRLTGANIIDEPLQQVIQFSLRDYVQYWYYTLS
DDESFLLEIRQTLQNALIQFATRSKEIDWQPYFTTRIVDDFGTHLRVFRKAQQKITEKDDQV
KGTAEDLVDTFFEVEVEMEKEVCRDLVCTSPKDEEGFLRDLCEVLLYLLLPPGDFQNKIM
RYFVREILARGILLPLINQLSDPDYINQYVIWMIRDSNCNYEAFMNIIKLSDNIGELEAVRD
KAAEELQYLRSLDTAGDDINTIKNQINSLLFVKKVCDSRIQRLQSGKEINTVKLAANFGKL
CTVPLDSILVDNVALQFFMDYMQQTGGQAHLFFWMTVEGYRVTAQQQLEVLLSRQRDG
KHQTNQTKGLLRAAAVGIYEQYLSEKASPRVTVDDYLVAKLADTLNHEDPTPEIFDDIQR
KVYELMLRDERFYPSFRQNALYVRMLAELDMLKDPSFRGSDDGDGESFNGSPTGSINLSL
DDLSNVSSDDSVQLHAYISDTVYADYDPYAVAGVCNDHGKTYALYAITVHRRNLNSEEM
WKTYRRYSDFHDFHMRITEQFESLSSILKLPGKKTFNNMDRDFLEKRKKDLNAYLQLLLA
PEMMKASPALAHYVYDFLENKAYSKGKGDFARKMDTFVNPLRNSMRNVSNAVKSLPDS
LAEGMTKMSDNMGKMSERLGQDIKQSFFKVPPLIPKTDSDPEHRRVSAQLDDNVDDNIPL
RVMLLLMDEVFDLKERNQWLRRNIKNLLQQLIRATYGDTINRKIVDHVDWMTSPEQVAD
SVKRFRDAFWPNGILAEAVPCRDKSIRMRTRVAGKTKLLAIMPDELKHIIGAETTRKGILRV
FEMFQHNQLNRRMVYVFLEGFLETLFPQYKFRELFNKLHSRSKQMQKYKQKLQTTQAPS
LQKR

>P30954 multipass membrane protein
MLLCFRFGNQSMKRENFTLITDFVFQGFSSFHEQQITLFGVFLALYILTLAGNIIIVTIIRMDL
HLHTPMYFFLSMLSTSETVYTLVILPRMLSSLVGMSQPISLAGCATQMFFFVTFGITNCFLL
TAMGYDRYVAICNPLRYMVIMNKRLRIQLVLGACSIGLIVAITQVTSVFRLPFCARKVPHFF
CDIRPVMKLSCIDTTVNEILTLIISVLVLVVPMGLVFISYVLIISTILKIASVEGRKKAFATCAS
HLTVVIVHYSCASIAYLKPKSENTREHDQLISVTYTVITPLLNPVVYTLRNKEVKDALCRA
VGGKFS

>Q12913 single-pass type I membrane protein
MKPAAREARLPPRSPGLRWALPLLLLLLRLGQILCAGGTPSPIPDPSVATVATGENGITQISS
TAESFHKQNGTGTPQVETNTSEDGESSGANDSLRTPEQGSNGTDGASQKTPSSTGPSPVFD
IKAVSISPTNVILTWKSNDTAASEYKYVVKHKMENEKTITVVHQPWCNITGLRPATSYVFSI
TPGIGNETWGDPRVIKVITEPIPVSDLRVALTGVRKAALSWSNGNGTASCRVLLESIGSHEE
LTQDSRLQVNISGLKPGVQYNINPYLLQSNKTKGDPLGTEGGLDASNTERSRAGSPTAPVH
DESLVGPVDPSSGQQSRDTEVLLVGLEPGTRYNATVYSQAANGTEGQPQAIEFRTNAIQVF
DVTAVNISATSLTLIWKVSDNESSSNYTYKIHVAGETDSSNLNVSEPRAVIPGLRSSTFYNIT
VCPVLGDIEGTPGFLQVHTPPVPVSDFRVTVVSTTEIGLAWSSHDAESFQMHITQEGAGNS
RVEITTNQSIIIGGLFPGTKYCFEIVPKGPNGTEGASRTVCNRTVPSAVFDIHVVYVTTTEM
WLDWKSPDGASEYVYHLVIESKHGSNHTSTYDKAITLQGLIPGTLYNITISPEVDHVWGDP
NSTAQYTRPSNVSNIDVSTNTTAATLSWQNFDDASPTYSYCLLIEKAGNSSNATQVVTDIGI
TDATVTELIPGSSYTVEIFAQVGDGIKSLEPGRKSFCTDPASMASFDCEVVPKEPALVLKWT
CPPGANAGFELEVSSGAWNNATHLESCSSENGTEYRTEVTYLNFSTSYNISITTVSCGKMA
APTRNTCTTGITDPPPPDGSPNITSVSHNSVKVKFSGFEASHGPIKAYAVILTTGEAGHPSAD
VLKYTYEDFKKGASDTYVTYLIRTEEKGRSQSLSEVLKYEIDVGNESTTLGYYNGKLEPL
GSYRACVAGFTNITFHPQNKGLIDGAESYVSFSRYSDAVSLPQDPGVICGAVFGCIFGALVI
VTVGGFIFWRKKRKDAKNNEVSFSQIKPKKSKLIRVENFEAYFKKQQADSNCGFAEEYED
LKLVGISQPKYAAELAENRGKNRYNNVLPYDISRVKLSVQTHSTDDYINANYMPGYHSKK
DFIATQGPLPNTLKDFWRMVWEKNVYAIIMLTKCVEQGRTKCEEYWPSKQAQDYGDITV
AMTSEIVLPEWTIRDFTVKNIQTSESHPLRQFHFTSWPDHGVPDTTDLLINFRYLVRDYMK
QSPPESPILVHCSAGVGRTGTFIAIDRLIYQIENENTVDVYGIVYDLRMHRPLMVQTEDQY
VFLNQCVLDIVRSQKDSKVDLIYQNTTAMTIYENLAPVTTFGKTNGYIA

>Q86UW7 peripheral membrane protein
MLDPSSSEEESDEGLEEESRDVLVAAGSSQRAPPAPTREGRRDAPGRAGGGGAARSVSPSP
SVLSEGRDEPQRQLDDEQERRIRLQLYVFVVRCIAYPFNAKQPTDMARRQQKLNKQQLQL
LKERFQAFLNGETQIVADEAFCNAVRSYYEVFLKSDRVARMVQSGGCSANDFREVFKKNI
EKRVRSLPEIDGLSKETVLSSWIAKYDAIYRGEEDLCKQPNRMALSAVSELILSKEQLYEM
FQQILGIKKLEHQLLYNACQLDNADEQAAQIRRELDGRLQLADKMAKERKFPKFIAKDM
ENMYIEELRSSVNLLMANLESLPVSKGGPEFKLQKLKRSQNSAFLDIGDENEIQLSKSDVV
LSFTLEIVIMEVQGLKSVAPNRIVYCTMEVEGEKLQTDQAEASRPQWGTQGDFTTTHPRP
VVKVKLFTESTGVLALEDKELGRVILYPTSNSSKSAELHRMVVPKNSQDSDLKIKLAVRM
DKPAHMKHSGYLYALGQKVWKRWKKRYFVLVQVSQYTFAMCSYREKKSEPQELMQLE
GYTVDYTDPHPGLQGGCMFFNAVKEGDTVIFASDDEQDRILWVQAMYRATGQSYKPVPA
IQTQKLNPKGGTLHADAQLSGKDADRFQKHGMDEFISANPCKLDHAFLFRILQRQTLDHR
LNDSYSCLGWFSPGQVFVLDEYCARYGVRGCHRHLCYLAELMEHSENGAVIDPTLLHYS
FAFCASHVHGNRPDGIGTVSVEEKERFEEIKERLSSLLENQISHFRYCFPFGRPEGALKATLS
LLERVLMKDIATPIPAEEVKKVVRKCLEKAALINYTRLTEYAKIEETMNQASPARKLEEILH
LAELCIEVLQQNEEHHAEGREAFAWWPDLLAEHAEKFWALFTVDMDTALEAQPQDSWD
SFPLFQLLNNFLRNDTLLCNGKFHKHLQEIFVPLVVRYVDLMESSIAQSIHRGFEQETWQP
VKNIANSLPNVALPKVPSLPLNLPQIPNISTASWMPSLYESTNGSATSEDLFWKLDALQMF
VFDLHWPEQEFAHHLEQRLKLMASDMLEACVKRTRTAFELKLQKASKTTDLRIPASVCT

MFNVLVDAKKQSTKLCALDGGQEQQYHSKIDDLIDNSVKEIISLLVSKFVSVLEGVLSKLS
RYDEGTFFSSILSFTVKAAAKYVDVPKPGMDLADTYIMFVRQNQDILREKVNEEMYIEKL
FDQWYSSSMKVICVWLTDRLDLQLHIYQLKTLIKIVKKTYRDFRLQGVLEGTLNSKTYDT
VHRRLTVEEATASVSEGGGLQGITMKDSDEEEEG

>Q14344 lipid-anchor
MADFLPSRSVLSVCFPGCLLTSGEAEQQRKSKEIDKCLSREKTYVKRLVKILLLGAGESGK
STFLKQMRIIHGQDFDQRAREEFRPTIYSNVIKGMRVLVDAREKLHIPWGDNSNQQHGDK
MMSFDTRAPMAAQGMVETRVFLQYLPAIRALWADSGIQNAYDRRREFQLGESVKYFLDN
LDKLGEPDYIPSQQDILLARRPTKGIHEYDFEIKNVPFKMVDVGGQRSERKRWFECFDSVT
SILFLVSSSEFDQVLMEDRLTNRLTESLNIFETIVNNRVFSNVSIILFLNKTDLLEEKVQIVSIK
DYFLEFEGDPHCLRDVQKFLVECFRNKRRDQQQKPLYHHFTTAINTENIRLVFRDVKDTIL
HDNLKQLMLQ

>Q08345 single-pass type I membrane protein
MGPEALSSLLLLLLVASGDADMKGHFDPAKCRYALGMQDRTIPDSDISASSSWSDSTAAR
HSRLESSDGDGAWCPAGSVFPKEEEYLQVDLQRLHLVALVGTQGRHAGGLGKEFSRSYRL
RYSRDGRRWMGWKDRWGQEVISGNEDPEGVVLKDLGPPMVARLVRFYPRADRVMSVCL
RVELYGCLWRDGLLSYTAPVGQTMYLSEAVYLNDSTYDGHTVGGLQYGGLGQLADGVV
GLDDFRKSQELRVWPGYDYVGWSNHSFSSGYVEMEFEFDRLRAFQAMQVHCNNMHTL
GARLPGGVECRFRRGPAMAWEGEPMRHNLGGNLGDPRARAVSVPLGGRVARFLQCRFLF
AGPWLLFSEISFISDVVNNSSPALGGTFPPAPWWPPGPPPTNFSSLELEPRGQQPVAKAEGS
PTAILIGCLVAIILLLLLIIALMLWRLHWRRLLSKAERRVLEEELTVHLSVPGDTILINNRPGP
REPPPYQEPRPRGNPPHSAPCVPNGSALLLSNPAYRLLLATYARPPRGPGPPTPAWAKPTNT
QAYSGDYMEPEKPGAPLLPPPPQNSVPHYAEADIVTLQGVTGGNTYAVPALPPGAVGDGP
PRVDFPRSRLRFKEKLGEGQFGEVHLCEVDSPQDLVSLDFPLNVRKGHPLLVAVKILRPDA
TKNARNDFLKEVKIMSRLKDPNIIRLLGVCVQDDPLCMITDYMENGDLNQFLSAHQLED
KAAEGAPGDGQAAQGPTISYPMLLHVAAQIASGMRYLATLNFVHRDLATRNCLVGENFTI
KIADFGMSRNLYAGDYYRVQGRAVLPIRWMAWECILMGKFTTASDVWAFGVTLWEVLM
LCRAQPFGQLTDEQVIENAGEFFRDQGRQVYLSRPPACPQGLYELMLRCWSRESEQRPPFS
QLHRFLAEDALNTV

>Q5VIR6 peripheral membrane protein
MMEEEELEFVEELEAVLQLTPEVQLAIEQVFPSQDPLDRADFNAVEYINTLFPTEQSLANID
EVVNKIRLKIRRLDDNIRTVVRGQTNVGQDGRQALEEAQKAIQQLFGKIKDIKDKAEKSE
QMVKEITRDIKQLDHAKRHLTTSITTLNHLHMLAGGVDSLEAMTRRRQYGEVANLLQGV
MNVLEHFHKYMGIPQIRQLSERVKAAQTELGQQILADFEEAFPSQGTKRPGGPSNVLRDA
CLVANILDPRIKQEIIKKFIKQHLSEYLVLFQENQDVAWLDKIDRRYAWIKRQLVDYEEKYG
RMFPREWCMAERIAVEFCHVTRAELAKIMRTRAKEIEVKLLLFAIQRTTNFEGFLAKRFSG
CTLTDGTLKKLESPPPSTNPFLEDEPTPEMEELATEKGDLDQPKKPKAPDNPFHGIVSKCFE
PHLYVVYIESQDKNLGELIDRFVADFKAQGPPKPNTDEGGAVLPSCADLFVYYKKCMVQCS
QLSTGEPMIALTTIFQKYLREYAWKILSGNLPKTTTSSGGLTISSLLKEKEGSEVAKFTLEEL
CLICNILSTAEYCLATTQQLEEKLKEKVDVSLIERINLTGEMDTFSTVISSSIQLLVQDLDAA
CDPALTAMSKMQWQNVEHVGDQSPYVTSVILHIKQNVPIIRDNLASTRKYFTQFCVKFAN

SFIPKFITHLFKCKPISMVGAEQVRWT

>O75460 single-pass type I membrane protein
MPARRLLLLLTLLLPGLGIFGSTSTVTLPETLLFVSTLDGSLHAVSKRTGSIKWTLKEDPVL
QVPTHVEEPAFLPDPNDGSLYTLGSKNNEGLTKLPFTIPELVQASPCRSSDGILYMGKKQDI
WYVIDLLTGEKQQTLSSAFADSLCPSTSLLYLGRTEYTITMYDTKTRELRWNATYFDYAAS
LPEDDVDYKMSHFVSNGDGLVVTVDSESGDVLWIQNYASPVVAFYVWQREGLRKVMHI
NVAVETLRYLTFMSGEVGRITKWKYPFPKETEAKSKLTPTLYVGKYSTSLYASPSMVHEGV
AVVPRGSTLPLLEGPQTDGVTIGDKGECVITPSTDVKFDPGLKSKNKLNYLRNYWLLIGH
HETPLSASTKMLERFPNNLPKHRENVIPADSEKKSFEEVINLVDQTSENAPTTVSRDVEEKP
AHAPARPEAPVDSMLKDMATIILSTFLLIGWVAFIITYPLSMHQQQQLQHQQFQKELEKIQ
LLQQQQQQLPFHPPGDTAQDGELLDTSGPYSESSGTSSPSTSPRASNHSLCSGSSASKAGSS
PSLEQDDGDEETSVVIVGKISFCPKDVLGHGAEGTIVYRGMFDNRDVAVKRILPECFSFAD
REVQLLRESDEHPNVIRYFCTEKDRQFQYIAIELCAATLQEYVEQKDFAHLGLEPITLLQQT
TSGLAHLHSLNIVHRDLKPHNILISMPNAHGKIKAMISDFGLCKKLAVGRHSFSRRSGVPG
TEGWIAPEMLSEDCKENPTYTVDIFSAGCVFYYVISEGSHPFGKSLQRQANILLGACSLDC
LHPEKHEDVIARELIEKMIAMDPQKRPSAKHVLKHPFFWSLEKQLQFFQDVSDRIEKESLD
GPIVKQLERGGRAVVKMDWRENITVPLQTDLRKFRTYKGGSVRDLLRAMRNKKHHYRE
LPAEVRETLGSLPDDFVCYFTSRFPHLLAHTYRAMELCSHERLFQPYYFHEPPEPQPPVTP
DAL

>P06340 single-pass type I membrane protein
MALRAGLVLGFHTLMTLLSPQEAGATKADHMGSYGPAFYQSYGASGQFTHEFDEEQLFS
VDLKKSEAVWRLPEFGDFARFDPQGGLAGIAAIKAHLDILVERSNRSRAINVPPRVTVLPK
SRVELGQPNILICIVDNIFPPVINITWLRNGQTVTEGVAQTSFYSQPDHLFRKFHYLPFVPSA
EDVYDCQVEHWGLDAPLLRHWELQVPIPPPDAMETLVCALGLAIGLVGFLVGTVLIIMGT
YVSSVPR

>Q8NGA1 multipass membrane protein
MEPRNQTSASQFILLGLSEKPEQETLLFSLFFCMYLVMVVGNLLIILAISIDSHLHTPMYFFL
ANLSLVDFCLATNTIPKMLVSLQTGSKAISYPCCLIQMYFFHFFGIVDSVIIAMMAYDRFVAI
CHPLHYAKIMSLRLCRLLVGALWAFSCFISLTHILLMARLVFCGSHEVPHYFCDLTPILRLSC
TDTSVNRIFILIVAGMVIATPFVCILASYARILVAIMKVPSAGGRKKAFSTCSSHLSVVALFY
GTTIGVYLCPSSVLTTVKEKASAVMYTAVTPMLNPFIYSLRNRDLKGALRKLVNRKITSSS

>P15151 single-pass type I membrane protein
MARAMAAAWPLLLVALLVLSWPPPGTGDVVVQAPTQVPGFLGDSVTLPCYLQVPNMEV
THVSQLTWARHGESGSMAVFHQTQGPSYSESKRLEFVAARLGAELRNASLRMFGLRVEDE
GNYTCLFVTFPQGSRSVDIWLRVLAKPQNTAEVQKVQLTGEPVPMARCVSTGGRPPAQIT
WHSDLGGMPNTSQVPGFLSGTVTVTSLWILVPSSQVDGKNVTCKVEHESFEKPQLLTVNL
TVYYPPEVSISGYDNNWYLGQNEATLTCDARSNPEPTGYNWSTTMGPLPPFAVAQGAQLL
IRPVDKPINTTLICNVTNALGARQAELTVQVKEGPPSEHSGISRNAIIFLVLGILVFLILLGIGI
YFYWSKCSREVLWHCHLCPSSTEHASASANGHVSYSAVSRENSSSQDPQTEGTR

>Q29983 single-pass type I membrane protein
MGLGPVFLLLAGIFPFAPPGAAAEPHSLRYNLTVLSWDGSVQSGFLTEVHLDGQPFLRCDR
QKCRAKPQGQWAEDVLGNKTWDRETRDLTGNGKDLRMTLAHIKDQKEGLHSLQEIRVC
EIHEDNSTRSSQHFYYDGELFLSQNLETKEWTMPQSSRAQTLAMNVRNFLKEDAMKTKT
HYHAMHADCLQELRRYLKSGVVLRRTVPPMVNVTRSEASEGNITVTCRASGFYPWNITL
SWRQDGVSLSHDTQQWGDVLPDGNGTYQTWVATRICQGEEQRFTCYMEHSGNHSTHPV
PSGKVLVLQSHWQTFHVSAVAAAAIFVIIIFYVRCCKKKTSAAEGPELVSLQVLDQHPVGT
SDHRDATQLGFQPLMSDLGSTGSTEGA

>Q8IUH5 multipass membrane protein
MQREEGFNTKMADGPDEYDTEAGCVPLLHPEEIKPQSHYNHGYGEPLGRKTHIDDYSTW
DIVKATQYGIYERCRELVEAGYDVRQPDKENVTLLHWAAINNRIDLVKYYISKGAIVDQL
GGDLNSTPLHWATRQGHLSMVVQLMKYGADPSLIDGEGCSCIHLAAQFGHTSIVAYLIAK
GQDVDMMDQNGMTPLMWAAYRTHSVDPTRLLLTFNVSVNLGDKYHKNTALHWAVLAG
NTTVISLLLEAGANVDAQNIKGESALDLAKQRKNVWMINHLQEARQAKGYDNPSFLRKL
KADKEFRQKVMLGTPFLVIWLVGFIADLNIDSWLIKGLMYGGVWATVQFLSKSFFDHSMH
SALPLGIYLATKFWMYVTWFFWFWNDLNFLFIHLPFLANSVALFYNFGKSWKSDPGIIKAT
EEQKKKTIVELAETGSLDLSIFCSTCLIRKPVRSKHCGVCNRCIAKFDHHCPWVGNCVGA
GNHRYFMGYLFFLLFMICWMIYGCISYWGLHCETTYTKDGFWTYITQIATCSPWMFWMF
LNSVFHFMWVAVLLMCQMYQISCLGITTNERMNARRYKHFKVTTTSIESPFNHGCVRNII
DFFEFRCCGLFRPVIVDWTRQYTIEYDQISGSGYQLV

>Q99466 single-pass type I membrane protein
MQPPSLLLLLLLLLLLLCVSVVRPRGLLCGSFPEPCANGGTCLSLSLGQGTCQCAPGFLGET
CQFPDPCQNAQLCQNGGSCQALLPAPLGLPSSPSPLTPSFLCTCLPGFTGERCQAKLEDPCP
PSFCSKRGRCHIQASGRPQCSCMPGWTGEQCQLRDFCSANPCVNGGVCLATYPQIQCHCP
PGFEGHACERDVNECFQDPGPCPKGTSCHNTLGSFQCLCPVGQEGPRCELRAGPCPPRGC
SNGGTCQLMPEKDSTFHLCLCPPGFIGPDCEVNPDNCVSHQCQNGGTCQDGLDTYTCLCP
ETWTGWDCSEDVDECETQGPPHCRNGGTCQNSAGSFHCVCVSGWGGTSCEENLDDCIA
ATCAPGSTCIDRVGSFSCLCPPGRTGLLCHLEDMCLSQPCHGDAQCSTNPLTGSTLCLCQP
GYSGPTCHQDLDECLMAQQGPSPCEHGGSCLNTPGSFNCLCPPGYTGSRCEADHNECLSQ
PCHPGSTCLDLLATFHCLCPPGLEGQLCEVETNECASAPCLNHADCHDLLNGFQCICLPGF
SGTRCEEDIDECRSSPCANGGQCQDQPGAFHCKCLPGFEGPRCQTEVDECLSDPCPVGAS
CLDLPGAFFCLCPSGFTGQLCEVPLCAPNLCQPKQICKDQKDKANCLCPDGSPGCAPPED
NCTCHHGHCQRSSCVCDVGWTGPECEAELGGCISAPCAHGGTCYPQPSGYNCTCPTGYT
GPTCSEEMTACHSGPCLNGGSCNPSPGGYYCTCPPSHTGPQCQTSTDYCVSAPCFNGGTC
VNRPGTFSCLCAMGFQGPRCEGKLRPSCADSPCRNRATCQDSPQGPRCLCPTGYTGGSCQ
TLMDLCAQKPCPRNSHCLQTGPSFHCLCLQGWTGPLCNLPLSSCQKAALSQGIDVSSLCH
NGGLCVDSGPSYFCHCPPGFQGSLCQDHVNPCESRPCQNGATCMAQPSGYLCQCAPGYD
GQNCSKELDACQSQPCHNHGTCTPKPGGFHCACPPGFVGLRCEGDVDECLDQPCHPTGT
AACHSLANAFYCQCLPGHTGQWCEVEIDPCHSQPCFHGGTCEATAGSPLGFICHCPKGFE
GPTCSHRAPSCGFHHCHHGGLCLPSPKPGFPPRCACLSGYGGPDCLTPPAPKGCGPPSPCL
YNGSCSETTGLGGPGFRCSCPHSSPGPRCQKPGAKGCEGRSGDGACDAGCSGPGGNWDG
GDCSLGVPDPWKGCPSHSRCWLLFRDGQCHPQCDSEECLFDGYDCETPPACTPAYDQYC

HDHFHNGHCEKGCNTAECGWDGGDCRPEDGDPEWGPSLALLVVLSPPALDQQLFALARV
LSLTLRVGLWVRKDRDGRDMVYPYPGARAEEKLGGTRDPTYQERAAPQTQPLGKETDSL
SAGFVVVMGVDLSRCGPDHPASRCPWDPGLLLRFLAAMAAVGALEPLLPGPLLAVHPHA
GTAPPANQLPWPVLCSPVAGVILLALGALLVLQLIRRRRREHGALWLPPGFTRRPRTQSAP
HRRRPPLGEDSIGLKALKPKAEVDEDGVVMCSGPEEGEEVGQAEETGPPSTCQLWSLSGG
CGALPQAAMLTPPQESEMEAPDLDTRGPDGVTPLMSAVCCGEVQSGTFQGAWLGCPEPW
EPLLDGGACPQAHTVGTGETPLHLAARFSRPTAARRLLEAGANPNQPDRAGRTPLHAAVA
ADAREVCQLLLRSRQTAVDARTEDGTTPLMLAARLAVEDLVEELIAAQADVGARDKWGK
TALHWAAAVNNARAARSLLQAGADKDAQDNREQTPLFLAAREGAVEVAQLLLGLGAAR
ELRDQAGLAPADVAHQRNHWDLLTLLEGAGPPEARHKATPGREAGPFPRARTVSVSVPPH
GGGALPRCRTLSAGAGPRGGGACLQARTWSVDLAARGGGAYSHCRSLSGVGAGGGPTP
RGRRFSAGMRGPRPNPAIMRGRYGVAAGRGGRVSTDDWPCDWVALGACGSASNIPIPPPC
LTPSPERGSPQLDCGPPALQEMPINQGGEGKK

>P08686 peripheral membrane protein
MLLLGLLLLPLLAGARLLWNWWKLRSLHLPPLAPGFLHLLQPDLPIYLLGLTQKFGPIYRL
HLGLQDVVVLNSKRTIEEAMVKKWADFAGRPEPLTYKLVSKNYPDLSLGDYSLLWKAHK
KLTRSALLLGIRDSMEPVVEQLTQEFCERMRAQPGTPVAIEEEFSLLTCSIICYLTFGDKIKD
DNLMPAYYKCIQEVLKTWSHWSIQIVDVIPFLRFFPNPGLRRLKQAIEKRDHIVEMQLRQH
KESLVAGQWRDMMDYMLQGVAQPSMEEGSGQLLEGHVHMAAVDLLIGGTETTANTLSW
AVVFLLHHPEIQQRLQEELDHELGPGASSSRVPYKDRARLPLLNATIAEVLRLRPVVPLALP
HRTTRPSSISGYDIPEGTVIIPNLQGAHLDETVWERPHEFWPDRFLEPGKNSRALAFGCGAR
VCLGEPLARLELFVVLTRLLQAFTLLPSGDALPSLQPLPHCSVILKMQPFQVRLQPRGMGA
HSPGQNQ

>Q8N1I0 peripheral membrane protein
MWIPTEHEKYGVVIASFRGTVPYGLSLEIGDTVQILEKCDGWYRGFALKNPNIKGIFPSSY
VHLKNACVKNKGQFEMVIPTEDSVITEMTSTLRDWGTMWKQLYVRNEGDLFHRLWHIM
NEILDLRRQVLVGHLTHDRMKDVKRHITARLDWGNEQLGLDLVPRKEYAMVDPEDISITE
LYRLMEHRHRKKDTPVQASSHHLFVQMKSLMCSNLGEELEVIFSLFDSKENRPISERFFLR
LNRNGLPKAPDKPERHCSLFVDLGSSELRKDIYITVHIIRIGRMGAGEKKNACSVQYRRPF
GCAVLSIADLLTGETKDDLILKVYMCNTESEWYQIHENIIKKLNARYNLTGSNAGLAVSLQ
LLHGDIEQIRREYSSVFSHGVSITRKLGFSNIIMPGEMRNDLYITIERGEFEKGGKSVARNVE
VTMFIVDSSGQTLKDFISFGSGEPPASEYHSFVLYHNNSPRWSELLKLPIPVDKFRGAHIRFE
FRHCSTKEKGEKKLFGFSFVPLMQEDGRTLPDGTHELIVHKCEENTNLQDTTRYLKLPFSK
GIFLGNNNQAMKATKESFCITSFLCSTKLTQNGDMLDLLKWRTHPDKITGCLSKLKEIDGS
EIVKFLQDTLDTLFGILDENSQKYGSKVFDSLVHIINLLQDSKFHHFKPVMDTYIESHFAGA
LAYRDLIKVLKWYVDRITEAERQEHIQEVLKAQEYIFKYIVQSRRLFSLATGGQNEEEFRC
CIQELLMSVRFFLSQESKGSGALSQSQAVFLSSFPAVYSELLKLFDVREVANLVQDTLGSLP
TILHVDDSLQAIKLQCIGKTVESQLYTNPDSRYILLPVVLHHLHIHLQEQKDLIMCARILSN
VFCLIKKNSSEKSVLEEIDVIVASLLDILLRTILEITSRPQPSSSAMRFQFQDVTGEFVACLLS
LLRQMTDRHYQQLLDSFNTKEELRDFLLQIFTVFRILIRPEMFPKDWTVMRLVANNVIITT
VLYLSDALRKNFLNENFDYKIWDSYFYLAVIFINQLCLQLEMFTPSKKKKVLEKYGDMRV
TMGCEIFSMWQNLGEHKLHFIPALIGPFLEVTLIPQPDLRNVMIPIFHDMMDWEQRRSGNF

KQVEAKLIDKLDSLMSEGKGDETYRELFNSILLKKIERETWRESGVSLIATVTRLMERLLD
YRDCMKMGEVDGKKIGCTVSLLNFYKTELNKEEMYIRYIHKLYDLHLKAQNFTEAAYTL
LLYDELLEWSDRPLREFLTYPMQTEWQRKEHLHLTIIQNFDRGKCWENGIILCRKIAEQYE
SYYDYRNLSKMRMMEASLYDKIMDQQRLEPEFFRVGFYGKKFPFFLRNKEFVCRGHDYE
RLEAFQQRMLNEFPHAIAMQHANQPDETIFQAEAQYLQIYAVTPIPESQEVLQREGVPDNI
KSFYKVNHIWKFRYDRPFHKGTKDKENEFKSLWVERTSLYLVQSLPGISRWFEVEKREVV
EMSPLENAIEVLENKNQQLKTLISQCQTRQMQNINPLTMCLNGVIDAAVNGGVSRYQEAF
FVKEYILSHPEDGEKIARLRELMLEQAQILEFGLAVHEKFVPQDMRPLHKKLVDQFFVMK
SSLGIQEFSACMQASPVHFPNGSPRVCRNSAPASVSPDGTRVIPRRSPLSYPAVNRYSSSSLS
SQASAEVSNITGQSESSDEVFNMQPSPSTSSLSSTHSASPNVTSSAPSSARASPLLSDKHKH
SRENSCLSPRERPCSAIYPTPVEPSQRMLFNHIGDGALPRSDPNLSAPEKAVNPTPSSWSLD
SGKEAKNMSDSGKLISPPVPPRPTQTASPARHTTSVSPSPAGRSPLKGSVQSFTPSPVEYHSP
GLISNSPVLSGSYSSGISSLSRCSTSETSGFENQVNEQSAPLPVPVPVPVPSYGGEEPVRKES
KTPPPYSVYERTLRRPVPLPHSLSIPVTSEPPALPPKPLAARSSHLENGARRTDPGPRPRPLP
RKVSQL

>Q96IZ2 multipass membrane protein
MTKTSTCIYHFLVLSWYTFLNYYISQEGKDEVKPKILANGARWKYMTLLNLLLQTIFYGV
TCLDDVLKRTKGGKDIKFLTAFRDLLFTTLAFPVSTFVFLAFWILFLYNRDLIYPKVLDTVI
PVWLNHAMHTFIFPITLAEVVLRPHSYPSKKTGLTLLAAASIAYISRILWLYFETGTWVYPV
FAKLSLLGLAAFFSLSYVFIASIYLLGEKLNHWKWGDMRQPRKKRK

>Q9P246 single-pass type I membrane protein
MLVLGLLVAGAADGCELVPRHLRGRRATGSAATAASSPAAAAGDSPALMTDPCMSLSPPC
FTEEDRFSLEALQTIHKQMDDDKDGGIEVEESDEFIREDMKYKDATNKHSHLHREDKHITI
EDLWKRWKTSEVHNWTLEDTLQWLIEFVELPQYEKNFRDNNVKGTTLPRIAVHEPSFMIS
QLKISDRSHRQKLQLKALDVVLFGPLTRPPHNWMKDFILTVSIVIGVGGCWFAYTQNKTS
KEHVAKMMKDLESLQTAEQSLMDLQERLEKAQEENRNVAVEKQNLERKMMDEINYAKE
EACRLRELREGAECELSRRQYAEQELEQVRMALKKAEKEFELRSSWSVPDALQKWLQLT
HEVEVQYYNIKRQNAEMQLAIAKDEAEKIKKKRSTVFGTLHVAHSSSLDEVDHKILEAKK
ALSELTTCLRERLFRWQQIEKICGFQIAHNSGLPSLTSSLYSDHSWVVMPRVSIPPYPIAGGV
DDLDEDTPPIVSQFPGTMAKPPGSLARSSSLCRSRRSIVPSSPQPQRAQLAPHAPHPSHPRH
PHHPQHTPHSLPSPDPDILSVSSCPALYRNEEEEEAIYFSAEKQWEVPDTASECDSLNSSIGR
KQSPPLSLEIYQTLSPRKISRDEVSLEDSSRGDSPVTVDVSWGSPDCVGLTETKSMIFSPASK
VYNGILEKSCSMNQLSSGIPVPKPRHTSCSSAGNDSKPVQEAPSVARISSIPHDLCHNGEKS
KKPSKIKSLFKKKSK

>P59534 multipass membrane protein
MLGRCFPPDTKEKQQLRMTKLCDPAESELSPFLITLILAVLLAEYLIGIIANGFIMAIHAAE
WVQNKAVSTSGRILVFLSVSRIALQSLMMLEITISSTSLSFYSEDAVYYAFKISFIFLNFCSLW
FAAWLSFFYFVKIANFSYPLFLKLRWRITGLIPWLLWLSVFISFSHSMFCINICTVYCNNSFP
IHSSNSTKKTYLSEINVVGLAFFFNLGIVTPLIMFILTATLLILSLKRHTLHMGSNATGSNDPS
MEAHMGAIKAISYFLILYIFNAVALFIYLSNMFDINSLWNNLCQIIMAAYPASHSILLIQDNP
GLRRAWKRLQLRLHLYPKEWTL

>Q70Z44 multipass membrane protein
MQKHSPGPPALALLSQSLLTTGNGDTLIINCPGFGQHRVDPAAFQAVFDRKAIGPVTNYSV
ATHVNISFTLSAIWNCYSRIHTFNCHHARPWHNQFVQWNPDECGGIKKSGMATENLWLS
DVFIEESVDQTPAGLMASMSIVKATSNTISQCGWSASANWTPSISPSMDRARAWRRMSRS
FQIHHRTSFRTRREWVLLGIQKRTIKVTVATNQYEQAIFHVAIRRRCRPSPYVVNFLVPSGIL
IAIDALSFYLPLESGNCAPFKMTVLLGYSVFLLMMNDLLPATSTSSHASLVAPLALMQTPLP
AGVYFALCLSLMVGSLLETIFITHLLHVATTQPLPLPRWLHSLLLHCTGQGRCCPTAPQKG
NKGPGLTPTHLPGVKEPEVSAGQMPGPGEAELTGGSEWTRAQREHEAQKQHSVELWVQF
SHAMDALLFRLYLLFMASSIITVICLWNT

>Q8N9A8 multipass membrane protein
MNSLEQAEDLKAFERRLTEYIHCLQPATGRWRMLLIVVSVCTATGAWNWLIDPETQKVSF
FTSLWNHPFFTISCITLIGLFFAGIHKRVVAPSIIAARCRTVLAEYNMSCDDTGKLILKPRPH
VQ

>Q2Y0W8 multipass membrane protein
MPAAGSNEPDGVLSYQRPDEEAVVDQGGTSTILNIHYEKEELEGHRTLYVGVRMPLGRQS
HRHHRTHGQKHRRRGRGKGASQGEEGLEALAHDTPSQRVQFILGTEEDEEHVPHELFTEL
DEICMKEGEDAEWKETARWLKFEEDVEDGGERWSKPYVATLSLHSLFELRSCLINGTVLL
DMHANSIEEISDLILDQQELSSDLNDSMRVKVREALLKKHHHQNEKKRNNLIPIVRSFAEV
GKKQSDPHLMDKHGQTVSPQSVPTTNLEVKNGVNCEHSPVDLSKVDLHFMKKIPTGAEA
SNVLVGEVDILDRPIVAFVRLSPAVLLSGLTEVPIPTRFLFILLGPVGKGQQYHEIGRSMATI
MTDEIFHDVAYKAKERDDLLAGIDEFLDQVTVLPPGEWDPSIRIEPPKNVPSQEKRKMPGV
PNGNVCHIEQEPHGGHSGPELQRTGRLFGGLVLDIKRKAPWYWSDYRDALSLQCLASFLF
LYCACMSPVITFGGLLGEATEGRISAIESLFGASMTGIAYSLFAGQALTILGSTGPVLVFEKIL
FKFCKDYALSYLSLRACIGLWTAFLCIVLVATDASSLVCYITRFTEEAFASLICIIFIYEAIEKLI
HLAETYPIHMHSQLDHLSLYYCRCTLPENPNNHTLQYWKDHNIVTAEVHWANLTVSECQ
EMHGEFMGSACGHHGPYTPDVLFWSCILFFTTFILSSTLKTFKTSRYFPTRVRSMVSDFAVF
LTIFTMVIIDFLIGVPSPKLQVPSVFKPTRDDRGWIINPIGPNPWWTVIAAIIPALLCTILIFMD
QQITAVIINRKEHKLKKGCGYHLDLLMVAIMLGVCSIMGLPWFVAATVLSITHVNSLKLES
ECSAPGEQPKFLGIREQRVTGLMIFVLMGCSVFMTAILKFIPMPVLYGVFLYMGVSSLQGI
QFFDRLKLFGMPAKHQPDFIYLRHVPLRKVHLFTLIQLTCLVLLWVIKASPAAIVFPMMVL
ALVFVRKVMDLCFSKRELSWLDDLMPESKKKKLDDAKKKAKEEEEAEKMLEIGGDKFP
LESRKLLSSPGKNISCRCDPSEINISDEMPKTTVWKALSMNSGNAKEKSLFN

>Q9Y3B3 single-pass type I membrane protein
MPRPGSAQRWAAVAGRWGCRLLALLLLVPGPGGASEITFELPDNAKQCFYEDIAQGTKCT
LEFQVITGGHYDVDCRLEDPDGKVLYKEMKKQYDSFTFTASKNGTYKFCFSNEFSTFTHK
TVYFDFQVGEDPPLFPSENRVSALTQMESACVSIHEALKSVIDYQTHFRLREAQGRSRAED
LNTRVAYWSVGEALILLVVSIGQVFLLKSFFSDKRTTTTRVGS

>Q8TEZ7 multipass membrane protein
MTTAILERLSTLSVSGQQLRRLPKILEDGLPKMPCTVPETDVPQLFREPYIRTGYRPTGHE

WRYYFFSLFQKHNEVVNVWTHLLAALAVLLRFWAFAEAEALPWASTHSLPLLLFILSSITY
LTCSLLAHLLQSKSELSHYTFYFVDYVGVSVYQYGSALAHFFYSSDQAWYDRFWLFFLPA
AAFCGWLSCAGCCYAKYRYRRPYPVMRKICQVVPAGLAFILDISPVAHRVALCHLAGCQE
QAAWYHTLQILFFLVSAYFFSCPVPEKYFPGSCDIVGHGHQIFHAFLSICTLSQLEAILLDYQ
GRQEIFLQRHGPLSVHMACLSFFFLAACSAATAALLRHKVKARLTKKDS

>Q14517 single-pass type I membrane protein
MGRHLALLLLLLLLFQHFGDSDGSQRLEQTPLQFTHLEYNVTVQENSAAKTYVGHPVKM
GVYITHPAWEVRYKIVSGDSENLFKAEEYILGDFCFLRIRTKGGNTAILNREVKDHYTLIVK
ALEKNTNVEARTKVRVQVLDTNDLRPLFSPTSYSVSLPENTAIRTSIARVSATDADIGTNGE
FYYSFKDRTDMFAIHPTSGVIVLTGRLDYLETKLYEMEILAADRGMKLYGSSGISSMAKLT
VHIEQANECAPVITAVTLSPSELDRDPAYAIVTVDDCDQGANGDIASLSIVAGDLLQQFRTV
RSFPGSKEYKVKAIGGIDWDSHPFGYNLTLQAKDKGTPPQFSSVKVIHVTSPQFKAGPVKF
EKDVYRAEISEFAPPNTPVVMVKAIPAYSHLRYVFKSTPGKAKFSLNYNTGLISILEPVKRQ
QAAHFELEVTTSDRKASTKVLVKVLGANSNPPEFTQTAYKAAFDENVPIGTTVMSLSAVD
PDEGENGYVTYSIANLNHVPFAIDHFTGAVSTSENLDYELMPRVYTLRIRASDWGLPYRRE
VEVLATITLNNLNDNTPLFEKINCEGTIPRDLGVGEQITTVSAIDADELQLVQYQIEAGNEL
DFFSLNPNSGVLSLKRSLMDGLGAKVSFHSLRITATDGENFATPLYINITVAASHKLVNLQC
EETGVAKMLAEKLLQANKLHNQGEVEDIFFDSHSVNAHIPQFRSTLPTGIQVKENQPVGSS
VIFMNSTDLDTGFNGKLVYAVSGGNEDSCFMIDMETGMLKILSPLDRETTDKYTLNITVY
DLGIPQKAAWRLLHVVVVDANDNPPEFLQESYFVEVSEDKEVHSEIIQVEATDKDLGPNG
HVTYSIVTDTDTFSIDSVTGVVNIARPLDRELQHEHSLKIEARDQAREEPQLFSTVVVKVS
LEDVNDNPPTFIPPNYRVKVREDLPEGTVIMWLEAHDPDLGQSGQVRYSLLDHGEGNFDV
DKLSGAVRIVQQLDFEKKQVYNLTVRAKDKGKPVSLSSTCYVEVEVVDVNENLHPPVFSS
FVEKGTVKEDAPVGSLVMTVSAHDEDARRDGEIRYSIRDGSGVGVFKIGEETGVIETSDRL
DRESTSHYWLTVFATDQGVVPLSSFIEIYIEVEDVNDNAPQTSEPVYYPEIMENSPKDVSVV
QIEAFDPDSSSNDKLMYKITSGNPQGFFSIHPKTGLITTTSRKLDREQQDEHILEVTVTDNG
SPPKSTIARVIVKILDENDNKPQFLQKFYKIRLPEREKPDRERNARREPLYHVIATDKDEGP
NAEISYSIEDGNEHGKFFIEPKTGVVSSKRFSAAGEYDILSIKAVDNGRPQKSSTTRLHIEWI
SKPKPSLEPISFEESFFTFTVMESDPVAHMIGVISVEPPGIPLWFDITGGNYDSHFDVDKGTG
TIIVAKPLDAEQKSNYNLTVEATDGTTTILTQVFIKVIDTNDHRPQFSTSKYEVVIPEDTAPE
TEILQISAVDQDEKNKLIYTLQSSRDPLSLKKFRLDPATGSLYTSEKLDHEAVHQHTLTVMV
RDQDVPVKRNFARIVVNVSDTNDHAPWFTASSYKGRVYESAAVGSVVLQVTALDKDKG
KNAEVLYSIESGNIGNSFMIDPVLGSIKTAKELDRSNQAEYDLMVKATDKGSPPMSEITSV
RIFVTIADNASPKFTSKEYSVELSETVSIGSFVGMVTAHSQSSVVYEIKDGNTGDAFDINPH
SGTIITQKALDFETLPIYTLIIQGTNMAGLSTNTTVLVHLQDENDNAPVFMQAEYTGLISES
ASINSVVLTDRNVPLVIRAADADKDSNALLVYHIVEPSVHTYFAIDSSTGAIHTVLSLDYEE
TSIFHFTVQVHDMGTPRLFAEYAANVTVHVIDINDCPPVFAKPLYEASLLLPTYKGVKVIT
VNATDADSSAFSQLIYSITEGNIGEKFSMDYKTGALTVQNTTQLRSRYELTVRASDGRFAG
LTSVKINVKESKESHLKFTQDVYSAVVKENSTEAETLAVITAIGNPINEPLFYHILNPDRRFK
ISRTSGVLSTTGTPFDREQQEAFDVVVEVTEEHKPSAVAHVVVKVIVEDQNDNAPVFVNL
PYYAVVKVDTEVGHVIRYVTAVDRDSGRNGEVHYYLKEHHEHFQIGPLGEISLKKQFELD
TLNKEYLVTVVAKDGGNPAFSAEVIVPITVMNKAMPVFEKPFYSAEIAESIQVHSPVVHVQ
ANSPEGLKVFYSITDGDPFSQFTINFNTGVINVIAPLDFEAHPAYKLSIRATDSLTGAHAEVF

VDIIVDDINDNPPVFAQQSYAVTLSEASVIGTSVVQVRATDSDSEPNRGISYQMFGNHSKSH
DHFHVDSSTGLISLLRTLDYEQSRQHTIFVRAVDGGMPTLSSDVIVTVDVTDLNDNPPLFE
QQIYEARISEHAPHGHFVTCVKAYDADSSDIDKLQYSILSGNDHKHFVIDSATGIITLSNLH
RHALKPFYSLNLSVSDGVFRSSTQVHVTVIGGNLHSPAFLQNEYEVELAENAPLHTLVME
VKTTDGDSGIYGHVTYHIVNDFAKDRFYINERGQIFTLEKLDRETPAEKVISVRLMAKDA
GGKVAFCTVNVILTDDNDNAPQFRATKYEVNIGSSAAKGTSVVKVLASDADEGSNADITY
AIEADSESVKENLEINKLSGVITTKESLIGLENEFFTFFVRAVDNGSPSKESVVLVYVKILPP
EMQLPKFSEPFYTFTVSEDVPIGTEIDLIRAEHSGTVLYSLVKGNTPESNRDESFVIDRQSGR
LKLEKSLDHETTKWYQFSILARCTQDDHEMVASVDVSIQVKDANDNSPVFESSPYEAFIV
ENLPGGSRVIQIRASDADSGTNGQVMYSLDQSQSVEVIESFAINMETGWITTLKELDHEKR
DNYQIKVVASDHGEKIQLSSTAIVDVTVTDVNDSPPRFTAEIYKGTVSEDDPQGGVIAILST
TDADSEEINRQVTYFITGGDPLGQFAVETIQNEWKVYVKKPLDREKRDNYLLTITATDGTF
SSKAIVEVKVLDANDNSPVCEKTLYSDTIPEDVLPGKLIMQISATDADIRSNAEITYTLLGS
GAEKFKLNPDTGELKTSTPLDREEQAVYHLLVRATDGGGRFCQASIVLTLEDVNDNAPEFS
ADPYAITVFENTEPGTLLTRVQATDADAGLNRKILYSLIDSADGQFSINELSGIIQLEKPLDR
ELQAVYTLSLKAVDQGLPRRLTATGTVIVSVLDINDNPPVFEYREYGATVSEDILVGTEVLQ
VYAASRDIEANAEITYSIISGNEHGKFSIDSKTGAVFIIENLDYESSHEYYLTVEATDGGTPSL
SDVATVNVNVTDINDNTPVFSQDTYTTVISEDAVLEQSVITVMADDADGPSNSHIHYSIIDG
NQGSSFTIDPVRGEVKVTKLLDRETISGYTLTVQASDNGSPPRVNTTTVNIDVSDVNDNAP
VFSRGNYSVIIQENKPVGFSVLQLVVTDEDSSHNGPPFFFTIVTGNDEKAFEVNPQGVLLTS
SAIKRKEKDHYLLQVKVADNGKPQLSSLTYIDIRVIEESIYPPAILPLEIFITSSGEEYSGGVIG
KIHATDQDVYDTLTYSLDPQMDNLFSVSSTGGKLIAHKKLDIGQYLLNVSVTDGKFTTVA
DITVHIRQVTQEMLNHTIAIRFANLTPEEFVGDYWRNFQRALRNILGVRRNDIQIVSLQSSE
PHPHLDVLLFVEKPGSAQISTKQLLHKINSSVTDIEEIIGVRILNVFQKLCAGLDCPWKFCD
EKVSVDESVMSTHSTARLSFVTPRHHRAAVCLCKEGRCPPVHHGCEDDPCPEGSECVSDP
WEEKHTCVCPSGRFGQCPGSSSMTLTGNSYVKYRLTENENKLEMKLTMRLRTYSTHAVV
MYARGTDYSILEIHHGRLQYKFDCGSGPGIVSVQSIQVNDGQWHAVALEVNGNYARLVLD
QVHTASGTAPGTLKTLNLDNYVFFGGHIRQQGTRHGRSPQVGNGFRGCMDSIYLNGQEL
PLNSKPRSYAHIEESVDVSPGCFLTATEDCASNPCQNGGVCNPSPAGGYYCKCSALYIGTH
CEISVNPCSSKPCLYGGTCVVDNGGFVCQCRGLYTGQRCQLSPYCKDEPCKNGGTCFDSL
DGAVCQCDSGFRGERCQSDIDECSGNPCLHGALCENTHGSYHCNCSHEYRGRHCEDAAP
NQYVSTPWNIGLAEGIGIVVFVAGIFLLVVVFVLCRKMISRKKKHQAEPKDKHLGPATAFL
QRPYFDSKLNKNIYSDIPPQVPVRPISYTPSIPSDSRNNLDRNSFEGSAIPEHPEFSTFNPESV
HGHRKAVAVCSVAPNLPPPPPSNSPSDSDSIQKPSWDFDYDTKVVDLDPCLSKKPLEEKPS
QPYSARESLSEVQSLSSFQSESCDDNGYHWDTSDWMPSVPLPDIQEFPNYEVIDEQTPLYS
ADPNAIDTDYYPGGYDIESDFPPPPEDFPAADELPPLPPEFSNQFESIHPPRDMPAAGSLGSS
SRNRQRFNLNQYLPNFYPLDMSEPQTKGTGENSTCREPHAPYPPGYQRHFEAPAVESMPM
SVYASTASCSDVSACCEVESEVMMSDYESGDDGHFEEVTIPPLDSQQHTEV

>Q96PE1 multipass membrane protein
MGAGGRRMRGAPARLLLPLLPWLLLLLAPEARGAPGCPLSIRSCKCSGERPKGLSGGVPG
PARRRVVCSGGDLPEPPEPGLLPNGTVTLLLSNNKITGLRNGSFLGLSLLEKLDLRNNIIST
VQPGAFLGLGELKRLDLSNNRIGCLTSETFQGLPRLLRLNISGNIFSSLQPGVFDELPALKV
VDLGTEFLTCDCHLRWLLPWAQNRSLQLSEHTLCAYPSALHAQALGSLQEAQLCCEGALE

LHTHHLIPSLRQVVFQGDRLPFQCSASYLGNDTRIRWYHNRAPVEGDEQAGILLAESLIHD
CTFITSELTLSHIGVWASGEWECTVSMAQGNASKKVEIVVLETSASYCPAERVANNRGDFR
WPRTLAGITAYQSCLQYPFTSVPLGGGAPGTRASRRCDRAGRWEPGDYSHCLYTNDITRV
LYTFVLMPINASNALTLAHQLRVYTAEAASFSDMMDVVYVAQMIQKFLGYVDQIKELVE
VMVDMASNLMLVDEHLLWLAQREDKACSRIVGALERIGGAALSPHAQHISVNARNVALE
AYLIKPHSYVGLTCTAFQRREGGVPGTRPGSPGQNPPPEPEPPADQQLRFRCTTGRPNVSLS
SFHIKNSVALASIQLPPSLFSSLPAALAPPVPPDCTLQLLVFRNGRLFHSHSNTSRPGAAGPG
KRRGVATPVIFAGTSGCGVGNLTEPVAVSLRHWAEGAEPVAAWWSQEGPGEAGGWTSEG
CQLRSSQPNVSALHCQHLGNVAVLMELSAFPREVGGAGAGLHPVVYPCTALLLLCLFATII
TYILNHSSIRVSRKGWHMLLNLCFHIAMTSAVFAGGITLTNYQMVCQAVGITLHYSSLSTL
LWMGVKARVLHKELTWRAPPPQEGDPALPTPSPMLRFYLIAGGIPLIICGITAAVNIHNYRD
HSPYCWLVWRPSLGAFYIPVALILLITWIYFLCAGLRLRGPLAQNPKAGNSRASLEAGEEL
RGSTRLRGSGPLLSDSGSLLATGSARVGTPGPPEDGDSLYSPGVQLGALVTTHFLYLAMWA
CGALAVSQRWLPRVVCSCLYGVAASALGLFVFTHHCARRRDVRASWRACCPPASPAAPH
APPRALPAAAEDGSPVFGEGPPSLKSSPSGSSGHPLALGPCKLTNLQLAQSQVCEAGAAAG
GEGEPEPAGTRGNLAHRHPNNVHHGRRAHKSRAKGHRAGEACGKNRLKALRGGAAGA
LELLSSESGSLHNSPTDSYLGSSRNSPGAGLQLEGEPMLTPSEGSDTSAAPLSEAGRAGQR
RSASRDSLKGGGALEKESHRRSYPLNAASLNGAPKGGKYDDVTLMGAEVASGGCMKTG
LWKSETTV

>O75023 single-pass type I membrane protein
MTLTLSVLICLGLSVGPRTCVQAGTLPKPTLWAEPASVIARGKPVTLWCQGPLETEEYRLD
KEGLPWARKRQNPLEPGAKAKFHIPSTVYDSAGRYRCYYETPAGWSEPSDPLELVATGFY
AEPTLLALPSPVVASGGNVTLQCDTLDGLLTFVLVEEEQKLPRTLYSQKLPKGPSQALFPV
GPVTPSCRWRFRCYYYYRKNPQVWSNPSDLLEILVPGVSRKPSLLIPQGSVVARGGSLTLQ
CRSDVGYDIFVLYKEGEHDLVQGSGQQPQAGLSQANFTLGPVSRSHGGQYRCYGAHNLS
PRWSAPSDPLDILIAGLIPDIPALSVQPGPKVASGENVTLLCQSWHQIDTFFLTKEGAAHPPL
CLKSKYQSYRHQAEFSMSPVTSAQGGTYRCYSAIRSYPYLLSSPSYPQELVVSGPSGDPSL
SPTGSTPTPGPEDQPLTPTGLDPQSGLGRHLGVVTGVSVAFVLLLFLLLFLLLRHRHQSKH
RTSAHFYRPAGAAGPEPKDQGLQKRASPVADIQEEILNAAVKDTQPKDGVEMDARAAAS
EAPQDVTYAQLHSLTLRREATEPPPSQEREPPAEPSIYAPLAIH

>Q9UPZ6 single-pass type I membrane protein
MGLQARRWASGSRGAAGPRRGVLQLLPLPLPLPLLLLLLLRPGAGRAAAQGEAEAPTLYL
WKTGPWGRCMGDECGPGGIQTRAVWCAHVEGWTTLHTNCKQAERPNNQQNCFKVCD
WHKELYDWRLGPWNQCQPVISKSLEKPLECIKGEEGIQVREIACIQKDKDIPAEDIICEYFE
PKPLLEQACLIPCQQDCIVSEFSAWSECSKTCGSGLQHRTRHVVAPPQFGGSGCPNLTEFQ
VCQSSPCEAEELRYSLHVGPWSTCSMPHSRQVRQARRRGKNKEREKDRSKGVKDPEARE
LIKKKRNRNRQNRQENKYWDIQIGYQTREVMCINKTGKAADLSFCQQEKLPMTFQSCVI
TKECQVSEWSEWSPCSKTCHDMVSPAGTRVRTRTIRQFPIGSEKECPEFEEKEPCLSQGDG
VVPCATYGWRTTEWTECRVDPLLSQQDKRRGNQTALCGGGIQTREVYCVQANENLLSQL
STHKNKEASKPMDLKLCTGPIPNTTQLCHIPCPTECEVSPWSAWGPCTYENCNDQQGKKG
FKLRKRRITNEPTGGSGVTGNCPHLLEAIPCEEPACYDWKAVRLGNCEPDNGKECGPGTQ
VQEVVCINSDGEEVDRQLCRDAIFPIPVACDAPCPKDCVLSTWSTWSSCSHTCSGKTTEGK

QIRARSILAYAGEEGGIRCPNSSALQEVRSCNEHPCTVYHWQTGPWGQCIEDTSVSSFNTT
TTWNGEASCSVGMQTRKVICVRVNVGQVGPKKCPESLRPETVRPCLLPCKKDCIVTPYSD
WTSCPSSCKEGDSSIRKQSRHRVIIQLPANGGRDCTDPLYEEKACEAPQACQSYRWKTHK
WRRCQLVPWSVQQDSPGAQEGCGPGRQARAITCRKQDGGQAGIHECLQYAGPVPALTQA
CQIPCQDDCQLTSWSKFSSCNGDCGAVRTRKRTLVGKSKKKEKCKNSHLYPLIETQYCPC
DKYNAQPVGNWSDCILPEGKVEVLLGMKVQGDIKECGQGYRYQAMACYDQNGRLVET
SRCNSHGYIEEACIIPCPSDCKLSEWSNWSRCSKSCGSGVKVRSKWLREKPYNGGRPCPK
LDHVNQAQVYEVVPCHSDCNQYLWVTEPWSICKVTFVNMRENCGEGVQTRKVRCMQN
TADGPSEHVEDYLCDPEEMPLGSRVCKLPCPEDCVISEWGPWTQCVLPCNQSSFRQRSAD
PIRQPADEGRSCPNAVEKEPCNLNKNCYHYDYNVTDWSTCQLSEKAVCGNGIKTRMLDC
VRSDGKSVDLKYCEALGLEKNWQMNTSCMVECPVNCQLSDWSPWSECSQTCGLTGKMI
RRRTVTQPFQGDGRPCPSLMDQSKPCPVKPCYRWQYGQWSPCQVQEAQCGEGTRTRNIS
CVVSDGSADDFSKVVDEEFCADIELIIDGNKNMVLEESCSQPCPGDCYLKDWSSWSLCQL
TCVNGEDLGFGGIQVRSRPVIIQELENQHLCPEQMLETKSCYDGQCYEYKWMASAWKGS
SRTVWCQRSDGINVTGGCLVMSQPDADRSCNPPCSQPHSYCSETKTCHCEEGYTEVMSSN
STLEQCTLIPVVVLPTMEDKRGDVKTSRAVHPTQPSSNPAGRGRTWFLQPFGPDGRLKTW
VYGVAAGAFVLLIFIVSMIYLACKKPKKPQRRQNNRLKPLTLAYDGDADM

>Q8TAC9 multipass membrane protein
MAEKVNNFPPLPKFIPLKPCFYQDFEADIPPQHVSMTKRLYYLWMLNSVTLAVNLVGCLA
WLIGGGGATNFGLAFLWLILFTPCSYVCWFRPIYKAFKTDSSFSFMAFFFTFMAQLVISIIQA
VGIPGWGVCGWIATISFFGTNIGSAVVMLIPTVMFTVMAVFSFIALSMVHKFYRGSGGSFS
KAQEEWTTGAWKNPHVQQAAQNAAMGAAQGAMNQPQTQYSATPNYTYSNEM

>O95866 single-pass type I membrane protein
MAVFLQLLPLLLSRAQGNPGASLDGRPGDRVNLSCGGVSHPIRWVWAPSFPACKGLSKGR
RPILWASSSGTPTVPPLQPFVGRLRSLDSGIRRLELLLSAGDSGTFFCKGRHEDESRTVLHV
LGDRTYCKAPGPTHGSVYPQLLIPLLGAGLVLGLGALGLVWWLHRRLPPQPIRPLPRFAPL
VKTEPQRPVKEEEPKIPGDLDQEPSLLYADLDHLALSRPRRLSTADPADASTIYAVVV

>Q8N1B4 peripheral membrane protein
MAAAATMAAAARELVLRAGTSDMEEEEGPLAGGPGLQEPLQLGELDITSDEFILDEVDVH
IQANLEDELVKEALKTGVDLRHYSKQVELELQQIEQKSIRDYIQESENIASLHNQITACDAV
LERMEQMLGAFQSDLSSISSEIRTLQEQSGAMNIRLRNRQAVRGKLGELVDGLVVPSALVT
AILEAPVTEPRFLEQLQELDAKAAAVREQEARGTAACADVRGVLDRLRVKAVTKIREFILQ
KIYSFRKPMTNYQIPQTALLKYRFFYQFLLGNERATAKEIRDEYVETLSKIYLSYYRSYLGR
LMKVQYEEVAEKDDLMGVEDTAKKGFFSKPSLRSRNTIFTLGTRGSVISPTELEAPILVPHT
AQRGEQRYPFEALFRSQHYALLDNSCREYLFICEFFVVSGPAAHDLFHAVMGRTLSMTLK
HLDSYLADCYDAIAVFLCIHIVLRFRNIAAKRDVPALDRYWEQVLALLWPRFELILEMNVQ
SVRSTDPQRLGGLDTRPHYITRRYAEFSSALVSINQTIPNERTMQLLGQLQVEVENFVLRVA
AEFSSRKEQLVFLINNYDMMLGVLMERAADDSKEVESFQQLLNARTQEFIEELLSPPFGGL
VAFVKEAEALIERGQAERLRGEEARVTQLIRGFGSSWKSSVESLSQDVMRSFTNFRNGTSII
QGALTQLIQLYHRFHRVLSQPQLRALPARAELINIHHLMVELKKHKPNF

>P50542 peripheral membrane protein
MAMRELVEAECGGANPLMKLAGHFTQDKALRQEGLRPGPWPPGAPASEAASKPLGVASE
DELVAEFLQDQNAPLVSRAPQTFKMDDLLAEMQQIEQSNFRQAPQRAPGVADLALSENW
AQEFLAAGDAVDVTQDYNETDWSQEFISEVTDPLSVSPARWAEEYLEQSEEKLWLGEPEG
TATDRWYDEYHPEEDLQHTASDFVAKVDDPKLANSEFLKFVRQIGEGQVSLESGAGSGRA
QAEQWAAEFIQQQGTSDAWVDQFTRPVNTSALDMEFERAKSAIESDVDFWDKLQAELEE
MAKRDAEAHPWLSDYDDLTSATYDKGYQFEEENPLRDHPQPFEEGLRRLQEGDLPNAVL
LFEAAVQQDPKHMEAWQYLGTTQAENEQELLAISALRRCLELKPDNQTALMALAVSFTN
ESLQRQACETLRDWLRYTPAYAHLVTPAEEGAGGAGLGPSKRILGSLLSDSLFLEVKELFL
AAVRLDPTSIDPDVQCGLGVLFNLSGEYDKAVDCFTAALSVRPNDYLLWNKLGATLANG
NQSEEAVAAYRRALELQPGYIRSRYNLGISCINLGAHREAVEHFLEALNMQRKSRGPRGEG
GAMSENIWSTLRLALSMLGQSDAYGAADARDLSTLLTMFGLPQ

>Q5SQ64 single-pass type I membrane protein
MAVLFLLLFLCGTPQAADNMQAIYVALGEAVELPCPSPPTLHGDEHLSWFCSPAAGSFTTL
VAQVQVGRPAPDPGKPGRESRLRLLGNYSLWLEGSKEEDAGRYWCAVLGQHHNYQNWR
VYDVLVLKGSQLSARAADGSPCNVLLCSVVPSRRMDSVTWQEGKGPVRGRVQSFWGSE
AALLLVCPGEGLSEPRSRRPRIIRCLMTHNKGVSFSLAASIDASPALCAPSTGWDMPWILM
LLLTMGQGVVILALSIVLWRQRVRGAPGRDASIPQFKPEIQVYENIHLARLGPPAHKPR

>Q9GZK4 multipass membrane protein
MVNQSSPMGFLLLGFSEHPALERTLFVVVFTSYLLTLVGNTLIILLSVLYPRLHSPMYFFLS
DLSFLDLCFTTSCVPQMLVNLWGPKKTISFLGCSVQLFIFLSLGTTECILLTVMAFDRYVAV
CQPLHYATIIHPRLCWQLASVAWVMSLVQSIVQTPSTLHLPFCPHQQIDDFLCEVPSLIRLSC
GDTSYNEIQLAVSSVIFVVVPLSLILASYGATAQAVLRINSATAWRKAFGTCSSHLTVVTLF
YSSVIAVYLQPKNPYAQGRGKFFGLFYAVGTPSLNPLVYTLRNKEIKRALRRLLGKERDSR
ESWRAA

>Q15599 peripheral membrane protein
MAAPEPLRPRLCRLVRGEQGYGFHLHGEKGRRGQFIRRVEPGSPAEAAALRAGDRLVEVN
GVNVEGETHHQVVQRIKAVEGQTRLLVVDQETDEELRRRQLTCTEEMAQRGLPPAHDPW
EPKPDWAHTGSHSSEAGKKDVSGPLRELRPRLCHLRKGPQGYGFNLHSDKSRPGQYIRSV
DPGSPAARSGLRAQDRLIEVNGQNVEGLRHAEVVASIKAREDEARLLVVDPETDEHFKRL
RVTPTEEHVEGPLPSPVTNGTSPAQLNGGSACSSRSDLPGSDKDTEDGSAWKQDPFQESGL
HLSPTAAEAKEKARAMRVNKRAPQMDWNRKREIFSNF

>P13473 single-pass type I membrane protein
MVCFRLFPVPGSGLVLVCLVLGAVRSYALELNLTDSENATCLYAKWQMNFTVRYETTNKT
YKTVTISDHGTVTYNGSICGDDQNGPKIAVQFGPGFSWIANFTKAASTYSIDSVSFSYNTG
DNTTFPDAEDKGILTVDELLAIRIPLNDLFRCNSLSTLEKNDVVQHYWDVLVQAFVQNGT
VSTNEFLCDKDKTSTVAPTIHTTVPSPTTTPTPKEKPEAGTYSVNNGNDTCLLATMGLQLN
ITQDKVASVININPNTTHSTGSCRSHTALLRLNSSTIKYLDFVFAVKNENRFYLKEVNISMY
LVNGSVFSIANNNLSYWDAPLGSSYMCNKEQTVSVSGAFQINTFDLRVQPFNVTQGKYST
AQDCSADDDNFLVPIAVGAALAGVLILVLLAYFIGLKHHHAGYEQF

>Q9UGF5 multipass membrane protein
MVNLTSMSGFLLMGFSDERKLQILHALVFLVTYLLALTGNLLIITIITVDRRLHSPMYYFLK
HLSLLDLCFISVTVPQSIANSLMGNGYISLVQCILQVFFFIALASSEVAILTVMSYDRYAAIC
QPLHYETIMDPRACRHAVIAVWIAGGLSGLMHAAINFSIPLCGKRVIHQFFCDVPQMLKLA
CSYEFINEIALAAFTTSAAFICLISIVLSYIRIFSTVLRIPSAEGRTKVFSTCLPHLFVATFFLSA
AGFEFLRLPSDSSSTVDLVFSVFYTVIPPTLNPVIYSLRNDSMKAALRKMLSKEELPQRKM
CLKAMFKL

>Q13561 peripheral membrane protein
MADPKYADLPGIARNEPDVYETSDLPEDDQAEFDAEELTSTSVEHIIVNPNAAYDKFKDKR
VGTKGLDFSDRIGKTKRTGYESGEYEMLGEGLGVKETPQQKYQRLLHEVQELTTEVEKIK
TTVKESATEEKLTPVLLAKQLAALKQQLVASHLEKLLGPDAAINLTDPDGALAKRLLLQLE
ATKNSKGGSGGKTTGTPPDSSLVTYELHSRPEQDKFSQAAKVAELEKRLTELETAVRCDQD
AQNPLSAGLQGACLMETVELLQAKVSALDLAVLDQVEARLQSVLGKVNEIAKHKASVED
ADTQSKVHQLYETIQRWSPIASTLPELVQRLVTIKQLHEQAMQFGQLLTHLDTTQQMIANS
LKDNTTLLTQVQTTMRENLATVEGNFASIDERMKKLGK

>O75674 peripheral membrane protein
MAFGKSHRDPYATSVGHLIEKATFAGVQTEDWGQFMHICDIINTTQDGPKDAVKALKKRI
SKNYNHKEIQLTLSLIDMCVQNCGPSFQSLIVKKEFVKENLVKLLNPRYNLPLDIQNRILNF
IKTWSQGFPGGVDVSEVKEVYLDLVKKGVQFPPSEAEAETARQETAQISSNPPTSVPTAPA
LSSVIAPKNSTVTLVPEQIGKLHSELDMVKMNVRVMSAILMENTPGSENHEDIELLQKLYK
TGREMQERIMDLLVVVENEDVTVELIQVNEDLNNAILGYERFTRNQQRILEQNKNQKEAT
NTTSEPSAPSQDLLDLSPSPRMPRATLGELNTMNNQLSGLNFSLPSSDVTNNLKPSLHPQM
NLLALENTEIPPFAQRTSQNLTSSHAYDNFLEHSNSVFLQPVSLQTIAAAPSNQSLPPLPSNH
PAMTKSDLQPPNYYEVMEFDPLAPAVTTEAIYEEIDAHQHKGAQNDGD

>Q8IXM6 multipass membrane protein
MAPALLLIPAALASFILAFGTGVEFVRFTSLRPLLGGIPESGGPDARQGWLAALQDRSILAP
LAWDLGLLLLFVGQHSLMAAERVKAWTSRYFGVLQRSLYVACTALALQLVMRYWEPIPK
GPVLWEARAEPWATWVPLLCFVLHVISWLLIFSILLVFDYAELMGLKQVYYHVLGLGEPL
ALKSPRALRLFSHLRHPVCVELLTVLWVVPTLGTDRLLLAFLLTLYLGLAHGLDQQDLRY
LRAQLQRKLHLLSRPQDGEAE

>P08173 multipass membrane protein
MANFTPVNGSSGNQSVRLVTSSSHNRYETVEMVFIATVTGSLSLVTVVGNILVMLSIKVNR
QLQTVNNYFLFSLACADLIIGAFSMNLYTVYIIKGYWPLGAVVCDLWLALDYVVSNASV
MNLLIISFDRYFCVTKPLTYPARRTTKMAGLMIAAAWVLSFVLWAPAILFWQFVVGKRTVP
DNQCFIQFLSNPAVTFGTAIAAFYLPVVIMTVLYIHISLASRSRVHKHRPEGPKEKKAKTLA
FLKSPLMKQSVKKPPPGEAAREELRNGKLEEAPPPALPPPPRPVADKDTSNESSSGSATQN
TKERPATELSTTEATTPAMPAPPLQPRALNPASRWSKIQIVTKQTGNECVTAIEIVPATPAGM
RPAANVARKFASIARNQVRKKRQMAARERKVTRTIFAILLAFILTWTPYNVMVLVNTFCQS
CIPDTVWSIGYWLCYVNSTINPACYALCNATFKKTFRHLLLCQYRNIGTAR

>O95867 GPI-anchor, lipid-anchor
MKALMLLTLSVLLCWVSADIRCHSCYKVPVLGCVDRQSCRLEPGQQCLTTHAYLGKMW
VFSNLRCGTPEEPCQEAFNQTNRKLGLTYNTTCCNKDNCNSAGPRPTPALGLVFLTSLAGL
GLWLLH

>Q99519 peripheral membrane protein
MTGERPSTALPDRRWGPRILGFWGGCRVWVFAAIFLLLSLAASWSKAENDFGLVQPLVTM
EQLLWVSGRQIGSVDTFRIPLITATPRGTLLAFAEARKMSSSDEGAKFIALRRSMDQGSTW
SPTAFIVNDGDVPDGLNLGAVVSDVETGVVFLFYSLCAHKAGCQVASTMLVWSKDDGVS
WSTPRNLSLDIGTEVFAPGPGSGIQKQREPRKGRLIVCGHGTLERDGVFCLLSDDHGASW
RYGSGVSGIPYGQPKQENDFNPDECQPYELPDGSVVINARNQNNYHCHCRIVLRSYDACD
TLRPRDVTFDPELVDPVVAAGAVVTSSGIVFFSNPAHPEFRVNLTLRWSFSNGTSWRKETV
QLWPGPSGYSSLATLEGSMDGEEQAPQLYVLYEKGRNHYTESISVAKISVYGTL

>Q9HBM0 multipass membrane protein
MTPEFDEEVVFENSPLYQYLQDLGHTDFEICSSLSPKTEKCTTEGQQKPPTRVLPKQGILLK
VAETIKSWIFFSQCNKKDDLLHKLDIGFRLDSLHTILQQEVLLQEDVELIELLDPSILSAGQS
QQQENGHLPTLCSLATPNIWDLSMLFAFISLLVMLPTWWIVSSWLVWGVILFVYLVIRALR
LWRTAKLQVTLKKYSVHLEDMATNSRAFTNLVRKALRLIQETEVISRGFTLVSAACPFNK
AGQHPSQHLIGLRKAVYRTLRANFQAARLATLYMLKNYPLNSESDNVTNYICVVPFKELG
LGLSEEQISEEEAHNFTDGFSLPALKVLFQLWVAQSSEFFRRLALLLSTANSPPGPLLTPALL
PHRILSDVTQGLPHAHSACLEELKRSYEFYRYFETQHQSVPQCLSKTQQKSRELNNVHTA
VRSLQLHLKALLNEVIILEDELEKLVCTKETQELVSEAYPILEQKLKLIQPHVQASNNCWEE
AISQVDKLLRRNTDKKGKPEIACENPHCTVVPLKQPTLHIADKDPIPEEQELEAYVDDIDID
SDFRKDDFYYLSQEDKERQKREHEESKRVLQELKSVLGFKASEAERQKWKQLLFSDHAV
LKSLSPVDPVEPISNSEPSMNSDMGKVSKNDTEEESNKSATTDNEISRTEYLCENSLEGKN
KDNSSNEVFPQGAEERMCYQCESEDEPQADGSGLTTAPPTPRDSLQPSIKQRLARLQLSPD
FTFTAGLAAEVAARSLSFTTMQEQTFGGEEEEQIIEENKNEIEEK

>P01903 single-pass type I membrane protein
MAISGVPVLGFFIIAVLMSAQESWAIKEEHVIIQAEFYLNPDQSGEFMFDFDGDEIFHVDMA
KKETVWRLEEFGRFASFEAQGALANIAVDKANLEIMTKRSNYTPITNVPPEVTVLTNSPVE
LREPNVLICFIDKFTPPVVNVTWLRNGKPVTTGVSETVFLPREDHLFRKFHYLPFLPSTED
VYDCRVEHWGLDEPLLKHWEFDAPSPLPETTENVVCALGLTVGLVGIIIGTIFIIKGVRKSN
AAERRGPL

>P01375 single-pass type II membrane protein
MSTESMIRDVELAEEALPKKTGGPQGSRRCLFLSLFSFLIVAGATTLFCLLHFGVIGPQREE
FPRDLSLISPLAQAVRSSSRTPSDKPVAHVVANPQAEGQLQWLNRRANALLANGVELRDN
QLVVPSEGLYLIYSQVLFKGQGCPSTHVLLTHTISRIAVSYQTKVNLLSAIKSPCQRETPEGA
EAKPWYEPIYLGGVFQLEKGDRLSAEINRPDYLDFAESGQVYFGIIAL

>P55808 single-pass type I membrane protein

MESWWGLPCLAFLCFLMHARGQRDFDLADALDDPEPTKKPNSDIYPKPKPPYYPQPENP
DSGGNIYPRPKPRPQPQPGNSGNSGGYFNDVDRDDGRYPPRPRPRPPAGGGGGGYSSYGN
SDNTHGGDHHSTYGNPEGNMVAKIVSPIVSVVVVTLLGAAASYFKLNNRRNCFRTHEPEN
V

>P28068 single-pass type I membrane protein
MITFLPLLLGLSLGCTGAGGFVAHVESTCLLDDAGTPKDFTYCISFNKDLLTCWDPEENK
MAPCEFGVLNSLANVLSQHLNQKDTLMQRLRNGLQNCATHTQPFWGSLTNRTRPPSVQV
AKTTPFNTREPVMLACYVWGFYPAEVTITWRKNGKLVMPHSSAHKTAQPNGDWTYQTL
SHLALTPSYGDTYTCVVEHIGAPEPILRDWTPGLSPMQTLKVSVSAVTLGLGLIIFSLGVIS
WRRAGHSSYTPLPGSNYSEGWHIS

>P27448 peripheral membrane protein
MSTRTPLPTVNERDTENHTSHGDGRQEVTSRTSRSGARCRNSIASCADEQPHIGNYRLLKT
IGKGNFAKVKLARHILTGREVAIKIIDKTQLNPTSLQKLFREVRIMKILNHPNIVKLFEVIET
EKTLYLIMEYASGGEVFDYLVAHGRMKEKEARSKFRQIVSAVQYCHQKRIVHRDLKAENL
LLDADMNIKIADFGFSNEFTVGGKLDTFCGSPPYAAPELFQGKKYDGPEVDVWSLGVILY
TLVSGSLPFDGQNLKELRERVLRGKYRIPFYMSTDCENLLKRFLVLNPIKRGTLEQIMKDR
WINAGHEEDELKPFVEPELDISDQKRIDIMVGMGYSQEEIQESLSKMKYDEITATYLLLGR
KSSELDASDSSSSSNLSLAKVRPSSDLNNSTGQSPHHKVQRSVFSSQKQRRYSDHAGPAIPS
VVAYPKRSQTSTADSDLKEDGISSRKSSGSAVGGKGIAPASPMLGNASNPNKADIPERKKS
STVPSSNTASGGMTRRNTYVCSERTTADRHSVIQNGKENSTIPDQRTPVASTHSISSAATPD
RIRFPRGTASRSTFHGQPRERRTATYNGPPASPSLSHEATPLSQTRSRGSTNLFSKLTSKLTR
RNMSFRFIKRLPTEYERNGRYEGSSRNVSAEQKDENKEAKPRSLRFTWSMKTTSSMDPGD
MMREIRKVLDANNCDYEQRERFLLFCVHGDGHAENLVQWEMEVCKLPRLSLNGVRFKR
ISGTSIAFKNIASKIANELKL

>P46695 single-pass type II membrane protein
MCHSRSCHPTMTILQAPTPAPSTIPGPRRGSGPEIFTFDPLPEPAAAPAGRPSASRGHRKRSR
RVLYPRVVRRQLPVEEPNPAKRLLFLLLTIVFCQILMAEEGVPAPLPPEDAPNAASLAPTPV
SAVLEPFNLTSEPSDYALDLSTFLQQHPAAF

>Q13336 multipass membrane protein
MEDSPTMVRVDSPTMVRGENQVSPCQGRRCFPKALGYVTGDMKELANQLKDKPVVLQF
IDWILRGISQVVFVNNPVSGILILVGLLVQNPWWALTGWLGTVVSTLMALLLSQDRSLIAS
GLYGYNATLVGVLMAVFSDKGDYFWWLLLPVCAMSMTCPIFSSALNSMLSKWDLPVFTL
PFNMALSMYLSATGHYNPFFPAKLVIPITTAPNISWSDLSALELLKSIPVGVGQIYGCDNPW
TGGIFLGAILLSSPLMCLHAAIGSLLGIAAGLSLSAPFEDIYFGLWGFNSSLACIAMGGMFM
ALTWQTHLLALGCALFTAYLGVGMANFMAEVGLPACTWPFCLATLLFLIMTTKNSNIYK
MPLSKVTYPEENRIFYLQAKKRMVESPL

>Q96RL6 single-pass type I membrane protein
MVPGQAQPQSPEMLLLPLLLPVLGAGSLNKDPSYSLQVQRQVPVPEGLCVIVSCNLSYPR
DGWDESTAAYGYWFKGRTSPKTGAPVATNNQSREVEMSTRDRFQLTGDPGKGSCSLVIRD

AQREDEAWYFFRVERGSRVRHSFLSNAFFLKVTALTKKPDVYIPETLEPGQPVTVICVFNW
AFKKCPAPSFSWTGAALSPRRTRPSTSHFSVLSFTPSPQDHDTDLTCHVDFSRKGVSAQRT
VRLRVAYAPKDLIISISHDNTSALELQGNVIYLEVQKGQFLRLLCAADSQPPATLSWVLQDR
VLSSSHPWGPRTLGLELRGVRAGDSGRYTCRAENRLGSQQQALDLSVQYPPENLRVMVS
QANRTVLENLGNGTSLPVLEGQSLRLVCVTHSSPPARLSWTRWGQTVGPSQPSDPGVLEL
PPIQMEHEGEFTCHAQHPLGSQHVSLSLSVHYPPQLLGPSCSWEAEGLHCSCSSQASPAPS
LRWWLGEELLEGNSSQGSFEVTPSSAGPWANSSLSLHGGLSSGLRLRCKAWNVHGAQSG
SVFQLLPGKLEHGGGLGLGAALGAGVAALLAFCSCLVVFRVKICRKEARKRAAAEQDVP
STLGPISQGHQHECSAGSSQDHPPPGAATYTPGKGEEQELHYASLSFQGLRLWEPADQEAP
STTEYSEIKIHTGQPLRGPGFGLQLEREMSGMVPK

>Q9UGF6 multipass membrane protein
MERKNQTAITEFIILGFSNLNELQFLLFTIFFLTYFCTLGGNILIILTTVTDPHLHTPMYYFLG
NLAFIDICYTTSNVPQMMVHLLSKKKSISYVGCVVQLFAFVFFVGSECLLLAAMAYDRYI
AICNPLRYSVILSKVLCNQLAASCWAAGFLNSVVHTVLTFCLPFCGNNQINYFFCDIPPLLI
LSCGNTSVNELALLSTGVFIGWTPFLCIVLSYICIISTILRIQSSEGRRKAFSTCASHLAIVFLF
YGSAIFTYVRPISTYSLKKDRLVSVLYSVVTPMLNPIIYTLRNKDIKEAVKTIGSKWQPPISS
LDSKLTY

>P24046 multipass membrane protein
MLAVPNMRFGIFLLWWGWVLATESRMHWPGREVHEMSKKGRPQRQRREVHEDAHKQV
SPILRRSPDITKSPLTKSEQLLRIDDHDFSMRPGFGGPAIPVGVDVQVESLDSISEVDMDFT
MTLYLRHYWKDERLSFPSTNNLSMTFDGRLVKKIWVPDMFFVHSKRSFIHDTTTDNVML
RVQPDGKVLYSLRVTVTAMCNMDFSRFPLDTQTCSLEIESYAYTEDDLMLYWKKGNDSL
KTDERISLSQFLIQEFHTTTKLAFYSSTGWYNRLYINFTLRRHIFFFLLQTYFPATLMVMLS
WVSFWIDRRAVPARVPLGITTVLTMSTIITGVNASMPRVSYIKAVDIYLWVSFVFVFLSVLE
YAAVNYLTTVQERKEQKLREKLPCTSGLPPPRTAMLDGNYSDGEVNDLDNYMPENGEKP
DRMMVQLTLASERSSPQRKSQRSSYVSMRIDTHAIDKYSRIIFPAAYILFNLIYWSIFS

>Q6IF82 multipass membrane protein
MEPRKNVTDFVLLGFTQNPKEQKVLFVMFLLFYILTMVGNLLIVVTVTVSETLGSPMYFF
LAGLSFIDIIYSSSISPRLISGLFFGNNSISFQSCMAQLFIEHIFGGSEVFLLLVMAYDCYVAIC
KPLHYLVIMRQWVCVVLLVVSWVGGFLHSVFQLSIIYGLPFCGPNVIDHFFCDMYPLLKL
VCTDTHAIGLLVVANGGLACTIVFLLLLISYGVILHSLKNLSQKGRQKALSTCSSHMTVVV
FFFVPCIFMYARPARTFPIDKSVSVFYTVITPMLNPLIYTLRNSEMTSAMKKLWRRDLISSST

>O94973 peripheral membrane protein
MPAVSKGDGMRGLAVFISDIRNCKSKEAEIKRINKELANIRSKFKGDKALDGYSKKKYVC
KLLFIFLLGHDIDFGHMEAVNLLSSNRYTEKQIGYLFISVLVNSNSELIRLINNAIKNDLASR
NPTFMGLALHCIASVGSREMAEAFAGEIPKVLVAGDTMDSVKQSAALCLLRLYRTSPDLV
PMGDWTSRVVHLLNDQHLGVVTAATSLITTLAQKNPEEFKTSVSLAVSRLSRIVTSASTDL
QDYTYYFVPAPWLSVKLLRLLQCYPPPDPAVRGRLTECLETILNKAQEPPKSKKVQHSNA
KNAVLFEAISLIIHHDSEPNLLVRACNQLGQFLQHRETNLRYLALESMCTLASSEFSHEAVK
THIETVINALKTERDVSVRQRAVDLLYAMCDRSNAPQIVAEMLSYLETADYSIREEIVLKVA

ILAEKYAVDYTWYVDTILNLIRIAGDYVSEEVWYRVIQIVINRDDVQGYAAKTVFEALQAP
ACHENLVKVGGYILGEFGNLIAGDPRSSPLIQFHLLHSKFHLCSVPTRALLLSTYIKFVNLF
PEVKPTIQDVLRSDSQLRNADVELQQRAVEYLRLSTVASTDILATVLEEMPPFPERESSILA
KLKKKKGPSTVTDLEDTKRDRSVDVNGGPEPAPASTSAVSTPSPSADLLGLGAAPPAPAGP
PPSSGGSGLLVDVFSDSASVVAPLAPGSEDNFARFVCKNNGVLFENQLLQIGLKSEFRQNL
GRMFIFYGNKTSTQFLNFTPTLICSDDLQPNLNLQTKPVDPTVEGGAQVQQVVNIECVSDF
TEAPVLNIQFRYGGTFQNVSVQLPITLNKFFQPTEMASQDFFQRWKQLSNPQQEVQNIFKA
KHPMDTEVTKAKIIGFGSALLEEVDPNPANFVGAGIIHTKTTQIGCLLRLEPNLQAQMYRL
TLRTSKEAVSQRLCELLSAQF

>Q86VD9 multipass membrane protein
MQICGSSVASVAAGTSFQVLGPVCWQQLDLKMAVRVLWGGLSLLRVLWCLLPQTGYVHP
DEFFQSPEVMAEDILGVQAARPWEFYPSSSCRSVLFPLLISGSTFWLLRLWEELGPWPGLV
SGYALLVGPRLLLTALSFALDGAVYHLAPPMGADRWNALALLSGSYVTLVFYTRTFSNTIE
GLLFTWLLVLVSSHVTWGPTRKEPAPGPRWRSWLLGGIVAAGFFNRPTFLAFAVVPLYLW
GTRGATNPGLKSLTREALVLLPGAALTAAVFVATDSWYFSSPATSRNLVLTPVNFLHYNLNP
QNLARHGTHARLTHLAVNGFLLFGVLHAQALQAAWQRLQVGLQASAQMGLLRALGARS
LLSSPRSYLLLLYFMPLALLSAFSHQEARFLIPLLVPLVLLCSPQTQPVPWKGTVVLFNALG
ALLFGCLHQGGLVPGLEYLEQVVHAPVLPSTPTHYTLLFTHTYMPPRHLLHLPGLGAPVE
VVDMGGTEDWALCQTLKSFTRQPACQVAGGPWLCRLFVVTPGTTRRAVEKCSFPFKNET
LLFPHLTLEDPPALSSLLSGAWRDHLSLHIVELGEET

>Q8IV36 lipid-anchor
MGSTDSKLNFRKAVIQLTTKTQPVEATDDAFWDQFWADTATSVQDVFALVPAAEIRAVRE
ESPSNLATLCYKAVEKLVQGAESGCHSEKEKQIVLNCSRLLTRVLPYIFEDPDWRGFFWST
VPGAGRGGQGEEDDEHARPLAESLLLAIADLLFCPDFTVQSHRRSTVDSAEDVHSLDSCE
YIWEAGVGFAHSPQPNYIHDMNRMELLKLLLTCFSEAMYLPPAPESGSTNPWVQFFCSTE
NRHALPLFTSLLNTVCAYDPVGYGIPYNHLLFSDYREPLVEEAAQVLIVTLDHDSASSASP
TVDGTTTGTAMDDADPPGPENLFVNYLSRIHREEDFQFILKGIARLLSNPLLQTYLPNSTK
KIQFHQELLVLFWKLCDFNKKFLFFVLKSSDVLDILVPILFFLNDARADQSRVGLMHIGVFI
LLLLSGERNFGVRLNKPYSIRVPMDIPVFTGTHADLLIVVFHKIITSGHQRLQPLFDCLLTIV
VNVSPYLKSLSMVTANKLLHLLEAFSTTWFLFSAAQNHHLVFFLLEVFNNIIQYQFDGNSN
LVYAIIRKRSIFHQLANLPTDPPTIHKALQRRRRTPEPLSRTGSQEGTSMEGSRPAAPAEPGT
LKTSLVATPGIDKLTEKSQVSEDGTLRSLEPEPQQSLEDGSPAKGEPSQAWREQRRPSTSSA
SGQWSPTPEWVLSWKSKLPLQTIMRLLQVLVPQVEKICIDKGLTDESEILRFLQHGTLVGL
LPVPHPILIRKYQANSGTAMWFRTYMWGVIYLRNVDPPVWYDTDVKLFEIQRV

>Q96RU3 peripheral membrane protein
MSWGTELWDQFDNLEKHTQWGIDILEKYIKFVKERTEIELSYAKQLRNLSKKYQPKKNSK
EEEEYKYTSCKAFISNLNEMNDYAGQHEVISENMASQIIVDLARYVQELKQERKSNFHDG
RKAQQHIETCWKQLESSKRRFERDCKEADRAQQYFEKMDADINVTKADVEKARQQAQI
RHQMAEDSKADYSSILQKFNHEQHEYYHTHIPNIFQKIQEMEERRIVRMGESMKTYAEVD
RQVIPIIGKCLDGIVKAAESIDQKNDSQLVIEAYKSGFEPPGDIEFEDYTQPMKRTVSDNSLS
NSRGEGKPDLKFGGKSKGKLWPFIKKNKLMSLLTSPHQPPPPPPASASPSAVPNGPQSPKQ

QKEPLSHRFNEFMTSKPKIHCFRSLKRGLSLKLGATPEDFSNLPPEQRRKKLQQKVDELNK
EIQKEMDQRDAITKMKDVYLKNPQMGDPASLDHKLAEVSQNIEKLRVETQKFEAWLAEV
EGRLPARSEQARRQSGLYDSQNPPTVNNCAQDRESPDGSYTEEQSQESEMKVLATDFDDE
FDDEEPLPAIGTCKALYTFEGQNEGTISVVEGETLYVIEEDKGDGWTRIRRNEDEEGYVPTS
YVEVCLDKNAKDS

>P58182 multipass membrane protein
MLNTTSVTEFLLLGVTDIQELQPFLFVVFLTIYFISVTGNGAVLMIVISDPRLHSLMYFFLGN
LSYLDICYSTVTLPKMLQNFLSTHKAISFLGCISQLHFFHSLGSTESMLFAVMAFDLSVAIC
KPLRYTVIMNPQLCTQMAITIWVIGFFHALLHSVMTSRLNFCGSNRIHHFLCDIKPLLKLA
CGNTELNQWLLSTVTGTIAMGPFFLTLLSYFYIITYLFFKTRSCSMLCKALSTCASHFMVVI
LFYAPVLFTYIHPALESFMDQDRIVAIMYTVVTPVLNPLIYTLRNKEVKGALGRVIRRL

>O15389 single-pass type I membrane protein
MLPLLLLPLLWGGSLQEKPVYELQVQKSVTVQEGLCVLVPCSFSYPWRSWYSSPPLYVY
WFRDGEIPYYAEVVATNNPDRRVKPETQGRFRLLGDVQKKNCSLSIGDARMEDTGSYFFR
VERGRDVKYSYQQNKLNLEVTALIEKPDIHFLEPLESGRPTRLSCSLPGSCEAGPPLTFSWT
GNALSPLDPETTRSSELTLTPRPEDHGTNLTCQMKRQGAQVTTERTVQLNVSYAPQTITIFR
NGIALEILQNTSYLPVLEGQALRLLCDAPSNPPAHLSWFQGSPALNATPISNTGILELRRVRS
AEEGGFTCRAQHPLGFLQIFLNLSVYSLPQLLGPSCSWEAEGLHCRCSFRARPAPSLCWRL
EEKPLEGNSSQGSFKVNSSSAGPWANSSLILHGGLSSDLKVSCKAWNIYGSQSGSVLLLQG
RSNLGTGVVPAALGGAGVMALLCICLCLIFFLIVKARRKQAAGRPEKMDDEDPIMGTITS
GSRKKPWPDSPGDQASPPGDAPPLEEQKELHYASLSFSEMKSREPKDQEAPSTTEYSEIKT
SK

>Q5H8A4 multipass membrane protein
MRLGSGTFATCCVAIEVLGIAVFLRGFFPAPVRSSARAEHGAEPPAPEPSAGASSNWTTLPP
PLFSKVVIVLIDALRDDFVFGSKGVKFMPYTTYLVEKGASHSFVAEAKPPTVTMPRIKALM
TGSLPGFVDVIRNLNSPALLEDSVIRQAKAAGKRIVFYGDETWVKLFPKHFVEYDGTTSFF
VSDYTEVDNNVTRHLDKVLKRGDWDILILHYLGLDHIGHISGPNSPLIGQKLSEMDSVLM
KIHTSLQSKERETPLPNLLVLCGDHGMSETGSHGASSTEEVNTPLILISSAFERKPGDIRHPK
HVQQTDVAATLAIALGLPIPKDSVGSLLFPVVEGRPMREQLRFLHLNTVQLSKLLQENVPS
YEKDPGFEQFKMSERLHGNWIRLYLEEKHSEVLFNLGSKVLRQYLDALKTLSLSLSAQVA
QYDIYSMMVGTVVVLEVLTLLLLSVPQALRRKAELEVPLSSPGFSLLFYLVILVLSAVHVIV
CTSAESSCYFCGLSWLAAGGVMVLASALLCVIVSVLTNVLVGGNTPRKNPMHPSSRWSEL
DLLILLGTAGHVLSLGASSFVEEEHQTWYFLVNTLCLALSQETYRNYFLGDDGEPPCGLC
VEQGHDGATAAWQDGPGCDVLERDKGHGSPSTSEVLRGREKWMVLASPWLILACCRLL
RSLNQTGVQWAHRPDLGHWLTSSDHKAELSVLAALSLLVVFVLVQRGCSPVSKAALALG
LLGVYCYRAAIGSVRFPWRPDSKDISKGIIEARFVYVFVLGILFTGTKDLLKSQVIAADFKL
KTVGLWEIYSGLVLLAALLFRPHNLPVLAFSLLIQTLMTKFIWKPLRHDAAEITVMHYWF
GQAFFYFQGNSNNIATVDISAGFVGLDTYVEIPAVLLTAFGTYAGPVLWASHLVHFLSSETR
SGSALSHACFCYALICSIPVFTYIVLVTSLRYHLFIWSVFSPKLLYEGMHLLITAAVCVFFTA
MDQTRLTQS

>Q03518 multipass membrane protein
MAELLASAGSACSWDFPRAPPSFPPPAASRGGLGGTRSFRPHRGAESPRPGRDRDGVRVP
MASSRCPAPRGCRCLPGASLAWLGTVLLLLADWVLLRTALPRIFSLLVPTALPLLRVWAVG
LSRWAVLWLGACGVLRATVGSKSENAGAQGWLAALKPLAAALGLALPGLALFRELISWG
APGSADSTRLLHWGSHPTAFVVSYAAALPAAALWHKLGSLWVPGGQGGSGNPVRRLLGC
LGSETRRLSLFLVLVVLSSLGEMAIPFFTGRLTDWILQDGSADTFTRNLTLMSILTIASAVLE
FVGDGIYNNTMGHVHSHLQGEVFGAVLRQETEFFQQNQTGNIMSRVTEDTSTLSDSLSEN
LSLFLWYLVRGLCLLGIMLWGSVSLTMVTLITLPLLFLLPKKVGKWYQLLEVQVRESLAK
SSQVAIEALSAMPTVRSFANEEGEAQKFREKLQEIKTLNQKEAVAYAVNSWTTSISGMLLK
VGILYIGGQLVTSGAVSSGNLVTFVLYQMQFTQAVEVLLSIYPRVQKAVGSSEKIFEYLDRT
PRCPPSGLLTPLHLEGLVQFQDVSFAYPNRPDVLVLQGLTFTLRPGEVTALVGPNGSGKSTV
AALLQNLYQPTGGQLLLDGKPLPQYEHRYLHRQVAAVGQEPQVFGRSLQENIAYGLTQKP
TMEEITAAAVKSGAHSFISGLPQGYDTEVDEAGSQLSGGQRQAVALARALIRKPCVLILDD
ATSALDANSQLQVEQLLYESPERYSRSVLLITQHLSLVEQADHILFLEGGAIREGGTHQQL
MEKKGCYWAMVQAPADAPE

>Q6P1K1 multipass membrane protein
MAPSRLQLGLRAAYSGISSVAGFSIFLVWTVVYRQPGTAAMGGLAGVLALWVLVTHVMY
MQDYWRTWLKGLRGFFFVGVLFSAVSIAAFCTFLVLAITRHQSLTDPTSYYLSSVWSFISF
KWAFLLSLYAHRYRADFADISILSDF

>Q0VD83 peripheral membrane protein
MDFLRLYLPGLHQALRGALDSLGTFVSYLLGDAVPTVEREAQAAEELGVVAVGKTGKIVE
EEAQEDLEGLRGSQNEGAGRLRGPGDDRRHEVGSSAVEQTWGWGDGSSHGSQAERQDS
GAGETAKAARCQEPSAHLEARKKSKAGSGACQDRSGQAQERQESHEQEVNREERLRSW
EQEEEEEEVRAREPGMARGAESEWTWHGETEGKAGAVGPKAAGDNREMEQGVREADA
GETEEPGAEGAGKGEEVVVVEKACESTRAWGTWGPGAEPEDWGILGREEARTTPGREEA
RAILDGEEARTISGGEEAETASGGEEAETASGGEEAGTASGGEEAGIASGGEAGTASGGEE
AGTASGGDEAWTTSGKEEADLLGVRQTEYGAVPGERLLEATGKVWVLEEEGDEEREAEV
SPFPKQAQVLGTERTEEAAESQTAGREAVGGQEAGESFEGQVDLRGKEAEMRQDLGIRA
DRARMEELVQAEEAQEERGSSRDPVAELPSDGEAEGTADLEATPEARPEEELTGEESEAAQ
TSCGLLGVEWGGLTHSVTKGQGPELMGGAQTPTKQPEEREAGEVELMGVLALSKEEQER
SLEAGPRHAGSVKPEASEAFPGAWENRTRKDMERGNTQEDAADGEQREEEETAGGQTLA
AEAEGDRESELSEVPEAGGEGLTTQDAGCGTEEGEASVSENQELDGSTGADAGPCPSLGE
AYARETEDEEAEADRTSRRGWRLQAVAVGLPDREDAQTGSVAAGIMGGDVVPHISAAGA
GEALEGALGQGWDSKEKEEAAAGEHAGGQEFGLEGSAEEEVTGRGSQVEAFESREGGP
WGGRVEAEESAGAEDSCGLDPAGSQTARAEGMGAMVEAGGLLEKWTLLEEEAVGWQE
REQREDSEGRCGDYHPEGEAPRLLDAEGLMVTGGRRAEAKETEPESLEHVRGQEEQPTH
QAPAEAAPESVGEAETAEAMGSARGGAANSWSEAPLPGSLLDVSVPRSRVHLSRSSSQRR
SRPSFRRTPAWEQQEEPPAPNPPEEELSAPEQRPLQLEEPLEPSPLRHDGTPVPARRRPLGHG
FGLAHPGMMQELQARLGRPKPQ

>Q08379 peripheral membrane protein
MWPQPRLPPRPAMSEETRQSKLAAAKKKLREYQQRNSPGVPTGAKKKKKIKNGSNPETT

TSGGCHSPEDTPKDNAATLQPSDDTVLPGGVPSPGASLTSMAASQNHDADNVPNLMDET
KTFSSTESLRQLSQQLNGLVCESATCVNGEGPASSANLKDLESRYQQLAVALDSSYVTNKQ
LNITIEKLKQQNQEITDQLEEEKKECHQKQGALREQLQVHIQTIGILVSEKAELQTALAHTQ
HAARQKEGESEDLASRLQYSRRRVGELERALSAVSTQQKKADRYNKELTKERDALRLELY
KNTQSNEDLKQEKSELEEKLRVLVTEKAGMQLNLEELQKKLEMTELLLQQFSSRCEAPDA
NQQLQQAMEERAQLEAHLGQVMESVRQLQMERDKYAENLKGESAMWRQRMQQMSEQ
VHTLREEKECSMSRVQELETSLAELRNQMAEPPPPEPPAGPSEVEQQLQAEAEHLRKELEG
LAGQLQAQVQDNEGLSRLNREQEERLLELERAAELWGEQAEARRQILETMQNDRTTISR
ALSQNRELKEQLAELQSGFVKLTNENMEITSALQSEQHVKRELGKKLGELQEKLSELKET
VELKSQEAQSLQQQRDQYLGHLQQYVAAYQQLTSEKEVLHNQLLLQTQLVDQLQQQEA
QGKAVAEMARQELQETQERLEAATQQNQQLRAQLSLMAHPGEGDGLDREEEEDEEEEEE
EAVAVPQPMPSIPEDLESREAMVAFFNSAVASAEEEQARLRGQLKEQRVRCRRLAHLLASA
QKEPEAAAPAPGTGGDSVCGETHRALQGAMEKLQSRFMELMQEKADLKERVEELEHRCI
QLSGETDTIGEYIALYQSQRAVLKERHREKEEYISRLAQDKEEMKVKLLELQELVLRLVGD
RNEWHGRFLAAAQNPADEPTSGAPAPQELGAANQQGDLCEVSLAGSVEPAQGEAREGSP
RDNPTAQQIMQLLREMQNPRERPGLGSNPCIPFFYRADENDEVKITVI

>O15547 multipass membrane protein
MCPQLAGAGSMGSPGATTGWGLLDYKTEKYVMTRNWRVGALQRLLQFGIVVYVVGWA
LLAKKGYQERDLEPQFSIITKLKGVSVTQIKELGNRLWDVADFVKPPQGENVFFLVTNFLV
TPAQVQGRCPEHPSVPLANCWVDEDCPEGEGGTHSHGVKTGQCVVFNGTHRTCEIWSW
CPVESGVVPSRPLLAQAQNFTLFIKNTVTFSKFNFSKSNALETWDPTYFKHCRYEPQFSPY
CPVFRIGDLVAKAGGTFEDLALLGGSVGIRVHWDCDLDTGDSGCWPHYSFQLQEKSYNFR
TATHWWEQPGVEARTLLKLYGIRFDILVTGQAGKFGLIPTAVTLGTGAAWLGVVTFFCDLL
LLYVDREAHFYWRTKYEEAKAPKATANSVWRELALASQARLAECLRRSSAPAPTATAAGS
QTQTPGWPCPSSDTHLPTHSGSL

>O76002 multipass membrane protein
MMIKKNASSEDFFILLGFSNWPQLEVVLFVVILIFYLMTLTGNLFIIILSYVDSHLHTPMYFF
LSNLSFLDLCHTTSSIPQLLVNLRGPEKTISYAGCMVQLYFVLALGIAECVLLVVMSYDRYV
AVCRPLHYTVLMHPRFCHLLAAASWVIGFTISALHSSFTFWVPLCGHRLVDHFFCEVPALL
RLSCVDTHANELTLMVMSSIFVLIPLILILTAYGAIARAVLSMQSTTGLQKVFRTCGAHLMV
VSLFFIPVMCMYLQPPSENSPDQGKFIALFYTVVTPSLNPLIYTLRNKHVKGAAKRLLGWE
WGK

>O14931 single-pass type I membrane protein
MAWMLLLILIMVHPGSCALWVSQPPEIRTLEGSSAFLPCSFNASQGRLAIGSVTWFRDEVV
PGKEVRNGTPEFRGRLAPLASSRFLHDHQAELHIRDVRGHDASIYVCRVEVLGLGVGTGN
GTRLVVEKEHPQLGAGTVLLLRAGFYAVSFLSVAVGSTVYYQGKCLTWKGPRRQLPAVVP
APLPPPCGSSAHLLPPVPGG

>Q15125 multipass membrane protein
MTTNAGPLHPYWPQHLRLDNFVPNDRPTWHILAGLFSVTGVLVVTTWLLSGRAAVVPLG
TWRRLSLCWFAVCGFIHLVIEGWFVLYYEDLLGDQAFLSQLWKEYAKGDSRYILGDNFTV

CMETITACLWGPLSLWVVIAFLRQHPLRFILQLVVSVGQIYGDVLYFLTEHRDGFQHGELG
HPLYFWFYFVFMNALWLVLPGVLVLDAVKHLTHAQSTLDAKATKAKSKKN

>Q16348 multipass membrane protein
MNPFQKNESKETLFSPVSIEEVPPRPPSPPKKPSPTICGSNYPLSIAFIVVNEFCERFSYYGM
KAVLILYFLYFLHWNEDTSTSIYHAFSSLCYFTPILGAAIADSWLGKFKTIIYLSLVYVLGHV
IKSLGALPILGGQVVHTVLSLIGLSLIALGTGGIKPCVAAFGGDQFEEKHAEERTRYFSVFY
LSINAGSLISTFITPMLRGDVQCFGEDCYALAFGVPGLLMVIALVVFAMGSKIYNKPPPEGN
IVAQVFKCIWFAISNRFKNRSGDIPKRQHWLDWAAEKYPKQLIMDVKALTRVLFLYIPLPM
FWALLDQQGSRWTLQAIRMNRNLGFFVLQPDQMQVLNPLLVLIFIPLFDFVIYRLVSKCGI
NFSSLRKMAVGMILACLAFAVAAAVEIKINEMAPAQPGPQEVFLQVLNLADDEVKVTVVG
NENNSLLIESIKSFQKTPHYSKLHLKTKSQDFHFHLKYHNLSLYTEHSVQEKNWYSLVIRE
DGNSISSMMVKDTESRTTNGMTTVRFVNTLHKDVNISLSTDTSLNVGEDYGVSAYRTVQ
RGEYPAVHCRTEDKNFSLNLGLLDFGAAYLFVITNNTNQGLQAWKIEDIPANKMSIAWQLP
QYALVTAGEVMFSVTGLEFSYSQAPSSMKSVLQAAWLLTIAVGNIIVLVVAQFSGLVQWAE
FILFSCLLLVICLIFSIMGYYYVPVKTEDMRGPADKHIPHIQGNMIKLETKKTKL

>O00461 single-pass type II membrane protein
MGNGMCSRKQKRIFQTLLLLTVVFGFLYGAMLYYELQTQLRKAEAVALKYQQHQESLSA
QLQVVYEHRSRLEKSLQKERLEHKKAKEDFLVYKLEAQETLNKGRQDSNSRYSALNVQH
QMLKSQHEELKKQHSDLEEEHRKQGEDFSRTFNDHKQKYLQLQQEKEQELSKLKETVYN
LREENRQLRKAHQDIHTQLQDVKQQHKNLLSEHEQLVVTLEDHKSALAAAQTQVAEYK
QLKDTLNRIPSLRKPDPAEQQNVTQVAHSPQGYNTAREKPTREVQEVSRNNDVWQNHEA
VPGRAEDTKLYAPTHKEAEFQAPPEPIQQEVERREPEEHQVEEEHRKALEEEEMEQVGQA
EHLEEEHDPSPEEQDREWKEQHEQREAANLLEGHARAEVYPSAKPMIKFQSPYEEQLEQ
QRLAVQQVEEAQQLREHQEALHQQRLQGHLLRQQEQQQQVAREMALQRQAELEEGRP
QHQEQLRQQAHYDAMDNDIVQGAEDQGIQGEEGAYERDNQHQDEAEGDPGNRHEPREQ
GPREADPESEADRAAVEDINPADDPNNQGEDEFEEAEQVREENLPDENEEQKQSNQKQEN
TEVEEHLVMAGNPDQQEDNVDEQYQEEAEEEVQEDLTEEKKRELEHNAEETYGENDENT
DDKNNDGEEQEVRDDNRPKGREEHYEEEEEEEEDGAAVAEKSHRRAEM

>Q30201 single-pass type I membrane protein
MGPRARPALLLLMLLQTAVLQGRLLRSHSLHYLFMGASEQDLGLSLFEALGYVDDQLFVF
YDHESRRVEPRTPWVSSRISSQMWLQLSQSLKGWDHMFTVDFWTIMENHNHSKESHTLQ
VILGCEMQEDNSTEGYWKYGYDGQDHLEFCPDTLDWRAAEPRAWPTKLEWERHKIRAR
QNRAYLERDCPAQLQQLLELGRGVLDQQVPPLVKVTHHVTSSVTTLRCRALNYYPQNIT
MKWLKDKQPMDAKEFEPKDVLPNGDGTYQGWITLAVPPGEEQRYTCQVEHPGLDQPLIV
IWEPSPSGTLVIGVISGIAVFVVILFIGILFIILRKRQGSRGAMGHYVLAERE

>Q9UKC9 lipid-anchor
MVFSNNDEGLINKKLPKELLLRIFSFLDIVTLCRCAQISKAWNILALDGSNWQRIDLFNFQT
DVEGRVVENISKRCGGFLRKLSLRGCIGVGDSSLKTFAQNCRNIEHLNLNGCTKITDSTCY
SLSRFCSKLKHLDLTSCVSITNSSLKGISEGCRNLEYLNLSWCDQITKDGIEALVRGCRGLK
ALLLRGCTQLEDEALKHIQNYCHELVSLNLQSCSRITDEGVVQICRGCHRLQALCLSGCSN

LTDASLTALGLNCPRLQILEAARCSHLTDAGFTLLARNCHELEKMDLEECILITDSTLIQLSI
HCPKLQALSLSHCELITDDGILHLSNSTCGHERLRVLELDNCLLITDVALEHLENCRGLERL
ELYDCQQVTRAGIKRMRAQLPHVKVHAYFAPVTPPTAVAGSGQRLCRCCVIL

>Q969T3 peripheral membrane protein
MHRGTQEGAMASRLLHRLRHALAGDGPGEAAASPEAEQFPESSELEDDDAEGLSSRLSG
TLSFTSAEDDEDDEDEDDEEAGPDQLPLGDGTSGEDAERSPPPDGQWGSQLLARQLQDF
WKKSRNTLAPQRLLFEVTSANVVKDPPSKYVLYTLAVIGPGPPDCQPAQISRRYSDFERLH
RNLQRQFRGPMAAISFPRKRLRRNFTAETIARRSRAFEQFLGHLQAVPELRHAPDLQDFFV
LPELRRAQSLTCTGLYREALALWANAWQLQAQLGTPSGPDRPLLTLAGLAVCHQELEDPG
EARACCEKALQLLGDKSLHPLLAPFLEAHVRLSWRLGLDKRQSEARLQALQEAGLTPTPP
PSLKELLIKEVLD

>Q13449 GPI-anchor, lipid-anchor
MVRRVQPDRKQLPLVLLRLLCLLPTGLPVRSVDFNRGTDNITVRQGDTAILRCVVEDKNS
KVAWLNRSGIIFAGHDKWSLDPRVELEKRHSLEYSLRIQKVDVYDEGSYTCSVQTQHEPK
TSQVYLIVQVPPKISNISSDVTVNEGSNVTLVCMANGRPEPVITWRHLTPTGREFEGEEEYL
EILGITREQSGKYECKAANEVSSADVKQVKVTVNYPPTITESKSNEATTGRQASLKCEASA
VPAPDFEWYRDDTRINSANGLEIKSTEGQSSLTVTNVTEEHYGNYTCVAANKLGVTNASL
VLFRPGSVRGINGSISLAVPLWLLAASLLCLLSKC

>Q96KK4 multipass membrane protein
MSANTSMVTEFLLLGFSHLADLQGLLFSVFLTIYLLTVAGNFLIVVLVSTDAALQSPMYFFL
RTLSALEIGYTSVTVPLLLHHLLTGRRHISRSGCALQMFFFLFFGATECCLLAAMAYDRYA
AICEPLRYPLLLSHRVCLQLAGSAWACGVLVGLGHTPFIFSLPFCGPNTIPQFFCEIQPVLQL
VCGDTSLNELQIILATALLILCPFGLILGSYGRILVTIFRIPSVAGRRKAFSTCSSHLIMVSLFY
GTALFIYIRPKASYDPATDPLVSLFYAVVTPILNPIIYSLRNTEVKAALKRTIQKTVPMEI

>O95622 multipass membrane protein
MSGSKSVSPPGYAAQKTAAPAPRGGPEHRSAWGEADSRANGYPHAPGGSARGSTKKPGG
AVTPQQQQRLASRWRSDDDDDPPLSGDDPLAGGFGFSFRSKSAWQERGGDDCGRGSRRQ
RRGAASGGSTRAPPAGGGGGSAAAAASAGGTEVRPRSVEVGLEERRGKGRAADELEAGA
VEGGEGSGDGGSSADSGSGAGPGAVLSLGACCLALLQIFRSKKFPSDKLERLYQRYFFRLN
QSSLTMLMAVLVLVCLVMLAFHAARPPLQLPYLAVLAAAVGVILIMAVLCNRAAFHQDH
MGLACYALIAVVLAVQVVGLLLPQPRSASEGIWWTVFFIYTIYTLLPVRMRAAVLSGVLLS
ALHLAIALRTNAQDQFLLKQLVSNVLIFSCTNIVGVCTHYPAEVSQRQAFQETRECIQARL
HSQRENQQQERLLLSVLPRHVAMEMKADINAKQEDMMFHKIYIQKHDNVSILFADIEGFT
SLASQCTAQELVMTLNELFARFDKLAAENHCLRIKILGDCYYCVSGLPEARADHAHCCVE
MGMDMIEAISLVREVTGVNVNMRVGIHSGRVHCGVLGLRKWQFDVWSNDVTLANHME
AGGKAGRIHITKATLNYLNGDYEVEPGCGGERNAYLKEHSIETFLILRCTQKRKEEKAMIA
KMNRQRTNSIGHNPPHWGAERPFYNHLGGNQVSKEMKRMGFEDPKDKNAQESANPEDE
VDEFLGRAIDARSIDRLRSEHVRKFLLTFREPDLEKKYSKQVDDRFGAYVACASLVFLFICF
VQITIVPHSIFMLSFYLTCSLLLTLVVFVSVIYSCVKLFPSPLQTLSRKIVRSKMNSTLVGVFT
ITLVFLAAFVNMFTCNSRDLLGCLAQEHNISASQVNACHVAESAVNYSLGDEQGFCGSPW

PNCNFPEYFTYSVLLSLLACSVFLQISCIGKLVLMLAIELIYVLIVEVPGVTLFDNADLLVTA
NAIDFFNNGTSQCPEHATKVALKVVTPIIISVFVLALYLHAQQVESTARLDFLWKLQATEEK
EEMEELQAYNRRLLHNILPKDVAAHFLARERRNDELYYQSCECVAVMFASIANFSEFYVEL
EANNEGVECLRLLNEIIADFDEIISEDRFRQLEKIKTIGSTYMAASGLNDSTYDKVGKTHIK
ALADFAMKLMDQMKYINEHSFNNFQMKIGLNIGPVVAGVIGARKPQYDIWGNTVNVASR
MDSTGVPDRIQVTTDMYQVLAANTYQLECRGVVKVKGKGEMMTYFLNGGPPLS

>O95674 multipass membrane protein
MTELRQRVAHEPVAPPEDKESESEAKVDGETASDSESRAESAPLPVSADDTPEVLNRALSN
LSSRWKNWWVRGILTLAMIAFFFIIIYLGPMVLMIIVMCVQIKCFHEIITIGYNVYHSYDLP
WFRTLSWYFLLCVNYFFYGETVTDYFFTLVQREEPLRILSKYHRFISFTLYLIGFCMFVLSL
VKKHYRLQFYMFGWTHVTLLIVVTQSHLVIHNLFEGMIWFIVPISCVICNDIMAYMFGFFF
GRTPLIKLSPKKTWEGFIGGFFATVVFGLLLSYVMSGYRCFVCPVEYNNDTNSFTVDCEPS
DLFRLQEYNIPGVIQSVIGWKTVRMYPFQIHSIALSTFASLIGPFGGFFASGFKRAFKIKDFA
NTIPGHGGIMDRFDCQYLMATFVNVYIASFIRGPNPSKLIQQFLTLRPDQQLHIFNTLRSHLI
DKGMLTSTTEDE

>P41217 single-pass type I membrane protein
MERLVIRMPFSHLSTYSLVWVMAAVVLCTAQVQVVTQDEREQLYTPASLKCSLQNAQEA
LIVTWQKKKAVSPENMVTFSENHGVVIQPAYKDKINITQLGLQNSTITFWNITLEDEGCYM
CLFNTFGFGKISGTACLTVYVQPIVSLHYKFSEDHLNITCSATARPAPMVFWKVPRSGIENS
TVTLSHPNGTTSVTSILHIKDPKNQVGKEVICQVLHLGTVTDFKQTVNKGYWFSVPLLLSI
VSLVILLVLISILLYWKRHRNQDRGELSQGVQKMT

>Q8N766 single-pass type I membrane protein
MAAEWASRFWLWATLLIPAAAVYEDQVGKFDWRQQYVGKVKFASLEFSPGSKKLVVATE
KNVIAALNSRTGEILWRHVDKGTAEGAVDAMLLHGQDVITVSNGGRIMRSWETNIGGLN
WEITLDSGSFQALGLVGLQESVRYIAVLKKTTLALHHLSSGHLKWVEHLPESDSIHYQMV
YSYGSGVVWALGVVPFSHVNIVKFNVEDGEIVQQVRVSTPWLQHLSGACGVVDEAVLVC
PDPSSRSLQTLALETEWELRQIPLQSLDLEFGSGFQPRVLPTQPNPVDASRAQFFLHLSPSH
YALLQYHYGTLSLLKNFPQTALVSFATTGEKTVAAVMACRNEVQKSSSSEDGSMGSFSEK
SSSKDSLACFNQTYTINLYLVETGRRLLDTTITFSLEQSGTRPERLYIQVFLKKDDSVGYRA
LVQTEDHLLLFLQQLAGKVVLWSREESLAEVVCLEMVDLPLTGAQAELEGEFGKKADGL
LGMFLKRLSSQLILLQAWTSHLWKMFYDARKPRSQIKNEINIDTLARDEFNLQKMMVMV
TASGKLFGIESSSGTILWKQYLPNVKPDSSFKLMVQRTTAHFPHPPQCTLLVKDKESGMSS
LYVFNPIFGKWSQVAPPVLKRPILQSLLLPVMDQDYAKVLLLIDDEYKVTAFPATRNVLRQ
LHELAPSIFFYLVDAEQGRLCGYRLRKDLTTELSWELTIPPEVQRIVKVKGKRSSEHVHSQ
GRVMGDRSVLYKSLNPNLLAVVTESTDAHHERTFIGIFLIDGVTGRIIHSSVQKKAKGPVHI
VHSENWVVYQYWNTKARRNEFTVLELYEGTEQYNATAFSSLDRPQLPQVLQQSYIFPSSIS
AMEATITERGITSRHLLIGLPSGAILSLPKALLDPRRPEIPTEQSREENLIPYSPDVQIHAERFI
NYNQTVSRMRGIYTAPSGLESTCLVVAYGLDIYQTRVYPSKQFDVLKDDYDYVLISSVLFG
LVFATMITKRLAQVKLLNRAWR

(2) Protein IDs, sequences and types in dataset S2

>P20645 single-pass type I membrane protein
MFPFYSCWRTGLLLLLLAVAVRESWQTEEKTCDLVGEKGKESEKELALVKRLKPLFNKSF
ESTVGQGSDTYIYIFRVCREAGNHTSGAGLVQINKSNGKETVVGRLNETHIFNGSNWIMLI
YKGGDEYDNHCGKEQRRAVVMISCNRHTLADNFNPVSEERGKVQDCFYLFEMDSSLACS
PEISHLSVGSILLVTFASLVAVYVVGGFLYQRLVVGAKGMEQFPHLAFWQDLGNLVADGC
DFVCRSKPRNVPAAYRGVGDDQLGEESEERDDHLLPM

>P13569 multipass membrane protein
MQRSPLEKASVVSKLFFSWTRPILRKGYRQRLELSDIYQIPSVDSADNLSEKLEREWDREL
ASKKNPKLINALRRCFFWRFMFYGIFLYLGEVTKAVQPLLLGRIIASYDPDNKEERSIAIYL
GIGLCLLFIVRTLLLHPAIFGLHHIGMQMRIAMFSLIYKKTLKLSSRVLDKISIGQLVSLLSN
NLNKFDEGLALAHFVWIAPLQVALLMGLIWELLQASAFCGLGFLIVLALFQAGLGRMMM
KYRDQRAGKISERLVITSEMIENIQSVKAYCWEEAMEKMIENLRQTELKLTRKAAYVRYF
NSSAFFFSGFFVVFLSVLPYALIKGIILRKIFTTISFCIVLRMAVTRQFPWAVQTWYDSLGAIN
KIQDFLQKQEYKTLEYNLTTTEVVMENVTAFWEEGFGELFEKAKQNNNNRKTSNGDDSL
FFSNFSLLGTPVLKDINFKIERGQLLAVAGSTGAGKTSLLMVIMGELEPSEGKIKHSGRISFC
SQFSWIMPGTIKENIIFGVSYDEYRYRSVIKACQLEEDISKFAEKDNIVLGEGGITLSGGQRA
RISLARAVYKDADLYLLDSPFGYLDVLTEKEIFESCVCKLMANKTRILVTSKMEHLKKAD
KILILHEGSSYFYGTFSELQNLQPDFSSKLMGCDSFDQFSAERRNSILTETLHRFSLEGDAP
VSWTETKKQSFKQTGEFGEKRKNSILNPINSIRKFSIVQKTPLQMNGIEEDSDEPLERRLSL
VPDSEQGEAILPRISVISTGPTLQARRRQSVLNLMTHSVNQGQNIHRKTTASTRKVSLAPQ
ANLTELDIYSRRLSQETGLEISEEINEEDLKECFFDDMESIPAVTTWNTYLRYITVHKSLIFV
LIWCLVIFLAEVAASLVVLWLLGNTPLQDKGNSTHSRNNSYAVIITSTSSYYVFYIYVGVAD
TLLAMGFFRGLPLVHTLITVSKILHHKMLHSVLQAPMSTLNTLKAGGILNRFSKDIAILDD
LLPLTIFDFIQLLLIVIGAIAVVAVLQPYIFVATVPVIVAFIMLRAYFLQTSQQLKQLESEGRSPI
FTHLVTSLKGLWTLRAFGRQPYFETLFHKALNLHTANWFLYLSTLRWFQMRIEMIFVIFFIA
VTFISILTTGEGEGRVGIILTLAMNIMSTLQWAVNSSIDVDSLMRSVSRVFKFIDMPTEGKPT
KSTKPYKNGQLSKVMIIENSHVKKDDIWPSGGQMTVKDLTAKYTEGGNAILENISFSISPG
QRVGLLGRTGSGKSTLLSAFLRLLNTEGEIQIDGVSWDSITLQQWRKAFGVIPQKVFIFSGT
FRKNLDPYEQWSDQEIWKVADEVGLRSVIEQFPGKLDFVLVDGGCVLSHGHKQLMCLAR
SVLSKAKILLLDEPSAHLDPVTYQIIRRTLKQAFADCTVILCEHRIEAMLECQQFLVIEENK
VRQYDSIQKLLNERSLFRQAISPSDRVKLFPHRNSSKCKSKPQIAALKEETEEEVQDTRL

>O14641 peripheral membrane protein
MAGSSTGGGGVGETKVIYHLDEEETPYLVKIPVPAERITLGDFKSVLQRPAGAKYFFKSM
DQDFGVVKEEISDDNARLPCFNGRVVSWLVSSDNPQPEMAPPVHEPRAELAPPAPPLPPLP
PERTSGIGDSRPPSFHPNVSSSHENLEPETETESVVSLRRERPRRRDSSEHGAGGHRTGGPS
RLERHLAGYESSSTLMTSELESTSLGDSDEEDTMSRFSSSTEQSSASRLLKRHRRRRKQRP
PRLERTSSFSSVTDSTMSLNIITVTLNMEKYNFLGISIVGQSNERGDGGIYIGSIMKGGAVAA
DGRIEPGDMLLQVNDMNFENMSNDDAVRVLRDIVHKPGPIVLTVAKCWDPSPQAYFTLPR
NEPIQPIDPAAWVSHSAALTGTFPAYPGSSSMSTITSGSSLPDGCEGRGLSVHTDMASVTKA
MAAPESGLEVRDRMWLKITIPNAFLGSDVVDWLYHHVEGFPERREARKYASGLLKAGLI
RHTVNKITFSEQCYYVFGDLSGGCESYLVNLSLNDNDGSSGASDQDTLAPLPGATPWPLL

PTFSYQYPAPHPYSPQPPPYHELSSYTYGGGSASSQHSEGSRSSGSTRSDGGAGRTGRPEER
APESKSGSGSESEPSSRGGSLRRGGEASGTSDGGPPPSRGSTGGAPNLRAHPGLHPYGPPP
GMALPYNPMMVVMMPPPPPPVPPAVQPPGAPPVRDLGSVPPELTASRQSFHMAMGNPSEF
FVDVM

>P78423 single-pass type I membrane protein
MAPISLSWLLRLATFCHLTVLLAGQHHGVTKCNITCSKMTSKIPVALLIHYQQNQASCGKR
AIILETRQHRLFCADPKEQWVKDAMQHLDRQAAALTRNGGTFEKQIGEVKPRTTPAAGG
MDESVVLEPEATGESSSLEPTPSSQEAQRALGTSPELPTGVTGSSGTRLPPTPKAQDGGPV
GTELFRVPPVSTAATWQSSAPHQPGPSLWAEAKTSEAPSTQDPSTQASTASSPAPEENAPSE
GQRVWGQGQSPRPENSLEREEMGPVPAHTDAFQDWGPGSMAHVSVVPVSSEGTPSREPV
ASGSWTPKAEEPIHATMDPQRLGVLITPVPDAQAATRRQAVGLLAFLGLLFCLGVAMFTY
QSLQGCPRKMAGEMAEGLRYIPRSCGSNSYVLVPV

>Q9P299 peripheral membrane protein
MQRPEAWPRPHPGEGAAAAQAGGPAPPARAGEPSGLRLQEPSLYTIKAVFILDNDGRRLL
AKYYDDTFPSMKEQMVFEKNVFNKTSRTESEIAFFGGMTIVYKNSIDLFLYVVGSSYENE
LMLMSVLTCLFESLNHMLRKNVEKRWLLENMDGAFLVLDEIVDGGVILESDPQQVIQKV
NFRADDGGLTEQSVAQVLQSAKEQIKWSLLK

>P26006 single-pass type I membrane protein
MGPGPSRAPRAPRLMLCALALMVAAGGCVVSAFNLDTRFLVVKEAGNPGSLFGYSVALH
RQTERQQRYLLLAGAPRELAVPDGYTNRTGAVYLCPLTAHKDDCERMNITVKNDPGHHII
EDMWLGVTVASQGPAGRVLVCAHRYTQVLWSGSEDQRRMVGKCYVRGNDLELDSSDD
WQTYHNEMCNSNTDYLETGMCQLGTSGGFTQNTVYFGAPGAYNWKGNSYMIQRKEWD
LSEYSYKDPEDQGNLYIGYTMQVGSFILHPKNITIVTGAPRHRHMGAVFLLSQEAGGDLRR
RQVLEGSQVGAYFGSAIALADLNNDGWQDLLVGAPYYFERKEEVGGAIYVFMNQAGTSF
PAHPSLLLHGPSGSAFGLSVASIGDINQDGFQDIAVGAPFEGLGKVYIYHSSSKGLLRQPQQ
VIHGEKLGLPGLATFGYSLSGQMDVDENFYPDLLVGSLSDHIVLLRARPVINIVHKTLVPRP
AVLDPALCTATSCVQVELCFAYNQSAGNPNYRRNITLAYTLEADRDRRPPRLRFAGSESAV
FHGFFSMPEMRCQKLELLLMDNLRDKLRPIIISMNYSLPLRMPDRPRLGLRSLDAYPILNQ
AQALENHTEVQFQKECGPDNKCESNLQMRAAFVSEQQQKLSRLQYSRDVRKLLLSINVT
NTRTSERSGEDAHEALLTLVVPPALLLSSVRPPGACQANETIFCELGNPFKRNQRMELLIAF
EVIGVTLHTRDLQVQLQLSTSSHQDNLWPMILTLLVDYTLQTSLSMVNHRLQSFFGGTVM
GESGMKTVEDVGSPLKYEFQVGPMGEGLVGLGTLVLGLEWPYEVSNGKWLLYPTEITVH
GNGSWPCRPPGDLINPLNLTLSDPGDRPSSPQRRRRQLDPGGGQGPPPVTLAAAKKAKSE
TVLTCATGRAHCVWLECPIPDAPVVTNVTVKARVWNSTFIEDYRDFDRVRVNGWATLFLR
TSIPTINMENKTTWFSVDIDSELVEELPAEIELWLVLVAVGAGLLLLGLIILLLWKCGFFKRA
RTRALYEAKRQKAEMKSQPSETERLTDDY

>P01730 single-pass type I membrane protein
MNRGVPFRHLLLVLQLALLPAATQGKKVVLGKKGDTVELTCTASQKKSIQFHWKNSNQI
KILGNQGSFLTKGPSKLNDRADSRRSLWDQGNFPLIIKNLKIEDSDTYICEVEDQKEEVQLL
VFGLTANSDTHLLQGQSLTLTLESPPGSSPSVQCRSPRGKNIQGGKTLSVSQLELQDSGTW

TCTVLQNQKKVEFKIDIVVLAFQKASSIVYKKEGEQVEFSFPLAFTVEKLTGSGELWWQA
ERASSSKSWITFDLKNKEVSVKRVTQDPKLQMGKKLPLHLTLPQALPQYAGSGNLTLALE
AKTGKLHQEVNLVVMRATQLQKNLTCEVWGPTSPKLMLSLKLENKEAKVSKREKAVWV
LNPEAGMWQCLLSDSGQVLLESNIKVLPTWSTPVQPMALIVLGGVAGLLLFIGLGIFFCVR
CRHRRRQAERMSQIKRLLSEKKTCQCPHRFQKTCSPI

>Q9NXS2 single-pass type I membrane protein
MRSGGRGRPRLRLGERGLMEPLLPPKRRLLPRVRLLPLLLALAVGSAFYTIWSGWHRRTE
ELPLGRELRVPLIGSLPEARLRRVVGQLDPQRLWSTYLRPLLVVRTPGSPGNLQVRKFLEA
TLRSLTAGWHVELDPFTASTPLGPVDFGNVVATLDPRAARHLTLACHYDSKLFPPGSTPFV
GATDSAVPCALLLELAQALDLELSRAKKQAAPVTLQLLFLDGEEALKEWGPKDSLYGSRH
LAQLMESIPHSPGPTRIQAIELFMLLDLLGAPNPTFYSHFPRTVRWFHRLRSIEKRLHRLNL
LQSHPQEVMYFQPGEPFGSVEDDHIPFLRRGVPVLHLISTPFPAVWHTPADTEVNLHPPTV
HNLCRILAVFLAEYLGL

>Q8NFJ5 multipass membrane protein
MATTVPDGCRNGLKSKYYRLCDKAEAWGIVLETVATAGVVTSVAFMLTLPILVCKVQDSN
RRKMLPTQFLFLLGVLGIFGLTFAFIIGLDGSTGPTRFFLFGILFSICFSCLLAHAVSLTKLVR
GRKPLSLLVILGLAVGFSLVQDVIAIEYIVLTMNRTNVNVFSELSAPRRNEDFVLLLTYVLF
LMALTFLMSSFTFCGSFTGWKRHGAHIYLTMLLSIAIWVAWITLLMLPDFDRRWDDTILSS
ALAANGWVFLLAYVSPEFWLLTKQRNPMDYPVEDAFCKPQLVKKSYGVENRAYSQEEIT
QGFEETGDTLYAPYSTHFQLQNQPPQKEFSIPRAHAWPSPYKDYEVKKEGS

>Q7KZN9 multipass membrane protein
MQRLLFPPLRALKGRQYLPLLAPRAAPRAQCDCIRRPLRPGQYSTISEVALQSGRGTVSLP
SKAAERVVGRWLLVCSGTVAGAVILGGVTRLTESGLSMVDWHLIKEMKPPTSQEEWEAEF
QRYQQFPEFKILNHDMTLTEFKFIWYMEYSHRMWGRLVGLVYILPAAYFWRKGWLSRGM
KGRVLALCGLVCFQGLLGWYMVKSGLEEKSDSHDIPRVSQYRLAAHLGSALVLYCASLW
TSLSLLLPPHKLPETHQLLQLRRFAHGTAGLVFLTALSGAFVAGLDAGLVYNSFPKMGESW
IPEDLFTFSPILRNVFENPTMVQFDHRILGITSVTAITVLYFLSRRIPLPRRTKMAAVTLLALA
YTQVGLGISTLLMYVPTPLAATHQSGSLALLTGALWLMNELRRVPK

>Q9NXJ0 multipass membrane protein
MMSSKPTSHAEVNETIPNPYPPSSFMAPGFQQPLGSINLENQAQGAQRAQPYGITSPGIFAS
SQPGQGNIQMINPSVGTAVMNFKEEAKALGVIQIMVGLMHIGFGIVLCLISFSFREVLGFAS
TAVIGGYPFWGGLSFIISGSLSVSASKELSRCLVKGSLGMNIVSSILAFIGVILLLVDMCINGV
AGQDYWAVLSGKGISATLMIFSLLEFFVACATAHFANQANTTTNMSVLVIPNMYESNPVTP
ASSSAPPRCNNYSANAPK

>P47872 multipass membrane protein
MRPHLSPPLQQLLLPVLLACAAHSTGALPRLCDVLQVLWEEQDQCLQELSREQTGDLGTE
QPVPGCEGMWDNISCWPSSVPGRMVEVECPRFLRMLTSRNGSLFRNCTQDGWSETFPRP
NLACGVNVNDSSNEKRHSYLLKLKVMYTVGYSSSLVMLLVALGILCAFRRLHCTRNYIH
MHLFVSFILRALSNFIKDAVLFSSDDVTYCDAHRAGCKLVMVLFQYCIMANYSWLLVEGL

YLHTLLAISFFSERKYLQGFVAFGWGSPAIFVALWAIARHFLEDVGCWDINANASIWWIIR
GPVILSILINFILFINILRILMRKLRTQETRGNEVSHYKRLARSTLLLIPLFGIHYIVFAFSPED
AMEIQLFFELALGSFQGLVVAVLYCFLNGEVQLEVQKKWQQWHLREFPLHPVASFSNSTK
ASHLEQSQGTCRTSII

>Q15311 peripheral membrane protein
MTECFLPPTSSPSEHRRVEHGSGLTRTPSSEEISPTKFPGLYRTGEPSPPHDILHEPPDVVSDD
EKDHGKKKGKFKKKEKRTEGYAAFQEDSSGDEAESPSKMKRSKGIHVFKKPSFSKKKEK
DFKIKEKPKEEKHKEEKHKEEKHKEKKSKDLTAADVVKQWKEKKKKKKPIQEPEVPQID
VPNLKPIFGIPLADAVERTMMYDGIRLPAVFRECIDYVEKYGMKCEGIYRVSGIKSKVDEL
KAAYDREESTNLEDYEPNTVASLLKQYLRDLPENLLTKELMPRFEEACGRTTETEKVQEF
QRLLKELPECNYLLISWLIVHMDHVIAKELETKMNIQNISIVLSPTVQISNRVLYVFFTHVQ
ELFGNVVLKQVMKPLRWSNMATMPTLPETQAGIKEEIRRQEFLLNCLHRDLQGGIKDLSK
EERLWEVQRILTALKRKLREAKRQECETKIAQEIASLSKEDVSKEEMNENEEVINILLAQE
NEILTEQEELLAMEQFLRRQIASEKEEIERLRAEIAEIQSRQQHGRSETEEYSSESESESEDEE
ELQIILEDLQRQNEELEIKNNHLNQAIHEEREAIIELRVQLRLLQMQRAKAEQQAQEDEEPE
WRGGAVQPPRDGVLEPKAAKEQPKAGKEPAKPSPSRDRKETSI

>Q9UBV7 single-pass type II membrane protein
MFPSRRKAAQLPWEDGRSGLLSGGLPRKCSVFHLFVACLSLGFFSLLWLQLSCSGDVARA
VRGQGQETSGPPRACPPEPPPEHWEEDASWGPHRLAVLVPFRERFEELLVFVPHMRRFLSR
KKIRHHIYVLNQVDHFRFNRAALINVGFLESSNSTDYIAMHDVDLLPLNEELDYGFPEAGP
FHVASPELHPLYHYKTYVGGILLLSKQHYRLCNGMSNRFWGWGREDDEFYRRIKGAGLQ
LFRPSGITTGYKTFRHLHDPAWRKRDQKRIAAQKQEQFKVDREGGLNTVKYHVASRTALS
VGGAPCTVLNIMLDCDKTATPWCTFS

>Q9BZR6 GPI-anchor, lipid-anchor
MKRASAGGSRLLAWVLWLQAWQVAAPCPGACVCYNEPKVTTSCPQQGLQAVPVGIPAA
SQRIFLHGNRISHVPAASFRACRNLTILWLHSNVLARIDAAAFTGLALLEQLDLSDNAQLR
SVDPATFHGLGRLHTLHLDRCGLQELGPGLFRGLAALQYLYLQDNALQALPDDTFRDLG
NLTHLFLHGNRISSVPERAFRGLHSLDRLLLHQNRVAHVHPHAFRDLGRLMTLYLFANNLS
ALPTEALAPLRALQYLRLNDNPWVCDCRARPLWAWLQKFRGSSSEVPCSLPQRLAGRDL
KRLAANDLQGCAVATGPYHPIWTGRATDEEPLGLPKCCQPDAADKASVLEPGRPASAGNA
LKGRVPPGDSPPGNGSGPRHINDSPFGTLPGSAEPPLTAVRPEGSEPPGFPTSGPRRRPGCSR
KNRTRSHCRLGQAGSGGGGTGDSEGSGALPSLTCSLTPLGLALVLWTVLGPC

>P43080 lipid-anchor
MGNVMEGKSVEELSSTECHQWYKKFMTECPSGQLTLYEFRQFFGLKNLSPSASQYVEQM
FETFDFNKDGYIDFMEYVAALSLVLKGKVEQKLRWYFKLYDVDGNGCIDRDELLTIIQAIR
AINPCSDTTMTAEEFTDTVFSKIDVNGDGELSLEEFIEGVQKDQMLLDTLTRSLDLTRIVRR
LQNGEQDEEGADEAAEAAG

>O75508 multipass membrane protein
MVATCLQVVGFVTSFVGWIGVIVTTSTNDWVVTCGYTIPTCRKLDELGSKGLWADCVMA

TGLYHCKPLVDILILPGYVQACRALMIAASVLGLPAILLLLTVLPCIRMGQEPGVAKYRRA
QLAGVLLILLALCALVATIWFPVCAHRETTIVSFGYSLYAGWIGAVLCLVGGCVILCCAGD
AQAFGENRFYYTAGSSSPTHAKSAHV

>Q969Z4 single-pass type I membrane protein
MKPSLLCRPLSCFLMLLPWPLATLTSTTLWQCPPGEEPDLDPGQGTLCRPCPPGTFSAAWG
SSPCQPHARCSLWRRLEAQVGMATRDTLCGDCWPGWFGPWGVPRVPCQPCSWAPLGTH
GCDEWGRRARRGVEVAAGASSGGETRQPGNGTRAGGPEETAAQYAVIAIVPVFCLMGLL
GILVCNLLKRKGYHCTAHKEVGPGPGGGGSGINPAYRTEDANEDTIGVLVRLITEKKENAA
ALEELLKEYHSKQLVQTSHRPVSKLPPAPPNVPHICPHRHHLHTVQGLASLSGPCCSRCSQ
KKWPEVLLSPEAVAATTPVPSLLPNPTRVPKAGAKAGRQGEITILSVGRFRVARIPEQRTSS
MVSEVKTITEAGPSWGDLPDSPQPGLPPEQQALLGSGGSRTKWLKPPAENKAEENRYVVR
LSESNLVI

>Q9NPH3 single-pass type I membrane protein
MTLLWCVVSLYFYGILQSDASERCDDWGLDTMRQIQVFEDEPARIKCPLFEHFLKFNYST
AHSAGLTLIWYWTRQDRDLEEPINFRLPENRISKEKDVLWFRPTLLNDTGNYTCMLRNTT
YCSKVAFPLEVVQKDSCFNSPMKLPVHKLYIEYGIQRITCPNVDGYFPSSVKPTITWYMGC
YKIQNFNNVIPEGMNLSFLIALISNNGNYTCVVTYPENGRTFHLTRTLTVKVVGSPKNAVPP
VIHSPNDHVVYEKEPGEELLIPCTVYFSFLMDSRNEVWWTIDGKKPDDITIDVTINESISHS
RTEDETRTQILSIKKVTSEDLKRSYVCHARSAKGEVAKAAKVKQKVPAPRYTVELACGFG
ATVLLVVILIVVYHVYWLEMVLFYRAHFGTDETILDGKEYDIYVSYARNAEEEEFVLLTLR
GVLENEFGYKLCIFDRDSLPGGIVTDETLSFIQKSRRLLVVLSPNYVLQGTQALLELKAGL
ENMASRGNINVILVQYKAVKETKVKELKRAKTVLTVIKWKGEKSKYPQGRFWKQLQVA
MPVKKSPRRSSSDEQGLSYSSLKNV

>Q68CJ9 single-pass type II membrane protein
MNTDLAAGKMASAACSMDPIDSFELLDLLFDRQDGILRHVELGEGWGHVKDQQVLPNP
DSDDFLSSILGSGDSLPSSPLWSPEGSDSGISEDLPSDPQDTPPRSGPATSPAGCHPAQPGKG
PCLSYHPGNSCSTTTPGPVIQVPEASVTIDLEMWSPGGRICAEKPADPVDLSPRCNLTVKDL
LLSGSSGDLQQHHLGASYLLRPGAGHCQELVLTEDEKKLLAKEGITLPTQLPLTKYEERVL
KKIRRKIRNKQSAQESRKKKKEYIDGLETRMSACTAQNQELQRKVLHLEKQNLSLLEQLK
KLQAIVVQSTSKSAQTGTCVAVLLLSFALIILPSISPFGPNKTESPGDFAPVRVFSRTLHNDA
ASRVAADAVPGSEAPGPRPEADTTREESPGSPGADWGFQDTANLTNSTEELDNATLVLRNA
TEGLGQVALLDWVAPGPSTGSGRAGLEAAGDEL

>Q9NUD9 multipass membrane protein
MWPQDPSRKEVLRFAVSCRILTLMLQALFNAIIPDHHAEAFSPPRLAPSGFVDQLVEGLLG
GLSHWDAEHFLFIAEHGYLYEHNFAFFPGFPLALLVGTELLRPLRGLLSLRSCLLISVASLN
FLFFMLAAVALHDLGCLVLHCPHQSFYAALLFCLSPANVFLAAGYSEALFALLTFSAMGQL
ERGRVWTSVLLFAFATGVRSNGLVSVGFLMHSQCQGFFSSLTMLNPLRQLFKLMASLFLS
VFTLGLPFALFQYYAYTQFCLPGSARPIPEPLVQLAVDKGYRIAEGNEPPWCFWDVPLIYSY
IQDVYWNVGFLKYYELKQVPNFLLAAPVAILVAWATWTYVTTHPWLCLTLGLQRSKNNK
TLEKPDLGFLSPQVFVYVVHAAVLLLFGGLCMHVQVLTRFLGSSTPIMYWFPAHLLQDQE

PLLRSLKTVPWKPLAEDSPPGQKVPRNPIMGLLYHWKTCSPVTRYILGYFLTYWLLGLLL
HCNFLPWT

>P20273 single-pass type I membrane protein
MHLLGPWLLLLVLEYLAFSDSSKWVFEHPETLYAWEGACVWIPCTYRALDGDLESFILFH
NPEYNKNTSKFDGTRLYESTKDGKVPSEQKRVQFLGDKNKNCTLSIHPVHLNDSGQLGLR
MESKTEKWMERIHLNVSERPFPPHIQLPPEIQESQEVTLTCLLNFSCYGYPIQLQWLLEGVP
MRQAAVTSTSLTIKSVFTRSELKFSPQWSHHGKIVTCQLQDADGKFLSNDTVQLNVKHTP
KLEIKVTPSDAIVREGDSVTMTCEVSSSNPEYTTVSWLKDGTSLKKQNTFTLNLREVTKD
QSGKYCCQVSNDVGPGRSEEVFLQVQYAPEPSTVQILHSPAVEGSQVEFLCMSLANPLPTN
YTWYHNGKEMQGRTEEKVHIPKILPWHAGTYSCVAENILGTGQRGPGAELDVQYPPKKV
TTVIQNPMPIREGDTVTLSCNYNSSNPSVTRYEWKPHGAWEEPSLGVLKIQNVGWDNTTI
ACAACNSWCSWASPVALNVQYAPRDVRVRKIKPLSEIHSGNSVSLQCDFSSSHPKEVQFF
WEKNGRLLGKESQLNFDSISPEDAGSYSCWVNNSIGQTASKAWTLEVLYAPRRLRVSMSP
GDQVMEGKSATLTCESDANPPVSHYTWFDWNNQSLPYHSQKLRLEPVKVQHSGAYWCQ
GTNSVGKGRSPLSTLTVYYSPETIGRRVAVGLGSCLAILILAICGLKLQRRWKRTQSQQGLQ
ENSSGQSFFVRNKKVRRAPLSEGPHSLGCYNPMMEDGISYTTLRFPEMNIPRTGDAESSE
MQRPPPDCDDTVTYSALHKRQVGDYENVIPDFPEDEGIHYSELIQFGVGERPQAQENVDY
VILKH

>P51787 multipass membrane protein
MAAASSPPRAERKRWGWGRLPGARRGSAGLAKKCPFSLELAEGGPAGGALYAPIAPGAP
GPAPPASPAAPAAPPVASDLGPRPPVSLDPRVSIYSTRRPVLARTHVQGRVYNFLERPTGWK
CFVYHFAVFLIVLVCLIFSVLSTIEQYAALATGTLFWMEIVLVVFFGTEYVVRLWSAGCRSK
YVGLWGRLRFARKPISIIDLIVVVASMVVLCVGSKGQVFATSAIRGIRFLQILRMLHVDRQG
GTWRLLGSVVFIHRQELITTLYIGFLGLIFSSYFVYLAEKDAVNESGRVEFGSYADALWWG
VVTVTTIGYGDKVPQTWVGKTIASCFSVFAISFFALPAGILGSGFALKVQQKQRQKHFNRQ
IPAAASLIQTAWRCYAAENPDSSTWKIYIRKAPRSHTLLSPSPKPKKSVVVKKKKFKLDKD
NGVTPGEKMLTVPHITCDPPEERRLDHFSVDGYDSSVRKSPTLLEVSMPHFMRTNSFAEDL
DLEGETLLTPITHISQLREHHRATIKVIRRMQYFVAKKKFQQARKPYDVRDVIEQYSQGHL
NLMVRIKELQRRLDQSIGKPSLFISVSEKSKDRGSNTIGARLNRVEDKVTQLDQRLALITD
MLHQLLSLHGGSTPGSGGPPREGGAHITQPCGSGGSVDPELFLPSNTLPTYEQLTVPRRGP
DEGS

>Q9GZP9 multipass membrane protein
MAYQSLRLEYLQIPPVSRAYTTACVLTTAAVQLELITPFQLYFNPELIFKHFQIWRLITNFLFF
GPVGFNFLFNMIFLYRYCRMLEEGSFRGRTADFVFMFLFGGFLMTLFGLFVSLVFLGQAFT
IMLVYVWSRRNPYVRMNFFGLLNFQAPFLPWVLMGFSLLLGNSIIVDLLGIAVGHIYFFLE
DVFPNQPGGIRILKTPSILKAIFDTPDEDPNYNPLPEERPGGFAWGEGQRLGG

>P19438 single-pass type I membrane protein
MGLSTVPDLLLPLVLLELLVGIYPSGVIGLVPHLGDREKRDSVCPQGKYIHPQNNSICCTKC
HKGTYLYNDCPGPGQDTDCRECESGSFTASENHLRHCLSCSKCRKEMGQVEISSCTVDRD
TVCGCRKNQYRHYWSENLFQCFNCSLCLNGTVHLSCQEKQNTVCTCHAGFFLRENECVS

CSNCKKSLECTKLCLPQIENVKGTEDSGTTVLLPLVIFFGLCLLSLLFIGLMYRYQRWKSKL
YSIVCGKSTPEKEGELEGTTTKPLAPNPSFSPTPGFTPTLGFSPVPSSTFTSSSTYTPGDCPNF
AAPRREVAPPYQGADPILATALASDPIPNPLQKWEDSAHKPQSLDTDDPATLYAVVENVPP
LRWKEFVRRLGLSDHEIDRLELQNGRCLREAQYSMLATWRRRTPRREATLELLGRVLRD
MDLLGCLEDIEEALCGPAALPPAPSLLR

>Q9NYQ7 multipass membrane protein
MMARRPPWRGLGGRSTPILLLLLLLSLFPLSQEELGGGGHQGWDPGLAATTGPRAHIGGGA
LALCPESSGVREDGGPGLGVREPIFVGLRGRRQSARNSRGPPEQPNEELGIEHGVQPLGSR
ERETGQGPGSVLYWRPEVSSCGRTGPLQRGSLSPGALSSGVPGSGNSSPLPSDFLIRHHGP
KPVSSQRNAGTGSRKRVGTARCCGELWATGSKGQGERATTSGAERTAPRRNCLPGASGSG
PELDSAPRTARTAPASGSAPRESRTAPEPAPKRMRSRGLFRCRFLPQRPGPRPPGLPARPEAR
KVTSANRARFRRAANRHPQFPQYNYQTLVPENEAAGTAVLRVVAQDPDAGEAGRLVYSL
AALMNSRSLELFSIDPQSGLIRTAAALDRESMERHYLRVTAQDHGSPRLSATTMVAVTVAD
RNDHSPVFEQAQYRETLRENVEEGYPILQLRATDGDAPPNANLRYRFVGPPAARAAAAA
AFEIDPRSGLISTSGRVDREHMESYELVVEASDQGQEPGPRSATVRVHITVLDENDNAPQFS
EKRYVAQVREDVRPHTVVLRVTATDRDKDANGLVHYNIISGNSRGHFAIDSLTGEIQVVAP
LDFEAEREYALRIRAQDAGRPPLSNNTGLASIQVVDINDHIPIFVSTPFQVSVLENAPLGHS
VIHIQAVDADHGENARLEYSLTGVAPDTPFVINSATGWVSVSGPLDRESVEHYFFGVEARD
HGSPPLSASASVTVTVLDVNDNRPEFTMKEYHLRLNEDAAVGTSVVSVTAVDRDANSAIS
YQITGGNTRNRFAISTQGGVGLVTLALPLDYKQERYFKLVLTASDRALHDHCYVHINITDA
NTHRPVFQSAHYSVSVNEDRPMGSTIVVISASDDDVGENARITYLLEDNLPQFRIDADSGA
ITLQAPLDYEDQVTYTLAITARDNGIPQKADTTYVEVMVNDVNDNAPQFVASHYTGLVSE
DAPPFTSVLQISATDRDAHANGRVQYTFQNGEDGDGDFTIEPTSGIVRTVRRLDREAVSVY
ELTAYAVDRGVPPLRTPVSIQVMVQDVNDNAPVFPAEEFEVRVKENSIVGSVVAQITAVDP
DEGPNAHIMYQIVEGNIPELFQMDIFSGELTALIDLDYEARQEYVIVVQATSAPLVSRATVH
VRLVDQNDNSPVLNNFQILFNNYVSNRSDTFPSGIIGRIPAYDPDVSDHLFYSFERGNELQL
LVVNQTSGELRLSRKLDNNRPLVASMLVTVTDGLHSVTAQCVLRVVIITEELLANSLTVRL
ENMWQERFLSPLLGRFLEGVAAVLATPAEDVFIFNIQNDTDVGGTVLNVSFSALAPRGAG
AGAAGPWFSSEELQEQLYVRRAALAARSLLDVLPFDDNVCLREPCENYMKCVSVLRFDS
SAPFLASASTLFRPIQPIAGLRCRCPPGFTGDFCETELDLCYSNPCRNGGACARREGGYTC
VCRPRFTGEDCELDTEAGRCVPGVCRNGGTCTDAPNGGFRCQCPAGGAFEGPRCEVAAR
SFPPSSFVMFRGLRQRFHLTLSLSFATVQQSGLLFYNGRLNEKHDFLALELVAGQVRLTYST
GESNTVVSPTVPGGLSDGQWHTVHLRYYNKPRTDALGGAQGPSKDKVAVLSVDDCDVAV
ALQFGAEIGNYSCAAAGVQTSSKKSLDLTGPLLLGGVPNLPENFPVSHKDFIGCMRDLHID
GRRVDMAAFVANNGTMAGCQAKLHFCDSGPCKNSGFCSERWGSFSCDCPVGFGGKDCQ
LTMAHPHHFRGNGTLSWNFGSDMAVSVPWYLGLAFRTRATQGVLMQVQAGPHSTLLCQ
LDRGLLSVTVTRGSGRASHLLLDQVTVSDGRWHDLRLELQEEPGGRRGHHVLMVSLDFS
LFQDTMAVGSELQGLKVKQLHVGGLPPGSAEEAPQGLVGCIQGVWLGSTPSGSPALLPPS
HRVNAEPGCVVTNACASGPCPPHADCRDLWQTFSCTCQPGYYGPGCVDACLLNPCQNQ
GSCRHLPGAPHGYTCDCVGGYFGHHCEHRMDQQCPRGWWGSPTCGPCNCDVHKGFDP
NCNKTNGQCHCKEFHYRPRGSDSCLPCDCYPVGSTSRSCAPHSGQCPCRPGALGRQCNS
CDSPFAEVTASGCRVLYDACPKSLRSGVWWPQTKFGVLATVPCPRGALGAAVRLCDEAQ
GWLEPDLFNCTSPAFRELSLLLDGLELNKTALDTMEAKKLAQRLREVTGHTDHYFSQDVR

VTARLLAHLLAFESHQQGFGLTATQDAHFNENLLWAGSALLAPETGDLWAALGQRAPGG
SPGSAGLVRHLEEYAATLARNMELTYLNPMGLVTPNIMLSIDRMEHPSSPRGARRYPRYHS
NLFRGQDAWDPHTHVLLPSQSPRPSPSEVLPTSSSIENSTTSSVVPPPAPPEPEPGISIIILLVY
RTLGGLLPAQFQAERRGARLPQNPVMNSPVVSVAVFHGRNFLRGILESPISLEFRLLQTANR
SKAICVQWDPPGLAEQHGVWTARDCELVHRNGSHARCRCSRTGTFGVLMDASPRERLEG
DLELLAVFTHVVVAVSVAALVLTAAILLSLRSLKSNVRGIHANVAAALGVAELLFLLGIHRT
HNQLVCTAVAILLHYFFLSTFAWLFVQGLHLYRMQVEPRNVDRGAMRFYHALGWGVPAV
LLGLAVGLDPEGYGNPDFCWISVHEPLIWSFAGPVVLVIVMNGTMFLLAARTSCSTGQRE
AKKTSALTLRSSFLLLLLVSASWLFGLLAVNHSILAFHYLHAGLCGLQGLAVLLLFCVLNA
DARAAWMPACLGRKAAPEEARPAPGLGPGAYNNTALFEESGLIRITLGASTVSSVSSARSG
RTQDQDSQRGRSYLRDNVLVRHGSAADHTDHSLQAHAGPTDLDVAMFHRDAGADSDSD
SDLSLEEERSLSIPSSESEDNGRTRGRFQRPLCRAAQSERLLTHPKDVDGNDLLSYWPALG
ECEAAPCALQTWGSERRLGLDTSKDAANNNQPDPALTSGDETSLGRAQRQRKGILKNRL
QYPLVPQTRGAPELSWCRAATLGHRAVPAASYGRIYAGGGTGSLSQPASRYSSREQLDLLL
RRQLSRERLEEAPAPVLRPLSRPGSQECMDAAPGRLEPKDRGSTLPRRQPPRDYPGAMAG
RFGSRDALDLGAPREWLSTLPPPRRTRDLDPQPPPLPLSPQRQLSRDPLLPSRPLDSLSRSS
NSREQLDQVPSRHPSREALGPLPQLLRAREDSVSGPSHGPSTEQLDILSSILASFNSSALSSV
QSSSTPLGPHTTATPSATASVLGPSTPRSATSHSISELSPDSEVPRSEGHS

>Q92521 multipass membrane protein
MRRPLSKCGMEPGGGDASLTLHGLQNRSHGKIKLRKRKSTLYFNTQEKSARRRGDLLGE
NIYLLLFTIALRILNCFLVQTSFVPDEYWQSLEVSHHMVFNYGYLTWEWTERLRSYTYPLI
FASIYKILHLLGKDSVQLLIWIPRLAQALLSAVADVRLYSLMKQLENQEVARWVFFCQLCS
WFTWYCCTRTLTNTMETVLTIIALFYYPLEGSKSMNSVKYSSLVALAFIIRPTAVILWTPLLF
RHFCQEPRKLDLILHHFLPVGFVTLSLSLMIDRIFFGQWTLVQFNFLKFNVLQNWGTFYGS
HPWHWYFSQGFPVILGTHLPFFIHGCYLAPKRYRILLVTVLWTLLVYSMLSHKEFRFIYPV
LPFCMVFCGYSLTHLKTWKKPALSFLFLSNLFLALYTGLVHQRGTLDVMSHIQKVCYNNP
NKSSASIFIMMPCHSTPYYSHVHCPLPMRFLQCPPDLTGKSHYLDEADVFYLNPLNWLHR
EFHDDASLPTHLITFSILEEEISAFLISSNYKRTAVFFHTHLPEGRIGSHIYVYERKLKGKFNM
KMKF

>Q9H902 multipass membrane protein
MVSWIISRLVVLIFGTLYPAYYSYKAVKSKDIKEYVKWMMYWIIFALFTTAETFTDIFLCWF
PFYYELKIAFVAWLLSPYTKGSSLLYRKFVHPTLSSKEKEIDDCLVQAKDRSYDALVHFGK
RGLNVAATAAVMAASKGQGALSERLRSFSMQDLTTIRGDGAPAPSGPPPPGSGRASGKHG
QPKMSRSASESASSSGTA

>P15907 single-pass type II membrane protein
MIHTNLKKKFSCCVLVFLLFAVICVWKEKKKGSYYDSFKLQTKEFQVLKSLGKLAMGSDS
QSVSSSSTQDPHRGRQTLGSLRGLAKAKPEASFQVWNKDSSSKNLIPRLQKIWKNYLSMN
KYKVSYKGPGPGIKFSAEALRCHLRDHVNVSMVEVTDFPFNTSEWEGYLPKESIRTKAGP
WGRCAVVSSAGSLKSSQLGREIDDHDAVLRFNGAPTANFQQDVGTKTTIRLMNSQLVTTE
KRFLKDSLYNEGILIVWDPSVYHSDIPKWYQNPDYNFFNNYKTYRKLHPNQPFYILKPQM
PWELWDILQEISPEEIQPNPPSSGMLGIIIMMTLCDQVDIYEFLPSKRKTDVCYYYQKFFDS

ACTMGAYHPLLYEKNLVKHLNQGTDEDIYLLGKATLPGFRTIHC

>P24394 single-pass type I membrane protein
MGWLCSGLLFPVSCLVLLQVASSGNMKVLQEPTCVSDYMSISTCEWKMNGPTNCSTELR
LLYQLVFLLSEAHTCIPENNGGAGCVCHLLMDDVVSADNYTLDLWAGQQLLWKGSFKPS
EHVKPRAPGNLTVHTNVSDTLLLTWSNPYPPDNYLYNHLTYAVNIWSENDPADFRIYNVT
YLEPSLRIAASTLKSGISYRARVRAWAQCYNTTWSEWSPSTKWHNSYREPFEQHLLLGVS
VSCIVILAVCLLCYVSITKIKKEWWDQIPNPARSRLVAIIIQDAQGSQWEKRSRGQEPAKCP
HWKNCLTKLLPCFLEHNMKRDEDPHKAAKEMPFQGSGKSAWCPVEISKTVLWPESISVV
RCVELFEAPVECEEEEEVEEEKGSFCASPESSRDDFQEGREGIVARLTESLFLDLLGEENGG
FCQQDMGESCLLPPSGSTSAHMPWDEFPSAGPKEAPPWGKEQPLHLEPSPPASPTQSPDNL
TCTETPLVIAGNPAYRSFSNSLSQSPCPRELGPDPLLARHLEEVEPEMPCVPQLSEPTTVPQP
EPETWEQILRRNVLQHGAAAAPVSAPTSGYQEFVHAVEQGGTQASAVVGLGPPGEAGYK
AFSSLLASSAVSPEKCGFGASSGEEGYKPFQDLIPGCPGDPAPVPVPLFTFGLDREPPRSPQS
SHLPSSSPEHLGLEPGEKVEDMPKPPLPQEQATDPLVDSLGSGIVYSALTCHLCGHLKQCH
GQEDGGQTPVMASPCCGCCCGDRSSPPTTPLRAPDPSPGGVPLEASLCPASLAPSGISEKS
KSSSSFHPAPGNAQSSSQTPKIVNFVSVGPTYMRVS

>O00398 multipass membrane protein
MANLDKYTETFKMGSNSTSTAEIYCNVTNVKFQYSLYATTYILIFIPGLLANSAALWVLCR
FISKKNKAIIFMINLSVADLAHVLSLPLRIYYYISHHWPFQRALCLLCFYLKYLNMYASICFL
TCISLQRCFFLLKPFRARDWKRRYDVGISAAIWIVVGTACLPFPILRSTDLNNNKSCFADLG
YKQMNAVALVGMITVAELAGFVIPVIIIAWCTWKTTISLRQPPMAFQGISERQKALRMVFM
CAAVFFICFTPYHINFIFYTMVKETIISSCPVVRIALYFHPFCLCLASLCCLLDPILYYFMASE
FRDQLSRHGSSVTRSRLMSKESGSSMIG

>P08138 single-pass type I membrane protein
MGAGATGRAMDGPRLLLLLLLLGVSLGGAKEACPTGLYTHSGECCKACNLGEGVAQPCG
ANQTVCEPCLDSVTFSDVVSATEPCKPCTECVGLQSMSAPCVEADDAVCRCAYGYYQDE
TTGRCEACRVCEAGSGLVFSCQDKQNTVCEECPDGTYSDEANHVDPCLPCTVCEDTERQ
LRECTRWADAECEEIPGRWITRSTPPEGSDSTAPSTQEPEAPPEQDLIASTVAGVVTTVMGS
SQPVVTRGTTDNLIPVYCSILAAVVVGLVAYIAFKRWNSCKQNKQGANSRPVNQTPPPEGE
KLHSDSGISVDSQSLHDQQPHTQTASGQALKGDGGLYSSLPPAKREEVEKLLNGSAGDTW
RHLAGELGYQPEHIDSFTHEACPVRALLASWATQDSATLDALLAALRRIQRADLVESLCSE
STATSPV

>Q9H2U9 single-pass type I membrane protein
MLPGCIFLMILLIPQVKEKFILGVEGQQLVRPKKLPLIQKRDTGHTHDDDILKTYEEELLYEI
KLNRKTLVLHLLRSREFLGSNYSETFYSMKGEAFTRHPQIMDHCFYQGSIVHEYDSAASIS
TCNGLRGFFRINDQRYLIEPVKYSDEGEHLVFKYNLRVPYGANYSCTELNFTRKTVPGDN
ESEEDSKIKGIHDEKYVELFIVADDTVYRRNGHPHNKLRNRIWGMVNFVNMIYKTLNIHV
TLVGIEIWTHEDKIELYSNIETTLLRFSFWQEKILKTRKDFDHVVLLSGKWLYSHVQGISYP
GGMCLPYYSTSIIKDLLPDTNIIANRMAHQLGHNLGMQHDEFPCTCPSGKCVMDSDGSIP
ALKFSKCSQNQYHQYLKDYKPTCMLNIPFPYNFHDFQFCGNKKLDEGEECDCGPAQECT

NPCCDAHTCVLKPGFTCAEGECCESCQIKKAGSICRPAKDECDFPEMCTGHSPACPKDQFR
VNGFPCKNSEGYCFMGKCPTREDQCSELFDDEAIESHDICYKMNTKGNKFGYCKNKENR
FLPCEEKDVRCGKIYCTGGELSSLLGEDKTYHLKDPQKNATVKCKTIFLYHDSTDIGLVAS
GTKCGEGMVCNNGECLNMEKVYISTNCPSQCNENPVDGHGLQCHCEEGQAPVACEETL
HVTNITILVVVLVLVIVGIGVLILLVRYRKCIKLKQVQSPPTETLGVENKGYFGDEQQIRTEP
ILPEIHFLNKPASKDSRGIADPNQSAK

>P21917 multipass membrane protein
MGNRSTADADGLLAGRGPAAGASAGASAGLAGQGAAALVGGVLLIGAVLAGNSLVCVS
VATERALQTPTNSFIVSLAAADLLLALLVLPLFVYSEVQGGAWLLSPRLCDALMAMDVML
CTASIFNLCAISVDRFVAVAVPLRYNRQGGSRRQLLLIGATWLLSAAVAAPVLCGLNDVRG
RDPAVCRLEDRDYVVYSSVCSFFLPCPLMLLLYWATFRGLQRWEVARRAKLHGRAPRRPS
GPGPPSPTPPAPRLPQDPCGPDCAPPAPGLPRGPCGPDCAPAAPGLPPDPCGPDCAPPAPGL
PQDPCGPDCAPPAPGLPRGPCGPDCAPPAPGLPQDPCGPDCAPPAPGLPPDPCGSNCAPPD
AVRAAALPPQTPPQTRRRRRAKITGRERKAMRVLPVVVGAFLLCWTPFFVVHITQALCPA
CSVPPRLVSAVTWLGYVNSALNPVIYTVFNAEFRNVFRKALRACC

>O94804 peripheral membrane protein
MAFANFRRILRLSTFEKRKSREYEHVRRDLDPNEVWEIVGELGDGAFGKVYKAKNKETG
ALAAAKVIETKSEEELEDYIVEIEILATCDHPYIVKLLGAYYHDGKLWIMIEFCPGGAVDAI
MLELDRGLTEPQIQVVCRQMLEALNFLHSKRIIHRDLKAGNVLMTLEGDIRLADFGVSAK
NLKTLQKRDSFIGTPYWMAPEVVMCETMKDTPYDYKADIWSLGITLIEMAQIEPPHHELN
PMRVLLKIAKSDPPTLLTPSKWSVEFRDFLKIALDKNPETRPSAAQLLEHPFVSSITSNKAL
RELVAEAKAEVMEEIEDGRDEGEEEDAVDAASTLENHTQNSSEVSPPSLNADKPLEESPST
PLAPSQSQDSVNEPCSQPSGDRSLQTTSPPVVAPGNENGLAVPVPLRKSRPVSMDARIQVA
QEKQVAEQGGDLSPAANRSQKASQSRPNSSALETLGGEKLANGSLEPPAQAAPGPSKRDS
DCSSLCTSESMDYGTNLSTDLSLNKEMGSLSIKDPKLYKKTLKRTRKFVVDGVEVSITTSK
IISEDEKKDEEMRFLRRQELRELRLLQKEEHRNQTQLSNKHELQLEQMHKRFEQEINAKK
KFFDTELENLERQQKQQVEKMEQDHAVRRREEARRIRLEQDRDYTRFQEQLKLMKKEVK
NEVEKLPRQQRKESMKQKMEEHTQKKQLLDRDFVAKQKEDLELAMKRLTTDNRREICD
KERECLMKKQELLRDREAALWEMEEHQLQERHQLVKQQLKDQYFLQRHELLRKHEKER
EQMQRYNQRMIEQLKVRQQQEKARLPKIQRSEGKTRMAMYKKSLHINGGGSAAEQREKI
KQFSQQEEKRQKSERLQQQQKHENQMRDMLAQCESNMSELQQLQNEKCHLLVEHETQK
LKALDESHNQNLKEWRDKLRPRKKALEEDLNQKKREQEMFFKLSEEAECPNPSTPSKAA
KFFPYSSADAS

>O43570 single-pass type I membrane protein
MPRRSLHAAAVLLLVVILKEQPSSPAPVNGSKWTYFGPDGENSWSKKYPSCGGLLQSPIDLH
SDILQYDASLTPLEFQGYNLSANKQFLLTNNGHSVKLNLPSDMHIQGLQSRYSATQLHLH
WGNPNDPHGSEHTVSGQHFAAELHIVHYNSDLYPDASTASNKSEGLAVLAVLIEMGSFNP
SYDKIFSHLQHVKYKGQEAFVPGFNIEELLPERTAEYYRYRGSLTTPPCNPTVLWTVFRNP
VQISQEQLLALETALYCTHMDDPSPREMINNFRQVQKFDERLVYTSFSQVQVCTAAGLSL
GIILSLALAGILGICIVVVVSIWLFRRKSIKKGDNKGVIYKPATKMETEAHA

>P56856 multipass membrane protein
MSTTTCQVVAFLLSILGLAGCIAATGMDMWSTQDLYDNPVTSVFQYEGLWRSCVRQSSGF
TECRPYFTILGLPAMLQAVRALMIVGIVLGAIGLLVSIFALKCIRIGSMEDSAKANMTLTSGI
MFIVSGLCAIAGVSVFANMLVTNFWMSTANMYTGMGGMVQTVQTRYTFGAALFVGWV
AGGLTLIGGVMMCIACRGLAPEETNYKAVSYHASGHSVAYKPGGFKASTGFGSNTKNKKI
YDGGARTEDEVQSYPSKHDYV

>Q9H6F2 multipass membrane protein
MELLSALSLGELALSFSRVPLFPVFDLSYFIVSILYLKYEPGAVELSRRHPIASWLCAMLHC
FGSYILADLLLGEPLIDYFSNNSSILLASAVWYLIFFCPLDLFYKCVCFLPVKLIFVAMKEVV
RVRKIAVGIHHAHHHYHHGWFVMIATGWVKGSGVALMSNFEQLLRGVWKPETNEILHM
SFPTKASLYGAILFTLQQTRWLPVSKASLIFIFTLFMVSCKVFLTATHSHSSPFDALEGYICP
VLFGSACGGDHHHDNHGGSHSGGGPGAQHSAMPAKSKEELSEGSRKKKAKKAD

>Q86WC4 single-pass type I membrane protein
MEPGPTAAQRRCSLPPWLPLGLLLWSGLALGALPFGSSPHRVFHDLLSEQQLLEVEDLSLS
LLQGGGLGPLSLPPDLPDLDPECRELLLDFANSSAELTGCLVRSARPVRLCQTCYPLFQQV
VSKMDNISRAAGNTSESQSCARSLLMADRMQIVVILSEFFNTTWQEANCANCLTNNSEEL
SNSTVYFLNLFNHTLTCFEHNLQGNAHSLLQTKNYSEVCKNCREAYKTLSSLYSEMQKM
NELENKAEPGTHLCIDVEDAMNITRKLWSRTFNCSVPCSDTVPVIAVSVFILFLPVVFYLSS
FLHSEQKKRKLILPKRLKSSTSFANIQENSN

>O60641 peripheral membrane protein
MSGQTLTDRIAAAQYSVTGSAVARAVCKATTHEVMGPKKKHLDYLIQATNETNVNIPQM
ADTLFERATNSSWVVVFKALVTTHHLMVHGNERFIQYLASRNTLFNLSNFLDKSGSHGY
DMSTFIRRYSRYLNEKAFSYRQMAFDFARVKKGADGVMRTMAPEKLLKSMPILQGQIDA
LLEFDVHPNELTNGVINAAFMLLFKDLIKLFACYNDGVINLLEKFFEMKKGQCKDALEIY
KRFLTRMTRVSEFLKVAEQVGIDKGDIPDLTQAPSSLMETLEQHLNTLEGKKPGNNEGSGA
PSPLSKSSPATTVTSPNSTPAKTIDTSPPVDLFATASAAVPVSTSKPSSDLLDLQPDFSSGGAA
AAAAPAPPPPAGGATAWGDLLGEDSLAALSSVPSEAQISDPFAPEPTPPTTTAEIATASASAS
TTTTVTAVTAEVDLFGDAFAASPGEAPAASEGAAAPATPTPVAAALDACSGNDPFAPSEGS
AEAAPELDLFAMKPPETSVPVVTPTASTAPPVPATAPSPAPAVAAAAAATTAATAAATTTTT
TSAATATTAPPALDIFGDLFESTPEVAAAPKPDAAPSIDLFSTDAFSSPPQGASPVPESSLTAD
LLSVDAFAAPSPATTASPAKVDSSGVIDLFGDAFGSSASEPQPASQAASSSSASADLLAGFG
GSFMAPSPSPVTPAQNNLLQPNFEAAFGTTPSTSSSSSFDPSVFDGLGDLLMPTMAPAGQP
APVSMVPPSPAMAASKALGSDLDSSLASLVGNLGISGTTTKKGDLQWNAGEKKLTGGAN
WQPKVAPATWSAGVPPSAPLQGAVPPTSSVPPVAGAPSVGQPGAGFGMPPAGTGMPMMP
QQPVMFAQPMMRPPFGAAAVPGTQLSPSPTPASQSPKKPPAKDPLADLNIKDFL

>O43264 peripheral membrane protein
MASFVTEVLAHSGRLEKEDLGTRISRLTRRVEEIKGEVCNMISKKYSEFLPSMQSAQGLIT
QVDKLSEDIDLLKSRIESEVRRDLHVSTGEFTDLKQQLERDSVVLSLLKQLQEFSTAIEEYN
CALTEKKYVTGAQRLEEAQKCLKLLKSRKCFDLKILKSLSMELTIQKQNILYHLGEEWQK
LIVWKFPPSKDTSSLESYLQTELHLYTEQSHKEEKTPMPPISSVLLAFSVLGELHSKLKSFG

QMLLKYILRPLASCPSLHAVIESQPNIVIIRFESIMTNLEYPSPSEVFTKIRLVLEVLQKQLLD
LPLDTDLENEKTSTVPLAEMLGDMIWEDLSECLIKNCLVYSIPTNSSKLQQYEEIIQSTEEF
ENALKEMRFLKGDTTDLLKYARNINSHFANKKCQDVIVAARNLMTSEIHNTVKIIPDSKIN
VPELPTPDEDNKLEVQKVSNTQYHEVMNLEPENTLDQHSFSLPTCRISESVKKLMELAYQ
TLLEATTSSDQCAVQLFYSVRNIFHLFHDVVPTYHKENLQKLPQLAAIHHNNCMYIAHHL
LTLGHQFRLRLAPILCDGTATFVDLVPGFRRLGTECFLAQMRAQKGELLERLSSARNFSNM
DDEENYSAASKAVRQVLHQLKRLGIVWQDVLPVNIYCKAMGTLLNTAISEVIGKITALEDI
STEDGDRLYSLCKTVMDEGPQVFAPLSEESKNKKYQEEVPVYVPKWMPFKELMMMLQA
SLQEIGDRWADGKGPLAAAFSSSEVKALIRALFQNTERRAAALAKIK

>P16144 single-pass type I membrane protein
MAGPRPSPWARLLLAALISVSLSGTLANRCKKAPVKSCTECVRVDKDCAYCTDEMFRDR
RCNTQAELLAAGCQRESIVVMESSFQITEETQIDTTLRRSQMSPQGLRVRLRPGEERHFEL
EVFEPLESPVDLYILMDFSNSMSDDLDNLKKMGQNLARVLSQLTSDYTIGFGKFVDKVSV
PQTDMRPEKLKEPWPNSDPPFSFKNVISLTEDVDEFRNKLQGERISGNLDAPEGGFDAILQ
TAVCTRDIGWRPDSTHLLVFSTESAFHYEADGANVLAGIMSRNDERCHLDTTGTYTQYRT
QDYPSVPTLVRLLAKHNIIPIFAVTNYSYSYYEKLHTYFPVSSLGVLQEDSSNIVELLEEAFN
RIRSNLDIRALDSPRGLRTEVTSKMFQKTRTGSFHIRRGEVGIYQVQLRALEHVDGTHVCQ
LPEDQKGNIHLKPSFSDGLKMDAGIICDVCTCELQKEVRSARCSFNGDFVCGQCVCSEGW
SGQTCNCSTGSLSDIQPCLREGEDKPCSGRGECQCGHCVCYGEGRYEGQFCEYDNFQCPR
TSGFLCNDRGRCSMGQCVCEPGWTGPSCDCPLSNATCIDSNGGICNGRGHCECGRCHCH
QQSLYTDTICEINYSAIHPGLCEDLRSCVQCQAWGTGEKKGRTCEECNFKVKMVDELKRA
EEVVVRCSFRDEDDDCTYSYTMEGDGAPGPNSTVLVHKKKDCPPGSFWWLIPLLLLLLPL
LALLLLLCWKYCACCKACLALLPCCNRGHMVGFKEDHYMLRENLMASDHLDTPMLRS
GNLKGRDVVRWKVTNNMQRPGFATHAASINPTELVPYGLSLRLARLCTENLLKPDTREC
AQLRQEVEENLNEVYRQISGVHKLQQTKFRQQPNAGKKQDHTIVDTVLMAPRSAKPALL
KLTEKQVEQRAFHDLKVAPGYYTLTADQDARGMVEFQEGVELVDVRVPLFIRPEDDDEK
QLLVEAIDVPAGTATLGRRLVNITIIKEQARDVVSFEQPEFSVSRGDQVARIPVIRRVLDGGK
SQVSYRTQDGTAQGNRDYIPVEGELLFQPGEAWKELQVKLLELQEVDSLLRGRQVRRFH
VQLSNPKFGAHLGQPHSTTIIIRDPDELDRSFTSQMLSSQPPPHGDLGAPQNPNAKAAGSR
KIHFNWLPPSGKPMGYRVKYWIQGDSESEAHLLDSKVPSVELTNLYPYCDYEMKVCAYG
AQGEGPYSSLVSCRTHQEVPSEPGRLAFNVVSSTVTQLSWAEPAETNGEITAYEVCYGLVN
DDNRPIGPMKKVLVDNPKNRMLLIENLRESQPYRYTVKARNGAGWGPEREAIINLATQPK
RPMSIPIIPDIPIVDAQSGEDYDSFLMYSDDVLRSPSGSQRPSVSDDTGCGWKFEPLLGEEL
DLRRVTWRLPPELIPRLSASSGRSSDAEAPHGPPDDGGAGGKGGSLPRSATPGPPGEHLVN
GRMDFAFPGSTNSLHRMTTTSAAAYGTHLSPHVPHRVLSTSSTLTRDYNSLTRSEHSHSTT
LPRDYSTLTSVSSHDSRLTAGVPDTPTRLVFSALGPTSLRVSWQEPRCERPLQGYSVEYQLL
NGGELHRLNIPNPAQTSVVVEDLLPNHSYVFRVRAQSQEGWGREREGVITIESQVHPQSPL
CPLPGSAFTLSTPSAPGPLVFTALSPDSLQLSWERPRRPNGDIVGYLVTCEMAQGGGPATAF
RVDGDSPESRLTVPGLSENVPYKFKVQARTTEGFGPEREGIITIESQDGGPFPQLGSRAGLF
QHPLQSEYSSITTTHTSATEPFLVDGLTLGAQHLEAGGSLTRHVTQEFVSRTLTTSGTLSTH
MDQQFFQT

>P18627 single-pass type I membrane protein

MWEAQFLGLLFLQPLWVAPVKPLQPGAEVPVVWAQEGAPAQLPCSPTIPLQDLSLLRRAG
VTWQHQPDSGPPAAAPGHPLAPGPHPAAPSSWGPRPRRYTVLSVGPGGLRSGRLPLQPRV
QLDERGRQRGDFSLWLRPARRADAGEYRAAVHLRDRALSCRLRLRLGQASMTASPPGSL
RASDWVILNCSFSRPDRPASVHWFRNRGQGRVPVRESPHHHLAESFLFLPQVSPMDSGPW
GCILTYRDGFNVSIMYNLTVLGLEPPTPLTVYAGAGSRVGLPCRLPAGVGTRSFLTAKWTPP
GGGPDLLVTGDNGDFTLRLEDVSQAQAGTYTCHIHLQEQQLNATVTLAIITVTPKSFGSPG
SLGKLLCEVTPVSGQERFVWSSLDTPSQRSFSGPWLEAQEAQLLSQPWQCQLYQGERLLG
AAVYFTELSSPGAQRSGRAPGALPAGHLLLFLILGVLSLLLLVTGAFGFHLWRRQWRPRRF
SALEQGIHPPQAQSKIEELEQEPEPEPEPEPEPEPEPEPEQL

>Q9NZD8 peripheral membrane protein
MGEIKVSPDYNWFRGTVPLKKIIVDDDDSKIWSLYDAGPRSIRCPLIFLPPVSGTADVFFRQ
ILALTGWGYRVIALQYPVYWDHLEFCDGFRKLLDHLQLDKVHLFGASLGGFLAQKFAEY
THKSPRVHSLILCNSFSDTSIFNQTWTANSFWLMPAFMLKKIVLGNFSSGPVDPMMADAID
FMVDRLESLGQSELASRLTLNCQNSYVEPHKIRDIPVTIMDVFDQSALSTEAKEEMYKLYP
NARRAHLKTGGNFPYLCRSAEVNLYVQIHLLQFHGTKYAAIDPSMVSAEELEVQKGSLGI
SQEEQ

>P49771 single-pass type I membrane protein
MTVLAPAWSPTTYLLLLLLLSSGLSGTQDCSFQHSPISSDFAVKIRELSDYLLQDYPVTVAS
NLQDEELCGGLWRLVLAQRWMERLKTVAGSKMQGLLERVNTEIHFVTKCAFQPPPSCLR
FVQTNISRLLQETSEQLVALKPWITRQNFSRCLELQCQPDSSTLPPPWSPRPLEATAPTAPQP
PLLLLLLLPVGLLLLAAAWCLHWQRTRRRTPRPGEQVPPVPSPQDLLLVEH

>Q08378 peripheral membrane protein
MDGASAEQDGLQEDRSHSGPSSLPEAPLKPPGPLVPPDQQDKVQCAEVNRASTEGESPDG
PGQGGLCQNGPTPPFPDPPSSLDPTTSPVGPDASPGVAGFHDNLRKSQGTSAEGSVRKEAL
QSLRLSLPMQETQLCSTDSPLPLEKEEQVRLQARKWLEEQLKQYRVKRQQERSSQPATKT
RLFSTLDPELMLNPENLPRASTLAMTKEYSFLRTSVPRGPKVGSLGLPAHPREKKTSKSSKI
RSLADYRTEDSNAGNSGGNVPAPDSTKGSLKQNRSSAASVVSEISLSPDTDDRLENTSLAG
DSVSEVDGNDSDSSSYSSASTRGTYGILSKTVGTQDTPYMVNGQEIPADTLGQFPSIKDVL
QAAAAEHQDQGQEVNGEVRSRRDSICSSVSLESSAAETQEEMLQVLKEKMRLEGQLEAL
SLEASQALKEKAELQAQLAALSTKLQAQVECSHSSQQRQDSLSSEVDTLKQSCWDLERA
MTDLQNMLEAKNASLASSNNDLQVAEEQYQRLMAKVEDMQRSMLSKDNTVHDLRQQ
MTALQSQLQQVQLERTTLTSKLKASQAEISSLQSVRQWYQQQLALAQEARVRLQGEMAH
IQVGQMTQAGLLEHLKLENVSLSQQLTETQHRSMKEKGRIAAQLQGIEADMLDQEAAFM
QIQEAKTMVEEDLQRRLEEFEGERERLQRMADSAASLEQQLEQVKLTLLQRDQQLEALQ
QEHLDLMKQLTLTQEALQSREQSLDALQTHYDELQARLGELQGEAASREDTICLLQNEKII
LEAALQAAKSGKEELDRGARRLEEGTEETSETLEKLREELAIKSGQVEHLQQETAALKKQ
MQKIKEQFLQQKVMVEAYRRDATSKDQLISELKATRKRLDSELKELRQELMQVHGEKRT
AEAELSRLHREVAQVRQHMADLEGHLQSAQKERDEMETHLQSLQFDKEQMVAVTEANE
ALKKQIEELQQEARKAITEQKQKMRRLGSDLTSAQKEMKTKHKAYENAVGILSRRLQEAL
AAKEAADAELGQLRAQGGSSDSSLALHERIQALEAELQAVSHSKTLLEKELQEVIALTSQE
LEESREKVLELEDELQESRGFRKKIKRLEESNKKLALELEHEKGKLTGLGQSNAALREHNS

ILETALAKREADLVQLNLQVQAVLQRKEEEDRQMKHLVQALQASLEKEKEKVNSLKEQV
AAAKVEAGHNRRHFKAASLELSEVKKELQAKEHLVQKLQAEADDLQIREGKHSQEIAQF
QAELAEARAQLQLLQKQLDEQLSKQPVGNQEMENLKWEVDQKEREIQSLKQQLDLTEQ
QGRKELEGLQQLLQNVKSELEMAQEDLSMTQKDKFMLQAKVSELKNNMKTLLQQNQQ
LKLDLRRGAAKTRKEPKGEASSSNPATPIKIPDCPVPASLLEELLRPPPAVSKEPLKNLNSCL
QQLKQEMDSLQRQMEEHALTVHESLSSWTPLEPATASPVPPGGHAGPRGDPQRHSQSRAS
KEGPGE

>P98172 single-pass type I membrane protein
MARPGQRWLGKWLVAMVVWALCRLATPLAKNLEPVSWSSLNPKFLSGKGLVIYPKIGDK
LDIICPRAEAGRPYEYYKLYLVRPEQAAACSTVLDPNVLVTCNRPEQEIRFTIKFQEFSPNY
MGLEFKKHHDYYITSTSNGSLEGLENREGGVCRTRTMKIIMKVGQDPNAVTPEQLTTSRPS
KEADNTVKMATQAPGSRGSLGDSDGKHETVNQEEKSGPGASGGSSGDPDGFFNSKVALF
AAVGAGCVIFLLIIIFLTVLLLKLRKRHRKHTQQRAAALSLSTLASPKGGSGTAGTEPSDIIIP
LRTTENNYCPHYEKVSGDYGHPVYIVQEMPPQSPANIYYKV

>Q92896 single-pass type I membrane protein
MAACGRVRRMFRLSAALHLLLLFAAGAEKLPGQGVHSQGQGPGANFVSFVGQAGGGGP
AGQQLPQLPQSSQLQQQQQQQQQQQQPQPPQPPFPAGGPPARRGGAGAGGGWKLAEEES
CREDVTRVCPKHTWSNNLAVLECLQDVREPENEISSDCNHLLWNYKLNLTTDPKFESVAR
EVCKSTITEIKECADEPVGKGYMVSCLVDHRGNITEYQCHQYITKMTAIIFSDYRLICGFM
DDCKNDINILKCGSIRLGEKDAHSQGEVVSCLEKGLVKEAEEREPKIQVSELCKKAILRVA
ELSSDDFHLDRHLYFACRDDRERFCENTQAGEGRVYKCLFNHKFEESMSEKCREALTTRQ
KLIAQDYKVSYSLAKSCKSDLKKYRCNVENLPRSREARLSYLLMCLESAVHRGRQVSSEC
QGEMLDYRRMLMEDFSLSPEIILSCRGEIEHHCSGLHRKGRTLHCLMKVVRGEKGNLGM
NCQQALQTLIQETDPGADYRIDRALNEACESVIQTACKHIRSGDPMILSCLMEHLYTEKMV
EDCEHRLLELQYFISRDWKLDPVLYRKCQGDASRLCHTHGWNETSEFMPQGAVFSCLYR
HAYRTEEQGRRLSRECRAEVQRILHQRAMDVKLDPALQDKCLIDLGKWCSEKTETGQEL
ECLQDHLDDLVVECRDIVGNLTELESEDIQIEALLMRACEPIIQNFCHDVADNQIDSGDLM
ECLIQNKHQKDMNEKCAIGVTHFQLVQMKDFRFSYKFKMACKEDVLKLCPNIKKKVDV
VICLSTTVRNDTLQEAKEHRVSLKCRRQLRVEELEMTEDIRLEPDLYEACKSDIKNFCSAV
QYGNAQIIECLKENKKQLSTRCHQKVFKLQETEMMDPELDYTLMRVCKQMIKRFCPEAD
SKTMLQCLKQNKNSELMDPKCKQMITKRQITQNTDYRLNPMLRKACKADIPKFCHGILT
KAKDDSELEGQVISCLKLRYADQRLSSDCEDQIRIIIQESALDYRLDPQLQLHCSDEISSLCA
EEAAAQEQTGQVEECLKVNLLKIKTELCKKEVLNMLKESKADIFVDPVLHTACALDIKHH
CAAITPGRGRQMSCLMEALEDKRVRLQPECKKRLNDRIEMWSYAAKVAPADGFSDLAMQ
VMTSPSKNYILSVISGSICILFLIGLMCGRITKRVTRELKDR

>Q9NX76 multipass membrane protein
MENGAVYSPTTEEDPGPARGPRSGLAAYFFMGRLPLLRRVLKGLQLLLSLLAFICEEVVSQ
CTLCGGLYFFEFVSCSAFLLSLLILIVYCTPFYERVDTTKVKSSDFYITLGTGCVFLLASIIFV
STHDRTSAEIAAIVFGFIASFMFLLDFITMLYEKRQESQLRKPENTTRAEALTEPLNA

>P03372 peripheral membrane protein, single-pass type I membrane protein

MTMTLHTKASGMALLHQIQGNELEPLNRPQLKIPLERPLGEVYLDSSKPAVYNYPEGAAY
EFNAAAAANAQVYGQTGLPYGPGSEAAAFGSNGLGGFPPLNSVSPSPLMLLHPPPQLSPF
LQPHGQQVPYYLENEPSGYTVREAGPPAFYRPNSDNRRQGGRERLASTNDKGSMAMESA
KETRYCAVCNDYASGYHYGVWSCEGCKAFFKRSIQGHNDYMCPATNQCTIDKNRRKSCQ
ACRLRKCYEVGMMKGGIRKDRRGGRMLKHKRQRDDGEGRGEVGSAGDMRAANLWPS
PLMIKRSKKNSLALSLTADQMVSALLDAEPPILYSEYDPTRPFSEASMMGLLTNLADRELV
HMINWAKRVPGFVDLTLHDQVHLLECAWLEILMIGLVWRSMEHPGKLLFAPNLLLDRNQ
GKCVEGMVEIFDMLLATSSRFRMMNLQGEEFVCLKSIILLNSGVYTFLSSTLKSLEEKDHI
HRVLDKITDTLIHLMAKAGLTLQQQHQRLAQLLLILSHIRHMSNKGMEHLYSMKCKNVV
PLYDLLLEMLDAHRLHAPTSRGGASVEETDQSHLATAGSTSSHSLQKYYITGEAEGFPATV

>Q8WUG5 multipass membrane protein
MASDPIFTLAPPLHCHYGAFPPNASGWEQPPNASGVSVASAALAASAASRVATSTDPSCSG
FAPPDFNHCLKDWDYNGLPVLTTNAIGQWDLVCDLGWQVILEQILFILGFASGYLFLGYPA
DRFGRRGIVLLTLGLVGPCGVGGAAAGSSTGVMALRFLLGFLLAGVDLGVYLMRLELCD
PTQRLRVALAGELVGVGGHFLFLGLALVSKDWRFLQRMITAPCILFLFYGWPGLFLESAR
WLIVKRQIEEAQSVLRILAERNRPHGQMLGEEAQEALQDLENTCPLPATSSFSFASLLNYR
NIWKNLLILGFTNFIAHAIRHCYQPVGGGGSPSDFYLCSLLASGTAALACVFLGVTVDRFG
RRGILLLSMTLTGIASLVLLGLWDCEHPIFPTVWAQQGNPNRDLNEAAITTFSVLGLFSSQA
AAILSTLLAAEVIPTTVRGRGLGLIMALGALGGLSGPAQRLHMGHGAFLQHVVLAACALL
CILSIMLLPETKRKLLPEVLRDGELCRRPSLLRQPPPTRCDHVPLLATPNPAL

>P22735 lipid-anchor
MMDGPRSDVGRWGGNPLQPPTTPSPEPEPEPDGRSRRGGGRSFWARCCGCCSCRNAADD
DWGPEPSDSRGRGSSSGTRRPGSRGSDSRRPVSRGSGVNAAGDGTIREGMLVVNGVDLLS
SRSDQNRREHHTDEYEYDELIVRRGQPFHMLLLLSRTYESSDRITLELLIGNNPEVGKGTH
VIIPVGKGGSGGWKAQVVKASGQNLNLRVHTSPNAIIGKFQFTVRTQSDAGEFQLPFDPR
NEIYILFNPWCPEDIVYVDHEDWRQEYVLNESGRIYYGTEAQIGERTWNYGQFDHGVLD
ACLYILDRRGMPYGGRGDPVNVSRVISAMVNSLDDNGVLIGNWSGDYSRGTNPSAWVGS
VEILLSYLRTGYSVPYGQCWVFAGVTTTVLRCLGLATRTVTNFNSAHDTDTSLTMDIYFDE
NMKPLEHLNHDSVWNFHVWNDCWMKRPDLPSGFDGWQVVDATPQETSSGIFCCGPCSV
ESIKNGLVYMKYDTPFIFAEVNSDKVYWQRQDDGSFKIVYVEEKAIGTLIVTKAISSNMRE
DITYLYKHPEGSDAERKAVETAAAHGSKPNVYANRGSAEDVAMQVEAQDAVMGQDLMV
SVMLINHSSSRRTVKLHLYLSVTFYTGVSGTIFKETKKEVELAPGASDRVTMPVAYKEYRP
HLVDQGAMLLNVSGHVKESGQVLAKQHTFRLRTPDLSLTLLGAAVVGQECEVQIVFKNP
LPVTLTNVVFRLEGSGLQRPKILNVGDIGGNETVTLRQSFVPVRPGPRQLIASLDSPQLSQV
HGVIQVDVAPAPGDGGFFSDAGGDSHLGETIPMASRGGA

>Q70J99 peripheral membrane protein
MATLLSHPQQRPPFLRQAIKIRRRRVRDLQDPPPQMAPEIQPPSHHFSPEQRALLYEDALYT
VLHRLGHPEPNHVTEASELLRYLQEAFHVEPEEHQQTLQRVRELEKPIFCLKATVKQAKGI
LGKDVSGFSDPYCLLGIEQGVGVPGGSPGSRHRQKAVVRHTIPEEETHRTQVITQTLNPVW
DETFILEFEDITNASFHLDMWDLDTVESVRQKLGELTDLHGLRRIFKEARKDKGQDDFLG
NVVLRLQDLRCREDQWYPLEPRTETYPDRGQCHLQFQLIHKRRATSASRSQPSYTVHLHL

LQQLVSHEVTQHEAGSTSWDGSLSPQAATVLFLHATQKDLSDFHQSMAQWLAYSRLYQS
LEFPSSCLLHPITSIEYQWIQGRLKAEQQEELAASFSSLLTYGLSLIRRFRSVFPLSVSDSPAR
LQSLLRVLVQMCKMKAFGELCPNTAPLPQLVTEALQTGTTEWFHLKQQHHQPMVQGIPE
AGKALLGLVQDVIGDLHQCQRTWDKIFHNTLKIHLFSMAFRELQWLVAKRVQDHTTVVG
DVVSPEMGESLFQLYISLKELCQLRMSSSERDGVLALDNFHRWFQPAIPSWLQKTYNEAL
ARVQRAVQMDELVPLGELTKHSTSAVDLSTCFAQISHTARQLDWPDPEEAFMITVKFVEDT
CRLALVYCSLIKARARELSSGQKDQGQAANMLCVVVNDMEQLRLVIGKLPAQLAWEALE
QRVGAVLEQGQLQNTLHAQLQSALAGLGHEIRTGVRTLAEQLEVGIAKHIQKLVGVRESV
LPEDAILPLMKFLEVELCYMNTNLVQENFSSLLTLLWTHTLTVLVEAAASQRSSSLASNRL
KIALQNLEICFHAEGCGLPPKALHTATFQALQRDLELQAASSRELIRKYFCSRIQQQAETTS
EELGAVTVKASYRASEQKLRVELLSASSLLPLDSNGSSDPFVQLTLEPRHEFPELAARETQ
KHKKDLHPLFDETFEFLVPAEPCRKAGACLLLTVLDYDTLGADDLEGEAFLPLREVPGLS
GSEEPGEVPQTRLPLTYPAPNGDPILQLLEGRKGDREAQVFVRLRRHRAKQASQHALRPA
P

>Q9ULW5 lipid-anchor
MSRKKTPKSKGASTPAASTLPTANGARPARSGTALSGPDAPPNGPLQPGRPSLGGGVDFY
DVAFKVMLVGDSGVGKTCLLVRFKDGAFLAGTFISTVGIDFRNKVLDVDGVKVKLQMW
DTAGQERFRSVTHAYYRDAHALLLLYDVTNKASFDNIQAWLTEIHEYAQHDVALMLLGN
KVDSAHERVVKREDGEKLAKEYGLPFMETSAKTGLNVDLAFTAIAKELKQRSMKAPSEP
RFRLHDYVKREGRGASCCRP

>Q9NTN9 single-pass type I membrane protein
MWGRLWPLLLSILTATAVPGPSLRRPSRELDATPRMTIPYEELSGTRHFKGQAQNYSTLLLE
EASARLLVGARGALFSLSANDIGDGAHKEIHWEASPEMQSKCHQKGKNNQTECFNHVRF
LQRLNSTHLYACGTHAFQPLCAAIDAEAFTLPTSFEEGKEKCPYDPARGFTGLIIDGGLYTA
TRYEFRSIPDIRRSRHPHSLRTEETPMHWLNDAEFVFSVLVRESKASAVGDDDKVYYFFTE
RATEEGSGSFTQSRSSHRVARVARVCKGDLGGKKILQKKWTSFLKARLICHIPLYETLRGV
CSLDAETSSRTHFYAAFTLSTQWKTLEASAICRYDLAEIQAVFAGPYMEYQDGSRRWGRY
EGGVPEPRPGSCITDSLRSQGYNSSQDLPSLVLDFVKLHPLMARPVVPTRGRPLLLKRNIR
YTHLTGTPVTTPAGPTYDLLFLGTADGWIHKAVVLGSGMHIIEETQVFRESQSVENLVISLL
QHSLYVGAPSGVIQLPLSSCSRYRSCYDCILARDPYCGWDPGTHACAAATTIANRTALIQDI
ERGNRGCESSRDTGPPPPLKTRSVLRGDDVLLPCDQPSNLARALWLLNGSMGLSDGQGG
YRVGVDGLLVTDAQPEHSGNYGCYAEENGLRTLLASYSLTVRPATPAPAPKAPATPGAQLA
PDVRLLYVLAIAALGGLCLILASSLLYVACLREGRRGRRRKYSLGRASRAGGSAVQLQTVS
GQCPGEEDEGDDEGAGGLEGSCLQIIPGEGAPAPPPPPPPPPPAELTNGLVALPSRLRRMNG
NSYVLLRQSNNGVPAGPCSFAEELSRILEKRKHTQLVEQLDESSV

>P18206 peripheral membrane protein
MPVFHTRTIESILEPVAQQISHLVIMHEEGEVDGKAIPDLTAPVAAVQAAVSNLVRVGKETV
QTTEDQILKRDMPPAFIKVENACTKLVQAAQMLQSDPYSVPARDYLIDGSRGILSGTSDLL
LTFDEAEVRKIIRVCKGILEYLTVAEVVETMEDLVTYTKNLGPGMTKMAKMIDERQQELT
HQEHRVMLVNSMNTVKELLPVLISAMKIFVTTKNSKNQGIEEALKNRNFTVEKMSAEINEI
IRVLQLTSWDEDAWASKDTEAMKRALASIDSKLNQAKGWLRDPSASPGDAGEQAIRQILD

EAGKVGELCAGKERREILGTCKMLGQMTDQVADLRARGQGSSPVAMQKAQQVSQGLDV
LTAKVENAARKLEAMTNSKQSIAKKIDAAQNWLADPNGGPEGEEQIRGALAEARKIAELC
DDPKERDDILRSLGEISALTSKLADLRRQGKGDSPEARALAKQVATALQNLQTKTNRAVA
NSRPAKAAVHLEGKIEQAQRWIDNPTVDDRGVGQAAIRGLVAEGHRLANVMMGPYRQD
LLAKCDRVDQLTAQLADLAARGEGESPQARALASQLQDSLKDLKARMQEAMTQEVSDV
FSDTTTPIKLLAVAATAPPDAPNREEVFDERAANFENHSGKLGATAEKAAAVGTANKSTVE
GIQASVKTARELTPQVVSAARILLRNPGNQAAYEHFETMKNQWIDNVEKMTGLVDEAIDT
KSLLDASEEAIKKDLDKCKVAMANIQPQMLVAGATSIARRANRILLVAKREVENSEDPKFR
EAVKAASDELSKTISPMVMDAKAVAGNISDPGLQKSFLDSGYRILGAVAKVREAFQPQEPD
FPPPPPDLEQLRLTDELAPPKPPLPEGEVPPPRPPPPEEKDEEFPEQKAGEVINQPMMMAAR
QLHDEARKWSSKPGIPAAEVGIGVVAEADAADAAGFPVPPDMEDDYEPELLLMPSNQPV
NQPILAAAQSLHREATKWSSKGNDIIAAAKRMALLMAEMSRLVRGGSGTKRALIQCAKDI
AKASDEVTRLAKEVAKQCTDKRIRTNLLQVCERIPTISTQLKILSTVKATMLGRTNISDEES
EQATEMLVHNAQNLMQSVKETVREAEAASIKIRTDAGFTLRWVRKTPWYQ

>Q9Y371 peripheral membrane protein
MNIMDFNVKKLAADAGTFLSRAVQFTEEKLGQAEKTELDAHLENLLSKAECTKIWTEKI
MKQTEVLLQPNPNARIEEFVYEKLDRKAPSRINNPELLGQYMIDAGTEFGPGTAYGNALIK
CGETQKRIGTADRELIQTSALNFLTPLRNFIEGDYKTIAKERKLLQNKRLDLDAAKTRLKK
AKAAETRNSSEQELRITQSEFDRQAEITRLLLEGISSTHAHHLRCLNDFVEAQMTYYAQCY
QYMLDLQKQLGSFPSNYLSNNNQTSVTPVPSVLPNAIGSSAMASTSGLVITSPSNLSDLKE
CSGSRKARVLYDYDAANSTELSLLADEVITVFSVVGMDSDWLMGERGNQKGKVPITYLE
LLN

>Q9NZ01 multipass membrane protein
MKHYEVEILDAKTREKLCFLDKVEPHATIAEIKNLFTKTHPQWYPARQSLRLDPKGKSLK
DEDVLQKLPVGTTATLYFRDLGAQISWVTVFLTEYAGPLFIYLLFYFRVPFIYGHKYDFTSS
RHTVVHLACICHSFHYIKRLLETLFVHRFSHGTMPLRNIFKNCTYYWGFAAWMAYYINHP
LYTPPTYGAQQVKLALAIFVICQLGNFSIHMALRDLRPAGSKTRKIPYPTKNPFTWLFLLVS
CPNYTYEVGSWIGFAIMTQCLPVALFSLVGFTQMTIWAKGKHRSYLKEFRDYPPLRMPIIPF
LL

>Q9Y5L4 peripheral membrane protein
MEGGFGSDFGGSGSGKLDPGLIMEQVKVQIAVANAQELLQRMTDKCFRKCIGKPGGSLD
NSEQKCIAMCMDRYMDAWNTVSRAYNSRLQRERANM

>Q13477 single-pass type I membrane protein
MDFGLALLLAGLLGLLLGQSLQVKPLQVEPPEPVVAVALGASRQLTCRLACADRGASVQ
WRGLDTSLGAVQSDTGRSVLTVRNASLSAAGTRVCVGSCGGRTFQHTVQLLVYAFPDQLT
VSPAALVPGDPEVACTAHKVTPVDPNALSFSLLVGGQELEGAQALGPEVQEEEEEPQGDE
DVLFRVTERWRLPPLGTPVPPALYCQATMRLPGLELSHRQAIPVLHSPTSPEPPDTTSPESP
DTTSPESPDTTSQEPPDTTSPEPPDKTSPEPAPQQGSTHTPRSPGSTRTRRPEISQAGPTQGE
VIPTGSSKPAGDQLPAALWTSSAVLGLLLLALPTYHLWKRCRHLAEDDTHPPASLRLLPQV
SAWAGLRGTGQVGISPS

>O95721 peripheral membrane protein
MSAYPKSYNPFDDDGEDEGARPAPWRDARDLPDGPDAPADRQQYLRQEVLRRAEATAAS
TSRSLALMYESEKVGVASSEELARQRGVLERTEKMVDKMDQDLKISQKHINSIKSVFGGL
VNYFKSKPVETPPEQNGTLTSQPNNRLKEAISTSKEQEAKYQASHPNLRKLDDTDPVPRG
AGSAMSTDAYPKNPHLRAYHQKIDSNLDELSMGLGRLKDIALGMQTEIEEQDDILDRLTT
KVDKLDVNIKSTERKVRQL

>P53007 multipass membrane protein
MPAPRAPRALAAAAPASGKAKLTHPGKAILAGGLAGGIEICITFPTEYVKTQLQLDERSHP
PRYRGIGDCVRQTVRSHGVLGLYRGLSSLLYGSIPKAAVRFGMFEFLSNHMRDAQGRLDS
TRGLLCGLGAGVAEAVVVVCPMETIKVKFIHDQTSPNPKYRGFFHGVREIVREQGLKGTY
QGLTATVLKQGSNQAIRFFVMTSLRNWYRGDNPNKPMNPLITGVFGAIAGAASVFGNTPL
DVIKTRMQGLEAHKYRNTWDCGLQILKKEGLKAFYKGTVPRLGRVCLDVAIVFVIYDEV
VKLLNKVWKTD

>Q96GD0 peripheral membrane protein
MARCERLRGAALRDVLGRAQGVLFDCDGVLWNGERAVPGAPELLERLARAGKAALFVS
NNSRRARPELALRFARLGFGGLRAEQLFSSALCAARLLRQRLPGPPDAPGAVFVLGGEGL
RAELRAAGLRLAGDPSAGDGAAPRVRAVLVGYDEHFSFAKLREACAHLRDPECLLVATDR
DPWHPLSDGSRTPGTGSLAAAVETASGRQALVVGKPSPYMFECITENFSIDPARTLMVGDR
LETDILFGHRCGMTTVLTLTGVSRLEEAQAYLAAGQHDLVPHYYVESIADLTEGLED

>Q9UH99 single-pass type II membrane protein
MSRRSQRLTRYSQGDDDGSSSSGGSSVAGSQSTLFKDSPLRTLKRKSSNMKRLSPAPQLGP
SSDAHTSYYSESLVHESWFPPRSSLEELHGDANWGEDLRVRRRRGTGGSESSRASGLVGR
KATEDFLGSSSGYSSEDDYVGYSDVDQQSSSSRLRSAVSRAGSLLWMVATSPGRLFRLLY
WWAGTTWYRLTTAASLLDVFVLTRRFSSLKTFLWFLLPLLLLTCLTYGAWYFYPYGLQTF
HPALVSWWAAKDSRRPDEGWEARDSSPHFQAEQRVMSRVHSLERRLEALAAEFSSNWQK
EAMRLERLELRQGAPGQGGGGGLSHEDTLALLEGLVSRREAALKEDFRRETAARIQEELS
ALRAEHQQDSEDLFKKIVRASQESEARIQQLKSEWQSMTQESFQESSVKELRRLEDQLAG
LQQELAALALKQSSVAEEVGLLPQQIQAVRDDVESQFPAWISQFLARGGGGRVGLLQREE
MQAQLRELESKILTHVAEMQGKSAREAAASLSLTLQKEGVIGVTEEQVHHIVKQALQRYS
EDRIGLADYALESGGASVISTRCSETYETKTALLSLFGIPLWYHSQSPRVILQPDVHPGNCW
AFQGPQGFAVVRLSARIRPTAVTLEHVPKALSPNSTISSAPKDFAIFGFDEDLQQEGTLLGK
FTYDQDGEPIQTFHFQAPTMATYQVVELRILTNWGHPEYTCIYRFRVHGEPAH

>Q92737 lipid-anchor
MGGSLRVAVLGAPGVGKTAIIRQFLFGDYPERHRPTDGPRLYRPAVLLDGAVYDLSIRDGD
VAGPGSSPGGPEEWPDAKDWSLQDTDAFVLVYDICSPDSFDYVKALRQRIAETRPAGAPE
APILVVGNKRDRQRLRFGPRRALAALVRRGWRCGYLECSAKYNWHVLRLFRELLRCALV
RARPAHPALRLQGALHPARCSLM

>Q9NST1 single-pass type II membrane protein

MYDAERGWSLSFAGCGFLGFYHVGATRCLSEHAPHLLRDARMLFGASAGALHCVGVLS
GIPLEQTLQVLSDLVRKARSRNIGIFHPSFNLSKFLRQGLCKCLPANVHQLISGKIGISLTRVS
DGENVLVSDFRSKDEVVDALVCSCFIPFYSGLIPPSFRGVRYVDGGVSDNVPFIDAKTTITV
SPFYGEYDICPKVKSTNFLHVDITKLSLRLCTGNLYLLSRAFVPPDLKVLGEICLRGYLDAF
RFLEEKGICNRPQPGLKSSSEGMDPEVAMPSWANMSLDSSPESAALAVRLEGDELLDHLR
LSILPWDESILDTLSPRLATALSEEMKDKGGYMSKICNLLPIRIMSYVMLPCTLPVESAIAIV
QRLVTWLPDMPDDVLWLQWVTSQVFTRVLMCLLPASRSQMPVSSQQASPCTPEQDWPC
WTPCSPKGCPAETKAEATPRSILRSSLNFFLGNKVPAGAEGLSTFPSFSLEKSL

>P14784 single-pass type I membrane protein
MAAPALSWRLPLLILLLPLATSWASAAVNGTSQFTCFYNSRANISCVWSQDGALQDTSCQ
VHAWPDRRRWNQTCELLPVSQASWACNLILGAPDSQKLTTVDIVTLRVLCREGVRWRVM
AIQDFKPFENLRLMAPISLQVVHVETHRCNISWEISQASHYFERHLEFEARTLSPGHTWEE
APLLTLKQKQEWICLETLTPDTQYEFQVRVKPLQGEFTTWSPWSQPLAFRTKPAALGKDTI
PWLGHLLVGLSGAFGFIILVYLLINCRNTGPWLKKVLKCNTPDPSKFFSQLSSEHGGDVQK
WLSSPFPSSSFSPGGLAPEISPLEVLERDKVTQLLLQQDKVPEPASLSSNHSLTSCFTNQGYF
FFHLPDALEIEACQVYFTYDPYSEEDPDEGVAGAPTGSSPQPLQPLSGEDDAYCTFPSRDD
LLLFSPSLLGGPSPPSTAPGGSGAGEERMPPSLQERVPRDWDPQPLGPPTPGVPDLVDFQPP
PELVLREAGEEVPDAGPREGVSFPWSRPPGQGEFRALNARLPLNTDAYLSLQELQGQDPT
HLV

>Q8TCT0 peripheral membrane protein
MGATGAAEPLQSVLWVKQQRCAVSLEPARALLRWWRSPGPGAGAPGADACSVPVSEIIA
VEETDVHGKHQGSGKWQKMEKPYAFTVHCVKRARRHRWKWAQVTFWCPEEQLCHLW
LQTLREMLEKLTSRPKHLLVFINPFGGKGQGKRIYERKVAPLFTLASITTDIIVTEHANQAK
ETLYEINIDKYDGIVCVGGDGMFSEVLHGLIGRTQRSAGVDQNHPRAVLVPSSLRIGIIPAG
STDCVCYSTVGTSDAETSALHIVVGDSLAMDVSSVHHNSTLLRYSVSLLGYGFYGDIIKDS
EKKRWLGLARYDFSGLKTFLSHHCYEGTVSFLPAQHTVGSPRDRKPCRAGCFVCRQSKQ
QLEEEQKKALYGLEAAEDVEEWQVVCGKFLAINATNMSCACRRSPRGLSPAAHLGDGSS
DLILIRKCSRFNFLRFLIRHTNQQDQFDFTFVEVYRVKKFQFTSKHMEDEDSDLKEGGKKR
FGHICSSHPSCCCTVSNSSWNCDGEVLHSPAIEVRVHCQLVRLFARGIEENPKPDSHS

>Q96EK6 peripheral membrane protein
MKPDETPMFDPSLLKEVDWSQNTATFSPAISPTHPGEGLVLRPLCTADLNRGFFKVLGQLT
ETGVVSPEQFMKSFEHMKKSGDYYVTVVEDVTLGQIVATATLIIEHKFIHSCAKRGRVEDV
VVSDECRGKQLGKLLLSTLTLLSKKLNCYKITLECLPQNVGFYKKFGYTVSEENYMCRRF
LK

>O95406 multipass membrane protein
MAFTFAAFCYMLALLLTAALIFFAIWHIIAFDELKTDYKNPIDQCNTLNPLVLPEYLIHAFFC
VMFLCAAEWLTLGLNMPLLAYHIWRYMSRPVMSGPGLYDPTTIMNADILAYCQKEGWC
KLAFYLLAFFYYLYGMIYVLVSS

>Q9NYT0 peripheral membrane protein

MEDGVLKEGFLVKRGHIVHNWKARWFILRQNTLVYYKLEGGRRVTPPKGRILLDGCTITC
PCLEYENRPLLIKLKTQTSTEYFLEACSREERDAWAFEITGAIHAGQPGKVQQLHSLRNSF
KLPPHISLHRIVDKMHDSNTGIRSSPNMEQGSTYKKTFLGSSLVDWLISNSFTASRLEAVTL
ASMLMEENFLRPVGVRSMGAIRSGDLAEQFLDDSTALYTFAESYKKKISPKEEISLSTVELS
GTVVKQGYLAKQGHKRKNWKVRRFVLRKDPAFLHYYDPSKEENRPVGGFSLRGSLVSA
LEDNGVPTGVKGNVQGNLFKVITKDDTHYYIQASSKAERAEWIEAIKKLT

>Q14973 multipass membrane protein
MEAHNASAPFNFTLPPNFGKRPTDLALSVILVFMLFFIMLSLGCTMEFSKIKAHLWKPKGL
AIALVAQYGIMPLTAFVLGKVFRLKNIEALAILVCGCSPGGNLSNVFSLAMKGDMNLSIVM
TTCSTFCALGMMPLLLYIYSRGIYDGDLKDKVPYKGIVISLVLVLIPCTIGIVLKSKRPQYM
RYVIKGGMIIILLCSVAVTVLSAINVGKSIMFAMTPLLIATSSLMPFIGFLLGYVLSALFCLN
GRCRRTVSMETGCQNVQLCSTILNVAFPPEVIGPLFFFPLLYMIFQLGEGLLLIAIFWCYEKF
KTPKDKTKMIYTAATTEETIPGALGNGTYKGEDCSPCTA

>P46663 multipass membrane protein
MASSWPPLELQSSNQSQLFPQNATACDNAPEAWDLLHRVLPTFIISICFFGLLGNLFVLLVF
LLPRRQLNVAEIYLANLAASDLVFVLGLPFWAENIWNQFNWPFGALLCRVINGVIKANLFI
SIFLVVAISQDRYRVLVHPMASRRQQRRRQARVTCVLIWVVGGLLSIPTFLLRSIQAVPDLN
ITACILLLPHEAWHFARIVELNILGFLLPLAAIVFFNYHILASLRTREEVSRTRCGGRKDSKT
TALILTLVVAFLVCWAPYHFFAFLEFLFQVQAVRGCFWEDFIDLGLQLANFFAFTNSSLNPV
IYVFVGRLFRTKVWELYKQCTPKSLAPISSSHRKEIFQLFWRN

>Q9UNN8 single-pass type I membrane protein
MLTTLLPILLLSGWAFCSQDASDGLQRLHMLQISYFRDPYHVWYQGNASLGGHLTHVLE
GPDTNTTIIQLQPLQEPESWARTQSGLQSYLLQFHGLVRLVHQERTLAFPLTIRCFLGCELPP
EGSRAHVFFEVAVNGSSFVSFRPERALWQADTQVTSGVVTFTLQQLNAYNRTRYELREFL
EDTCVQYVQKHISAENTKGSQTSRSYTSLVLGVLVGSFIIAGVAVGIFLCTGGRRC

>Q96BD0 multipass membrane protein
MPLHQLGDKPLTFPSPNSAMENGLDHTPPSRRASPGTPLSPGSLRSAAHSPLDTSKQPLCQ
LWAEKHGARGTHEVRYVSAGQSVACGWWAFAPPCLQVLNTPKGILFFLCAAAFLQGMTV
NGFINTVITSLERRYDLHSYQSGLIASSYDIAACLCLTFVSYFGGSGHKPRWLGWGVLLMG
TGSLVFALPHFTAGRYEVELDAGVRTCPANPGAVCADSTSGLSRYQLVFMLGQFLHGVGA
TPLYTLGVTYLDENVKSSCSPVYIAIFYTAAILGPAAGYLIGGALLNIYTEMGRRTELTTESP
LWVGAWWVGFLGSGAAAFFTAVPILGYPRQLPGSQRYAVMRAAEMHQLKDSSRGEASNP
DFGKTIRDLPLSIWLLLKNPTFILLCLAGATEATLITGMSTFSPKFLESQFSLSASEAATLFGY
LVVPAGGGGTFLGGFFVNKLRLRGSAVIKFCLFCTVVSLLGILVFSLHCPSVPMAGVTASY
GGSLLPEGHLNLTAPCNAACSCQPEHYSPVCGSDGLMYFSLCHAGCPAATETNVDGQKV
YRDCSCIPQNLSSGFGHATAGKCTSTCQRKPLLLVFIFVVIFFTFLSSIPALTATLRCVRDPQR
SFALGIQWIVVRILGGIPGPIAFGWVIDKACLLWQDQCGQQGSCLVYQNSAMSRYILIMGL
LYKVLGVLFFAIACFLYKPLSESSDGLETCLPSQSSAPDSATDSQLQSSV

>Q9NQ40 multipass membrane protein

MAFLMHLLVCVFGMGSWVTINGLWVELPLLVMELPEGWYLPSYLTVVIQLANIGPLLVTL
LHHFRPSCLSEVPIIFTLLGVGTVTCIIFAFLWNMTSWVLDGHHSIAFLVLTFFLALVDCTSS
VTFLPFMSRLPTYYLTTFFVGEGLSGLLPALVALAQGSGLTTCVNVTEISDSVPSPVPTRET
DIAQGVPRALVSALPGMEAPLSHLESRYLPAHFSPLVFFLLLSIMMACCLVAFFVLQRQPRC
WEASVEDLLNDQVTLHSIRPREENDLGPAGTVDSSQGQGYLEEKAAPCCPAHLAFIYTLV
AFVNALTNGMLPSVQTYSCLSYGPVAYHLAATLSIVANPLASLVSMFLPNRSLLFLGVLSV
LGTCFGGYNMAMAVMSPCPLLQGHWGGEVLIVASWVLFSGCLSYVKVMLGVVLRDLSR
SALLWCGAAVQLGSLLGALLMFPLVNVLRLFSSADFCNLHCPA

>Q9BQL6 peripheral membrane protein
MLSSTDFTFASWELVVRVDHPNEEQQKDVTLRVSGDLHVGGVMLKLVEQINISQDWSDF
ALWWEQKHCWLLKTHWTLDKYGVQADAKLLFTPQHKMLRLRLPNLKMVRLRVSFSAV
VFKAVSDICKILNIRRSEELSLLKPSGDYFKKKKKKDKNNKEPIIEDILNLESSPTASGSSVSP
GLYSKTMTPIYDPINGTPASSTMTWFSDSPLTEQNCSILAFSQPPQSPEALADMYQPRSLVD
KAKLNAGWLDSSRSLMEQGIQEDEQLLLRFKYYSFFDLNPKYDAVRINQLYEQARWAILL
EEIDCTEEEMLIFAALQYHISKLSLSAETQDFAGESEVDEIEAALSNLEVTLEGGKADSLLE
DITDIPKLADNLKLFRPKKLLPKAFKQYWFIFKDTSIAYFKNKELEQGEPLEKLNLRGCEV
VPDVNVAGRKFGIKLLIPVADGMNEMYLRCDHENQYAQWMAACMLASKGKTMADSSY
QPEVLNILSFLRMKNRNSASQVASSLENMDMNPECFVSPRCAKRHKSKQLAARILEAHQN
VAQMPLVEAKLRFIQAWQSLPEFGLTYYLVRFKGSKKDDILGVSYNRLIKIDAATGIPVTT
WRFTNIKQWNVNWETRQVVIEFDQNVFTAFTCLSADCKIVHEYIGGYIFLSTRSKDQNET
LDEDLFHKLTGGQD

>Q9HDC9 single-pass type II membrane protein
MSEADGLRQRRPLRPQVVTDDDGQAPEAKDGSSFSGRVFRVTFLMLAVSLTVPLLGAMM
LLESPIDPQPLSFKEPPLLLGVLHPNTKLRQAERLFENQLVGPESIAHIGDVMFTGTADGRV
VKLENGEIETIARFGSGPCKTRDDEPVCGRPLGIRAGPNGTLFVADAYKGLFEVNPWKRE
VKLLLSSETPIEGKNMSFVNDLTVTQDGRKIYFTDSSSKWQRRDYLLLVMEGTDDGRLLE
YDTVTREVKVLLDQLRFPNGVQLSPAEDFVLVAETTMARIRRVYVSGLMKGGADLFVEN
MPGFPDNIRPSSSGGYWVGMSTIRPNPGFSMLDFLSERPWIKRMIFKLFSQETVMKFVPRY
SLVLELSDSGAFRRSLHDPDGLVATYISEVHEHDGHLYLGSFRSPFLCRLSLQAV

>P08842 multipass membrane protein
MPLRKMKIPFLLLFFLWEAESHAASRPNIILVMADDLGIGDPGCYGNKTIRTPNIDRLASGG
VKLTQHLAASPLCTPSRAAFMTGRYPVRSGMASWSRTGVFLFTASSGGLPTDEITFAKLLK
DQGYSTALIGKWHLGMSCHSKTDFCHHPLHHGFNYFYGISLTNLRDCKPGEGSVFTTGFK
RLVFLPLQIVGVTLLTLAALNCLGLLHVPLGVFFSLLFLAALILTLFLGFLHYFRPLNCFMM
RNYEIIQQPMSYDNLTQRLTVEAAQFIQRNTETPFLLVLSYLHVHTALFSSKDFAGKSQHG
VYGDAVEEMDWSVGQILNLLDELRLANDTLIYFTSDQGAHVEEVSSKGEIHGGSNGIYKG
GKANNWEGGIRVPGILRWPRVIQAGQKIDEPTSNMDIFPTVAKLAGAPLPEDRIIDGRDLM
PLLEGKSQRSDHEFLFHYCNAYLNAVRWHPQNSTSIWKAFFFTPNFNPVGSNGCFATHVCF
CFGSYVTHHDPPLLFDISKDPRERNPLTPASEPRFYEILKVMQEAADRHTQTLPEVPDQFS
WNNFLWKPWLQLCCPSTGLSCQCDREKQDKRLSR

>P51841 single-pass type I membrane protein
MFLGLGRFSRLVLWFAAFRKLLGHHGLASAKFLWCLCLLSVMSLPQQVWTLPYKIGVVG
PWACDSLFSKALPEVAARLAIERINRDPSFDLSYSFEYVILNEDCQTSRALSSFISHHQMAS
GFIGPTNPGYCEAASLLGNSWDKGIFSWACVNYELDNKISYPTFSRTLPSPIRVLVTVMKYF
QWAHAGVISSDEDIWVHTANRVASALRSHGLPVGVVLTTGQDSQSMRKALQRIHQADRIR
IIIMCMHSALIGGETQMHLLECAHDLKMTDGTYVFVPYDALLYSLPYKHTPYRVLRNNPK
LREAYDAVLTITVESQEKTFYQAFTEAAARGEIPEKLEFDQVSPLFGTIYNSIYFIAQAMNN
AMKENGQAGAASLVQHSRNMQFHGFNQLMRTDSNGNGISEYVILDTNLKEWELHSTYT
VDMEMELLRFGGTPIHFPGGRPPRADAKCWFAEGKICHGGIDPAFAMMVCLTLLIALLSIN
GFAYFIRRRINKIQLIKGPNRILLTLEDVTFINPHFGSKRGSRASVSFQITSEVQSGRSPRLSFS
SGSLTPATYENSNIAIYEGDWVWLKKFSLGDFGDLKSIKSRASDVFEMMKDLRHENINPLL
GFFYDSGMFAIVTEFCSRGSLEDILTNQDVKLDWMFKSSLLLDLIKGMKYLHHREFVHGR
LKSRNCVVDGRFVLKVTDYGFNDILEMLRLSEEESSMEELLWTAPELLRAPRGSRLGSFA
GDVYSFAIIMQEVMVRGTPFCMMDLPAQEIINRLKKPPPVYRPVVPPEHAPPECLQLMKQ
CWAEAAEQRPTFDEIFNQFKTFNKGKKTNIIDSMLRMLEQYSSNLEDLIRERTEELEIEKQK
TEKLLTQMLPPSVAESLKKGCTVEPEGFDLVTLYFSDIVGFTTISAMSEPIEVVDLLNDLYTL
FDAIIGSHDVYKVETIGDAYMVASGLPKRNGSRHAAEIANMSLDILSSVGTFKMRHMPEV
PVRIRIGLHSGPVVAGVVGLTMPRYCLFGDTVNTASRMESTGLPYRIHVSLSTVTILQNLSE
GYEVELRGRTELKGKGTEETFWLIGKKGFMKPLPVPPPVDKDGQVGHGLQPVEIAAFQR
RKAERQLVRNKP

>P33897 multipass membrane protein
MPVLSRPRPWRGNTLKRTAVLLALAAYGAHKVYPLVRQCLAPARGLQAPAGEPTQEASG
VAAAKAGMNRVFLQRLLWLLRLLFPRVLCRETGLLALHSAALVSRTFLSVYVARLDGRLA
RCIVRKDPRAFGWQLLQWLLIALPATFVNSAIRYLEGQLALSFRSRLVAHAYRLYFSQQTY
YRVSNMDGRLRNPDQSLTEDVVAFAASVAHLYSNLTKPLLDVAVTSYTLLRAARSRGAGT
AWPSAIAGLVVFLTANVLRAFSPKFGELVAEEARRKGELRYMHSRVVANSEEIAFYGGHEV
ELALLQRSYQDLASQINLILLERLWYVMLEQFLMKYVWSASGLLMVAVPIITATGYSESDA
EAVKKAALEKKEEELVSERTEAFTIARNLLTAAADAIERIMSSYKEVTELAGYTARVHEMF
QVFEDVQRCHFKRPRELEDAQAGSGTIGRSGVRVEGPLKIRGQVVDVEQGIICENIPIVTPS
GEVVVASLNIRVEEGMHLLITGPNGCGKSSLFRILGGLWPTYGGVLYKPPPQRMFYIPQRP
YMSVGSLRDQVIYPDSVEDMQRKGYSEQDLEAILDVVHLHHILQREGGWEAMCDWKDV
LSGGEKQRIGMARMFYHRPKYALLDECTSAVSIDVEGKIFQAAKDAGIALLSITHRPSLWK
YHTHLLQFDGEGGWKFEKLDSAARLSLTEEKQRLEQQLAGIPKMQRRLQELCQILGEAVA
PAHVPAPSPQGPGGLQGAST

>Q13585 multipass membrane protein
MGPTLAVPTPYGCIGCKLPQPEYPPALIIFMFCAMVITIVVDLIGNSMVILAVTKNKKLRNS
GNIFVVSLSVADMLVAIYPYPLMLHAMSIGGWDLSQLQCQMVGFITGLSVVGSIFNIVAIAI
NRYCYICHSLQYERIFSVRNTCIYLVITWIMTVLAVLPNMYIGTIEYDPRTYTCIFNYLNNPV
FTVTIVCIHFVLPLLIVGFCYVRIWTKVLAARDPAGQNPDNQLAEVRNFLTMFVIFLLFAVC
WCPINVLTVLVAVSPKEMAGKIPNWLYLAAYFIAYFNSCLNAVIYGLLNENFRREYWTIFH
AMRHPIIFFSGLISDIREMQEARTLARARAHARDQAREQDRAHACPAVEETPMNVRNVPL
PGDAAAGHPDRASGHPKPHSRSSSAYRKSASTHHKSVFSHSKAASGHLKPVSGHSKPASG

HPKSATVYPKPASVHFKADSVHFKGDSVHFKPDSVHFKPASSNPKPITGHHVSAGSHSKSA
FSAATSHPKPTTGHIKPATSHAEPTTADYPKPATTSHPKPTAADNPELSASHCPEIPAIAHPVS
DDSDLPESASSPAAGPTKPAASQLESDTIADLPDPTVVTTSTNDYHDVVVIDVEDDPDEMA
V

>O75695 lipid-anchor
MGCFFSKRRKADKESRPENEEERPKQYSWDQREKVDPKDYMFSGLKDETVGRLPGTVA
GQQFLIQDCENCNIYIFDHSATVTIDDCTNCIIFLGPVKGSVFFRNCRDCKCTLACQQFRVR
DCRKLEVFLCCATQPIIESSSNIKFGCFQWYYPELAFQFKDAGLSIFNNTWSNIHDFTPVSG
ELNWSLLPEDAVVQDYVPIPTTEELKAVRVSTEANRSIVPISRGQRQKSSDESCLVVLFAGD
YTIANARKLIDEMVGKGFFLVQTKEVSMKAEDAQRVFREKAPDFLPLLNKGPVIALEFNG
DGAVEVCQLIVNEIFNGTKMFVSESKETASGDVDSFYNFADIQMGI

>Q9NV92 multipass membrane protein
MARRRSQRVCASGPSMLNSARGAPELLRGTATNAEVSAAAAGATGSEELPPGDRGCRNG
GGRGPAATTSSTGVAVGAEHGEDSLSRKPDPEPGRMDHHQPGTGRYQVLLNEEDNSESSA
IEQPPTSNPAPQIVQAASSAPALETDSSPPPYSSITVEVPTTSDTEVYGEFYPVPPPYSVATSL
PTYDEAEKAKAAAMAAAAAETSQRIQEEECPPRDDFSDADQLRVGNDGIFMLAFFMAFIF
NWLGFCLSFCITNTIAGRYGAICGFGLSLIKWILIVRFSDYFTGYFNGQYWLWWIFLVLGL
LLFFRGFVNYLKVRNMSESMAAAHRTRYFFLL

>O43193 multipass membrane protein
MGSPWNGSDGPEGAREPPWPALPPCDERRCSPFPLGALVPVTAVCLCLFVVGVSGNVVTV
MLIGRYRDMRTTTNLYLGSMAVSDLLILLGLPFDLYRLWRSRPWVFGPLLCRLSLYVGEG
CTYATLLHMTALSVERYLAICRPLRARVLVTRRRVRALIAVLWAVALLSAGPFLFLVGVEQ
DPGISVVPGLNGTARIASSPLASSPPLWLSRAPPPSPPSGPETAEAAALFSRECRPSPAQLGA
LRVMLWVTTAYFFLPFLCLSILYGLIGRELWSSRRPLRGPAASGRERGHRQTVRVLLVVVL
AFIICWLPFHVGRIIYINTEDSRMMYFSQYFNIVALQLFYLSASINPILYNLISKKYRAAAFK
LLLARKSRPRGFHRSRDTAGEVAGDTGGDTVGYTETSANVKTMG

>Q9Y342 multipass membrane protein
MAEFPSKVSTRTSSPAQGAEASVSALRPDLGFVRSRLGALMLLQLVLGLLVWALIADTPY
HLYPAYGWVMFVAVFLWLVTIVLFNLYLFQLHMKLYMVPWPLVLMIFNISATVLYITAFIA
CSAAVDLTSLRGTRPYNQRAAASFFACLVMIAYGVSAFFSYQAWRGVGSNAATSQMAGG
YA

>P51511 single-pass type I membrane protein
MGSDPSAPGRPGWTGSLLGDREEAARPRLLPLLLVLLGCLGLGVAAEDAEVHAENWLRL
YGYLPQPSRHMSTMRSAQILASALAEMQRFYGIPVTGVLDEETKEWMKRPRCGVPDQFG
VRVKANLRRRRKRYALTGRKWNNHHLTFSIQNYTEKLGWYHSMEAVRRAFRVWEQATPL
VFQEVPYEDIRLRRQKEADIMVLFASGFHGDSSPFDGTGGFLAHAYFPGPGLGGDTHFDA
DEPWTFSSTDLHGNNLFLVAVHELGHALGLEHSSNPNAIMAPFYQWKDVDNFKLPEDDL
RGIQQLYGTPDGQPQPTQPLPTVTPRRPGRPDHRPPRPPQPPPPGGKPERPPKPGPPVQPRA
TERPDQYGPNICDGDFDTVAMLRGEMFVFKGRWFWRVRHNRVLDNYPMPIGHFWRGLP

GDISAAYERQDGRFVFFKGDRYWLFREANLEPGYPQPLTSYGLGIPYDRIDTAIWWEPTGH
TFFFQEDRYWRFNEETQRGDPGYPKPISVWQGIPASPKGAFLSNDAAYTYFYKGTKYWKF
DNERLRMEPGYPKSILRDFMGCQEHVEPGPRWPDVARPPFNPHGGAEPGADSAEGDVGD
GDGDFGAGVNKDGGSRVVVQMEEVARTVNVVMVLVPLLLLLCVLGLTYALVQMQRKG
APRVLLYCKRSLQEWV

>Q9NY59 lipid-anchor
MVLYTTPFPNSCLSALHCVSWALIFPCYWLVDRLAASFIPTTYEKRQRADDPCCLQLLCTA
LFTPIYLALLVASLPFAFLGFLFWSPLQSARRPYIYSRLEDKGLAGGAALLSEWKGTGPGKS
FCFATANVCLLPDSLARVNNLFNTQARAKEIGQRIRNGAARPQIKIYIDSPTNTSISAASFSS
LVSPQGGDGVARAVPGSIKRTASVEYKGDGGRHPGDEAANGPASGDPVDSSSPEDACIVRI
GGEEGGRPPEADDPVPGGQARNGAGGGPRGQTPNHNQQDGDSGSLGSPSASRESLVKGR
AGPDTSASGEPGANSKLLYKASVVKKAAARRRRHPDEAFDHEVSAFFPANLDFLCLQEVF
DKRAATKLKEQLHGYFEYILYDVGVYGCQGCCSFKCLNSGLLFASRYPIMDVAYHCYPNK
CNDDALASKGALFLKVQVGSTPQDQRIVGYIACTHLHAPQEDSAIRCGQLDLLQDWLAD
FRKSTSSSSAANPEELVAFDVVCGDFNFDNCSSDDKLEQQHSLFTHYRDPCRLGPGEEKP
WAIGTLLDTNGLYDEDVCTPDNLQKVLESEEGRREYLAFPTSKSSGQKGRKELLKGNGRR
IDYMLHAEEGLCPDWKAEVEEFSFITQLSGLTDHLPVAMRLMVSSGEEEA

>Q7L5A8 multipass membrane protein
MAPAPPPAASFSPSEVQRRLAAGACWVRRGARLYDLSSFVRHHPGGEQLLRARAGQDISA
DLDGPPHRHSANARRWLEQYYVGELRGEQQGSMENEPVALEETQKTDPAMEPRFKVVD
WDKDLVDWRKPLLWQVGHLGEKYDEWVHQPVTRPIRLFHSDLIEGLSKTVWYSVPIIWV
PLVLYLSWSYYRTFAQGNVRLFTSFTTEYTVAVPKSMFPGLFMLGTFLWSLIEYLIHRFLFH
MKPPSDSYYLIMLHFVMHGQHHKAPFDGSRLVFPPVPASLVIGVFYLCMQLILPEAVGGTV
FAGGLLGYVLYDMTHYYLHFGSPHKGSYLYSLKAHHVKHHFAHQKSGFGISTKLWDYCF
HTLTPEKPHLKTQ

>P49815 peripheral membrane protein
MAKPTSKDSGLKEKFKILLGLGTPRPNPRSAEGKQTEFIITAEILRELSMECGLNNRIRMIG
QICEVAKTKKFEEHAVEALWKAVADLLQPERPLEARHAVLALLKAIVQGQGERLGVLRAL
FFKVIKDYPSNEDLHERLEVFKALTDNGRHITYLEEELADFVLQWMDVGLSSEFLLVLVNL
VKFNSCYLDEYIARMVQMICLLCVRTASSVDIEVSLQVLDAVVCYNCLPAESLPLFIVTLC
RTINVKELCEPCWKLMRNLLGTHLGHSAIYNMCHLMEDRAYMEDAPLLRGAVFFVGMA
LWGAHRLYSLRNSPTSVLPSFYQAMACPNEVVSYEIVLSITRLIKKYRKELQVVAWDILLN
IIERLLQQLQTLDSPELRTIVHDLLTTVEELCDQNEFHGSQERYFELVERCADQRPESSLLNL
ISYRAQSIHPAKDGWIQNLQALMERFFRSESRGAVRIKVLDVLSFVLLINRQFYEEELINSV
VISQLSHIPEDKDHQVRKLATQLLVDLAEGCHTHHFNSLLDIIEKVMARSLSPPPELEERDV
AAYSASLEDVKTAVLGLLVILQTKLYTLPASHATRVYEMLVSHIQLHYKHSYTLPIASSIRL
QAFDFLLLLRADSLHRLGLPNKDGVVRFSPYCVCDYMEPERGSEKKTSGPLSPPTGPPGPA
PAGPAVRLGSVPYSLLFRVLLQCLKQESDWKVLKLVLGRLPESLRYKVLIFTSPCSVDQLC
SALCSMLSGPKTLERLRGAPEGFSRTDLHLAVVPVLTALISYHNYLDKTKQREMVYCLEQ
GLIHRCASQCVVALSICSVEMPDIIIKALPVLVVKLTHISATASMAVPLLEFLSTLARLPHLYR
NFAAEQYASVFAISLPYTNPSKFNQYIVCLAHHVIAMWFIRCRLPFRKDFVPFITKGLRSNV

LLSFDDTPEKDSFRARSTSLNERPKSLRIARPPKQGLNNSPPVKEFKESSAAEAFRCRSISVS
EHVVRSRIQTSLTSASLGSADENSVAQADDSLKNLHLELTETCLDMMARYVFSNFTAVPKR
SPVGEFLLAGGRTKTWLVGNKLVTVTTSVGTGTRSLLGLDSGELQSGPESSSSPGVHVRQ
TKEAPAKLESQAGQQVSRGARDRVRSMSGGHGLRVGALDVPASQFLGSATSPGPRTAPAA
KPEKASAGTRVPVQEKTNLAAYVPLLTQGWAEILVRRPTGNTSWLMSLENPLSPFSSDINN
MPLQELSNALMAAERFKEHRDTALYKSLSVPAASTAKPPPLPRSNTVASFSSLYQSSCQGQ
LHRSVSWADSAVVMEEGSPGEVPVLVEPPGLEDVEAALGMDRRTDAYSRSSSVSSQEEKS
LHAEELVGRGIPIERVVSSEGGRPSVDLSFQPSQPLSKSSSSPELQTLQDILGDPGDKADVG
RLSPEVKARSQSGTLDGESAAWSASGEDSRGQPEGPLPSSSPRSPSGLRPRGYTISDSAPSR
RGKRVERDALKSRATASNAEKVPGINPSFVFLQLYHSPFFGDESNKPILLPNESQSFERSVQ
LLDQIPSYDTHKIAVLYVGEGQSNSELAILSNEHGSYRYTEFLTGLGRLIELKDCQPDKVYL
GGLDVCGEDGQFTYCWHDDIMQAVFHIATLMPTKDVDKHRCDKKRHLGNDFVSIVYND
SGEDFKLGTIKGQFNFVHVIVTPLDYECNLVSLQCRKDMEGLVDTSVAKIVSDRNLPFVAR
QMALHANMASQVHHSRSNPTDIYPSKWIARLRHIKRLRQRICEEAAYSNPSLPLVHPPSHS
KAPAQTPAEPTPGYEVGQRKRLISSVEDFTEFV

>O75783 multipass membrane protein
MGRVEDGGTTEELEDWDPGTSALPAPGIKQGPREQTGTGPLSQKCWEPEPDAPSQPGPAL
WSRGRARTQALAGGSSLQQLDPENTGFIGADTFTGLVHSHELPLDPAKLDMLVALAQSNE
QGQVCYQELVDLISSKRSSSFKRAIANGQRALPRDGPLDEPGLGVYKRFVRYVAYEILPCE
VDRRWYFYRHRSCPPPVFMASVTLAQIIVFLCYGARLNKWVLQTYHPEYMKSPLVYHPG
HRARAWRFLTYMFMHVGLEQLGFNALLQLMIGVPLEMVHGLLRISLLYLAGVLAGSLTV
SITDMRAPVVGGSGGVYALCSAHLANVVMNWAGMRCPYKLLRMVLALVCMSSEVGRA
VWLRFSPPLPASGPQPSFMAHLAGAVVGVSMGLTILRSYEERLRDQCGWWVVLLAYGTF
LLFAVFWNVFAYDLLGAHIPPPP

>Q05996 single-pass type I membrane protein
MACRQRGGSWSPSGWFNAGWSTYRSISLFFALVTSGNSIDVSQLVNPAFPGTVTCDEREIT
VEFPSSPGTKKWHASVVDPLGLDMPNCTYILDPEKLTLRATYDNCTRRVHGGHQMTIRV
MNNSAALRHGAVMYQFFCPAMQVEETQGLSASTICQKDFMSFSLPRVFSGLADDSKGTK
VQMGWSIEVGDGARAKTLTLPEAMKEGFSLLIDNHRMTFHVPFNATGVTHYVQGNSHLY
MVSLKLTFISPGQKVIFSSQAICAPDPVTCNATHMTLTIPEFPGKLKSVSFENQNIDVSQLHD
NGIDLEATNGMKLHFSKTLLKTKLSEKCLLHQFYLASLKLTFLLRPETVSMVIYPECLCESP
VSIVTGELCTQDGFMDVEVYSYQTQPALDLGTLRVGNSSCQPVFEAQSQGLVRFHIPLNG
CGTRYKFEDDKVVYENEIHALWTDFPPSKISRDSEFRMTVKCSYSRNDMLLNINVESLTPP
VASVKLGPFTLILQSYPDNSYQQPYGENEYPLVRFLRQPIYMEVRVLNRDDPNIKLVLDDC
WATSTMDPDSFPQWNVVVDGCAYDLDNYQTTFHPVGSSVTHPDHYQRFDMKAFAFVSE
AHVLSSLVYFHCSALICNRLSPDSPLCSVTCPVSSRHRRATGATEAEKMTVSLPGPILLLSD
DSSFRGVGSSDLKASGSSGEKSRSETGEEVGSRGAMDTKGHKTAGDVGSKAVAAVAAFA
GVVATLGFIYYLYEKRTVSNH

>O94778 multipass membrane protein
MSGEIAMCEPEFGNDKAREPSVGGRWRVSWYERFVQPCLVELLGSALFIFIGCLSVIENGT
DTGLLQPALAHGLALGLVIATLGNISGGHFNPAVSLAAMLIGGLNLVMLLPYWVSQLLGG

MLGAALAKAVSPEERFWNASGAAFVTVQEQGQVAGALVAEIILTTLLALAVCMGAINEKT
KGPLAPFSIGFAVTVDILAGGPVSGGCMNPARAFGPAVVANHWNFHWIYWLGPLLAGLLV
GLLIRCFIGDGKTRLILKAR

>Q9P253 peripheral membrane protein
MASILDEYENSLSRSAVLQPGCPSVGIPHSGYVNAQLEKEVPIFTKQRIDFTPSERITSLVVS
SNQLCMSLGKDTLLRIDLGKANEPNHVELGRKDDAKVHKMFLDHTGSHLLIALSSTEVL
YVNRNGQKVRPLARWKGQLVESVGWNKALGTESSTGPILVGTAQGHIFEAELSASEGGLF
GPAPDLYFRPLYVLNEEGGPAPVCSLEAERGPDGRSFVIATTRQRLFQFIGRAAEGAEAQGF
SGLFAAYTDHPPPFREFPSNLGYSELAFYTPKLRSAPRAFAWMMGDGVLYGALDCGRPDS
LLSEERVWEYPEGVGPGASPPLAIVLTQFHFLLLLADRVEAVCTLTGQVVLRDHFLEKFGP
LKHMVKDSSTGQLWAYTERAVFRYHVQREARDVWRTYLDMNRFDLAKEYCRERPDCLD
TVLAREADFCFRQRRYLESARCYALTQSYFEEIALKFLEARQEEALAEFLQRKLASLKPAE
RTQATLLTTWLTELYLSRLGALQGDPEALTLYRETKECFRTFLSSPRHKEWLFASRASIHEL
LASHGDTEHMVYFAVIMQDYERVVAYHCQHEAYEEALAVLARHRDPQLFYKFSPILIRHIP
RQLVDAWIEMGSRLDARQLIPALVNYSQGGEVQQVSQAIRYMEFCVNVLGETEQAIHNYL
LSLYARGRPDSLLAYLEQAGASPHRVHYDLKYALRLCAEHGHHRACVHVYKVLELYEEA
VDLALQVDVDLAKQCADLPEEDEELRKKLWLKIARHVVQEEEDVQTAMACLASCPLLKI
EDVLPFFPDFVTIDHFKEAICSSLKAYNHHIQELQREMEEATASAQRIRRDLQELRGRYGTV
EPQDKCATCDFPLLNRPFYLFLCGHMFHADCLLQAVRPGLPAYKQARLEELQRKLGAAPP
PAKGSARAKEAEGGAATAGPSREQLKADLDELVAAECVYCGELMIRSIDRPFIDPQRYEEE
QLSWL

>Q9UL45 peripheral membrane protein
MSVPGPSSPDGALTRPPYCLEAGEPTPGLSDTSPDEGLIEDLTIEDKAVEQLAEGLLSHYLP
DLQRSKQALQELTQNQVVLLDTLEQEISKFKECHSMLDINALFAEAKHYHAKLVNIRKEM
LMLHEKTSKLKKRALKLQQKRQKEELEREQQREKEFEREKQLTARPAKRM

>O43909 single-pass type II membrane protein
MTGYTMLRNGGAGNGGQTCMLRWSNRIRLTWLSFTLFVILVFFPLIAHYYLTTLDEADEA
GKRIFGPRVGNELCEVKHVLDLCRIRESVSEELLQLEAKRQELNSEIAKLNLKIEACKKSIE
NAKQDLLQLKNVISQTEHSYKELMAQNQPKLSLPIRLLPEKDDAGLPPPKATRGCRLHNC
FDYSRCPLTSGFPVYVYDSDQFVFGSYLDPLVKQAFQATARANVYVTENADIACLYVILVG
EMQEPVVLRPAELEKQLYSLPHWRTDGHNHVIINLSRKSDTQNLLYNVSTGRAMVAQSTF
YTVQYRPGFDLVVSPLVHAMSEPNFMEIPPQVPVKRKYLFTFQGEKIESLRSSLQEARSFEE
EMEGDPPADYDDRIIATLKAVQDSKLDQVLVEFTCKNQPKPSLPTEWALCGEREDRLELLK
LSTFALIITPGDPRLVISSGCATRLFEALEVGAVPVVLGEQVQLPYQDMLQWNEAALVVPK
PRVTEVHFLLRSLSDSDLLAMRRQGRFLWETYFSTADSIFNTVLAMIRTRIQIPAAPIREEAA
AEIPHRSGKAAGTDPNMADNGDLDLGPVETEPPYASPRYLRNFTLTVTDFYRSWNCAPGP
FHLFPHTPFDPVLPSEAKFLGSGTGFRPIGGGAGGSGKEFQAALGGNVPREQFTVVMLTYE
REEVLMNSLERLNGLPYLNKVVVVWNSPKLPSEDLLWPDIGVPIMVVRTEKNSLNNRFLP
WNEIETEAILSIDDDAHLRHDEIMFGFRVWREARDRIVGFPGRYHAWDIPHQSWLYNSNY
SCELSMVLTGAAFFHKYYAYLYSYVMPQAIRDMVDEYINCEDIAMNFLVSHITRKPPIKVT
SRWTFRCPGCPQALSHDDSHFHERHKCINFFVKVYGYMPLLYTQFRVDSVLFKTRLPHDK

TKCFKFI

>P37268 multipass membrane protein
MEFVKCLGHPEEFYNLVRFRIGGKRKVMPKMDQDSLSSSLKTCYKYLNQTSRSFAAVIQA
LDGEMRNAVCIFYLVLRALDTLEDDMTISVEKKVPLLHNFHSFLYQPDWRFMESKEKDRQ
VLEDFPTISLEFRNLAEKYQTVIADICRRMGIGMAEFLDKHVTSEQEWDKYCHYVAGLVG
IGLSRLFSASEFEDPLVGEDTERANSMGLFLQKTNIIRDYLEDQQGGREFWPQEVWSRYV
KKLGDFAKPENIDLAVQCLNELITNALHHIPDVITYLSRLRNQSVFNFCAIPQVMAIATLAA
CYNNQQVFKGAVKIRKGQAVTLMMDATNMPAVKAIIYQYMEEIYHRIPDSDPSSSKTRQII
STIRTQNLPNCQLISRSHYSPIYLSFVMLLAALSWQYLTTLSQVTEDYVQTGEH

>Q8TB36 multipass membrane protein
MAERQEEQRGSPPLRAEGKADAEVKLILYHWTHSFSSQKVRLVIAEKALKCEEHDVSLPL
SEHNEPWFMRLNSTGEVPVLIHGENIICEATQIIDYLEQTFLDERTPRLMPDKESMYYPRVQ
HYRELLDSLPMDAYTHGCILHPELTVDSMIPAYATTRIRSQIGNTESELKKLAEENPDLQEA
YIAKQKRLKSKLLDHDNVKYLKKILDELEKVLDQVETELQRRNEETPEEGQQPWLCGESF
TLADVSLAVTLHRLKFLGFARRNWGNGKRPNLETYYERVLKRKTFNKVLGHVNNILISAV
LPTAFRVAKKRAPKVLGTTLVVGLLAGVGYFAFMLFRKRLGSMILAFRPRPNYF

>Q93045 peripheral membrane protein
MAKTAMAYKEKMKELSMLSLICSCFYPEPRNINIYTYDDMEVKQINKRASGQAFELILKP
PSPISEAPRTLASPKKKDLSLEEIQKKLEAAEERRKSQEAQVLKQLAEKREHEREVLQKAL
EENNNFSKMAEEKLILKMEQIKENREANLAAIIERLQEKERHAAEVRRNKELQVELSG

>O00220 single-pass type I membrane protein
MAPPPARVHLGAFLAVTPNPGSAASGTEAAAATPSKVWGSSAGRIEPRGGGRGALPTSMG
QHGPSARARAGRAPGPRPAREASPRLRVHKTFKFVVVGVLLQVVPSSAATIKLHDQSIGT
QQWEHSPLGELCPPGSHRSEHPGACNRCTEGVGYTNASNNLFACLPCTACKSDEEERSPC
TTTRNTACQCKPGTFRNDNSAEMCRKCSRGCPRGMVKVKDCTPWSDIECVHKESGNGH
NIWVILVVTLVVPLLLVAVLIVCCCIGSGCGGDPKCMDRVCFWRLGLLRGPGAEDNAHNEI
LSNADSLSTFVSEQQMESQEPADLTGVTVQSPGEAQCLLGPAEAEGSQRRRLLVPANGAD
PTETLMLFFDKFANIVPFDSWDQLMRQLDLTKNEIDVVRAGTAGPGDALYAMLMKWVN
KTGRNASIHTLLDALERMEERHAREKIQDLLVDSGKFIYLEDGTGSAVSLE

>Q8IY17 single-pass type I membrane protein
MGTSSHGLATNSSGAKVAERDGFQDVLAPGEGSAGRICGAQPVPFVPQVLGVMIGAGVA
VVVTAVLILLVVRRLRVPKTPAPDGPRYRFRKRDKVLFYGRKIMRKVSQSTSSLVDTSVSA
TSRPRMRKKLKMLNIAKKILRIQKETPTLQRKEPPPAVLEADLTEGDLANSHLPSEVLYML
KNVRVLGHFEKPLFLELCRHMVFQRLGQGDYVFRPGQPDASIYVVQDGLLELCLPGPDG
KECVVKEVVPGDSVNSLLSILDVITGHQHPQRTVSARAARDSTVLRLPVEAFSAVFTKYPE
SLVRVVQIIMVRLQRVTFLALHNYLGLTNELFSHEIQPLRLFPSPGLPTRTSPVRGSKRMVS
TSATDEPRETPGRPPDPTGAPLPGPTGDPVKPTSLETPSAPLLSRCVSMPGDISGLQGGPRS
DFDMAYERGRISVSLQEEASGGSLAAPARTPTQEPREQPAGACEYSYCEDESATGGCPFGP
YQGRQTSSIFEAAKQELAKLMRIEDPSLLNSRVLLHHAKAGTIIARQGDQDVSLHFVLWG

CLHVYQRMIDKAEDVCLFVAQPGELVGQLAVLTGEPLIFTLRAQRDCTFLRISKSDFYEIM
RAQPSVVLSAAHTVAARMSPFVRQMDFAIDWTAVEAGRALYRQGDRSDCTYIVLNGRLR
SVIQRGSGKKELVGEYGRGDLIGVVEALTRQPRATTVHAVRDTELAKLPEGTLGHIKRRYP
QVVTRLIHLLSQKILGNLQQLQGPFPAGSGLGVPPHSELTNPASNLATVAILPVCAEVPMVA
FTLELQHALQAIGPTLLLNSDIIRARLGASALDSIQEFRLSGWLAQQEDAHRIVLYQTDASL
TPWTVRCLRQADCILIVGLGDQEPTLGQLEQMLENTAVRALKQLVLLHREEGAGPTRTVE
WLNMRSWCSGHLHLRCPRRLFSRRSPAKLHELYEKVFSRRADRHSDFSRLARVLTGNTIA
LVLGGGGARGCSHIGVLKALEEAGVPVDLVGGTSIGSFIGALYAEERSASRTKQRAREWA
KSMTSVLEPVLDLTYPVTSMFTGSAFNRSIHRVFQDKQIEDLWLPYFNVTTDITASAMRVH
KDGSLWRYVRASMTLSGYLPPLCDPKDGHLLMDGGYINNLPADIARSMGAKTVIAIDVG
SQDETDLSTYGDSLSGWWLLWKRLNPWADKVKVPDMAEIQSRLAYVSCVRQLEVVKSS
SYCEYLRPPIDCFKTMDFGKFDQIYDVGYQYGKAVFGGWSRGNVIEKMLTDRRSTDLNES
RRADVLAFPSSGFTDLAEIVSRIEPPTSYVSDGCADGEESDCLTEYEEDAGPDCSRDEGGSP
EGASPSTASEMEEEKSILRQRRCLPQEPPGSATDA

>Q96HA9 multipass membrane protein
MASLSGLASALESYRGRDRLIRVLGYCCQLVGGVLVEQCPARSEVGTRLLVVSTQLSHCR
TILRLFDDLAMFVYTKQYGLGAQEEDAFVRCVSVLGNLADQLYYPCEHVAWAADARVL
HVDSSRWWTLSTTLWALSLLLGVARSLWMLLKLRQRLRSPTAPFTSPLPRGKRRAMEAQ
MQSEALSLLSNLADLANAVHWLPRGVLWAGRFPPWLVGLMGTISSILSMYQAARAGGQA
EATTP

>P78329 peripheral membrane protein
MSQLSLSWLGLWPVAASPWLLLLLVGASWLLAHVLAWTYAFYDNCRRLRCFPQPPRRN
WFWGHQGMVNPTEEGMRVLTQLVATYPQGFKVWMGPISPLLSLCHPDIIRSVINASAAIAP
KDKFFYSFLEPWLGDGLLLSAGDKWSRHRRMLTPAFHFNILKPYMKIFNESVNIMHAKW
QLLASEGSACLDMFEHISLMTLDSLQKCVFSFDSHCQEKPSEYIAAILELSALVSKRHHEIL
LHIDFLYYLTPDGQRFRRACRLVHDFTDAVIQERRRTLPSQGVDDFLQAKAKSKTLDFIDV
LLLSKDEDGKKLSDEDIRAEADTFMFEGHDTTASGLSWVLYHLAKHPEYQERCRQEVQE
LLKDREPKEIEWDDLAHLPFLTMCMKESLRLHPPVPVISRHVTQDIVLPDGRVIPKGIICLIS
VFGTHHNPAVWPDPEVYDPFRFDPENIKERSPLAFIPFSAGPRNCIGQTFAMAEMKVVLAL
TLLRFRVLPDHTEPRRKPELVLRAEGGLWLRVEPLS

>P51693 single-pass type I membrane protein
MGPASPAARGLSRRPGQPPLPLLLPLLLLLLRAQPAIGSLAGGSPGAAEAPGSAQVAGLCG
RLTLHRDLRTGRWEPDPQRSRRCLRDPQRVLEYCRQMYPELQIARVEQATQAIPMERWCG
GSRSGSCAHPHHQVVPFRCLPGEFVSEALLVPEGCRFLHQERMDQCESSTRRHQEAQEAC
SSQGLILHGSGMLLPCGSDRFRGVEYVCCPPPGTPDPSGTAVGDPSTRSWPPGSRVEGAED
EEEEESFPQPVDDYFVEPPQAEEEEETVPPPSSHTLAVVGKVTPTPRPTDGVDIYFGMPGEI
SEHEGFLRAKMDLEERRMRQINEVMREWAMADNQSKNLPKADRQALNEHFQSILQTLEE
QVSGERQRLVETHATRVIALINDQRRALEGFLAALQADPPQAERVLLALRRYLRAEQKE
QRHTLRHYQHVAAVDPEKAQQMRFQVHTHLQVIEERVNQSLGLLDQNPHLAQELRPQIQ
ELLHSEHLGPSELEAPAPGGSSEDKGGLQPPDSKDDTPMTLPKGSTEQDAASPEKEKMNP
LEQYERKVNASVPRGFPFHSSEIQRDELAPAGTGVSREAVSGLLIMGAGGGSLIVLSMLLL

RRKKPYGAISHGVVEVDPMLTLEEQQLRELQRHGYENPTYRFLEERP

>O75871 single-pass type I membrane protein
MGPPSAAPRGGHRPWQGLLITASLLTFWHPPTTVQFTIEALPSSAAEGKDVLLLACNISETI
QAYYWHKGKTAEGSPLIAGYITDIQANIPGAAYSGRETVYPNGSLLFQNITLEDAGSYTLR
TINASYDSDQATGQLHVHQNNVPGLPVGAVAGIVTGVLVGVALVAALVCFLLLSRTGRASI
QRDLREQPPPASTPGHGPSHRSTFSAPLPSPRTATPIYEELLYSDANIYCQIDHKADVVS

>P11912 single-pass type I membrane protein
MPGGPGVLQALPATIFLLFLLSAVYLGPGCQALWMHKVPASLMVSLGEDAHFQCPHNSSN
NANVTWWRVLHGNYTWPPEFLGPGEDPNGTLIIQNVNKSHGGIYVCRVQEGNESYQQSC
GTYLRVRQPPPRPFLDMGEGTKNRIITAEGIILLFCAVVPGTLLLFRKRWQNEKLGLDAGD
EYEDENLYEGLNLDDCSMYEDISRGLQGTYQDVGSLNIGDVQLEKP

>P55344 multipass membrane protein
MYSFMGGGLFCAWVGTILLVVAMATDHWMQYRLSGSFAHQGLWRYCLGNKCYLQTDSI
AYWNATRAFMILSALCAISGIIMGIMAFAHQPTFSRISRPFSAGIMFFSSTLFVVLALAIYTG
VTVSFLGRRFGDWRFSWSYILGWVAVLMTFFAGIFYMCAYRVHECRRLSTPR

>Q16617 multipass membrane protein
MELCRSLALLGGSLGLMFCLIALSTDFWFEAVGPTHSAHSGLWPTGHGDIISGYIHVTQTF
SIMAVLWALVSVSFLVLSCFPSLFPPGHGPLVSTTAAFAAAISMVVAMAVYTSERWDQPPHP
QIQTFFSWSFYLGWVSAILLLCTGALSLGAHCGGPRPGYETL

>Q9UMF0 single-pass type I membrane protein
MPGPSPGLRRALLGLWAALGLGLFGLSAVSQEPFWADLQPRVAFVERGGSLWLNCSTNCP
RPERGGLETSLRRNGTQRGLRWLARQLVDIREPETQPVCFFRCARRTLQARGLIRTFQRPD
RVELMPLPPWQPVGENFTLSCRVPGAGPRASLTLTLLRGAQELIRRSFAGEPPRARGAVLTA
TVLARREDHGANFSCRAELDLRPHGLGLFENSSAPRELRTFSLSPDAPRLAAPRLLEVGSE
RPVSCTLDGLFPASEARVYLALGDQNLSPDVTLEGDAFVATATATASAEQEGARQLVCNVT
LGGENRETRENVTIYSFPAPLLTLSEPSVSEGQMVTVTCAAGAQALVTLEGVPAAVPGQPA
QLQLNATENDDRRSFFCDATLDVDGETLIKNRSAELRVLYAPRLDDSDCPRSWTWPEGPE
QTLRCEARGNPEPSVHCARSDGGAVLALGLLGPVTRALSGTYRCKAANDQGEAVKDVTL
TVEYAPALDSVGCPERITWLEGTEASLSCVAHGVPPPDVICVRSGELGAVIEGLLRVAREHA
GTYRCEATNPRGSAAKNVAVTVEYGPRFEEPSCPSNWTWVEGSGRLFSCEVDGKPQPSVK
CVGSGGATEGVLLPLAPPDPSPRAPRIPRVLAPGIYVCNATNRHGSVAKTVVVSAESPPEM
DESTCPSHQTWLEGAEASALACAARGRPSPGVRCSREGIPWPEQQRVSREDAGTYHCVAT
NAHGTDSRTVTVGVEYRPVVAELAASPPGGVRPGGNFTLTCRAEAWPPAQISWRAPPGAL
NIGLSSNNSTLSVAGAMGSHGGEYECAATNAHGRHARRITVRVAGPWLWVAVGGAAGGA
ALLAAGAGLAFYVQSTACKKGEYNVQEAESSGEAVCLNGAGGGAGGAAGAEGGPEAAG
GAAESPAEGEVFAIQLTSA

>P06731 GPI-anchor, lipid-anchor
MESPSAPPHRWCIPWQRLLLTASLLTFWNPPTTAKLTIESTPFNVAEGKEVLLLVHNLPQHL

FGYSWYKGERVDGNRQIIGYVIGTQQATPGPAYSGREIIYPNASLLIQNIIQNDTGFYTLHVI
KSDLVNEEATGQFRVYPELPKPSISSNNSKPVEDKDAVAFTCEPETQDATYLWWVNNQSLP
VSPRLQLSNGNRTLTLFNVTRNDTASYKCETQNPVSARRSDSVILNVLYGPDAPTISPLNTS
YRSGENLNLSCHAASNPPAQYSWFVNGTFQQSTQELFIPNITVNNSGSYTCQAHNSDTGL
NRTTVTTITVYAEPPKPFITSNNSNPVEDEDAVALTCEPEIQNTTYLWWVNNQSLPVSPRLQ
LSNDNRTLTLLSVTRNDVGPYECGIQNKLSVDHSDPVILNVLYGPDDPTISPSYTYYRPGV
NLSLSCHAASNPPAQYSWLIDGNIQQHTQELFISNITEKNSGLYTCQANNSASGHSRTTVK
TITVSAELPKPSISSNNSKPVEDKDAVAFTCEPEAQNTTYLWWVNGQSLPVSPRLQLSNGN
RTLTLFNVTRNDARAYVCGIQNSVSANRSDPVTLDVLYGPDTPIISPPDSSYLSGANLNLSC
HSASNPSPQYSWRINGIPQQHTQVLFIAKITPNNNGTYACFVSNLATGRNNSIVKSITVSAS
GTSPGLSAGATVGIMIGVLVGVALI

>P19235 single-pass type I membrane protein
MDHLGASLWPQVGSLCLLLAGAAWAPPPNLPDPKFESKAALLAARGPEELLCFTERLEDL
VCFWEEAASAGVGPGNYSFSYQLEDEPWKLCRLHQAPTARGAVRFWCSLPTADTSSFVPL
ELRVTAASGAPRYHRVIHINEVVLLDAPVGLVARLADESGHVVLRWLPPPETPMTSHIRYE
VDVSAGNGAGSVQRVEILEGRTECVLSNLRGRTRYTFAVRARMAEPSFGGFWSAWSEPVS
LLTPSDLDPLILTLSLILVVILVLLTVLALLSHRRALKQKIWPGIPSPESEFEGLFTTHKGNFQ
LWLYQNDGCLWWSPCTPFTEDPPASLEVLSERCWGTMQAVEPGTDDEGPLLEPVGSEHA
QDTYLVLDKWLLPRNPPSEDLPGPGGSVDIVAMDEGSEASSCSSALASKPSPEGASAASFE
YTILDPSSQLLRPWTLCPELPPTPPHLKYLYLVVSDSGISTDYSSGDSQGAQGGLSDGPYSN
PYENSLIPAAEPLPPSYVACS

>Q92911 multipass membrane protein
MEAVETGERPTFGAWDYGVFALMLLVSTGIGLWVGLARGGQRSAEDFFTGGRRLAALPV
GLSLSASFMSAVQVLGVPSEAYRYGLKFLWMCLGQLLNSVLTALLFMPVFYRLGLTSTYE
YLEMRFSRAVRLCGTLQYIVATMLYTGIVIYAPALILNQVTGLDIWASLLSTGIICTFYTAVG
GMKAVVWTDVFQVVVMLSGFWVVLARGVMLVGGPRQVLTLAQNHSRINLMDFNPDPR
SRYTFWTFVVGGTLVWLSMYGVNQAQVQRYVACRTEKQAKLALLINQVGLFLIVSSAAC
CGIVMFVFYTDCDPLLLGRISAPDQYMPLLVLDIFEDLPGVPGLFLACAYSGTLSTASTSIN
AMAAVTVEDLIKPRLRSLAPRKLVIISKGLSLIYGSACLTVAALSSLLGGGVLQGSFTVMG
VISGPLLGAFILGMFLPACNTPGVLAGLGAGLALSLWVALGATLYPPSEQTMRVLPSSAAR
CVALSVNASGLLDPALLPANDSSRAPSSGMDASRPALADSFYAISYLYYGALGTLTTVLCG
ALISCLTGPTKRSTLAPGLLWWDLARQTASVAPKEEVAILDDNLVKGPEELPTGNKKPPGF
LPTNEDRLFFLGQKELEGAGSWTPCVGHDGGRDQQETNL

>Q9NZ42 multipass membrane protein
MNLERVSNEEKLNLCRKYYLGGFAFLPFLWLVNIFWFFREAFLVPAYTEQSQIKGYVWRS
AVGFLFWVIVLTSWITIFQIYRPRWGALGDYLSFTIPLGTP

>O00322 multipass membrane protein
MASAAAAEAEKGSPVVVGLLVVGNIIILLSGLSLFAETIWVTADQYRVYPLMGVSGKDDV
FAGAWIAIFCGFSFFMVASFGVGAALCRRRSMVLTYLVLMLIVYIFECASCITSYTHRDYM
VSNPSLITKQMLTFYSADTDQGQELTRLWDRVMIEQECCGTSGPMDWVNFTSAFRAATPE

VVFPWPPLCCRRTGNFIPLNEEGCRLGHMDYLFTKGCFEHIGHAIDSYTWGISWFGFAILM
WTLPVMLIAMYFYTML

>Q9BVK8 multipass membrane protein
MTLFHFGNCFALAYFPYFITYKCSGLSEYNAFWKCVQAGVTYLFVQLCKMLFLATFFPTW
EGGIYDFIGEFMKASVDVADLIGLNLVMSRNAGKGEYKIMVAALGWATAELIMSRCIPLW
VGARGIEFDWKYIQMSIDSNISLVHYIVASAQVWMITRYDLYHTFRPAVLLLMFLSVYKAF
VMETFVHLCSLGSWAALLARAVVTGLLALSTLALYVAVVNVHS

>Q9HB55 peripheral membrane protein
MDLIPNFAMETWVLVATSLVLLYIYGTHSHKLFKKLGIPGPTPLPFLGTILFYLRGLWNFDR
ECNEKYGEMWGLYEGQQPMLVIMDPDMIKTVLVKECYSVFTNQMPLGPMGFLKSALSFA
EDEEWKRIRTLLSPAFTSVKFKEMVPIISQCGDMLVRSLRQEAENSKSINLKDFFGAYTMD
VITGTLFGVNLDSLNNPQDPFLKNMKKLLKLDFLDPFLLLISLFPFLTPVFEALNIGLFPKD
VTHFLKNSIERMKESRLKDKQKHRVDFFQQMIDSQNSKETKSHKALSDLELVAQSIIIIFAA
YDTTSTTLPFIMYELATHPDVQQKLQEEIDAVLPNKAPVTYDALVQMEYLDMVVNETLRL
FPVVSRVTRVCKKDIEINGVFIPKGLAVMVPIYALHHDPKYWTEPEKFCPERFSKKNKDSID
LYRYIPFGAGPRNCIGMRFALTNIKLAVIRALQNFSFKPCKETQIPLKLDNLPILQPEKPIVLK
VHLRDGITSGP

>Q15165 peripheral membrane protein
MGRLVAVGLLGIALALLGERLLALRNRLKASREVESVDLPHCHLIKGIEAGSEDIDILPNGL
AFFSVGLKFPGLHSFAPDKPGGILMMDLKEEKPRARELRISRGFDLASFNPHGISTFIDNDD
TVYLFVVNHPEFKNTVEIFKFEEAENSLLHLKTVKHELLPSVNDITAVGPAHFYATNDHYF
SDPFLKYLETYLNLHWANVVYYSPNEVKVVAEGFDSANGINISPDDKYIYVADILAHEIHV
LEKHTNMNLTQLKVLELDTLVDNLSIDPSSGDIWVGCHPNGQKLFVYDPNNPPSSEVLRIQ
NILCEKPTVTTVYANNGSVLQGSSVASVYDGKLLIGTLYHRALYCEL

>P26012 single-pass type I membrane protein
MCGSALAFFTAAFVCLQNDRRGPASFLWAAWVFSLVLGLGQGEDNRCASSNAASCARCL
ALGPECGWCVQEDFISGGSRSERCDIVSNLISKGCSVDSIEYPSVHVIIPTENEINTQVTPGE
VSIQLRPGAEANFMLKVHPLKKYPVDLYYLVDVSASMHNNIEKLNSVGNDLSRKMAFFS
RDFRLGFGSYVDKTVSPYISIHPERIHNQCSDYNLDCMPPHGYIHVLSLTENITEFEKAVHR
QKISGNIDTPEGGFDAMLQAAVCESHIGWRKEAKRLLLVMTDQTSHLALDSKLAGIVVPN
DGNCHLKNNVYVKSTTMEHPSLGQLSEKLIDNNINVIFAVQGKQFHWYKDLLPLLPGTIA
GEIESKAANLNNLVVEAYQKLISEVKVQVENQVQGIYFNITAICPDGSRKPGMEGCRNVTS
NDEVLFNVTVTMKKCDVTGGKNYAIIKPIGFNETAKIHIHRNCSCQCEDNRGPKGKCVDE
TFLDSKCFQCDENKCHFDEDQFSSESCKSHKDQPVCSGRGVCVCGKCSCHKIKLGKVYG
KYCEKDDFSCPYHHGNLCAGHGECEAGRCQCFSGWEGDRCQCPSAAAQHCVNSKGQV
CSGRGTCVCGRCECTDPRSIGRFCEHCPTCYTACKENWNCMQCLHPHNLSQAILDQCKTS
CALMEQQHYVDQTSECFSSPSYLRIFFIIFIVTFLIGLLKVLIIRQVILQWNSNKIKSSSDYRV
SASKKDKLILQSVCTRAVTYRREKPEEIKMDISKLNAHETFRCNF

>P51636 peripheral membrane protein

MGLETEKADVQLFMDDDSYSHHSGLEYADPEKFADSDQDRDPHRLNSHLKLGFEDVIAE
PVTTHSFDKVWICSHALFEISKYVMYKFLTVFLAIPLAFIAGILFATLSCLHIWILMPFVKTC
LMVLPSVQTIWKSVTDVIIAPLCTSVGRCFSSVSLQLSQD

>Q9Y5X2 peripheral membrane protein
MTGRAMDPLPAAAVGAAAEAEADEEADPPASDLPTPQAIEPQAIVQQVPAPSRMQMPQG
NPLLLSHTLQELLARDTVQVELIPEKKGLFLKHVEYEVSSQRFKSSVYRRYNDFVVFQEM
LLHKFPYRMVPALPPKRMLGADREFIEARRRALKRFVNLVARHPLFSEDVVLKLFLSFSGS
DVQNKLKESAQCVGDEFLNCKLATRAKDFLPADIQAQFAISRELIRNIYNSFHKLRDRAER
IASRAIDNAADLLIFGKELSAIGSDTTPLPSWAALNSSTWGSLKQALKGLSVEFALLADKA
AQQGKQEENDVVEKLNLFLDLLQSYKDLCERHEKGVLHKHQRALHKYSLMKRQMMSA
TAQNREPESVEQLESRIVEQENAIQTMELRNYFSLYCLHQETQLIHVYLPLTSHILRAFVNS
QIQGHKEMSKVWNDLRPKLSCLFAGPHSTLTPPCSPPEDGLCPH

>Q9UP52 single-pass type II membrane protein
MERLWGLFQRAQQLSPRSSQTVYQRVEGPRKGHLEEEEEDGEEGAETLAHFCPMELRGP
EPLGSRPRQPNLIPWAAAGRRAAPYLVLTALLIFTGAFLLGYVAFRGSCQACGDSVLVVSE
DVNYEPDLDFHQGRLYWSDLQAMFLQFLGEGRLEDTIRQTSLRERVAGSAGMAALTQDIR
AALSRQKLDHVWTDTHYVGLQFPDPAHPNTLHWVDEAGKVGEQLPLEDPDVYCPYSAI
GNVTGELVYAHYGRPEDLQDLRARGVDPVGRLLLVRVGVISFAQKVTNAQDFGAQGVLI
YPEPADFSQDPPKPSLSSQQAVYGHVHLGTGDPYTPGFPSFNQTQFPPVASSGLPSIPAQPIS
ADIASRLLRKLKGPVAPQEWQGSLLGSPYHLGPGPRLRLVVNNHRTSTPINNIFGCIEGRSE
PDHYVVIGAQRDAWGPGAAKSAVGTAILLELVRTFSSMVSNGFRPRRSLLFISWDGGDFG
SVGSTEWLEGYLSVLHLKAVVYVSLDNAVLGDDKFHAKTSPLLTSLIESVLKQVDSPNHS
GQTLYEQVVFTNPSWDAEVIRPLPMDSSAYSFTAFVGVPAVEFSFMEDDQAYPFLHTKEDT
YENLHKVLQGRLPAVAQAVAQLAGQLLIRLSHDRLLPLDFGRYGDVVLRHIGNLNEFSGD
LKARGLTLQWVYSARGDYIRAAEKLRQEIYSSEERDERLTRMYNVRIMRVEFYFLSQYVS
PADSPFRHIFMGRGDHTLGALLDHLRLLRSNSSGTPGATSSTGFQESRFRRQLALLTWTLQ
GAANALSGDVWNIDNNF

>O60568 peripheral membrane protein
MTSSGPGPRFLLLLPLLLPPAASASDRPRGRDPVNPEKLLVITVATAETEGYLRFLRSAEFFN
YTVRTLGLGEEWRGGDVARTVGGGQKVRWLKKEMEKYADREDMIIMFVDSYDVILAGS
PTELLKKFVQSGSRLLFSAESFCWPEWGLAEQYPEVGTGKRFLNSGGFIGFATTIHQIVRQ
WKYKDDDDDQLFYTRLYLDPGLREKLSLNLDHKSRIFQNLNGALDEVVLKFDRNRVRIR
NVAYDTLPIVVHGNGPTKLQLNYLGNYVPNGWTPEGGCGFCNQDRRTLPGGQPPPRVFLA
VFVEQPTPFLPRFLQRLLLLDYPPDRVTLFLHNNEVFHEPHIADSWPQLQDHFSAVKLVGP
EEALSPGEARDMAMDLCRQDPECEFYFSLDADAVLTNLQTLRILIEENRKVIAPMLSRHG
KLWSNFWGALSPDEYYARSEDYVELVQRKRVGVWNVPYISQAYVIRGDTLRMELPQRDV
FSGSDTDPDMAFCKSFRDKGIFLHLSNQHEFGRLLATSRYDTEHLHPDLWQIFDNPVDWK
EQYIHENYSRALEGEGIVEQPCPDVYWFPLLSEQMCDELVAEMEHYGQWSGGRHEDSRL
AGGYENVPTVDIHMKQVGYEDQWLQLLRTYVGPMTESLFPGYHTKARAVMNFVVRYRP
DEQPSLRPHHDSSTFTLNVALNHKGLDYEGGGCRFLRYDCVISSPRKGWALLHPGRLTHY
HEGLPTTWGTRYIMVSFVDP

>O15498 lipid-anchor
MKLYSLSVLYKGEAKVVLLKAAYDVSSFSFFQRSSVQEFMTFTSQLIVERSSKGTRASVKE
QDYLCHVYYVRNDSLAGVVIADNEYPSRVAFTLLEKVLDEFSKQVDRIDWPVGSPATIHYP
ALDGHLSRYQNPREADPMTKVQAELDETKIILHNTMESLLERGEKLDDLVSKSEVLGTQS
KAFYKTARKQNSCCAIM

>P32971 single-pass type II membrane protein
MDPGLQQALNGMAPPGDTAMHVPAGSVASHLGTTSRSYFYLTTATLALCLVFTVATIMVL
VVQRTDSIPNSPDNVPLKGGNCSEDLLCILKRAPFKKSWAYLQVAKHLNKTKLSWNKDGI
LHGVRYQDGNLVIQFPGLYFIICQLQFLVQCPNNSVDLKLELLINKHIKKQALVTVCESGM
QTKHVYQNLSQFLLDYLQVNTTISVNVDTFQYIDTSTFPLENVLSIFLYSNSD

>Q9HBL7 multipass membrane protein
MGFIFSKSMNESMKNQKEFMLMNARLQLERQLIMQSEMRERQMAMQIAWSREFLKYFG
TFFGLAAISLTAGAIKKKKPAFLVPIVPLSFILTYQYDLGYGTLLERMKGEAEDILETEKSKL
QLPRGMITFESIEKARKEQSRFFIDK

>O43174 peripheral membrane protein
MGLPALLASALCTFVLPLLLFLAAIKLWDLYCVSGRDRSCALPLPPGTMGFPFFGETLQMV
LQRRKFLQMKRRKYGFIYKTHLFGRPTVRVMGADNVRRILLGEHRLVSVHWPASVRTILG
SGCLSNLHDSSHKQRKKVIMRAFSREALECYVPVITEEVGSSLEQWLSCGERGLLVYPEV
KRLMFRIAMRILLGCEPQLAGDGDSEQQLVEAFEEMTRNLFSLPIDVPFSGLYRGMKARN
LIHARIEQNIRAKICGLRASEAGQGCKDALQLLIEHSWERGERLDMQALKQSSTELLFGGH
ETTASAATSLITYLGLYPHVLQKVREELKSKGLLCKSNQDNKLDMEILEQLKYIGCVIKET
LRLNPPVPGGFRVALKTFELNGYQIPKGWNVIYSICDTHDVAEIFTNKEEFNPDRFMLPHPE
DASRFSFIPFGGGLRSCVGKEFAKILLKIFTVELARHCDWQLLNGPPTMKTSPTVYPVDNL
PARFTHFHGEI

>P36894 single-pass type I membrane protein
MPQLYIYIRLLGAYLFIISRVQGQNLDSMLHGTGMKSDSDQKKSENGVTLAPEDTLPFLKC
YCSGHCPDDAINNTCITNGHCFAIIEEDDQGETTLASGCMKYEGSDFQCKDSPKAQLRRTI
ECCRTNLCNQYLQPTLPPVVIGPFFDGSIRWLVLLISMAVCIIAMIIFSSCFCYKHYCKSISSR
RRYNRDLEQDEAFIPVGESLKDLIDQSQSSGSGSGLPLLVQRTIAKQIQMVRQVGKGRYGE
VWMGKWRGEKVAVKVFFTTEEASWFRETEIYQTVLMRHENILGFIAADIKGTGSWTQLY
LITDYHENGSLYDFLKCATLDTRALLKLAYSAACGLCHLHTEIYGTQGKPAIAHRDLKSKN
ILIKKNGSCCIADLGLAVKFNSDTNEVDVPLNTRVGTKRYMAPEVLDESLNKNHFQPYIM
ADIYSFGLIIWEMARRCITGGIVEEYQLPYYNMVPSDPSYEDMREVVCVKRLRPIVSNRW
NSDECLRAVLKLMSECWAHNPASRLTALRIKKTLAKMVESQDVKI

>Q9BWM7 multipass membrane protein
MESKMGELPLDINIQEPRWDQSTFLGRARHFFTVTDPRNLLLSGAQLEASRNIVQNYRAG
VVTPGITEDQLWRAKYVYDSAFHPDTGEKVVLIGRMSAQVPMNMTITGCMLTFYRKTPT
VVFWQWVNQSFNAIVNYSNRSGDTPITVRQLGTAYVSATTGAVATALGLKSLTKHLPPLV

GRFVPFAAVAAANCINIPLMRQRELQVGIPVADEAGQRLGYSVTAAKQGIFQVVISRICMAI
PAMAIPPLIMDTLEKKDFLKRRPWLGAPLQVGLVGFCLVFATPLCCALFPQKSSIHISNLEP
ELRAQIHEQNPSVEVVYYNKGL

>Q93086 multipass membrane protein
MGQAGCKGLCLSLFDYKTEKYVIAKNKKVGLLYRLLQASILAYLVVWVFLIKKGYQDVD
TSLQSAVITKVKGVAFTNTSDLGQRIWDVADYVIPAQGENVFFVVTNLIVTPNQRQNVCAE
NEGIPDGACSKDSDCHAGEAVTAGNGVKTGRCLRRENLARGTCEIFAWCPLETSSRPEEPF
LKEAEDFTIFIKNHIRFPKFNFSKSNVMDVKDRSFLKSCHFGPKNHYCPIFRLGSVIRWAGS
DFQDIALEGGVIGINIEWNCDLDKAASECHPHYSFSRLDNKLSKSVSSGYNFRFARYYRDA
AGVEFRTLMKAYGIRFDVMVNGKGAFFCDLVLIYLIKKREFYRDKKYEEVRGLEDSSQEA
EDEASGLGLSEQLTSGPGLLGMPEQQELQEPPEAKRGSSSQKGNGSVCPQLLEPHRST

>O14907 peripheral membrane protein
MSYIPGQPVTAVVQRVEIHKLRQGENLILGFSIGGGIDQDPSQNPFSEDKTDKGIYVTRVSE
GGPAEIAGLQIGDKIMQVNGWDMTMVTHDQARKRLTKRSEEVVRLLVTRQSLQKAVQQS
MLS

>Q02978 multipass membrane protein
MAATASAGAGGIDGKPRTSPKSVKFLFGGLAGMGATVFVQPLDLVKNRMQLSGEGAKTR
EYKTSFHALTSILKAEGLRGIYTGLSAGLLRQATYTTTRLGIYTVLFERLTGADGTPPGFLL
KAVIGMTAGATGAFVGTPAEVALIRMTADGRLPADQRRGYKNVFNALIRITREEGVLTLW
RGCIPTMARAVVVNAAQLASYSQSKQFLLDSGYFSDNILCHFCASMISGLVTTAASMPVDI
AKTRIQNMRMIDGKPEYKNGLDVLFKVVRYEGFFSLWKGFTPYYARLGPHTVLTFIFLEQ
MNKAYKRLFLSG

>Q9Y272 lipid-anchor
MKLAAMIKKMCPSDSELSIPAKNCYRMVILGSSKVGKTAIVSRFLTGRFEDAYTPTIEDFH
RKFYSIRGEVYQLDILDTSGNHPFPAMRRLSILTGDVFILVFSLDNRDSFEEVQRLRQQILDT
KSCLKNKTKENVDVPLVICGNKGDRDFYREVDQREIEQLVGDDPQRCAYFEISAKKNSSL
DQMFRALFAMAKLPSEMSPDLHRKVSVQYCDVLHKKALRNKKLLRAGSGGGGGDPGD
AFGIVAPFARRPSVHSDLMYIREKASAGSQAKDKERCVIS

>O00623 multipass membrane protein
MAEHGAHFTAASVADDQPSIFEVVAQDSLMTAVRPALQHVVKVLAESNPTHYGFLWRWF
DEIFTLLDLLLQQHYLSRTSASFSENFYGLKRIVMGDTHKSQRLASAGLPKQQLWKSIMFL
VLLPYLKVKLEKLVSSLREEDEYSIHPPSSRWKRFYRAFLAAYPFVNMAWEGWFLVQQLR
YILGKAQHHSPLLRLAGVQLGRLTVQDIQALEHKPAKASMMQQPARSVSEKINSALKKAV
GGVALSLSTGLSVGVFFLQFLDWWYSSENQETIKSLTALPTPPPPVHLDYNSDSPLLPKMK
TVCPLCRKTRVNDTVLATSGYVFCYRCVFHYVRSHQACPITGYPTEVQHLIKLYSPEN

>Q06432 multipass membrane protein
MSQTKMLKVRVTLFCILAGIVLAMTAVVTDHWAVLSPHMEHHNTTCEAAHFGLWRICTK
RIPMDDSKTCGPITLPGEKNCSYFRHFNPGESSEIFEFTTQKEYSISAAAIAIFSLGFIILGSLC

VLLSLGKKRDYLLRPASMFYAFAGLCILVSVEVMRQSVKRMIDSEDTVWIEYYYSWSFAC
ACAAFILLFLGGLALLLFSLPRMPRNPWESCMDAEPEH

>Q15768 single-pass type I membrane protein
MGPPHSGPGGVRVGALLLLGVLGLVSGLSLEPVYWNSANKRFQAEGGYVLYPQIGDRLD
LLCPRARPPGPHSSPNYEFYKLYLVGGAQGRRCEAPPAPNLLLTCDRPDLDLRFTIKFQEYS
PNLWGHEFRSHHDYYIIATSDGTREGLESLQGGVCLTRGMKVLLRVGQSPRGGAVPRKPV
SEMPMERDRGAAHSLEPGKENLPGDPTSNATSRGAEGPLPPPSMPAVAGAAGGLALLLLG
VAGAGGAMCWRRRRAKPSESRHPGPGSFGRGGSLGLGGGGGMGPREAEPGELGIALRG
GGAADPPFCPHYEKVSGDYGHPVYIVQDGPPQSPPNIYYKV

>P28907 single-pass type II membrane protein
MANCEFSPVSGDKPCCRLSRRAQLCLGVSILVLILVVVLAVVVPRWRQQWSGPGTTKRFP
ETVLARCVKYTEIHPEMRHVDCQSVWDAFKGAFISKHPCNITEEDYQPLMKLGTQTVPC
NKILLWSRIKDLAHQFTQVQRDMFTLEDTLLGYLADDLTWCGEFNTSKINYQSCPDWRK
DCSNNPVSVFWKTVSRRFAEAACDVVHVMLNGSRSKIFDKNSTFGSVEVHNLQPEKVQT
LEAWVIHGGREDSRDLCQDPTIKELESIISKRNIQFSCKNIYRPDKFLQCVKNPEDSSCTSEI

>P30968 multipass membrane protein
MANSASPEQNQNHCSAINNSIPLMQGNLPTLTLSGKIRVTVTFFLFLLSATFNASFLLKLQK
WTQKKEKGKKLSRMKLLLKHLTLANLLETLIVMPLDGMWNITVQWYAGELLCKVLSYL
KLFSMYAPAFMMVVISLDRSLAITRPLALKSNSKVGQSMVGLAWILSSVFAGPQLYIFRMI
HLADSSGQTKVFSQCVTHCSFSQWWHQAFYNFFTFSCLFIIPLFIMLICNAKIIFTLTRVLHQ
DPHELQLNQSKNNIPRARLKTLKMTVAFATSFTVCWTPYYVLGIWYWFDPEMLNRLSDP
VNHFFFLFAFLNPCFDPLIYGYFSL

>Q9UHA4 peripheral membrane protein
MADDLKRFLYKKLPSVEGLHAIVVSDRDGVPVIKVANDNAPEHALRPGFLSTFALATDQG
SKLGLSKNKSIICYYNTYQVVQFNRLPLVVSFIASSSANTGLIVSLEKELAPLFEELRQVVE
VS

>O76024 multipass membrane protein
MDSNTAPLGPSCPQPPPAPQPQARSRLNATASLEQERSERPRAPGPQAGPGPGVRDAAAPA
EPQAQHTRSRERADGTGPTKGDMEIPFEEVLERAKAGDPKAQTEVGKHYLQLAGDTDEE
LNSCTAVDWLVLAAKQGRREAVKLLRRCLADRRGITSENEREVRQLSSETDLERAVRKAA
LVMYWKLNPKKKKQVAVAELLENVGQVNEHDGGAQPGPVPKSLQKQRRMLERLVSSES
KNYIALDDFVEITKKYAKGVIPSSLFLQDDEDDDELAGKSPEDLPLRLKVVKYPLHAIMEI
KEYLIDMASRAGMHWLSTIIPTHHINALIFFFIVSNLTIDFFAFFIPLVIFYLSFISMVICTLKV
FQDSKAWENFRTLTDLLLRFEPNLDVEQAEVNFGWNHLEPYAHFLLSVFFVIFSFPIASKD
CIPCSELAVITGFFTVTSYLSLSTHAEPYTRRALATEVTAGLLSLLPSMPLNWPYLKVLGQT
FITVPVGHLVVLNVSVPCLLYVYLLYLFFRMAQLRNFKGTYCYLVPYLVCFMWCELSVVI
LLESTGLGLLRASIGYFLFLFALPILVAGLALVGVLQFARWFTSLELTKIAVTVAVCSVPLLL
RWWTKASFSVVGMVKSLTRSSMVKLILVWLTAIVLFCWFYVYRSEGMKVYNSTLTWQQ
YGALCGPRAWKETNMARTQILCSHLEGHRVTWTGRFKYVRVTDIDNSAESAINMLPFFIG

DWMRCLYGEAYPACSPGNTSTAEEELCRLKLLAKHPCHIKKFDRYKFEITVGMPFSSGAD
GSRSREEDDVTKDIVLRASSEFKSVLLSLRQGSLIEFSTILEGRLGSKWPVFELKAISCLNC
MAQLSPTRRHVKIEHDWRSTVHGAVKFAFDFFFFPFLSAA

>P27701 multipass membrane protein
MGSACIKVTKYFLFLFNLIFFILGAVILGFGVWILADKSSFISVLQTSSSSLRMGAYVFIGVG
AVTMLMGFLGCIGAVNEVRCLLGLYFAFLLLILIAQVTAGALFYFNMGKLKQEMGGIVTE
LIRDYNSSREDSLQDAWDYVQAQVKCCGWVSFYNWTDNAELMNRPEVTYPCSCEVKGE
EDNSLSVRKGFCEAPGNRTQSGNHPEDWPVYQEGCMEKVQAWLQENLGIILGVGVGVAII
ELLGMVLSICLCRHVHSEDYSKVPKY

>Q11206 single-pass type II membrane protein
MVSKSRWKLLAMLALVLVVMVWYSISREDRYIELFYFPIPEKKEPCLQGEAESKASKLFG
NYSRDQPIFLRLEDYFWVKTPSAYELPYGTKGSEDLLLRVLAITSSSIPKNIQSLRCRRCVV
VGNGHRLRNSSLGDAINKYDVVIRLNNAPVAGYEGDVGSKTTMRLFYPESAHFDPKVEN
NPDTLLVLVAFKAMDFHWIETILSDKKRVRKGFWKQPPLIWDVNPKQIRILNPFFMEIAAD
KLLSLPMQQPRKIKQKPTTGLLAITLALHLCDLVHIAGFGYPDAYNKKQTIHYYEQITLKS
MAGSGHNVSQEALAIKRMLEMGAIKNLTSF

>Q13651 single-pass type I membrane protein
MLPCLVVLLAALLSLRLGSDAHGTELPSPPSVWFEAEFFHHILHWTPIPNQSESTCYEVALL
RYGIESWNSISNCSQTLSYDLTAVTLDLYHSNGYRARVRAVDGSRHSNWTVTNTRFSVDE
VTLTVGSVNLEIHNGFILGKIQLPRPKMAPANDTYESIFSHFREYEIAIRKVPGNFTFTHKKV
KHENFSLLTSGEVGEFCVQVKPSVASRSNKGMWSKEECISLTRQYFTVTNVIIFFAFVLLLS
GALAYCLALQLYVRRRKKLPSVLLFKKPSPFIFISQRPSPETQDTIHPLDEEAFLKVSPELKN
LDLHGSTDSGFGSTKPSLQTEEPQFLLPDPHPQADRTLGNREPPVLGDSCSSGSSNSTDSGI
CLQEPSLSPSTGPTWEQQVGSNSRGQDDSGIDLVQNSEGRAGDTQGGSALGHHSPPEPEV
PGEEDPAAVAFQGYLRQTRCAEEKATKTGCLEEESPLTDGLGPKFGRCLVDEAGLHPPALA
KGYLKQDPLEMTLASSGAPTGQWNQPTEEWSLLALSSCSDLGISDWSFAHDLAPLGCVA
APGGLLGSFNSDLVTLPLISSLQSSE

>Q8IY34 multipass membrane protein
MPAPRAREQPRVPGERQPLLPRGARGPRRWRRAAGAAVLLVEMLERAAFFGVTANLVLY
LNSTNFNWTGEQATRAALVFLGASYLLAPVGGWLADVYLGRYRAVALSLLLYLAASGLL
PATAFPDGRSSFCGEMPASPLGPACPSAGCPRSSPSPYCAPVLYAGLLLLGLAASSVRSNLTS
FGADQVMDLGRDATRRFFNWFYWSINLGAVLSLLVVAFIQQNISFLLGYSIPVGCVGLAFF
IFLFATPVFITKPPMGSQVSSMLKLALQNCCPQLWQRHSARDRQCARVLADERSPQPGASP
QEDIANFQVLVKILPVMVTLVPYWMVYFQMQSTYVLQGLHLHIPNIFPANPANISVALRAQ
GSSYTIPEAWLLLANVVVVLILVPLKDRLIDPLLLRCKLLPSALQKMALGMFFGFTSVIVA
GVLEMERLHYIHHNETVSQQIGEVLYNAAPLSIWWQIPQYLLIGISEIFASIPGLEFAYSEAP
RSMQGAIMGIFFCLSGVGSLLGSSLVALLSLPGGWLHCPKDFGNINNCRMDLYFFLLAGIQ
AVTALLFVWIAGRYERASQGPASHSRFSRDRG

>Q96PD7 multipass membrane protein

MKTLIAAYSGVLRGERQAEADRSQRSHGGPALSREGSGRWGTGSSILSALQDLFSVTWLN
RSKVEKQLQVISVLQWVLSFLVLGVACSAILMYIFCTDCWLIAVLYFTWLVFDWNTPKKG
GRRSQWVRNWAVWRYFRDYFPIQLVKTHNLLTTRNYIFGYHPHGIMGLGAFCNFSTEATE
VSKKFPGIRPYLATLAGNFRMPVLREYLMSGGICPVSRDTIDYLLSKNGSGNAIIIVVGGA
AESLSSMPGKNAVTLRNRKGFVKLALRHGADLVPIYSFGENEVYKQVIFEEGSWGRWVQ
KKFQKYIGFAPCIFHGRGLFSSDTWGLVPYSKPITTVVGEPITIPKLEHPTQQDIDLYHTMY
MEALVKLFDKHKTKFGLPETEVLEVN

>P21583 single-pass type I membrane protein
MKKTQTWILTCIYLQLLLFNPLVKTEGICRNRVTNNVKDVTKLVANLPKDYMITLKYVPG
MDVLPSHCWISEMVVQLSDSLTDLLDKFSNISEGLSNYSIIDKLVNIVDDLVECVKENSSK
DLKKSFKSPEPRLFTPEEFFRIFNRSIDAFKDFVVASETSDCVVSSTLSPEKDSRVSVTKPFM
LPPVAASSLRNDSSSSNRKAKNPPGDSSLHWAAMALPALFSLIIGFAFGALYWKKRQPSLT
RAVENIQINEEDNEISMLQEKEREFQEV

>Q00325 multipass membrane protein
MFSSVAHLARANPFNTPHLQLVHDGLGDLRSSSPGPTGQPRRPRNLAAAAVEEQYSCDYG
SGRFFILCGLGGIISCGTTHTALVPLDLVKCRMQVDPQKYKGIFNGFSVTLKEDGVRGLAK
GWAPTFLGYSMQGLCKFGFYEVFKVLYSNMLGEENTYLWRTSLYLAASASAEFFADIALA
PMEAAKVRIQTQPGYANTLRDAAPKMYKEEGLKAFYKGVAPLWMRQIPYTMMKFACFE
RTVEALYKFVVPKPRSECSKPEQLVVTFVAGYIAGVFCAIVSHPADSVVSVLNKEKGSSAS
LVLKRLGFKGVWKGLFARIIMIGTLTALQWFIYDSVKVYFRLPRPPPPEMPESLKKKLGLT
Q

>Q07108 single-pass type II membrane protein
MSSENCFVAENSSLHPESGQENDATSPHFSTRHEGSFQVPVLCAVMNVVFITILIIALIALSV
GQYNCPGQYTFSMPSDSHVSSCSEDWVGYQRKCYFISTVKRSWTSAQNACSEHGATLAVI
DSEKDMNFLKRYAGREEHWVGLKKEPGHPWKWSNGKEFNNWFNVTGSDKCVFLKNTE
VSSMECEKNLYWICNKPYK

>P37088 multipass membrane protein
MEGNKLEEQDSSPPQSTPGLMKGNKREEQGLGPEPAAPQQPTAEEEALIEFHRSYRELFEF
FCNNTTIHGAIRLVCSQHNRMKTAFWAVLWLCTFGMMYWQFGLLFGEYFSYPVSLNINL
NSDKLVFPAVTICTLNPYRYPEIKEELEELDRITEQTLFDLYKYSSFTTLVAGSRSRRDLRGT
LPHPLQRLRVPPPPHGARRARSVASSLRDNNPQVDWKDWKIGFQLCNQNKSDCFYQTYS
SGVDAVREWYRFHYINILSRLPETLPSLEEDTLGNFIFACRFNQVSCNQANYSHFHHPMYG
NCYTFNDKNNSNLWMSSMPGINNGLSLMLRAEQNDFIPLLSTVTGARVMVHGQDEPAFM
DDGGFNLRPGVETSISMRKETLDRLGGDYGDCTKNGSDVPVENLYPSKYTQQVCIHSCFQ
ESMIKECGCAYIFYPRPQNVEYCDYRKHSSWGYCYYKLQVDFSSDHLGCFTKCRKPCSVT
SYQLSAGYSRWPSVTSQEWVFQMLSRQNNYTVNNKRNGVAKVNIFFKELNYKTNSESPS
VTMVTLLSNLGSQWSLWFGSSVLSVVEMAELVFDLLVIMFLMLLRRFRSRYWSPGRGGR
GAQEVASTLASSPPSHFCPHPMSLSLSQPGPAPSPALTAPPPAYATLGPRPSPGGSAGASSST
CPLGGP

>P36941 single-pass type I membrane protein
MLLPWATSAPGLAWGPLVLGLFGLLAASQPQAVPPYASENQTCRDQEKEYYEPQHRICCS
RCPPGTYVSAKCSRIRDTVCATCAENSYNEHWNYLTICQLCRPCDPVMGLEEIAPCTSKRK
TQCRCQPGMFCAAWALECTHCELLSDCPPGTEAELKDEVGKGNNHCVPCKAGHFQNTSS
PSARCQPHTRCENQGLVEAAPGTAQSDTTCKNPLEPLPPEMSGTMLMLAVLLPLAFFLLLA
TVFSCIWKSHPSLCRKLGSLLKRRPQGEGPNPVAGSWEPPKAHPYFPDLVQPLLPISGDVSP
VSTGLPAAPVLEAGVPQQQSPLDLTREPQLEPGEQSQVAHGTNGIHVTGGSMTITGNIYIY
NGPVLGGPPGPGDLPATPEPPYPIPEEGDPGPPGLSTPHQEDGKAWHLAETEHCGATPSNR
GPRNQFITHD

>Q93070 GPI-anchor, lipid-anchor
MGPLINRCKKILLPTTVPPATMRIWLLGGLLPFLLLLSGLQRPTEGSEVAIKIDFDFAPGSFD
DQYQGCSKQVMEKLTQGDYFTKDIEAQKNYFRMWQKAHLAWLNQGKVLPQNMTTTHA
VAILFYTLNSNVHSDFTRAMASVARTPQQYERSFHFKYLHYYLTSAIQLLRKDSIMENGTL
CYEVHYRTKDVHFNAYTGATIRFGQFLSTSLLKEEAQEFGNQTLFTIFTCLGAPVQYFSLK
KEVLIPPYELFKVINMSYHPRGNWLQLRSTGNLSTYNCQLLKASSKKCIPDPIAIASLSFLT
SVIIFSKSRV

>Q9Y2I9 peripheral membrane protein
MDVLPTGGGRPGLRTELEFRGGGGEARLESQEEETIPAAPPAPRLRGAAERPRRSRDTWD
GDEDTEPGEACGGRTSRTASLVSGLLNELYSCTEEEEAAGGGRGAEGRRRRRDSLDSSTE
ASGSDVVLGGRSGAGDSRVLQELQERPSQRHQMLYLRQKDANELKTILRELKYRIGIQSA
KLLRHLKQKDRLLHKVQRNCDIVTACLQAVSQKRRVDTKLKFTLEPSLGQNGFQQWYDA
LKAVARLSTGIPKEWRRKVWLTLADHYLHSIAIDWDKTMRFTFNERSNPDDDSMGIQIVK
DLHRTGCSSYCGQEAEQDRVVLKRVLLAYARWNKTVGYCQGFNILAALILEVMEGNEGD
ALKIMIYLIDKVLPESYFVNNLRALSVDMAVFRDLLRMKLPELSQHLDTLQRTANKESGG
GYEPPLTNVFTMQWFLTLFATCLPNQTVLKIWDSVFFEGSEIILRVSLAIWAKLGEQIECCET
ADEFYSTMGRLTQEMLENDLLQSHELMQTVYSMAPFPFPQLAELREKYTYNITPFPATVK
PTSVSGRHSKARDSDEENDPDDEDAVVNAVGCLGPFSGFLAPELQKYQKQIKEPNEEQSL
RSNNIAELSPGAINSCRSEYHAAFNSMMMERMTTDINALKRQYSRIKKKQQQQVHQVYI
RADKGPVTSILPSQVNSSPVINHLLLGKKMKMTNRAAKNAVIHIPGHTGGKISPVPYEDLK
TKLNSPWRTHIRVHKKNMPRTKSHPGCGDTVGLIDEQNEASKTNGLGAAEAFPSGCTATA
GREGSSPEGSTRRTIEGQSPEPVFGDADVDVSAVQAKLGALELNQRDAAAETELRVHPPC
QRHCPEPPSAPEENKATSKAPQGSNSKTPIFSPFPSVKPLRKSATARNLGLYGPTERTPTVHF
PQMSRSFSKPGGGNSGTKKR

>Q8WUD6 multipass membrane protein
MAAGAGAGSAPRWLRALSEPLSAAQLRRLEEHRYSAAGVSLLEPPLQLYWTWLLQWIPL
WMAPNSITLLGLAVNVVTTLVLISYCPTATEEAPYWTYLLCALGLFIYQSLDAIDGKQARR
TNSCSPLGELFDHGCDSLSTVFMAVGASIAARLGTYPDWFFFCSFIGMFVFYCAHWQTYV
SGMLRFGKVDVTEIQIALVIVFVLSAFGGATMWDYTIPILEIKLKILPVLGFLGGVIFSCSNY
FHVILHGGVGKNGSTIAGTSVLSPGLHIGLIIILAIMIYKKSATDVFEKHPCLYILMFGCVFA
KVSQKLVVAHMTKSELYLQDTVFLGPGLLFLDQYFNNFIDEYVVLWMAMVISSFDMVIYF
SALCLQISRHLHLNIFKTACHQAPEQVQVLSSKSHQNNMD

>Q9Y3E0 multipass membrane protein
MISLTDTQKIGMGLTGFGVFFLFFGMILFFDKALLAIGNVLFVAGLAFVIGLERTFRFFFQK
HKMKATGFFLGGVFVVLIGWPLIGMIFEIYGFFLLFRGFFPVVVGFIRRVPVLGSLLNLPGI
RSFVDKVGESNNMV

>O00294 peripheral membrane protein
MPLRDETLREVWASDSGHEEESLSPEAPRRPKQRPAPAQRLRKKRTEAPESPCPTGSKPRK
PGAGRTGRPREEPSPDPAQARAPQTVYARFLRDPEAKKRDPRETFLVARAPDAEDEEEEEE
EDEEDEEEEAEEKKEKILLPPKKPLREKSSADLKERRAKAQGPRGDLGSPDPPPKPLRVRN
KEAPAGEGTKMRKTKKKGSGEADKDPSGSPASARKSPAAMFLVGEGSPDKKALKKKGTP
KGARKEEEEEEEAATVIKKSNQKGKAKGKGKKKAKEERAPSPPVEVDEPREFVLRPAPQG
RTVRCRLTRDKKGMDRGMYPSYFLHLDTEKKVFLLAGRKRKRSKTANYLISIDPTNLSRG
GENFIGKLRSNLLGNRFTVFDNGQNPQRGYSTNVASLRQELAAVIYETNVLGFRGPRRMT
VIIPGMSAENERVPIRPRNASDGLLVRWQNKTLESLIELHNKPPVWNDDSGSYTLNFQGRV
TQASVKNFQIVHADDPDYIVLQFGRVAEDAFTLDYRYPLCALQAFAIALSSFDGKLACE

>Q9BZJ6 multipass membrane protein
MVFSAVLTAFHTGTSNTTFVVYENTYMNITLPPPFQHPDLSPLLRYSFETMAPTGLSSLTVN
STAVPTTPAAFKSLNLPLQITLSAIMIFILFVSFLGNLVVCLMVYQKAAMRSAINILLASLAF
ADMLLAVLNMPFALVTILTTRWIFGKFFCRVSAMFFWLFVIEGVAILLIISIDRFLIIVQRQDK
LNPYRAKVLIAVSWATSFCVAFPLAVGNPDLQIPSRAPQCVFGYTTNPGYQAYVILISLISFFI
PFLVILYSFMGILNTLRHNALRIHSYPEGICLSQASKLGLMSLQRPFQMSIDMGFKTRAFTTI
LILFAVFIVCWAPFTTYSLVATFSKHFYYQHNFFEISTWLLWLCYLKSALNPLIYYWRIKKF
HDACLDMMPKSFKFLPQLPGHTKRRIRPSAVYVCGEHRTVV

>Q86WB7 multipass membrane protein
MDRSLRNVLVVSFGFLLLFTAYGGLQSLQSSLYSEEGLGVTALSTLYGGMLLSSMFLPPLLI
ERLGCKGTIILSMCGYVAFSVGNFFASWYTLIPTSILLGLGAAPLWSAQCTYLTITGNTHAE
KAGKRGKDMVNQYFGIFFLIFQSSGVWGNLISSLVFGQTPSQETLPEEQLTSCGASDCLMA
TTTTNSTQRPSQQLVYTLLGIYTGSGVLAVLMIAAFLQPIRDVQRESEGEKKSVPFWSTLLS
TFKLYRDKRLCLLILLPLYSGLQQGFLSSEYTRSYVTCTLGIQFVGYVMICFSATDALCSVL
YGKVSQYTGRAVLYVLGAVTHVSCMIALLLWRPRADHLAVFFVFSGLWGVADAVWQTQ
NNALYGVLFEKSKEAAFANYRLWEALGFVIAFGYSMFLCVHVKLYILLGVLSLTMVAYGL
VECVESKNPIRPHAPGQVNQAEDEEIQTKM

>P23942 multipass membrane protein
MALLKVKFDQKKRVKLAQGLWLMNWFSVLAGIIIFSLGLFLKIELRKRSDVMNNSESHFV
PNSLIGMGVLSCVFNSLAGKICYDALDPAKYARWKPWLKPYLAICVLFNIILFLVALCCFLL
RGSLENTLGQGLKNGMKYYRDTDTPGRCFMKKTIDMLQIEFKCCGNNGFRDWFEIQWIS
NRYLDFSSKEVKDRIKSNVDGRYLVDGVPFSCCNPSSPRPCIQYQITNNSAHYSYDHQTEE
LNLWVRGCRAALLSYYSSLMNSMGVVTLLIWLFEVTITIGLRYLQTSLDGVSNPEESESES
EGWLLEKSVPETWKAFLESVKKLGKGNQVEAEGAGAGQAPEAG

>Q13308 single-pass type I membrane protein
MGAARGSPARPRRLPLLSVLLLPLLGGTQTAIVFIKQPSSQDALQGRRALLRCEVEAPGPV
HVYWLLDGAPVQDTERRFAQGSSLSFAAVDRLQDSGTFQCVARDDVTGEEARSANASFNI
KWIEAGPVVLKHPASEAEIQPQTQVTLRCHIDGHPRPTYQWFRDGTPLSDGQSNHTVSSK
ERNLTLRPAGPEHSGLYSCCAHSAFGQACSSQNFTLSIADESFARVVLAPQDVVVARYEEA
MFHCQFSAQPPPSLQWLFEDETPITNRSRPPHLRRATVFANGSLLLTQVRPRNAGIYRCIGQ
GQRGPPIILEATLHLAEIEDMPLFEPRVFTAGSEERVTCLPPKGLPEPSVWWEHAGVRLPTH
GRVYQKGHELVLANIAESDAGVYTCHAANLAGQRRQDVNITVATVPSWLKKPQDSQLEE
GKPGYLDCLTQATPKPTVVWYRNQMLISEDSRFEVFKNGTLRINSVEVYDGTWYRCMSS
TPAGSIEAQARVQVLEKLKFTPPPQPQQCMEFDKEATVPCSATGREKPTIKWERADGSSLP
EWVTDNAGTLHFARVTRDDAGNYTCIASNGPQGQIRAHVQLTVAVFITFKVEPERTTVYQ
GHTALLQCEAQGDPKPLIQWKGKDRILDPTKLGPRMHIFQNGSLVIHDVAPEDSGRYTCIA
GNSCNIKHTEAPLYVVDKPVPEESEGPGSPPPYKMIQTIGLSVGAAVAYIIAVLGLMFYCKK
RCKAKRLQKQPEGEEPEMECLNGGPLQNGQPSAEIQEEVALTSLGSGPAATNKRHSTSDK
MHFPRSSLQPITTLGKSEFGEVFLAKAQGLEEGVAETLVLVKSLQSKDEQQQLDFRRELEM
FGKLNHANVVRLLGLCREAEPHYMVLEYVDLGDLKQFLRISKSKDEKLKSQPLSTKQKV
ALCTQVALGMEHLSNNRFVHKDLAARNCLVSAQRQVKVSALGLSKDVYNSEYYHFRQA
WVPLRWMSPEAILEGDFSTKSDVWAFGVLMWEVFTHGEMPHGGQADDEVLADLQAGK
ARLPQPEGCPSKLYRLMQRCWALSPKDRPSFSEIASALGDSTVDSKP

>Q16819 single-pass type I membrane protein
MAWIRSTCILFFTLLFAHIAAVPIKYLPEENVHDADFGEQKDISEINLAAGLDLFQGDILLQ
KSRNGLRDPNTRWTFPIPYILADNLGLNAKGAILYAFEMFRLKSCVDFKPYEGESSYIIFQQ
FDGCWSEVGDQHVGQNISIGQGCAYKAIIEHEILHALGFYHEQSRTDRDDYVNIWWDQIL
SGYQHNFDTYDDSLITDLNTPYDYESLMHYQPFSFNKNASVPTITAKIPEFNSIIGQRLDFS
AIDLERLNRMYNCTTTHTLLDHCTFEKANICGMIQGTRDDTDWAHQDSAQAGEVDHTLL
GQCTGAGYFMQFSTSSGSAEEAALLESRILYPKRKQQCLQFFYKMTGSPSDRLVVWVRRD
DSTGNVRKLVKVQTFQGDDDHNWKIAHVVLKEEQKFRYLFQGTKGDPQNSTGGIYLDDI
TLTETPCPTGVWTVRNFSQVLENTSKGDKLQSPRFYNSEGYGFGVTLYPNSRESSGYLRL
AFHVCSGENDAILEWPVENRQVIITILDQEPDVRNRMSSSMVFTTSKSHTSPAINDTVIWD
RPSRVGTYHTDCNCFRSIDLGWSGFISHQMLKRRSFLKNDDLIIFVDFEDITHLSQTEVPTK
GKRLSPQGLILQGQEQQVSEEGSGKAMLEEALPVSLSQGQPSRQKRSVENTGPLEDHNWP
QYFRDPCDPNPCQNDGICVNVKGMASCRCISGHAFFYTGERCQAVQVHGSVLGMVIGGT
AGVIFLTFSIIAILSQRPRK

>Q99884 multipass membrane protein
MKKLQGAHLRKPVTPDLLMTPSDQGDVDLDVDFAAHRGNWTGKLDFLLSCIGYCVGLG
NVWRFPYRAYTNGGGAFLVPYFLMLAICGIPLFFLELSLGQFSSLGPLAVWKISPLFKGAG
AAMLLIVGLVAIYYNMIIAYVLFYLFASLTSDLPWEHCGNWWNTELCLEHRVSKDGNGAL
PLNLTCTVSPSEEYWSRYVLHIQGSQGIGSPGEIRWNLCLCLLLAWVIVFLCILKGVKSSGK
VVYFTATFPYLILLMLLVRGVTLPGAWKGIQFYLTPQFHHLLSSKVWIEAALQIFYSLGVGF
GGLLTFASYNTFHQNIYRDTFIVTLGNAITSILAGFAIFSVLGYMSQELGVPVDQVAKAGPG
LAFVVYPQAMTMLPLSPFWSFLFFFMLLTLGLDSQFAFLETIVTAVTDEFPYYLRPKKAVFS
GLICVAMYLMGLILTTDGGMYWLVLLDDYSASFGLMVVVITTCLAVTRVYGIQRFCRDIH

MMLGFKPGLYFRACWLFLSPATLLALMVYSIVKYQPSEYGSYRFPPWAELLGILMGLLSC
LMIPAGMLVAVLREEGSLWERLQQASRPAMDWGPSLEENRTGMYVATLAGSQSPKPLMV
HMRKYGGITSFENTAIEVDREIAEEEESMM

>P10912 single-pass type I membrane protein
MDLWQLLLTLALAGSSDAFSGSEATAAILSRAPWSLQSVNPGLKTNSSKEPKFTKCRSPER
ETFSCHWTDEVHHGTKNLGPIQLFYTRRNTQEWTQEWKECPDYVSAGENSCYFNSSFTSI
WIPYCIKLTSNGGTVDEKCFSVDEIVQPDPPIALNWTLLNVSLTGIHADIQVRWEAPRNADI
QKGWMVLEYELQYKEVNETKWKMMDPILTTSVPVYSLKVDKEYEVRVRSKQRNSGNY
GEFSEVLYVTLPQMSQFTCEEDFYFPWLLIIIFGIFGLTVMLFVFLFSKQQRIKMLILPPVPVP
KIKGIDPDLLKEGKLEEVNTILAIHDSYKPEFHSDDSWVEFIELDIDEPDEKTEESDTDRLLS
SDHEKSHSNLGVKDGDSGRTSCCEPDILETDFNANDIHEGTSEVAQPQRLKGEADLLCLD
QKNQNNSPYHDACPATQQPSVIQAEKNKPQPLPTEGAESTHQAAHIQLSNPSSLSNIDFYA
QVSDITPAGSVVLSPGQKNKAGMSQCDMHPEMVSLCQENFLMDNAYFCEADAKKCIPVA
PHIKVESHIQPSLNQEDIYITTESLTTAAGRPGTGEHVPGSEMPVPDYTSIHIVQSPQGLILNA
TALPLPDKEFLSSCGYVSTDQLNKIMP

>Q99075 single-pass type I membrane protein
MKLLPSVVLKLFLAAVLSALVTGESLERLRRGLAAGTSNPDPPTVSTDQLLPLGGGRDRK
VRDLQEADLDLLRVTLSSKPQALATPNKEEHGKRKKKGKGLGKKRDPCLRKYKDFCIHG
ECKYVKELRAPSCICHPGYHGERCHGLSLPVENRLYTYDHTTILAVVAVVLSSVCLLVIVG
LLMFRYHRRGGYDVENEEKVKLGMTNSH

>Q9UIQ6 single-pass type II membrane protein
MEPFTNDRLQLPRNMIENSMFEEEPDVVDLAKEPCLHPLEPDEVEYEPRGSRLLVRGLGE
HEMEEDEEDYESSAKLLGMSFMNRSSGLRNSATGYRQSPDGACSVPSARTMVVCAFVIV
VAVSVIMVIYLLPRCTFTKEGCHKKNQSIGLIQPFATNGKLFPWAQIRLPTAVVPLRYELSLH
PNLTSMTFRGSVTISVQALQVTWNIILHSTGHNISRVTFMSAVSSQEKQAEILEYAYHGQIAI
VAPEALLAGHNYTLKIEYSANISSSYYGFYGFSYTDESNEKKYFAATQFEPLAARSAFPCF
DEPAFKATFIIKIIRDEQYTALSNMPKKSSVVLDDGLVQDEFSESVKMSTYLVAFIVGEMKN
LSQDVNGTLVSIYAVPEKIGQVHYALETTVKLLEFFQNYFEIQYPLKKLDLVAIPDFEAGAM
ENWGLLTFREETLLYDSNTSSMADRKLVTKIIAHELAHQWFGNLVTMKWWNDLWLNEGF
ATFMEYFSLEKIFKELSSYEDFLDARFKTMKKDSLNSSHPISSSVQSSEQIEEMFDSLSYFK
GSSLLLMLKTYLSEDVFQHAVVLYLHNHSYASIQSDDLWDSFNEVTNQTLDVKRMMKTW
TLQKGFPLVTVQKKGKELFIQQERFFLNMKPEIQPSDTSYLWHIPLSYVTEGRNYSKYQSV
SLLDKKSGVINLTEEVLWVKVNINMNGYYIVHYADDDWEALIHQLKINPYVLSDKDRAN
LINNIFELAGLGKVPLKRAFDLINYLGNENHTAPITEALFQTDLIYNLLEKLGYMDLASRLV
TRVFKLLQNQIQQQTWTDEGTPSMRELRSALLEFACTHNLGNCSTTAMKLFDDWMASNG
TQSLPTDVMTTVFKVGAKTDKGWSFLLGKYISIGSEAEKNKILEALASSEDVRKLYWLMK
SSLNGDNFRTQKLSFIIRTVGRHFPGHLLAWDFVKENWNKLVQKFPLGSYTIQNIVAGSTY
LFSTKTHLSEVQAFFENQSEATFRLRCVQEALEVIQLNIQWMEKNLKSLTWWL

>Q9NPG4 single-pass type I membrane protein
MMQLLQLLLGLLGPGGYLFLLGDCQEVTTLTVKYQVSEEVPSGTVIGKLSQELGREERRR

QAGAAFQVLQLPQALPIQVDSEEGLLSTGRRLDREQLCRQWDPCLVSFDVLATGDLALIH
VEIQVLDINDHQPRFPKGEQELEISESASLRTRIPLDRALDPDTGPNTLHTYTLSPSEHFALD
VIVGPDETKHAELIVVKELDREIHSFFDLVLTAYDNGNPPKSGTSLVKVNVLDSNDNSPAFA
ESSLALEIQEDAAPGTLLIKLTATDPDQGPNGEVEFFLSKHMPPEVLDTFSIDAKTGQVILR
RPLDYEKNPAYEVDVQARDLGPNPIPAHCKVLIKVLDVNDNIPSIHVTWASQPSLVSEALP
KDSFIALVMADDLDSGHNGLVHCWLSQELGHFRLKRTNGNTYMLLTNATLDREQWPKYT
LTLLAQDQGLQPLSAKKQLSIQISDINDNAPVFEKSRYEVSTRENNLPSLHLITIKAHDADL
GINGKVSYRIQDSPVAHLVAIDSNTGEVTAQRSLNYEEMAGFEFQVIAEDSGQPMLASSVS
VWVSLLDANDNAPEVVQPVLSDGKASLSVLVNASTGHLLVPIETPNGLGPAGTDTPPLAT
HSSRPFLLTTIVARDADSGANGEPLYSIRSGNEAHLFILNPHTGQLFVNVTNASSLIGSEWE
LEIVVEDQGSPPLQTRALLRVMFVTSVDHLRDSARKPGALSMSMLTVICLAVLLGIFGLIL
ALFMSICRTEKKDNRAYNCREAESTYRQQPKRPQKHIQKADIHLVPVLRGQAGEPCEVGQ
SHKDVDKEAMMEAGWDPCLQAPFHLTPTLYRTLRNQGNQGAPAESREVLQDTVNLLFN
HPRQRNASRENLNLPEPQPATGQPRSRPLKVAGSPTGRLAGDQGSEEAPQRPPASSATLRR
QRHLNGKVSPEKESGPRQILRSLVRLSVAAFAERNPVEELTVDSPPVQQISQLLSLLHQGQF
QPKPNHRGNKYLAKPGGSRSAIPDTDGPSARAGGQTDPEQEEGPLDPEEDLSVKQLLEEE
LSSLLDPSTGLALDRLSAPDPAWMARLSLPLTTNYRDNVISPDAAATEEPRTFQTFGKAEA
PELSPTGTRLASTFVSEMSSLLEMLLEQRSSMPVEAASEALRRLSVCGRTLSLDLATSAAS
GMKVQGDPGGKTGTEGKSRGSSSSSRCL

>O75694 peripheral membrane protein
MPSSLLGAAMPASTSAAALQEALENAGRLIDRQLQEDRMYPDLSELLMVSAPNNPTVSG
MSDMDYPLQGPGLLSVPNLPEISSIRRVPLPPELVEQFGHMQCNCMMGVFPPISRAWLTID
SDIFMWNYEDGGDLAYFDGLSETILAVGLVKPKAGIFQPHVRHLLVLATPVDIVILGLSYA
NLQTGSGVLNDSLSGGMQLLPDPLYSLPTDNTYLLTITSTDNGRIFLAGKDGCLYEVAYQA
EAGWFSQRCRKINHSKSSLSFLVPSLLQFTFSEDDPILQIAIDNSRNILYTRSEKGVIQVYDL
GQDGQGMSRVASVSQNAIVSAAGNIARTIDRSVFKPIVQIAVIENSESLDCQLLAVTHAGVR
LYFSTCPFRQPLARPNTLTLVHVRLPPGFSASSTVEKPSKVHRALYSKGILLMAASENEDN
DILWCVNHDTFPFQKPMMETQMTAGVDGHSWALSAIDELKVDKIITPLNKDHIPITDSPVV
VQQHMLPPKKFVLLSAQGSLMFHKLRPVDQLRHLLVSNVGGDGEEIERFFKLHQEDQAC
ATCLILACSTAACDREVSAWATRAFFRYGGEAQMRFPTTLPPPSNVGPILGSPVYSSSPVPS
GSPYPNPSFLGTPSHGIQPPAMSTPVCALGNPATQATNMSCVTGPEIVYSGKHNGICIYFSRI
MGNIWDASLVVERIFKSGNREITAIESSVPCQLLESVLQELKGLQEFLDRNSQFAGGPLGNP
NTTAKVQQRLIGFMRPENGNPQQMQQELQRKFHEAQLSEKISLQAIQQLVRKSYQALAL
WKLLCEHQFTIIVAELQKELQEQLKITTFKDLVIRDKELTGALIASLINCYIRDNAAVDGISL
HLQDICPLLYSTDDAICSKANELLQRSRQVQNKTEKERMLRESLKEYQKISNQVDLSNVC
AQYRQVRFYEGVVELSLTAAEKKDPQGLGLHFYKHGEPEEDIVGLQAFQERLNSYKCITD
TLQELVNQSKAAPQSPSVPKKPGPPVLSSDPNMLSNEEAGHHFEQMLKLSQRSKDELFSIA
LYNWLIQVDLADKLLQVASPFLEPHLVRMAKVDQNRVRYMDLLWRYYEKNRSFSNAAR
VLSRLADMHSTEISLQQRLEYIARAILSAKSSTAISSIAADGEFLHELEEKMEVARIQLQIQE
TLQRQYSHHSSVQDAVSQLDSELMDITKLYGEFADPFKLAECKLAIIHCAGYSDPILVQTL
WQDIIEKELSDSVTLSSSDRMHALSLKIVLLGKIYAGTPRFFPLDFIVQFLEQQVCTLNWDV
GFVIQTMNEIGVPLPRLLEVYDQLFKSRDPFWNRMKKPLHLLDCIHVLLIRYVENPSQVLN
CERRRFTNLCLDAVCGYLVELQSMSSSVAVQAITGNFKSLQAKLERLH

>P22760 single-pass type II membrane protein
MGRKSLYLLIVGILIAYYIYTPLPDNVEEPWRMMWINAHLKTIQNLATFVELLGLHHFMDS
FKVVGSFDEVPPTSDENVTVTETKFNNILVRVYVPKRKSEALRRGLFYIHGGGWCVGSAA
LSGYDLLSRWTADRLDAVVVSTNYRLAPKYHFPIQFEDVYNALRWFLRKKVLAKYGVNP
ERIGISGDSAGGNLAAAVTQQLLDDPDVKIKLKIQSLIYPALQPLDVDLPSYQENSNFLFLS
KSLMVRFWSEYFTTDRSLEKAMLSRQHVPVESSHLFKFVNWSSLLPERFIKGHVYNNPNY
GSSELAKKYPGFLDVRAAPLLADDNKLRGLPLTYVITCQYDLLRDDGLMYVTRLRNTGV
QVTHNHVEDGFHGAFSFLGLKISHRLINQYIEWLKENL

>P30414 peripheral membrane protein
MGAQDRPQCHFDIEINREPVGRIMFQLFSDICPKTCKNFLCLCSGEKGLGKTTGKKLCYK
GSTFHRVVKNFMIQGGDFSEGNGKGGESIYGGYFKDENFILKHDRAFLLSMANRGKHTN
GSQFFITTKPAPHLDGVHVVFGLVISGFEVIEQIENLKTDAASRPYADVRVIDCGVLATKSIK
DVFEKKRKKPTHSEGSDSSSNSSSSSESSSESELEHERSRRRKHKRRPKVKRSKKRRKEAS
SSEEPRNKHAMNPKGHSERSDTNEKRSVDSSAKREKPVVRPEEIPPVPENRFLLRRDMPV
VTAEPEPKIPDVAPIVSDQKPSVSKSGRKIKGRGTIRYHTPPRSRSCSESDDDDSSETPPHWK
EEMQRLRAYRPPSGEKWSKGDKLSDPCSSRWDERSLSQRSRSWSYNGYYSDLSTARHSG
HHKKRRKEKKVKHKKKGKKQKHCRRHKQTKKRRILIPSDIESSKSSTRRMKSSCDRERSS
RSSSLSSHHSSKRDWSKSDKDVQSSLTHSSRDSYRSKSHSQSYSRGSSRSRTASKSSSHSRS
RSKSRSSSKSGHRKRASKSPRKTASQLSENKPVKTEPLRATMAQNENVVVQPVVAENIPVI
PLSDSPPPSRWKPGQKPWKPSYERIQEMKAKTTHLLPIQSTYSLANIKETGSSSSYHKREK
NSESDQSTYSKYSDRSSESSPRSRSRSSRSRSYSRSYTRSRSLASSHSRSRSPSSRSHSRNKY
SDHSQCSRSSSYTSISSDDGRRAKRRLRSSGKKNSVSHKKHSSSSEKTLHSKYVKGRDRSS
CVRKYSESRSSLDYSSDSEQSSVQATQSAQEKEKQGQMERTHNKQEKNRGEEKSKSEREC
PHSKKRTLKENLSDHLRNGSKPKRKNYAGSKWDSESNSERDVTKNSKNDSHPSSDKEEG
EATSDSESEVSEIHIKVKPTTKSSTNTSLPDDNGAWKSSKQRTSTSDSEGSCSNSENNRGKP
QKHKHGSKENLKREHTKKVKEKLKGKKDKKHKAPKRKQAFHWQPPLEFGEEEEEEIDD
KQVTQESKEKKVSENNETIKDNILKTEKSSEEDLSGKHDTVTVSSDLDQFTKDDSKLSISPT
ALNTEENVACLQNIQHVEESVPNGVEDVLQTDDNMEICTPDRSSPAKVEETSPLGNARLD
TPDINIVLKQDMATEHPQAEVVKQESSMSESKVLGEVGKQDSSSASLASAGESTGKKEVA
EKSQINLIDKKWKPLQGVGNLAAPNAATSSAVEVKVLTTVPEMKPQGLRIEIKSKNKVRP
GSLFDEVRKTARLNRRPRNQESSSDEQTPSRDDDSQSRSPSRSRSKSETKSRHRTRSVSYSH
SRSRSRSSTSSYRSRSYSRSRSRGWYSRGRTRSRSSSYRSYKSHRTSSRSRSRSSSYDPHSRS
RSYTYDSYYSRSRSRSRSQRSDSYHRGRSYNRRSRSCRSYGSDSESDRSYSHHRSPSESSR
YS

>P39210 multipass membrane protein
MALWRAYQRALAAHPWKVQVLTAGSLMGLGDIISQQLVERRGLQEHQRGRTLTMVSLG
CGFVGPVVGGWYKVLDRFIPGTTKVDALKKMLLDQGGFAPCFLGCFLPLVGALNGLSAQ
DNWAKLQRDYPDALITNYYLWPAVQLANFYLVPLHYRLAVVQCVAVIWNSYLSWKAHRL

>Q15036 peripheral membrane protein
MHFSIPETESRSGDSGGSAYVAYNIHVNGVLHCRVRYSQLLGLHEQLRKEYGANVLPAFPP

KKLFSLTPAEVEQRREQLEKYMQAVRQDPLLGSSETFNSFLRRAQQETQQVPTEEVSLEVL
LSNGQKVLVNVLTSDQTEDVLEAVAAKLDLPDDLIGYFSLFLVREKEDGAFSFVRKLQEFE
LPYVSVTSLRSQEYKIVLRKSYWDSAYDDDVMENRVGLNLLYAQTVSDIERGWILVTKEQ
HRQLKSLQEKVSKKEFLRLAQTLRHYGYLRFDACVADFPEKDCPVVVSAGNSELSLQLRL
PGQQLREGSFRVTRMRCWRVTSSVPLPSGSTSSPGRGRGEVRLELAFEYLMSKDRLQWVT
ITSPQAIMMSICLQSMVDELMVKKSGGSIRKMLRRRVGGTLRRSDSQQAVKSPPLLESPDA
TRESMVKLSSKLSAVSLRGIGSPSTDASASDVHGNFAFEGIGDEDL

>O95996 peripheral membrane protein
MASSVAPYEQLVRQVEALKAENSHLRQELRDNSSHLSKLETETSGMKEVLKHLQGKLEQ
EARVLVSSGQTEVLEQLKALQMDITSLYNLKFQPPTLGPEPAARTPEGSPVHGSGPSKDSF
GELSRATIRLLEELDRERCFLLNEIEKEEKEKLWYYSQLQGLSKRLDELPHVETQFSMQMD
LIRQQLEFEAQHIRSLMEERFGTSDEMVQRAQIRASRLEQIDKELLEAQDRVQQTEPQALL
AVKSVPVDEDPETEVPTHPEDGTPQPGNSKVEVVFWLLSMLATRDQEDTARTLLAMSSSP
ESCVAMRRSGCLPLLLQILHGTEAAAGGRAGAPGAPGAKDARMRANAALHNIVFSQPDQ
GLARKEMRVLHVLEQIRAYCETCWDWLQARDGGPEGGGAGSAPIPIEPQICQATCAVMKL
SFDEEYRRAMNELGGLQAVAELLQVDYEMHKMTRDPLNLALRRYAGMTLTNLTFGDVA
NKATLCARRGCMEAIVAQLASDSEELHQVVSSILRNLSWRADINSKKVLREAGSVTALVQ
CVLRATKESTLKSVLSALWNLSAHSTENKAAICQVDGALGFLVSTLTYKCQSNSLAIIESG
GGILRNVSSLVATREDYRQVLRDHNCLQTLLQHLTSHSLTIVSNACGTLWNLSARSARDQE
LLWDLGAVGMLRNLVHSKHKMIAMGSAAALRNLLAHRPAKHQAAATAVSPGSCVPSLYV
RKQRALEAELDARHLAQALEHLEKQGPPAAEAATKKPLPPLRHLDGLAQDYASDSGCFD
DDDAPSSLAAAAATGEPASPAALSLFLGSPFLQGQALARTPPTRRGGKEAEKDTSGEAAVA
AKAKAKLALAVARIDQLVEDISALHTSSDDSFSLSSGDPGQEAPREGRAQSCSPCRGPEGG
RREAGSRAHPLLRLKAAHASLSNDSLNSGSASDGYCPREHMLPCPLAALASRREDPRCGQ
PRPSRLDLDLPGCQAEPPAREATSADARVRTIKLSPTYQHVPLLEGASRAGAEPLAGPGISP
GARKQAWLPADHLSKVPEKLAAAPLSVASKALQKLAAQEGPLSLSRCSSLSSLSSAGRPG
PSEGGDLDDSDSSLEGLEEAGPSEAELDSTWRAPGATSLPVAIPAPRRNRGRGLGVEDATP
SSSSENYVQETPLVLSRCSSVSSLGSFESPSIASSIPSEPCSGQGSGTISPSELPDSPGQTMPPS
RSKTPPLAPAPQGPPEATQFSLQWESYVKRFLDIADCRERCRLPSELDAGSVRFTVEKPDE
NFSCASSLSALALHEHYVQQDVELRLLPSACPERGGGAGGAGLHFAGHRRREEGPAPTGS
RPRGAADQELELLRECLGAAVPARLRKVASALVPGRRALPVPVYMLVPAPAPAQEDDSCT
DSAEGTPVNFSSAASLSDETLQGPPRDQPGGPAGRQRPTGRPTSARQAMGHRHKAGGAG
RSAEQSRGAGKNRAGLELPLGRPPSAPADKDGSKPGRTRGDGALQSLCLTTPTEEAVYCF
YGNDSDEEPPAAAPTPTHRRTSAIPRAFTRERPQGRKEAPAPSKAAPAAPPPARTQPSLIAD
ETPPCYSLSSSASSLSEPEPSEPPAVHPRGREPAVTKDPGPGGGRDSSPSPRAAEELLQRCISS
ALPRRRPPVSGLRRRKPRATRLDERPAEGSRERGEEAAGSDRASDLDSVEWRAIQEGANSI
VTWLHQAAAATREASSESDSILSFVSGLSVGSTLQPPKHRKGRQAEGEMGSARRPEKRGA
ASVKTSGSPRSPAGPEKPRGTQKTTPGVPAVLRGRTVIYVPSPAPRAQPKGTPGPRATPRKV
APPCLAQPAAPAKVPSPGQQRSRSLHRPAKTSELATLSQPPRSATPPARLAKTPSSSSSQTSP
ASQPLPRKRPPVTQAAGALPGPGASPVPKTPARTLLAKQHKTQRSPVRIPFMQRPARRGPP
PLARAVPEPGPRGRAGTEAGPGARGGRLGLVRVASALSSGSESSDRSGFRRQLTFIKESPGL
RRRRSELSSAESAASAPQGASPRRGRPALPAVFLCSSRCEELRAAPRQGPAPARQRPPAARP
SPGERPARRTTSESPSRLPVRAPAARPETVKRYASLPHISVARRPDGAVPAAPASADAARRS

SDGEPRPLPRVAAPGTTWRRIRDEDVPHILRSTLPATALPLRGSTPEDAPAGPPPRKTSDAV
VQTEEVAAPKTNSSTSPSLETREPPGAPAGGQLSLLGSDVDGPSLAKAPISAPFVHEGLGVA
VGGFPASRHGSPSRSARVPPFNYVPSPMVVAATTDSAAEKAPATASATLLE

>Q13724 single-pass type II membrane protein
MARGERRRRAVPAEGVRTAERAARGGPGRRDGRGGGPRSTAGGVALAVVVLSLALGMS
GRWVLAWYRARRAVTLHSAPPVLPADSSSPAVAPDLFWGTYRPHVYFGMKTRSPKPLLTG
LMWAQQGTTPGTPKLRHTCEQGDGVGPYGWEFHDGLSFGRQHIQDGALRLTTEFVKRPG
GQHGGDWSWRVTVEPQDSGTSALPLVSLFFYVVTDGKEVLLPEVGAKGQLKFISGHTSEL
GDFRFTLLPPTSPGDTAPKYGSYNVFWTSNPGLPLLTEMVKSRLNSWFQHRPPGAPPERYL
GLPGSLKWEDRGPSGQGQGQFLIQQVTLKIPISIEFVFESGSAQAGGNQALPRLAGSLLTQ
ALESHAEGFRERFEKTFQLKEKGLSSGEQVLGQAALSGLLGGIGYFYGQGLVLPDIGVEGS
EQKVDPALFPPVPLFTAVPSRSFFPRGFLWDEGFHQLVVQRWDPSLTREALGHWLGLLNA
DGWIGREQILGDEARARVPPEFLVQRAVHANPPTLLLPVAHMLEVGDPDDLAFLRKALPR
LHAWFSWLHQSQAGPLPLSYRWRGRDPALPTLLNPKTLPSGLDDYPRASHPSVTERHLDL
RCWVALGARVLTRLAEHLGEAEVAAELGPLAASLEAAESLDELHWAPELGVFADFGNHT
KAVQLKPRPPQGLVRVVGRPQPQLQYVDALGYVSLFPLLLRLLDPTSSRLGPLLDILADSR
HLWSPFGLRSLAASSSFYGQRNSEHDPPYWRGAVWLNVNYLALGALHHYGHLEGPHQA
RAAKLHGELRANVVGNVWRQYQATGFLWEQYSDRDGRGMGCRPFHGWTSLVLLAMAE
DY

>O43813 peripheral membrane protein
MAQRAFPNPYADYNKSLAEGYFDAAGRLTPEFSQRLTNKIRELLQQMERGLKSADPRDGT
GYTGWAGIAVLYLHLYDVFGDPAYLQLAHGYVKQSLNCLTKRSITFLCGDAGPLAVAAVL
YHKMNNEKQAEDCITRLIHLNKIDPHAPNEMLYGRIGYIYALLFVNKNFGVEKIPQSHIQQI
CETILTSGENLARKRNFTAKSPLMYEWYQEYYVGAAHGLAGIYYYLMQPSLQVSQGKLH
SLVKPSVDYVCQLKFPSGNYPPCIGDNRDLLVHWCHGAPGVIYMLIQAYKVFREEKYLCD
AYQCADVIWQYGLLKKGYGLCHGSAGNAYAFLTLYNLTQDMKYLYRACKFAEWCLEYG
EHGCRTPDTPFSLFEGMAGTIYFLADLLVPTKARFPAFEL

>O60928 multipass membrane protein
MDSSNCKVIAPLLSQRYRRMVTKDGHSTLQMDGAQRGLAYLRDAWGILMDMRWRWM
MLVFSASFVVHWLVFAVLWYVLAEMNGDLELDHDAPPENHTICVKYITSFTAAFSFSLET
QLTIGYGTMFPSGDCPSAIALLAIQMLLGLMLEAFITGAFVAKIARPKNRAFSIRFTDTAVV
AHMDGKPNLIFQVANTRPSPLTSVRVSAVLYQERENGKLYQTSVDFHLDGISSDECPFFIFP
LTYYHSITPSSPLATLLQHENPSHFELVVFLSAMQEGTGEICQRRTSYLPSEIMLHHCFASLL
TRGSKGEYQIKMENFDKTVPEFPTPLVSKSPNRTDLDIHINGQSIDNFQISETGLTE

>P38435 multipass membrane protein
MAVSAGSARTSPSSDKVQKDKAELISGPRQDSRIGKLLGFEWTDLSSWRRLVTLLNRPTDP
ASLAVFRFLFGFLMVLDIPQERGLSSLDRKYLDGLDVCRFPLLDALRPLPLDWMYLVYTI
MFLGALGMMLGLCYRISCVLFLLPYWYVFLLDKTSWNNHSYLYGLLAFQLTFMDANHY
WSVDGLLNAHRRNAHVPLWNYAVLRGQIFIVYFIAGVKKLDADWVEGYSMEYLSRHWL
FSPFKLLLSEELTSLLVVHWGGLLLDLSAGFLLFFDVSRSIGLFFVSYFHCMNSQLFSIGMF

SYVMLASSPLFCSPEWPRKLVSYCPRRLQQLLPLKAAPQPSVSCVYKRSRGKSGQKPGLR
HQLGAAFTLLYLLEQLFLPYSHFLTQGYNNWTNGLYGYSWDMMVHSRSHQHVKITYRD
GRTGELGYLNPGVFTQSRRWKDHADMLKQYATCLSRLLPKYNVTEPQIYFDIWVSINDRF
QQRIFDPRVDIVQAAWSPFQRTSWVQPLLMDLSPWRAKLQEIKSSLDNHTEVVFIADFPGL
HLENFVSEDLGNTSIQLLQGEVTVELVAEQKNQTLREGEKMQLPAGEYHKVYTTSPSPSC
YMYVYVNTTELALEQDLAYLQELKEKVENGSETGPLPPELQPLLEGEVKGGPEPTPLVQT
FLRRQQRLQEIERRRNTPFHERFFRFLLRKLYVFRRSFLMTCISLRNLILGRPSLEQLAQEVT
YANLRPFEAVGELNPSNTDSSHSNPPESNPDPVHSEF

>Q01638 single-pass type I membrane protein
MGFWILAILTILMYSTAAKFSKQSWGLENEALIVRCPRQGKPSYTVDWYYSQTNKSIPTQE
RNRVFASGQLLKFLPAAVADSGIYTCIVRSPTFNRTGYANVTIYKKQSDCNVPDYLMYSTV
SGSEKNSKIYCPTIDLYNWTAPLEWFKNCQALQGSRYRAHKSFLVIDNVMTEDAGDYTCK
FIHNENGANYSVTATRSFTVKDEQGFSLFPVIGAPAQNEIKEVEIGKNANLTCSACFGKGTQ
FLAAVLWQLNGTKITDFGEPRIQQEEGQNQSFSNGLACLDMVLRIADVKEEDLLLQYDCL
ALNLHGLRRHTVRLSRKNPIDHHSIYCIIAVCSVFLMLINVLVIILKMFWIEATLLWRDIAKP
YKTRNDGKLYDAYVVYPRNYKSSTDGASRVEHFVHQILPDVLENKCGYTLCIYGRDMLP
GEDVVTAVETNIRKSRRHIFILTPQITHNKEFAYEQEVALHCALIQNDAKVILIEMEALSELD
MLQAEALQDSLQHLMKVQGTIKWREDHIANKRSLNSKFWKHVRYQMPVPSKIPRKASSL
TPLAAQKQ

>Q13478 single-pass type I membrane protein
MNCRELPLTLWVLISVSTAESCTSRPHITVVEGEPFYLKHCSCSLAHEIETTTKSWYKSSGS
QEHVELNPRSSSRIALHDCVLEFWPVELNDTGSYFFQMKNYTQKWKLNVIRRNKHSCFT
ERQVTSKIVEVKKFFQITCENSYYQTLVNSTSLYKNCKKLLLENNKNPTIKKNAEFEDQGY
YSCVHFLHHNGKLFNITKTFNITIVEDRSNIVPVLLGPKLNHVAVELGKNVRLNCSALLNE
EDVIYWMFGEENGSDPNIHEEKEMRIMTPEGKWHASKVLRIENIGESNLNVLYNCTVAST
GGTDTKSFILVRKADMADIPGHVFTRGMIIAVLILVAVVCLVTVCVIYRVDLVLFYRHLTRR
DETLTDGKTYDAFVSYLKECRPENGEEHTFAVEILPRVLEKHFGYKLCIFERDVVPGGAVV
DEIHSLIEKSRRLIIVLSKSYMSNEVRYELESGLHEALVERKIKIILIEFTPVTDFTFLPQSLKL
LKSHRVLKWKADKSLSYNSRFWKNLLYLMPAKTVKPGRDEPEVLPVLSES

>Q9H8Y8 lipid-anchor, peripheral membrane protein
MGSSQSVEIPGGGTEGYHVLRVQENSPGHRAGLEPFFDFIVSINGSRLNKDNDTLKDLLKA
NVEKPVKMLIYSSKTLELRETSVTPSNLWGGQGLLGVSIRFCSFDGANENVWHVLEVESN
SPAALAGLRPHSDYIIGADTVMNESEDLFSLIETHEAKPLKLYVYNTDTDNCREVIITPNSA
WGGEGSLGCGIGYGYLHRIPTRPFEEGKKISLPGQMAGTPITPLKDGFTEVQLSSVNPPSLS
PPGTTGIEQSLTGLSISSTPPAVSSVLSTGVPTVPLLPPQVNQSLTSVPPMNPATTLPGLMPLP
AGLPNLPNLNLNLPAPHIMPGVGLPELVNPGLPPLPSMPPRNLPGIAPLPLPSEFLPSFPLVP
ESSSAASSGELLSSLPPTSNAPSDPATTTAKADAASSLTVDVTPPTAKAPTTVEDRVGDSTP
VSEKPVSAAVDANASESP

>P43007 multipass membrane protein
MEKSNETNGYLDSAQAGPAAGPGAPGTAAGRARRCAGFLRRQALVLLTVSGVLAGAGL

GAALRGLSLSRTQVTYLAFPGEMLLRMLRMIILPLVVCSLVSGAASLDASCLGRLGGIAVA
YFGLTTLSASALAVALAFIIKPGSGAQTLQSSDLGLEDSGPPPVPKETVDSFLDLARNLFPSN
LVVAAFRTYATDYKVVTQNSSSGNVTHEKIPIGTEIEGMNILGLVLFALVLGVALKKLGSEG
EDLIRFFNSLNEATMVLVSWIMWYVPVGIMFLVGSKIVEMKDIIVLVTSLGKYIFASILGHV
IHGGIVLPLIYFVFTRKNPFRFLLGLLAPFATAFATCSSSATLPSMMKCIEENNGVDKRISRFI
LPIGATVNMDGAAIFQCVAAVFIAQLNNVELNAGQIFTILVTATASSVGAAGVPAGGVLTIA
IILEAIGLPTHDLPLILAVDWIVDRTTTVVNVEGDALGAGILHHLNQKATKKGEQELAEVK
VEAIPNCKSEEETSPLVTHQNPAGPVASAPELESKESVL

>Q969F8 multipass membrane protein
MHTVATSGPNASWGAPANASGCPGCGANASDGPVPSPRAVDAWLVPLFFAALMLLGLVG
NSLVIYVICRHKPMRTVTNFYIANLAATDVTFLLCCVPFTALLYPLPGWVLGDFMCKFVN
YIQQVSVQATCATLTAMSVDRWYVTVFPLRALHRRTPRLALAVSLSIWVGSAAVSAPVLA
LHRLSPGPRAYCSEAFPSRALERAFALYNLLALYLLPLLATCACYAAMLRHLGRVAVRPAP
ADSALQGQVLAERAGAVRAKVSRLVAAVVLLFAACWGPIQLFLVLQALGPAGSWHPRSY
AAYALKTWAHCMSYSNSALNPLLYAFLGSHFRQAFRRVCPCAPRRPRRPRRPGPSDPAAP
HAELLRLGSHPAPARAQKPGSSGLAARGLCVLGEDNAPL

>P15313 peripheral membrane protein
MAMEIDSRPGGLPGSSCNLGAAREHMQAVTRNYITHPRVTYRTVCSVNGPLVVLDRVKFA
QYAEIVHFTLPDGTQRSGQVLEVAGTKAIVQVFEGTSGIDARKTTCEFTGDILRTPVSEDM
LGRVFNGSGKPIDKGPVVMAEDFLDINGQPINPHSRIYPEEMIQTGISPIDVMNSIARGQKIP
IFSAAGLPHNEIAAQICRQAGLVKKSKAVLDYHDDNFAIVFAAMGVNMETARFFKSDFEQ
NGTMGNVCLFLNLANDPTIERIITPRLALTTAEFLAYQCEKHVLVILTDMSSYAEALREVSA
AREEVPGRRGFPGYMYTDLATIYERAGRVEGRGGSITQIPILTMPNDDITHPIPDLTGFITEG
QIYVDRQLHNRQIYPPINVLPSLSRLMKSAIGEGMTRKDHGDVSNQLYACYAIGKDVQAM
KAVVGEEALTSEDLLYLEFLQKFEKNFINQGPYENRSVFESLDLGWKLLRIFPKEMLKRIP
QAVIDEFYSREGALQDLAPDTAL

>P51797 multipass membrane protein
MAGCRGSLCCCCRWCCCCGERETRTPEELTILGETQEEEDEILPRKDYESLDYDRCINDPY
LEVLETMDNKKGRRYEAVKWMVVFAIGVCTGLVGLFVDFFVRLFTQLKFGVVQTSVEEC
SQKGCLALSLLELLGFNLTFVFLASLLVLIEPVAAGSGIPEVKCYLNGVKVPGIVRLRTLLC
KVLGVLFSVAGGLFVEKEGPMIHSGSVVGAGLPQFQSISLRKIQFNFPYFRSDRDKRDFVS
AGAAAGVAAAFGAPIGGTLFSLEEGSSFWNQGLTWKVLFCSMSATFTLNFFRSGIQFGSW
GSFQLPGLLNFGEFKCSDSDKKCHLWTAMDLGFFVVMGVIGGLLGATFNCLNKRLAKYR
MRNVHPKPKLVRVLESLLVSLVTTVVVFVASMVLGECRQMSSSSQIGNDSFQLQVTEDVN
SSIKTFFCPNDTYNDMATLFFNPQESAILQLFHQDGTFSPVTLALFFVLYFLLACWTYGISV
PSGLFVPSLLCGAAFGRLVANVLKSYIGLGHIYSGTFALIGAAAFLGGVVRMTISLTVILIES
TNEITYGLPIMVTLMVAKWTGDFFNKGIYDIHVGLRGVPLLEWETEVEMDKLRASDIMEP
NLTYVYPHTRIQSLVSILRTTVHHAFPVVTENRGNEKEFMKGNQLISNNIKFKKSSILTRAG
EQRKRSQSMKSYPSSELRNMCDEHIASEEPAEKEDLLQQMLERRYTPYPNLYPDQSPSED
WTMEERFRPLTFHGLILRSQLVTLLVRGVCYSESQSSASQPRLSYAEMAEDYPRYPDIHDL
DLTLLNPRMIVDVTPYMNPSPFTVSPNTHVSQVFNLFRTMGLRHLPVVNAVGEIVGIITRH

NLTYEFLQARLRQHYQTI

>Q9BXS4 single-pass type I membrane protein
MAAPKGSLWVRTQLGLPPLLLLTMALAGGSGTASAEAFDSVLGDTASCHRACQLTYPLHT
YPKEEELYACQRGCRLFSICQFVDDGIDLNRTKLECESACTEAYSQSDEQYACHLGCQNQL
PFAELRQEQLMSLMPKMHLLFPLTLVRSFWSDMMDSAQSFITSSWTFYLQADDGKIVIFQS
KPEIQYAPHLEQEPTNLRESSLSKMSYLQMRNSQAHRNFLEDGESDGFLRCLSLNSGWILT
TTLVLSVMVLLWICCATVATAVEQYVPSEKLSIYGDLEFMNEQKLNRYPASSLVVVRSKTE
DHEEAGPLPTKVNLAHSEI

>O95140 multipass membrane protein
MSLLFSRCNSIVTVKKNKRHMAEVNASPLKHFVTAKKKINGIFEQLGAYIQESATFLEDTY
RNAELDPVTTEEQVLDVKGYLSKVRGISEVLARRHMKVAFFGRTSNGKSTVINAMLWDK
VLPSGIGHTTNCFLRVEGTDGHEAFLLTEGSEEKRSAKTVNQLAHALHQDKQLHAGSLVS
VMWPNSKCPLLKDDLVLMDSPGIDVTTELDSWIDKFCLDADVFVLVANSESTLMQTEKH
FFHKVSERLSRPNIFILNNRWDASASEPEYMEEVRRQHMERCTSFLVDELGVVDRSQAGD
RIFFVSAKEVLNARIQKAQGMPEGGGALAEGFQVRMFEFQNFERRFEECISQSAVKTKFEQ
HTVRAKQIAEAVRLIMDSLHMAAREQQVYCEEMREERQDRLKFIDKQLELLAQDYKLRI
KQITEEVERQVSTAMAEEIRRLSVLVDDYQMDFHPSPVVLKVYKNELHRHIEEGLGRNMS
DRCSTAITNSLQTMQQDMIDGLKPLLPVSVRSQIDMLVPRQCFSLNYDLNCDKLCADFQE
DIEFHFSLGWTMLVNRFLGPKNSRRALMGYNDQVQRPIPLTPANPSMPPLPQGSLTQEEFM
VSMVTGLASLTSRTSMGILVVGGVVWKAVGWRLIALSFGLYGLLYVYERLTWTTKAKER
AFKRQFVEHASEKLQLVISYTGSNCSHQVQQELSGTFAHLCQQVDVTRENLEQEIAAMNK
KIEVLDSLQSKAKLLRNKAGWLDSELNMFTHQYLQPSR

>Q9NTN3 multipass membrane protein
MAEVHRRQHARVKGEAPAKSSTLRDEEELGMASAETLTVFLKLLAAGFYGVSSFLIVVVN
KSVLTNYRFPSSLCVGLGQMVATVAVLWVGKALRVVKFPDLDRNVPRKTFPLPLLYFGNQ
ITGLFSTKKLNLPMFTVLRRFSILFTMFAEGVLLKKTFSWGIKMTVFAMIIGAFVAASSDLA
FDLEGYAFILINDVLTAANGAYVKQKLDSKELGKYGLLYYNALFMILPTLAIAYFTGDAQK
AVEFEGWADTLFLLQFTLSCVMGFILMYATVLCTQYNSALTTTIVGCIKNILITYIGMVFGG
DYIFTWTNFIGLNISIAGSLVYSYITFTEEQLSKQSEANNKLDIKGKGAV

>O95971 GPI-anchor, lipid-anchor
MLLEPGRGCCALAILLAIVDIQSGGCINITSSASQEGTRLNLICTVWHKKEEAEGFVVFLCK
DRSGDCSPETSLKQLRLKRDPGIDGVGEISSQLMFTISQVTPLHSGTYQCCARSQKSGIRLQ
GHFFSILFTETGNYTVTGLKQRQHLEFSHNEGTLSSGFLQEKVWVMLVTSLVALQAL

>Q99437 multipass membrane protein
MTGLALLYSGVFVAFWACALAVGVCYTIFDLGFRFDVAWFLTETSPFMWSNLGIGLAISLS
VVGAAWGIYITGSSIIGGGVKAPRIKTKNLVSIIFCEAVAIYGIIMAIVISNMAEPFSATDPKAI
GHRNYHAGYSMFGAGLTVGLSNLFCGVCVGIVGSGAALADAQNPSLFVKILIVEIFGSAIG
LFGVIVAILQTSRVKMGD

>O60779 multipass membrane protein
MDVPGPVSRRAAAAAATVLLRTARVRRECWFLPTALLCAYGFFASLRPSEPFLTPYLLGPD
KNLTEREVFNEIYPVWTYSYLVLLFPVFLATDYLRYKPVVLLQGLSLIVTWFMLLYAQGLL
AIQFLEFFYGIATATEIAYYSYIYSVVDLGMYQKVTSYCRSATLVGFTVGSVLGQILVSVAG
WSLFSLNVISLTCVSVAFAVAWFLPMPQKSLFFHHIPSTCQRVNGIKVQNGGIVTDTPASNH
LPGWEDIESKIPLNMEEPPVEEPEPKPDRLLVLKVLWNDFLMCYSSRPLLCWSVWWALST
CGYFQVVNYTQGLWEKVMPSRYAAIYNGGVEAVSTLLGAVAVFAVGYIKISWSTWGEMT
LSLFSLLIAAAVYIMDTVGNIWVCYASYVVFRIIYMLLITIATFQIAANLSMERYALVFGVN
TFIALALQTLLTLIVVDASGLGLEITTQFLIYASYFALIAVVFLASGAVSVMKKCRKLEDPQS
SSQVTTS

>Q9H204 peripheral membrane protein
MAAPLGGMFSGQPPGPPQAPPGLPGQASLLQAAPGAPRPSSSTLVDELESSFEACFASLVS
QDYVNGTDQEEIRTGVDQCIQKFLDIARQTECFFLQKRLQLSVQKPEQVIKEDVSELRNEL
QRKDALVQKHLTKLRHWQQVLEDINVQHKKPADIPQGSLAYLEQASANIPAPLKPT

>O00237 multipass membrane protein
MWLKLFFLLLYFLVLFVLARFFEAIVWYETGIFATQLVDPVALSFKKLKTILECRGLGYSGL
PEKKDVRELVEKSGDLMEGELYSALKEEEASESVSSTNFSGEMHFYELVEDTKDGIWLVQ
VIANDRSPLVGKIHWEKMVKKVSRFGIRTGTFNCSSDPRYCRRRGWVRSTLIMSVPQTSTS
KGKVMLKEYSGRKIEVEHIFKWITAHAASRIKTIYNAEHLKEEWNKSDQYWLKIYLFANL
DQPPAFFSALSIKFTGRVEFIFVNVENWDNKSYMTDIGIYNMPSYILRTPEGIYRYGNHTGE
FISLQAMDSFLRSLQPEVNDLFVLSLVLVNLMAWMDLFITQGATIKRFVVLISTLGTYNSLL
IISWLPVLGFLQLPYLDSFYEYSLKLLRYSNTTTLASWVRADWMFYSSHPALFLSTYLGHG
LLIDYFEKKRRRNNNNDEVNANNLEWLSSLWDWYTSYLFHPIASFQNFPVESDWDEDPD
LFLERLAFPDLWLHPLIPTDYIKNLPMWRFKCLGVQSEEEMSEGSQDTENDSESENTDTLS
SEKEVFEDKQSVLHNSPGTASHCDAEACSCANKYCQTSPCERKGRSYGSYNTNEDMEPD
WLTWPADMLHCTECVVCLENFENGCLLMGLPCGHVFHQNCIVMWLAGGRHCCPVCRW
PSYKKKQPYAQHQPLSNDVPS

>Q9UNQ0 multipass membrane protein
MSSSNVEVFIPVSQGNTNGFPATASNDLKAFTEGAVLSFHNICYRVKLKSGFLPCRKPVEK
EILSNINGIMKPGLNAILGPTGGGKSSLLDVLAARKDPSGLSGDVLINGAPRPANFKCNSG
YVVQDDVVMGTLTVRENLQFSAALRLATTMTNHEKNERINRVIQELGLDKVADSKVGTQ
FIRGVSGGERKRTSIGMELITDPSILFLDEPTTGLDSSTANAVLLLLKRMSKQGRTIIFSIHQP
RYSIFKLFDSLTLLASGRLMFHGPAQEALGYFESAGYHCEAYNNPADFFLDIINGDSTAVAL
NREEDFKATEIIEPSKQDKPLIEKLAEIYVNSSFYKETKAELHQLSGGEKKKKITVFKEISYT
TSFCHQLRWVSKRSFKNLLGNPQASIAQIIVTVVLGLVIGAIYFGLKNDSTGIQNRAGVLFF
LTTNQCFSSVSAVELFVVEKKLFIHEYISGYYRVSSYFLGKLLSDLLPMRMLPSIIFTCIVYF
MLGLKPKADAFFVMMFTLMMVAYSASSMALAIAAGQSVVSVATLLMTICFVFMMIFSGL
LVNLTTIASWLSWLQYFSIPRYGFTALQHNEFLGQNFCPGLNATGNNPCNYATCTGEEYLV
KQGIDLSPWGLWKNHVALACMIVIFLTIAYLKLLFLKKYS

>O75351 peripheral membrane protein

MSSTSPNLQKAIDLASKAAQEDKAGNYEEALQLYQHAVQYFLHVVKYEAQGDKAKQSIR
AKCTEYLDRAEKLKEYLKNKEKKAQKPVKEGQPSPADEKGNDSDGEGESDDPEKKKLQ
NQLQGAIVIERPNVKWSDVAGLEGAKEALKEAVILPIKFPHLFTGKRTPWRGILLFGPPGT
GKSYLAKAVATEANNSTFFSISSSDLVSKWLGESEKLVKNLFQLARENKPSIIFIDEIDSLCG
SRSENESEAARRIKTEFLVQMQGVGVDNDGILVLGATNIPWVLDSAIRRRFEKRIYIPLPEP
HARAAMFKLHLGTTQNSLTEADFRELGRKTDGYSGADISIIVRDALMQPVRKVQSATHFK
KVRGPSRADPNHLVDDLLTPCSPGDPGAIEMTWMDVPGDKLLEPVVSMSDMLRSLSNTK
PTVNEHDLLKLKKFTEDFGQEG

>Q9UPI3 multipass membrane protein
MVNEGPNQEESDDTPVPESALQADPSVSVHPSVSVHPSVSINPSVSVHPSSSAHPSALAQP
SGLAHPSSSGPEDLSVIKVSRRRWAVVLVFSCYSMCNSFQWIQYGSINNIFMHFYGVSAFAI
DWLSMCYMLTYIPLLLPVAWLLEKFGLRTIALTGSALNCLGAWVKLGSLKPHLFPVTVVG
QLICSVAQVFILGMPSRIASVWFGANEVSTACSVAVFGNQLGIAIGFLVPPVLVPNIEDRDEL
AYHISIMFYIIGGVATLLLILVIIVFKEKPKYPPSRAQSLSYALTSPDASYLGSIARLFKNLNFV
LLVITYGLNAGAFYALSTLLNRMVIWHYPGEEVNAGRIGLTIVIAGMLGAVISGIWLDRSK
TYKETTLVVYIMTLVGMVVYTFTLNLGHLWVVFITAGTMGFFMTGYLPLGFEFAVELTYP
ESEGISSGLLNISAQVFGIIFTISQGQIIDNYGTKPGNIFLCVFLTLGAALTAFIKADLRRQKA
NKETLENKLQEEEEESNTSKVPTAVSEDHL

>Q15743 multipass membrane protein
MGNITADNSSMSCTIDHTIHQTLAPVVYVTVLVVGFPANCLSLYFGYLQIKARNELGVYLC
NLTVADLFYICSLPFWLQYVLQHDNWSHGDLSCQVCGILLYENIYISVGFLCCISVDRYLAV
AHPFRFHQFRTLKAAVGVSVVIWAKELLTSIYFLMHEEVIEDENQHRVCFEHYPIQAWQRA
INYYRFLVGFLFPICLLLASYQGILRAVRRSHGTQKSRKDQIQRLVLSTVVIFLACFLPYHVL
LLVRSVWEASCDFAKGVFNAYHFSLLLTSFNCVADPVLYCFVSETTHRDLARLRGACLAFL
TCSRTGRAREAYPLGAPEASGKSGAQGEEPELLTKLHPAFQTPNSPGSGGFPTGRLA

>Q9BSR8 multipass membrane protein
MQPPGPPPAYAPTNGDFTFVSSADAEDLSGSIASPDVKLNLGGDFIKESTATTFLRQRGYG
WLLEVEDDDPEDNKPLLEELDIDLKDIYYKIRCVLMPMPSLGFNRQVVRDNPDFWGPLAV
VLFFSMISLYGQFRVVSWIITIWIFGSLTIFLLARVLGGEVAYGQVLGVIGYSLLPLIVIAPVL
LVVGSFEVVSTLIKLFGVFWAAYSAASLLVGEEFKTKKPLLIYPIFLLYIYFLSLYTGV

>O60242 multipass membrane protein
MKAVRNLLIYIFSTYLLVMFGFNAAQDFWCSTLVKGVIYGSYSVSEMFPKNFTNCTWTLE
NPDPTKYSIYLKFSKKDLSCSNFSLLAYQFDHFSHEKIKDLLRKNHSIMQLCNSKNAFVFL
QYDKNFIQIRRVFPTNFPGLQKKGEEDQKSFFEFLVLNKVSPSQFGCHVLCTWLESCLKSE
NGRTESCGIMYTKCTCPQHLGEWGIDDQSLILLNNVVLPLNEQTEGCLTQELQTTQVCNL
TREAKRPPKEEFGMMGDHTIKSQRPRSVHEKRVPQEQADAAKFMAQTGESGVEEWSQW
STCSVTCGQGSQVRTRTCVSPYGTHCSGPLRESRVCNNTALCPVHGVWEEWSPWSLCSFT
CGRGQRTRTRSCTPPQYGGRPCEGPETHHKPCNIALCPVDGQWQEWSSWSQCSVTCSNG
TQQRSRQCTAAAHGGSECRGPWAESRECYNPECTANGQWNQWGHWSGCSKSCDGGWE
RRIRTCQGAVITGQQCEGTGEEVRRCNEQRCPAPYEICPEDYLMSMVWKRTPAGDLAFNQ

CPLNATGTTSRRCSLSLHGVAFWEQPSFARCISNEYRHLQHSIKEHLAKGQRMLAGDGMS
QVTKTLLDLTQRKNFYAGDLLMSVEILRNVTDTFKRASYIPASDGVQNFFQIVSNLLDEEN
KEKWEDAQQIYPGSIELMQVIEDFIHIVGMGMMDFQNSYLMTGNVVASIQKLPAASVLTD
INFPMKGRKGMVDWARNSEDRVVIPKSIFTPVSSKELDESSVFVLGAVLYKNLDLILPTLR
NYTVINSKIIVVTIRPEPKTTDSFLEIELAHLANGTLNPYCVLWDDSKTNESLGTWSTQGCK
TVLTDASHTKCLCDRLSTFAILAQQPREIIMESSGTPSVTLIVGSGLSCLALITLAVVYAALW
RYIRSERSIILINFCLSIISSNILILVGQTQTHNKSICTTTTAFLHFFFLASFCWVLTEAWQSYM
AVTGKIRTRLIRKRFLCLGWGLPALVVATSVGFTRTKGYGTDHYCWLSLEGGLLYAFVGPA
AAVVLVNMVIGILVFNKLVSRDGILDKKLKHRAGQMSEPHSGLTLKCAKCGVVSTTALSA
TTASNAMASLWSSCVVLPLLALTWMSAVLAMTDKRSILFQILFAVFDSLQGFVIVMVHCIL
RREVQDAFRCRLRNCQDPINADSSSSFPNGHAQIMTDFEKDVDIACRSVLHKDIGPCRAAT
ITGTLSRISLNDDEEEKGTNPEGLSYSTLPGNVISKVIIQQPTGLHMPMSMNELSNPCLKKE
NSELRRTVYLCTDDNLRGADMDIVHPQERMMESDYIVMPRSSVNNQPSMKEESKMNIG
METLPHERLLHYKVNPEFNMNPPVMDQFNMNLEQHLAPQEHMQNLPFEPRTAVKNFMA
SELDDNAGLSRSETGSTISMSSLERRKSRYSDLDFEKVMHTRKRHMELFQELNQKFQTLD
RFRDIPNTSSMENPAPNKNPWDTFKNPSEYPHYTTINVLDTEAKDALELRPAEWEKCLNLP
LDVQEGDFQTEV

>P49683 multipass membrane protein
MASSTTRGPRVSDLFSGLPPAVTTPANQSAEASAGNGSVAGADAPAVTPFQSLQLVHQLKG
LIVLLYSVVVVVGLVGNCLLVLVIARVRRLHNVTNFLIGNLALSDVLMCTACVPLTLAYAF
EPRGWVFGGGLCHLVFFLQPVTVYVSVFTLTTIAVDRYVVLVHPLRRRISLRLSAYAVLAIW
ALSAVLALPAAVHTYHVELKPHDVRLCEEFWGSQERQRQLYAWGLLLVTYLLPLLVILLS
YVRVSVKLRNRVVPGCVTQSQADWDRARRRRTFCLLVVIVVVFAVCWLPLHVFNLLRDL
DPHAIDPYAFGLVQLLCHWLAMSSACYNPFIYAWLHDSFREELRKLLVAWPRKIAPHGQN
MTVSVVI

>Q9BXI2 multipass membrane protein
MKSGPGIQAAIDLTAGAAGGTACVLTGQPFDTIKVKMQTFPDLYKGLTDCFLKTYAQVGL
RGFYKGTGPALMAYVAENSVLFMCYGFCQQFVRKVAGMDKQAKLSDLQTAAAGSFASA
FAALALCPTELVKCRLQTMYEMEMSGKIAKSHNTIWSVVKGILKKDGPLGFYHGLSSTLL
QEVPGYFFFFGGYELSRSFFASGRSKDELGPVHLMLSGGVAGICLWLVVFPVDCIKSRIQV
LSMYGKQAGFIGTLLSVVRNEGIVALYSGLKATMIRAIPANGALFVAYEYSRKMMMKQLE
AY

>O14788 single-pass type II membrane protein
MRRASRDYTKYLRGSEEMGGGPGAPHEGPLHAPPPPAPHQPPAASRSMFVALLGLGLGQ
VVCSVALFFYFRAQMDPNRISEDGTHCIYRILRLHENADFQDTTLESQDTKLIPDSCRRIKQ
AFQGAVQKELQHIVGSQHIRAEKAMVDGSWLDLAKRSKLEAQPFAHLTINATDIPSGSHK
VSLSSWYHDRGWAKISNMTFSNGKLIVNQDGFYYLYANICFRHHETSGDLATEYLQLMV
YVTKTSIKIPSSHTLMKGGSTKYWSGNSEFHFYSINVGGFFKLRSGEEISIEVSNPSLLDPD
QDATYFGAFKVRDID

>P26718 single-pass type II membrane protein

MGWIRGRRSRHSWEMSEFHNYNLDLKKSDFSTRWQKQRCPVVKSKCRENASPFFFCCFI
AVAMGIRFIIMVAIWSAVFLNSLFNQEVQIPLTESYCGPCPKNWICYKNNCYQFFDESKNW
YESQASCMSQNASLLKVYSKEDQDLLKLVKSYHWMGLVHIPTNGSWQWEDGSILSPNLL
TIIEMQKGDCALYASSFKGYIENCSTPNTYICMQRTV

>Q15842 multipass membrane protein
MLARKSIIPEEYVLARIAAENLRKPRIRDRLPKARFIAKSGACNLAHKNIREQGRFLQDIFT
TLVDLKWRHTLVIFTMSFLCSWLLFAIMWWLVAFAHGDIYAYMEKSGMEKSGLESTVCVT
NVRSFTSAFLFSIEVQVTIGFGGRMMTEECPLAITVLILQNIVGLIINAVMLGCIFMKTAQA
HRRAETLIFSRHAVIAVRNGKLCFMFRVGDLRKSMIISASVRIQVVKKTTTPEGEVVPIHQL
DIPVDNPIESNNIFLVAPLIICHVIDKRSPLYDISATDLANQDLEVIVILEGVVETTGITTQART
SYIAEEIQWGHRFVSIVTEEEGVYSVDYSKFGNTVKVAAPRCSARELDEKPSILIQTLQKSE
LSHQNSLRKRNSMRRNNSMRRNNSIRRNNSSLMVPKVQFMTPEGNQNTSES

>Q9NYW3 multipass membrane protein
MADKVQTTLLFLAVGEFSVGILGNAFIGLVNCMDWVKKRKIASIDLILTSLAISRICLLCVIL
LDCFILVLYPDVYATGKEMRIIDFFWTLTNHLSIWFATCLSIYYFFKIGNFFHPLFLWMKWRI
DRVISWILLGCVVLSVFISLPATENLNADFRFCVKAKRKTNLTWSCRVNKTQHASTKLFLN
LATLLPFCVCLMSFFLLILSLRRHIRRMQLSATGCRDPSTEAHVRALKAVISFLLLFIAYYLS
FLIATSSYFMPETELAVIFGESIALIYPSSHSFILILGNNKLRHASLKVIWKVMSILKGRKFQQ
HKQI

>Q9Y5Y5 multipass membrane protein
MEKLRLLGLRYQEYVTRHPAATAQLETAVRGFSYLLAGRFADSHELSELVYSASNLLVLLN
DGILRKELRKKLPVSLSQQKLLTWLSVLECVEVFMEMGAAKVWGEVGRWLVIALVQLA
KAVLRMLLLLWFKAGLQTSPPIVPLDRETQAQPPDGDHSPGNHEQSYVGKRSNRVVRTLQ
NTPSLHSRHWGAPQQREGRQQQHHEELSATPTPLGLQETIAEFLYIARPLLHLLSLGLWGQ
RSWKPWLLAGVVDVTSLSLLSDRKGLTRRERRELRRRTILLLYYLLRSPFYDRFSEARILFL
LQLLADHVPGVGLVTRPLMDYLPTWQKIYFYSWG

>Q9Y6H8 multipass membrane protein
MGDWSFLGRLLENAQEHSTVIGKVWLTVLFIFRILVLGAAAEDVWGDEQSDFTCNTQQP
GCENVCYDRAFPISHIRFWALQIIFVSTPTLIYLGHVLHIVRMEEKKKEREEEEQLKRESPSP
KEPPQDNPSSRDDRGRVRMAGALLRTYVFNIIFKTLFEVGFIAGQYFLYGFELKPLYRCDR
WPCPNTVDCFISRPTEKTIFIIFMLAVACASLLLNMLEIYHLGWKKLKQGVTSRLGPDASE
APLGTADPPPLPPSSRPPAVAIGFPPYYAHTAAPLGQARAVGYPGAPPPAADFKLLALTEAR
GKGQSAKLYNGHHHLLMTEQNWANQAAERQPPALKAYPAASTPAAPSPVGSSSPPLAHE
AEAGAAPLLLDGSGSSLEGSALAGTPEEEEQAVTTAAQMHQPPLPLGDPGRASKASRASS
GRARPEDLAI

>Q92847 multipass membrane protein
MWNATPSEEPGFNLTLADLDWDASPGNDSLGDELLQLFPAPLLAGVTATCVALFVVGIAG
NLLTMLVVSRFRELRTTTNLYLSSMAFSDLLIFLCMPLDLVRLWQYRPWNFGDLLCKLFQF
VSESCTYATVLTITALSVERYFAICFPLRAKVVVTKGRVKLVIFVIWAVAFCSAGPIFVLVGV

EHENGTDPWDTNECRPTEFAVRSGLLTVMVWVSSIFFFLPVFCLTVLYSLIGRKLWRRRRG
DAVVGASLRDQNHKQTVKMLAVVVFAFILCWLPFHVGRYLFSKSFEPGSLEIAQISQYCNL
VSFVLFYLSAAINPILYNIMSKKYRVAVFRLLGFEPFSQRKLSTLKDESSRAWTESSINT

>P27037 single-pass type I membrane protein
MGAAAKLAFAVFLISCSSGAILGRSETQECLFFNANWEKDRTNQTGVEPCYGDKDKRRHC
FATWKNISGSIEIVKQGCWLDDINCYDRTDCVEKKDSPEVYFCCCEGNMCNEKFSYFPEM
EVTQPTSNPVTPKPPYYNILLYSLVPLMLIAGIVICAFWVYRHHKMAYPPVLVPTQDPGPPP
PSPLLGLKPLQLLEVKARGRFGCVWKAQLLNEYVAVKIFPIQDKQSWQNEYEVYSLPGM
KHENILQFIGAEKRGTSVDVDLWLITAFHEKGSLSDFLKANVVSWNELCHIAETMARGLA
YLHEDIPGLKDGHKPAISHRDIKSKNVLLKNNLTACIADFGLALKFEAGKSAGDTHGQVG
TRRYMAPEVLEGAINFQRDAFLRIDMYAMGLVLWELASRCTAADGPVDEYMLPFEEEIGQ
HPSLEDMQEVVVHKKKRPVLRDYWQKHAGMAMLCETIEECWDHDAEARLSAGCVGER
ITQMQRLTNIITTEDIVTVVTMVTNVDFPPKESSL

>P36888 single-pass type I membrane protein
MPALARDGGQLPLLVVFSAMIFGTITNQDLPVIKCVLINHKNNDSSVGKSSSYPMVSESPE
DLGCALRPQSSGTVYEAAAVEVDVSASITLQVLVDAPGNISCLWVFKHSSLNCQPHFDLQ
NRGVVSMVILKMTETQAGEYLLFIQSEATNYTILFTVSIRNTLLYTLRRPYFRKMENQDAL
VCISESVPEPIVEWVLCDSQGESCKEESPAVVKKEEKVLHELFGTDIRCCARNELGRECTR
LFTIDLNQTPQTTLPQLFLKVGEPLWIRCKAVHVNHGFGLTWELENKALEEGNYFEMSTY
STNRTMIRILFAFVSSVARNDTGYYTCSSSKHPSQSALVTIVEKGFINATNSSEDYEIDQYEE
FCFSVRFKAYPQIRCTWTFSRKSFPCEQKGLDNGYSISKFCNHKHQPGEYIFHAENDDAQF
TKMFTLNIRRKPQVLAEASASQASCFSDGYPLPSWTWKKCSDKSPNCTEEITEGVWNRK
ANRKVFGQWVSSSTLNMSEAIKGFLVKCCAYNSLGTSCETILLNSPGPFPFIQDNISFYATIG
VCLLFIVVLTLLICHKYKKQFRYESQLQMVQVTGSSDNEYFYVDFREYEYDLKWEFPREN
LEFGKVLGSGAFGKVMNATAYGISKTGVSIQVAVKMLKEKADSSEREALMSELKMMTQL
GSHENIVNLLGACTLSGPIYLIFEYCCYGDLLNYLRSKREKFHRTWTEIFKEHNFSFYPTFQ
SHPNSSMPGSREVQIHPDSDQISGLHGNSFHSEDEIEYENQKRLEEEEDLNVLTFEDLLCFA
YQVAKGMEFLEFKSCVHRDLAARNVLVTHGKVVKICDFGLARDIMSDSNYVVRGNARLP
VKWMAPESLFEGIYTIKSDVWSYGILLWEIFSLGVNPYPGIPVDANFYKLIQNGFKMDQPF
YATEEIYIIMQSCWAFDSRKRPSFPNLTSFLGCQLADAEEAMYQNVDGRVSECPHTYQNRR
PFSREMDLGLLSPQAQVEDS

>O60896 single-pass type I membrane protein
METGALRRPQLLPLLLLLCGGCPRAGGCNETGMLERLPLCGKAFADMMGKVDVWKWC
NLSEFIVYYESFTNCTEMEANVVGCYWPNPLAQGFITGIHRQFFSNCTVDRVHLEDPPDEV
LIPLIVIPVVLTVAMAGLVVWRSKRTDTLL

>P09496 peripheral membrane protein
MAELDPFGAPAGAPGGPALGNGVAGAGEEDPAAAFLAQQESEIAGIENDEAFAILDGGAP
GPQPHGEPPGGPDAVDGVMNGEYYQESNGPTDSYAAISQVDRLQSEPESIRKWREEQMER
LEALDANSRKQEAEWKEKAIKELEEWYARQDEQLQKTKANNRVADEAFYKQPFADVIGY
VTNINHPCYSLEQAAEEAFVNDIDESSPGTEWERVARLCDFNPKSSKQAKDVSRMRSVLIS

LKQAPLVH

>P26992 GPI-anchor, lipid-anchor
MAAPVPWACCAVLAAAAAVVYAQRHSPQEAPHVQYERLGSDVTLPCGTANWDAAVTW
RVNGTDLAPDLLNGSQLVLHGLELGHSGLYACFHRDSWHLRHQVLLHVGLPPREPVLSCR
SNTYPKGFYCSWHLPTPTYIPNTFNVTVLHGSKIMVCEKDPALKNRCHIRYMHLFSTIKYK
VSISVSNALGHNATAITFDEFTIVKPDPPENVVARPVPSNPRRLEVTWQTPSTWPDPESFPL
KFFLRYRPLILDQWQHVELSDGTAHTITDAYAGKEYIIQVAAKDNEIGTWSDWSVAAHATP
WTEEPRHLTTEAQAAETTTSTTSSLAPPPTTKICDPGELGSGGGPSAPFLVSVPITLALAAA
AATASSLLI

>P48960 multipass membrane protein
MGGRVFLAFCVWLTLPGAETQDSRGCARWCPQNSSCVNATACRCNPGFSSFSEIITTPTET
CDDINECATPSKVSCGKFSDCWNTEGSYDCVCSPGYEPVSGAKTFKNESENTCQDVDECQ
QNPRLCKSYGTCVNTLGSYTCQCLPGFKFIPEDPKVCTDVNECTSGQNPCHSSTHCLNNV
GSYQCRCRPGWQPIPGSPNGPNNTVCEDVDECSSGQHQCDSSTVCFNTVGSYSCRCRPG
WKPRHGIPNNQKDTVCEDMTFSTWTPPPGVHSQTLSRFFDKVQDLGRDSKTSSAEVTIQN
VIKLVDELMEAPGDVEALAPPVRHLIATQLLSNLEDIMRILAKSLPKGPFTYISPSNTELTL
MIQERGDKNVTMGQSSARMKLNWAVAAGAEDPGPAVAGILSIQNMTTLLANASLNLHSK
KQAELEEIYESSIRGVQLRRLSAVNSIFLSHNNTKELNSPILFAFSHLESSDGEAGRDPPAKD
VMPGPRQELLCAFWKSDSDRGGHWATEGCQVLGSKNGSTTCQCSHLSSFAILMAHYDVE
DWKLTLITRVGLALSLFCLLLCILTFLLVRPIQGSRTTIHLHLCICLFVGSTIFLAGIENEGGQ
VGLRCRLVAGLLHYCFLAAFCWMSLEGLELYFLVVRVFQGQGLSTRWLCLIGYGVPLLIV
GVSAAIYSKGYGRPRYCWLDFEQGFLWSFLGPVTFIILCNAVIFVTTVWKLTQKFSEINPD
MKKLKKARALTITAIAQLFLLGCTWVFGLFIFDDRSLVLTYVFTILNCLQGAFLYLLHCLLN
KKVREEYRKWACLVAGGSKYSEFTSTTSGTGHNQTRALRASESGI

>Q9BY08 multipass membrane protein
MGAEWELGAEAGGSLLLCAALLAAGCALGLRLGRGQGAADRGALIWLCYDALVHFALE
GPFVYLSLVGNVANSDGLIASLWKEYGKADARWVYFDPTIVSVEILTVALDGSLALFLIYAI
VKEKYYRHFLQITLCVCELYGCWMTFLPEWLTRSPNLNTSNWLYCWLYLFFFNGVWVLIP
GLLLWQSWLELKKMHQKETSSVKKFQ

>Q53FV1 multipass membrane protein
MNVGVAHSEVNPNTRVMNSRGIWLAYIILVGLLHMVLLSIPFFSIPVVWTLTNVIHNLATY
VFLHTVKGTPFETPDQGKARLLTHWEQMDYGLQFTSSRKFLSISPIVLYLLASFYTKYDAA
HFLINTASLLSVLLPKLPQFHGVRVFGINKY

>Q07954 peripheral membrane protein, single-pass type I membrane protein
MLTPPLLLLLPLLSALVAAAIDAPKTCSPKQFACRDQITCISKGWRCDGERDCPDGSDEAPE
ICPQSKAQRCQPNEHNCLGTELCVPMSRLCNGVQDCMDGSDEGPHCRELQGNCSRLGCQ
HHCVPTLDGPTCYCNSSFQLQADGKTCKDFDECSVYGTCSQLCTNTDGSFICGCVEGYLL
QPDNRSCKAKNEPVDRPPVLLIANSQNILATYLSGAQVSTITPTSTRQTTAMDFSYANETV
CWVHVGDSAAQTQLKCARMPGLKGFVDEHTINISLSLHHVEQMAIDWLTGNFYFVDDID

DRIFVCNRNGDTCVTLLDLELYNPKGIALDPAMGKVFFTDYGQIPKVERCDMDGQNRTKL
VDSKIVFPHGITLDLVSRLVYWADAYLDYIEVVDYEGKGRQTIIQGILIEHLYGLTVFENYL
YATNSDNANAQQKTSVIRVNRFNSTEYQVVTRVDKGGALHIYHQRRQPRVRSHACENDQ
YGKPGGCSDICLLANSHKARTCRCRSGFSLGSDGKSCKKPEHELFLVYGKGRPGIIRGMD
MGAKVPDEHMIPIENLMNPRALDFHAETGFIYFADTTSYLIGRQKIDGTERETILKDGIHN
VEGVAVDWMGDNLYWTDDGPKKTISVARLEKAAQTRKTLIEGKMTHPRAIVVDPLNGW
MYWTDWEEDPKDSRRGRLERAWMDGSHRDIFVTSKTVLWPNGLSLDIPAGRLYWVDAF
YDRIETILLNGTDRKIVYEGPELNHAFGLCHHGNYLFWTEYRSGSVYRLERGVGGAPPTV
TLLRSERPPIFEIRMYDAQQQQVGTNKCRVNNGGCSSLCLATPGSRQCACAEDQVLDADG
VTCLANPSYVPPPQCQPGEFACANSRCIQERWKCDGDNDCLDNSDEAPALCHQHTCPSDR
FKCENNRCIPNRWLCDGDNDCGNSEDESNATCSARTCPPNQFSCASGRCIPISWTCDLDDD
CGDRSDESASCAYPTCFPLTQFTCNNGRCININWRCDNDNDCGDNSDEAGCSHSCSSTQF
KCNSGRCIPEHWTCDGDNDCGDYSDETHANCTNQATRPPGGCHTDEFQCRLDGLCIPLR
WRCDGDTDCMDSSDEKSCEGVTHVCDPSVKFGCKDSARCISKAWVCDGDNDCEDNSDE
ENCESLACRPPSHPCANNTSVCLPPDKLCDGNDDCGDGSDEGELCDQCSLNNGGCSHNC
SVAPGEGIVCSCPLGMELGPDNHTCQIQSYCAKHLKCSQKCDQNKFSVKCSCYEGWVLEP
DGESCRSLDPFKPFIIFSNRHEIRRIDLHKGDYSVLVPGLRNTIALDFHLSQSALYWTDVVE
DKIYRGKLLDNGALTSFEVVIQYGLATPEGLAVDWIAGNIYWVESNLDQIEVAKLDGTLRT
TLLAGDIEHPRAIALDPRDGILFWTDWDASLPRIEAASMSGAGRRTVHRETGSGGWPNGL
TVDYLEKRILWIDARSDAIYSARYDGSGHMEVLRGHEFLSHPFAVTLYGGEVYWTDWRT
NTLAKANKWTGHNVTVVQRTNTQPFDLQVYHPSRQPMAPNPCEANGGQGPCSHLCLIN
YNRTVSCACPHLMKLHKDNTTCYEFKKFLLYARQMEIRGVDLDAPYYNYIISFTVPDIDN
VTVLDYDAREQRVYWSDVRTQAIKRAFINGTGVETVVSADLPNAHGLAVDWVSRNLFW
TSYDTNKKQINVARLDGSFKNAVVQGLEQPHGLVVHPLRGKLYWTDGDNISMANMDGS
NRTLLFSGQKGPVGLAIDFPESKLYWISSGNHTINRCNLDGSGLEVIDAMRSQLGKATALAI
MGDKLWWADQVSEKMGTCSKADGSGSVVLRNSTTLVMHMKVYDESIQLDHKGTNPCS
VNNGDCSQLCLPTSETTRSCMCTAGYSLRSGQQACEGVGSFLLYSVHEGIRGIPLDPNDKS
DALVPVSGTSLAVGIDFHAENDTIYWVDMGLSTISRAKRDQTWREDVVTNGIGRVEGIAV
DWIAGNIYWTDQGFDVIEVARLNGSFRYVVISQGLDKPRAITVHPEKGYLFWTEWGQYPR
IERSRLDGTERVVLVNVSISWPNGISVDYQDGKLYWCDARTDKIERIDLETGENREVVLSS
NNMDMFSVSVFEDFIYWSDRTHANGSIKRGSKDNATDSVPLRTGIGVQLKDIKVFNRDRQ
KGTNVCAVANGGCQQLCLYRGRGQRACACAHGMLAEDGASCREYAGYLLYSERTILKSI
HLSDERNLNAPVQPFEDPEHMKNVIALAFDYRAGTSPGTPNRIFFSDIHFGNIQQINDDGS
RRITIVENVGSVEGLAYHRGWDTLYWTSYTTSTITRHTVDQTRPGAFERETVITMSGDDHP
RAFVLDECQNLMFWTNWNEQHPSIMRAALSGANVLTLIEKDIRTPNGLAIDHRAEKLYFS
DATLDKIERCEYDGSHRYVILKSEPVHPFGLAVYGEHIFWTDWVRRAVQRANKHVGSNM
KLLRVDIPQQPMGIIAVANDTNSCELSPCRINNGGCQDLCLLTHQGHVNCSCRGGRILQDD
LTCRAVNSSCRAQDEFECANGECINFSLTCDGVPHCKDKSDEKPSYCNSRRCKKTFRQCS
NGRCVSNMLWCNGADDCGDGSDEIPCNKTACGVGEFRCRDGTCIGNSSRCNQFVDCEDA
SDEMNCSATDCSSYFRLGVKGVLFQPCERTSLCYAPSWVCDGANDCGDYSDERDCPGVK
RPRCPLNYFACPSGRCIPMSWTCDKEDDCEHGEDETHCNKFCSEAQFECQNHRCISKQWL
CDGSDDCGDGSDEAAHCEGKTCGPSSFSCPGTHVCVPERWLCDGDKDCADGADESIAAG
CLYNSTCDDREFMCQNRQCIPKHFVCDHDRDCADGSDESPECEYPTCGPSEFRCANGRCL
SSRQWECDGENDCHDQSDEAPKNPHCTSQEHKCNASSQFLCSSGRCVAEALLCNGQDDC

GDSSDERGCHINECLSRKLSGCSQDCEDLKIGFKCRCRPGFRLKDDGRTCADVDECSTTFP
CSQRCINTHGSYKCLCVEGYAPRGGDPHSCKAVTDEEPFLIFANRYYLRKLNLDGSNYTLL
KQGLNNAVALDFDYREQMIYWTDVTTQGSMIRRMHLNGSNVQVLHRTGLSNPDGLAVD
WVGGNLYWCDKGRDTIEVSKLNGAYRTVLVSSGLREPRALVVDVQNGYLYWTDWGDHS
LIGRIGMDGSSRSVIVDTKITWPNGLTLDYVTERIYWADAREDYIEFASLDGSNRHVVLSQ
DIPHIFALTLFEDYVYWTDWETKSINRAHKTTGTNKTLLISTLHRPMDLHVFHALRQPDVP
NHPCKVNNGGCSNLCLLSPGGGHKCACPTNFYLGSDGRTCVSNCTASQFVCKNDKCIPF
WWKCDTEDDCGDHSDEPPDCPEFKCRPGQFQCSTGICTNPAFICDGDNDCQDNSDEANC
DIHVCLPSQFKCTNTNRCIPGIFRCNGQDNCGDGEDERDCPEVTCAPNQFQCSITKRCIPRV
WVCDRDNDCVDGSDEPANCTQMTCGVDEFRCKDSGRCIPARWKCDGEDDCGDGSDEPK
EECDERTCEPYQFRCKNNRCVPGRWQCDYDNDCGDNSDEESCTPRPCSESEFSCANGRCI
AGRWKCDGDHDCADGSDEKDCTPRCDMDQFQCKSGHCIPLRWRCDADADCMDGSDEE
ACGTGVRTCPLDEFQCNNTLCKPLAWKCDGEDDCGDNSDENPEECARFVCPPNRPFRCK
NDRVCLWIGRQCDGTDNCGDGTDEEDCEPPTAHTTHCKDKKEFLCRNQRCLSSSLRCNM
FDDCGDGSDEEDCSIDPKLTSCATNASICGDEARCVRTEKAAYCACRSGFHTVPGQPGCQ
DINECLRFGTCSQLCNNTKGGHLCSCARNFMKTHNTCKAEGSEYQVLYIADDNEIRSLFP
GHPHSAYEQAFQGDESVRIDAMDVHVKAGRVYWTNWHTGTISYRSLPPAAPPTTSNRHR
RQIDRGVTHLNISGLKMPRGIAIDWVAGNVYWTDSGRDVIEVAQMKGENRKTLISGMIDE
PHAIVVDPLRGTMYWSDWGNHPKIETAAMDGTLRETLVQDNIQWPTGLAVDYHNERLY
WADAKLSVIGSIRLNGTDPIVAADSKRGLSHPFSIDVFEDYIYGVTYINNRVFKIHKFGHSPL
VNLTGGLSHASDVVLYHQHKQPEVTNPCDRKKCEWLCLLSPSGPVCTCPNGKRLDNGTC
VPVPSPTPPPDAPRPGTCNLQCFNGGSCFLNARRQPKCRCQPRYTGDKCELDQCWEHCRN
GGTCAASPSGMPTCRCPTGFTGPKCTQQVCAGYCANNSTCTVNQGNQPQCRCLPGFLGD
RCQYRQCSGYCENFGTCQMAADGSRQCRCTAYFEGSRCEVNKCSRCLEGACVVNKQSG
DVTCNCTDGRVAPSCLTCVGHCSNGGSCTMNSKMMPECQCPPHMTGPRCEEHVFSQQQP
GHIASILIPLLLLLLLVLVAGVVFWYKRRVQGAKGFQHQRMTNGAMNVEIGNPTYKMYE
GGEPDDVGGLLDADFALDPDKPTNFTNPVYATLYMGGHGSRHSLASTDEKRELLGRGPE
DEIGDPLA

>Q14627 single-pass type I membrane protein
MAFVCLAIGCLYTFLISTTFGCTSSSDTEIKVNPPQDFEIVDPGYLGYLYLQWQPPLSLDHF
KECTVEYELKYRNIGSETWKTIITKNLHYKDGFDLNKGIEAKIHTLLPWQCTNGSEVQSS
WAETTYWISPQGIPETKVQDMDCVYYNWQYLLCSWKPGIGVLLDTNYNLFYWYEGLDH
ALQCVDYIKADGQNIGCRFPYLEASDYKDFYICVNGSSENKPIRSSYFTFQLQNIVKPLPPV
YLTFTRESSCEIKLKWSIPLGPIPARCFDYEIEIREDDTTLVTATVENETYTLKTTNETRQLCF
VVRSKVNIYCSDDGIWSEWSDKQCWEGEDLSKKTLLRFWLPFGFILILVIFVTGLLLRKPN
TYPKMIPEFFCDT

>P61619 multipass membrane protein
MAIKFLEVIKPFCVILPEIQKPERKIQFKEKVLWTAITLFIFLVCCQIPLFGIMSSDSADPFYW
MRVILASNRGTLMELGISPIVTSGLIMQLLAGAKIIEVGDTPKDRALFNGAQKLFGMIITIG
QSIVYVMTGMYGDPSEMGAGICLLITIQLFVAGLIVLLLDELLQKGYGLGSGISLFIATNICE
TIVWKAFSPTTVNTGRGMEFEGAIIALFHLLATRTDKVRALREAFYRQNLPNLMNLIATIF
VFAVVIYFQGFRVDLPIKSARYRGQYNTYPIKLFYTSNIPIILQSALVSNLYVISQMLSARFSG

NLLVSLLGTWSDTSSGGPARAYPVGGLCYYLSPPESFGSVLEDPVHAVVYIVFMLGSCAFF
SKTWIEVSGSSAKDVAKQLKEQQMVMRGHRETSMVHELNRYIPTAAAFGGLCIGALSVL
ADFLGAIGSGTGILLAVTIIYQYFEIFVKEQSEVGSMGALLF

>Q9NP90 lipid-anchor
MSGKSLLLKVILLGDGGVGKSSLMNRYVTNKFDSQAFHTIGVEFLNRDLEVDGRFVTLQI
WDTAGQERFKSLRTPFYRGADCCLLTFSVDDRQSFENLGNWQKEFIYYADVKDPEHFPFV
VLGNKVDKEDRQVTTEEAQTWCMENGDYPYLETSAKDDTNVTVAFEEAVRQVLAVEEQ
LEHCMLGHTIDLNSGSKAGSSCC

>P57729 lipid-anchor
MQAPHKEHLYKLLVIGDLGVGKTSIIKRYVHQNFSSHYRATIGVDFALKVLHWDPETVVR
LQLWDIAGQERFGNMTRVYYREAMGAFIVFDVTRPATFEAVAKWKNDLDSKLSLPNGKP
VSVVLLANKCDQGKDVLMNNGLKMDQFCKEHGFVGWFETSAKENINIDEASRCLVKHIL
ANECDLMESIEPDVVKPHLTSTKVASCSGCAKS

>Q9NYM4 multipass membrane protein
MVPHLLLLCLLPLVRATEPHEGRADEQSAEAALAVPNASHFFSWNNYTFSDWQNFVGRR
RYGAESQNPTVKALLIVAYSFIIVFSLFGNVLVCHVIFKNQRMHSATSLFIVNLAVADIMITL
LNTPFTLVRFVNSTWIFGKGMCHVSRFAQYCSLHVSALTLTAIAVDRHQVIMHPLKPRISIT
KGVIYIAVIWTMATFFSLPHAICQKLFTFKYSEDIVRSLCLPDFPEPADLFWKYLDLATFILL
YILPLLIISVAYARVAKKLWLCNMIGDVTTEQYFALRRKKKKTIKMLMLVVVLFALCWFPL
NCYVLLLSSKVIRTNNALYFAFHWFAMSSTCYNPFIYCWLNENFRIELKALLSMCQRPPKP
QEDRPPSPVPSFRVAWTEKNDGQRAPLANNLLPTSQLQSGKTDLSSVEPIVTMS

>Q8N2H4 multipass membrane protein
MAGQFRSYVWDPLLILSQIVLMQTVYYGSLGLWLALVDGLVRSSPSLDQMFDAEILGFST
PPGRLSMMSFILNALTCALGLLYFIRRGKQCLDFTVTVHFFHLLGCWFYSSRFPSALTWWL
VQAVCIALMAVIGEYLCMRTELKEIPLNSAPKSNV

>Q01113 single-pass type I membrane protein
MGLGRCIWEGWTLESEALRRDMGTWLLACICICTCVCLGVSVTGEGQGPRSRTFTCLTNN
ILRIDCHWSAPELGQGSSPWLLFTSNQAPGGTHKCILRGSECTVVLPPEAVLVPSDNFTITF
HHCMSGREQVSLVDPEYLPRRHVKLDPPSDLQSNISSGHCILTWSISPALEPMTTLLSYELA
FKKQEEAWEQAQHRDHIVGVTWLILEAFELDPGFIHEARLRVQMATLEDDVVEEERYTG
QWSEWSQPVCFQAPQRQGPLIPPWGWPGNTLVAVSIFLLLTGPTYLLFKLSPRVKRIFYQN
VPSPAMFFQPLYSVHNGNFQTWMGAHGAGVLLSQDCAGTPQGALEPCVQEATALLTCGP
ARPWKSVALEEEQEGPGTRLPGNLSSEDVLPAGCTEWRVQTLAYLPQEDWAPTSLTRPAPP
DSEGSRSSSSSSSSNNNNNYCALGCYGGWHLSALPGNTQSSGPIPALACGLSCDHQGLETQ
QGVAWVLAGHCQRPGLHEDLQGMLLPSVLSKARSWTF

>O95274 GPI-anchor, lipid-anchor
MDPARKAGAQAMIWTAGWLLLLLLRGGAQALECYSCVQKADDGCSPNKMKTVKCAPG
VDVCTEAVGAVETIHGQFSLAVRGCGSGLPGKNDRGLDLHGLLAFIQLQQCAQDRCNAK

LNLTSRALDPAGNESAYPPNGVECYSCVGLSREACQGTSPPVVSCYNASDHVYKGCFDGN
VTLTAANVTVSLPVRGCVQDEFCTRDGVTGPGFTLSGSCCQGSRCNSDLRNKTYFSPRIPP
LVRLPPPEPTTVASTTSVTTSTSAPVRPTSTTKPMPAPTSQTPRQGVEHEASRDEEPRLTGG
AAGHQDRSNSGQYPAKGGPQQPHNKGCVAPTAGLAALLLAVAAGVLL

>Q13410 single-pass type I membrane protein
MAVFPSSGLPRCLLTLILLQLPKLDSAPFDVIGPPEPILAVVGEDAELPCRLSPNASAEHLEL
RWFRKKVSPAVLVHRDGREQEAEQMPEYRGRATLVQDGIAKGRVALRIRGVRVSDDGEY
TCFFREDGSYEEALVHLKVAALGSDPHISMQVQENGEICLECTSVGWYPEPQVQWRTSKG
EKFPSTSESRNPDEEGLFTVAASVIIRDTSAKNVSCYIQNLLLGQEKKVEISIPASSLPRLTPW
IVAVAVILMVLGLLTIGSIFFTWRLYNERPRERRNEFSSKERLLEELKWKKATLHAVDVTLD
PDTAHPHLFLYEDSKSVRLEDSRQKLPEKTERFDSWPCVLGRETFTSGRHYWEVEVGDRT
DWAIGVCRENVMKKGFDPMTPENGFWAVELYGNGYWALTPLRTPLPLAGPPRRVGIFLDY
ESGDISFYNMNDGSDIYTFSNVTFSGPLRPFFCLWSSGKKPLTICPIADGPERVTVIANAQDL
SKEIPLSPMGEDSAPRDADTLHSKLIPTQPSQGAP

>Q14916 multipass membrane protein
MQMDNRLPPKKVPGFCSFRYGLSFLVHCCNVIITAQRACLNLTMVVMVNSTDPHGLPNTS
TKKLLDNIKNPMYNWSPDIQGIILSSTSYGVIIIQVPVGYFSGIYSTKKMIGFALCLSSVLSL
LIPPAAGIGVAWVVVCRAVQGAAQGIVATAQFEIYVKWAPPLERGRLTSMSTSGFLLGPFIV
LLVTGVICESLGWPMVFYIFGACGCAVCLLWFVLFYDDPKDHPCISISEKEYITSSLVQQVS
SSRQSLPIKAILKSLPVWAISTGSFTFFWSHNIMTLYTPMFINSMLHVNIKENGFLSSLPYLF
AWICGNLAGQLSDFFLTRNILSVIAVRKLFTAAGFLLPAIFGVCLPYLSSTFYSIVIFLILAGA
TGSFCLGGVFINGLDIAPRYFGFIKACSTLTGMIGGLIASTLTGLILKQDPESAWFKTFILMA
AINVTGLIFYLIVATAEIQDWAKEKQHTRL

>Q5T3U5 multipass membrane protein
MERLLAQLCGSSAAWPLPLWEGDTTGHCFTQLVLSALPHALLAVLSACYLGTPRSPDYILP
CSPGWRLRLAASFLLSVFPLLDLLPVALPPGAGPGPIGLEVLAGCVAAVAWISHSLALWVL
AHSPHGHSRGPLALALVALLPAPALVLTVLWHCQRGTLLPPLLPGPMARLCLLILQLAALL
AYALGWAAPGGPREPWAQEPLLPEDQEPEVAEDGESWLSRFSYAWLAPLLARGACGELR
QPQDICRLPHRLQPTYLARVFQAHWQEGARLWRALYGAFGRCYLALGLLKLVGTMLGFS
GPLLLSLLVGFLEEGQEPLSHGLLYALGLAGGAVLGAVLQNQYGYEVYKVTLQARGAVLN
ILYCKALQLGPSRPPTGEALNLLGTDSERLLNFAGSFHEAWGLPLQLAITLYLLYQQVGVA
FVGGLILALLLVPVNKVIATRIMASNQEMLQHKDARVKLVTELLSGIRVIKFCGWEQALGA
RVEACRARELGRLRVIKYLDAACVYLWAALPVVISIVIFITYVLMGHQLTATKVFTALALV
RMLILPLNNFPWVINGLLEAKVSLDRIQLFLDLPNHNPQAYYSPDPPAEPSTVLELHGALFS
WDPVGTSLETFISHLEVKKGMLVGIVGKVGCGKSSLLAAIAGELHRLRGHVAVRGLSKGF
GLATQEPWIQFATIRDNILFGKTFDAQLYKEVLEACALNDDLSILPAGDQTEVGEKGVTLS
GGQRARIALARAVYQEKELYLLDDPLAAVDADVANHLLHRCILGMLSYTTRLLCTHRTEY
LERADAVLLMEAGRLIRAGPPSEILPLVQAVPKAWAENGQESDSATAQSVQNPEKTKEGLE
EEQSTSGRLLQEESKKEGAVALHVYQAYWKAVGQGLALAILFSLLLMQATRNAADWWLS
HWISQLKAENSSQEAQPSTSPASMGLFSPQLLLFSPGNLYIPVFPLPKAAPNGSSDIRFYLTV
YATIAGVNSLCTLLRAVLFAAGTLQAAATLHRRLLHRVLMAPVTFFNATPTGRILNRFSSDV

ACADDSLPFILNILLANAAGLLGLLAVLGSGLPWLLLLLPPLSIMYYHVQRHYRASSRELR
RLGSLTLSPLYSHLADTLAGLSVLRATGATYRFEEENLRLLELNQRCQFATSATMQWLDIR
LQLMGAAVVSAIAGIALVQHQQGLANPGLVGLSLSYALSLTGLLSGLVSSFTQTEAMLVSV
ERLEEYTCDLPQEPQGQPLQLGTGWLTQGGVEFQDVVLAYRPGLPNALDGVTFCVQPGE
KLGIVGRTGSGKSSLLLVLFRLLEPSSGRVLLDGVDTSQLELAQLRSQLAIIPQEPFLFSGTV
RENLDPQGLHKDRALWQALKQCHLSEVITSMGGLDGELGEGGRSLSLGQRQLLCLARAL
LTDAKILCIDEATASVDQKTDQLLQQTICKRFANKTVLTIAHRLNTILNSDRVLVLQAGRVV
ELDSPATLRNQPHSLFQQLLQSSQQGVPASLGGP

>P43307 single-pass type I membrane protein
MRLLPRLLLLLLLVFPATVLFRGGPRGLLAVAQDLTEDEETVEDSIIEDEDDEAEVEEDEPT
DLVEDKEEEDVSGEPEASPSADTTILFVKGEDFPANNIVKFLVGFTNKGTEDFIVESLDASF
RYPQDYQFYIQNFTALPLNTVVPPQRQATFEYSFIPAEPMGGRPFGLVINLNYKDLNGNVF
QDAVFNQTVTVIEREDGLDGETIFMYMFLAGLGLLVIVGLHQLLESRKRKRPIQKVEMGT
SSQNDVDMSWIPQETLNQINKASPRRLPRKRAQKRSVGSDE

>O14944 single-pass type I membrane protein
MTAGRRMEMLCAGRVPALLLCLGFHLLQAVLSTTVIPSCIPGESSDNCTALVQTEDNPRVA
QVSITKCSSDMNGYCLHGQCIYLVDMSQNYCRCEVGYTGVRCEHFFLTVHQPLSKEYVAL
TVILIILFLITVVGSTYYFCRWYRNRKSKEPKKEYERVTSGDPELPQV

>Q9P0I2 multipass membrane protein
MAGPELLLDSNIRLWVVLPIVIITFFVGMIRHYVSILLQSDKKLTQEQVSDSQVLIRSRVLRE
NGKYIPKQSFLTRKYYFNNPEDGFFKKTKRKVVPPSPMTDPTMLTDMMKGNVTNVLPMI
LIGGWINMTFSGFVTTKVPFPLTLRFKPMLQQGIELLTLDASWVSSASWYFLNVFGLRSIY
SLILGQDNAADQSRMMQEQMTGAAMAMPADTNKAFKTEWEALELTDHQWALDDVEEE
LMAKDLHFEGMFKKELQTSIF

>Q12908 multipass membrane protein
MNDPNSCVDNATVCSGASCVVPESNFNNILSVVLSTVLTILLALVMFSMGCNVEIKKFLGH
IKRPWGICVGFLCQFGIMPLTGFILSVAFDILPLQAVVVLIIGCCPGGTASNILAYWVDGDM
DLSVSMTTCSTLLALGMMPLCLLIYTKMWVDSGSIVIPYDNIGTSLVSLVVPVSIGMFVNH
KWPQKAKIILKIGSIAGAILIVLIAVVGGILYQSAWIIAPKLWIIGTIFPVAGYSLGFLLARIAG
LPWYRCRTVAFETGMQNTQLCSTIVQLSFTPEELNVVFTFPLIYSIFQLAFAAIFLGFYVAYK
KCHGKNKAEIPESKENGTEPESSFYKANGGFQPDEK

>O76082 multipass membrane protein
MRDYDEVTAFLGEWGPFQRLIFFLLSASIIPNGFTGLSSVFLIATPEHRCRVPDAANLSSAW
RNHTVPLRLRDGREVPHSCRRYRLATIANFSALGLEPGRDVDLGQLEQESCLDGWEFSQD
VYLSTIVTEWNLVCEDDWKAPLTISLFFVGVLLGSFISGQLSDRFGRKNVLFVTMGMQTG
FSFLQIFSKNFEMFVVLFVLVGMGQISNYVAAFVLGTEILGKSVRIIFSTLGVCIFYAFGYM
VLPLFAYFIRDWRMLLVALTMPGVLCVALWWFIPESPRWLISQGRFEEAEVIIRKAAKANGI
VVPSTIFDPSELQDLSSKKQQSHNILDLLRTWNIRMVTIMSIMLWMTISVGYFGLSLDTPNL
HGDIFVNCFLSAMVEVPAYVLAWLLLQYLPRRYSMATALFLGGSVLLFMQLVPPDLYYLA

TVLVMVGKFGVTAAFSMVYVYTAELYPTVVRNMGVGVSSTASRLGSILSPYFVYLGAYD
RFLPYILMGSLTILTAILTLFLPESFGTPLPDTIDQMLRVKGMKHRKTPSHTRMLKDGQERP
TILKSTAF

>Q9NZ52 peripheral membrane protein
MAEAEGESLESWLNKATNPSNRQEDWEYIIGFCDQINKELEGPQIAVRLLAHKIQSPQEWE
ALQALTVLEACMKNCGRRFHNEVGKFRFLNELIKVVSPKYLGDRVSEKVKTKVIELLYSW
TMALPEEAKIKDAYHMLKRQGIVQSDPPIPVDRTLIPSPPPRPKNPVFDDEEKSKLLAKLLK
SKNPDDLQEANKLIKSMVKEDEARIQKVTKRLHTLEEVNNNVRLLSEMLLHYSQEDSSD
GDRELMKELFDQCENKRRTLFKLASETEDNDNSLGDILQASDNLSRVINSYKTIIEGQVIN
GEVATLTLPDSEGNSQCSNQGTLIDLAELDTTNSLSSVLAPAPTPPSSGIPILPPPPQASGPPR
SRSSSQAEATLGPSSTSNALSWLDEELLCLGLADPAPNVPPKESAGNSQWHLLQREQSDL
DFFSPRPGTAACGASDAPLLQPSAPSSSSSQAPLPPPFPAPVVPASVPAPSAGSSLFSTGVAPA
LAPKVEPAVPGHHGLALGNSALHHLDALDQLLEEAKVTSGLVKPTTSPLIPTTTPARPLLPF
STGPGSPLFQPLSFQSQGSPPKGPELSLASIHVPLESIKPSSALPVTAYDKNGFRILFHFAKEC
PPGRPDVLVVVVSMLNTAPLPVKSIVLQAAVPKSMKVKLQPPSGTELSPFSPIQPPAAITQV
MLLANPLKEKVRLRYKLTFALGEQLSTEVGEVDQFPPVEQWGNL

>P41273 single-pass type II membrane protein
MEYASDASLDPEAPWPPAPRARACRVLPWALVAGLLLLLLLAAACAVFLACPWAVSGARA
SPGSAASPRLREGPELSPDDPAGLLDLRQGMFAQLVAQNVLLIDGPLSWYSDPGLAGVSLT
GGLSYKEDTKELVVAKAGVYYVFFQLELRRVVAGEGSGSVSLALHLQPLRSAAGAAALA
LTVDLPPASSEARNSAFGFQGRLLHLSAGQRLGVHLHTEARARHAWQLTQGATVLGLFRV
TPEIPAGLPSPRSE

>P32970 single-pass type II membrane protein
MPEEGSGCSVRRRPYGCVLRAALVPLVAGLVICLVVCIQRFAQAQQQLPLESLGWDVAEL
QLNHTGPQQDPRLYWQGGPALGRSFLHGPELDKGQLRIHRDGIYMVHIQVTLAICSSTTAS
RHHPTTLAVGICSPASRSISLLRLSFHQGCTIASQRLTPLARGDTLCTNLTGTLLPSRNTDET
FFGVQWVRP

>O43557 single-pass type II membrane protein
MEESVVRPSVFVVDGQTDIPFTRLGRSHRRQSCSVARVGLGLLLLLMGAGLAVQGWFLL
QLHWRLGEMVTRLPDGPAGSWEQLIQERRSHEVNPAAHLTGANSSLTGSGGPLLWETQL
GLAFLRGLSYHDGALVVTKAGYYYIYSKVQLGGVGCPLGLASTITHGLYKRTPRYPEELE
LLVSQQSPCGRATSSSRVWWDSSFLGGVVHLEAGEKVVVRVLDERLVRLRDGTRSYFGAF
MV

>Q92797 peripheral membrane protein
MASGSGDSVTRRSVASQFFTQEEGPGIDGMTTSERVVDLLNQAALITNDSKITVLKQVQEL
IINKDPTLLDNFLDEIIAFQADKSIEVRKFVIGFIEEACKRDIELLLKLIANLNMLLRDENVN
VVKKAILTMTQLYKVALQWMVKSRVISELQEACWDMVSAMAGDIILLLDSDNDGIRTHAI
KFVEGLIVTLSPRMADSEIPRRQEHDISLDRIPRDHPYIQYNVLWEEGKAALEQLLKFMVH
PAISSINLTTALGSLANIARQRPMFMSEVIQAYETLHANLPPTLAKSQVSSVRKNLKLHLLS

VLKHPASLEFQAQITTLLVDLGTPQAEIARNMPSSKDTRKRPRDDSDSTLKKMKLEPNLGE
DDEDKDLEPGPSGTSKASAQISGQSDTDITAEFLQPLLTPDNVANLVLISMVYLPEAMPASF
QAIYTPVESAGTEAQIKHLARLMATQMTAAGLGPGVEQTKQCKEEPKEEKVVKTESVLIK
RRLSAQGQAISVVGSLSSMSPLEEEAPQAKRRPEPIIPVTQPRLAGAGGRKKIFRLSDVLKP
LTDAQVEAMKLGAVKRILRAEKAVACSGAAQVRIKILASLVTQFNSGLKAEVLSFILEDVR
ARLDLAFAWLYQEYNAYLAAGASGSLDKYEDCLIRLLSGLQEKPDQKDGIFTKVVLEAPL
ITESALEVVRKYCEDESRTYLGMSTLRDLIFKRPSRQFQYLHVLLDLSSHEKDKVRSQALL
FIKRMYEKEQLREYVEKFALNYLQLLVHPNPPSVLFGADKDTEVAAPWTEETVKQCLYLY
LALLPQNHKLIHELAAVYTEAIADIKRTVLRVIEQPIRGMGMNSPELLLLVENCPKGAETLV
TRCLHSLTDKVPPSPELVKRVRDLYHKRLPDVRFLIPVLNGLEKKEVIQALPKLIKLNPIVV
KEVFNRLLGTQHGEGNSALSPLNPGELLIALHNIDSVKCDMKSIIKATNLCFAERNVYTSE
VLAVVMQQLMEQSPLPMLLMRTVIQSLTMYPRLGGFVMNILSRLIMKQVWKYPKVWEG
FIKCCQRTKPQSFQVILQLPPQQLGAVFDKCPELREPLLAHVRSFTPHQQAHIPNSIMTILEA
SGKQEPEAKEAPAGPLEEDDLEPLTLAPAPAPRPPQDLIGLRLAQEKALKRQLEEEQKLKP
GGVGAPSSSSPSPSPSARPGPPPSEEAMDFREEGPECETPGIFISMDDDSGLTEAALLDSSLE
GPLPKETAAGGLTLKEERSPQTLAPVGEDAMKTPSPAAEDAREPEAKGNS

>Q9NPY3 single-pass type I membrane protein
MATSMGLLLLLLLLLLTQPGAGTGADTEAVVCVGTACYTAHSGKLSAAEAQNHCNQNGG
NLATVKSKEEAQHVQRVLAQLLRREAALTARMSKFWIGLQREKGKCLDPSLPLKGFSWV
GGGEDTPYSNWHKELRNSCISKRCVSLLLDLSQPLLPSRLPKWSEGPCGSPGSPGSNIEGF
VCKFSFKGMCRPLALGGPGQVTYTTPFQTTSSSLEAVPFASAANVACGEGDKDETQSHYF
LCKEKAPDVFDWGSSGPLCVSPKYGCNFNNGGCHQDCFEGGDGSFLCGCRPGFRLLDDL
VTCASRNPCSSSPCRGGATCVLGPHGKNYTCRCPQGYQLDSSQLDCVDVDECQDSPCAQ
ECVNTPGGFRCECWVGYEPGGPGEGACQDVDECALGRSPCAQGCTNTDGSFHCSCEEGY
VLAGEDGTQCQDVDECVGPGGPLCDSLCFNTQGSFHCGCLPGWVLAPNGVSCTMGPVS
LGPPSGPPDEEDKGEKEGSTVPRAATASPTRGPEGTPKATPTTSRPSLSSDAPITSAPLKML
APSGSPGVWREPSIHHATAASGPQEPAGGDSSVATQNNDGTDGQKLLLFYILGTVVAILLL
LALALGLLVYRKRRAKREEKKEKKPQNAADSYSWVPERAESRAMENQYSPTPGTDC

>Q9NUS5 peripheral membrane protein
MVHAFLIHTLRAPNTEDTGLCRVLYSCVFGAEKSPDDPRPHGAERDRLLRKEQILAVARQ
VESMCRLQQQASGRPPMDLQPQSSDEQVPLHEAPRGAFRLAAENPFQEPRTVVWLGVLS
LGFALVLDAHENLLLAEGTLRLLTRLLLDHLRLLAPSTSLLLRADRIEGILTRFLPHGQLLFL
NDQFVQGLEKEFSAAWPR

>Q9UJQ1 single-pass type I membrane protein
MDLQGRGVPSIDRLRVLLMLFHTMAQIMAEQEVENLSGLSTNPEKDIFVVRENGTTCLM
AEFAAKFIVPYDVWASNYVDLITEQADIALTRGAEVKGRCGHSQSELQVFWVDRAYALK
MLFVKESHNMSKGPEATWRLSKVQFVYDSSEKTHFKDAVSAGKHTANSHHLSALVTPAG
KSYECQAQQTISLASSDPQKTVTMILSAVHIQPFDIISDFVFSEEHKCPVDEREQLEETLPLIL
GLILGLVIMVTLAIYHVHHKMTANQVQIPRDRSQYKHMG

>O95977 multipass membrane protein

MNATGTPVAPESCQQLAAGGHSRLIVLHYNHSGRLAGRGGPEDGGLGALRGLSVAASCL
VVLENLLVLAAITSHMRSRRWVYYCLVNITLSDLLTGAAYLANVLLSGARTFRLAPAQWF
LREGLLFTALAASTFSLLFTAGERFATMVRPVAESGATKTSRVYGFIGLCWLLAALLGMLP
LLGWNCLCAFDRCSSLLPLYSKRYILFCLVIFAGVLATIMGLYGAIFRLVQASGQKAPRPAA
RRKARRLLKTVLMILLAFLVCWGPLFGLLLADVFGSNLWAQEYLRGMDWILALAVLNSA
VNPIIYSFRSREVCRAVLSFLCCGCLRLGMRGPGDCLARAVEAHSGASTTDSSLRPRDSFR
GSRSLSFRMREPLSSISSVRSI

>Q9H665 single-pass type I membrane protein
MGPGRCLLTALLLLALAPPPEASQYCGRLEYWNPDNKCCSSCLQRFGPPPCPDYEFRENC
GLNDHGDFVTPPFRKCSSGQCNPDGAELCSPCGGGAVTPTPAAGGGRTPWRCRERPVPAK
GHCPLTPGNPGAPSSQERSSPASSIAWRTPEPVPQQAWPNFLPLVVLVLLLTLAVIAILLFILL
WHLCWPKEKADPYPYPGLVCGVPNTHTPSSSHLSSPGALETGDTWKEASLLPLLSRELSS
LASQPLSRLLDELEVLEELIVLLDPEPGPGGGMAHGTTRHLAARYGLPAAWSTFAYSLRPS
RSPLRALIEMVVAREPSASLGQLGTHLAQLGRADALRVLSKLGSSGVCWA

>O15552 multipass membrane protein
MLPDWKSSLILMAYIIIFLTGLPANLLALRAFVGRIRQPQPAPVHILLLSLTLADLLLLLLLPF
KIIEAASNFRWYLPKVVCALTSFGFYSSIYCSTWLLAGISIERYLGVAFPVQYKLSRRPLYG
VIAALVAWVMSFGHCTIVIIVQYLNTTEQVRSGNEITCYENFTDNQLDVVLPVRLELCLVL
FFIPMAVTIFCYWRFVWIMLSQPLVGAQRRRRAVGLAVVTLLNFLVCFGPYNVSHLVGYH
QRKSPWWRSIAVVFSSLNASLDPLLFYFSSSVVRRAFGRGLQVLRNQGSSLLGRRGKDTA
EGTNEDRGVGQGEGMPSSDFTTE

>Q9UBK5 single-pass type I membrane protein
MIHLGHILFLLLLPVAAAQTTPGERSSLPAFYPGTSGSCSGCGSLSLPLLAGLVAADAVASLL
IVGAVFLCARPRRSPAQEDGKVYINMPGRG

>O14842 multipass membrane protein
MDLPPQLSFGLYVAAFALGFPLNVLAIRGATAHARLRLTPSLVYALNLGCSDLLLTVSLPLK
AVEALASGAWPLPASLCPVFAVAHFFPLYAGGGFLAALSAGRYLGAAFPLGYQAFRRPCYS
WGVCAAIWALVLCHLGLVFGLEAPGGWLDHSNTSLGINTPVNGSPVCLEAWDPASAGPA
RFSLSLLLFFLPLAITAFCYVGCLRALARSGLTHRRKLRAAWVAGGALLTLLLCVGPYNAS
NVASFLYPNLGGSWRKLGLITGAWSVVLNPLVTGYLGRGPGLKTVCAARTQGGKSQK

>P32248 multipass membrane protein
MDLGKPMKSVLVVALLVIFQVCLCQDEVTDDYIGDNTTVDYTLFESLCSKKDVRNFKAW
FLPIMYSIICFVGLLGNGLVVLTYIYFKRLKTMTDTYLLNLAVADILFLLTLPFWAYSAAKS
WVFGVHFCKLIFAIYKMSFFSGMLLLLCISIDRYVAIVQAVSAHRHRARVLLISKLSCVGIW
ILATVLSIPELLYSDLQRSSSEQAMRCSLITEHVEAFITIQVAQMVIGFLVPLLAMSFCYLVII
RTLLQARNFERNKAIKVIIAVVVVFIVFQLPYNGVVLAQTVANFNITSSTCELSKQLNIAYD
VTYSLACVRCCVNPFLYAFIGVKFRNDLFKLFKDLGCLSQEQLRQWSSCRHIRRSSMSVE
AETTTTFSP

>P10301 lipid-anchor
MSSGAASGTGRGRPRGGGPGPGDPPPSETHKLVVVGGGGVGKSALTIQFIQSYFVSDYDP
TIEDSYTKICSVDGIPARLDILDTAGQEEFGAMREQYMRAGHGFLLVFAINDRQSFNEVGK
LFTQILRVKDRDDFPVVLVGNKADLESQRQVPRSEASAFGASHHVAYFEASAKLRLNVDE
AFEQLVRAVRKYQEQELPPSPPSAPRKKGGGCPCVLL

>O14669 single-pass type I membrane protein
MRGHPSLLLLYMALTTCLDTSPSEETDQEVFLGPPEAQSFLSSHTRIPRANHWDLELLTPG
NLERECLEERCSWEEAREYFEDNTLTERFWESYIYNGKGGRGRVDVASLAVGLTGGILLIV
LAGLGAFWYLRWRQHRGQQPCPQEAGLISPLSPLNPLGPPTPLPPPPPPPPGLPTYEQALA
ASGVHDAPPPPYTSLRRPH

>P78381 multipass membrane protein
MAAVGAGGSTAAPGPGAVSAGALEPGTASAAHRRLKYISLAVLVVQNASLILSIRYARTLP
GDRFFATTAVVMAEVLKGLTCLLLLFAQKRGNVKHLVLFLHEAVLVQYVDTLKLAVPSLIY
TLQNNLQYVAISNLPAATFQVTYQLKILTTALFSVLMLNRSLSRLQWASLLLLFTGVAIVQA
QQAGGGGPRPLDQNPGAGLAAVVASCLSSGFAGVYFEKILKGSSGSVWLRNLQLGLFGTA
LGLVGLWWAEGTAVATRGFFFGYTPAVWGVVLNQAFGGLLVAVVVKYADNILKGFATSLSI
VLSTVASIRLFGFHVDPLFALGAGLVIGAVYLYSLPRGAAKAIASASASASGPCVHQQPPGQ
PPPPQLSSHRGDLITEPFLPKLLTKVKGS

>Q9BX95 multipass membrane protein
MSLRQRLAQLVGRLQDPQKVARFQRLCGVEAPPRRSADRREDEKAEAPLAGDPRLRGRQ
PGAPGGPQPPGSDRNQCPAKPDGGGAPNGVRNGLAAELGPASPRRAGALRRNSLTGEEG
QLARVSNWPLYCLFCFGTELGNELFYILFFPFWIWNLDPLVGRRLVVIWVLVMYLGQCTK
DIIRWPRPASPPVVKLEVFYNSEYSMPSTHAMSGTAIPISMVLLTYGRWQYPLIYGLILIPC
WCSLVCLSRIYMGMHSILDIIAGFLYTILILAVFYPFVDLIDNFNQTHKYAPFIIIGLHLALGIF
SFTLDTWSTSRGDTAEILGSGAGIACGSHVTYNMGLVLDPSLDTLPLAGPPITVTLFGKAIL
RILIGMVFVLIIRDVMKKITIPLACKIFNIPCDDIRKARQHMEVELPYRYITYGMVGFSITFF
VPYIFFFIGIS

>P22794 single-pass type I membrane protein
MPTDMEHTGHYLHLAFLMTTVFSLSPGTKANYTRLWANSTSSWDSVIQNKTGRNQNENI
NTNPITPEVDYKGNSTNMPETSHIVALTSKSEQELYIPSVVSNSPSTVQSIENTSKSHGEIFK
KDVCAENNNNMAMLICLIIIAVLFLICTFLFLSTVVLANKVSSLRRSKQVGKRQPRSNGDF
LASGLWPAESDTWKRTKQLTGPNLVMQSTGVLTATRERKDEEGTEKLTNKQIG

>P23515 GPI-anchor, lipid-anchor
MEYQILKMSLCLFILLFLTPGILCICPLQCICTERHRHVDCSGRNLSTLPSGLQENIIHLNLSY
NHFTDLHNQLTQYTNLRTLDISNNRLESLPAHLPRSLWNMSAANNNIKLLDKSDTAYQWN
LKYLDVSKNMLEKVVLIKNTLRSLEVLNLSSNKLWTVPTNMPSKLHIVDLSNNSLTQILPG
TLINLTNLTHLYLHNNKFTFIPDQSFDQLFQLQEITLYNNRWSCDHKQNITYLLKWMMETK
AHVIGTPCSTQISSLKEHNMYPTPSGFTSSLFTVSGMQTVDTINSLSVVTQPKVTKIPKQYR
TKETTFGATLSKDTTFTSTDKAFVPYPEDTSTETINSHEAAAATLTIHLQDGMVTNTSLTSS

TKSSPTPMTLSITSGMPNNFSEMPQQSTTLNLWREETTTNVKTPLPSVANAWKVNASFLLL
LNVVVMLAV

>Q9BQI0 peripheral membrane protein
MSGELSNRFQGGKAFGLLKARQERRLAEINREFLCDQKYSDEENLPEKLTAFKEKYMEFD
LNNEGEIDLMSLKRMMEKLGVPKTHLEMKKMISEVTGGVSDTISYRDFVNMMLGKRSA
VLKLVMMFEGKANESSPKPVGPPPERDIASLP

>P19075 multipass membrane protein
MAGVSACIKYSMFTFNFLFWLCGILILALAIWVRVSNDSQAIFGSEDVGSSSYVAVDILIAV
GAIIMILGFLGCCGAIKESRCMLLLFFIGLLLILLLQVATGILGAVFKSKSDRIVNETLYENTK
LLSATGESEKQFQEAIIVFQEEFKCCGLVNGAADWGNNFQHYPELCACLDKQRPCQSYNG
KQVYKETCISFIKDFLAKNLIIVIGISFGLAVIEILGLVFSMVLYCQIGNK

>O95178 peripheral membrane protein
MSALTRLASFARVGGRLFRSGCARTAGDGGVRHAGGGVHIEPRYRQFPQLTRSQVFQSEF
FSGLMWFWILWRFWHDSEEVLGHFPYPDPSQWTDEELGIPPDDED

>Q9NYW5 multipass membrane protein
MLRLFYFSAIIASVILNFVGIIMNLFITVVNCKTWVKSHRISSSDRILFSLGITRFLMLGLFLV
NTIYFVSSNTERSVYLSAFFVLCFMFLDSSSVWFVTLLNILYCVKITNFQHSVFLLLKRNISP
KIPRLLLACVLISAFTTCLYITLSQASPFPELVTTRNNTSFNISEGILSLVVSLVLSSSLQFIINV
TSASLLIHSLRRHIQKMQKNATGFWNPQTEAHVGAMKLMVYFLILYIPYSVATLVQYLPFY
AGMDMGTKSICLIFATLYSPGHSVLIIITHPKLKTTAKKILCFKK

>Q9NYW4 multipass membrane protein
MLSAGLGLLMLVAVVEFLIGLIGNGSLVVWSFREWIRKFNWSSYNLIILGLAGCRFLLQWL
IILDLSLFPLFQSSRWLRYLSIFWVLVSQASLWFATFLSVFYCKKITTFDRPAYLWLKQRAYN
LSLWCLLGYFIINLLLTVQIGLTFYHPPQGNSSIRYPFESWQYLYAFQLNSGSYLPLVVFLVS
SGMLIVSLYTHHKKMKVHSAGRRDVRAKAHITALKSLGCFLLLHLVYIMASPFSITSKTYP
PDLTSVFIWETLMAAYPSLHSLILIMGIPRVKQTCQKILWKTVCARRCWGP

>Q96RI0 multipass membrane protein
MWGRLLLWPLVLGFSLSGGTQTPSVYDESGSTGGGDDSTPSILPAPRGYPGQVCANDSDT
LELPDSSRALLLGWVPTRLVPALYGLVLVVGLPANGLALWVLATQAPRLPSTMLLMNLAA
ADLLLALALPPRIAYHLRGQRWPFGEAACRLATAALYGHMYGSVLLLAAVSLDRYLALVH
PLRARALRGRRLALGLCMAAWLMAAALALPLTLQRQTFRLARSDRVLCHDALPLDAQAS
HWQPAFTCLALLGCFLPLLAMLLCYGATLHTLAASGRRYGHALRLTAVVLASAVAFFVPS
NLLLLLHYSDPSPSAWGNLYGAYVPSLALSTLNSCVDPFIYYYVSAEFRDKVRAGLFQRSP
GDTVASKASAEGGSRGMGTHSSLLQ

>Q96S21 lipid-anchor
MGSQGSPVKSYDYLLKFLLVGDSDVGKGEILESLQDGAAESPYAYSNGIDYKTTTILLDGR
RVKLELWDTSGQGRFCTIFRSYSRGAQGILLVYDITNRWSFDGIDRWIKEIDEHAPGVPRIL

VGNRLHLAFKRQVPTEQARAYAEKNCMTFFEVSPLCNFNVIESFTELSRIVLMRHGMEKI
WRPNRVFSLQDLCCRAIVSCTPVHLIDKLPLPVTIKSHLKSFSMANGMNAVMMHGRSYSL
ASGAGGGGSKGNSLKRSKSIRPPQSPPQNCSRSNCKIS

>Q9P2W3 lipid-anchor
MEEWDVPQMKKEVESLKYQLAFQREMASKTIPELLKWIEDGIPKDPFLNPDLMKNNPWV
EKGKCTIL

>Q9BV81 multipass membrane protein
MAAVVAKREGPPFISEAAVRGNAAVLDYCRTSVSALSGATAGILGLTGLYGFIFYLLASVLL
SLLLILKAGRRWNKYFKSRRPLFTGGLIGGLFTYVLFWTFLYGMVHVY

>P63211 lipid-anchor
MPVINIEDLTEKDKLKMEVDQLKKEVTLERMLVSKCCEEVRDYVEERSGEDPLVKGIPED
KNPFKELKGGCVIS

>P19440 single-pass type II membrane protein
MKKKLVVLGLLAVVLVLVIVGLCLWLPSASKEPDNHVYTRAAVAADAKQCSKIGRDALR
DGGSAVDAAIAALLCVGLMNAHSMGIGGGLFLTIYNSTTRKAEVINAREVAPRLAFATMF
NSSEQSQKGGLSVAVPGEIRGYELAHQRHGRLPWARLFQPSIQLARQGFPVGKGLAAALE
NKRTVIEQQPVLCEVFCRDRKVLREGERLTLPQLADTYETLAIEGAQAFYNGSLTAQIVKD
IQAAGGIVTAEDLNNYRAELIEHPLNISLGDVVLYMPSAPLSGPVLALILNILKGYNFSRES
VESPEQKGLTYHRIVEAFRFAYAKRTLLGDPKFVDVTEVVRNMTSEFFAAQLRAQISDDTT
HPISYYKPEFYTPDDGGTAHLSVVAEDGSAVSATSTINLYFGSKVRSPVSGILFNNEMDDFS
SPSITNEFGVPPSPANFIQPGKQPLSSMCPTIMVGQDGQVRMVVGAAGGTQITTATALAIIY
NLWFGYDVKRAVEEPRLHNQLLPNVTTVERNIDQAVTAALETRHHHTQIASTFIAVVQAIV
RTAGGWAAASDSRKGGEPAGY

>Q00587 peripheral membrane protein
MPGPQGGRGAATMSLGKLSPVGWVSSSQGKRRLTADMISHPLGDFRHTMHVGRGGDVF
GDTSFLSNHGGSSGSTHRSPRSFLAKKLQLVRRVGAPPRRMASPPAPSPAPPAISPIIKNAISL
PQLNQAAYDSLVVGKLSFDSSPTSSTDGHSSYGLDSGFCTISRLPRSEKPHDRDRDGSFPSE
PGLRRSDSLLSFRLDLDLGPSLLSELLGVMSLPEAPAAETPAPAANPPAPTANPTGPAANPPA
TTANPPAPAANPSAPAATPTGPAANPPAPAASSTPHGHCPNGVTAGLGPVAEVKSSPVGGG
PRGPAGPALGRHWGAGWDGGHHYPEMDARQERVEVLPQARASWESLDEEWRAPQAGS
RTPVPSTVQANTFEFADAEEDDEVKV

>Q99705 multipass membrane protein
MSVGAMKKGVGRAVGLGGGSGCQATEEDPLPNCGACAPGQGGRRWRLPQPAWVEGSSA
RLWEQATGTGWMDLEASLLPTGPNASNTSDGPDNLTSAGSPPRTGSISYINIIMPSVFGTIC
LLGIIGNSTVIFAVVKKSKLHWCNNVPDIFIINLSVVDLLFLLGMPFMIHQLMGNGVWHFG
ETMCTLITAMDANSQFTSTYILTAMAIDRYLATVHPISSTKFRKPSVATLVICLLWALSFISIT
PVWLYARLIPFPGGAVGCGIRLPNPDTDLYWFTLYQFFLAFALPFVVITAAYVRILQRMTSS
VAPASQRSIRLRTKRVTRTAIAICLVFFVCWAPYYVLQLTQLSISRPTLTFVYLYNAAISLGY

ANSCLNPFVYIVLCETFRKRLVLSVKPAAQGQLRAVSNAQTADEERTESKGT

>Q9NYV7 multipass membrane protein
MIPIQLTVFFMIIYVLESLTIIVQSSLIVAVLGREWLQVRRLMPVDMILISLGISRFCLQWASM
LNNFCSYFNLNYVLCNLTITWEFFNILTFWLNSLLTVFYCIKVSSFTHHIFLWLRWRILRLFP
WILLGSLMITCVTIIPSAIGNYIQIQLLTMEHLPRNSTVTDKLENFHQYQFQAHTVALVIPFIL
FLASTIFLMASLTKQIQHHSTGHCNPSMKARFTALRSLAVLFIVFTSYFLTILITIIGTLFDKR
CWLWVWEAFVYAFILMHSTSLMLSSPTLKRILKGKC

>Q9HBW1 single-pass type I membrane protein
MKLLWQVTVHHHTWNAILLPFVYLTAQVWILCAAIAAAASAGPQNCPSVCSCSNQFSKV
VCTRRGLSEVPQGIPSNTRYLNLMENNIQMIQADTFRHLHHLEVLQLGRNSIRQIEVGAFN
GLASLNTLELFDNWLTVIPSGAFEYLSKLRELWLRNNPIESIPSYAFNRVPSLMRLDLGELK
KLEYISEGAFEGLFNLKYLNLGMCNIKDMPNLTPLVGLEELEMSGNHFPEIRPGSFHGLSSL
KKLWVMNSQVSLIERNAFDGLASLVELNLAHNNLSSLPHDLFTPLRYLVELHLHHNPWNC
DCDILWLAWWLREYIPTNSTCCGRCHAPMHMRGRYLVEVDQASFQCSAPFIMDAPRDLN
ISEGRMAELKCRTPPMSSVKWLLPNGTVLSHASRHPRISVLNDGTLNFSHVLLSDTGVYT
CMVTNVAGNSNASAYLNVSTAELNTSNYSFFTTVTVETTEISPEDTTRKYKPVPTTSTGYQ
PAYTTSTTVLIQTTRVPKQVAVPATDTTDKMQTSLDEVMKTTKIIIGCFVAVTLLAAAMLIV
FYKLRKRHQQRSTVTAARTVEIIQVDEDIPAATSAAATAAPSGVSGEGAVVLPTIHDHINYN
TYKPAHGAHWTENSLGNSLHPTVTTISEPYIIQTHTKDKVQETQI

>Q99835 multipass membrane protein
MAAARPARGPELPLLGLLLLLLLLGDPGRGAASSGNATGPGPRSAGGSARRSAAVTGPPPPL
SHCGRAAPCEPLRYNVCLGSVLPYGATSTLLAGDSDSQEEAHGKLVLWSGLRNAPRCWA
VIQPLLCAVYMPKCENDRVELPSRTLCQATRGPCAIVERERGWPDFLRCTPDRFPEGCTNE
VQNIKFNSSGQCEVPLVRTDNPKSWYEDVEGCGIQCQNPLFTEAEHQDMHSYIAAFGAVT
GLCTLFTLATFVADWRNSNRYPAVILFYVNACFFVGSIGWLAQFMDGARREIVCRADGTM
RLGEPTSNETLSCVIIFVIVYYALMAGVVWFVVLTYAWHTSFKALGTTYQPLSGKTSYFHL
LTWSLPFVLTVAILAVAQVDGDSVSGICFVGYKNYRYRAGFVLAPIGLVLIVGGYFLIRGV
MTLFSIKSNHPGLLSEKAASKINETMLRLGIFGFLAFGFVLITFSCHFYDFFNQAEWERSFR
DYVLCQANVTIGLPTKQPIPDCEIKNRPSLLVEKINLFAMFGTGIAMSTWVWTKATLLIWR
RTWCRLTGQSDDEPKRIKKSKMIAKAFSKRHELLQNPGQELSFSMHTVSHDGPVAGLAFD
LNEPSADVSSAWAQHVTKMVARRGAILPQDISVTPVATPVPPEEQANLWLVEAEISPELQK
RLGRKKKRRKRKKEVCPLAPPPELHPPAPAPSTIPRLPQLPRQKCLVAAGAWGAGDSCRQ
GAWTLVSNPFCPEPSPPQDPFLPSAPAPVAWAHGRRQGLGPIHSRTNLMDTELMDADSDF

>O00161 lipid-anchor, peripheral membrane protein
MDNLSSEEIQQRAHQITDESLESTRRILGLAIESQDAGIKTITMLDEQKEQLNRIEEGLDQIN
KDMRETEKTLTELNKCCGLCVCPCNRTKNFESGKAYKTTWGDGGENSPCNVVSKQPGPV
TNGQLQQPTTGAASGGYIKRITNDAREDEMEENLTQVGSILGNLKDMALNIGNEIDAQNP
QIKRITDKADTNRDRIDIANARAKKLIDS

>Q9NWW5 multipass membrane protein

MEATRRRQHLGATGGPGAQLGASFLQARHGSVSADEAARTAPFHLDLWFYFTLQNWVL
DFGRPIAMLVFPLEWFPLNKPSVGDYFHMAYNVITPFLLLKLIERSPRTLPRSITYVSIIIFIM
GASIHLVGDSVNHRLLFSGYQHHLSVRENPIIKNLKPETLIDSFELLYYYDEYLGHCMWYI
PFFLILFMYFSGCFTASKAESLIPGPALLLVAPSGLYYWYLVTEGQIFILFIFTFFAMLALVLH
QKRKRLFLDSNGLFLFSSFALTLLLVALWVAWLWNDPVLRKKYPGVIYVPEPWAFYTLHV
SSRH

>Q9NPB9 multipass membrane protein
MALEQNQSTDYYYEENEMNGTYDYSQYELICIKEDVREFAKVFLPVFLTIVFVIGLAGNS
MVVAIYAYYKKQRTKTDVYILNLAVADLLLLFTLPFWAVNAVHGWVLGKIMCKITSALYT
LNFVSGMQFLACISIDRYVAVTKVPSQSGVGKPCWIICFCVWMAAILLSIPQLVFYTVNDN
ARCIPIFPRYLGTSMKALIQMLEICIGFVVPFLIMGVCYFITARTLMKMPNIKISRPLKVLLT
VVIVFIVTQLPYNIVKFCRAIDIIYSLITSCNMSKRMDIAIQVTESIALFHSCLNPILYVFMGA
SFKNYVMKVAKKYGSWRRQRQSVEEFPFDSEGPTEPTSTFSI

>P53618 peripheral membrane protein
MTAAENVCYTLINVPMDSEPPSEISLKNDLEKGDVKSKTEALKKVIIMILNGEKLPGLLMT
IIRFVLPLQDHTIKKLLLVFWEIVPKTTPDGRLLHEMILVCDAYRKDLQHPNEFIRGSTLRFL
CKLKEAELLEPLMPAIRACLEHRHSYVRRNAVLAIYTIYRNFEHLIPDAPELIHDFLVNEKD
ASCKRNAFMMLIHADQDRALDYLSTCIDQVQTFGDILQLVIVELIYKVCHANPSERARFIR
CIYNLLQSSSPAVKYEAAGTLVTLSSAPTAIKAAAQCYIDLIIKESDNNVKLIVLDRLIELKE
HPAHERVLQDLVMDILRVLSTPDLEVRKKTLQLALDLVSSRNVEELVIVLKKEVIKTNNVS
EHEDTDKYRQLLVRTLHSCSVRFPDMAANVIPVLMEFLSDNNEAAAADVLEFVREAIQRF
DNLRMLIVEKMLEVFHAIKSVKIYRGALWILGEYCSTKEDIQSVMTEIRRSLGEIPIVESEIK
KEAGELKPEEEITVGPVQKLVTEMGTYATQSALSSSRPTKKEEDRPPLRGFLLDGDFFVAA
SLATTLTKIALRYVALVQEKKKQNSFVAEAMLLMATILHLGKSSLPKKPITDDDVDRISLCL
KVLSECSPLMNDIFNKECRQSLSHMLSAKLEEEKLSQKKESEKRNVTVQPDDPISFMQLTA
KNEMNCKEDQFQLSLLAAMGNTQRKEAADPLASKLNKVTQLTGFSDPVYAEAYVHVNQ
YDIVLDVLVVNQTSDTLQNCTLELATLGDLKLVEKPSPLTLAPHDFANIKANVKVASTENG
IIFGNIVYDVSGAASDRNCVVLSDIHIDIMDYIQPATCTDAEFRQMWAEFEWENKVTVNTN
MVDLNDYLQHILKSTNMKCLTPEKALSGYCGFMAANLYARSIFGEDALANVSIEKPIHQG
PDAAVTGHIRIRAKSQGMALSLGDKINLSQKKTSI

>P34810 single-pass type I membrane protein
MRLAVLFSGALLGLLAAQGTGNDCPHKKSATLLPSFTVTPTVTESTGTTSHRTTKSHKTTT
HRTTTTGTTSHGPTTATHNPTTTSHGNVTVHPTSNSTATSQGPSTATHSPATTSHGNATVHP
TSNSTATSPGFTSSAHPEPPPPSPSPSPTSKETIGDYTWTNGSQPCVHLQAQIQIRVMYTTQG
GGEAWGISVLNPNKTKVQGSCEGAHPHLLLSFPYGHLSFGFMQDLQQKVVYLSYMAVEY
NVSFPHAAQWTFSAQNASLRDLQAPLGQSFSCSNSSIILSPAVHLDLLSLRLQAAQLPHTG
VFGQSFSCPSDRSILLPLIIGLILLGLLALVLIAFCIIRRRPSAYQAL

>P14415 single-pass type II membrane protein
MVIQKEKKSCGQVVEEWKEFVWNPRTHQFMGRTGTSWAFILLFYLVFYGFLTAMFTLTM
WVMLQTVSDHTPKYQDRLATPGLMIRPKTENLDVIVNVSDTESWDQHVQKLNKFLEPYN

DSIQAQKNDVCRPGRYYEQPDNGVLNYPKRACQFNRTQLGNCSGIGDSTHYGYSTGQPC
VFIKMNRVINFYAGANQSMNVTCAGKRDEDAENLGNFVMFPANGNIDLMYFPYYGKKF
HVNYTQPLVAVKFLNVTPNVEVNVECRINAANIATDDERDKFAGRVAFKLRINKT

>Q9Y6Q5 peripheral membrane protein
MSASAVFILDVKGKPLISRNYKGDVAMSKIEHFMPLLVQREEEGALAPLLSHGQVHFLWIK
HSNLYLVATTSKNANASLVYSFLYKTIEVFCEYFKELEEESIRDNFVIVYELLDELMDFGFP
QTTDSKILQEYITQQSNKLETGKSRVPPTVTNAVSWRSEGIKYKKNEVFIDVIESVNLLVNA
NGSVLLSEIVGTIKLKVFLSGMPELRLGLNDRVLFELTGRSKNKSVELEDVKFHQCVRLSR
FDNDRTISFIPPDGDFELMSYRLSTQVKPLIWIESVIEKFSHSRVEIMVKAKGQFKKQSVAN
GVEISVPVPSDADSPRFKTSVGSAKYVPERNVVIWSIKSFPGGKEYLMRAHFGLPSVEKEE
VEGRPPIGVKFEIPYFTVSGIQVRYMKIIEKSGYQALPWVRYITQSGDYQLRTS

>Q92843 peripheral membrane protein
MATPASAPDTRALVADFVGYKLRQKGYVCGAGPGEGPAADPLHQAMRAAGDEFETRFRR
TFSDLAAQLHVTPGSAQQRFTQVSDELFQGGPNWGRLVAFFVFGAALCAESVNKEMEPL
VGQVQEWMVAYLETQLADWIHSSGGWAEFTALYGDGALEEARRLREGNWASVRTVLTG
AVALGALVTVGAFFASK

>P52961 GPI-anchor, lipid-anchor
MQMPAMMSLLLVSVGLMEALQAQSHPITRRDLFSQEIQLDMALASFDDQYAGCAAAMT
AALPDLNHTEFQANQVYADSWTLASSQWQERQARWPEWSLSPTRPSPPPLGFRDEHGVA
LLAYTANSPLHKEFNAAVREAGRSRAHYLHHFSFKTLHFLLTEALQLLGSGQRPPRCHQV
FRGVHGLRFRPAGPRATVRLGGFASASLKHVAAQQFGEDTFFGIWTCLGAPIKGYSFFPGE
EEVLIPPFETFQVINASRLAQGPARIYLRALGKHSTYNCEYIKDKKCKSGPCHLDNSAMGQ
SPLSAVWSLLLLLWFLVVRAFPDGPGLL

>P49257 single-pass type I membrane protein
MAGSRQRGLRARVRPLFCALLLSLGRFVRGDGVGGDPAVALPHRRFEYKYSFKGPHLVQS
DGTVPFWAHAGNAIPSSDQIRVAPSLKSQRGSVWTKTKAAFENWEVEVTFRVTGRGRIGA
DGLAIWYAENQGLEGPVFGSADLWNGVGIFFDSFDNDGKKNNPAIVIIGNNGQIHYDHQN
DGASQALASCQRDFRNKPYPVRAKITYYQNTLTVMINNGFTPDKNDYEFCAKVENMIIPA
QGHFGISAATGGLADDHDVLSFLTFQLTEPGKEPPTPDKEISEKEKEKYQEEFEHFQQELD
KKKEEFQKGHPDLQGQPAEEIFESVGDRELRQVFEGQNRIHLEIKQLNRQLDMILDEQRRY
VSSLTEEISKRGAGMPGQHGQITQQELDTVVKTQHEILRQVNEMKNSMSETVRLVSGMQ
HPGSAGGVYETTQHFIDIKEHLHIVKRDIDNLVQRNMPSNEKPKCPELPPFPSCLSTVHFIIF
VVVQTVLFIGYIMYRSQQEAAAKKFF

>Q8TDJ6 peripheral membrane protein
MHLHQVLTGAVNPGDNCYSVGSVGDVPFTAYGSGCDIVILANDFECVQIIPGAKHGNIQV
SCVECSNQQGRIAASYGNAVCIFEPLGINSHKRNCQLKCQWLKTGQFFLSSVTYNLAWDP
QDNRLLTATDSIQLWAPPGDDILEEEEEIDNTVPPVLNDWKCVWQCKTSVSVHLMEWSPD
GEYFATAGKDDCLLKVWYPMTGWKSSIIPQDHHEVKRRQSSTQFSFVYLAHPRAVTGFS
WRKTSKYMPRGSVCNVLLTSCHDGVCRLWAETLLPEDCLLGEQICETTTSSIASSLSHAGR

HKDRIQHALETIHHLKNLRKGQRRSSVLVTHAELMPDQTAMHEVQRHISHHANALCHFHI
AASINPATDIPNVLVGTAFNVDDGNGGFVVHWLNNKEFHFTSSTEVFMHQLRKLSDKQV
DHENDDADREDEEHSQEDRERGLHMKLDHDLSLDRESEAGTGSSEHEDGEREGSPRTYS
RLSVPMPLPTVLLDRKIETLLTEWNKNPDMLFTIHPVDGTFLVWHVKYLDEYNPGIFRQV
QVSFSSRIPVAFPSGDASSLSKNIMMYACINATKDSHHTLLHQEGMSVGSPHGSQPHSRSH
STHMNILAPTVMMISKHIDGSLNQWAVTFADKSAFTTVLTVSHKFRYCGHRFHLNDLACH
SVLPLLLTSSHHNALLTPELDCQWDSDNKLSRLMDPVKHIKGSSKQPLRNAATRTFHDPN
AIYSELILWRVDPIGPLSYTGGVSELARINSLHTSAFSNVAWLPTLIPSYCLGTYCNSASACF
VASDGKNLRLYQAVVDARKLLDELSDPESSKLIGEVFNIVSQQSTARPGCIIELDAITNQCG
SNTQLLHVFQEDFIIGYKPHKEDMEKKETEIFFQPSQGYRPPPFSEKFFLVVIEKDSNNNSIL
HMWHLHLKSVQACLAKASEGASSESLLSVPGQKNVDSSPETSPSVSPMPHSSSIANLQTAS
KLILSSRLVYSQPLDLPESVEVIRATPSAGHLSSSSIYPVCLAPYLVVTTCSDNKVRFWKCC
MEANPECNKSDEKEIYHWKRWPLMNDEGEDNSSTVSIVGRPVAVSCSYTGRLAVAYKQPI
HHNGFVSKEFSMHVCIFECESTGGSEWVLEQTIHLDDLVKVGSVLDSRVSVDSNLFVYSK
SDALLSKDRYLIPNIKHLVHLDWVSKEDGSHILTVGVGANIFMYGRLSGIVTEQTNSKDGV
AVITLPLGGSIKQGVKSRWVLLRSIDLVSSVDGTPSLPVSLSWVRDGILVVGMDCEMHVYA
QWKHAVKFGDTEADSSNAEEAAMQDHSTFKSNMLARKSVVEGTAISDDVFCSPTVIQDG
GLFEAAHVLSPTLPQYHPTQLLELMDLGKVRRAKAILSHLVKCIAGEVAIVRDPDAGEGT
KRHLSRTISVSGSTAKETVTVGKDGTRDYTEIDSIPPLPLYALLAADQDTSYRISEESTKIPQ
SYEDQTVSQPEDQYSELFQIQDIPTDDIDLEPEKRENKSKVINLSQYGPAYFGQEHARVLSS
HLMHSSLPGLTRLEQMFLVALADTVATTSTELDESRDKSCSGRDTLDECGLRYLLAMRLH
TCLLTSLPPLYRVQLLHQGVSTCHFAWAFHSEAEEELINMIPAIQRGDPQWSELRAMGIGW
WVRNINTLRRCIEKVAKASFQRNNDALDAALFYLSMKKKAVVWGLFRSQHDEKMTTFFS
HNFNEDRWRKAALKNAFSLLGKQRFEQSAAFFLLAGSLKDAIEVCLEKMEDIQLAMVIA
RLYESEFETSSTYISILNQKILGCQKDGSGFSCKRLHPDPFLRSLAYWVMKDYTRALDTLL
EQTPKEDDEHQVIIKSCNPVAFSFYNYLRTHPLLIRRNLASPEGTLATLGLKTEKNFVDKIN
LIERKLFFTTANAHFKVGCPVLALEVLSKIPKVTKTSALSAKKDQPDFISHRMDDVPSHSK
ALSDGNGSSGIEWSNVTSSQYDWSQPIVKVDEEPLNLDWGEDHDSALDEEEDDAVGLVM
KSTDAREKDKQSDQKASDPNMLLTPQEEDDPEGDTEVDVIAEQLKFRACLKILMTELRTL
ATGYEVDGGKLRFQLYNWLEKEIAALHEICNHESVIKEYSSKTYSKVESDLLDQEEMVDK
PDIGSYERHQIERRRLQAKREHAERRKSWLQKNQDLLRVFLSYCSLHGAQGGGLASVRM
ELKFLLQESQQETTVKQLQSPLPLPTTLPLLSASIASTKTVIANPVLYLNNHIHDILYTIVQM
KTPPHPSIEDVKVHTLHSLAASLSASIYQALCDSHSYSQTEGNQFTGMAYQGLLLSDRRRL
RTESIEEHATPNSSPAQWPGVSSLINLLSSAQDEDQPKLNILLCEAVVAVYLSLLIHALATNS
SSELFRLAAHPLNNRMWAAVFGGGVKLVVKPRRQSENISAPPVLSEDIDKHRRRFNMRML
VPGRPVKDATPPPVPAERPSYKEKFIPPELSMWDYFVAKPFLPLSDSGVIYDSDESIHSDEE
DDAFFSDTQIQEHQDPNSYSWALLHLTMVKLALHNVKNFFPIAGLEFSELPVTSPLGIAVIK
NLENWEQILQEKMDQFEGPPPNYINTYPTDLSVGAGPAILRNKAMLEPENTPFKSRDSSAF
PVKRLWHFLVKQEVLQETFIRYIFTKKRKQSEVEADLGYPGGKAKVIHKESDMIMAFSVN
KANCNEIVLASTHDVQELDVTSLLACQSYIWIGEEYDRESKSSDDVDYRGSTTTLYQPSAT
SYSASQVHPPSSLPWLGTGQTSTGASVLMKRNLHNVKRMTSHPVHQYYLTGAQDGSVR
MFEWTRPQQLVCFRQAGNARVTRLYFNSQGNKCGVADGEGFLSIWQVNQTASNPKPYMS
WQCHSKATSDFAFITSSSLVATSGHSNDNRNVCLWDTLISPGNSLIHGFTCHDHGATVLQY
APKQQLLISGGRKGHVCIFDIRQRQLIHTFQAHDSAIKALALDPYEEYFTTGSAEGNIKVW

RLTGHGLIHSFKSEHAKQSIFRNIGAGVMQIDIIQGNRLFSCGADGTLKTRVLPNAFNIPNRI
LDIL

>Q14028 multipass membrane protein
MLGWVQRVLPQPPGTPRKTKMQEEEEVEPEPEMEAEVEPEPNPEEAETESESMPPEESFK
EEEVAVADPSPQETKEAALTSTISLRAQGAEISEMNSPSRRVLTWLMKGVEKVIPQPVHSIT
EDPAQILGHGSTGDTGCTDEPNEALEAQDTRPGLRLLLWLEQNLERVLPQPPKSSEVWRD
EPAVATGAASDPAPPGRPQEMGPKLQARETPSLPTPIPLQPKEEPKEAPAPEPQPGSQAQTSS
LPPTRDPARLVAWVLHRLEMALPQPVLHGKIGEQEPDSPGICDVQTISILPGGQVEPDLVLE
EVEPPWEDAHQDVSTSPQGTEVVPAYEEENKAVEKMPRELSRIEEEKEDEEEEEEEEEEE
EEEVTEVLLDSCVVSQVGVGQSEEDGTRPQSTSDQKLWEEVGEEAKKEAEEKAKEEAEE
VAEEEAEKEPQDWAETKEEPEAEAEAASSGVPATKQHPEVQVEDTDADSCPLMAEENPPS
TVLPPPSPAKSDTLIVPSSASGTHRKKLPSEDDEAEELKALSPAESPVVAWSDPTTPKDTDG
QDRAASTASTNSAIINDRLQELVKLFKERTEKVKEKLIDPDVTSDEESPKPSPAKKAPEPAP
DTKPAEAEPVEEEHYCDMLCCKFKHRPWKKYQFPQSIDPLTNLMYVLWLFFVVMAWNW
NCWLIPVRWAFPYQTPDNIHHWLLMDYLCDLIYFLDITVFQTRLQFVRGGDIITDKKDMR
NNYLKSRRFKMDLLSLLPLDFLYLKVGVNPLLRLPRCLKYMAFFEFNSRLESILSKAYVYR
VIRTTAYLLYSLHLNSCLYYWASAYQGLGSTHWVYDGVGNSYIRCYYFAVKTLITIGGLPD
PKTLFEIVFQLLNYFTGVFAFSVMIGQMRDVVGAATAGQTYYRSCMDSTVKYMNFYKIP
KSVQNRVKTWYEYTWHSQGMLDESELMVQLPDKMRLDLAIDVNYNIVSKVALFQGCDR
QMIFDMLKRLRSVVYLPNDYVCKKGEIGREMYIIQAGQVQVLGGPDGKSVLVTLKAGSV
FGEISLLAVGGGNRRTANVVAHGFTNLFILDKKDLNEILVHYPESQKLLRKKARRMLRSNN
KPKEEKSVLILPPRAGTPKLFNAALAMTGKMGGKGAKGGKLAHLRARLKELAALEAAA
KQQELVEQAKSSQDVKGEEGSAAPDQHTHPKEAATDPPAPRTPPEPPGSPPSSPPPASLGRP
EGEEEGPAEPEEHSVRICMSPGPEPGEQILSVKMPEEREEKAE

>A0FGR8 multipass membrane protein
MTANRDAALSSHRHPGCAQRPRTPTFASSSQRRSAFGFDDGNFPGLGERSHAPGSRLGAR
RRAKTARGLRGHRQRGAGAGLSRPGSARAPSPPRPGGPENPGGVLSVELPGLLAQLARSF
ALLLPVYALGYLGLSFSWVLLALALLAWCRRSRGLKALRLCRALALLEDEERVVRLGVR
ACDLPAWVHFPDTERAEWLNKTVKHMWPFICQFIEKLFRETIEPAVRGANTHLSTFSFTKV
DVGQQPLRINGVKVYTENVDKRQIILDLQISFVGNCEIDLEIKRYFCRAGVKSIQIHGTMRV
ILEPLIGDMPLVGALSIFFLRKPLLEINWTGLTNLLDVPGLNGLSDTIILDIISNYLVLPNRITV
PLVSEVQIAQLRFPVPKGVLRIHFIEAQDLQGKDTYLKGLVKGKSDPYGIIRVGNQIFQSRVI
KENLSPKWNEVYEALVYEHPGQELEIELFDEDPDKDDFLGSLMIDLIEVEKERLLDEWFTL
DEVPKGKLHLRLEWLTLMPNASNLDKVLTDIKADKDQANDGLSSALLILYLDSARNLPSG
KKISSNPNPVVQMSVGHKAQESKIRYKTNEPVWEENFTFFIHNPKRQDLEVEVRDEQHQC
SLGNLKVPLSQLLTSEDMTVSQRFQLSNSGPNSTIKMKIALRVLHLEKRERPPDHQHSAQV
KRPSVSKEGRKTSIKSHMSGSPGPGGSNTAPSTPVIGGSDKPGMEEKAQPPEAGPQGLHDL
GRSSSSLLASPGHISVKEPTPSIASDISLPIATQELRQRLRQLENGTTLGQSPLGQIQLTIRHSS
QRNKLIVVVHACRNLIAFSEDGSDPYVRMYLLPDKRRSGRRKTHVSKKTLNPVFDQSFDF
SVSLPEVQRRTLDVAVKNSGGFLSKDKGLLGKVLVALASEELAKGWTQWYDLTEDGTRP
QAMT

>O95219 peripheral membrane protein
MEQAPPDPERQLQPAPLEPLGSPDAGLGAAVGKEAEGAGEESSGVDTMTHNNFWLKKIEI
SVSEAEKRTGRNAMNMQETYTAYLIETRSVEHTDGQSVLTDSLWRRYSEFELLRSYLLVY
YPHIVVPPLPEKRAEFVWHKLSADNMDPDFVERRRIGLENFLLRIASHPILCRDKIFYLFLT
QEGNWKETVNETGFQLKADSRLKALNATFRVKNPDKRFTDLKHYSDELQSVISHLLRVR
ARVADRLYGVYKVHGNYGRVFSEWSAIEKEMGDGLQSAGHHMDVYASSIDDILEDEEHY
ADQLKEYLFYAEALRAVCRKHELMQYDLEMAAQDLASKKQQCEELVTGTVRTFSLKGM
TTKLFGQETPEQREARIKVLEEQINEGEQQLKSKNLEGREFVKNAWADIERFKEQKNRDL
KEALISYAVMQISMCKKGIQVWTNAKECFSKM

>Q99816 peripheral membrane protein
MAVSESQLKKMVSKYKYRDLTVRETVNVITLYKDLKPVLDSYVFNDGSSRELMNLTGTIP
VPYRGNTYNIPICLWLLDTYPYNPPICFVKPTSSMTIKTGKHVDANGKIYLPYLHEWKHPQ
SDLLGLIQVMIVVFGDEPPVFSRPISASYPPYQATGPPNTSYMPGMPGGISPYPSGYPPNPS
GYPGCPYPPGGPYPATTSSQYPSQPPVTTVGPSRDGTISEDTIRASLISAVSDKLRWRMKEE
MDRAQAELNALKRTEEDLKKGHQKLEEMVTRLDQEVAEVDKNIELLKKKDEELSSALEK
MENQSENNDIDEVIIPTAPLYKQILNLYAEENAIEDTIFYLGEALRRGVIDLDVFLKHVRLLS
RKQFQLRALMQKARKTAGLSDLY

>O43678 peripheral membrane protein
MAAAAASRGVGAKLGLREIRIHLCQRSPGSQGVRDFIEKRYVELKKANPDLPILIRECSDV
QPKLWARYAFGQETNVPLNNFSADQVTRALENVLSGKA

>Q92692 single-pass type I membrane protein
MARAAALLPSRSPPTPLLWPLLLLLLLETGAQDVRVQVLPEVRGQLGGTVELPCHLLPPV
PGLYISLVTWQRPDAPANHQNVAAFHPKMGPSFPSPKPGSERLSFVSAKQSTGQDTEAELQ
DATLALHGLTVEDEGNYTCEFATFPKGSVRGMTWLRVIAKPKNQAEAQKVTFSQDPTTVA
LCISKEGRPPARISWLSSLDWEAKETQVSGTLAGTVTVTSRFTLVPSGRADGVTVTCKVEH
ESFEEPALIPVTLSVRYPPEVSISGYDDNWYLGRTDATLSCDVRSNPEPTGYDWSTTSGTFP
TSAVAQGSQLVIHAVDSLFNTTFVCTVTNAVGMGRAEQVIFVRETPNTAGAGATGGIIGGII
AAIIATAVAATGILICRQQRKEQTLQGAEEDEDLEGPPSYKPPTPKAKLEAQEMPSQLFTLG
ASEHSPLKTPYFDAGASCTEQEMPRYHELPTLEERSGPLHPGATSLGSPIPVPPGPPAVEDV
SLDLEDEEGEEEEEYLDKINPIYDALSYSSPSDSYQGKGFVMSRAMYV

>O96008 multipass membrane protein
MGNVLAASSPPAGPPPPPAPALVGLPPPPPSPPGFTLPPLGGSLGAGTSTSRSSERTPGAATA
SASGAAEDGACGCLPNPGTFEECHRKCKELFPIQMEGVKLTVNKGLSNHFQVNHTVALST
IGESNYHFGVTYVGTKQLSPTEAFPVLVGDMDNSGSLNAQVIHQLGPGLRSKMAIQTQQS
KFVNWQVDGEYRGSDFTAAVTLGNPDVLVGSGILVAHYLQSITPCLALGGELVYHRRPGE
EGTVMSLAGKYTLNNWLATVTLGQAGMHATYYHKASDQLQVGVEFEASTRMQDTSVSF
GYQLDLPKANLLFKGSVDSNWIVGATLEKKLPPLPLTLALGAFLNHRKNKFQCGFGLTIG

>Q9BYF1 single-pass type I membrane protein
MSSSSWLLLSLVAVTAAQSTIEEQAKTFLDKFNHEAEDLFYQSSLASWNYNTNITEENVQN

MNNAGDKWSAFLKEQSTLAQMYPLQEIQNLTVKLQLQALQQNGSSVLSEDKSKRLNTIL
NTMSTIYSTGKVCNPDNPQECLLLEPGLNEIMANSLDYNERLWAWESWRSEVGKQLRPLY
EEYVVLKNEMARANHYEDYGDYWRGDYEVNGVDGYDYSRGQLIEDVEHTFEEIKPLYE
HLHAYVRAKLMNAYPSYISPIGCLPAHLLGDMWGRFWTNLYSLTVPFGQKPNIDVTDAM
VDQAWDAQRIFKEAEKFFVSVGLPNMTQGFWENSMLTDPGNVQKAVCHPTAWDLGKGD
FRILMCTKVTMDDFLTAHHEMGHIQYDMAYAAQPFLLRNGANEGFHEAVGEIMSLSAATP
KHLKSIGLLSPDFQEDNETEINFLLKQALTIVGTLPFTYMLEKWRWMVFKGEIPKDQWMK
KWWEMKREIVGVVEPVPHDETYCDPASLFHVSNDYSFIRYYTRTLYQFQFQEALCQAAK
HEGPLHKCDISNSTEAGQKLFNMLRLGKSEPWTLALENVVGAKNMNVRPLLNYFEPLFT
WLKDQNKNSFVGWSTDWSPYADQSIKVRISLKSALGDKAYEWNDNEMYLFRSSVAYAM
RQYFLKVKNQMILFGEEDVRVANLKPRISFNFFVTAPKNVSDIIPRTEVEKAIRMSRSRIND
AFRLNDNSLEFLGIQPTLGPPNQPPVSIWLIVFGVVMGVIVVGIVILIFTGIRDRKKKNKAR
SGENPYASIDISKGENNPGFQNTDDVQTSF

>Q10589 GPI-anchor, lipid-anchor, single-pass type II membrane protein
MASTSYDYCRVPMEDGDKRCKLLLGIGILVLLIIVILGVPLIIFTIKANSEACRDGLRAVME
CRNVTHLLQQELTEAQKGFQDVEAQAATCNHTVMALMASLDAEKAQGQKKVEELEGEI
TTLNHKLQDASAEVERLRRENQVLSVRIADKKYYPSSQDSSSAAAPQLLIVLLGLSALLQ

>P04201 multipass membrane protein
MDGSNVTSFVVEEPTNISTGRNASVGNAHRQIPIVHWVIMSISPVGFVENGILLWFLCFRM
RRNPFTVYITHLSIADISLLFCIFILSIDYALDYELSSGHYYTIVTLSVTFLFGYNTGLYLLTAI
SVERCLSVLYPIWYRCHRPKYQSALVCALLWALSCLVTTMEYVMCIDREEESHSRNDCRA
VIIFIAILSFLVFTPLMLVSSTILVVKIRKNTWASHSSKLYIVIMVTIIIFLIFAMPMRLLYLLYY
EYWSTFGNLHHISLLFSTINSSANPFIYFFVGSSKKKRFKESLKVVLTRAFKDEMQPRRQK
DNCNTVTVETVV

>Q5FVE4 peripheral membrane protein
MTGTPKTQEGAKDLEVDMNKTEVTPRLWTTCRDGEVLLRLSKHGPGHETPMTIPEFFRES
VNRFGTYPALASKNGKKWEILNFNQYYEACRKAAKSLIKLGLERFHGVGILGFNSAEWFI
TAVGAILAGGLCVGIYATNSAEVCQYVITHAKVNILLVENDQQLQKILSIPQSSLEPLKAIIQ
YRLPMKKNNNLYSWDDFMELGRSIPDTQLEQVIESQKANQCAVLIYTSGTTGIPKGVMLS
HDNITWIAGAVTKDFKLTDKHETVVSYLPLSHIAAQMMDIWVPIKIGALTYFAQADALKG
TLVSTLKEVKPTVFIGVPQIWEKIHEMVKKNSAKSMGLKKKAFVWARNIGFKVNSKKML
GKYNTPVSYRMAKTLVFSKVKTSLGLDHCHSFISGTAPLNQETAEFFLSLDIPIGELYGLSE
SSGPHTISNQNNYRLLSCGKILTGCKNMLFQQNKDGIGEICLWGRHIFMGYLESETETTEAI
DDEGWLHSGDLGQLDGLGFLYVTGHIKEILITAGGENVPPIPVETLVKKKIPIISNAMLVGD
KLKFLSMLLTLKCEMNQMSGEPLDKLNFEAINFCRGLGSQASTVTEIVKQQDPLVYKAIQ
[truncated: 488,397 more chars]
